# Species Distribution Modelling: Contrasting presence-only models with plot abundance data

**DOI:** 10.1038/s41598-017-18927-1

**Published:** 2018-01-17

**Authors:** Vitor H. F. Gomes, Stéphanie D. IJff, Niels Raes, Iêda Leão Amaral, Rafael P. Salomão, Luiz de Souza Coelho, Francisca Dionízia de Almeida Matos, Carolina V. Castilho, Diogenes de Andrade Lima Filho, Dairon Cárdenas López, Juan Ernesto Guevara, William E. Magnusson, Oliver L. Phillips, Florian Wittmann, Marcelo de Jesus Veiga Carim, Maria Pires Martins, Mariana Victória Irume, Daniel Sabatier, Jean-François Molino, Olaf S. Bánki, José Renan da Silva Guimarães, Nigel C. A. Pitman, Maria Teresa Fernandez Piedade, Abel Monteagudo Mendoza, Bruno Garcia Luize, Eduardo Martins Venticinque, Evlyn Márcia Moraes de Leão Novo, Percy Núñez Vargas, Thiago Sanna Freire Silva, Angelo Gilberto Manzatto, John Terborgh, Neidiane Farias Costa Reis, Juan Carlos Montero, Katia Regina Casula, Beatriz S. Marimon, Ben-Hur Marimon, Euridice N. Honorio Coronado, Ted R. Feldpausch, Alvaro Duque, Charles Eugene Zartman, Nicolás Castaño Arboleda, Timothy J. Killeen, Bonifacio Mostacedo, Rodolfo Vasquez, Jochen Schöngart, Rafael L. Assis, Marcelo Brilhante Medeiros, Marcelo Fragomeni Simon, Ana Andrade, William F. Laurance, José Luís Camargo, Layon O. Demarchi, Susan G. W. Laurance, Emanuelle de Sousa Farias, Henrique Eduardo Mendonça Nascimento, Juan David Cardenas Revilla, Adriano Quaresma, Flavia R. C. Costa, Ima Célia Guimarães Vieira, Bruno Barçante Ladvocat Cintra, Hernán Castellanos, Roel Brienen, Pablo R. Stevenson, Yuri Feitosa, Joost F. Duivenvoorden, Gerardo A. Aymard C., Hugo F. Mogollón, Natalia Targhetta, James A. Comiskey, Alberto Vicentini, Aline Lopes, Gabriel Damasco, Nállarett Dávila, Roosevelt García-Villacorta, Carolina Levis, Juliana Schietti, Priscila Souza, Thaise Emilio, Alfonso Alonso, David Neill, Francisco Dallmeier, Leandro Valle Ferreira, Alejandro Araujo-Murakami, Daniel Praia, Dário Dantas do Amaral, Fernanda Antunes Carvalho, Fernanda Coelho de Souza, Kenneth Feeley, Luzmila Arroyo, Marcelo Petratti Pansonato, Rogerio Gribel, Boris Villa, Juan Carlos Licona, Paul V. A. Fine, Carlos Cerón, Chris Baraloto, Eliana M. Jimenez, Juliana Stropp, Julien Engel, Marcos Silveira, Maria Cristina Peñuela Mora, Pascal Petronelli, Paul Maas, Raquel Thomas-Caesar, Terry W. Henkel, Doug Daly, Marcos Ríos Paredes, Tim R. Baker, Alfredo Fuentes, Carlos A. Peres, Jerome Chave, Jose Luis Marcelo Pena, Kyle G. Dexter, Miles R. Silman, Peter Møller Jørgensen, Toby Pennington, Anthony Di Fiore, Fernando Cornejo Valverde, Juan Fernando Phillips, Gonzalo Rivas-Torres, Patricio von Hildebrand, Tinde R. van Andel, Ademir R. Ruschel, Adriana Prieto, Agustín Rudas, Bruce Hoffman, César I. A. Vela, Edelcilio Marques Barbosa, Egleé L. Zent, George Pepe Gallardo Gonzales, Hilda Paulette Dávila Doza, Ires Paula de Andrade Miranda, Jean-Louis Guillaumet, Linder Felipe Mozombite Pinto, Luiz Carlos de Matos Bonates, Natalino Silva, Ricardo Zárate Gómez, Stanford Zent, Therany Gonzales, Vincent A. Vos, Yadvinder Malhi, Alexandre A. Oliveira, Angela Cano, Bianca Weiss Albuquerque, Corine Vriesendorp, Diego Felipe Correa, Emilio Vilanova Torre, Geertje van der Heijden, Hirma Ramirez-Angulo, José Ferreira Ramos, Kenneth R. Young, Maira Rocha, Marcelo Trindade Nascimento, Maria Natalia Umaña Medina, Milton Tirado, Ophelia Wang, Rodrigo Sierra, Armando Torres-Lezama, Casimiro Mendoza, Cid Ferreira, Cláudia Baider, Daniel Villarroel, Henrik Balslev, Italo Mesones, Ligia Estela Urrego Giraldo, Luisa Fernanda Casas, Manuel Augusto Ahuite Reategui, Reynaldo Linares-Palomino, Roderick Zagt, Sasha Cárdenas, William Farfan-Rios, Adeilza Felipe Sampaio, Daniela Pauletto, Elvis H. Valderrama Sandoval, Freddy Ramirez Arevalo, Isau Huamantupa-Chuquimaco, Karina Garcia-Cabrera, Lionel Hernandez, Luis Valenzuela Gamarra, Miguel N. Alexiades, Susamar Pansini, Walter Palacios Cuenca, William Milliken, Joana Ricardo, Gabriela Lopez-Gonzalez, Edwin Pos, Hans ter Steege

**Affiliations:** 1 Coordenação de Botânica, Museu Paraense Emílio Goeldi, Av. Magalhães Barata 376, C.P. 399, Belém, PA 66040-170 Brazil; 2 Programa de Pós-Graduação em Ciência Ambientais, Universidade Federal do Pará, Rua Augusto Corrêa 01, Belém, PA 66075-110 Brazil; 3 Biodiversity Dynamics, Naturalis Biodiversity Center, PO Box 9517, Leiden, 2300 RA The Netherlands; 4 Marine and Coastal Management, Deltares, Boussinesqweg 1, Delft 2629 HV The Netherlands; 5 Coordenação de Biodiversidade, Instituto Nacional de Pesquisas da Amazônia – INPA, Av. André Araújo 2936, Petrópolis, Manaus, AM 69067-375 Brazil; 6 EMBRAPA – Centro de Pesquisa Agroflorestal de Roraima, BR 174 km 8, Distrito Industrial, Boa Vista, RR 69301-970 Brazil; 7 Herbario Amazónico Colombiano, Instituto SINCHI, Calle 20 No 5-44, Bogotá, DC Colombia; 8 Department of Integrative Biology University of California, Berkeley, CA 94720-3140 USA; 9 Universidad San Francisco de Quito, Colegio de Ciencias Biológicas, Diego de Robles y Vía Interoceánica, Quito, Pichincha Ecuador; 10 Coordenação de Pesquisas em Ecologia, Instituto Nacional de Pesquisas da Amazônia – INPA, Av. André Araújo 2936, Petrópolis, Manaus, AM 69067-375 Brazil; 11 School of Geography, University of Leeds, Woodhouse Lane, Leeds LS2 9JT UK; 12 Department of Wetland Ecology, Institute of Geography and Geoecology, Karlsruhe Institute of Technology – KIT, Josefstr 1, Rastatt, D-76437 Germany; 13 Biogeochemistry, Max Planck Institute for Chemistry, Hahn-Meitner Weg 1, Mainz, 55128 Germany; 14 Departamento de Botânica, Instituto de Pesquisas Científicas e Tecnológicas do Amapá – IEPA, Rodovia JK Km 10, Campus do IEPA da Fazendinha, Amapá, 68901-025 Brazil; 15 AMAP, IRD, Cirad, CNRS, INRA, Université de Montpellier, TA A-51/PS2, Bd. de la Lironde, Montpellier, 34398 France; 16 Science and Education, The Field Museum, 1400 S. Lake Shore Drive, Chicago, IL 60605-2496 USA; 17 Coordenação de Dinâmica Ambiental, Instituto Nacional de Pesquisas da Amazônia – INPA, Av. André Araújo 2936, Petrópolis, Manaus, AM 69067-375 Brazil; 18 Jardín Botánico de Missouri, Oxapampa, Pasco Peru; 19 Departamento de Ecologia, Universidade Estadual Paulista – UNESP, Instituto de Biociências – IB, Av. 24 A 1515, Bela Vista, Rio Claro, SP 13506-900 Brazil; 20 Centro de Biociências, Departamento de Ecologia, Universidade Federal do Rio Grande do Norte, Av. Senador Salgado Filho 3000, Natal, RN 59072-970 Brazil; 21 Divisao de Sensoriamento Remoto – DSR, Instituto Nacional de Pesquisas Espaciais – INPE, Av. dos Astronautas 1758, Jardim da Granja, São José dos Campos, SP 12227-010 Brazil; 22 Herbario Vargas, Universidad Nacional de San Antonio Abad del Cusco, Avenida de la Cultura, Nro 733, Cusco, Cuzco Peru; 23 Departamento de Geografia, Universidade Estadual Paulista – UNESP, Instituto de Geociências e Ciências Extas – IGCE, Bela Vista, Rio Claro, SP 13506-900 Brazil; 24 Departamento de Biologia, Universidade Federal de Rondônia, Rodovia BR 364 s/n Km 9.5, Rural, Porto Velho, RO 76.824-027 Brazil; 25 Center for Tropical Conservation, Duke University, Nicholas School of the Environment, Durham, NC 27708 USA; 26 Programa de Pós-Graduação em Biodiversidade e Biotecnologia - PPG-Bionorte, Universidade Federal de Rondônia, Campus Porto Velho, Km 9.5, Rural, Porto Velho, RO 76.824-027 Brazil; 27 Instituto Boliviano de Investigacion Forestal, Av. 6 de agosto 28 Km 14, Doble via La Guardia Casilla, 6204, Santa Cruz, Santa Cruz, Bolivia; 28 Programa de Pós-Graduação em Ecologia e Conservação, Universidade do Estado de Mato Grosso, Nova Xavantina, MT Brazil; 29 Instituto de Investigaciones de la Amazonía Peruana – IIAP, Av. A. Quiñones km 2.5, Iquitos, Loreto 784 Peru; 30 Geography College of Life and Environmental Sciences, University of Exeter, Exeter, EX4 4RJ UK; 31 Departamento de Ciencias Forestales, Universidad Nacional de Colombia, Calle 64 x Cra 65, Medellín, Antioquia 1027 Colombia; 32 Agteca-Amazonica, Santa Cruz, Bolivia; 33 Facultad de Ciencias Agrícolas Universidad Autónoma Gabriel René Moreno Santa Cruz, Santa Cruz, Bolivia; 34 Prédio da Botânica e Ecologia Embrapa Recursos Genéticos e Biotecnologia Parque Estação Biológica, Av. W5 Norte, Brasilia, DF 70770-917 Brazil; 35 Projeto Dinâmica Biológica de Fragmentos Florestais, Instituto Nacional de Pesquisas da Amazônia - INPA, Av. André Araújo 2936, Petrópolis, Manaus, AM 69067-375 Brazil; 36 Centre for Tropical Environmental and Sustainability Science and College of Science and Engineering James Cook University, Cairns, Queensland 4870 Australia; 37 Laboratório de Ecologia de Doenças Transmissíveis da Amazônia (EDTA), Instituto Leônidas e Maria Deane, Fiocruz, Rua Terezina 476, Adrianópolis, Manaus, AM 69060-001 Brazil; 38 Programa de Pós-graduação em Biodiversidade e Saúde Instituto Oswaldo Cruz - IOC/FIOCRUZ Pav. Arthur Neiva – Térreo Av. Brasil 4365 – Manguinhos, Rio de Janeiro, RJ 21040-360 Brazil; 39 Centro de Investigaciones Ecológicas de Guayana Universidad Nacional Experimental de Guayana, Calle Chile, urbaniz Chilemex, Puerto Ordaz, Bolivar, Venezuela; 40 Laboratorio de Ecología de Bosques Tropicales y Primatología Universidad de los Andes Carrera 1 # 18a- 10, Bogotá, DC 111711 Colombia; 41 Programa de Pós-Graduação em Biologia (Botânica) Instituto Nacional de Pesquisas da Amazônia - INPA, Av. André Araújo 2936, Petrópolis, Manaus, AM 69067-375 Brazil; 42 Institute of Biodiversity and Ecosystem Dynamics University of Amsterdam, Sciencepark 904, Amsterdam, 1098 XH The Netherlands; 43 Programa de Ciencias del Agro y el Mar Herbario Universitario (PORT) UNELLEZ-Guanare, Guanare, Portuguesa 3350 Venezuela; 44 Endangered Species Coalition, 8530 Geren Rd, Silver Spring, MD 20901 USA; 45 MAUA Working Group Instituto Nacional de Pesquisas da Amazônia - INPA Av. André Araújo 2936, Petrópolis, Manaus, AM 69067-375 Brazil; 46 Inventory and Monitoring Program National Park Service 120 Chatham Lane, Fredericksburg, VA 22405 USA; 47 Center for Conservation Education and Sustainability Smithsonian Conservation Biology Institute, 1100 Jefferson Dr. SW, Suite 3123, Washington, DC 20560-0705 USA; 48 Biologia Vegetal, Universidade Estadual de Campinas, Caixa Postal 6109, Campinas, SP 13.083-970 Brazil; 49 Institute of Molecular Plant Sciences University of Edinburgh, Mayfield Rd, Edinburgh, EH3 5LR UK; 50 Royal Botanic Garden Edinburgh 20a Inverleith Row, Edinburgh, Scotland EH3 5LR UK; 51 Programa de Pós-Graduação em Ecologia, Instituto Nacional de Pesquisas da Amazônia - INPA, Av. André Araújo 2936, Petrópolis, Manaus, AM 69067-375 Brazil; 52 Forest Ecology and Forest Management Group Wageningen University Wageningen University & Research, Droevendaalsesteeg 3, Wageningen P.O. Box 47, 6700 AA The Netherlands; 53 Natural Capital and Plant Health Royal Botanic Gardens, Kew, Richmond, Surrey TW9 3AB UK; 54 Ecosistemas Biodiversidad y Conservación de Especies Universidad Estatal Amazónica, Km. 2 1/2 vía a Tena (Paso Lateral), Puyo, Pastaza Ecuador; 55 Museo de Historia Natural Noel Kempff Mercado Universidad Autónoma Gabriel Rene Moreno, Avenida Irala 565 Casilla Postal 2489, Santa Cruz, Santa Cruz, Bolivia; 56 Centro de Biociências, Departamento de Botânica e Zoologia, Universidade Federal do Rio Grande do Norte, Campus Universitário, Lagoa Nova, Natal, RN 59078-970 Brazil; 57 Department of Biology University of Miami Coral, Gables, FL 33146 USA; 58 Fairchild Tropical Botanic Garden Coral, Gables, FL 33156 USA; 59 Instituto de Biociências - Dept. Ecologia, Universidade de Sao Paulo - USP, Rua do Matão, Trav. 14, no. 321, Cidade Universitária, São Paulo, SP 05508-090 Brazil; 60 Diretoria de Pesquisas Científicas, Instituto de Pesquisas Jardim Botânico do Rio de Janeiro, Rio de Janeiro, RJ Brazil; 61 Escuela de Biología Herbario Alfredo Paredes Universidad Central, Ap. Postal 17.01.2177, Quito, Pichincha Ecuador; 62 International Center for Tropical Botany (ICTB) Department of Biological Sciences Florida International University, 11200 SW 8th Street, OE 243, Miami, FL 33199 USA; 63 Grupo de Ecología de Ecosistemas Terrestres Tropicales, Universidad Nacional de Colombia Sede Amazonía, Leticia, Amazonas Colombia; 64 Institute of Biological and Health Sciences, Federal University of Alagoas, Av. Lourival Melo Mota, s/n, Tabuleiro do Martins, Maceio, AL 57072-970 Brazil; 65 Museu Universitário/Centro de Ciências Biológicas e da Natureza/Laboratório de Botânica e Ecologia Vegetal, Universidade Federal do Acre, Rio Branco, AC 69915-559 Brazil; 66 Universidad Regional Amazónica IKIAM, Km 7 via Muyuna, Tena, Napo Ecuador; 67 Cirad UMR Ecofog AgrosParisTech CNRS INRA Univ Guyane, Campus agronomique, Kourou Cedex, 97379 France; 68 Iwokrama International Programme for Rainforest Conservation, Georgetown, Guyana; 69 Department of Biological Sciences, Humboldt State University, 1 Harpst Street, Arcata, CA 95521 USA; 70 New York Botanical Garden 2900 Southern Blvd, Bronx, New York, NY 10458-5126 USA; 71 Servicios de Biodiversidad EIRL, Jr. Independencia 405, Iquitos, Loreto 784 Peru; 72 Herbario Nacional de Bolivia, Universitario UMSA, Casilla 10077 Correo Central, La Paz, La Paz, Bolivia; 73 Missouri Botanical Garden, P.O. Box 299, St. Louis, MO 63166-0299 USA; 74 School of Environmental Sciences, University of East Anglia, Norwich, NR4 7TJ UK; 75 Laboratoire Evolution et Diversité Biologique CNRS and Université Paul Sabatier UMR 5174 EDB, Toulouse, 31000 France; 76 Department of Forestry Management, Universidad Nacional Agraria La Molina, Avenido La Molina, Apdo. 456, La Molina, Lima Peru; 77 School of Geosciences University of Edinburgh, 201 Crew Building, King’s Buildings, Edinburgh, EH9 3JN UK; 78 Biology Department and Center for Energy, Environment and Sustainability, Wake Forest University, 1834 Wake Forest Rd, Winston Salem, NC 27106 USA; 79 Department of Anthropology University of Texas at Austin, SAC 5.150, 2201 Speedway Stop C3200, Austin, TX 78712 USA; 80 Andes to Amazon Biodiversity Program, Madre de Dios, Madre de Dios, Peru; 81 Fundación Puerto Rastrojo, Cra 10 No. 24-76 Oficina 1201, Bogotá, DC Colombia; 82 Colegio de Ciencias Biológicas y Ambientales-COCIBA & Galapagos Institute for the Arts and Sciences-GAIAS, Universidad San Francisco de Quito-USFQ, Quito, Pichincha Ecuador; 83 Department of Wildlife Ecology and Conservation University of Florida, 110 Newins-Ziegler Hall, Gainesville, FL 32611 USA; 84 Fundación Estación de Biología, Cra 10 No. 24-76 Oficina 1201, Bogotá, DC Colombia; 85 Embrapa Amazônia Oriental, Trav. Dr. Enéas Pinheiro s/nº, Belém, PA 66095-100 Brazil; 86 Instituto de Ciencias Naturales, Universidad Nacional de Colombia, Apartado, 7945 Bogotá, DC Colombia; 87 Amazon Conservation Team, Doekhieweg Oost #24, Paramaribo, Suriname; 88 Facultad de Ciencias Forestales y Medio Ambiente, Universidad Nacional de San Antonio Abad del Cusco, Jirón San Martín 451, Puerto Maldonado, Madre de Dios Peru; 89 Laboratory of Human Ecology, Instituto Venezolano de Investigaciones Científicas - IVIC, Ado 20632, Caracas, Caracas, 1020A Venezuela; 90 Departement EV, Muséum national d’histoire naturelle de Paris, 16 rue Buffon, Paris, 75005 France; 91 Instituto de Ciência Agrárias, Universidade Federal Rural da Amazônia, Av. Presidente Tancredo Neves 2501, Belém, PA 66.077-901 Brazil; 92 PROTERRA, Instituto de Investigaciones de la Amazonía Peruana (IIAP), Av. A. Quiñones km 2 5, Iquitos, Loreto 784 Peru; 93 ACEER Foundation, Jirón Cusco N° 370, Puerto Maldonado, Madre de Dios, Peru; 94 Universidad Autónoma del Beni José Ballivián, Campus Universitario Final Av. Ejercito, Riberalta, Beni Bolivia; 95 Regional Norte Amazónico, Centro de Investigación y Promoción del Campesinado, C/Nicanor Gonzalo Salvatierra N° 362, Riberalta, Beni Bolivia; 96 Environmental Change Institute, Oxford University Centre for the Environment, Dyson Perrins Building, South Parks Road, Oxford, England OX1 3QY UK; 97 School of Agriculture and Food Sciences - ARC Centre of Excellence for Environmental Decisions CEED, The University of Queensland, St. Lucia, QLD 4072 Australia; 98 Instituto de Investigaciones para el Desarrollo Forestal (INDEFOR), Universidad de los Andes, Conjunto Forestal, C.P. 5101, Mérida, Mérida, Venezuela; 99 School of Environmental and Forest Sciences, University of Washington, Seattle, WA 98195-2100 USA; 100 University of Nottingham, University Park, Nottingham, NG7 2RD UK; 101 Geography and the Environment, University of Texas at Austin, 305 E. 23rd Street, CLA building, Austin, TX 78712 USA; 102 Laboratório de Ciências Ambientais, Universidade Estadual do Norte Fluminense, Av. Alberto Lamego 2000, Campos dos Goyatacazes, RJ 28013-620 Brazil; 103 Department of Biology, University of Maryland, College Park, MD 20742 USA; 104 GeoIS, El Día 369 y El Telégrafo, 3° Piso, Quito, Pichincha Ecuador; 105 Environmental Science and Policy, Northern Arizona University, Flagstaff, AZ 86011 USA; 106 FOMABO, Manejo Forestal en las Tierras Tropicales de Bolivia, Sacta, Cochabamba Bolivia; 107 Escuela de Ciencias Forestales (ESFOR), Universidad Mayor de San Simon (UMSS), Sacta, Cochabamba Bolivia; 108 Agricultural Services, Ministry of Agro-Industry and Food Security, Agricultural Services, Ministry of Agro-Industry and Food Security, The Mauritius Herbarium, Reduit, Mauritius; 109 Department of Bioscience, Aarhus University, Building 1540 Ny Munkegade, Aarhus C, Aarhus, DK-8000 Denmark; 110 Ciencias Biológicas, Universidad de Los Andes, Carrera 1 # 18a- 10, Bogotá, DC 111711 Colombia; 111 Medio Ambiente, PLUSPRETOL, Iquitos, Loreto Peru; 112 Center for Conservation Education and Sustainability, Smithsonian’s National Zoo & Conservation Biology Institute, National Zoological Park, 3001 Connecticut Ave, Washington, DC 20008 USA; 113 Tropenbos International, Lawickse Allee 11 PO Box 232, Wageningen, 6700 AE The Netherlands; 114 Instituto de Biodiversidade e Floresta, Universidade Federal do Oeste do Pará, Rua Vera Paz, Campus Tapajós, Santarém, PA 68015-110 Brazil; 115 Department of Biology, University of Missouri, St. Louis, MO 63121 USA; 116 Facultad de Biologia, Universidad Nacional de la Amazonia Peruana, Pevas 5ta cdra, Iquitos, Loreto Peru; 117 School of Anthropology and Conservation, University of Kent, Marlowe Building, Canterbury, Kent CT2 7NR UK; 118 Herbario Nacional del Ecuador, Universidad Técnica del Norte, Quito, Pichincha Ecuador; 119 Ecology & Biodiversity Group, Utrecht University, Padualaan 8, Utrecht, 3584 CH The Netherlands; 120 Systems Ecology, Free University, De Boelelaan 1087, Amsterdam, 1081 HV Netherlands

## Abstract

Species distribution models (SDMs) are widely used in ecology and conservation. Presence-only SDMs such as MaxEnt frequently use natural history collections (NHCs) as occurrence data, given their huge numbers and accessibility. NHCs are often spatially biased which may generate inaccuracies in SDMs. Here, we test how the distribution of NHCs and MaxEnt predictions relates to a spatial abundance model, based on a large plot dataset for Amazonian tree species, using inverse distance weighting (IDW). We also propose a new pipeline to deal with inconsistencies in NHCs and to limit the area of occupancy of the species. We found a significant but weak positive relationship between the distribution of NHCs and IDW for 66% of the species. The relationship between SDMs and IDW was also significant but weakly positive for 95% of the species, and sensitivity for both analyses was high. Furthermore, the pipeline removed half of the NHCs records. Presence-only SDM applications should consider this limitation, especially for large biodiversity assessments projects, when they are automatically generated without subsequent checking. Our pipeline provides a conservative estimate of a species’ area of occupancy, within an area slightly larger than its extent of occurrence, compatible to e.g. IUCN red list assessments.

## Introduction

Species distribution models (SDMs) are widely used in the fields of macroecology, biogeography and biodiversity research for modelling species geographic distributions based on correlations between known occurrence records and the environmental conditions at occurrence localities^1,2^. SDMs generate geographical maps of a species’ environmental suitability, its likelihood of being collected, and its local abundance^3^. Their application includes selecting conservation areas, predicting the effects of climate change on species ranges and determining the risk of species invasions^4,5^. The wide use of SDMs in ecological and conservation research can partly be explained by the growing availability of georeferenced species records (e.g. GBIF, SpeciesLink) and environmental data (e.g. WorldClim, CliMond)^6,7^ on the web, together with the user-friendly character of some of the modelling methods.

One of the most commonly used SDMs is MaxEnt, which has become increasingly popular since its introduction^8^. This machine-learning algorithm estimates a species’ probability distribution that has maximum entropy (closest to uniform), subject to a set of constraints based upon our knowledge of the environmental conditions at known occurrence sites^1^. MaxEnt is a presence-only model, enabling scientists to utilize the abundant data sources of natural history collections (NHCs), avoiding the high costs of sampling the species throughout their extent of occurrence. Presence data are abundant, but absence data are hard to obtain and often unreliable due to insufficient survey effort. To counter the lack of absences, MaxEnt uses a background sample to contrast the distribution of presences along environmental gradients against the distribution background points, randomly drawing from the study area.

NHCs, however, may not be independently drawn from the investigated populations due to the non-random nature of collecting^9,10^. Because collectors aim to collect as many species as possible, rare species are often overrepresented in herbaria, whereas common species are underrepresented, producing collectors’ bias^11^. Therefore, the relative number of specimens per species in herbaria is not a good representation of the species’ relative abundance in the field. Additionally, NHCs have spatial bias due to geographical differences in survey effort, data storage and mobilization^9,10,12^. This may have negative impacts on the performance of presence-only SDMs if this results in environmentally biased sampling^12–15^. Negative impact of spatial bias is not always present, however^16,17^.

MaxEnt has shown to outperform other SDMs in several studies^18–22^. Nevertheless, some drawbacks have been identified. For example, MaxEnt may underestimate the probability of occurrence within areas of observed presence, while overestimating it in areas beyond the species’ known extent of occurence^23^. Like other SDMs, one essential assumption of MaxEnt is that the presence-data are an independent sample from the species’ unknown probability distribution of occurrence over the study area^1^. Given the shortcomings of NHCs due to collectors’ bias mentioned above, this assumption may not be met.

With a large set of plots with quantitative data, species abundances may be estimated by a spatial interpolation of local species’ abundances^24^. Based on plot data, where all species are collected (regardless of commonness or rarity), the interpolation method arguably suffers less from the collectors’ bias and is exclusively based on location. The abundance maps may serve as the species’ estimated probability distribution and a higher local abundance implies a higher probability of collecting. That is, the chance of encountering a species is higher in a region where the relative abundance of that species is high, than where the relative abundance is low. With spatially interpolated abundances we may thus test whether NHCs can actually be considered a random sample of the unknown probability distribution.

Here we test how the geographic distribution of NHCs relates to the species relative abundance. To achieve this we address the following questions: (1) Do NHCs represent an independently drawn sample from the unknown probability distribution of a species? And (2) how does MaxEnt’s predicted environmental suitability compare to plot abundance data and spatial interpolation of species abundances? To answer these questions, we used NHCs and abundance plot data of 227 hyperdominant Amazonian tree species, which are the most common tree species that together make up half of all trees with a diameter (dbh) over 10 cm in Amazonia^24,25^, the most biodiverse rainforest on Earth. We used NHCs and MaxEnt to construct presence-only SDMs for all 227 species and constructed the abundance maps by spatial interpolation of the plot abundance data for all species as well. To answer the first question, we compared the collection records to the interpolated abundance maps for each species. Secondly, we compared MaxEnt’s predicted environmental suitability maps to the same interpolated abundance maps for each of the 227 species.

## Results

### NHCs data distribution and relative abundance analysis

The analysis testing our first question, whether NHCs are an independent draw from the unknown probability distribution, resulted in a significant (*P* < 0.05), but very weak positive relationship for 149 (66%) species of the 227. For these species the chance of being collected indeed increased slightly with higher interpolated relative abundance. For the other 78 species (34%), this relationship was non-significant or negative (Appendix S1).

### Predicted environmental suitability compared to species relative abundances

**Figure 1:**
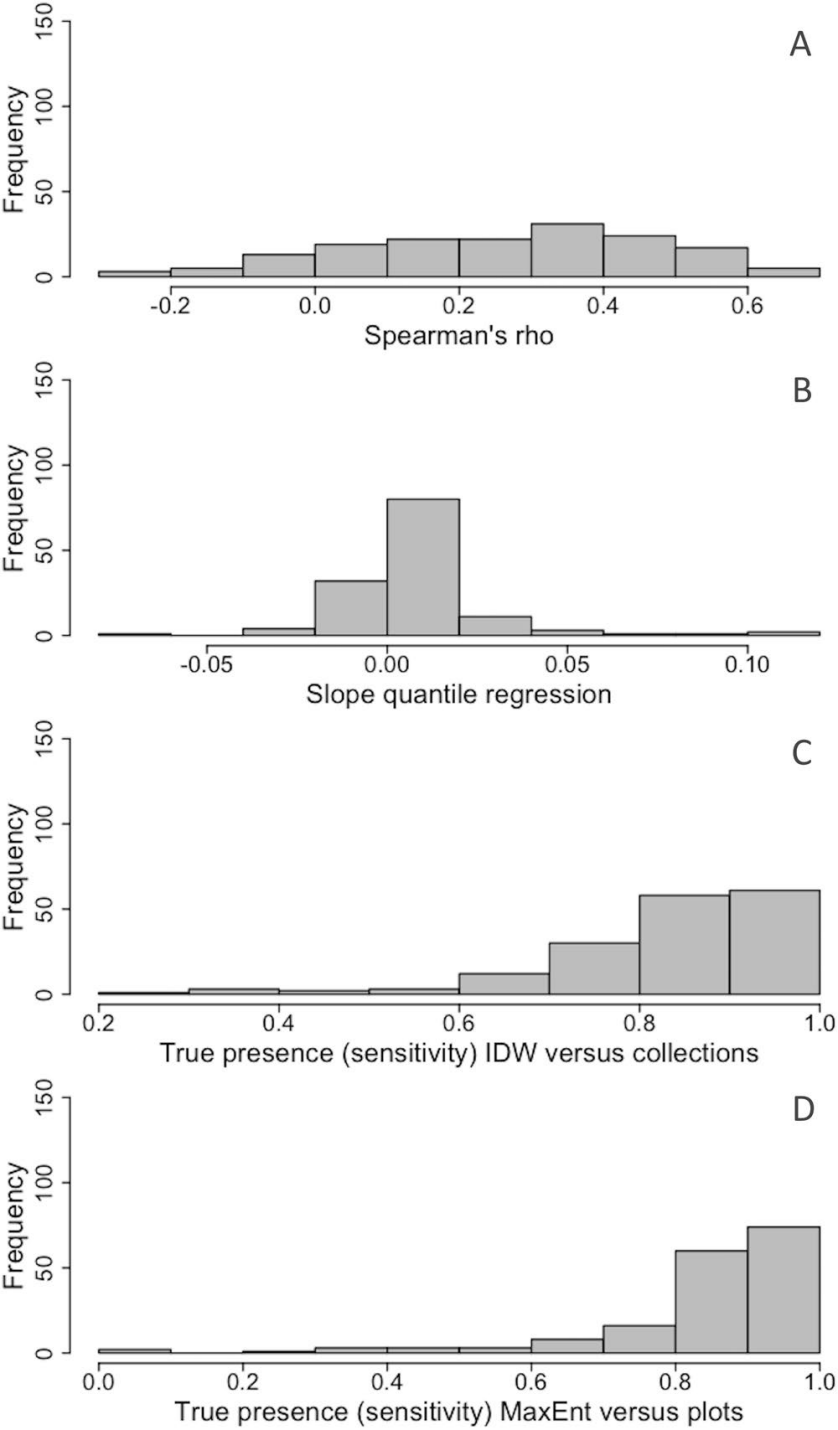
Frequency distributions for 189 significant hyperdominant Amazonian tree species of (**A**) the Spearman’s correlation index rho between MaxEnt’s predicted environmental suitability and relative local abundance of the plots; (**B**) The slopes of the linear 90^th^ percentile quantile regression between MaxEnt’s predicted environmental suitability and the relative local abundance of the plots; (**C**) The true presence (sensitivity) of the distribution predicted by the IDW maps compared to the collection localities; and (**D**) The true presence (sensitivity) of the distribution predicted by the MaxEnt maps compared to the plot presence.

Further analyses were carried out using only 170 species. Species, that had MaxEnt’s predicted environmental suitability not significantly different from a random expectation tested with bias corrected null models, were excluded (57 species). For 161 of the 170 species (95%), MaxEnt’s predicted environmental suitability was also significantly correlated with interpolated abundance (*P* < 0.05). The correlations and, thus the biological significance, were low however, with a mean rho (Spearman rank correlation) of 0.26 (Fig. 1A). A linear 90^th^ quantile regression revealed that for 135 (79%) of the 175 species, the logistic output of MaxEnt could significantly (*P* < 0.05) predict the highest 10% of the local relative abundance values. The slope of the regression and thus the biological significance was very low, with a mean slope of only 0.01 (Fig. 1B).

We also investigated the performance of the IDW output against NHCs data and the MaxEnt output against plot presence (sensitivity), to check whether the models were accurate references to the occurrence data of each other (Appendix S2). Approximately 87% of the grid cells with species’ NHCs were correctly predicted as present by the IDW maps with a median true positive rate of 0.87 (Fig. 1C). The same analyses for MaxEnt showed that 88% of the grid cells with plot presence were correctly predicted by MaxEnt maps, with a median true positive rate of 0.88 (Fig. 1D). Sensitivity for both analyses was high.

**Figure 2:**
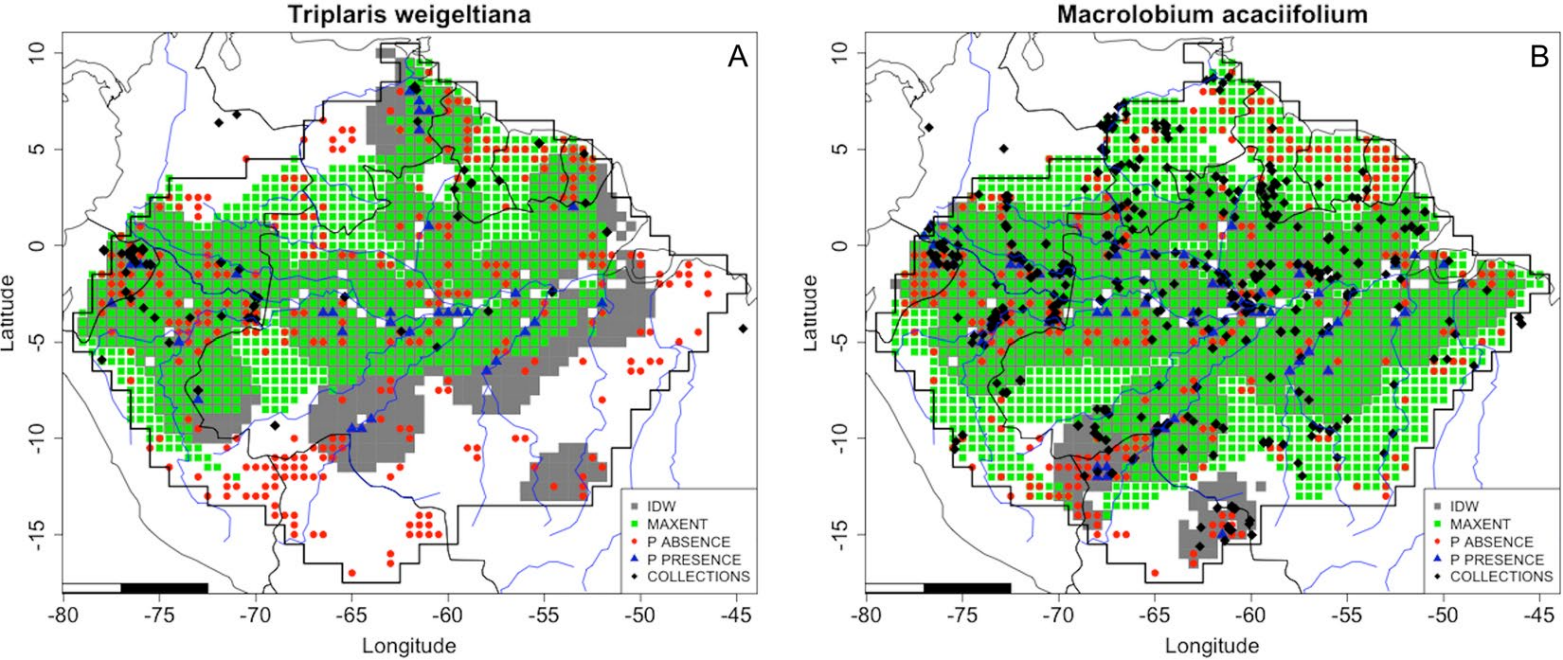
The predicted area of occupancy by MaxEnt (green) and the IDW map (grey) of (**A**) *Triplaris weigeltiana*; and (**B**) *Macrolobium acaciifolium*. The localities of the collections, presence and absence plots are also indicated. Maps created with custom R script. Base map source (country.shp, rivers.shp): ESRI (http://www.esri.com/data/basemaps, © Esri, DeLorme Publishing Company).

We provide maps (combined MaxEnt and IDW maps [as in Fig. 2]) for all species in the Supplementary Material S3. The predicted environmentally suitable region and the abundance distribution were similar for very abundant species with a large extent of occurence, such as *Brosimum rubescens* (Fig. S3_14A), *Conceveiba guianensis* (Fig. S3_32A) and *Eschweilera coriacea* (Fig. S3_49A). The same was true in the case of the species *Clathrotropis glaucophylla* (Fig. S3_30A) and *Cenostigma tocantinum* (Fig. S3_26A), despite the fact that neither species has a wide extent of occurence.

Moreover, MaxEnt also correctly predicted the environmental unsuitability of non-forested savanna areas, which are located in the north (Brazil, Guianas and Venezuela) and south of the map (northern Bolivia). These close matches apply to very abundant species with a large extent of occurence, such as *Licania micrantha* (Fig. S3_87A), and *Ocotea aciphylla* (Fig. S3_111A).

For *Triplaris weigeltiana*, a species with a northern Amazonian distribution, MaxEnt also correctly predicted its absence in these northern non-forested areas (Fig. 2A, S3_160A,B). In this case MaxEnt was able to establish a relationship between species distribution and vegetation type, based on climate variables (temperature and precipitation) and species occurrence. For *Macrolobium acaciifolium*, a riverine species, the IDW presented limitations. This species is rarely recorded in plots, because the plots are mostly far from river edges. Thus, the species was found only in plots near to major rivers such as the Amazon. In this case NHCs provided better information about species occurrence, as collectors can reach areas closer to other smaller rivers aiming to collect more species. In such a case, MaxEnt maps presented a wider distribution for the species (Fig. 2B, S3_92A–C).

IDW maps predicted widespread distributions for palms, for which the MaxEnt estimates were in sharp disagreement. Palms species are more difficult to collect, which can result in a lack of specimens in NHCs^24^. IDW maps appear to be more accurate for these species, because all species are recorded inside plots. In eastern Amazonia this was particularly severe because NHCs showed a large lack of occurrence in comparison with plot data but also proper locations were rejected by a kernel density estimate (KDE), because of the huge amount of palm occurrence data from the Aarhus University Palm Transect Database in western Amazonia. Some of the species affected were *Attalea butyracea* (Fig. S3_8A), *Euterpe precatoria* (Fig. S3_60A), *Iriartea deltoidea* (Fig. S3_72A), *Oenocarpus bacaba* (Fig. S3_113A), *Oenocarpus bataua* (Fig. S3_114A) and *Socratea exorrhiza* (Fig. S3_150A).

### NHCs data cleaning treatment and MaxEnt map building

All 227 hyperdominant species had records excluded by the data cleaning treatment, the consequence of records that either lack geographic information, are duplicates at the used grid cell resolution of 0.5 degree or were outliers based on a kernel-density estimate (Appendix S4). An average of 50% of the records was excluded. The first twelve species with the most excluded records were palms, with a mean of 96% of excluded records. The total average of excluded records decreased to 43% when palms were taken out of the analyses (Appendix S4). This high percentage is due to the huge amount of palm occurrence data of the Aarhus University Palm Transect Database^26^. At this moment this database contains 543,000 records, all available in GBIF. Most of these records represent observations in many plots inside the same grid cell, thus these records were removed and considered as a single observation.

**Figure 3:**
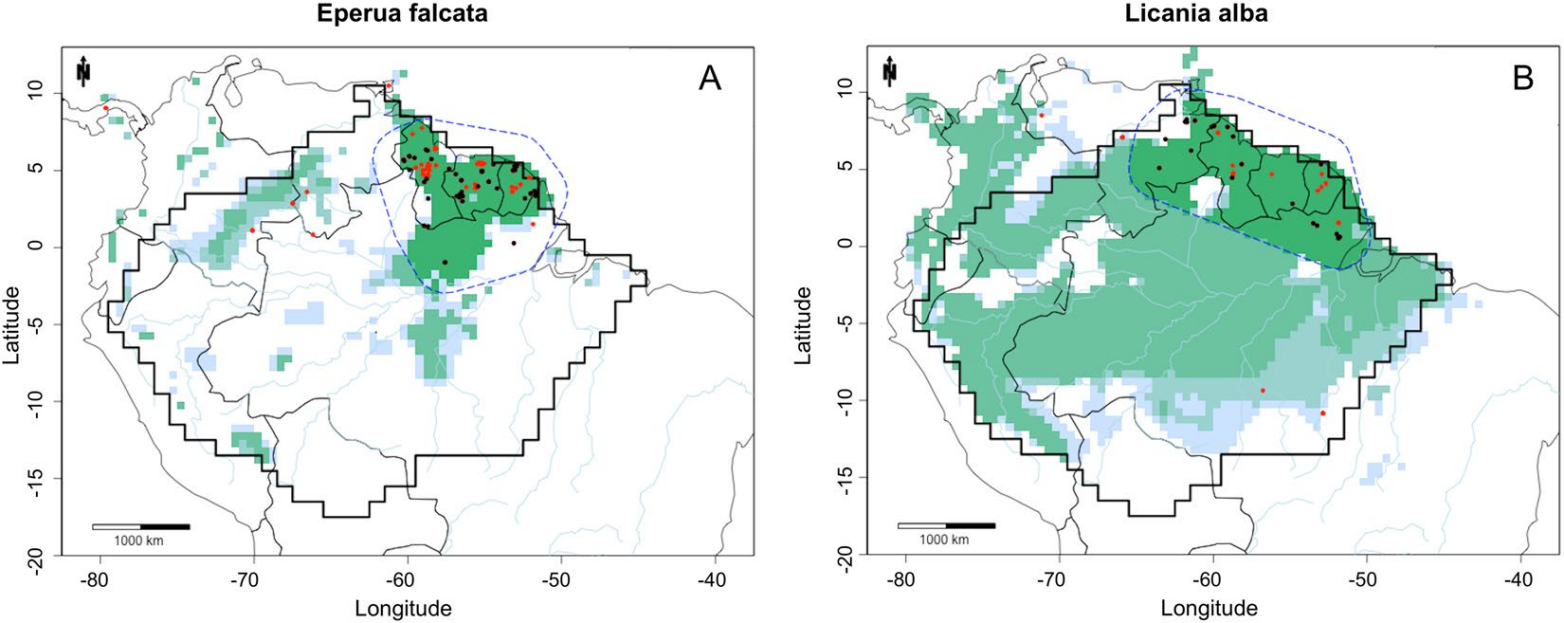
MaxEnt environmental suitability maps for (**A**) *Eperua falcata*; (**B**) *Licania alba*. MaxEnt maps constructed using GBIF records, cleaned GBIF records, kernel-density estimate GBIF records, and kernel-density estimate GBIF records plus the buffer clip. ***Black dots***: GBIF records. ***Red dots***: GBIF records after the use of the cleaning pipeline. ***Dashed blue line***: buffer based on a convex hull around species cleaned collections. ***Light blue***: predicted environmental suitability using GBIF records. ***Light green***: predicted environmental suitability using cleaned GBIF records. ***Medium green***: predicted environmental suitability using kernel density estimate GBIF records. ***Dark green***: predicted environmental suitability using kernel density estimate GBIF records and the buffer clip, resulting in the final predicted area of occupancy. Maps created with custom R script. Base map source (country.shp, rivers.shp): ESRI (http://www.esri.com/data/basemaps, © Esri, DeLorme Publishing Company).

After the kernel density estimate treatment the average of excluded records was 57%, presenting an increment of 6.7% in the total amount of records excluded. *Eperua purpurea* and *Eperua leucantha* collections were in good agreement with plot data distribution, after outliers were excluded by the kernel density estimate (Appendix S5). In the case of *Eperua falcata*, some occurrences in Colombia and Venezuela were in fact misidentifications of *Eperua leucantha*, since this species occurs only in the Guianas (H. ter Steege, pers. obs.). The kernel density estimate function correctly removed these occurrences outside the *E. falcata* cluster observed in the Guianas (Fig. 3A). Some occurrences of *Licania alba* in southeast Amazonia, an area with no plot data, were also removed by the kernel-density estimate function (Fig. 3B).

Because of the use of the buffer treatment to limit MaxEnt predictions around the species’ extent of occurrence, MaxEnt maps predicted an area of occupancy close to that of the IDW maps., The median value for MaxEnt’s area of occupancy was 1354 0.5-degree grid cells, and the median for IDW was 1217. For 98 (58%) of the 170 species, MaxEnt predicted an area of occupancy bigger than the that predicted by IDW, and for 115 (42%) of the species IDW had a predicted area of occupancy bigger than MaxEnt. In 15% of the cases (26 species) the size difference in area of occupancy was smaller than 5% (Appendix S2).

## Discussion

### Using NHCs for presence-only SDMs

Collection density was weakly related to relative abundance in most tree species, and for 34% there was no positive relationship between the chance of being collected and local abundance, violating the assumption of MaxEnt that collection localities are an independently drawn sample from a species’ unknown probability distribution^1^. The differences between the distribution of NHCs and local abundance could limit the ability of presence-only SDMs to predict species probability of distribution as predicted by spatial interpolation of local species’ abundance.

MaxEnt’s premise that species occurrences are drawn randomly from the unknown probability distribution^1^ may not be met for two reasons: (1) collections are spatially biased with regard to environmental conditions^13^; (2) and collections are spatially biased with regard to areas of high abundance, with underrepresentation of in areas of high abundance and overrepresentation in areas of low abundance. Much attention has been given to the possible impacts of spatial bias on the performance of presence-only SDMs, with some showing a negative impact on these models^13–15^, and others arguing for the robustness of MaxEnt against spatial bias^17,27^. However, little attention has been given to the second issue. With our plot dataset, we addressed the relationship between collection localities and the predicted spatial abundance distribution.

In 66% of the cases we found that a higher local relative abundance indeed increased the chance of being collected, although the correlations were very weak (Fig. 1A), and the majority of collections originated from areas with a low relative abundance due to the large areas where a given species’ abundance is low. Even hyperdominant species are usually only dominant in one or two of the six regions of Amazonia, most hyperdominant species have a large geographic extent of occurrence but are habitat specialists^24^. Steege *et al*.^25^ also found that abundance is a poor predictor for the number of collections of a species compared to the size of its extent of occurrence. Additionally, herbaria are characterized by the earlier discussed collectors’ bias^11^. Although we addressed the spatial bias of survey effort by including a bias based background file in our MaxEnt modelling, the lack of a significant positive relationship between relative abundance estimated by IDW and collection density for many species suggests that this assumption of MaxEnt is not met because of the way species are collected.

### MaxEnt maps vs. IDW maps

We also asked if MaxEnt maps would be a close match to the IDW maps. In general, environmental suitability does not reflect a species abundance. Presence-only SDMs, such as MaxEnt, are based on correlations between species presence and environmental conditions, predicting the environmental suitability for a species, and not their realized distribution^5^. Relative abundance in the other hand is based solely on abundance, estimating the number of trees belonging to each species in the grid cells^28^. The Spearman’s rank correlation and the linear 90^th^ percentile quantile regression showed a very weak positive relationship between MaxEnt’s predicted environmental suitability and IDW relative abundance prediction at plot localities, contrary to the results of VanDerWal *et al*.^29^, who found a strong relationship between the two. Their research differs in that they modelled a biogeographical region with tropical and subtropical rainforests, and also drier and warmer environments. The relationship between environmental suitability and local abundance is likely to be stronger when more (extreme) divergent conditions are included, such as areas from different biomes. Perhaps Amazonia’s less divergent conditions, representing perhaps a one single biome, in a much larger area, are potentially responsible for this weak relationship.

To test their further predictive performances we also converted both outputs to binary maps. Some studies have addressed questions about the transformation of SDM predictions into discrete representations such as binary maps, aiming to estimate area of occupancy, species richness and others applications^30–33^. Binary maps can add more uncertainties to model predictions, especially because it is necessary to set a threshold to distinguish between species presence and absence, which can be selected arbitrarily or without taking into account the context of the study. However, we avoided thresholds based on specificity (prediction of absences) because of the lack of absence data^34^. In many cases our MaxEnt binary maps presented an area of occupancy close to those made with IDW, presenting a high median sensitivity (88%). Moreover our MaxEnt binary maps also correctly predicted absence in naturally non-forested areas in northern Amazonia for many species (Appendix S3).

MaxEnt’s environmental suitability mostly predicted much larger area of occupancy than those predicted with the IDW relative abundance. We reduced this effect estimating species extent of occurrence using a convex hull around each species records, plus a buffer of 300 km. This approach minimized MaxEnt’s overestimation of the area of occupancy beyond the species’ known geographical range (extent of occurrence), over climatically suitable areas, by restricting the species’ predicted suitable habitat, providing a more conservative estimate for the species’ area of occupancy (Appendix S5)^35,36^.

The IDW relative abundance models showed an opposite behaviour, underpredicting areas where collections are present but where no plots have recorded the species. The high sensitivity of the MaxEnt compared to those of the IDW is in agreement with a previous study^37^, where models fit to presence-only data yielded higher sensitivity but a lower specificity than presence-absence models. Nevertheless, in our case, the IDW relative abundance yielded sensitivity rates based on collection localities that were as high as the sensitivity rates of MaxEnt’s predicted environmental suitability based on plot presence localities (87%). Thus, both models function similarly in predicting species presences.

### Collections versus abundance plot data

In some cases, collections were located outside the species’ extent of occurrence predicted by the IDW maps. This divergence follows from the methodical differences between collections and plot assessments. The distributions as predicted by the IDW do not always cover the whole species’ extent of occurrence. Because there are only 550 individuals (on average) in one plot, and 16,000 tree species in Amazonia^24^, one plot obviously cannot contain all species that are present in the surrounding area. Furthermore, many plots, lacking a given species, are within the extent of occurrence predicted by IDW, and many plots with absences are located in close proximity to plots with presence data. This results in low specificity values. NHCs comprise a species’ range including areas of low abundance; while plot data have information on abundance, but may miss areas of low abundance, and, thus, may miss rare species more easily.

### Environmental suitability versus dispersal limitation

The second large difference between the two models is the theoretical principles they are based upon. MaxEnt is based on environmental suitability, which is appropriate since correlations between species’ distributions and climate are evident^5,36^. Nevertheless, predicting actual (realized) distributions also requires information on biotic interactions, dispersal limitation, and other environmental variables, which are beyond presence-only SDM^5^. IDW, on the other hand, is based on location only. Thus, both models cover only one of the three explanatory variables for species distributions. Again, it will depend on the aim of the research which type of model is most suitable. In either case predicted species distributions need to be interpreted with caution.

### Collection data and cleaning pipeline

We propose a cleaning pipeline to remove possible inconsistencies in collection data. Unlike species-specific approaches, many studies use large numbers of species, lacking correction because of the great number of references and specialists to be consulted^38^. Collection data available in global datacentres, such as GBIF, cannot carry out thorough data-correction procedures, and the quality of the records has been debated and tested in some cases^39^. Some records have no locality information, or coordinates are based on cities close to the observed distribution, and may contain duplicated data or zeros as information^38–40^.

We used a pipeline that cleans collection data by removing records with a lack of geographic information^38^, and we strongly recommend the use of analytical tools to correct inconsistencies present in global databases. The cleaning process also removed coordinates considered spatial outliers by a kernel-density estimate, omitting locations too far from the central part of the distribution, which we assume to be misidentifications.

Our results suggest that half of the species records are likely inconsistent, missing geographical information, such as latitude, longitude or locality. Palms were the most impacted species, because the huge amount of records available with high levels of redundancy.

We used a kernel density estimate (KDE) to remove geographical outliers of the NHCs. This function removed e.g. occurrences outside the *Eperua falcata* cluster observed in the Guianas (Fig. 3A), and *Licania alba* in southeast Amazonia (Fig. 3B). Although the KDE excluded only a small number of records compared to the previous cleaning step, it was able to identify some isolated occurrences, which we considered likely misidentifications. The KDE, however, showed limitations with palm species, removing some eastern Amazonia records, simply caused by the great number of collections in the Aarhus University Palm Transect Database in western Amazonia.

## Conclusion

We have shown that the NHCs violate the assumption of MaxEnt that collection localities are an independently drawn sample from a species’ unknown probability distribution. Although we found a relationship between NHCs and relative abundance for some species, it was very weak. Additionally, we found that the majority of MaxEnt’s predicted environmental suitability values differ from those of the IDW relative abundance values, and its results cannot be interpreted as an abundance estimate. Nevertheless, MaxEnt predicts probability of occurrence well, and both models largely overlap and predict similar areas of occupancy, showing high sensitivities. Furthermore, NHCs data should undergo cleaning processes before being used to represent occurrences in species distribution models. We showed that, half of the species records are likely inconsistent, missing geographical information, such as latitude, longitude or locality, and it also may represents misidentifications of the species. We therefore conclude that distribution maps as generated by MaxEnt should be used with caution. Their application should not be based solely on unsupervised models, especially because their easily constructed distribution maps are tempting to utilize without indication of probable errors. This outcome is particularly important for biodiversity assessments, for which SDMs of a large number of species are automatically generated without subsequent checking. Our pipeline provides a conservative means to do so. As our pipeline removes inconsistencies from NHCs data and estimates area of occupancy in an area slightly larger than the extent of occurrence of a species, compatible with IUCN red list assessments^35,41^.

## Methods

### Species

We focused our analysis on 227 hyperdominant Amazonian tree species. The hyperdominant species are the most common tree species in Amazon, and together make up half of all trees with a diameter (dbh) over 10 cm^24^. We chose only hyperdominant species to reduce the emergence of too many ‘false absences’ when plot data are interpolated into abundance maps. They present the largest probability of occurrence in the plots where they are present in the surrounding area.

### Collections

Species collections were downloaded from GBIF (August 2017, www.gbif.org). We used data from the species’ complete extent of occurrence to prevent deficiencies that are associated with SDMs based on a species’ partial geographic range, such as under-prediction^42^. All individuals were assigned to species level; intraspecific levels were ignored.

Taxonomic names were checked with the Taxonomic Name Resolution Service (TNRS, http://tnrs.iplantcollaborative.org/). Although misidentification may represent a major problem in tree plots, we assume it is less severe in common species such as the hyperdominants; which are better represented in herbaria and more likely to be collected fertile^25^. We assume that misidentification is within acceptable limits.

### Collections cleaning pipeline

The cleaning pipeline consisted of a two-step process to remove inconsistencies from GBIF downloaded data (GBIF records). The first step consisted of removing all records with missing latitude, longitude or locality information (imprecise georeferences)^39^ and all duplicates at 0.5-degree spatial resolution^40^. With the GeoClean function from speciesgeocodeR R Package^38^ we also removed coordinates assigned to capital cities, coordinates with latitude equal to longitude, coordinates equal to exactly zero; coordinates based on centroids of provinces, and corrected country references (cleaned GBIF records).

In the second step we used a kernel-density estimate function to remove spatial outliers from the cleaned GBIF data, assuming that these are misidentifications or incorrect coordinates not filtered by the step described above. This function calculates a fixed-bandwidth kernel-density estimate of the point process density function that produced the point patterns^43^, using the density.ppp function from spatstat R Package^44^ to generate a kernel-density estimate. Outliers were identified and removed based on the kernel-density values for each species coordinate, using a threshold based on a quantile function from stats R Package^45^ (kernel-density estimate GBIF records).

The quantile threshold was set according to the number of Amazonian regions in which a species occurred, six in total as defined by ter Steege *et al*.^24^. The quantile threshold was larger for species with narrow distribution (occurring in one to three Amazonian regions) and smaller for species with wide distribution (occurring in more than three Amazonian regions). As some hyperdominants are very widely distributed in Amazonia a larger quantile threshold cuts off too many occurrences, removing not only outliers, but also potential correct occurrence or entire occurrence clusters. Both steps reduced the number of species collection records (Appendix S4), and the predicted area of occupancy (Appendix S5).

### Plot abundance data

Abundance maps were constructed using 1675 1-ha tree inventory plots well distributed across Amazonia (defined as the tropical rain forest of the Amazon basin and the Guyana Shield) from the Amazon Tree Diversity Network (ATDN) (http://atdn.myspecies.info/). All individuals with ≥10 cm diameter at breast height (dbh) were recorded within the plots^24^. Because a relatively small number of collections from these plots have been deposited in herbaria, they constitute a dataset nearly independent from the NHCs.

### Constructing abundance maps

Inverse distance weighting (IDW) interpolation was used to create abundance maps from the plot abundance data. First, Amazonia was divided into 2193 0.5-degree grid cells. We then constructed the inverse distance weighting (IDW) models based on relative abundance following ter Steege *et al*.^28^. Then, the relative abundance (RA) for each cell was defined as RA_i_ = n_i_/N, where: n_i_ = the number of individuals of species i, and N = the total number of trees. IDW models were based on the nearest 150 plots within a limit of 300 km distance. Each plot weight was calculated by taking the square root of the distance in degrees. The 150 plots that were taken into account ensured that within an area consisting of absence plots only, the species is predicted to be absent. In addition, the 3-degree distance limit causes the model to predict the absence of a species when no occurrence plots are present within a radius of 3 degrees. This setting is based on the notion that within a non-environmental model a species’ extent of occurrence is restricted by dispersal limitation only^46^. The maximum dispersal distance has been optimized to a 3-degree distance by determining the best match between the IDW maps and the Fisher’s Alpha diversity map of all species^47^.

### Constructing presence-only SDMs using MaxEnt

We used MaxEnt version 3.3.3 k^1,48^, to construct presence-only SDMs for all the 227 species. Data of 19 environmental variables were downloaded from WorldClim^6^. These included variables related to temperature and precipitation. Since collinearity, the non-independence of predictor variables, potentially leads to the wrong identification of relevant predictors for the model, we used the common Spearman’s rank correlation coefficient threshold of |rho| >0.7 to identify correlated variables^49^.

Subsequently, we selected least correlated variables (|rho| <0.7) based on biological relevance and their loadings in a principal component analysis (PCA). The PCA consisted of all environmental variables for all collection localities of the 227 species. For temperature, we selected isothermality, temperature seasonality, and maximum temperature of warmest month. For precipitation we chose annual precipitation, wettest month precipitation and driest month precipitation. All the environmental variables were cropped to the extent of the Neotropics^42^, and aggregated to a 0.5-degree spatial resolution, using the function ‘mean’ from R package ‘raster’^50^. We used precipitation and temperature variables to assess MaxEnt’s predicted environmental suitability based on climate only. In the MaxEnt feature settings we excluded the product, threshold and hinge features given their lack of biological justification with the variables used^34,36^.

Correcting for geographical sampling bias has been found to improve the predictive performance of MaxEnt^14^. Also, environmental bias can be assessed by environmental filtering, which improves MaxEnt discriminatory ability^51^. We produced a bias file to employ the target-group background method recommended by Phillips and Dudík^52^, an option which is implemented in MaxEnt. The bias file consisted of a binary raster grid based on all Amazon tree species collections^25^, at each grid cell downloaded from GBIF, which reflects local survey effort. This is an essential step in the analysis, given MaxEnt’s assumption that the occurrences are independently drawn from the unknown probability distribution of the species. Without a bias file, sampling bias could severely reduce models accuracy. We used the bias file to produce a background file according the efforts of collection. Finally we used a convex hull around cleaned occurrences (kernel-density estimate GBIF records) of each species to estimate their extent of occurrence sensu^35^, plus a buffer of 300 km, equal to the buffer set for the IDW analysis, to crop the area of predicted environmental suitability^29,41^. The latter is our predicted area of occupancy.

### Data analysis

We compared collection presences and absences to IDW relative abundance to answer our first question whether NHCs are independent drawn from the unknown probability distribution. A binomial generalized linear model (logit regression) was used to determine if a significant positive relationship existed between the probability of being collected and predicted local relative abundance.

To answer the second question, how MaxEnt’s predicted environmental suitability compares to IDW relative abundance, we first tested which species’ MaxEnt maps were significantly different from random expectation with a bias corrected null-model^53^. For each species, 99 null-models were generated by randomly drawing *n* collection localities without replacement from the same spatial grid as the environmental layers, with *n* being the number of geographically unique collections for that species. Using an upper one-sided 95% confidence interval, we determined the probability value of the observed AUC as calculated by MaxEnt against those generated by the null distribution. If the species’ observed AUC value ranks 95 or above, the chance that a random set of *n* points could generate an equally good model is less than 5%, hence considered significantly different from random expectation. All species for which the SDM prediction did not deviate significantly from random expectation were excluded from further analysis.

Second, a Spearman Rank Correlation test was used to test the relationship between MaxEnt logistic output and IDW relative abundance at plot localities. Additionally, following VanDerWal *et al*.^29^, we determined the linear 90th percentile quantile regression between the IDW relative abundance and MaxEnt logistic outputs at plot localities. The confidence intervals of the linear quantile regressions were calculated with the Markov chain marginal bootstrap method as suggested by Kocherginsky *et al*.^54^. We computed the correlations and regressions for all plots separately, even if multiple plots were present in one grid square.

Third, we tested the predictive performance of MaxEnt and IDW. For MaxEnt, its logistic output was transformed into binary maps with a 10% training presence threshold. Although the maximum sum of sensitivity and specificity is considered to be the best threshold method for presence-only SDMs by Liu *et al*.^55^, we followed the advice of Merow *et al*.^34^ to avoid measures with specificity because they are based on absences that are unknown in this analysis. Then we tested its sensitivity by calculating true positive rate of the binary maps against plot presence. That is, the fraction of the grid cells with a plot for which MaxEnt predicted the species correctly to be present. Finally we calculated the median predicted area of occupancy.

For IDW, its output was transformed into binary maps by converting the grids cells with RA >0 into 1. Last, naturally non-forested areas were excluded from the maps based on Soares-Filho *et al*.^56^. We then calculated its output true positive rate against collections presences and absences. That is, the fraction of the grid cells with a collection for which the IDW relative abundance predicted the species correctly to be present. Finally we also calculated the median predicted area of occupancy for IDW.

All calculations and analyses were performed with R version 3.0.3^3^, including the R packages raster^50^, rgdal^57^, gstat^58^, dismo^59^, vegan^60^, quantreg^61^, sp^62^, rJava^63^ and SDMTools^64^.

## Electronic supplementary material

How does abundance affect the chance of being found?

Comparing the results of modelling the area of occupancy with MaxEnt and with inverse distance weighting (IDW).

Maps of combined MaxEnt IDW predictions.

Cleaning pipeline results for all 227 hyperdominant species.

Area of occupancy predicted by MaxEnt for each step of the modelling pipeline.
