# Supplementary material for: Species Distribution Modelling: Contrasting presence-only models with plot abundance data
Source: Sci Rep. 2018 Jan 17;8:1003. doi: 10.1038/s41598-017-18927-1 (PMC5772443; doi:10.1038/s41598-017-18927-1)
Supplement: Supplementary file 1 — How does abundance affect the chance of being found? [file 41598_2017_18927_MOESM1_ESM.pdf]

Supplemental Material

for

Species Distribution Modelling: Contrasting presence-only models with plot abundance data

Vitor F. Gomes*, Stéphanie D. IJff, Niels Raes, Iêda Leão Amaral, Rafael P. Salomão, Luiz de Souza Coelho, Francisca Dionízia de Almeida Matos, Carolina V. Castilho, Diogenes de Andrade Lima Filho, Dairon Cárdenas López, Juan Ernesto Guevara, William E. Magnusson, Oliver L. Phillips, Florian Wittmann, Marcelo de Jesus Veiga Carim, Maria Pires Martins, Mariana Victória Irume, Daniel Sabatier, Jean-François Molino, Olaf S. Bánki, José Renan da Silva Guimarães, Nigel C.A. Pitman, Maria Teresa Fernandez Piedade, Abel Monteagudo Mendoza, Bruno Garcia Luize, Eduardo Martins Venticinque, Evlyn Márcia Moraes de Leão Novo, Percy Núñez Vargas, Thiago Sanna Freire Silva, Angelo Gilberto Manzatto, John Terborgh, Neidiane Farias Costa Reis, Juan Carlos Montero, Katia Regina Casula, Beatriz S. Marimon, Ben-Hur Marimon Jr., Euridice N. Honorio Coronado, Ted R. Feldpausch, Alvaro Duque, Charles Eugene Zartman, Nicolás Castaño Arboleda, Timothy J. Killeen, Bonifacio Mostacedo, Rodolfo Vasquez, Jochen Schöngart, Rafael L. Assis, Marcelo Brilhante Medeiros, Marcelo Fragomeni Simon, Ana Andrade, William F. Laurance, José Luís Camargo, Layon O. Demarchi, Susan G.W. Laurance, Emanuelle de Sousa Farias, Henrique Eduardo Mendonça Nascimento, Juan David Cardenas Revilla, Adriano Quaresma, Flávia R.C. Costa, Ima Célia Guimarães Vieira, Bruno Barçante Ladvocat Cintra, Hernán Castellanos, Roel Brienen, Pablo R. Stevenson, Yuri Feitosa, Joost F. Duivenvoorden, Gerardo A. Aymard C., Hugo F. Mogollón, Natalia Targhetta, James A. Comiskey, Alberto Vicentini, Aline Lopes, Gabriel Damasco, Nállarett Dávila, Roosevelt García-Villacorta, Carolina Levis, Juliana Schietti, Priscila Souza, Thaise Emilio, Alfonso Alonso, David Neill, Francisco Dallmeier, Leandro Valle Ferreira, Alejandro Araujo-Murakami, Daniel Praia, Dário Dantas do Amaral, Fernanda Antunes Carvalho, Fernanda Coelho de Souza, Kenneth Feeley, Luzmila Arroyo, Marcelo Petratti Pansonato, Rogerio Gribel, Boris Villa, Juan Carlos Licona,

Paul V.A. Fine, Carlos Cerón, Chris Baraloto, Eliana M. Jimenez, Juliana Stropp, Julien Engel, Julien Engel, Marcos Silveira, Maria Cristina Peñuela Mora, Pascal Petronelli, Paul Maas, Raquel Thomas-Caesar, Terry W. Henkel, Doug Daly, Marcos Ríos Paredes, Tim R. Baker, Alfredo Fuentes, Carlos A. Peres, Jerome Chave, Jose Luis Marcelo Pena, Kyle G. Dexter, Miles R. Silman, Peter Møller Jørgensen, Toby Pennington, Anthony Di Fiore, Fernando Cornejo Valverde, Juan Fernando Phillips, Gonzalo Rivas-Torres, Patricio von Hildebrand, Tinde R. van Andel, Ademir R. Ruschel, Adriana Prieto, Agustín Rudas, Bruce Hoffman, César I.A. Vela, Edelcilio Marques Barbosa, Egleé L. Zent, George Pepe Gallardo Gonzales, Hilda Paulette Dávila Doza, Ires Paula de Andrade Miranda, Jean-Louis Guillaumet, Linder Felipe Mozombite Pinto, Luiz Carlos de Matos Bonates, Natalino Silva, Ricardo Zárate Gómez, Stanford Zent, Therany Gonzales, Vincent A. Vos, Yadvinder Malhi, Alexandre A. Oliveira, Angela Cano, Bianca Weiss Albuquerque, Corine Vriesendorp, Diego Felipe Correa, Emilio Vilanova Torre, Geertje van der Heijden, Hirma Ramirez-Angulo, José Ferreira Ramos, Kenneth R. Young, Maira Rocha, Marcelo Trindade Nascimento, Maria Natalia Umaña Medina, Milton Tirado, Ophelia Wang, Rodrigo Sierra, Armando Torres-Lezama, Casimiro Mendoza, Cid Ferreira, Cláudia Baider, Daniel Villarroel, Henrik Balslev, Italo Mesones, Ligia Estela Urrego Giraldo, Luisa Fernanda Casas, Manuel Augusto Ahuite Reategui, Reynaldo Linares-Palomino, Roderick Zagt, Sasha Cárdenas, William Farfan-Rios, Adeilza Felipe Sampaio, Daniela Pauletto, Elvis H. Valderrama Sandoval, Freddy Ramirez Arevalo, Isau Huamantupa-Chuquimaco, Karina Garcia-Cabrera, Lionel Hernandez, Luis Valenzuela Gamarra, Miguel N. Alexiades, Susamar Pansini, Walter Palacios Cuenca, William Milliken, Joana Ricardo, Gabriela Lopez-Gonzalez, Edwin Pos, Hans ter Steege*

*Correspondence and requests for materials should be addressed to H.T.S. (email: hans.tersteege@naturalis.nl)

Appendix S1: How does abundance affect the chance of being found?

Comparison of the collection localities to the probability distribution as predicted by an inverse distance weighting model (IDW), based on the abundance of species in tree inventory plots. A binomial generalized linear model (logit regression) was used to determine if a significant positive relationship existed between the probability of being collected and predicted local relative abundance for all 227 hyperdominant Amazonian tree species.

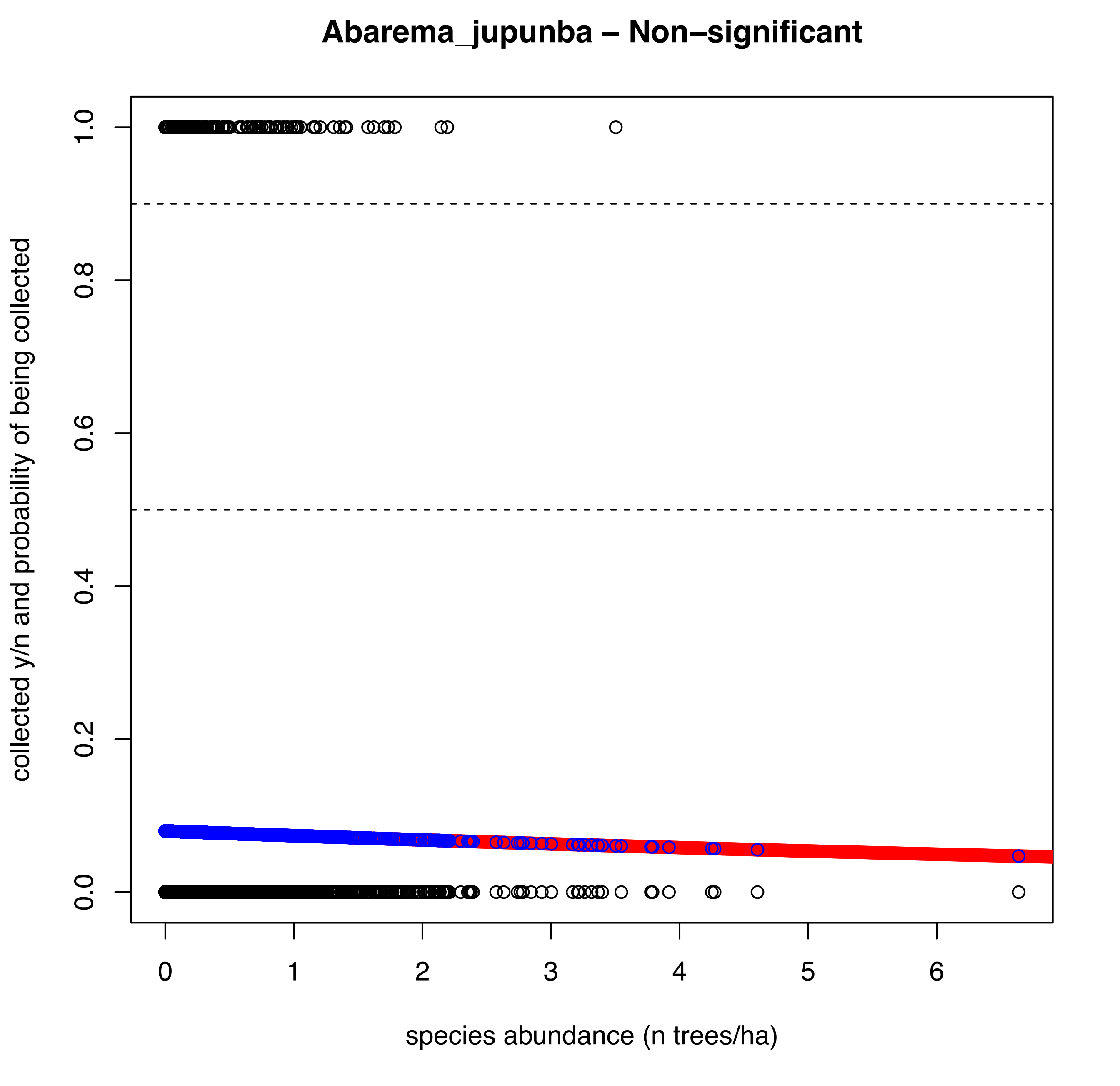

Abarema_jupunba – Non–significant
collected y/n and probability of being collected
species abundance (n trees/ha)
0.0
0.2
0.4
0.6
0.8
1.0
0
1
2
3
4
5
6

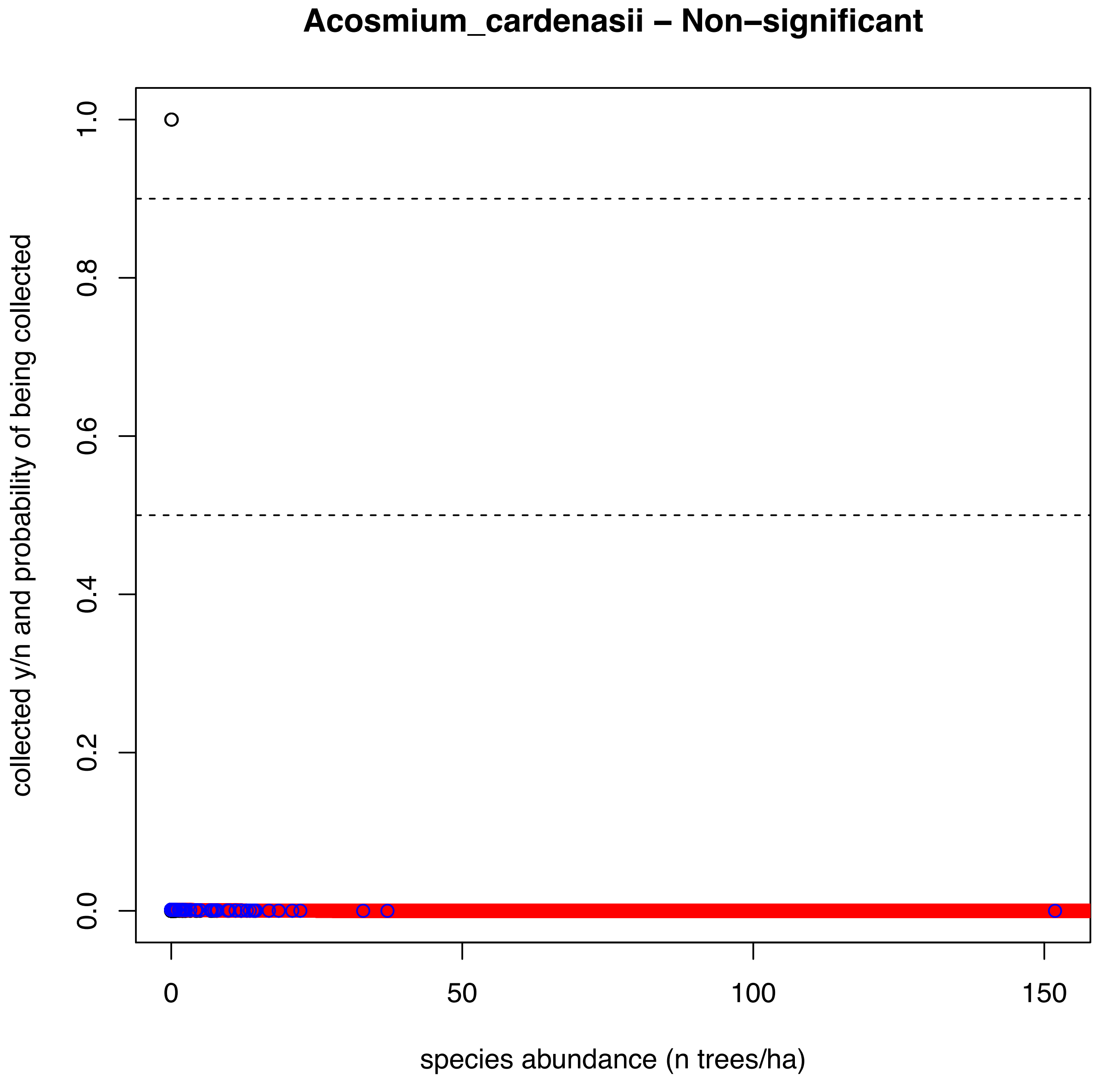

Acosmium_cardenasii – Non–significant
collected y/n and probability of being collected
species abundance (n trees/ha)
0.0
0.2
0.4
0.6
0.8
1.0
0
50
100
150

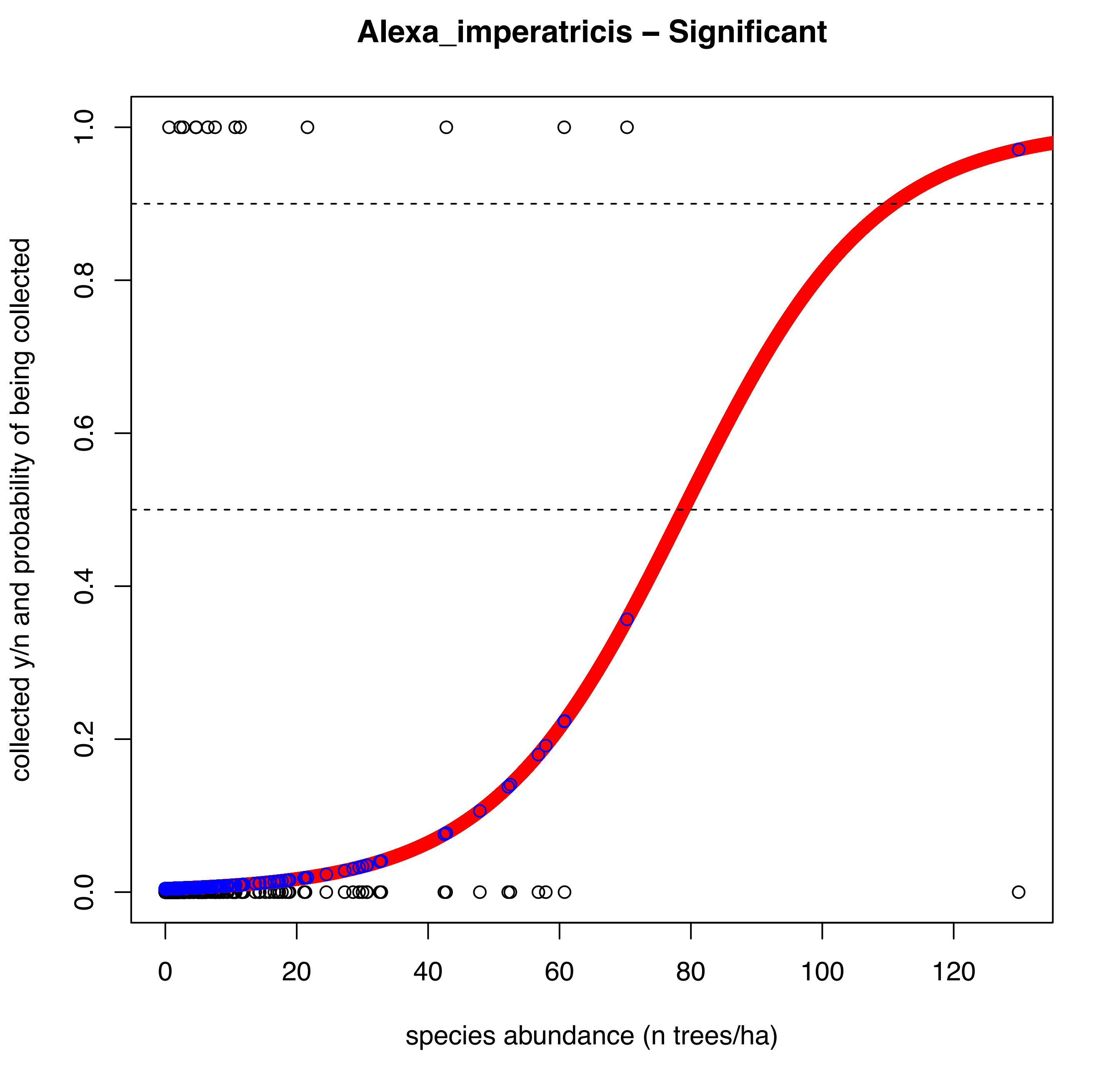

Alexa_imperatricis – Significant
collected y/n and probability of being collected
species abundance (n trees/ha)
0.0
0.2
0.4
0.6
0.8
1.0
0
20
40
60
80
100
120

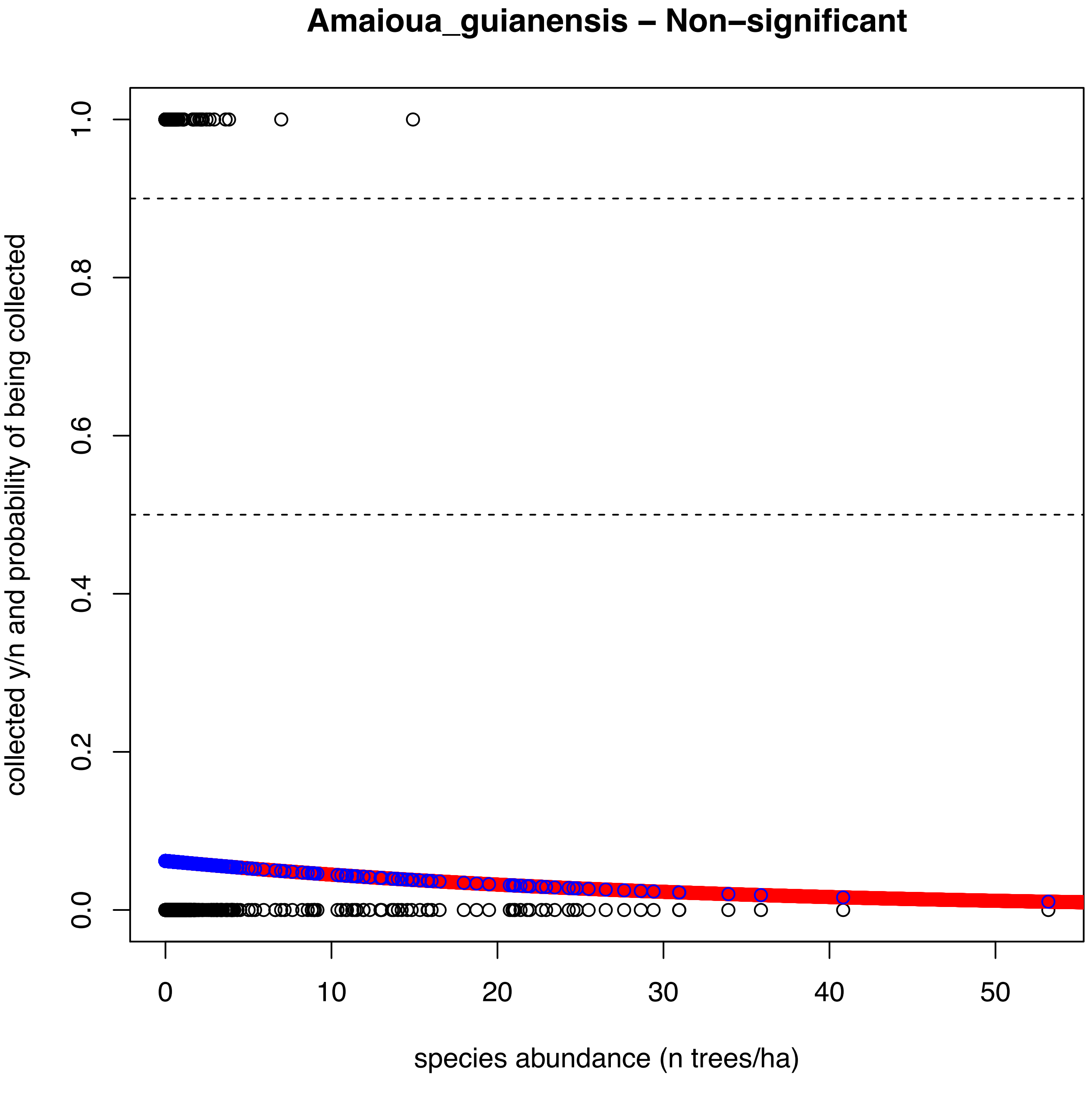

Amaioua_guianensis – Non–significant
collected y/n and probability of being collected
species abundance (n trees/ha)
0.0
0.2
0.4
0.6
0.8
1.0
0
10
20
30
40
50

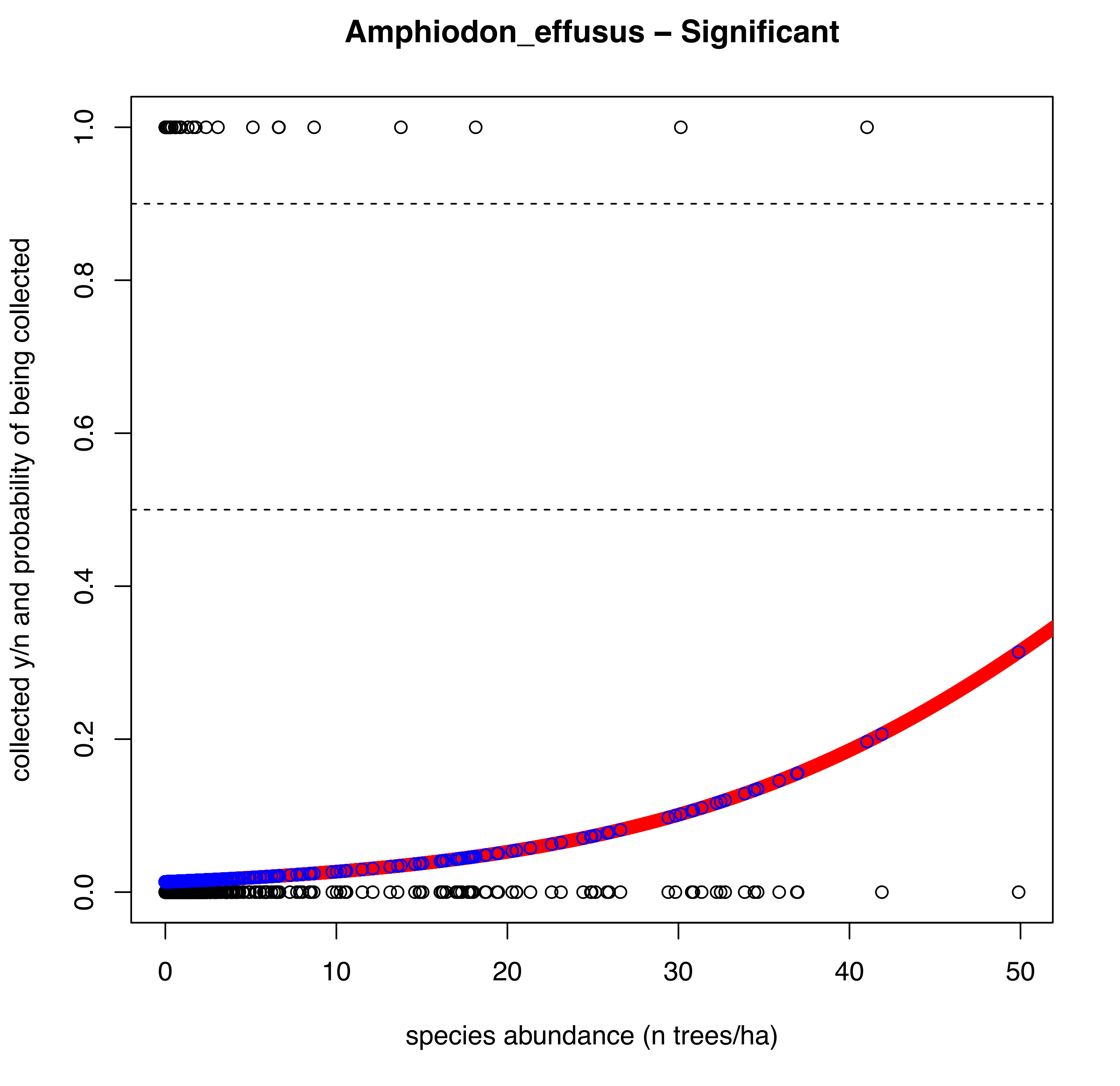
Amphiodon_effusus – Significant
collected y/n and probability of being collected
species abundance (n trees/ha)
0.0
0.2
0.4
0.6
0.8
1.0
0
10
20
30
40
50

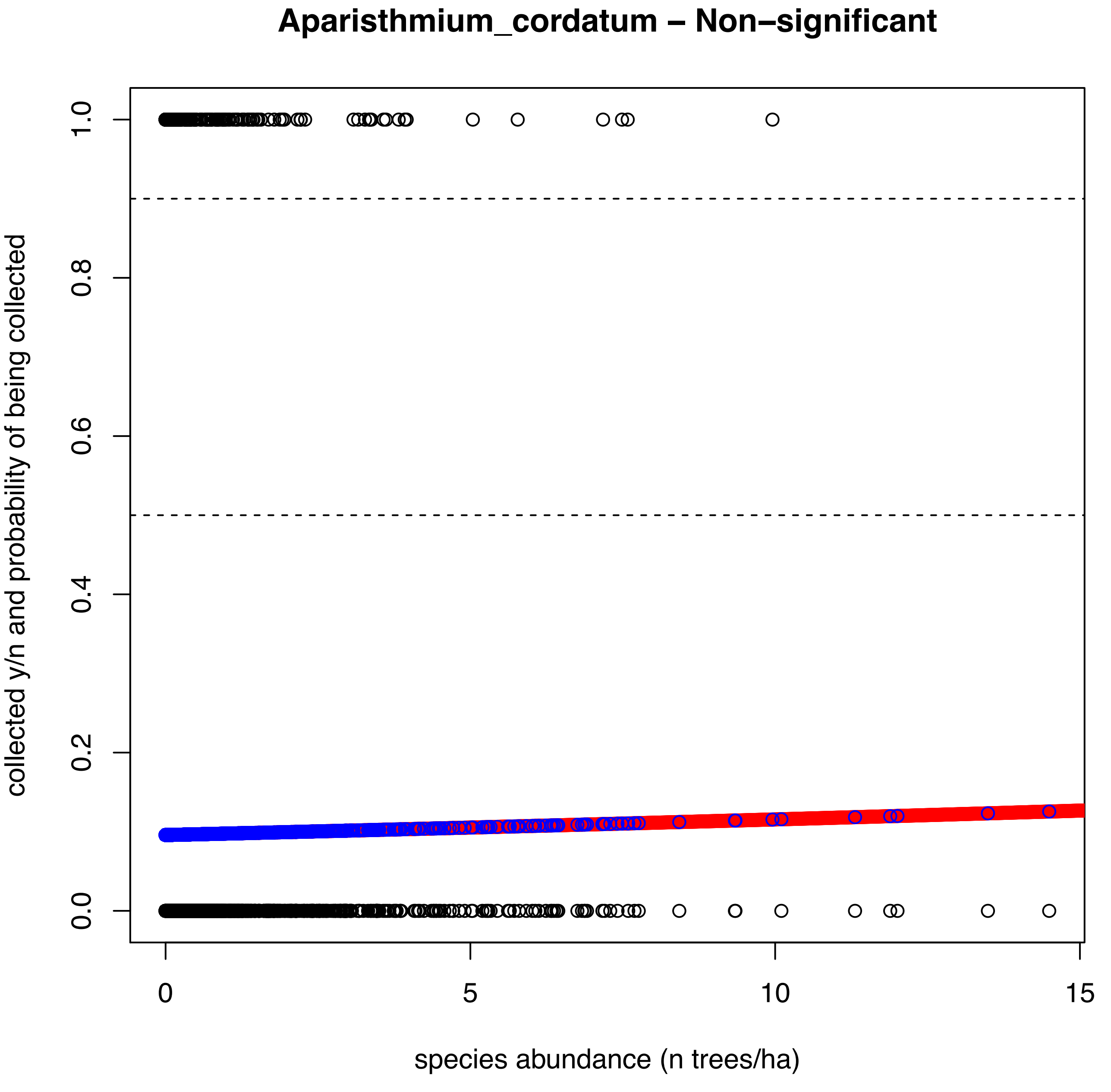
Aparisthmium_cordatum – Non–significant
collected y/n and probability of being collected
species abundance (n trees/ha)
0.0
0.2
0.4
0.6
0.8
1.0
0
5
10
15

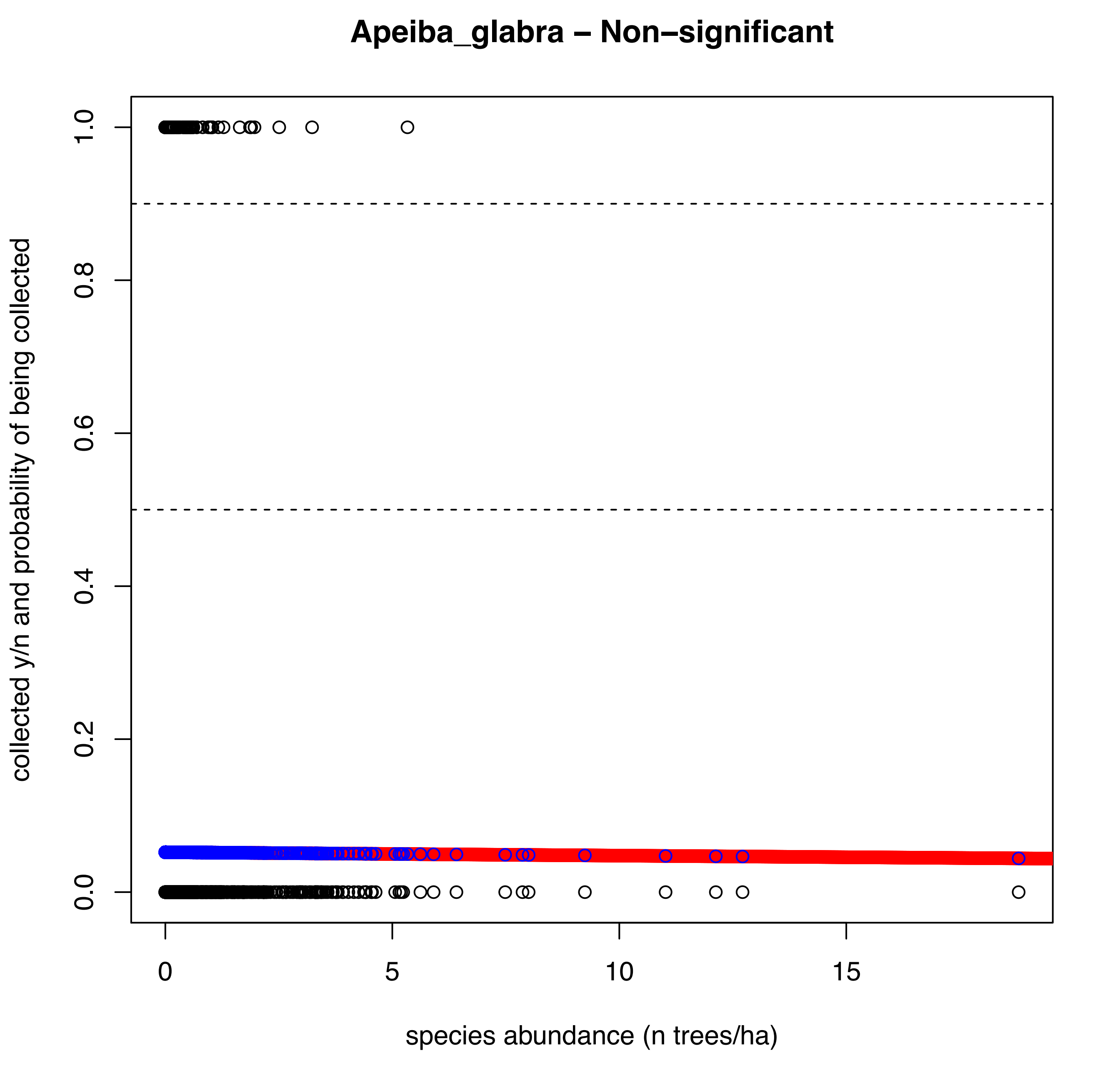

Apeiba_glabra – Non–significant
collected y/n and probability of being collected
species abundance (n trees/ha)
0.0
0.2
0.4
0.6
0.8
1.0
0
5
10
15

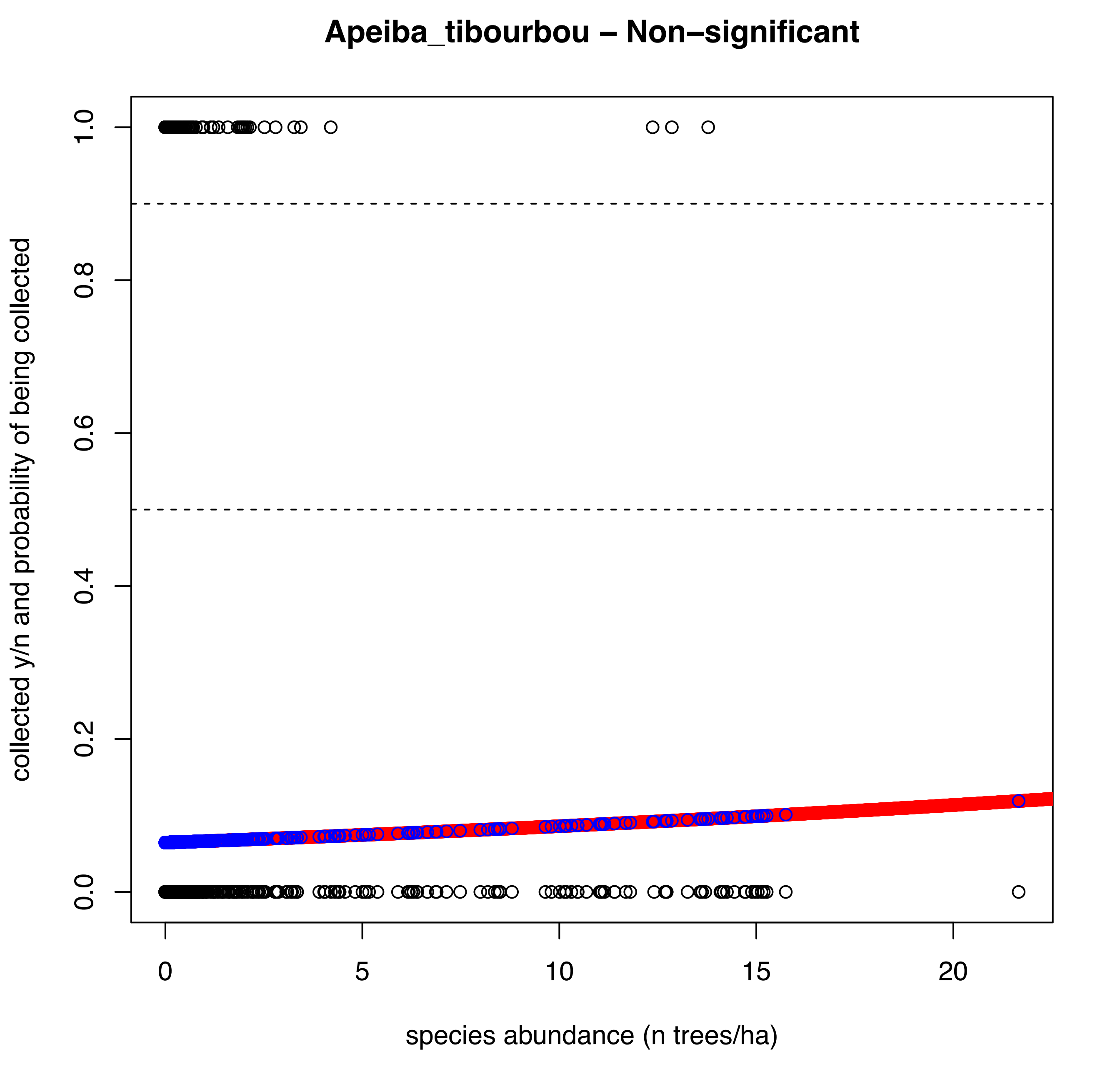
Apeiba_tibourbou – Non–significant
collected y/n and probability of being collected
species abundance (n trees/ha)
0.0
0.2
0.4
0.6
0.8
1.0
0
5
10
15
20

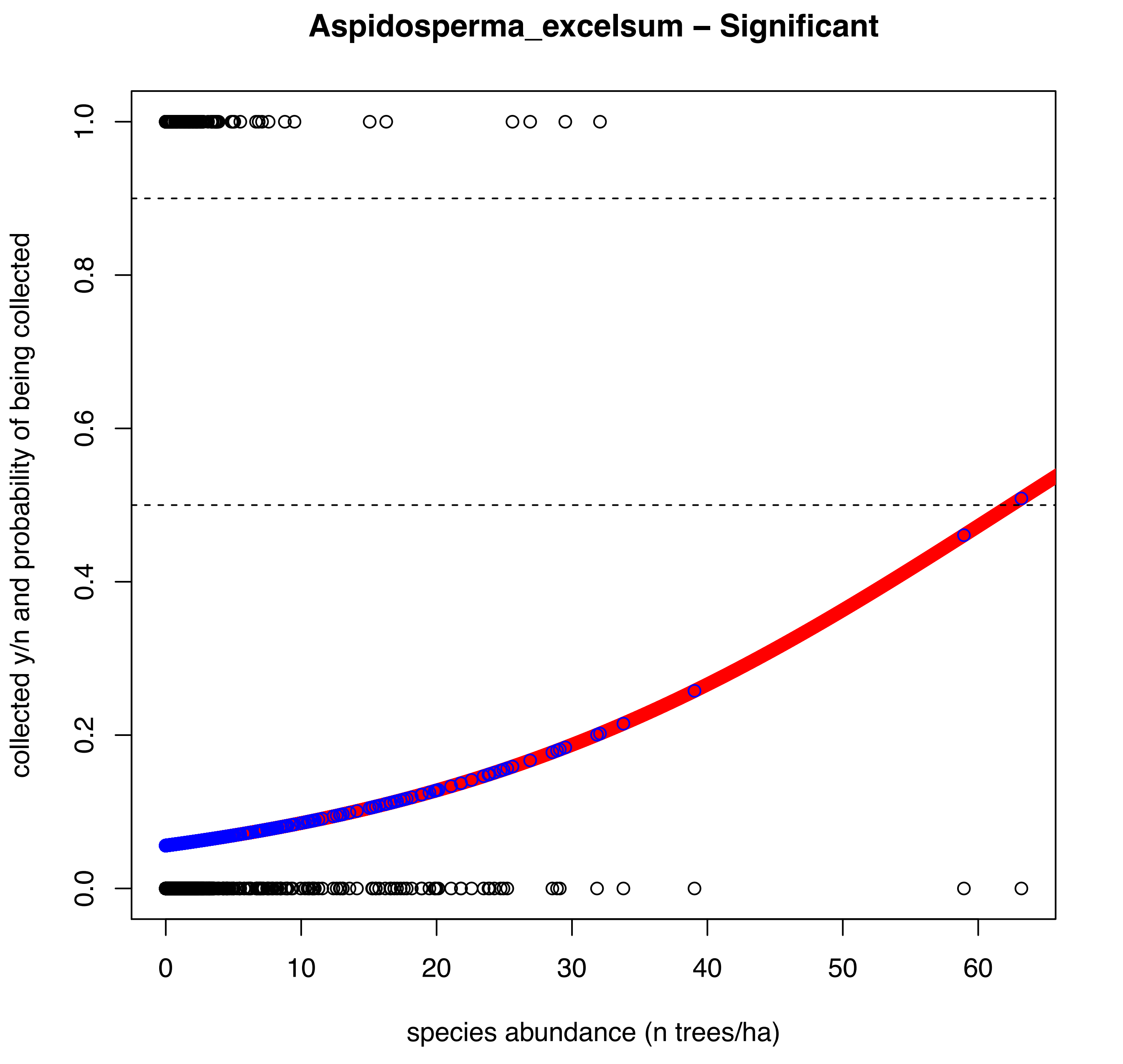
Aspidosperma_excelsum – Significant
collected y/n and probability of being collected
species abundance (n trees/ha)
0.0
0.2
0.4
0.6
0.8
1.0
0
10
20
30
40
50
60

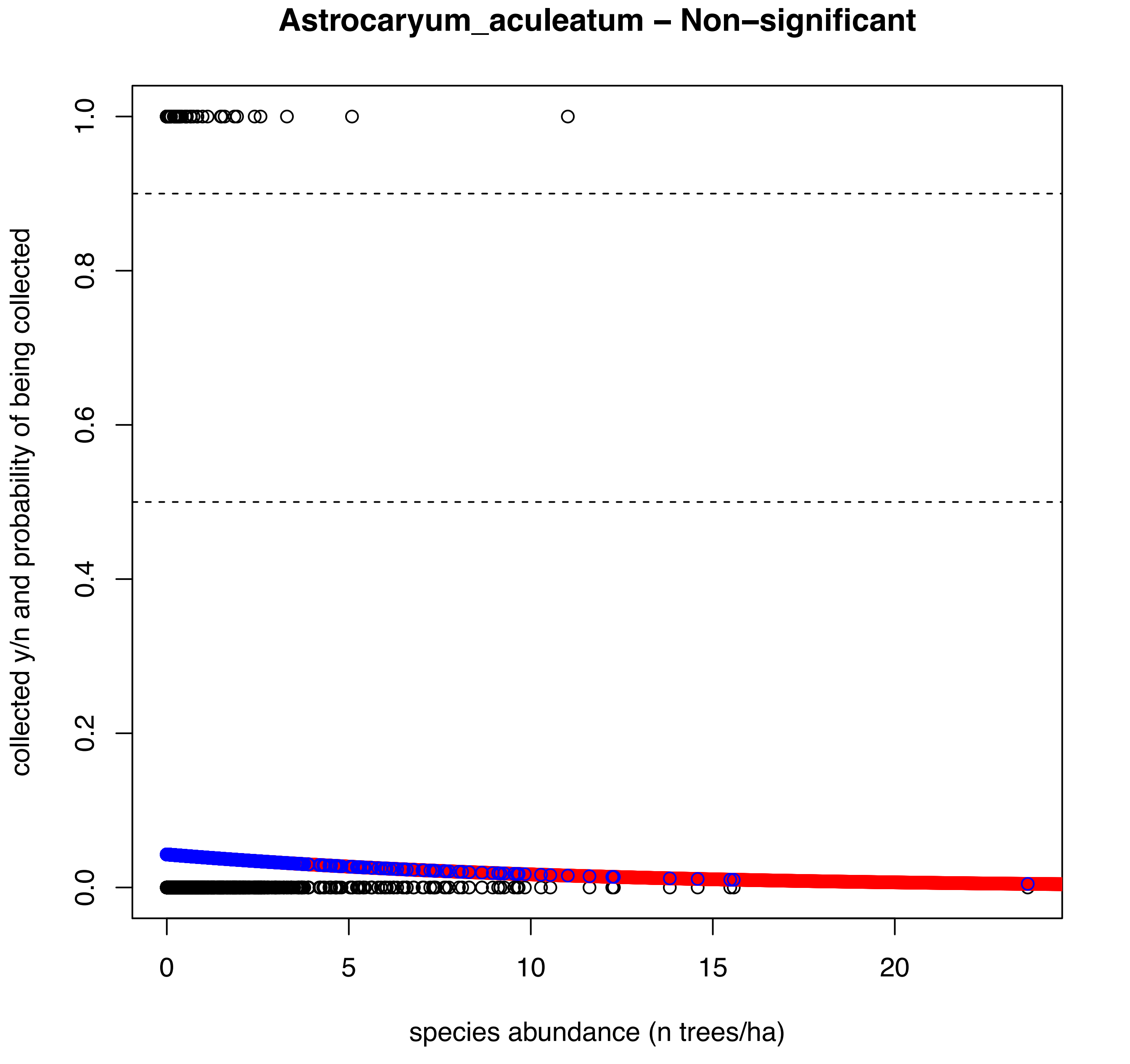
Astrocaryum_aculeatum – Non–significant
collected y/n and probability of being collected
species abundance (n trees/ha)
0.0
0.2
0.4
0.6
0.8
1.0
0
5
10
15
20

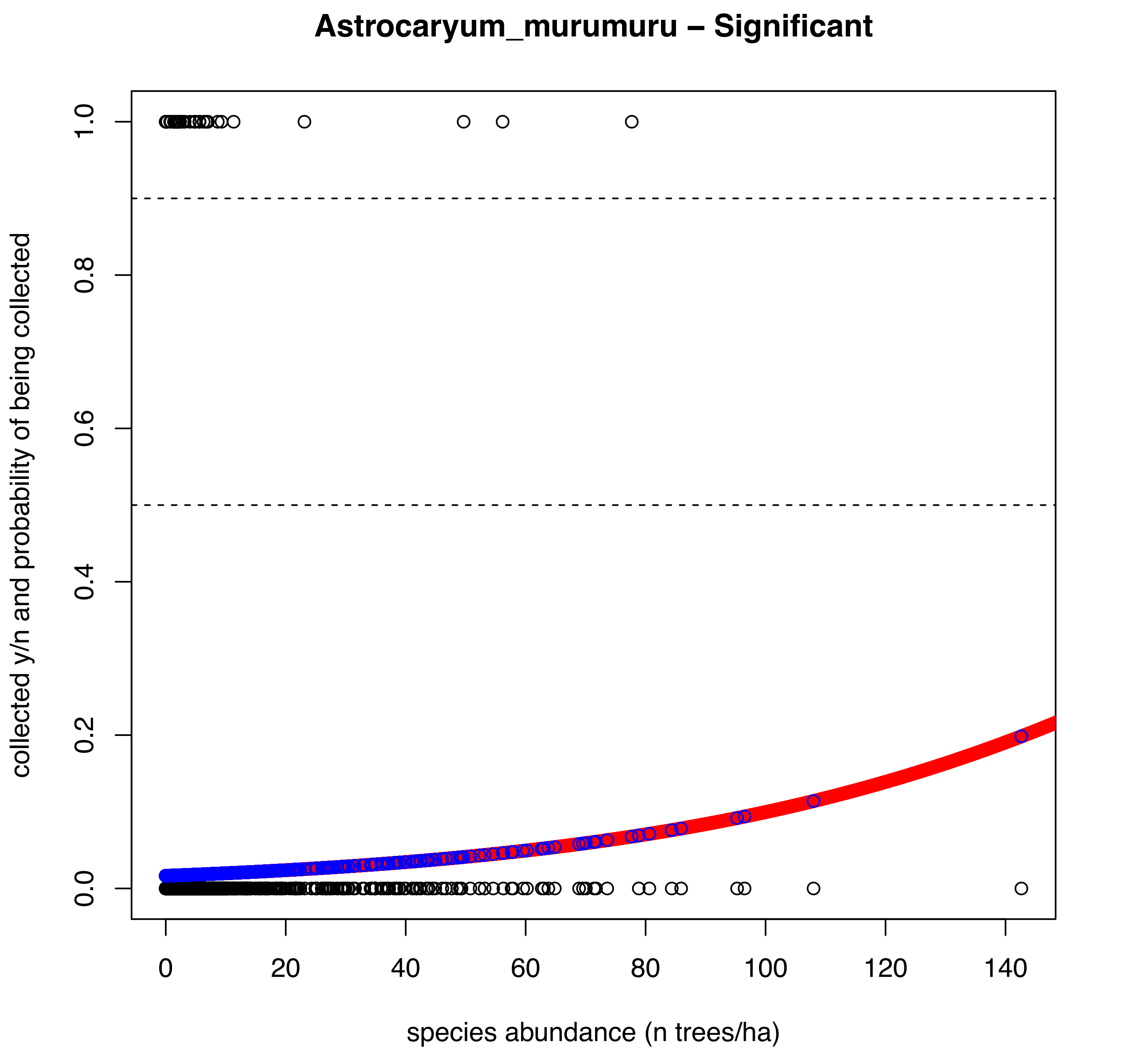
Astrocaryum_murumuru – Significant
collected y/n and probability of being collected
species abundance (n trees/ha)
0.0
0.2
0.4
0.6
0.8
1.0
0
20
40
60
80
100
120
140

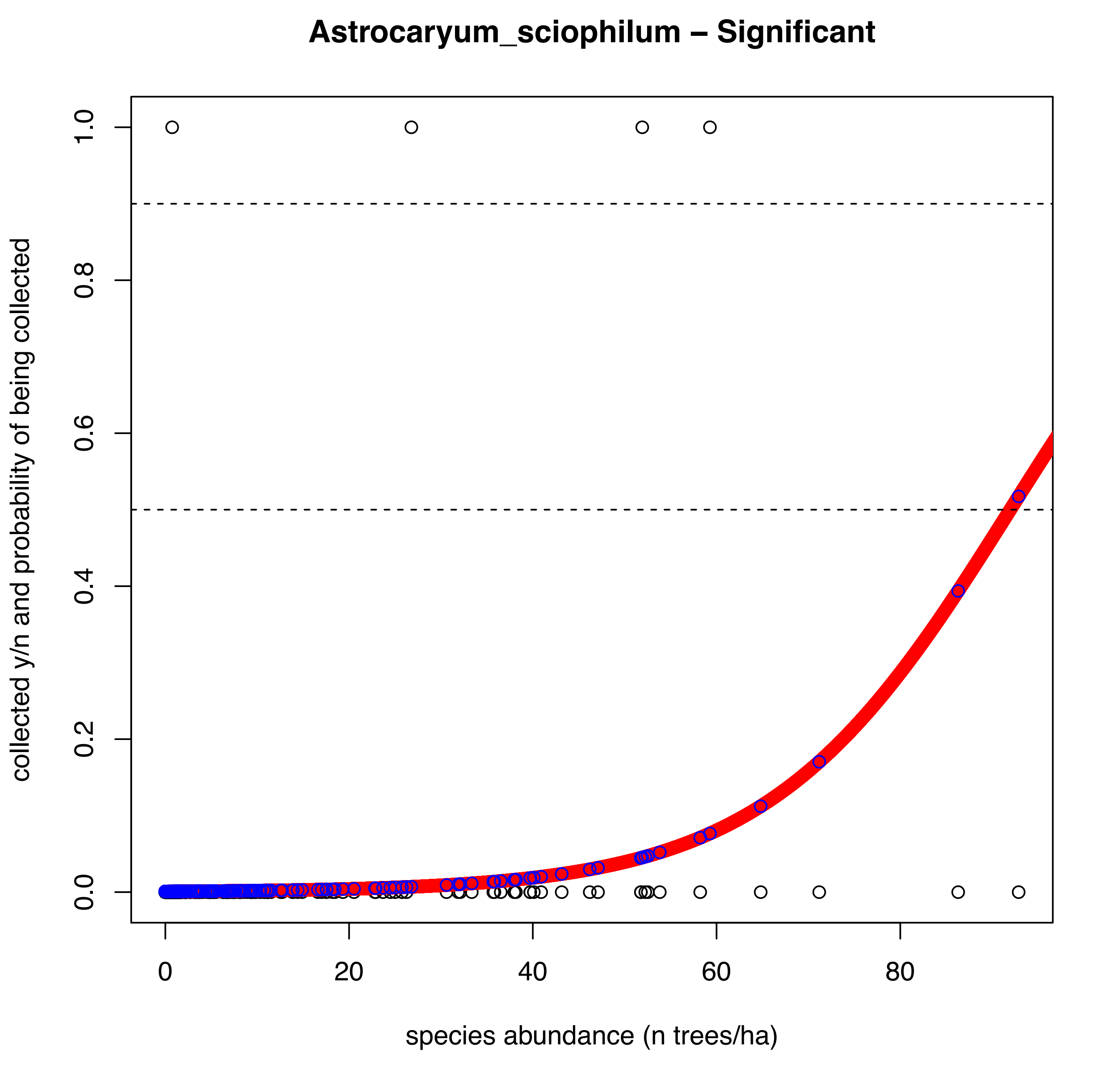
Astrocaryum_sciophilum – Significant
collected y/n and probability of being collected
species abundance (n trees/ha)
0.0
0.2
0.4
0.6
0.8
1.0
0
20
40
60
80

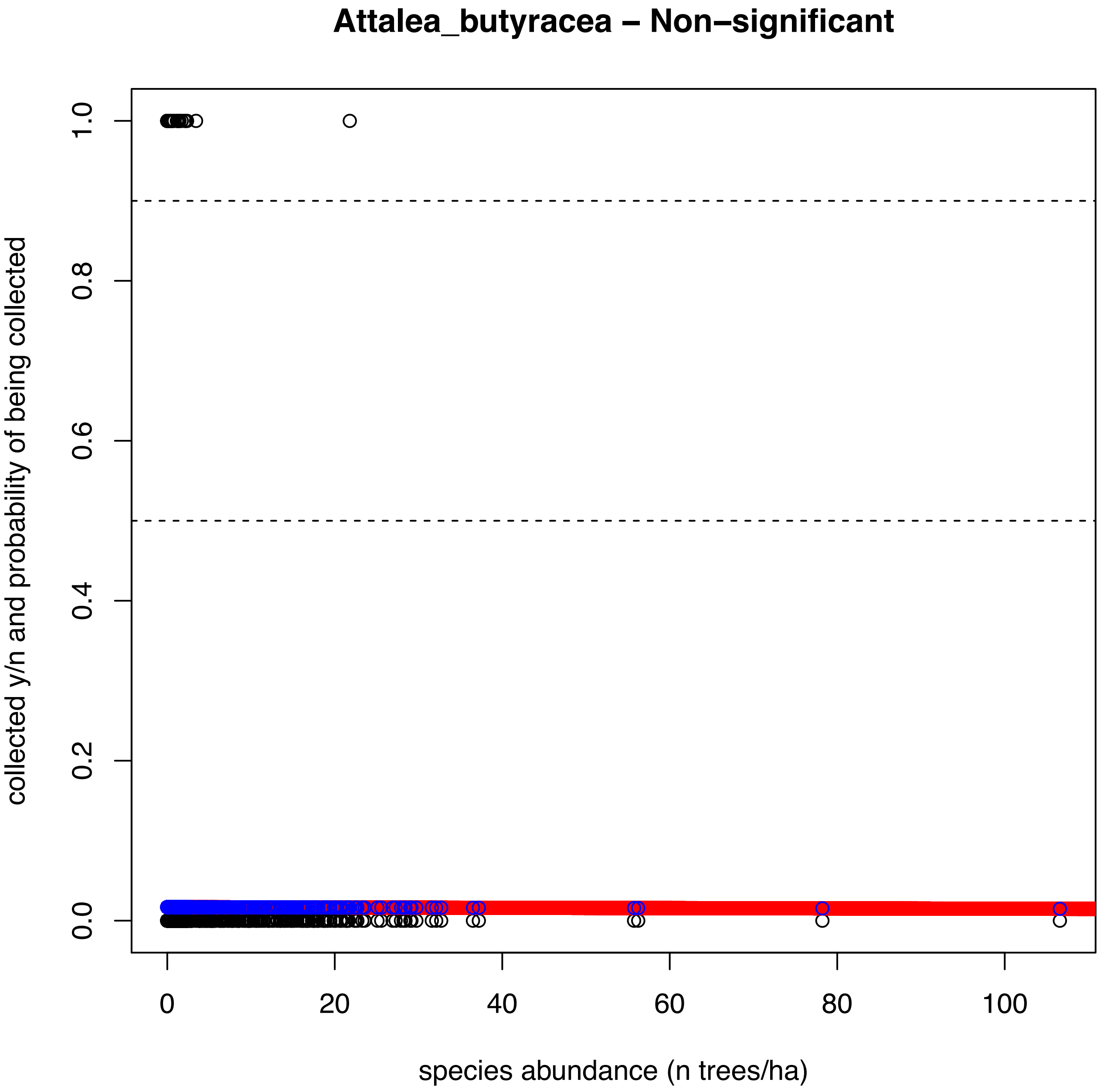
Attalea_butyracea – Non–significant
collected y/n and probability of being collected
species abundance (n trees/ha)
0.0
0.2
0.4
0.6
0.8
1.0
0
20
40
60
80
100

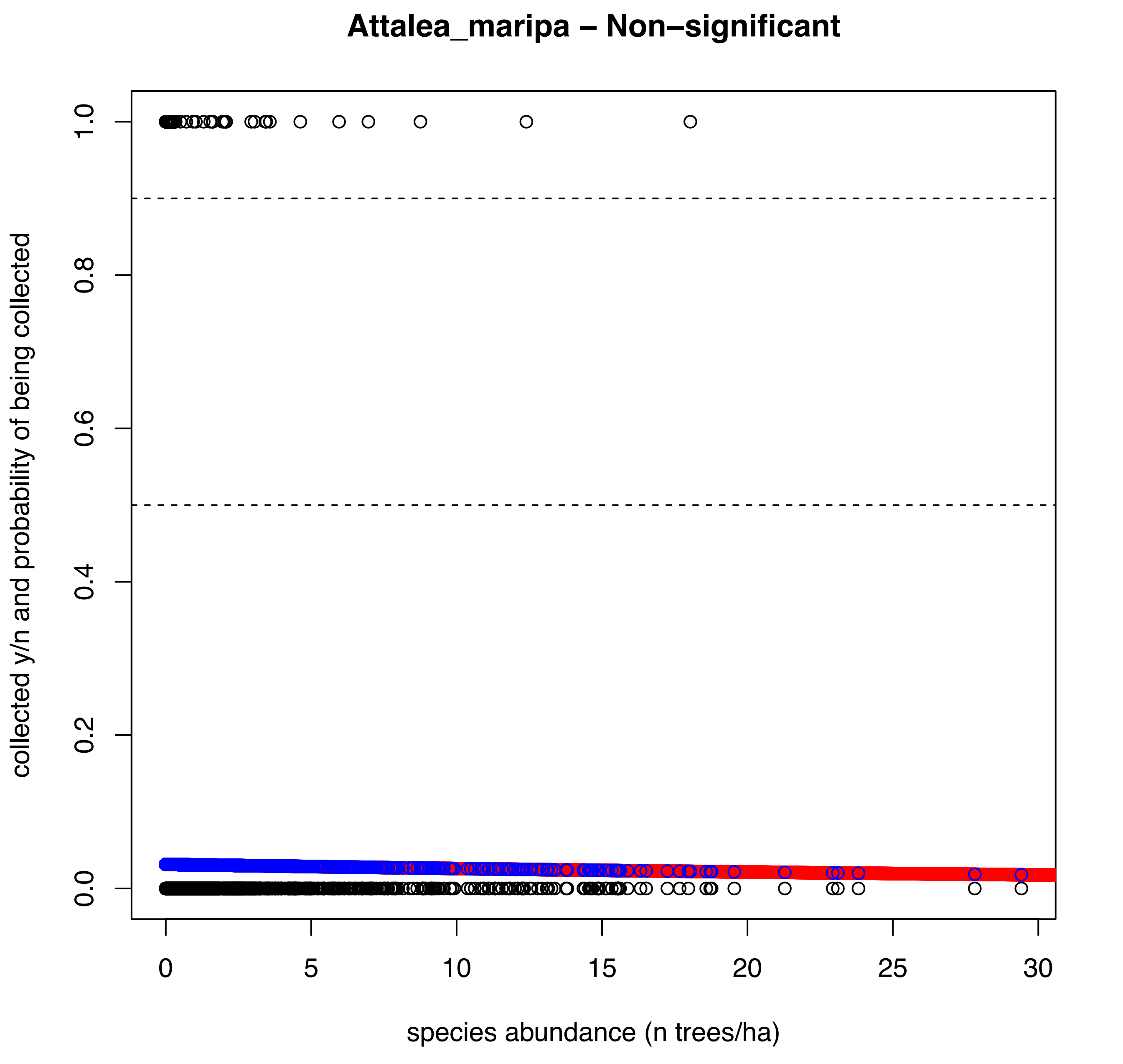
Attalea_maripa – Non–significant
collected y/n and probability of being collected
species abundance (n trees/ha)
0.0
0.2
0.4
0.6
0.8
1.0
0
5
10
15
20
25
30

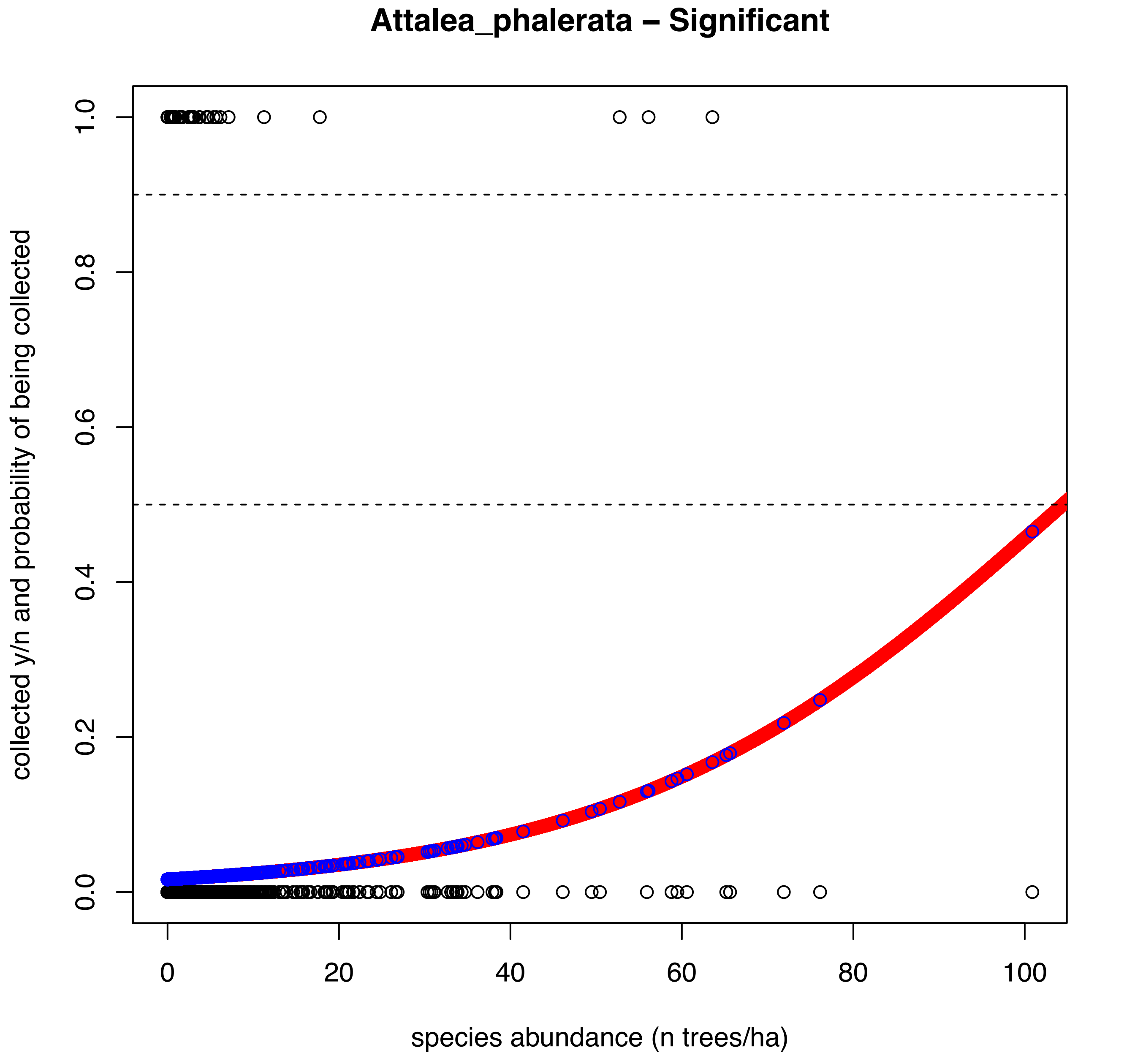
Attalea_phalerata – Significant
collected y/n and probability of being collected
species abundance (n trees/ha)
0.0
0.2
0.4
0.6
0.8
1.0
0
20
40
60
80
100

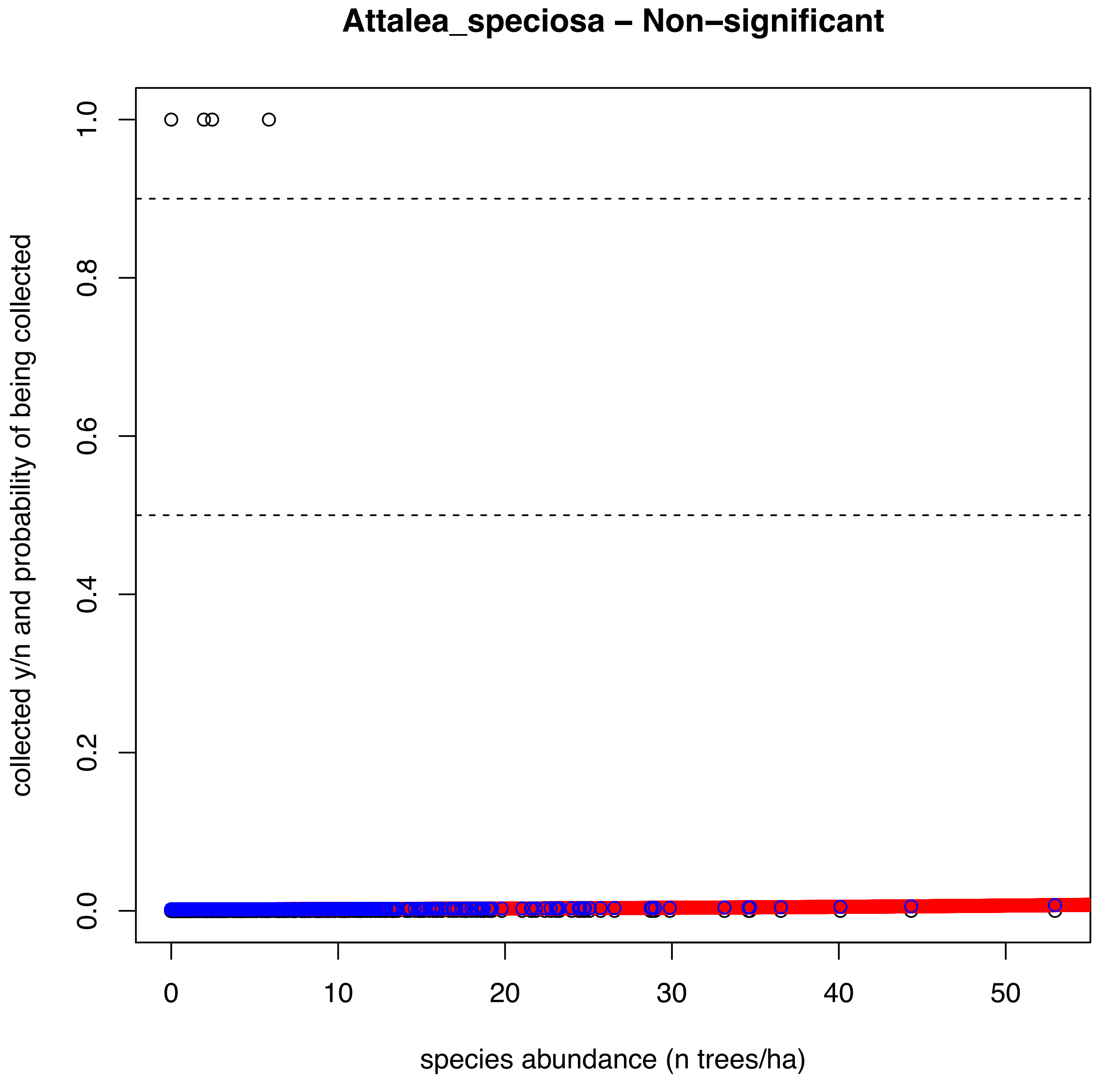
Attalea_speciosa – Non–significant
collected y/n and probability of being collected
species abundance (n trees/ha)
0.0
0.2
0.4
0.6
0.8
1.0
0
10
20
30
40
50

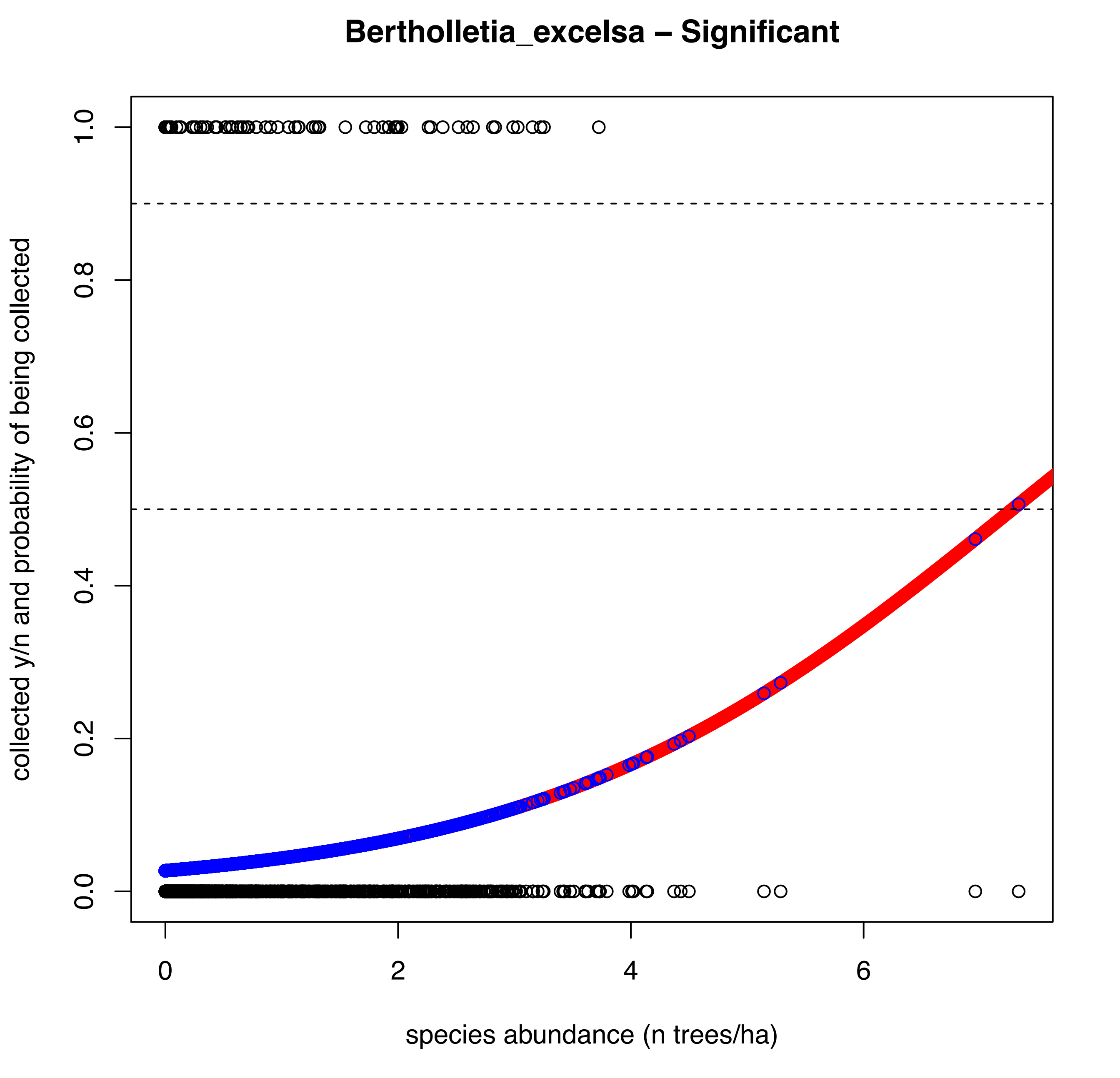
Bertholletia_excelsa – Significant
collected y/n and probability of being collected
species abundance (n trees/ha)
0.0
0.2
0.4
0.6
0.8
1.0
0
2
4
6

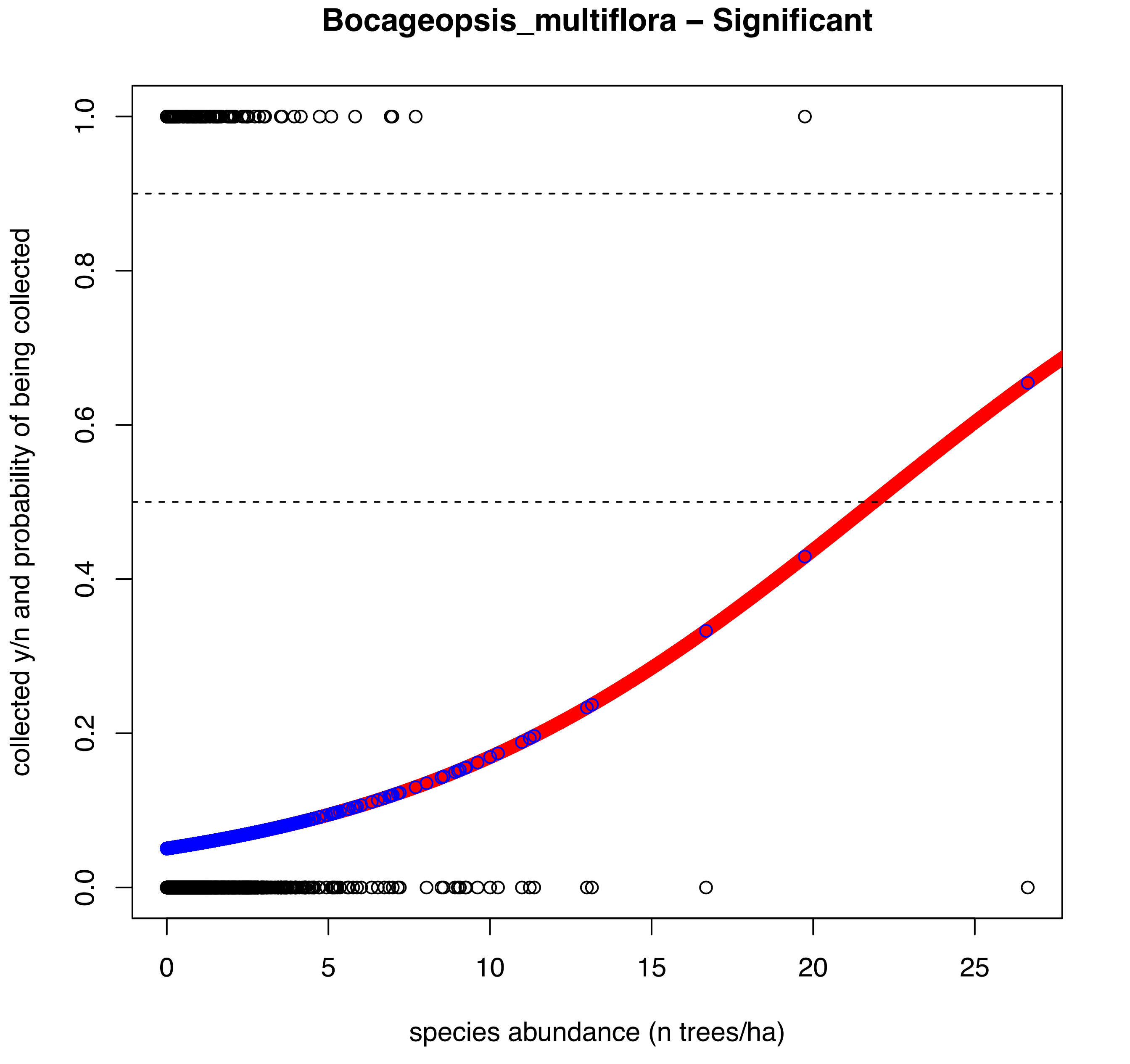
Bocageopsis_multiflora – Significant
collected y/n and probability of being collected
species abundance (n trees/ha)
0.0
0.2
0.4
0.6
0.8
1.0
0
5
10
15
20
25

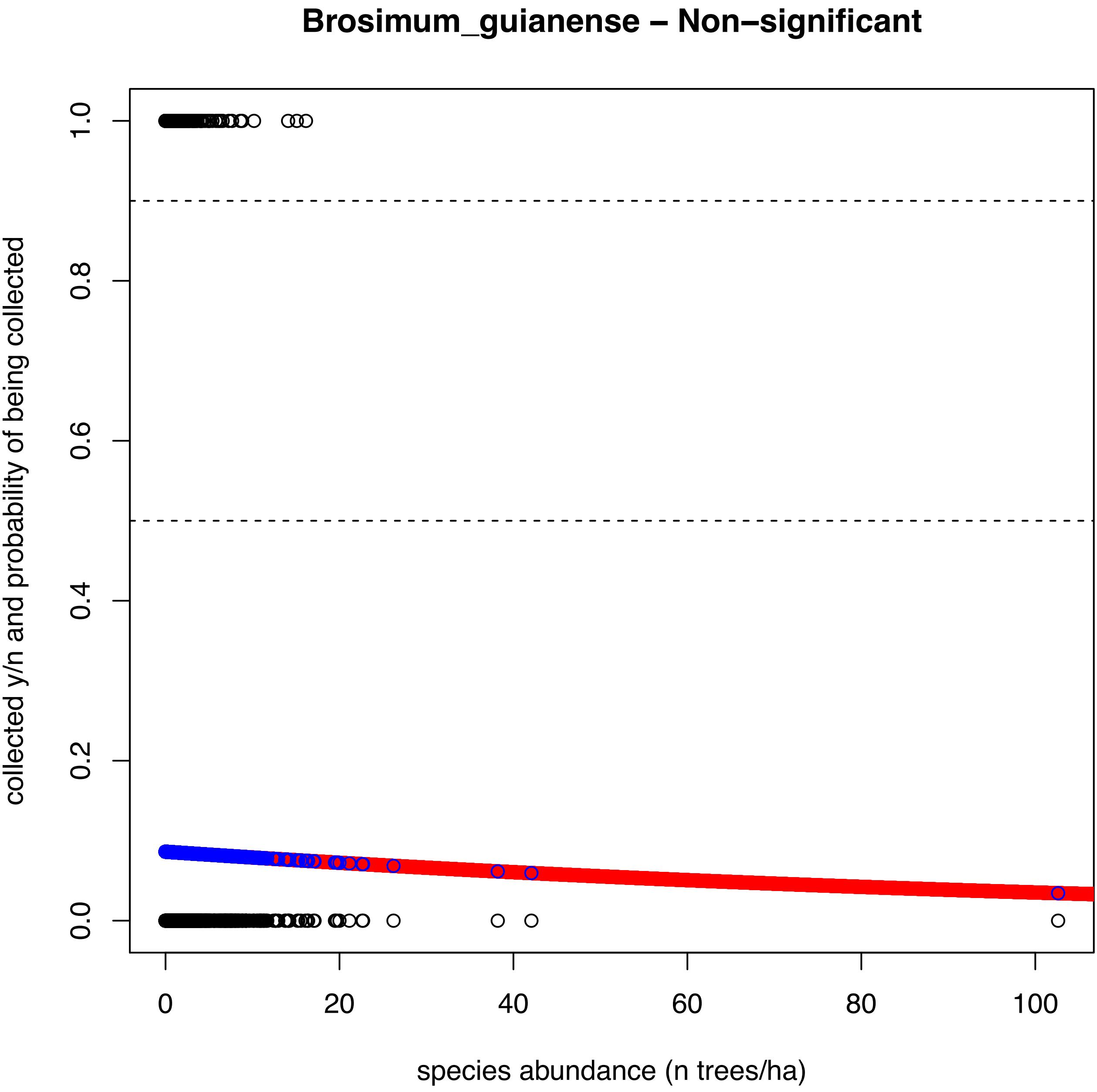

Brosimum_guianense – Non–significant
collected y/n and probability of being collected
species abundance (n trees/ha)
0.0
0.2
0.4
0.6
0.8
1.0
0
20
40
60
80
100

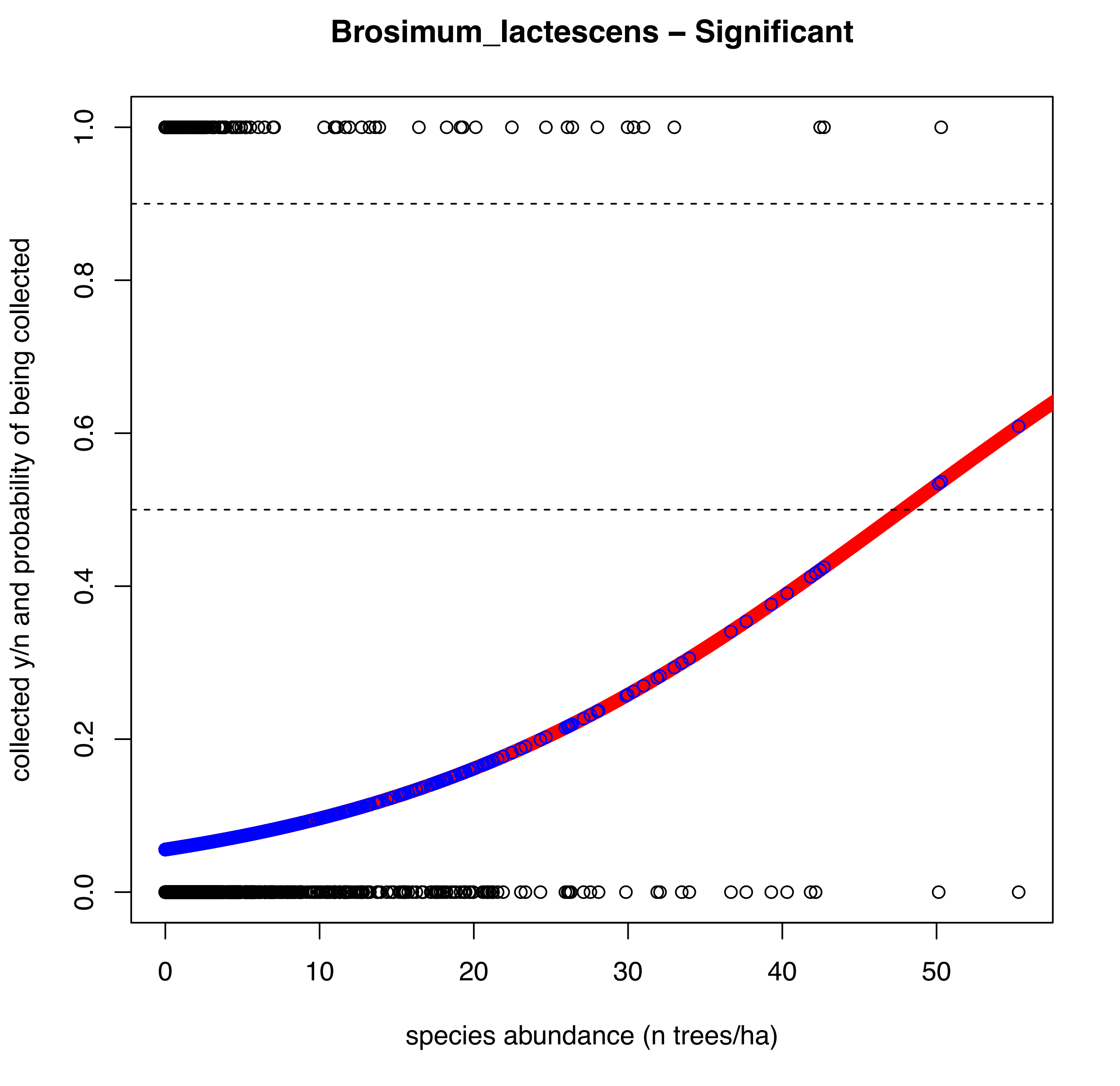
Brosimum_lactescens – Significant
collected y/n and probability of being collected
species abundance (n trees/ha)
0.0
0.2
0.4
0.6
0.8
1.0
0
10
20
30
40
50

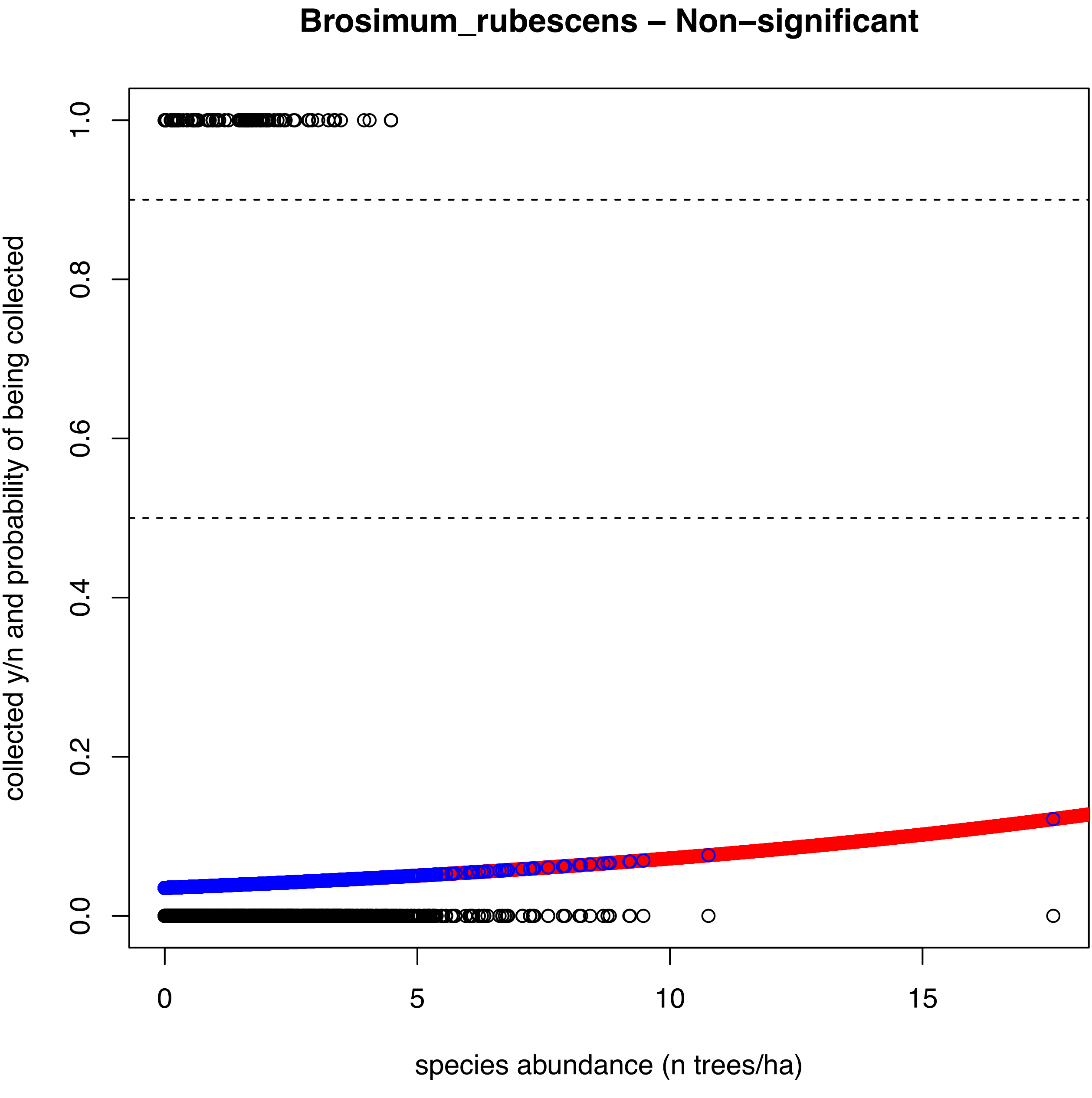
Brosimum_rubescens – Non–significant
collected y/n and probability of being collected
species abundance (n trees/ha)
0.0
0.2
0.4
0.6
0.8
1.0
0
5
10
15

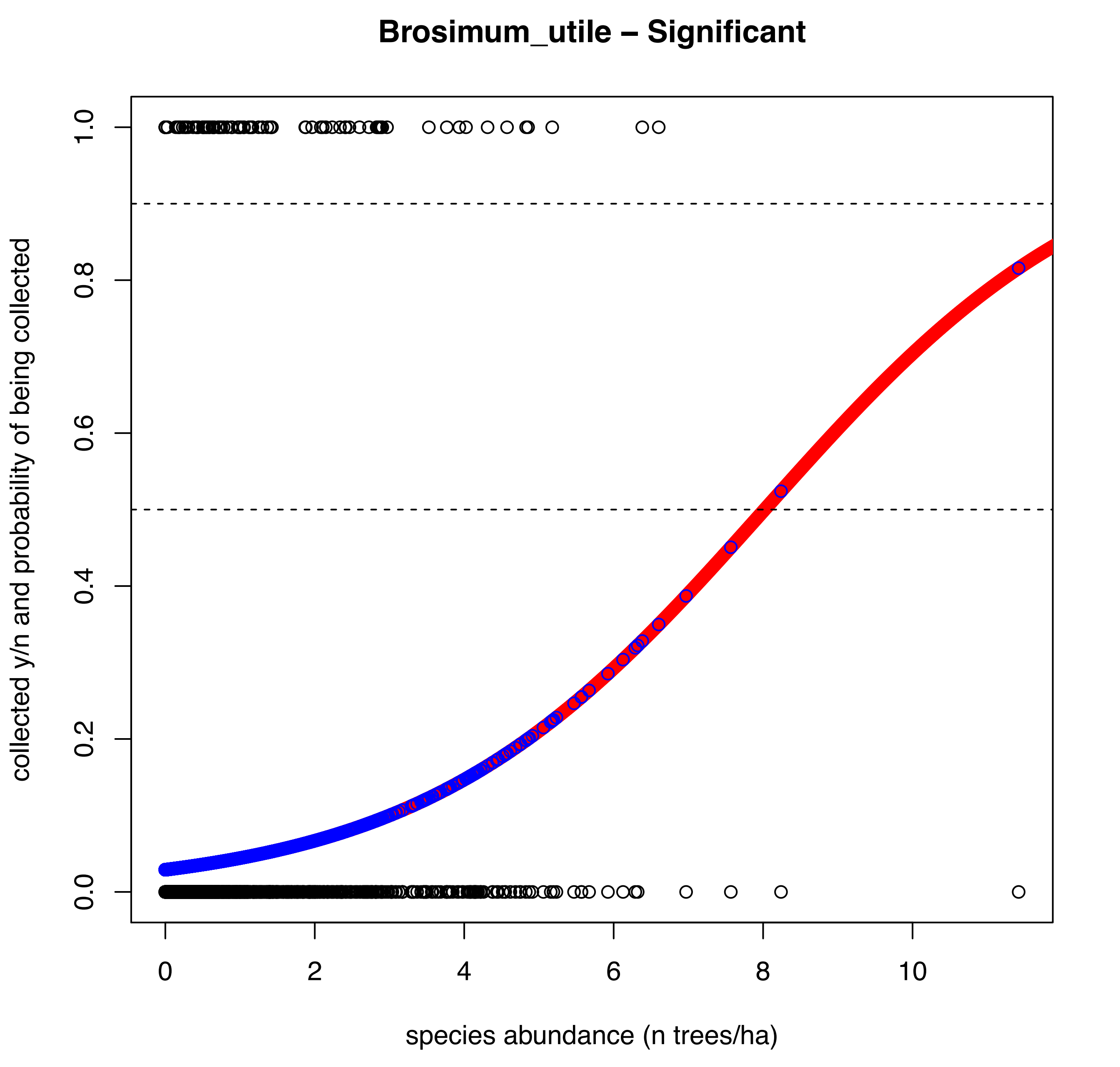
Brosimum_utile – Significant
collected y/n and probability of being collected
species abundance (n trees/ha)
0.0
0.2
0.4
0.6
0.8
1.0
0
2
4
6
8
10

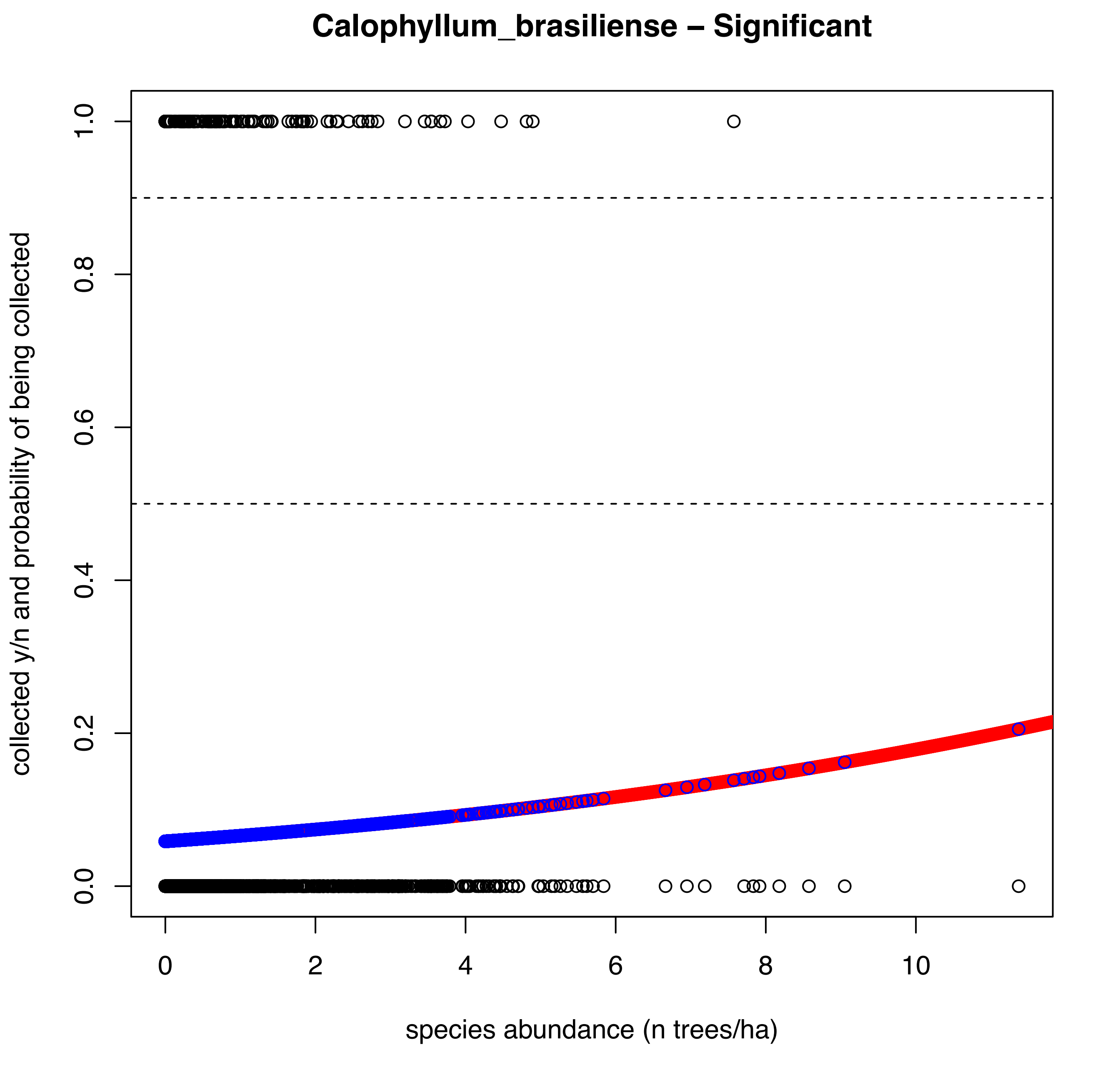

Calophyllum_brasiliense – Significant
collected y/n and probability of being collected
species abundance (n trees/ha)
0.0
0.2
0.4
0.6
0.8
1.0
0
2
4
6
8
10

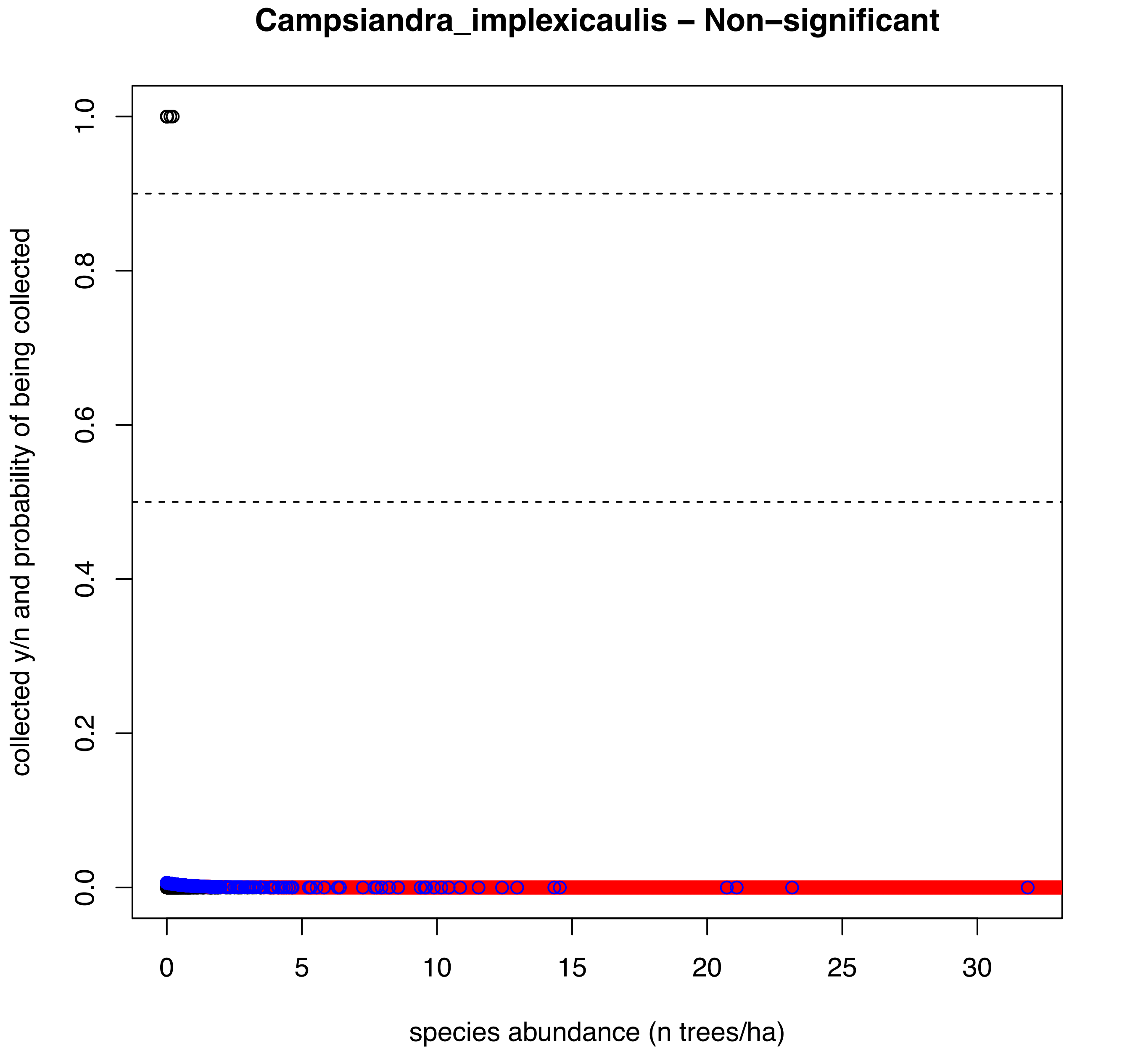

Campsiandra_implexicaulis – Non–significant
collected y/n and probability of being collected
species abundance (n trees/ha)
0.0
0.2
0.4
0.6
0.8
1.0
0
5
10
15
20
25
30

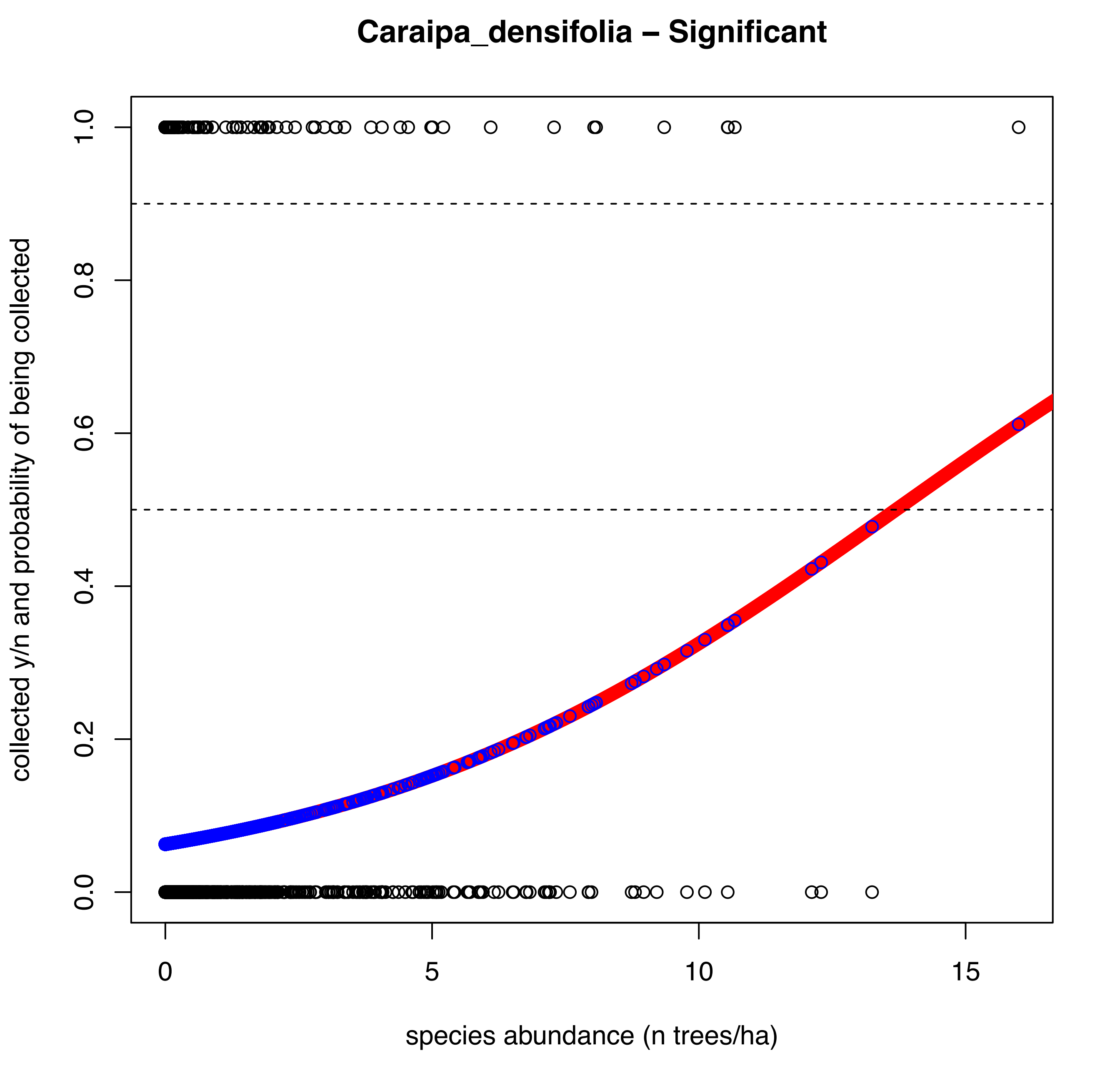
Caraipa_densifolia – Significant
collected y/n and probability of being collected
0.0
0.2
0.4
0.6
0.8
1.0
0
5
10
15
species abundance (n trees/ha)

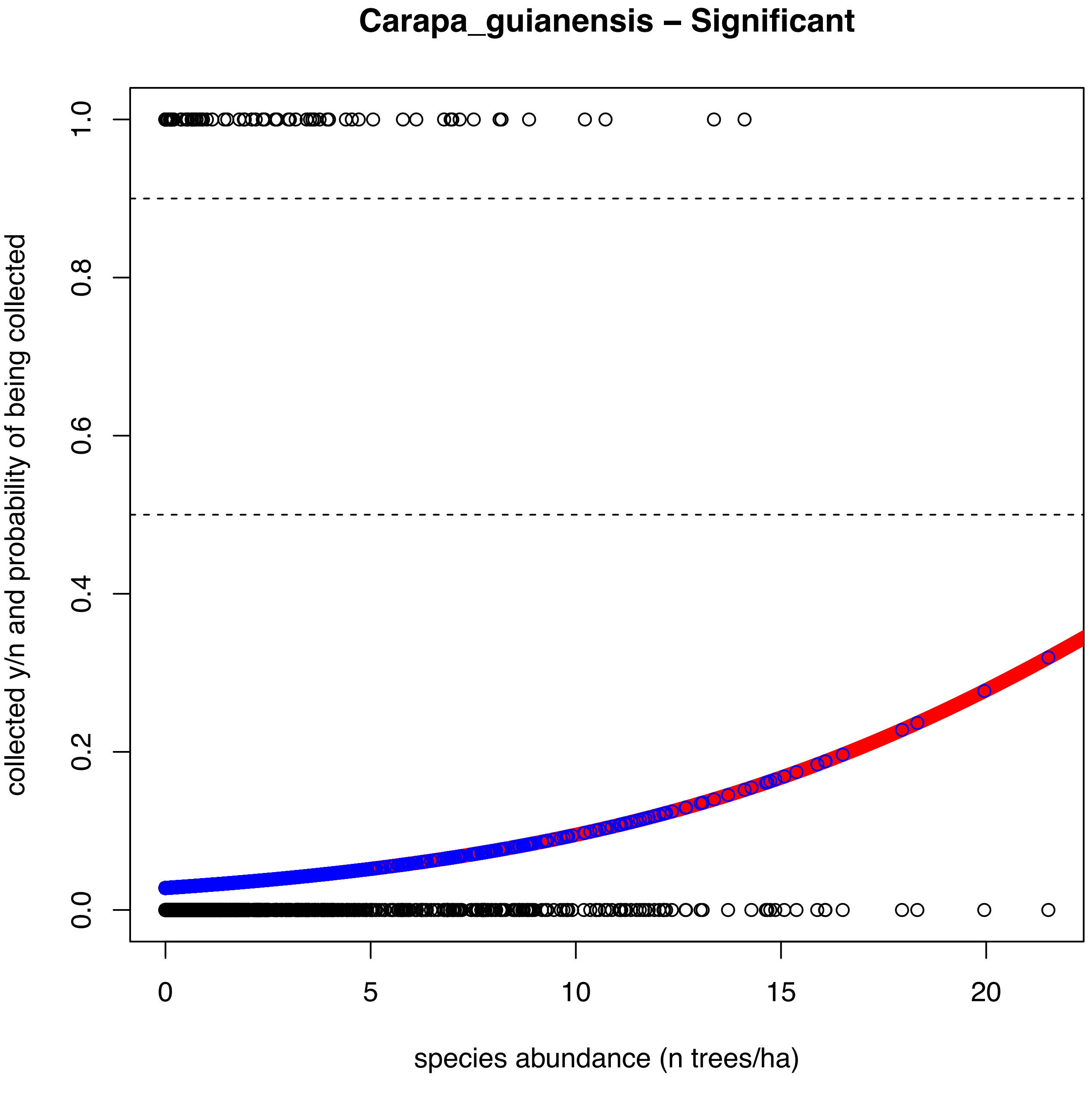

Carapa_guianensis – Significant
collected y/n and probability of being collected
species abundance (n trees/ha)
0.0
0.2
0.4
0.6
0.8
1.0
0
5
10
15
20

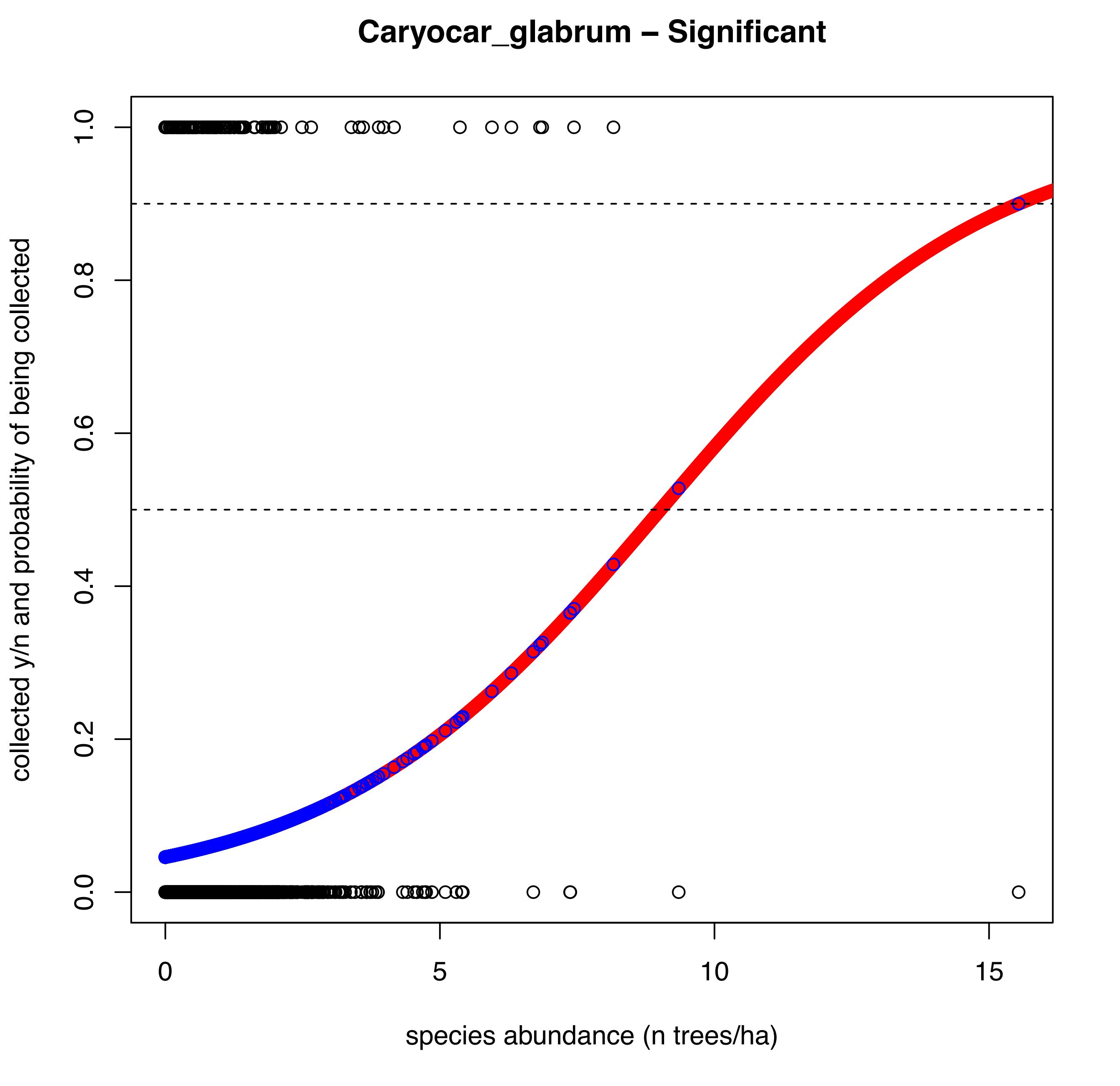

Caryocar_glabrum – Significant
1.0
0.8
0.6
0.4
0.2
0.0
collected y/n and probability of being collected
0
5
10
15
species abundance (n trees/ha)

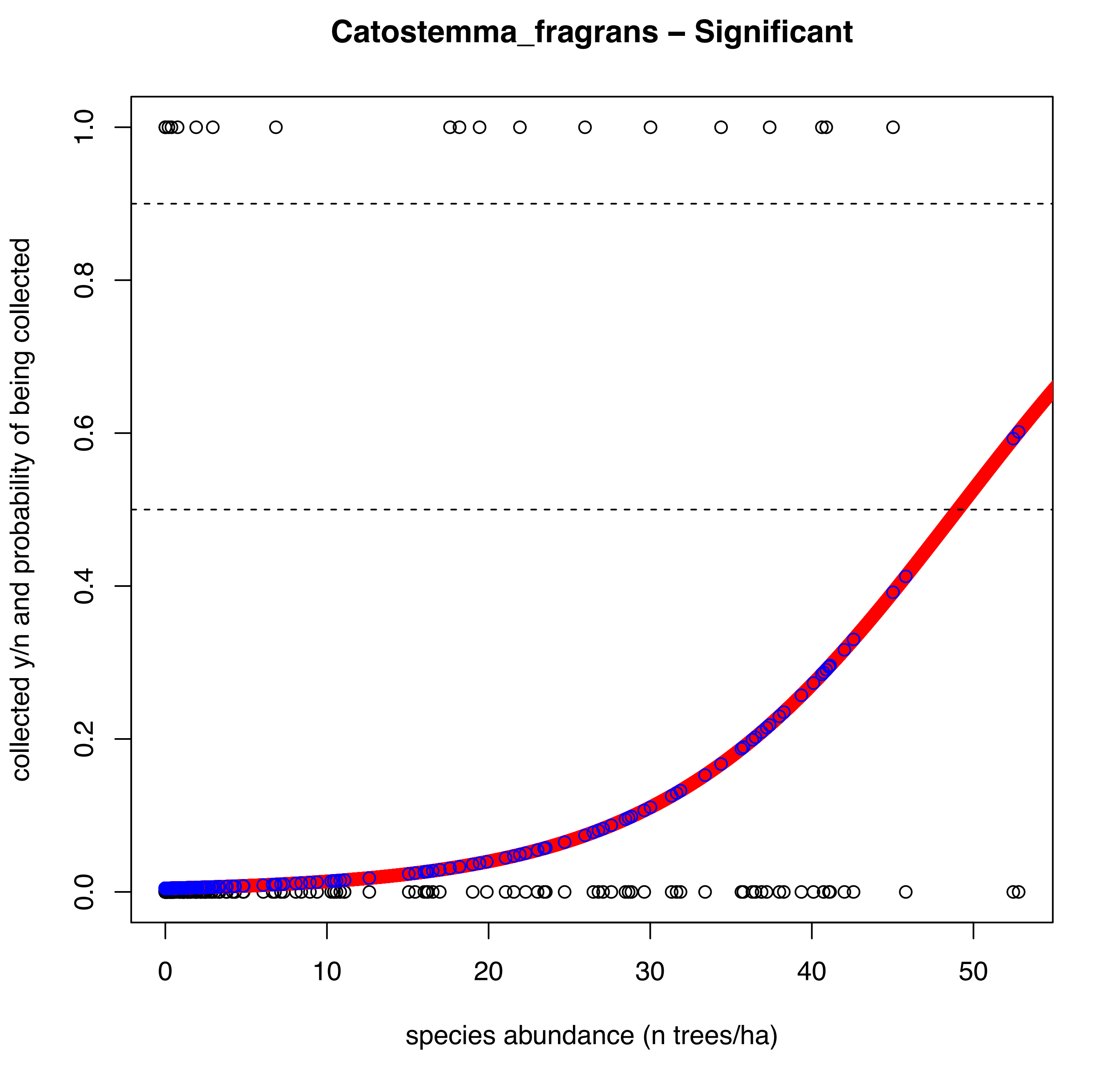
Catostemma_fragrans – Significant
collected y/n and probability of being collected
species abundance (n trees/ha)
0.0
0.2
0.4
0.6
0.8
1.0
0
10
20
30
40
50

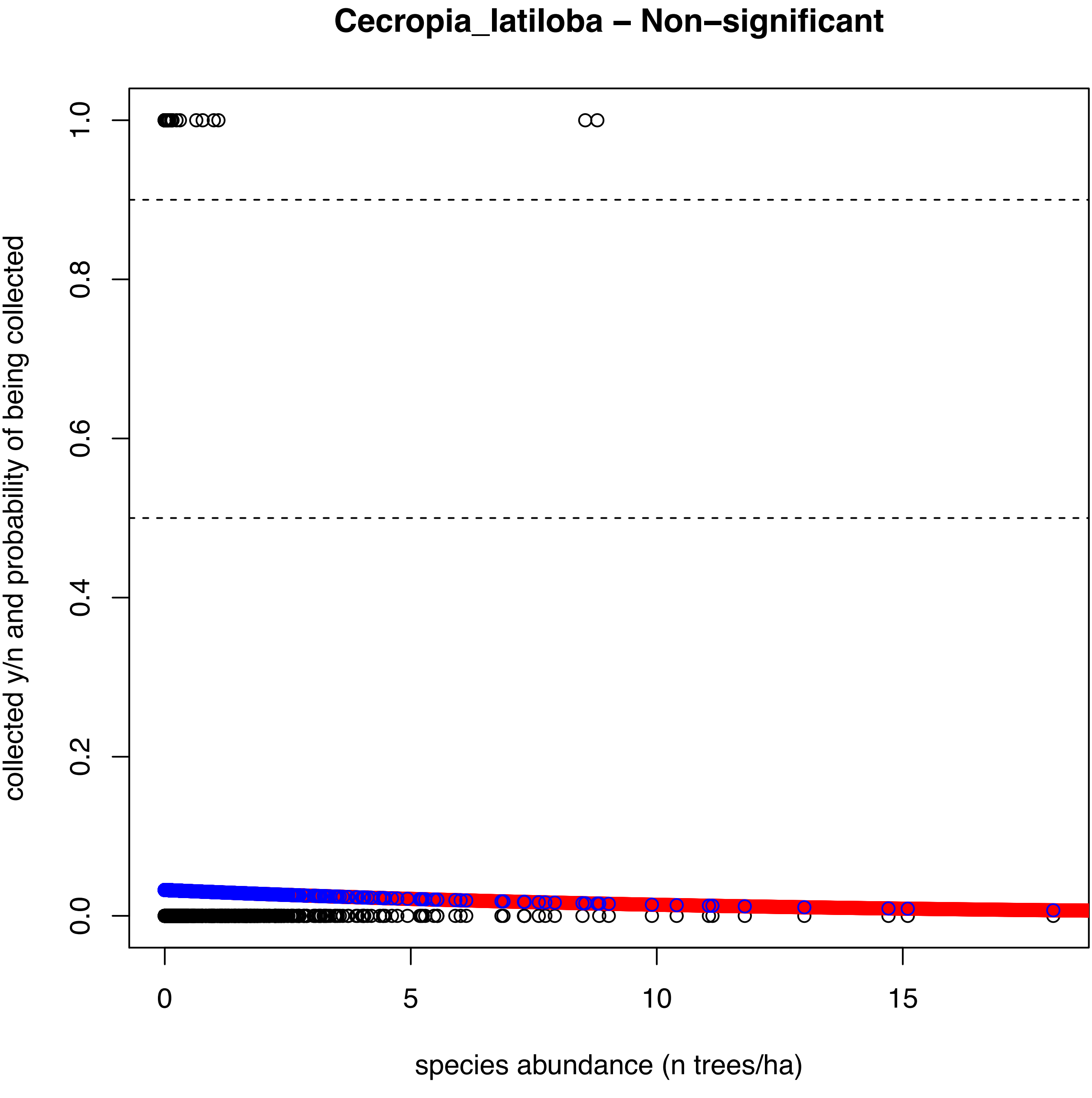

Cecropia_latiloba – Non–significant
collected y/n and probability of being collected
species abundance (n trees/ha)
0.0
0.2
0.4
0.6
0.8
1.0
0
5
10
15

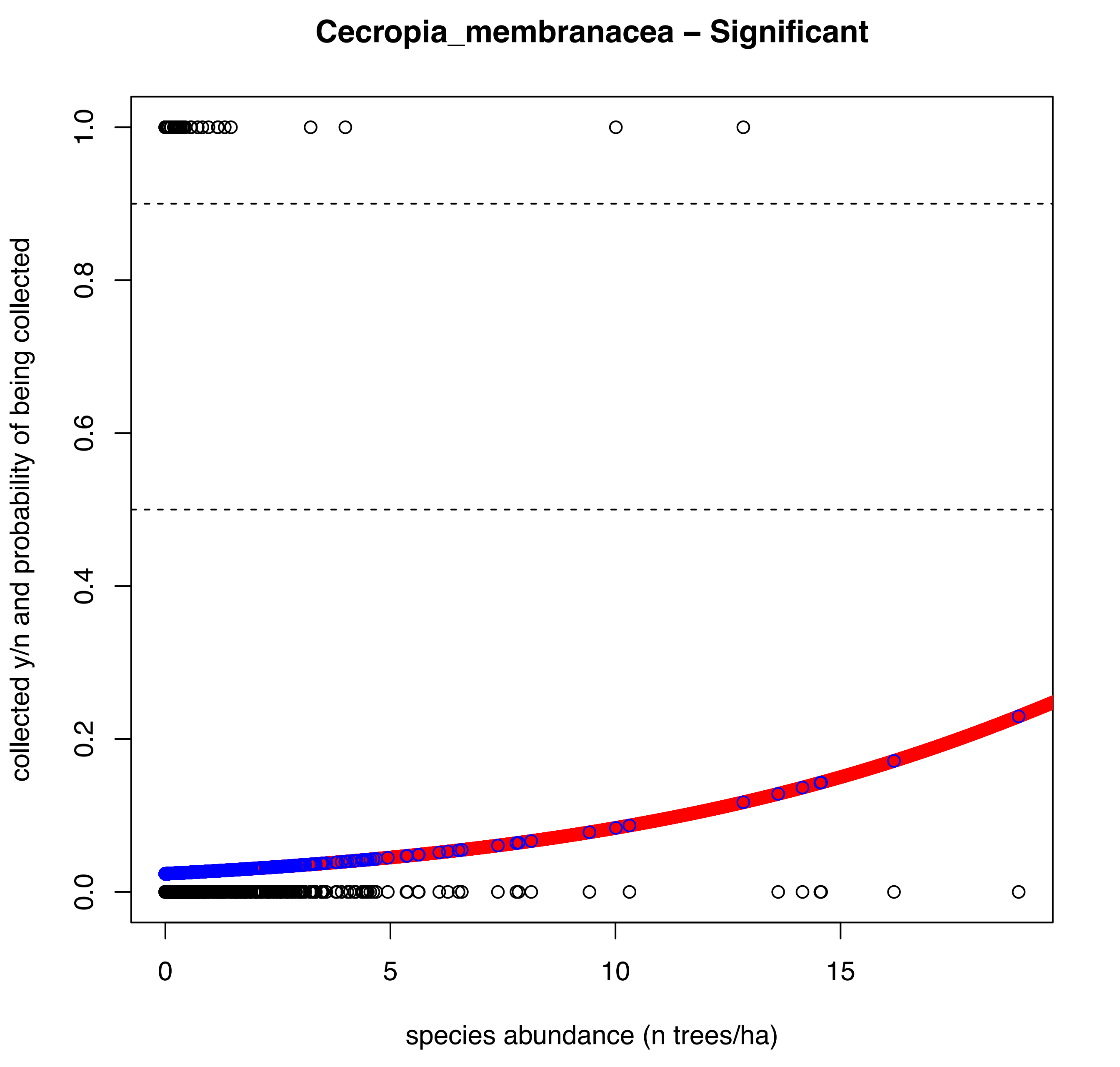
Cecropia_membranacea – Significant
collected y/n and probability of being collected
species abundance (n trees/ha)
0.0
0.2
0.4
0.6
0.8
1.0
0
5
10
15

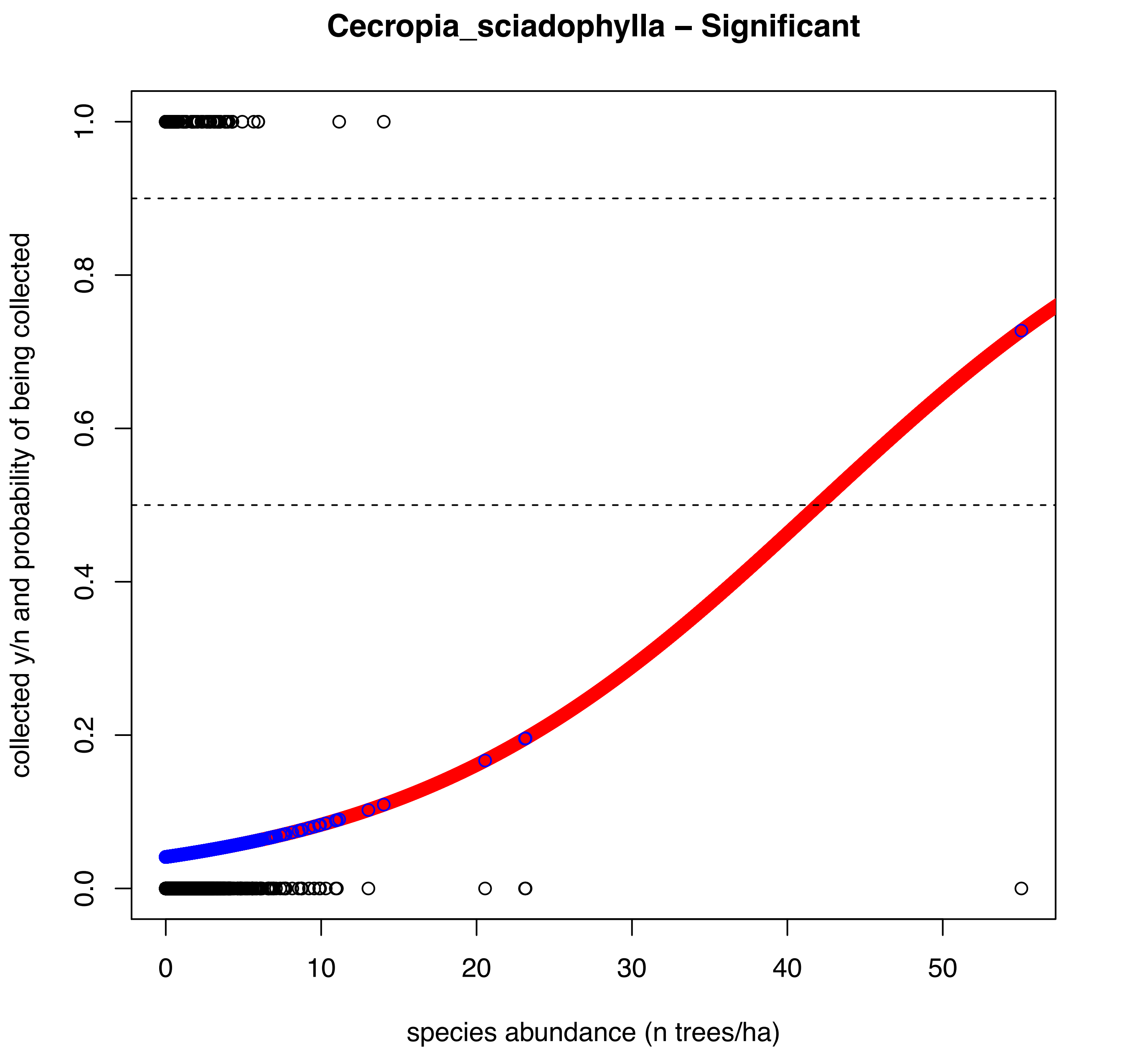

Cecropia_sciadophylla – Significant
collected y/n and probability of being collected
0.0
0.2
0.4
0.6
0.8
1.0
0
10
20
30
40
50
species abundance (n trees/ha)

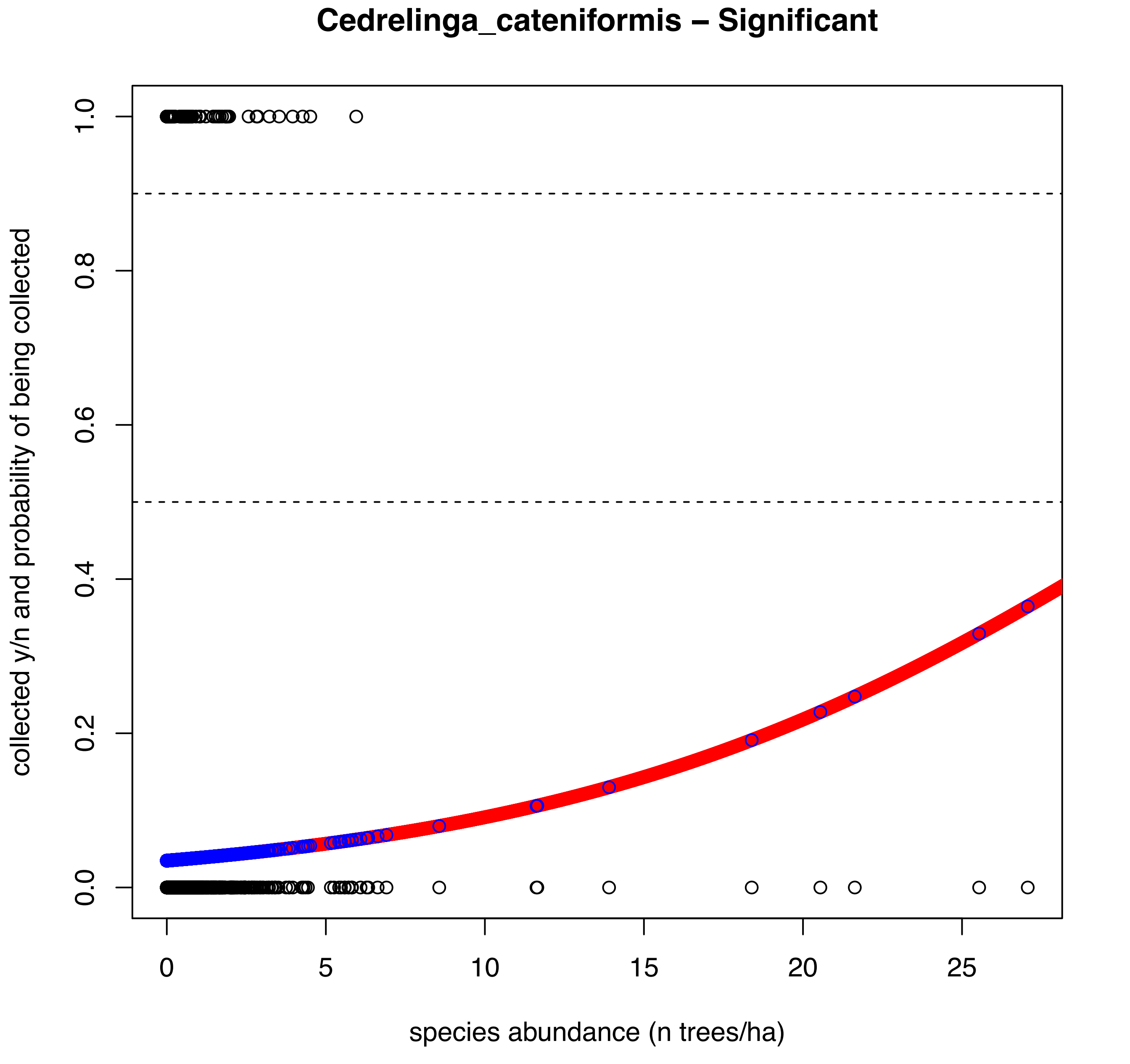

Cedrelinga_cateniformis – Significant
collected y/n and probability of being collected
species abundance (n trees/ha)
0.0
0.2
0.4
0.6
0.8
1.0
0
5
10
15
20
25

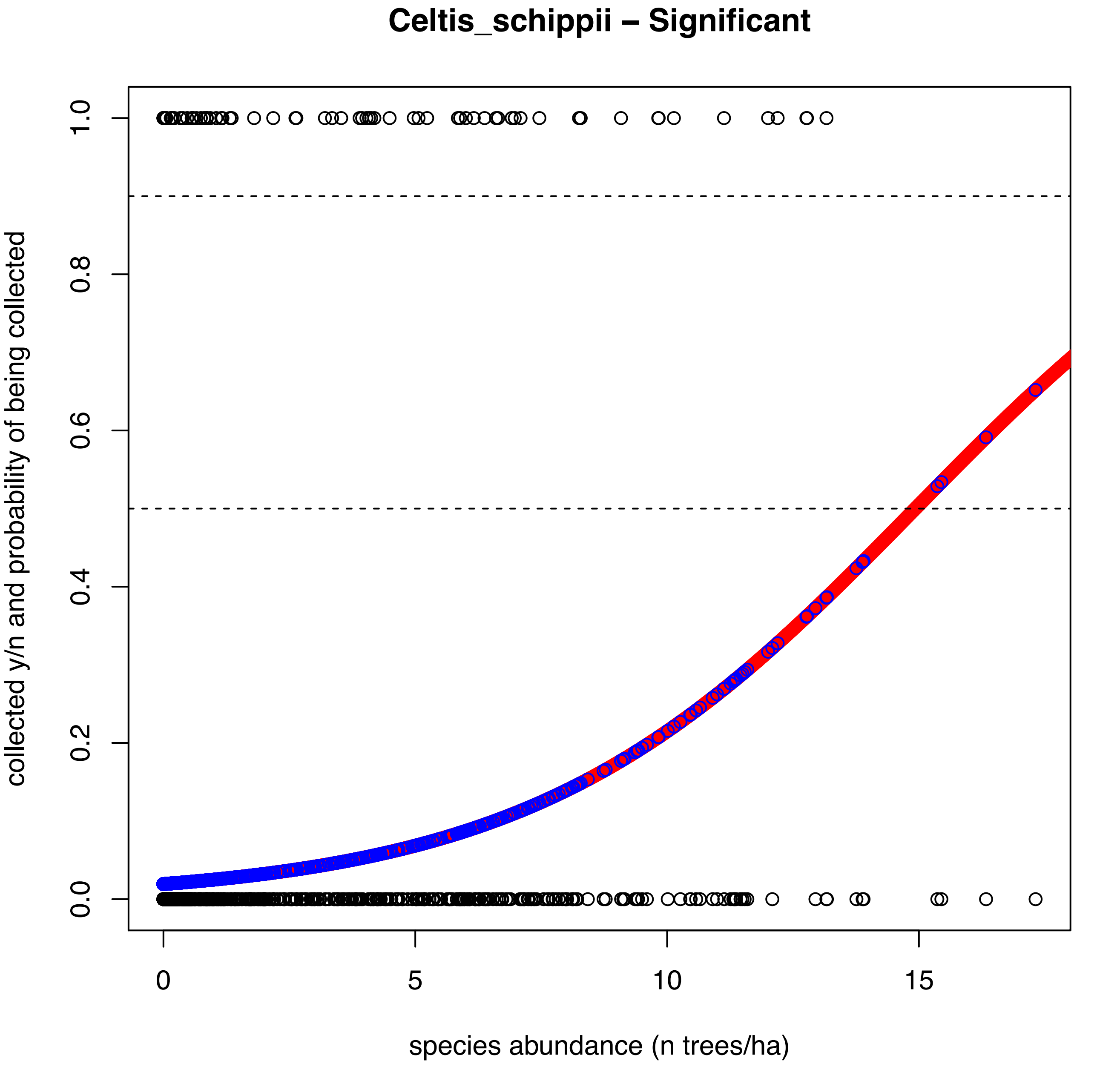
Celtis_schippii – Significant
collected y/n and probability of being collected
species abundance (n trees/ha)
0.0
0.2
0.4
0.6
0.8
1.0
0
5
10
15

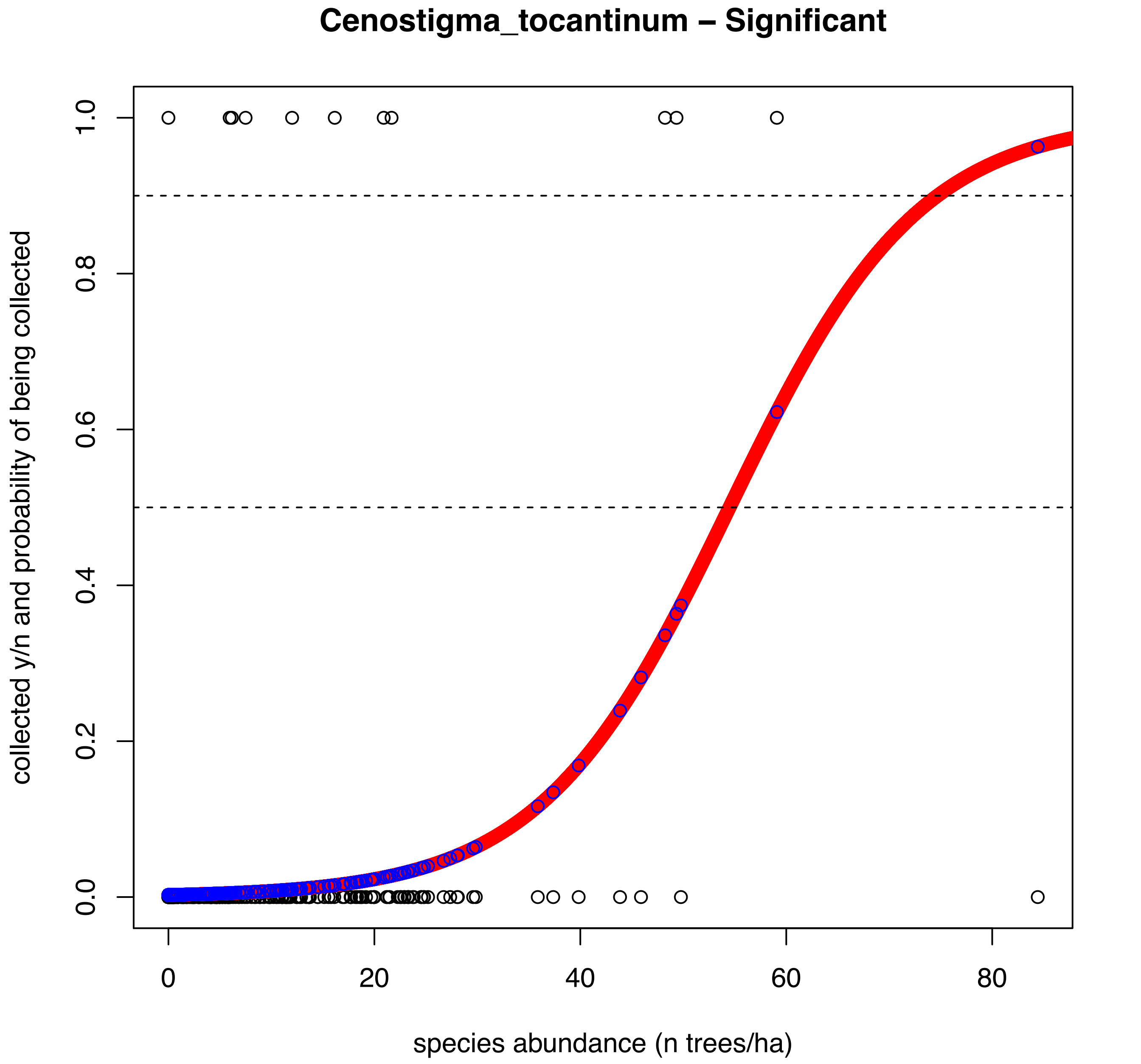

Cenostigma_tocantinum – Significant
collected y/n and probability of being collected
species abundance (n trees/ha)
0.0
0.2
0.4
0.6
0.8
1.0
0
20
40
60
80

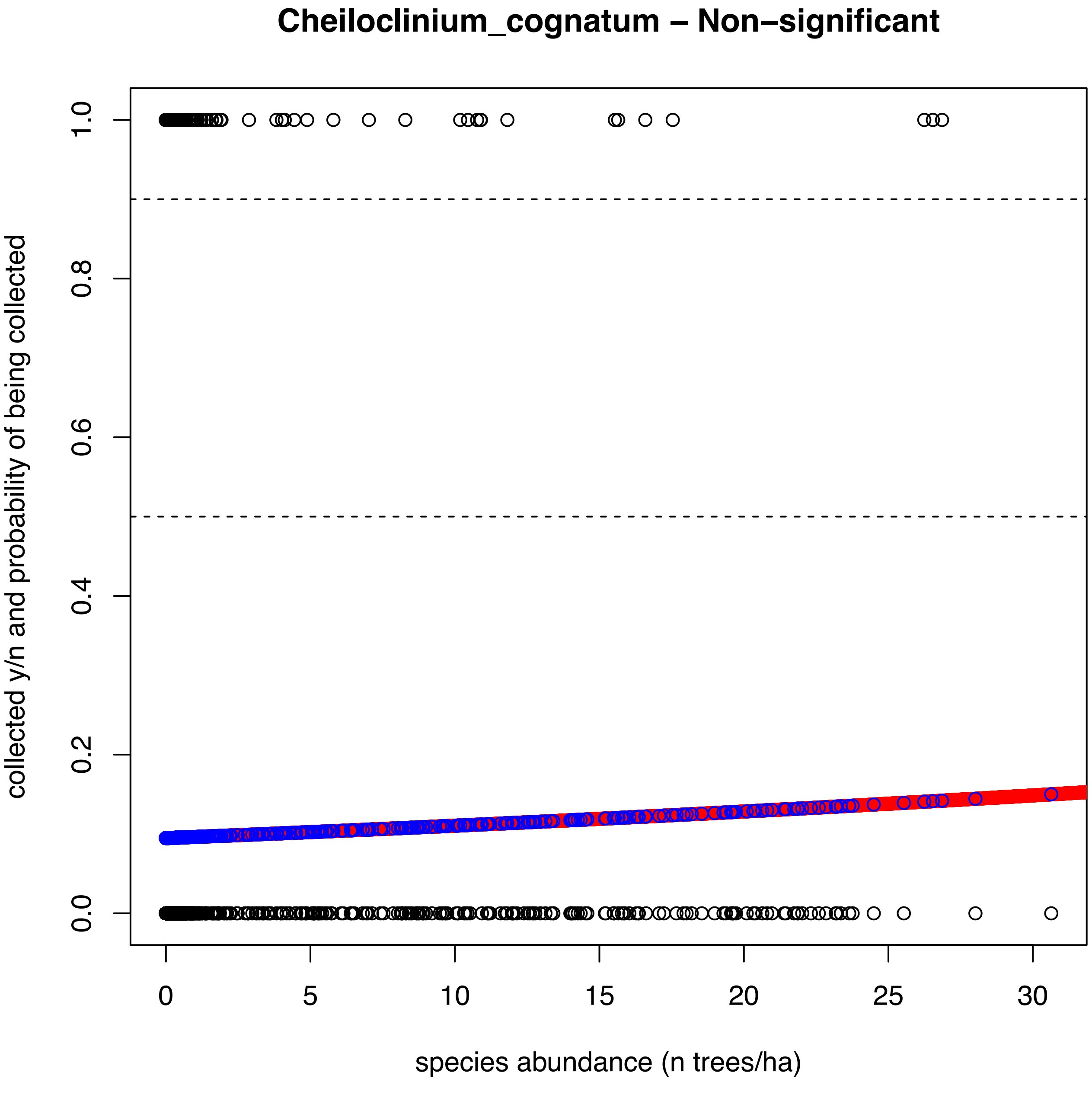

Cheiloclinium_cognatum – Non–significant
collected y/n and probability of being collected
species abundance (n trees/ha)
0.0
0.2
0.4
0.6
0.8
1.0
0
5
10
15
20
25
30

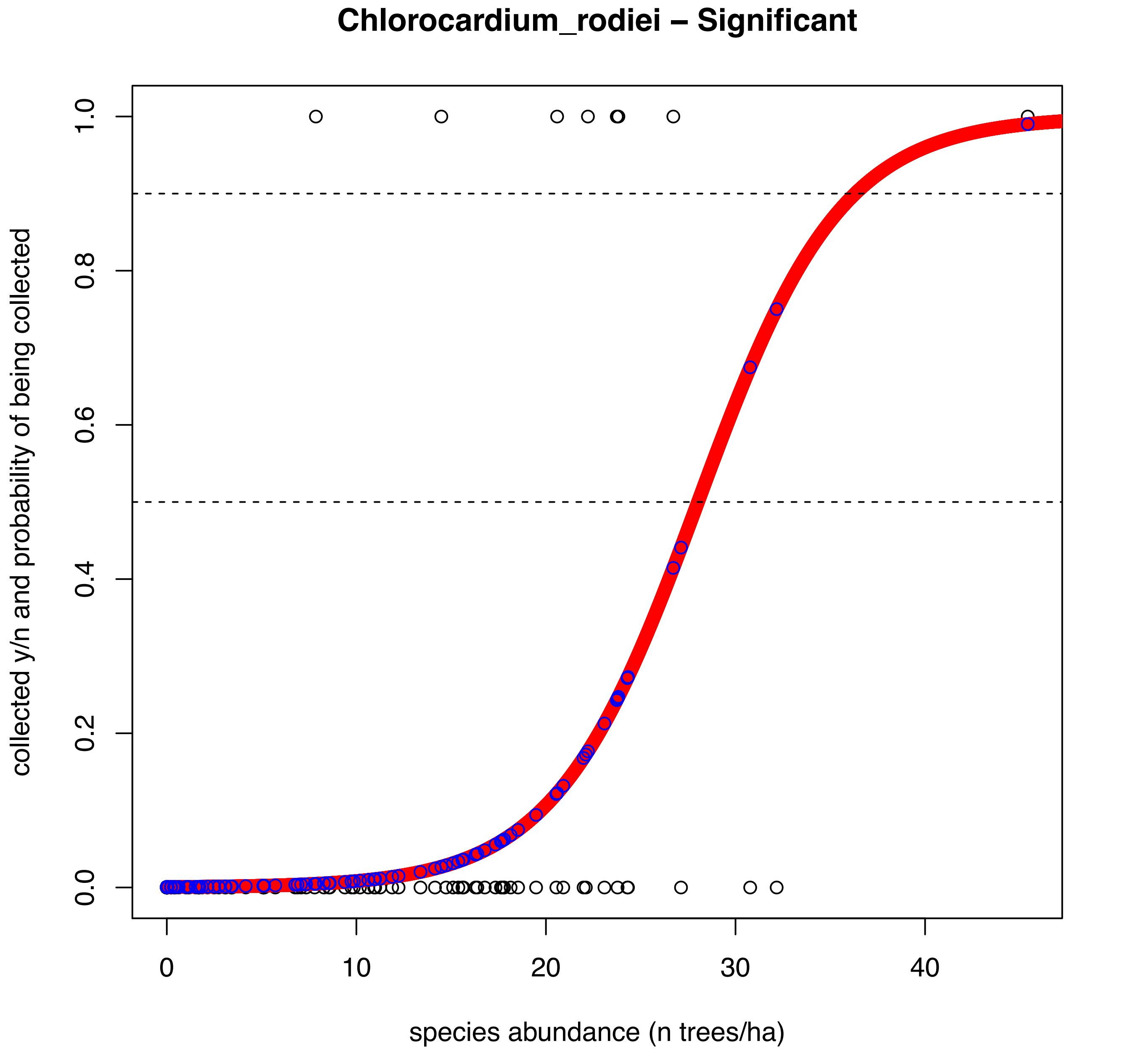

Chlorocardium_rodiei – Significant
collected y/n and probability of being collected
species abundance (n trees/ha)
0.0
0.2
0.4
0.6
0.8
1.0
0
10
20
30
40

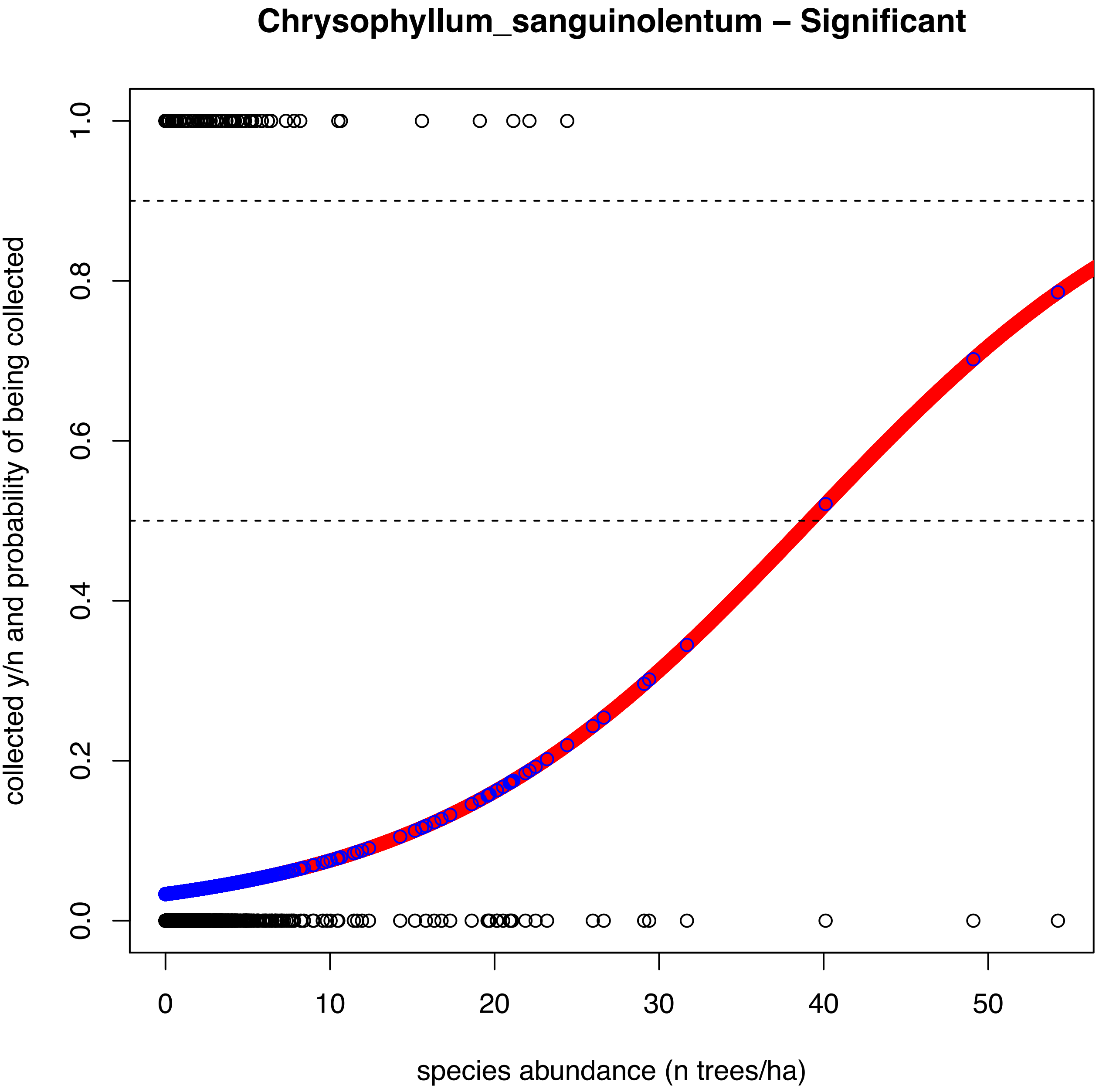

Chrysophyllum_sanguinolentum – Significant
collected y/n and probability of being collected
0.0
0.2
0.4
0.6
0.8
1.0
0
10
20
30
40
50
species abundance (n trees/ha)

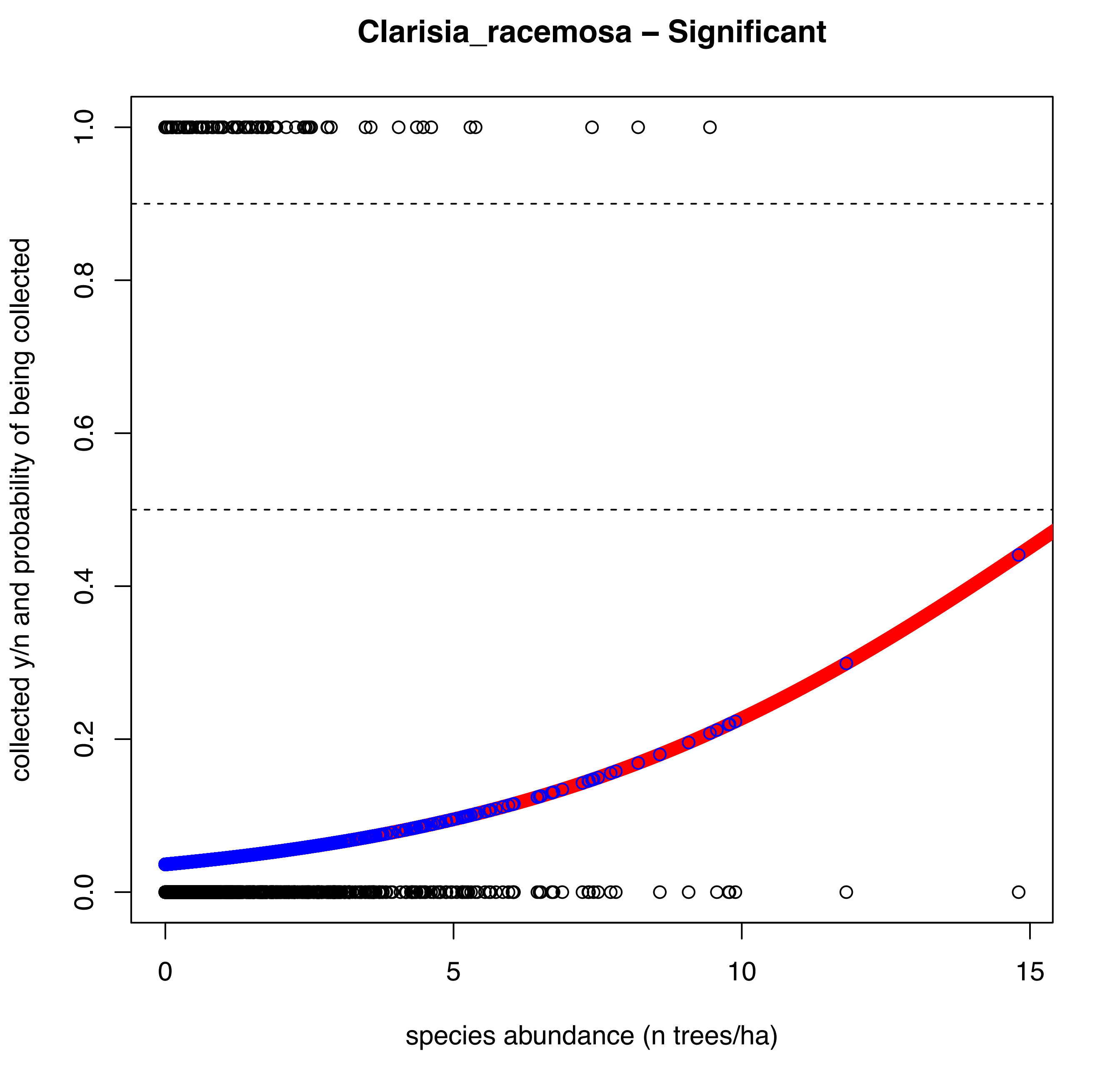
Clarisia_racemosa – Significant
collected y/n and probability of being collected
species abundance (n trees/ha)
0.0
0.2
0.4
0.6
0.8
1.0
0
5
10
15

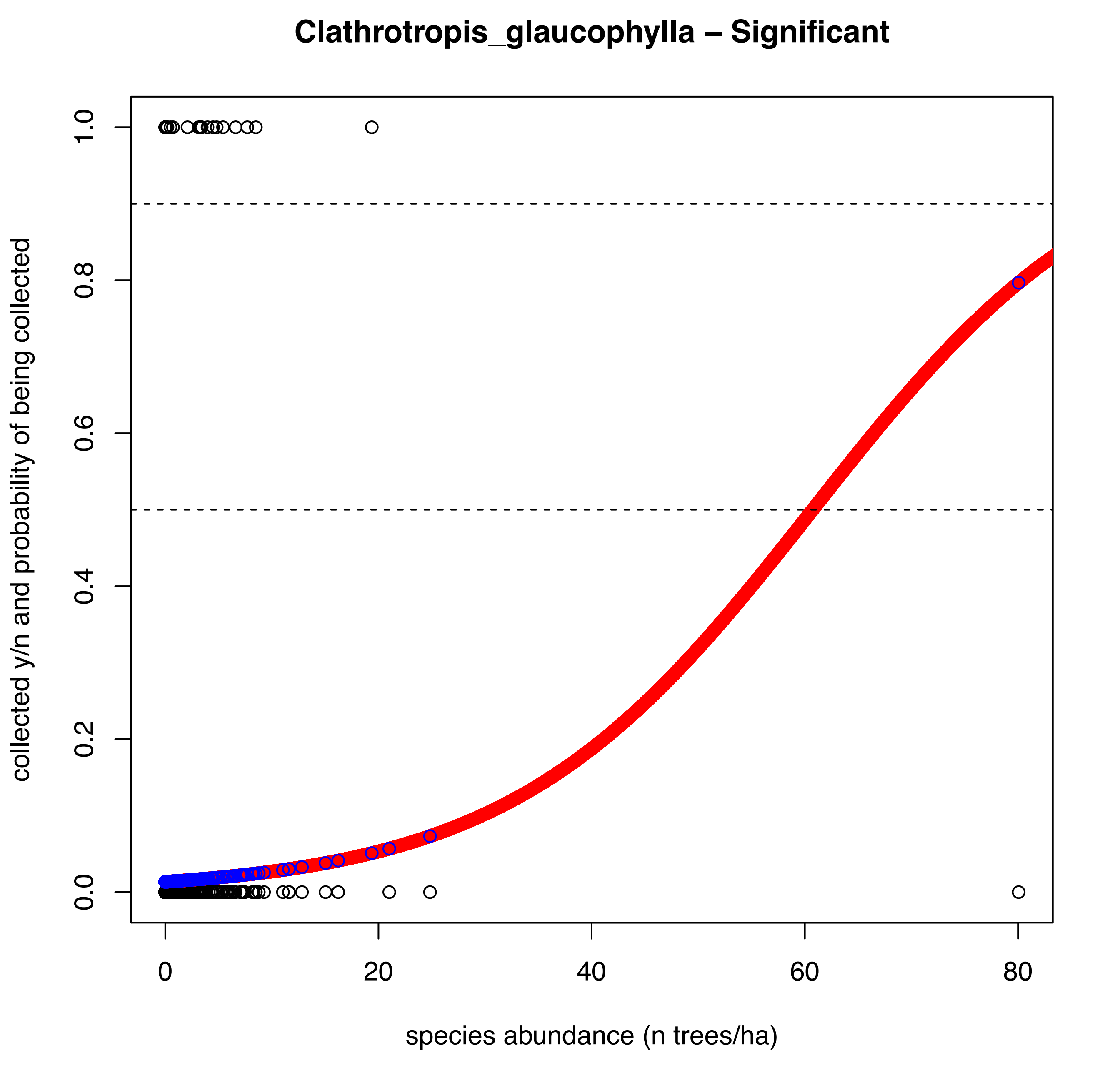
Clathrotropis_glaucophylla – Significant
collected y/n and probability of being collected
0.0
0.2
0.4
0.6
0.8
1.0
0
20
40
60
80
species abundance (n trees/ha)

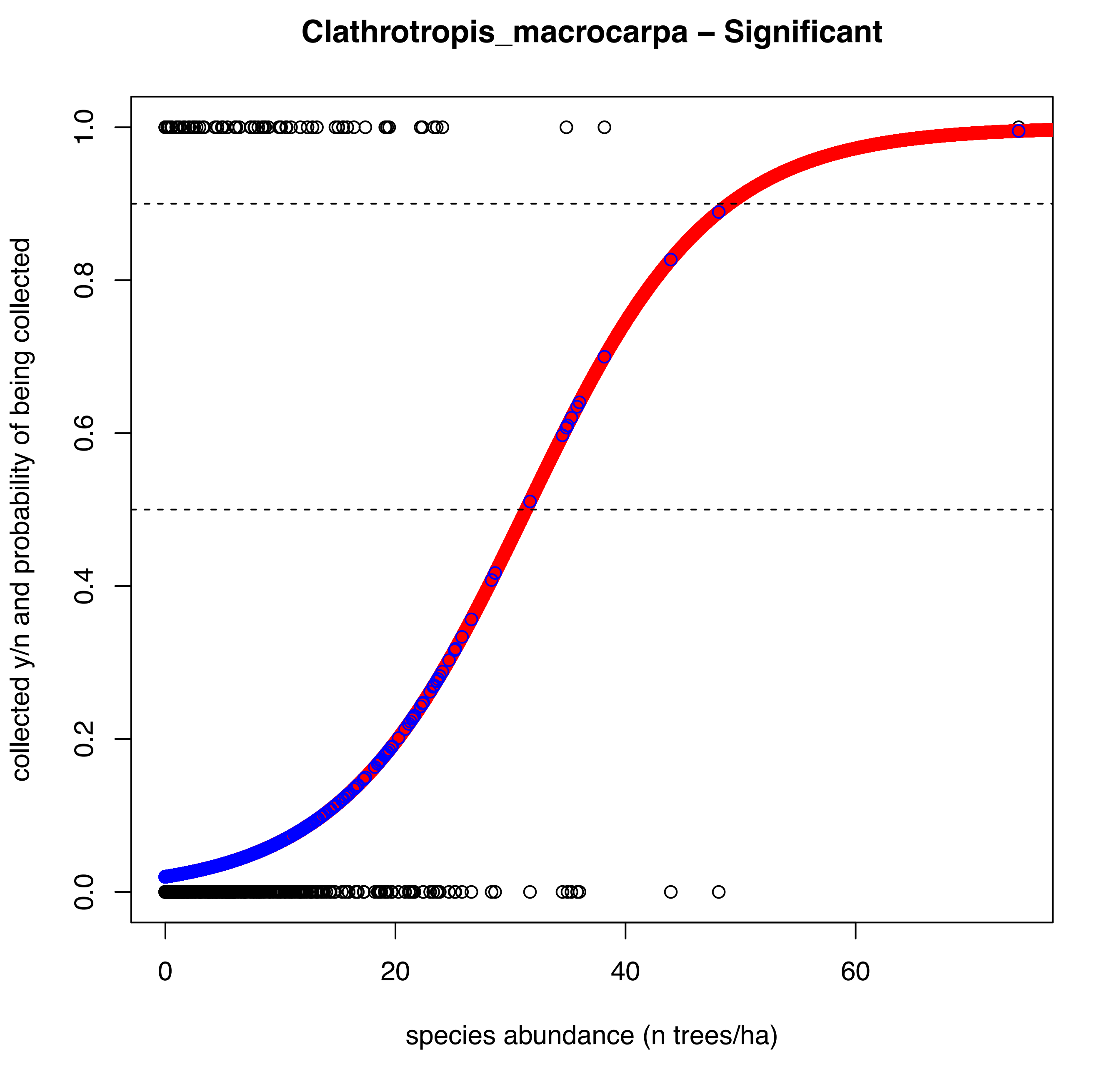
Clathrotropis_macrocarpa – Significant
collected y/n and probability of being collected
0.0
0.2
0.4
0.6
0.8
1.0
0
20
40
60
species abundance (n trees/ha)

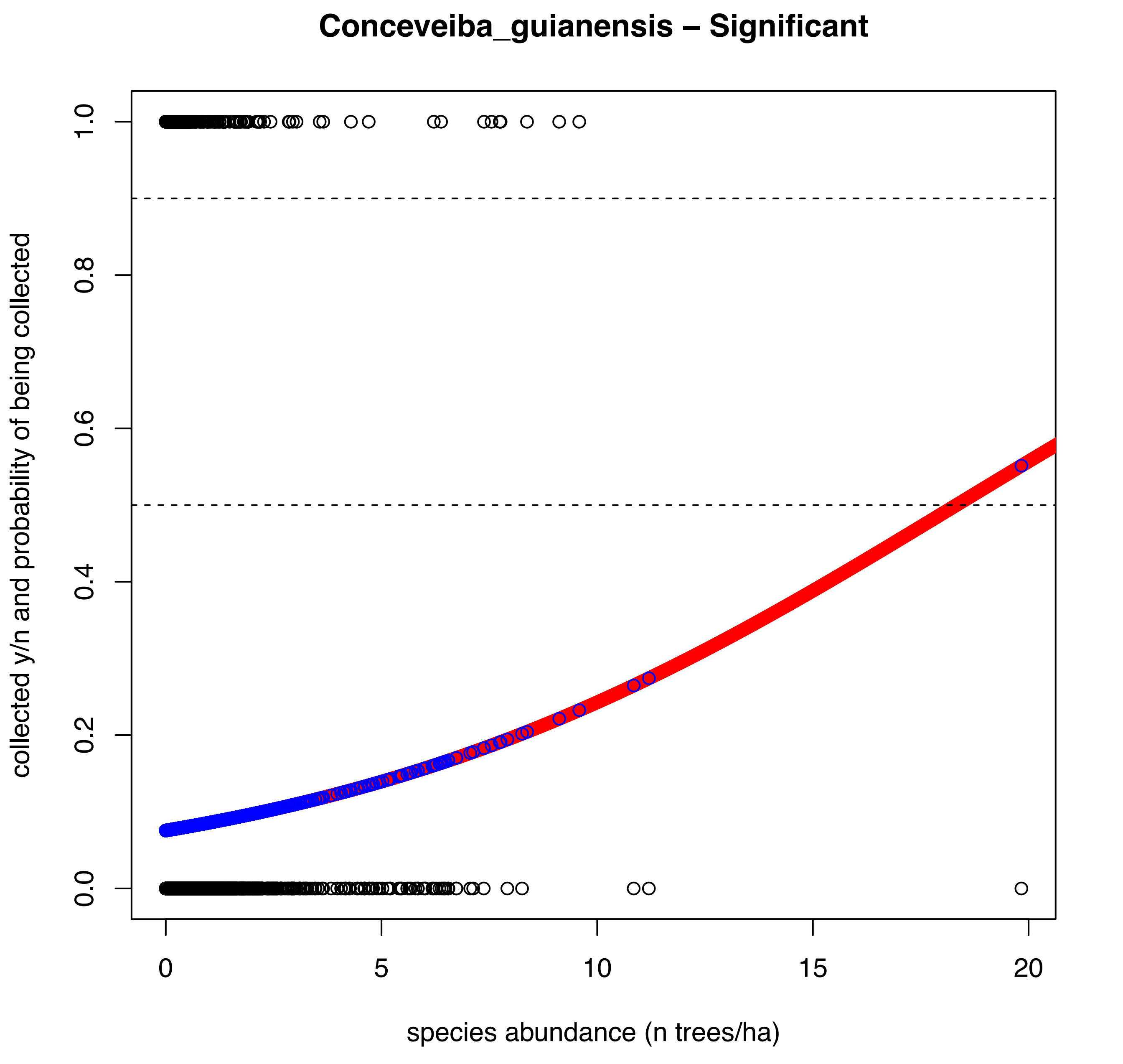

Conceveiba_guianensis – Significant
collected y/n and probability of being collected
species abundance (n trees/ha)
0.0
0.2
0.4
0.6
0.8
1.0
0
5
10
15
20

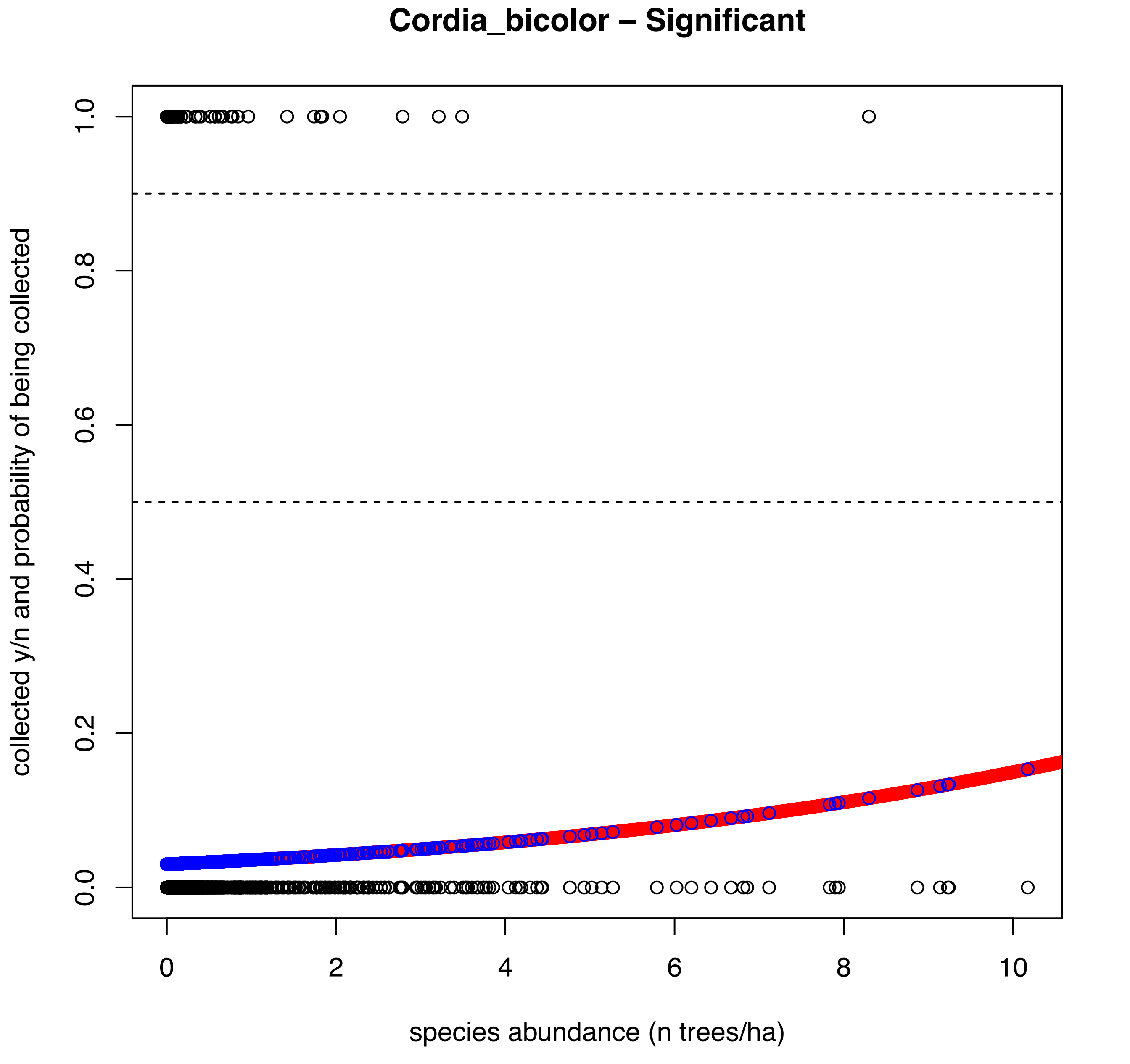
Cordia_bicolor – Significant
collected y/n and probability of being collected
species abundance (n trees/ha)
0.0
0.2
0.4
0.6
0.8
1.0
0
2
4
6
8
10

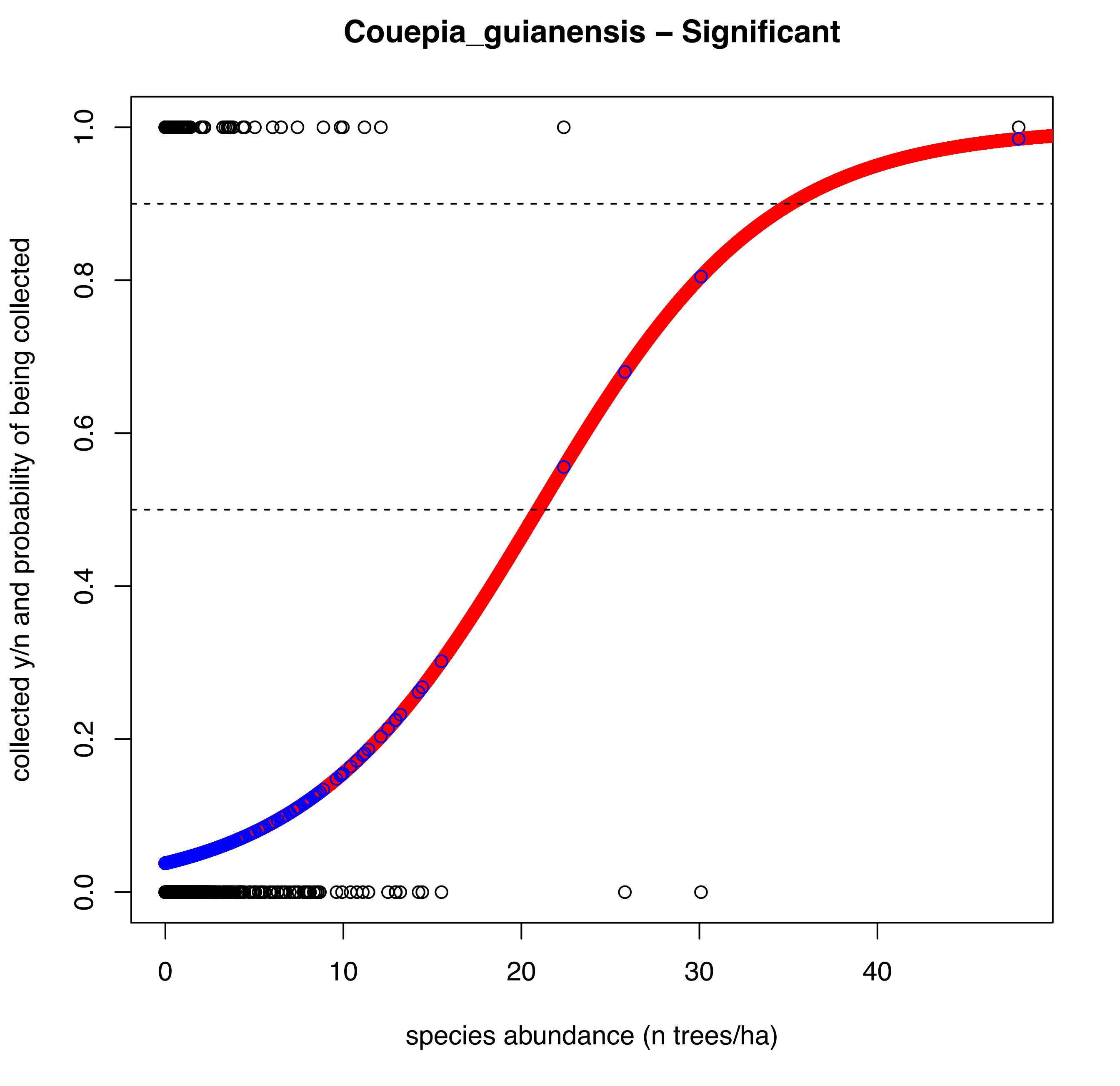
Couepia_guianensis – Significant
collected y/n and probability of being collected
species abundance (n trees/ha)
0.0
0.2
0.4
0.6
0.8
1.0
0
10
20
30
40

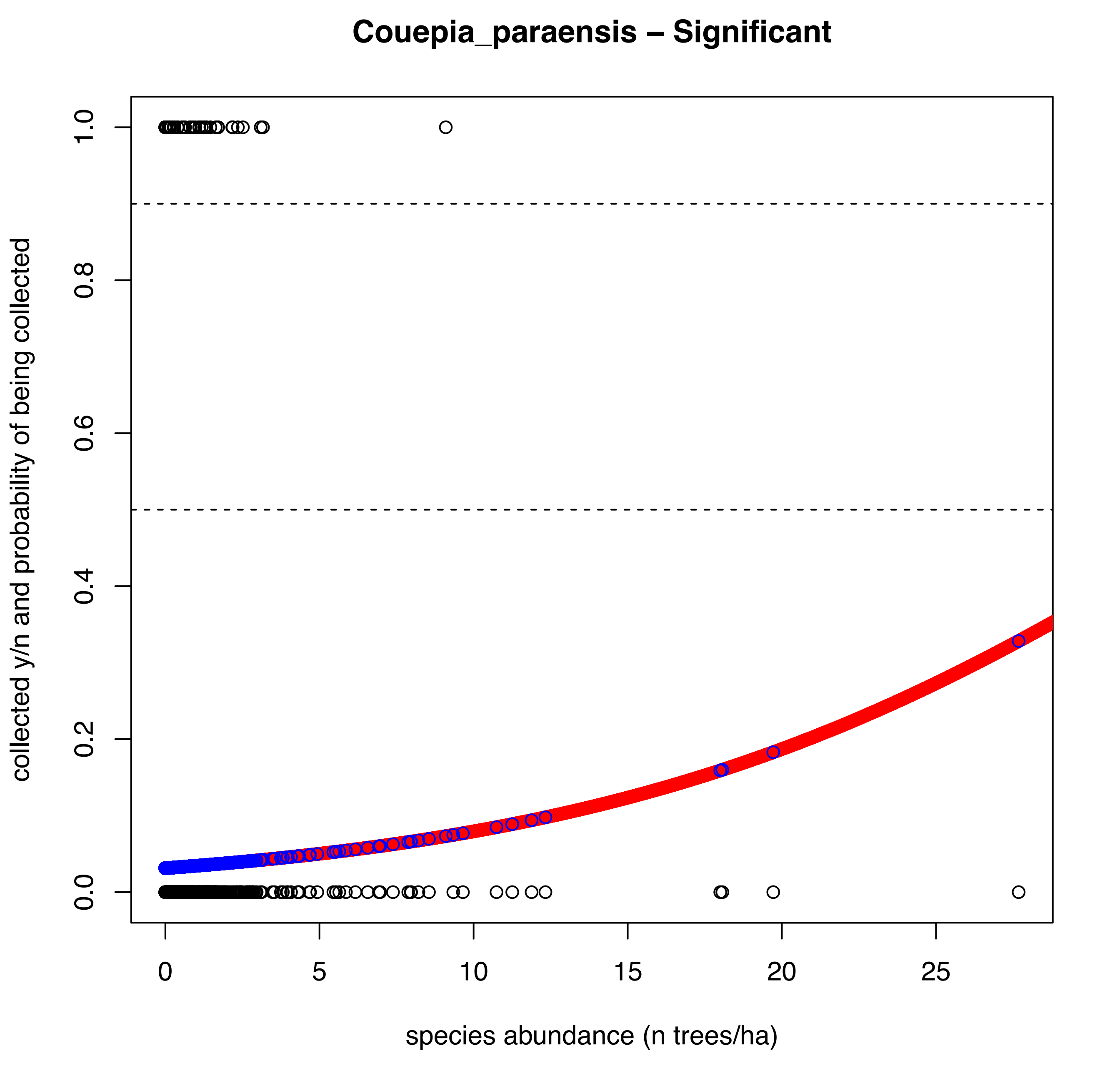
Couepia_paraensis – Significant
collected y/n and probability of being collected
0.0
0.2
0.4
0.6
0.8
1.0
0
5
10
15
20
25
species abundance (n trees/ha)

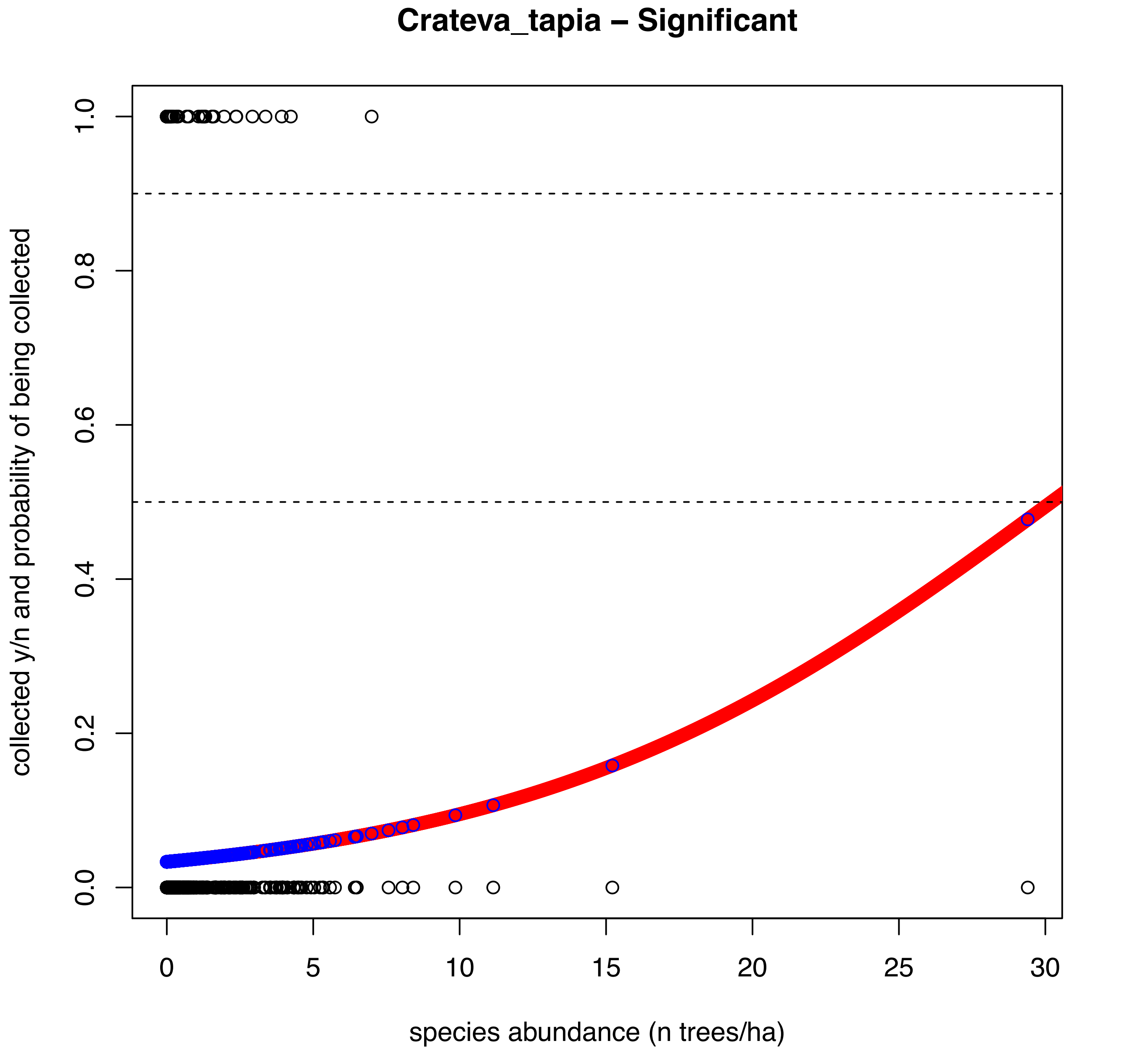

Crateva_tapia – Significant
collected y/n and probability of being collected
0.0
0.2
0.4
0.6
0.8
1.0
0
5
10
15
20
25
30
species abundance (n trees/ha)

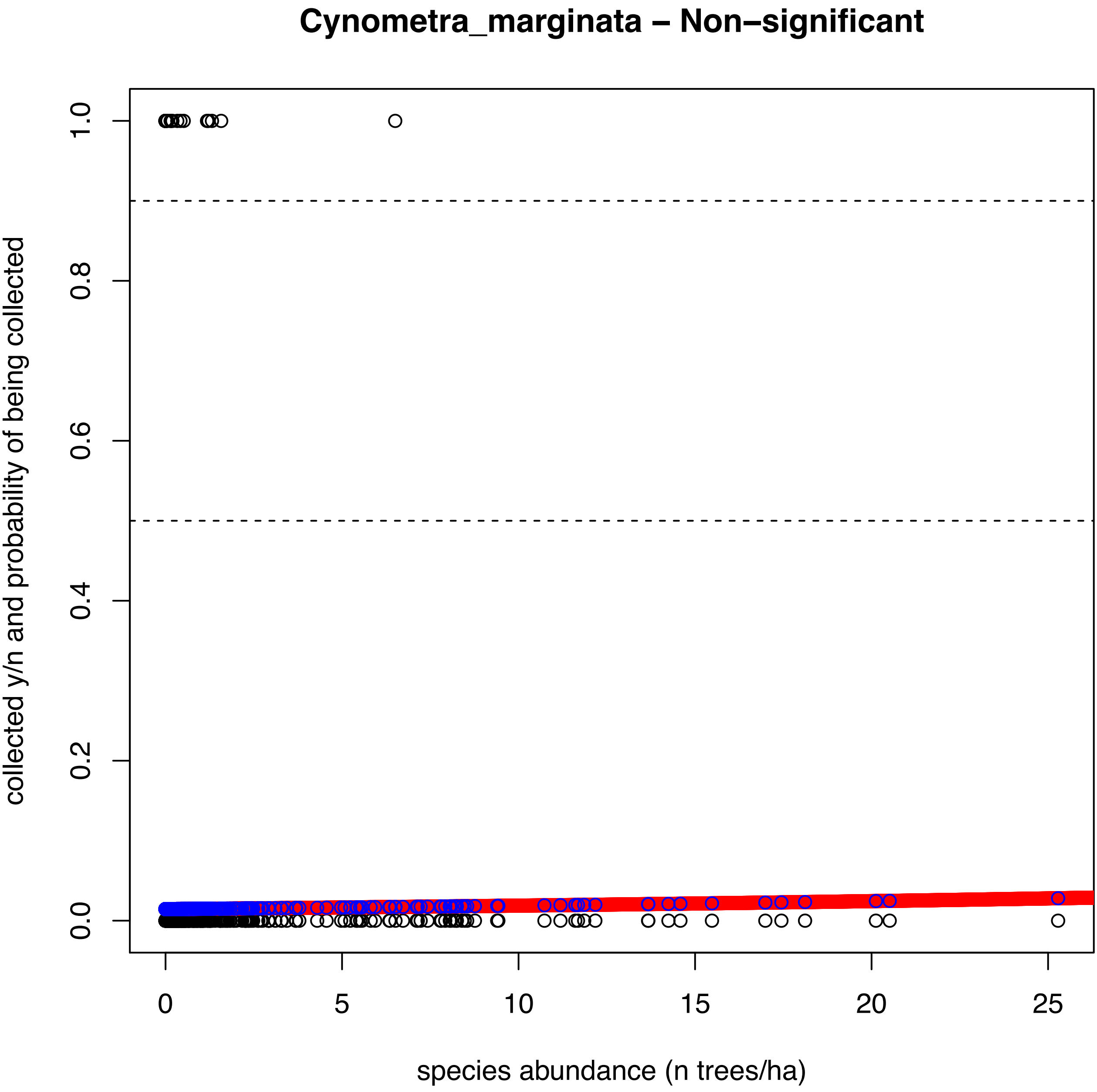

Cynometra_marginata – Non–significant
collected y/n and probability of being collected
1.0
0.8
0.6
0.4
0.2
0.0
0
5
10
15
20
25
species abundance (n trees/ha)

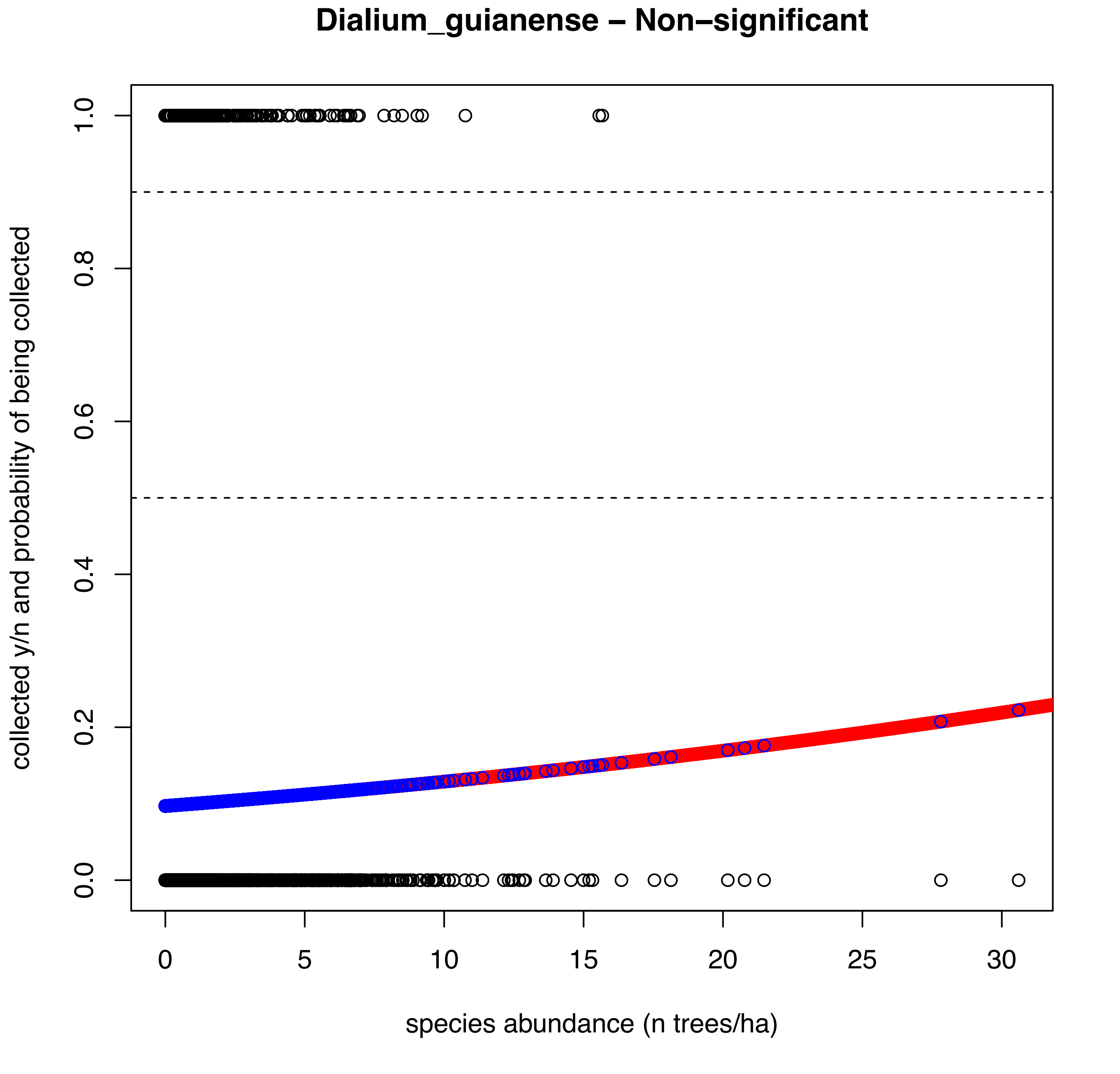

Dialium_guianense – Non–significant
collected y/n and probability of being collected
species abundance (n trees/ha)
0.0
0.2
0.4
0.6
0.8
1.0
0
5
10
15
20
25
30

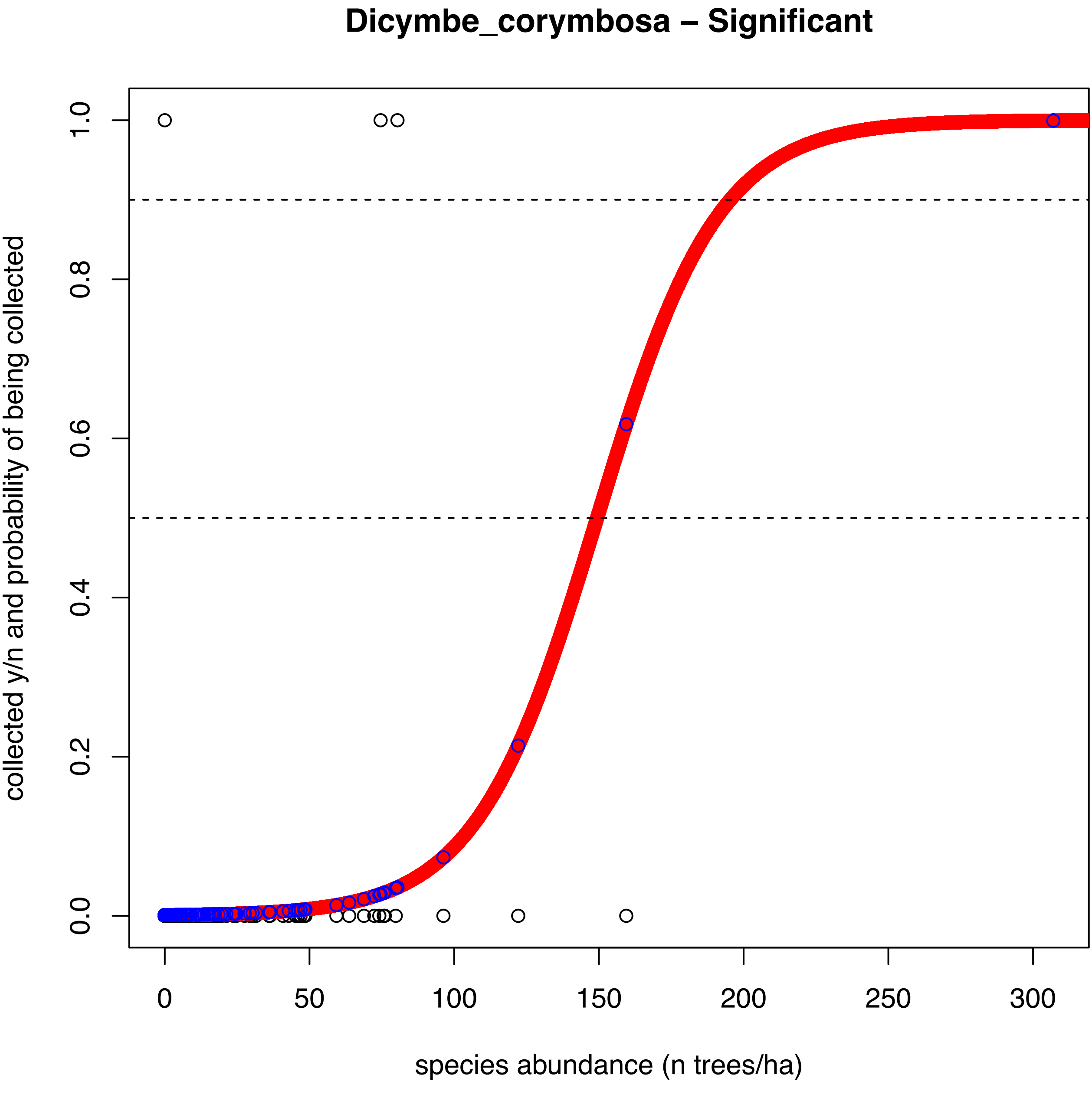
Dicymbe_corymbosa – Significant
collected y/n and probability of being collected
species abundance (n trees/ha)
0.0
0.2
0.4
0.6
0.8
1.0
0
50
100
150
200
250
300

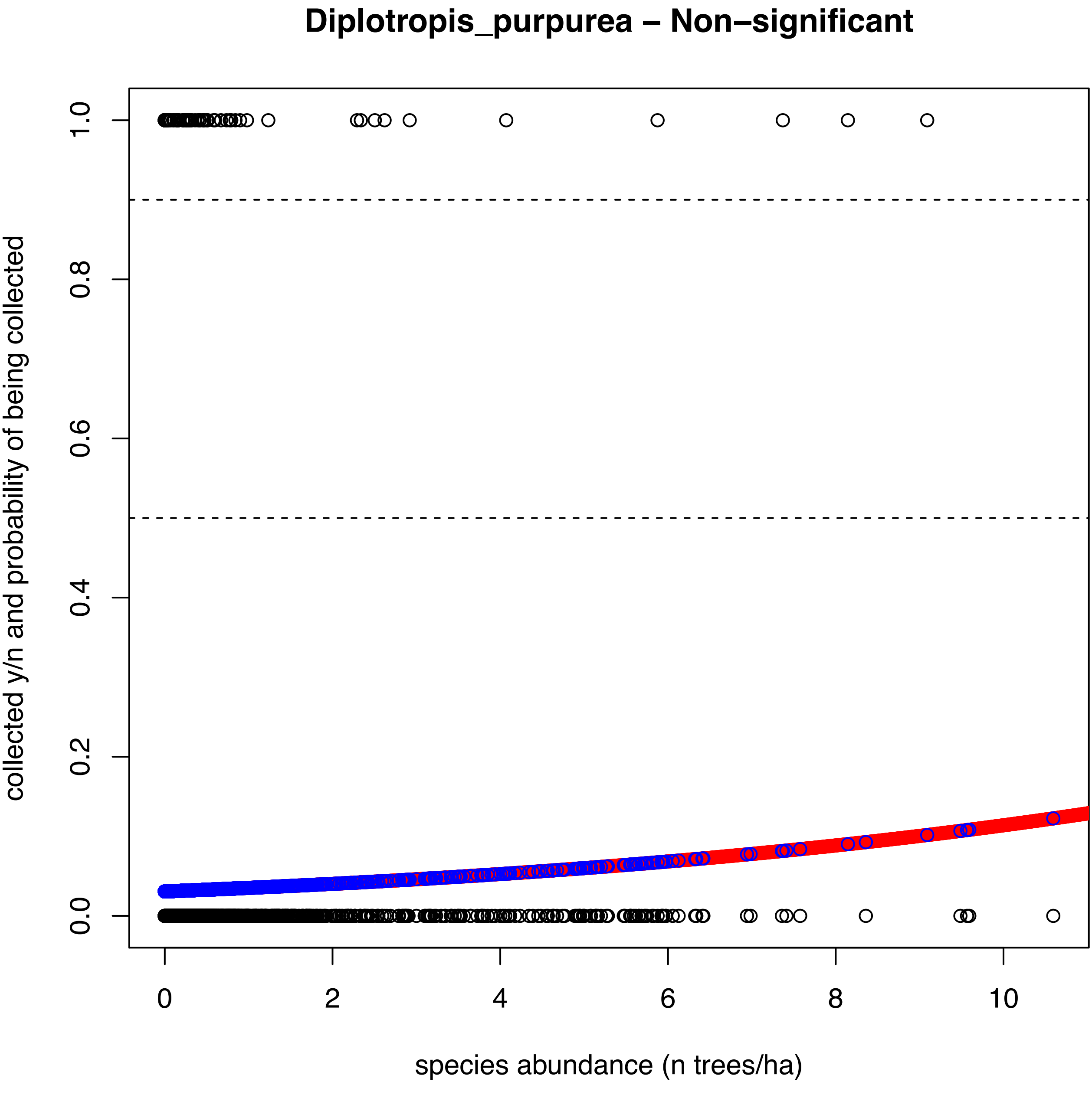

Diplotropis_purpurea – Non–significant
collected y/n and probability of being collected
species abundance (n trees/ha)
0.0
0.2
0.4
0.6
0.8
1.0
0
2
4
6
8
10

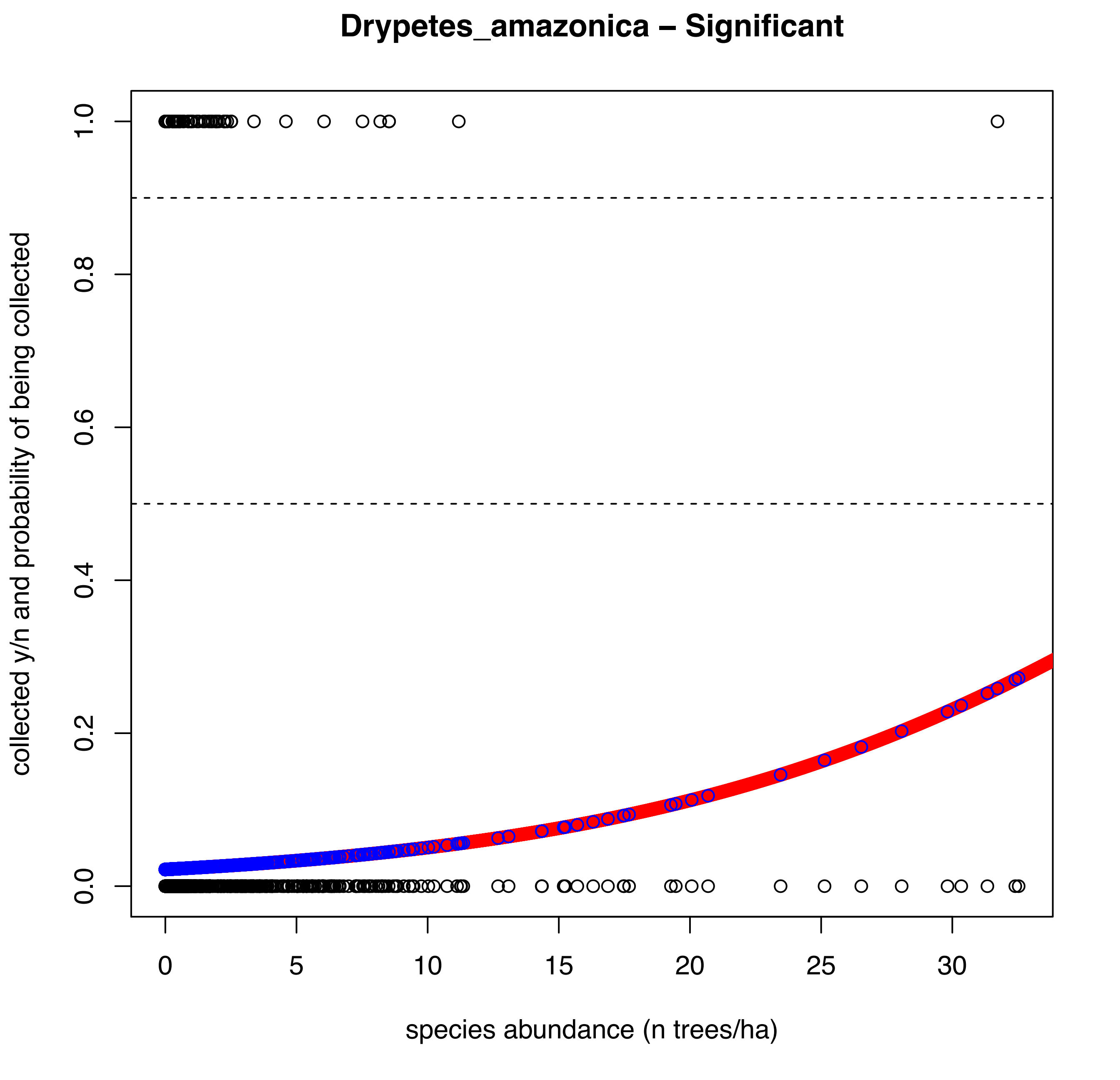

Drypetes_amazonica – Significant
collected y/n and probability of being collected
species abundance (n trees/ha)
0.0
0.2
0.4
0.6
0.8
1.0
0
5
10
15
20
25
30

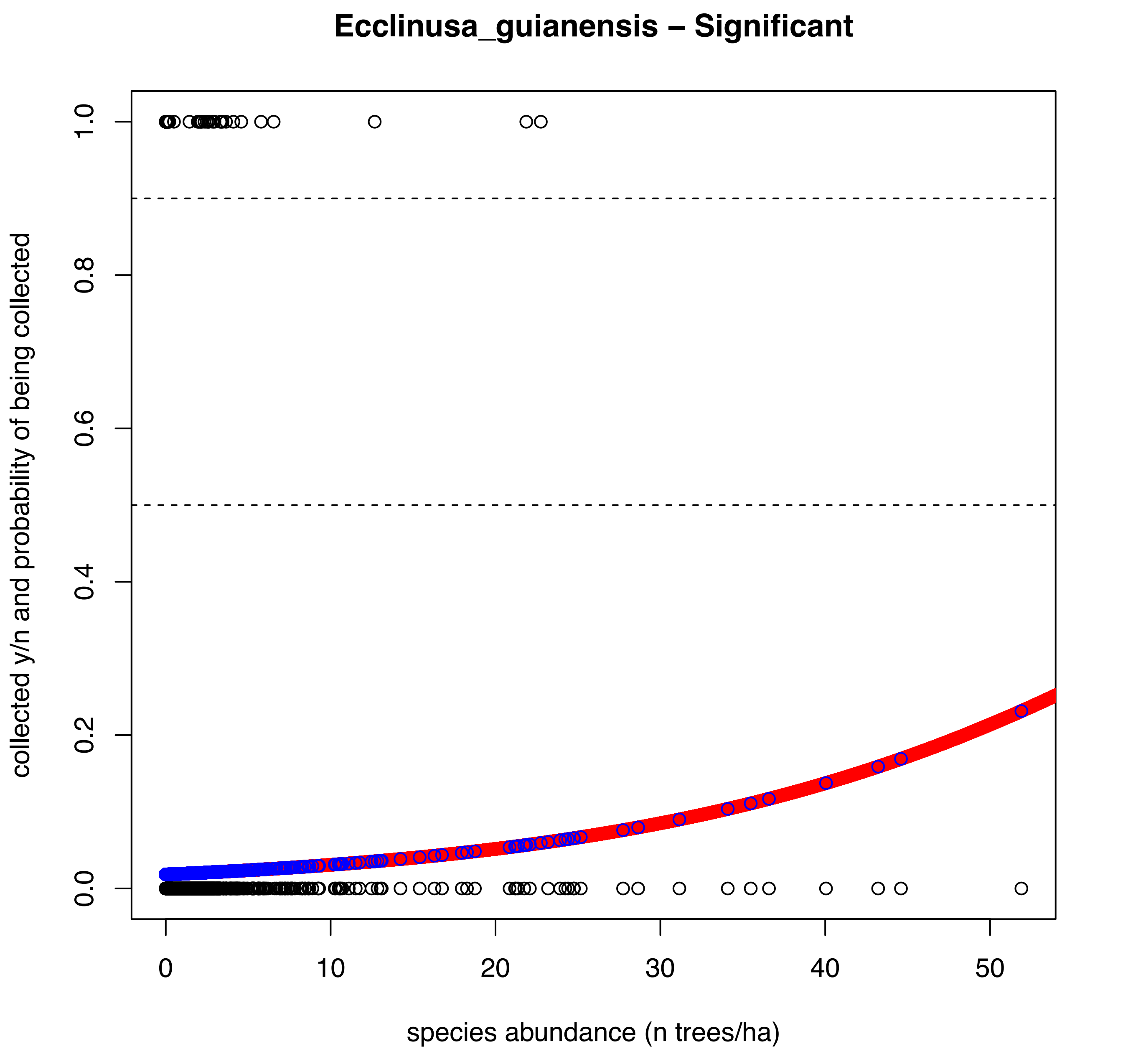

Ecclinusa_guianensis – Significant
collected y/n and probability of being collected
0.0
0.2
0.4
0.6
0.8
1.0
0
10
20
30
40
50
species abundance (n trees/ha)

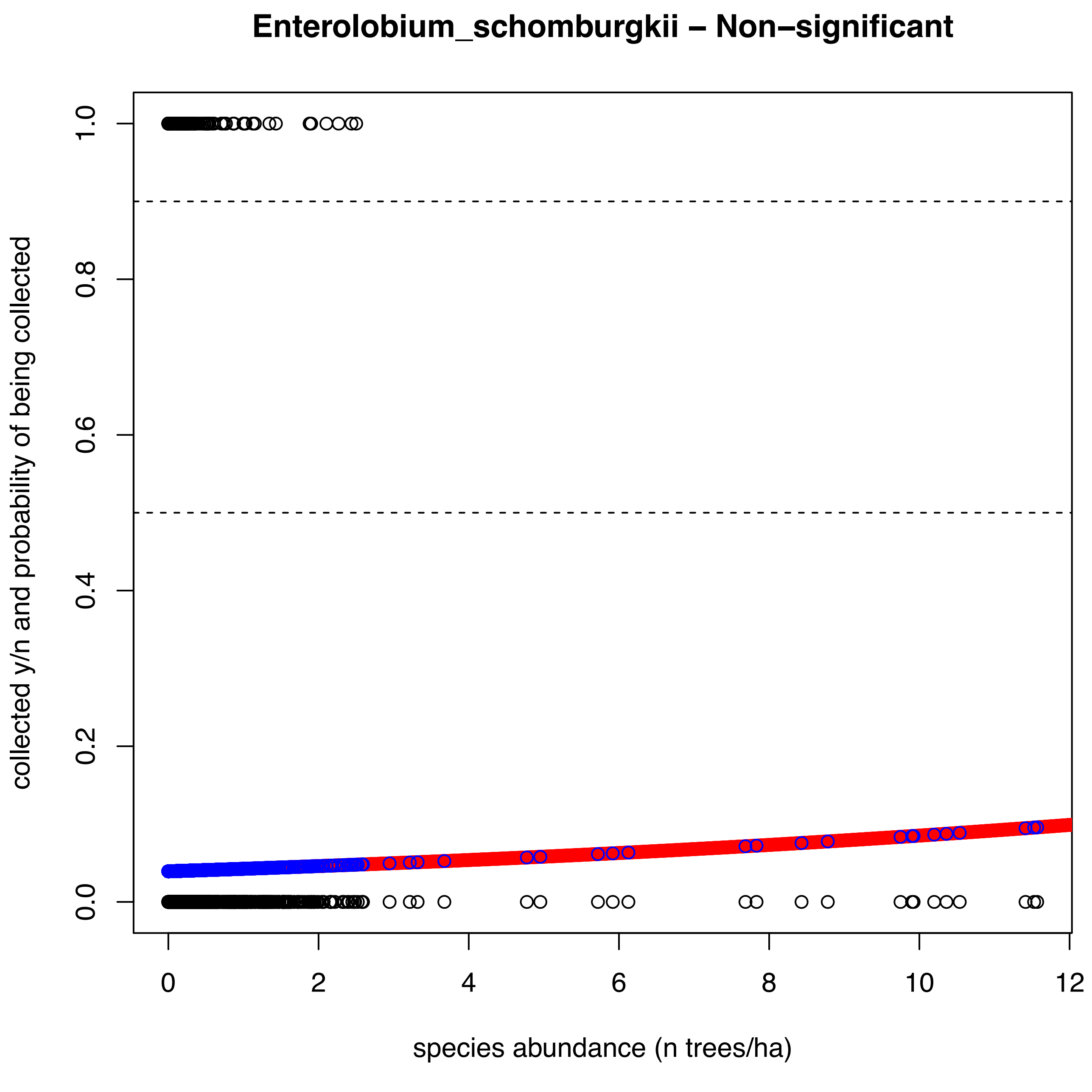
Enterolobium_schomburgkii – Non–significant
collected y/n and probability of being collected
species abundance (n trees/ha)
0.0
0.2
0.4
0.6
0.8
1.0
0
2
4
6
8
10
12

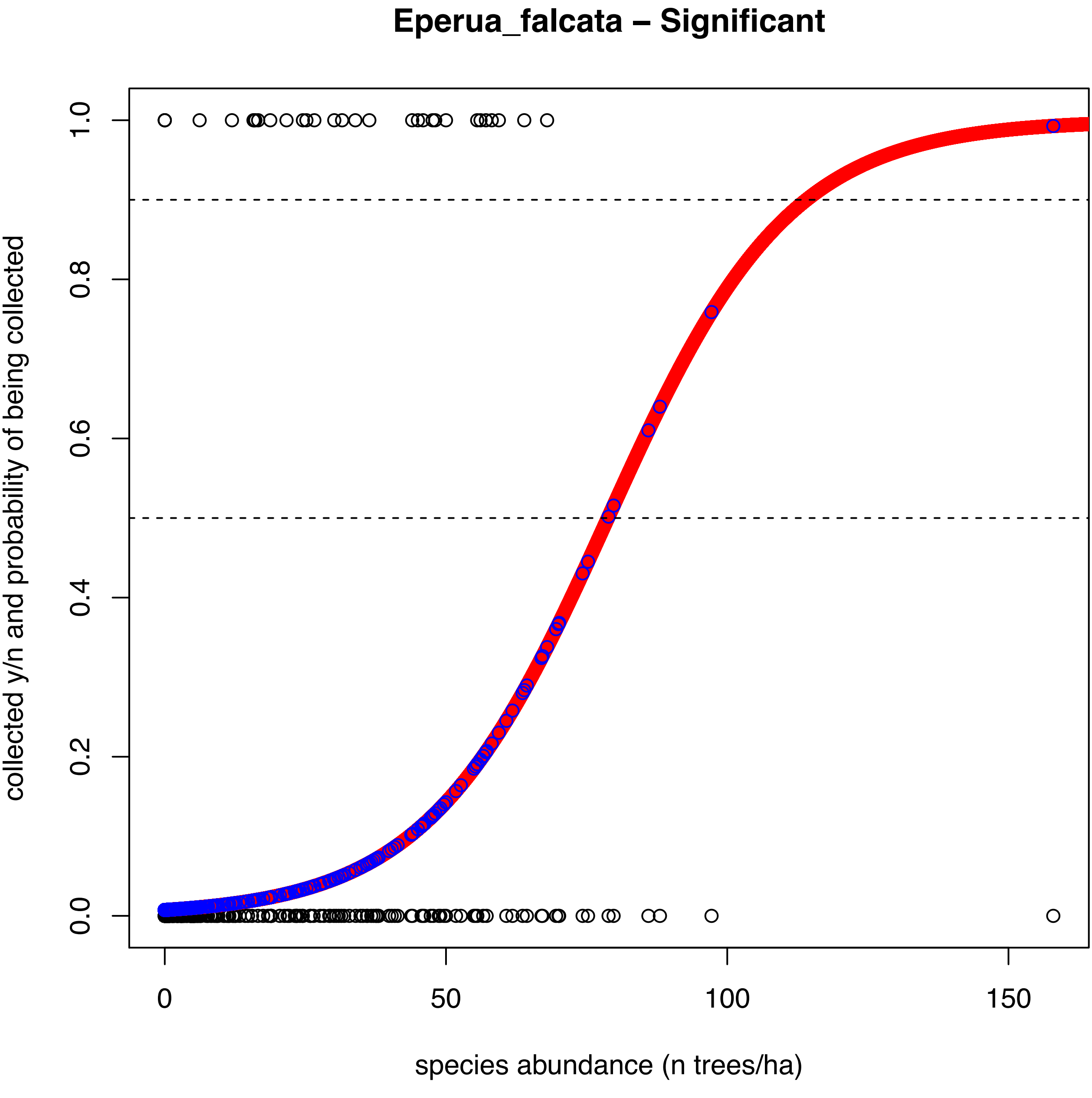

Eperua_falcata – Significant
collected y/n and probability of being collected
species abundance (n trees/ha)
0.0
0.2
0.4
0.6
0.8
1.0
0
50
100
150

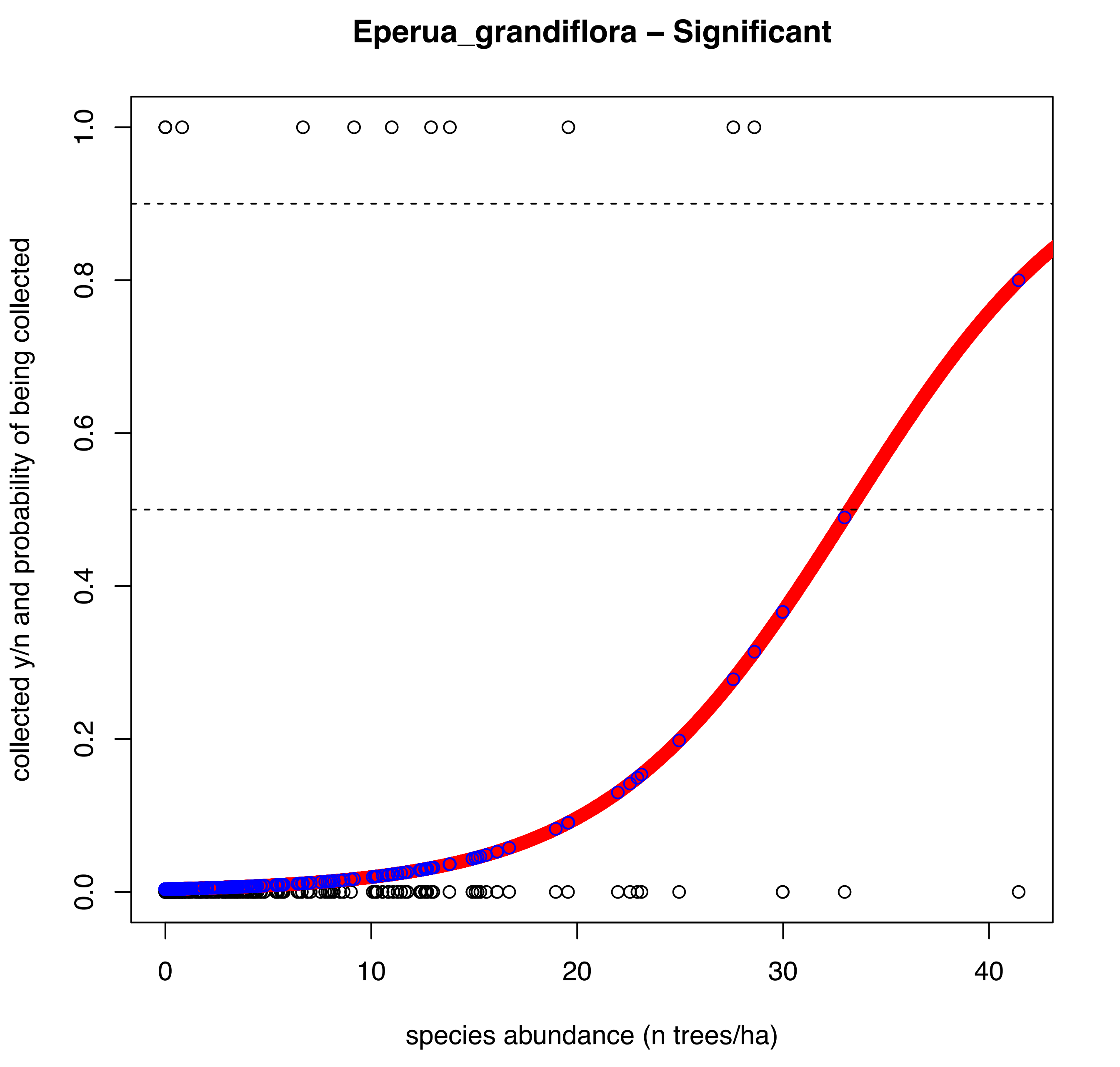
Eperua_grandiflora – Significant
collected y/n and probability of being collected
species abundance (n trees/ha)
0.0
0.2
0.4
0.6
0.8
1.0
0
10
20
30
40

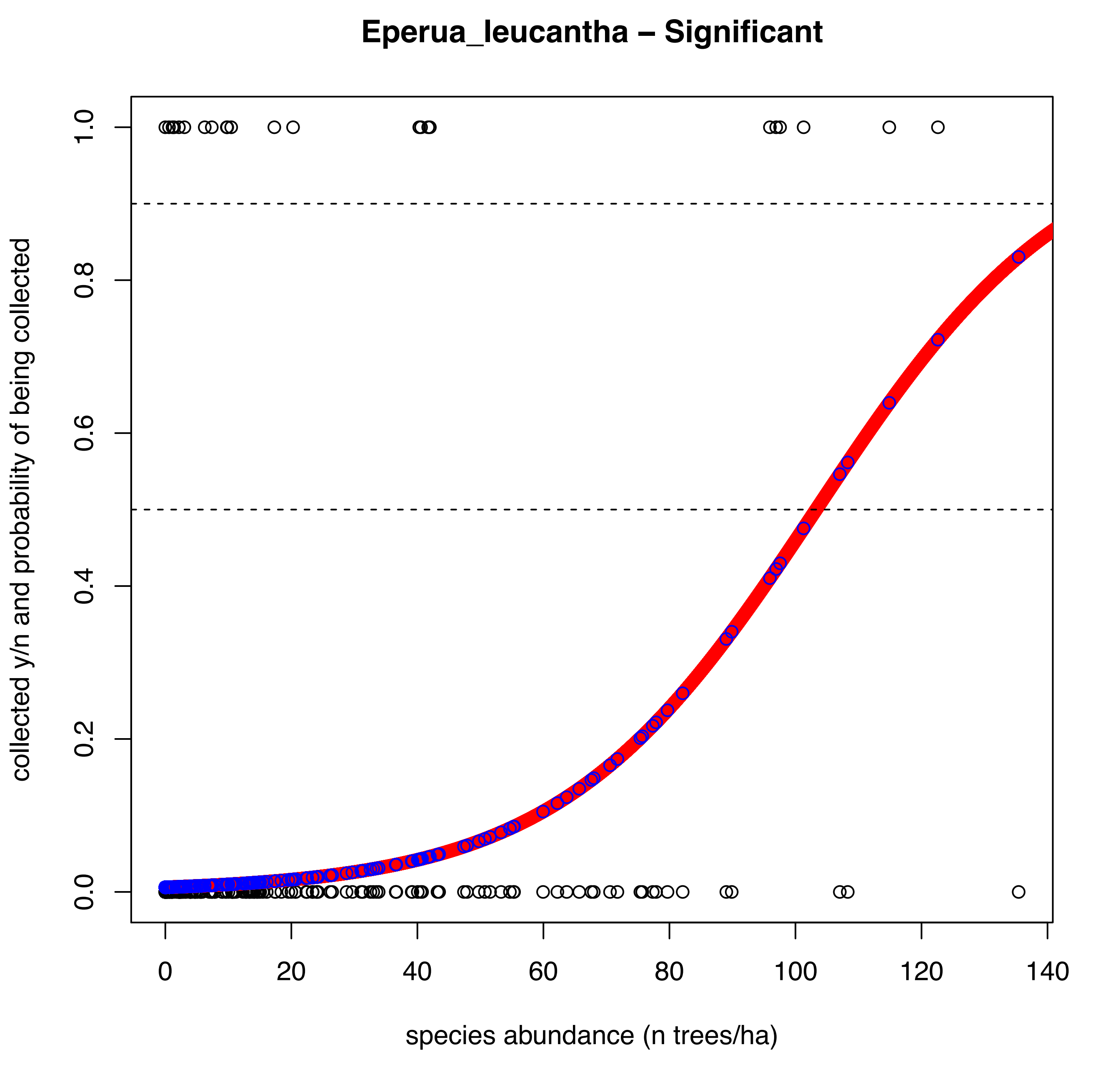

Eperua_leucantha – Significant
collected y/n and probability of being collected
species abundance (n trees/ha)
0.0
0.2
0.4
0.6
0.8
1.0
0
20
40
60
80
100
120
140

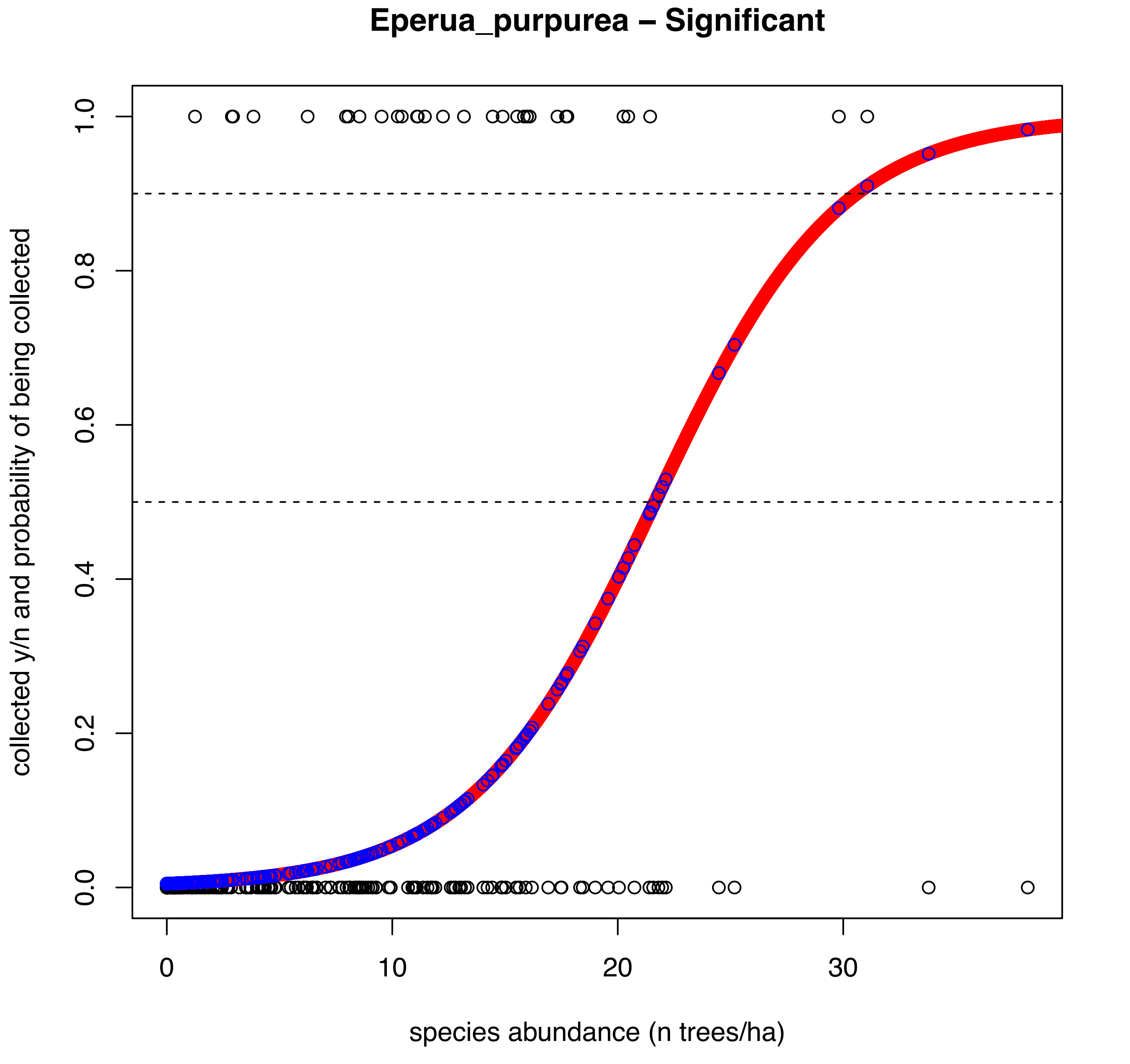
Eperua_purpurea – Significant
collected y/n and probability of being collected
species abundance (n trees/ha)
0.0
0.2
0.4
0.6
0.8
1.0
0
10
20
30

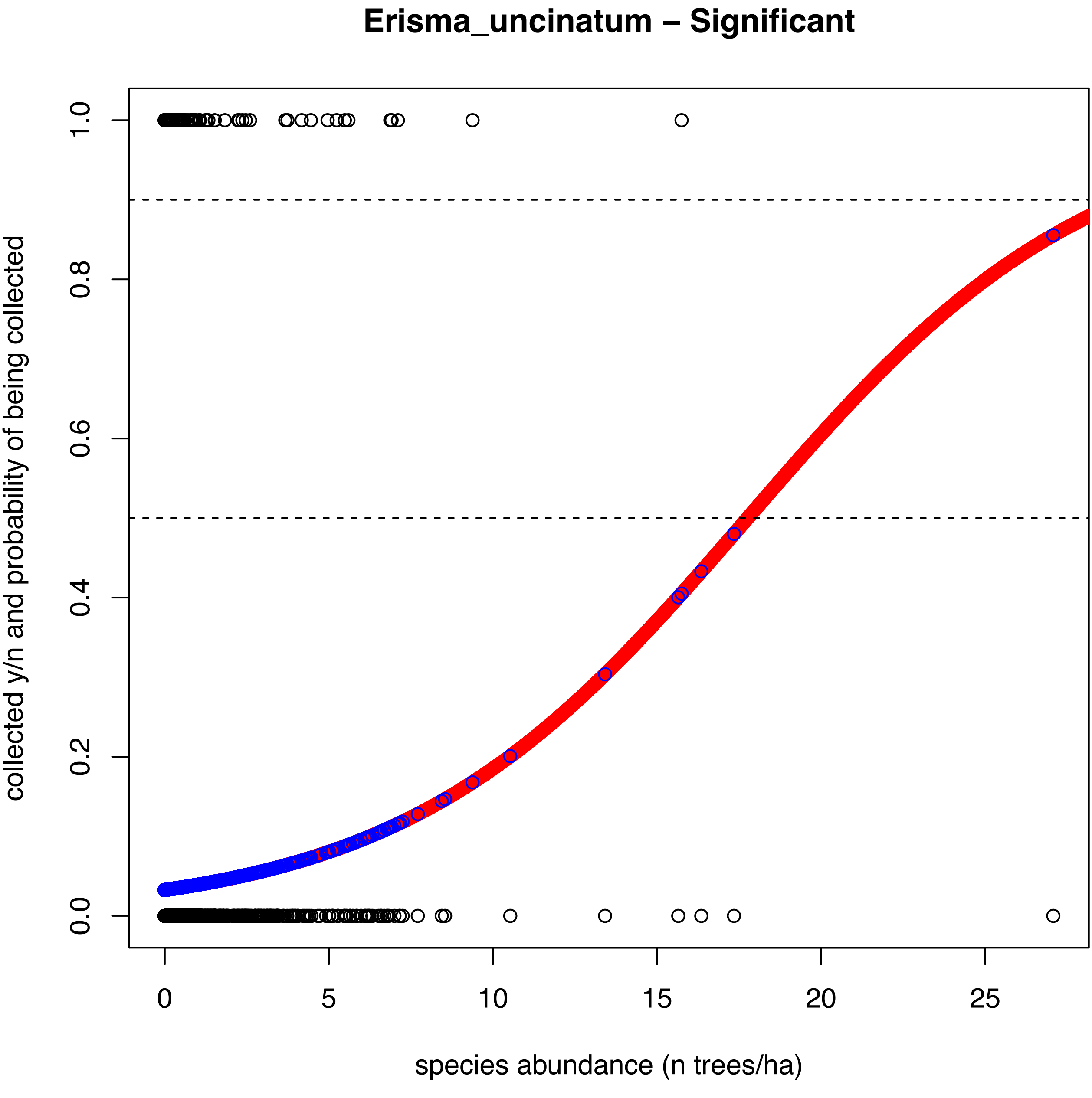
Erisma_uncinatum – Significant
collected y/n and probability of being collected
species abundance (n trees/ha)
0.0
0.2
0.4
0.6
0.8
1.0
0
5
10
15
20
25

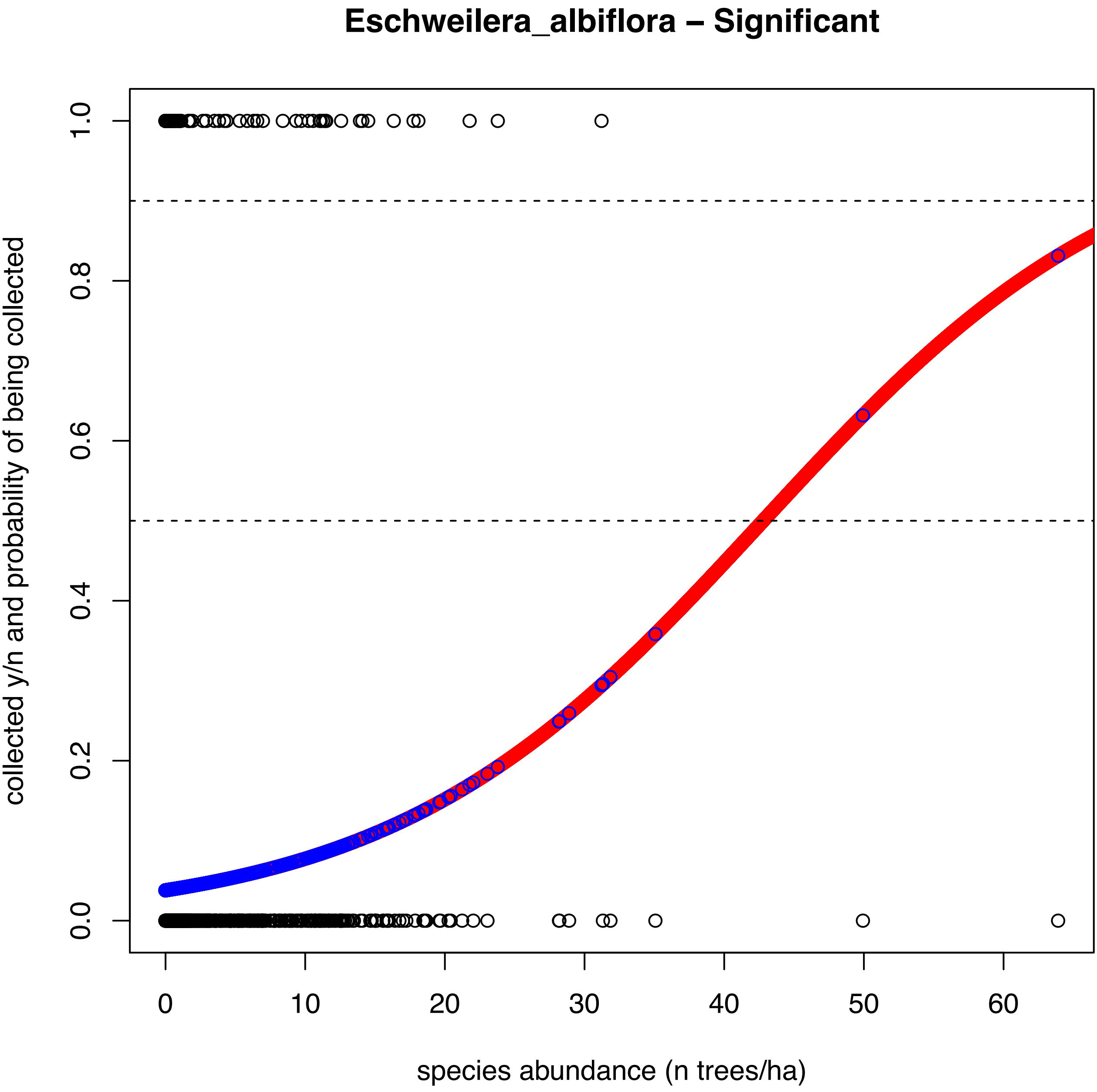
Eschweilera_albiflora – Significant
collected y/n and probability of being collected
species abundance (n trees/ha)
0.0
0.2
0.4
0.6
0.8
1.0
0
10
20
30
40
50
60

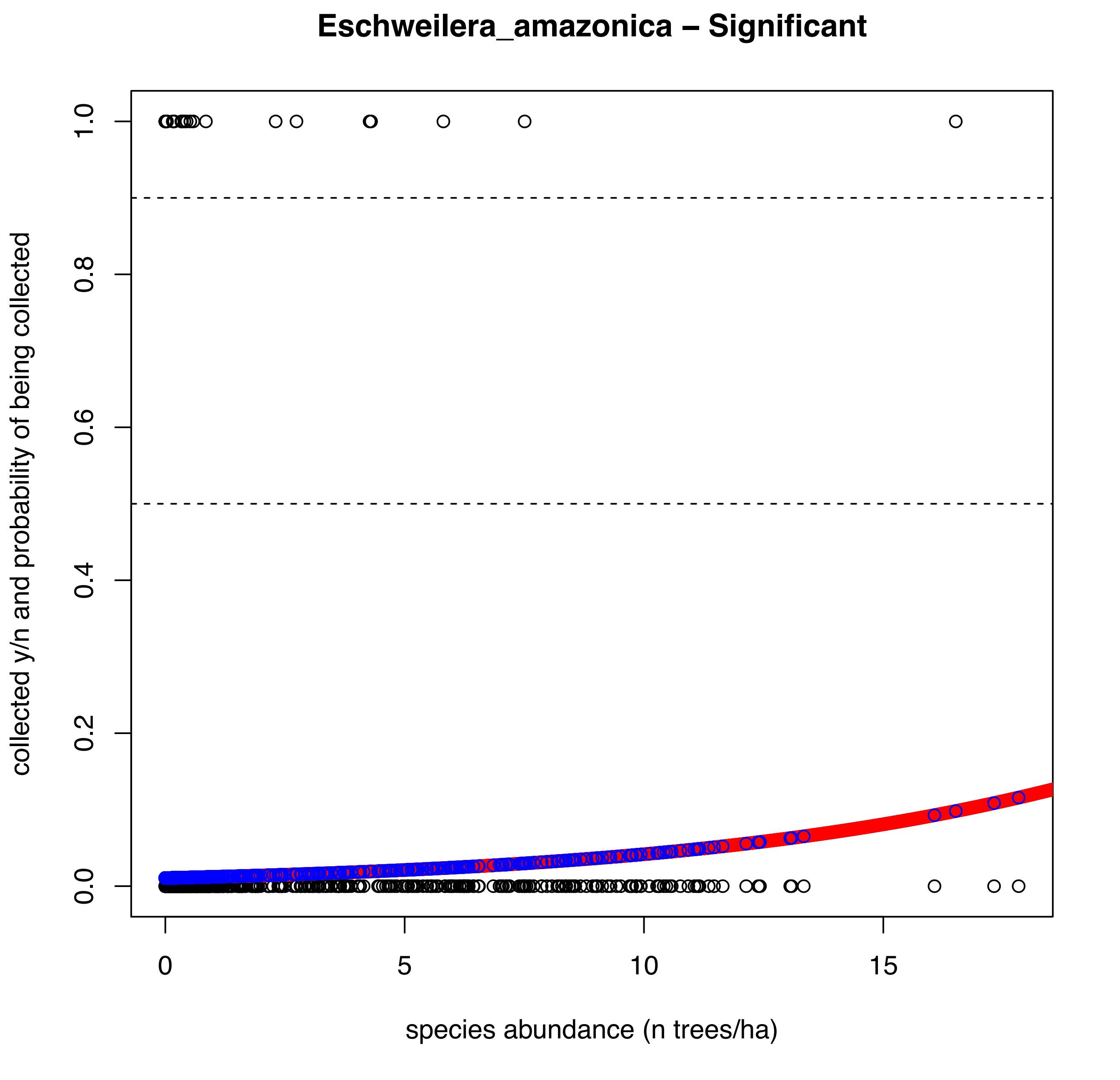

Eschweilera_amazonica – Significant
collected y/n and probability of being collected
species abundance (n trees/ha)
0.0
0.2
0.4
0.6
0.8
1.0
0
5
10
15

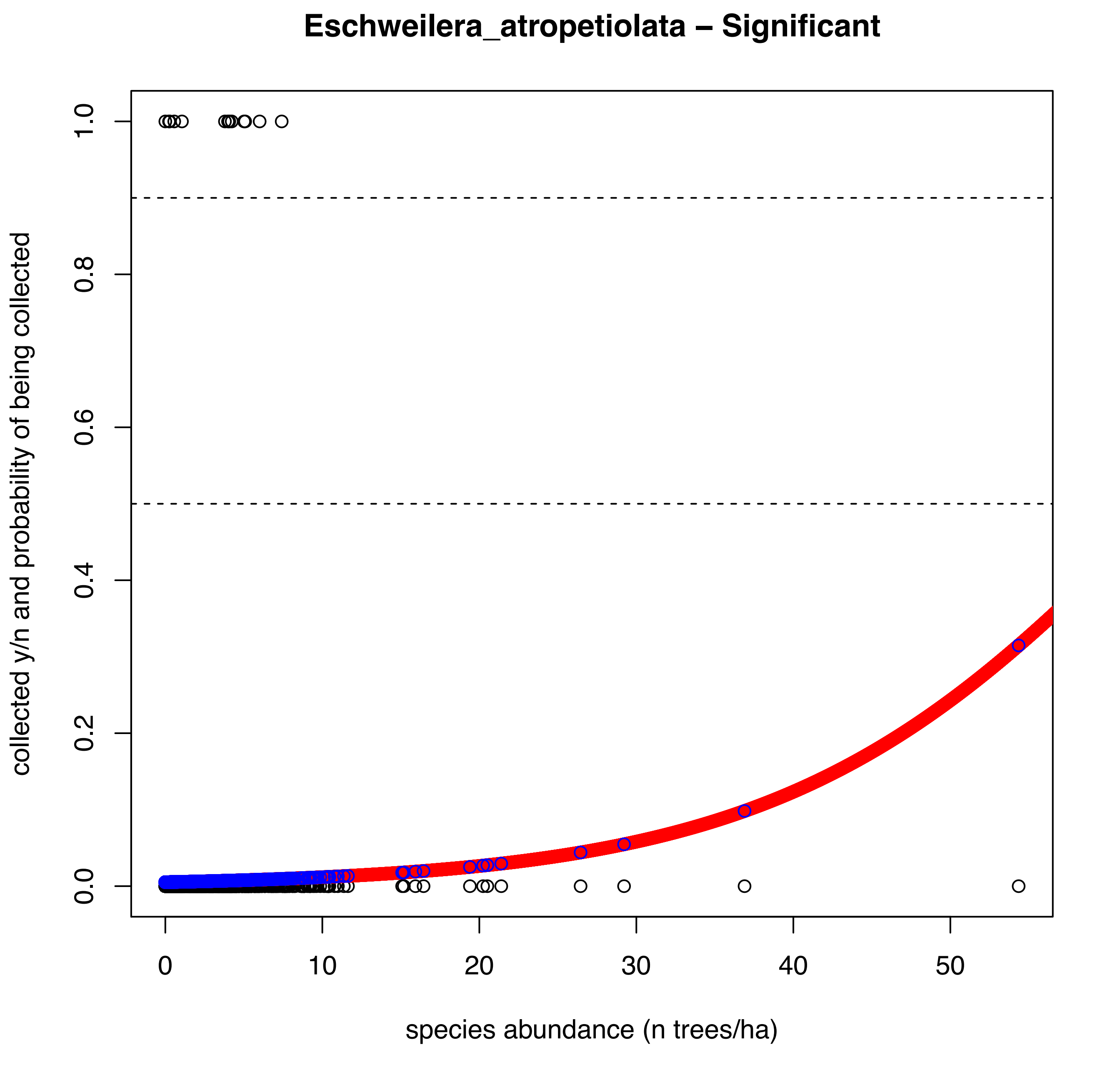

Eschweilera_atropetiolata – Significant
collected y/n and probability of being collected
species abundance (n trees/ha)
0.0
0.2
0.4
0.6
0.8
1.0
0
10
20
30
40
50

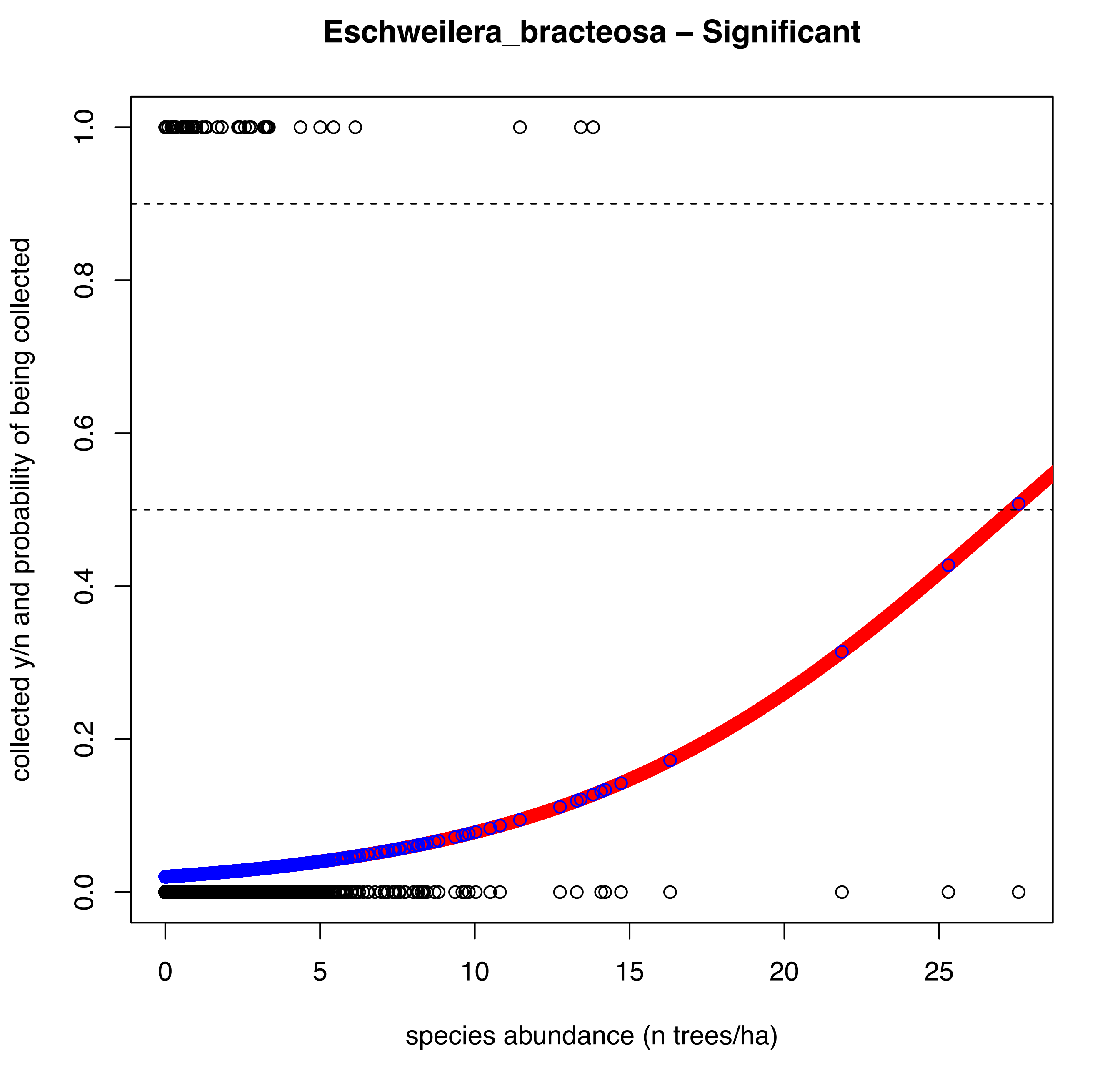
Eschweilera_bracteosa – Significant
collected y/n and probability of being collected
species abundance (n trees/ha)
0.0
0.2
0.4
0.6
0.8
1.0
0
5
10
15
20
25

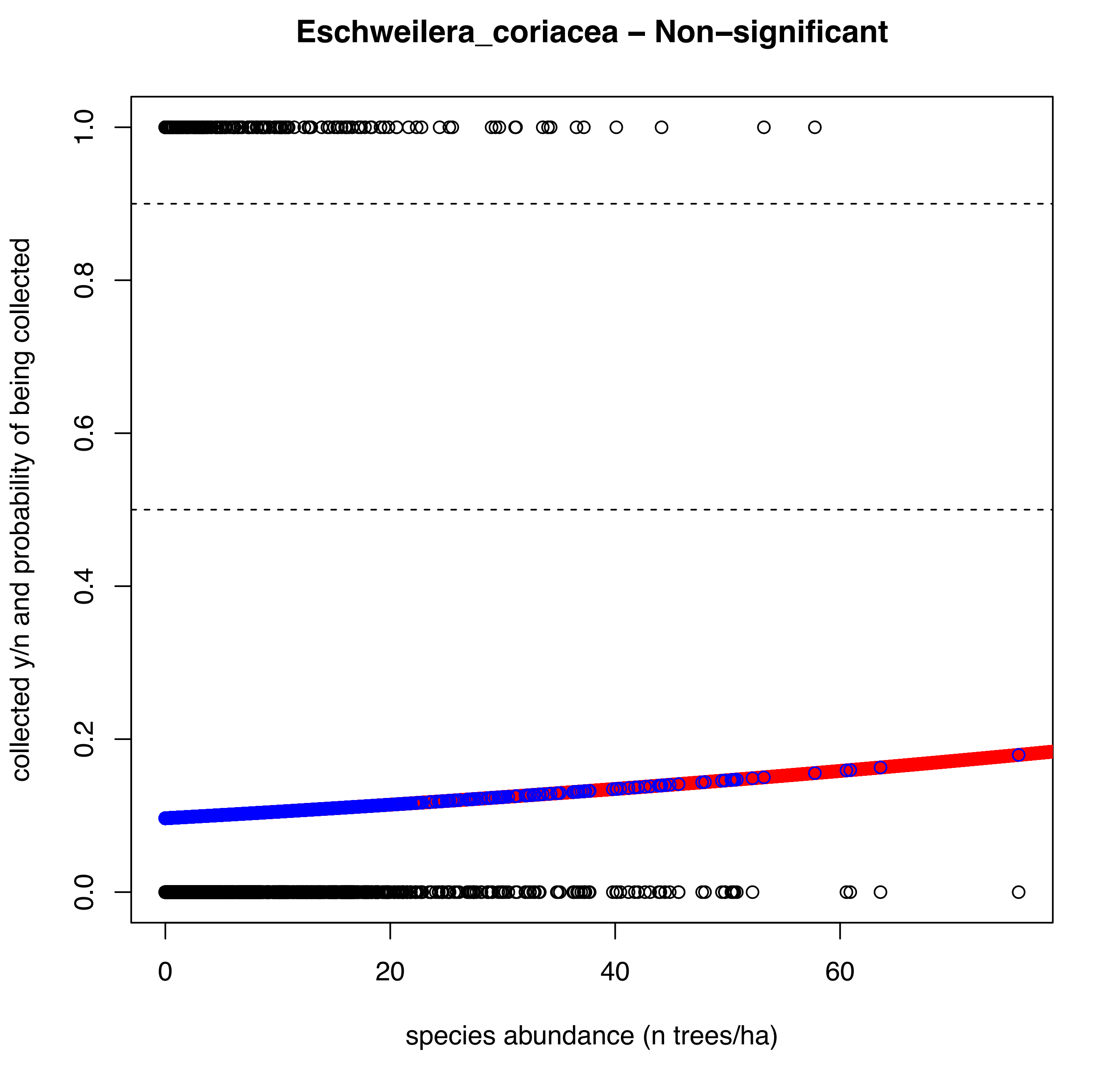

Eschweilera_coriacea – Non-significant
collected y/n and probability of being collected
species abundance (n trees/ha)
0.0
0.2
0.4
0.6
0.8
1.0
0
20
40
60

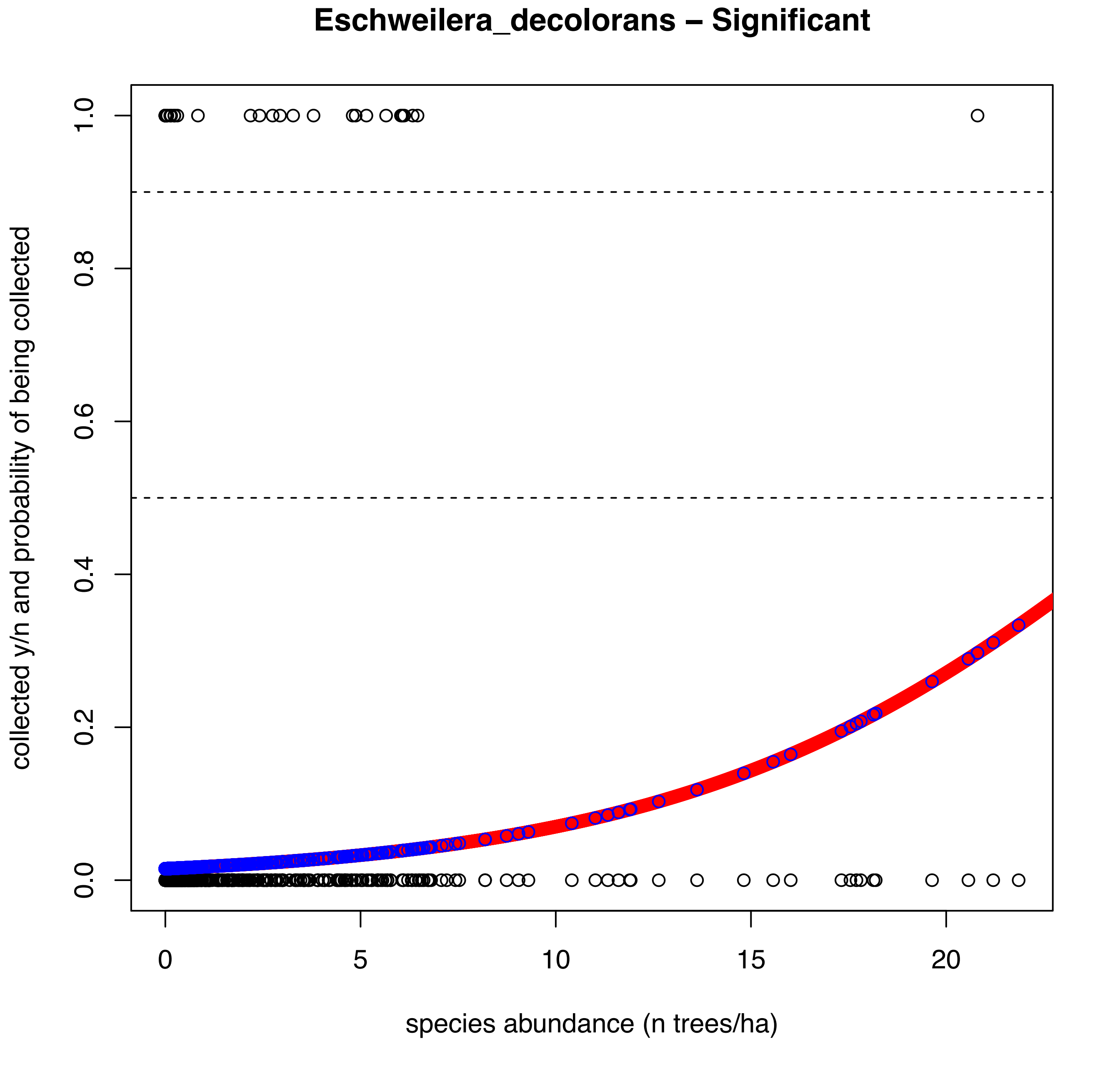

Eschweilera_decolorans – Significant
collected y/n and probability of being collected
species abundance (n trees/ha)
0.0
0.2
0.4
0.6
0.8
1.0
0
5
10
15
20

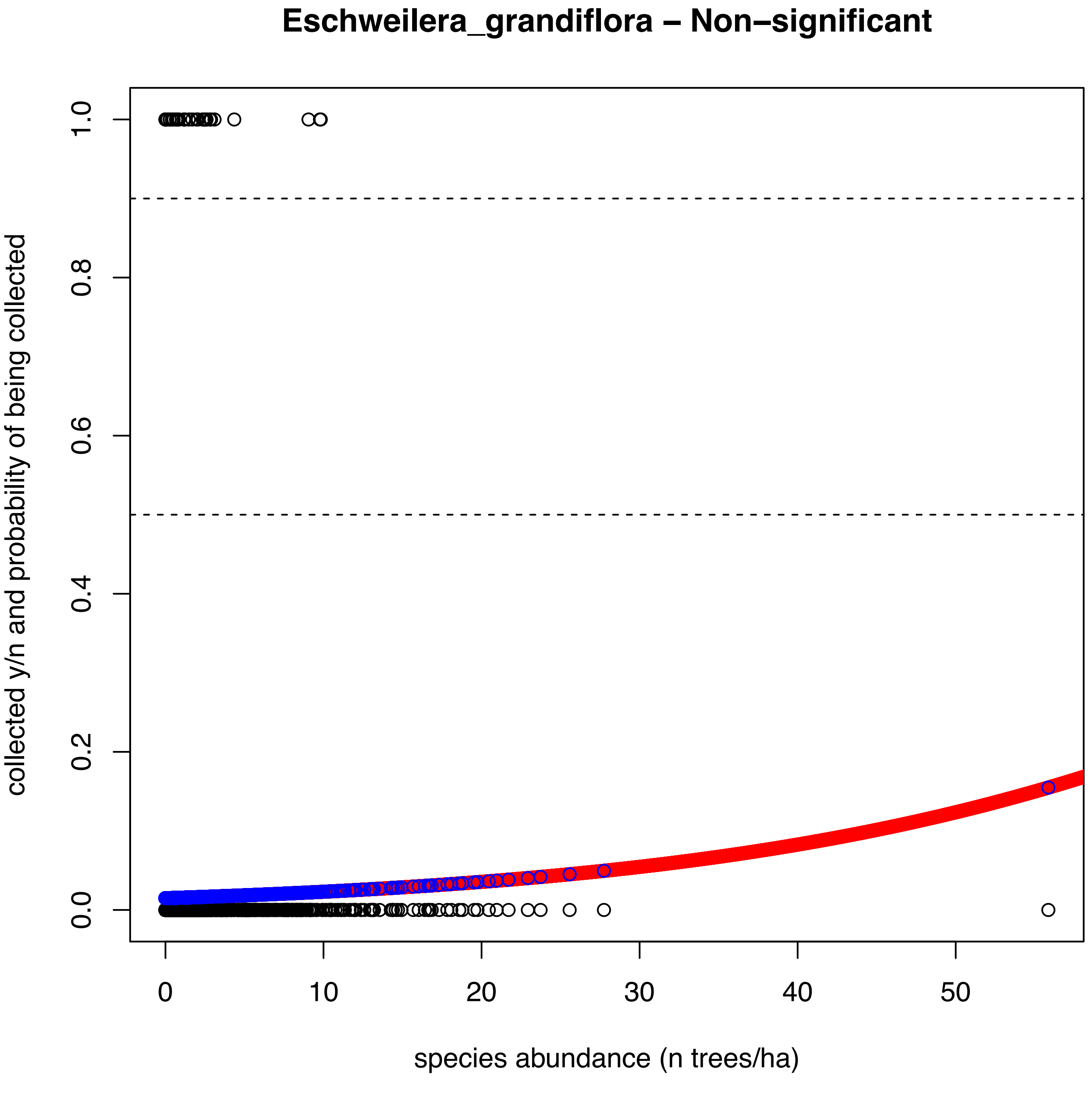
Eschweilera_grandiflora – Non–significant
collected y/n and probability of being collected
species abundance (n trees/ha)
0.0
0.2
0.4
0.6
0.8
1.0
0
10
20
30
40
50

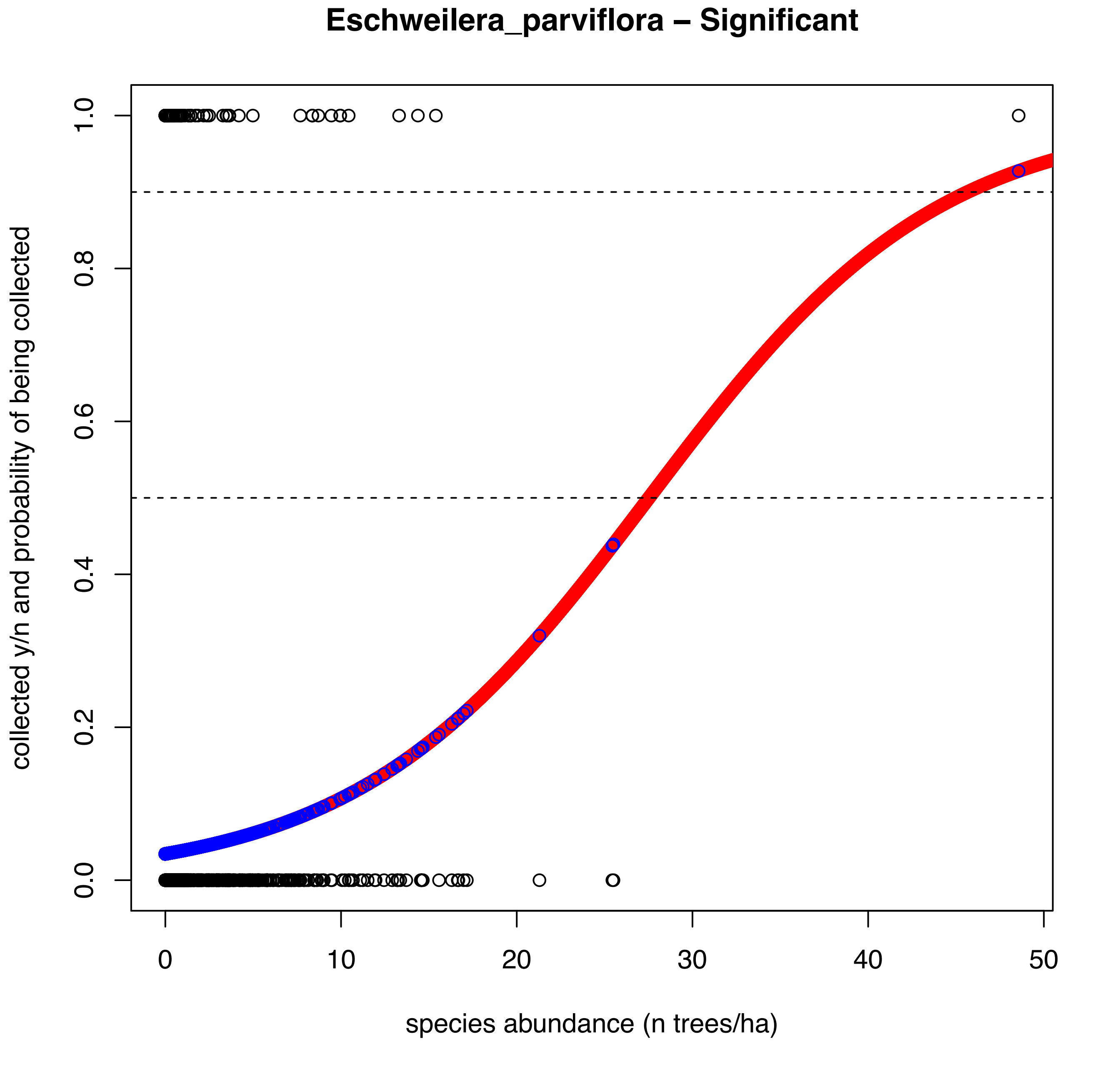

Eschweilera_parviflora – Significant
collected y/n and probability of being collected
species abundance (n trees/ha)
0.0
0.2
0.4
0.6
0.8
1.0
0
10
20
30
40
50

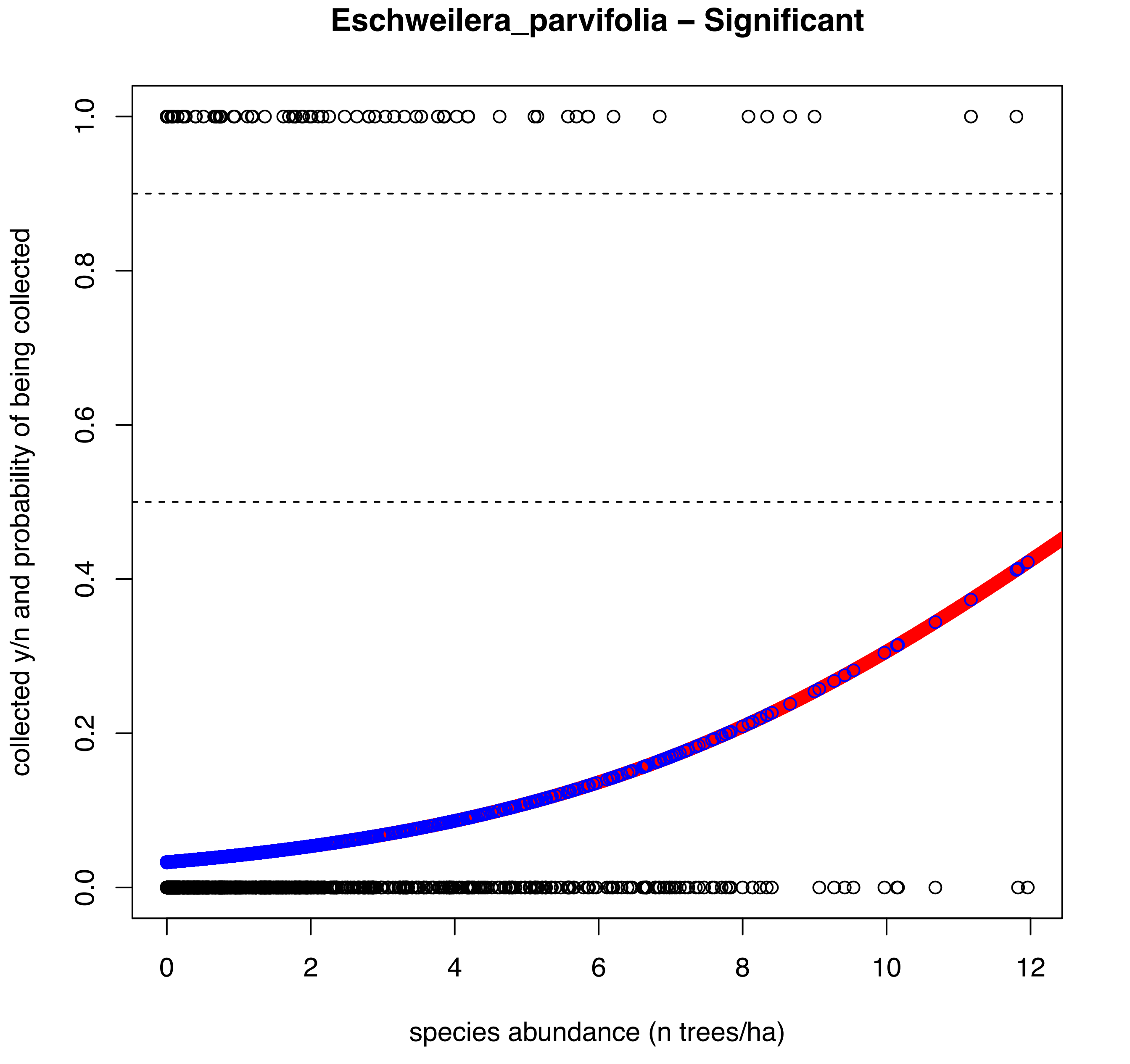

Eschweilera_parvifolia – Significant
collected y/n and probability of being collected
species abundance (n trees/ha)
0.0
0.2
0.4
0.6
0.8
1.0
0
2
4
6
8
10
12

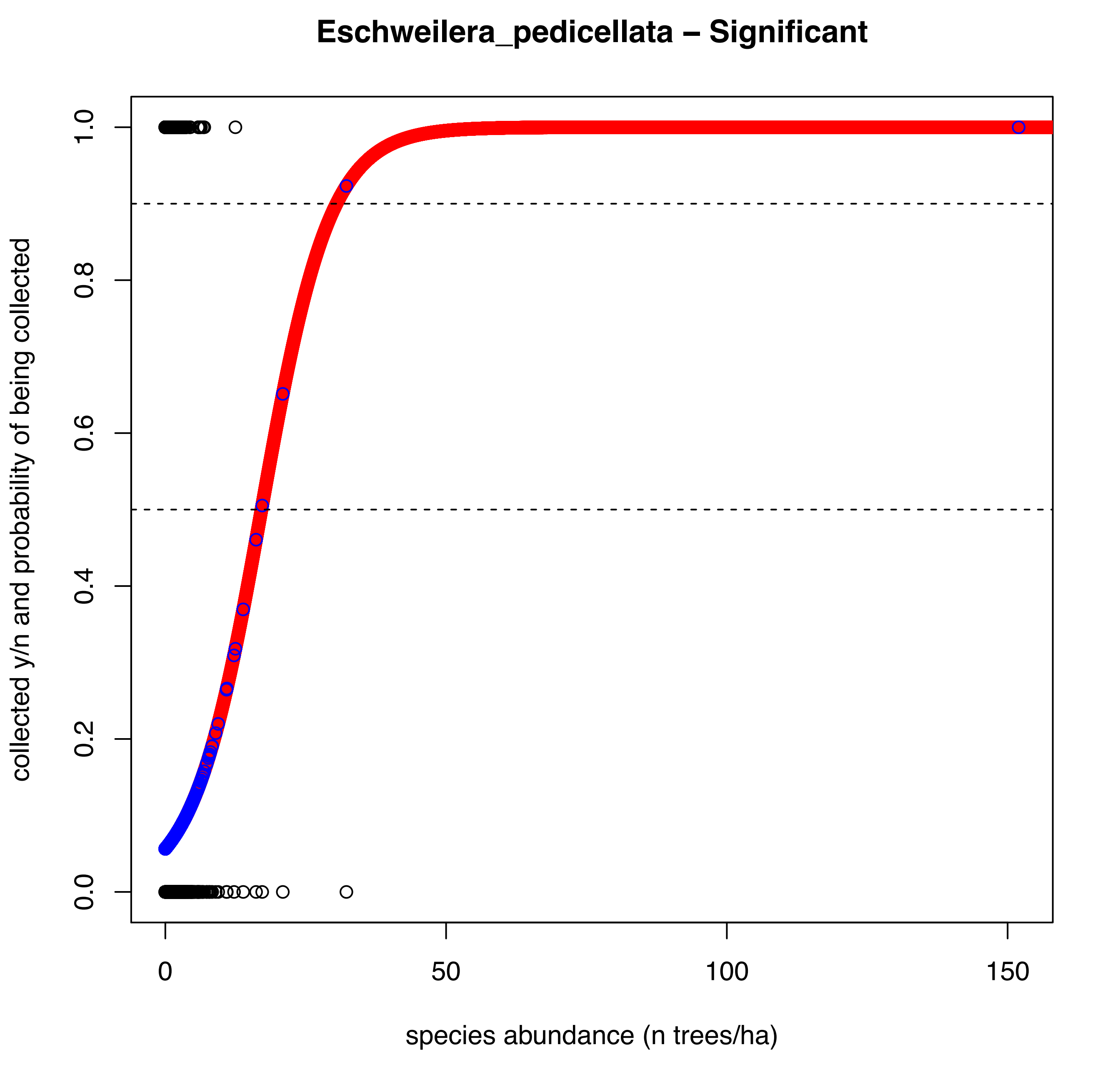
Eschweilera_pedicellata – Significant
collected y/n and probability of being collected
species abundance (n trees/ha)
0.0
0.2
0.4
0.6
0.8
1.0
0
50
100
150

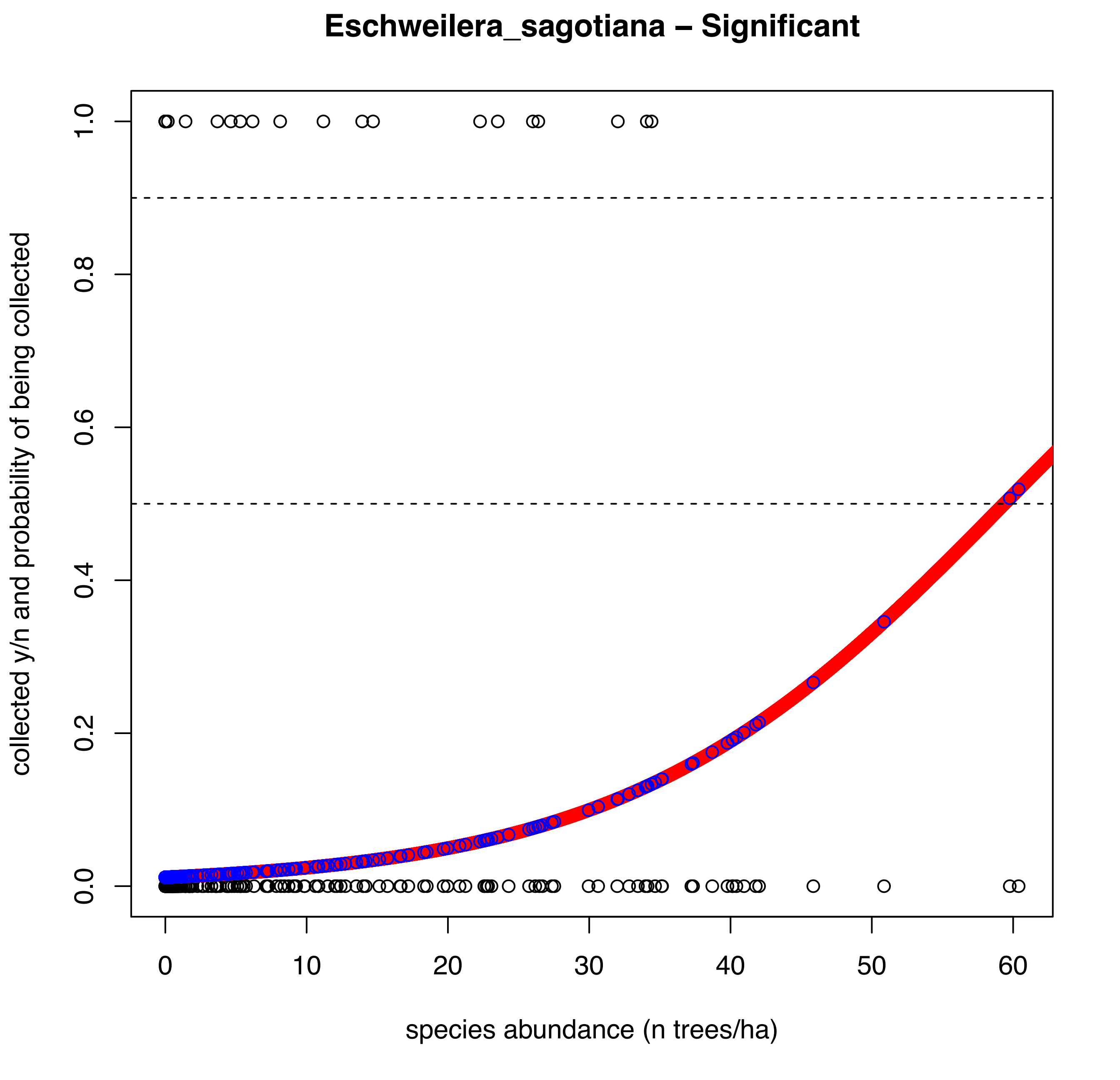

Eschweilera_sagotiana – Significant
collected y/n and probability of being collected
species abundance (n trees/ha)
0.0
0.2
0.4
0.6
0.8
1.0
0
10
20
30
40
50
60

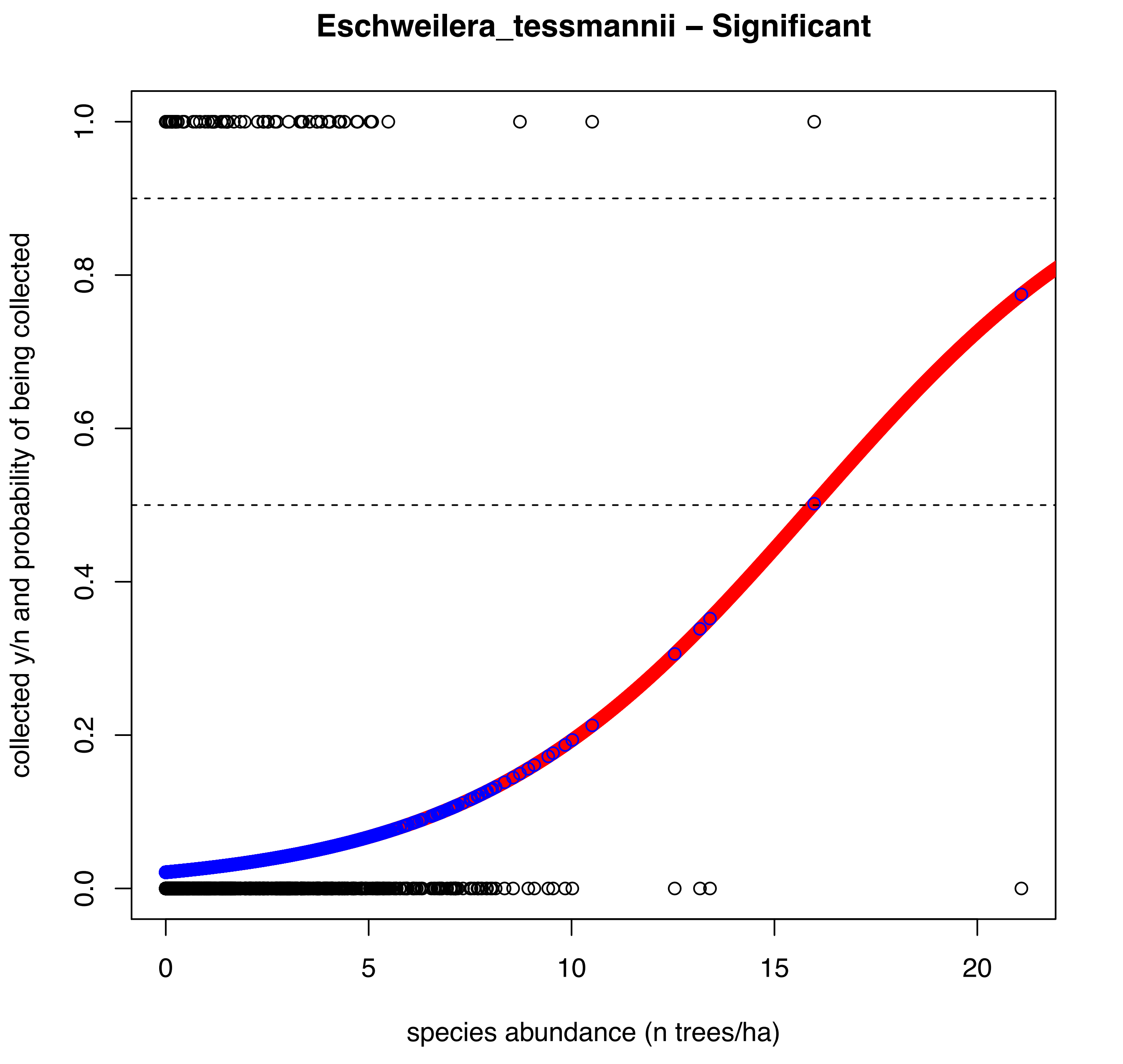

Eschweilera_tessmannii – Significant
collected y/n and probability of being collected
species abundance (n trees/ha)
0.0
0.2
0.4
0.6
0.8
1.0
0
5
10
15
20

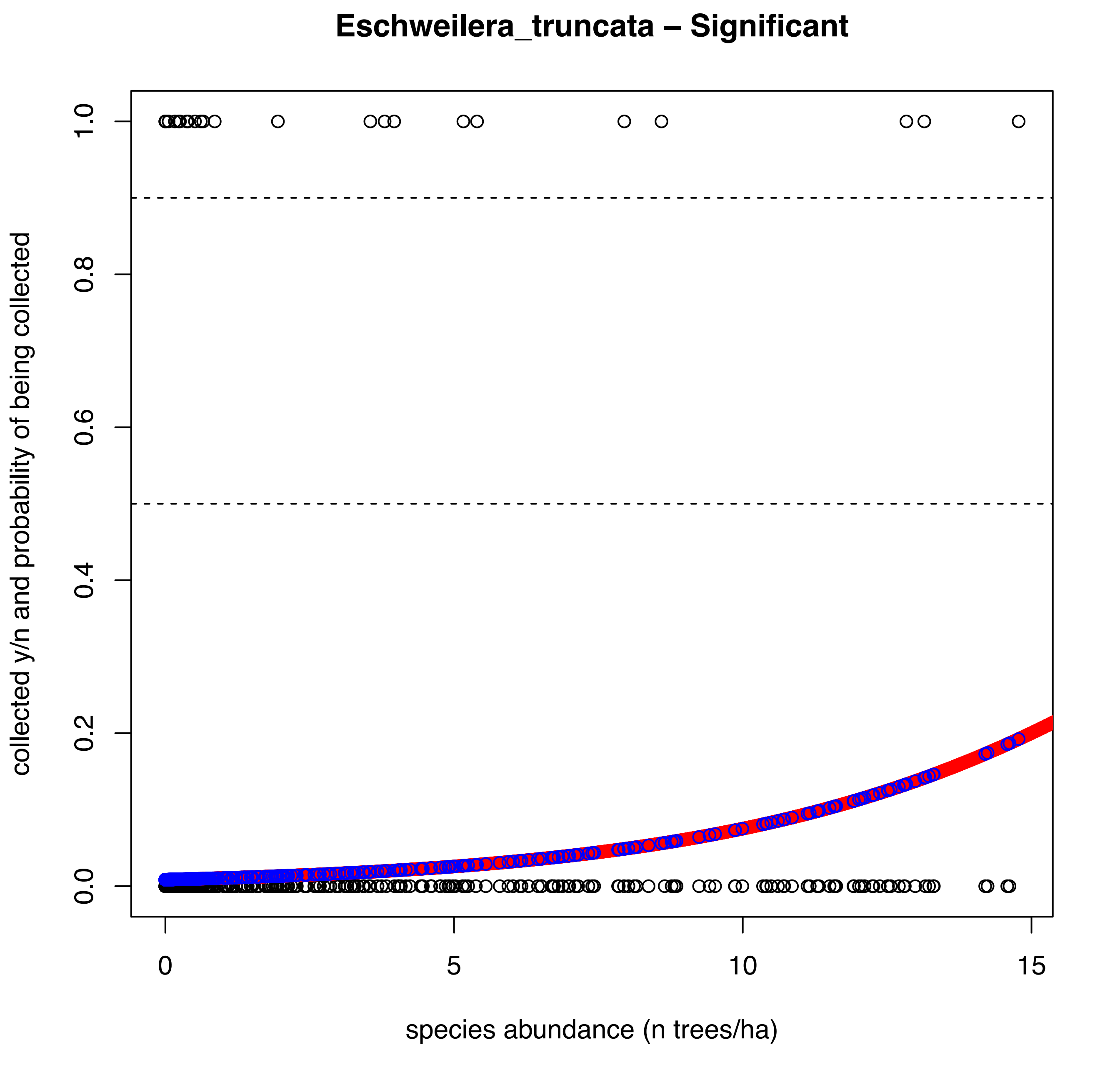
Eschweilera_truncata – Significant
collected y/n and probability of being collected
species abundance (n trees/ha)
0.0
0.2
0.4
0.6
0.8
1.0
0
5
10
15

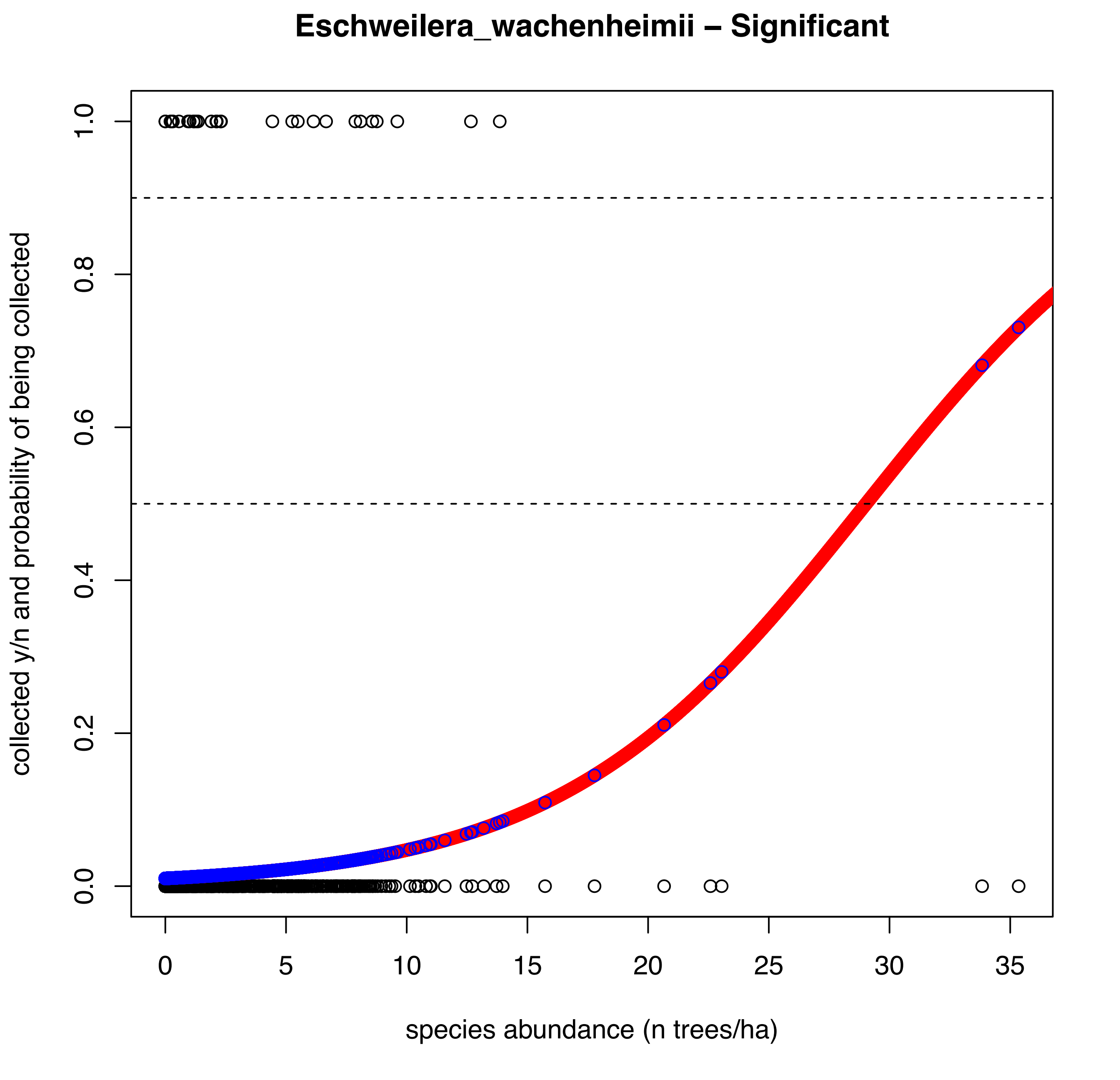
Eschweilera_wachenheimii – Significant
collected y/n and probability of being collected
species abundance (n trees/ha)
0.0
0.2
0.4
0.6
0.8
1.0
0
5
10
15
20
25
30
35

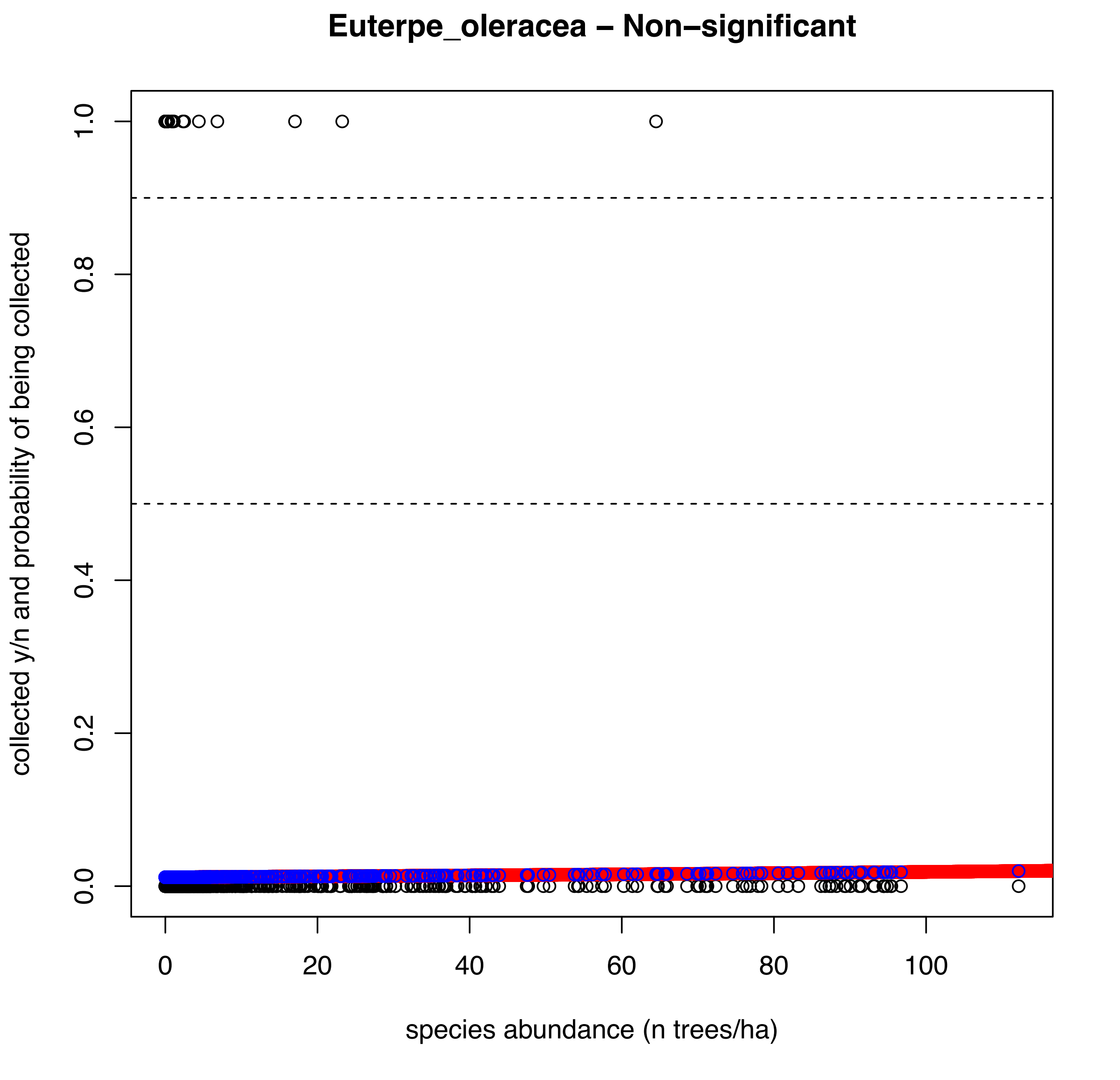

Euterpe_oleracea – Non–significant
collected y/n and probability of being collected
species abundance (n trees/ha)
0.0
0.2
0.4
0.6
0.8
1.0
0
20
40
60
80
100

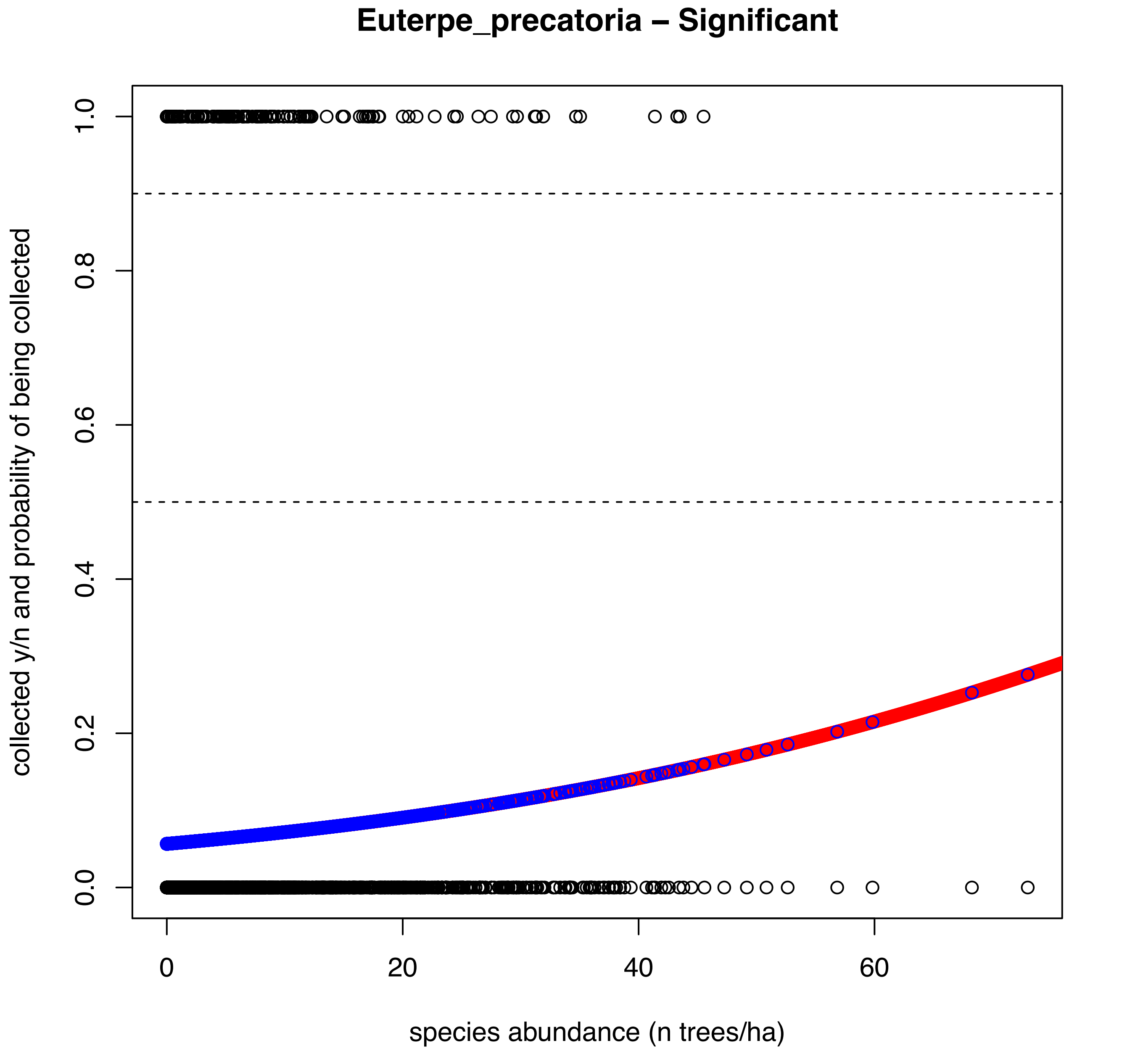
Euterpe_precatoria – Significant
collected y/n and probability of being collected
species abundance (n trees/ha)
0.0
0.2
0.4
0.6
0.8
1.0
0
20
40
60

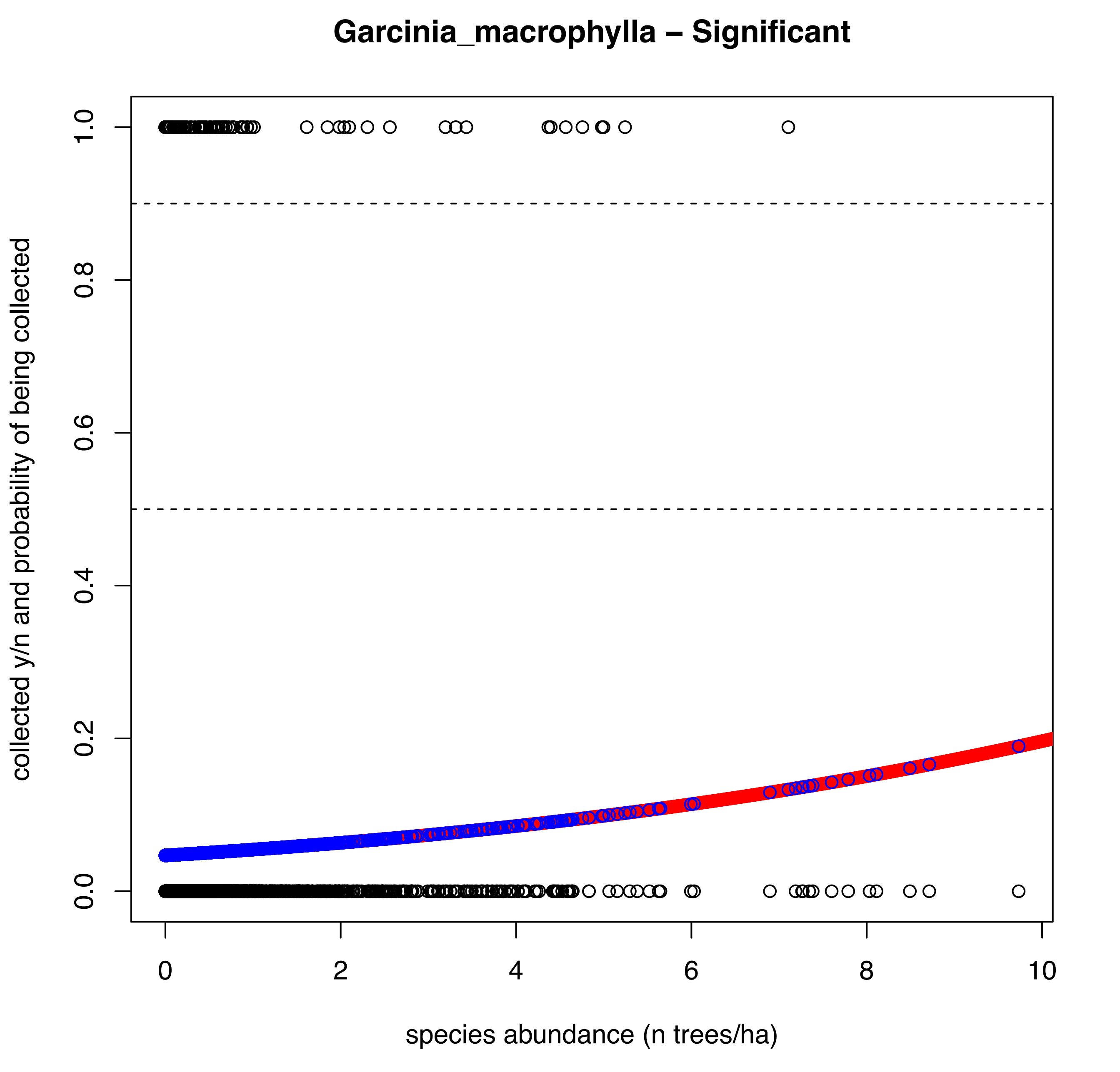

Garcinia_macrophylla – Significant
collected y/n and probability of being collected
0.0
0.2
0.4
0.6
0.8
1.0
0
2
4
6
8
10
species abundance (n trees/ha)

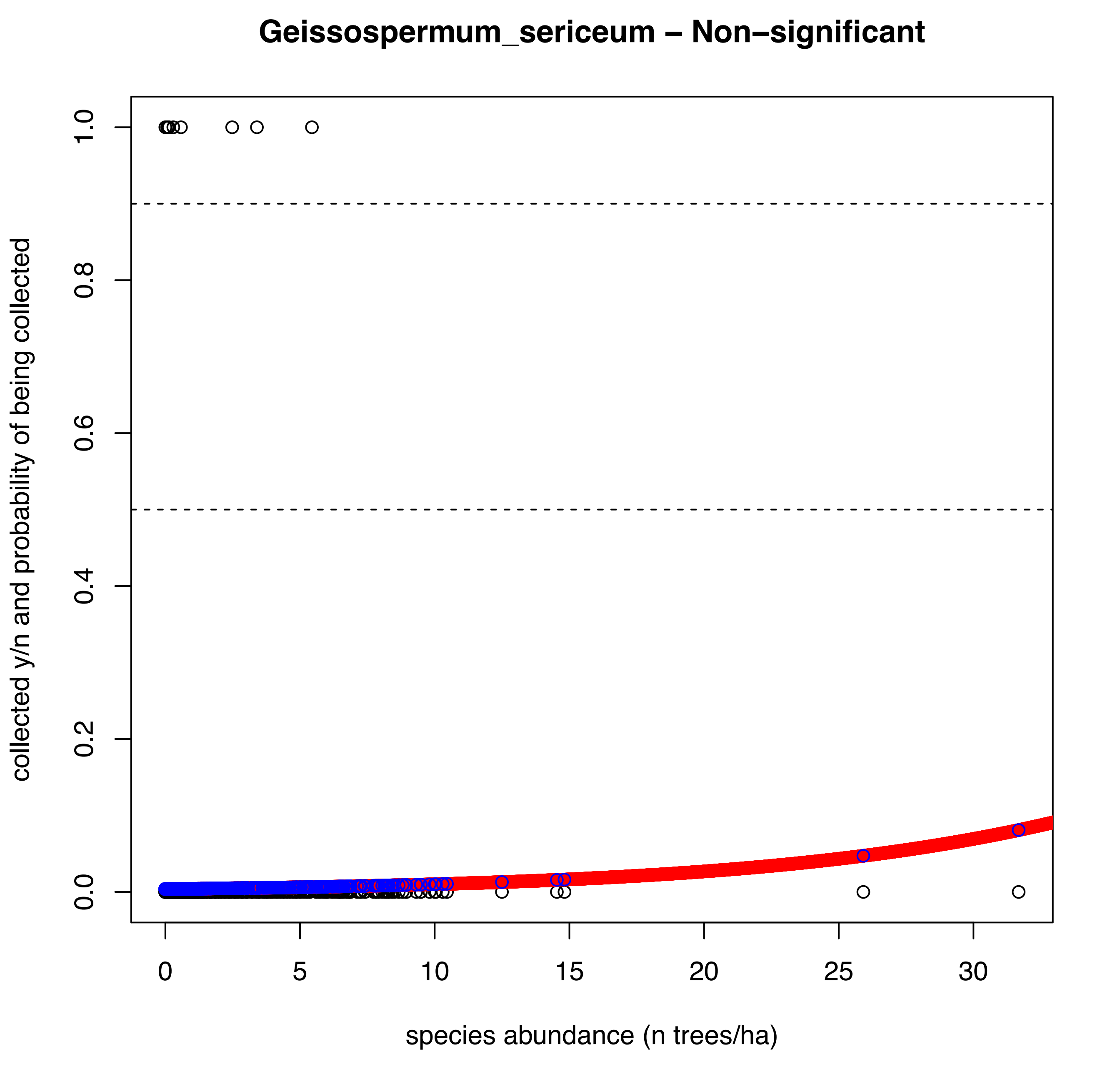

Geissospermum_sericeum – Non–significant
collected y/n and probability of being collected
species abundance (n trees/ha)
0.0
0.2
0.4
0.6
0.8
1.0
0
5
10
15
20
25
30

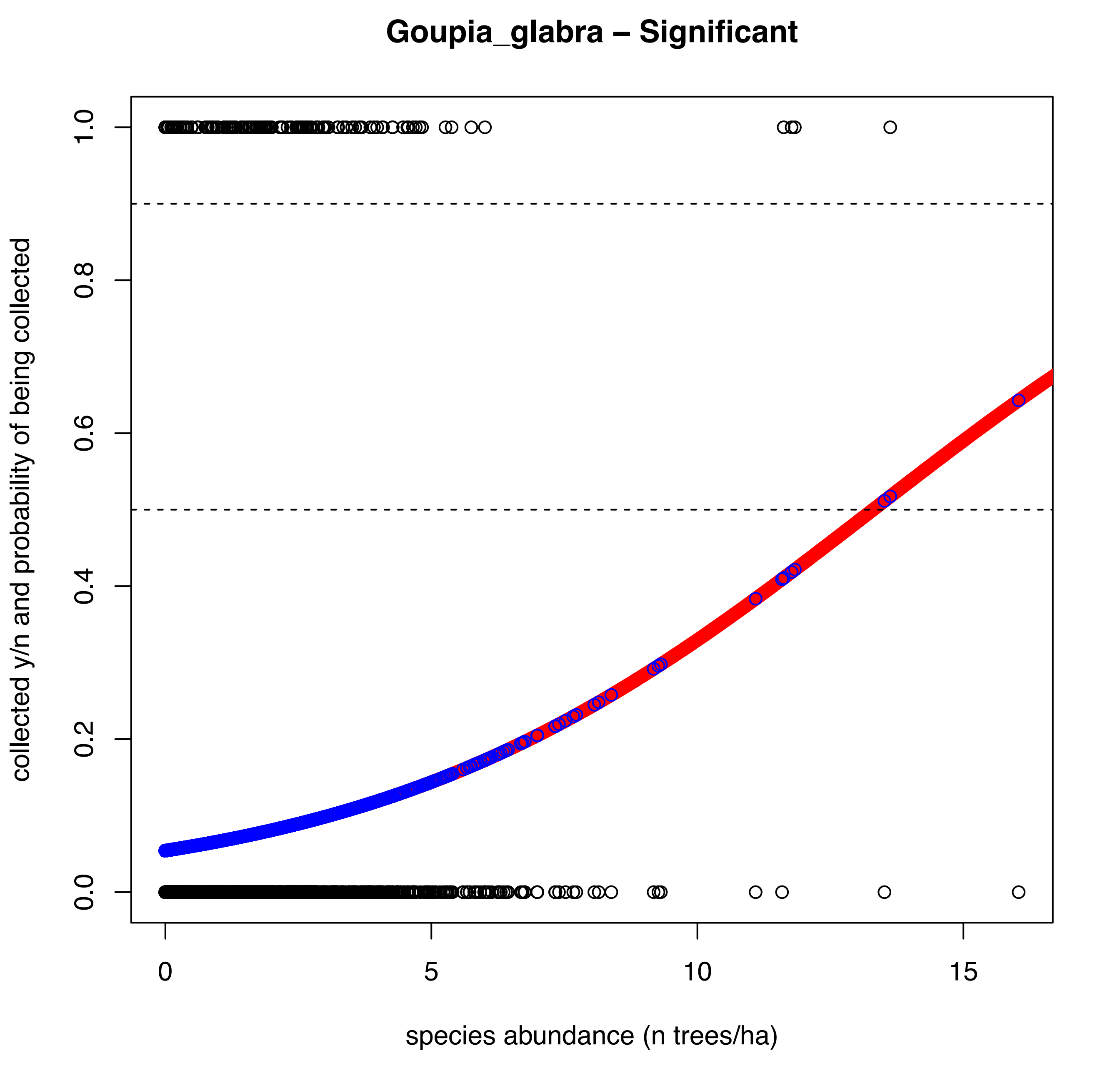

Goupia_glabra – Significant
collected y/n and probability of being collected
species abundance (n trees/ha)
0.0
0.2
0.4
0.6
0.8
1.0
0
5
10
15

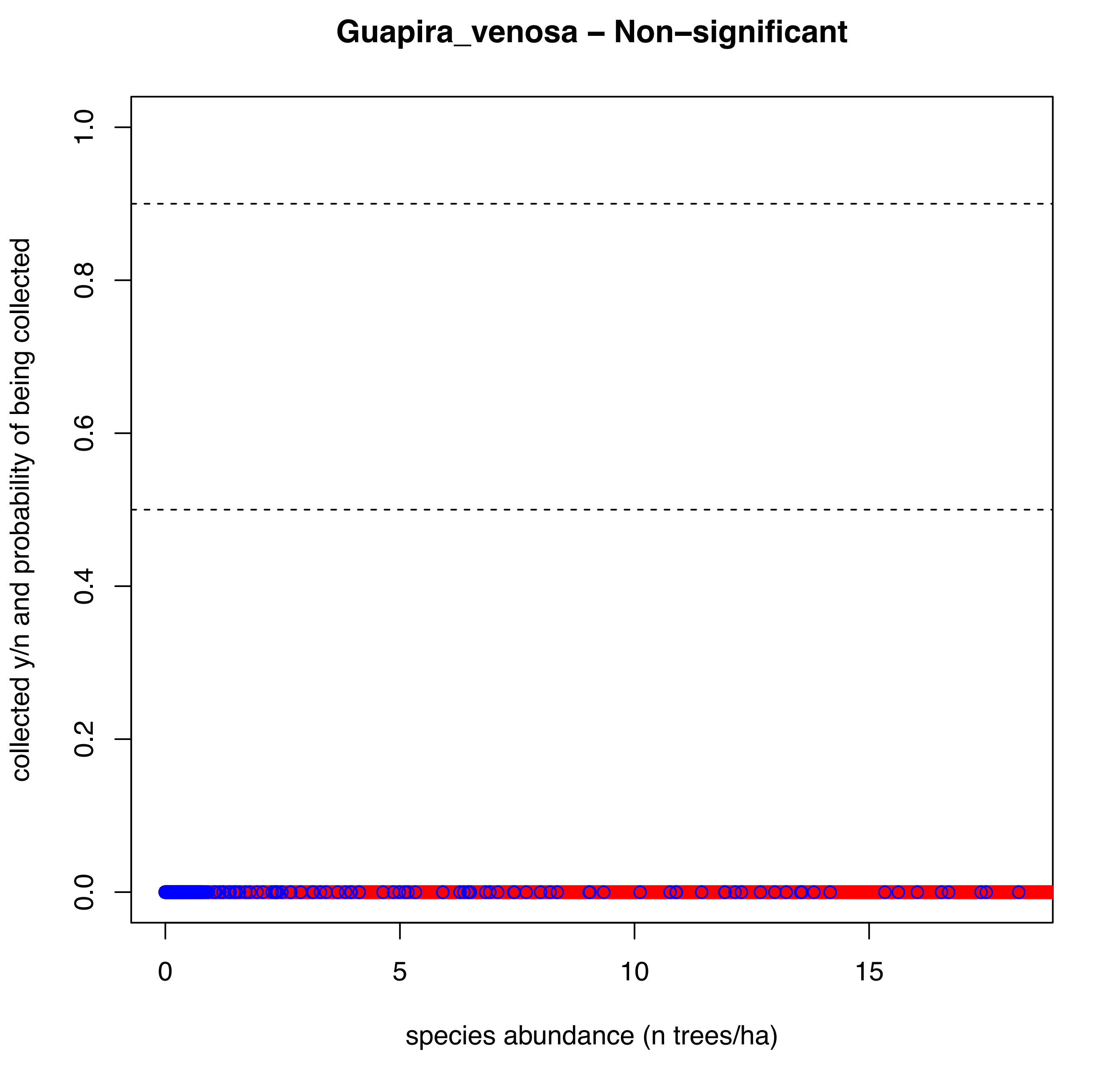
Guapira_venosa – Non–significant
collected y/n and probability of being collected
0.0
0.2
0.4
0.6
0.8
1.0
0
5
10
15
species abundance (n trees/ha)

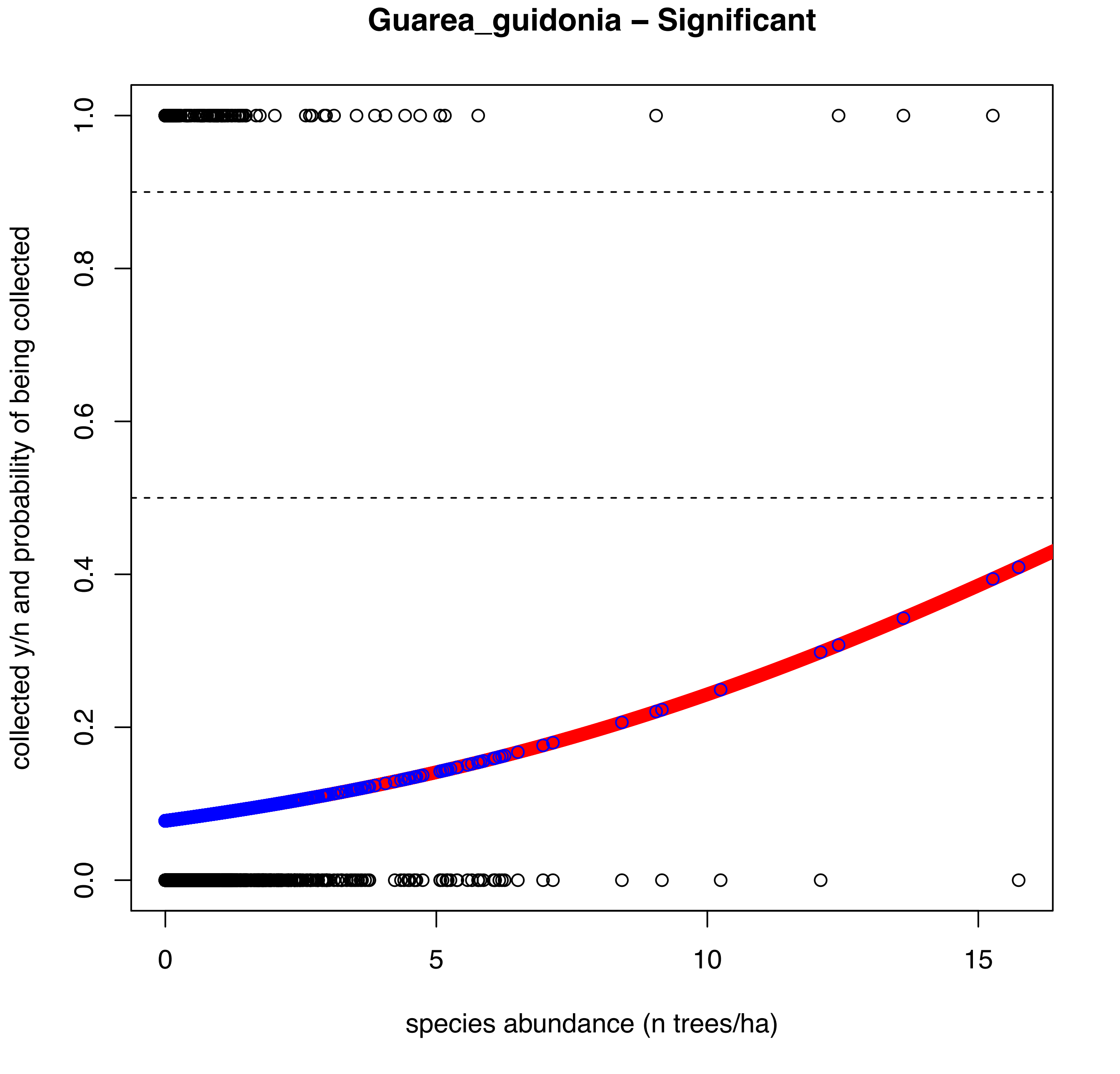

Guarea_guidonia – Significant
collected y/n and probability of being collected
species abundance (n trees/ha)
0.0
0.2
0.4
0.6
0.8
1.0
0
5
10
15

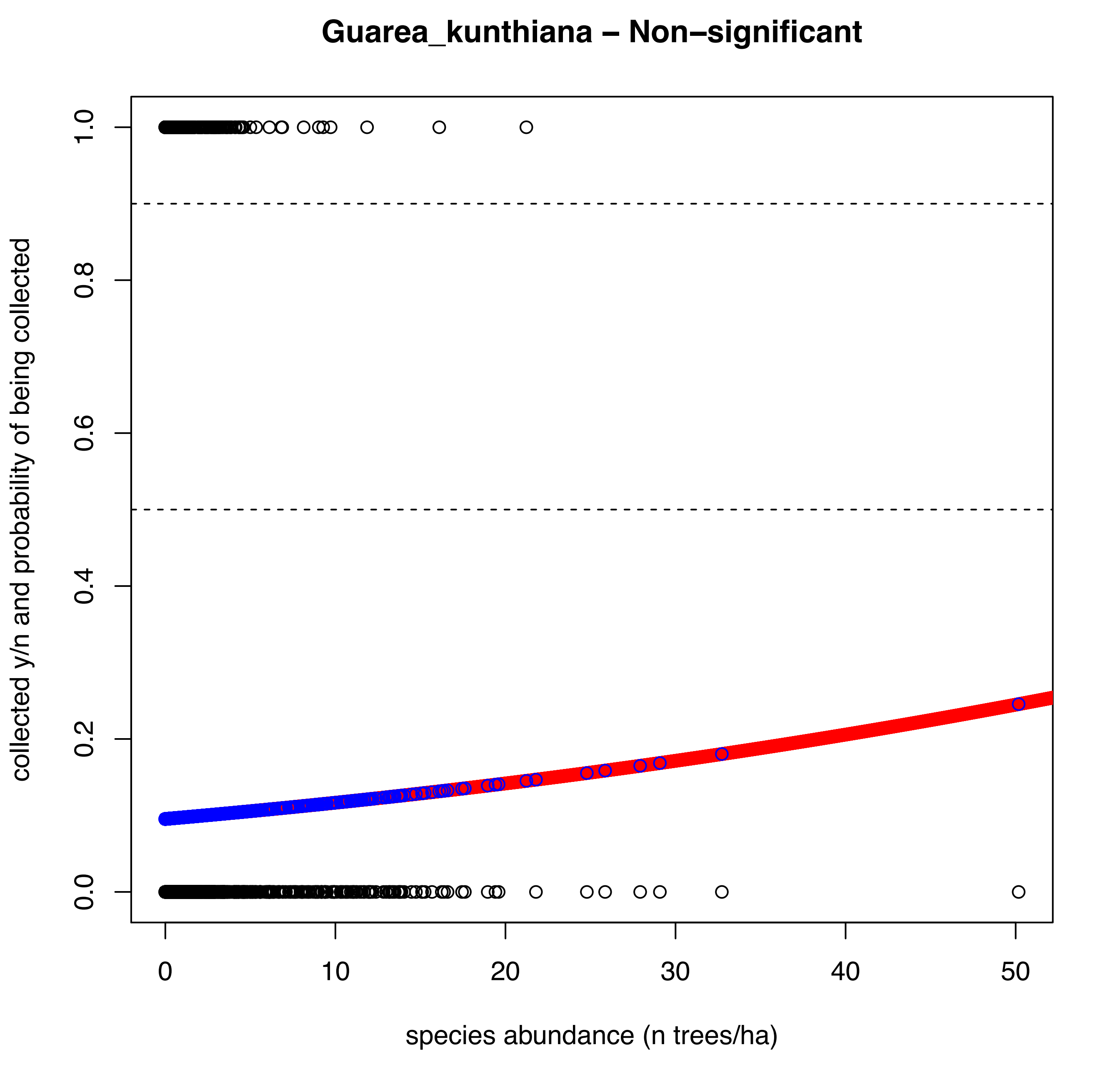
Guarea_kunthiana – Non–significant
collected y/n and probability of being collected
species abundance (n trees/ha)
0.0
0.2
0.4
0.6
0.8
1.0
0
10
20
30
40
50

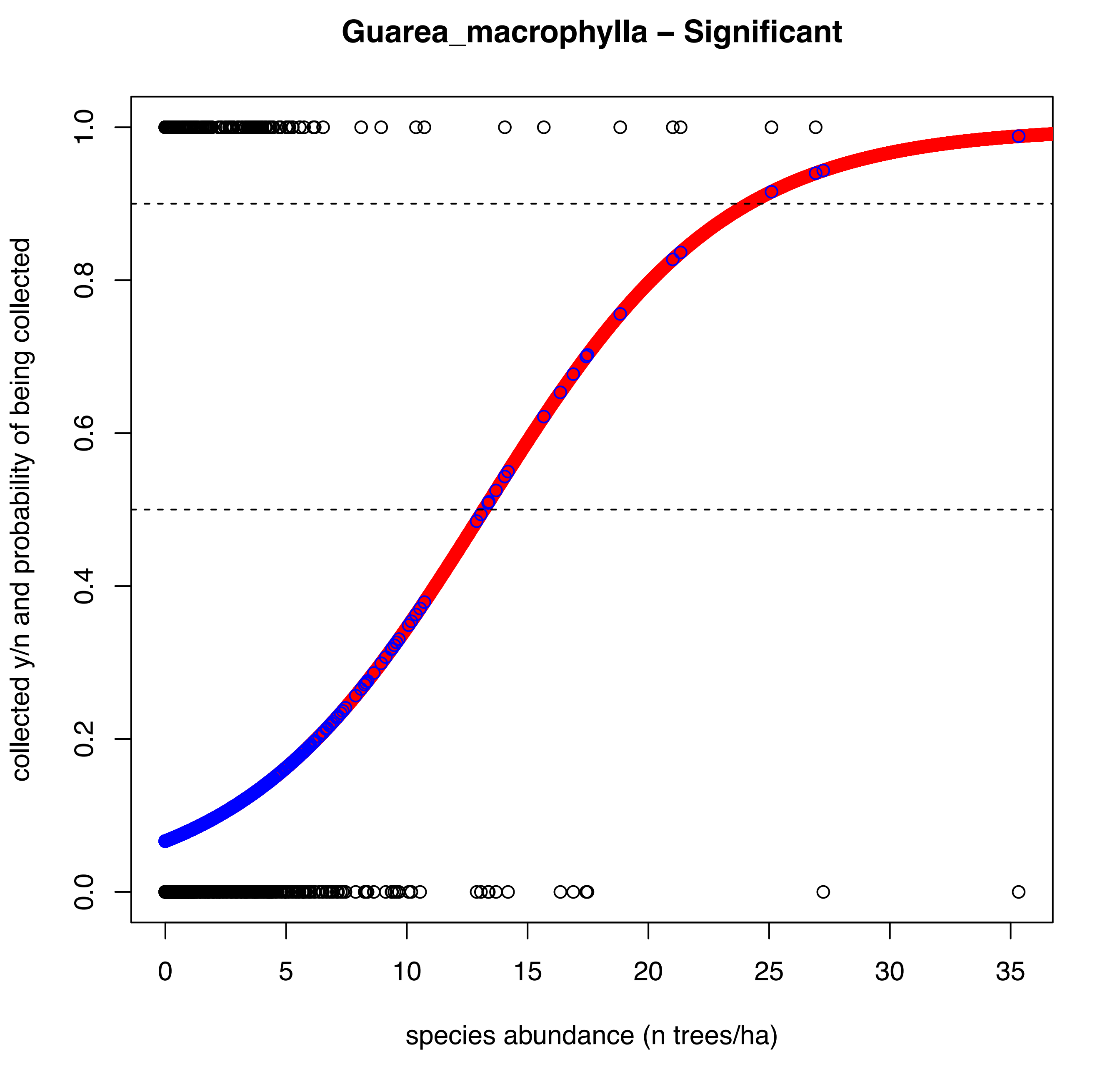
Guarea_macrophylla – Significant
collected y/n and probability of being collected
species abundance (n trees/ha)
0.0
0.2
0.4
0.6
0.8
1.0
0
5
10
15
20
25
30
35

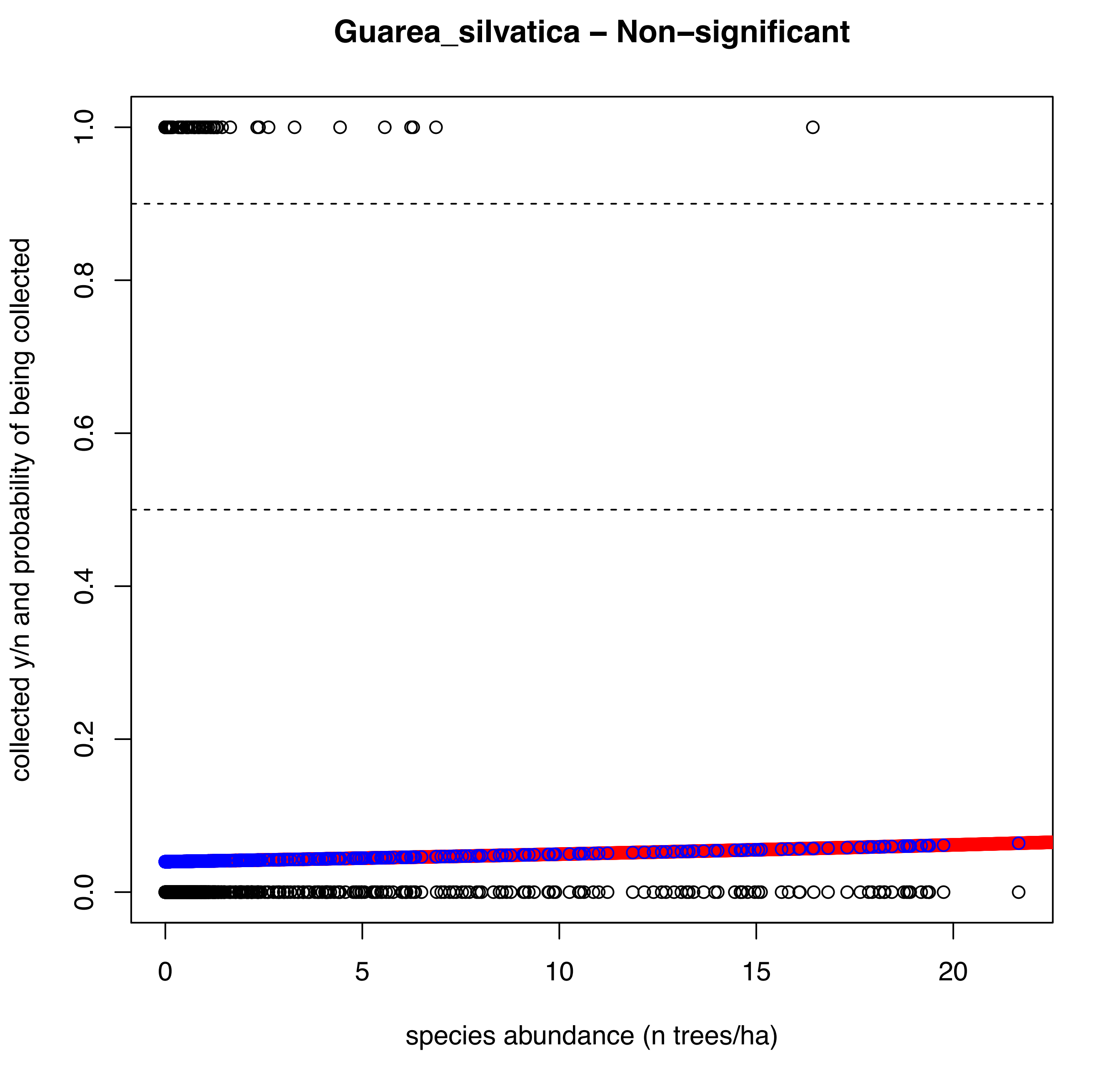
Guarea_silvatica – Non–significant
collected y/n and probability of being collected
species abundance (n trees/ha)
0.0
0.2
0.4
0.6
0.8
1.0
0
5
10
15
20

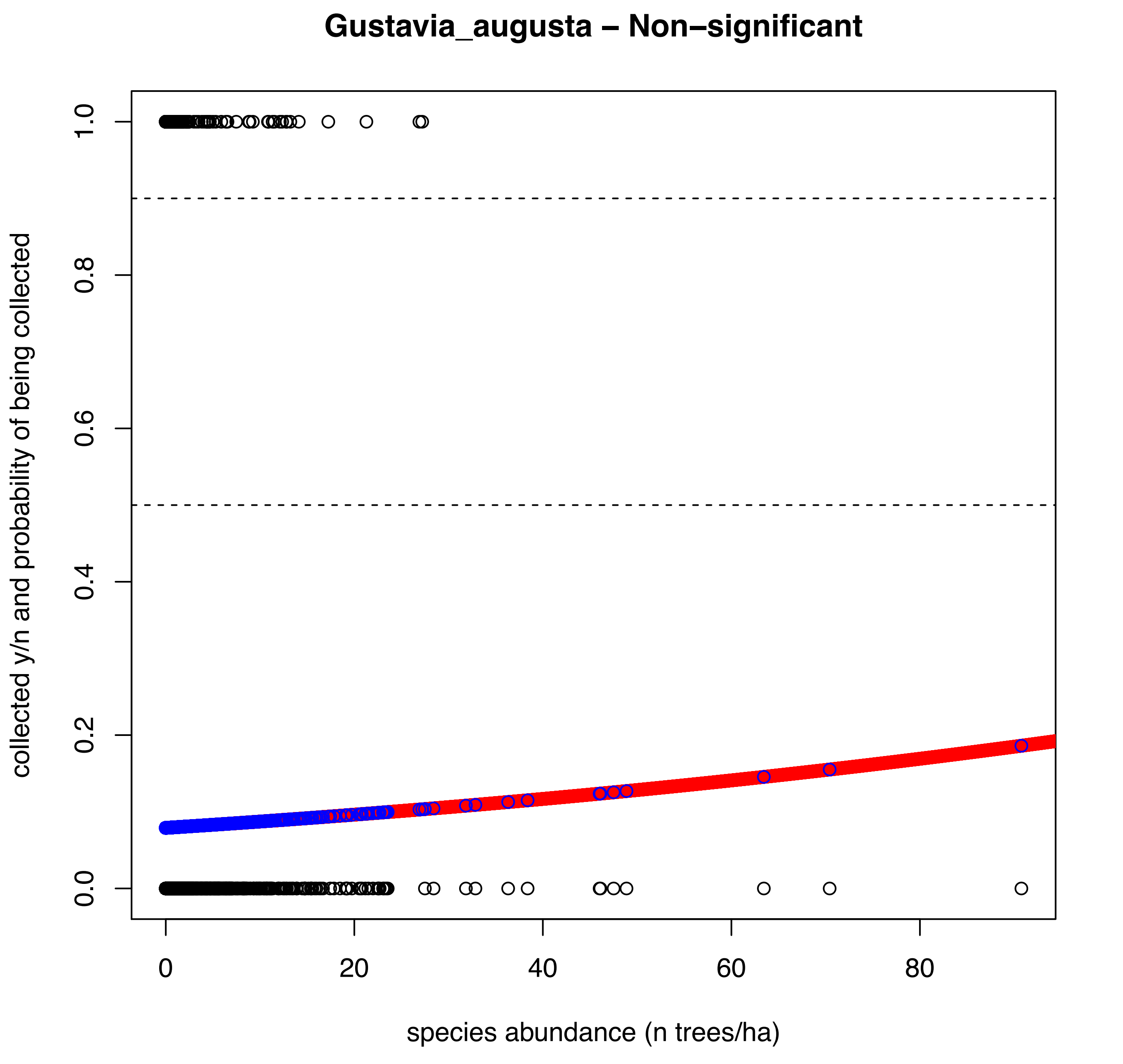
Gustavia_augusta – Non–significant
collected y/n and probability of being collected
species abundance (n trees/ha)
0.0
0.2
0.4
0.6
0.8
1.0
0
20
40
60
80

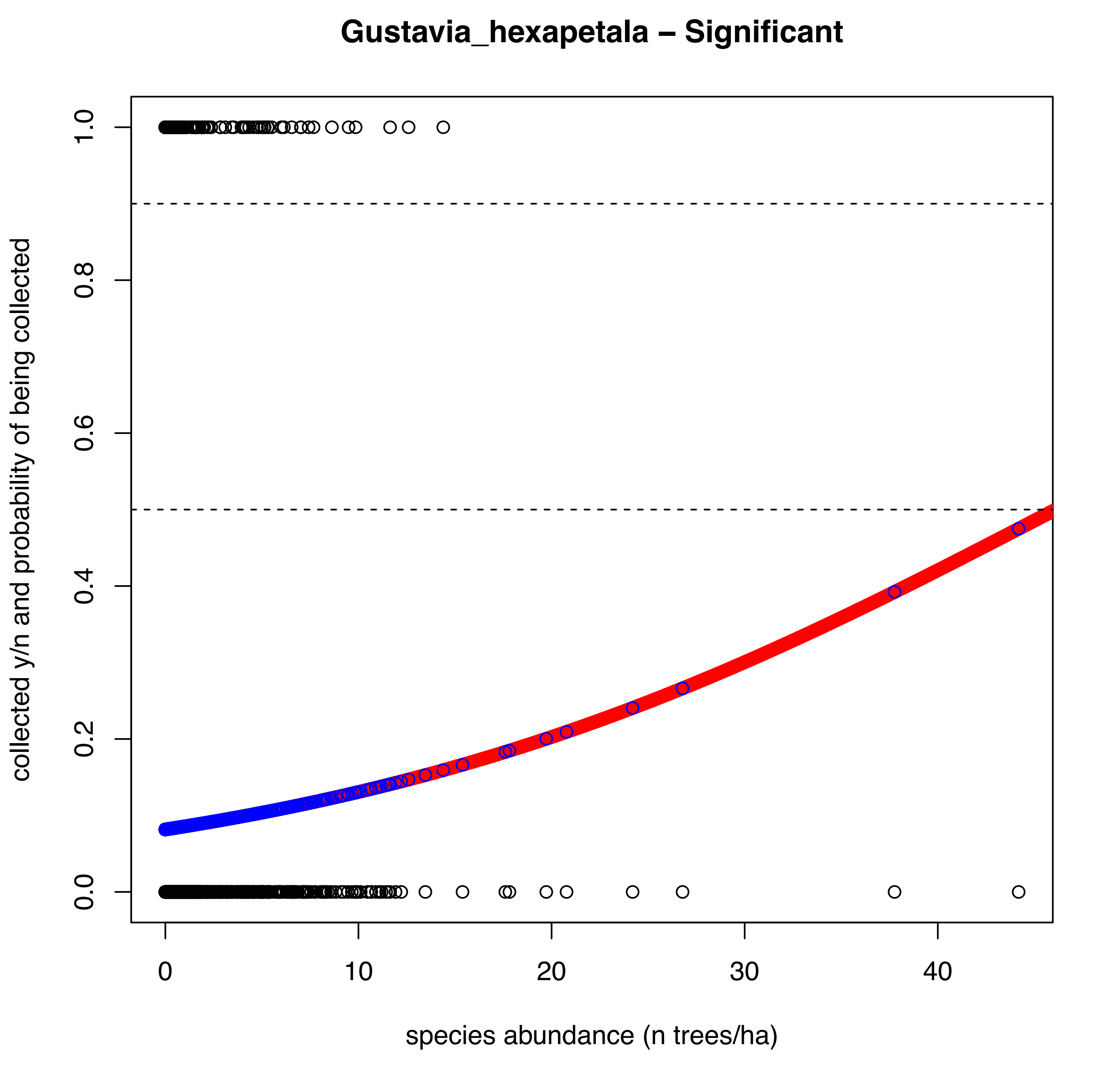
Gustavia_hexapetala – Significant
collected y/n and probability of being collected
0.0
0.2
0.4
0.6
0.8
1.0
0
10
20
30
40
species abundance (n trees/ha)

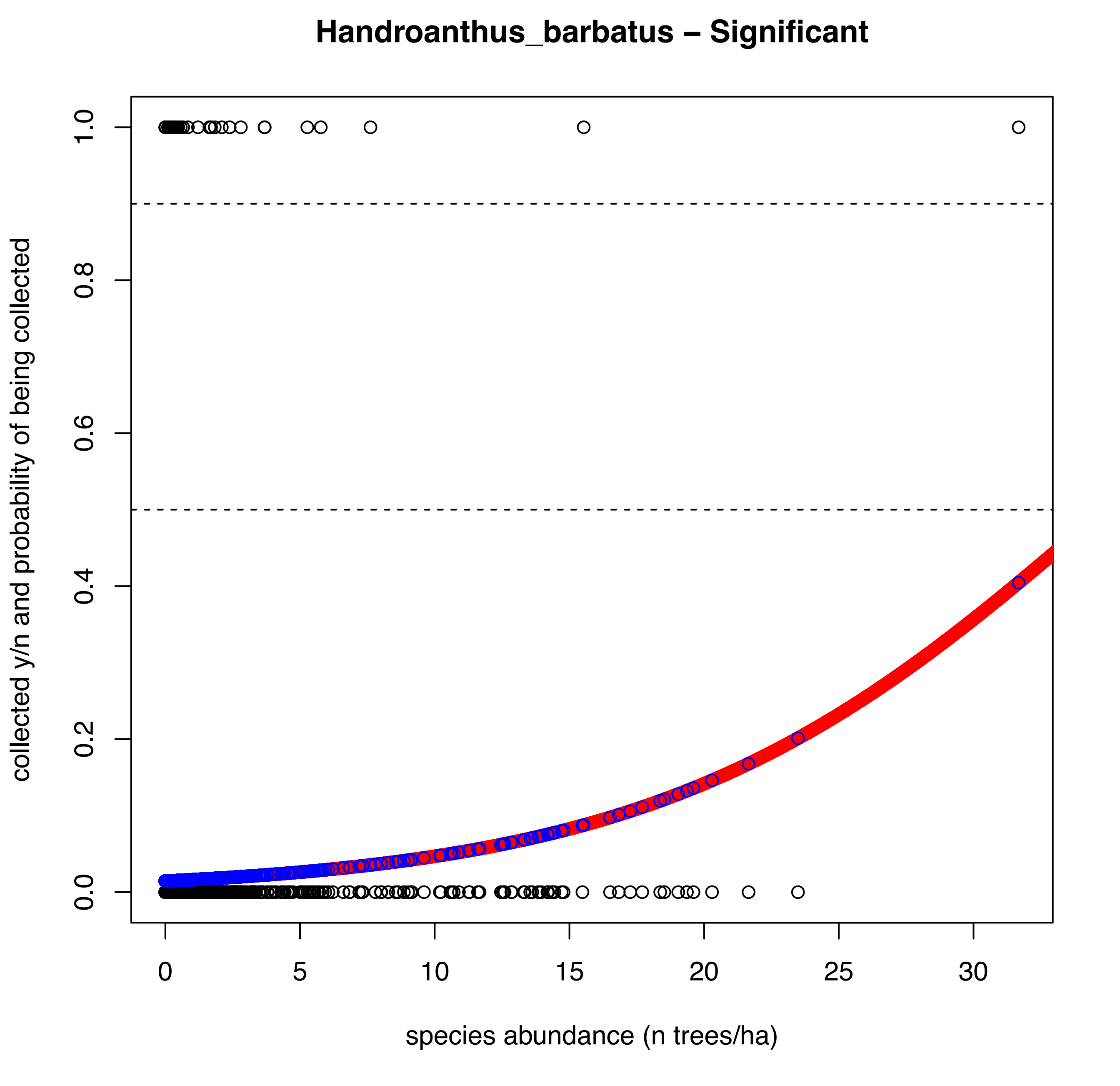
Handroanthus_barbatus – Significant
collected y/n and probability of being collected
species abundance (n trees/ha)
0.0
0.2
0.4
0.6
0.8
1.0
0
5
10
15
20
25
30

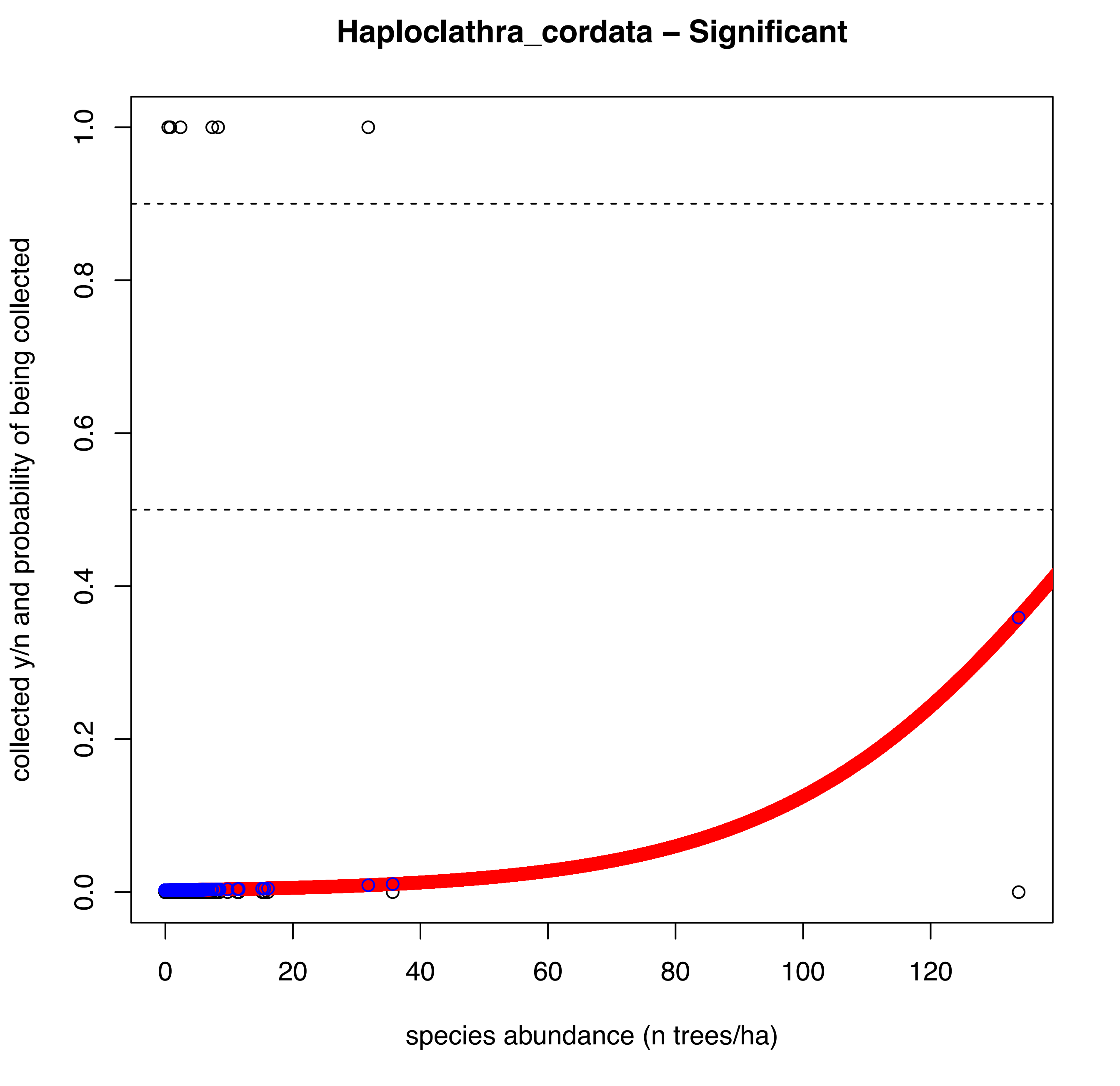
Haploclathra_cordata – Significant
collected y/n and probability of being collected
0.0
0.2
0.4
0.6
0.8
1.0
0
20
40
60
80
100
120
species abundance (n trees/ha)

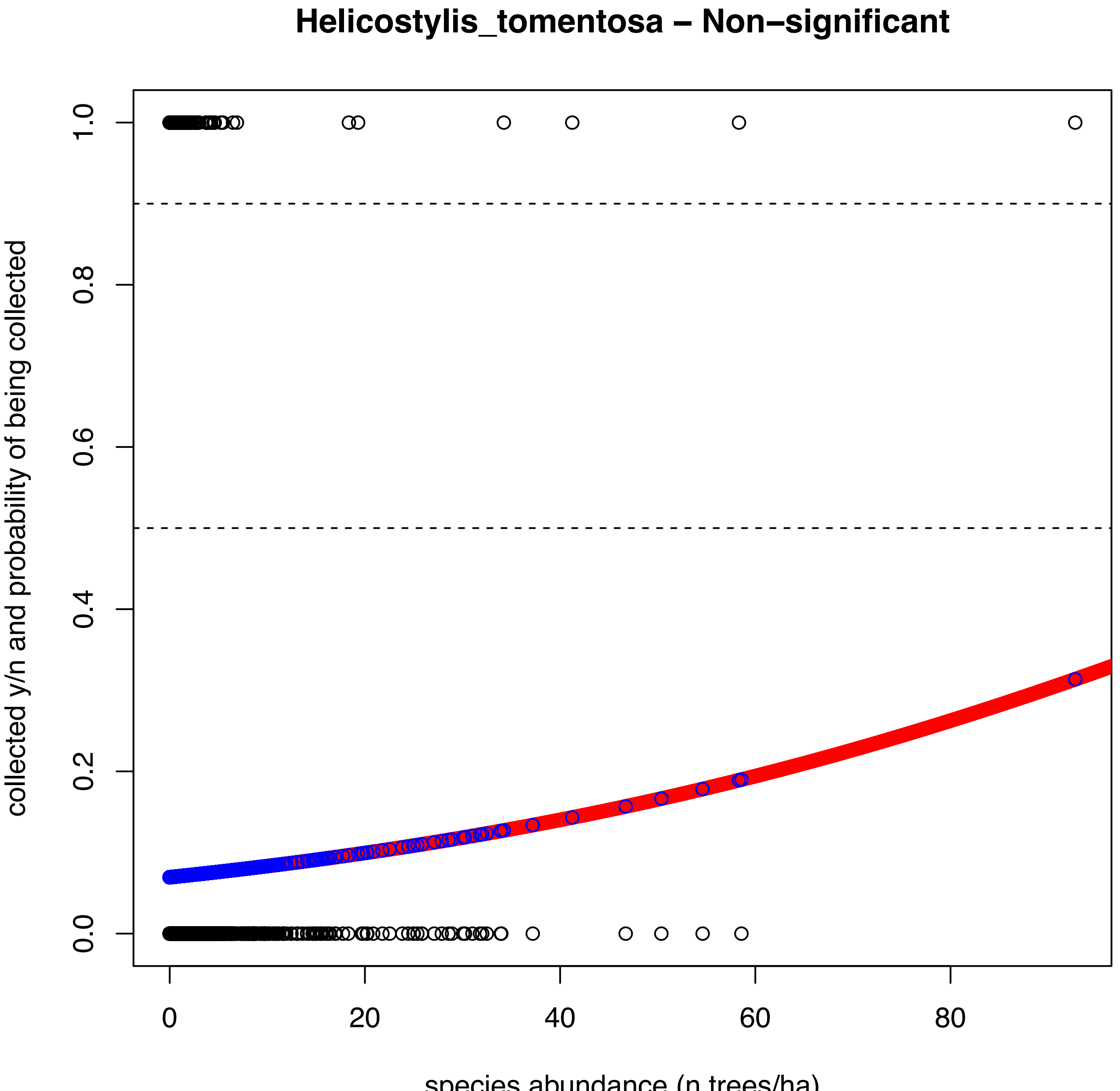
Helicostylis_tomentosa – Non–significant
collected y/n and probability of being collected
0.0
0.2
0.4
0.6
0.8
1.0
0
20
40
60
80
species abundance (n trees/ha)

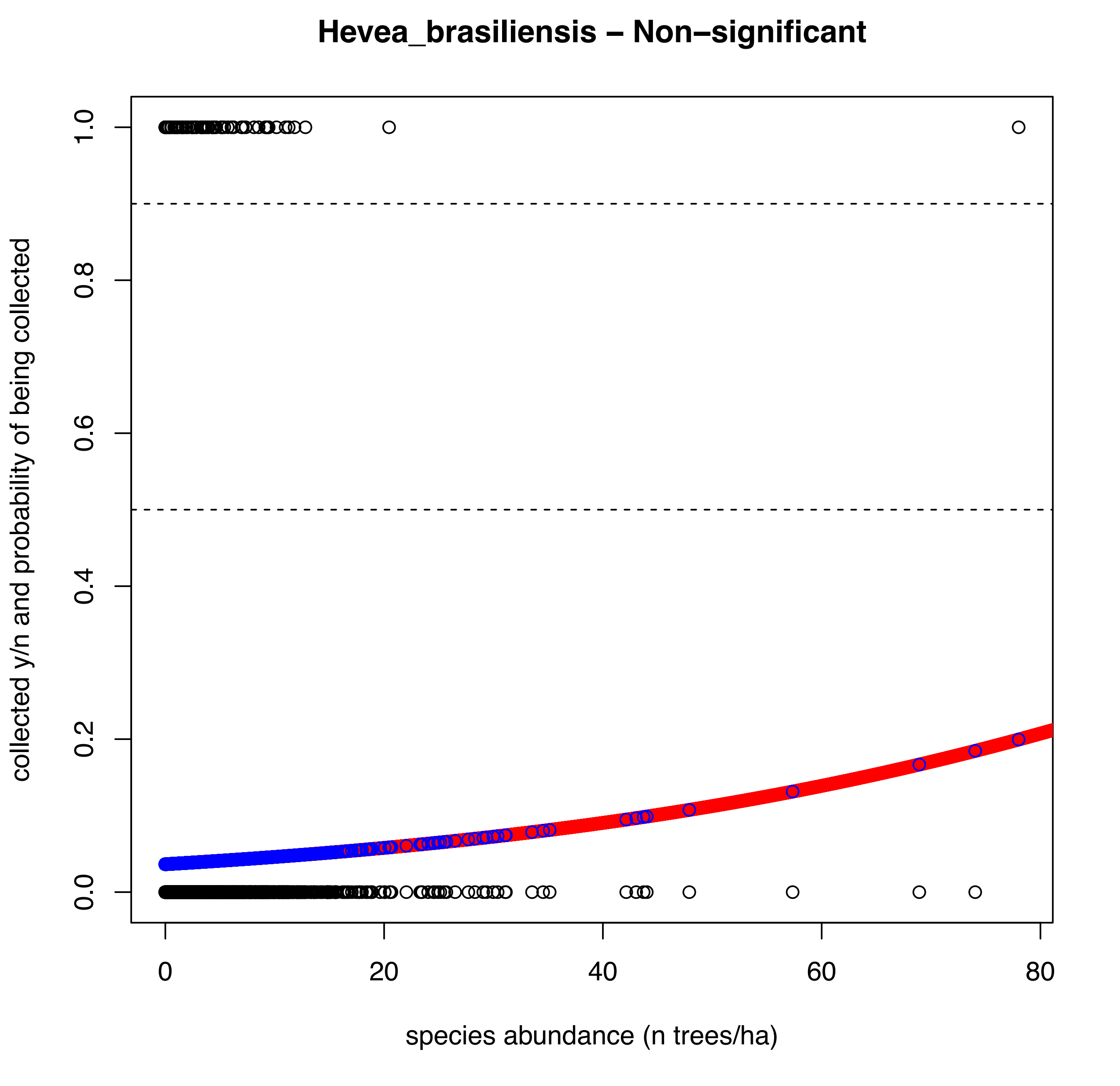
Hevea_brasiliensis – Non–significant
collected y/n and probability of being collected
species abundance (n trees/ha)
0.0
0.2
0.4
0.6
0.8
1.0
0
20
40
60
80

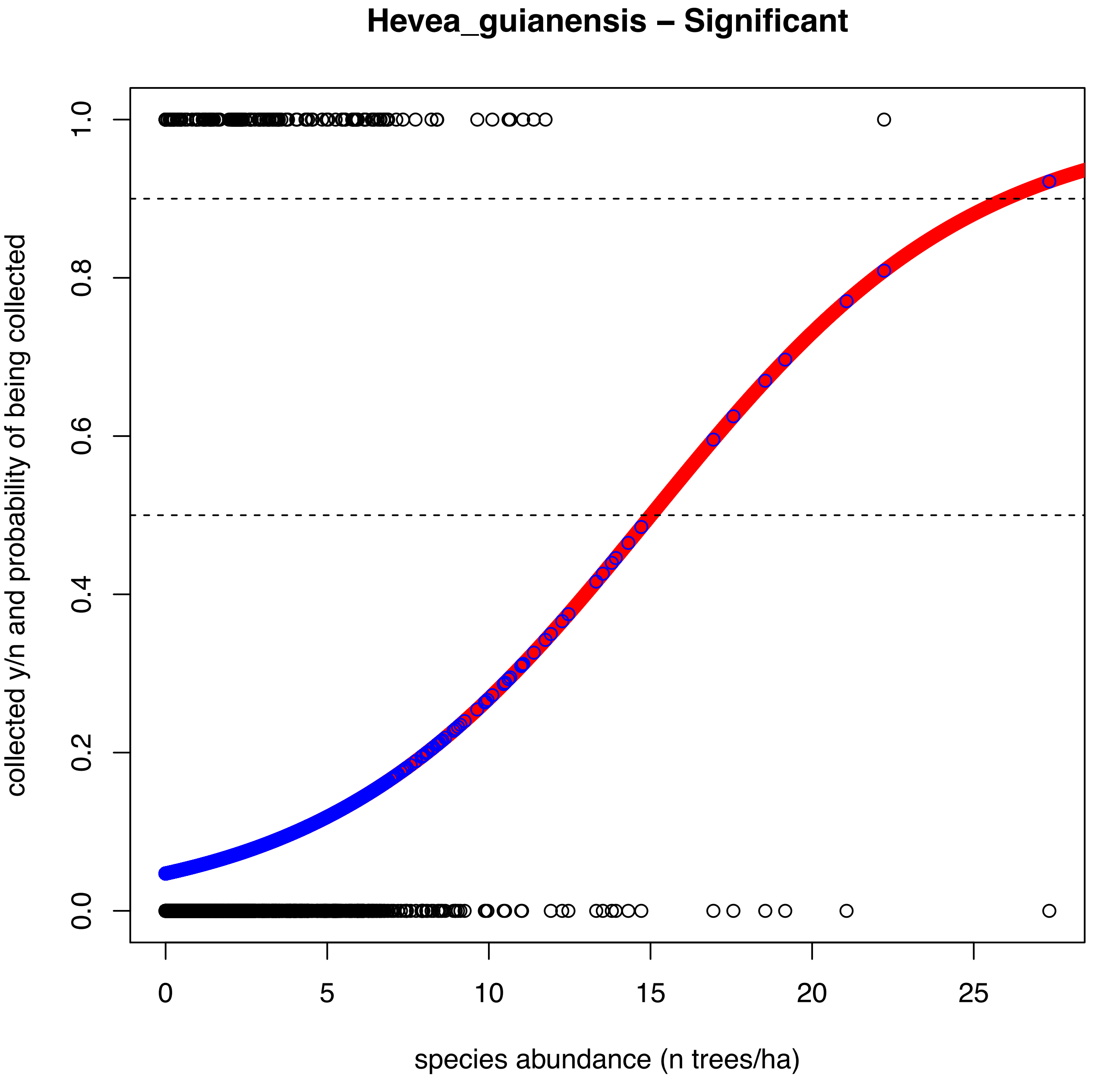

Hevea_guianensis – Significant
collected y/n and probability of being collected
species abundance (n trees/ha)
0.0
0.2
0.4
0.6
0.8
1.0
0
5
10
15
20
25

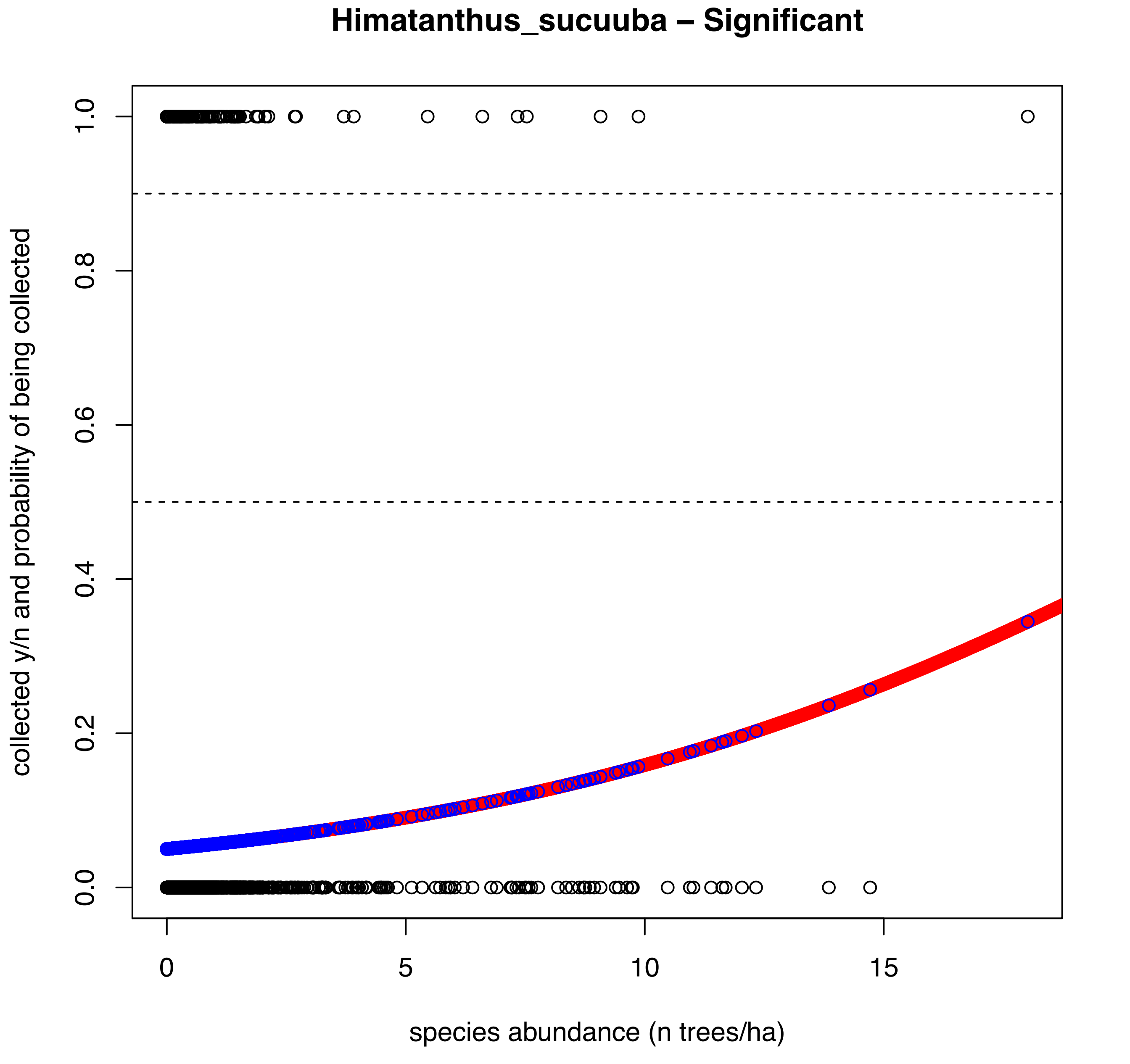
Himatanthus_sucuuba – Significant
collected y/n and probability of being collected
species abundance (n trees/ha)
0.0
0.2
0.4
0.6
0.8
1.0
0
5
10
15

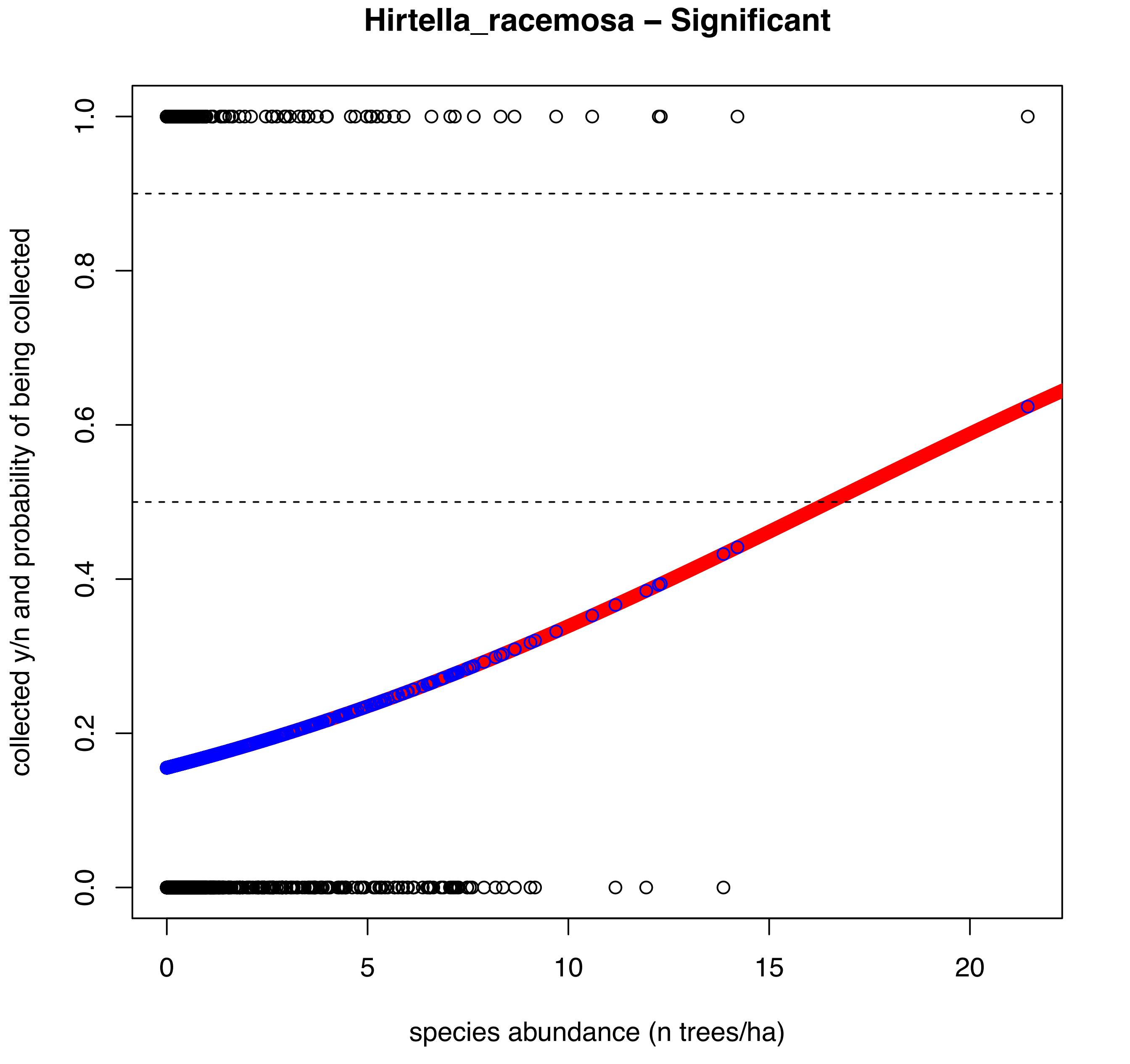

Hirtella_racemosa – Significant
collected y/n and probability of being collected
species abundance (n trees/ha)
0.0
0.2
0.4
0.6
0.8
1.0
0
5
10
15
20

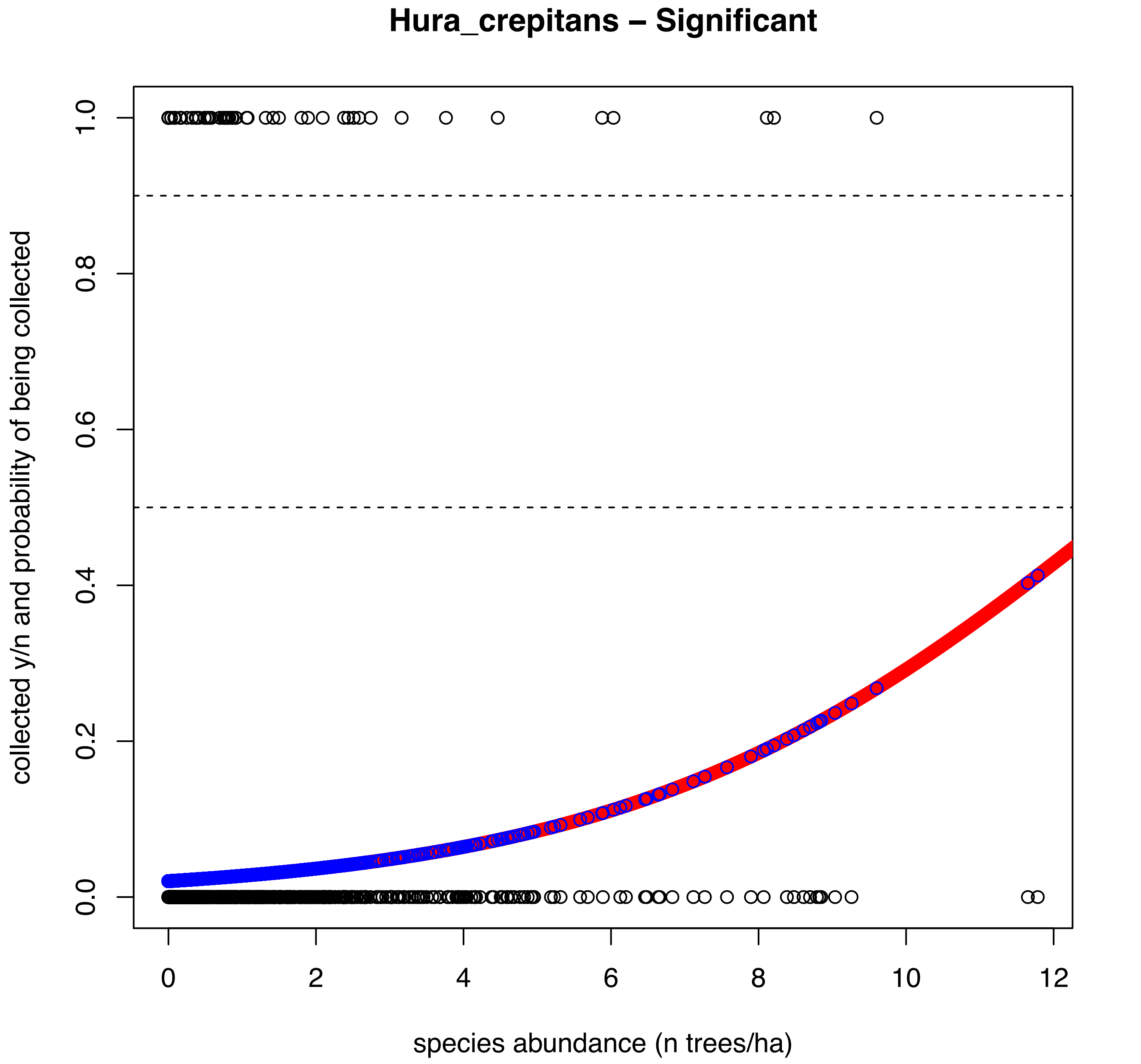

Hura_crepitans – Significant
collected y/n and probability of being collected
species abundance (n trees/ha)
0.0
0.2
0.4
0.6
0.8
1.0
0
2
4
6
8
10
12

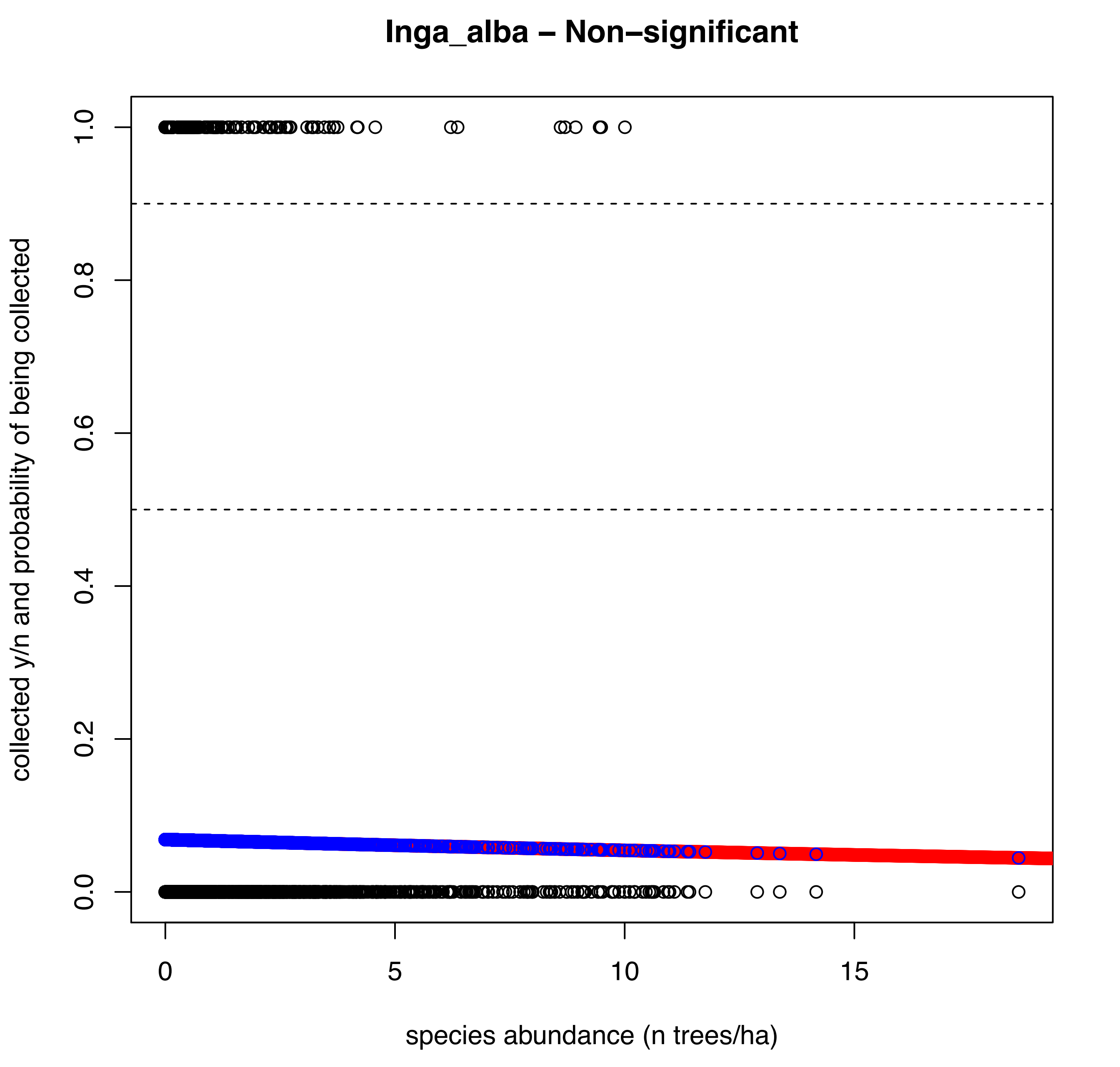
Inga_alba – Non–significant
collected y/n and probability of being collected
species abundance (n trees/ha)
0.0
0.2
0.4
0.6
0.8
1.0
0
5
10
15

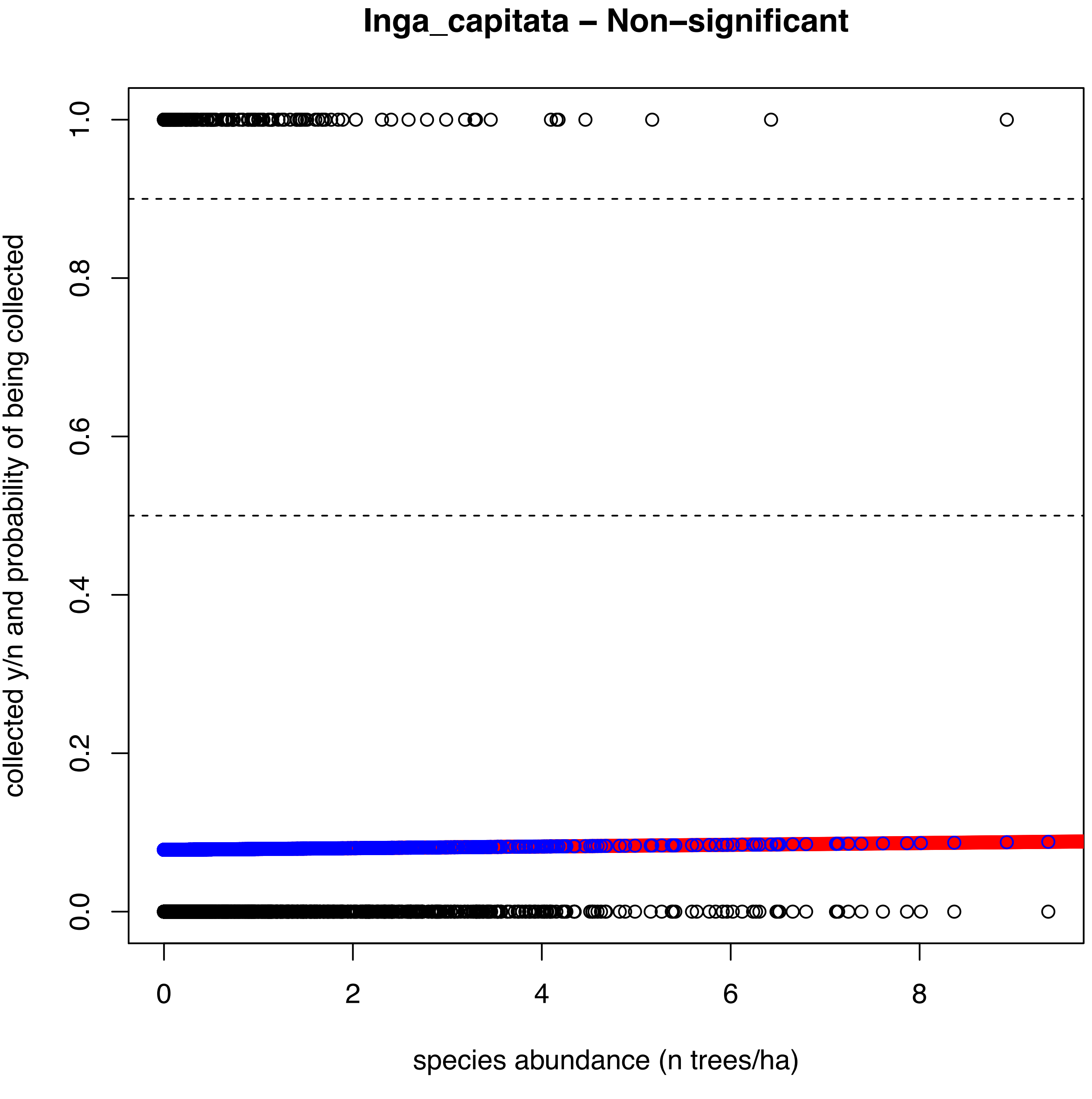
Inga_capitata – Non–significant
collected y/n and probability of being collected
species abundance (n trees/ha)
0.0
0.2
0.4
0.6
0.8
1.0
0
2
4
6
8

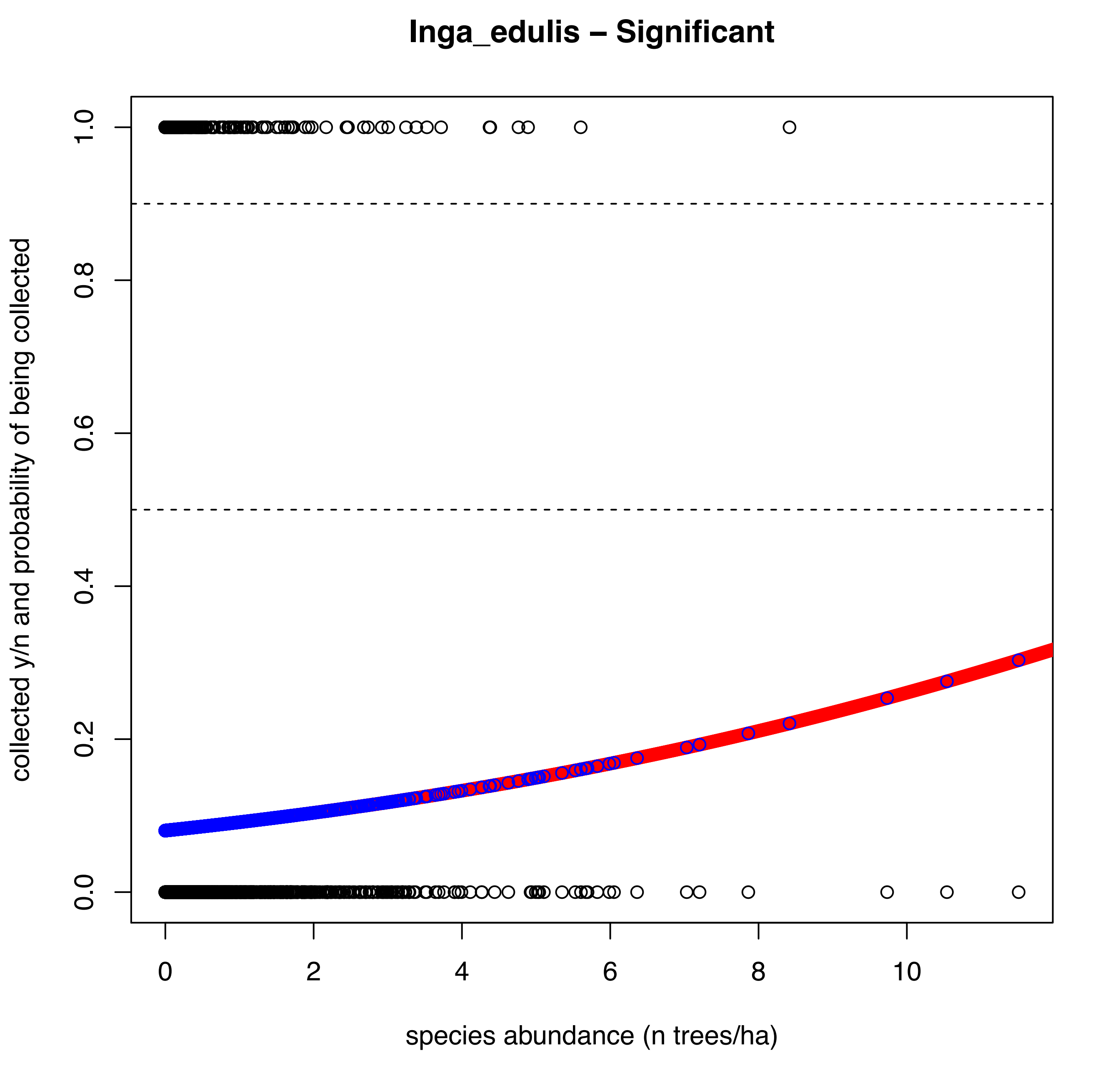
Inga_edulis – Significant
collected y/n and probability of being collected
0.0
0.2
0.4
0.6
0.8
1.0
0
2
4
6
8
10
species abundance (n trees/ha)

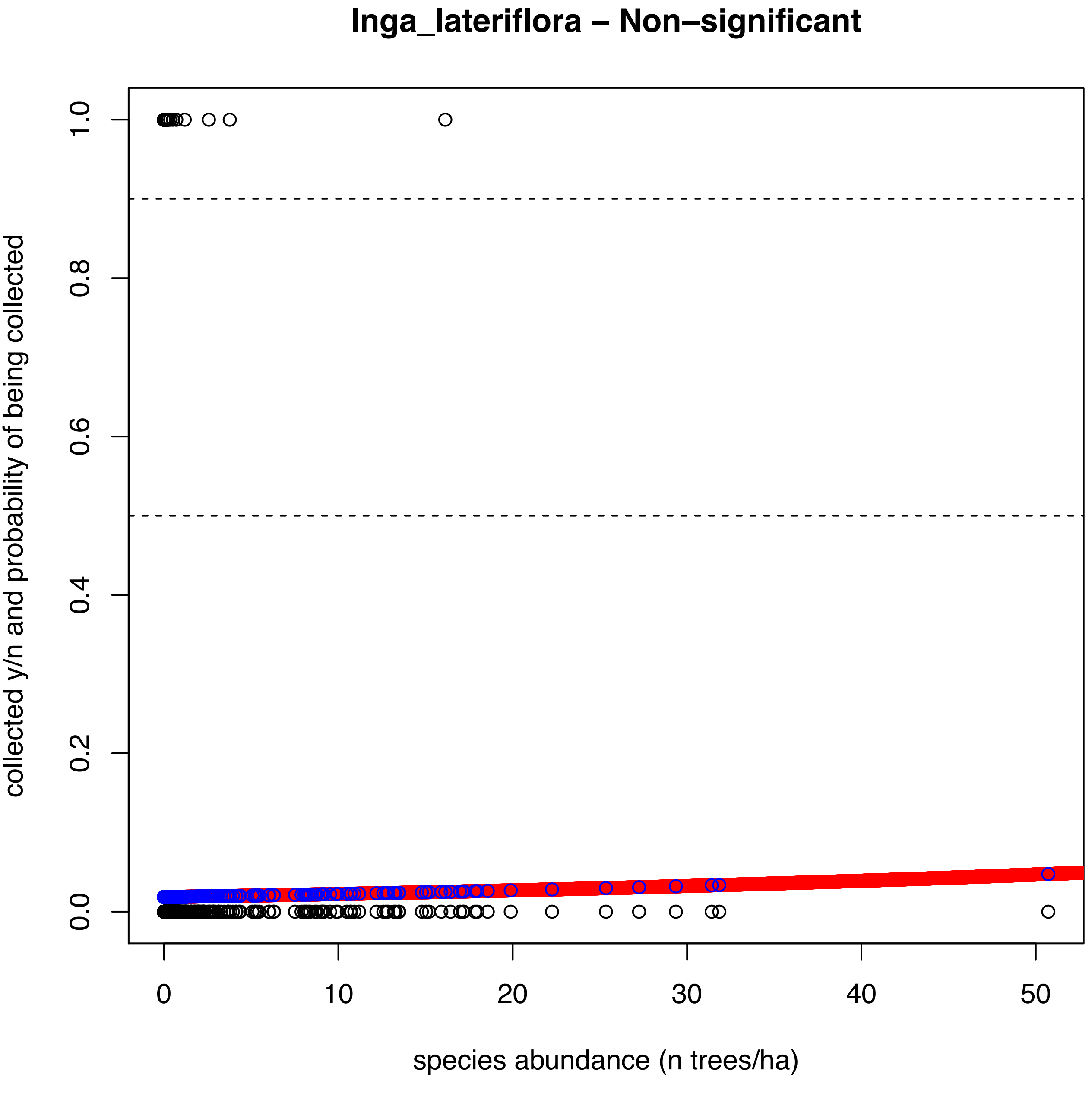

Inga_lateriflora – Non–significant
collected y/n and probability of being collected
0.0
0.2
0.4
0.6
0.8
1.0
0
10
20
30
40
50
species abundance (n trees/ha)

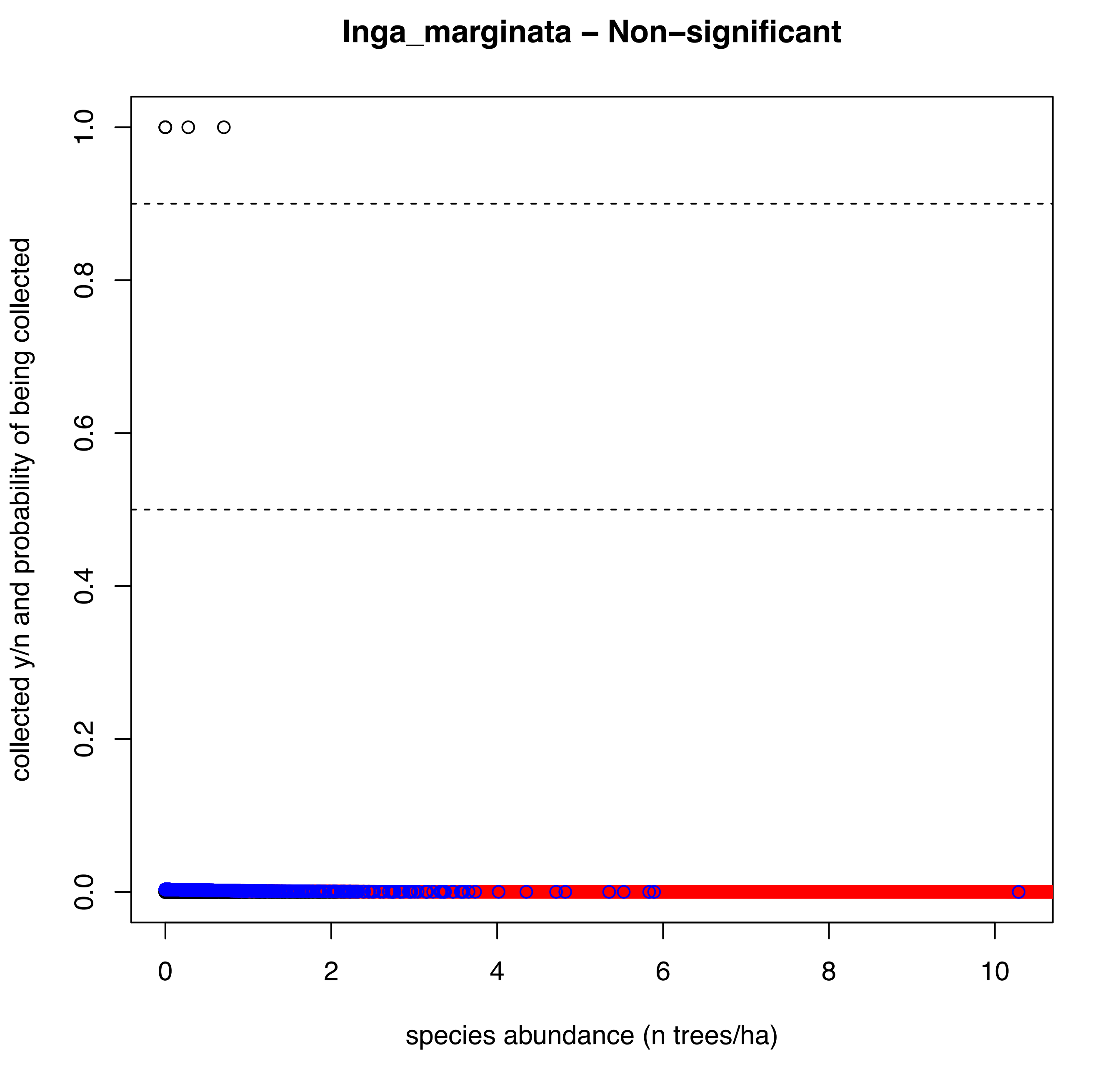

Inga_marginata – Non–significant
collected y/n and probability of being collected
1.0
0.8
0.6
0.4
0.2
0.0
0
2
4
6
8
10
species abundance (n trees/ha)

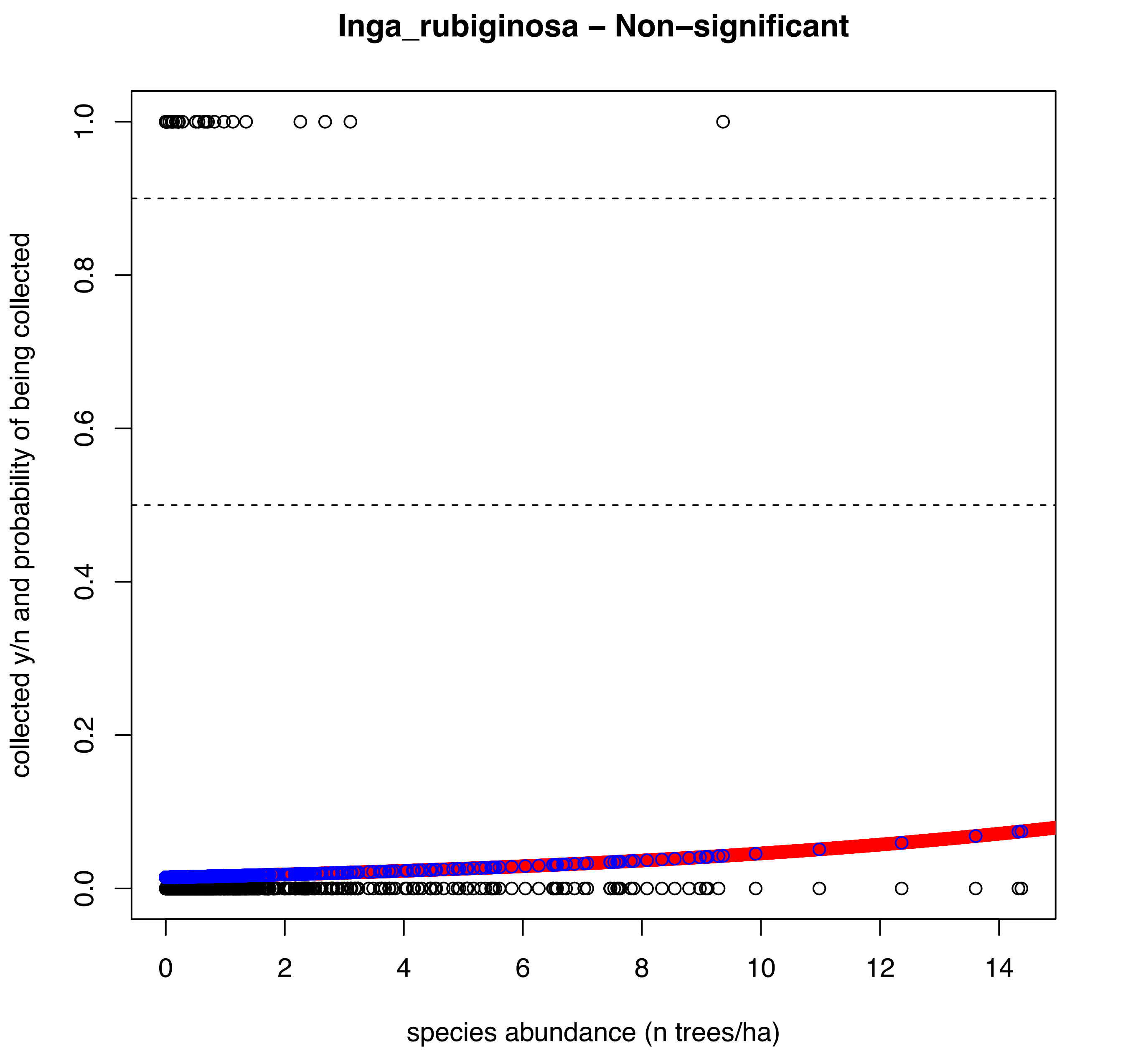
Inga_rubiginosa – Non–significant
collected y/n and probability of being collected
species abundance (n trees/ha)
0.0
0.2
0.4
0.6
0.8
1.0
0
2
4
6
8
10
12
14

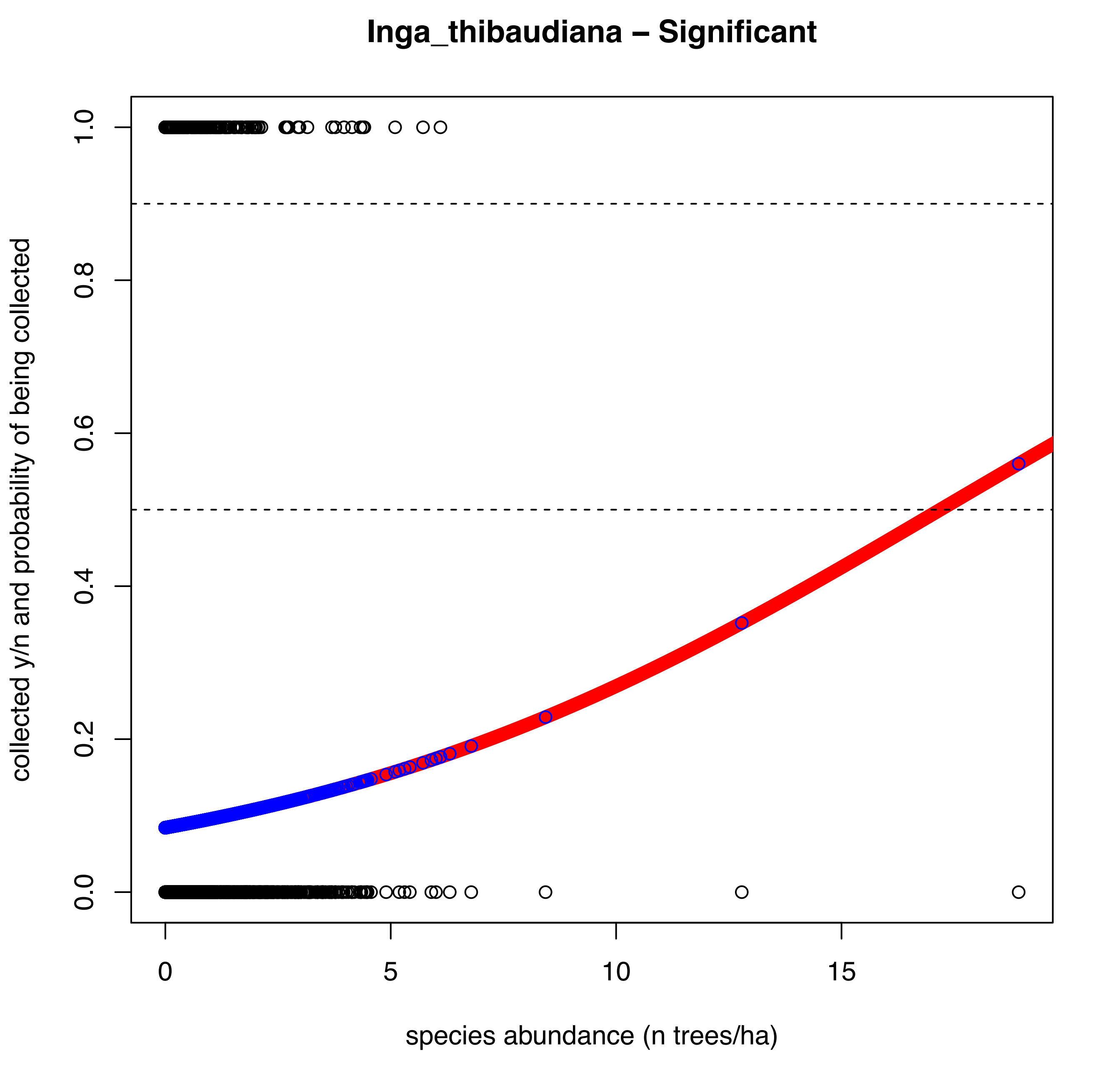

Inga_thibaudiana – Significant
collected y/n and probability of being collected
species abundance (n trees/ha)
0.0
0.2
0.4
0.6
0.8
1.0
0
5
10
15

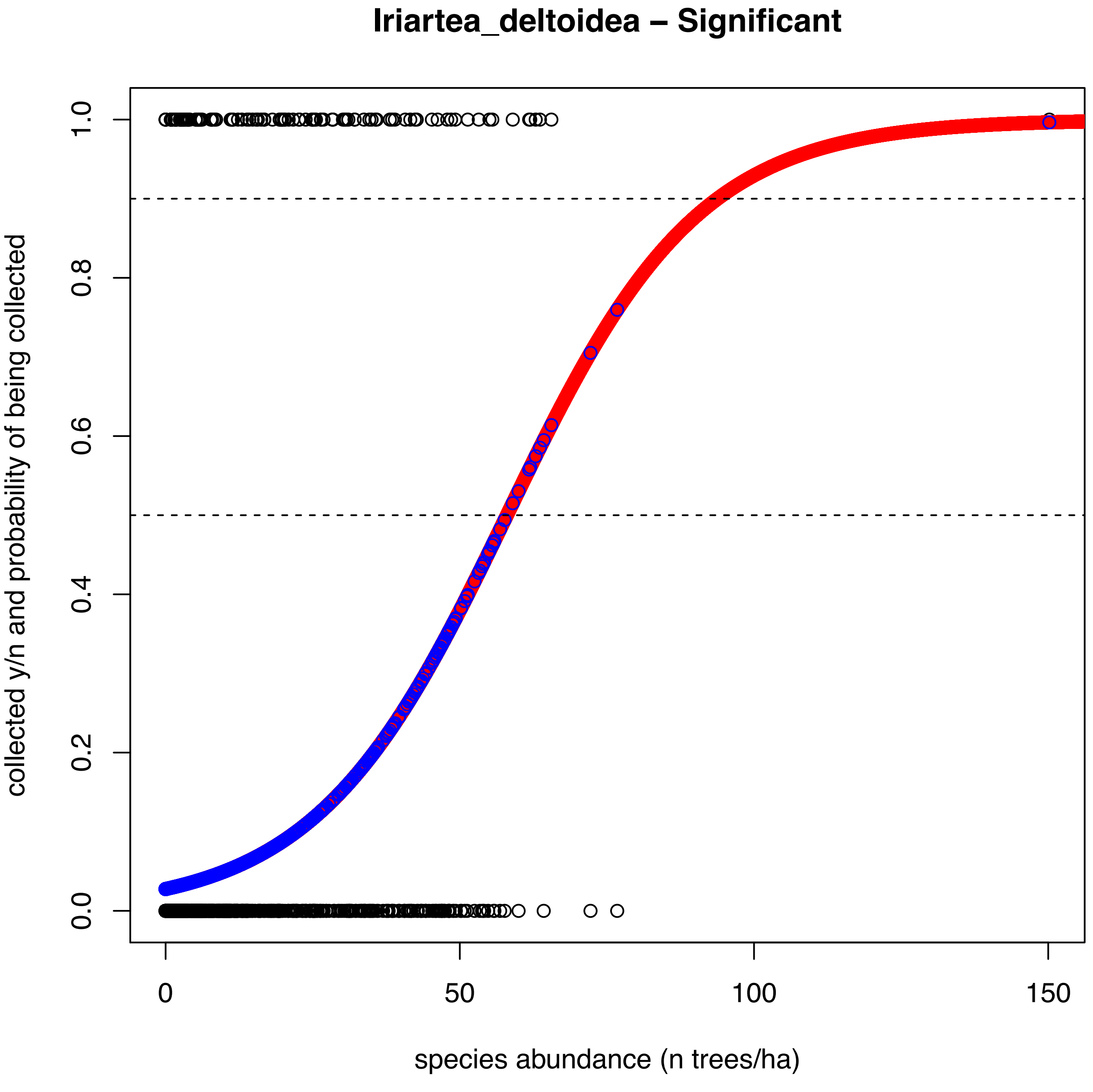
Iriartea_deltoidea – Significant
collected y/n and probability of being collected
species abundance (n trees/ha)
0.0
0.2
0.4
0.6
0.8
1.0
0
50
100
150

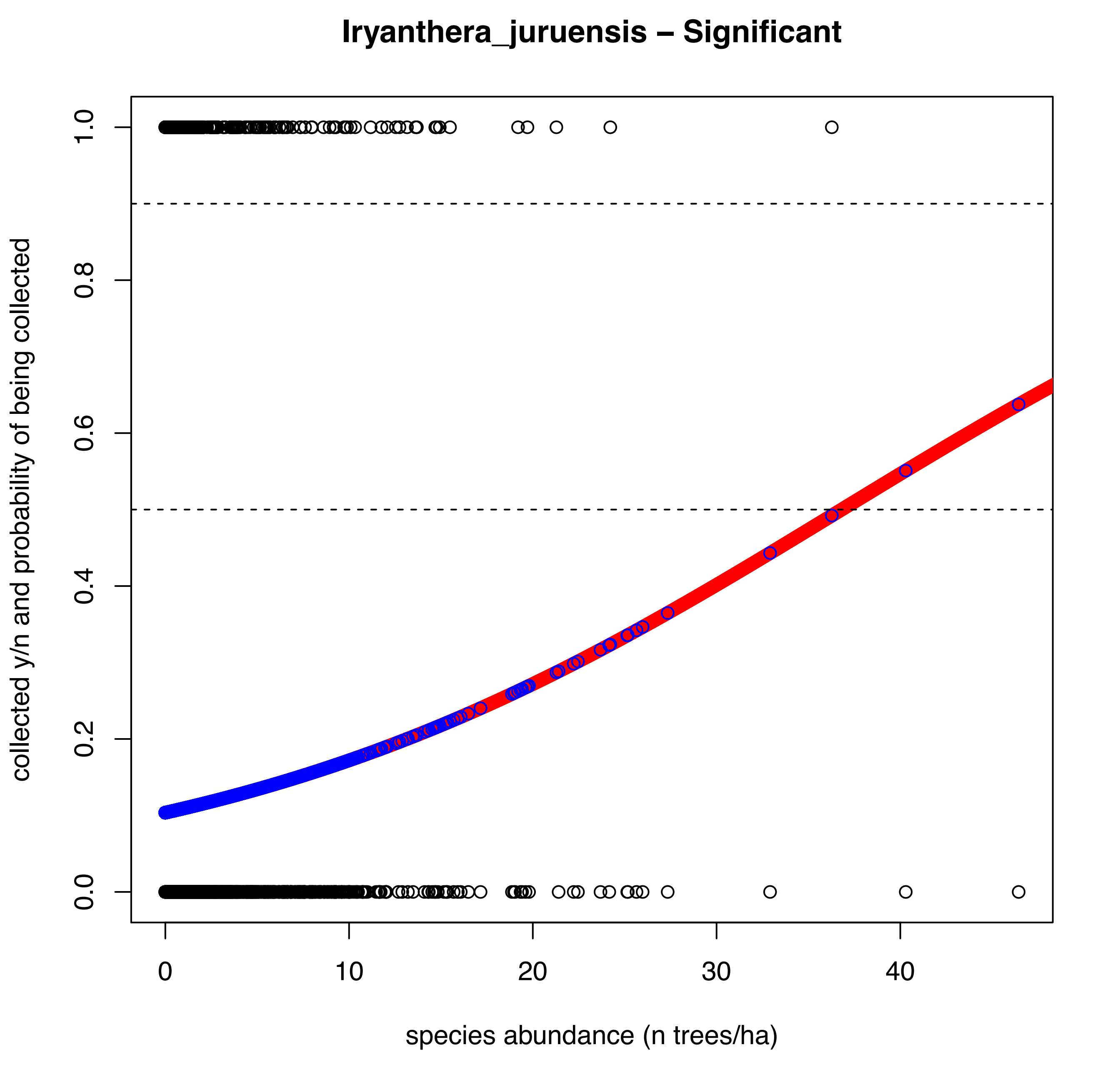

Iryanthera_juruensis – Significant
collected y/n and probability of being collected
species abundance (n trees/ha)
0.0
0.2
0.4
0.6
0.8
1.0
0
10
20
30
40

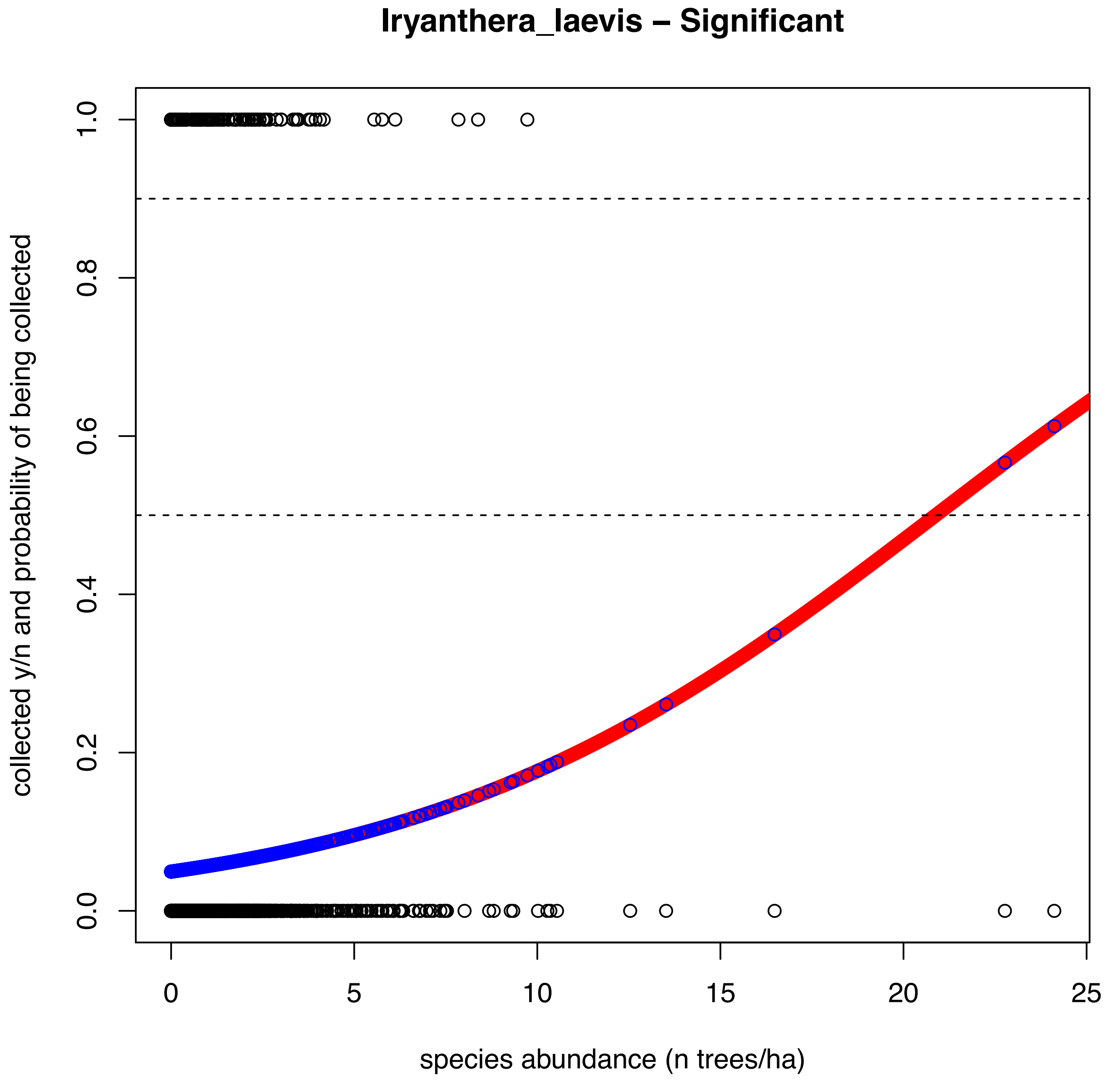

Iryanthera_laevis – Significant
collected y/n and probability of being collected
species abundance (n trees/ha)
0.0
0.2
0.4
0.6
0.8
1.0
0
5
10
15
20
25

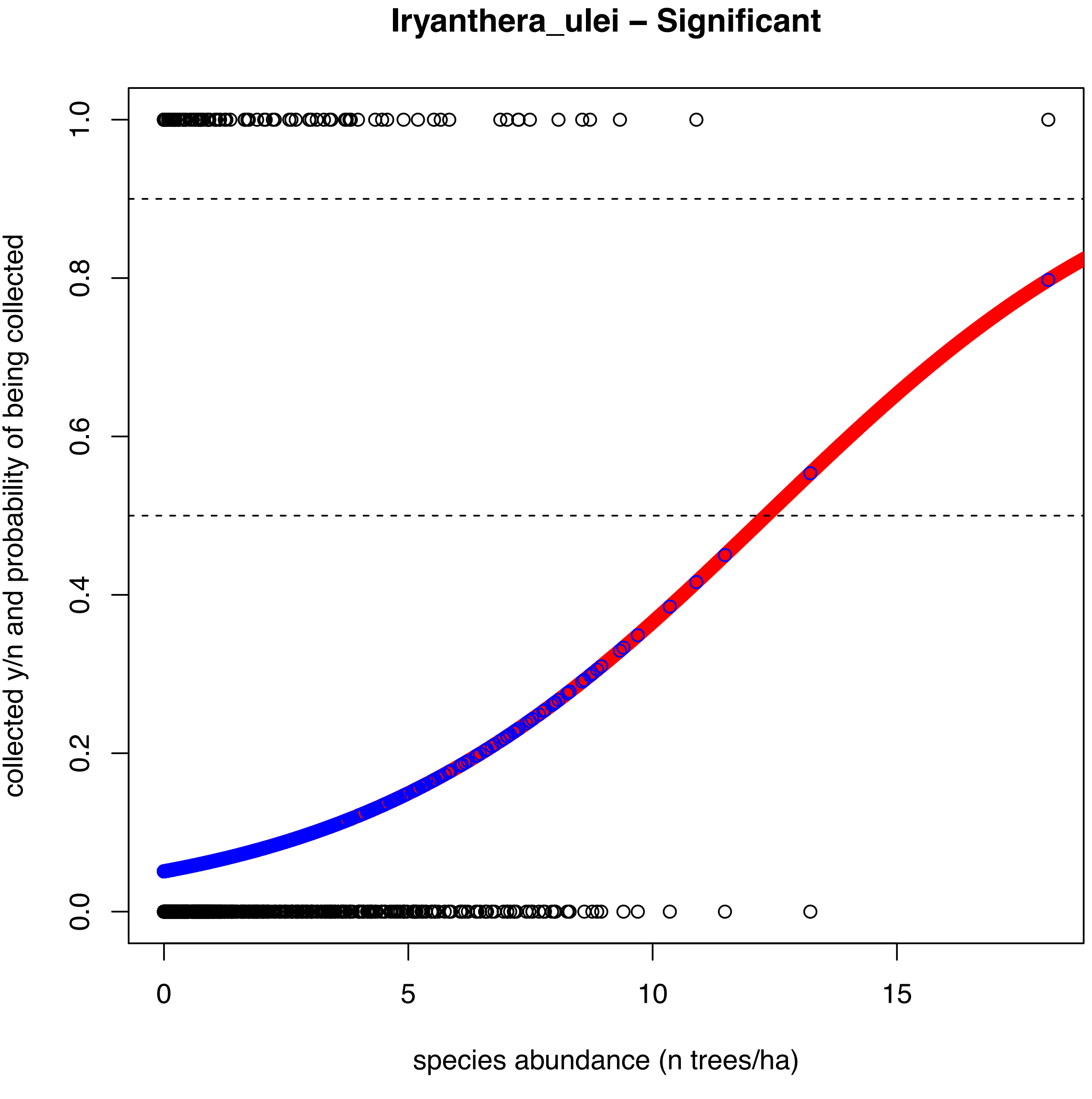

Iryanthera_ulei – Significant
collected y/n and probability of being collected
species abundance (n trees/ha)
0.0
0.2
0.4
0.6
0.8
1.0
0
5
10
15

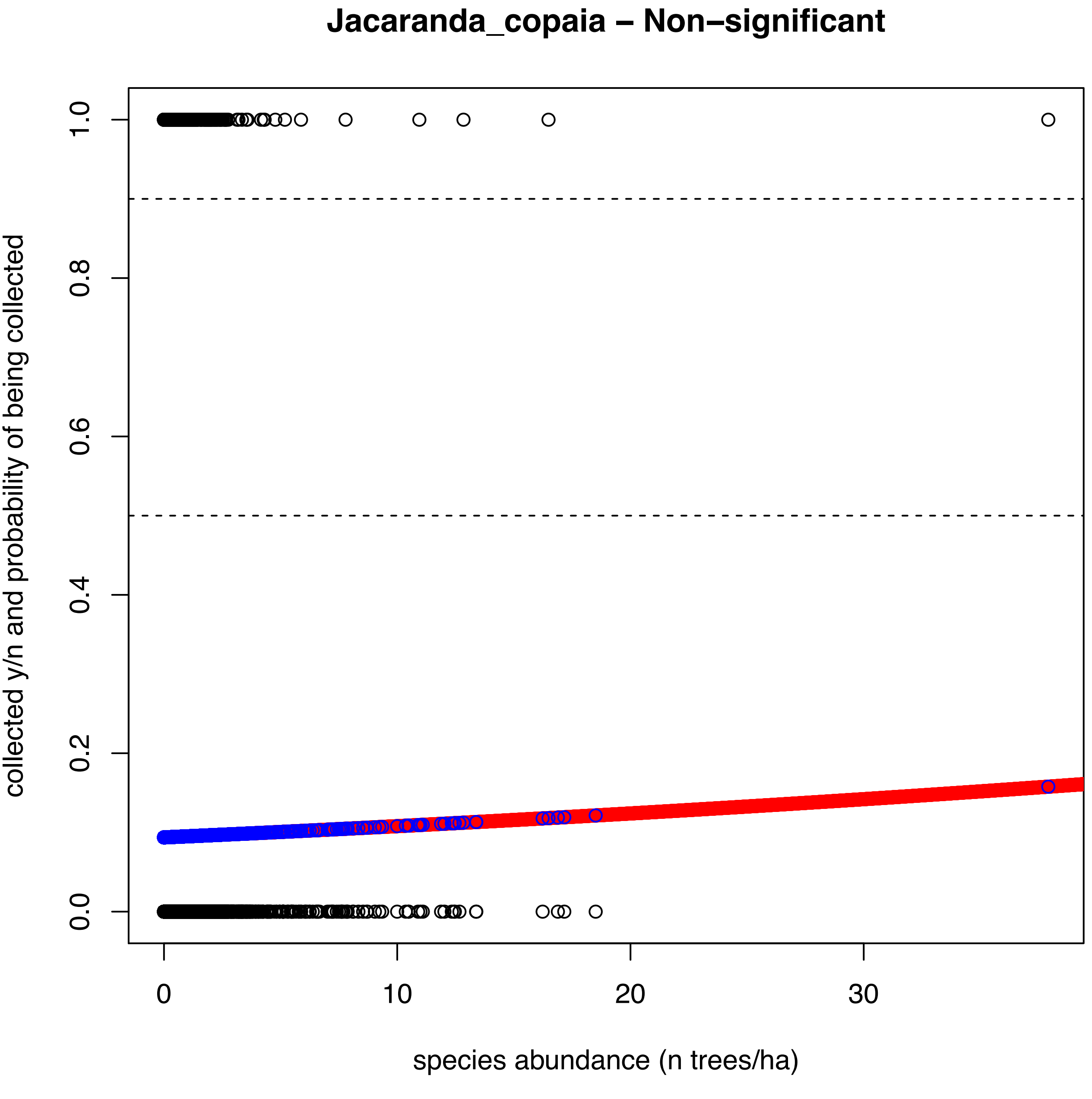

Jacaranda_copaia – Non–significant
collected y/n and probability of being collected
0.0
0.2
0.4
0.6
0.8
1.0
0
10
20
30
species abundance (n trees/ha)

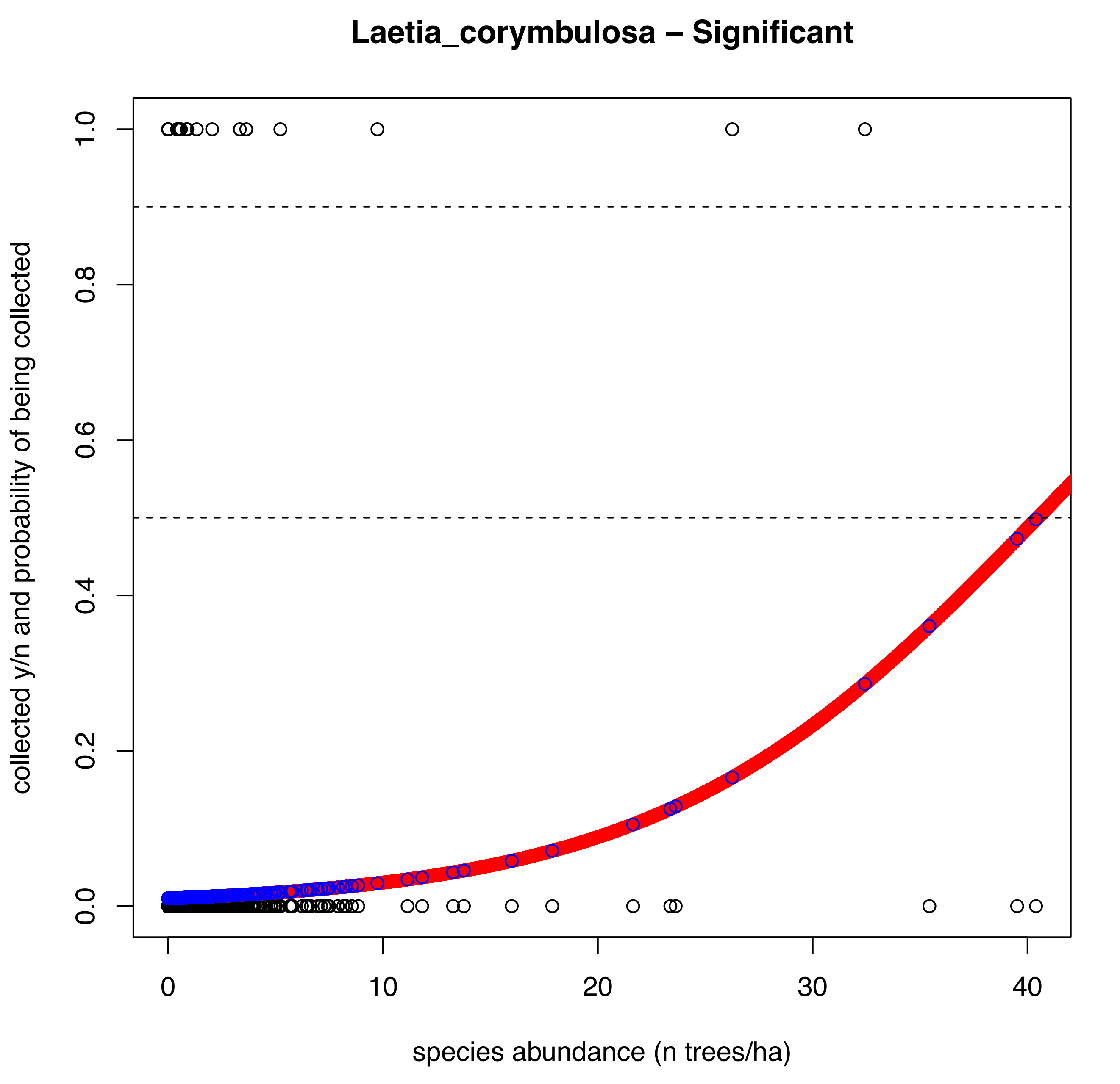

Laetia_corymbulosa – Significant
collected y/n and probability of being collected
species abundance (n trees/ha)
0.0
0.2
0.4
0.6
0.8
1.0
0
10
20
30
40

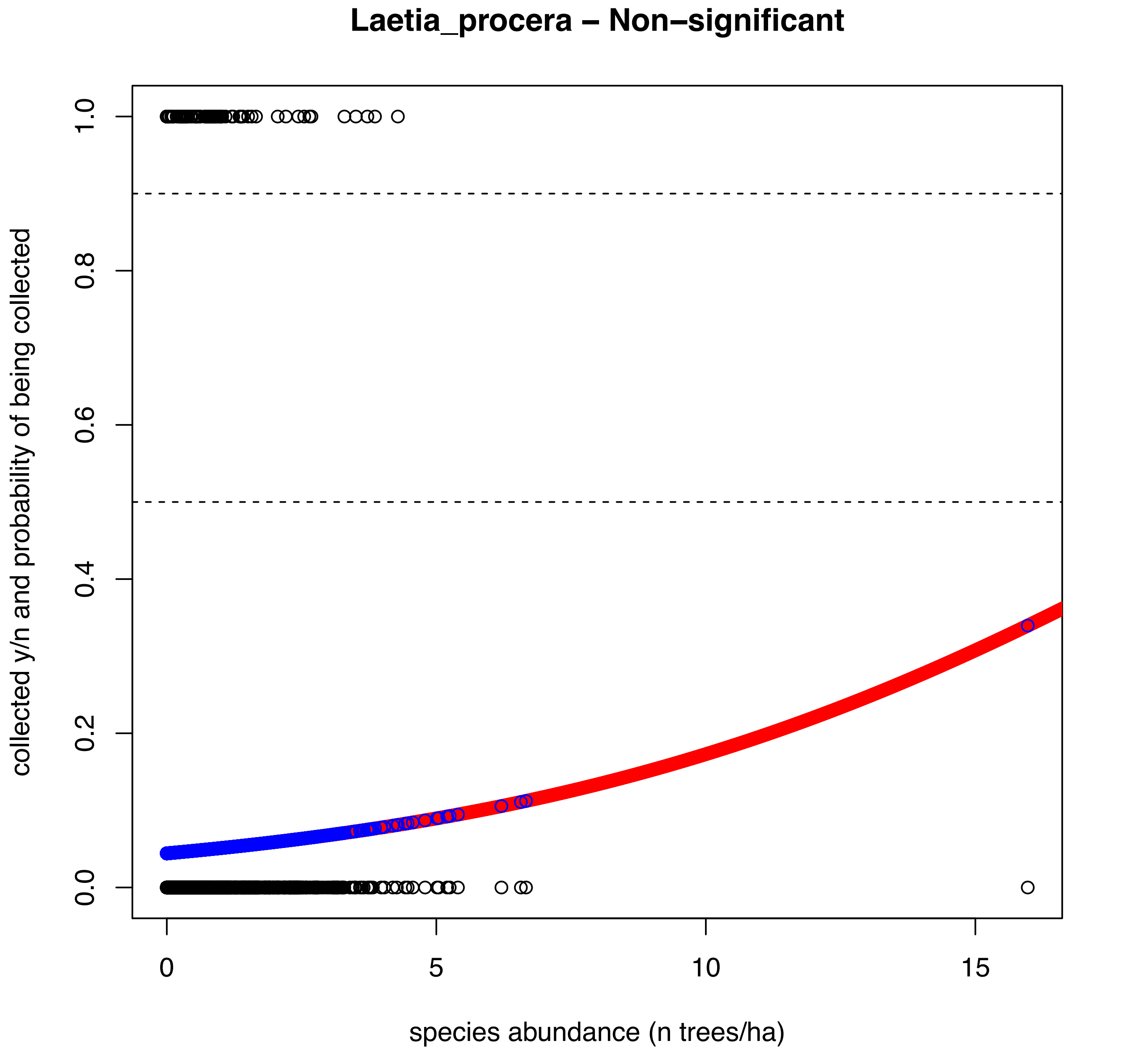

Laetia_procera – Non–significant
collected y/n and probability of being collected
species abundance (n trees/ha)
0.0
0.2
0.4
0.6
0.8
1.0
0
5
10
15

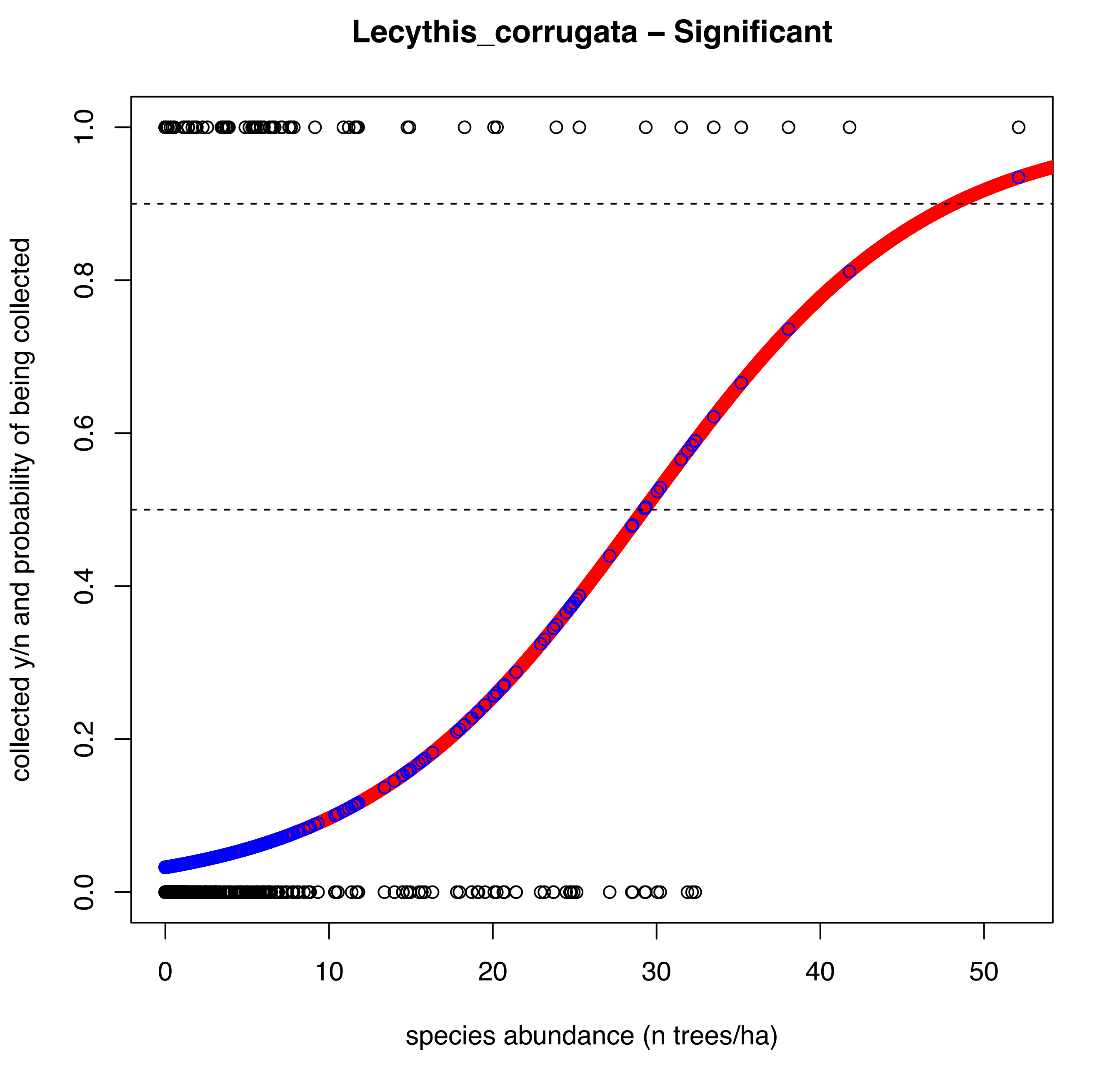

Lecythis_corrugata – Significant
collected y/n and probability of being collected
species abundance (n trees/ha)
0.0
0.2
0.4
0.6
0.8
1.0
0
10
20
30
40
50

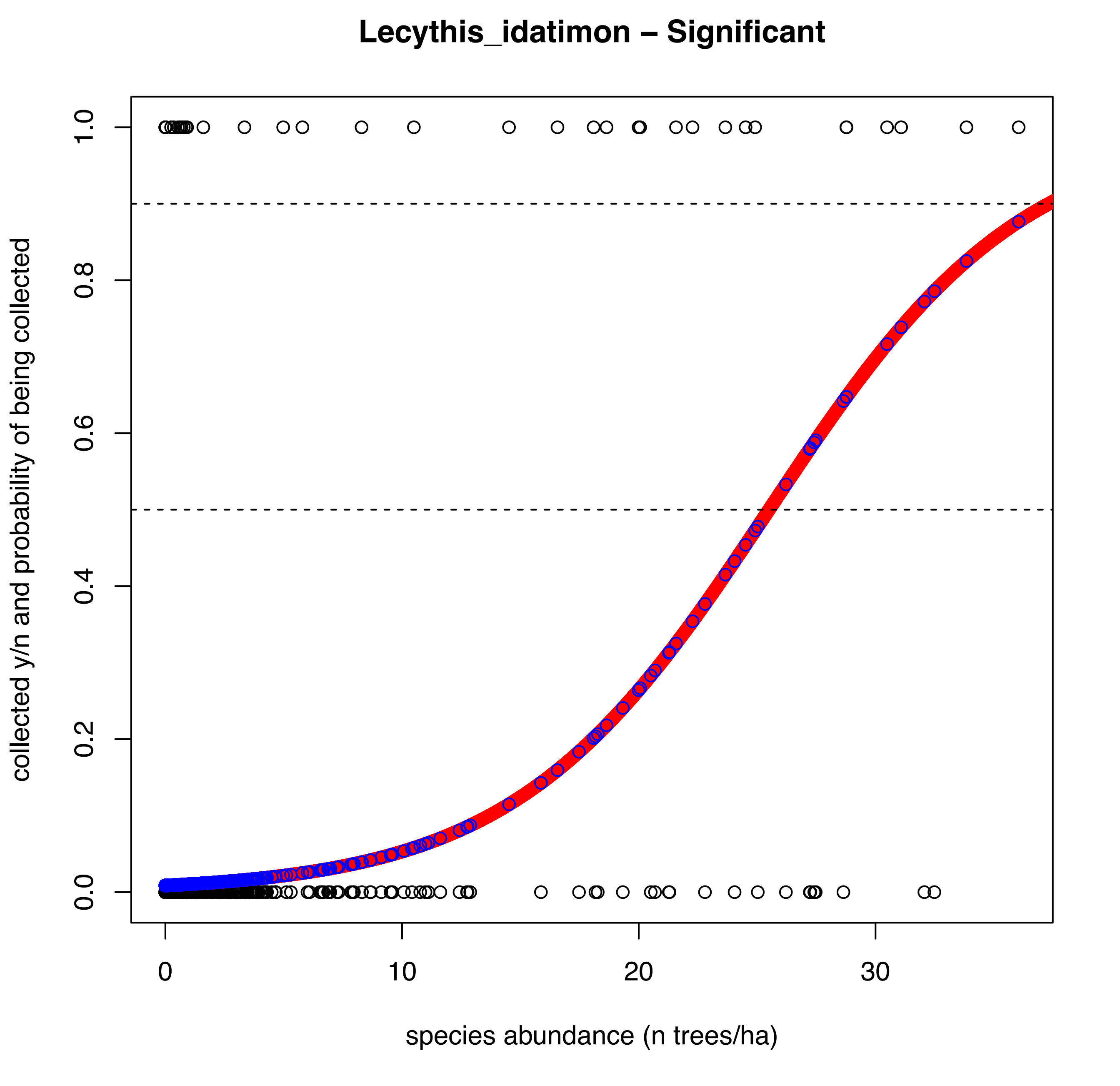
Lecythis_idatimon – Significant
1.0
0.8
0.6
0.4
0.2
0.0
0
10
20
30
collected y/n and probability of being collected
species abundance (n trees/ha)

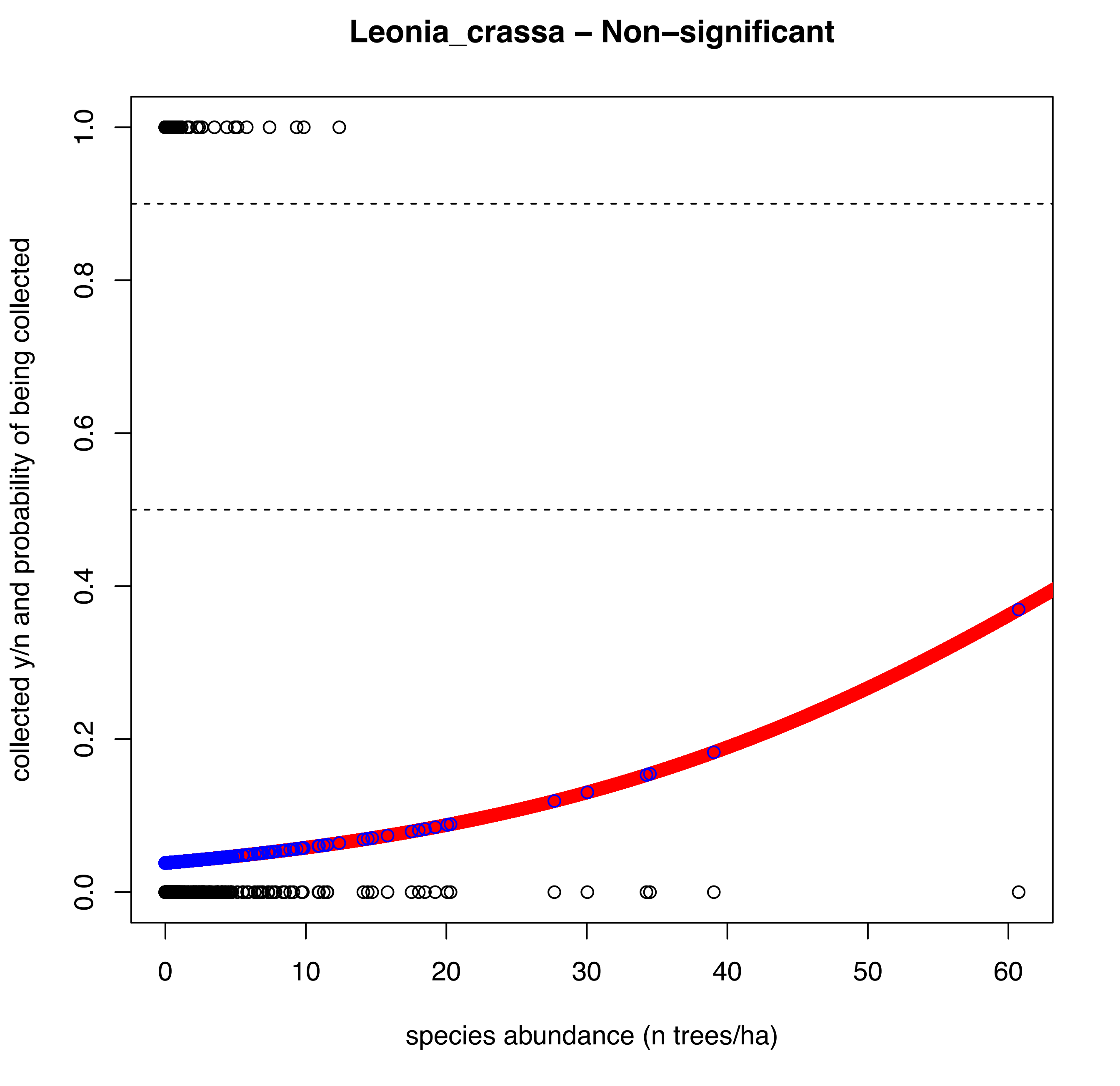
Leonia_crassa – Non–significant
collected y/n and probability of being collected
species abundance (n trees/ha)
0.0
0.2
0.4
0.6
0.8
1.0
0
10
20
30
40
50
60

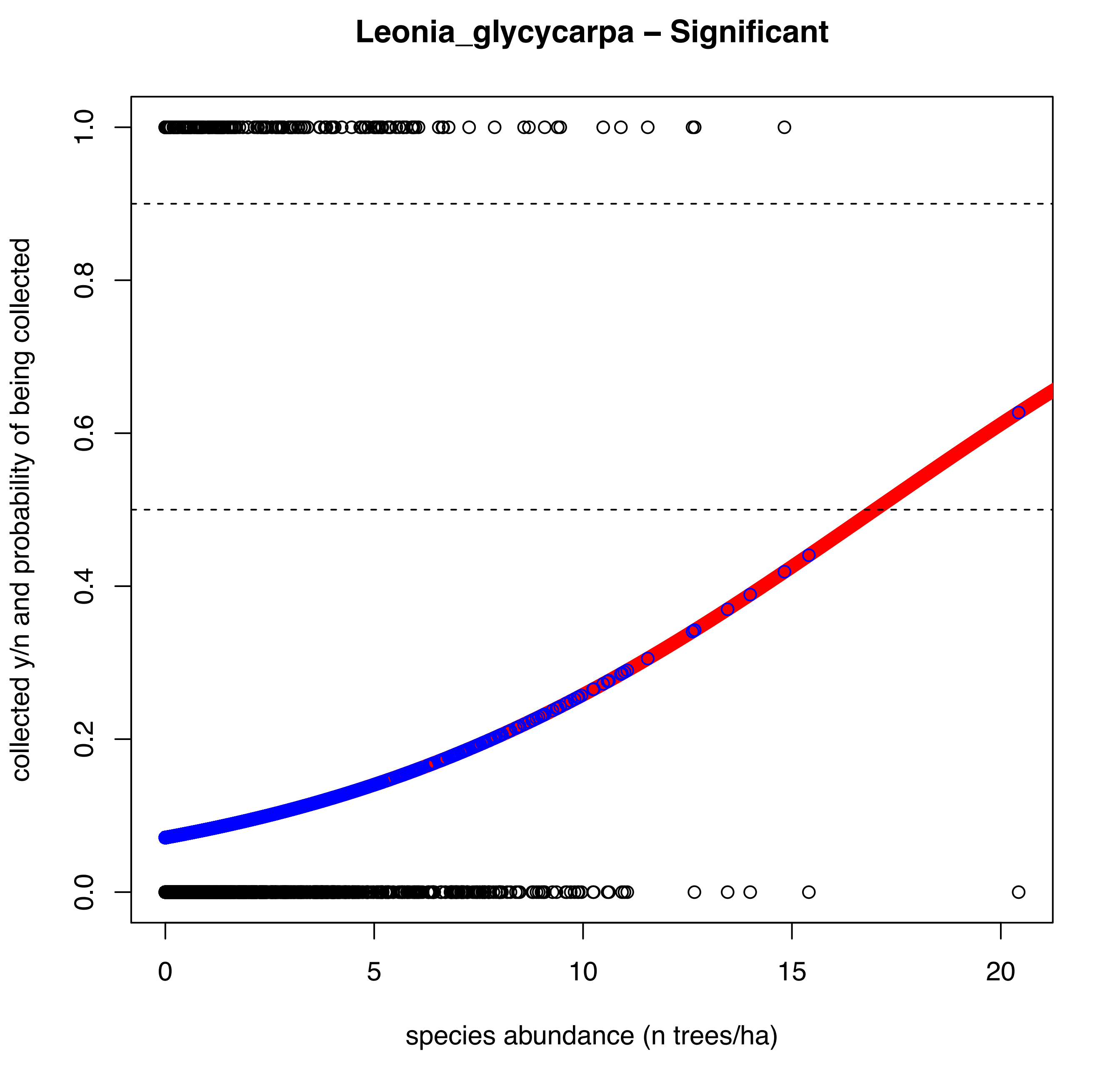

Leonia_glycycarpa – Significant
collected y/n and probability of being collected
species abundance (n trees/ha)
0.0
0.2
0.4
0.6
0.8
1.0
0
5
10
15
20

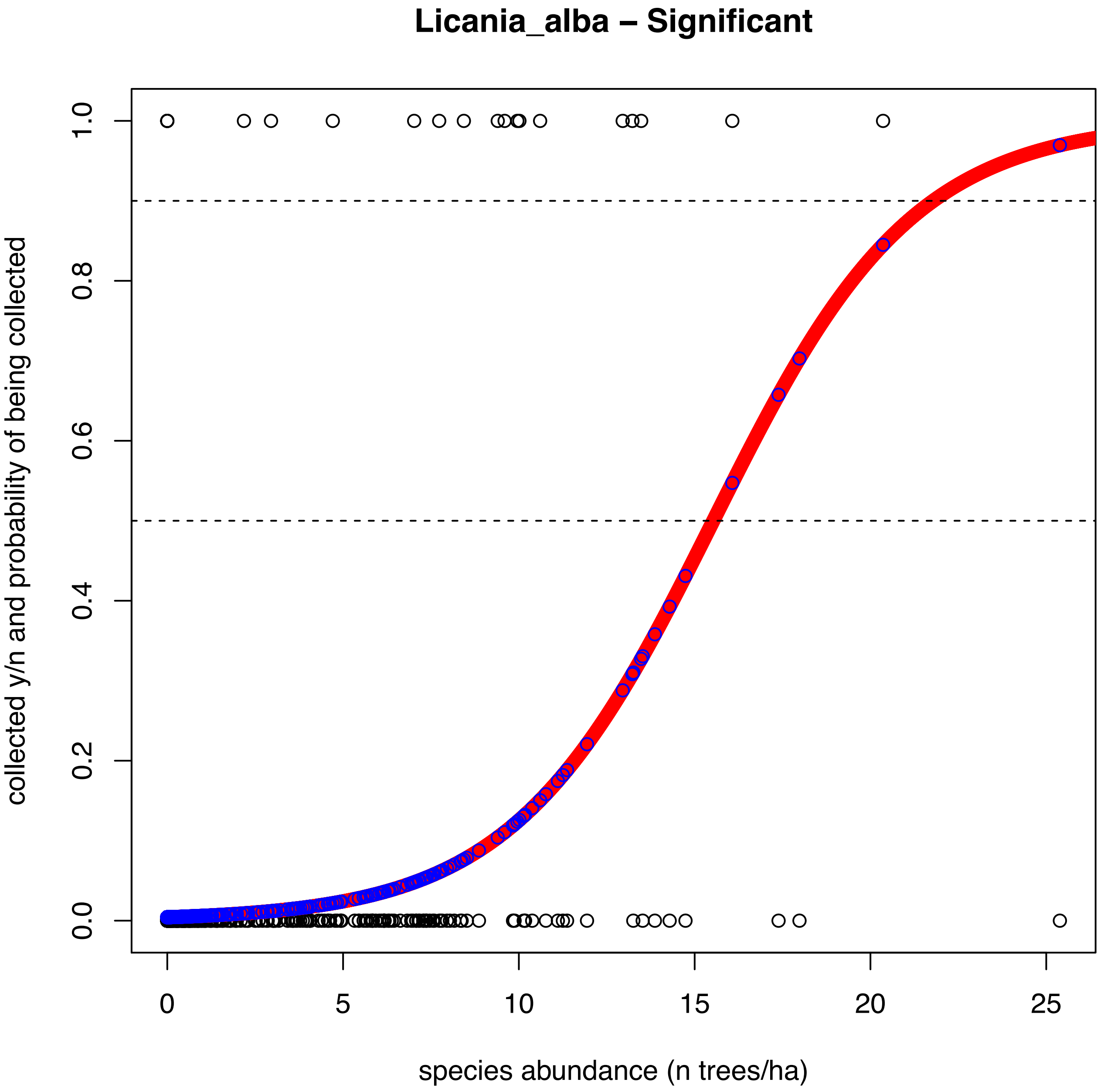

Licania_alba – Significant
collected y/n and probability of being collected
species abundance (n trees/ha)
0.0
0.2
0.4
0.6
0.8
1.0
0
5
10
15
20
25

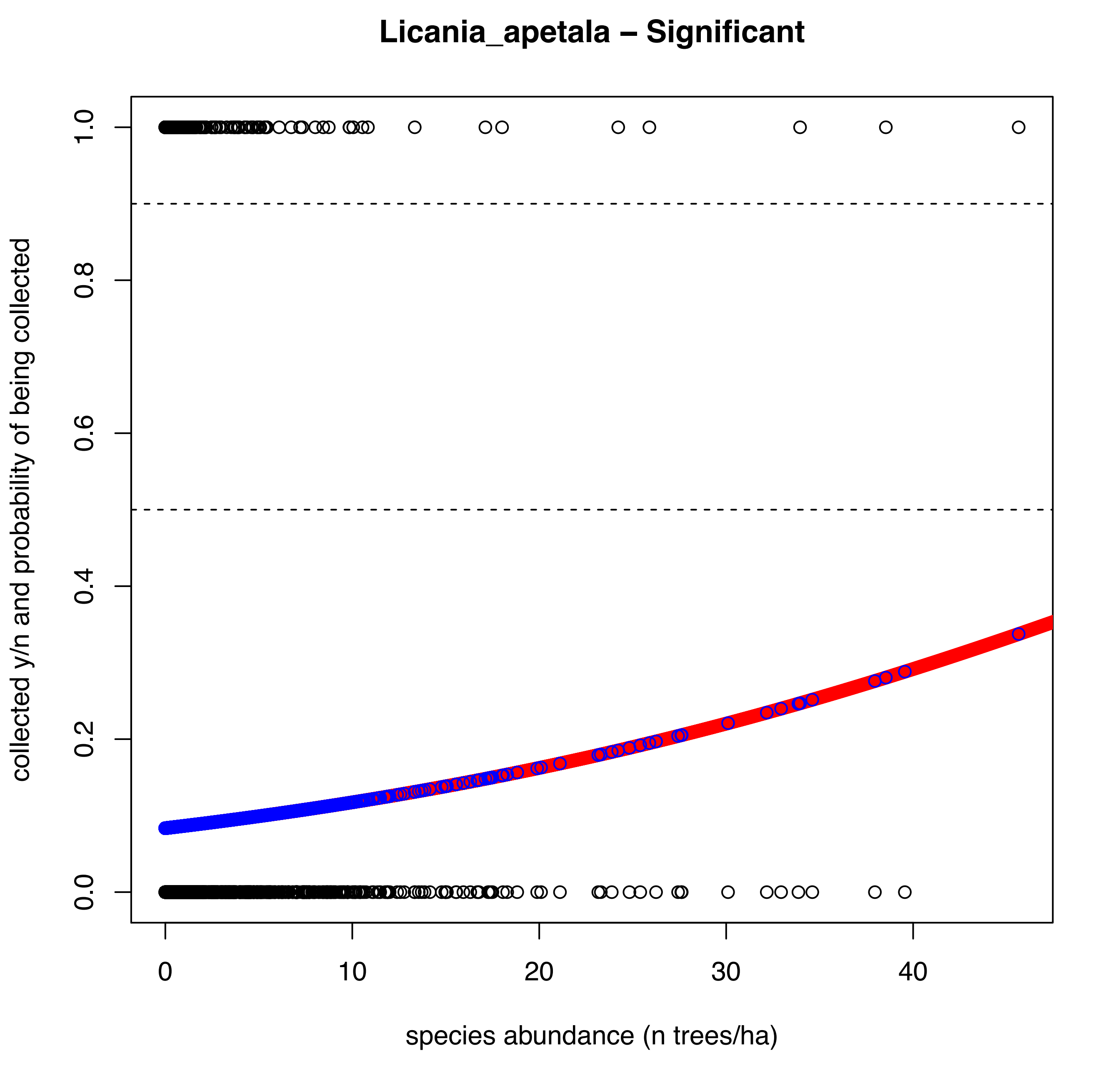
Licania_apetala – Significant
collected y/n and probability of being collected
species abundance (n trees/ha)
0.0
0.2
0.4
0.6
0.8
1.0
0
10
20
30
40

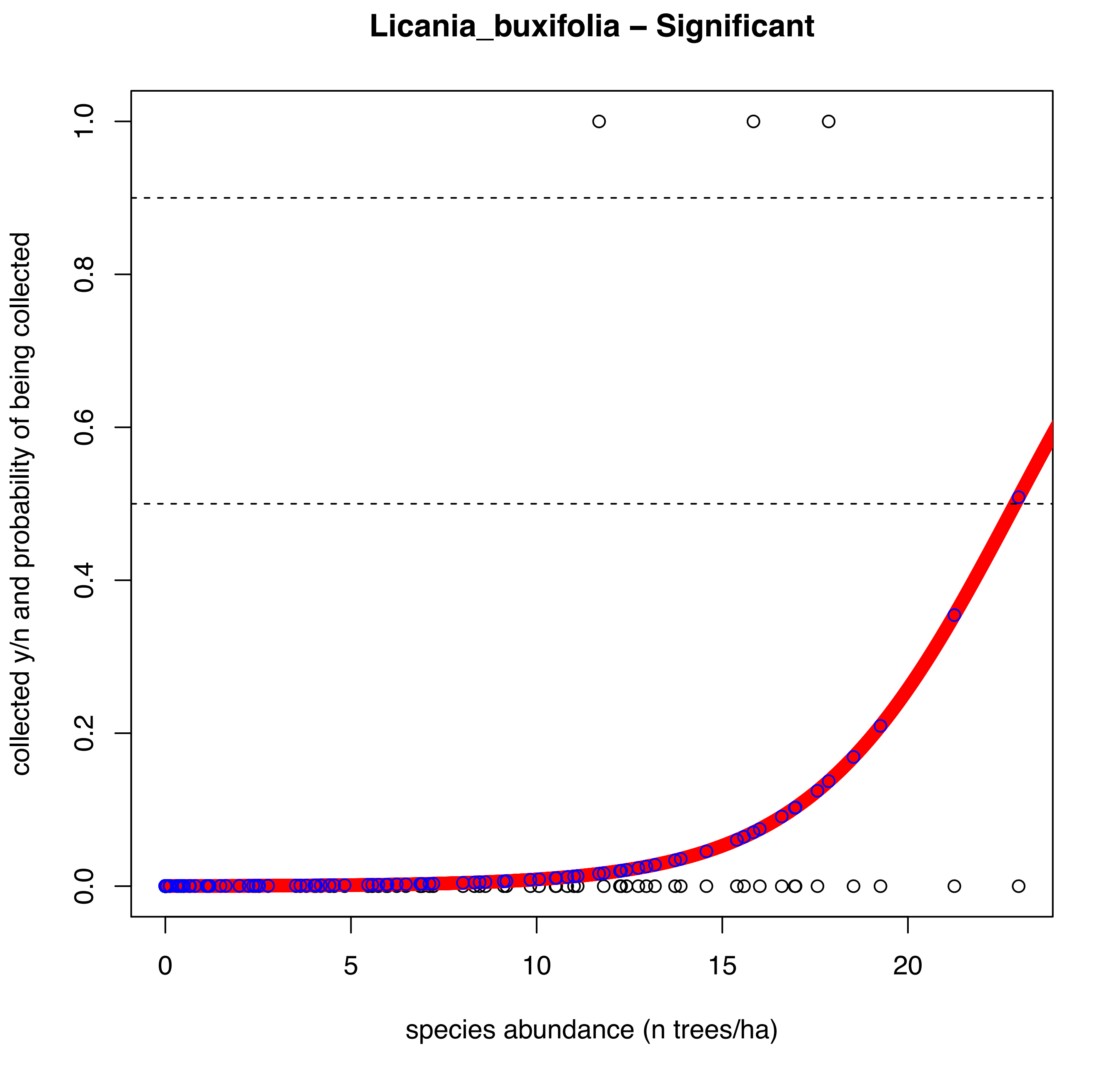
Licania_buxifolia – Significant
collected y/n and probability of being collected
species abundance (n trees/ha)
0.0
0.2
0.4
0.6
0.8
1.0
0
5
10
15
20

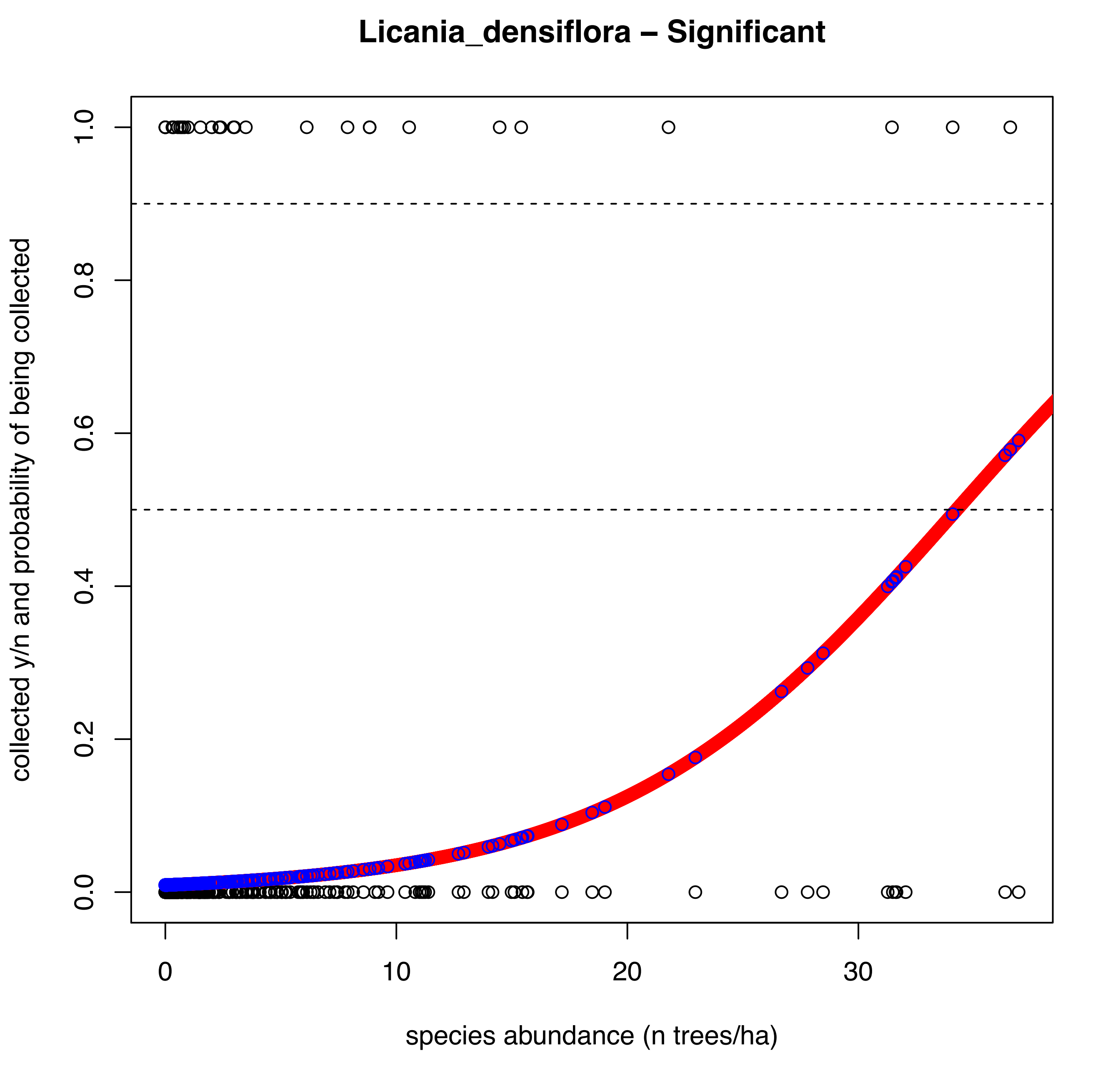

Licania_densiflora – Significant
collected y/n and probability of being collected
species abundance (n trees/ha)
0.0
0.2
0.4
0.6
0.8
1.0
0
10
20
30

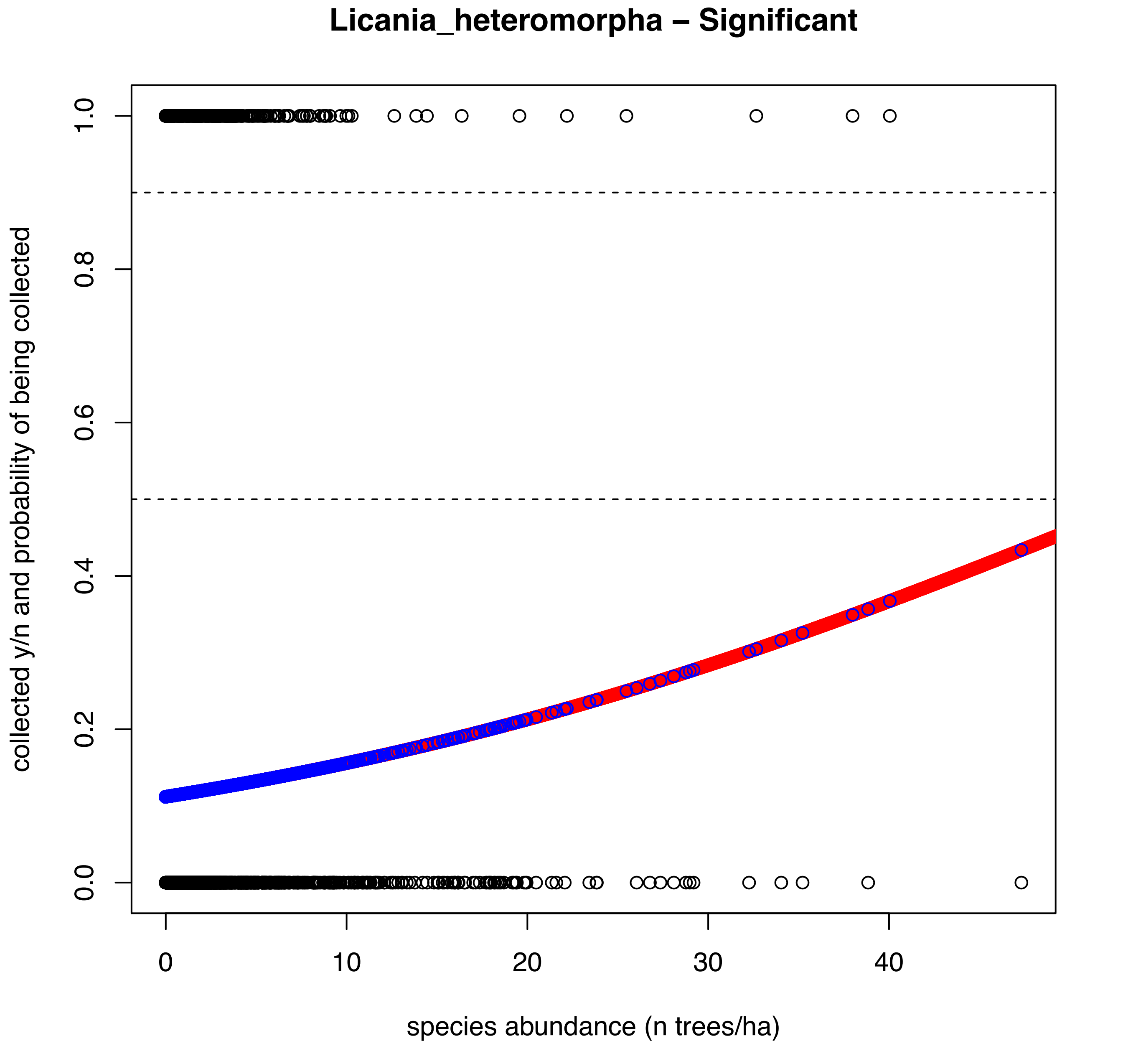
Licania_heteromorpha – Significant
collected y/n and probability of being collected
species abundance (n trees/ha)
0.0
0.2
0.4
0.6
0.8
1.0
0
10
20
30
40

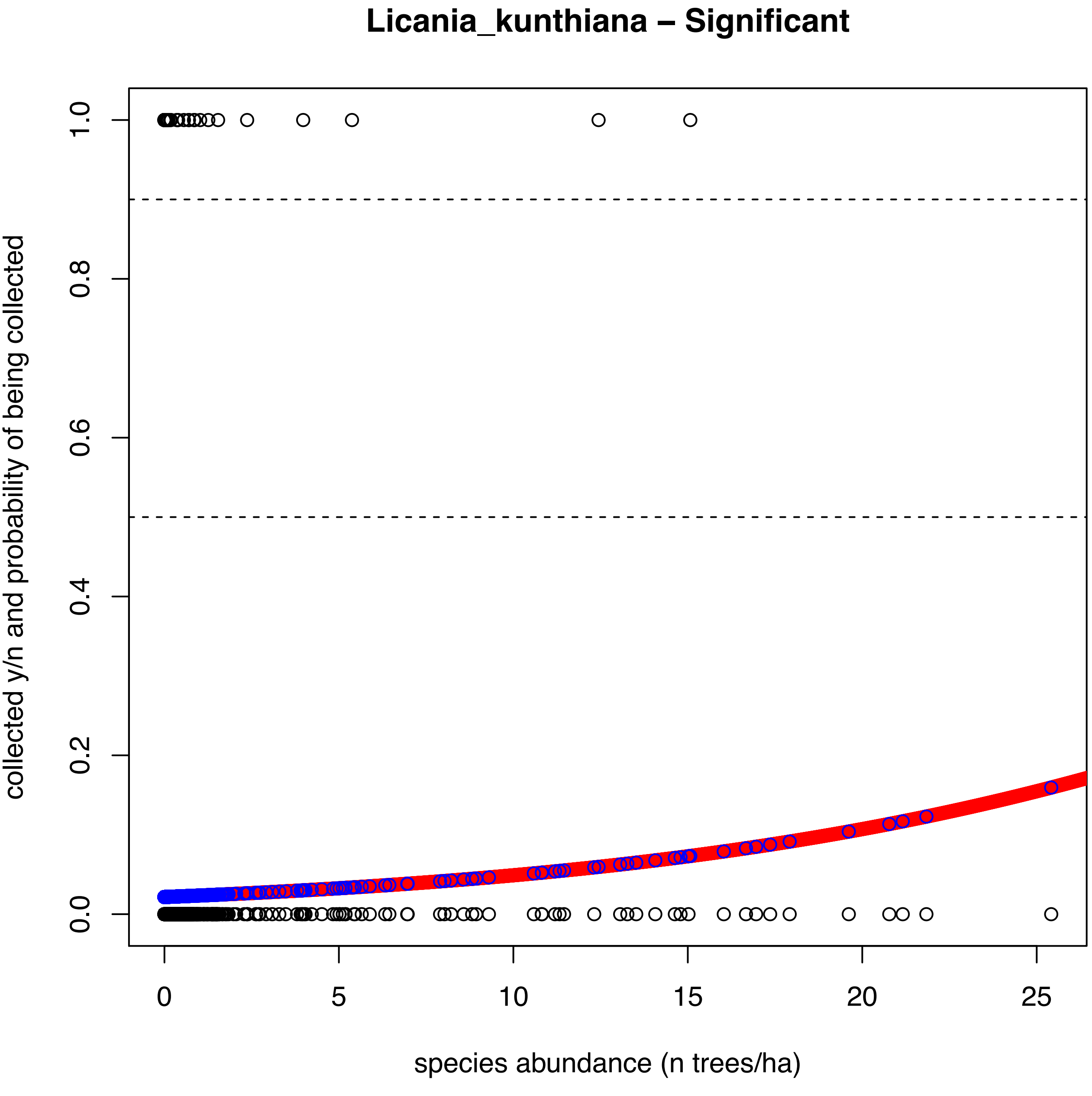
Licania_kunthiana – Significant
collected y/n and probability of being collected
species abundance (n trees/ha)
0.0
0.2
0.4
0.6
0.8
1.0
0
5
10
15
20
25

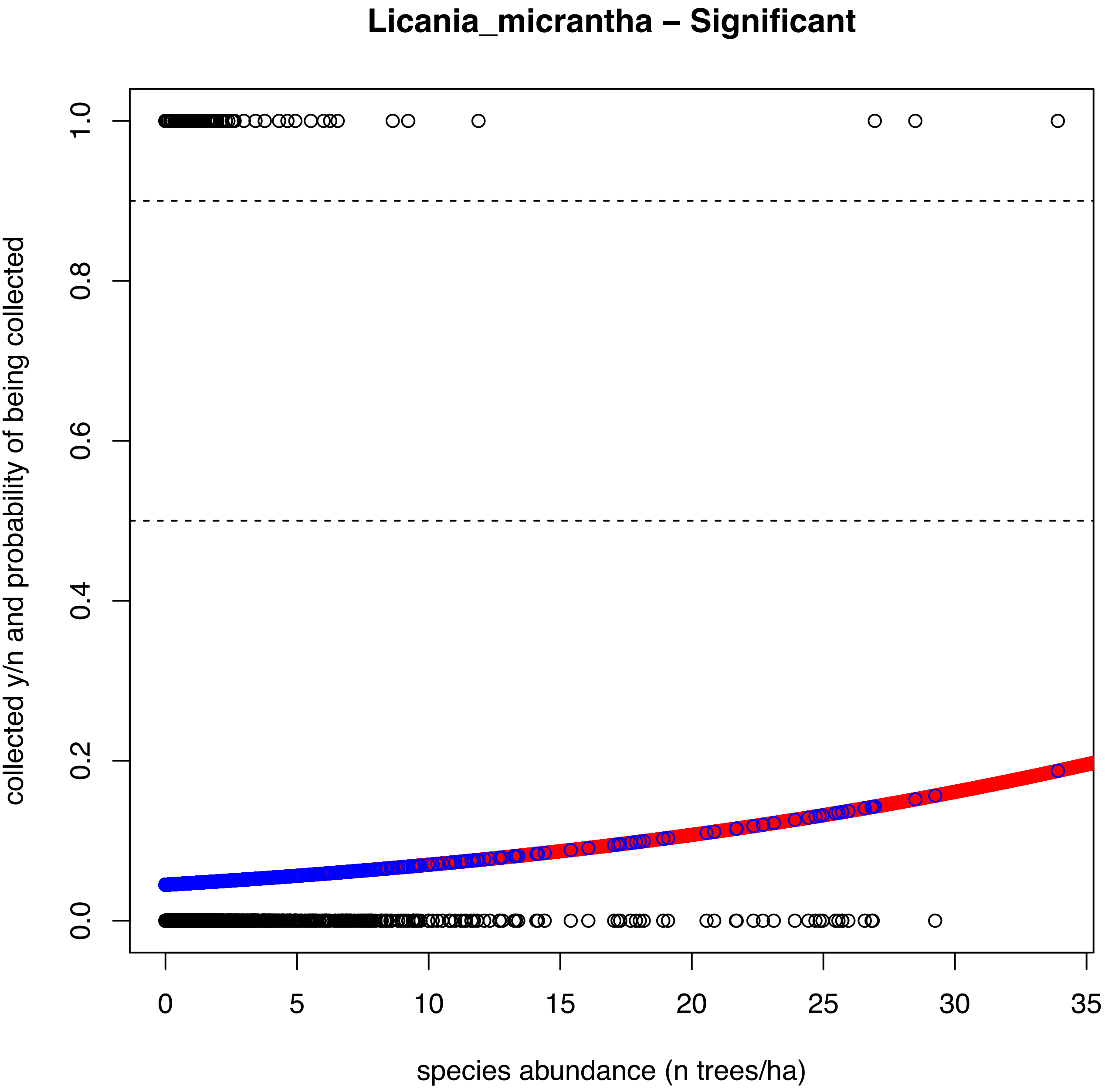
Licania_micrantha – Significant
collected y/n and probability of being collected
species abundance (n trees/ha)
0.0
0.2
0.4
0.6
0.8
1.0
0
5
10
15
20
25
30
35

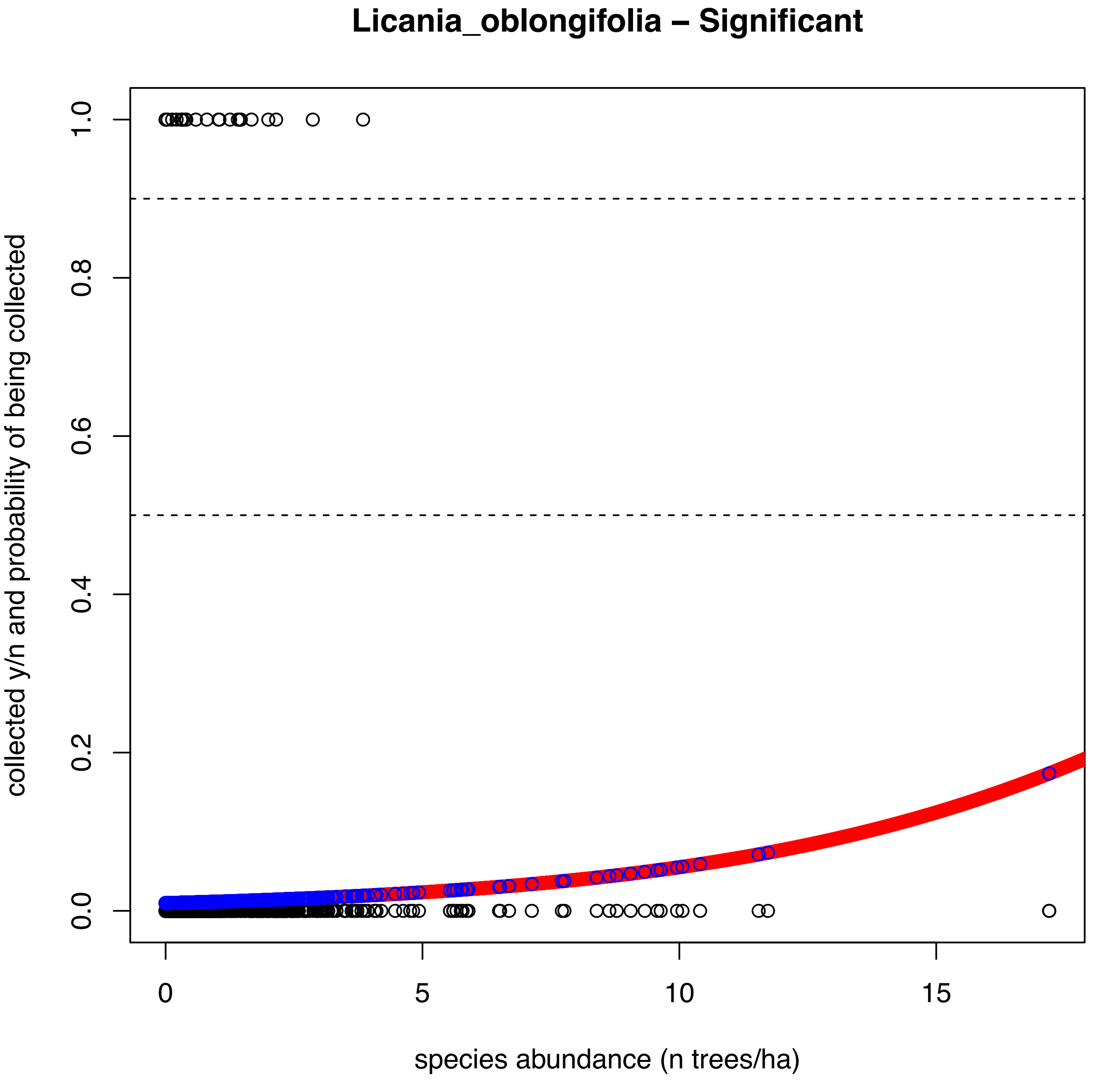
Licania_oblongifolia – Significant
collected y/n and probability of being collected
species abundance (n trees/ha)
0.0
0.2
0.4
0.6
0.8
1.0
0
5
10
15

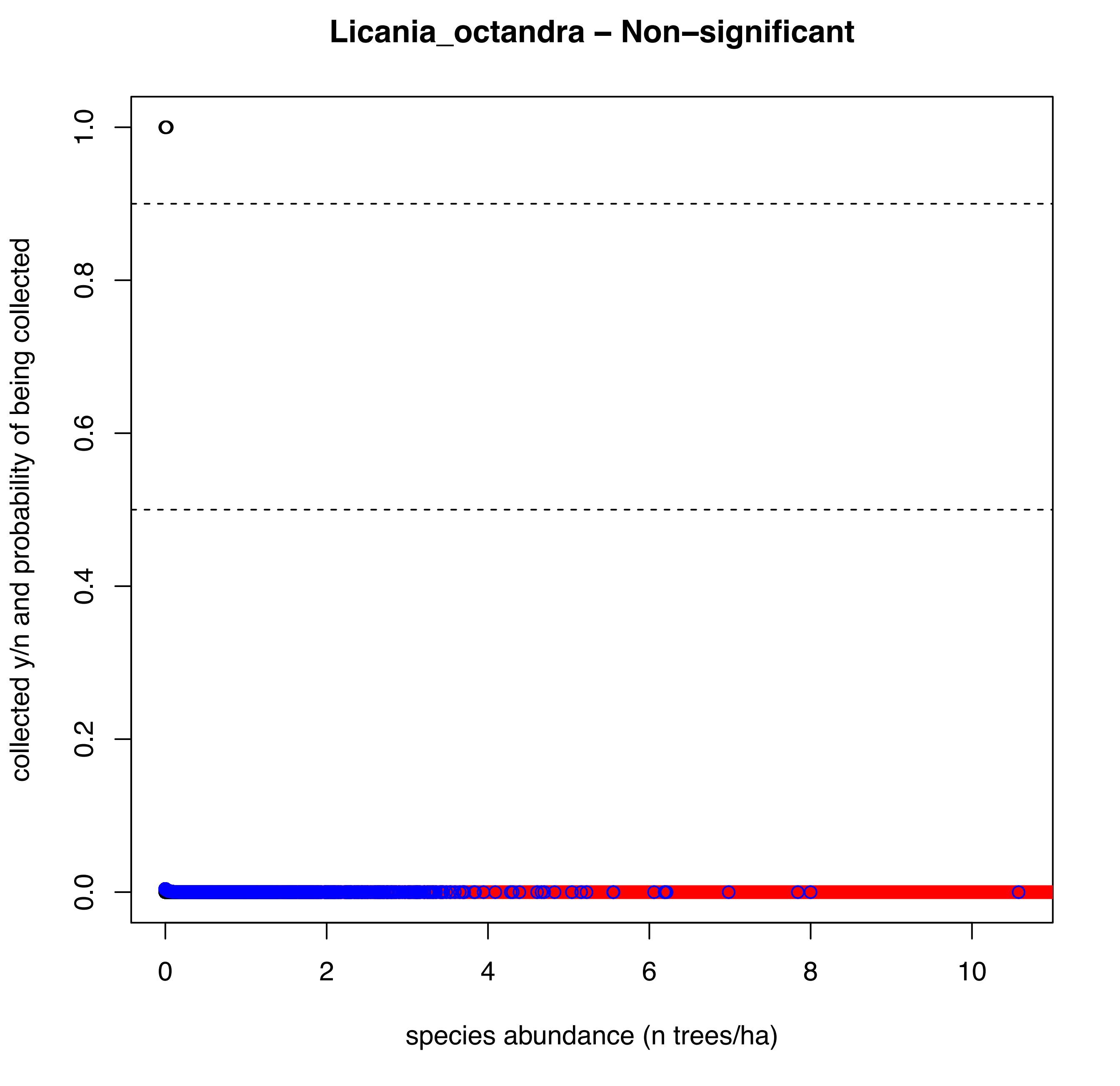

Licania_octandra – Non–significant
collected y/n and probability of being collected
species abundance (n trees/ha)
0.0
0.2
0.4
0.6
0.8
1.0
0
2
4
6
8
10

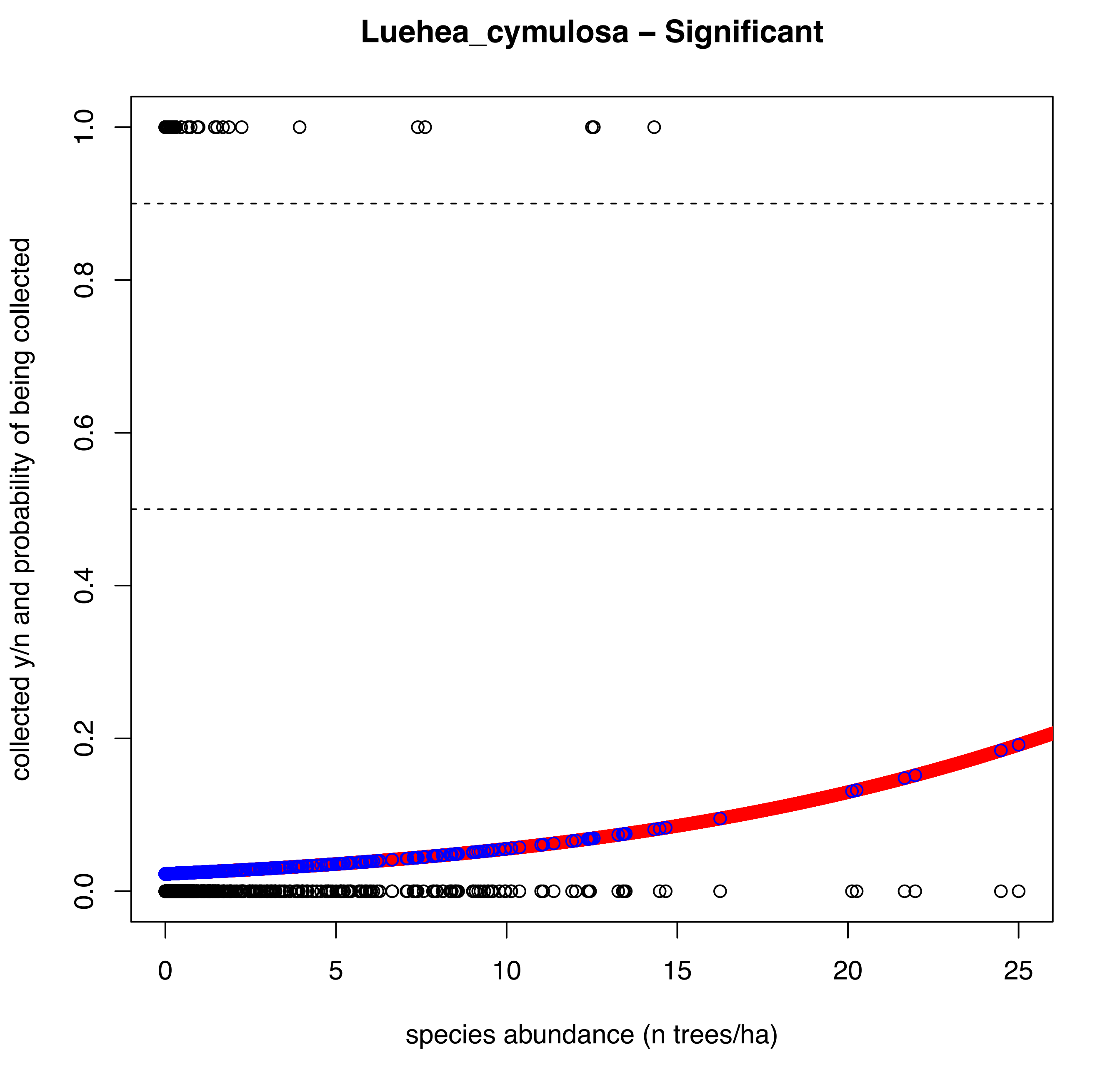

Luehea_cymulosa – Significant
collected y/n and probability of being collected
species abundance (n trees/ha)
0.0
0.2
0.4
0.6
0.8
1.0
0
5
10
15
20
25

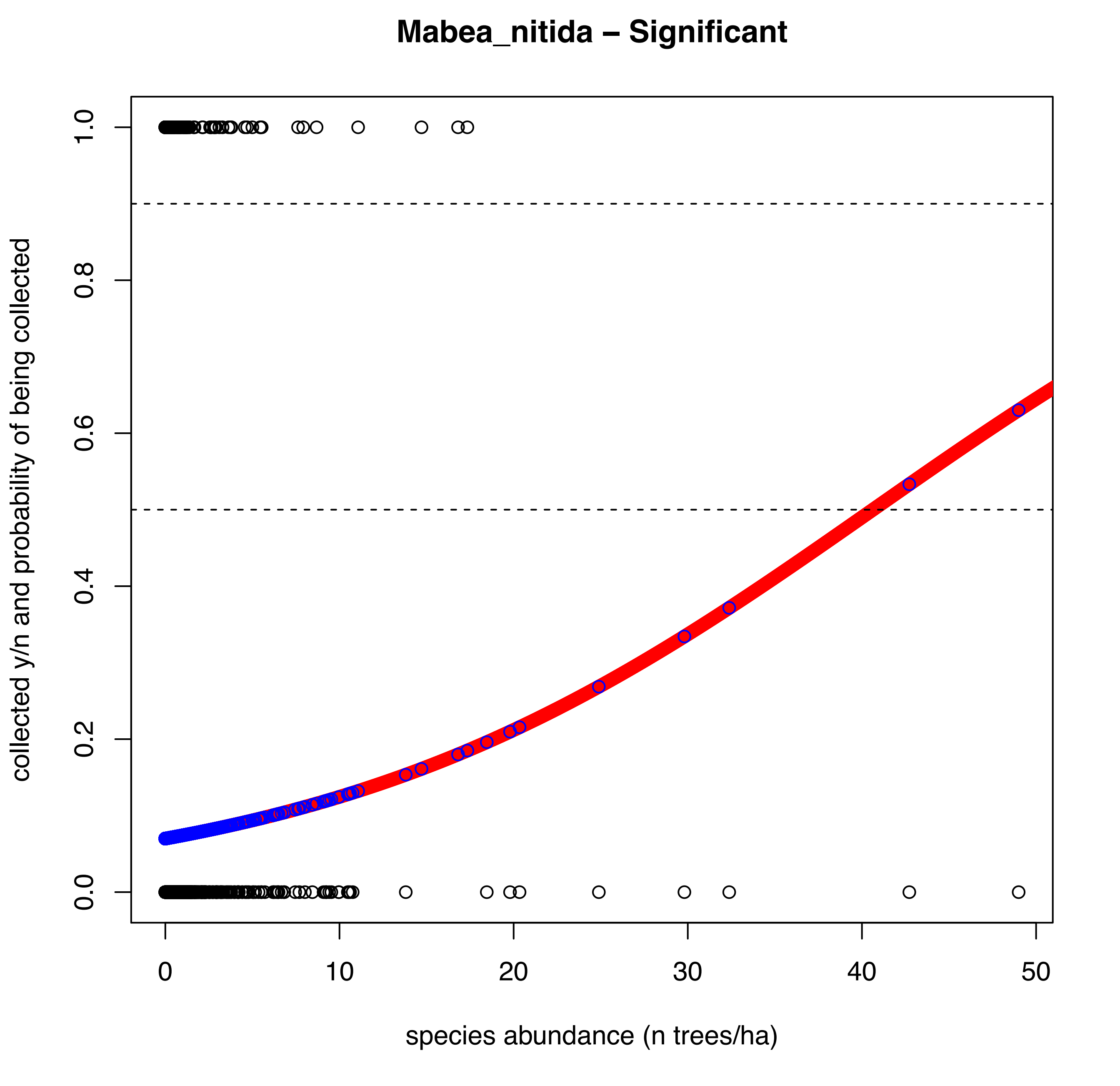
Mabea_nitida – Significant
collected y/n and probability of being collected
0.0
0.2
0.4
0.6
0.8
1.0
0
10
20
30
40
50
species abundance (n trees/ha)

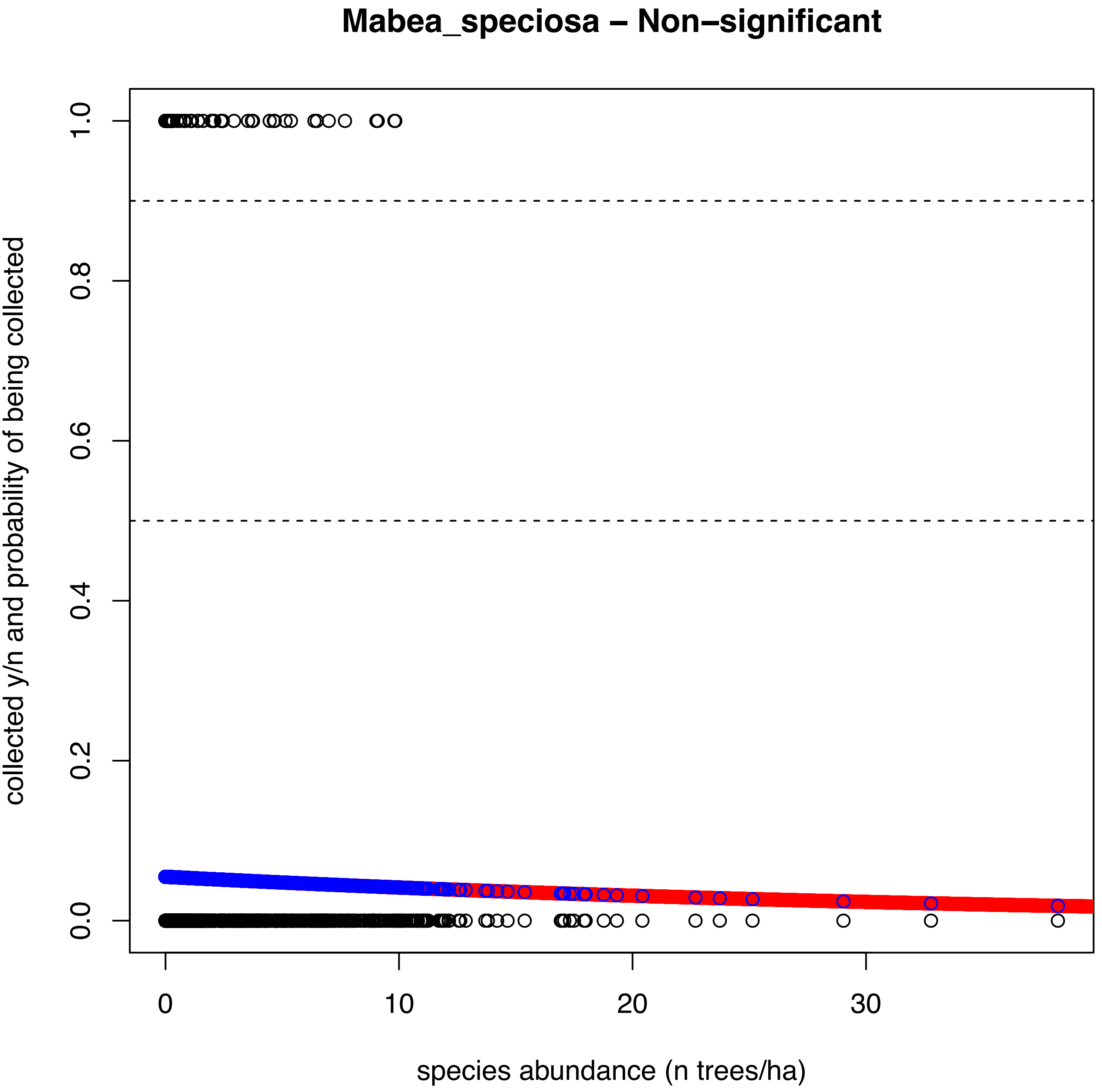

Mabea_speciosa – Non–significant
collected y/n and probability of being collected
species abundance (n trees/ha)
0.0
0.2
0.4
0.6
0.8
1.0
0
10
20
30

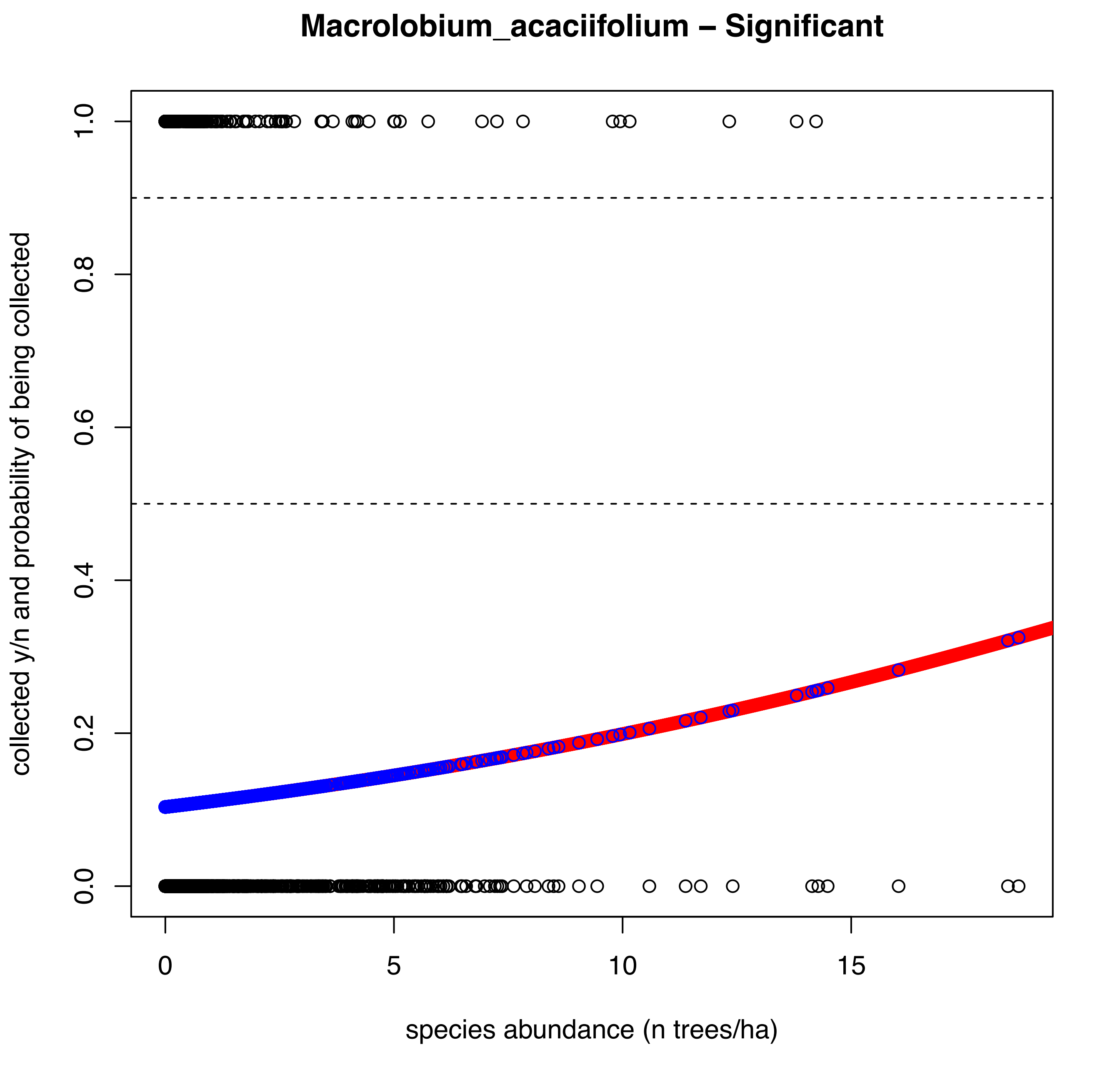
Macrolobium_acaciifolium – Significant
collected y/n and probability of being collected
species abundance (n trees/ha)
0.0
0.2
0.4
0.6
0.8
1.0
0
5
10
15

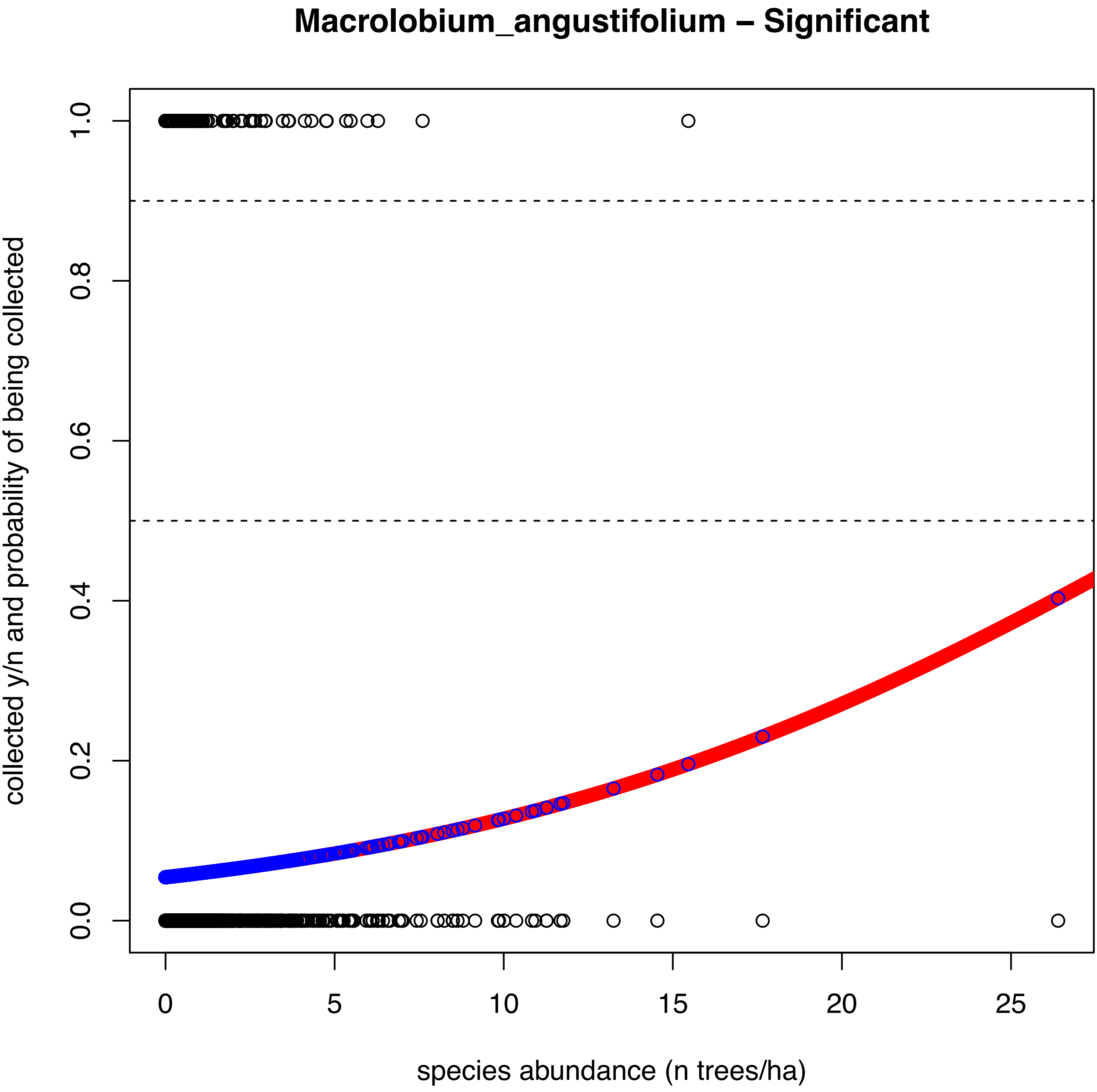

Macrolobium_angustifolium – Significant
collected y/n and probability of being collected
species abundance (n trees/ha)
0.0
0.2
0.4
0.6
0.8
1.0
0
5
10
15
20
25

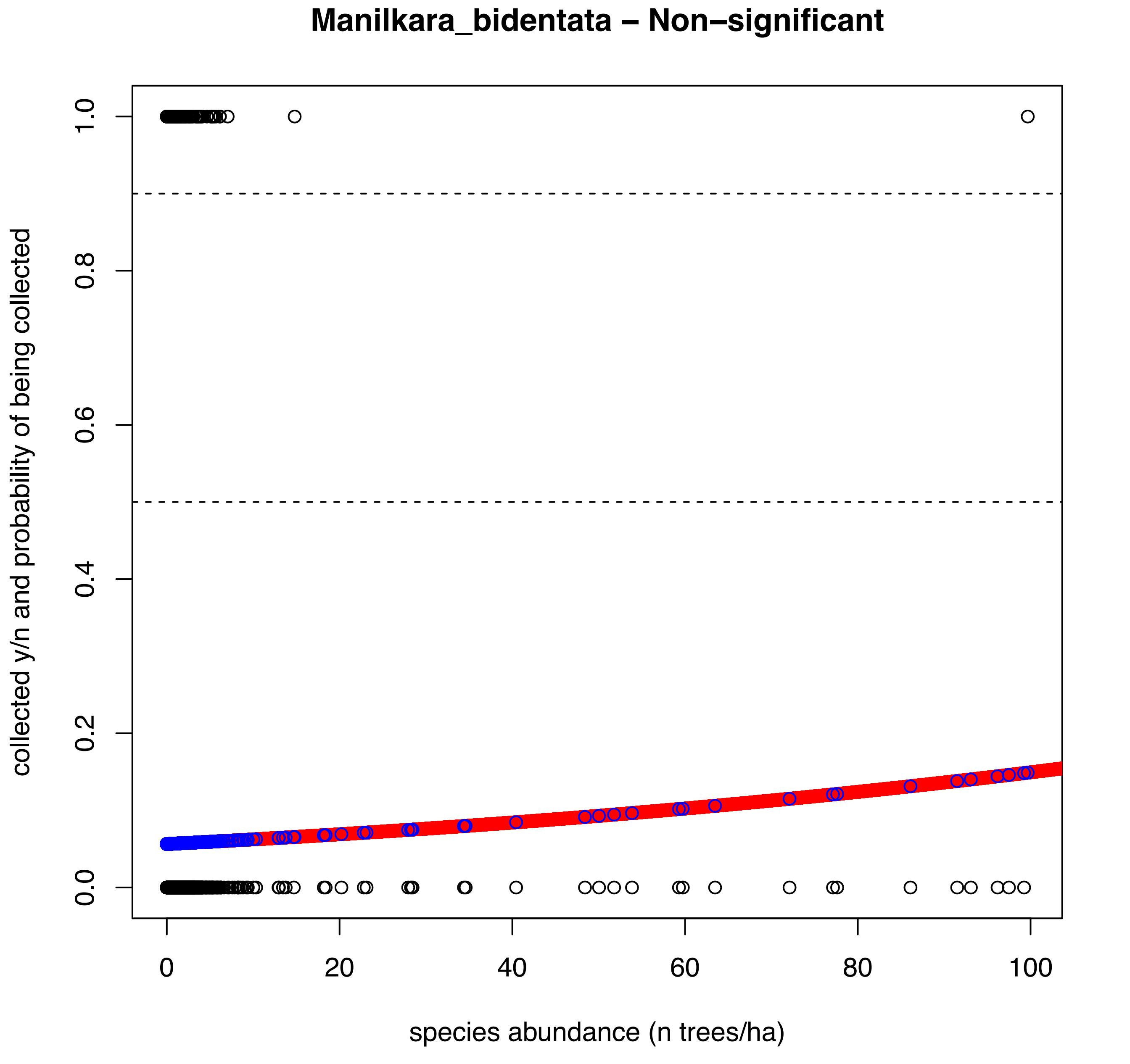

Manilkara_bidentata – Non−significant
collected y/n and probability of being collected
species abundance (n trees/ha)
0.0
0.2
0.4
0.6
0.8
1.0
0
20
40
60
80
100

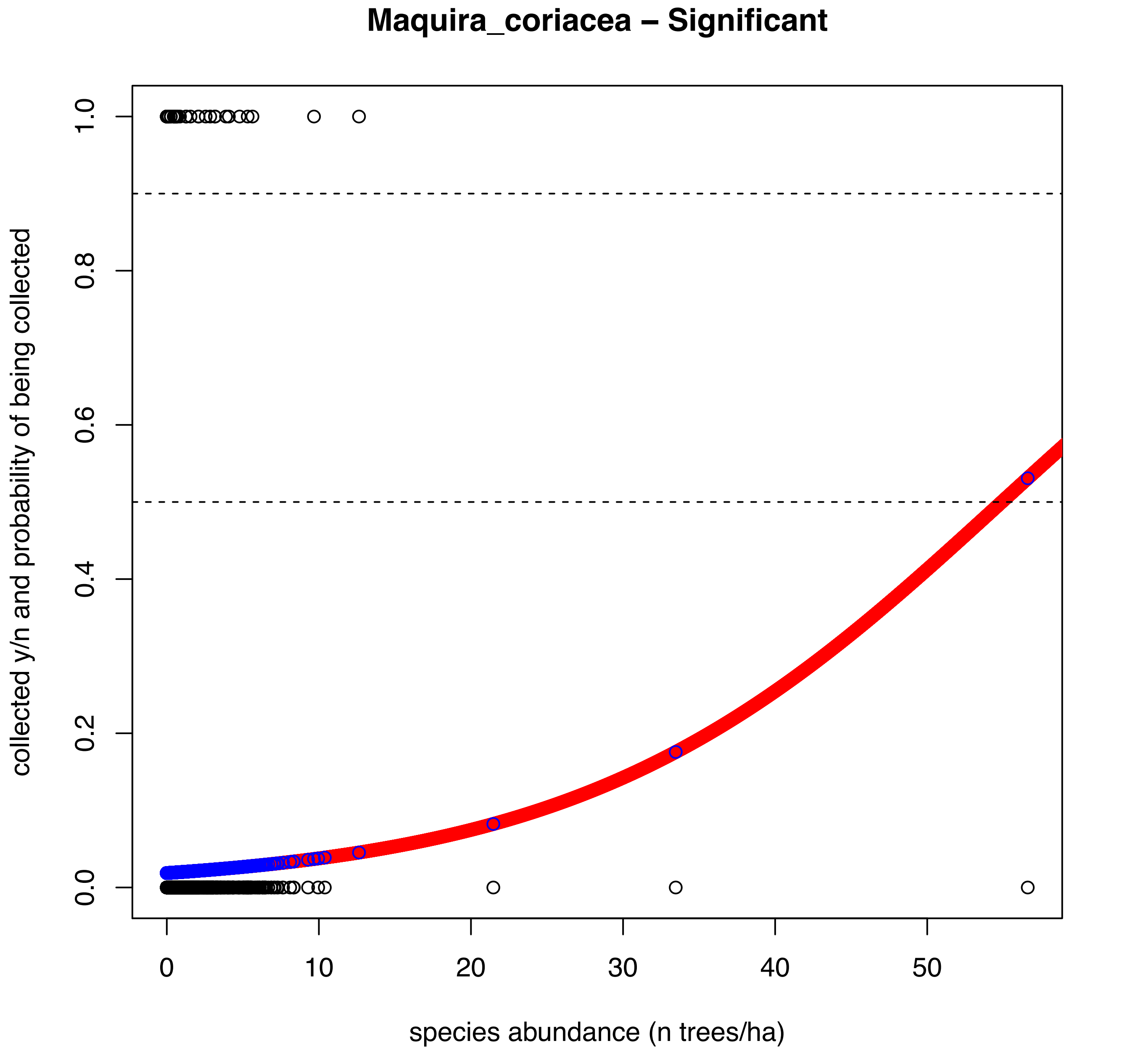

Maquira_coriacea – Significant
collected y/n and probability of being collected
species abundance (n trees/ha)
0.0
0.2
0.4
0.6
0.8
1.0
0
10
20
30
40
50

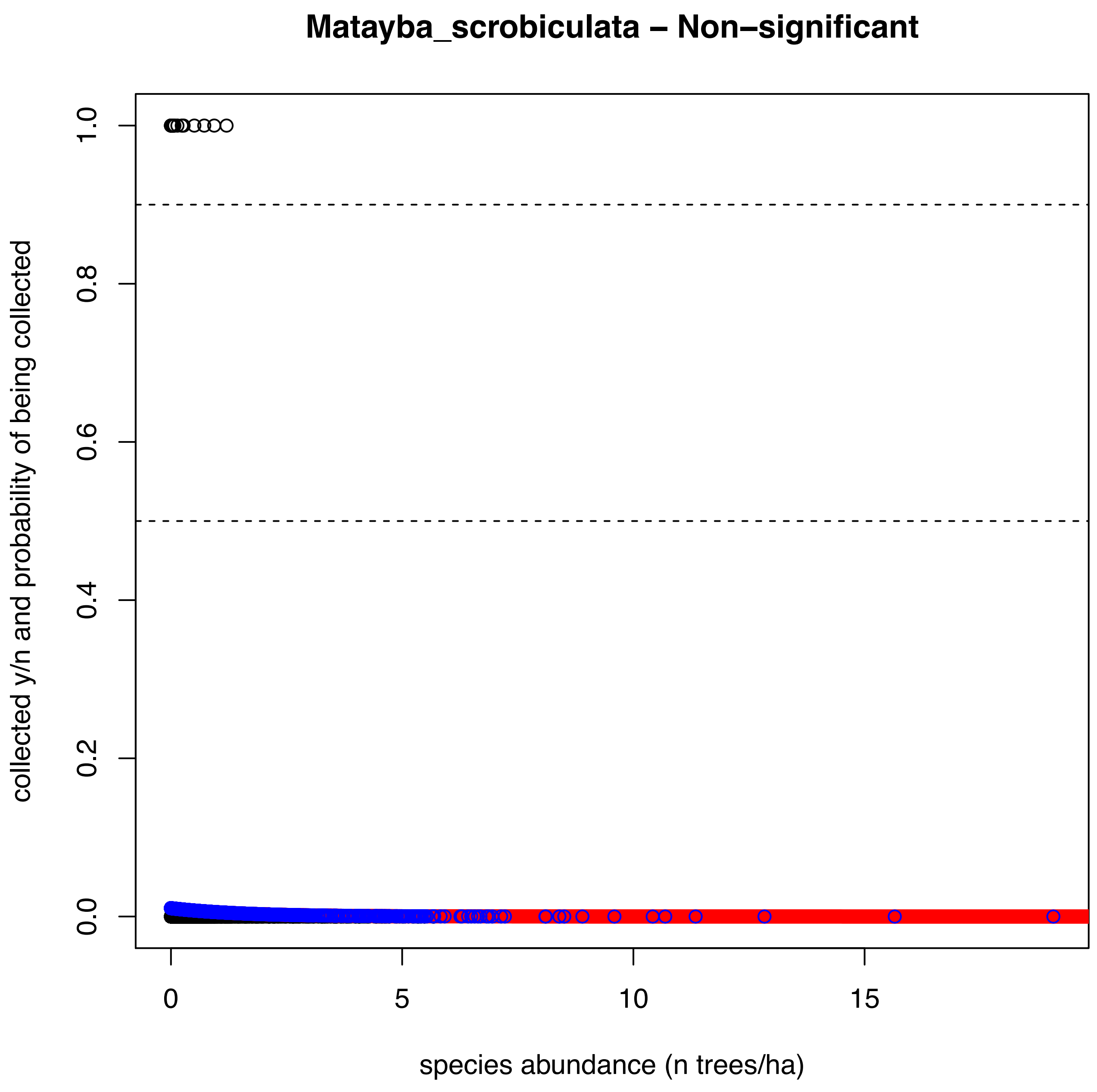
Matayba_scrobiculata – Non–significant
collected y/n and probability of being collected
species abundance (n trees/ha)
0.0
0.2
0.4
0.6
0.8
1.0
0
5
10
15

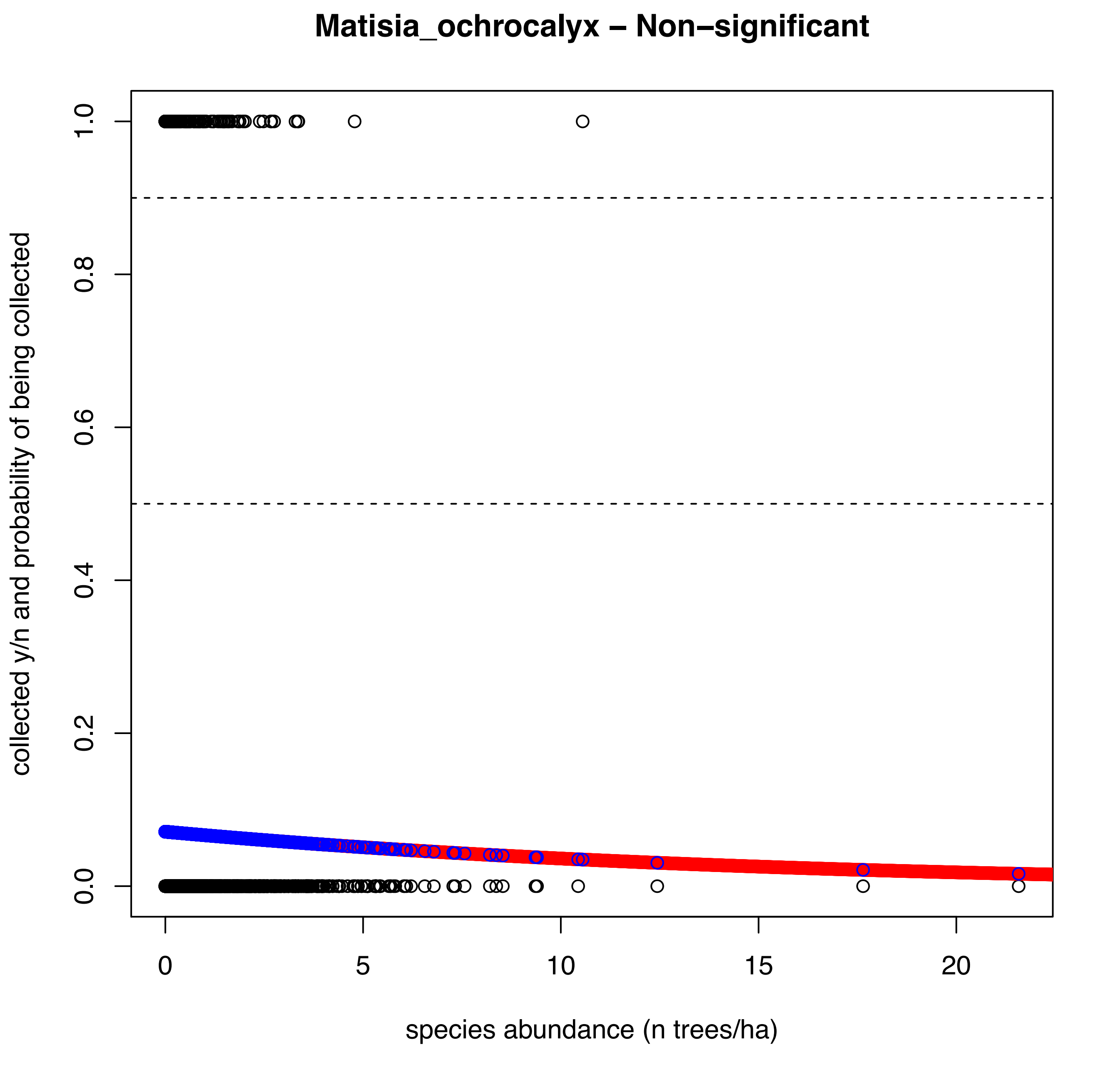
Matisia_ochrocalyx – Non–significant
collected y/n and probability of being collected
0.0
0.2
0.4
0.6
0.8
1.0
0
5
10
15
20
species abundance (n trees/ha)

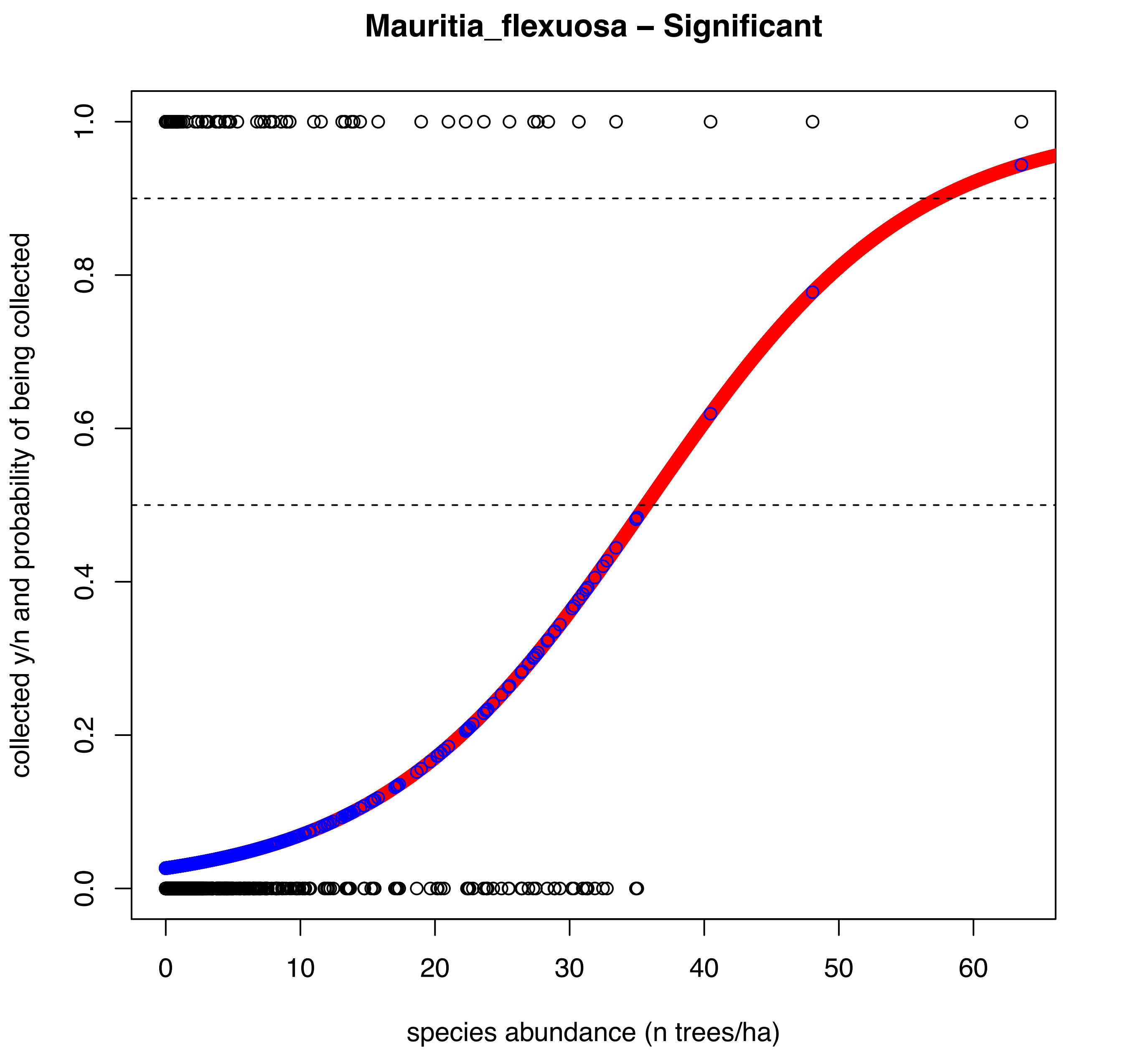

Mauritia_flexuosa – Significant
collected y/n and probability of being collected
species abundance (n trees/ha)
0.0
0.2
0.4
0.6
0.8
1.0
0
10
20
30
40
50
60

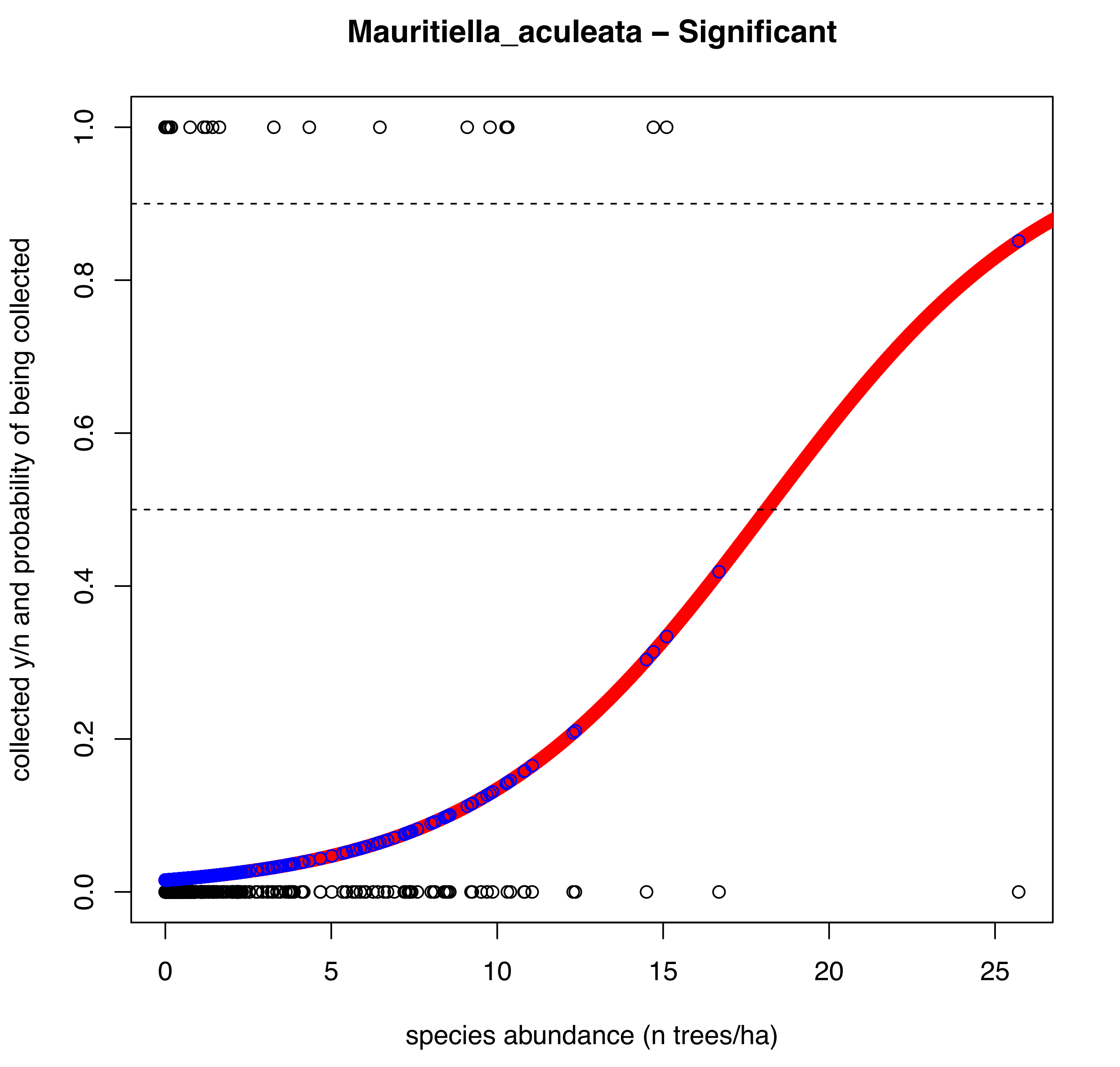

Mauritiella_aculeata – Significant
collected y/n and probability of being collected
species abundance (n trees/ha)
0.0
0.2
0.4
0.6
0.8
1.0
0
5
10
15
20
25

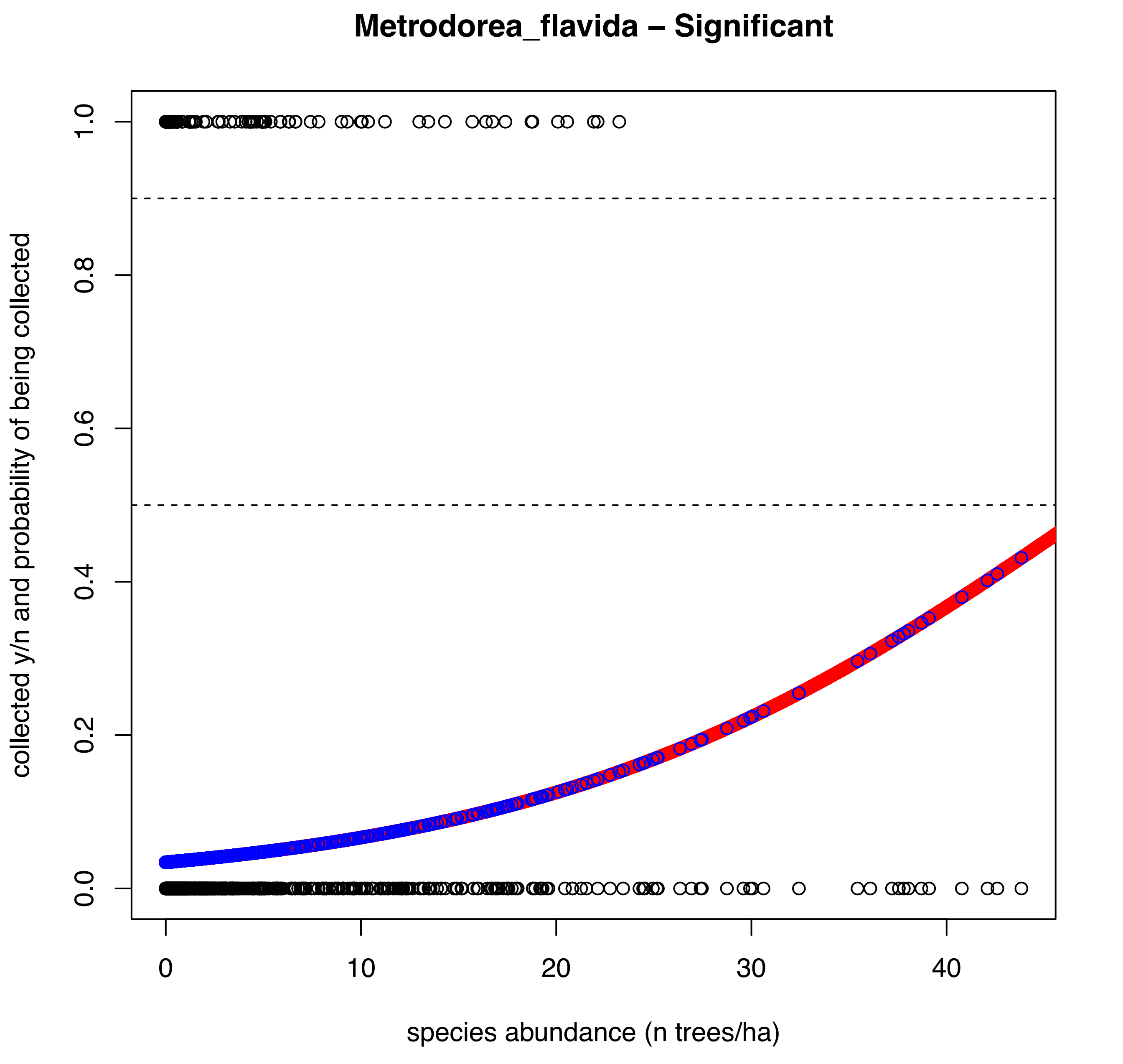

Metrodorea_flavida – Significant
collected y/n and probability of being collected
species abundance (n trees/ha)
0.0
0.2
0.4
0.6
0.8
1.0
0
10
20
30
40

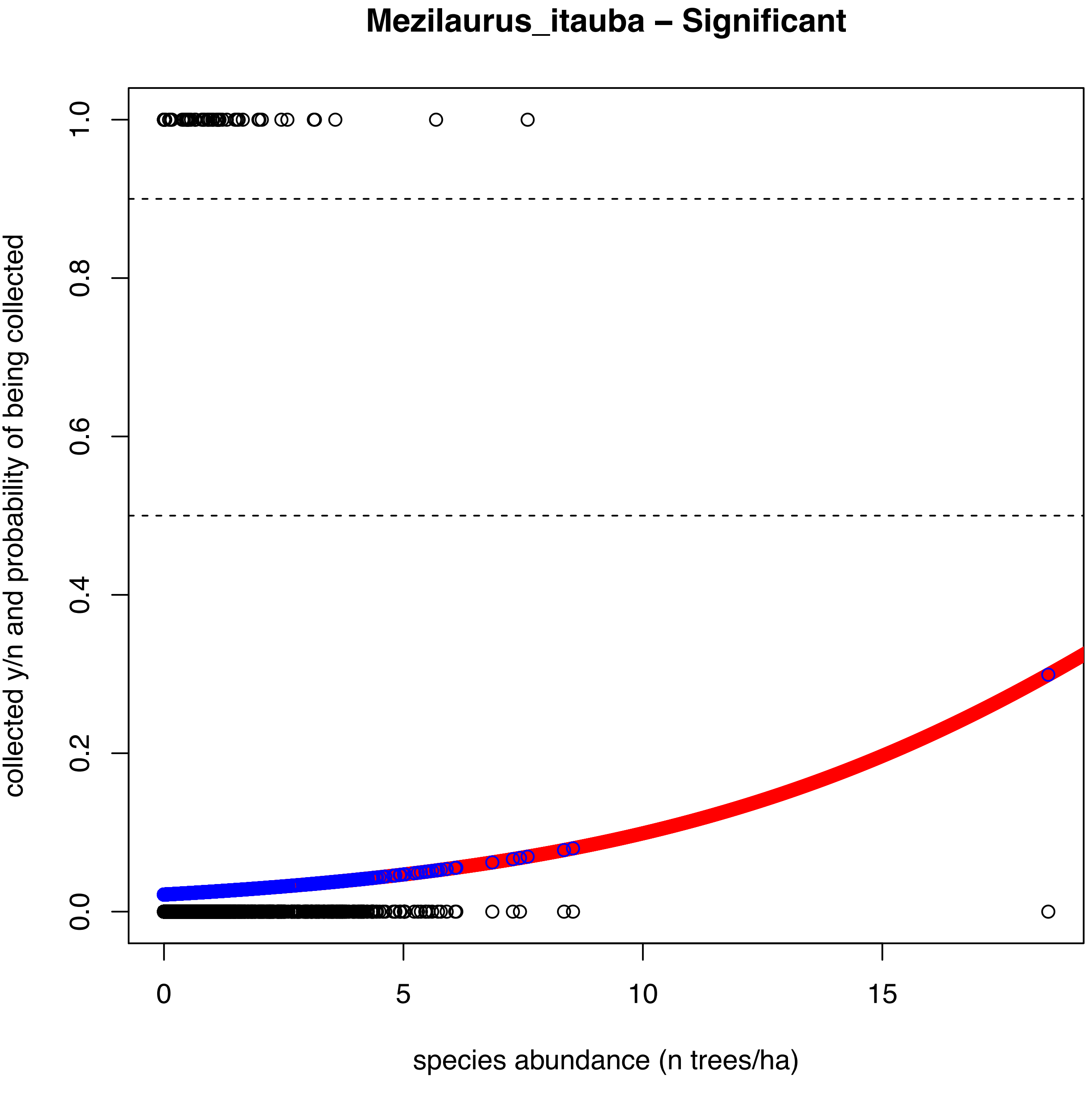
Mezilaurus_itauba – Significant
collected y/n and probability of being collected
species abundance (n trees/ha)
0.0
0.2
0.4
0.6
0.8
1.0
0
5
10
15

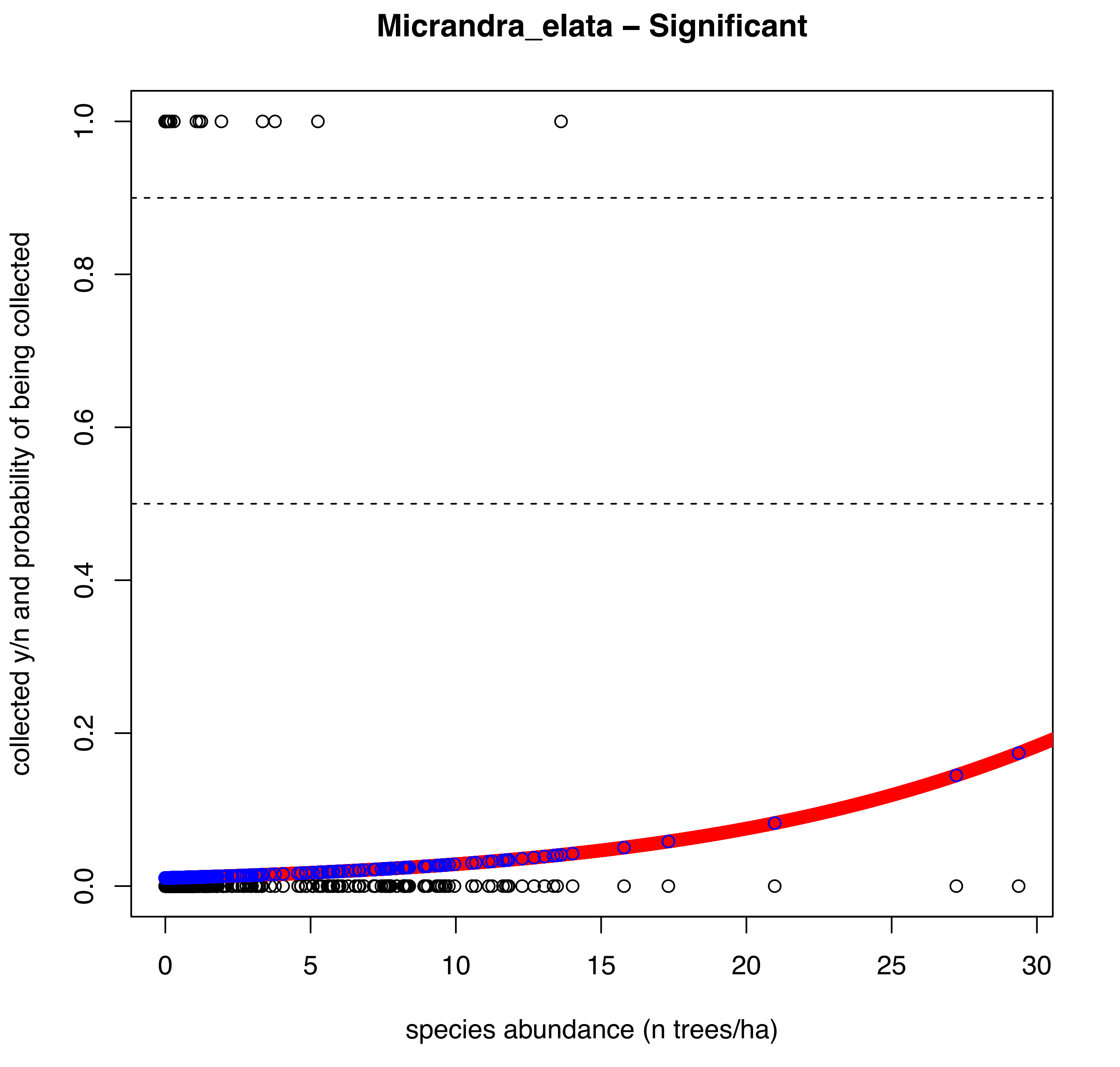

Micrandra_elata – Significant
collected y/n and probability of being collected
species abundance (n trees/ha)
0.0
0.2
0.4
0.6
0.8
1.0
0
5
10
15
20
25
30

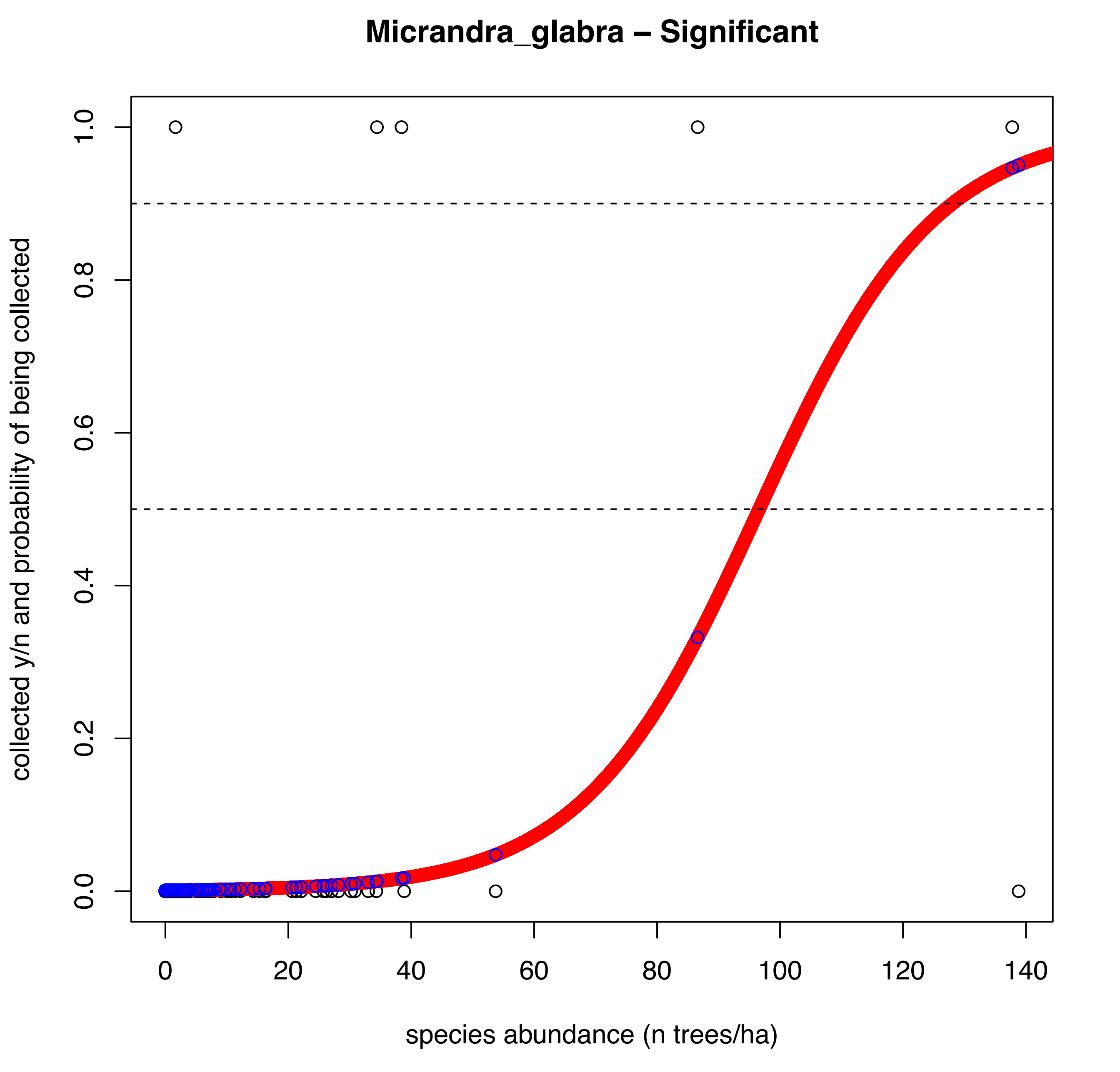
Micrandra_glabra – Significant
collected y/n and probability of being collected
species abundance (n trees/ha)
0.0
0.2
0.4
0.6
0.8
1.0
0
20
40
60
80
100
120
140

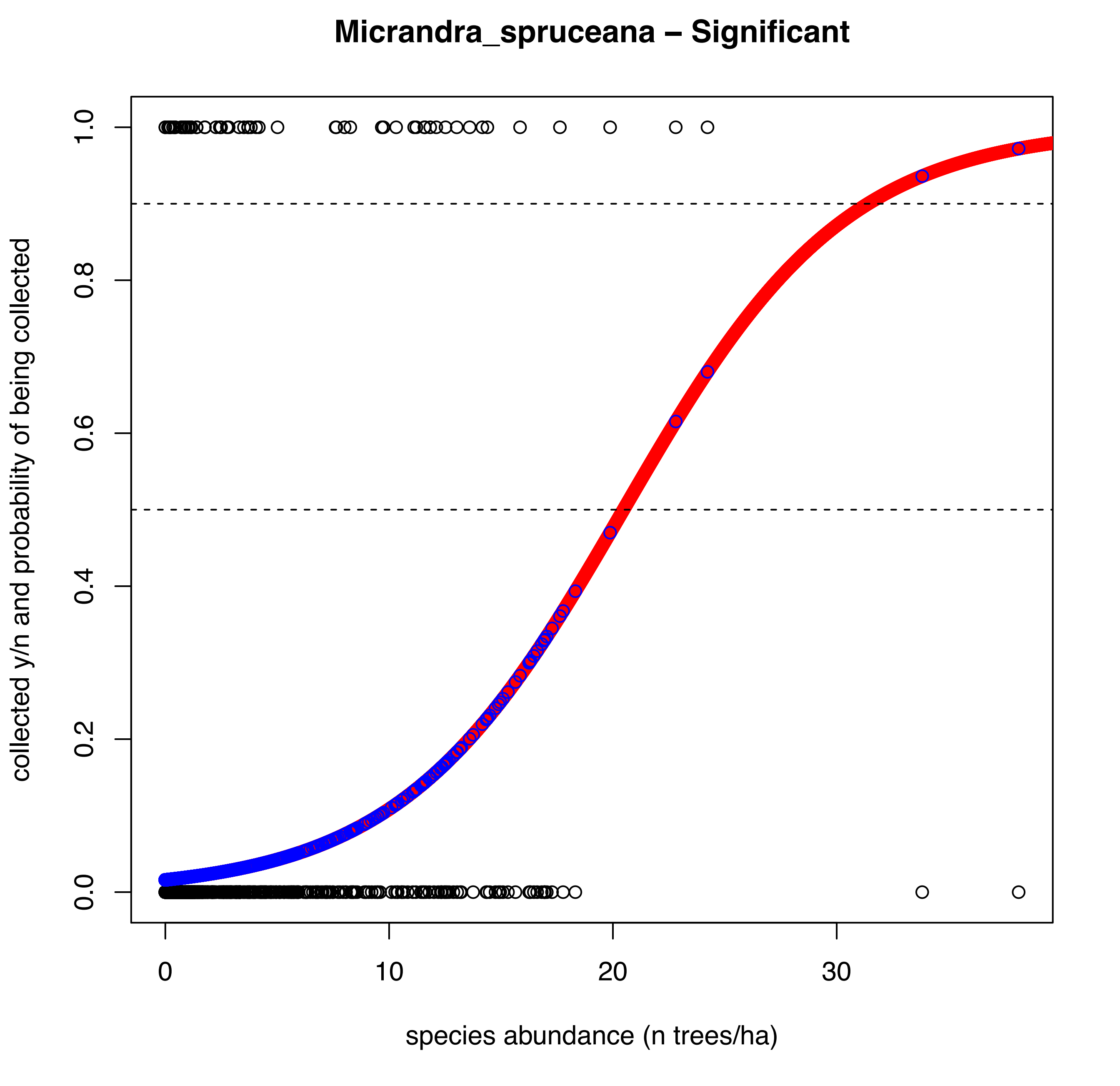
Micrandra_spruceana – Significant
collected y/n and probability of being collected
species abundance (n trees/ha)
0.0
0.2
0.4
0.6
0.8
1.0
0
10
20
30

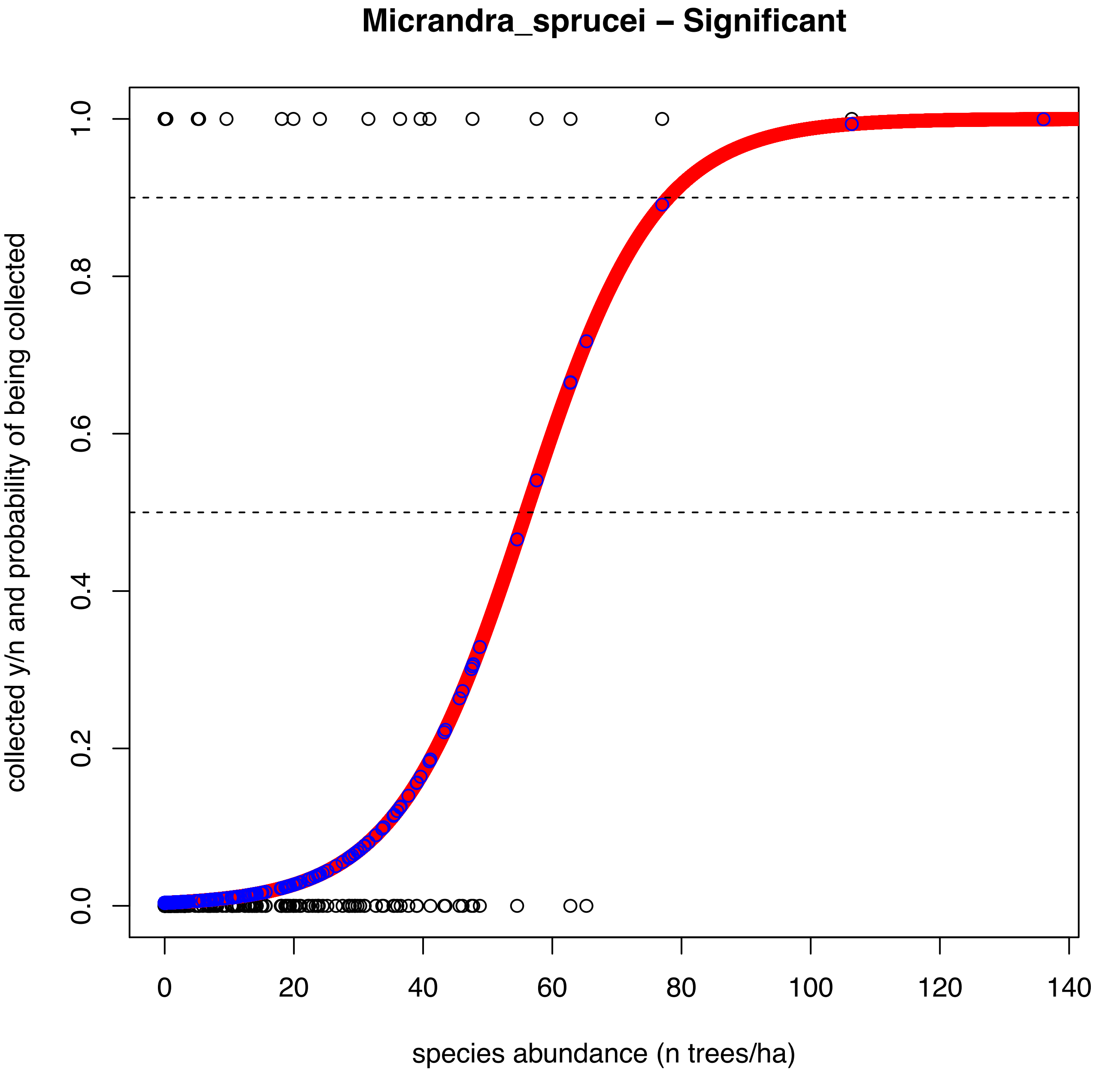

Micrandra_sprucei – Significant
collected y/n and probability of being collected
species abundance (n trees/ha)
0.0
0.2
0.4
0.6
0.8
1.0
0
20
40
60
80
100
120
140

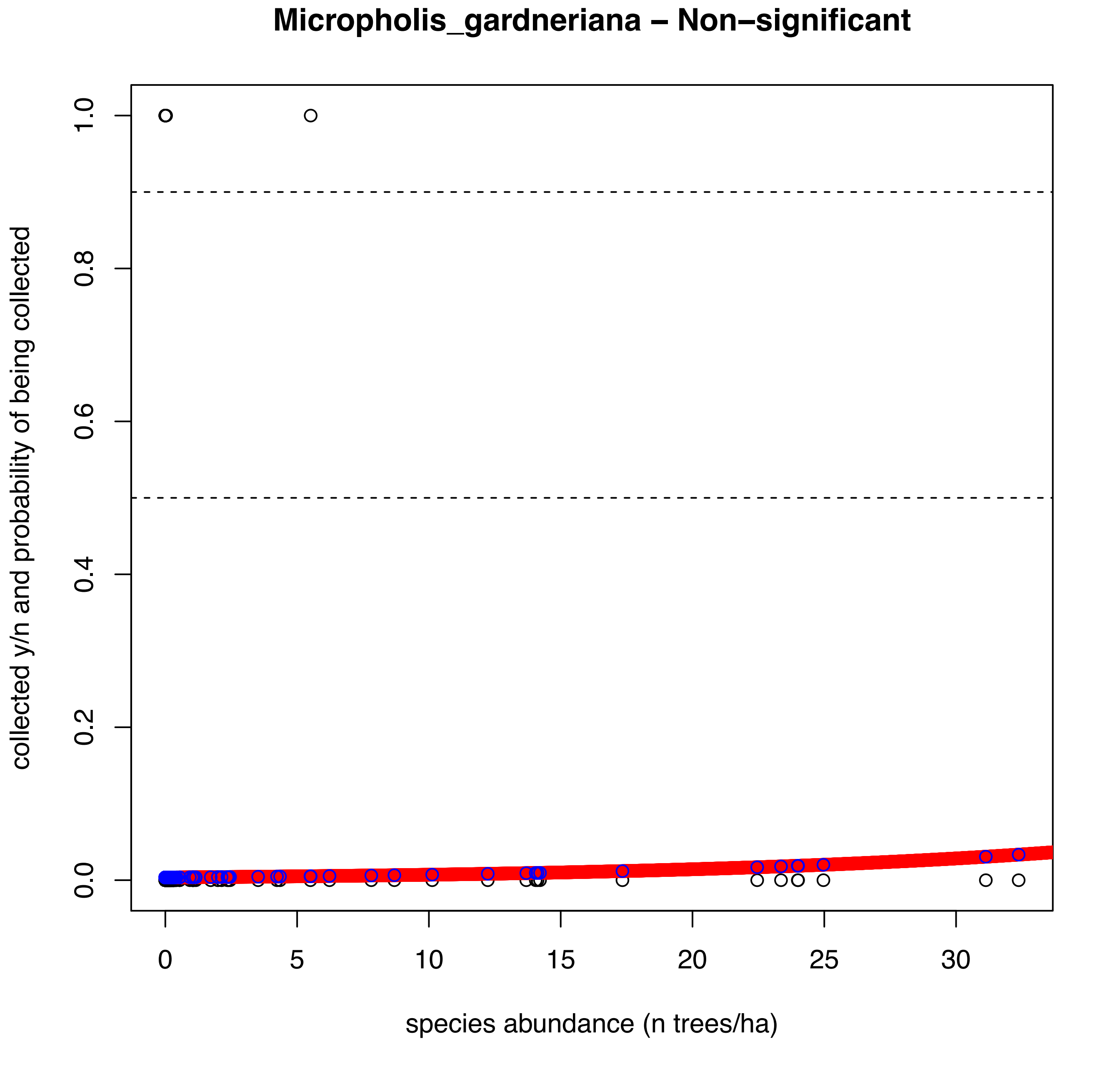
Micropholis_gardneriana – Non−significant
collected y/n and probability of being collected
species abundance (n trees/ha)
0.0
0.2
0.4
0.6
0.8
1.0
0
5
10
15
20
25
30

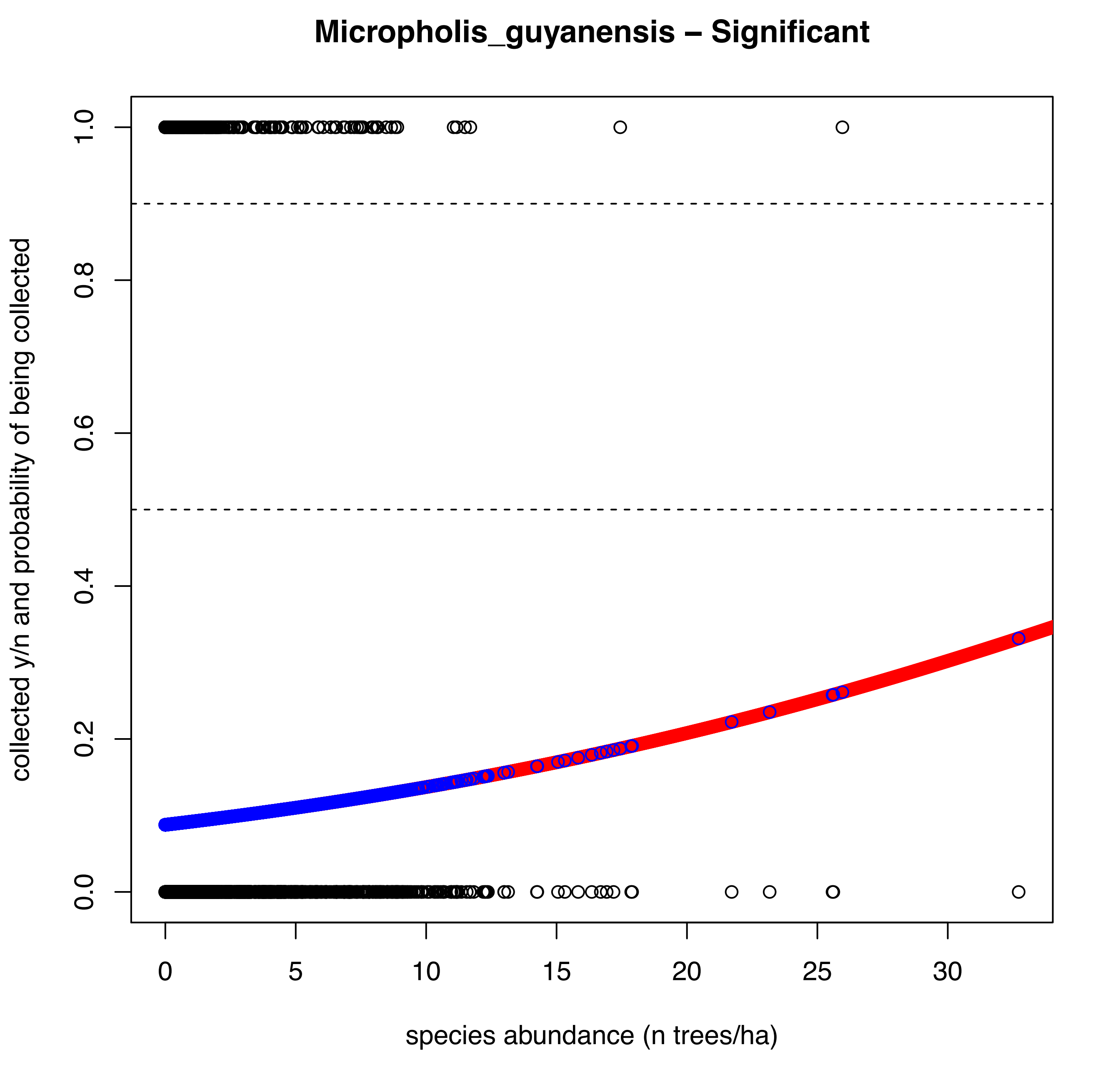
Micropholis_guyanensis – Significant
collected y/n and probability of being collected
0.0
0.2
0.4
0.6
0.8
1.0
0
5
10
15
20
25
30
species abundance (n trees/ha)

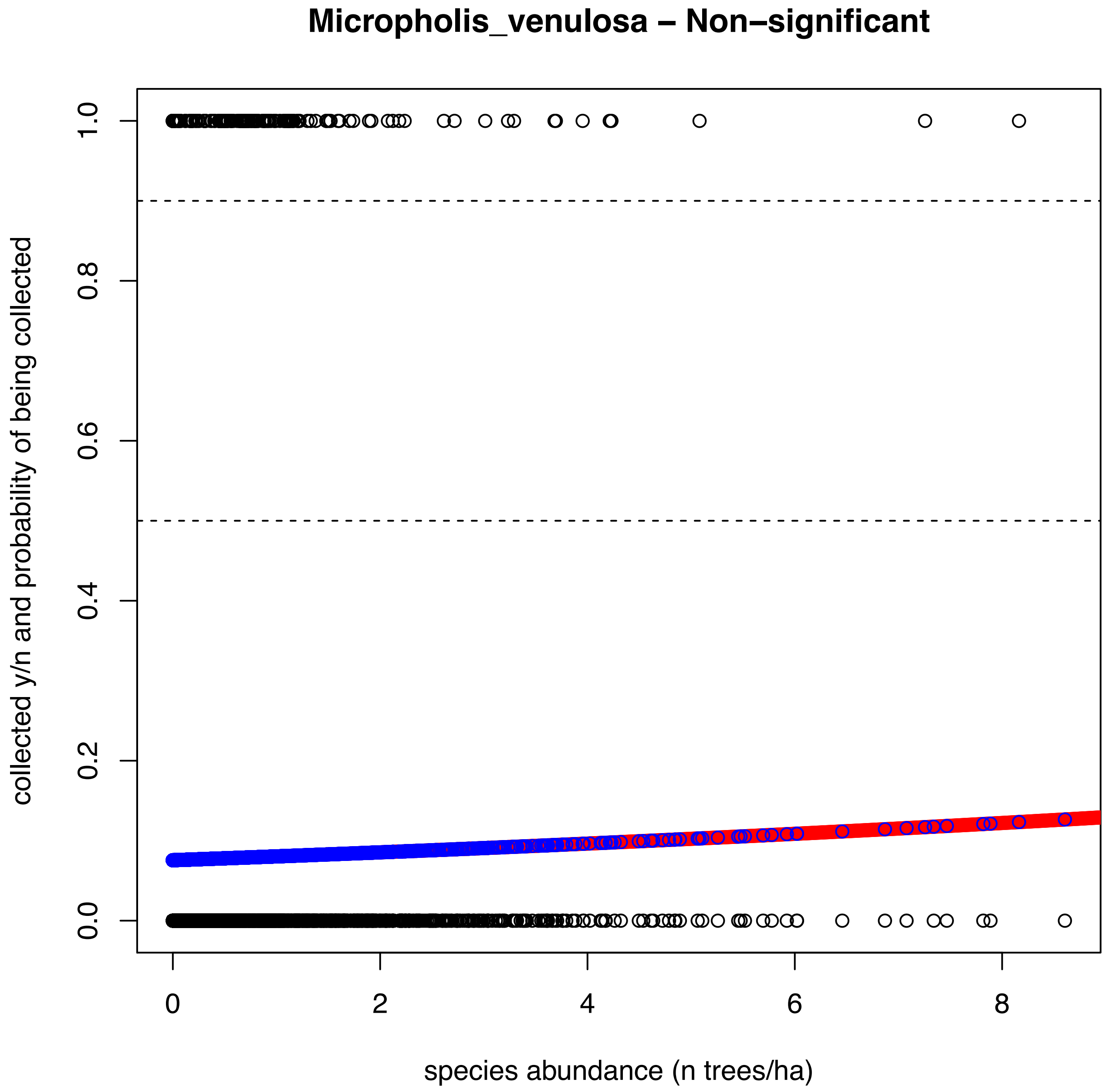
Micropholis_venulosa – Non–significant
collected y/n and probability of being collected
species abundance (n trees/ha)
0.0
0.2
0.4
0.6
0.8
1.0
0
2
4
6
8

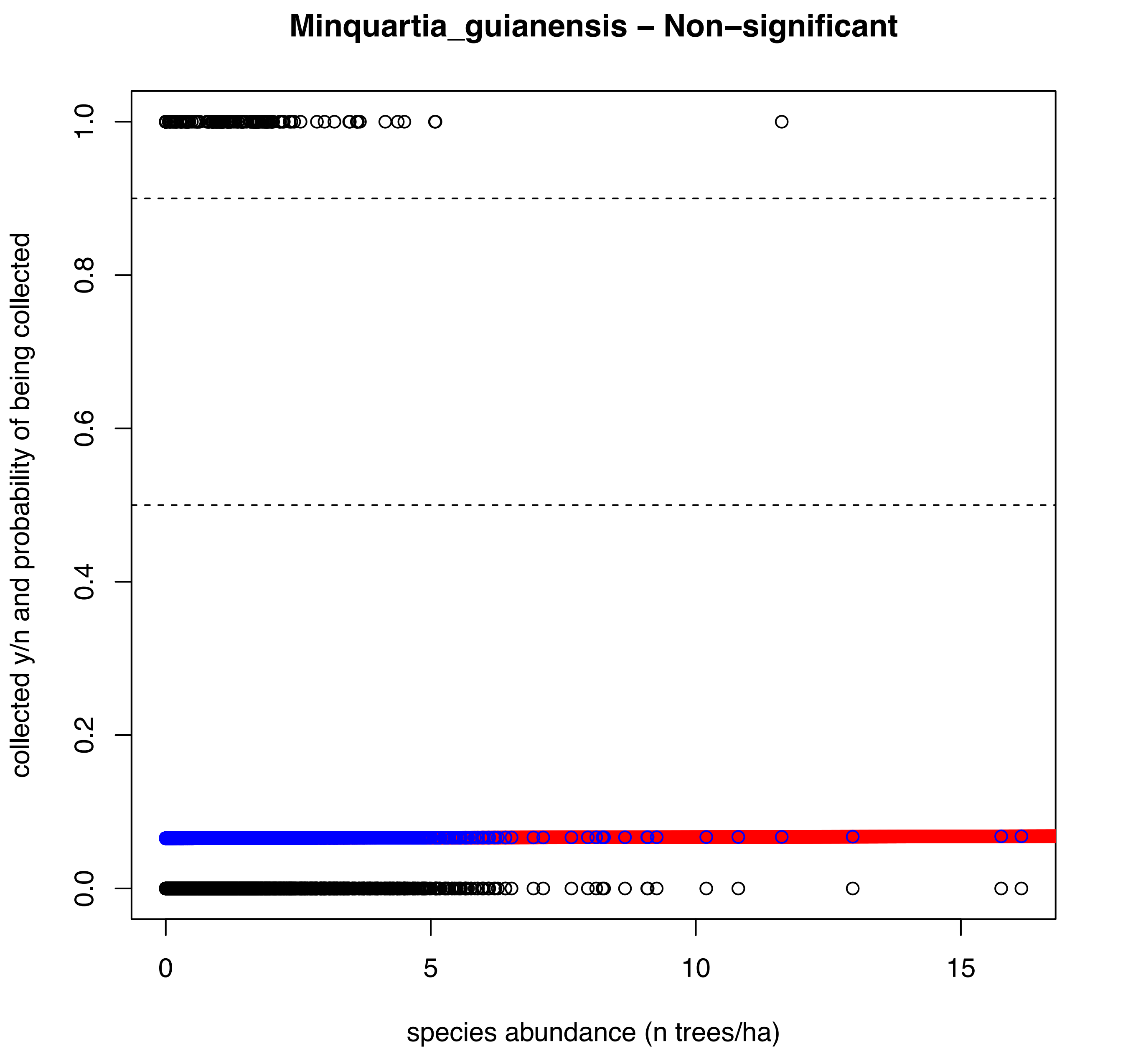

Minquartia_guianensis – Non–significant
collected y/n and probability of being collected
species abundance (n trees/ha)
0.0
0.2
0.4
0.6
0.8
1.0
0
5
10
15

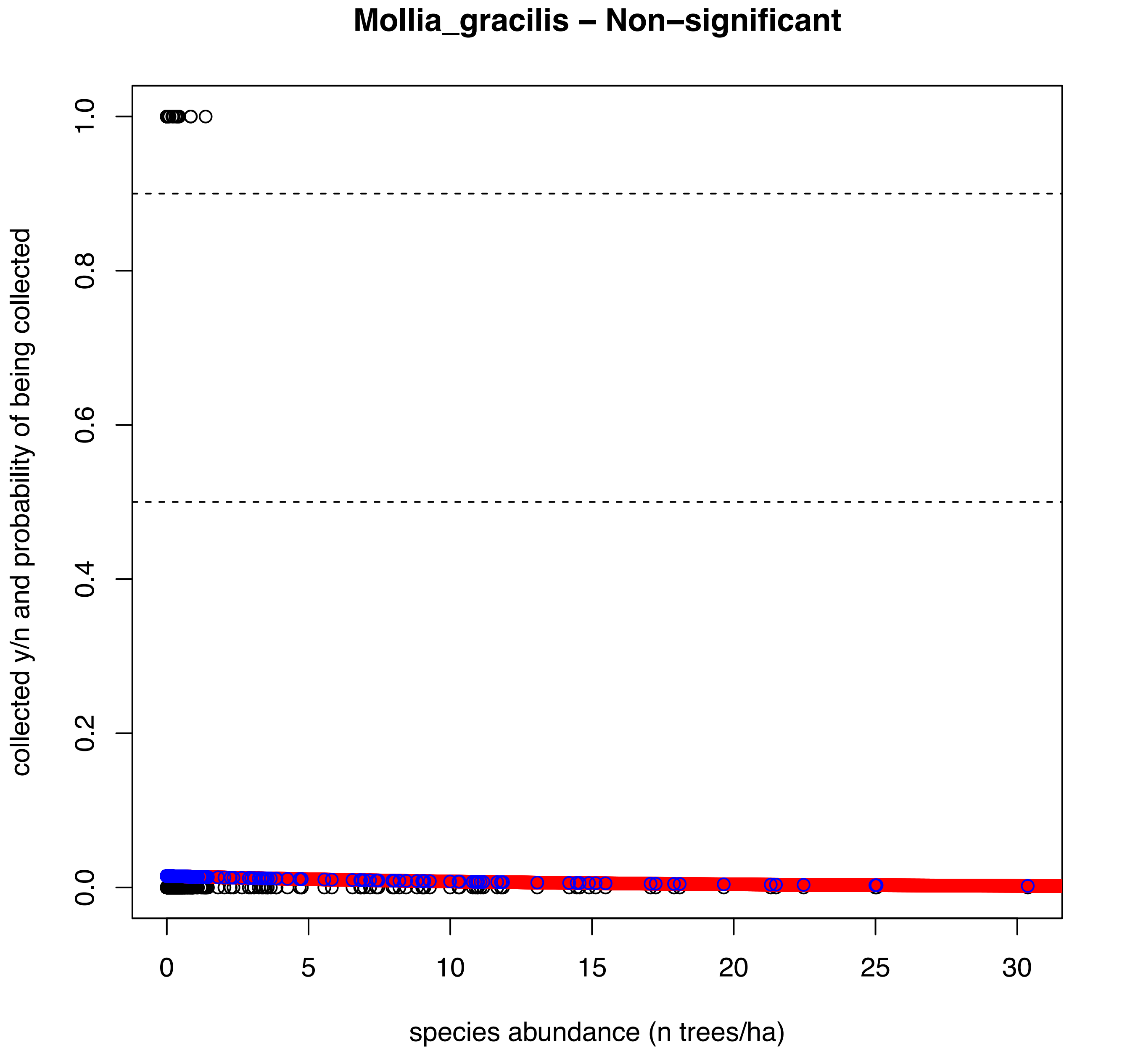
Mollia_gracilis – Non–significant
collected y/n and probability of being collected
species abundance (n trees/ha)
0.0
0.2
0.4
0.6
0.8
1.0
0
5
10
15
20
25
30

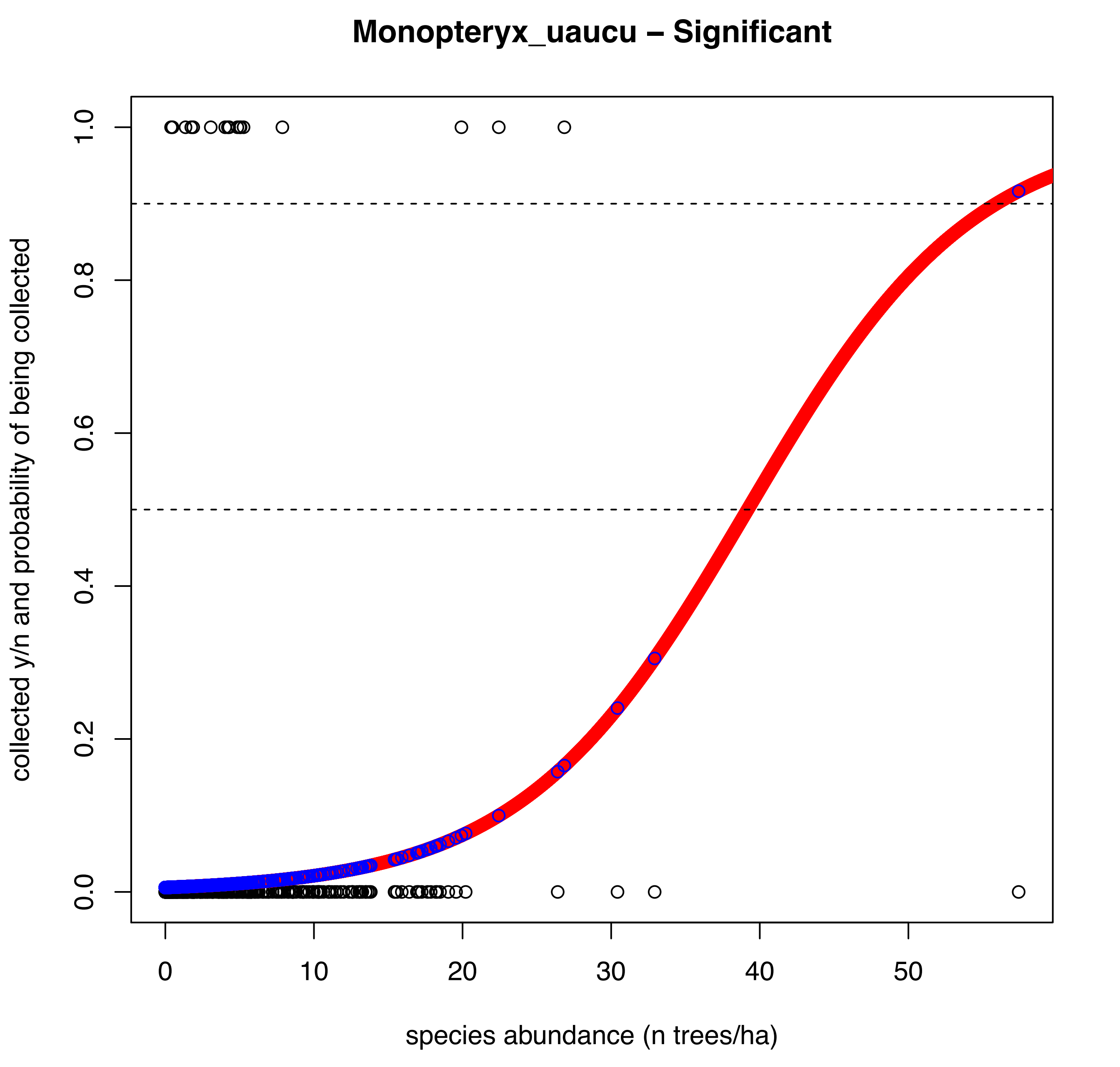
Monopteryx_uaucu – Significant
collected y/n and probability of being collected
species abundance (n trees/ha)
0.0
0.2
0.4
0.6
0.8
1.0
0
10
20
30
40
50

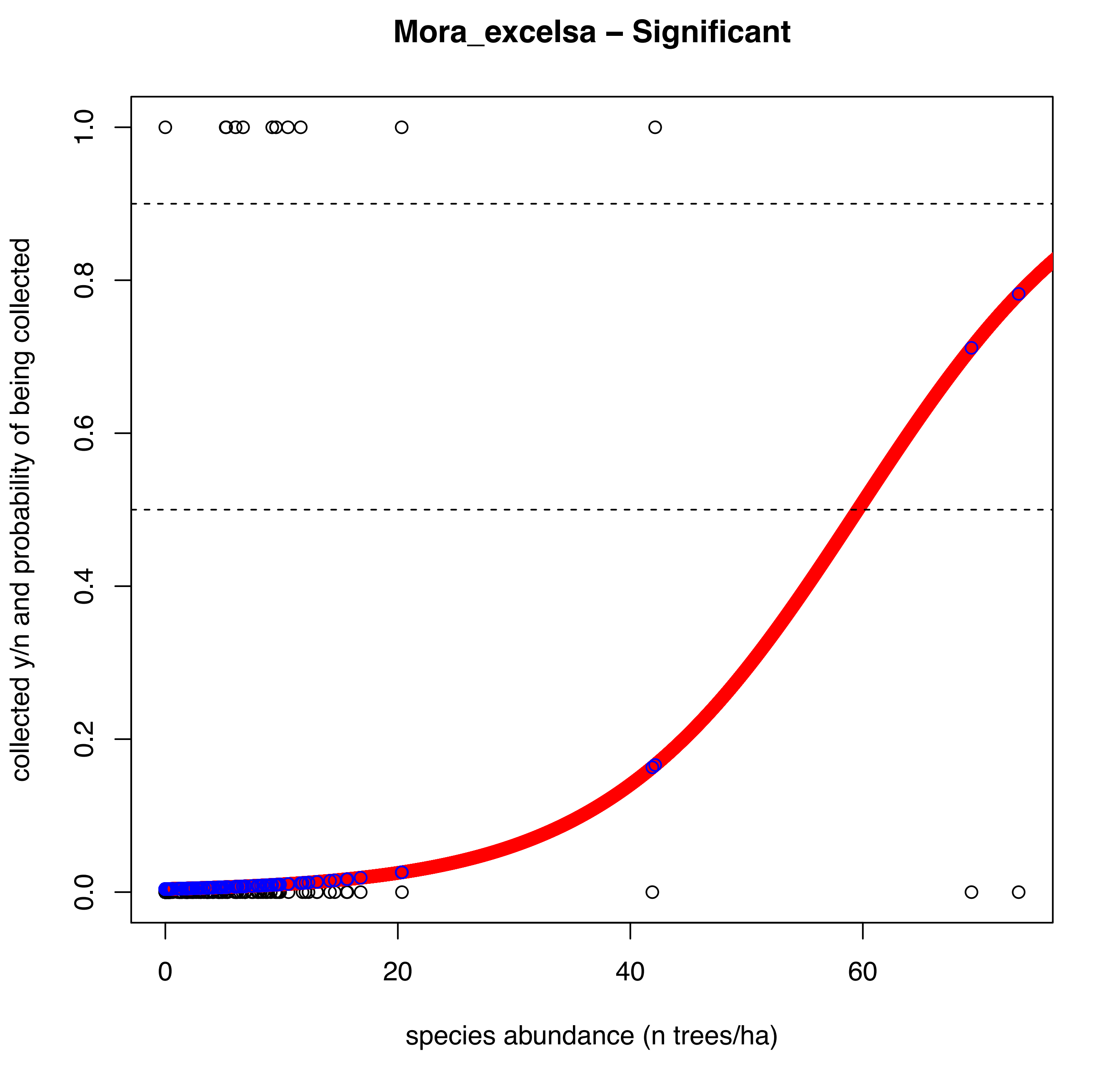
Mora_excelsa – Significant
collected y/n and probability of being collected
species abundance (n trees/ha)
0.0
0.2
0.4
0.6
0.8
1.0
0
20
40
60

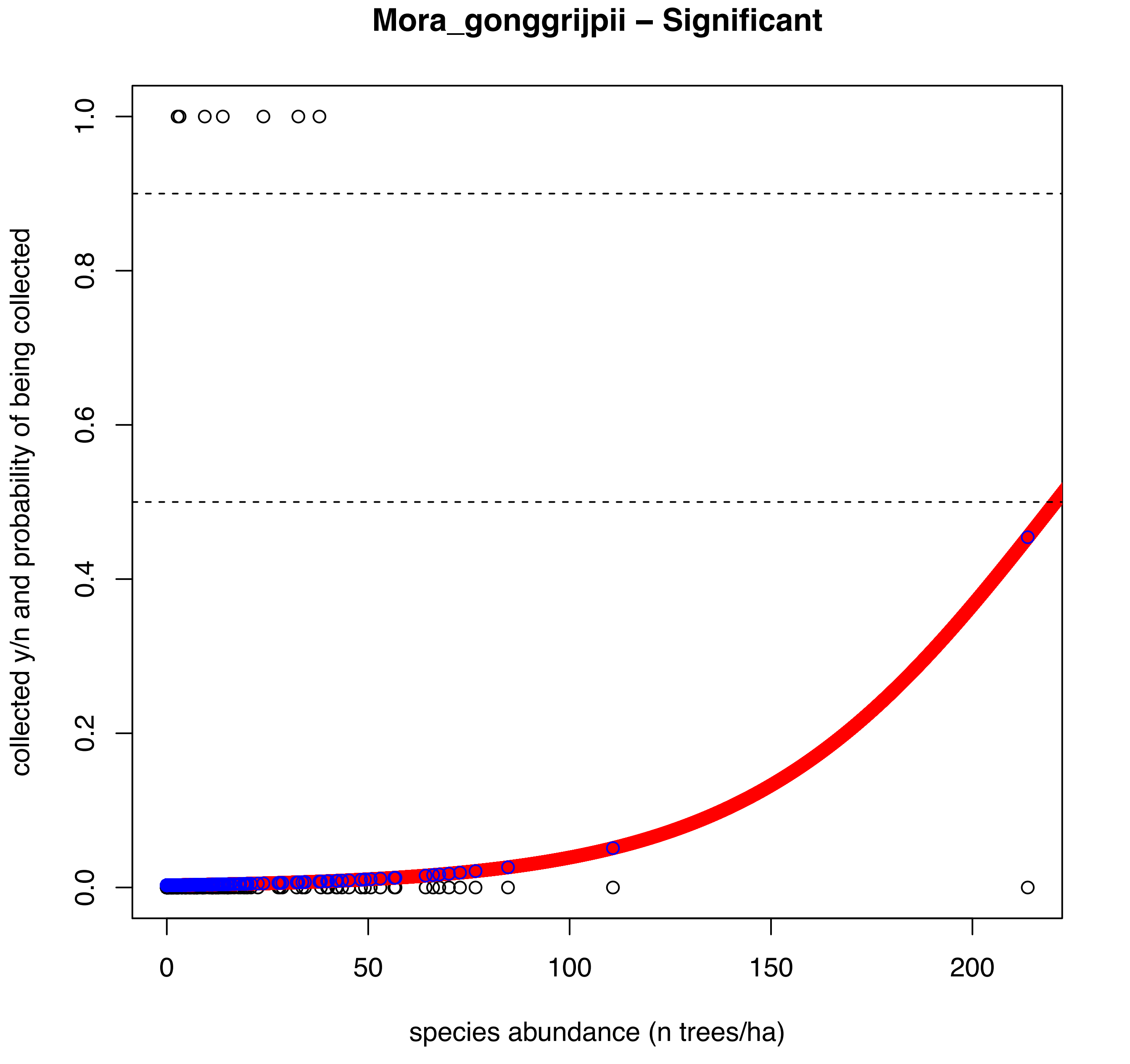

Mora_gonggrijpii – Significant
collected y/n and probability of being collected
species abundance (n trees/ha)
0.0
0.2
0.4
0.6
0.8
1.0
0
50
100
150
200

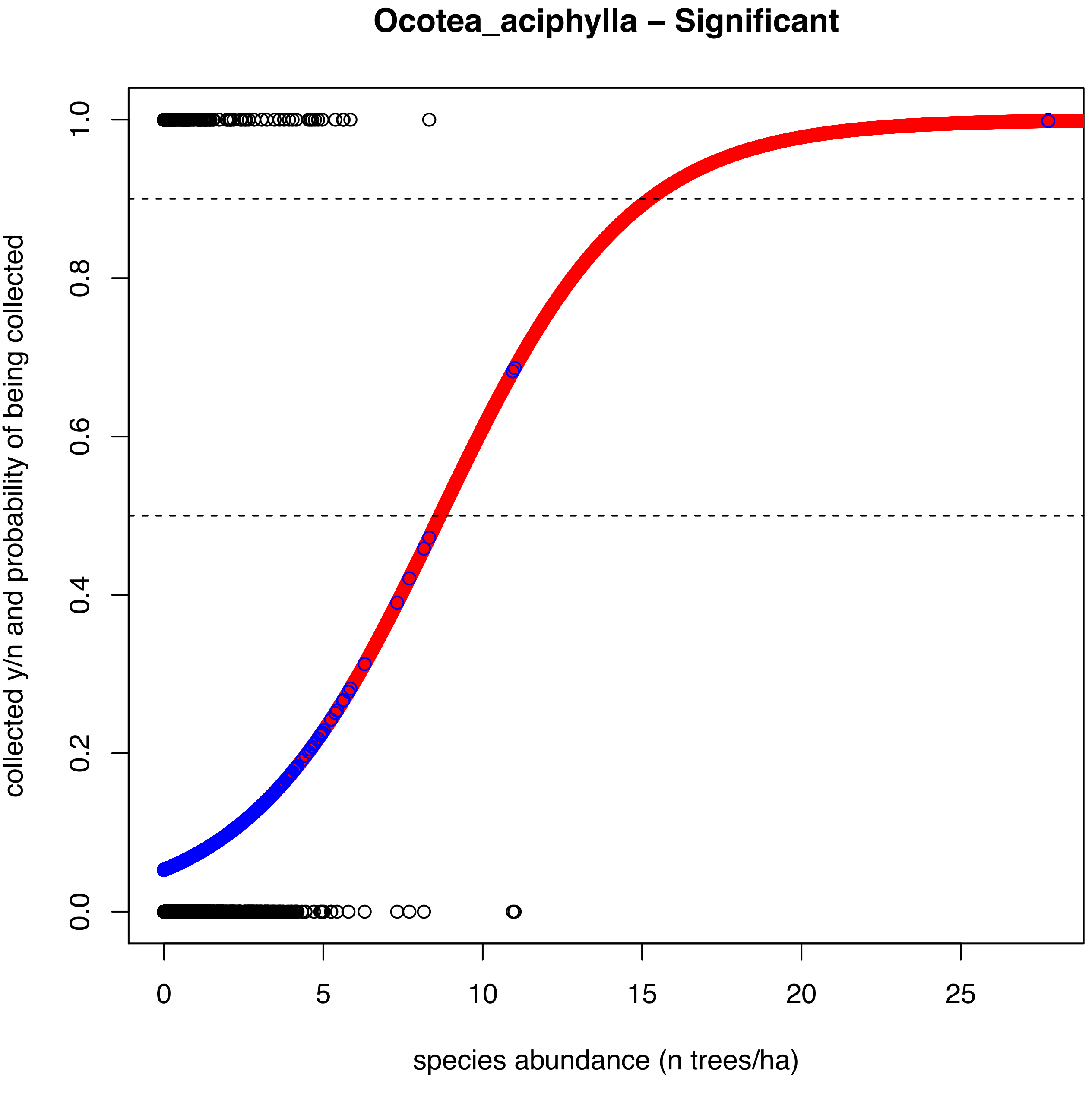
Ocotea_aciphylla – Significant
collected y/n and probability of being collected
species abundance (n trees/ha)
0.0
0.2
0.4
0.6
0.8
1.0
0
5
10
15
20
25

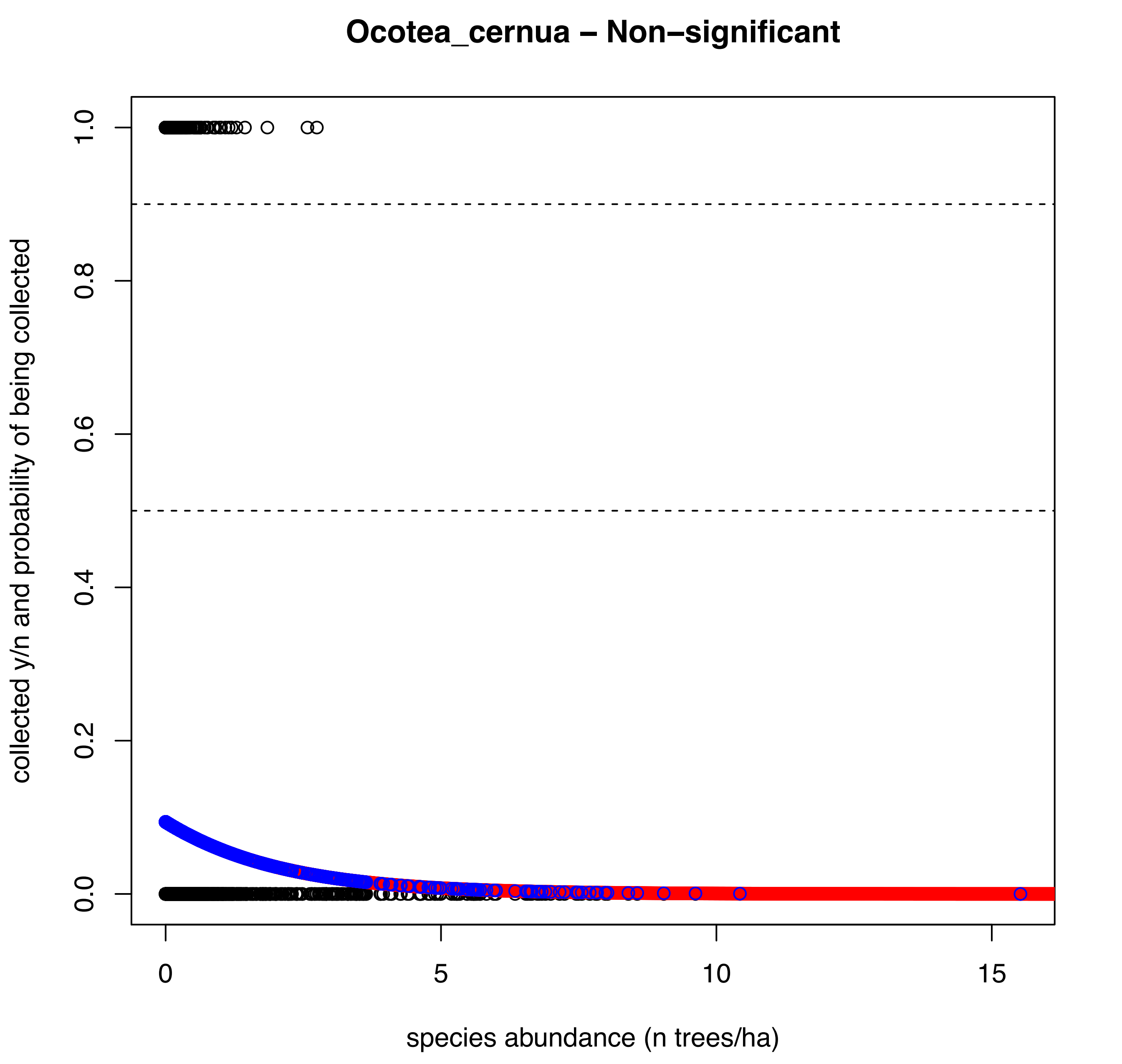

Ocotea_cernua – Non–significant
collected y/n and probability of being collected
species abundance (n trees/ha)
0.0
0.2
0.4
0.6
0.8
1.0
0
5
10
15

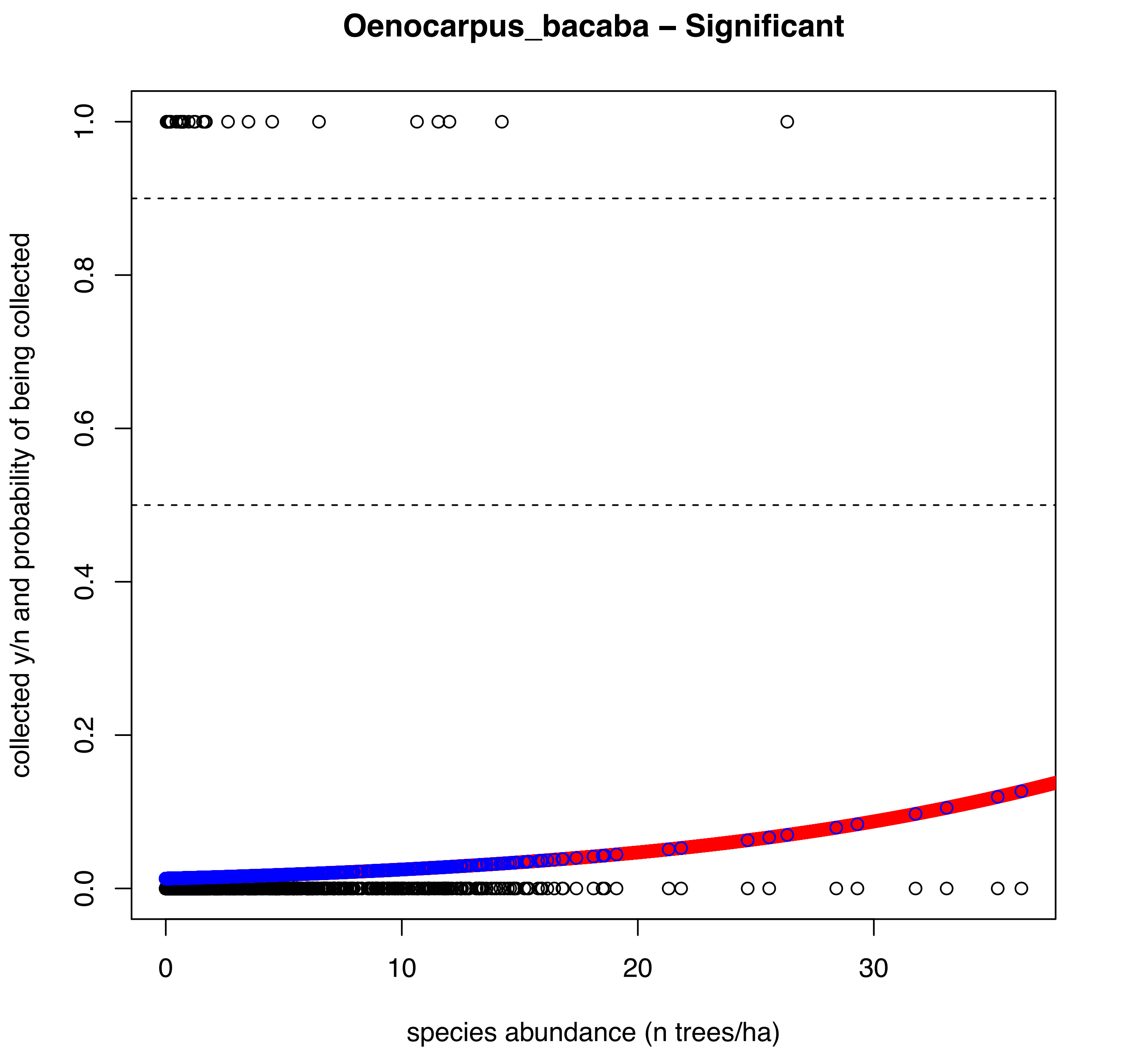

Oenocarpus_bacaba – Significant
collected y/n and probability of being collected
species abundance (n trees/ha)
0.0
0.2
0.4
0.6
0.8
1.0
0
10
20
30

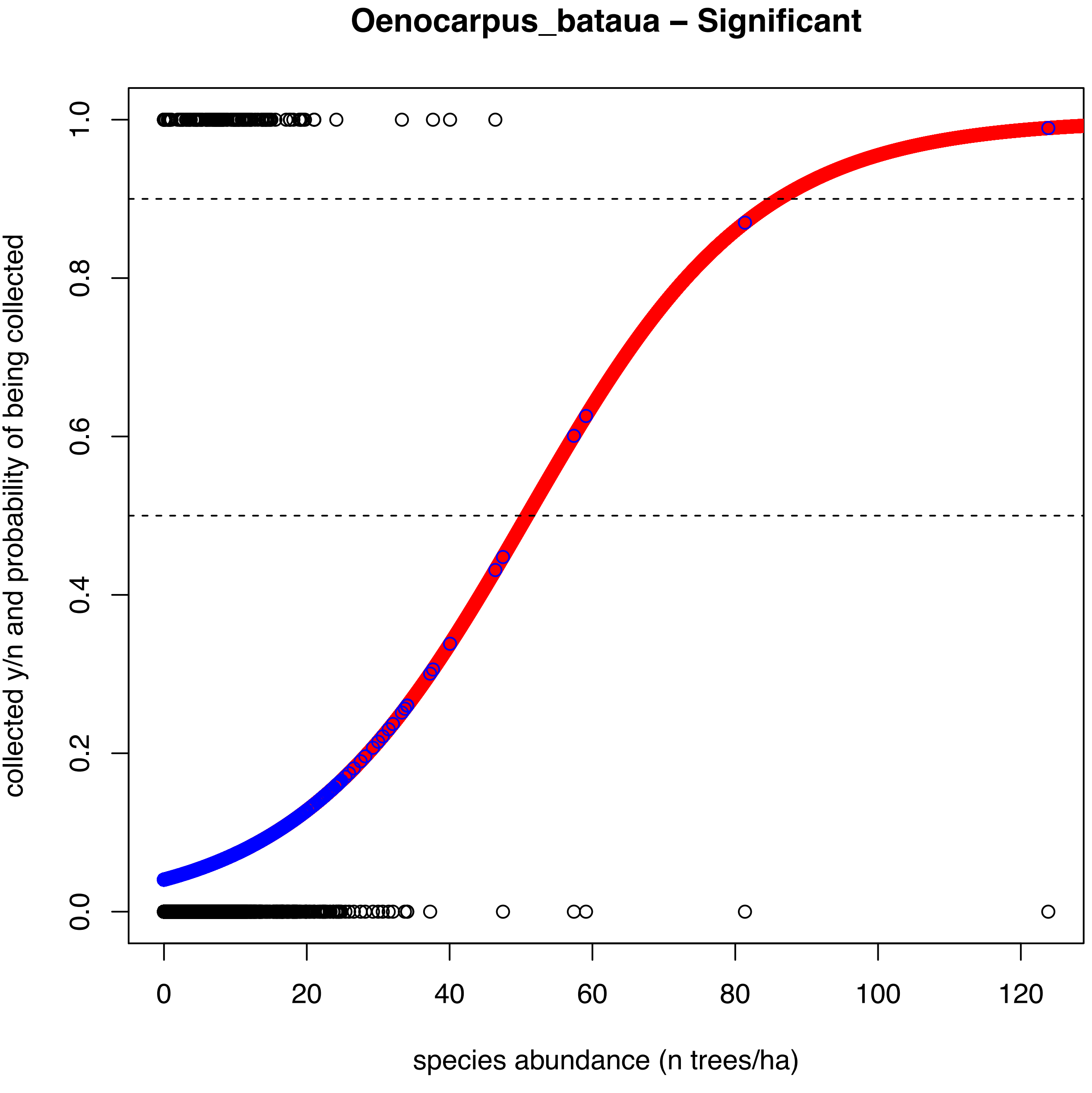
Oenocarpus_bataua – Significant
collected y/n and probability of being collected
species abundance (n trees/ha)
0.0
0.2
0.4
0.6
0.8
1.0
0
20
40
60
80
100
120

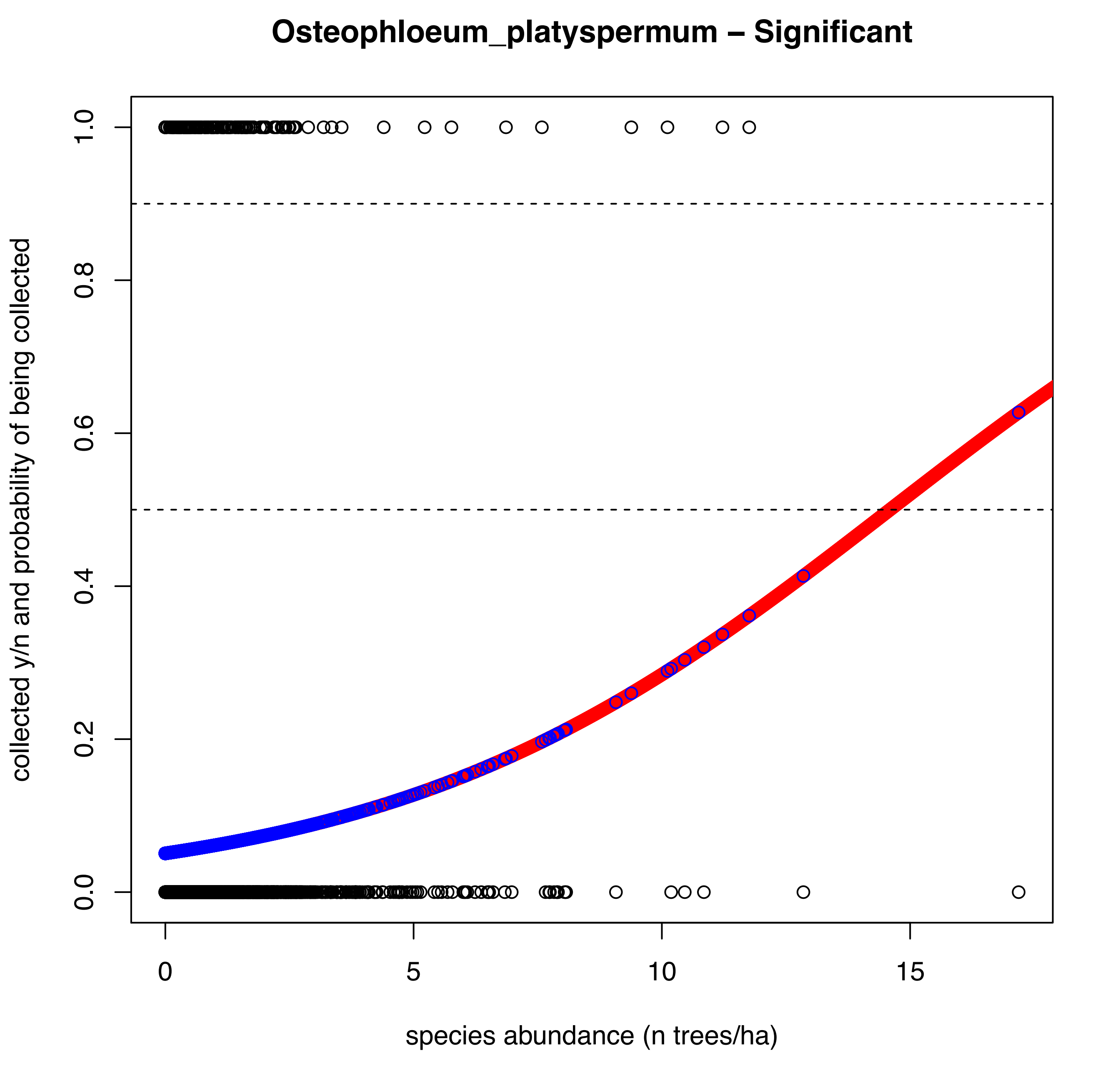
Osteophloeum_platyspermum – Significant
collected y/n and probability of being collected
species abundance (n trees/ha)
0.0
0.2
0.4
0.6
0.8
1.0
0
5
10
15

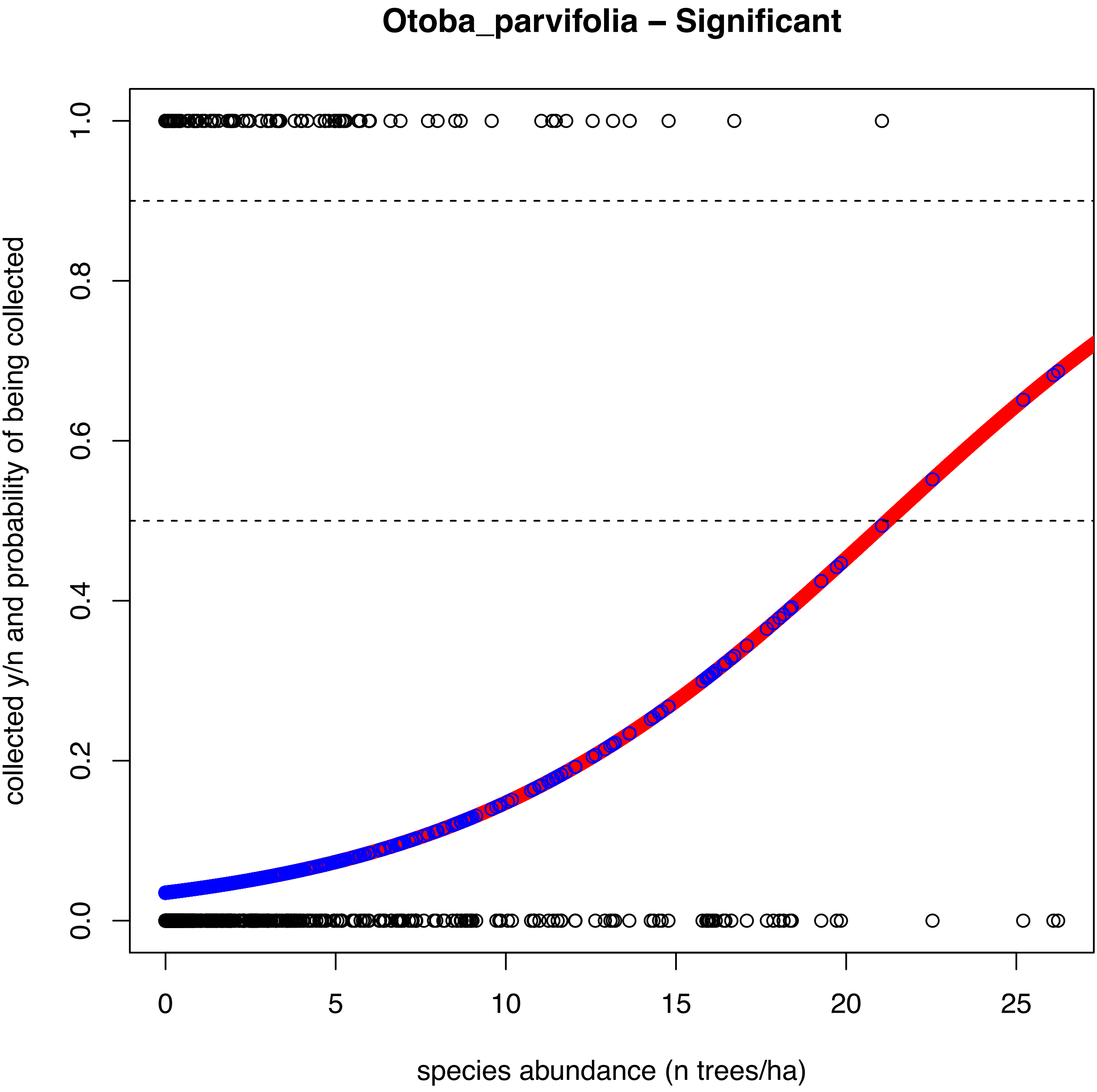
Otoba_parvifolia – Significant
collected y/n and probability of being collected
species abundance (n trees/ha)
0.0
0.2
0.4
0.6
0.8
1.0
0
5
10
15
20
25

Oxandra_polyantha – Significant

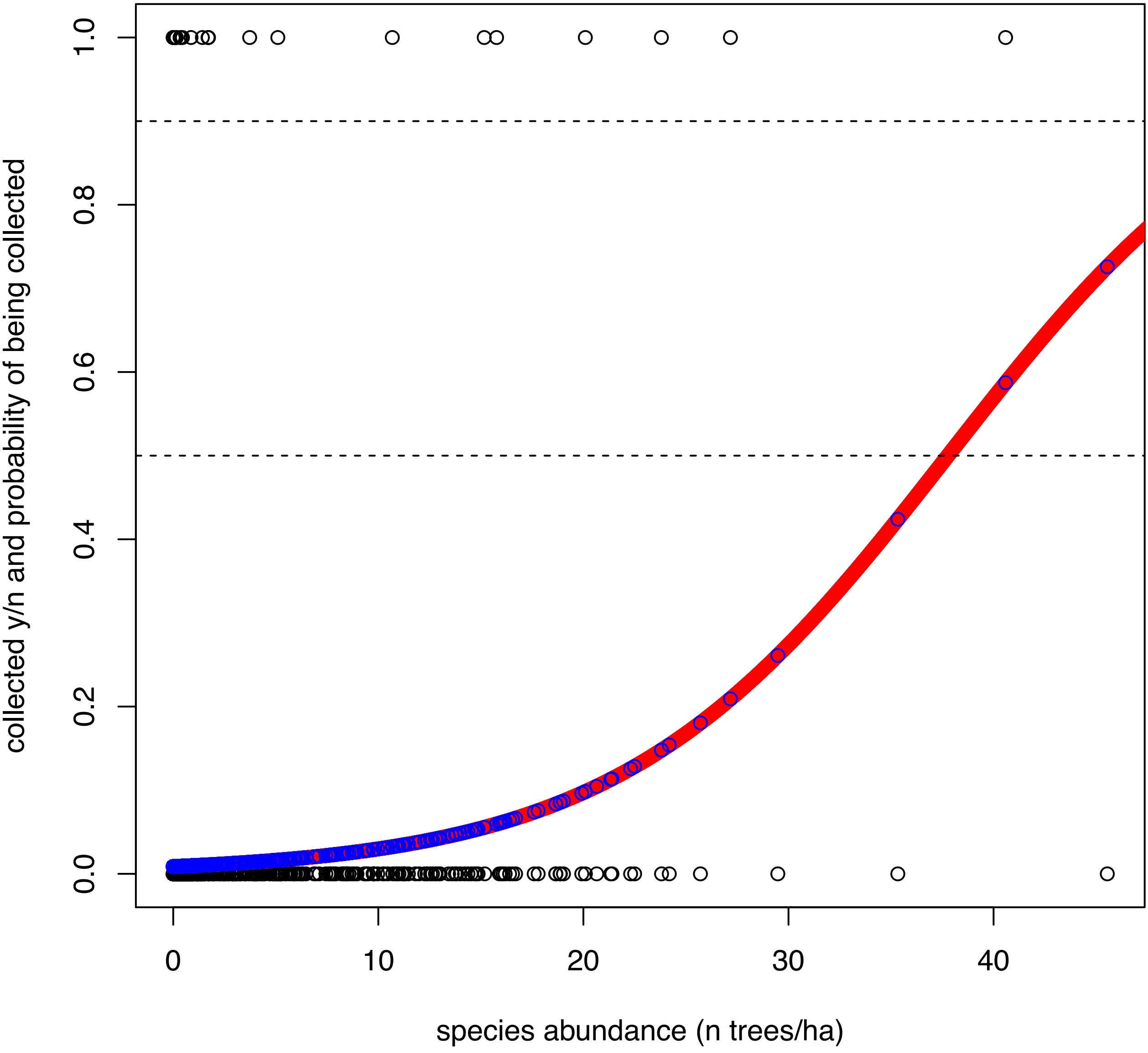

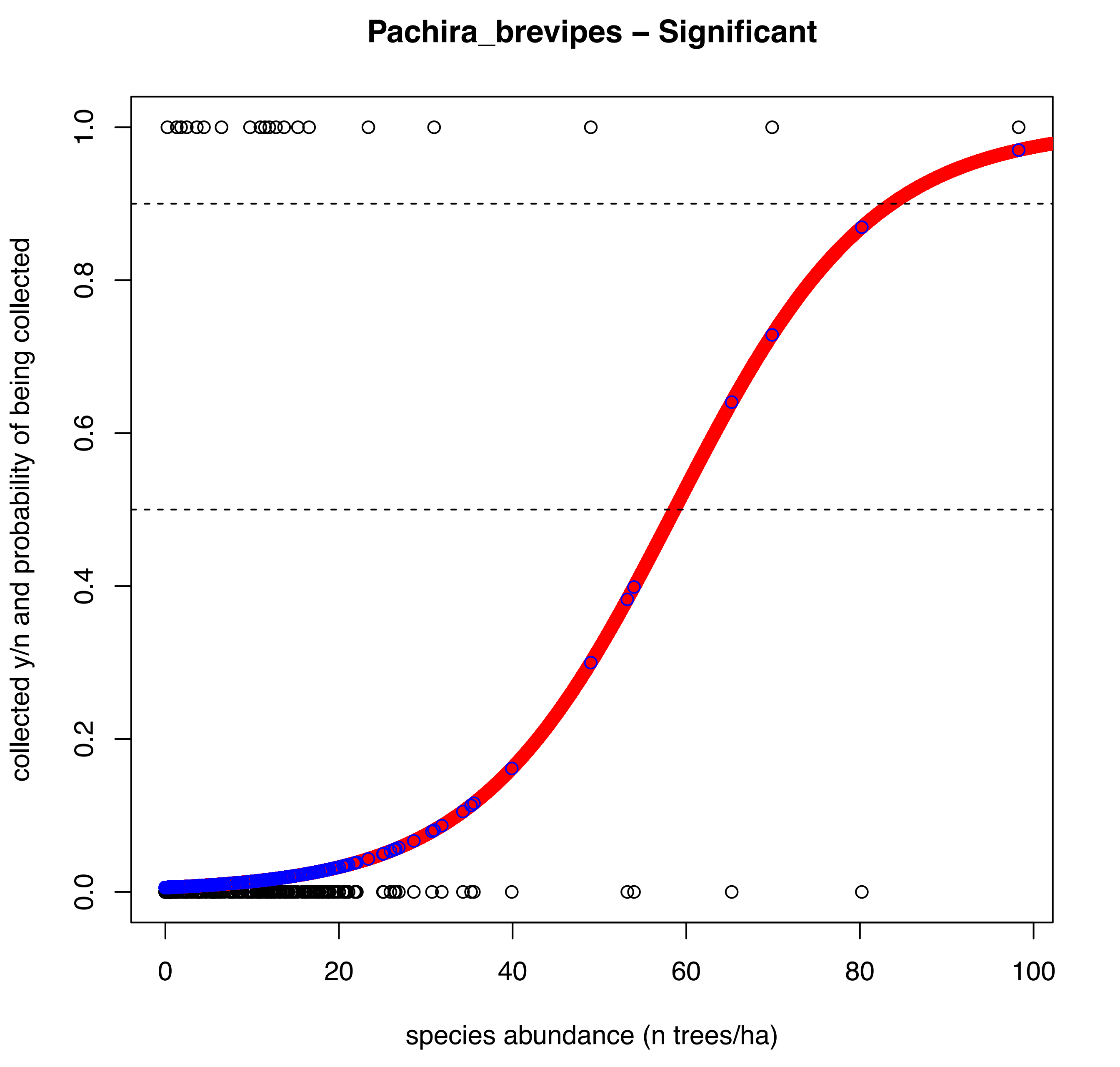

Pachira_brevipes – Significant
collected y/n and probability of being collected
species abundance (n trees/ha)
0.0
0.2
0.4
0.6
0.8
1.0
0
20
40
60
80
100

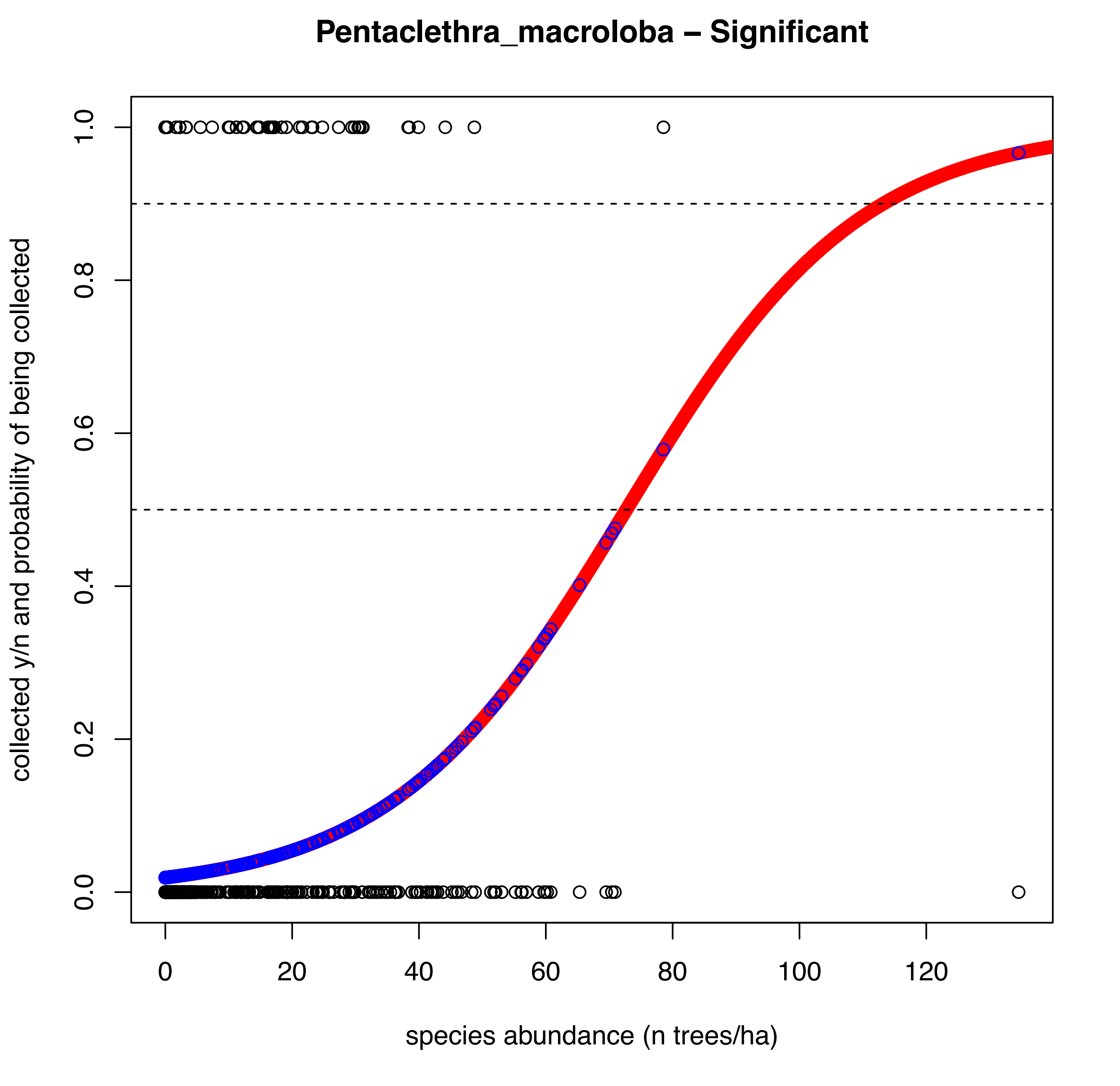

Pentaclethra_macroloba – Significant
collected y/n and probability of being collected
species abundance (n trees/ha)
0.0
0.2
0.4
0.6
0.8
1.0
0
20
40
60
80
100
120

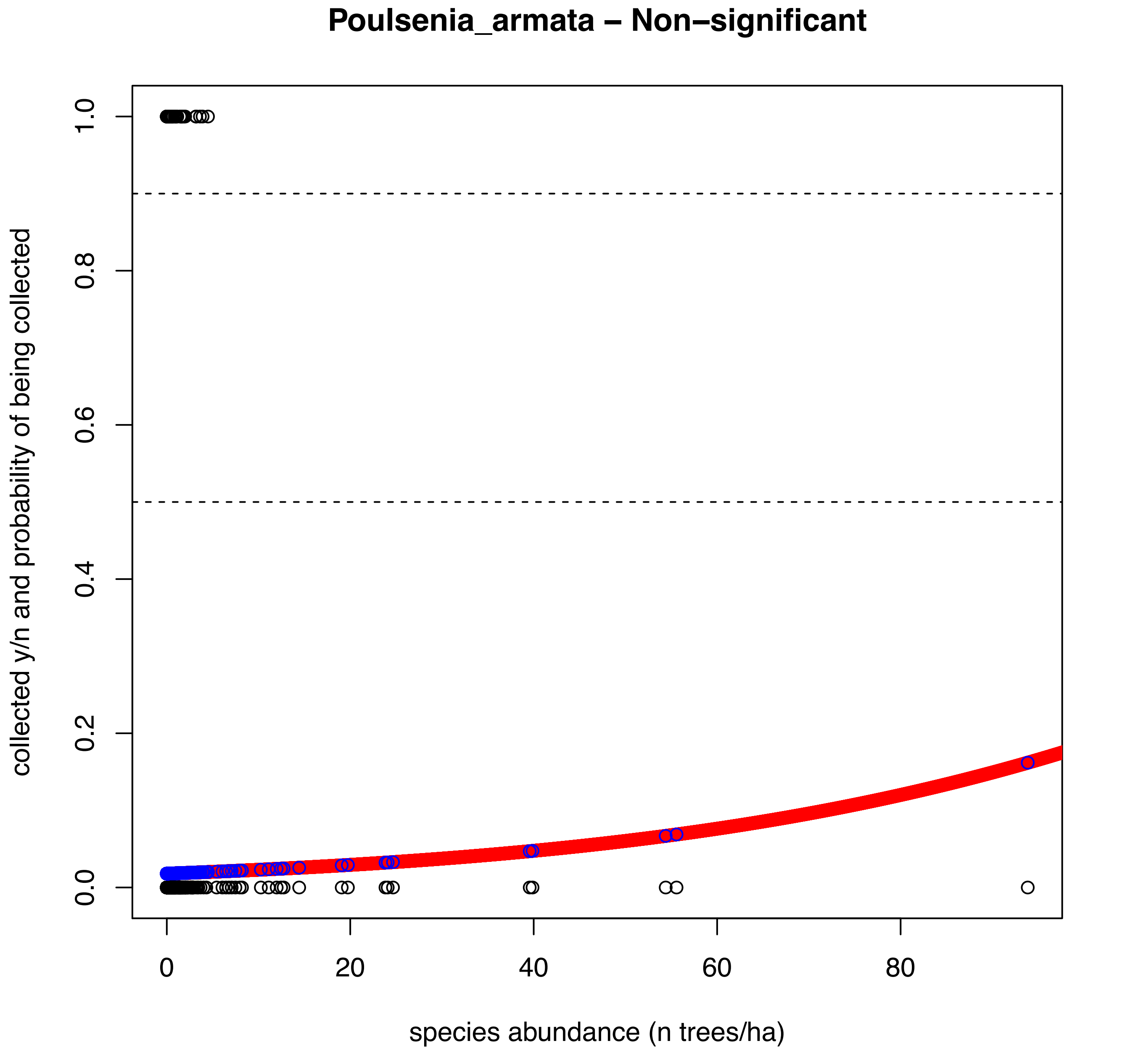

Poulsenia_armata – Non–significant
collected y/n and probability of being collected
species abundance (n trees/ha)
0.0
0.2
0.4
0.6
0.8
1.0
0
20
40
60
80

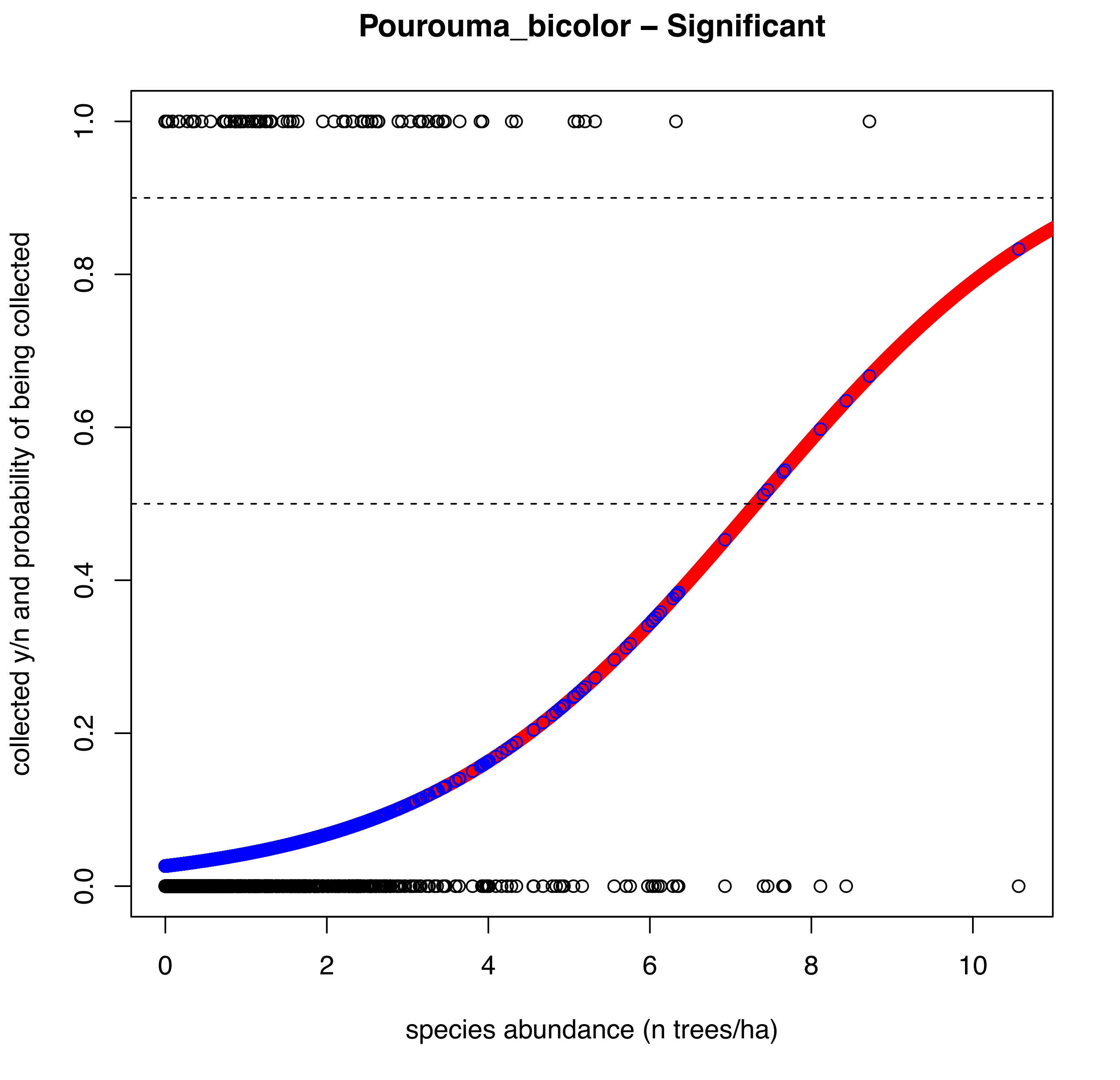
Pourouma_bicolor – Significant
collected y/n and probability of being collected
species abundance (n trees/ha)
0.0
0.2
0.4
0.6
0.8
1.0
0
2
4
6
8
10

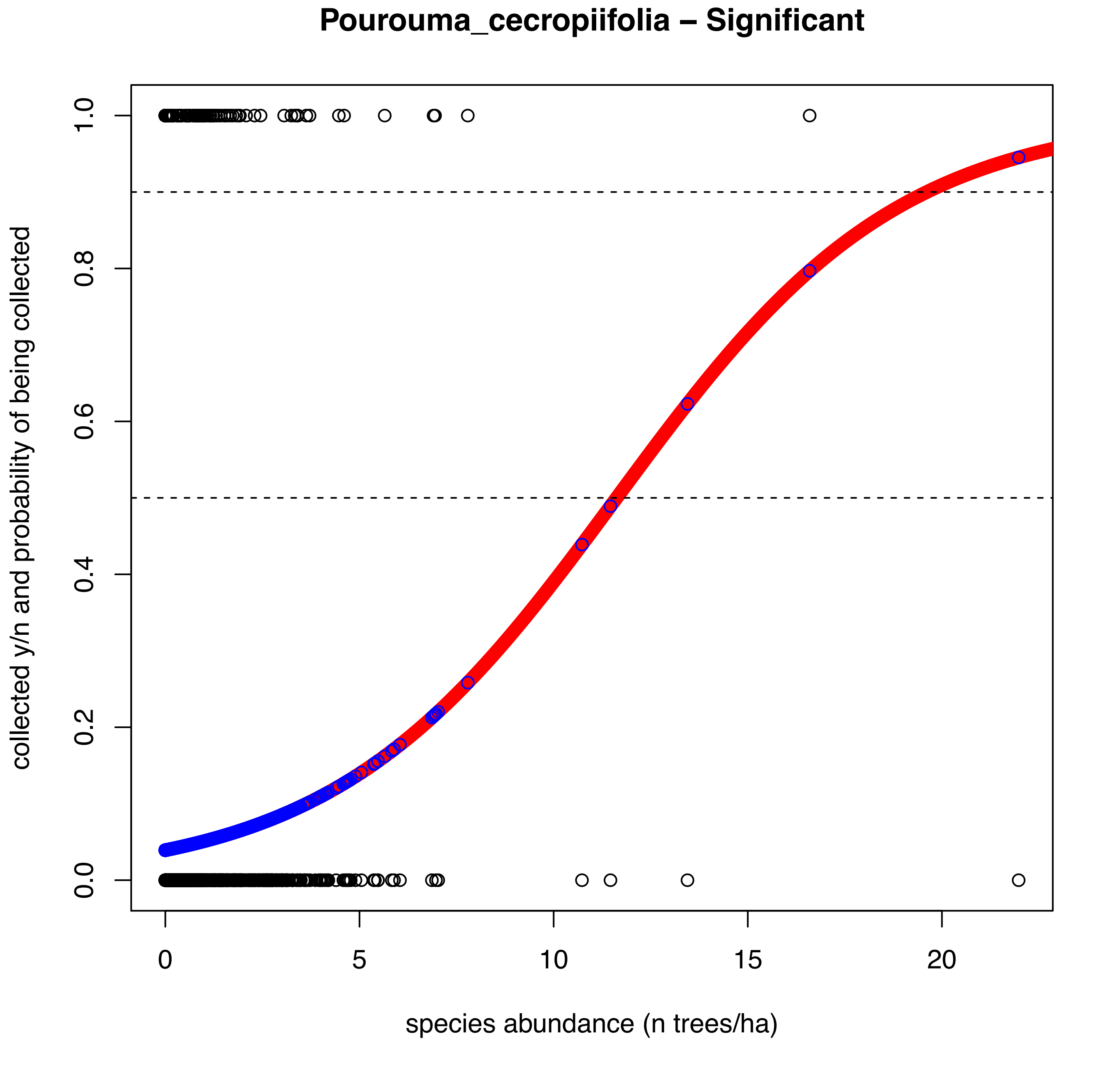

Pourouma_cecropiifolia – Significant
collected y/n and probability of being collected
species abundance (n trees/ha)
0.0
0.2
0.4
0.6
0.8
1.0
0
5
10
15
20

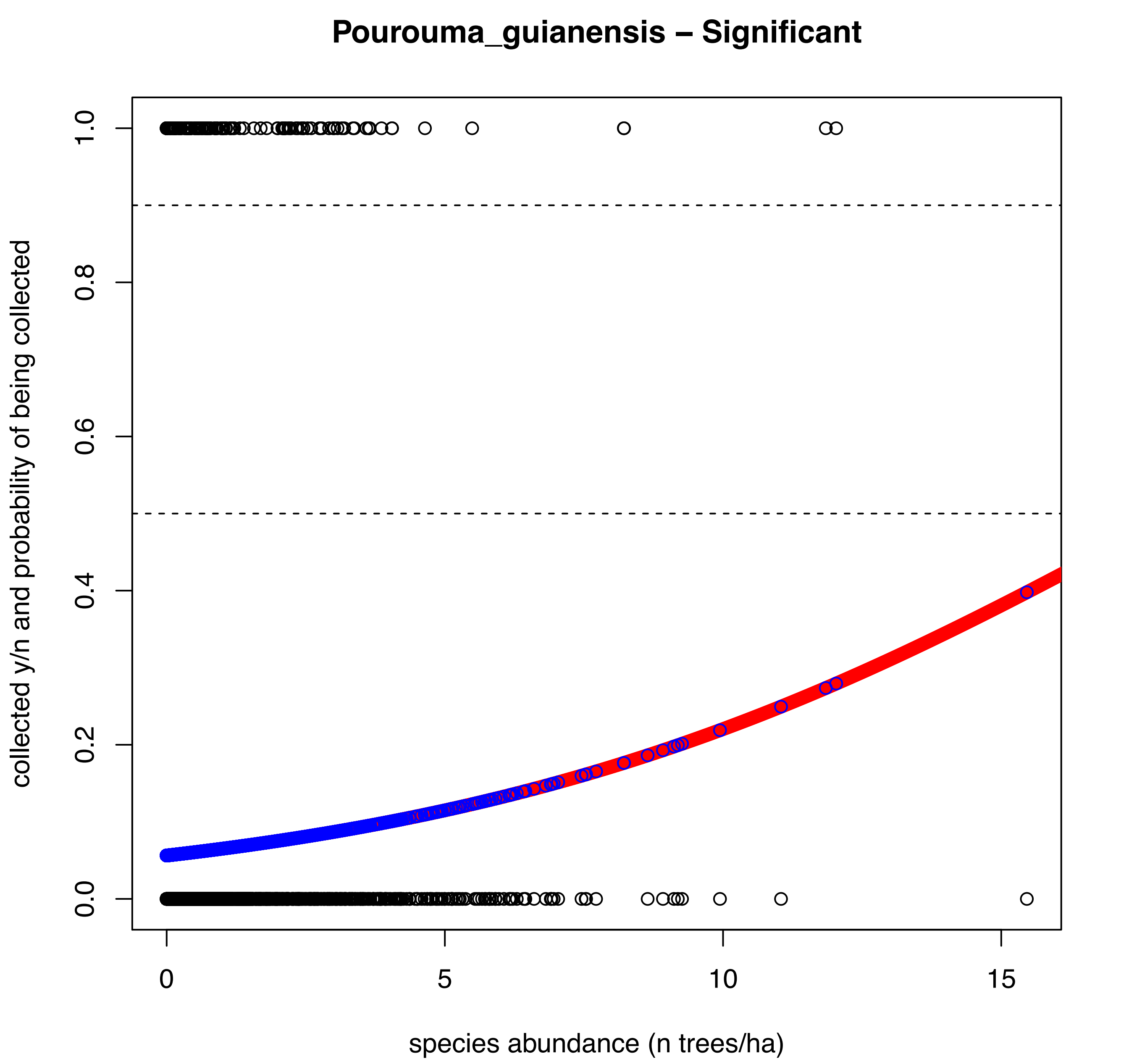

Pourouma_guianensis – Significant
collected y/n and probability of being collected
species abundance (n trees/ha)
0.0
0.2
0.4
0.6
0.8
1.0
0
5
10
15

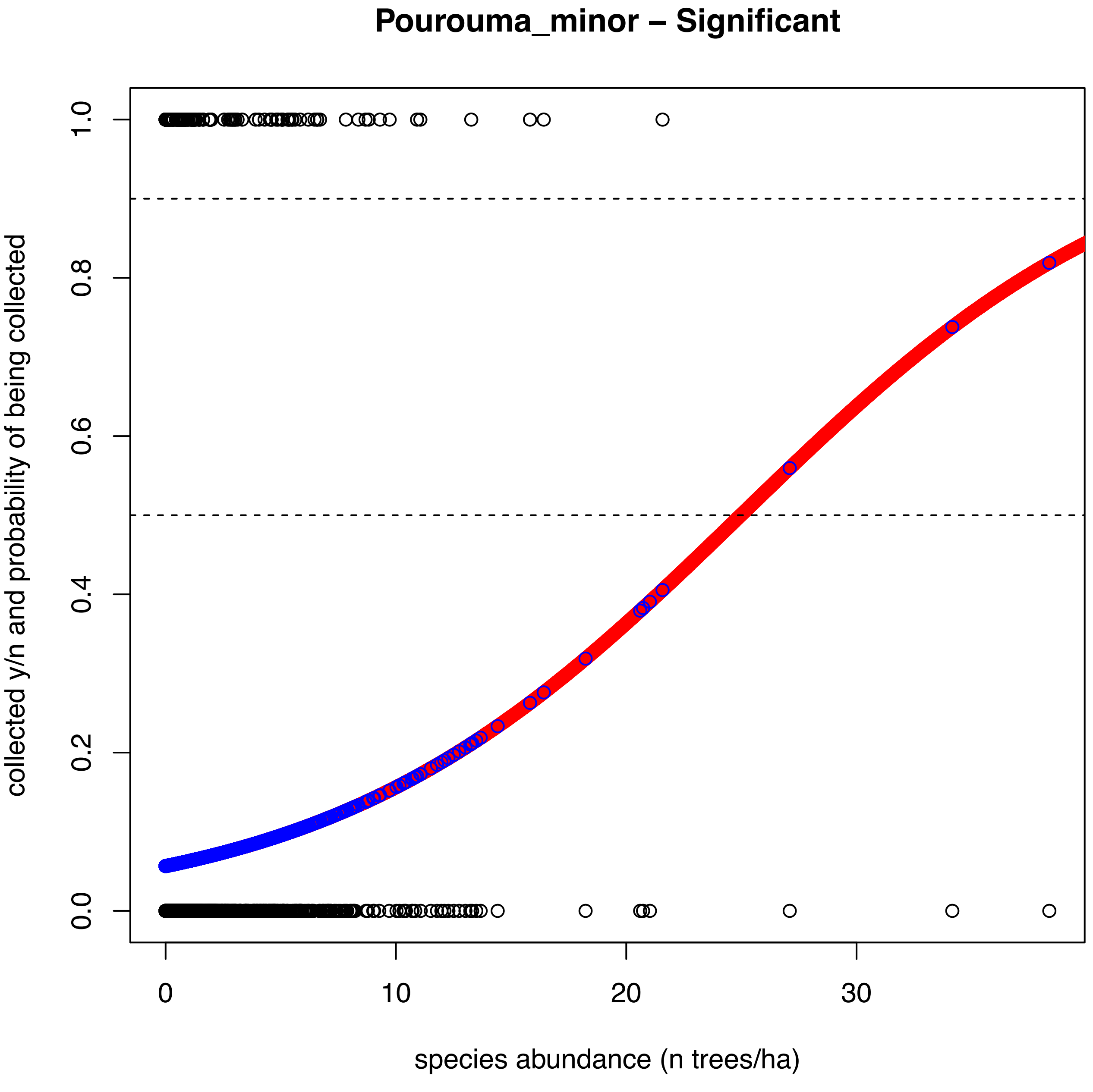
Pourouma_minor – Significant
collected y/n and probability of being collected
species abundance (n trees/ha)
0.0
0.2
0.4
0.6
0.8
1.0
0
10
20
30

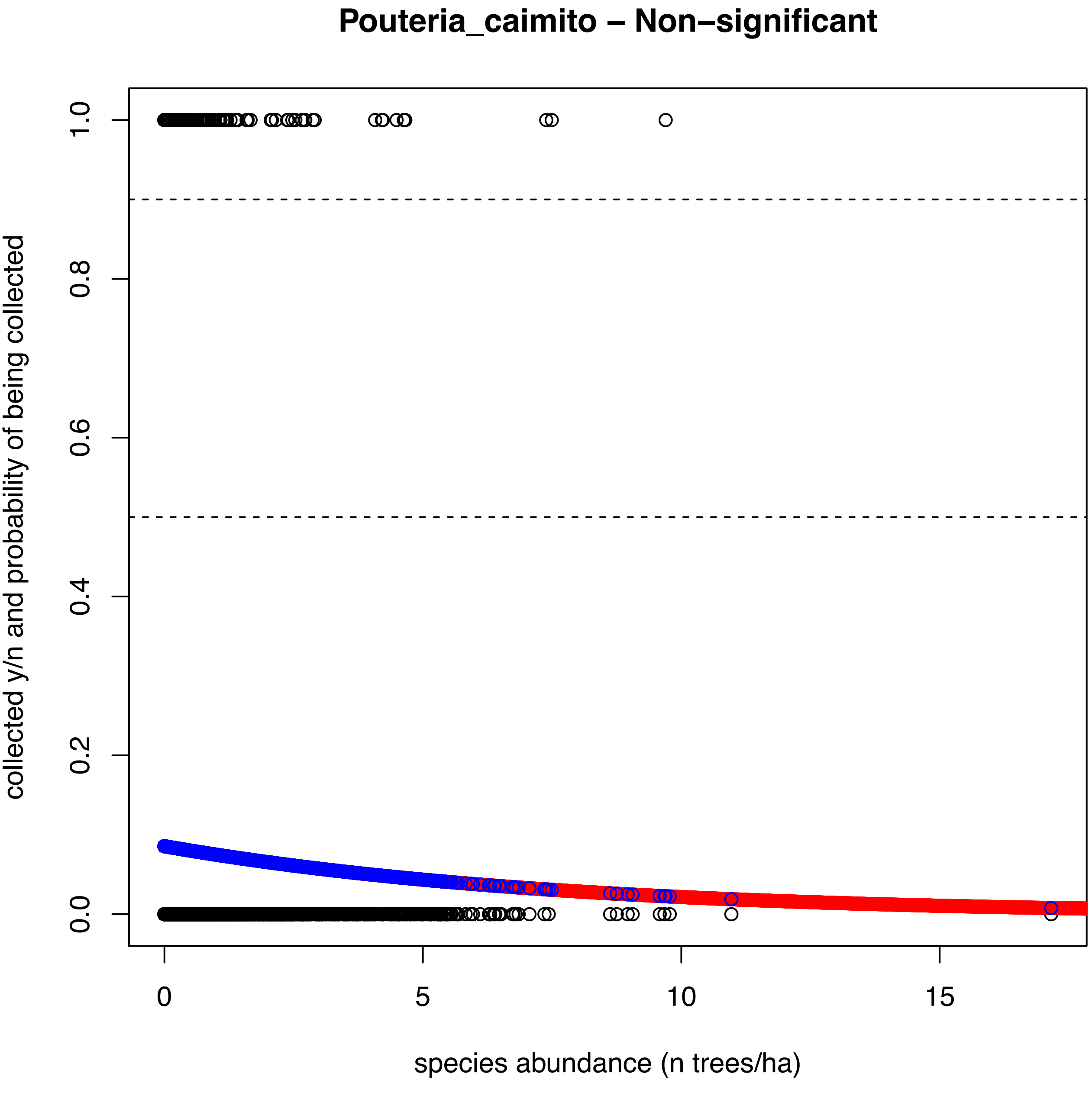
Pouteria_caimito – Non–significant
collected y/n and probability of being collected
species abundance (n trees/ha)
0.0
0.2
0.4
0.6
0.8
1.0
0
5
10
15

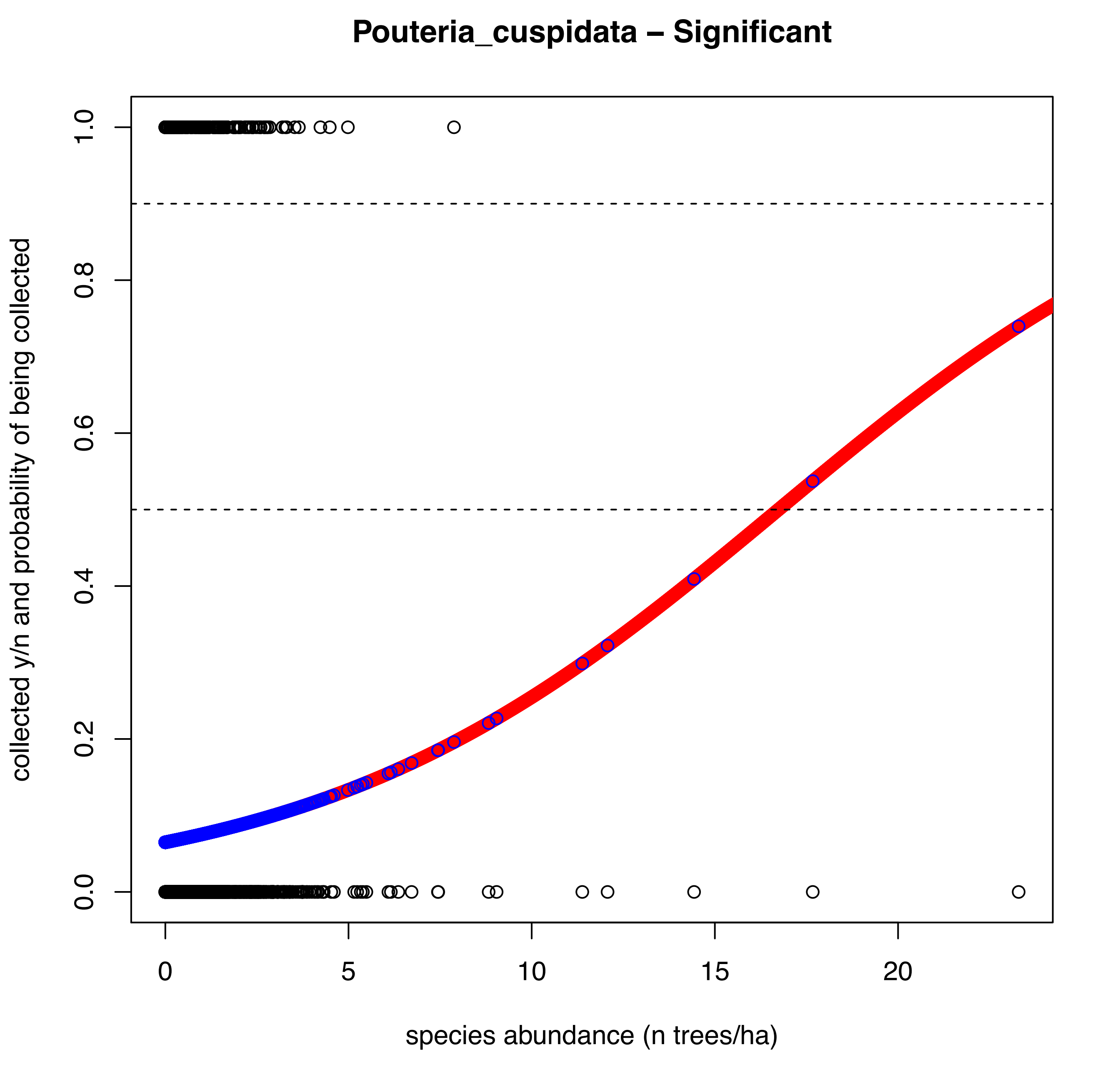

Pouteria_cuspidata – Significant
collected y/n and probability of being collected
species abundance (n trees/ha)
0.0
0.2
0.4
0.6
0.8
1.0
0
5
10
15
20

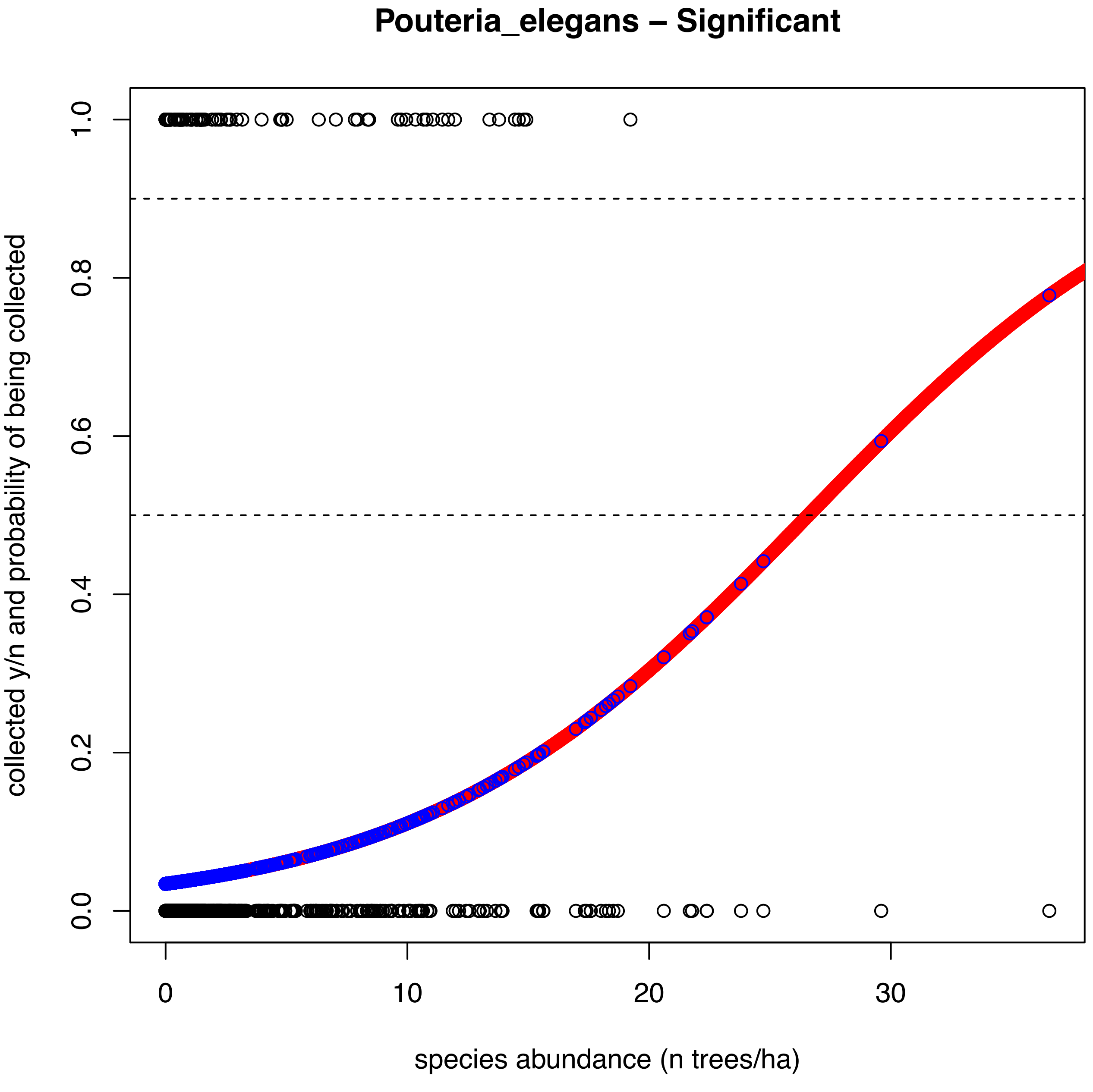

Pouteria_elegans – Significant
collected y/n and probability of being collected
species abundance (n trees/ha)
0.0
0.2
0.4
0.6
0.8
1.0
0
10
20
30

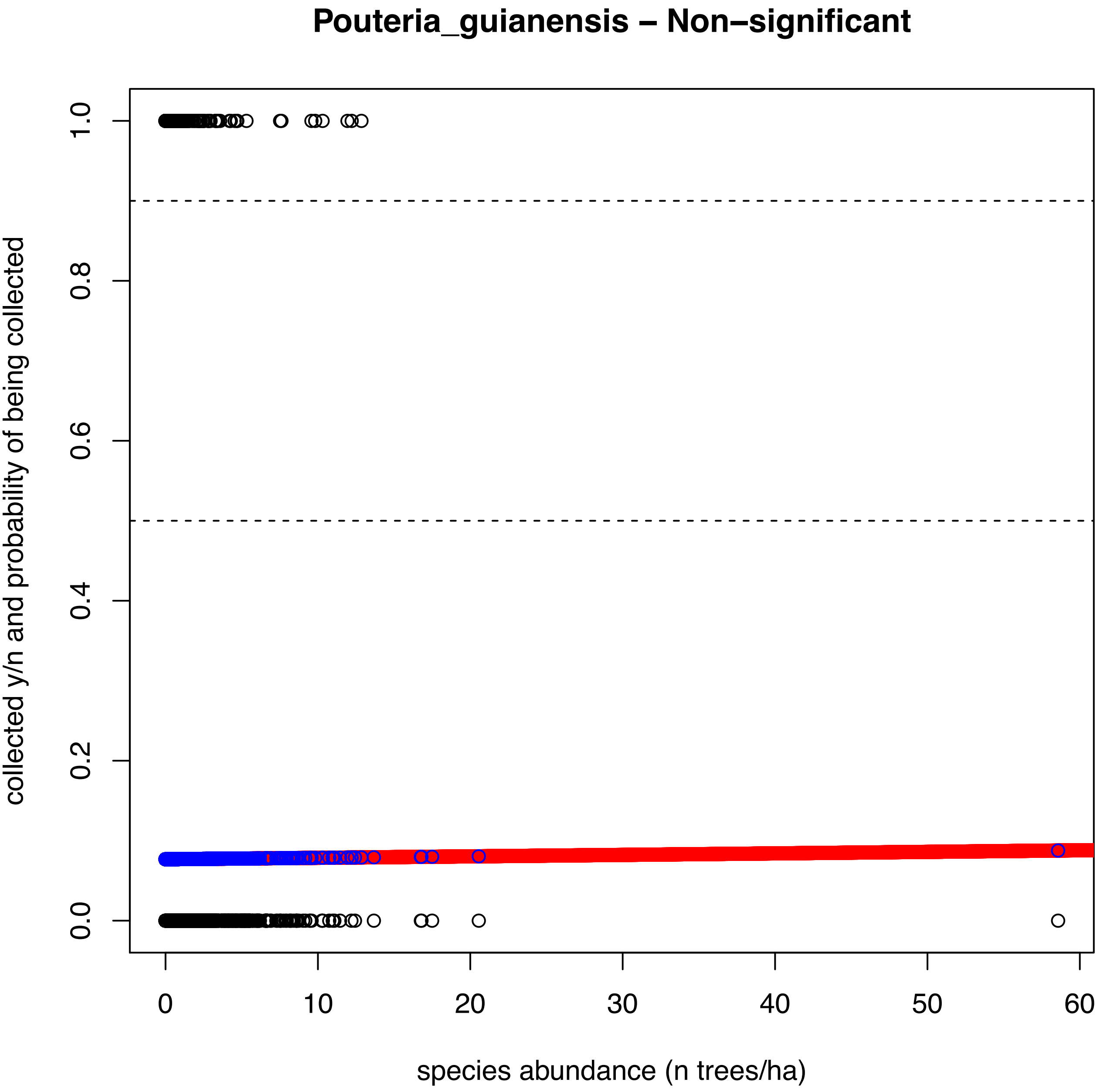

Pouteria_guianensis – Non–significant
collected y/n and probability of being collected
0.0
0.2
0.4
0.6
0.8
1.0
0
10
20
30
40
50
60
species abundance (n trees/ha)

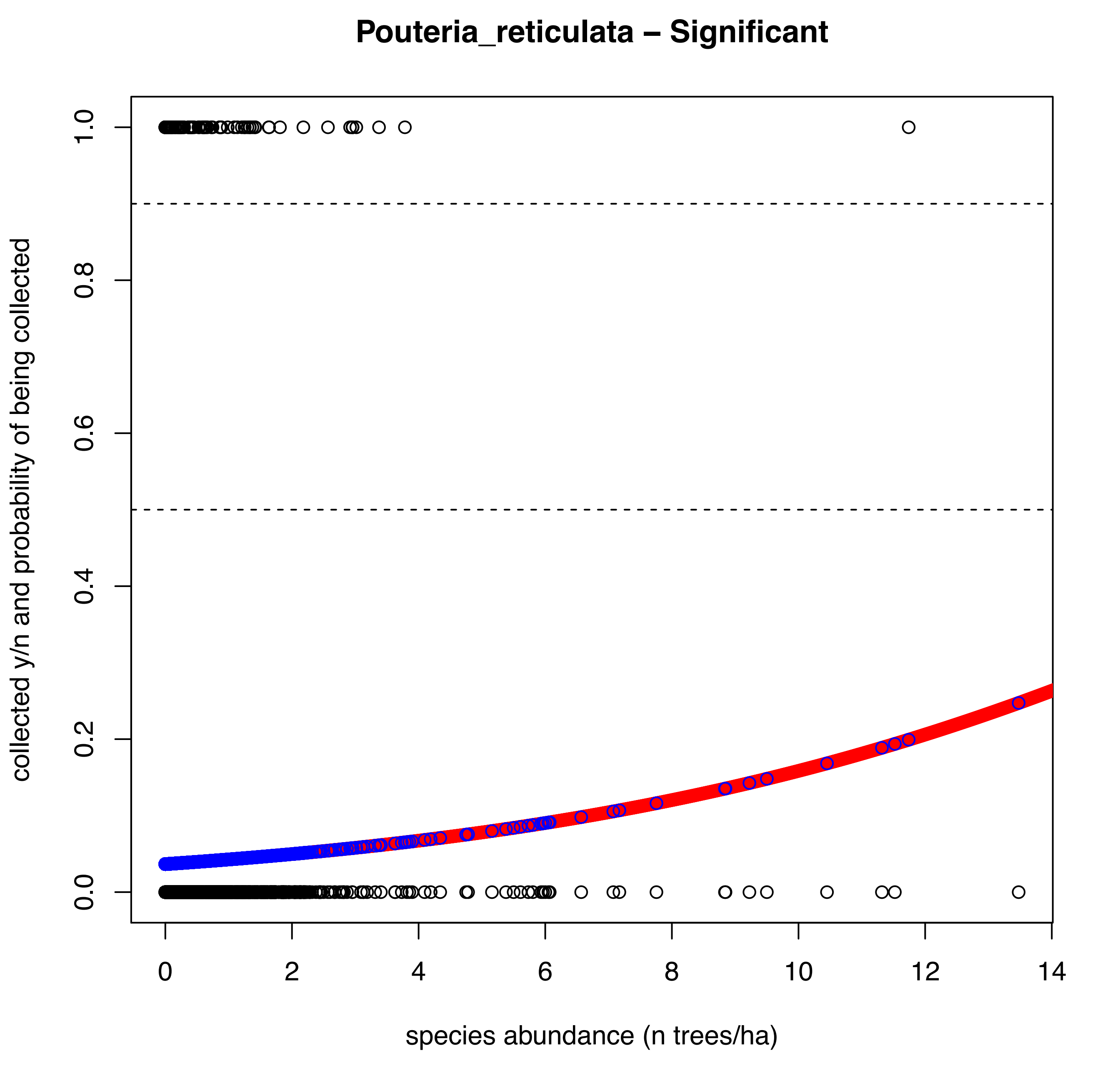
Pouteria_reticulata – Significant
collected y/n and probability of being collected
species abundance (n trees/ha)
0.0
0.2
0.4
0.6
0.8
1.0
0
2
4
6
8
10
12
14

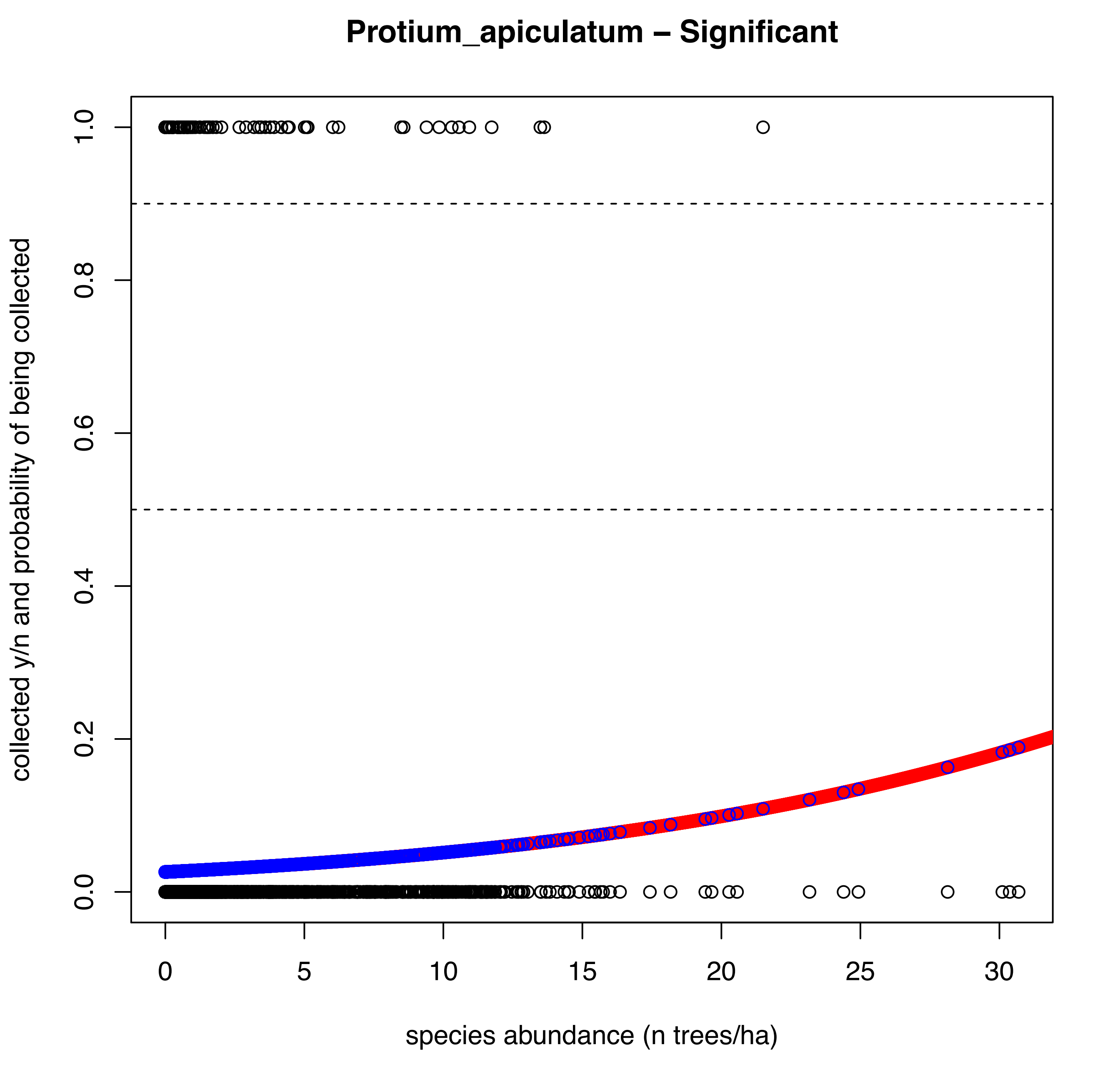

Protium_apiculatum – Significant
collected y/n and probability of being collected
0.0
0.2
0.4
0.6
0.8
1.0
0
5
10
15
20
25
30
species abundance (n trees/ha)

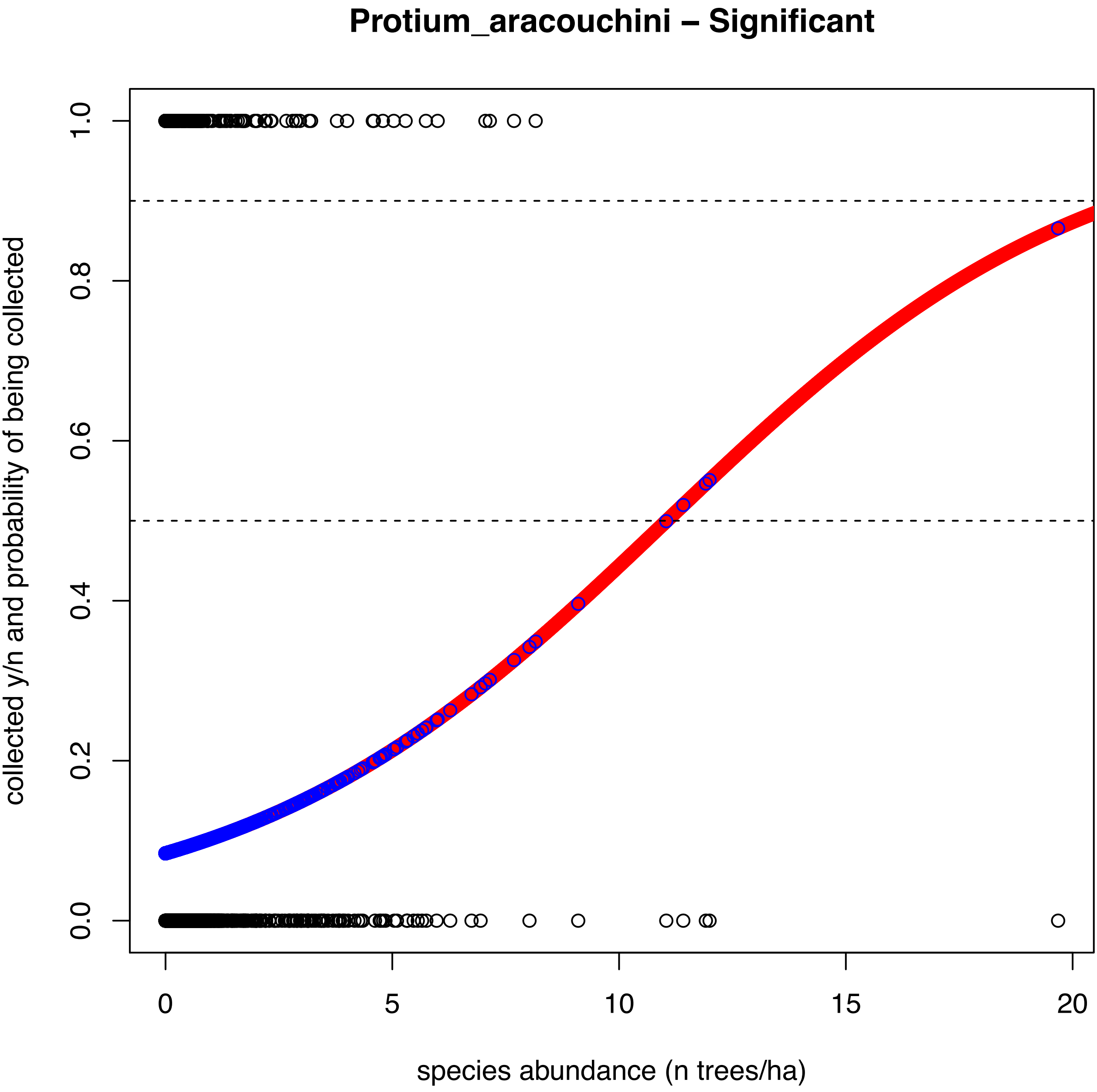
Protium_aracouchini – Significant
collected y/n and probability of being collected
species abundance (n trees/ha)
0.0
0.2
0.4
0.6
0.8
1.0
0
5
10
15
20

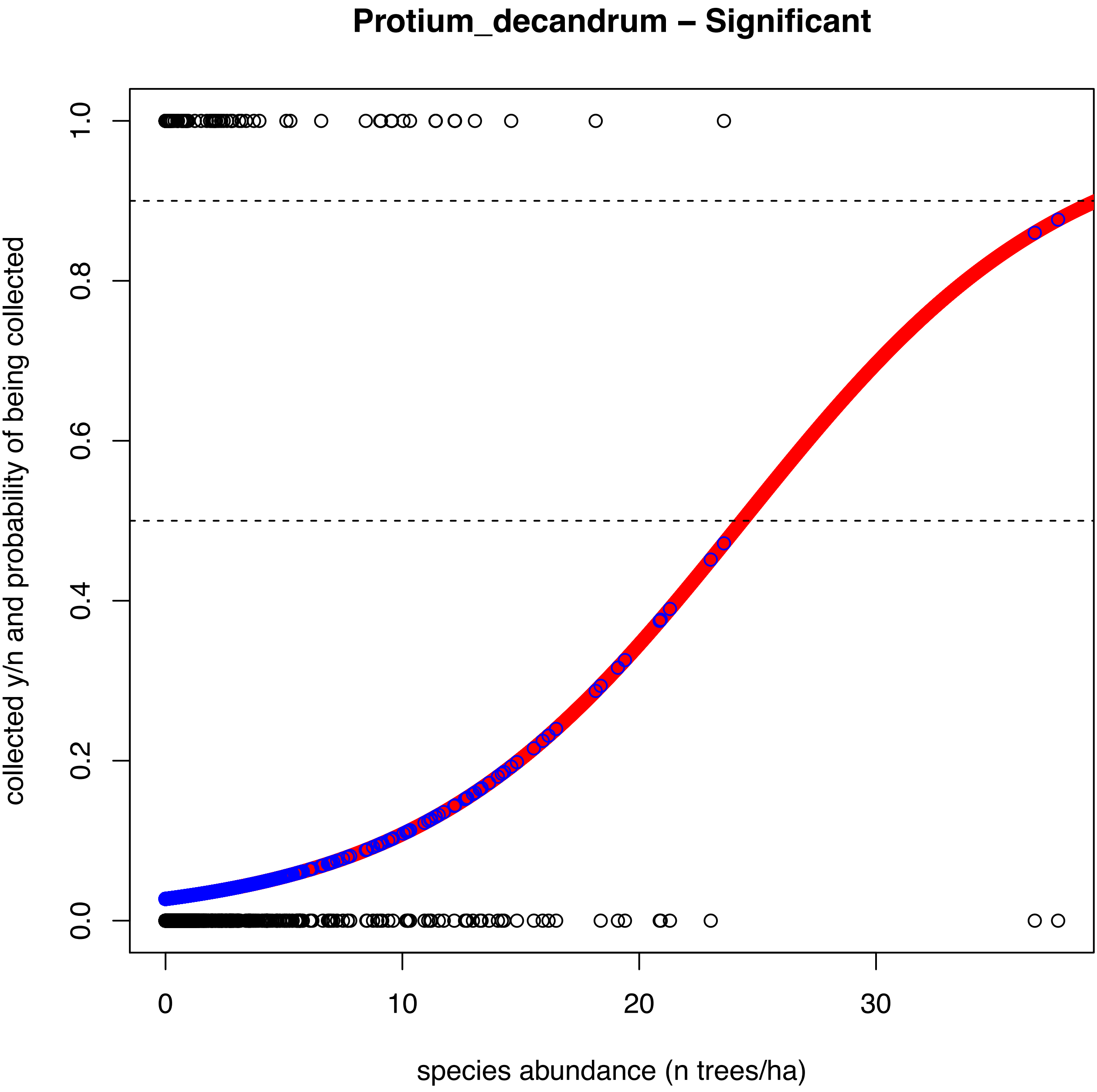

Protium_decandrum – Significant
collected y/n and probability of being collected
species abundance (n trees/ha)
0.0
0.2
0.4
0.6
0.8
1.0
0
10
20
30

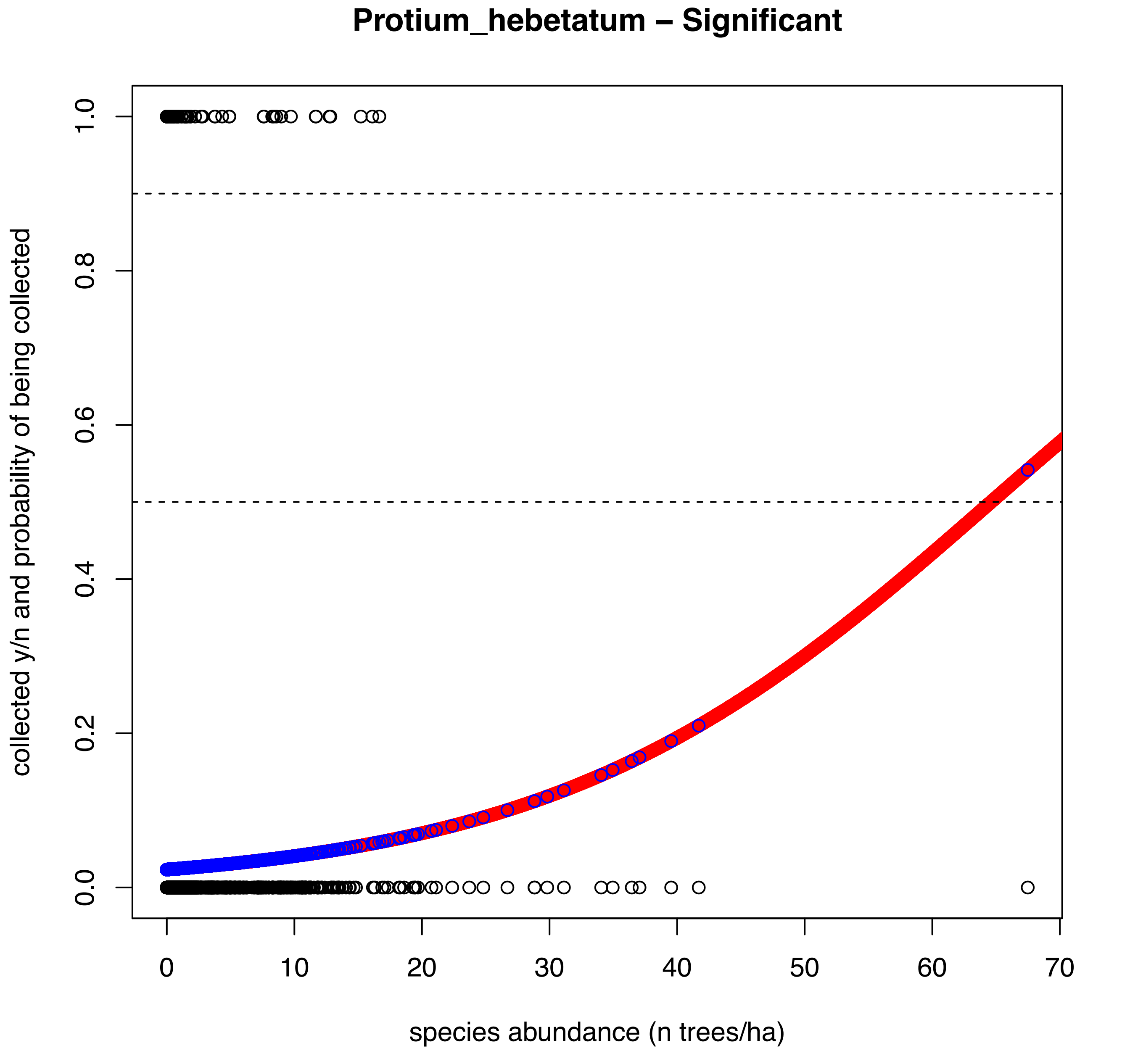

Protium_hebetatum – Significant
collected y/n and probability of being collected
species abundance (n trees/ha)
0.0
0.2
0.4
0.6
0.8
1.0
0
10
20
30
40
50
60
70

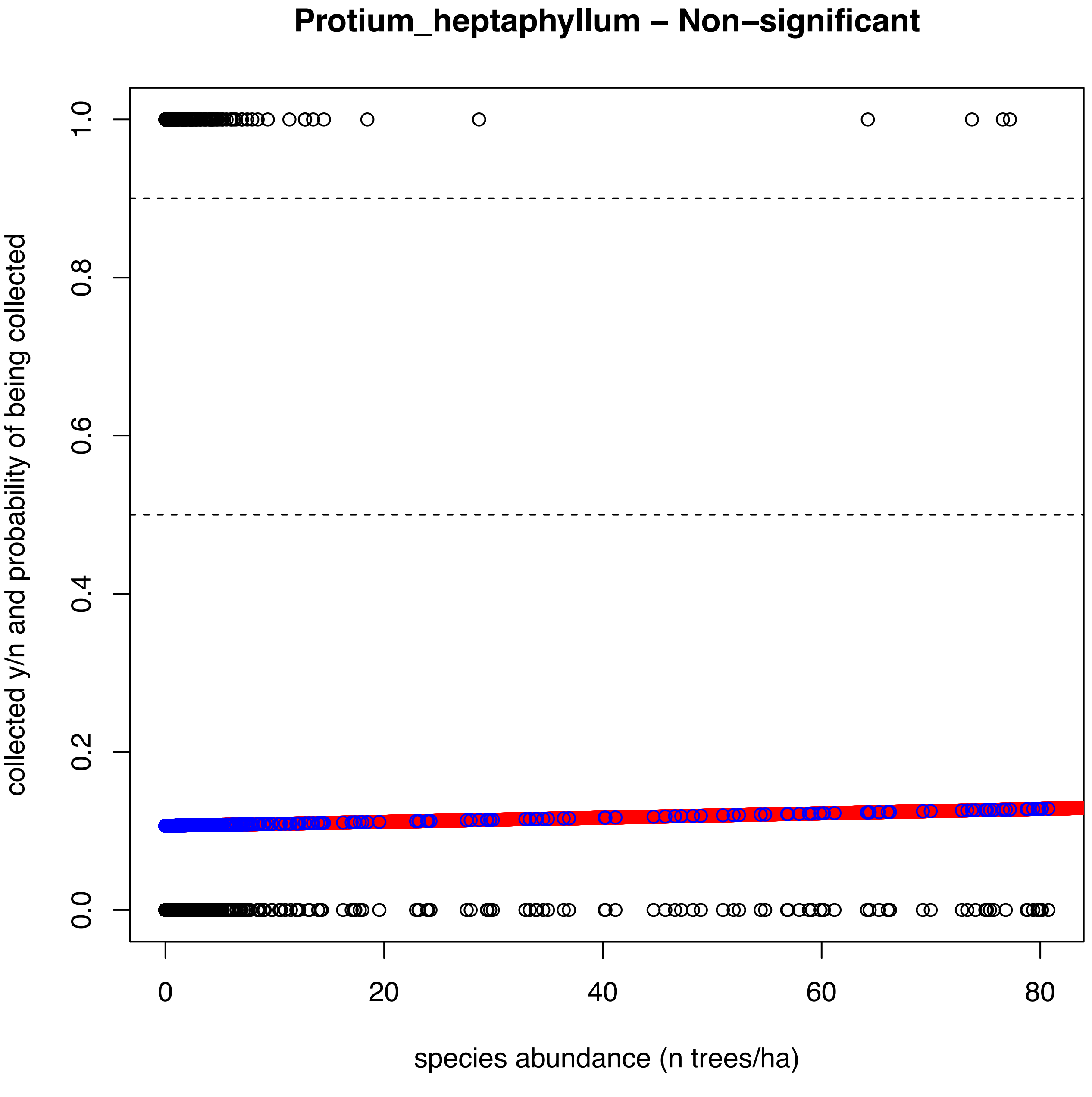
Protium_heptaphyllum – Non–significant
collected y/n and probability of being collected
species abundance (n trees/ha)
0.0
0.2
0.4
0.6
0.8
1.0
0
20
40
60
80

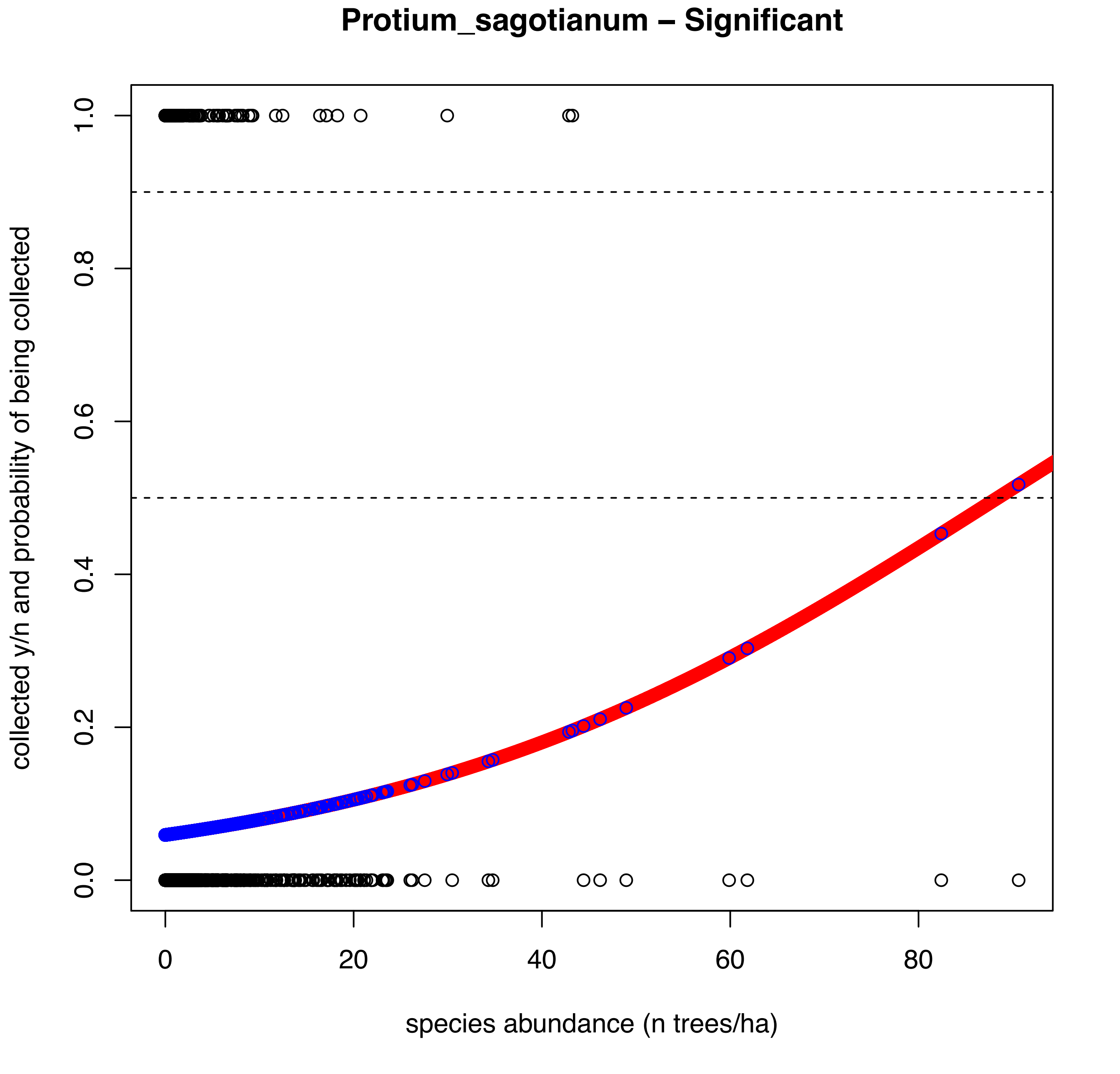
Protium_sagotianum – Significant
collected y/n and probability of being collected
species abundance (n trees/ha)
0.0
0.2
0.4
0.6
0.8
1.0
0
20
40
60
80

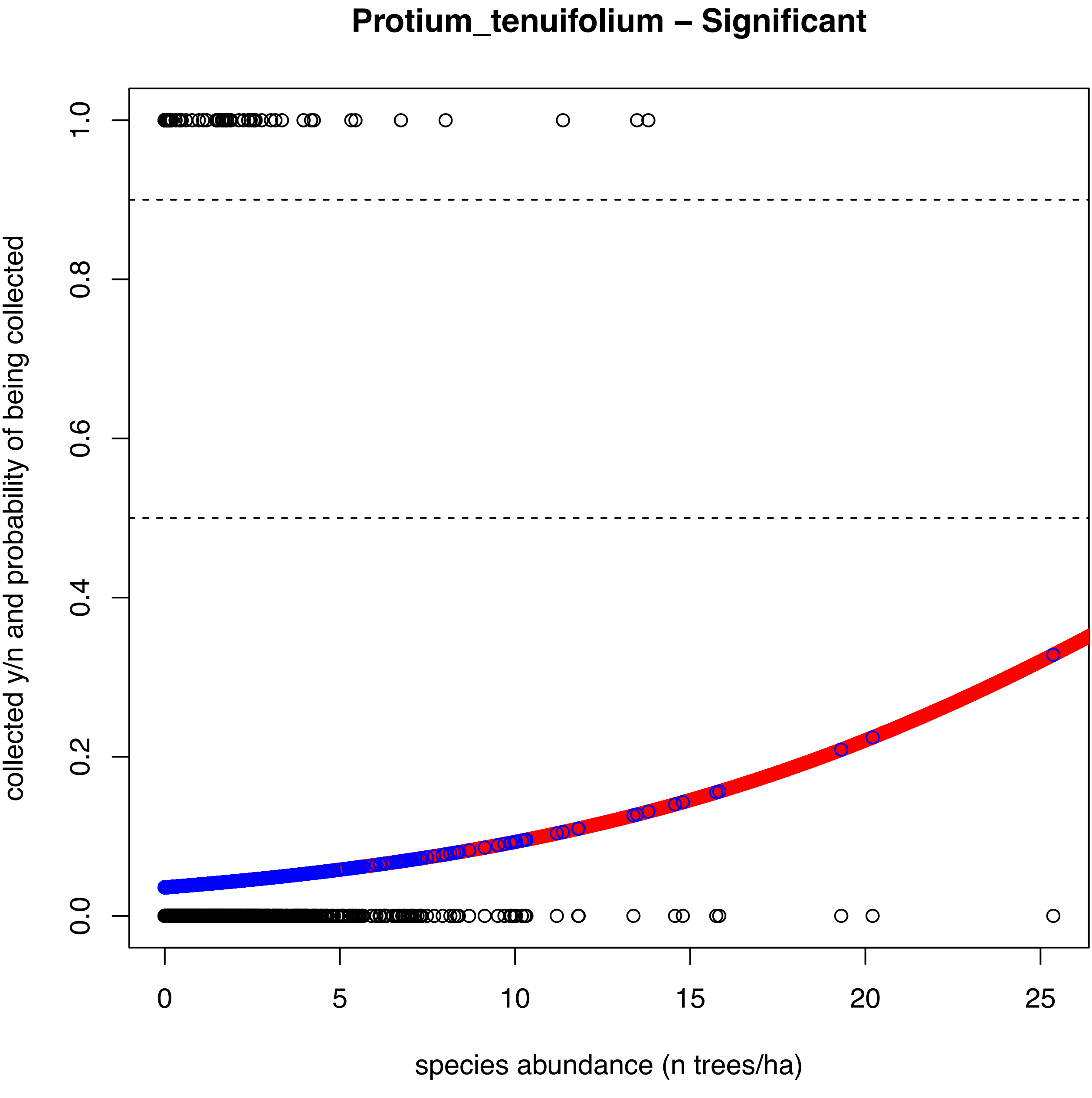

Protium_tenuifolium – Significant
collected y/n and probability of being collected
species abundance (n trees/ha)
0.0
0.2
0.4
0.6
0.8
1.0
0
5
10
15
20
25

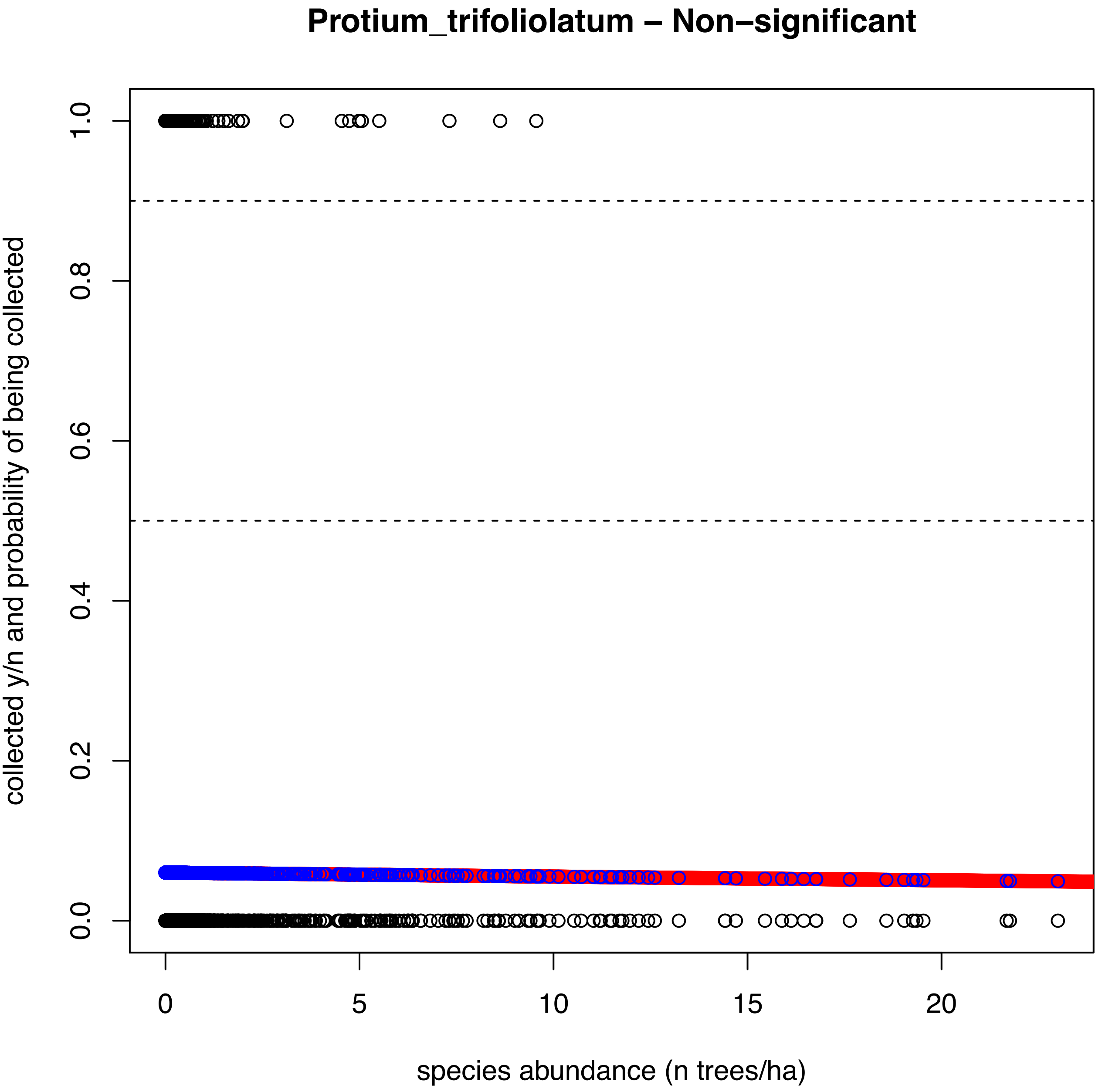
Protium_trifoliolatum – Non−significant
collected y/n and probability of being collected
species abundance (n trees/ha)
0.0
0.2
0.4
0.6
0.8
1.0
0
5
10
15
20

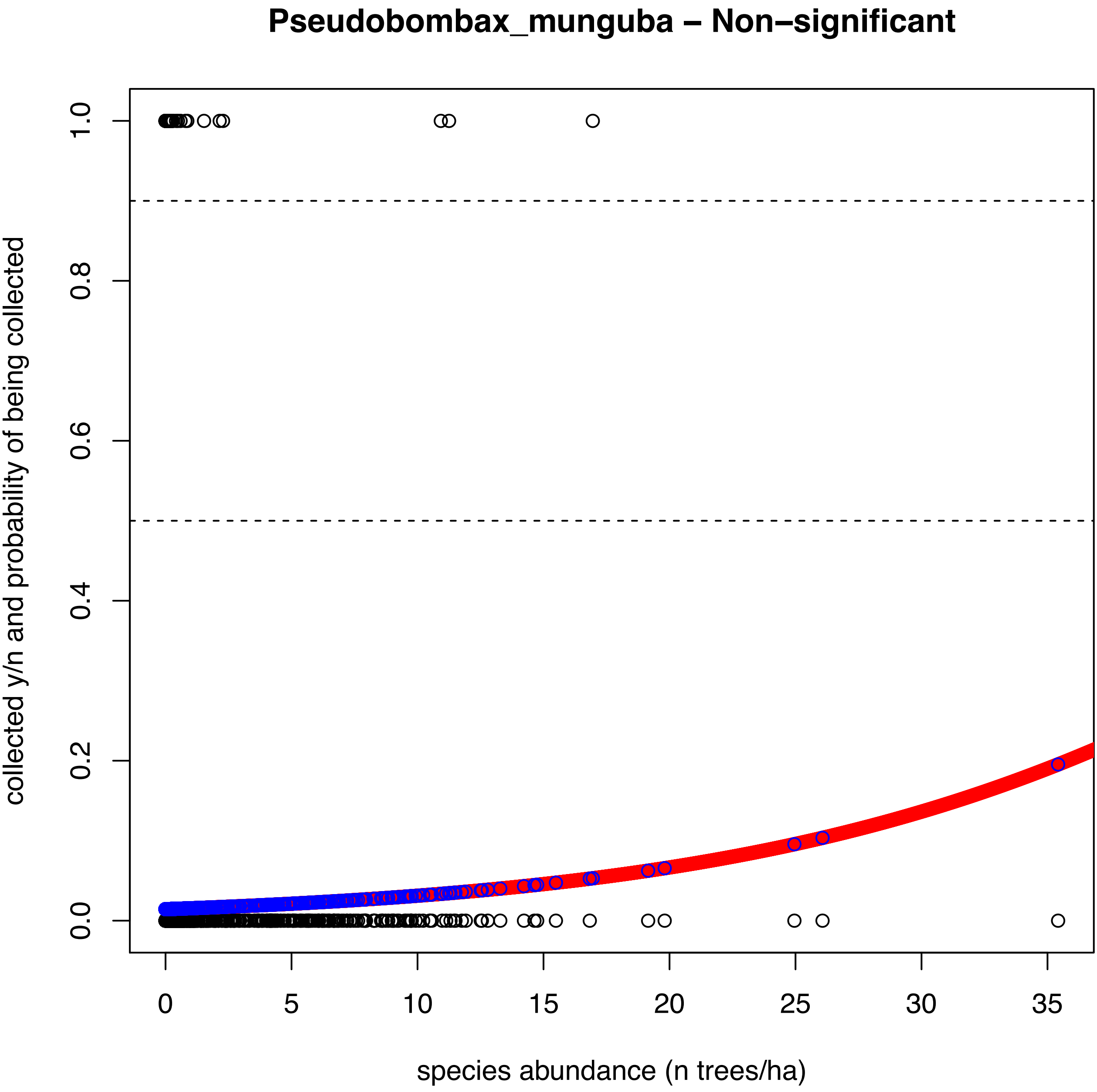
Pseudobombax_munguba – Non–significant
collected y/n and probability of being collected
species abundance (n trees/ha)
0.0
0.2
0.4
0.6
0.8
1.0
0
5
10
15
20
25
30
35

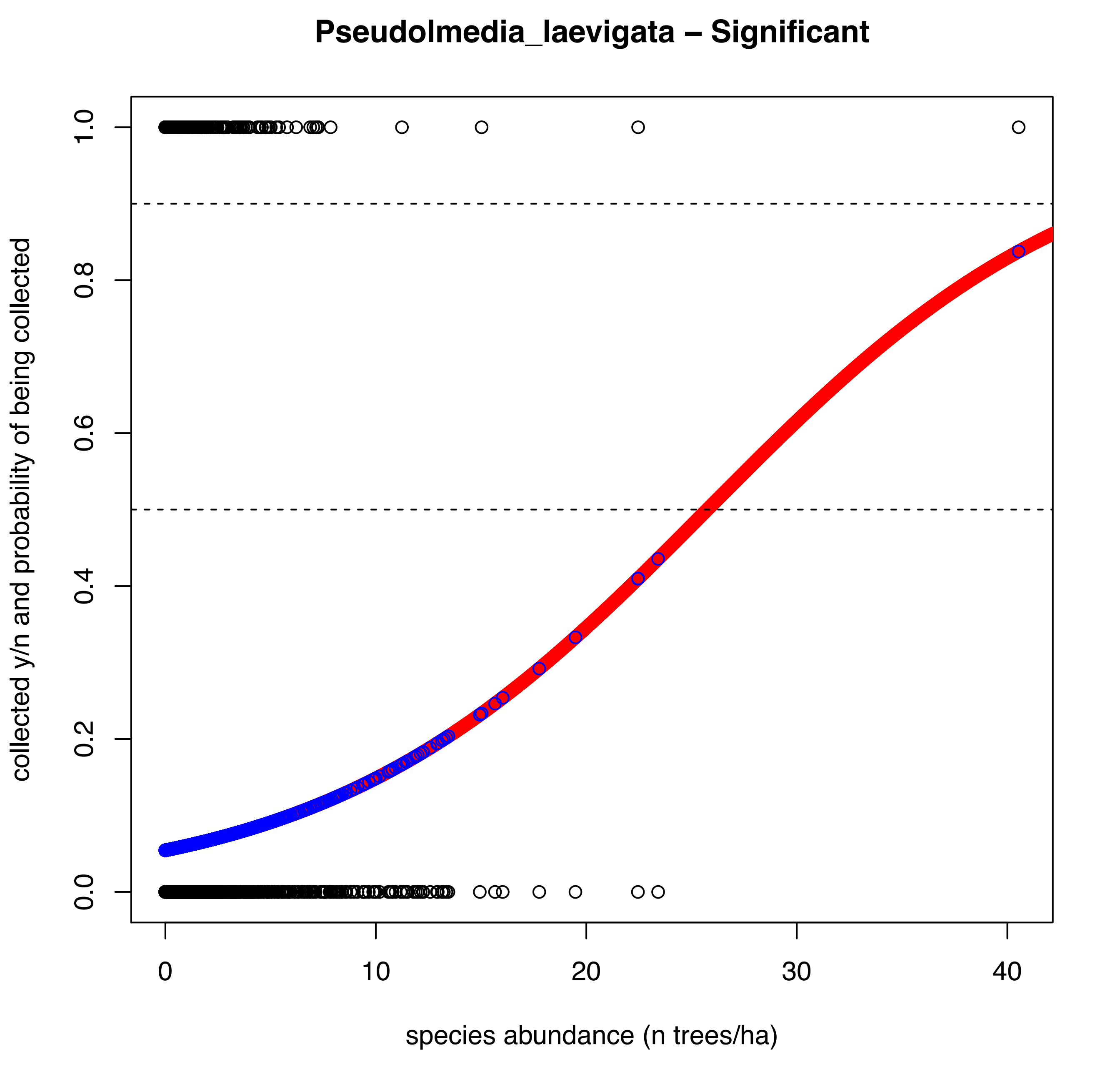
Pseudolmedia_laevigata – Significant
collected y/n and probability of being collected
species abundance (n trees/ha)
0.0
0.2
0.4
0.6
0.8
1.0
0
10
20
30
40

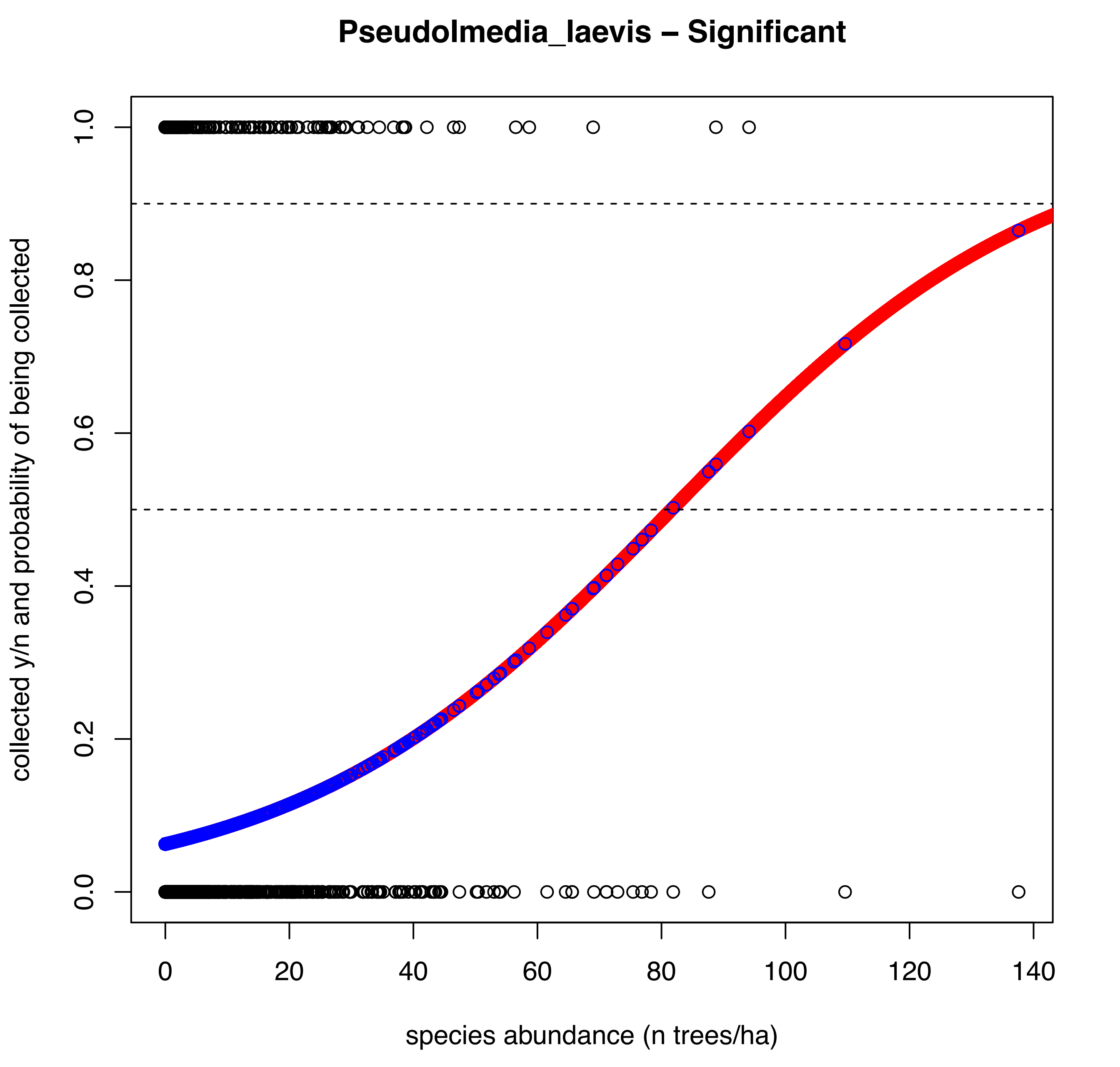

Pseudolmedia_laevis – Significant
collected y/n and probability of being collected
species abundance (n trees/ha)
0.0
0.2
0.4
0.6
0.8
1.0
0
20
40
60
80
100
120
140

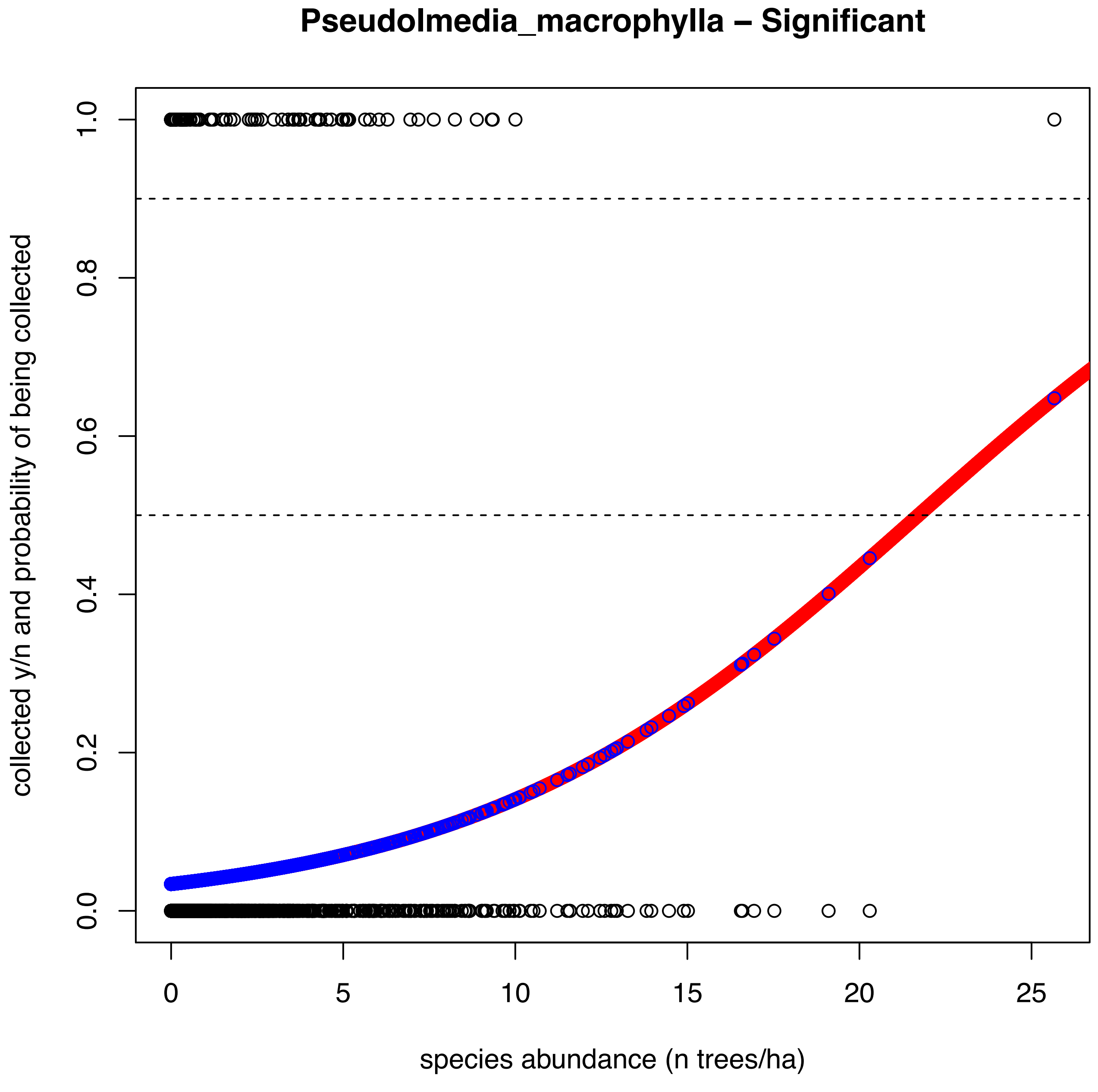

Pseudolmedia_macrophylla – Significant
collected y/n and probability of being collected
species abundance (n trees/ha)
0.0
0.2
0.4
0.6
0.8
1.0
0
5
10
15
20
25

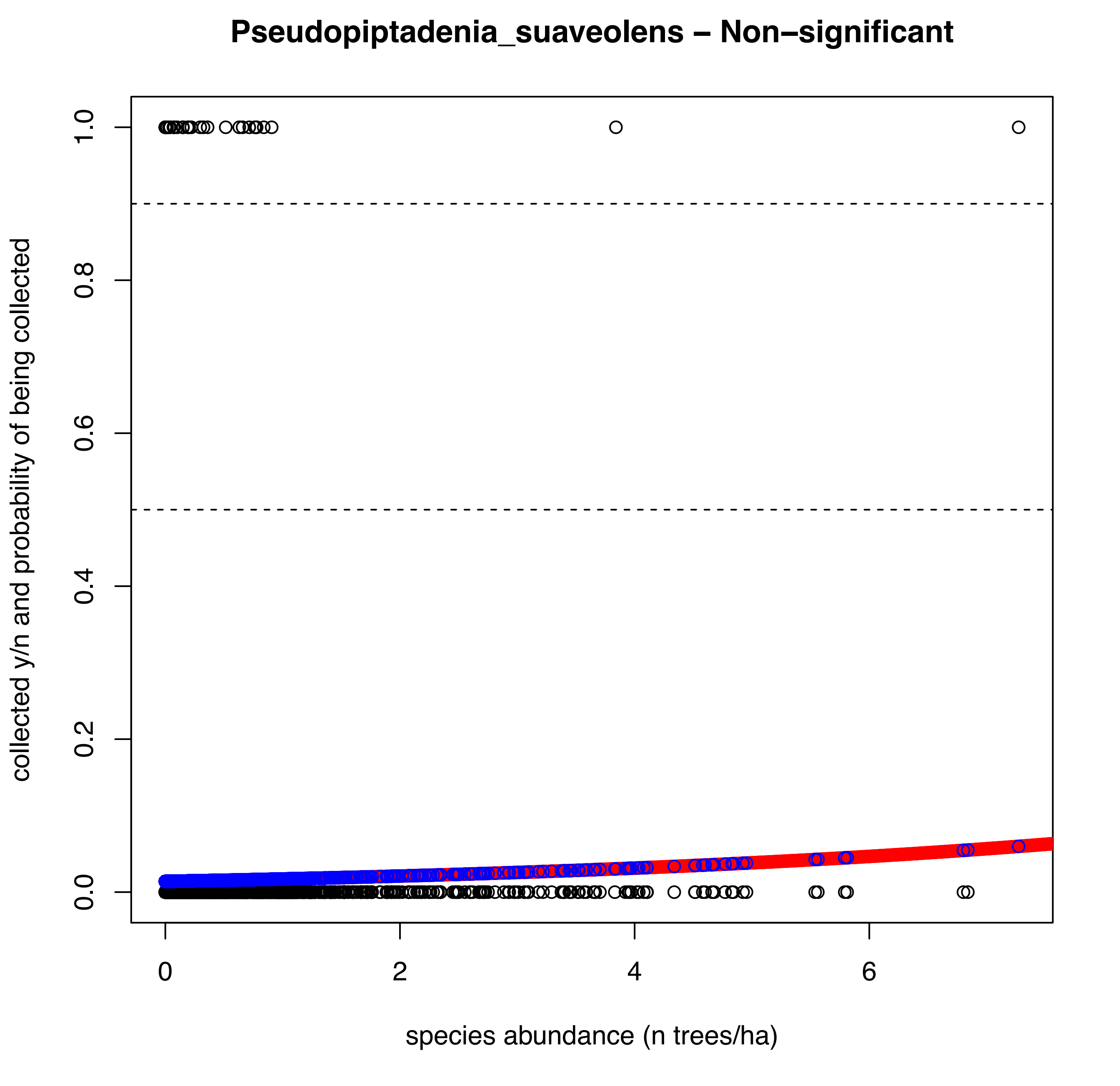
Pseudopiptadenia_suaveolens – Non–significant
collected y/n and probability of being collected
species abundance (n trees/ha)
0.0
0.2
0.4
0.6
0.8
1.0
0
2
4
6

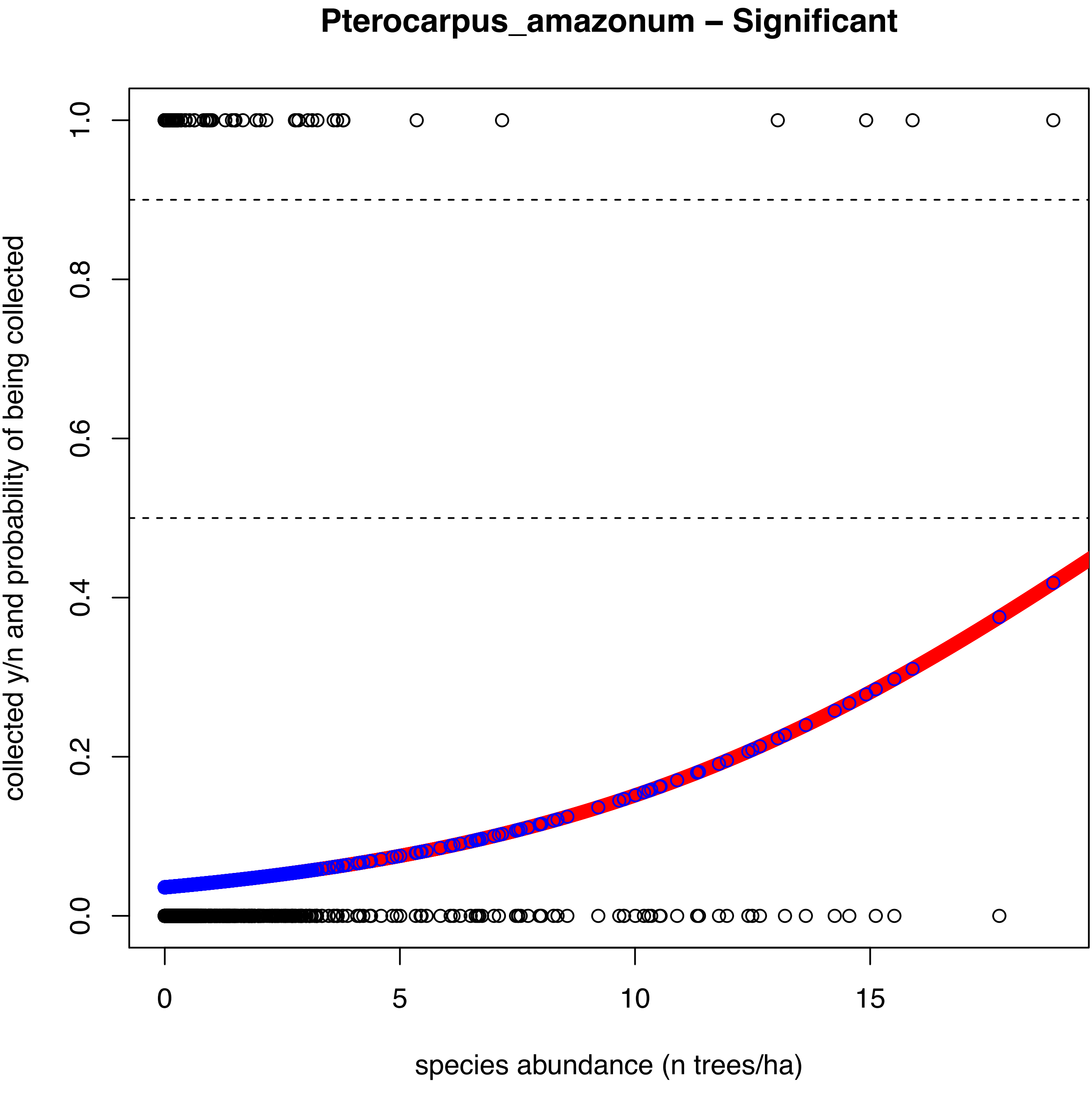
Pterocarpus_amazonum – Significant
collected y/n and probability of being collected
species abundance (n trees/ha)
0.0
0.2
0.4
0.6
0.8
1.0
0
5
10
15

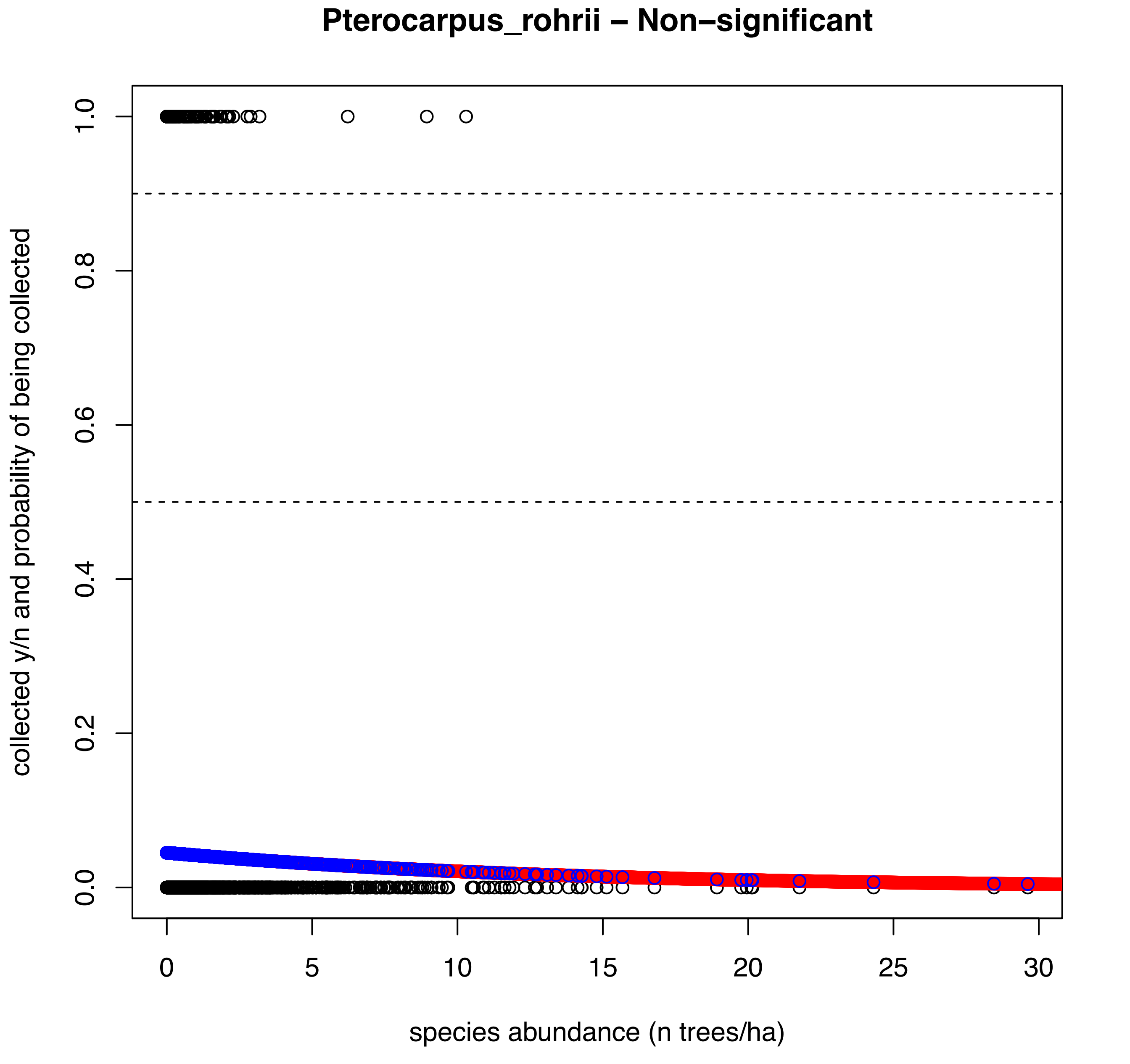
Pterocarpus_rohrii – Non–significant
collected y/n and probability of being collected
0.0
0.2
0.4
0.6
0.8
1.0
0
5
10
15
20
25
30
species abundance (n trees/ha)

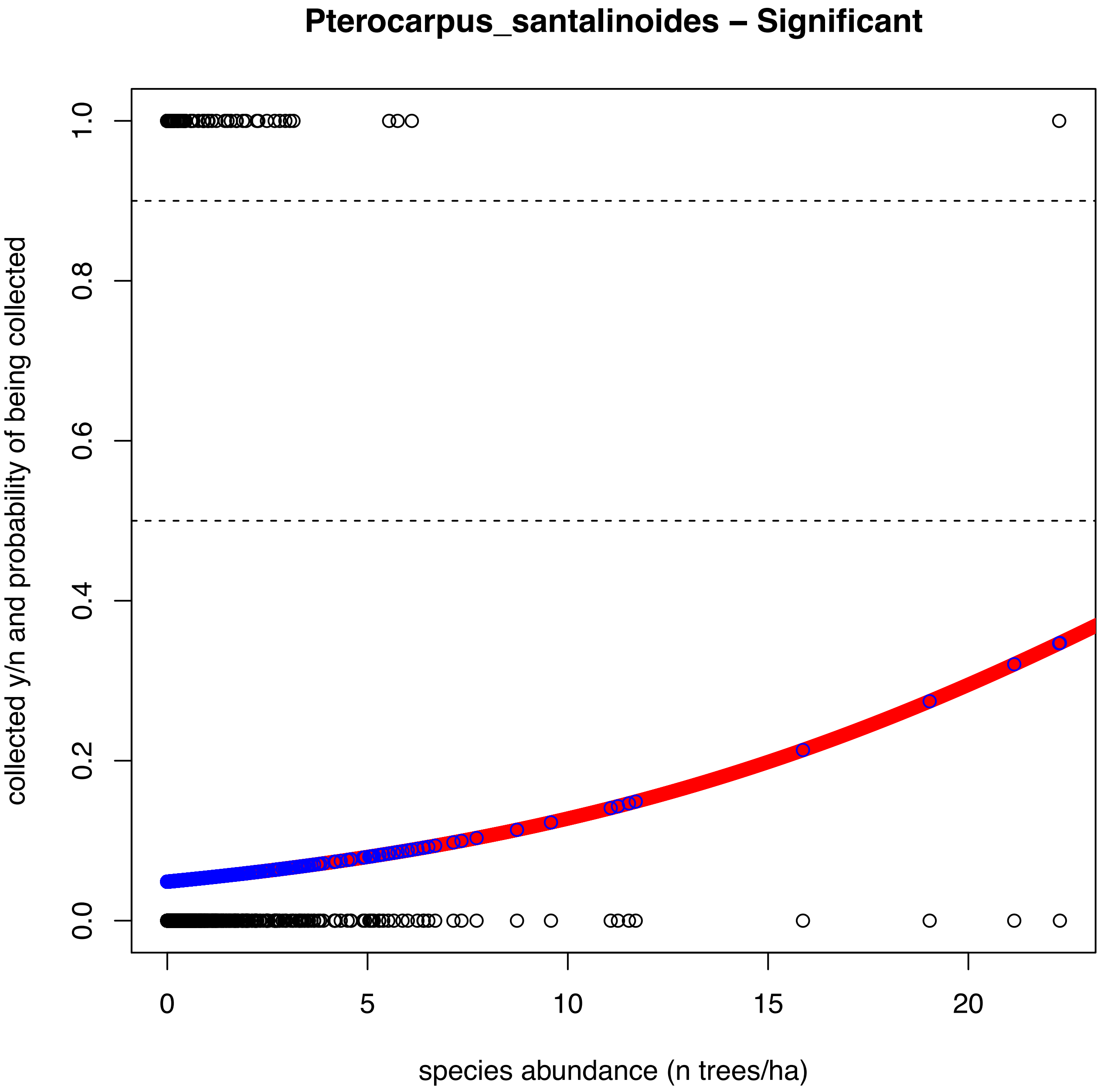
Pterocarpus_santalinoides – Significant
collected y/n and probability of being collected
species abundance (n trees/ha)
0.0
0.2
0.4
0.6
0.8
1.0
0
5
10
15
20

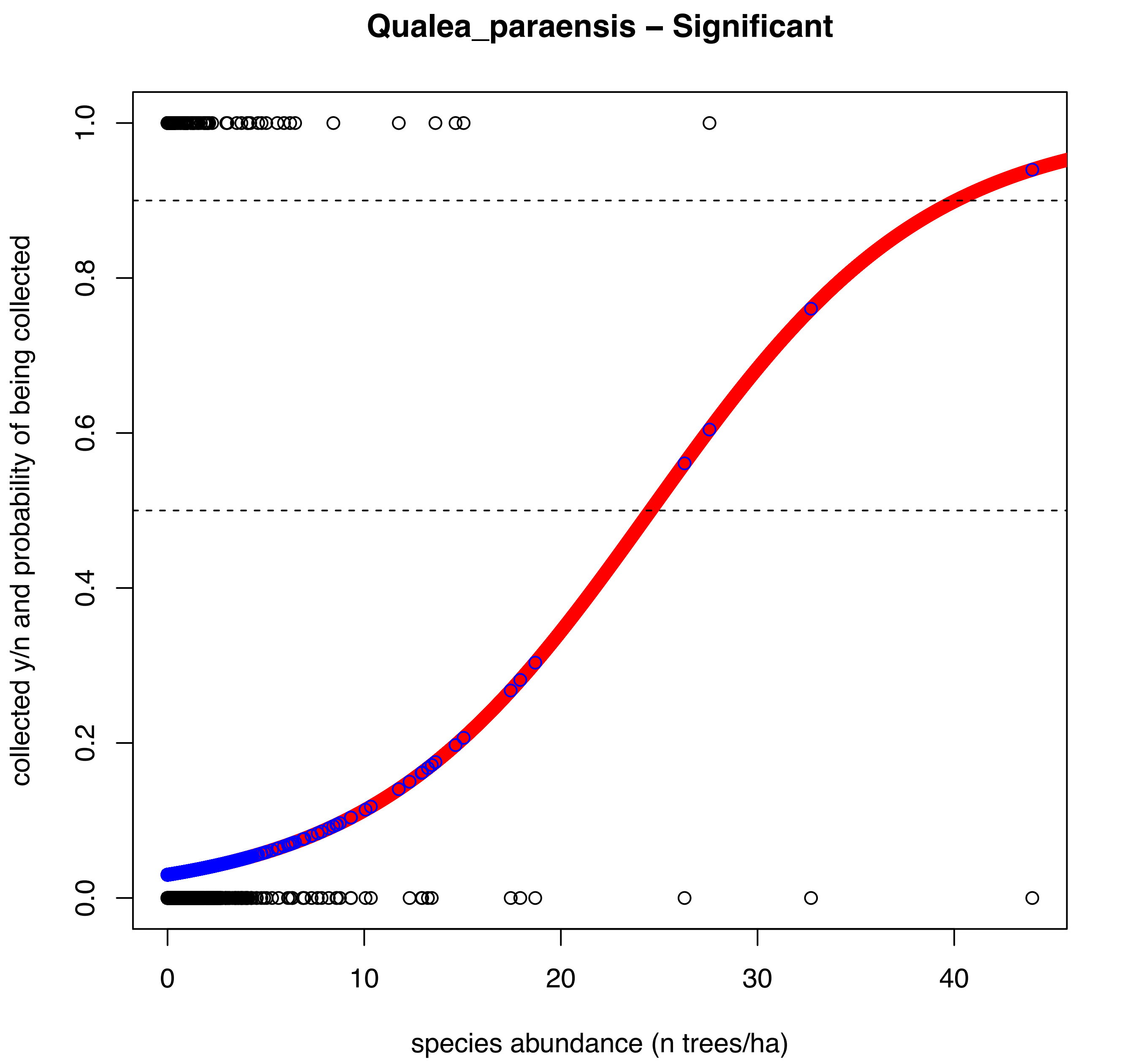
Qualea_paraensis – Significant
collected y/n and probability of being collected
species abundance (n trees/ha)
0.0
0.2
0.4
0.6
0.8
1.0
0
10
20
30
40

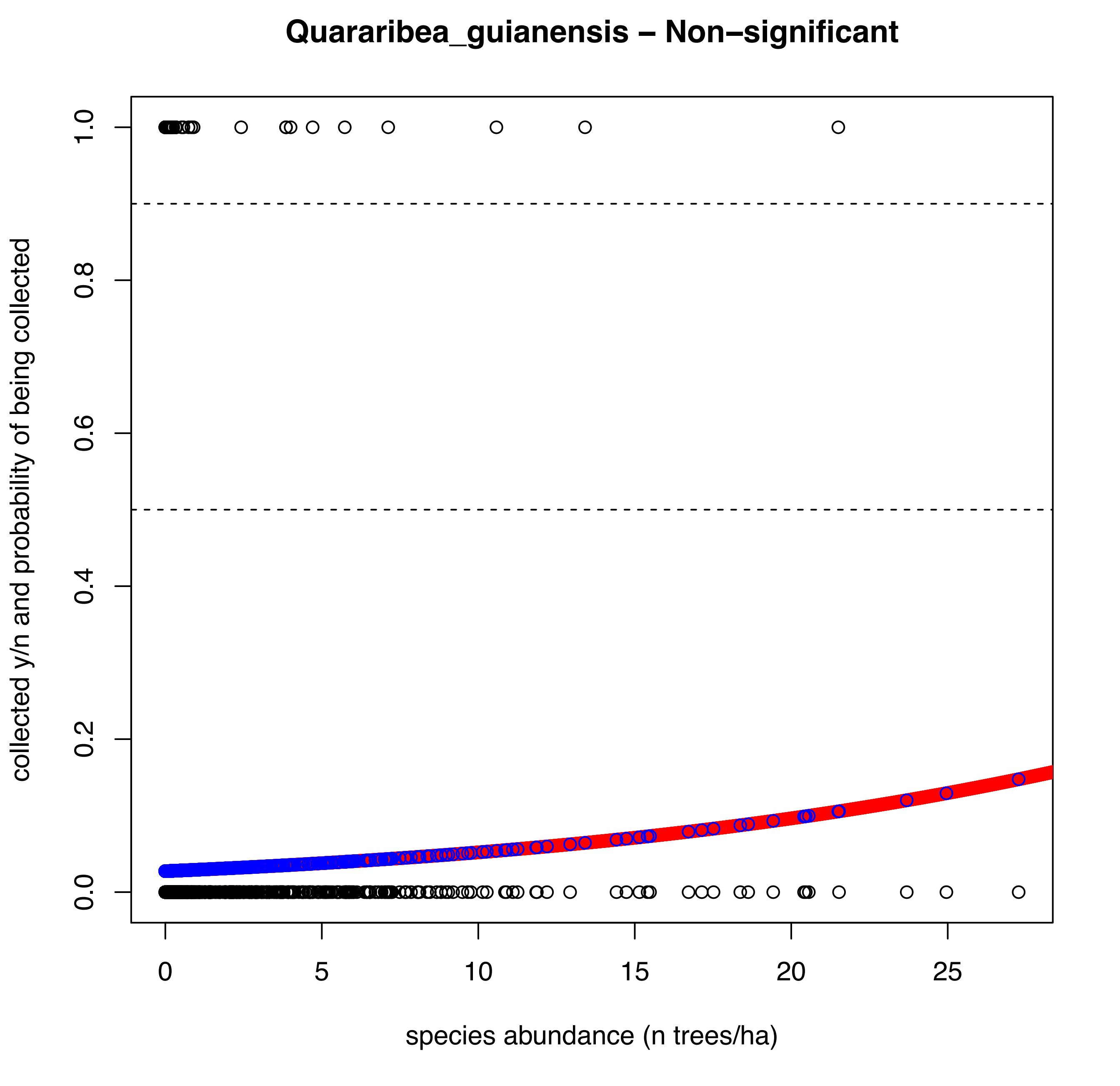

Quararibea_guianensis – Non–significant
collected y/n and probability of being collected
species abundance (n trees/ha)
0.0
0.2
0.4
0.6
0.8
1.0
0
5
10
15
20
25

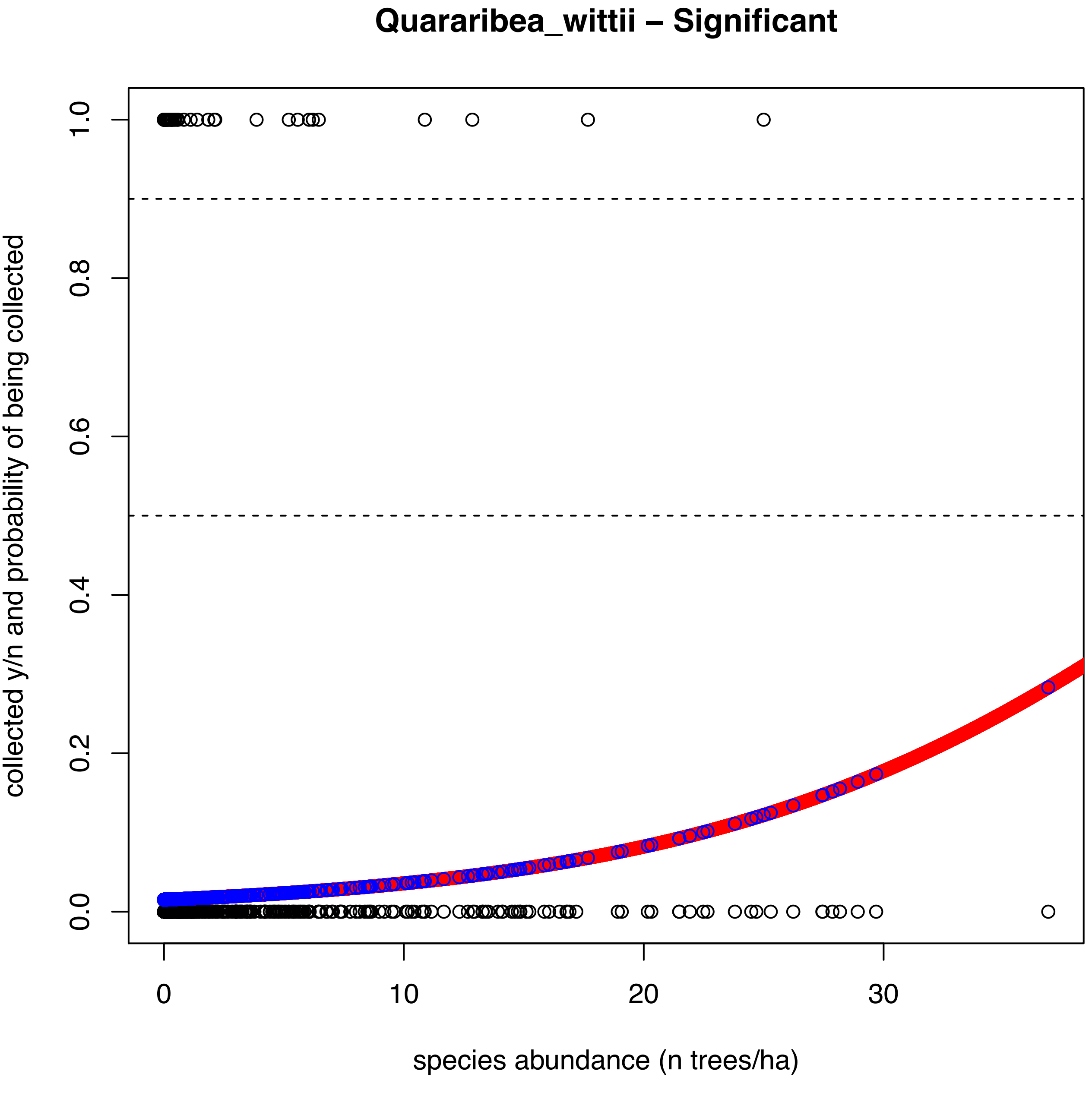

Quararibea_wittii – Significant
collected y/n and probability of being collected
species abundance (n trees/ha)
0.0
0.2
0.4
0.6
0.8
1.0
0
10
20
30

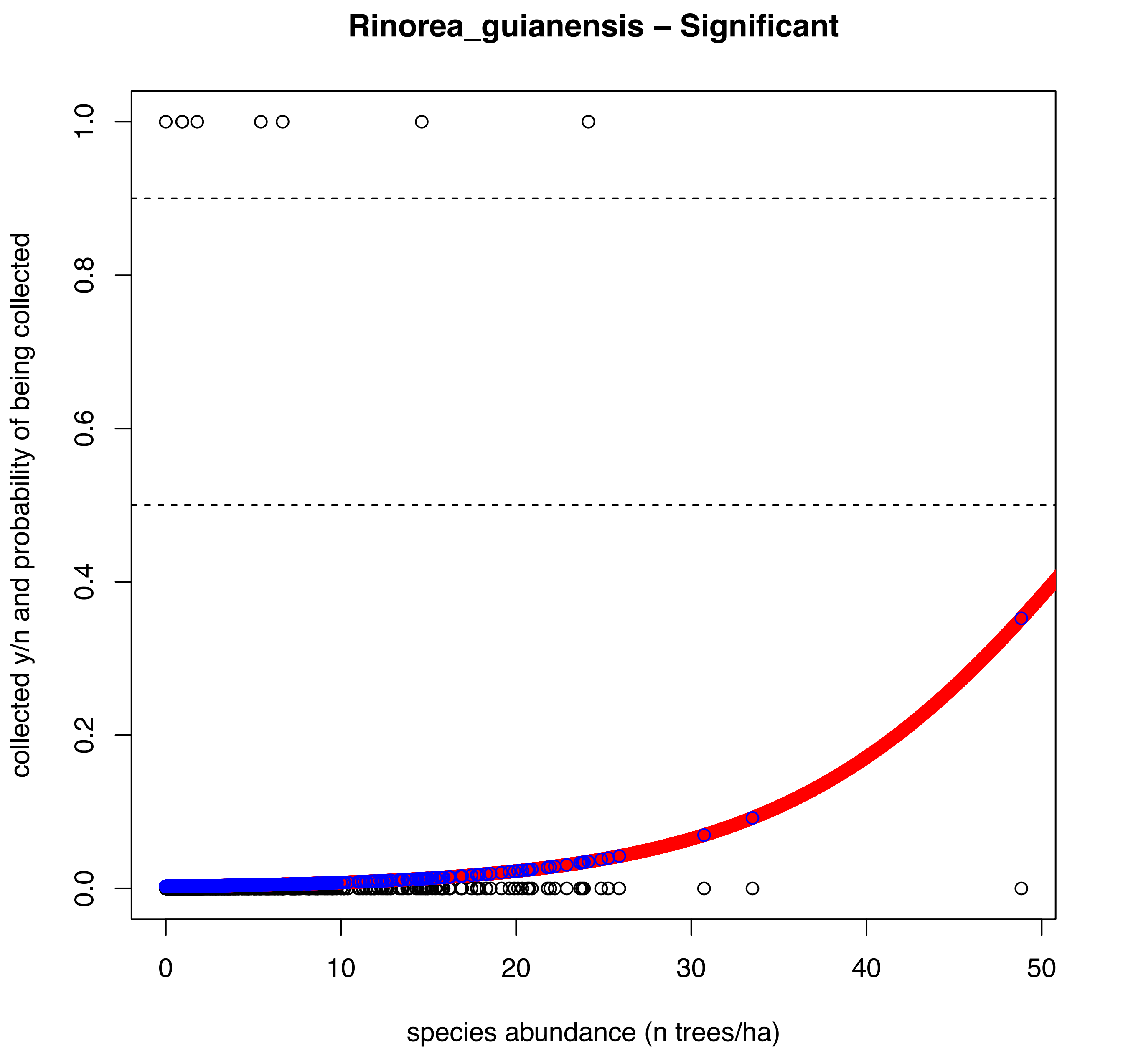

Rinorea_guianensis – Significant
collected y/n and probability of being collected
species abundance (n trees/ha)
0.0
0.2
0.4
0.6
0.8
1.0
0
10
20
30
40
50

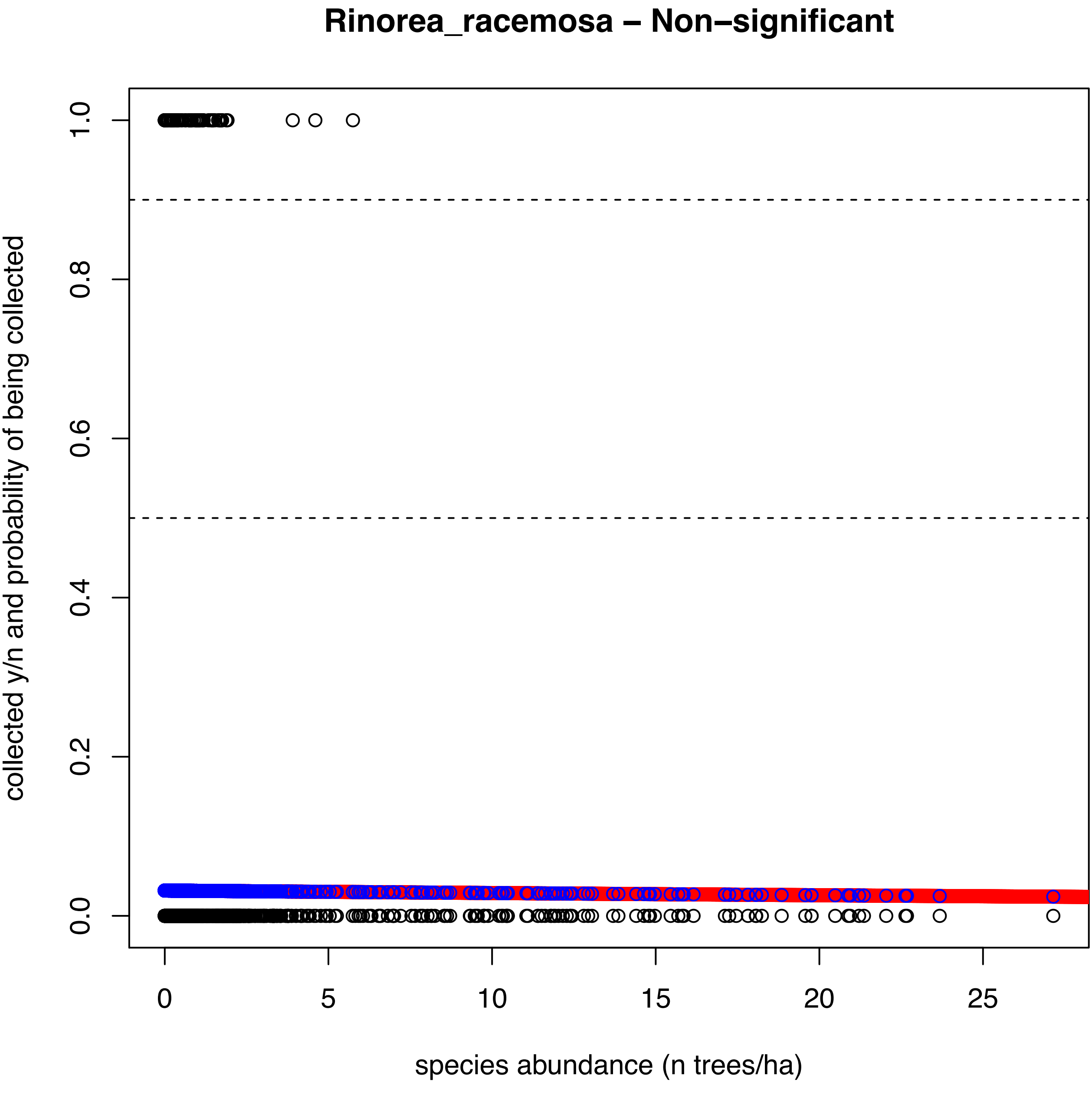

Rinorea_racemosa – Non–significant
collected y/n and probability of being collected
0.0
0.2
0.4
0.6
0.8
1.0
0
5
10
15
20
25
species abundance (n trees/ha)

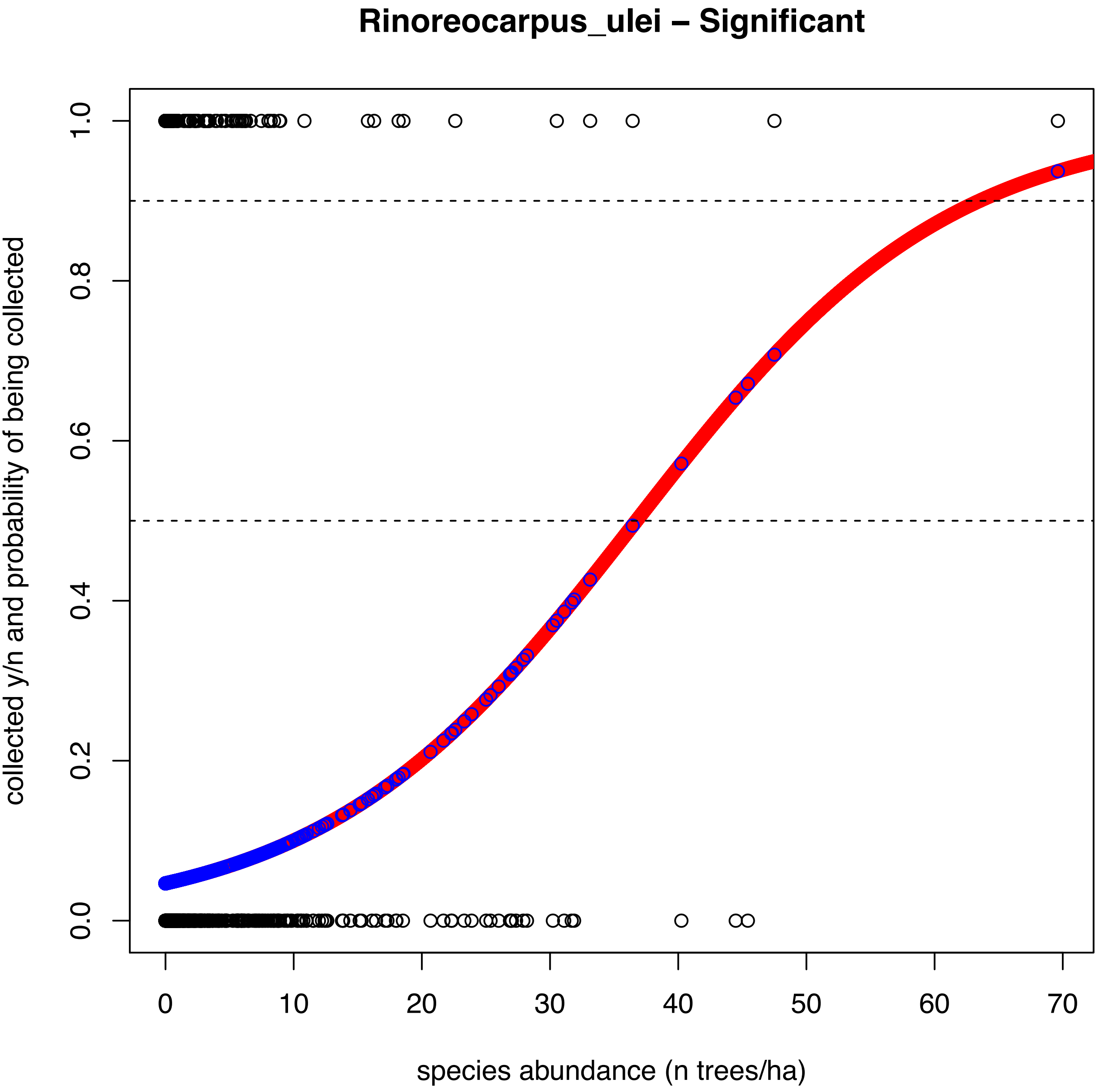

Rinoreocarpus_ulei – Significant
collected y/n and probability of being collected
species abundance (n trees/ha)
0.0
0.2
0.4
0.6
0.8
1.0
0
10
20
30
40
50
60
70

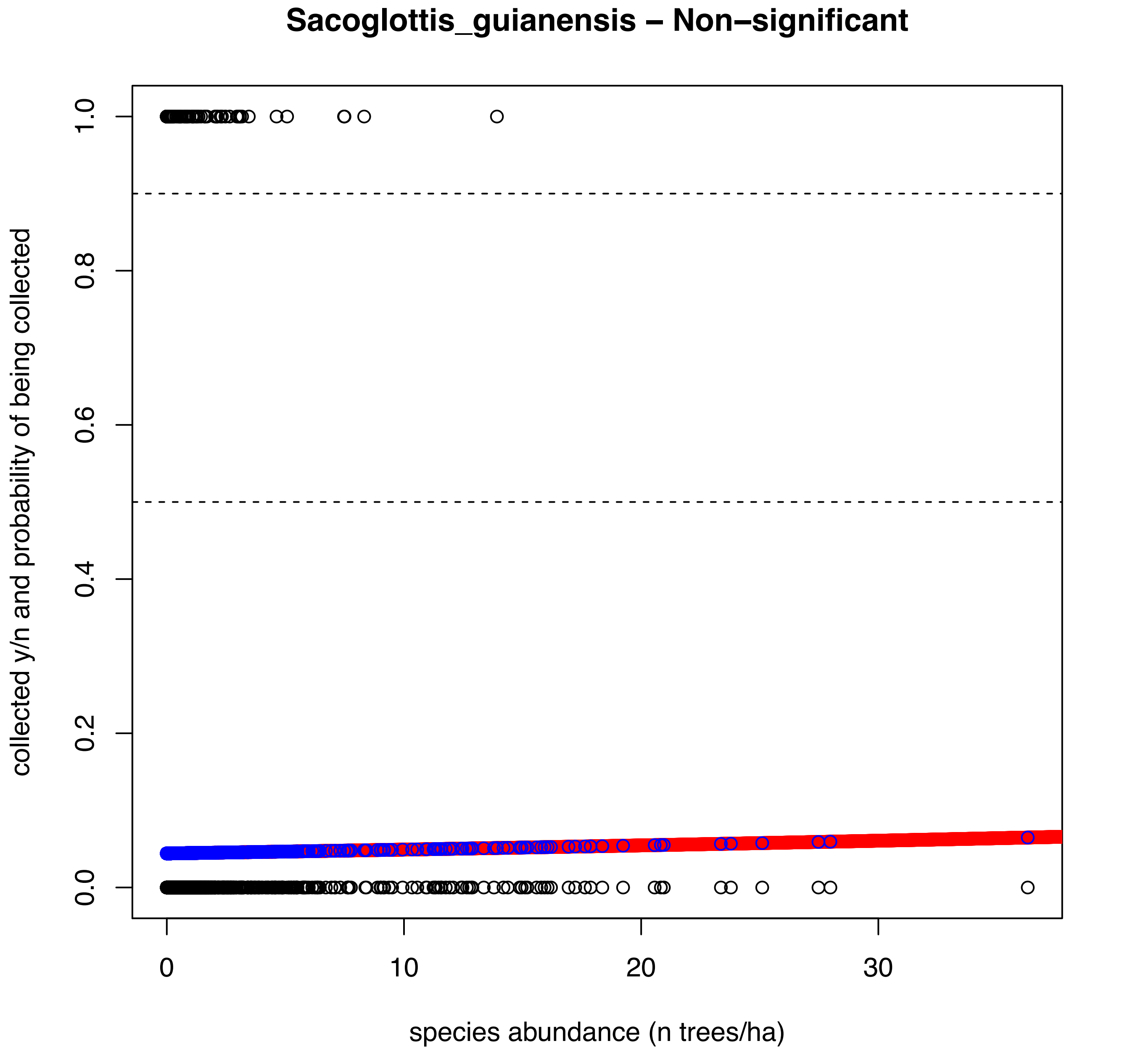

Sacoglottis_guianensis – Non–significant
collected y/n and probability of being collected
species abundance (n trees/ha)
0.0
0.2
0.4
0.6
0.8
1.0
0
10
20
30

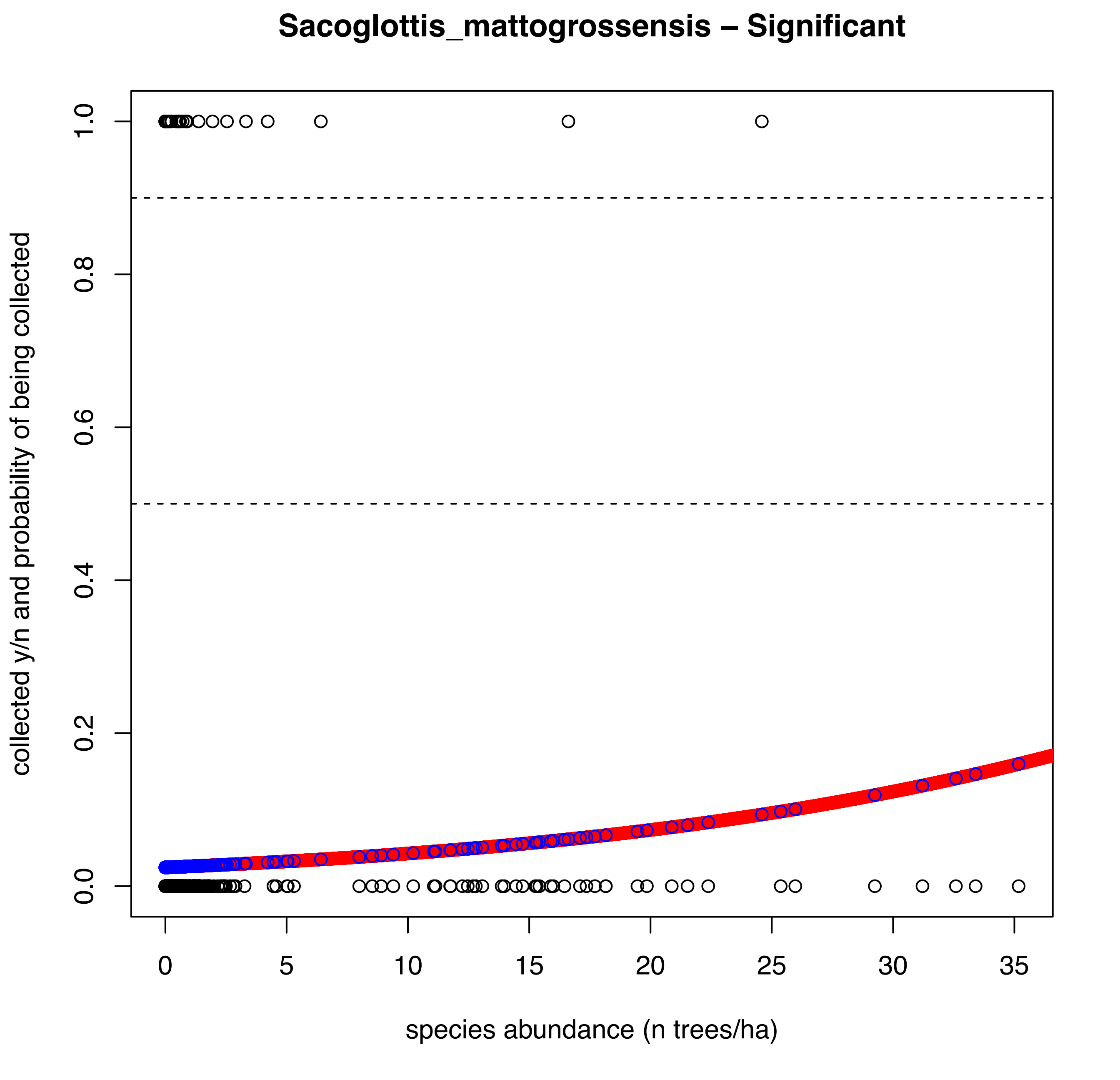

Sacoglottis_mattogrossensis – Significant
collected y/n and probability of being collected
species abundance (n trees/ha)
0.0
0.2
0.4
0.6
0.8
1.0
0
5
10
15
20
25
30
35

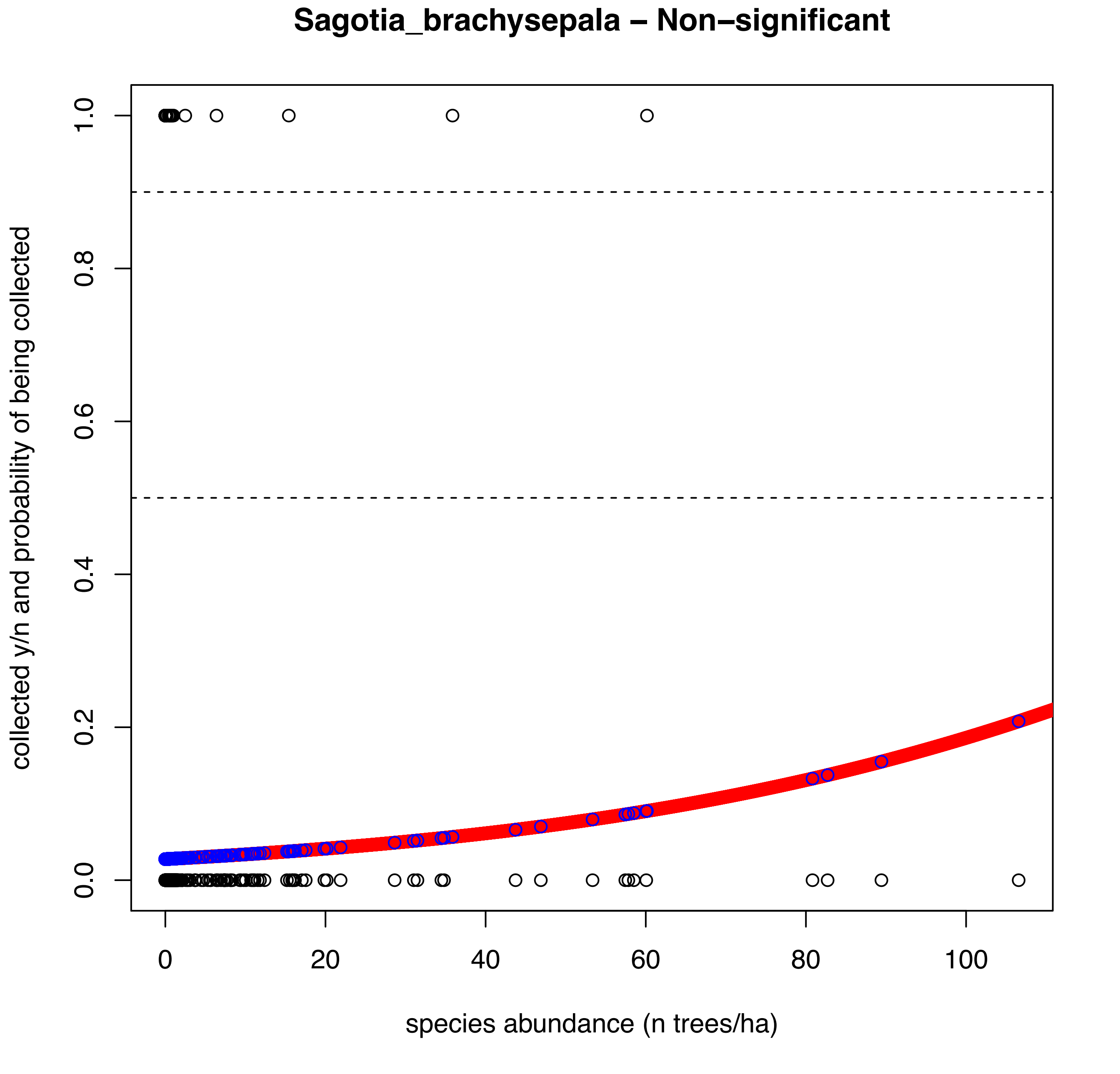

Sagotia_brachysepala – Non–significant
collected y/n and probability of being collected
species abundance (n trees/ha)
0.0
0.2
0.4
0.6
0.8
1.0
0
20
40
60
80
100

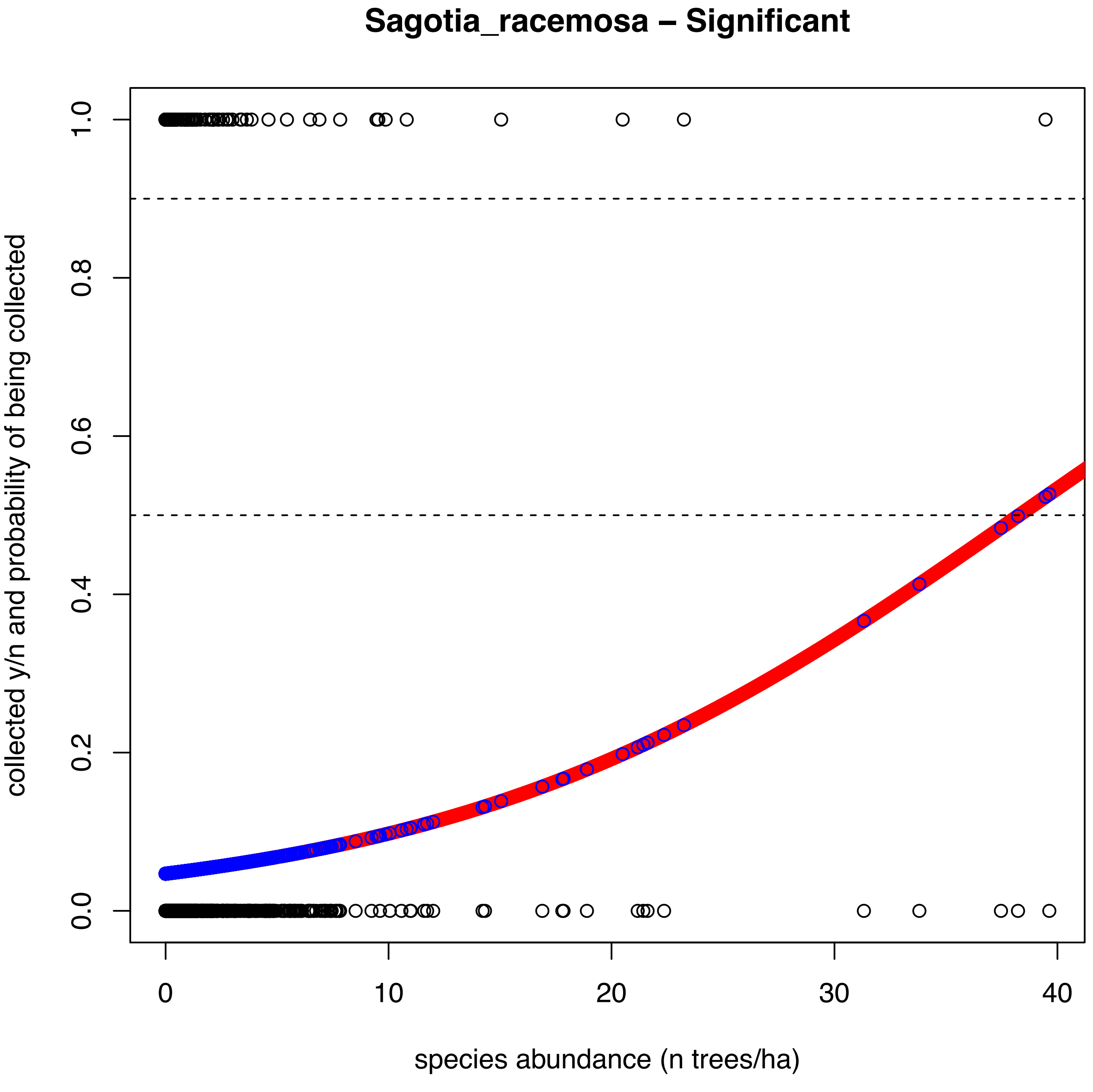

Sagotia_racemosa – Significant
collected y/n and probability of being collected
species abundance (n trees/ha)
0.0
0.2
0.4
0.6
0.8
1.0
0
10
20
30
40

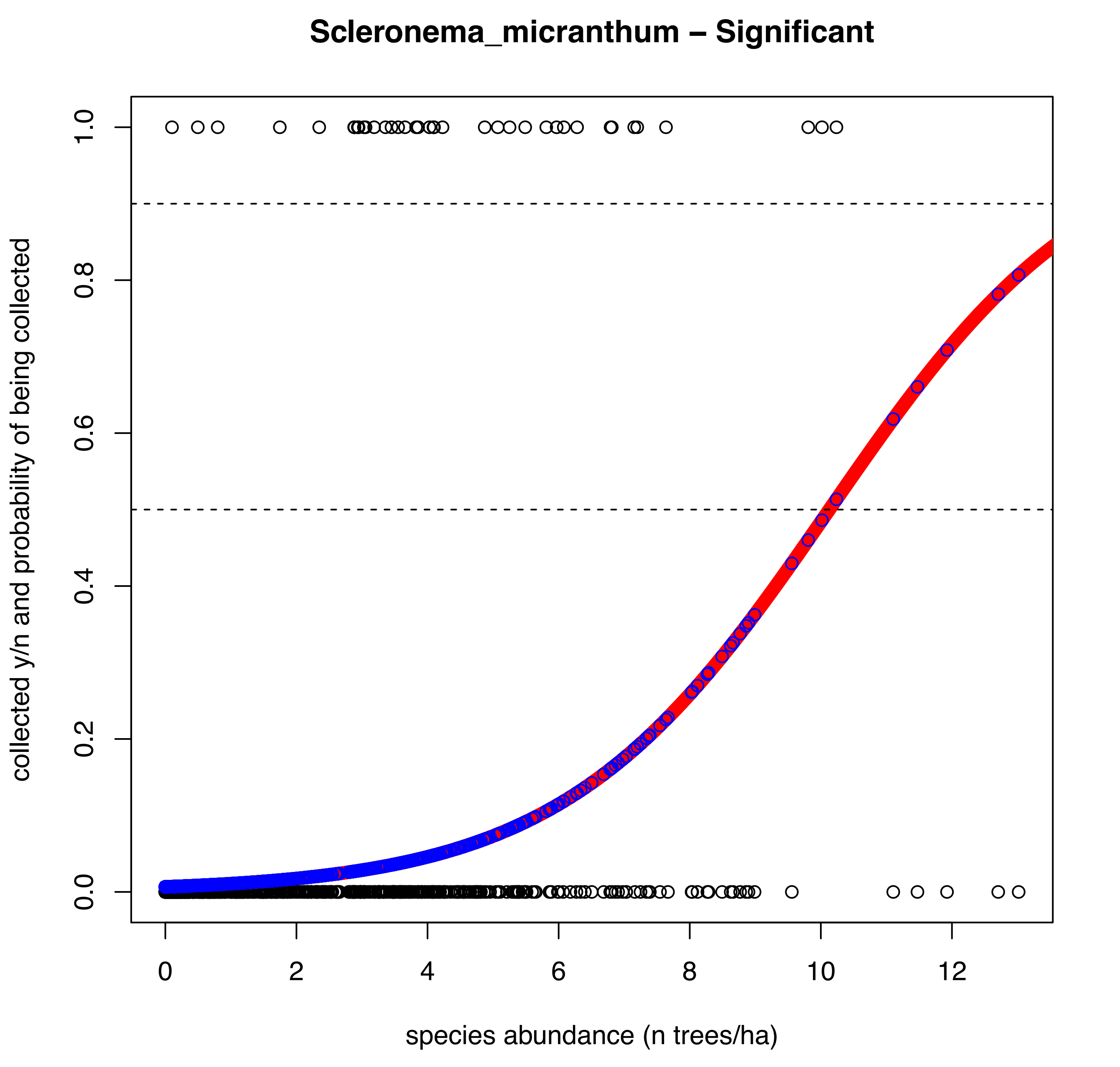

Scleronema_micranthum – Significant
collected y/n and probability of being collected
species abundance (n trees/ha)
0.0
0.2
0.4
0.6
0.8
1.0
0
2
4
6
8
10
12

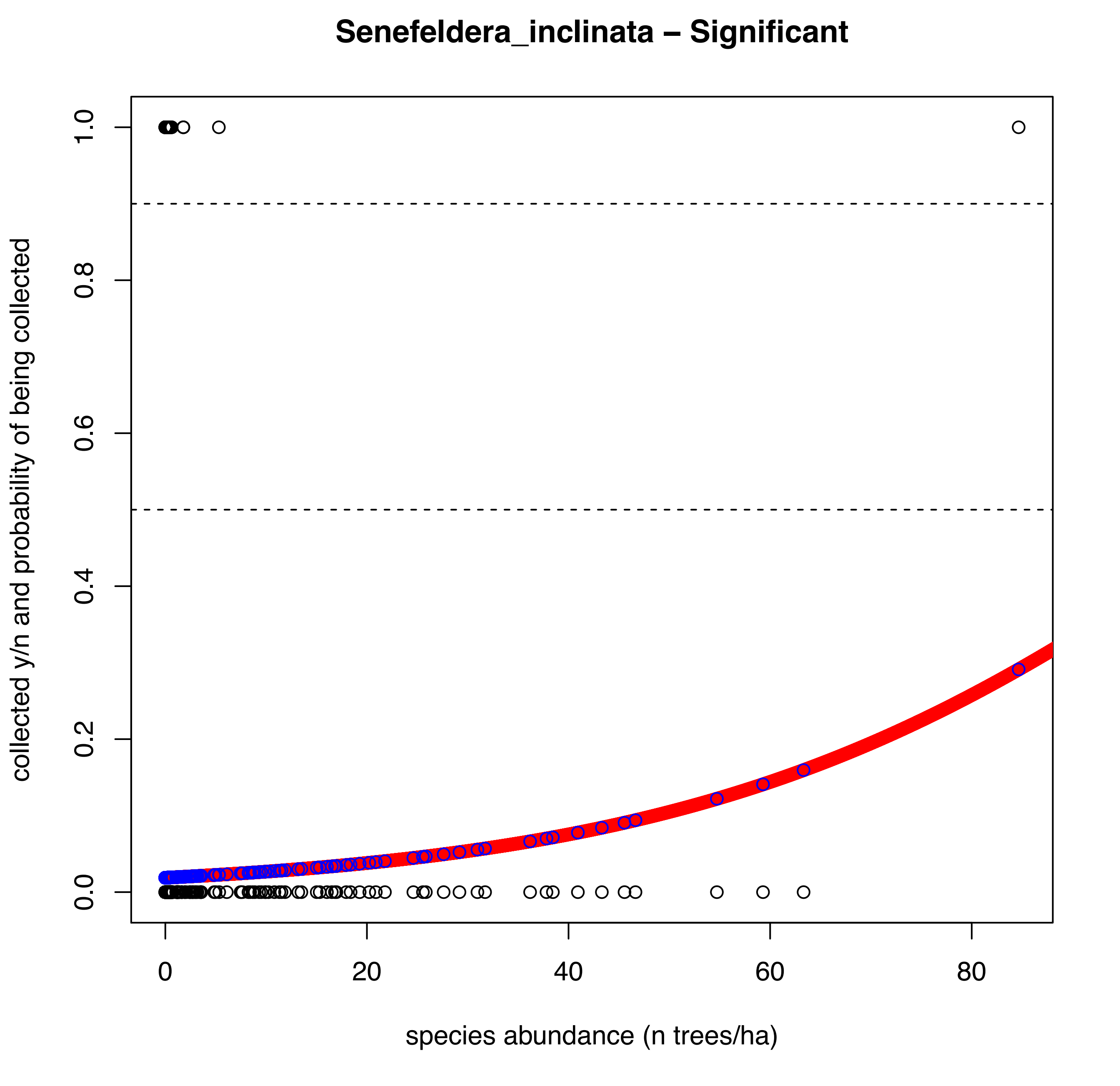
Senefeldera_inclinata – Significant
collected y/n and probability of being collected
species abundance (n trees/ha)
0.0
0.2
0.4
0.6
0.8
1.0
0
20
40
60
80

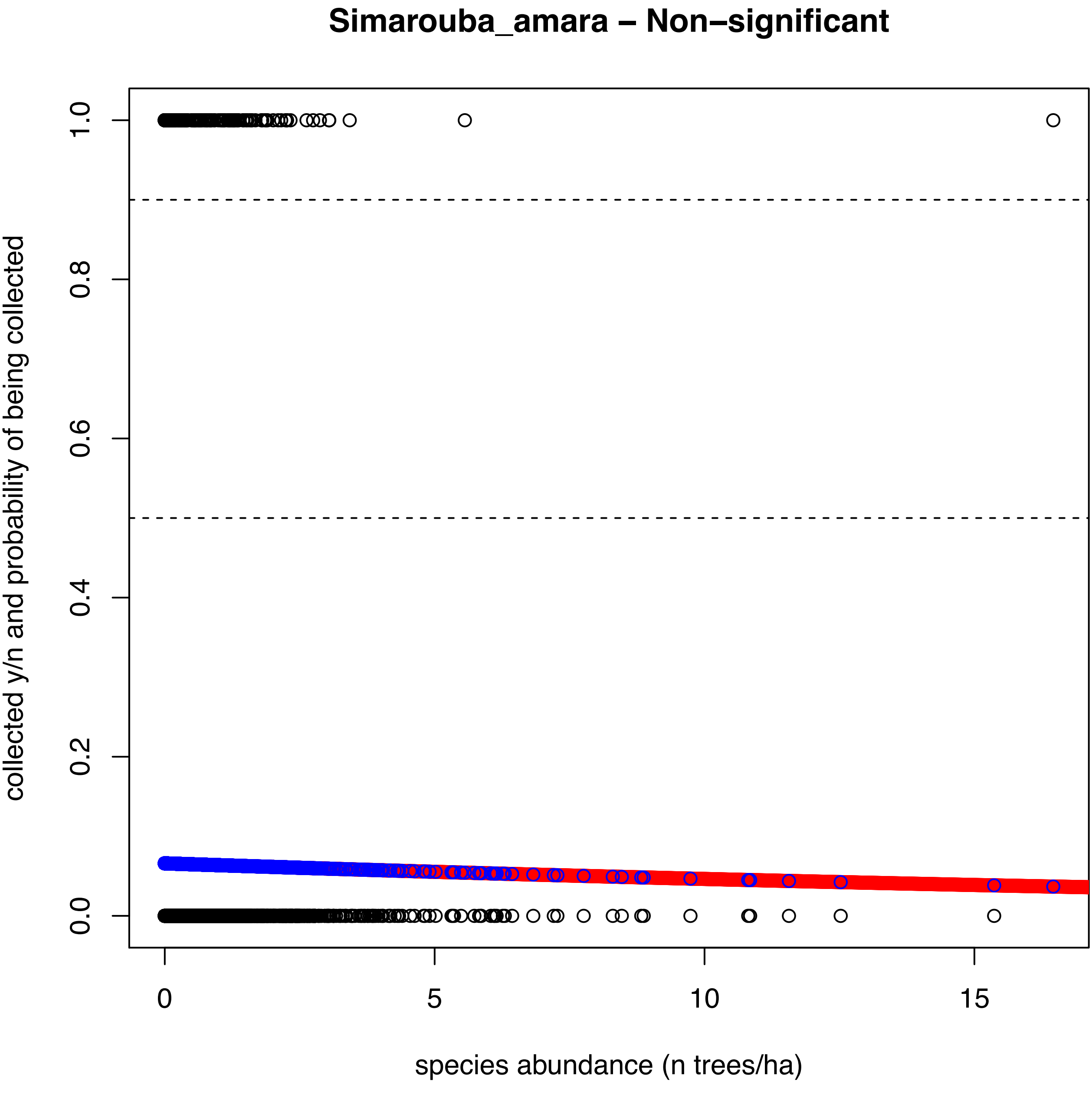
Simarouba_amara – Non–significant
collected y/n and probability of being collected
species abundance (n trees/ha)
0.0
0.2
0.4
0.6
0.8
1.0
0
5
10
15

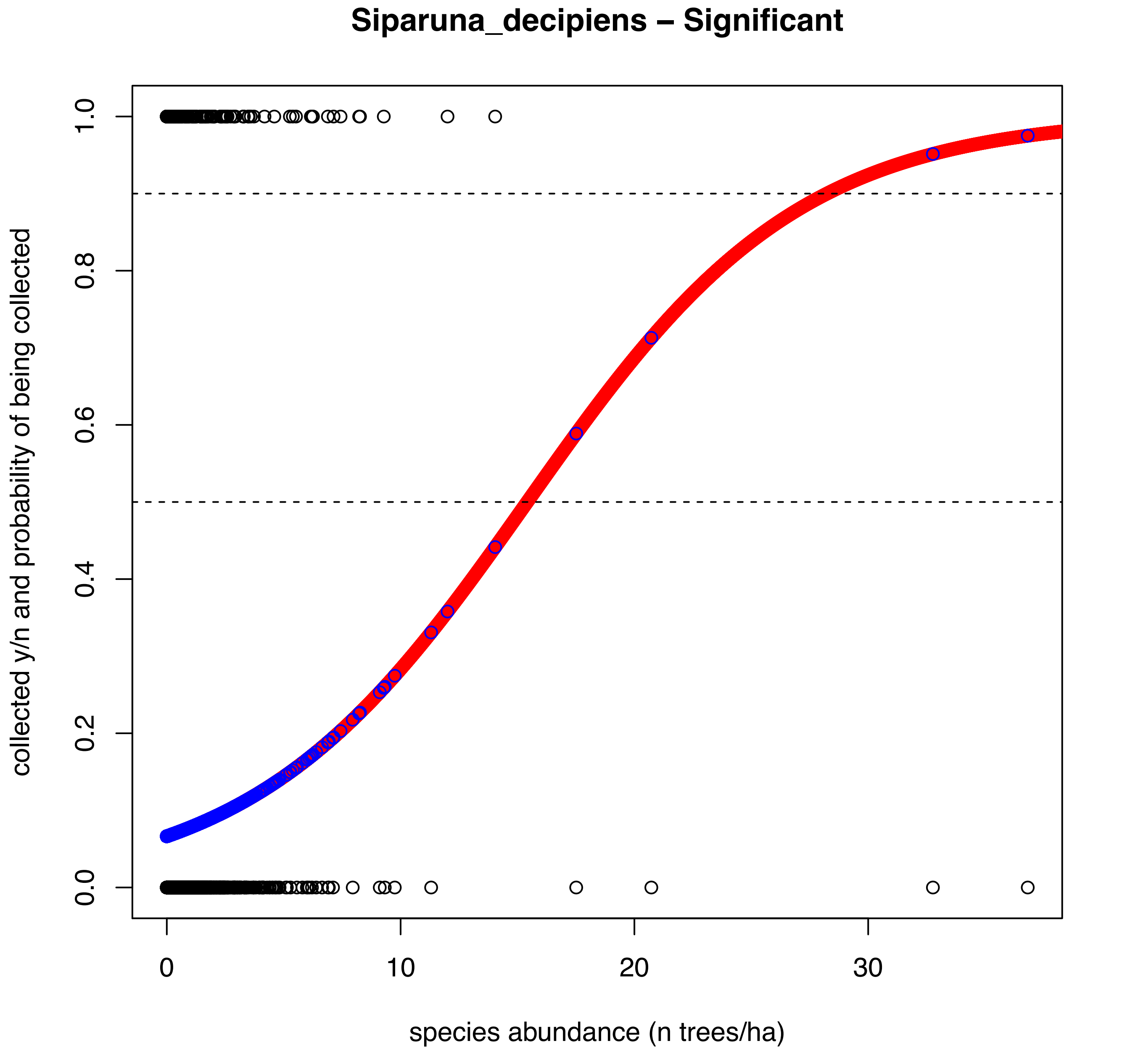

Siparuna_decipiens – Significant
collected y/n and probability of being collected
species abundance (n trees/ha)
0.0
0.2
0.4
0.6
0.8
1.0
0
10
20
30

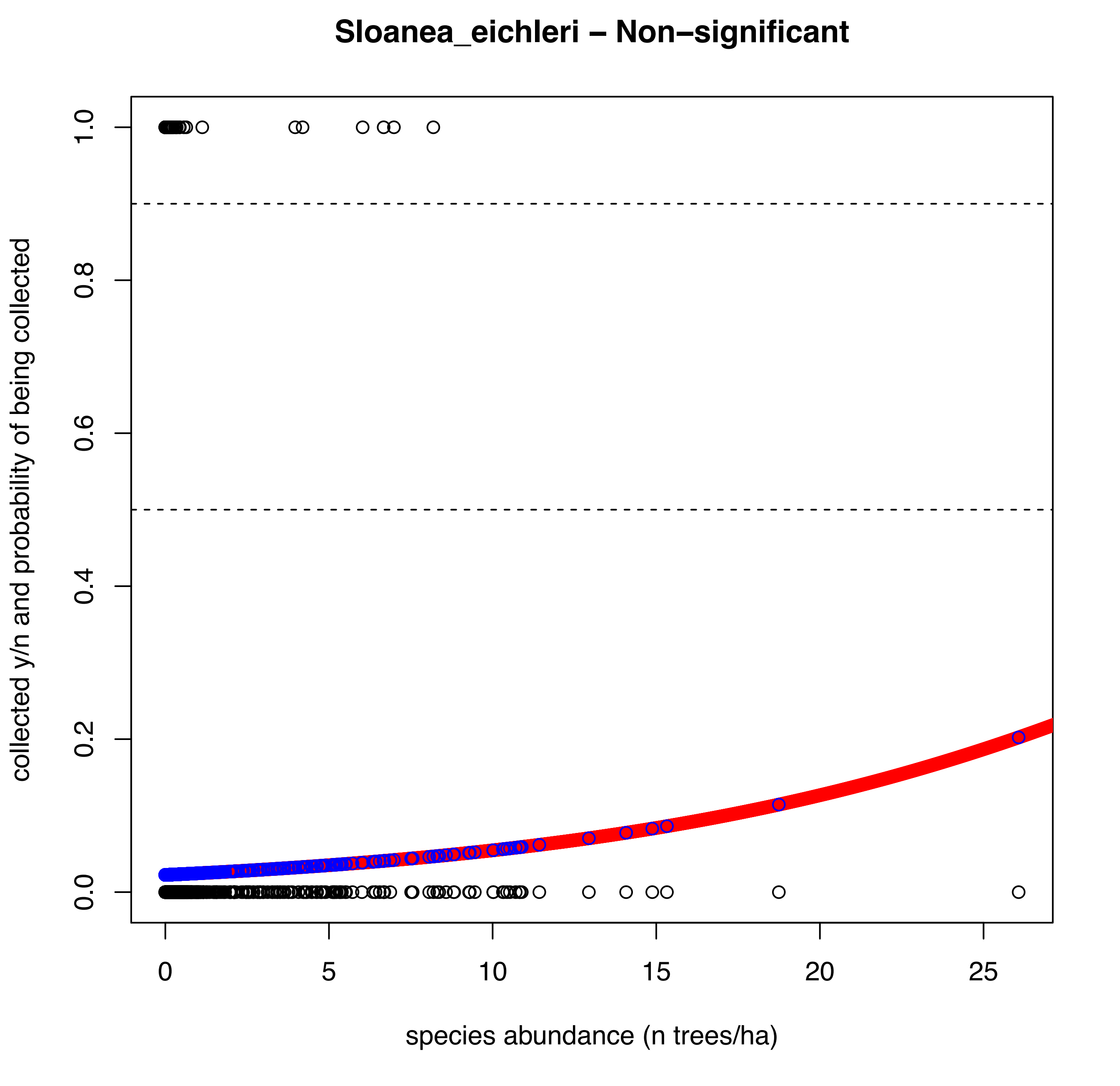

Sloanea_eichleri – Non–significant
collected y/n and probability of being collected
species abundance (n trees/ha)
0.0
0.2
0.4
0.6
0.8
1.0
0
5
10
15
20
25

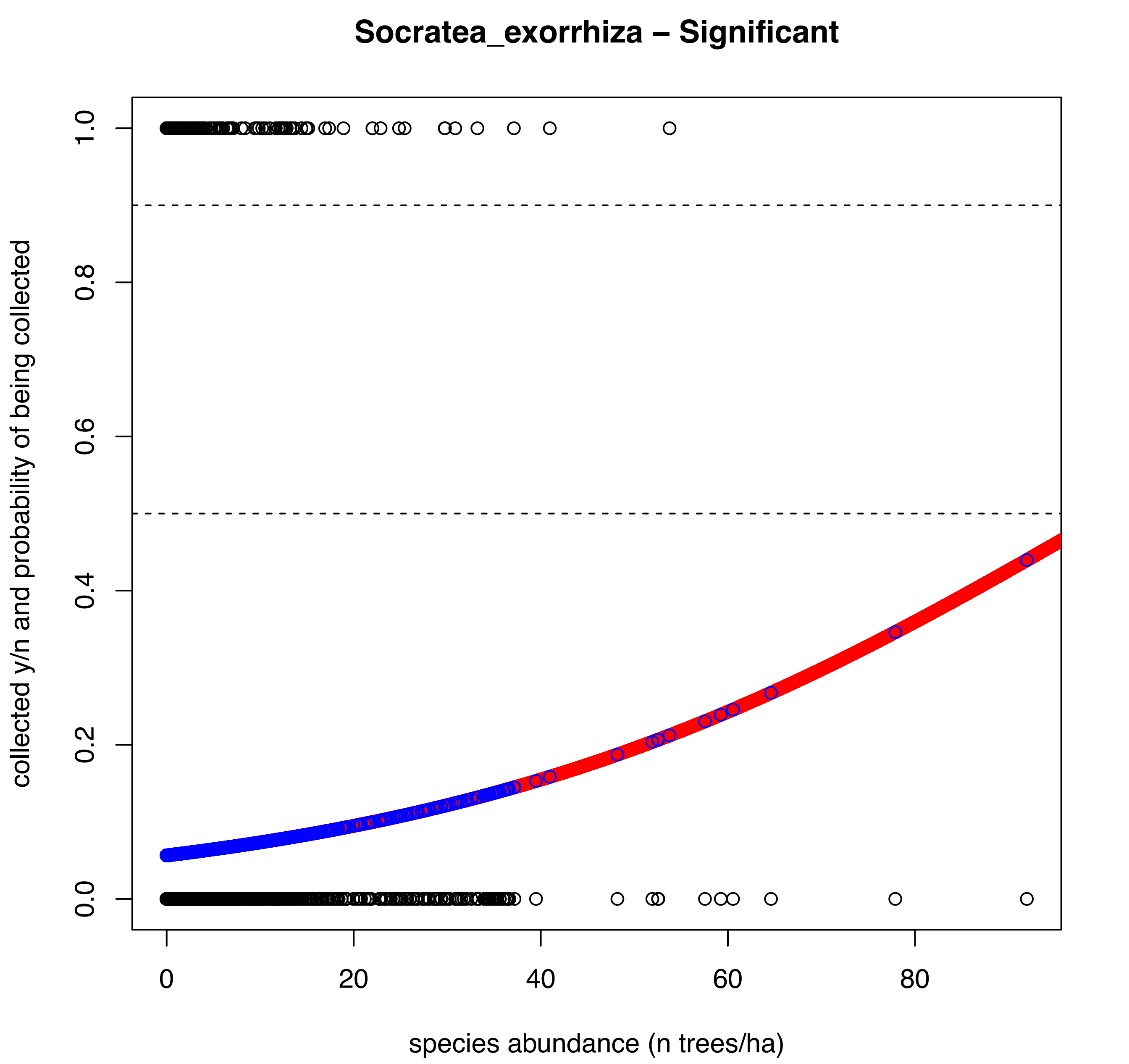
Socratea_exorrhiza – Significant
collected y/n and probability of being collected
species abundance (n trees/ha)
0.0
0.2
0.4
0.6
0.8
1.0
0
20
40
60
80

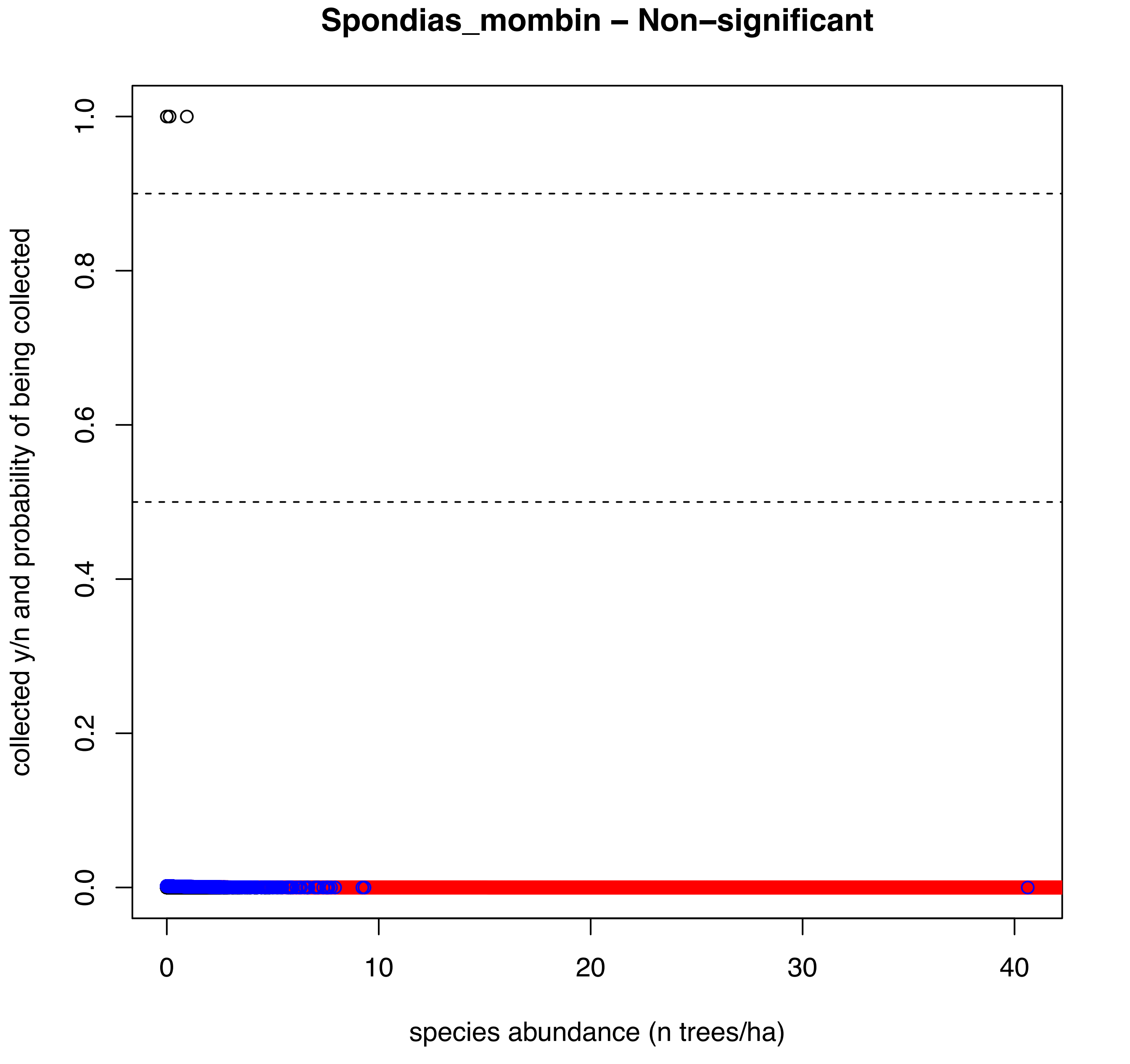
Spondias_mombin – Non–significant
collected y/n and probability of being collected
0.0
0.2
0.4
0.6
0.8
1.0
0
10
20
30
40
species abundance (n trees/ha)

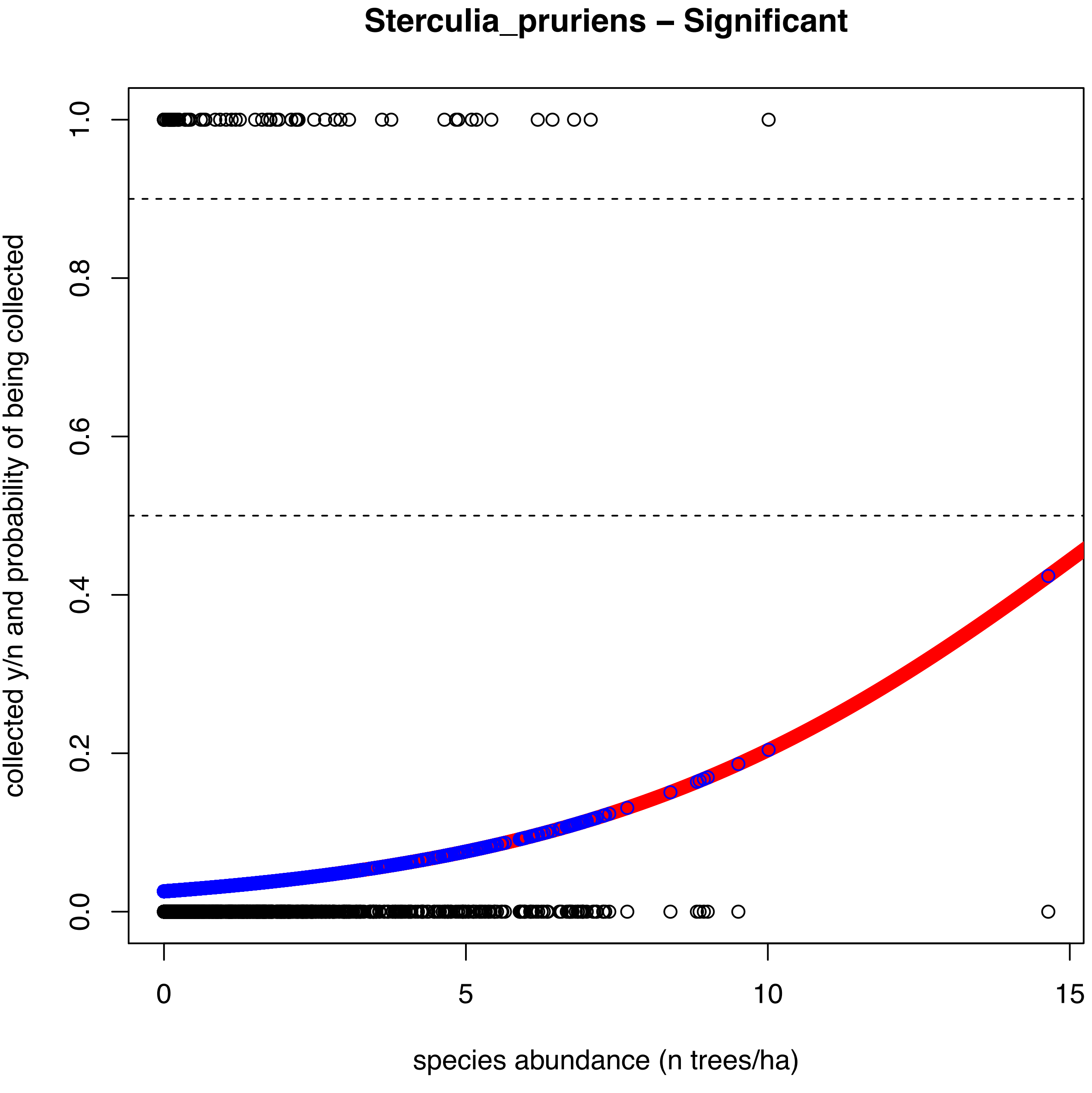

Sterculia_pruriens – Significant
collected y/n and probability of being collected
0.0
0.2
0.4
0.6
0.8
1.0
0
5
10
15
species abundance (n trees/ha)

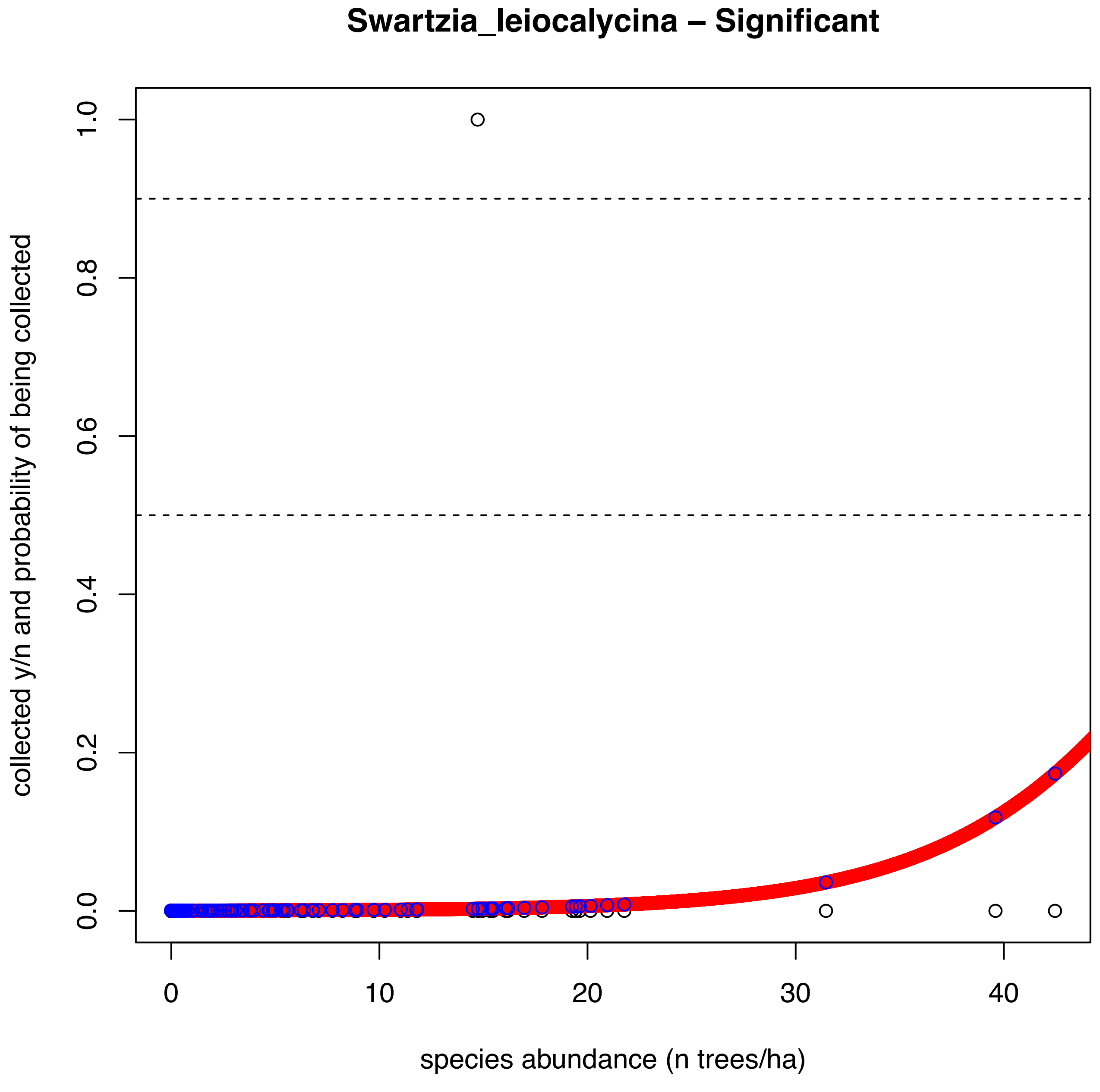
Swartzia_leiocalycina – Significant
collected y/n and probability of being collected
species abundance (n trees/ha)
0.0
0.2
0.4
0.6
0.8
1.0
0
10
20
30
40

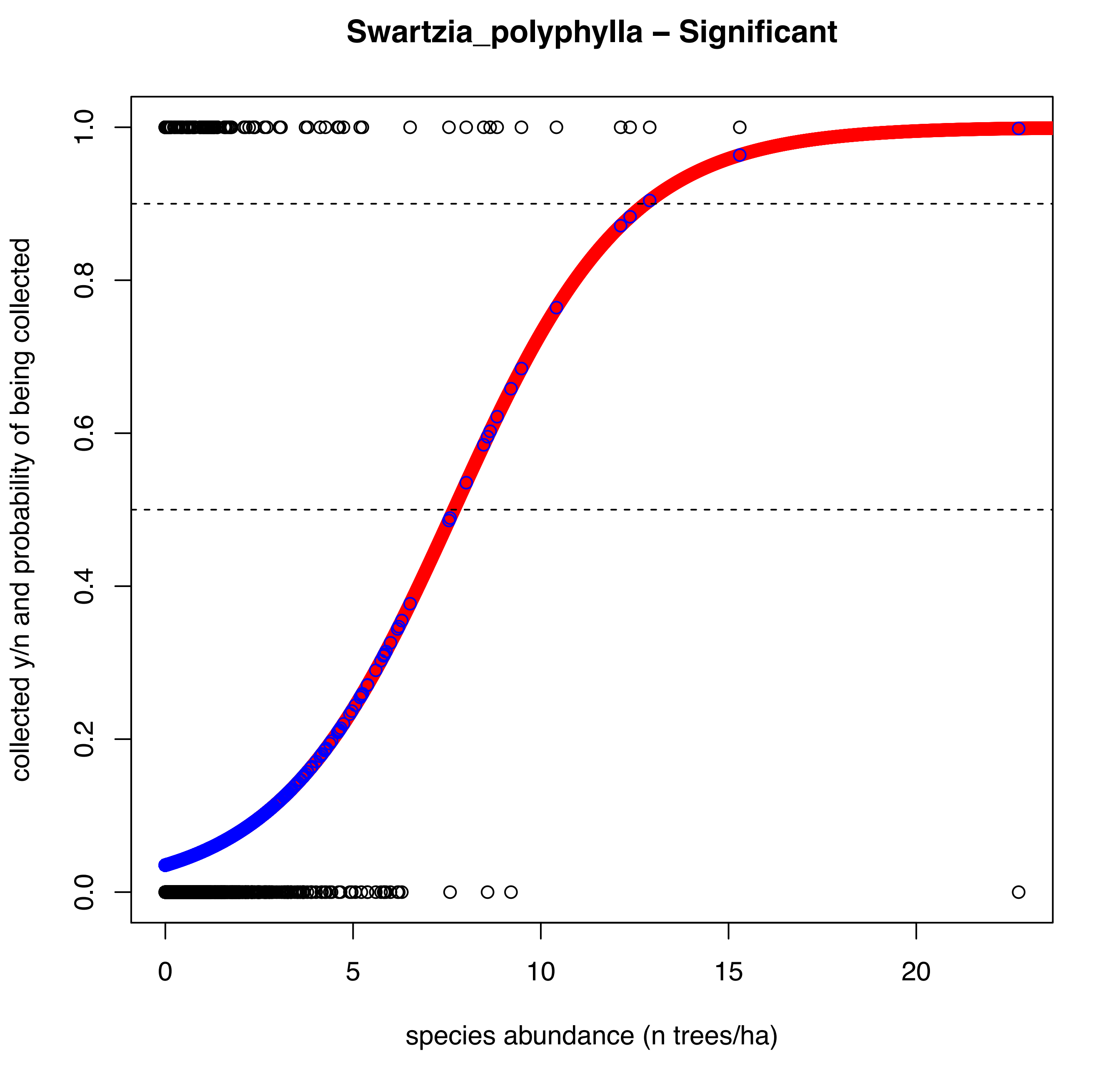

Swartzia_polyphylla – Significant
collected y/n and probability of being collected
species abundance (n trees/ha)
0.0
0.2
0.4
0.6
0.8
1.0
0
5
10
15
20

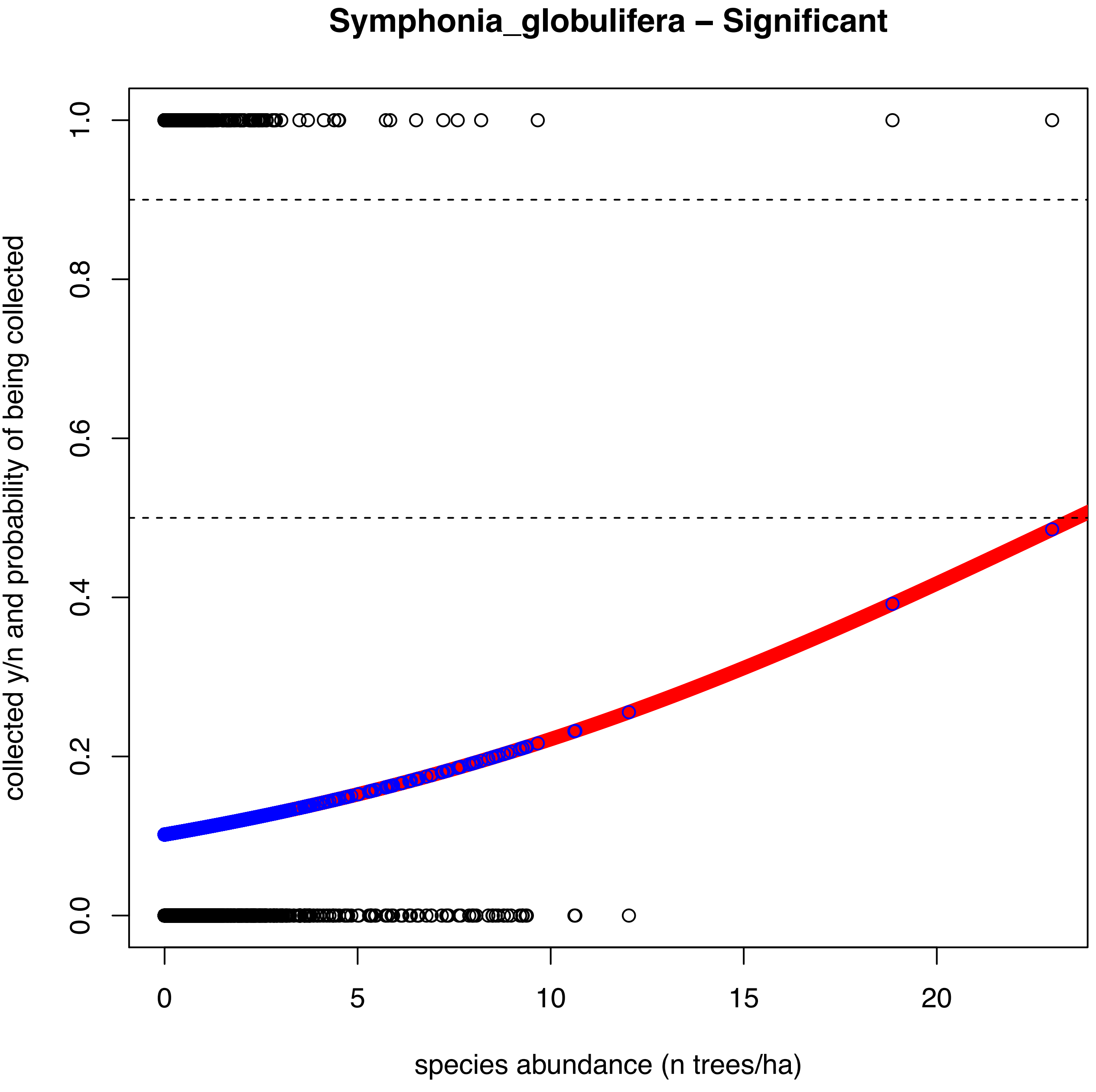

Symphonia_globulifera – Significant
collected y/n and probability of being collected
0.0
0.2
0.4
0.6
0.8
1.0
0
5
10
15
20
species abundance (n trees/ha)

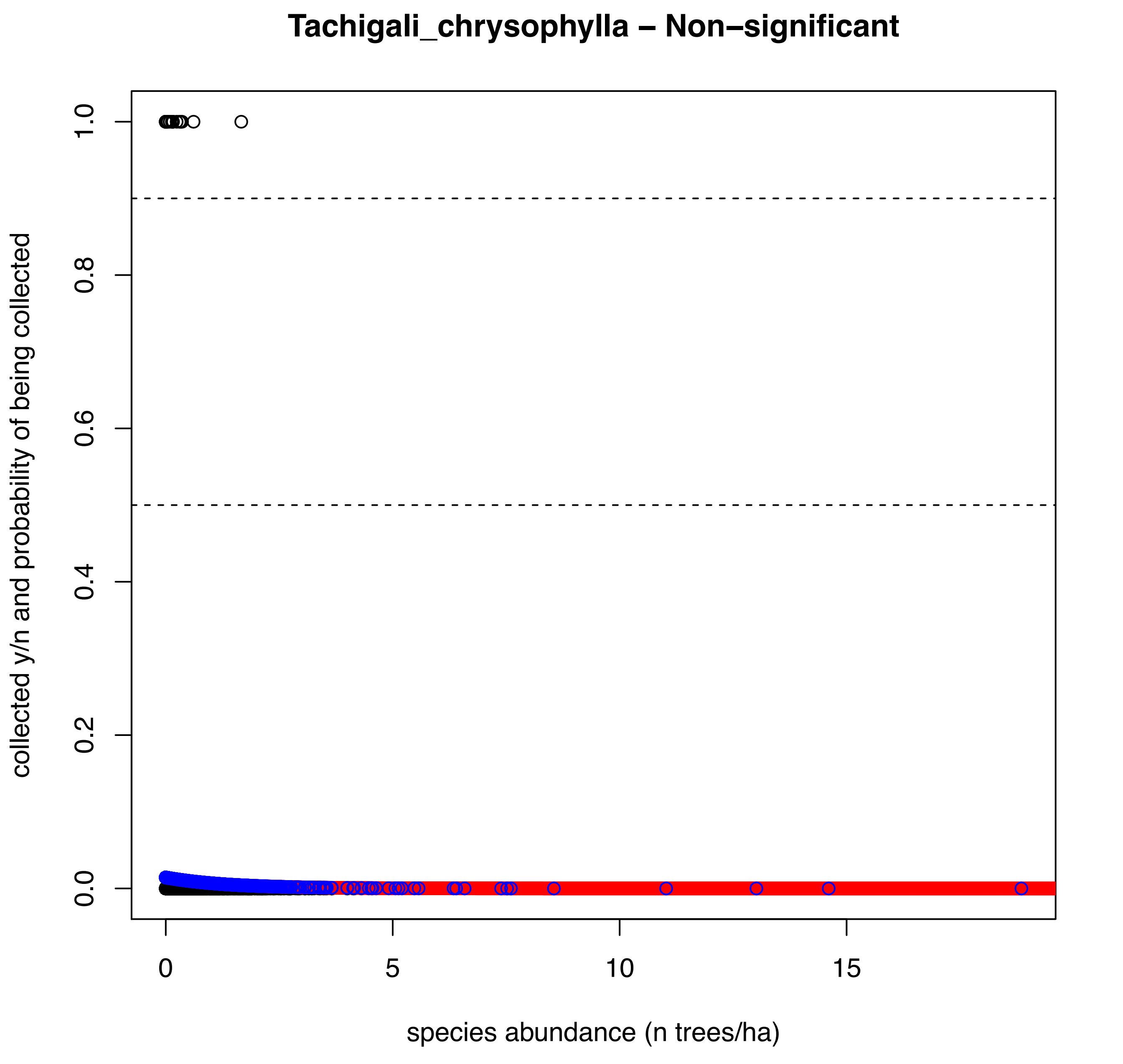

Tachigali_chrysophylla – Non–significant
collected y/n and probability of being collected
species abundance (n trees/ha)
0.0
0.2
0.4
0.6
0.8
1.0
0
5
10
15

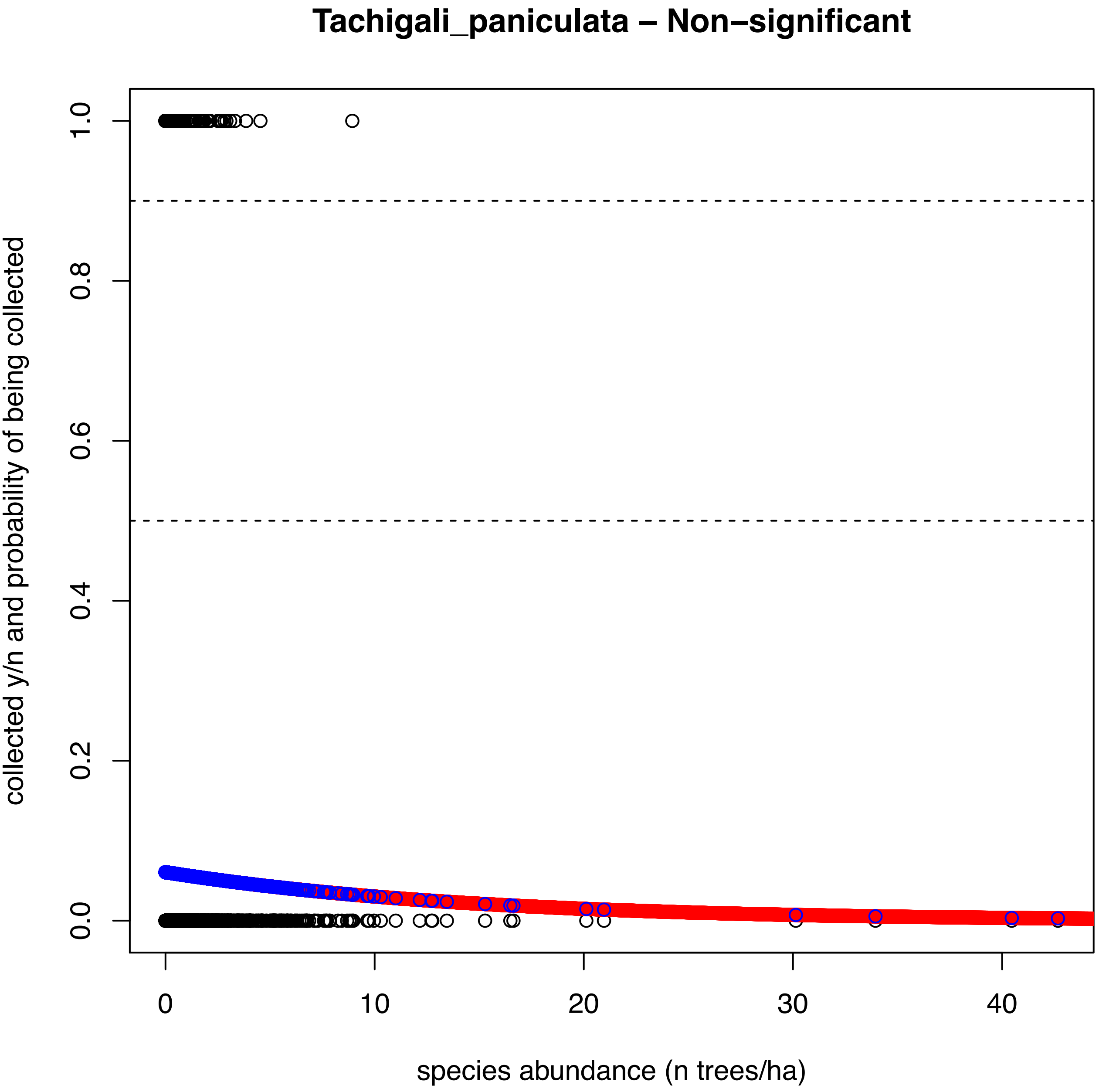

Tachigali_paniculata – Non–significant
collected y/n and probability of being collected
0.0
0.2
0.4
0.6
0.8
1.0
0
10
20
30
40
species abundance (n trees/ha)

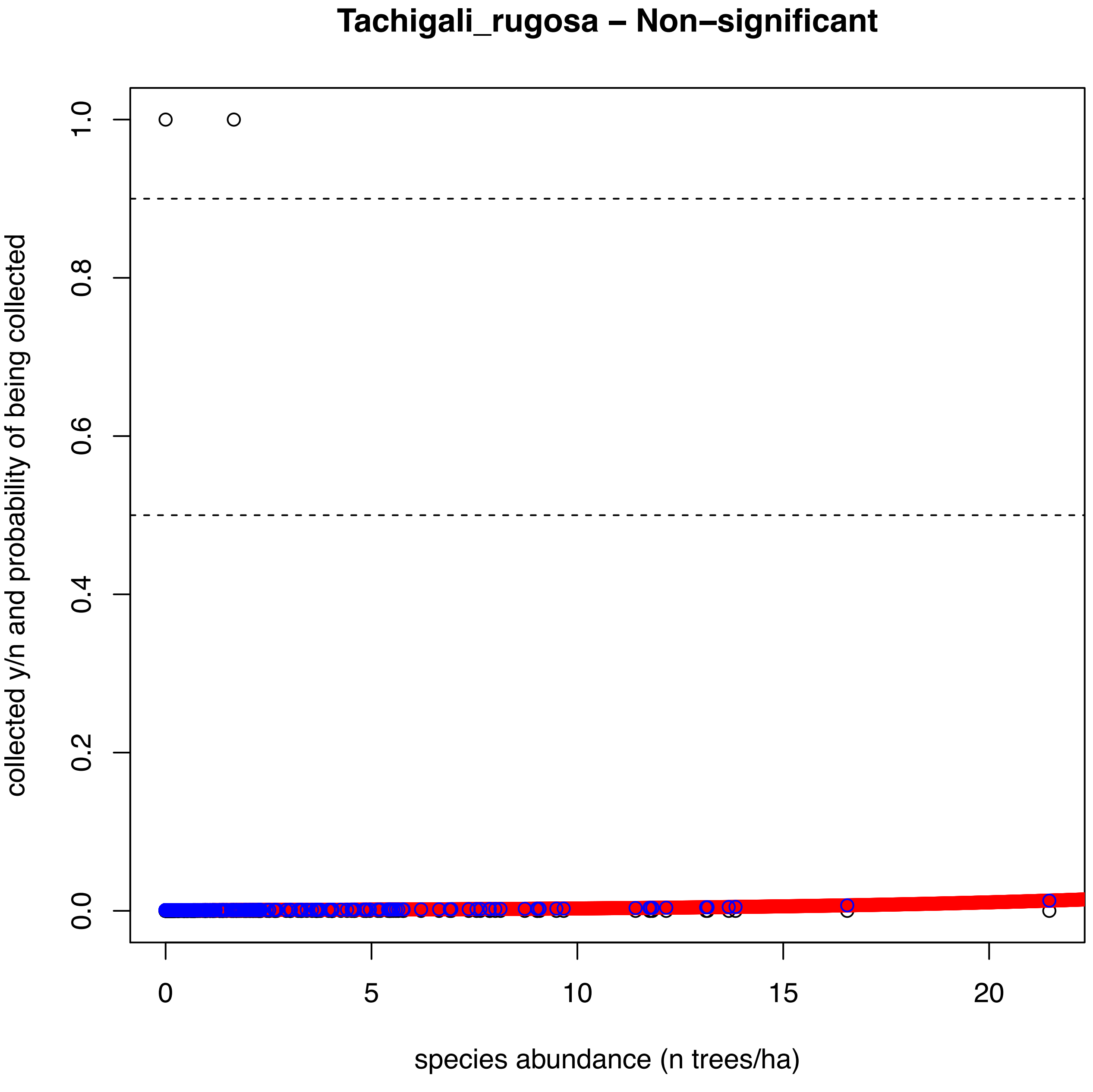
Tachigali_rugosa – Non–significant
collected y/n and probability of being collected
species abundance (n trees/ha)
0.0
0.2
0.4
0.6
0.8
1.0
0
5
10
15
20

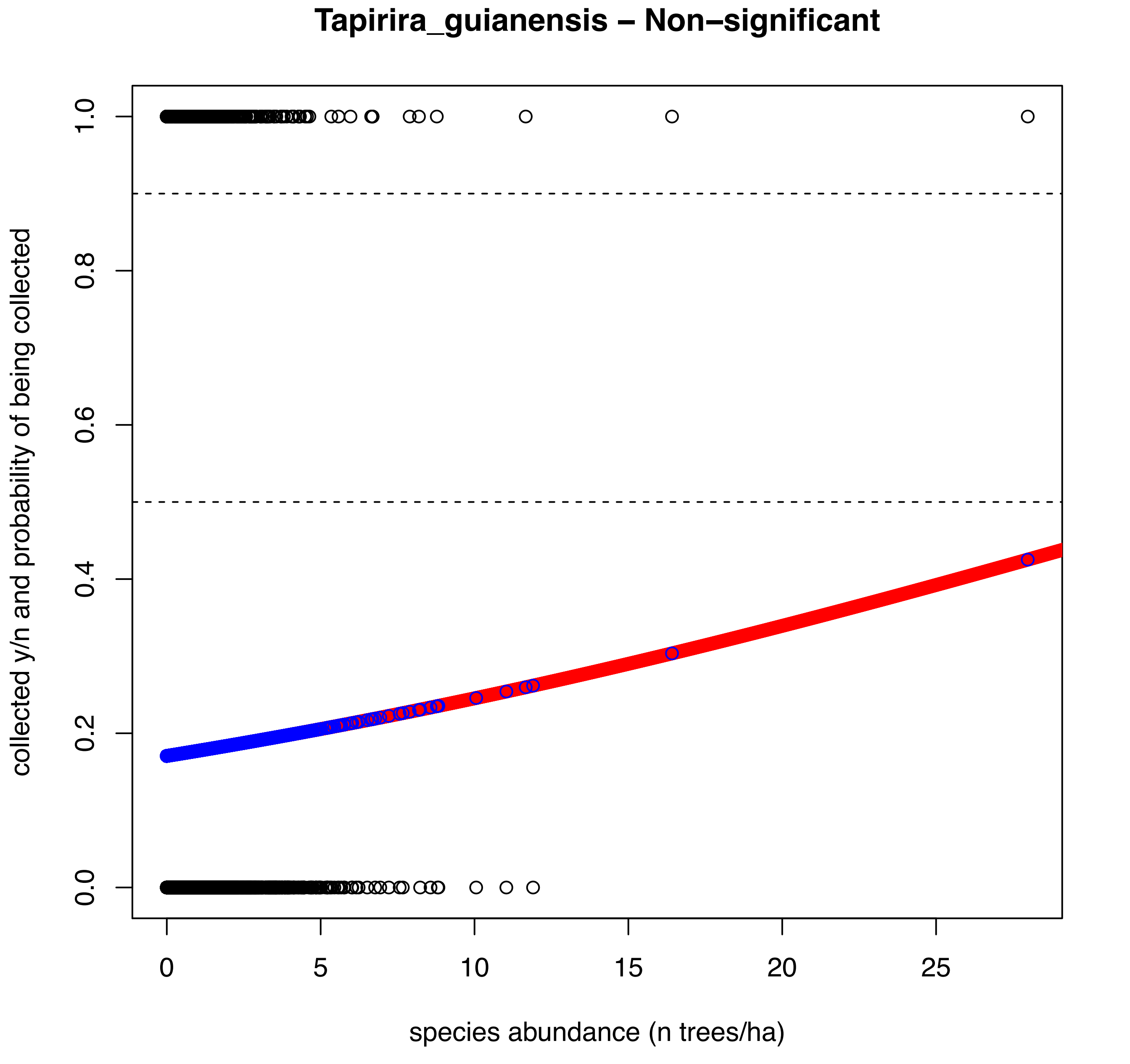
Tapirira_guianensis – Non–significant
collected y/n and probability of being collected
species abundance (n trees/ha)
0.0
0.2
0.4
0.6
0.8
1.0
0
5
10
15
20
25

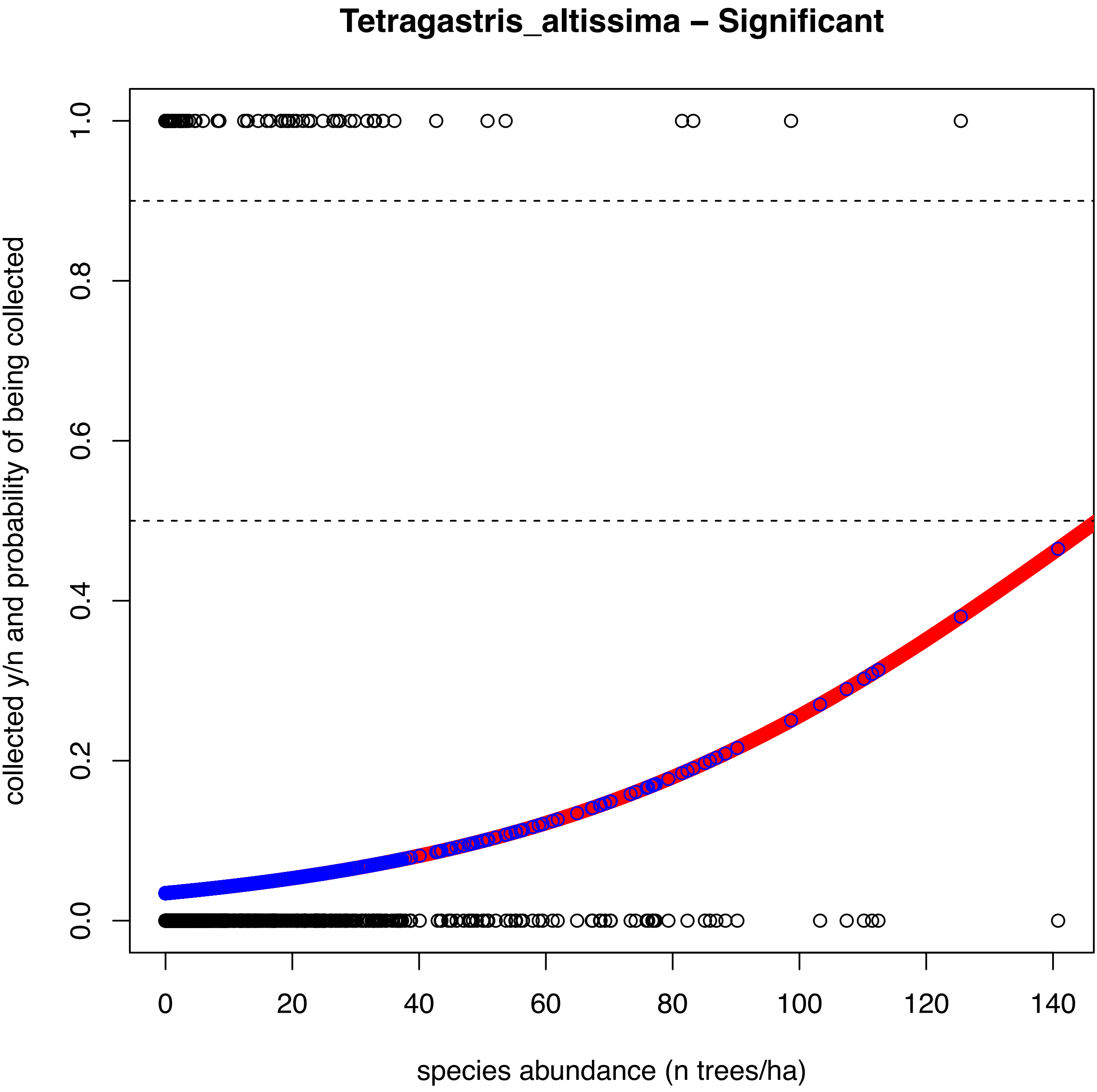
Tetragastris_altissima – Significant
collected y/n and probability of being collected
species abundance (n trees/ha)
0.0
0.2
0.4
0.6
0.8
1.0
0
20
40
60
80
100
120
140

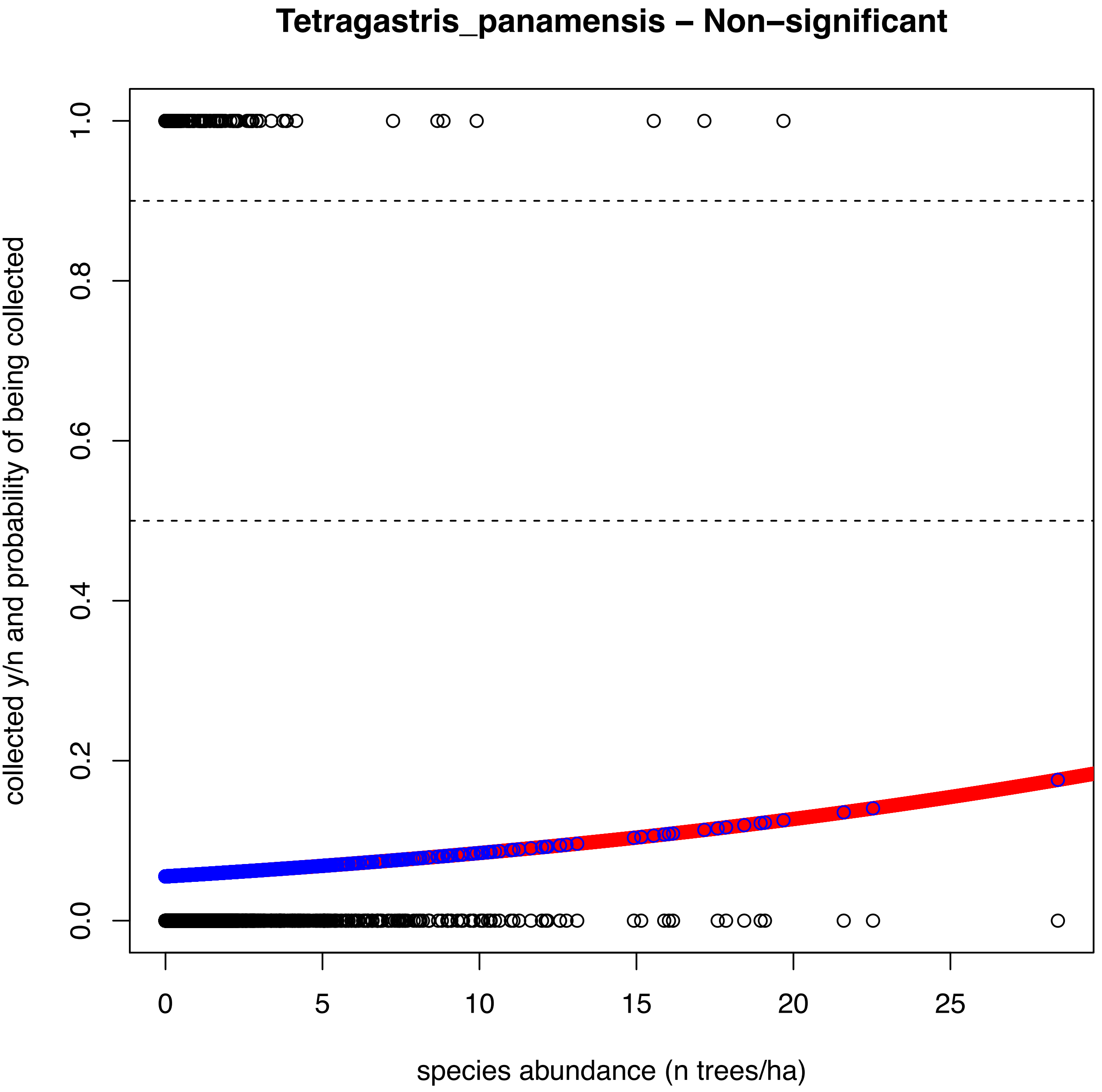

Tetragastris_panamensis – Non–significant
collected y/n and probability of being collected
species abundance (n trees/ha)
0.0
0.2
0.4
0.6
0.8
1.0
0
5
10
15
20
25

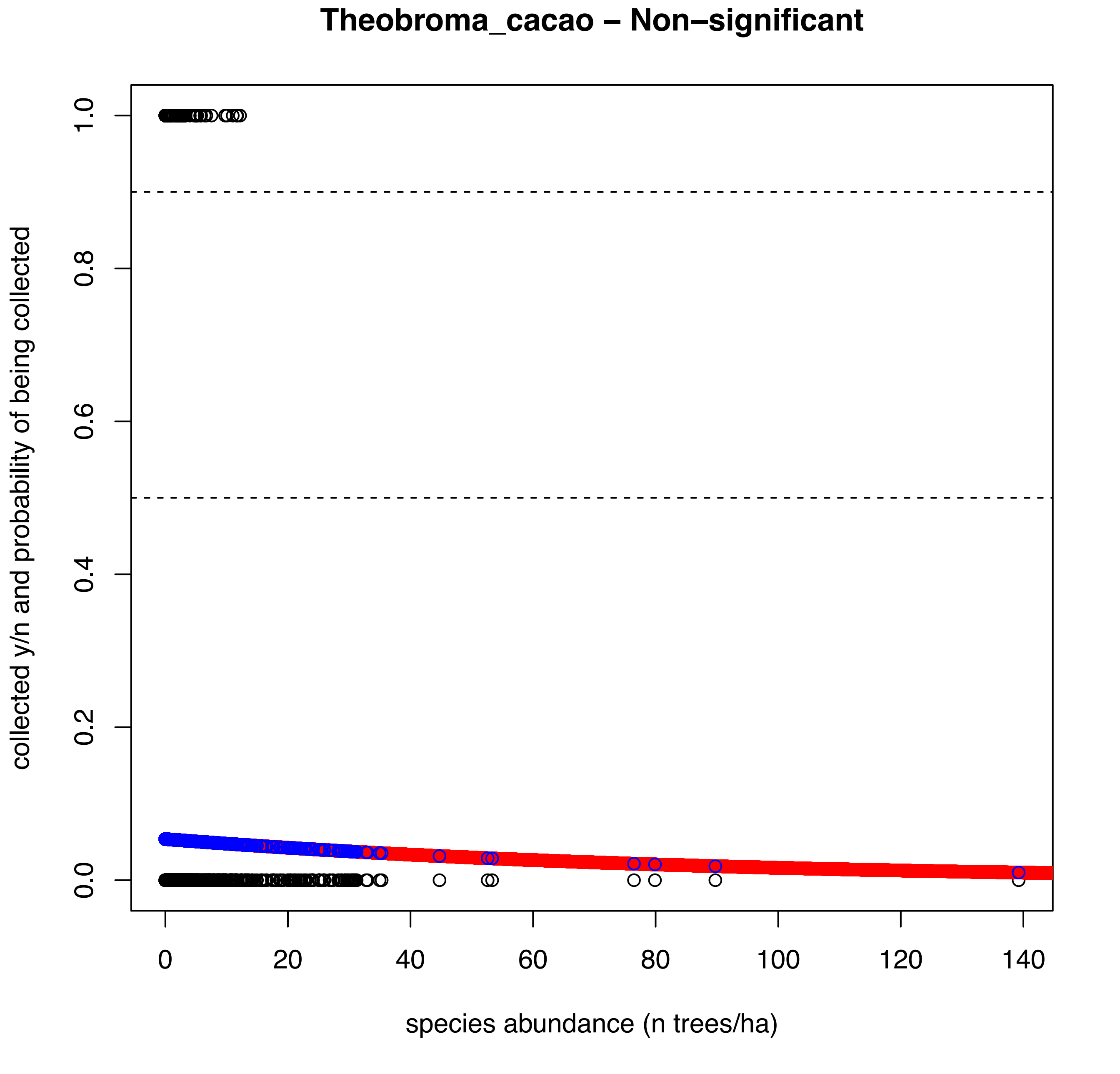

Theobroma_cacao – Non–significant
collected y/n and probability of being collected
species abundance (n trees/ha)
0.0
0.2
0.4
0.6
0.8
1.0
0
20
40
60
80
100
120
140

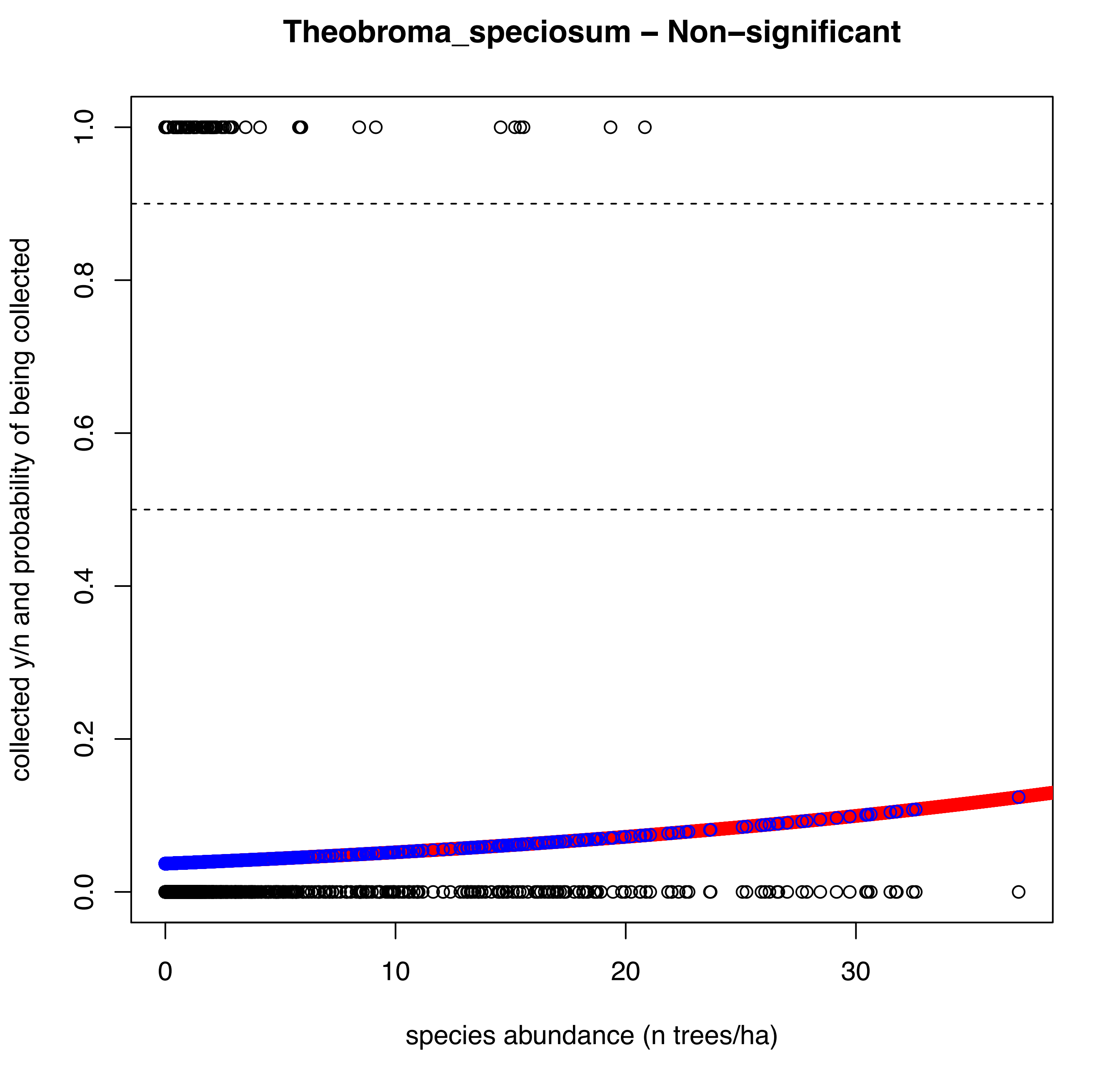
Theobroma_speciosum – Non–significant
collected y/n and probability of being collected
species abundance (n trees/ha)
0.0
0.2
0.4
0.6
0.8
1.0
0
10
20
30

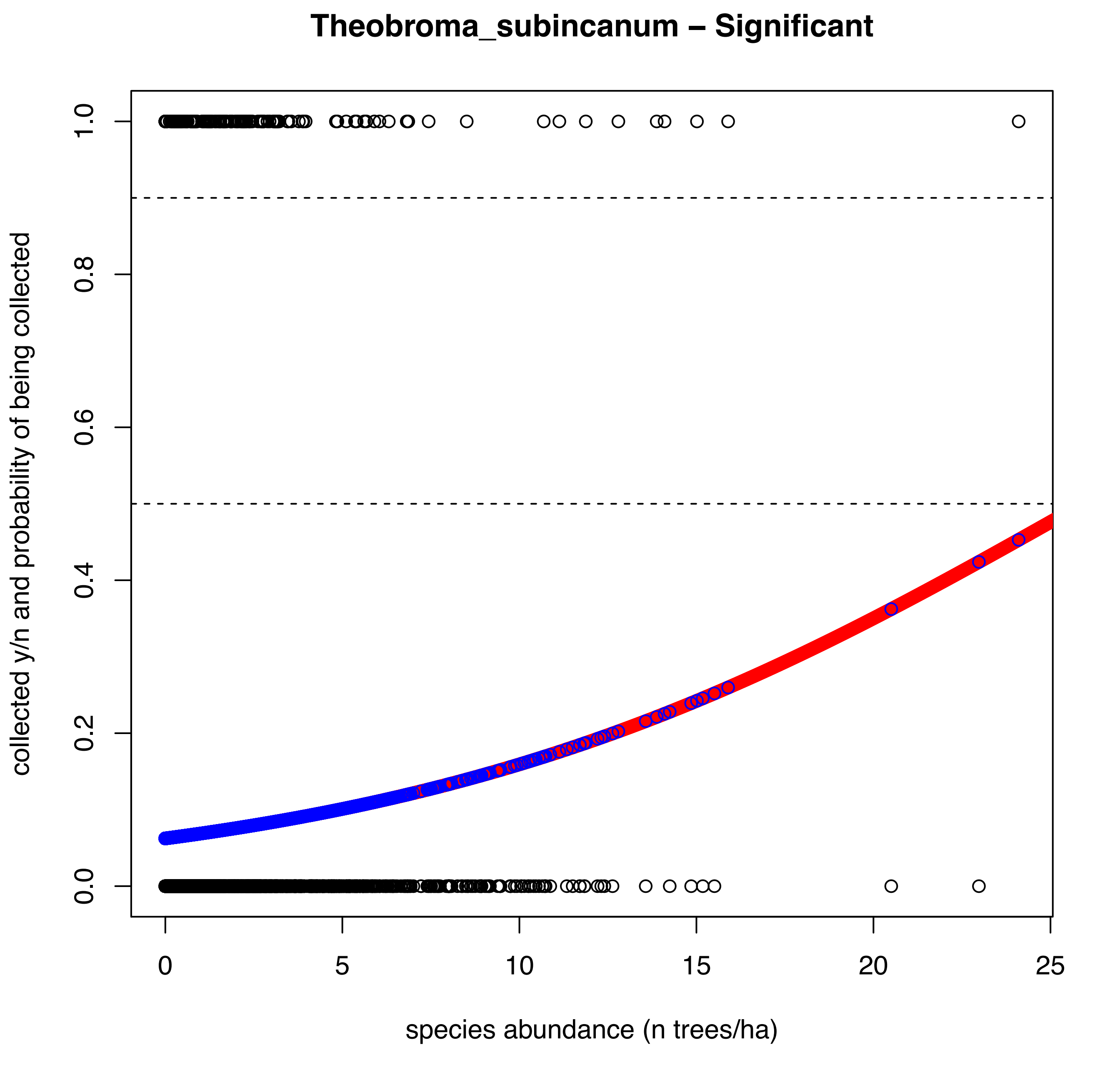
Theobroma_subincanum – Significant
collected y/n and probability of being collected
0.0
0.2
0.4
0.6
0.8
1.0
0
5
10
15
20
25
species abundance (n trees/ha)

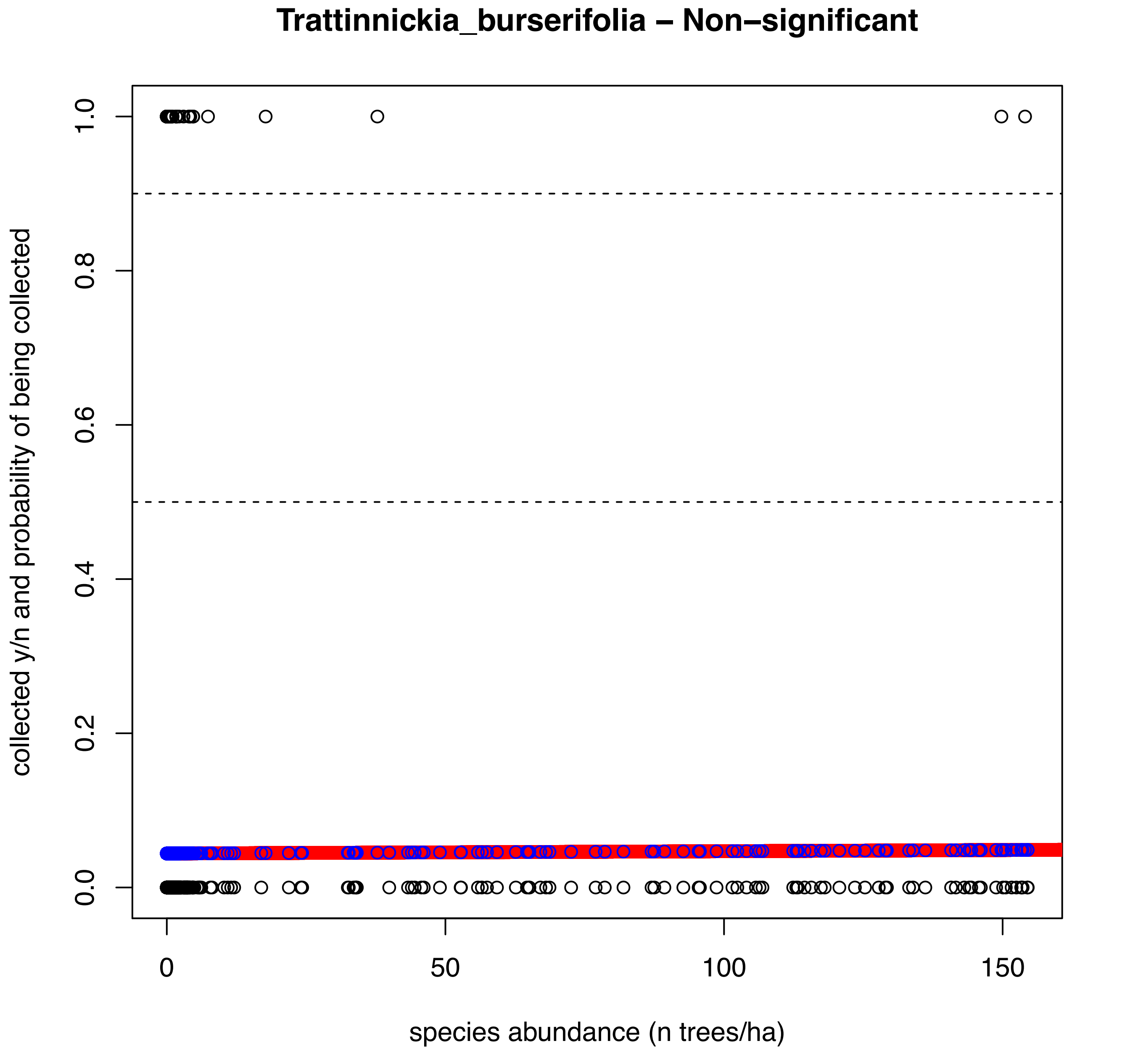
Trattinnickia_burserifolia – Non−significant
collected y/n and probability of being collected
0.0
0.2
0.4
0.6
0.8
1.0
0
50
100
150
species abundance (n trees/ha)

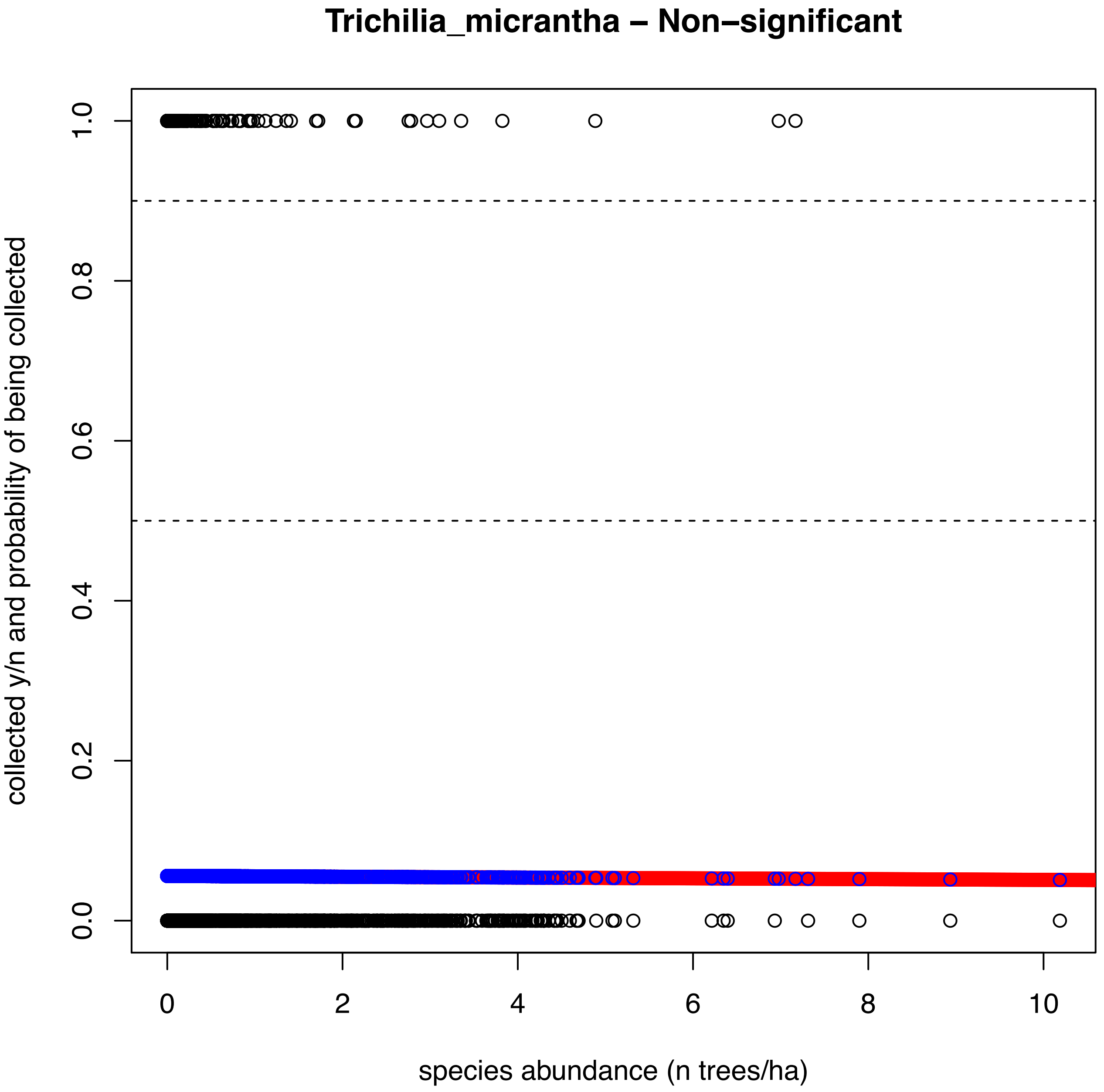

Trichilia_micrantha – Non–significant
collected y/n and probability of being collected
0.0
0.2
0.4
0.6
0.8
1.0
0
2
4
6
8
10
species abundance (n trees/ha)

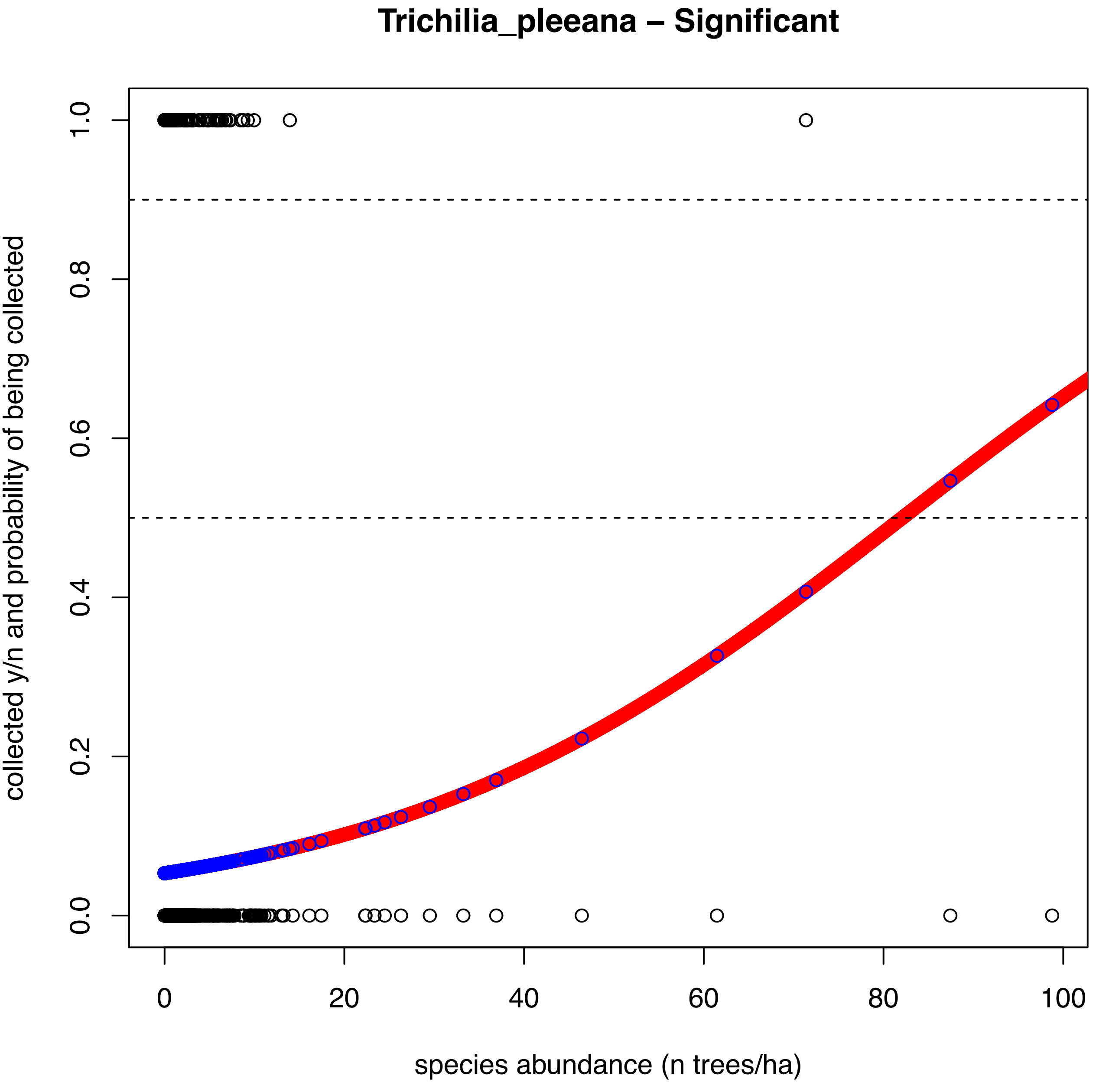
Trichilia_pleeana – Significant
collected y/n and probability of being collected
0.0
0.2
0.4
0.6
0.8
1.0
0
20
40
60
80
100
species abundance (n trees/ha)

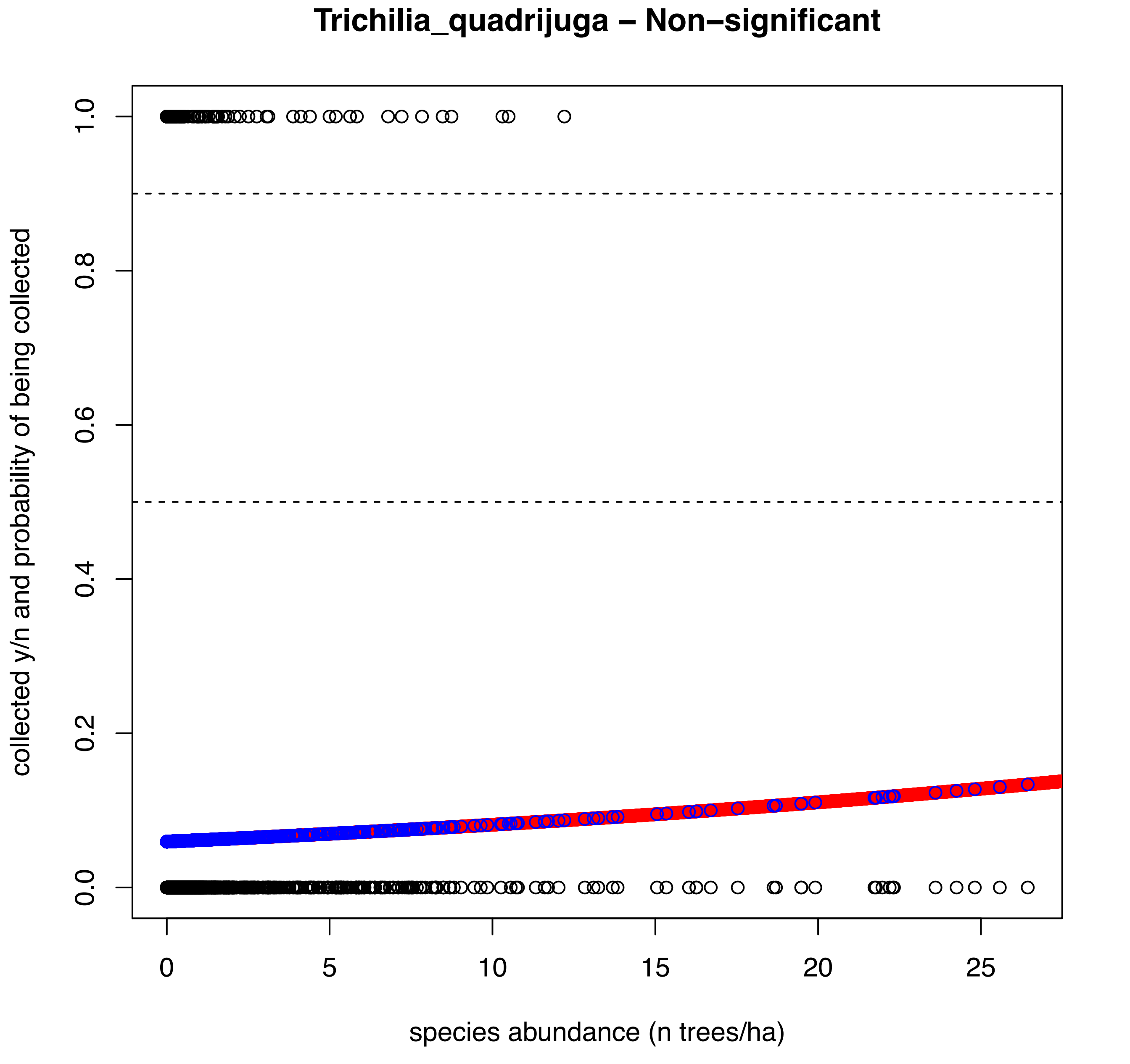
Trichilia_quadrijuga – Non–significant
collected y/n and probability of being collected
0.0
0.2
0.4
0.6
0.8
1.0
0
5
10
15
20
25
species abundance (n trees/ha)

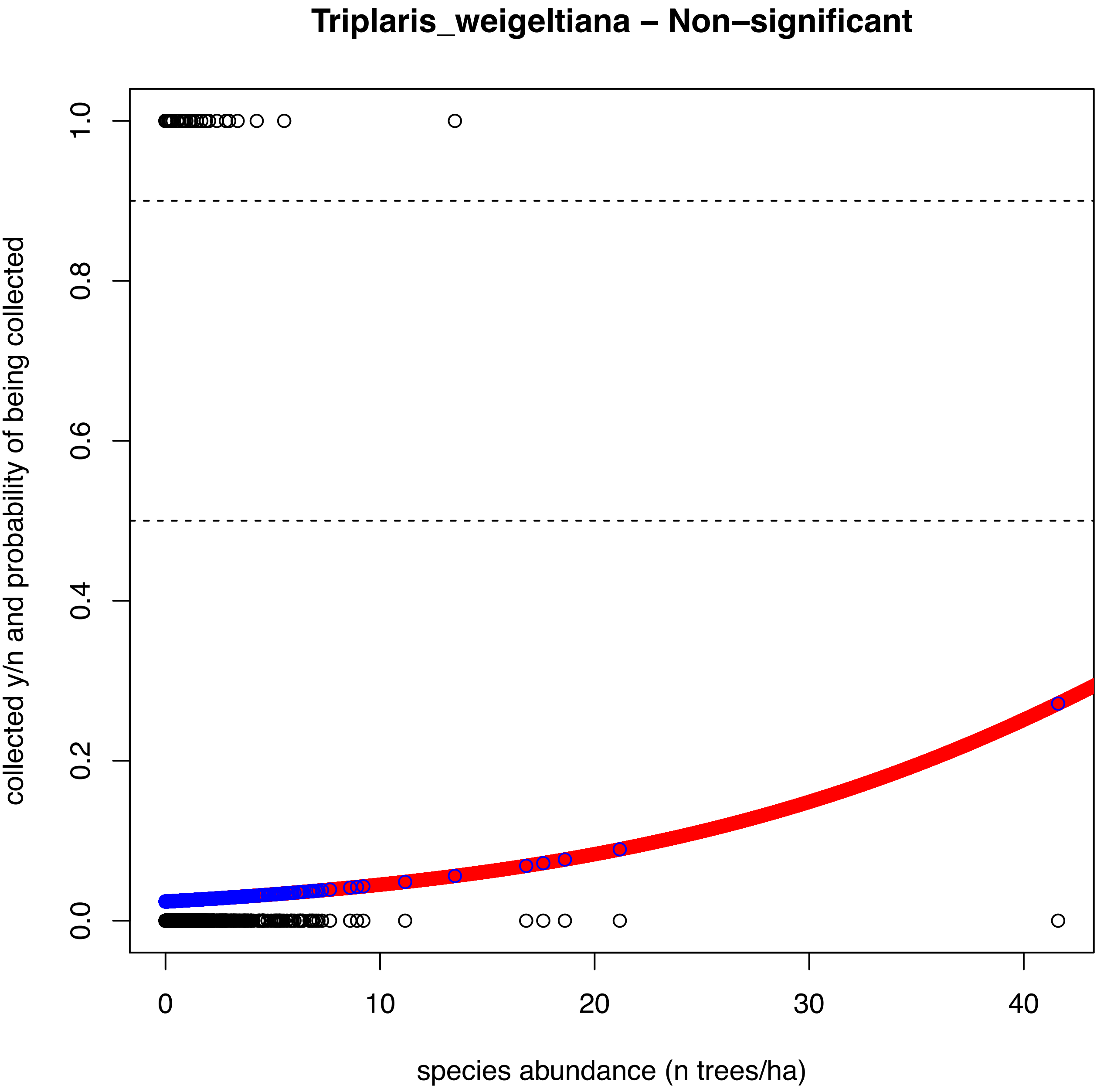

Triplaris_weigeltiana – Non–significant
collected y/n and probability of being collected
0.0
0.2
0.4
0.6
0.8
1.0
0
10
20
30
40
species abundance (n trees/ha)

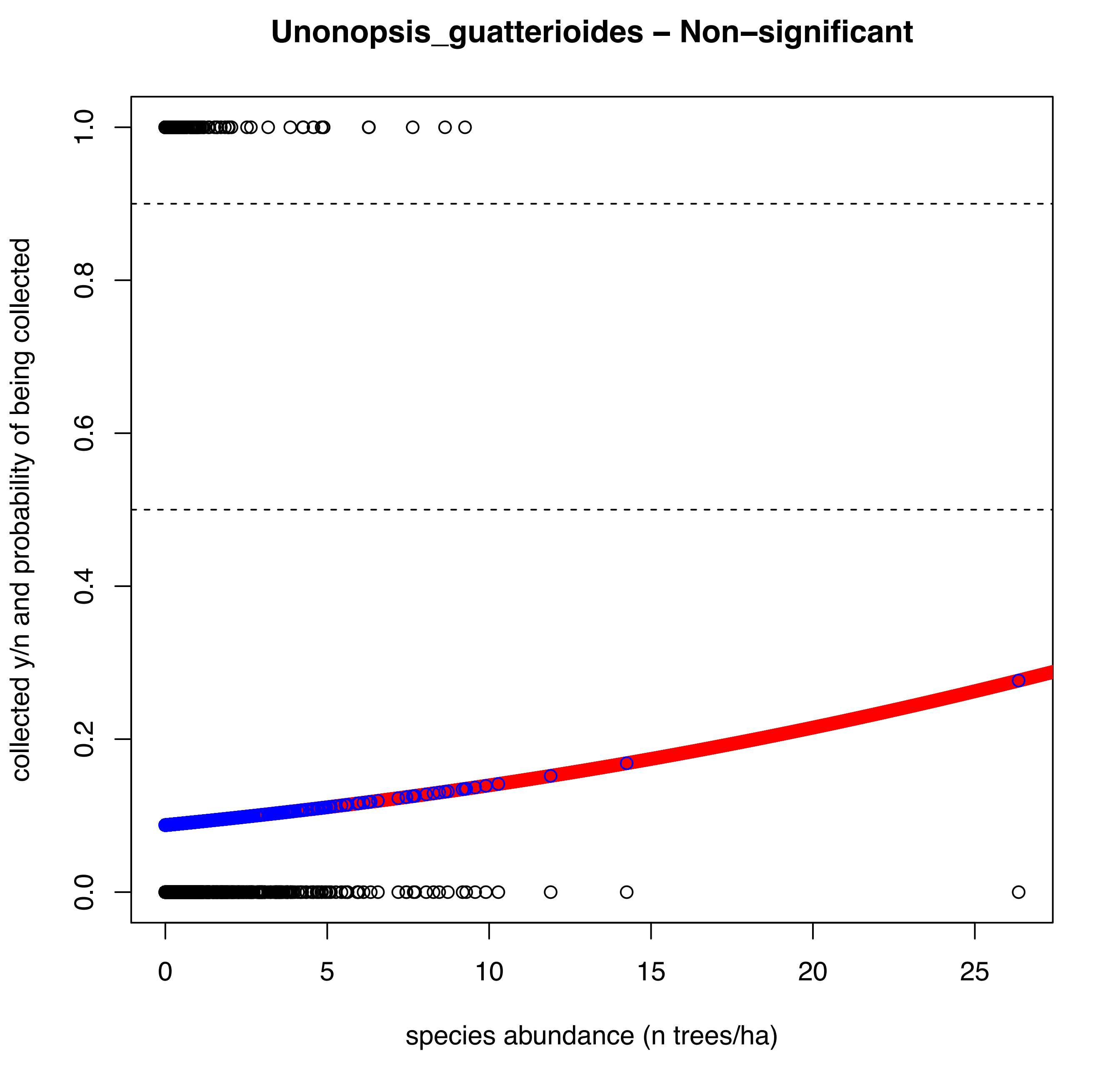
Unonopsis_guatterioides – Non–significant
collected y/n and probability of being collected
0.0
0.2
0.4
0.6
0.8
1.0
0
5
10
15
20
25
species abundance (n trees/ha)

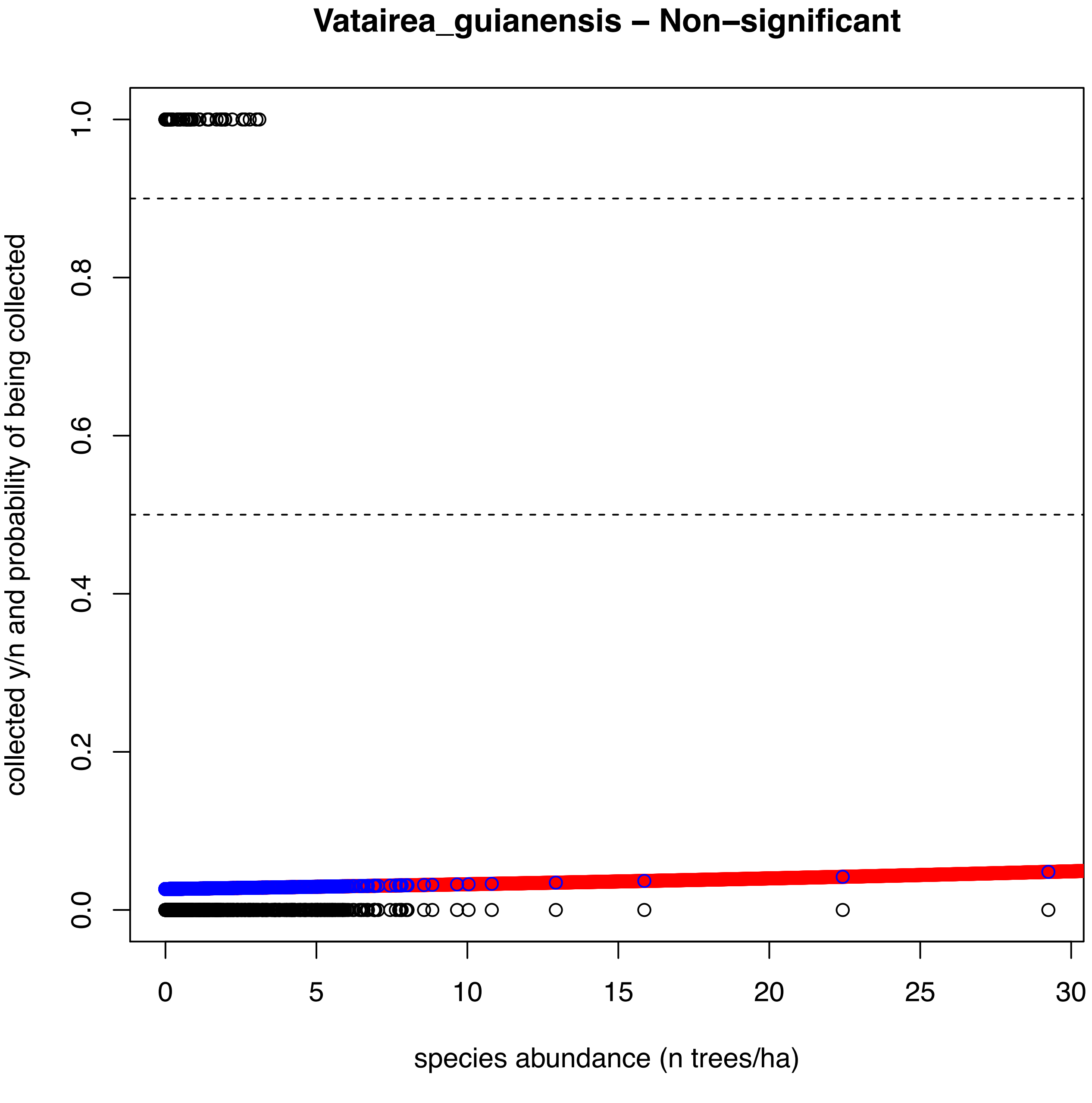

Vatairea_guianensis – Non–significant
collected y/n and probability of being collected
species abundance (n trees/ha)
0.0
0.2
0.4
0.6
0.8
1.0
0
5
10
15
20
25
30

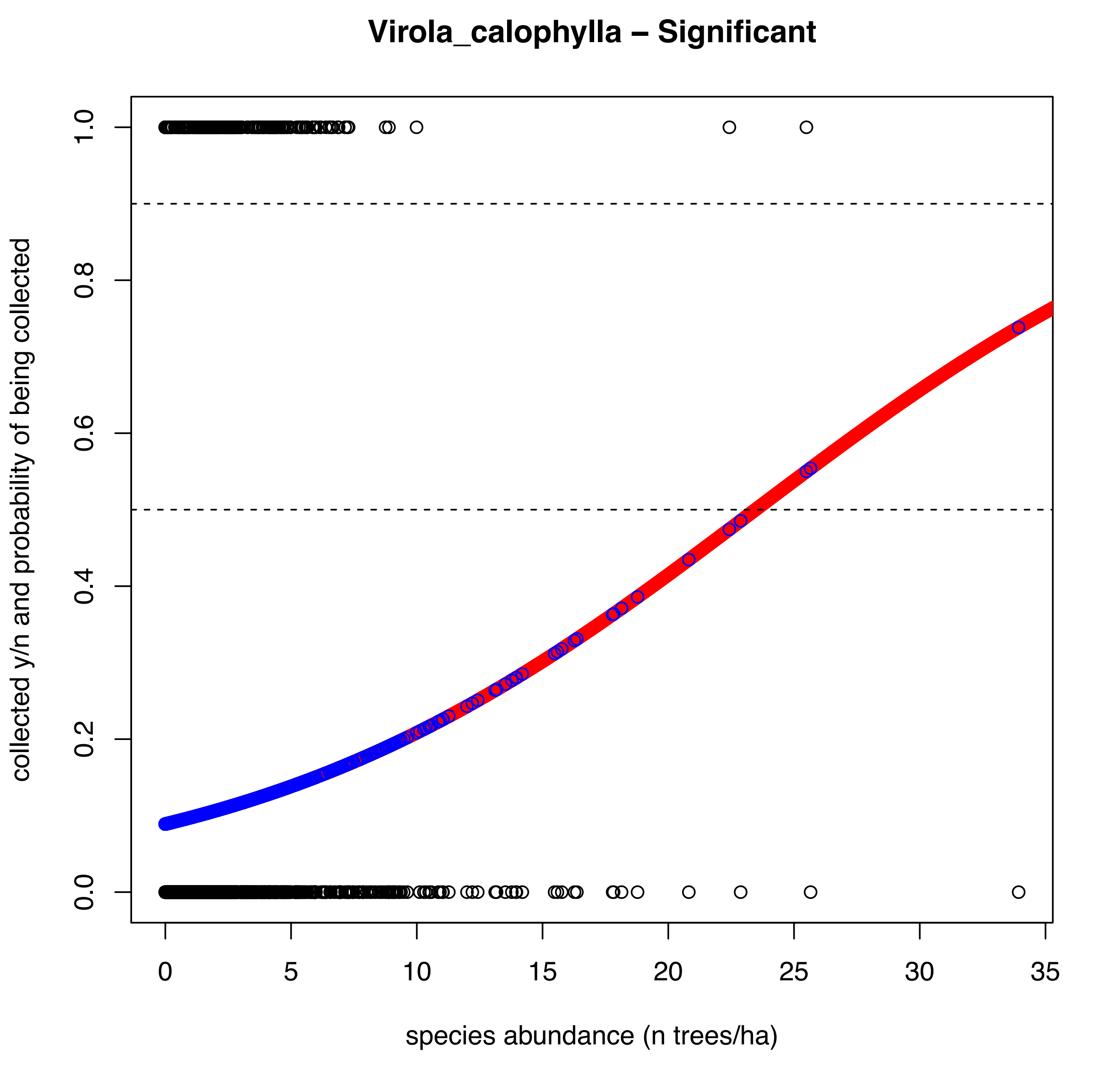

Virola_calophylla – Significant
collected y/n and probability of being collected
species abundance (n trees/ha)
0.0
0.2
0.4
0.6
0.8
1.0
0
5
10
15
20
25
30
35

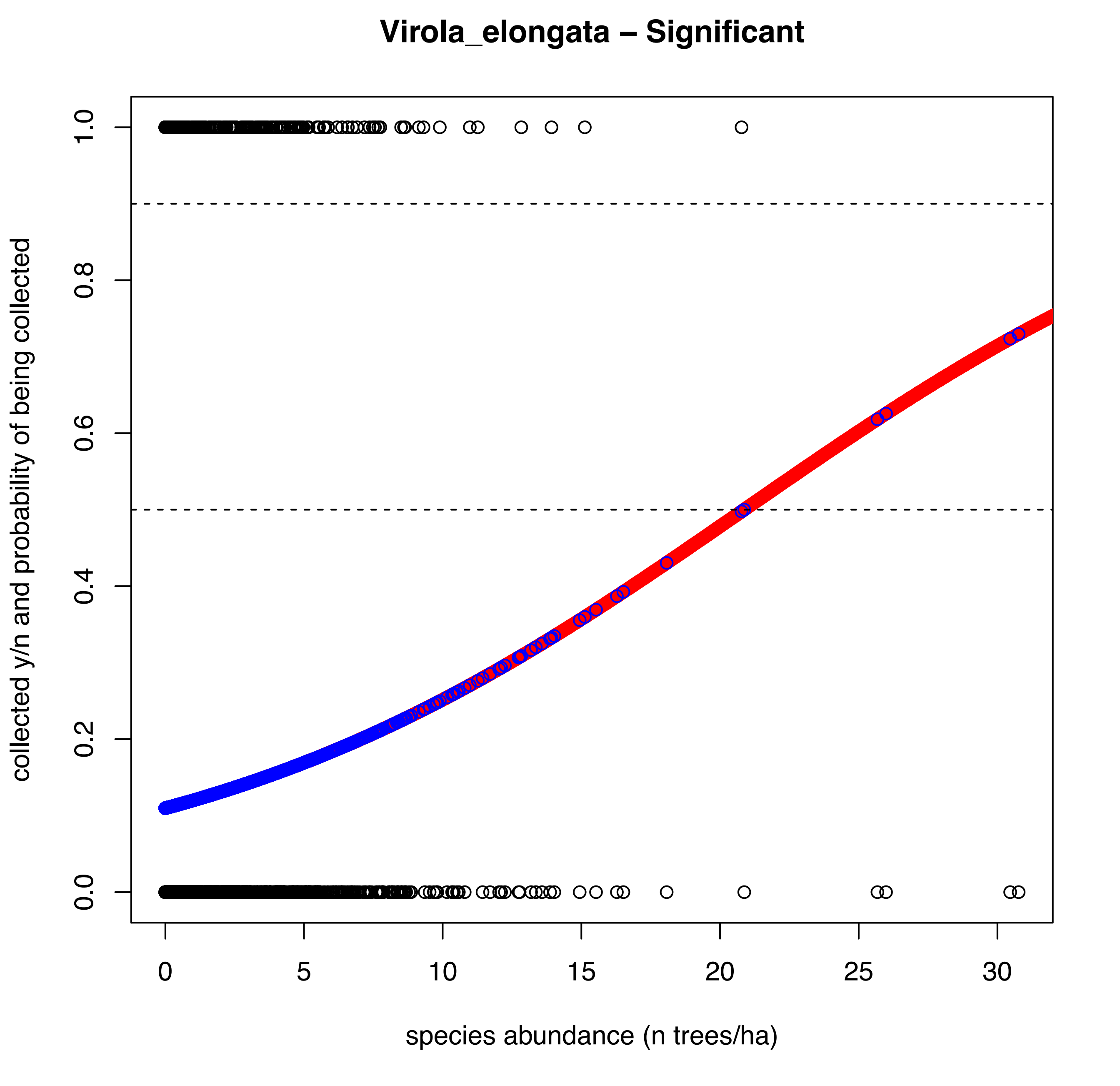

Virola_elongata – Significant
collected y/n and probability of being collected
0.0
0.2
0.4
0.6
0.8
1.0
0
5
10
15
20
25
30
species abundance (n trees/ha)

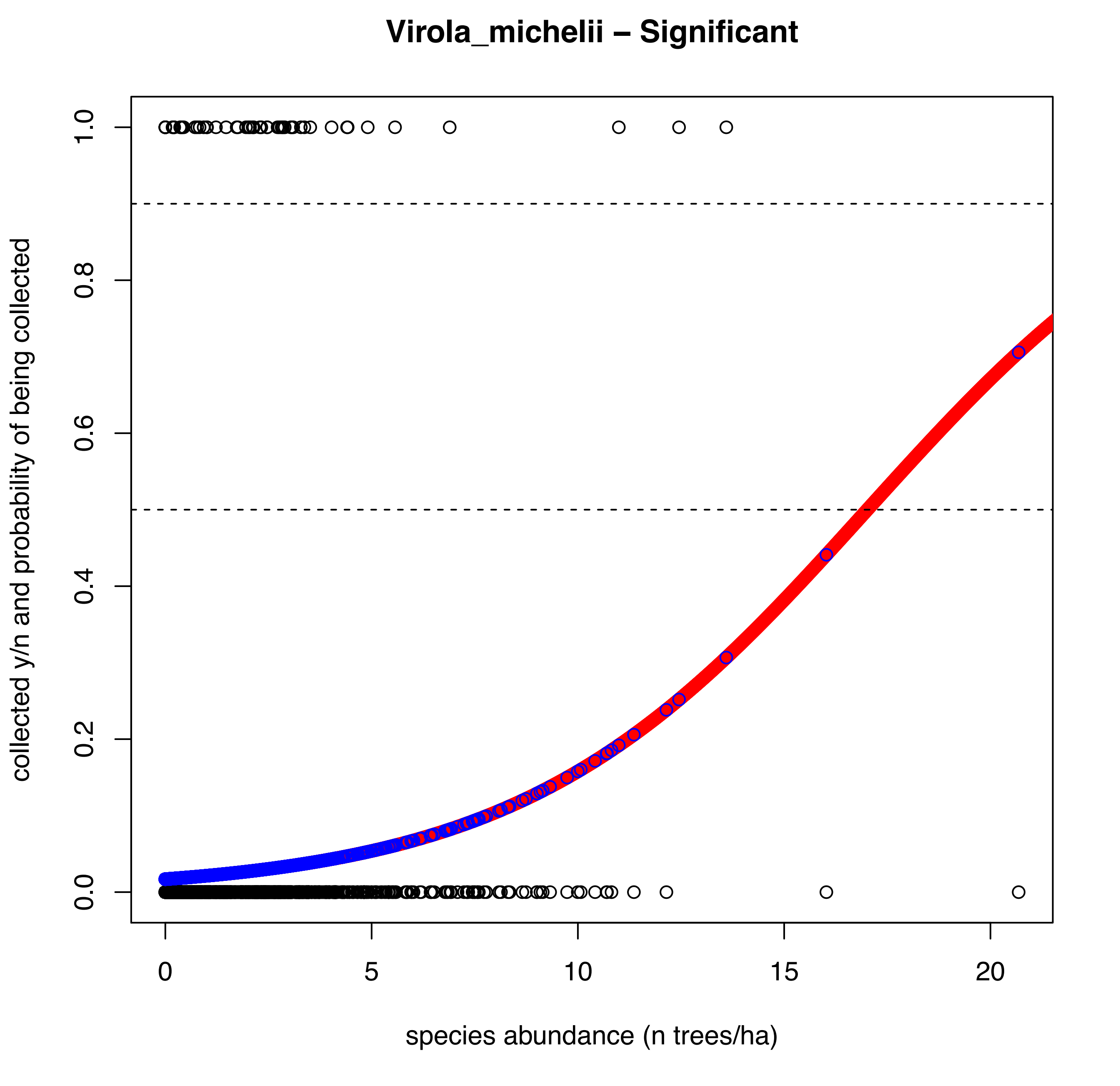
Virola_michelii – Significant
collected y/n and probability of being collected
species abundance (n trees/ha)
0.0
0.2
0.4
0.6
0.8
1.0
0
5
10
15
20

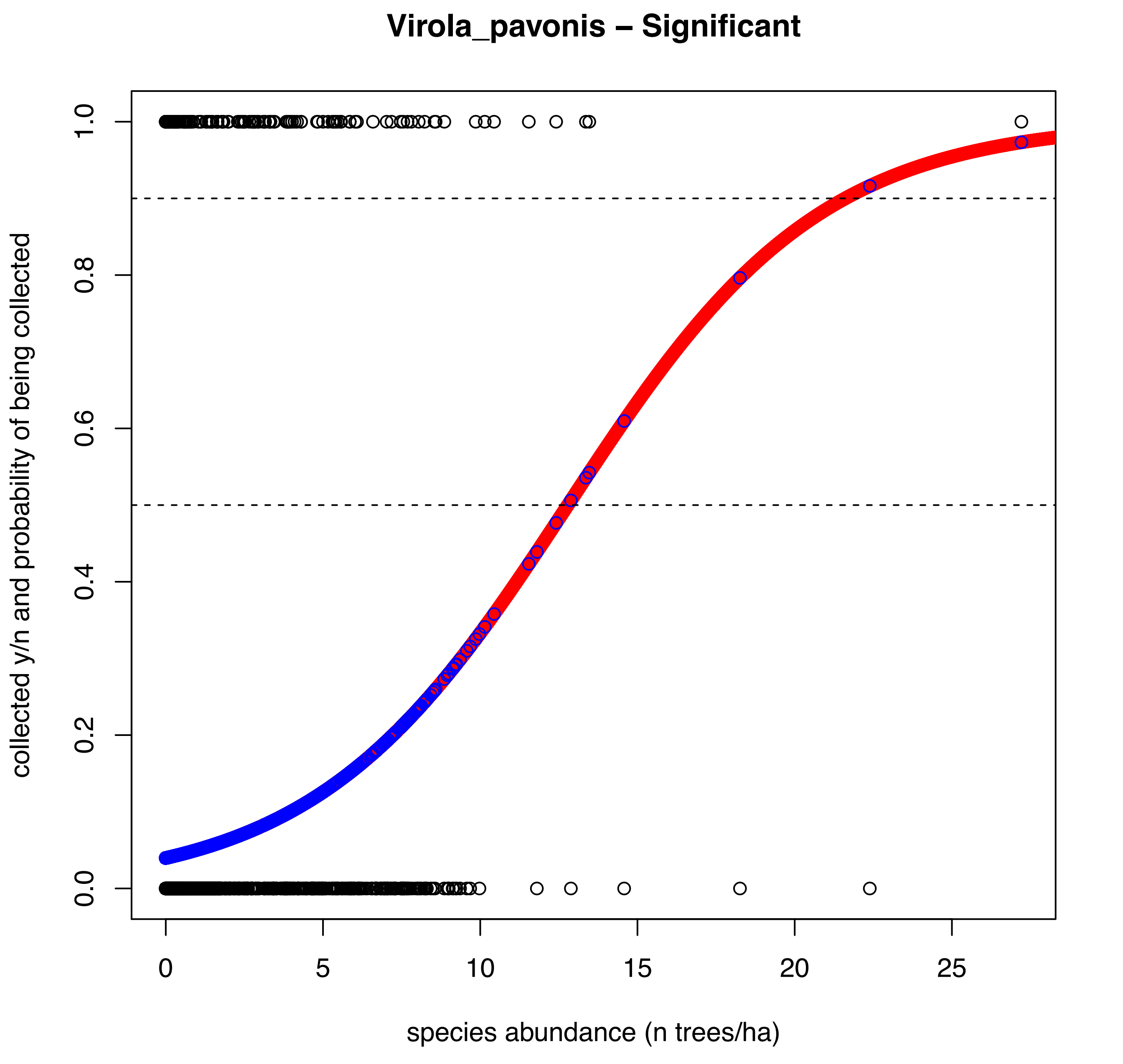

Virola_pavonis – Significant
collected y/n and probability of being collected
species abundance (n trees/ha)
0.0
0.2
0.4
0.6
0.8
1.0
0
5
10
15
20
25

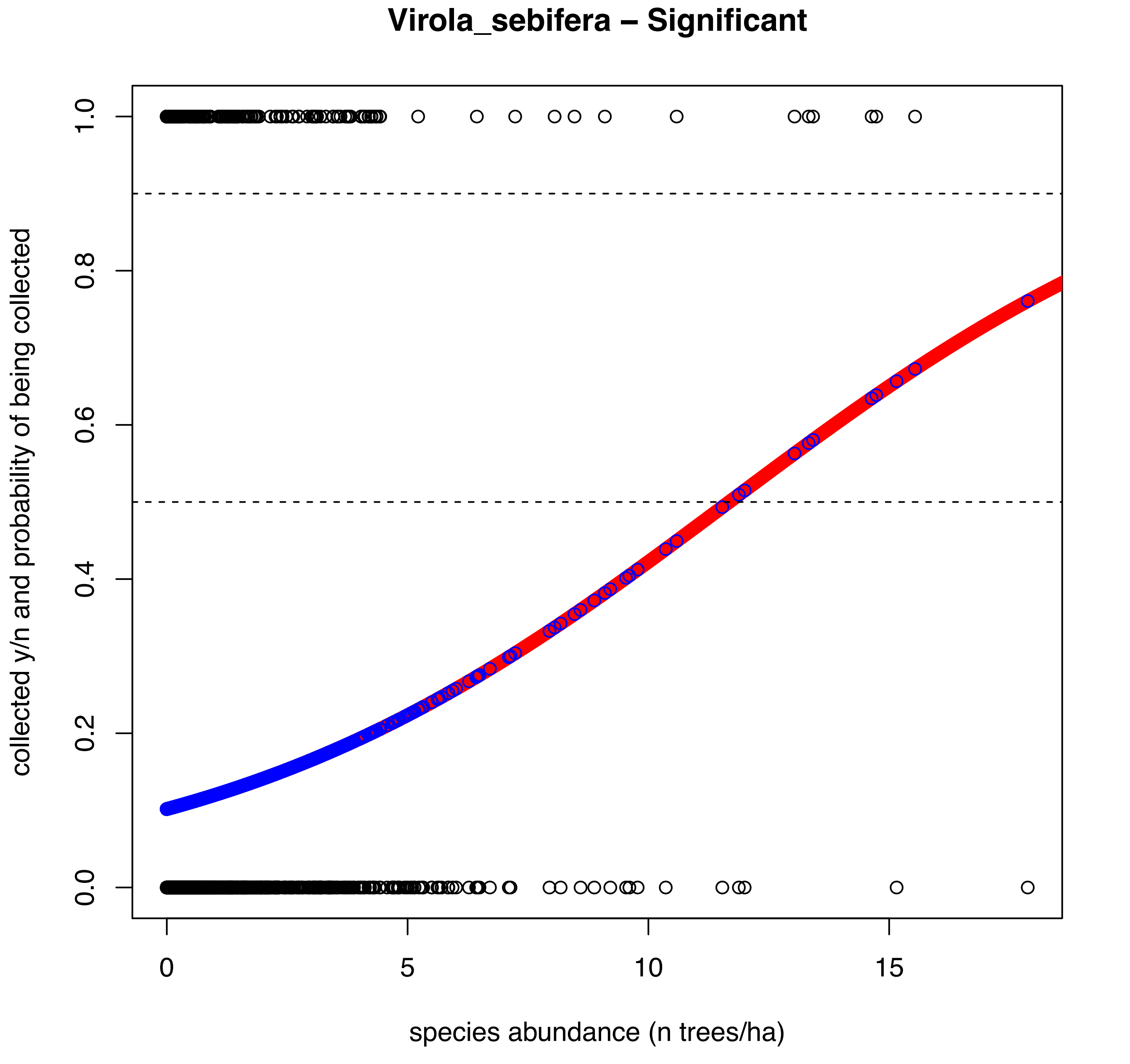
Virola_sebifera – Significant
collected y/n and probability of being collected
species abundance (n trees/ha)
0.0
0.2
0.4
0.6
0.8
1.0
0
5
10
15

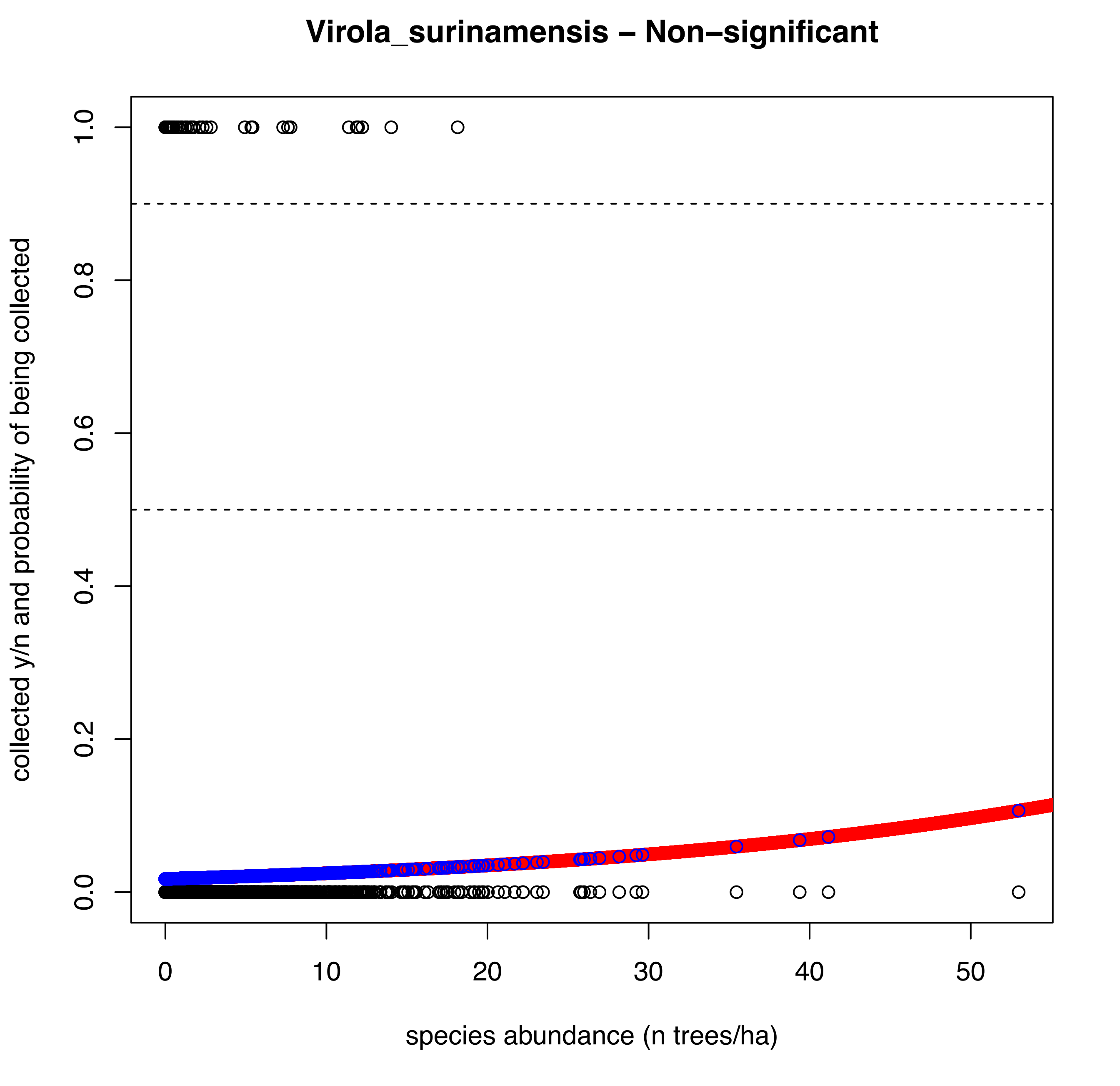

Virola_surinamensis – Non–significant
collected y/n and probability of being collected
0.0
0.2
0.4
0.6
0.8
1.0
0
10
20
30
40
50
species abundance (n trees/ha)

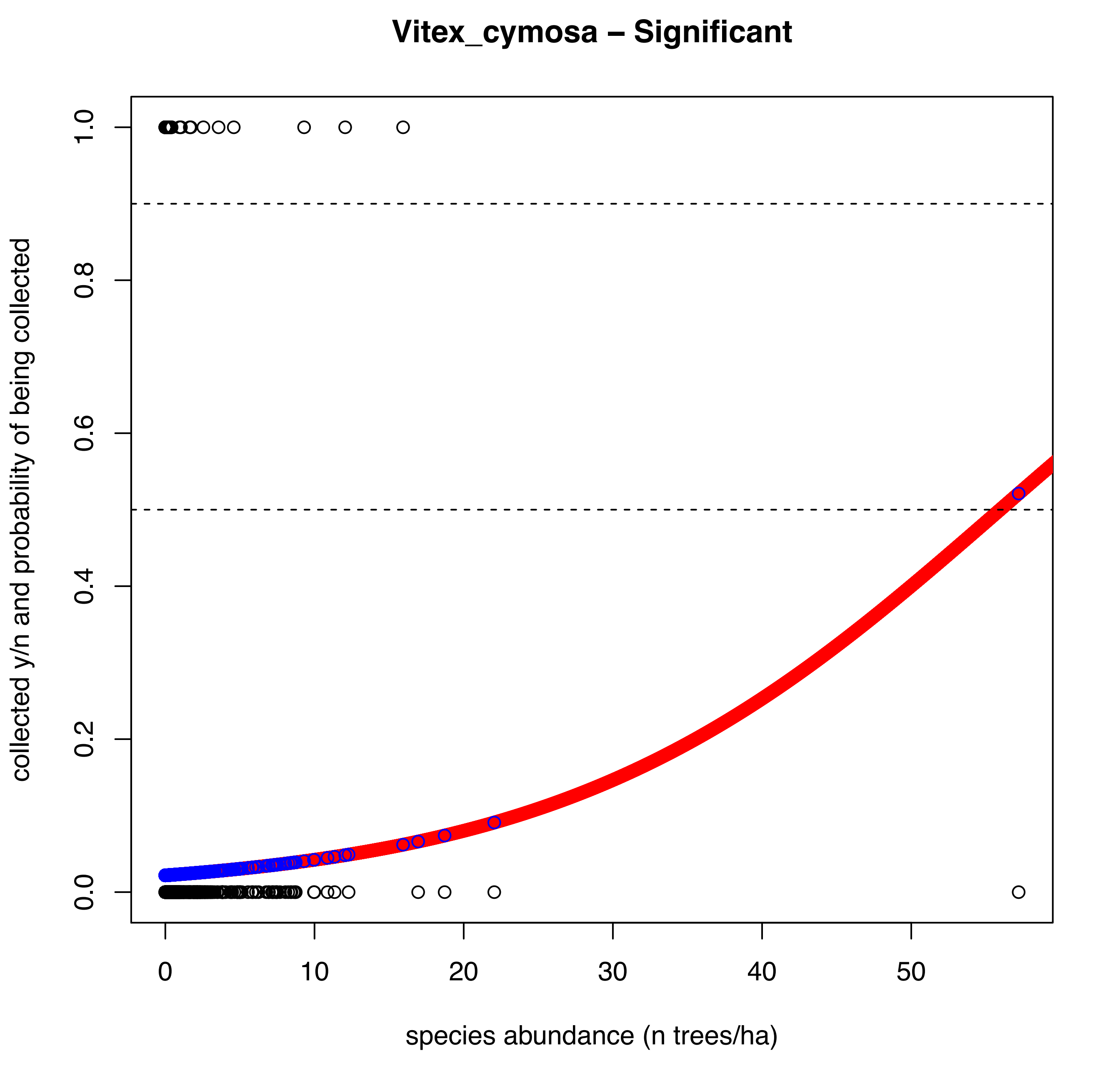

Vitex_cymosa – Significant
collected y/n and probability of being collected
species abundance (n trees/ha)
0.0
0.2
0.4
0.6
0.8
1.0
0
10
20
30
40
50

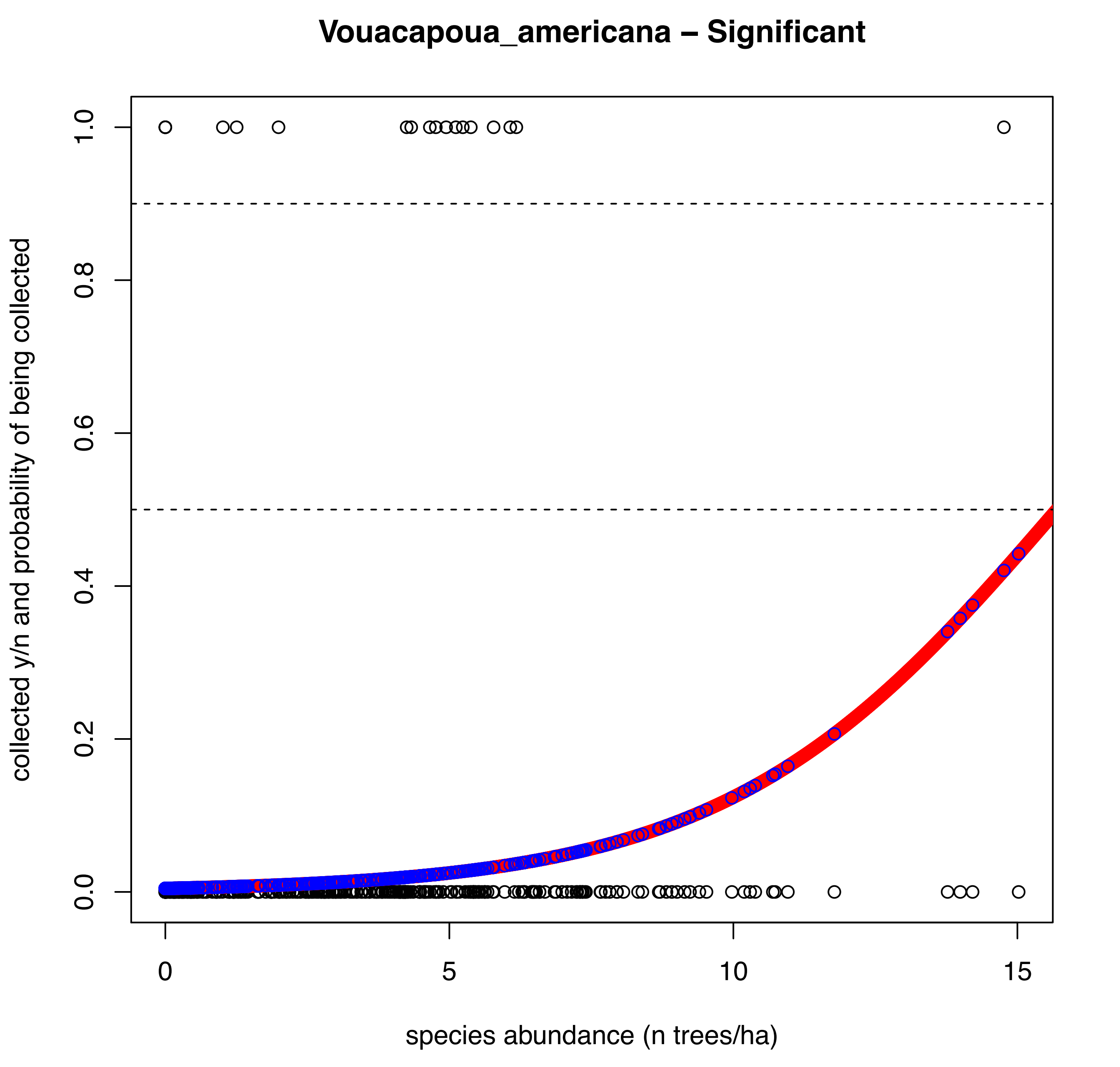
Vouacapoua_americana – Significant
collected y/n and probability of being collected
1.0
0.8
0.6
0.4
0.2
0.0
0
5
10
15
species abundance (n trees/ha)

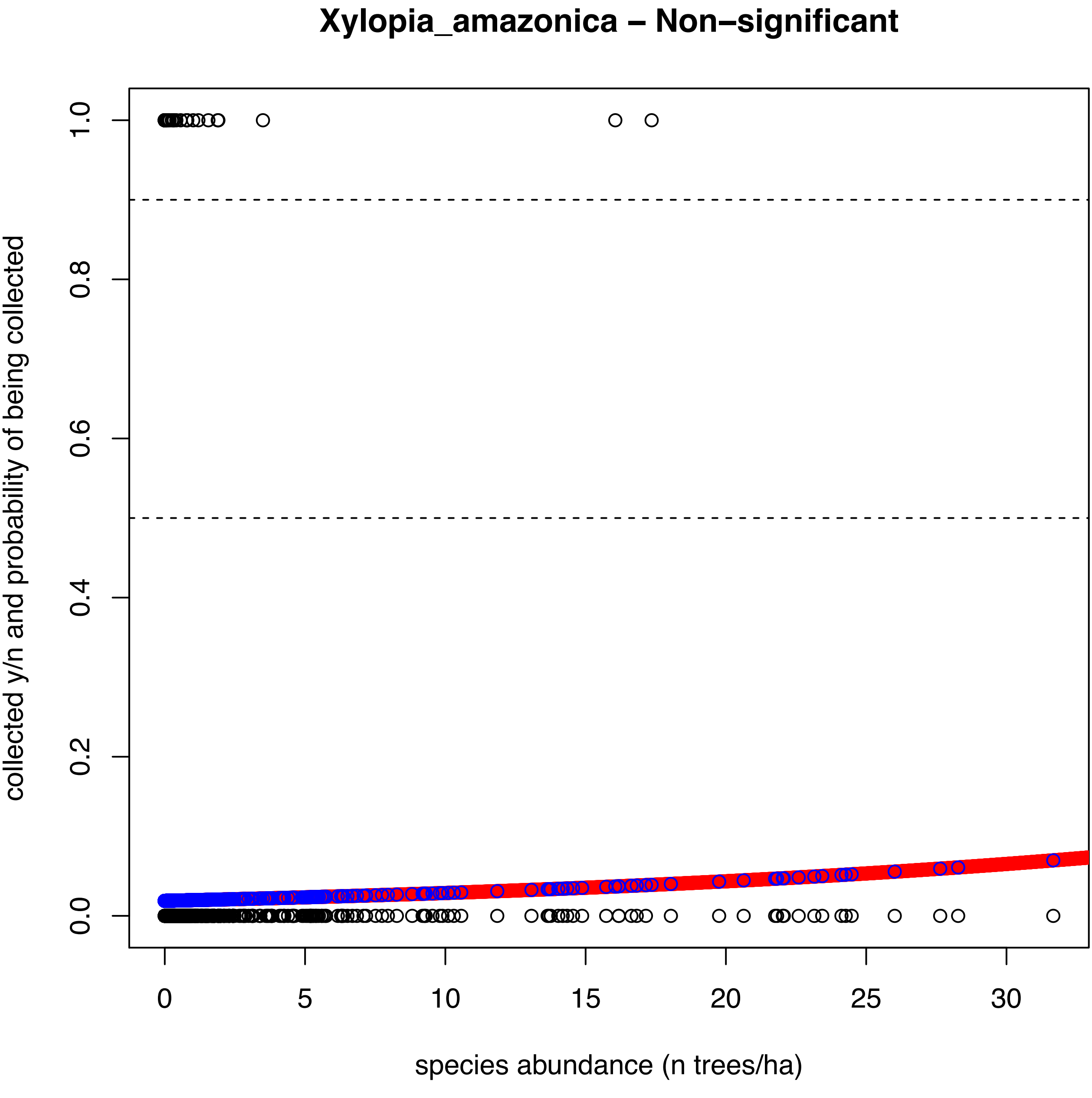

Xylopia_amazonica – Non–significant
collected y/n and probability of being collected
species abundance (n trees/ha)
0.0
0.2
0.4
0.6
0.8
1.0
0
5
10
15
20
25
30

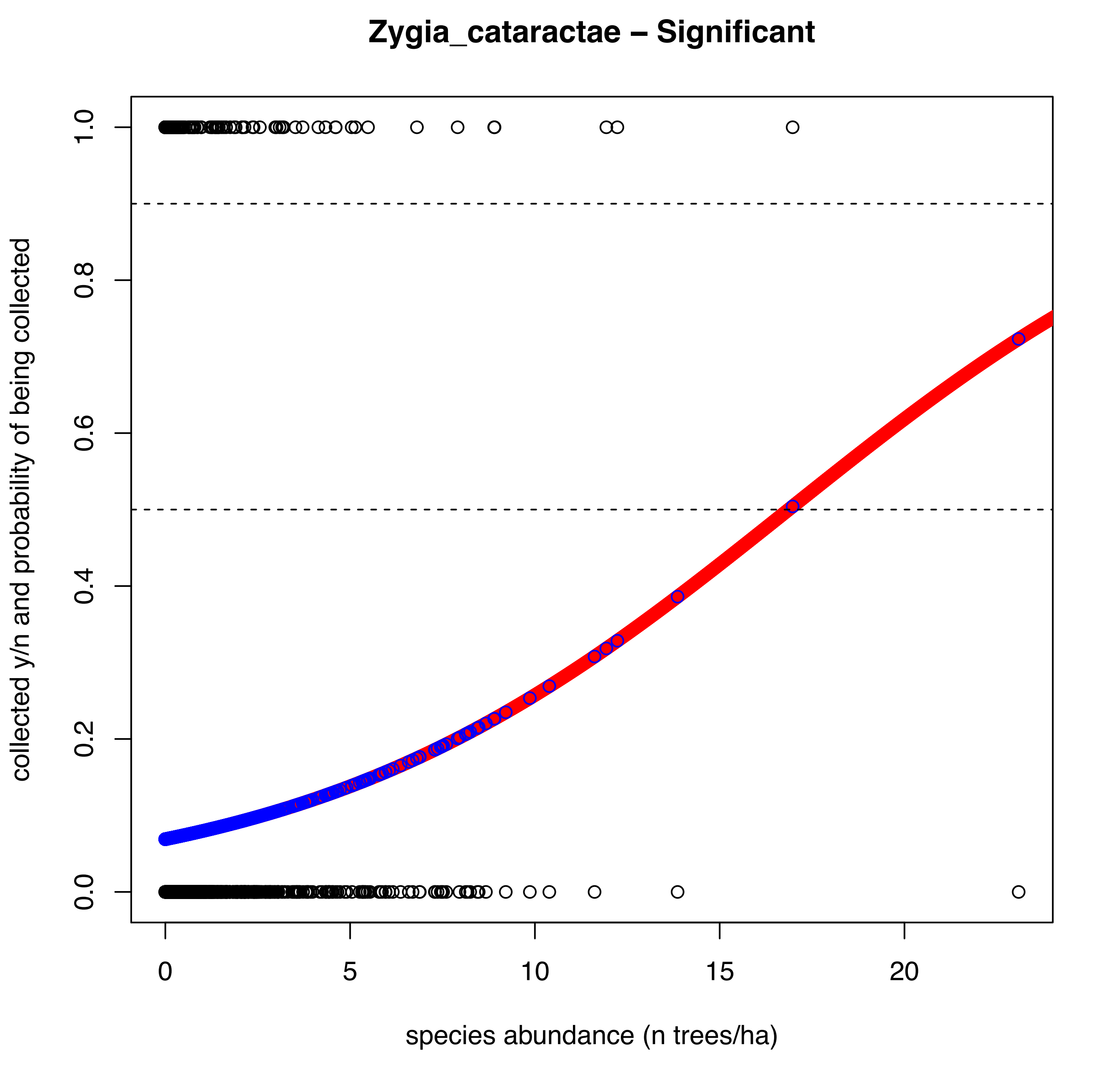

Zygia_cataractae – Significant
collected y/n and probability of being collected
species abundance (n trees/ha)
0.0
0.2
0.4
0.6
0.8
1.0
0
5
10
15
20

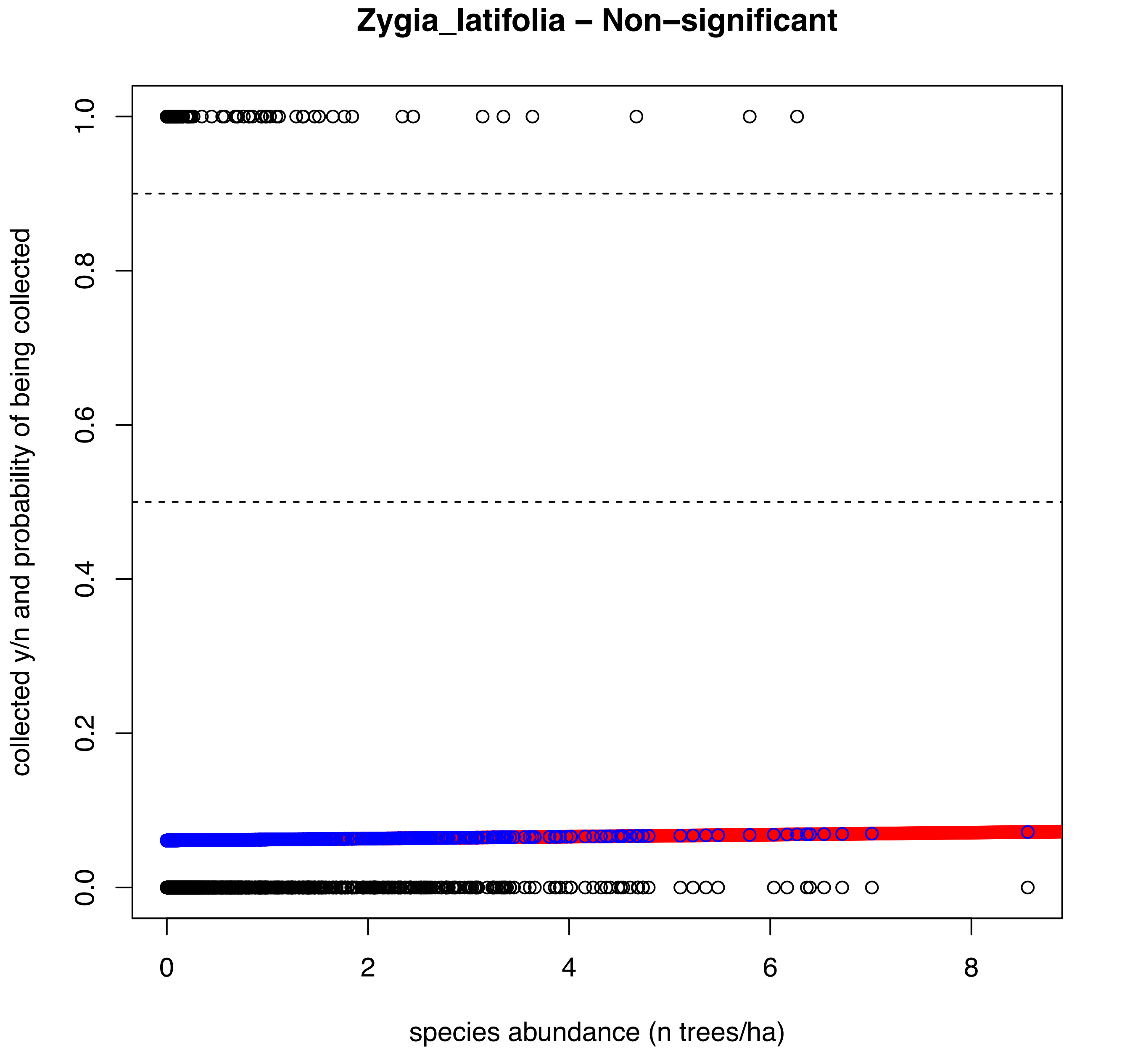

Zygia_latifolia – Non–significant
collected y/n and probability of being collected
species abundance (n trees/ha)
0.0
0.2
0.4
0.6
0.8
1.0
0
2
4
6
8

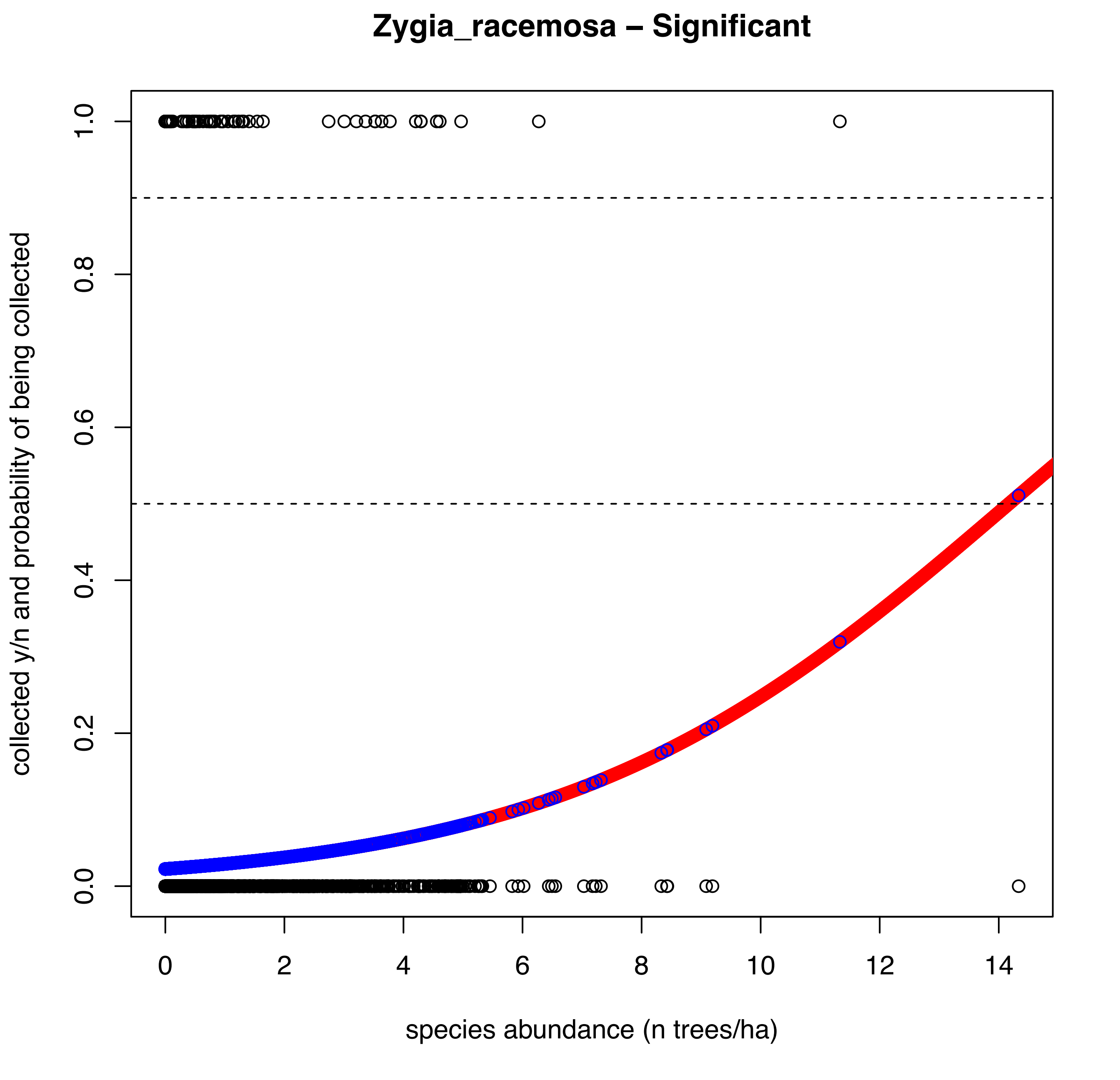
Zygia_racemosa – Significant
collected y/n and probability of being collected
species abundance (n trees/ha)
0.0
0.2
0.4
0.6
0.8
1.0
0
2
4
6
8
10
12
14
