# Supplementary material for: Species Distribution Modelling: Contrasting presence-only models with plot abundance data
Source: Sci Rep. 2018 Jan 17;8:1003. doi: 10.1038/s41598-017-18927-1 (PMC5772443; doi:10.1038/s41598-017-18927-1)
Supplement: Supplementary file 2 — Comparing the results of modelling the area of occupancy with MaxEnt and with inverse distance weighting (IDW). [file 41598_2017_18927_MOESM2_ESM.pdf]

Supplemental Material

for

Species Distribution Modelling: Contrasting presence-only models with plot abundance data

Vitor F. Gomes*, Stéphanie D. IJff, Niels Raes, Iêda Leão Amaral, Rafael P. Salomão, Luiz de Souza Coelho, Francisca Dionízia de Almeida Matos, Carolina V. Castilho, Diogenes de Andrade Lima Filho, Dairon Cárdenas López, Juan Ernesto Guevara, William E. Magnusson, Oliver L. Phillips, Florian Wittmann, Marcelo de Jesus Veiga Carim, Maria Pires Martins, Mariana Victória Irume, Daniel Sabatier, Jean-François Molino, Olaf S. Bánki, José Renan da Silva Guimarães, Nigel C.A. Pitman, Maria Teresa Fernandez Piedade, Abel Monteagudo Mendoza, Bruno Garcia Luize, Eduardo Martins Venticinque, Evlyn Márcia Moraes de Leão Novo, Percy Núñez Vargas, Thiago Sanna Freire Silva, Angelo Gilberto Manzatto, John Terborgh, Neidiane Farias Costa Reis, Juan Carlos Montero, Katia Regina Casula, Beatriz S. Marimon, Ben-Hur Marimon Jr., Euridice N. Honorio Coronado, Ted R. Feldpausch, Alvaro Duque, Charles Eugene Zartman, Nicolás Castaño Arboleda, Timothy J. Killeen, Bonifacio Mostacedo, Rodolfo Vasquez, Jochen Schöngart, Rafael L. Assis, Marcelo Brilhante Medeiros, Marcelo Fragomeni Simon, Ana Andrade, William F. Laurance, José Luís Camargo, Layon O. Demarchi, Susan G.W. Laurance, Emanuelle de Sousa Farias, Henrique Eduardo Mendonça Nascimento, Juan David Cardenas Revilla, Adriano Quaresma, Flávia R.C. Costa, Ima Célia Guimarães Vieira, Bruno Barçante Ladvocat Cintra, Hernán Castellanos, Roel Brienen, Pablo R. Stevenson, Yuri Feitosa, Joost F. Duivenvoorden, Gerardo A. Aymard C., Hugo F. Mogollón, Natalia Targhetta, James A. Comiskey, Alberto Vicentini, Aline Lopes, Gabriel Damasco, Nállarett Dávila, Roosevelt García-Villacorta, Carolina Levis, Juliana Schietti, Priscila Souza, Thaise Emilio, Alfonso Alonso, David Neill, Francisco Dallmeier, Leandro Valle Ferreira, Alejandro Araujo-Murakami, Daniel Praia, Dário Dantas do Amaral, Fernanda Antunes Carvalho, Fernanda Coelho de Souza, Kenneth Feeley, Luzmila Arroyo, Marcelo Petratti Pansonato, Rogerio Gribel, Boris Villa, Juan Carlos Licona,

Paul V.A. Fine, Carlos Cerón, Chris Baraloto, Eliana M. Jimenez, Juliana Stropp, Julien Engel, Julien Engel, Marcos Silveira, Maria Cristina Peñuela Mora, Pascal Petronelli, Paul Maas, Raquel Thomas-Caesar, Terry W. Henkel, Doug Daly, Marcos Ríos Paredes, Tim R. Baker, Alfredo Fuentes, Carlos A. Peres, Jerome Chave, Jose Luis Marcelo Pena, Kyle G. Dexter, Miles R. Silman, Peter Møller Jørgensen, Toby Pennington, Anthony Di Fiore, Fernando Cornejo Valverde, Juan Fernando Phillips, Gonzalo Rivas-Torres, Patricio von Hildebrand, Tinde R. van Andel, Ademir R. Ruschel, Adriana Prieto, Agustín Rudas, Bruce Hoffman, César I.A. Vela, Edelcilio Marques Barbosa, Egleé L. Zent, George Pepe Gallardo Gonzales, Hilda Paulette Dávila Doza, Ires Paula de Andrade Miranda, Jean-Louis Guillaumet, Linder Felipe Mozombite Pinto, Luiz Carlos de Matos Bonates, Natalino Silva, Ricardo Zárate Gómez, Stanford Zent, Therany Gonzales, Vincent A. Vos, Yadvinder Malhi, Alexandre A. Oliveira, Angela Cano, Bianca Weiss Albuquerque, Corine Vriesendorp, Diego Felipe Correa, Emilio Vilanova Torre, Geertje van der Heijden, Hirma Ramirez-Angulo, José Ferreira Ramos, Kenneth R. Young, Maira Rocha, Marcelo Trindade Nascimento, Maria Natalia Umaña Medina, Milton Tirado, Ophelia Wang, Rodrigo Sierra, Armando Torres-Lezama, Casimiro Mendoza, Cid Ferreira, Cláudia Baider, Daniel Villarroel, Henrik Balslev, Italo Mesones, Ligia Estela Urrego Giraldo, Luisa Fernanda Casas, Manuel Augusto Ahuite Reategui, Reynaldo Linares-Palomino, Roderick Zagt, Sasha Cárdenas, William Farfan-Rios, Adeilza Felipe Sampaio, Daniela Pauletto, Elvis H. Valderrama Sandoval, Freddy Ramirez Arevalo, Isau Huamantupa-Chuquimaco, Karina Garcia-Cabrera, Lionel Hernandez, Luis Valenzuela Gamarra, Miguel N. Alexiades, Susamar Pansini, Walter Palacios Cuenca, William Milliken, Joana Ricardo, Gabriela Lopez-Gonzalez, Edwin Pos, Hans ter Steege*

*Correspondence and requests for materials should be addressed to H.T.S. (email: hans.tersteege@naturalis.nl)

Appendix S2: Comparing the results of modelling the area of occupancy with MaxEnt and with inverse distance weighting (IDW).

Analysis results for the predicted area of occupancy as calculated by Maxent and IDW methods. The analyses were conducted for 170 of all 227 hyperdominant species that had MaxEnt's predicted environmental suitability significantly different from a null model. The following results are shown: Spearman's correlation and 90th quantile, testing the relationship between the methods at plot localities; sensitivity to test their predictive performance; AUC values for MaxEnt, AUC values for 95th ranked null model; and the thresholded predicted area (of occupancy) for both methods measured by the number of grid cells. Additionally, the number of Amazonian regions in which each species collections is distributed.

Species	Spearman's Correlation	90th Quantile	IDW Sensitivity	MaxEnt Sensitivity	MaxEnt AUC	Null Model AUC 95th Rank	Area MaxEnt	Area IDW	N of Amazonian Regions
Acosmium_cardenasii	0.428	0.001	1.000	0.333	0.898	0.840	200	203	2
Alexa_imperatricis	0.198	0.003	1.000	0.733	0.942	0.824	161	392	1
Amphiodon_effusus	0.422	0.009	0.912	0.829	0.881	0.795	858	933	5
Apeiba_glabra	0.160	0.002	0.789	0.800	0.835	0.759	1395	1518	6
Aspidosperma_excelsum	0.293	0.022	0.910	0.950	0.799	0.764	2000	1801	6
Astrocaryum_aculeatum	-0.089	0.000	0.640	0.904	0.805	0.784	1808	1321	6
Astrocaryum_murumuru	0.431	-0.007	0.900	0.614	0.882	0.803	1046	1244	5
Attalea_butyracea	0.269	-0.008	0.784	0.324	0.901	0.792	513	761	2
Attalea_maripa	-0.204	-0.008	0.851	0.546	0.867	0.781	1195	1743	5
Attalea_phalerata	0.095	0.014	0.878	0.649	0.837	0.795	614	778	4
Attalea_speciosa	0.560	0.046	0.750	0.447	0.889	0.851	450	796	4
Bertholletia_excelsa	0.136	-0.001	0.854	0.880	0.801	0.787	1634	1147	6
Bocageopsis_multiflora	0.387	0.009	0.904	0.873	0.848	0.767	1311	1434	6
Brosimum_rubescens	0.225	0.002	0.941	0.993	0.792	0.772	1959	1819	6
Brosimum_utile	0.406	0.006	0.904	0.806	0.838	0.768	1444	1395	5
Campsiandra_implexicaulis	0.358	0.003	0.333	0.000	0.935	0.845	254	425	4
Caraipa_densifolia	0.275	0.003	0.631	0.975	0.773	0.755	2007	1209	6
Carapa_guianensis	0.300	0.014	0.844	0.944	0.790	0.768	1707	1388	6
Caryocar_glabrum	0.368	0.002	0.895	0.953	0.832	0.765	1619	1661	6
Catostemma_fragrans	0.403	0.049	0.895	0.947	0.969	0.840	231	165	2
Cecropia_latiloba	0.268	0.003	0.464	0.962	0.811	0.782	1679	1078	6
Cecropia_membranacea	0.598	0.012	0.750	0.867	0.884	0.773	762	982	5
Cecropia_sciadophylla	0.049	-0.002	0.899	0.786	0.814	0.775	1574	1756	6
Cedrelinga_cateniformis	0.387	-0.001	0.864	0.732	0.869	0.782	1095	1433	6
Celtis_schippii	0.417	0.014	0.943	0.841	0.836	0.779	754	806	4
Cenostigma_tocantinum	0.338	0.017	0.909	1.000	0.926	0.836	293	204	2
Chlorocardium_rodiei	0.159	0.000	1.000	1.000	0.930	0.875	49	89	1
Chrysophyllum_sanguinolentum	0.600	0.015	0.942	0.849	0.878	0.778	1000	1308	5
Clarisia_racemosa	-0.071	-0.014	0.867	0.849	0.765	0.761	1649	1756	6
Clathrotropis_glaucophylla	0.401	0.001	0.774	0.714	0.946	0.810	377	290	3
Clathrotropis_macrocarpa	0.470	0.028	0.930	0.771	0.924	0.788	568	668	4
Conceveiba_guianensis	0.312	0.007	0.903	0.903	0.811	0.756	1694	1806	6
Couepia_guianensis	0.494	0.006	0.928	0.940	0.849	0.785	1410	1362	6
Couepia_paraensis	0.400	0.003	0.653	0.917	0.835	0.785	1544	849	6
Cynometra_marginata	0.134	0.001	0.406	0.867	0.872	0.809	1329	738	5
Diplotropis_purpurea	-0.062	-0.003	0.784	0.884	0.804	0.788	1723	1642	6
Drypetes_amazonica	0.564	0.013	0.944	0.827	0.852	0.787	843	1196	4
Ecclinusa_guianensis	0.164	0.012	0.841	0.909	0.800	0.793	1718	1558	5
Enterolobium_schomburgkii	0.125	-0.005	0.843	0.957	0.770	0.767	1927	1652	6
Eperua_falcata	0.393	0.118	0.903	0.882	0.963	0.810	218	297	3

Species	Spearman's Correlation	90th Quantile	IDW Sensitivity	MaxEnt Sensitivity	MaxEnt AUC	Null Model AUC 95th Rank	Area MaxEnt	Area IDW	N of Amazonian Regions
Eperua_grandiflora	0.320	0.011	0.692	1.000	0.901	0.824	229	203	3
Eperua_leucantha	0.391	0.017	0.957	0.889	0.979	0.830	154	270	3
Eperua_purpurea	0.326	0.029	1.000	0.818	0.983	0.817	125	215	3
Erisma_uncinatum	0.000	-0.006	0.880	0.857	0.784	0.780	2009	1564	6
Eschweilera_albiflora	0.577	0.030	0.814	0.952	0.862	0.785	1426	1209	6
Eschweilera_amazonica	0.667	0.035	0.778	0.857	0.892	0.809	811	674	5
Eschweilera_atropetiolata	0.451	0.018	0.923	0.469	0.932	0.836	230	892	3
Eschweilera_bracteosa	0.435	0.012	0.925	0.633	0.936	0.791	508	1115	5
Eschweilera_coriacea	0.287	0.093	0.902	0.929	0.790	0.749	1784	1813	6
Eschweilera_decolorans	0.282	0.005	0.846	0.829	0.850	0.804	1175	1040	5
Eschweilera_grandiflora	0.541	0.009	0.943	0.875	0.876	0.800	1194	1215	6
Eschweilera_parviflora	0.419	0.009	0.739	0.947	0.853	0.780	1408	977	6
Eschweilera_parvifolia	0.504	0.016	0.735	0.872	0.856	0.774	1432	976	5
Eschweilera_pedicellata	0.682	0.008	0.774	0.984	0.858	0.766	1258	1040	6
Eschweilera_sagotiana	0.525	0.028	0.688	0.944	0.904	0.809	695	296	3
Eschweilera_tessmannii	0.555	0.016	0.970	0.821	0.926	0.781	707	1108	5
Eschweilera_truncata	0.435	0.032	0.885	0.824	0.902	0.807	605	456	5
Eschweilera_wachenheimii	0.697	0.028	0.931	0.839	0.907	0.817	584	729	4
Euterpe_oleracea	0.018	-0.007	0.731	0.787	0.843	0.780	1392	927	5
Euterpe_precatoria	0.241	-0.063	0.953	0.612	0.841	0.752	903	1774	5
Garcinia_macrophylla	0.188	-0.002	0.842	0.931	0.776	0.772	1876	1785	6
Goupia_glabra	0.306	0.008	0.952	0.869	0.830	0.762	1495	1601	6
Guarea_macrophylla	0.417	0.030	0.877	0.849	0.735	0.727	1259	1337	6
Guarea_silvatica	0.345	0.001	0.865	0.904	0.844	0.783	1470	1114	6
Gustavia_augusta	0.192	-0.001	0.819	0.935	0.755	0.750	1966	1496	6
Gustavia_hexapetala	0.346	0.014	0.746	0.977	0.789	0.753	1710	1259	6
Handroanthus_barbatus	0.384	0.007	0.838	0.739	0.916	0.793	613	772	5
Haploclathra_cordata	0.508	0.010	1.000	0.800	0.950	0.909	191	259	2
Hevea_guianensis	0.493	0.016	0.911	0.936	0.847	0.758	1303	1526	6
Inga_lateriflora	0.035	0.001	0.683	0.889	0.810	0.793	1751	1218	6
Inga_marginata	0.268	0.003	0.333	0.419	0.895	0.783	537	1507	1
Iriartea_deltoidea	0.338	0.112	0.957	0.762	0.865	0.769	629	1200	4
Iryanthera_juruensis	0.243	-0.036	0.931	0.939	0.781	0.743	1764	1895	6
Iryanthera_laevis	0.273	0.006	0.944	0.865	0.833	0.772	1336	1600	5
Iryanthera_ulei	0.337	0.008	0.786	0.950	0.849	0.762	1173	969	5
Jacaranda_copaia	-0.198	-0.004	0.929	0.902	0.762	0.742	1995	1961	6
Laetia_corymbulosa	0.232	0.005	0.640	0.846	0.881	0.802	1076	580	5
Laetia_procera	0.078	0.005	0.879	0.939	0.802	0.772	1913	1717	6
Lecythis_corrugata	0.642	0.016	0.789	0.907	0.881	0.775	790	774	5
Lecythis_idatimon	0.495	0.027	0.865	0.846	0.924	0.790	353	514	3

Species	Spearman's Correlation	90th Quantile	IDW Sensitivity	MaxEnt Sensitivity	MaxEnt AUC	Null Model AUC 95th Rank	Area MaxEnt	Area IDW	N of Amazonian Regions
Leonia_crassa	0.562	0.004	0.767	0.871	0.890	0.780	816	695	5
Leonia_glycycarpa	0.237	0.008	0.893	0.944	0.779	0.753	1797	1723	6
Licania_alba	0.329	0.013	0.762	0.840	0.890	0.836	361	322	2
Licania_apetala	0.292	0.020	0.833	0.981	0.753	0.744	2094	1794	6
Licania_densiflora	0.421	0.003	0.893	0.542	0.918	0.809	323	467	2
Licania_heteromorpha	0.518	0.020	0.900	0.937	0.807	0.740	1725	1785	6
Licania_micrantha	0.352	0.002	0.907	0.970	0.806	0.772	1763	1568	6
Licania_oblongifolia	0.411	0.006	0.917	0.861	0.853	0.841	1112	942	5
Luehea_cymulosa	0.525	0.007	0.778	0.931	0.847	0.787	1454	824	6
Mabea_nitida	0.531	0.007	0.764	0.939	0.846	0.762	1227	949	5
Mabea_speciosa	0.186	0.009	0.767	0.694	0.867	0.772	1133	1318	6
Macrolobium_acaciifolium	0.170	-0.002	0.685	0.906	0.787	0.746	1903	1201	6
Macrolobium_angustifolium	0.325	0.006	0.806	0.952	0.847	0.768	1533	1372	6
Manilkara_bidentata	0.219	0.010	0.913	0.871	0.781	0.760	1725	1659	6
Maquira_coriacea	0.073	0.012	0.818	0.897	0.798	0.783	1831	1262	6
Matisia_ochrocalyx	0.041	-0.011	0.852	0.845	0.828	0.761	1437	1514	6
Mauritia_flexuosa	0.493	0.038	0.817	0.706	0.832	0.771	1363	1366	5
Mauritiella_aculeata	0.521	0.003	0.512	1.000	0.880	0.793	1164	611	5
Metrodorea_flavida	0.584	0.016	0.934	0.828	0.845	0.774	798	959	5
Mezilaurus_itauba	0.077	0.001	0.855	0.929	0.799	0.795	1837	1541	6
Micrandra_elata	0.523	0.006	0.680	1.000	0.821	0.802	1355	585	6
Micrandra_glabra	-0.087	0.000	1.000	0.333	0.930	0.911	31	161	1
Micrandra_spruceana	0.624	0.017	0.966	0.833	0.920	0.787	676	840	5
Micrandra_sprucei	0.296	0.077	0.850	1.000	0.981	0.824	110	176	3
Micropholis_gardneriana	0.106	0.000	0.250	0.000	0.869	0.783	270	150	5
Micropholis_guyanensis	0.231	0.008	0.907	0.968	0.761	0.748	1956	1902	6
Minquartia_guianensis	0.198	-0.015	0.917	0.863	0.830	0.757	1571	1794	6
Mollia_gracilis	0.539	0.001	0.656	0.750	0.915	0.802	646	461	3
Monopteryx_uaucu	0.353	0.014	1.000	0.812	0.966	0.828	204	334	3
Mora_excelsa	0.359	0.003	0.909	0.857	0.975	0.882	102	118	1
Ocotea_aciphylla	0.406	0.010	0.808	0.974	0.761	0.746	1759	1549	5
Ocotea_cernua	0.144	-0.004	0.769	0.947	0.741	0.739	1906	1661	6
Oenocarpus_bacaba	0.070	0.034	1.000	0.273	0.918	0.809	571	1527	3
Oenocarpus_bataua	0.392	0.006	0.945	0.636	0.842	0.762	986	1753	5
Osteophloeum_platyspermum	0.398	0.003	0.912	0.925	0.861	0.768	1439	1417	6
Otoba_parvifolia	0.482	0.014	0.931	0.717	0.890	0.775	623	751	4
Oxandra_polyantha	0.433	0.008	0.885	0.765	0.901	0.809	713	605	5
Pachira_brevipes	0.564	0.057	1.000	0.833	0.966	0.824	251	333	2
Pentaclethra_macroloba	0.146	0.010	0.810	0.833	0.850	0.774	972	520	5
Poulsenia_armata	0.324	0.002	0.850	0.667	0.859	0.785	550	540	3

Species	Spearman's Correlation	90th Quantile	IDW Sensitivity	MaxEnt Sensitivity	MaxEnt AUC	Null Model AUC 95th Rank	Area MaxEnt	Area IDW	N of Amazonian Regions
Pourouma_bicolor	0.328	0.013	0.880	0.667	0.859	0.747	881	1533	5
Pourouma_cecropiifolia	0.351	0.009	0.865	0.786	0.837	0.772	965	1278	5
Pourouma_minor	0.242	-0.006	0.878	0.911	0.803	0.757	1656	1708	6
Pouteria_cuspidata	0.423	0.004	0.914	0.941	0.787	0.754	2009	1729	6
Pouteria_elegans	0.330	0.014	0.798	0.887	0.874	0.785	1463	1059	6
Protium_apiculatum	0.253	0.016	0.868	0.935	0.837	0.788	1489	1476	6
Protium_aracouchini	0.114	0.002	0.846	0.960	0.756	0.743	1972	1526	6
Protium_decandrum	0.516	0.007	0.947	0.936	0.855	0.788	1323	1378	6
Protium_hebetatum	0.534	0.043	0.897	0.840	0.906	0.778	826	1098	5
Protium_sagotianum	0.002	0.012	0.899	0.956	0.759	0.755	1987	1804	6
Protium_tenuifolium	0.316	0.009	0.784	0.929	0.772	0.772	1802	1437	6
Protium_trifoliolatum	0.447	0.005	0.809	0.928	0.844	0.766	1515	1354	6
Pseudobombax_munguba	0.294	0.007	0.794	0.848	0.856	0.809	1197	989	6
Pseudolmedia_laevigata	0.259	-0.001	0.918	0.900	0.791	0.752	1717	1738	5
Pseudolmedia_laevis	0.275	-0.067	0.918	0.825	0.781	0.751	1565	1820	5
Pseudopiptadenia_suaveolens	0.167	-0.003	0.853	0.916	0.821	0.800	1646	1548	6
Pterocarpus_amazonum	0.455	0.014	0.744	0.958	0.824	0.784	1701	1015	6
Pterocarpus_santalinoides	0.312	0.004	0.726	0.975	0.765	0.761	2099	1313	6
Qualea_paraensis	0.241	-0.003	0.936	0.933	0.817	0.782	1929	1615	6
Quararibea_guianensis	0.045	0.000	0.812	0.938	0.805	0.773	1641	990	6
Quararibea_wittii	0.544	0.014	0.895	0.885	0.856	0.782	526	520	3
Rinorea_racemosa	0.345	-0.010	0.928	0.820	0.908	0.788	848	1114	5
Rinoreocarpus_ulei	0.254	0.011	0.717	0.976	0.812	0.777	1486	834	6
Sacoglottis_guianensis	0.401	0.011	0.776	0.989	0.781	0.773	1947	1562	6
Sagotia_brachysepala	0.374	0.002	0.323	1.000	0.835	0.787	1538	632	6
Sagotia_racemosa	0.274	0.006	0.743	0.921	0.839	0.771	1514	1111	6
Scleronema_micranthum	0.561	0.021	1.000	0.857	0.933	0.795	506	778	4
Senefeldera_inclinata	0.439	0.001	0.535	0.833	0.927	0.792	635	316	4
Siparuna_decipiens	0.301	-0.002	0.874	0.957	0.807	0.757	1586	1470	5
Socratea_exorrhiza	0.135	-0.004	0.951	0.528	0.832	0.756	980	1929	5
Sterculia_pruriens	0.332	0.007	0.849	0.953	0.820	0.779	1746	1560	6
Swartzia_polyphylla	0.552	0.007	0.868	0.959	0.875	0.769	1383	1243	6
Symphonia_globulifera	0.220	0.004	0.882	0.929	0.757	0.738	1861	1798	6
Tachigali_paniculata	0.255	-0.001	0.816	0.878	0.788	0.766	1857	1708	6
Tetragastris_panamensis	-0.018	-0.022	0.899	0.941	0.770	0.748	1917	1664	6
Theobroma_cacao	0.120	0.000	0.765	0.896	0.766	0.743	1725	1362	6
Theobroma_speciosum	0.142	0.001	0.908	0.886	0.784	0.780	1500	1365	6
Theobroma_subincanum	0.334	-0.002	0.916	0.878	0.840	0.756	1592	1671	6
Trattinnickia_burserifolia	0.484	0.007	0.649	0.940	0.796	0.774	1861	1346	6
Triplaris_weigeltiana	0.349	0.014	0.709	0.882	0.852	0.780	1221	1143	6

Species	Spearman's Correlation	90th Quantile	IDW Sensitivity	MaxEnt Sensitivity	MaxEnt AUC	Null Model AUC 95th Rank	Area MaxEnt	Area IDW	N of Amazonian Regions
Vatairea_guianensis	0.519	0.007	0.881	0.838	0.886	0.783	1082	1270	6
Virola_calophylla	0.392	0.016	0.940	0.897	0.807	0.751	1640	1599	6
Virola_elongata	0.489	0.014	0.933	0.954	0.808	0.739	1730	1706	5
Virola_michelii	0.611	0.015	0.887	0.736	0.907	0.795	661	1251	4
Virola_pavonis	0.566	0.021	0.932	0.871	0.873	0.769	1031	1291	5
Virola_surinamensis	-0.005	0.005	0.905	0.908	0.822	0.784	1812	1689	6
Vouacapoua_americana	0.286	0.019	0.833	0.880	0.925	0.840	707	314	4
Xylopia_amazonica	0.371	0.004	0.558	0.933	0.838	0.793	1703	1034	6
Zygia_cataractae	-0.106	0.006	0.675	0.938	0.782	0.750	1933	1180	6
Zygia_racemosa	0.521	0.012	0.828	0.892	0.859	0.783	1353	1217	6
