# Supplementary material for: Species Distribution Modelling: Contrasting presence-only models with plot abundance data
Source: Sci Rep. 2018 Jan 17;8:1003. doi: 10.1038/s41598-017-18927-1 (PMC5772443; doi:10.1038/s41598-017-18927-1)
Supplement: Supplementary file 4 — Cleaning pipeline results for all 227 hyperdominant species. [file 41598_2017_18927_MOESM4_ESM.pdf]

**Supplemental Material**

**for**

**Species Distribution Modelling: Contrasting presence-only models with plot abundance data**

Vitor F. Gomes*, Stéphanie D. IJff, Niels Raes, Iêda Leão Amaral, Rafael P. Salomão, Luiz de Souza Coelho, Francisca Dionízia de Almeida Matos, Carolina V. Castilho, Diogenes de Andrade Lima Filho, Dairon Cárdenas López, Juan Ernesto Guevara, William E. Magnusson, Oliver L. Phillips, Florian Wittmann, Marcelo de Jesus Veiga Carim, Maria Pires Martins, Mariana Victória Irume, Daniel Sabatier, Jean-François Molino, Olaf S. Bánki, José Renan da Silva Guimarães, Nigel C.A. Pitman, Maria Teresa Fernandez Piedade, Abel Monteagudo Mendoza, Bruno Garcia Luize, Eduardo Martins Venticinque, Evlyn Márcia Moraes de Leão Novo, Percy Núñez Vargas, Thiago Sanna Freire Silva, Angelo Gilberto Manzatto, John Terborgh, Neidiane Farias Costa Reis, Juan Carlos Montero, Katia Regina Casula, Beatriz S. Marimon, Ben-Hur Marimon Jr., Euridice N. Honorio Coronado, Ted R. Feldpausch, Alvaro Duque, Charles Eugene Zartman, Nicolás Castaño Arboleda, Timothy J. Killeen, Bonifacio Mostacedo, Rodolfo Vasquez, Jochen Schöngart, Rafael L. Assis, Marcelo Brilhante Medeiros, Marcelo Fragomeni Simon, Ana Andrade, William F. Laurance, José Luís Camargo, Layon O. Demarchi, Susan G.W. Laurance, Emanuelle de Sousa Farias, Henrique Eduardo Mendonça Nascimento, Juan David Cardenas Revilla, Adriano Quaresma, Flávia R.C. Costa, Ima Célia Guimarães Vieira, Bruno Barçante Ladvocat Cintra, Hernán Castellanos, Roel Brienen, Pablo R. Stevenson, Yuri Feitosa, Joost F. Duivenvoorden, Gerardo A. Aymard C., Hugo F. Mogollón, Natalia Targhetta, James A. Comiskey, Alberto Vicentini, Aline Lopes, Gabriel Damasco, Nállarett Dávila, Roosevelt García-Villacorta, Carolina Levis, Juliana Schietti, Priscila Souza, Thaise Emilio, Alfonso Alonso, David Neill, Francisco Dallmeier, Leandro Valle Ferreira, Alejandro Araujo-Murakami, Daniel Praia, Dário Dantas do Amaral, Fernanda Antunes Carvalho, Fernanda Coelho de Souza, Kenneth Feeley, Luzmila Arroyo, Marcelo Petratti Pansonato, Rogerio Gribel, Boris Villa, Juan Carlos Licona,

Paul V.A. Fine, Carlos Cerón, Chris Baraloto, Eliana M. Jimenez, Juliana Stropp, Julien Engel, Julien Engel, Marcos Silveira, Maria Cristina Peñuela Mora, Pascal Petronelli, Paul Maas, Raquel Thomas-Caesar, Terry W. Henkel, Doug Daly, Marcos Ríos Paredes, Tim R. Baker, Alfredo Fuentes, Carlos A. Peres, Jerome Chave, Jose Luis Marcelo Pena, Kyle G. Dexter, Miles R. Silman, Peter Møller Jørgensen, Toby Pennington, Anthony Di Fiore, Fernando Cornejo Valverde, Juan Fernando Phillips, Gonzalo Rivas-Torres, Patricio von Hildebrand, Tinde R. van Andel, Ademir R. Ruschel, Adriana Prieto, Agustín Rudas, Bruce Hoffman, César I.A. Vela, Edelcilio Marques Barbosa, Egleé L. Zent, George Pepe Gallardo Gonzales, Hilda Paulette Dávila Doza, Ires Paula de Andrade Miranda, Jean-Louis Guillaumet, Linder Felipe Mozombite Pinto, Luiz Carlos de Matos Bonates, Natalino Silva, Ricardo Zárate Gómez, Stanford Zent, Therany Gonzales, Vincent A. Vos, Yadvinder Malhi, Alexandre A. Oliveira, Angela Cano, Bianca Weiss Albuquerque, Corine Vriesendorp, Diego Felipe Correa, Emilio Vilanova Torre, Geertje van der Heijden, Hirma Ramirez-Angulo, José Ferreira Ramos, Kenneth R. Young, Maira Rocha, Marcelo Trindade Nascimento, Maria Natalia Umaña Medina, Milton Tirado, Ophelia Wang, Rodrigo Sierra, Armando Torres-Lezama, Casimiro Mendoza, Cid Ferreira, Cláudia Baider, Daniel Villarroel, Henrik Balslev, Italo Mesones, Ligia Estela Urrego Giraldo, Luisa Fernanda Casas, Manuel Augusto Ahuite Reategui, Reynaldo Linares-Palomino, Roderick Zagt, Sasha Cárdenas, William Farfan-Rios, Adeilza Felipe Sampaio, Daniela Pauletto, Elvis H. Valderrama Sandoval, Freddy Ramirez Arevalo, Isau Huamantupa-Chuquimaco, Karina Garcia-Cabrera, Lionel Hernandez, Luis Valenzuela Gamarra, Miguel N. Alexiades, Susamar Pansini, Walter Palacios Cuenca, William Milliken, Joana Ricardo, Gabriela Lopez-Gonzalez, Edwin Pos, Hans ter Steege*

*Correspondence and requests for materials should be addressed to H.T.S. (email: hans.tersteege@naturalis.nl)

**Appendix S4: Cleaning pipeline results for all 227 hyperdominant species.**

Cleaning pipeline results and percentages for all 227 hyperdominant species. The cleaning pipeline removed GBIF data inconsistencies. First, the total number of records download from GBIF is presented (GBIF records). Then, we present the total number of records after been removed those with no latitude, longitude and locality; duplicates at 0.5-degree spatial resolution; coordinates located in capitals; coordinates with latitude equal to longitude or equal to exactly zero; and corrected country references (cleaned GBIF records). Finally, we present the total number of records after treatment with kernel density estimate function over coordinate point patterns to remove spatial outliers (kernel-density estimate GBIF records).

| Species | N GBIF records | N cleaned GBIF records | N cleaned GBIF records (% GBIF records) | N kernel-density estimate GBIF records | kernel density estimate GBIF records (% GBIF records) |
|---|---|---|---|---|---|
| Abarema_jupunba | 751 | 521 | 69.37 | 503 | 66.98 |
| Acosmium_cardenasii | 69 | 58 | 84.06 | 53 | 76.81 |
| Alexa_imperatricis | 24 | 10 | 41.67 | 9 | 37.50 |
| Amaioua_guianensis | 825 | 428 | 51.88 | 380 | 46.06 |
| Amphiodon_effusus | 89 | 62 | 69.66 | 61 | 68.54 |
| Aparisthmium_cordatum | 1239 | 733 | 59.16 | 670 | 54.08 |
| Apeiba_glabra | 476 | 313 | 65.76 | 289 | 60.71 |
| Apeiba_tibourbou | 1554 | 976 | 62.81 | 662 | 42.60 |
| Aspidosperma_excelsum | 450 | 280 | 62.22 | 260 | 57.78 |
| Astrocaryum_aculeatum | 1774 | 174 | 9.81 | 170 | 9.58 |
| Astrocaryum_murumuru | 143 | 73 | 51.05 | 69 | 48.25 |
| Astrocaryum_sciophilum | 28 | 7 | 25.00 | 6 | 21.43 |
| Attalea_butyracea | 12821 | 198 | 1.54 | 184 | 1.44 |
| Attalea_maripa | 2946 | 232 | 7.88 | 226 | 7.67 |
| Attalea_phalerata | 14961 | 289 | 1.93 | 279 | 1.86 |
| Attalea_speciosa | 95 | 55 | 57.89 | 49 | 51.58 |
| Bertholletia_excelsa | 266 | 130 | 48.87 | 124 | 46.62 |
| Bocageopsis_multiflora | 397 | 242 | 60.96 | 239 | 60.20 |
| Brosimum_guianense | 249 | 172 | 69.08 | 121 | 48.59 |
| Brosimum_lactescens | 977 | 504 | 51.59 | 385 | 39.41 |
| Brosimum_rubescens | 455 | 213 | 46.81 | 203 | 44.62 |
| Brosimum_utile | 437 | 221 | 50.57 | 171 | 39.13 |
| Calophyllum_brasiliense | 1733 | 1075 | 62.03 | 686 | 39.58 |
| Campsiandra_implexicaulis | 34 | 22 | 64.71 | 20 | 58.82 |
| Caraipa_densifolia | 473 | 319 | 67.44 | 314 | 66.38 |
| Carapa_guianensis | 3084 | 333 | 10.80 | 197 | 6.39 |
| Caryocar_glabrum | 439 | 286 | 65.15 | 283 | 64.46 |
| Catostemma_fragrans | 35 | 20 | 57.14 | 18 | 51.43 |
| Cecropia_latiloba | 176 | 74 | 42.05 | 74 | 42.05 |
| Cecropia_membranacea | 238 | 111 | 46.64 | 104 | 43.70 |
| Cecropia_sciadophylla | 347 | 178 | 51.30 | 175 | 50.43 |
| Cedrelinga_cateniformis | 258 | 155 | 60.08 | 150 | 58.14 |

| Species | N GBIF records | N cleaned GBIF records | N cleaned GBIF records (% GBIF records) | N kernel-density estimate GBIF records | kernel density estimate GBIF records (% GBIF records) |
|---|---|---|---|---|---|
| Celtis_schippii | 423 | 244 | 57.68 | 188 | 44.44 |
| Cenostigma_tocantinum | 73 | 28 | 38.36 | 21 | 28.77 |
| Cheiloclinium_cognatum | 1596 | 805 | 50.44 | 716 | 44.86 |
| Chlorocardium_rodiei | 47 | 13 | 27.66 | 13 | 27.66 |
| Chrysophyllum_sanguinolentum | 708 | 213 | 30.08 | 206 | 29.10 |
| Clarisia_racemosa | 623 | 302 | 48.48 | 267 | 42.86 |
| Clathrotropis_glaucophylla | 50 | 44 | 88.00 | 40 | 80.00 |
| Clathrotropis_macrocarpa | 350 | 160 | 45.71 | 148 | 42.29 |
| Conceveiba_guianensis | 555 | 304 | 54.77 | 297 | 53.51 |
| Cordia_bicolor | 475 | 295 | 62.11 | 198 | 41.68 |
| Couepia_guianensis | 345 | 173 | 50.14 | 158 | 45.80 |
| Couepia_paraensis | 275 | 187 | 68.00 | 185 | 67.27 |
| Crateva_tapia | 1573 | 1001 | 63.64 | 350 | 22.25 |
| Cynometra_marginata | 121 | 52 | 42.98 | 49 | 40.50 |
| Dialium_guianense | 1551 | 867 | 55.90 | 624 | 40.23 |
| Dicymbe_corymbosa | 6 | 5 | 83.33 | 4 | 66.67 |
| Diplotropis_purpurea | 204 | 120 | 58.82 | 112 | 54.90 |
| Drypetes_amazonica | 381 | 169 | 44.36 | 162 | 42.52 |
| Ecclinusa_guianensis | 155 | 75 | 48.39 | 72 | 46.45 |
| Enterolobium_schomburgkii | 406 | 236 | 58.13 | 181 | 44.58 |
| Eperua_falcata | 234 | 62 | 26.50 | 57 | 24.36 |
| Eperua_grandiflora | 90 | 20 | 22.22 | 18 | 20.00 |
| Eperua_leucantha | 127 | 70 | 55.12 | 64 | 50.39 |
| Eperua_purpurea | 107 | 66 | 61.68 | 61 | 57.01 |
| Erisma_uncinatum | 277 | 134 | 48.38 | 130 | 46.93 |
| Eschweilera_albiflora | 383 | 226 | 59.01 | 221 | 57.70 |
| Eschweilera_amazonica | 108 | 50 | 46.30 | 47 | 43.52 |
| Eschweilera_atropetiolata | 194 | 51 | 26.29 | 47 | 24.23 |
| Eschweilera_bracteosa | 218 | 132 | 60.55 | 125 | 57.34 |
| Eschweilera_coriacea | 2066 | 725 | 35.09 | 687 | 33.25 |
| Eschweilera_decolorans | 128 | 64 | 50.00 | 63 | 49.22 |
| Eschweilera_grandiflora | 167 | 73 | 43.71 | 67 | 40.12 |

| Species | N GBIF records | N cleaned GBIF records | N cleaned GBIF records (% GBIF records) | N kernel-density estimate GBIF records | kernel density estimate GBIF records (% GBIF records) |
|---|---|---|---|---|---|
| Eschweilera_parviflora | 265 | 162 | 61.13 | 161 | 60.75 |
| Eschweilera_parvifolia | 418 | 274 | 65.55 | 267 | 63.88 |
| Eschweilera_pedicellata | 640 | 312 | 48.75 | 308 | 48.13 |
| Eschweilera_sagotiana | 136 | 53 | 38.97 | 52 | 38.24 |
| Eschweilera_tessmannii | 446 | 192 | 43.05 | 187 | 41.93 |
| Eschweilera_truncata | 221 | 77 | 34.84 | 75 | 33.94 |
| Eschweilera_wachenheimii | 156 | 59 | 37.82 | 56 | 35.90 |
| Euterpe_oleracea | 2868 | 105 | 3.66 | 76 | 2.65 |
| Euterpe_precatoria | 24604 | 953 | 3.87 | 853 | 3.47 |
| Garcinia_macrophylla | 482 | 365 | 75.73 | 291 | 60.37 |
| Geissospermum_sericeum | 35 | 23 | 65.71 | 22 | 62.86 |
| Goupia_glabra | 641 | 304 | 47.43 | 294 | 45.87 |
| Guapira_venosa | 51 | 26 | 50.98 | 22 | 43.14 |
| Guarea_guidonia | 2263 | 1137 | 50.24 | 949 | 41.94 |
| Guarea_kunthiana | 2532 | 1278 | 50.47 | 1082 | 42.73 |
| Guarea_macrophylla | 2617 | 1285 | 49.10 | 915 | 34.96 |
| Guarea_silvatica | 381 | 245 | 64.30 | 242 | 63.52 |
| Gustavia_augusta | 838 | 431 | 51.43 | 391 | 46.66 |
| Gustavia_hexapetala | 969 | 391 | 40.35 | 382 | 39.42 |
| Handroanthus_barbatus | 118 | 91 | 77.12 | 86 | 72.88 |
| Haploclathra_cordata | 45 | 13 | 28.89 | 13 | 28.89 |
| Helicostylis_tomentosa | 1170 | 552 | 47.18 | 530 | 45.30 |
| Hevea_brasiliensis | 645 | 287 | 44.50 | 199 | 30.85 |
| Hevea_guianensis | 901 | 330 | 36.63 | 321 | 35.63 |
| Himatanthus_sucuuba | 355 | 259 | 72.96 | 246 | 69.30 |
| Hirtella_racemosa | 2514 | 1409 | 56.05 | 1004 | 39.94 |
| Hura_crepitans | 966 | 506 | 52.38 | 212 | 21.95 |
| Inga_alba | 798 | 429 | 53.76 | 364 | 45.61 |
| Inga_capitata | 1319 | 721 | 54.66 | 656 | 49.73 |
| Inga_edulis | 1509 | 851 | 56.39 | 677 | 44.86 |
| Inga_lateriflora | 132 | 60 | 45.45 | 57 | 43.18 |
| Inga_marginata | 118 | 71 | 60.17 | 21 | 17.80 |

| Species | N GBIF records | N cleaned GBIF records | N cleaned GBIF records (% GBIF records) | N kernel-density estimate GBIF records | kernel density estimate GBIF records (% GBIF records) |
|---|---|---|---|---|---|
| Inga_rubiginosa | 114 | 54 | 47.37 | 51 | 44.74 |
| Inga_thibaudiana | 1627 | 950 | 58.39 | 723 | 44.44 |
| Iriartea_deltoidea | 20270 | 573 | 2.83 | 498 | 2.46 |
| Iryanthera_juruensis | 1585 | 717 | 45.24 | 699 | 44.10 |
| Iryanthera_laevis | 597 | 274 | 45.90 | 267 | 44.72 |
| Iryanthera_ulei | 1026 | 456 | 44.44 | 434 | 42.30 |
| Jacaranda_copaia | 816 | 478 | 58.58 | 420 | 51.47 |
| Laetia_corymbulosa | 77 | 40 | 51.95 | 37 | 48.05 |
| Laetia_procera | 640 | 265 | 41.41 | 195 | 30.47 |
| Lecythis_corrugata | 353 | 197 | 55.81 | 187 | 52.97 |
| Lecythis_idatimon | 251 | 88 | 35.06 | 71 | 28.29 |
| Leonia_crassa | 356 | 231 | 64.89 | 226 | 63.48 |
| Leonia_glycycarpa | 1029 | 509 | 49.47 | 501 | 48.69 |
| Licania_alba | 87 | 26 | 29.89 | 25 | 28.74 |
| Licania_apetala | 898 | 448 | 49.89 | 446 | 49.67 |
| Licania_buxifolia | 11 | 5 | 45.45 | 4 | 36.36 |
| Licania_densiflora | 81 | 33 | 40.74 | 30 | 37.04 |
| Licania_heteromorpha | 1303 | 621 | 47.66 | 603 | 46.28 |
| Licania_kunthiana | 364 | 222 | 60.99 | 193 | 53.02 |
| Licania_micrantha | 379 | 226 | 59.63 | 205 | 54.09 |
| Licania_oblongifolia | 84 | 35 | 41.67 | 34 | 40.48 |
| Licania_octandra | 42 | 31 | 73.81 | 23 | 54.76 |
| Luehea_cymulosa | 185 | 120 | 64.86 | 119 | 64.32 |
| Mabea_nitida | 591 | 344 | 58.21 | 335 | 56.68 |
| Mabea_speciosa | 367 | 216 | 58.86 | 213 | 58.04 |
| Macrolobium_acaciifolium | 903 | 525 | 58.14 | 519 | 57.48 |
| Macrolobium_angustifolium | 366 | 277 | 75.68 | 272 | 74.32 |
| Manilkara_bidentata | 767 | 290 | 37.81 | 246 | 32.07 |
| Maquira_coriacea | 255 | 93 | 36.47 | 91 | 35.69 |
| Matayba_scrobiculata | 95 | 36 | 37.89 | 29 | 30.53 |
| Matisia_ochrocalyx | 789 | 440 | 55.77 | 367 | 46.51 |
| Mauritia_flexuosa | 3357 | 230 | 6.85 | 223 | 6.64 |

| Species | N GBIF records | N cleaned GBIF records | N cleaned GBIF records (% GBIF records) | N kernel-density estimate GBIF records | kernel density estimate GBIF records (% GBIF records) |
|---|---|---|---|---|---|
| Mauritiella_aculeata | 816 | 59 | 7.23 | 58 | 7.11 |
| Metrodorea_flavida | 392 | 229 | 58.42 | 215 | 54.85 |
| Mezilaurus_itauba | 194 | 98 | 50.52 | 97 | 50.00 |
| Micrandra_elata | 104 | 43 | 41.35 | 40 | 38.46 |
| Micrandra_glabra | 27 | 9 | 33.33 | 9 | 33.33 |
| Micrandra_spruceana | 319 | 116 | 36.36 | 114 | 35.74 |
| Micrandra_sprucei | 82 | 37 | 45.12 | 34 | 41.46 |
| Micropholis_gardneriana | 257 | 130 | 50.58 | 121 | 47.08 |
| Micropholis_guyanensis | 1291 | 607 | 47.02 | 558 | 43.22 |
| Micropholis_venulosa | 980 | 525 | 53.57 | 492 | 50.20 |
| Minquartia_guianensis | 786 | 404 | 51.40 | 321 | 40.84 |
| Mollia_gracilis | 135 | 67 | 49.63 | 63 | 46.67 |
| Monopteryx_uaucu | 55 | 43 | 78.18 | 40 | 72.73 |
| Mora_excelsa | 42 | 13 | 30.95 | 10 | 23.81 |
| Mora_gonggrijpii | 13 | 6 | 46.15 | 6 | 46.15 |
| Ocotea_aciphylla | 1154 | 570 | 49.39 | 508 | 44.02 |
| Ocotea_cernua | 1399 | 737 | 52.68 | 476 | 34.02 |
| Oenocarpus_bacaba | 3076 | 85 | 2.76 | 83 | 2.70 |
| Oenocarpus_bataua | 52272 | 565 | 1.08 | 546 | 1.04 |
| Osteophloeum_platyspermum | 772 | 364 | 47.15 | 340 | 44.04 |
| Otoba_parvifolia | 973 | 389 | 39.98 | 372 | 38.23 |
| Oxandra_polyantha | 139 | 66 | 47.48 | 62 | 44.60 |
| Pachira_brevipes | 187 | 46 | 24.60 | 42 | 22.46 |
| Pentaclethra_macroloba | 399 | 237 | 59.40 | 113 | 28.32 |
| Poulsenia_armata | 489 | 280 | 57.26 | 175 | 35.79 |
| Pourouma_bicolor | 3171 | 518 | 16.34 | 386 | 12.17 |
| Pourouma_cecropiifolia | 410 | 258 | 62.93 | 248 | 60.49 |
| Pourouma_guianensis | 802 | 406 | 50.62 | 383 | 47.76 |
| Pourouma_minor | 733 | 379 | 51.71 | 340 | 46.38 |
| Pouteria_caimito | 905 | 521 | 57.57 | 474 | 52.38 |
| Pouteria_cuspidata | 738 | 376 | 50.95 | 353 | 47.83 |
| Pouteria_elegans | 250 | 169 | 67.60 | 168 | 67.20 |

| Species | N GBIF records | N cleaned GBIF records | N cleaned GBIF records (% GBIF records) | N kernel-density estimate GBIF records | kernel density estimate GBIF records (% GBIF records) |
|---|---|---|---|---|---|
| Pouteria_guianensis | 947 | 497 | 52.48 | 480 | 50.69 |
| Pouteria_reticulata | 1081 | 553 | 51.16 | 261 | 24.14 |
| Protium_apiculatum | 401 | 175 | 43.64 | 172 | 42.89 |
| Protium_aracouchini | 1121 | 616 | 54.95 | 556 | 49.60 |
| Protium_decandrum | 314 | 133 | 42.36 | 125 | 39.81 |
| Protium_hebetatum | 412 | 141 | 34.22 | 138 | 33.50 |
| Protium_heptaphyllum | 2753 | 1484 | 53.90 | 1342 | 48.75 |
| Protium_sagotianum | 573 | 345 | 60.21 | 338 | 58.99 |
| Protium_tenuifolium | 469 | 252 | 53.73 | 154 | 32.84 |
| Protium_trifoliolatum | 417 | 238 | 57.07 | 229 | 54.92 |
| Pseudobombax_munguba | 78 | 58 | 74.36 | 57 | 73.08 |
| Pseudolmedia_laevigata | 958 | 449 | 46.87 | 431 | 44.99 |
| Pseudolmedia_laevis | 1076 | 442 | 41.08 | 437 | 40.61 |
| Pseudolmedia_macrophylla | 374 | 216 | 57.75 | 216 | 57.75 |
| Pseudopiptadenia_suaveolens | 107 | 65 | 60.75 | 47 | 43.93 |
| Pterocarpus_amazonum | 285 | 201 | 70.53 | 199 | 69.82 |
| Pterocarpus_rohrii | 1062 | 528 | 49.72 | 318 | 29.94 |
| Pterocarpus_santalinoides | 727 | 431 | 59.28 | 182 | 25.03 |
| Qualea_paraensis | 323 | 179 | 55.42 | 160 | 49.54 |
| Quararibea_guianensis | 143 | 93 | 65.03 | 87 | 60.84 |
| Quararibea_wittii | 222 | 109 | 49.10 | 100 | 45.05 |
| Rinorea_guianensis | 30 | 23 | 76.67 | 21 | 70.00 |
| Rinorea_racemosa | 360 | 186 | 51.67 | 181 | 50.28 |
| Rinoreocarpus_ulei | 420 | 253 | 60.24 | 250 | 59.52 |
| Sacoglottis_guianensis | 302 | 177 | 58.61 | 169 | 55.96 |
| Sacoglottis_mattogrossensis | 379 | 221 | 58.31 | 185 | 48.81 |
| Sagotia_brachysepala | 183 | 98 | 53.55 | 90 | 49.18 |
| Sagotia_racemosa | 356 | 220 | 61.80 | 208 | 58.43 |
| Scleronema_micranthum | 418 | 151 | 36.12 | 139 | 33.25 |
| Senefeldera_inclinata | 109 | 73 | 66.97 | 69 | 63.30 |
| Simarouba_amara | 1330 | 782 | 58.80 | 605 | 45.49 |
| Siparuna_decipiens | 919 | 383 | 41.68 | 373 | 40.59 |

| Species | N GBIF records | N cleaned GBIF records | N cleaned GBIF records (% GBIF records) | N kernel-density estimate GBIF records | kernel density estimate GBIF records (% GBIF records) |
|---|---|---|---|---|---|
| Sloanea_eichleri | 174 | 116 | 66.67 | 111 | 63.79 |
| Socratea_exorrhiza | 19519 | 775 | 3.97 | 710 | 3.64 |
| Spondias_mombin | 406 | 245 | 60.34 | 78 | 19.21 |
| Sterculia_pruriens | 174 | 83 | 47.70 | 82 | 47.13 |
| Swartzia_leiocalycina | 18 | 18 | 100.00 | 16 | 88.89 |
| Swartzia_polyphylla | 475 | 262 | 55.16 | 250 | 52.63 |
| Symphonia_globulifera | 335 | 106 | 31.64 | 68 | 20.30 |
| Tachigali_chrysophylla | 87 | 48 | 55.17 | 46 | 52.87 |
| Tachigali_paniculata | 474 | 232 | 48.95 | 226 | 47.68 |
| Tachigali_rugosa | 95 | 54 | 56.84 | 50 | 52.63 |
| Tapirira_guianensis | 5272 | 2541 | 48.20 | 2237 | 42.43 |
| Tetragastris_altissima | 412 | 259 | 62.86 | 255 | 61.89 |
| Tetragastris_panamensis | 1165 | 577 | 49.53 | 378 | 32.45 |
| Theobroma_cacao | 1172 | 476 | 40.61 | 330 | 28.16 |
| Theobroma_speciosum | 293 | 178 | 60.75 | 175 | 59.73 |
| Theobroma_subincanum | 829 | 471 | 56.82 | 466 | 56.21 |
| Trattinnickia_burserifolia | 294 | 155 | 52.72 | 151 | 51.36 |
| Trichilia_micrantha | 392 | 233 | 59.44 | 229 | 58.42 |
| Trichilia_pleeana | 823 | 426 | 51.76 | 368 | 44.71 |
| Trichilia_quadrijuga | 707 | 393 | 55.59 | 286 | 40.45 |
| Triplaris_weigeltiana | 140 | 78 | 55.71 | 71 | 50.71 |
| Unonopsis_guatterioides | 1121 | 615 | 54.86 | 604 | 53.88 |
| Vatairea_guianensis | 145 | 97 | 66.90 | 92 | 63.45 |
| Virola_calophylla | 1449 | 636 | 43.89 | 628 | 43.34 |
| Virola_elongata | 1736 | 848 | 48.85 | 826 | 47.58 |
| Virola_michelii | 283 | 106 | 37.46 | 98 | 34.63 |
| Virola_pavonis | 866 | 392 | 45.27 | 377 | 43.53 |
| Virola_sebifera | 2604 | 1283 | 49.27 | 1030 | 39.55 |
| Virola_surinamensis | 691 | 376 | 54.41 | 316 | 45.73 |
| Vitex_cymosa | 315 | 215 | 68.25 | 189 | 60.00 |
| Vouacapoua_americana | 91 | 32 | 35.16 | 26 | 28.57 |
| Xylopia_amazonica | 113 | 81 | 71.68 | 79 | 69.91 |

| Species | N GBIF records | N cleaned GBIF records | N cleaned GBIF records (% GBIF records) | N kernel-density estimate GBIF records | kernel density estimate GBIF records (% GBIF records) |
|---|---|---|---|---|---|
| Zygia_cataractae | 524 | 359 | 68.51 | 349 | 66.60 |
| Zygia_latifolia | 514 | 360 | 70.04 | 285 | 55.45 |
| Zygia_racemosa | 242 | 129 | 53.31 | 124 | 51.24 |
