# Supplementary material for: Species Distribution Modelling: Contrasting presence-only models with plot abundance data
Source: Sci Rep. 2018 Jan 17;8:1003. doi: 10.1038/s41598-017-18927-1 (PMC5772443; doi:10.1038/s41598-017-18927-1)

Supplemental Material

for

Species Distribution Modelling: Contrasting presence-only models with plot abundance data

Vitor F. Gomes*, Stéphanie D. IJff, Niels Raes, Iêda Leão Amaral, Rafael P. Salomão, Luiz de Souza Coelho, Francisca Dionízia de Almeida Matos, Carolina V. Castilho, Diogenes de Andrade Lima Filho, Dairon Cárdenas López, Juan Ernesto Guevara, William E. Magnusson, Oliver L. Phillips, Florian Wittmann, Marcelo de Jesus Veiga Carim, Maria Pires Martins, Mariana Victória Irume, Daniel Sabatier, Jean-François Molino, Olaf S. Bánki, José Renan da Silva Guimarães, Nigel C.A. Pitman, Maria Teresa Fernandez Piedade, Abel Monteagudo Mendoza, Bruno Garcia Luize, Eduardo Martins Venticinque, Evlyn Márcia Moraes de Leão Novo, Percy Núñez Vargas, Thiago Sanna Freire Silva, Angelo Gilberto Manzatto, John Terborgh, Neidiane Farias Costa Reis, Juan Carlos Montero, Katia Regina Casula, Beatriz S. Marimon, Ben-Hur Marimon Jr., Euridice N. Honorio Coronado, Ted R. Feldpausch, Alvaro Duque, Charles Eugene Zartman, Nicolás Castaño Arboleda, Timothy J. Killeen, Bonifacio Mostacedo, Rodolfo Vasquez, Jochen Schöngart, Rafael L. Assis, Marcelo Brilhante Medeiros, Marcelo Fragomeni Simon, Ana Andrade, William F. Laurance, José Luís Camargo, Layon O. Demarchi, Susan G.W. Laurance, Emanuelle de Sousa Farias, Henrique Eduardo Mendonça Nascimento, Juan David Cardenas Revilla, Adriano Quaresma, Flávia R.C. Costa, Ima Célia Guimarães Vieira, Bruno Barçante Ladvocat Cintra, Hernán Castellanos, Roel Brienen, Pablo R. Stevenson, Yuri Feitosa, Joost F. Duivenvoorden, Gerardo A. Aymard C., Hugo F. Mogollón, Natalia Targhetta, James A. Comiskey, Alberto Vicentini, Aline Lopes, Gabriel Damasco, Nállarett Dávila, Roosevelt García-Villacorta, Carolina Levis, Juliana Schietti, Priscila Souza, Thaise Emilio, Alfonso Alonso, David Neill, Francisco Dallmeier, Leandro Valle Ferreira, Alejandro Araujo-Murakami, Daniel Praia, Dário Dantas do Amaral, Fernanda Antunes Carvalho, Fernanda Coelho de Souza, Kenneth Feeley, Luzmila Arroyo, Marcelo Petratti Pansonato, Rogerio Gribel, Boris Villa, Juan Carlos Licona,

Paul V.A. Fine, Carlos Cerón, Chris Baraloto, Eliana M. Jimenez, Juliana Stropp, Julien Engel, Julien Engel, Marcos Silveira, Maria Cristina Peñuela Mora, Pascal Petronelli, Paul Maas, Raquel Thomas-Caesar, Terry W. Henkel, Doug Daly, Marcos Ríos Paredes, Tim R. Baker, Alfredo Fuentes, Carlos A. Peres, Jerome Chave, Jose Luis Marcelo Pena, Kyle G. Dexter, Miles R. Silman, Peter Møller Jørgensen, Toby Pennington, Anthony Di Fiore, Fernando Cornejo Valverde, Juan Fernando Phillips, Gonzalo Rivas-Torres, Patricio von Hildebrand, Tinde R. van Andel, Ademir R. Ruschel, Adriana Prieto, Agustín Rudas, Bruce Hoffman, César I.A. Vela, Edelcilio Marques Barbosa, Egleé L. Zent, George Pepe Gallardo Gonzales, Hilda Paulette Dávila Doza, Ires Paula de Andrade Miranda, Jean-Louis Guillaumet, Linder Felipe Mozombite Pinto, Luiz Carlos de Matos Bonates, Natalino Silva, Ricardo Zárate Gómez, Stanford Zent, Therany Gonzales, Vincent A. Vos, Yadvinder Malhi, Alexandre A. Oliveira, Angela Cano, Bianca Weiss Albuquerque, Corine Vriesendorp, Diego Felipe Correa, Emilio Vilanova Torre, Geertje van der Heijden, Hirma Ramirez-Angulo, José Ferreira Ramos, Kenneth R. Young, Maira Rocha, Marcelo Trindade Nascimento, Maria Natalia Umaña Medina, Milton Tirado, Ophelia Wang, Rodrigo Sierra, Armando Torres-Lezama, Casimiro Mendoza, Cid Ferreira, Cláudia Baider, Daniel Villarroel, Henrik Balslev, Italo Mesones, Ligia Estela Urrego Giraldo, Luisa Fernanda Casas, Manuel Augusto Ahuite Reategui, Reynaldo Linares-Palomino, Roderick Zagt, Sasha Cárdenas, William Farfan-Rios, Adeilza Felipe Sampaio, Daniela Pauletto, Elvis H. Valderrama Sandoval, Freddy Ramirez Arevalo, Isau Huamantupa-Chuquimaco, Karina Garcia-Cabrera, Lionel Hernandez, Luis Valenzuela Gamarra, Miguel N. Alexiades, Susamar Pansini, Walter Palacios Cuenca, William Milliken, Joana Ricardo, Gabriela Lopez-Gonzalez, Edwin Pos, Hans ter Steege*

*Correspondence and requests for materials should be addressed to H.T.S. (email: hans.tersteege@naturalis.nl)

Appendix S5: Area of occupancy predicted by MaxEnt for each step of the modelling pipeline.

MaxEnt's environmental suitability maps for all 227 hyperdominant species for each step of the cleaning pipeline. *Red dots:* excluded records from original GBIF data using 'GeoClean' function and kernel density estimate. *Dashed blue line*: convex hull including all accepted collection localities plus a 300 km buffer. *Light blue*: Predicted area of occupancy using GBIF untreated data (GBIF records). *Light green*: Predicted area of occupancy using GBIF data with GeoClean treatment (cleaned GBIF records). *Medium green*: Predicted area of occupancy using GBIF data with 'GeoClean' function and kernel density estimate treatments (kernel density). *Dark green*: Final, accepted, area of occupancy using 'GeoClean' function, kernel density estimate, convex hull and buffer treatments (kernel density estimate GBIF records). Maps created with custom R script. Base map source (country.shp, rivers.shp): ESRI (http://www.esri.com/data/basemaps, © Esri, DeLorme Publishing Company).

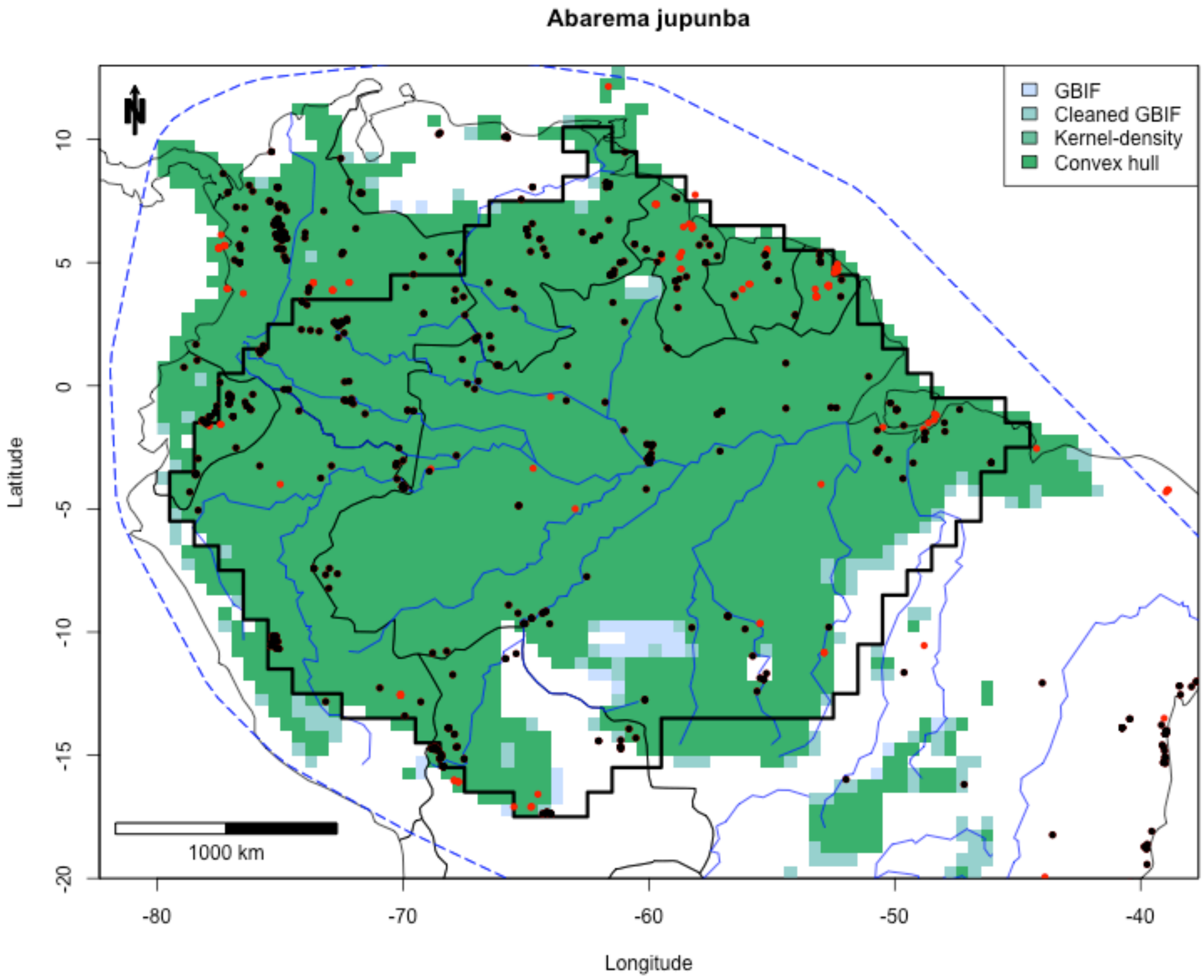

Abarema jupunba
GBIF
Cleaned GBIF
Kernel-density
Convex hull
N
1000 km
Latitude
Longitude
10
5
0
-5
-10
-15
-20
-80
-70
-60
-50
-40

Acosmium cardenasii

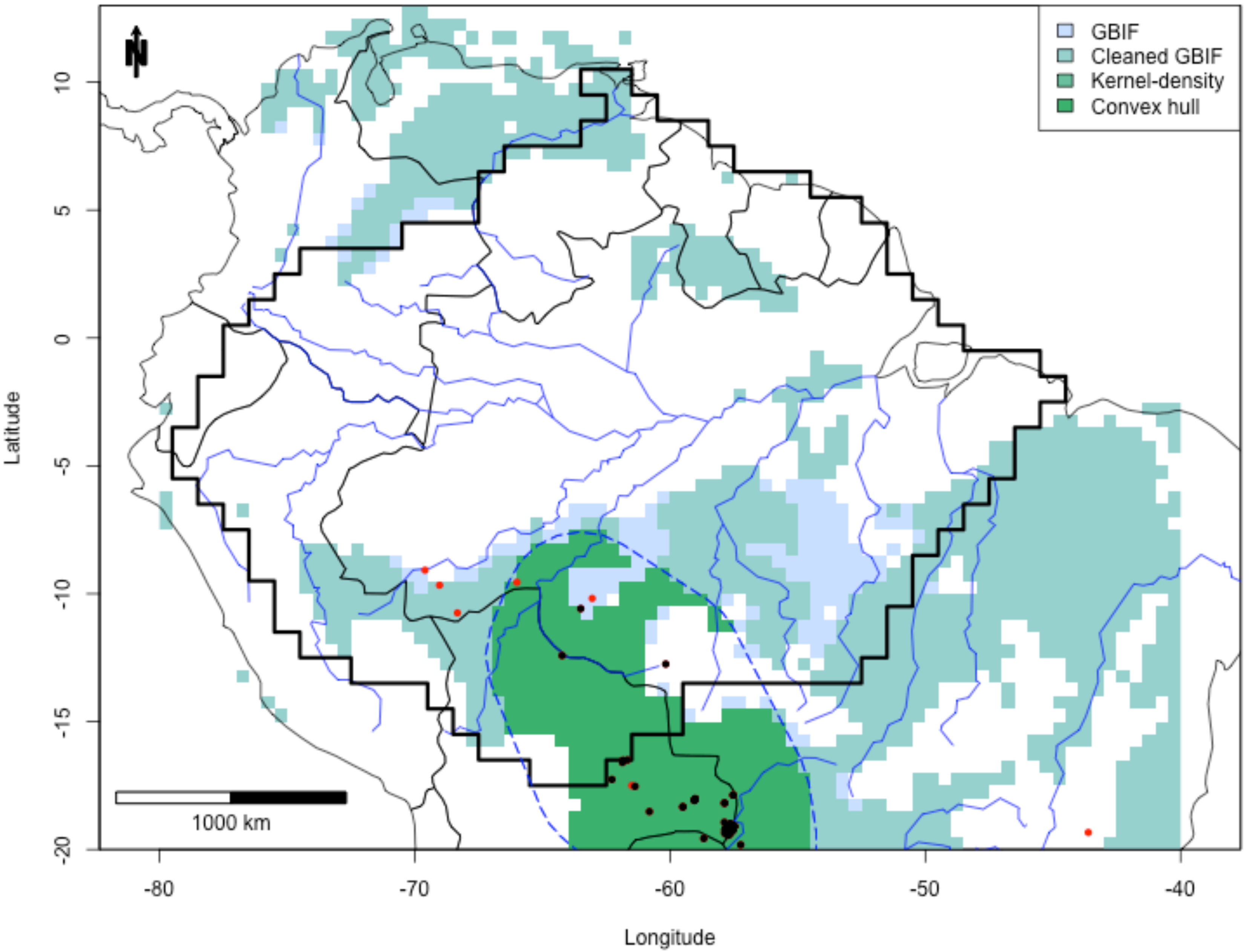

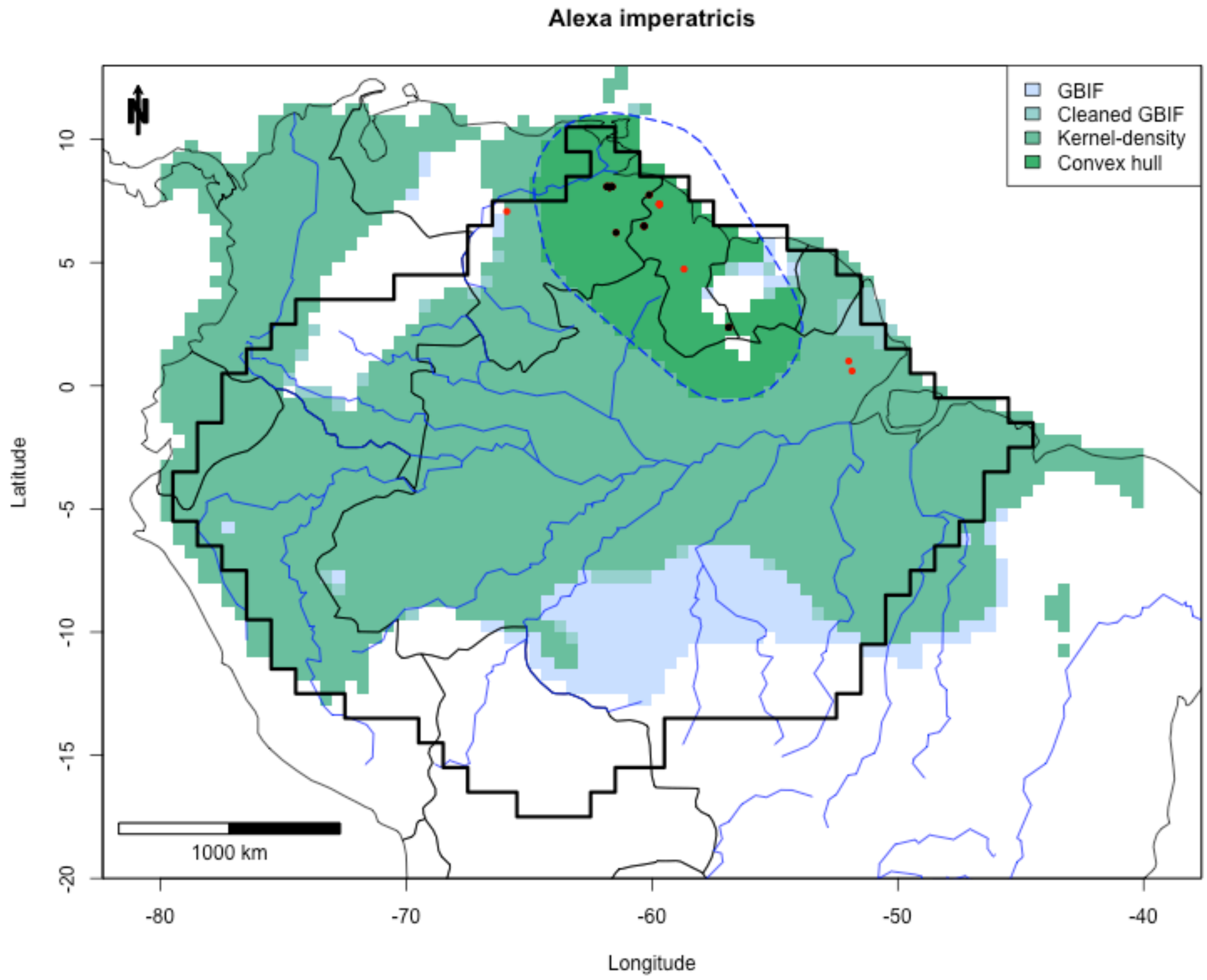

Alexa imperatricis
GBIF
Cleaned GBIF
Kernel-density
Convex hull
N
1000 km
Latitude
Longitude
10
5
0
-5
-10
-15
-20
-80
-70
-60
-50
-40

Amaioua guianensis

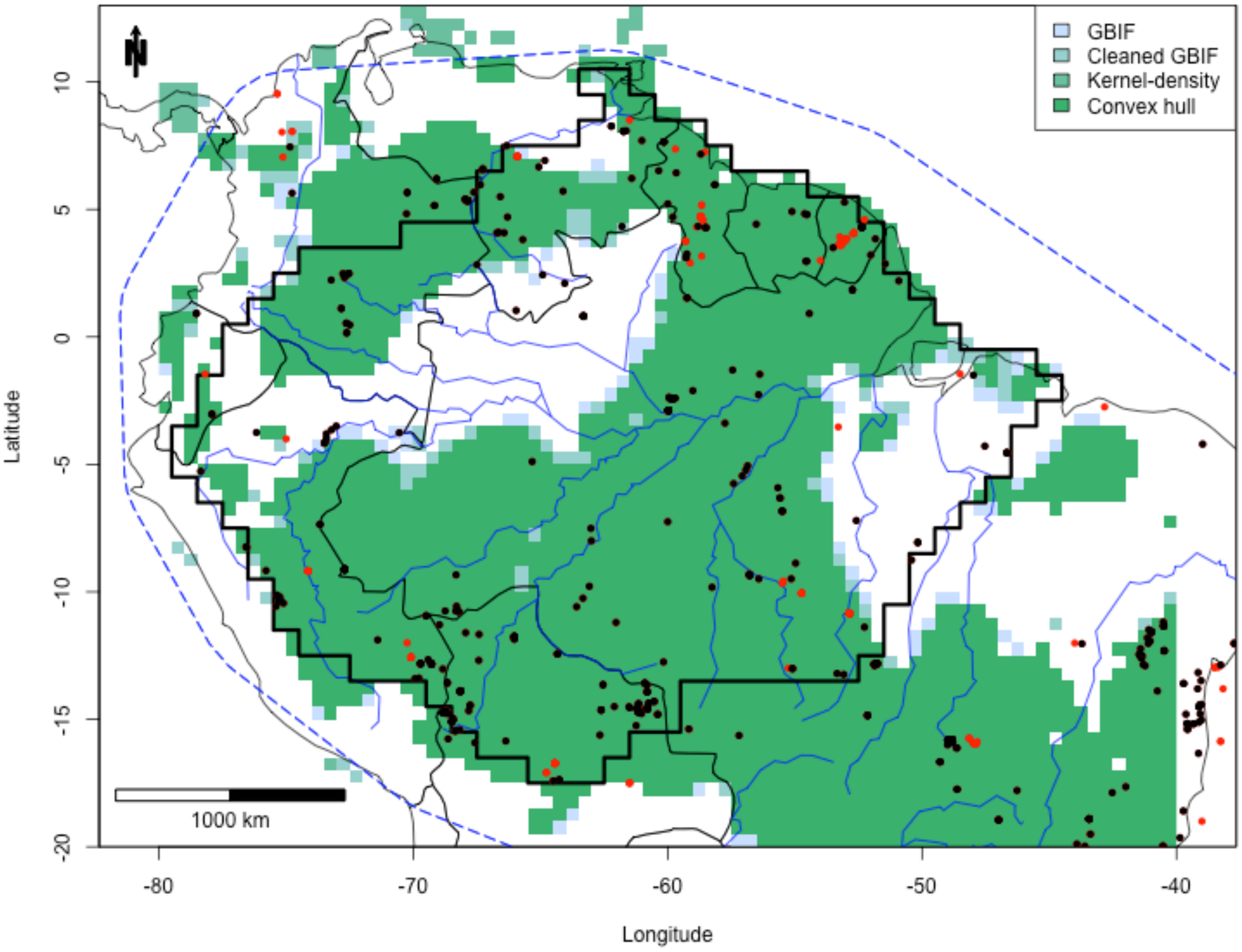

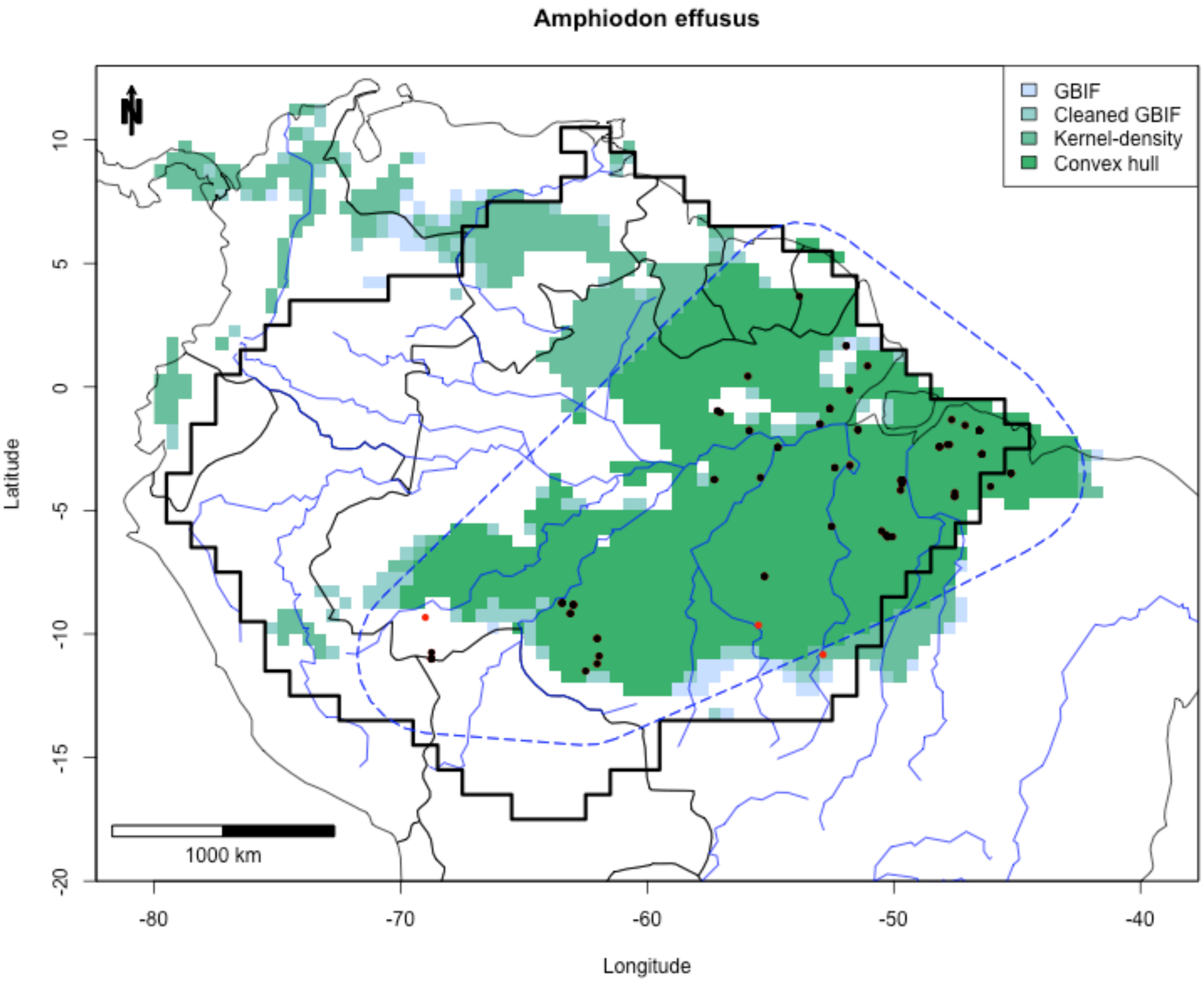

Amphiodon effusus
GBIF
Cleaned GBIF
Kernel-density
Convex hull
N
1000 km
10
5
0
-5
-10
-15
-20
-80
-70
-60
-50
-40
Latitude
Longitude

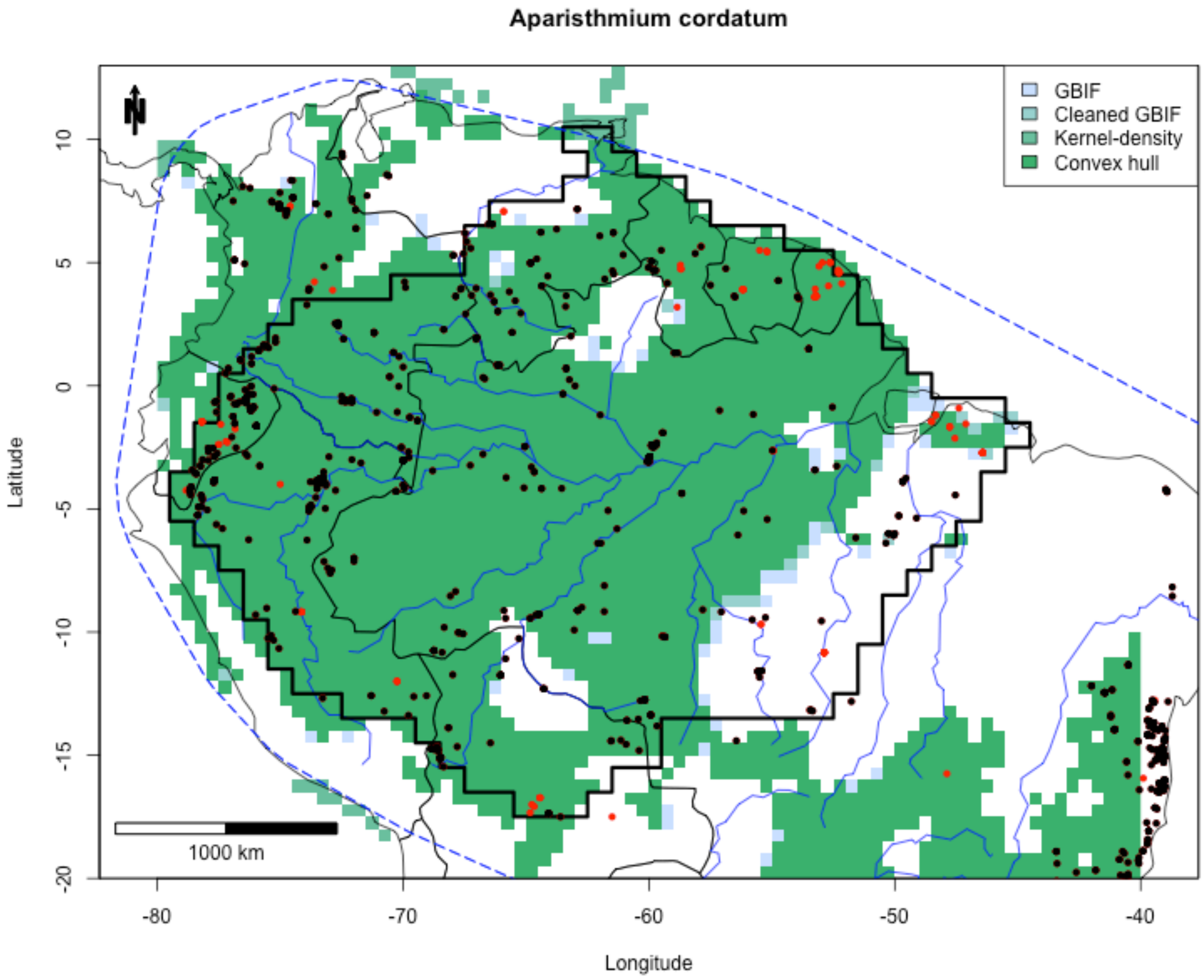
Aparisthmium cordatum
GBIF
Cleaned GBIF
Kernel-density
Convex hull
N
1000 km
Latitude
Longitude
10
5
0
-5
-10
-15
-20
-80
-70
-60
-50
-40

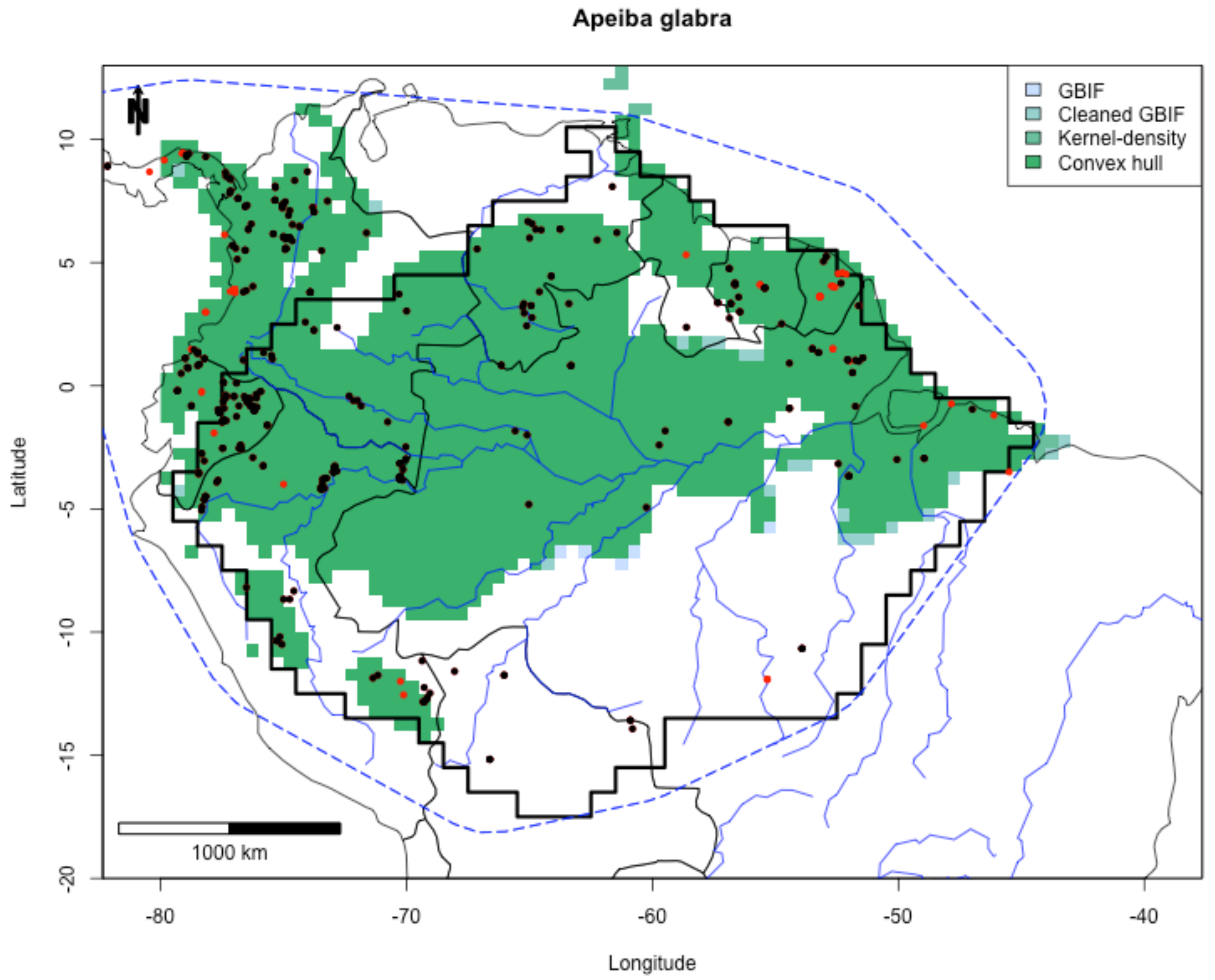

Apeiba glabra
GBIF
Cleaned GBIF
Kernel-density
Convex hull
N
1000 km
Latitude
Longitude
10
5
0
-5
-10
-15
-20
-80
-70
-60
-50
-40

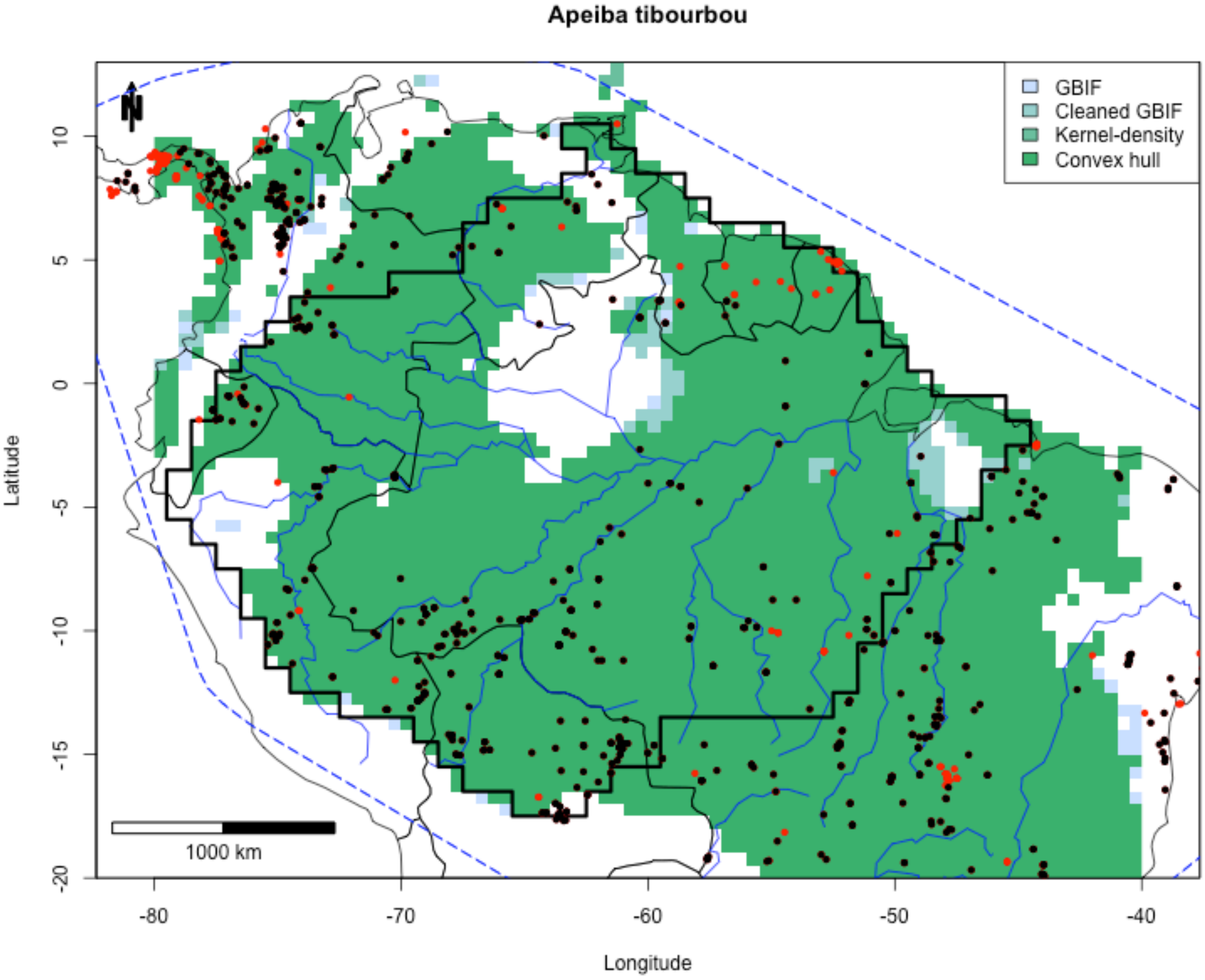
Apeiba tibourbou
GBIF
Cleaned GBIF
Kernel-density
Convex hull
N
1000 km
Latitude
Longitude
10
5
0
-5
-10
-15
-20
-80
-70
-60
-50
-40

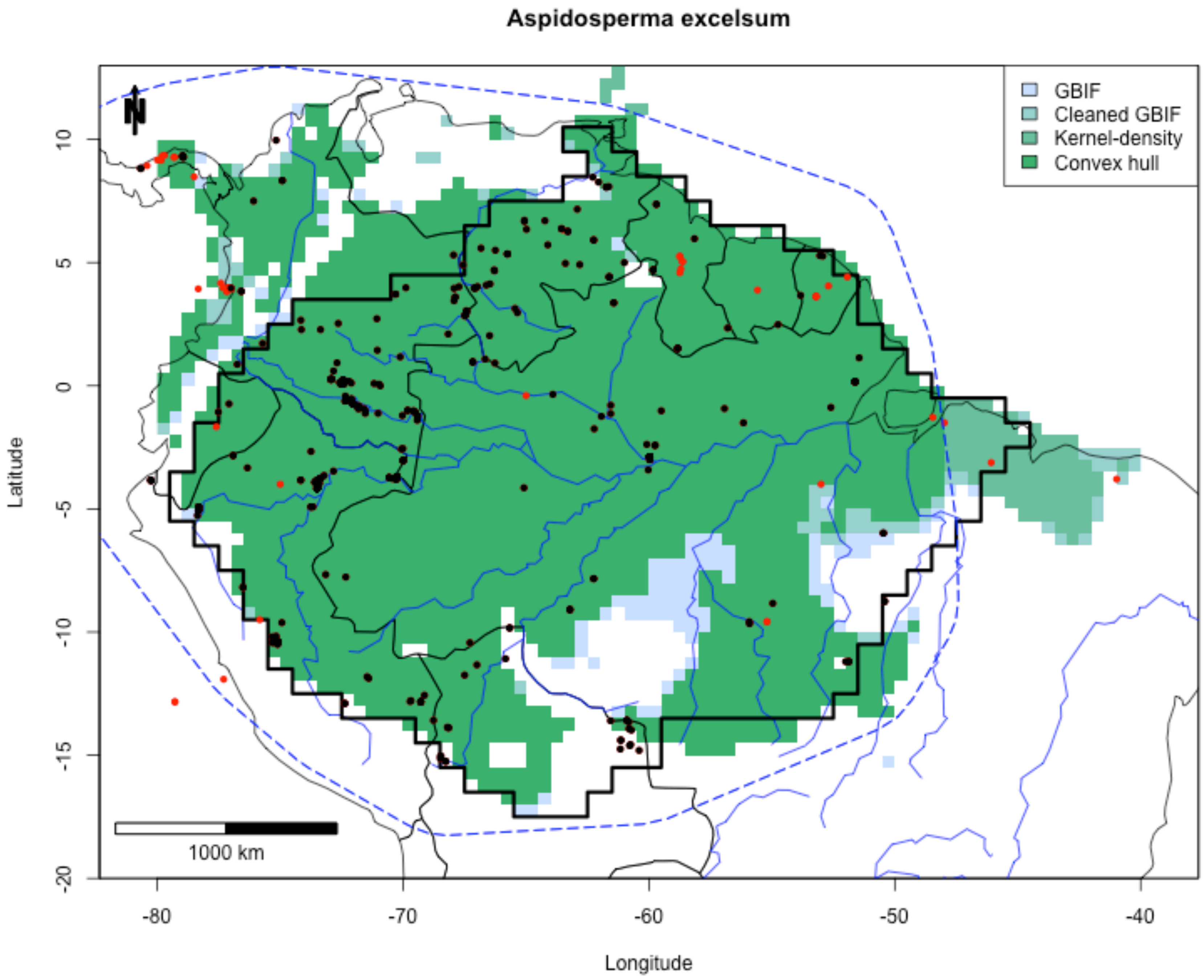

Aspidosperma excelsum
GBIF
Cleaned GBIF
Kernel-density
Convex hull
N
1000 km
Latitude
Longitude
10
5
0
-5
-10
-15
-20
-80
-70
-60
-50
-40

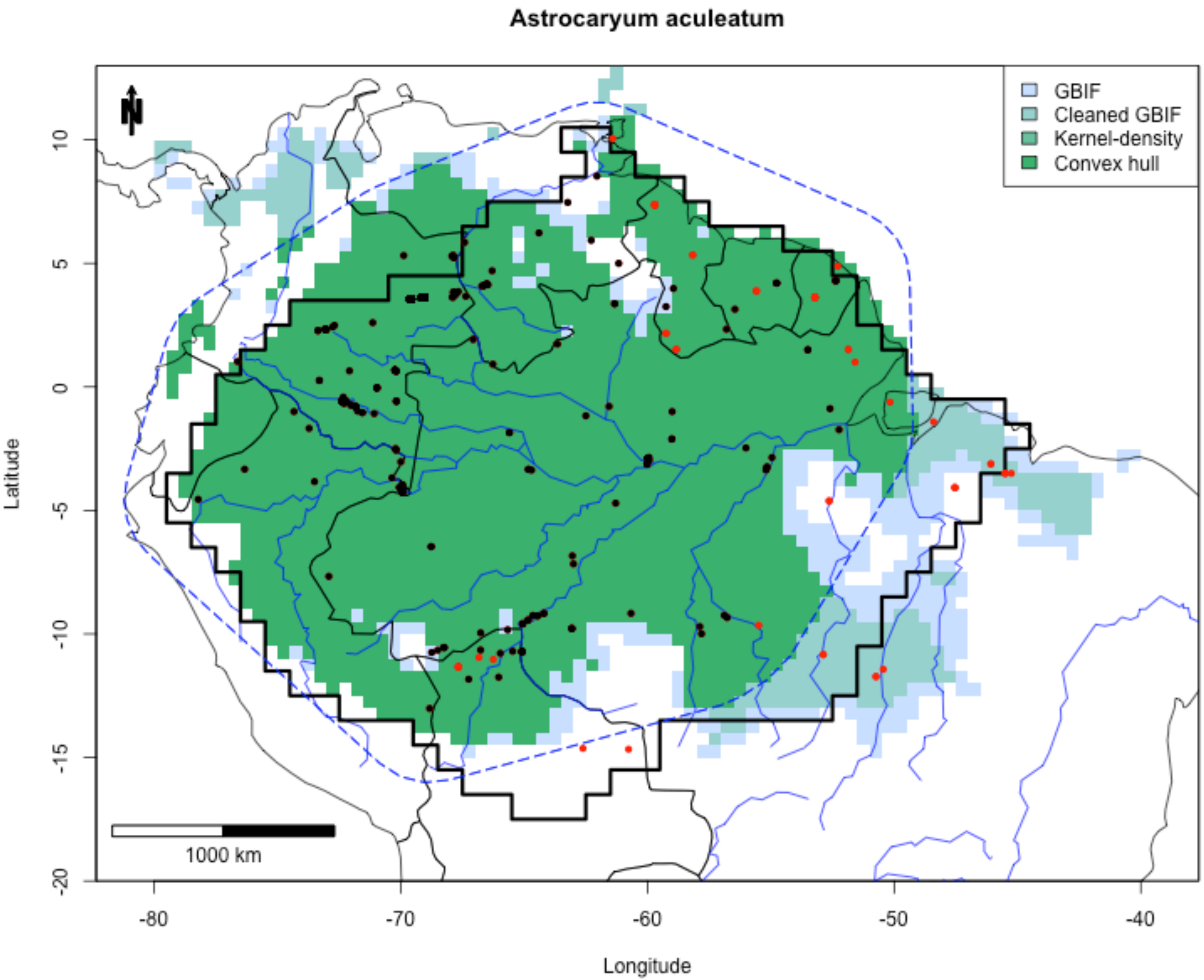

Astrocaryum aculeatum
GBIF
Cleaned GBIF
Kernel-density
Convex hull
N
1000 km
Latitude
Longitude
10
5
0
-5
-10
-15
-20
-80
-70
-60
-50
-40

Astrocaryum murumuru

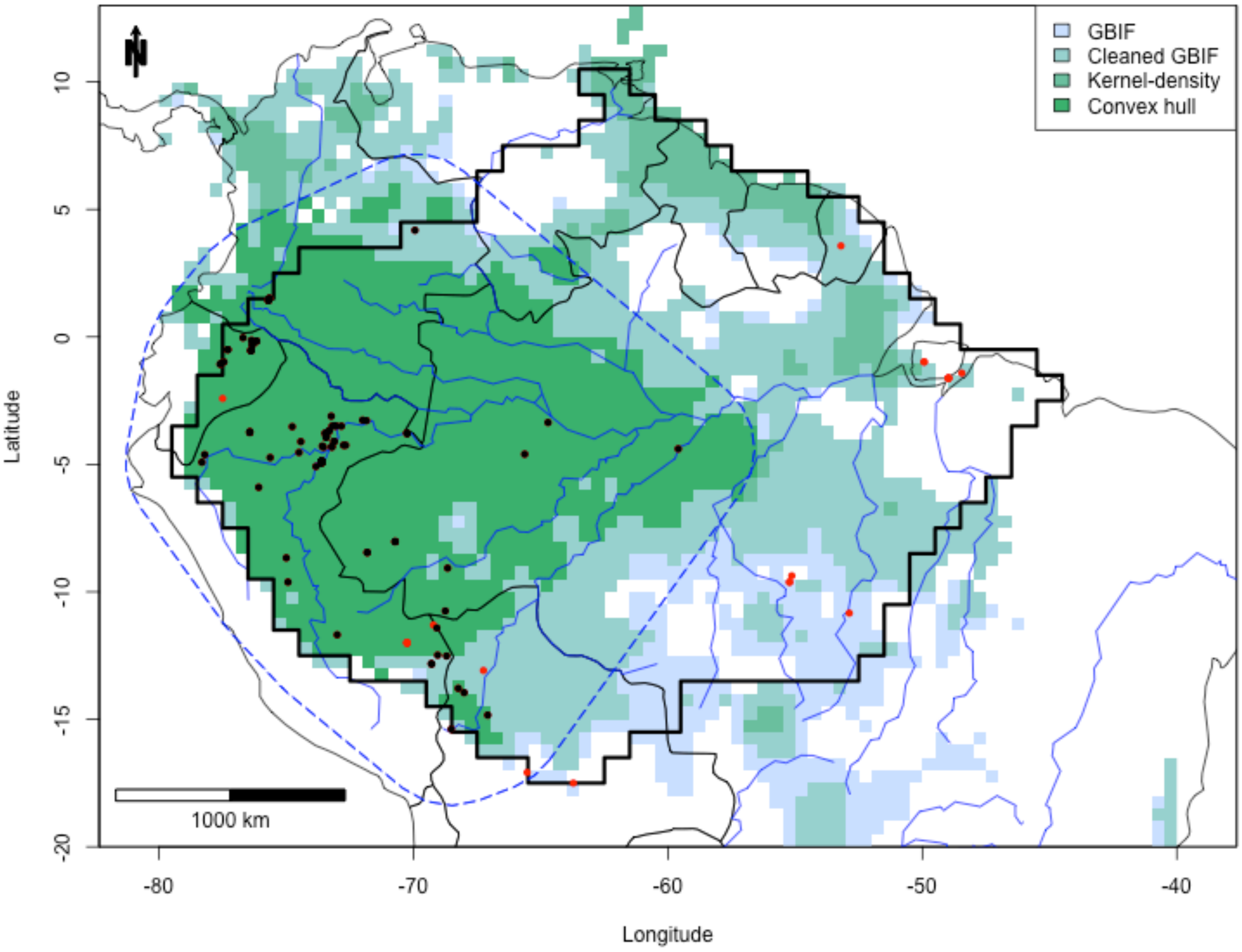

Astrocaryum sciophilum

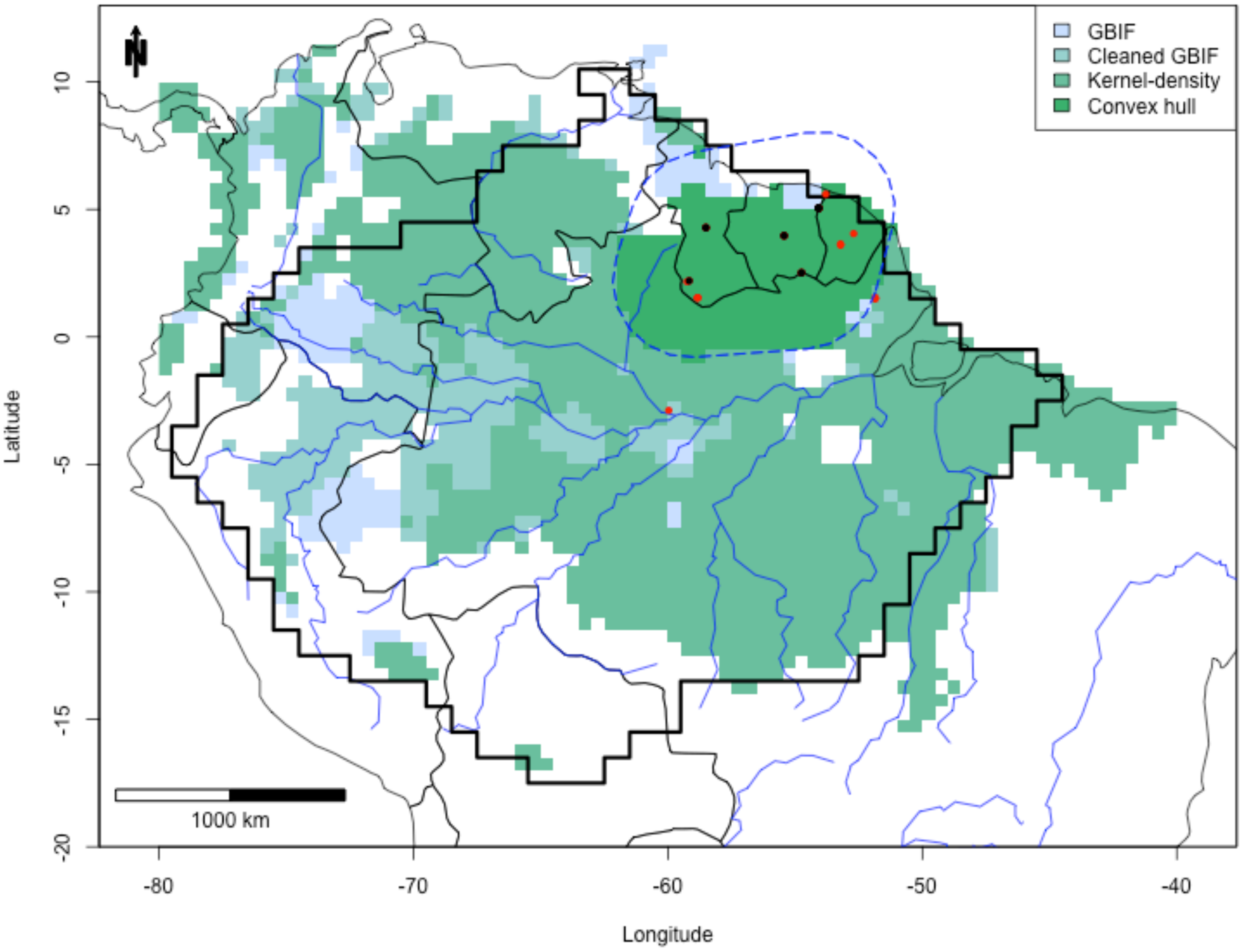

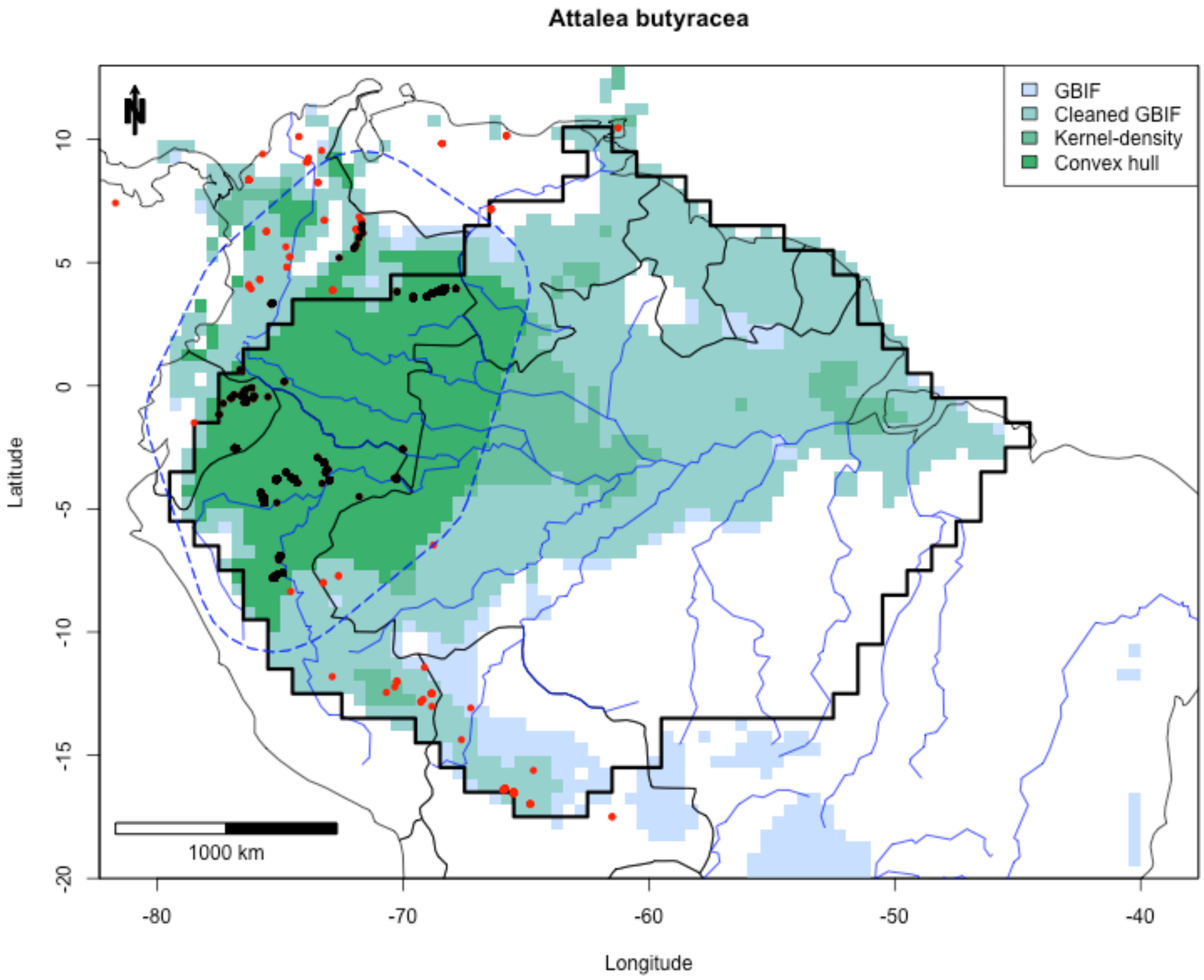

Attalea butyracea
GBIF
Cleaned GBIF
Kernel-density
Convex hull
N
1000 km
Latitude
Longitude
10
5
0
-5
-10
-15
-20
-80
-70
-60
-50
-40

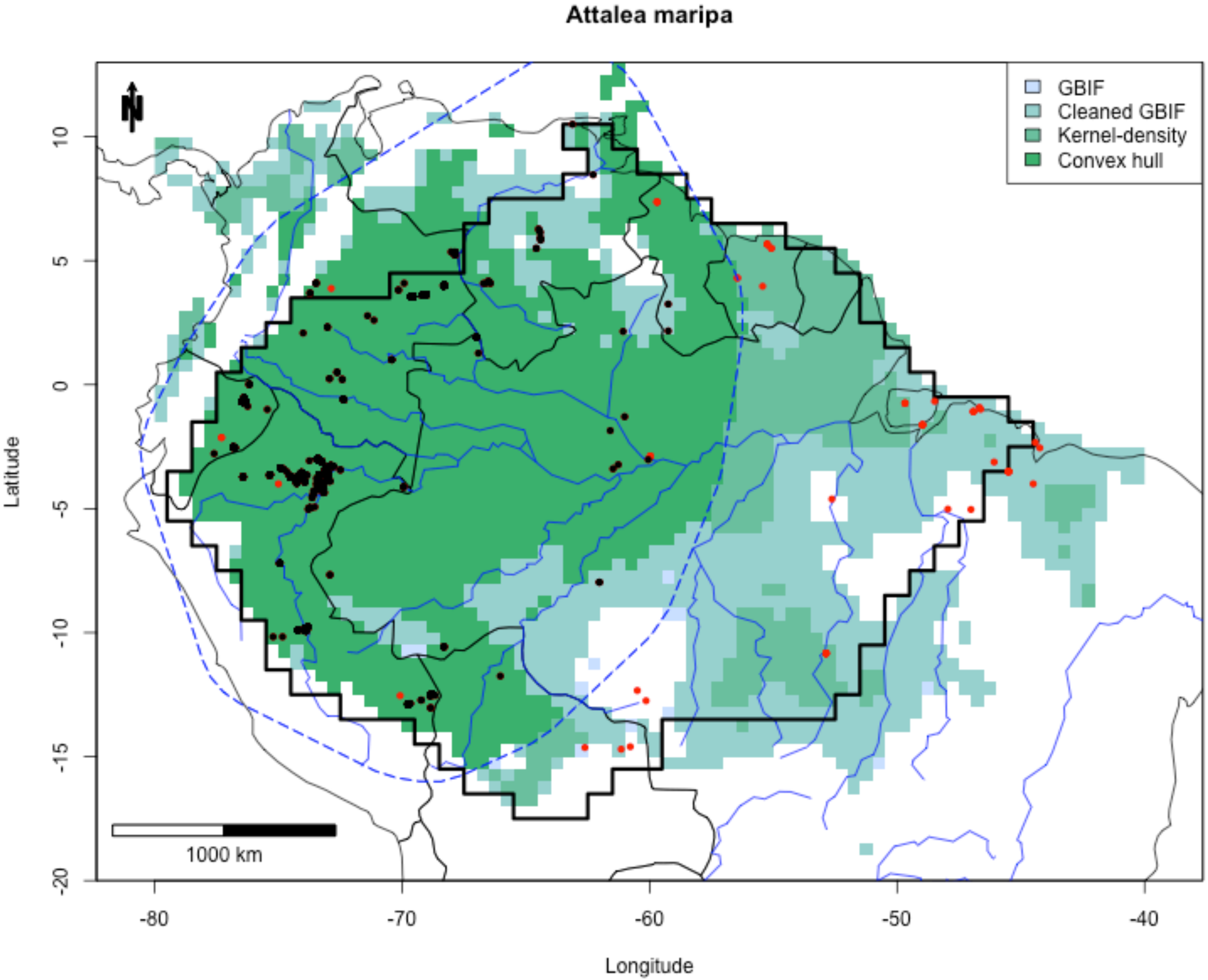
Attalea maripa
GBIF
Cleaned GBIF
Kernel-density
Convex hull
N
1000 km
Latitude
Longitude
10
5
0
-5
-10
-15
-20
-80
-70
-60
-50
-40

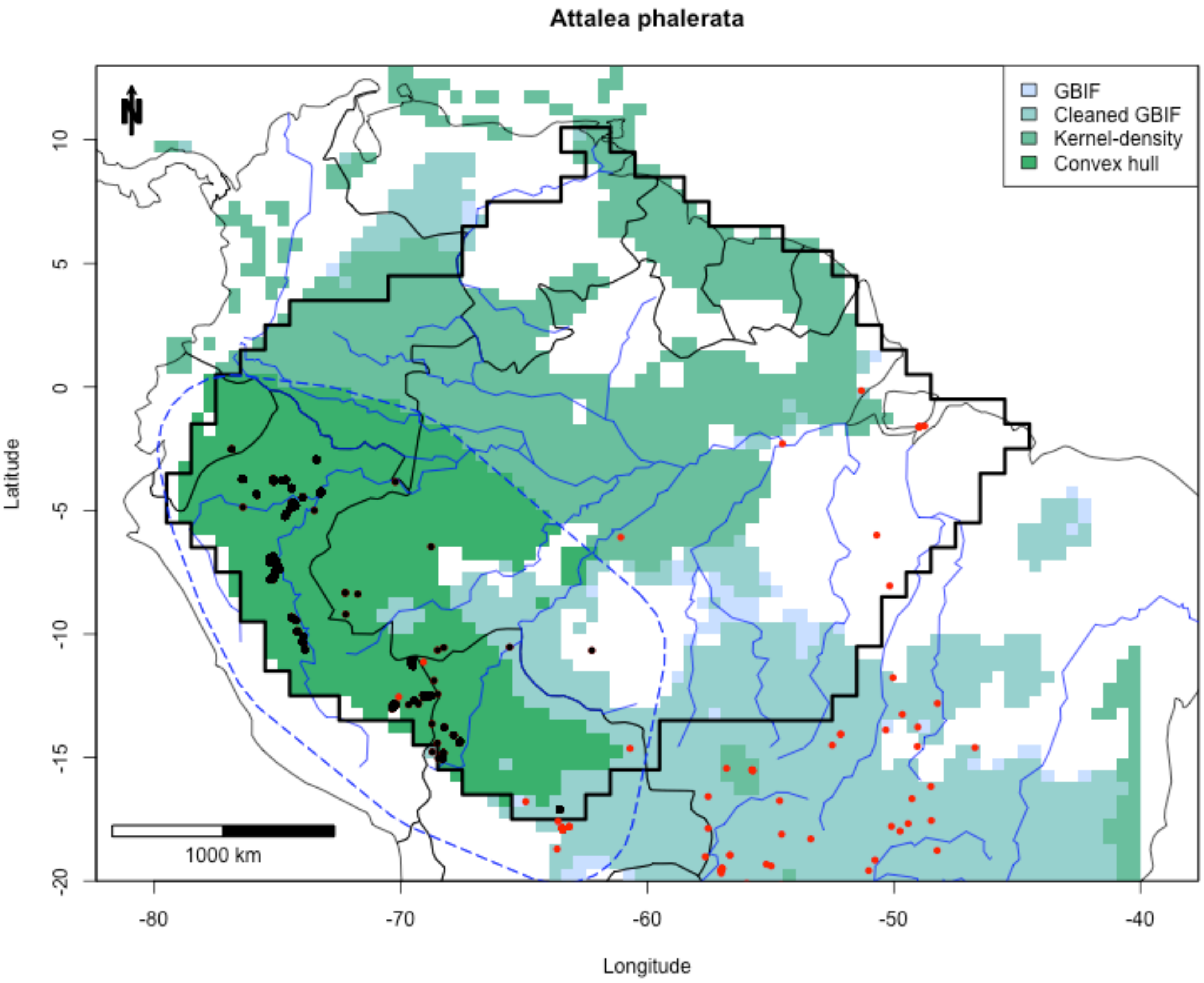

Attalea phalerata
GBIF
Cleaned GBIF
Kernel-density
Convex hull
N
1000 km
Latitude
Longitude
10
5
0
-5
-10
-15
-20
-80
-70
-60
-50
-40

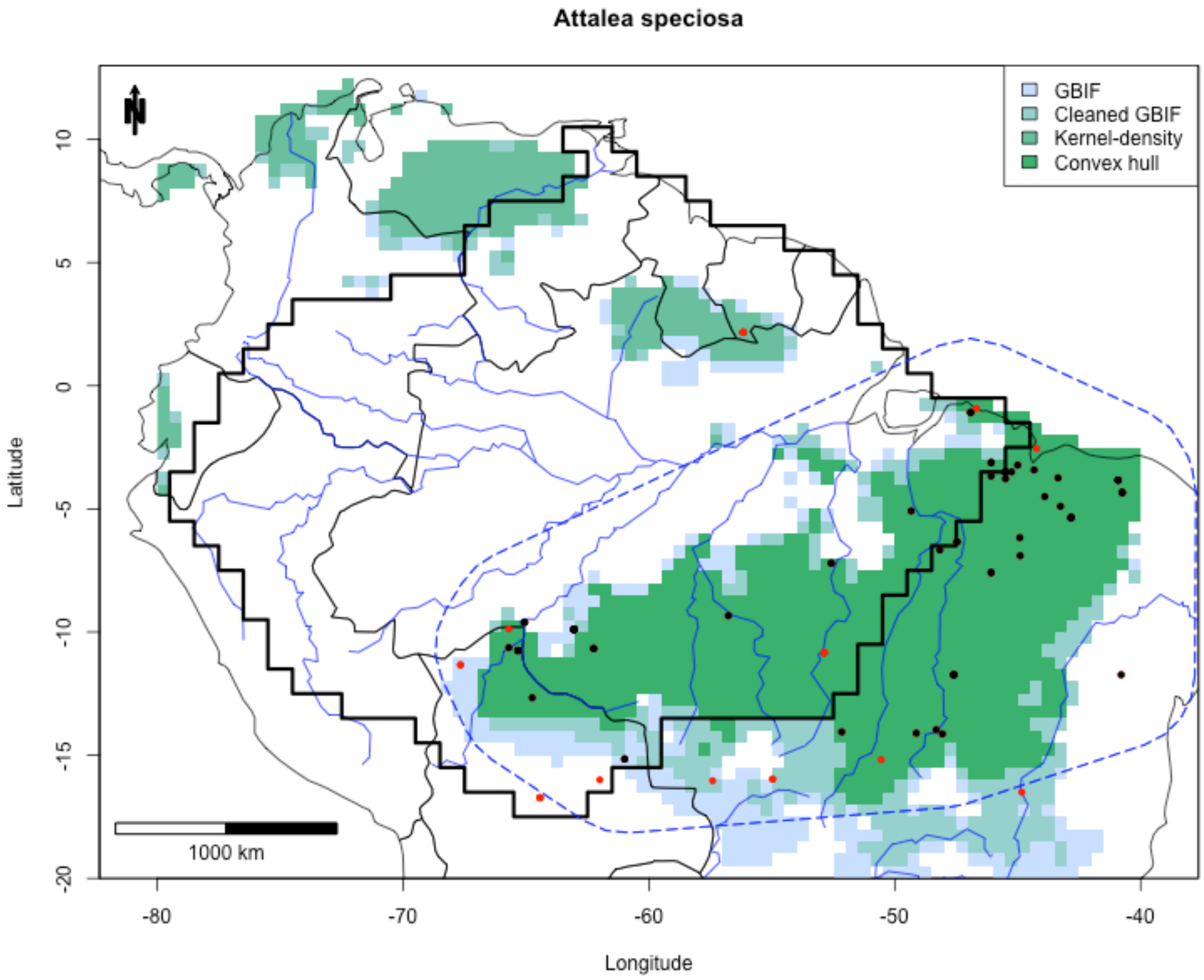

Attalea speciosa
GBIF
Cleaned GBIF
Kernel-density
Convex hull
N
1000 km
Latitude
Longitude
10
5
0
-5
-10
-15
-20
-80
-70
-60
-50
-40

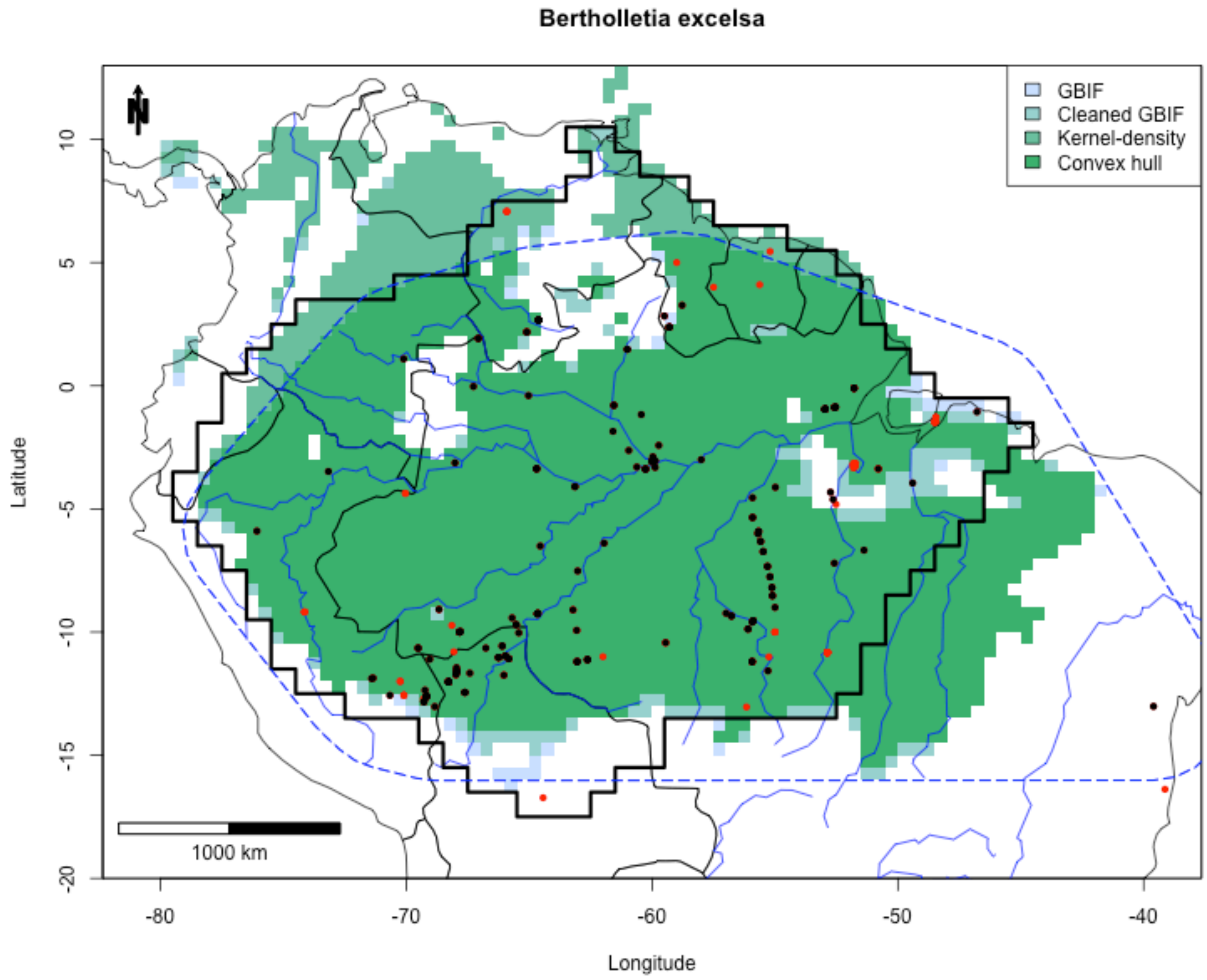

Bertholletia excelsa
GBIF
Cleaned GBIF
Kernel-density
Convex hull
N
1000 km
Latitude
Longitude
10
5
0
-5
-10
-15
-20
-80
-70
-60
-50
-40

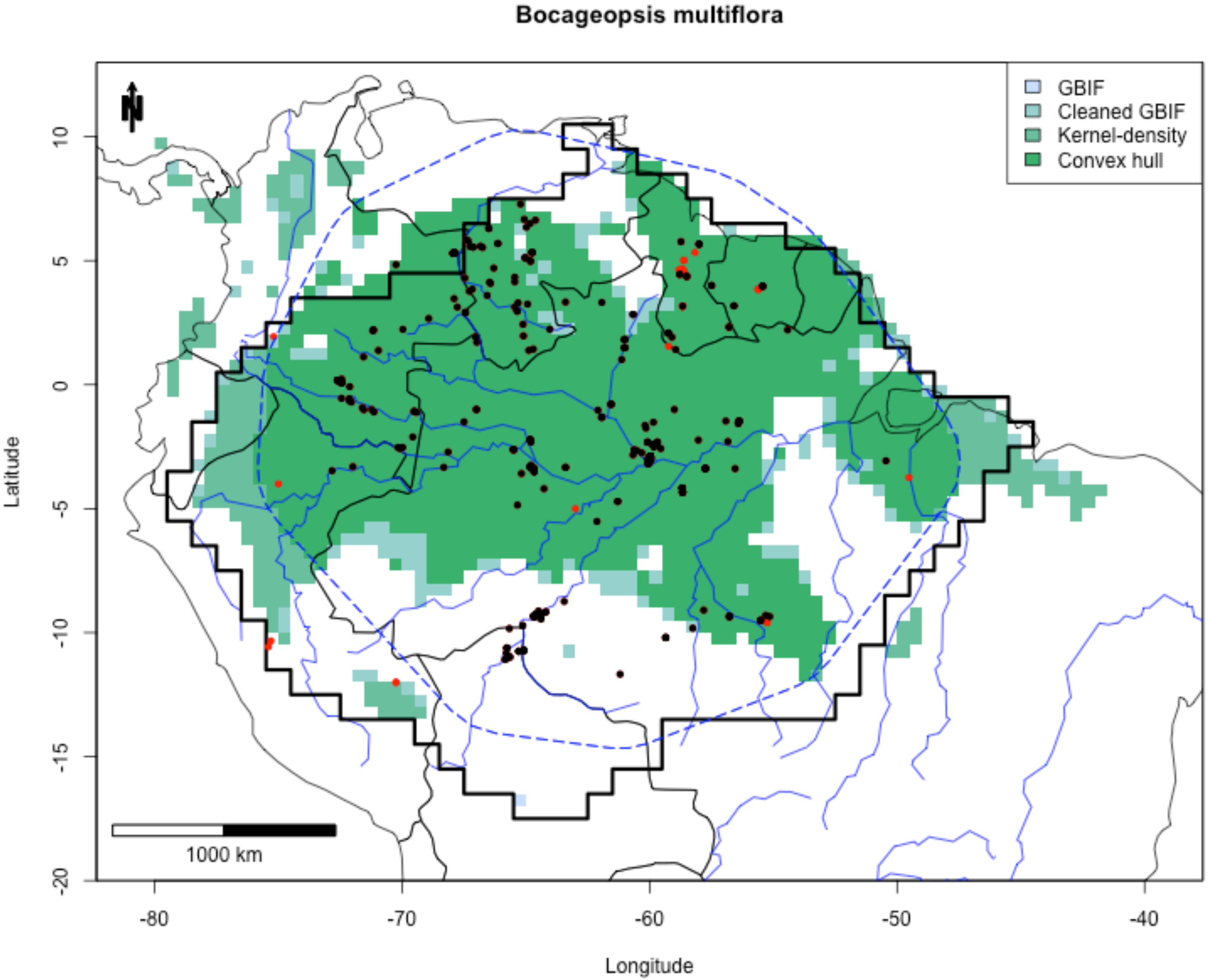
Bocageopsis multiflora
GBIF
Cleaned GBIF
Kernel-density
Convex hull
N
1000 km
Latitude
Longitude
10
5
0
-5
-10
-15
-20
-80
-70
-60
-50
-40

Brosimum guianense

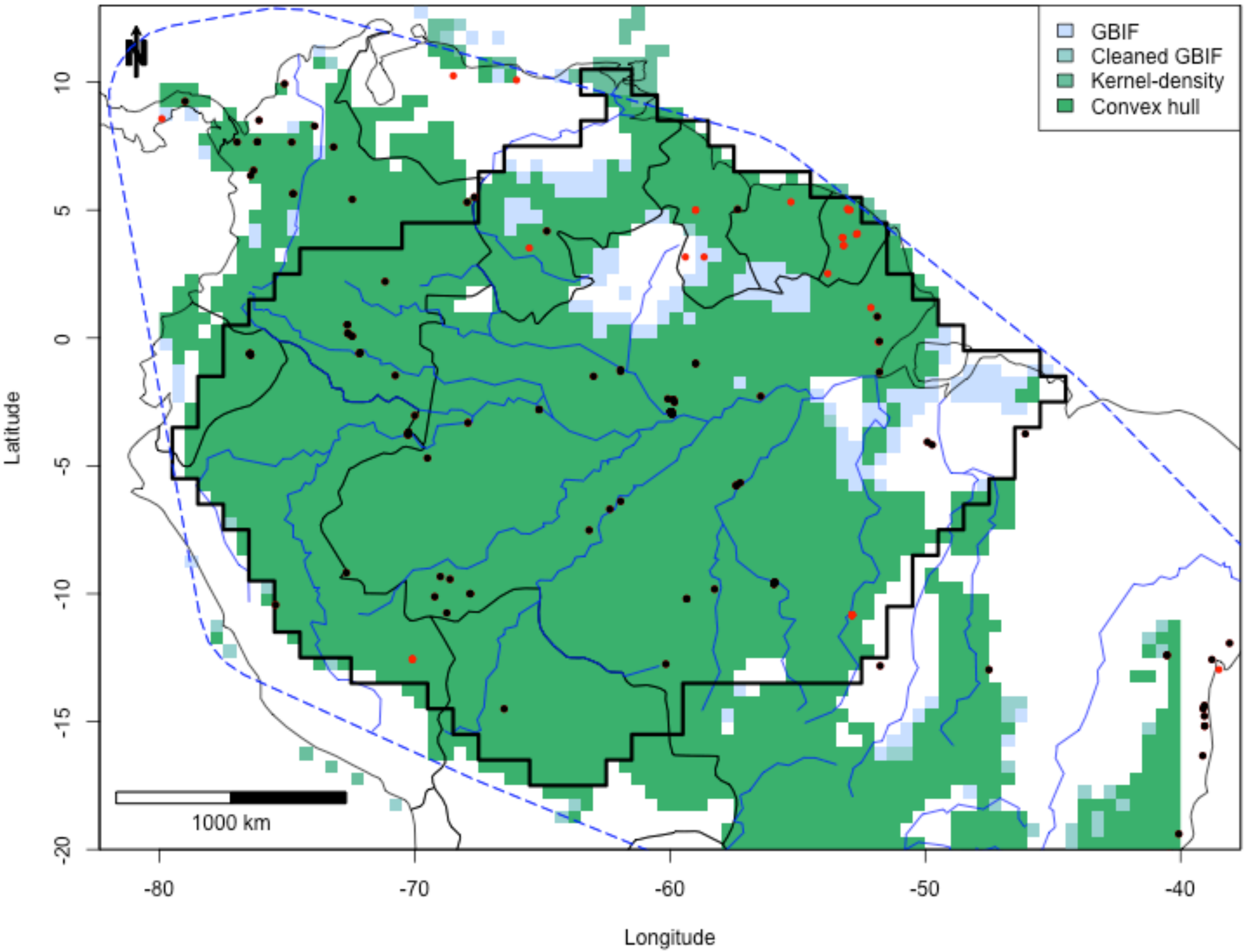

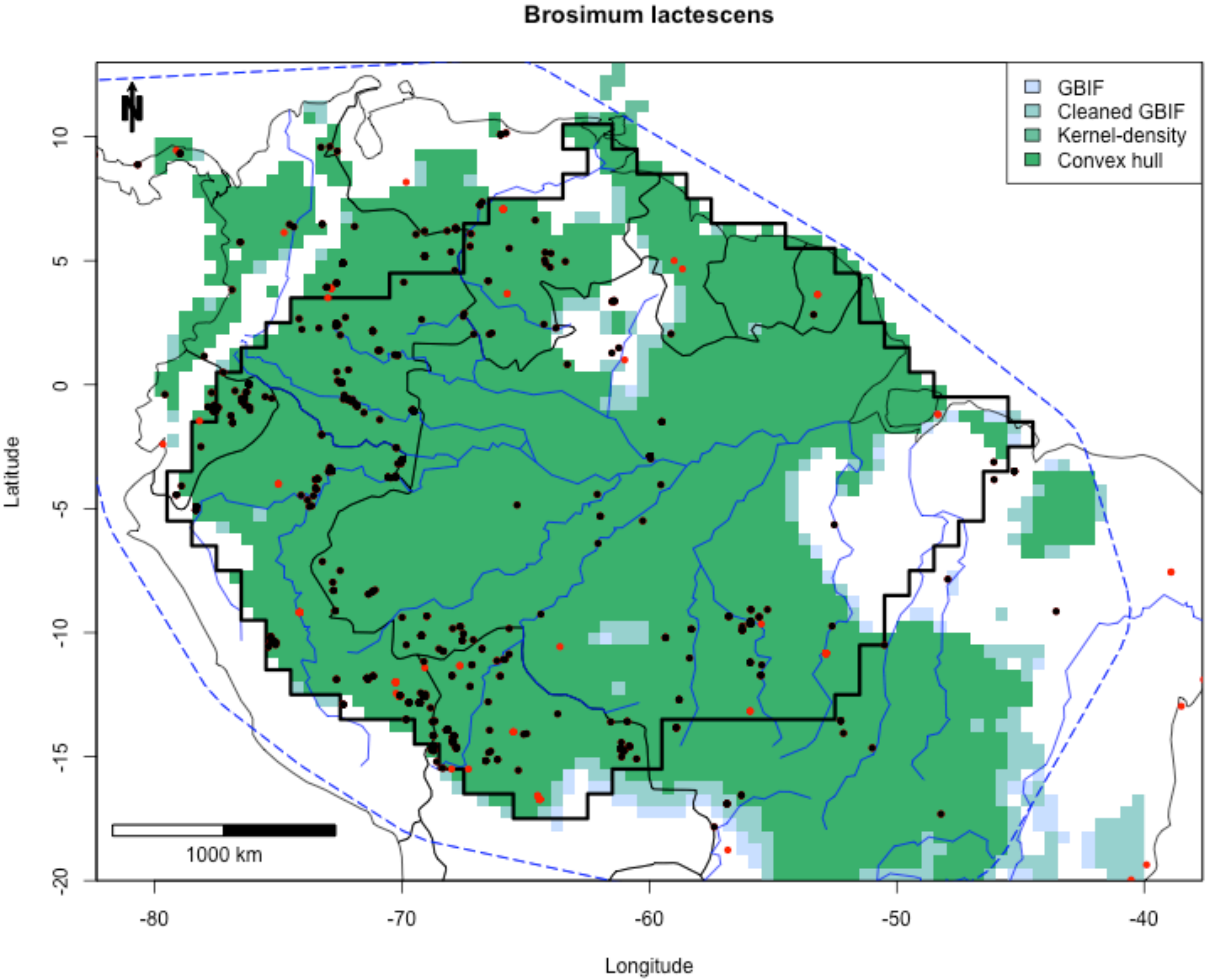
Brosimum lactescens
GBIF
Cleaned GBIF
Kernel-density
Convex hull
N
1000 km
Latitude
Longitude
10
5
0
-5
-10
-15
-20
-80
-70
-60
-50
-40

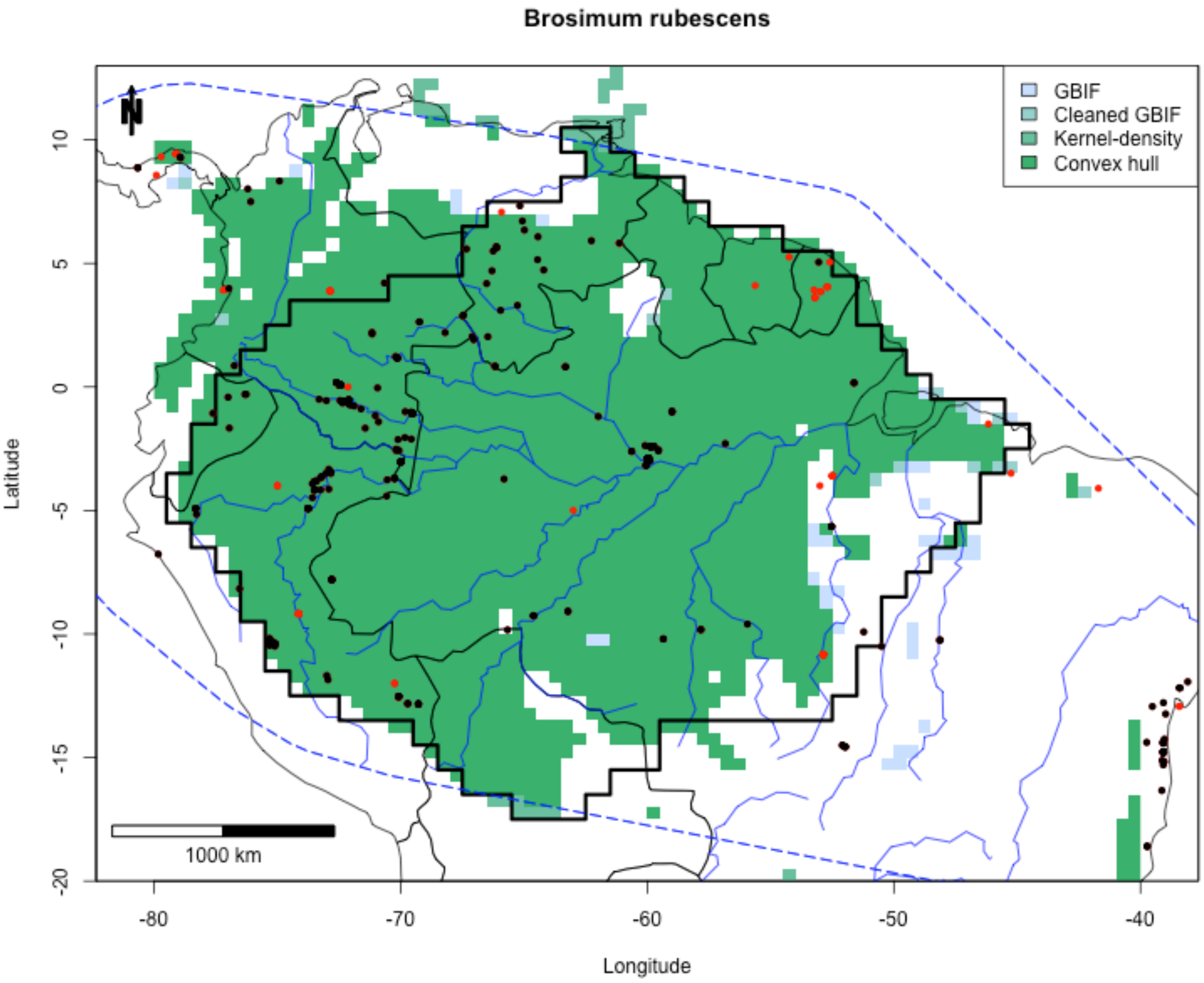
Brosimum rubescens
GBIF
Cleaned GBIF
Kernel-density
Convex hull
N
1000 km
Latitude
Longitude
10
5
0
-5
-10
-15
-20
-80
-70
-60
-50
-40

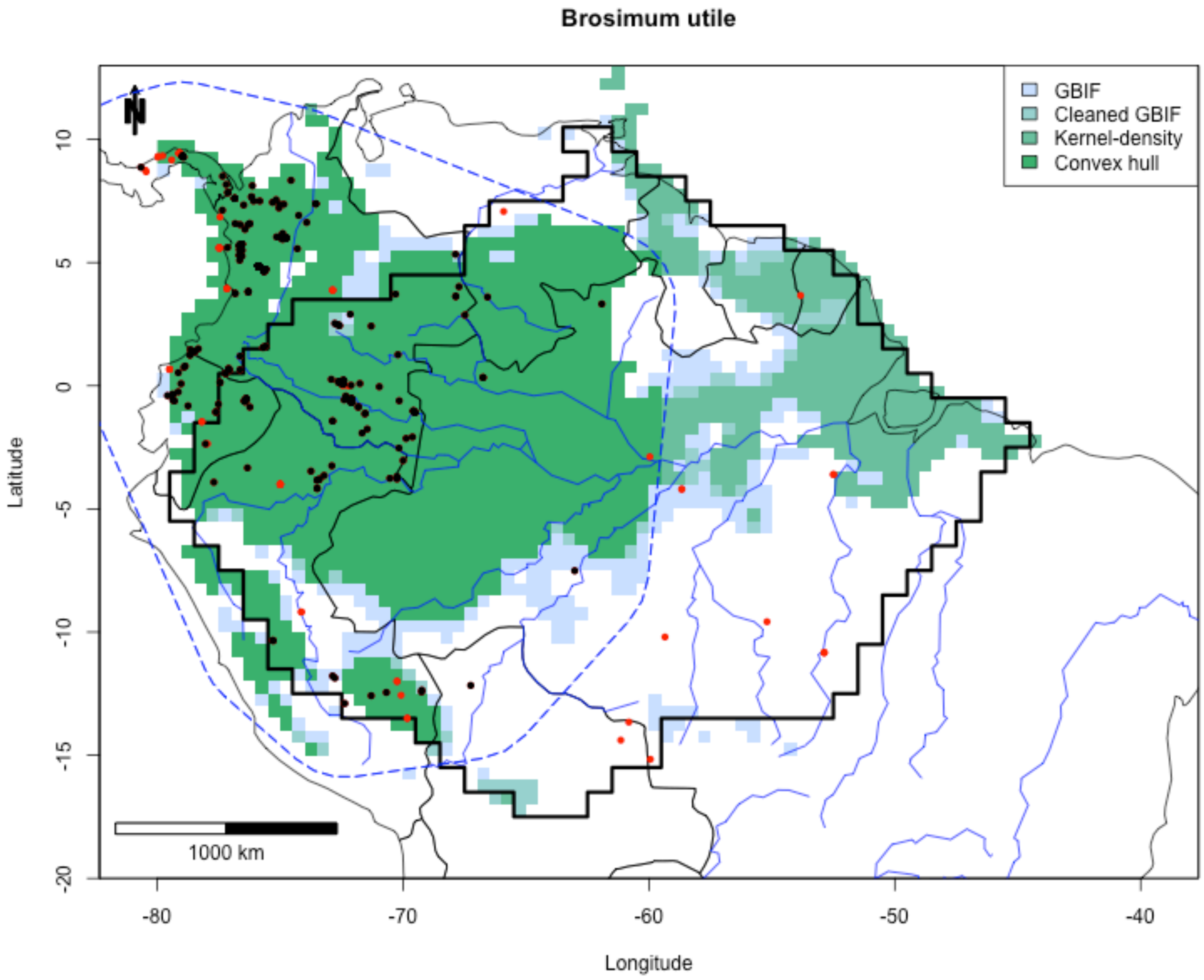
Brosimum utile
GBIF
Cleaned GBIF
Kernel-density
Convex hull
N
1000 km
Latitude
Longitude
10
5
0
-5
-10
-15
-20
-80
-70
-60
-50
-40

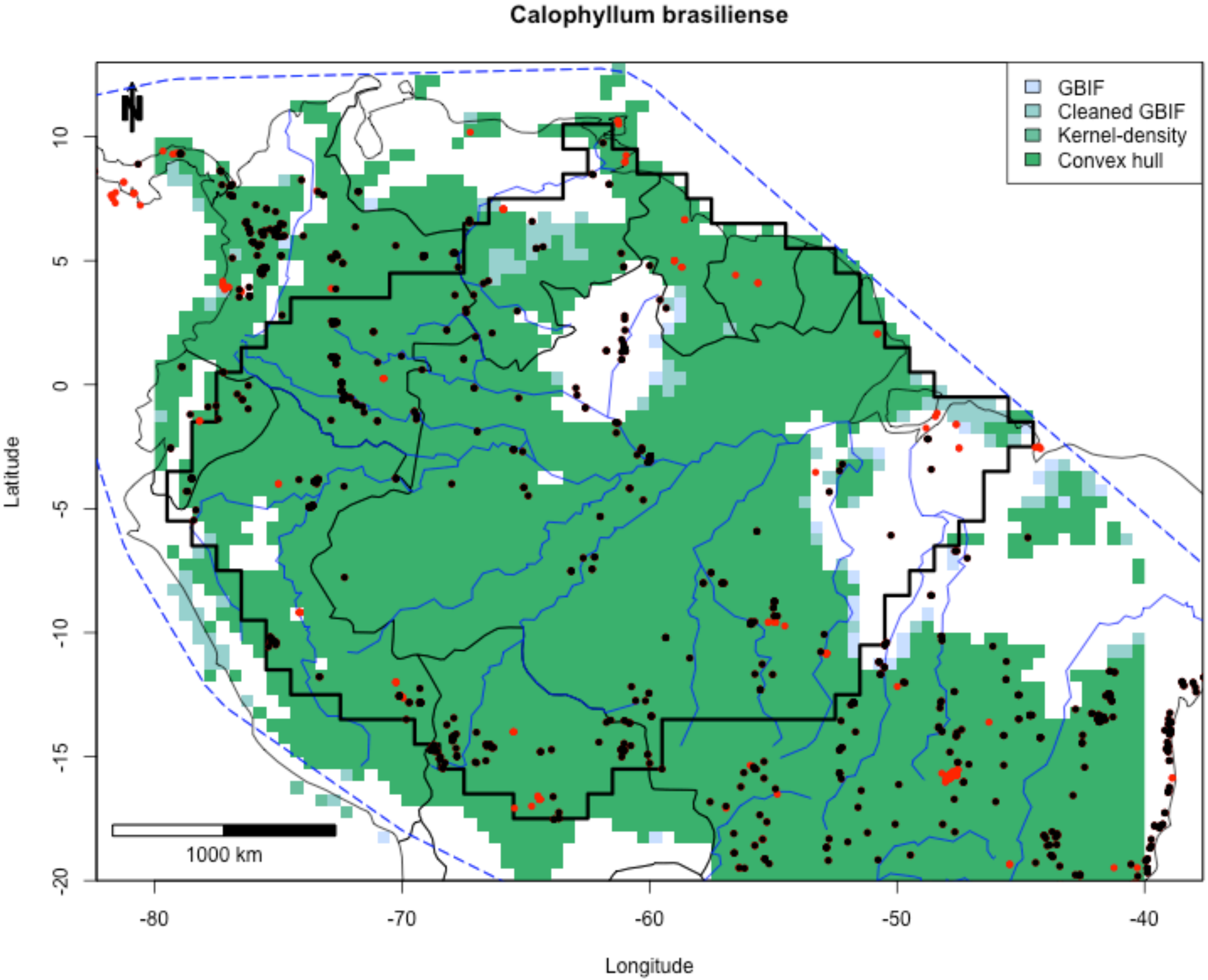
Calophyllum brasiliense
GBIF
Cleaned GBIF
Kernel-density
Convex hull
N
1000 km
Latitude
Longitude
10
5
0
-5
-10
-15
-20
-80
-70
-60
-50
-40

Campsiandra implexicaulis

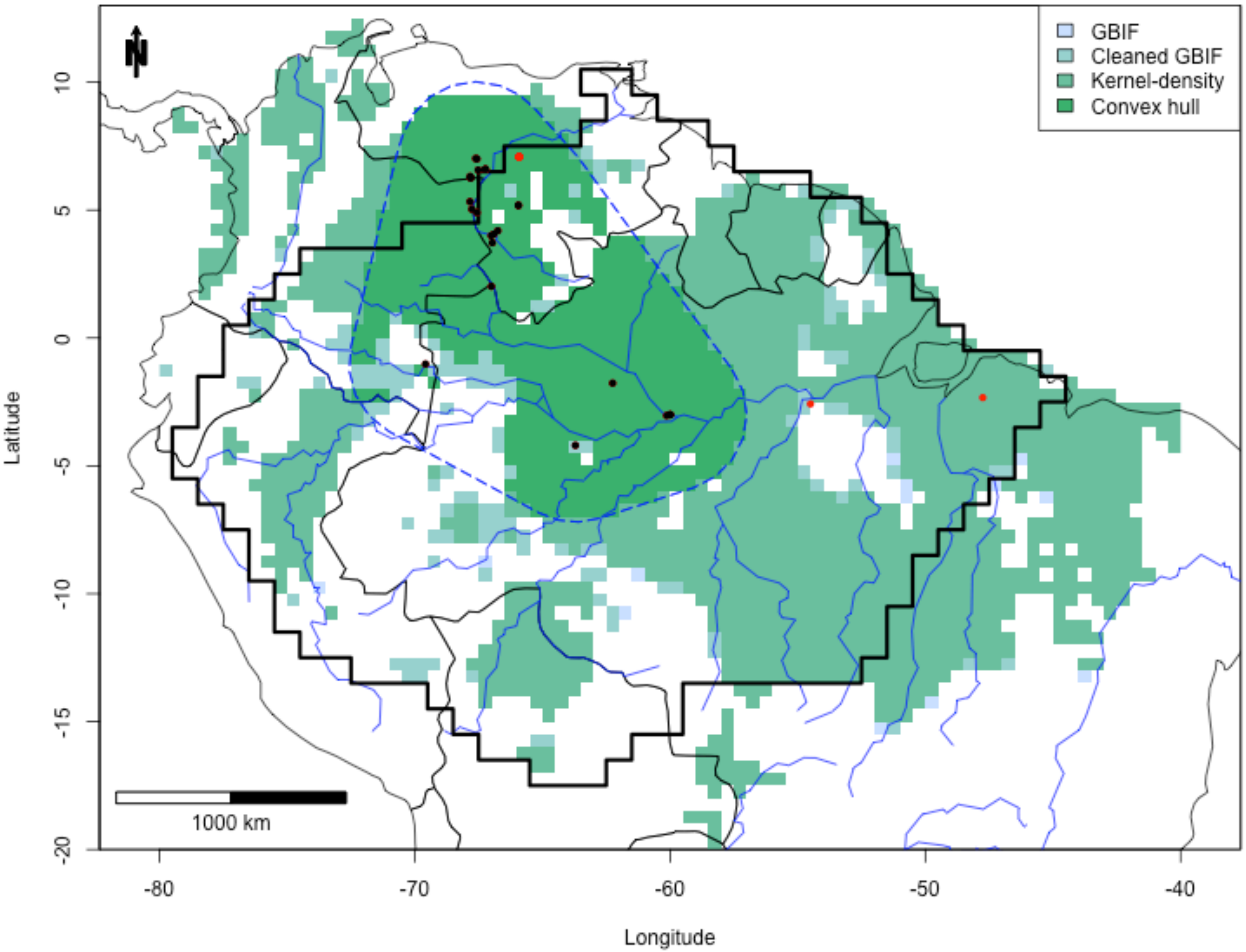

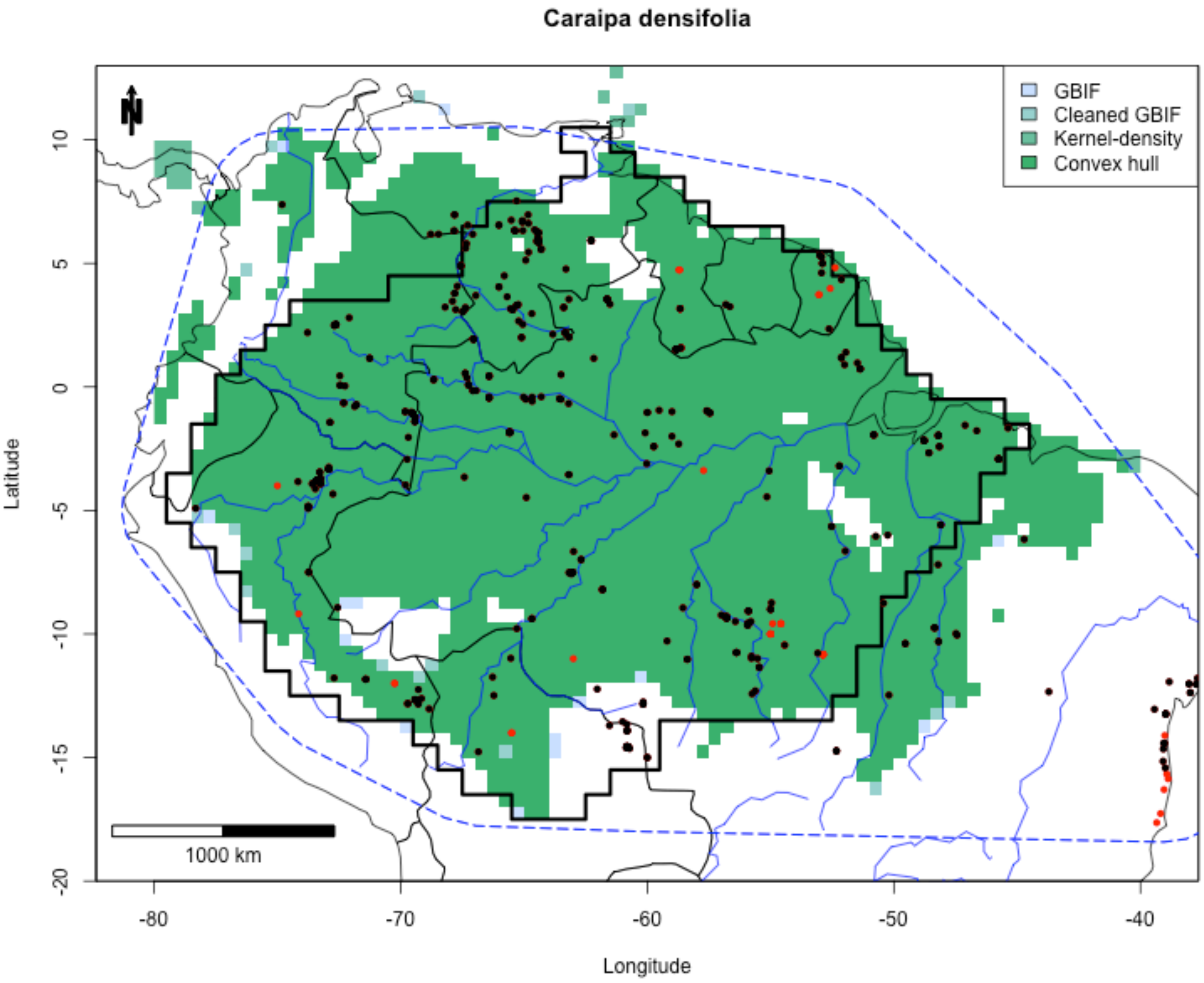

Caraipa densifolia
GBIF
Cleaned GBIF
Kernel-density
Convex hull
1000 km
Latitude
Longitude
10
5
0
-5
-10
-15
-20
-80
-70
-60
-50
-40

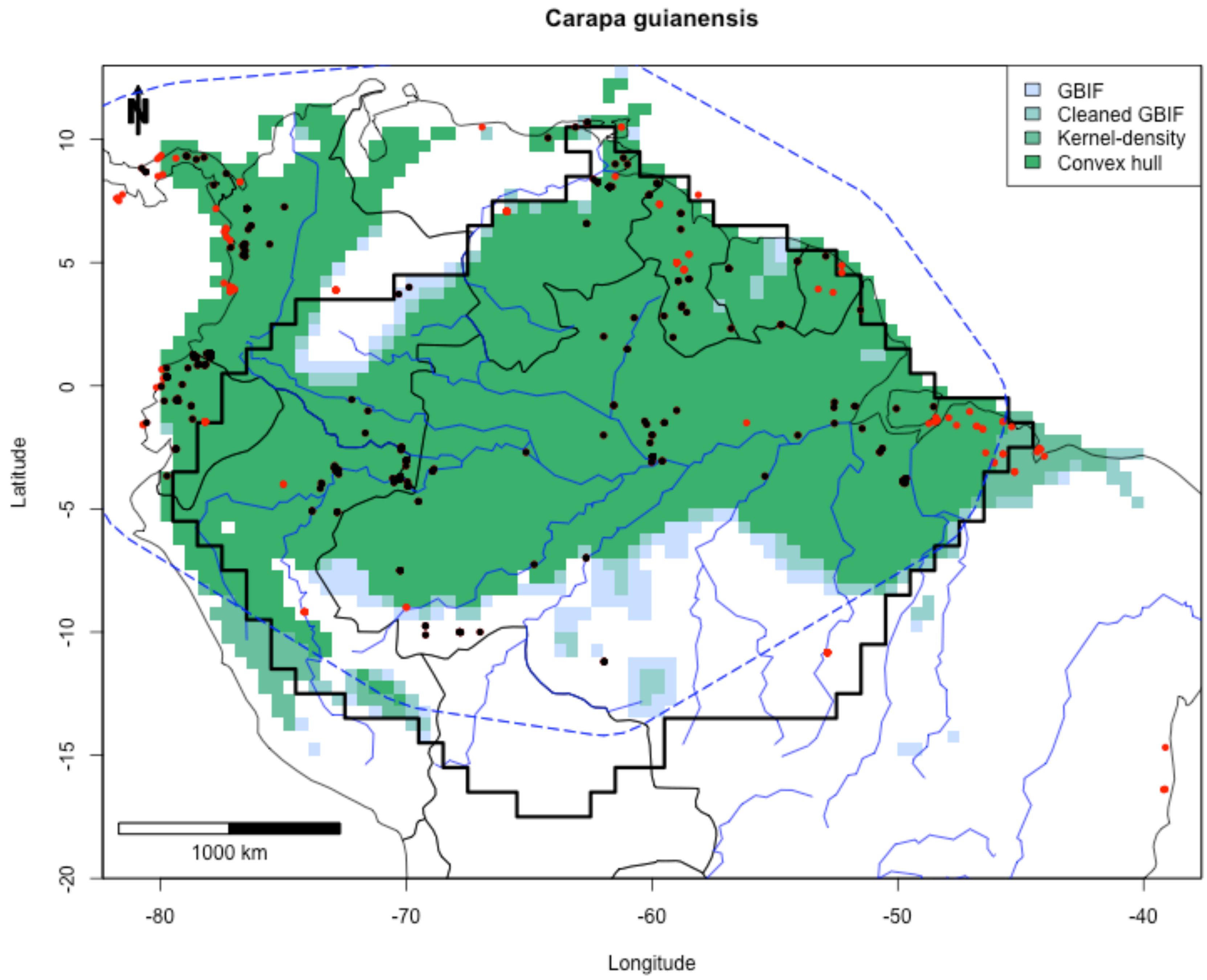

Carapa guianensis
GBIF
Cleaned GBIF
Kernel-density
Convex hull
1000 km
Latitude
Longitude
10
5
0
-5
-10
-15
-20
-80
-70
-60
-50
-40
N

Caryocar glabrum

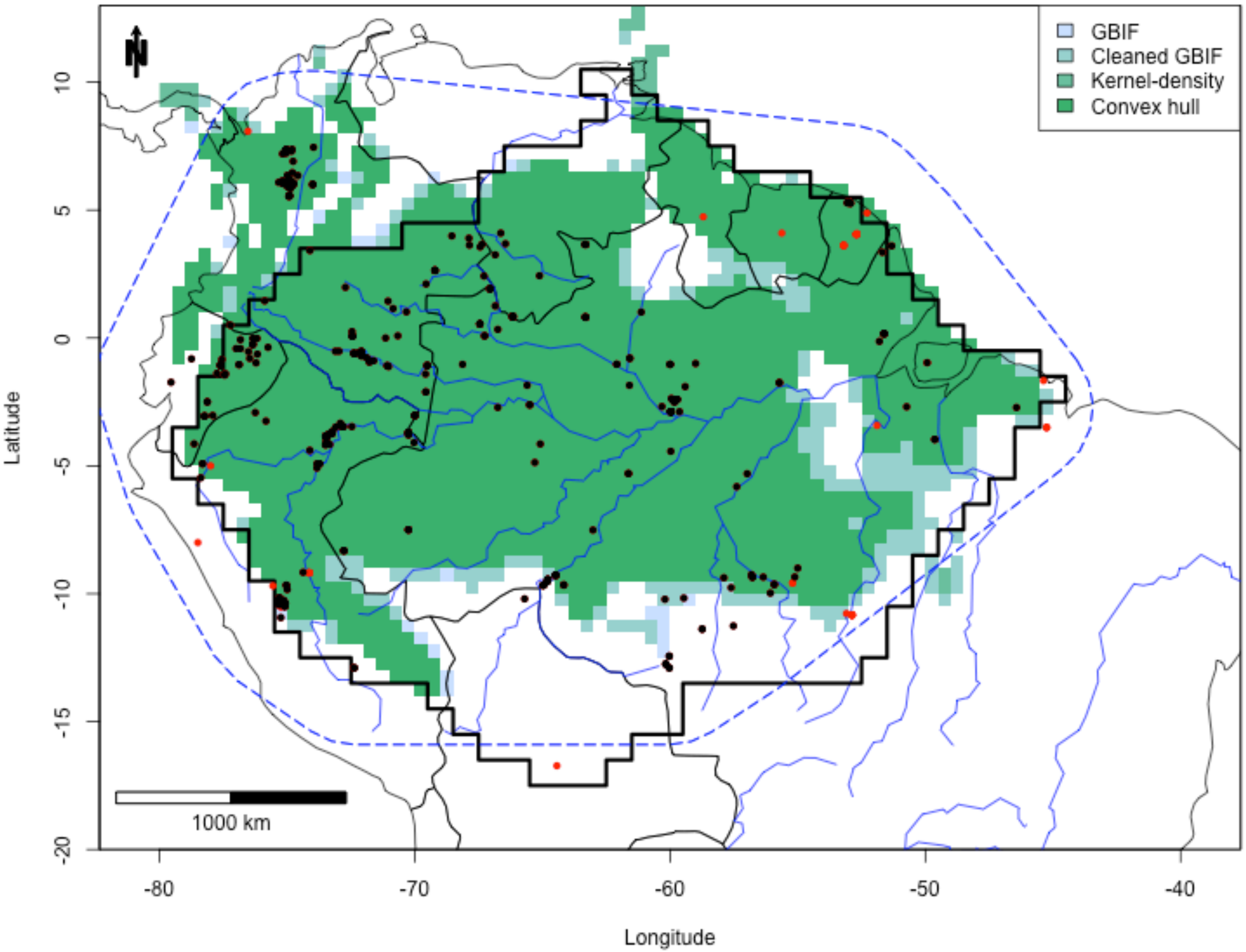

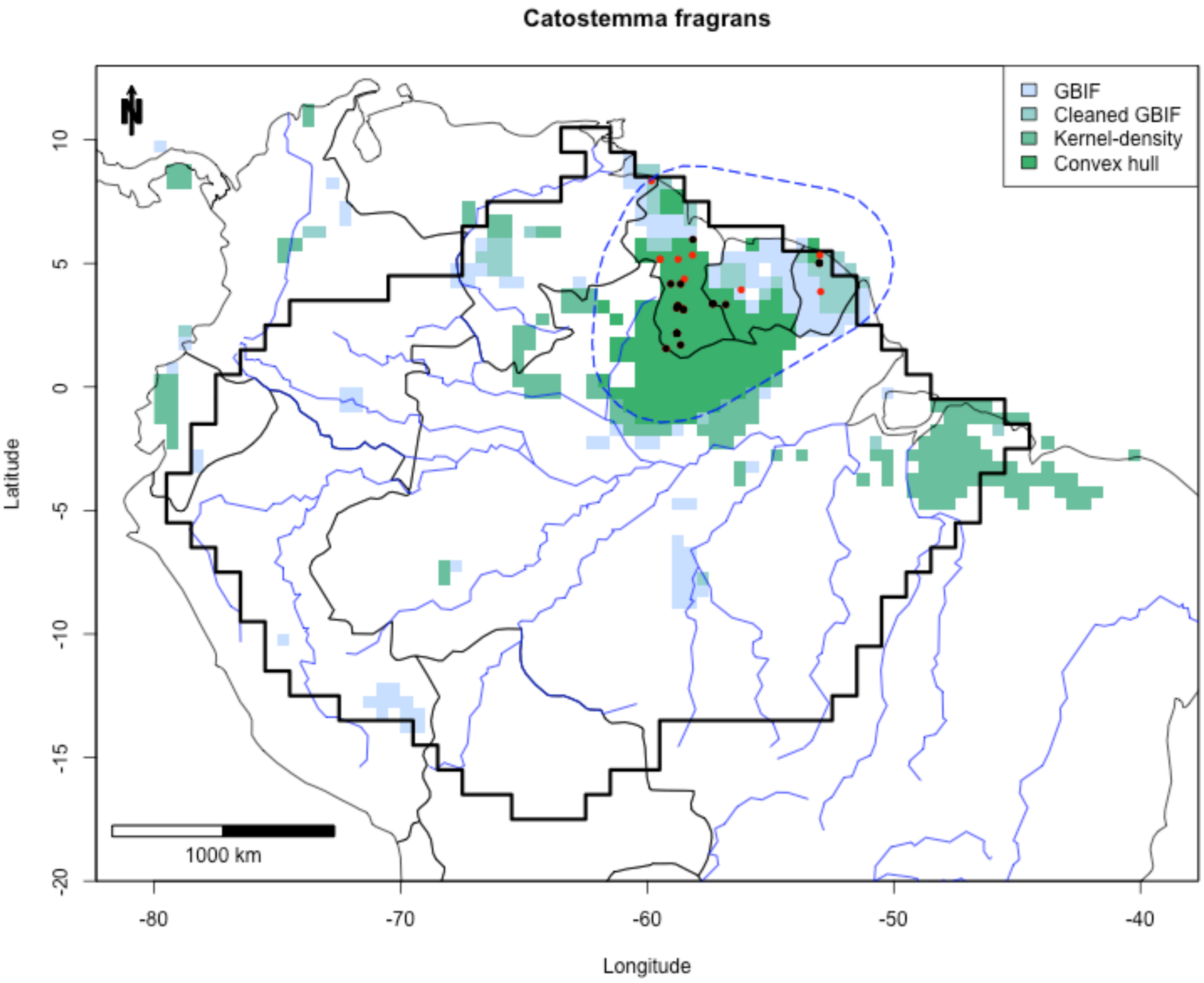
Catostemma fragrans
GBIF
Cleaned GBIF
Kernel-density
Convex hull
N
1000 km
Latitude
Longitude
10
5
0
-5
-10
-15
-20
-80
-70
-60
-50
-40

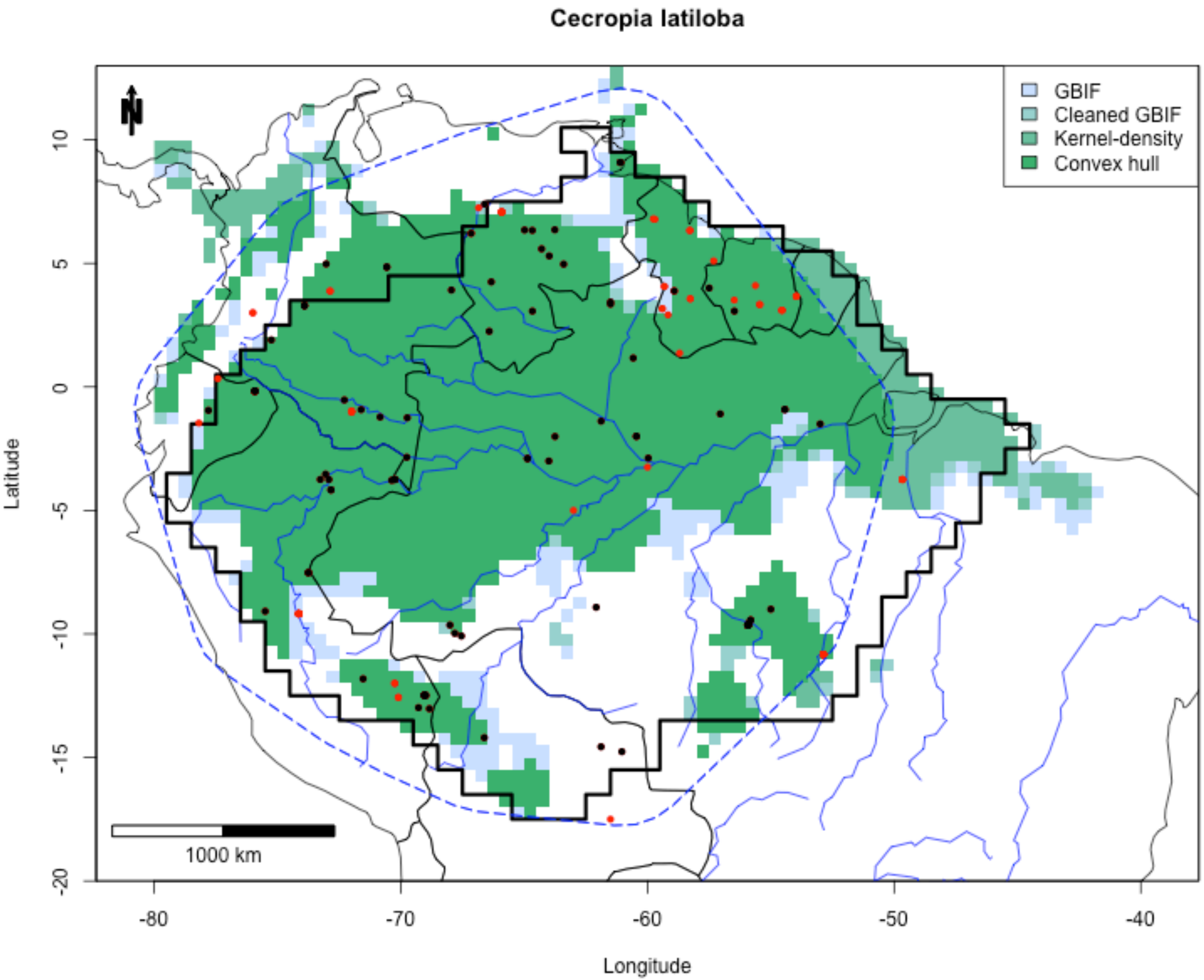

Cecropia latiloba
GBIF
Cleaned GBIF
Kernel-density
Convex hull
N
1000 km
Latitude
Longitude
10
5
0
-5
-10
-15
-20
-80
-70
-60
-50
-40

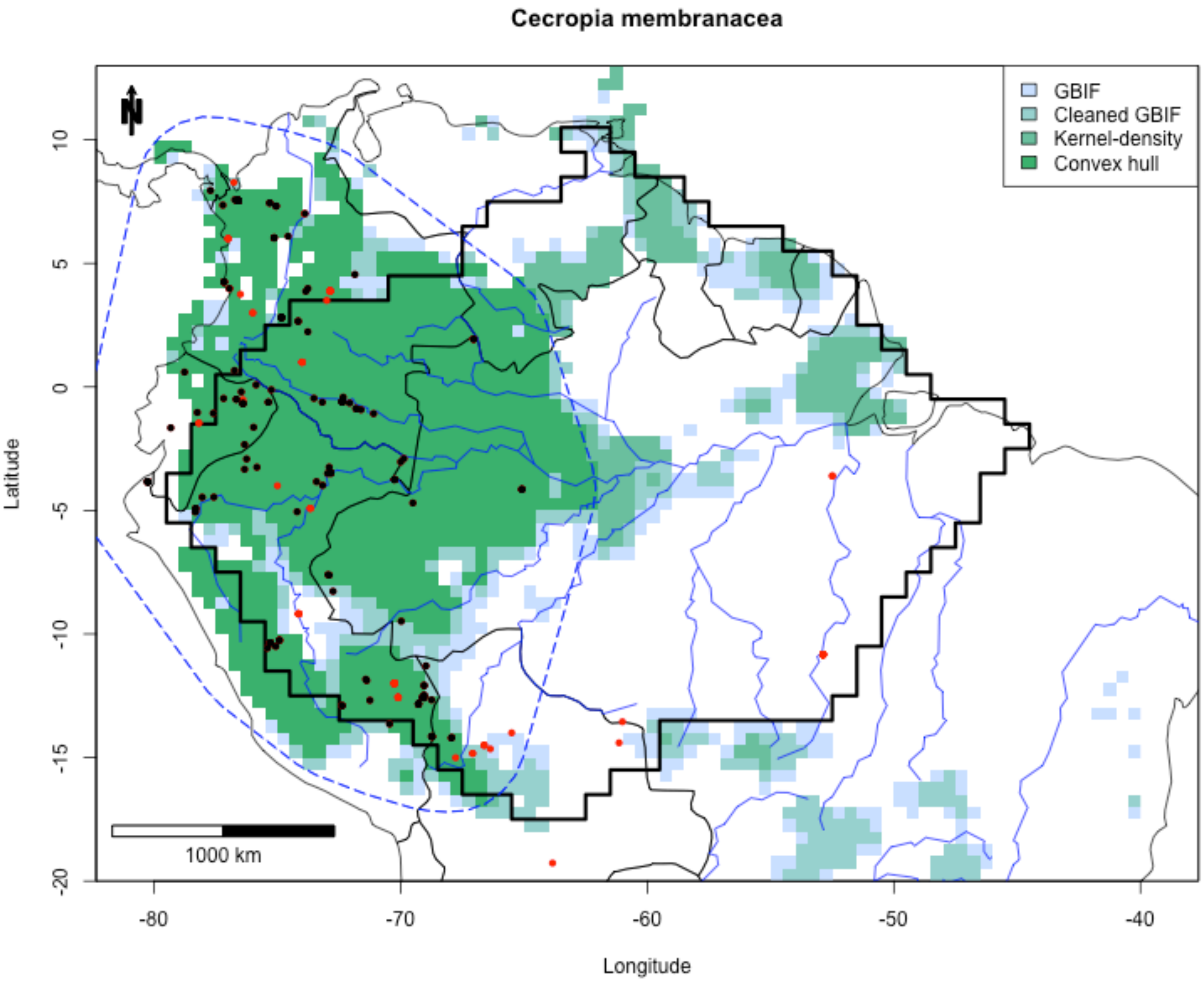

Cecropia membranacea
GBIF
Cleaned GBIF
Kernel-density
Convex hull
1000 km
Latitude
Longitude
10
5
0
-5
-10
-15
-20
-80
-70
-60
-50
-40

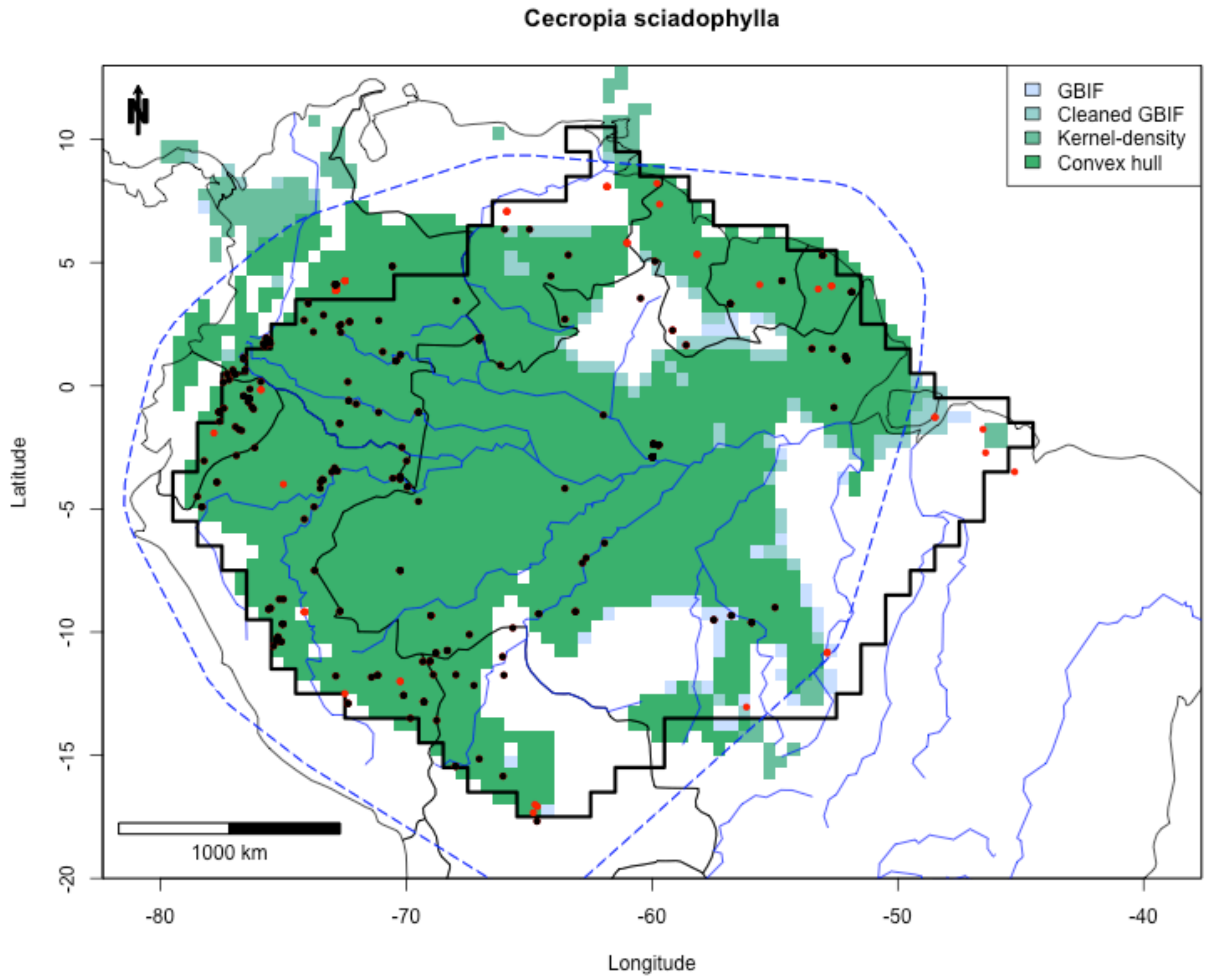
Cecropia sciadophylla
GBIF
Cleaned GBIF
Kernel-density
Convex hull
N
1000 km
Latitude
Longitude
10
5
0
-5
-10
-15
-20
-80
-70
-60
-50
-40

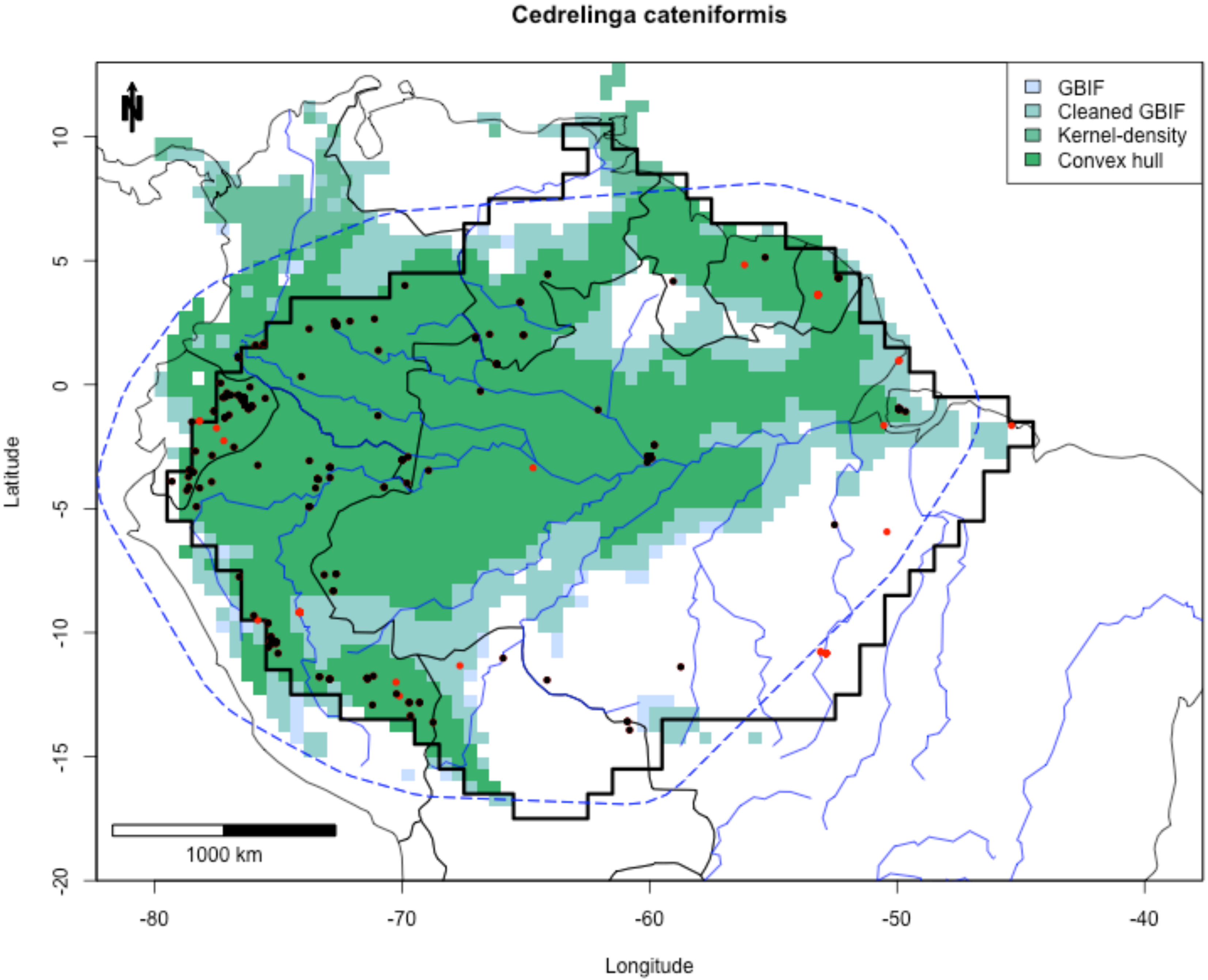

Cedrelinga cateniformis
GBIF
Cleaned GBIF
Kernel-density
Convex hull
N
1000 km
Latitude
Longitude
10
5
0
-5
-10
-15
-20
-80
-70
-60
-50
-40

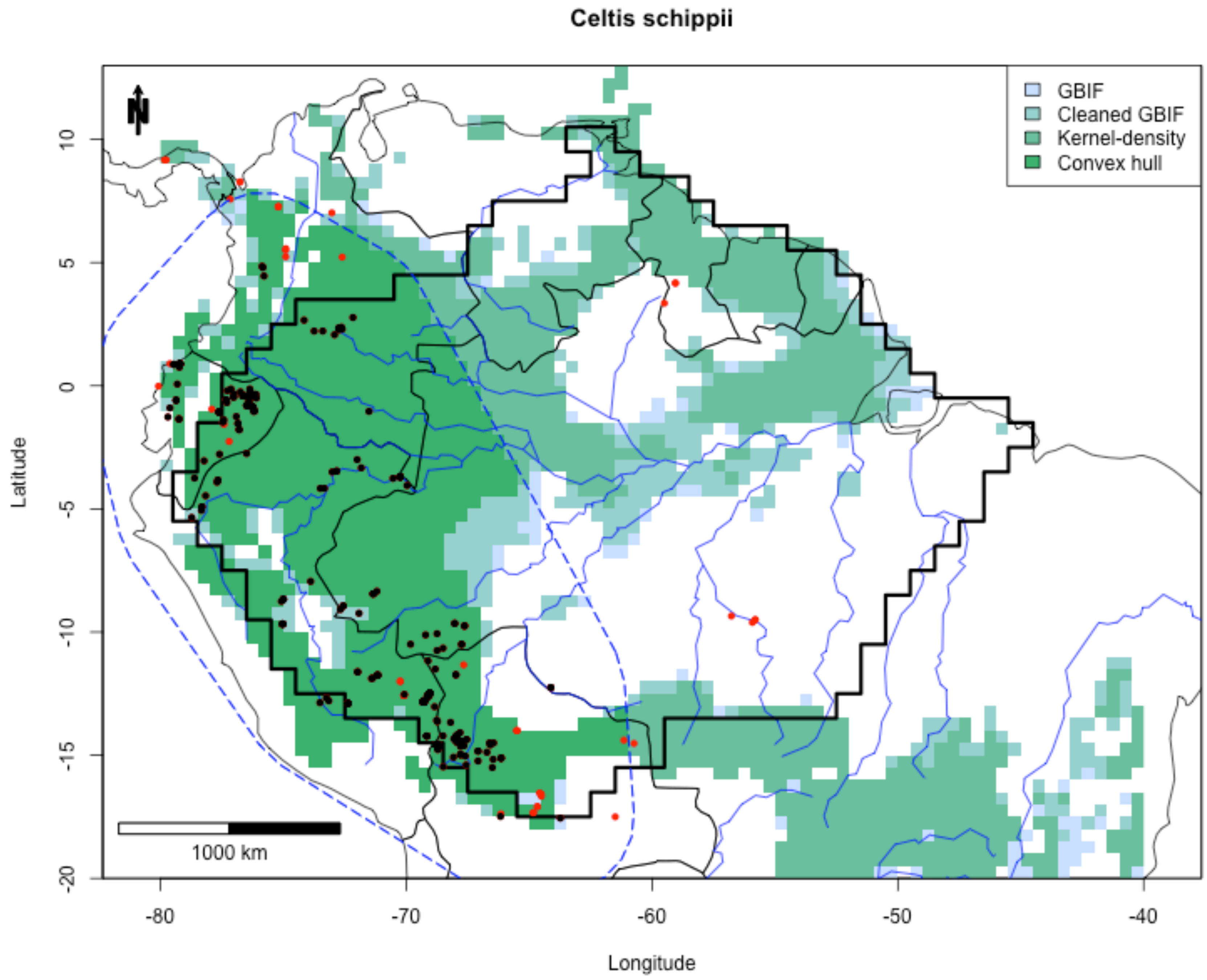
Celtis schippii
GBIF
Cleaned GBIF
Kernel-density
Convex hull
N
1000 km
Latitude
Longitude
10
5
0
-5
-10
-15
-20
-80
-70
-60
-50
-40

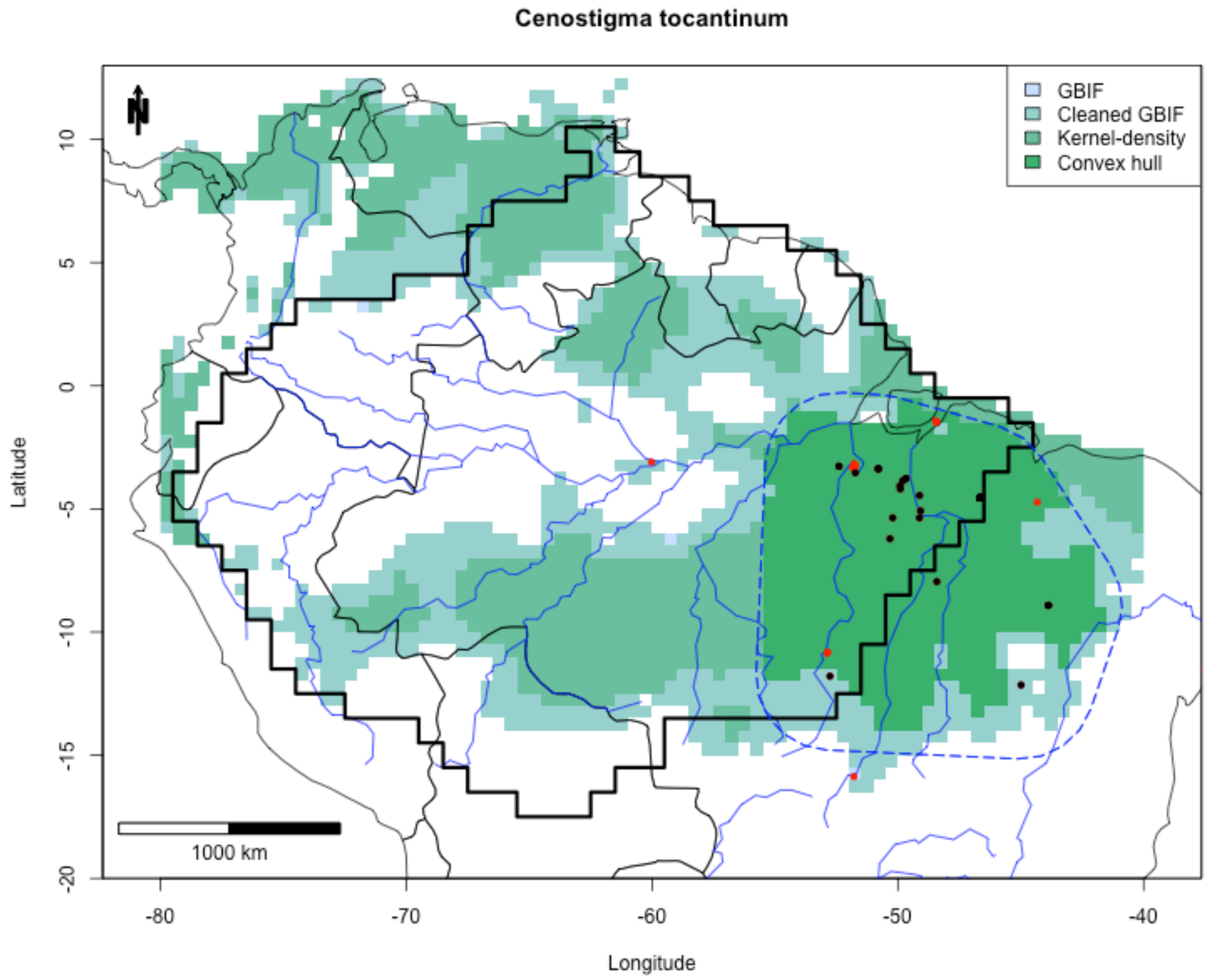
Cenostigma tocantinum
GBIF
Cleaned GBIF
Kernel-density
Convex hull
N
1000 km
Latitude
Longitude
10
5
0
-5
-10
-15
-20
-80
-70
-60
-50
-40

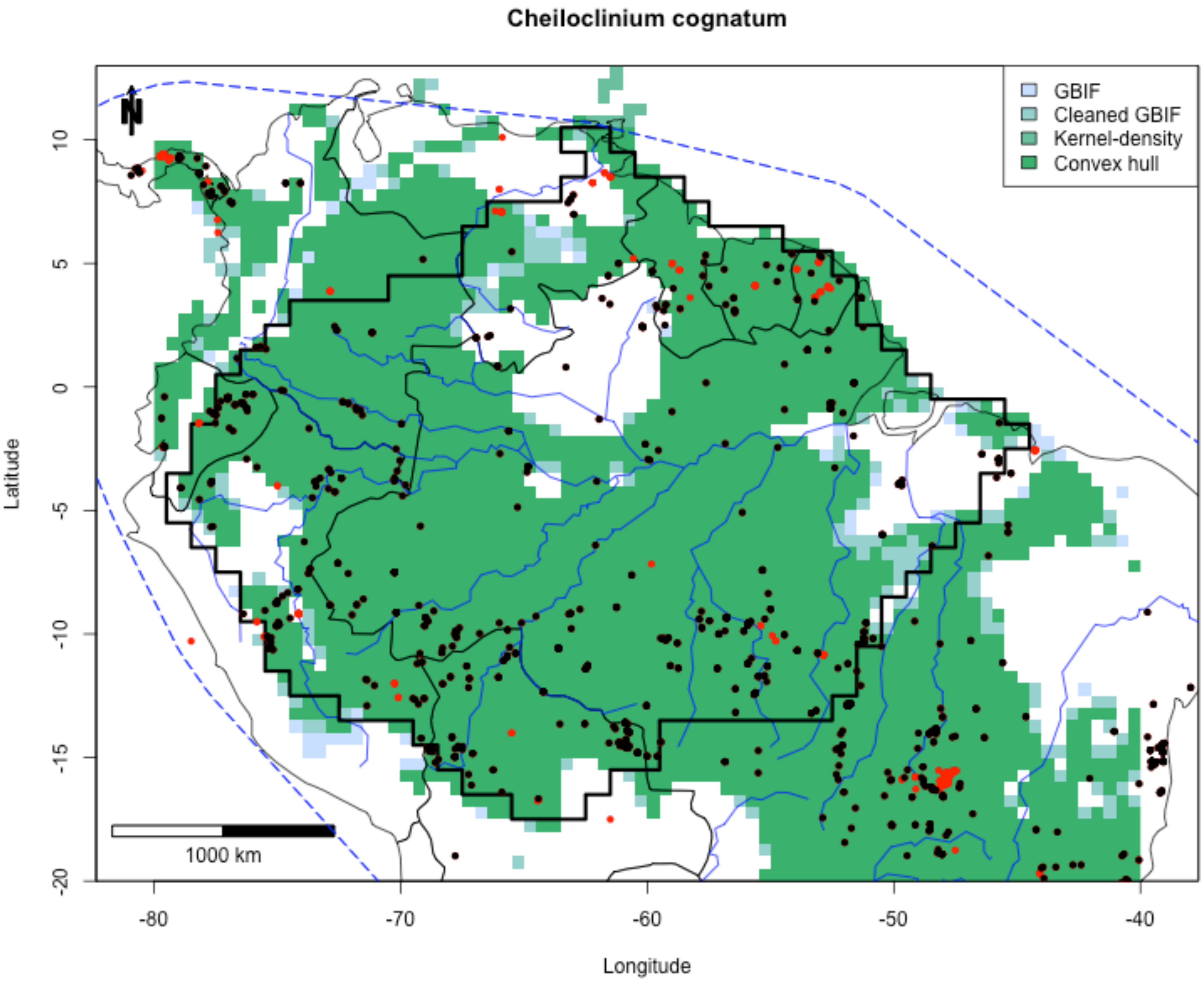

Cheiloclinium cognatum
GBIF
Cleaned GBIF
Kernel-density
Convex hull
N
1000 km
Latitude
Longitude
10
5
0
-5
-10
-15
-20
-80
-70
-60
-50
-40

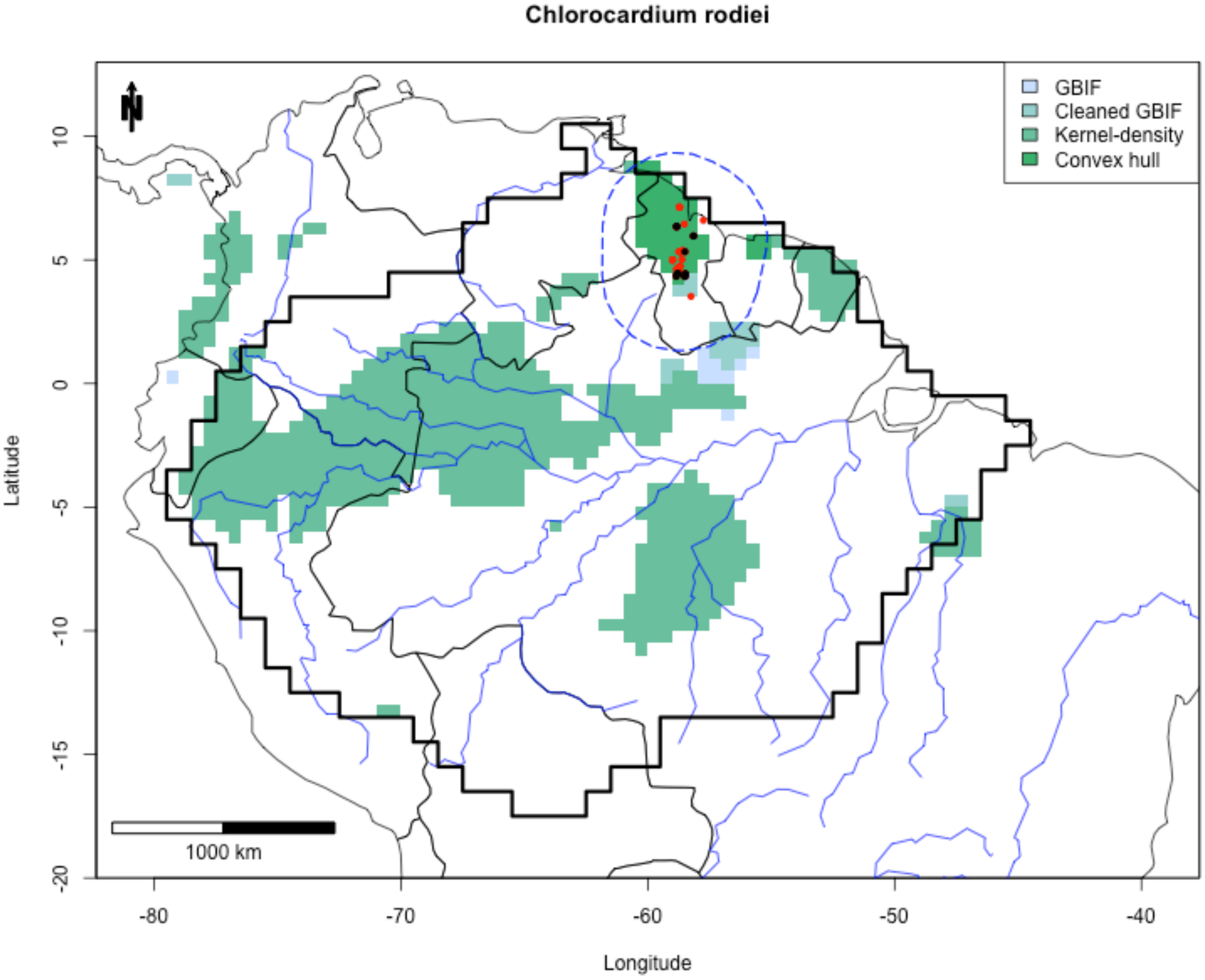

Chlorocardium rodiei
GBIF
Cleaned GBIF
Kernel-density
Convex hull
N
1000 km
Latitude
Longitude
10
5
0
-5
-10
-15
-20
-80
-70
-60
-50
-40

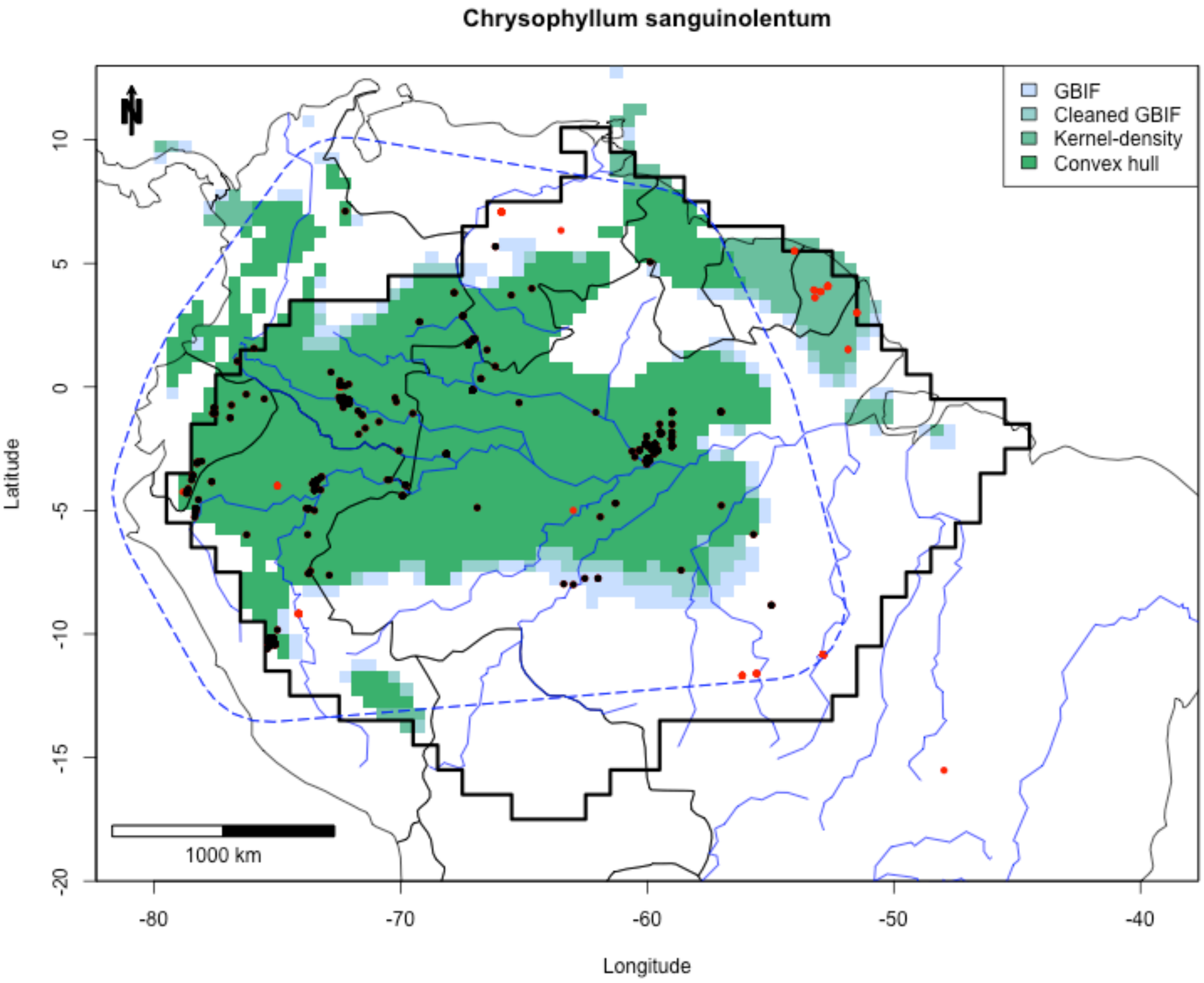

Chrysophyllum sanguinolentum
GBIF
Cleaned GBIF
Kernel-density
Convex hull
1000 km
Latitude
Longitude
10
5
0
-5
-10
-15
-20
-80
-70
-60
-50
-40

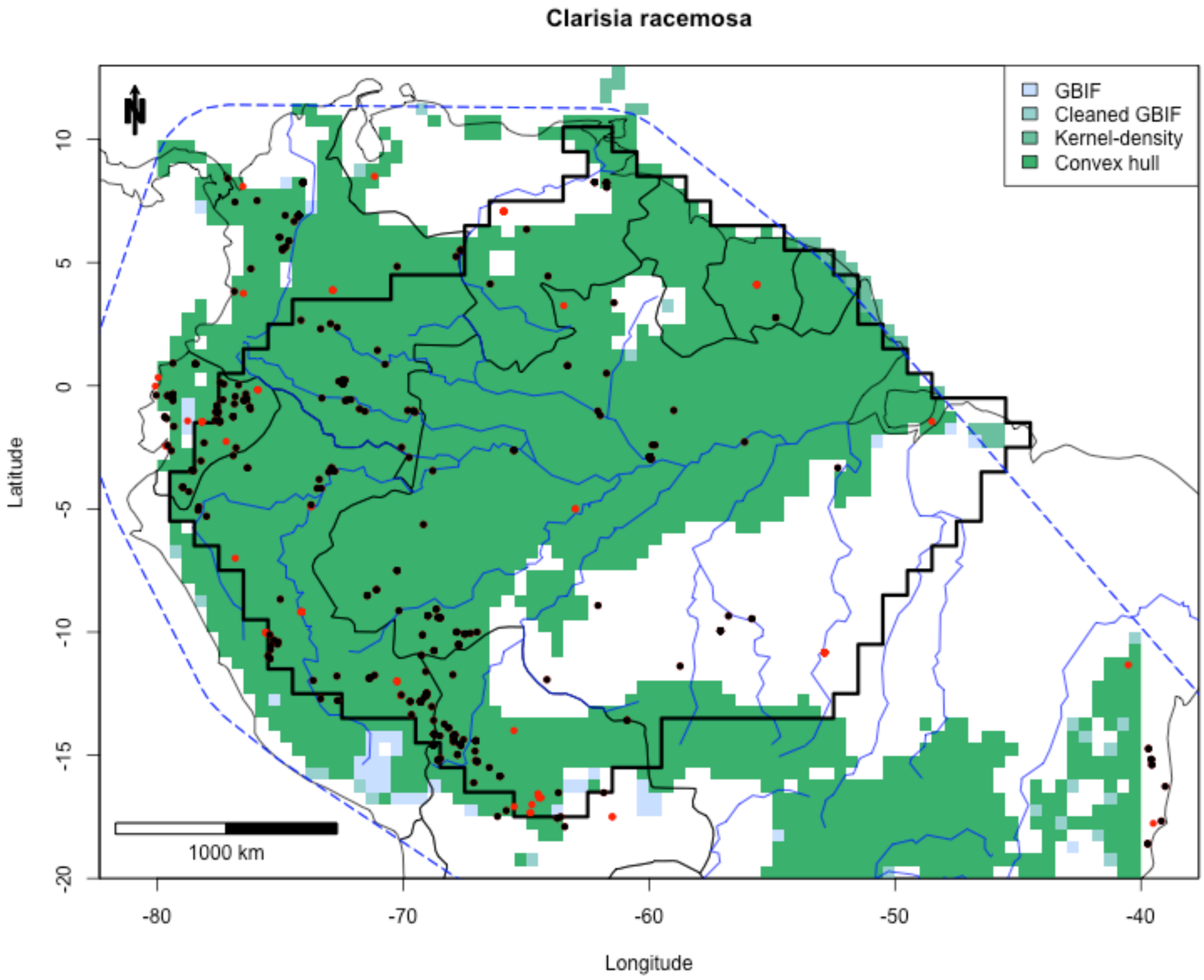
Clarisia racemosa
GBIF
Cleaned GBIF
Kernel-density
Convex hull
N
1000 km
Latitude
Longitude
10
5
0
-5
-10
-15
-20
-80
-70
-60
-50
-40

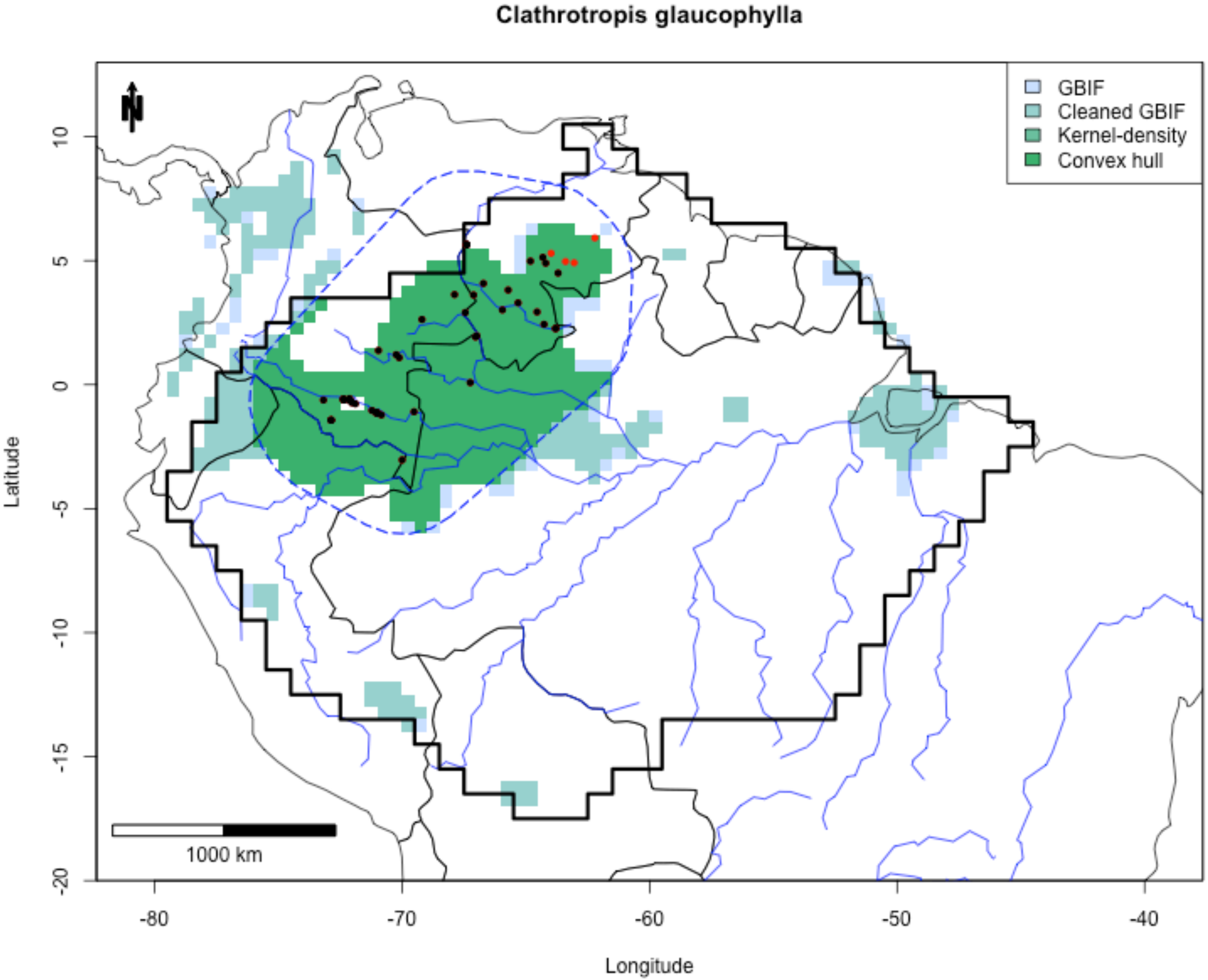

Clathrotropis glaucophylla
GBIF
Cleaned GBIF
Kernel-density
Convex hull
N
1000 km
Latitude
Longitude
10
5
0
-5
-10
-15
-20
-80
-70
-60
-50
-40

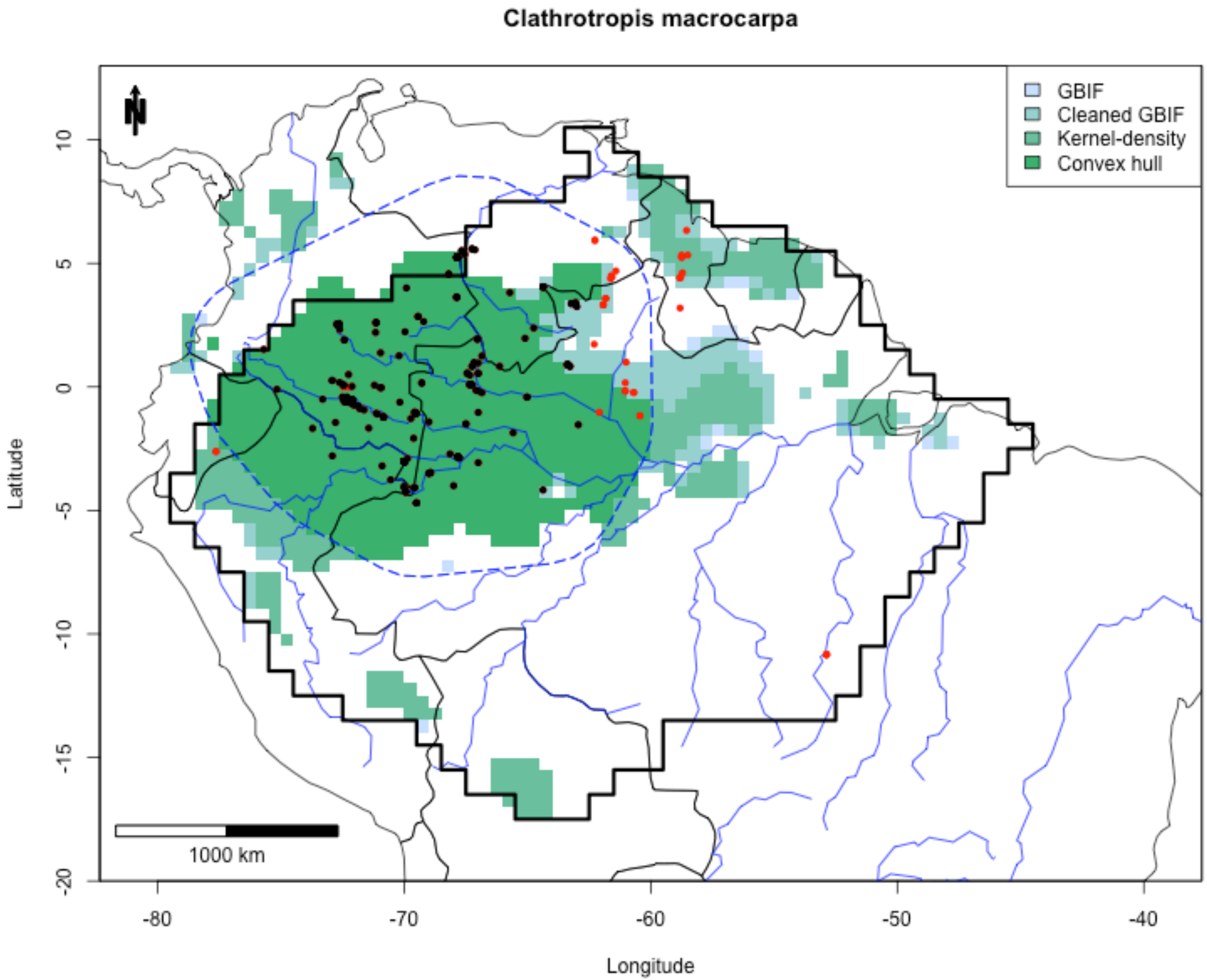

Clathrotropis macrocarpa
GBIF
Cleaned GBIF
Kernel-density
Convex hull
N
1000 km
Latitude
Longitude
10
5
0
-5
-10
-15
-20
-80
-70
-60
-50
-40

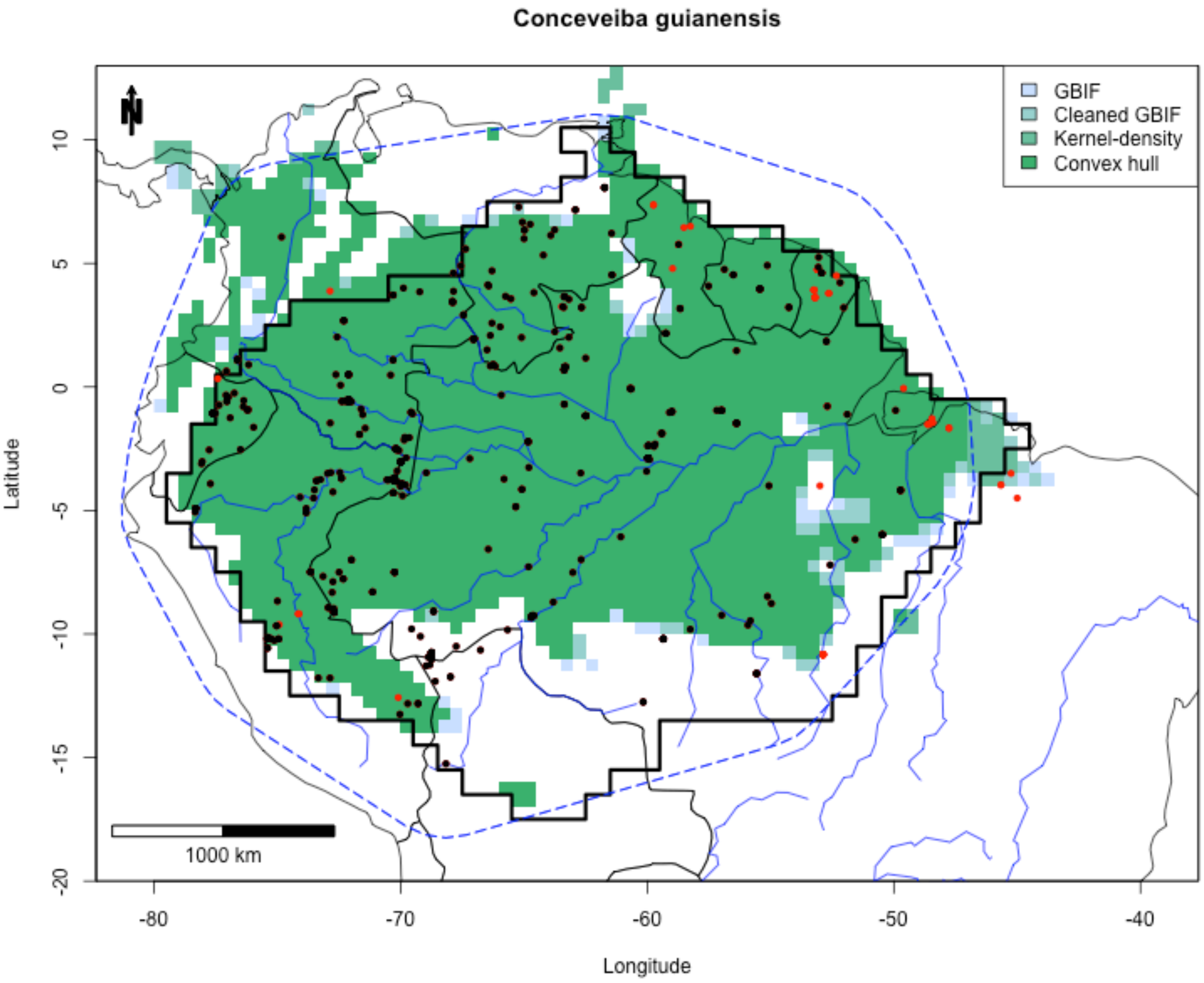

Conceveiba guianensis
GBIF
Cleaned GBIF
Kernel-density
Convex hull
N
1000 km
Latitude
Longitude
10
5
0
-5
-10
-15
-20
-80
-70
-60
-50
-40

Cordia bicolor

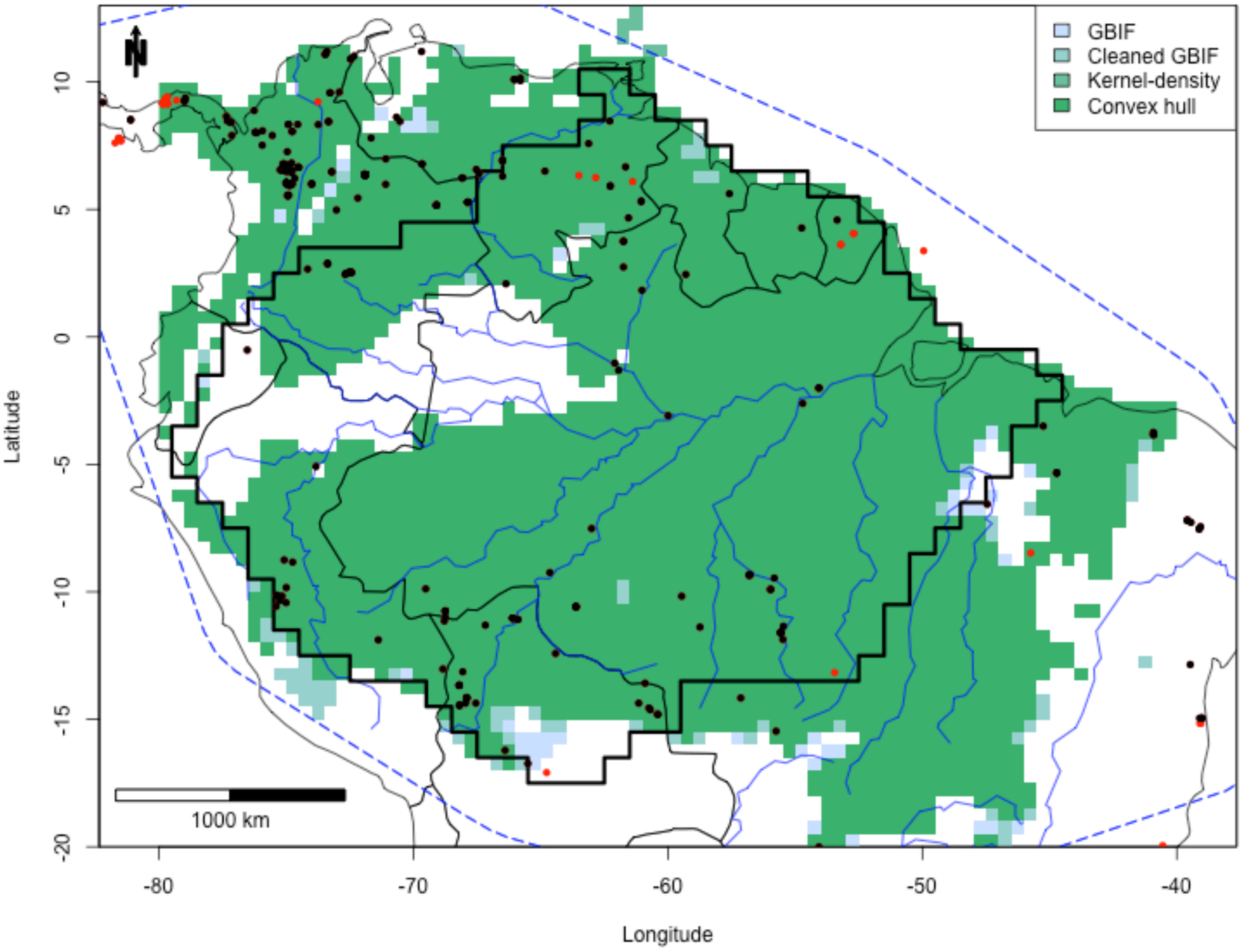

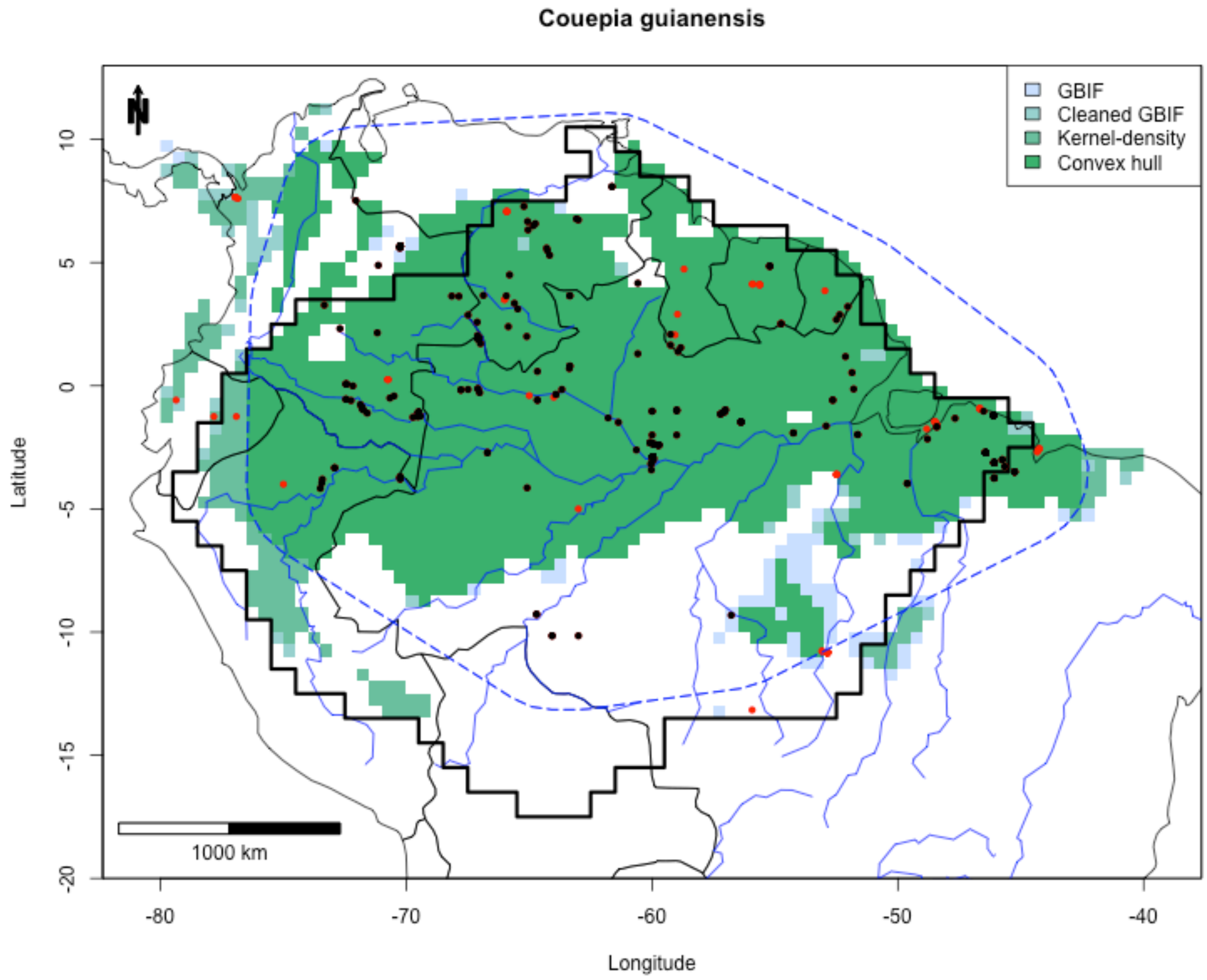
Couepia guianensis
GBIF
Cleaned GBIF
Kernel-density
Convex hull
N
1000 km
Latitude
Longitude
10
5
0
-5
-10
-15
-20
-80
-70
-60
-50
-40

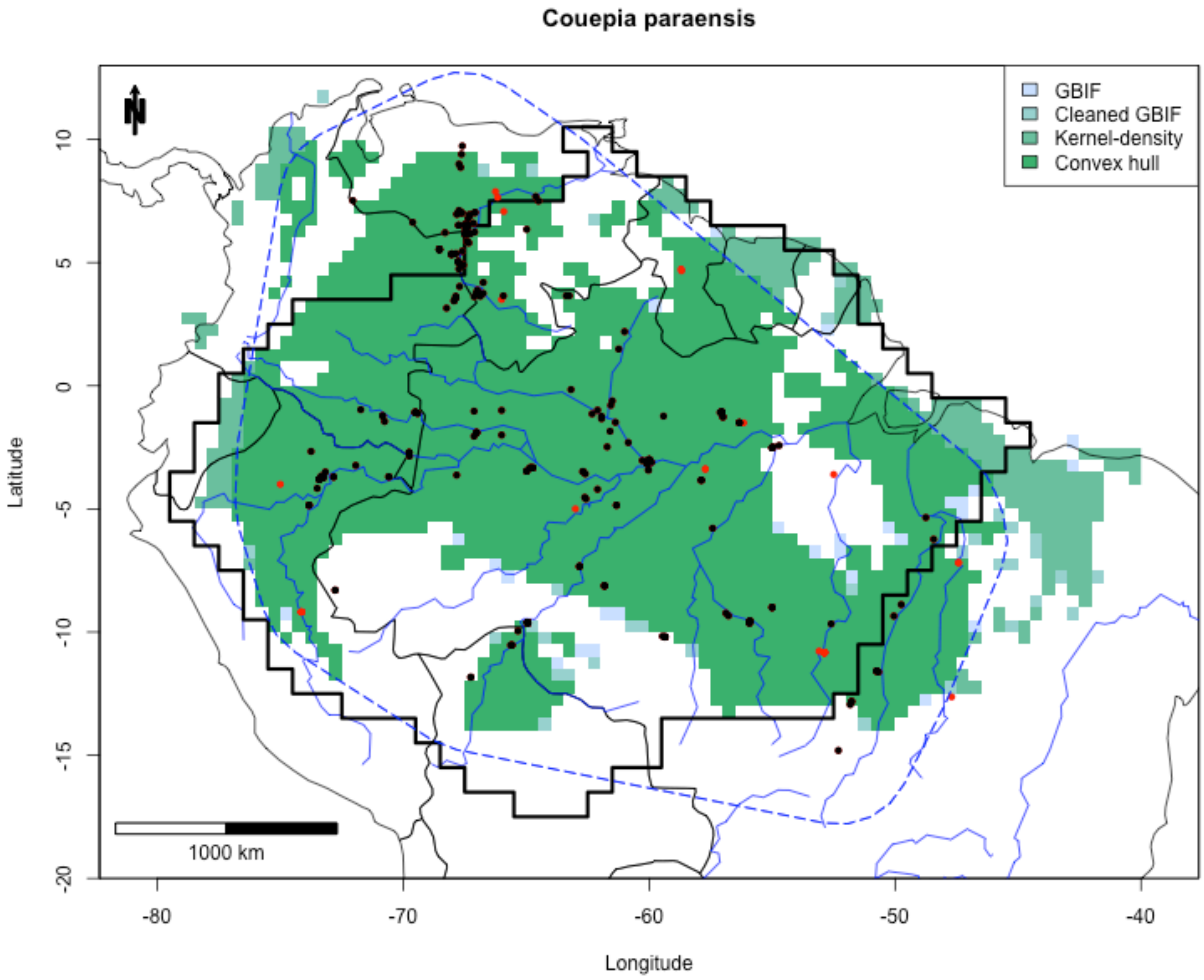
Couepia paraensis
GBIF
Cleaned GBIF
Kernel-density
Convex hull
N
1000 km
Latitude
Longitude
10
5
0
-5
-10
-15
-20
-80
-70
-60
-50
-40

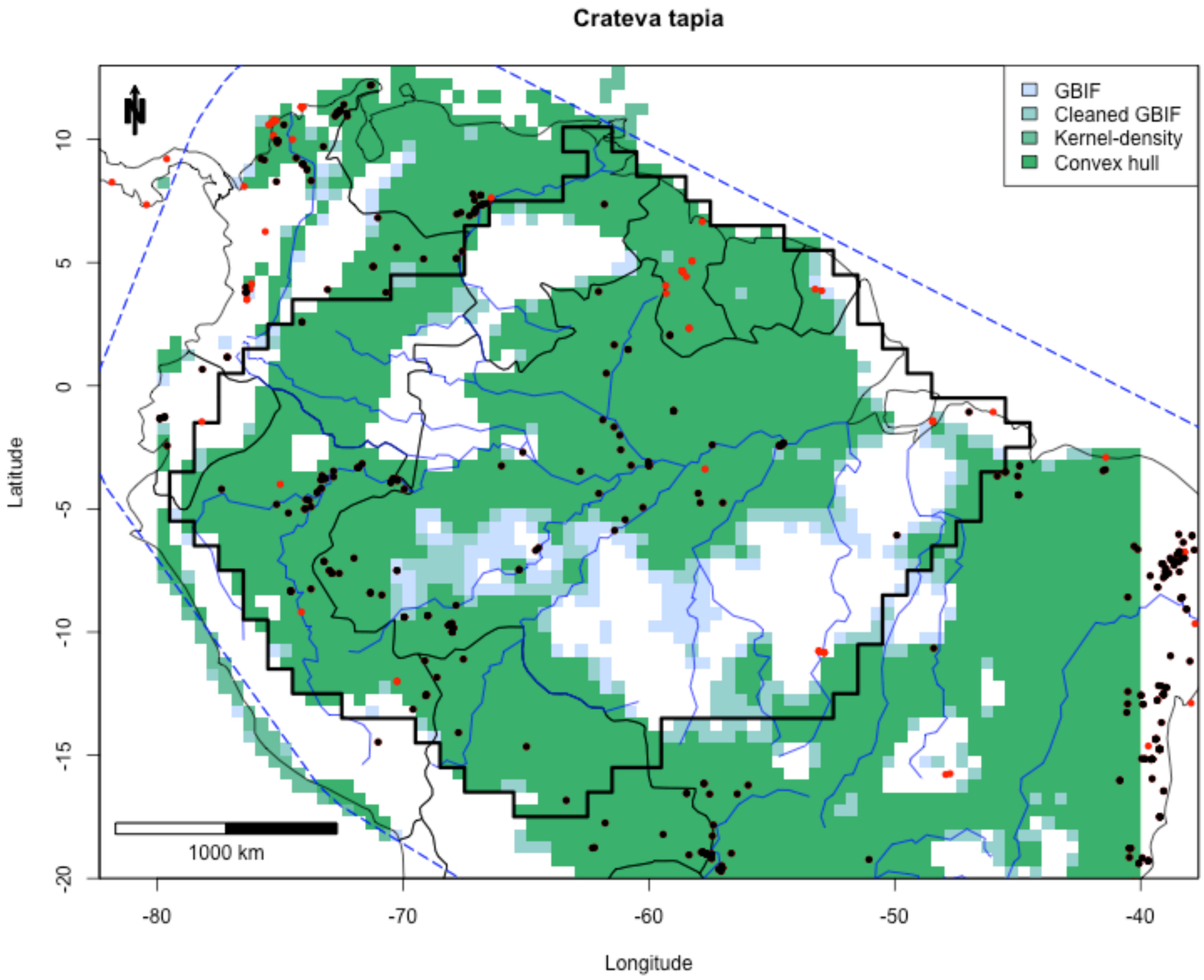

Crateva tapia
GBIF
Cleaned GBIF
Kernel-density
Convex hull
N
1000 km
Latitude
Longitude
10
5
0
-5
-10
-15
-20
-80
-70
-60
-50
-40

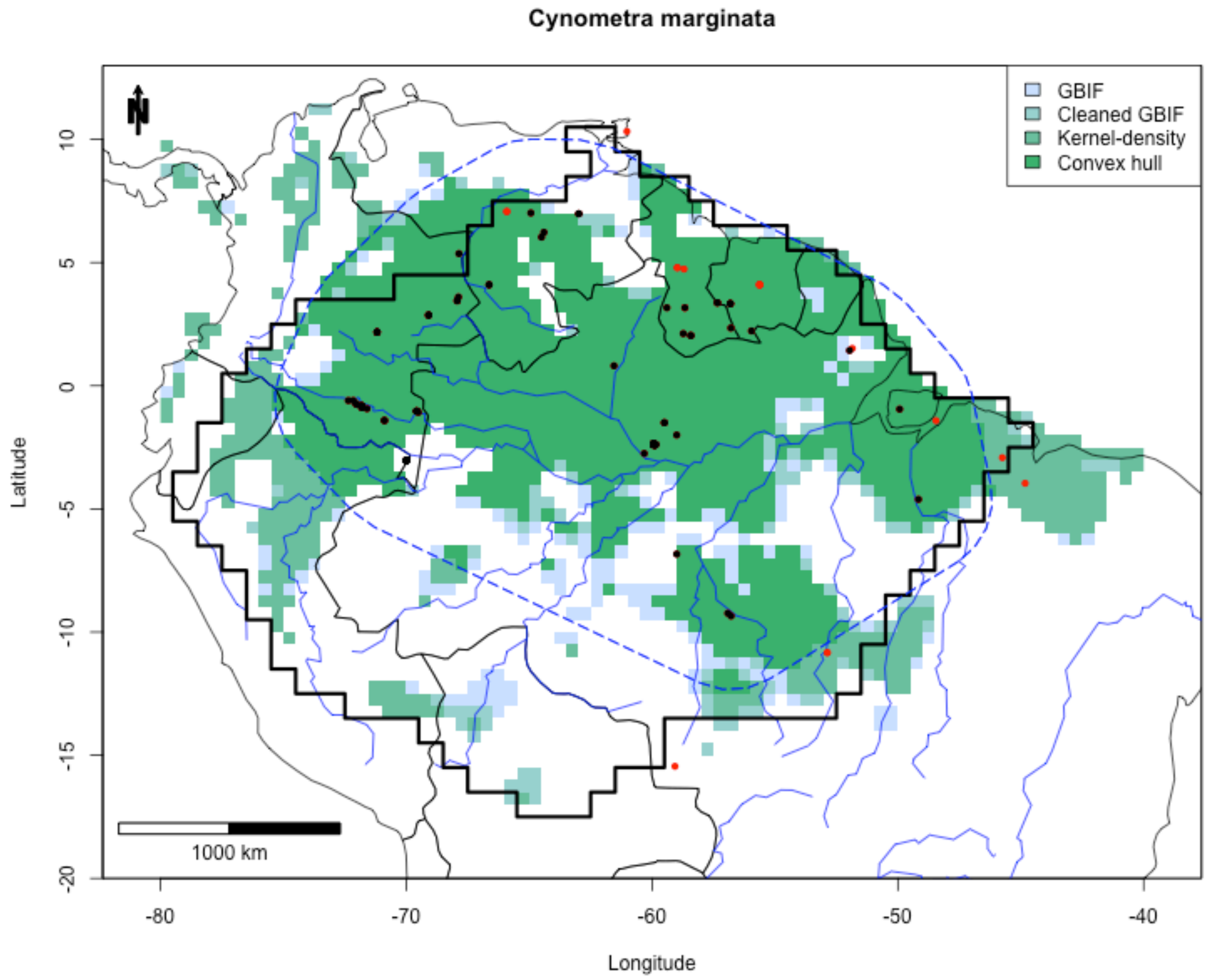
Cynometra marginata
GBIF
Cleaned GBIF
Kernel-density
Convex hull
N
1000 km
Latitude
Longitude
10
5
0
-5
-10
-15
-20
-80
-70
-60
-50
-40

Dialium guianense

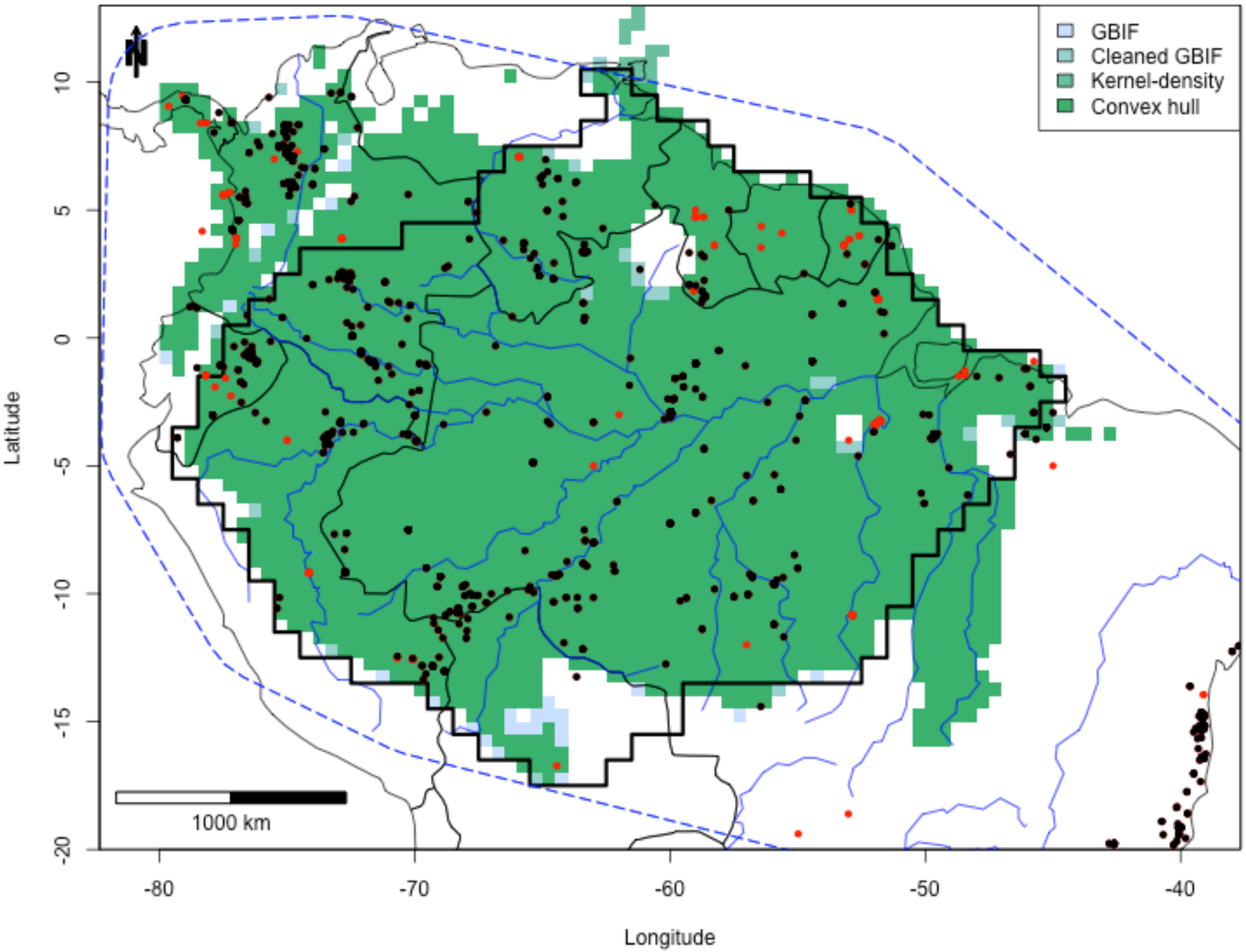

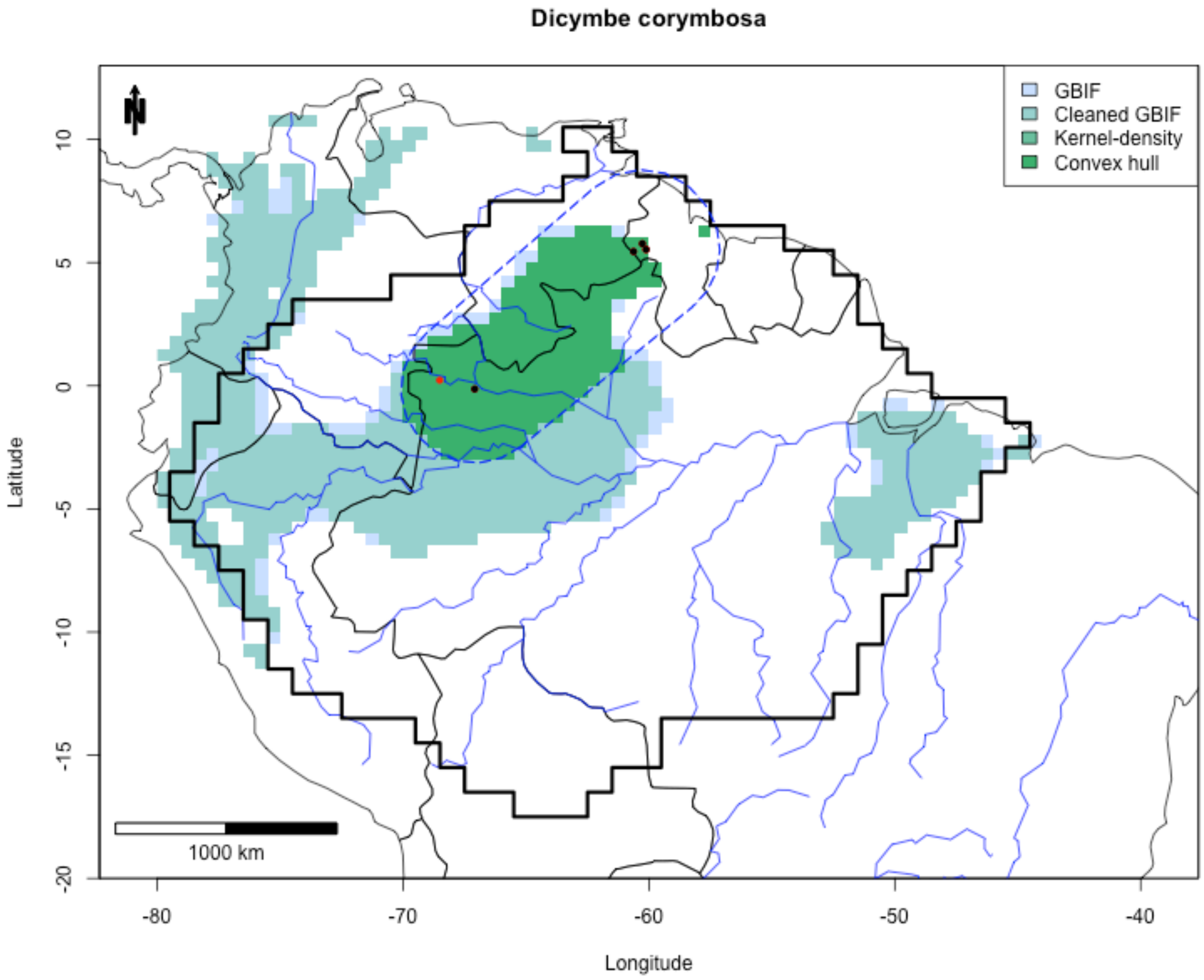

Dicymbe corymbosa
GBIF
Cleaned GBIF
Kernel-density
Convex hull
N
1000 km
Latitude
Longitude
10
5
0
-5
-10
-15
-20
-80
-70
-60
-50
-40

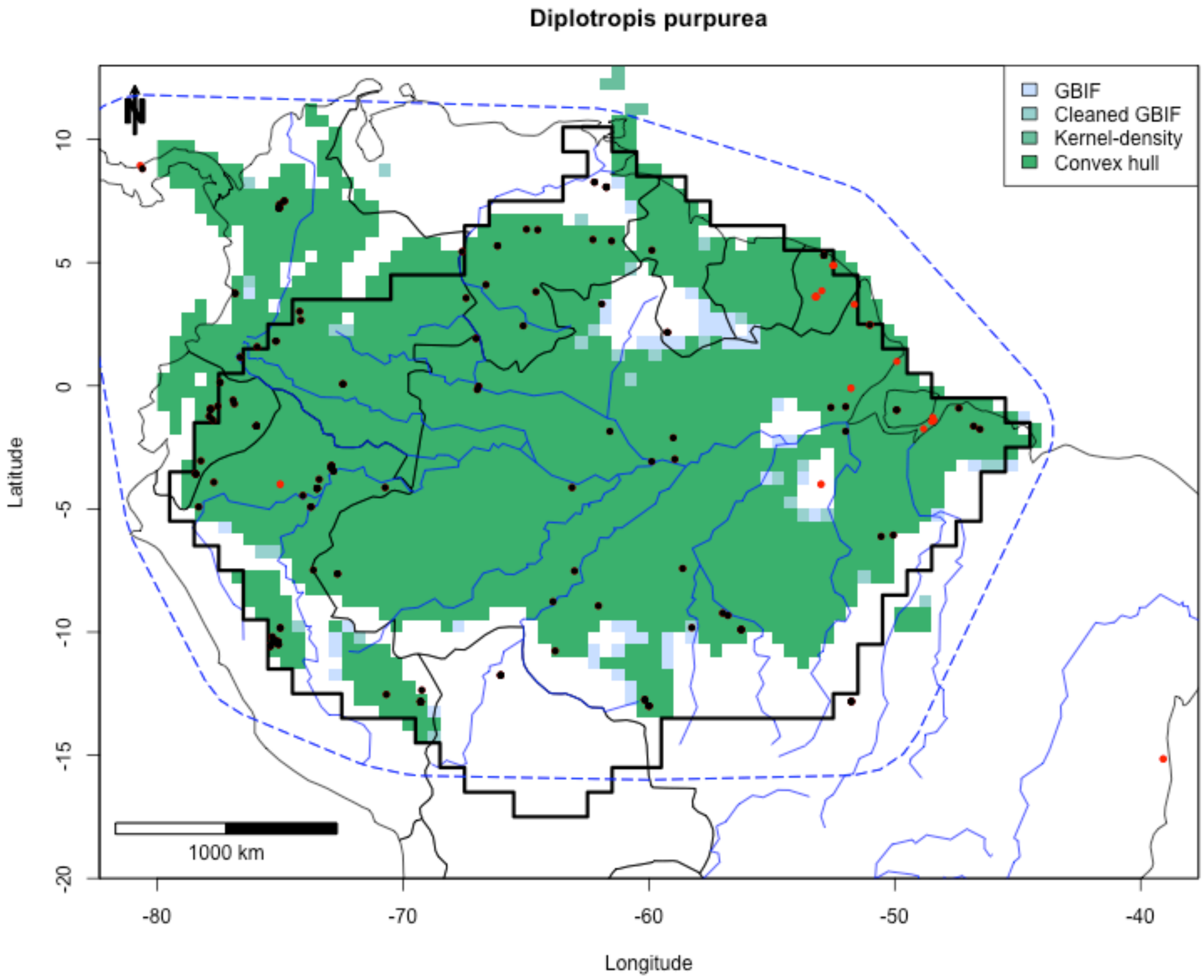
Diplotropis purpurea
GBIF
Cleaned GBIF
Kernel-density
Convex hull
N
1000 km
Latitude
Longitude
10
5
0
-5
-10
-15
-20
-80
-70
-60
-50
-40

Drypetes amazonica

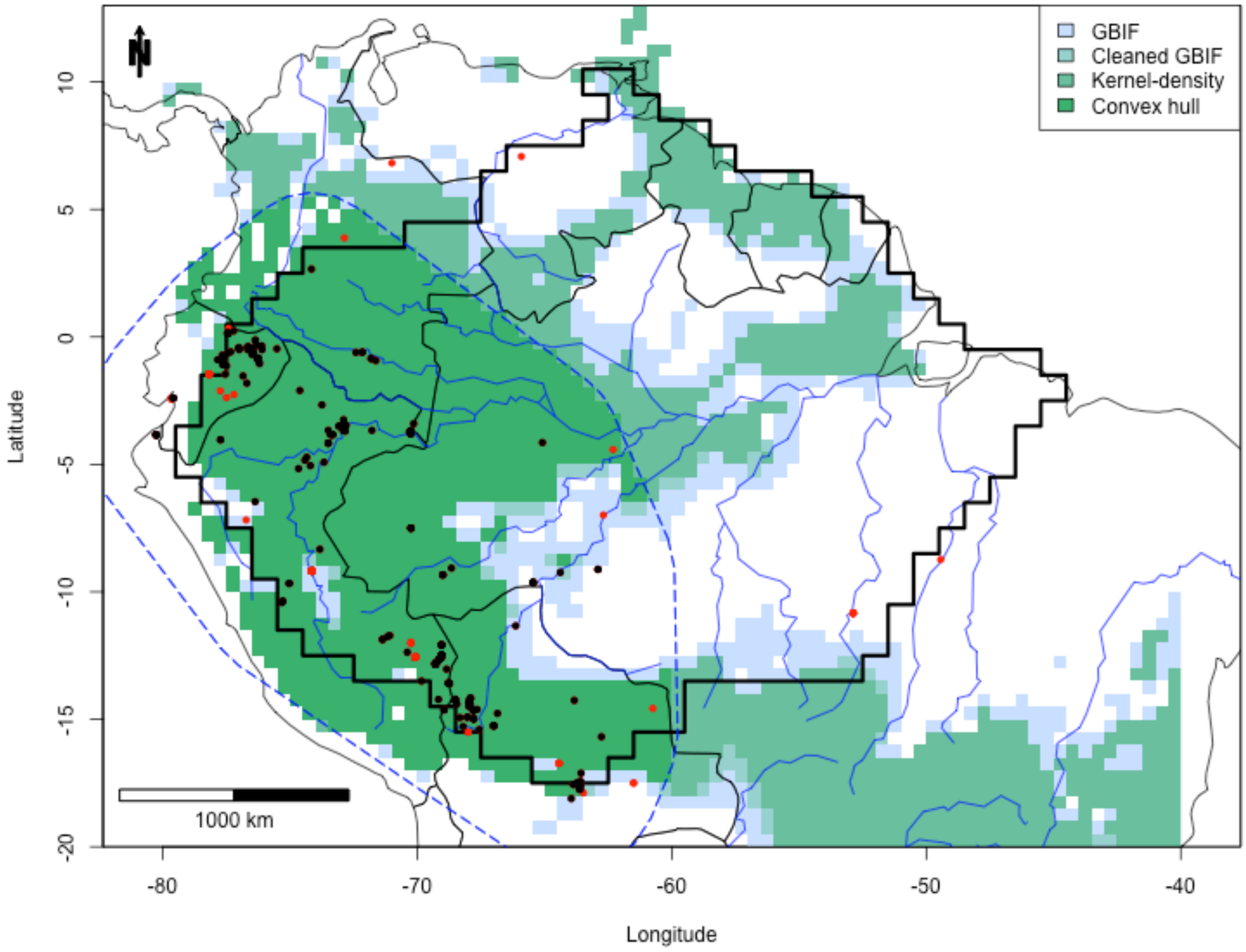

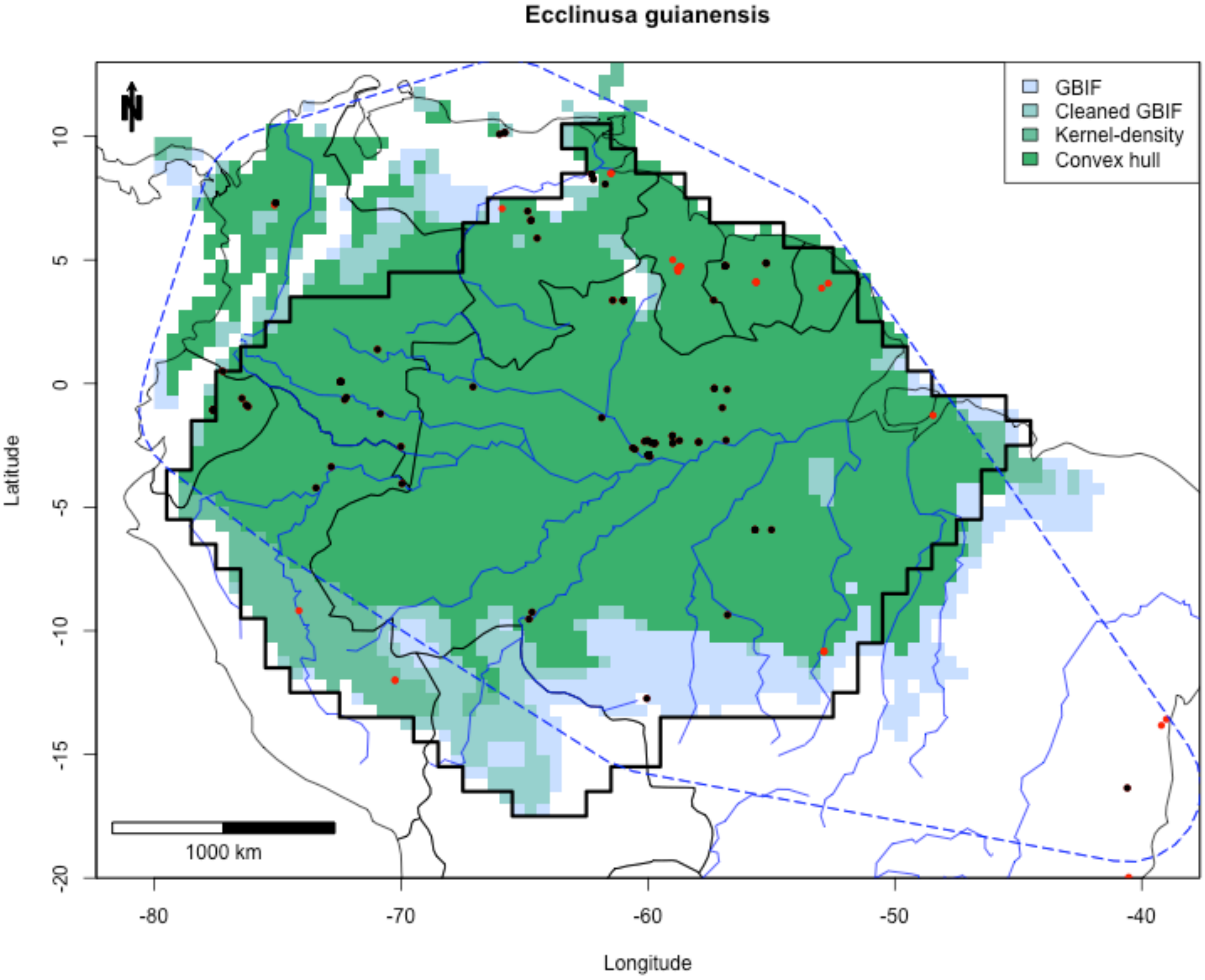
Ecclinusa guianensis
GBIF
Cleaned GBIF
Kernel-density
Convex hull
N
1000 km
Latitude
Longitude
10
5
0
-5
-10
-15
-20
-80
-70
-60
-50
-40

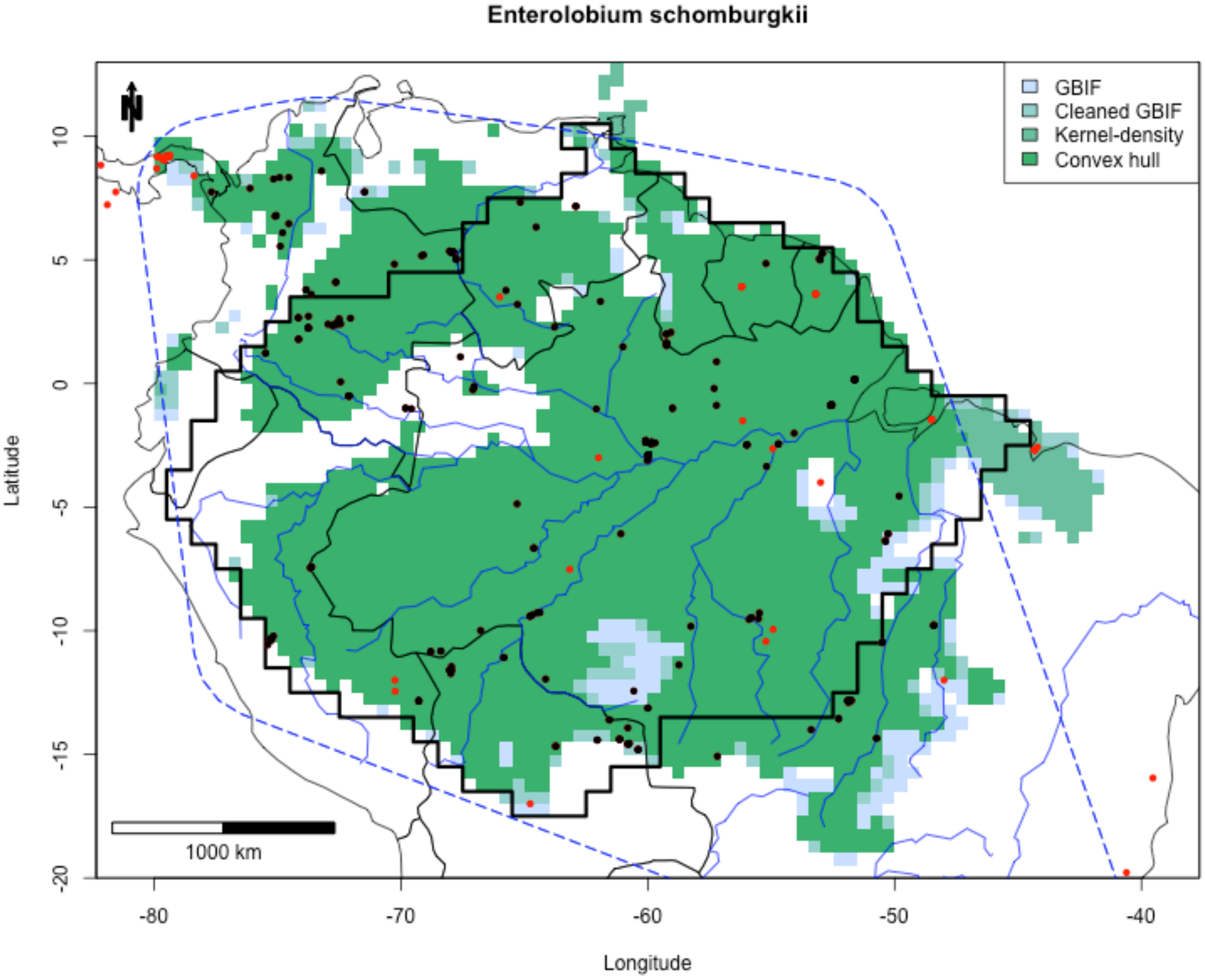

Enterolobium schomburgkii
GBIF
Cleaned GBIF
Kernel-density
Convex hull
N
1000 km
Latitude
Longitude
10
5
0
-5
-10
-15
-20
-80
-70
-60
-50
-40

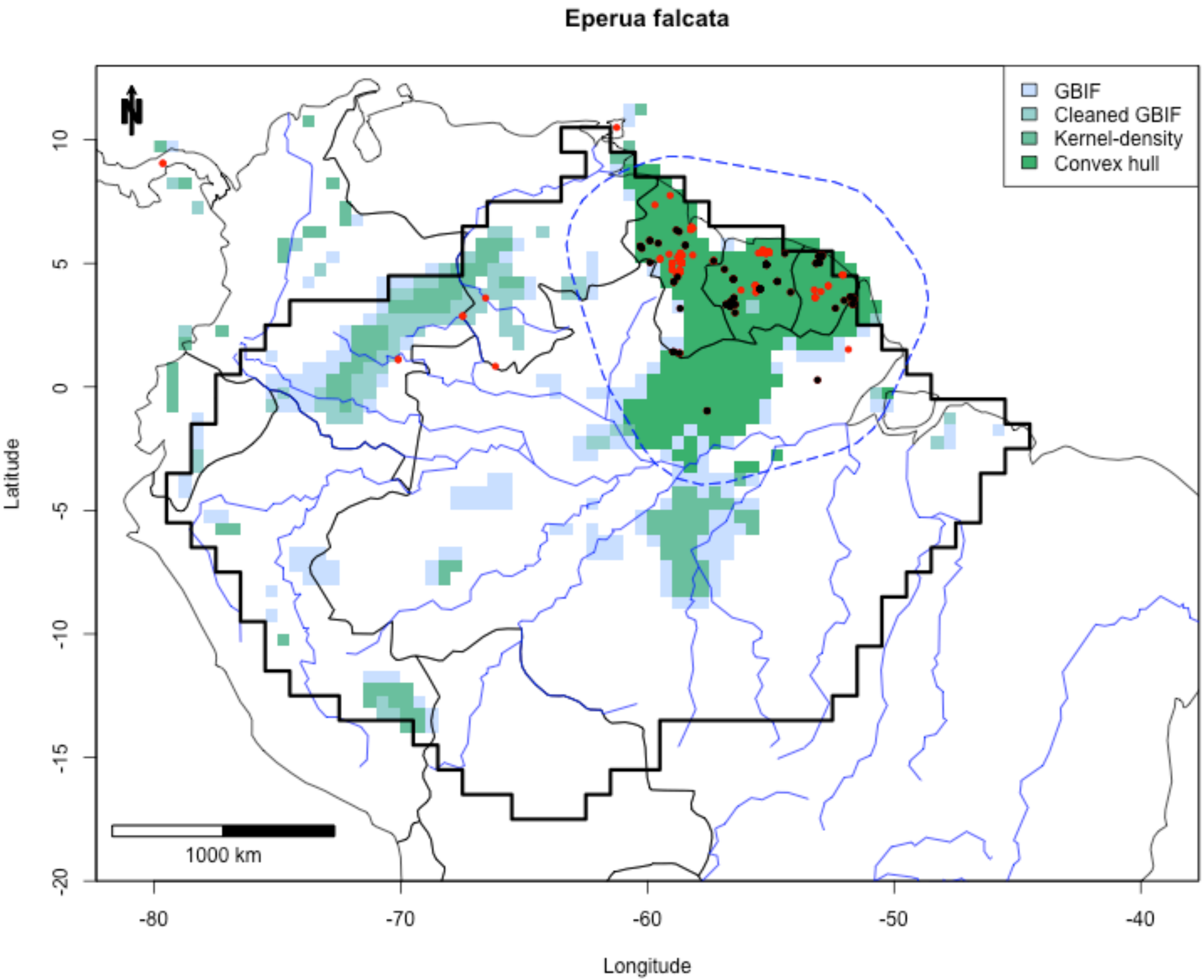

Eperua falcata
GBIF
Cleaned GBIF
Kernel-density
Convex hull
N
1000 km
Latitude
Longitude
10
5
0
-5
-10
-15
-20
-80
-70
-60
-50
-40

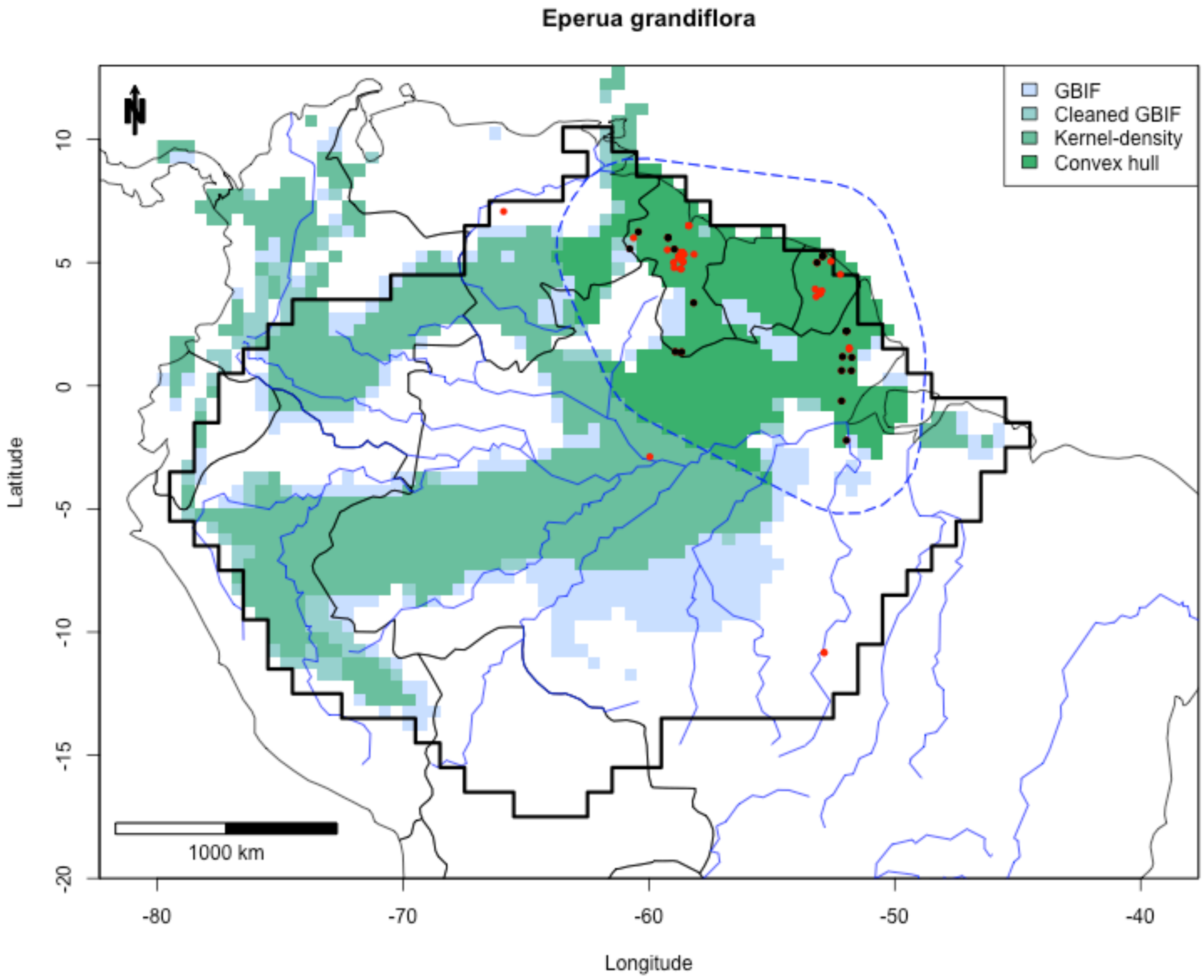
Eperua grandiflora
GBIF
Cleaned GBIF
Kernel-density
Convex hull
N
1000 km
Latitude
Longitude
10
5
0
-5
-10
-15
-20
-80
-70
-60
-50
-40

Eperua leucantha

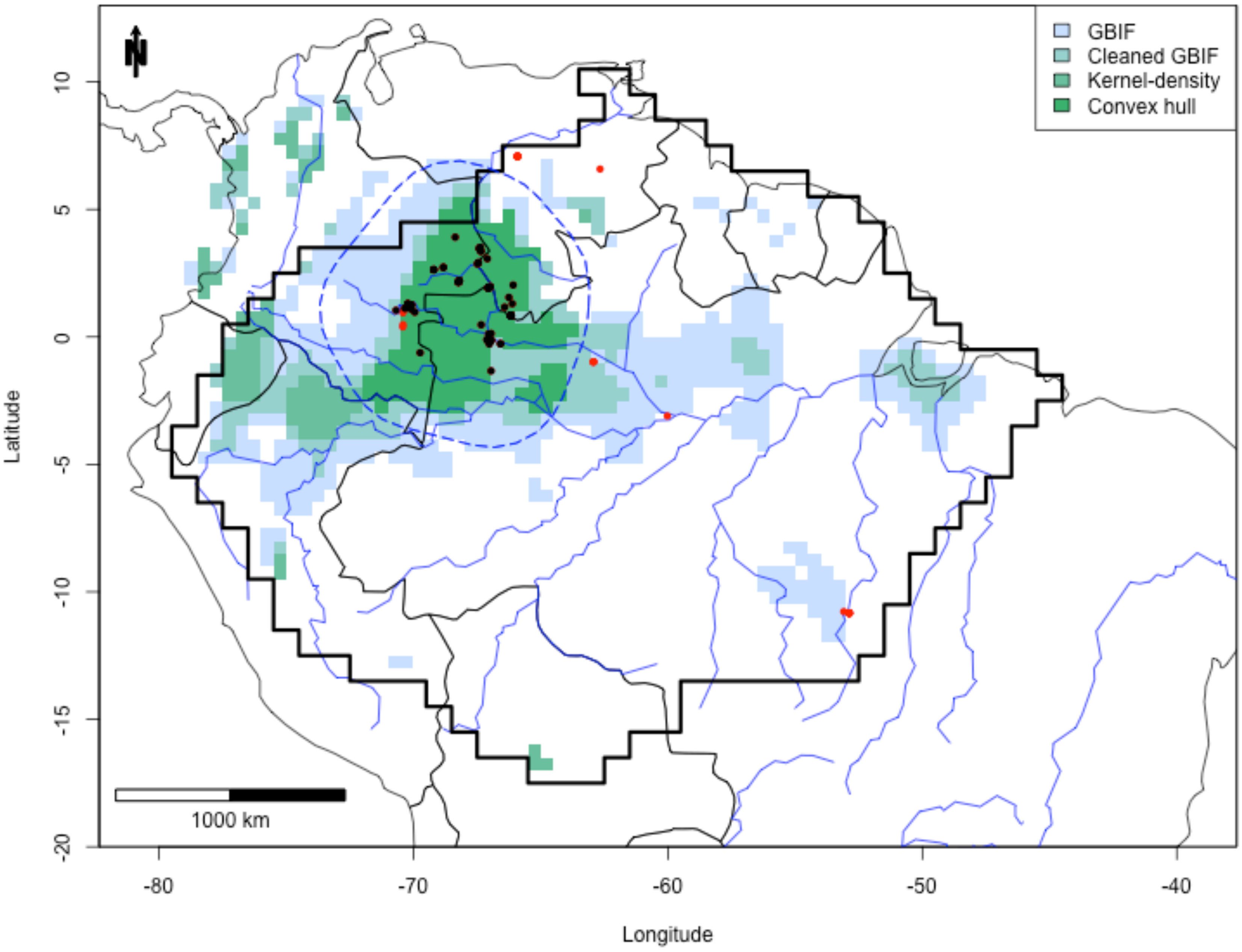

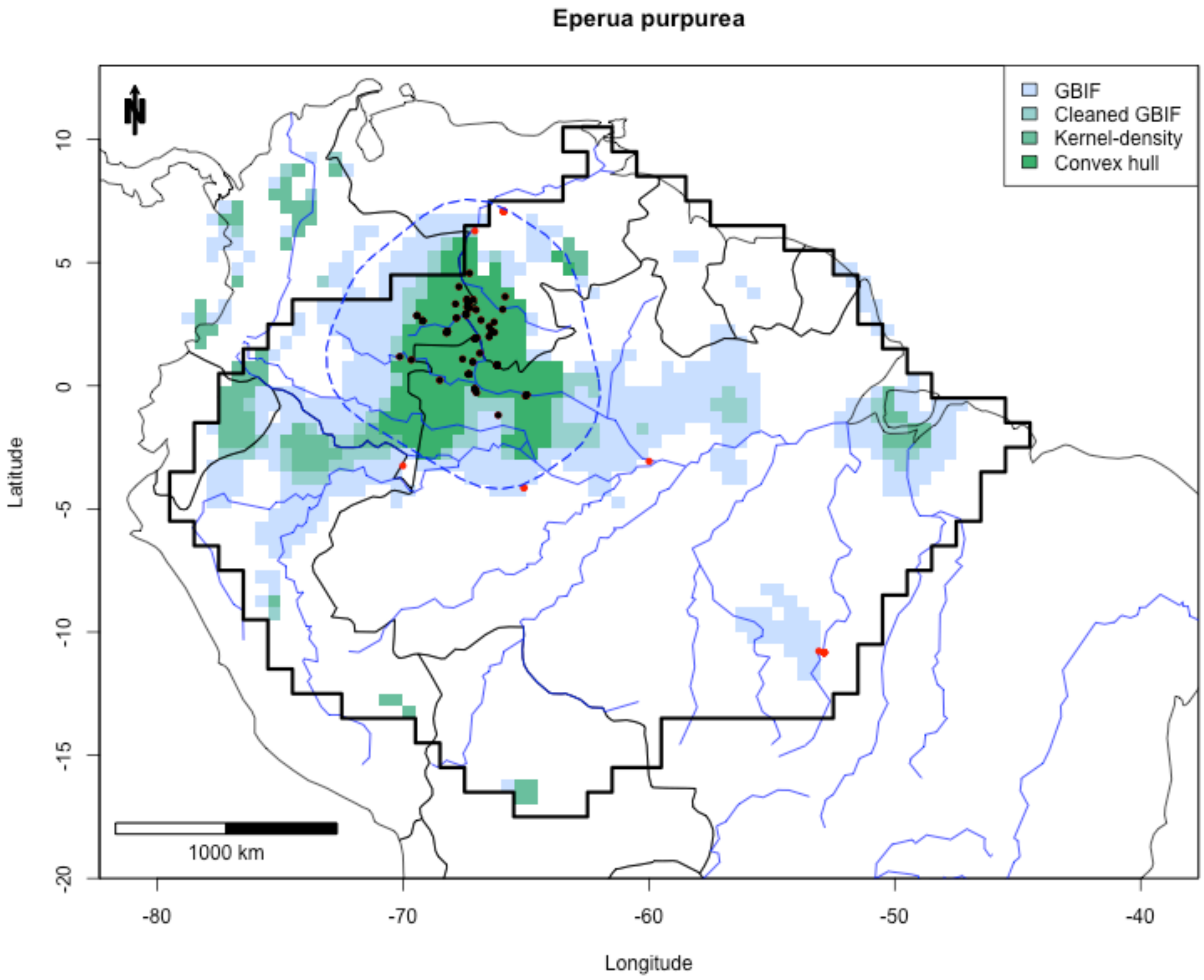
Eperua purpurea
GBIF
Cleaned GBIF
Kernel-density
Convex hull
N
10
5
0
-5
-10
-15
-20
Latitude
-80
-70
-60
-50
-40
Longitude
1000 km

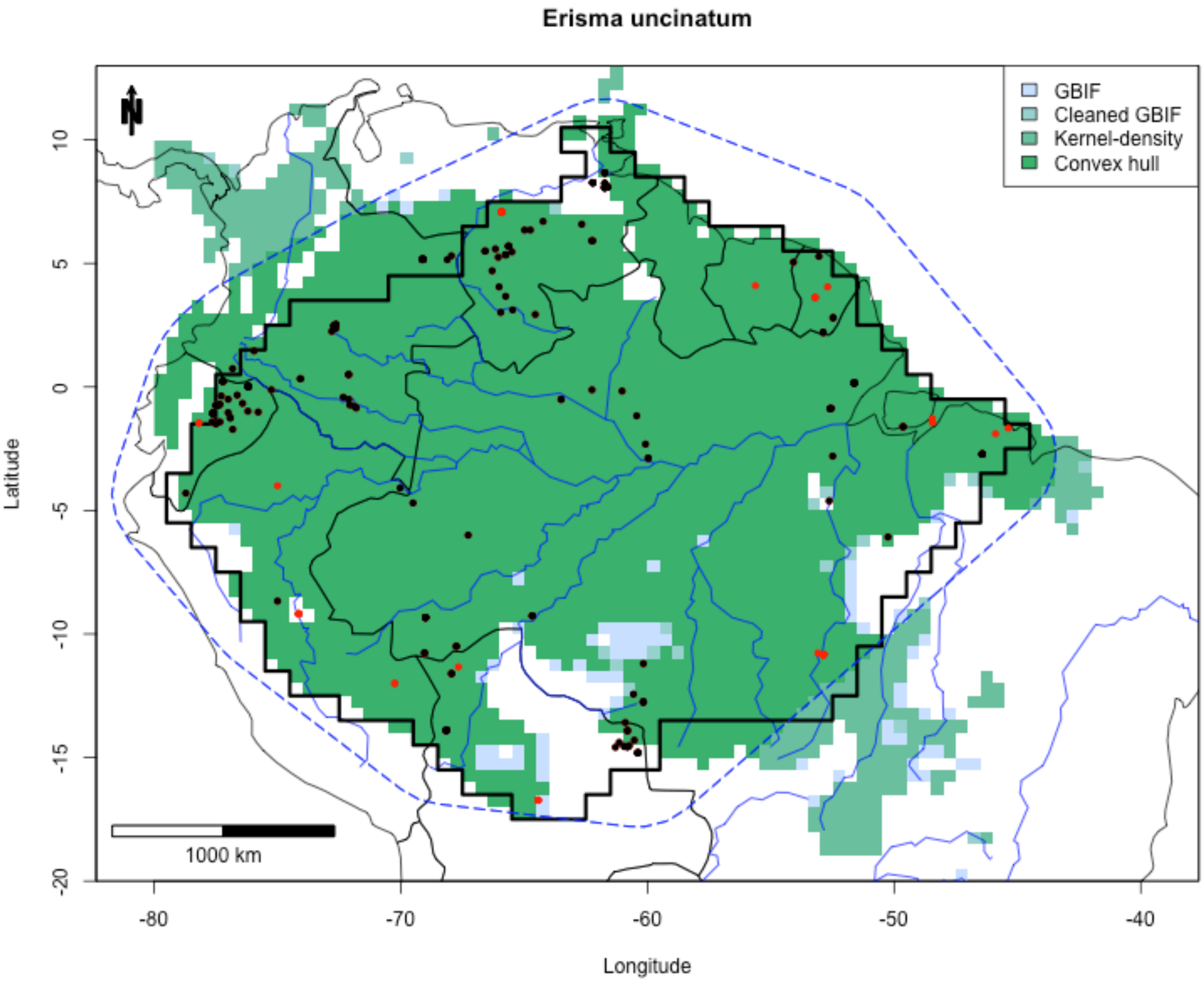

Erisma uncinatum
GBIF
Cleaned GBIF
Kernel-density
Convex hull
1000 km
Latitude
Longitude
10
5
0
-5
-10
-15
-20
-80
-70
-60
-50
-40

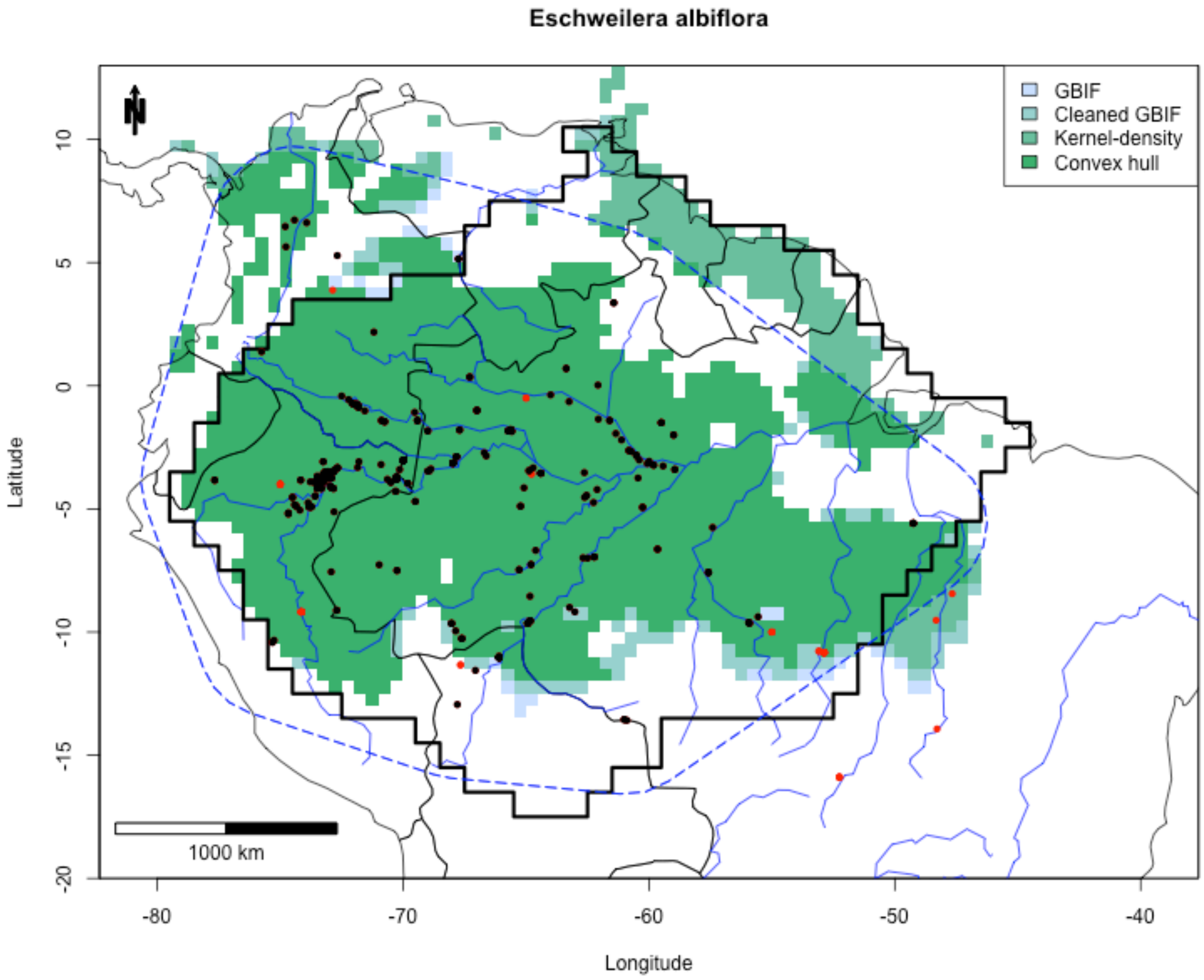
Eschweilera albiflora
GBIF
Cleaned GBIF
Kernel-density
Convex hull
10
5
0
-5
-10
-15
-20
Latitude
-80
-70
-60
-50
-40
Longitude
1000 km
N

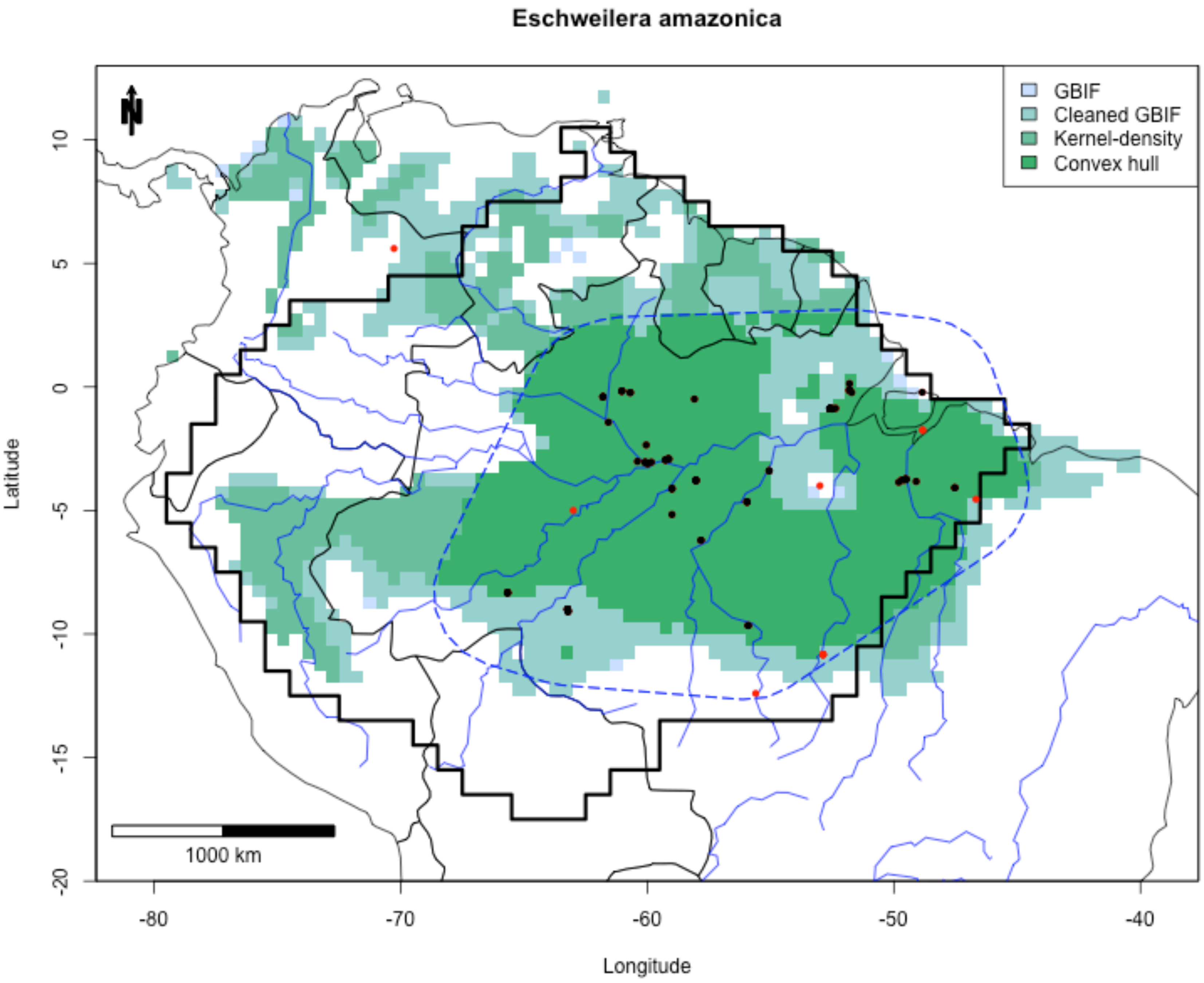
Eschweilera amazonica
GBIF
Cleaned GBIF
Kernel-density
Convex hull
1000 km
Latitude
Longitude
10
5
0
-5
-10
-15
-20
-80
-70
-60
-50
-40

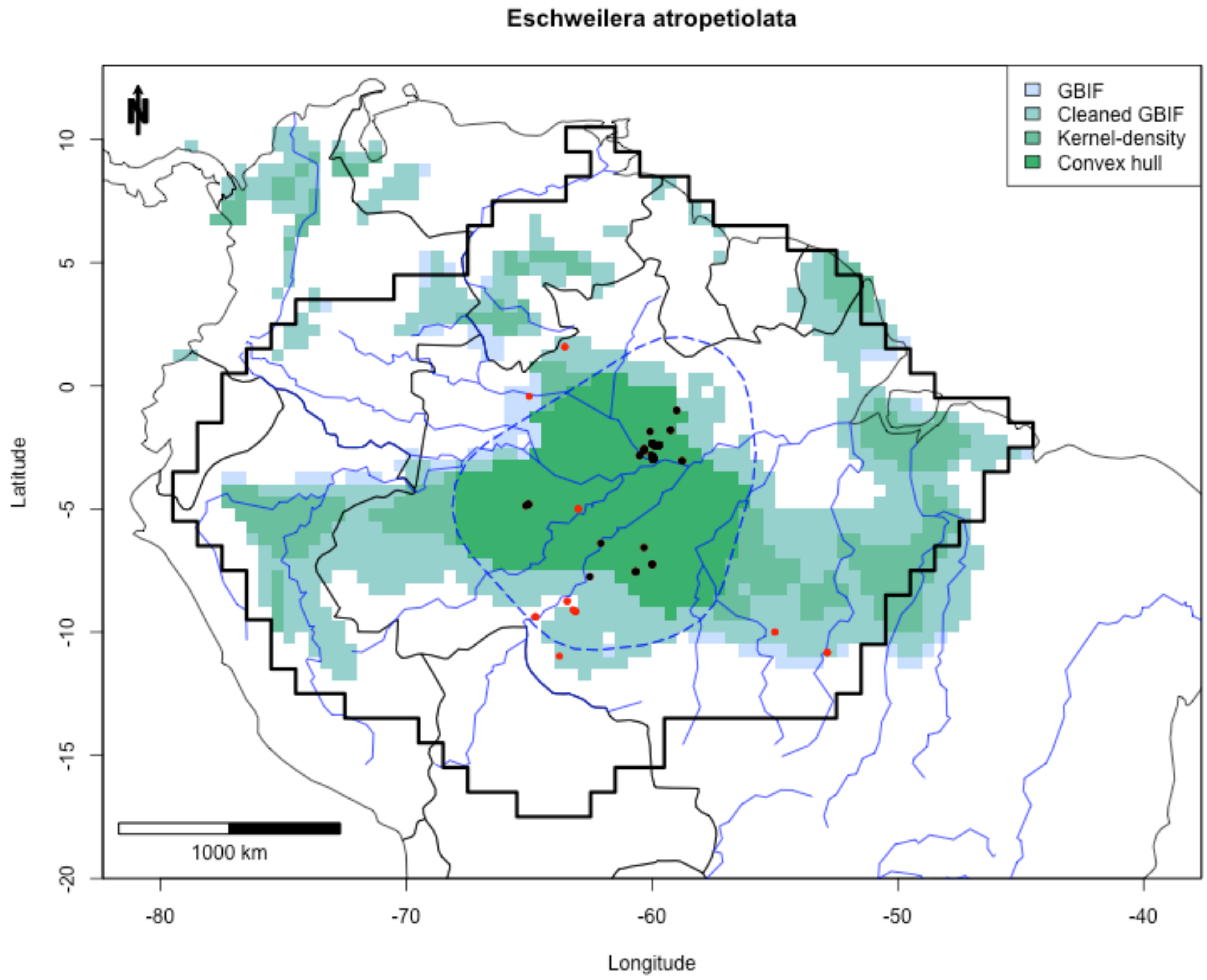
Eschweilera atropetiolata
GBIF
Cleaned GBIF
Kernel-density
Convex hull
N
1000 km
Latitude
Longitude
10
5
0
-5
-10
-15
-20
-80
-70
-60
-50
-40

Eschweilera bracteosa

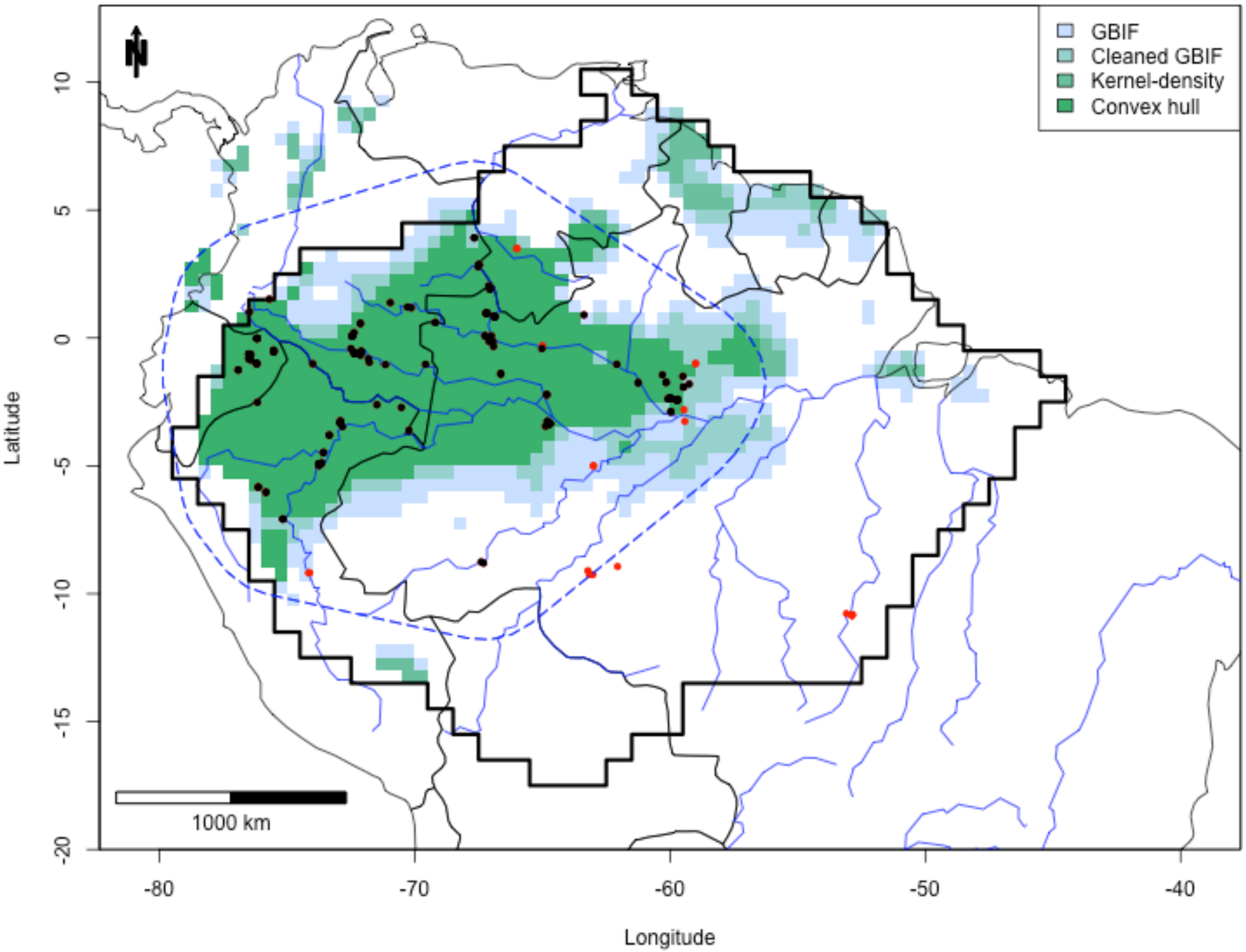

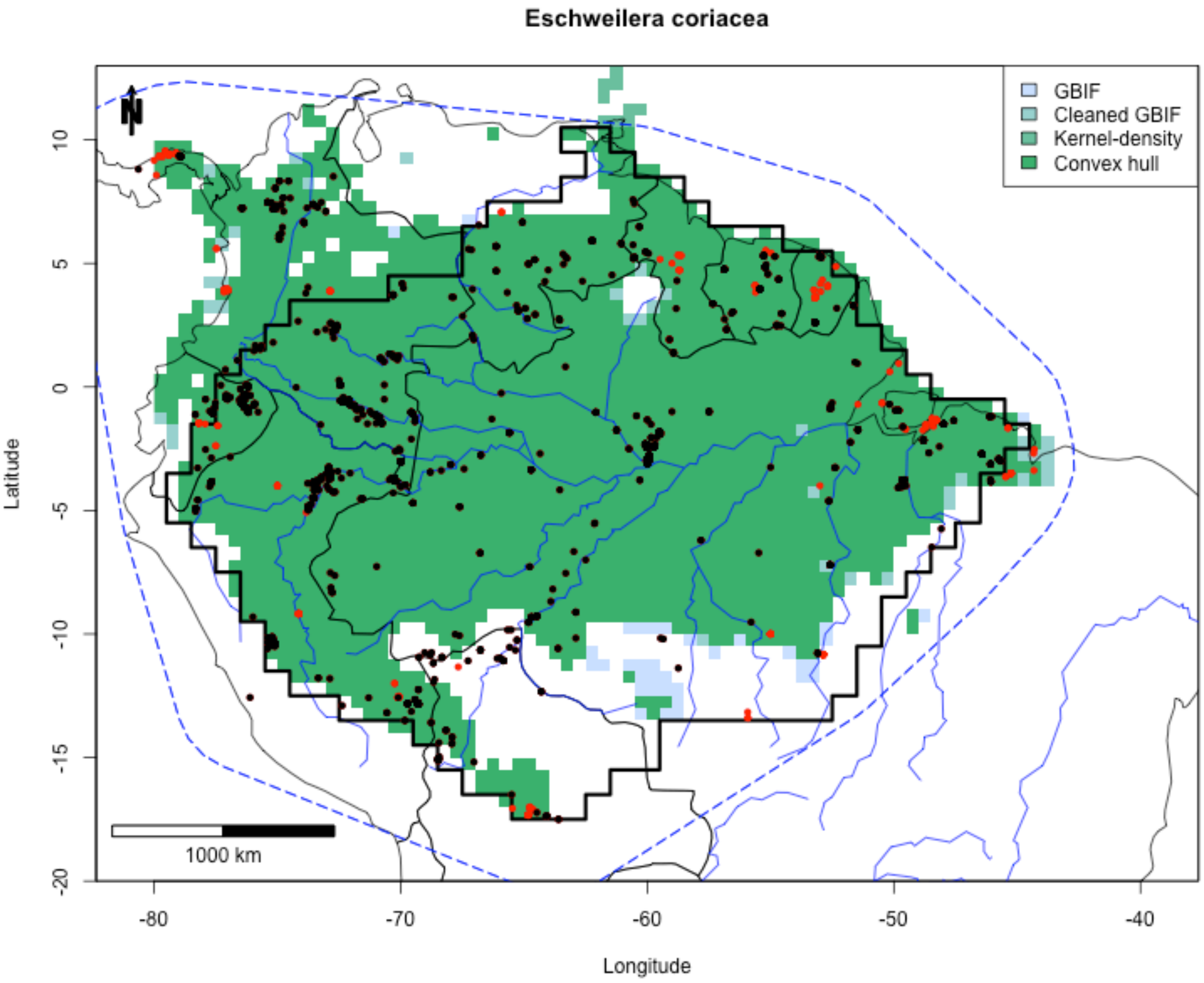
Eschweilera coriacea
GBIF
Cleaned GBIF
Kernel-density
Convex hull
N
1000 km
Latitude
Longitude
10
5
0
-5
-10
-15
-20
-80
-70
-60
-50
-40

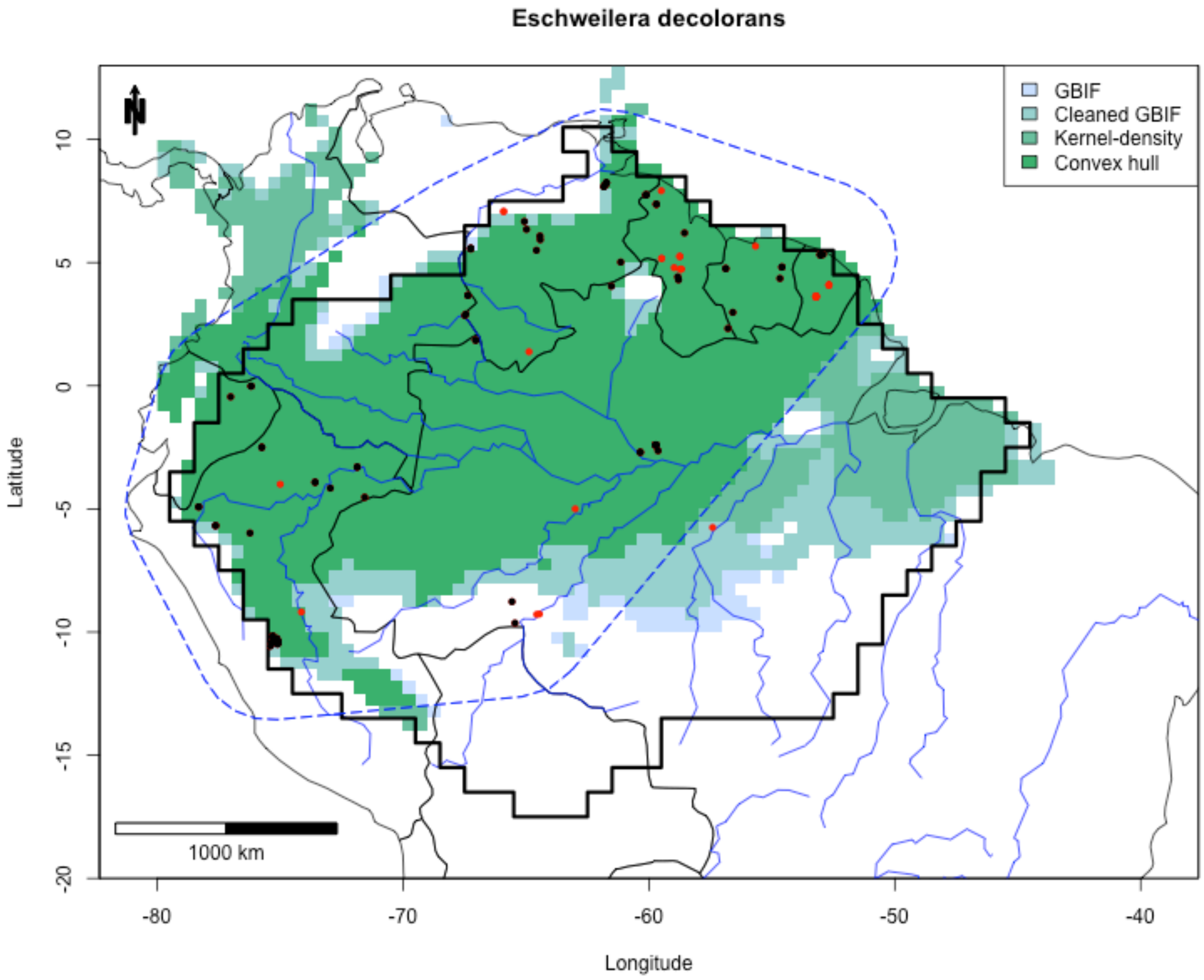

Eschweilera decolorans
GBIF
Cleaned GBIF
Kernel-density
Convex hull
N
1000 km
Latitude
Longitude
10
5
0
-5
-10
-15
-20
-80
-70
-60
-50
-40

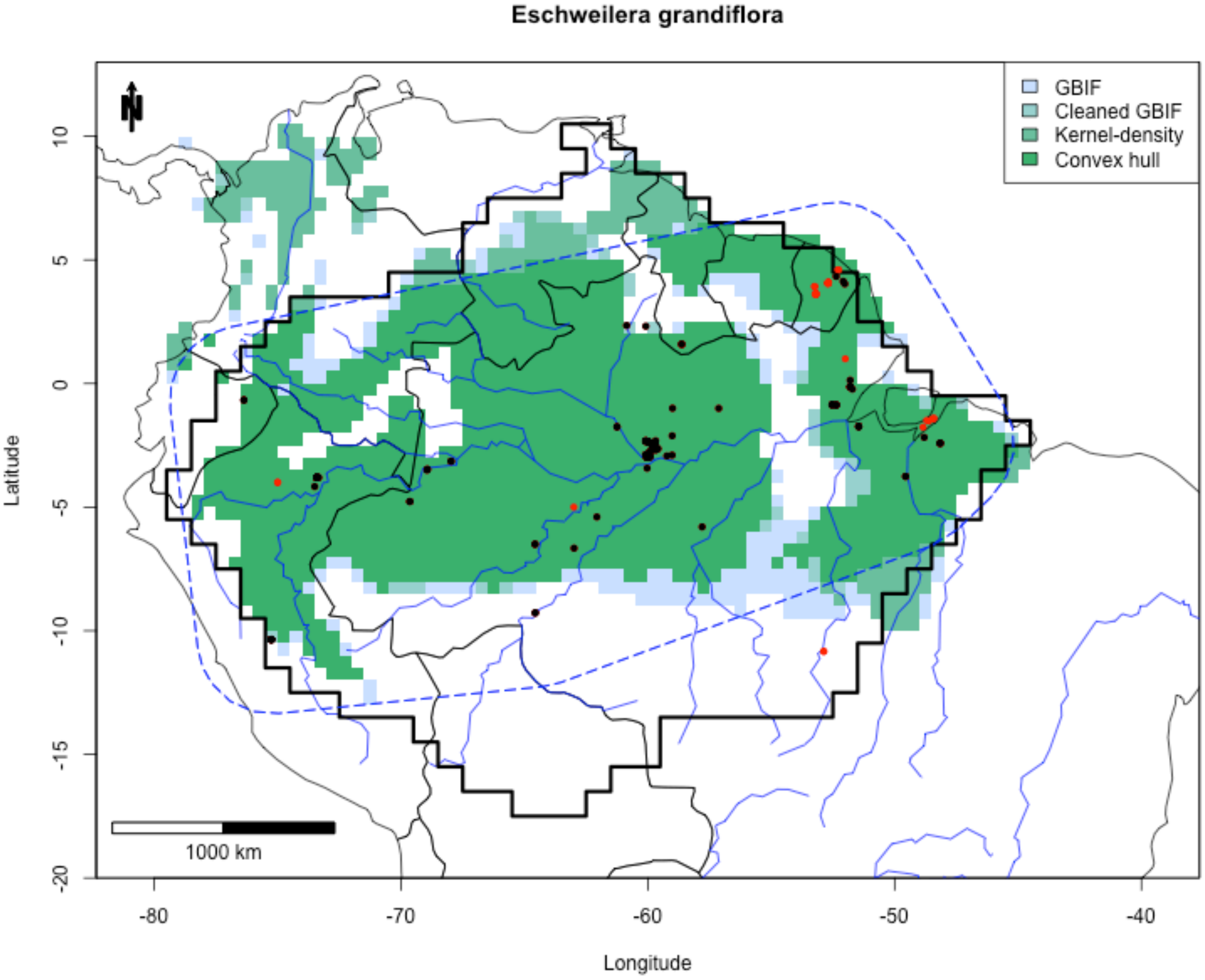

Eschweilera grandiflora
GBIF
Cleaned GBIF
Kernel-density
Convex hull
N
1000 km
Latitude
Longitude
10
5
0
-5
-10
-15
-20
-80
-70
-60
-50
-40

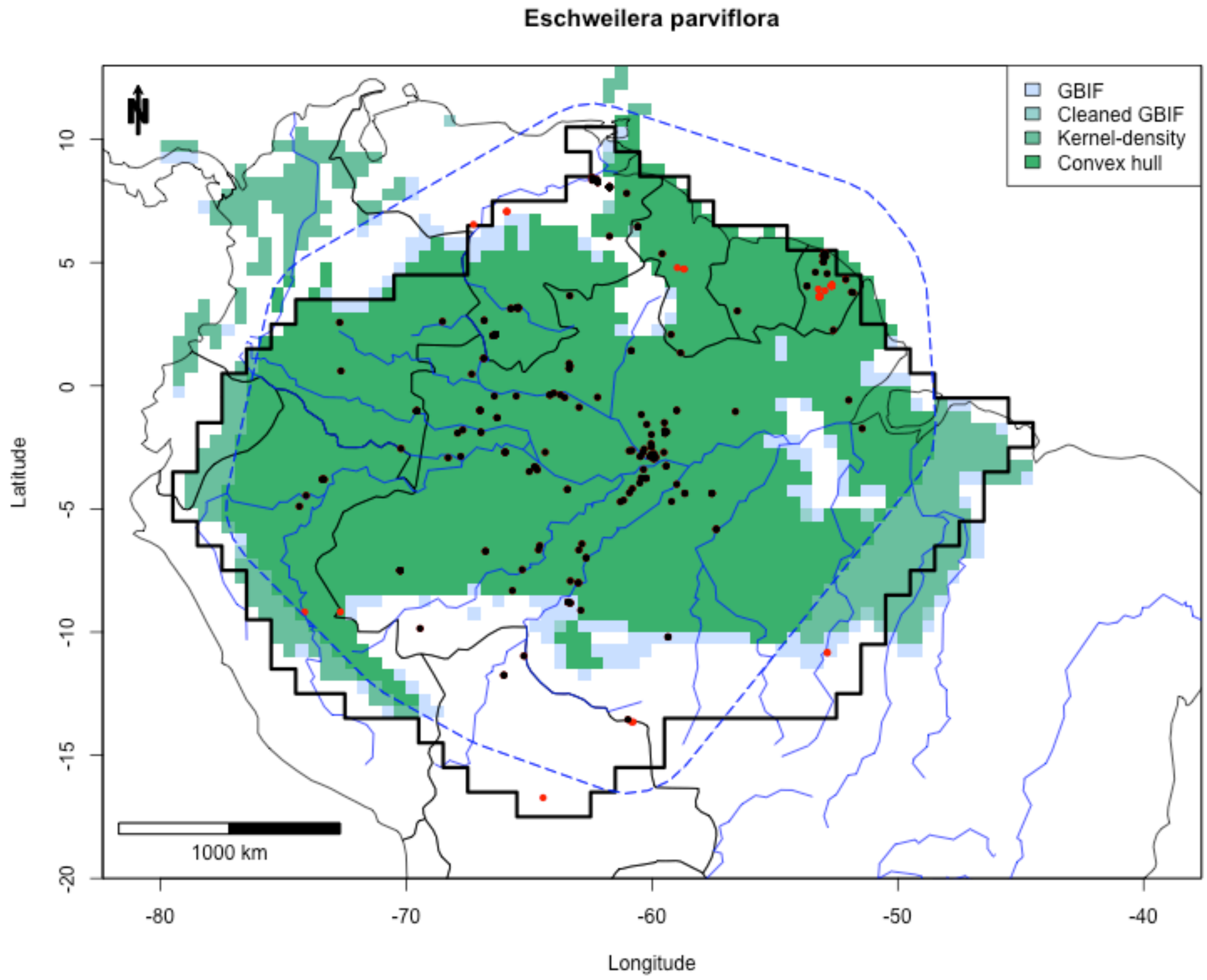

Eschweilera parviflora
GBIF
Cleaned GBIF
Kernel-density
Convex hull
N
1000 km
10
5
0
-5
-10
-15
-20
-80
-70
-60
-50
-40
Latitude
Longitude

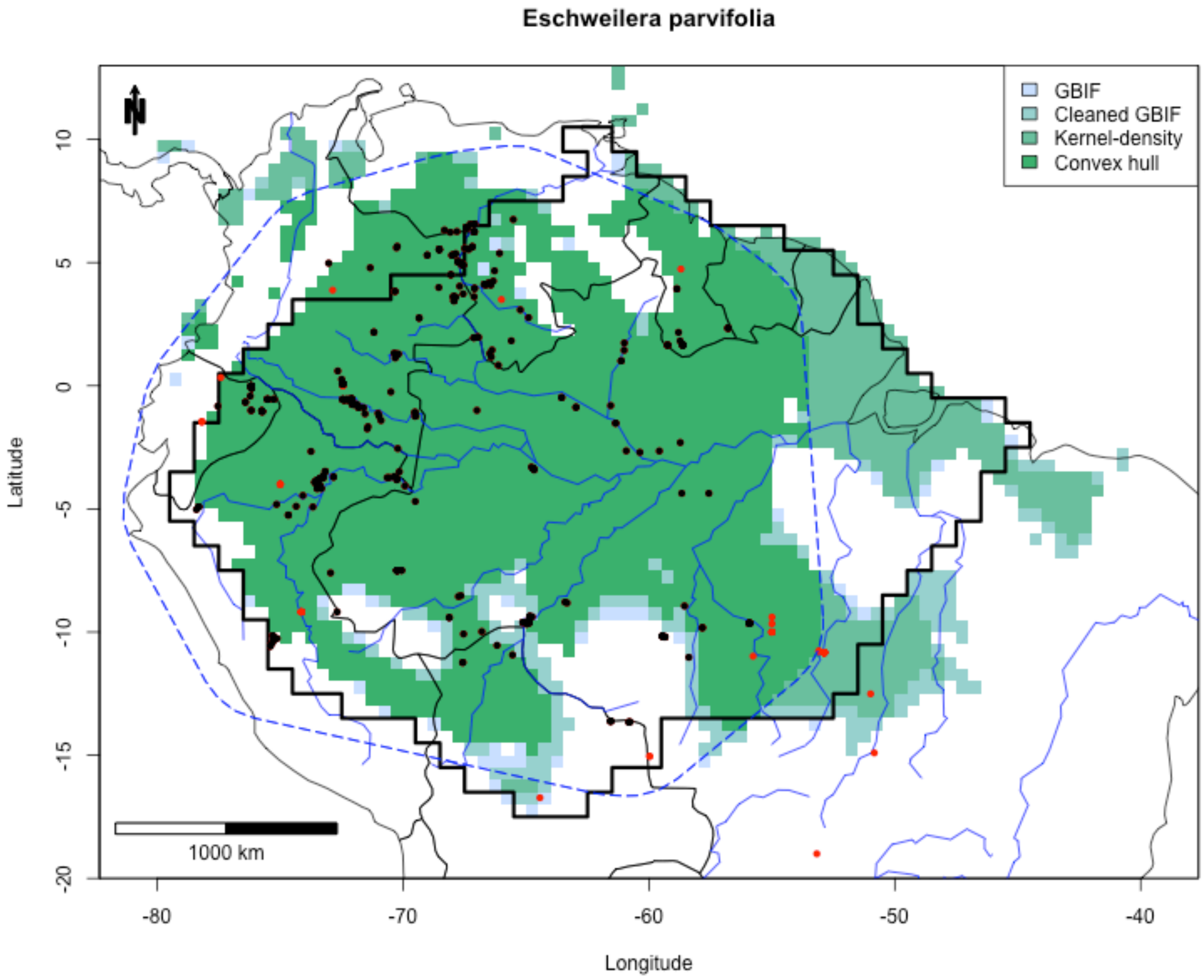

Eschweilera parvifolia
GBIF
Cleaned GBIF
Kernel-density
Convex hull
N
1000 km
Latitude
Longitude
10
5
0
-5
-10
-15
-20
-80
-70
-60
-50
-40

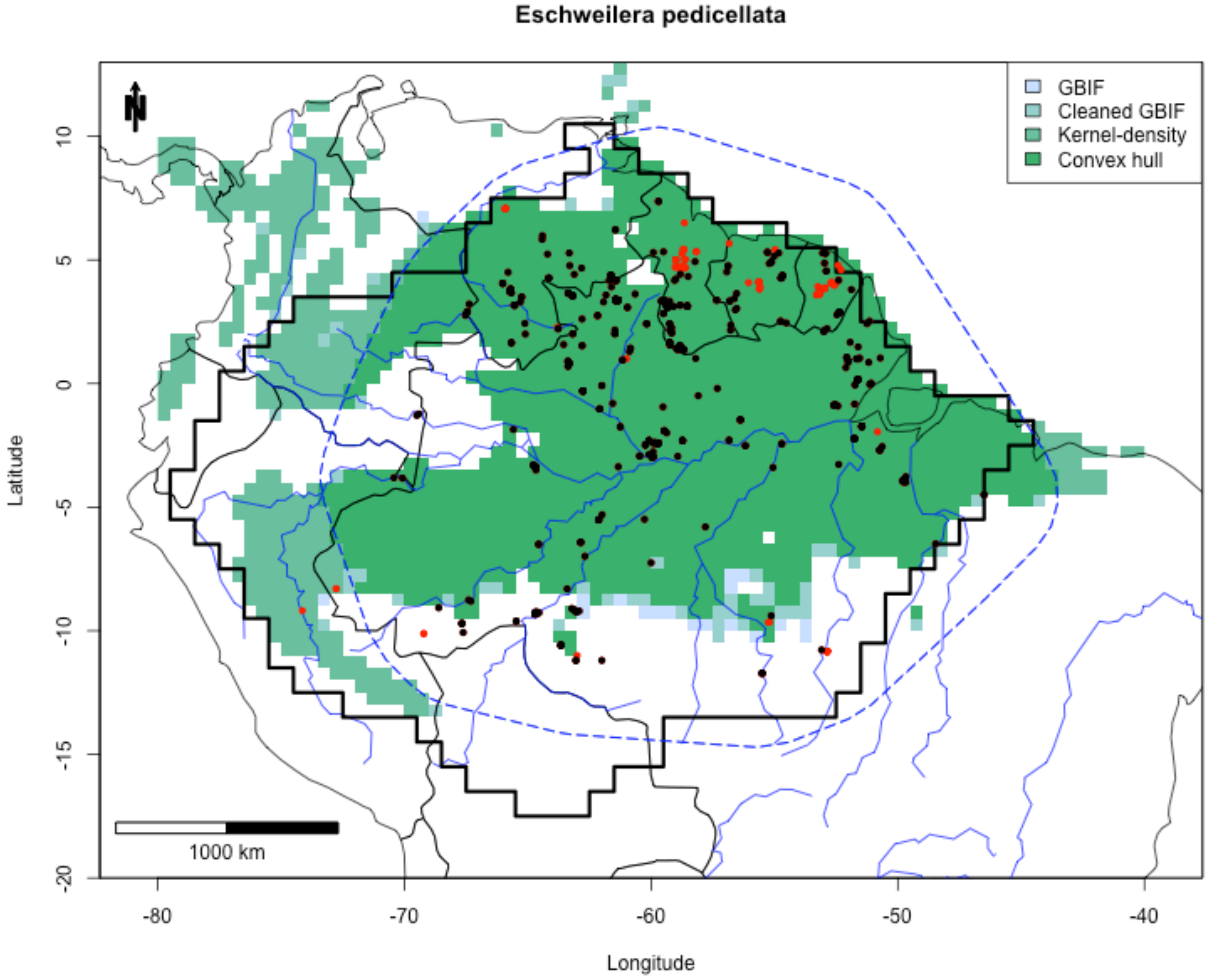

Eschweilera pedicellata
GBIF
Cleaned GBIF
Kernel-density
Convex hull
N
1000 km
Latitude
Longitude
10
5
0
-5
-10
-15
-20
-80
-70
-60
-50
-40

Eschweilera sagotiana

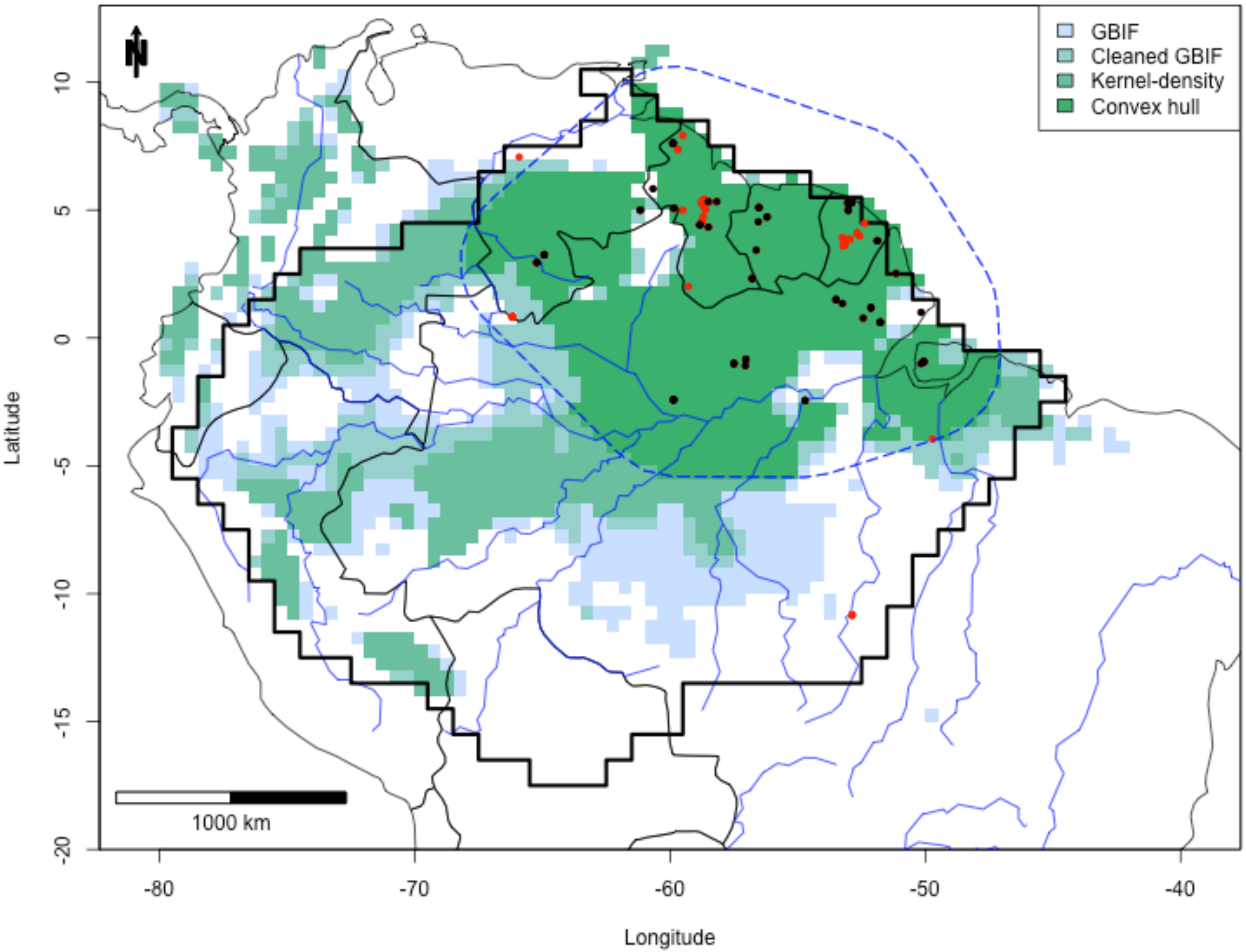

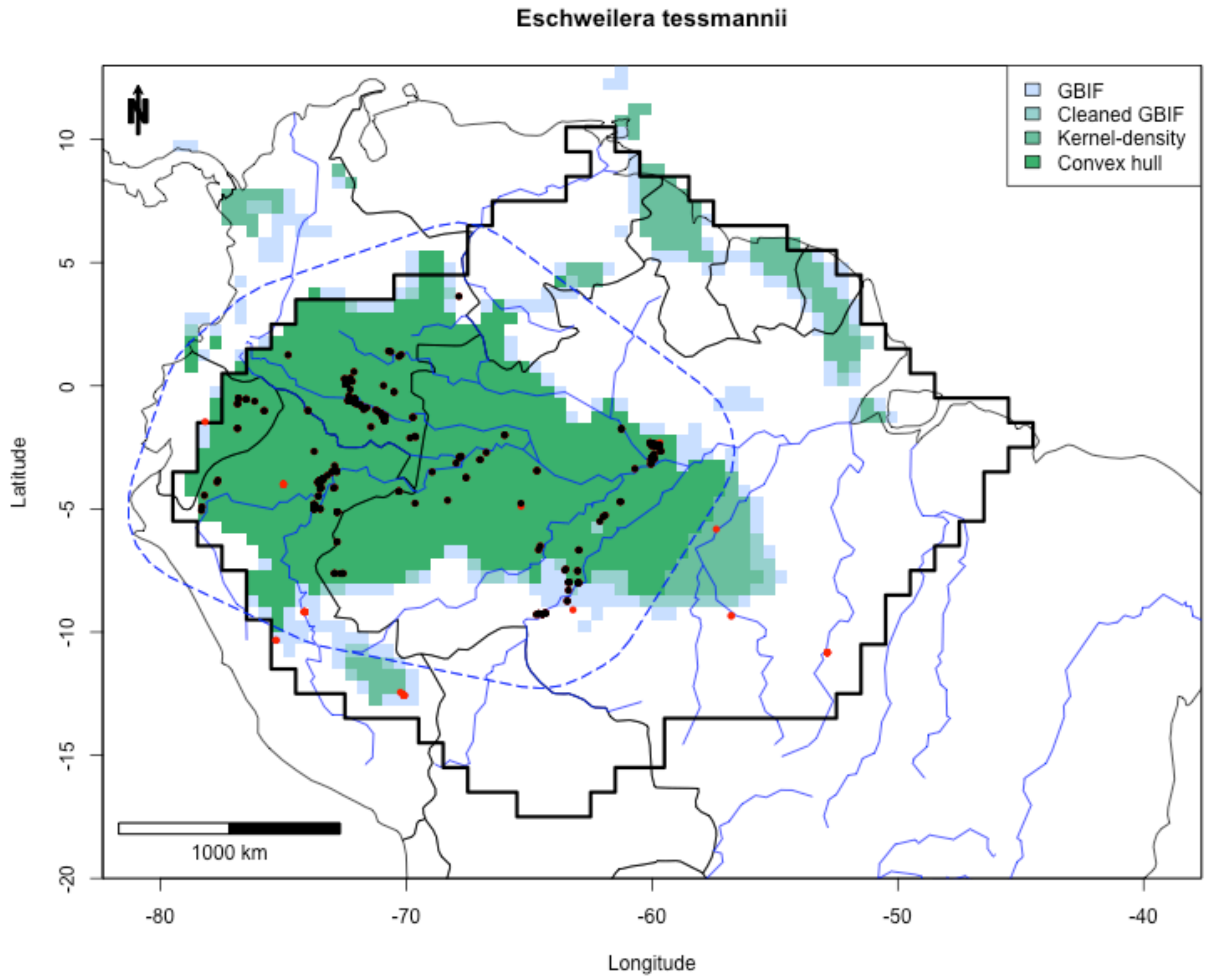

Eschweilera tessmannii
GBIF
Cleaned GBIF
Kernel-density
Convex hull
1000 km
Latitude
Longitude
10
5
0
-5
-10
-15
-20
-80
-70
-60
-50
-40

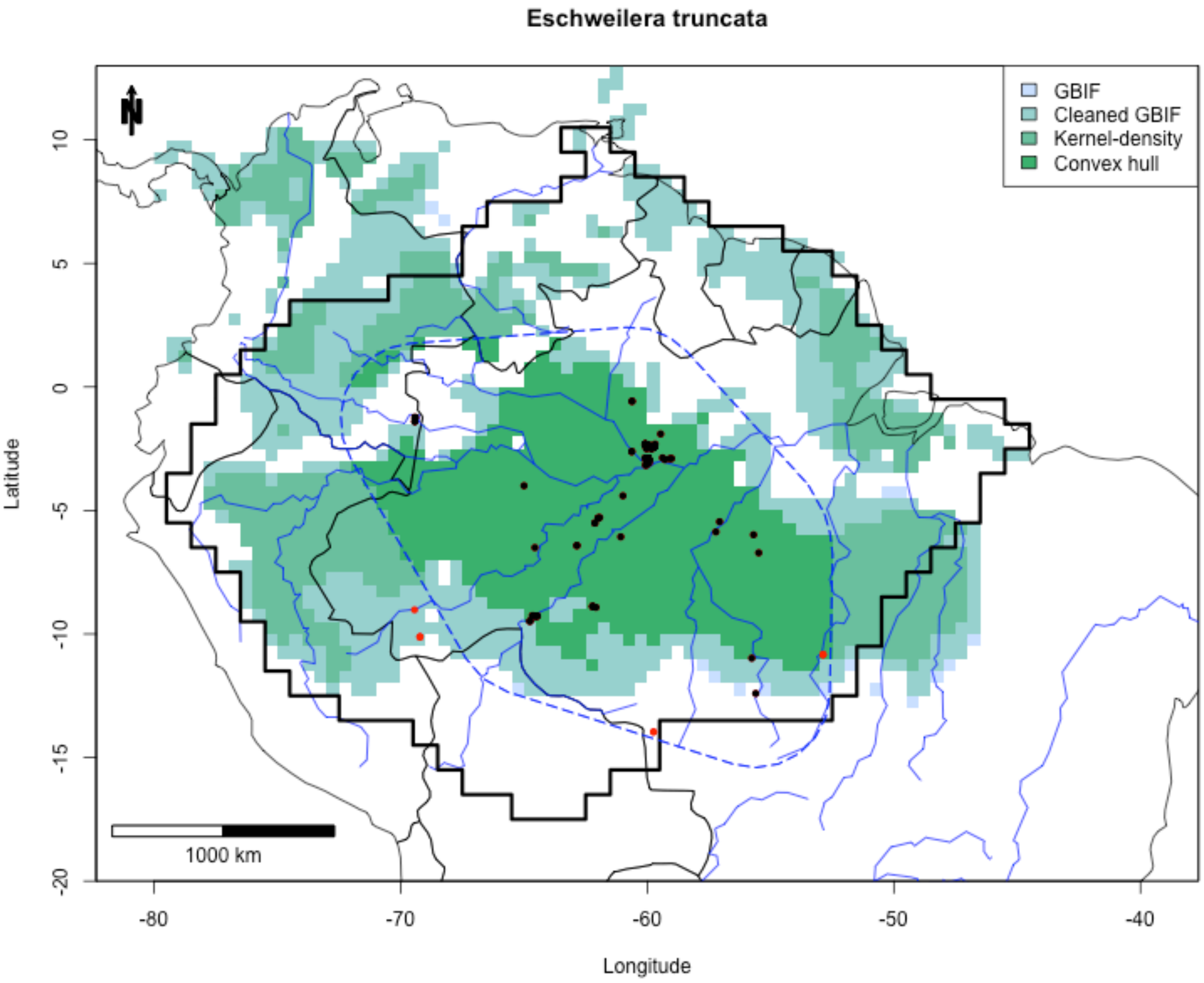
Eschweilera truncata
GBIF
Cleaned GBIF
Kernel-density
Convex hull
N
1000 km
Latitude
Longitude
10
5
0
-5
-10
-15
-20
-80
-70
-60
-50
-40

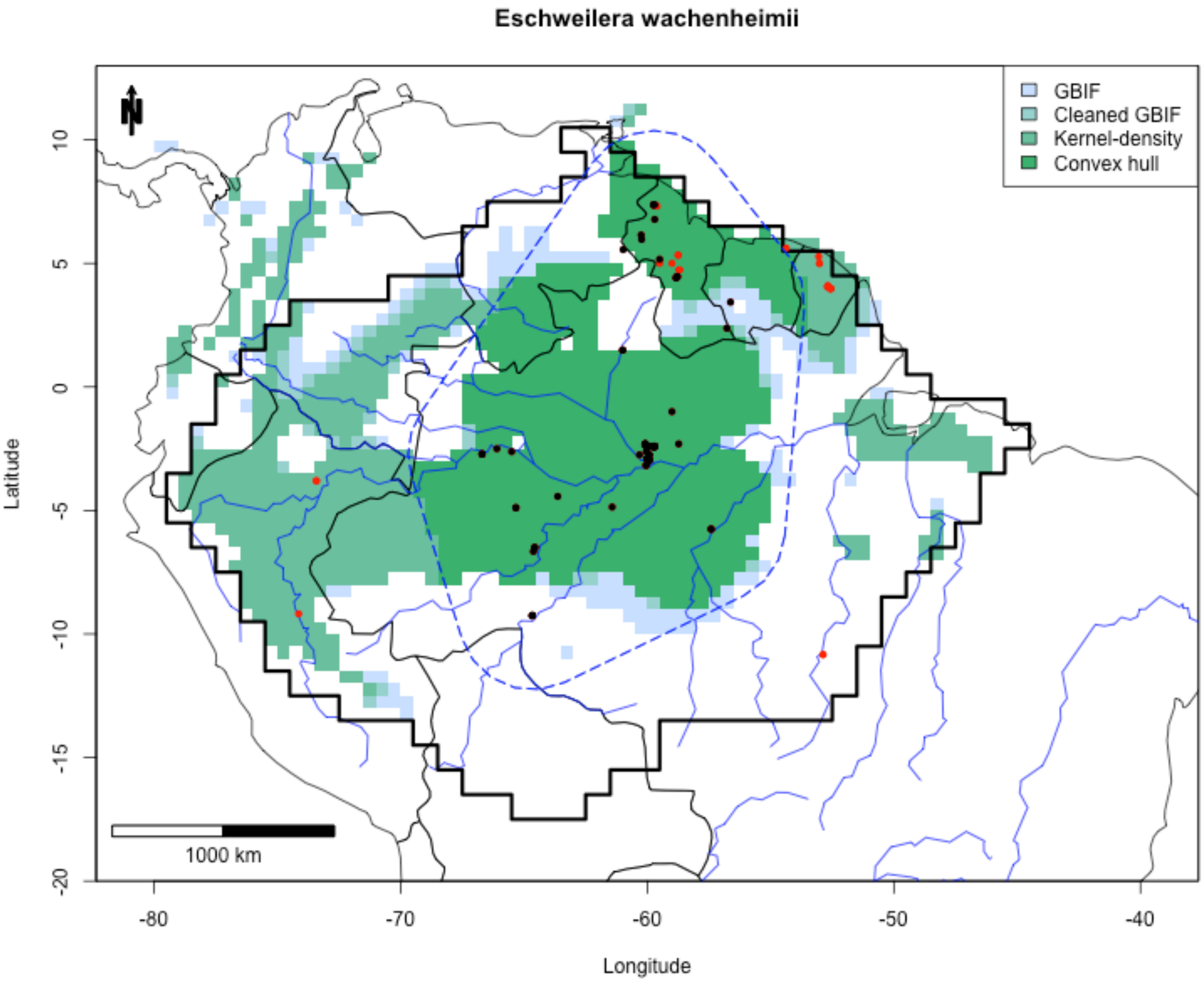

Eschweilera wachenheimii
GBIF
Cleaned GBIF
Kernel-density
Convex hull
N
1000 km
Latitude
Longitude
10
5
0
-5
-10
-15
-20
-80
-70
-60
-50
-40

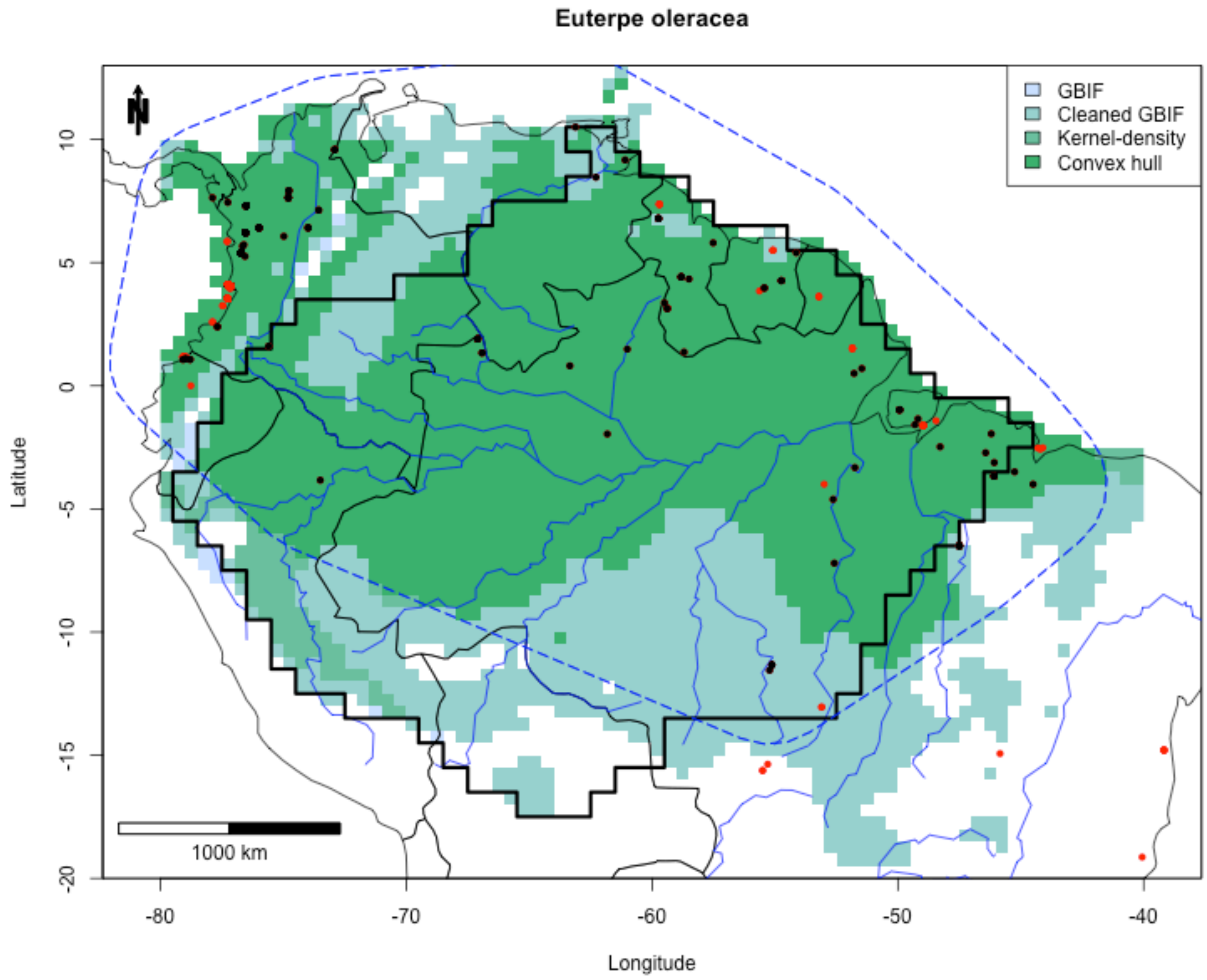
Euterpe oleracea
GBIF
Cleaned GBIF
Kernel-density
Convex hull
N
1000 km
Latitude
Longitude
10
5
0
-5
-10
-15
-20
-80
-70
-60
-50
-40

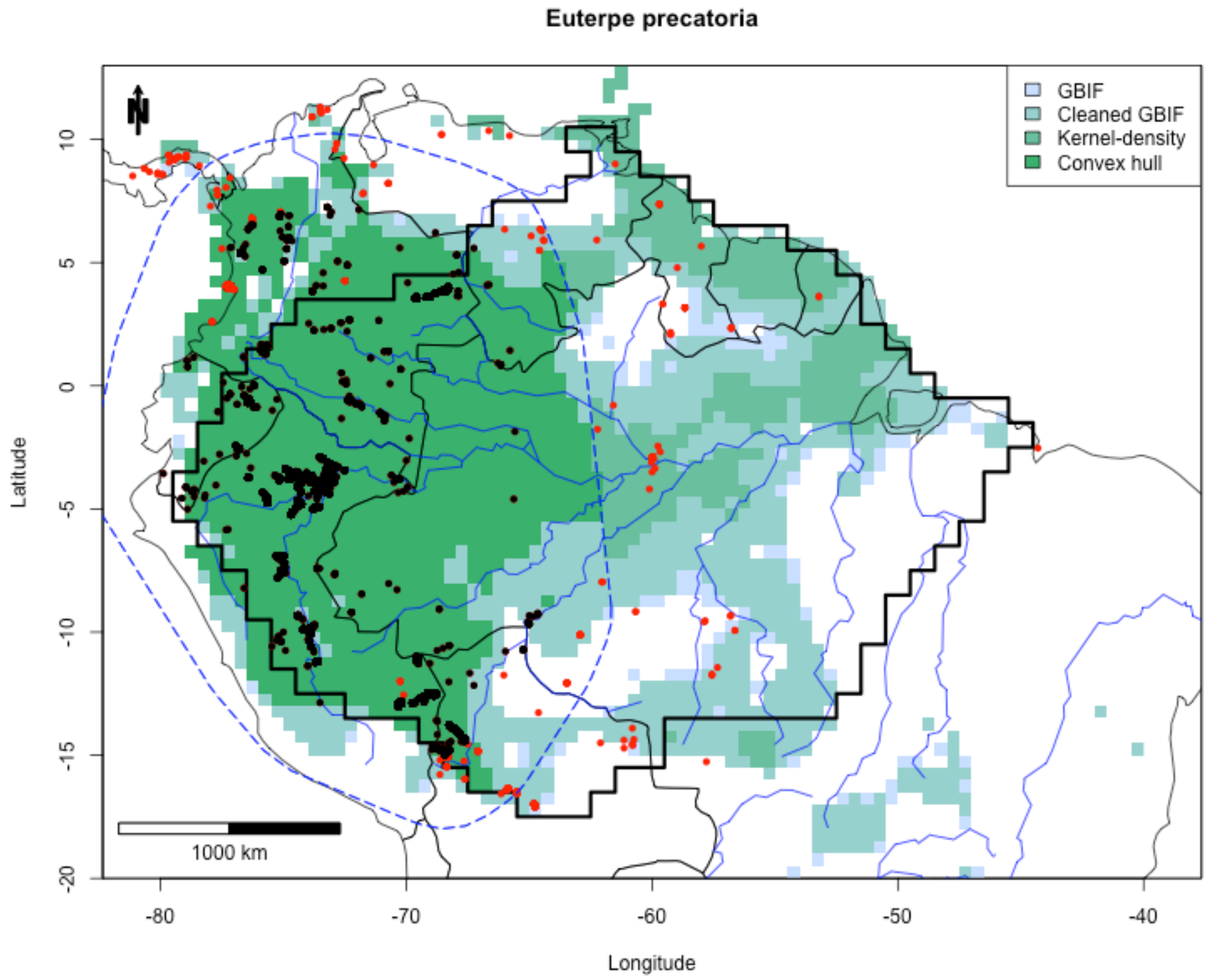

Euterpe precatoria
GBIF
Cleaned GBIF
Kernel-density
Convex hull
1000 km
Latitude
Longitude
10
5
0
-5
-10
-15
-20
-80
-70
-60
-50
-40

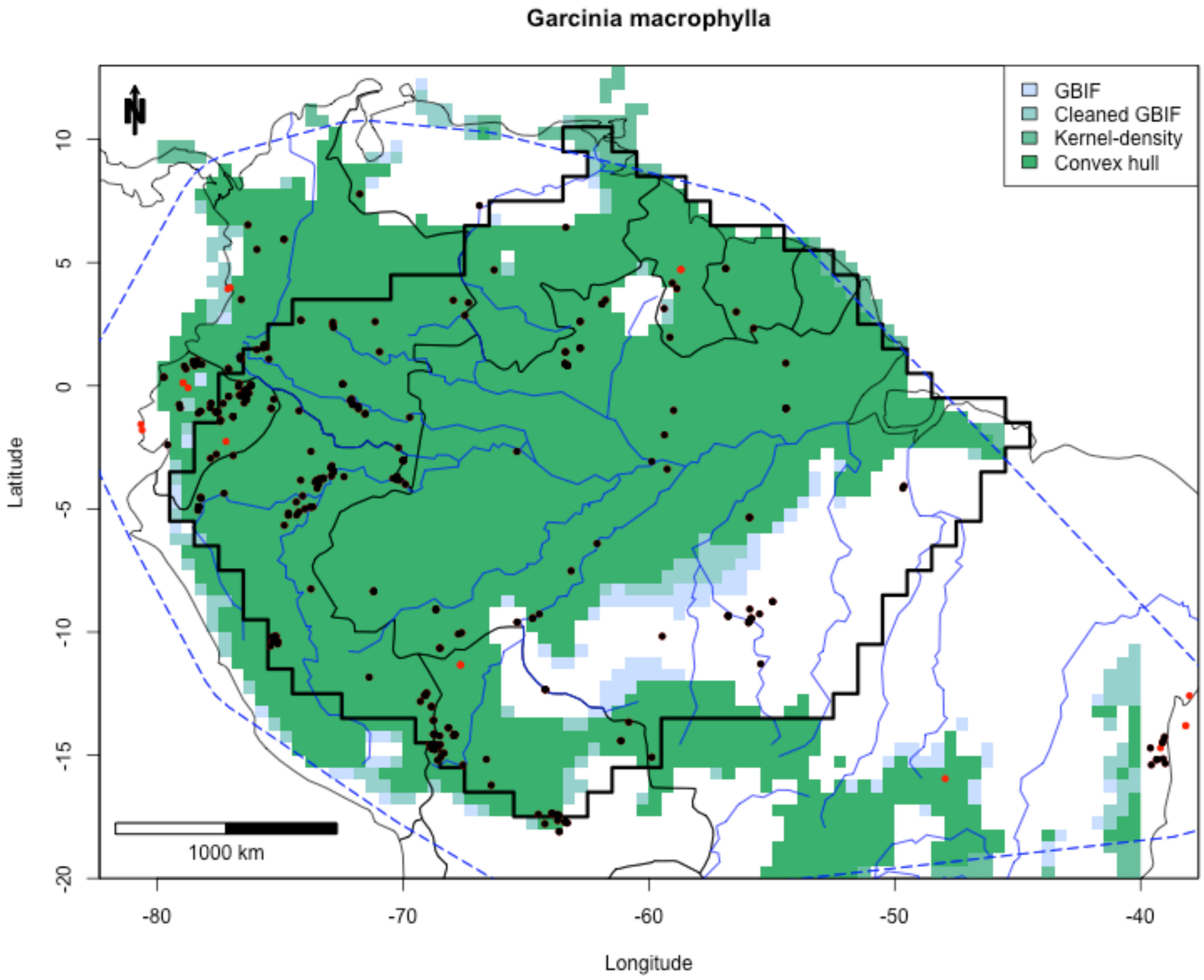
Garcinia macrophylla
GBIF
Cleaned GBIF
Kernel-density
Convex hull
1000 km
Latitude
Longitude
10
5
0
-5
-10
-15
-20
-80
-70
-60
-50
-40

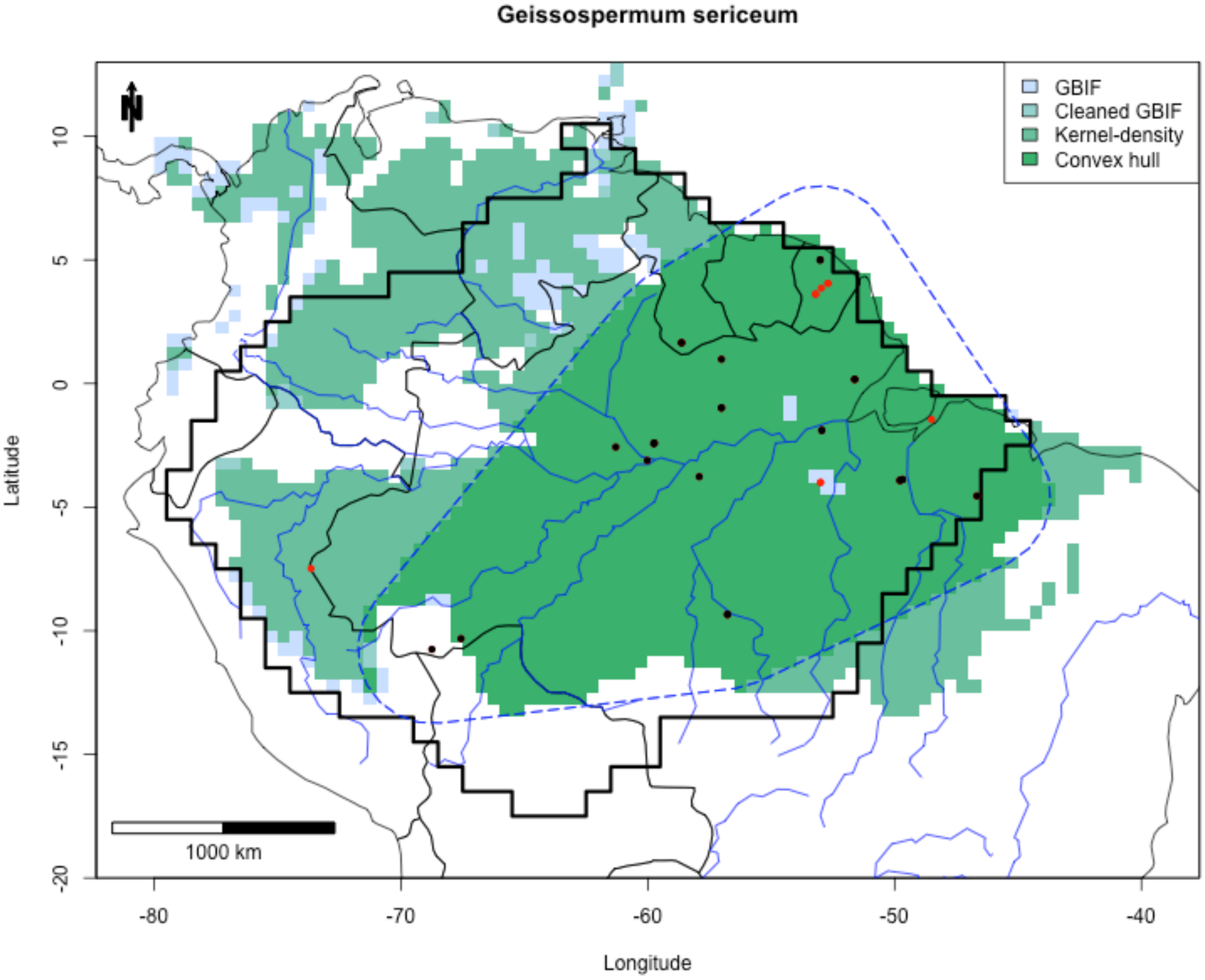
Geissospermum sericeum
GBIF
Cleaned GBIF
Kernel-density
Convex hull
N
1000 km
Latitude
Longitude
10
5
0
-5
-10
-15
-20
-80
-70
-60
-50
-40

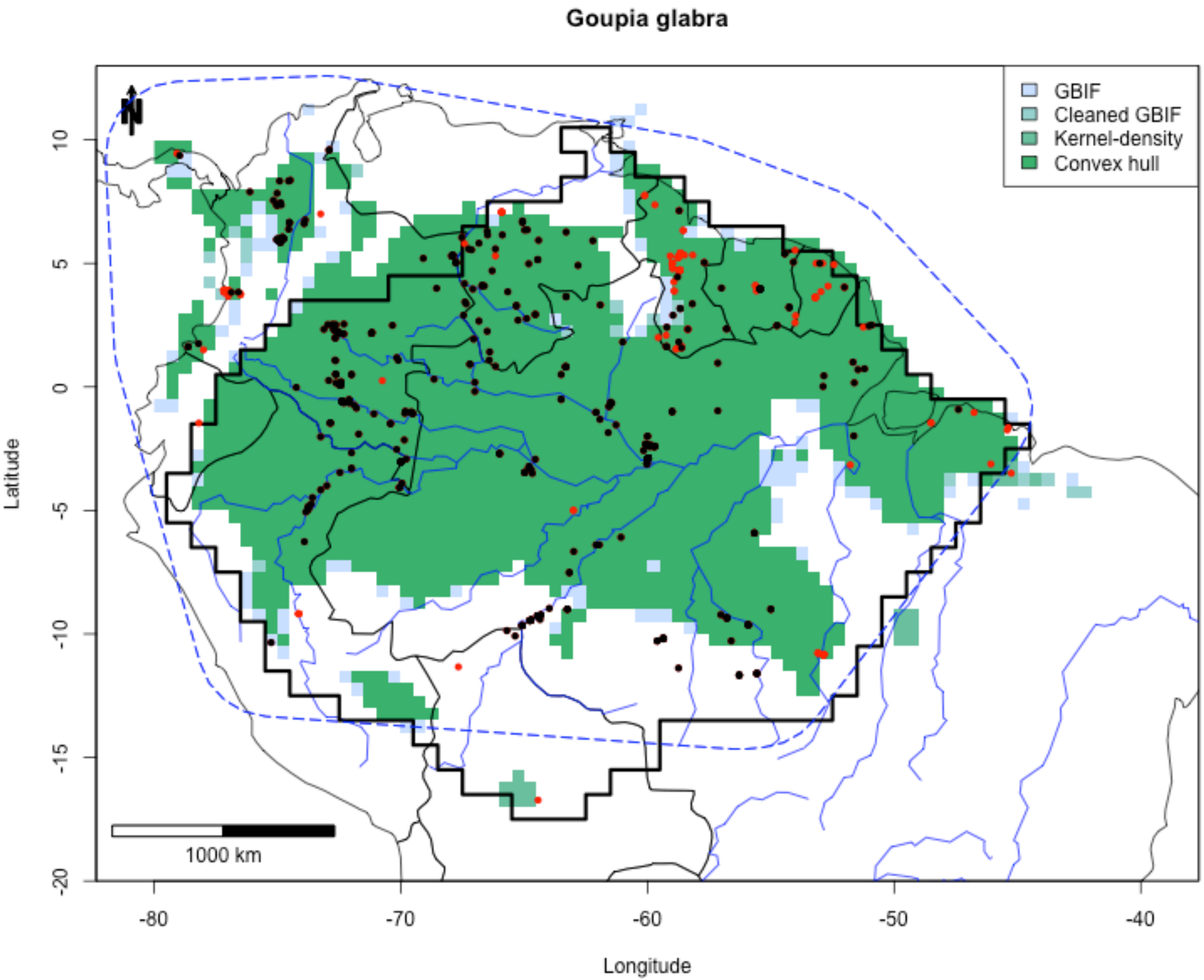
Goupia glabra
GBIF
Cleaned GBIF
Kernel-density
Convex hull
N
1000 km
Latitude
Longitude
10
5
0
-5
-10
-15
-20
-80
-70
-60
-50
-40

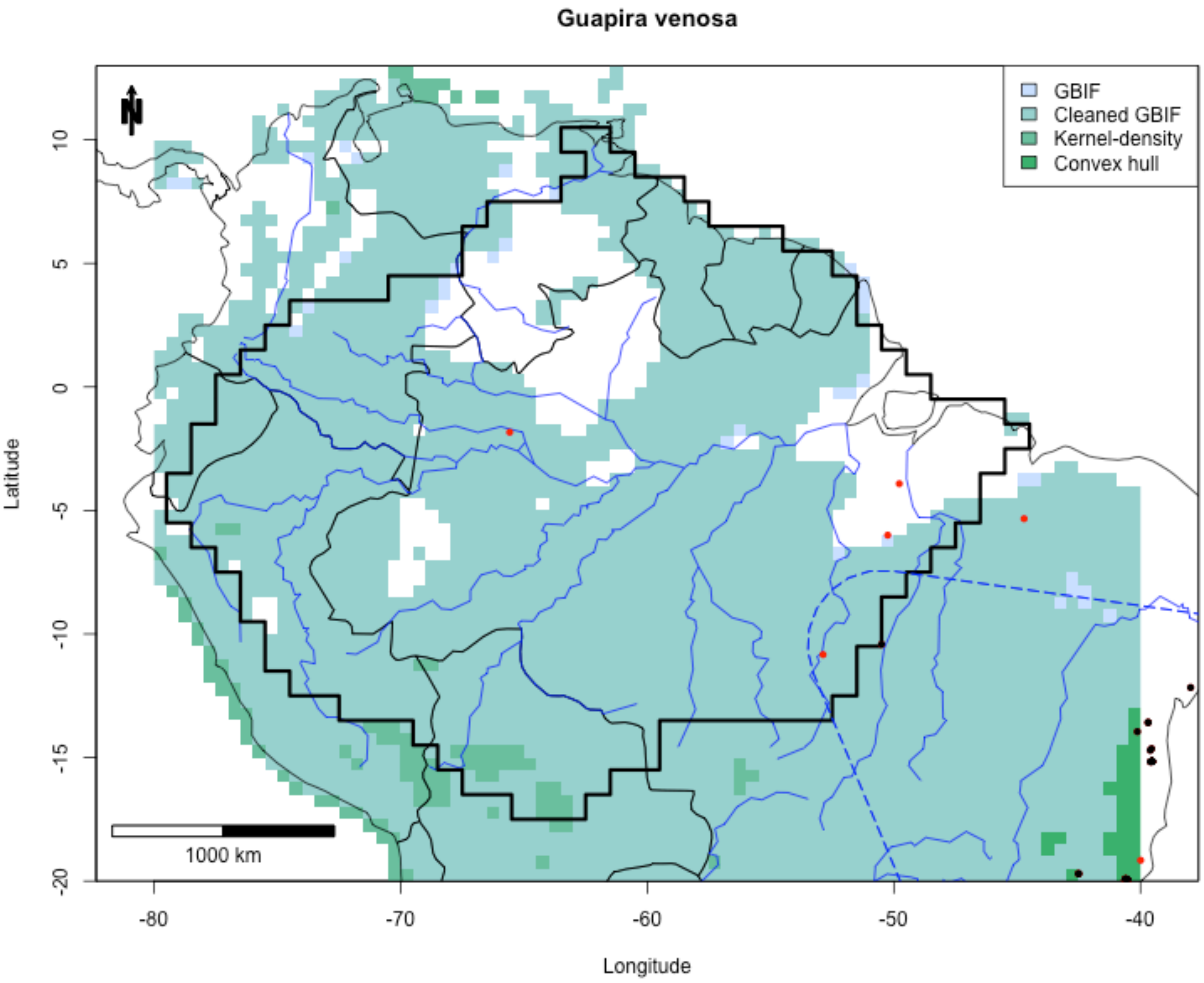

Guapira venosa
GBIF
Cleaned GBIF
Kernel-density
Convex hull
N
1000 km
Latitude
10
5
0
-5
-10
-15
-20
-80
-70
-60
-50
-40
Longitude

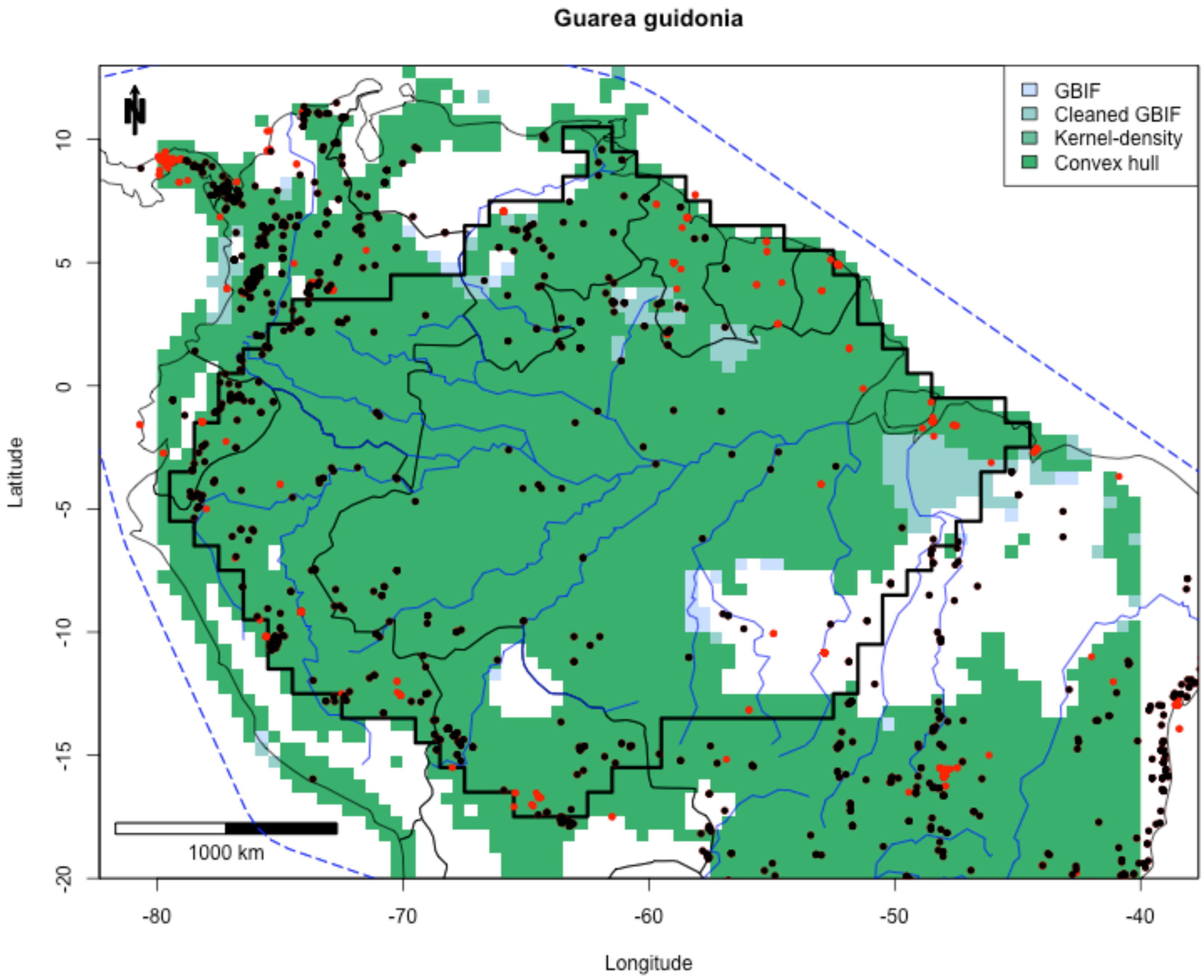

Guarea guidonia
GBIF
Cleaned GBIF
Kernel-density
Convex hull
1000 km
Latitude
Longitude
10
5
0
-5
-10
-15
-20
-80
-70
-60
-50
-40

Guarea kunthiana

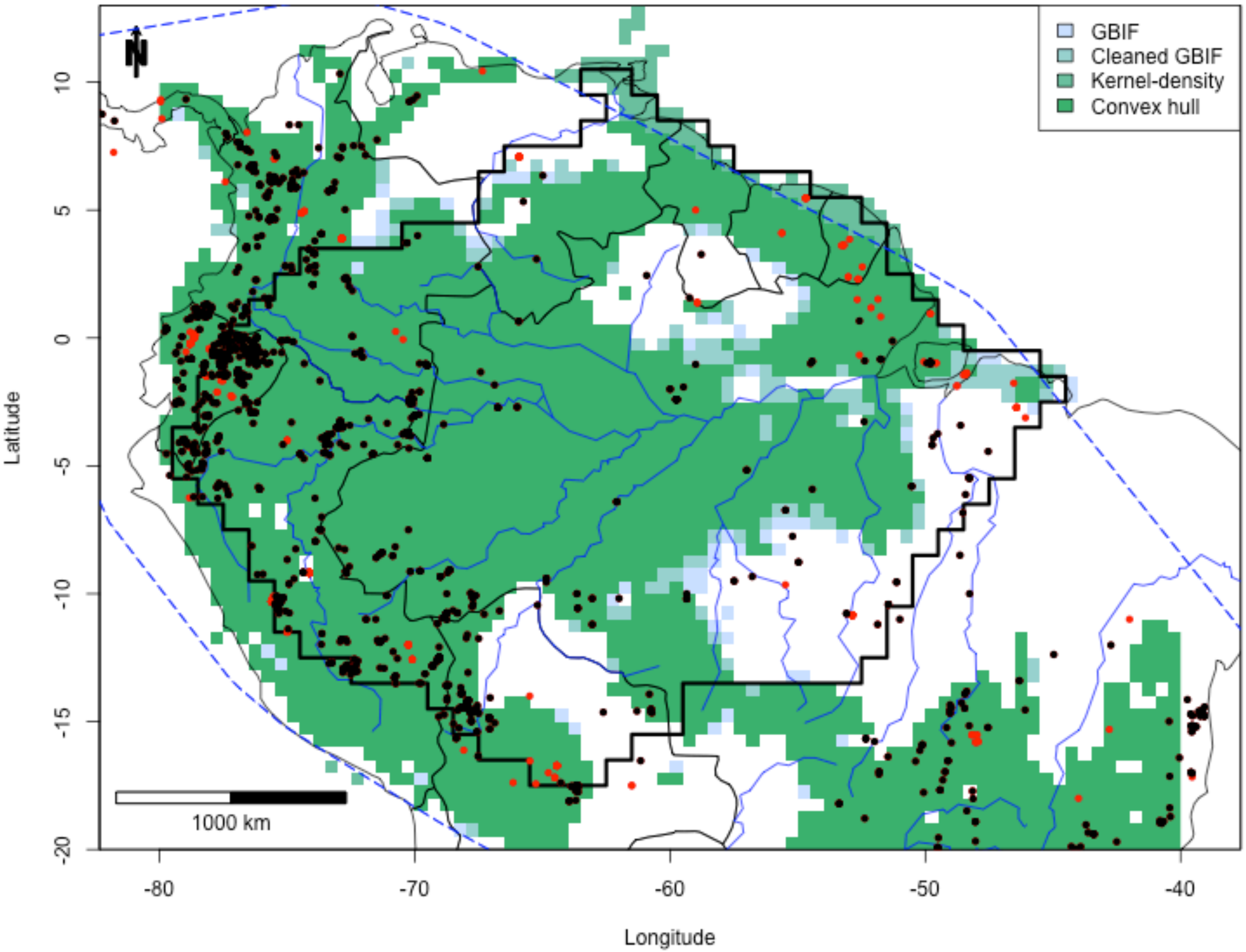

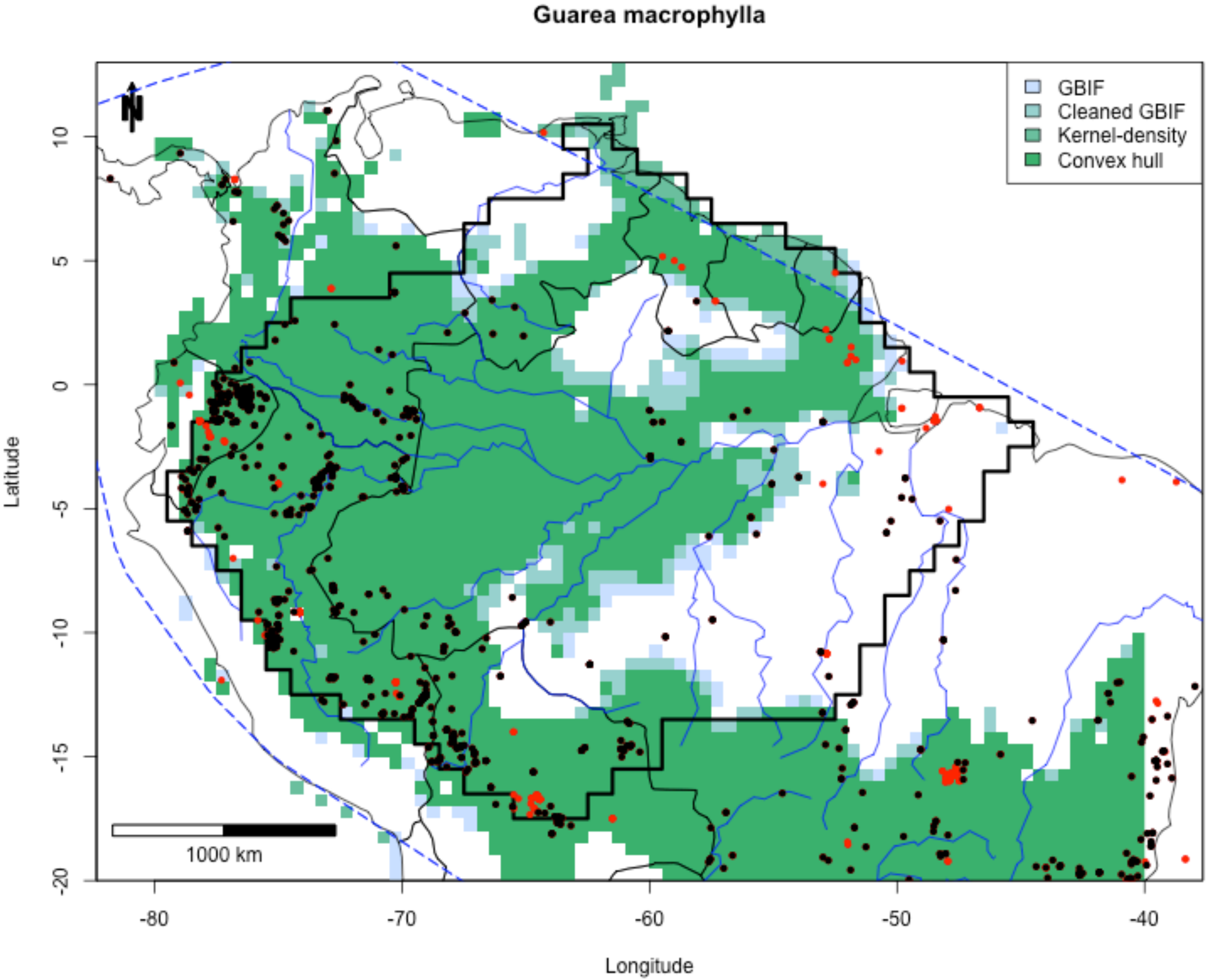

Guarea macrophylla
GBIF
Cleaned GBIF
Kernel-density
Convex hull
N
1000 km
Latitude
Longitude
10
5
0
-5
-10
-15
-20
-80
-70
-60
-50
-40

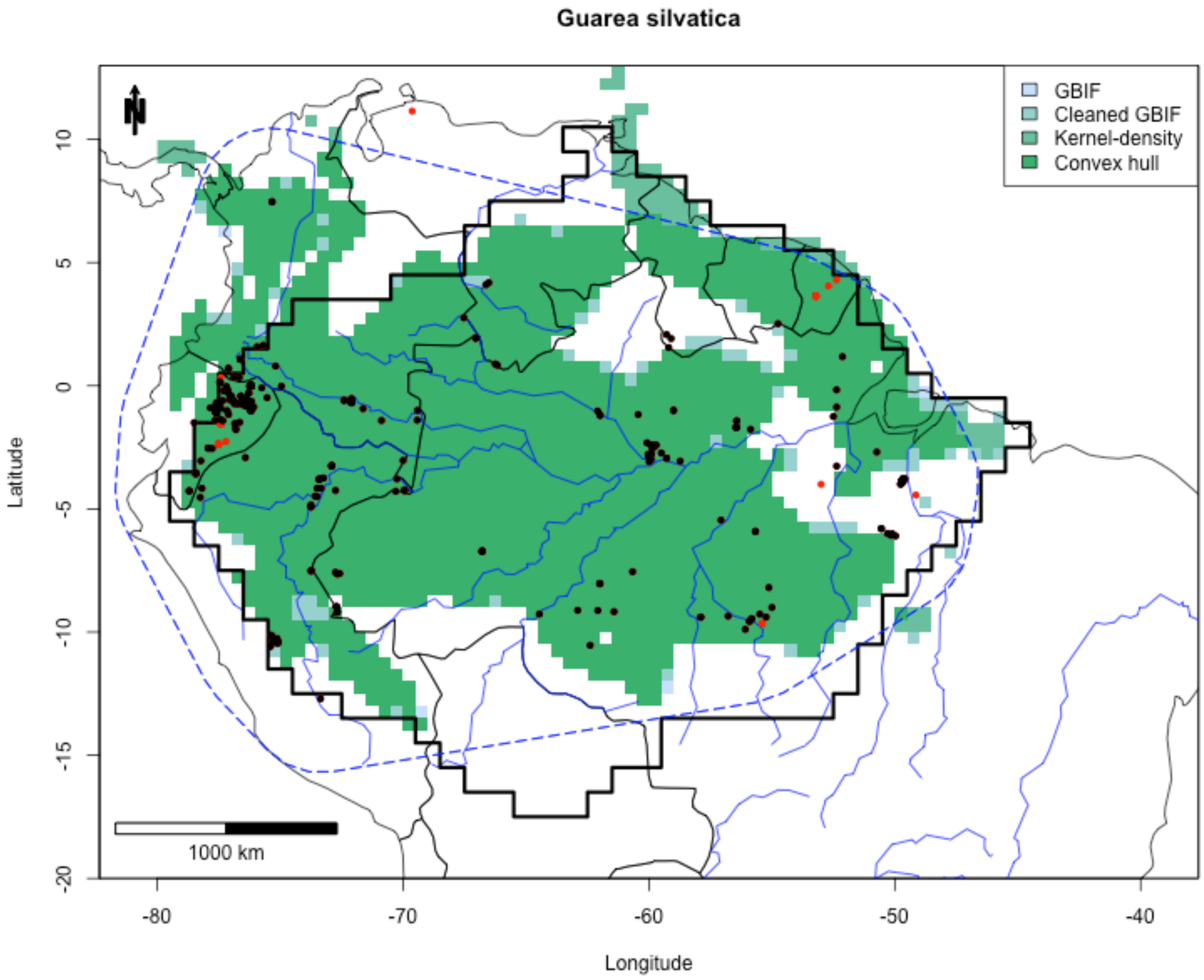
Guarea silvatica
GBIF
Cleaned GBIF
Kernel-density
Convex hull
N
1000 km
Latitude
Longitude
10
5
0
-5
-10
-15
-20
-80
-70
-60
-50
-40

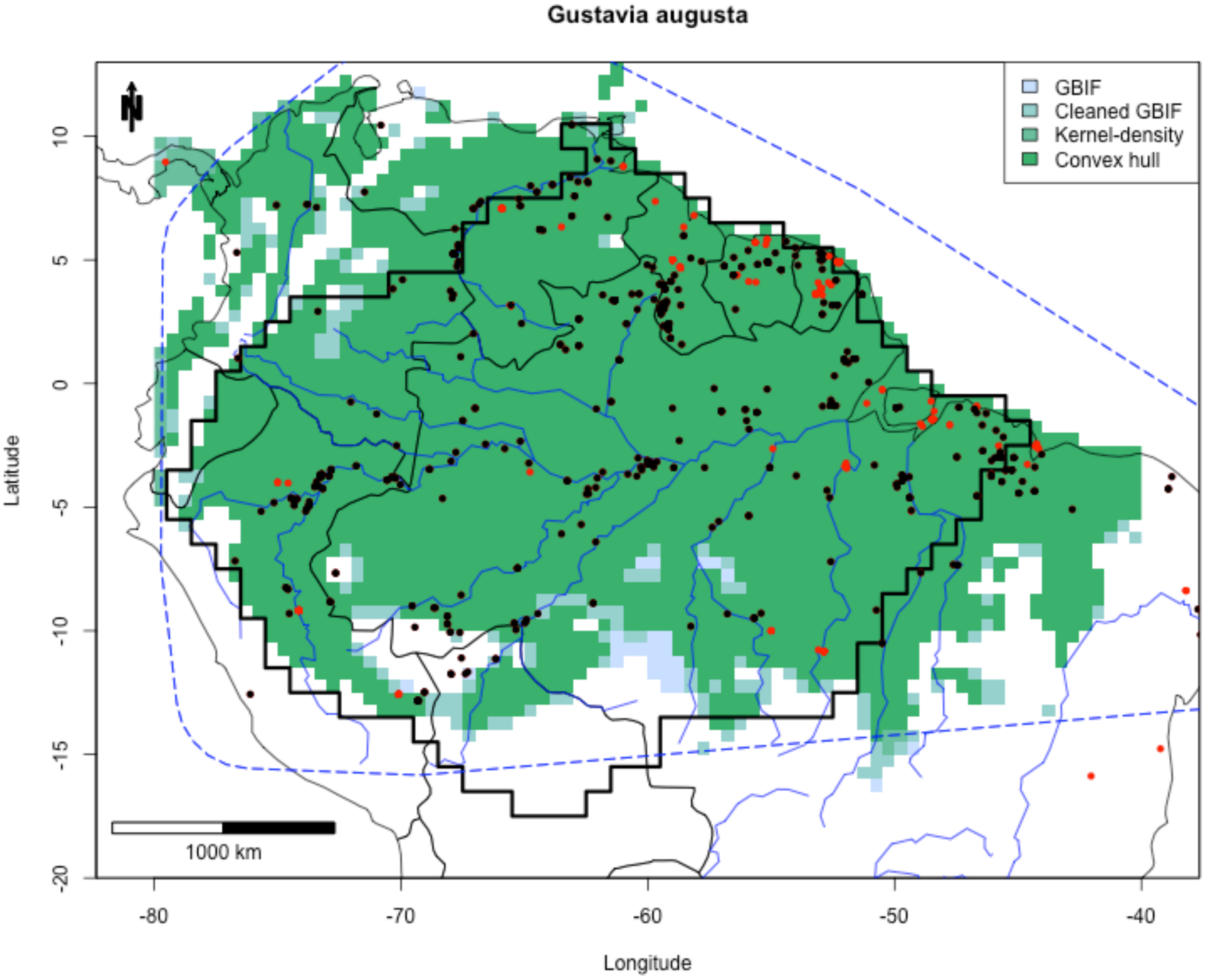

Gustavia augusta
GBIF
Cleaned GBIF
Kernel-density
Convex hull
N
1000 km
10
5
0
-5
-10
-15
-20
Latitude
-80
-70
-60
-50
-40
Longitude

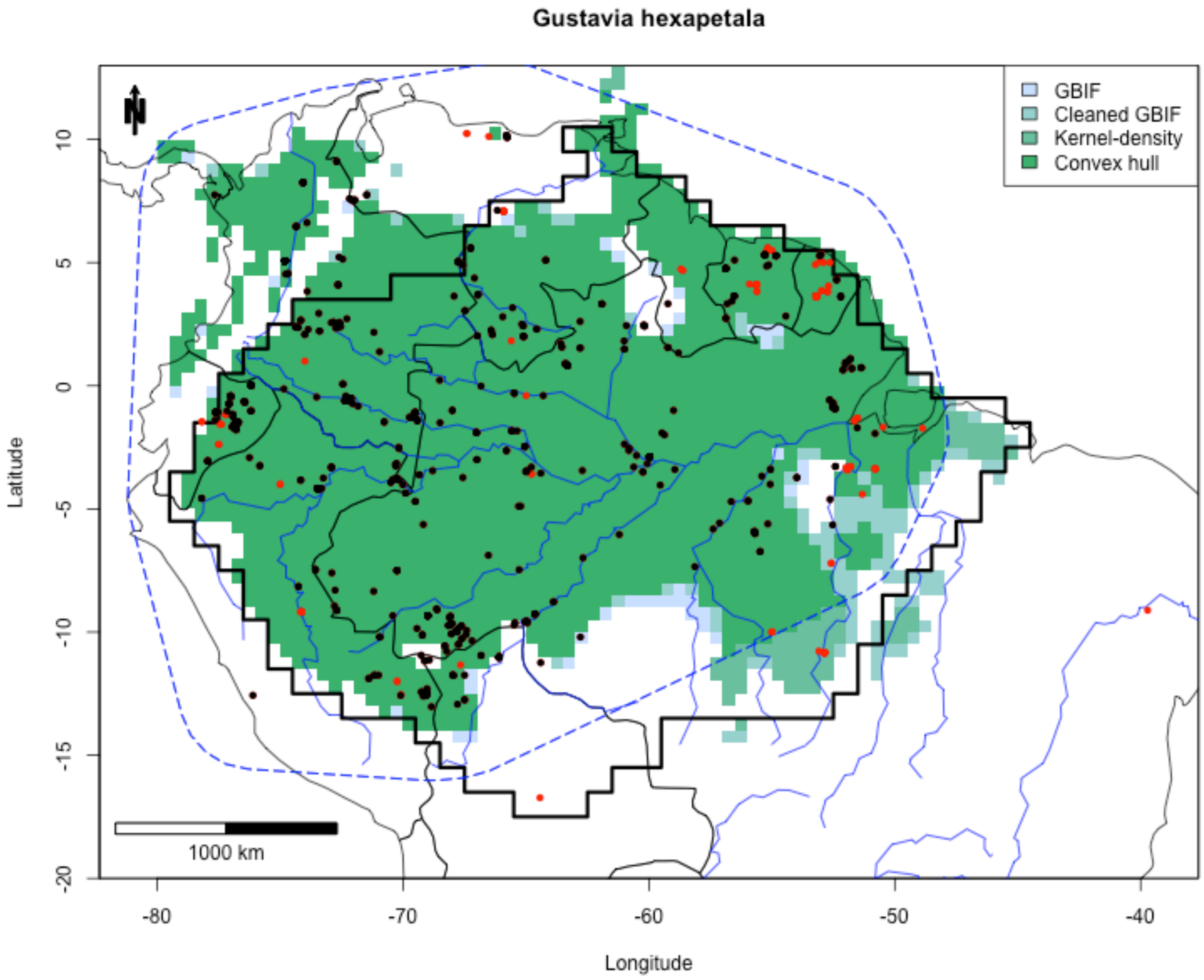

Gustavia hexapetala
GBIF
Cleaned GBIF
Kernel-density
Convex hull
1000 km
Latitude
Longitude
-80
-70
-60
-50
-40
10
5
0
-5
-10
-15
-20

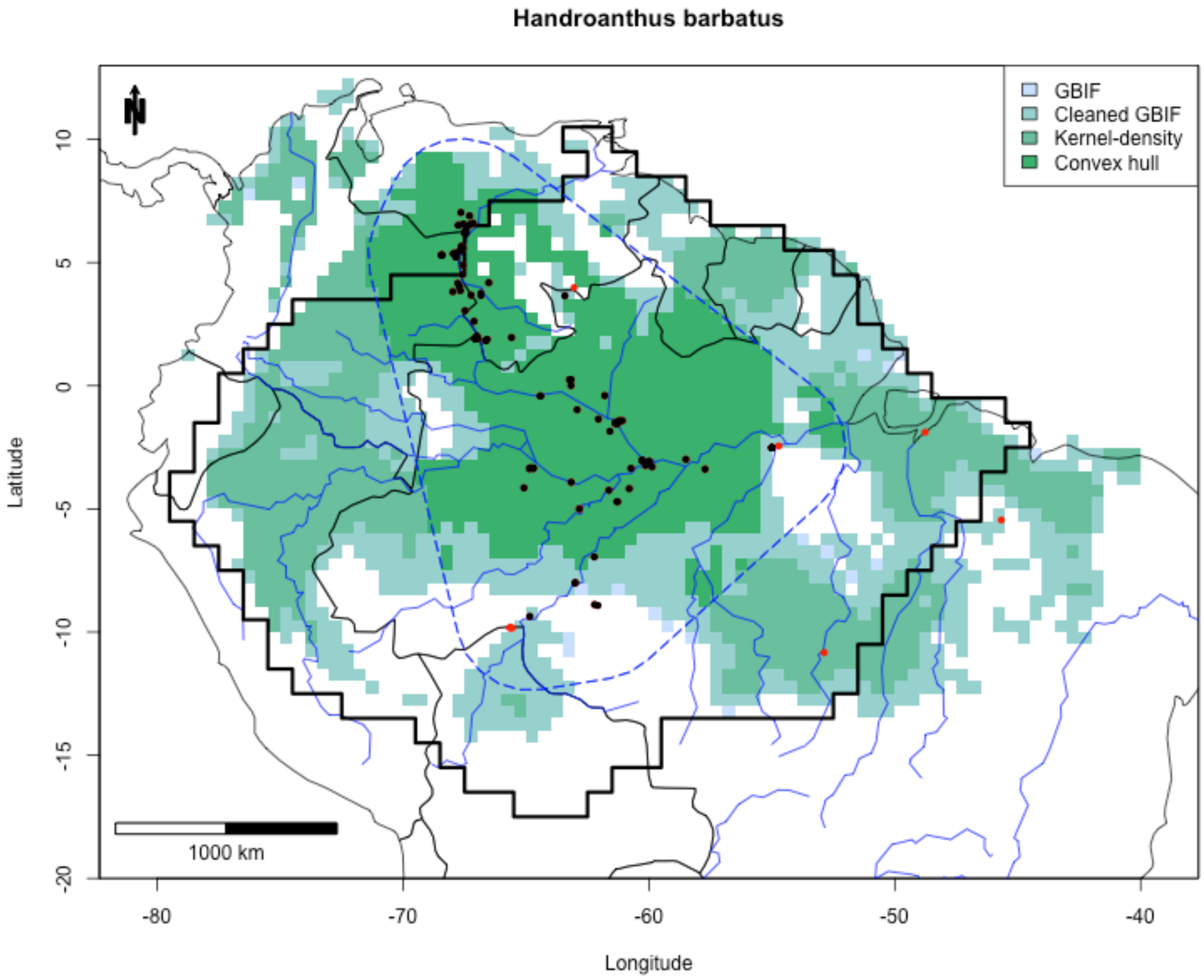

Handroanthus barbatus
GBIF
Cleaned GBIF
Kernel-density
Convex hull
N
1000 km
Latitude
Longitude
10
5
0
-5
-10
-15
-20
-80
-70
-60
-50
-40

Haploclathra cordata

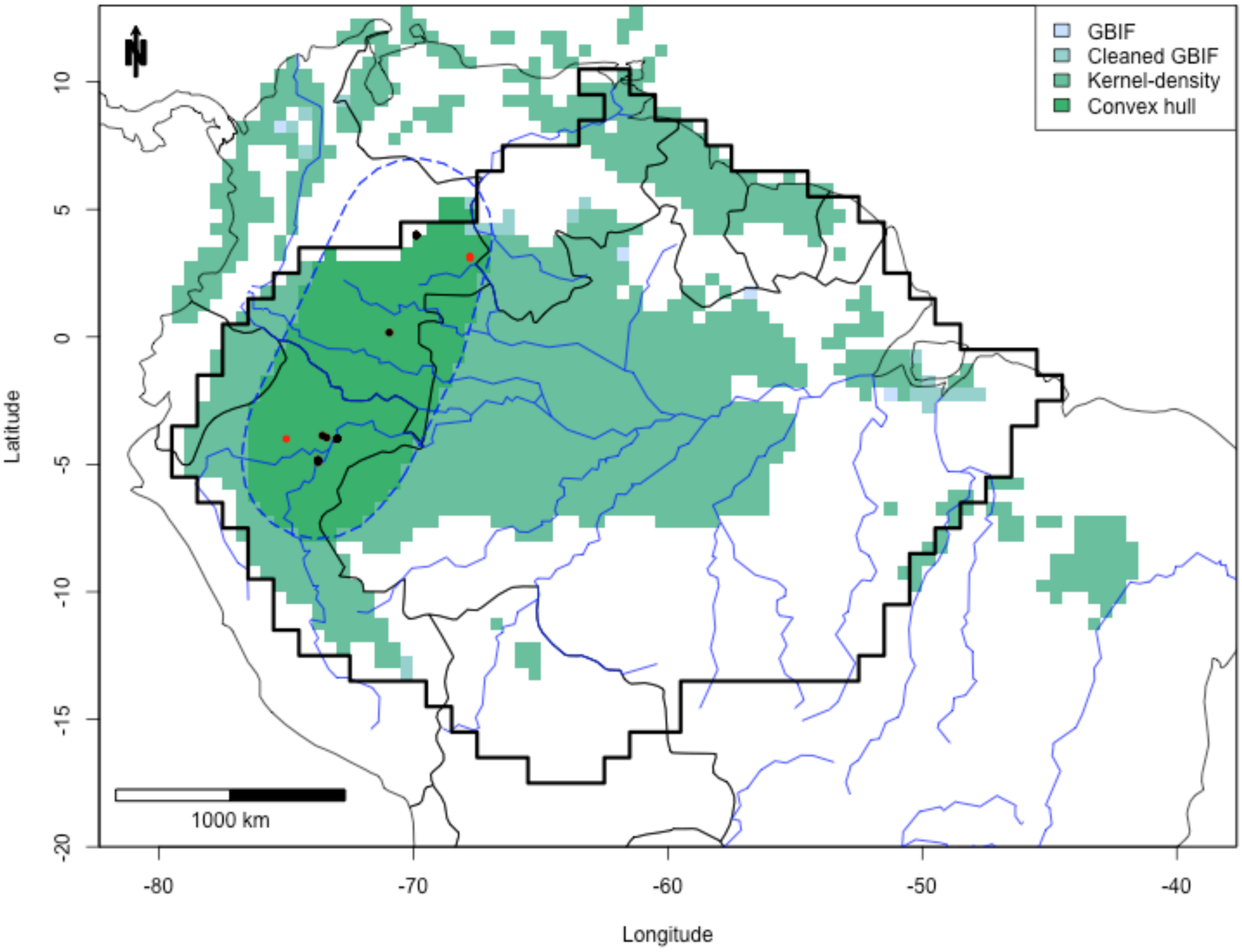

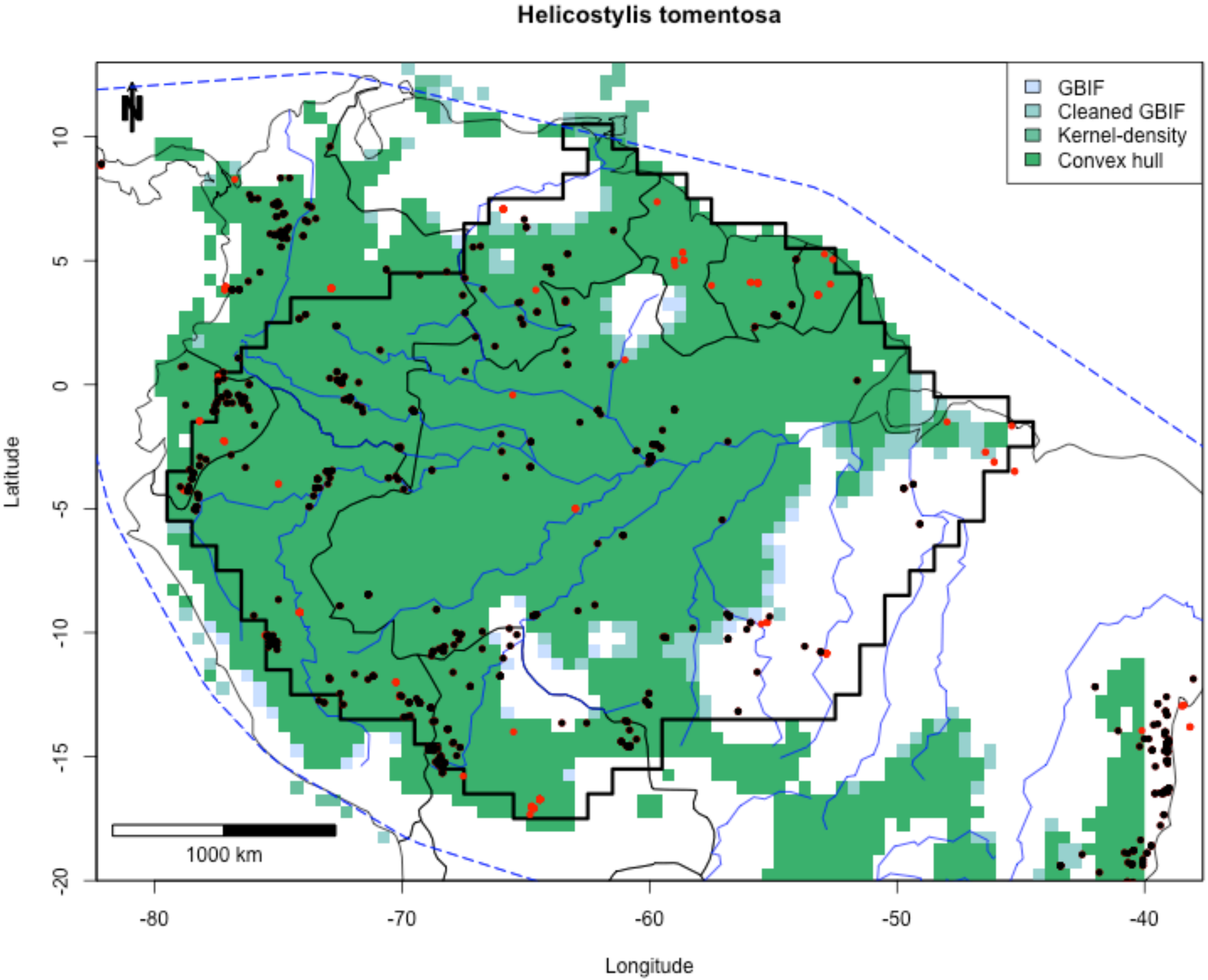
Helicostylis tomentosa
GBIF
Cleaned GBIF
Kernel-density
Convex hull
N
1000 km
Latitude
Longitude
10
5
0
-5
-10
-15
-20
-80
-70
-60
-50
-40

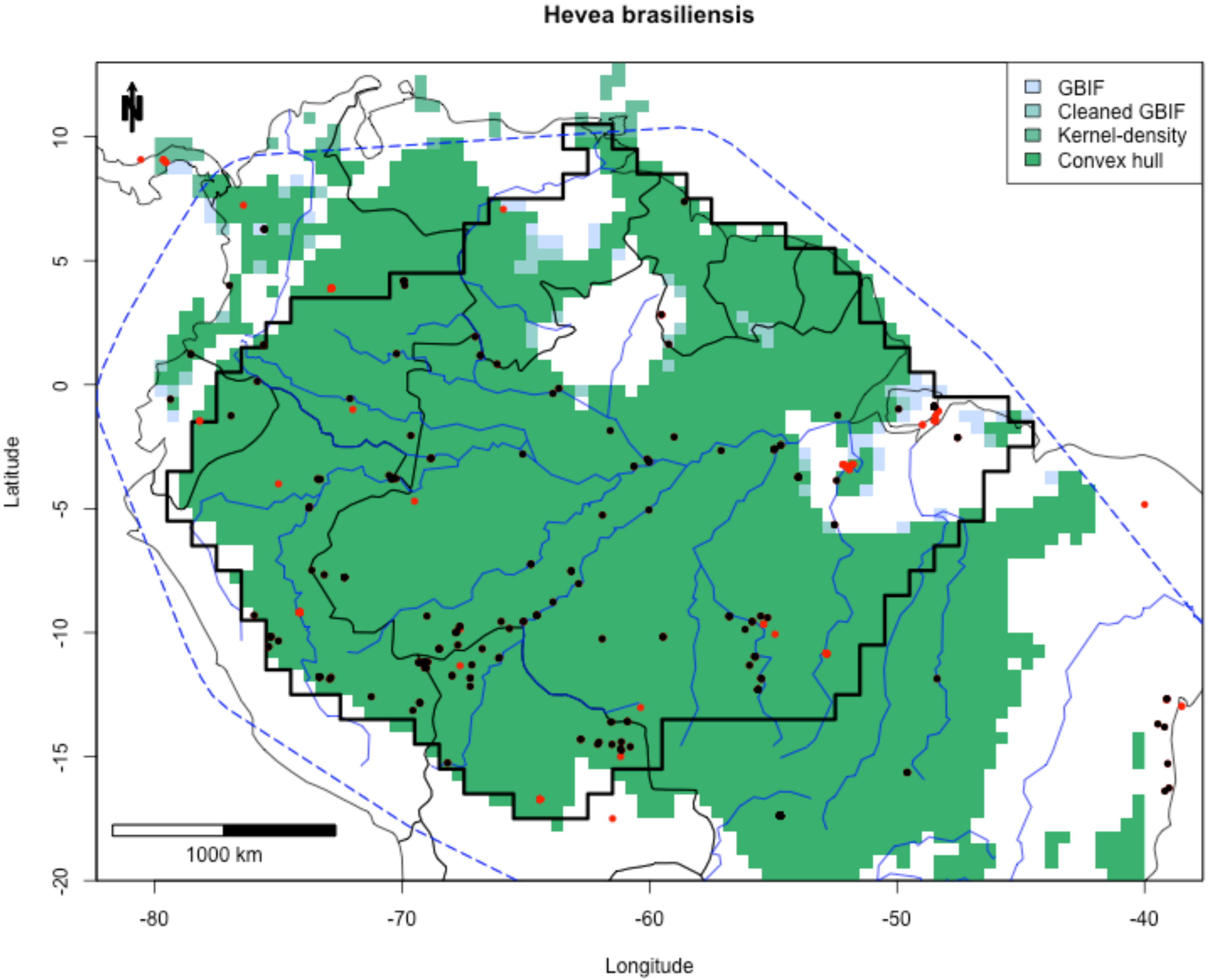

Hevea brasiliensis
GBIF
Cleaned GBIF
Kernel-density
Convex hull
N
1000 km
Latitude
Longitude
10
5
0
-5
-10
-15
-20
-80
-70
-60
-50
-40

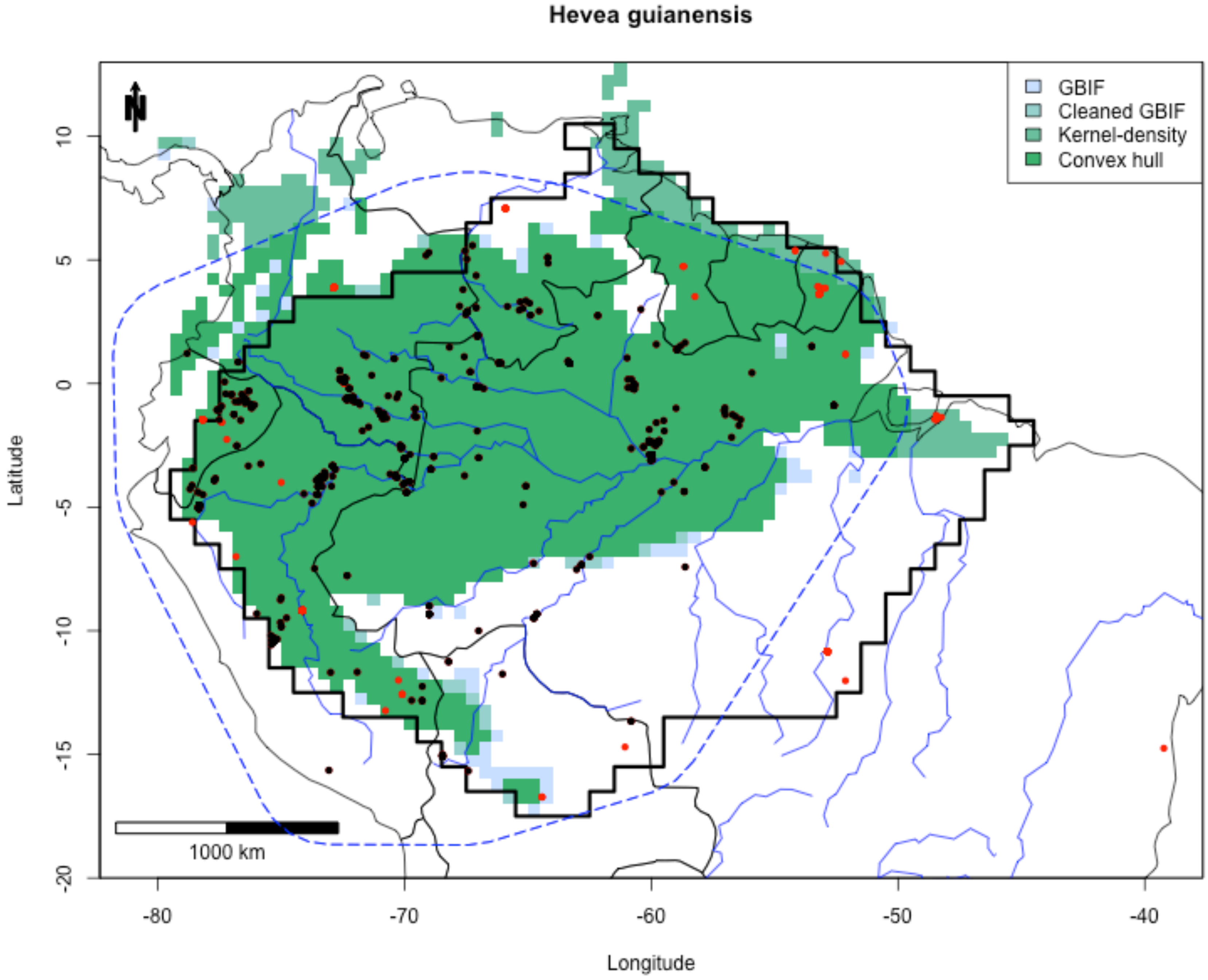

Hevea guianensis
GBIF
Cleaned GBIF
Kernel-density
Convex hull
N
1000 km
Latitude
Longitude
10
5
0
-5
-10
-15
-20
-80
-70
-60
-50
-40

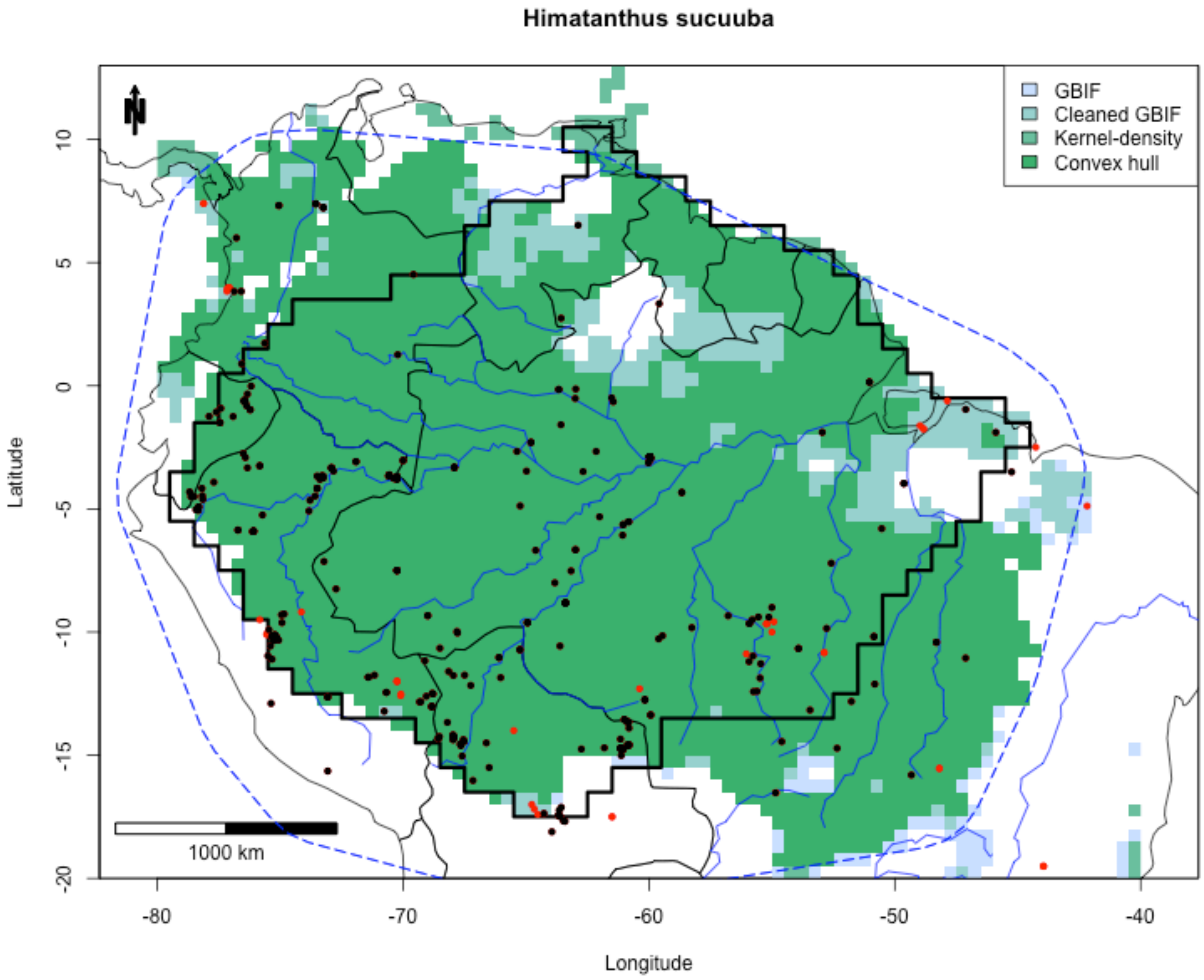
Himatanthus sucuuba
GBIF
Cleaned GBIF
Kernel-density
Convex hull
1000 km
Latitude
Longitude
10
5
0
-5
-10
-15
-20
-80
-70
-60
-50
-40

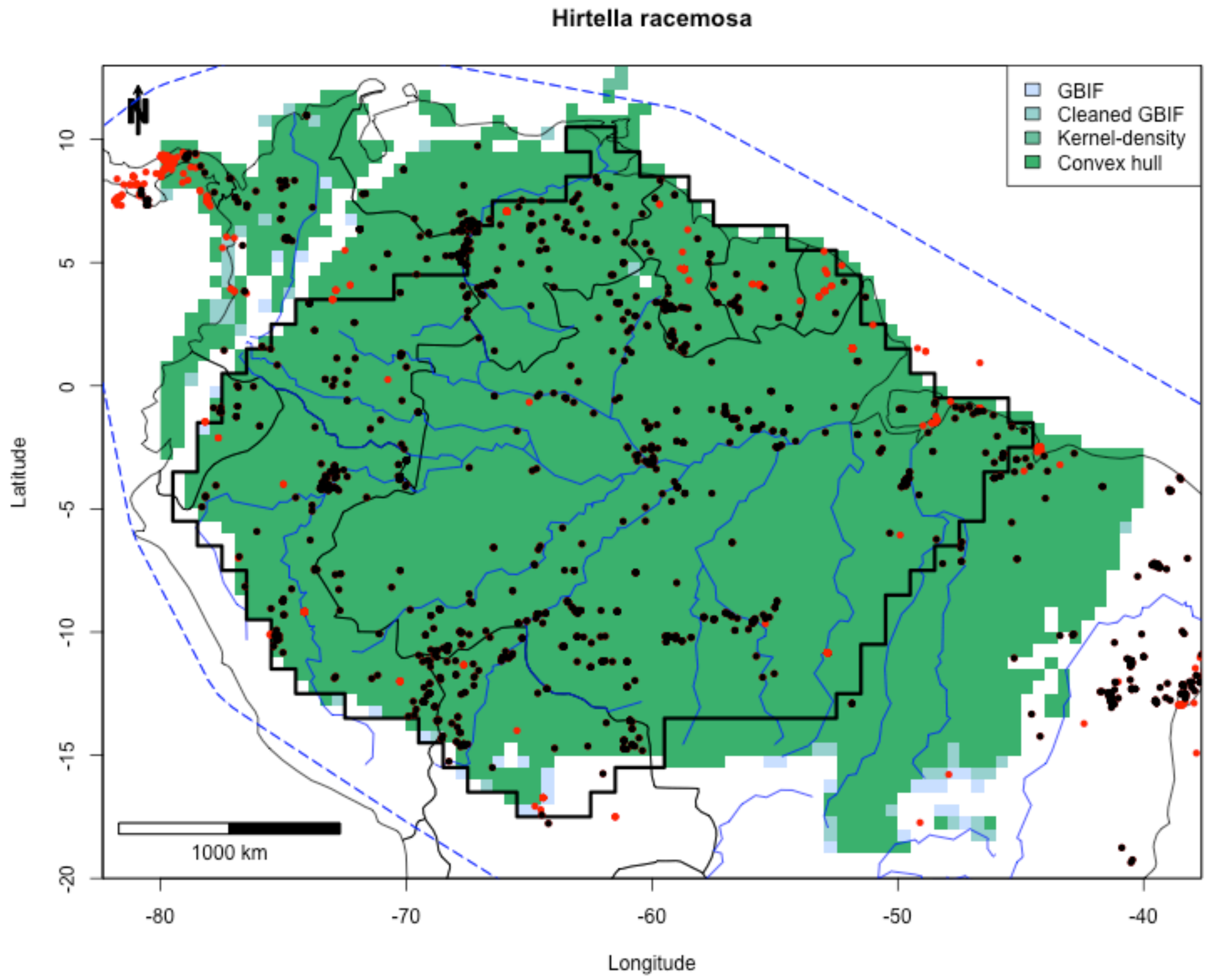

Hirtella racemosa
GBIF
Cleaned GBIF
Kernel-density
Convex hull
N
1000 km
Latitude
Longitude
10
5
0
-5
-10
-15
-20
-80
-70
-60
-50
-40

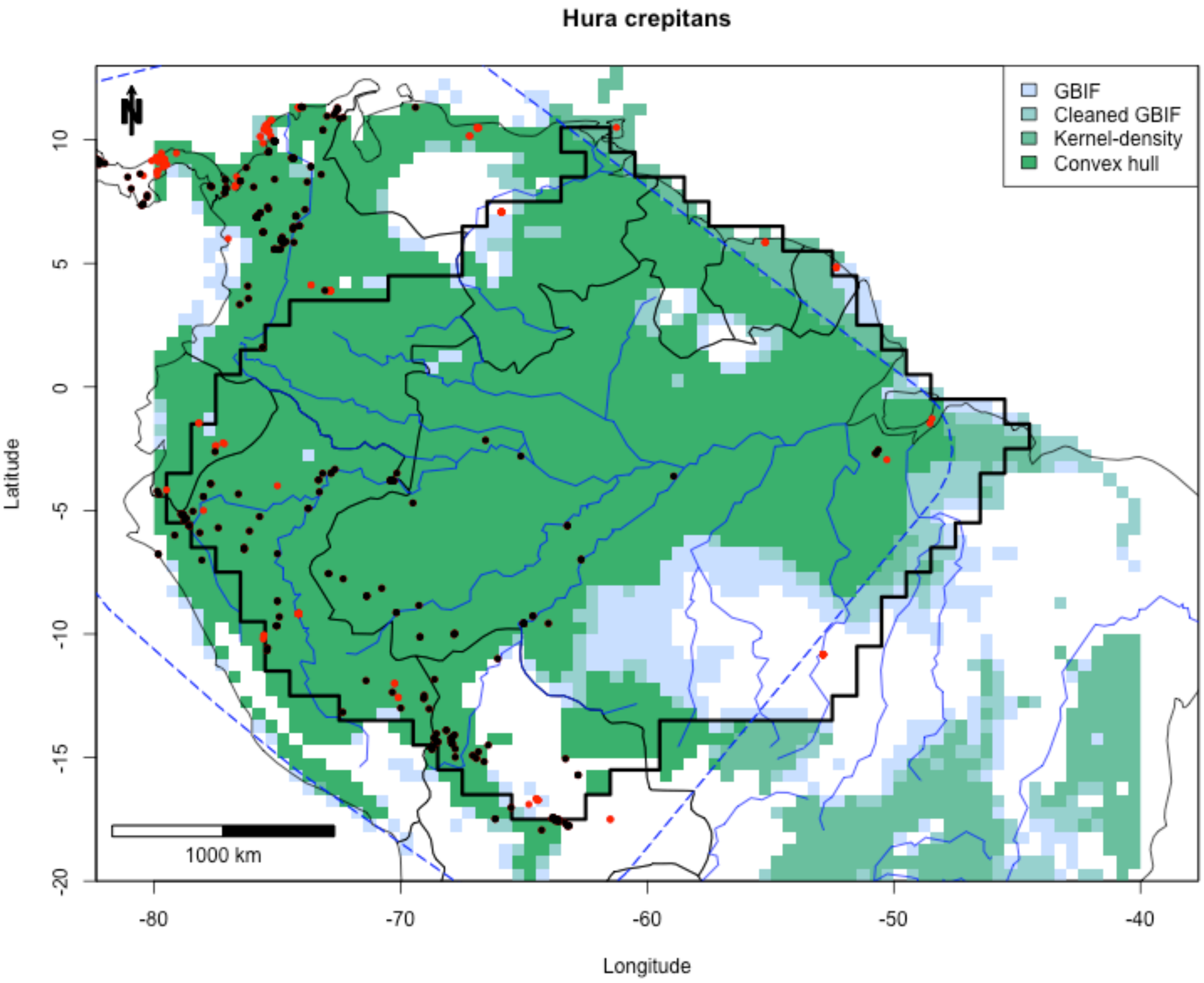
Hura crepitans
GBIF
Cleaned GBIF
Kernel-density
Convex hull
N
1000 km
Latitude
Longitude
10
5
0
-5
-10
-15
-20
-80
-70
-60
-50
-40

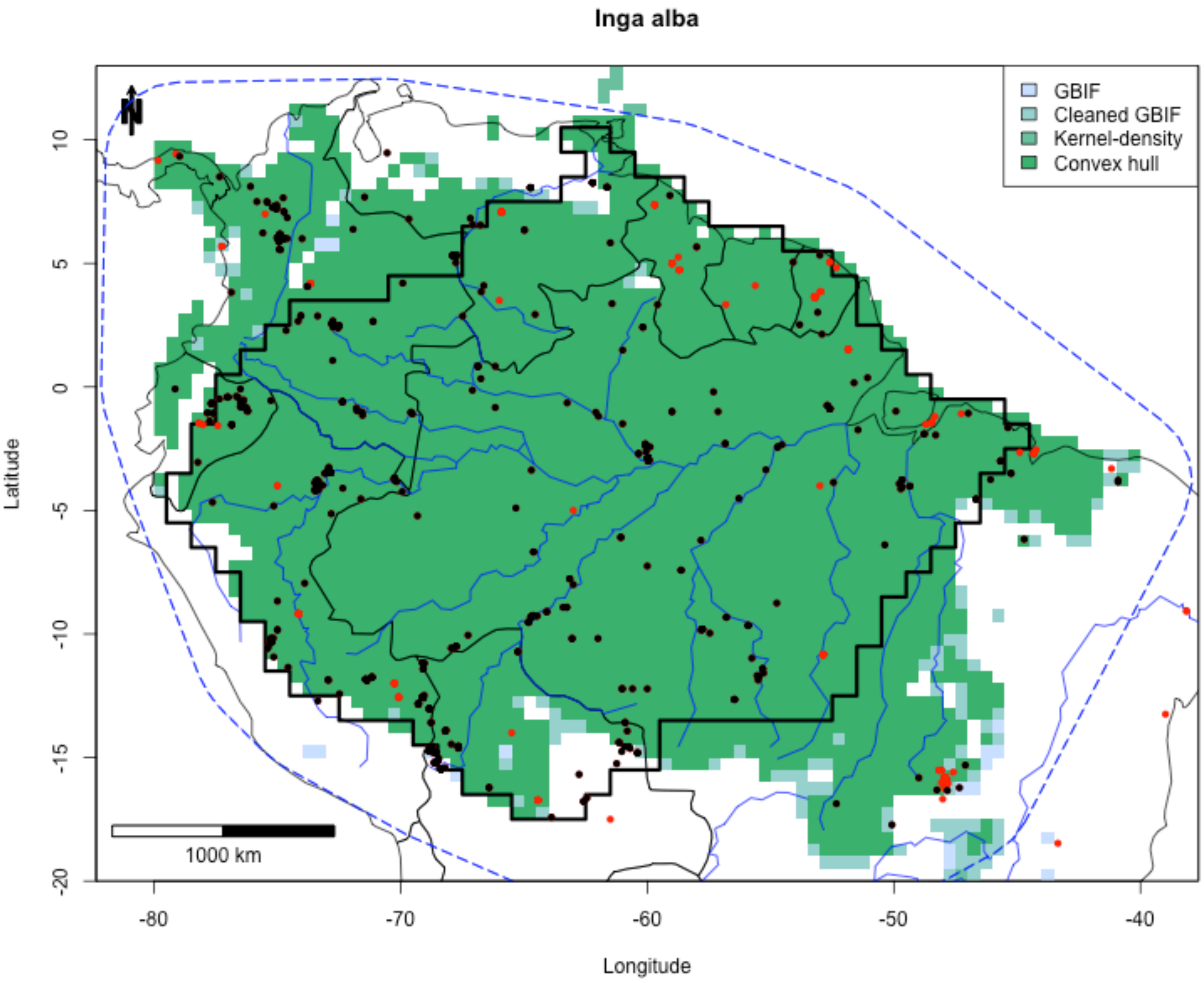
Inga alba
GBIF
Cleaned GBIF
Kernel-density
Convex hull
N
1000 km
Latitude
Longitude
10
5
0
-5
-10
-15
-20
-80
-70
-60
-50
-40

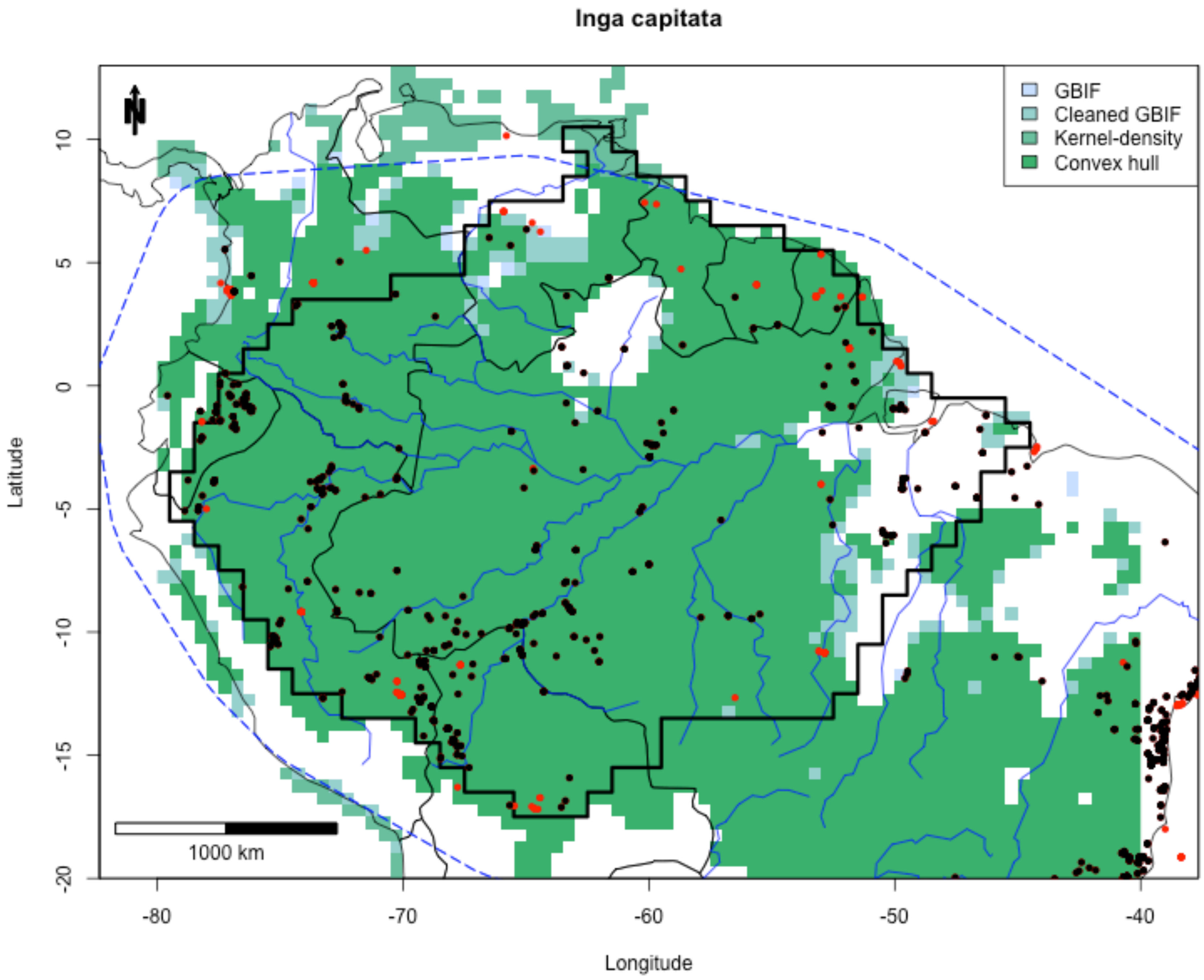

Inga capitata
GBIF
Cleaned GBIF
Kernel-density
Convex hull
N
1000 km
Latitude
Longitude
10
5
0
-5
-10
-15
-20
-80
-70
-60
-50
-40

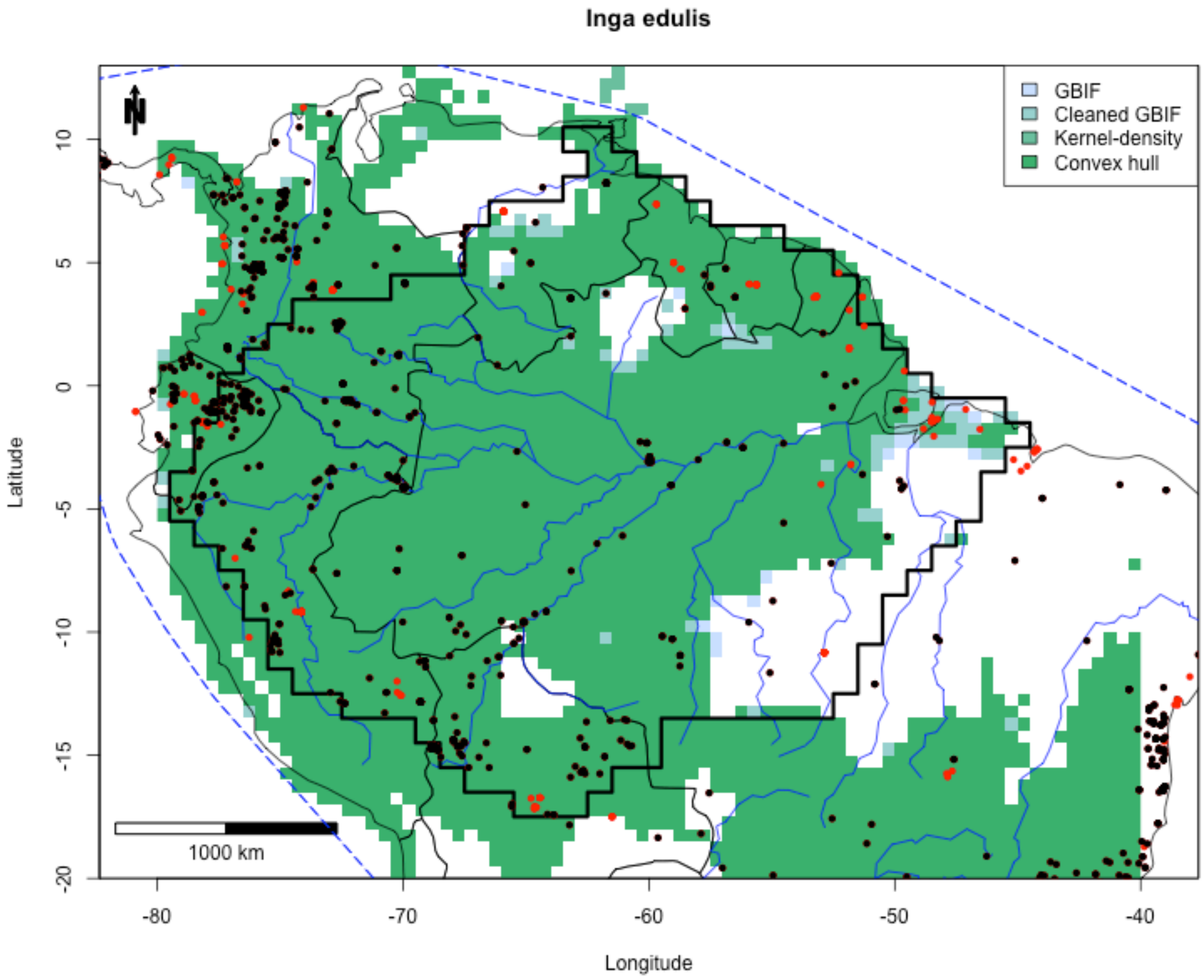
Inga edulis
GBIF
Cleaned GBIF
Kernel-density
Convex hull
N
1000 km
Latitude
Longitude
10
5
0
-5
-10
-15
-20
-80
-70
-60
-50
-40

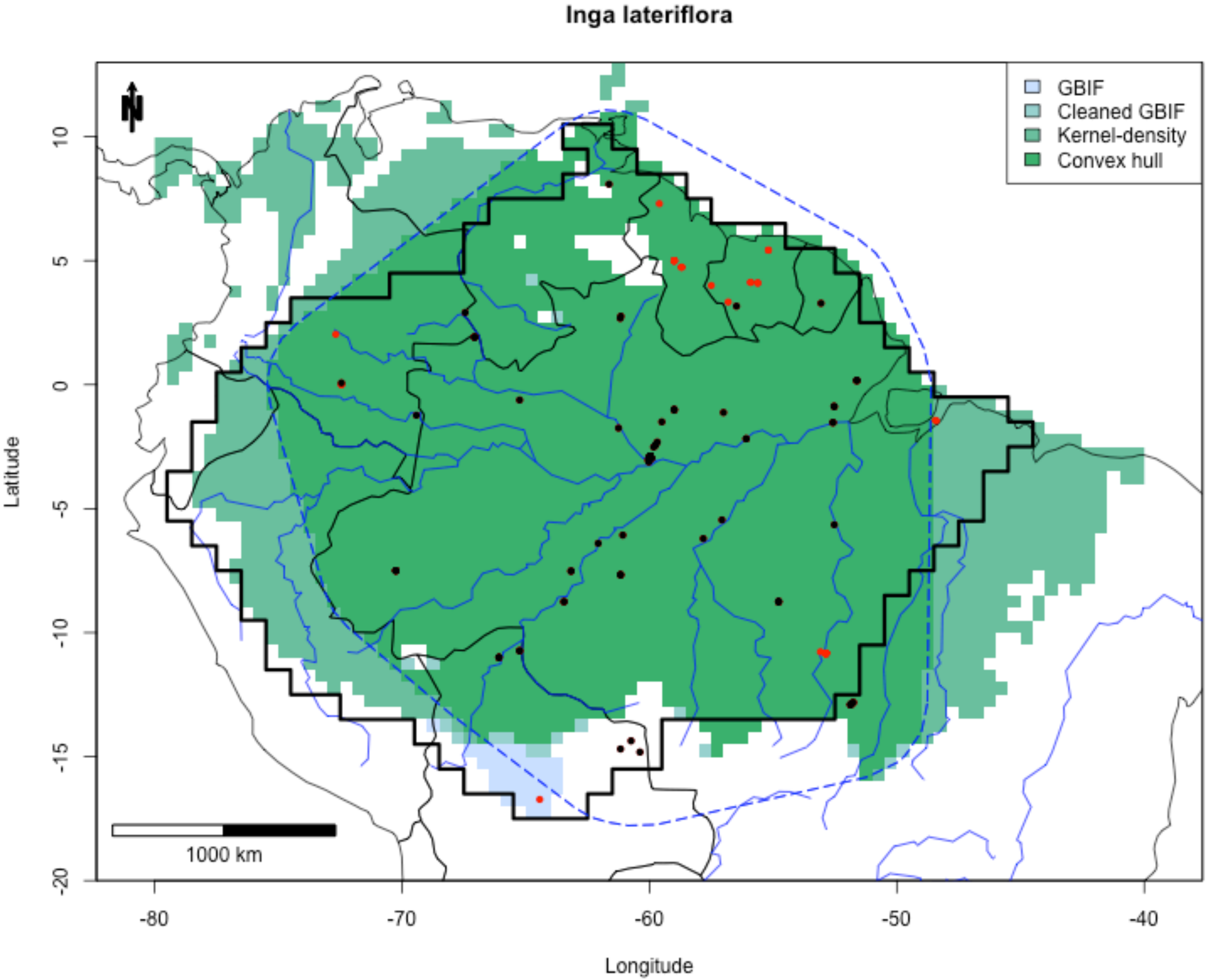

Inga lateriflora
GBIF
Cleaned GBIF
Kernel-density
Convex hull
N
1000 km
Latitude
Longitude
10
5
0
-5
-10
-15
-20
-80
-70
-60
-50
-40

Inga marginata

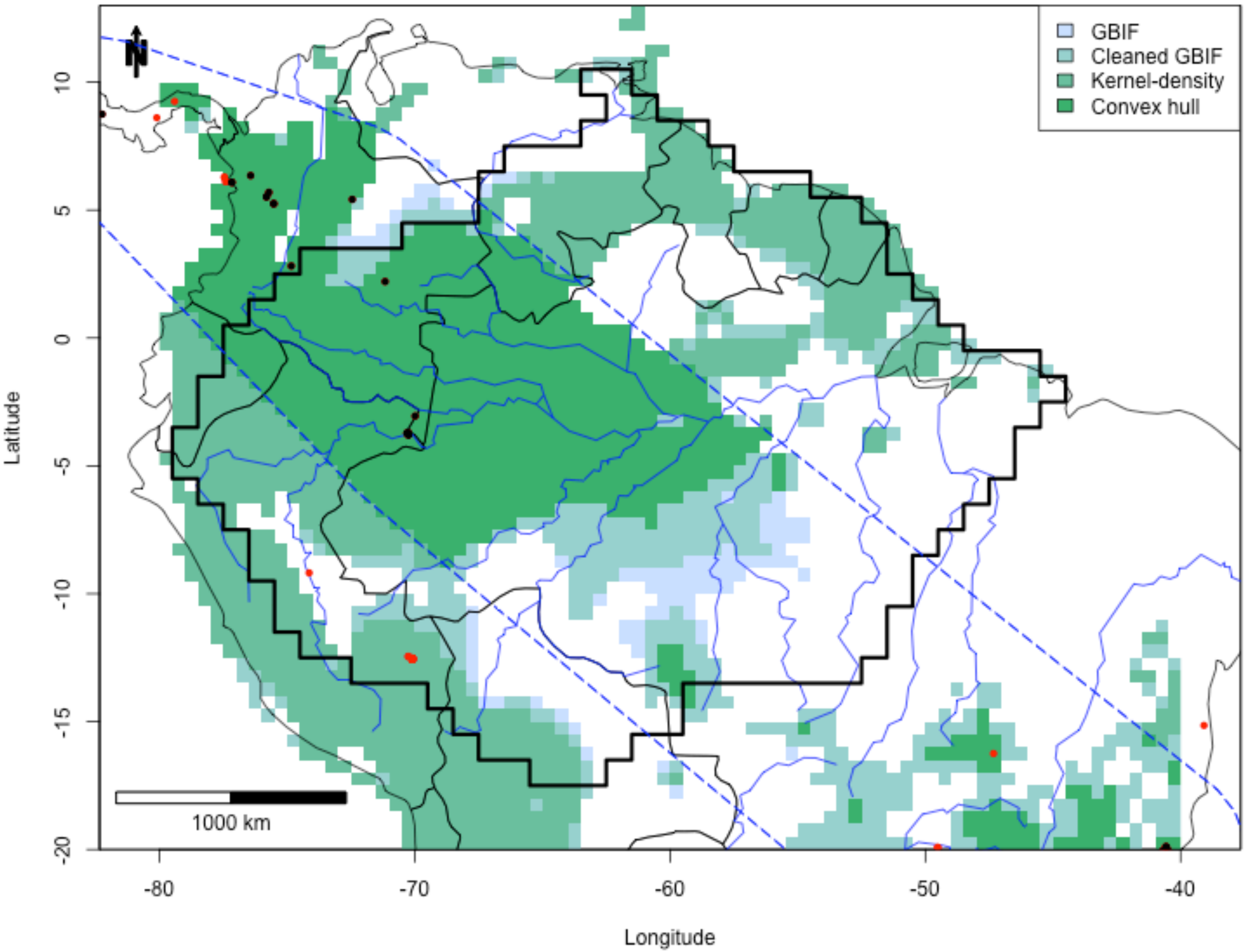

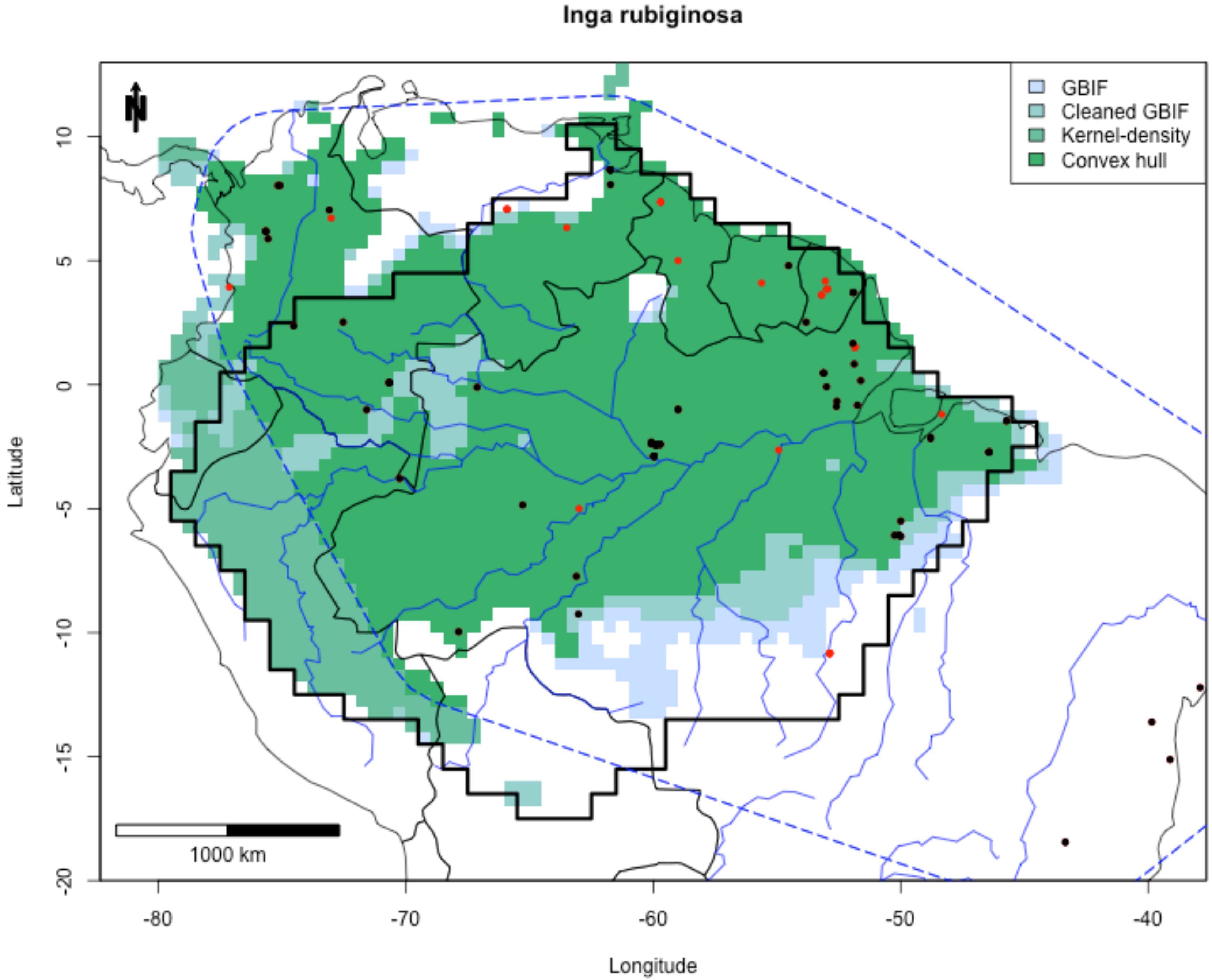
Inga rubiginosa
GBIF
Cleaned GBIF
Kernel-density
Convex hull
N
1000 km
Latitude
Longitude
10
5
0
-5
-10
-15
-20
-80
-70
-60
-50
-40

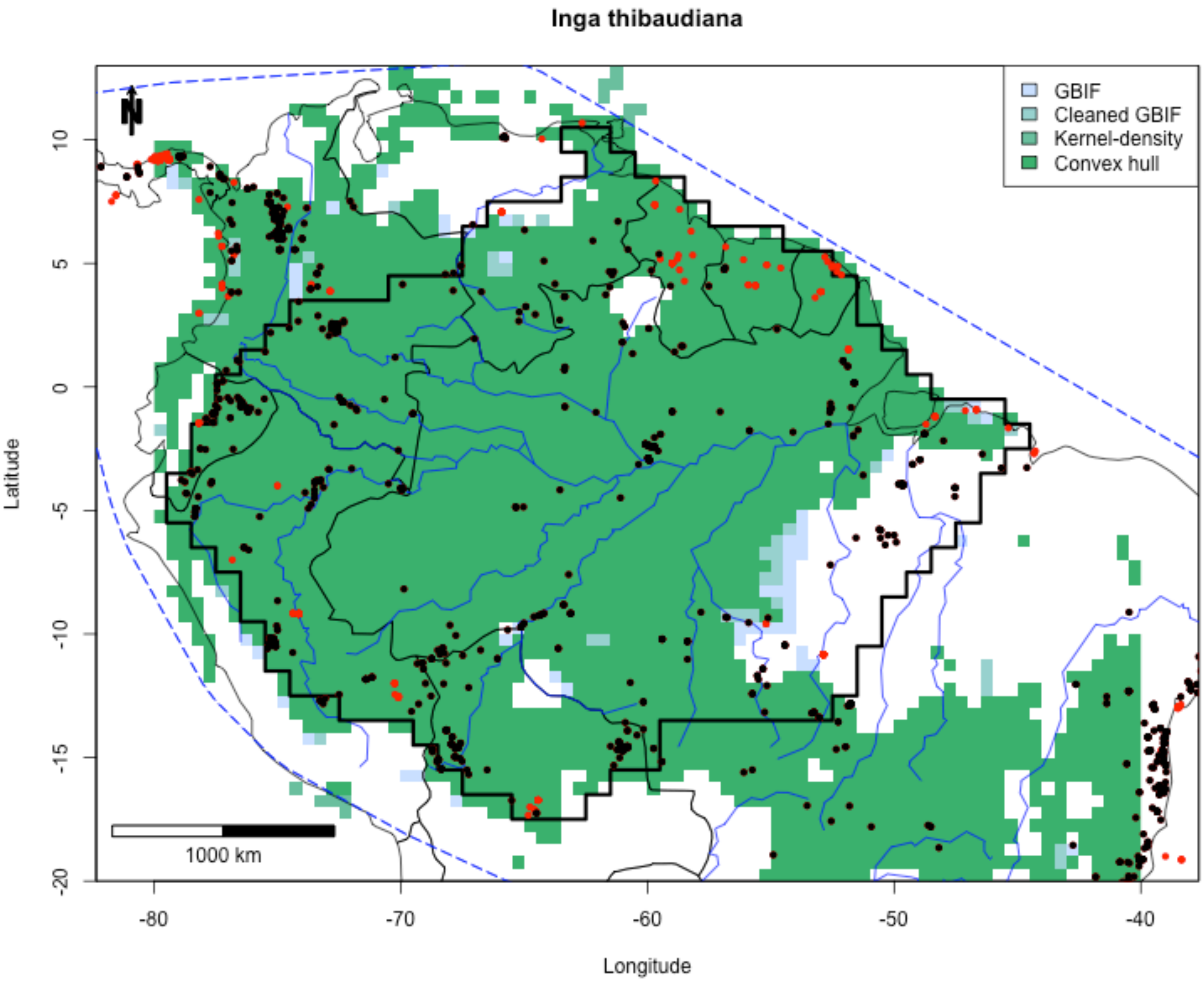
Inga thibaudiana
GBIF
Cleaned GBIF
Kernel-density
Convex hull
1000 km
N
Latitude
Longitude
10
5
0
-5
-10
-15
-20
-80
-70
-60
-50
-40

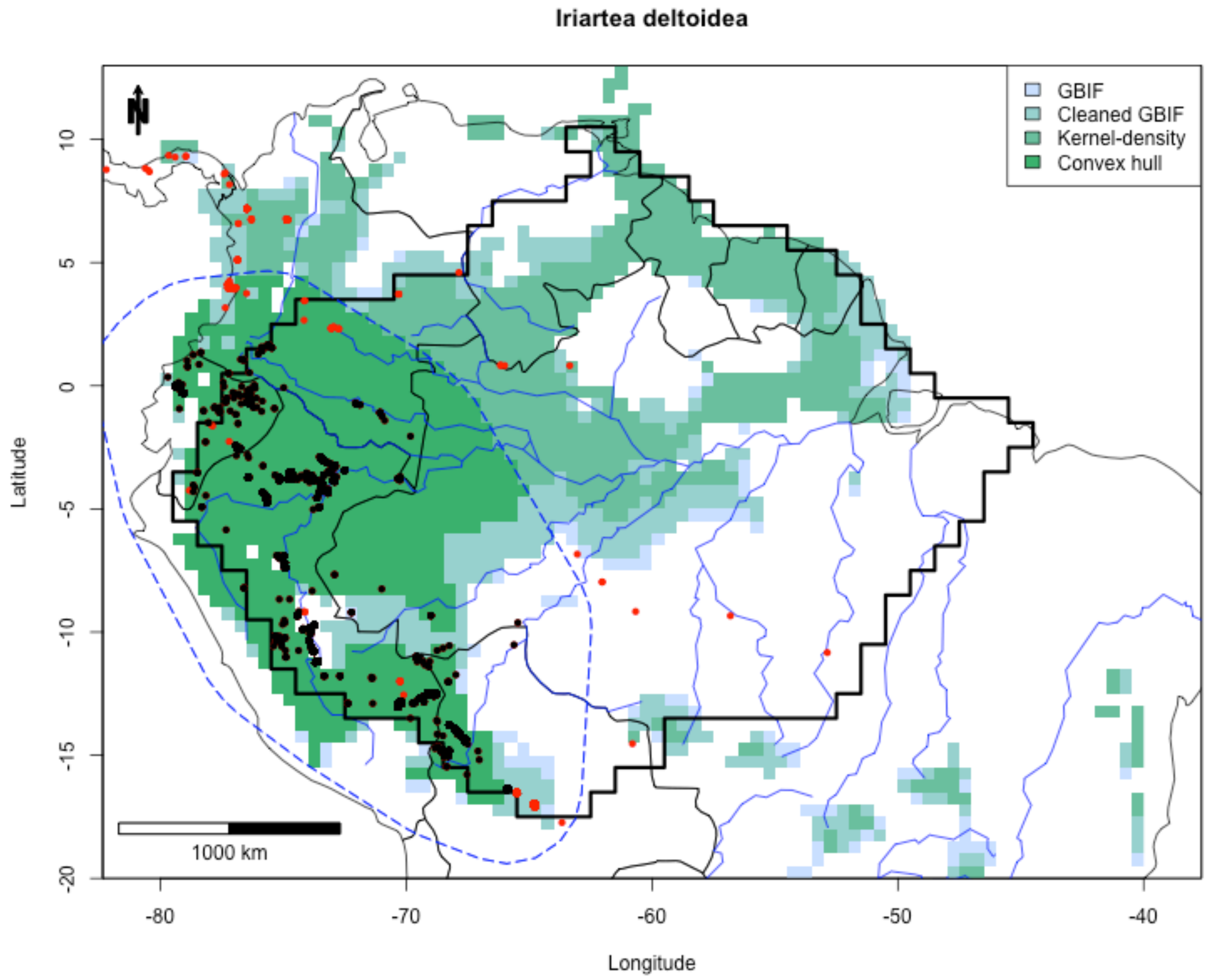

Iriartea deltoidea
GBIF
Cleaned GBIF
Kernel-density
Convex hull
N
1000 km
Latitude
Longitude
10
5
0
-5
-10
-15
-20
-80
-70
-60
-50
-40

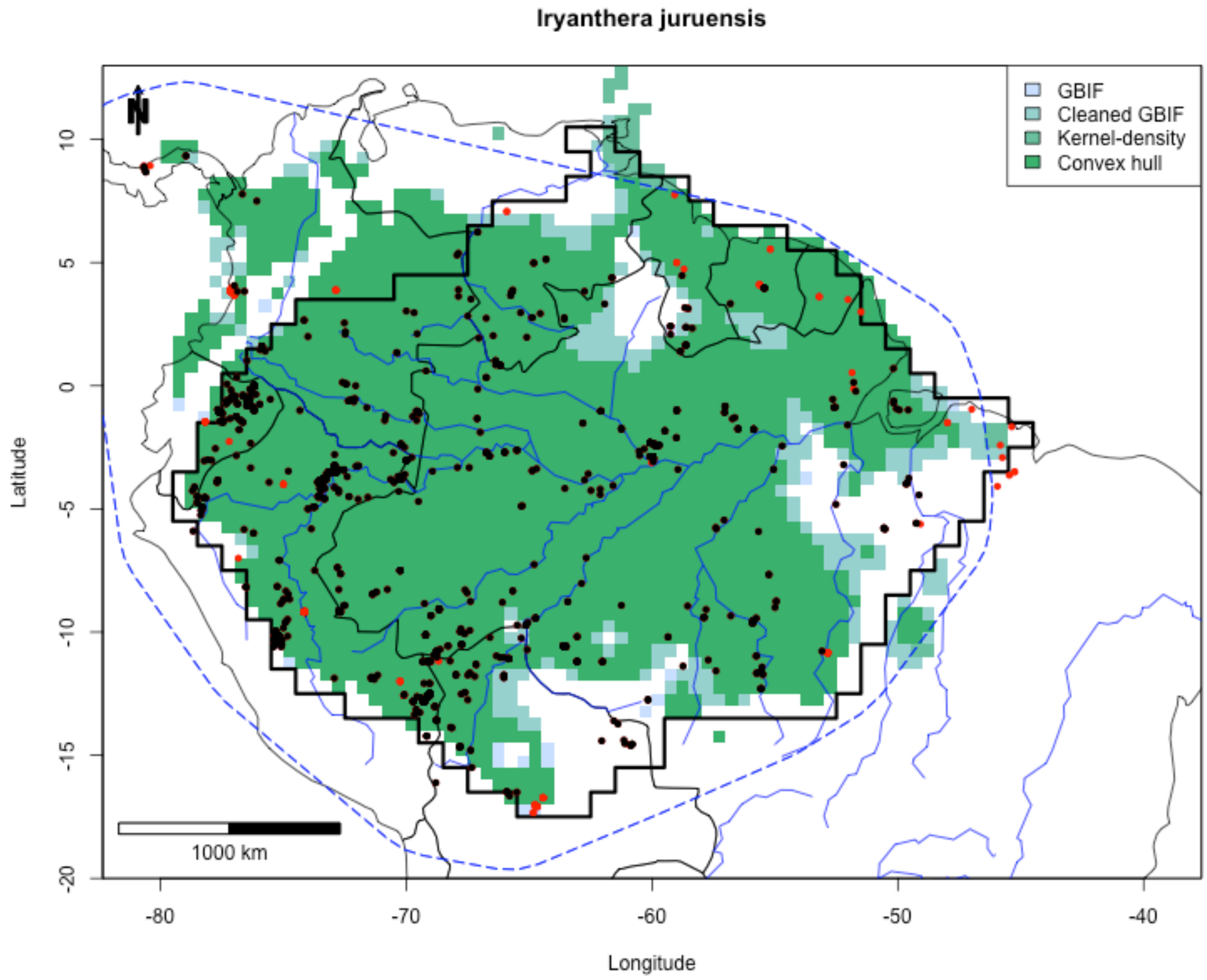
Iryanthera juruensis
GBIF
Cleaned GBIF
Kernel-density
Convex hull
N
1000 km
Latitude
10
5
0
-5
-10
-15
-20
-80
-70
-60
-50
-40
Longitude

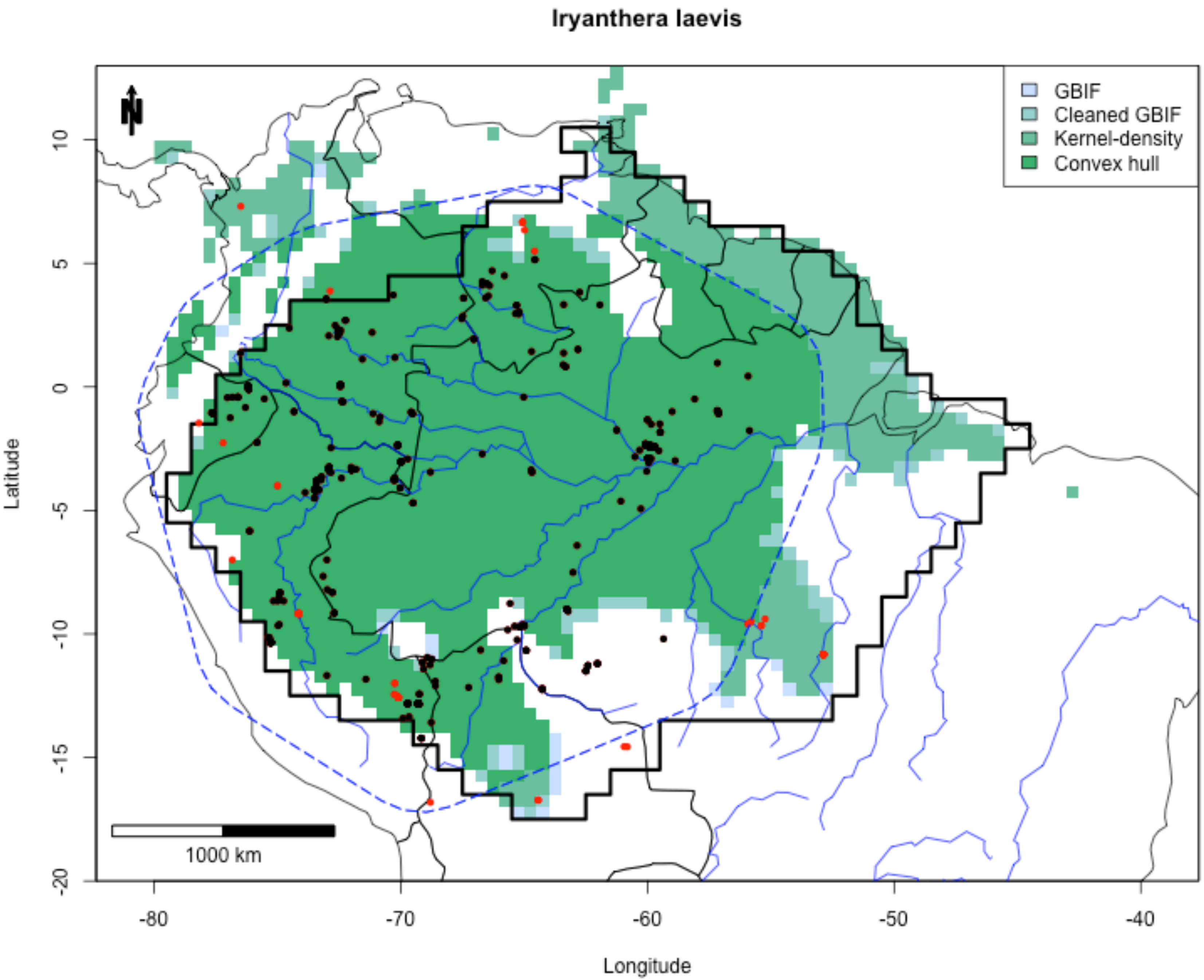
Iryanthera laevis
GBIF
Cleaned GBIF
Kernel-density
Convex hull
N
1000 km
Latitude
Longitude
10
5
0
-5
-10
-15
-20
-80
-70
-60
-50
-40

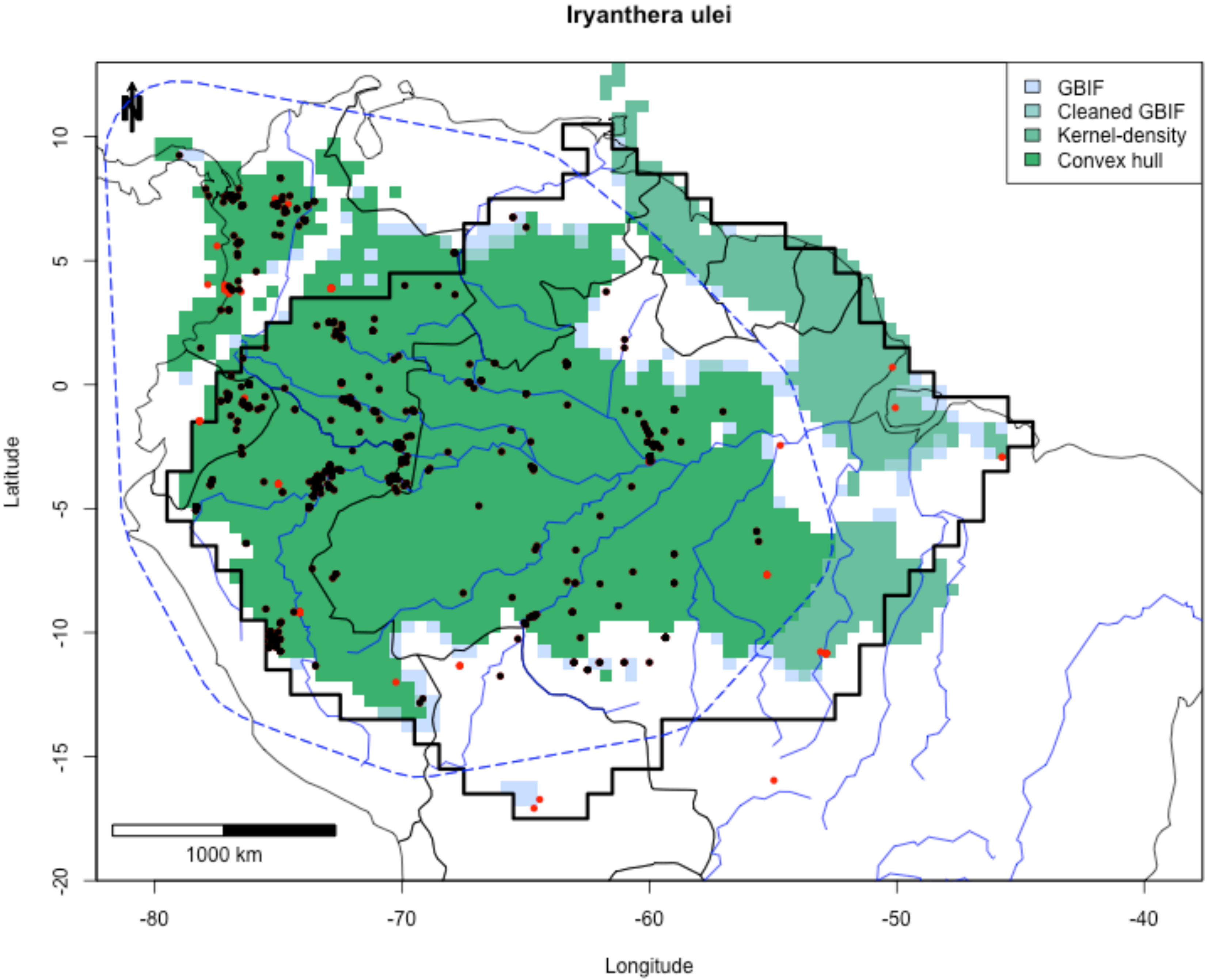

Iryanthera ulei
GBIF
Cleaned GBIF
Kernel-density
Convex hull
1000 km
Latitude
Longitude
10
5
0
-5
-10
-15
-20
-80
-70
-60
-50
-40

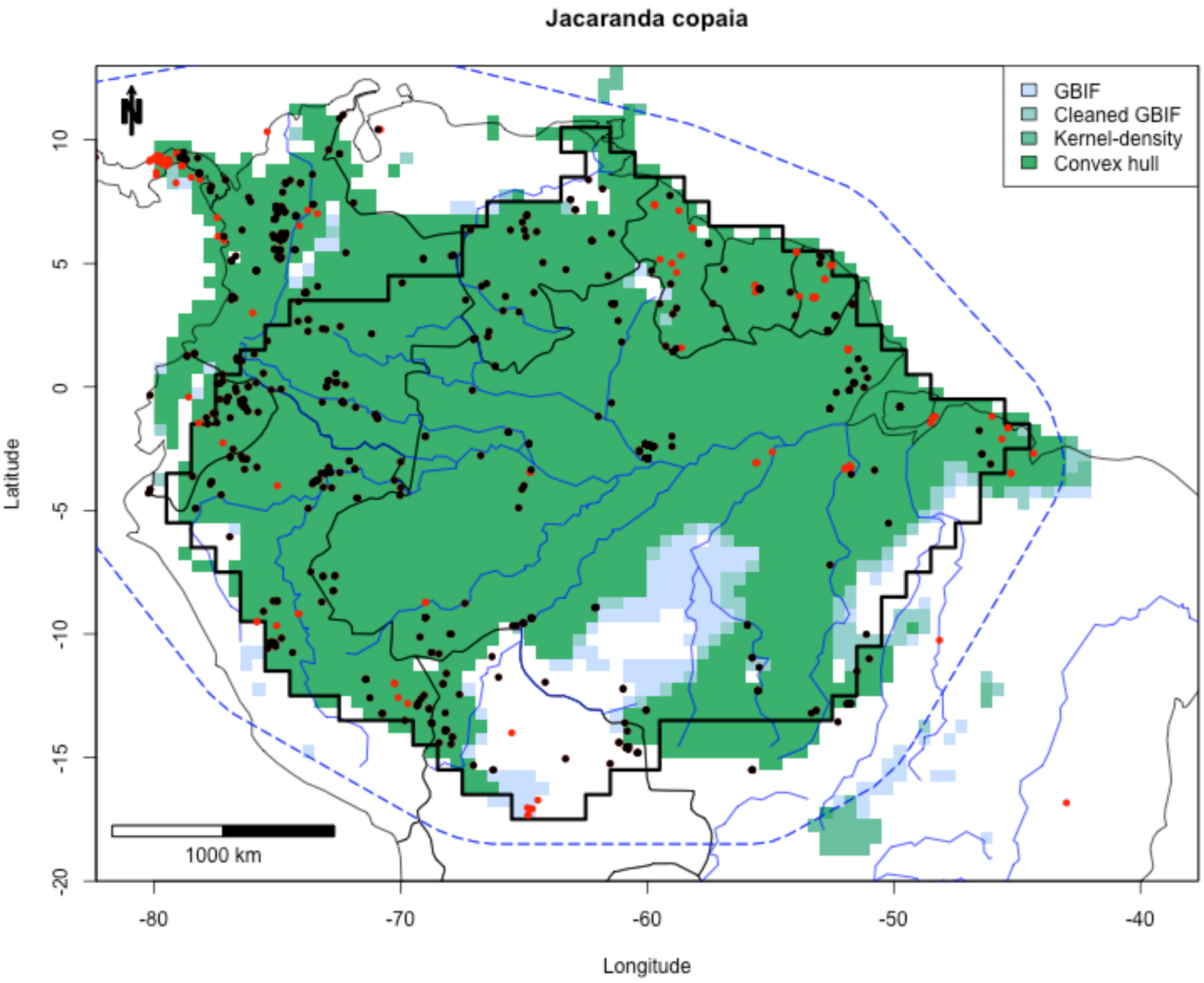

Jacaranda copaia
GBIF
Cleaned GBIF
Kernel-density
Convex hull
N
1000 km
Latitude
Longitude
10
5
0
-5
-10
-15
-20
-80
-70
-60
-50
-40

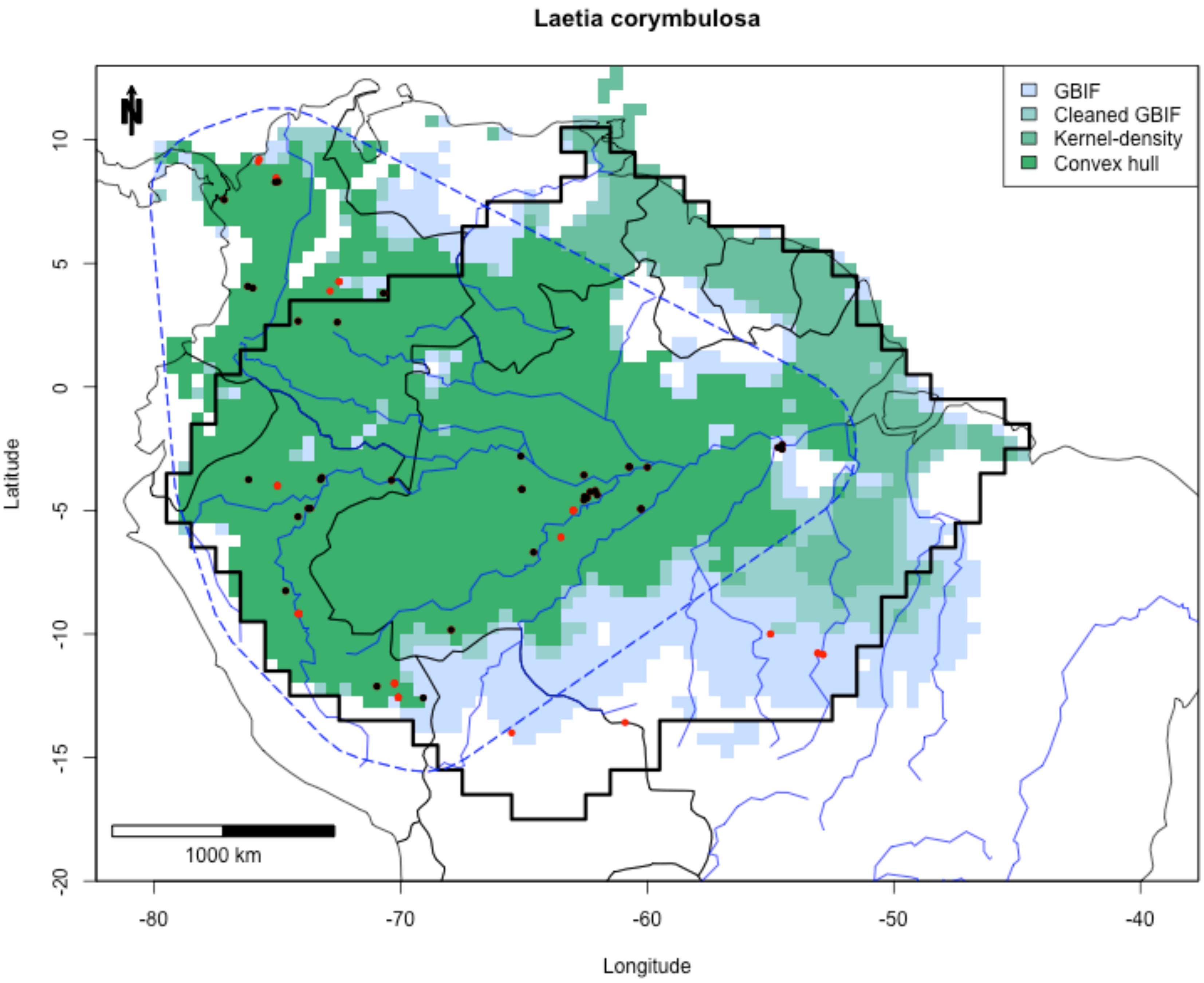

Laetia corymbulosa
GBIF
Cleaned GBIF
Kernel-density
Convex hull
N
1000 km
Latitude
Longitude
10
5
0
-5
-10
-15
-20
-80
-70
-60
-50
-40

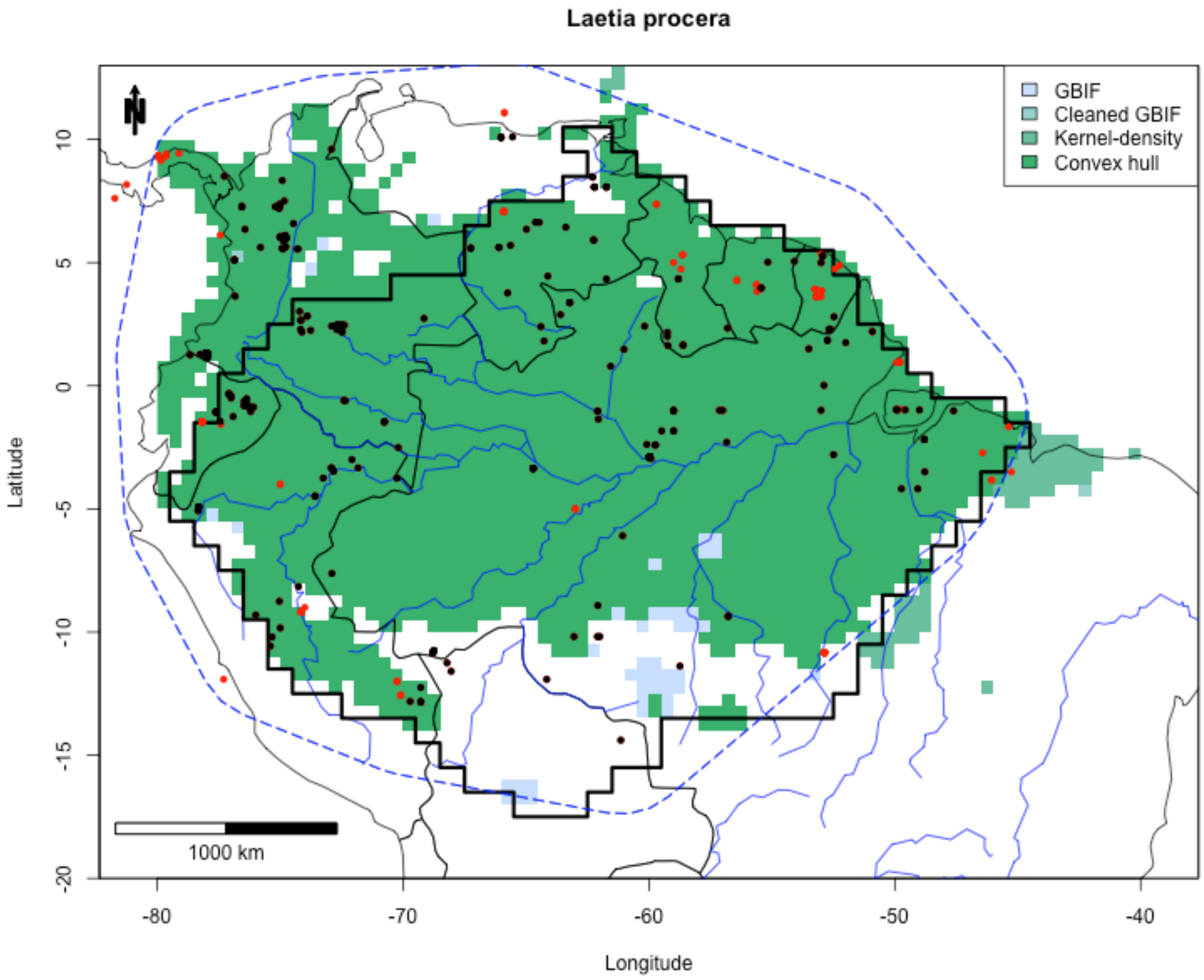
Laetia procera
GBIF
Cleaned GBIF
Kernel-density
Convex hull
N
1000 km
Latitude
Longitude
10
5
0
-5
-10
-15
-20
-80
-70
-60
-50
-40

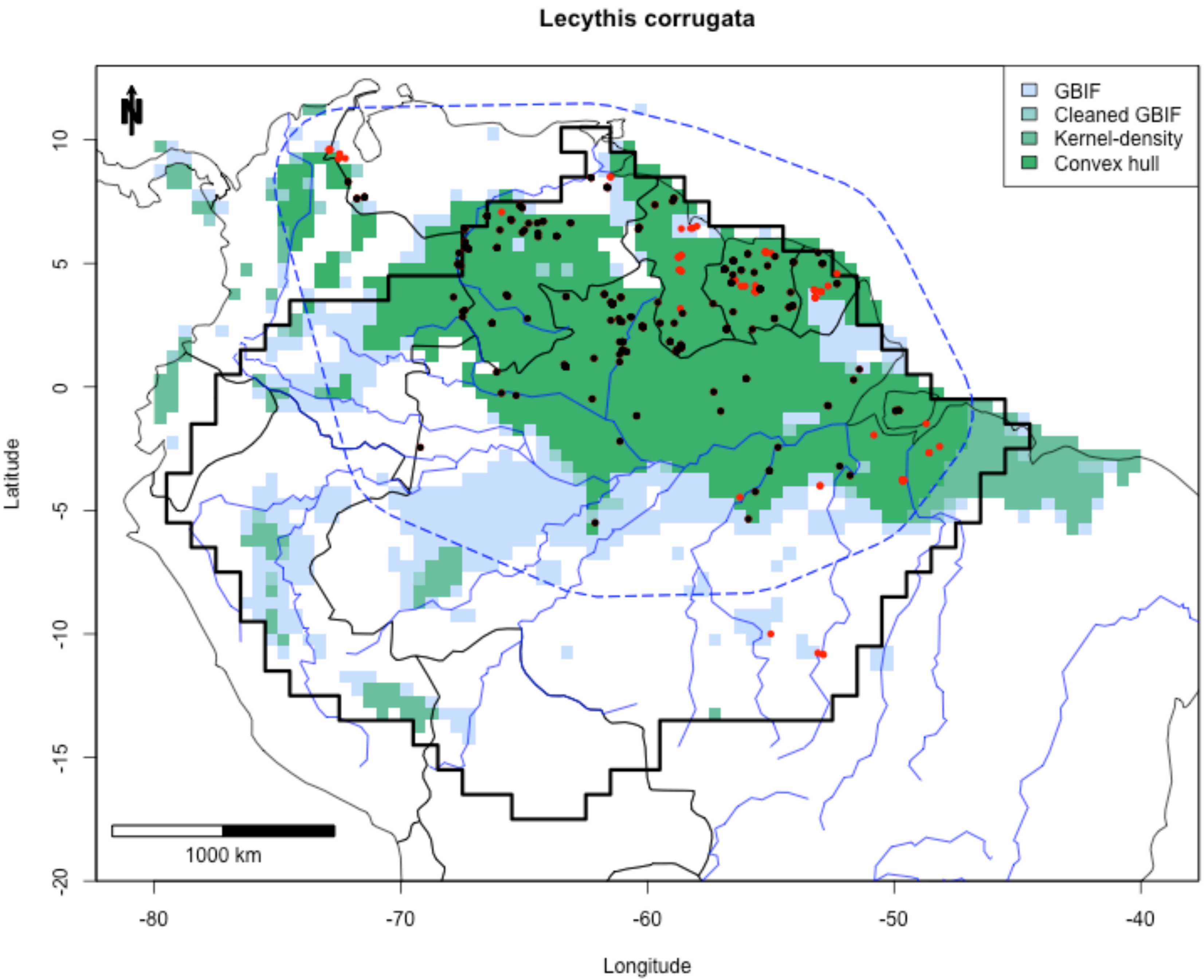

Lecythis corrugata
GBIF
Cleaned GBIF
Kernel-density
Convex hull
N
1000 km
Latitude
Longitude
10
5
0
-5
-10
-15
-20
-80
-70
-60
-50
-40

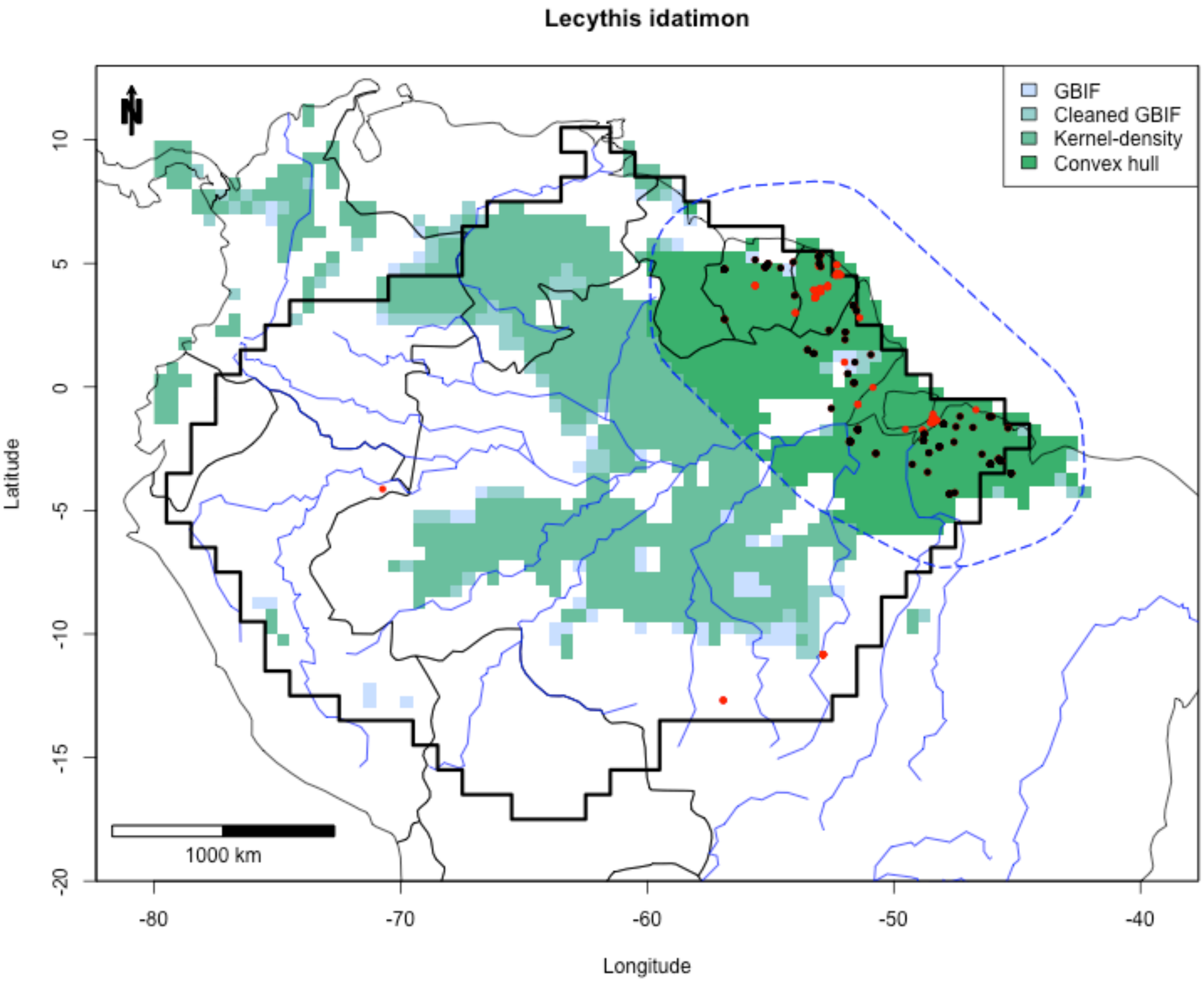

Lecythis idatimon
GBIF
Cleaned GBIF
Kernel-density
Convex hull
N
1000 km
Latitude
Longitude
10
5
0
-5
-10
-15
-20
-80
-70
-60
-50
-40

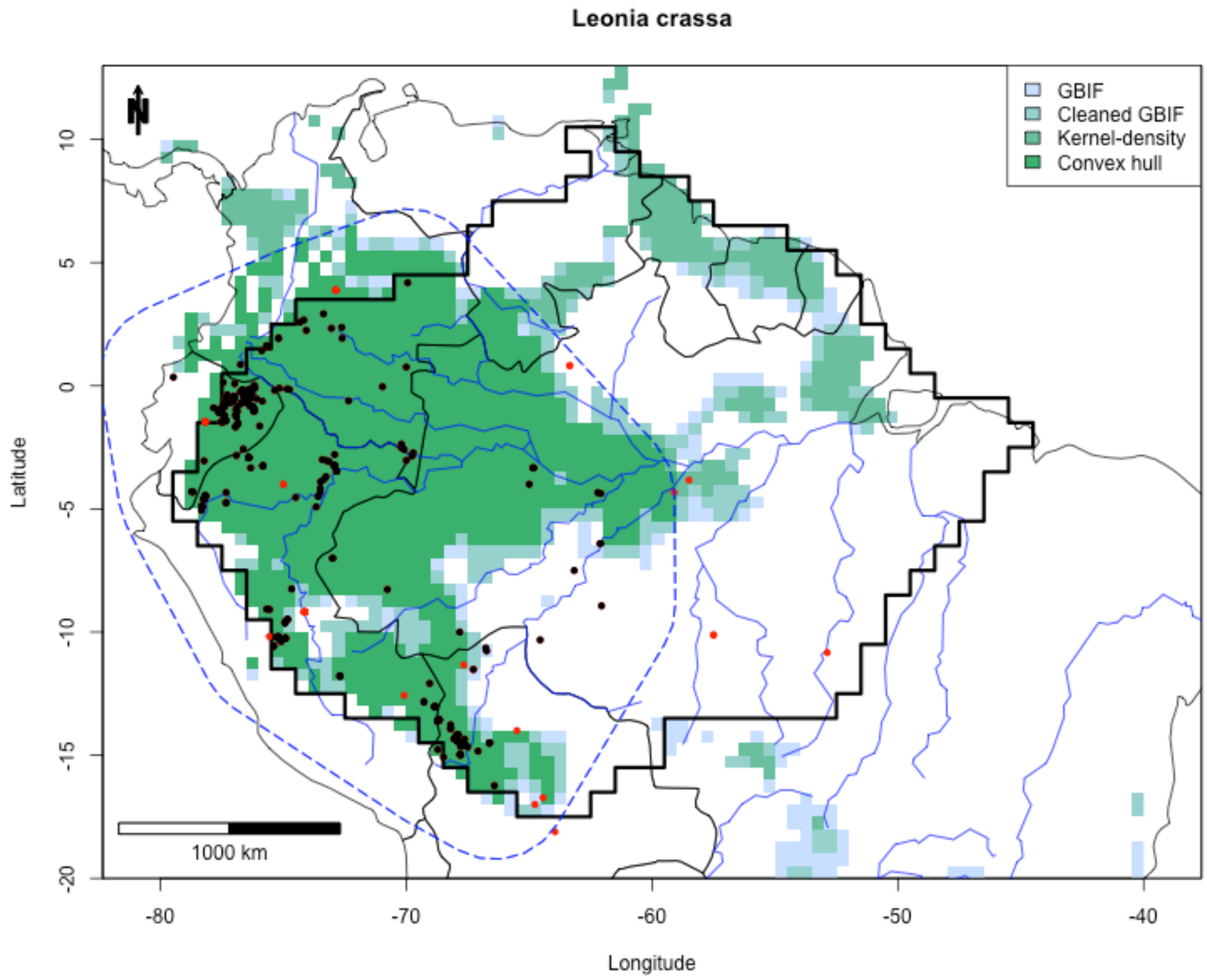
Leonia crassa
N
GBIF
Cleaned GBIF
Kernel-density
Convex hull
10
5
0
-5
-10
-15
-20
Latitude
1000 km
-80
-70
-60
-50
-40
Longitude

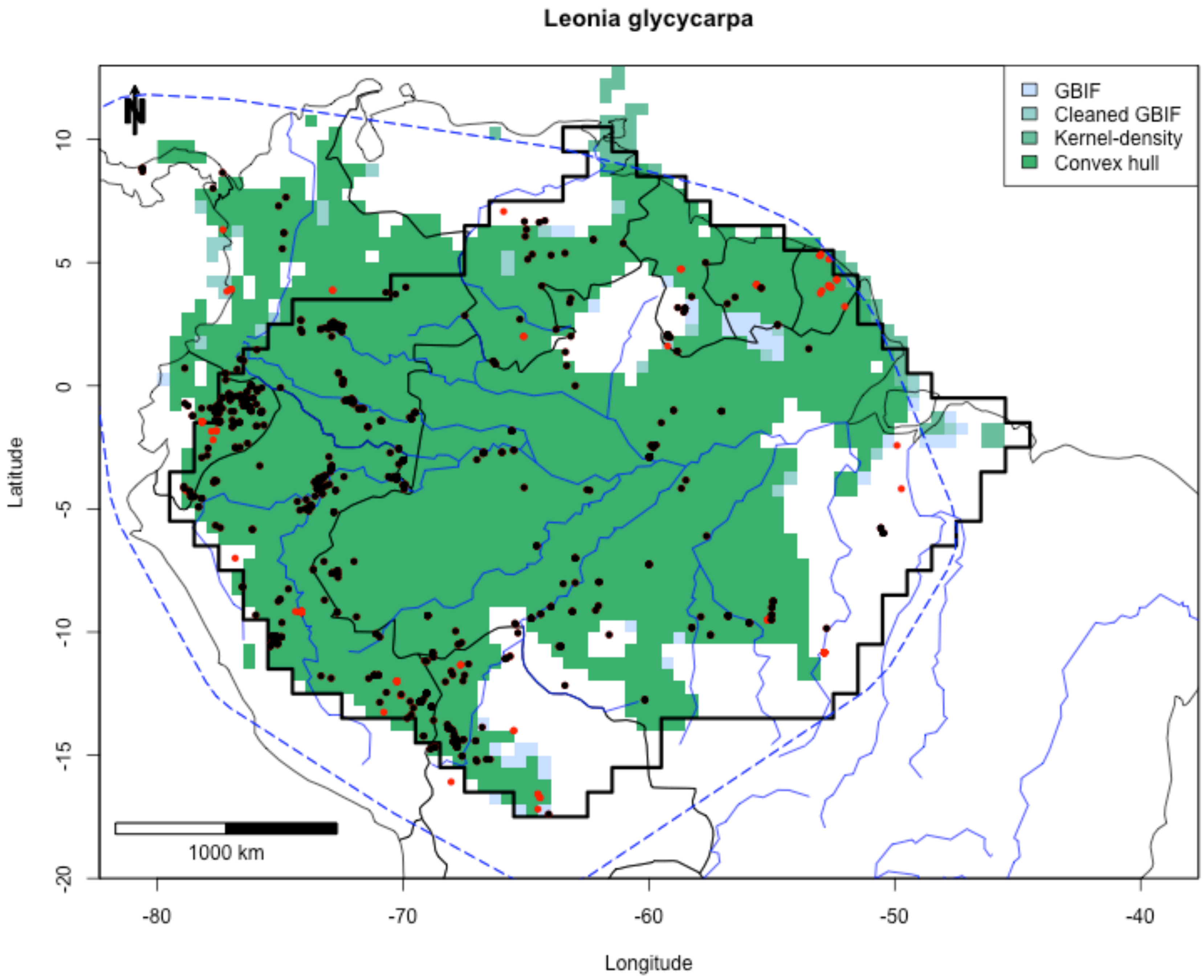

Leonia glycycarpa
GBIF
Cleaned GBIF
Kernel-density
Convex hull
N
1000 km
Latitude
Longitude
10
5
0
-5
-10
-15
-20
-80
-70
-60
-50
-40

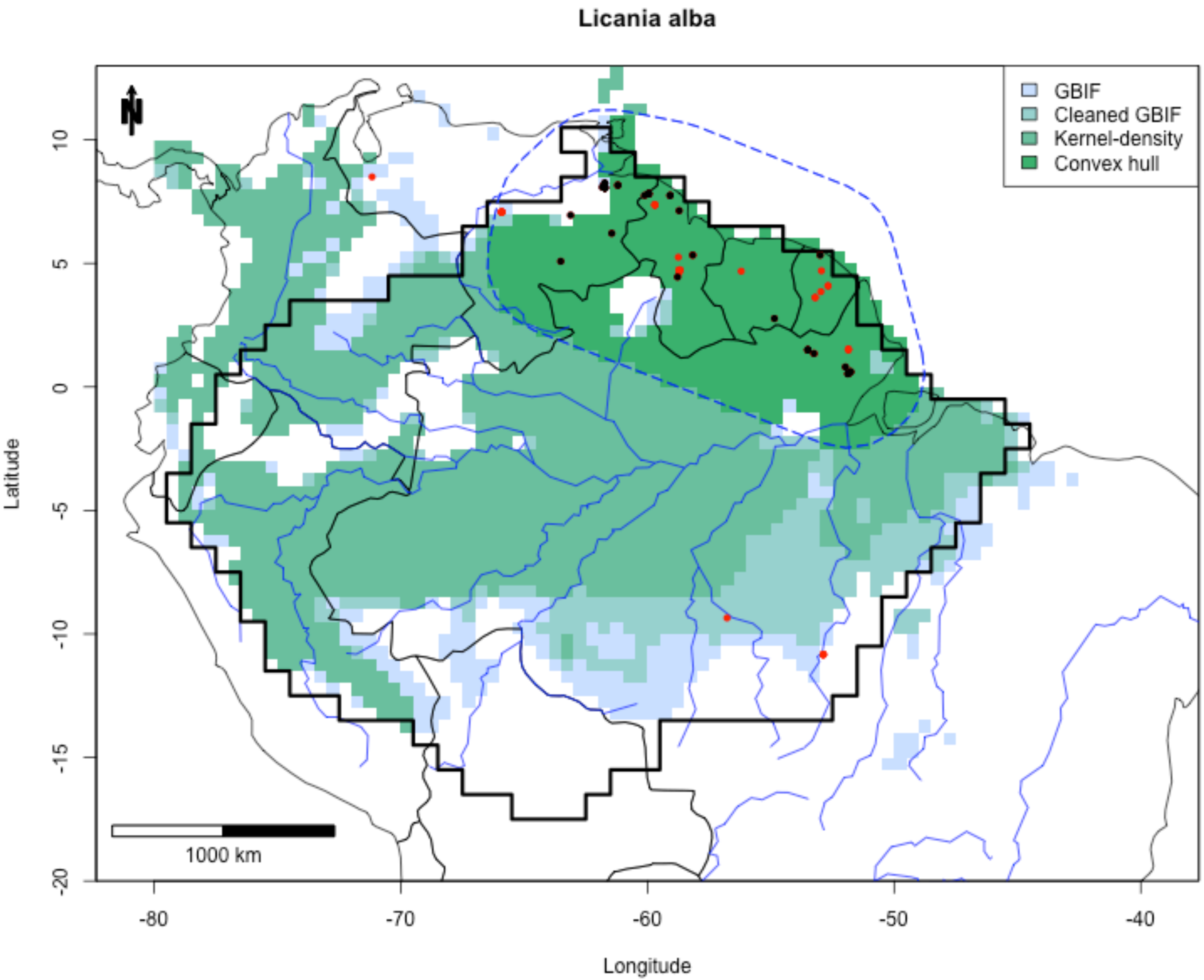

Licania alba
GBIF
Cleaned GBIF
Kernel-density
Convex hull
N
1000 km
Latitude
Longitude
10
5
0
-5
-10
-15
-20
-80
-70
-60
-50
-40

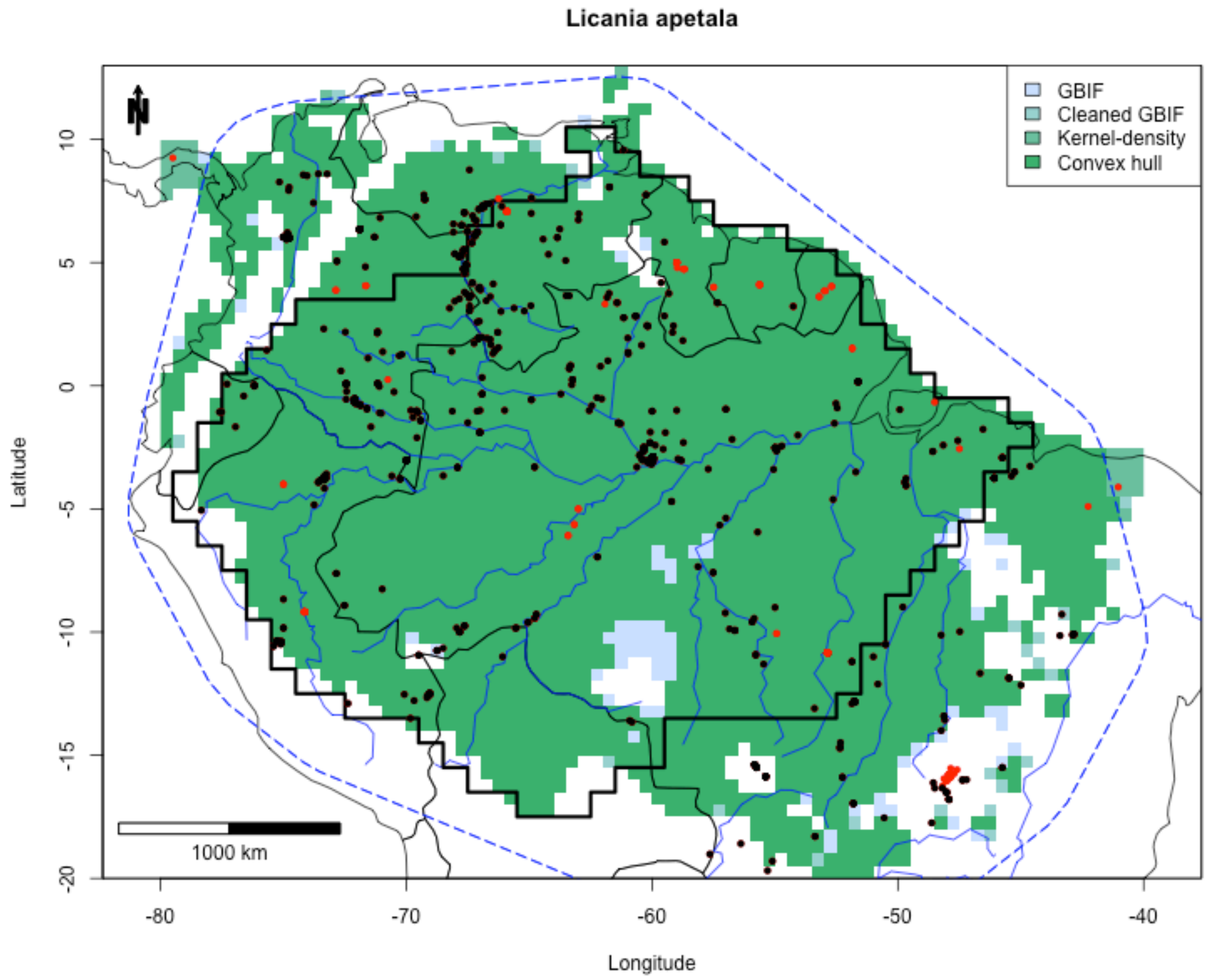

Licania apetala
GBIF
Cleaned GBIF
Kernel-density
Convex hull
1000 km
Latitude
Longitude
10
5
0
-5
-10
-15
-20
-80
-70
-60
-50
-40

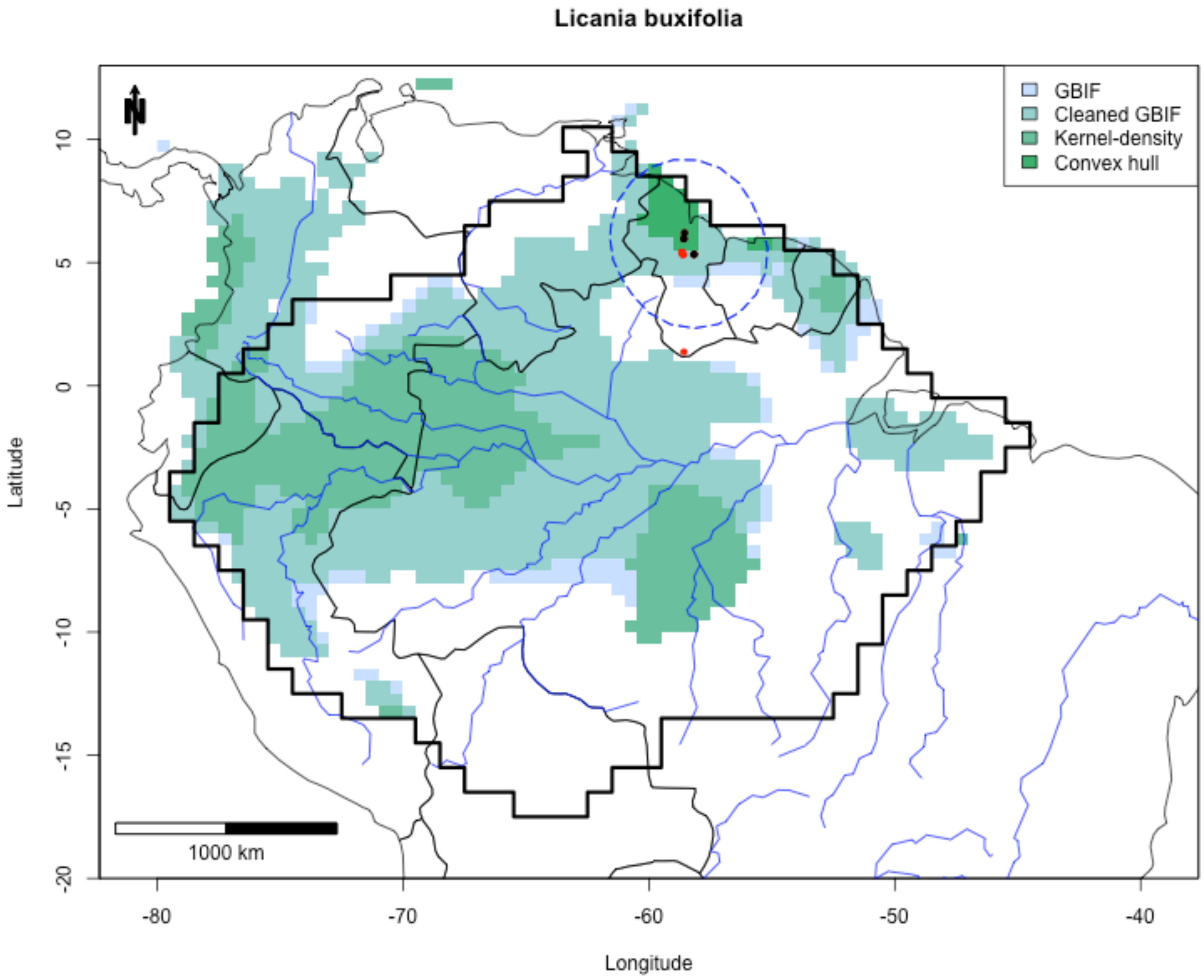
Licania buxifolia
GBIF
Cleaned GBIF
Kernel-density
Convex hull
N
1000 km
Latitude
Longitude
10
5
0
-5
-10
-15
-20
-80
-70
-60
-50
-40

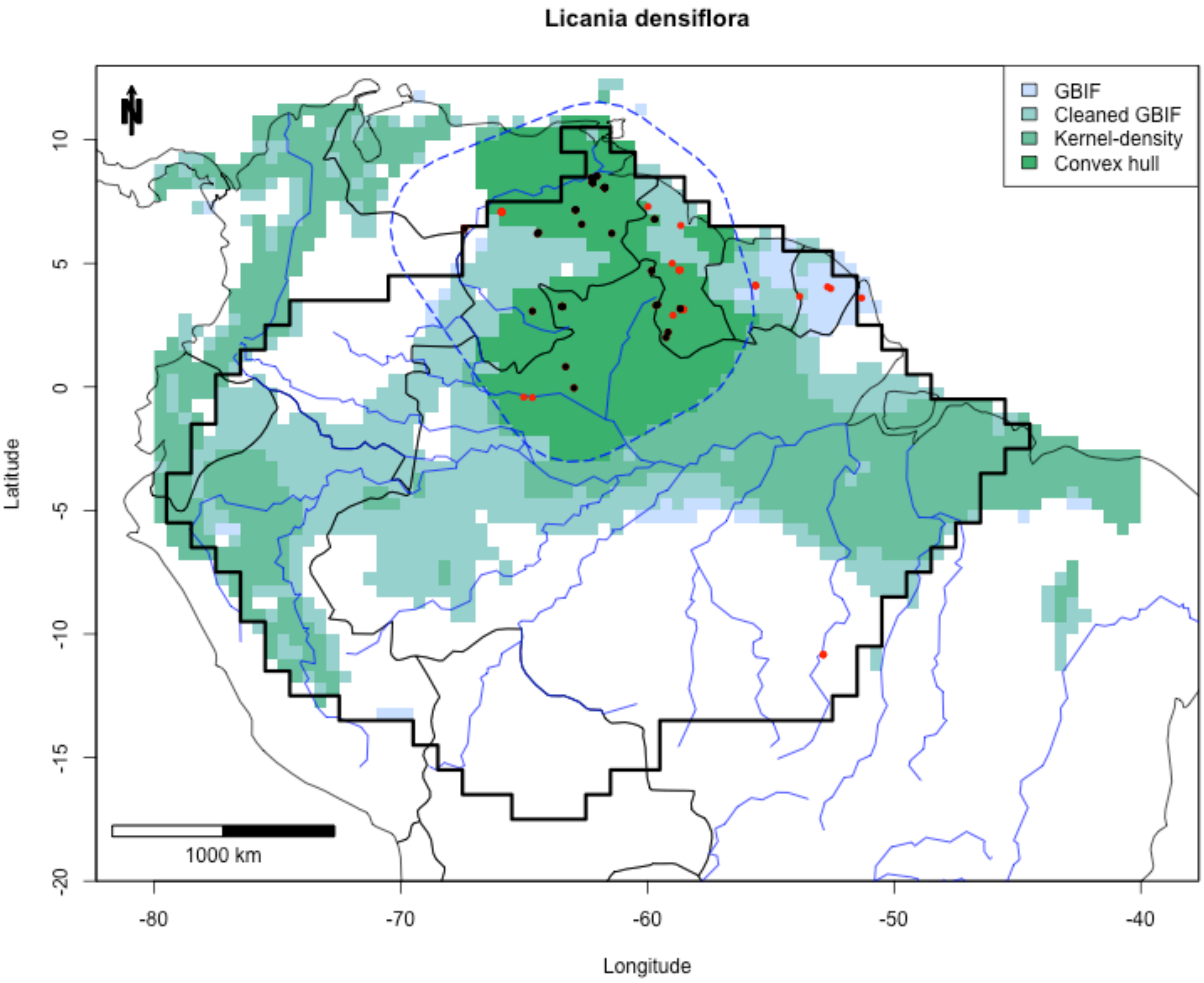
Licania densiflora
GBIF
Cleaned GBIF
Kernel-density
Convex hull
N
1000 km
Latitude
Longitude
10
5
0
-5
-10
-15
-20
-80
-70
-60
-50
-40

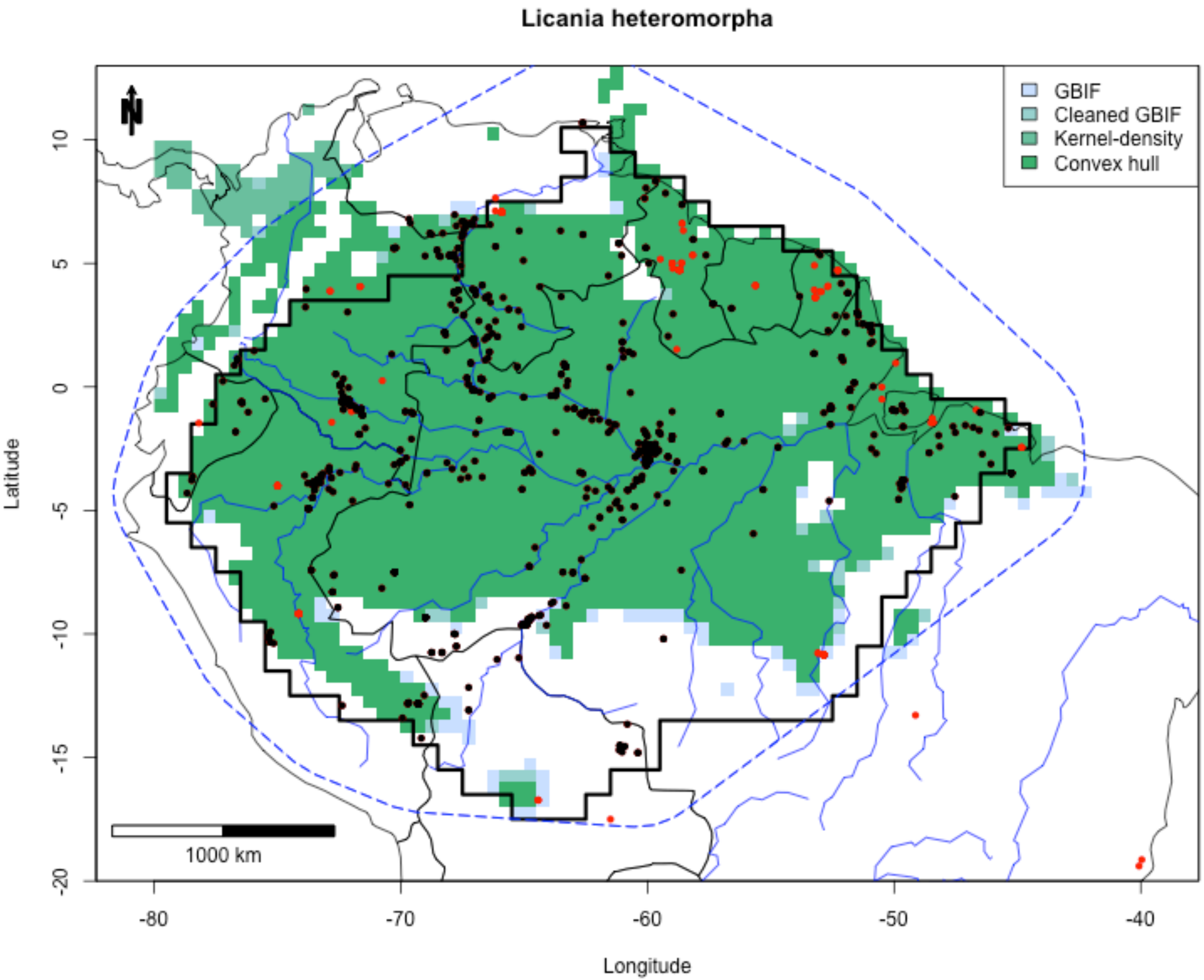

Licania heteromorpha
GBIF
Cleaned GBIF
Kernel-density
Convex hull
N
1000 km
Latitude
Longitude
10
5
0
-5
-10
-15
-20
-80
-70
-60
-50
-40

Licania kunthiana

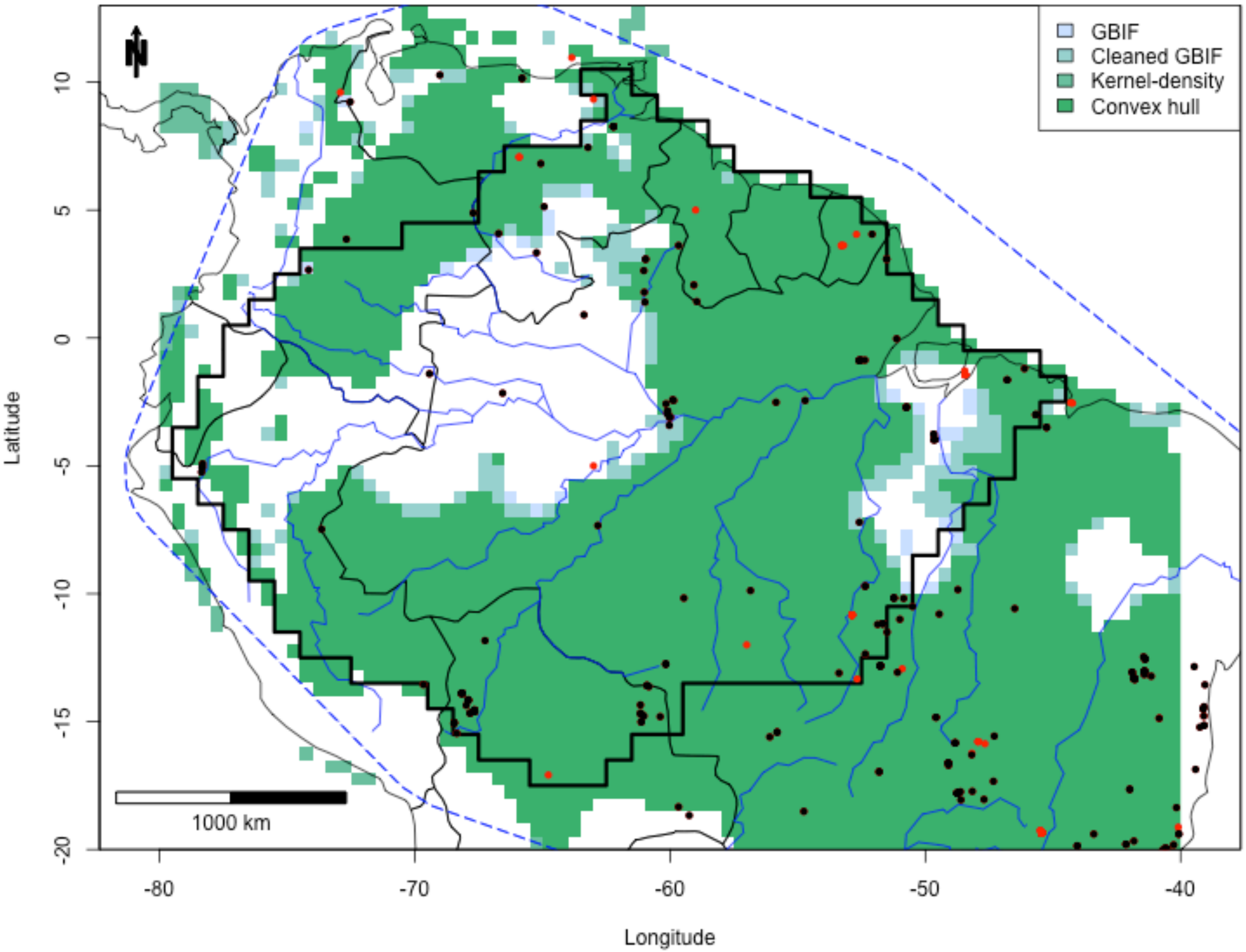

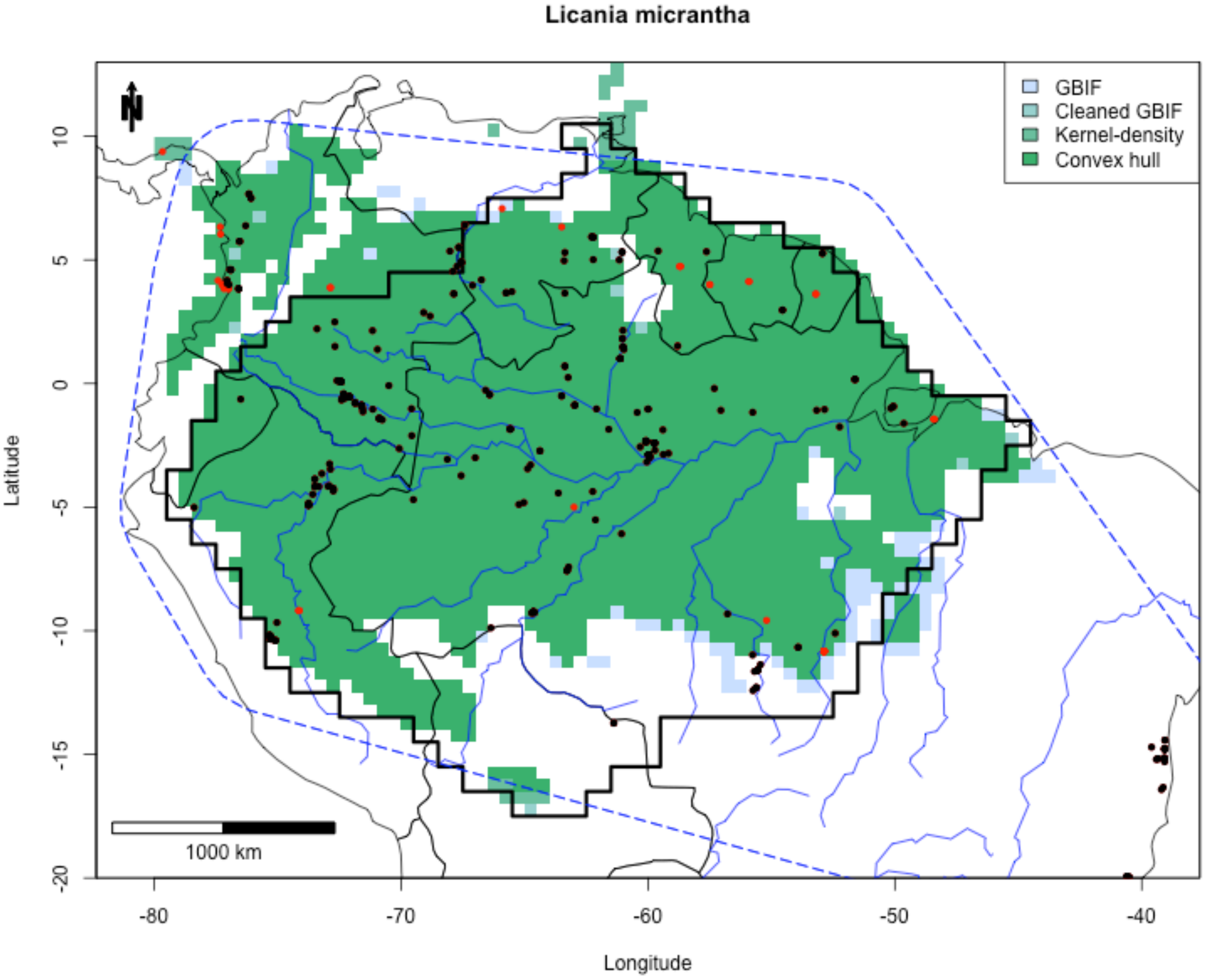

Licania micrantha
GBIF
Cleaned GBIF
Kernel-density
Convex hull
N
1000 km
Latitude
Longitude
10
5
0
-5
-10
-15
-20
-80
-70
-60
-50
-40

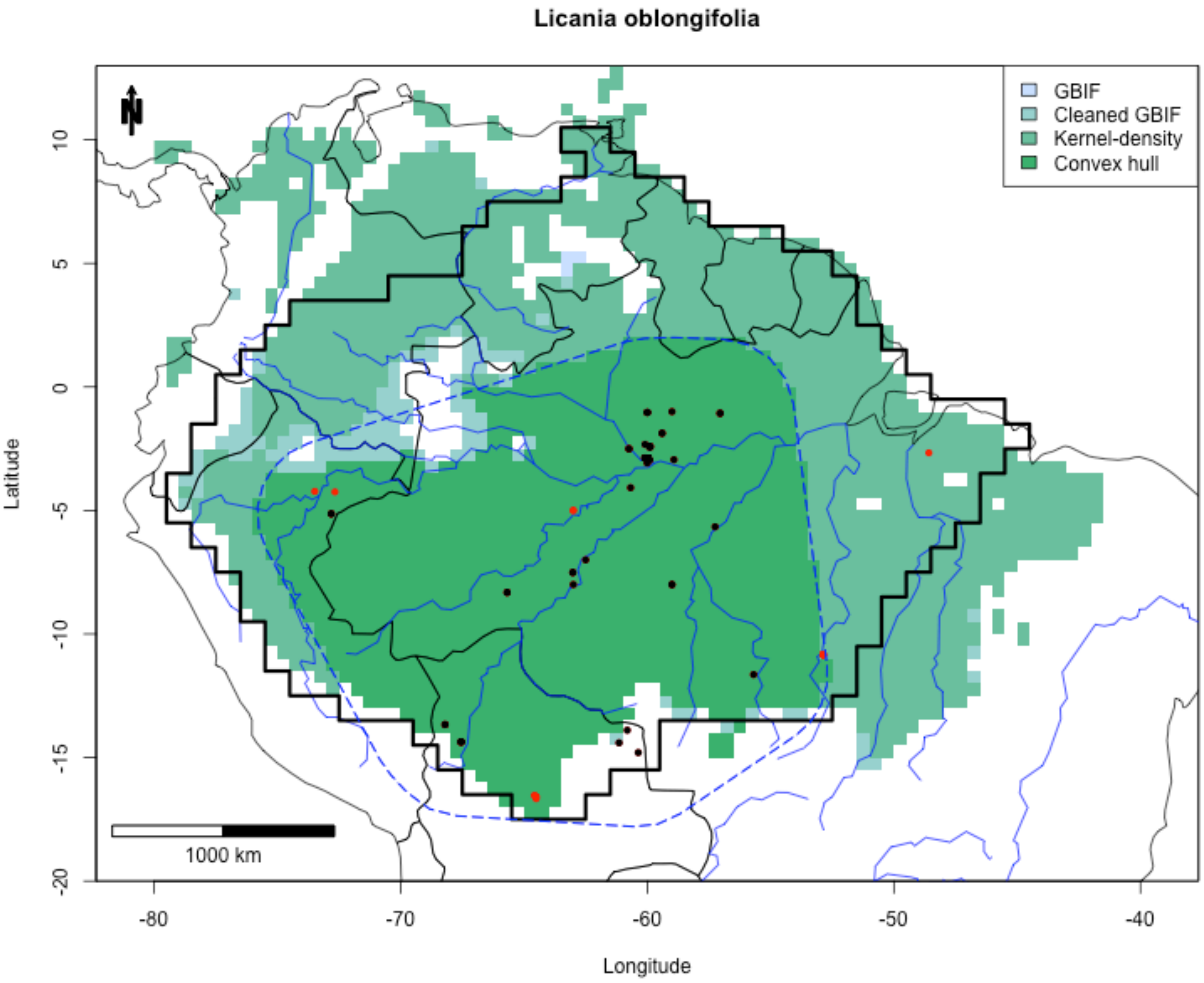

Licania oblongifolia
N
GBIF
Cleaned GBIF
Kernel-density
Convex hull
10
5
0
-5
-10
-15
-20
Latitude
-80
-70
-60
-50
-40
Longitude
1000 km

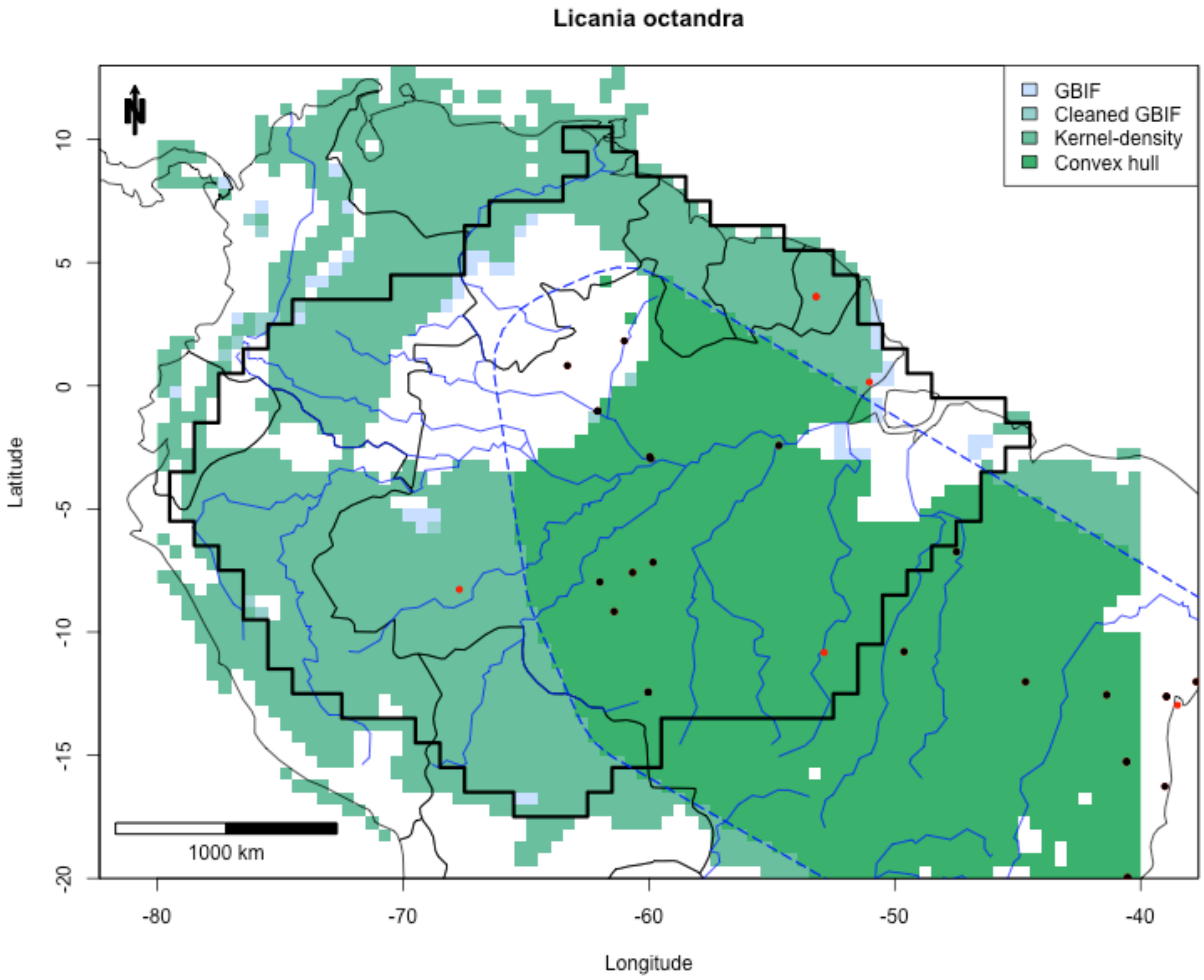

Licania octandra
GBIF
Cleaned GBIF
Kernel-density
Convex hull
N
1000 km
10
5
0
-5
-10
-15
-20
Latitude
-80
-70
-60
-50
-40
Longitude

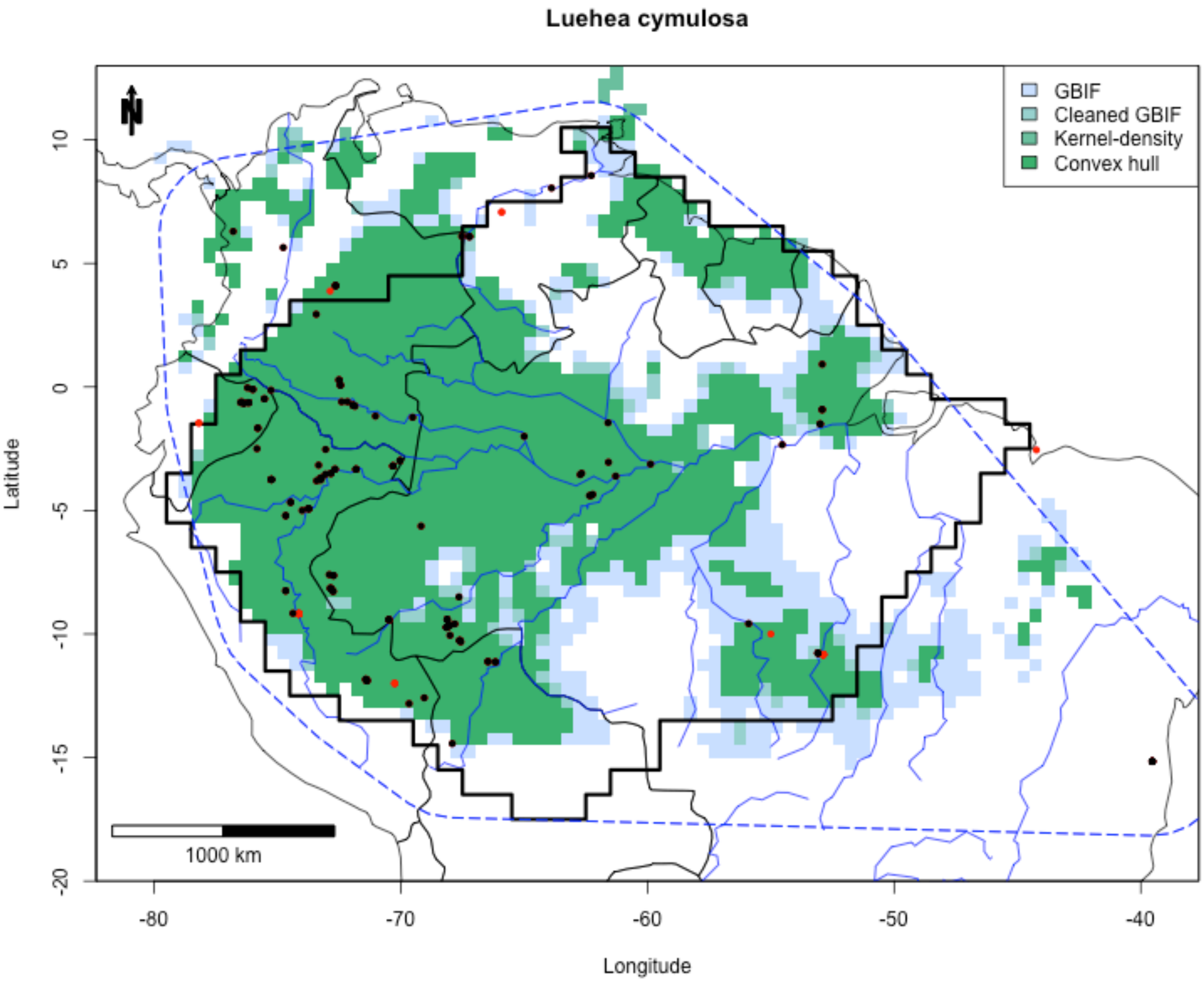

Luehea cymulosa
GBIF
Cleaned GBIF
Kernel-density
Convex hull
N
1000 km
Latitude
Longitude
10
5
0
-5
-10
-15
-20
-80
-70
-60
-50
-40

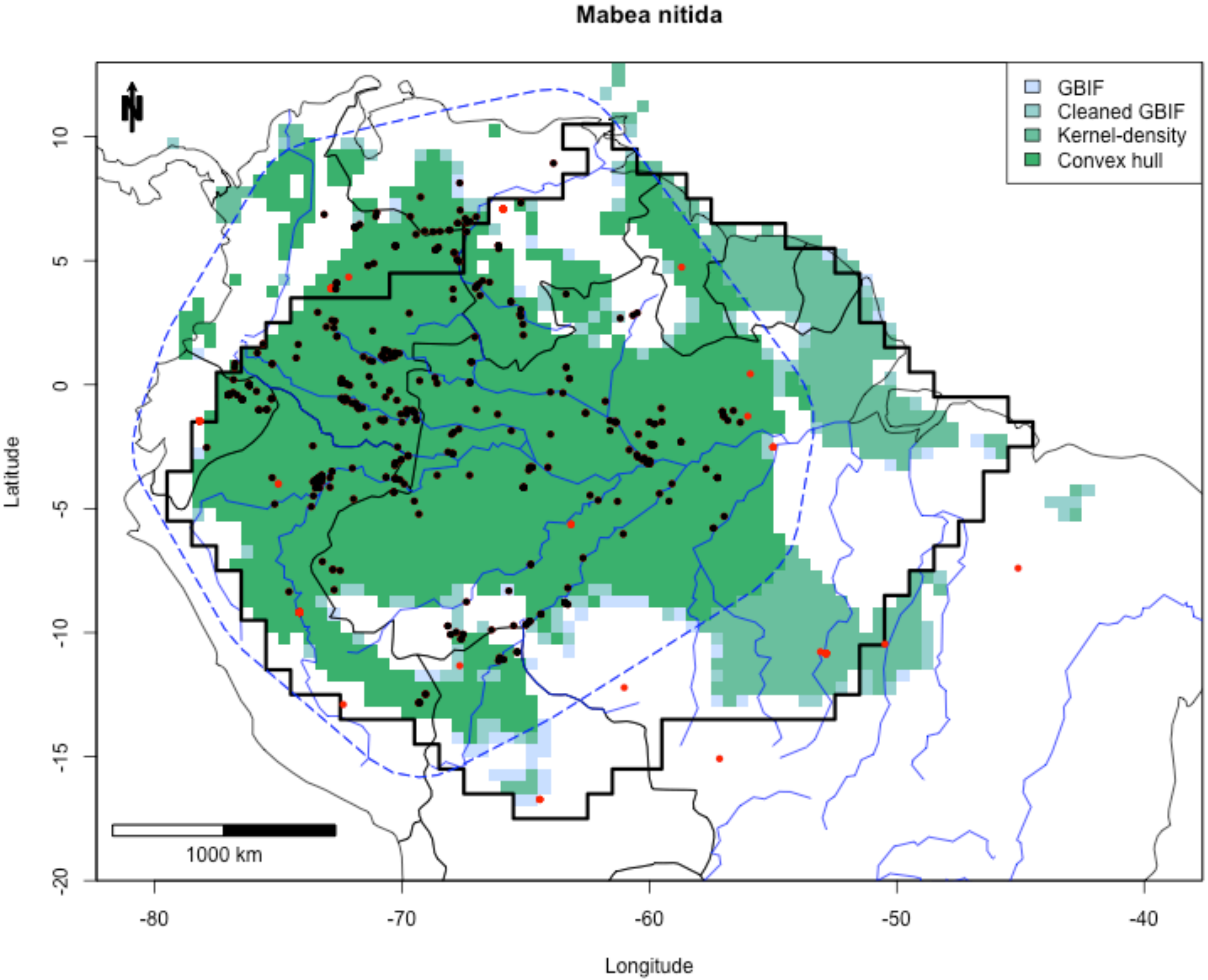
Mabea nitida
GBIF
Cleaned GBIF
Kernel-density
Convex hull
N
1000 km
Latitude
Longitude
10
5
0
-5
-10
-15
-20
-80
-70
-60
-50
-40

Mabea speciosa

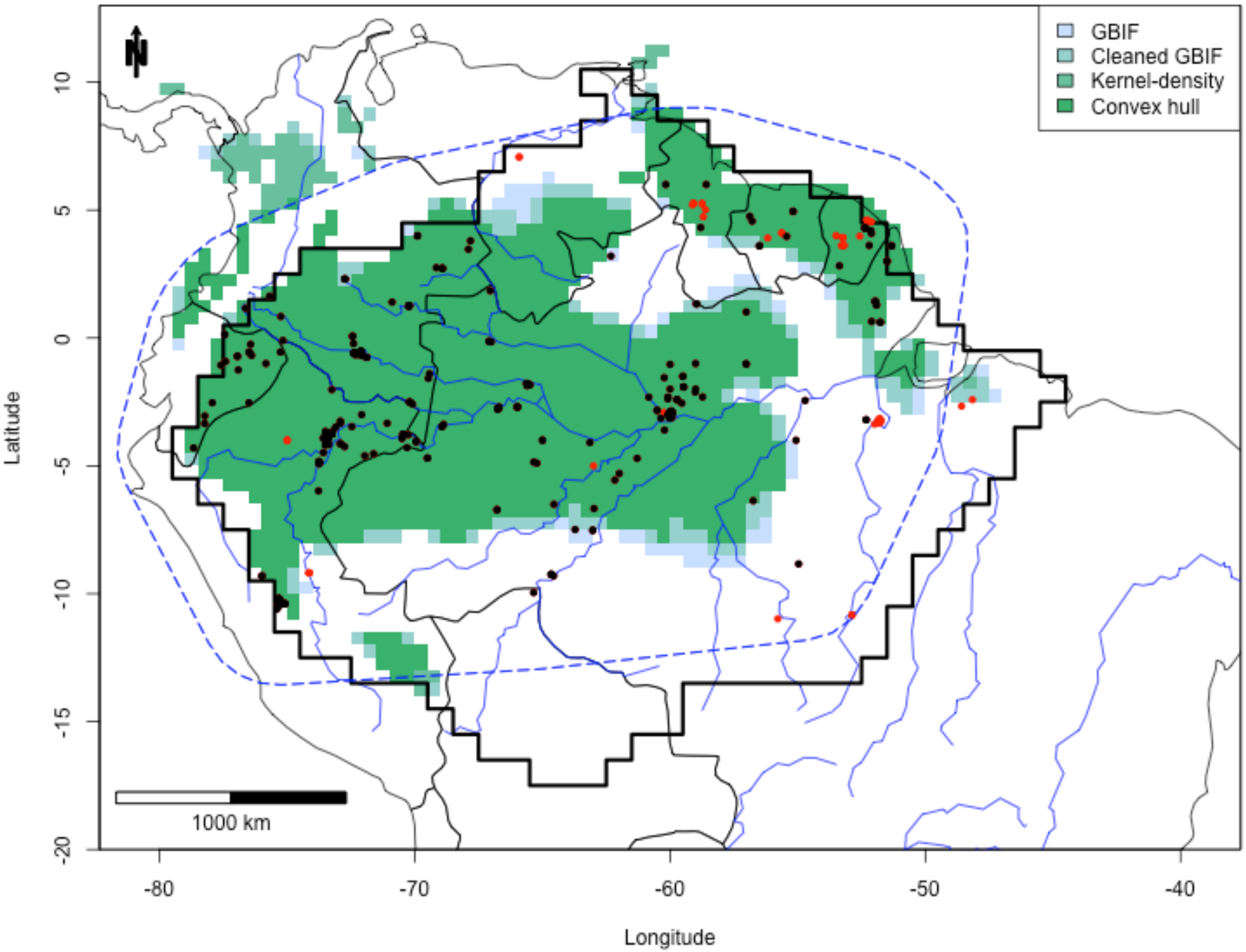

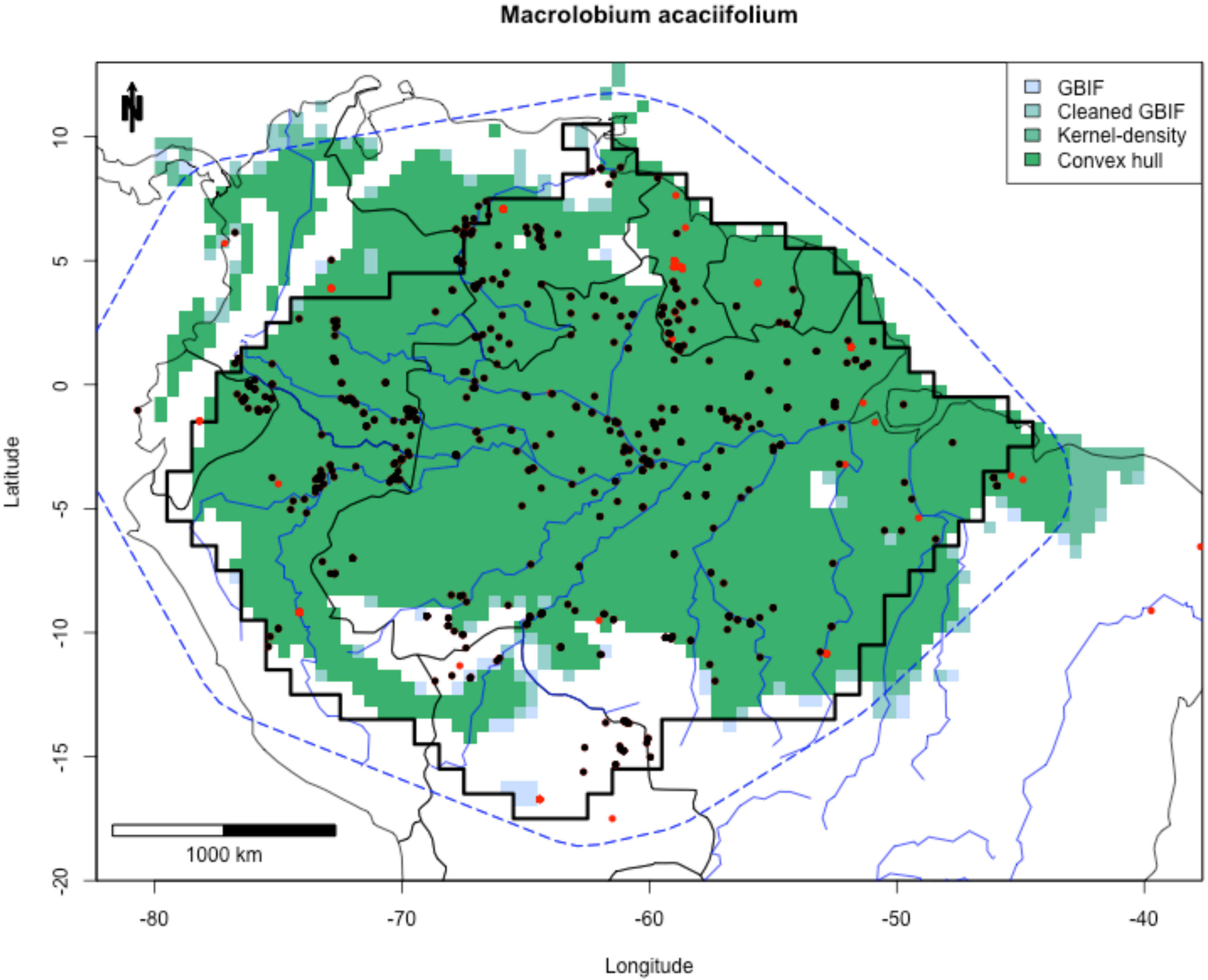
Macrolobium acaciifolium
GBIF
Cleaned GBIF
Kernel-density
Convex hull
N
1000 km
Latitude
Longitude
10
5
0
-5
-10
-15
-20
-80
-70
-60
-50
-40

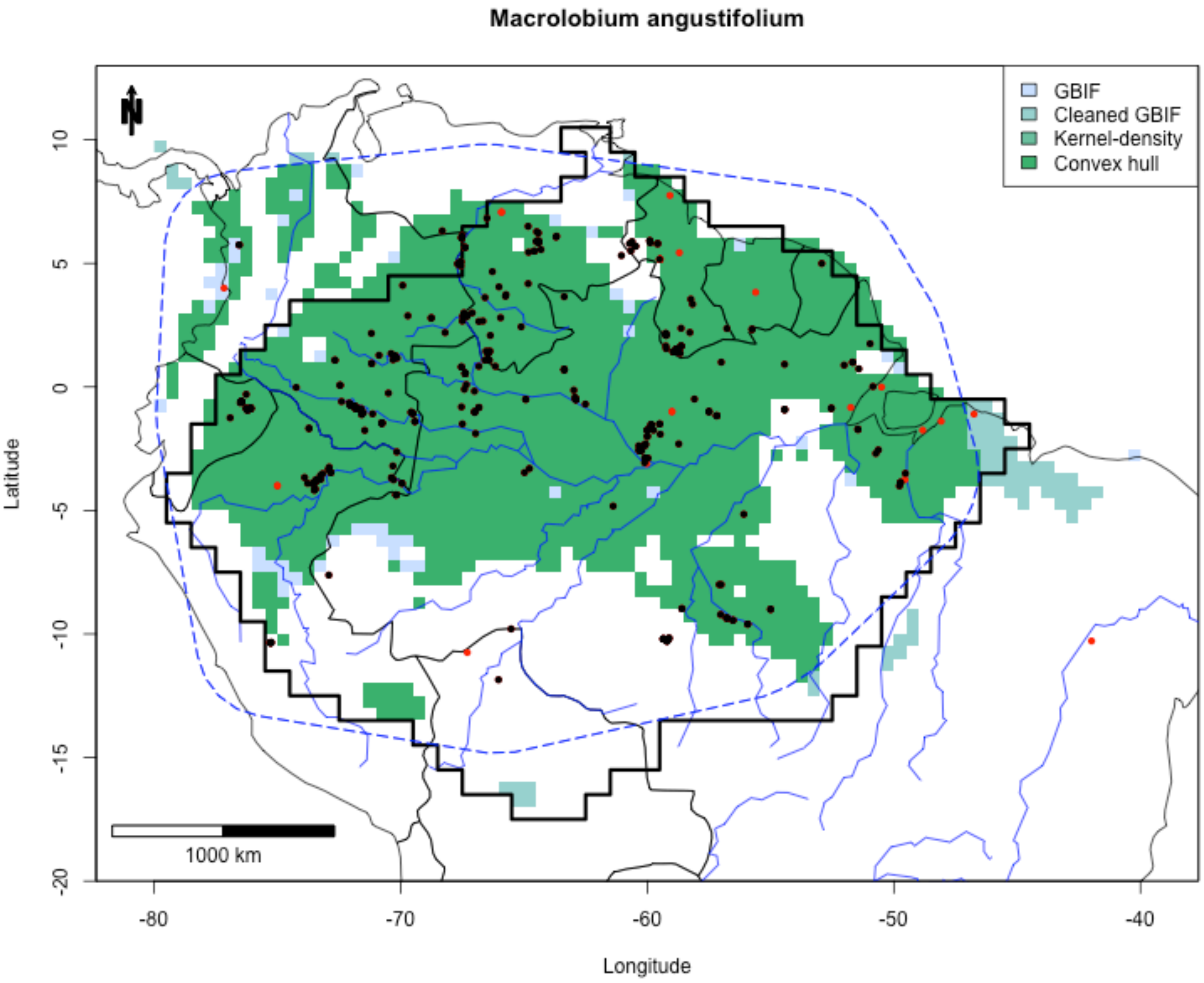

Macrolobium angustifolium
GBIF
Cleaned GBIF
Kernel-density
Convex hull
N
1000 km
Latitude
Longitude
10
5
0
-5
-10
-15
-20
-80
-70
-60
-50
-40

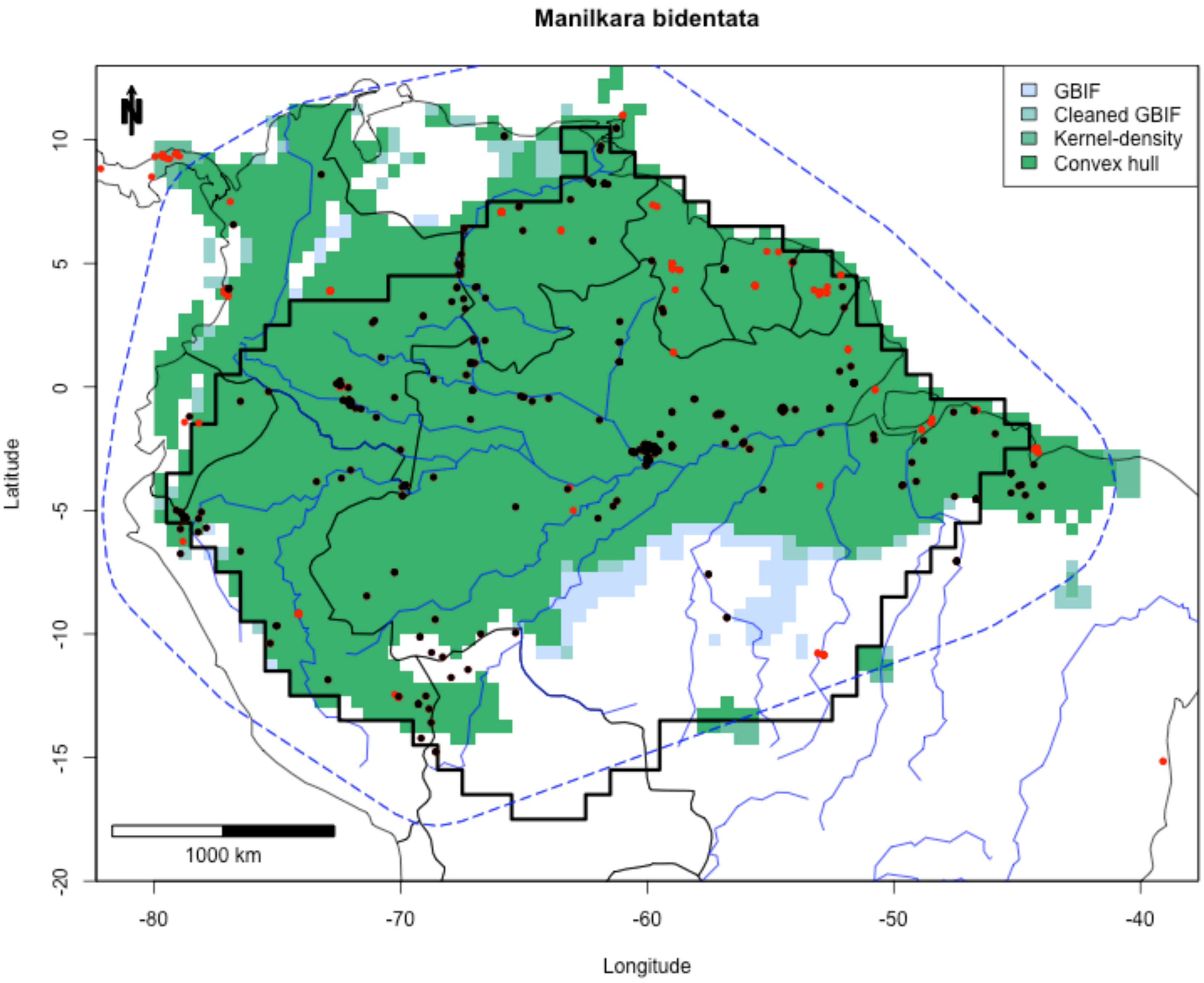

Manilkara bidentata
GBIF
Cleaned GBIF
Kernel-density
Convex hull
1000 km
N
Latitude
Longitude
10
5
0
-5
-10
-15
-20
-80
-70
-60
-50
-40

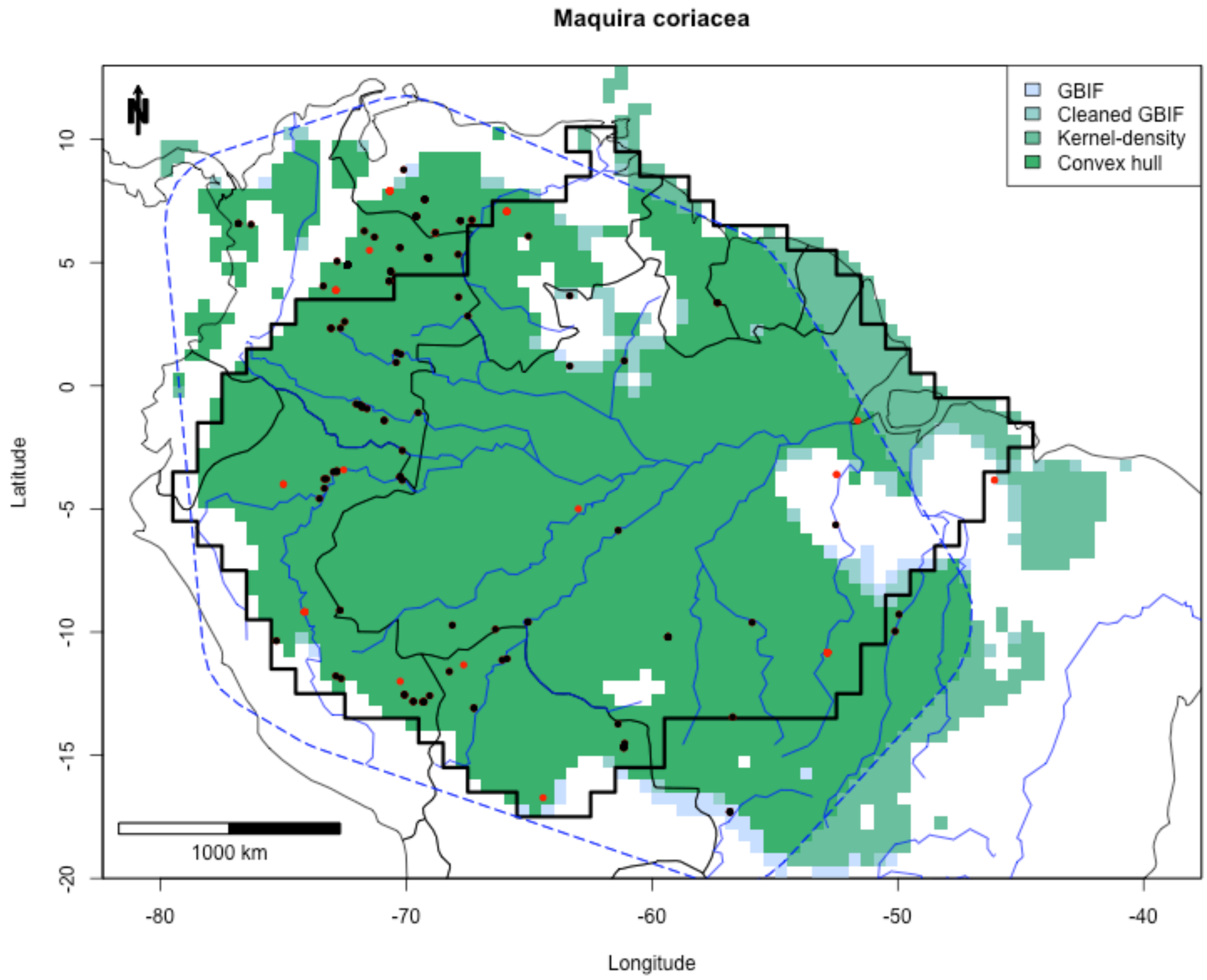
Maquira coriacea
GBIF
Cleaned GBIF
Kernel-density
Convex hull
1000 km
Latitude
Longitude
10
5
0
-5
-10
-15
-20
-80
-70
-60
-50
-40

Matayba scrobiculata

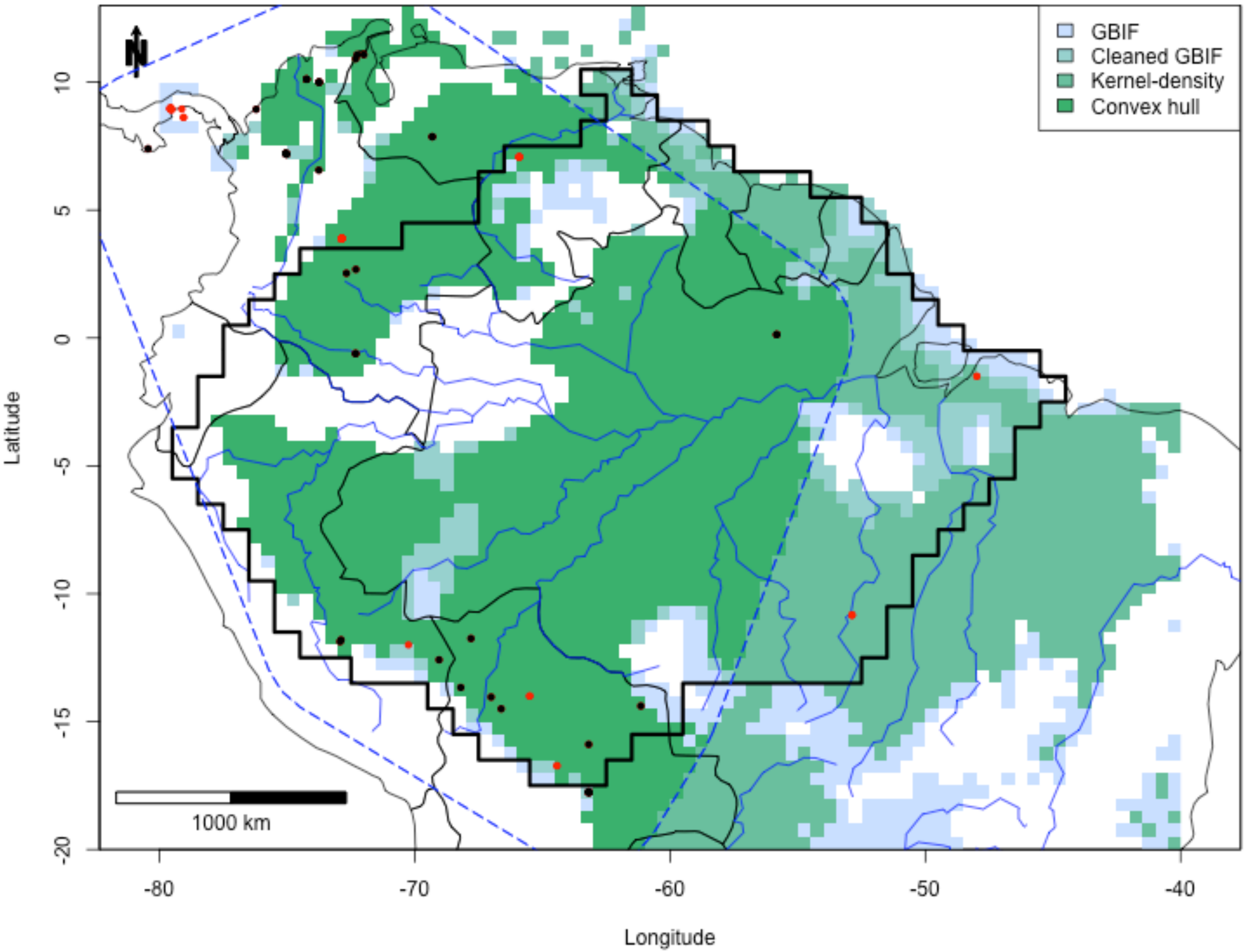

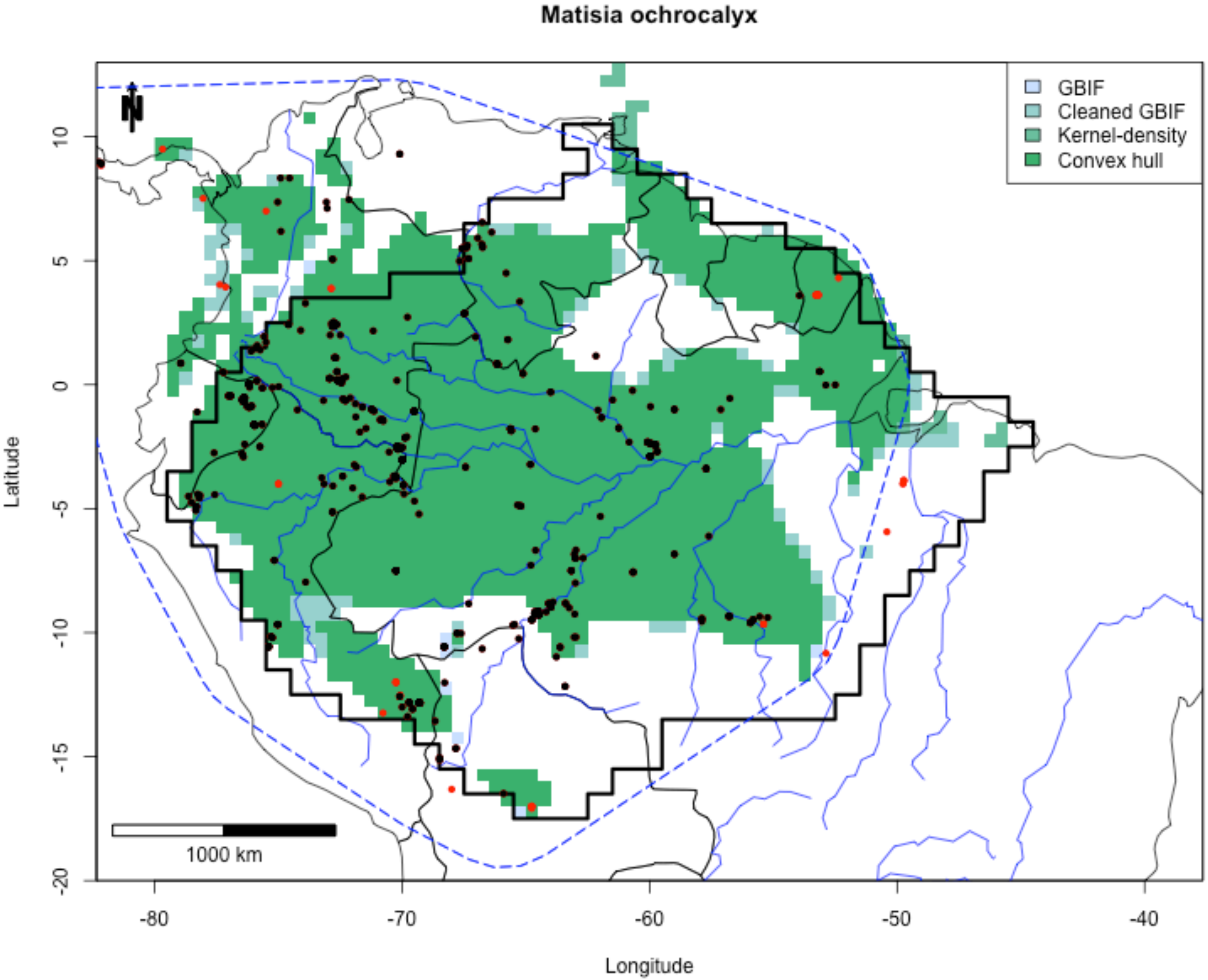
Matisia ochrocalyx
GBIF
Cleaned GBIF
Kernel-density
Convex hull
N
1000 km
Latitude
Longitude
10
5
0
-5
-10
-15
-20
-80
-70
-60
-50
-40

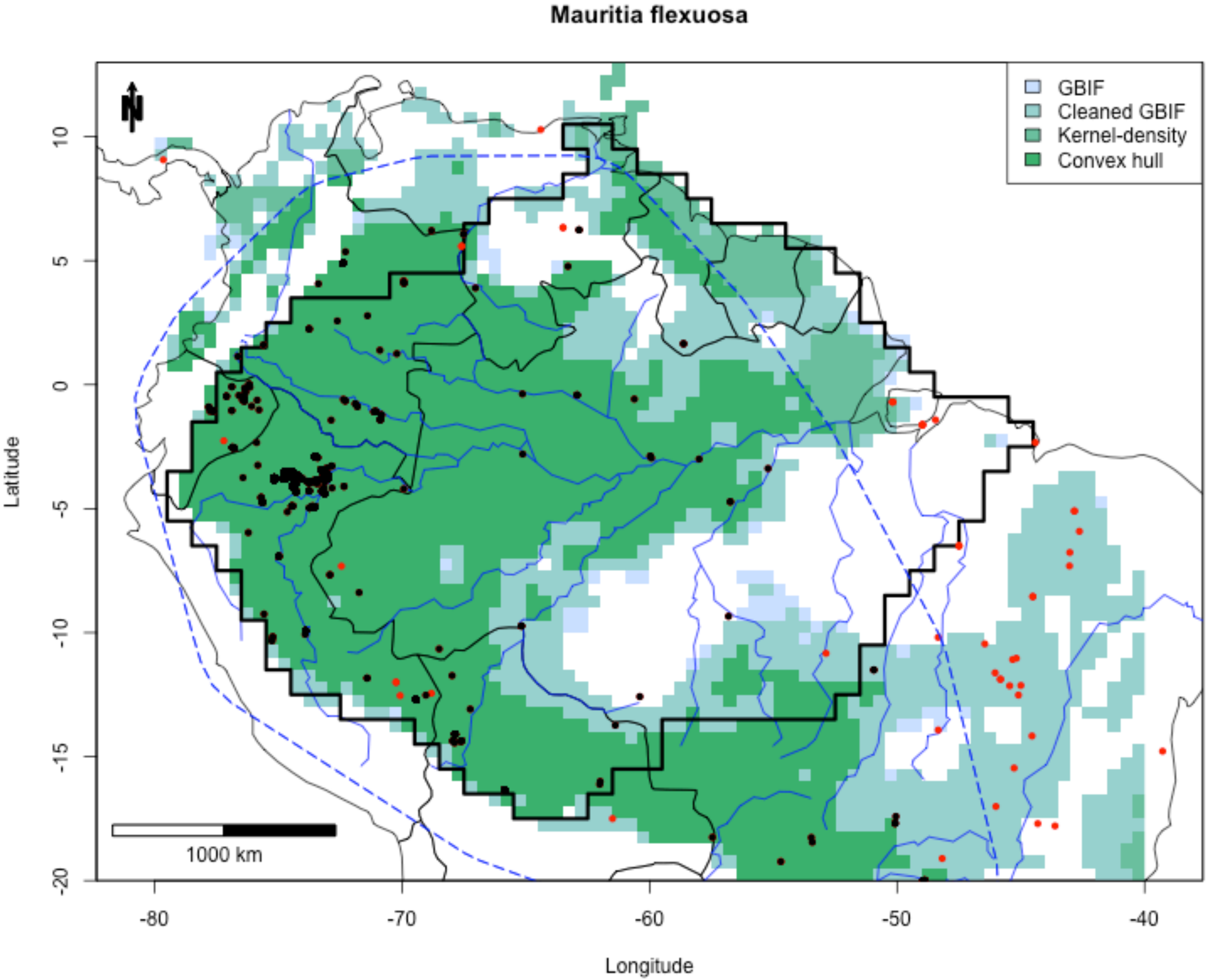
Mauritia flexuosa
GBIF
Cleaned GBIF
Kernel-density
Convex hull
N
1000 km
Latitude
Longitude
10
5
0
-5
-10
-15
-20
-80
-70
-60
-50
-40

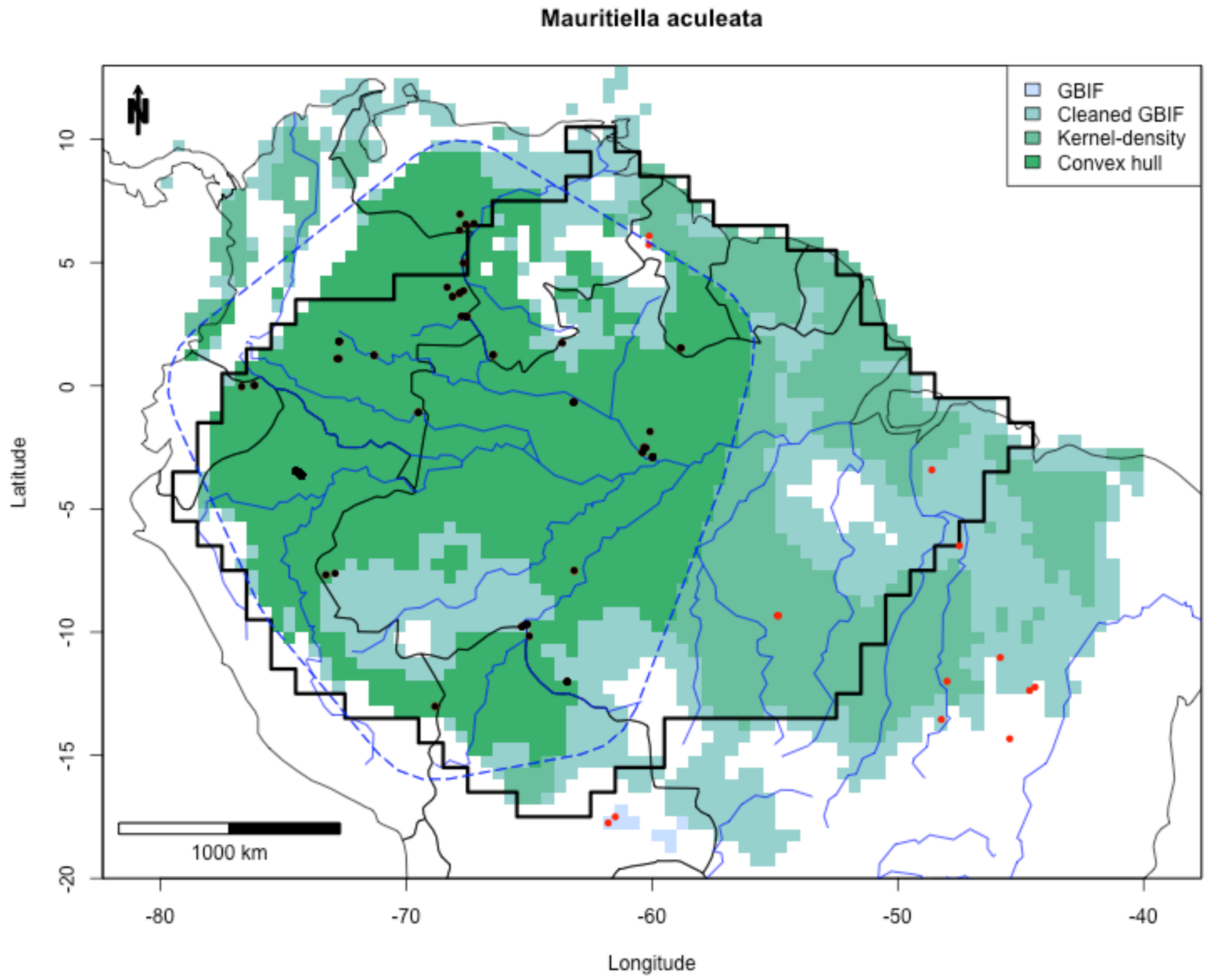
Mauritiella aculeata
GBIF
Cleaned GBIF
Kernel-density
Convex hull
N
1000 km
Latitude
Longitude
10
5
0
-5
-10
-15
-20
-80
-70
-60
-50
-40

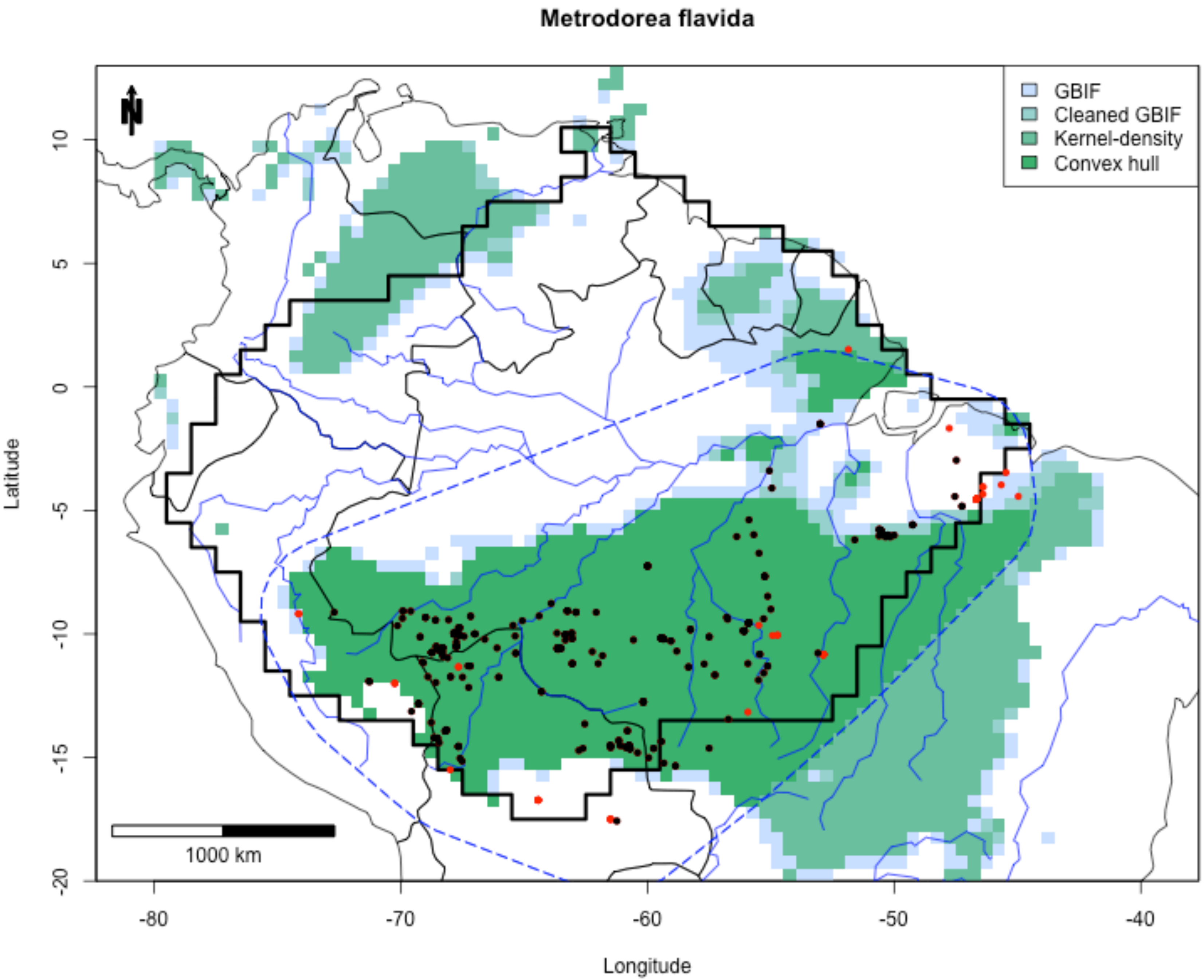

Metrodorea flavida
GBIF
Cleaned GBIF
Kernel-density
Convex hull
N
1000 km
Latitude
Longitude
10
5
0
-5
-10
-15
-20
-80
-70
-60
-50
-40

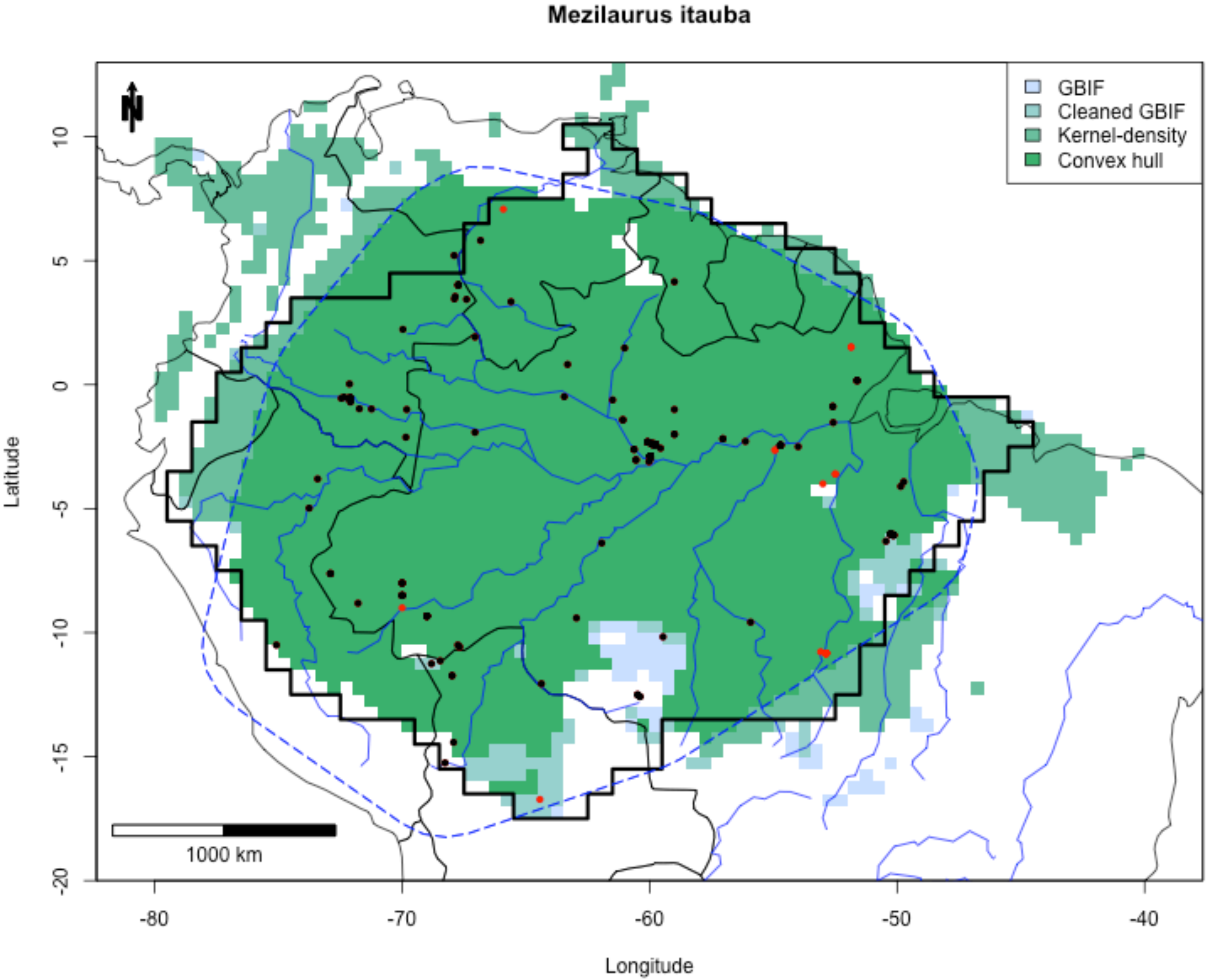

Mezilaurus itauba
GBIF
Cleaned GBIF
Kernel-density
Convex hull
N
1000 km
Latitude
Longitude
10
5
0
-5
-10
-15
-20
-80
-70
-60
-50
-40

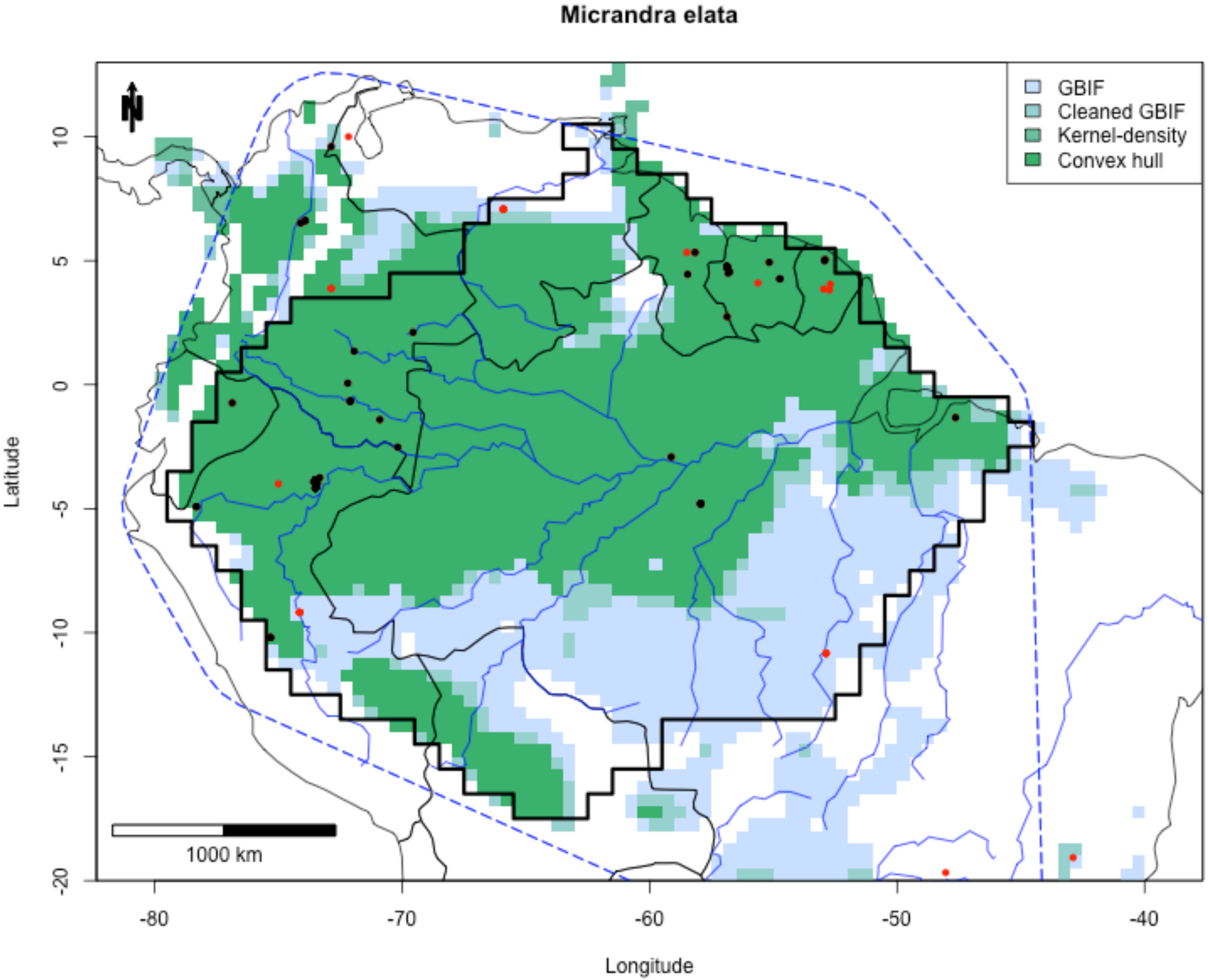

Micrandra elata
GBIF
Cleaned GBIF
Kernel-density
Convex hull
N
1000 km
Latitude
Longitude
10
5
0
-5
-10
-15
-20
-80
-70
-60
-50
-40

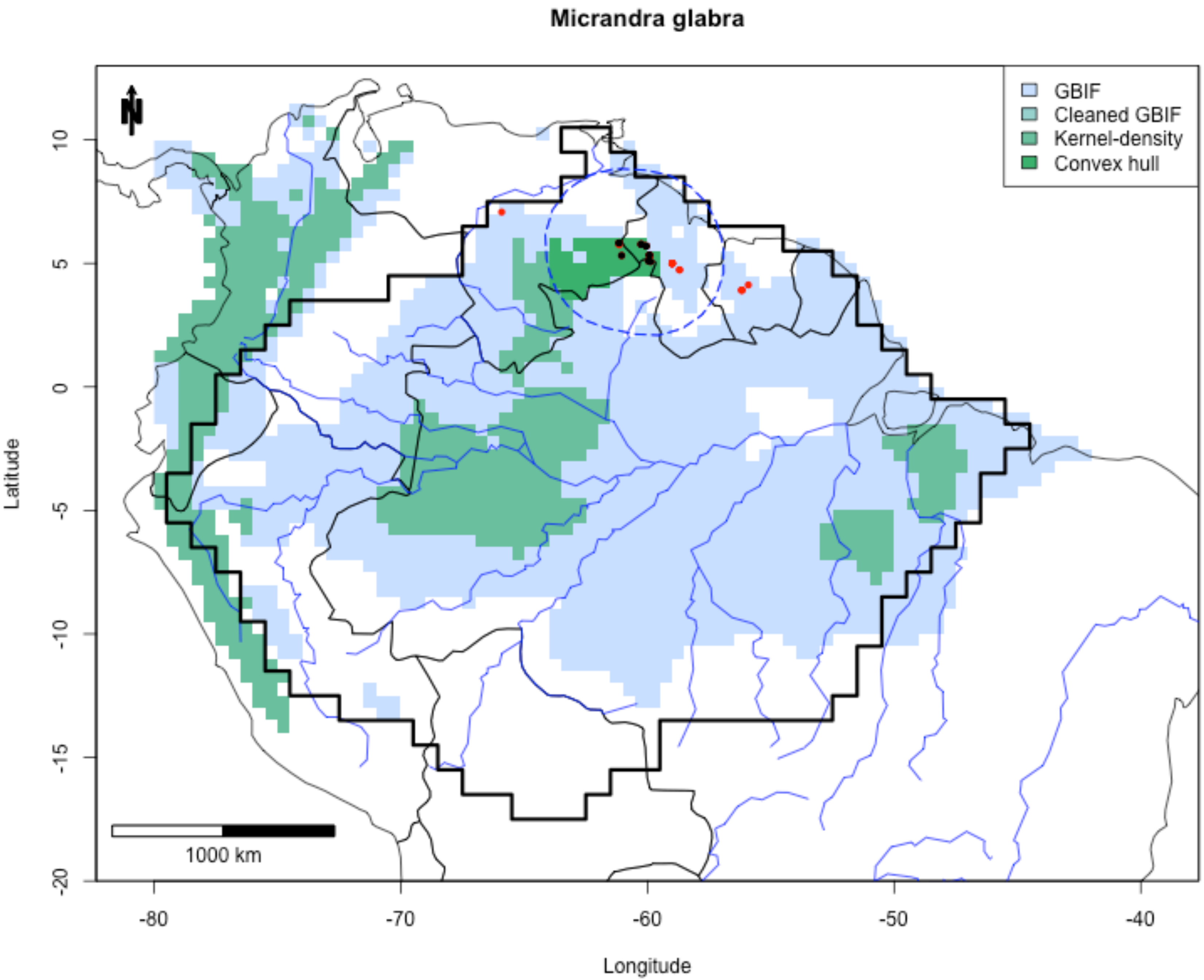

Micrandra glabra
GBIF
Cleaned GBIF
Kernel-density
Convex hull
N
1000 km
Latitude
Longitude
10
5
0
-5
-10
-15
-20
-80
-70
-60
-50
-40

Micrandra spruceana

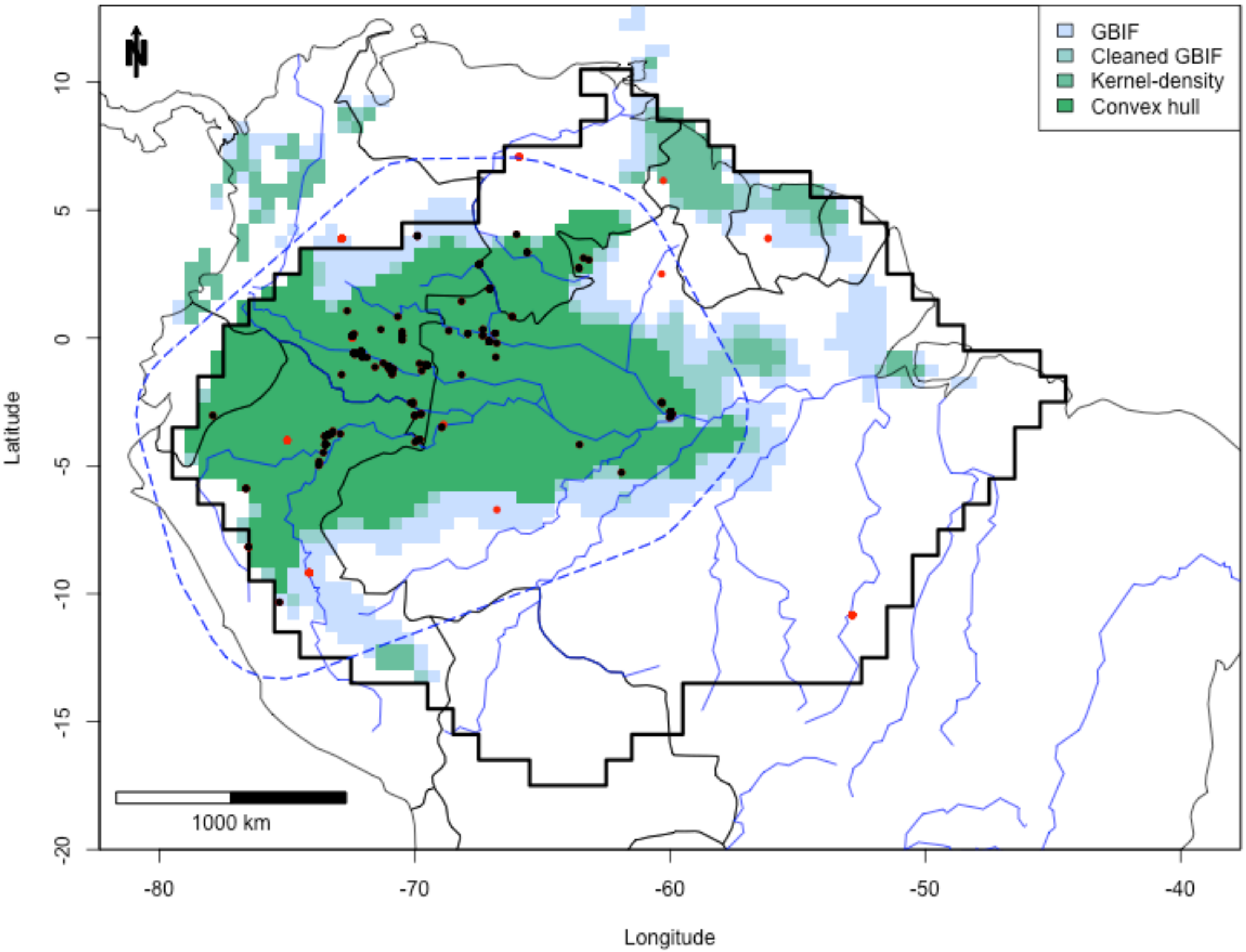

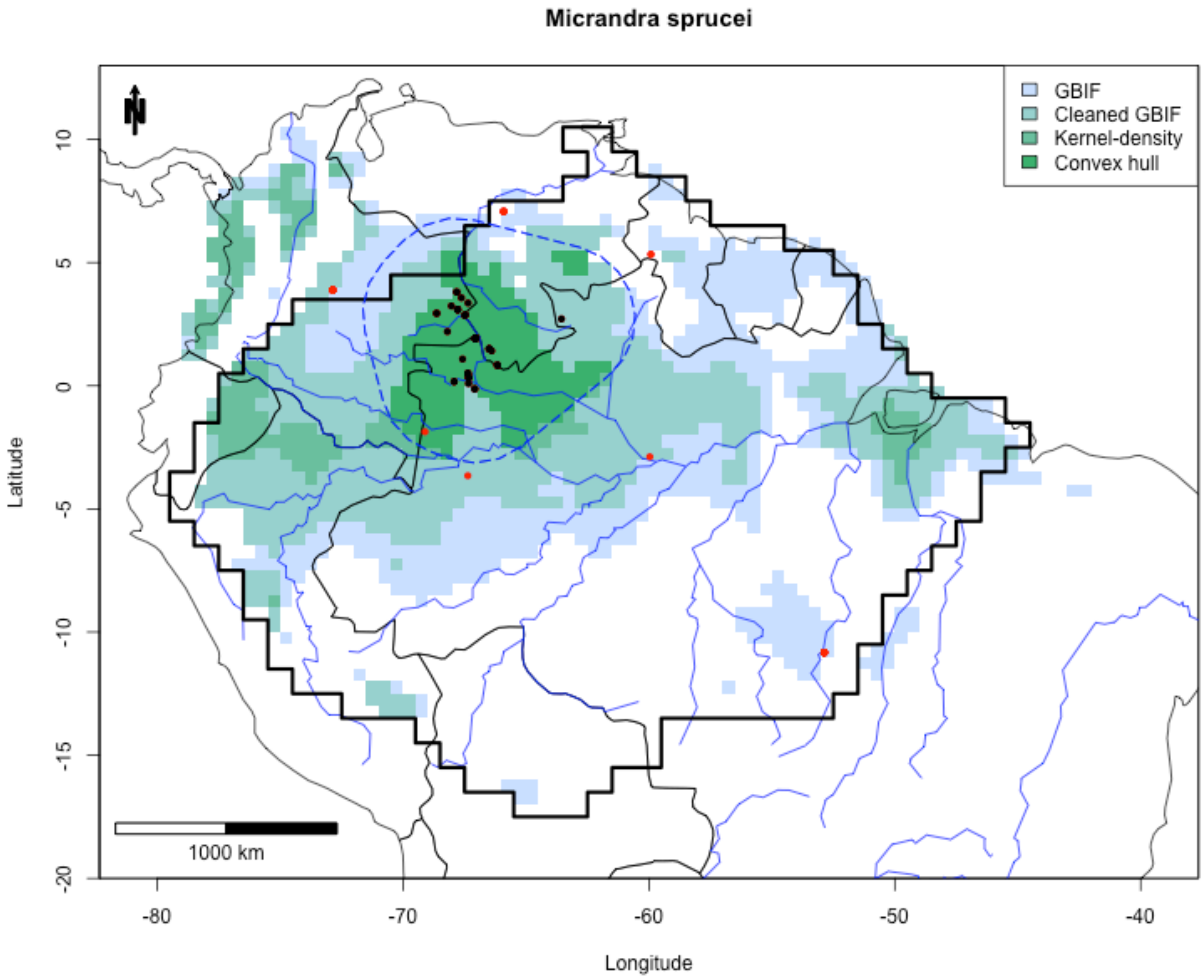
Micrandra sprucei
GBIF
Cleaned GBIF
Kernel-density
Convex hull
N
1000 km
Latitude
Longitude
10
5
0
-5
-10
-15
-20
-80
-70
-60
-50
-40

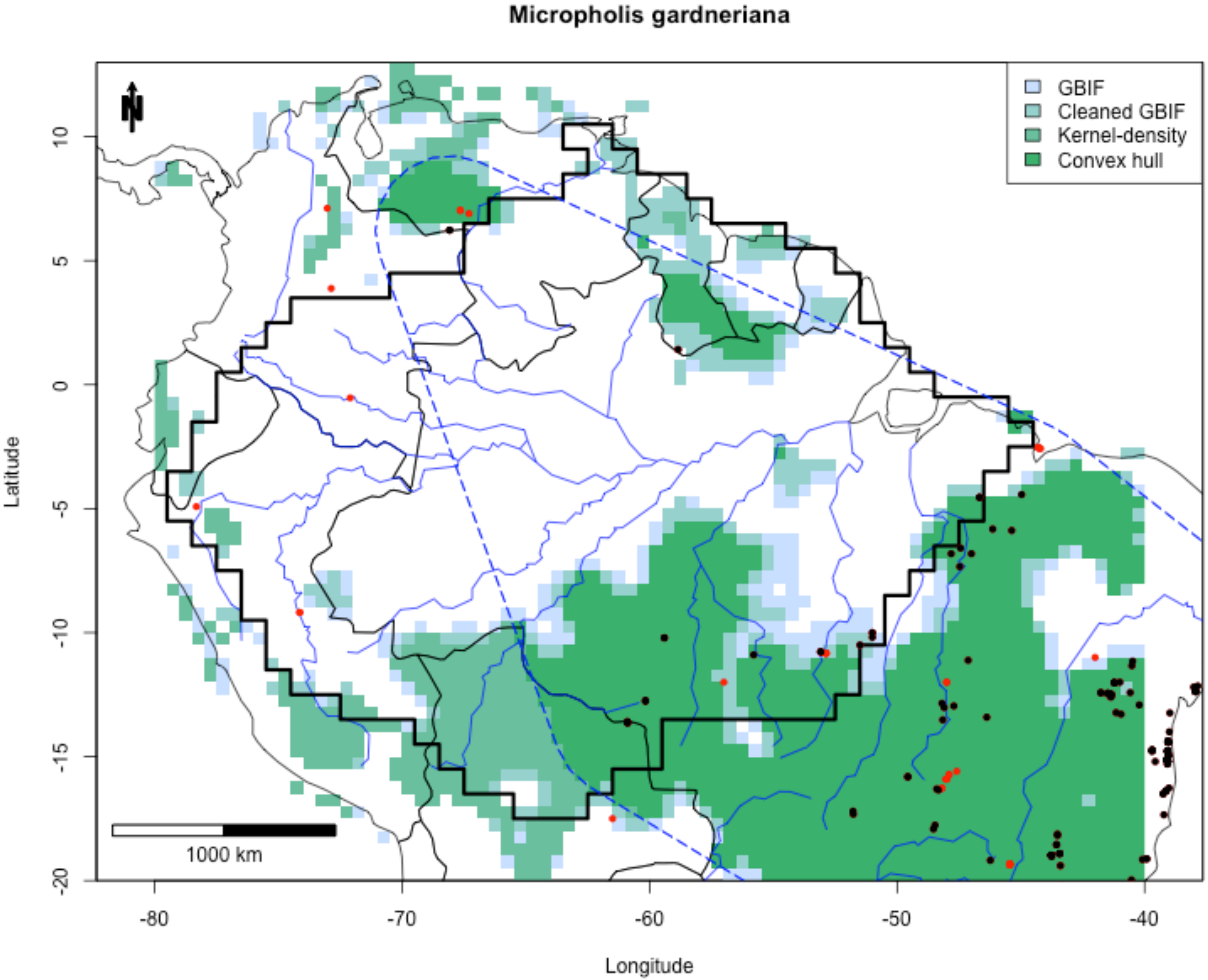

Micropholis gardneriana
GBIF
Cleaned GBIF
Kernel-density
Convex hull
N
1000 km
Latitude
Longitude
10
5
0
-5
-10
-15
-20
-80
-70
-60
-50
-40

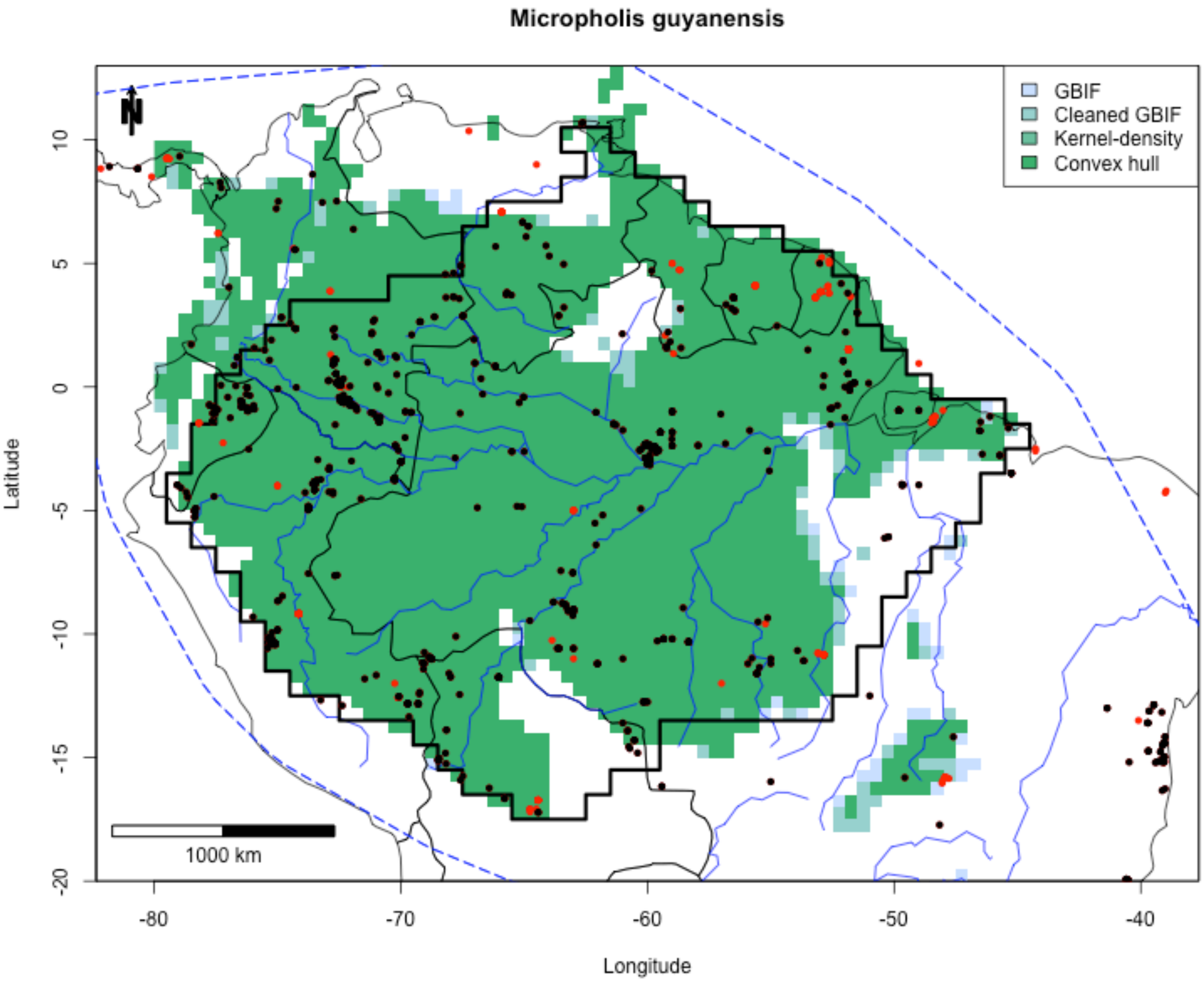

Micropholis guyanensis
GBIF
Cleaned GBIF
Kernel-density
Convex hull
1000 km
Latitude
Longitude
10
5
0
-5
-10
-15
-20
-80
-70
-60
-50
-40

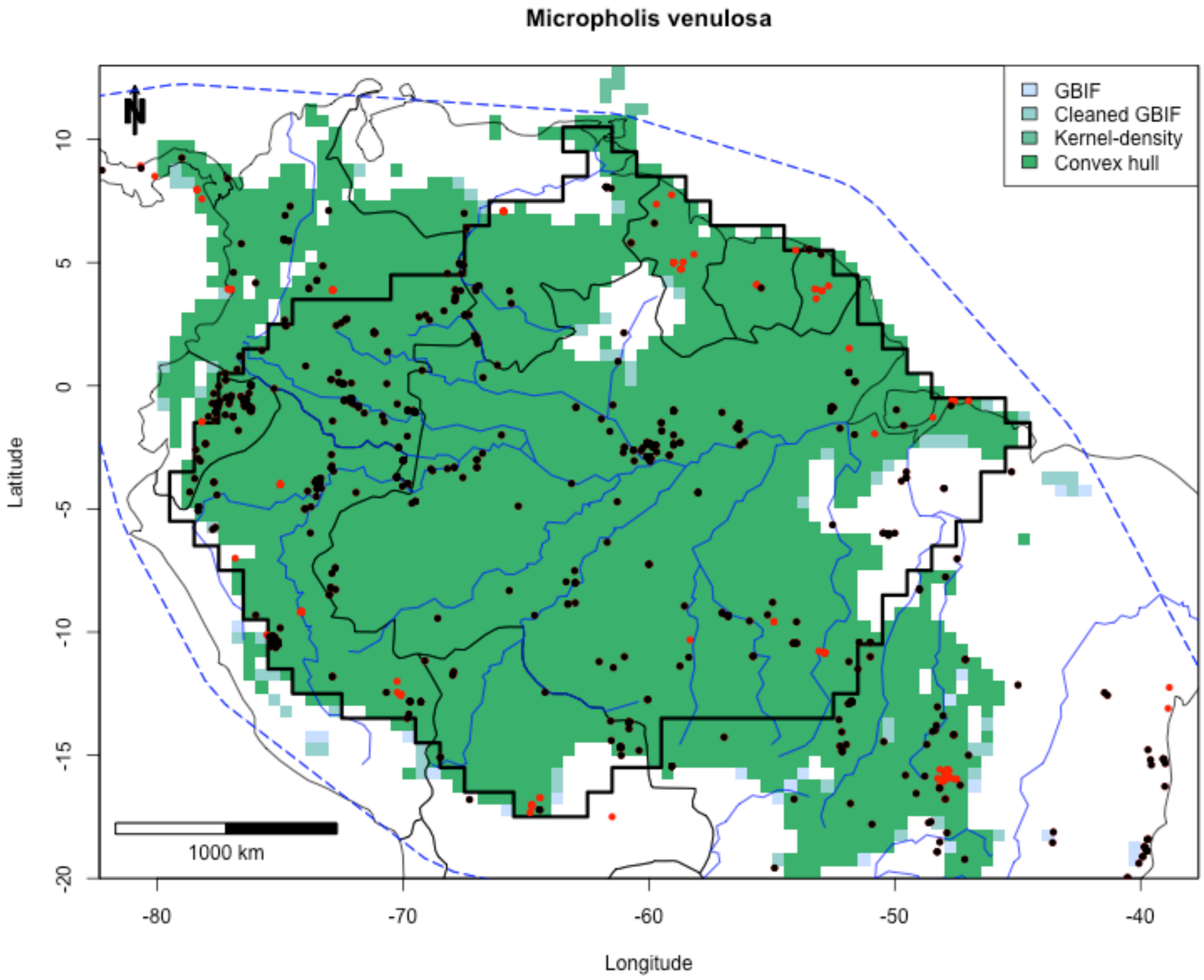
Micropholis venulosa
GBIF
Cleaned GBIF
Kernel-density
Convex hull
N
1000 km
Latitude
Longitude
10
5
0
-5
-10
-15
-20
-80
-70
-60
-50
-40

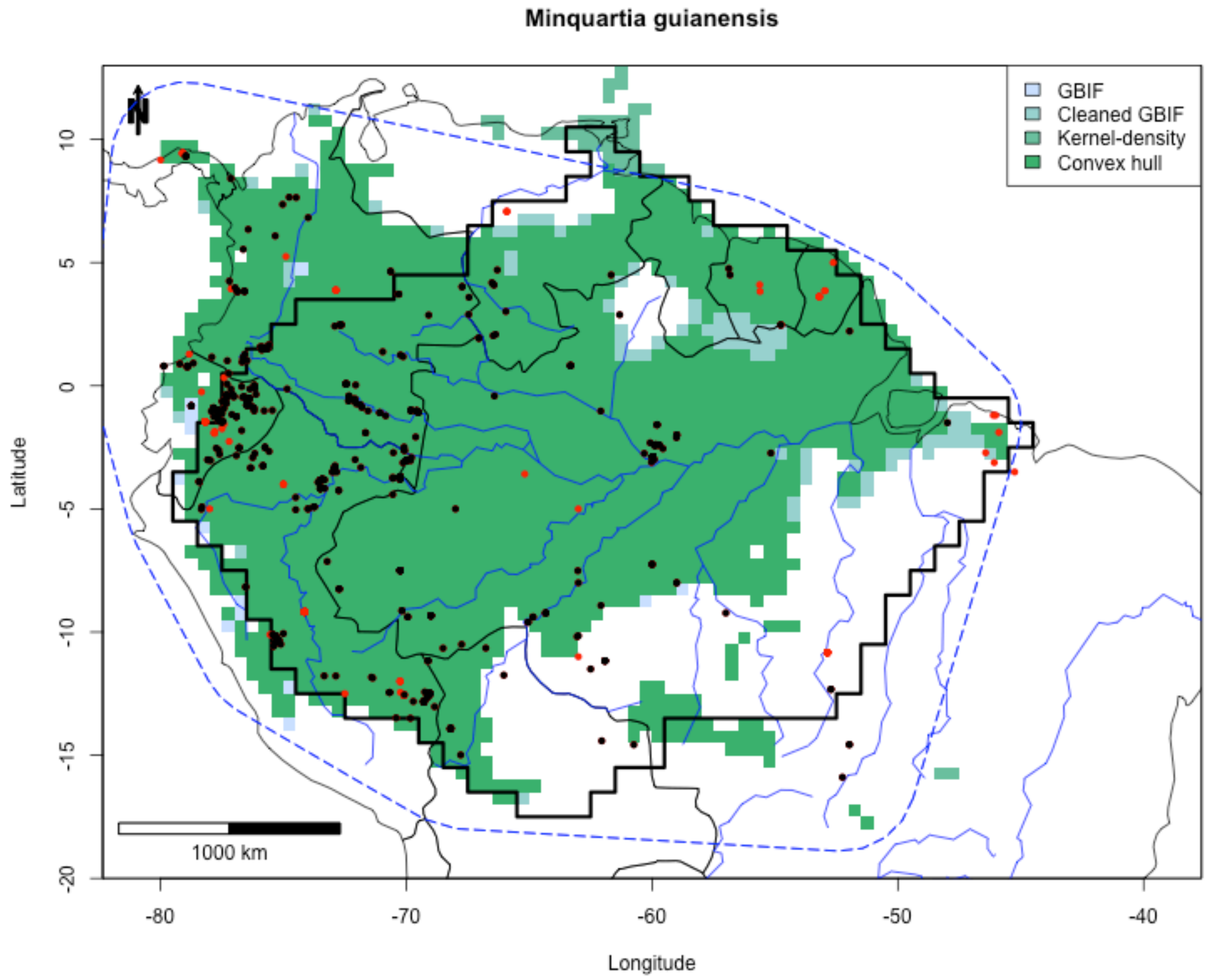
Minquartia guianensis
GBIF
Cleaned GBIF
Kernel-density
Convex hull
N
1000 km
Latitude
Longitude
10
5
0
-5
-10
-15
-20
-80
-70
-60
-50
-40

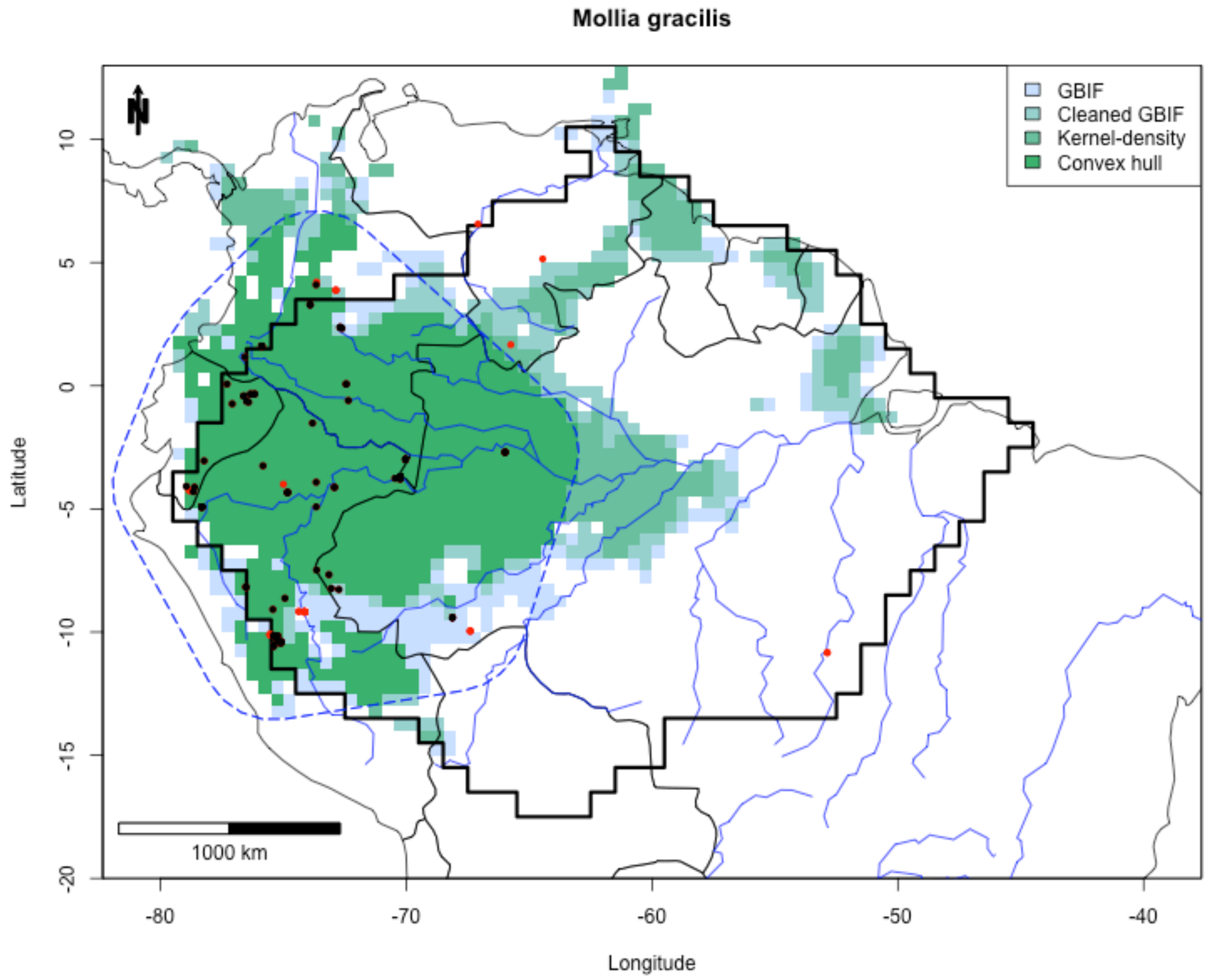

Mollia gracilis
GBIF
Cleaned GBIF
Kernel-density
Convex hull
Latitude
Longitude
1000 km
N
10
5
0
-5
-10
-15
-20
-80
-70
-60
-50
-40

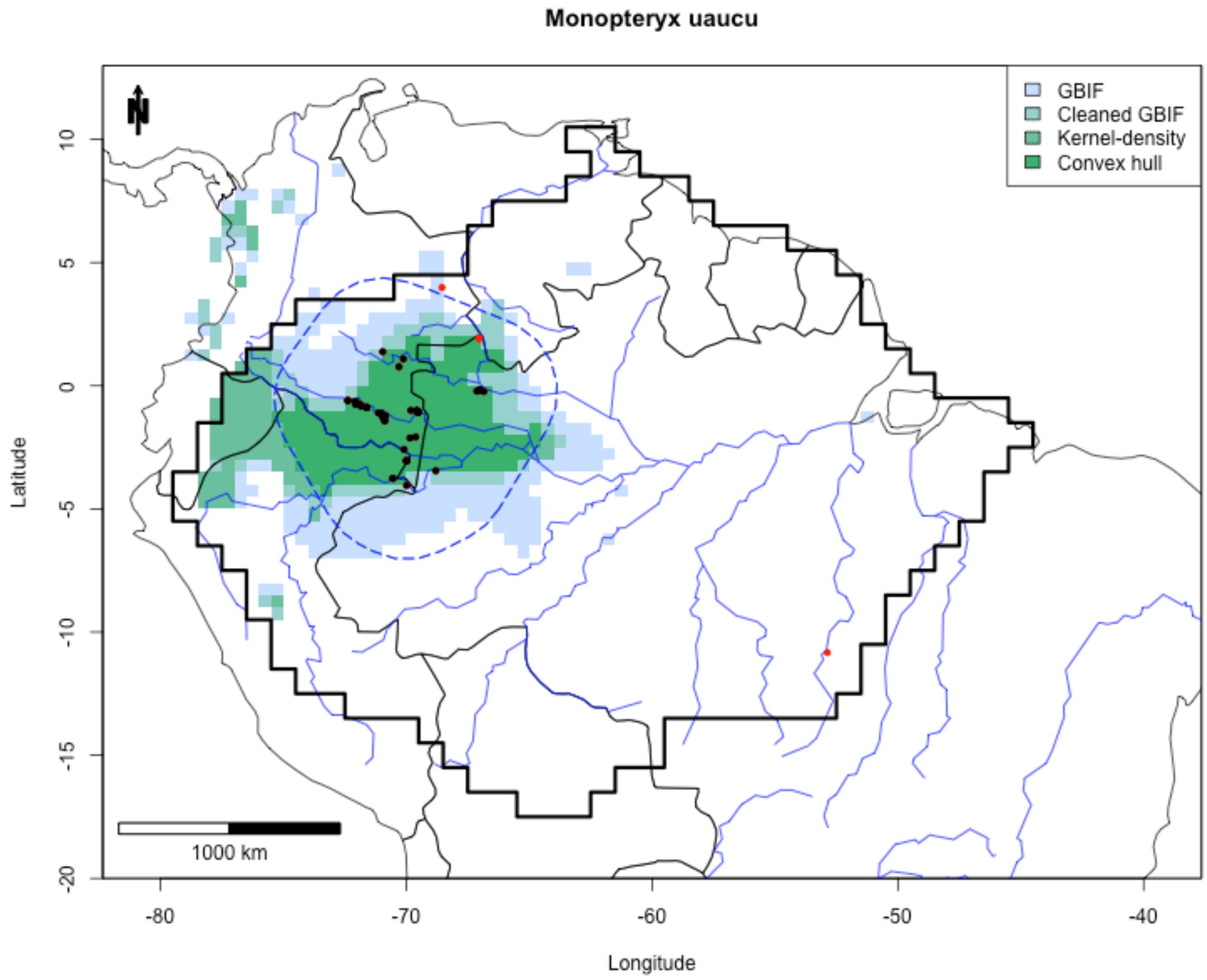

Monopteryx uaucu
GBIF
Cleaned GBIF
Kernel-density
Convex hull
N
1000 km
Latitude
Longitude
10
5
0
-5
-10
-15
-20
-80
-70
-60
-50
-40

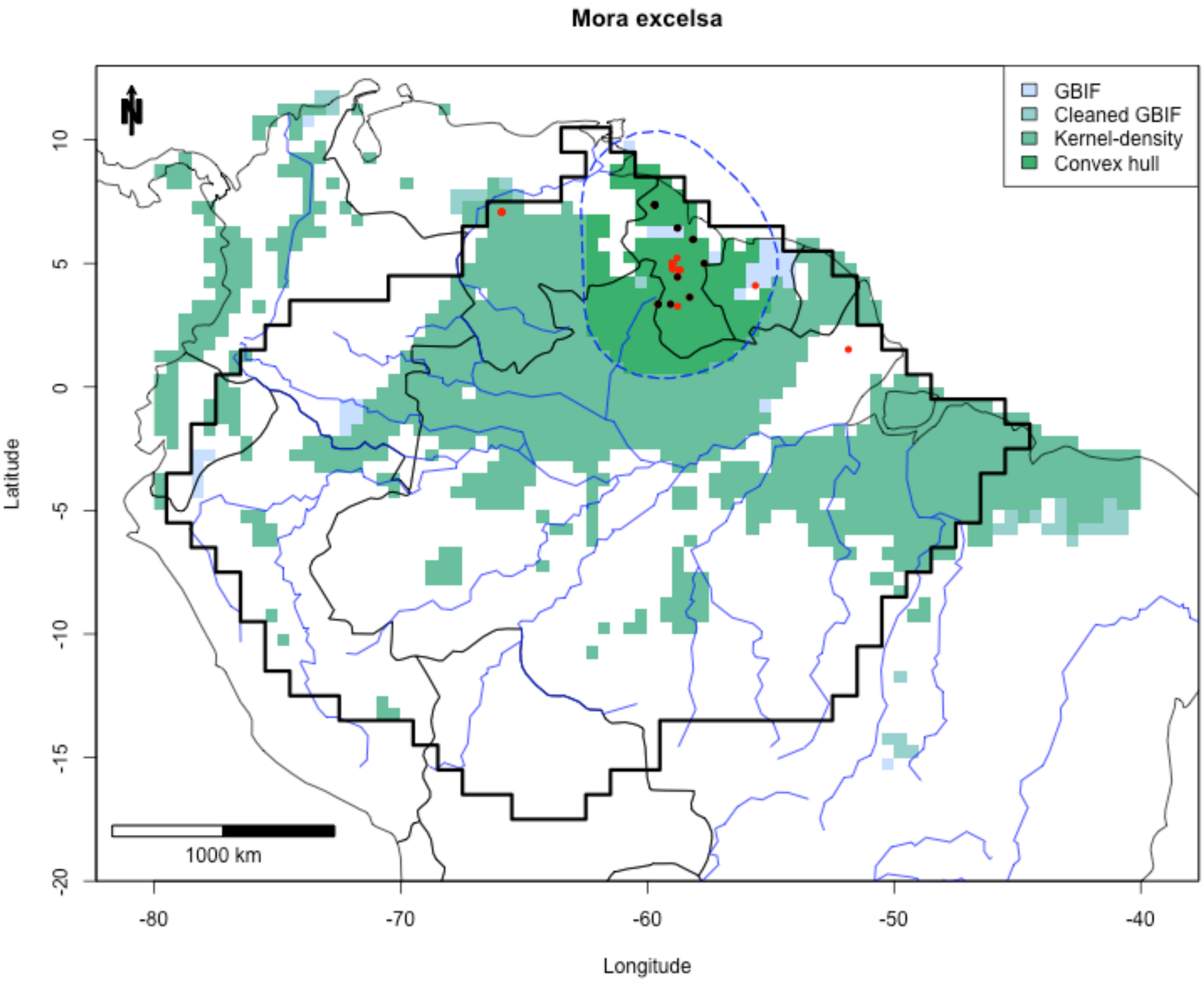

Mora excelsa
GBIF
Cleaned GBIF
Kernel-density
Convex hull
N
1000 km
Latitude
Longitude
10
5
0
-5
-10
-15
-20
-80
-70
-60
-50
-40

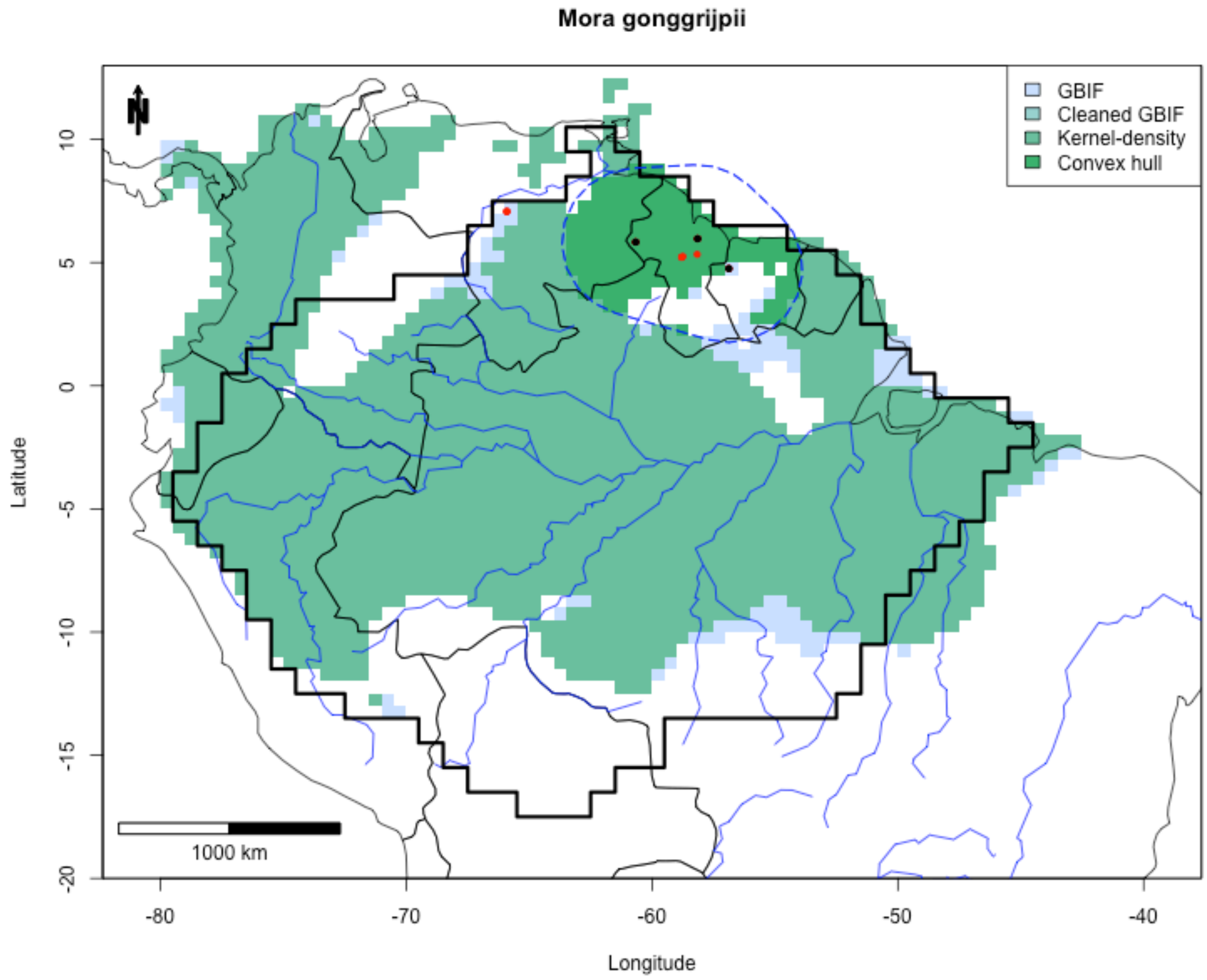
Mora gonggrijpii
GBIF
Cleaned GBIF
Kernel-density
Convex hull
N
1000 km
Latitude
Longitude
10
5
0
-5
-10
-15
-20
-80
-70
-60
-50
-40

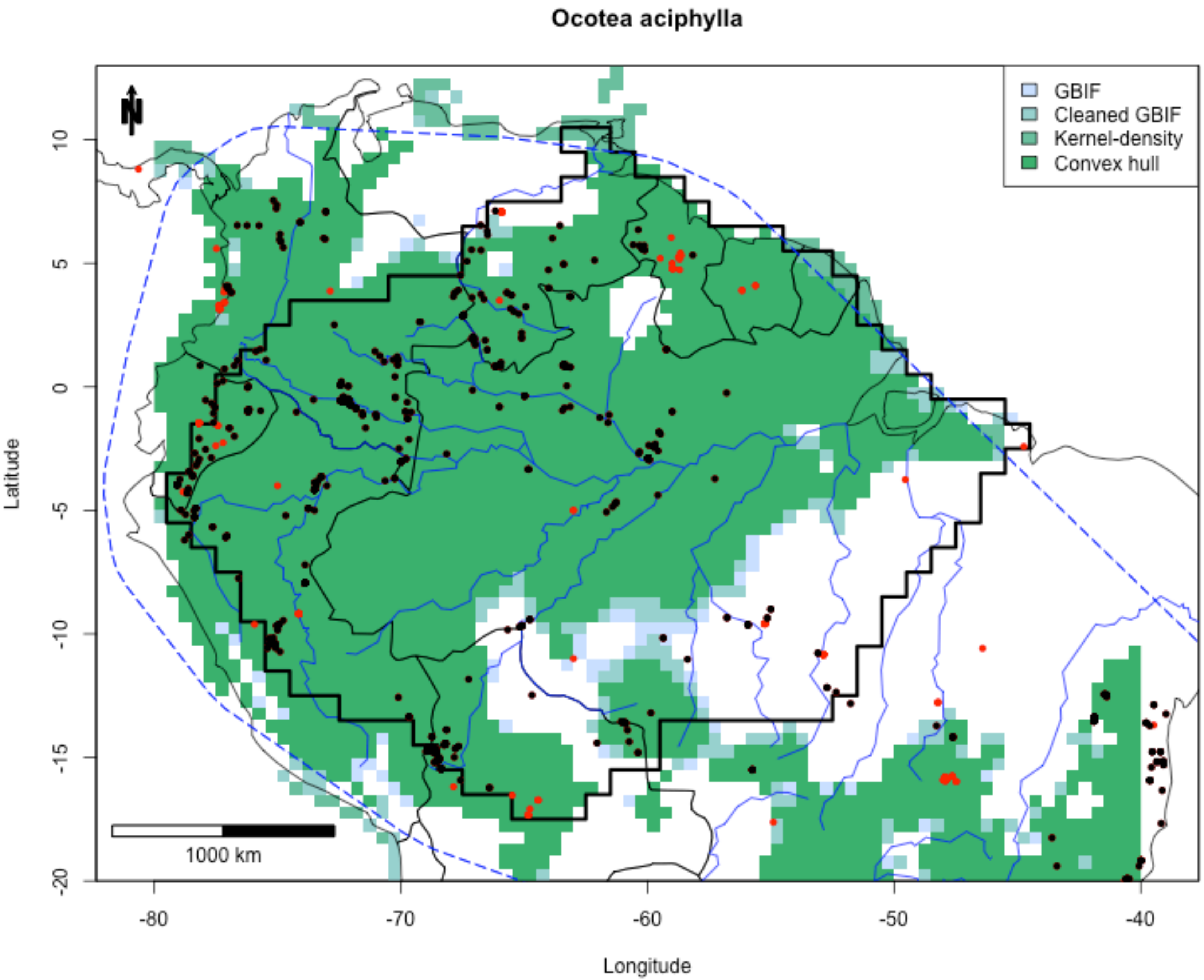

Ocotea aciphylla
GBIF
Cleaned GBIF
Kernel-density
Convex hull
N
1000 km
Latitude
Longitude
10
5
0
-5
-10
-15
-20
-80
-70
-60
-50
-40

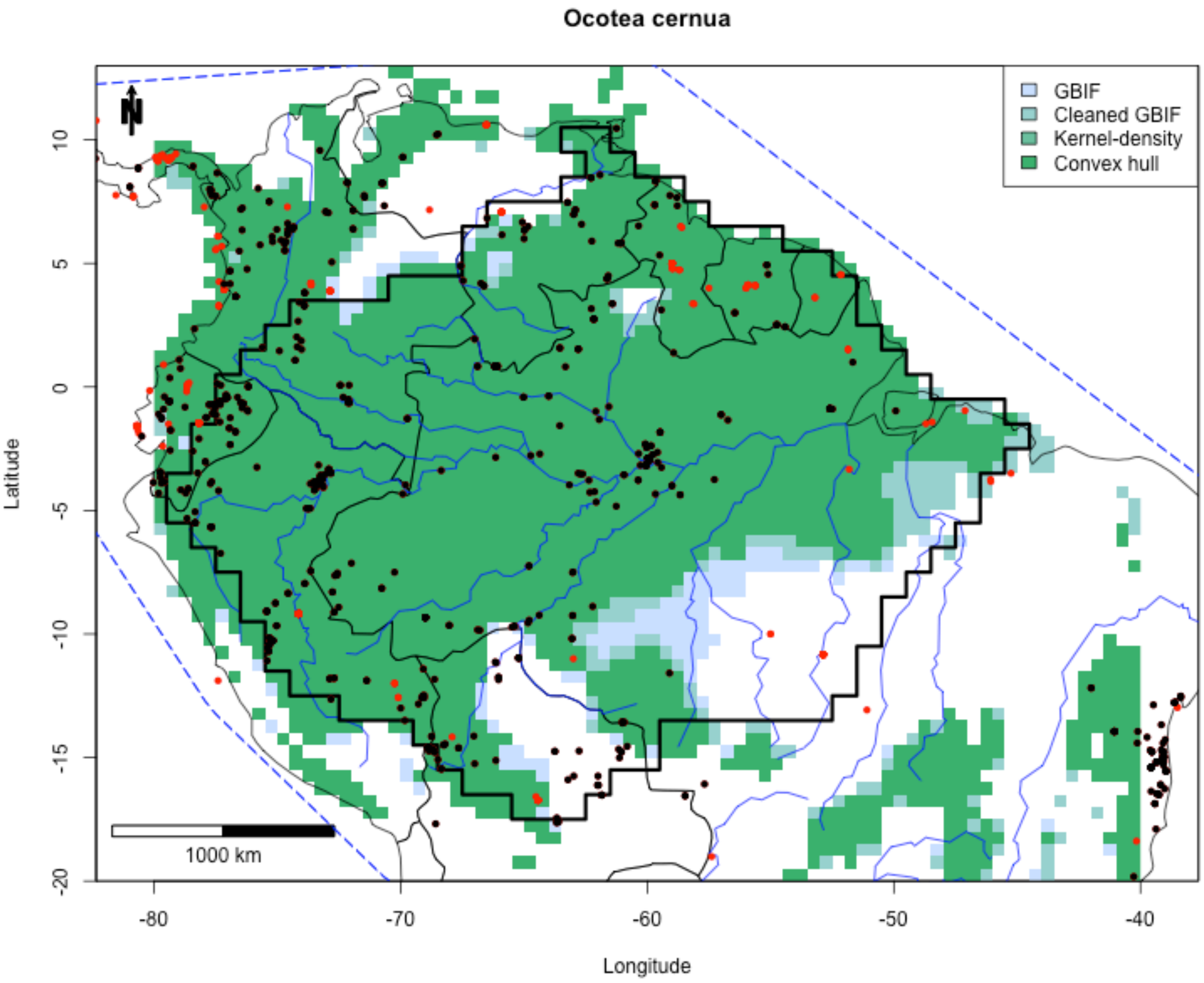

Ocotea cernua
GBIF
Cleaned GBIF
Kernel-density
Convex hull
N
1000 km
Latitude
Longitude
10
5
0
-5
-10
-15
-20
-80
-70
-60
-50
-40

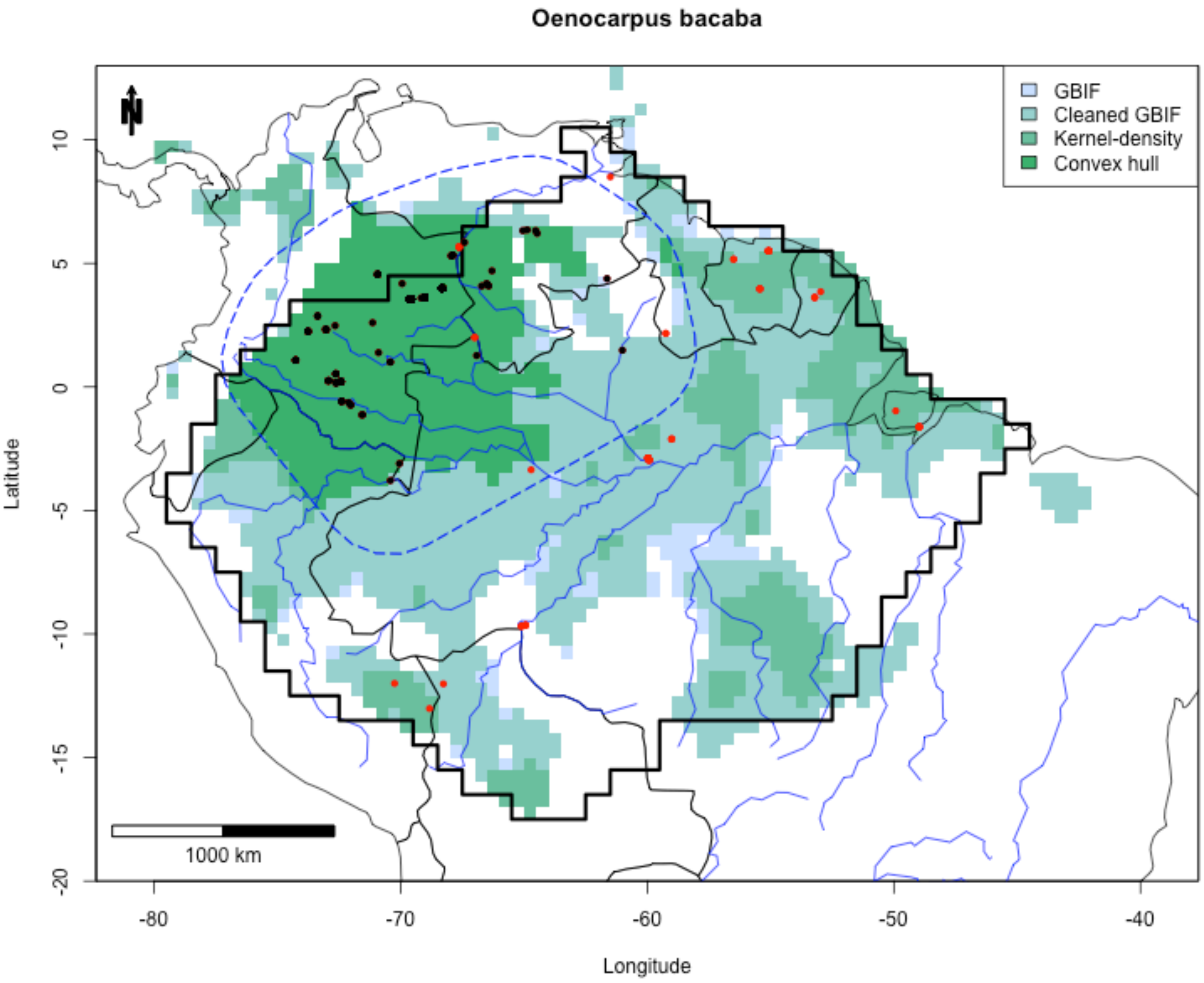

Oenocarpus bacaba
GBIF
Cleaned GBIF
Kernel-density
Convex hull
N
1000 km
Latitude
Longitude
10
5
0
-5
-10
-15
-20
-80
-70
-60
-50
-40

Oenocarpus bataua

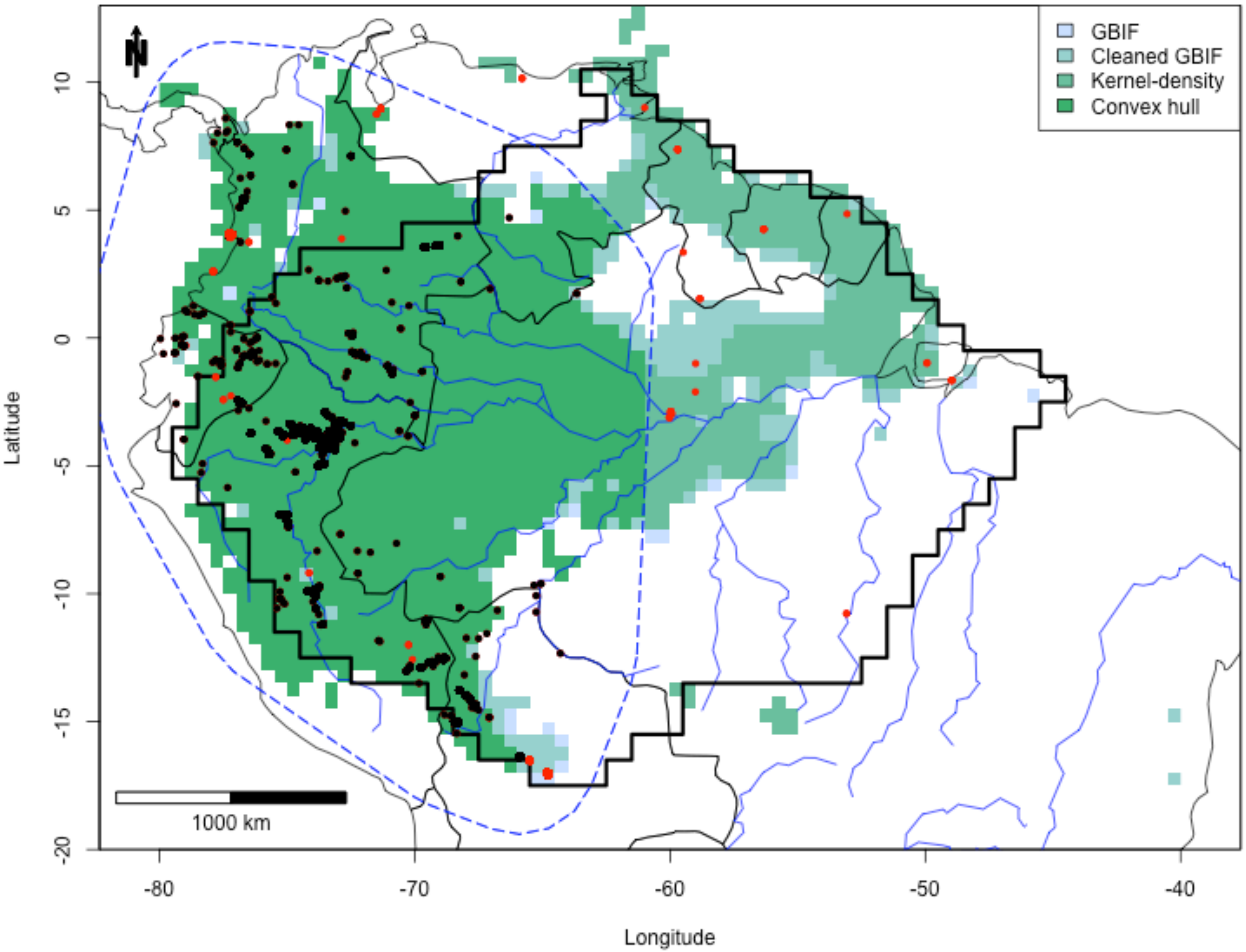

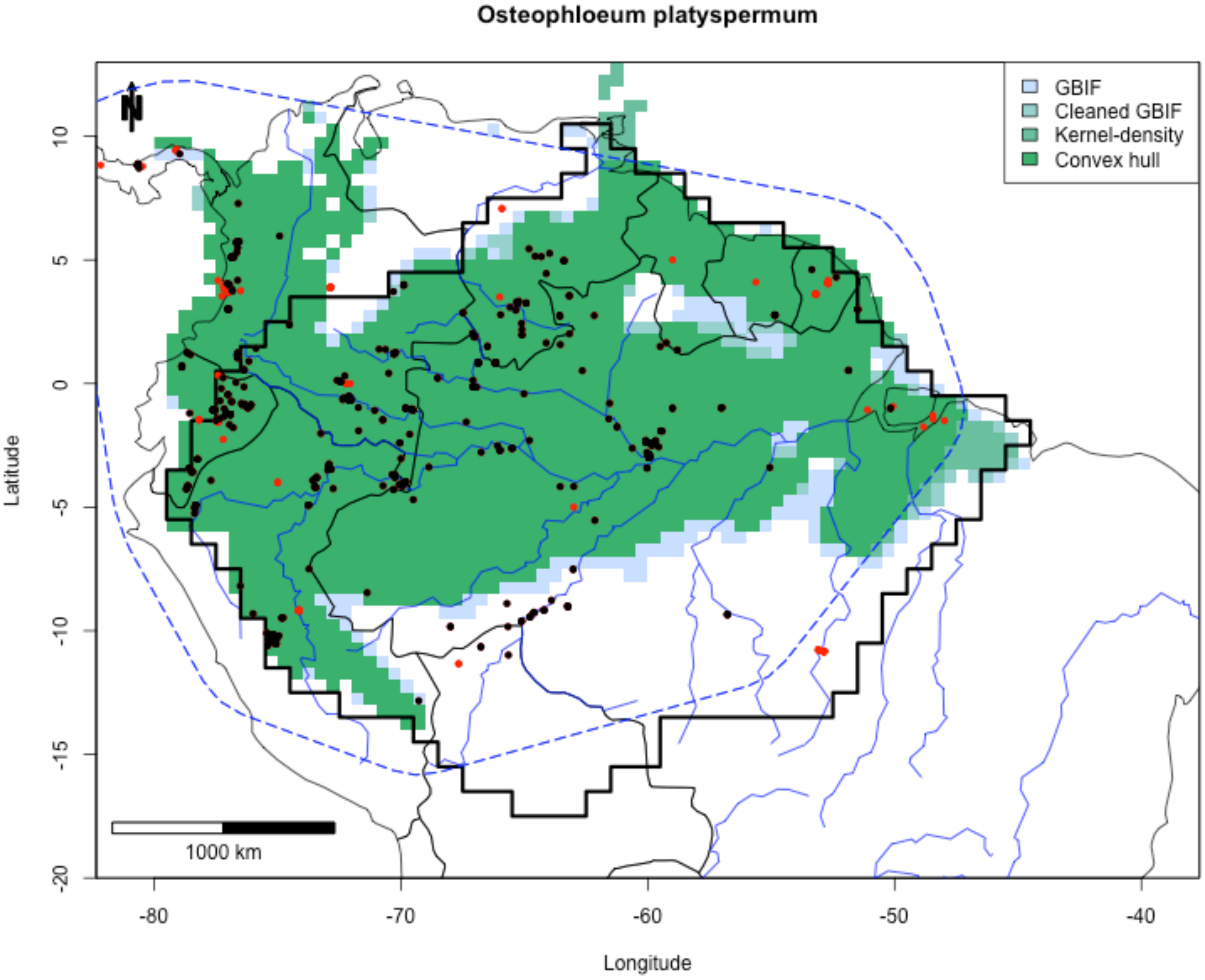

Osteophloeum platyspermum
GBIF
Cleaned GBIF
Kernel-density
Convex hull
N
1000 km
Latitude
Longitude
10
5
0
-5
-10
-15
-20
-80
-70
-60
-50
-40

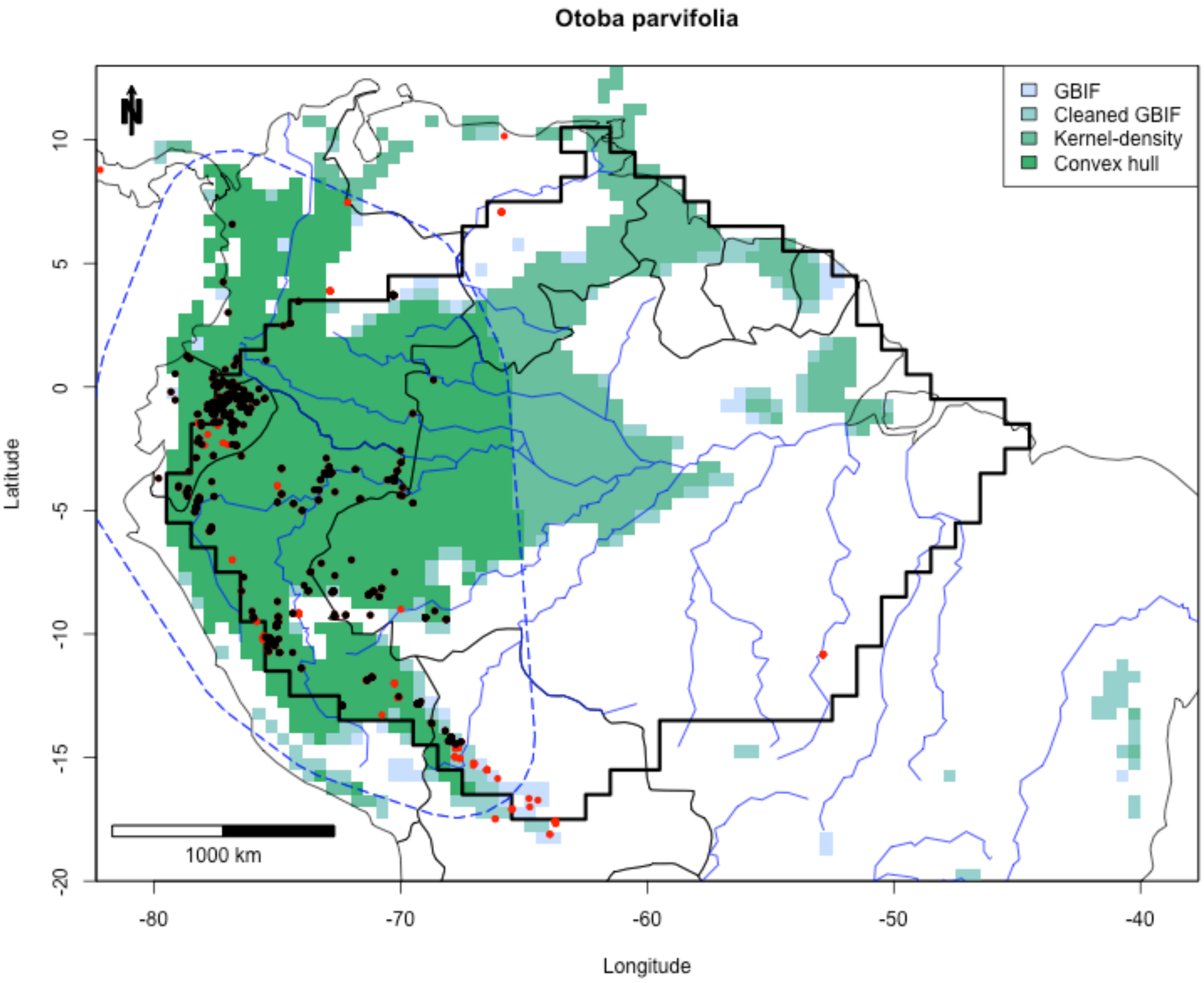

Otoba parvifolia
GBIF
Cleaned GBIF
Kernel-density
Convex hull
N
1000 km
Latitude
Longitude
10
5
0
-5
-10
-15
-20
-80
-70
-60
-50
-40

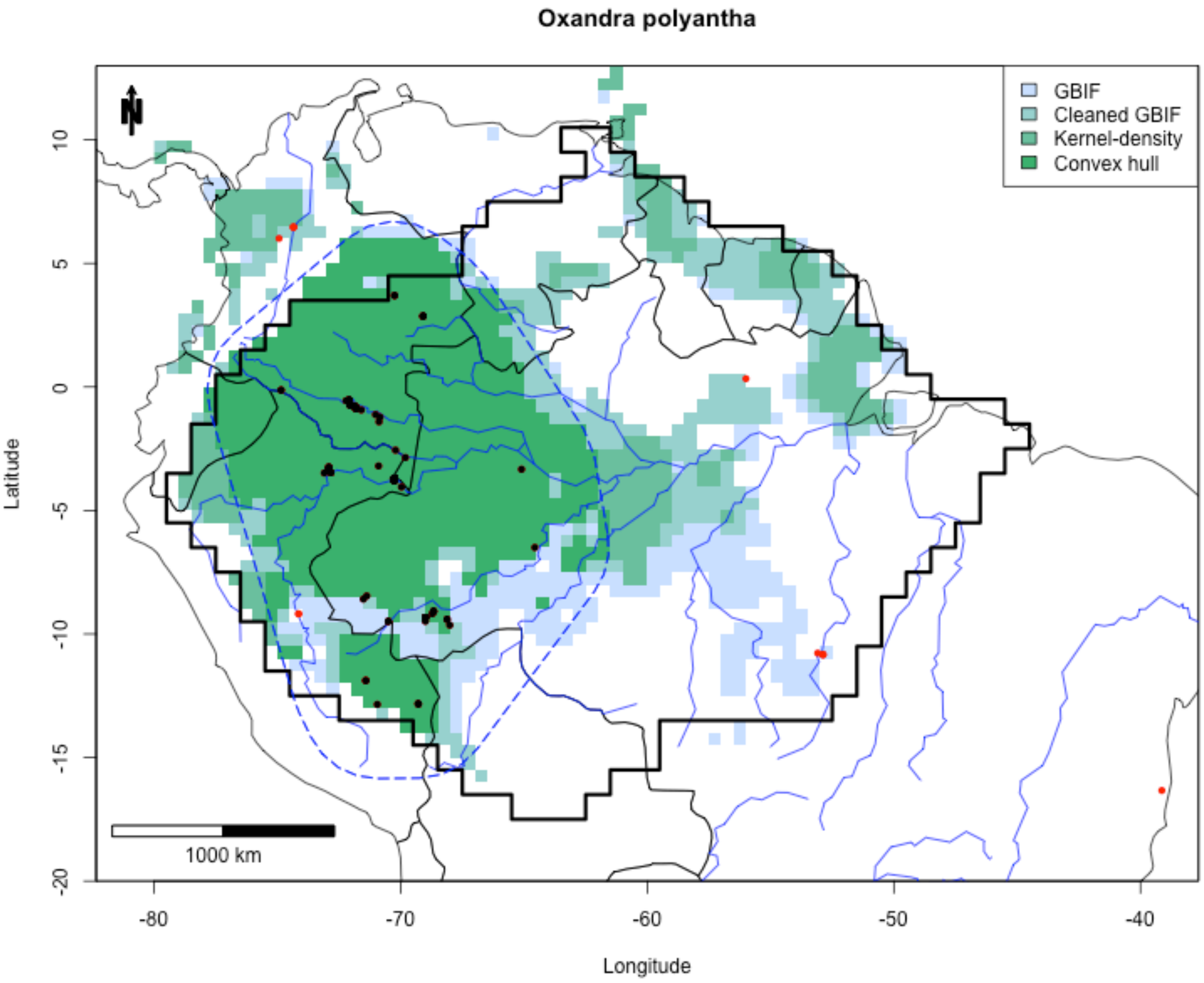
Oxandra polyantha
GBIF
Cleaned GBIF
Kernel-density
Convex hull
1000 km
N
Latitude
Longitude
10
5
0
-5
-10
-15
-20
-80
-70
-60
-50
-40

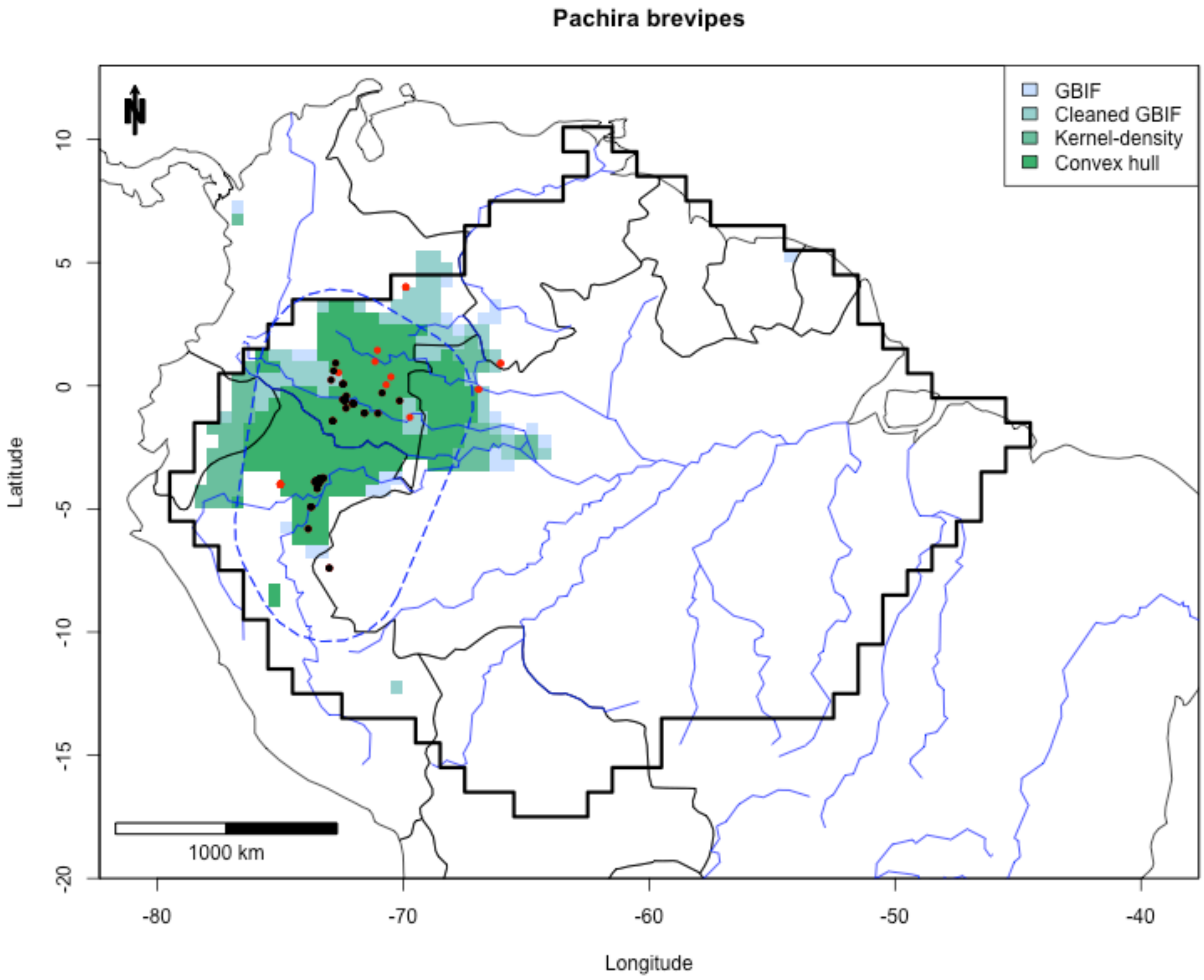

Pachira brevipes
GBIF
Cleaned GBIF
Kernel-density
Convex hull
N
1000 km
Latitude
Longitude
10
5
0
-5
-10
-15
-20
-80
-70
-60
-50
-40

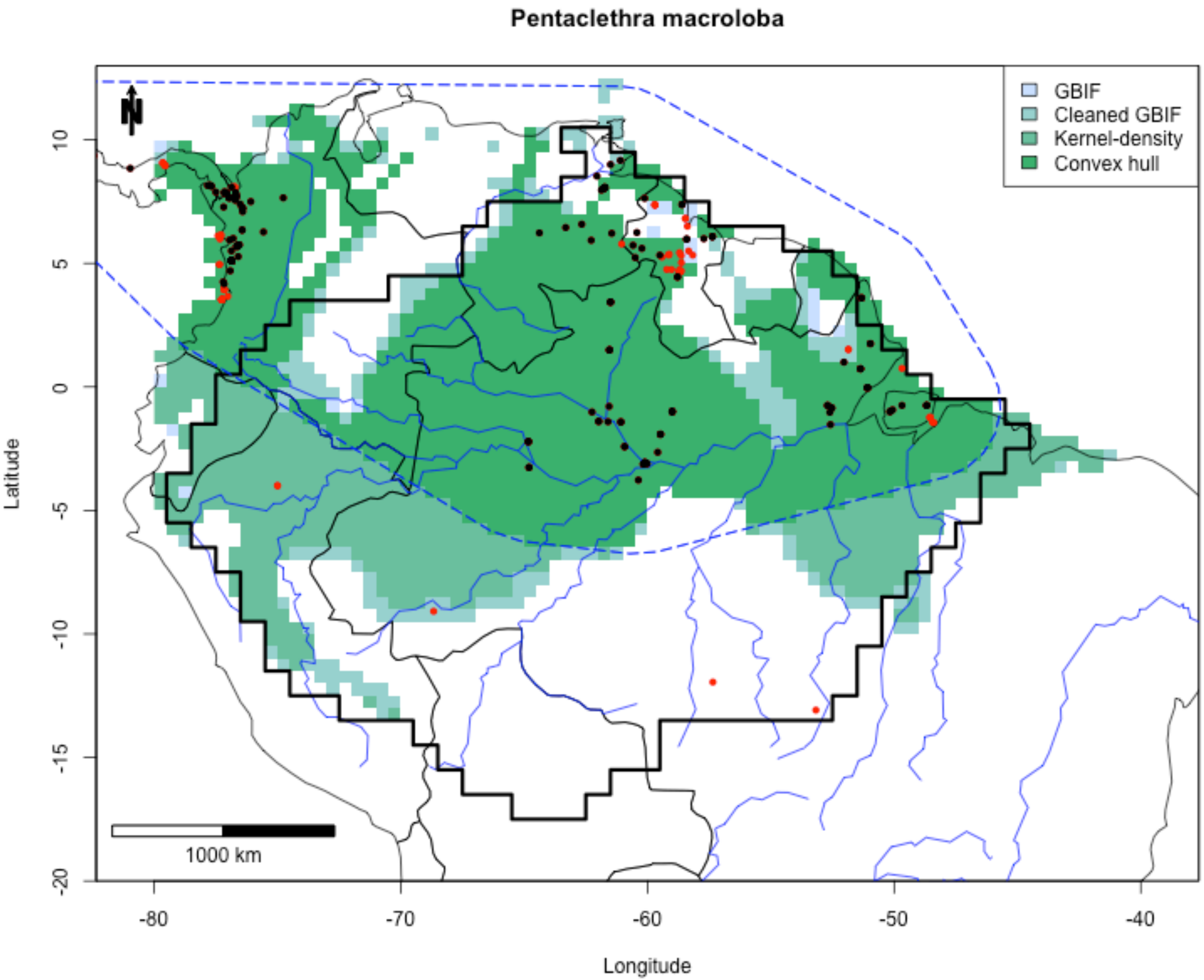

Pentaclethra macroloba
GBIF
Cleaned GBIF
Kernel-density
Convex hull
N
1000 km
Latitude
Longitude
10
5
0
-5
-10
-15
-20
-80
-70
-60
-50
-40

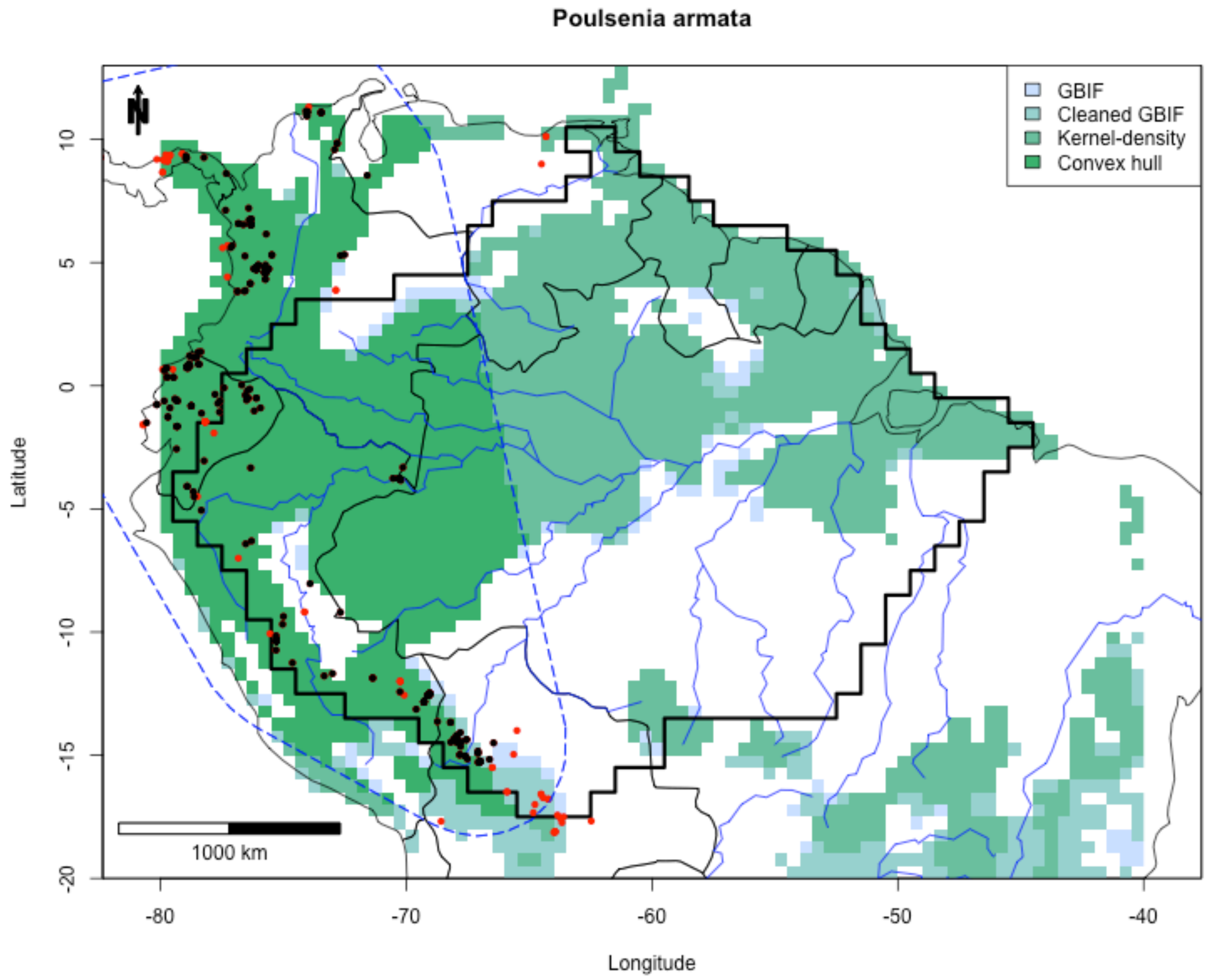

Poulsenia armata
GBIF
Cleaned GBIF
Kernel-density
Convex hull
1000 km
Latitude
Longitude
10
5
0
-5
-10
-15
-20
-80
-70
-60
-50
-40

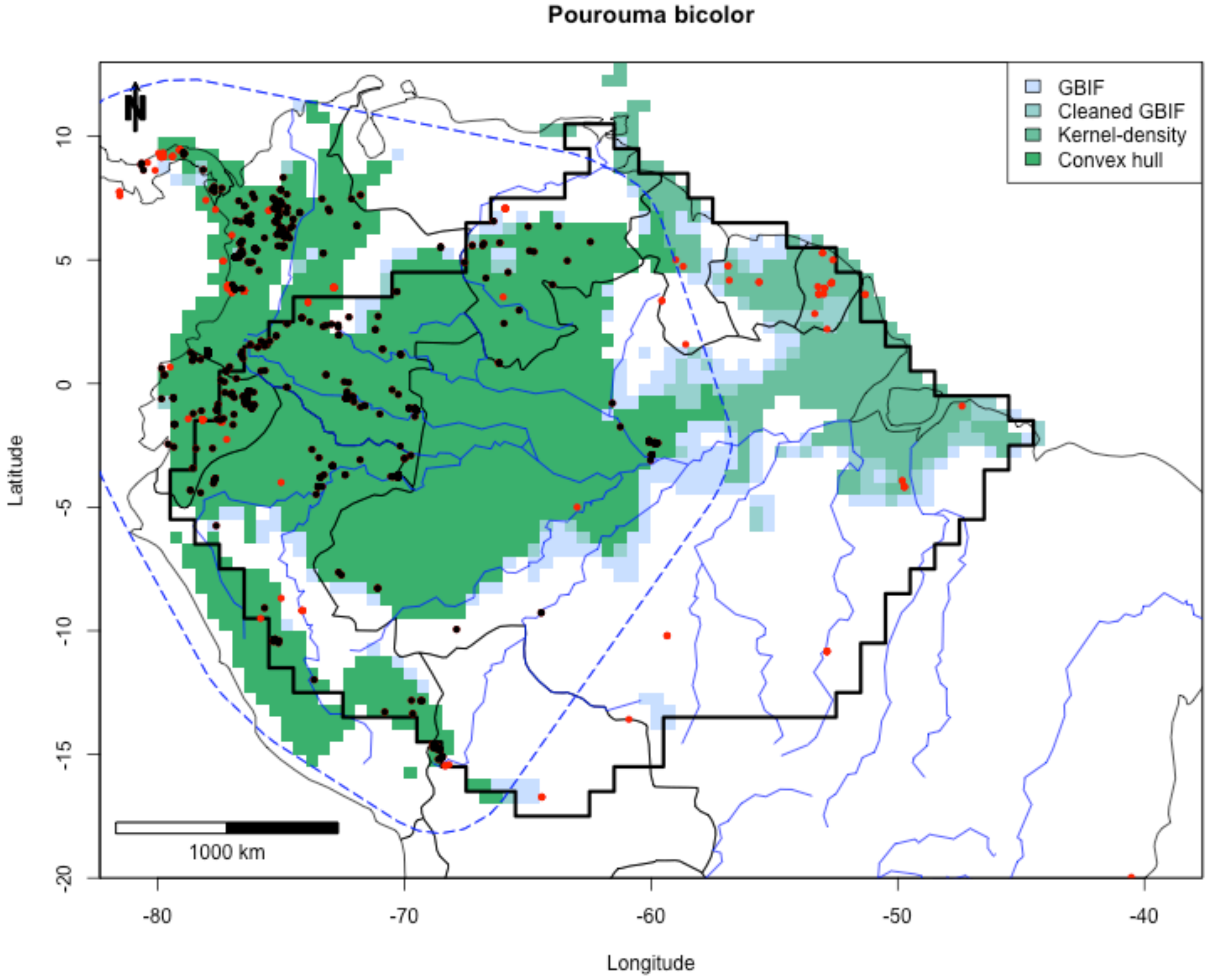

Pourouma bicolor
GBIF
Cleaned GBIF
Kernel-density
Convex hull
N
1000 km
Latitude
Longitude
10
5
0
-5
-10
-15
-20
-80
-70
-60
-50
-40

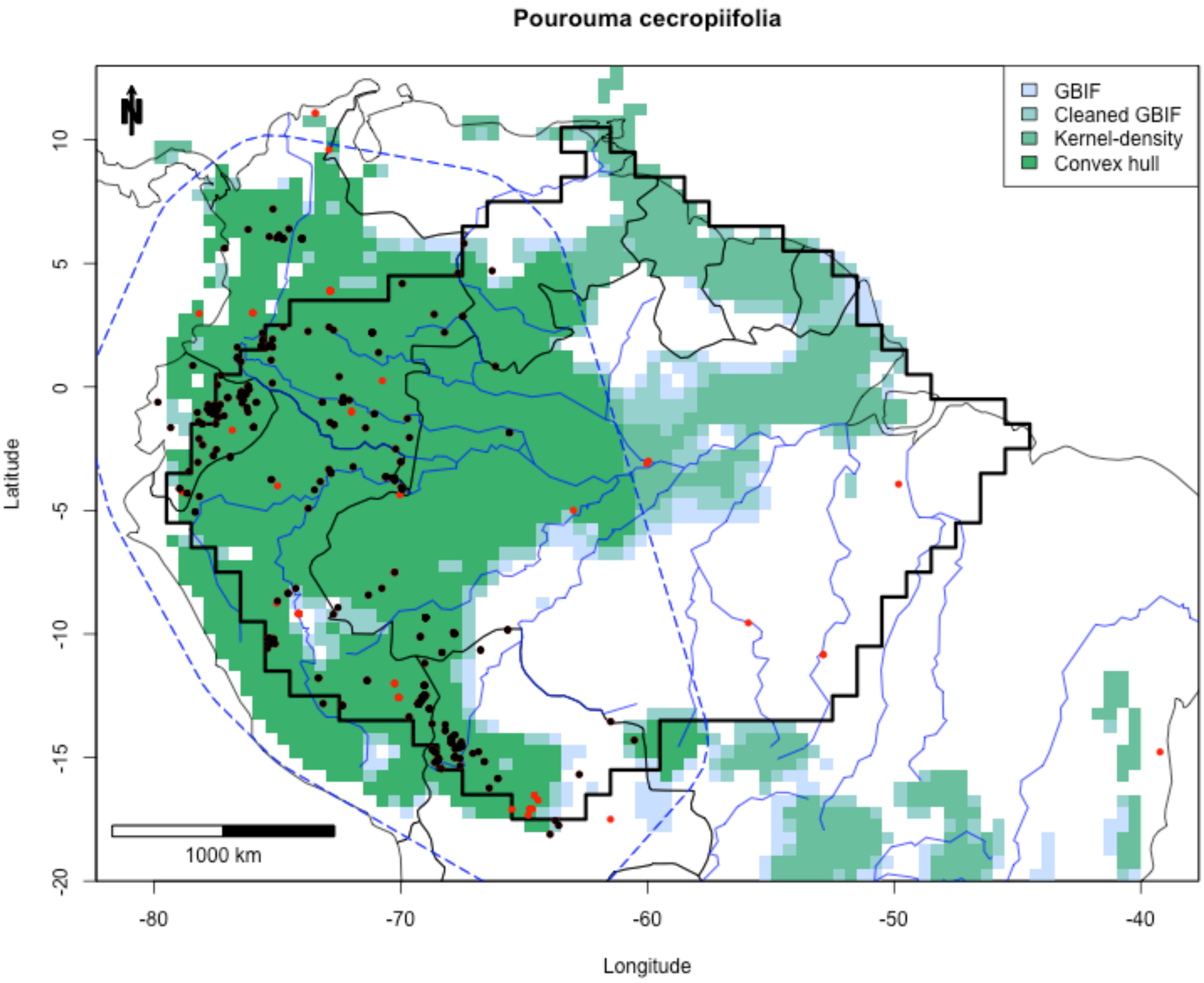

Pourouma cecropiifolia
N
GBIF
Cleaned GBIF
Kernel-density
Convex hull
1000 km
10
5
0
-5
-10
-15
-20
-80
-70
-60
-50
-40
Latitude
Longitude

Pourouma guianensis

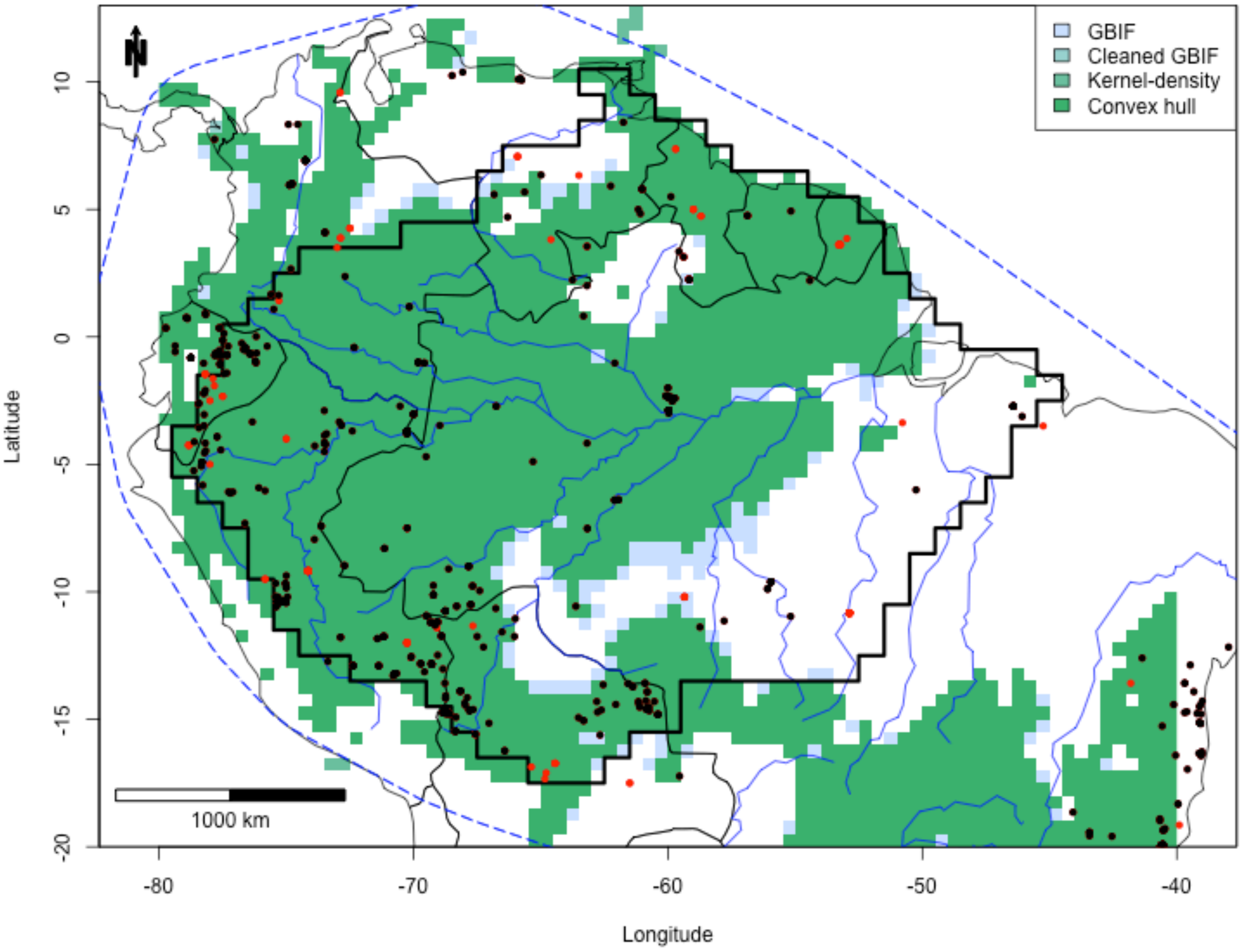

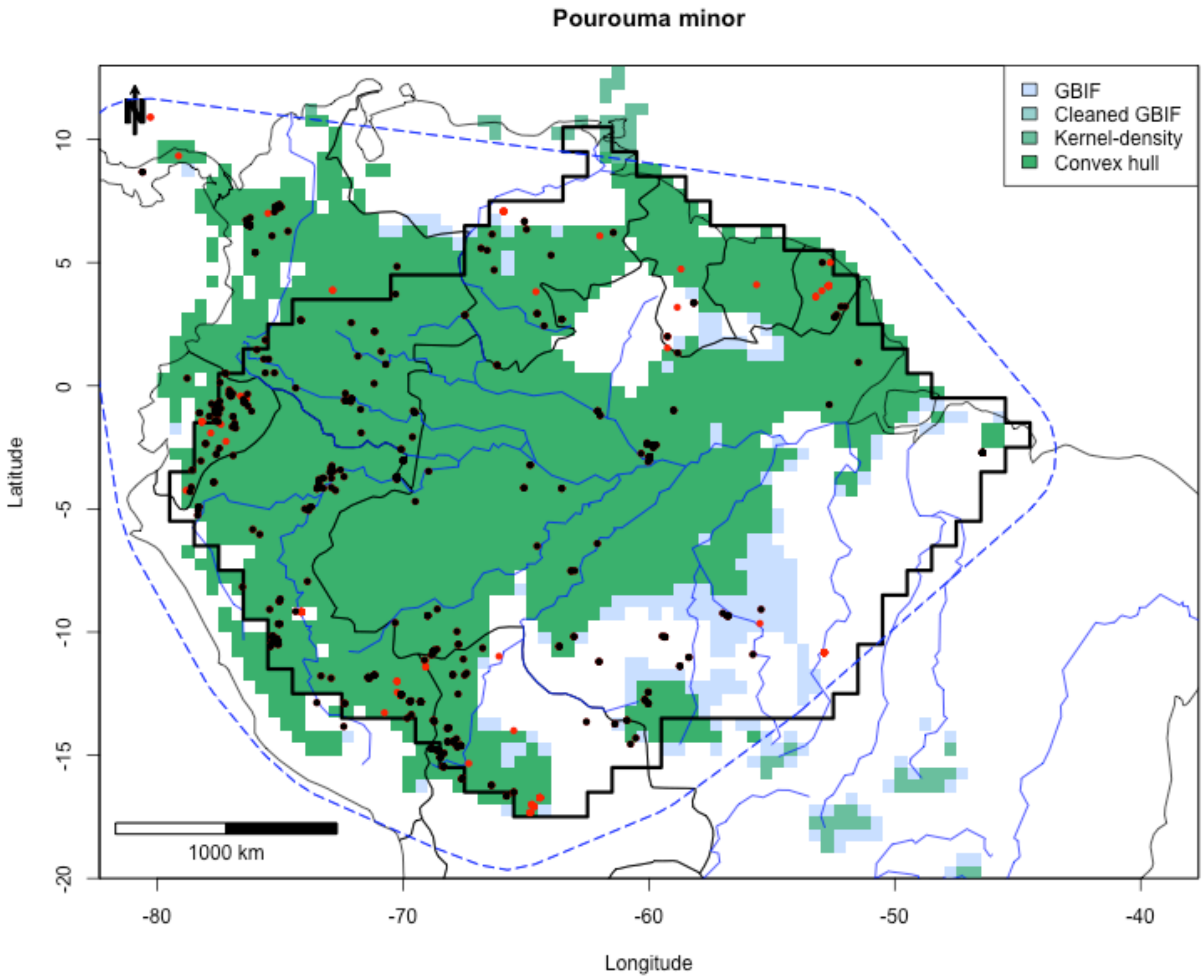
Pourouma minor
GBIF
Cleaned GBIF
Kernel-density
Convex hull
N
1000 km
Latitude
Longitude
10
5
0
-5
-10
-15
-20
-80
-70
-60
-50
-40

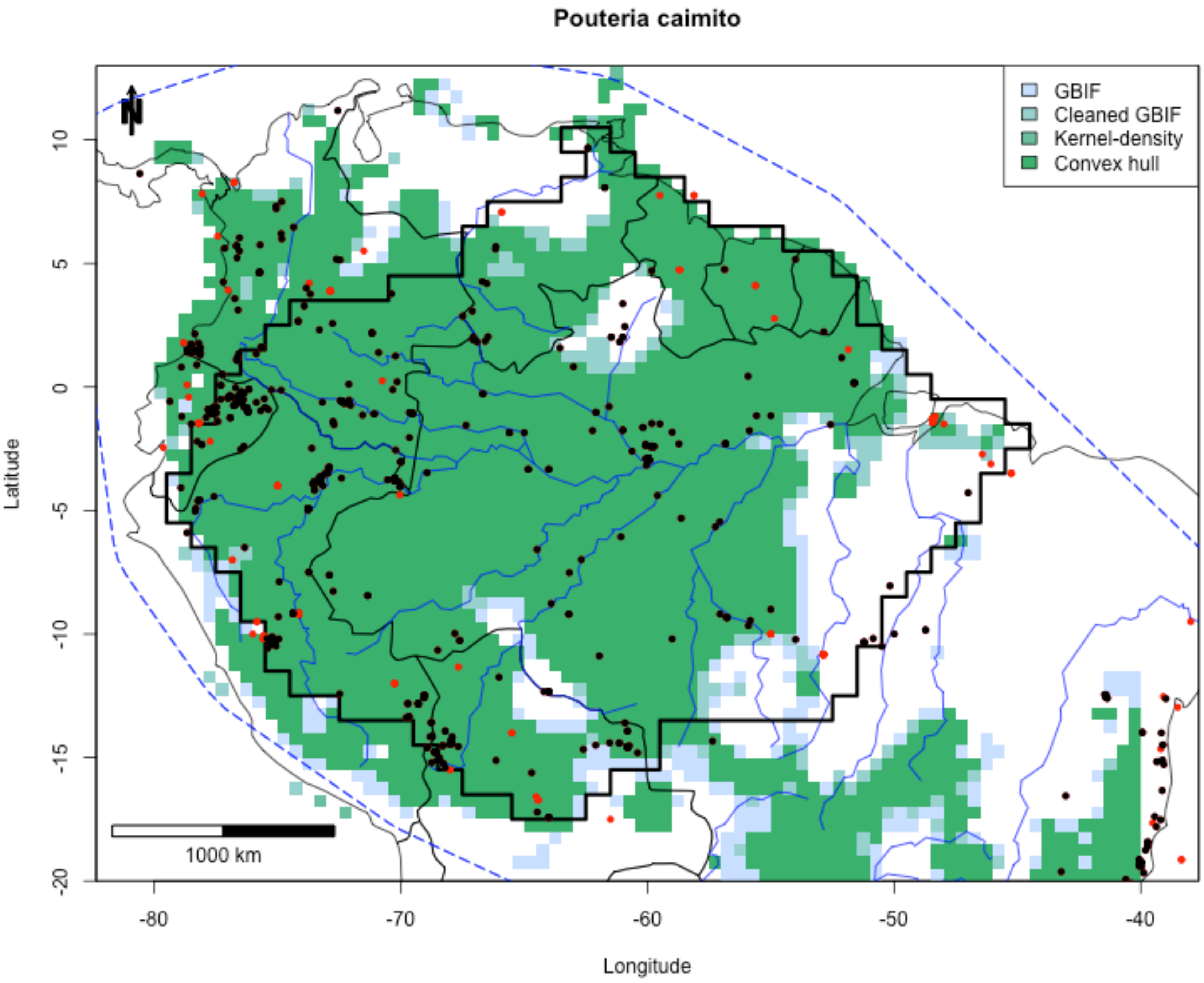
Pouteria caimito
GBIF
Cleaned GBIF
Kernel-density
Convex hull
N
1000 km
Latitude
Longitude
10
5
0
-5
-10
-15
-20
-80
-70
-60
-50
-40

Pouteria cuspidata

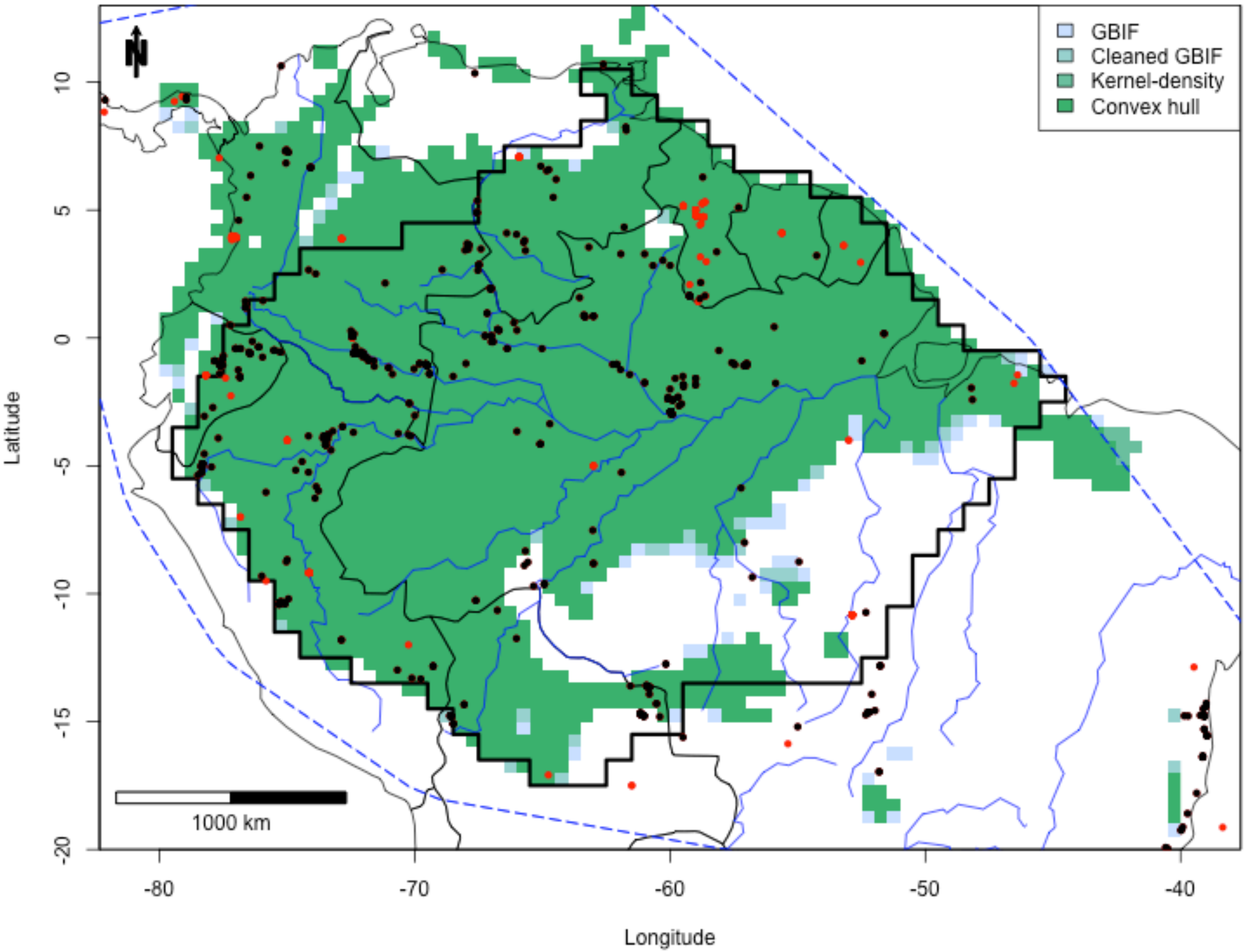

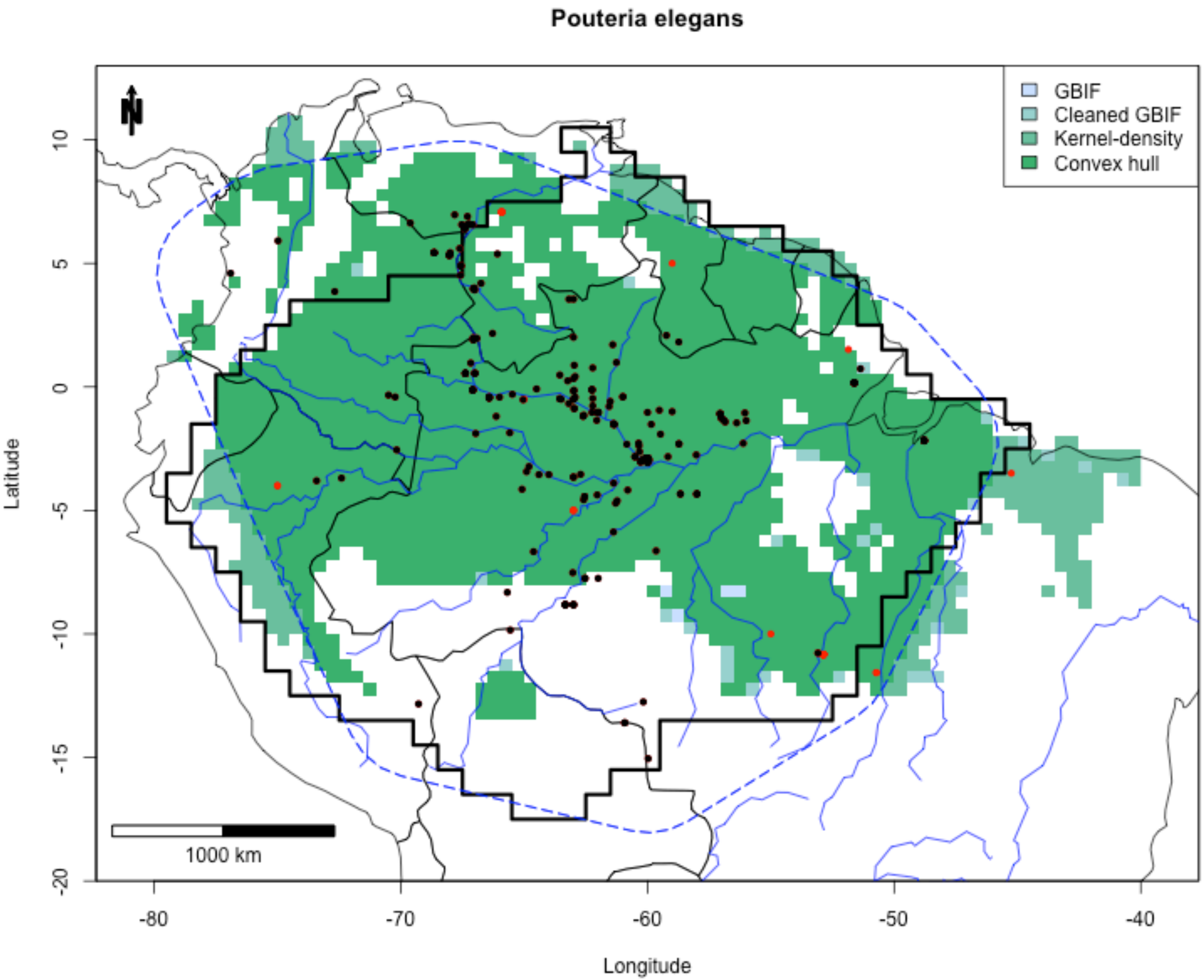

Pouteria elegans
N
GBIF
Cleaned GBIF
Kernel-density
Convex hull
1000 km
10
5
0
-5
-10
-15
-20
-80
-70
-60
-50
-40
Latitude
Longitude

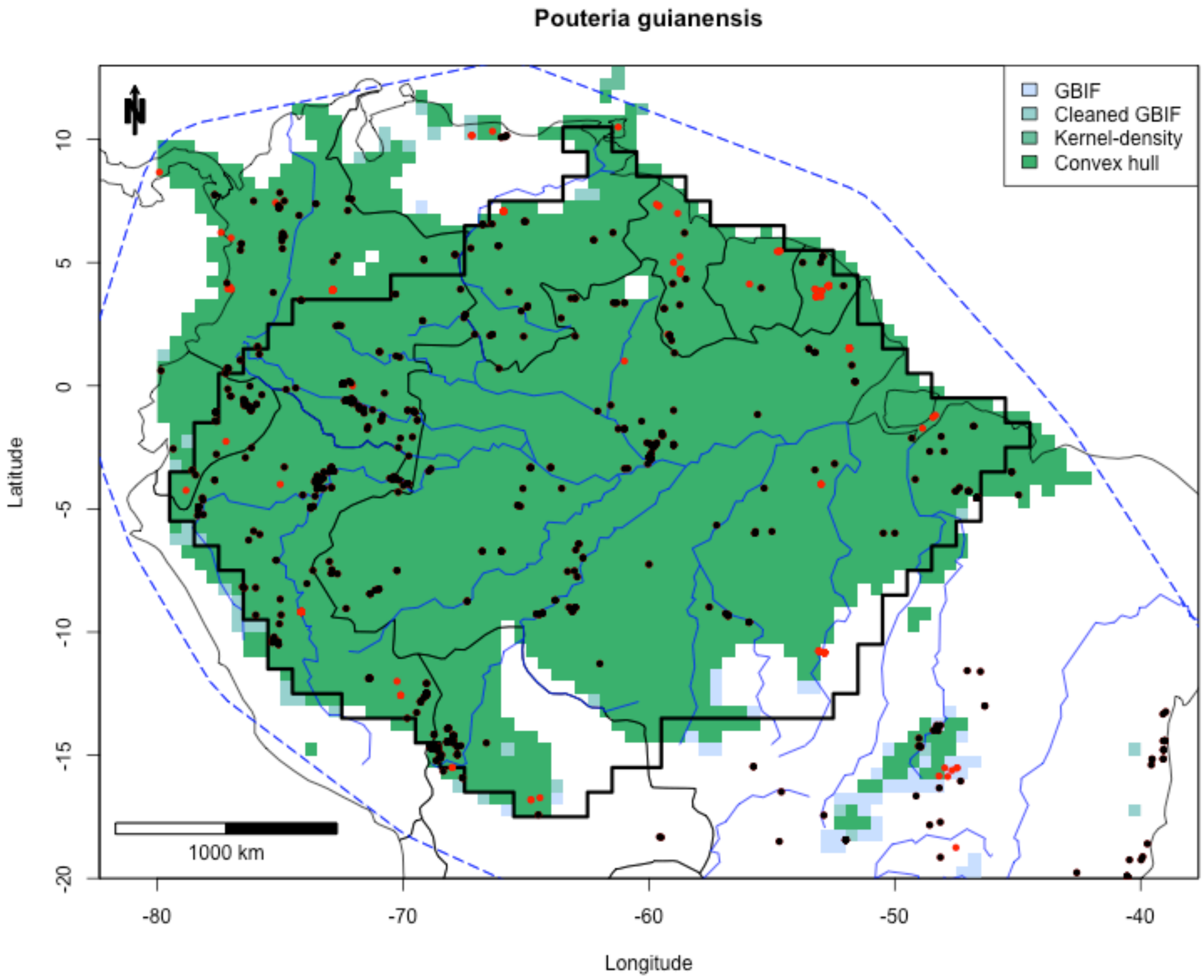

Pouteria guianensis
N
GBIF
Cleaned GBIF
Kernel-density
Convex hull
10
5
0
-5
-10
-15
-20
Latitude
1000 km
-80
-70
-60
-50
-40
Longitude

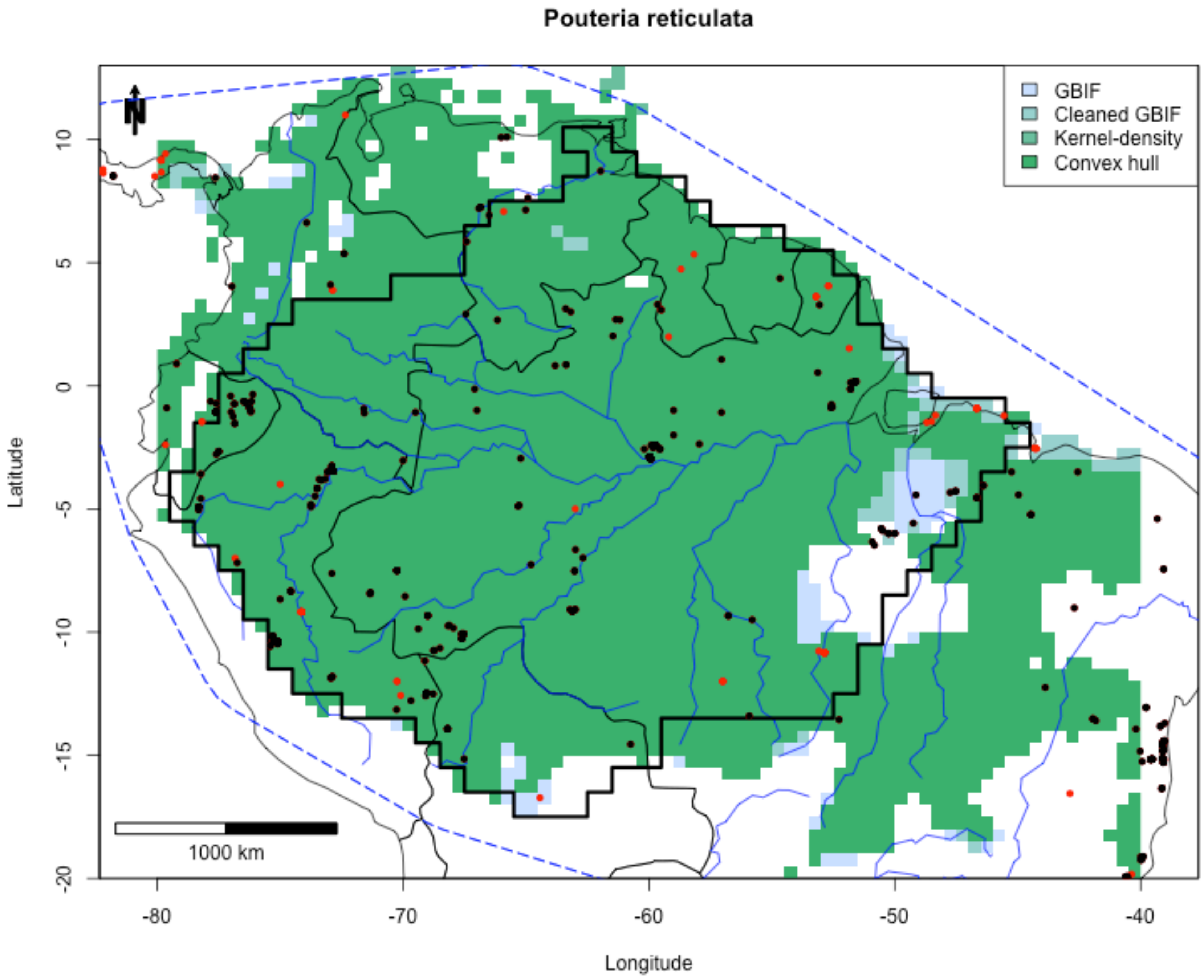

Pouteria reticulata
GBIF
Cleaned GBIF
Kernel-density
Convex hull
N
1000 km
Latitude
Longitude
10
5
0
-5
-10
-15
-20
-80
-70
-60
-50
-40

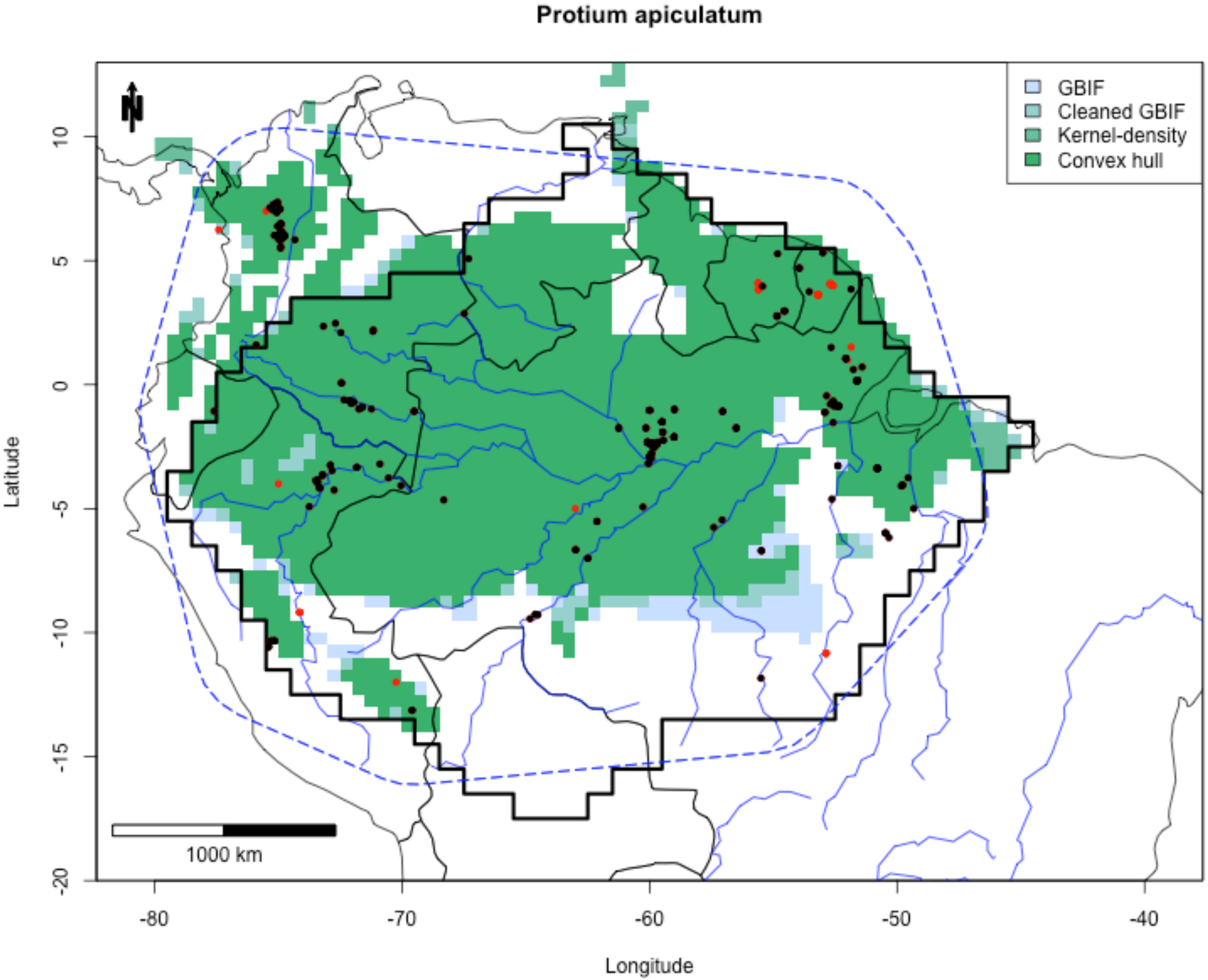

Protium apiculatum
GBIF
Cleaned GBIF
Kernel-density
Convex hull
1000 km
Latitude
Longitude
10
5
0
-5
-10
-15
-20
-80
-70
-60
-50
-40

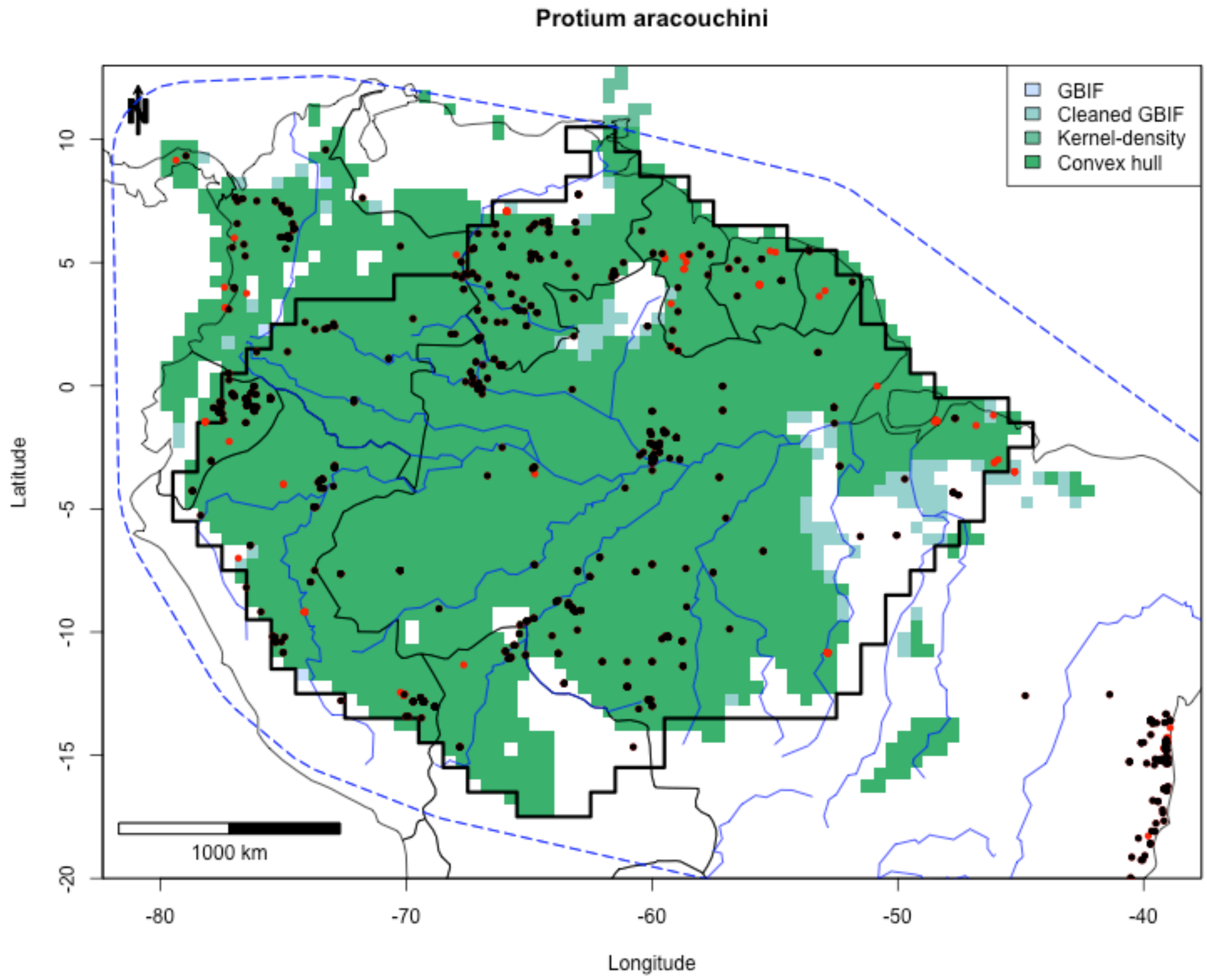

Protium aracouchini
GBIF
Cleaned GBIF
Kernel-density
Convex hull
N
1000 km
Latitude
10
5
0
-5
-10
-15
-20
Longitude
-80
-70
-60
-50
-40

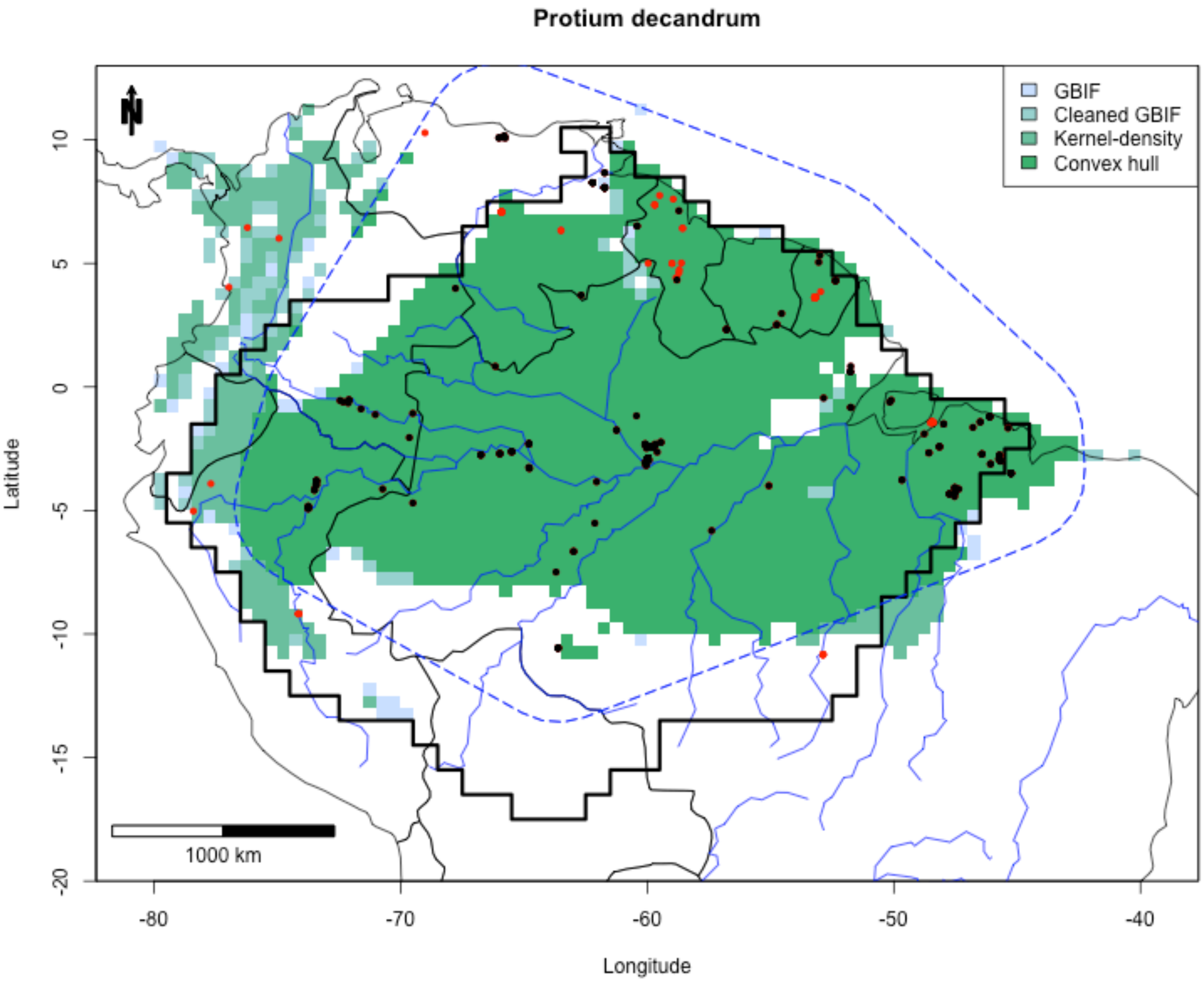
Protium decandrum
GBIF
Cleaned GBIF
Kernel-density
Convex hull
N
1000 km
Latitude
Longitude
10
5
0
-5
-10
-15
-20
-80
-70
-60
-50
-40

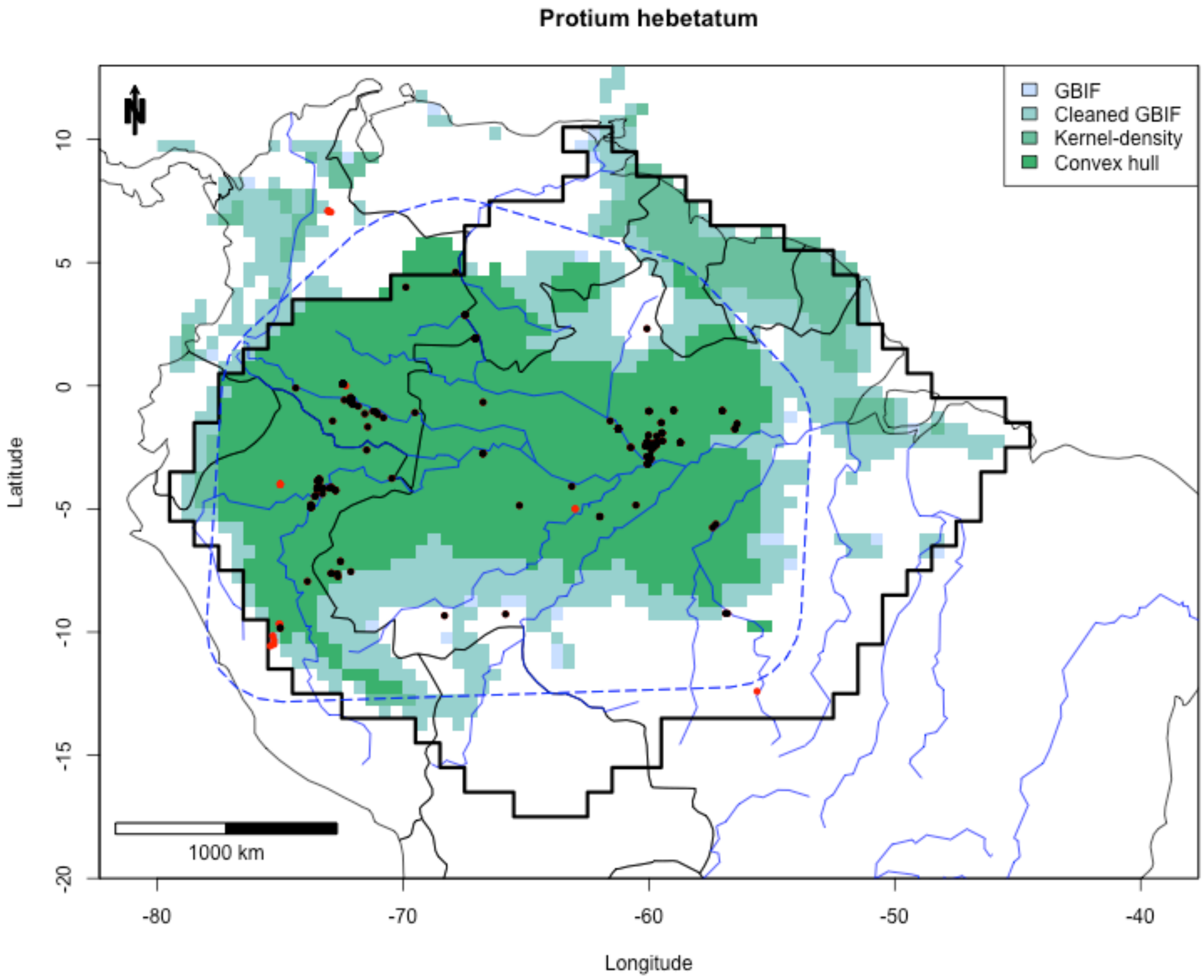

Protium hebetatum
GBIF
Cleaned GBIF
Kernel-density
Convex hull
N
1000 km
Latitude
Longitude
10
5
0
-5
-10
-15
-20
-80
-70
-60
-50
-40

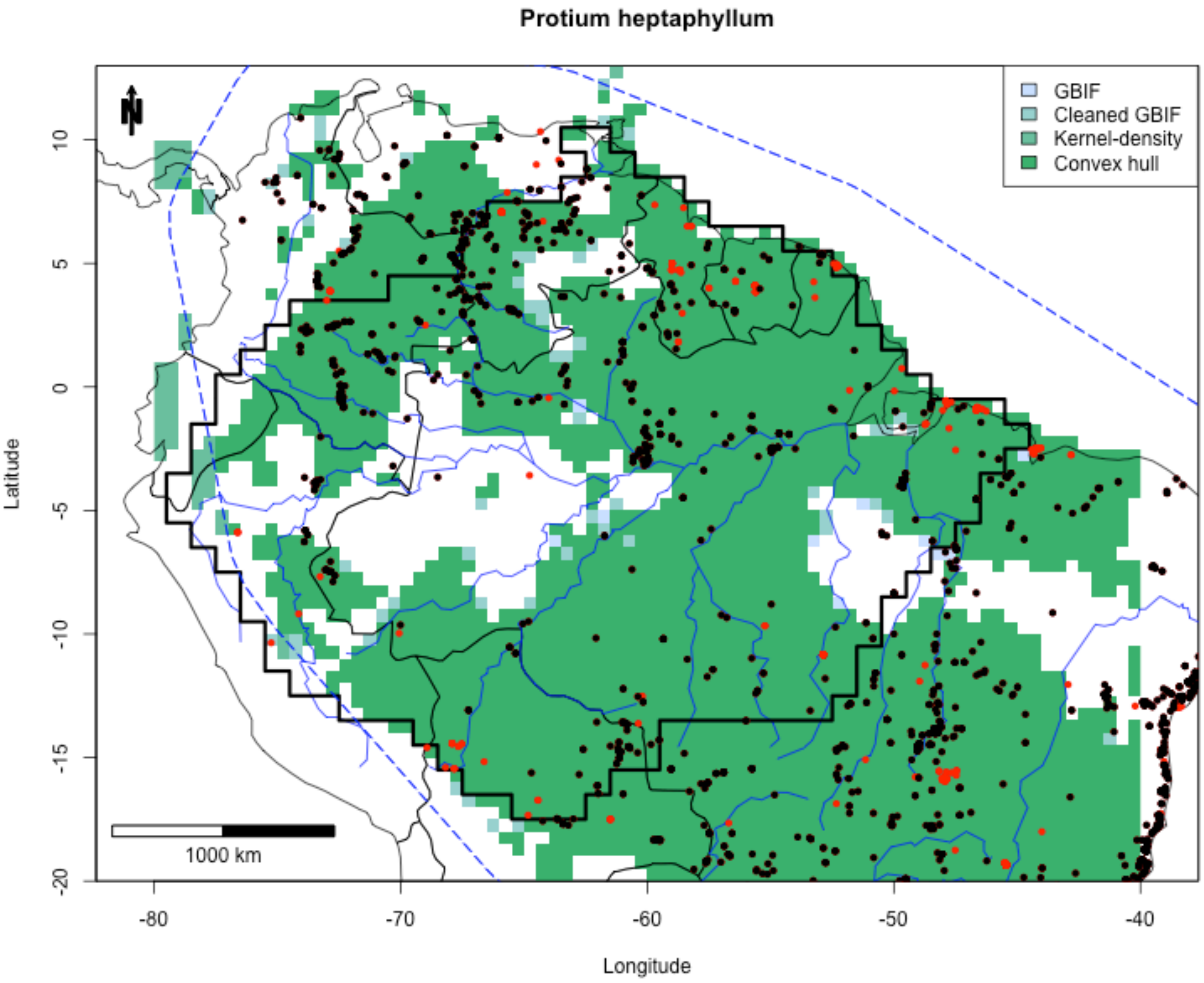

Protium heptaphyllum
GBIF
Cleaned GBIF
Kernel-density
Convex hull
N
1000 km
Latitude
Longitude
10
5
0
-5
-10
-15
-20
-80
-70
-60
-50
-40

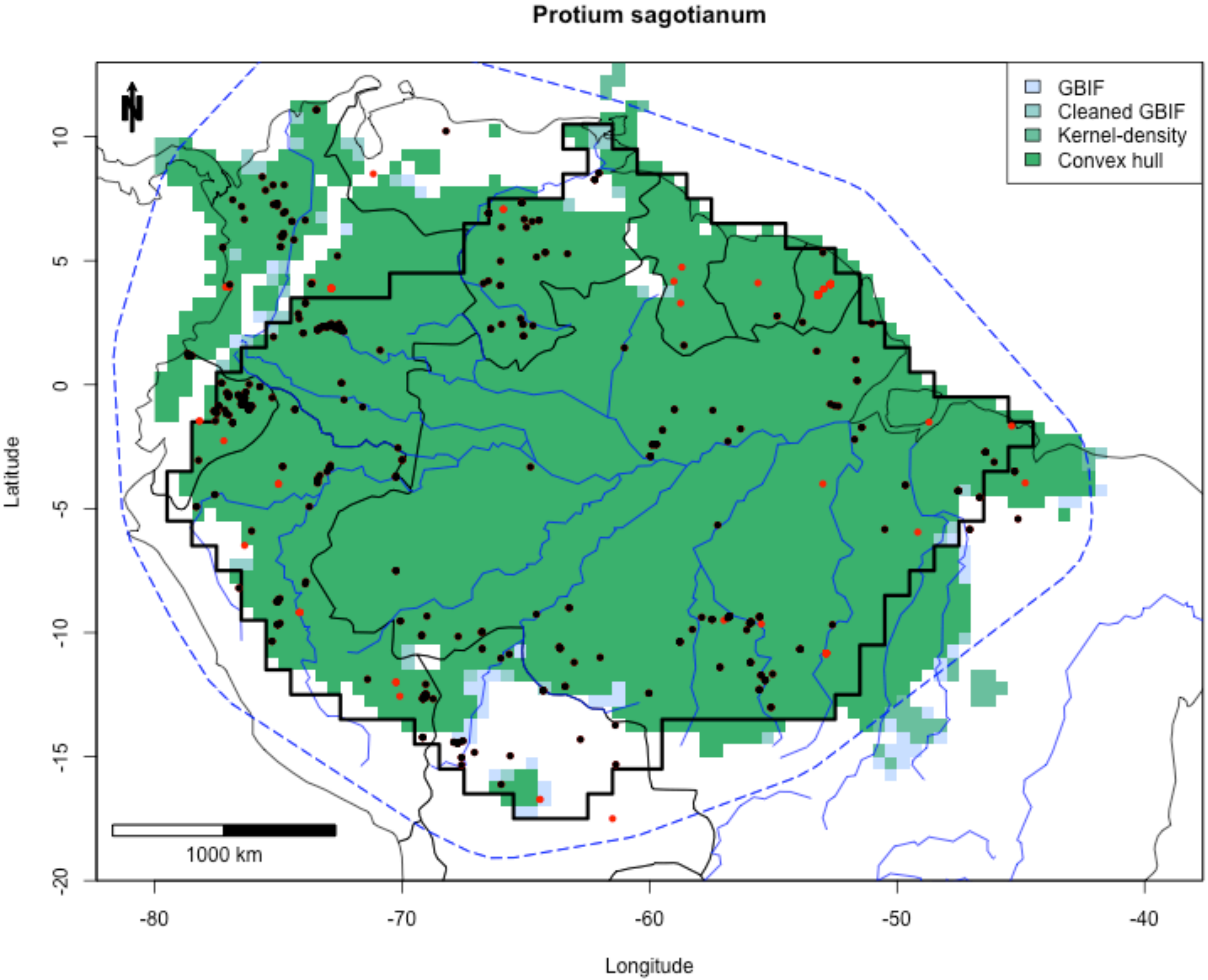

Protium sagotianum
GBIF
Cleaned GBIF
Kernel-density
Convex hull
1000 km
Latitude
Longitude
10
5
0
-5
-10
-15
-20
-80
-70
-60
-50
-40

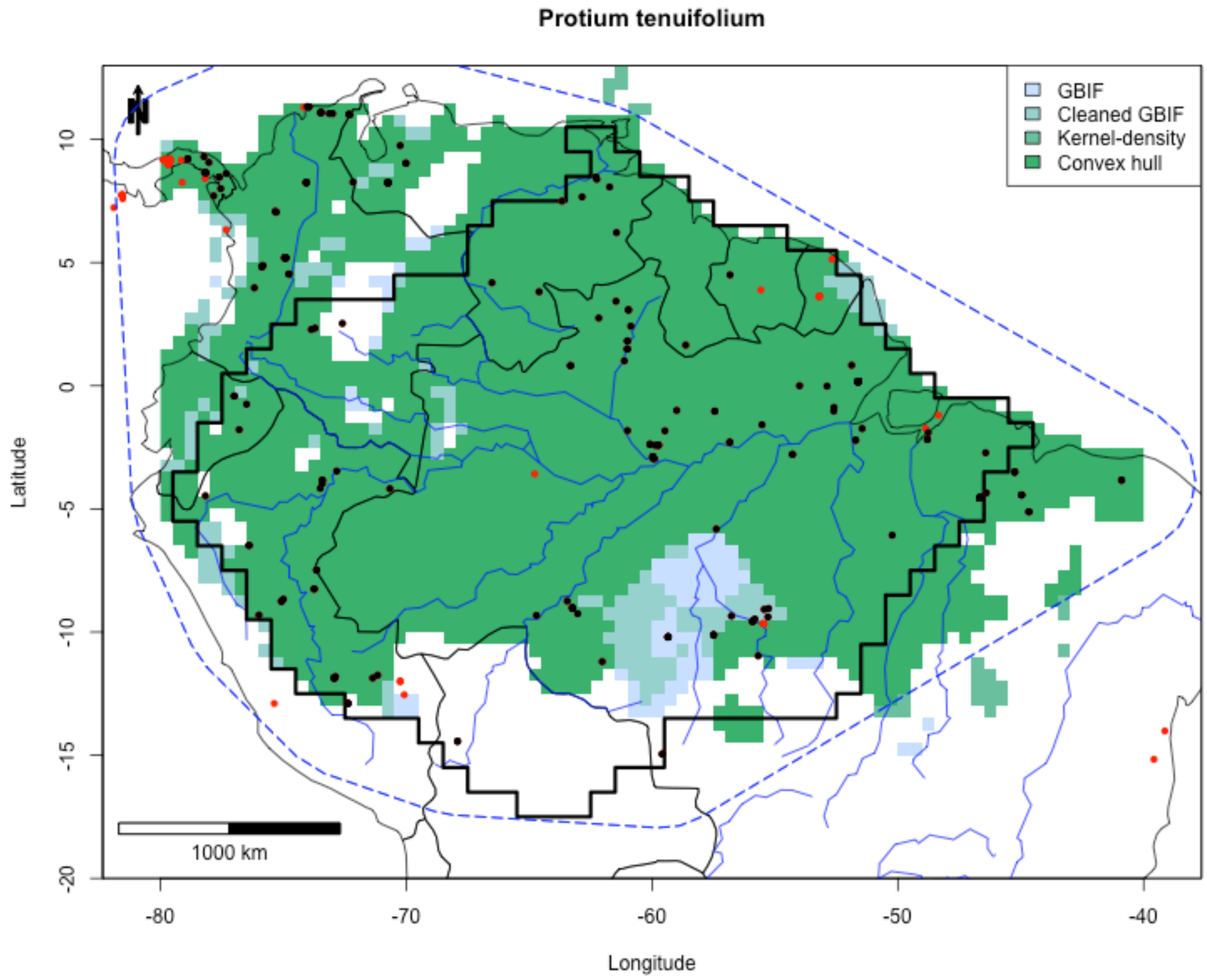
Protium tenuifolium
GBIF
Cleaned GBIF
Kernel-density
Convex hull
1000 km
Latitude
Longitude
-80
-70
-60
-50
-40
10
5
0
-5
-10
-15
-20
N

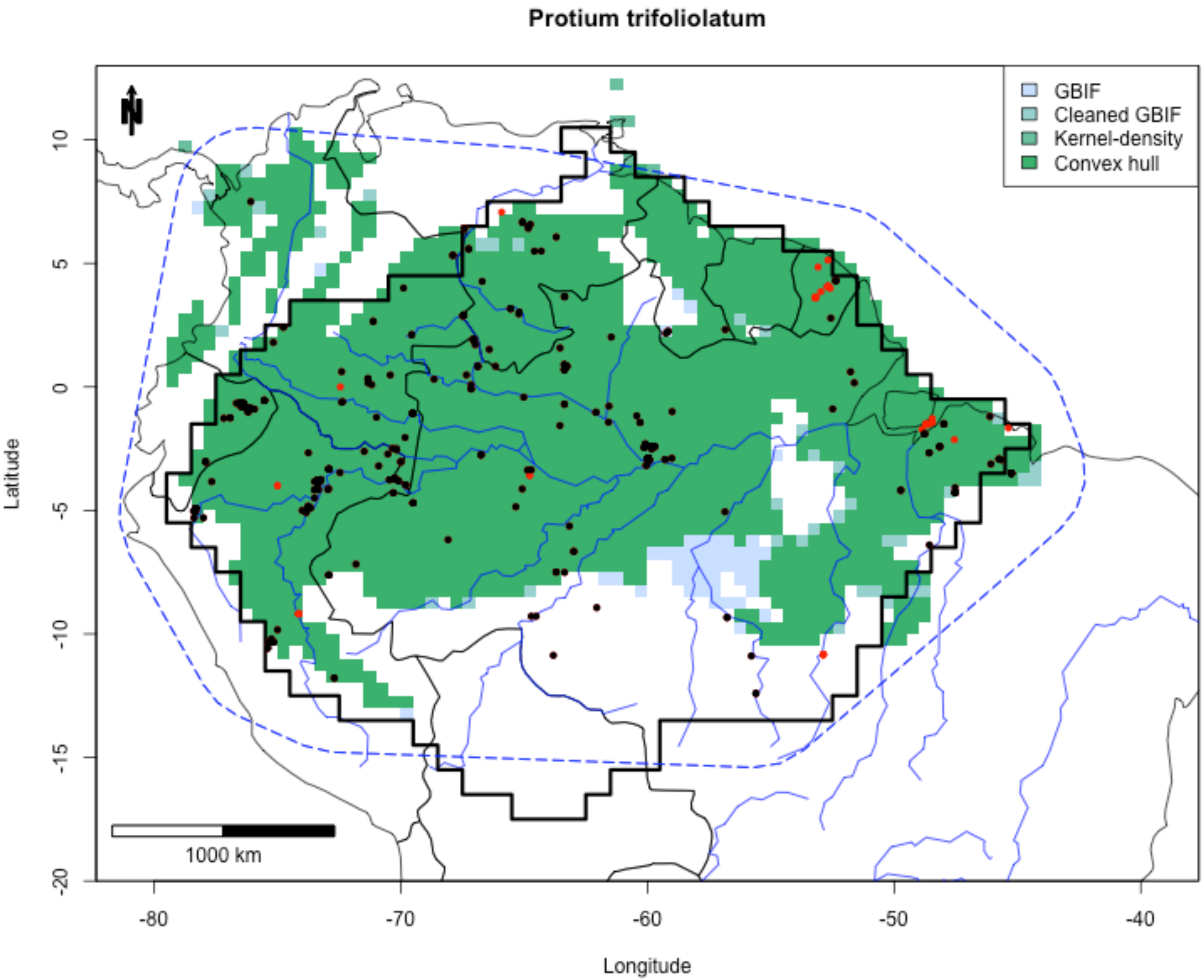

Protium trifoliolatum
GBIF
Cleaned GBIF
Kernel-density
Convex hull
N
1000 km
Latitude
Longitude
10
5
0
-5
-10
-15
-20
-80
-70
-60
-50
-40

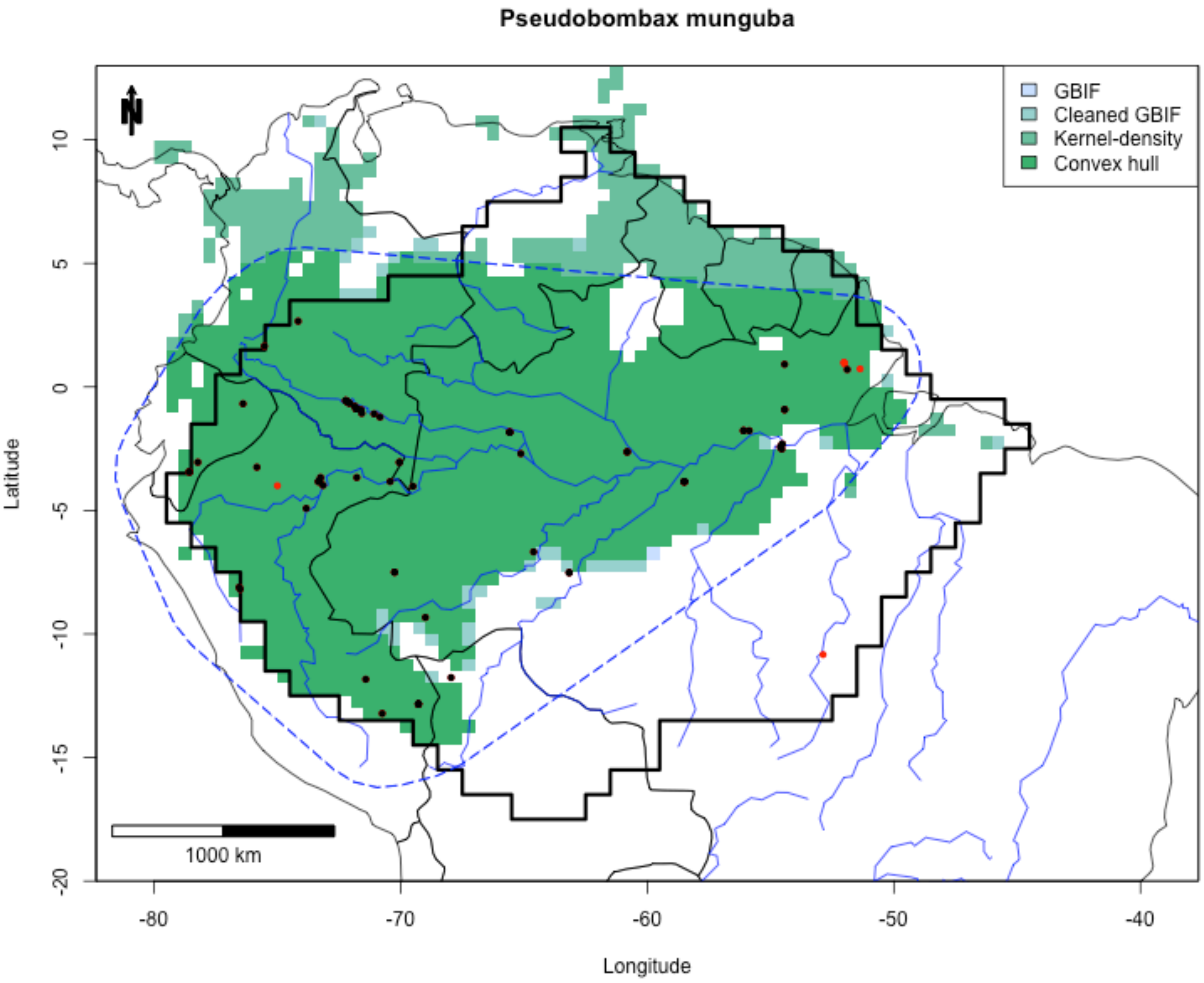

Pseudobombax munguba
GBIF
Cleaned GBIF
Kernel-density
Convex hull
N
1000 km
Latitude
Longitude
10
5
0
-5
-10
-15
-20
-80
-70
-60
-50
-40

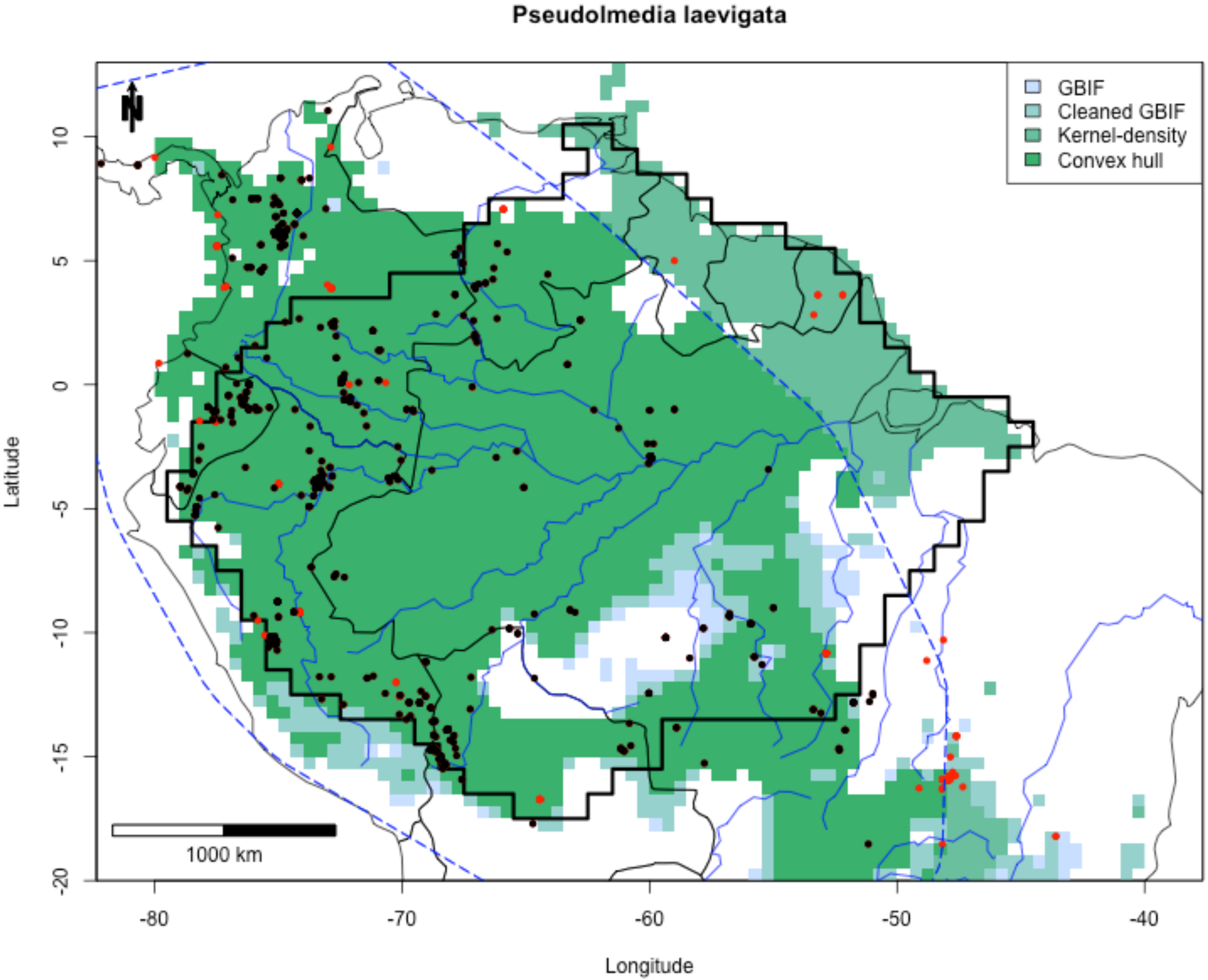

Pseudolmedia laevigata
GBIF
Cleaned GBIF
Kernel-density
Convex hull
N
1000 km
Latitude
Longitude
10
5
0
-5
-10
-15
-20
-80
-70
-60
-50
-40

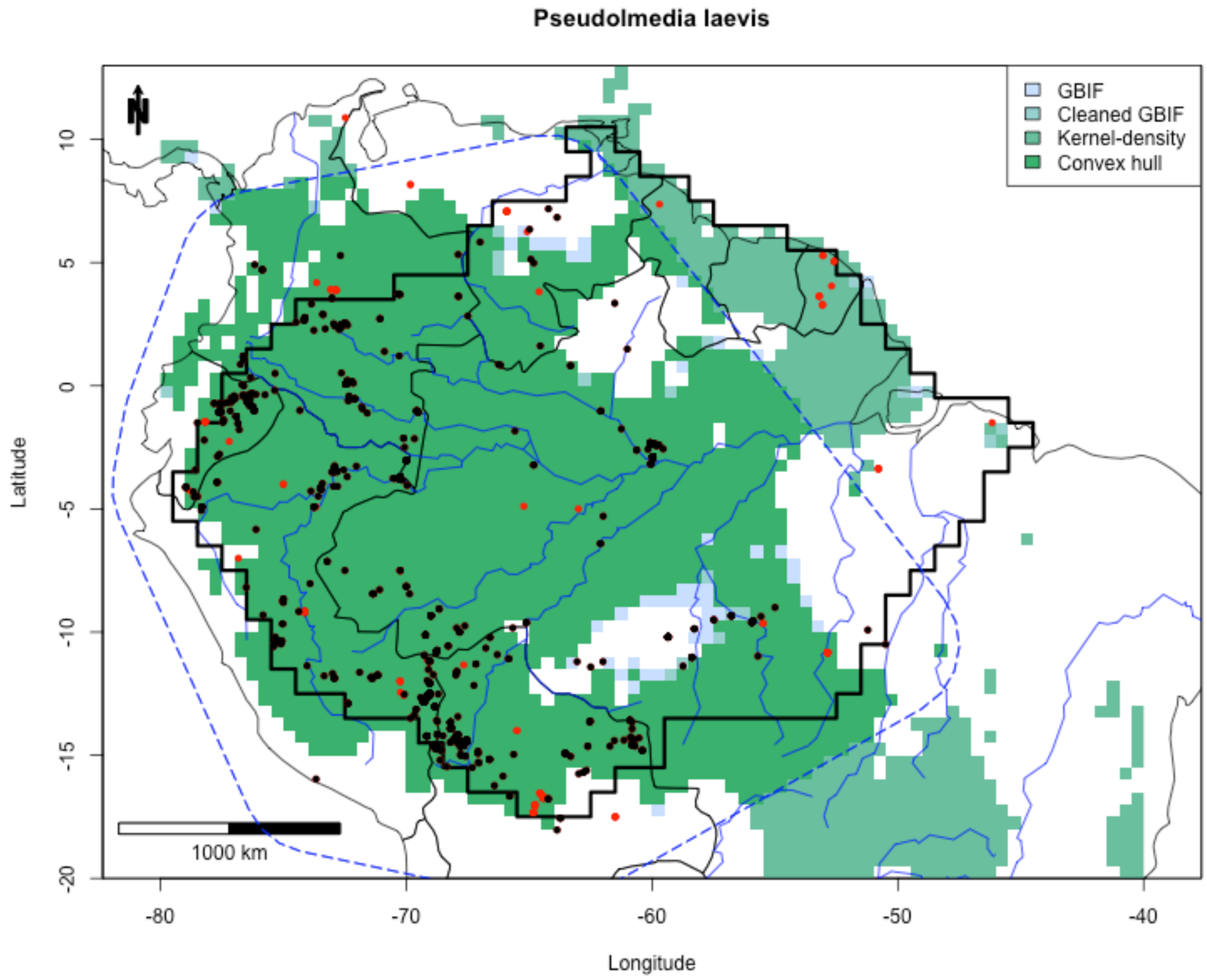

Pseudolmedia laevis
GBIF
Cleaned GBIF
Kernel-density
Convex hull
1000 km
Latitude
Longitude
10
5
0
-5
-10
-15
-20
-80
-70
-60
-50
-40

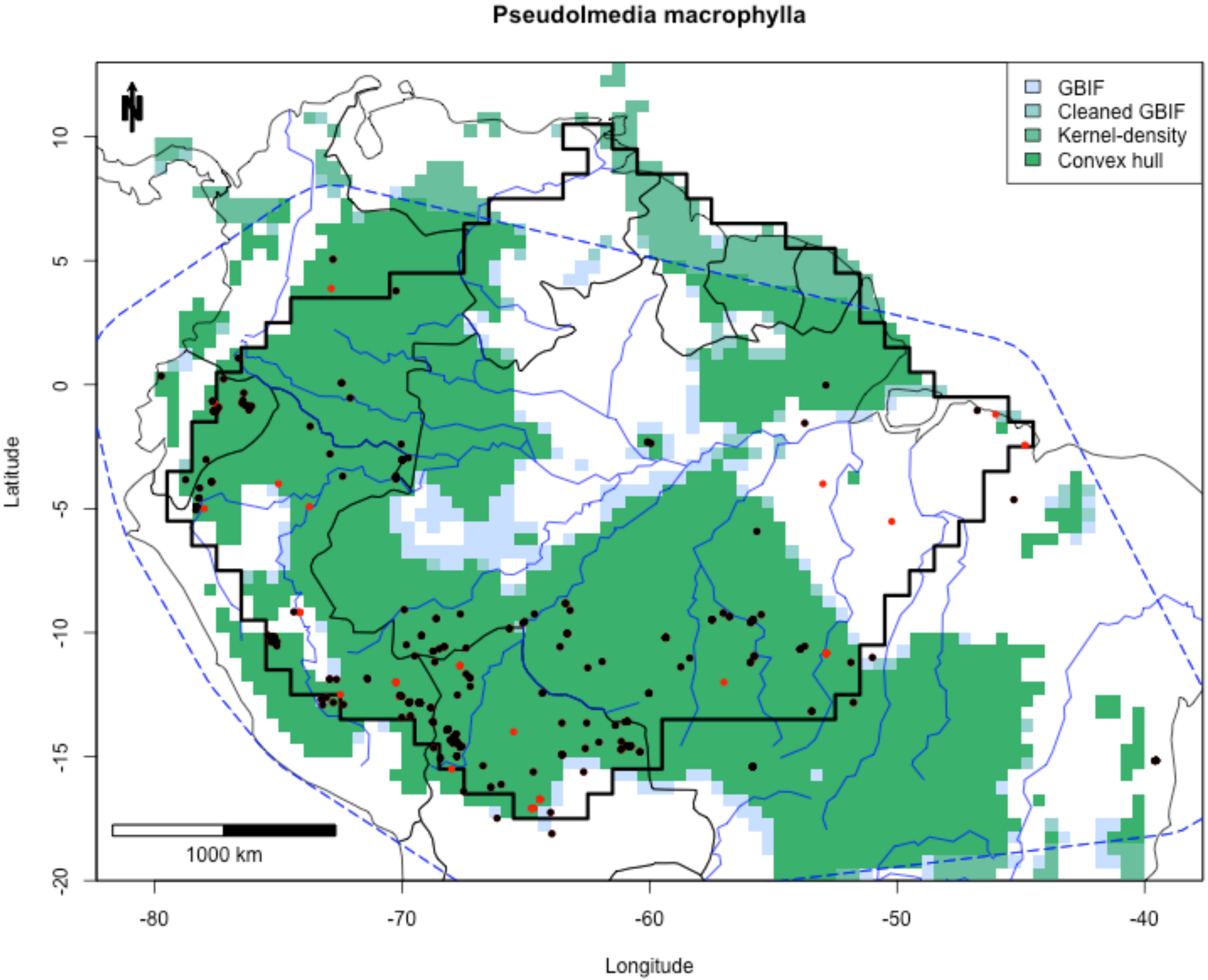
Pseudolmedia macrophylla
GBIF
Cleaned GBIF
Kernel-density
Convex hull
N
1000 km
Latitude
Longitude
10
5
0
-5
-10
-15
-20
-80
-70
-60
-50
-40

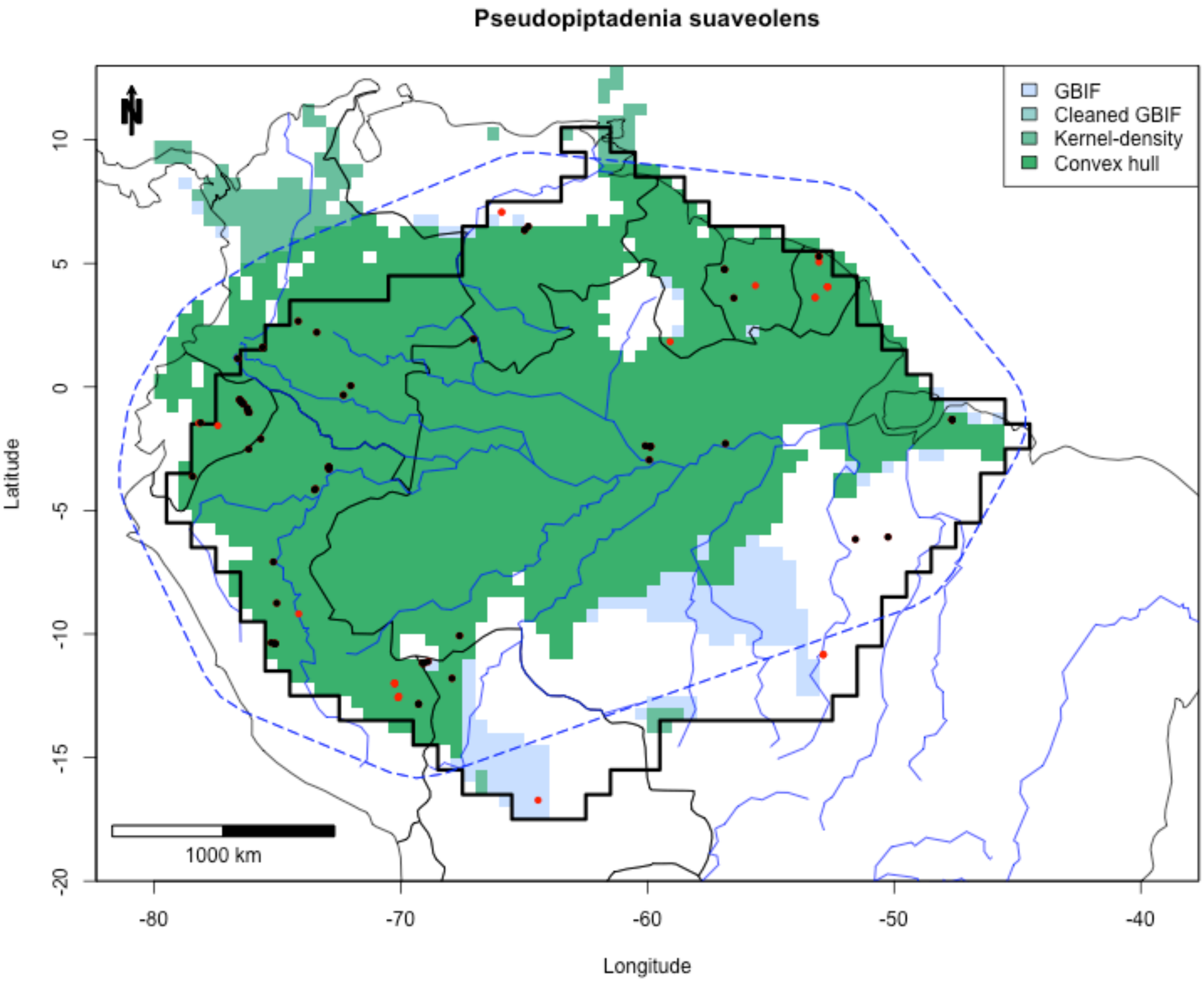
Pseudopiptadenia suaveolens
GBIF
Cleaned GBIF
Kernel-density
Convex hull
N
1000 km
Latitude
Longitude
10
5
0
-5
-10
-15
-20
-80
-70
-60
-50
-40

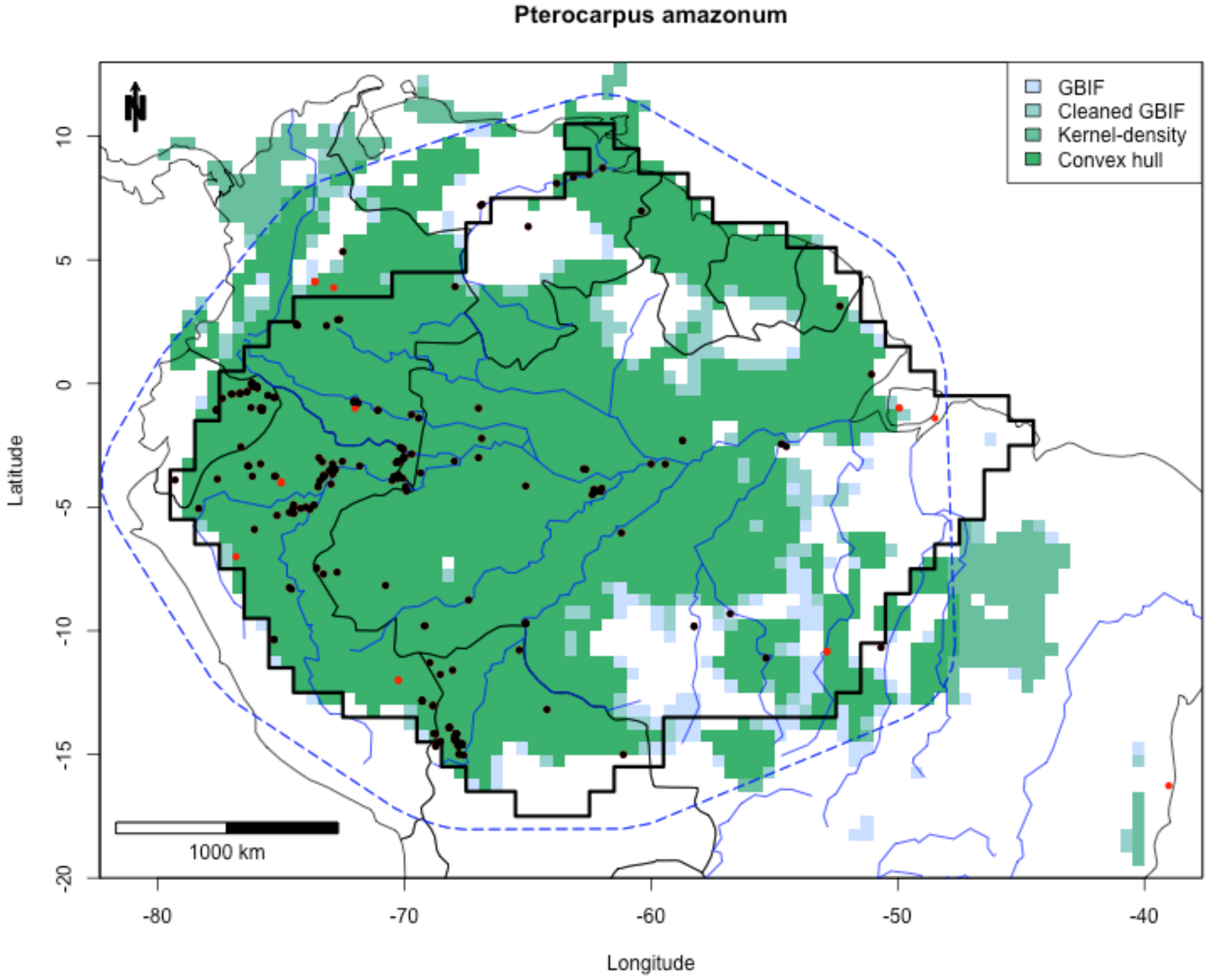

Pterocarpus amazonum
GBIF
Cleaned GBIF
Kernel-density
Convex hull
N
1000 km
Latitude
Longitude
10
5
0
-5
-10
-15
-20
-80
-70
-60
-50
-40

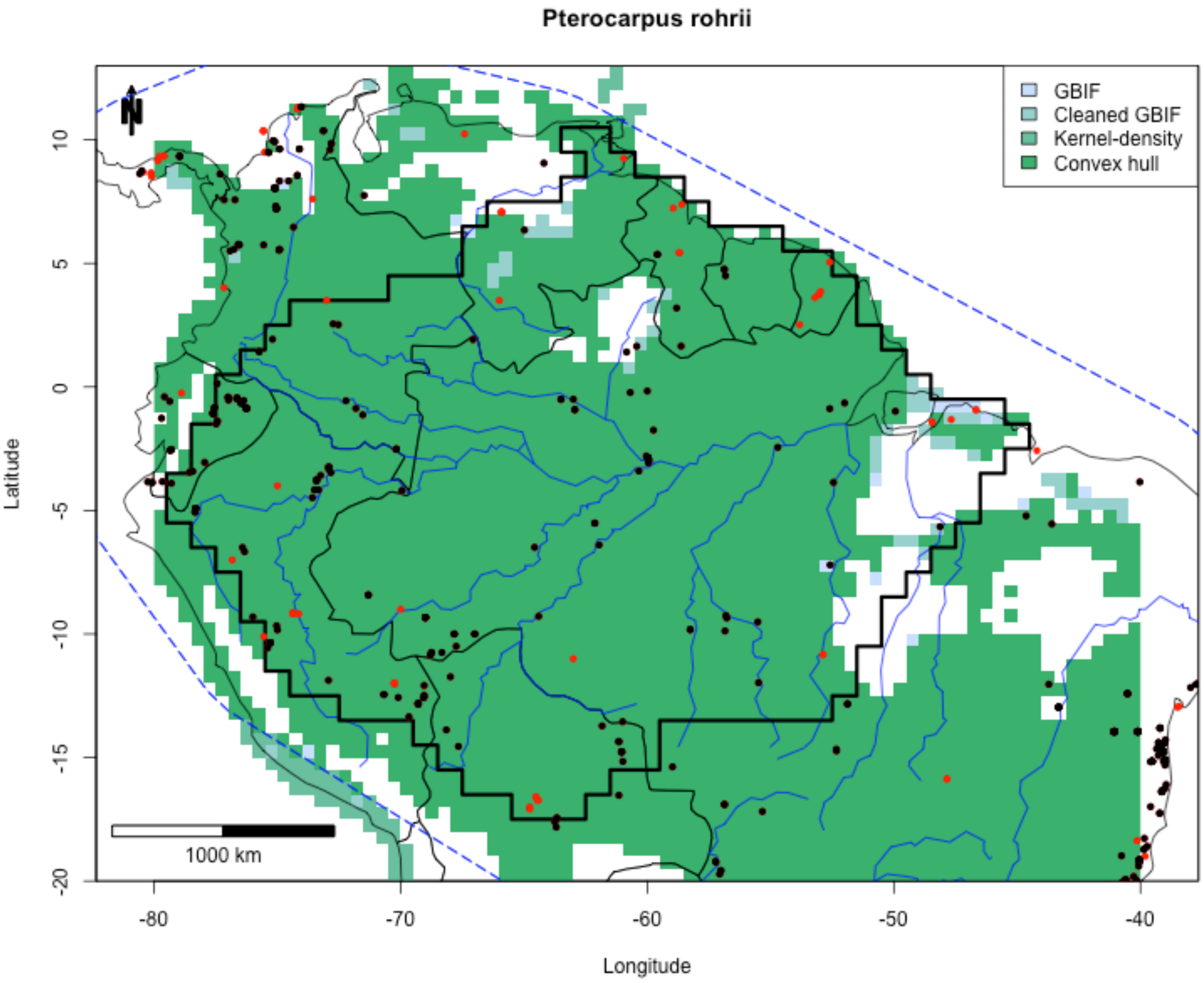

Pterocarpus rohrii
GBIF
Cleaned GBIF
Kernel-density
Convex hull
N
1000 km
Latitude
Longitude
10
5
0
-5
-10
-15
-20
-80
-70
-60
-50
-40

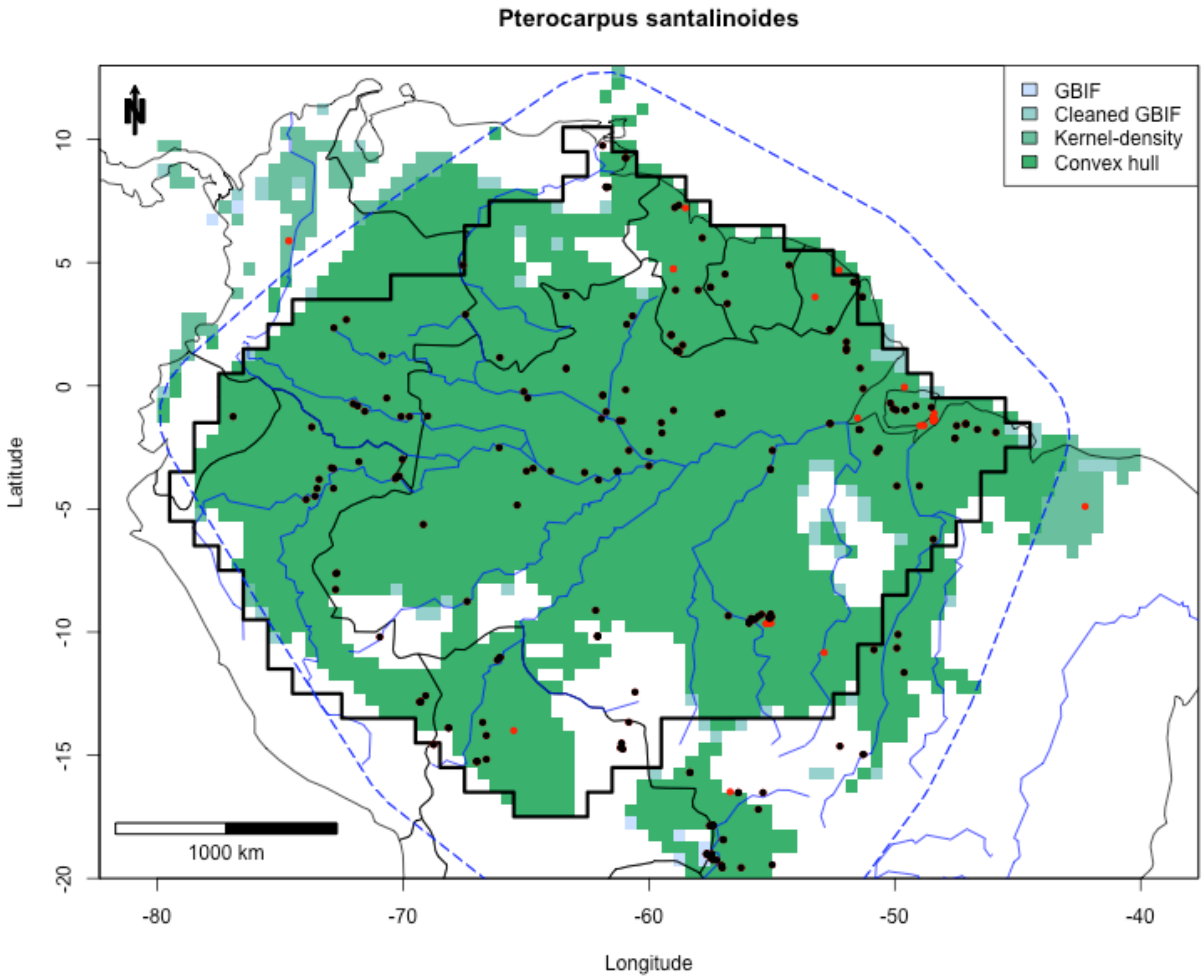

Pterocarpus santalinoides
GBIF
Cleaned GBIF
Kernel-density
Convex hull
N
1000 km
Latitude
Longitude
10
5
0
-5
-10
-15
-20
-80
-70
-60
-50
-40

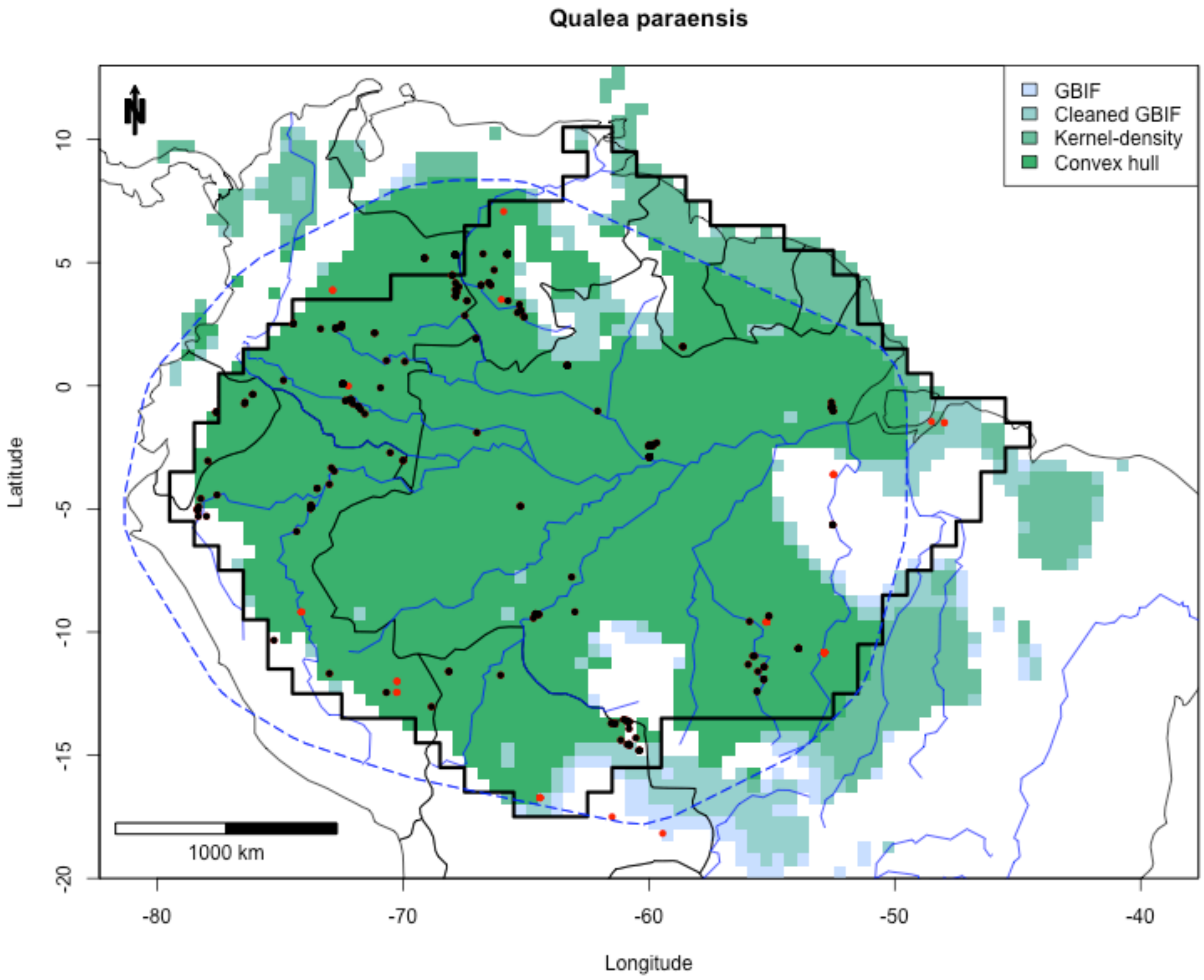
Qualea paraensis
GBIF
Cleaned GBIF
Kernel-density
Convex hull
N
1000 km
Latitude
Longitude
10
5
0
-5
-10
-15
-20
-80
-70
-60
-50
-40

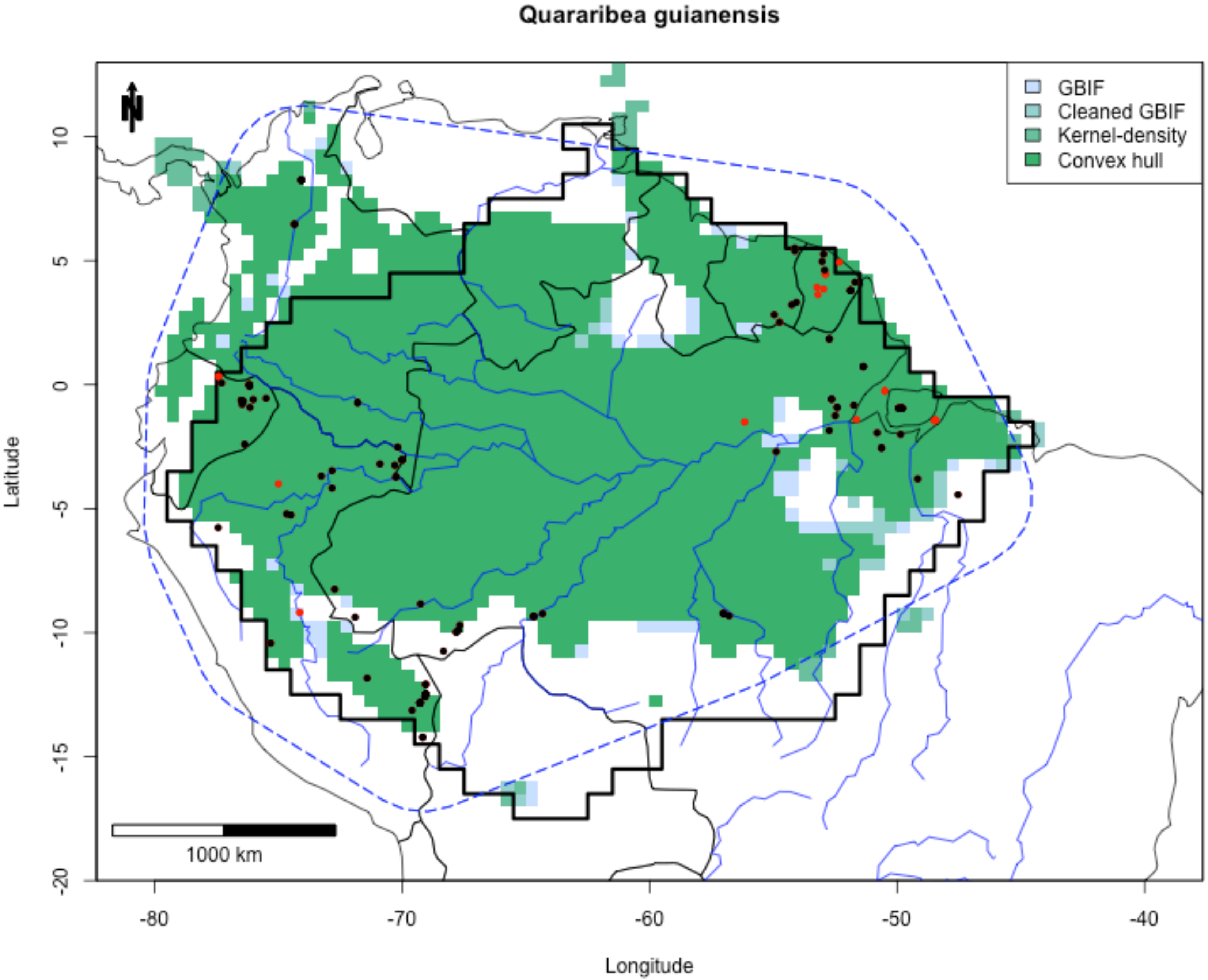

Quararibea guianensis
GBIF
Cleaned GBIF
Kernel-density
Convex hull
N
10
5
0
-5
-10
-15
-20
Latitude
-80
-70
-60
-50
-40
Longitude
1000 km

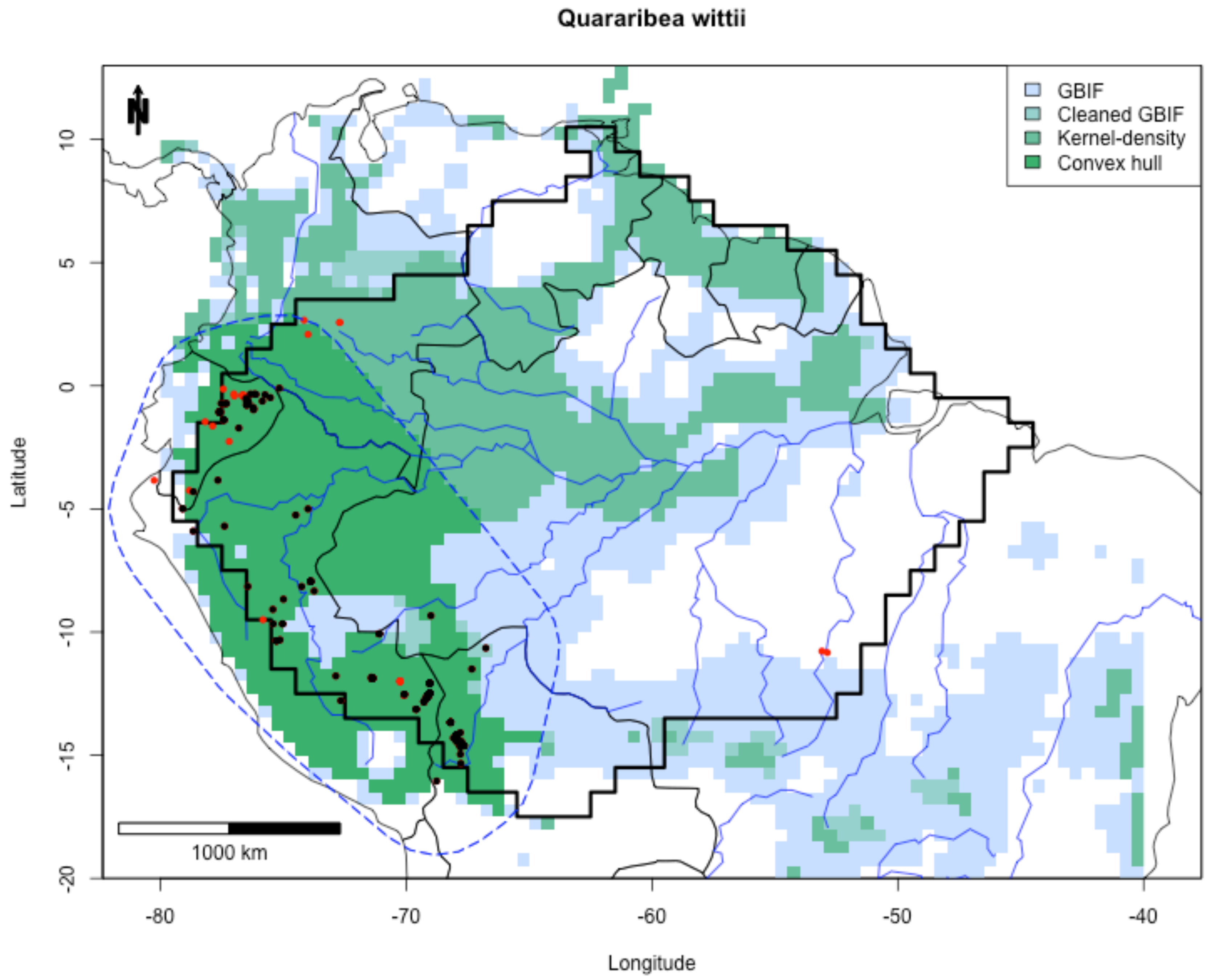
Quararibea wittii
GBIF
Cleaned GBIF
Kernel-density
Convex hull
N
1000 km
Latitude
Longitude
10
5
0
-5
-10
-15
-20
-80
-70
-60
-50
-40

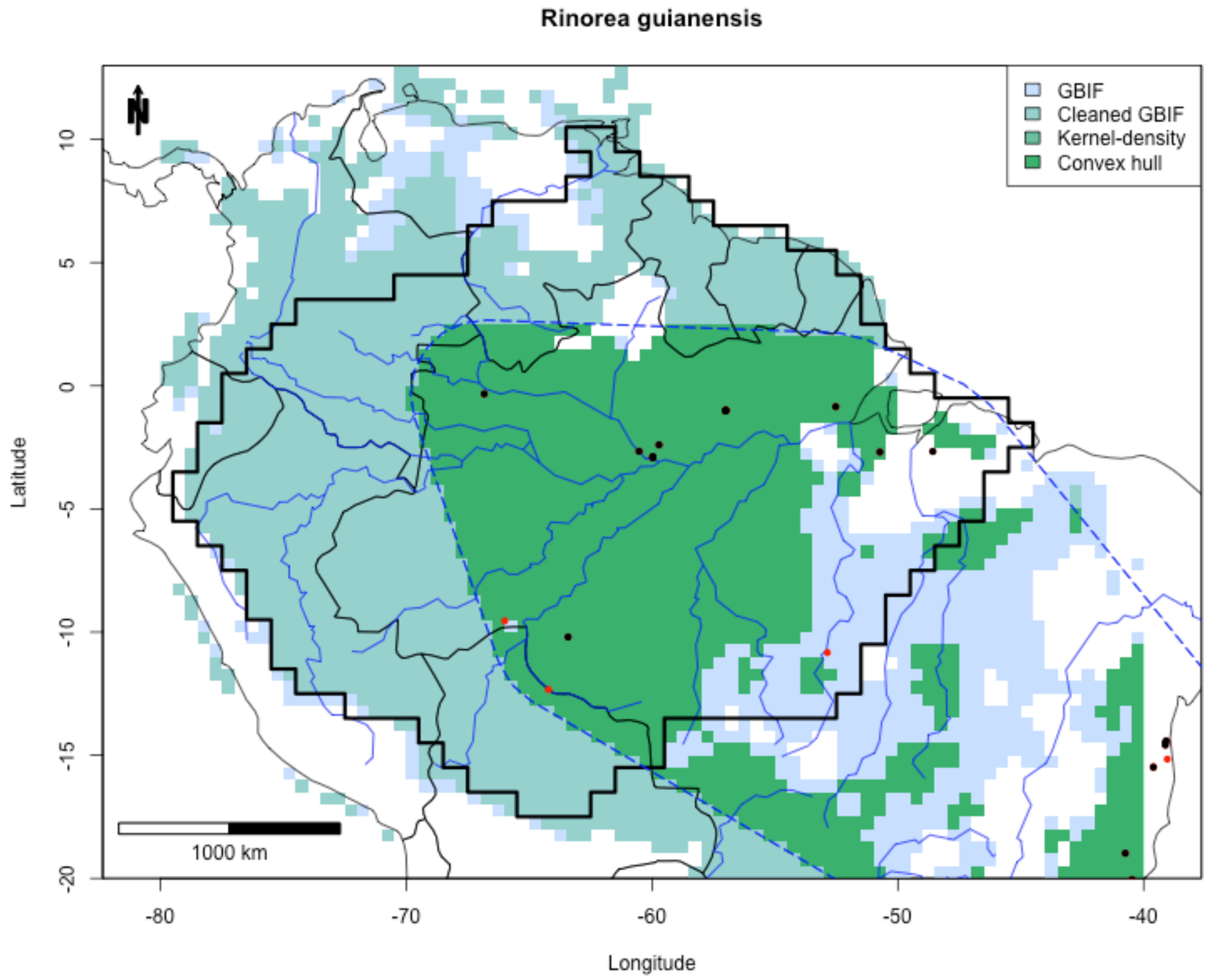

Rinorea guianensis
GBIF
Cleaned GBIF
Kernel-density
Convex hull
N
1000 km
Latitude
Longitude
10
5
0
-5
-10
-15
-20
-80
-70
-60
-50
-40

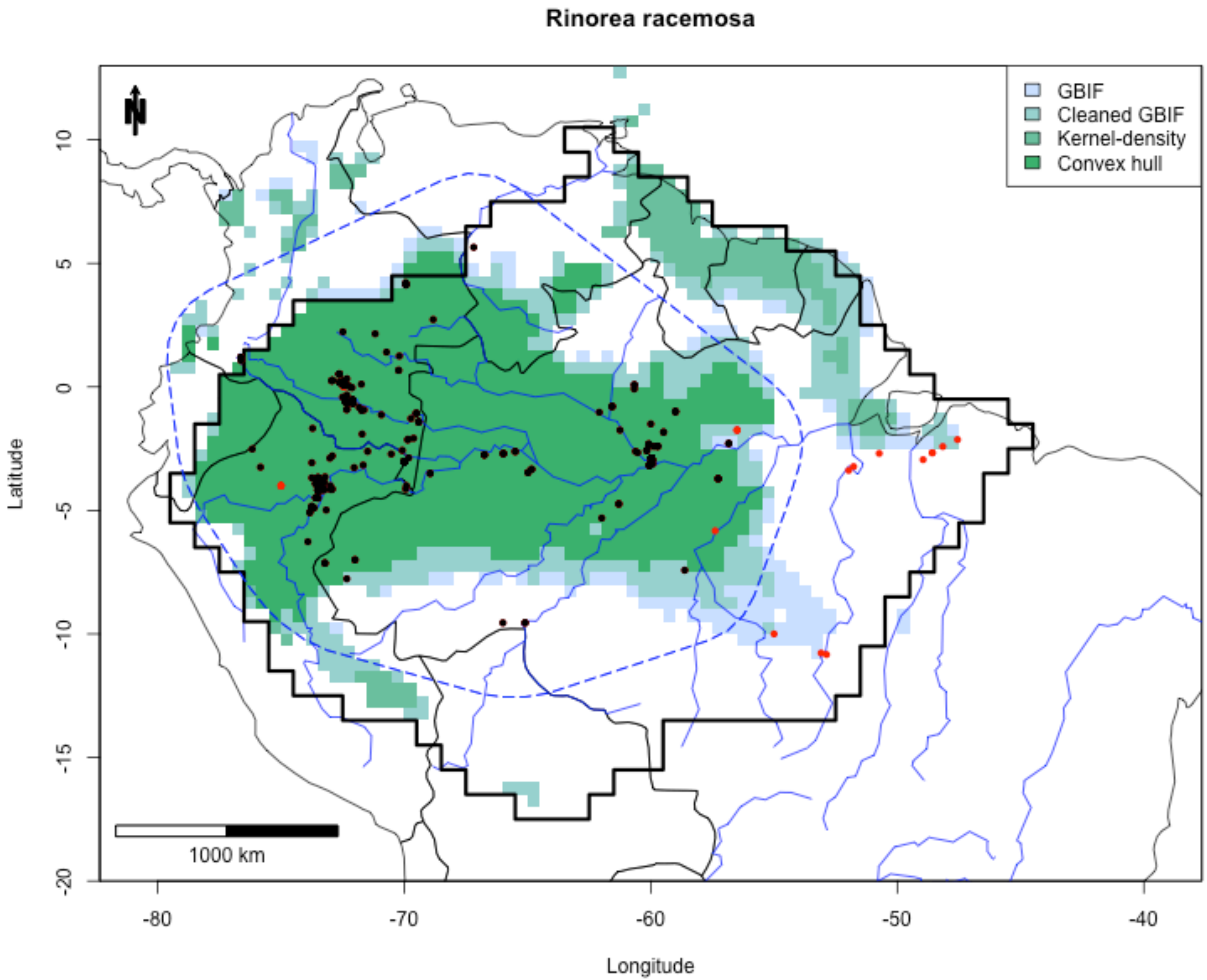

Rinorea racemosa
GBIF
Cleaned GBIF
Kernel-density
Convex hull
N
1000 km
Latitude
Longitude
10
5
0
-5
-10
-15
-20
-80
-70
-60
-50
-40

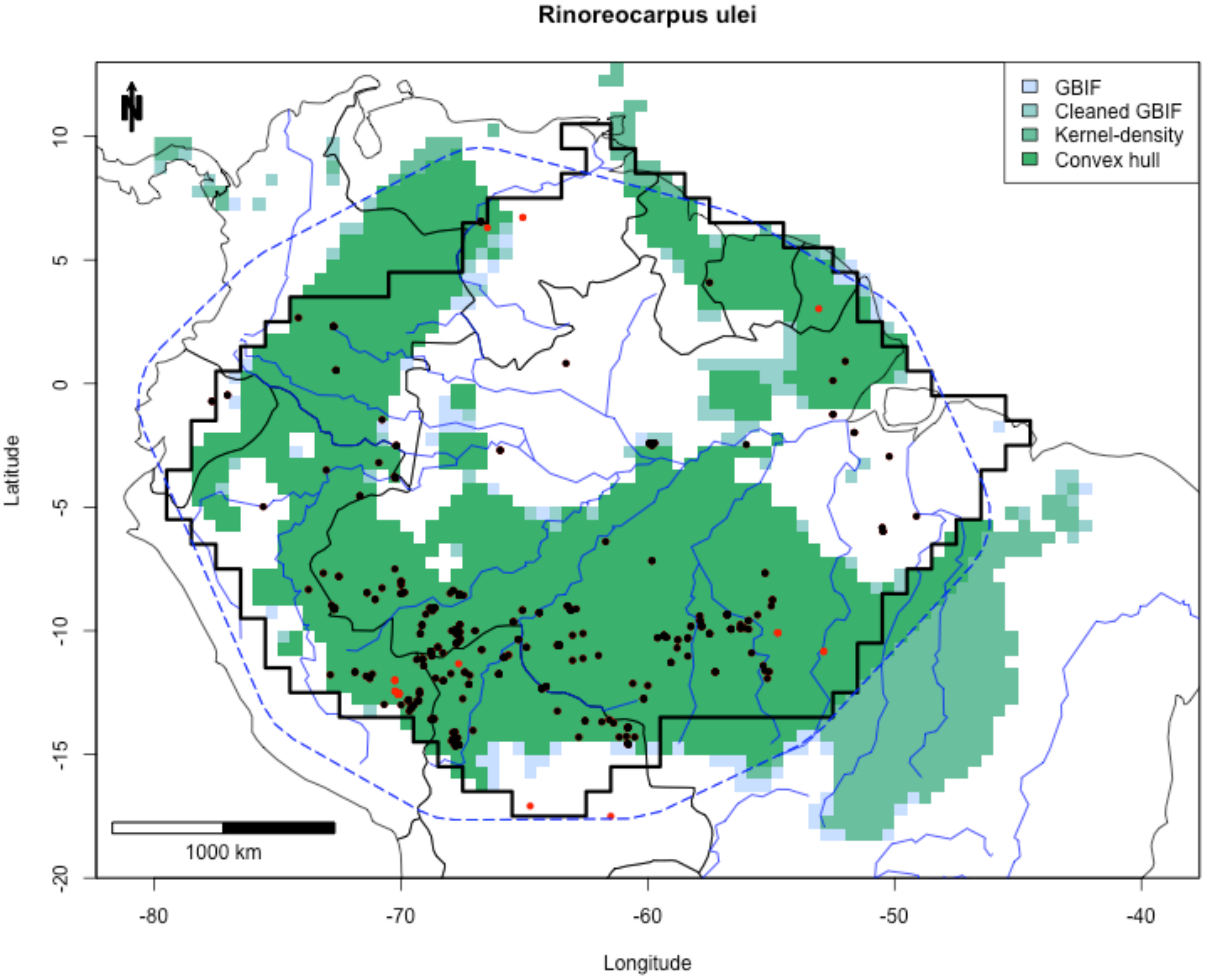

Rinoreocarpus ulei
GBIF
Cleaned GBIF
Kernel-density
Convex hull
1000 km
Latitude
Longitude
10
5
0
-5
-10
-15
-20
-80
-70
-60
-50
-40

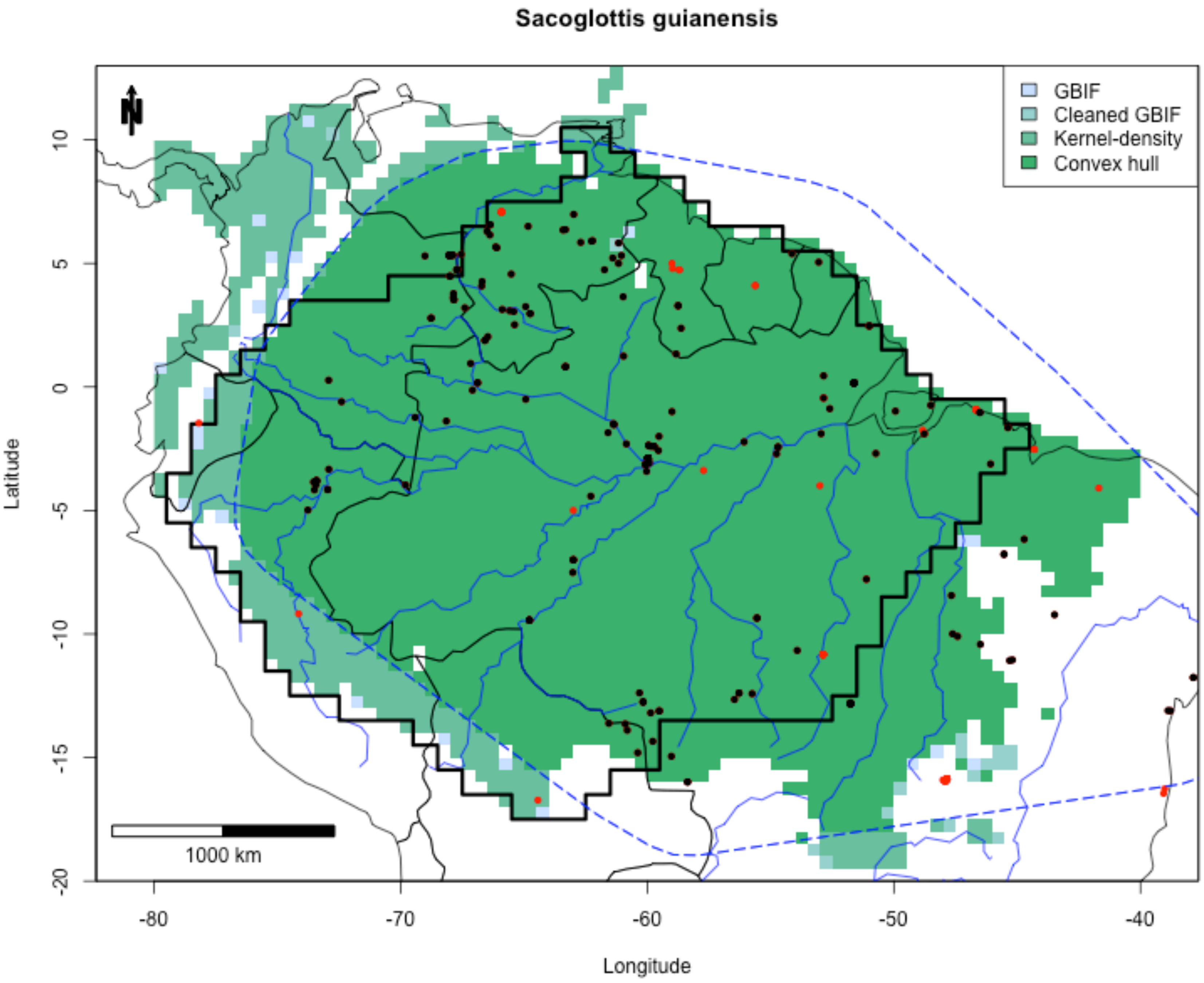
Sacoglottis guianensis
GBIF
Cleaned GBIF
Kernel-density
Convex hull
1000 km
Latitude
Longitude
10
5
0
-5
-10
-15
-20
-80
-70
-60
-50
-40

Sacoglottis mattogrossensis

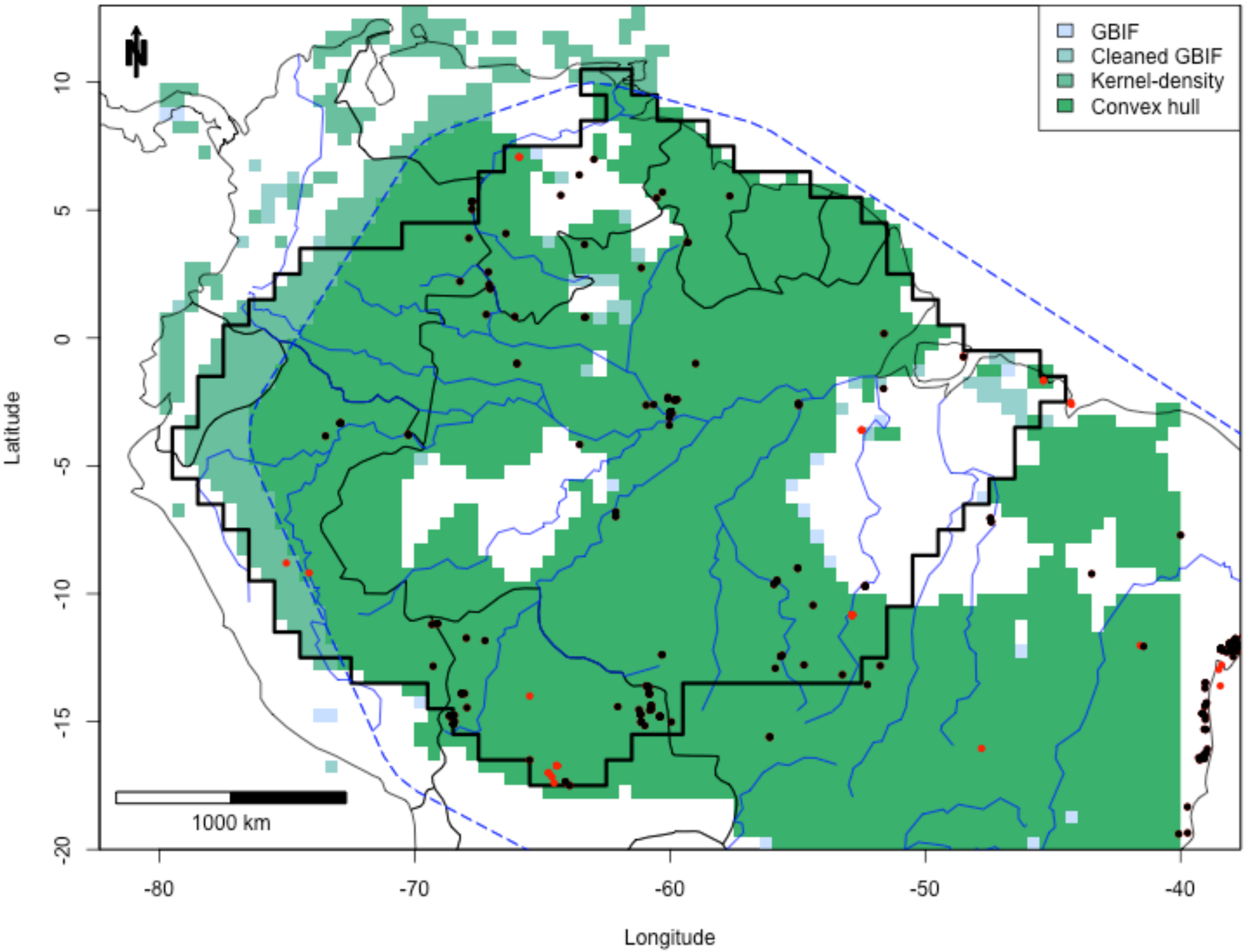

Sagotia brachysepala

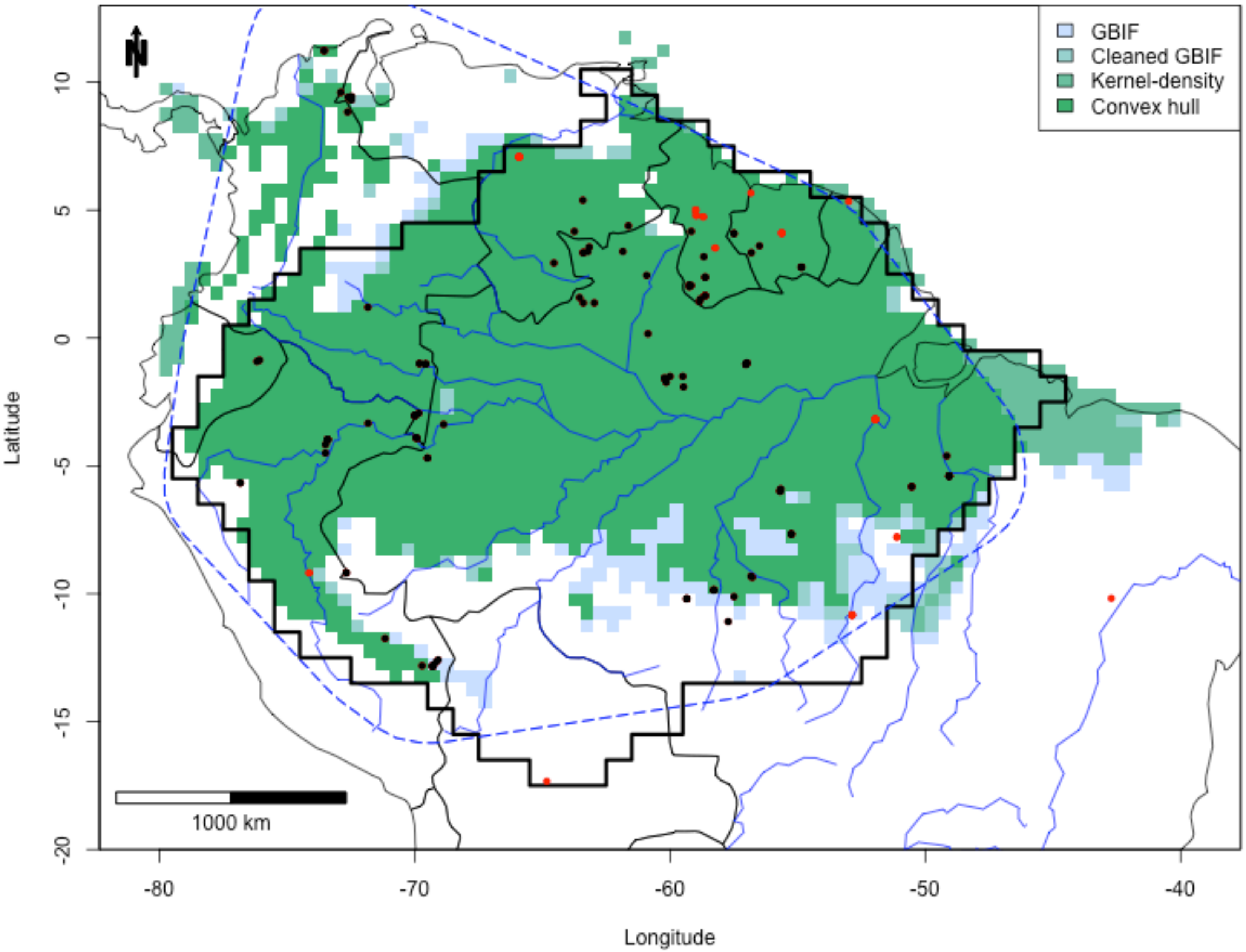

Sagotia racemosa

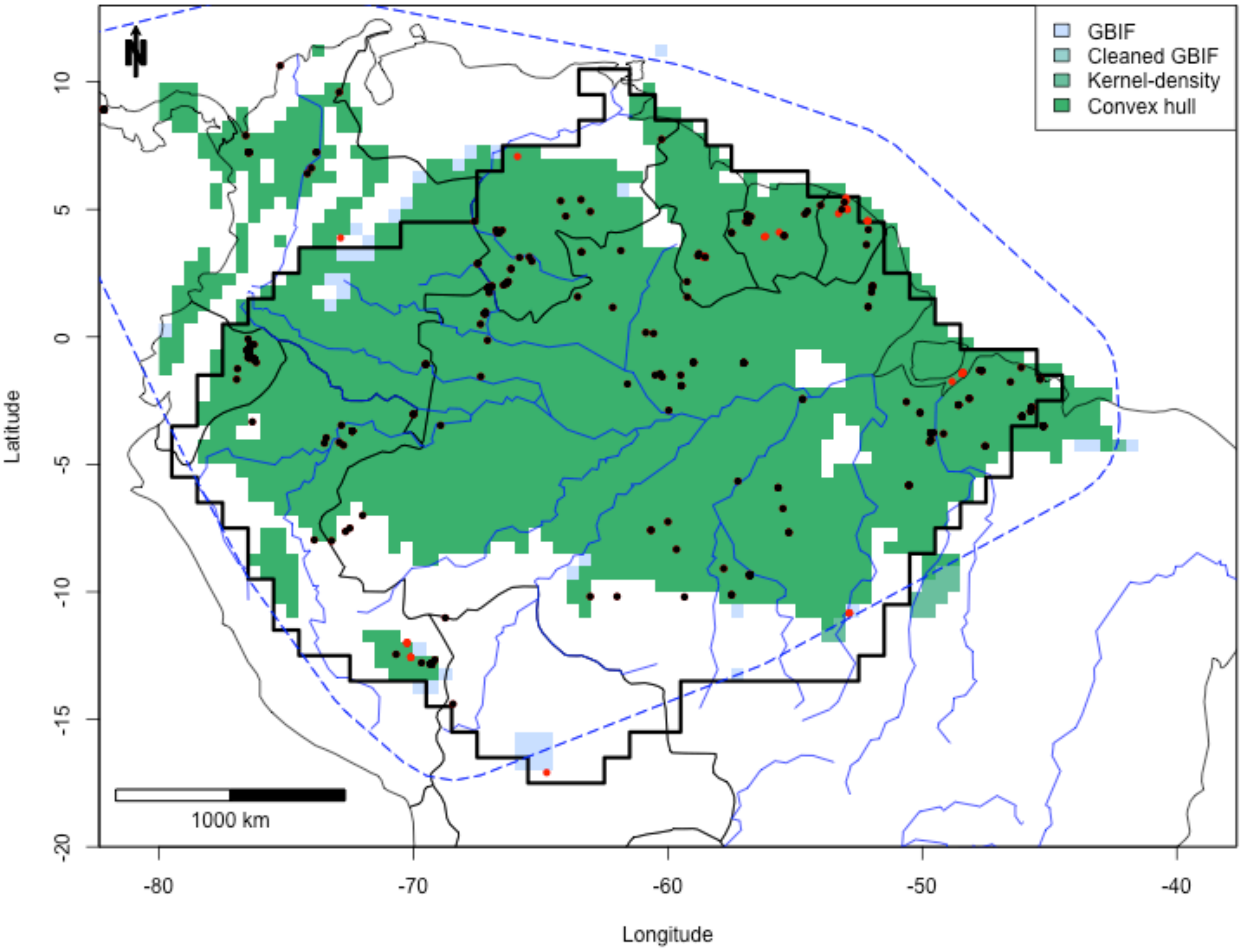

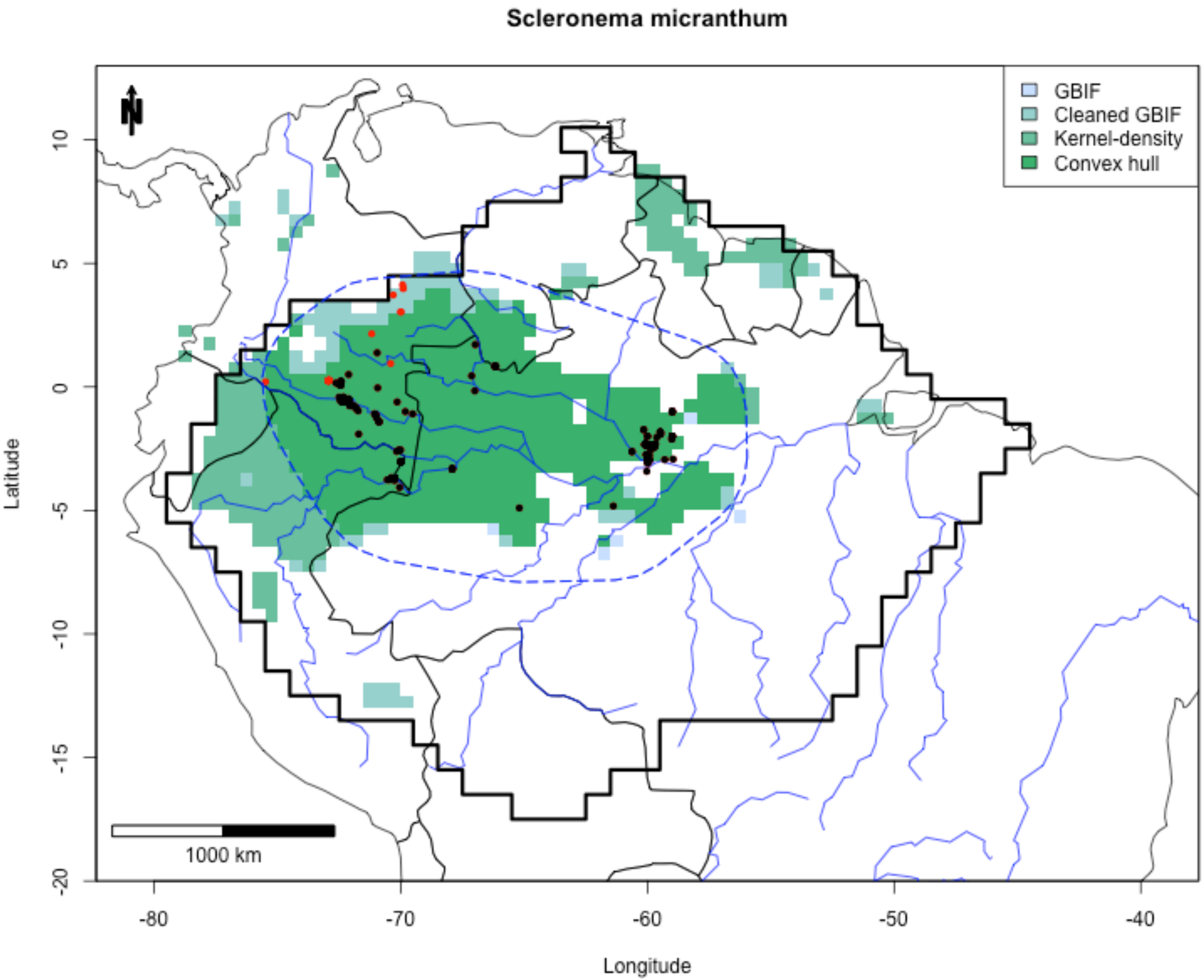

Scleronema micranthum
GBIF
Cleaned GBIF
Kernel-density
Convex hull
N
1000 km
Latitude
Longitude
10
5
0
-5
-10
-15
-20
-80
-70
-60
-50
-40

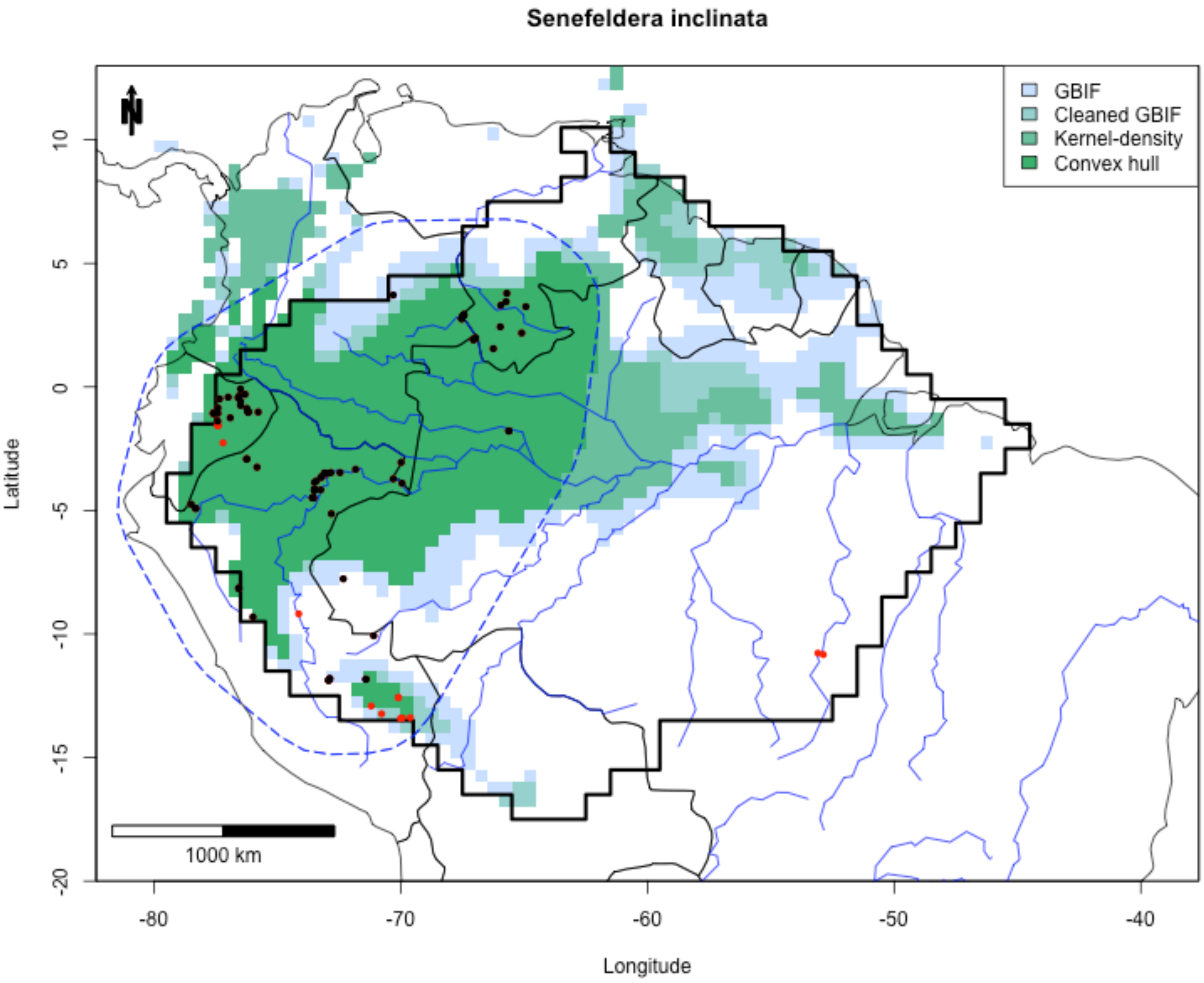

Senefeldera inclinata
GBIF
Cleaned GBIF
Kernel-density
Convex hull
N
1000 km
Latitude
Longitude
10
5
0
-5
-10
-15
-20
-80
-70
-60
-50
-40

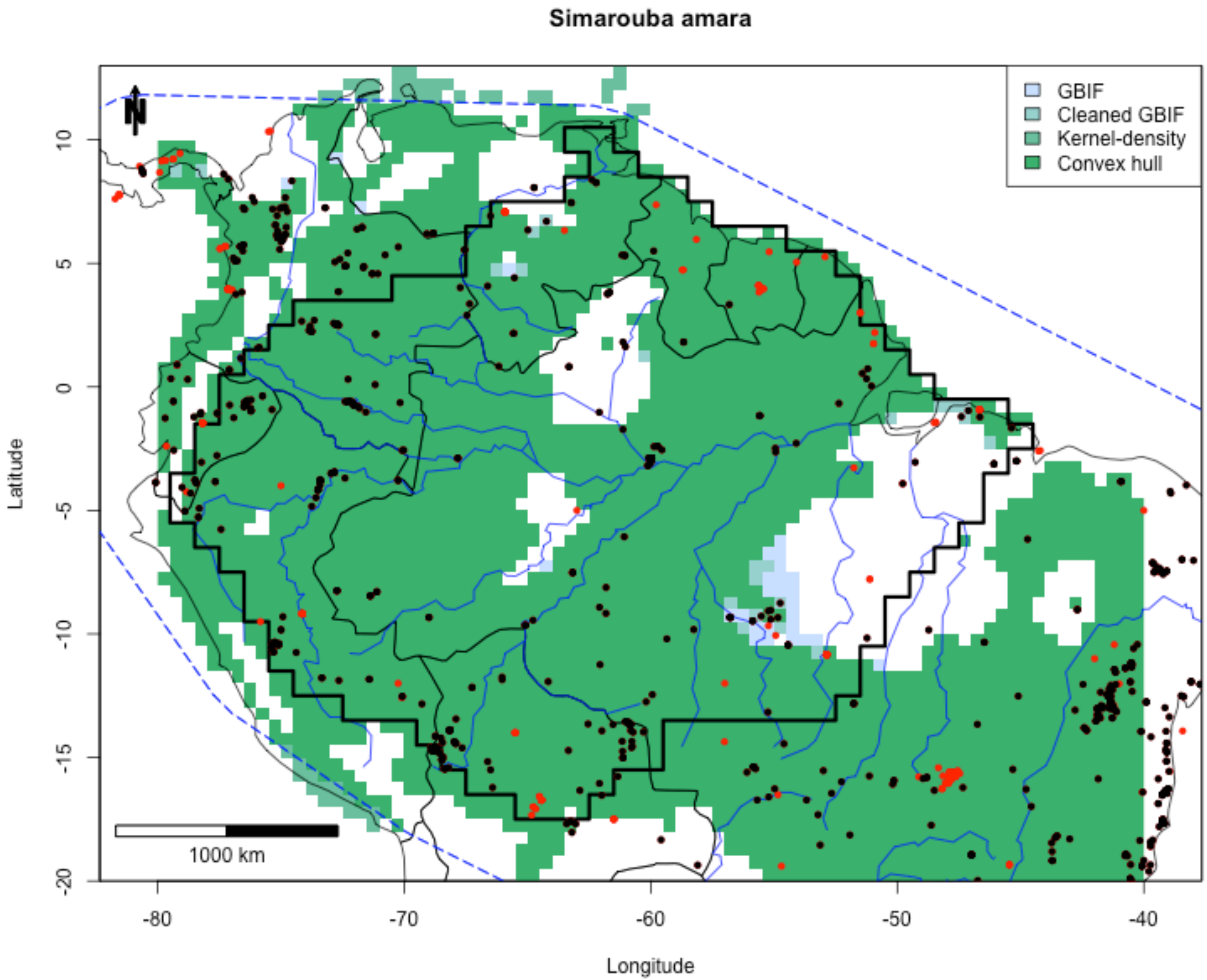

Simarouba amara
GBIF
Cleaned GBIF
Kernel-density
Convex hull
N
1000 km
Latitude
Longitude
10
5
0
-5
-10
-15
-20
-80
-70
-60
-50
-40

Siparuna decipiens

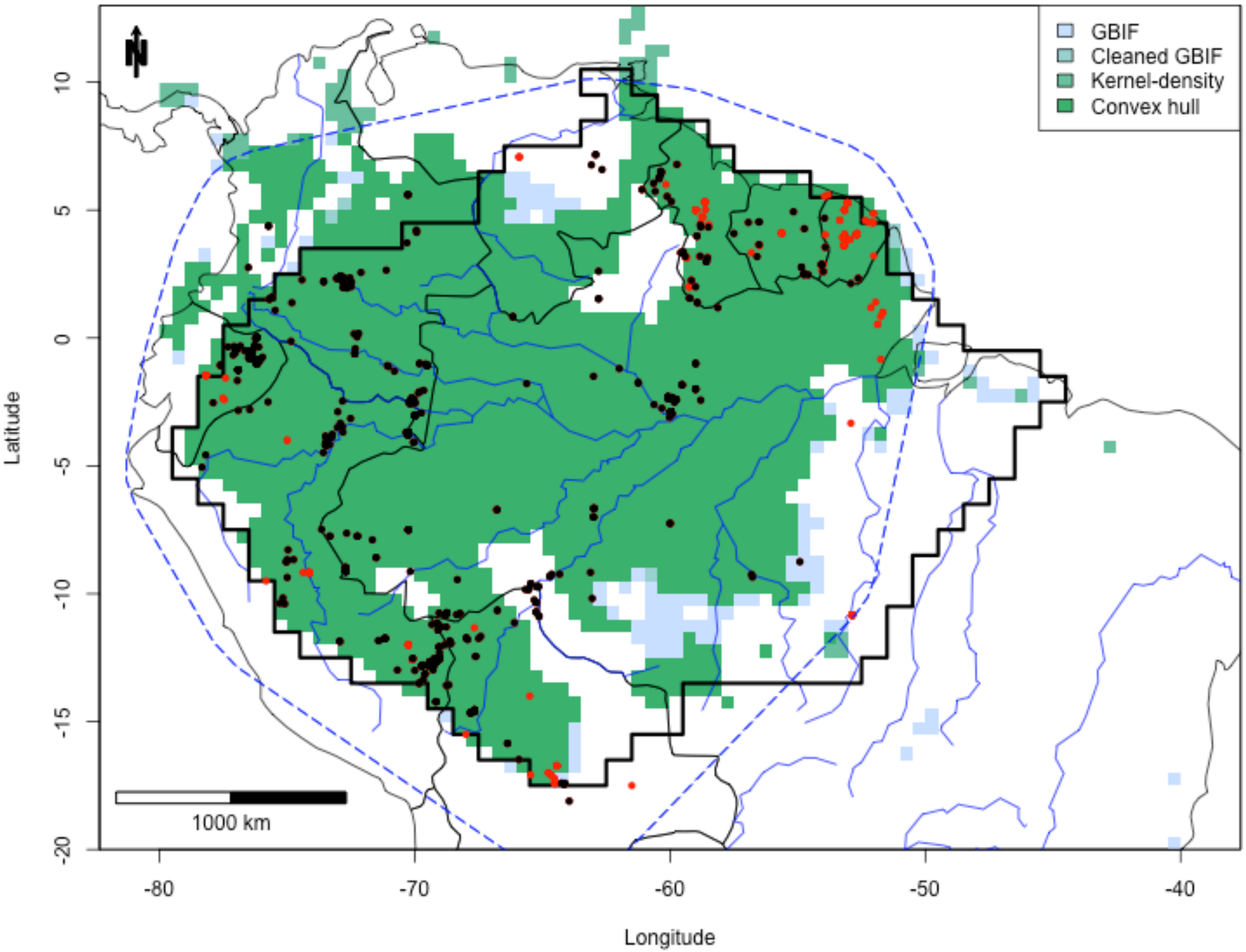

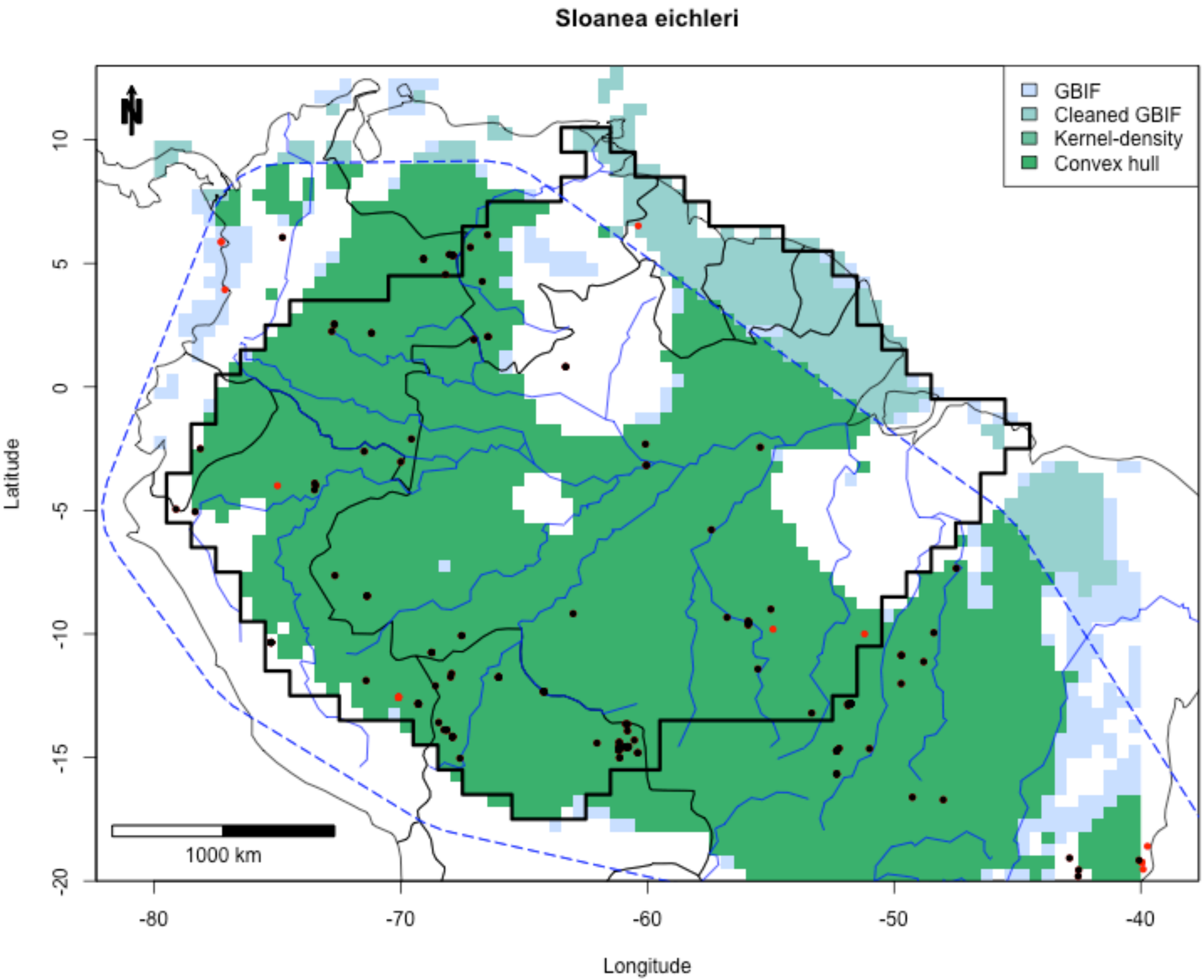
Sloanea eichleri
GBIF
Cleaned GBIF
Kernel-density
Convex hull
N
1000 km
Latitude
Longitude
10
5
0
-5
-10
-15
-20
-80
-70
-60
-50
-40

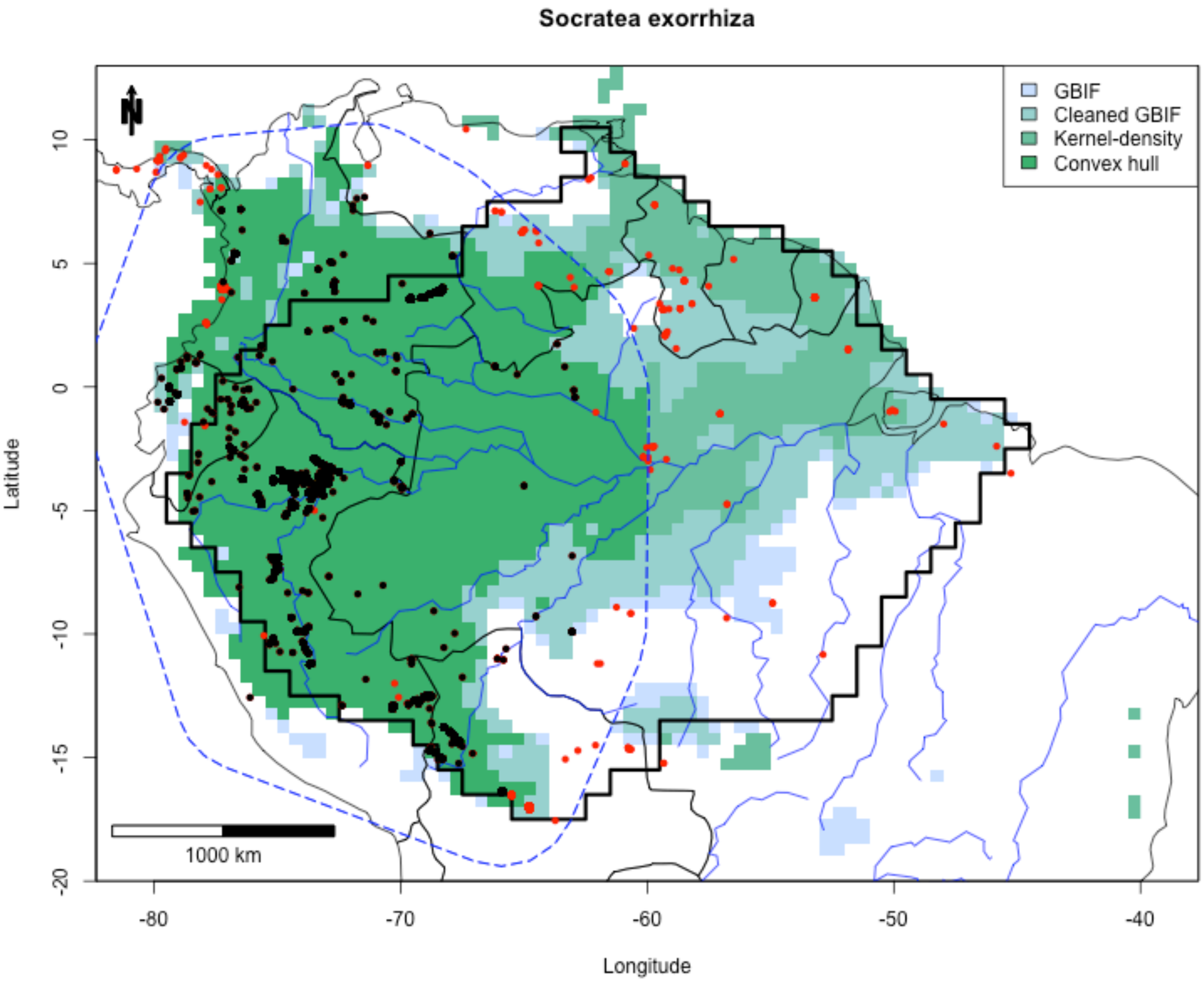

Socratea exorrhiza
GBIF
Cleaned GBIF
Kernel-density
Convex hull
N
1000 km
Latitude
Longitude
10
5
0
-5
-10
-15
-20
-80
-70
-60
-50
-40

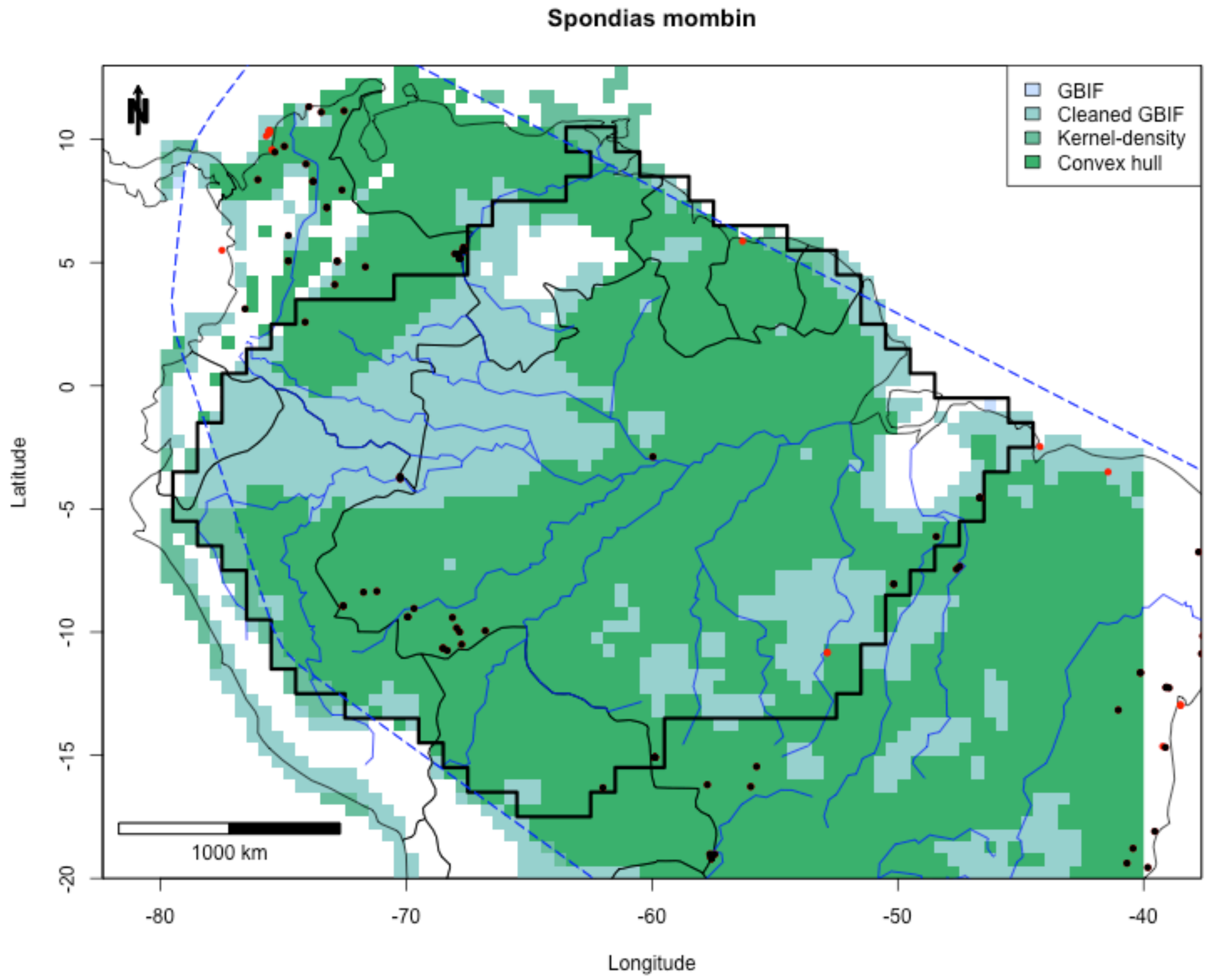
Spondias mombin
GBIF
Cleaned GBIF
Kernel-density
Convex hull
Latitude
Longitude
10
5
0
-5
-10
-15
-20
-80
-70
-60
-50
-40
1000 km
N

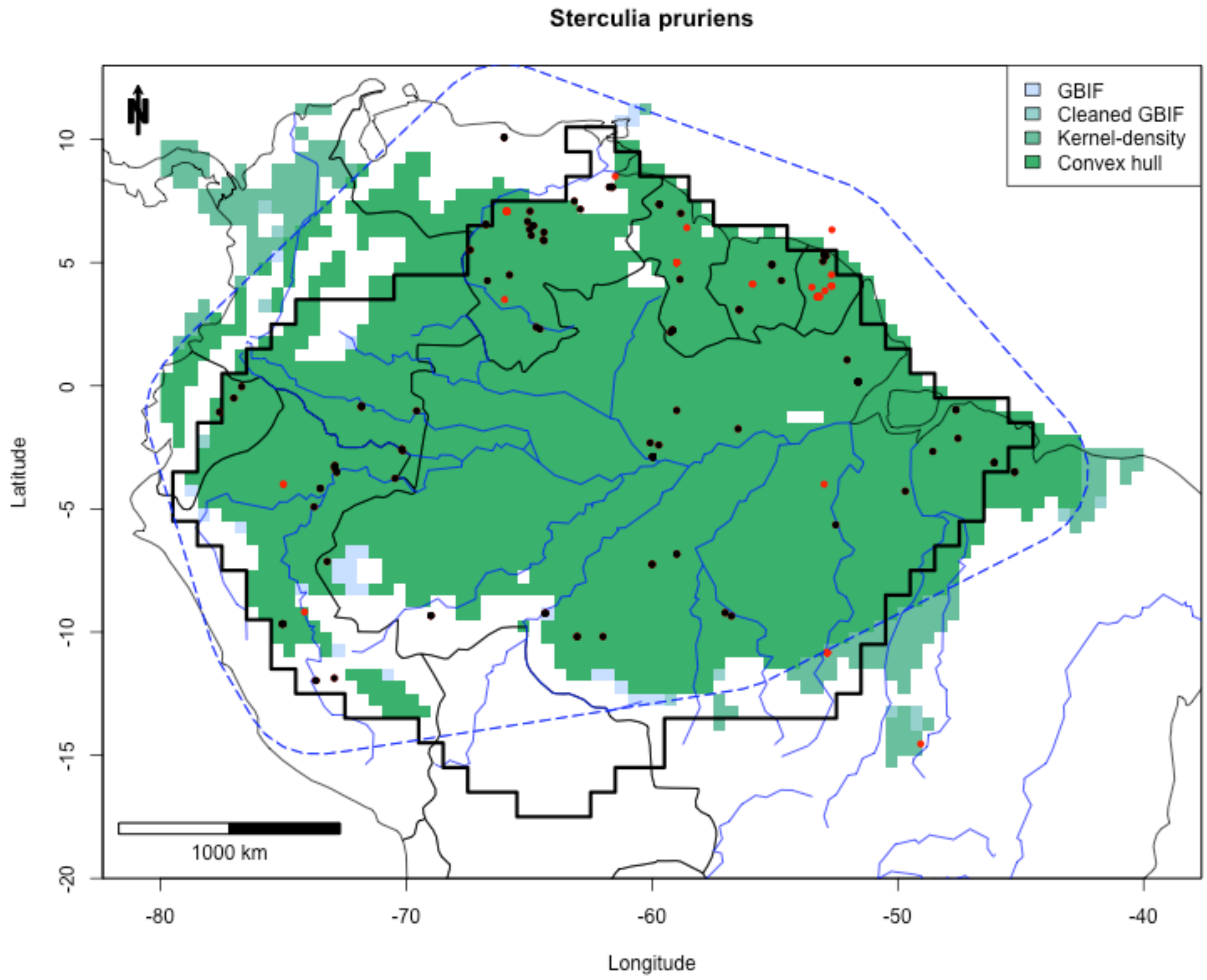

Sterculia pruriens
GBIF
Cleaned GBIF
Kernel-density
Convex hull
N
1000 km
Latitude
Longitude
10
5
0
-5
-10
-15
-20
-80
-70
-60
-50
-40

Swartzia leiocalycina

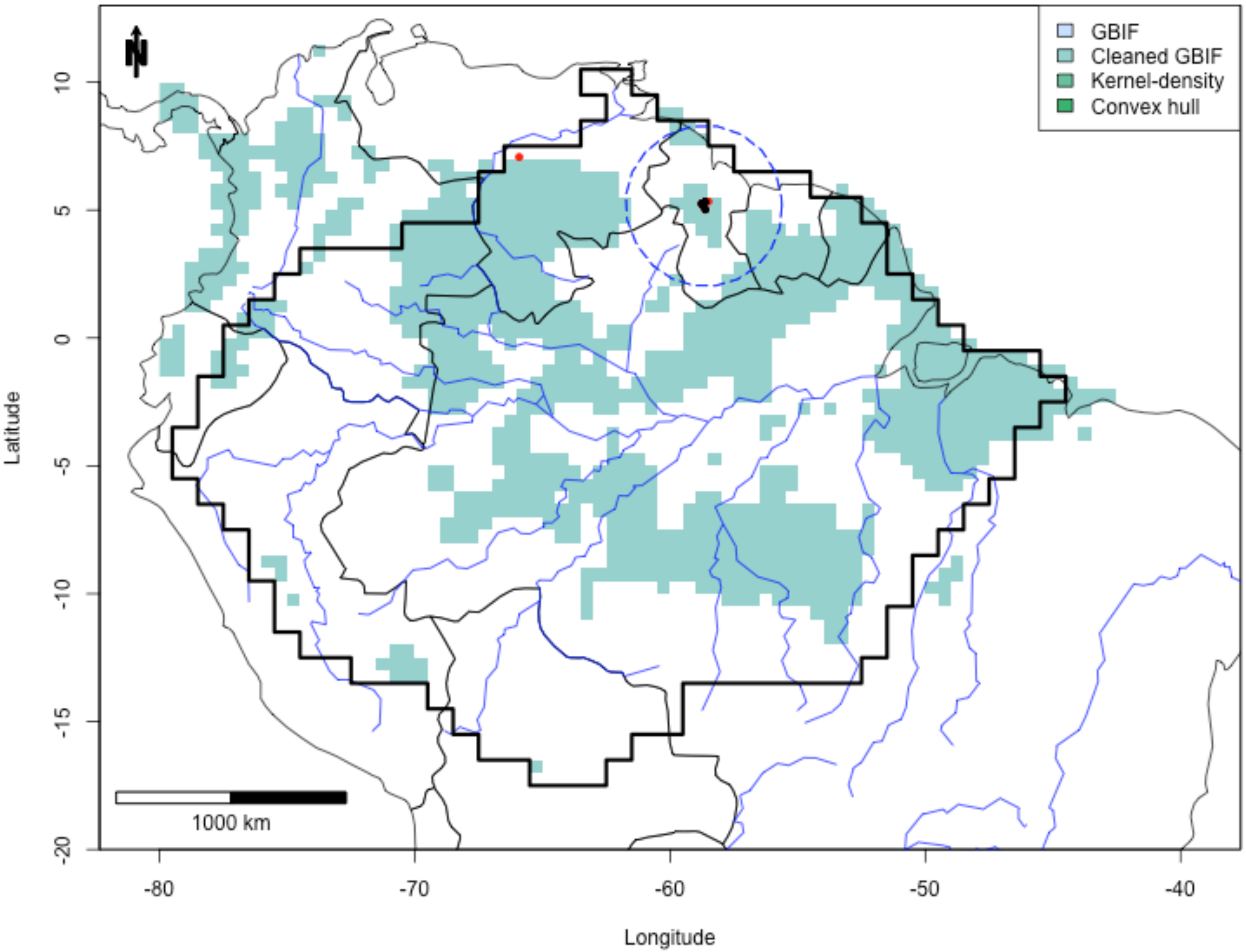

Swartzia polyphylla

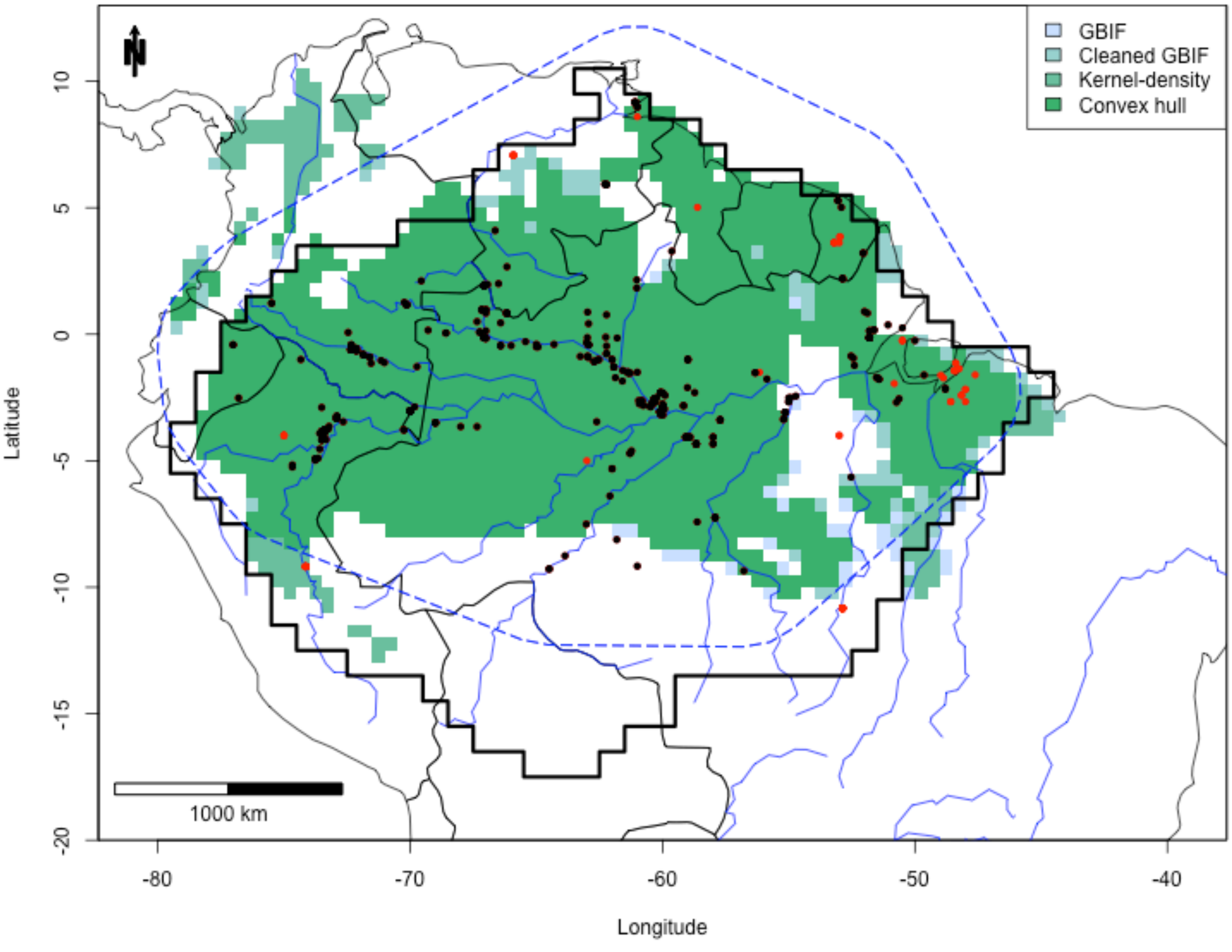

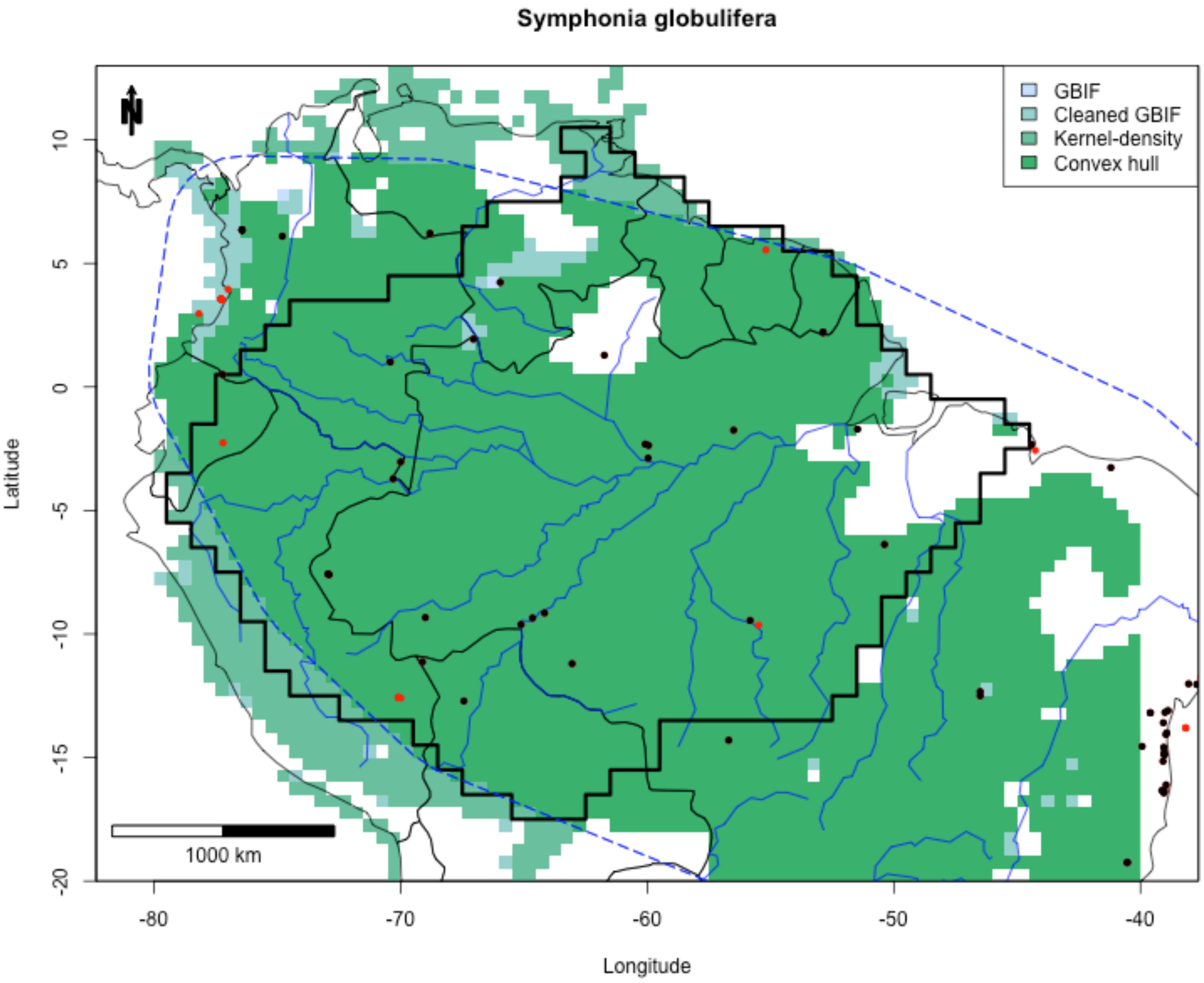

Symphonia globulifera
GBIF
Cleaned GBIF
Kernel-density
Convex hull
N
1000 km
Latitude
Longitude
10
5
0
-5
-10
-15
-20
-80
-70
-60
-50
-40

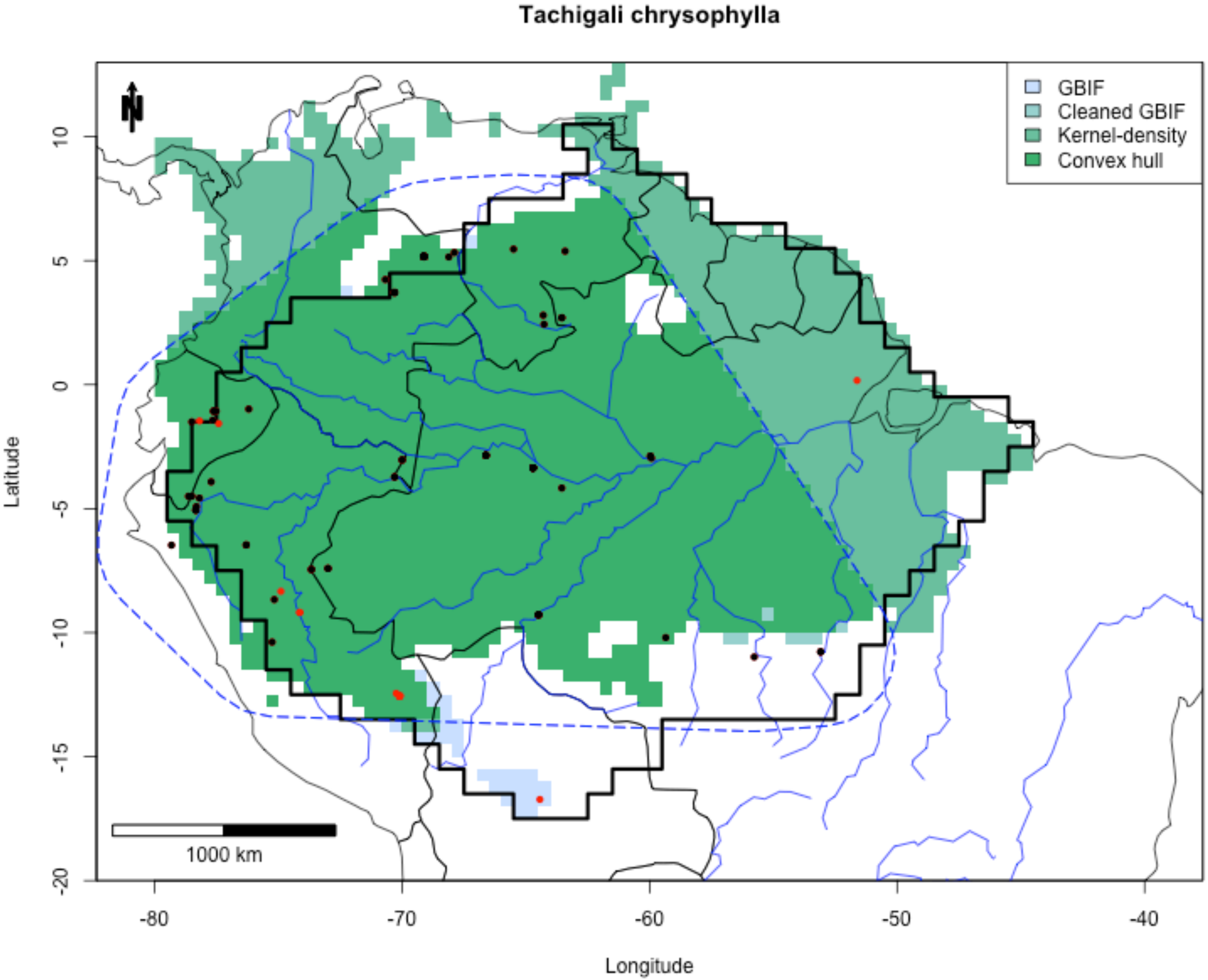
Tachigali chrysophylla
GBIF
Cleaned GBIF
Kernel-density
Convex hull
1000 km
Latitude
Longitude
10
5
0
-5
-10
-15
-20
-80
-70
-60
-50
-40
N

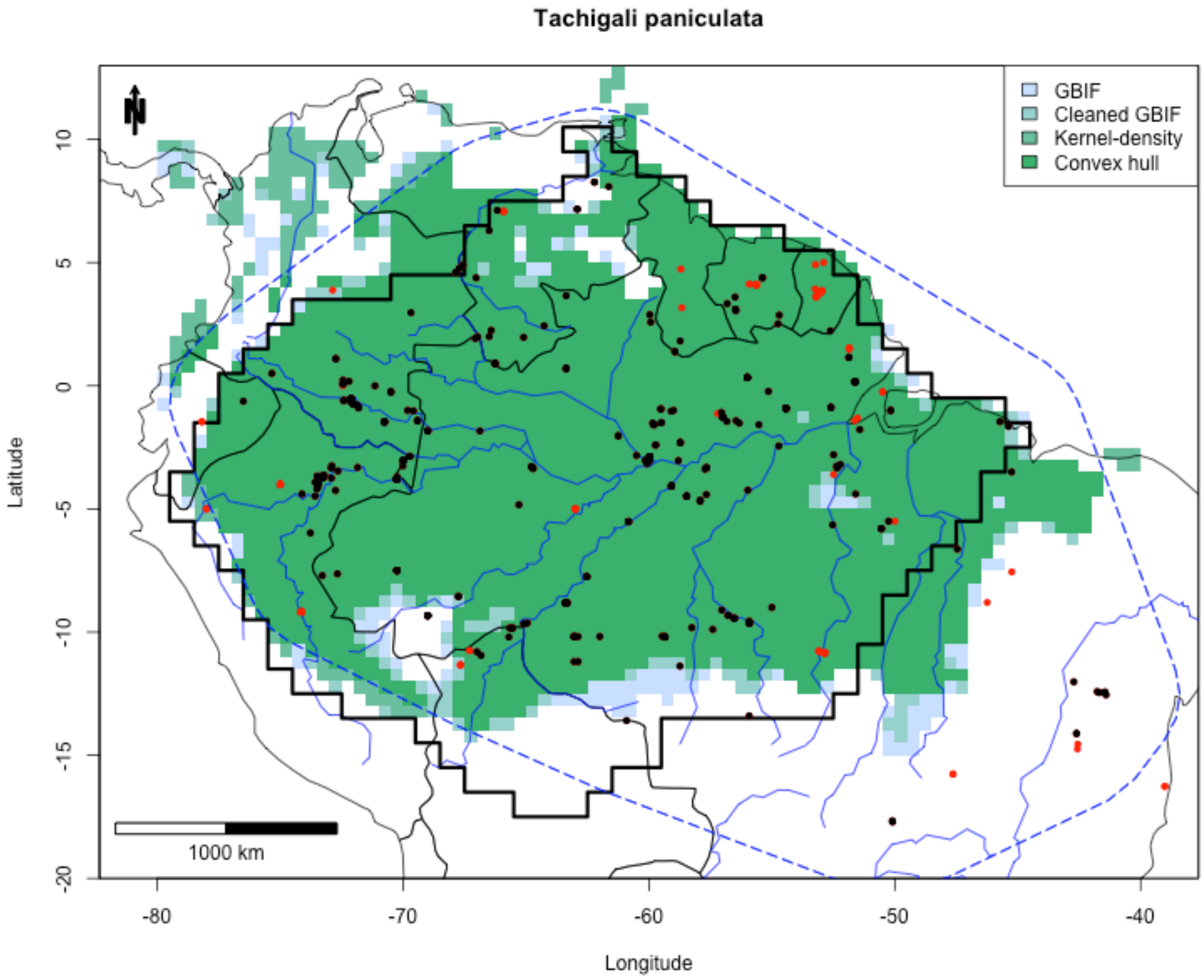
Tachigali paniculata
GBIF
Cleaned GBIF
Kernel-density
Convex hull
1000 km
Latitude
Longitude
10
5
0
-5
-10
-15
-20
-80
-70
-60
-50
-40

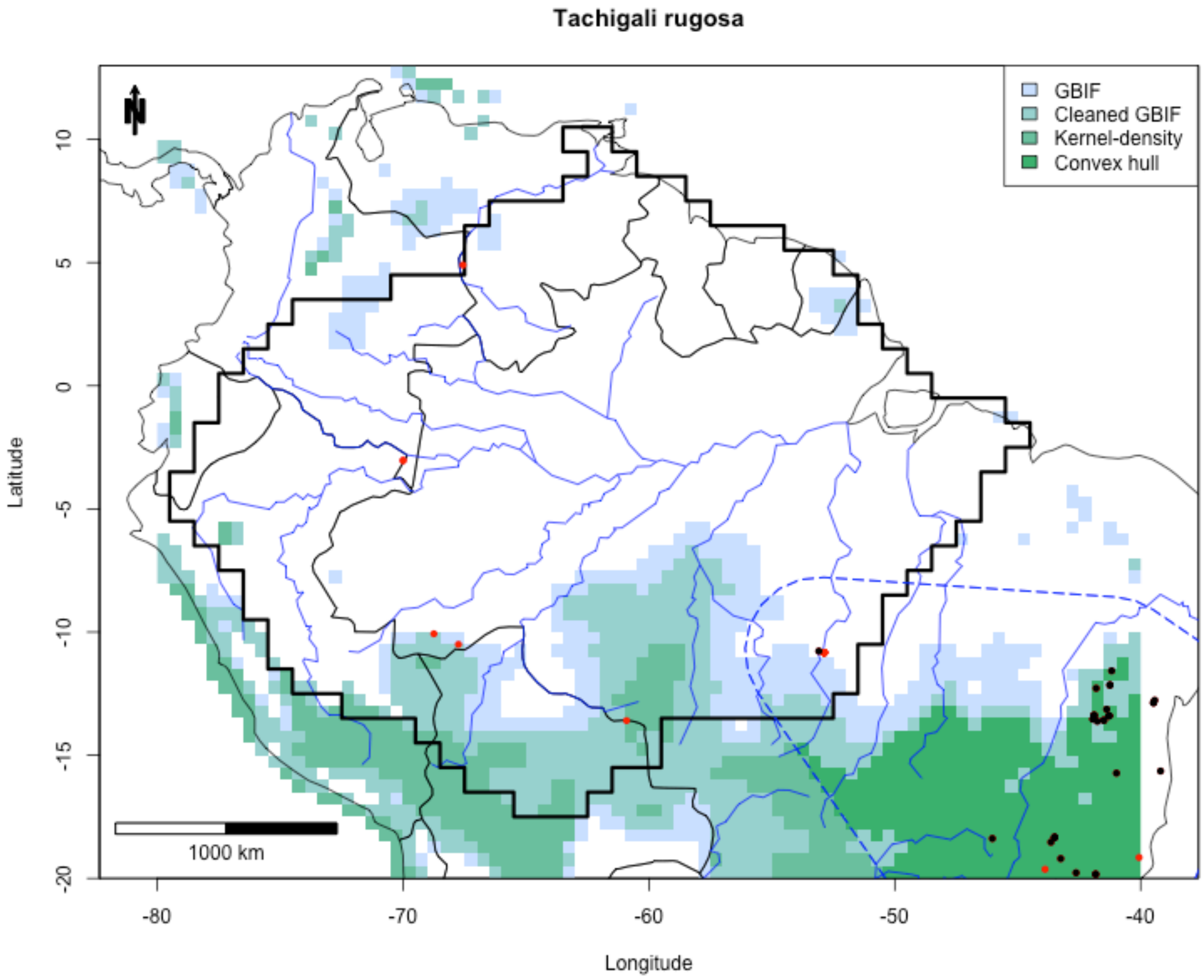

Tachigali rugosa
GBIF
Cleaned GBIF
Kernel-density
Convex hull
N
10
5
0
-5
-10
-15
-20
Latitude
-80
-70
-60
-50
-40
Longitude
1000 km

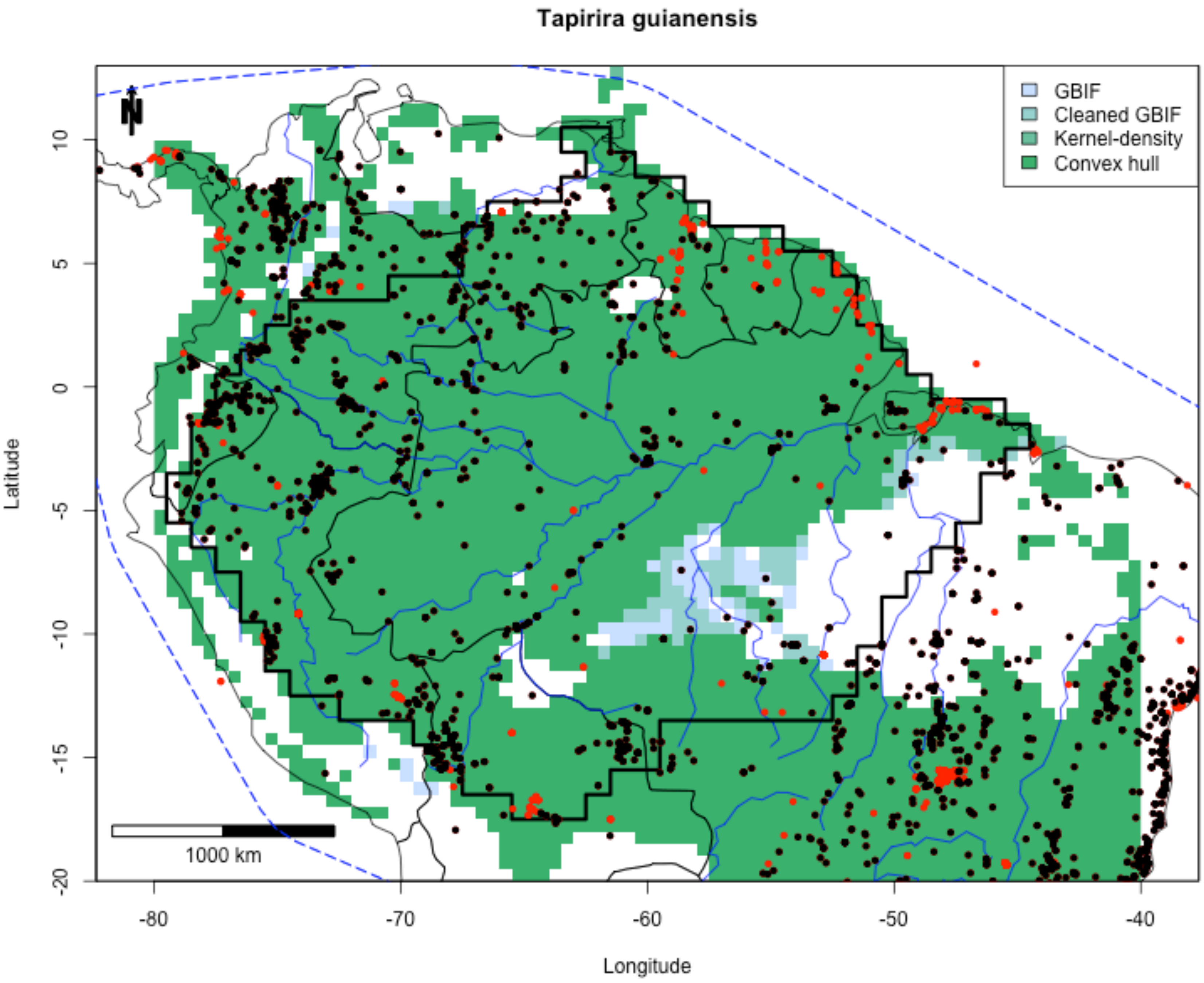
Tapirira guianensis
GBIF
Cleaned GBIF
Kernel-density
Convex hull
N
1000 km
Latitude
Longitude
10
5
0
-5
-10
-15
-20
-80
-70
-60
-50
-40

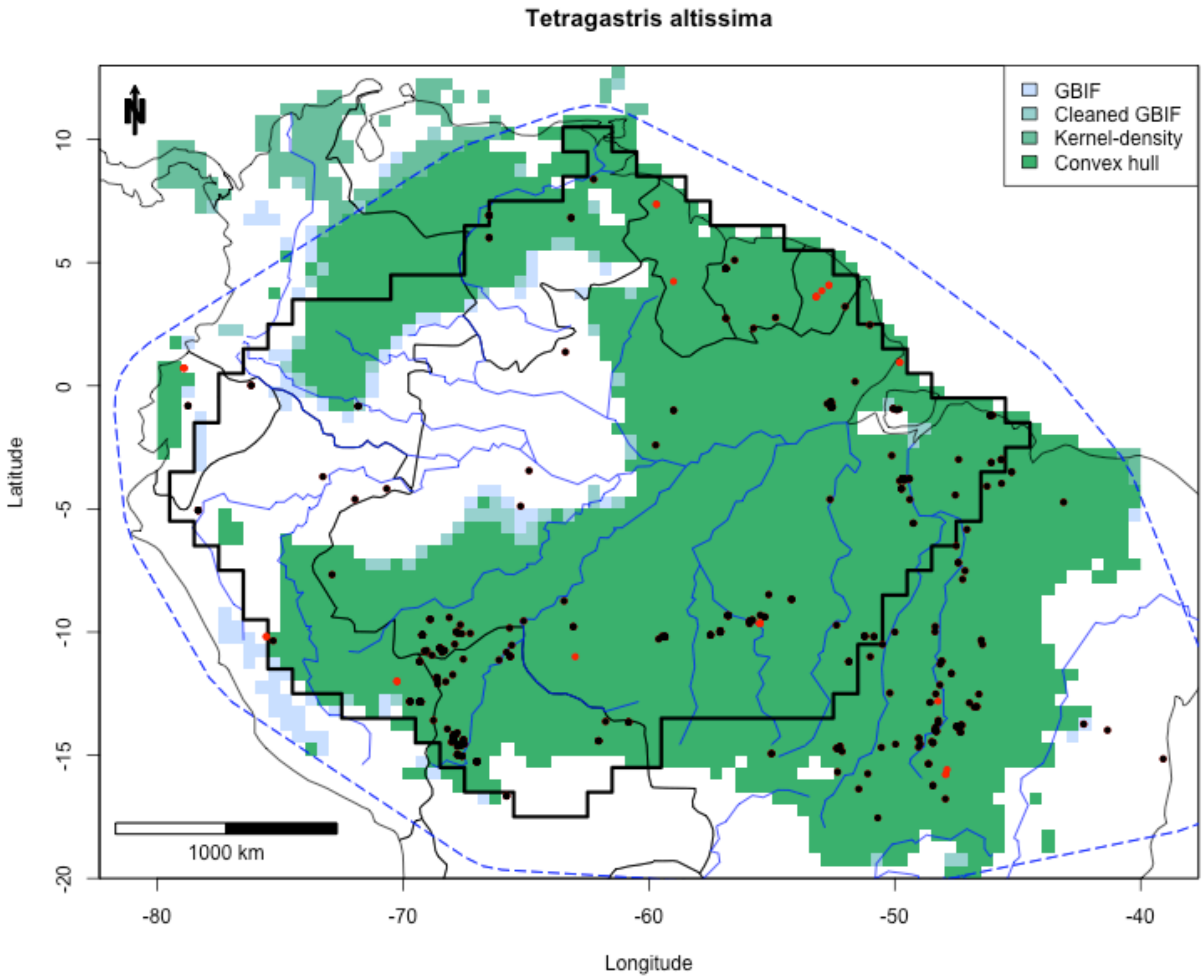

Tetragastris altissima
GBIF
Cleaned GBIF
Kernel-density
Convex hull
N
1000 km
Latitude
Longitude
10
5
0
-5
-10
-15
-20
-80
-70
-60
-50
-40

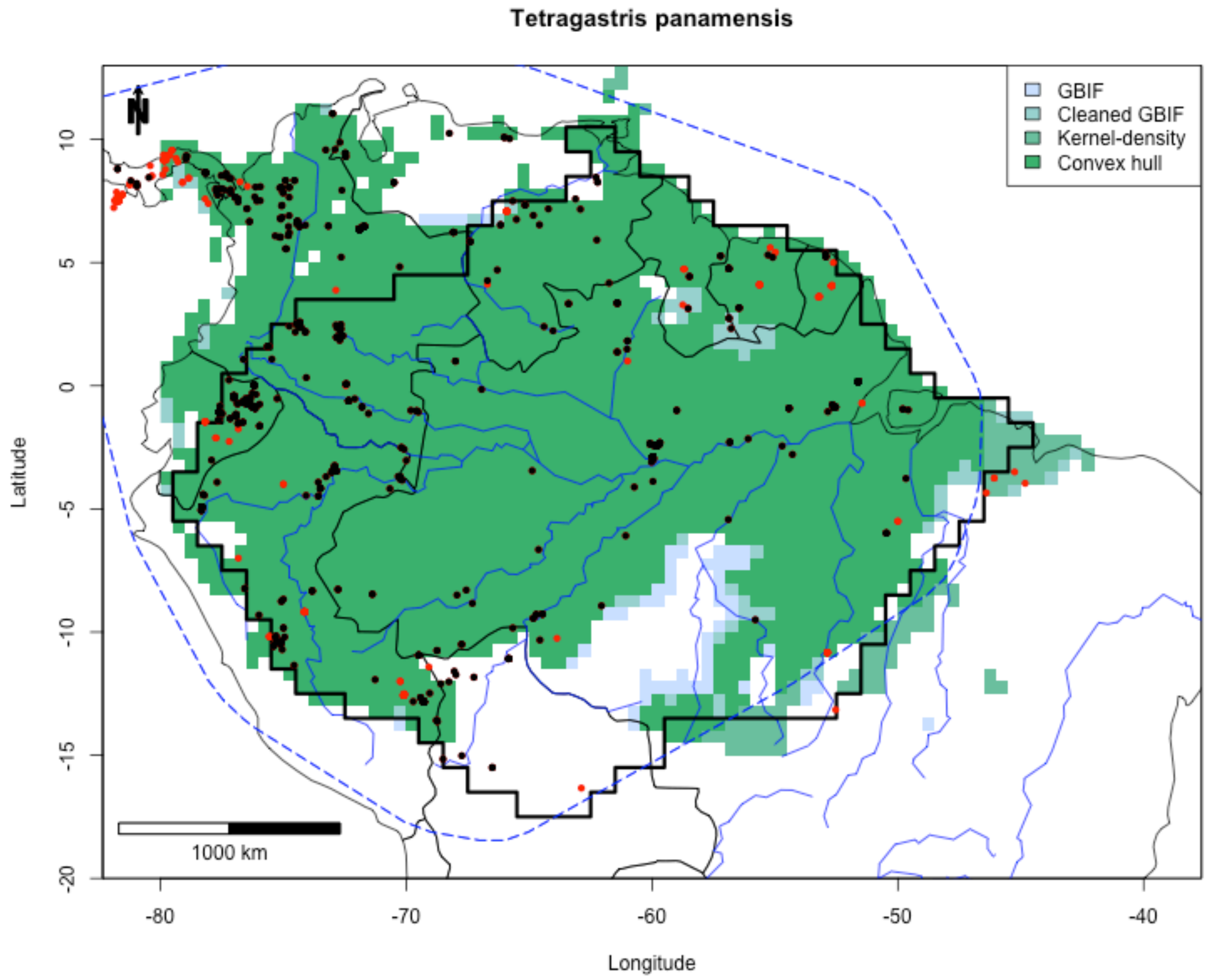

Tetragastris panamensis
GBIF
Cleaned GBIF
Kernel-density
Convex hull
1000 km
Latitude
Longitude
10
5
0
-5
-10
-15
-20
-80
-70
-60
-50
-40

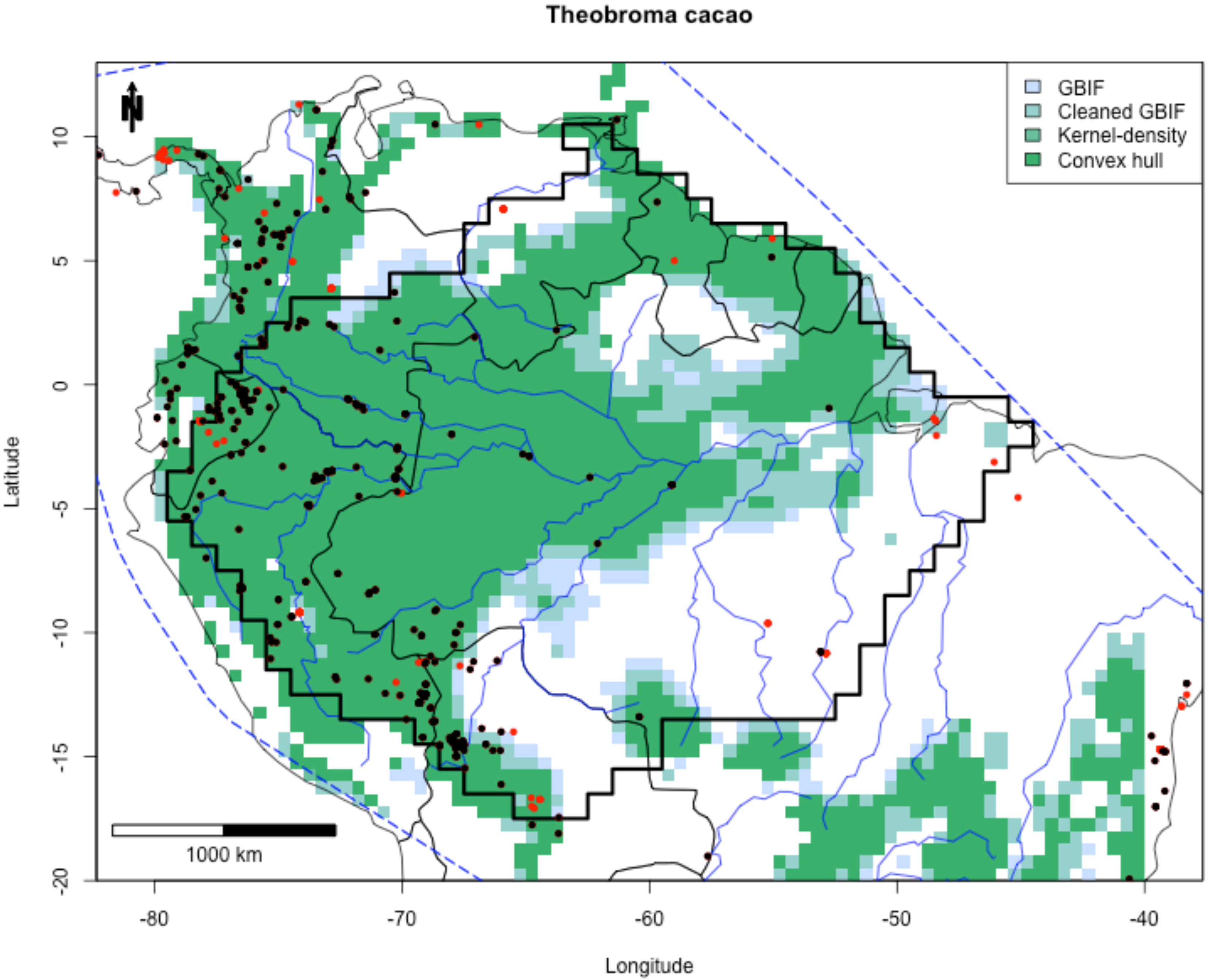

Theobroma cacao
GBIF
Cleaned GBIF
Kernel-density
Convex hull
N
1000 km
Latitude
Longitude
10
5
0
-5
-10
-15
-20
-80
-70
-60
-50
-40

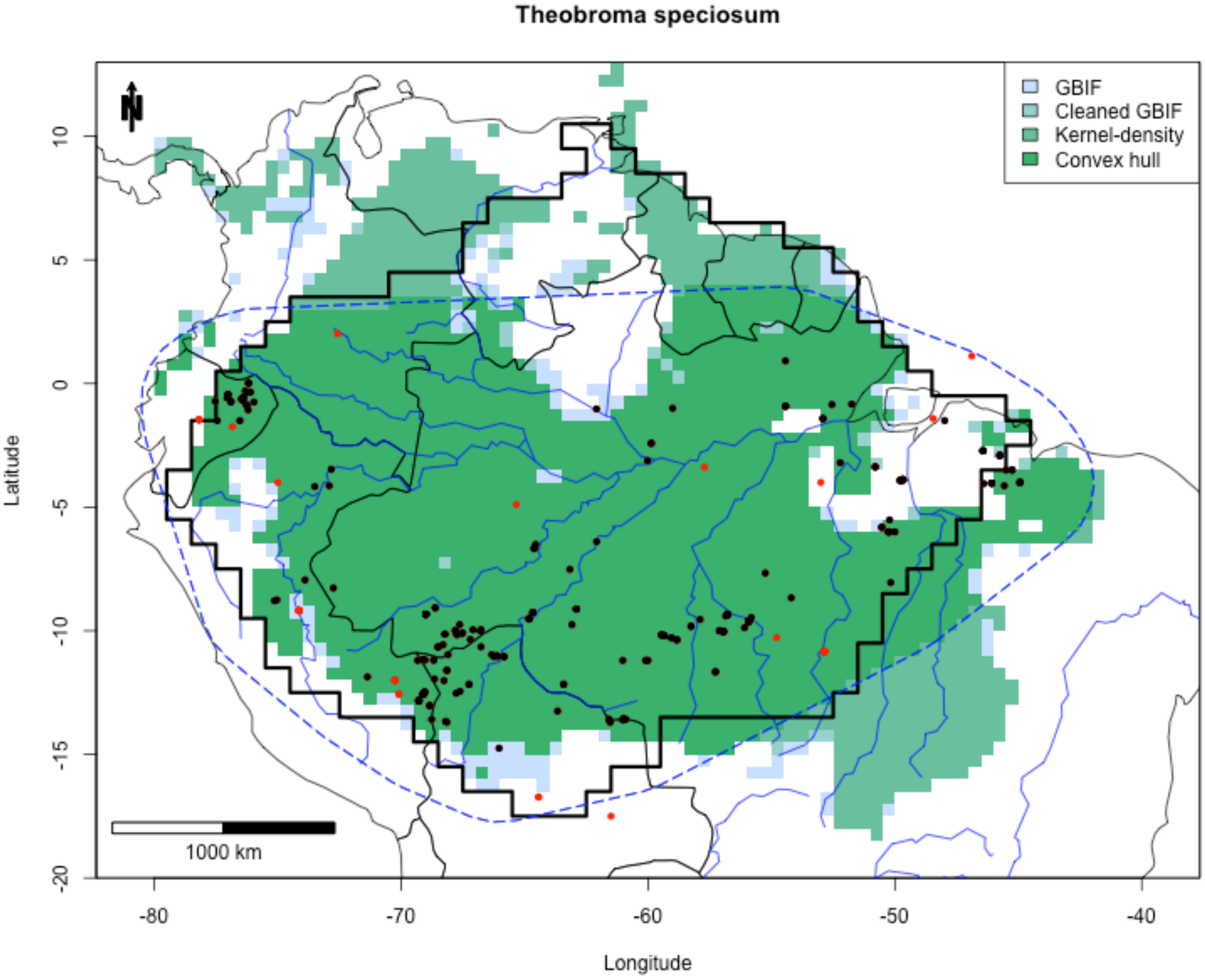

Theobroma speciosum
GBIF
Cleaned GBIF
Kernel-density
Convex hull
10
5
0
-5
-10
-15
-20
Latitude
-80
-70
-60
-50
-40
Longitude
1000 km
N

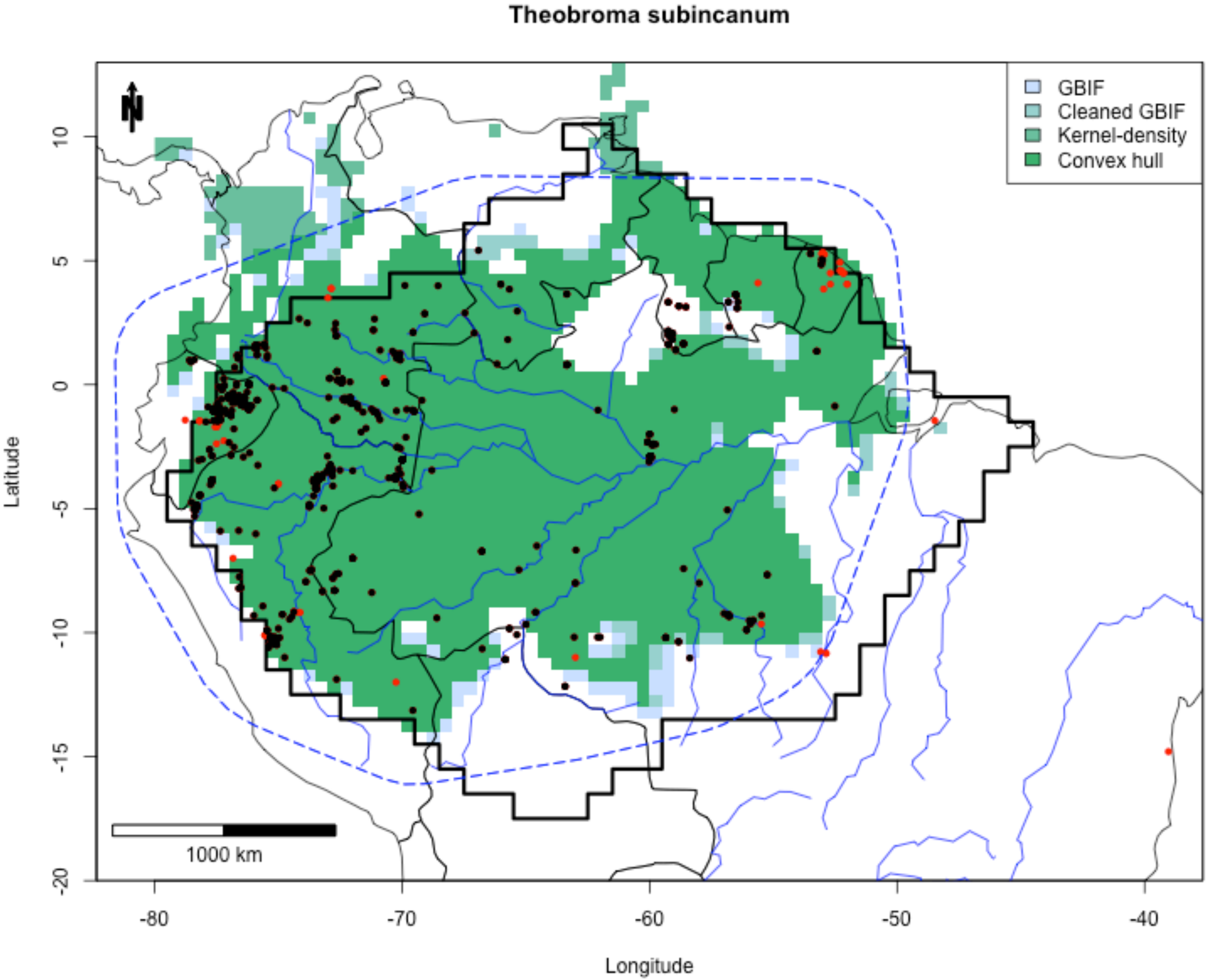
Theobroma subincanum
GBIF
Cleaned GBIF
Kernel-density
Convex hull
N
1000 km
Latitude
Longitude
10
5
0
-5
-10
-15
-20
-80
-70
-60
-50
-40

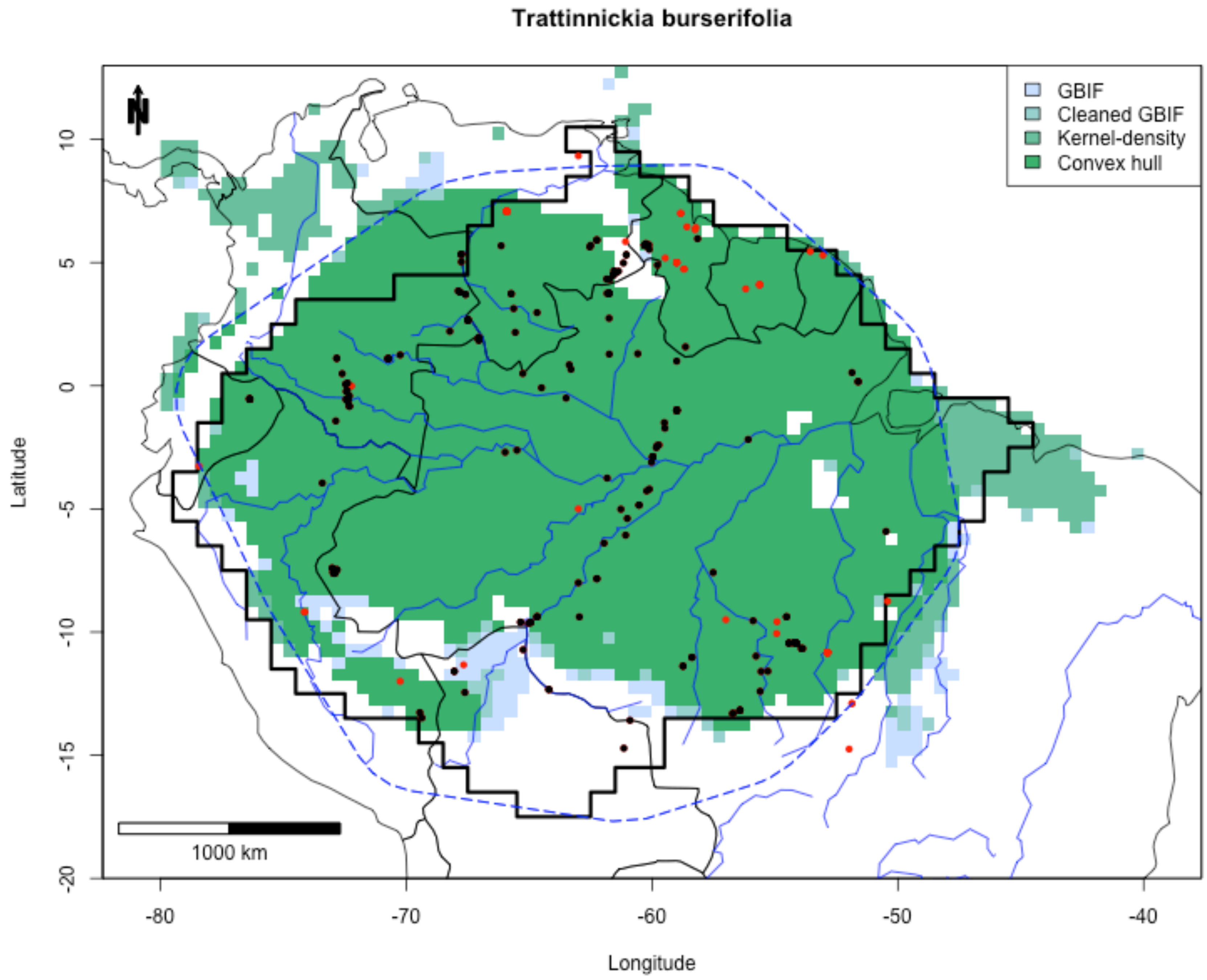

Trattinnickia burserifolia
N
GBIF
Cleaned GBIF
Kernel-density
Convex hull
1000 km
Latitude
Longitude
10
5
0
-5
-10
-15
-20
-80
-70
-60
-50
-40

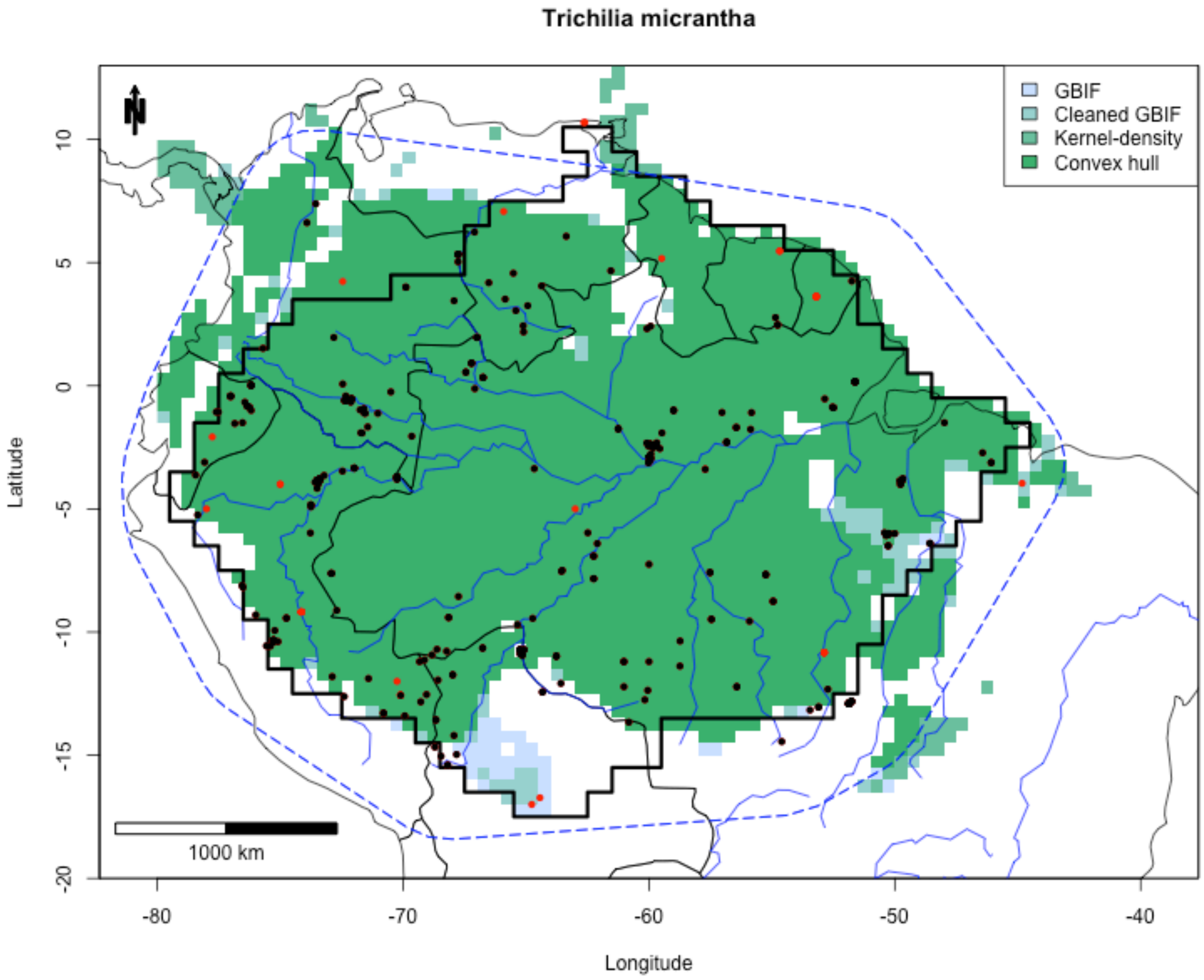

Trichilia micrantha
GBIF
Cleaned GBIF
Kernel-density
Convex hull
N
1000 km
Latitude
Longitude
10
5
0
-5
-10
-15
-20
-80
-70
-60
-50
-40

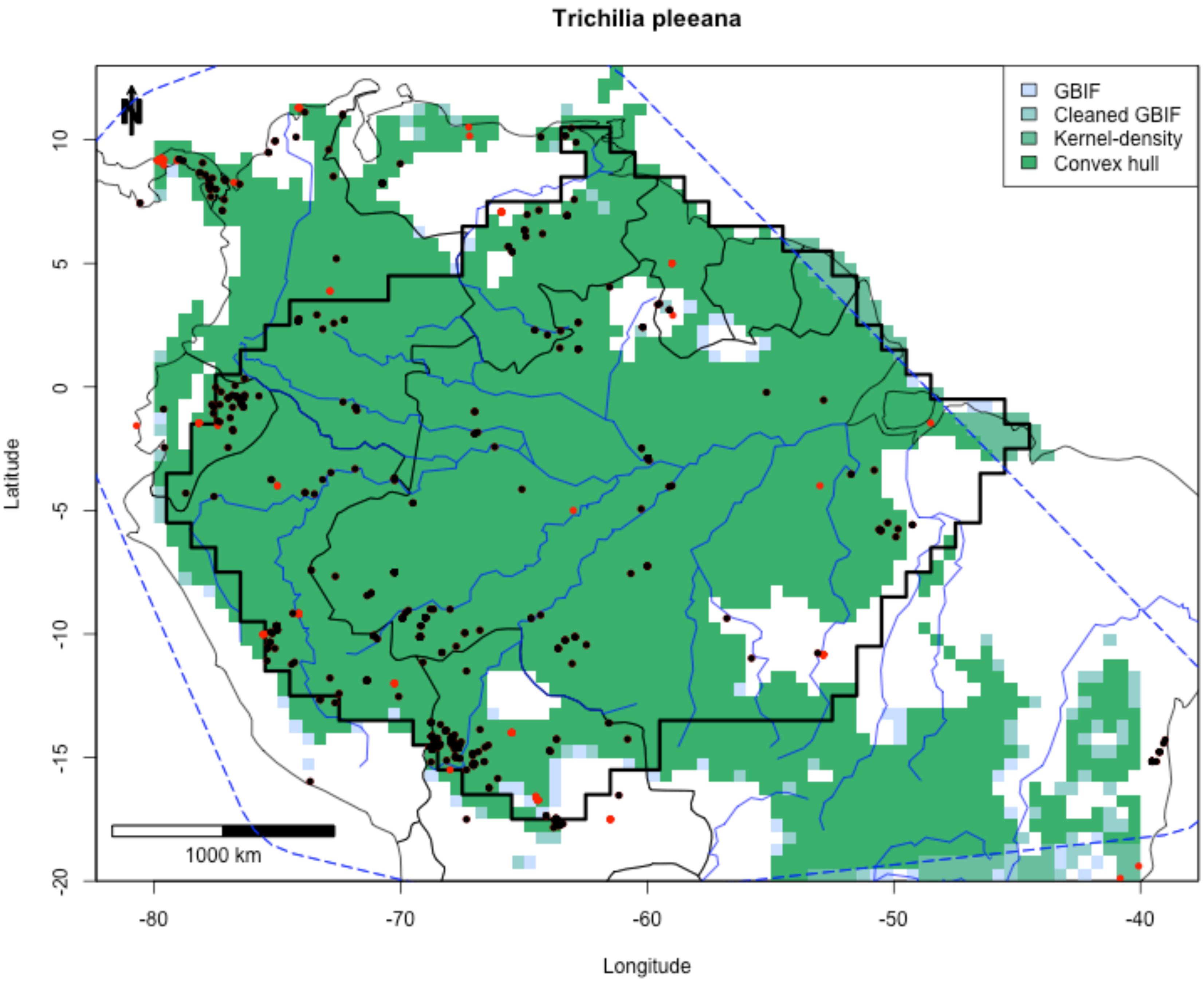
Trichilia pleeana
GBIF
Cleaned GBIF
Kernel-density
Convex hull
N
1000 km
Latitude
Longitude
10
5
0
-5
-10
-15
-20
-80
-70
-60
-50
-40

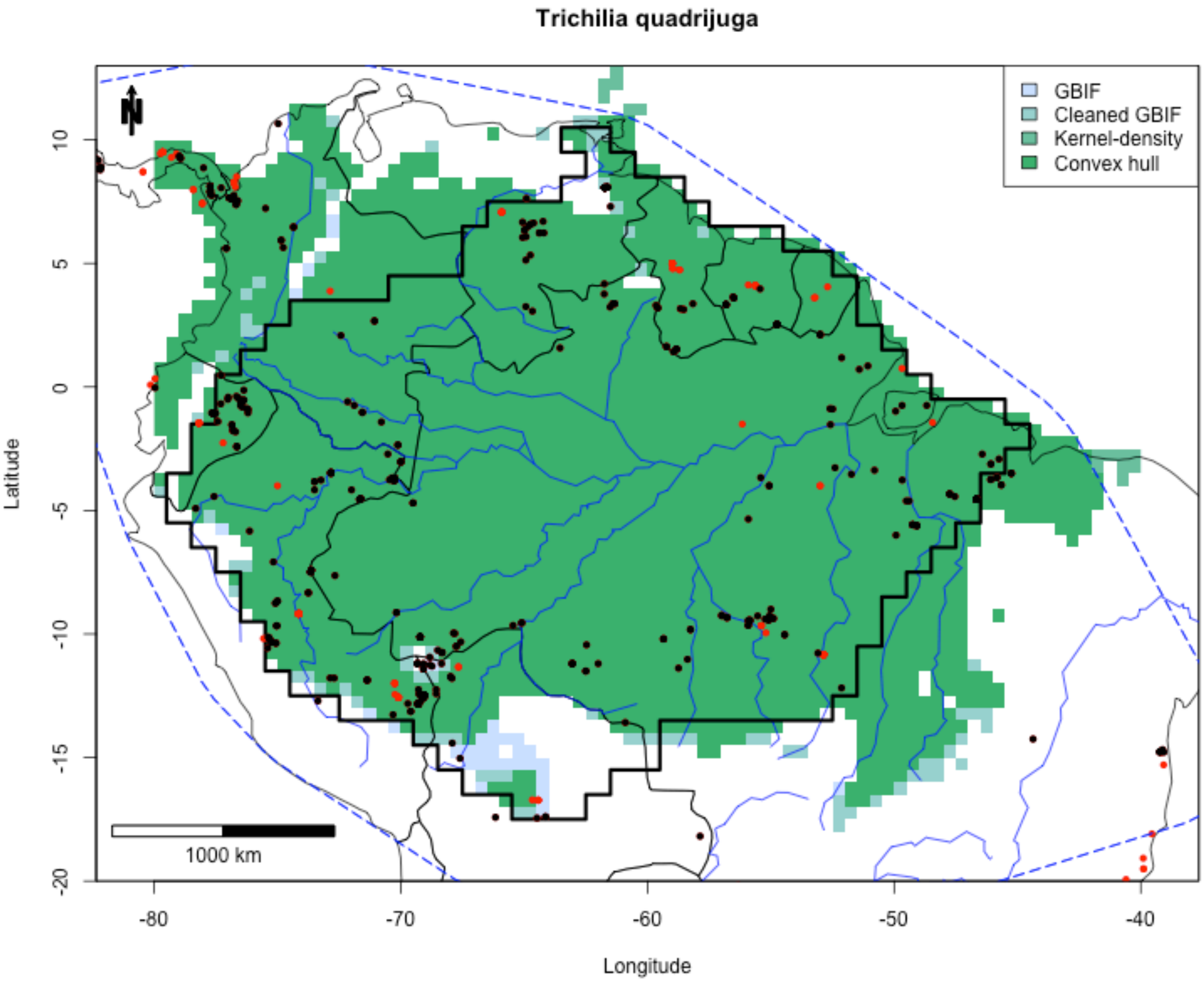
Trichilia quadrijuga
GBIF
Cleaned GBIF
Kernel-density
Convex hull
N
1000 km
Latitude
Longitude
10
5
0
-5
-10
-15
-20
-80
-70
-60
-50
-40

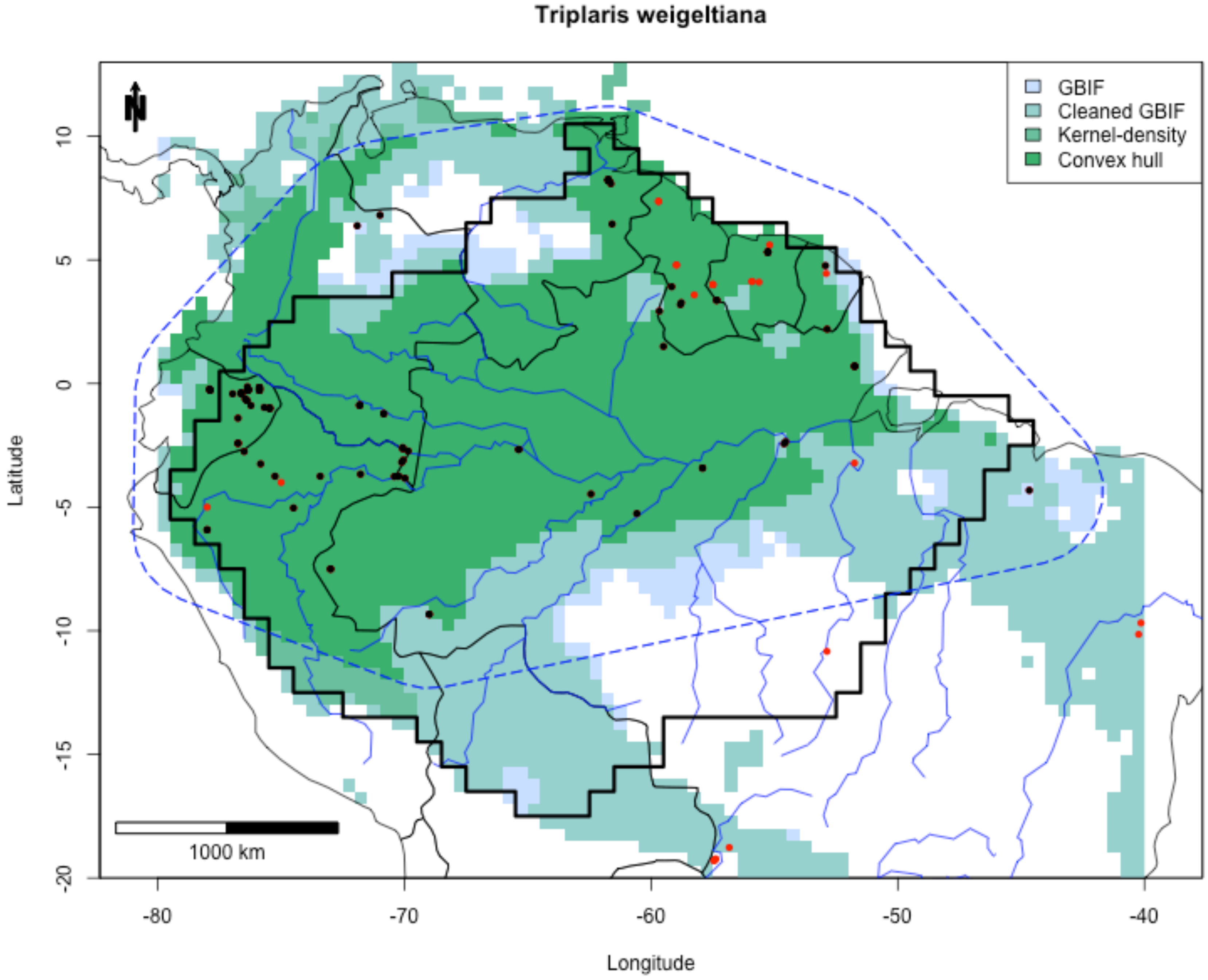
Triplaris weigeltiana
GBIF
Cleaned GBIF
Kernel-density
Convex hull
N
1000 km
Latitude
Longitude
10
5
0
-5
-10
-15
-20
-80
-70
-60
-50
-40

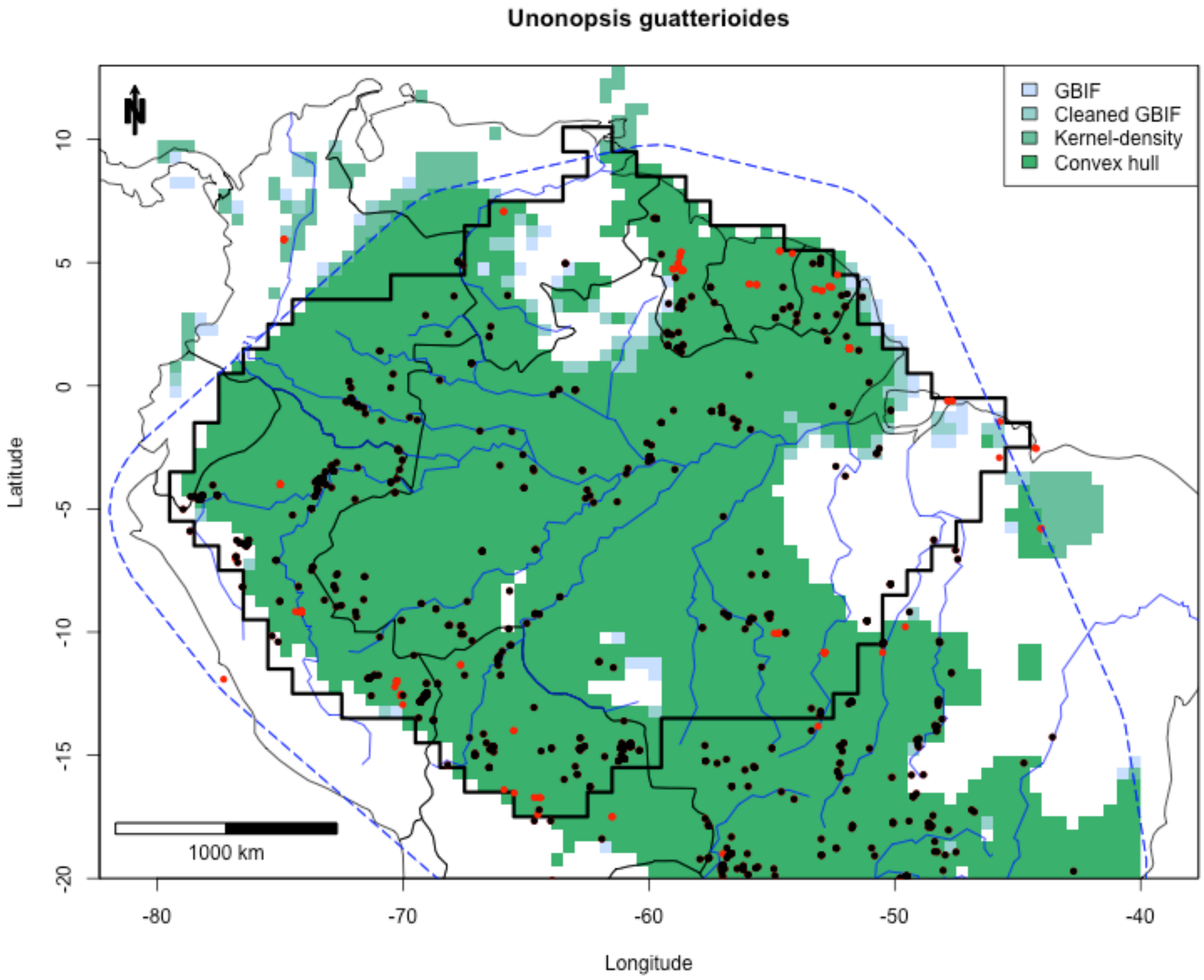

Unonopsis guatterioides
GBIF
Cleaned GBIF
Kernel-density
Convex hull
N
1000 km
Latitude
Longitude
10
5
0
-5
-10
-15
-20
-80
-70
-60
-50
-40

Vatairea guianensis

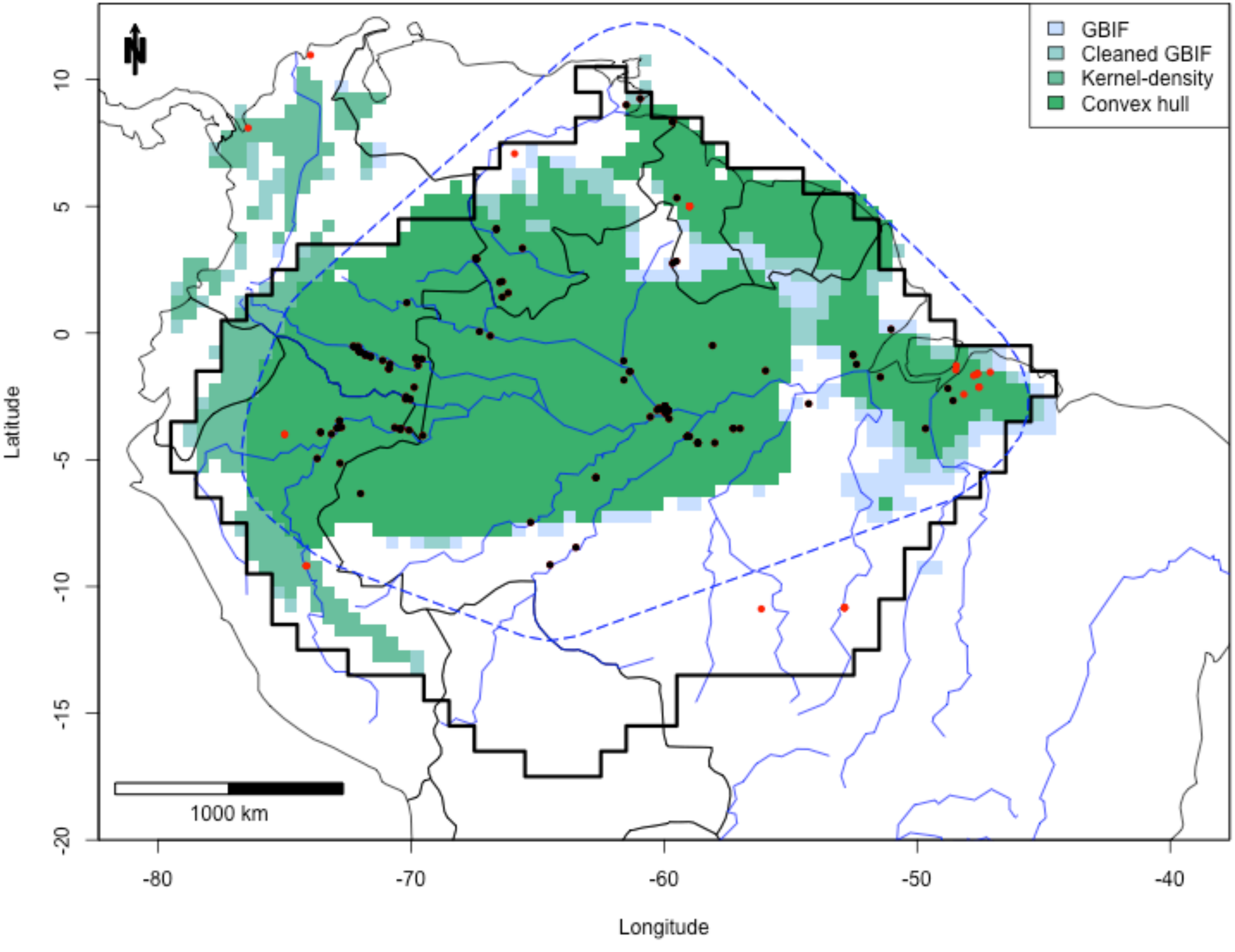

Virola calophylla

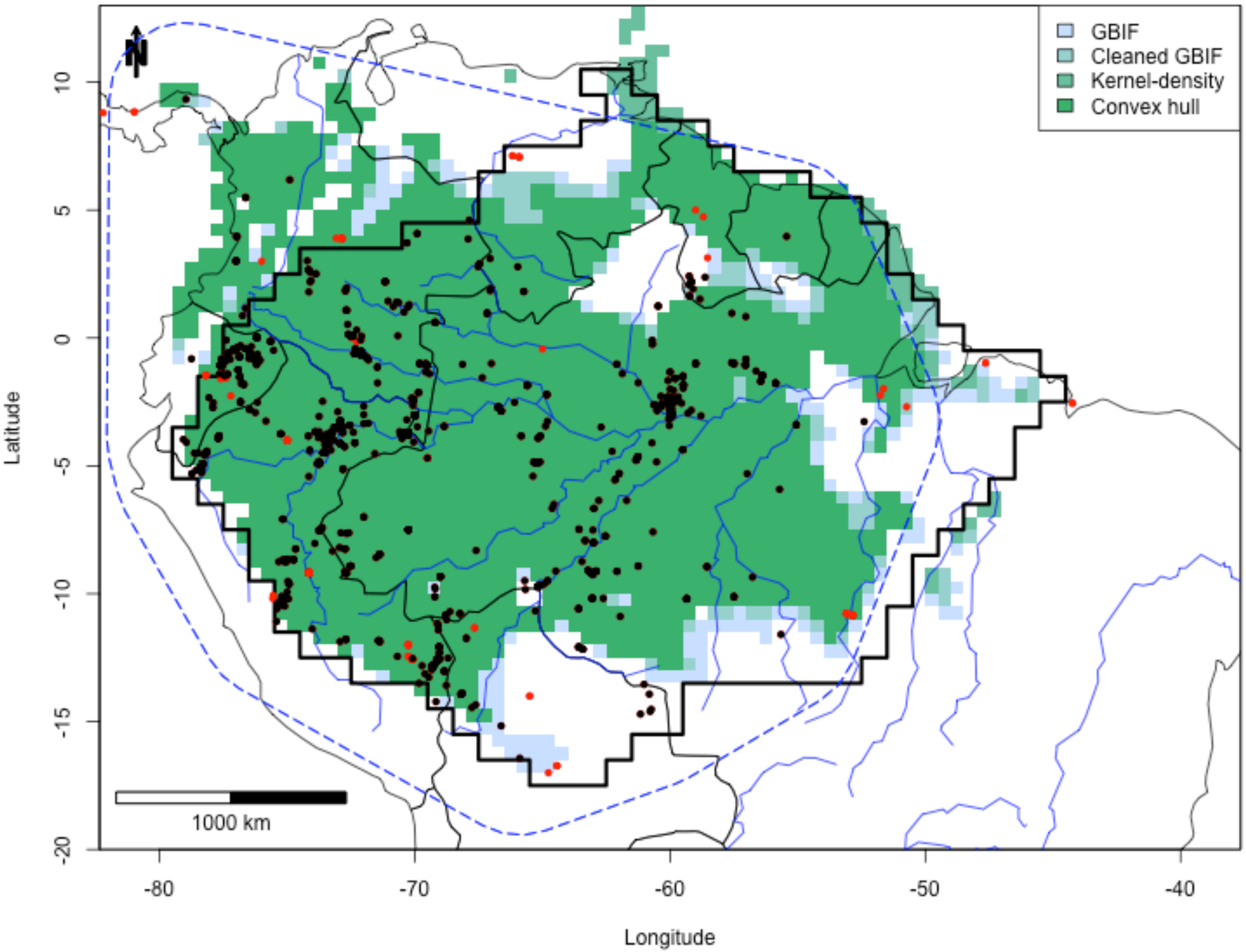

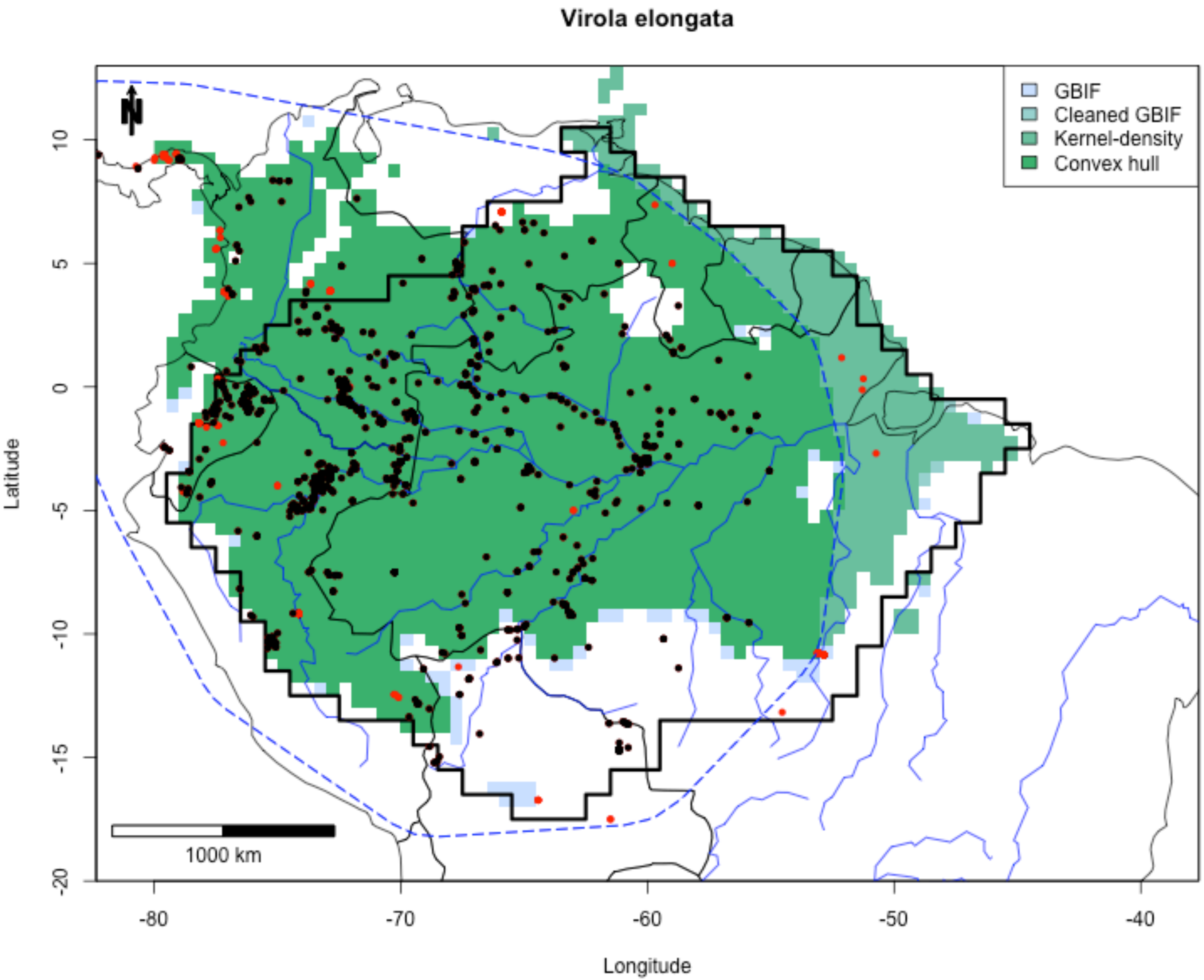

Virola elongata
GBIF
Cleaned GBIF
Kernel-density
Convex hull
1000 km
Latitude
Longitude
10
5
0
-5
-10
-15
-20
-80
-70
-60
-50
-40

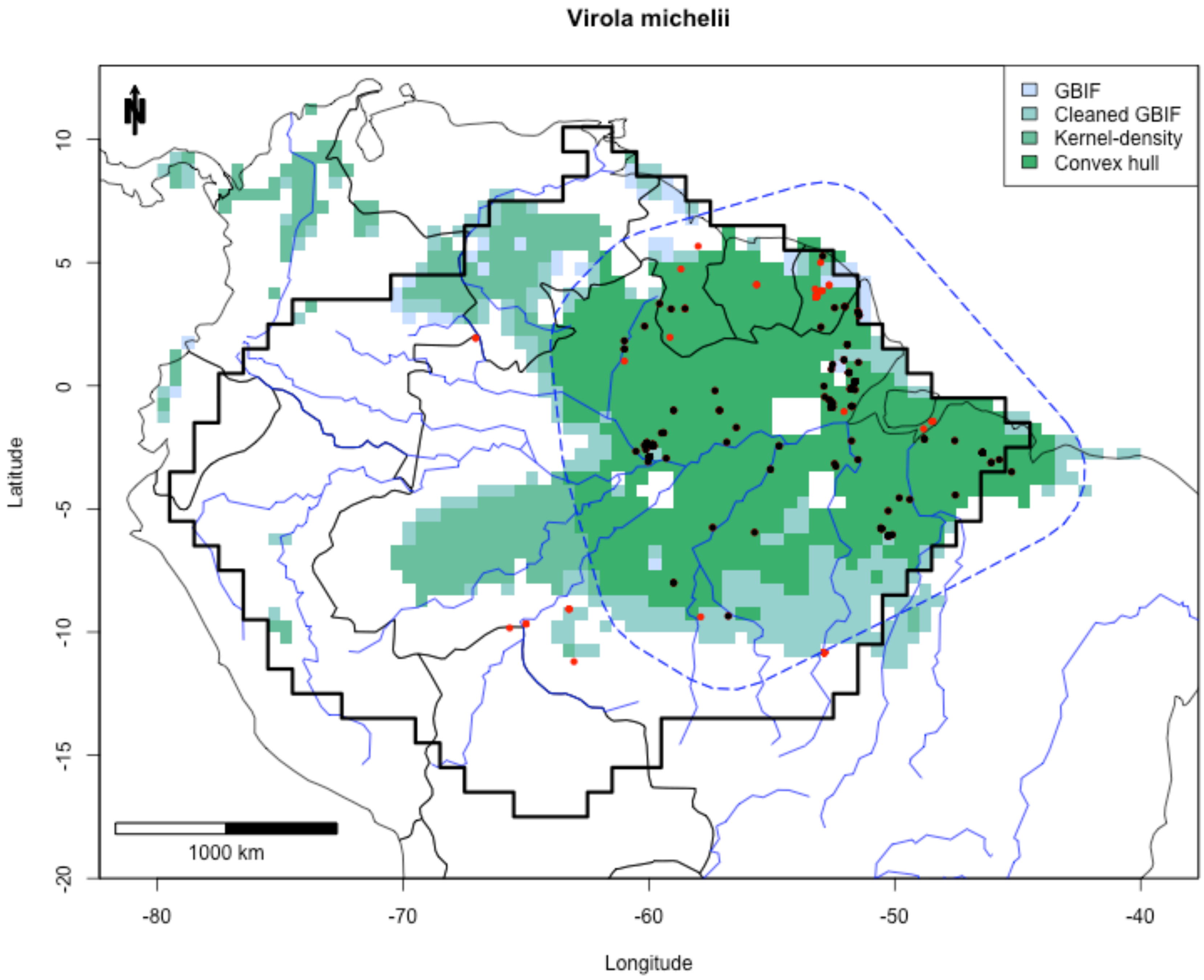

Virola michelii
GBIF
Cleaned GBIF
Kernel-density
Convex hull
N
1000 km
Latitude
Longitude
10
5
0
-5
-10
-15
-20
-80
-70
-60
-50
-40

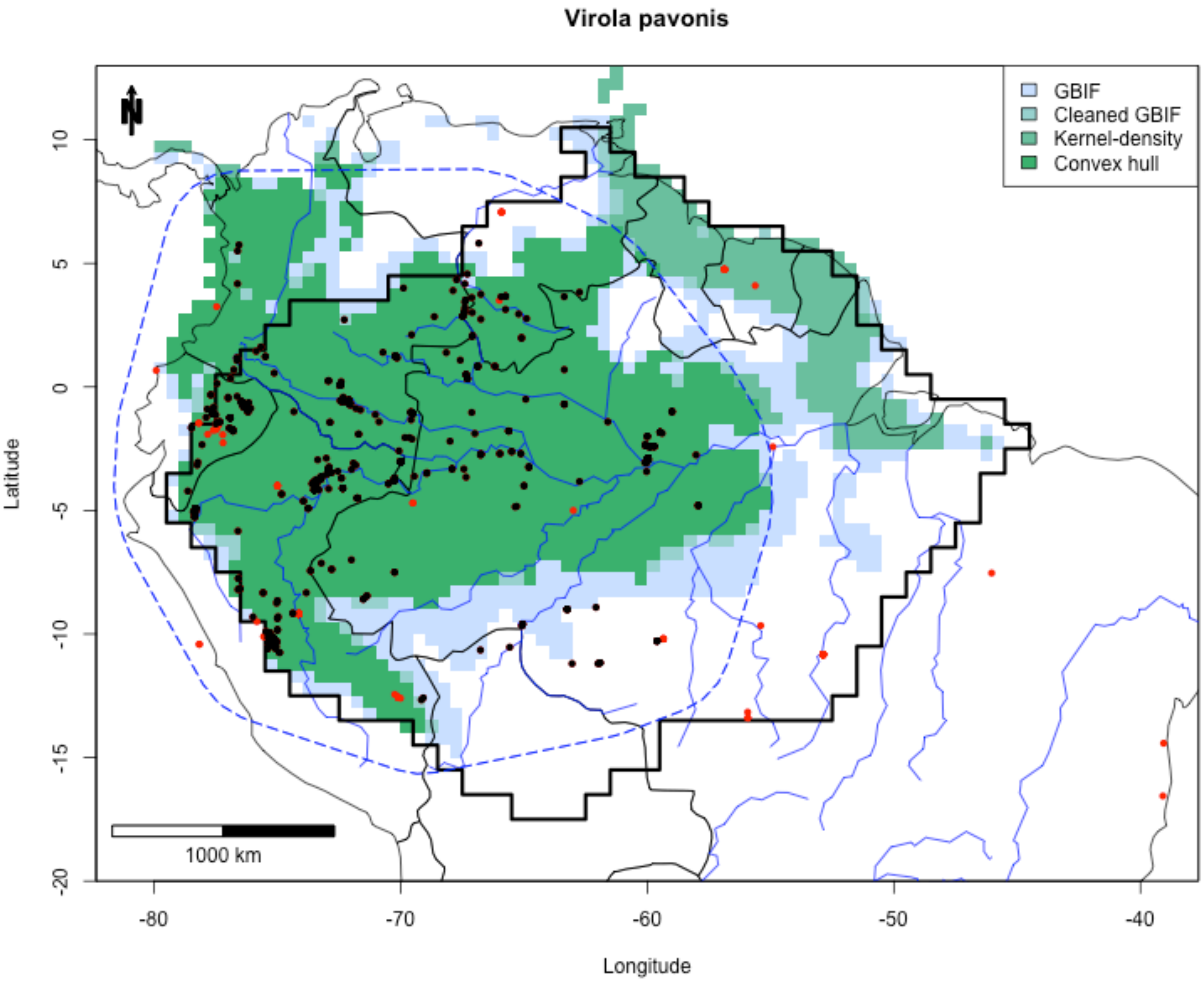
Virola pavonis
GBIF
Cleaned GBIF
Kernel-density
Convex hull
1000 km
Latitude
Longitude
10
5
0
-5
-10
-15
-20
-80
-70
-60
-50
-40
N

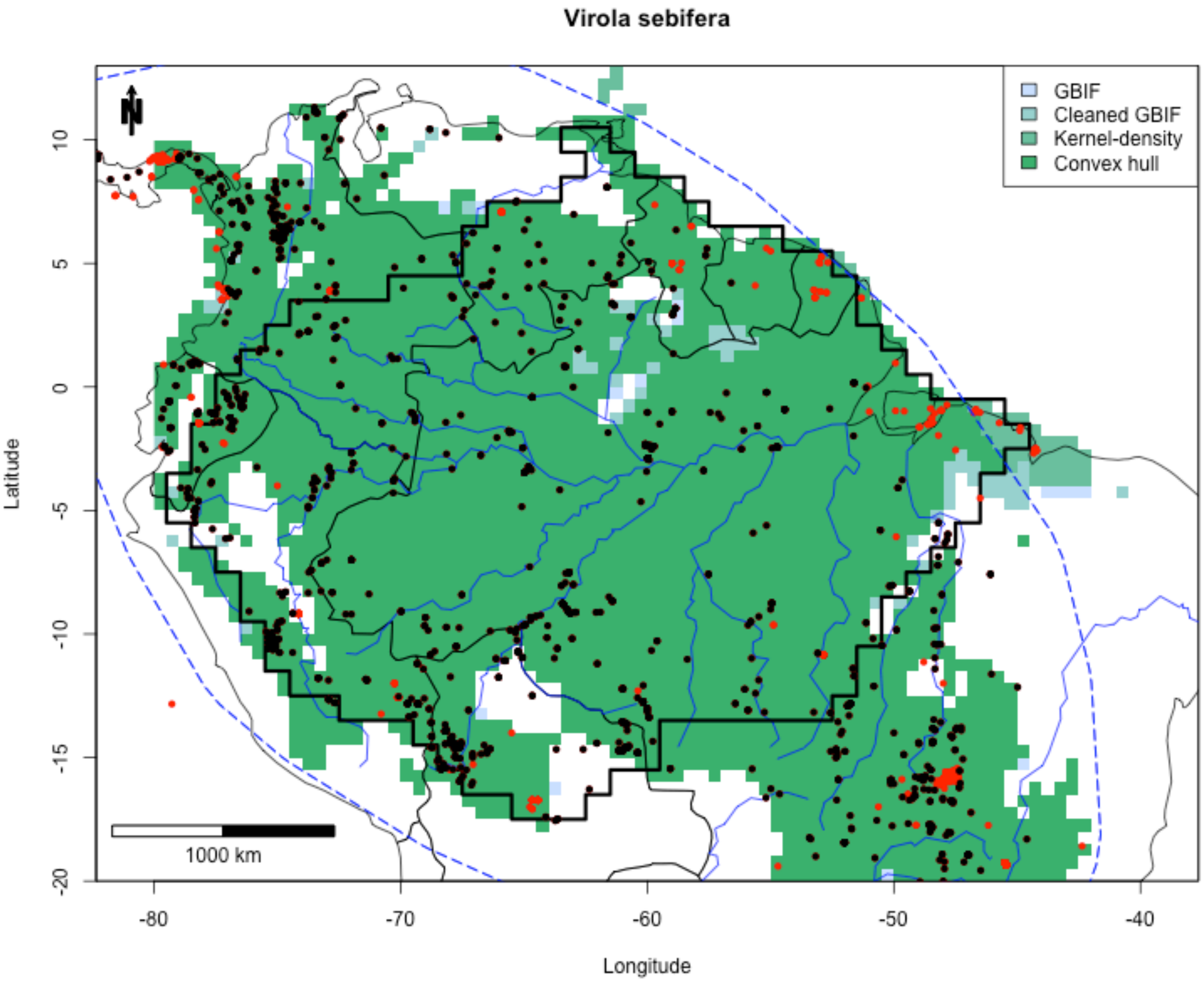

Virola sebifera
GBIF
Cleaned GBIF
Kernel-density
Convex hull
N
1000 km
Latitude
Longitude
10
5
0
-5
-10
-15
-20
-80
-70
-60
-50
-40

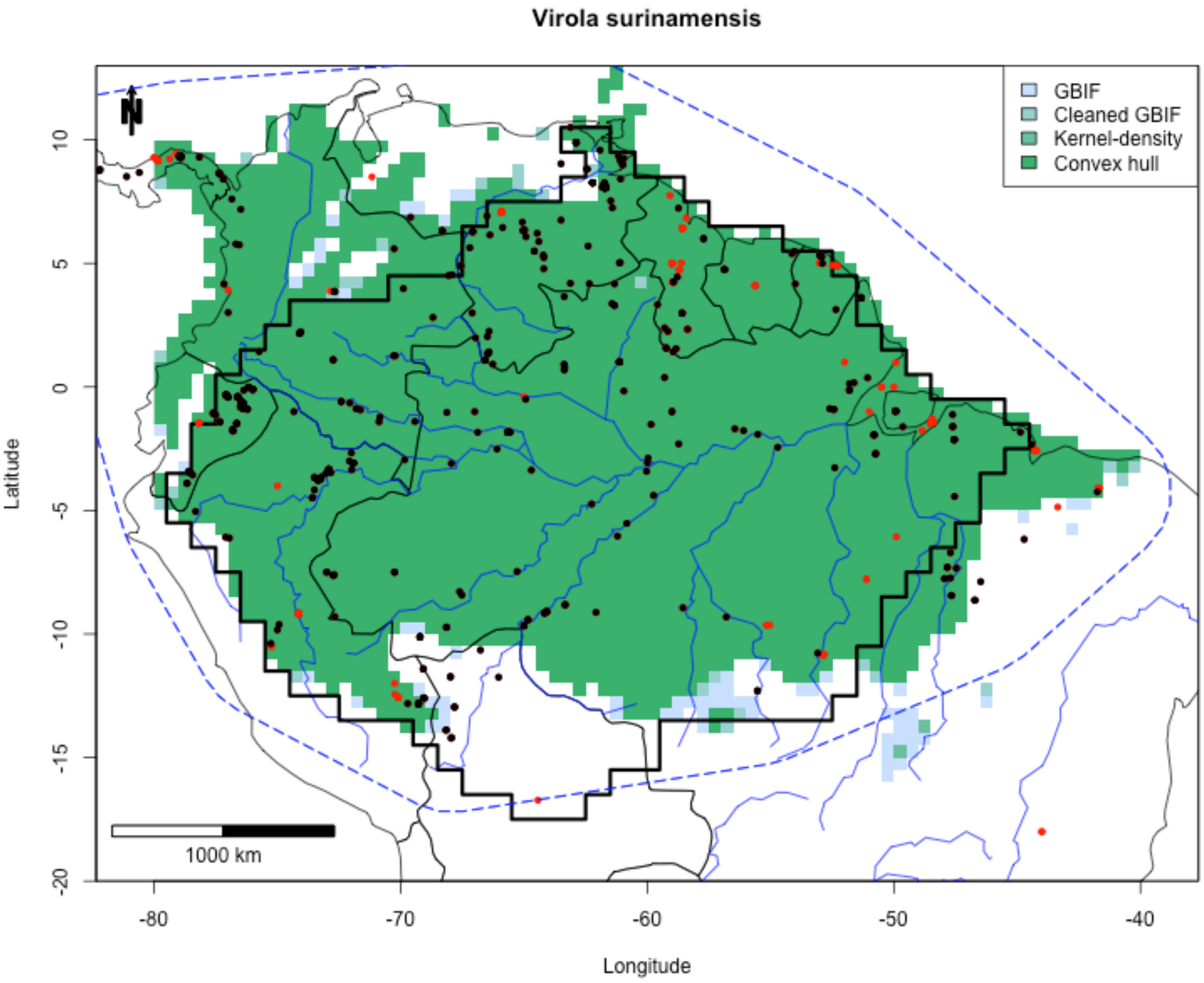

Virola surinamensis
GBIF
Cleaned GBIF
Kernel-density
Convex hull
N
1000 km
Latitude
Longitude
10
5
0
-5
-10
-15
-20
-80
-70
-60
-50
-40

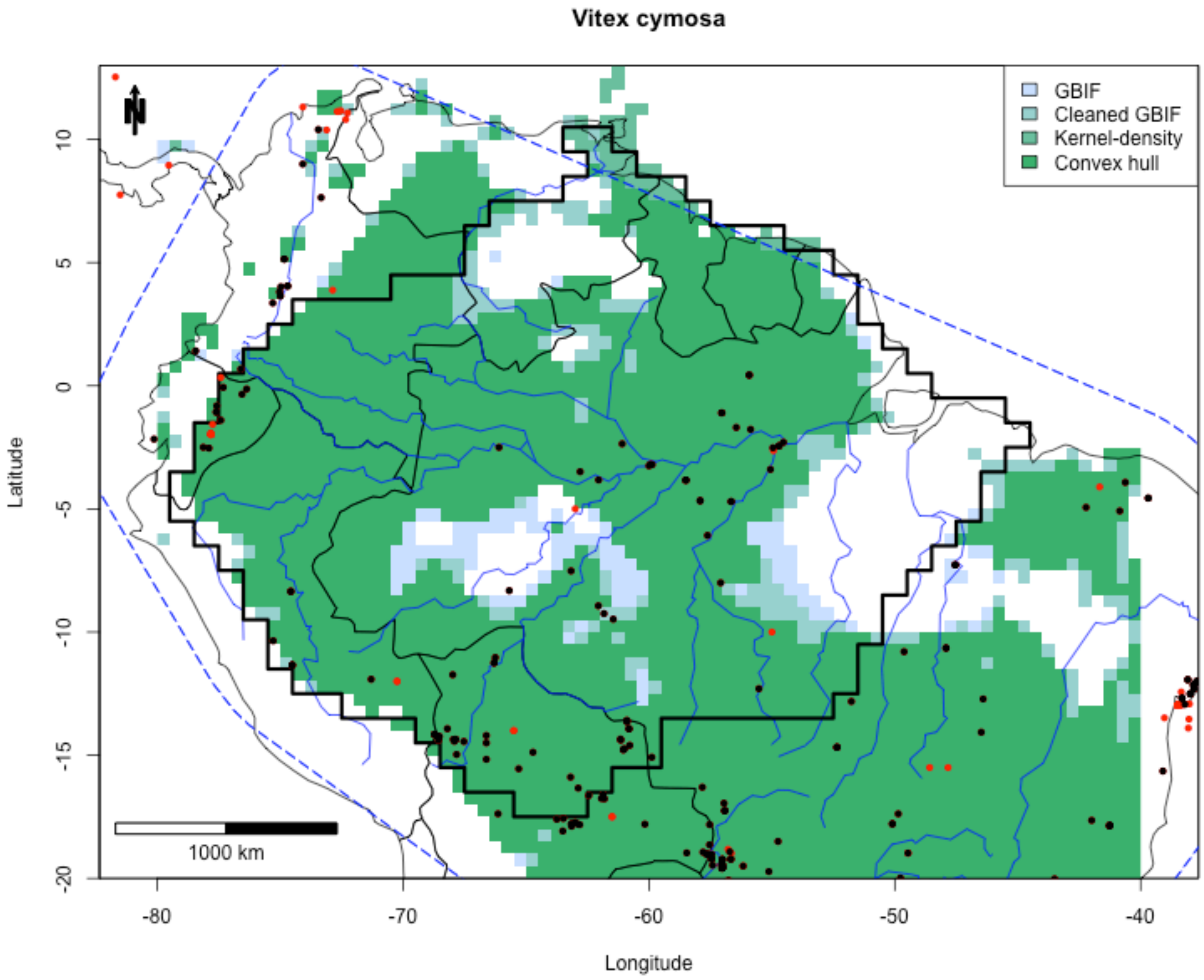
Vitex cymosa
GBIF
Cleaned GBIF
Kernel-density
Convex hull
1000 km
Latitude
Longitude
10
5
0
-5
-10
-15
-20
-80
-70
-60
-50
-40

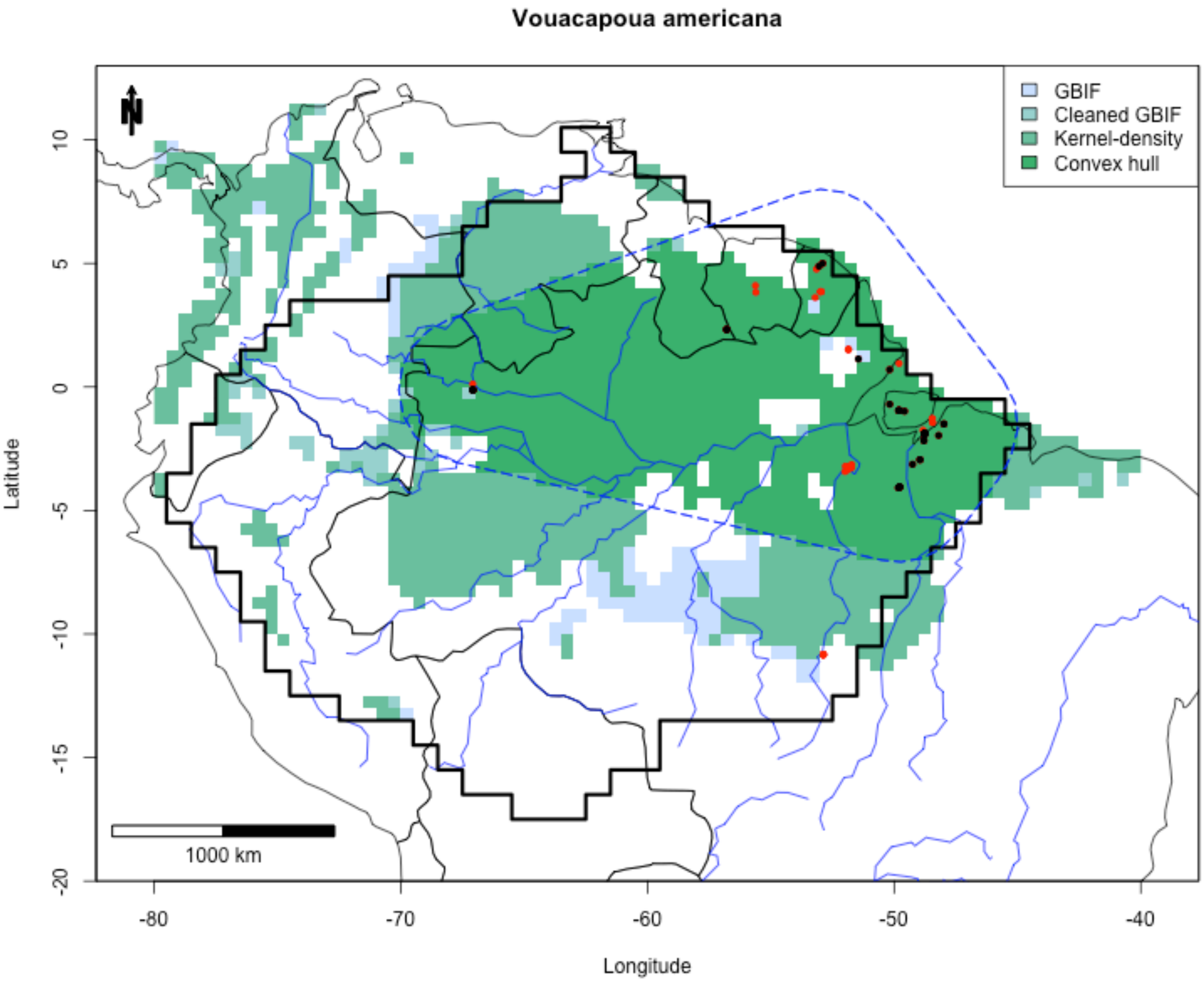
Vouacapoua americana
GBIF
Cleaned GBIF
Kernel-density
Convex hull
N
1000 km
Latitude
Longitude
10
5
0
-5
-10
-15
-20
-80
-70
-60
-50
-40

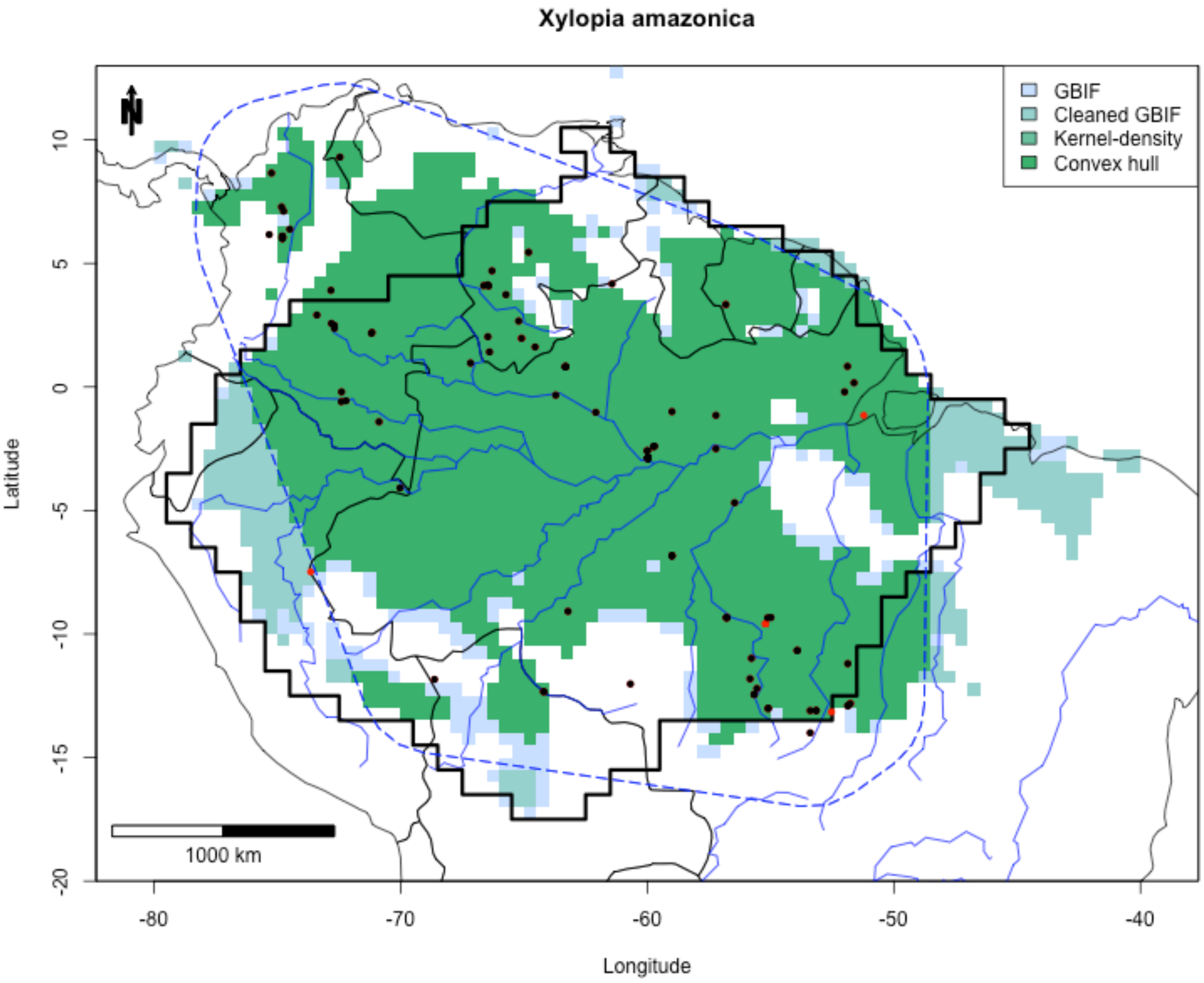

Xylopia amazonica
GBIF
Cleaned GBIF
Kernel-density
Convex hull
1000 km
Latitude
Longitude
10
5
0
-5
-10
-15
-20
-80
-70
-60
-50
-40

Zygia cataractae

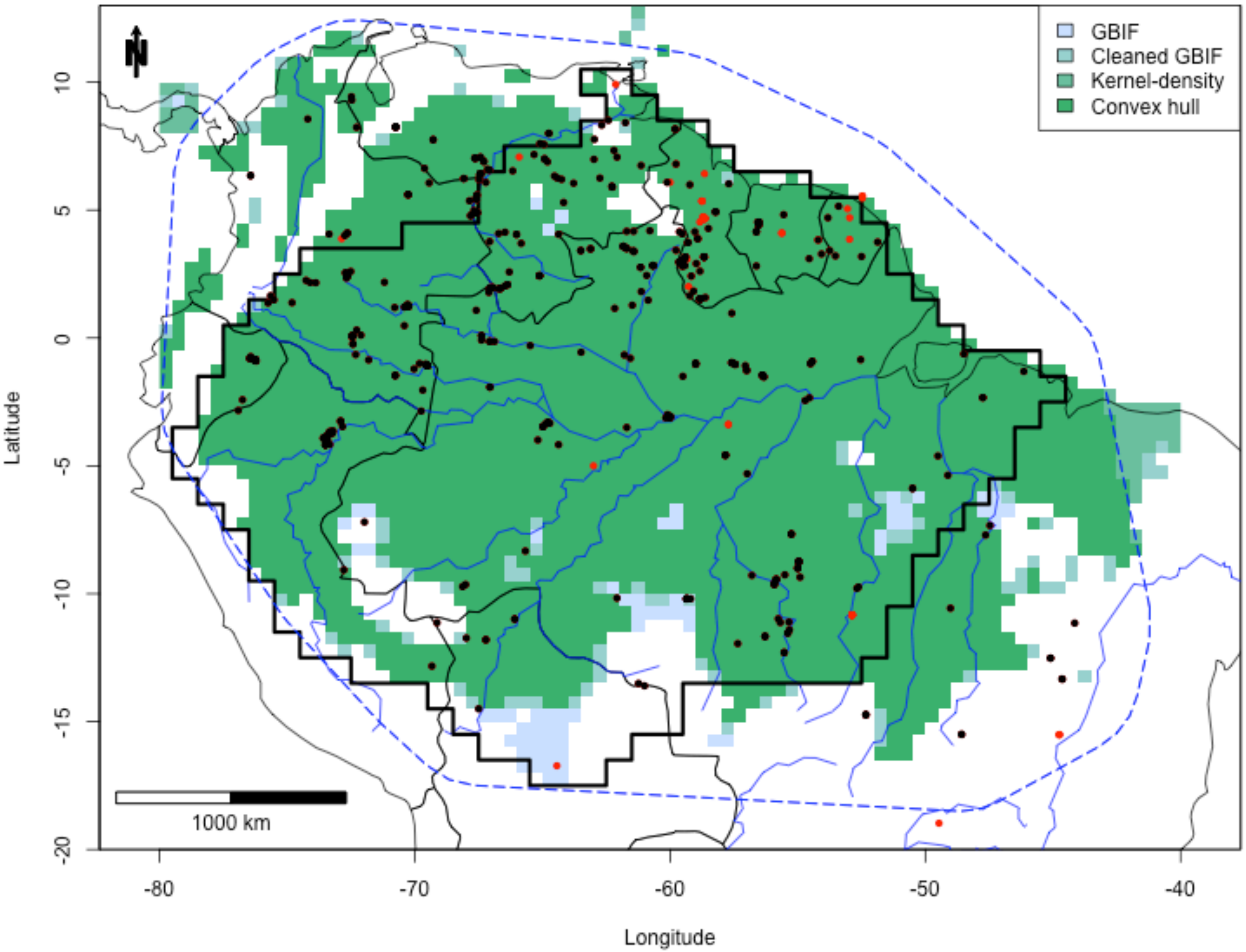

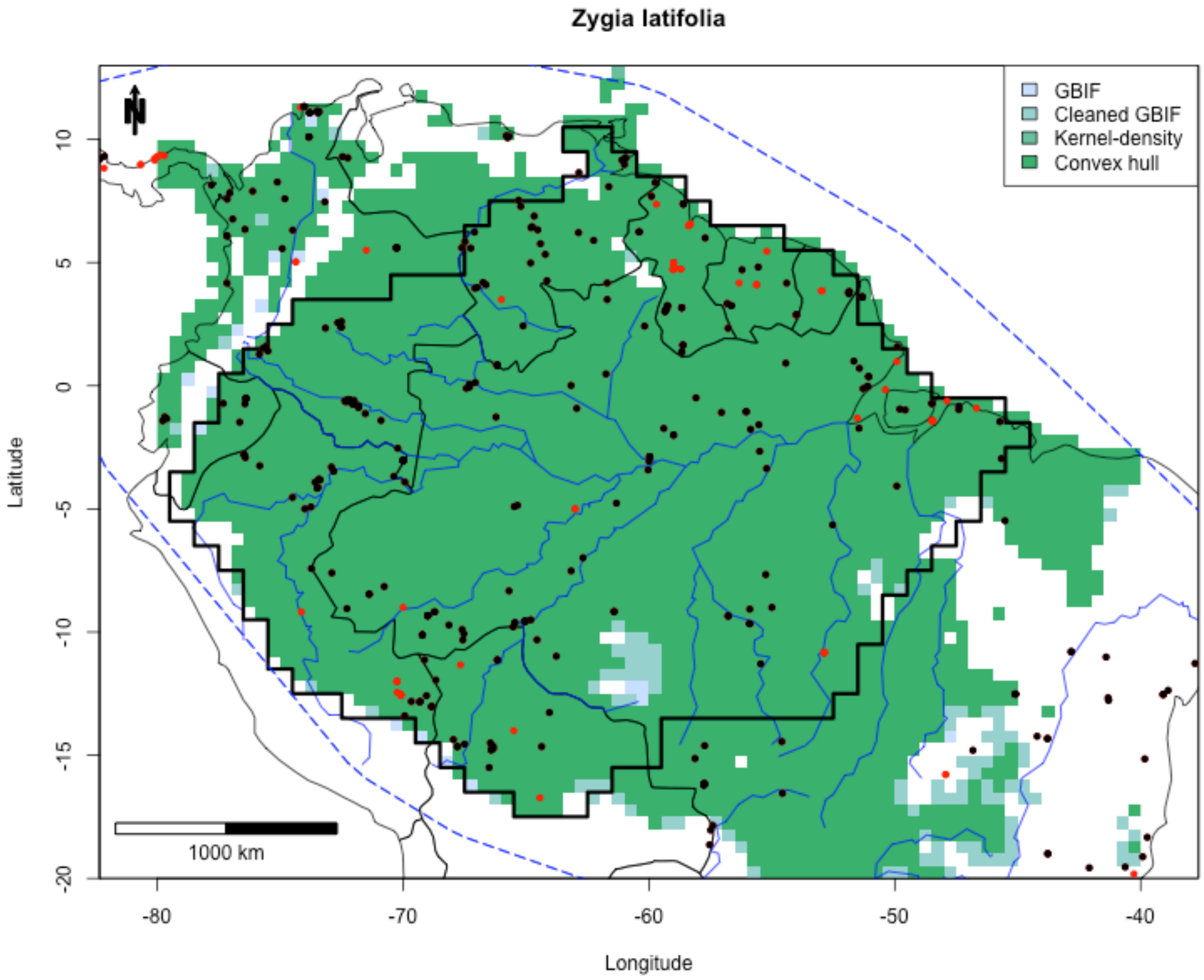

Zygia latifolia
GBIF
Cleaned GBIF
Kernel-density
Convex hull
1000 km
Latitude
Longitude
10
5
0
-5
-10
-15
-20
-80
-70
-60
-50
-40

Zygia racemosa

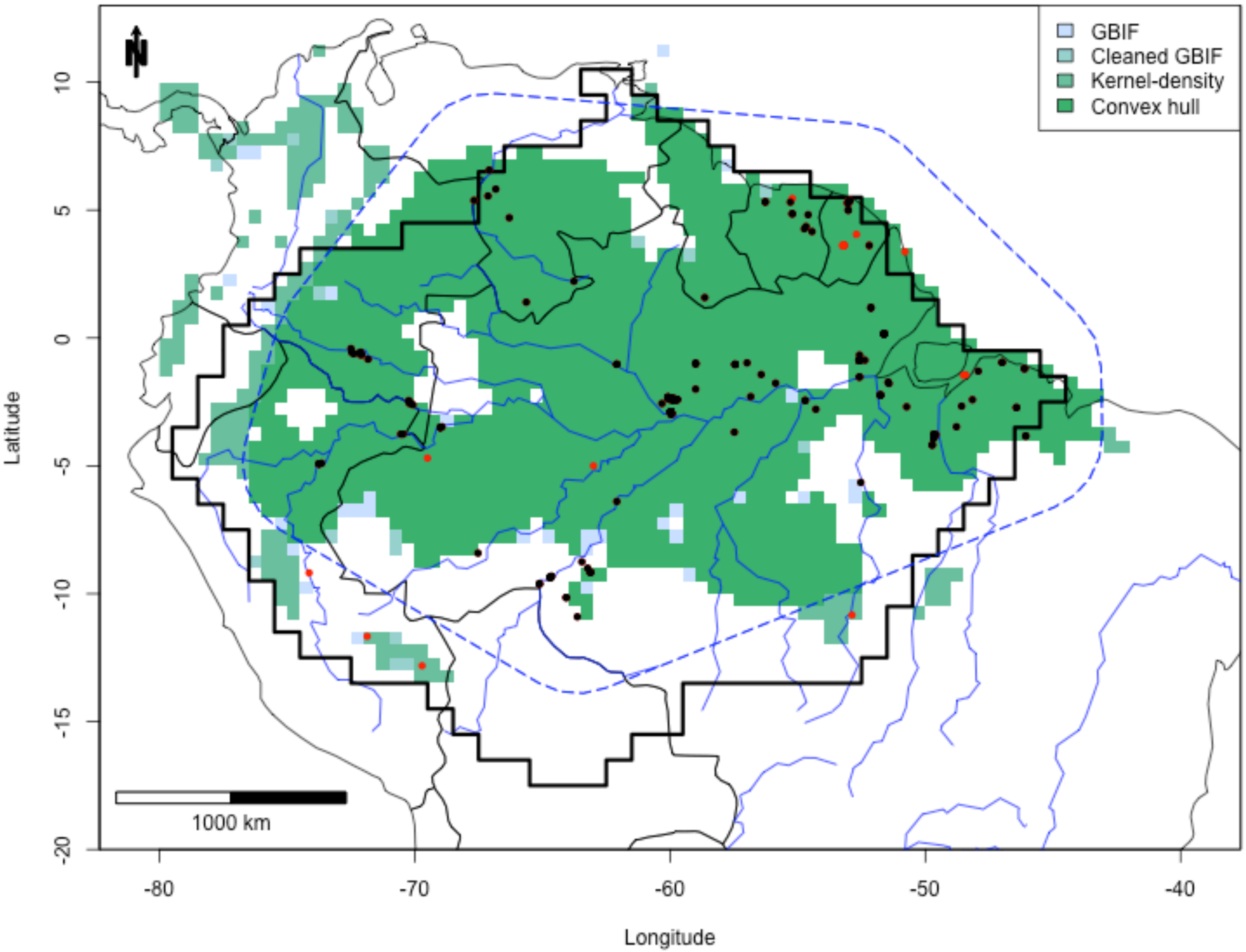

Supplement: Supplementary file 5 — Area of occupancy predicted by MaxEnt for each step of the modelling pipeline. [file 41598_2017_18927_MOESM5_ESM.pdf]
