# Supplementary material for: Species Distribution Modelling: Contrasting presence-only models with plot abundance data
Source: Sci Rep. 2018 Jan 17;8:1003. doi: 10.1038/s41598-017-18927-1 (PMC5772443; doi:10.1038/s41598-017-18927-1)

Supplemental Material

for

Species Distribution Modelling: Contrasting presence-only models with plot abundance data

Vitor F. Gomes*, Stéphanie D. IJff, Niels Raes, Iêda Leão Amaral, Rafael P. Salomão, Luiz de Souza Coelho, Francisca Dionízia de Almeida Matos, Carolina V. Castilho, Diogenes de Andrade Lima Filho, Dairon Cárdenas López, Juan Ernesto Guevara, William E. Magnusson, Oliver L. Phillips, Florian Wittmann, Marcelo de Jesus Veiga Carim, Maria Pires Martins, Mariana Victória Irume, Daniel Sabatier, Jean-François Molino, Olaf S. Bánki, José Renan da Silva Guimarães, Nigel C.A. Pitman, Maria Teresa Fernandez Piedade, Abel Monteagudo Mendoza, Bruno Garcia Luize, Eduardo Martins Venticinque, Evlyn Márcia Moraes de Leão Novo, Percy Núñez Vargas, Thiago Sanna Freire Silva, Angelo Gilberto Manzatto, John Terborgh, Neidiane Farias Costa Reis, Juan Carlos Montero, Katia Regina Casula, Beatriz S. Marimon, Ben-Hur Marimon Jr., Euridice N. Honorio Coronado, Ted R. Feldpausch, Alvaro Duque, Charles Eugene Zartman, Nicolás Castaño Arboleda, Timothy J. Killeen, Bonifacio Mostacedo, Rodolfo Vasquez, Jochen Schöngart, Rafael L. Assis, Marcelo Brilhante Medeiros, Marcelo Fragomeni Simon, Ana Andrade, William F. Laurance, José Luís Camargo, Layon O. Demarchi, Susan G.W. Laurance, Emanuelle de Sousa Farias, Henrique Eduardo Mendonça Nascimento, Juan David Cardenas Revilla, Adriano Quaresma, Flávia R.C. Costa, Ima Célia Guimarães Vieira, Bruno Barçante Ladvocat Cintra, Hernán Castellanos, Roel Brienen, Pablo R. Stevenson, Yuri Feitosa, Joost F. Duivenvoorden, Gerardo A. Aymard C., Hugo F. Mogollón, Natalia Targhetta, James A. Comiskey, Alberto Vicentini, Aline Lopes, Gabriel Damasco, Nállarett Dávila, Roosevelt García-Villacorta, Carolina Levis, Juliana Schietti, Priscila Souza, Thaise Emilio, Alfonso Alonso, David Neill, Francisco Dallmeier, Leandro Valle Ferreira, Alejandro Araujo-Murakami, Daniel Praia, Dário Dantas do Amaral, Fernanda Antunes Carvalho, Fernanda Coelho de Souza, Kenneth Feeley, Luzmila Arroyo, Marcelo Petratti Pansonato, Rogerio Gribel, Boris Villa, Juan Carlos Licona,

Paul V.A. Fine, Carlos Cerón, Chris Baraloto, Eliana M. Jimenez, Juliana Stropp, Julien Engel, Julien Engel, Marcos Silveira, Maria Cristina Peñuela Mora, Pascal Petronelli, Paul Maas, Raquel Thomas-Caesar, Terry W. Henkel, Doug Daly, Marcos Ríos Paredes, Tim R. Baker, Alfredo Fuentes, Carlos A. Peres, Jerome Chave, Jose Luis Marcelo Pena, Kyle G. Dexter, Miles R. Silman, Peter Møller Jørgensen, Toby Pennington, Anthony Di Fiore, Fernando Cornejo Valverde, Juan Fernando Phillips, Gonzalo Rivas-Torres, Patricio von Hildebrand, Tinde R. van Andel, Ademir R. Ruschel, Adriana Prieto, Agustín Rudas, Bruce Hoffman, César I.A. Vela, Edelcilio Marques Barbosa, Egleé L. Zent, George Pepe Gallardo Gonzales, Hilda Paulette Dávila Doza, Ires Paula de Andrade Miranda, Jean-Louis Guillaumet, Linder Felipe Mozombite Pinto, Luiz Carlos de Matos Bonates, Natalino Silva, Ricardo Zárate Gómez, Stanford Zent, Therany Gonzales, Vincent A. Vos, Yadvinder Malhi, Alexandre A. Oliveira, Angela Cano, Bianca Weiss Albuquerque, Corine Vriesendorp, Diego Felipe Correa, Emilio Vilanova Torre, Geertje van der Heijden, Hirma Ramirez-Angulo, José Ferreira Ramos, Kenneth R. Young, Maira Rocha, Marcelo Trindade Nascimento, Maria Natalia Umaña Medina, Milton Tirado, Ophelia Wang, Rodrigo Sierra, Armando Torres-Lezama, Casimiro Mendoza, Cid Ferreira, Cláudia Baider, Daniel Villarroel, Henrik Balslev, Italo Mesones, Ligia Estela Urrego Giraldo, Luisa Fernanda Casas, Manuel Augusto Ahuite Reategui, Reynaldo Linares-Palomino, Roderick Zagt, Sasha Cárdenas, William Farfan-Rios, Adeilza Felipe Sampaio, Daniela Pauletto, Elvis H. Valderrama Sandoval, Freddy Ramirez Arevalo, Isau Huamantupa-Chuquimaco, Karina Garcia-Cabrera, Lionel Hernandez, Luis Valenzuela Gamarra, Miguel N. Alexiades, Susamar Pansini, Walter Palacios Cuenca, William Milliken, Joana Ricardo, Gabriela Lopez-Gonzalez, Edwin Pos, Hans ter Steege*

*Correspondence and requests for materials should be addressed to H.T.S. (email: hans.tersteege@naturalis.nl)

Appendix S3: Maps of combined MaxEnt IDW predictions.

Combined MaxEnt's environmental suitability map and relative IDW abundance map for all 170 significant species. **(A)** Combined map of area of occupancy as predicted by MaxEnt and IDW. **(B)** MaxEnt's environmental suitability map. **(C)** Abundance map as predicted by IDW. In this map the size of the black dot represents the relative abundance of the species in the plot. The grey color signifies the interpolated values. Ranges of relative abundance of plots and interpolation are given in the figure title. Maps created with custom R script. Base map source (country.shp, rivers.shp): ESRI (http://www.esri.com/data/basemaps, © Esri, DeLorme Publishing Company).

Figure S3_1A Combined map of Acosmium_cardenasii

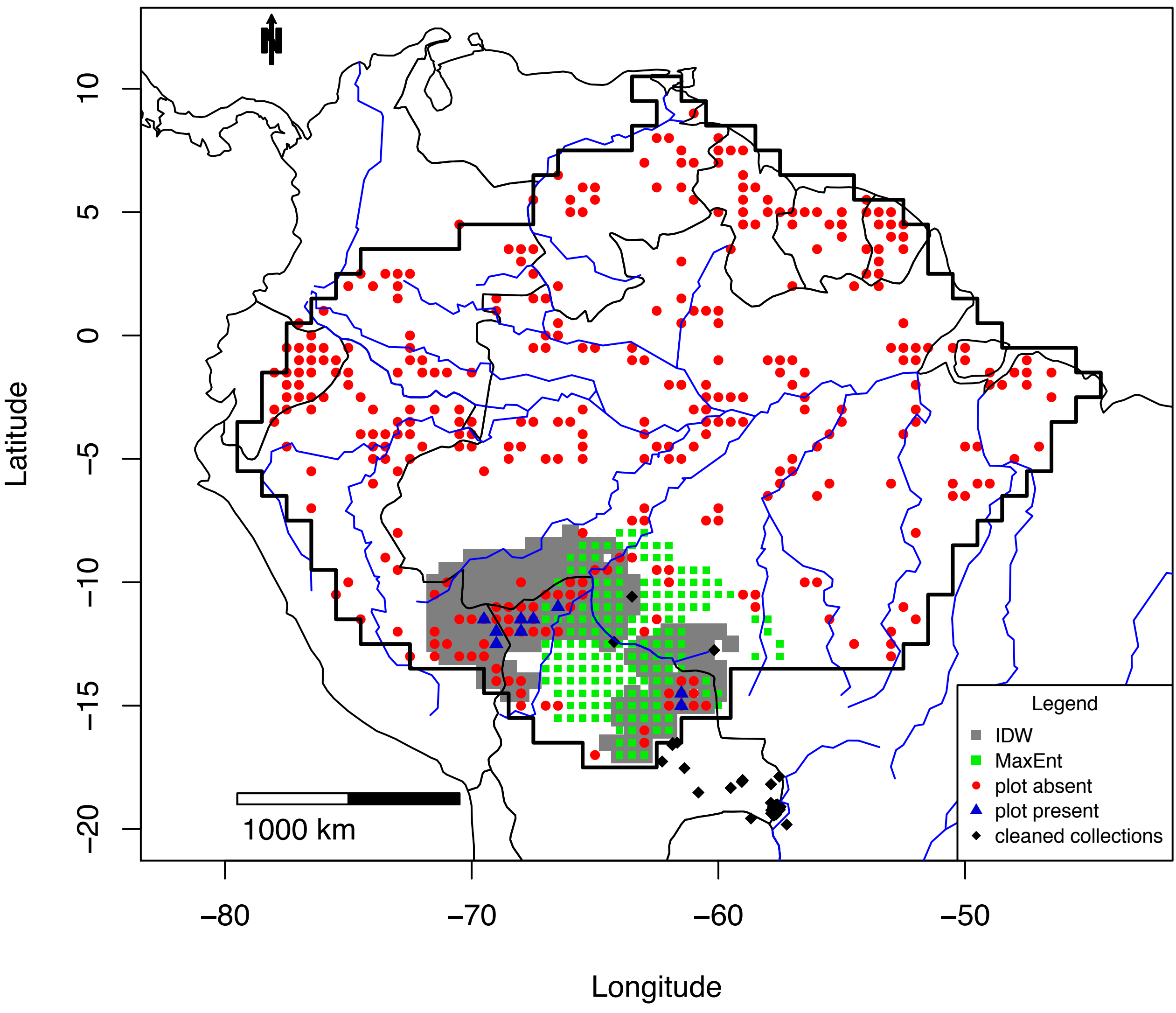

Figure S3_1B MaxEnt map of Acosmium_cardenasii

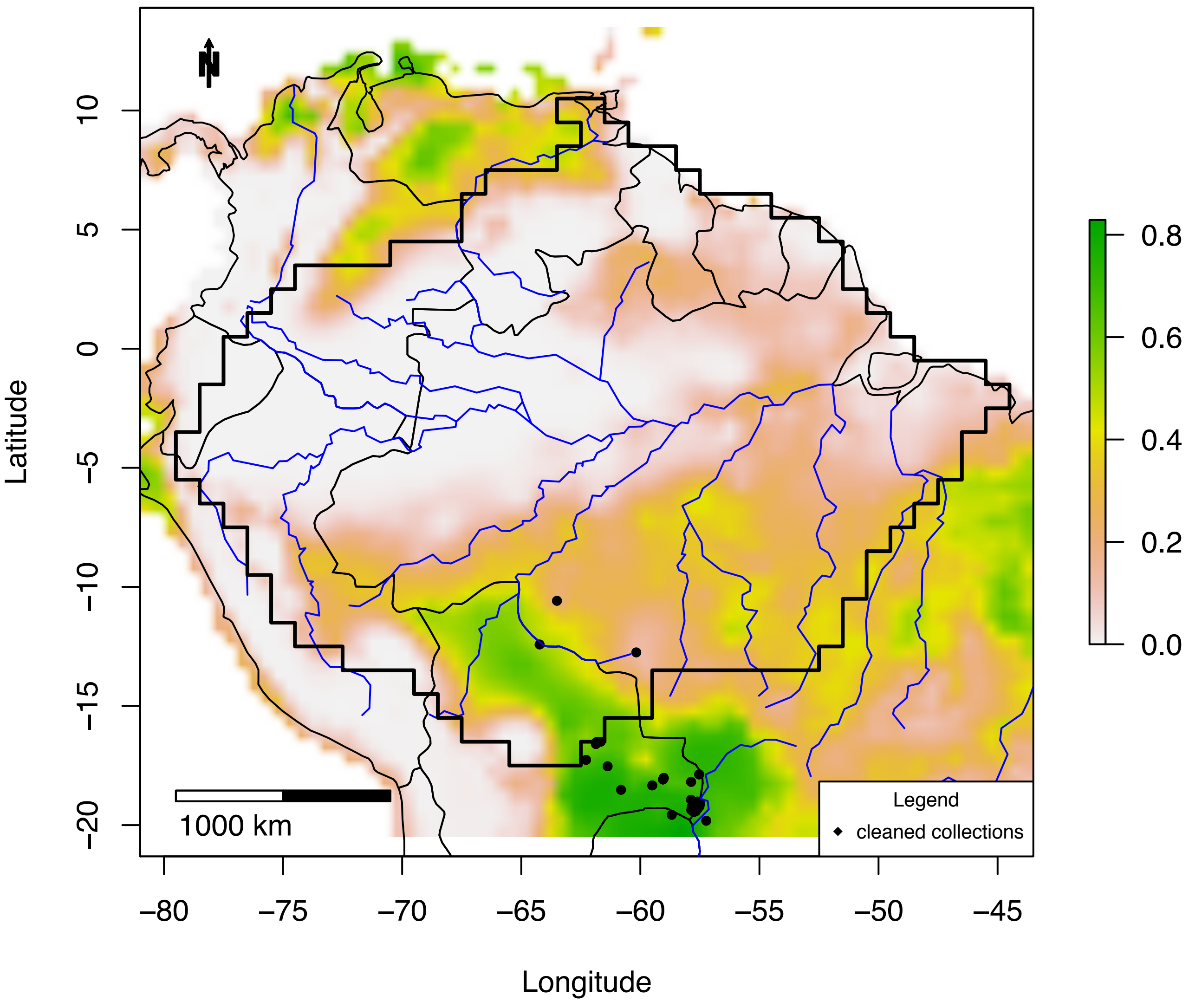

Figure S3_1C IDW map of Acosmium_cardenasii: plots =0 – 0.37; IDW: 0 – 0.27

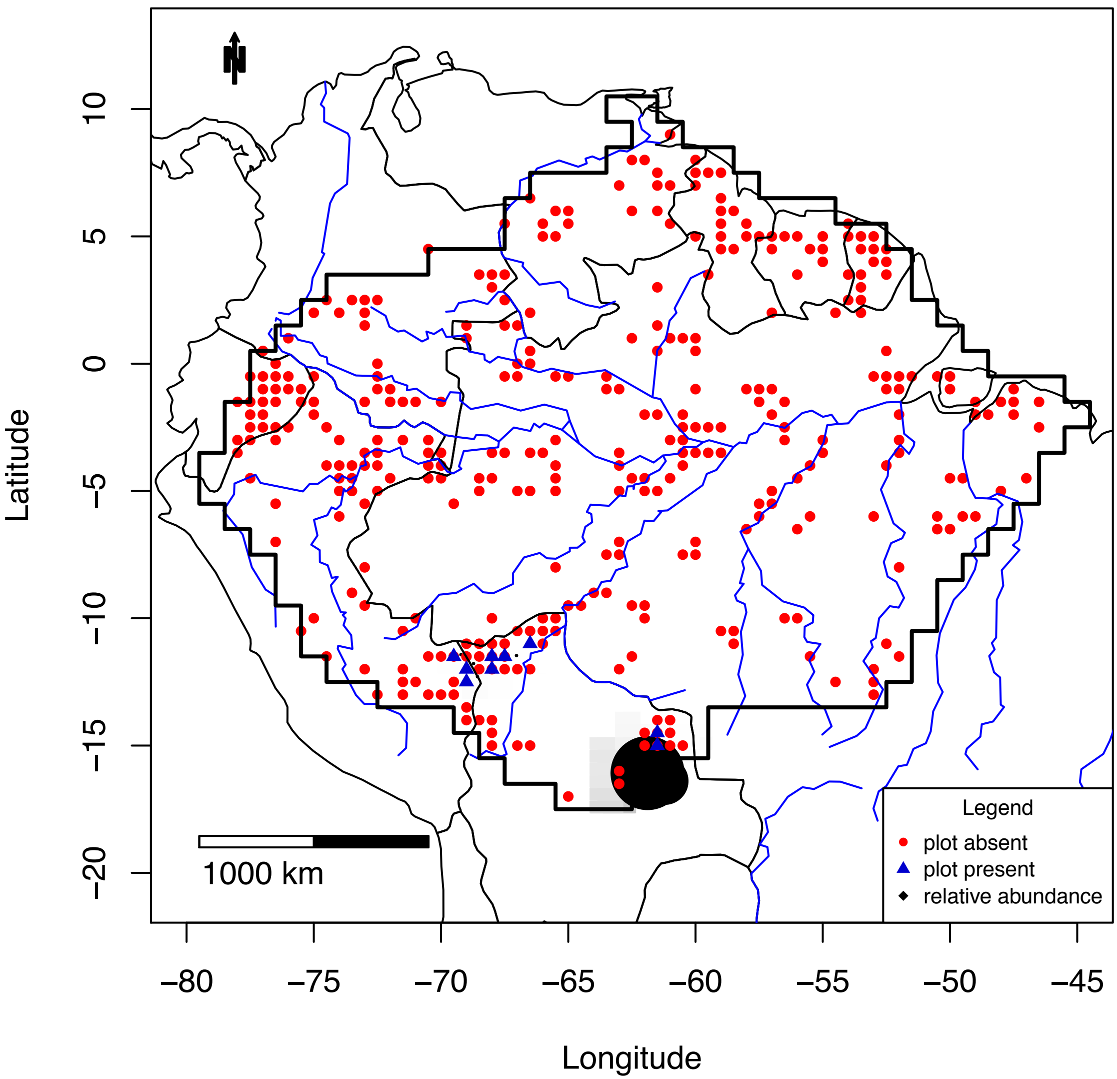

Figure S3_2A Combined map of Alexa_imperatricis

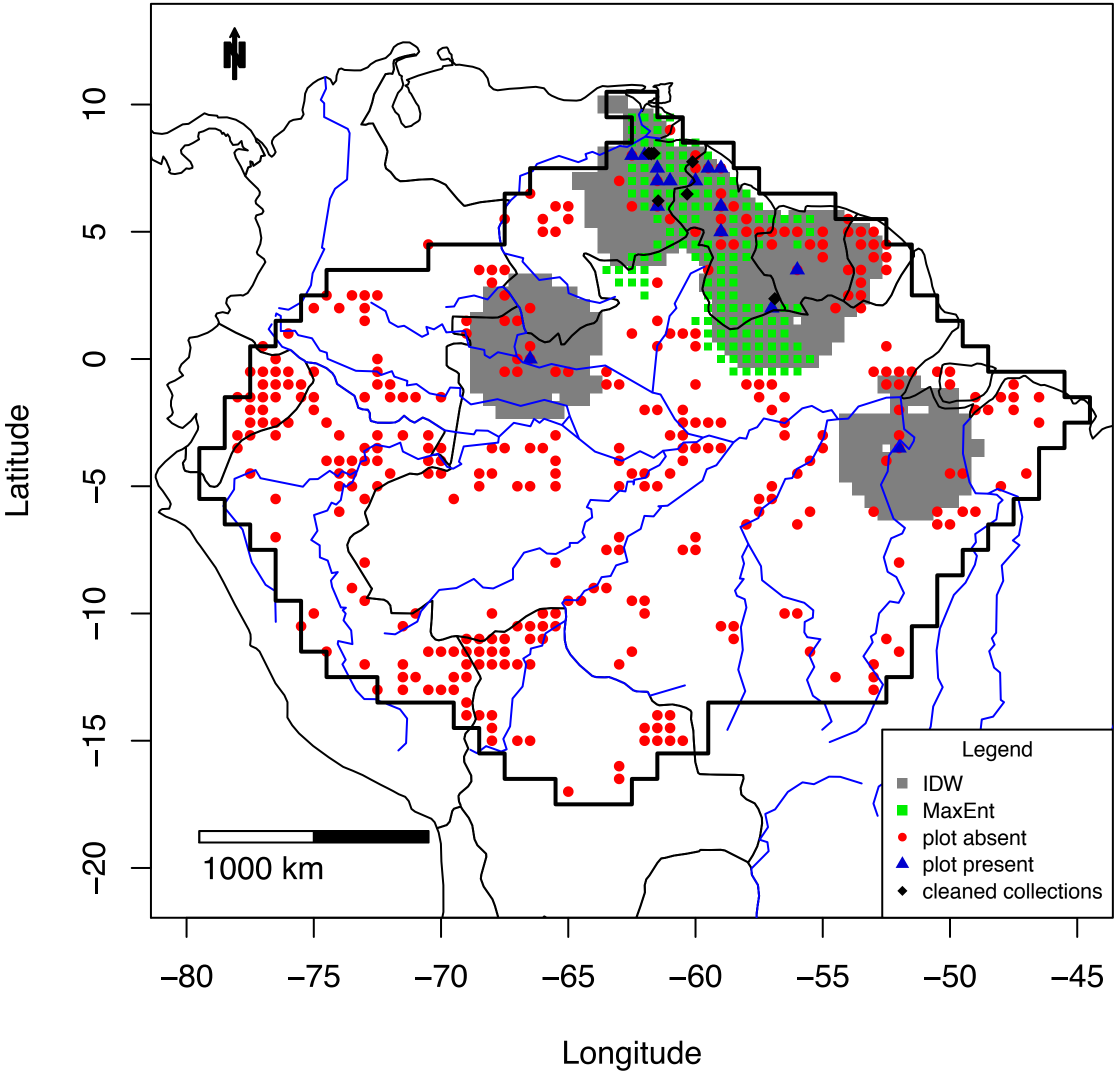

Figure S3_2B MaxEnt map of Alexa_imperatricis

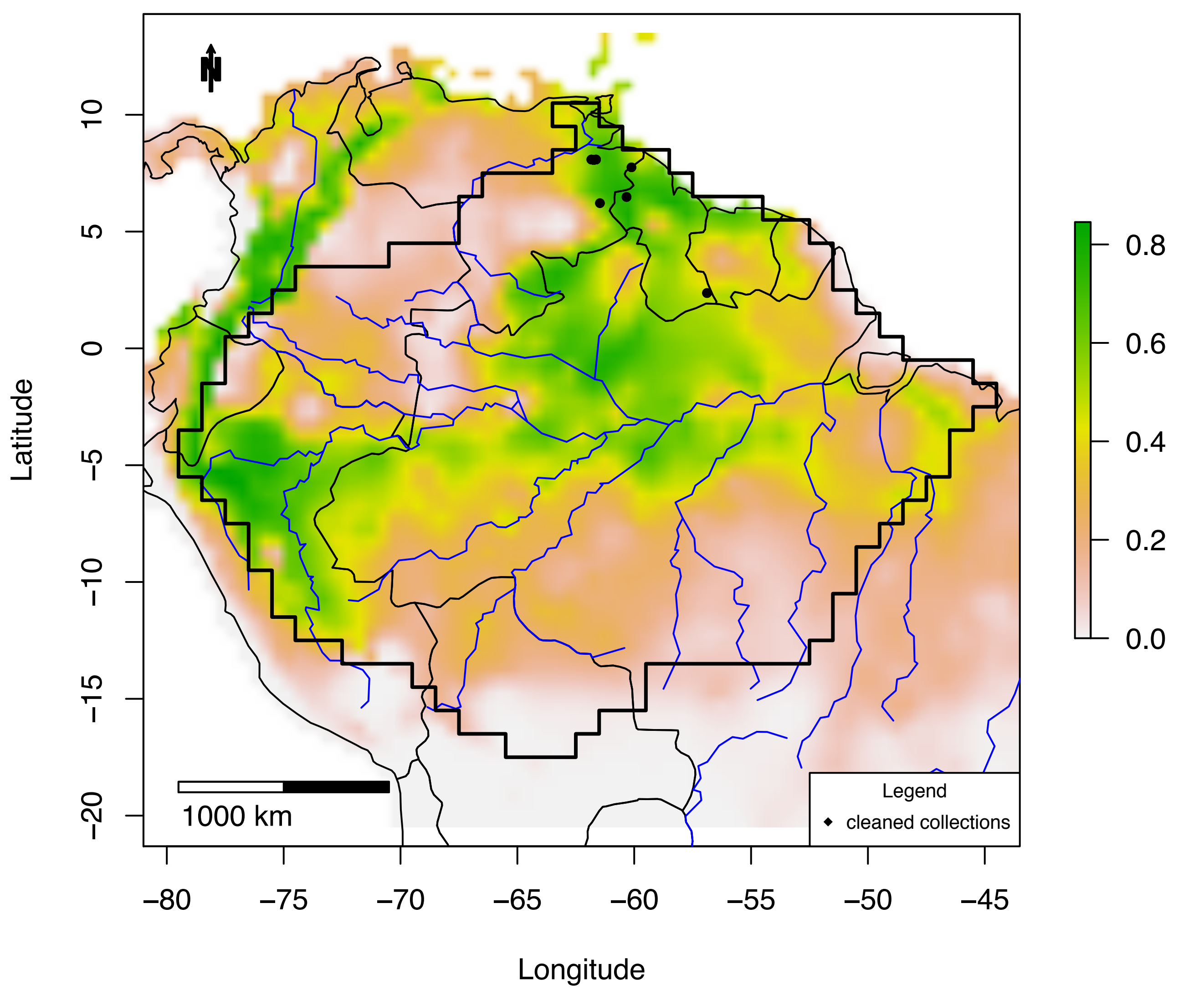

# Figure S3_2C IDW map of Alexa_imperatricis:
plots =0 – 0.29; IDW: 0 – 0.23

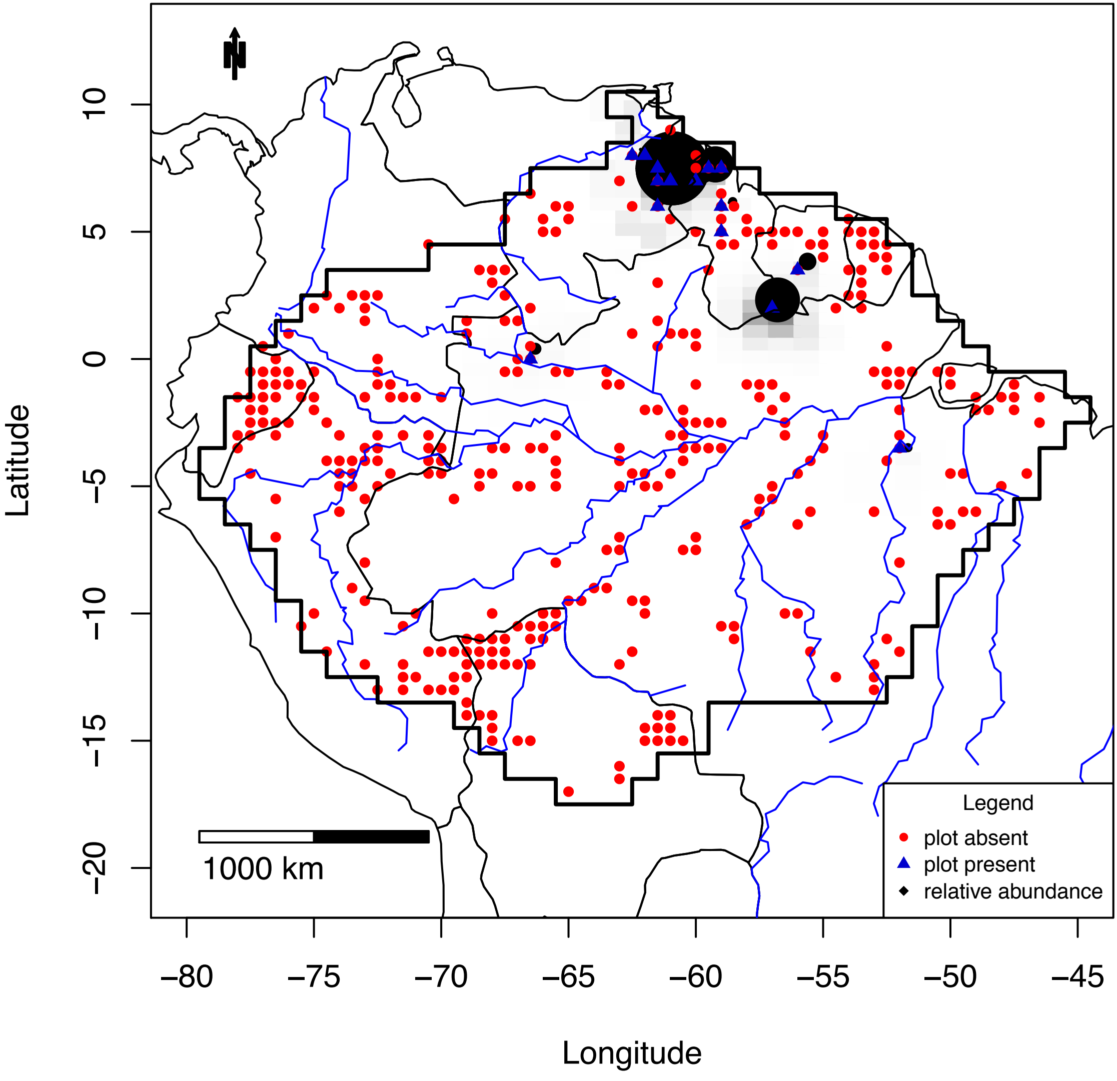

Figure S3_3A Combined map of Amphiodon_effusus

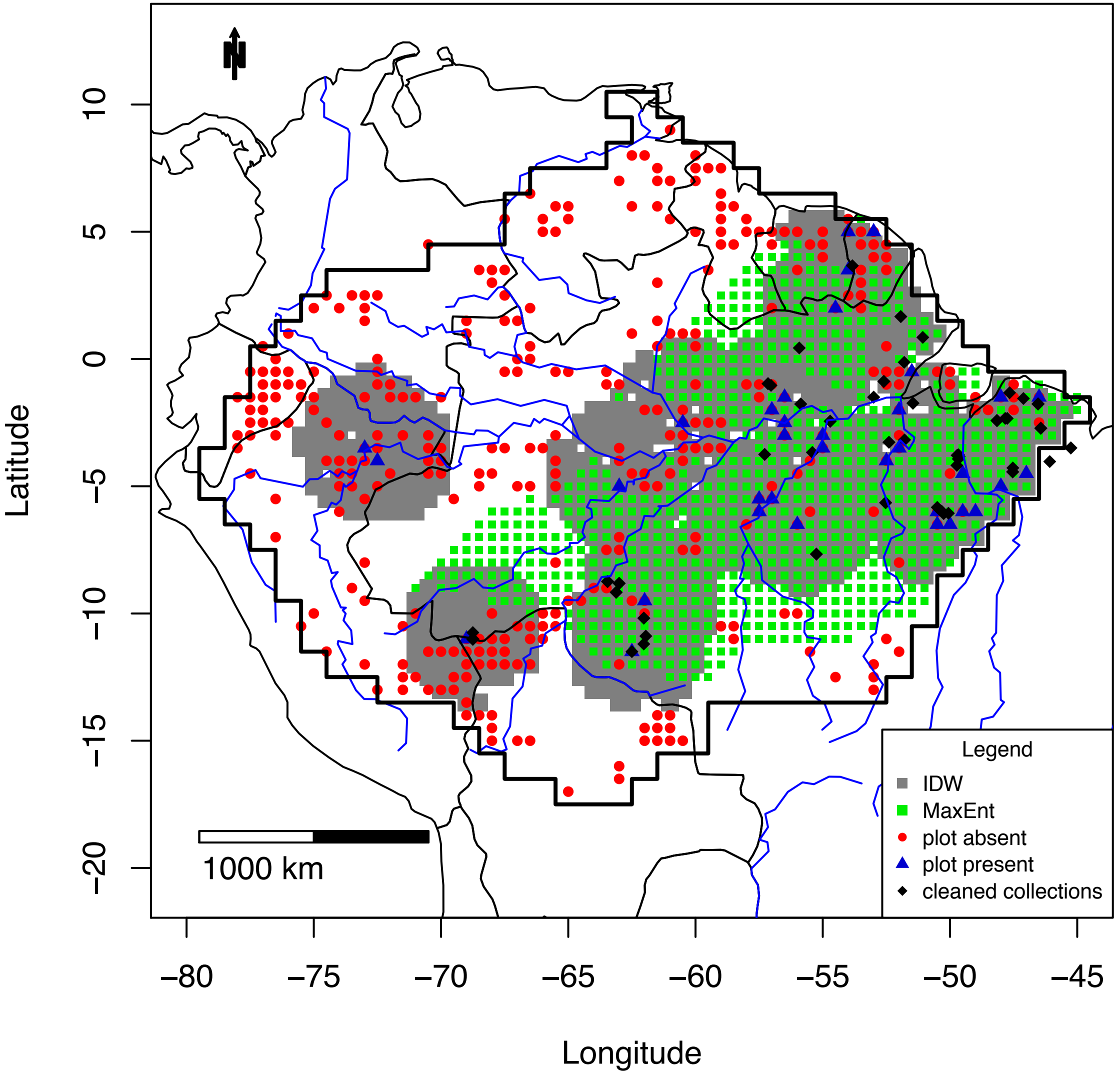

Figure S3_3B MaxEnt map of Amphiodon_effusus

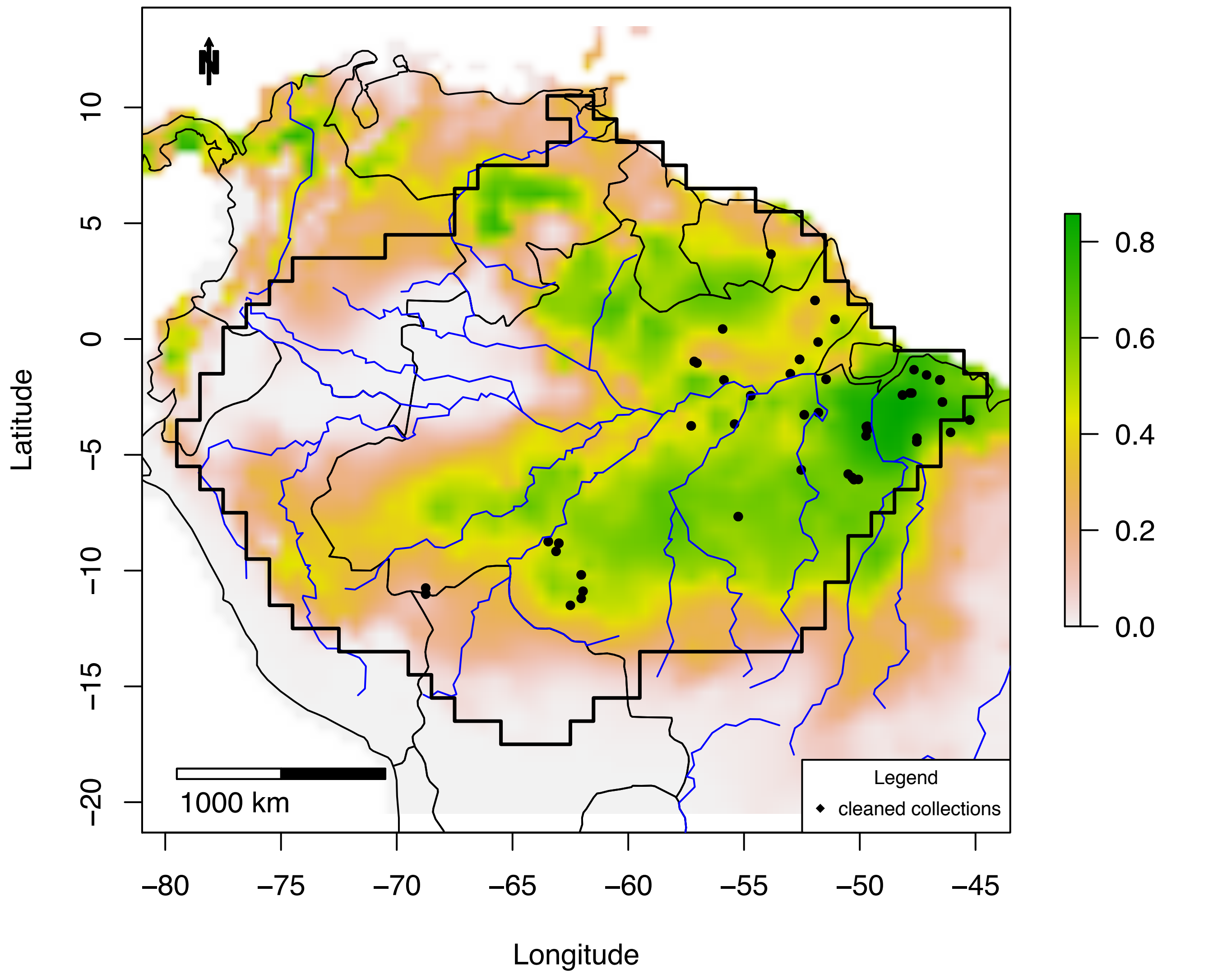

Figure S3_3C IDW map of Amphiodon_effusus: plots =0 – 0.24; IDW: 0 – 0.09

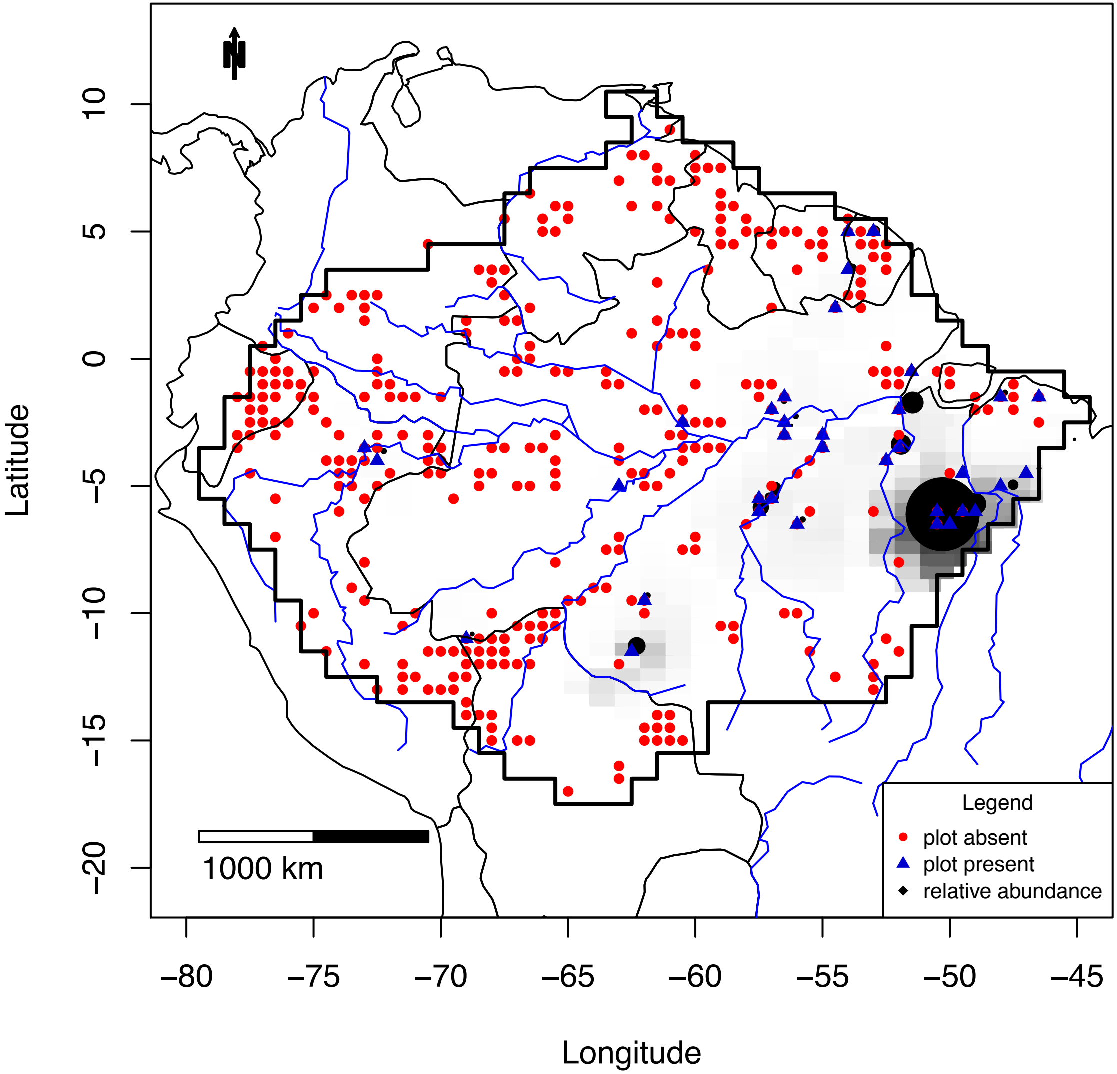

Figure S3_4A Combined map of Apeiba_glabra

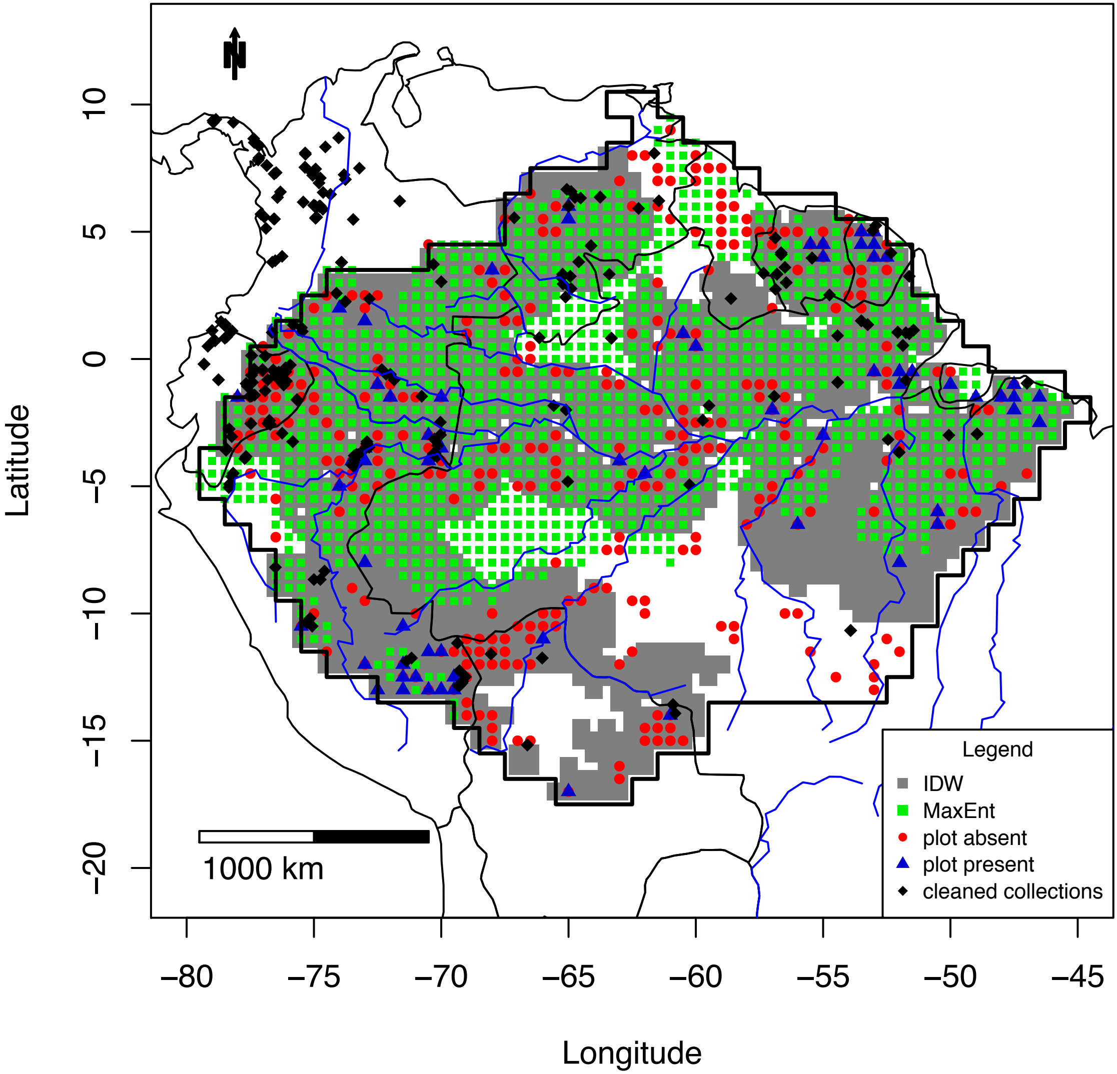

Figure S3_4B MaxEnt map of Apeiba_glabra

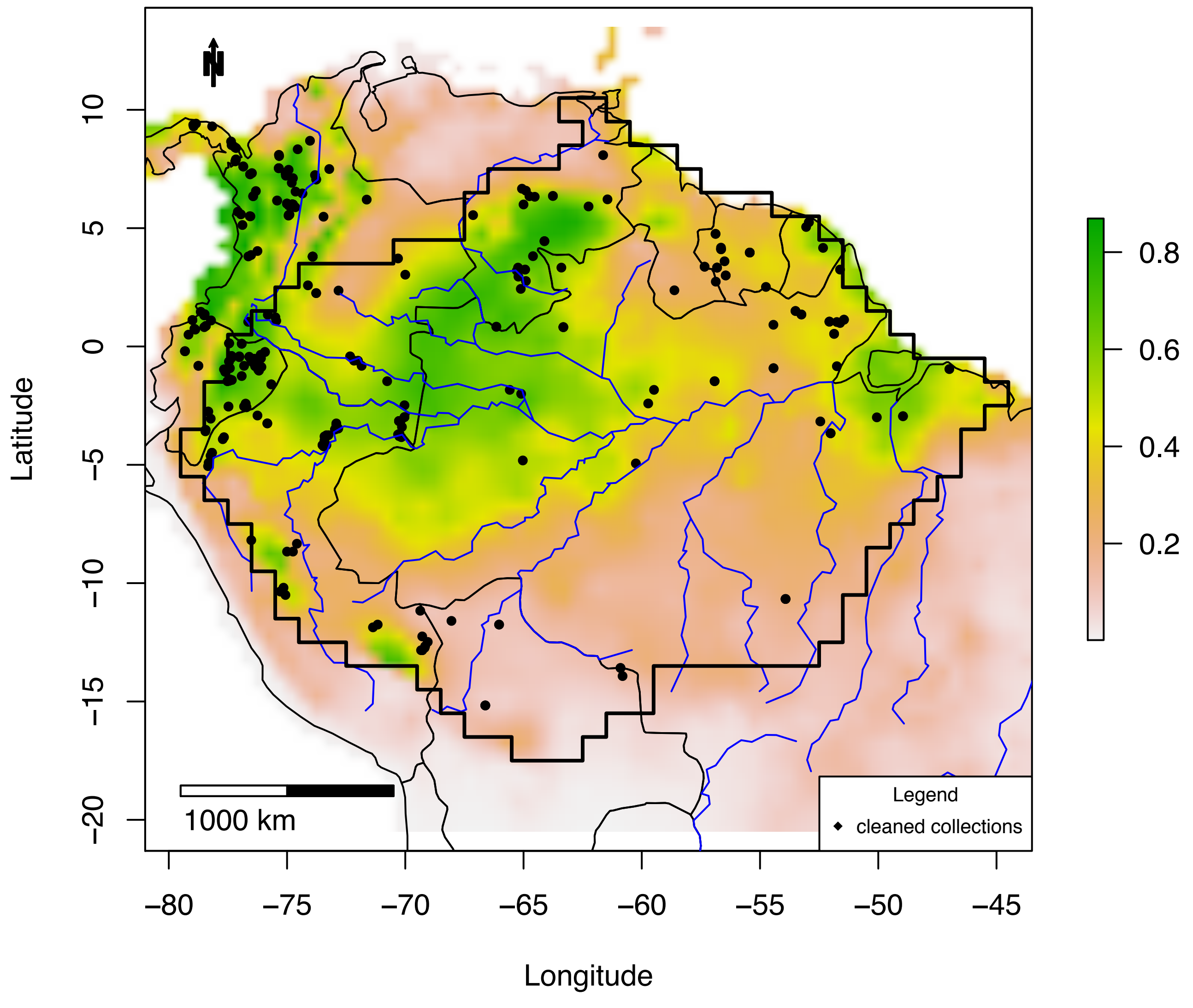

Figure S3_4C IDW map of Apeiba_glabra:
plots =0 – 0.04; IDW: 0 – 0.03

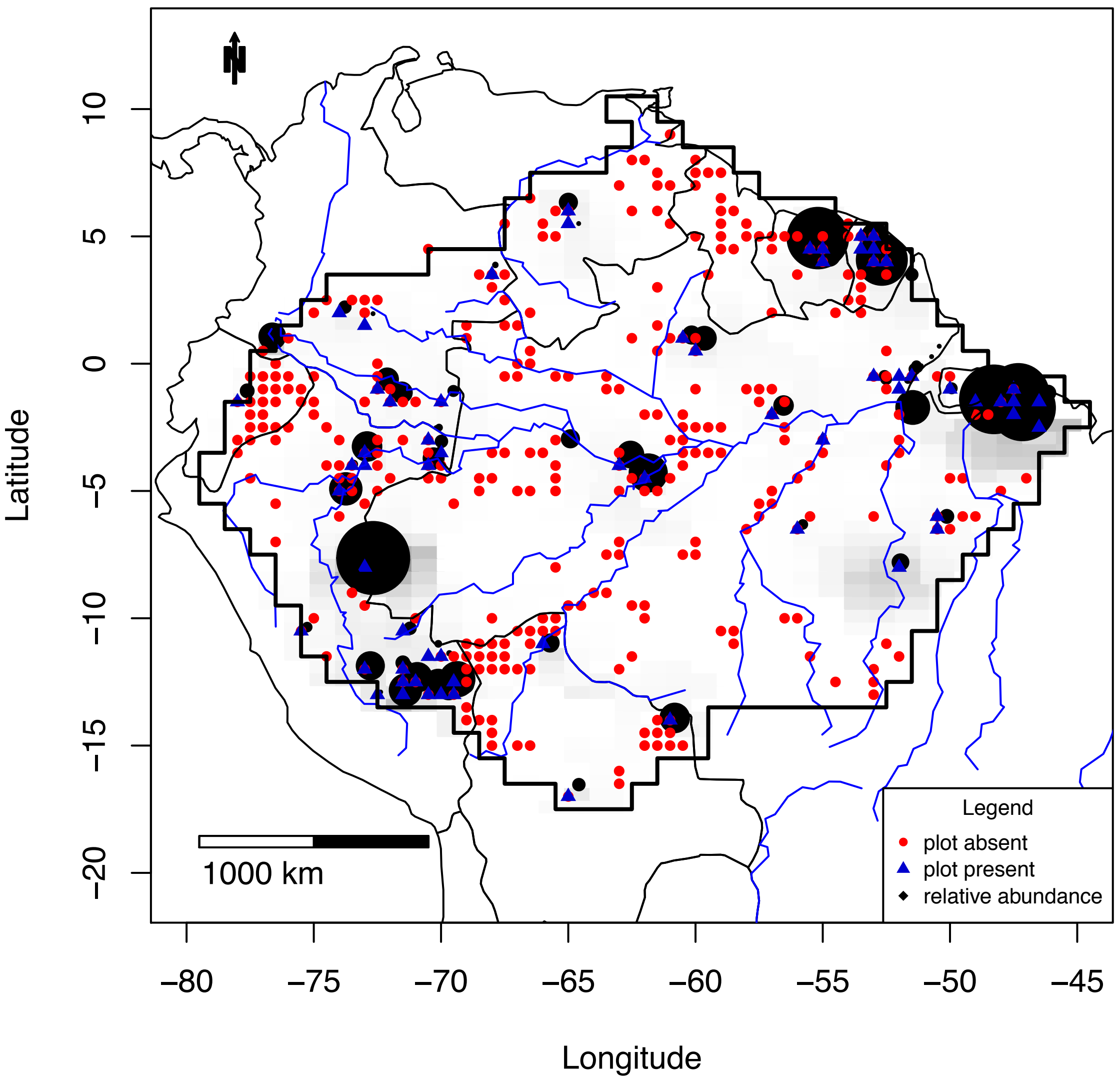

Figure S3_5A Combined map of Aspidosperma_excelsum

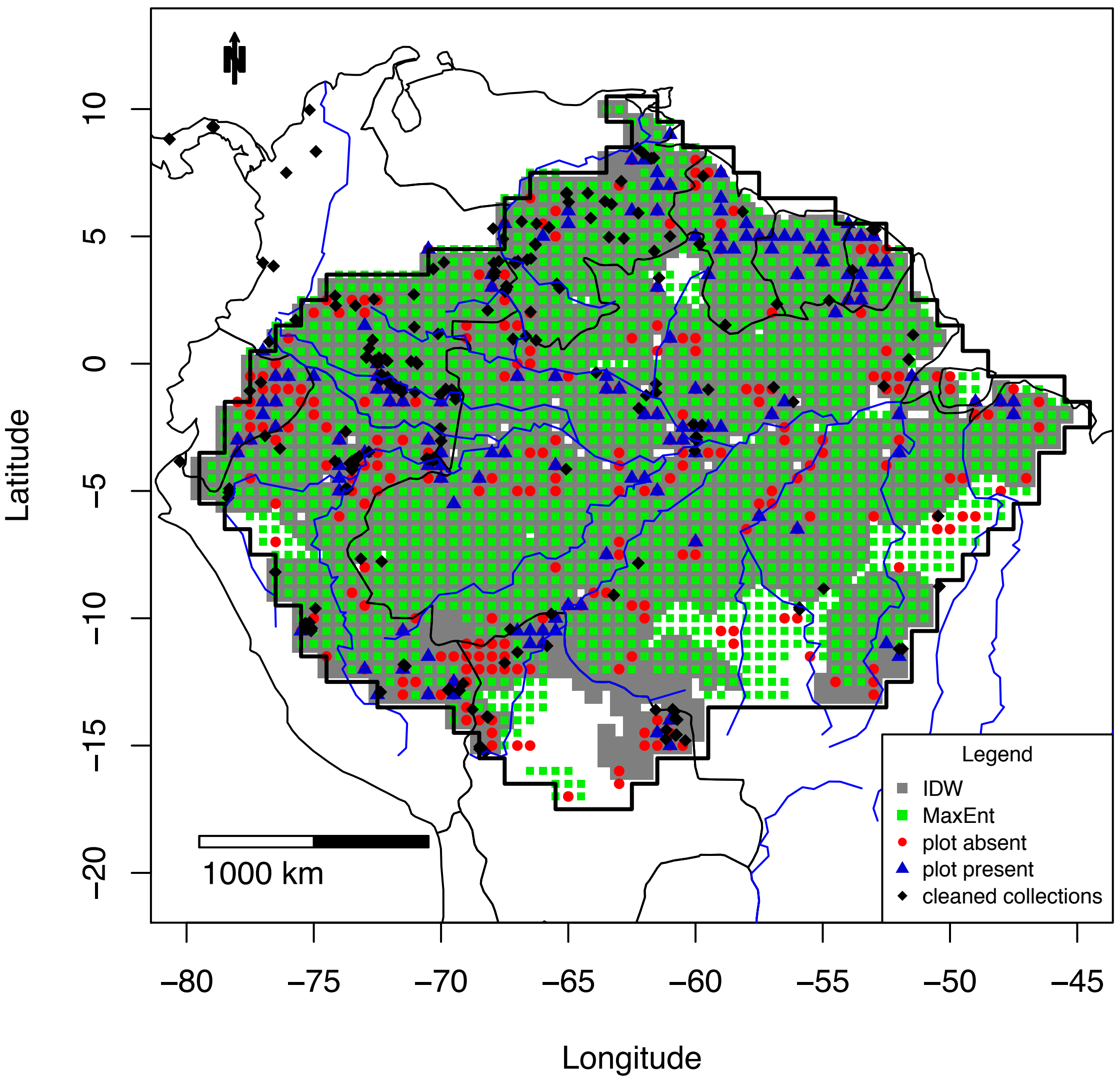

Figure S3_5B MaxEnt map of Aspidosperma_excelsum

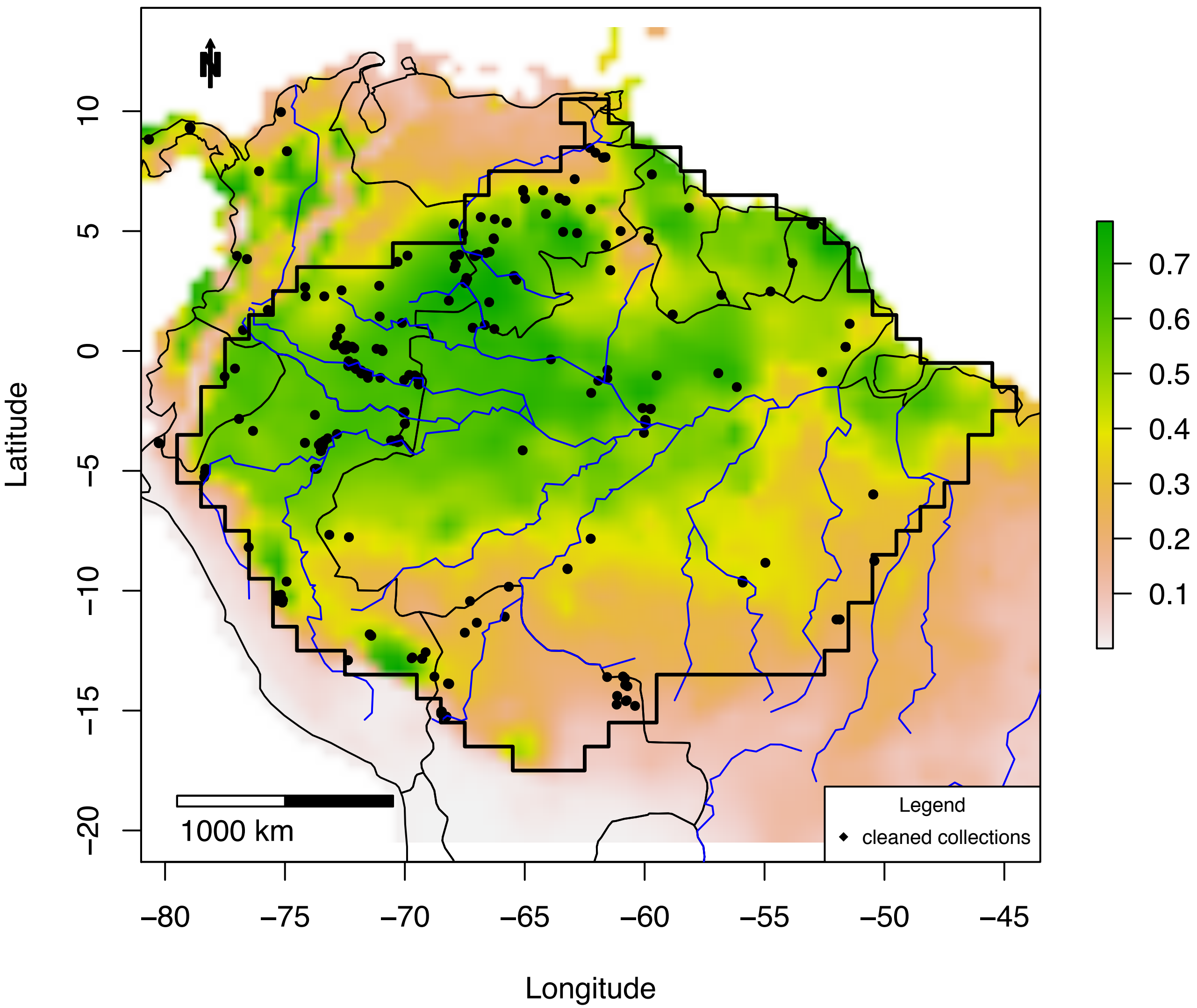

Figure S3_5C IDW map of Aspidosperma_excelsum: plots =0 – 0.28; IDW: 0 – 0.11

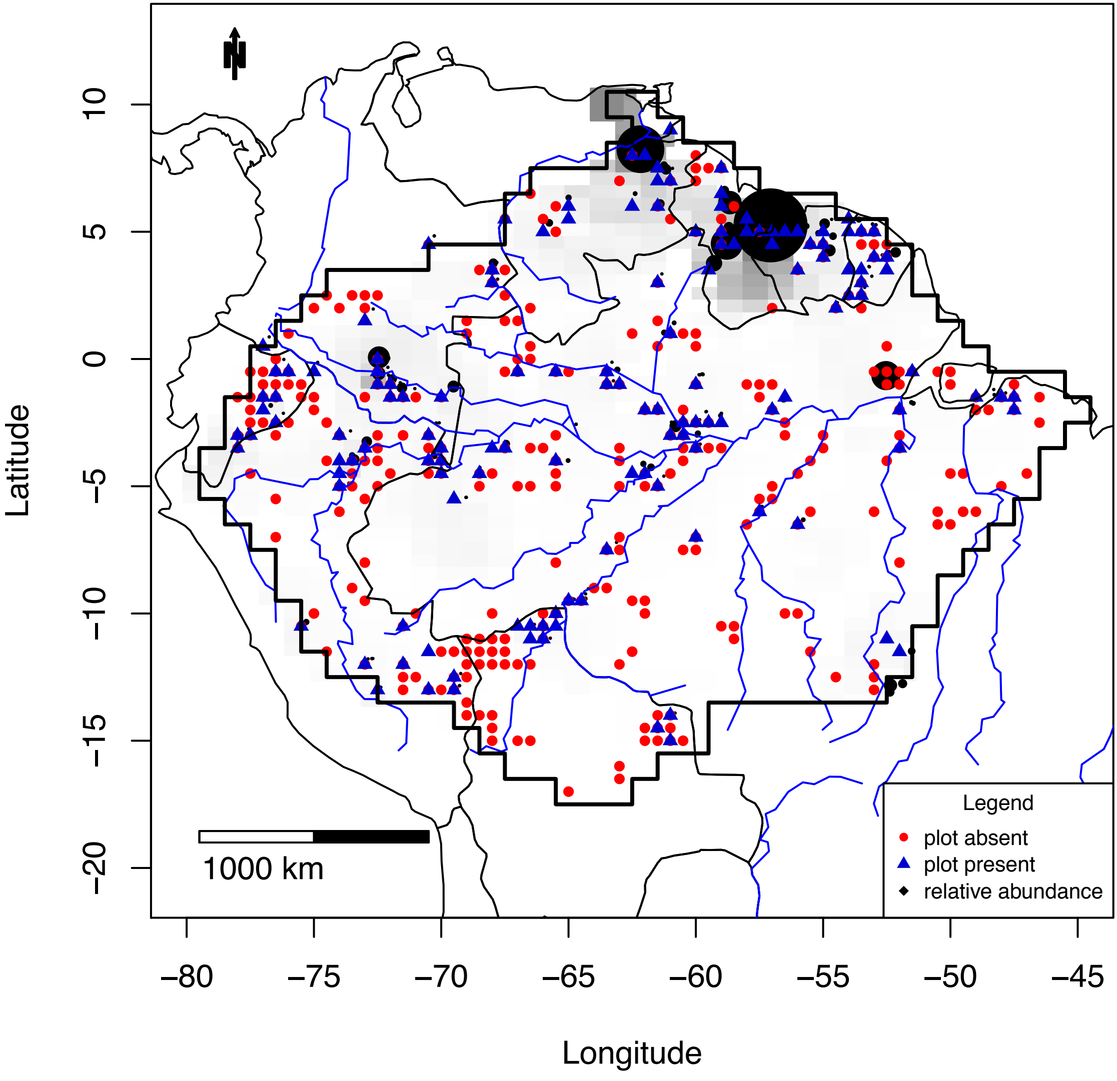

Figure S3_6A Combined map of Astrocaryum_aculeatum

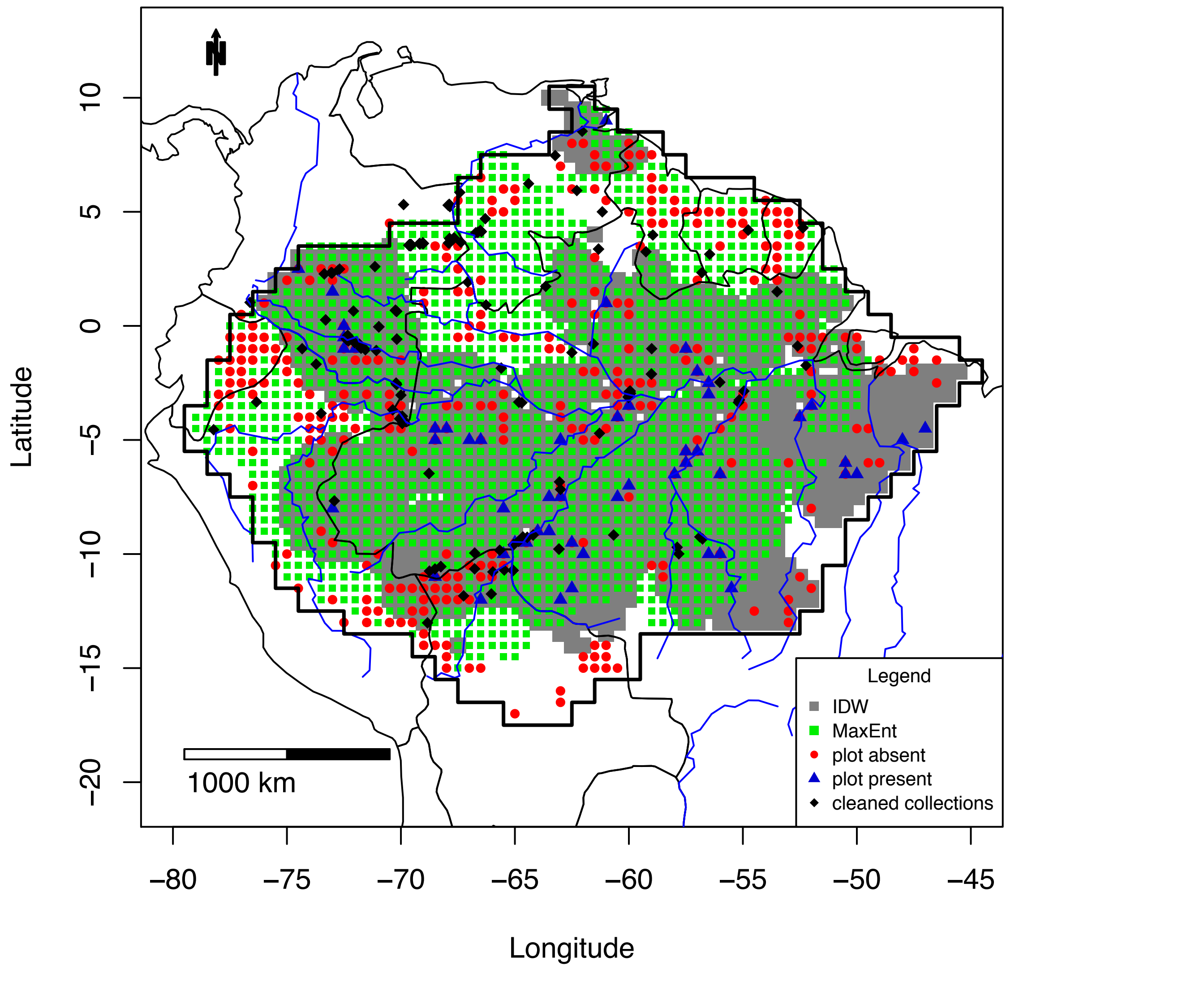

Figure S3_6B MaxEnt map of Astrocaryum_aculeatum

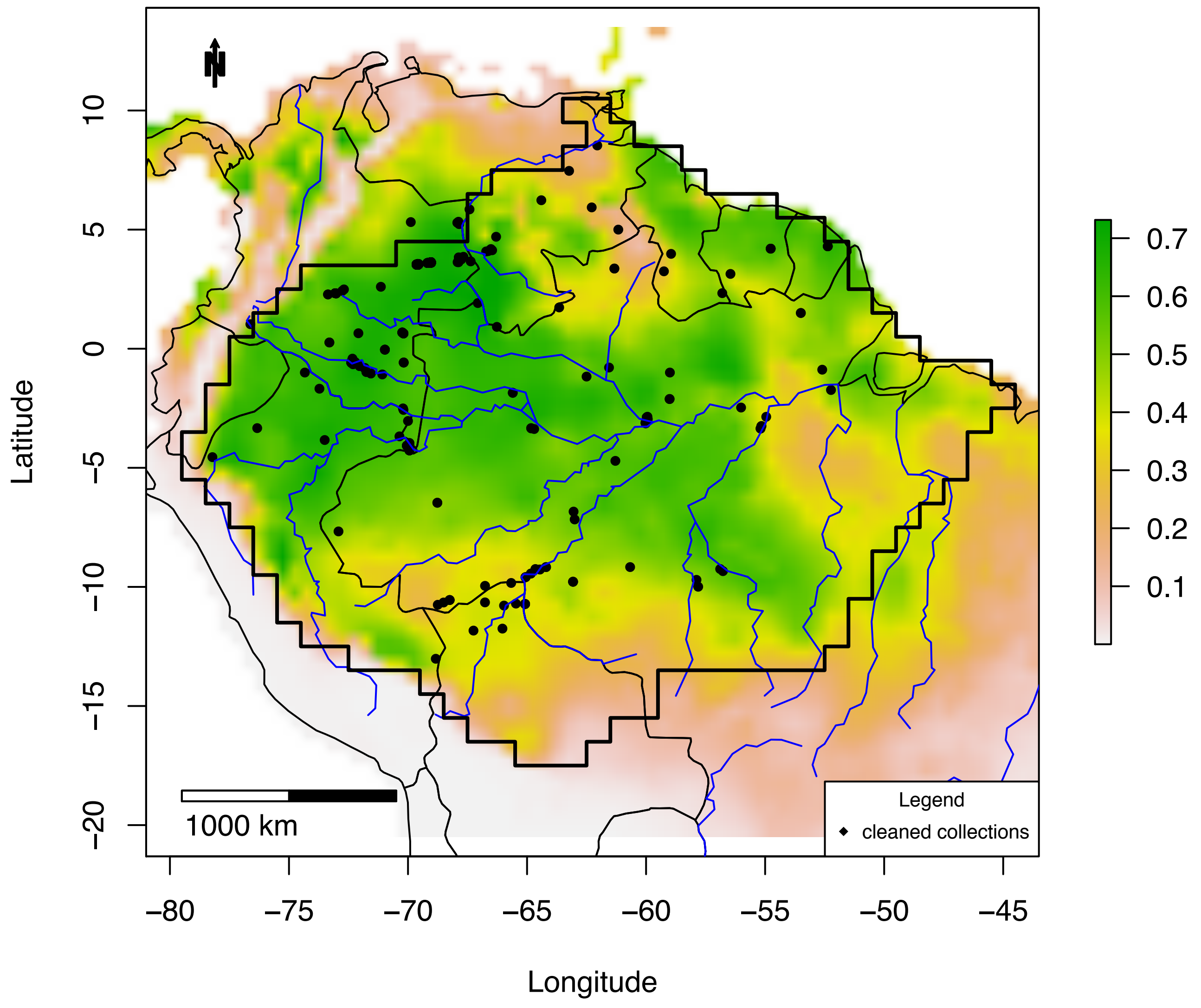

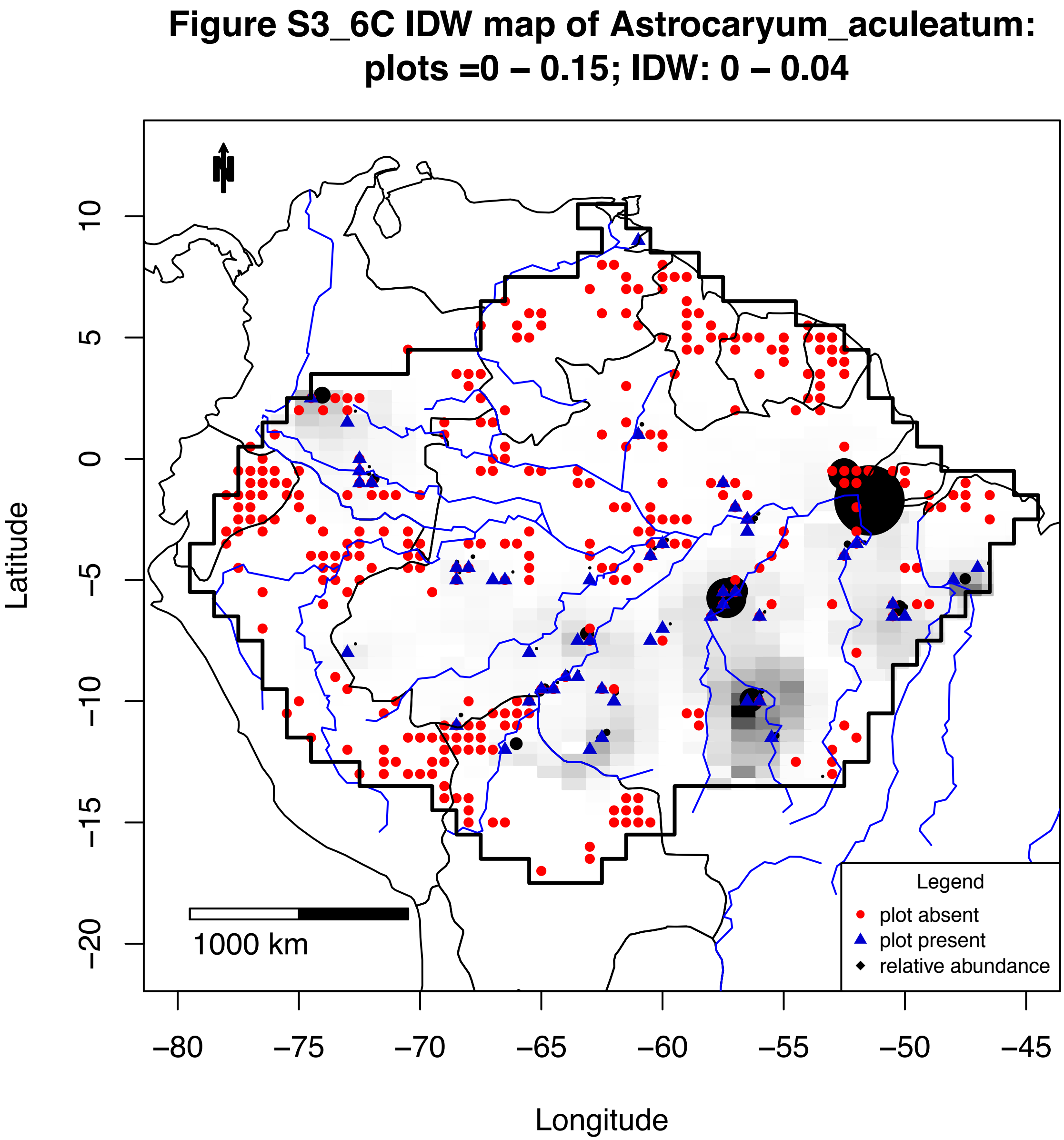
Figure S3_6C IDW map of Astrocaryum_aculeatum:
plots =0 – 0.15; IDW: 0 – 0.04
N
Latitude
10
5
0
−5
−10
−15
−20
Longitude
−80
−75
−70
−65
−60
−55
−50
−45
1000 km
Legend
plot absent
plot present
relative abundance

Figure S3_7A Combined map of Astrocaryum_murumuru

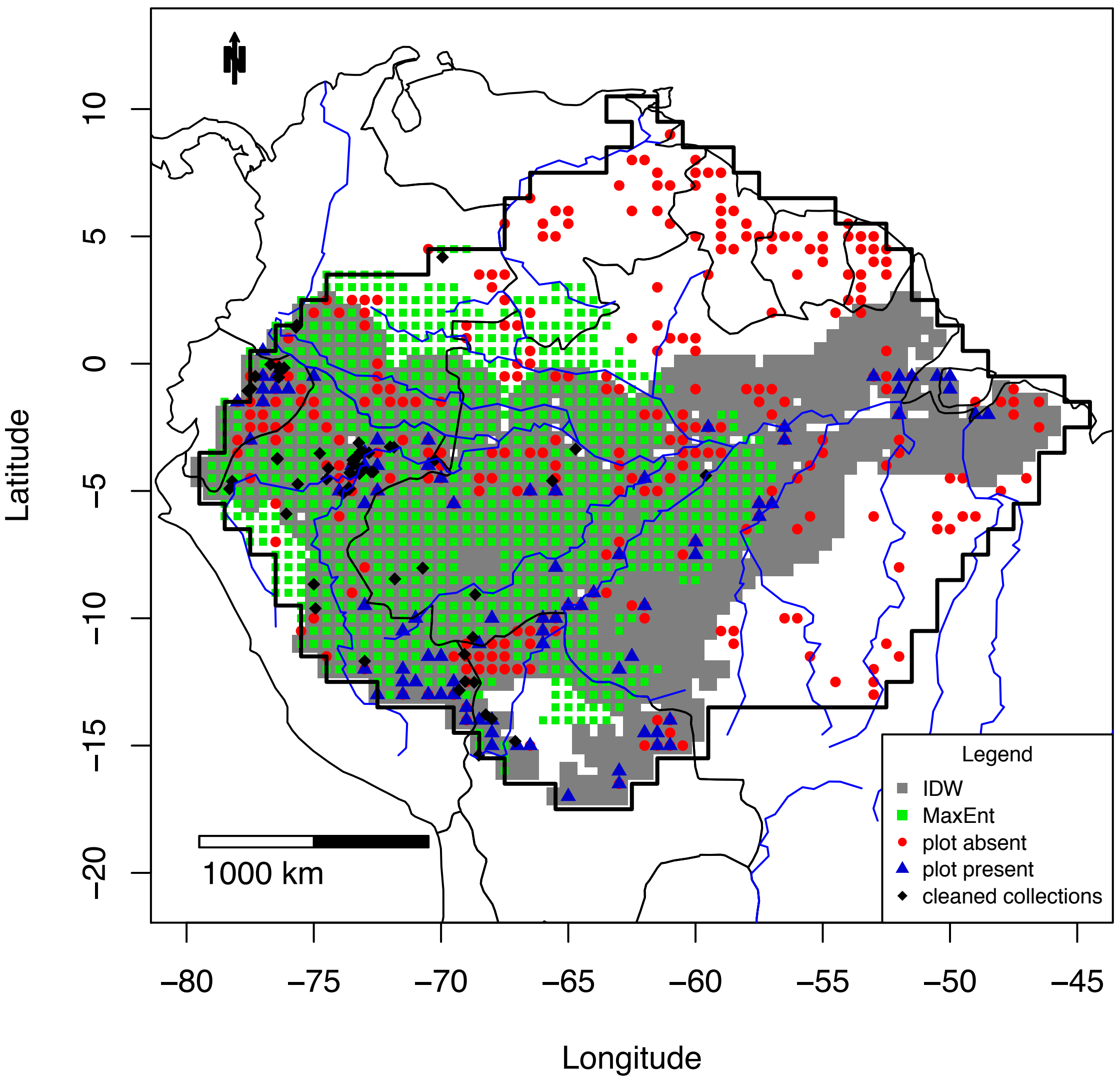

Figure S3_7B MaxEnt map of Astrocaryum_murumuru

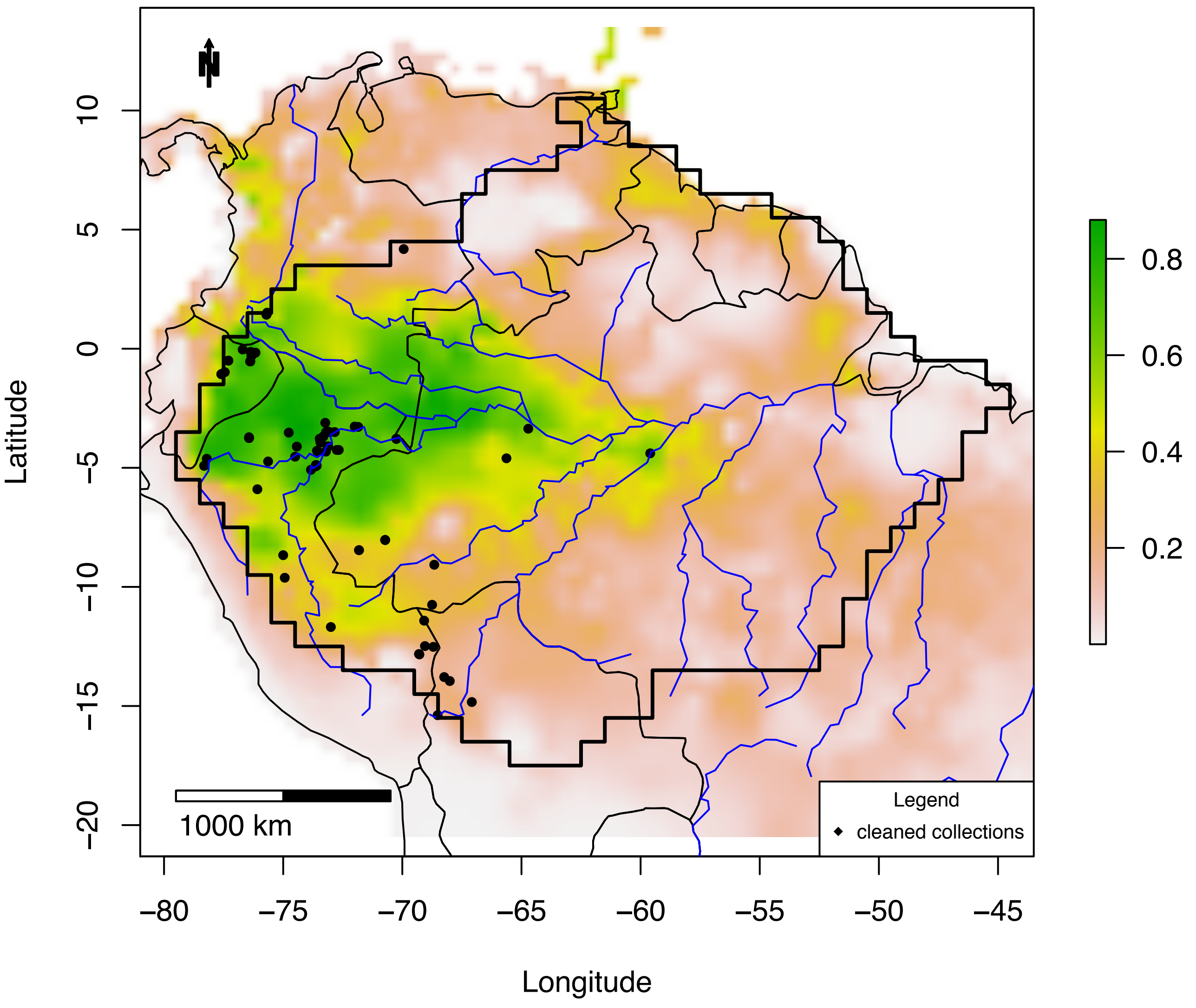

Figure S3_7C IDW map of Astrocaryum_murumuru: plots =0 – 0.52; IDW: 0 – 0.25

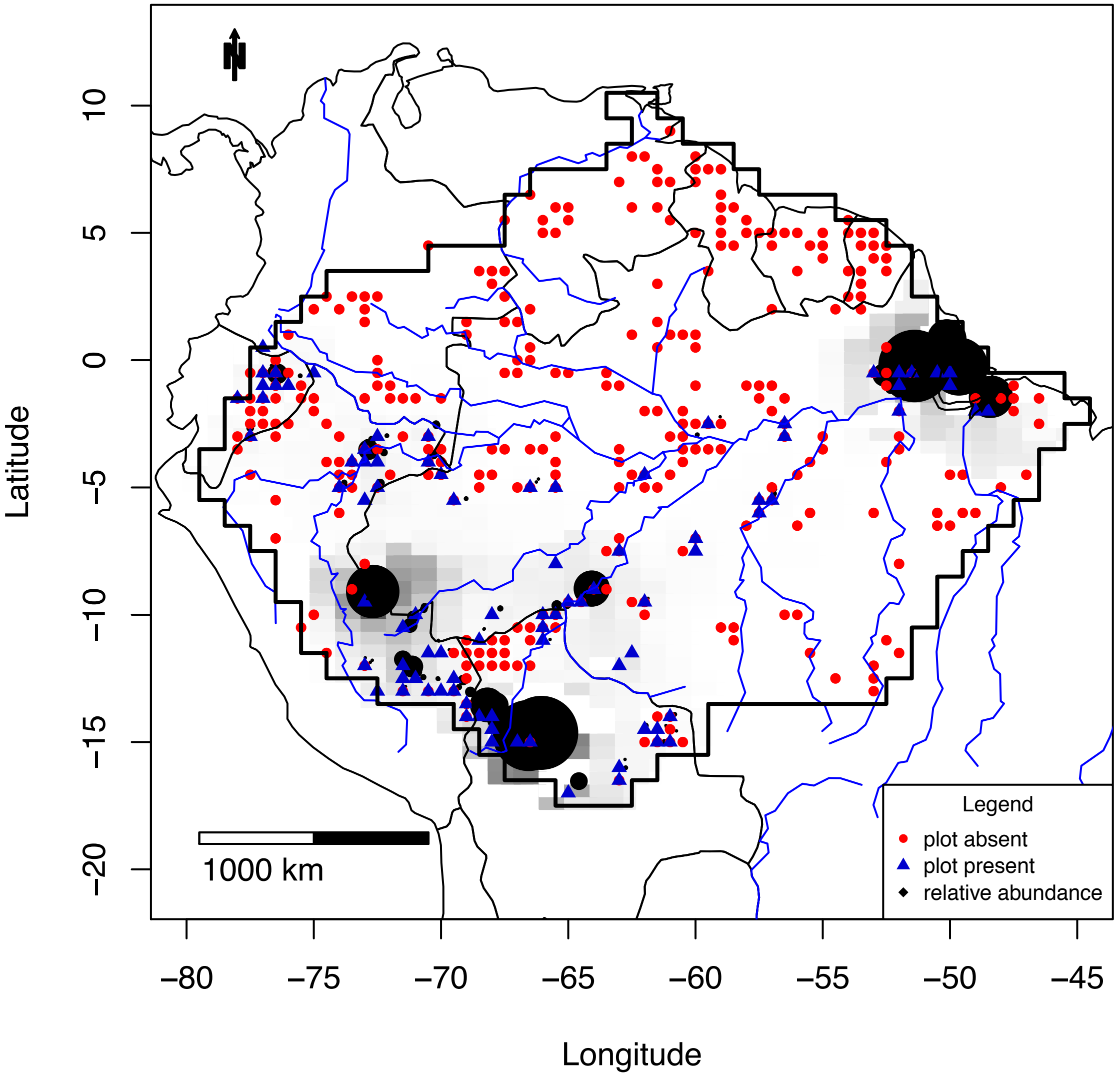

Figure S3_8A Combined map of Attalea_butyracea

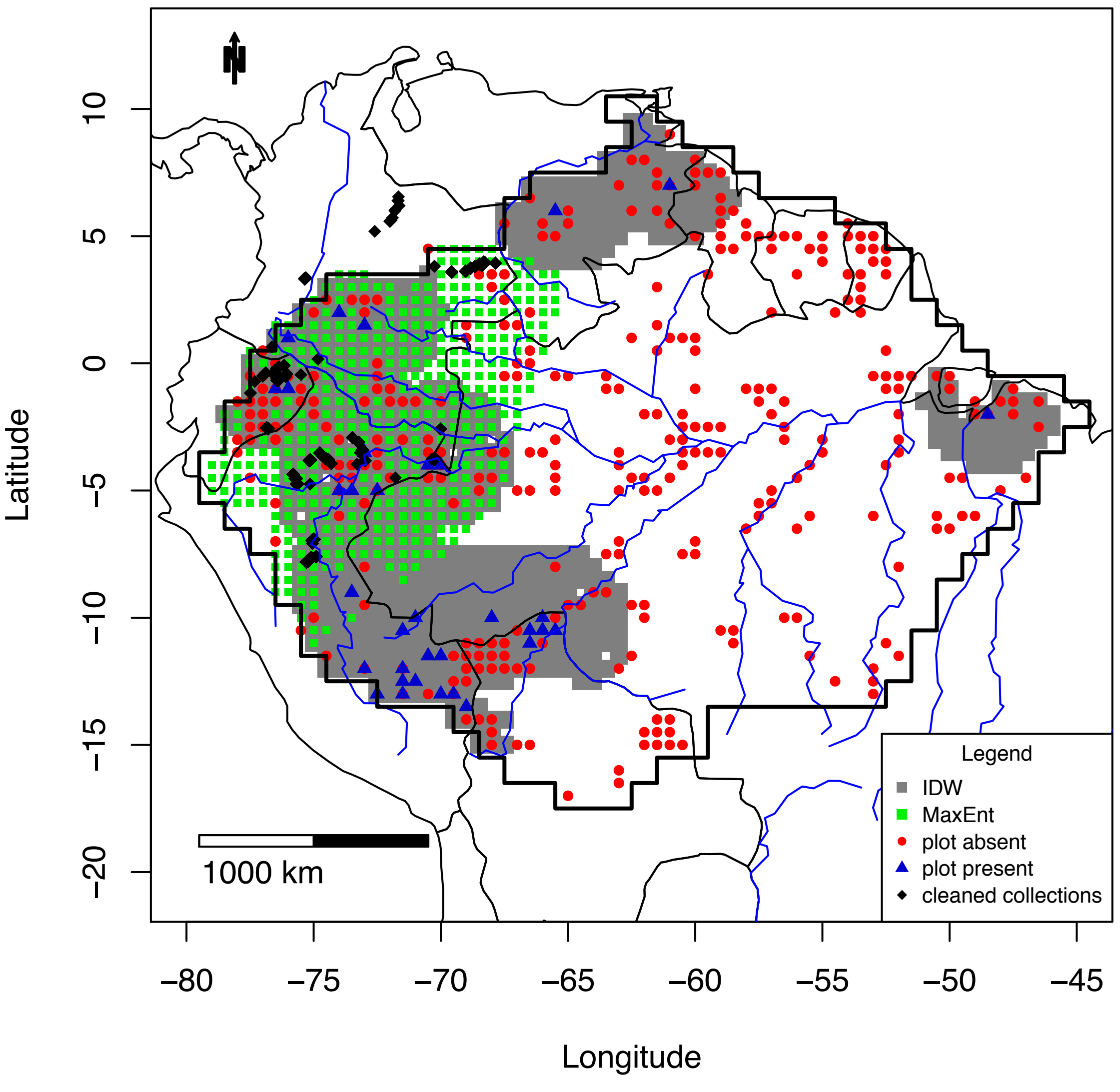

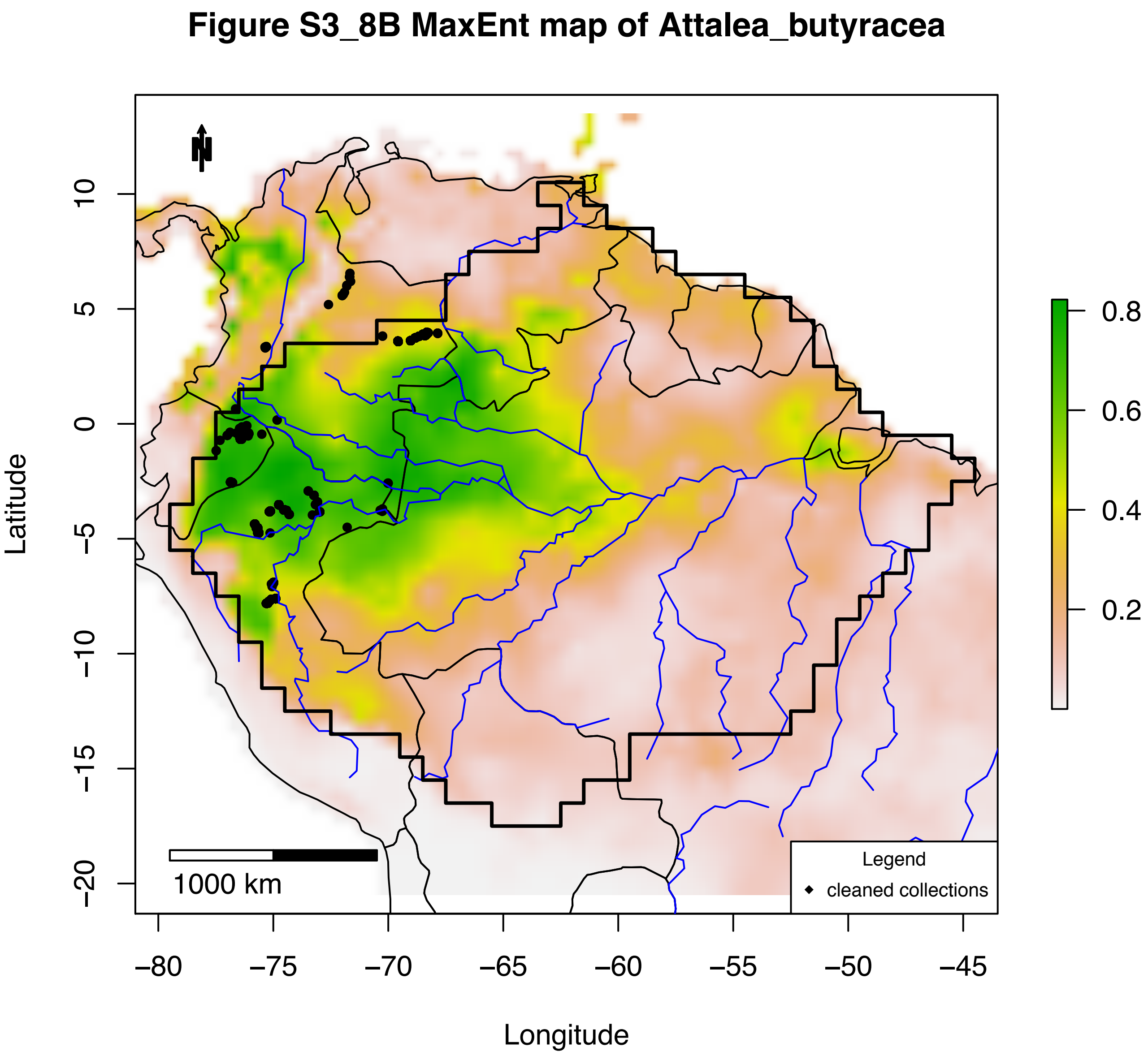

Figure S3_8B MaxEnt map of Attalea_butyracea
N
1000 km
Legend
cleaned collections
Latitude
Longitude
10
5
0
−5
−10
−15
−20
−80
−75
−70
−65
−60
−55
−50
−45
0.8
0.6
0.4
0.2

Figure S3_8C IDW map of Attalea_butyracea: plots =0 – 0.33; IDW: 0 – 0.19

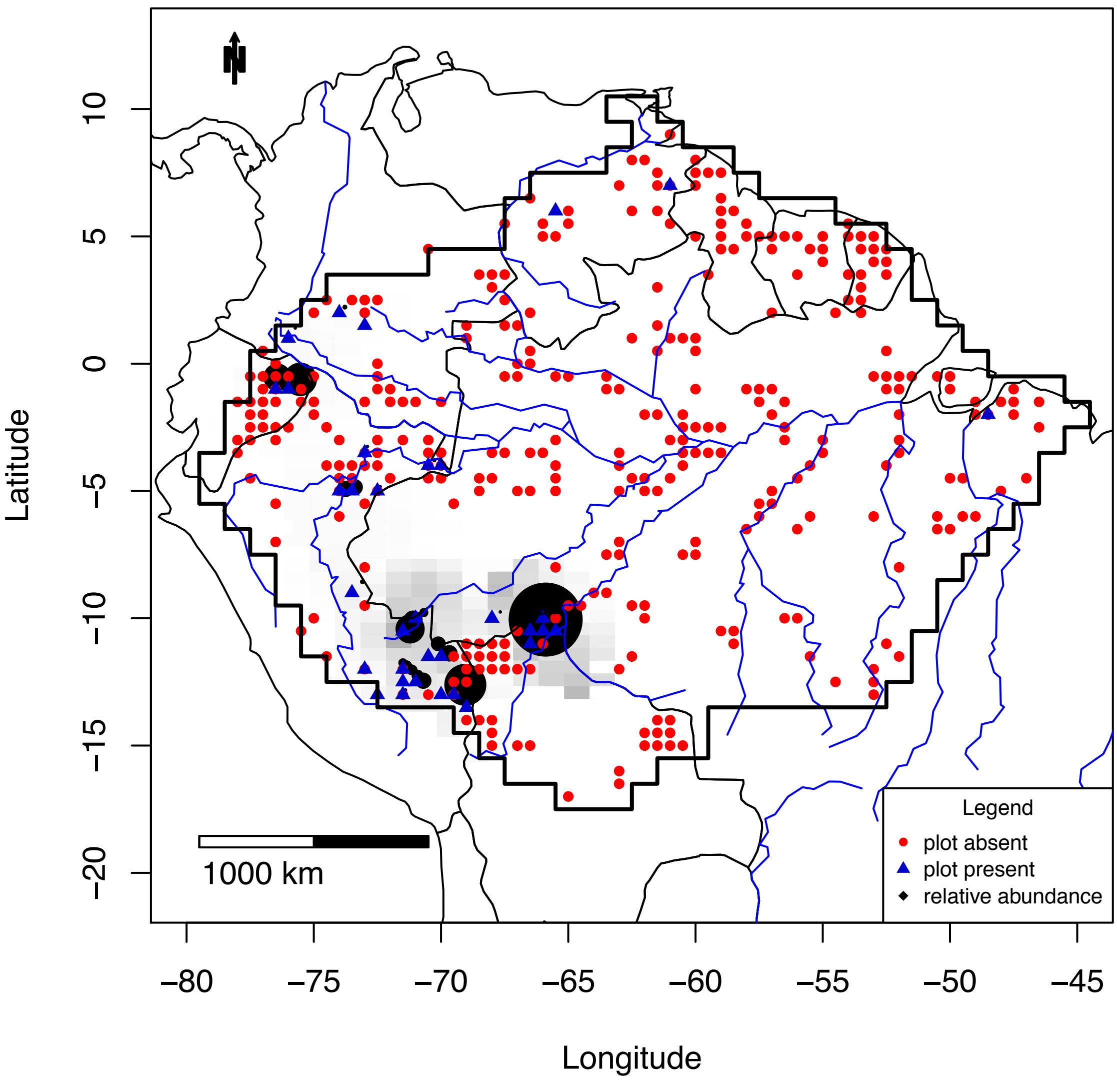

Figure S3_9A Combined map of Attalea_maripa

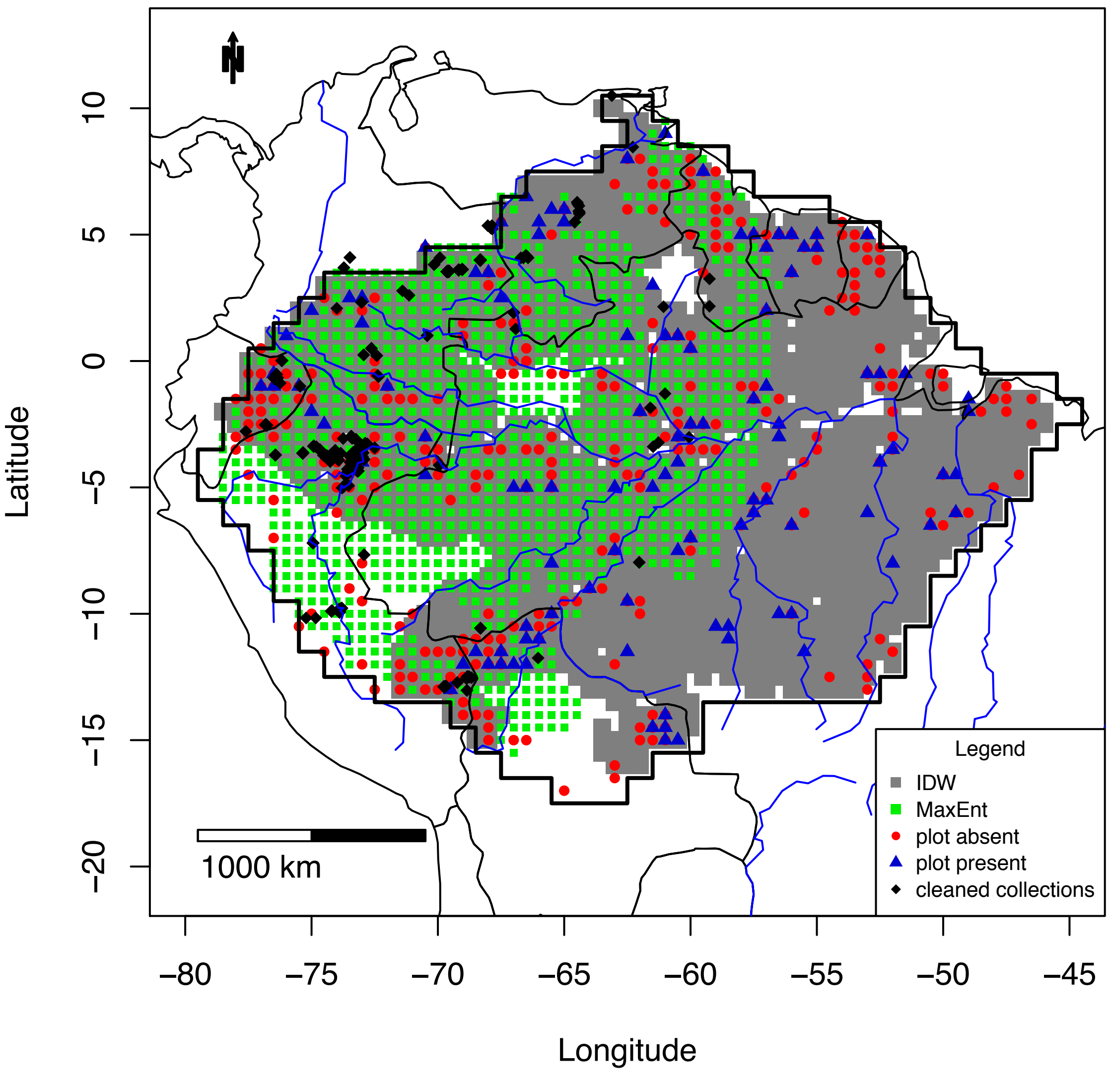

Figure S3_9B MaxEnt map of Attalea_maripa

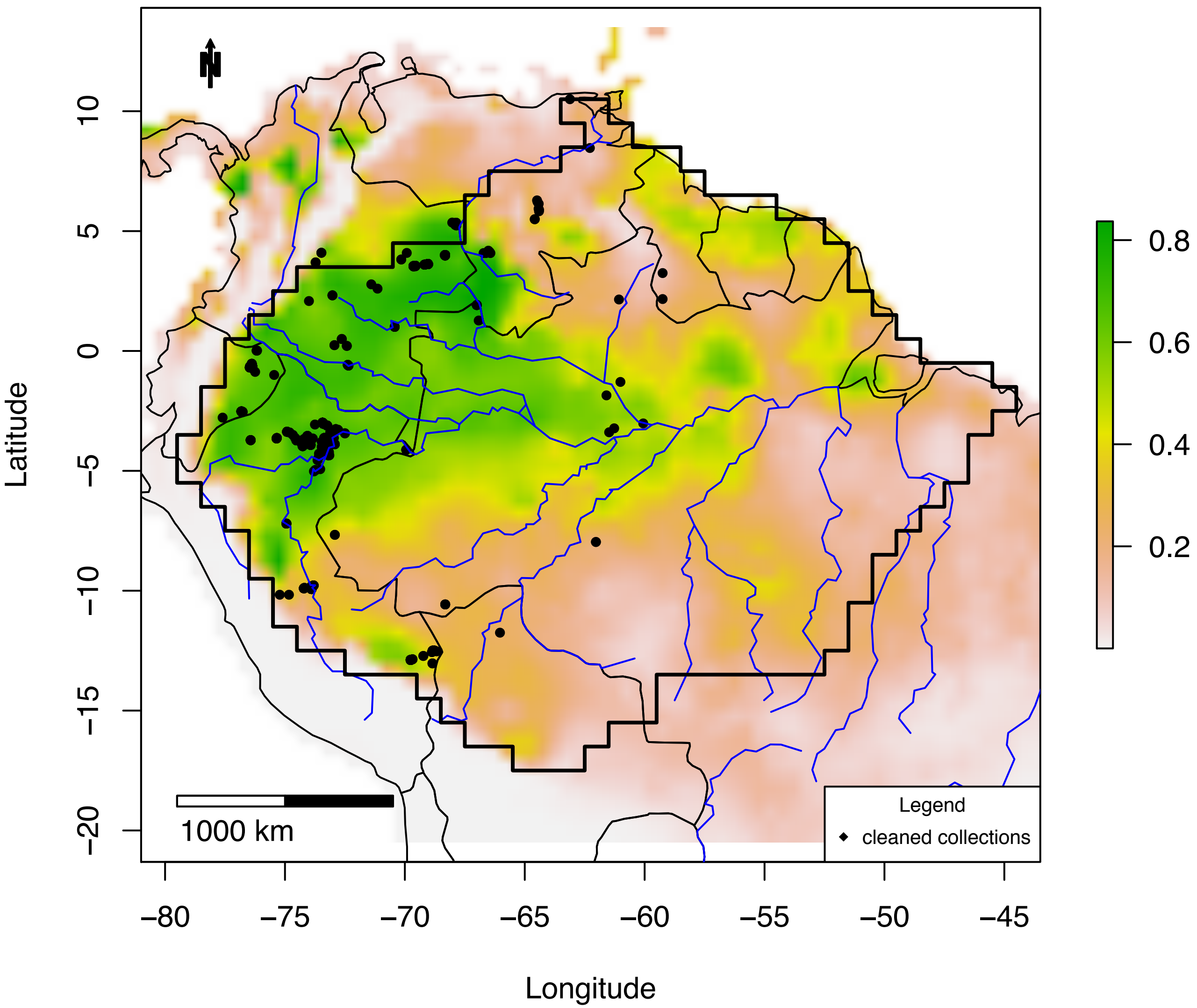

Figure S3_9C IDW map of Attalea_maripa: plots =0 – 0.13; IDW: 0 – 0.05

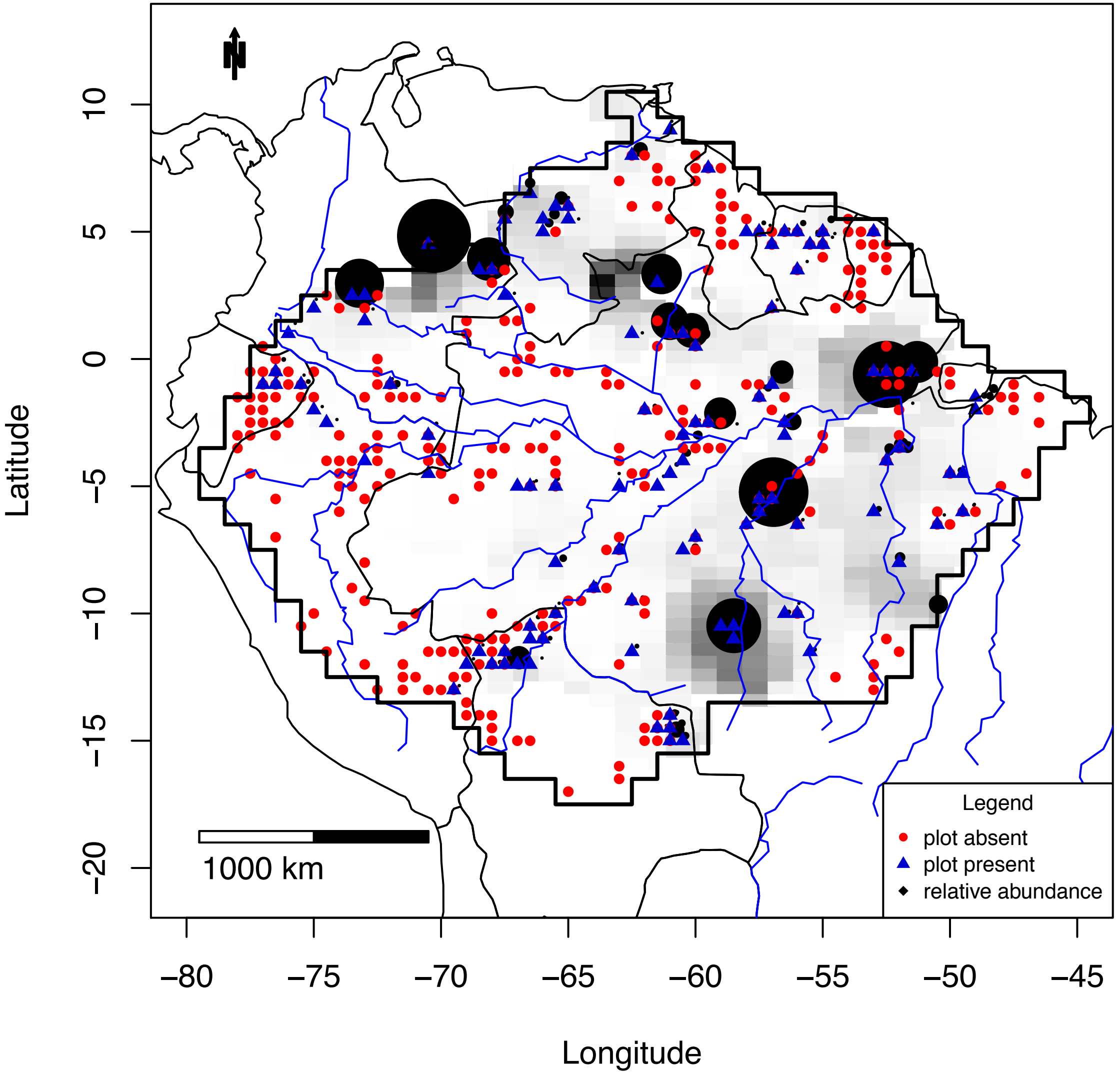

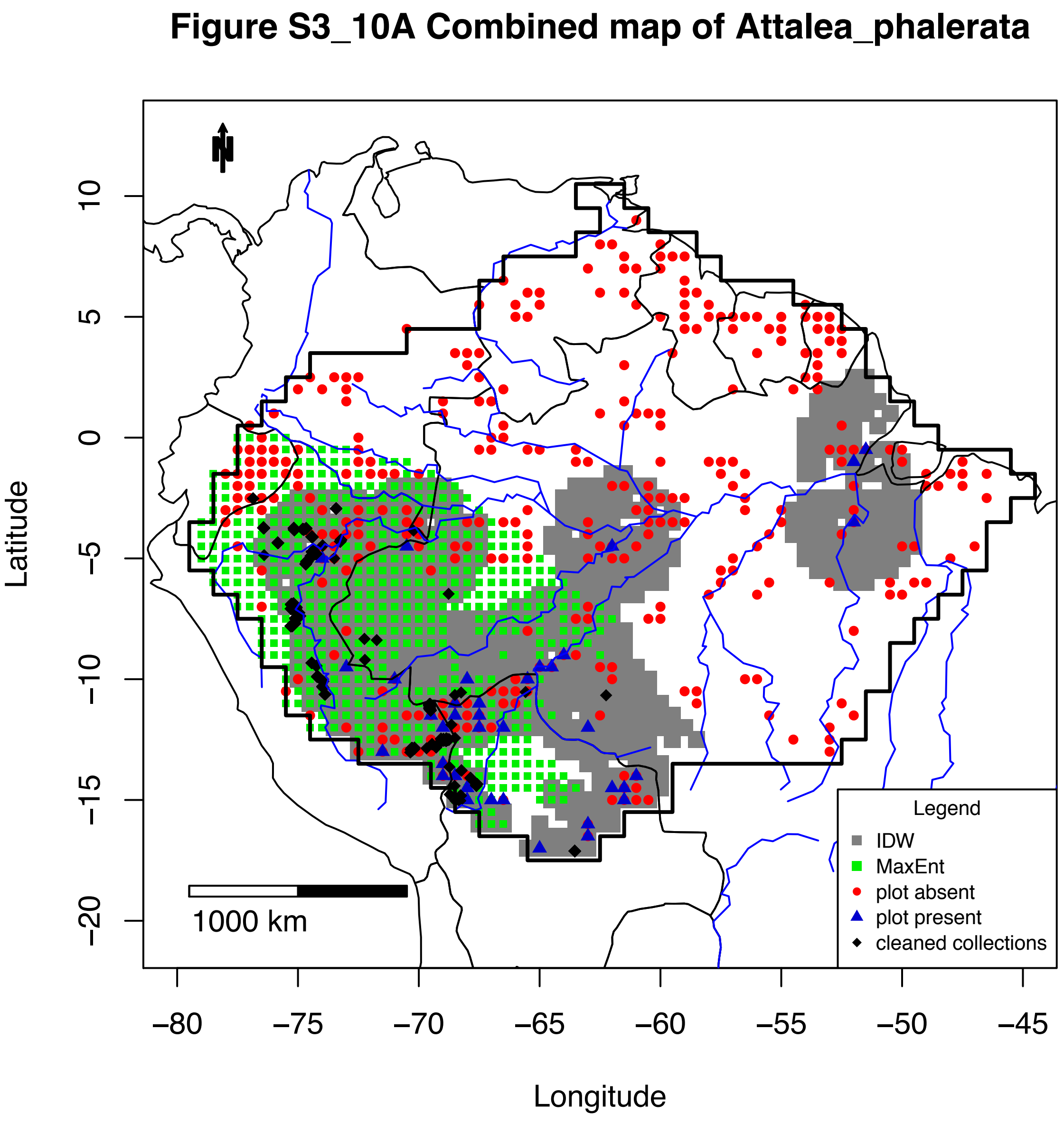

Figure S3_10A Combined map of Attalea_phalerata
Latitude
Longitude
10
5
0
−5
−10
−15
−20
−80
−75
−70
−65
−60
−55
−50
−45
N
1000 km
Legend
IDW
MaxEnt
plot absent
plot present
cleaned collections

Figure S3_10B MaxEnt map of Attalea_phalerata

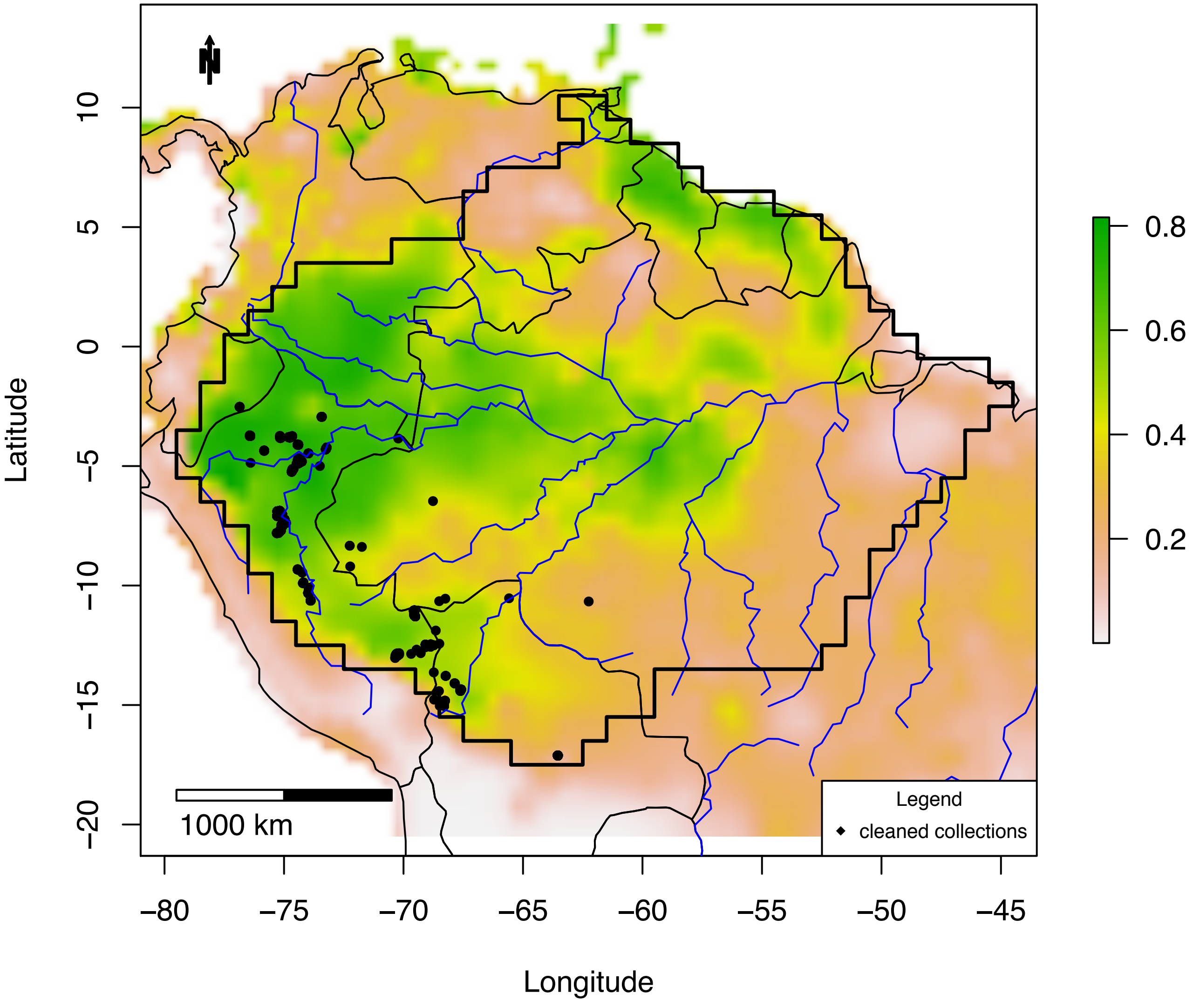

Figure S3_10C IDW map of Attalea_phalerata: plots =0 – 0.45; IDW: 0 – 0.18

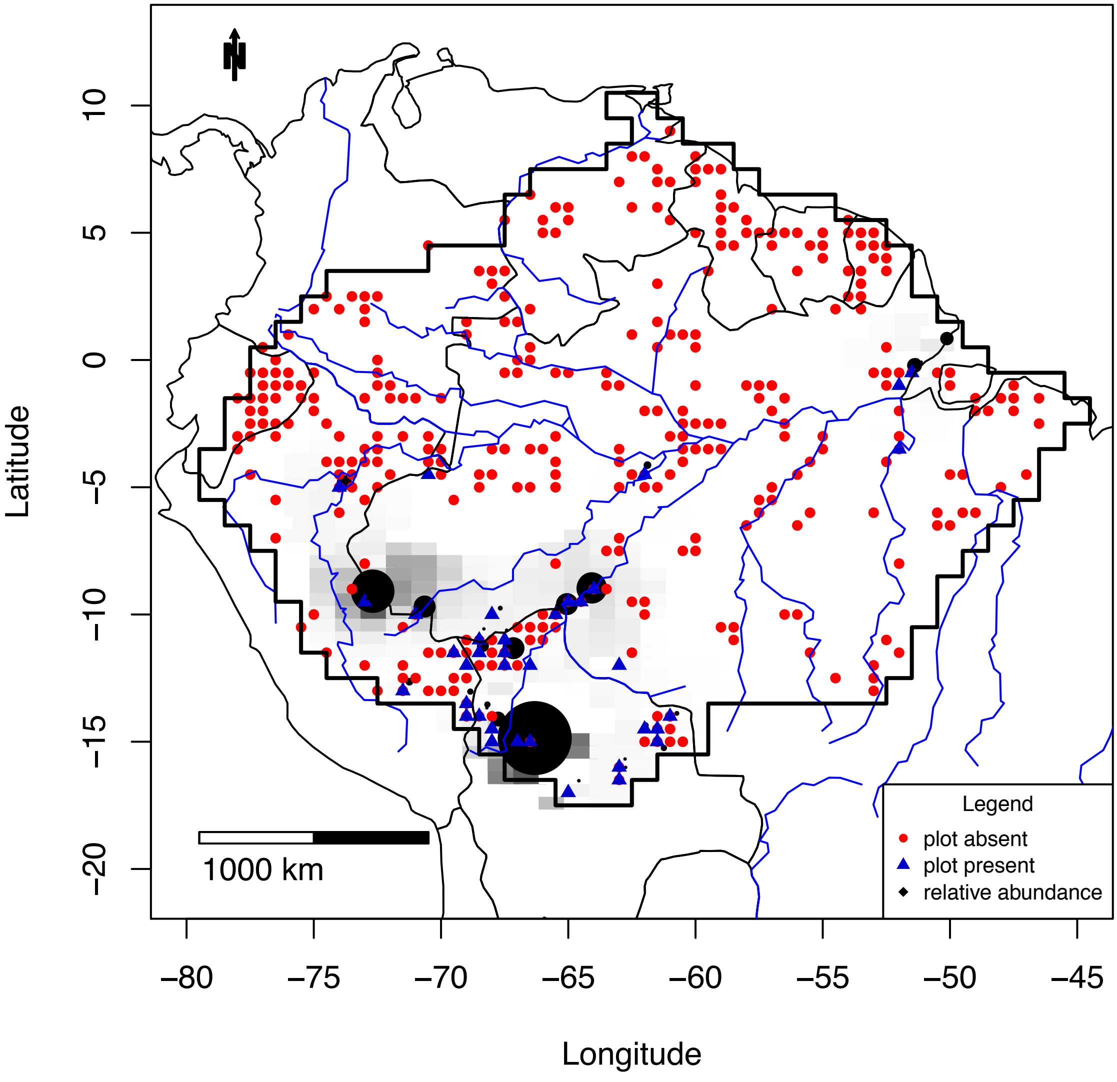

Figure S3_11A Combined map of Attalea_speciosa

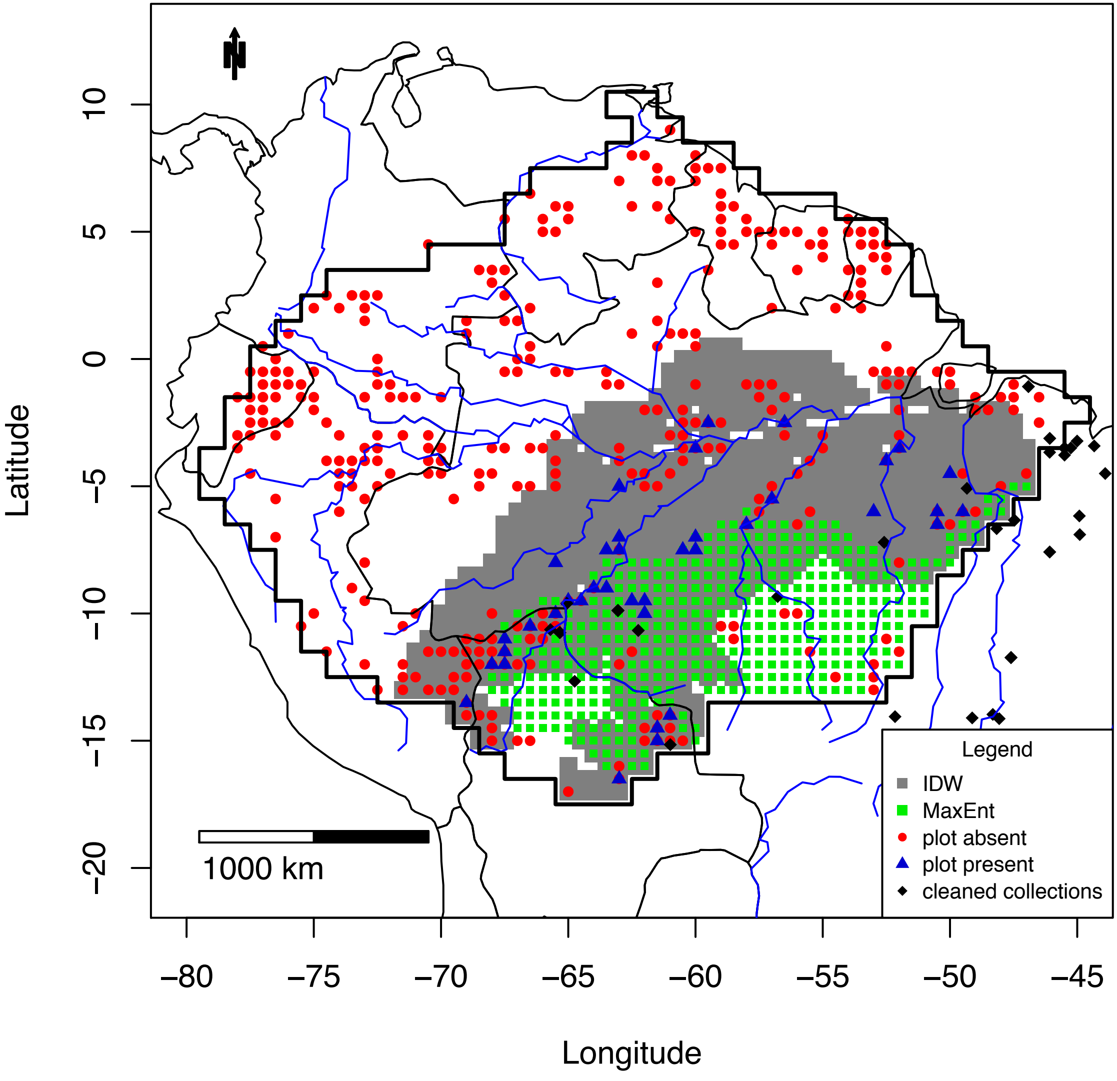

Figure S3_11B MaxEnt map of Attalea_speciosa

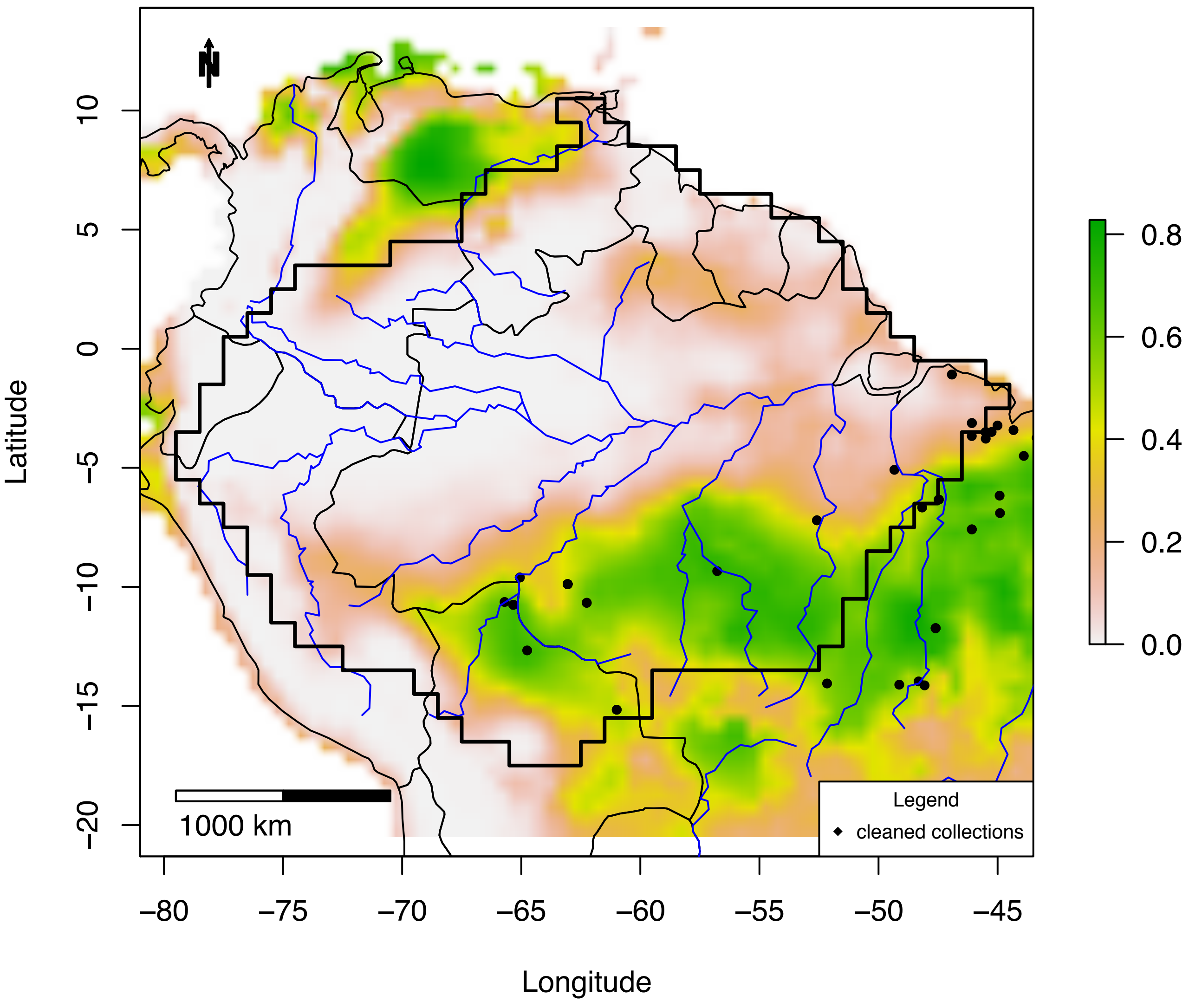

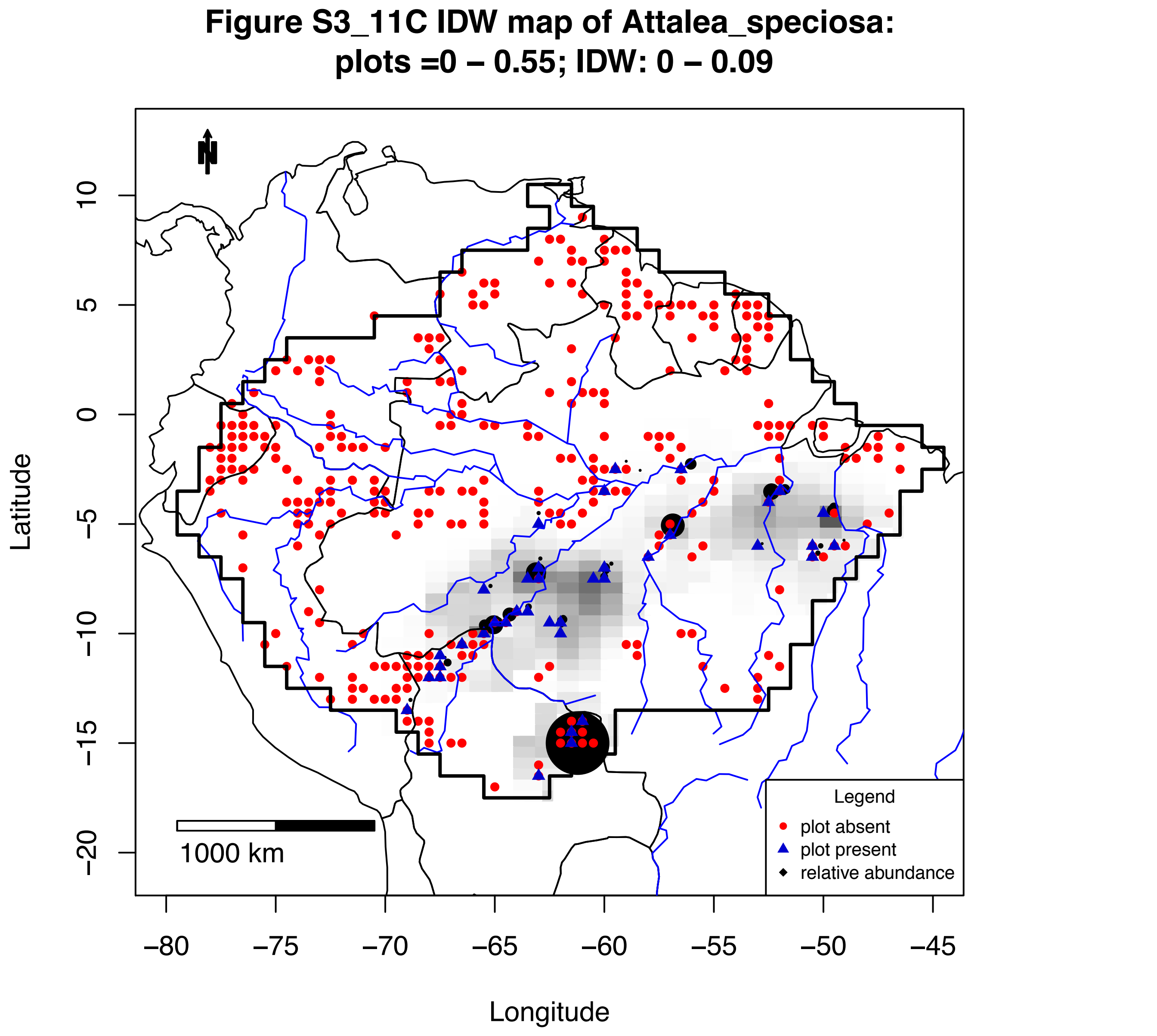

Figure S3_11C IDW map of Attalea_speciosa:
plots =0 – 0.55; IDW: 0 – 0.09
N
1000 km
Legend
plot absent
plot present
relative abundance
Latitude
Longitude
10
5
0
−5
−10
−15
−20
−80
−75
−70
−65
−60
−55
−50
−45

Figure S3_12A Combined map of Bertholletia_excelsa

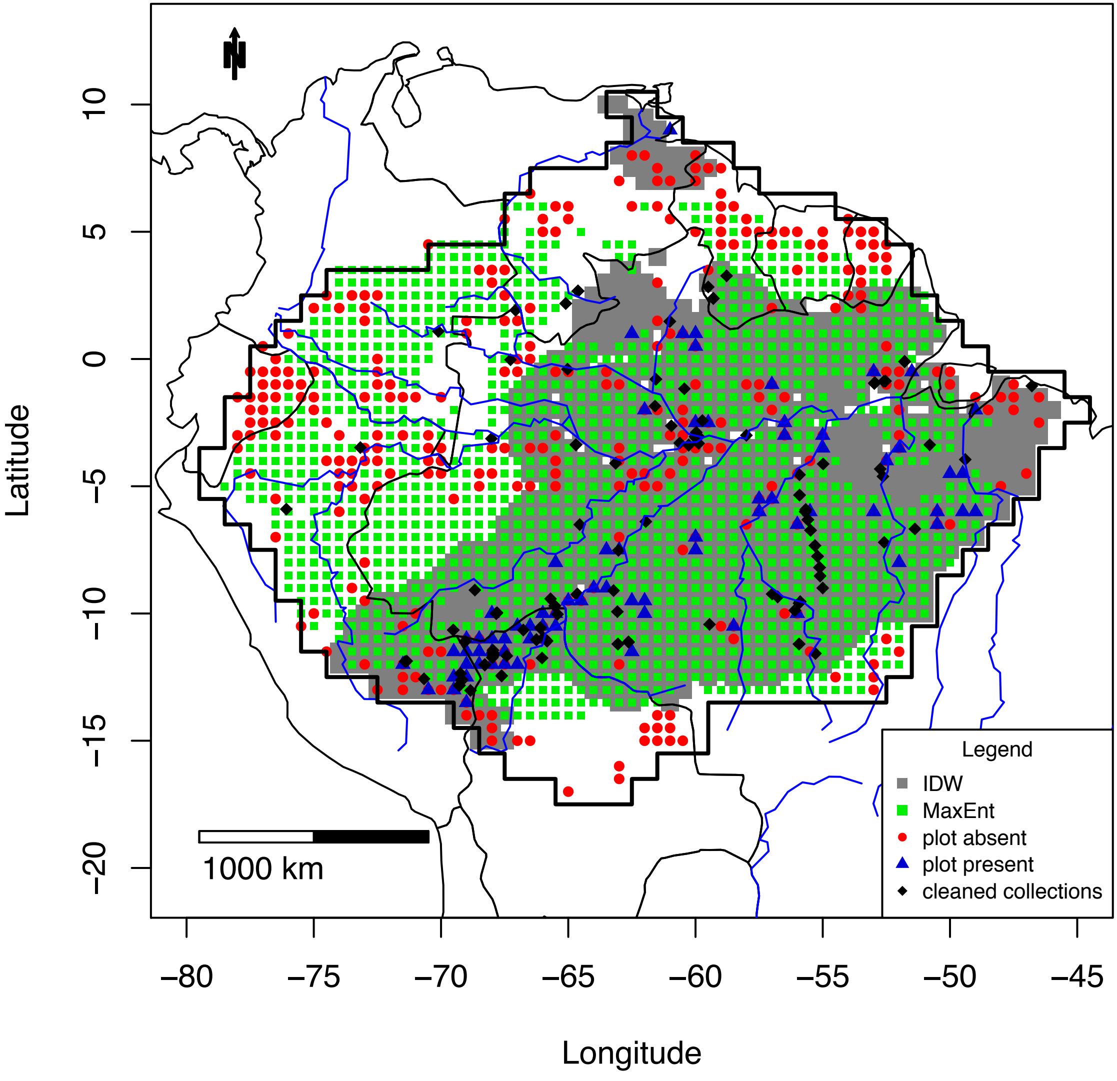

Figure S3_12B MaxEnt map of Bertholletia_excelsa

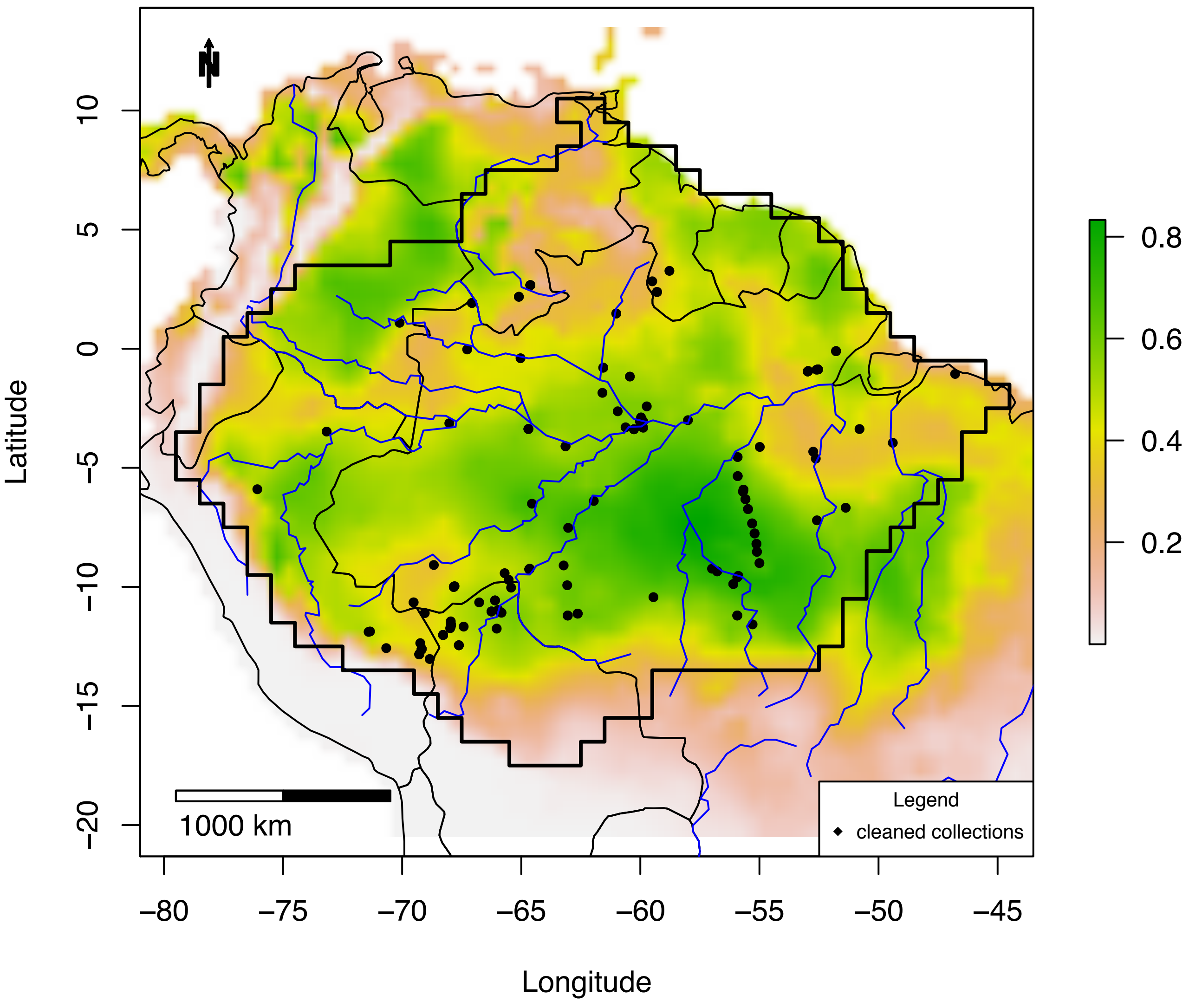

Figure S3_12C IDW map of Bertholletia_excelsa: plots =0 – 0.07; IDW: 0 – 0.01

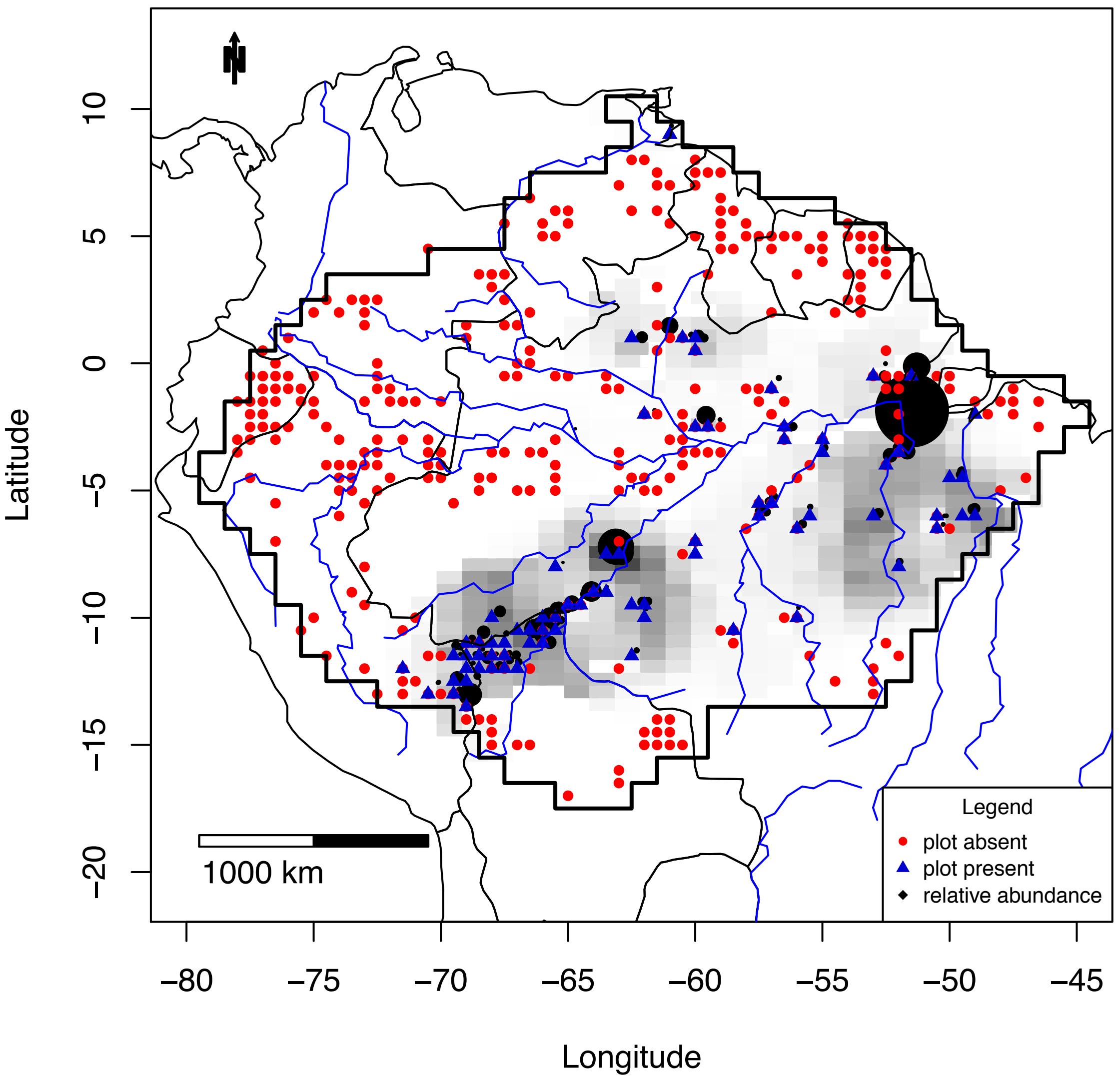

Figure S3_13A Combined map of Bocageopsis_multiflora

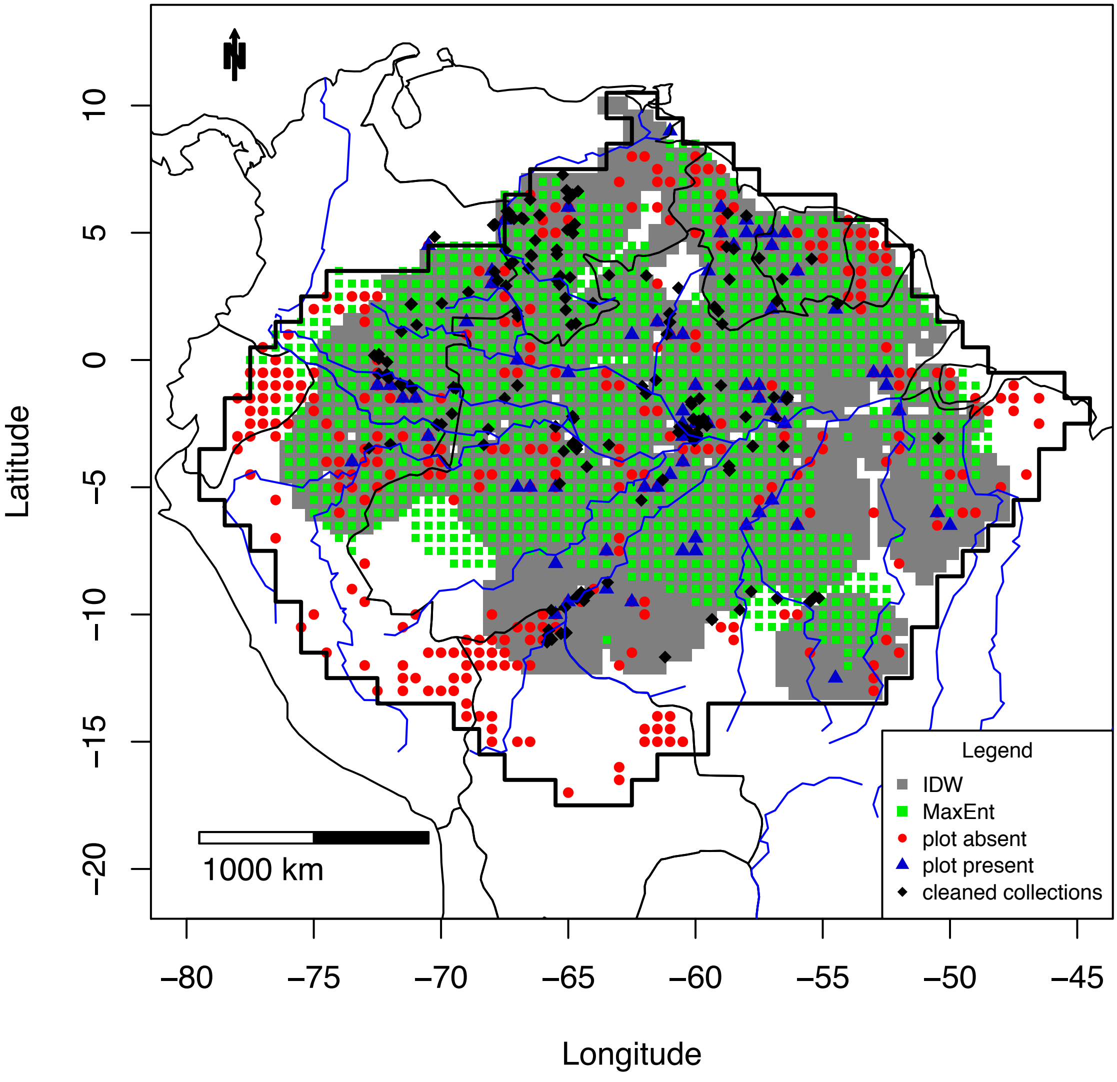

Figure S3_13B MaxEnt map of Bocageopsis_multiflora

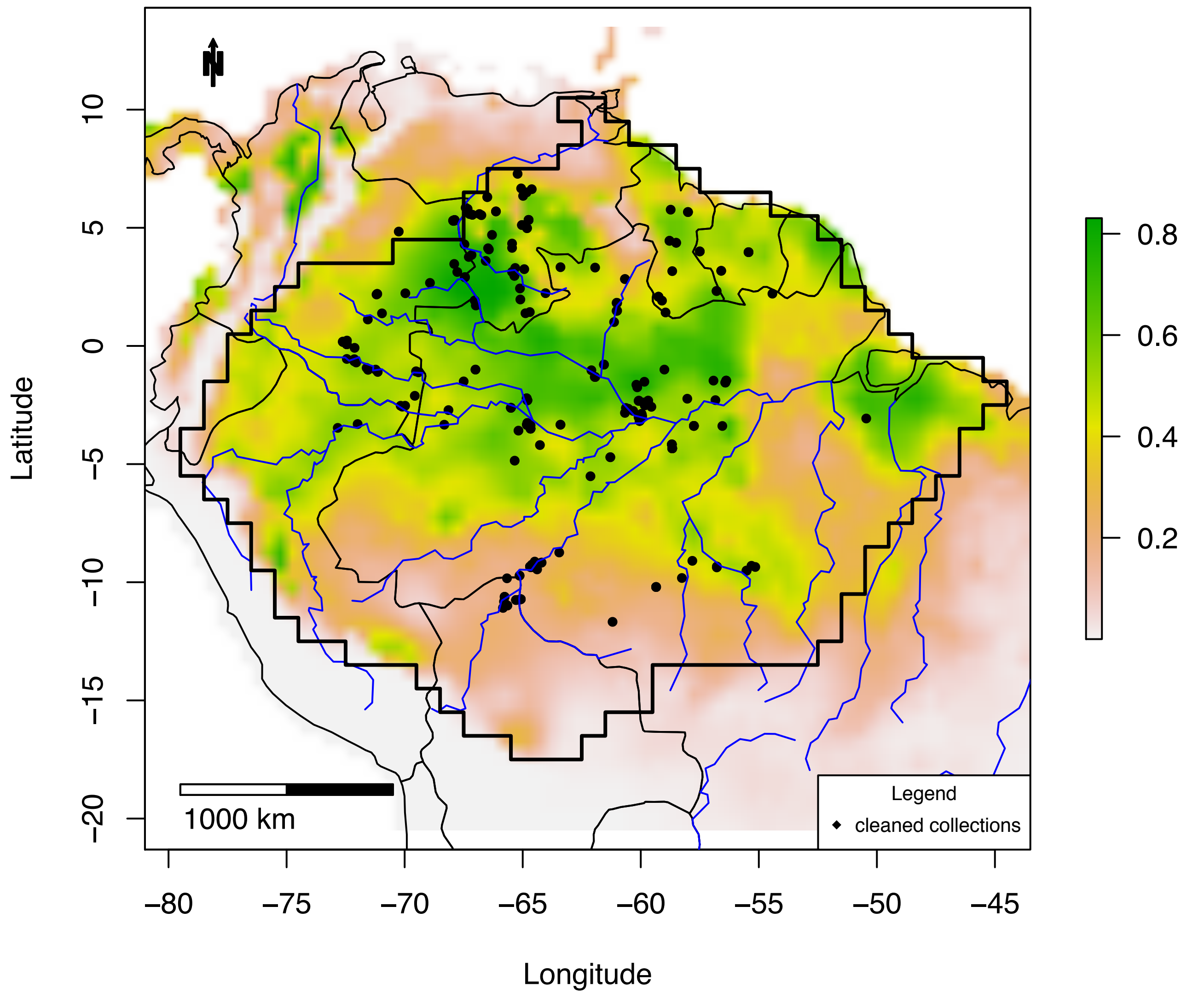

Figure S3_13C IDW map of Bocageopsis_multiflora: plots =0 – 0.15; IDW: 0 – 0.05

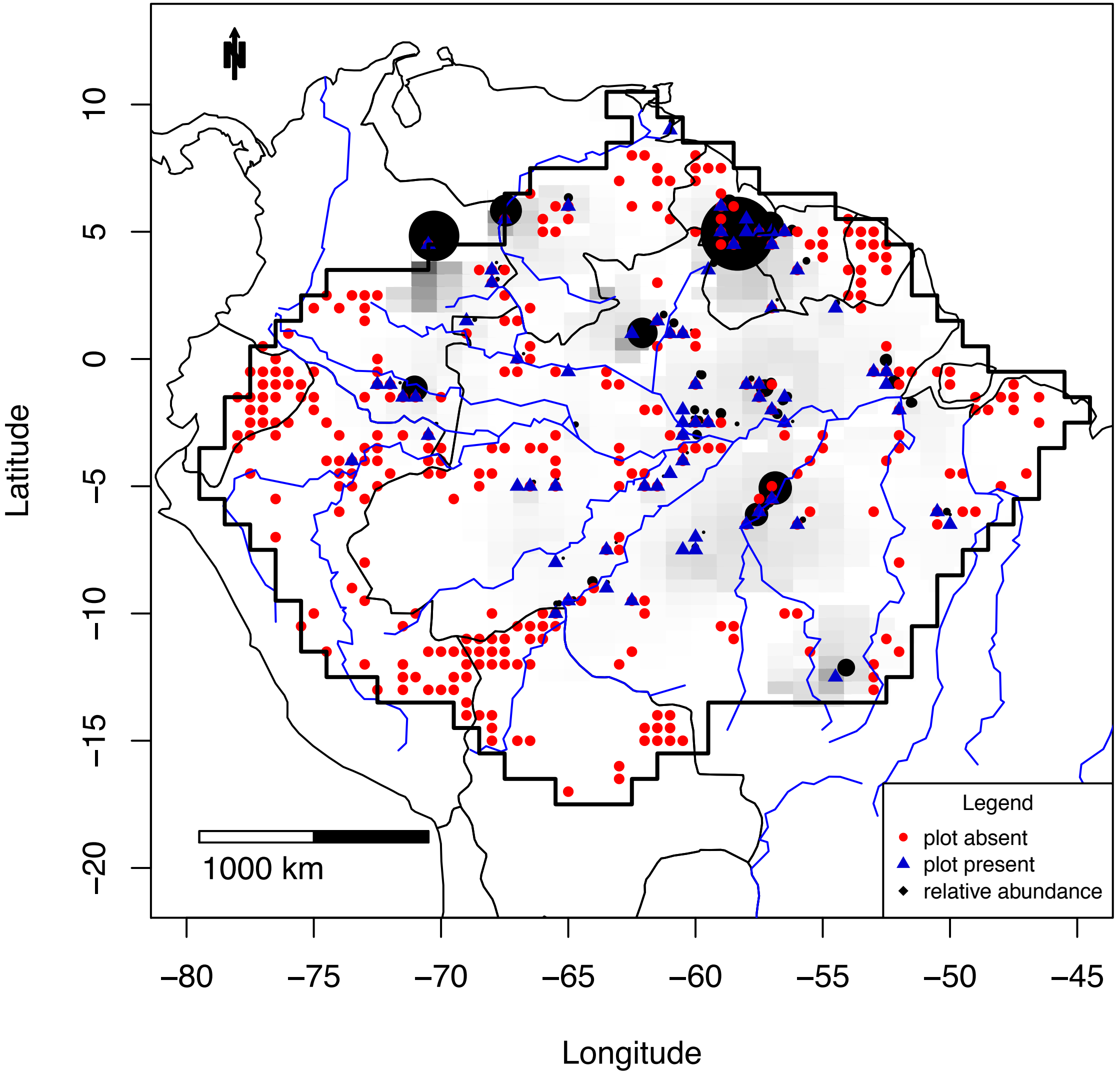

Figure S3_14A Combined map of Brosimum_rubescens

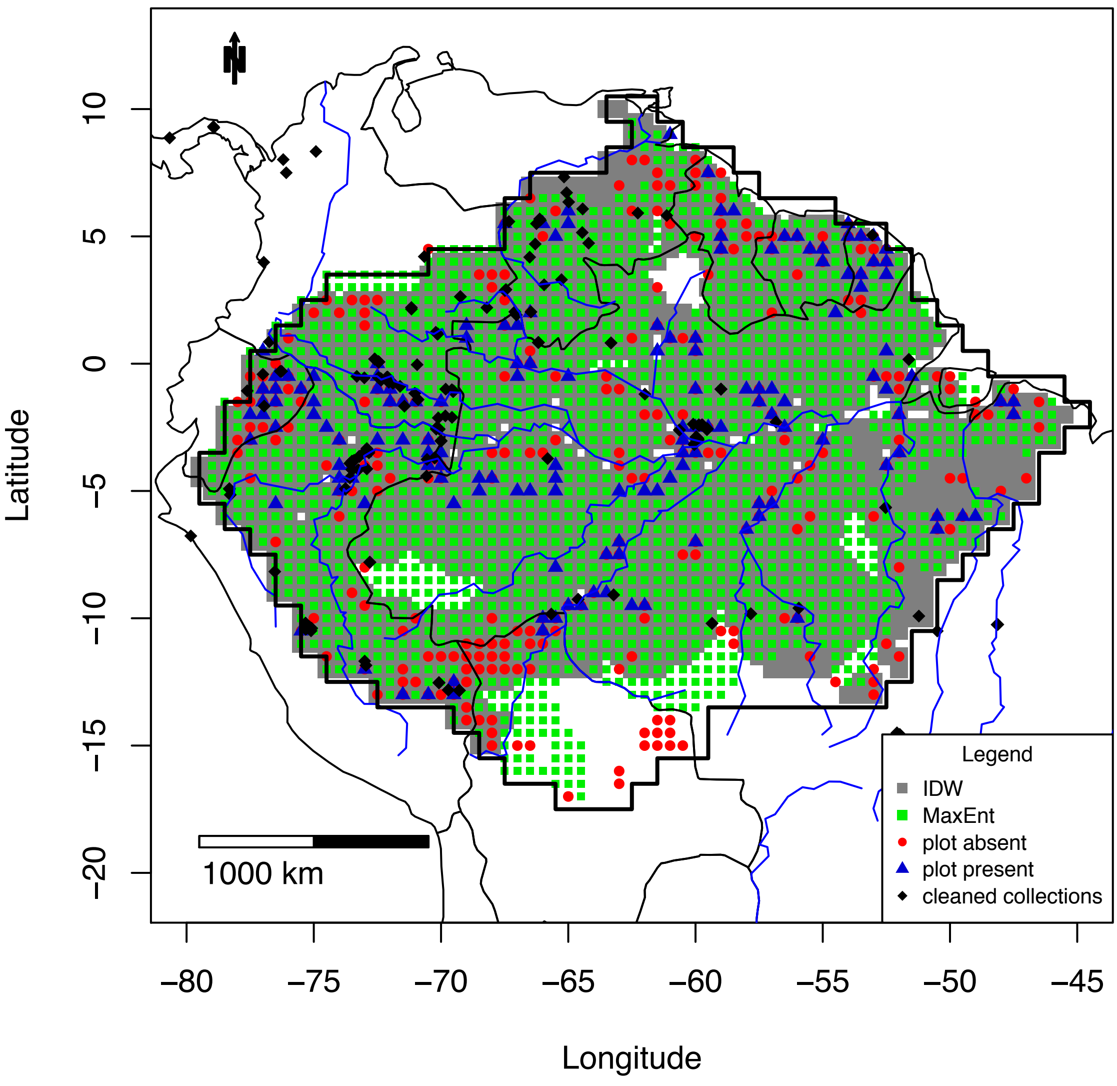

Figure S3_14B MaxEnt map of Brosimum_rubescens

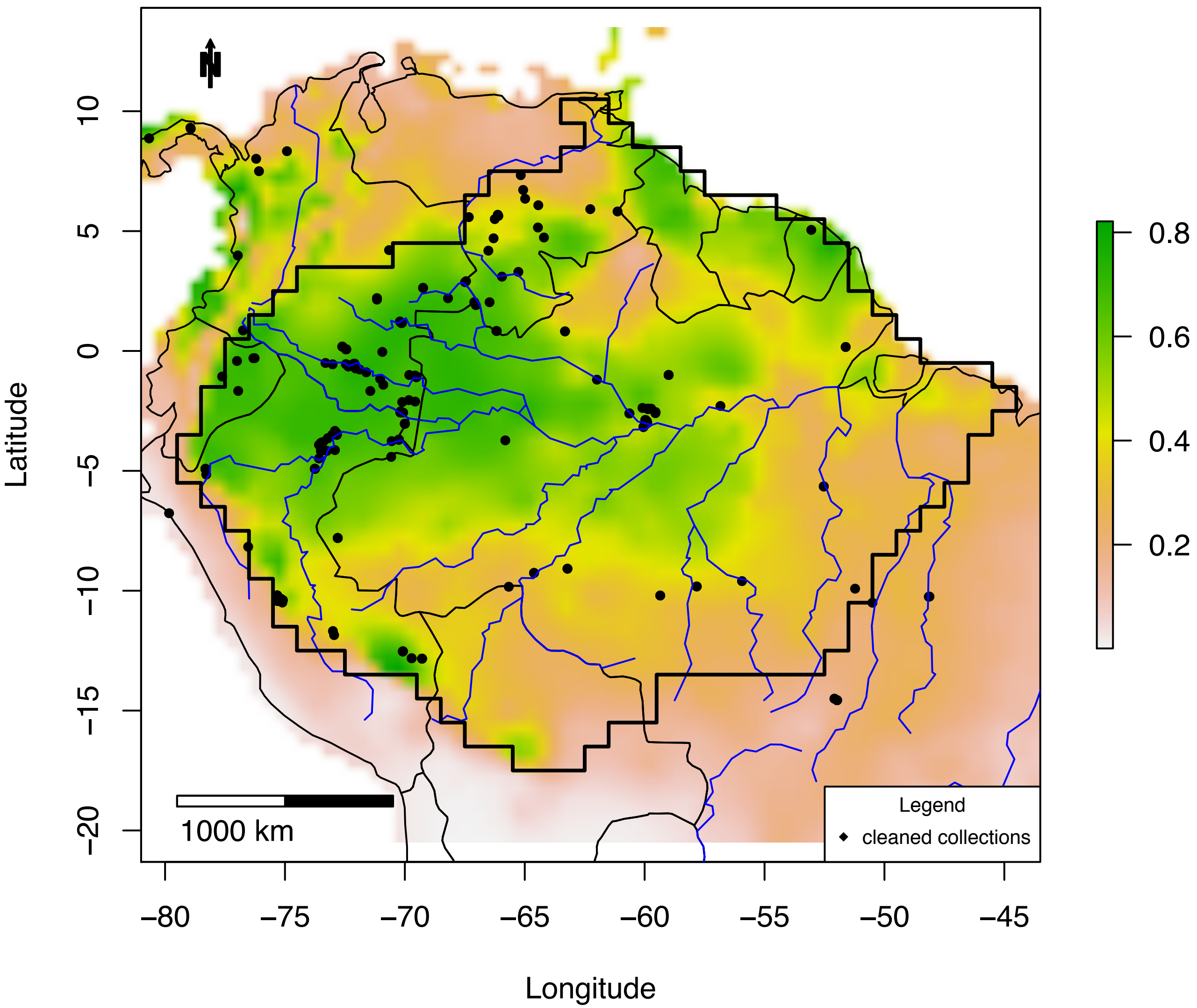

Figure S3_14C IDW map of Brosimum_rubescens:
plots =0 – 0.42; IDW: 0 – 0.03

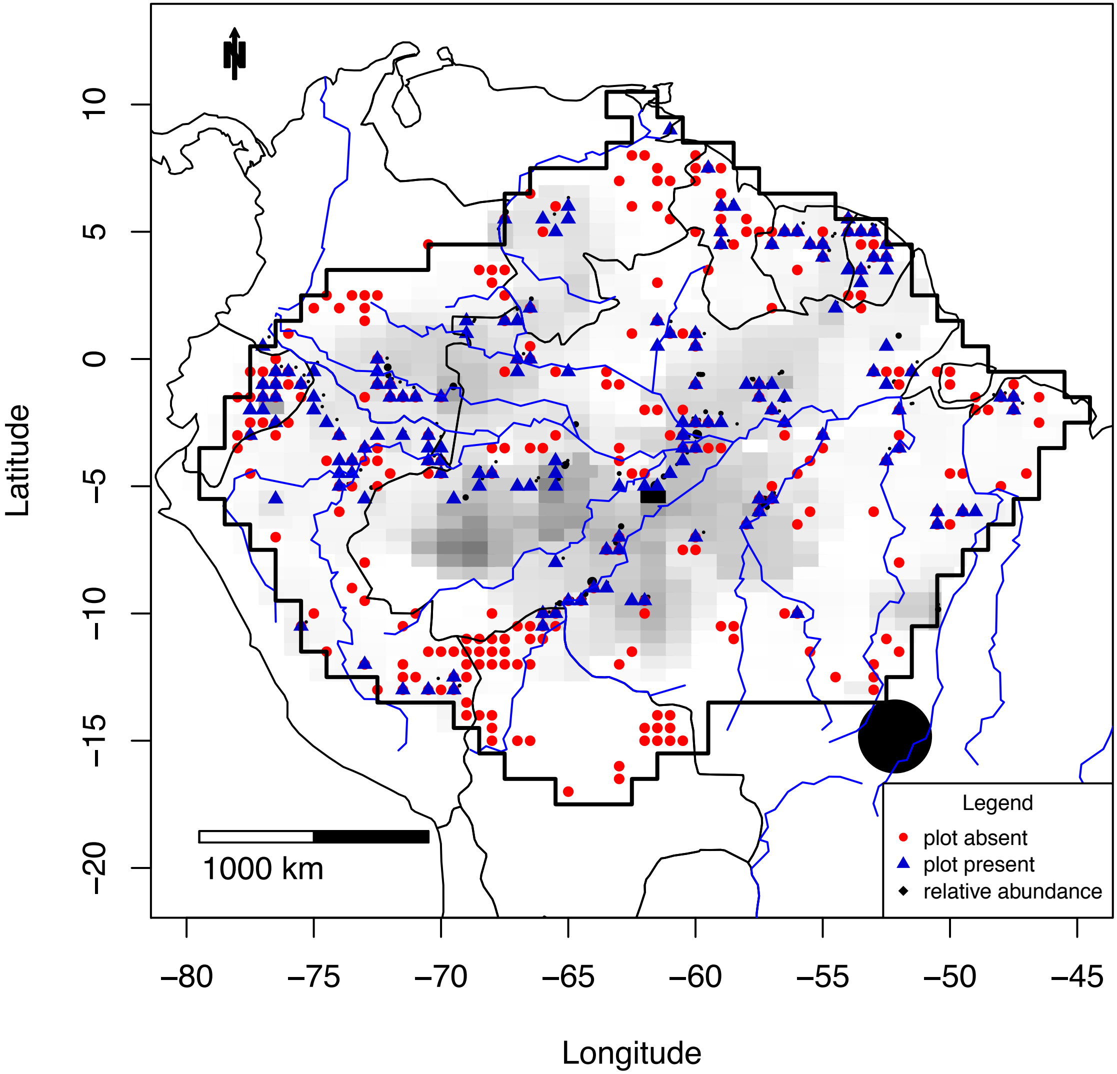

Figure S3_15A Combined map of Brosimum_utile

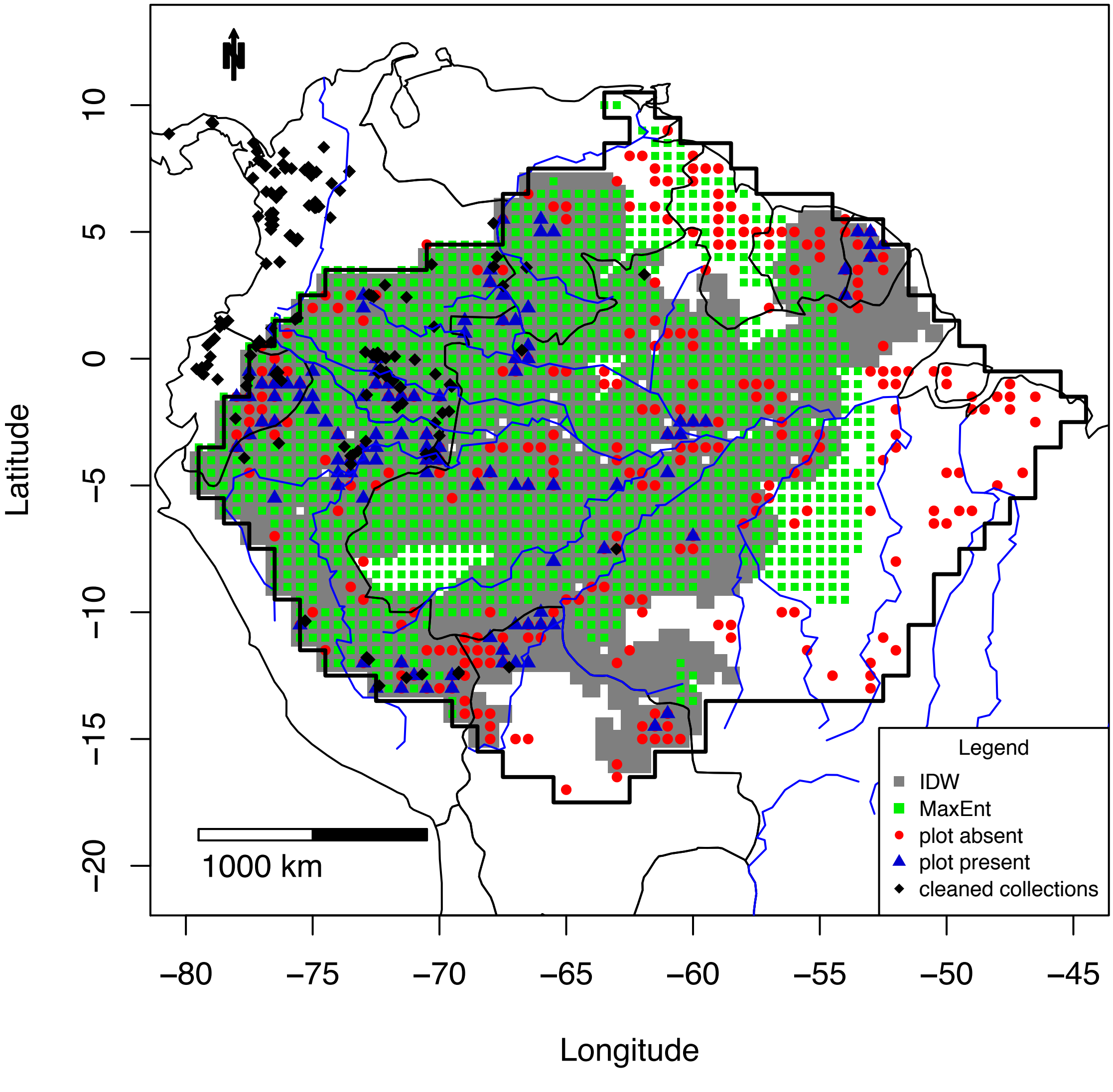

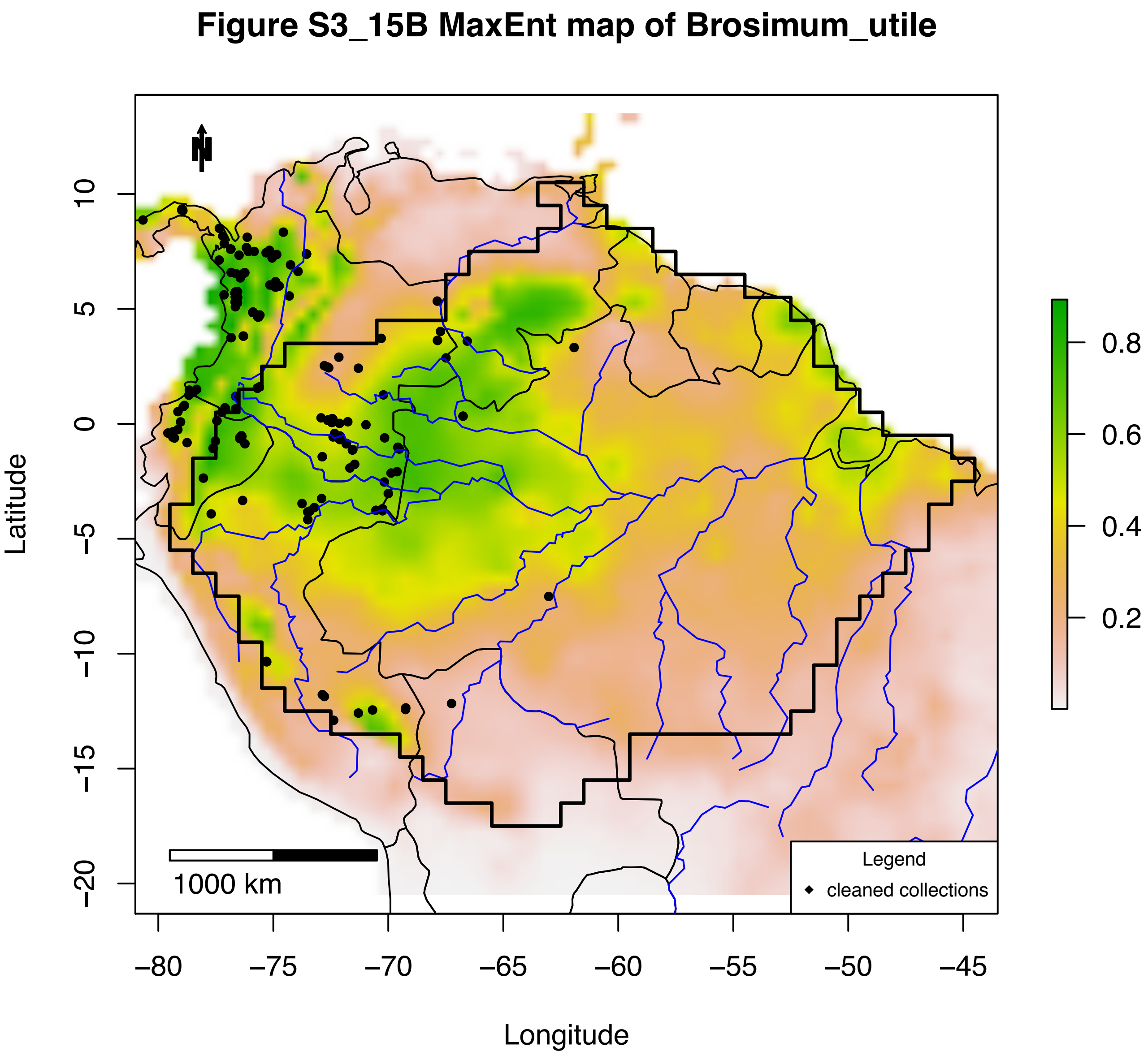
Figure S3_15B MaxEnt map of Brosimum_utile
N
Latitude
Longitude
10
5
0
−5
−10
−15
−20
−80
−75
−70
−65
−60
−55
−50
−45
0.8
0.6
0.4
0.2
1000 km
Legend
cleaned collections

Figure S3_15C IDW map of Brosimum_utile: plots =0 – 0.06; IDW: 0 – 0.02

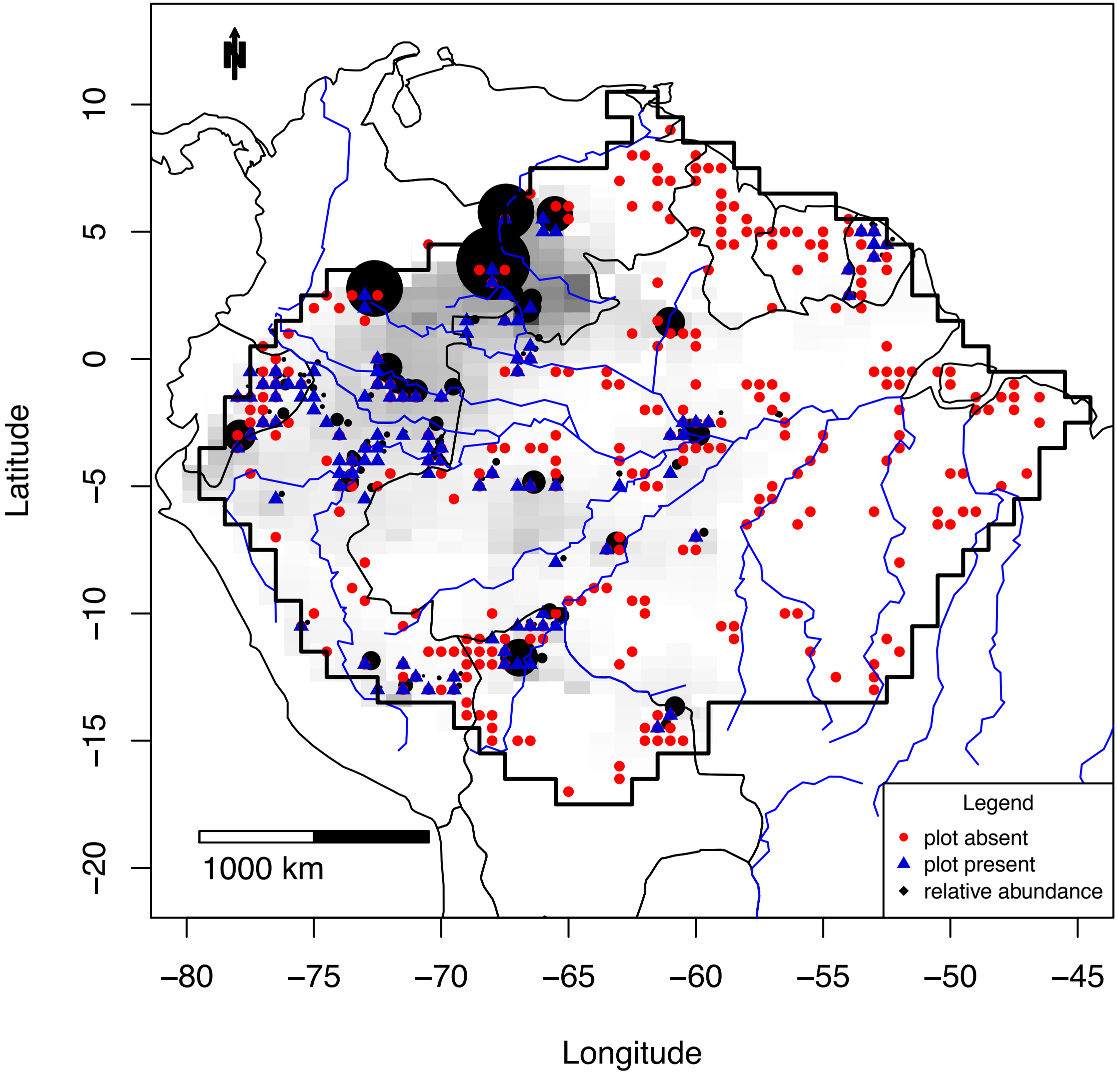

Figure S3_16A Combined map of Campsiandra_implexicaulis

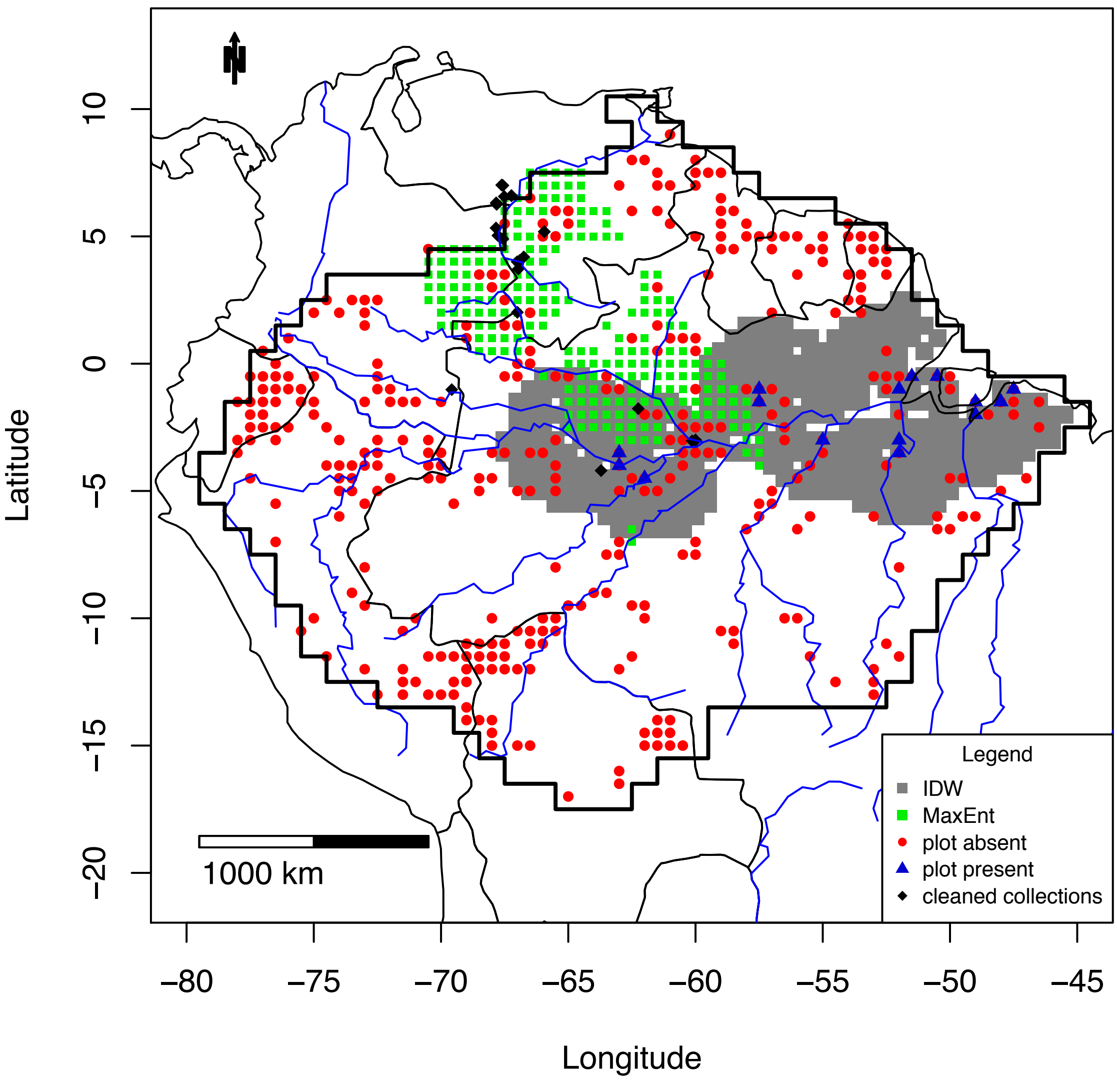

Figure S3_16B MaxEnt map of Campsiandra_implexicaulis

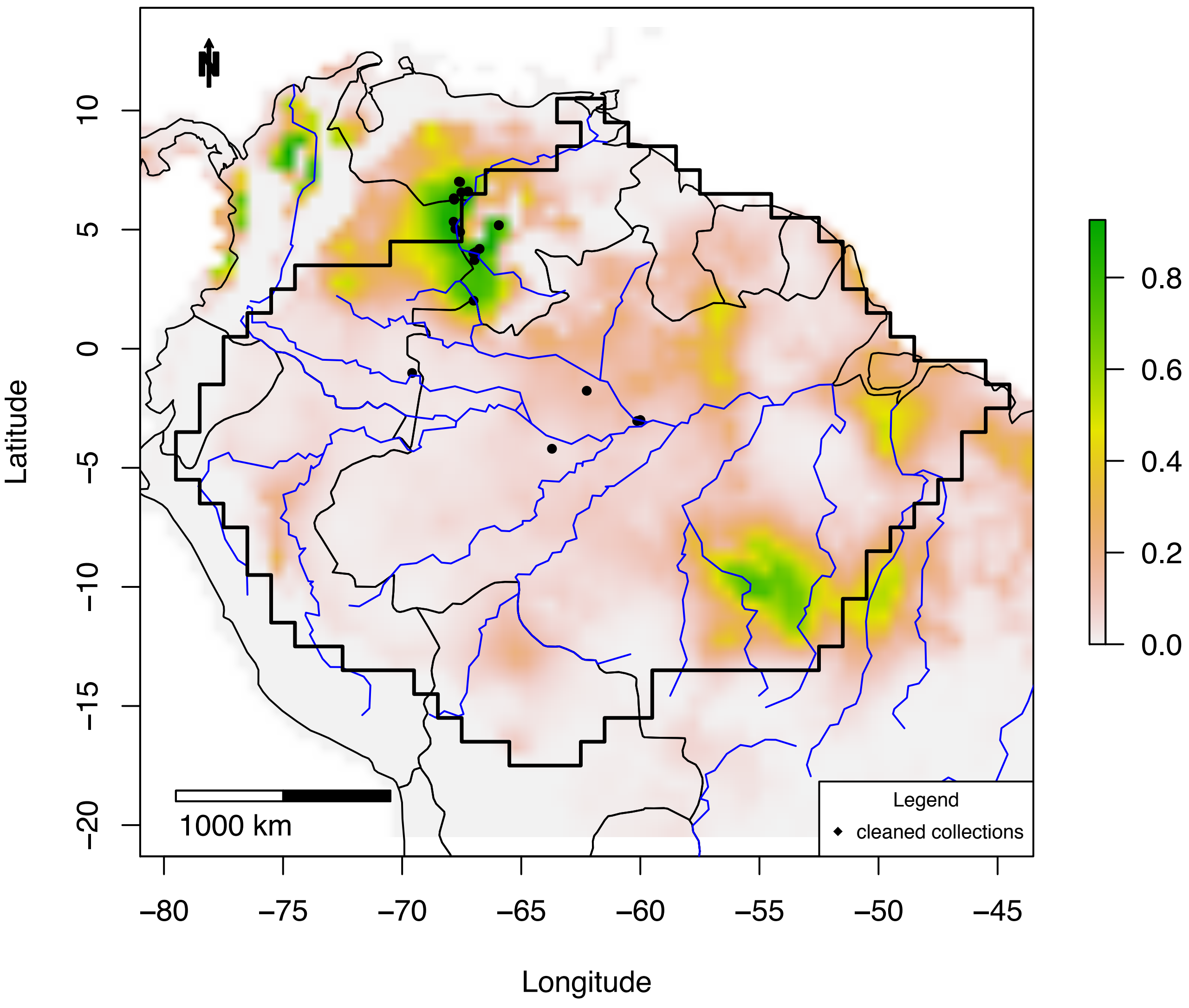

Figure S3_16C IDW map of Campsiandra_implexicaulis: plots =0 – 0.34; IDW: 0 – 0.06

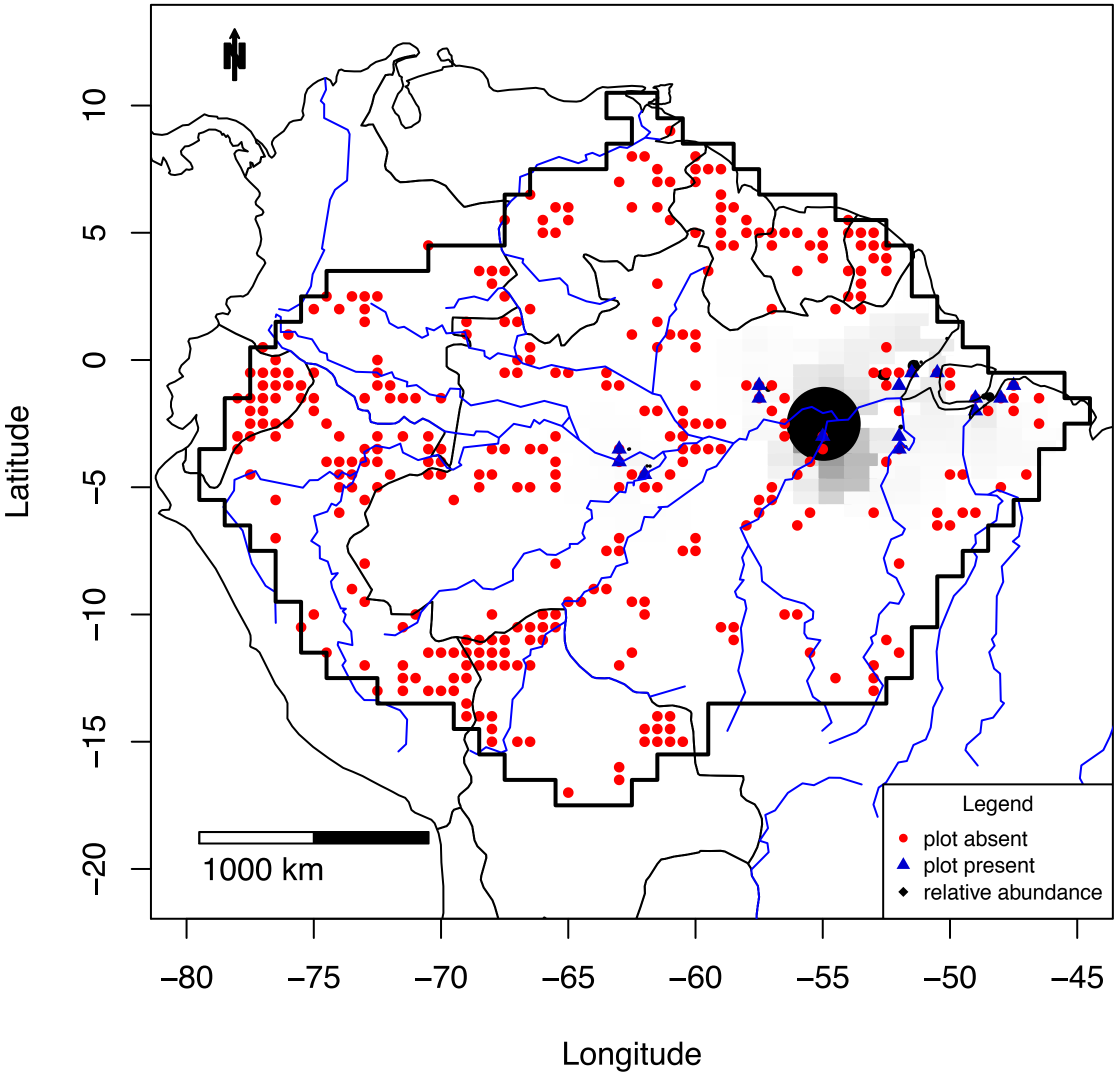

Figure S3_17A Combined map of Caraipa_densifolia

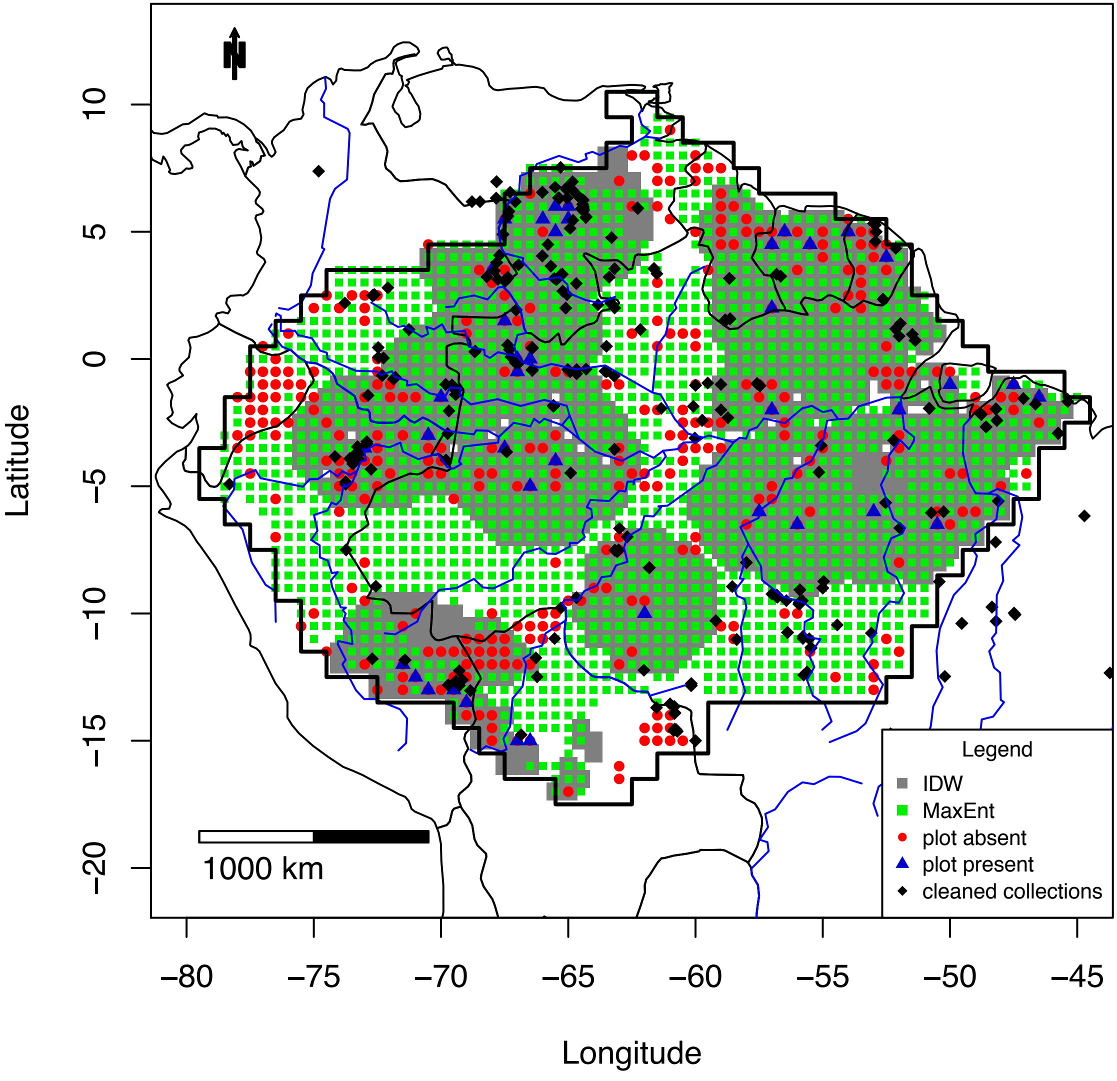

Figure S3_17B MaxEnt map of Caraipa_densifolia

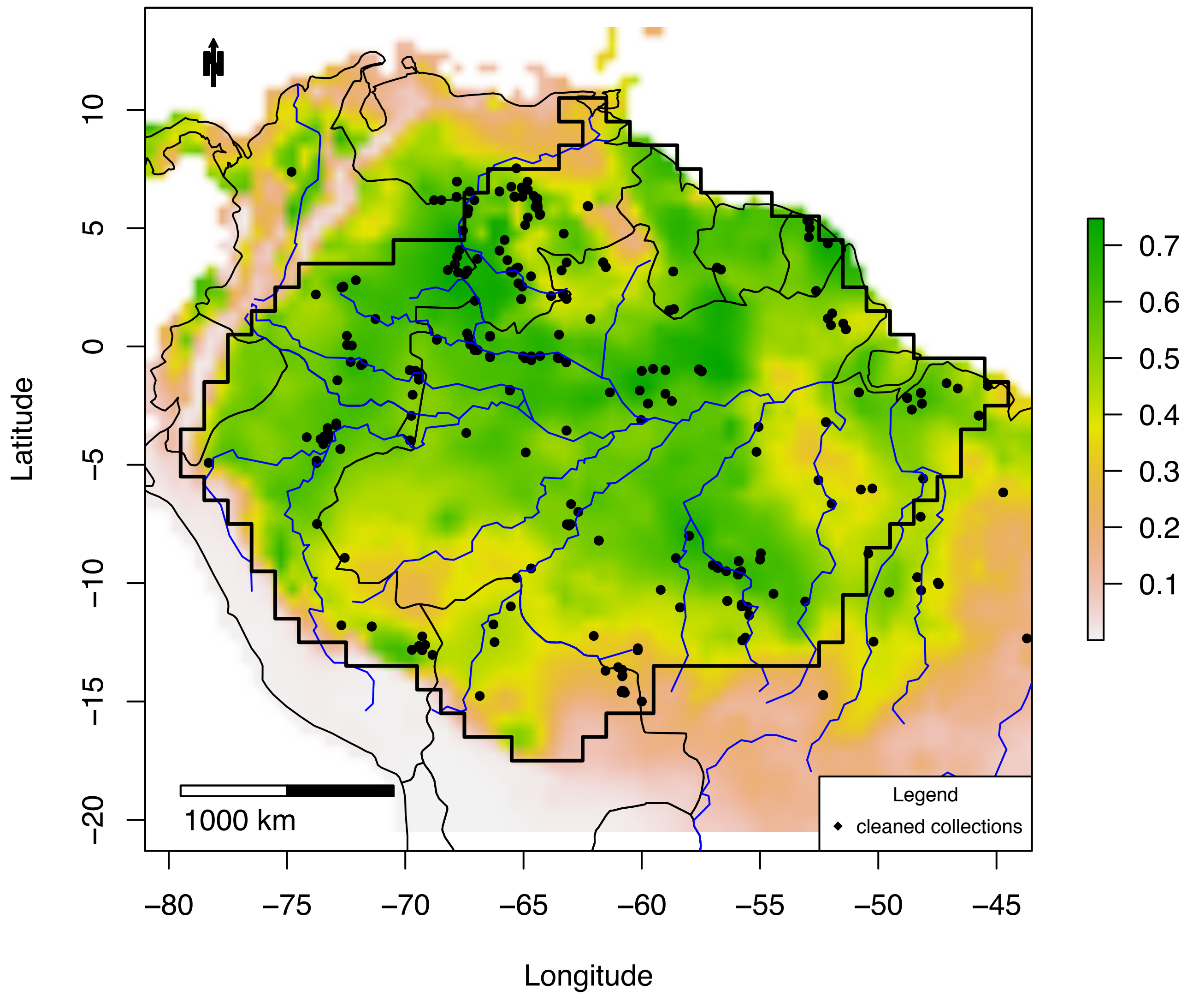

Figure S3_17C IDW map of Caraipa_densifolia: plots =0 – 0.14; IDW: 0 – 0.03

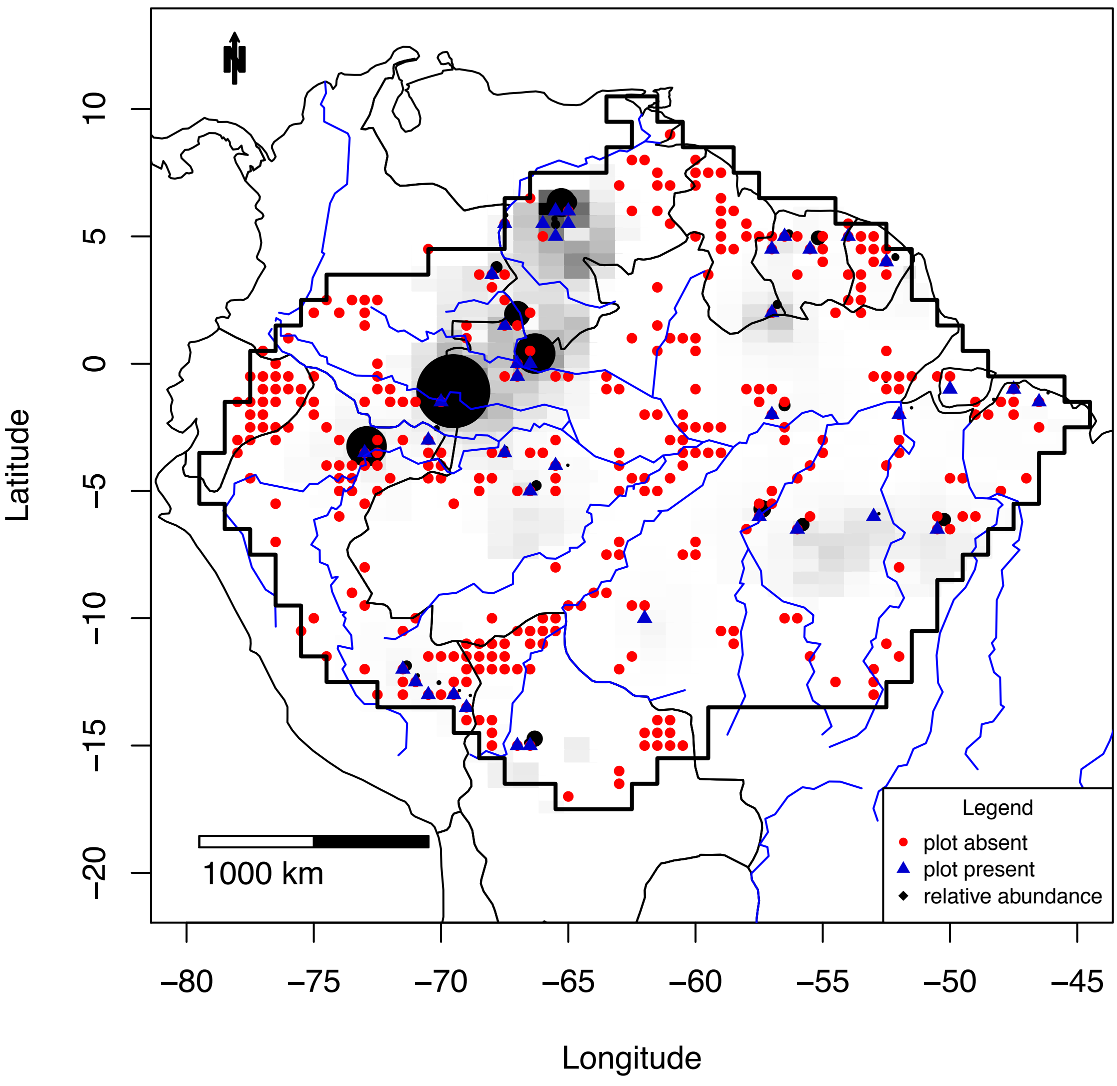

Figure S3_18A Combined map of Carapa_guianensis

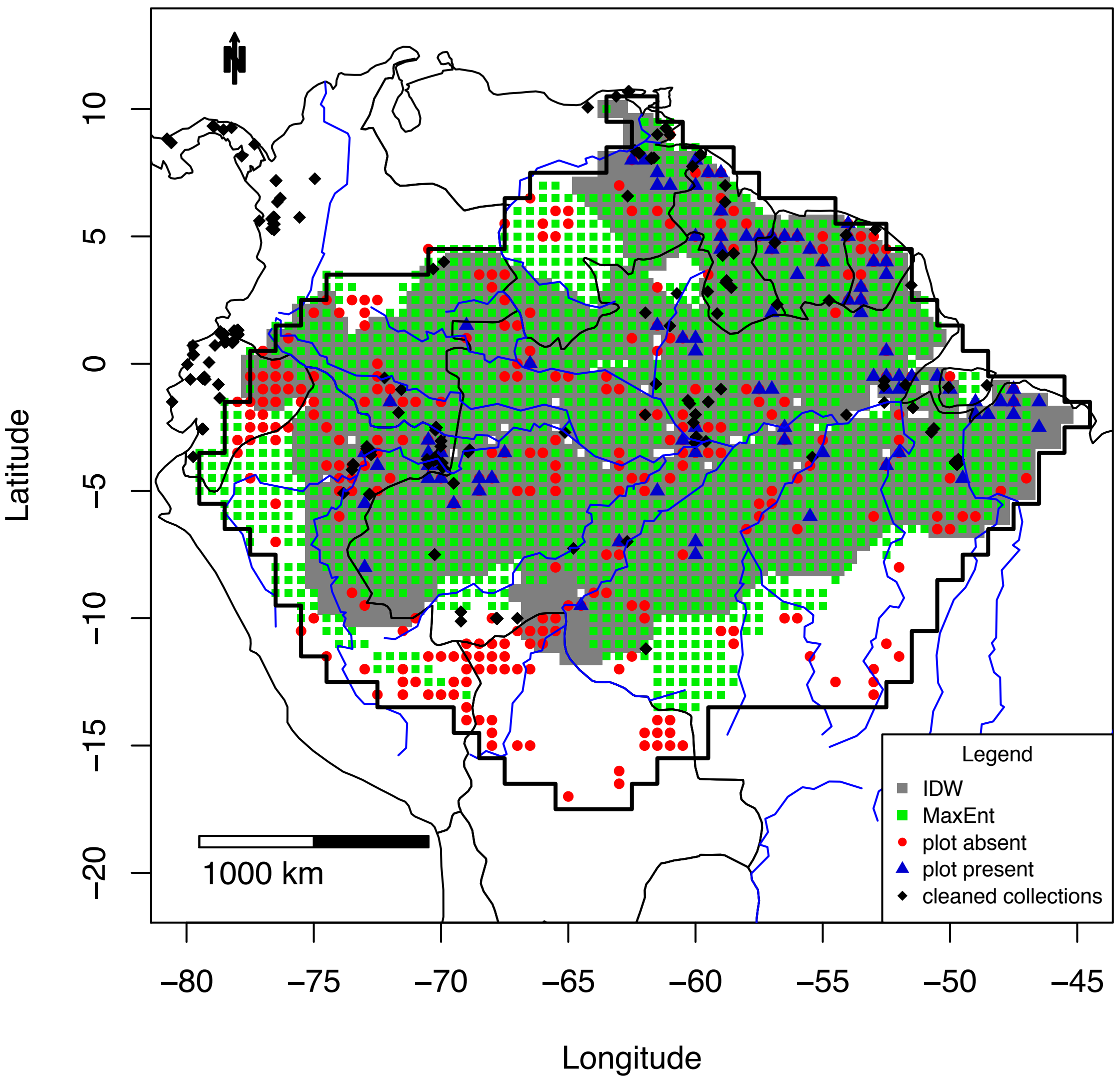

Figure S3_18B MaxEnt map of Carapa_guianensis

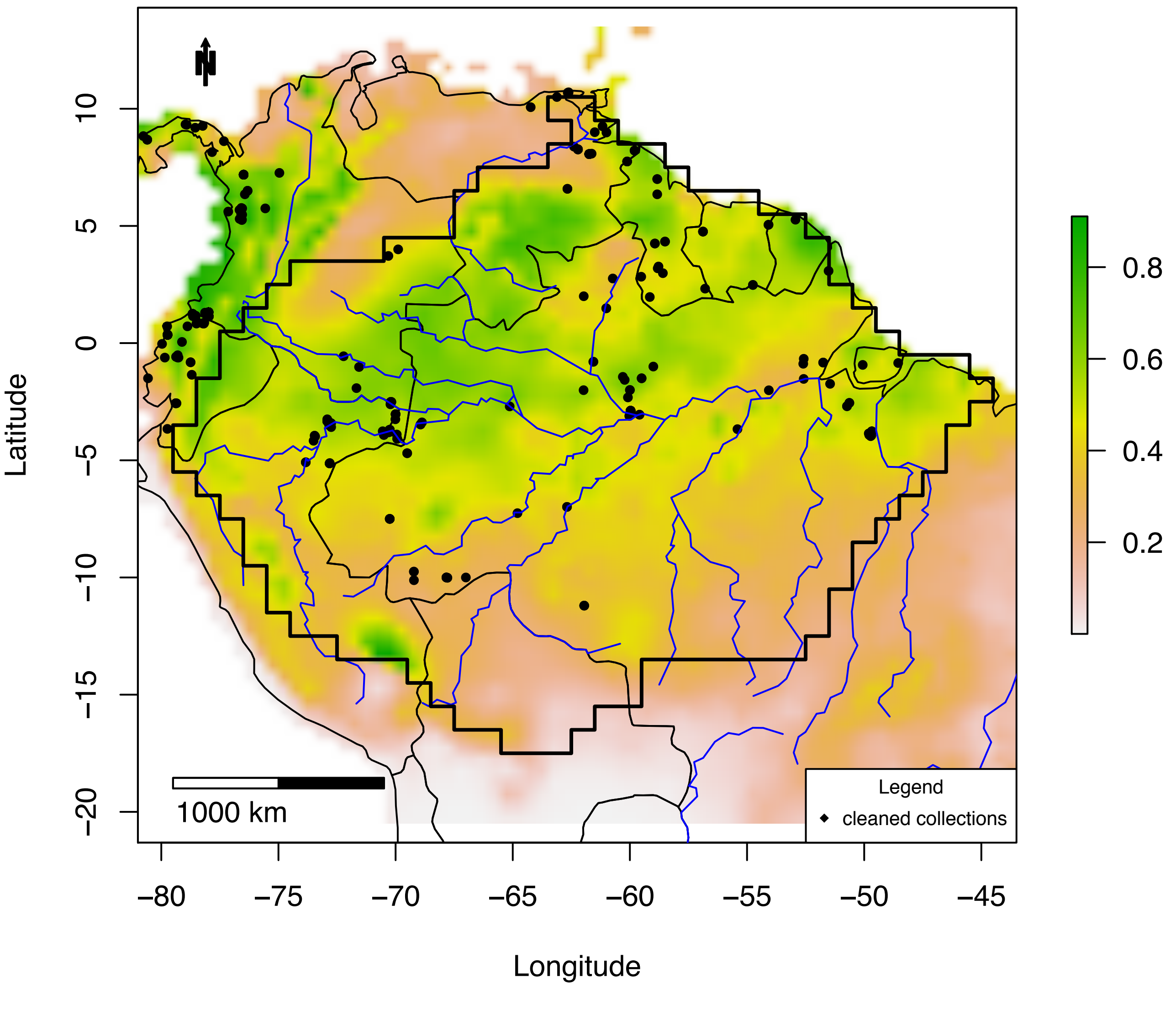

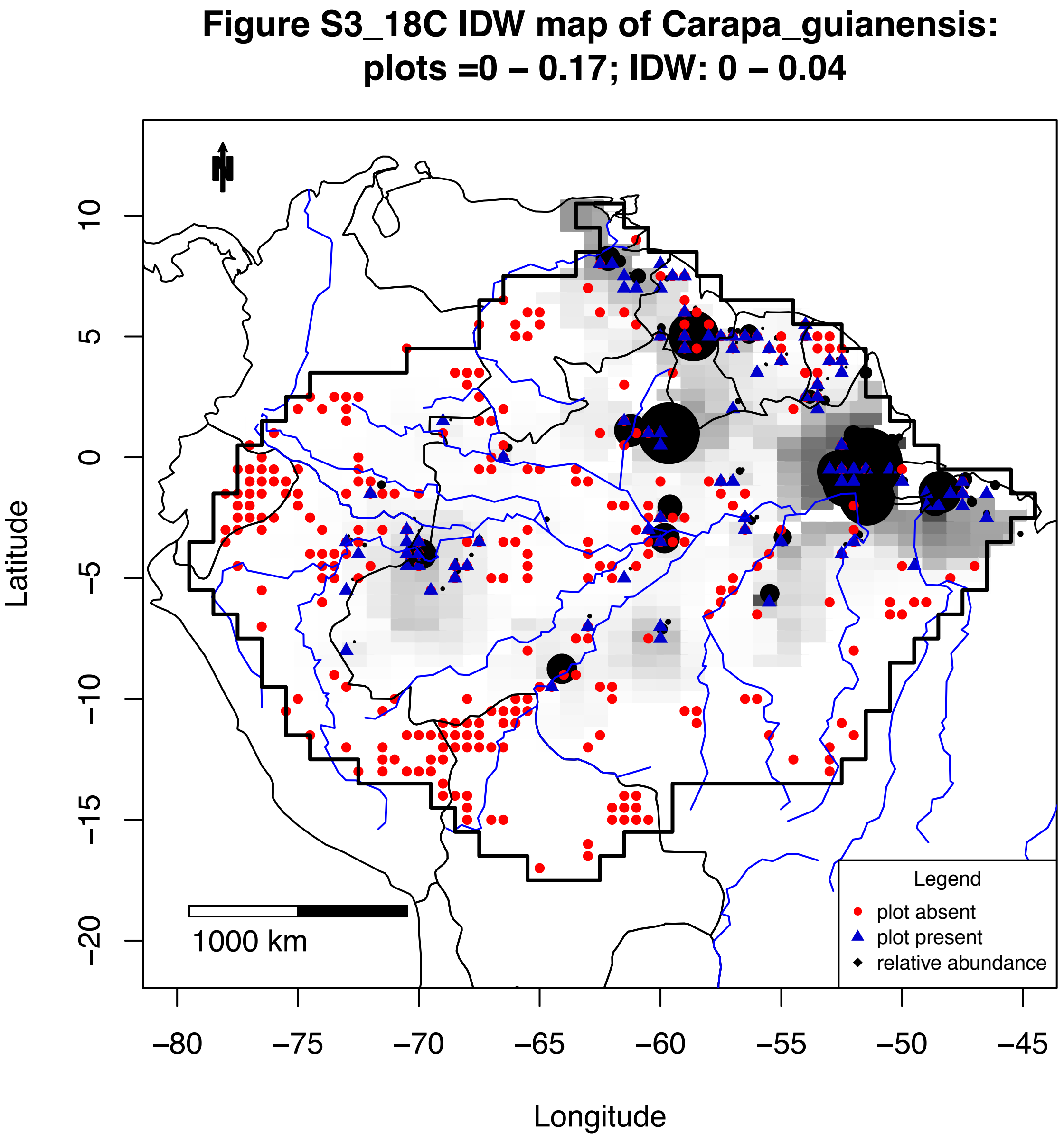

Figure S3_18C IDW map of Carapa_guianensis:
plots =0 – 0.17; IDW: 0 – 0.04
Latitude
Longitude
10
5
0
−5
−10
−15
−20
−80
−75
−70
−65
−60
−55
−50
−45
N
1000 km
Legend
plot absent
plot present
relative abundance

Figure S3_19A Combined map of Caryocar_glabrum

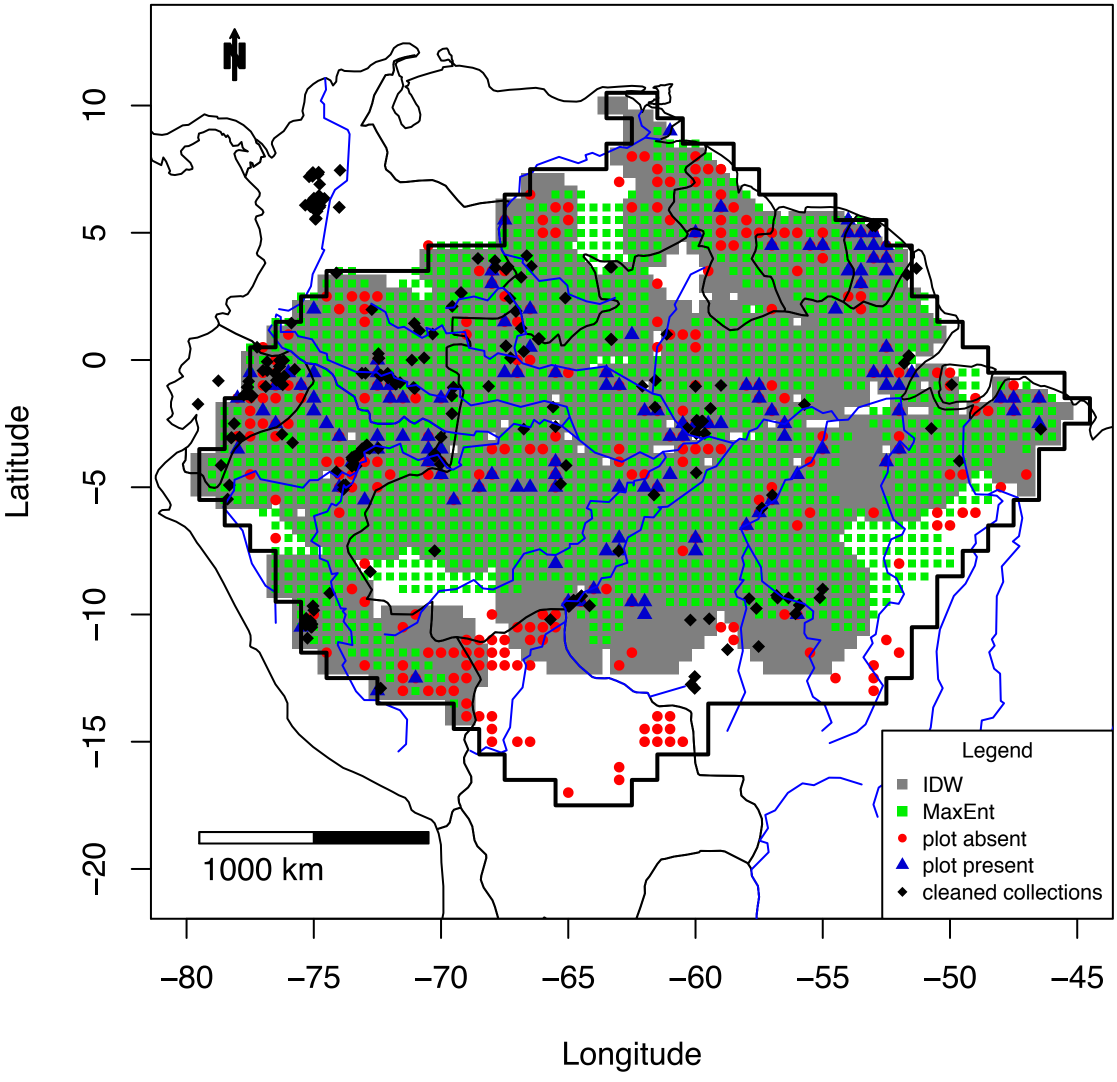

Figure S3_19B MaxEnt map of Caryocar_glabrum

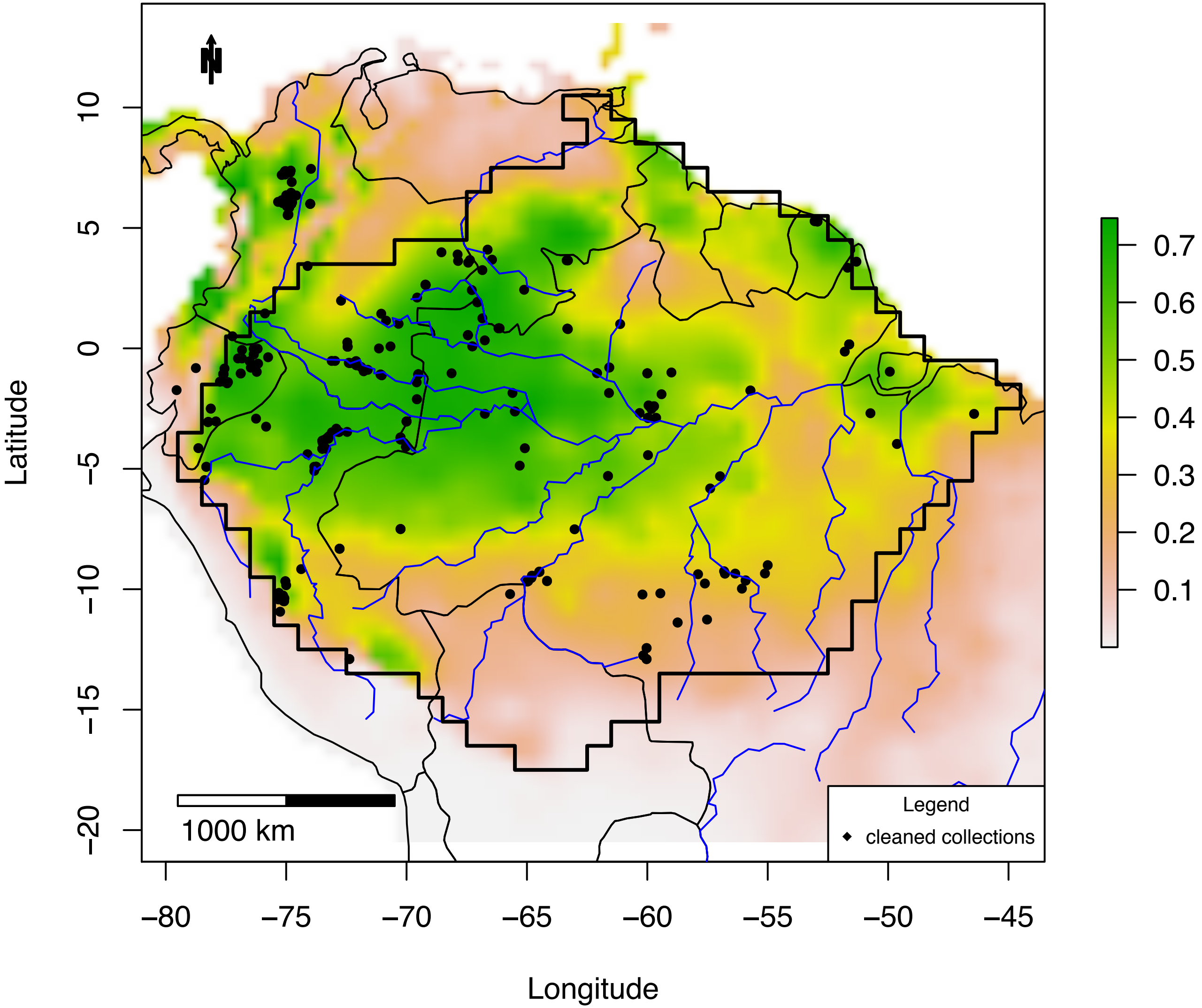

Figure S3_19C IDW map of Caryocar_glabrum:
plots =0 – 0.06; IDW: 0 – 0.03

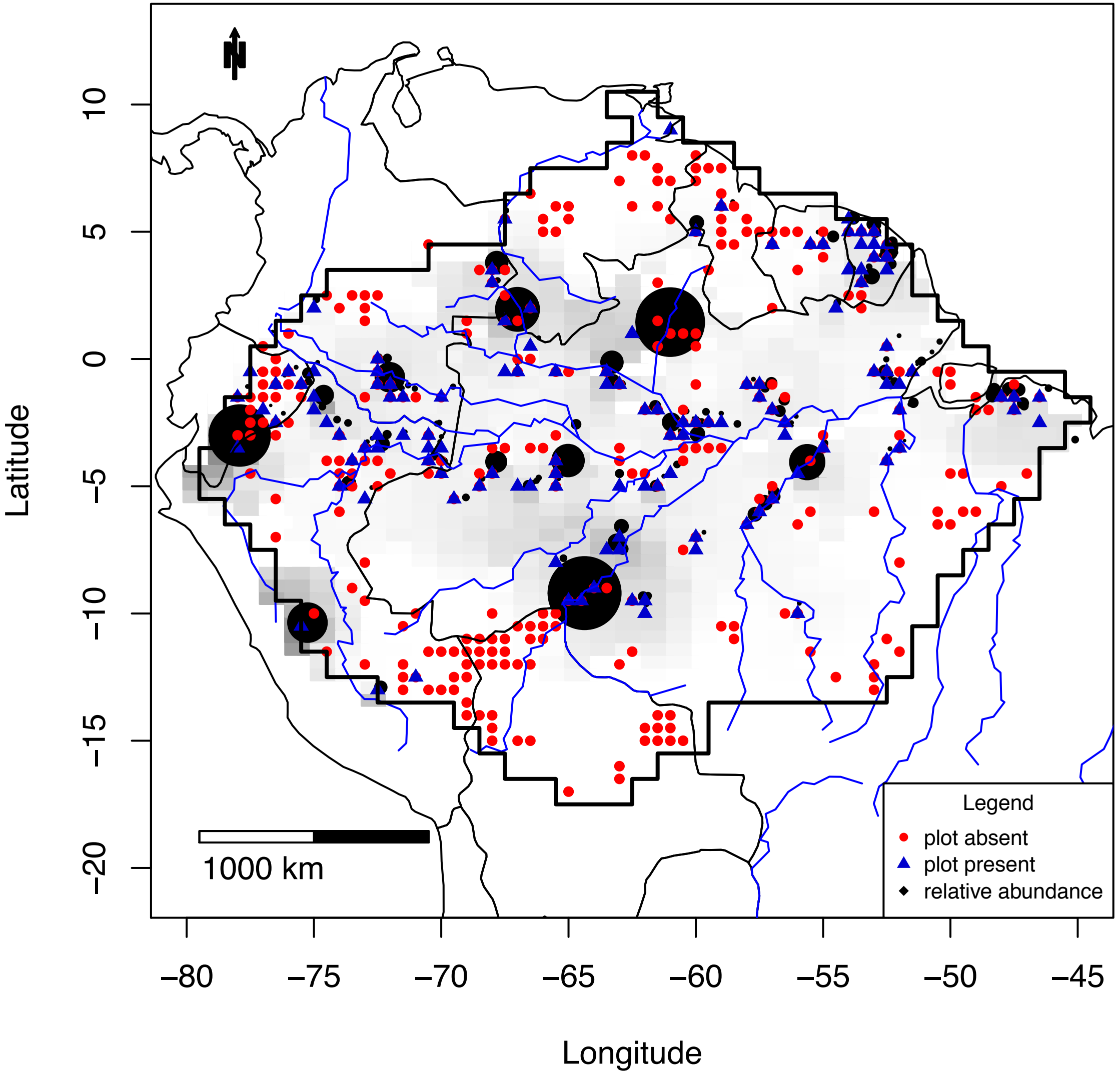

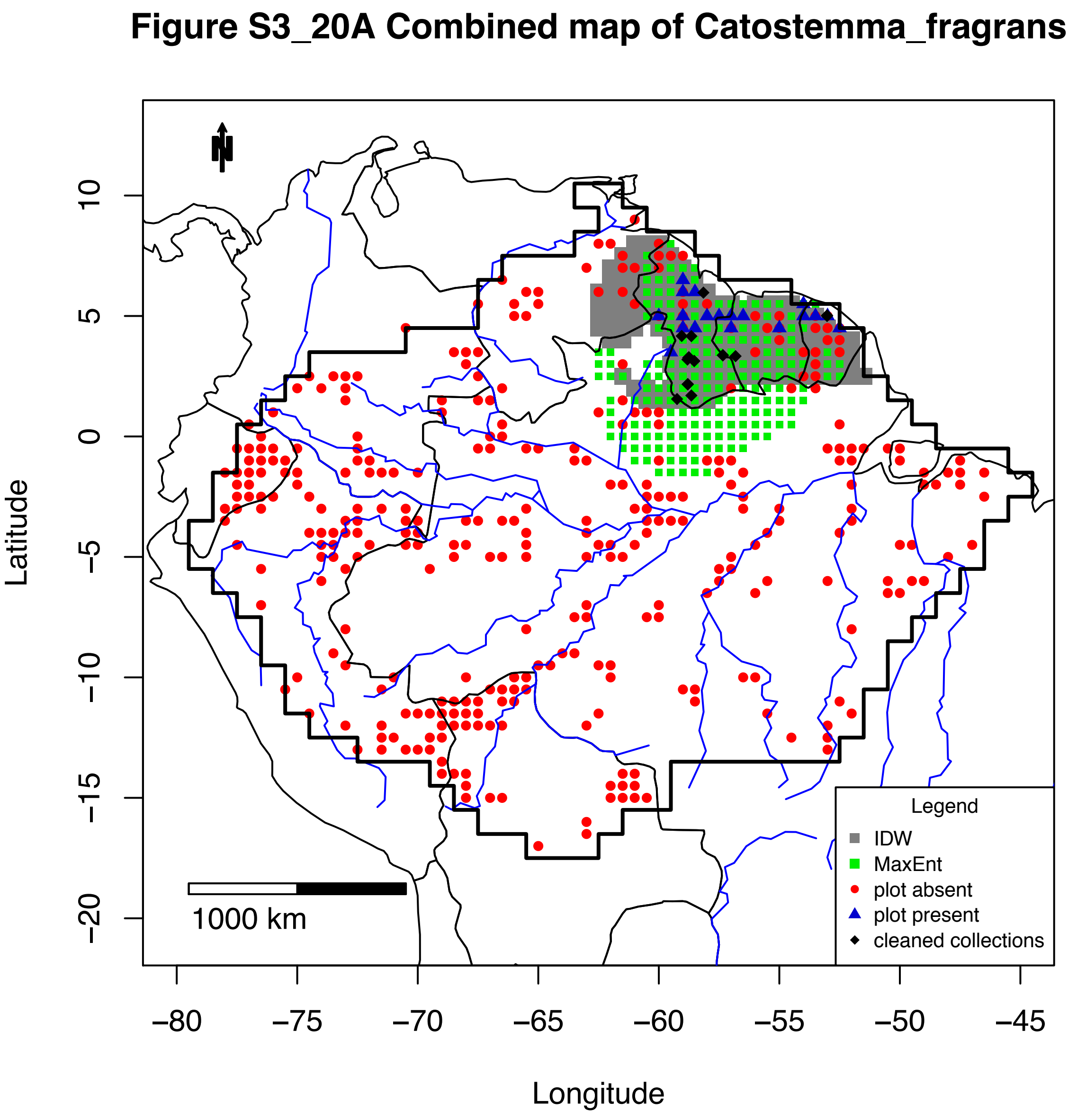

Figure S3_20A Combined map of Catostemma_fragrans
Latitude
Longitude
10
5
0
−5
−10
−15
−20
−80
−75
−70
−65
−60
−55
−50
−45
N
1000 km
Legend
IDW
MaxEnt
plot absent
plot present
cleaned collections

Figure S3_20B MaxEnt map of Catostemma_fragrans

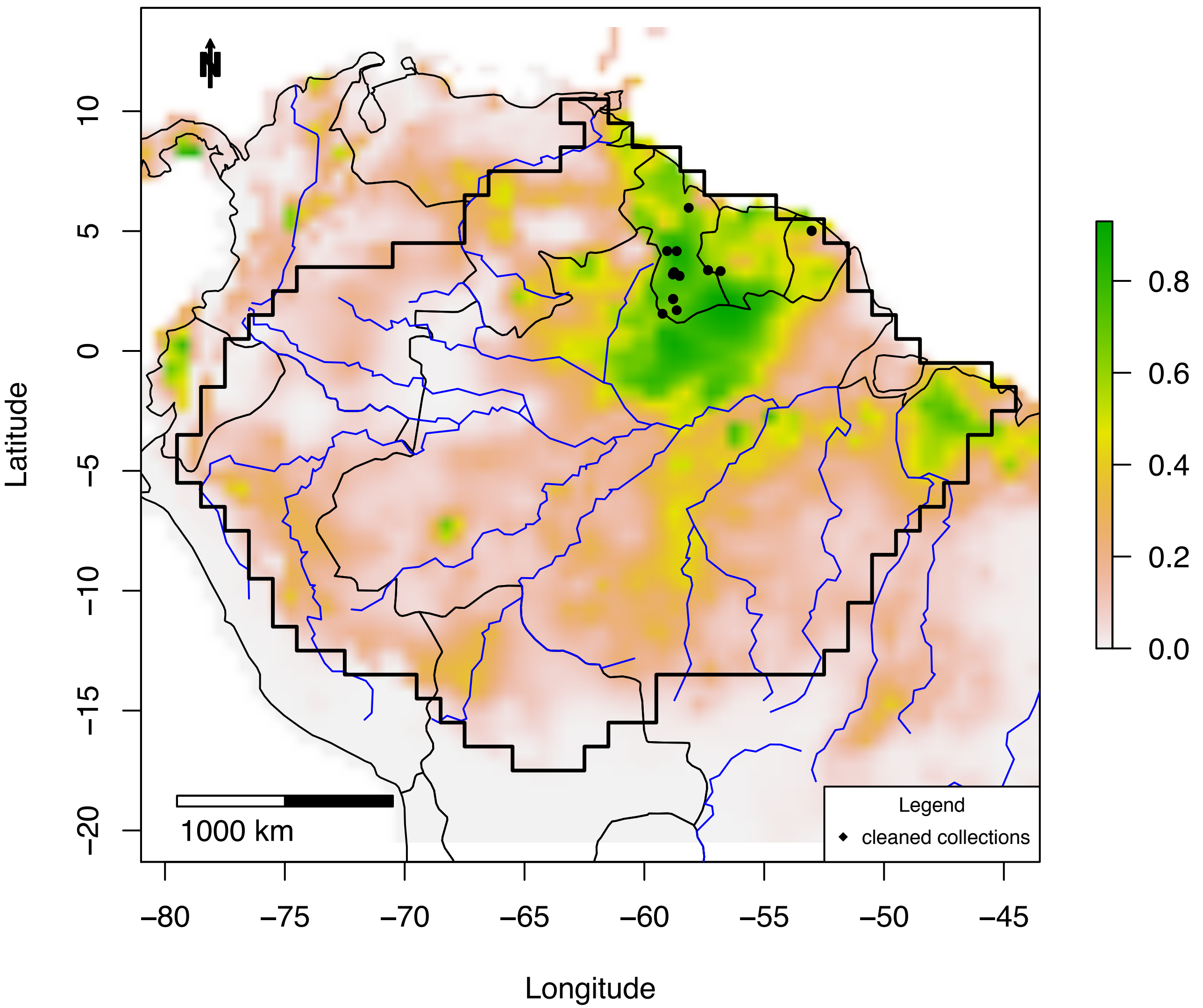

# Figure S3_20C IDW map of Catostemma_fragrans:
plots =0 – 0.33; IDW: 0 – 0.09

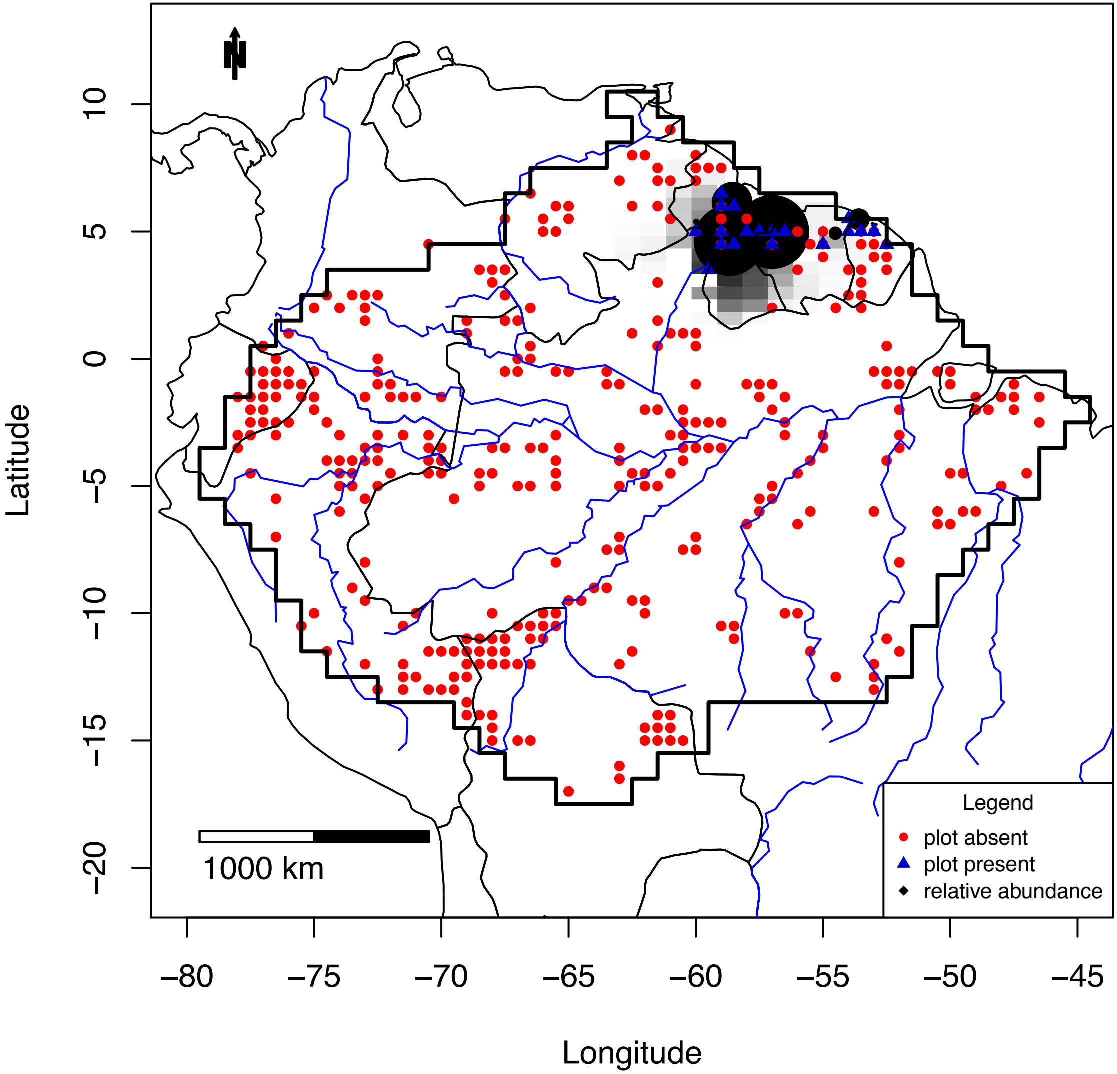

Figure S3_21A Combined map of Cecropia_latiloba

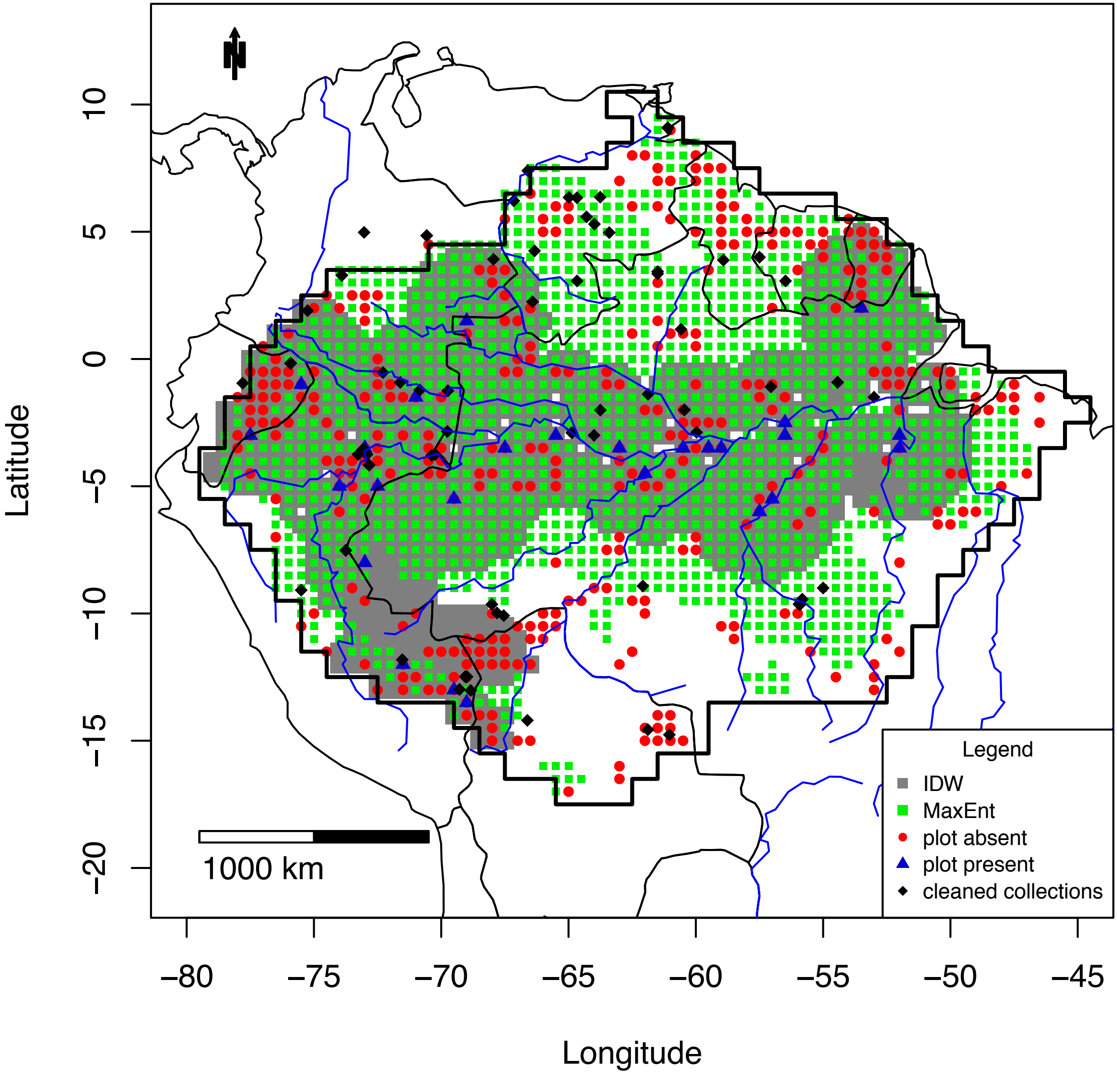

Figure S3_21B MaxEnt map of Cecropia_latiloba

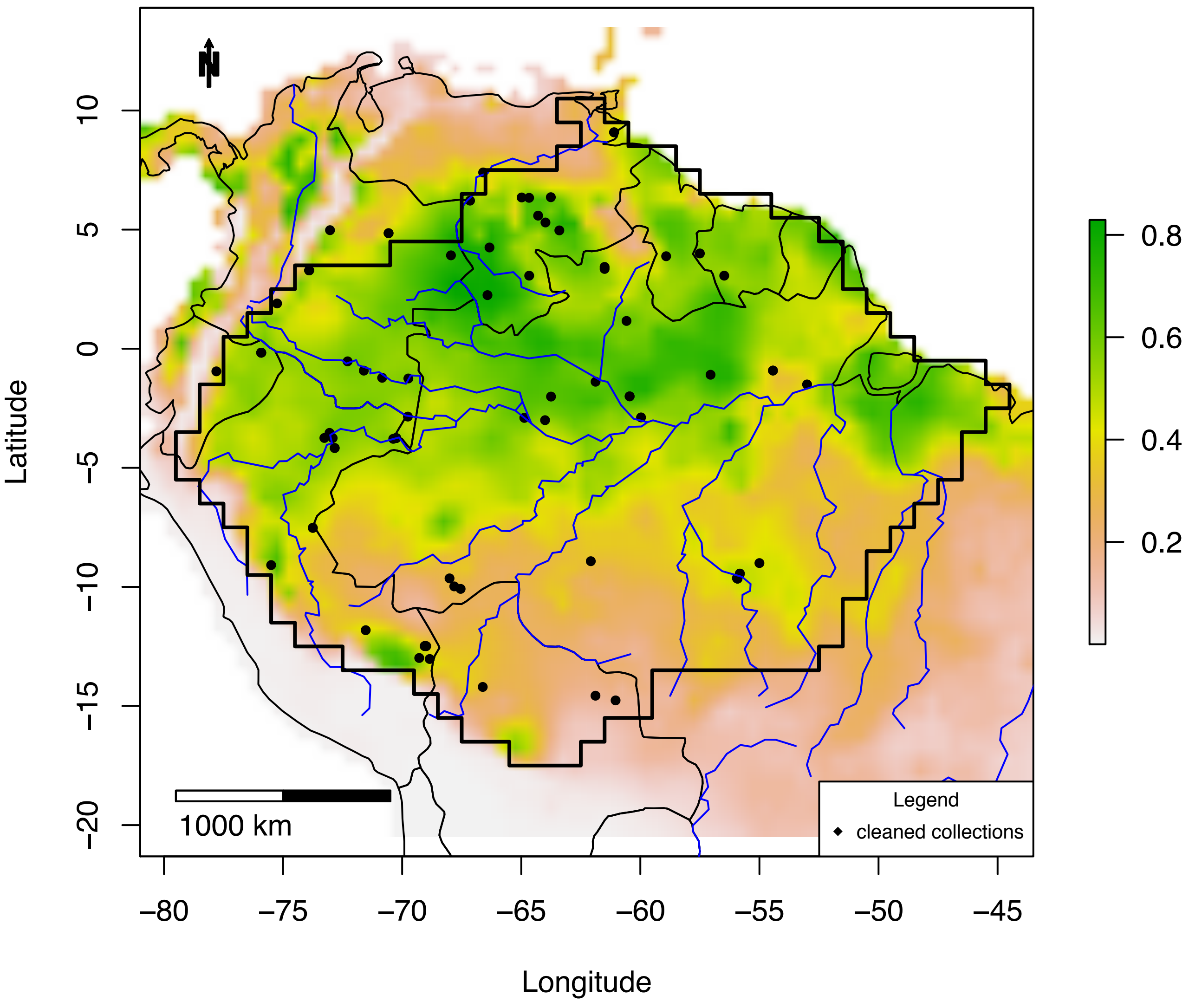

Figure S3_21C IDW map of Cecropia_latiloba: plots =0 – 0.32; IDW: 0 – 0.03

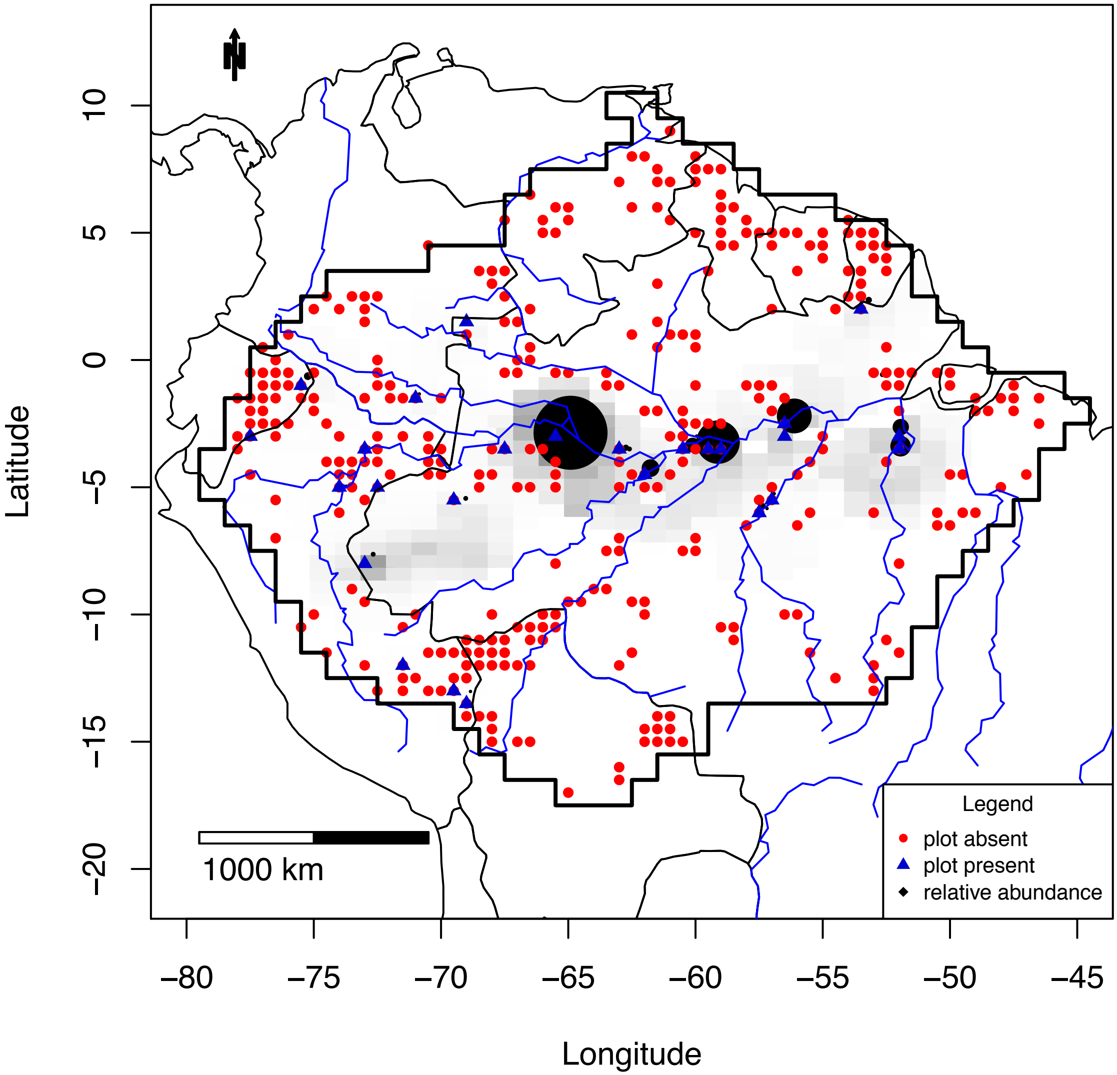

Figure S3_22A Combined map of Cecropia_membranacea

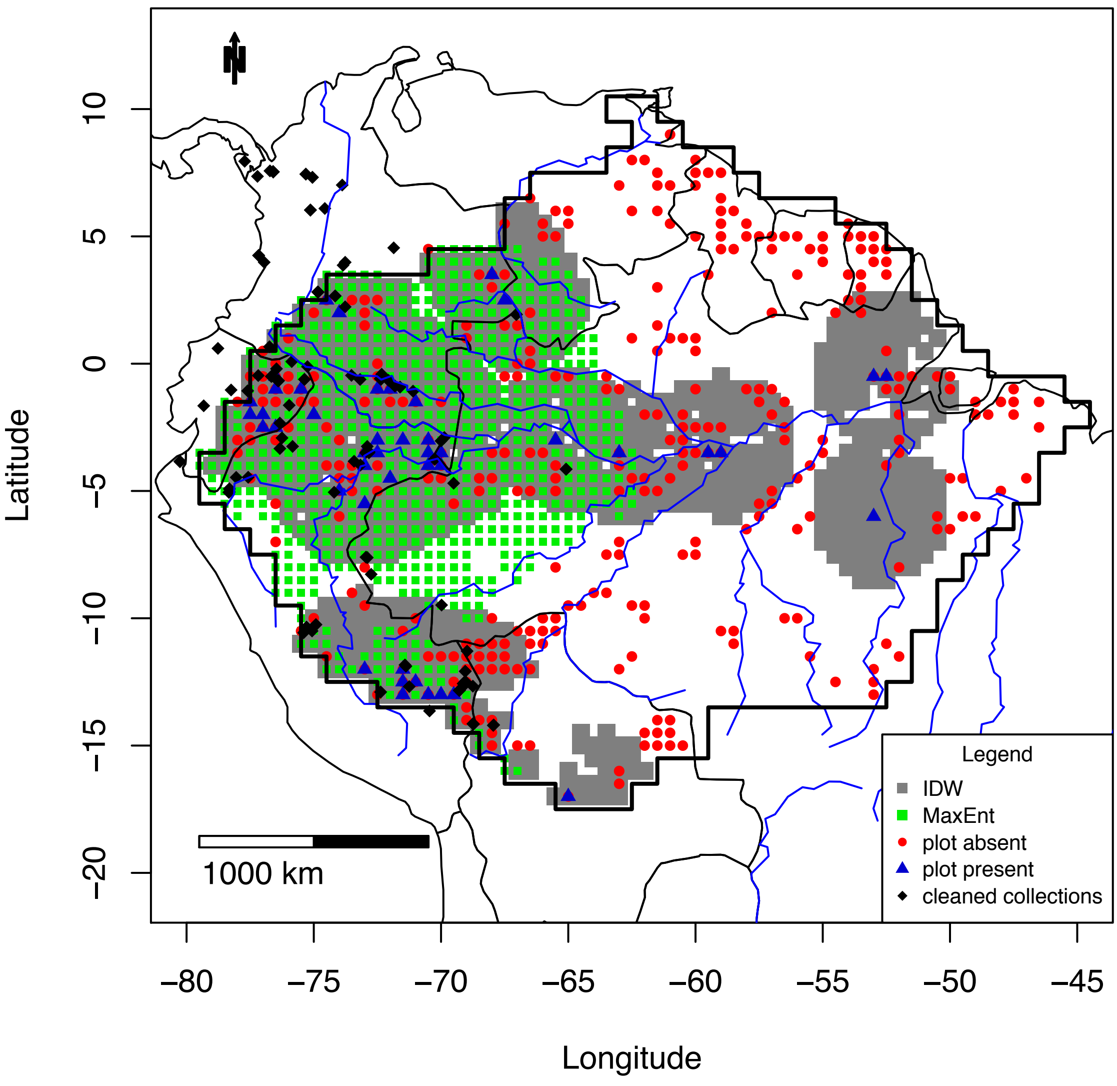

Figure S3_22B MaxEnt map of Cecropia_membranacea

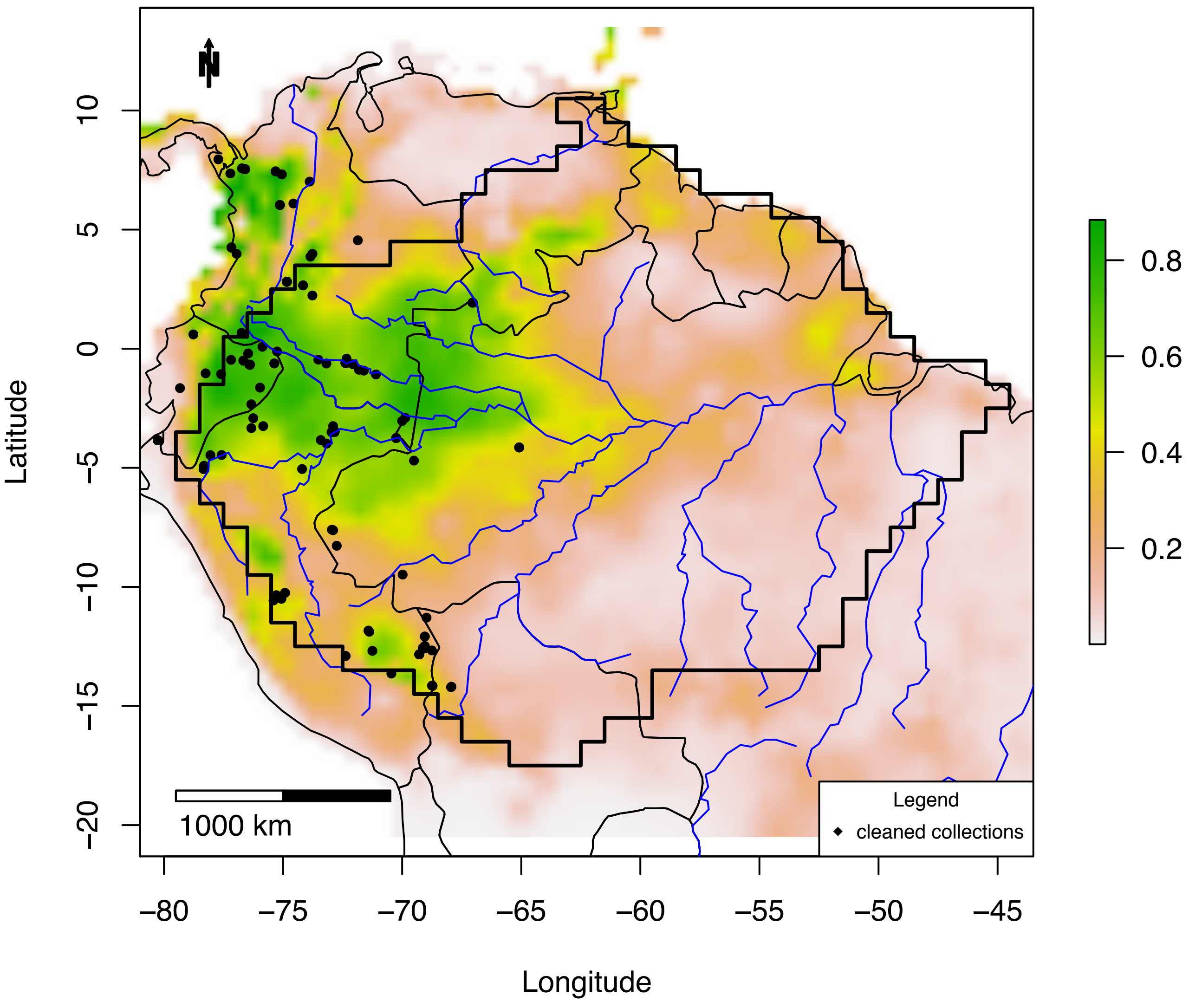

Figure S3_22C IDW map of Cecropia_membranacea: plots =0 – 0.41; IDW: 0 – 0.03

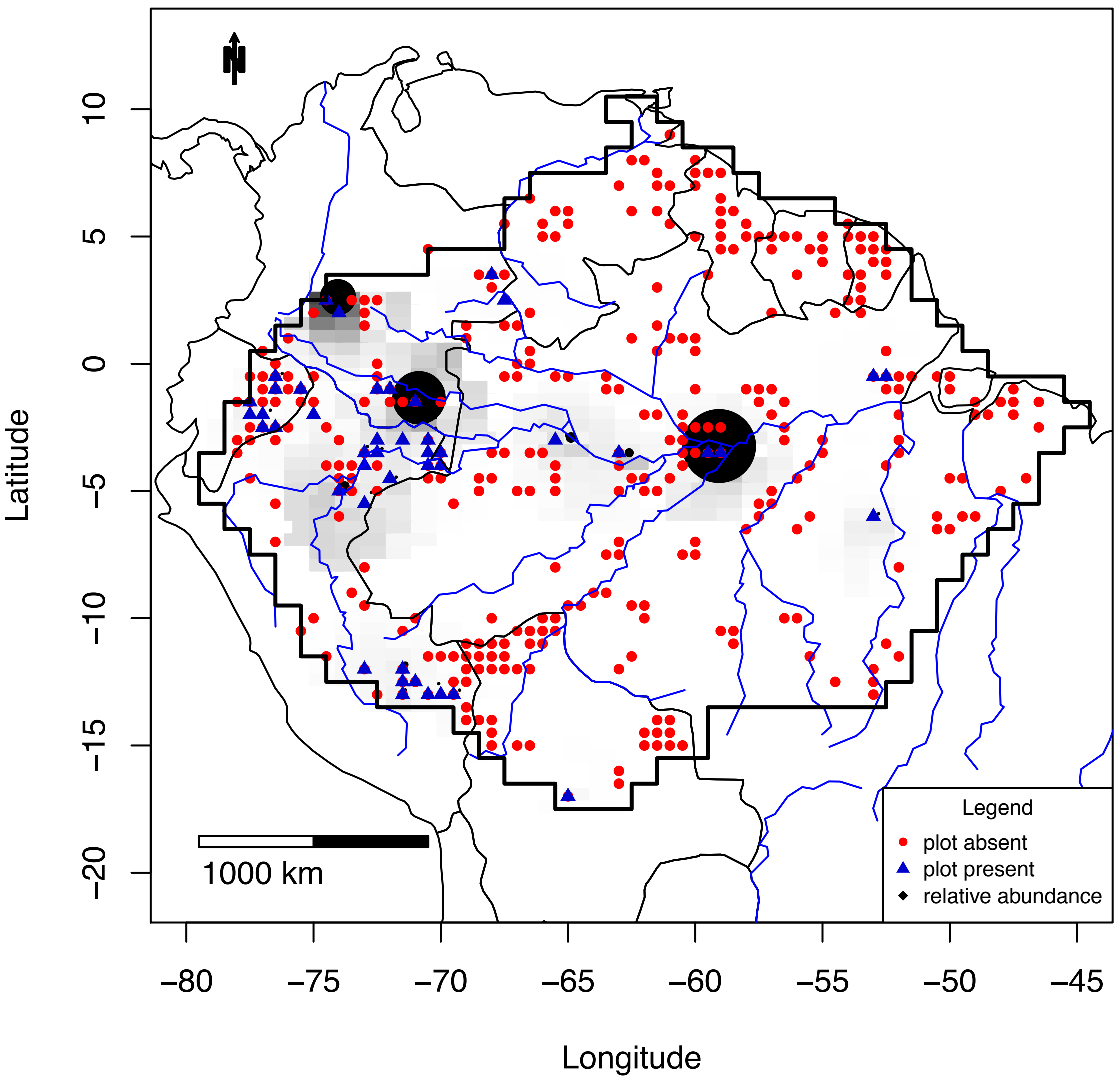

Figure S3_23A Combined map of Cecropia_sciadophylla

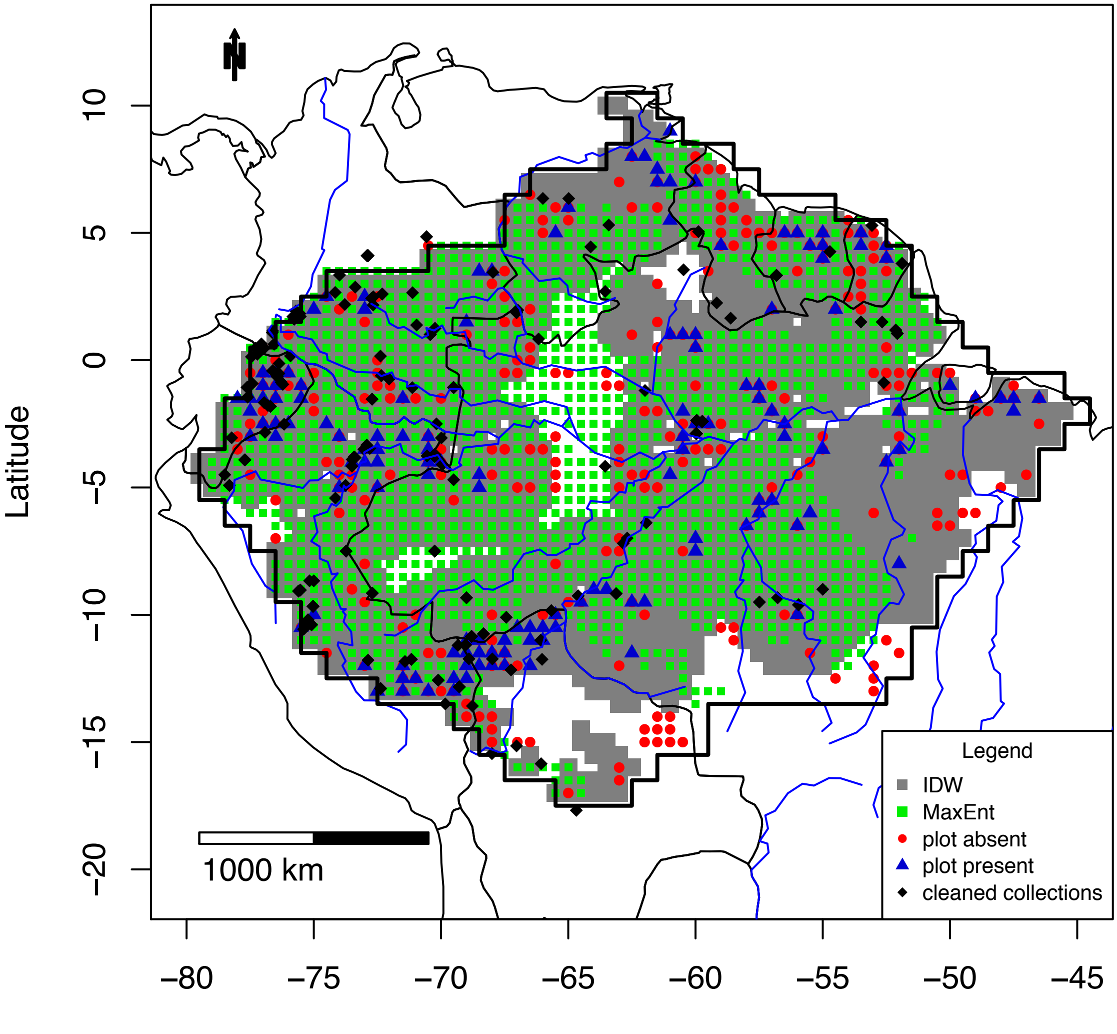

Figure S3_23B MaxEnt map of Cecropia_sciadophylla

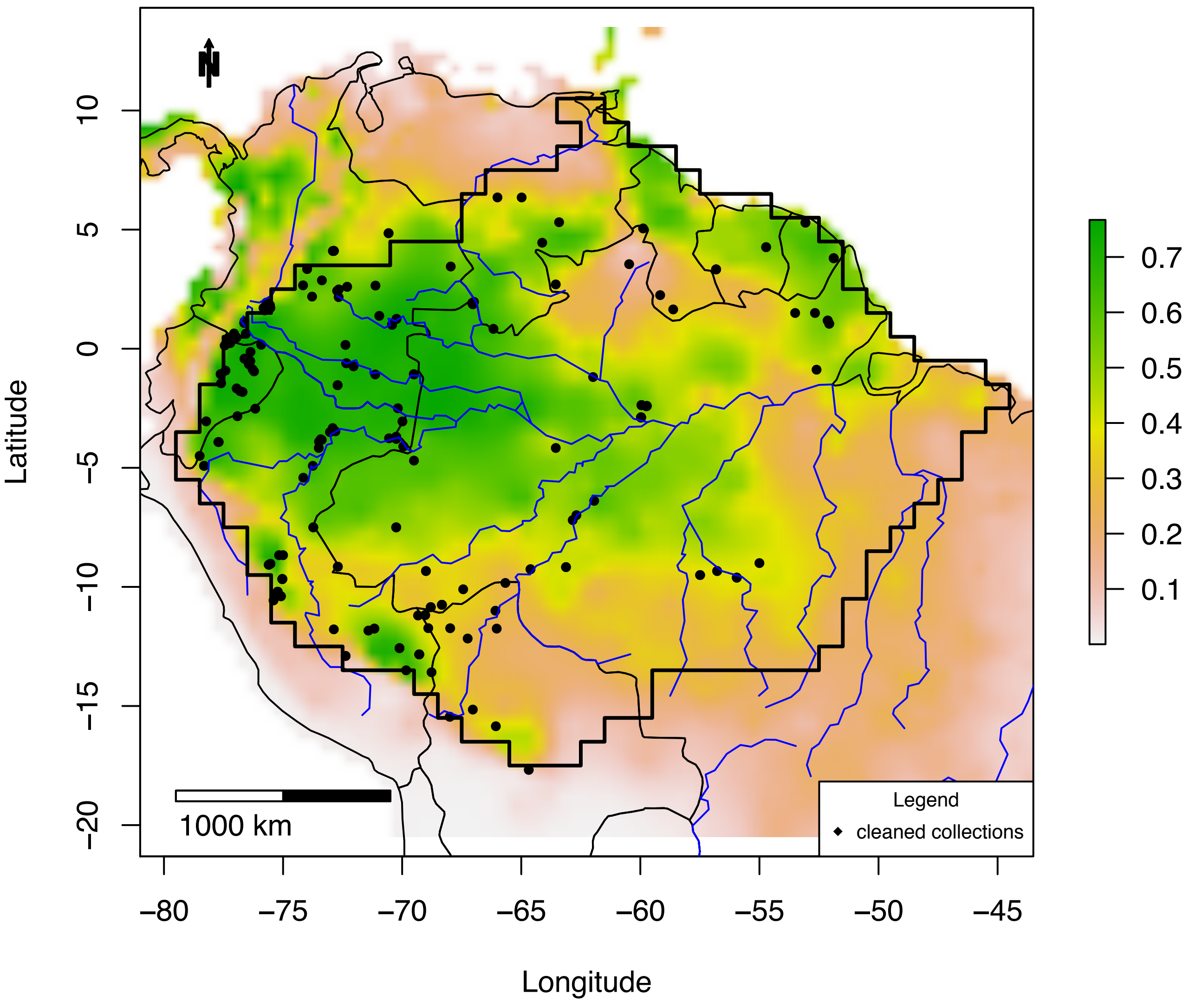

Figure S3_23C IDW map of Cecropia_sciadophylla: plots =0 – 0.21; IDW: 0 – 0.1

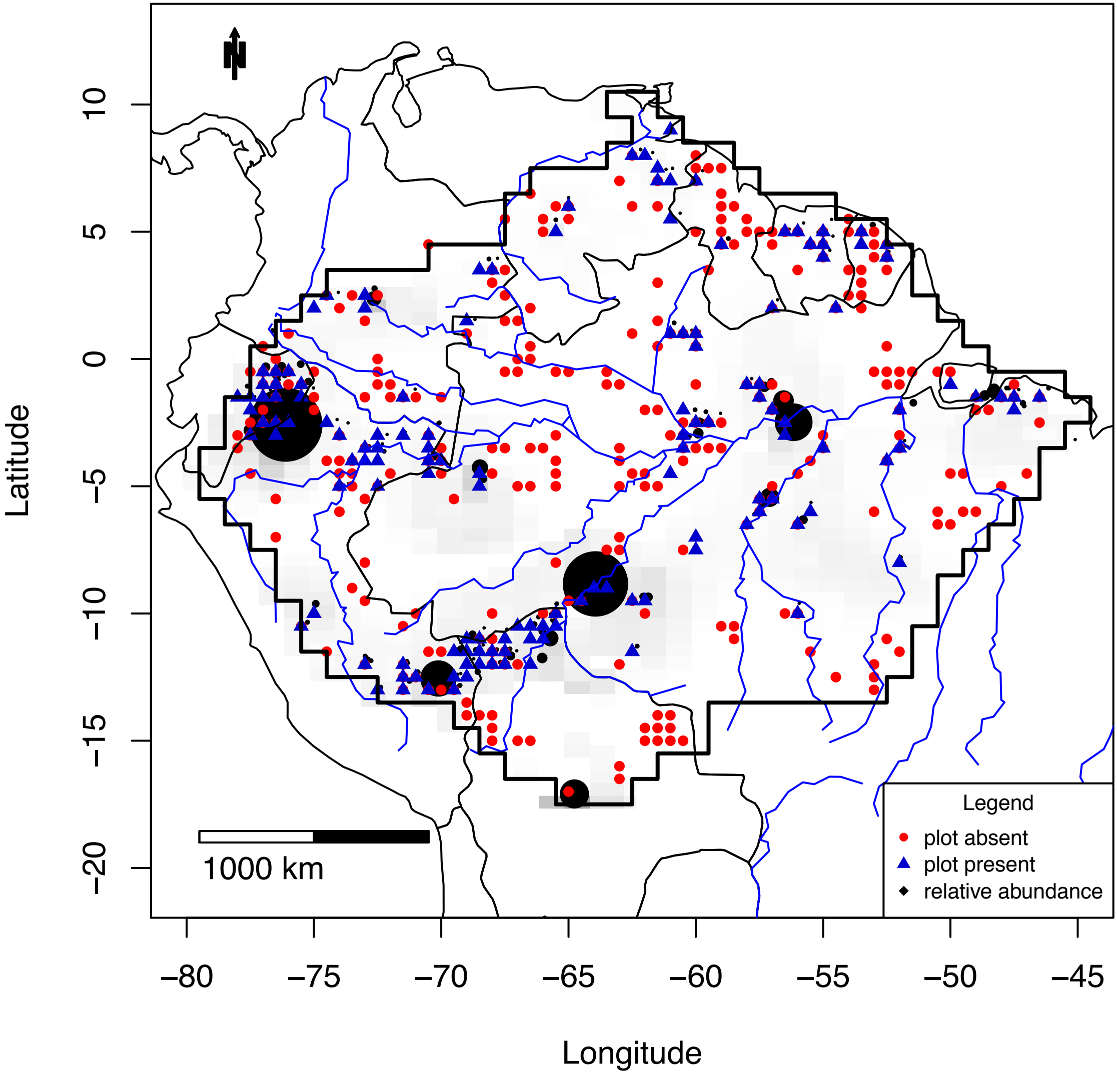

Figure S3_24A Combined map of Cedrelinga_cateniformis

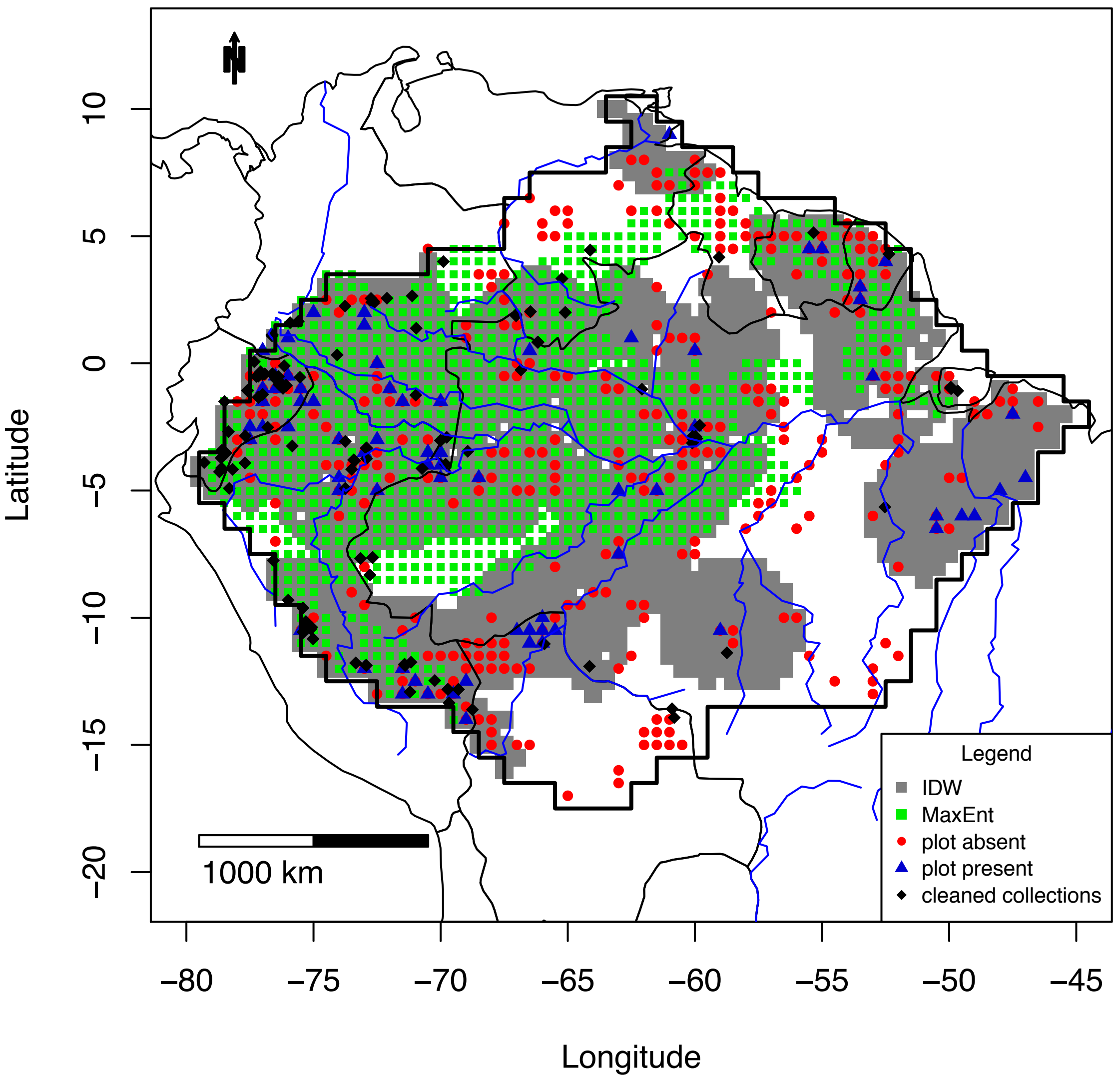

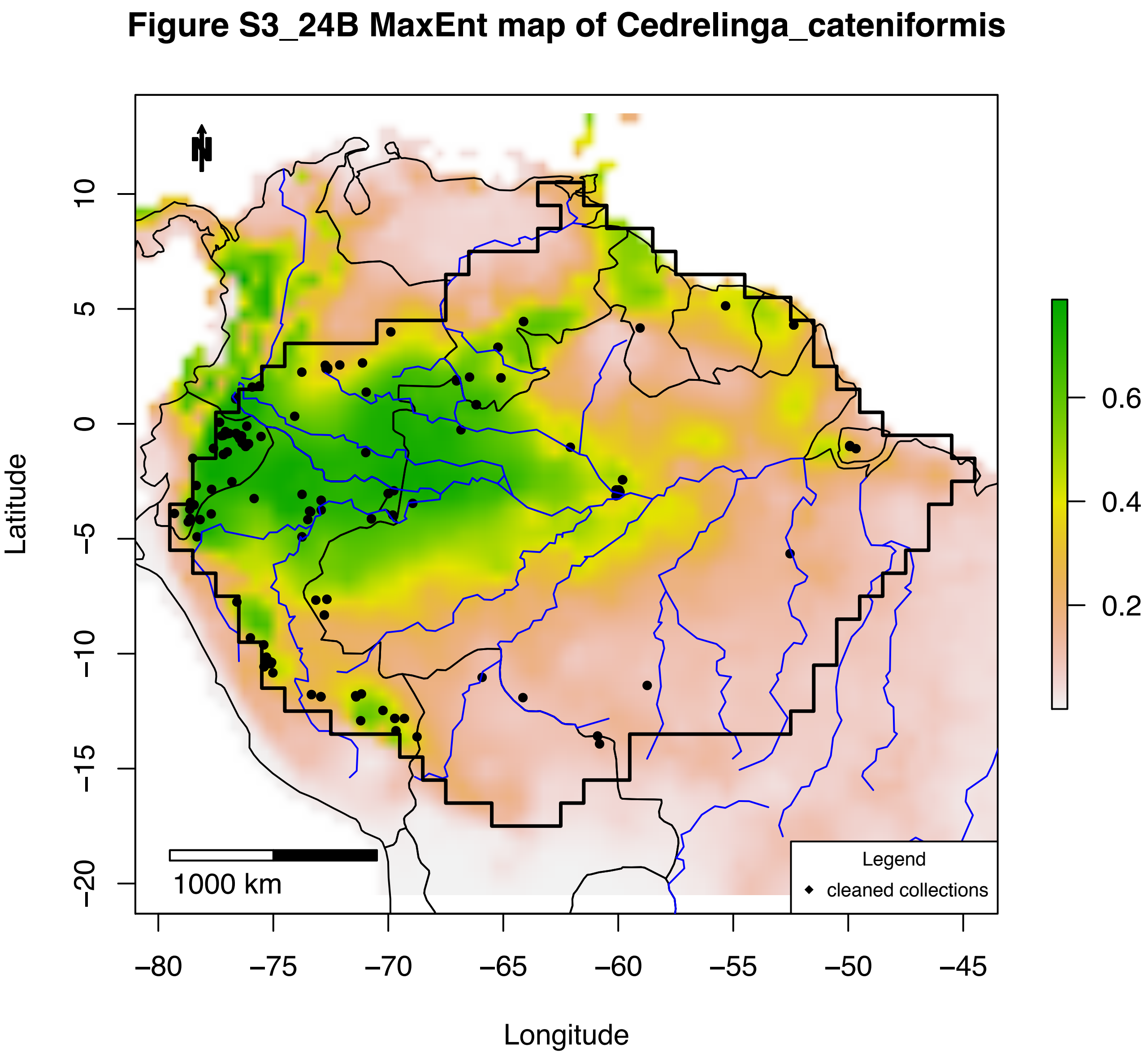

Figure S3_24B MaxEnt map of Cedrelinga_cateniformis
N
10
5
0
−5
−10
−15
−20
Latitude
−80
−75
−70
−65
−60
−55
−50
−45
Longitude
0.6
0.4
0.2
1000 km
Legend
cleaned collections

Figure S3_24C IDW map of Cedrelinga_cateniformis: plots =0 – 0.09; IDW: 0 – 0.05

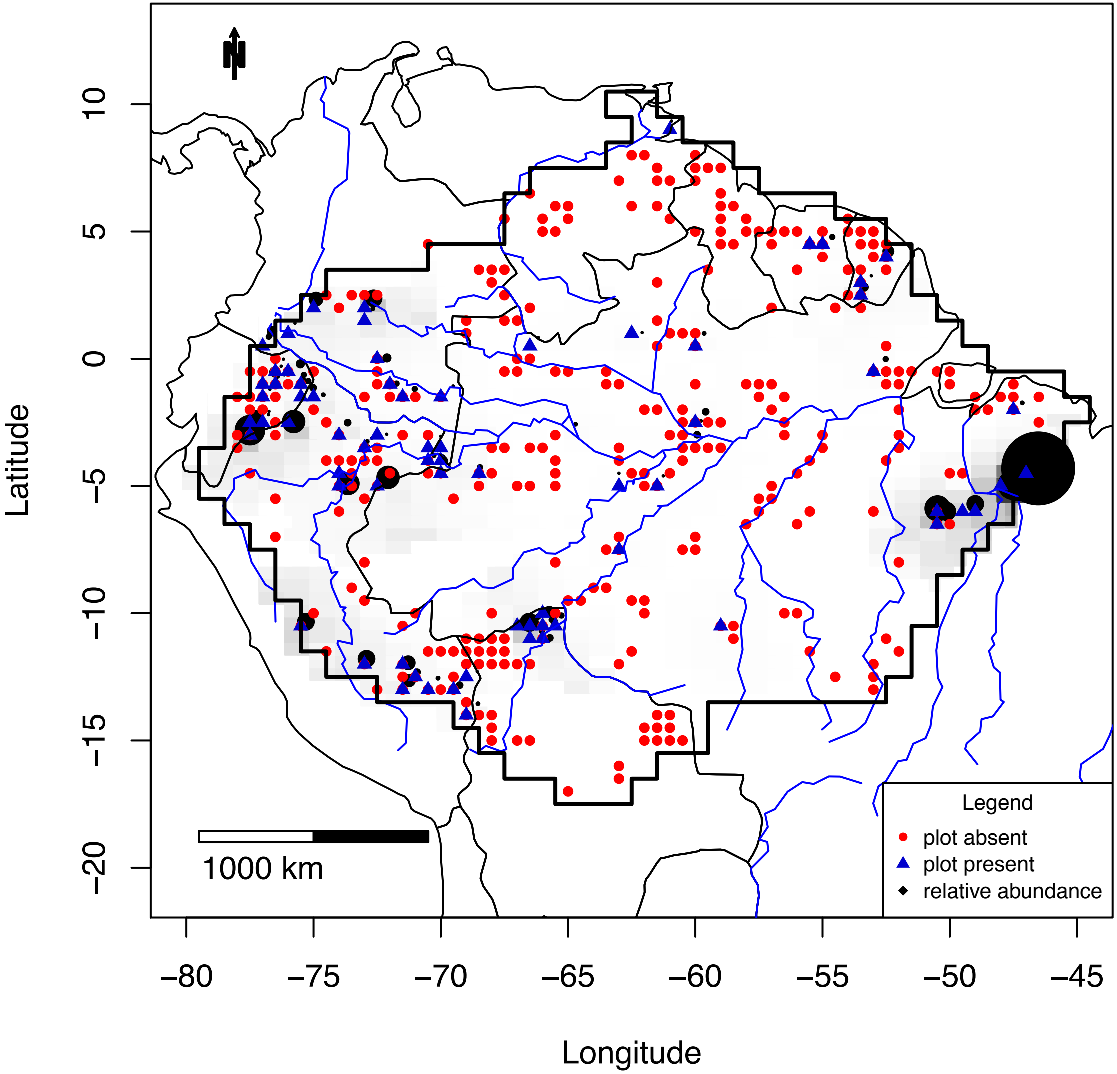

Figure S3_25A Combined map of Celtis_schippii

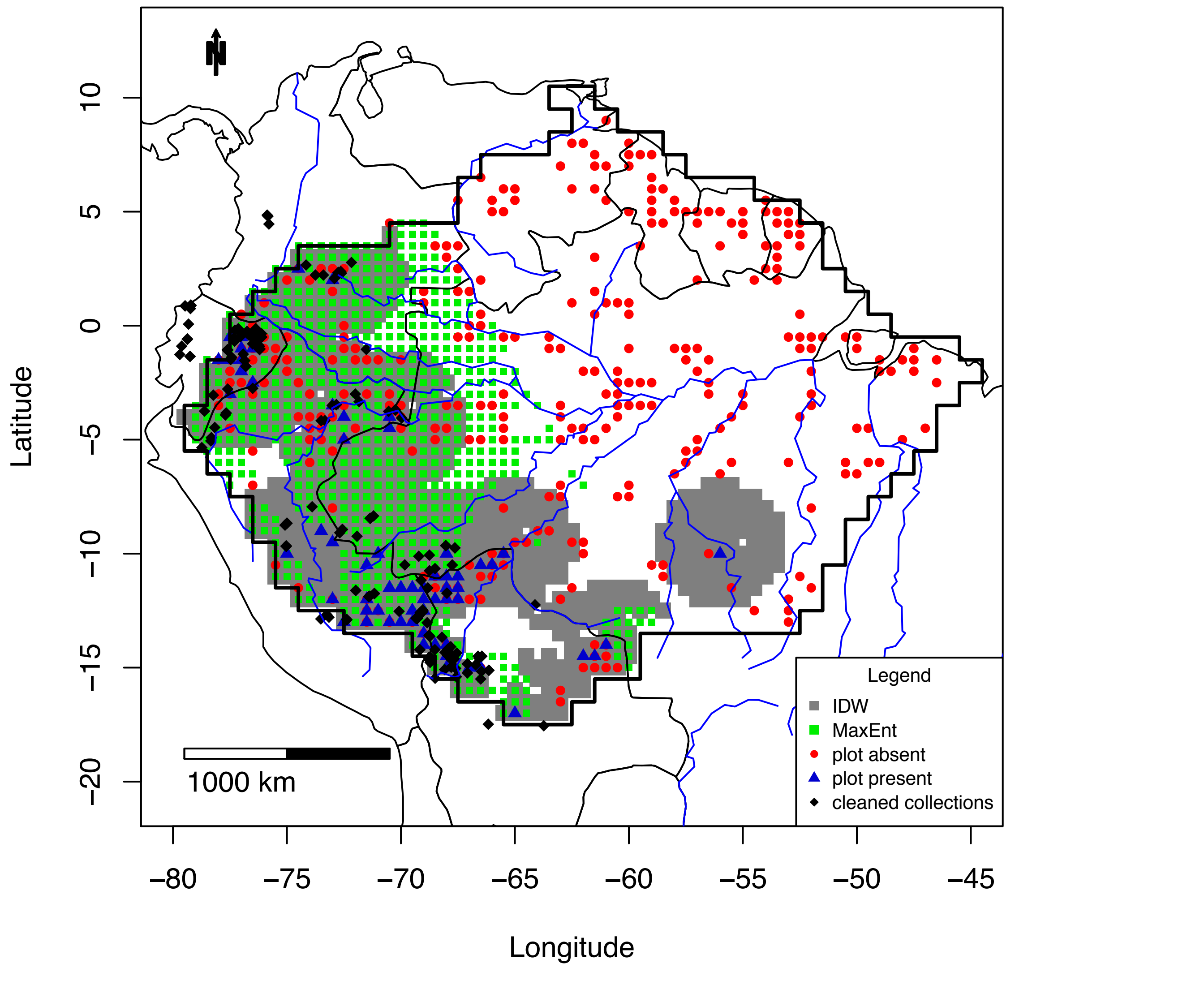

Figure S3_25B MaxEnt map of Celtis_schippii

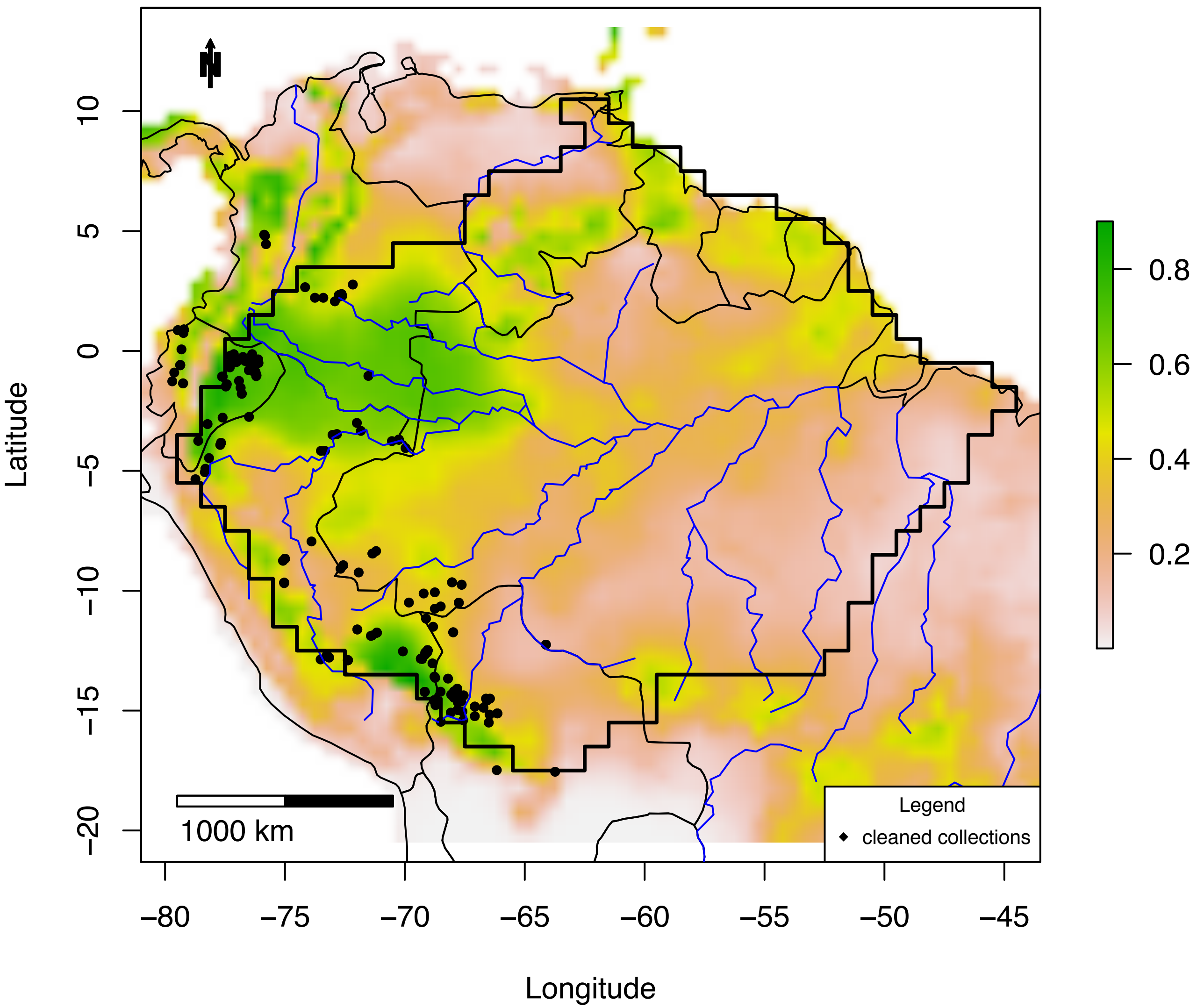

Figure S3_25C IDW map of Celtis_schippii: plots =0 – 0.11; IDW: 0 – 0.03

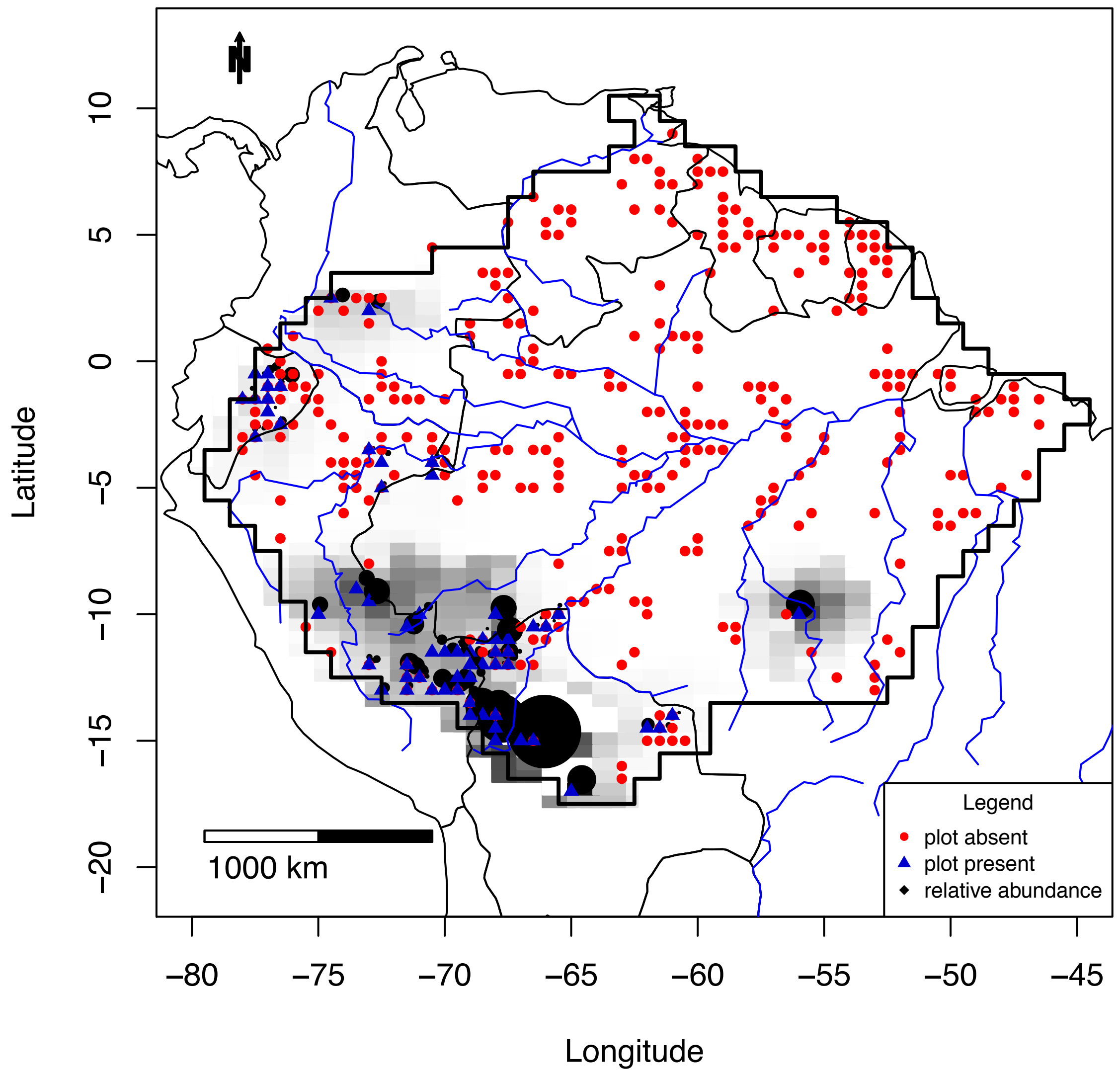

Figure S3_26A Combined map of Cenostigma_tocantinum

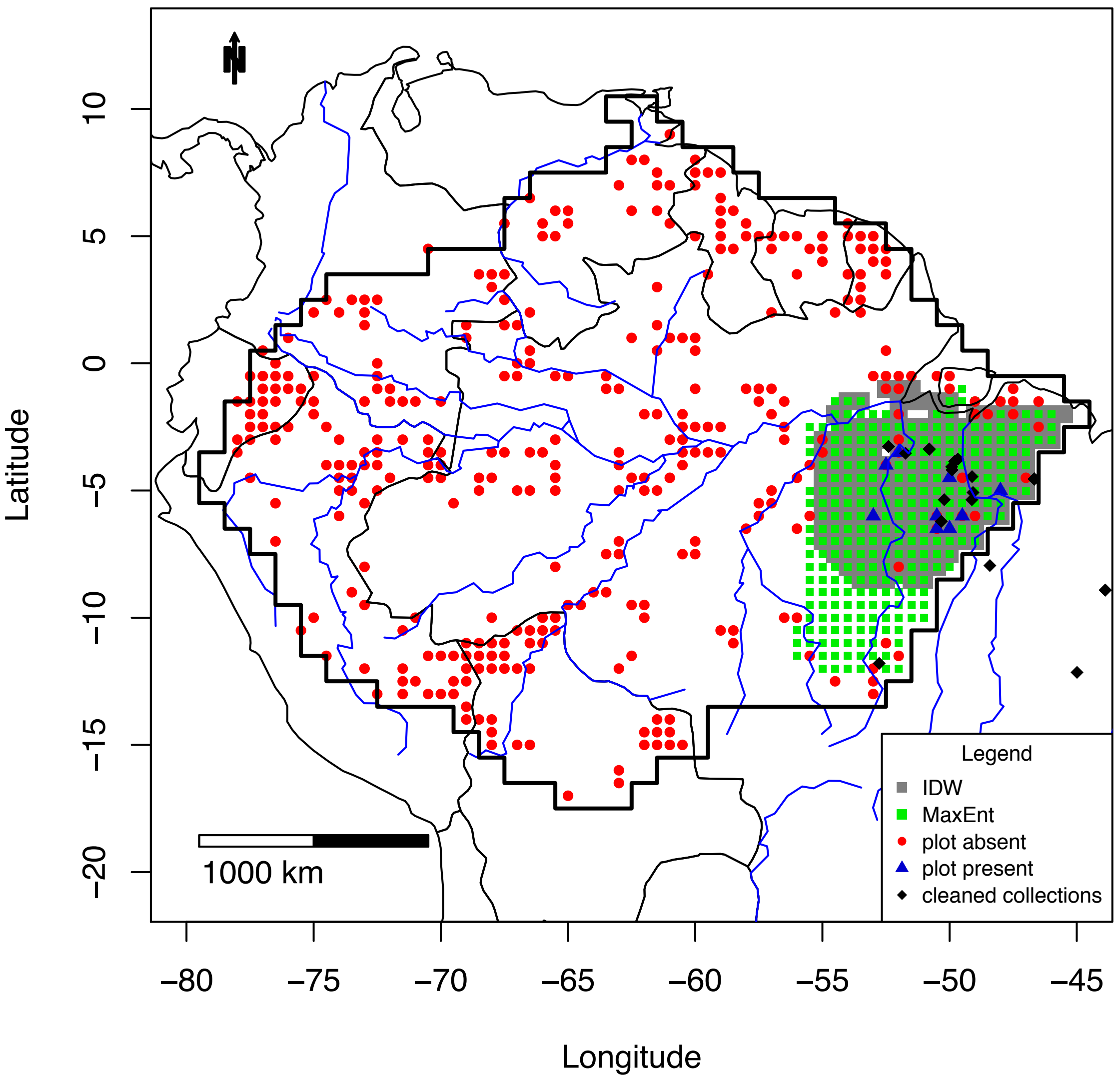

Figure S3_26B MaxEnt map of Cenostigma_tocantinum

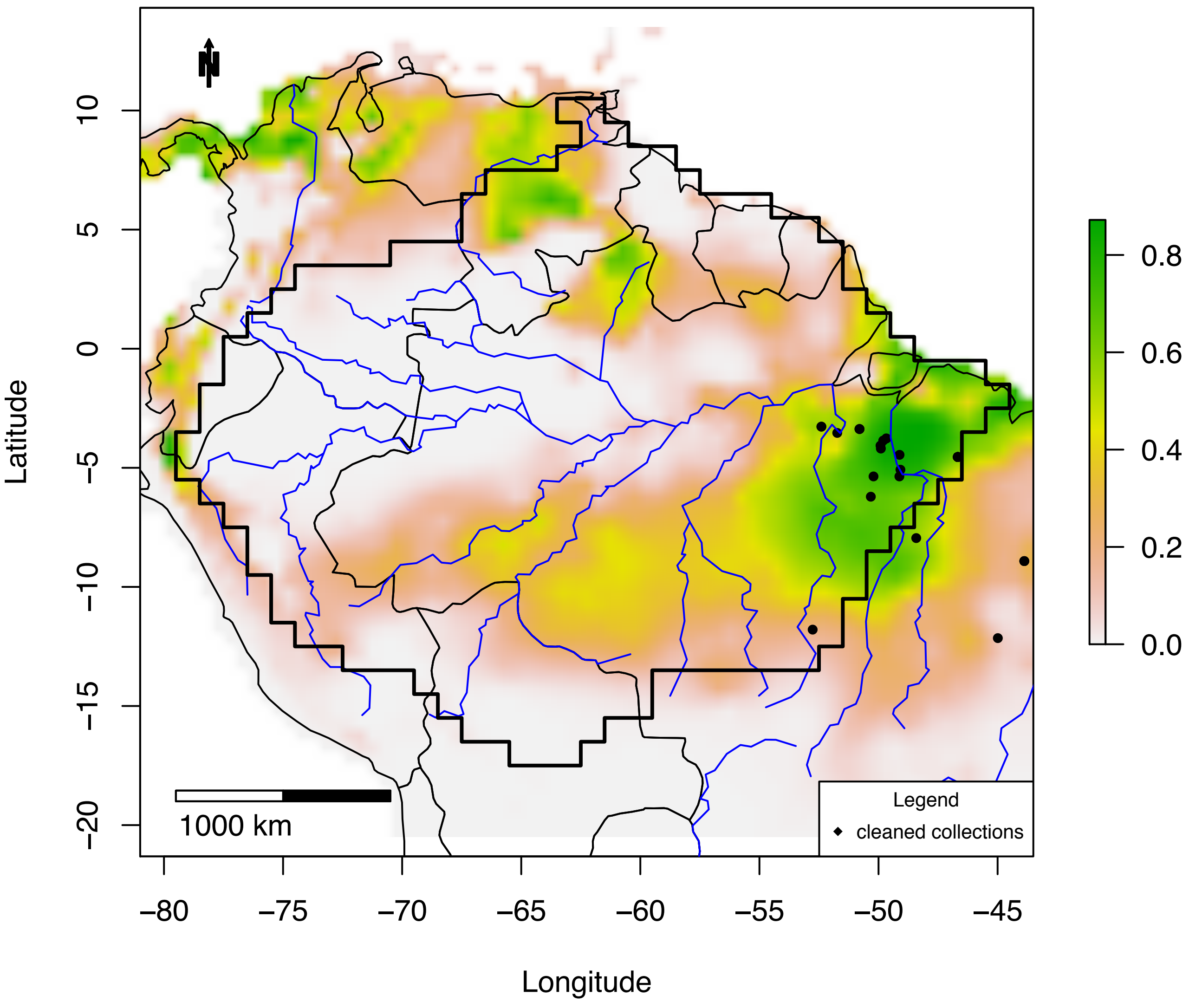

Figure S3_26C IDW map of Cenostigma_tocantinum: plots =0 – 0.35; IDW: 0 – 0.15

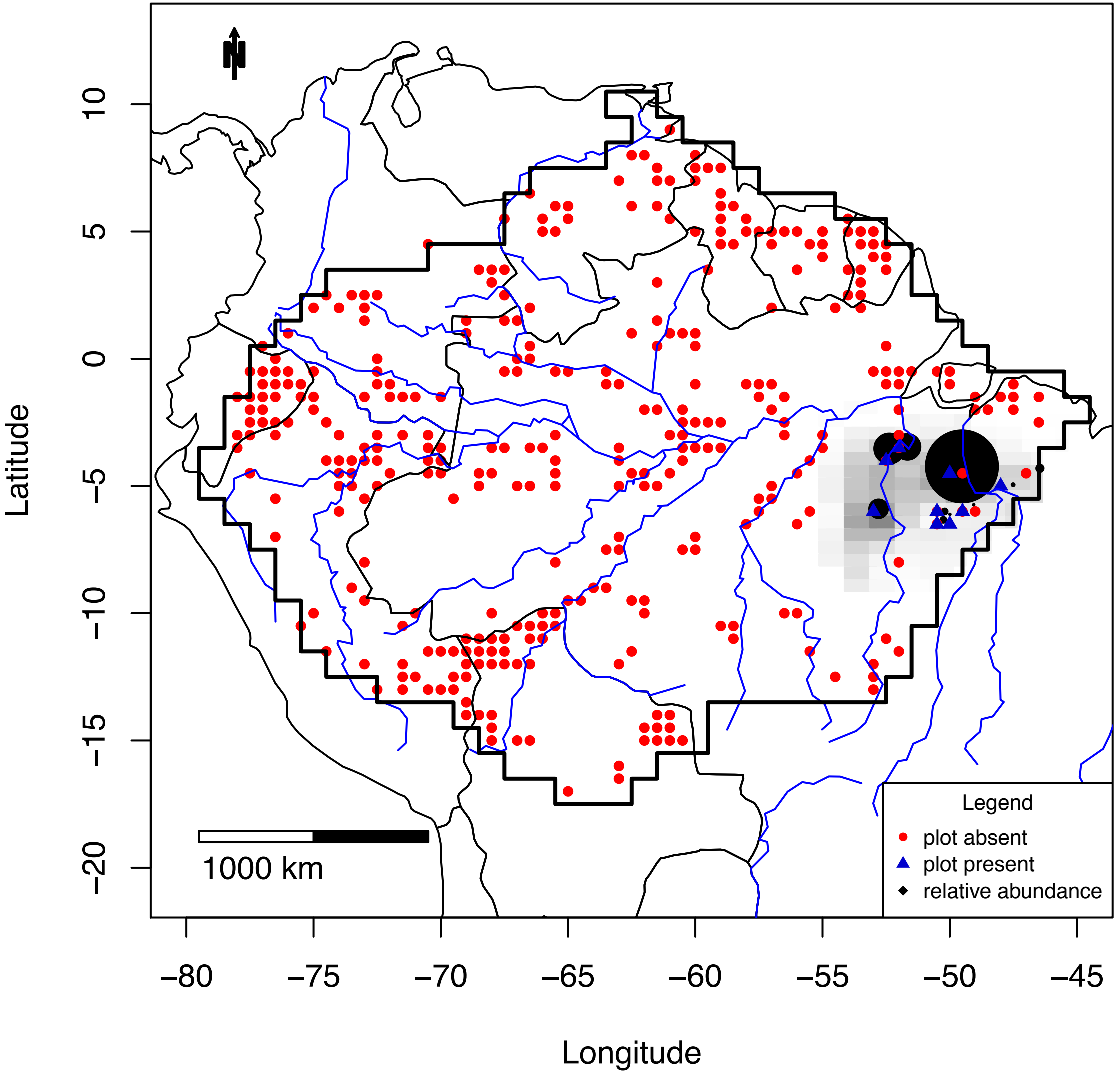

Figure S3_27A Combined map of Chlorocardium_rodiei

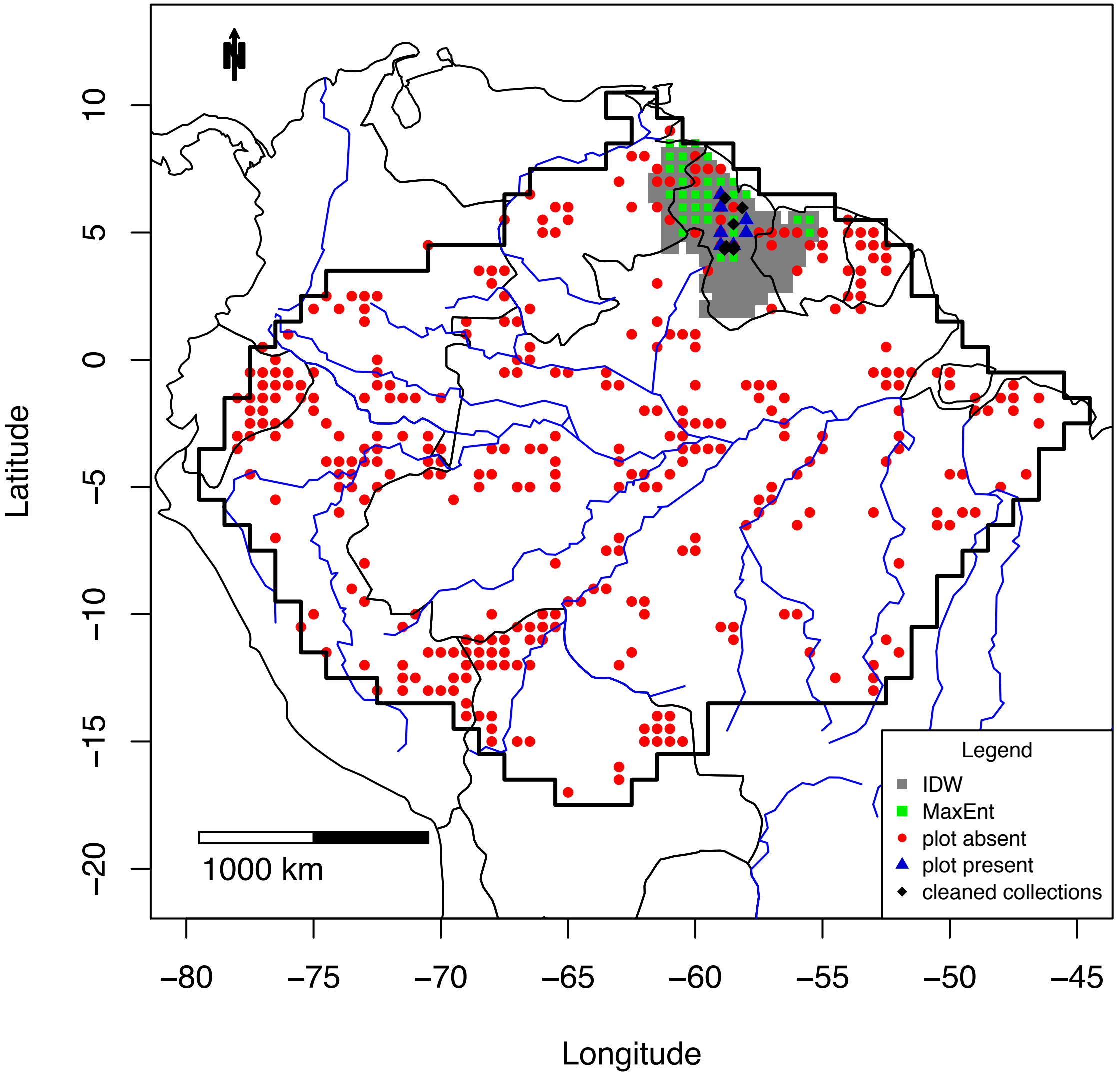

Figure S3_27B MaxEnt map of Chlorocardium_rodiei

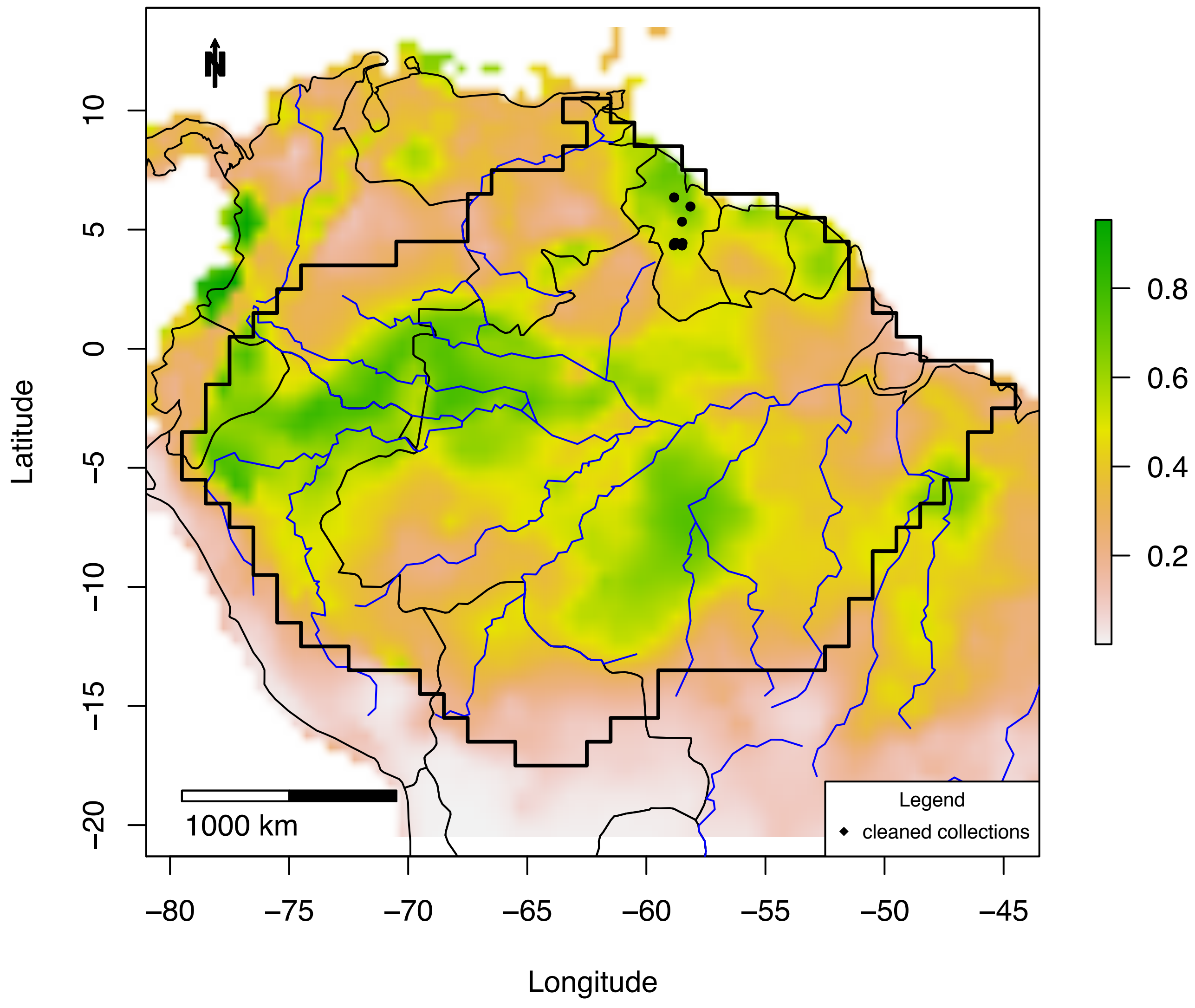

Figure S3_27C IDW map of Chlorocardium_rodiei: plots =0 – 0.22; IDW: 0 – 0.08

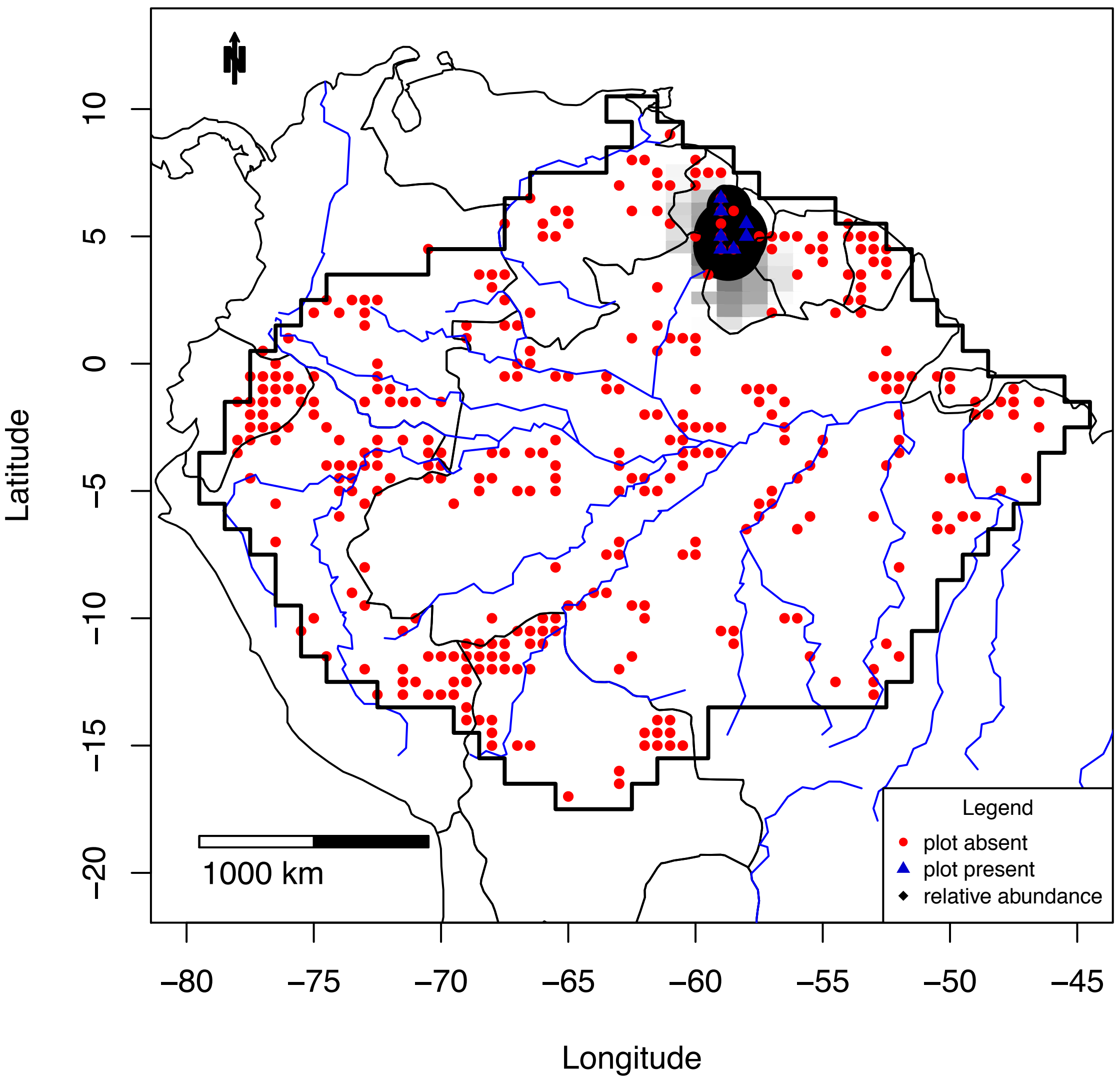

Figure S3_28A Combined map of Chrysophyllum_sanguinolentum

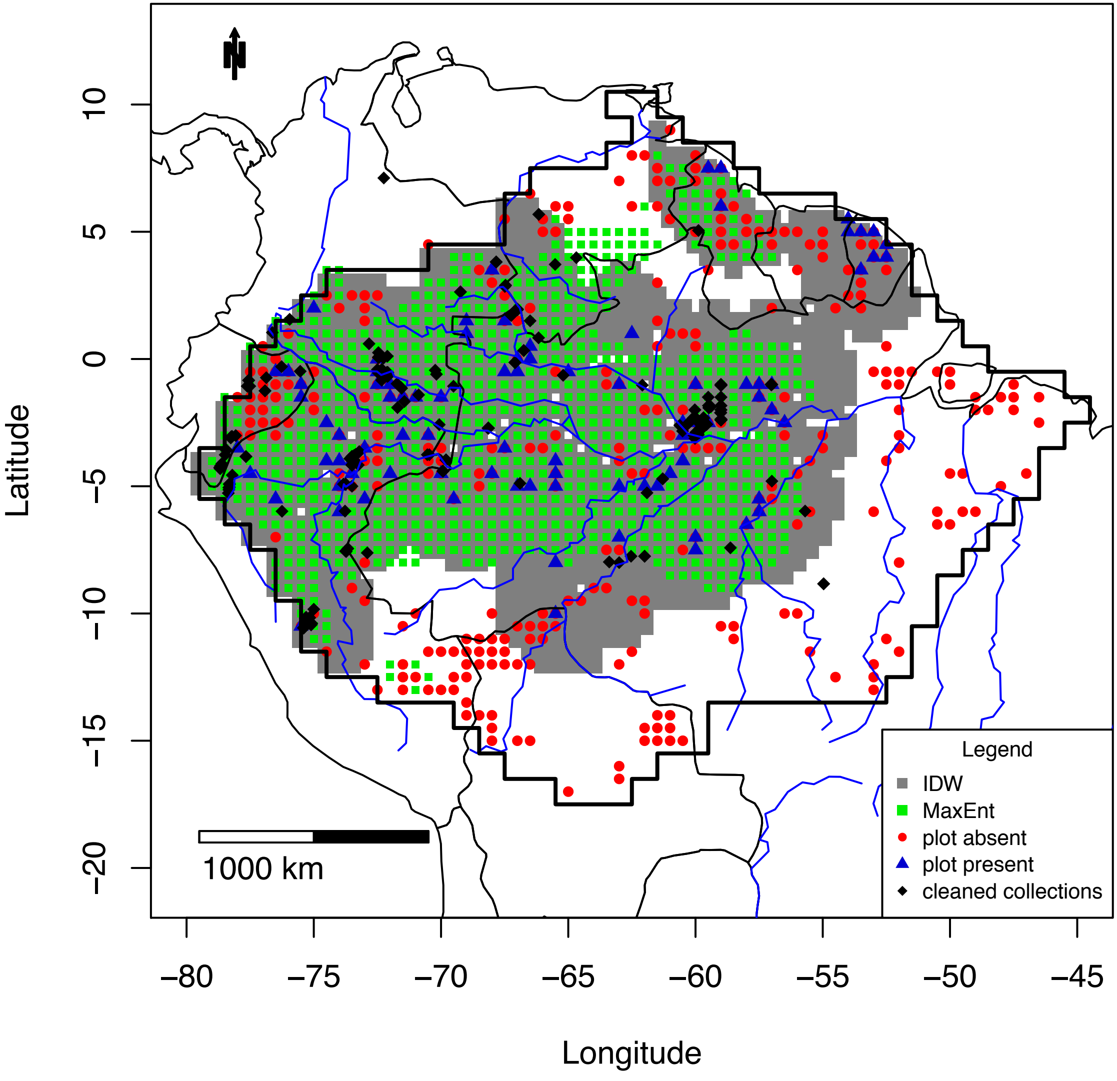

Figure S3_28B MaxEnt map of Chrysophyllum_sanguinolentum

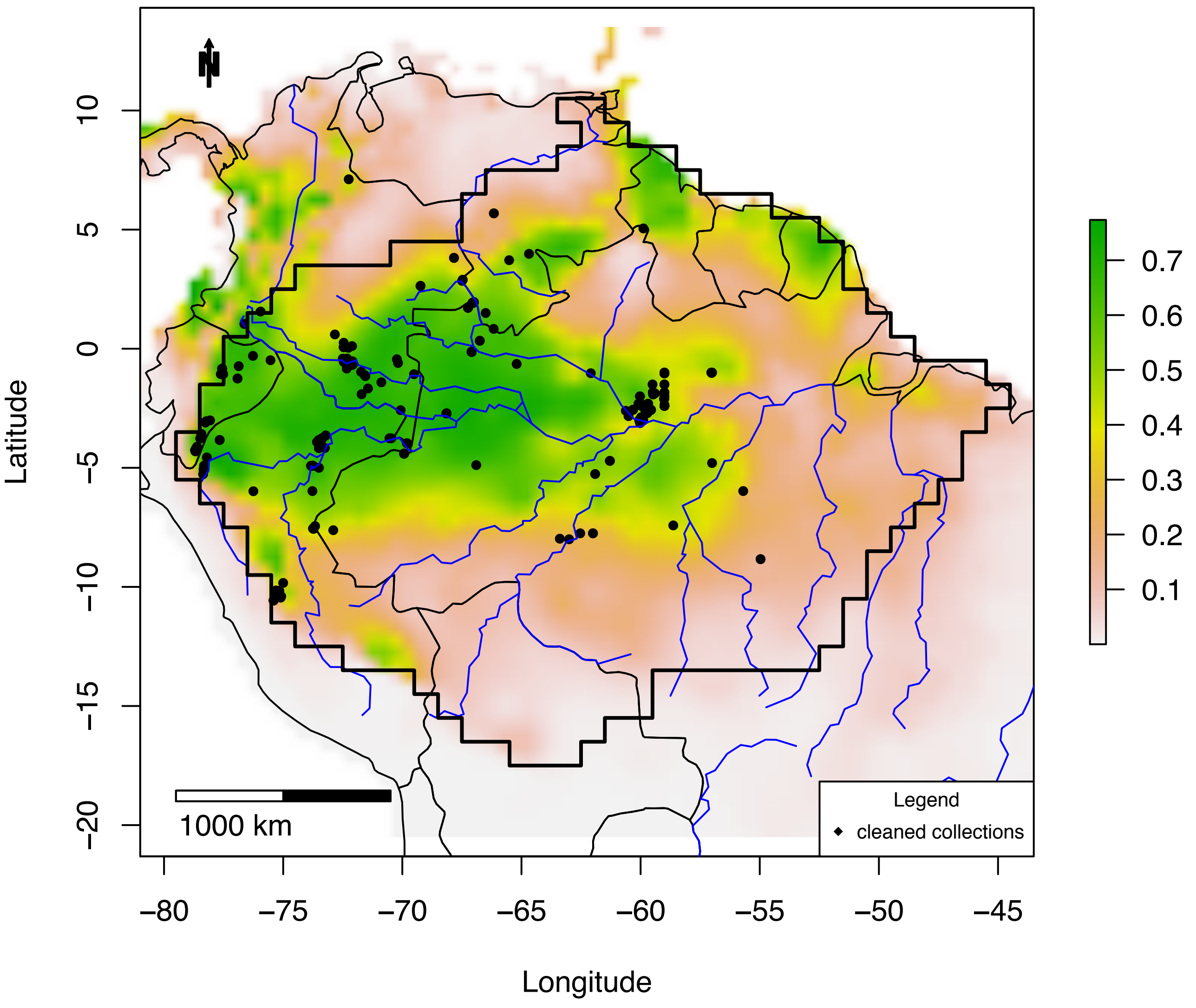

Figure S3_28C IDW map of Chrysophyllum_sanguinolentum: plots =0 – 0.2; IDW: 0 – 0.1

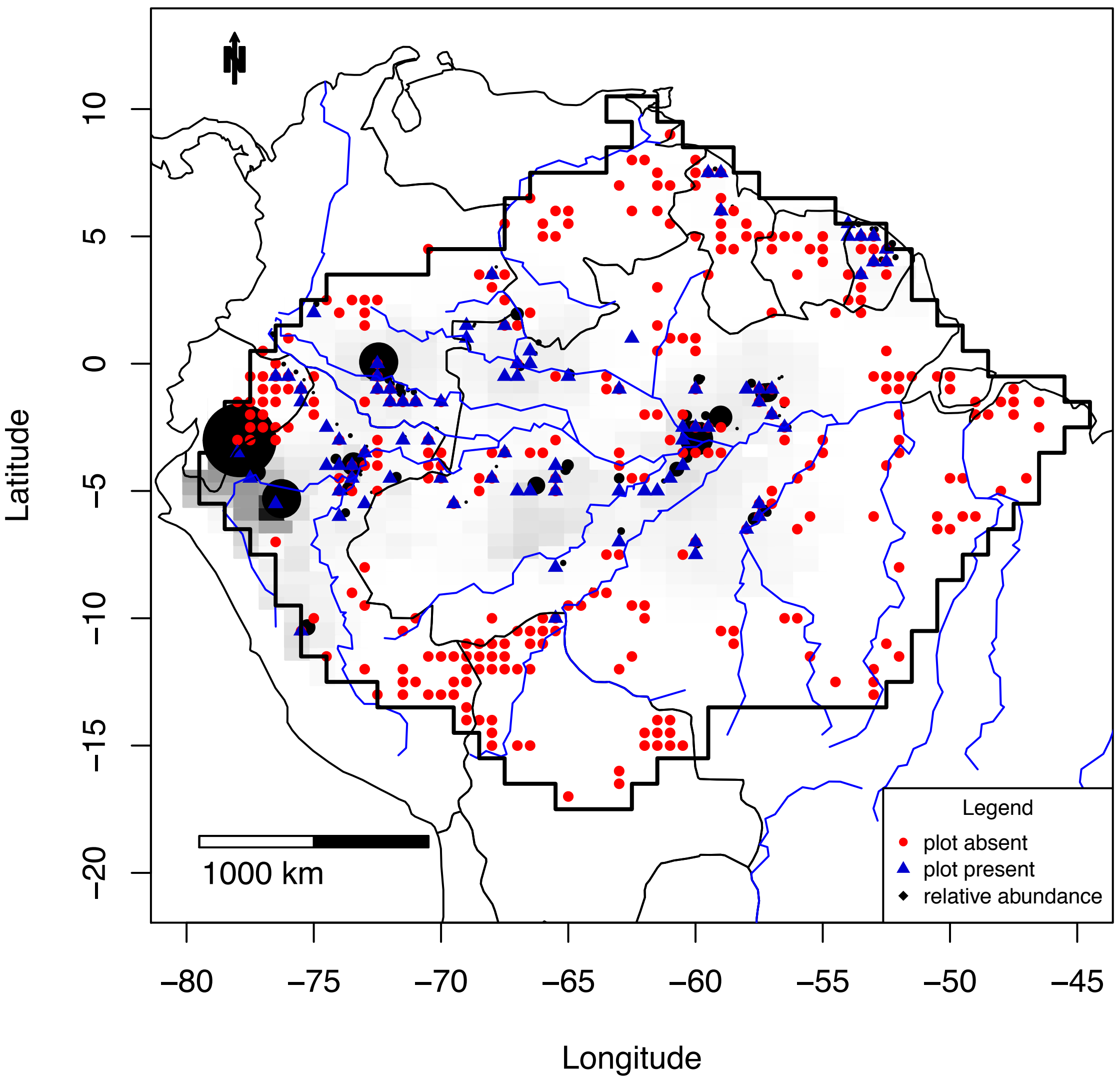

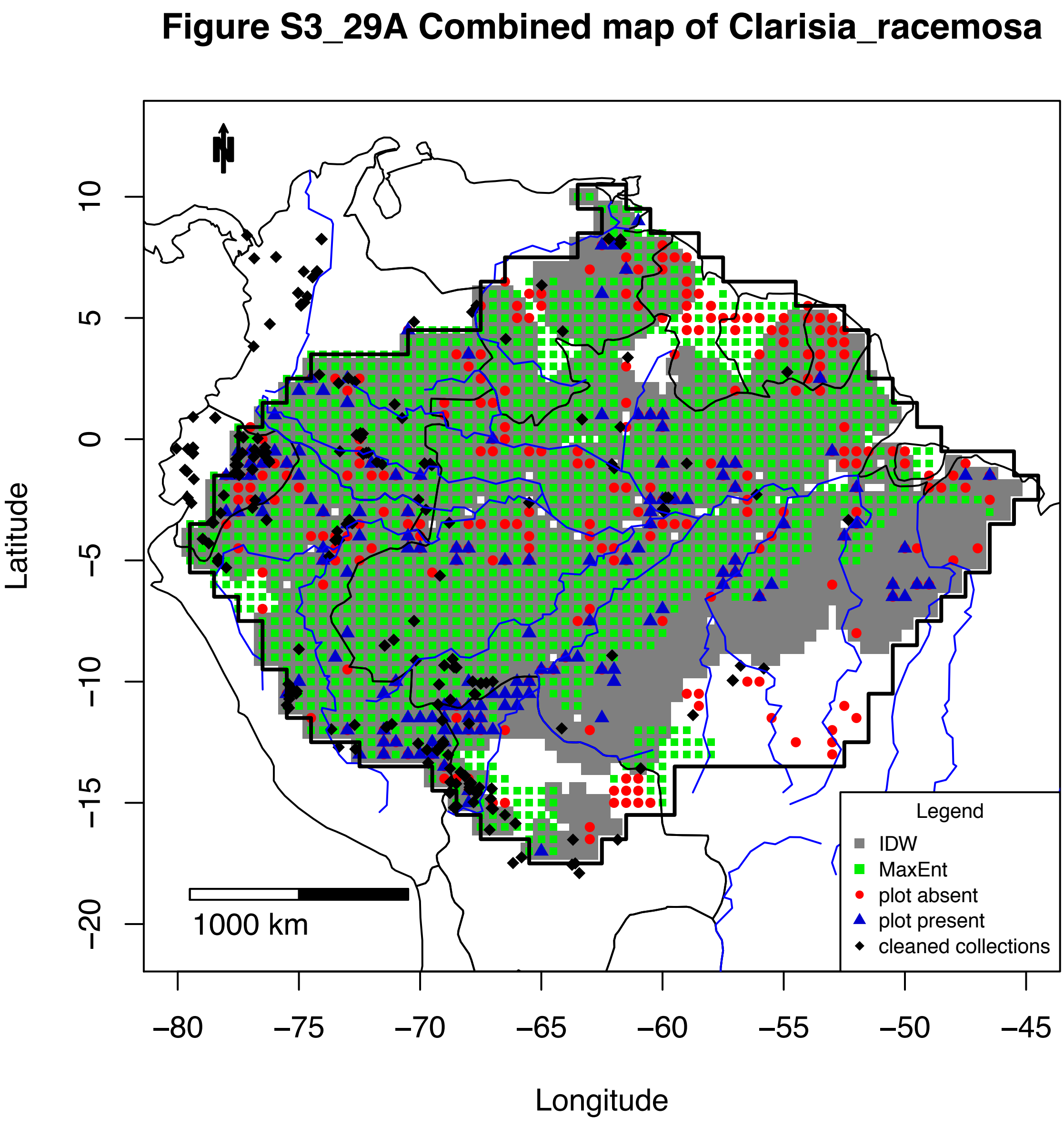
Figure S3_29A Combined map of Clarisia_racemosa
Latitude
Longitude
10
5
0
−5
−10
−15
−20
−80
−75
−70
−65
−60
−55
−50
−45
N
1000 km
Legend
IDW
MaxEnt
plot absent
plot present
cleaned collections

Figure S3_29B MaxEnt map of Clarisia_racemosa

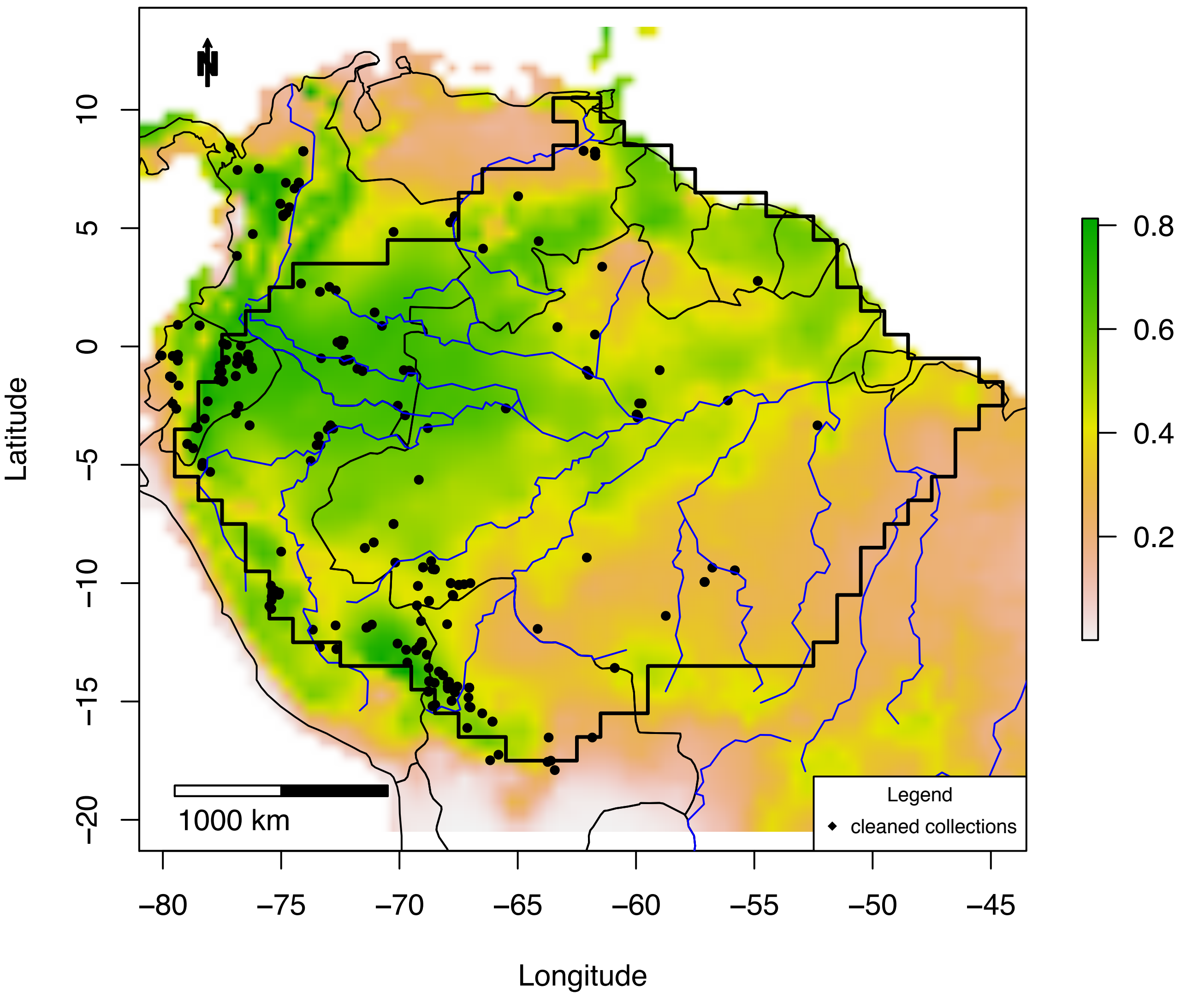

Figure S3_29C IDW map of Clarisia_racemosa: plots =0 – 0.04; IDW: 0 – 0.03

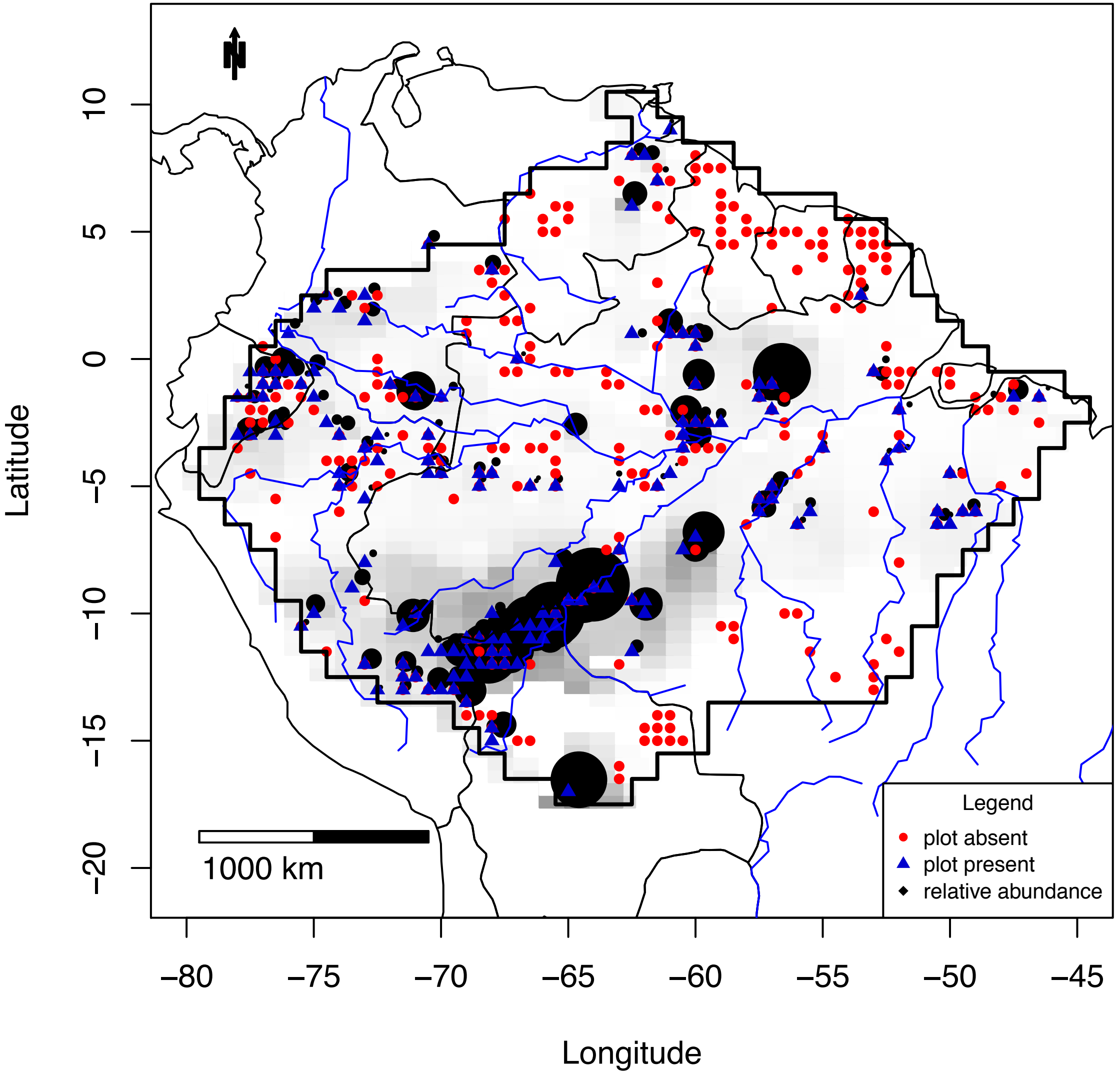

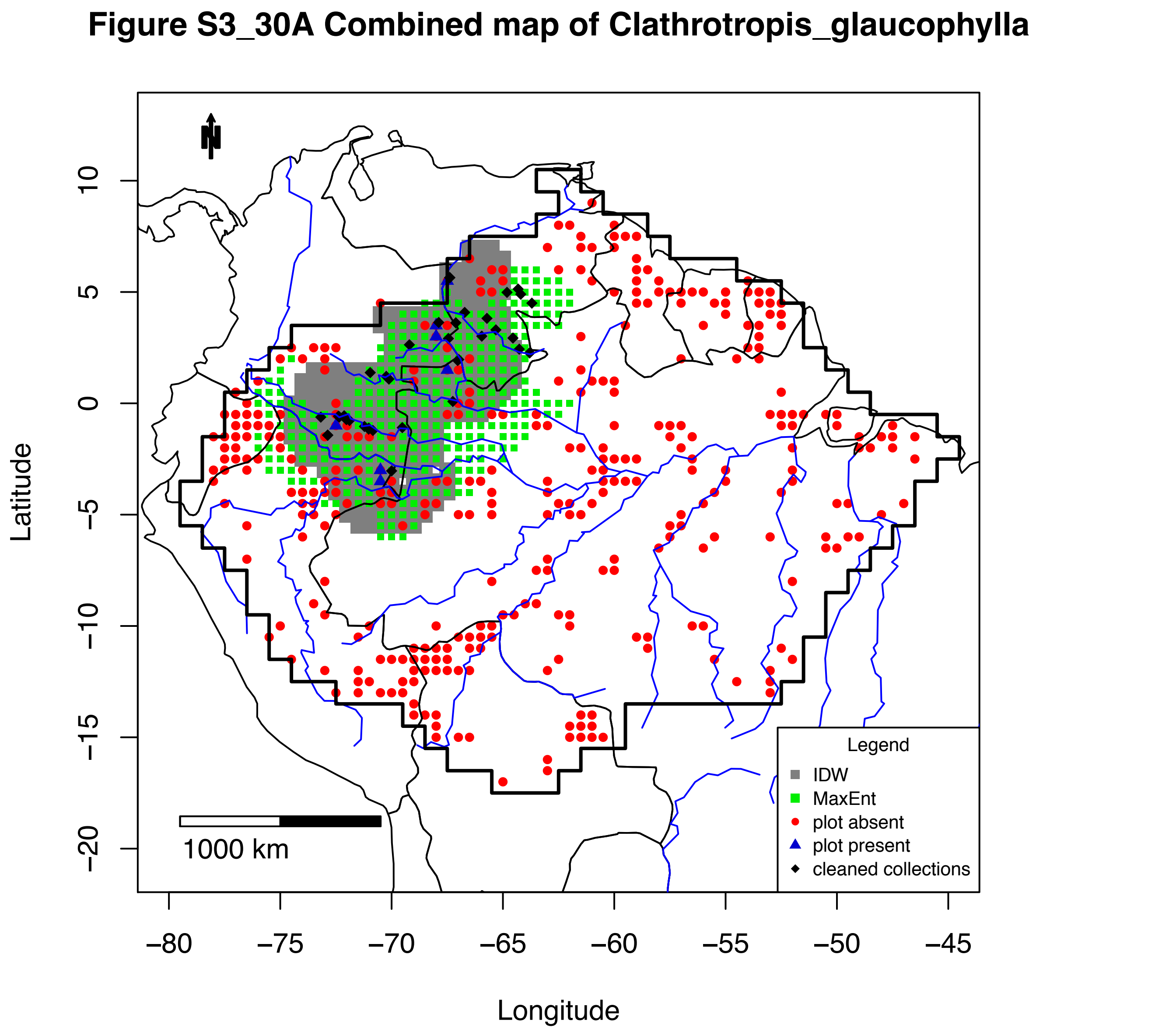

Figure S3_30A Combined map of Clathrotropis_glaucophylla
Latitude
Longitude
10
5
0
−5
−10
−15
−20
−80
−75
−70
−65
−60
−55
−50
−45
N
1000 km
Legend
IDW
MaxEnt
plot absent
plot present
cleaned collections

Figure S3_30B MaxEnt map of Clathrotropis_glaucophylla

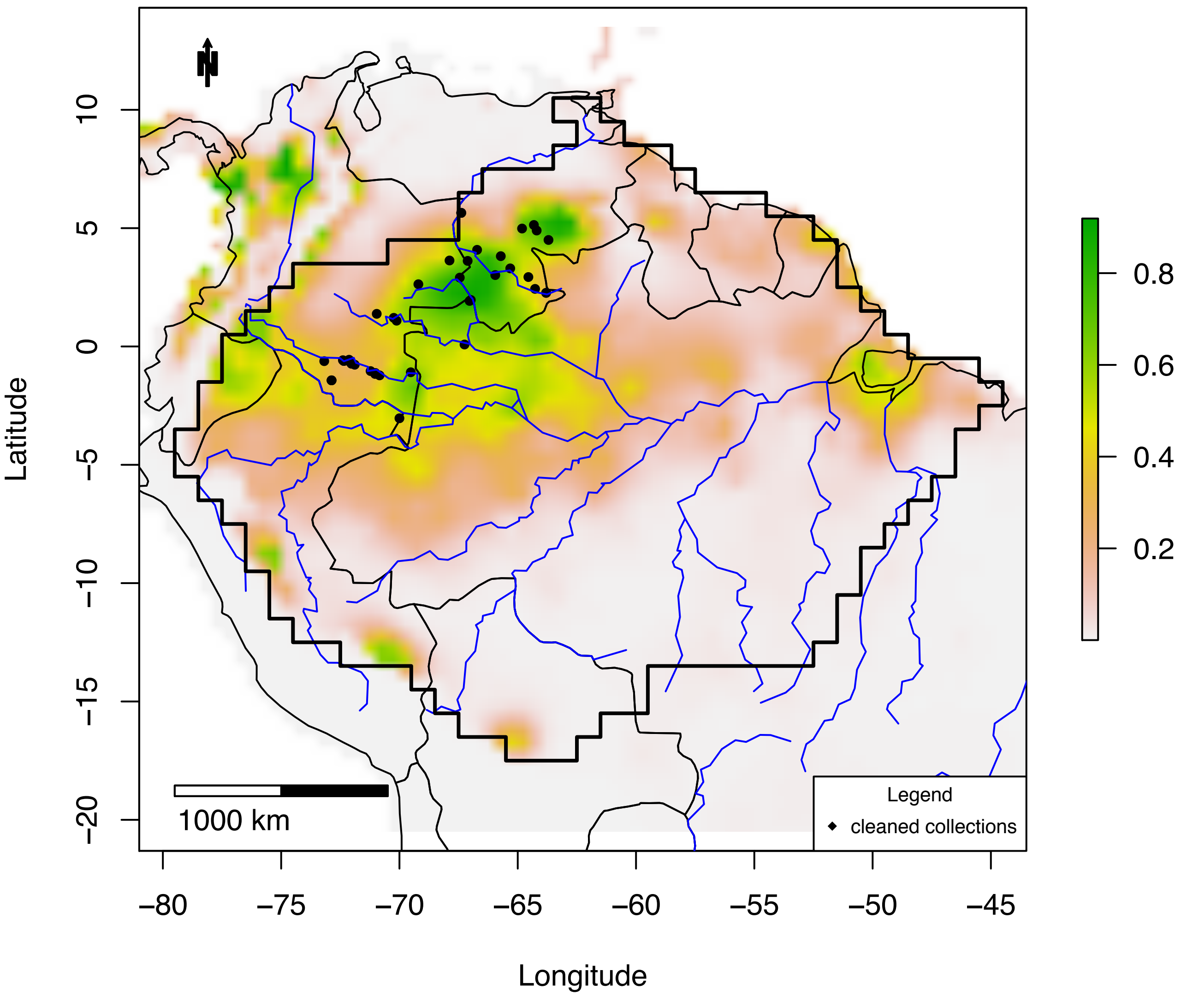

Figure S3_30C IDW map of Clathrotropis_glaucophylla: plots =0 – 0.43; IDW: 0 – 0.14

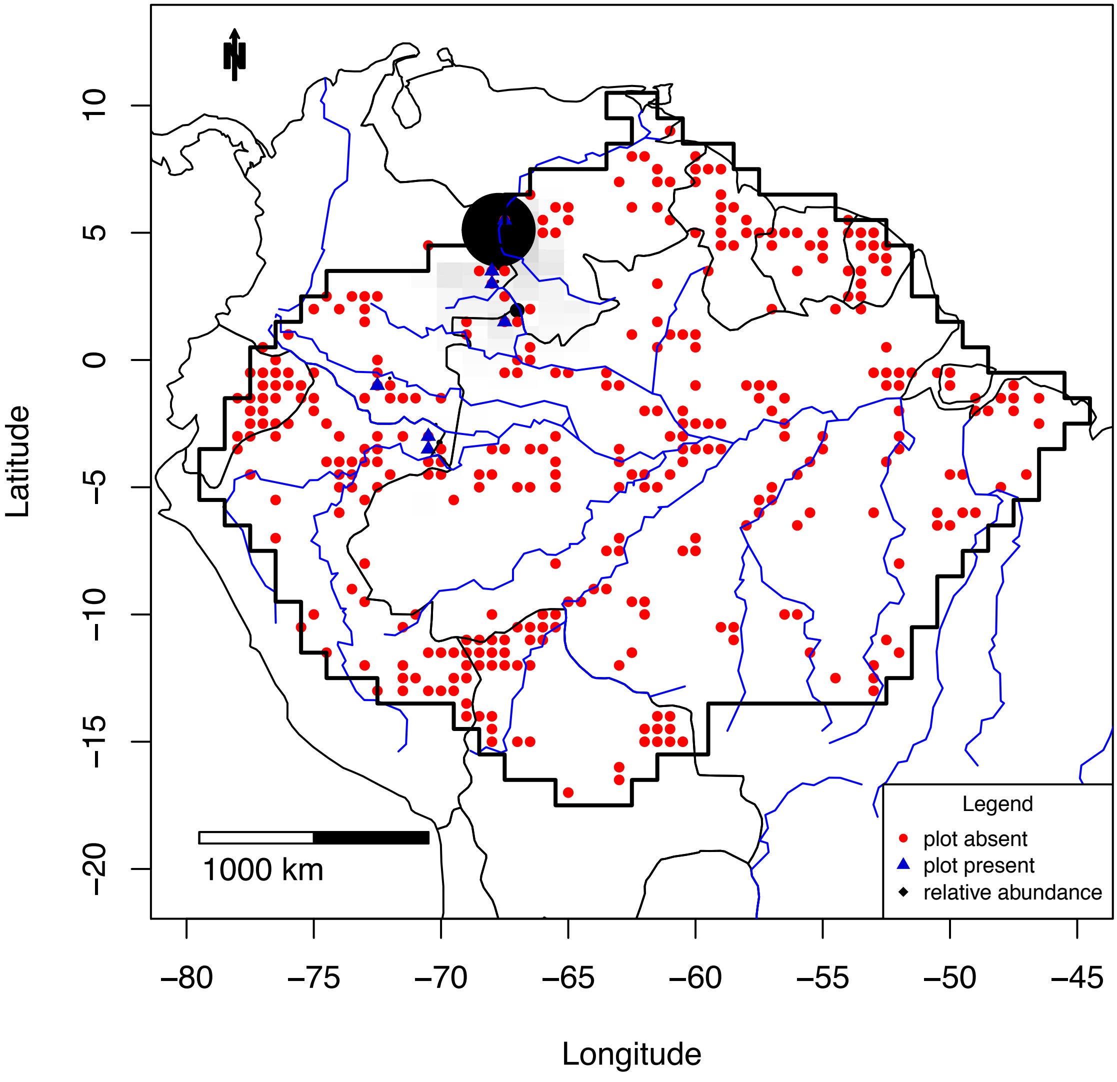

Figure S3_31A Combined map of Clathrotropis_macrocarpa

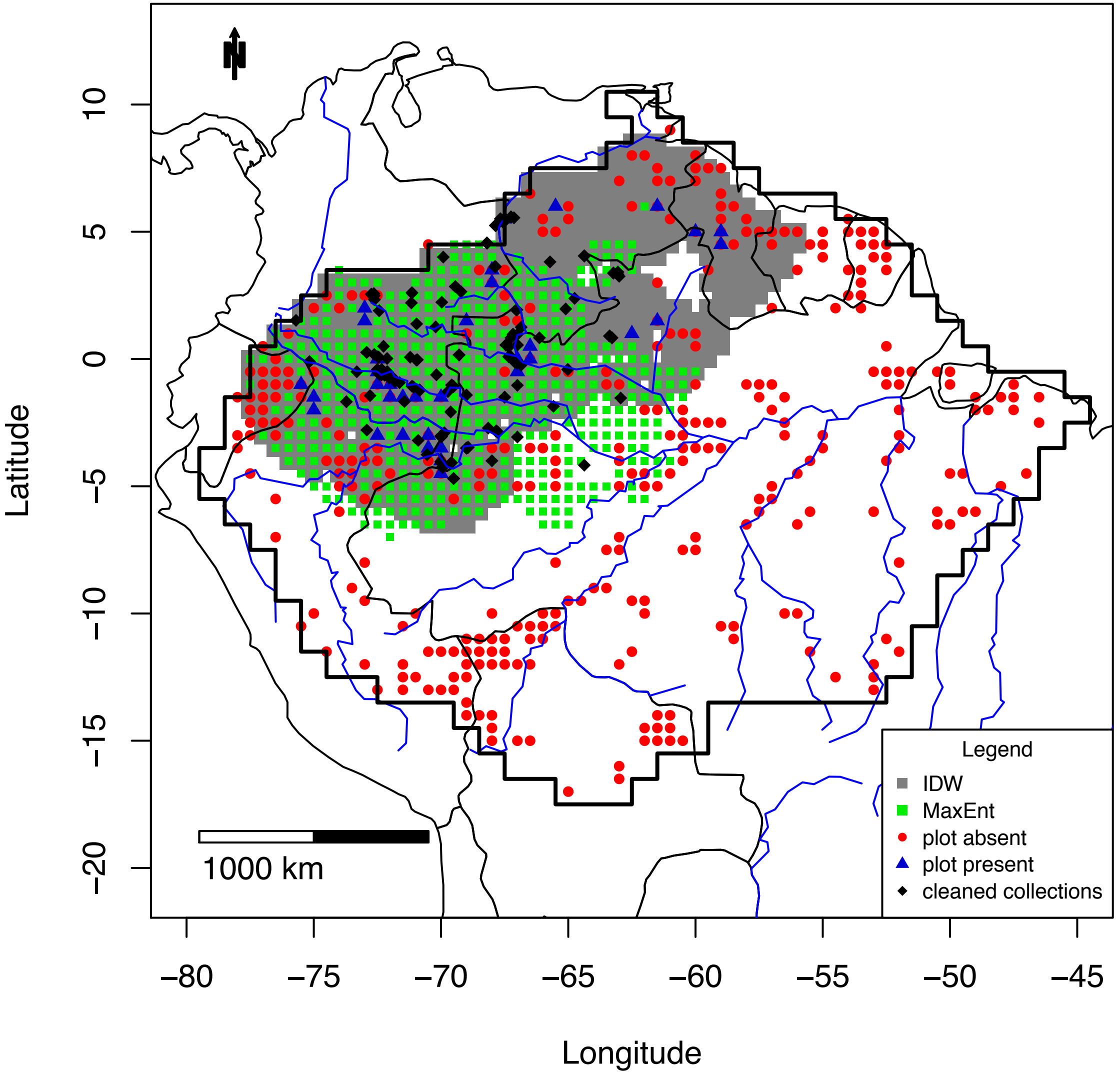

Figure S3_31B MaxEnt map of Clathrotropis_macrocarpa

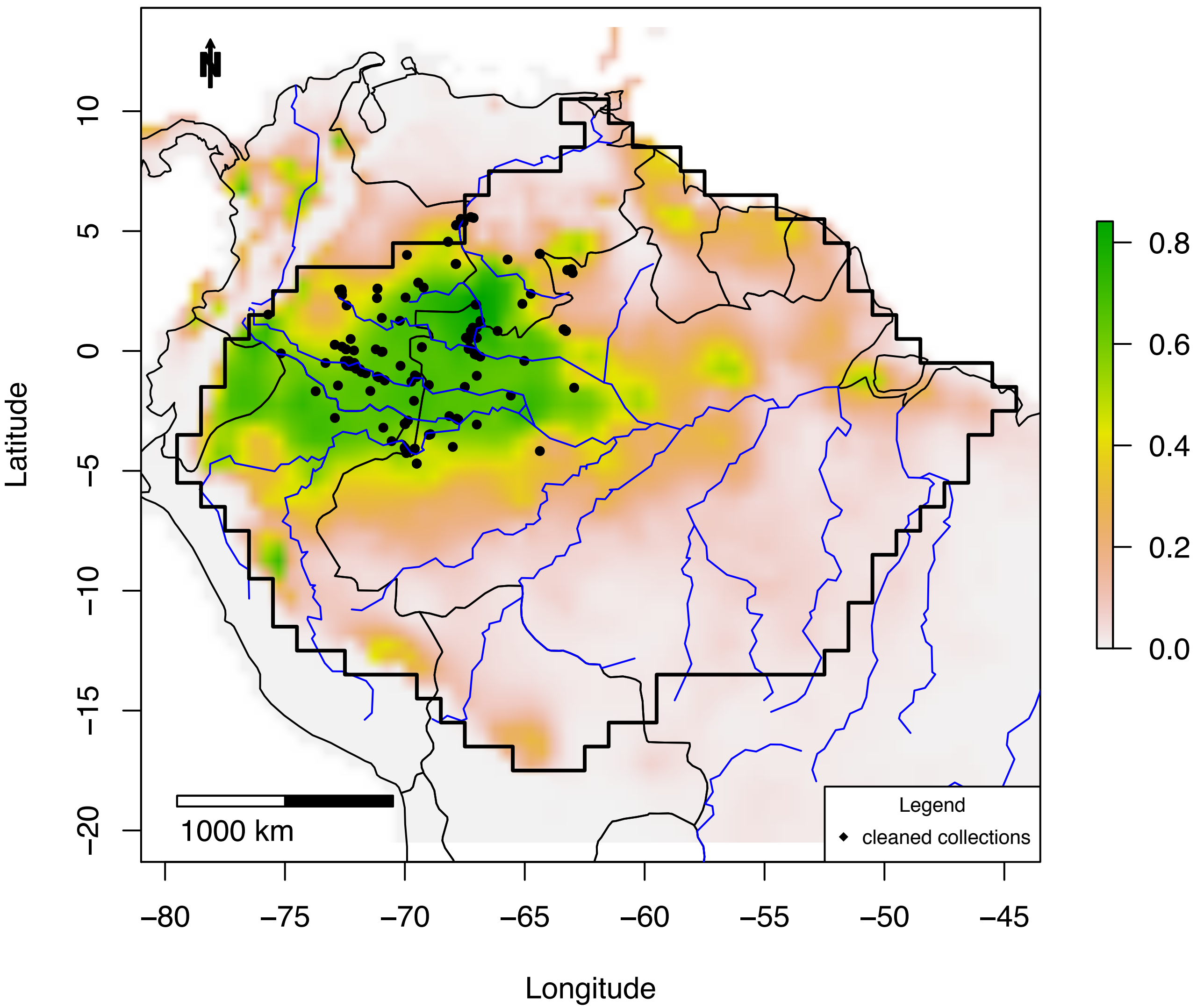

Figure S3_31C IDW map of Clathrotropis_macrocarpa: plots =0 – 0.27; IDW: 0 – 0.13

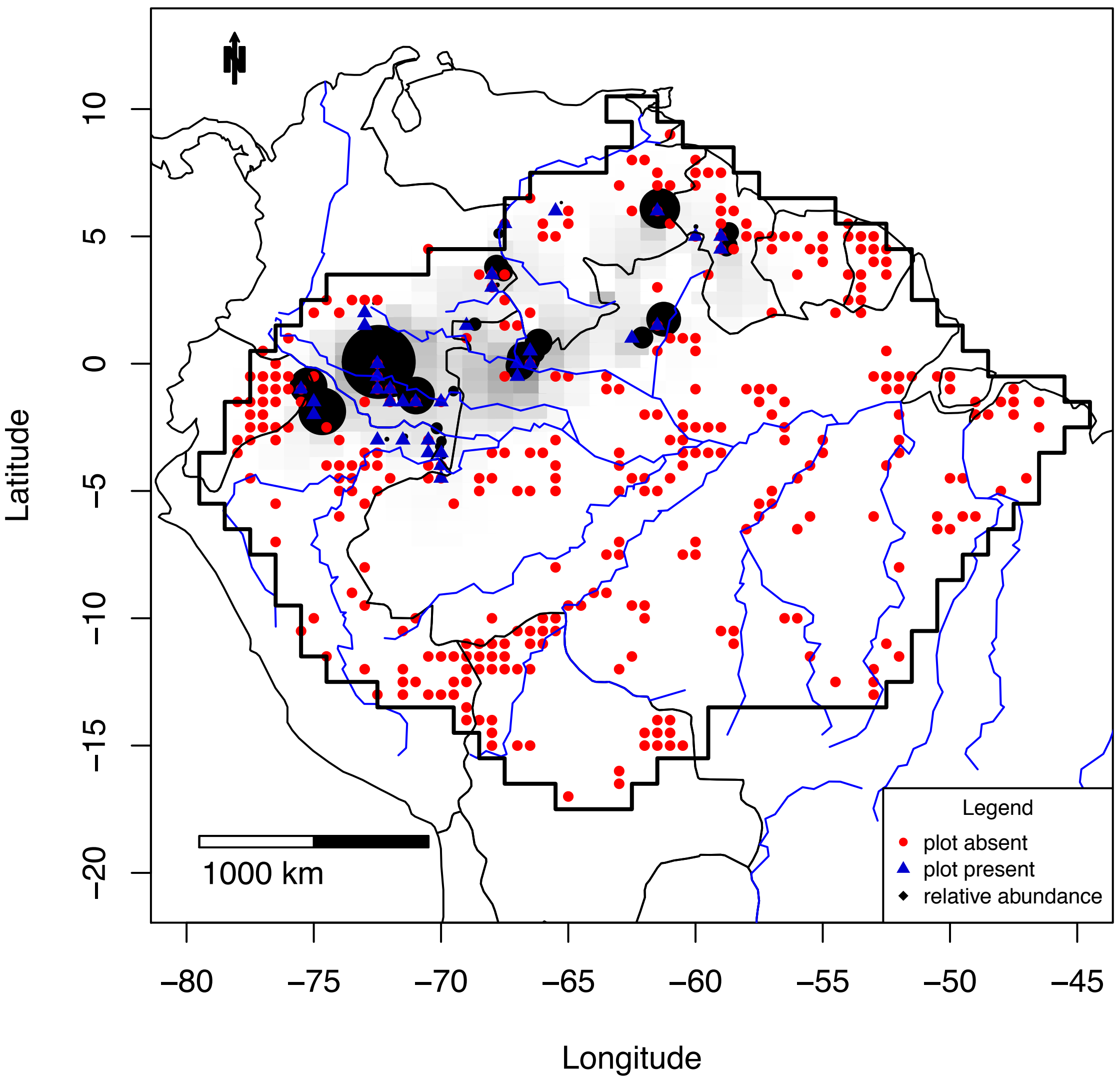

Figure S3_32A Combined map of Conceveiba_guianensis

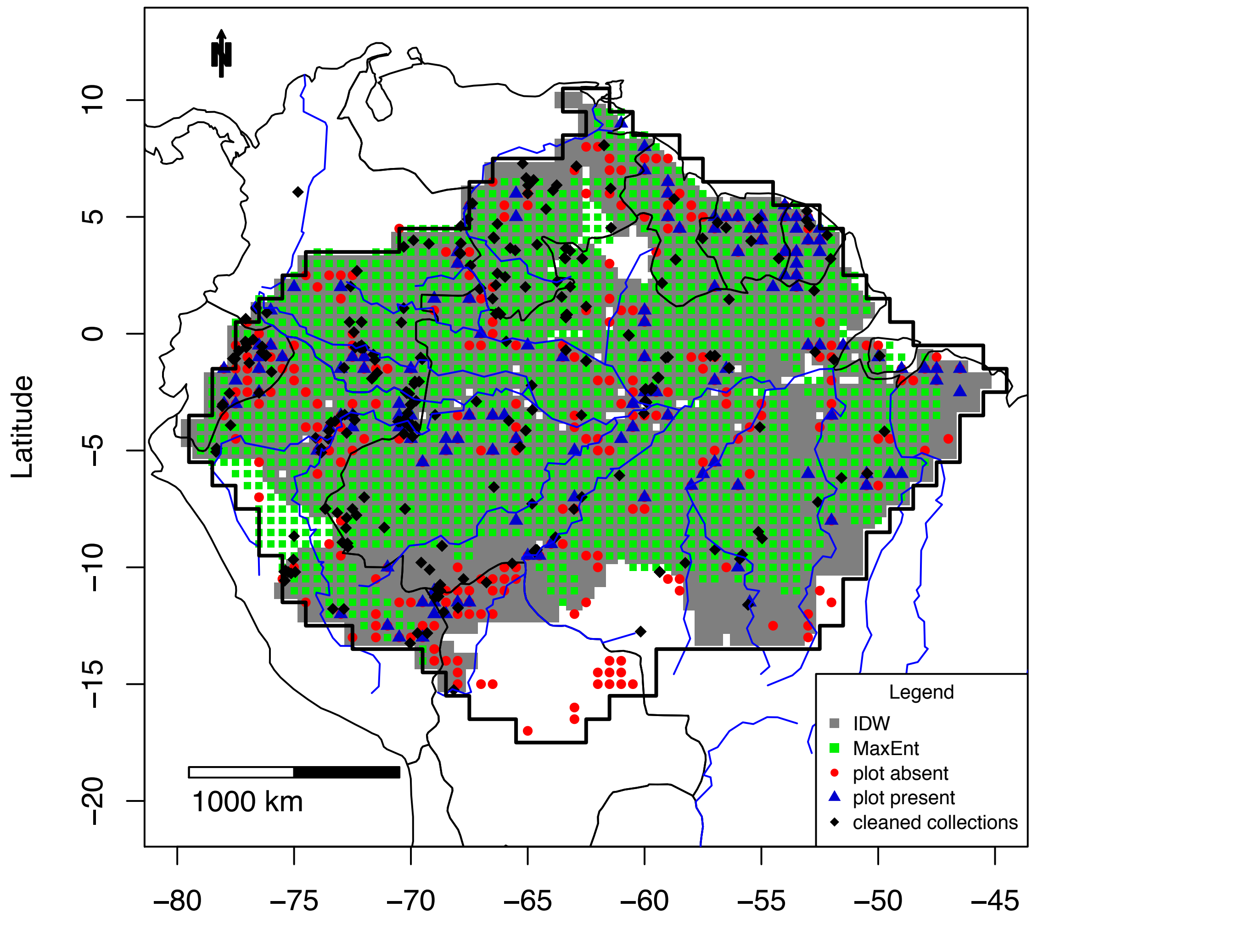

Figure S3_32B MaxEnt map of Conceveiba_guianensis

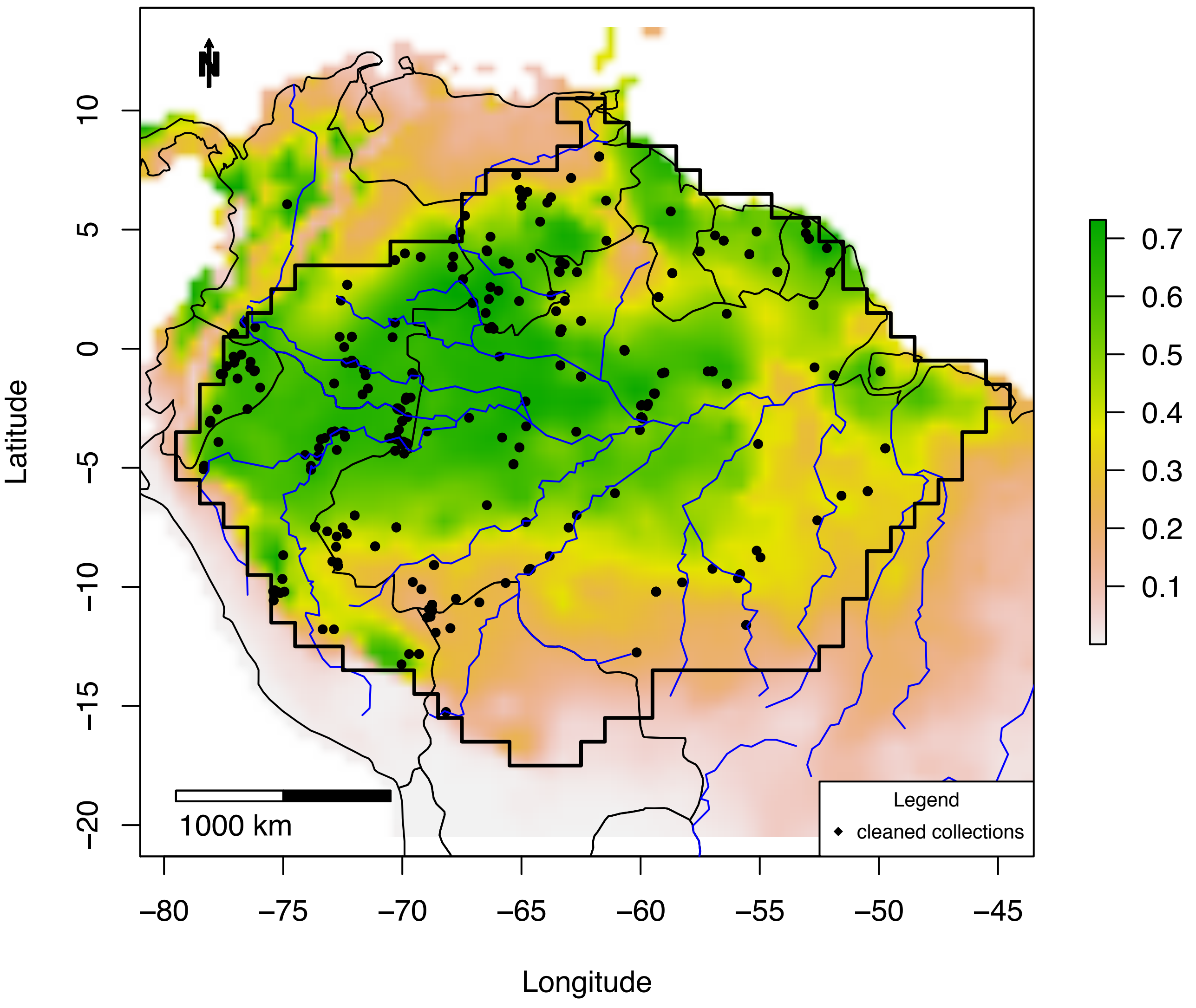

Figure S3_32C IDW map of Conceveiba_guianensis: plots =0 – 0.04; IDW: 0 – 0.04

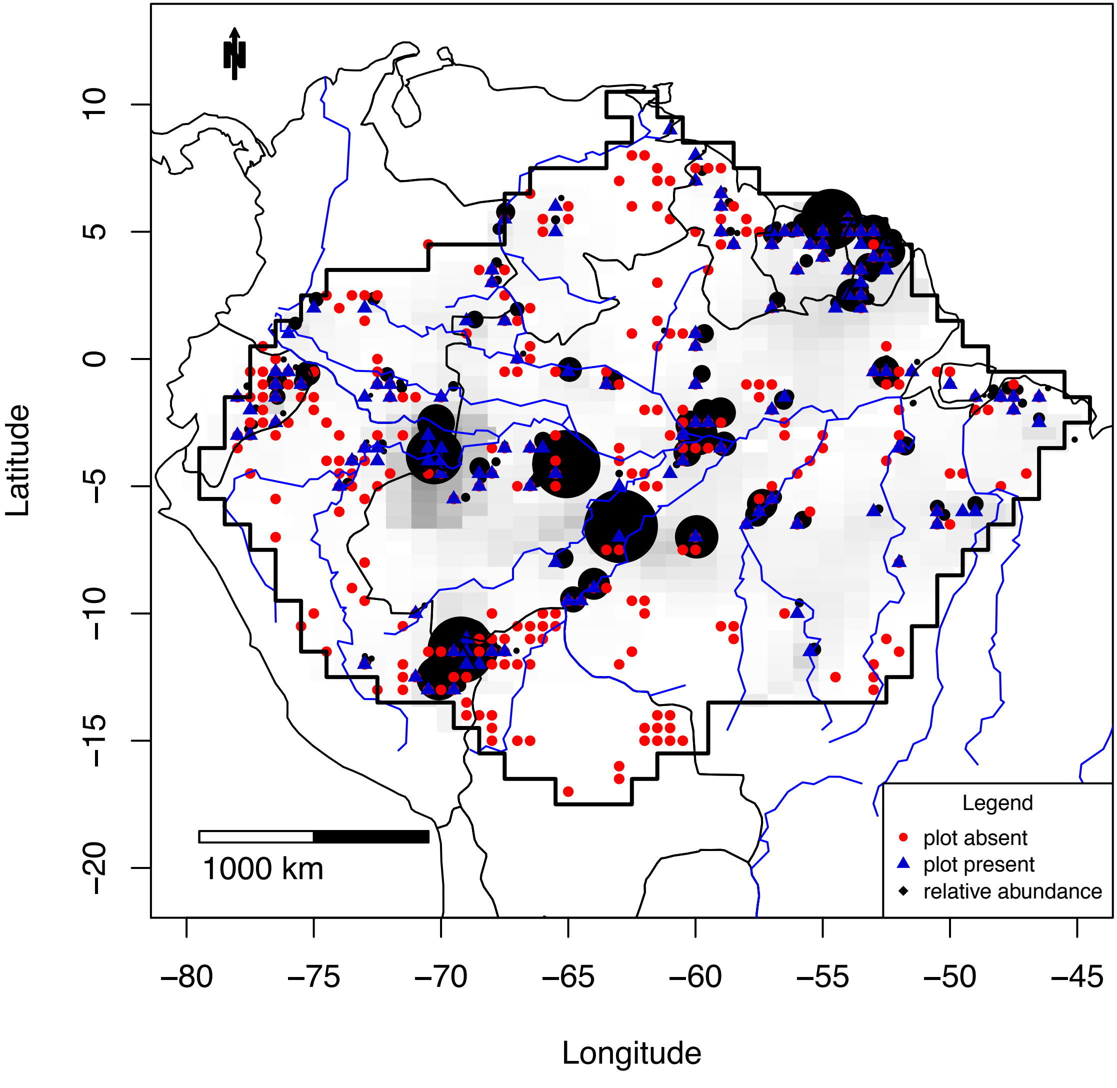

Figure S3_33A Combined map of Couepia_guianensis

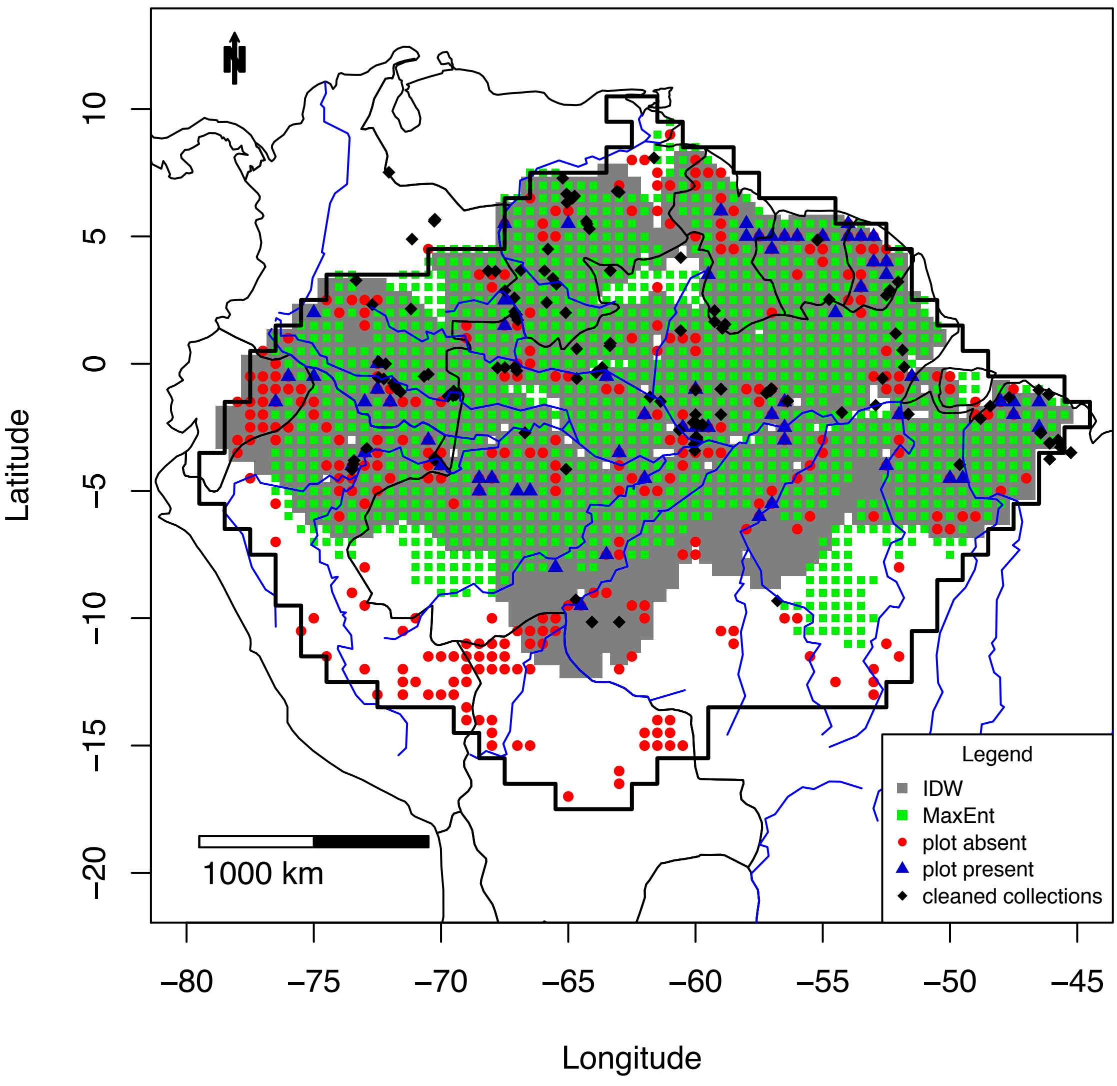

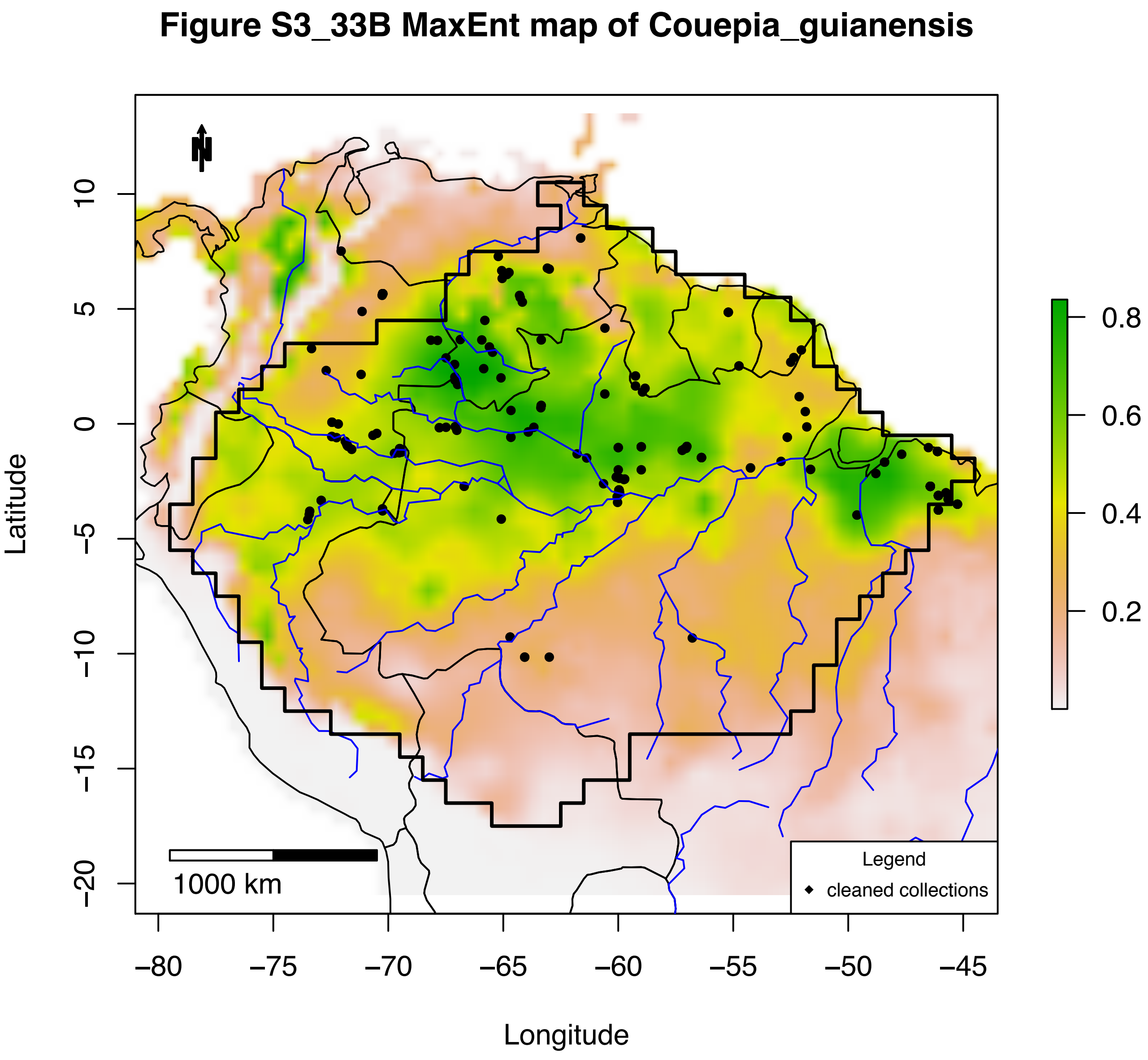
Figure S3_33B MaxEnt map of Couepia_guianensis
N
10
5
0
−5
−10
−15
−20
Latitude
−80
−75
−70
−65
−60
−55
−50
−45
Longitude
1000 km
Legend
cleaned collections
0.8
0.6
0.4
0.2

Figure S3_33C IDW map of Couepia_guianensis: plots =0 – 0.17; IDW: 0 – 0.08

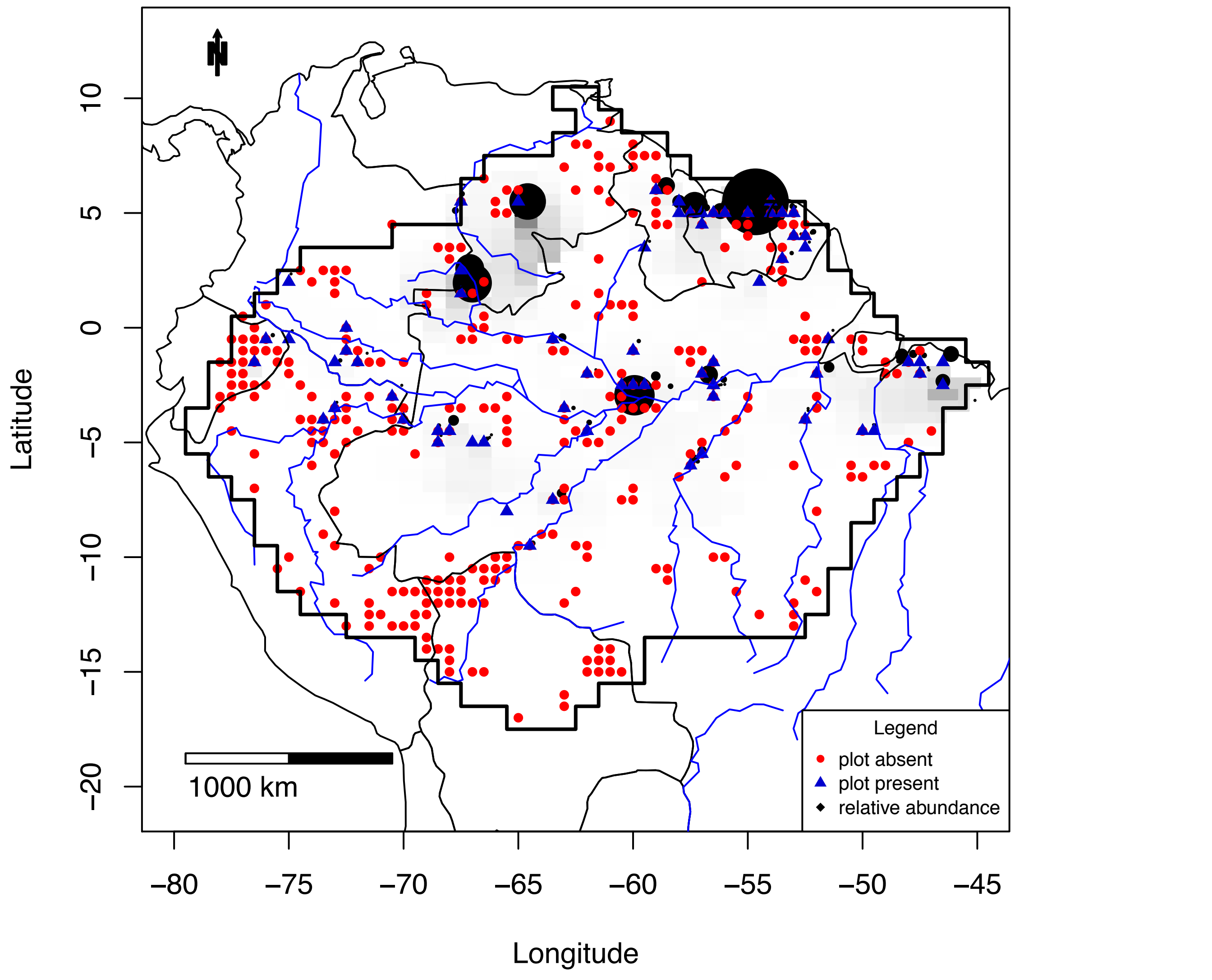

Figure S3_34A Combined map of Couepia_paraensis

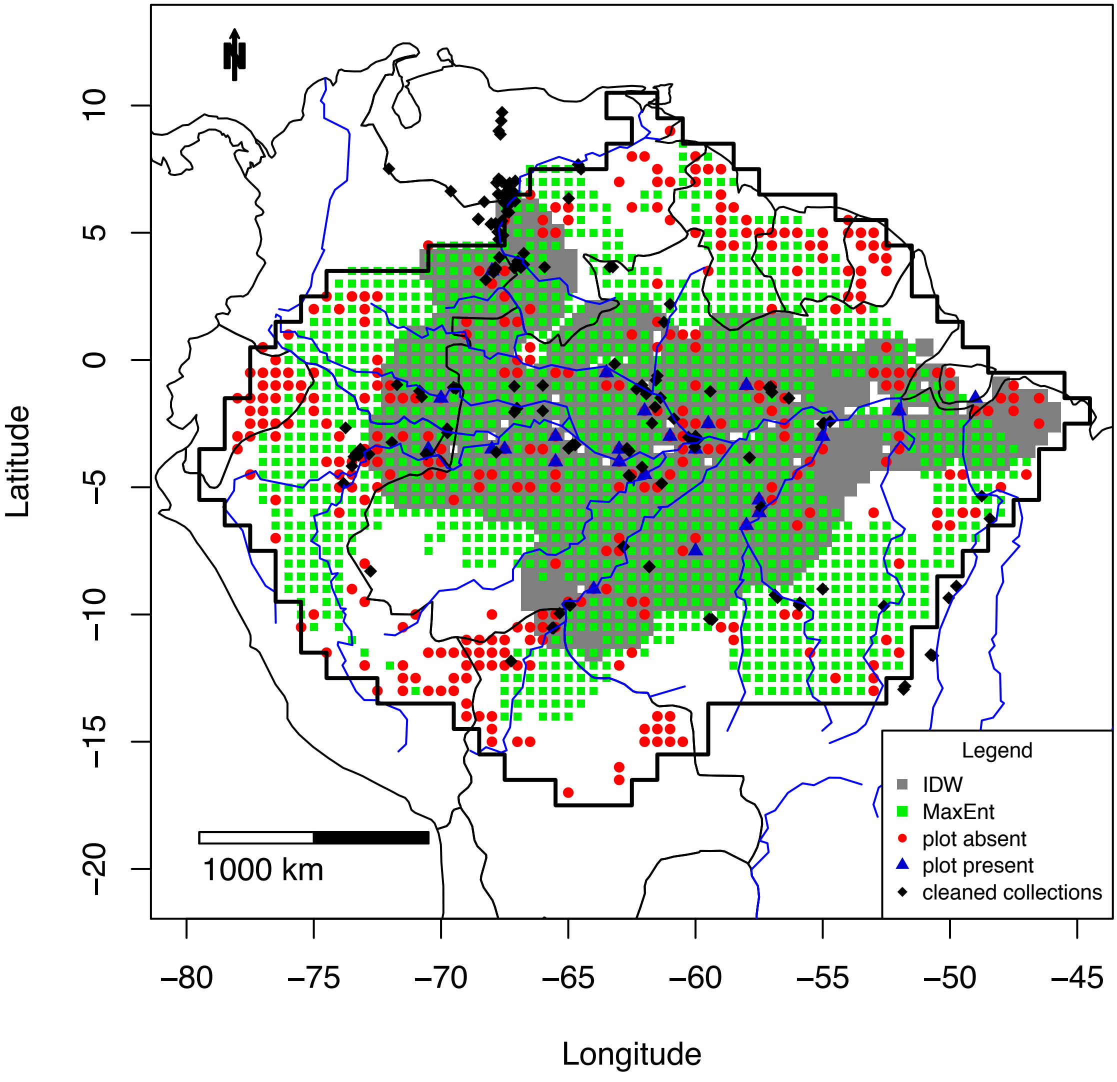

Figure S3_34B MaxEnt map of Couepia_paraensis

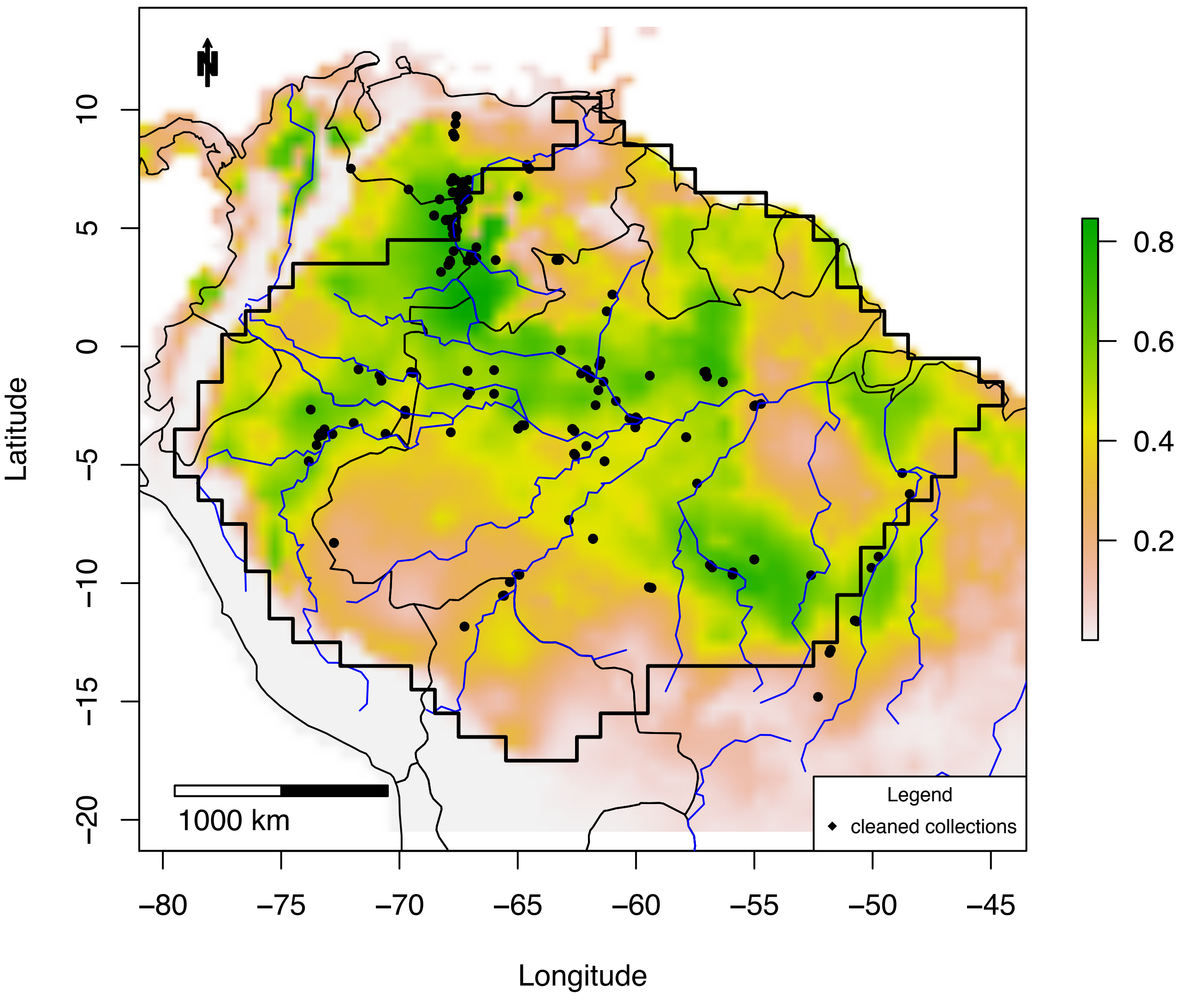

Figure S3_34C IDW map of Couepia_paraensis: plots =0 – 0.34; IDW: 0 – 0.05

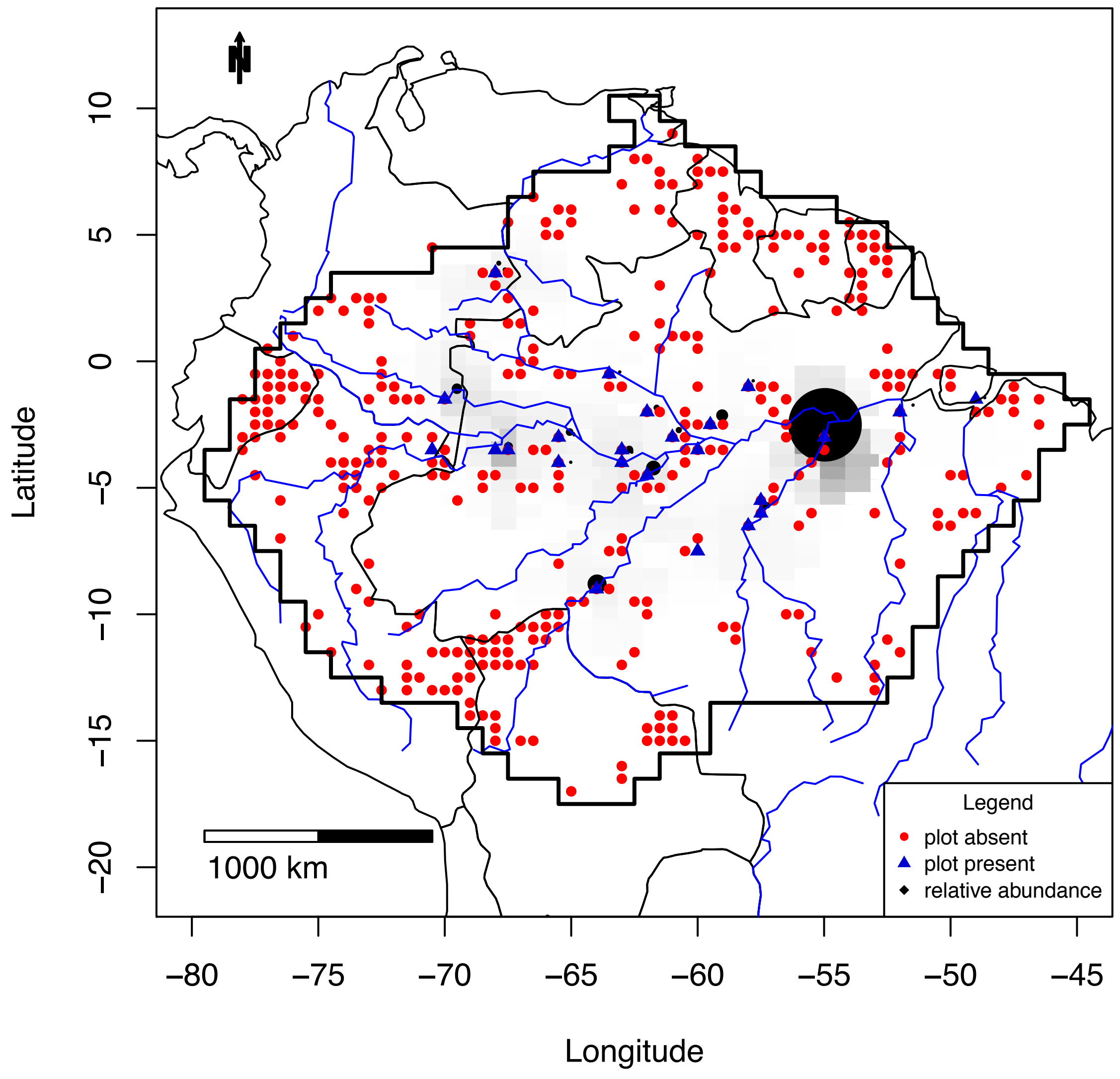

Figure S3_35A Combined map of Cynometra_marginata

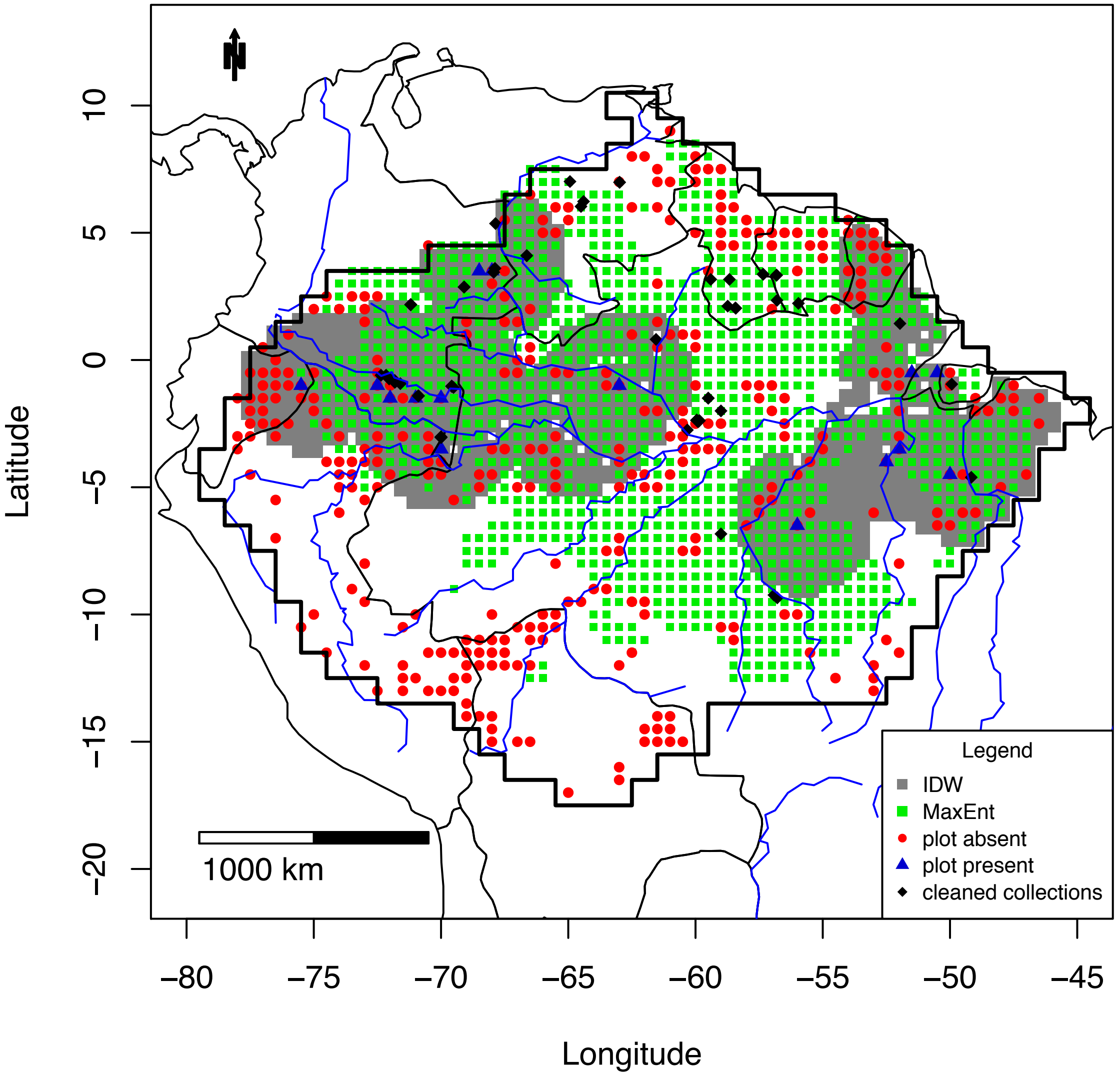

Figure S3_35B MaxEnt map of Cynometra_marginata

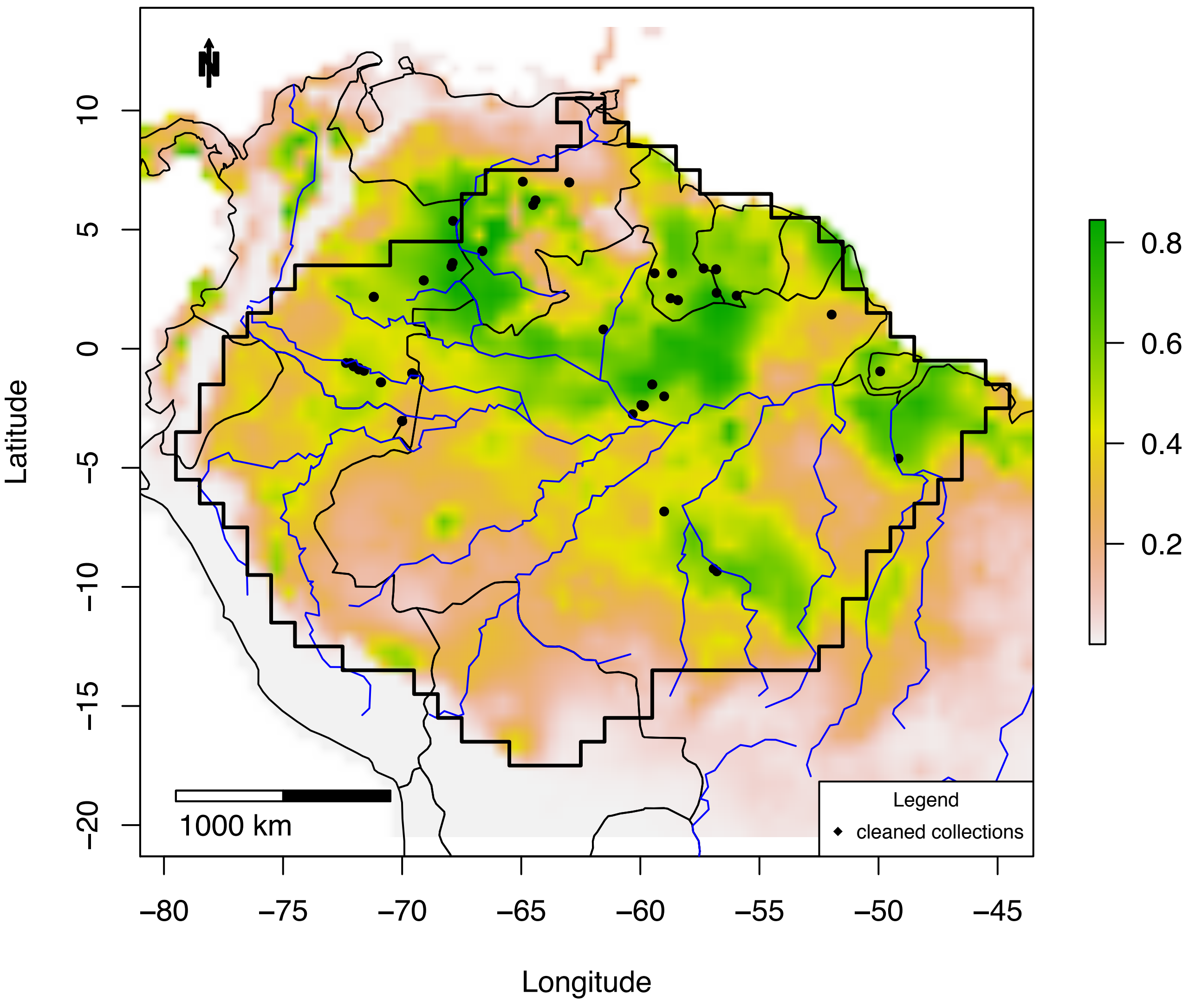

Figure S3_35C IDW map of Cynometra_marginata: plots =0 – 0.2; IDW: 0 – 0.04

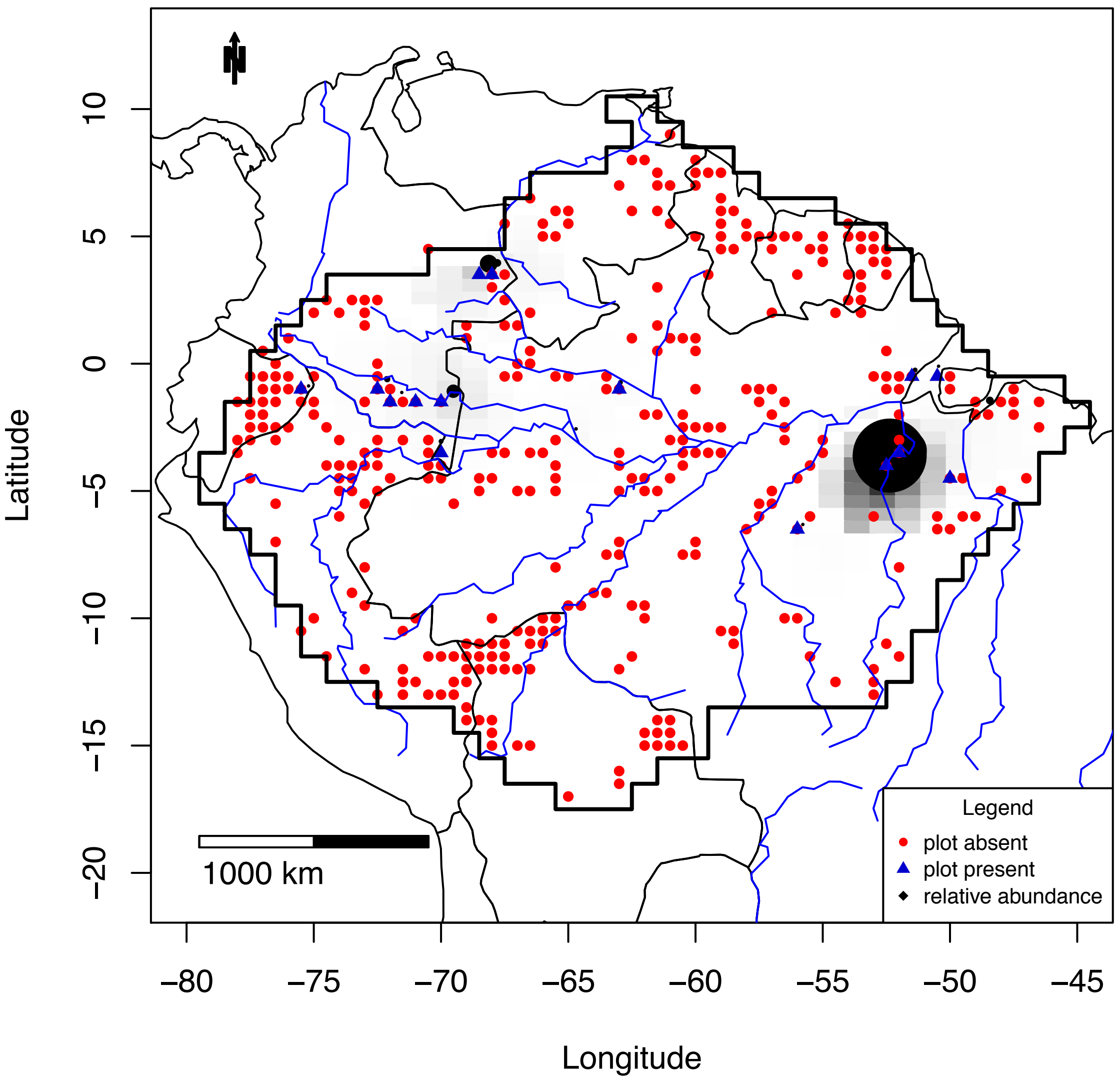

Figure S3_36A Combined map of Diplotropis_purpurea

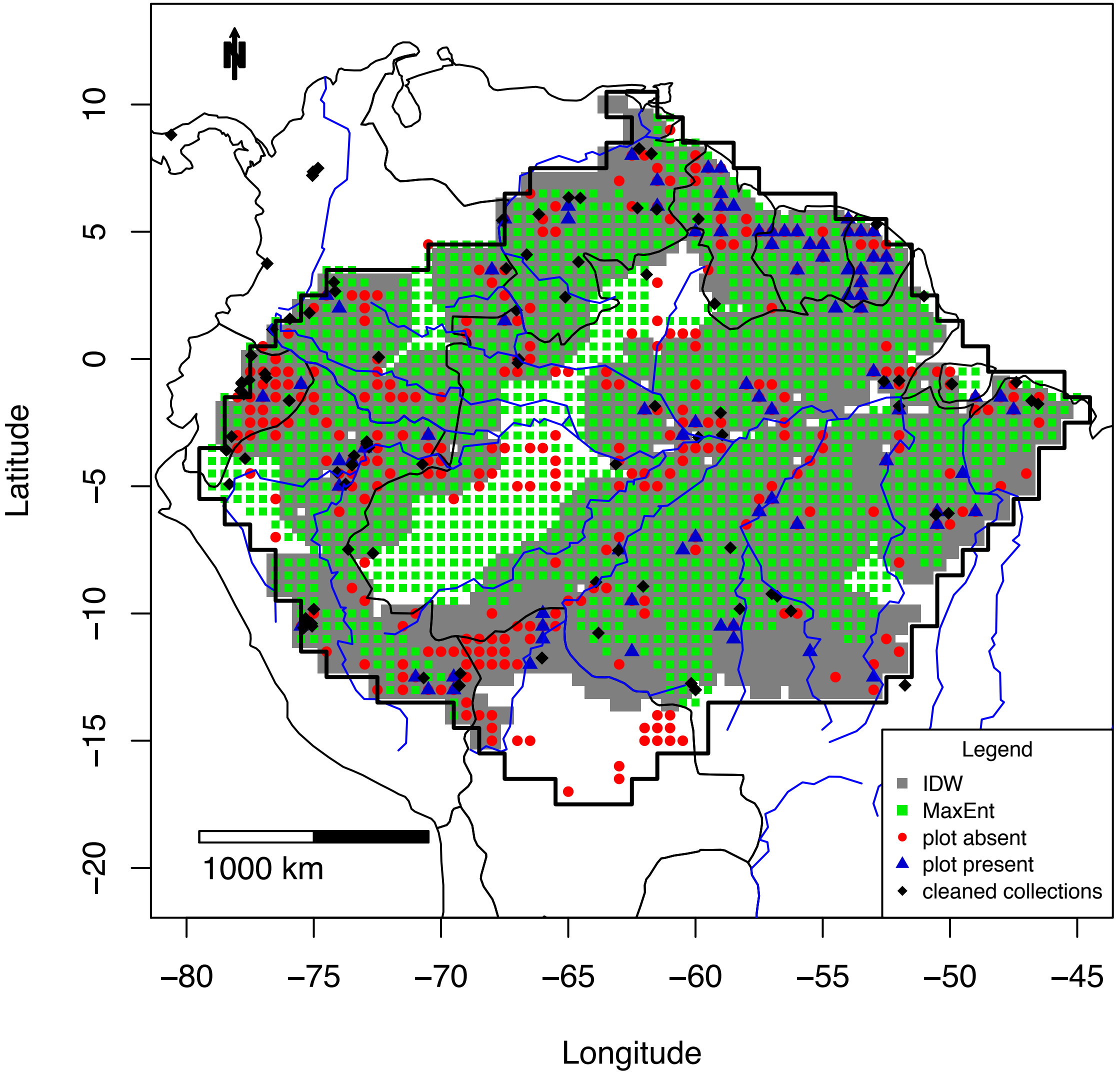

Figure S3_36B MaxEnt map of Diplotropis_purpurea

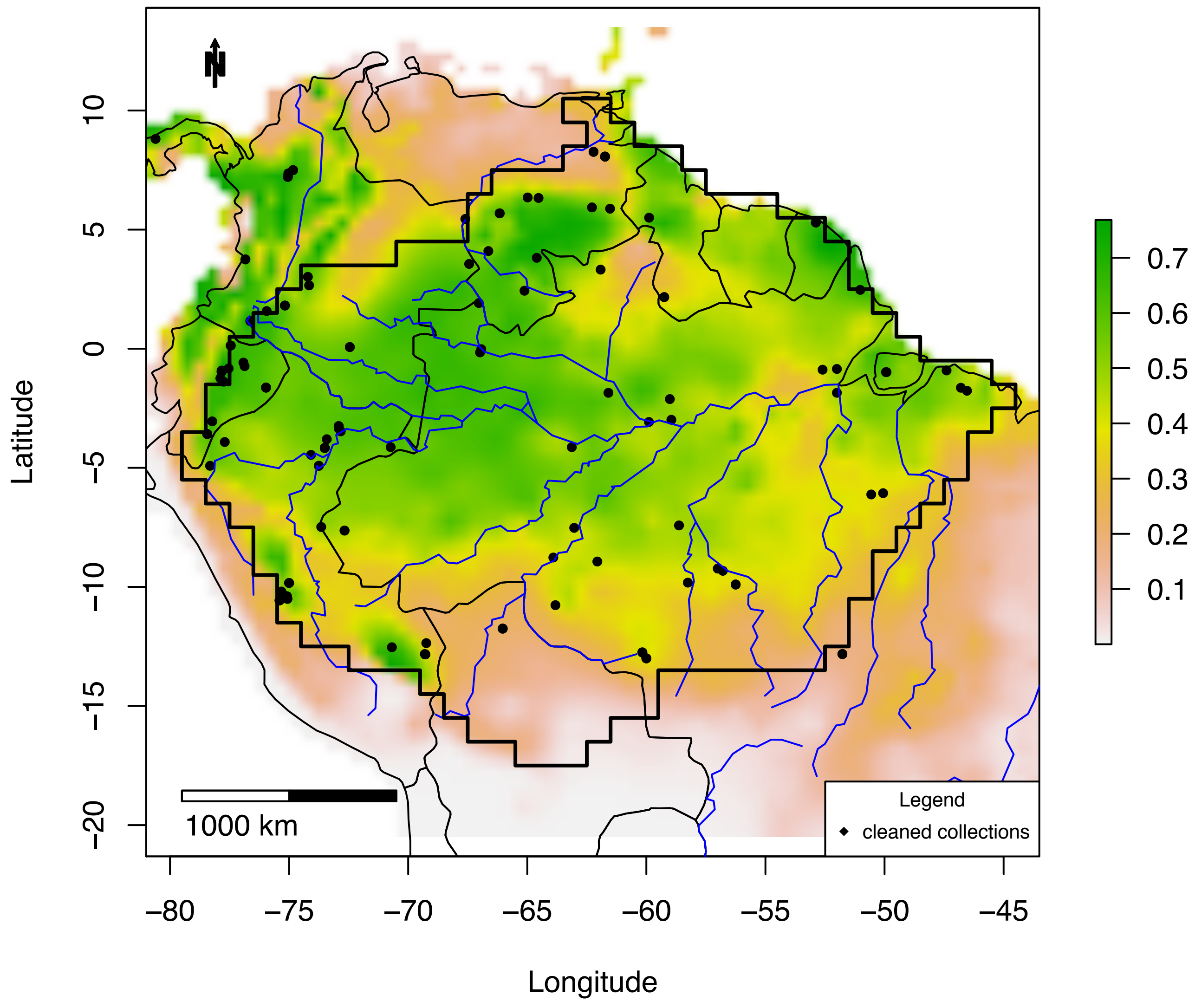

Figure S3_36C IDW map of Diplotropis_purpurea: plots =0 – 0.06; IDW: 0 – 0.02

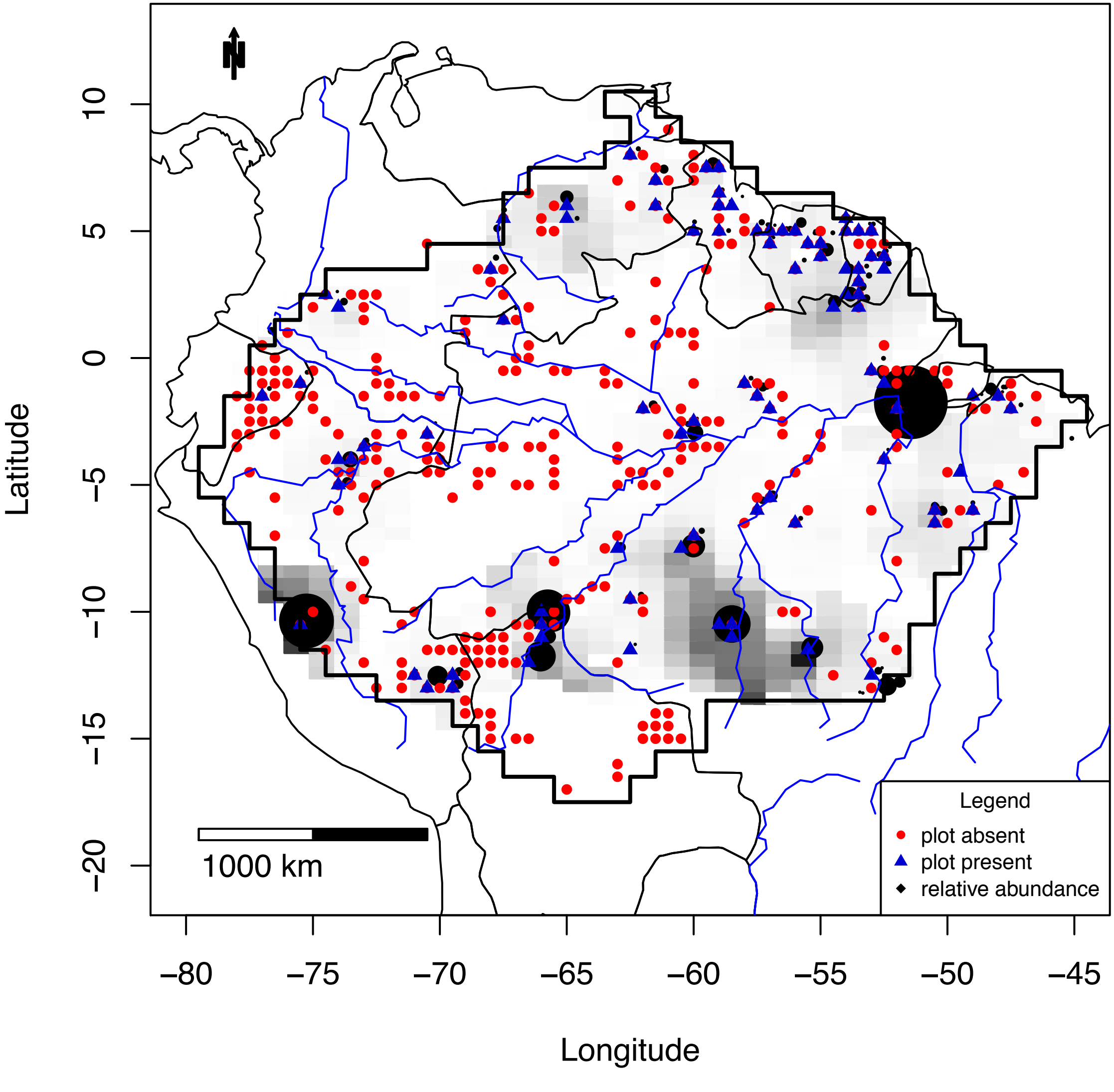

Figure S3_37A Combined map of Drypetes_amazonica

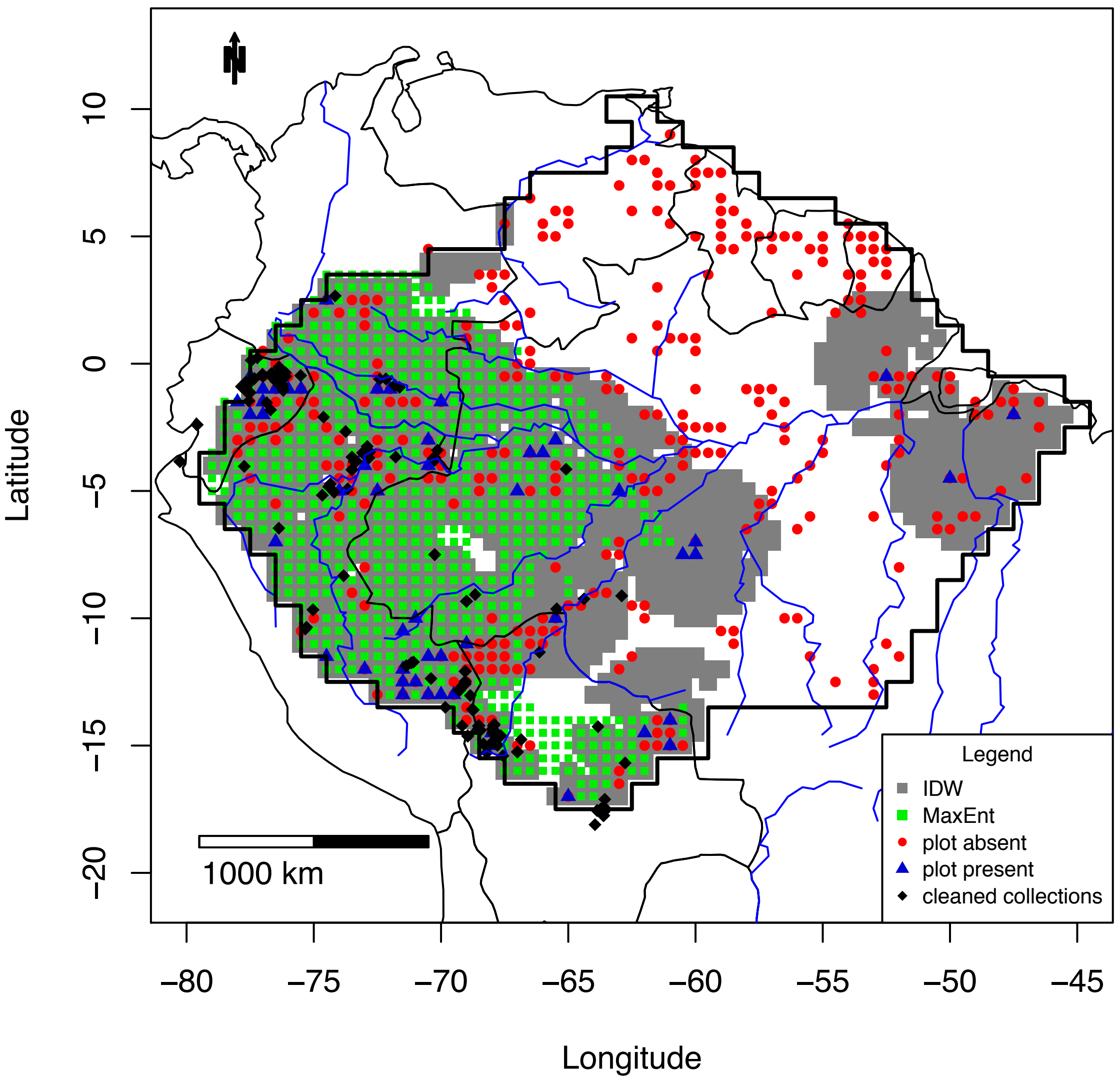

Figure S3_37B MaxEnt map of Drypetes_amazonica

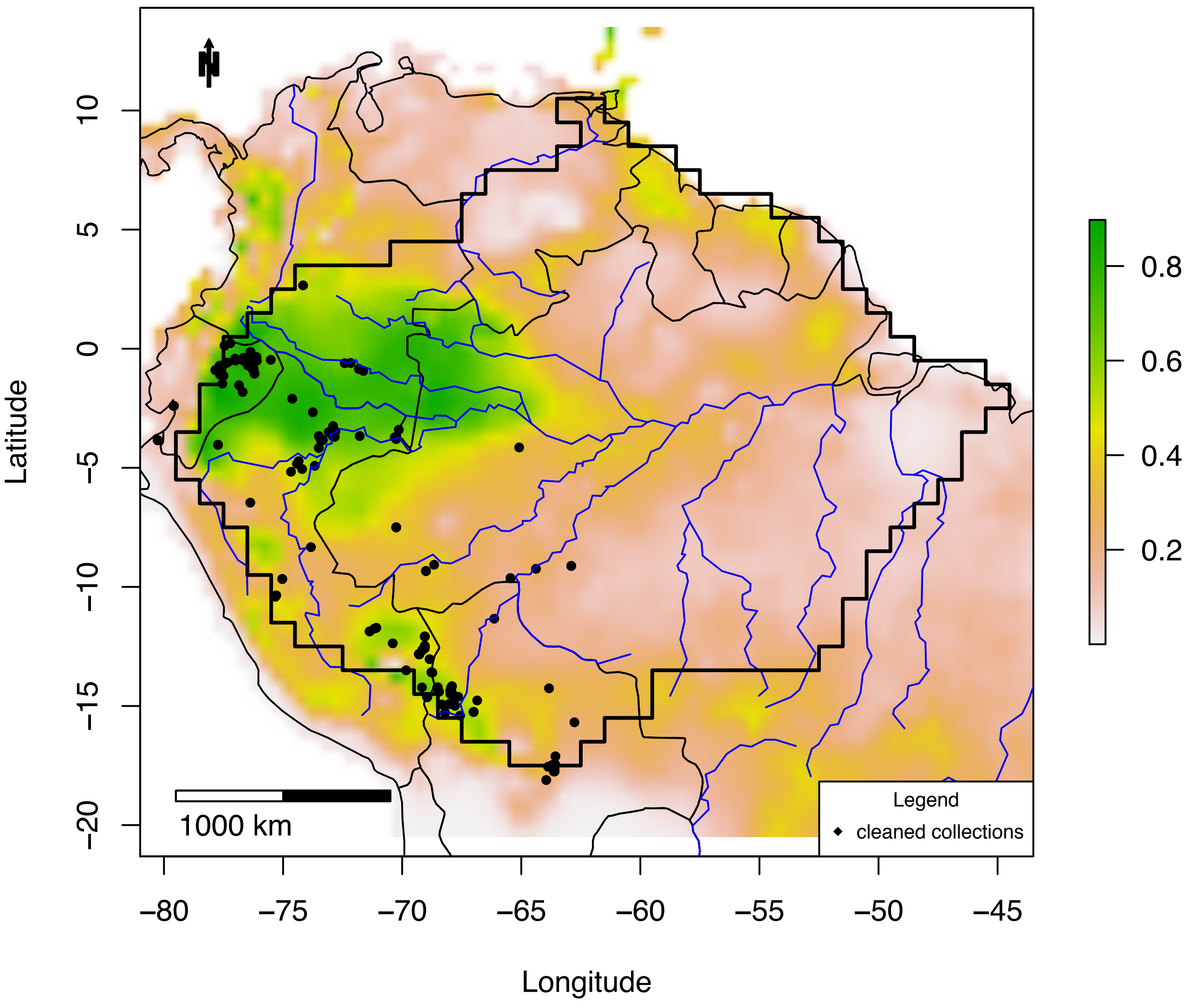

Figure S3_37C IDW map of Drypetes_amazonica: plots =0 – 0.12; IDW: 0 – 0.06

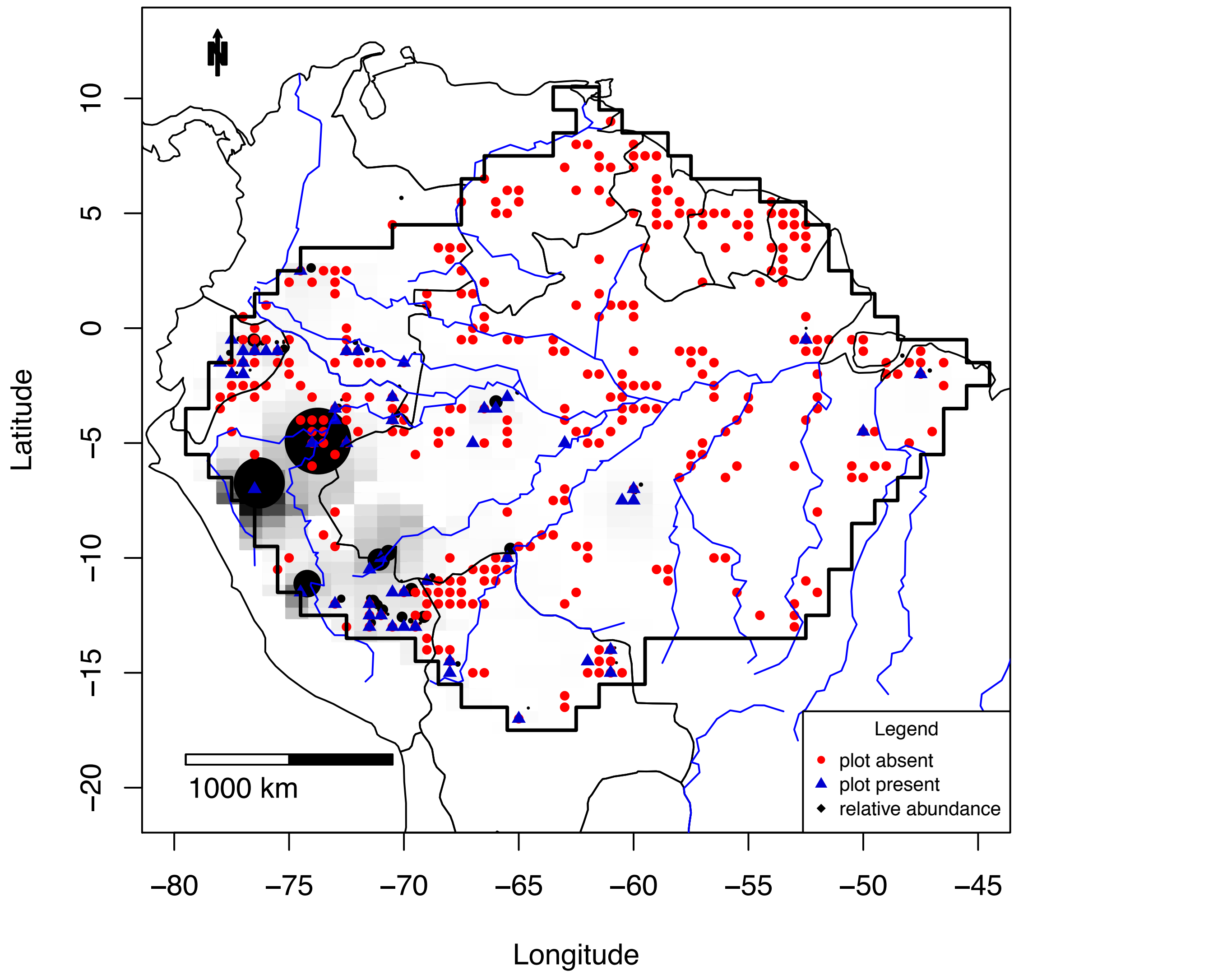

Figure S3_38A Combined map of Ecclinusa_guianensis

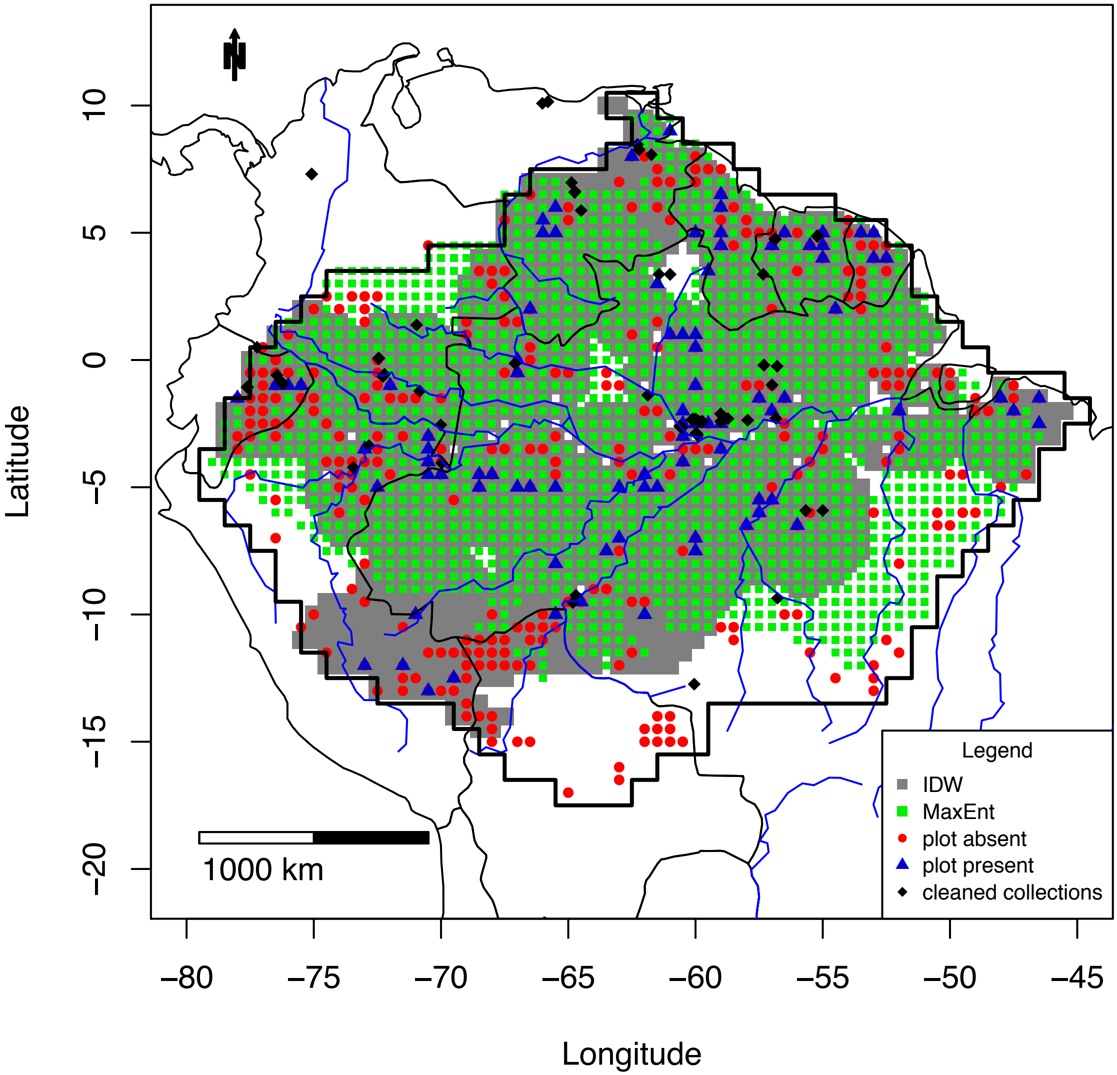

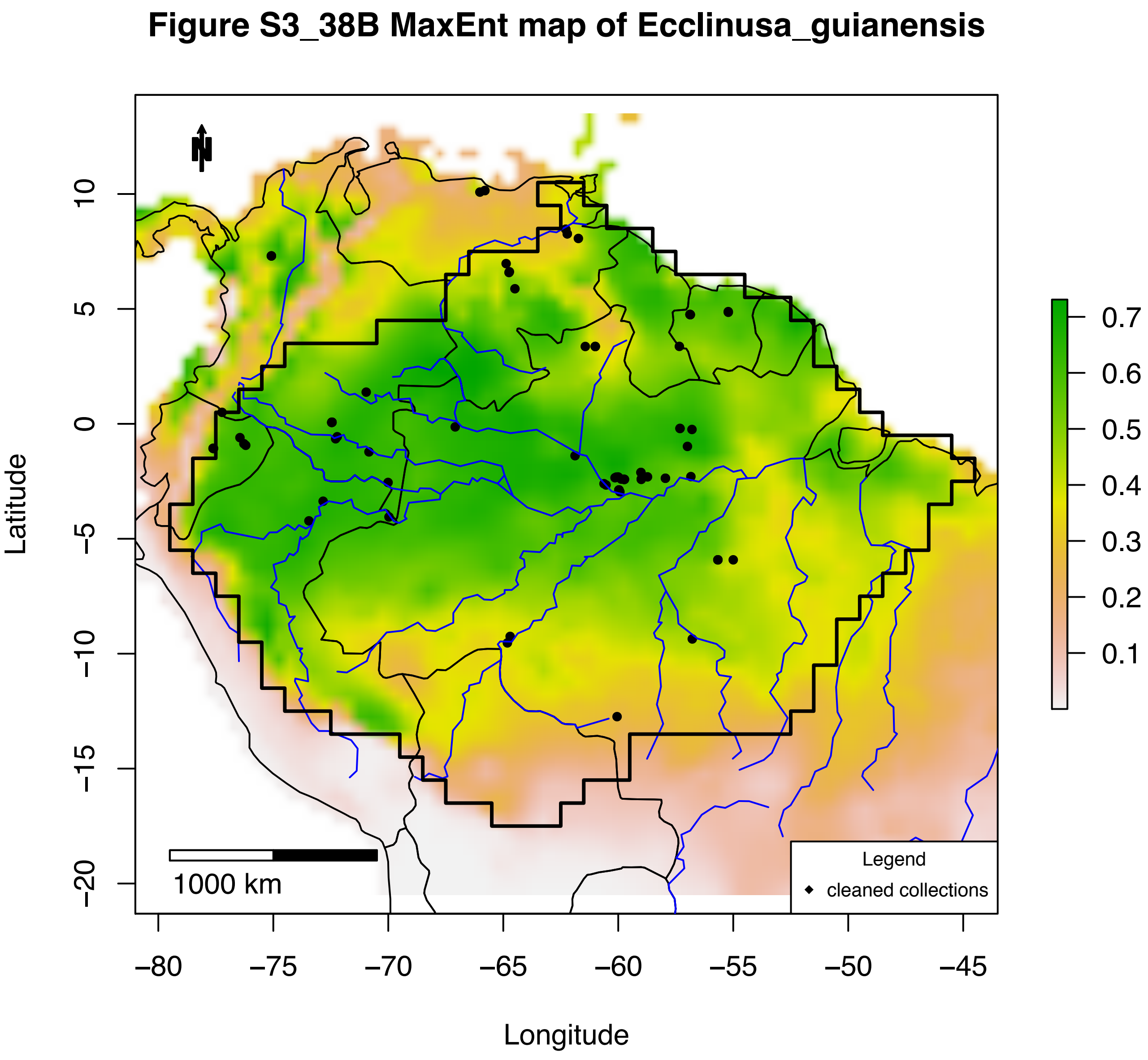
Figure S3_38B MaxEnt map of Ecclinusa_guianensis
N
10
5
0
−5
−10
−15
−20
Latitude
−80
−75
−70
−65
−60
−55
−50
−45
Longitude
1000 km
Legend
cleaned collections
0.7
0.6
0.5
0.4
0.3
0.2
0.1

Figure S3_38C IDW map of Ecclinusa_guianensis: plots =0 – 0.17; IDW: 0 – 0.09

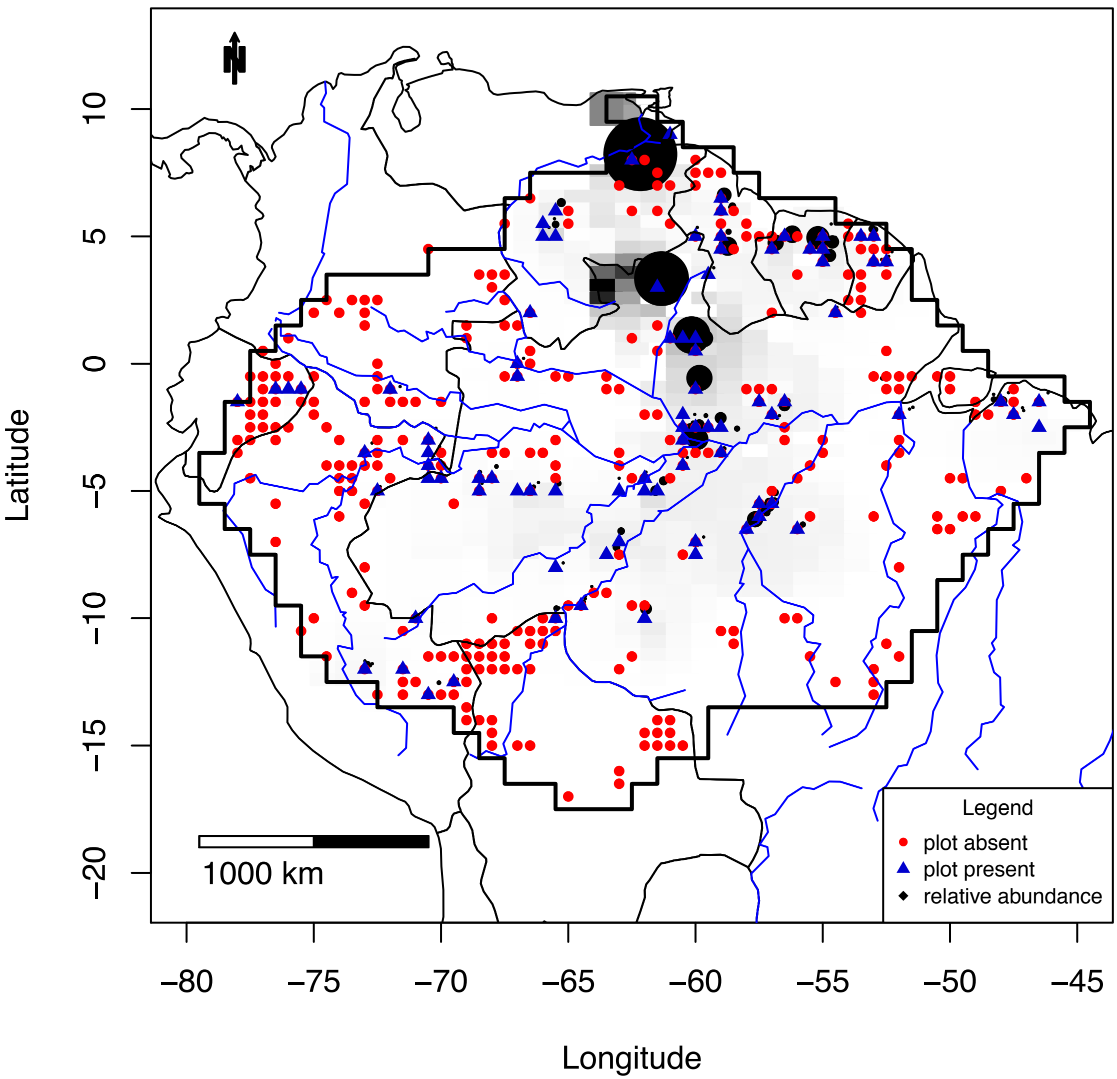

Figure S3_39A Combined map of Enterolobium_schomburgkii

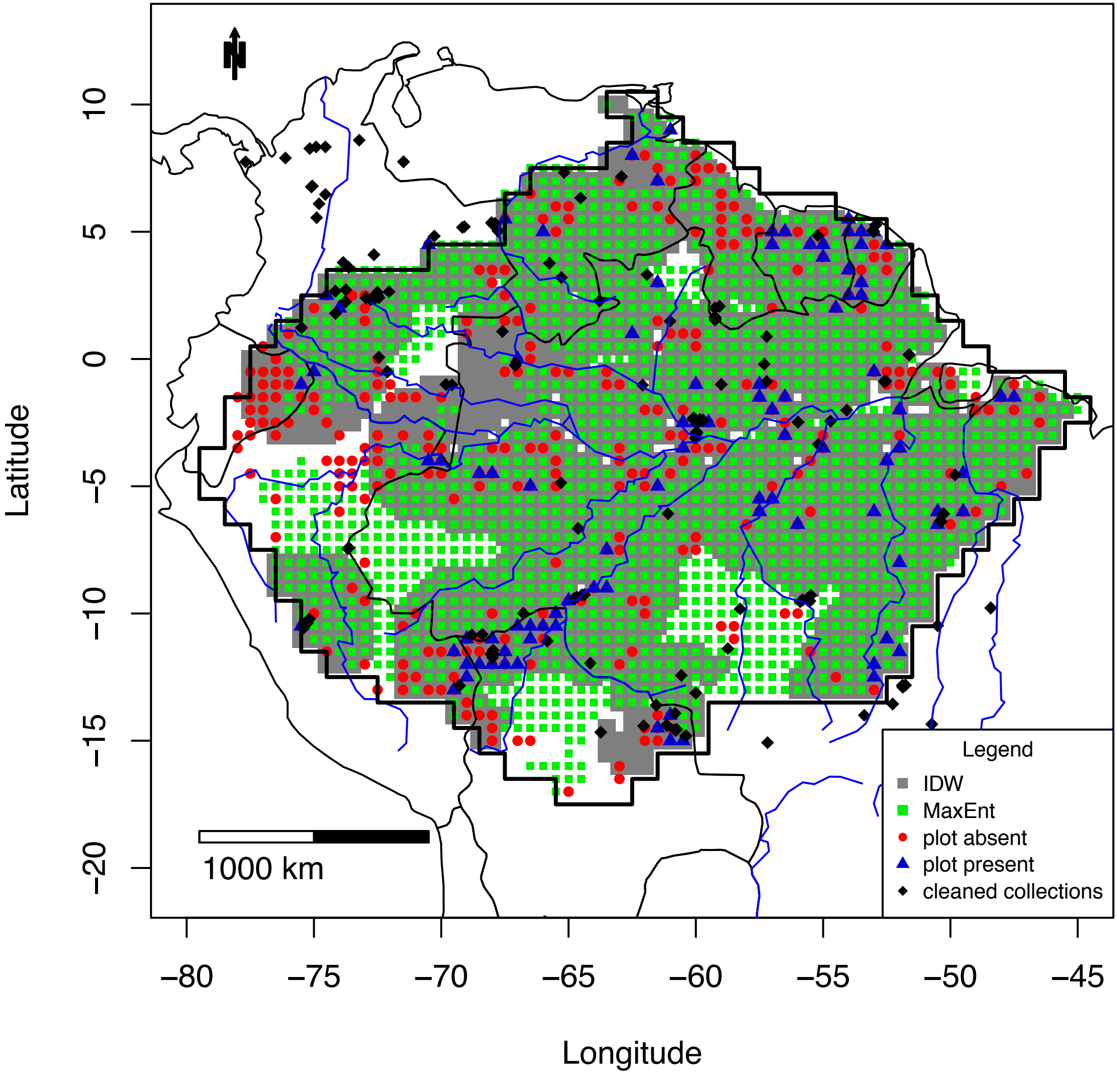

Figure S3_39B MaxEnt map of Enterolobium_schomburgkii

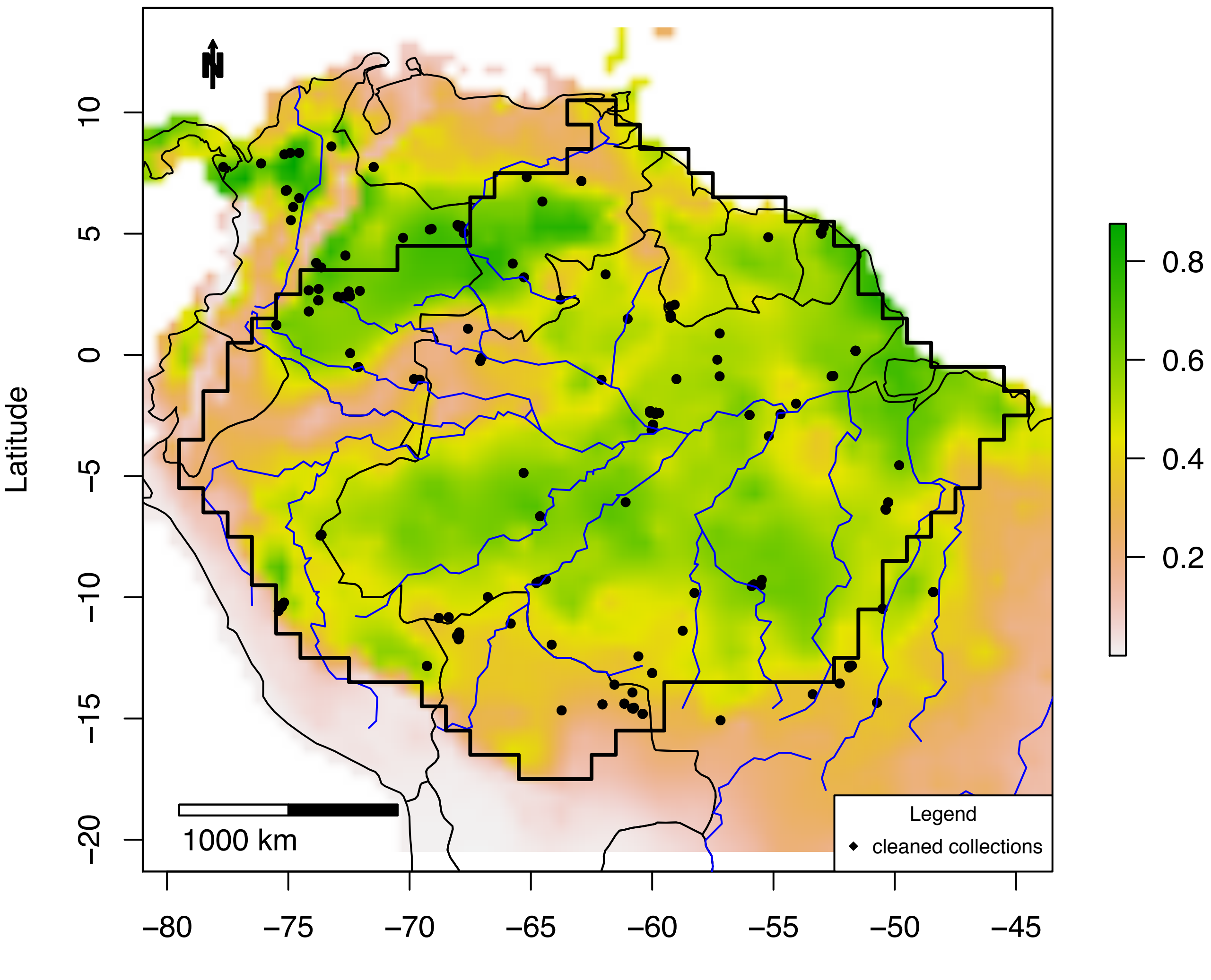

Figure S3_39C IDW map of Enterolobium_schomburgkii: plots =0 – 0.09; IDW: 0 – 0.02

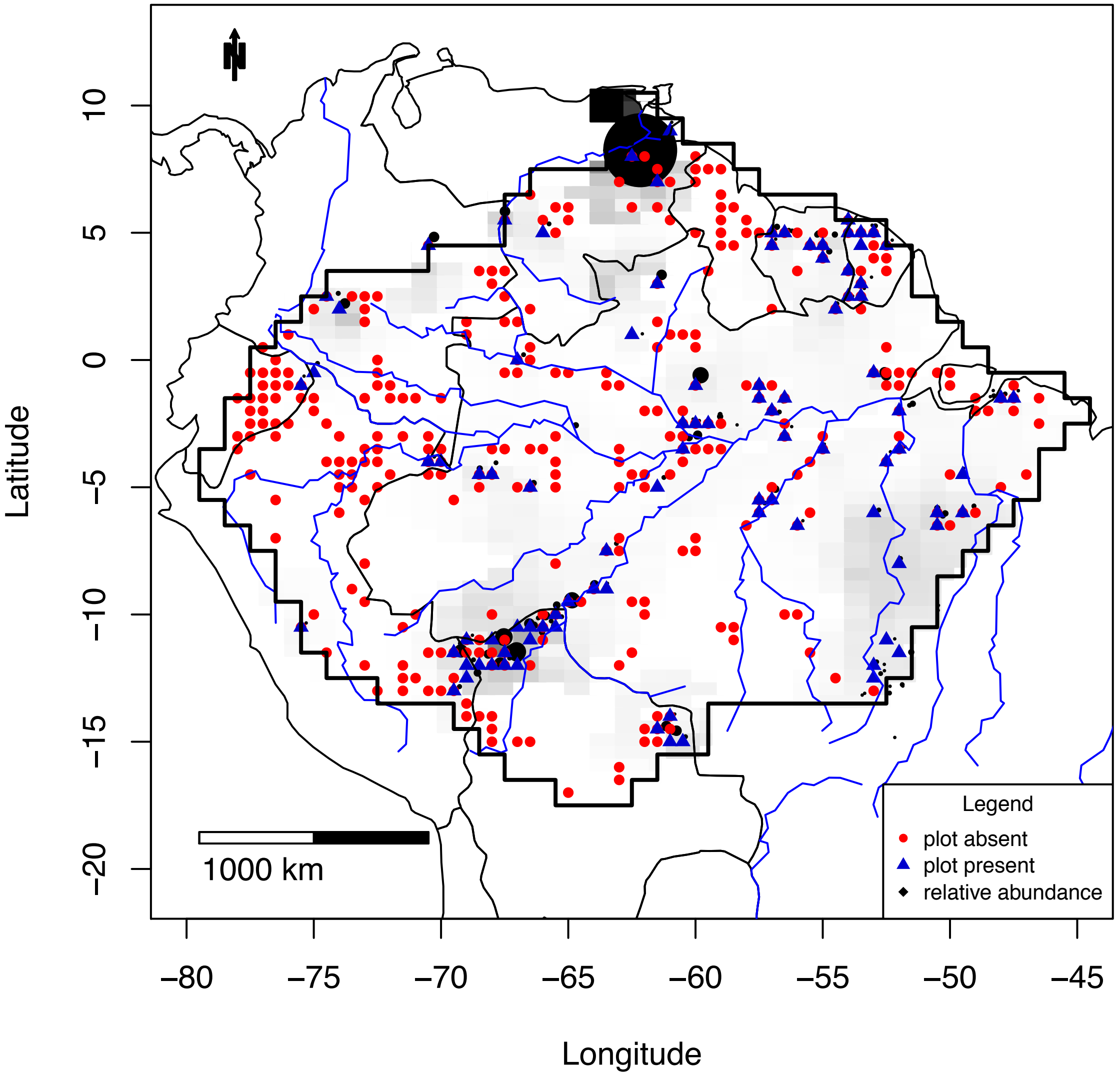

Figure S3_40A Combined map of Eperua_falcata

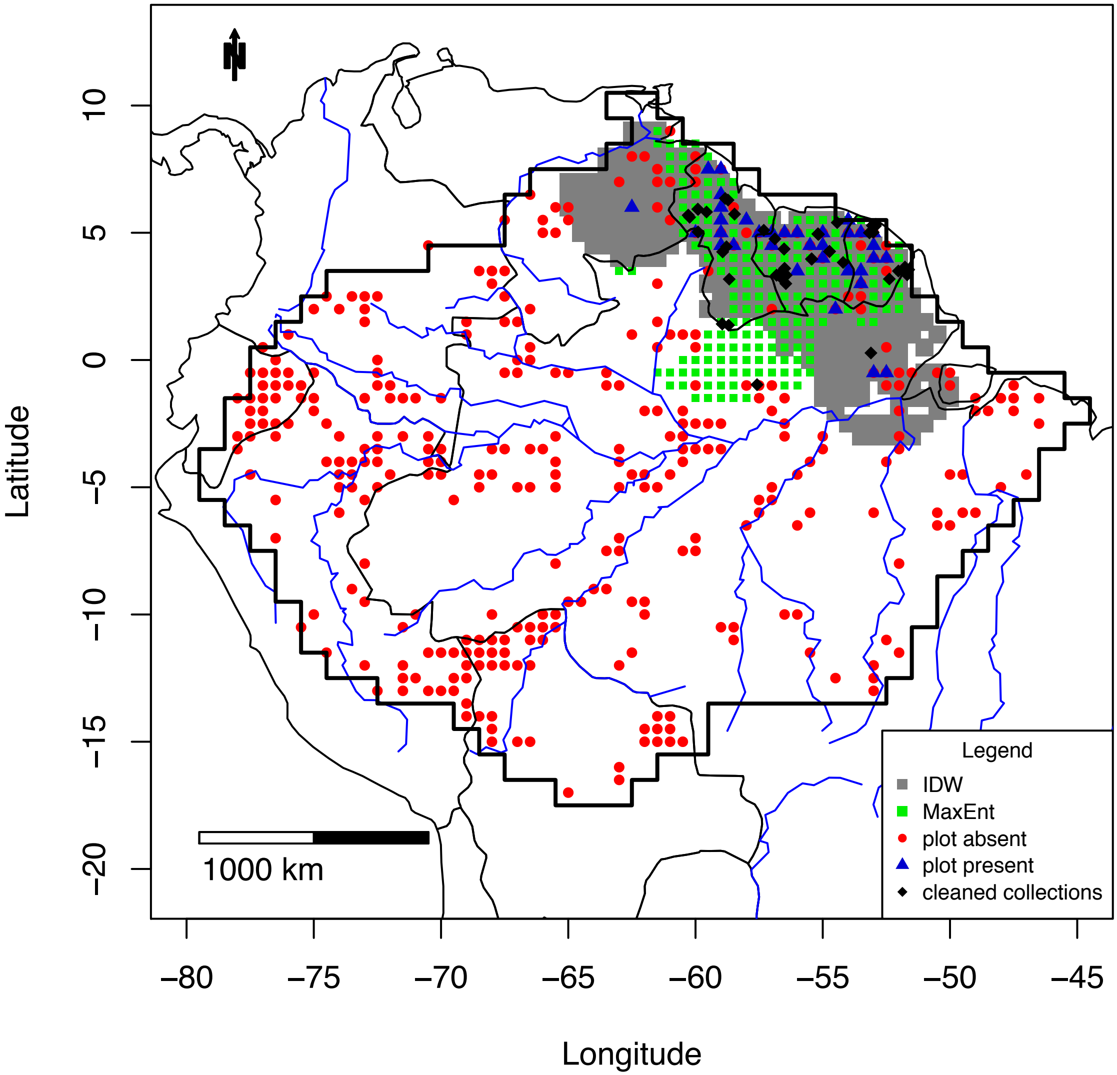

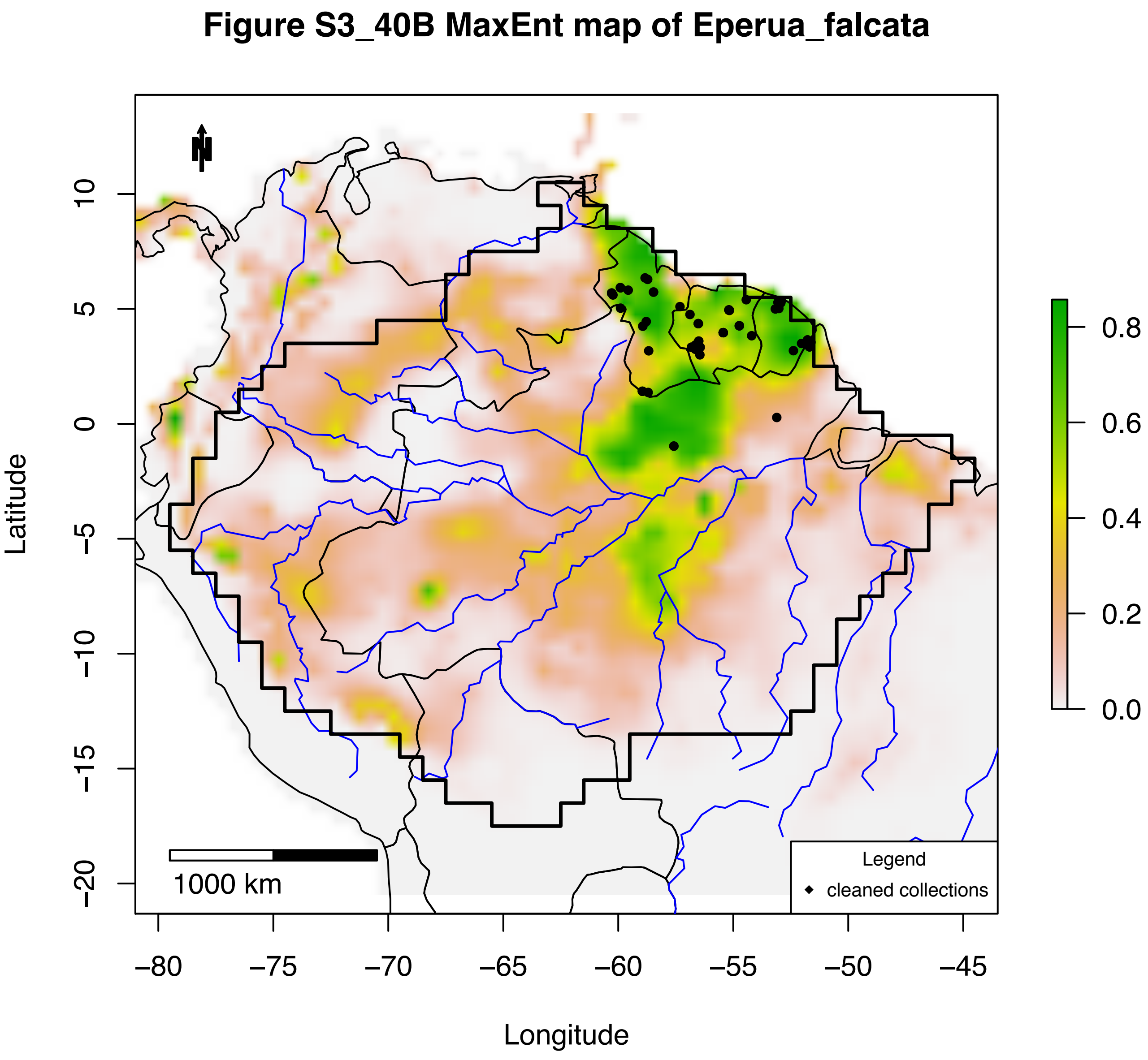

Figure S3_40B MaxEnt map of Eperua_falcata
N
10
5
0
−5
−10
−15
−20
Latitude
−80
−75
−70
−65
−60
−55
−50
−45
Longitude
1000 km
Legend
cleaned collections
0.8
0.6
0.4
0.2
0.0

Figure S3_40C IDW map of Eperua_falcata: plots =0 – 0.73; IDW: 0 – 0.28

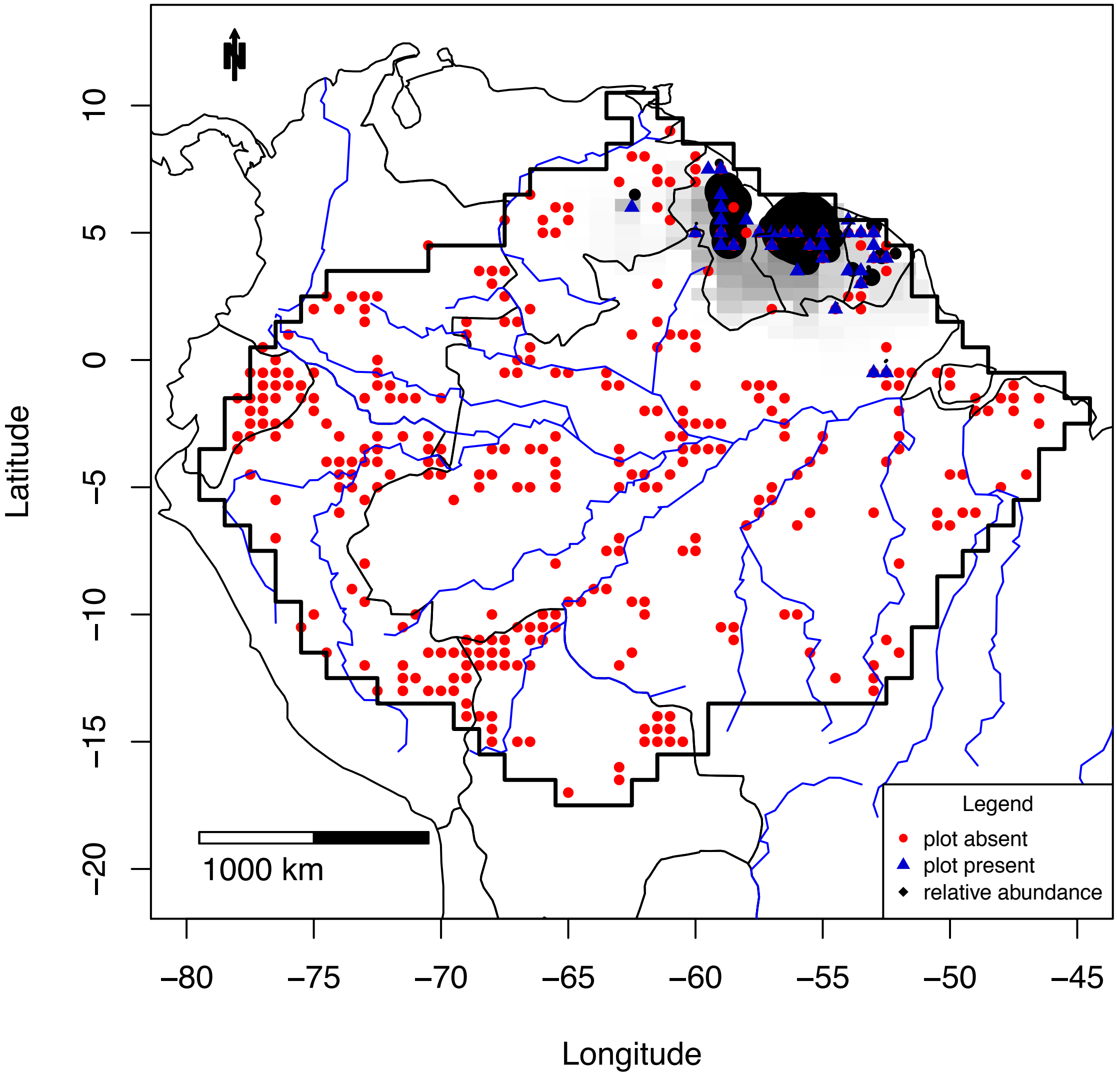

Figure S3_41A Combined map of Eperua_grandiflora

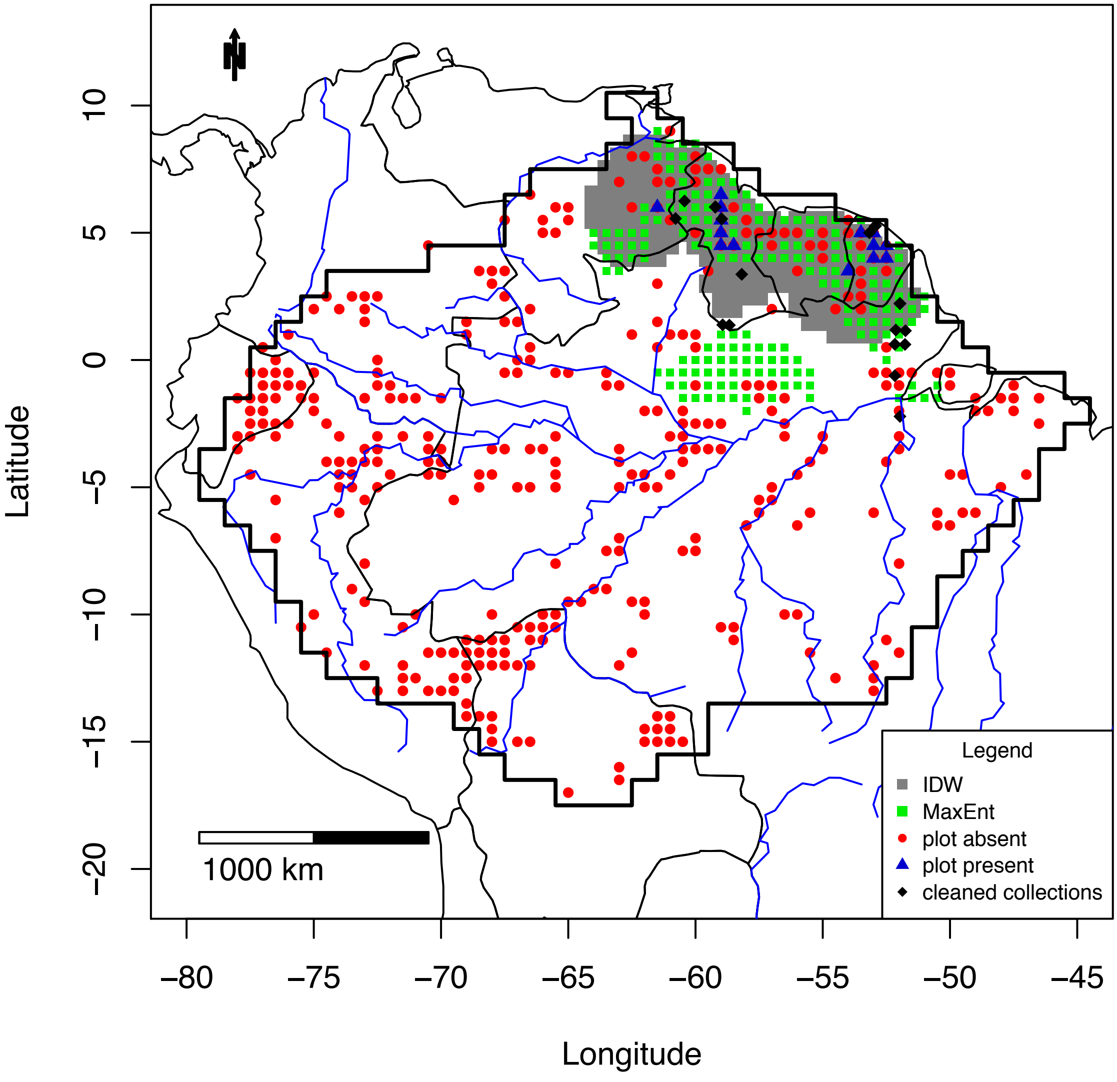

Figure S3_41B MaxEnt map of Eperua_grandiflora

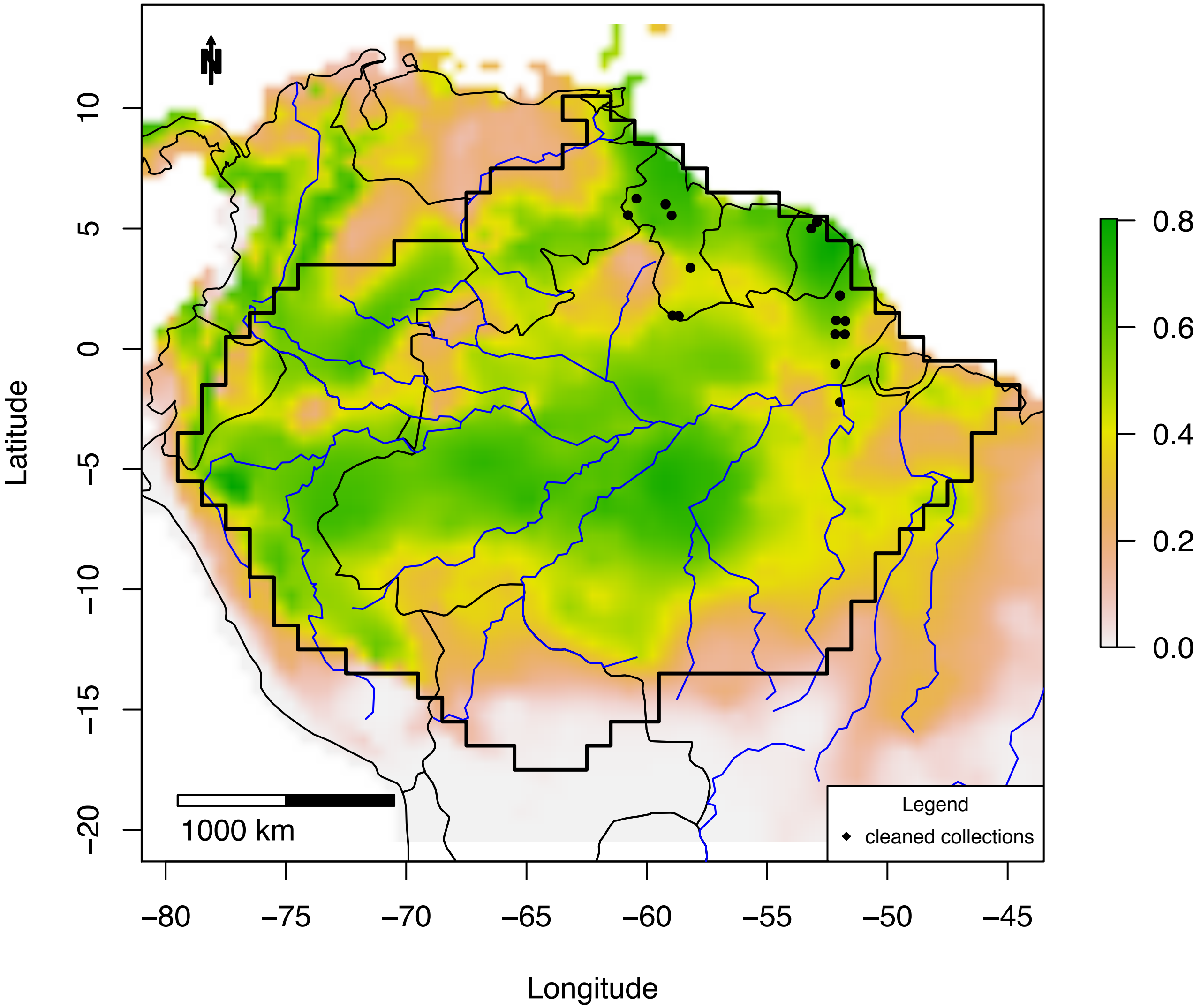

Figure S3_41C IDW map of Eperua_grandiflora: plots =0 – 0.47; IDW: 0 – 0.07

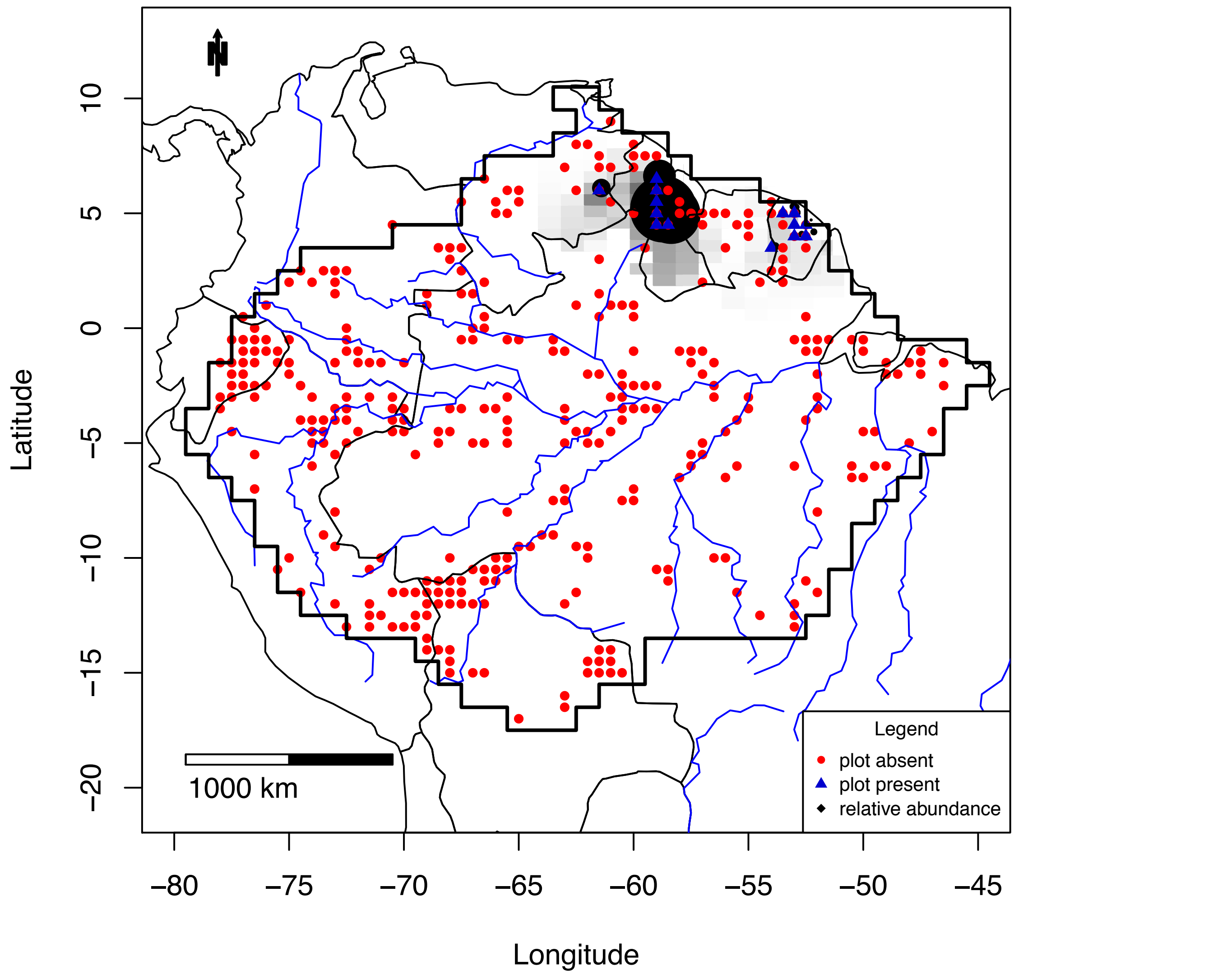

Figure S3_42A Combined map of Eperua_leucantha

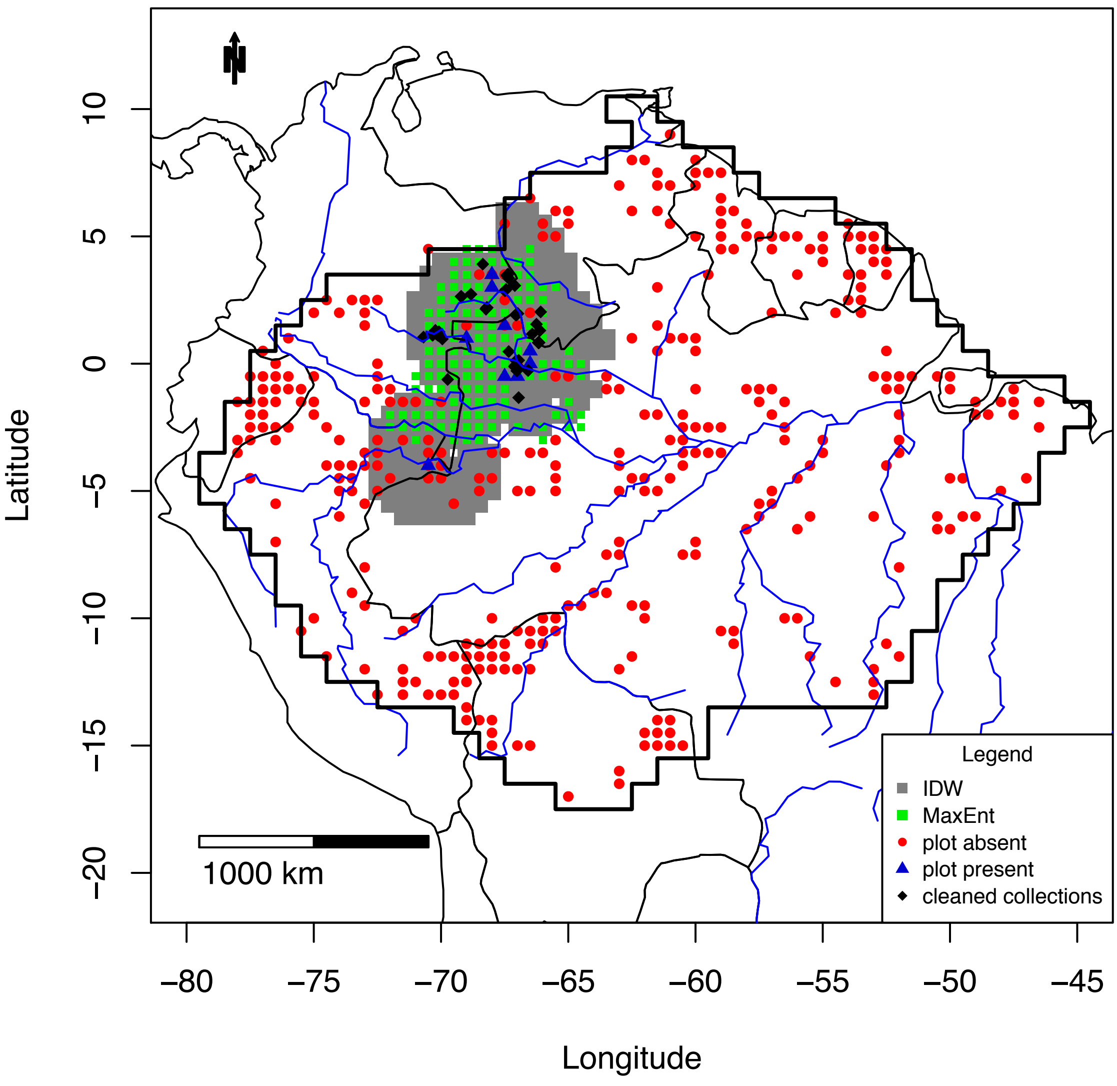

Figure S3_42B MaxEnt map of Eperua_leucantha

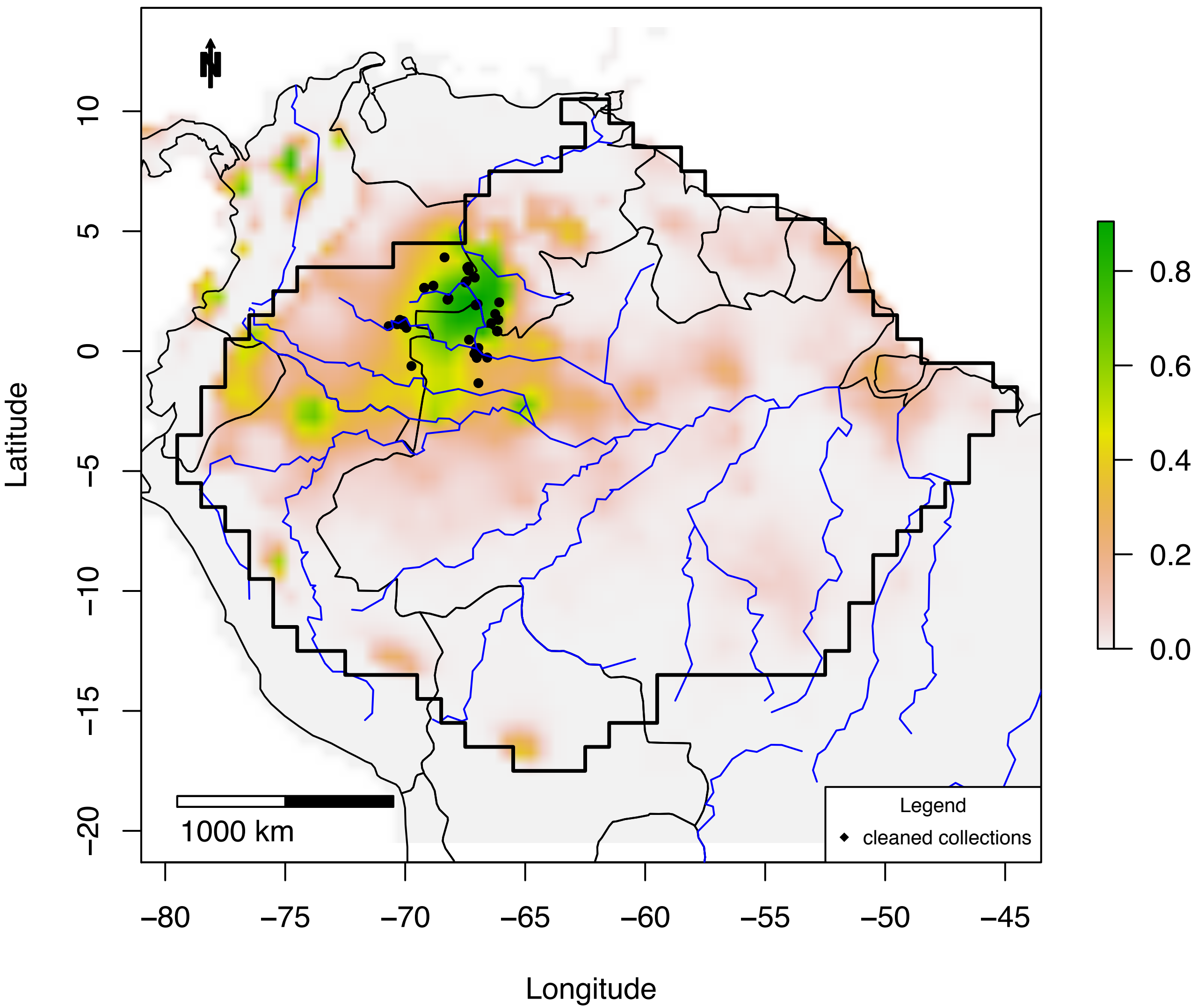

Figure S3_42C IDW map of Eperua_leucantha:
plots =0 – 0.49; IDW: 0 – 0.24

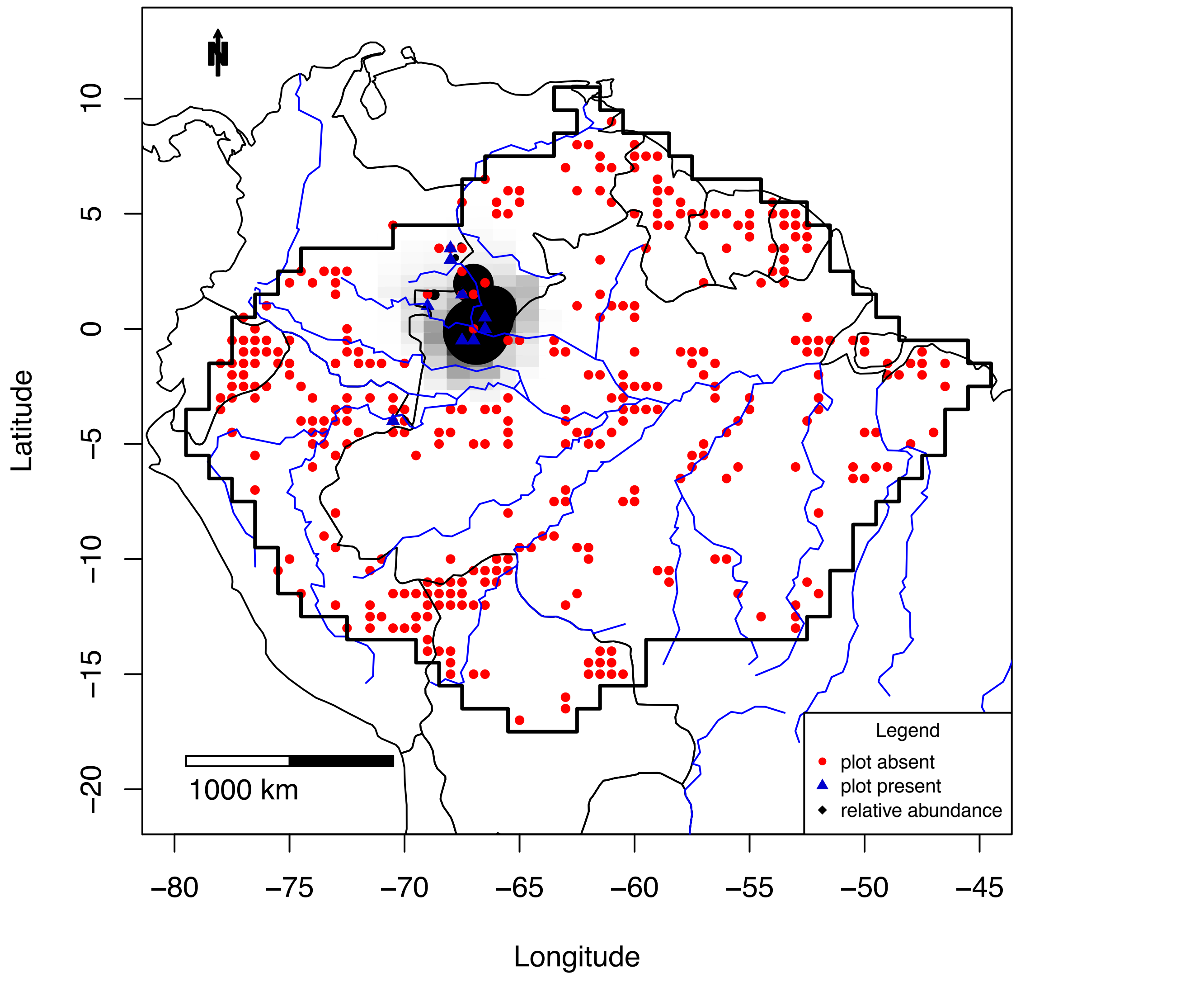

Figure S3_43A Combined map of Eperua_purpurea

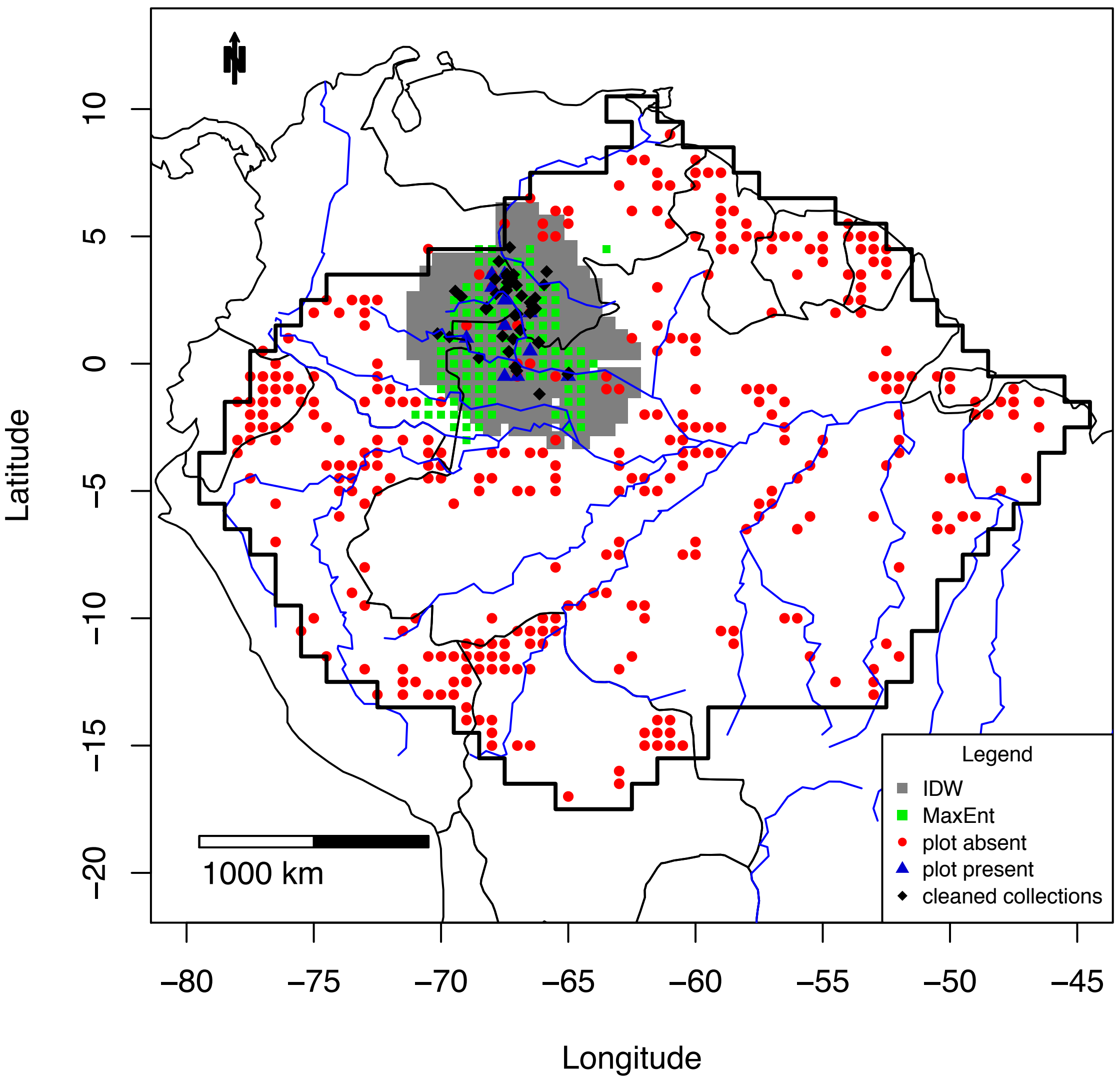

Figure S3_43B MaxEnt map of Eperua_purpurea

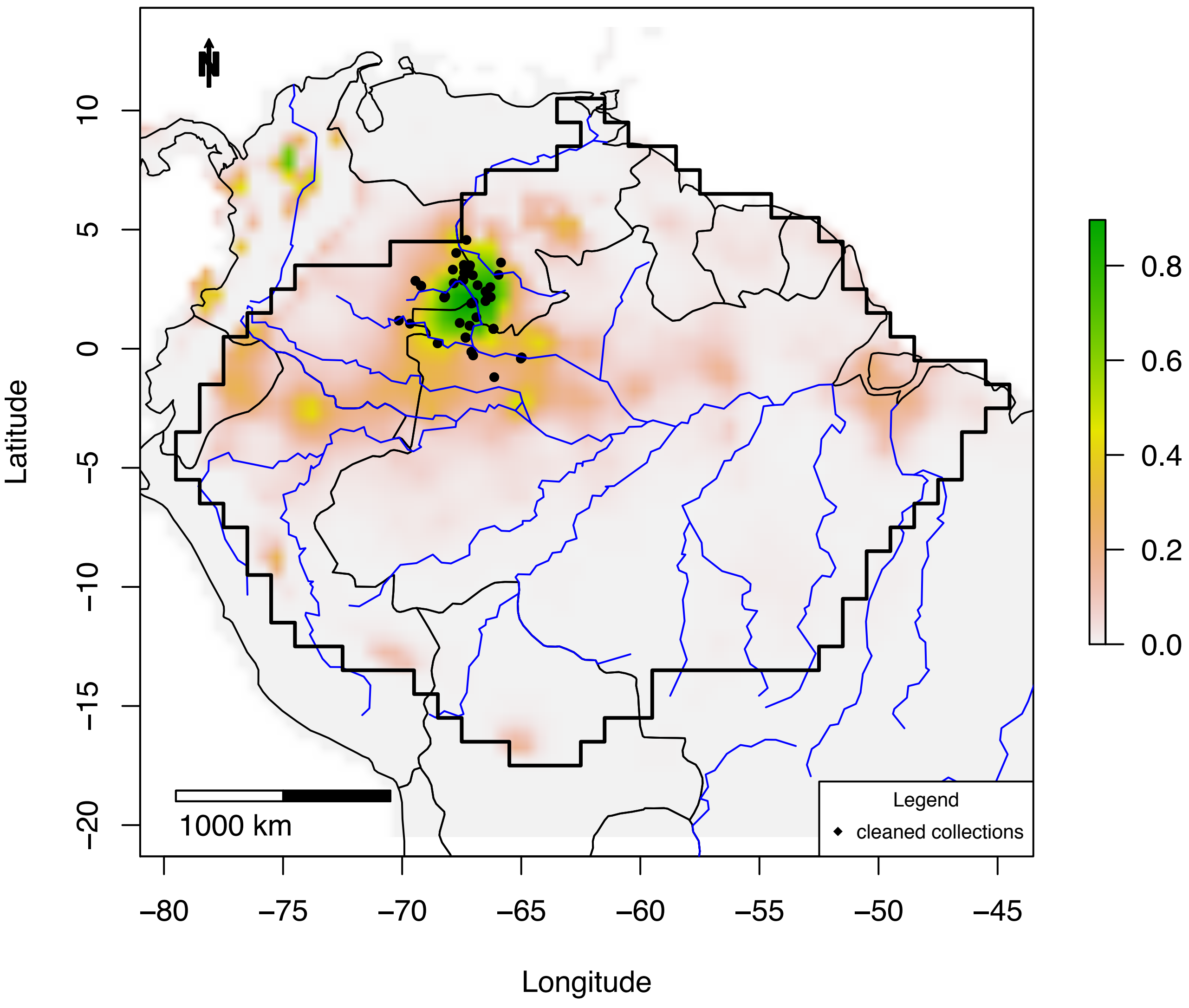

Figure S3_43C IDW map of Eperua_purpurea: plots =0 – 0.2; IDW: 0 – 0.07

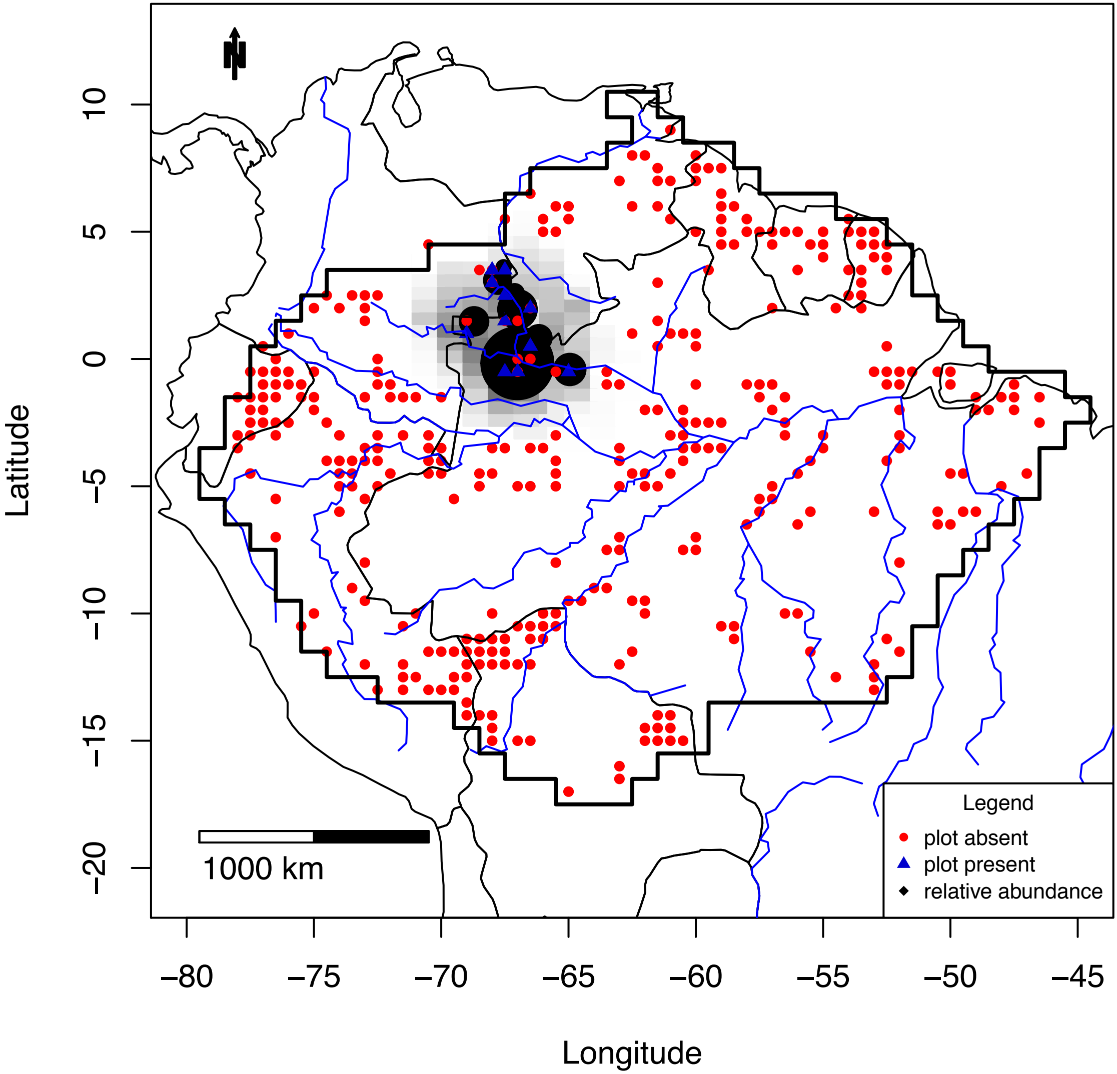

Figure S3_44A Combined map of Erisma_uncinatum

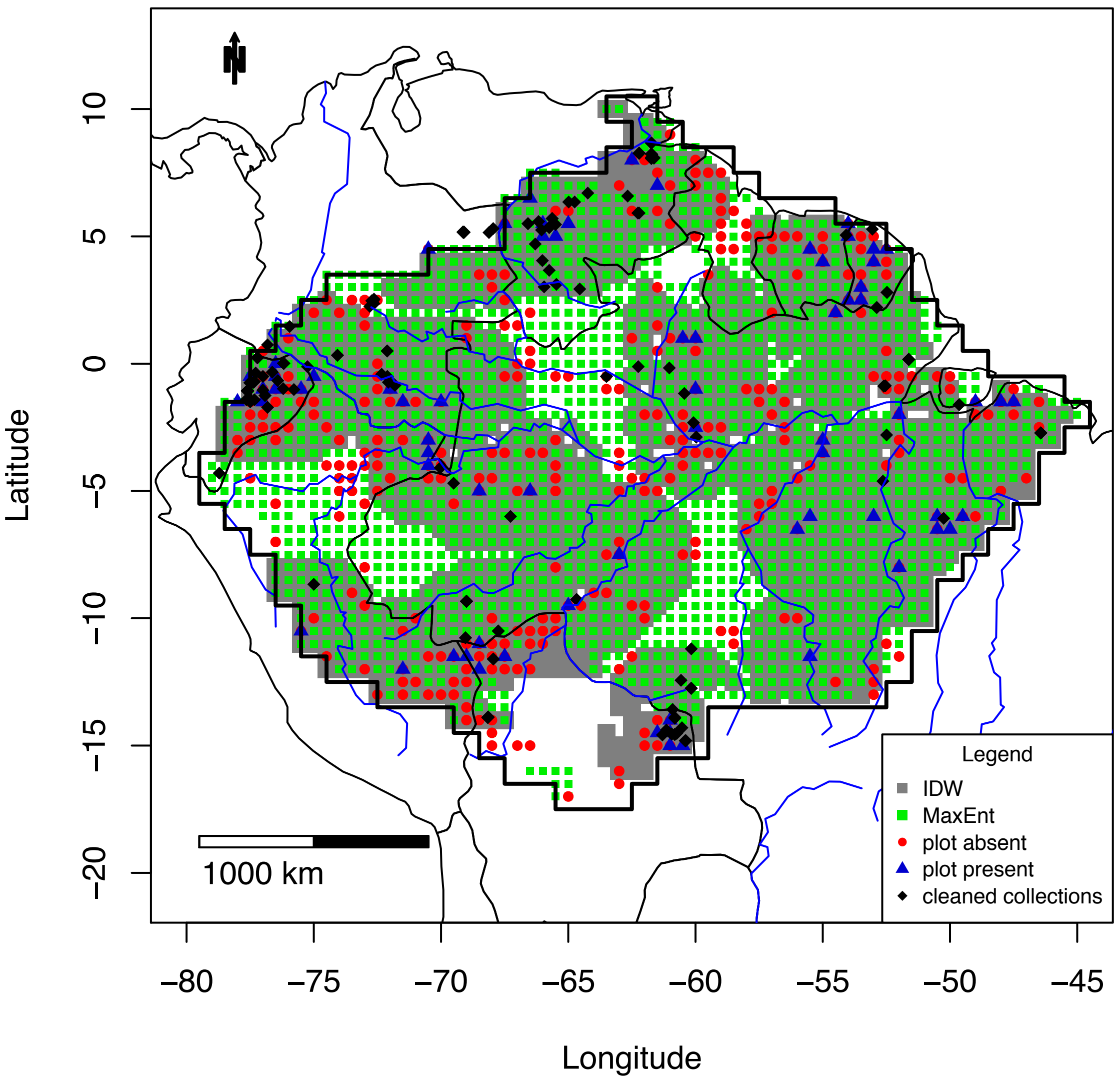

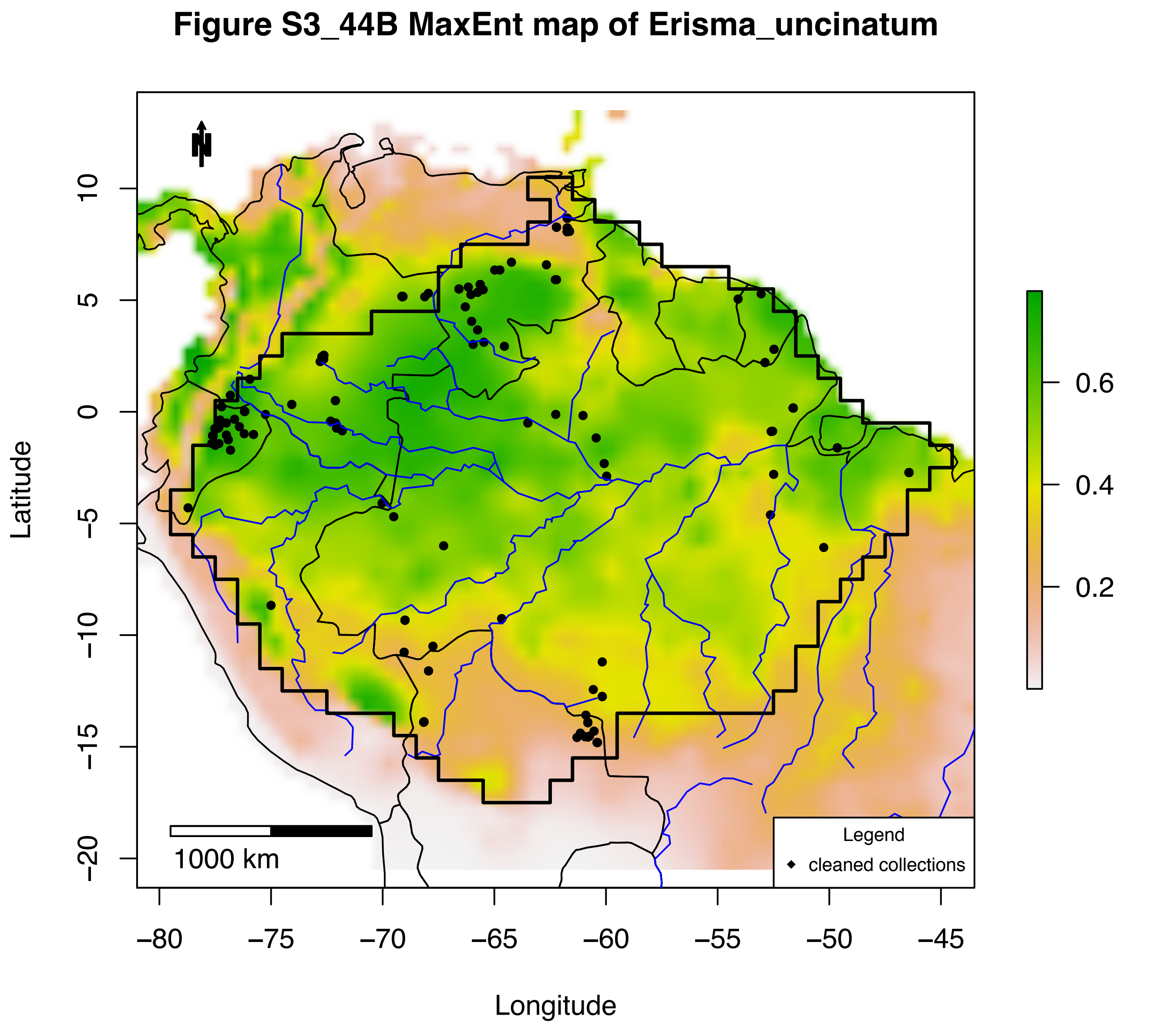

Figure S3_44B MaxEnt map of Erisma_uncinatum
N
10
5
0
−5
−10
−15
−20
Latitude
−80
−75
−70
−65
−60
−55
−50
−45
Longitude
0.6
0.4
0.2
1000 km
Legend
cleaned collections

Figure S3_44C IDW map of Erisma_uncinatum:
plots =0 – 0.11; IDW: 0 – 0.05

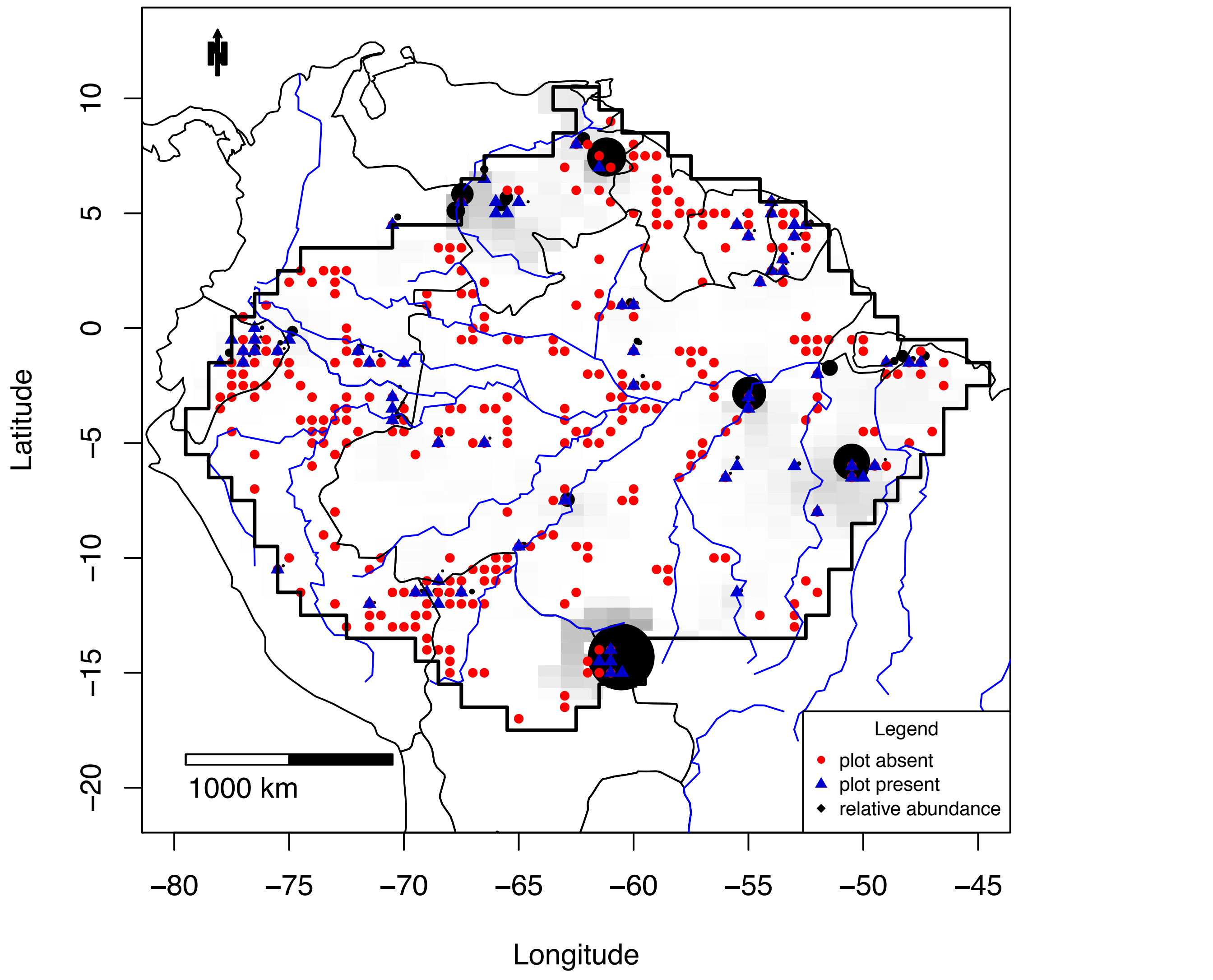

Figure S3_45A Combined map of Eschweilera_albiflora

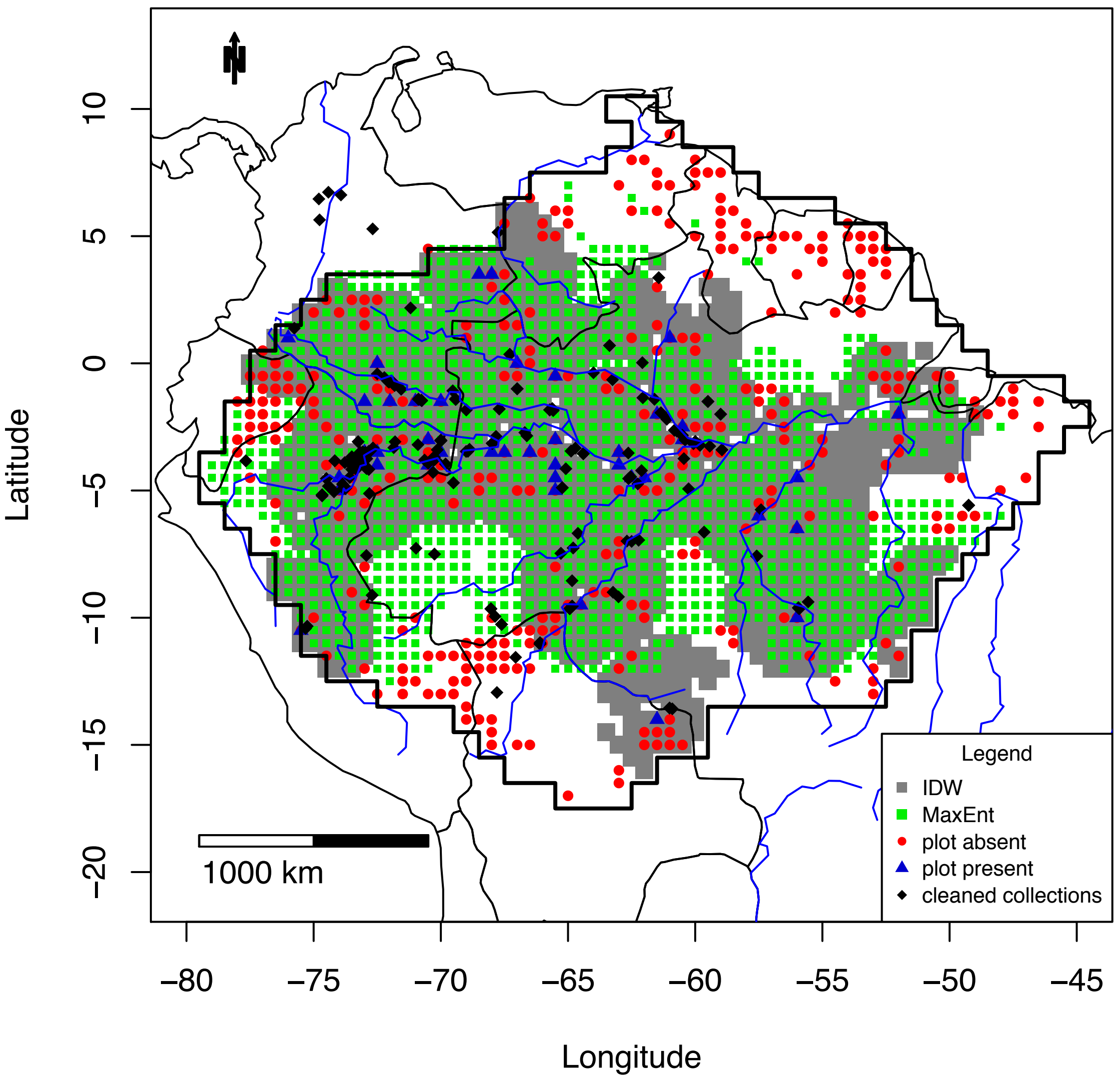

Figure S3_45B MaxEnt map of Eschweilera_albiflora

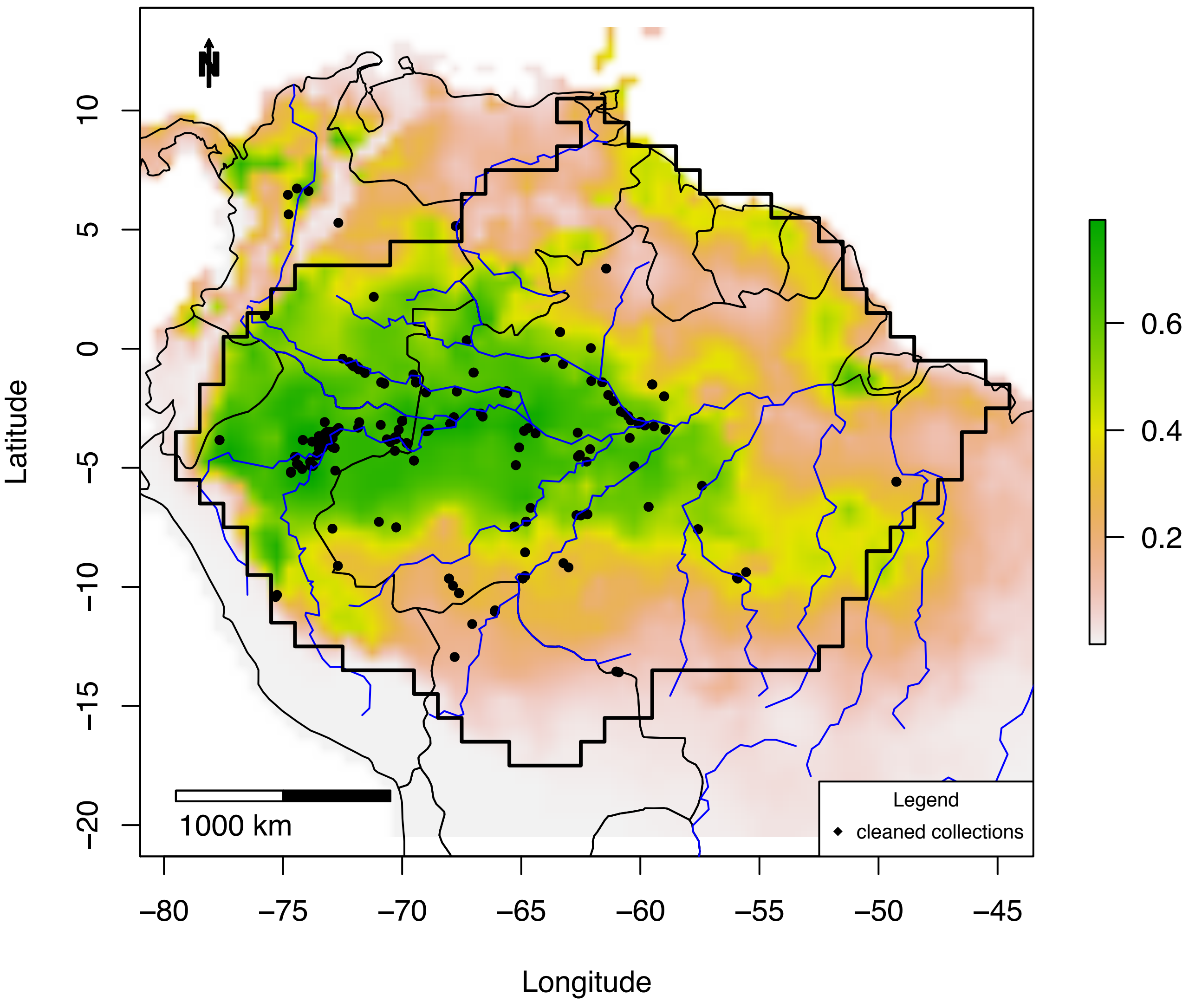

Figure S3_45C IDW map of Eschweilera_albiflora: plots =0 – 0.22; IDW: 0 – 0.11

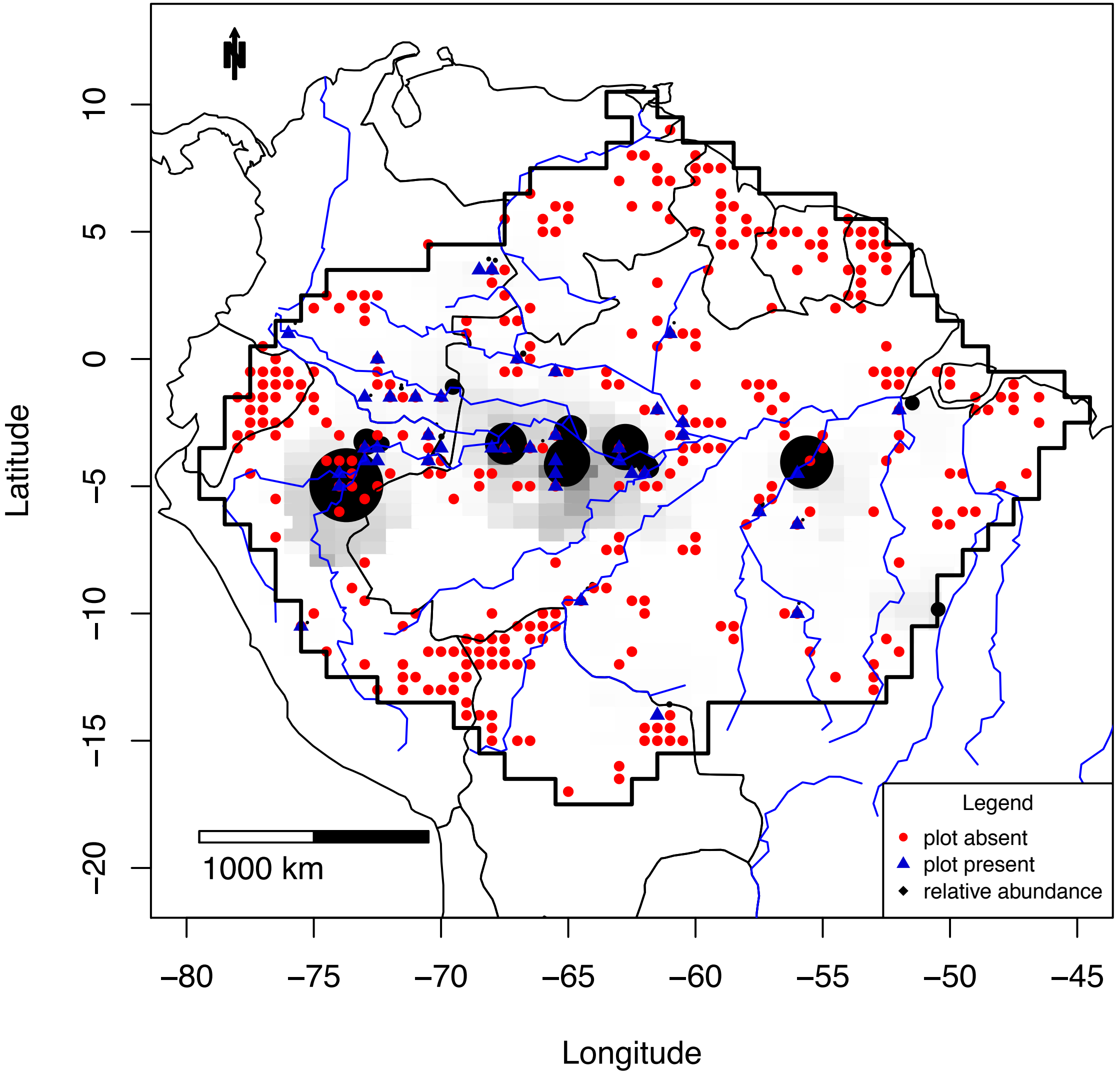

Figure S3_46A Combined map of Eschweilera_amazonica

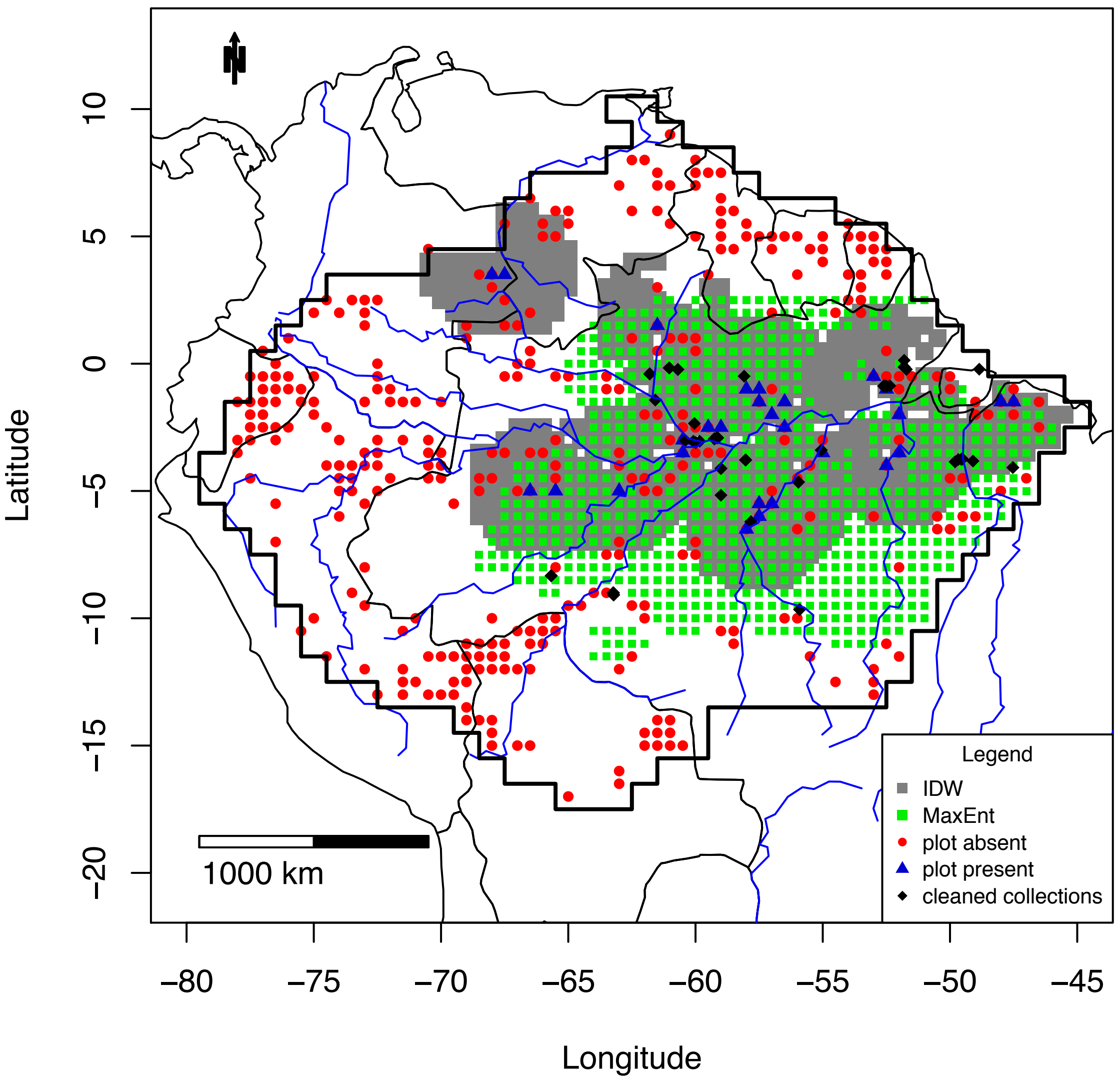

Figure S3_46B MaxEnt map of Eschweilera_amazonica

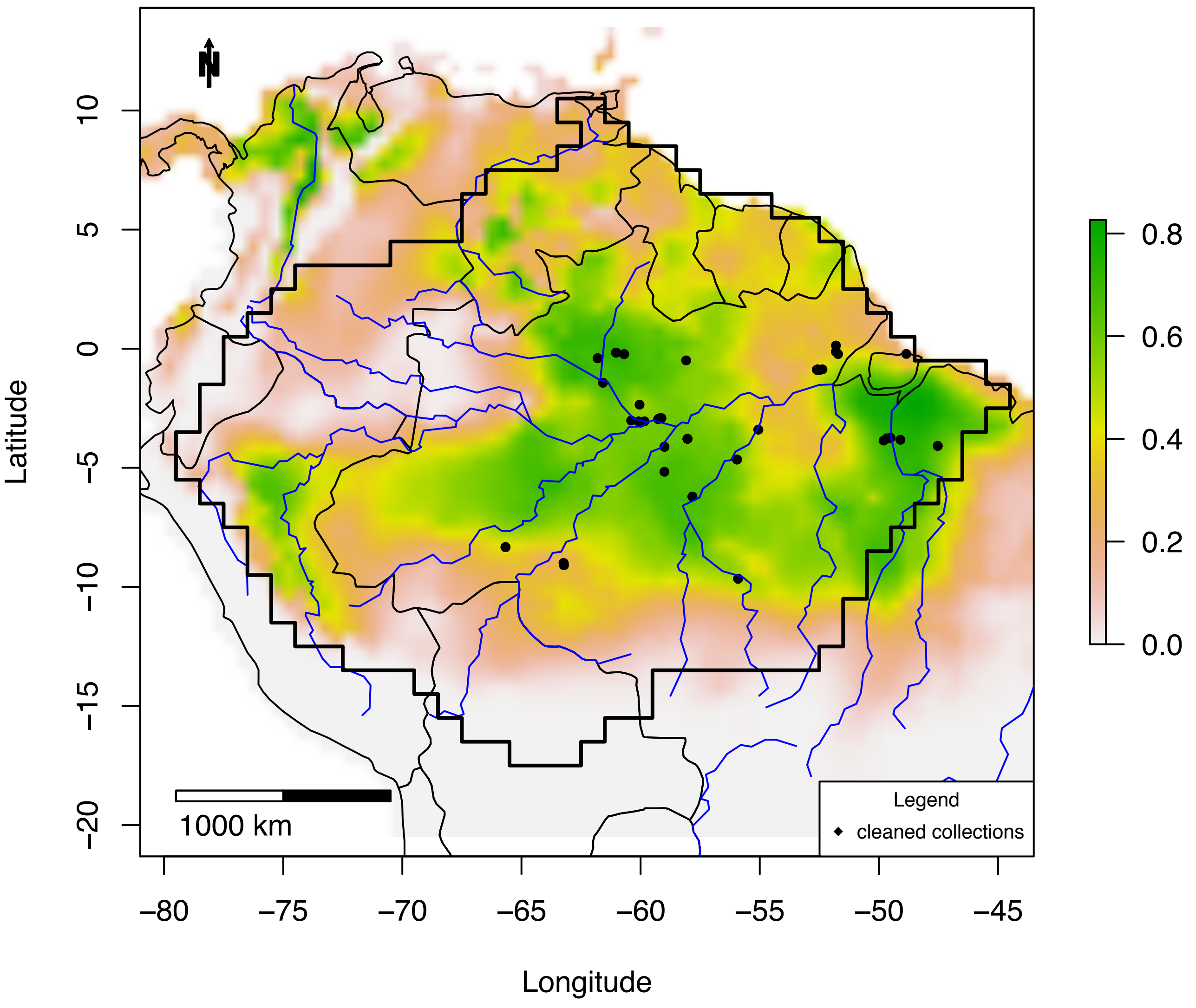

Figure S3_46C IDW map of Eschweilera_amazonica: plots =0 – 0.16; IDW: 0 – 0.03

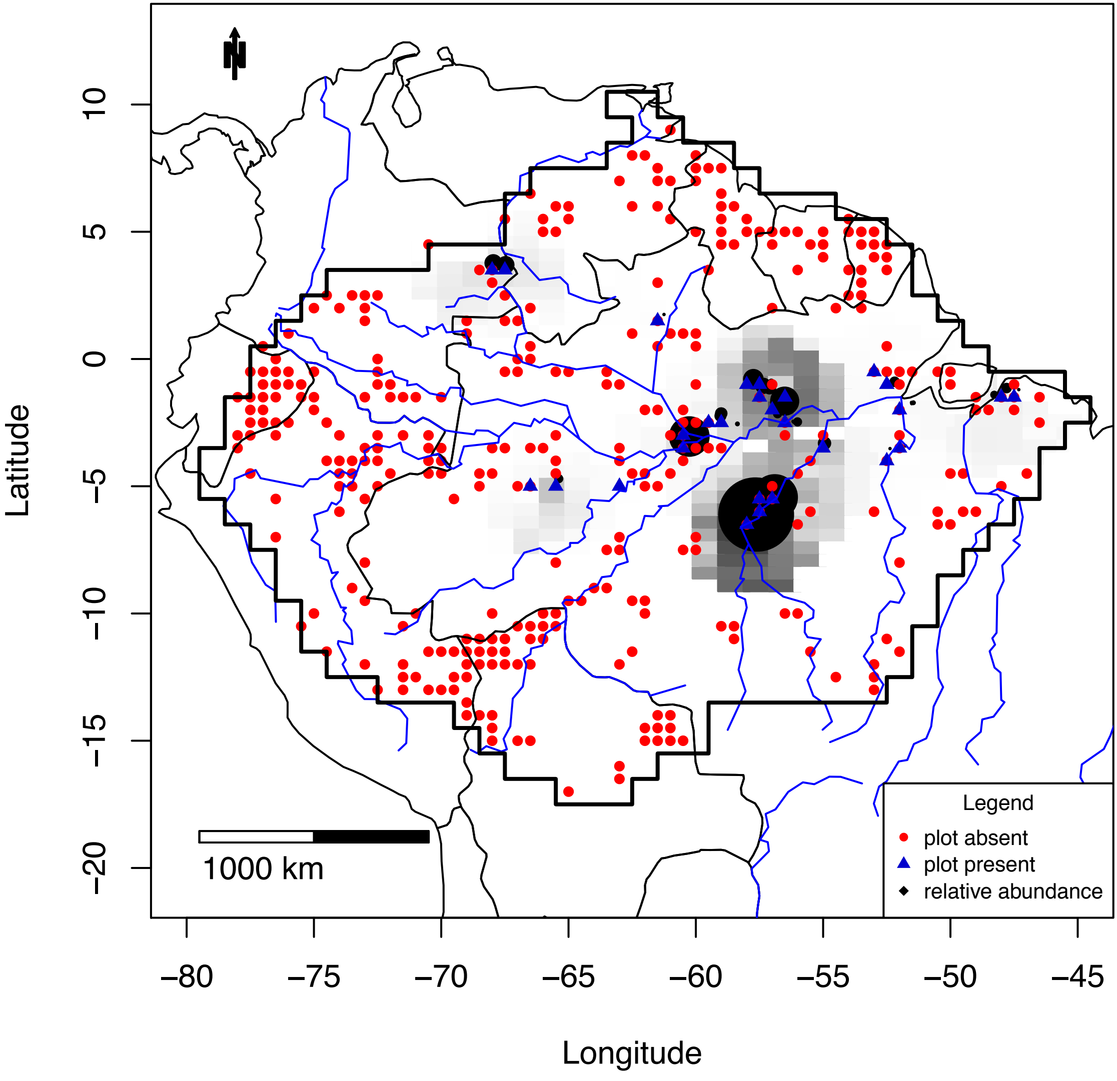

Figure S3_47A Combined map of Eschweilera_atropetiolata

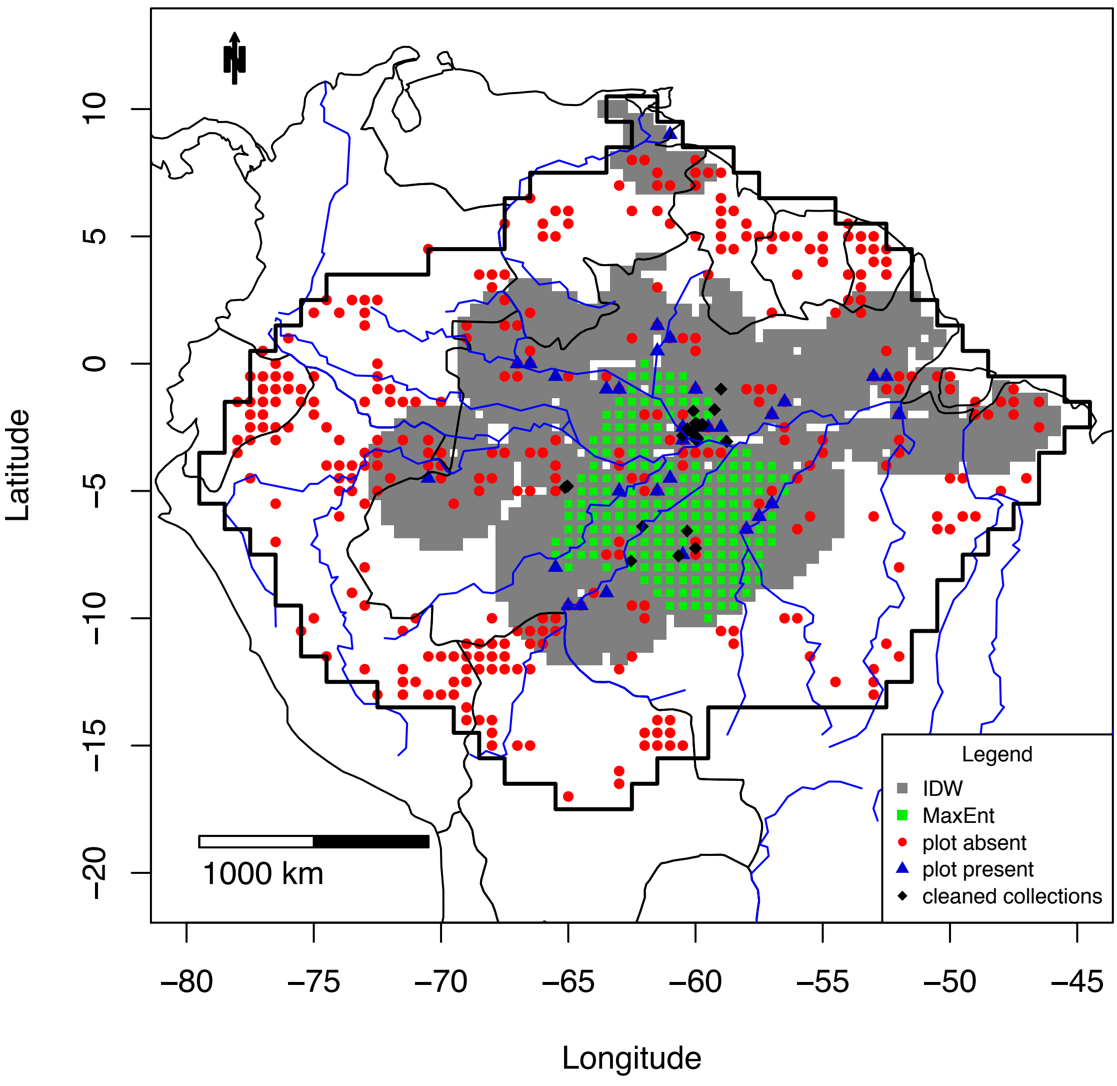

Figure S3_47B MaxEnt map of Eschweilera_atropetiolata

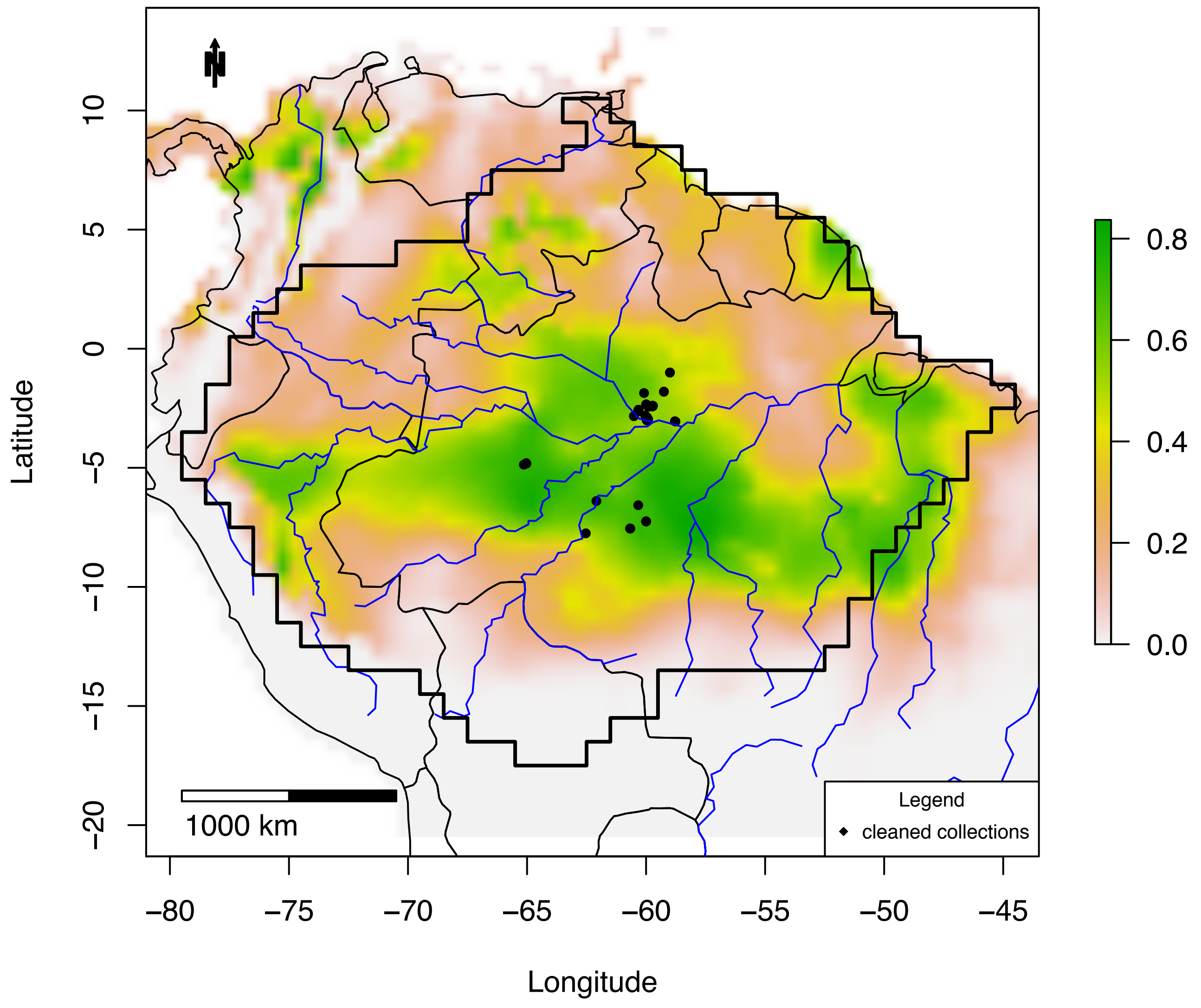

Figure S3_47C IDW map of Eschweilera_atropetiolata: plots =0 – 0.3; IDW: 0 – 0.1

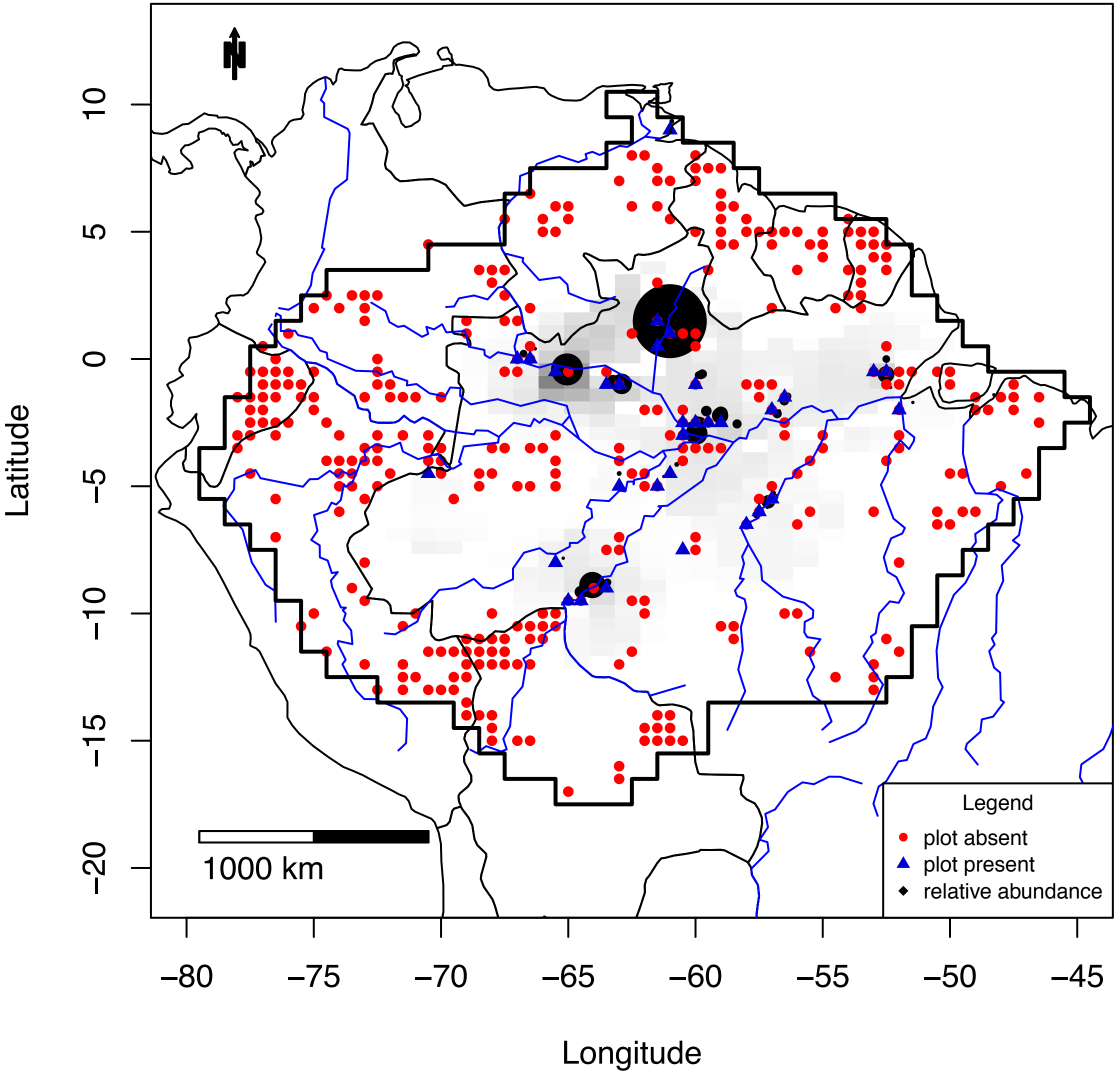

Figure S3_48A Combined map of Eschweilera_bracteosa

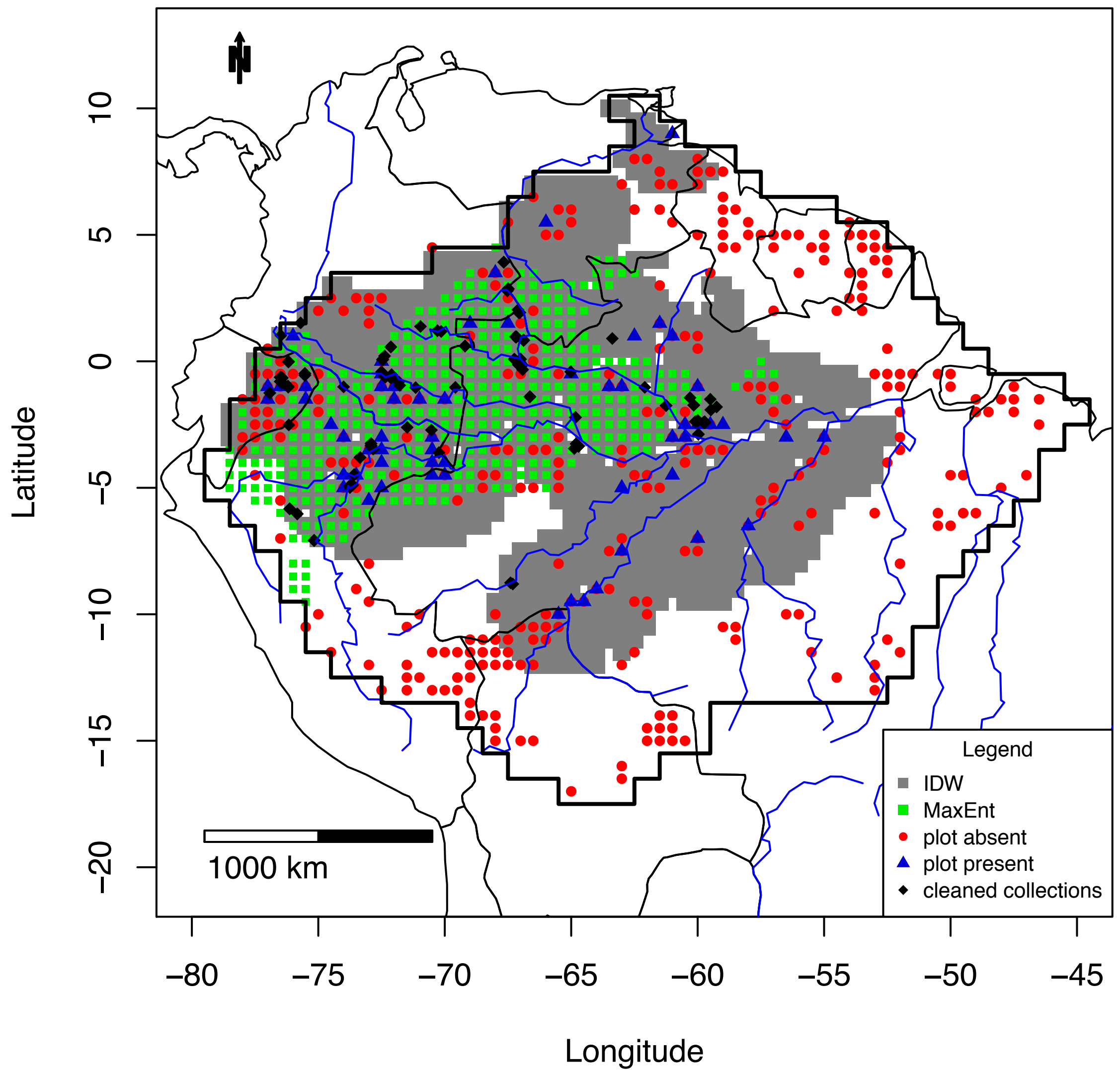

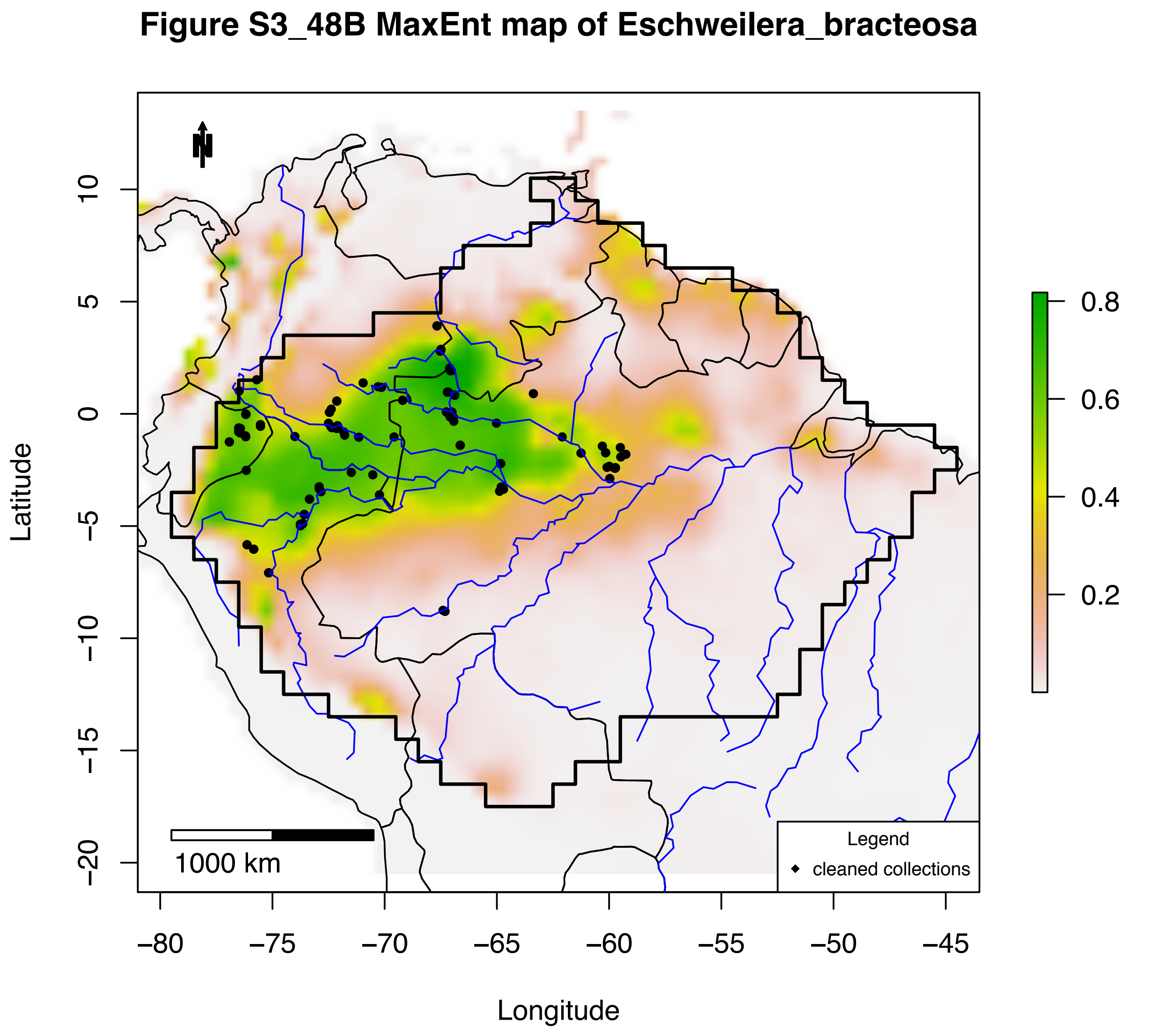
Figure S3_48B MaxEnt map of Eschweilera_bracteosa
N
10
5
0
−5
−10
−15
−20
Latitude
−80
−75
−70
−65
−60
−55
−50
−45
Longitude
1000 km
Legend
cleaned collections
0.8
0.6
0.4
0.2

Figure S3_48C IDW map of Eschweilera_bracteosa: plots =0 – 0.15; IDW: 0 – 0.05

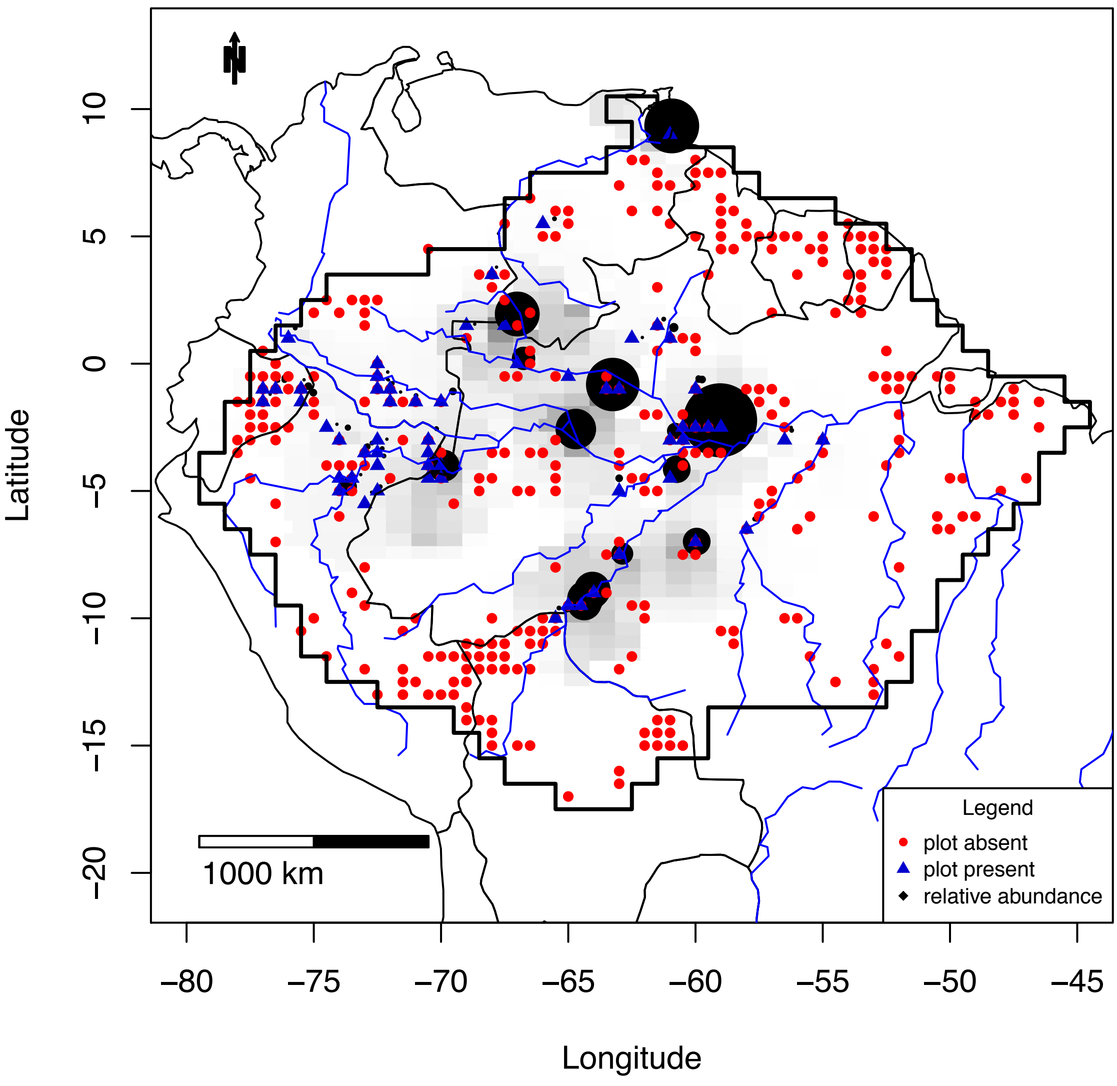

Figure S3_49A Combined map of Eschweilera_coriacea

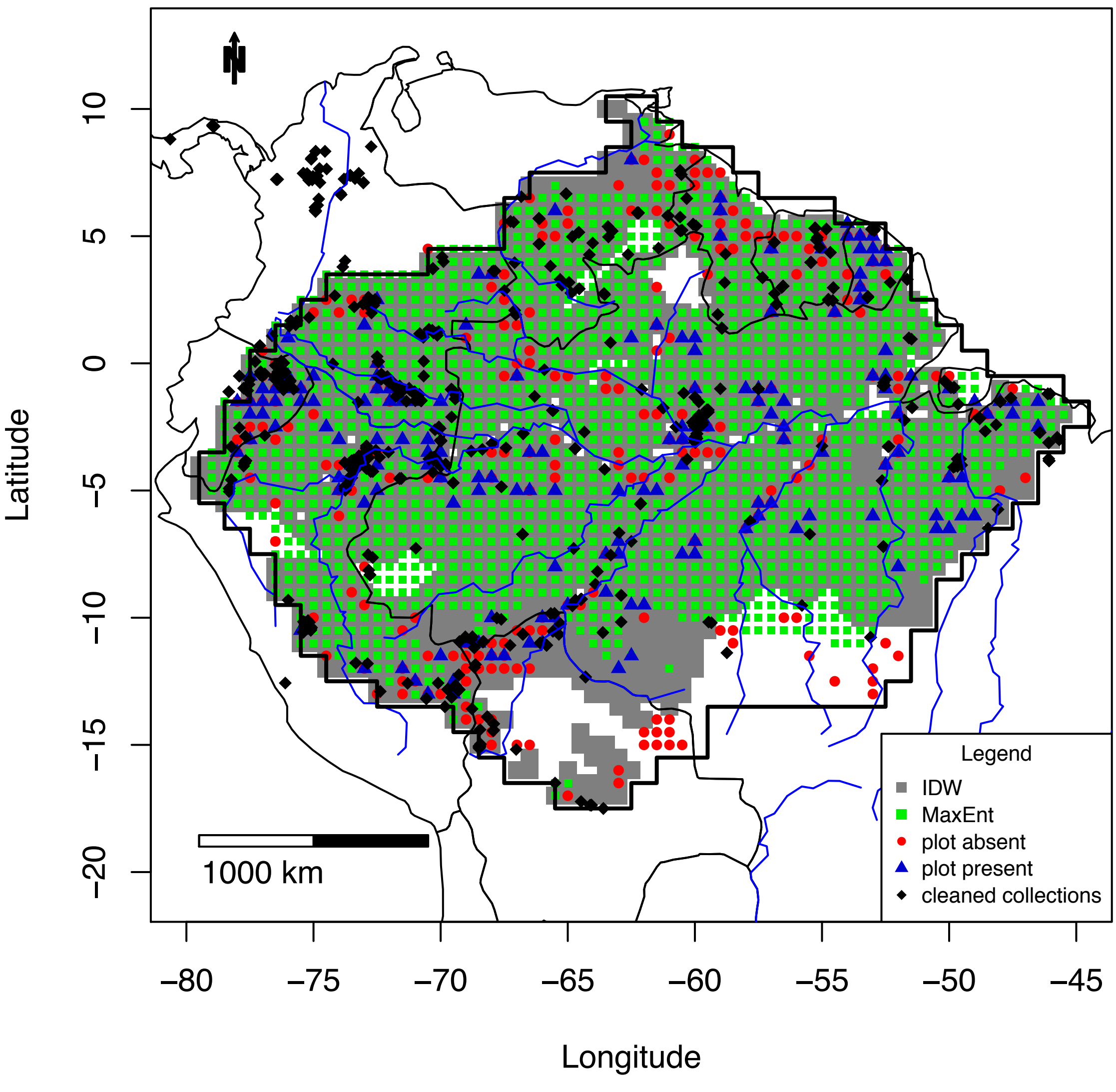

Figure S3_49B MaxEnt map of Eschweilera_coriacea

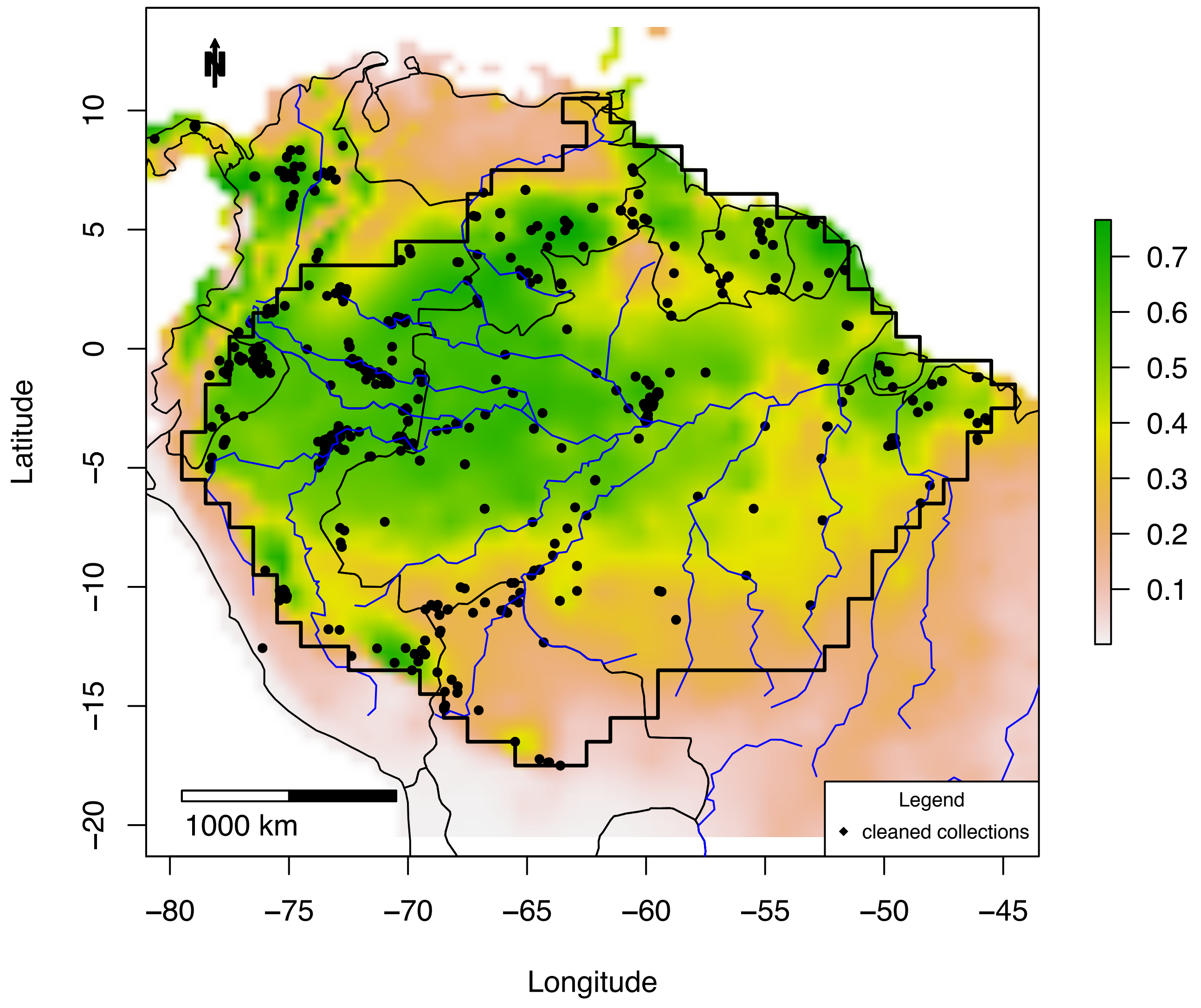

Figure S3_49C IDW map of Eschweilera_coriacea: plots =0 – 0.22; IDW: 0 – 0.13

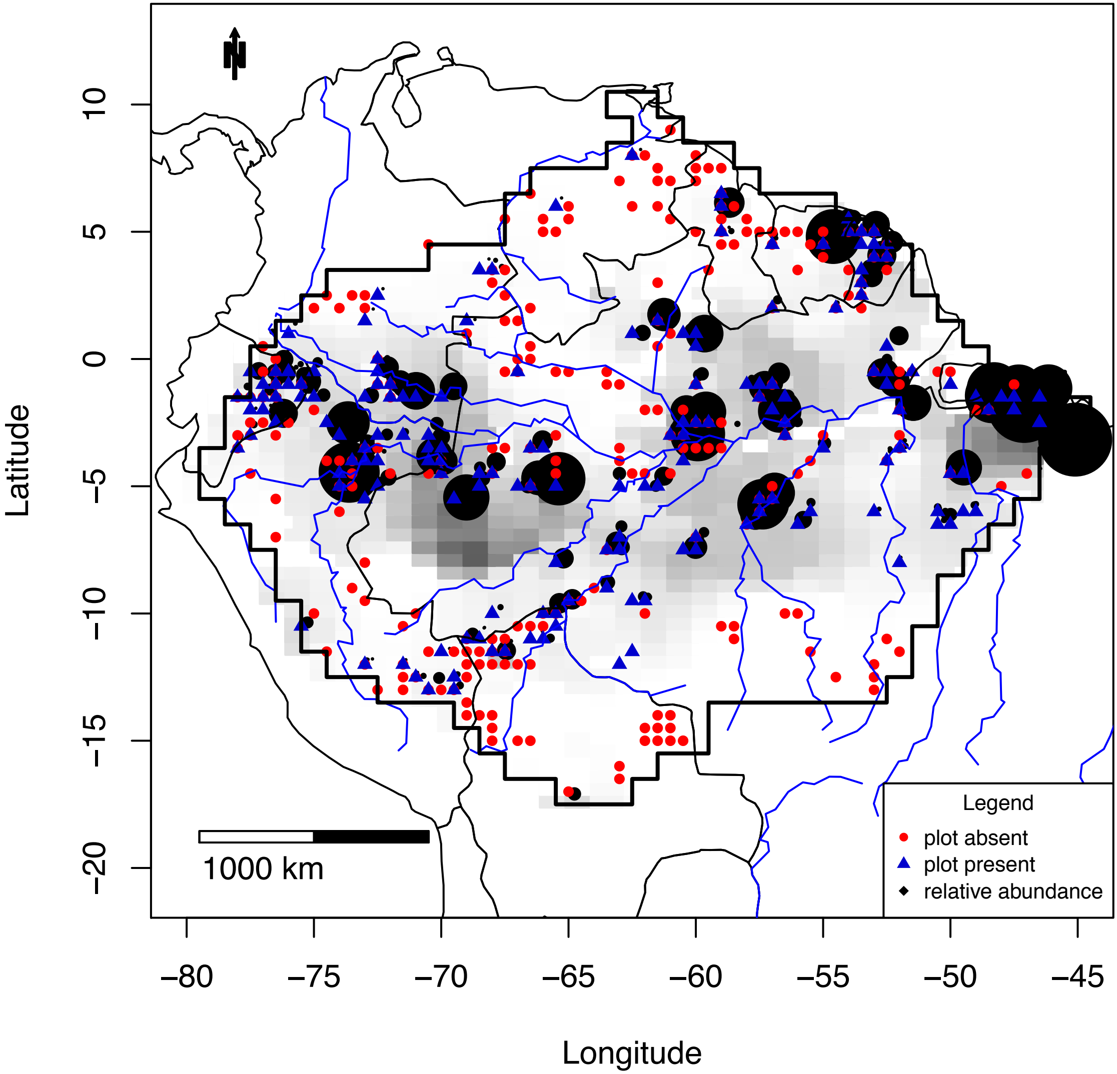

Figure S3_50A Combined map of Eschweilera_decolorans

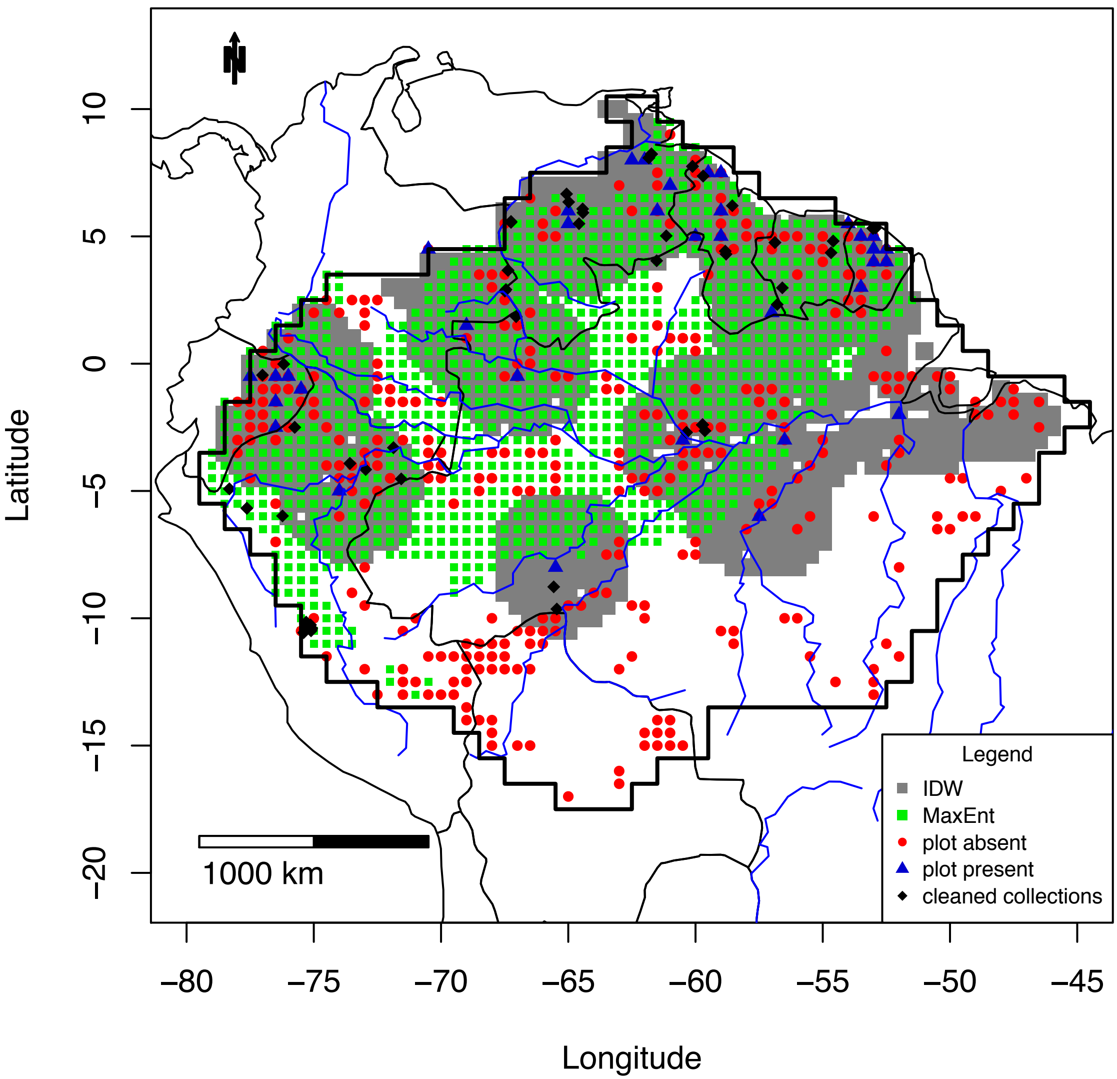

Figure S3_50B MaxEnt map of Eschweilera_decolorans

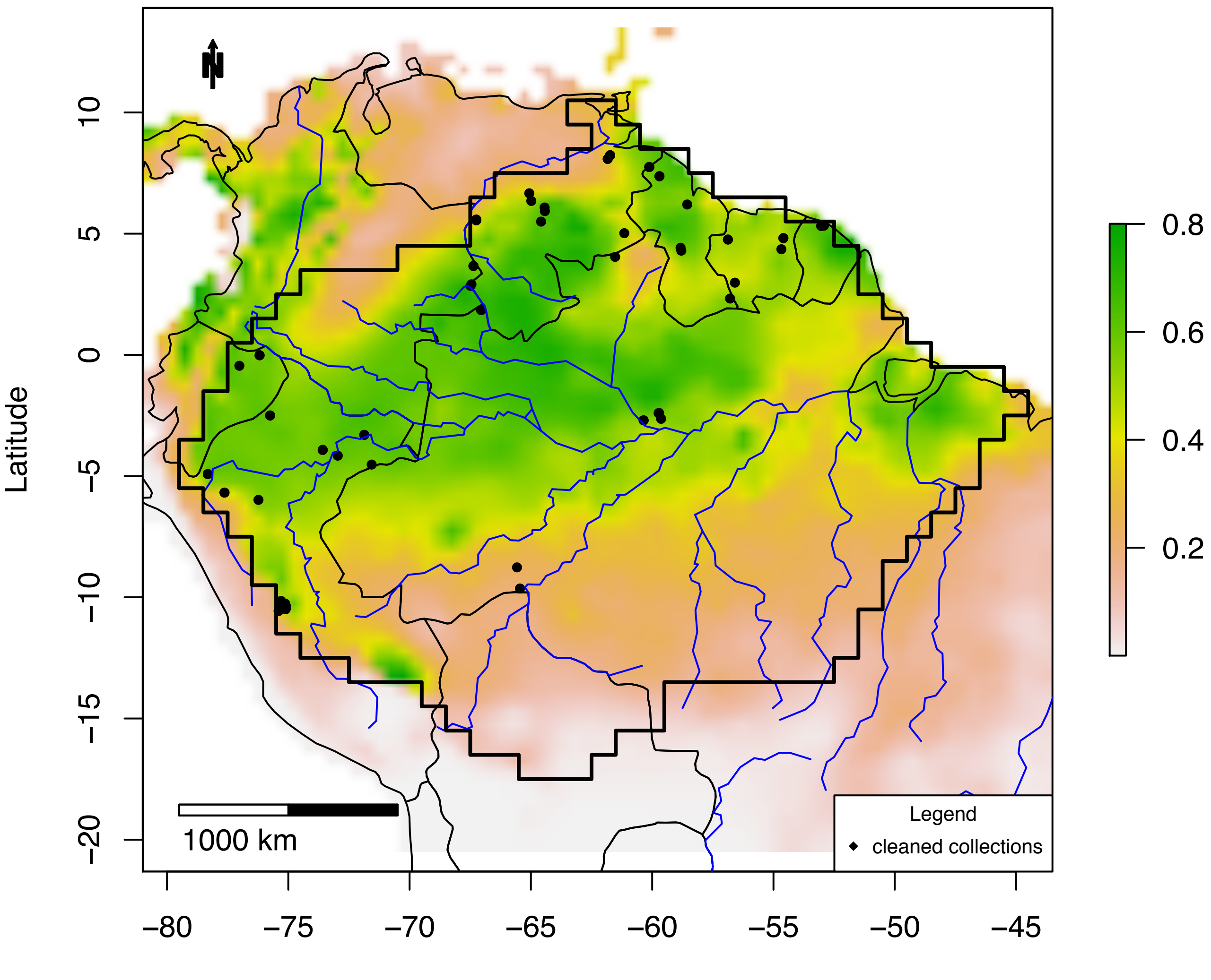

Figure S3_50C IDW map of Eschweilera_decolorans: plots =0 – 0.14; IDW: 0 – 0.04

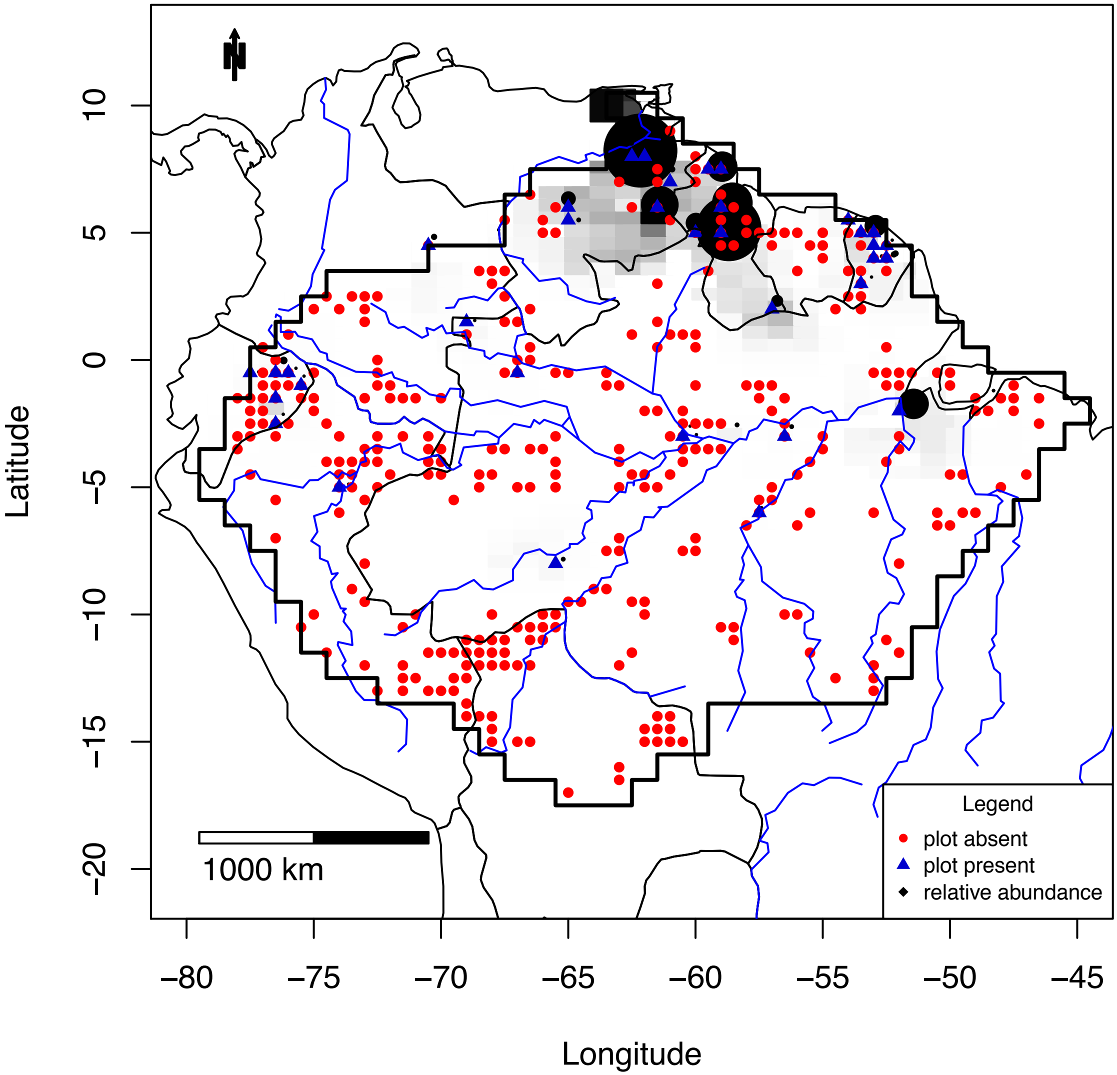

Figure S3_51A Combined map of Eschweilera_grandiflora

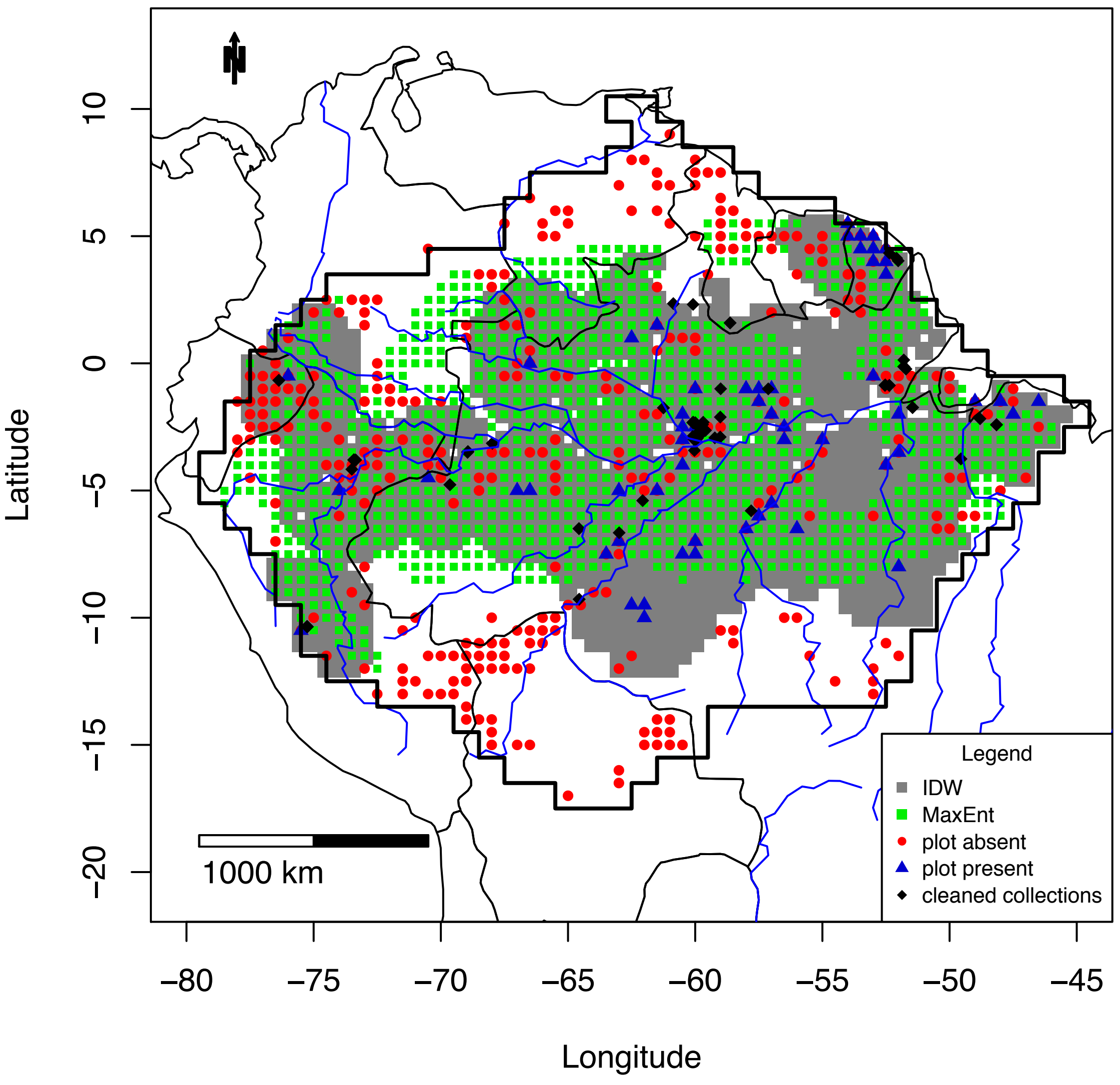

Figure S3_51B MaxEnt map of Eschweilera_grandiflora

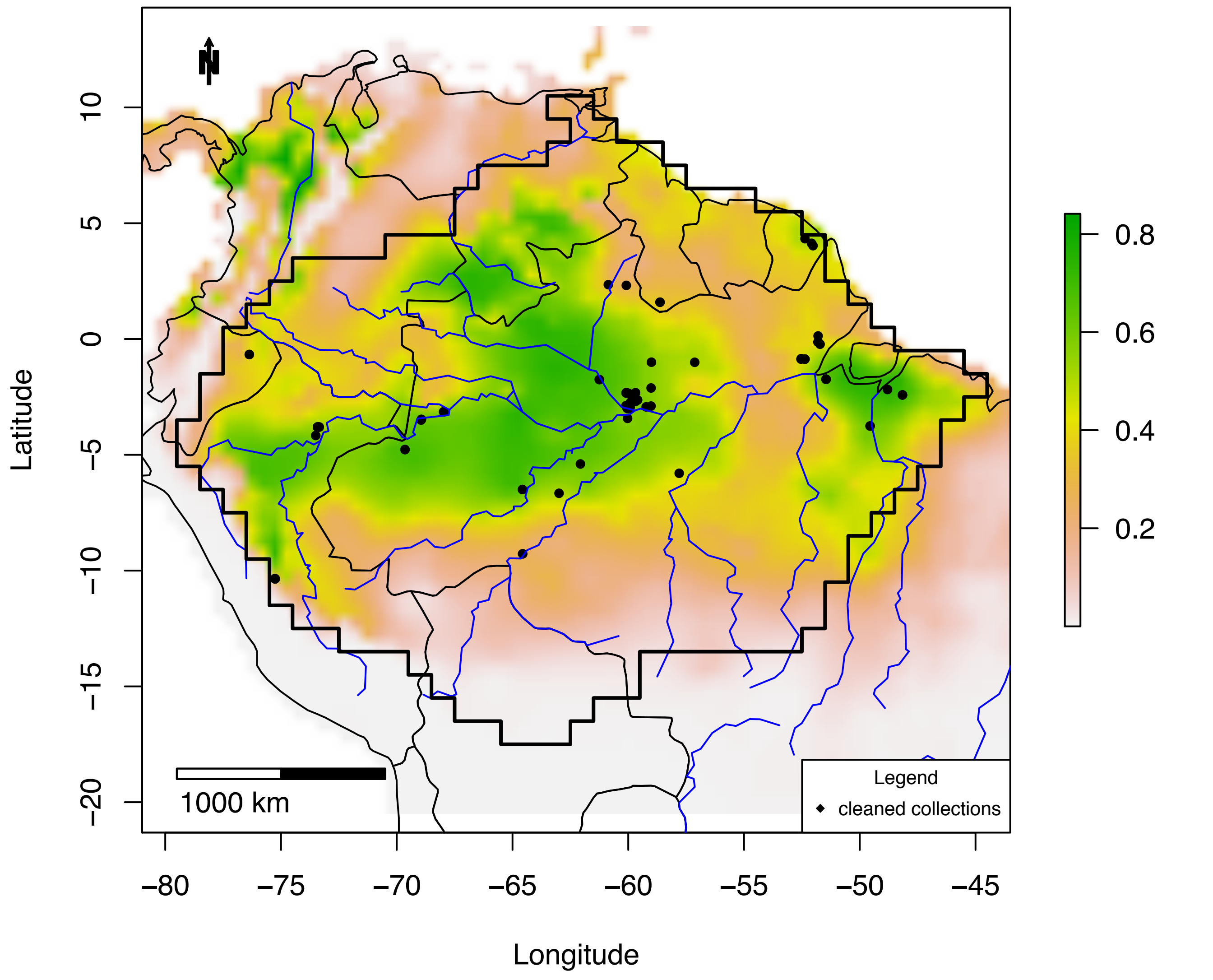

Figure S3_51C IDW map of Eschweilera_grandiflora: plots =0 – 0.12; IDW: 0 – 0.1

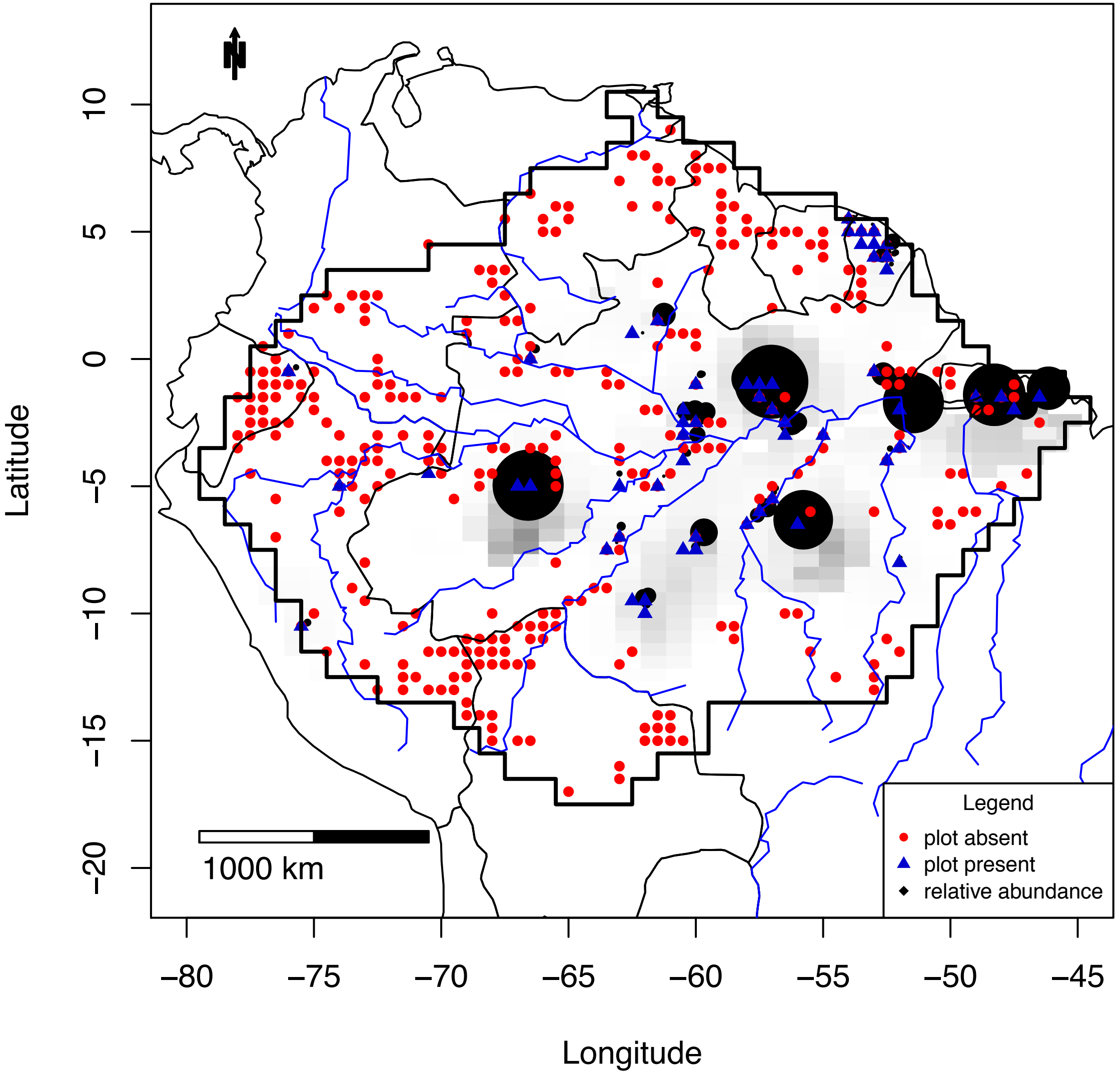

Figure S3_52A Combined map of Eschweilera_parviflora

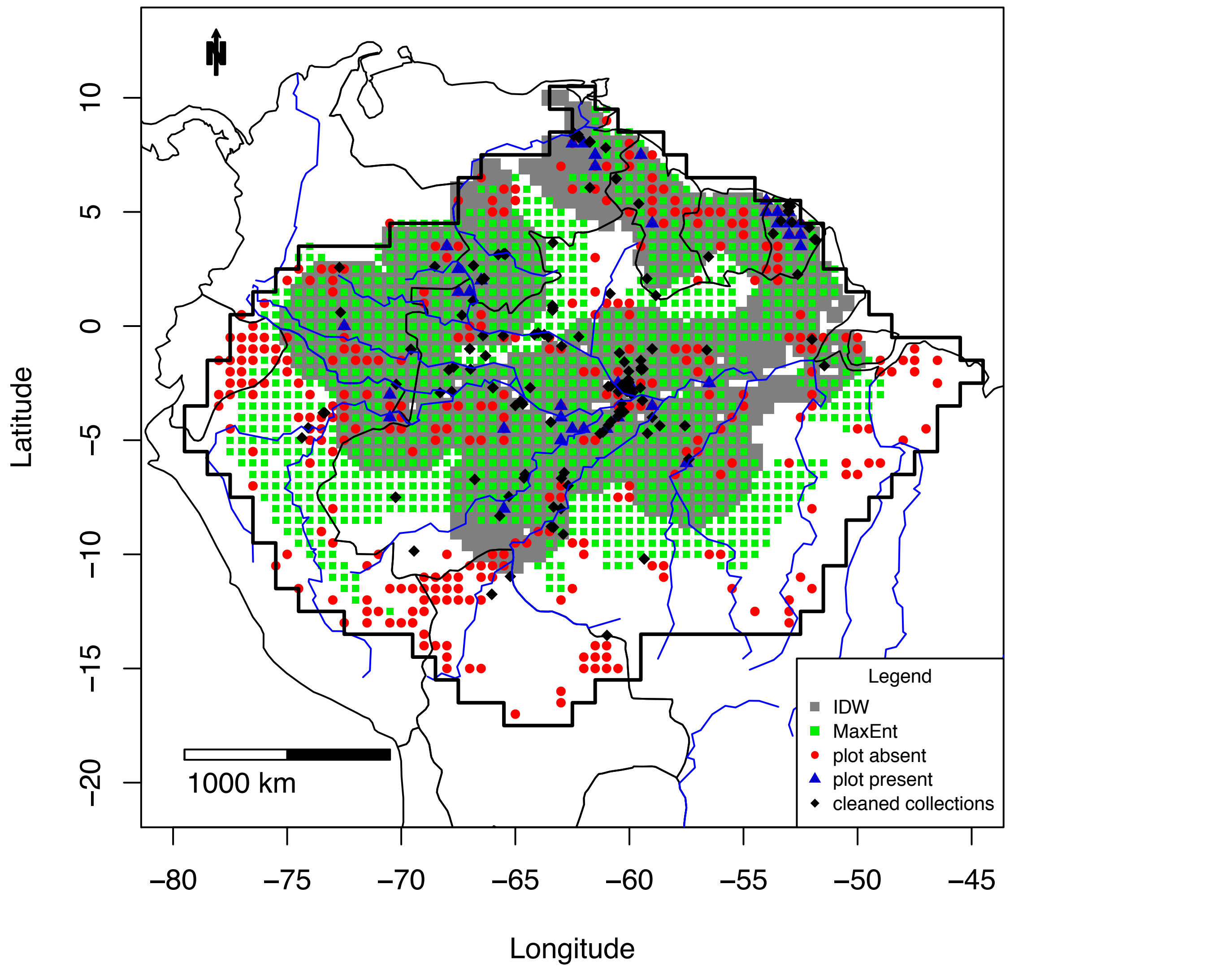

Figure S3_52B MaxEnt map of Eschweilera_parviflora

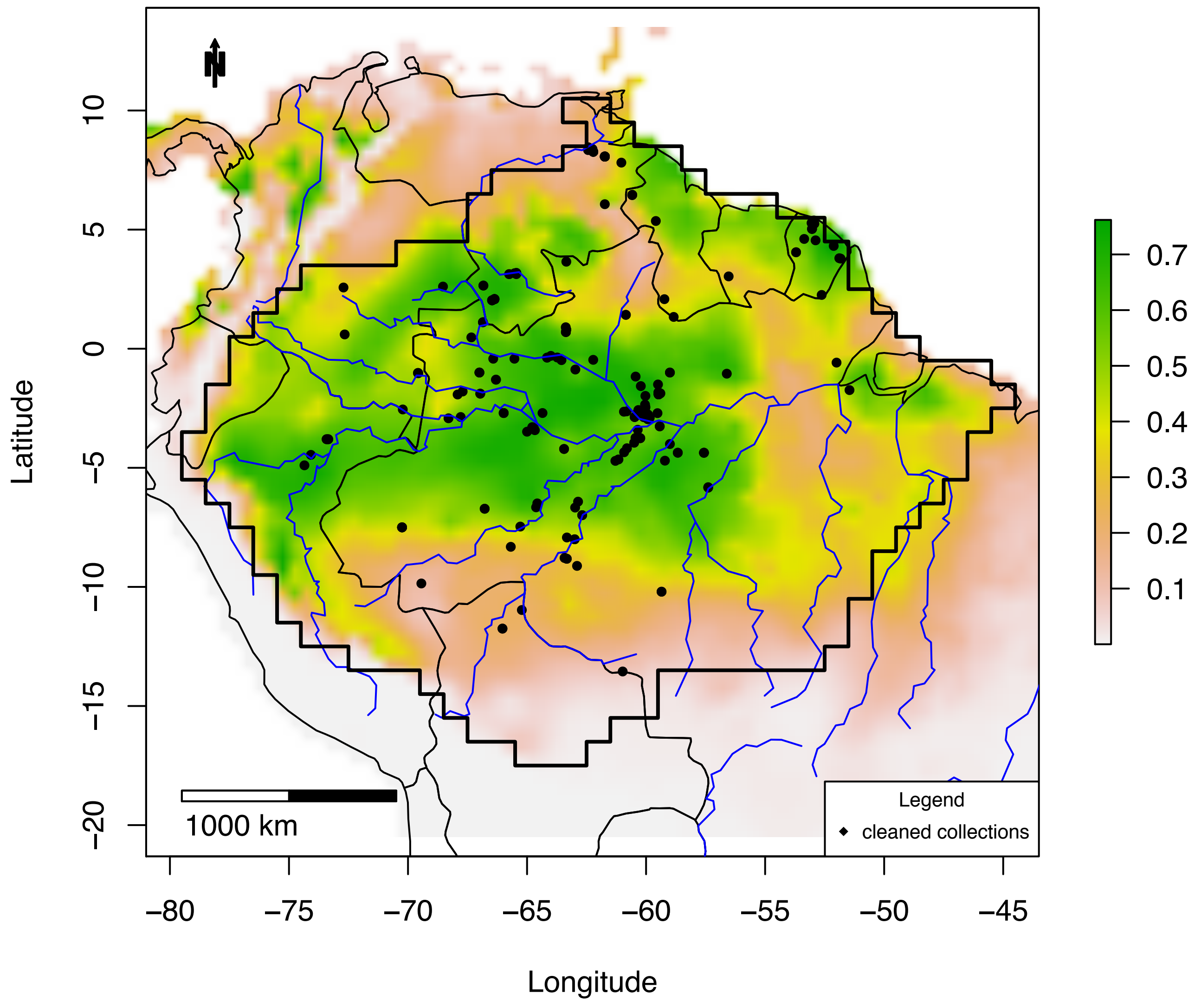

Figure S3_52C IDW map of Eschweilera_parviflora: plots =0 – 0.16; IDW: 0 – 0.09

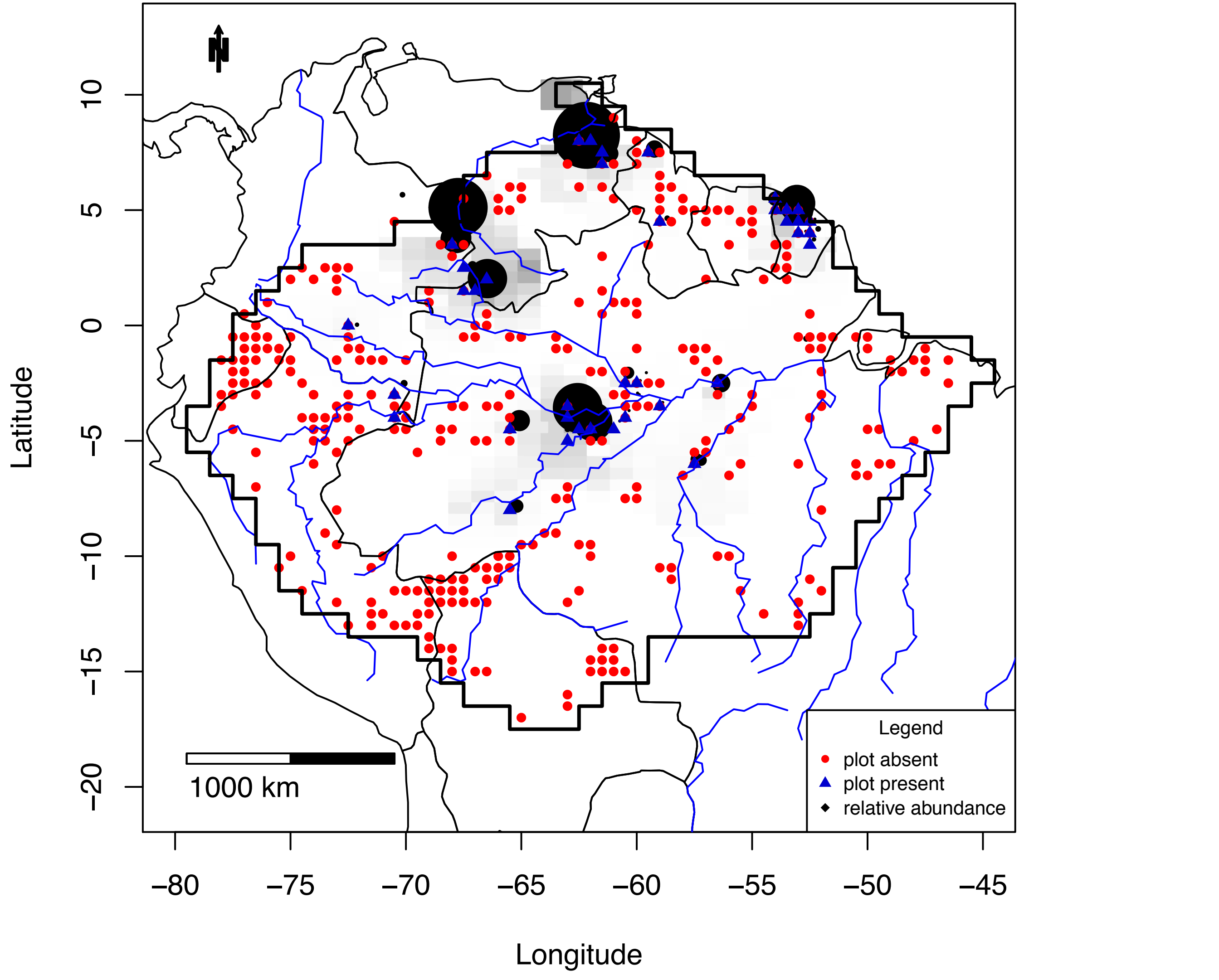

Figure S3_53A Combined map of Eschweilera_parvifolia

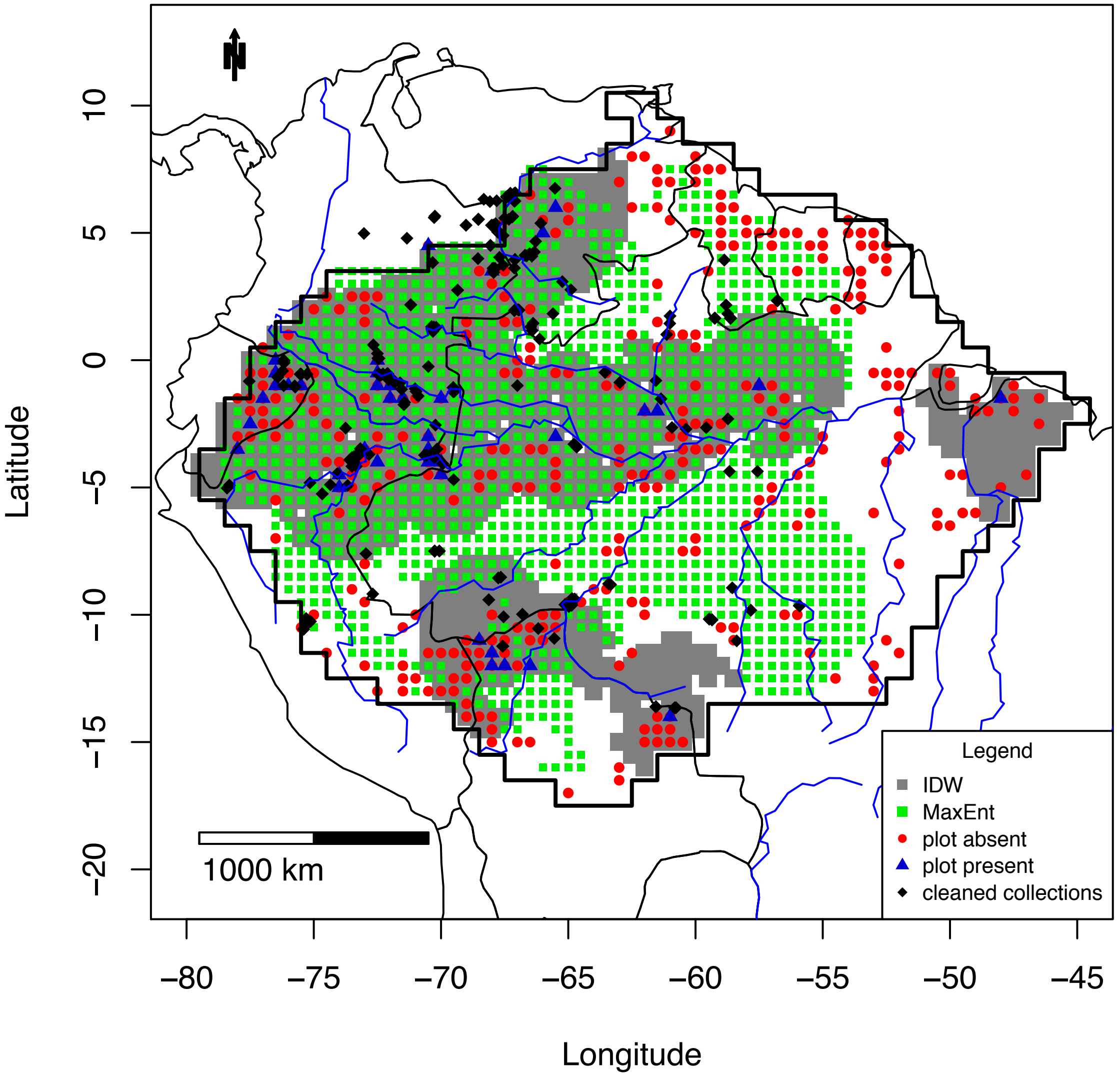

Figure S3_53B MaxEnt map of Eschweilera_parvifolia

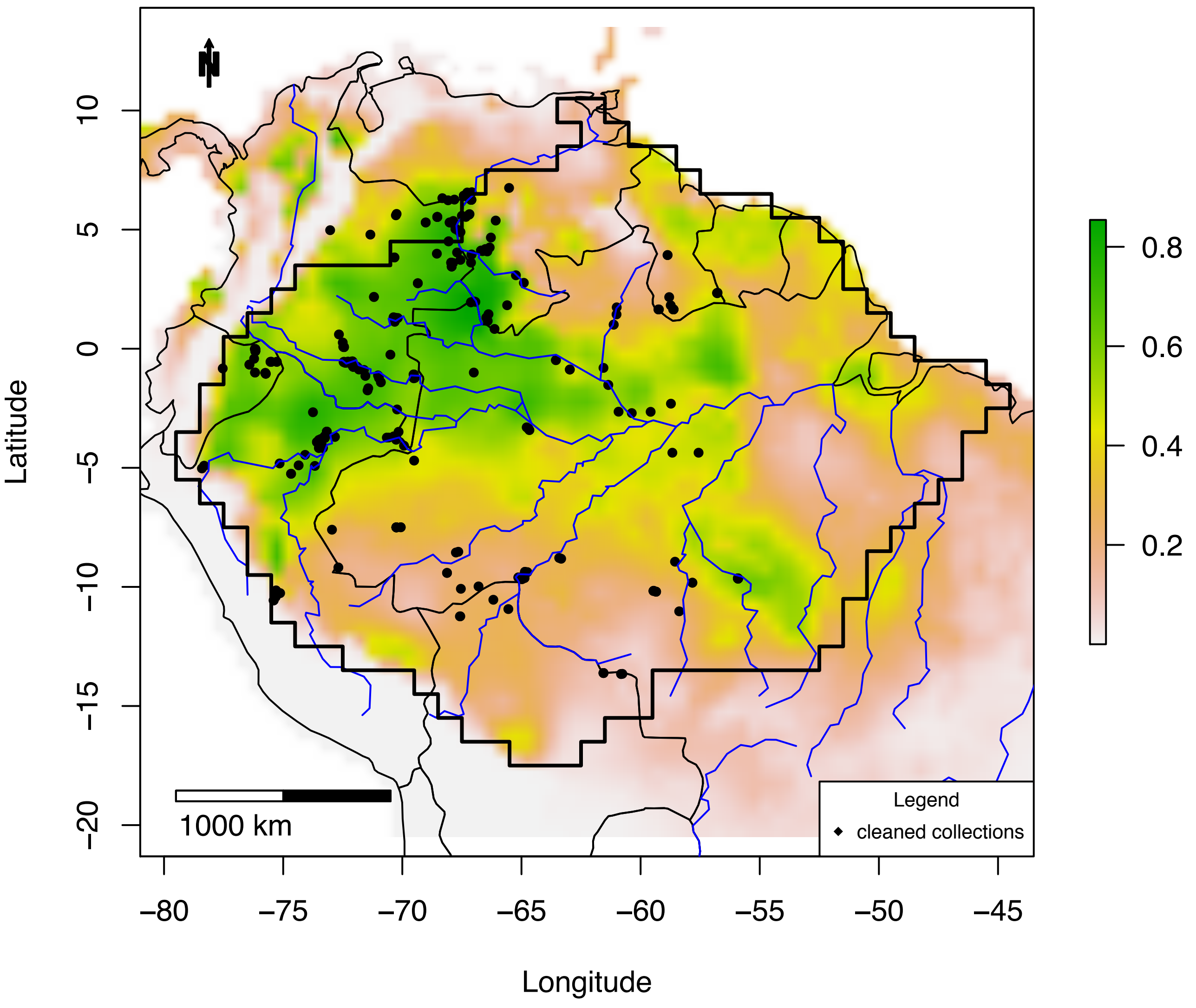

Figure S3_53C IDW map of Eschweilera_parvifolia: plots =0 – 0.16; IDW: 0 – 0.02

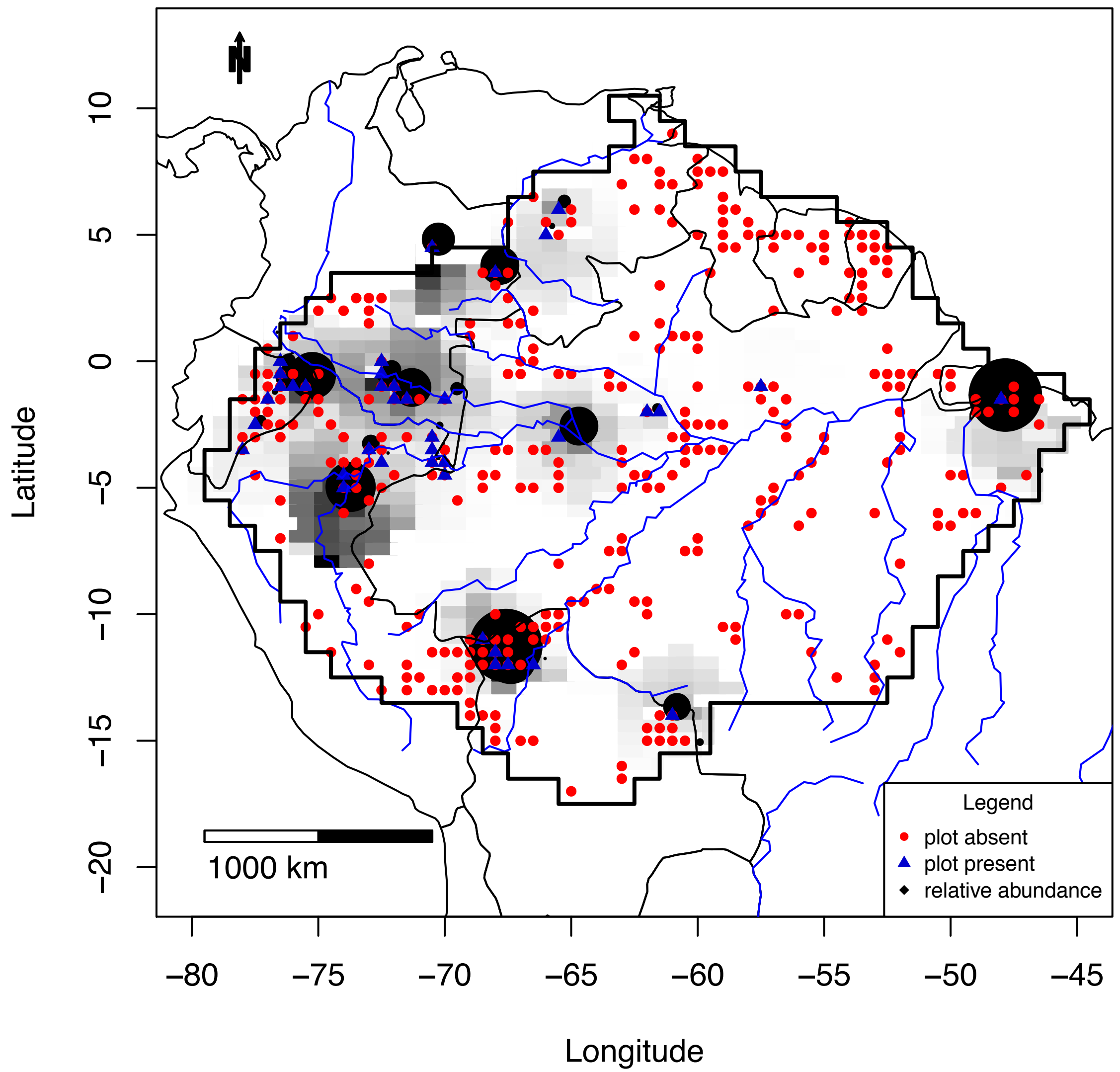

Figure S3_54A Combined map of Eschweilera_pedicellata

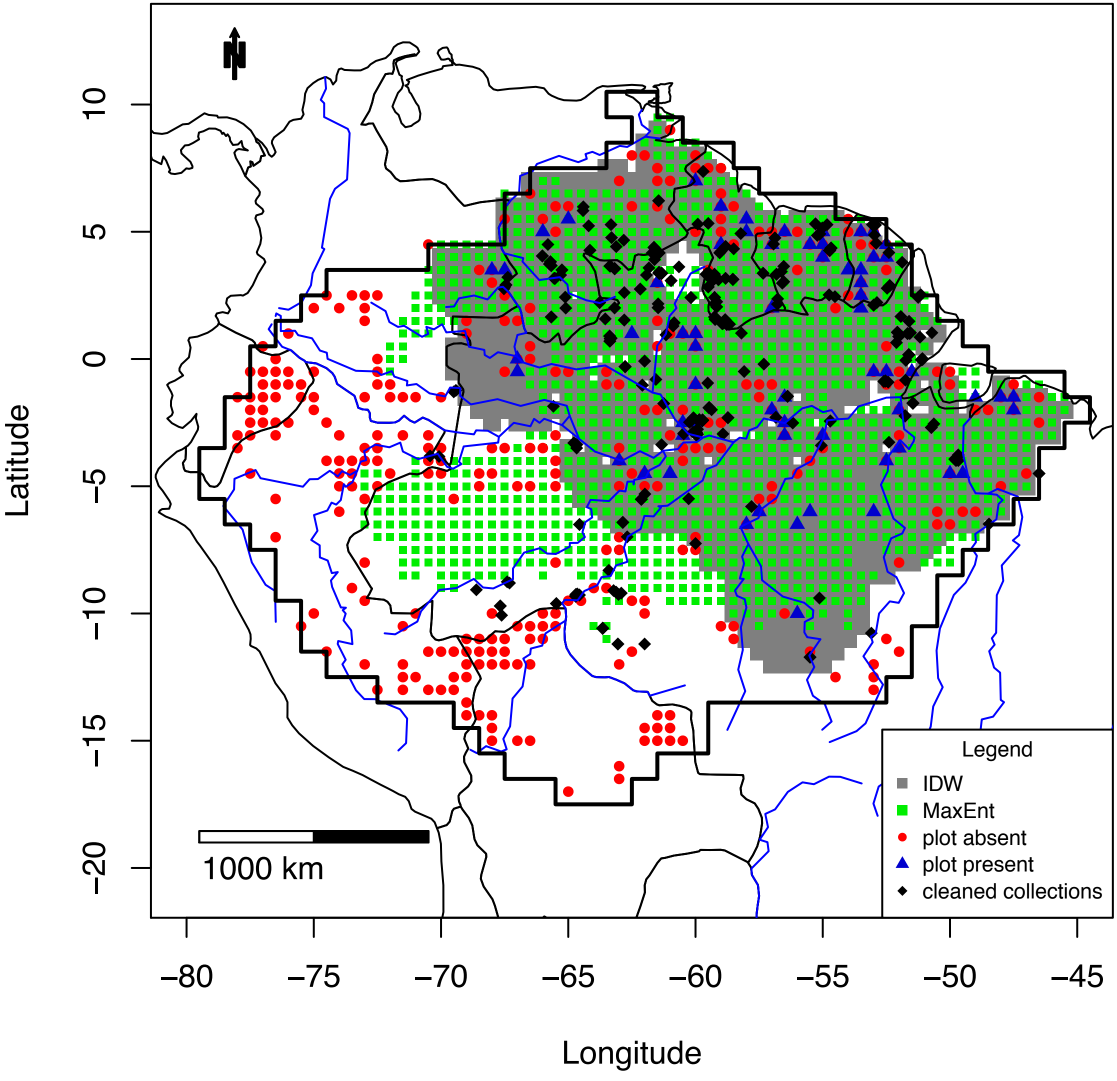

Figure S3_54B MaxEnt map of Eschweilera_pedicellata

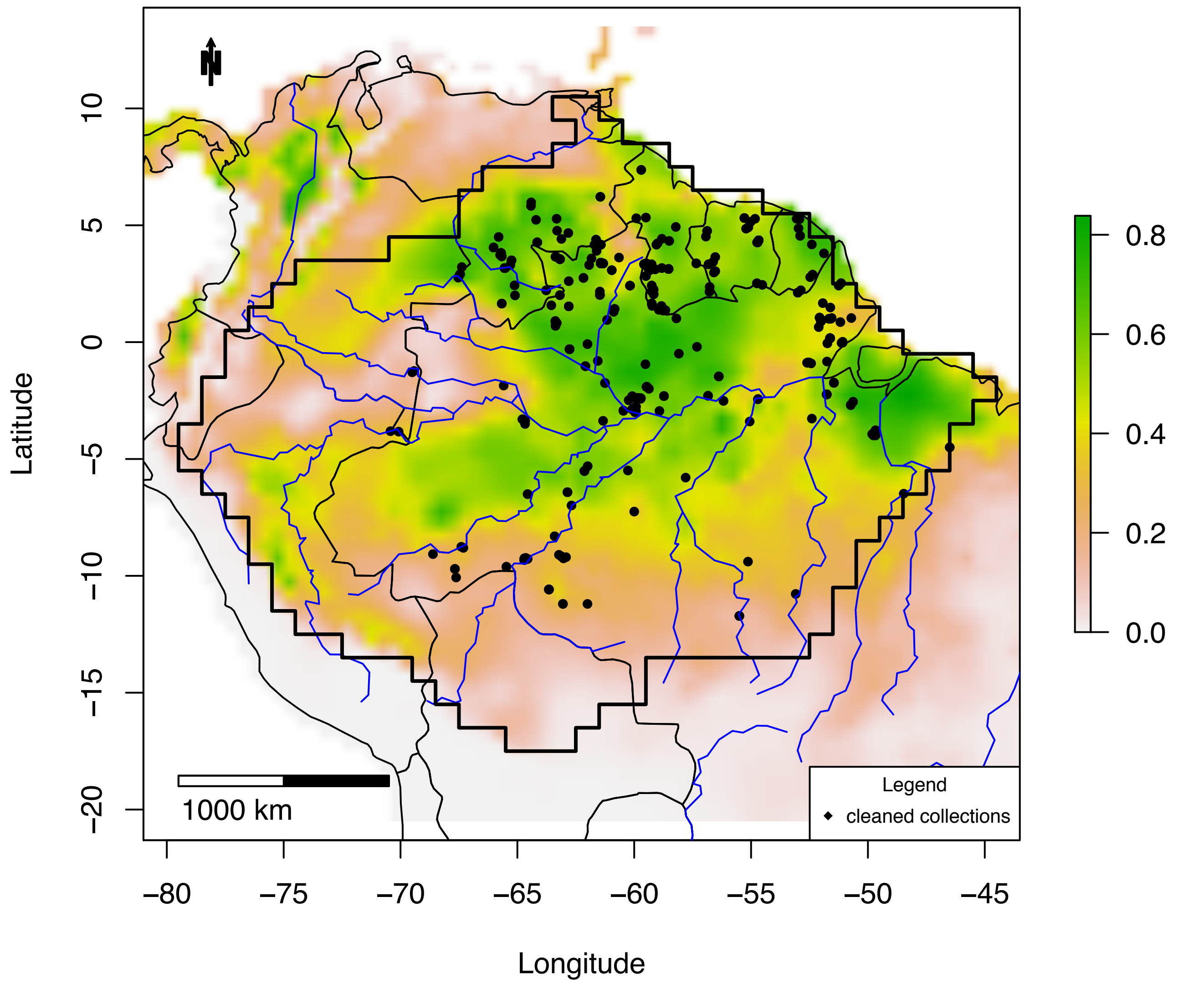

Figure S3_54C IDW map of Eschweilera_pedicellata: plots =0 – 0.3; IDW: 0 – 0.27

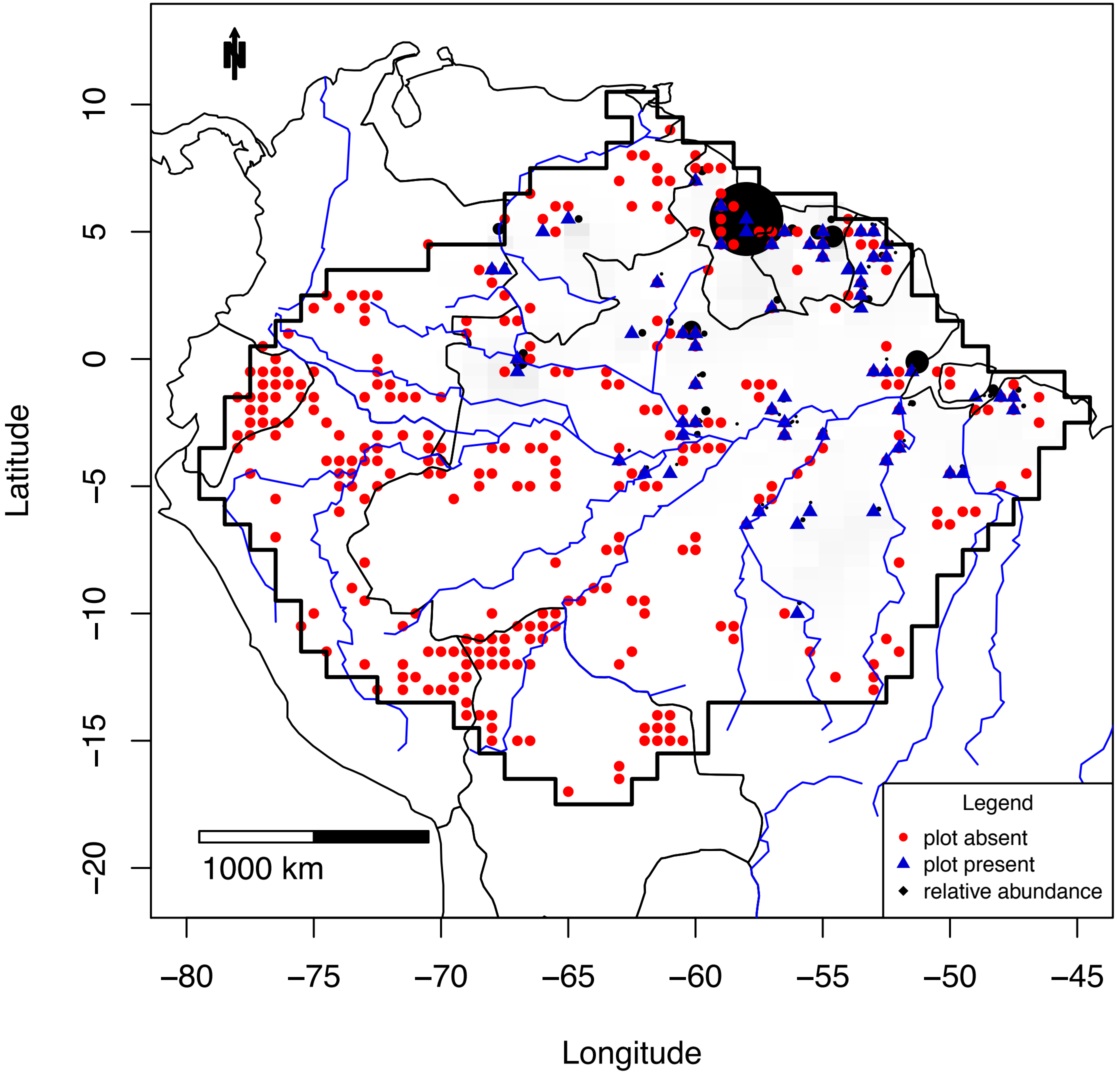

Figure S3_55A Combined map of Eschweilera_sagotiana

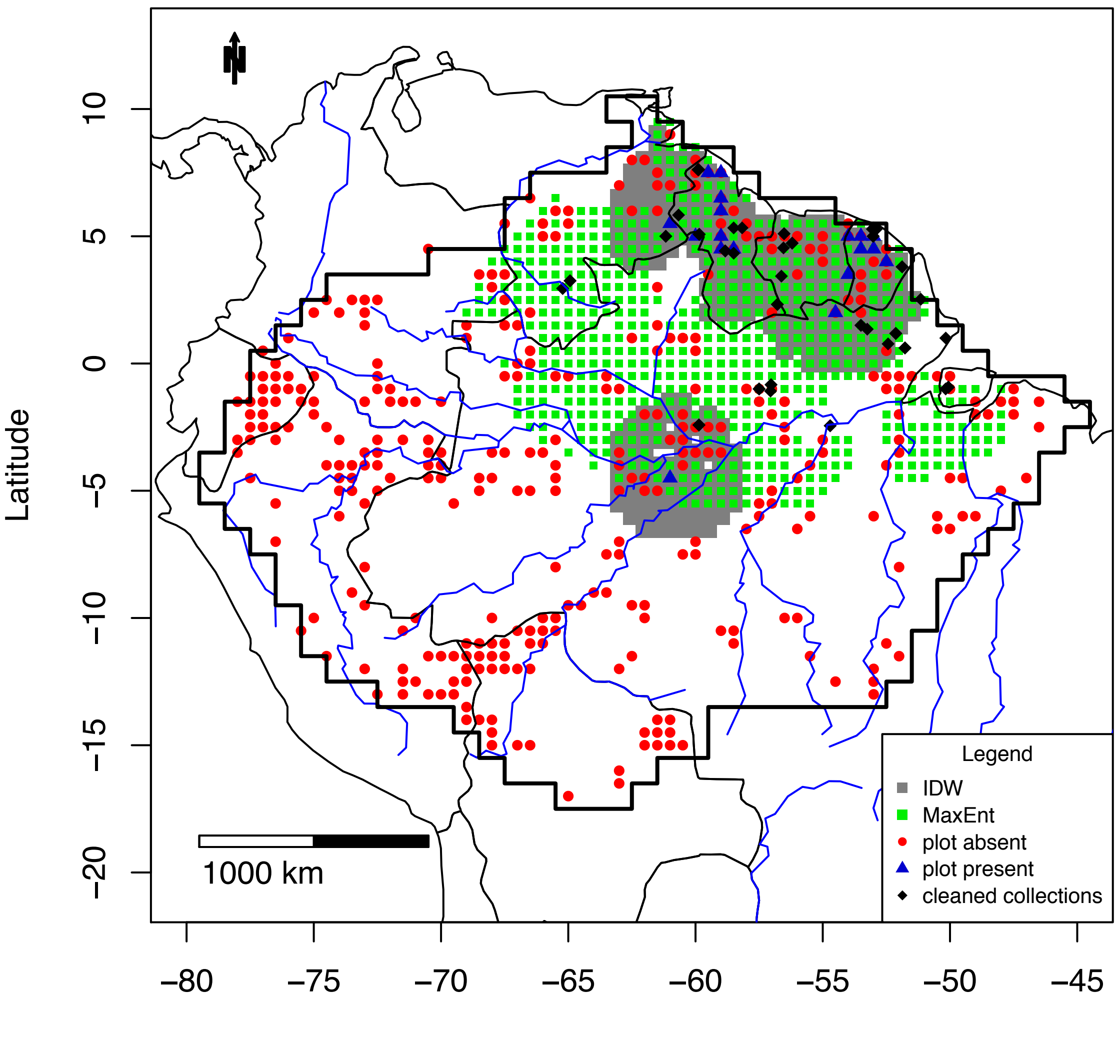

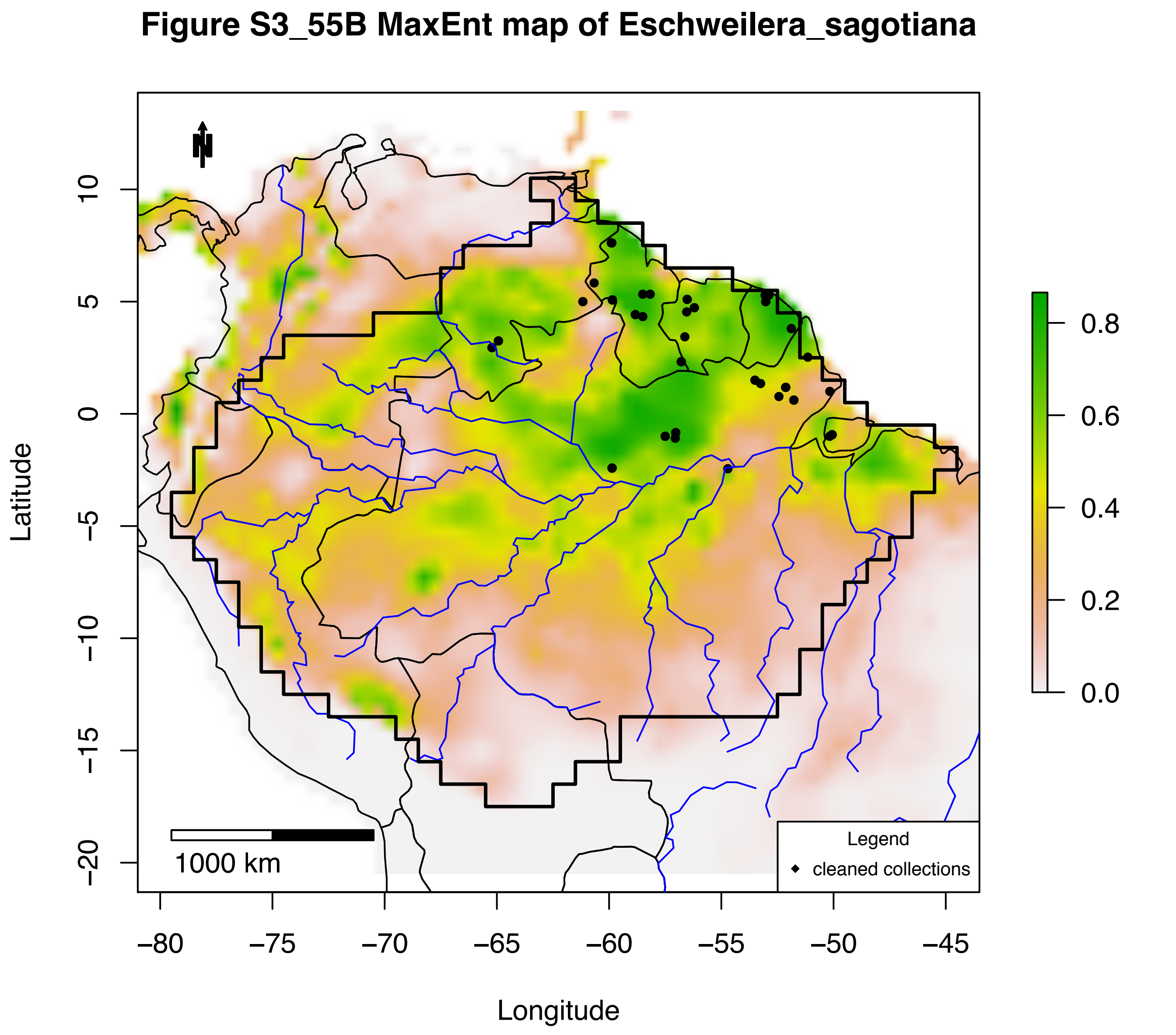

Figure S3_55B MaxEnt map of Eschweilera_sagotiana
N
10
5
0
−5
−10
−15
−20
Latitude
−80
−75
−70
−65
−60
−55
−50
−45
Longitude
0.8
0.6
0.4
0.2
0.0
1000 km
Legend
cleaned collections

Figure S3_55C IDW map of Eschweilera_sagotiana: plots =0 – 0.32; IDW: 0 – 0.11

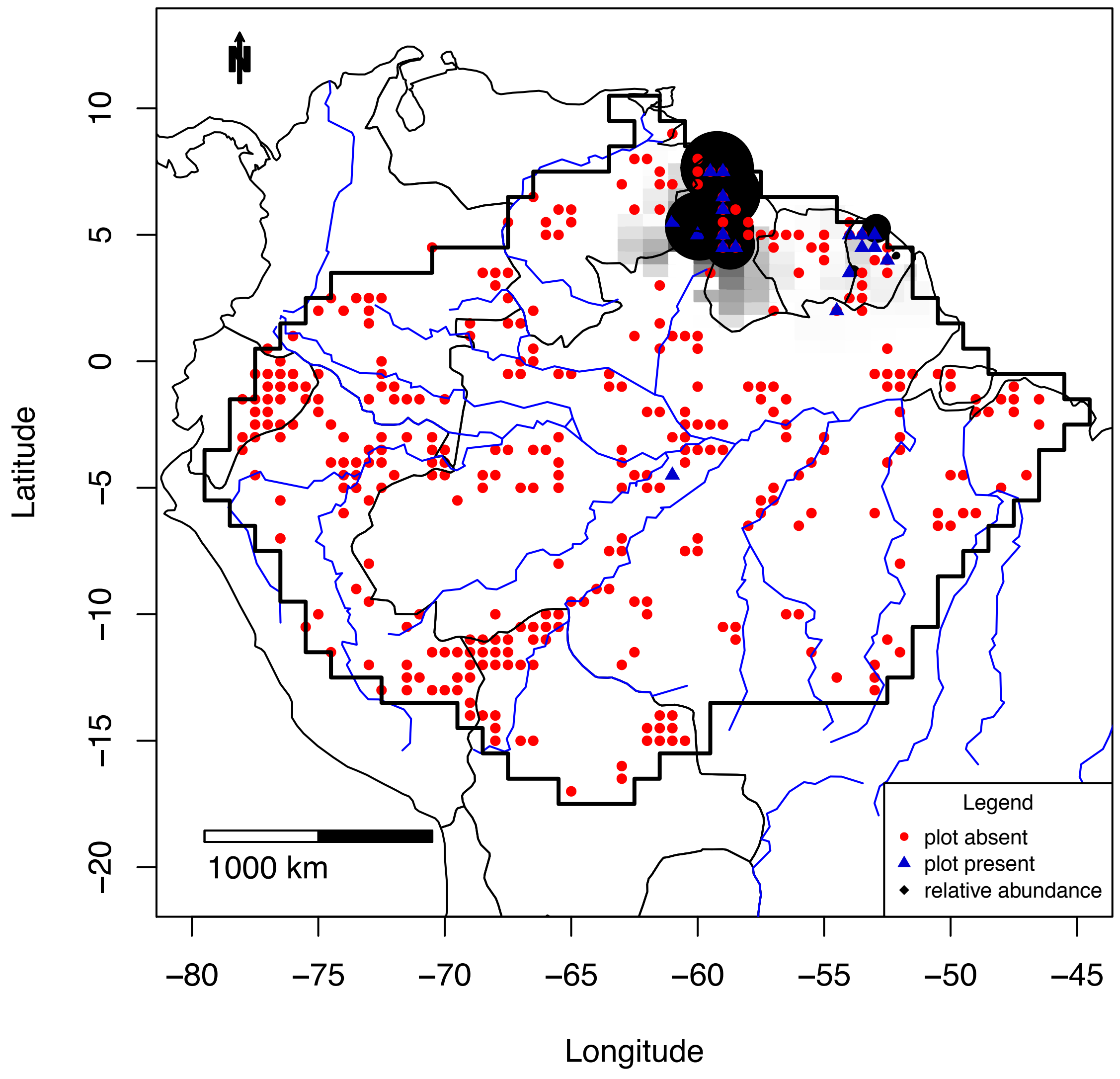

Figure S3_56A Combined map of Eschweilera_tessmannii

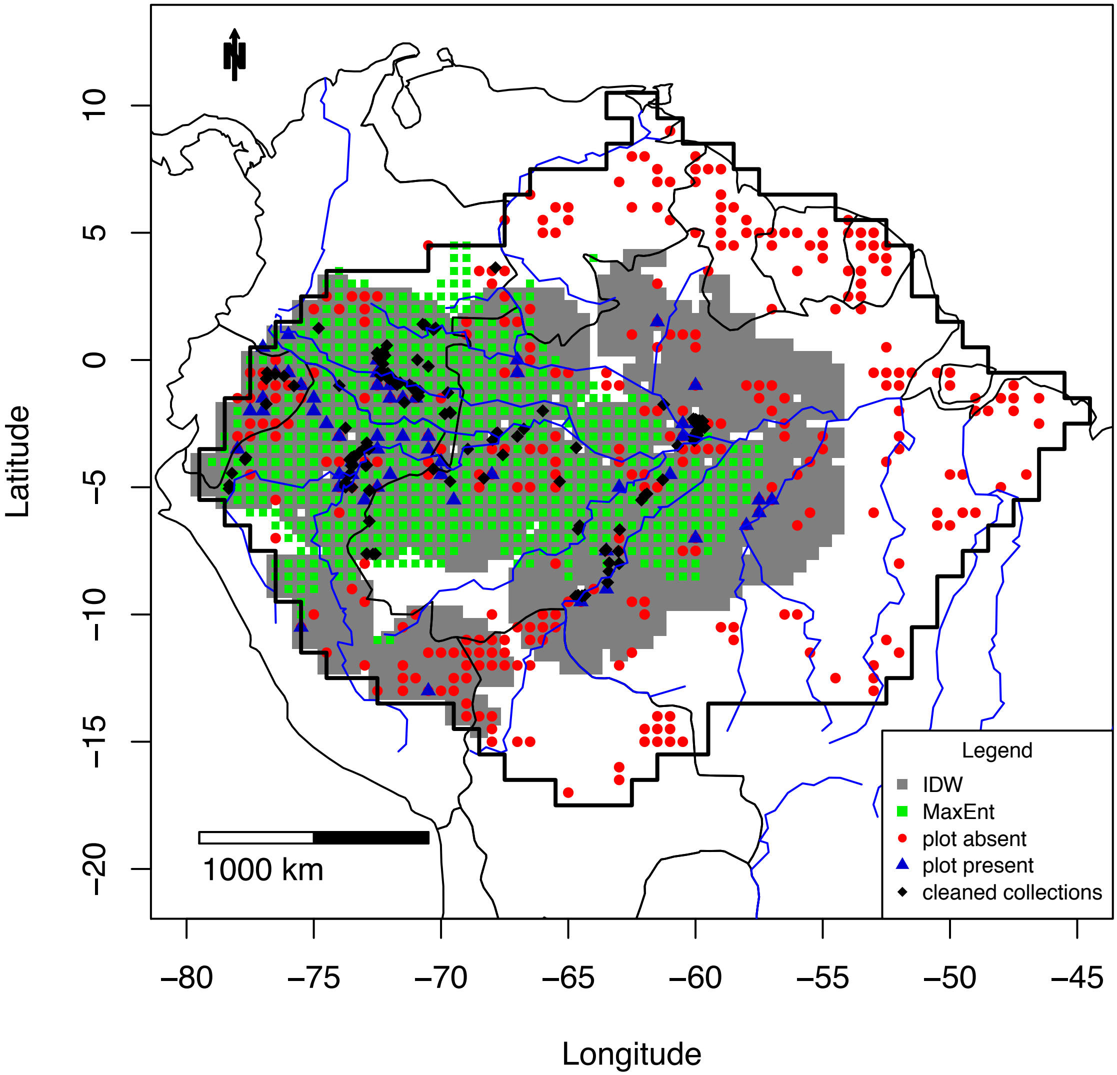

Figure S3_56B MaxEnt map of Eschweilera_tessmannii

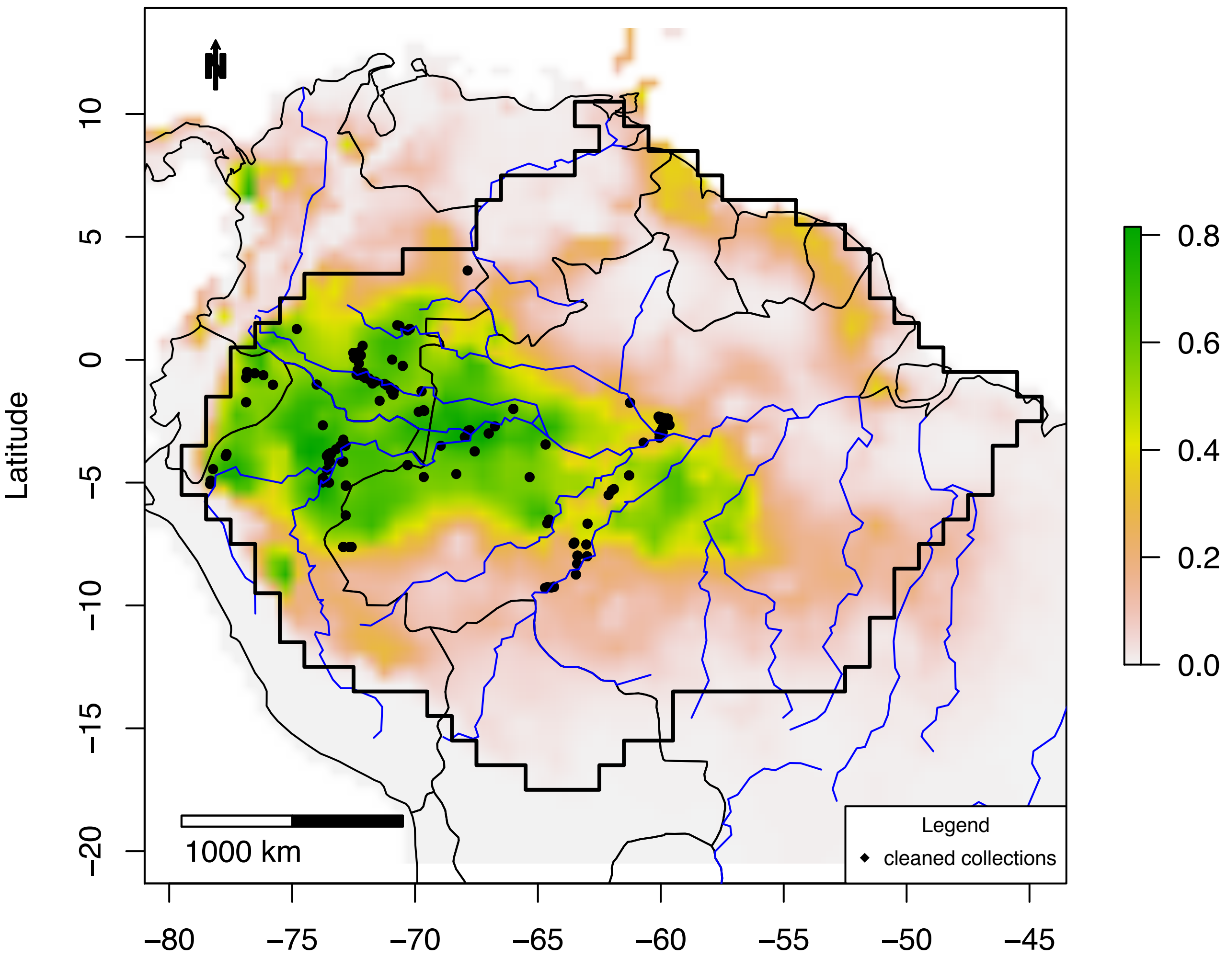

Figure S3_56C IDW map of Eschweilera_tessmannii: plots =0 – 0.25; IDW: 0 – 0.04

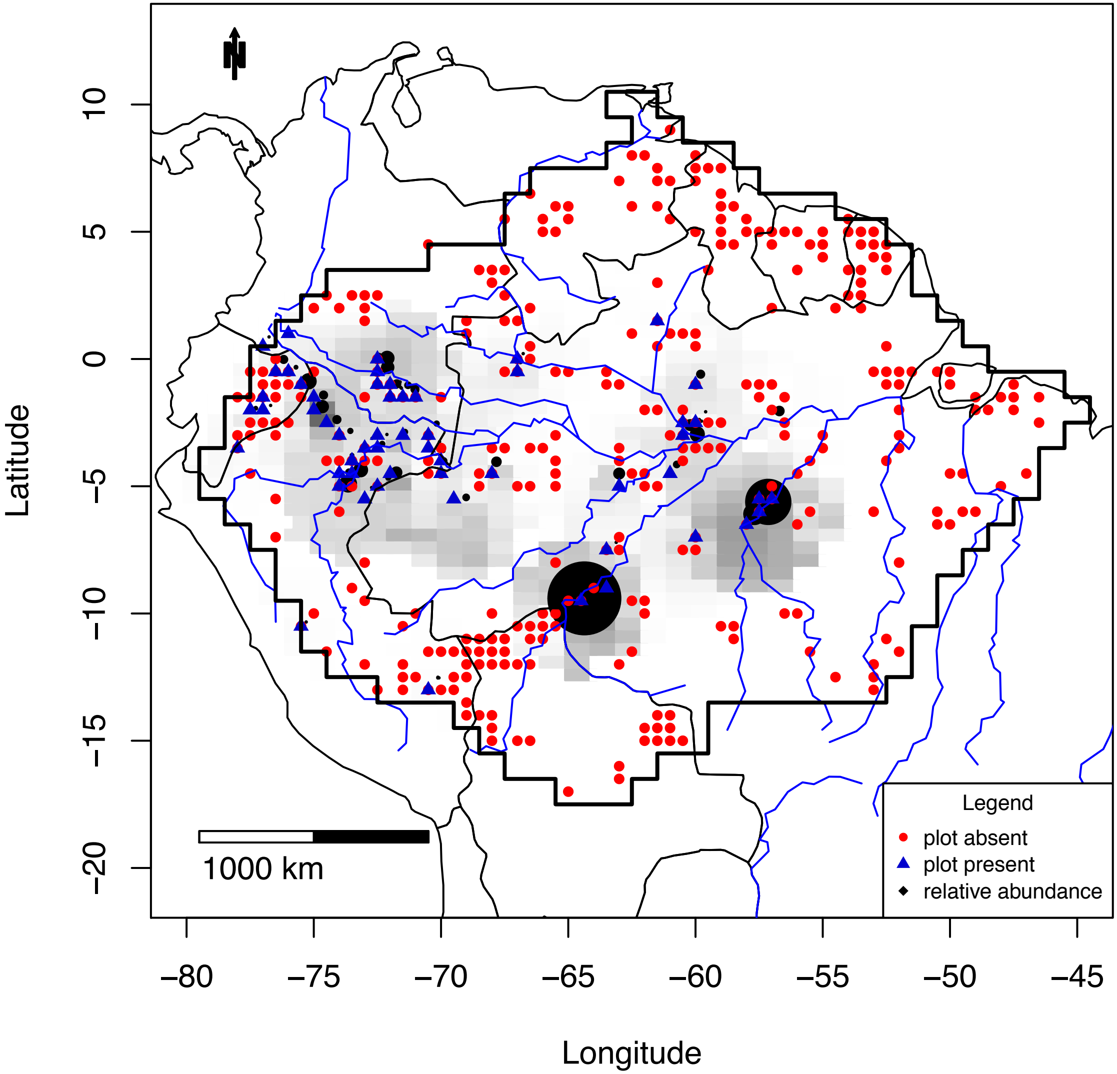

Figure S3_57A Combined map of Eschweilera_truncata

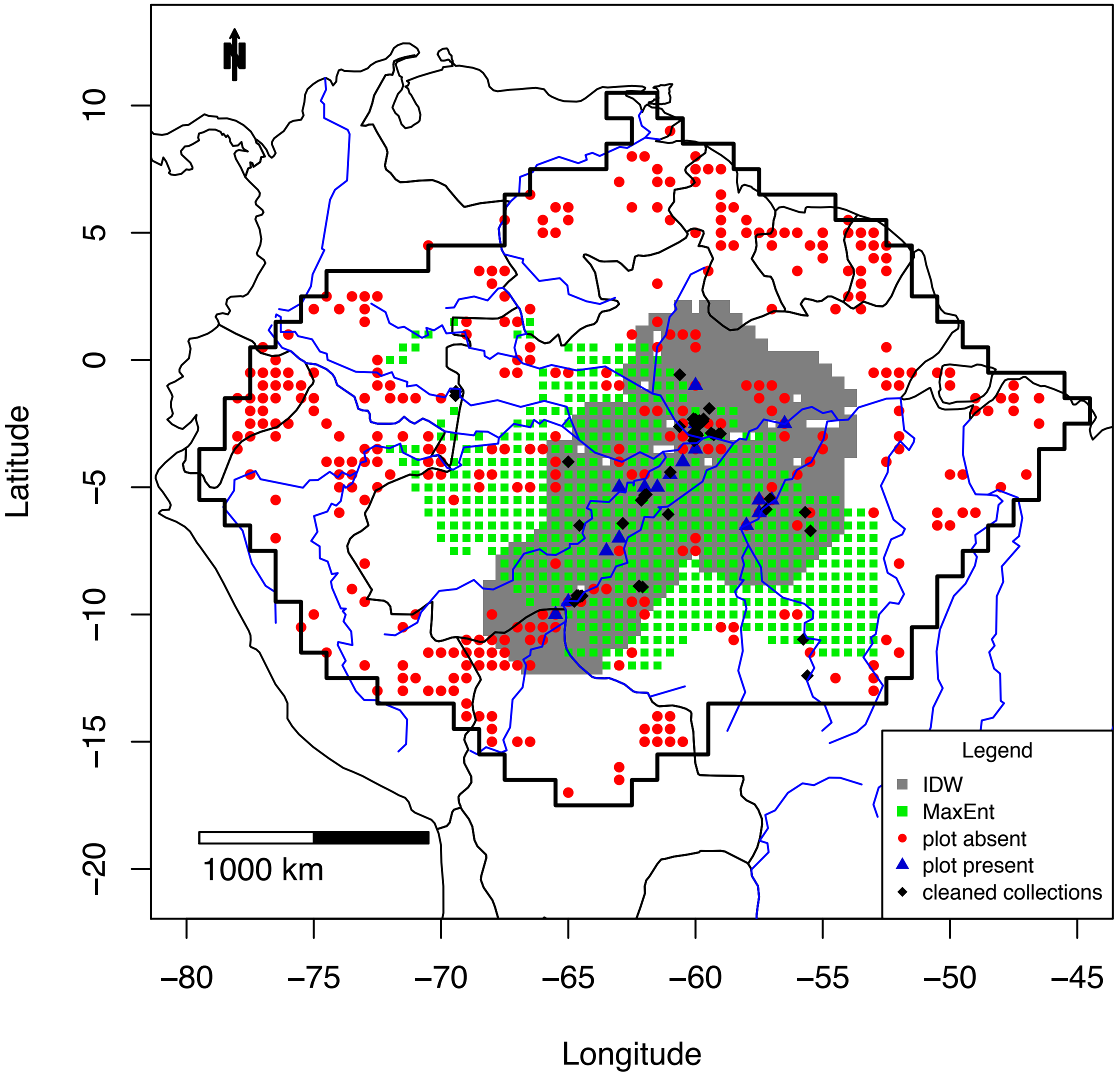

Figure S3_57B MaxEnt map of Eschweilera_truncata

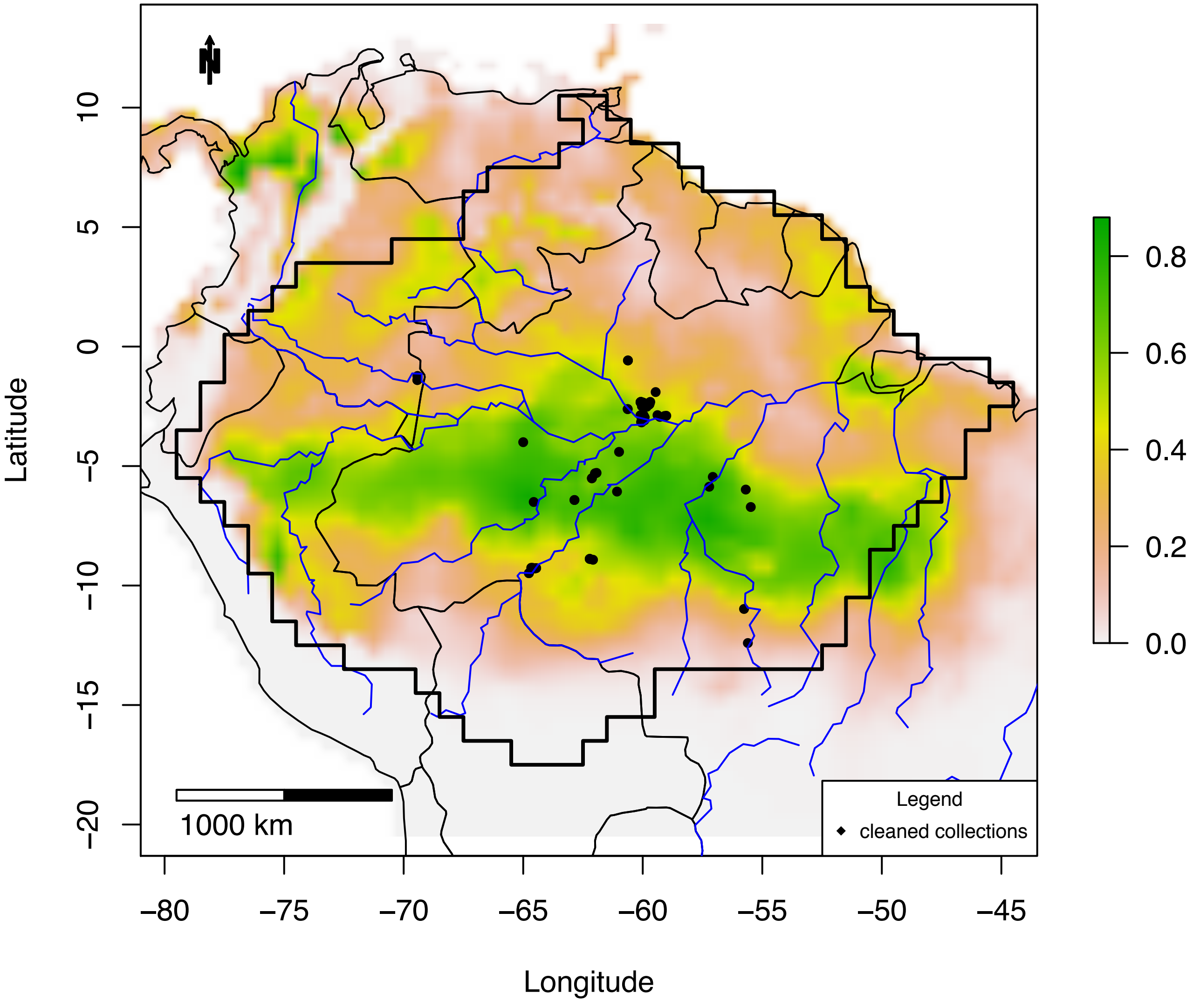

Figure S3_57C IDW map of Eschweilera_truncata: plots =0 – 0.13; IDW: 0 – 0.03

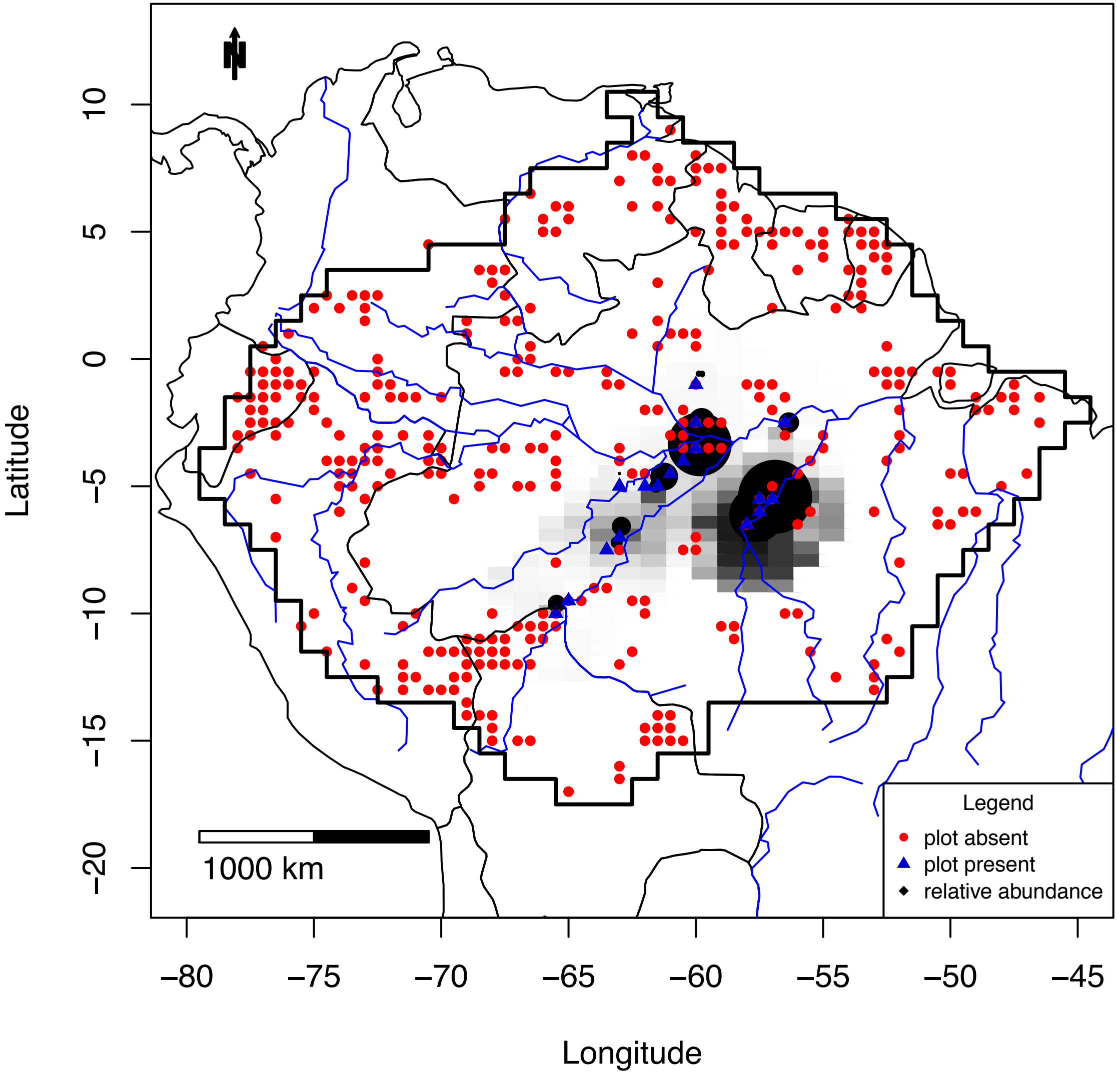

Figure S3_58A Combined map of Eschweilera_wachenheimii

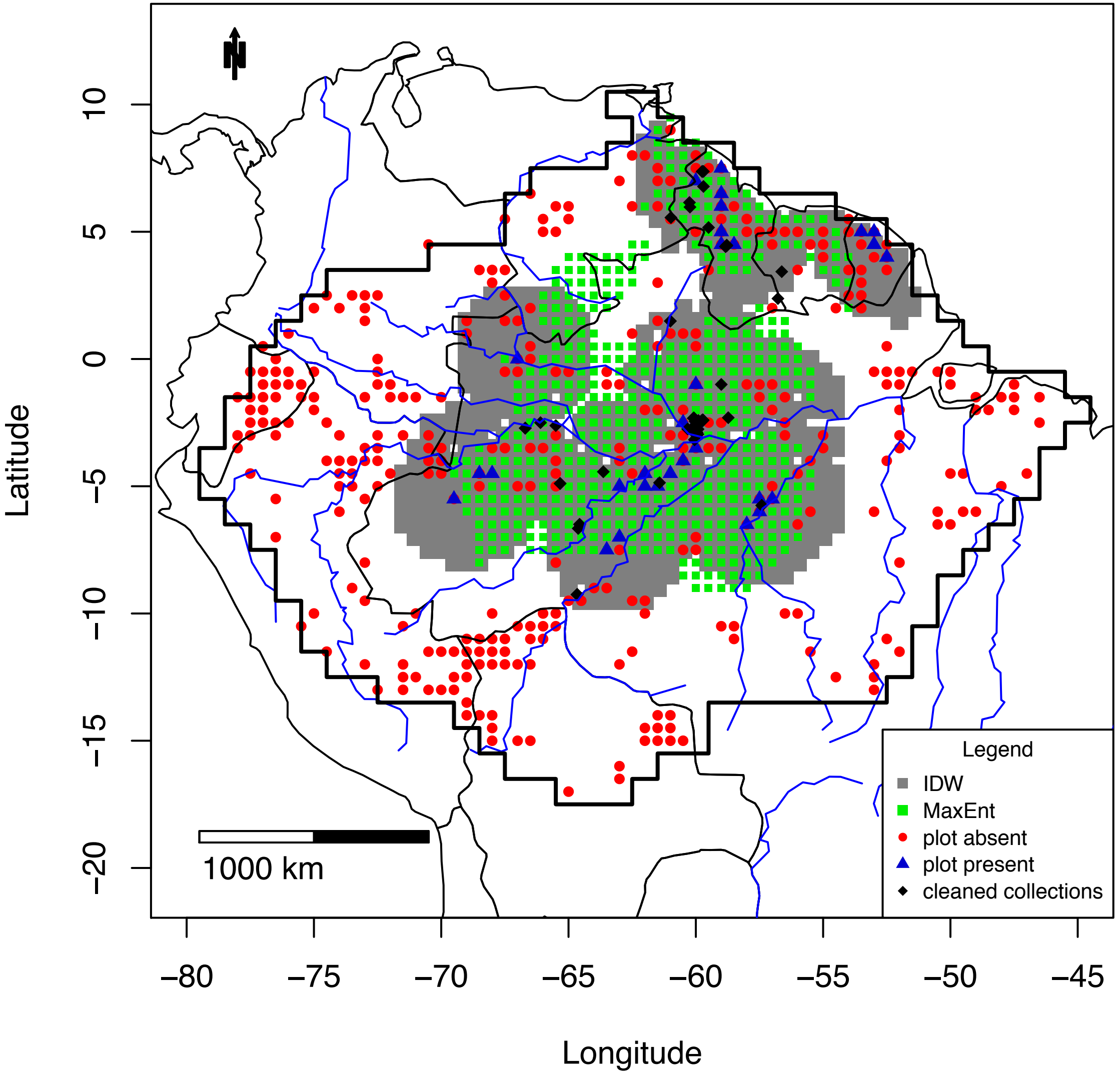

Figure S3_58B MaxEnt map of Eschweilera_wachenheimii

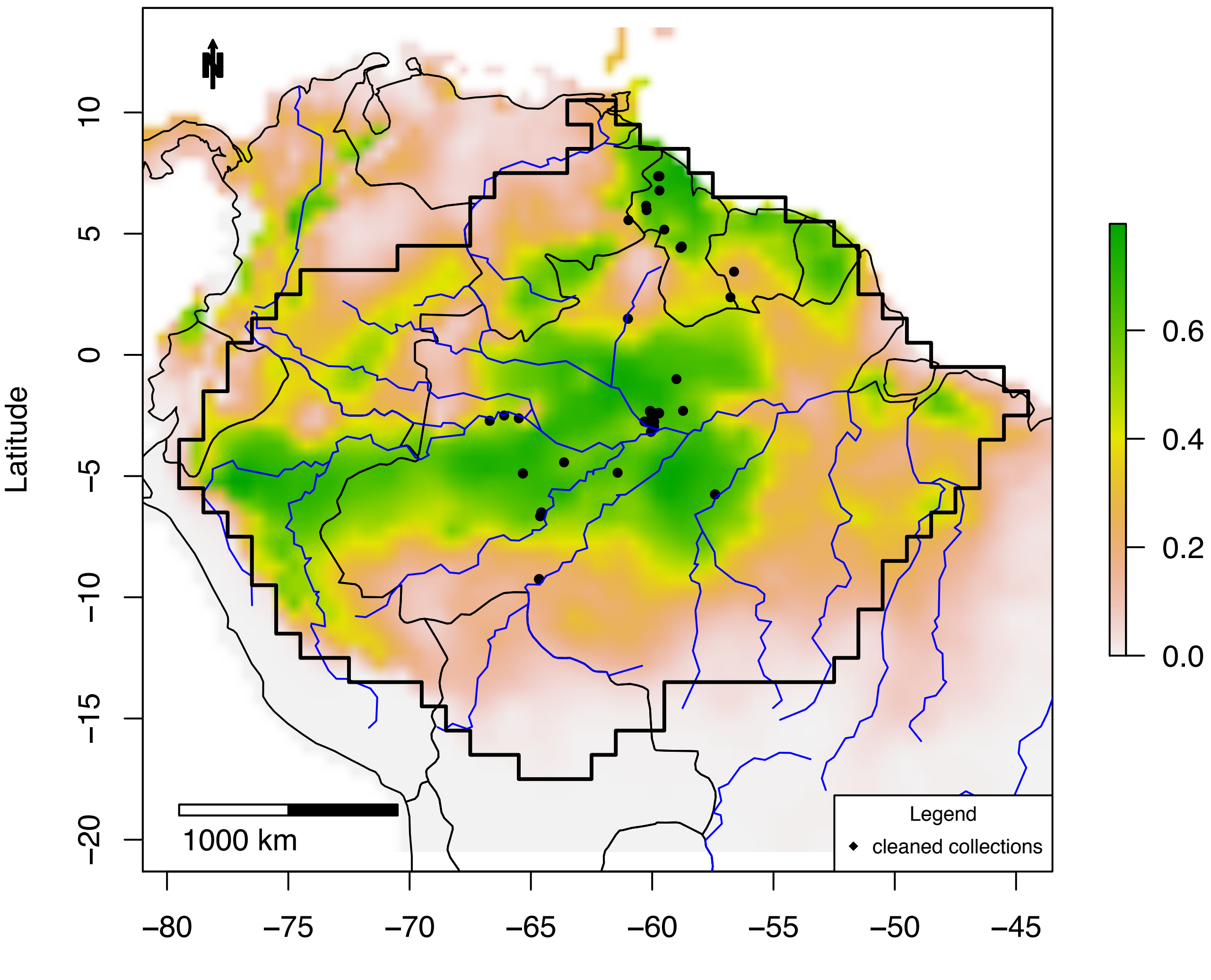

Figure S3_58C IDW map of Eschweilera_wachenheimii: plots =0 – 0.13; IDW: 0 – 0.06

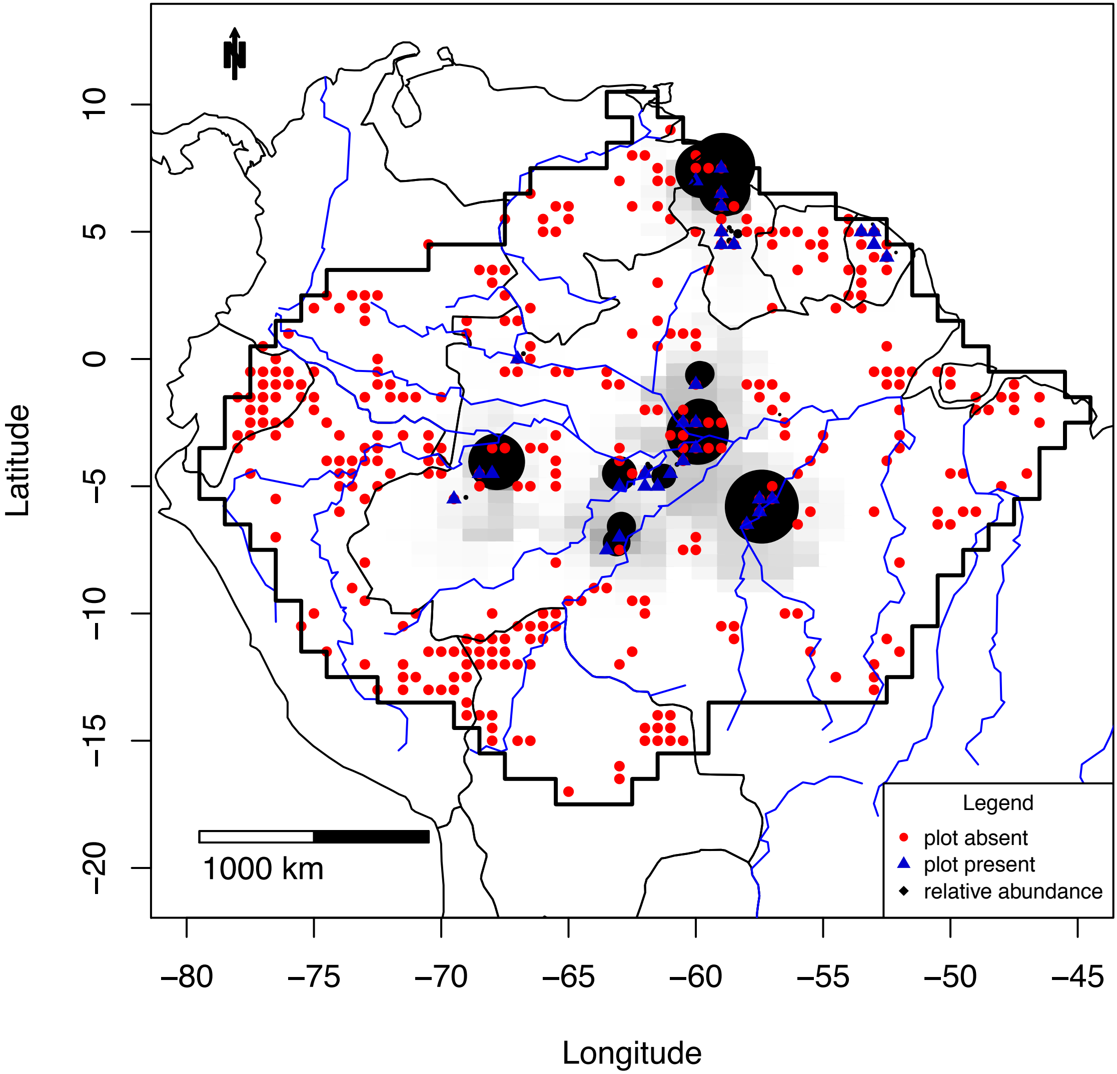

Figure S3_59A Combined map of Euterpe_oleracea

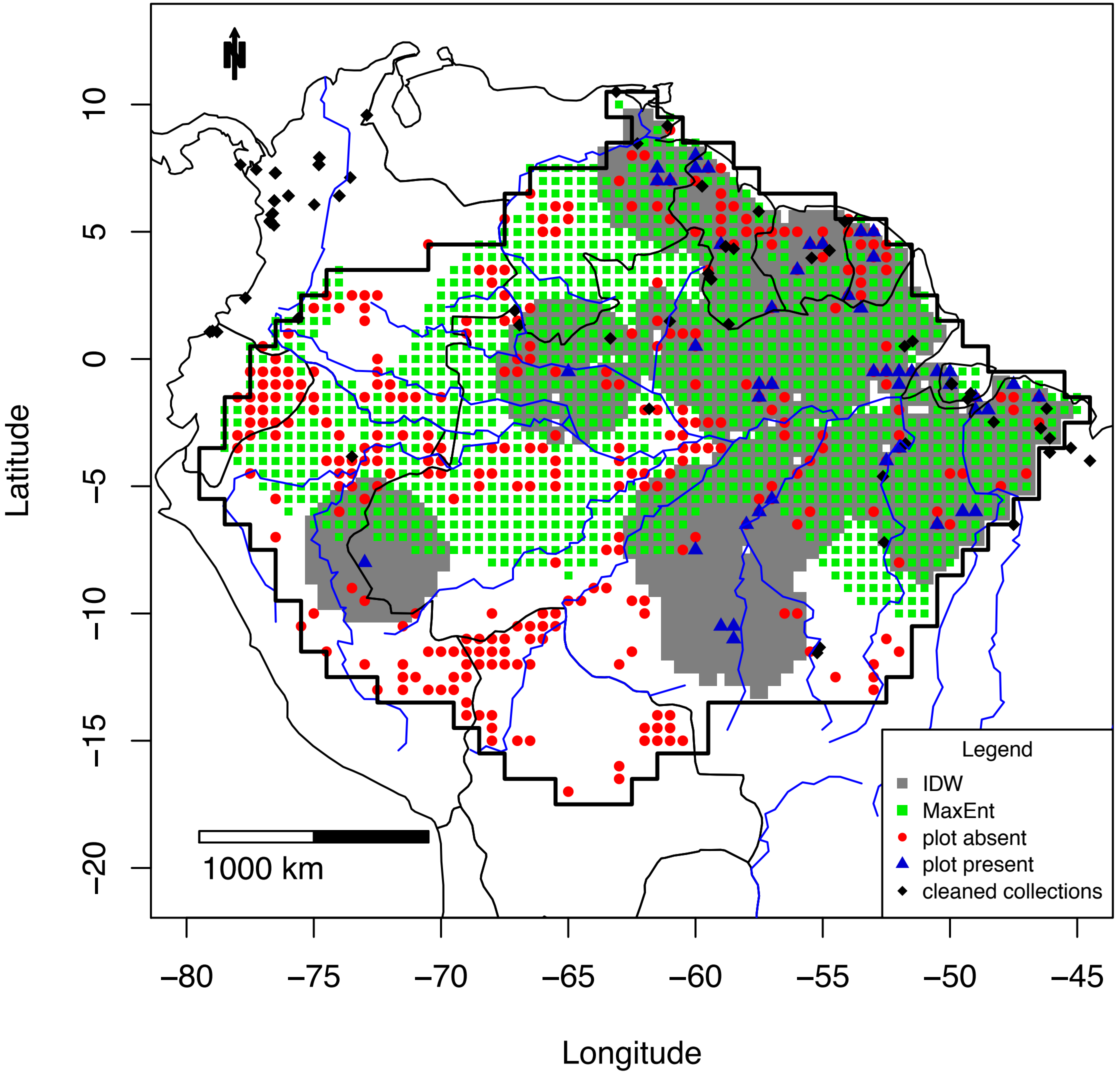

Figure S3_59B MaxEnt map of Euterpe_oleracea

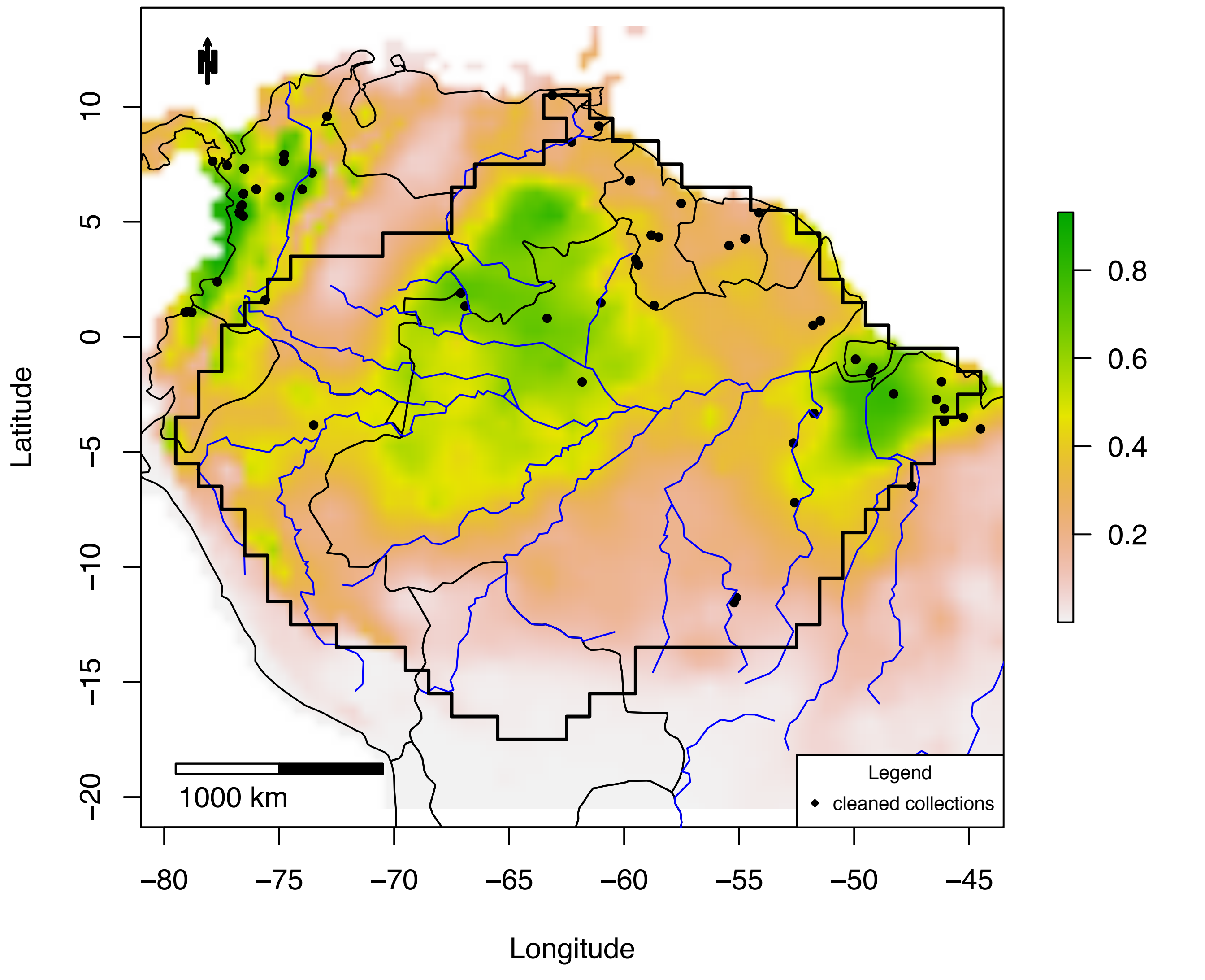

Figure S3_59C IDW map of Euterpe_oleracea: plots =0 – 0.59; IDW: 0 – 0.2

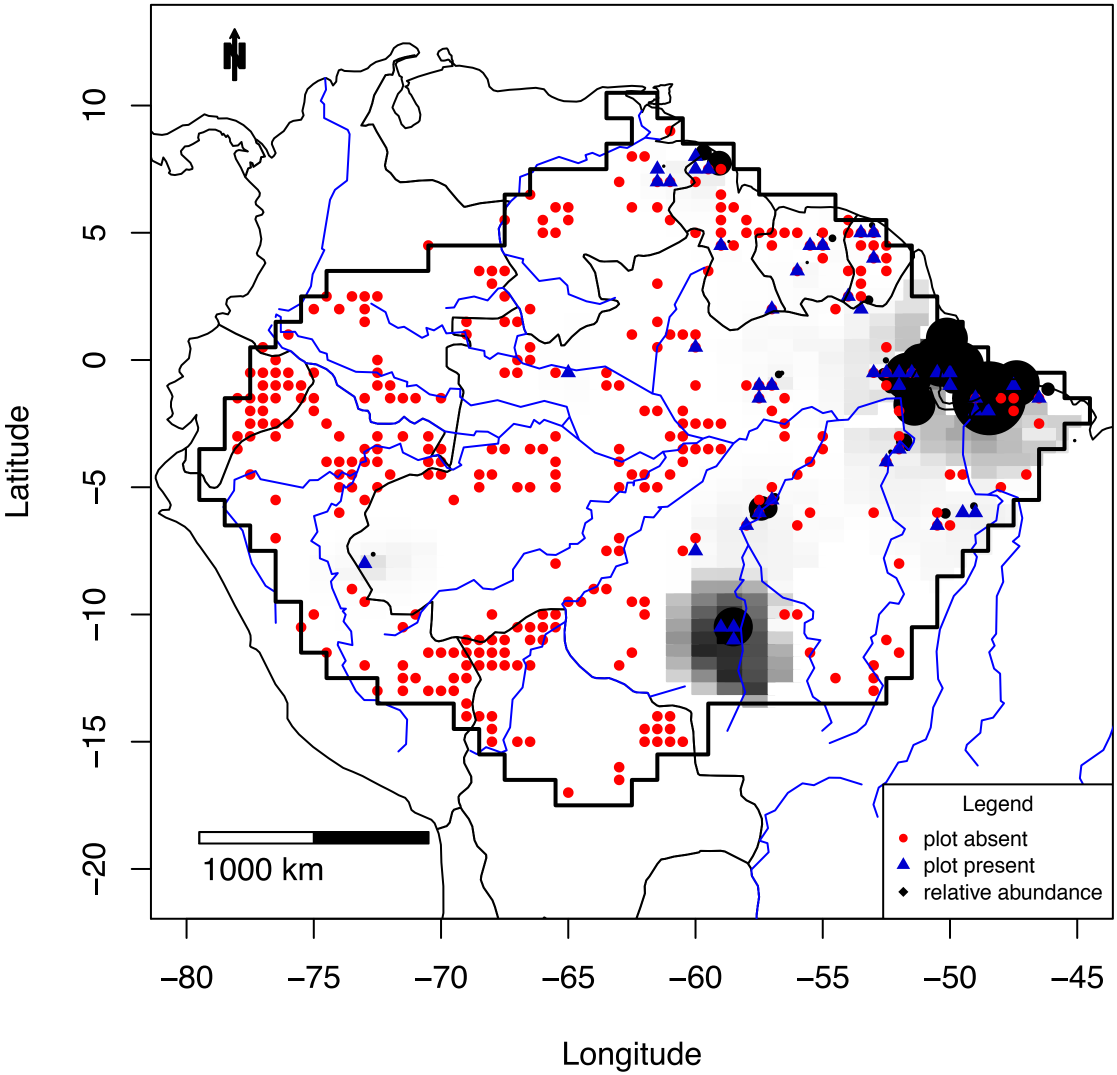

Figure S3_60A Combined map of Euterpe_precatoria

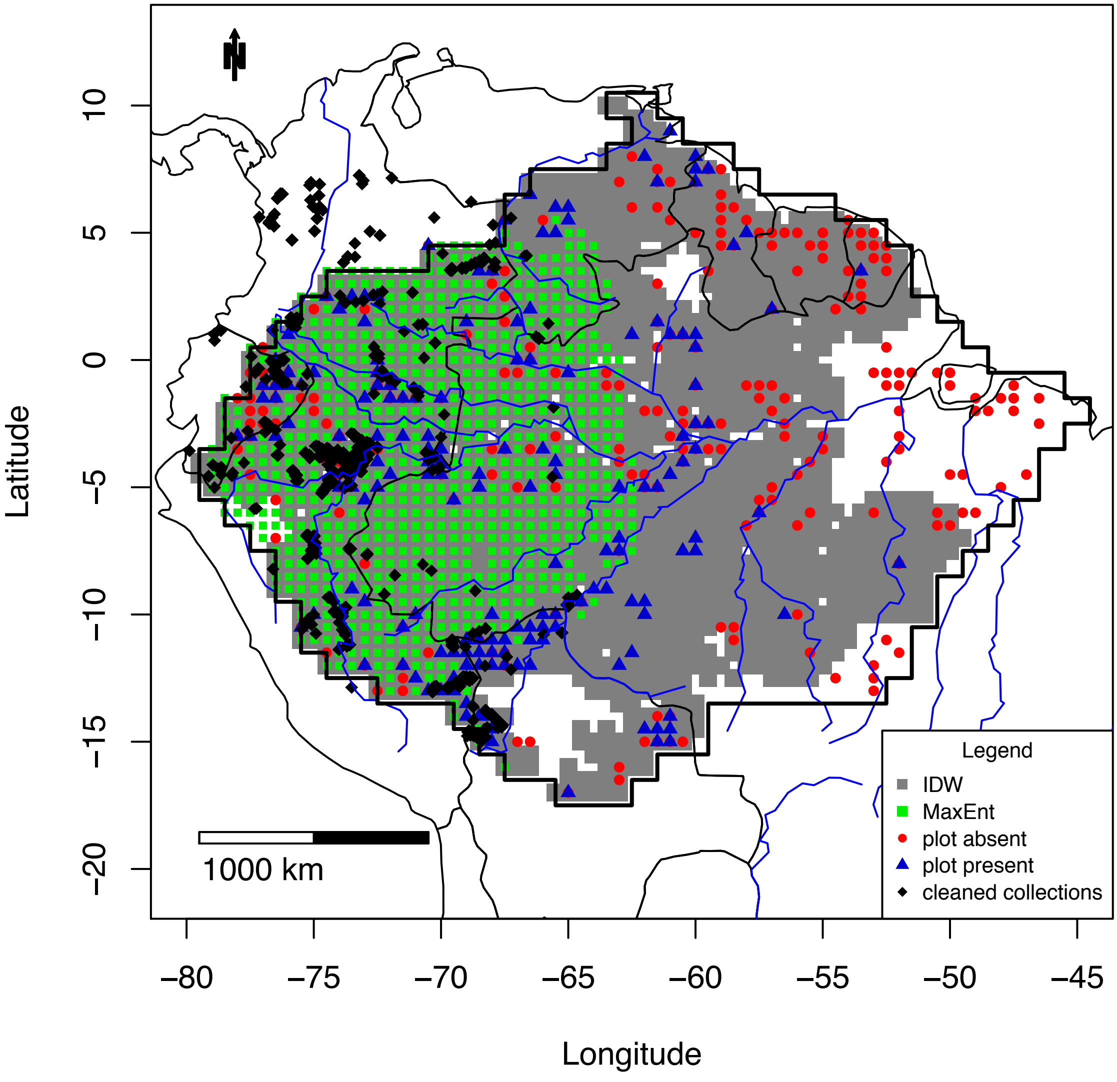

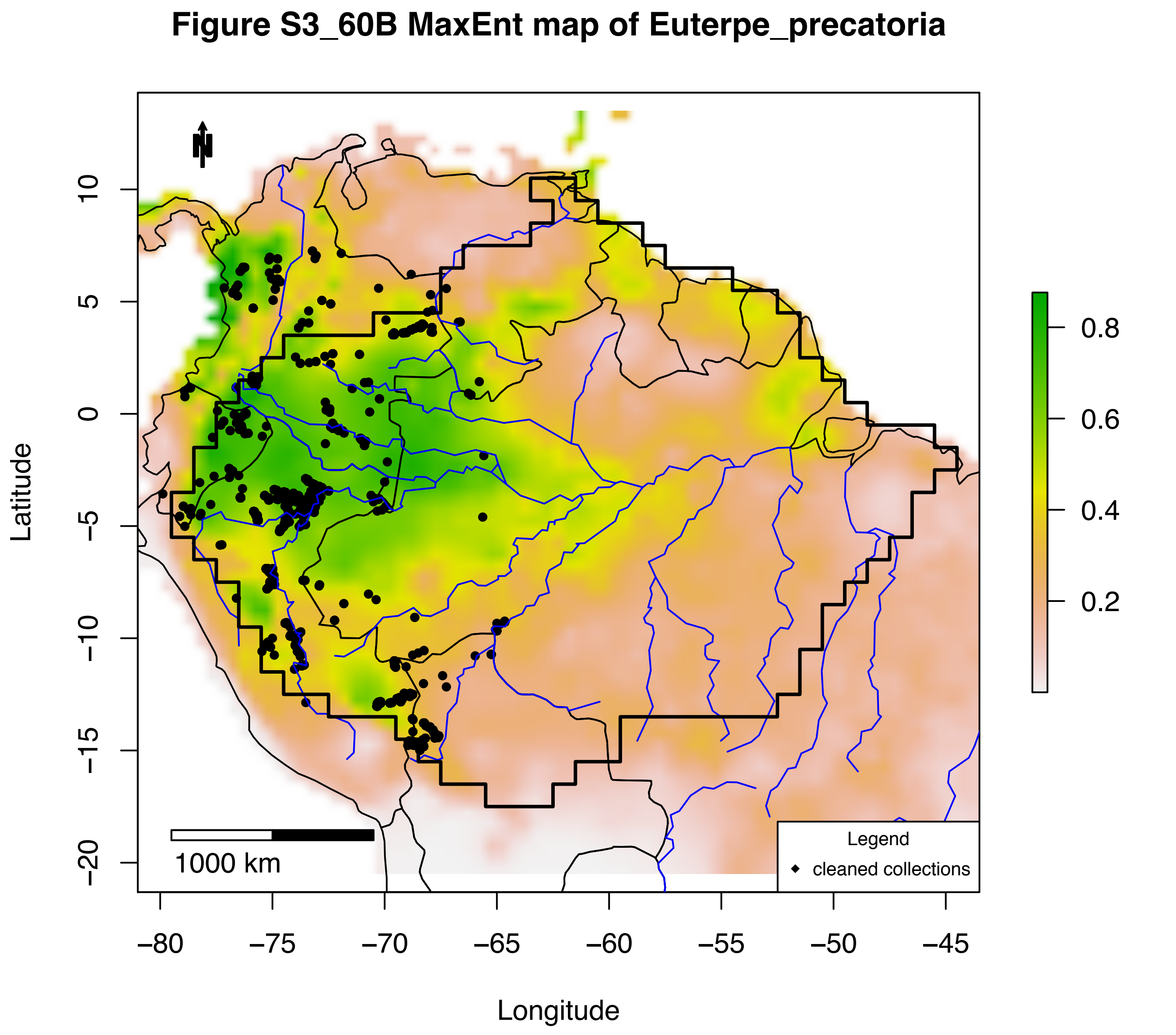

Figure S3_60B MaxEnt map of Euterpe_precatoria
Latitude
Longitude
10
5
0
−5
−10
−15
−20
−80
−75
−70
−65
−60
−55
−50
−45
0.8
0.6
0.4
0.2
N
1000 km
Legend
cleaned collections

Figure S3_60C IDW map of Euterpe_precatoria: plots =0 – 0.36; IDW: 0 – 0.13

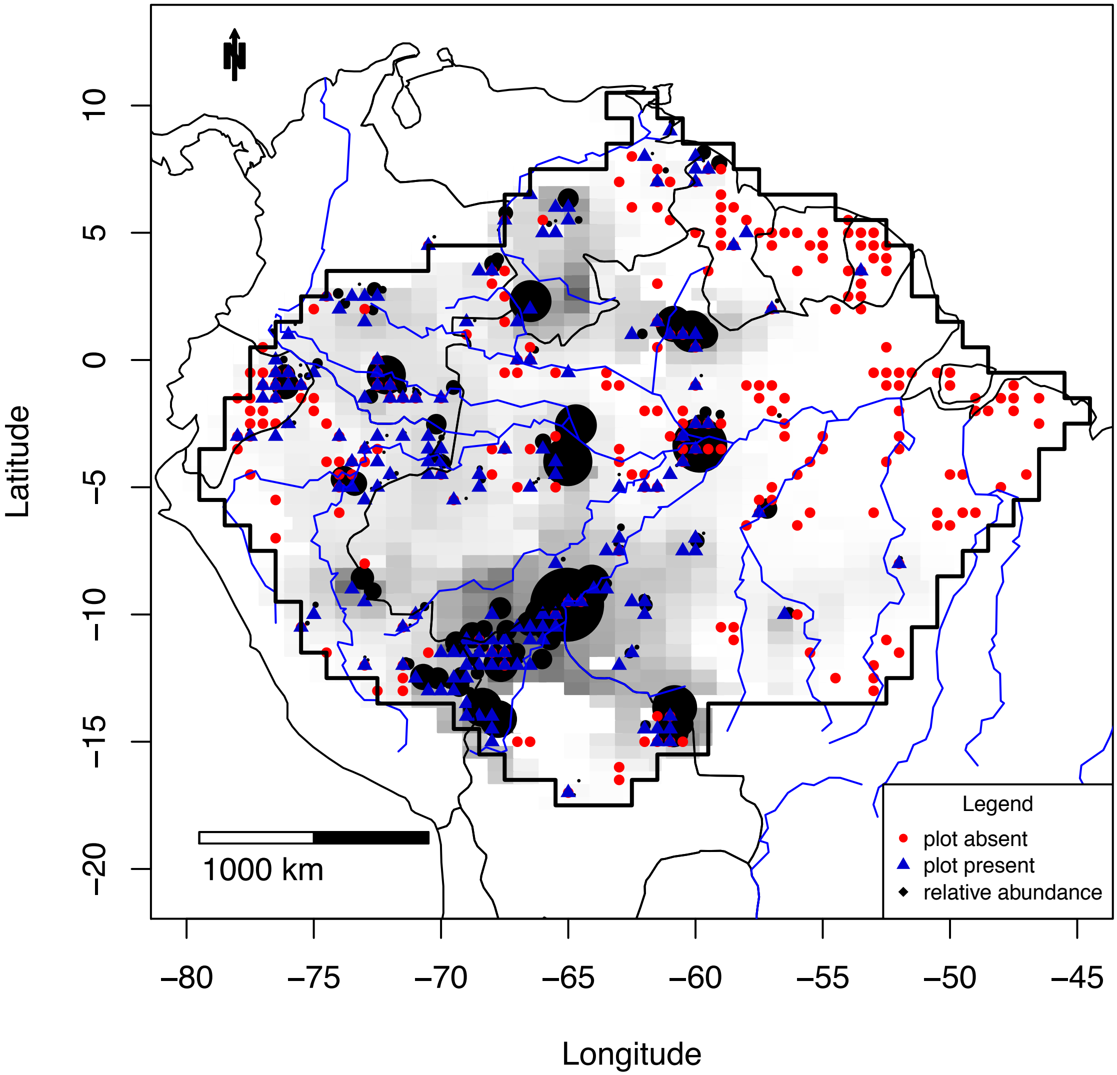

Figure S3_61A Combined map of Garcinia_macrophylla

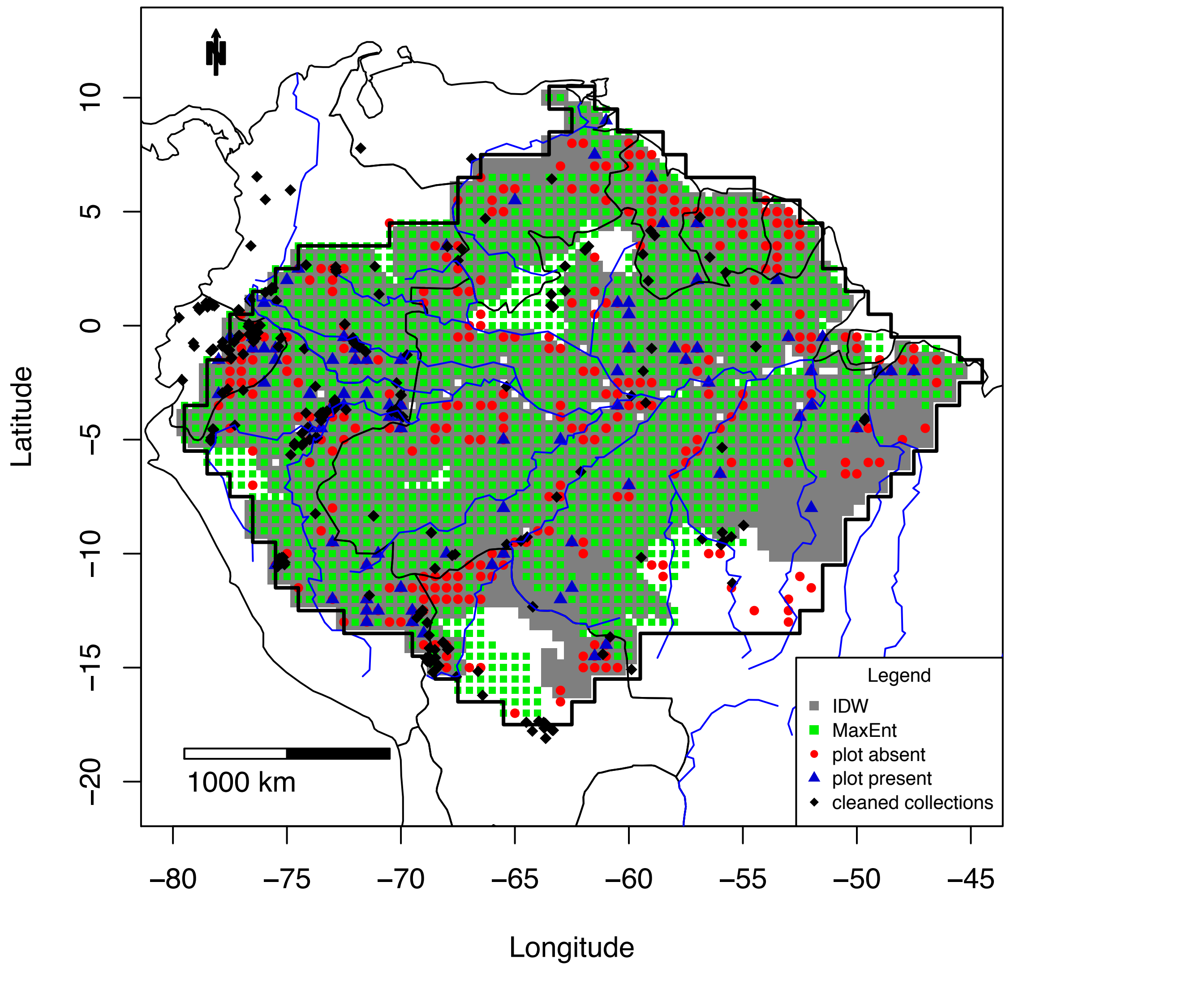

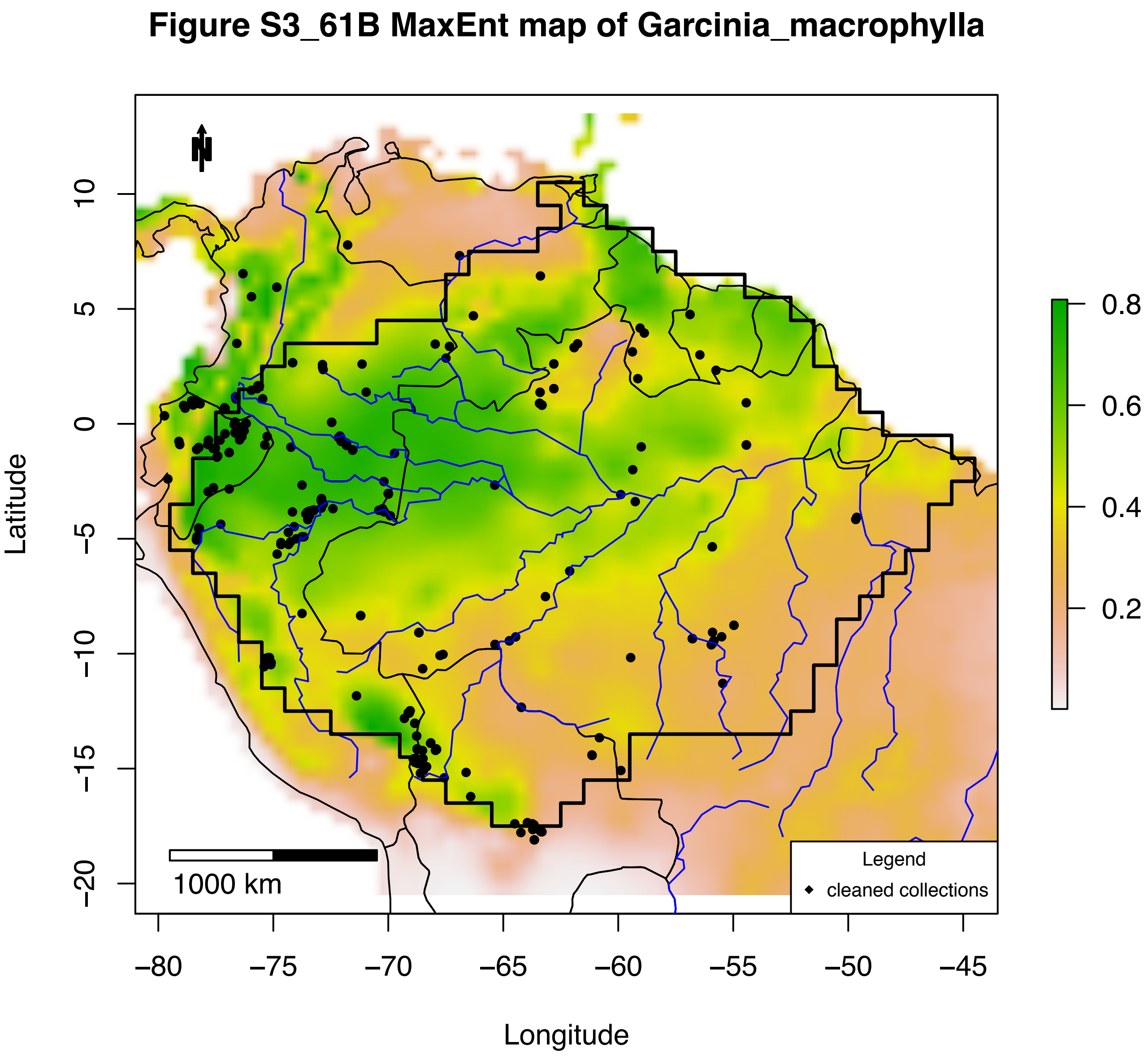

Figure S3_61B MaxEnt map of Garcinia_macrophylla
N
Latitude
Longitude
10
5
0
−5
−10
−15
−20
−80
−75
−70
−65
−60
−55
−50
−45
0.8
0.6
0.4
0.2
1000 km
Legend
cleaned collections

Figure S3_61C IDW map of Garcinia_macrophylla: plots =0 – 0.08; IDW: 0 – 0.02

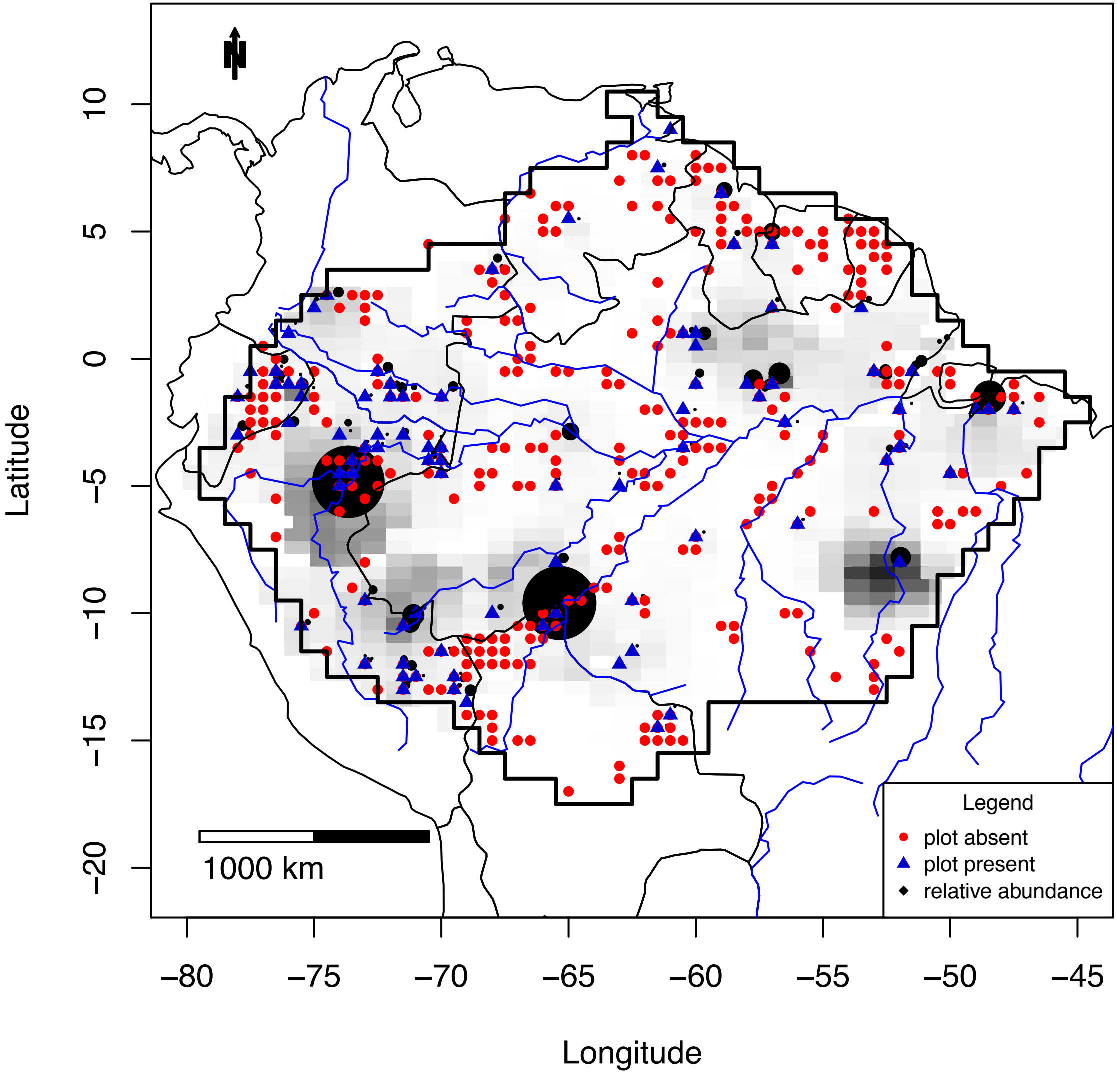

Figure S3_62A Combined map of Goupia_glabra

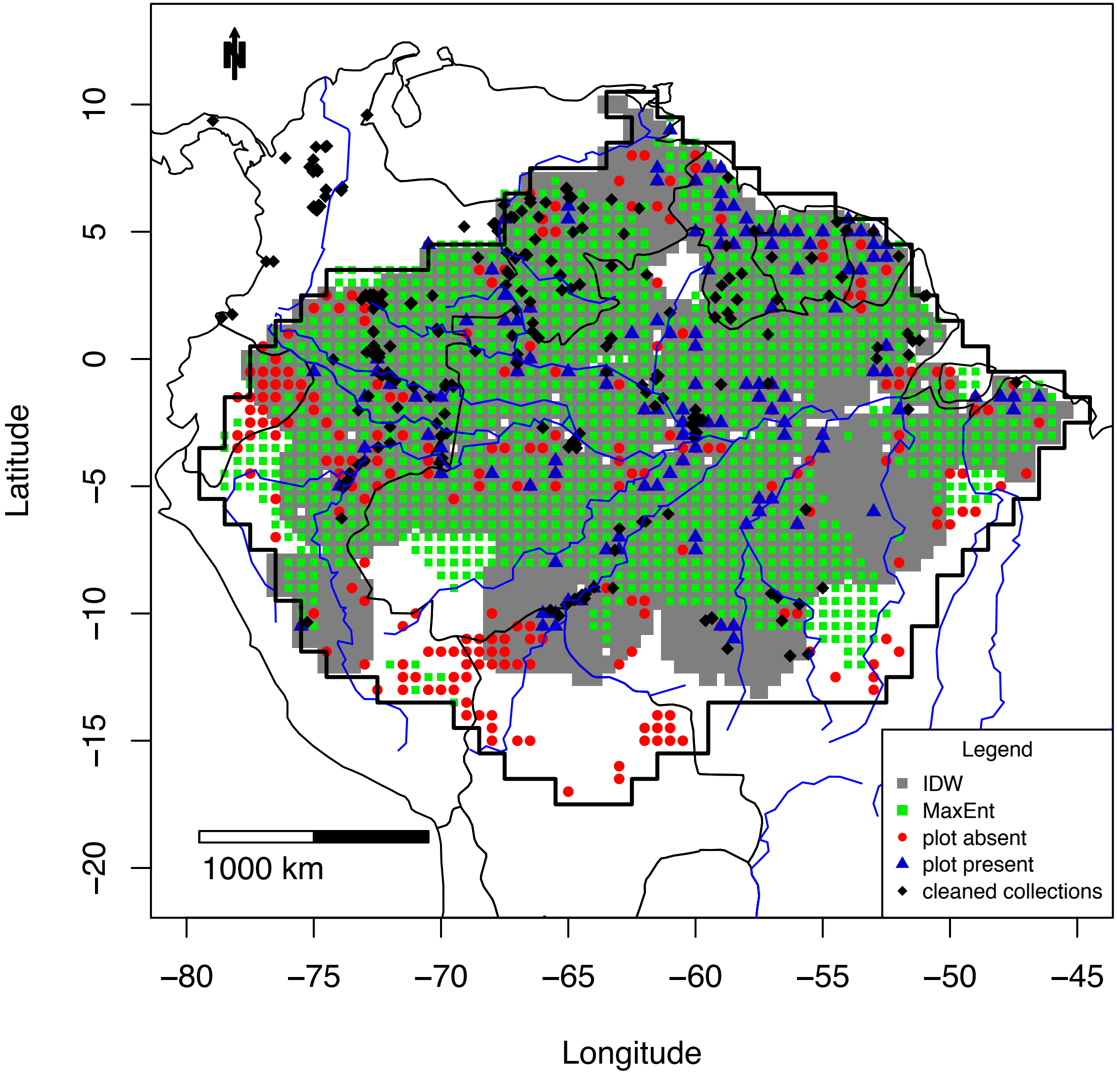

Figure S3_62B MaxEnt map of Goupia_glabra

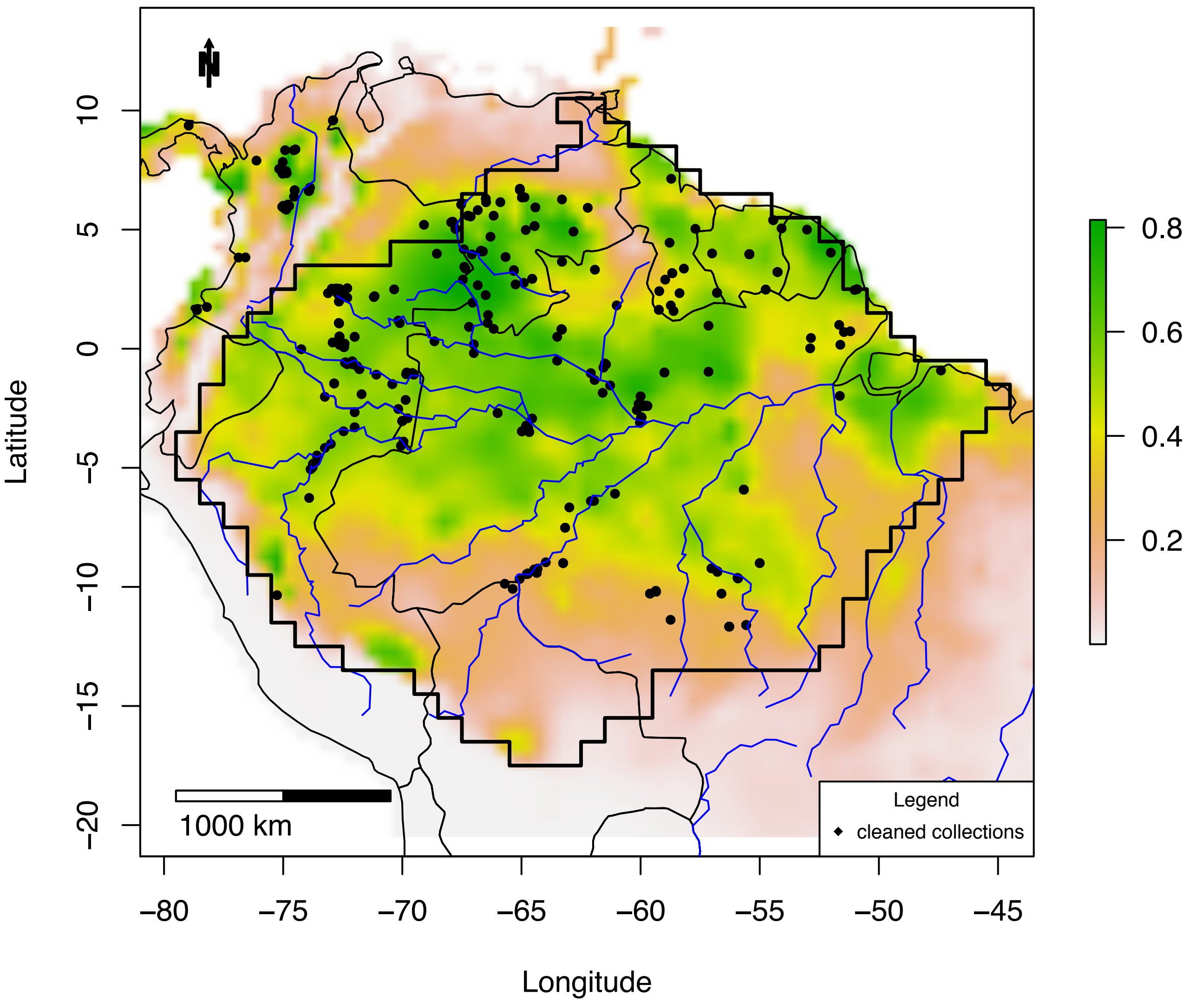

Figure S3_62C IDW map of Goupia_glabra: plots =0 – 0.13; IDW: 0 – 0.03

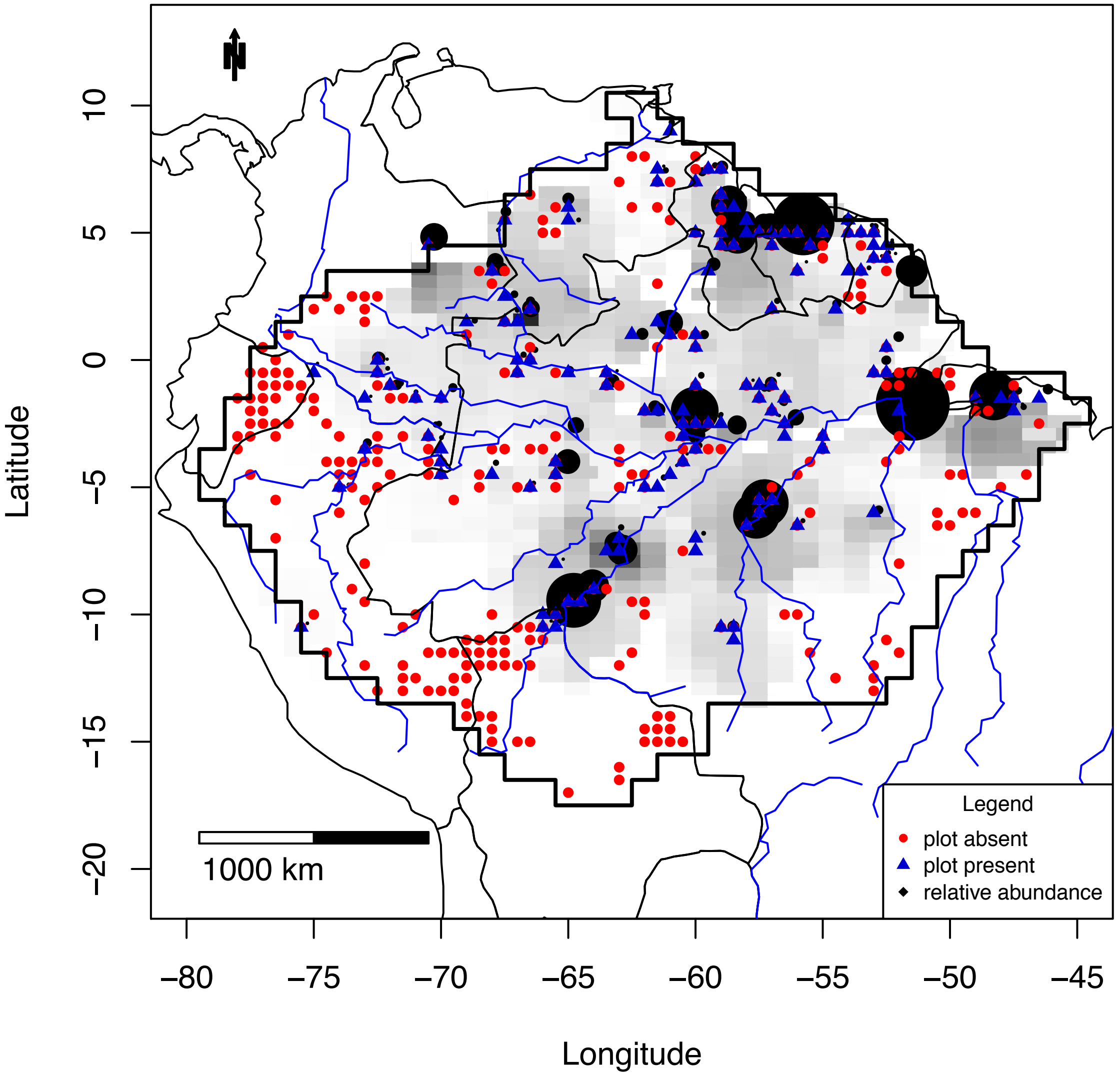

Figure S3_63A Combined map of Guarea_macrophylla

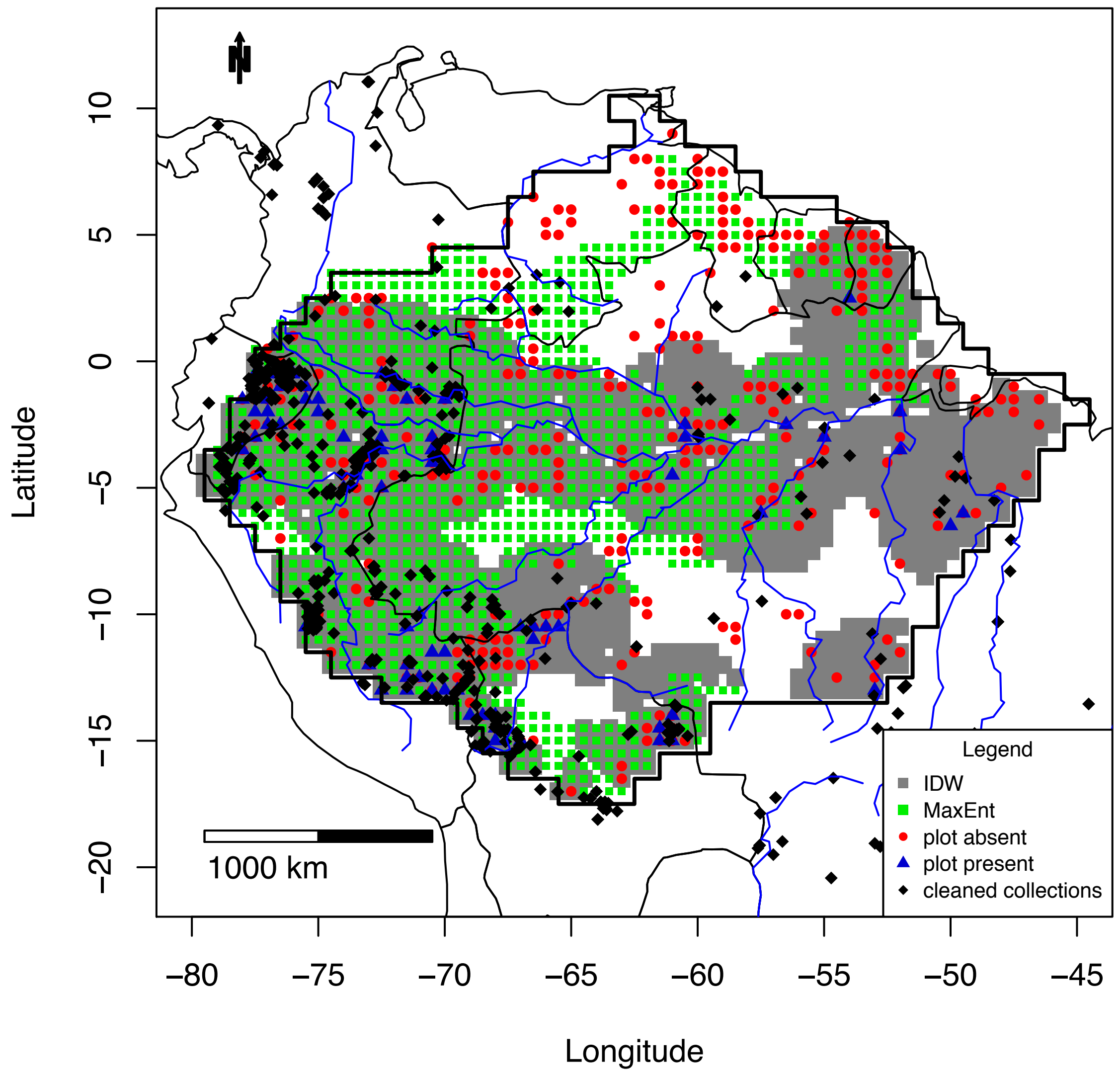

Figure S3_63B MaxEnt map of Guarea_macrophylla

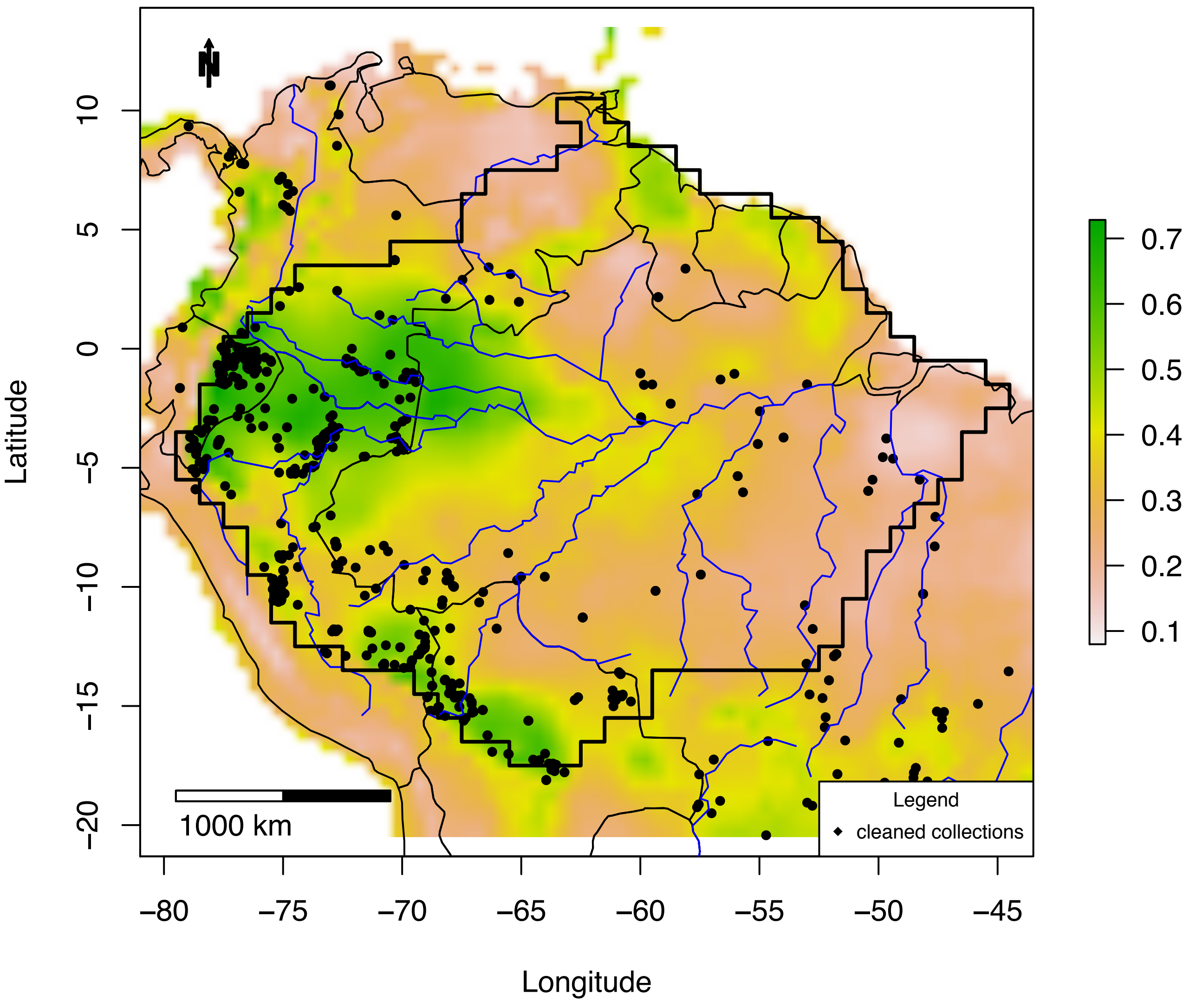

Figure S3_63C IDW map of Guarea_macrophylla: plots =0 – 0.22; IDW: 0 – 0.06

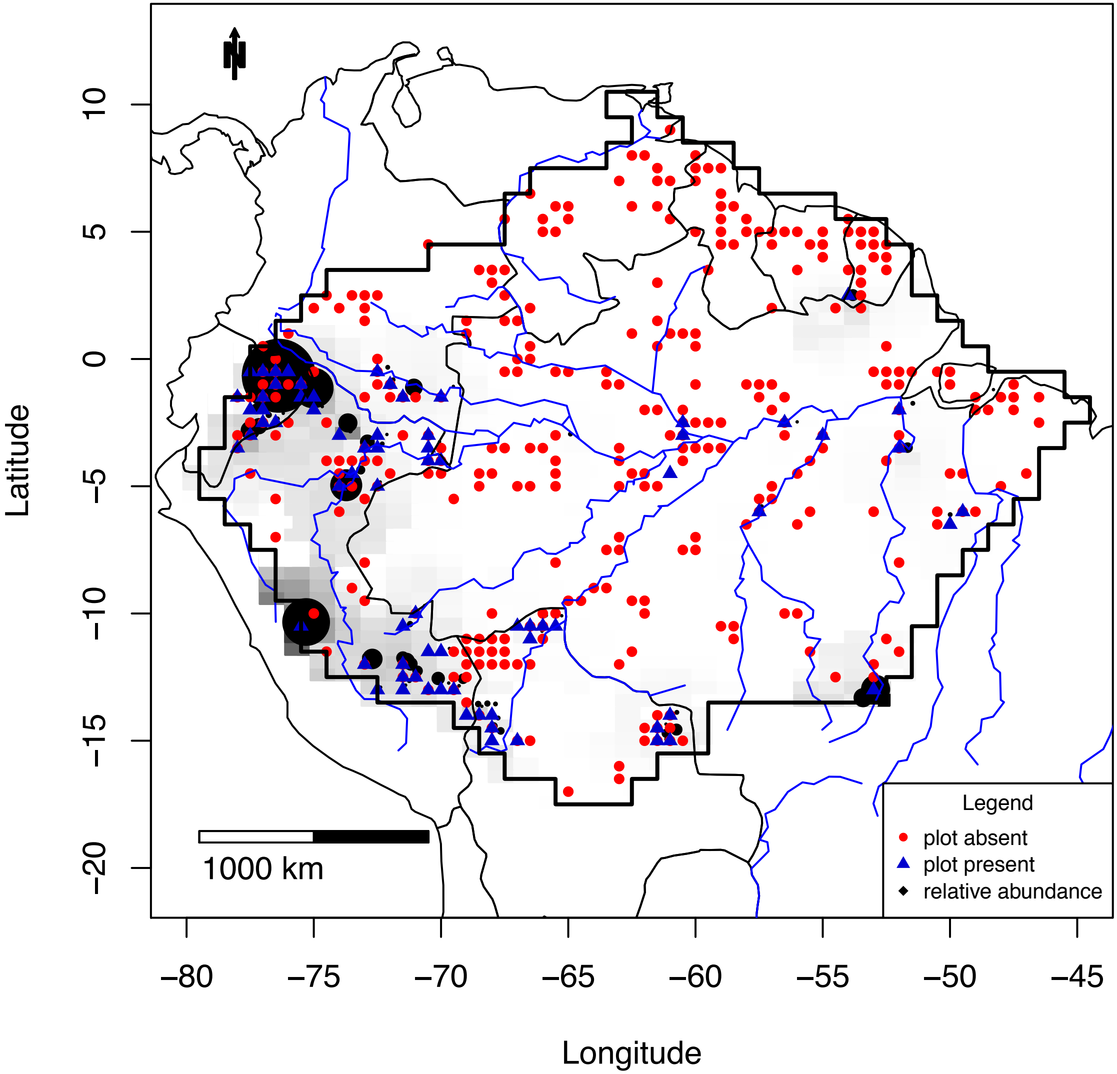

Figure S3_64A Combined map of Guarea_silvatica

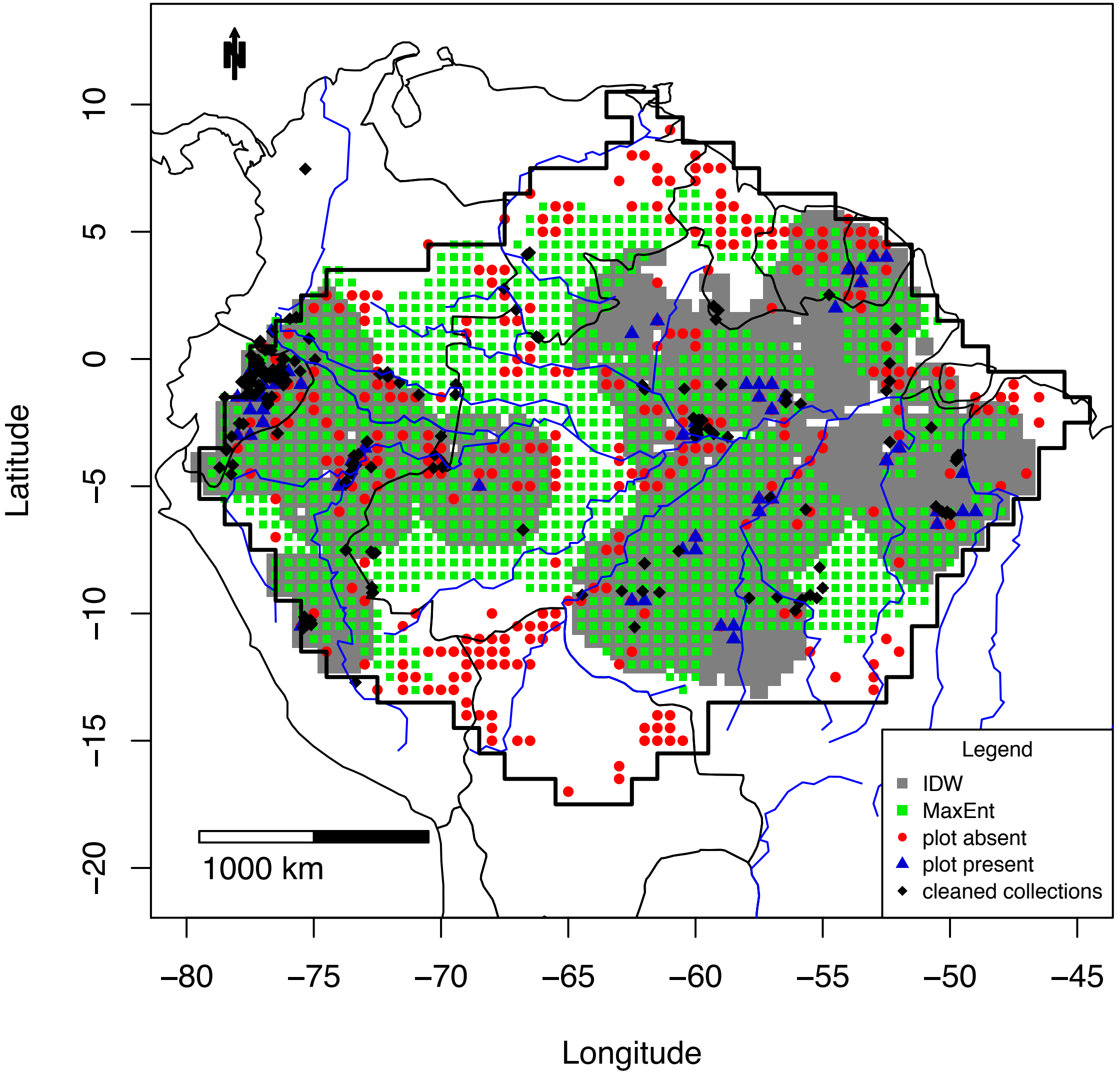

Figure S3_64B MaxEnt map of Guarea_silvatica

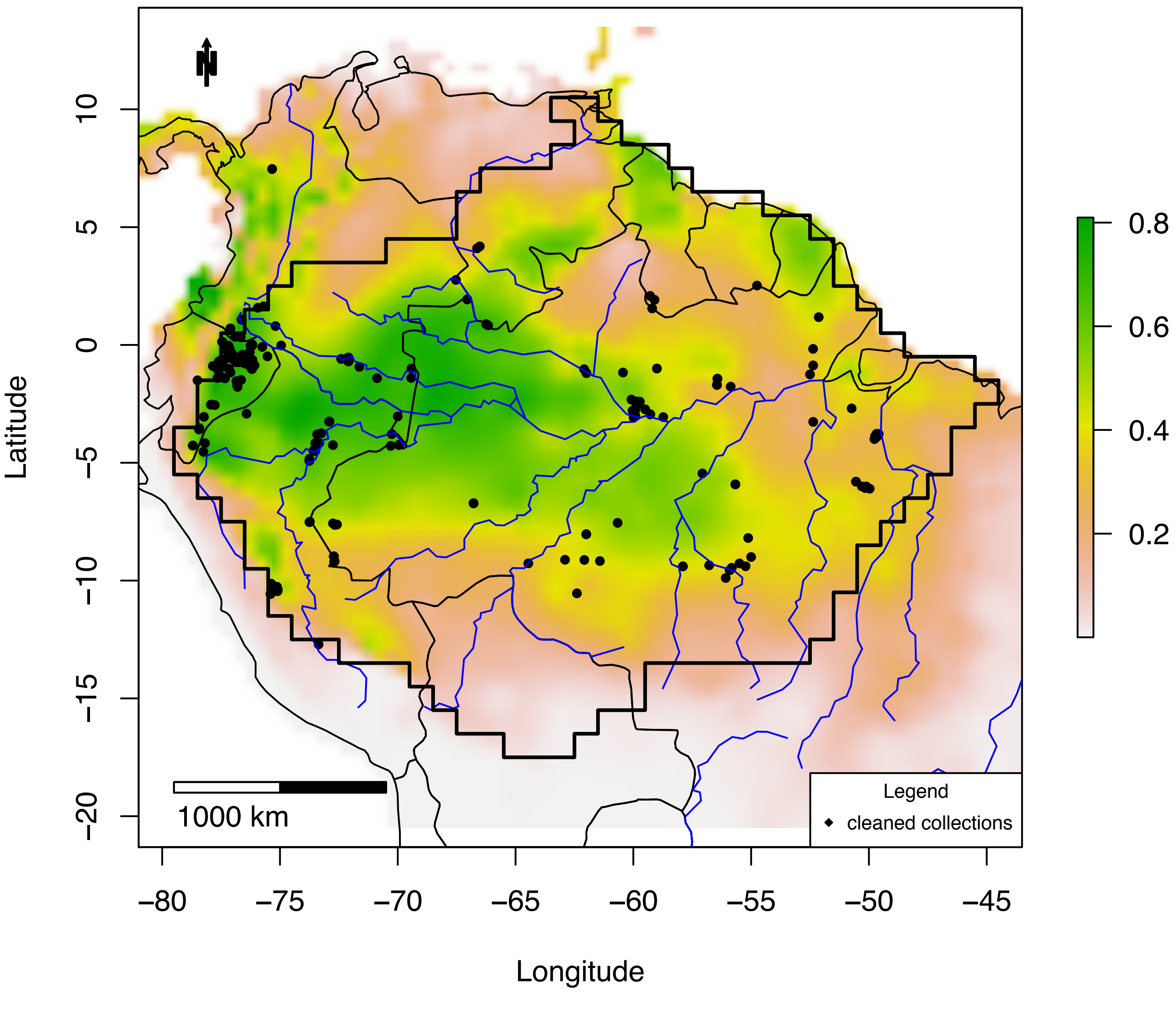

Figure S3_64C IDW map of Guarea_silvatica: plots =0 – 0.08; IDW: 0 – 0.04

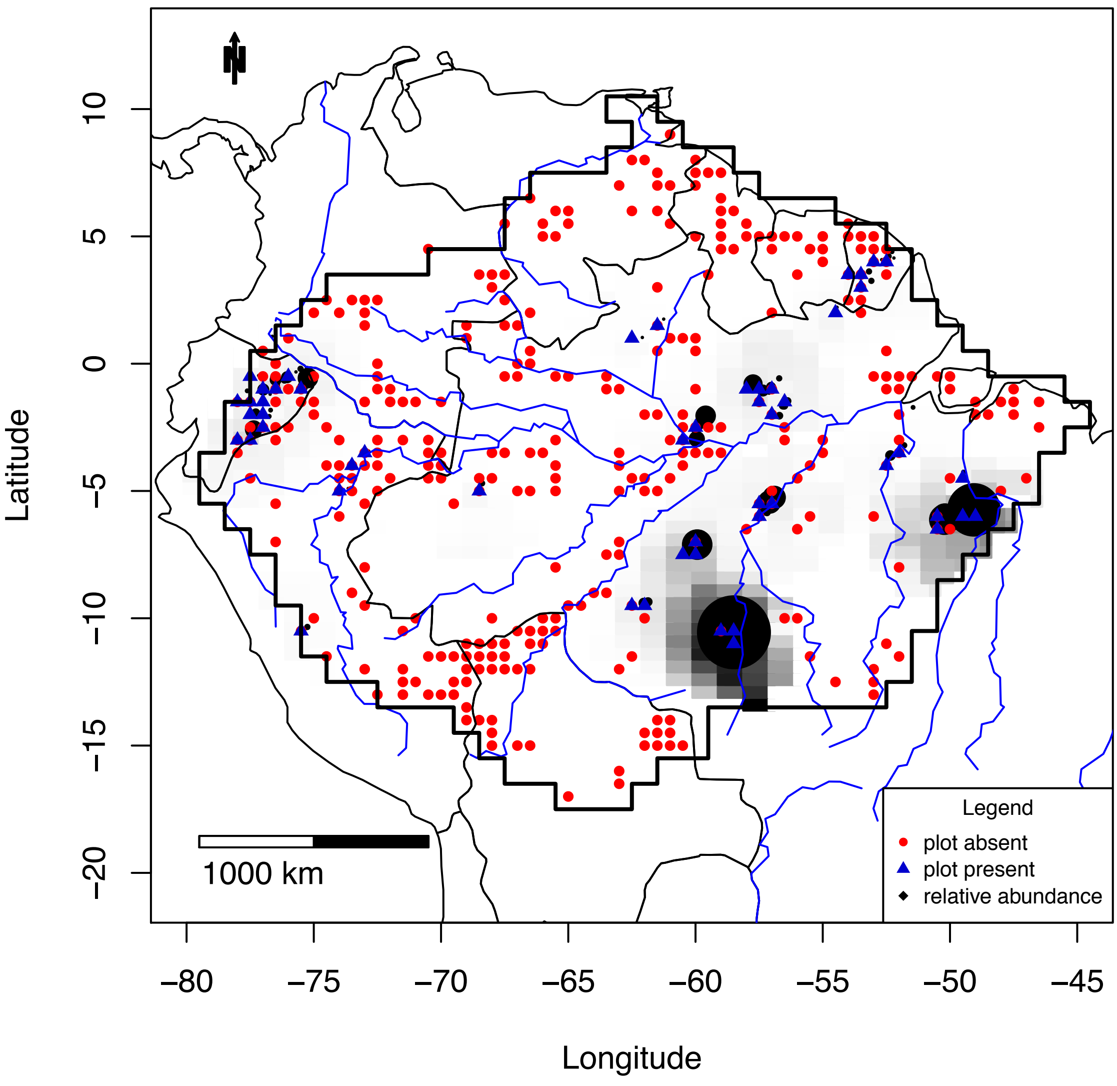

Figure S3_65A Combined map of Gustavia_augusta

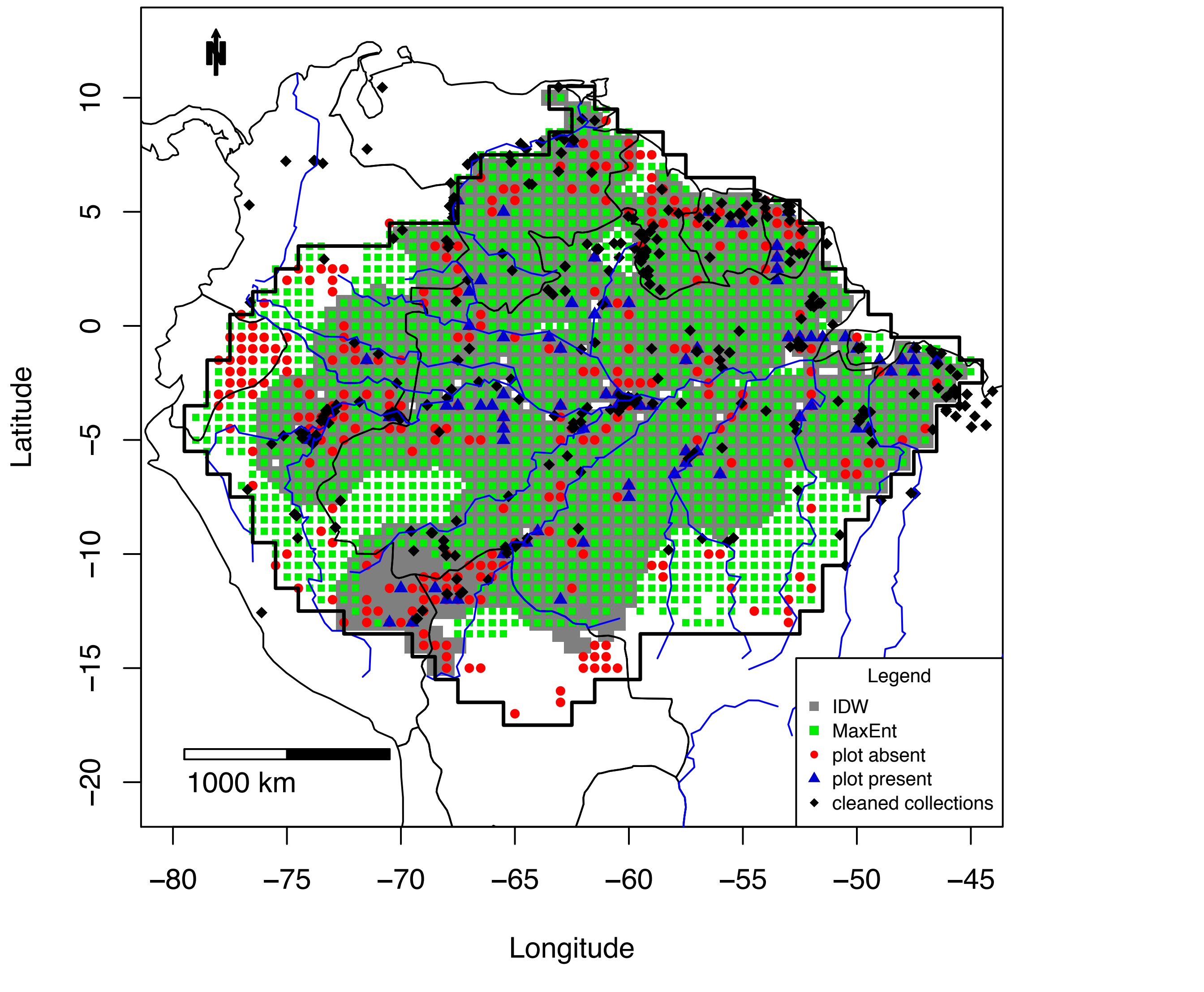

Figure S3_65B MaxEnt map of Gustavia_augusta

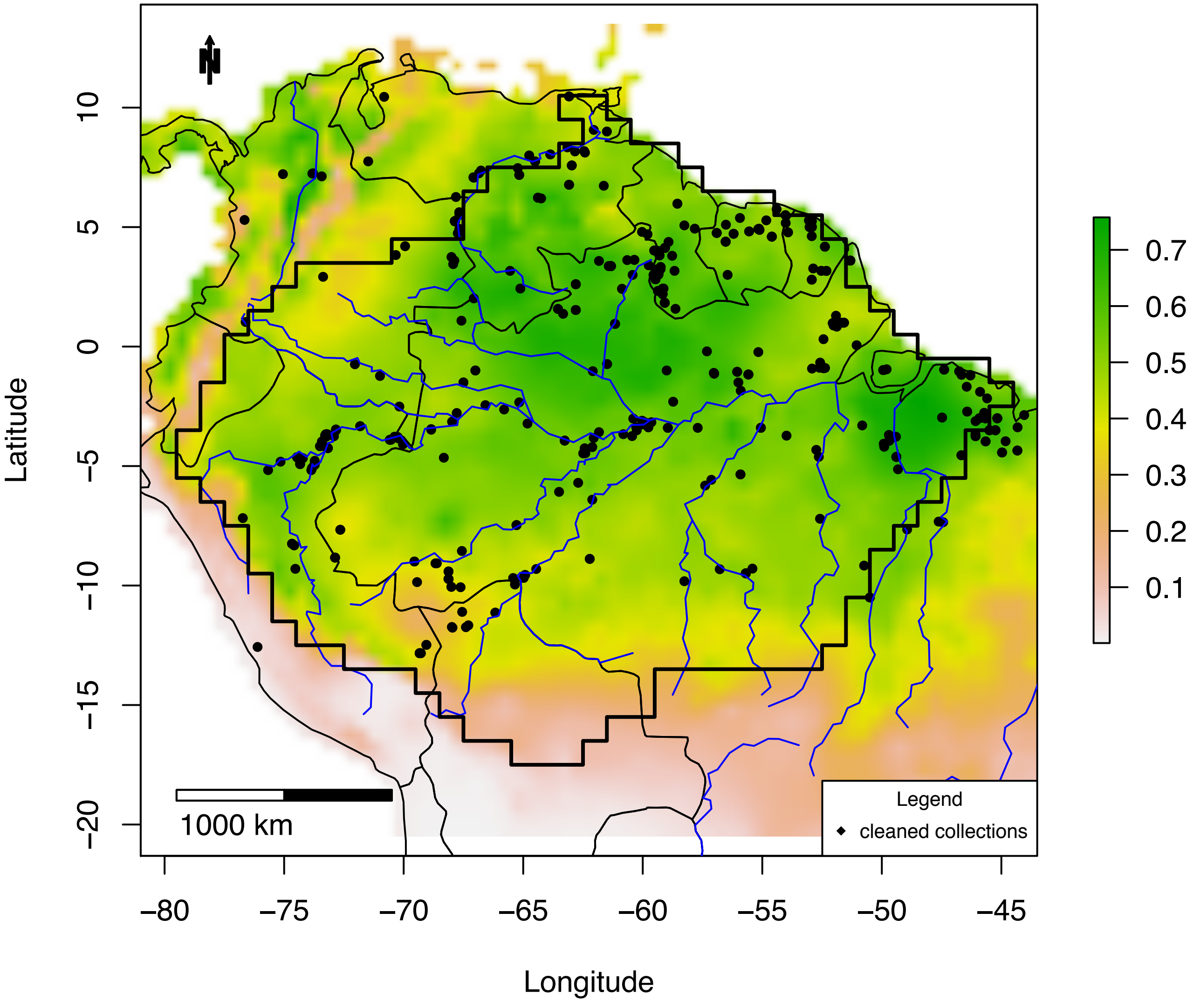

Figure S3_65C IDW map of Gustavia_augusta: plots =0 – 0.29; IDW: 0 – 0.16

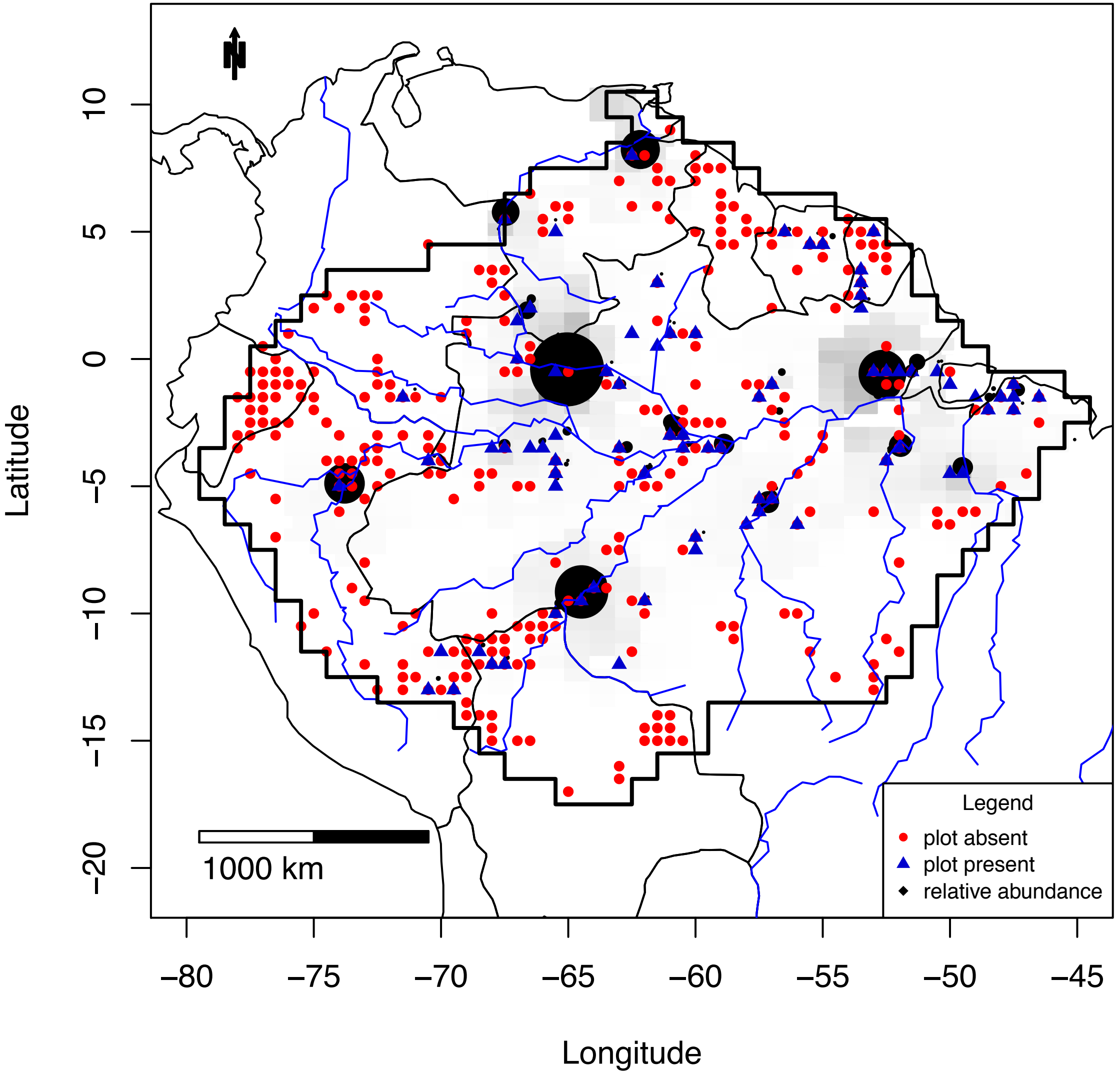

Figure S3_66A Combined map of Gustavia_hexapetala

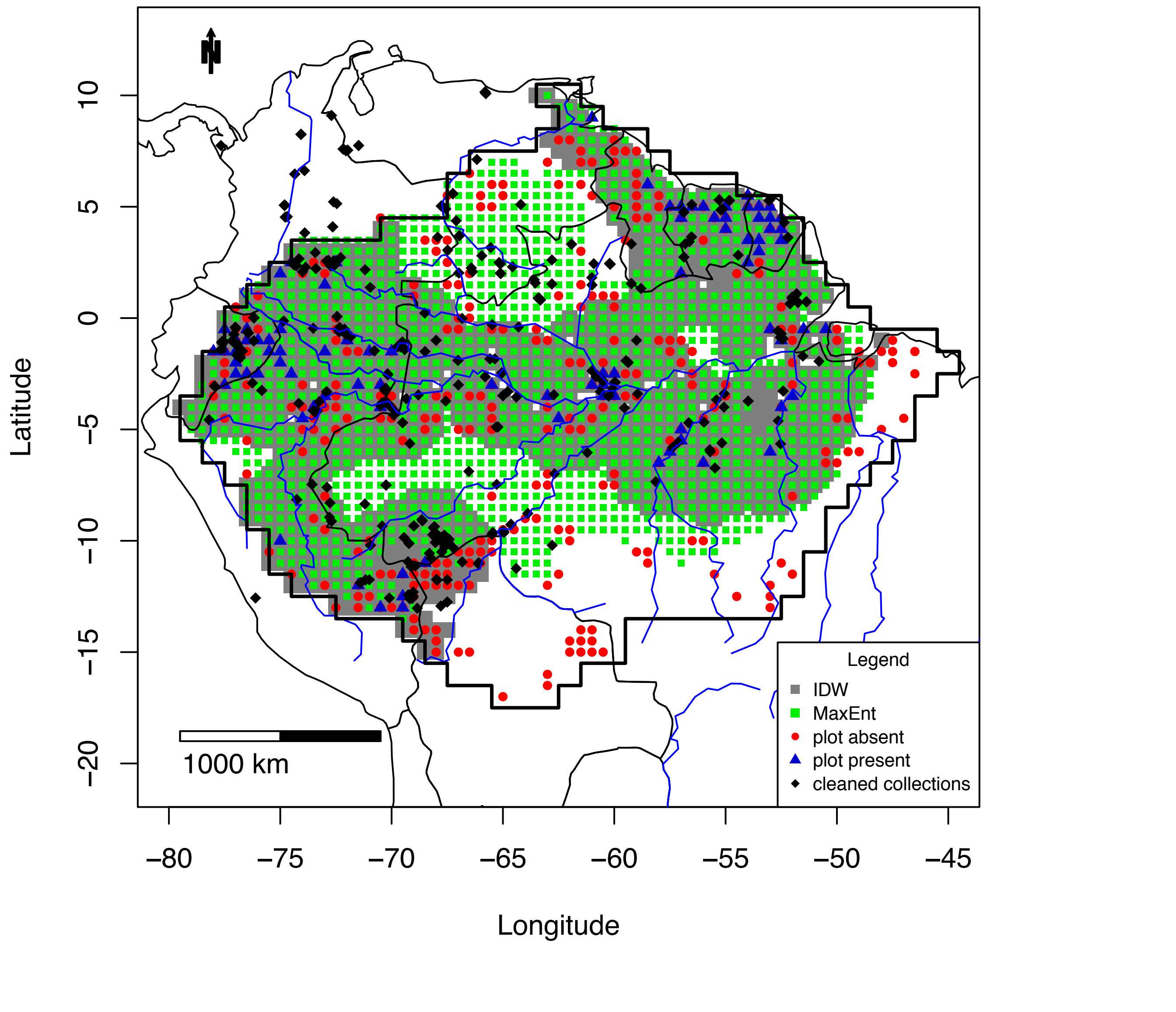

Figure S3_66B MaxEnt map of Gustavia_hexapetala

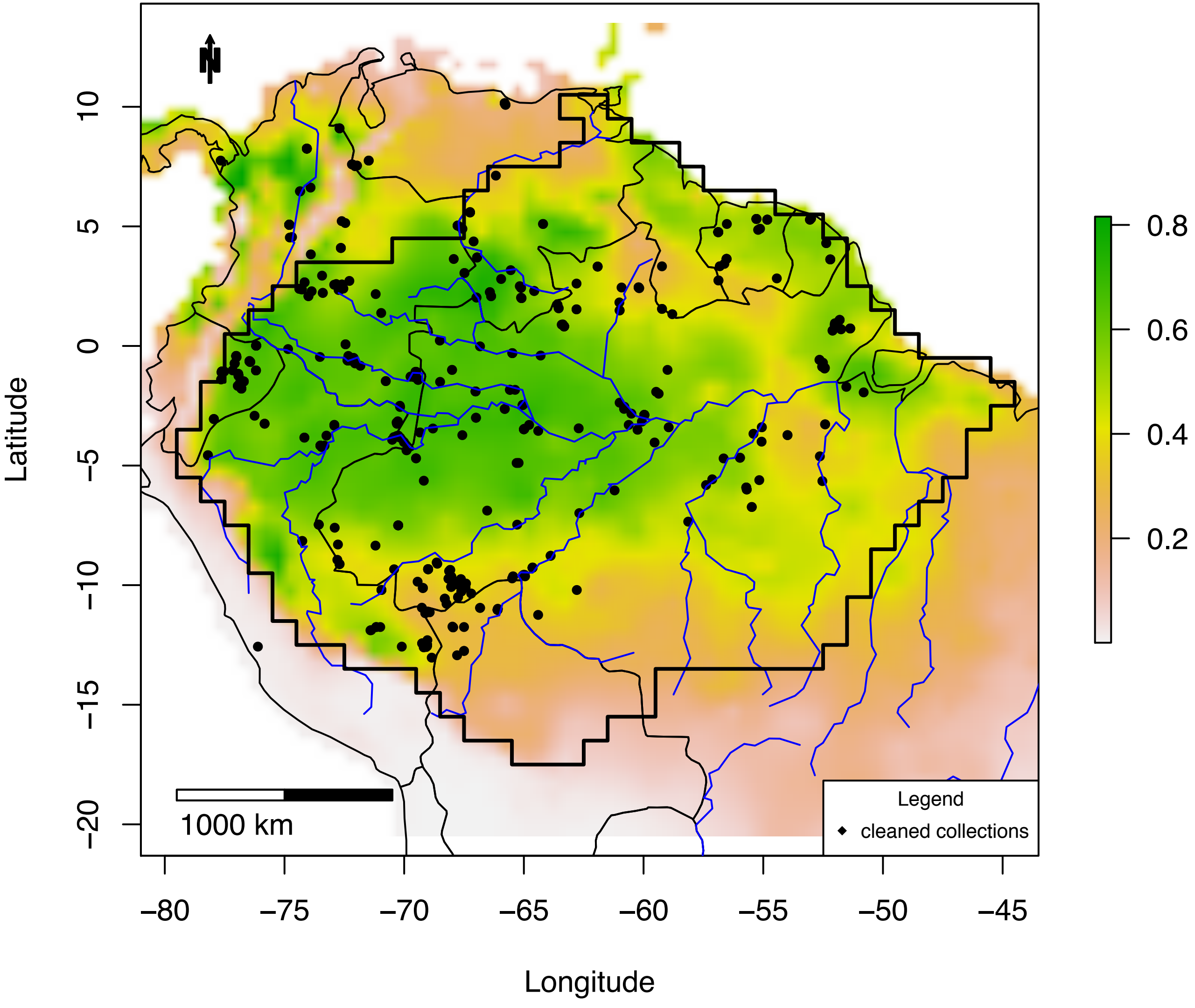

Figure S3_66C IDW map of Gustavia_hexapetala: plots =0 – 0.2; IDW: 0 – 0.08

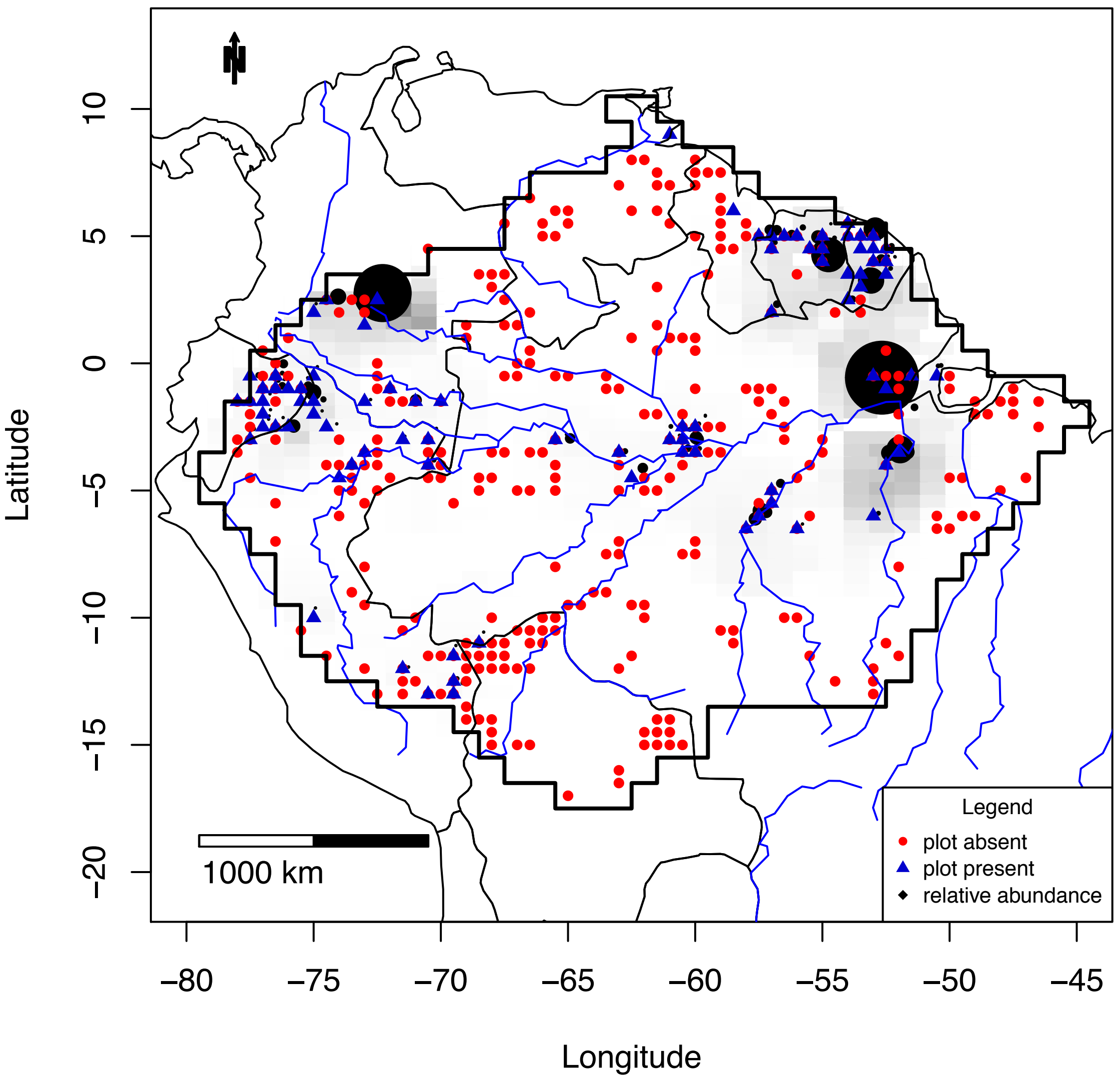

Figure S3_67A Combined map of Handroanthus_barbatus

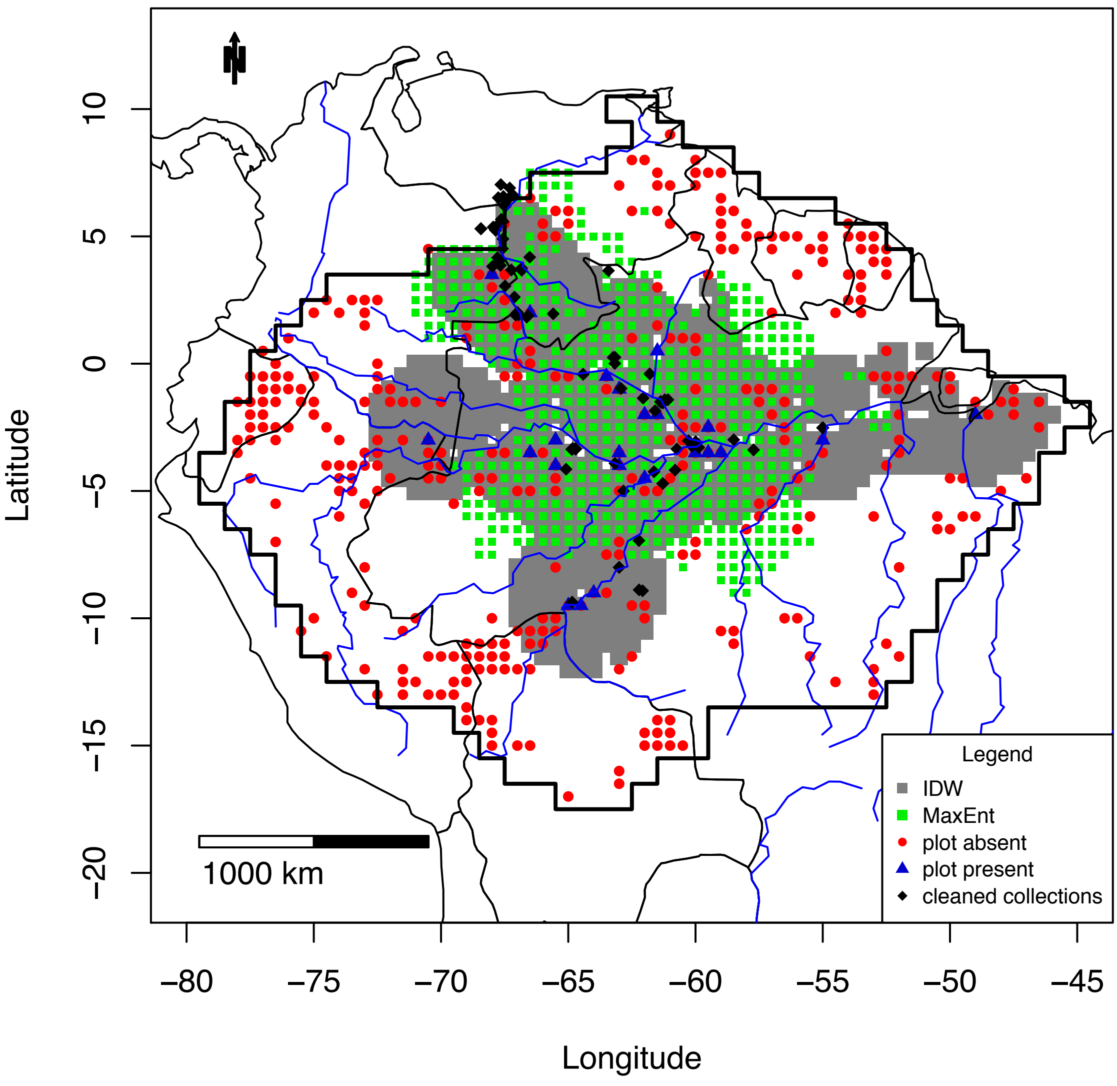

Figure S3_67B MaxEnt map of Handroanthus_barbatus

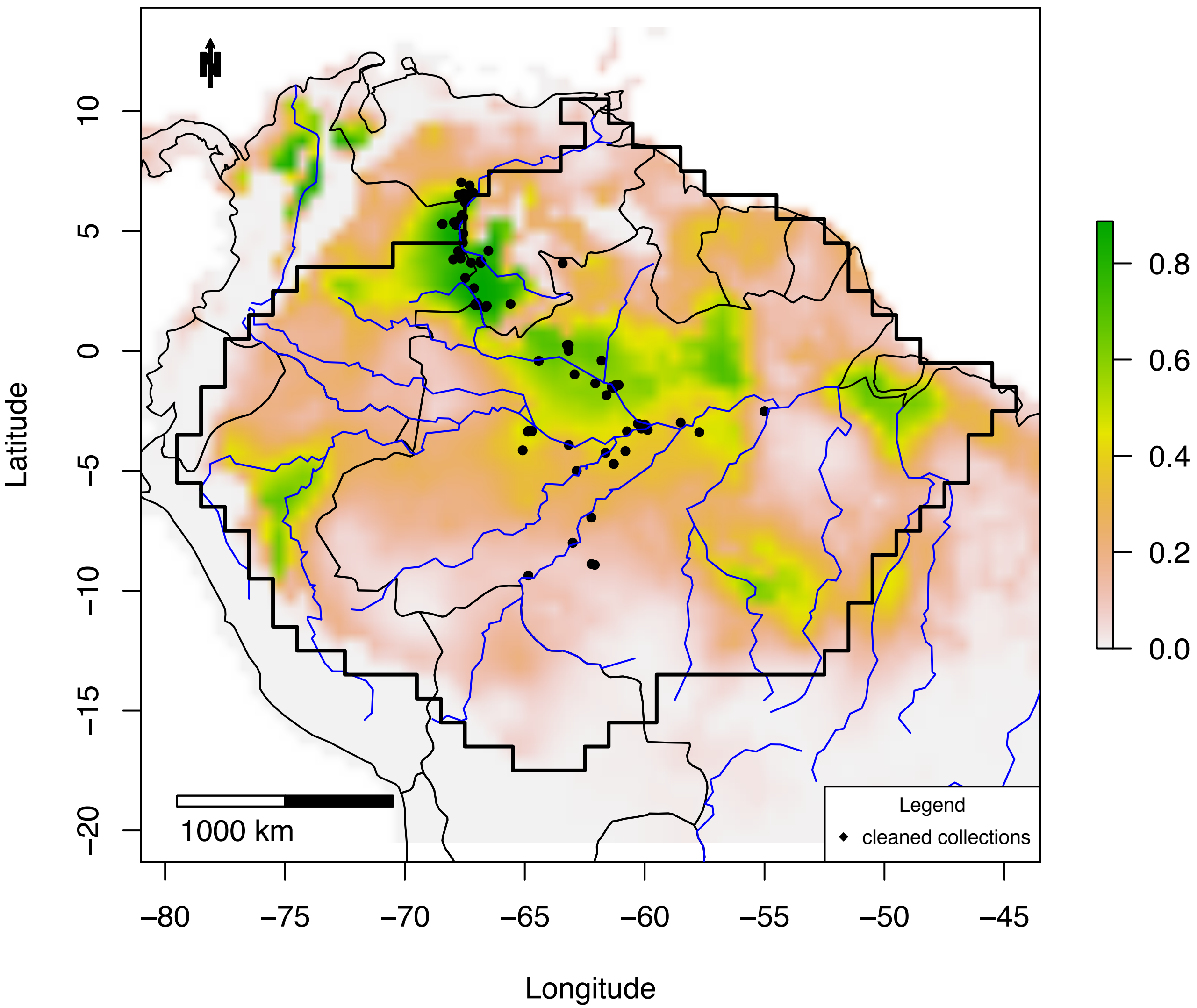

Figure S3_67C IDW map of Handroanthus_barbatus: plots =0 – 0.3; IDW: 0 – 0.06

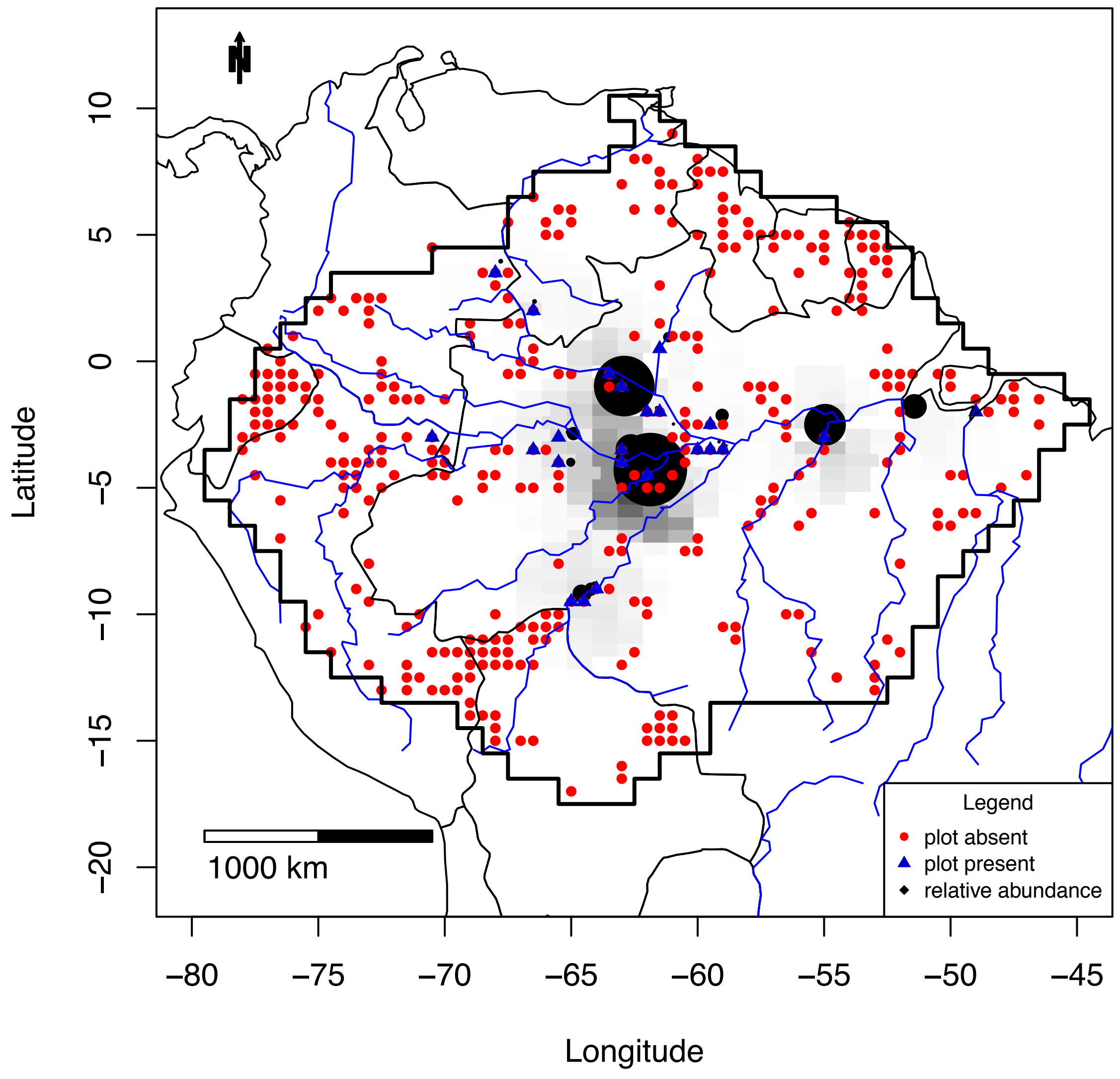

Figure S3_68A Combined map of Haploclathra_cordata

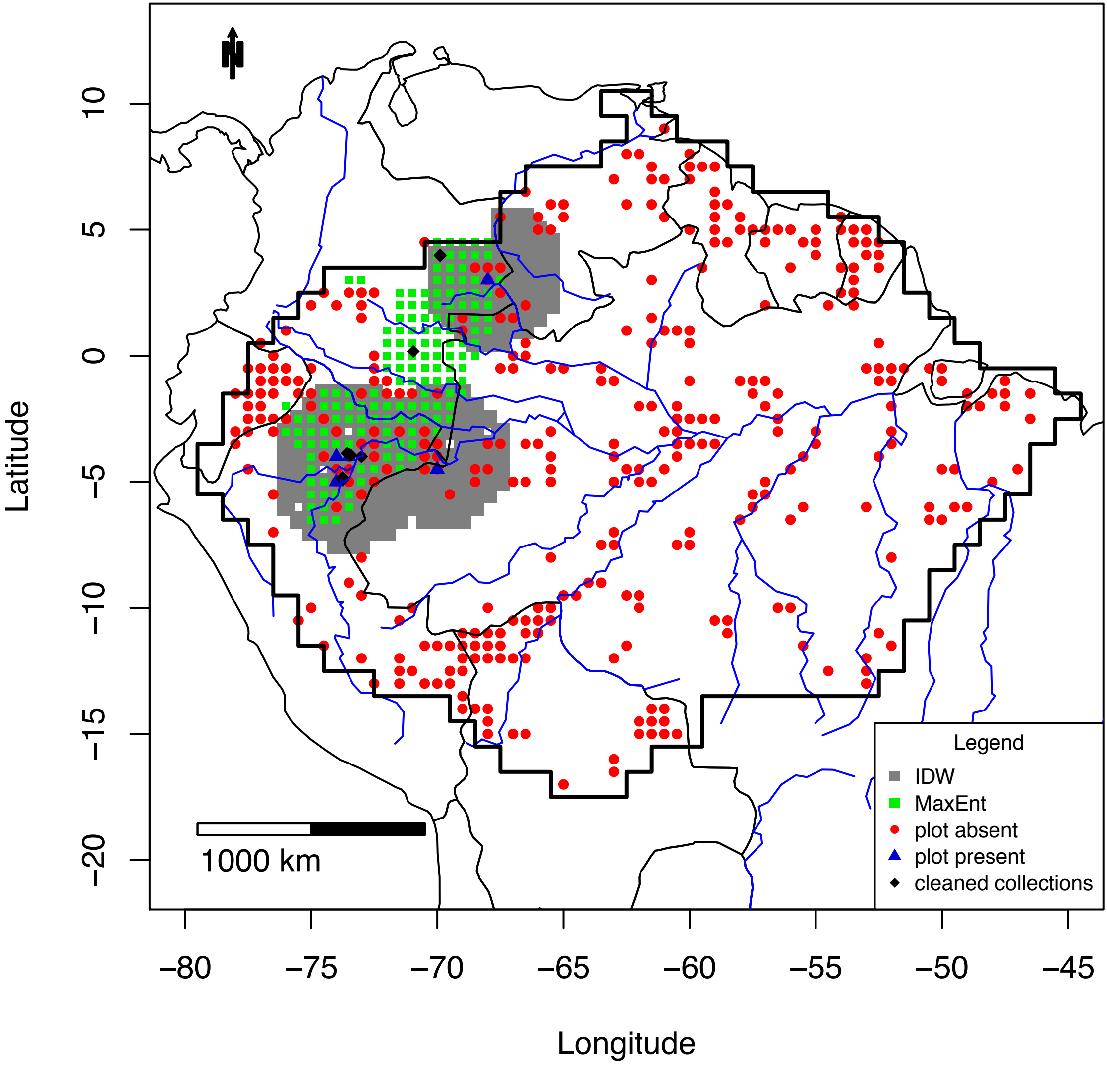

Figure S3_68B MaxEnt map of Haploclathra_cordata

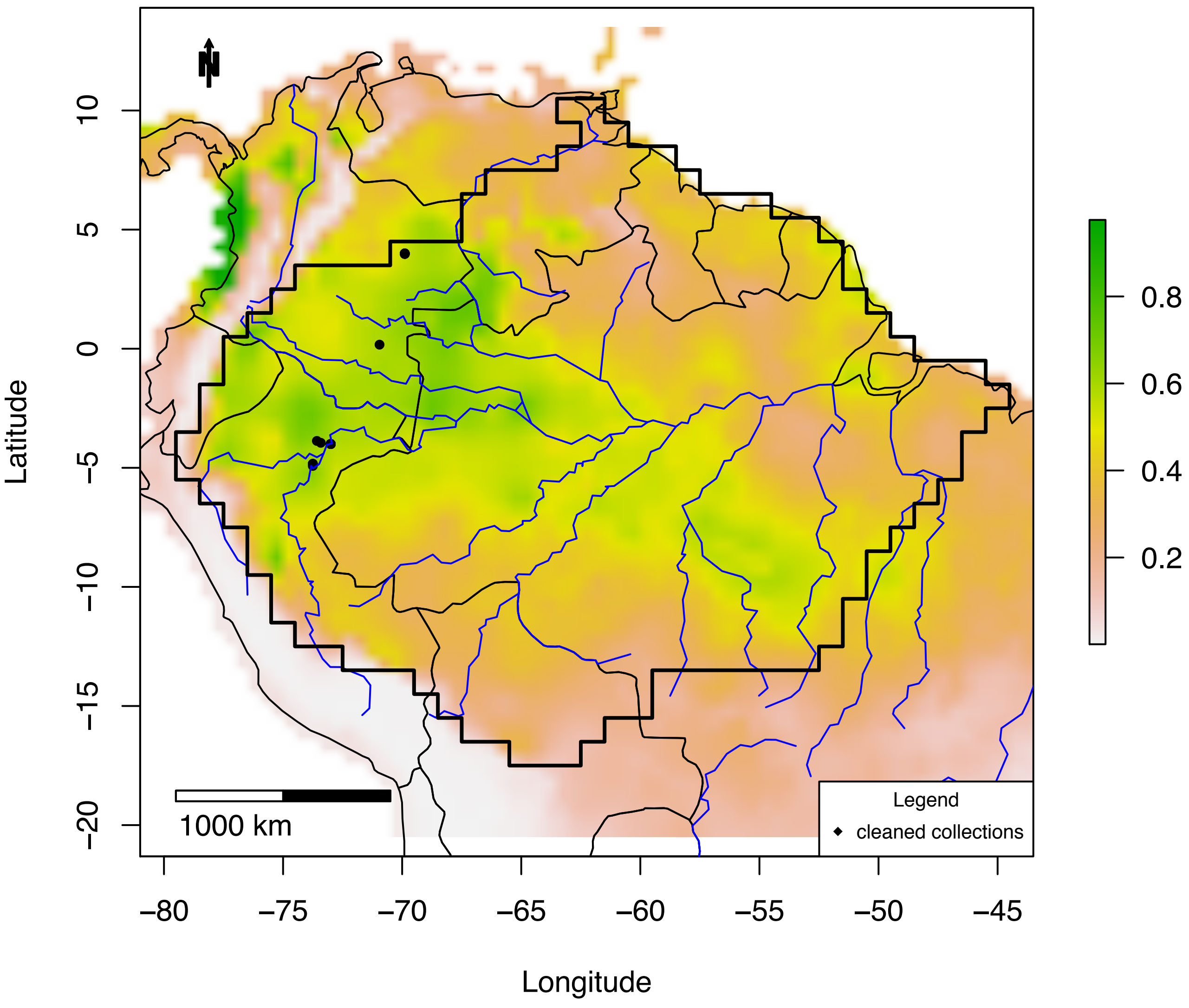

Figure S3_68C IDW map of Haploclathra_cordata: plots =0 – 0.42; IDW: 0 – 0.24

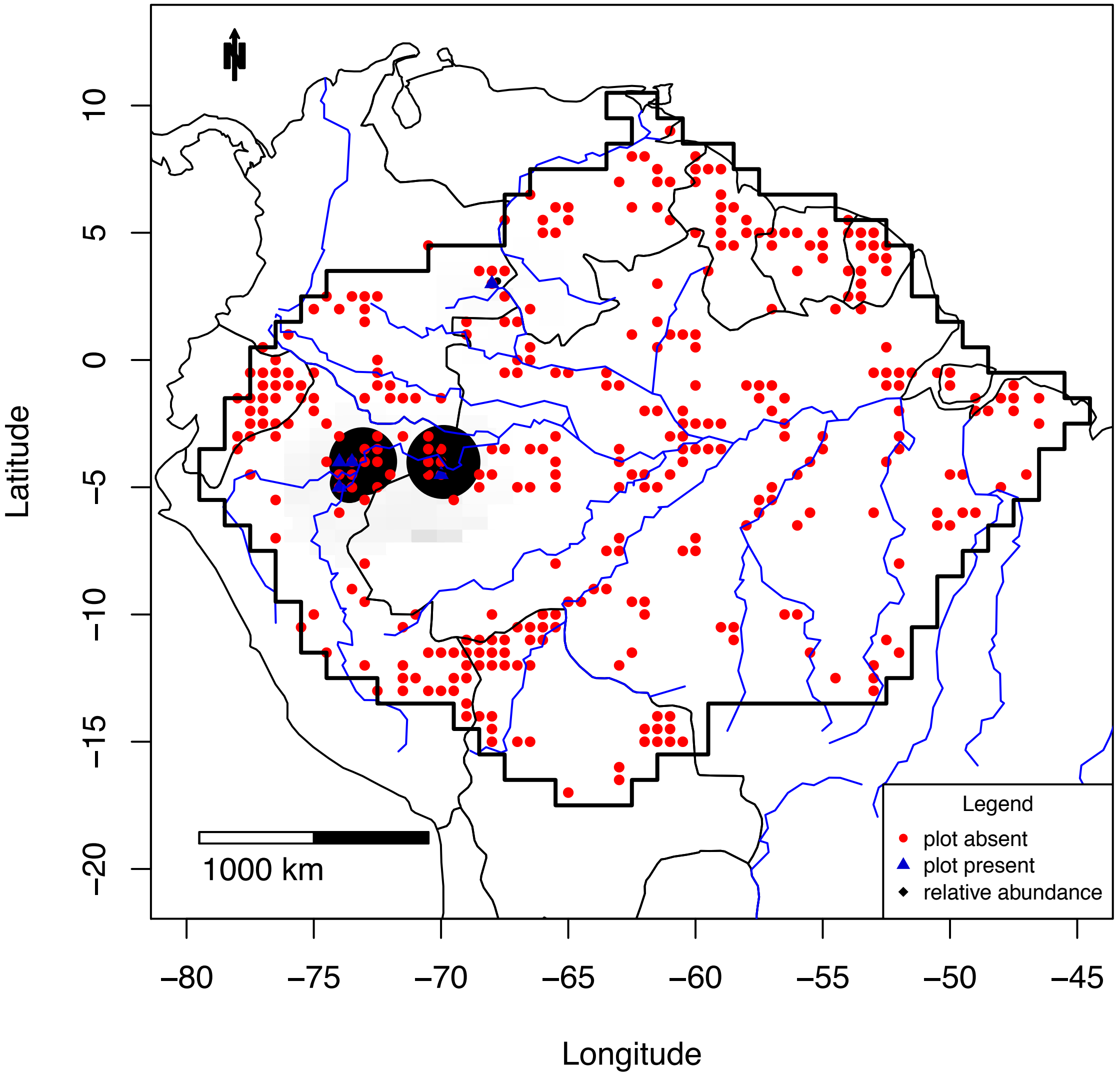

Figure S3_69A Combined map of Hevea_guianensis

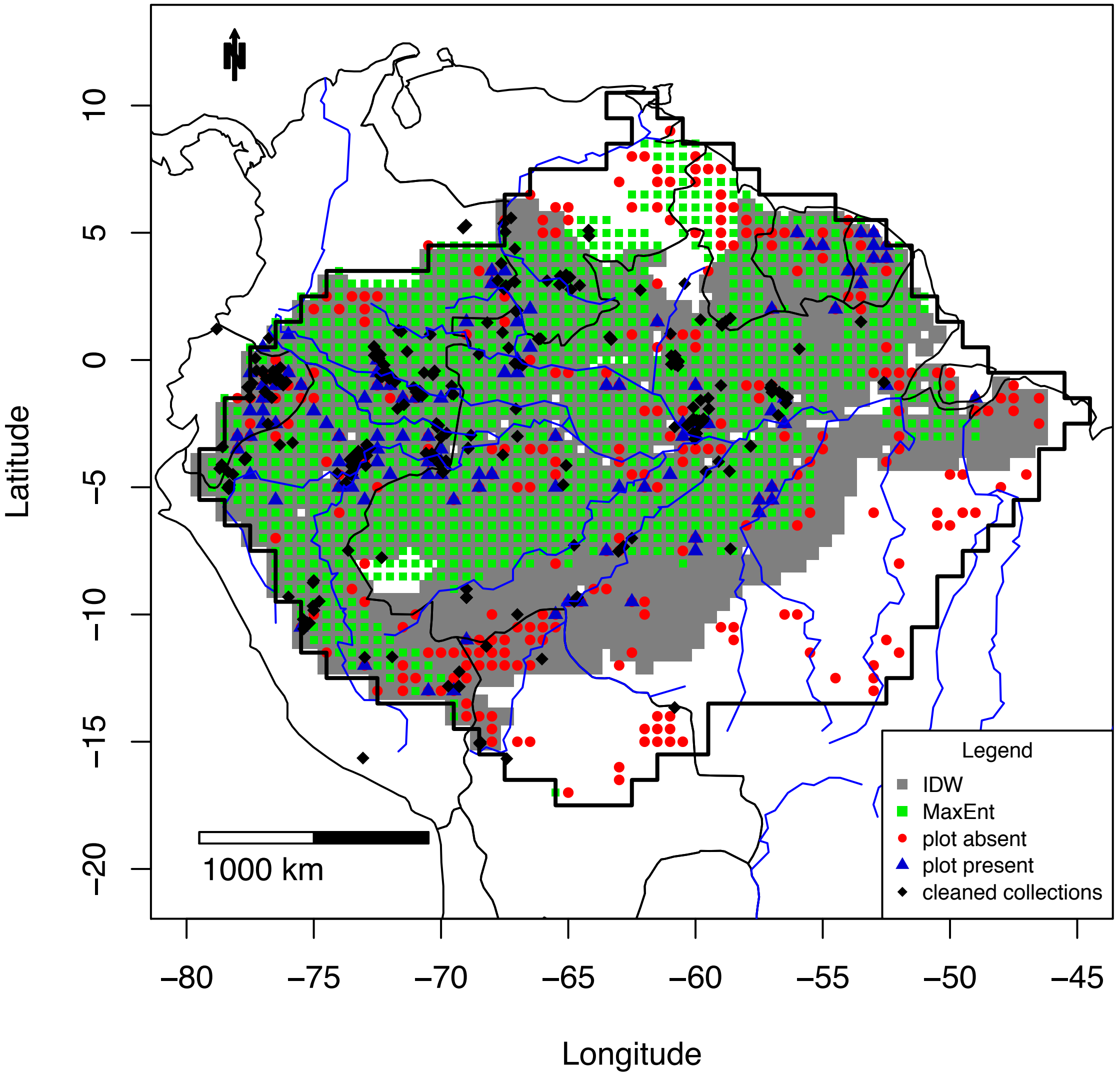

Figure S3_69B MaxEnt map of Hevea_guianensis

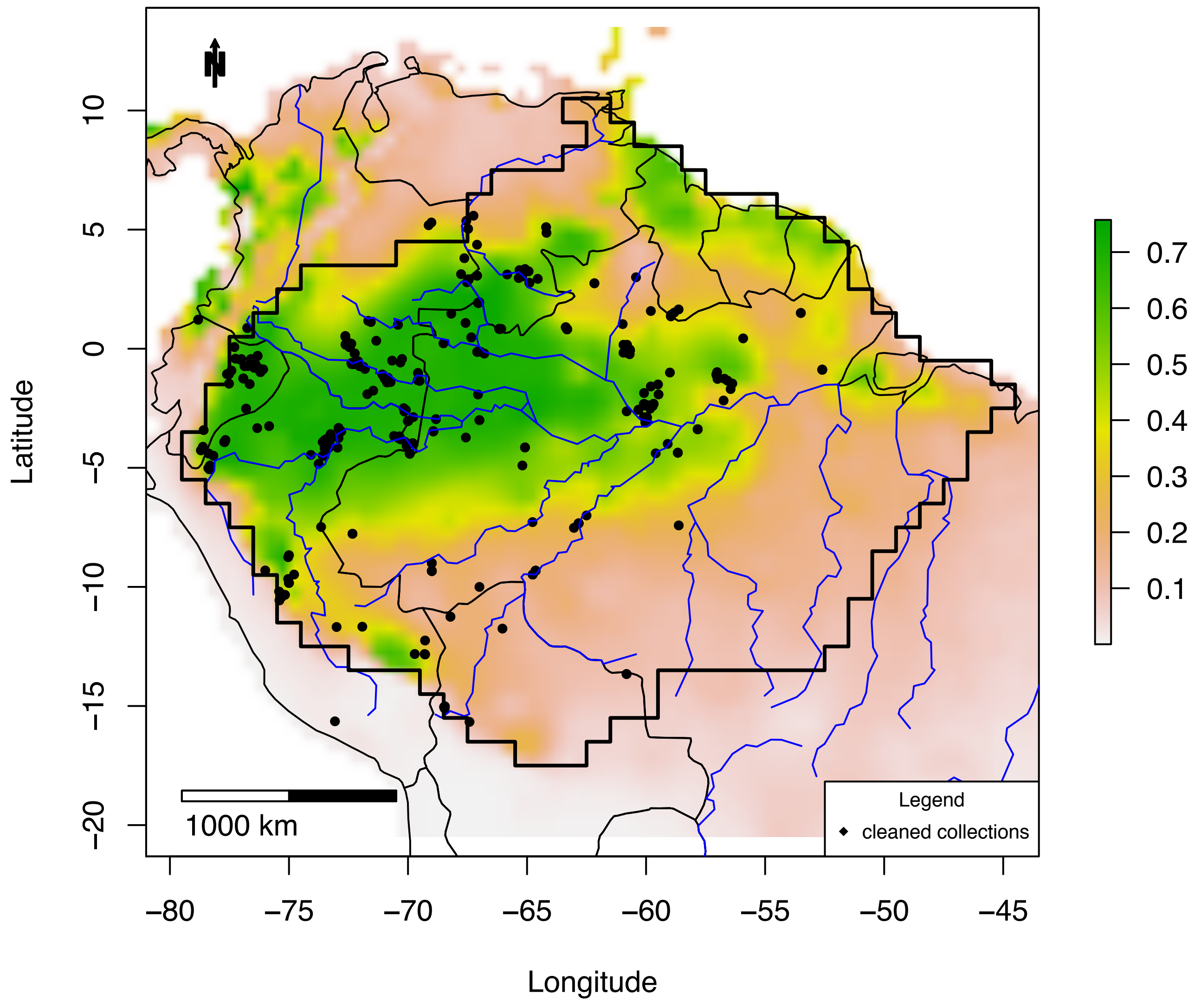

Figure S3_69C IDW map of Hevea_guianensis: plots =0 – 0.08; IDW: 0 – 0.05

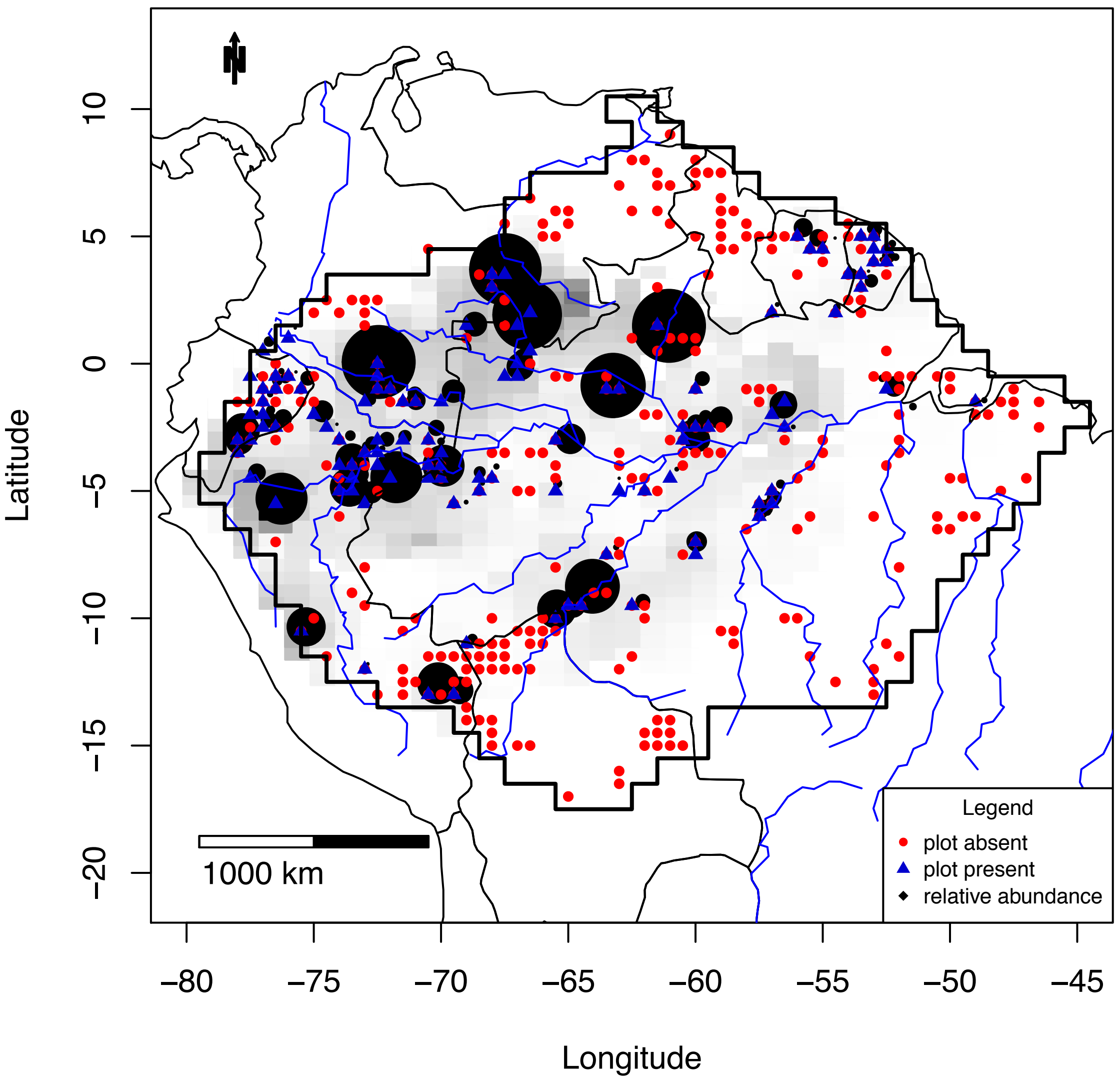

Figure S3_70A Combined map of Inga_lateriflora

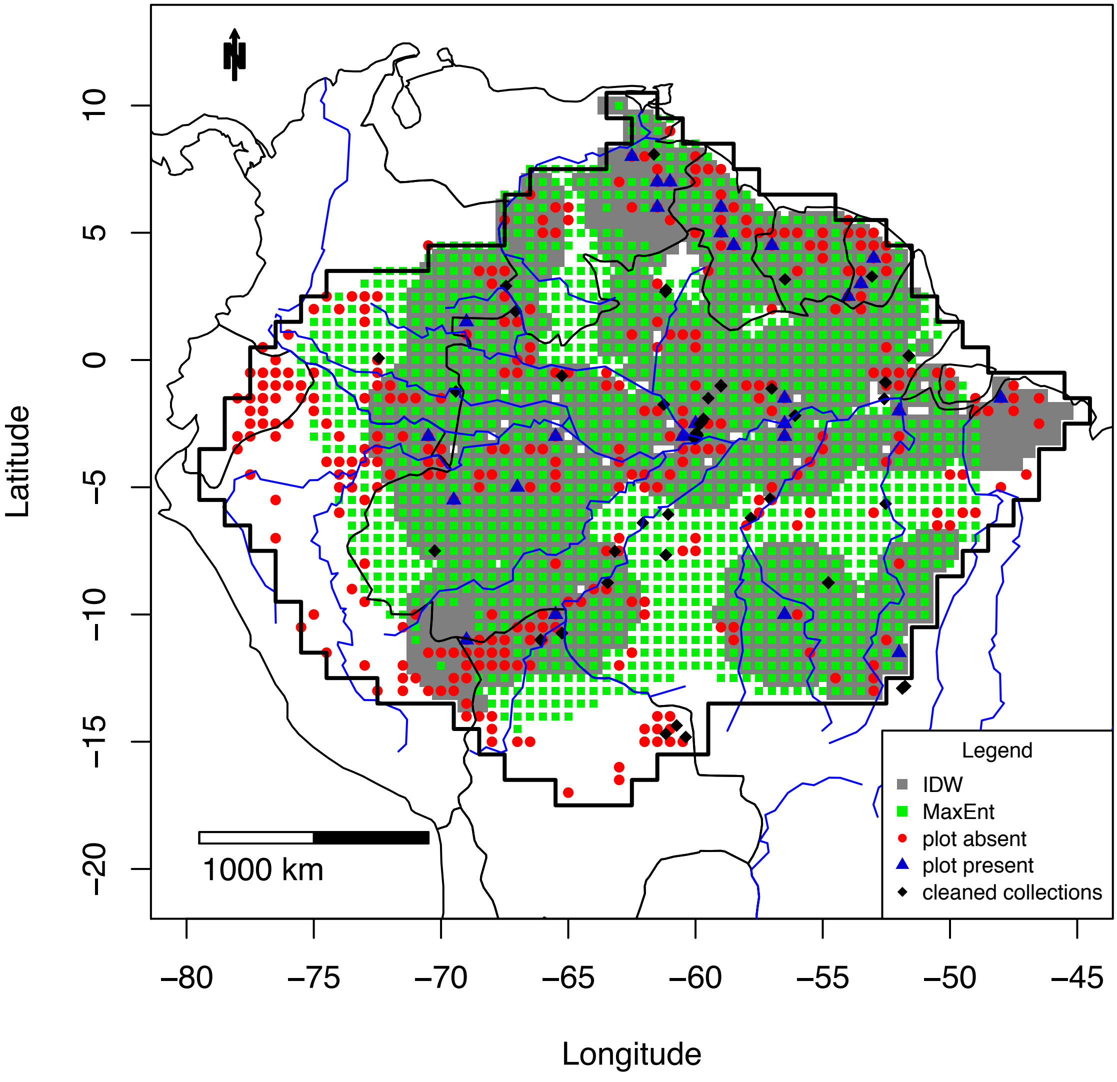

Figure S3_70B MaxEnt map of Inga_lateriflora

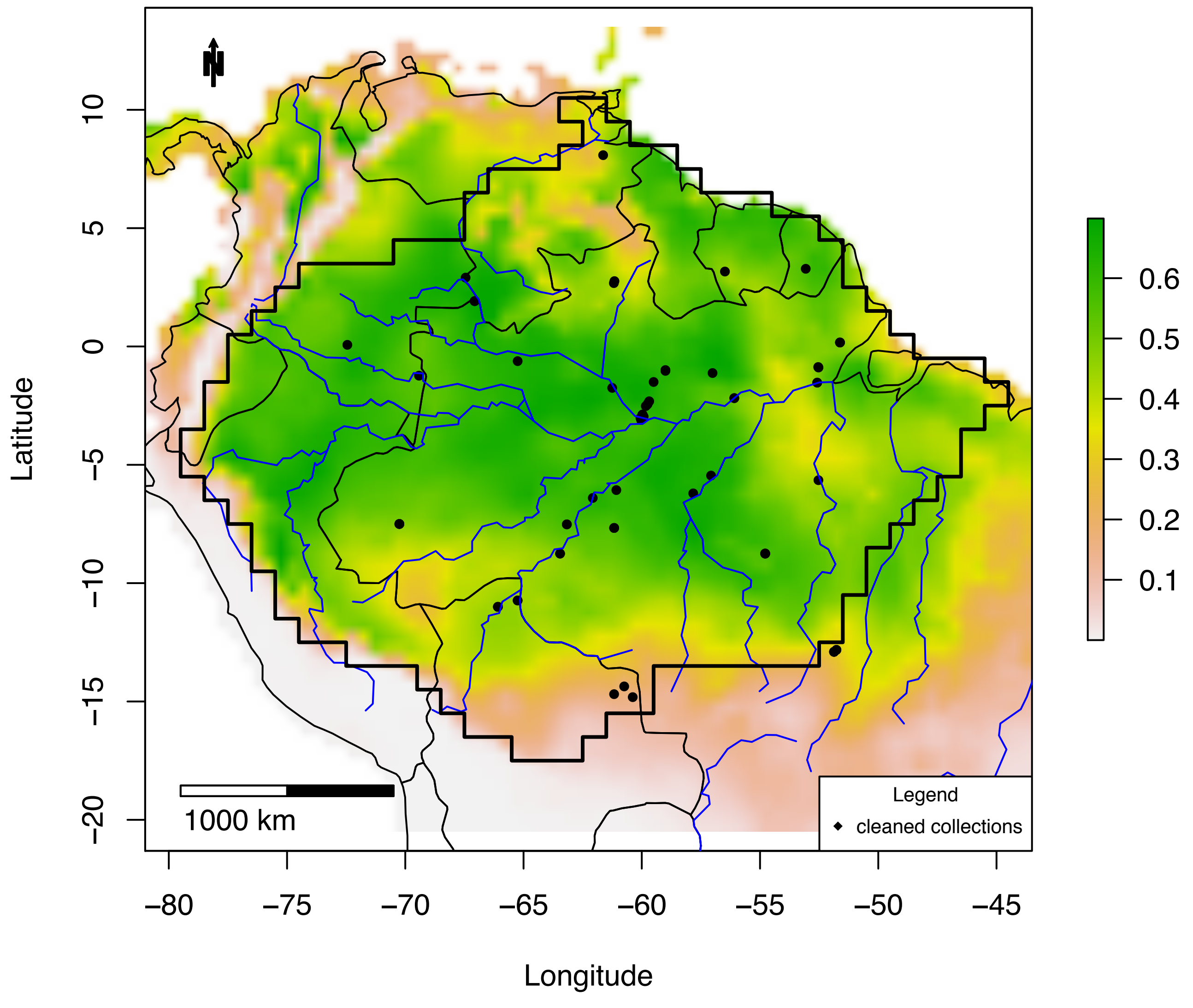

Figure S3_70C IDW map of Inga_lateriflora:
plots =0 – 0.12; IDW: 0 – 0.09

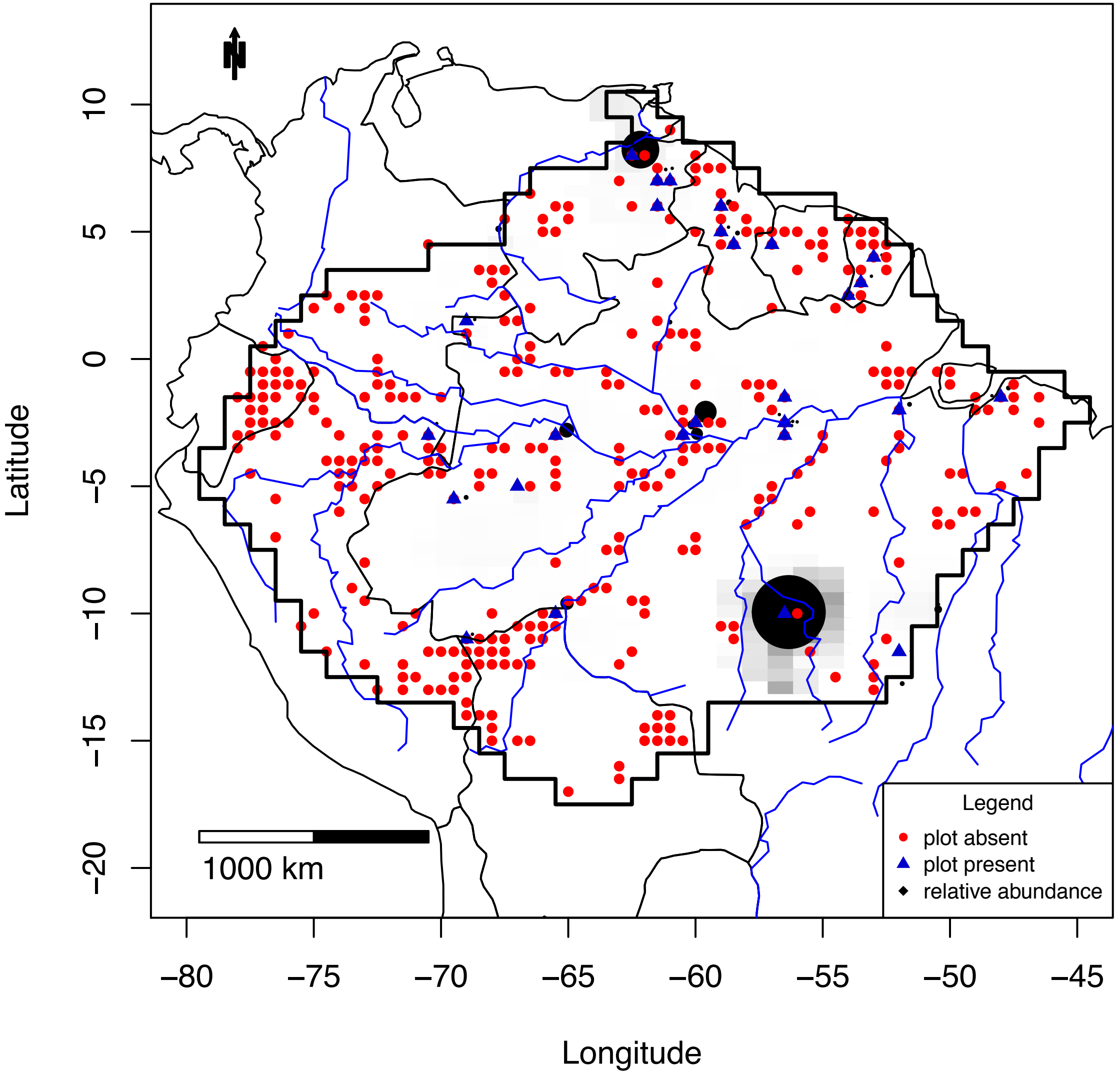

Figure S3_71A Combined map of Inga_marginata

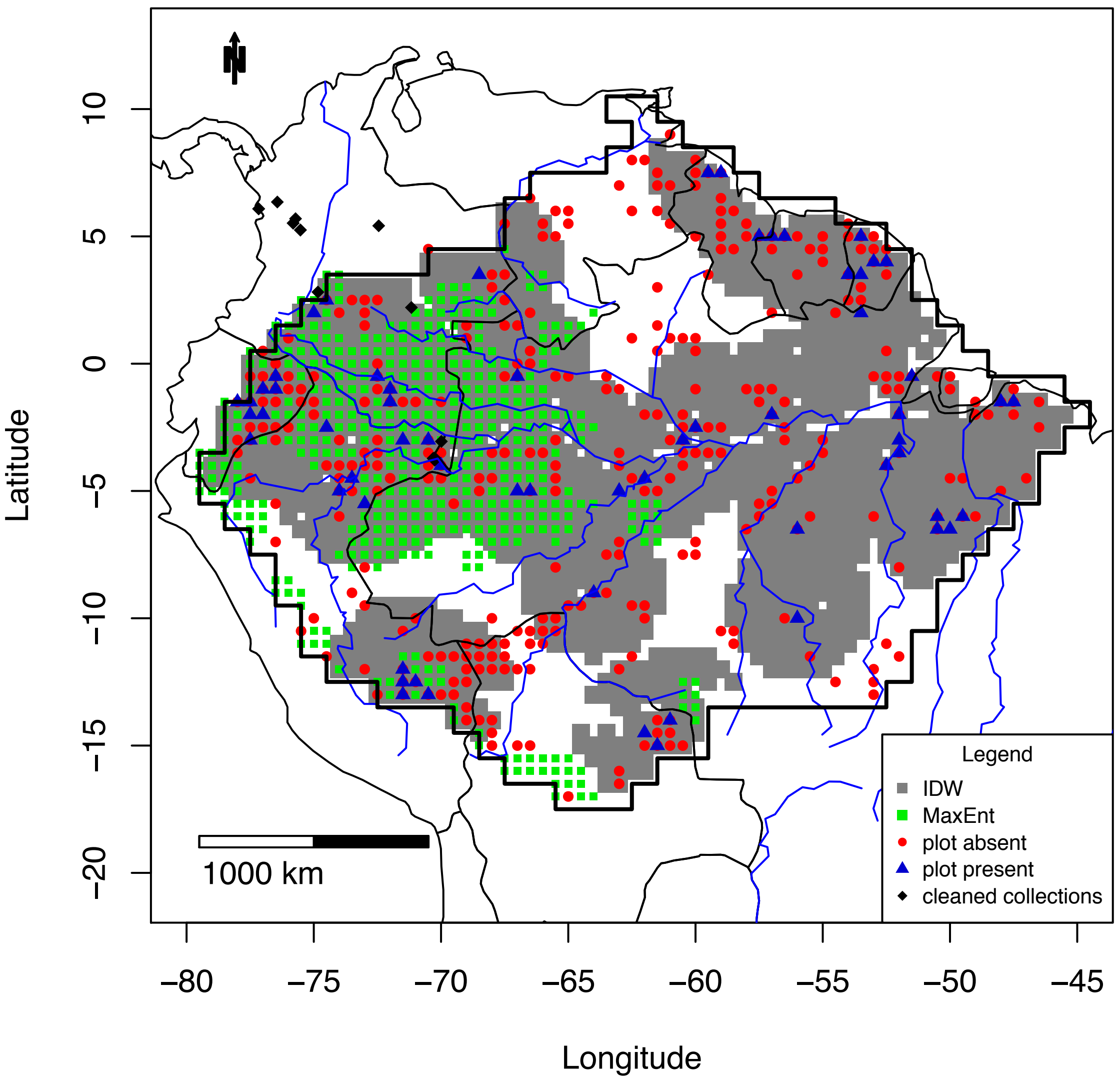

Figure S3_71B MaxEnt map of Inga_marginata

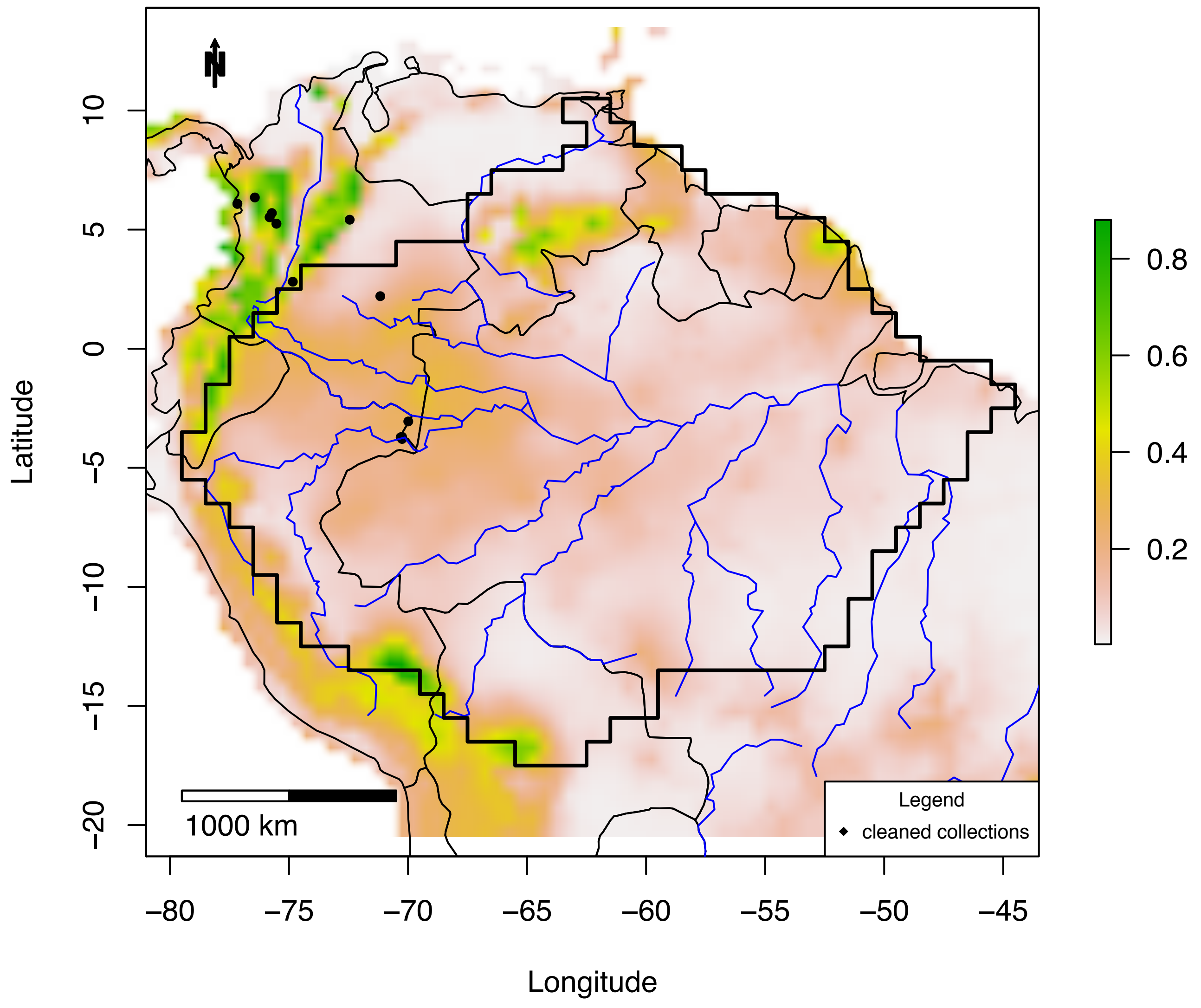

Figure S3_71C IDW map of Inga_marginata: plots =0 – 0.1; IDW: 0 – 0.02

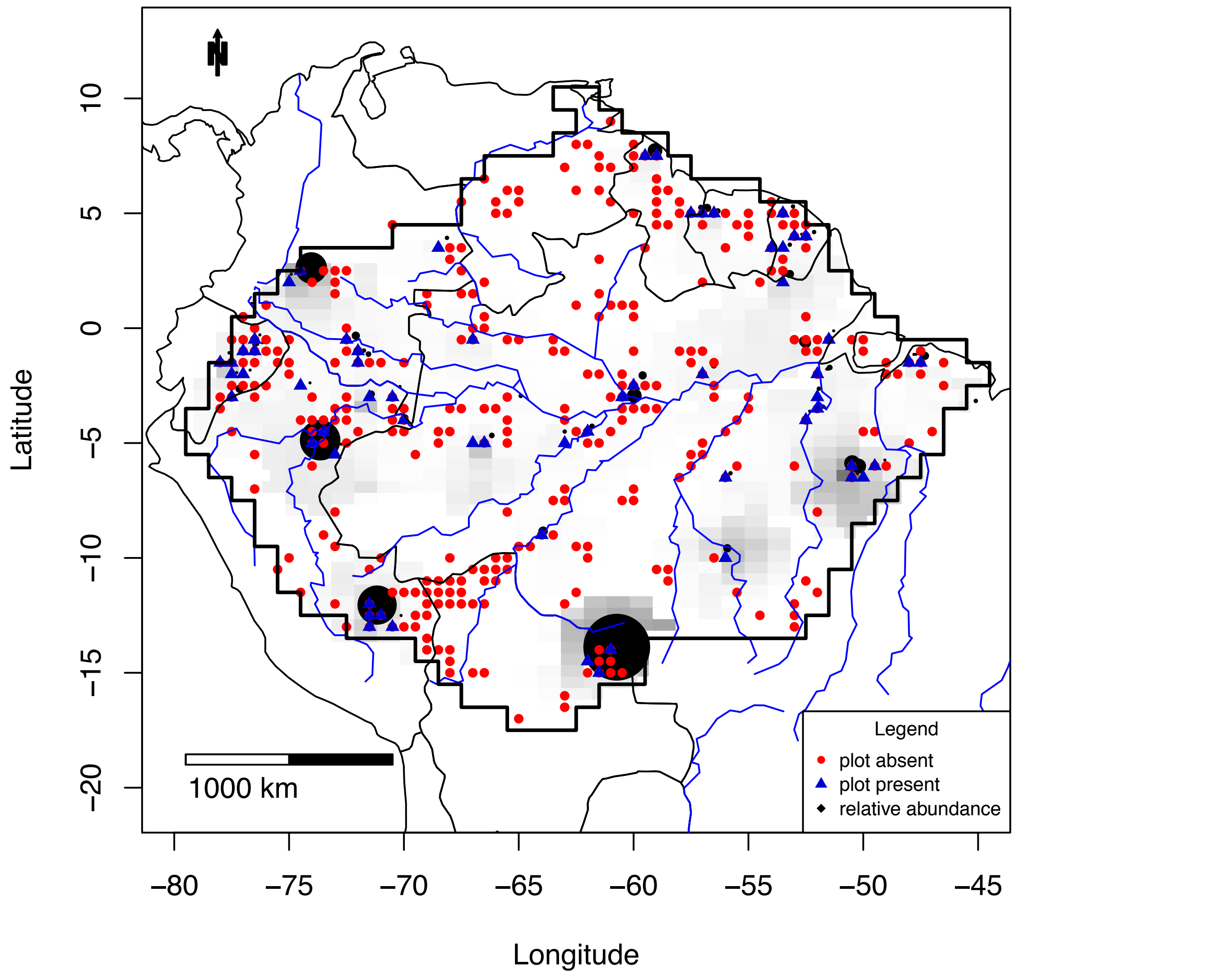

Figure S3_72A Combined map of Iriartea_deltoidea

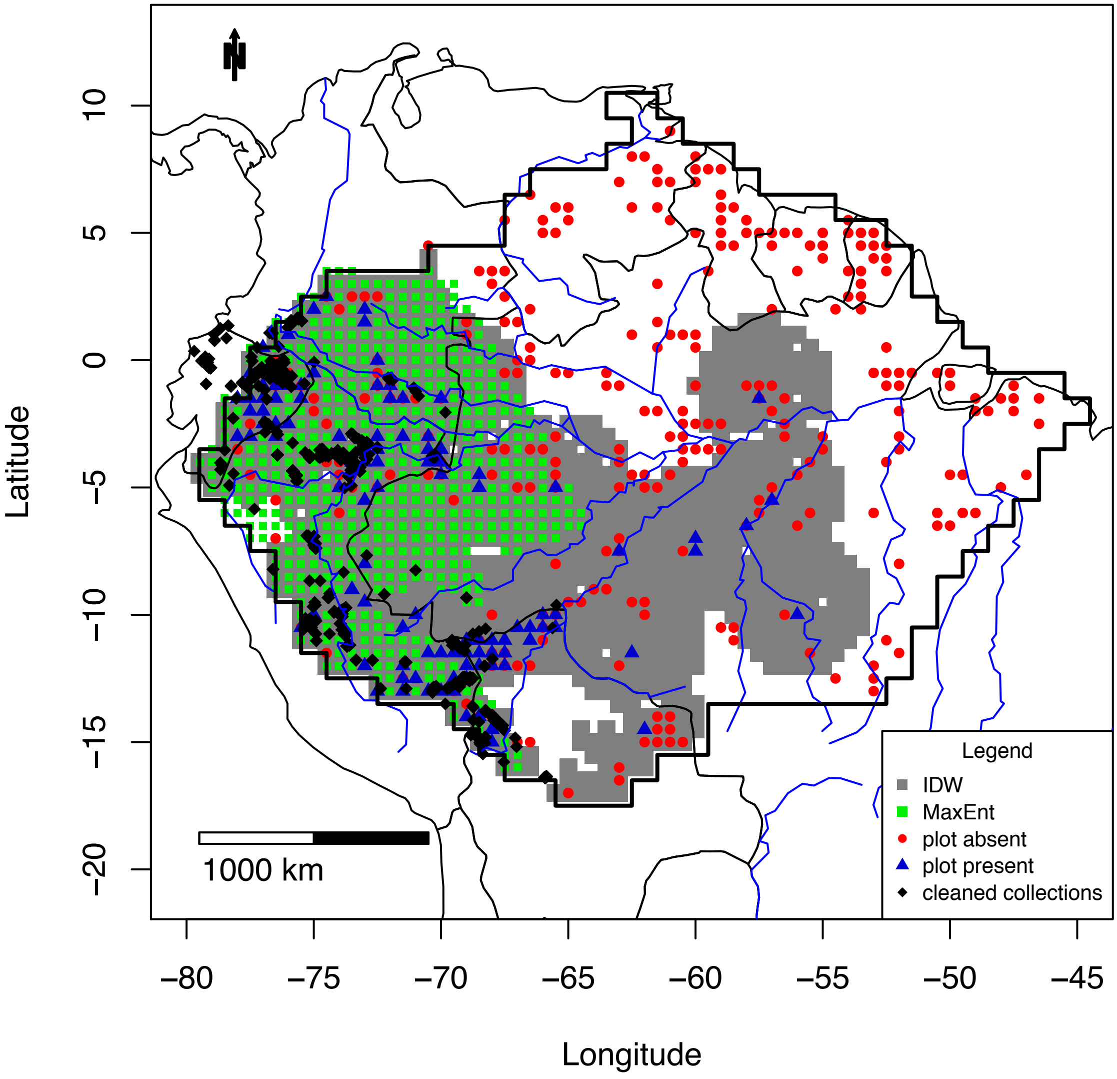

Figure S3_72B MaxEnt map of Iriartea_deltoidea

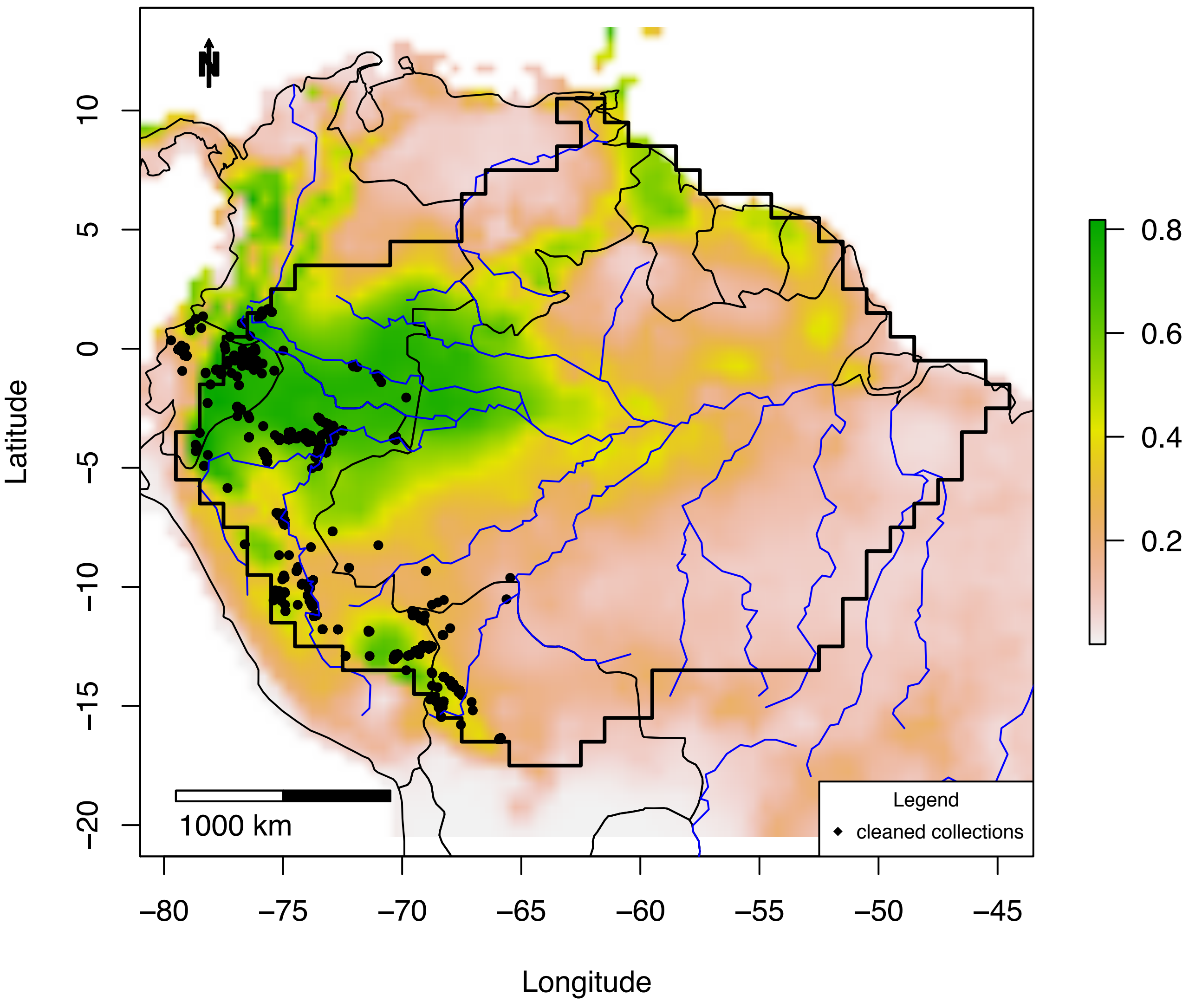

Figure S3_72C IDW map of Iriartea_deltoidea: plots =0 – 0.39; IDW: 0 – 0.27

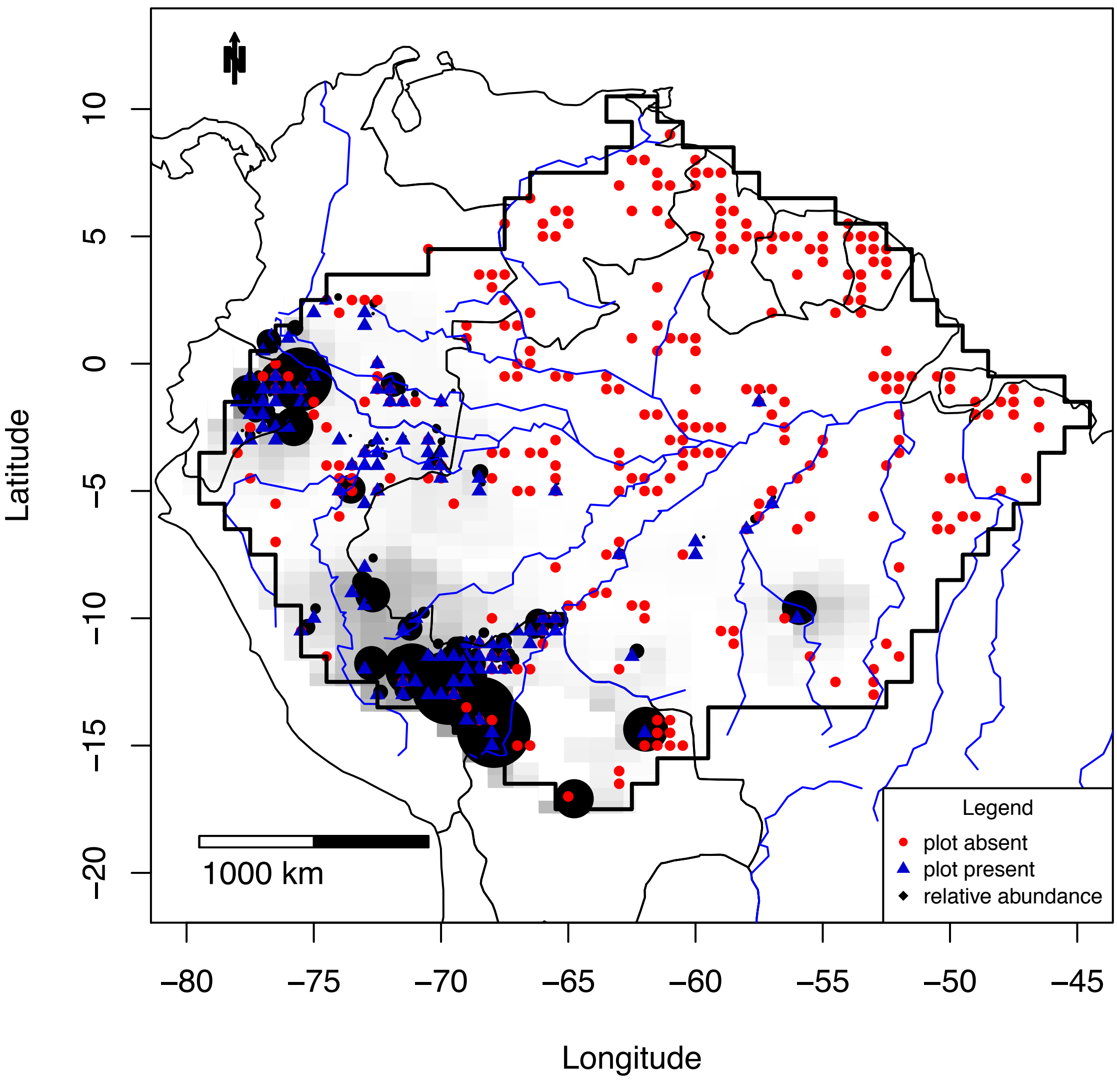

Figure S3_73A Combined map of Iryanthera_juruensis

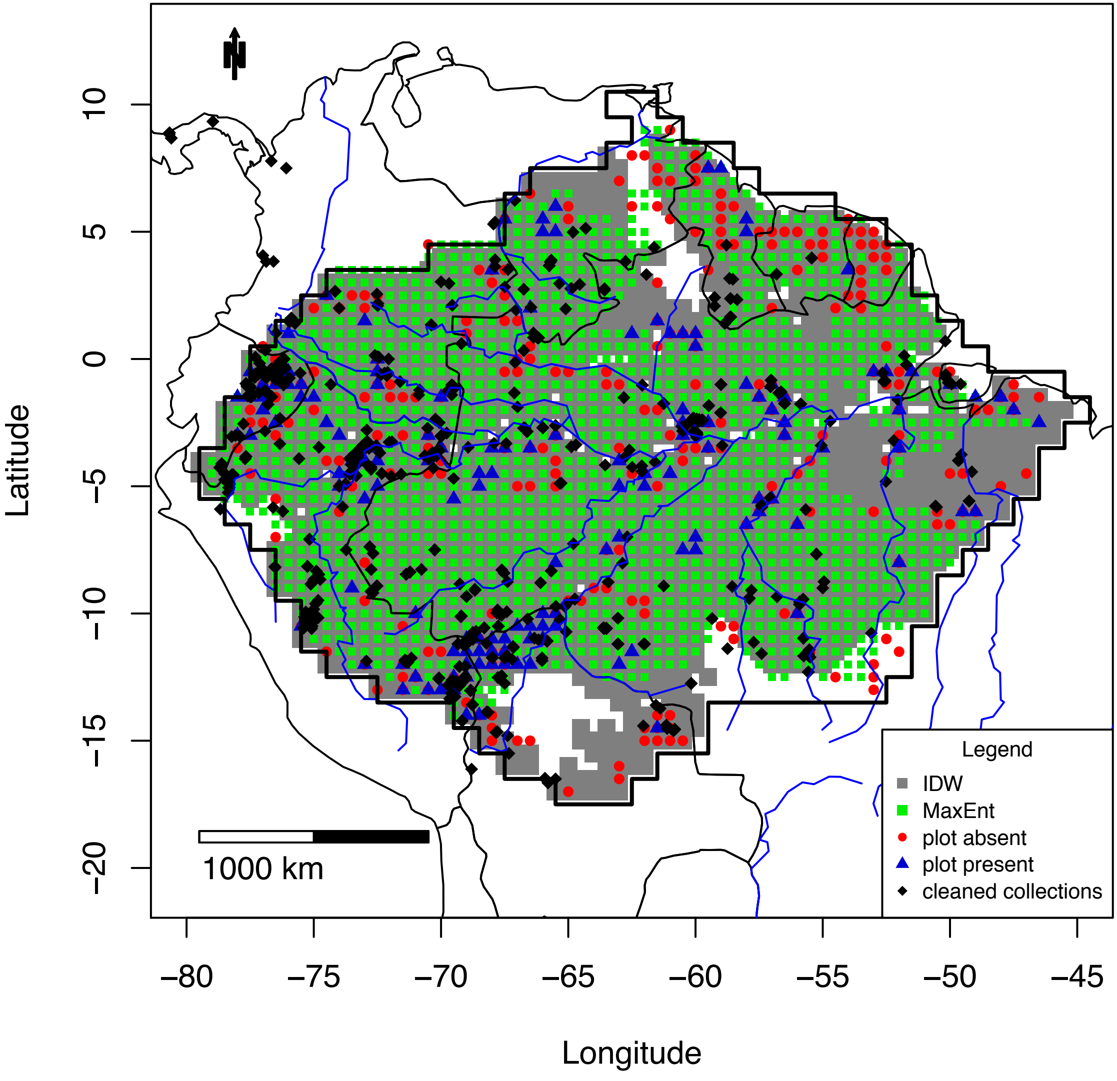

Figure S3_73B MaxEnt map of Iryanthera_juruensis

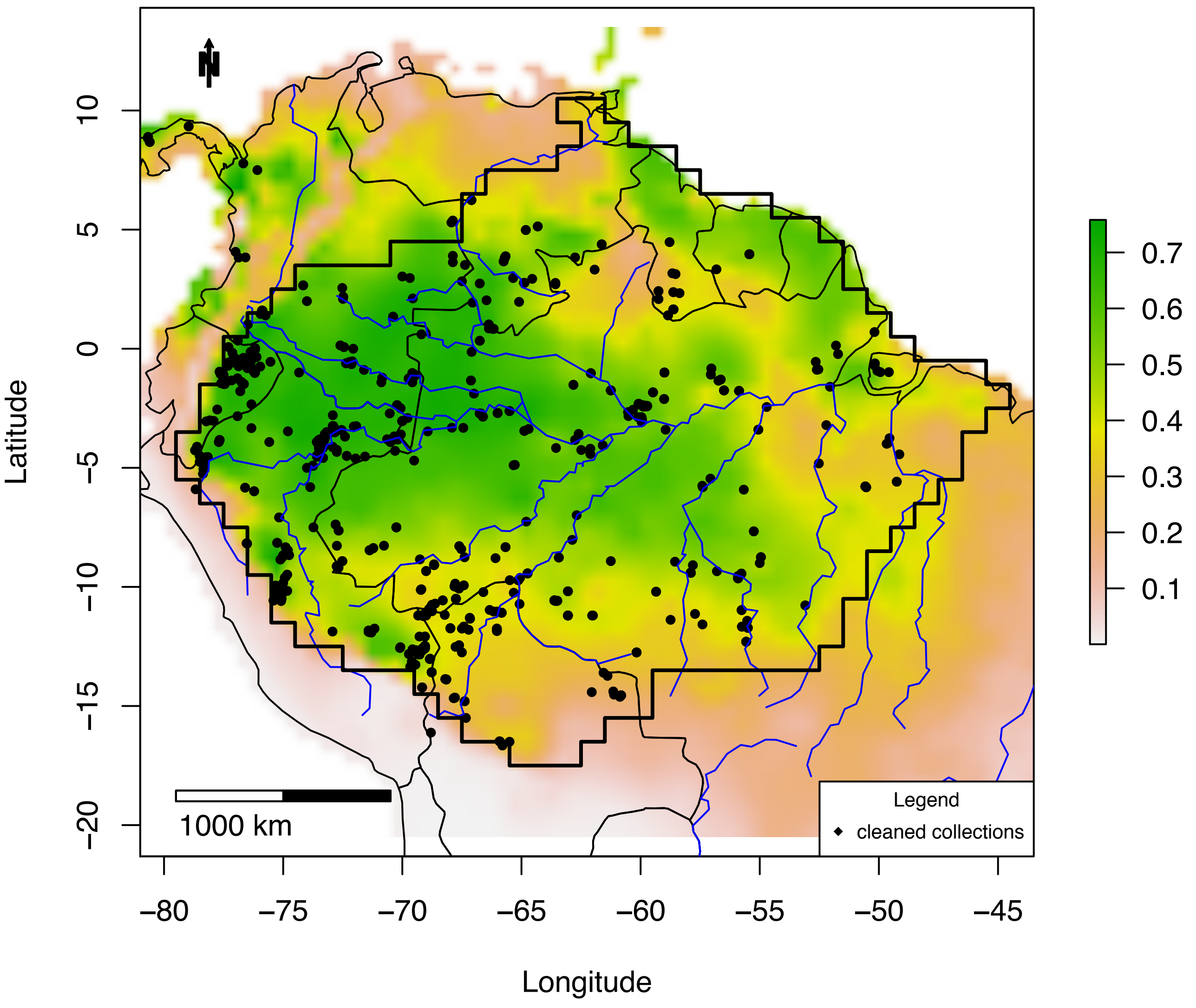

Figure S3_73C IDW map of Iryanthera_juruensis: plots =0 – 0.15; IDW: 0 – 0.08

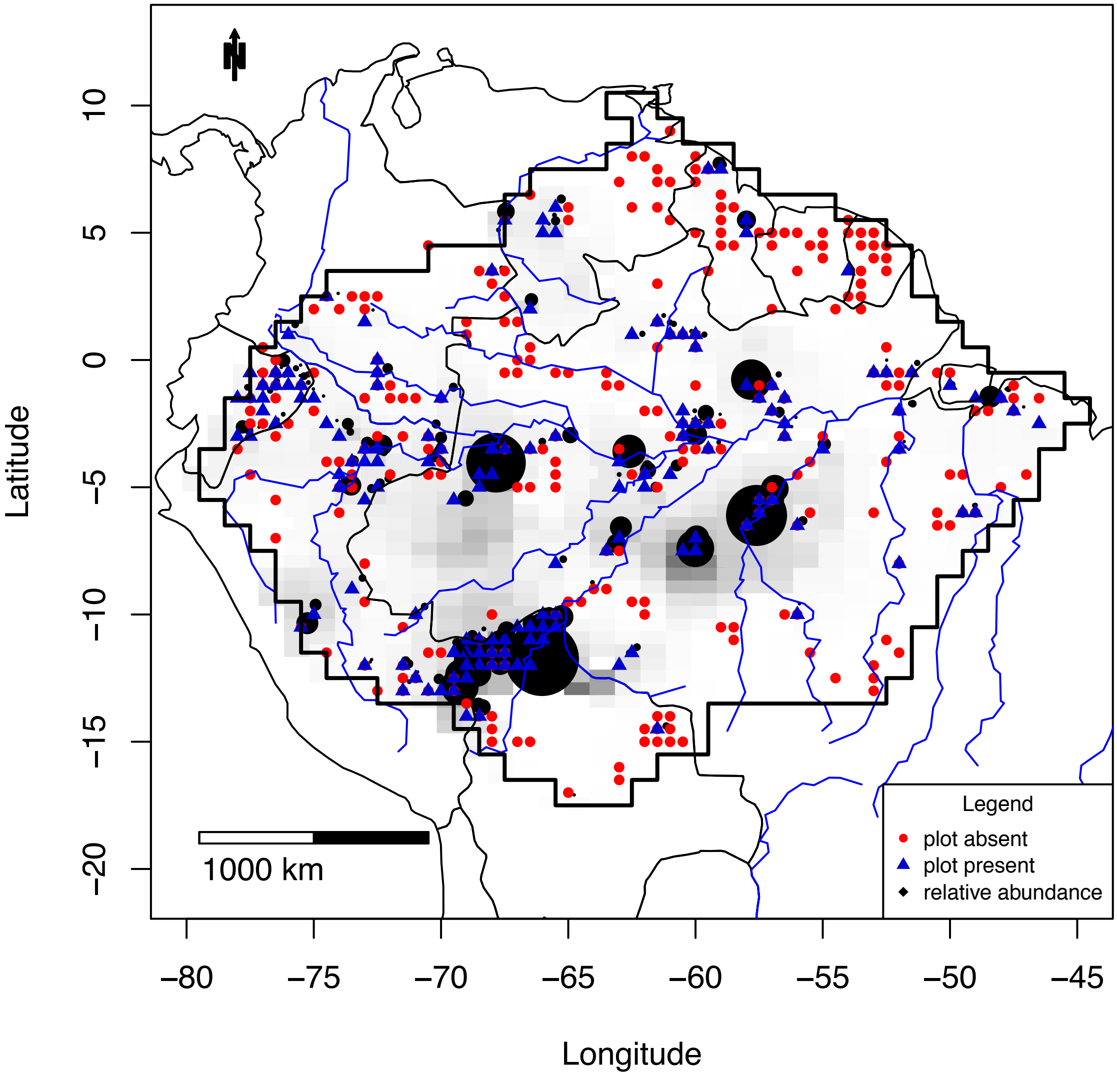

Figure S3_74A Combined map of Iryanthera_laevis

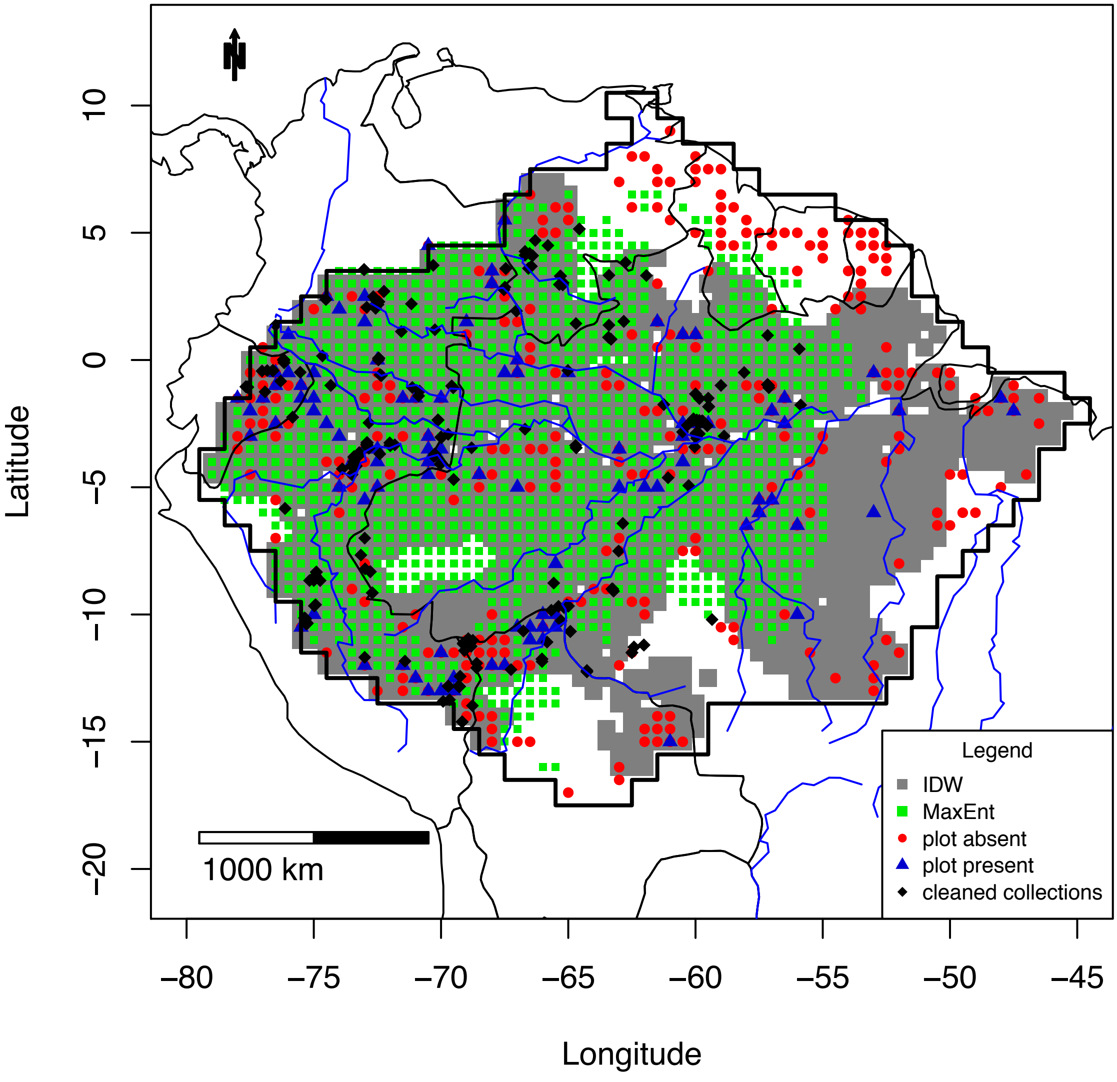

Figure S3_74B MaxEnt map of Iryanthera_laevis

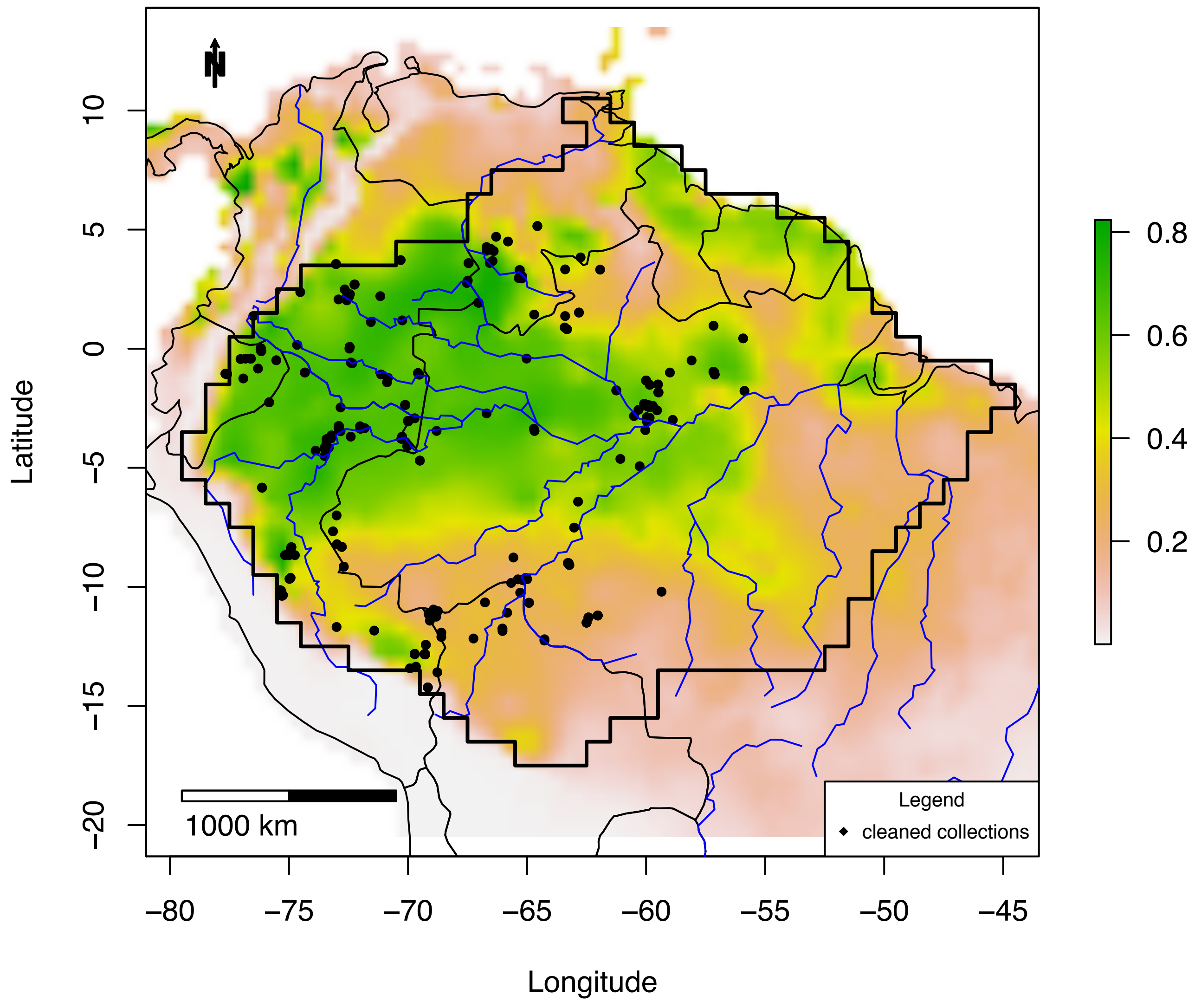

Figure S3_74C IDW map of Iryanthera_laevis:
plots =0 – 0.13; IDW: 0 – 0.04

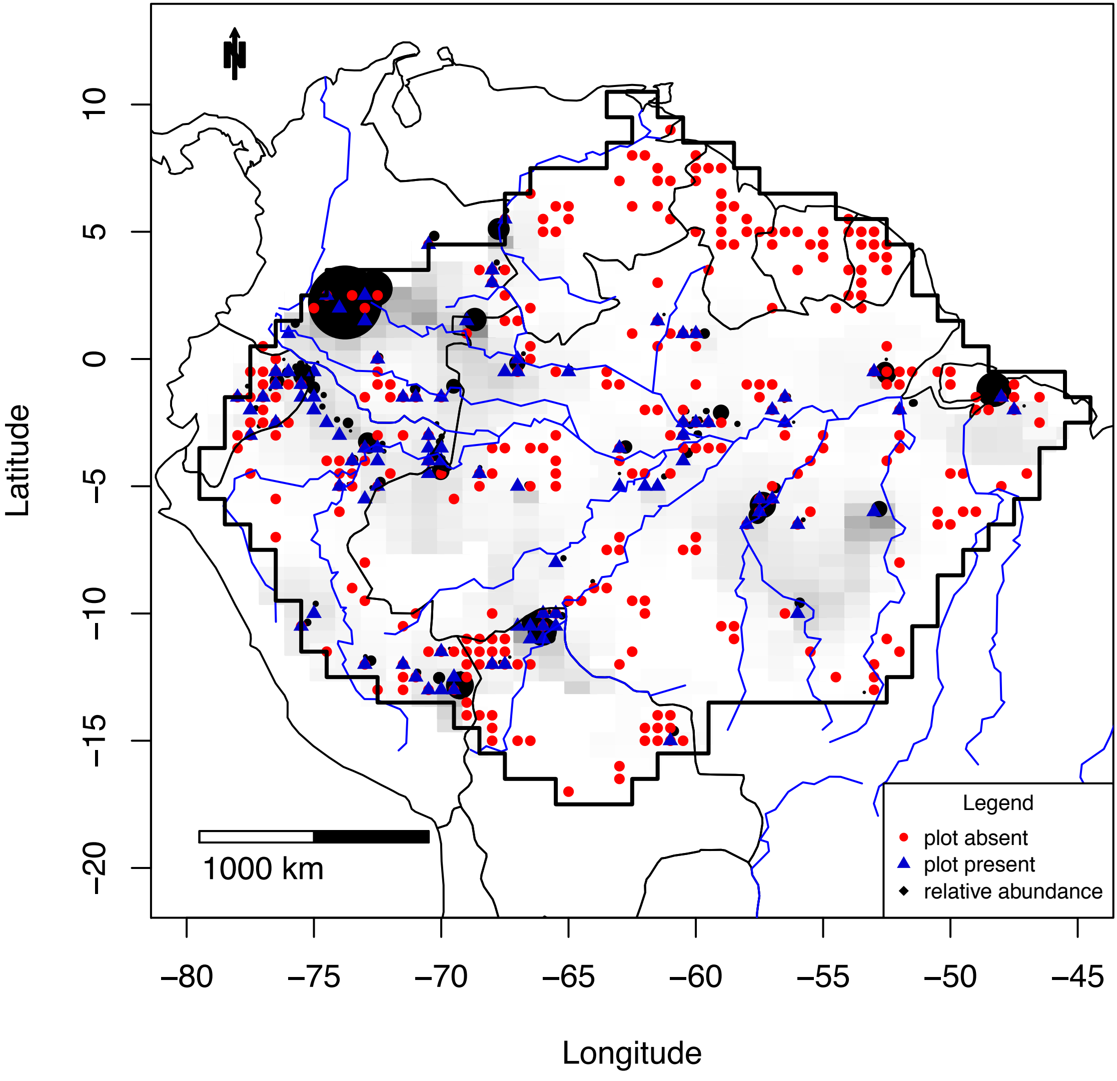

Figure S3_75A Combined map of Iryanthera_ulei

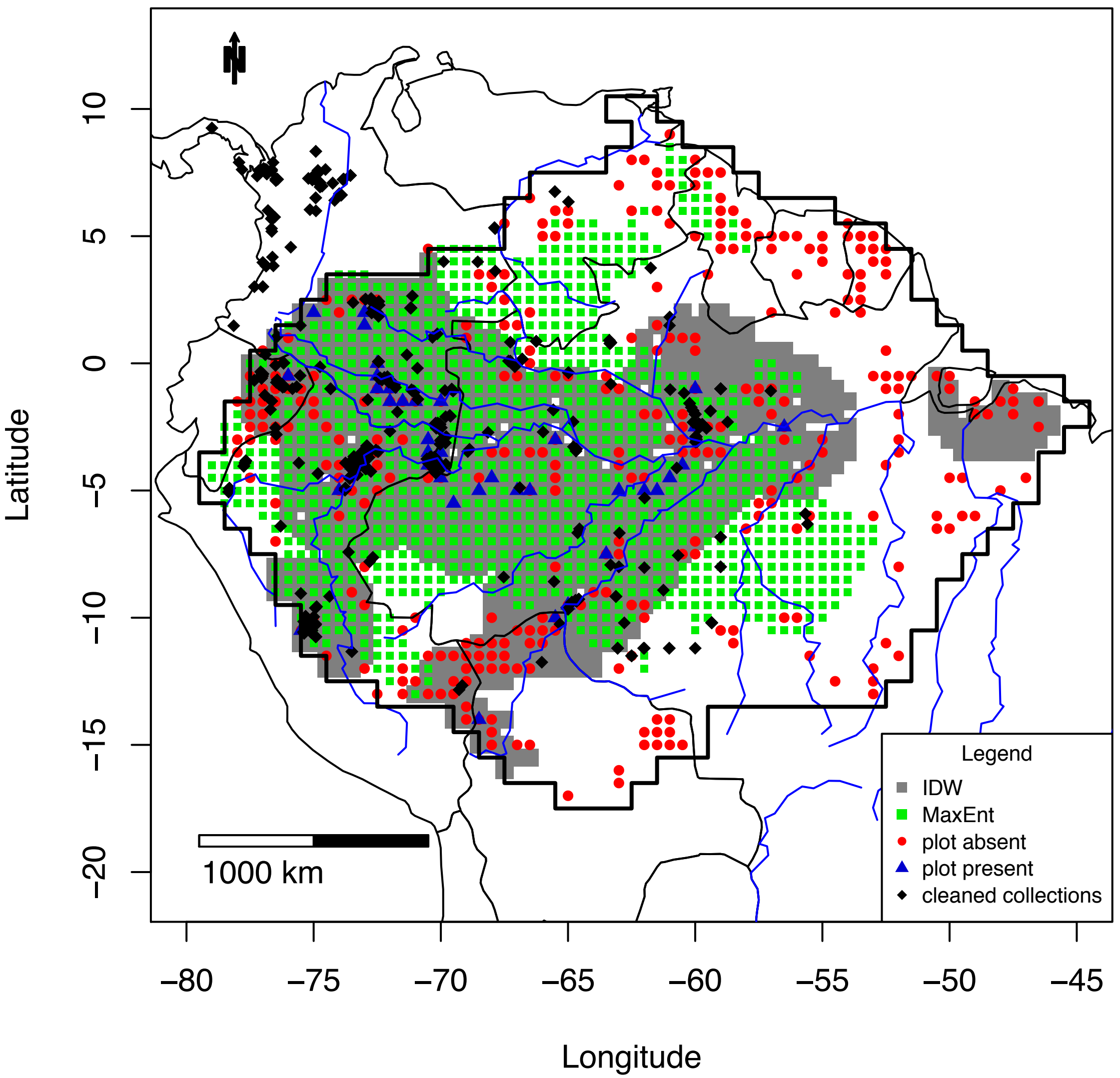

Figure S3_75B MaxEnt map of Iryanthera_ulei

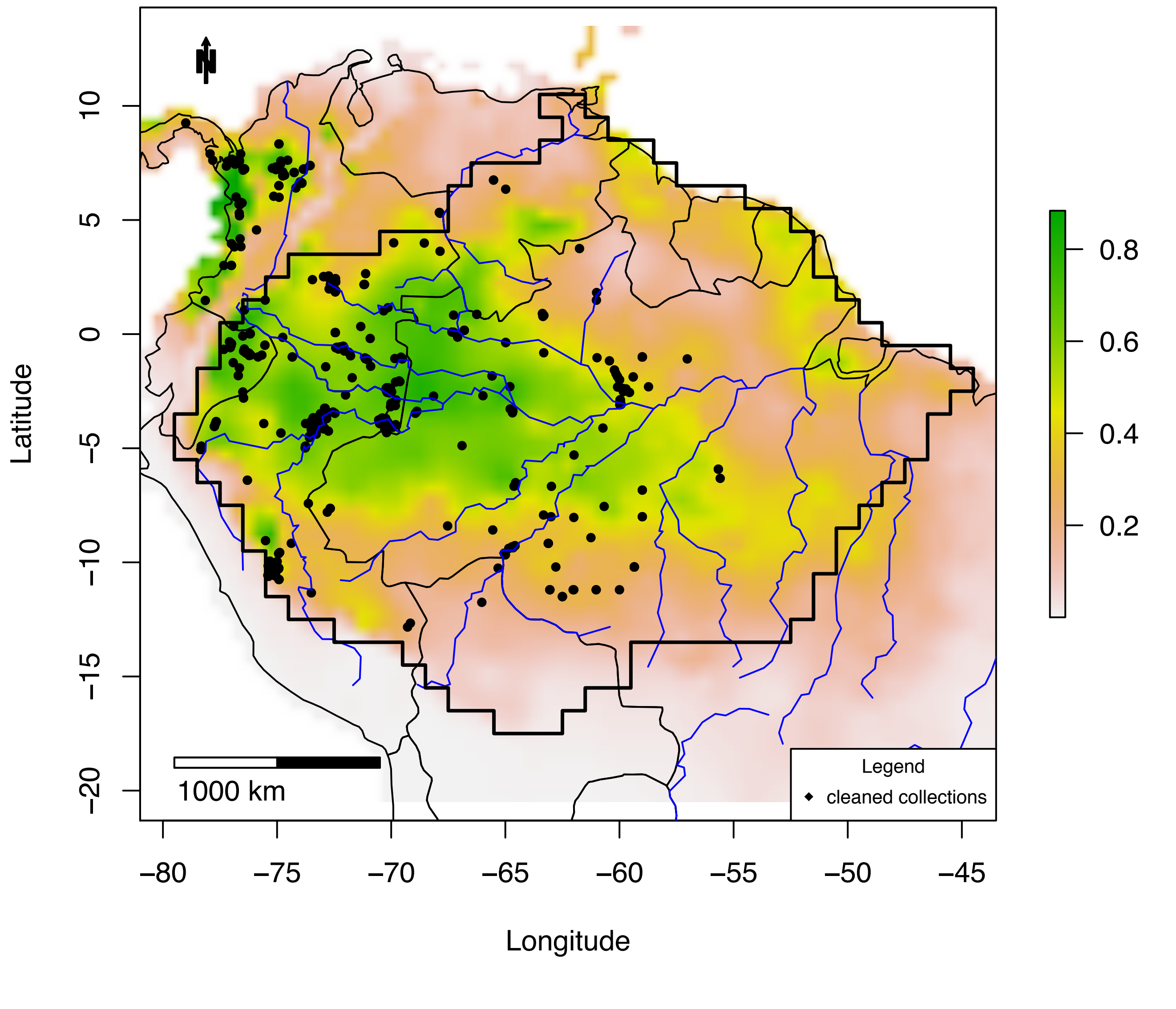

Figure S3_75C IDW map of Iryanthera_ulei: plots =0 – 0.18; IDW: 0 – 0.03

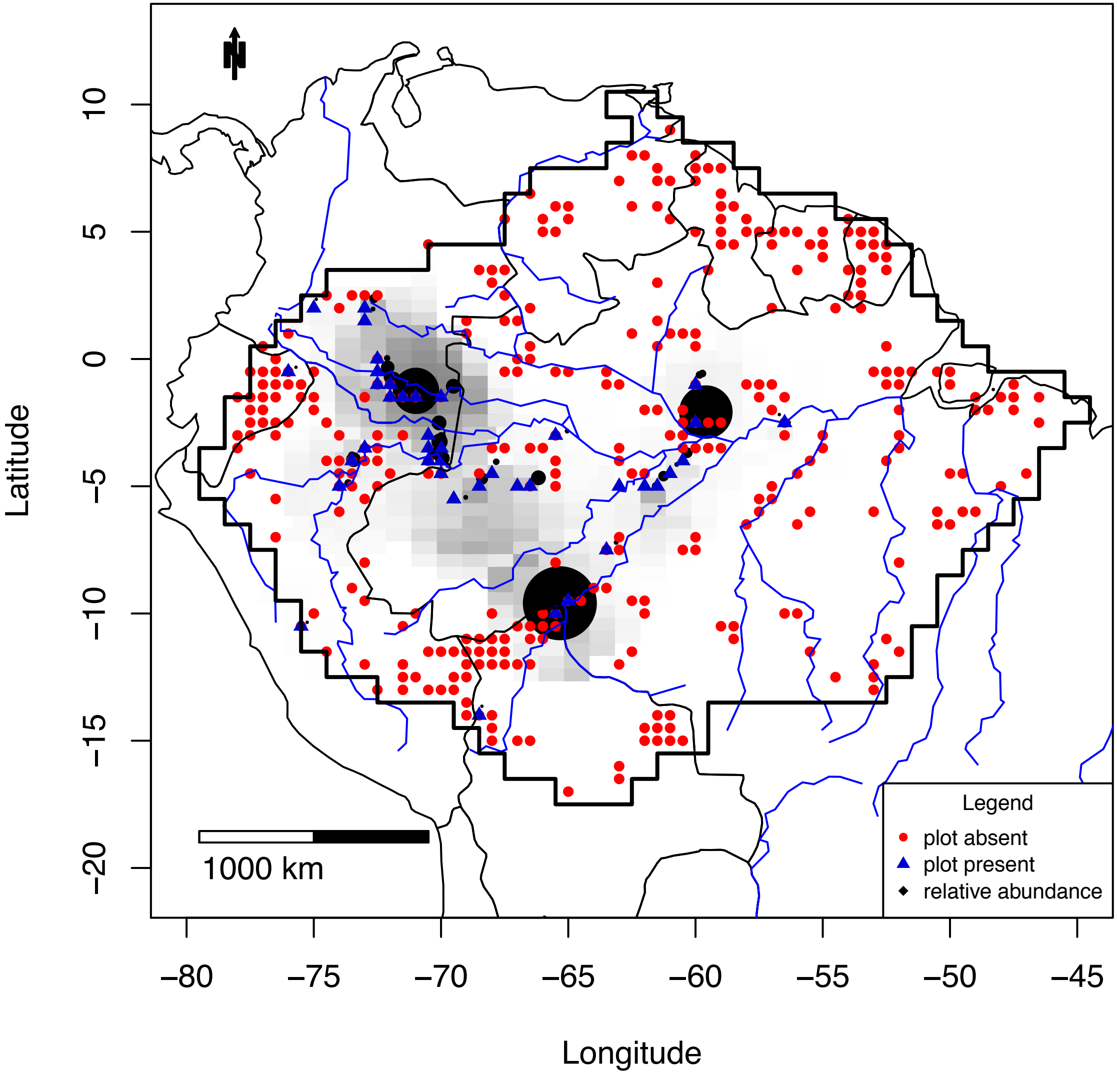

Figure S3_76A Combined map of Jacaranda_copaia

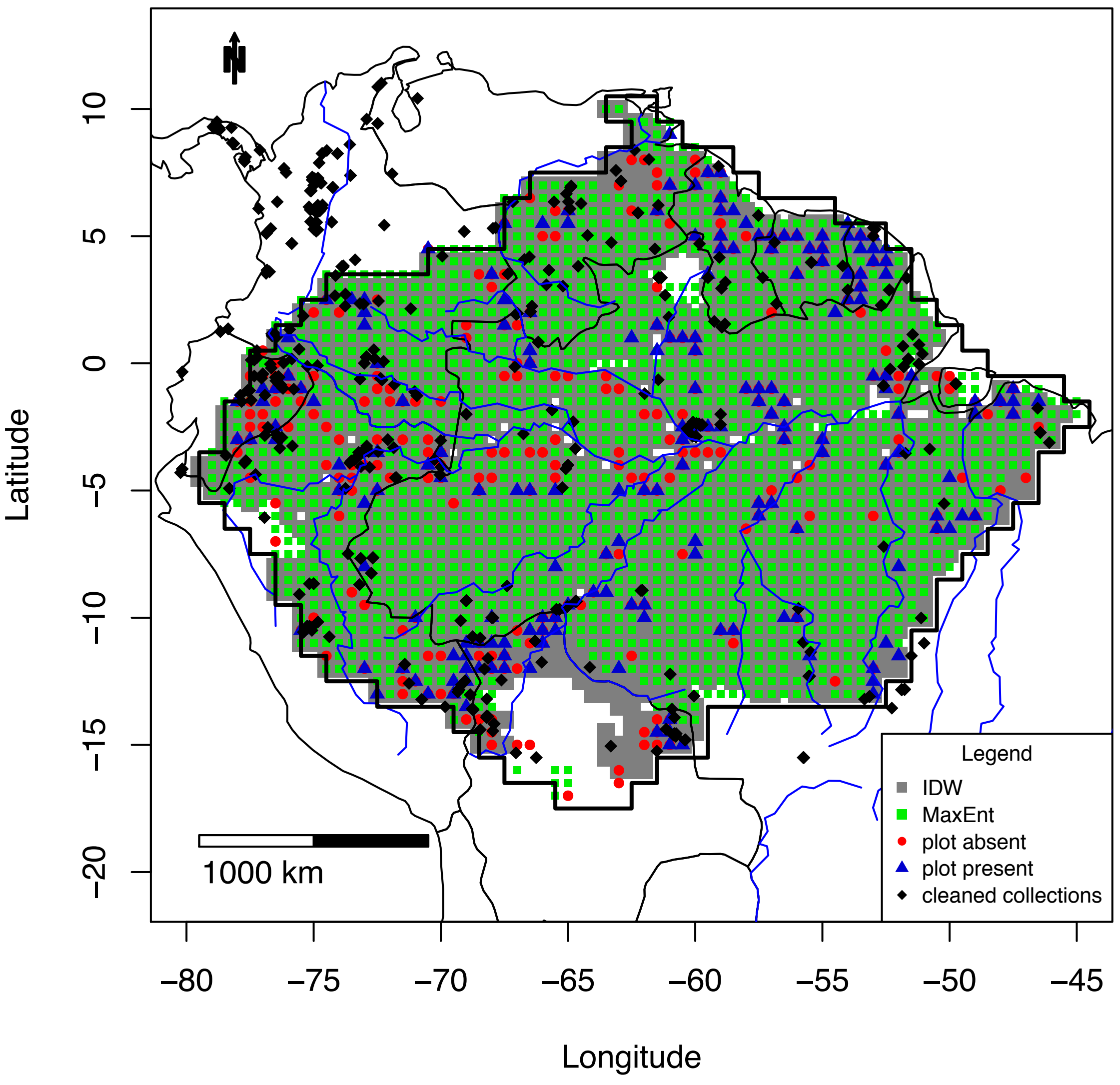

Figure S3_76B MaxEnt map of Jacaranda_copaia

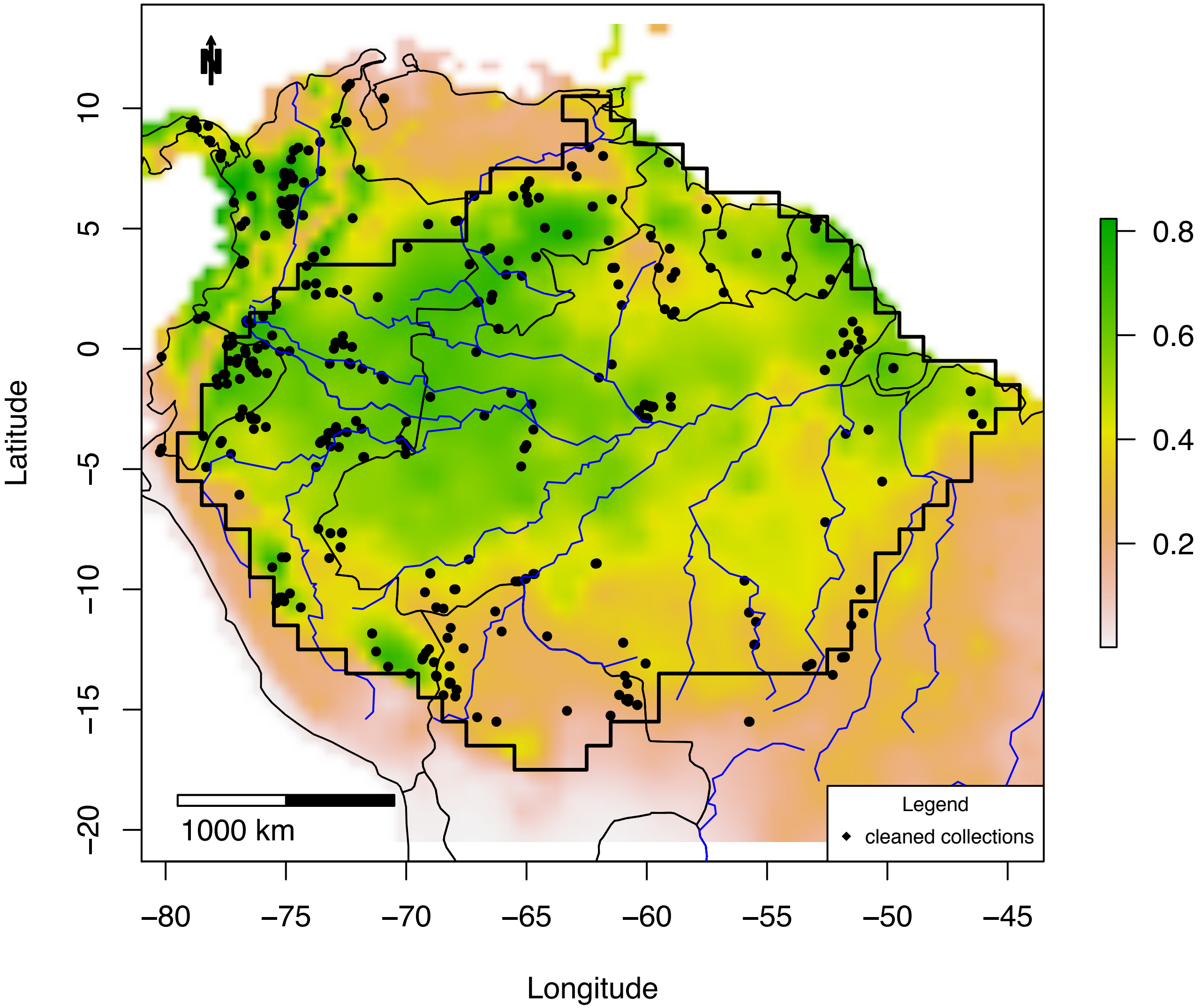

Figure S3_76C IDW map of Jacaranda_copaia: plots =0 – 0.5; IDW: 0 – 0.07

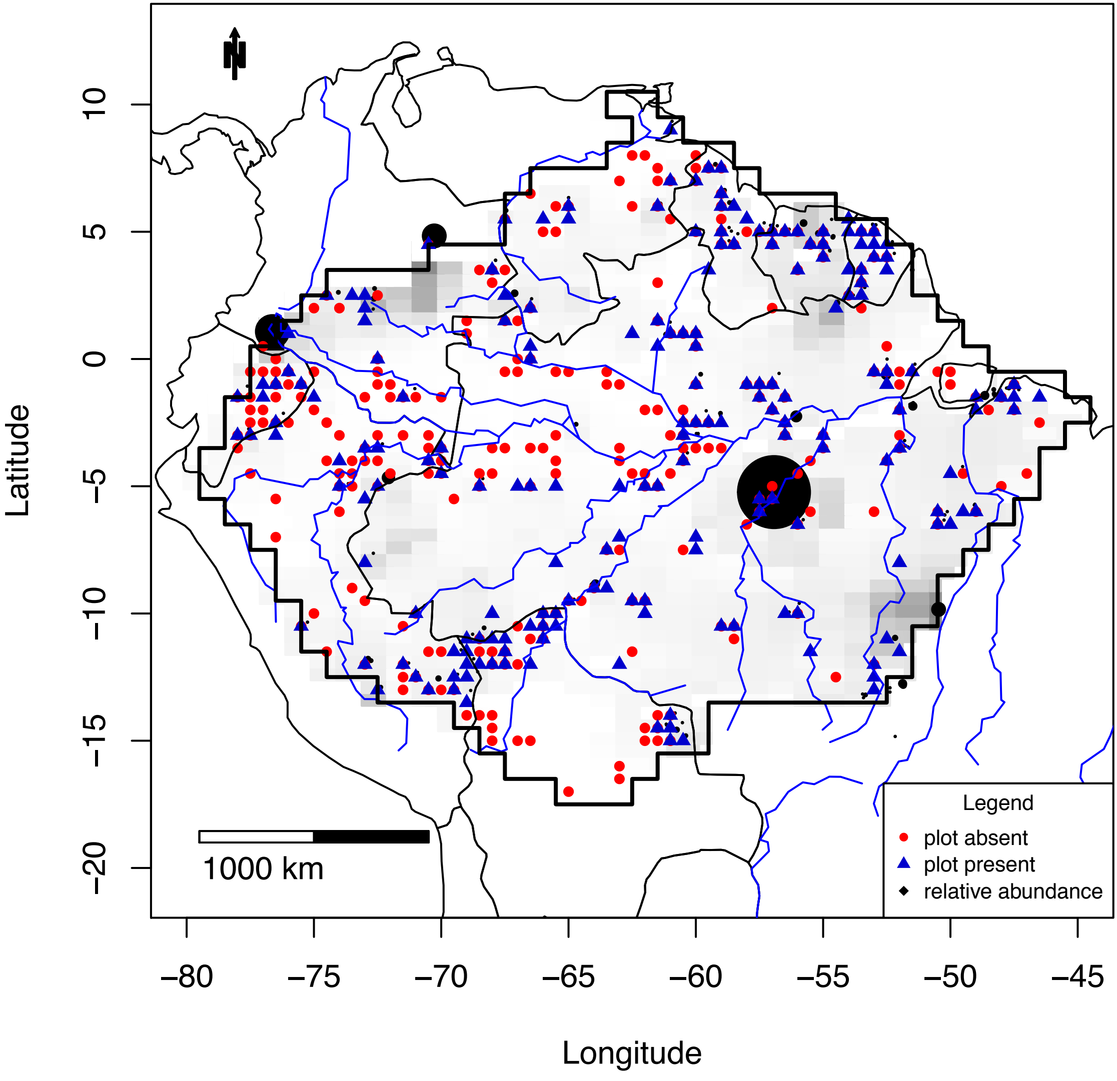

Figure S3_77A Combined map of Laetia_corymbulosa

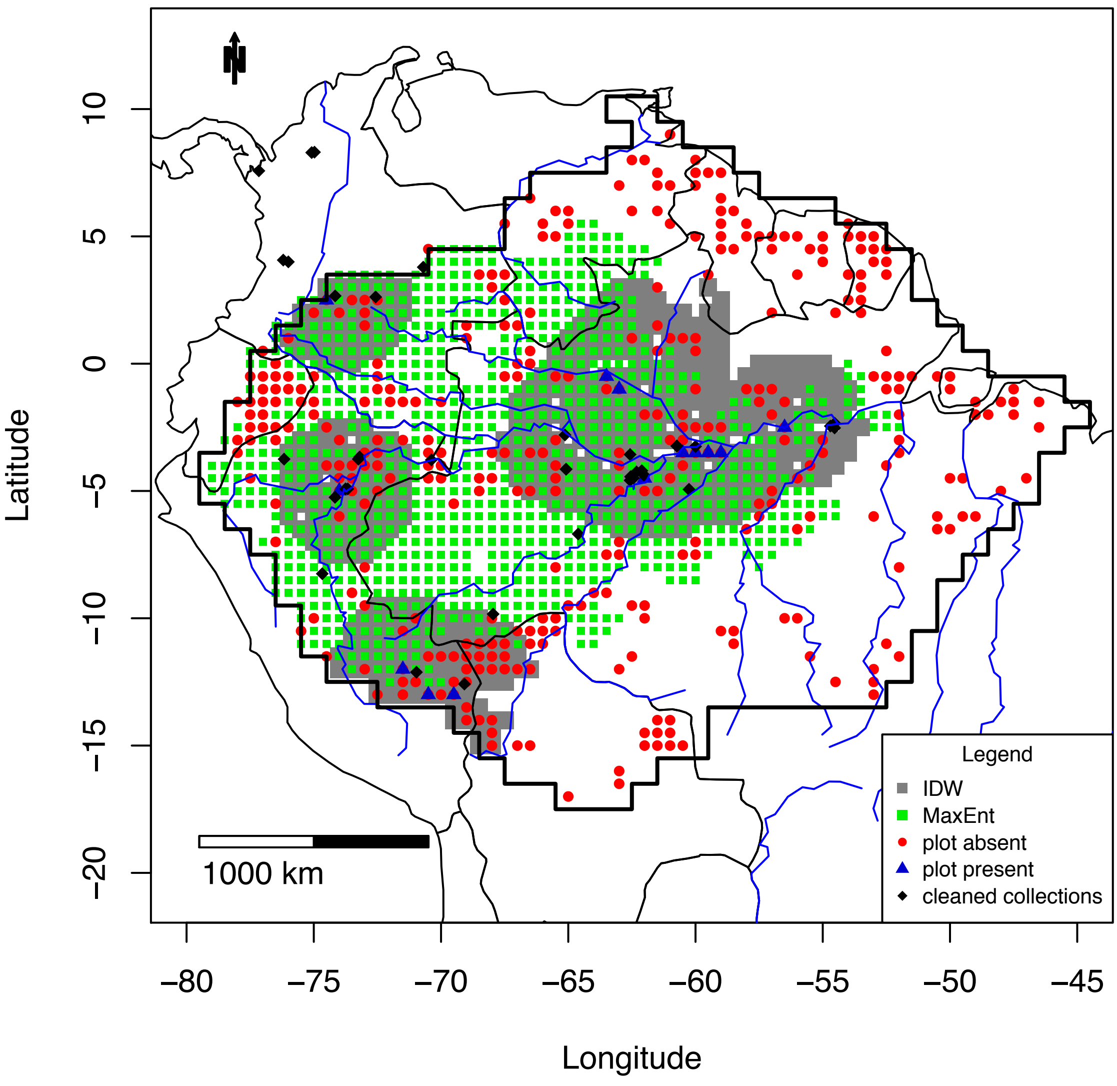

Figure S3_77B MaxEnt map of Laetia_corymbulosa

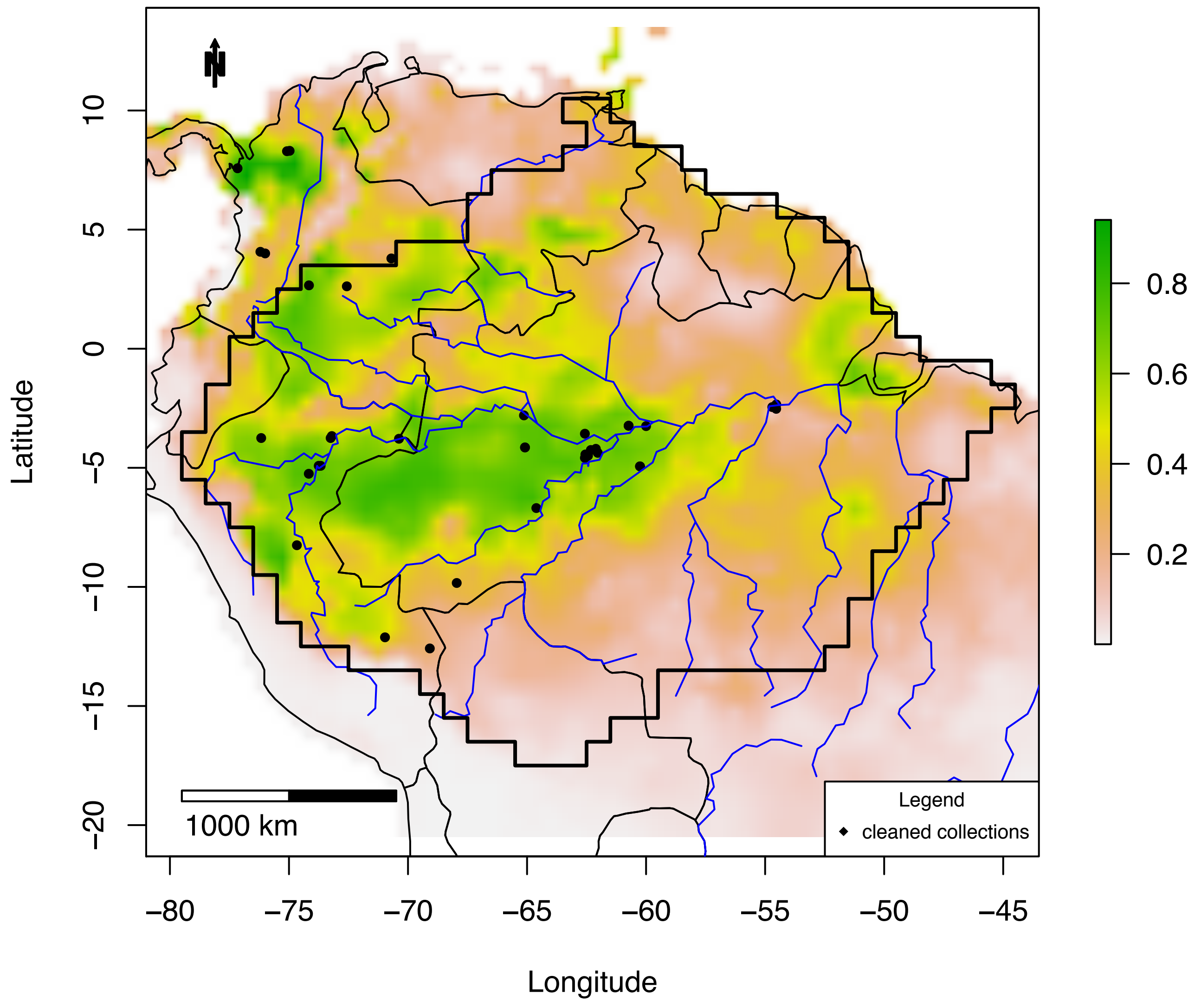

Figure S3_77C IDW map of Laetia_corymbulosa: plots =0 – 0.47; IDW: 0 – 0.07

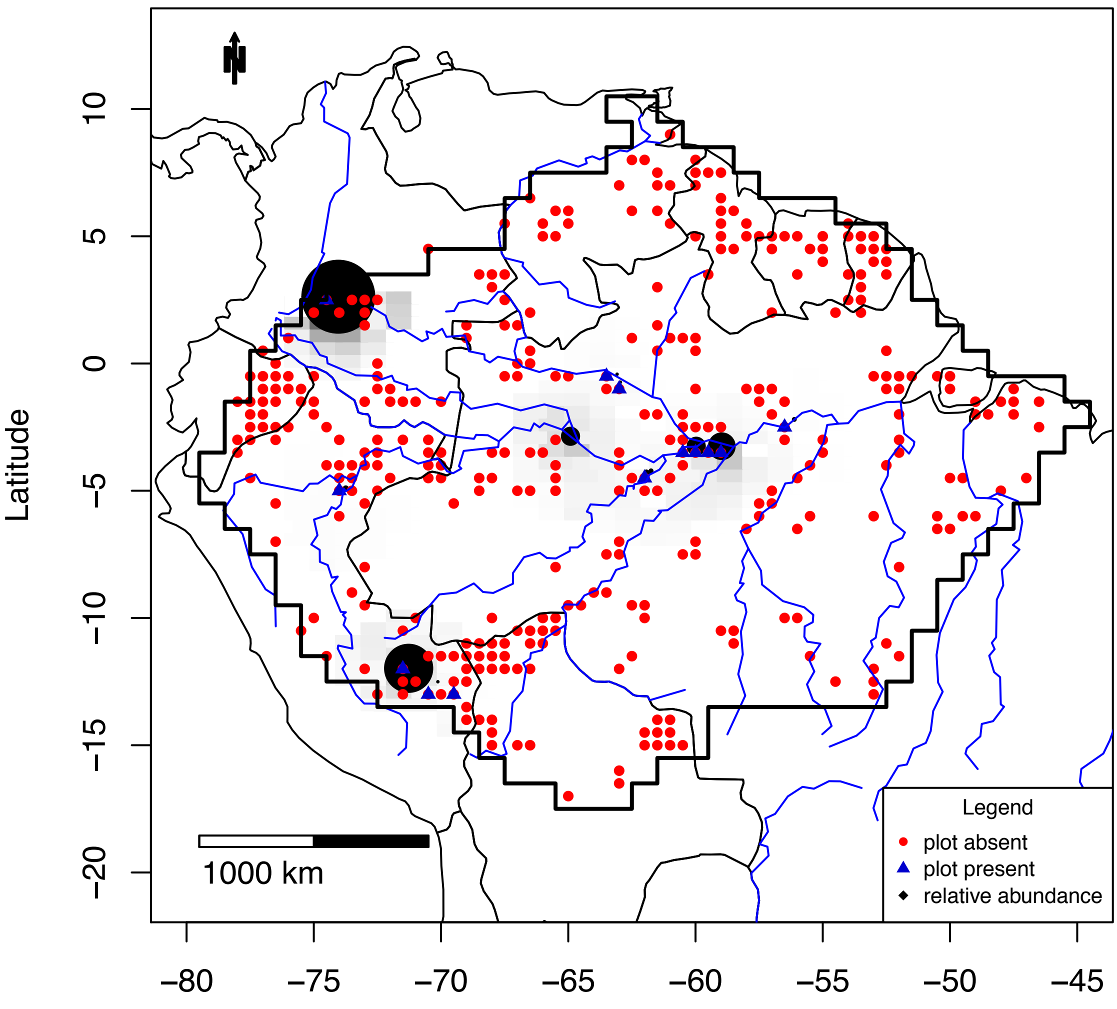

Figure S3_78A Combined map of Laetia_procera

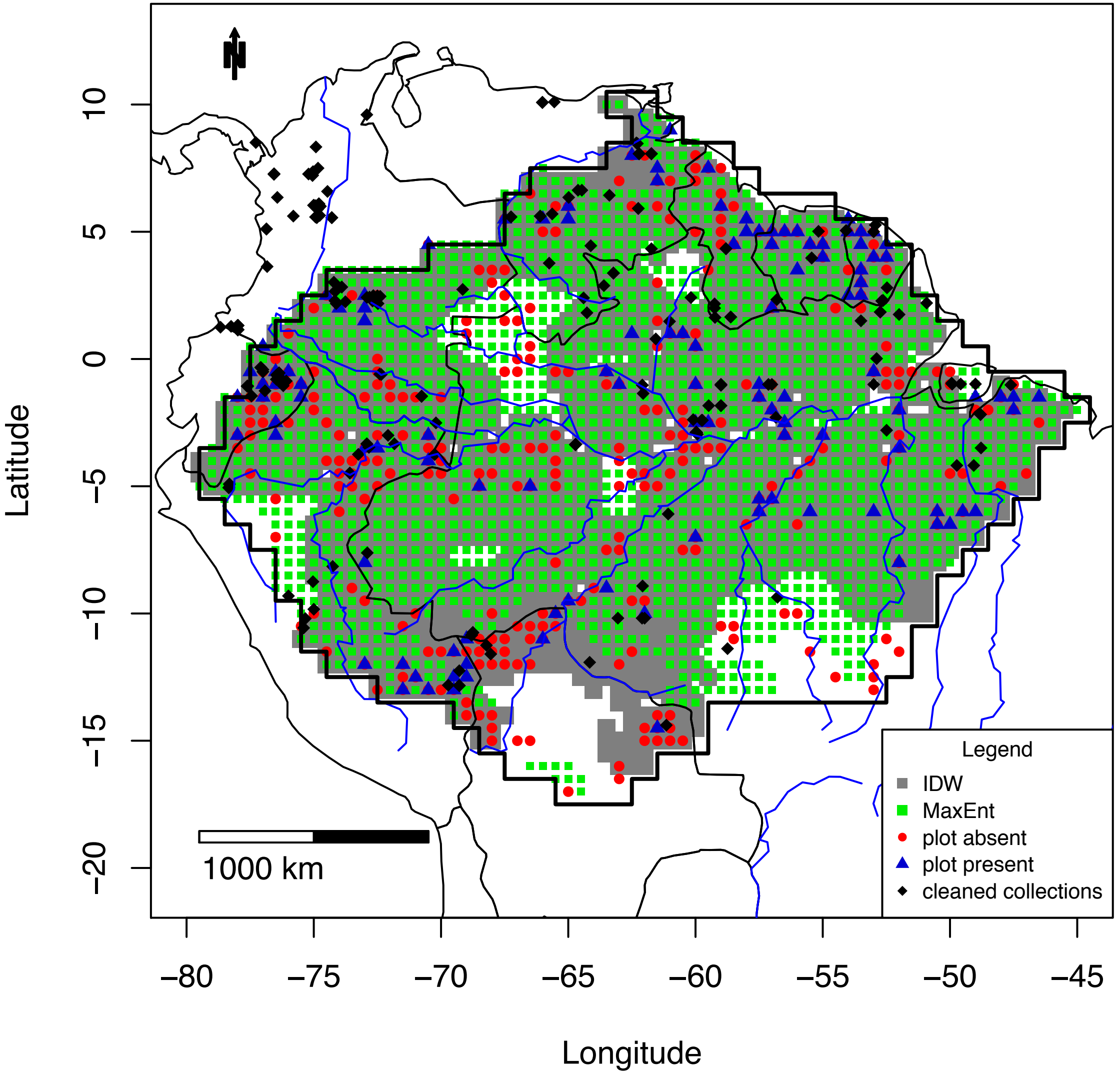

Figure S3_78B MaxEnt map of Laetia_procera

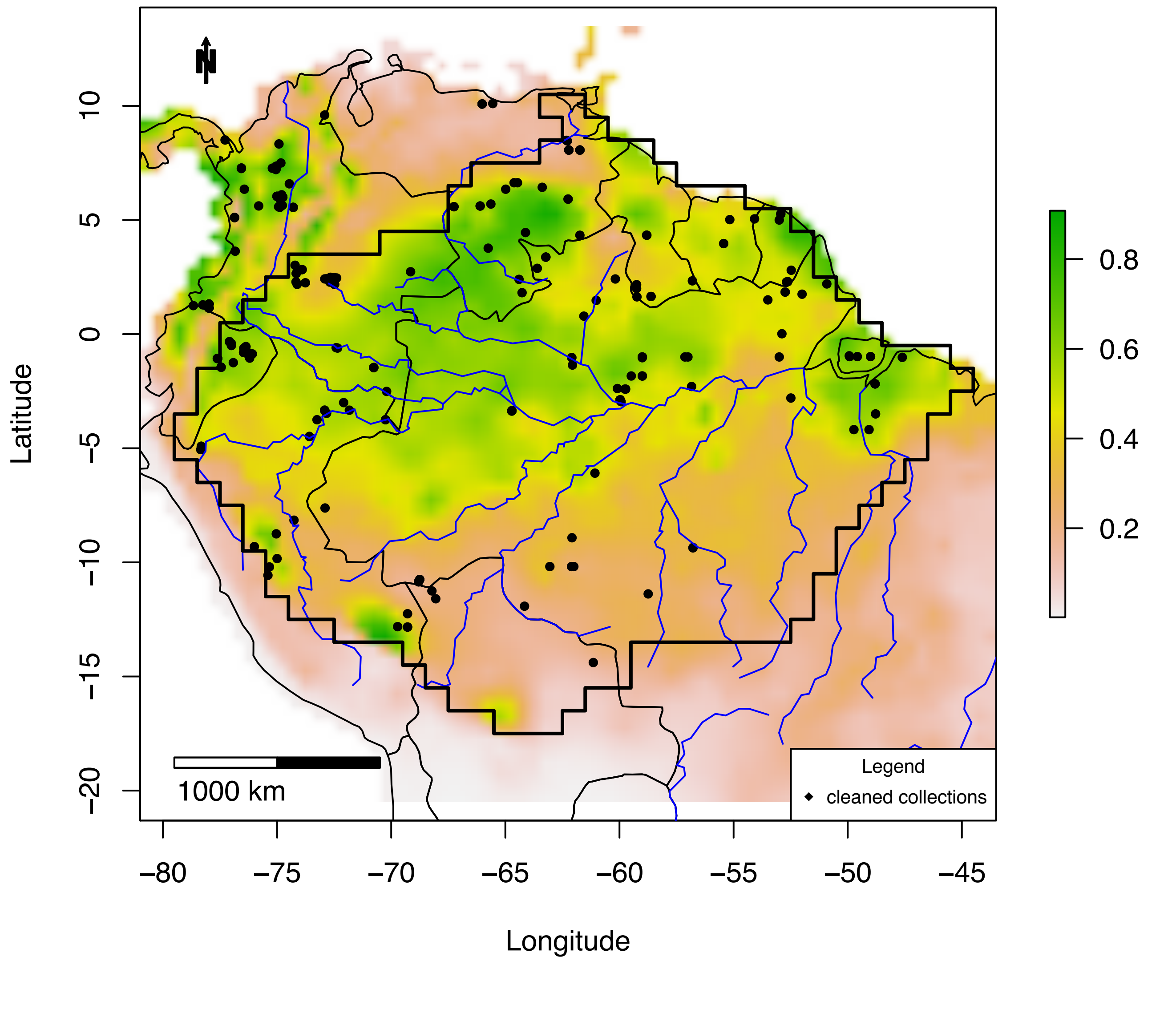

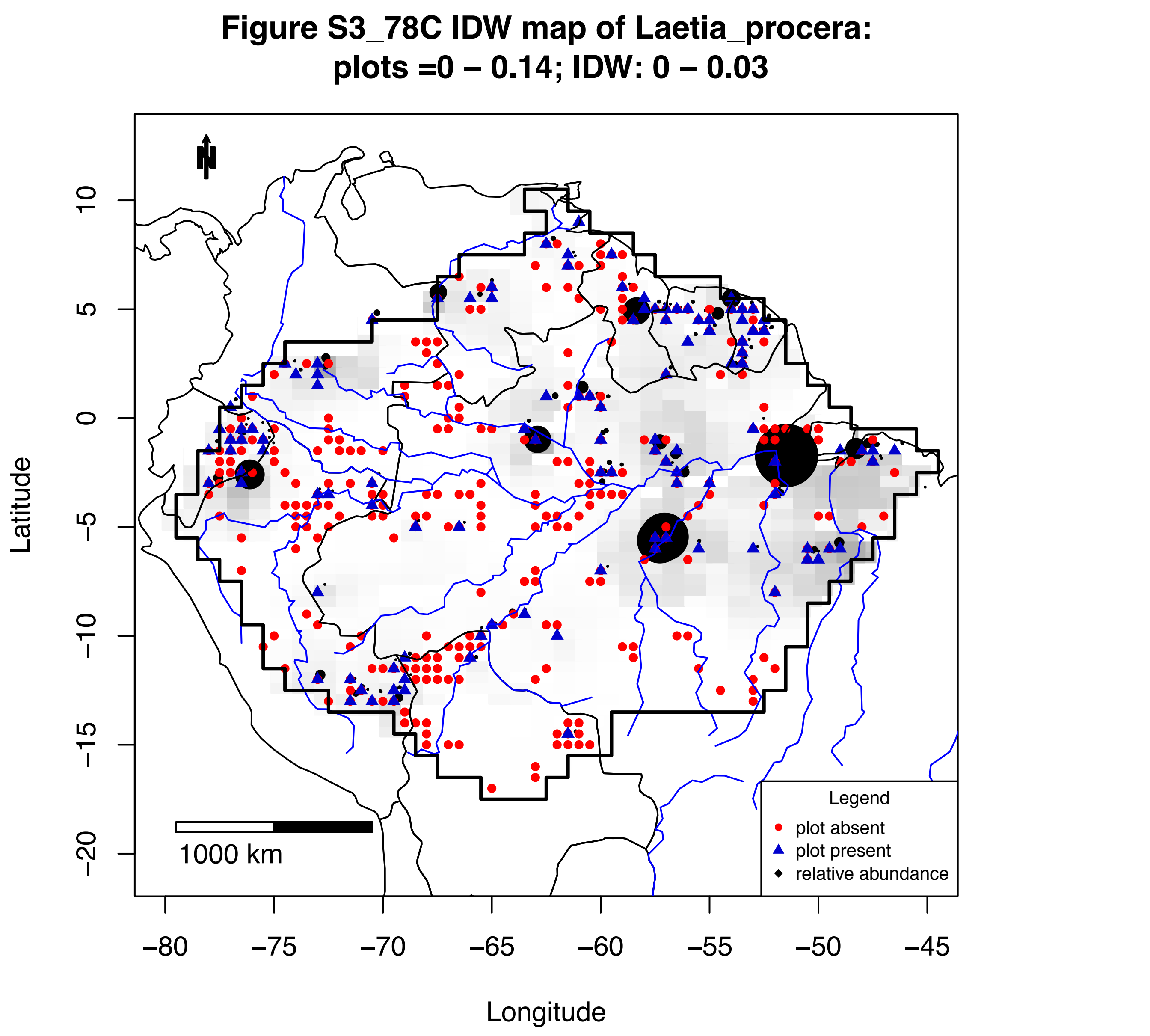
Figure S3_78C IDW map of Laetia_procera:
plots =0 – 0.14; IDW: 0 – 0.03
Latitude
Longitude
10
5
0
−5
−10
−15
−20
−80
−75
−70
−65
−60
−55
−50
−45
N
1000 km
Legend
plot absent
plot present
relative abundance

Figure S3_79A Combined map of Lecythis_corrugata

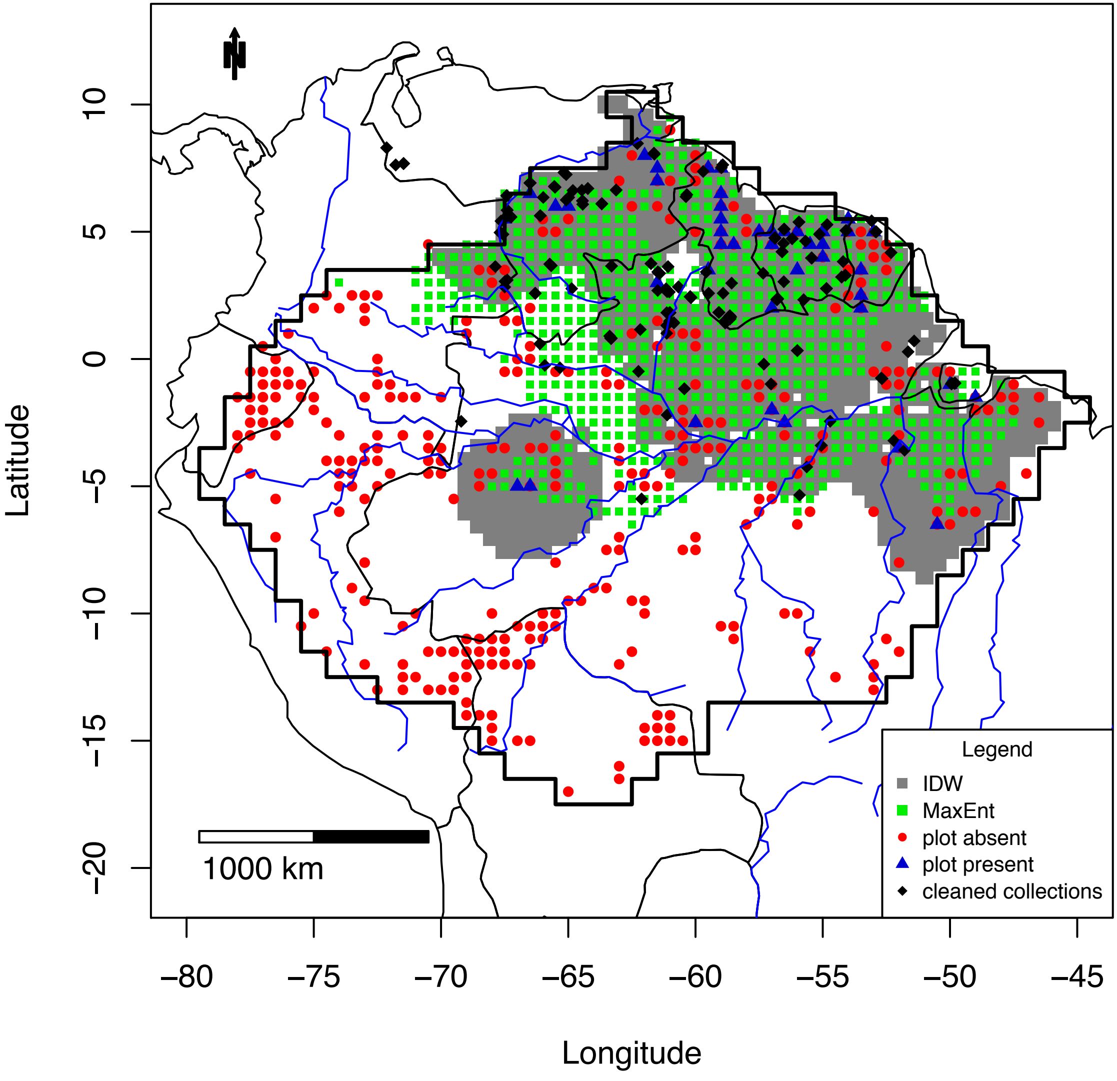

Figure S3_79B MaxEnt map of Lecythis_corrugata

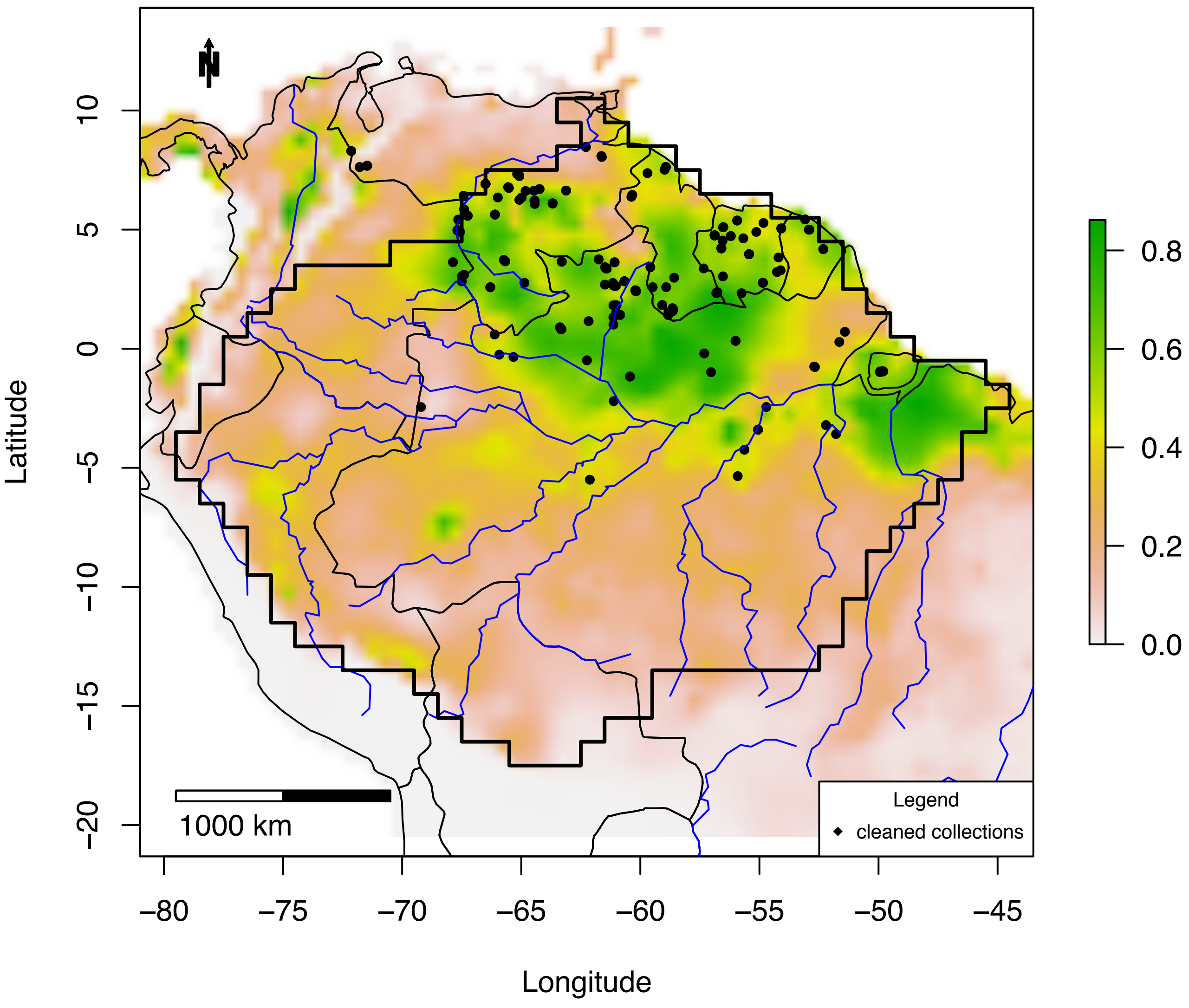

Figure S3_79C IDW map of Lecythis_corrugata: plots =0 – 0.17; IDW: 0 – 0.09

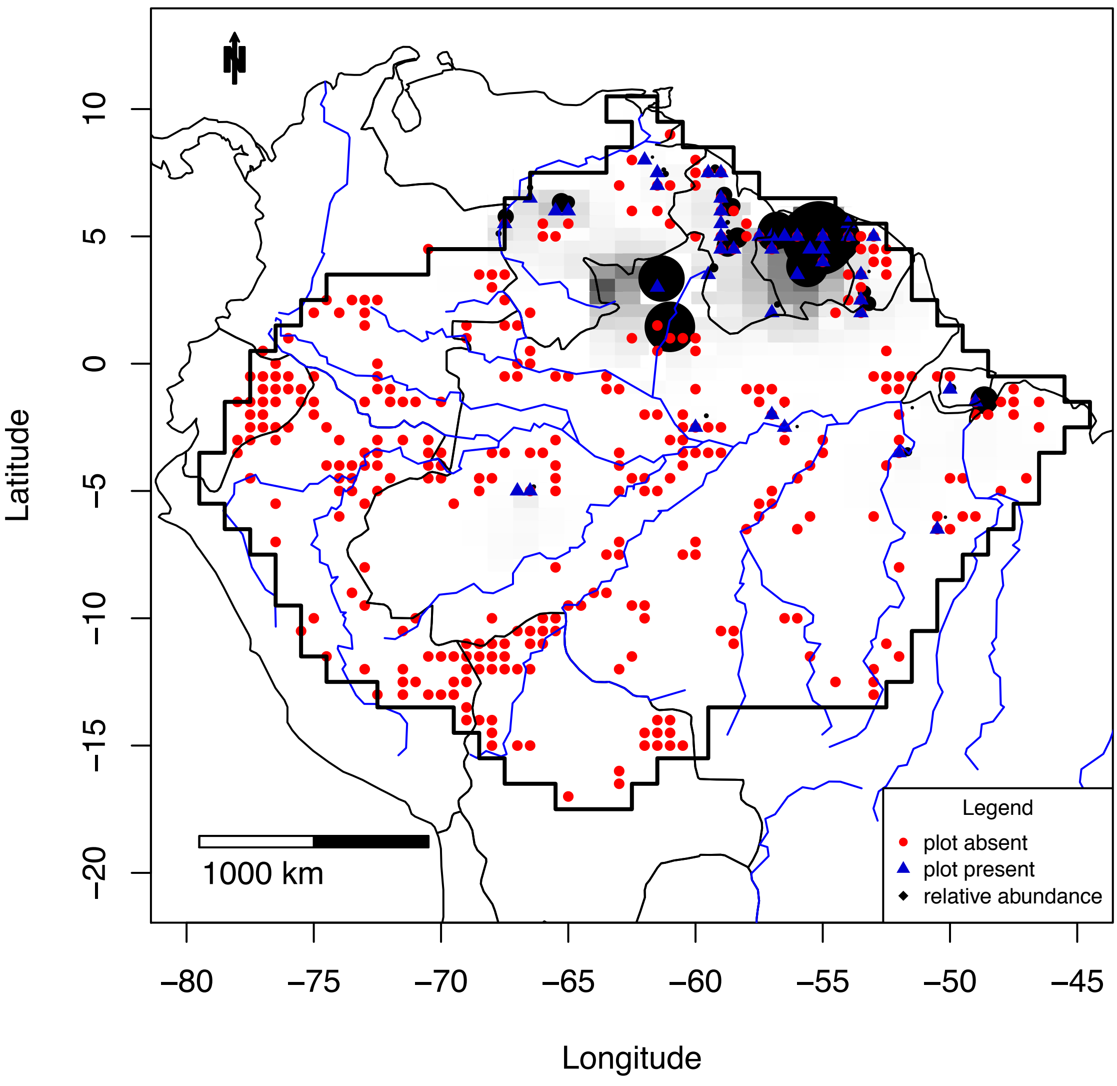

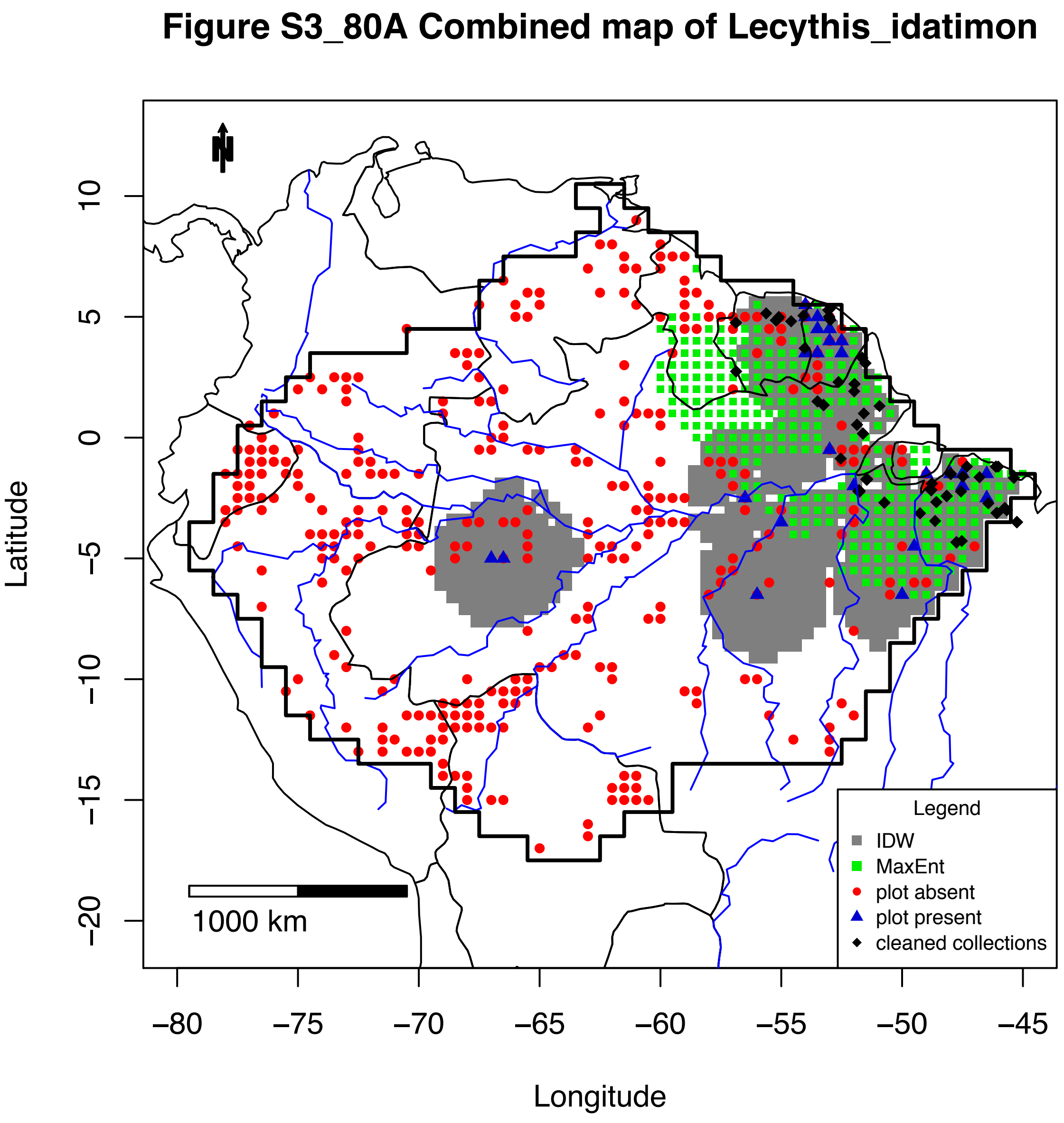
Figure S3_80A Combined map of Lecythis_idatimon
Latitude
Longitude
10
5
0
−5
−10
−15
−20
−80
−75
−70
−65
−60
−55
−50
−45
1000 km
Legend
IDW
MaxEnt
plot absent
plot present
cleaned collections

Figure S3_80B MaxEnt map of Lecythis_idatimon

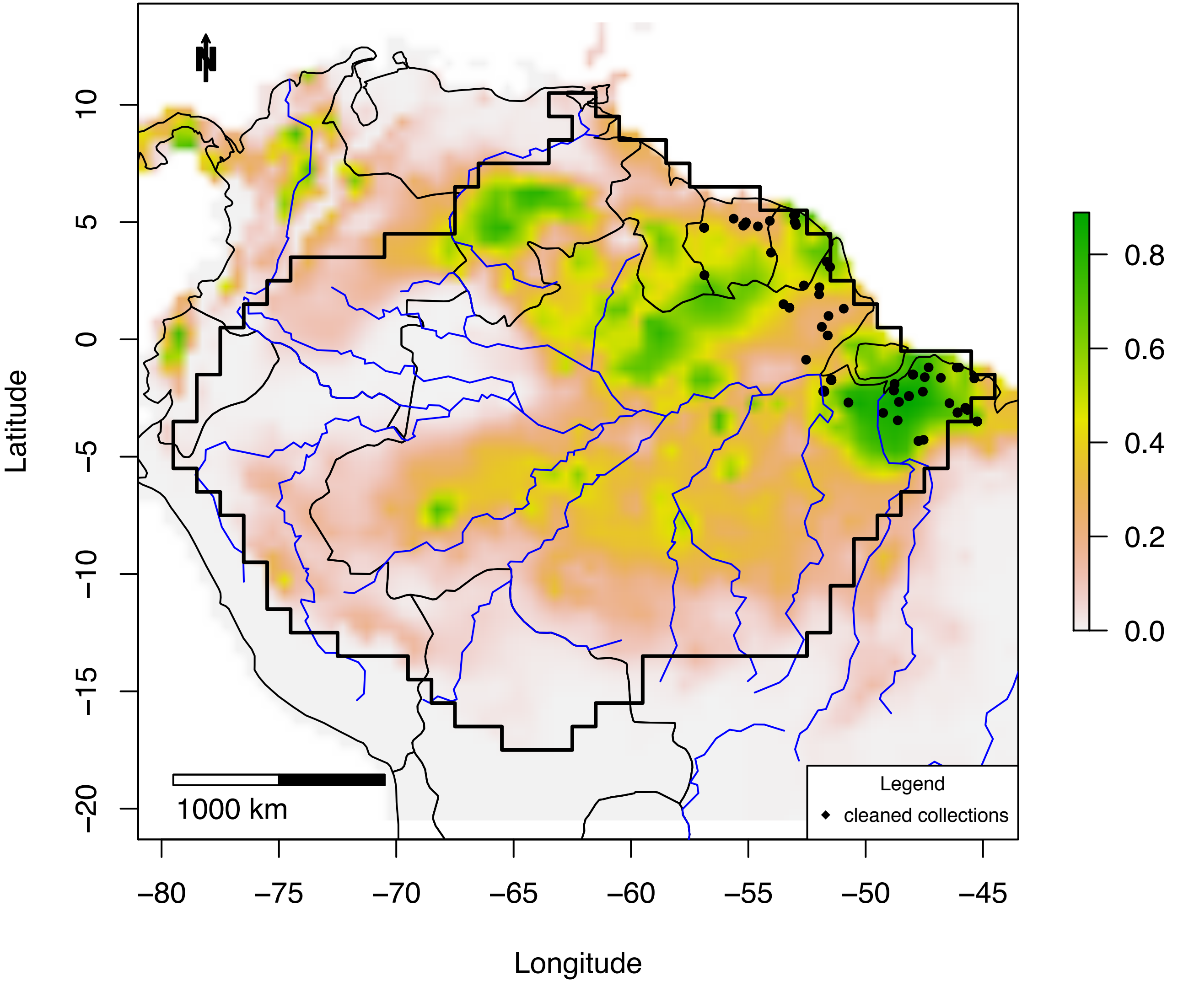

Figure S3_80C IDW map of Lecythis_idatimon: plots =0 – 0.21; IDW: 0 – 0.06

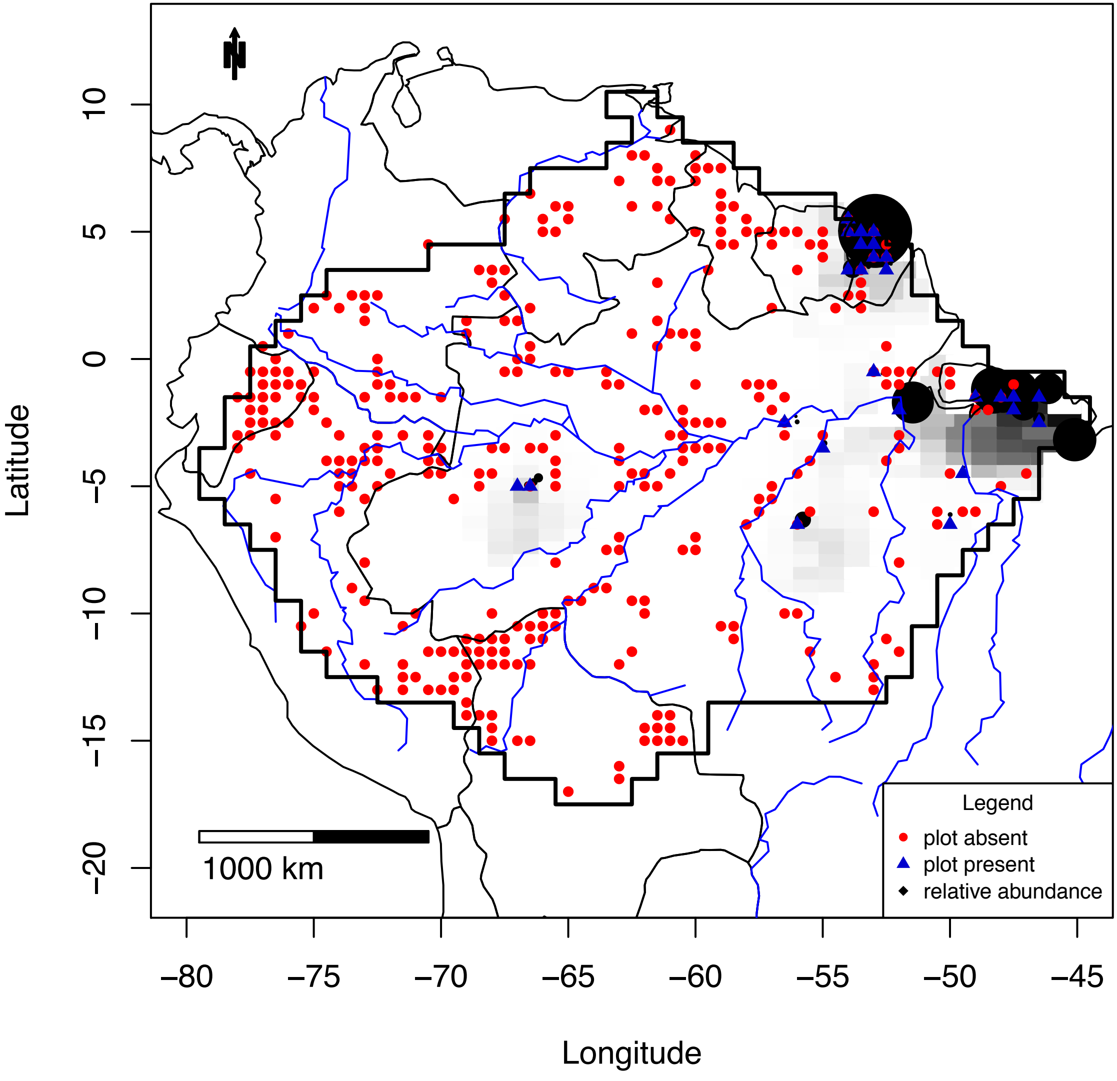

Figure S3_81A Combined map of Leonia_crassa

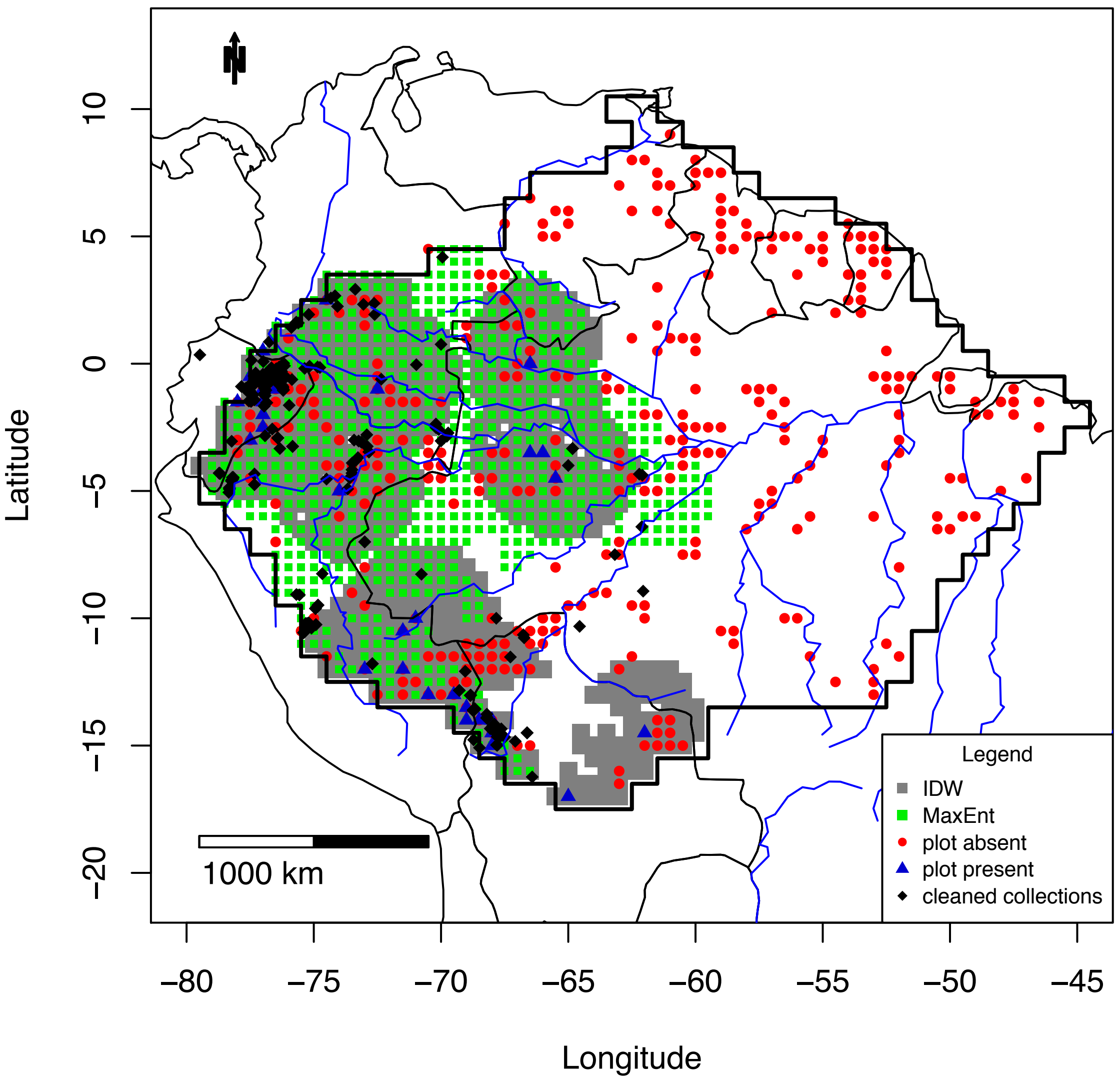

Figure S3_81B MaxEnt map of Leonia_crassa

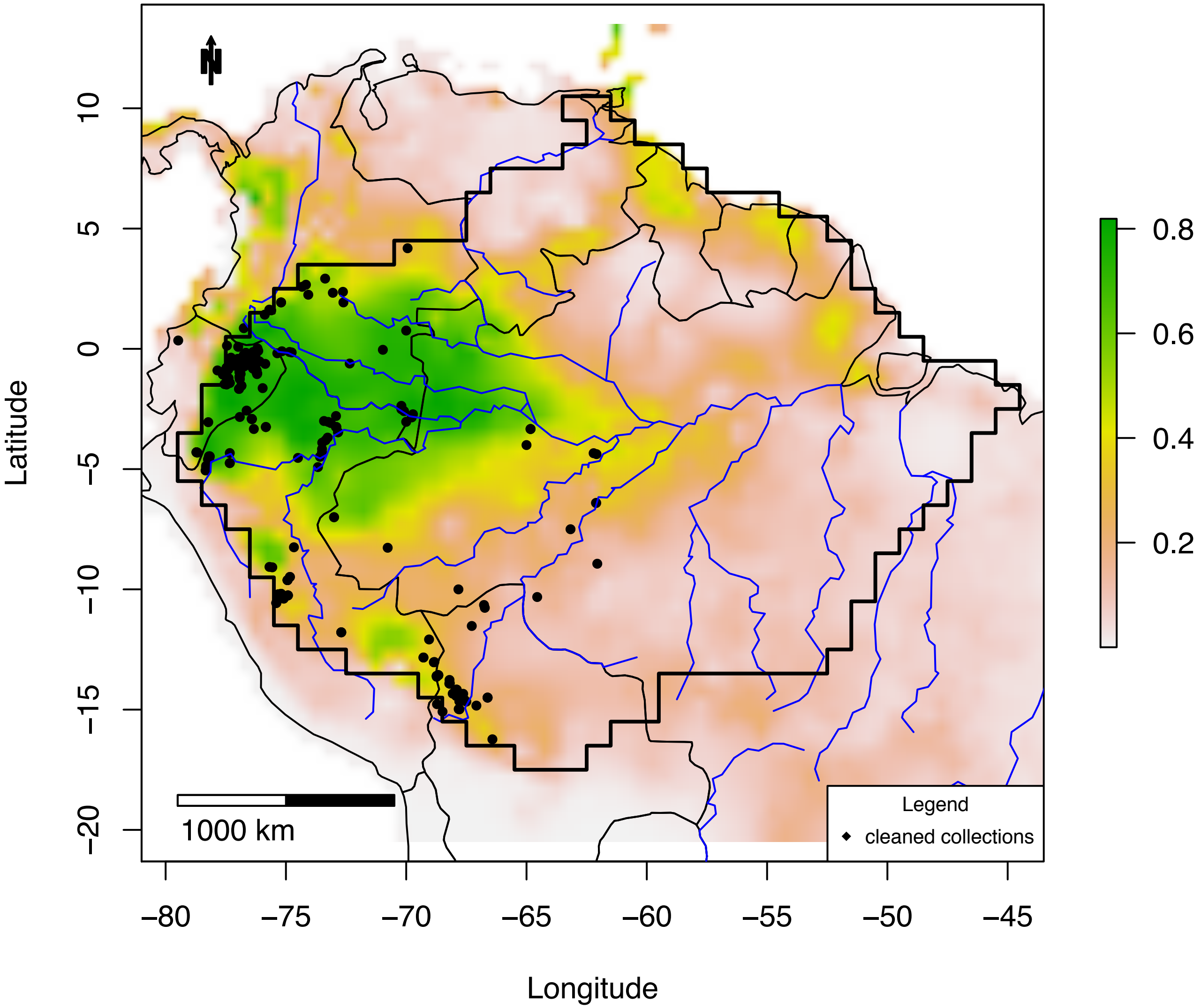

Figure S3_81C IDW map of Leonia_crassa: plots =0 – 0.16; IDW: 0 – 0.11

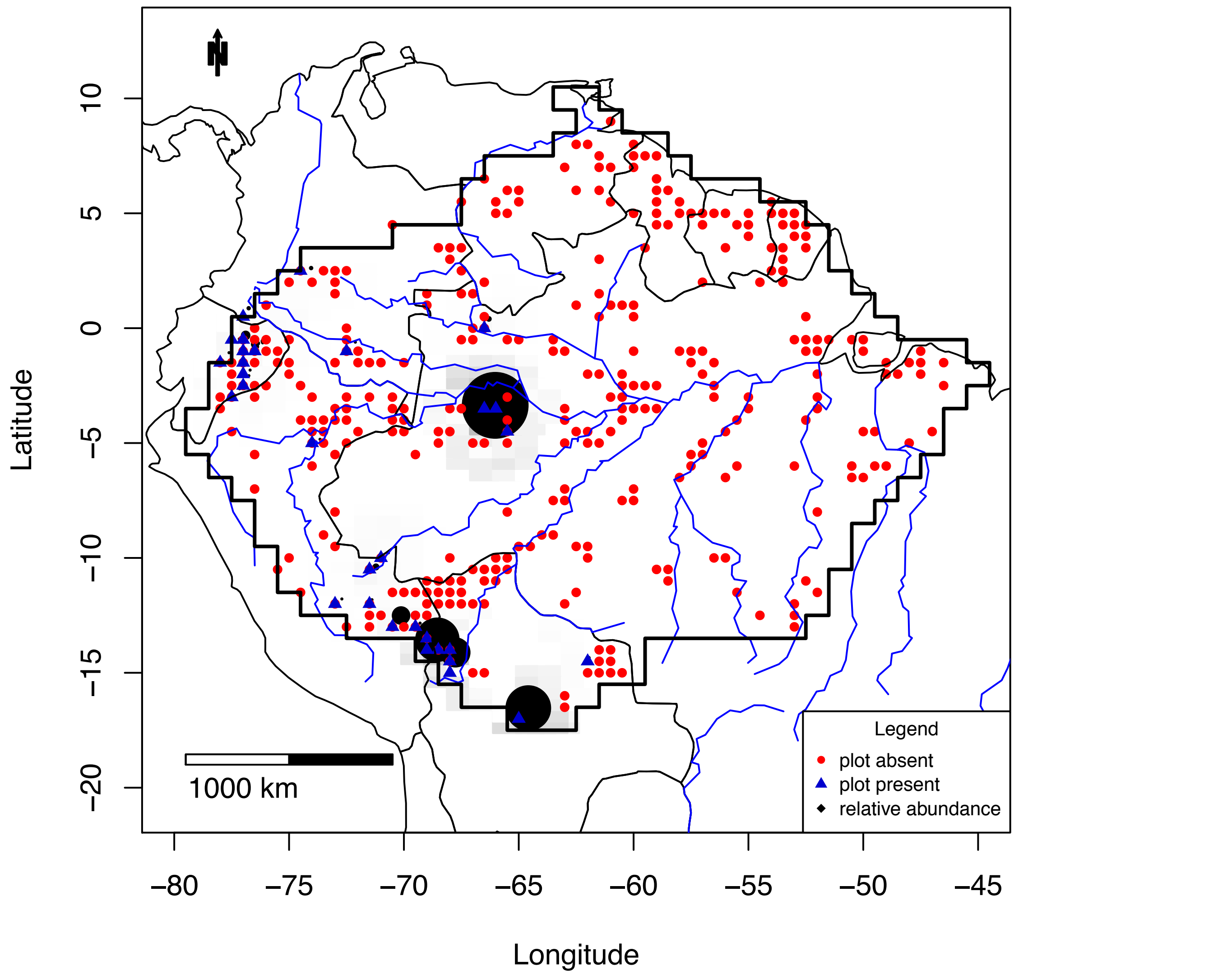

Figure S3_82A Combined map of Leonia_glycycarpa

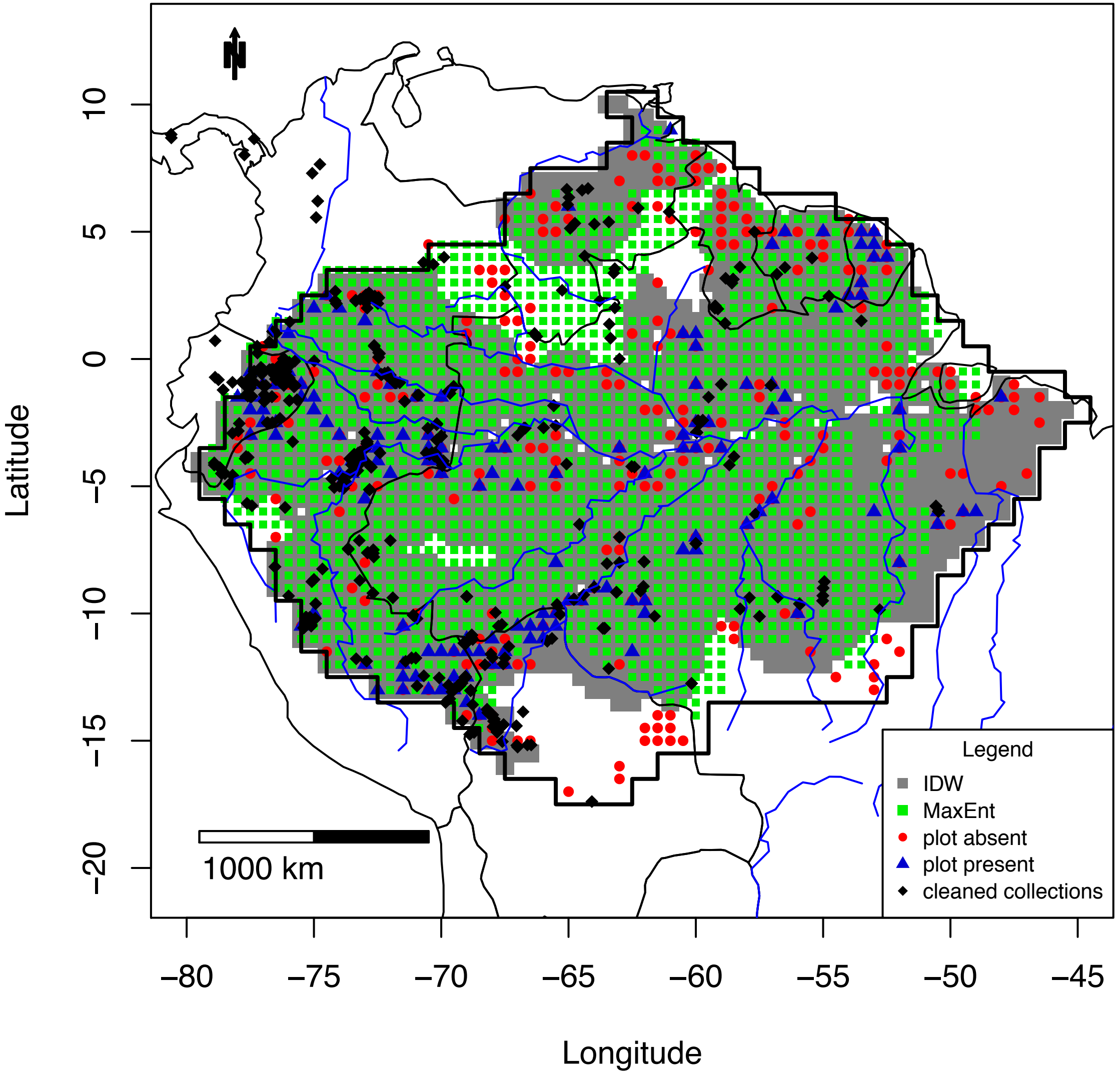

Figure S3_82B MaxEnt map of Leonia_glycycarpa

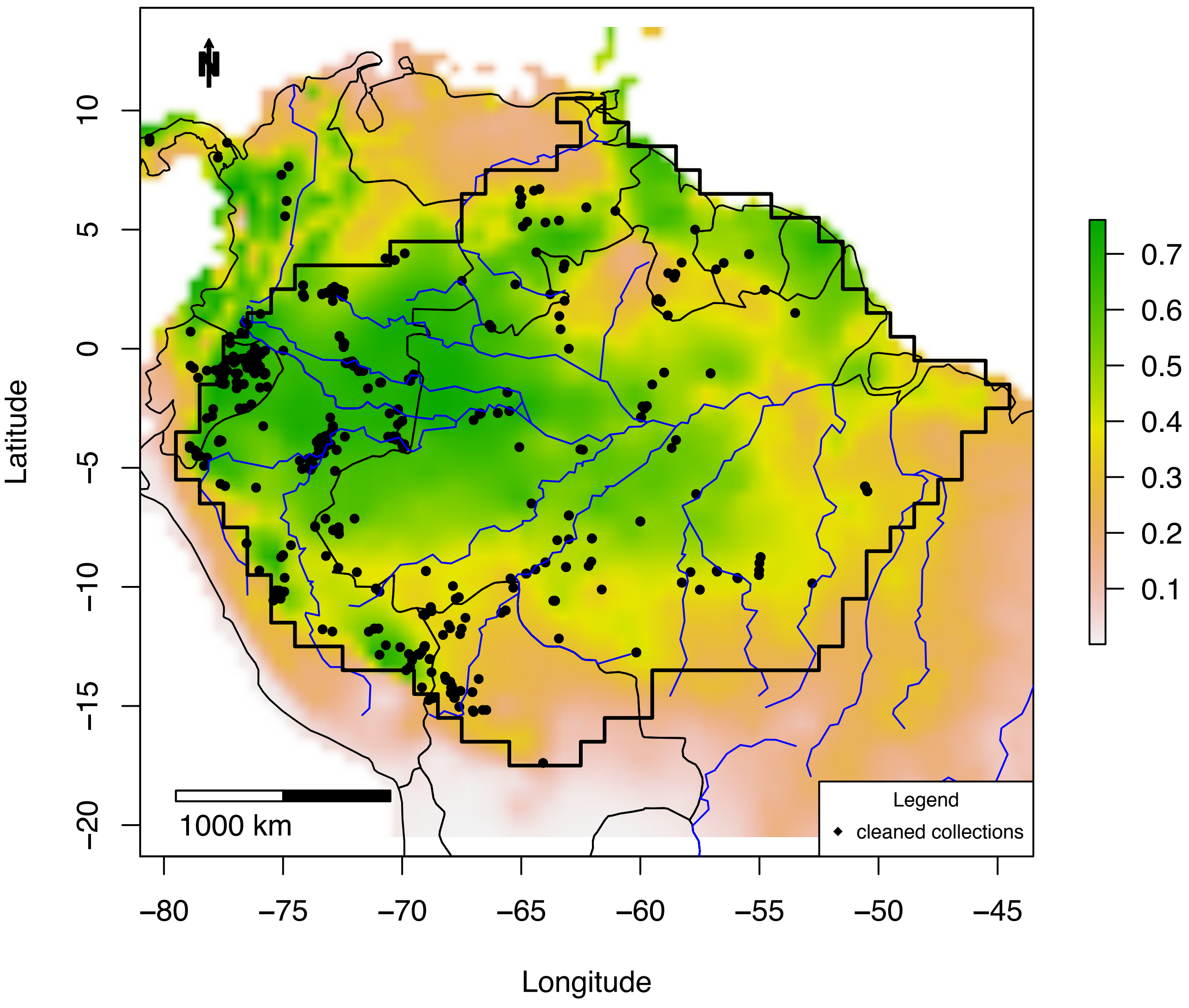

Figure S3_82C IDW map of Leonia_glycycarpa: plots =0 – 0.57; IDW: 0 – 0.04

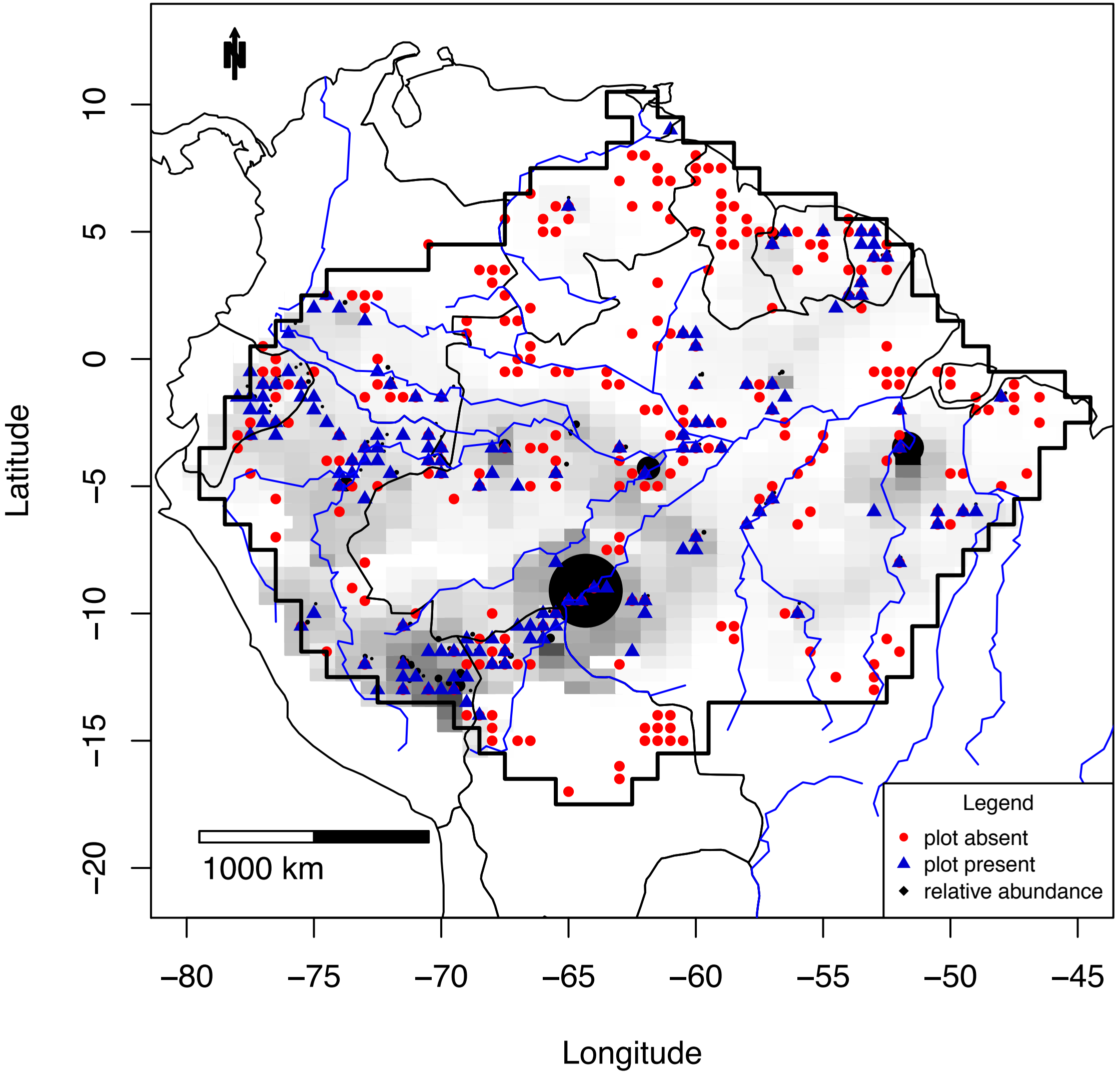

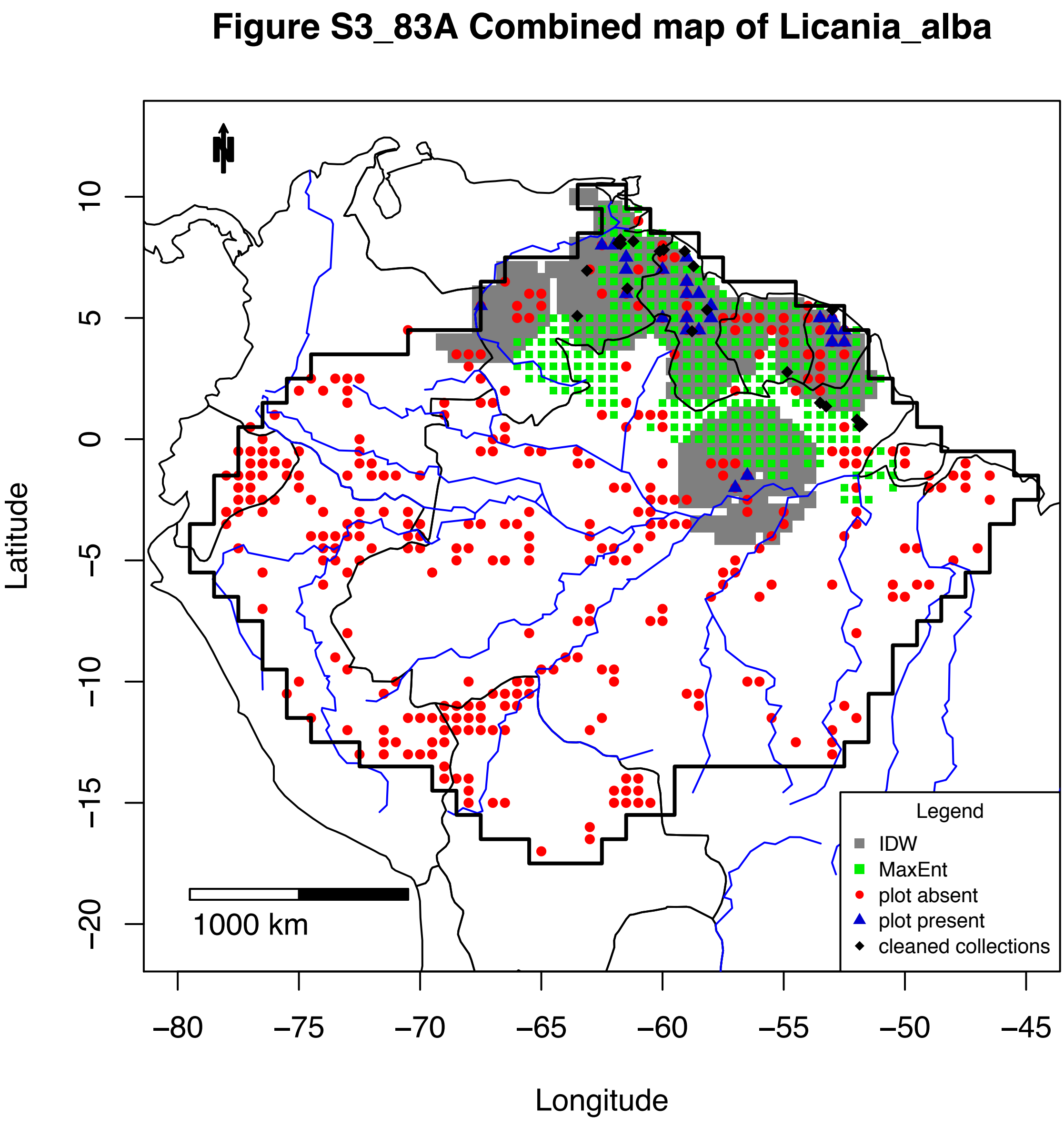
Figure S3_83A Combined map of Licania_alba
N
Latitude
Longitude
10
5
0
−5
−10
−15
−20
−80
−75
−70
−65
−60
−55
−50
−45
1000 km
Legend
IDW
MaxEnt
plot absent
plot present
cleaned collections

Figure S3_83B MaxEnt map of Licania_alba

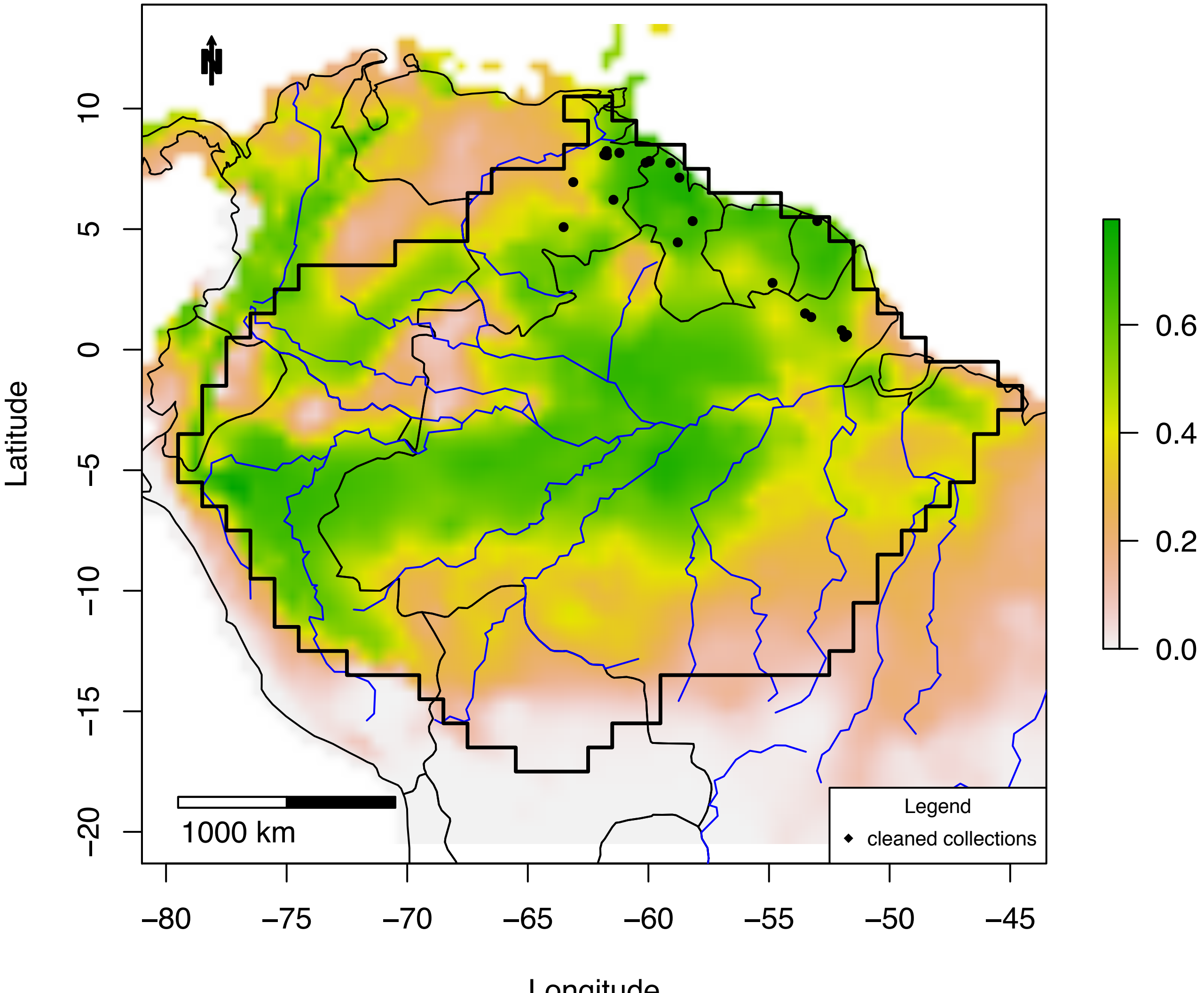

Figure S3_83C IDW map of Licania_alba: plots =0 – 0.12; IDW: 0 – 0.04

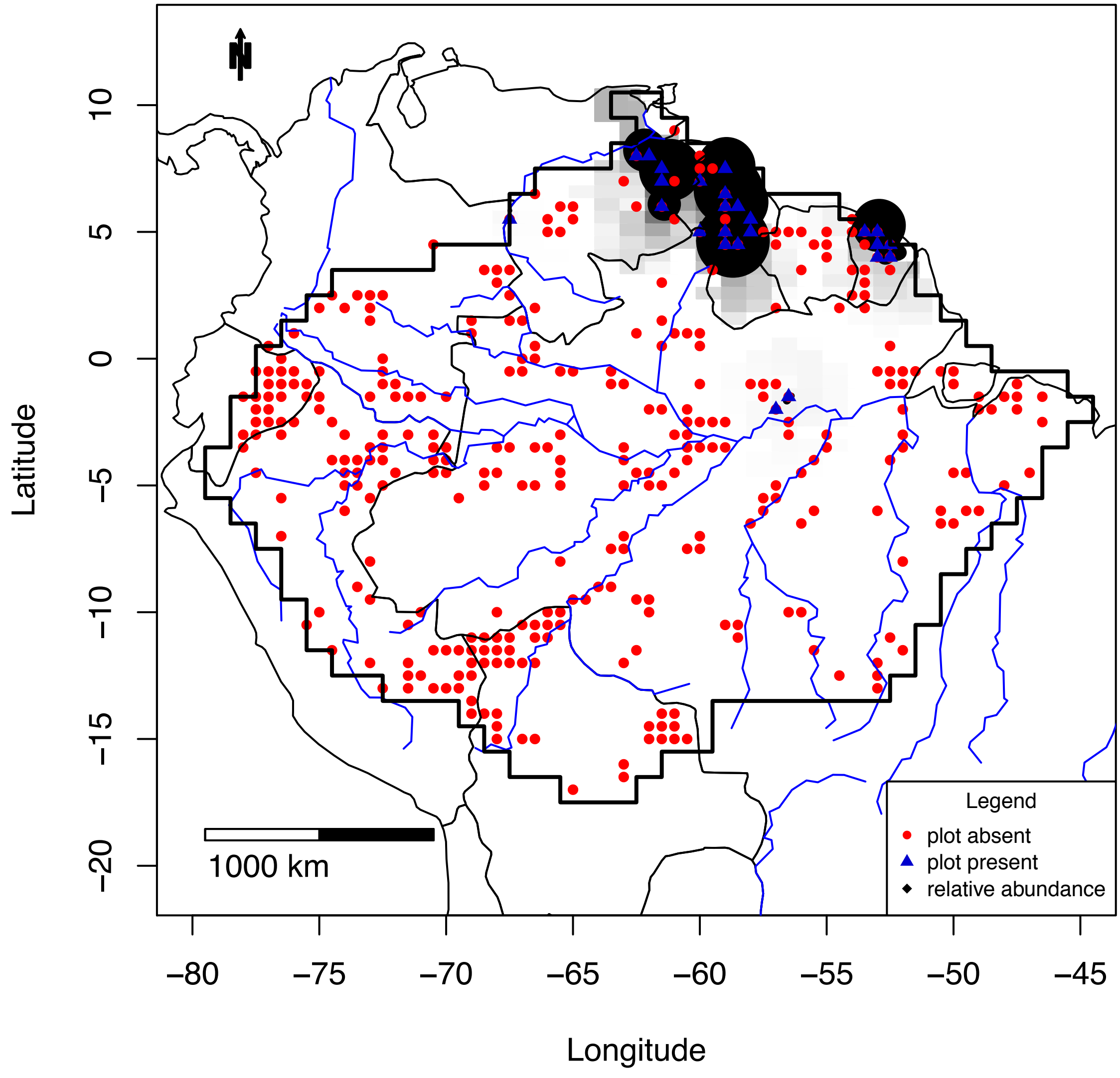

Figure S3_84A Combined map of Licania_apetala

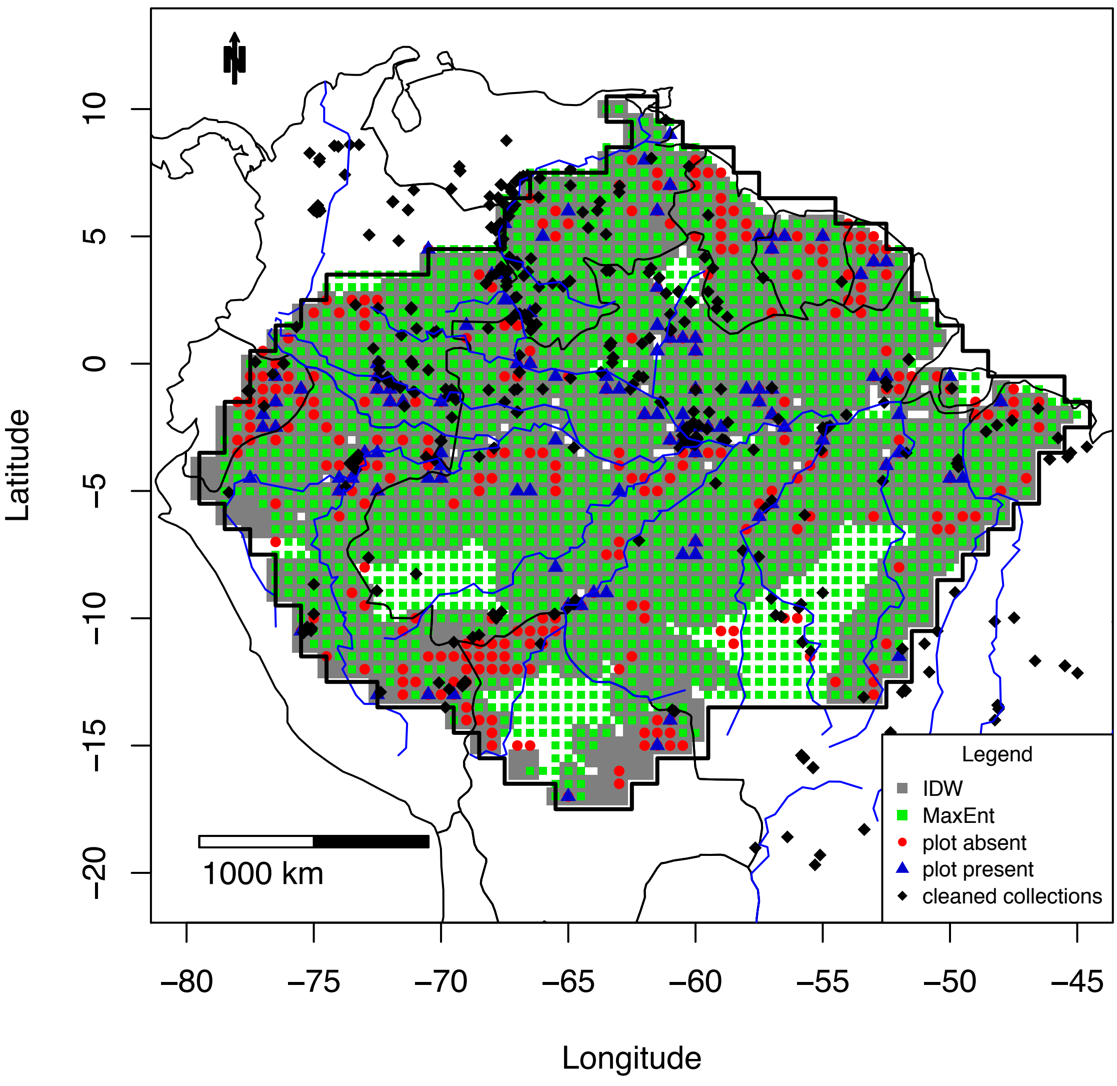

Figure S3_84B MaxEnt map of Licania_apetala

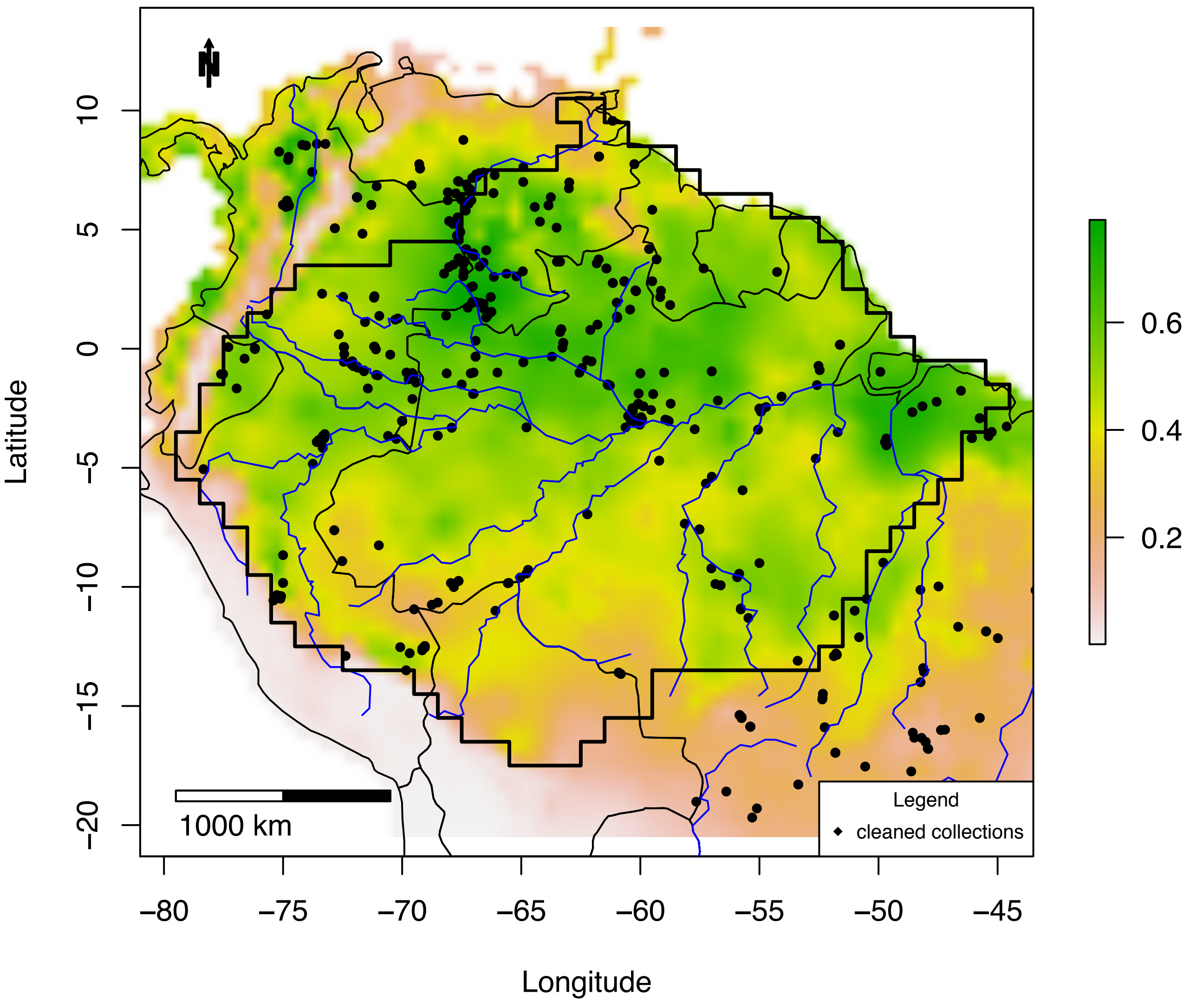

Figure S3_84C IDW map of Licania_apetala: plots =0 – 0.44; IDW: 0 – 0.08

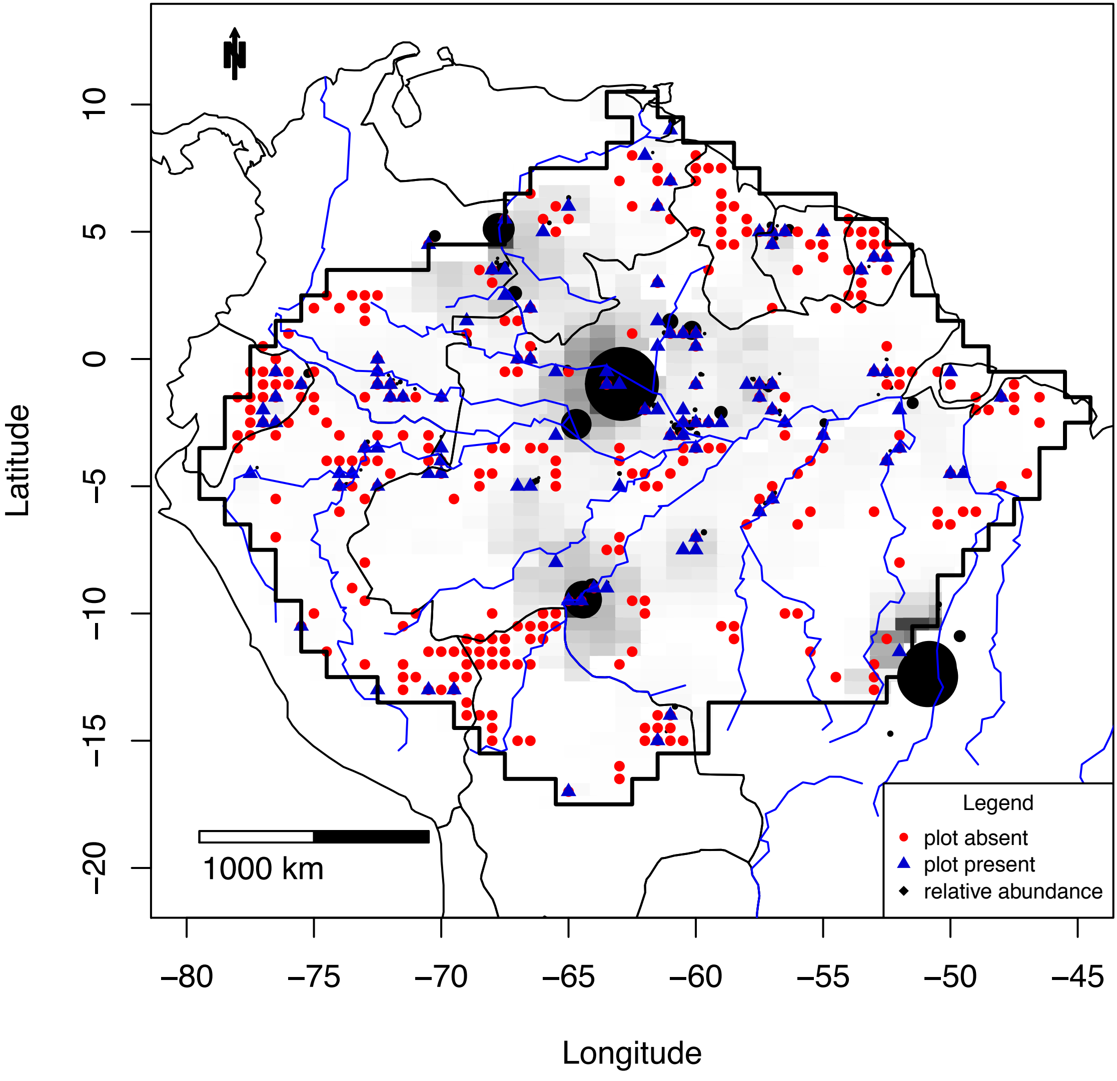

Figure S3_85A Combined map of Licania_densiflora

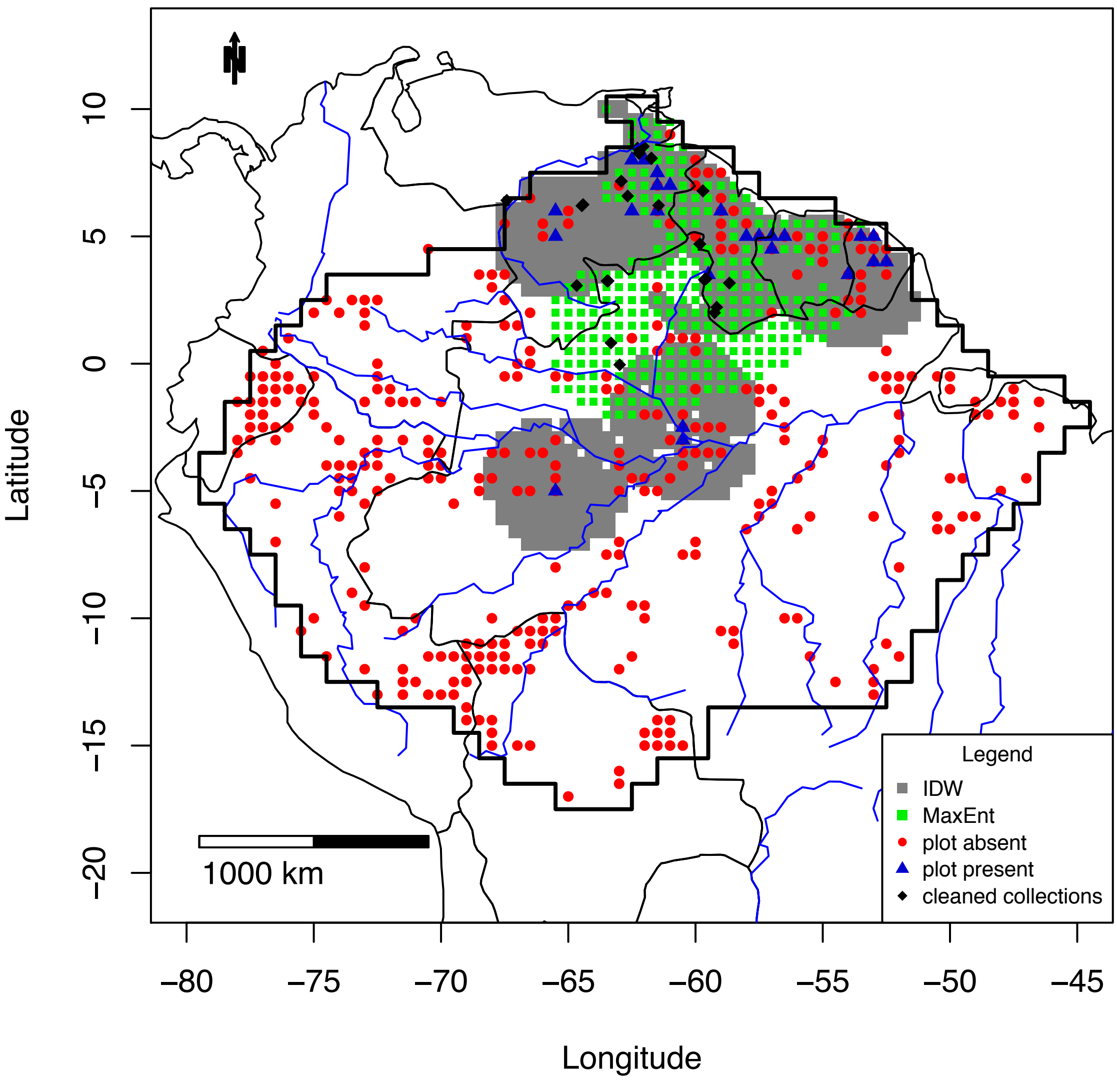

Figure S3_85B MaxEnt map of Licania_densiflora

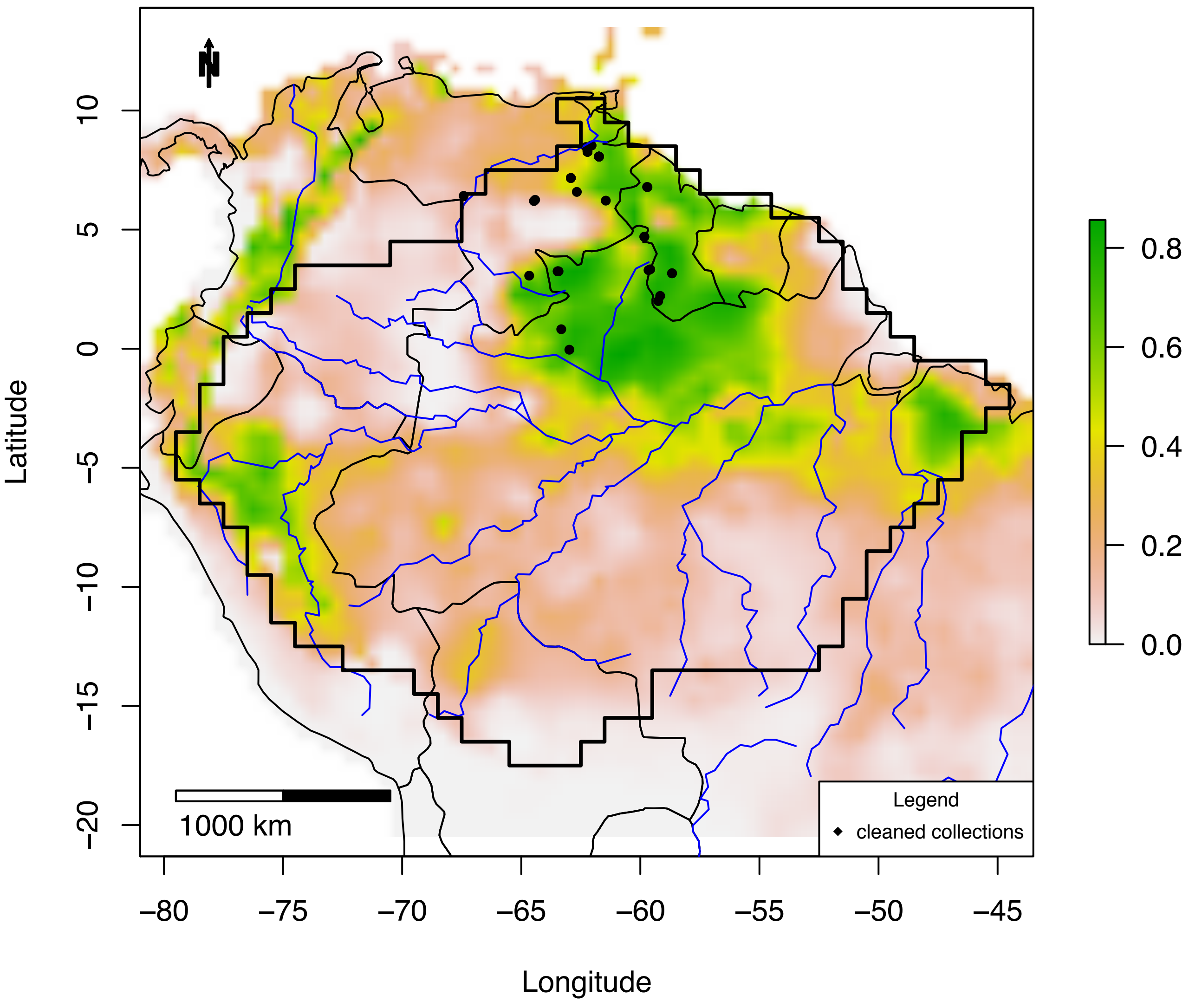

Figure S3_85C IDW map of Licania_densiflora: plots =0 – 0.45; IDW: 0 – 0.07

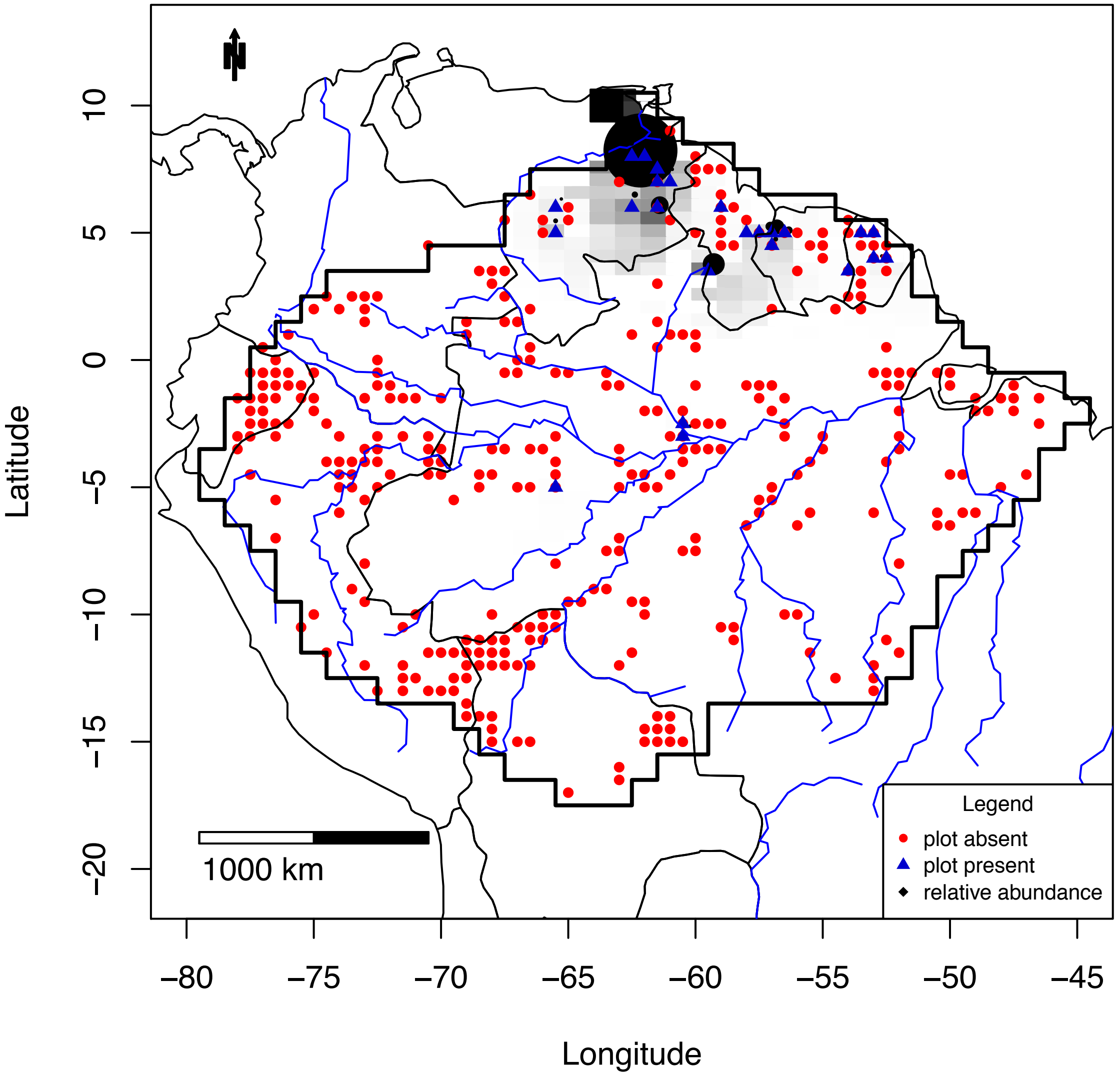

Figure S3_86A Combined map of Licania_heteromorpha

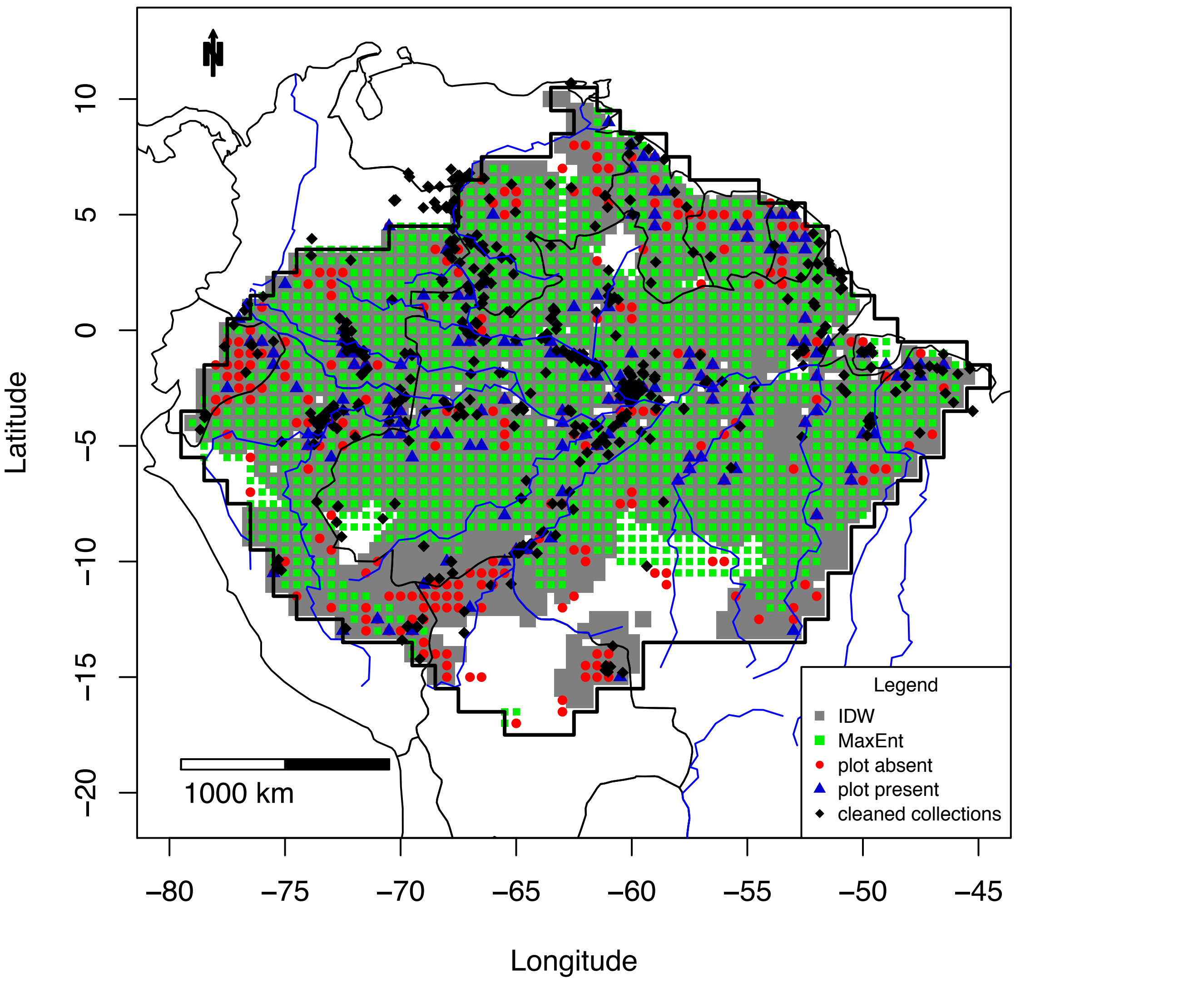

Figure S3_86B MaxEnt map of Licania_heteromorpha

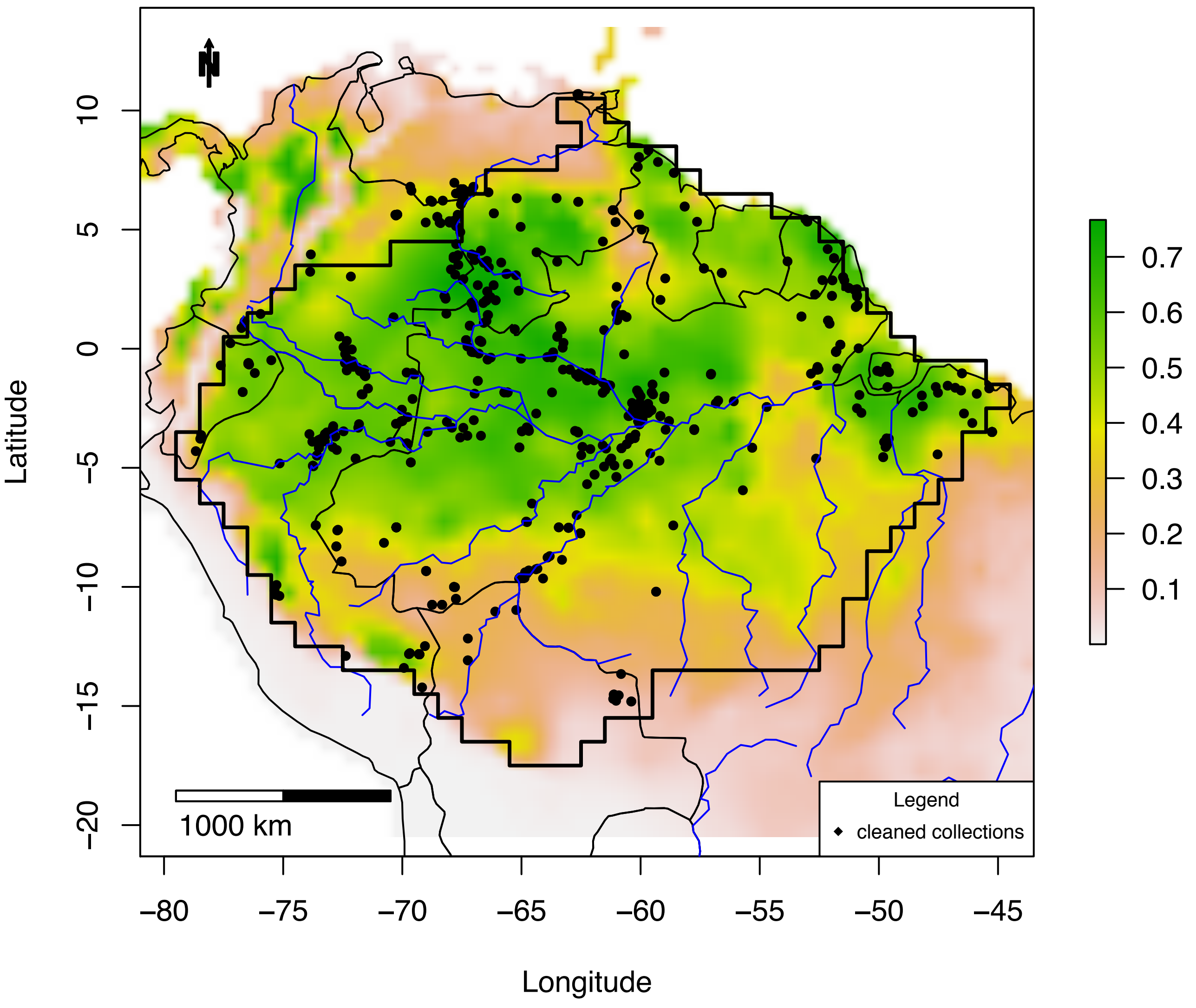

Figure S3_86C IDW map of Licania_heteromorpha: plots =0 – 0.28; IDW: 0 – 0.08

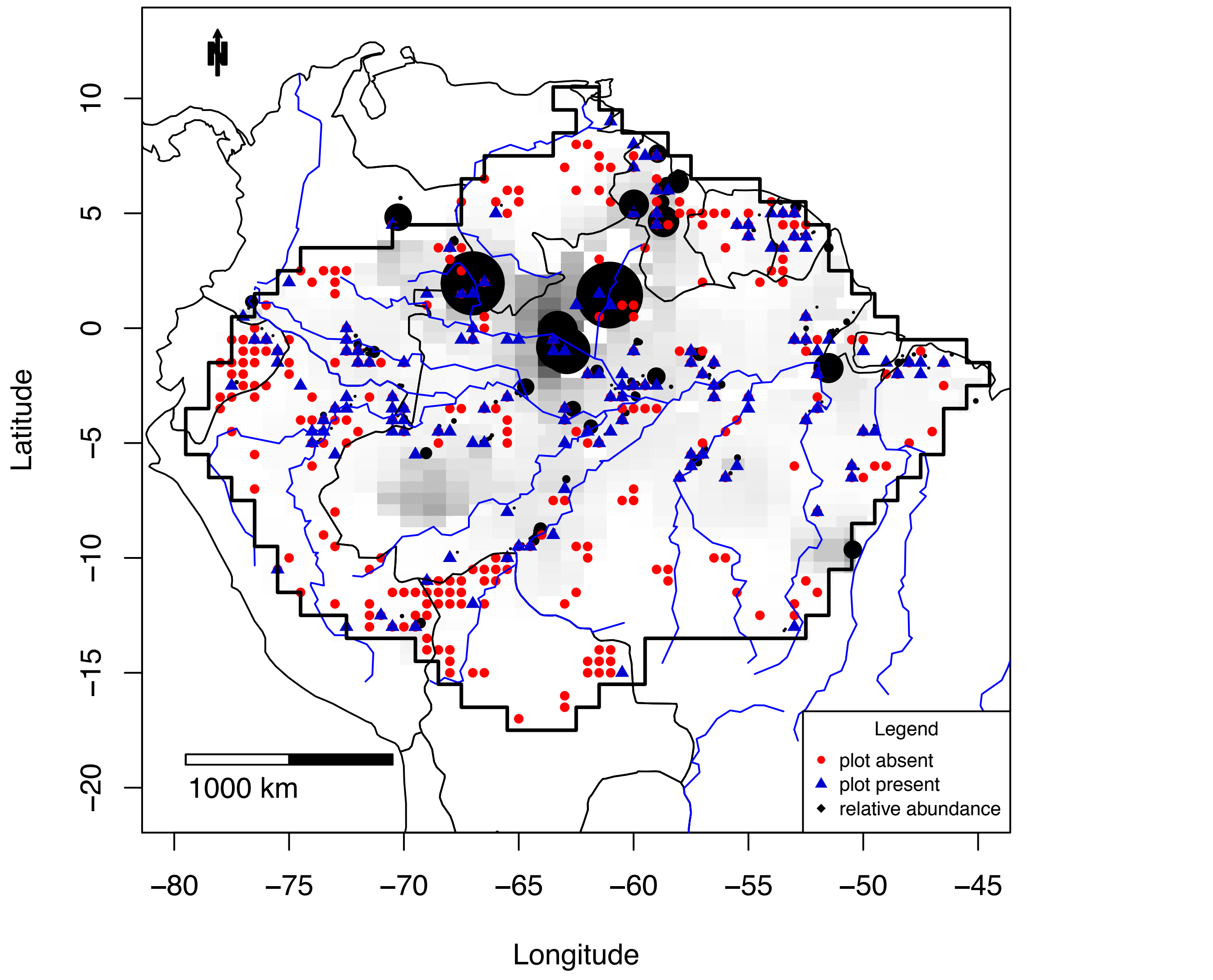

Figure S3_87A Combined map of Licania_micrantha

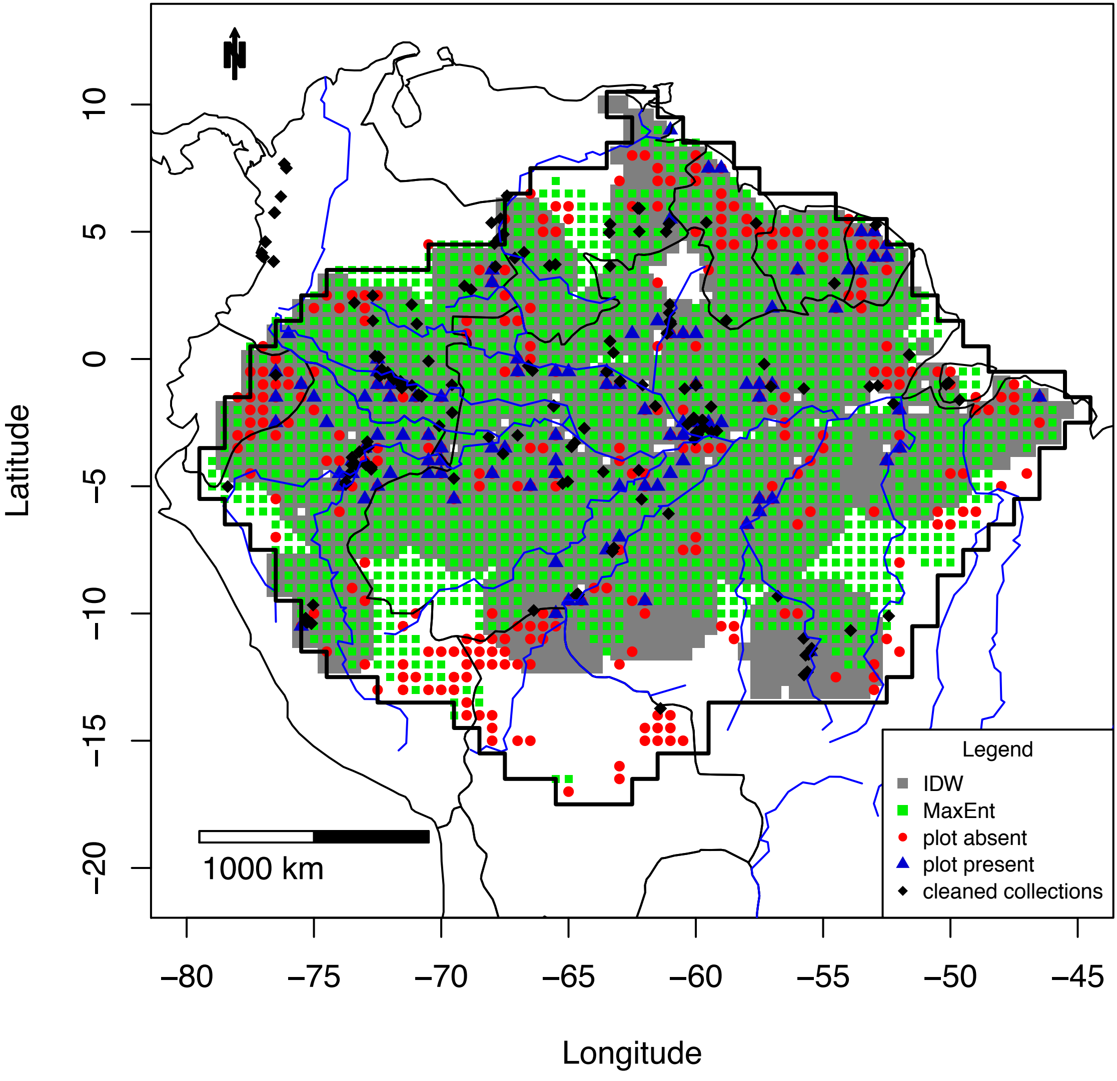

Figure S3_87B MaxEnt map of Licania_micrantha

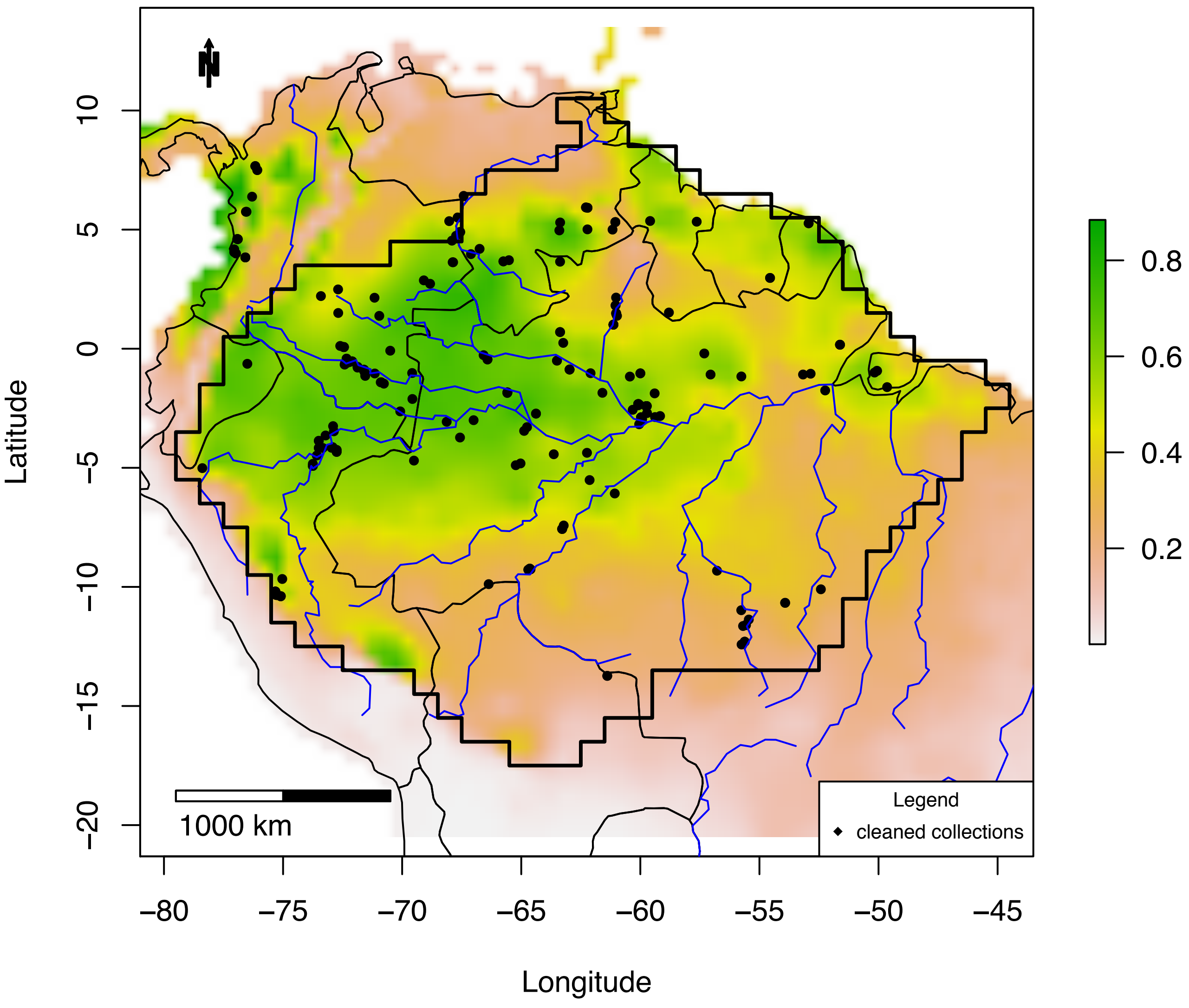

Figure S3_87C IDW map of Licania_micrantha: plots =0 – 0.26; IDW: 0 – 0.06

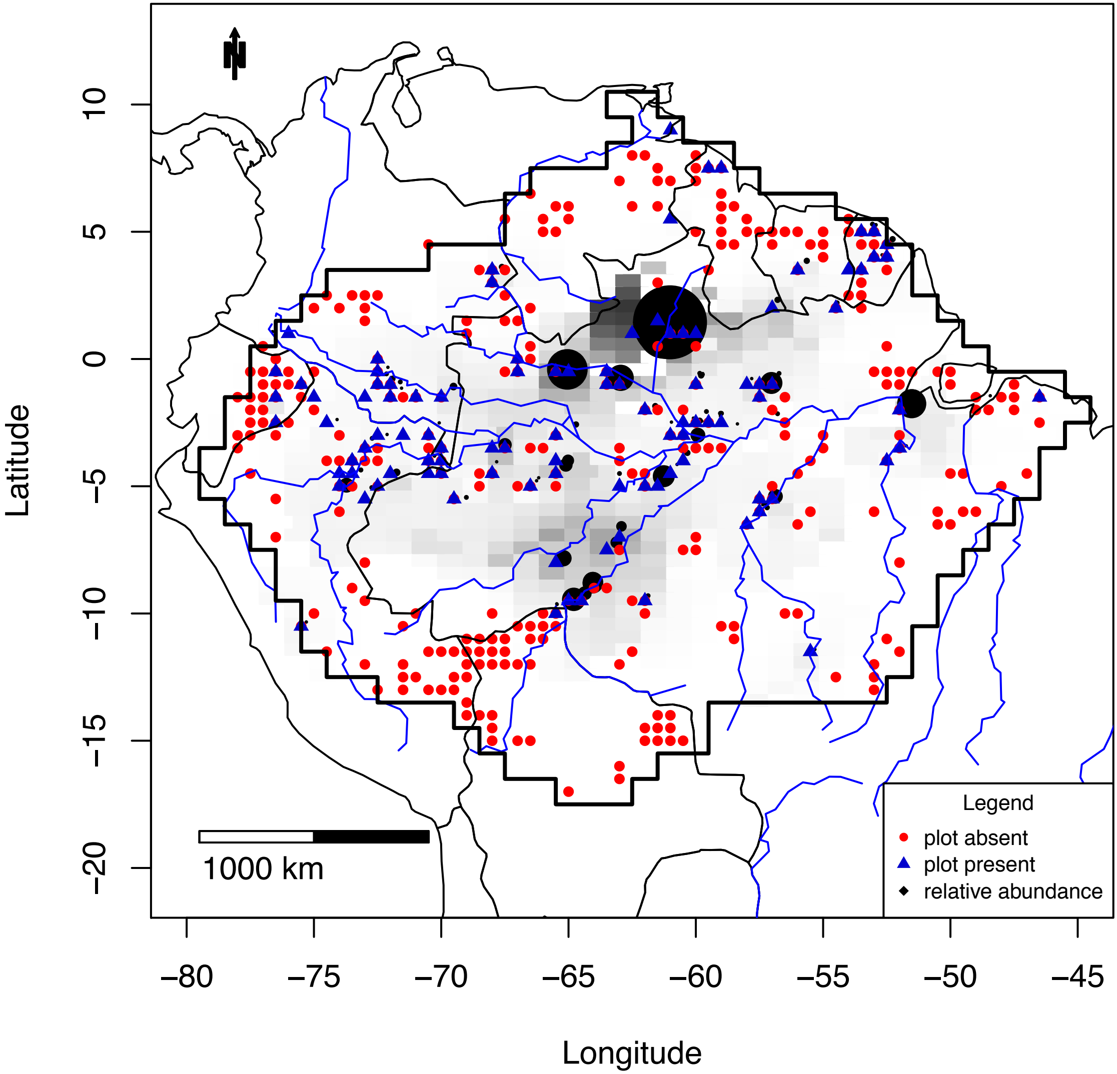

Figure S3_88A Combined map of Licania_oblongifolia

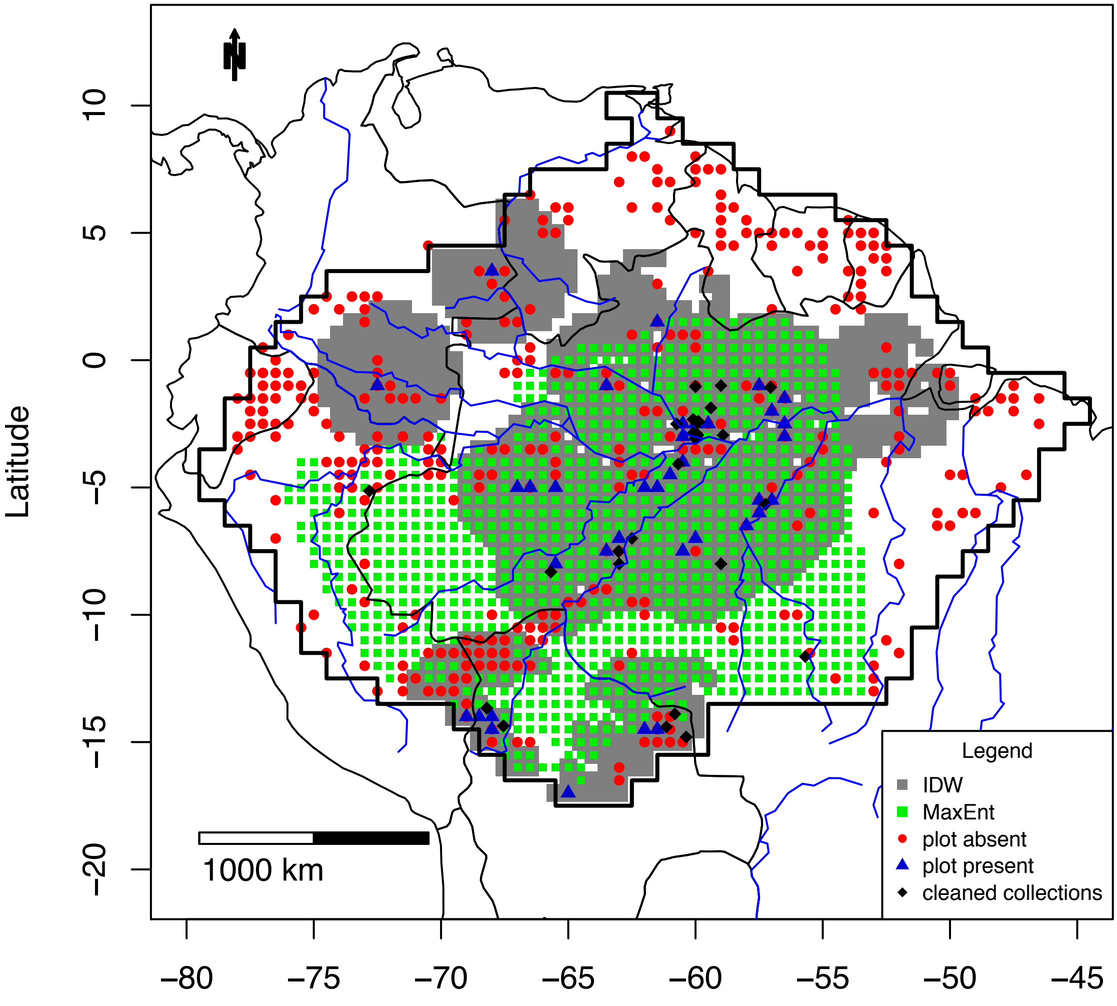

Figure S3_88B MaxEnt map of Licania_oblongifolia

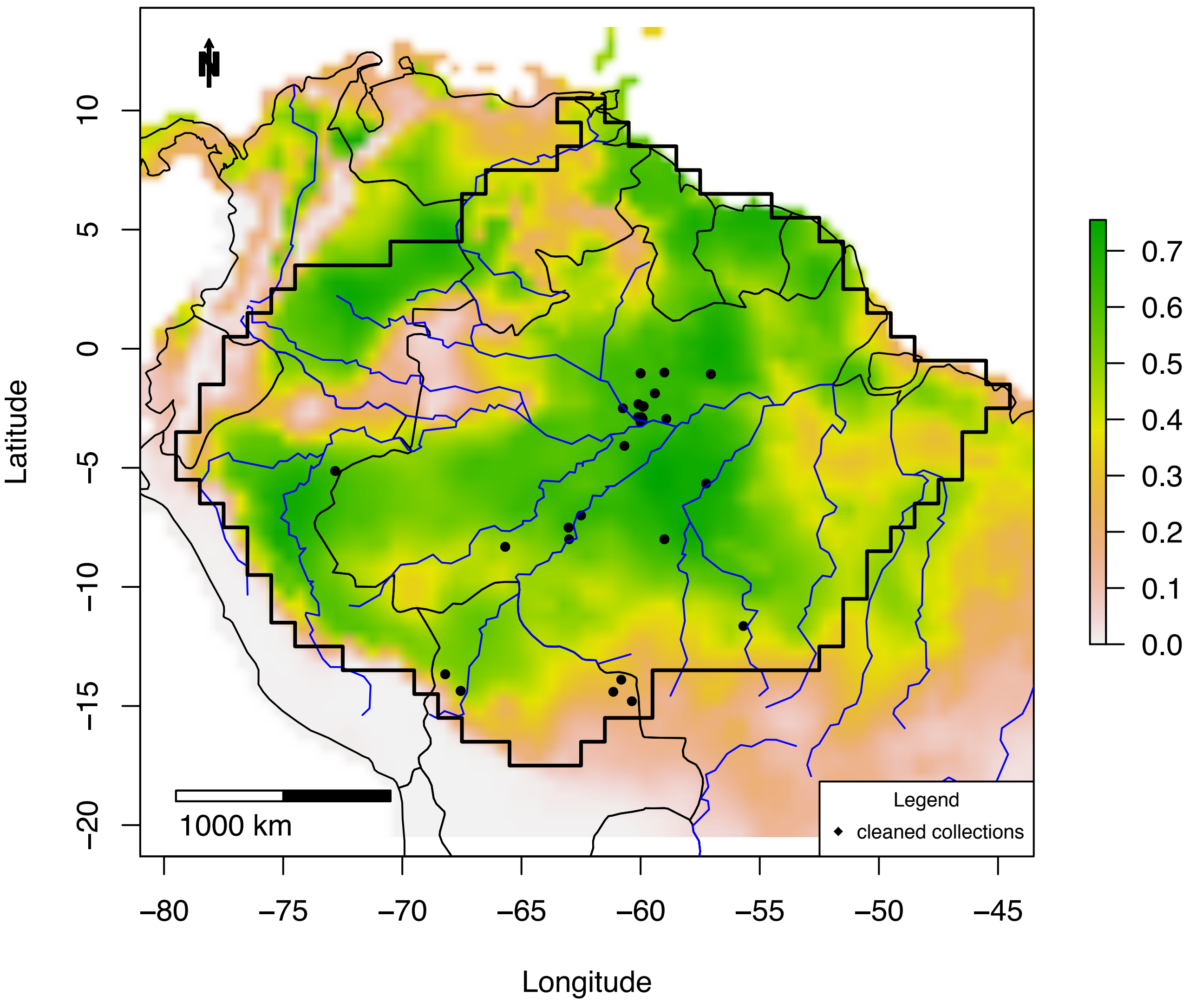

Figure S3_88C IDW map of Licania_oblongifolia: plots =0 – 0.07; IDW: 0 – 0.03

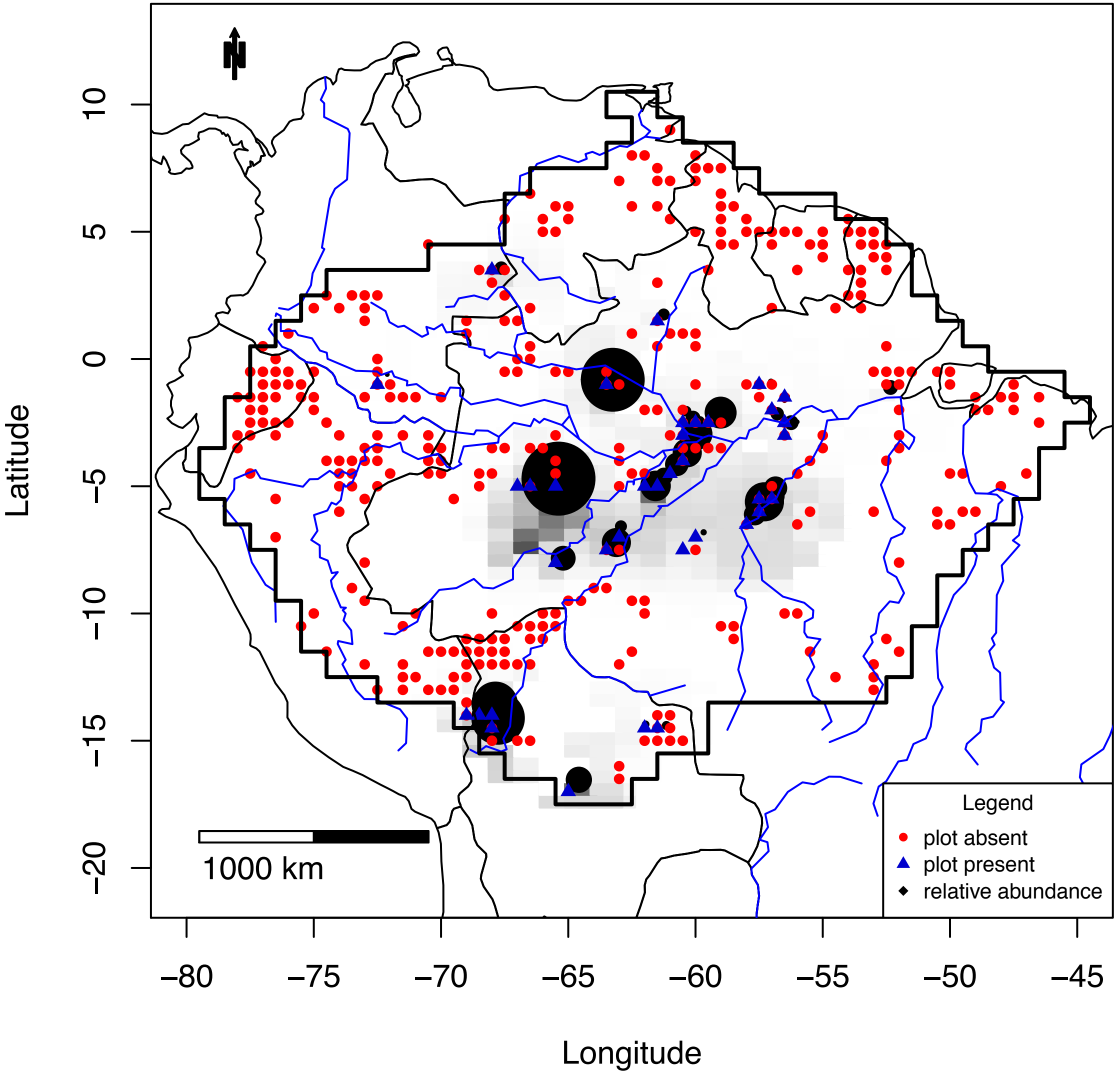

Figure S3_89A Combined map of Luehea_cymulosa

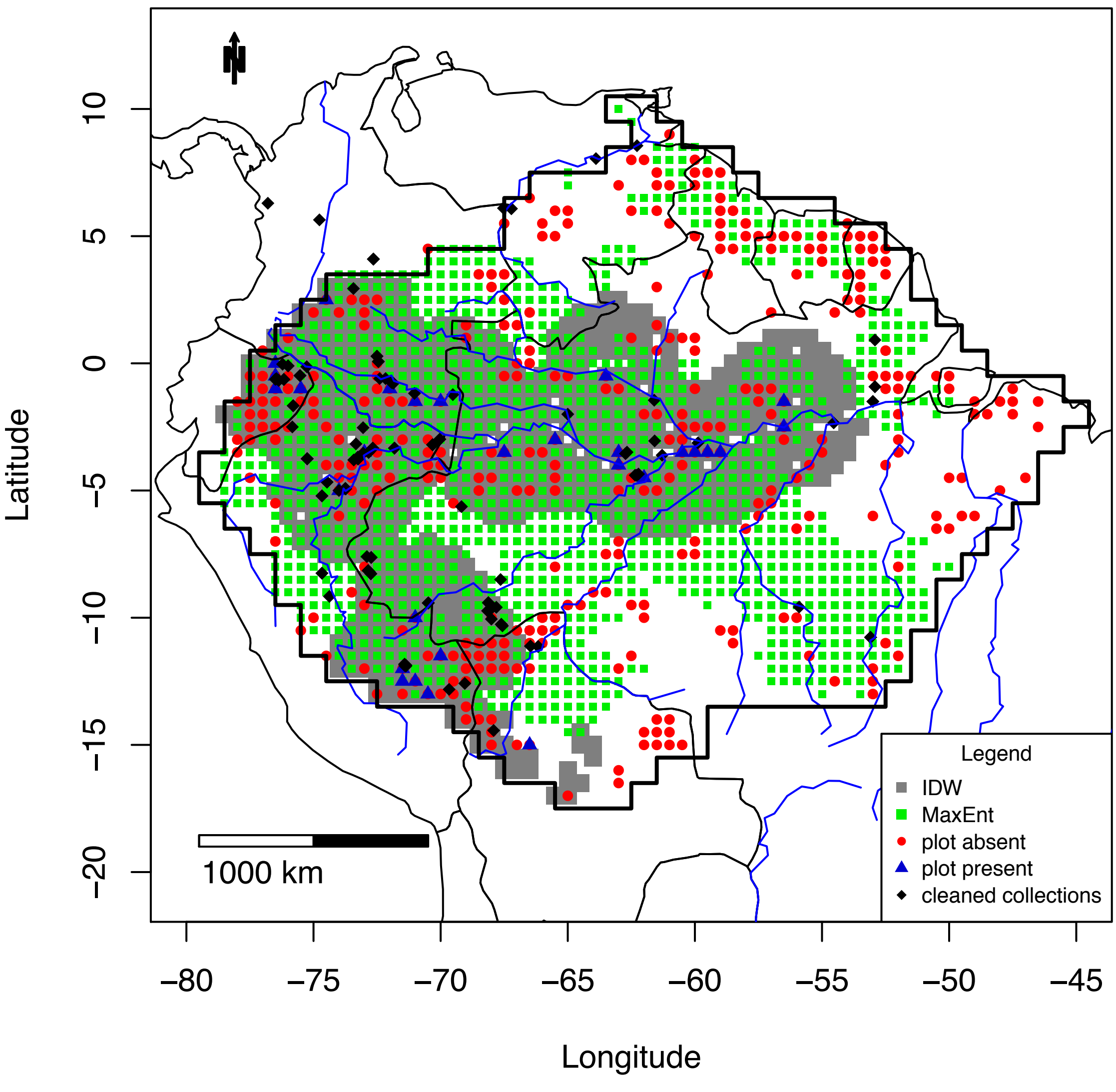

Figure S3_89B MaxEnt map of Luehea_cymulosa

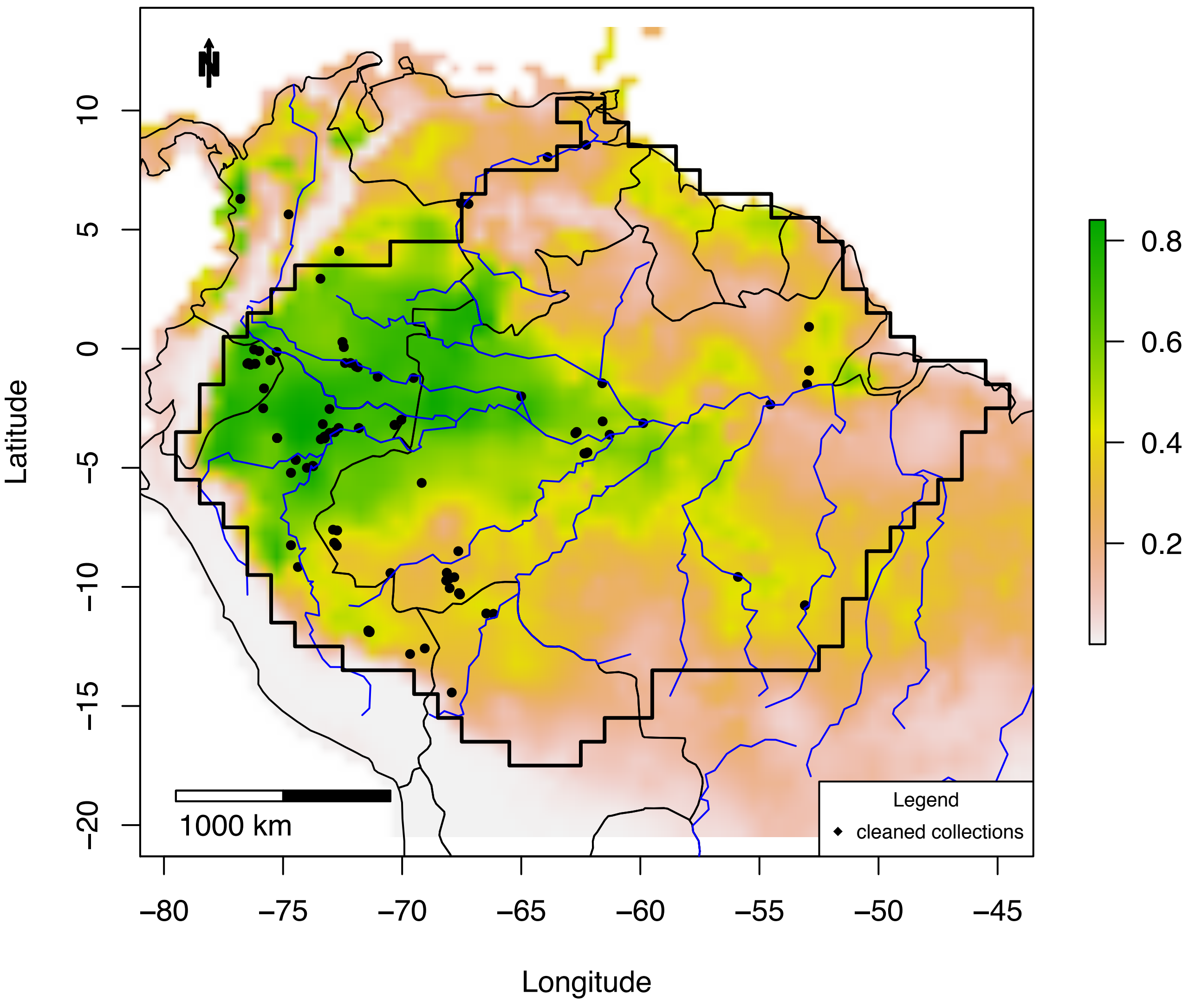

Figure S3_89C IDW map of Luehea_cymulosa:
plots =0 – 0.44; IDW: 0 – 0.04

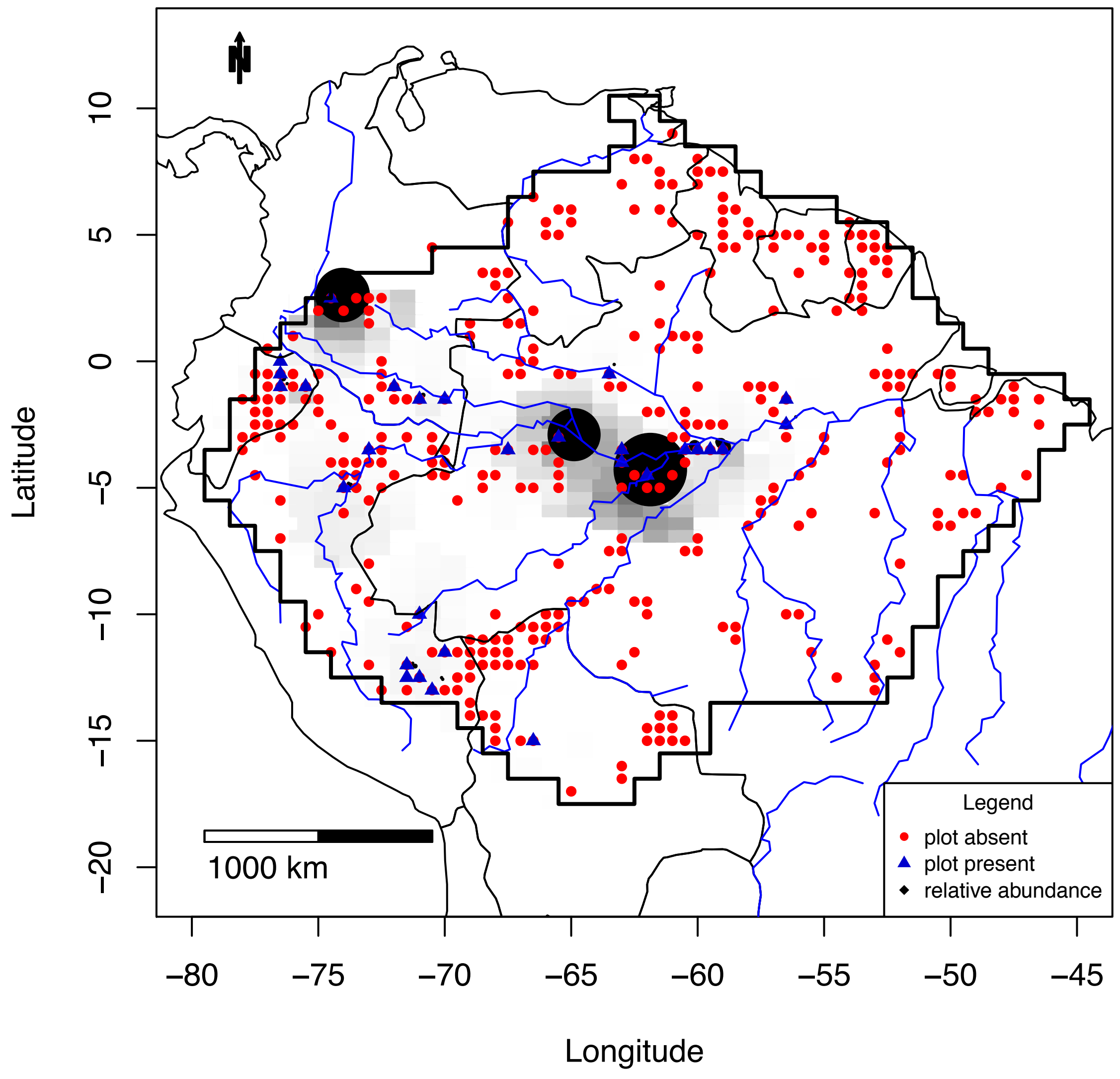

Figure S3_90A Combined map of Mabea_nitida

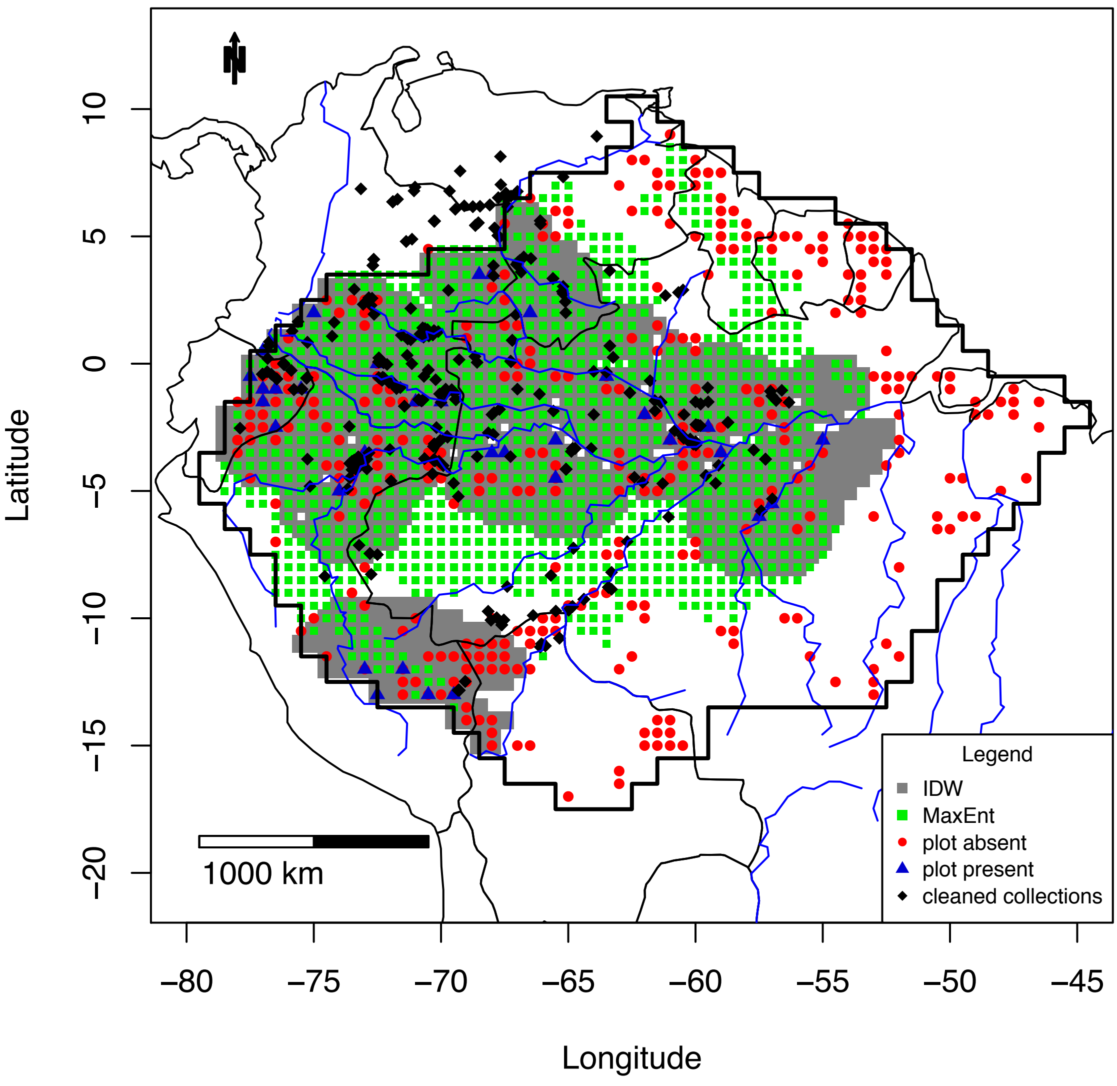

Figure S3_90B MaxEnt map of Mabea_nitida

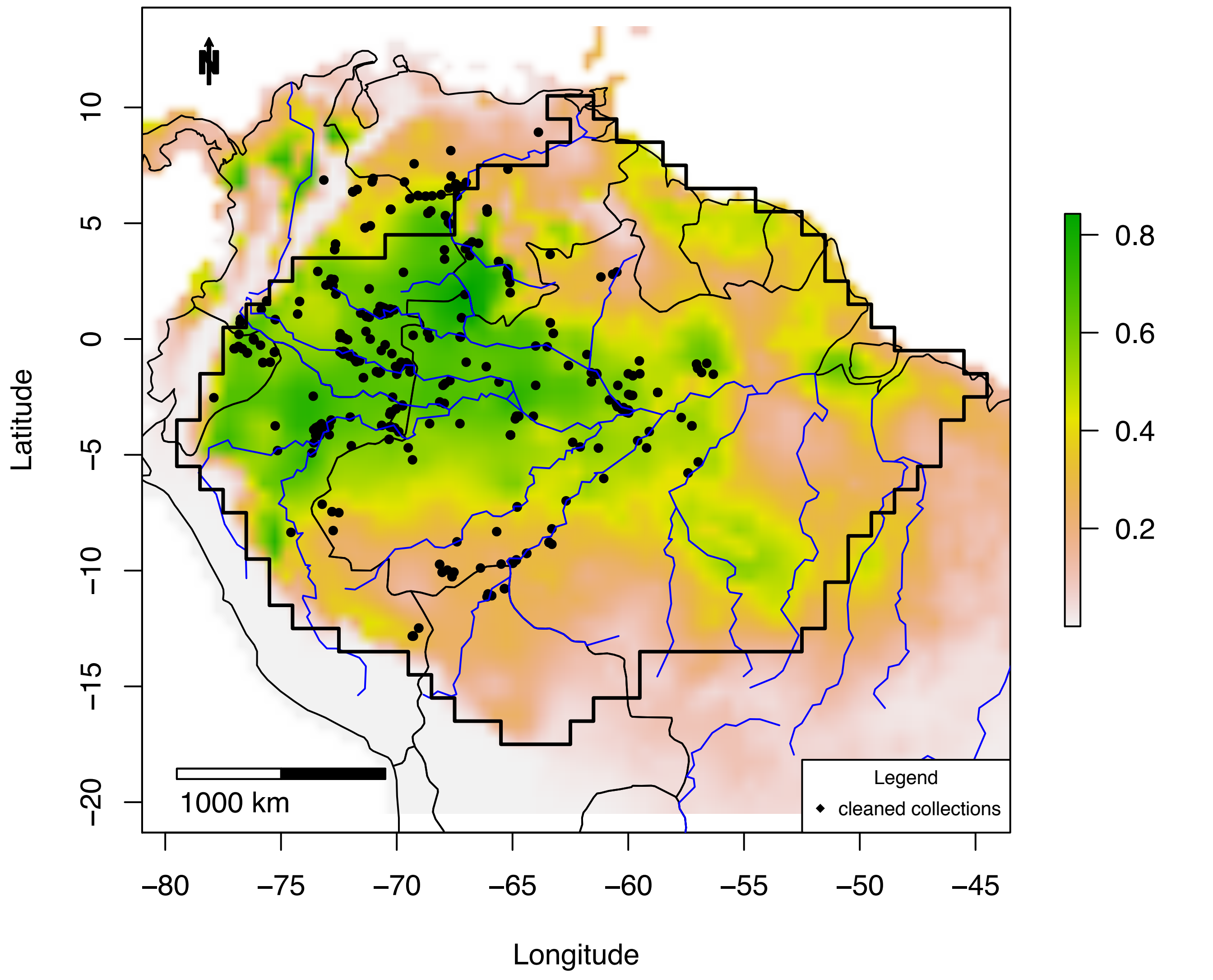

Figure S3_90C IDW map of Mabea_nitida: plots =0 – 0.19; IDW: 0 – 0.09

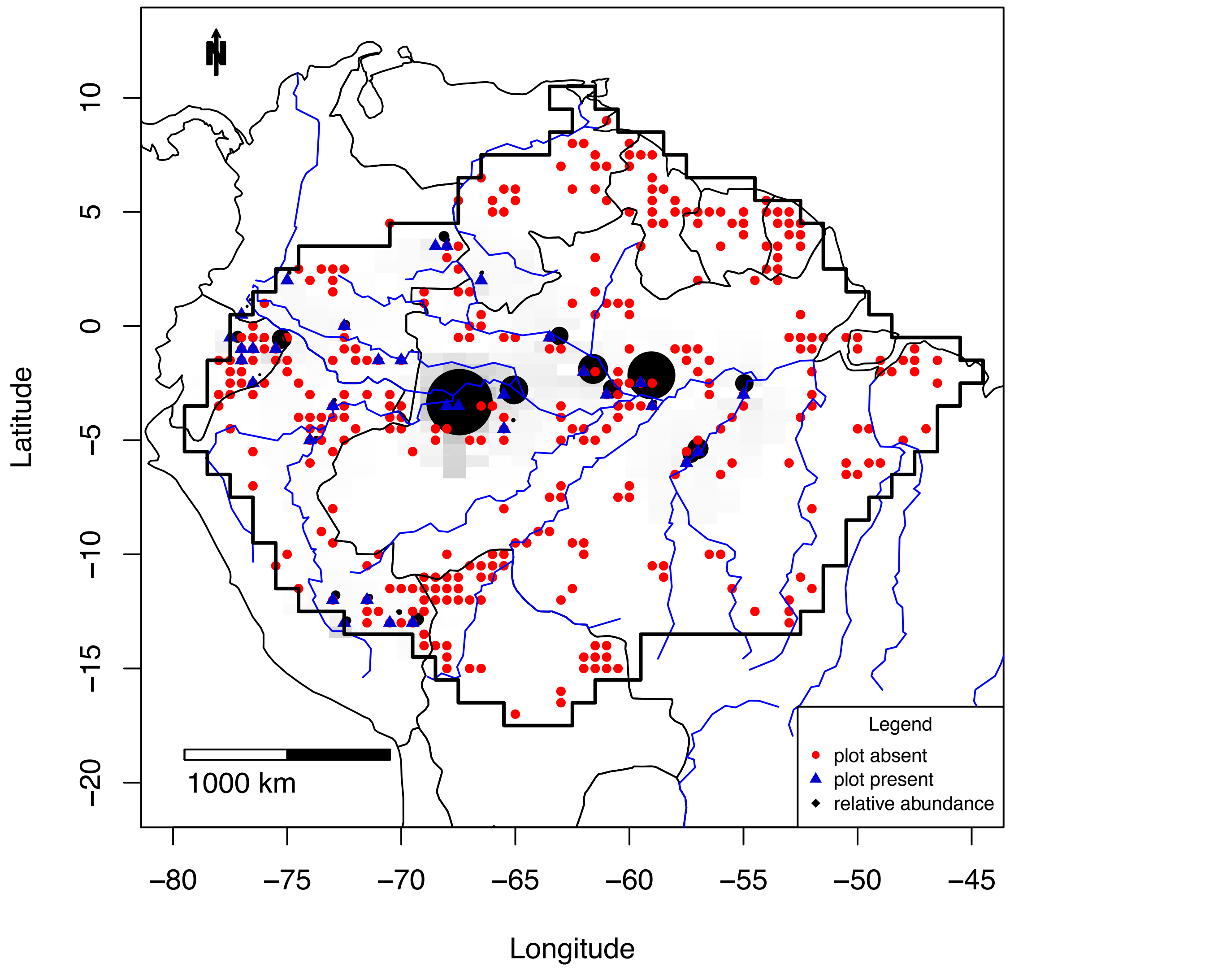

Figure S3_91A Combined map of Mabea_speciosa

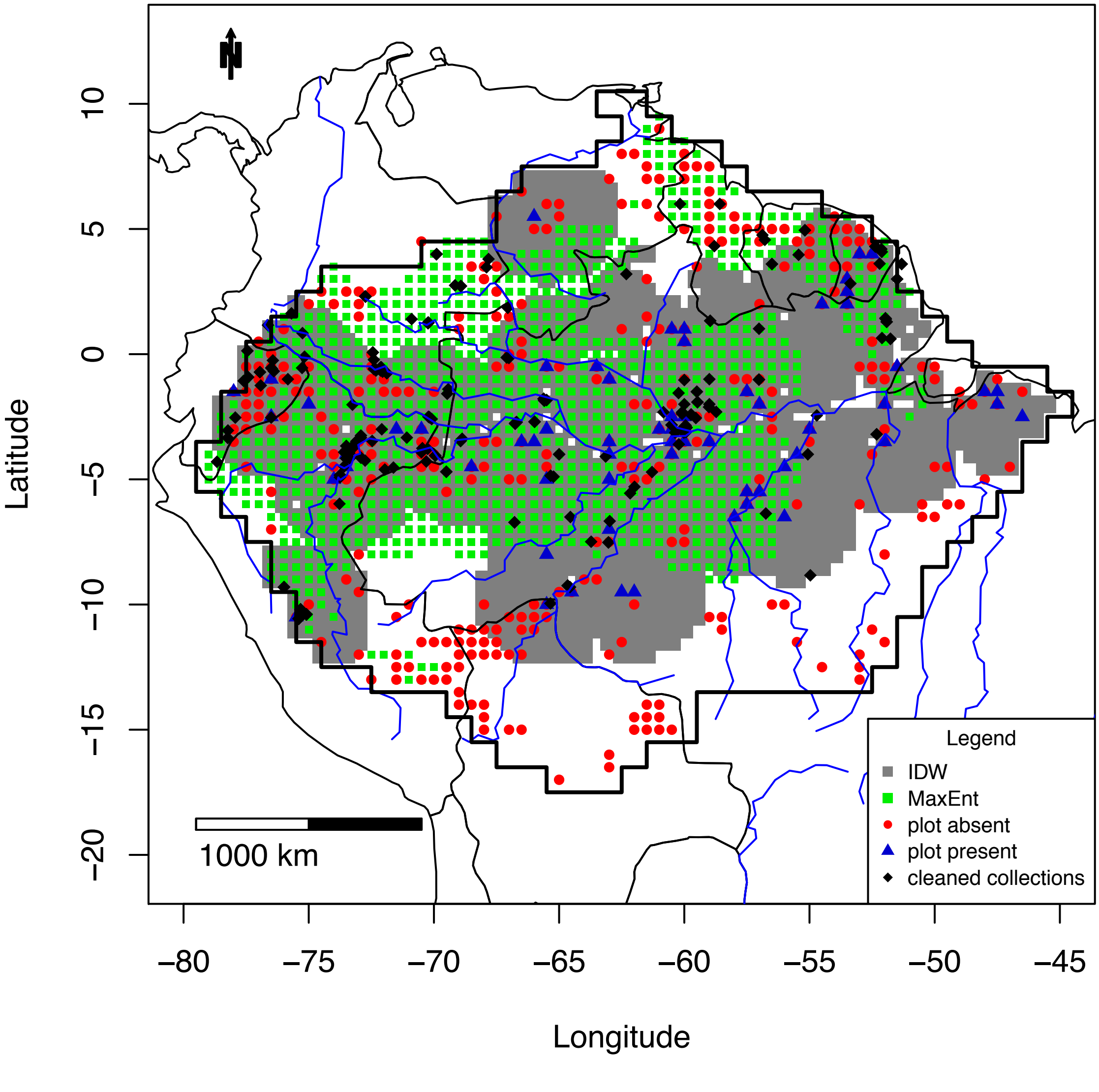

Figure S3_91B MaxEnt map of Mabea_speciosa

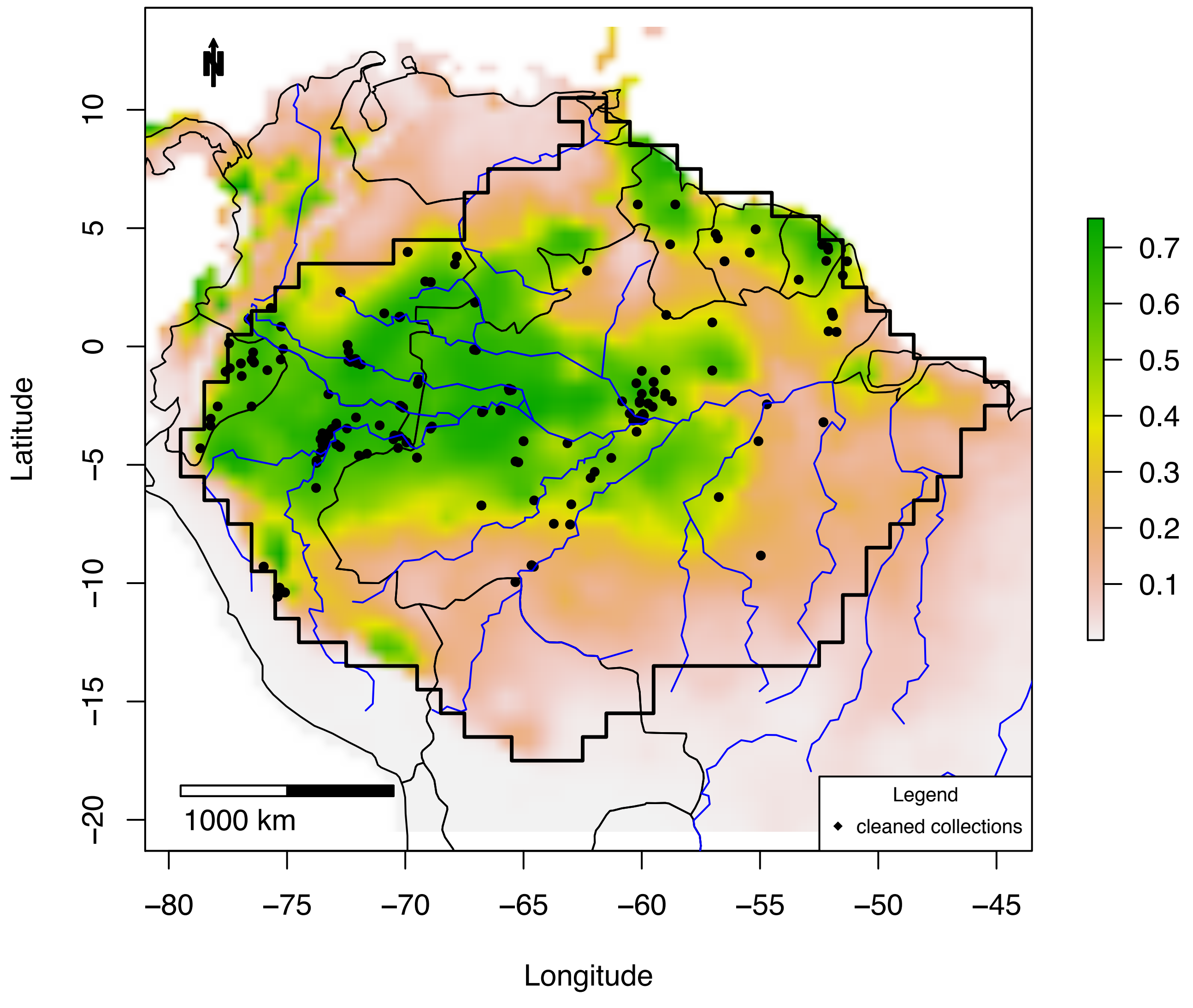

Figure S3_91C IDW map of Mabea_speciosa: plots =0 – 0.33; IDW: 0 – 0.07

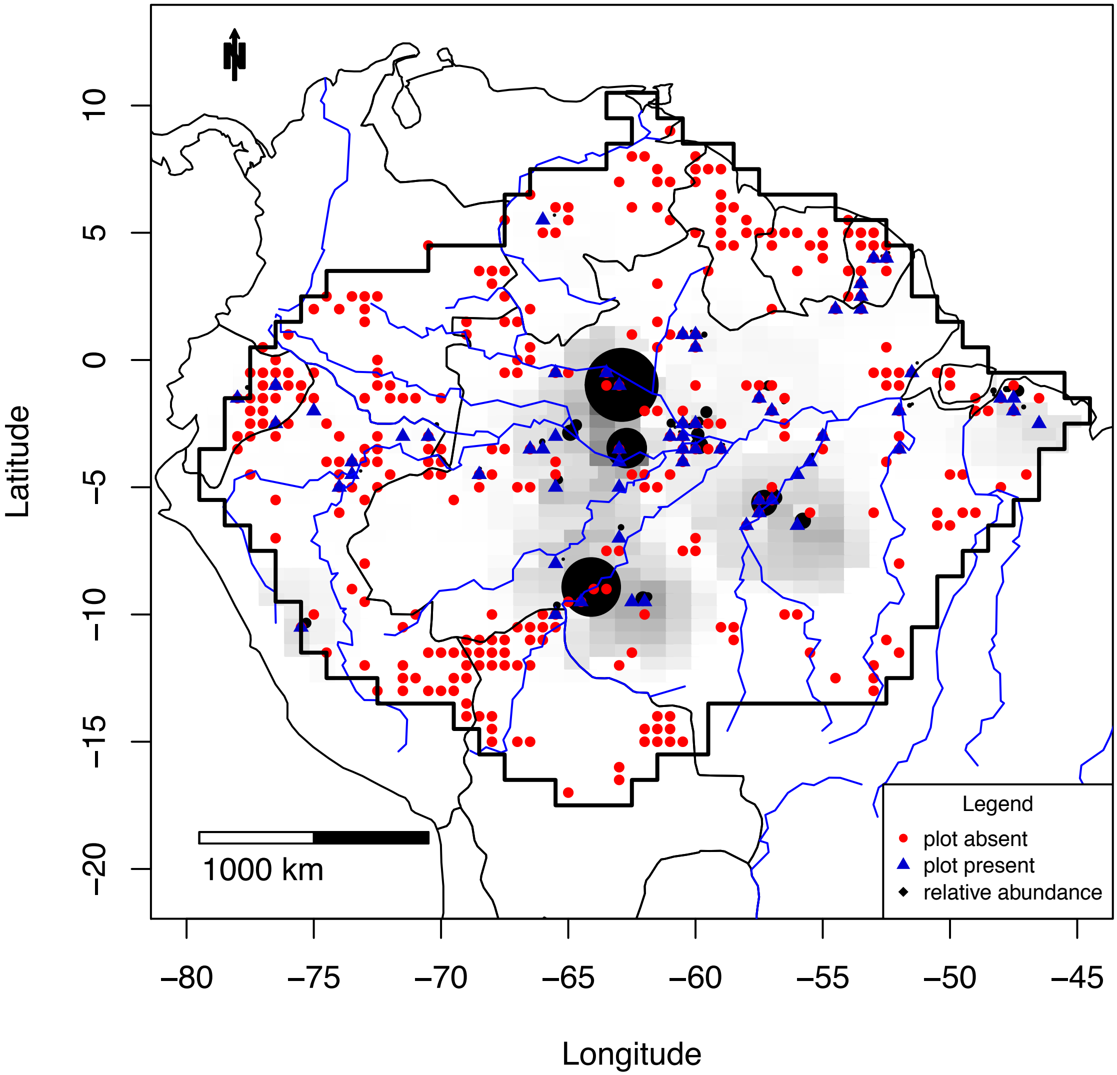

Figure S3_92A Combined map of Macrolobium_acaciifolium

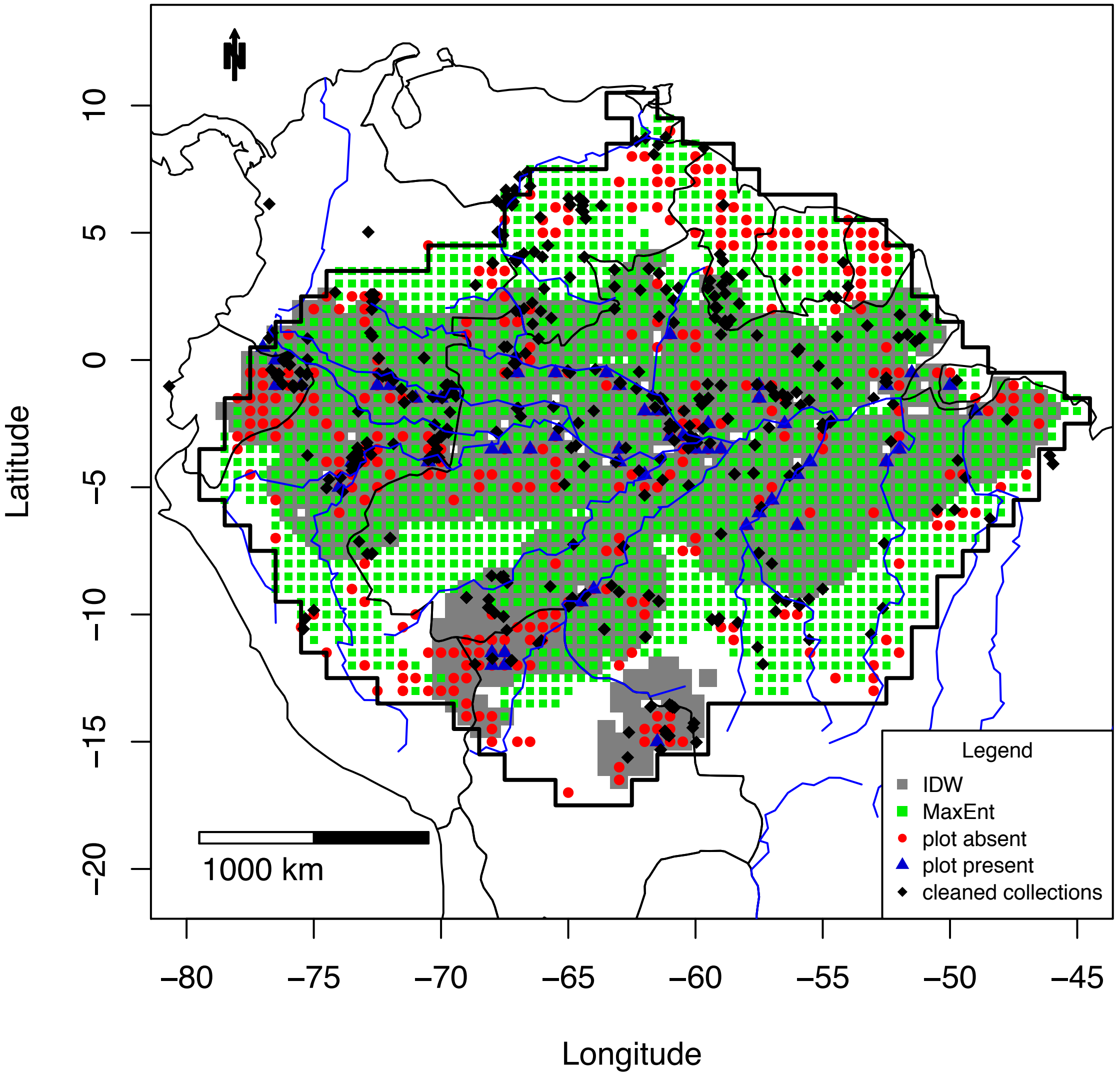

Figure S3_92B MaxEnt map of Macrolobium_acaciifolium

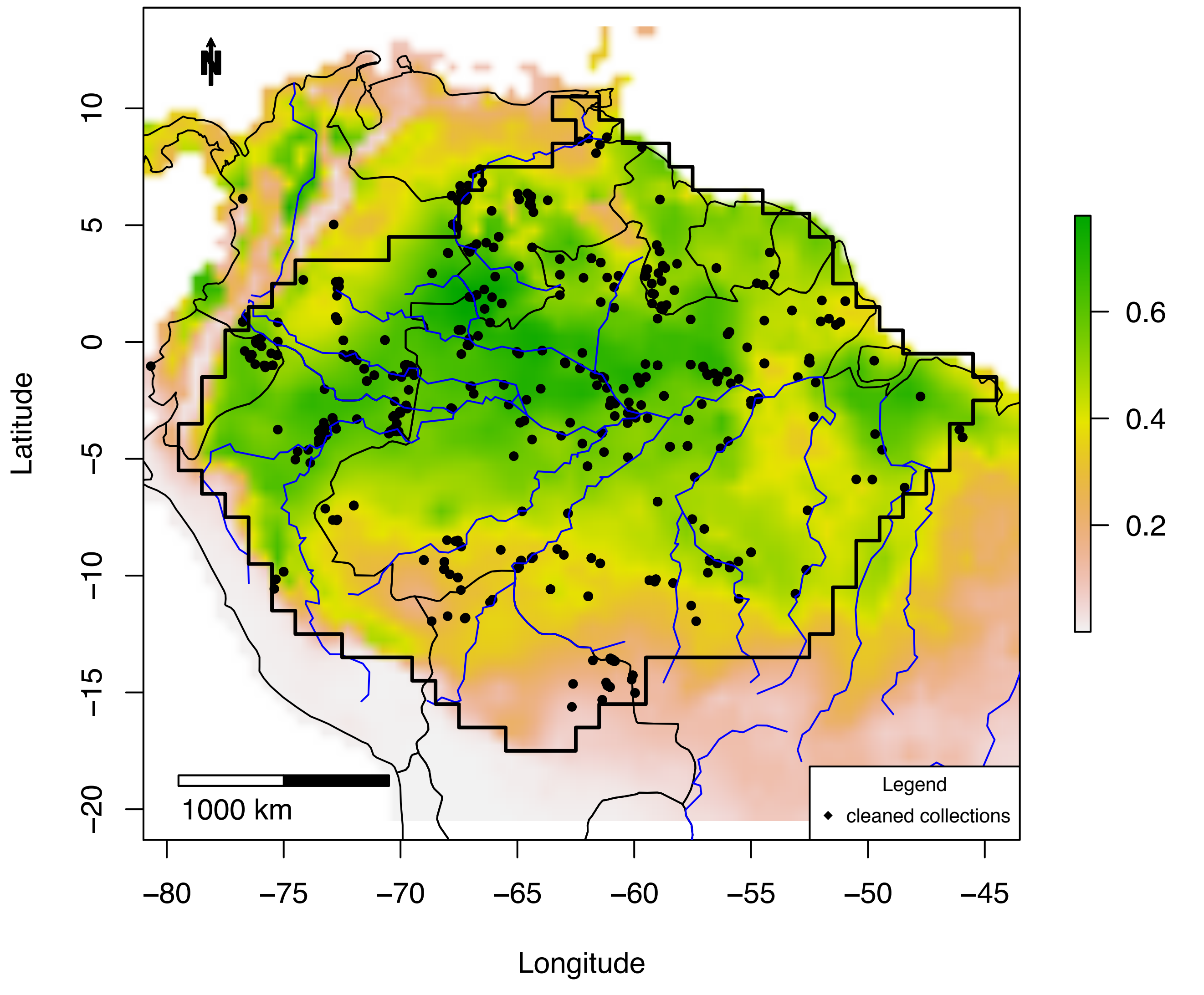

Figure S3_92C IDW map of Macrolobium_acaciifolium: plots =0 – 0.19; IDW: 0 – 0.03

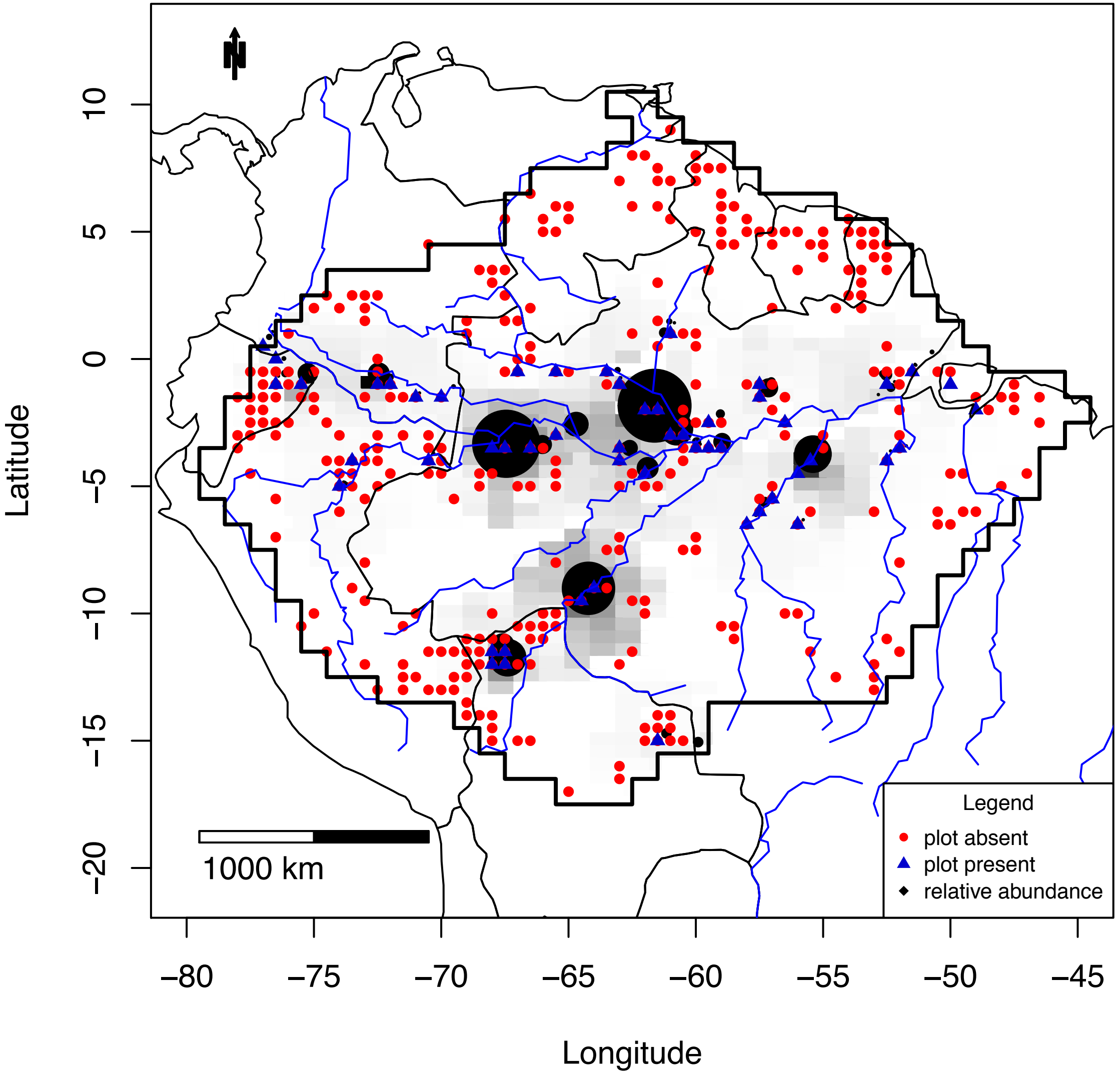

Figure S3_93A Combined map of Macrolobium_angustifolium

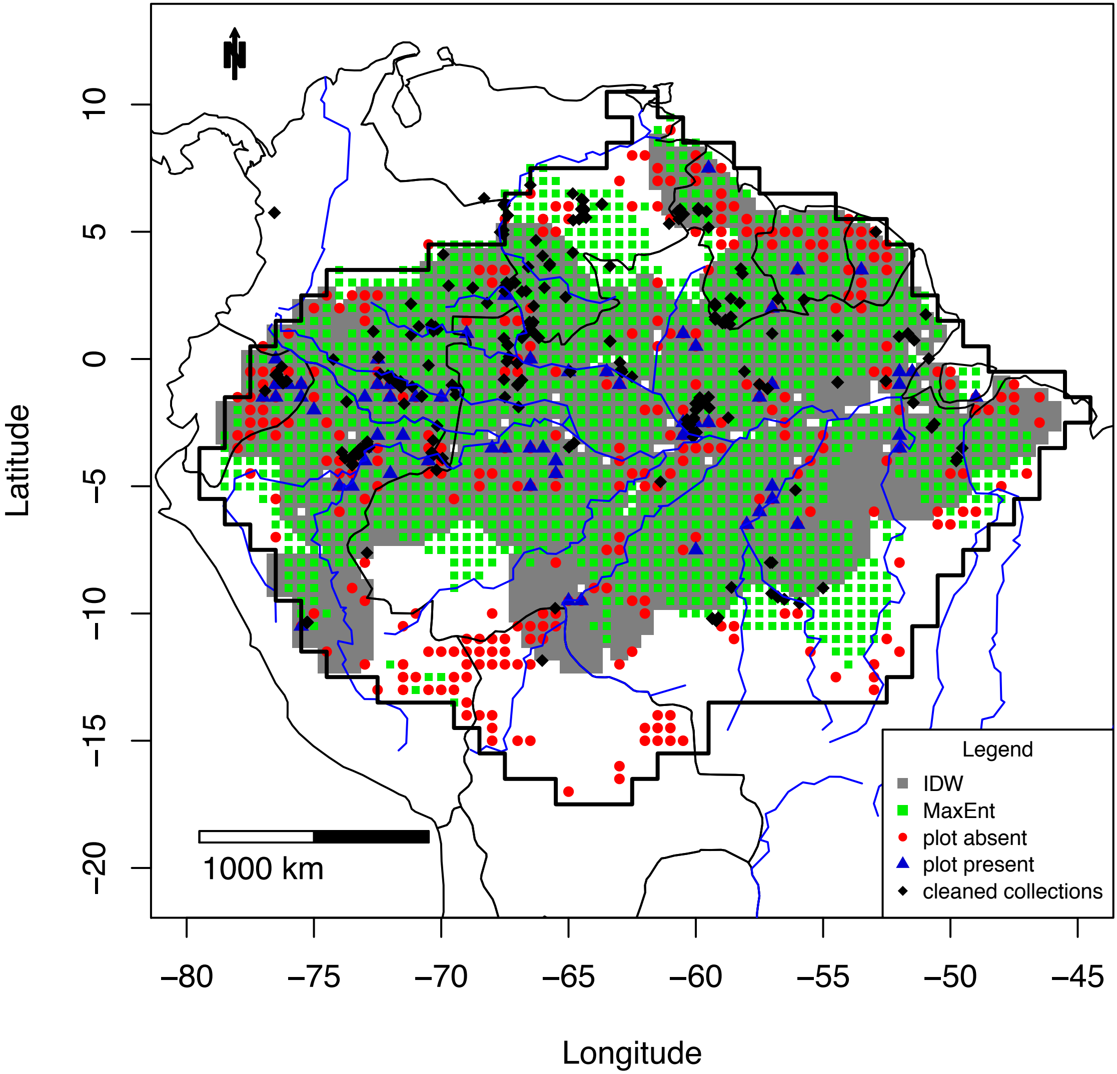

Figure S3_93B MaxEnt map of Macrolobium_angustifolium

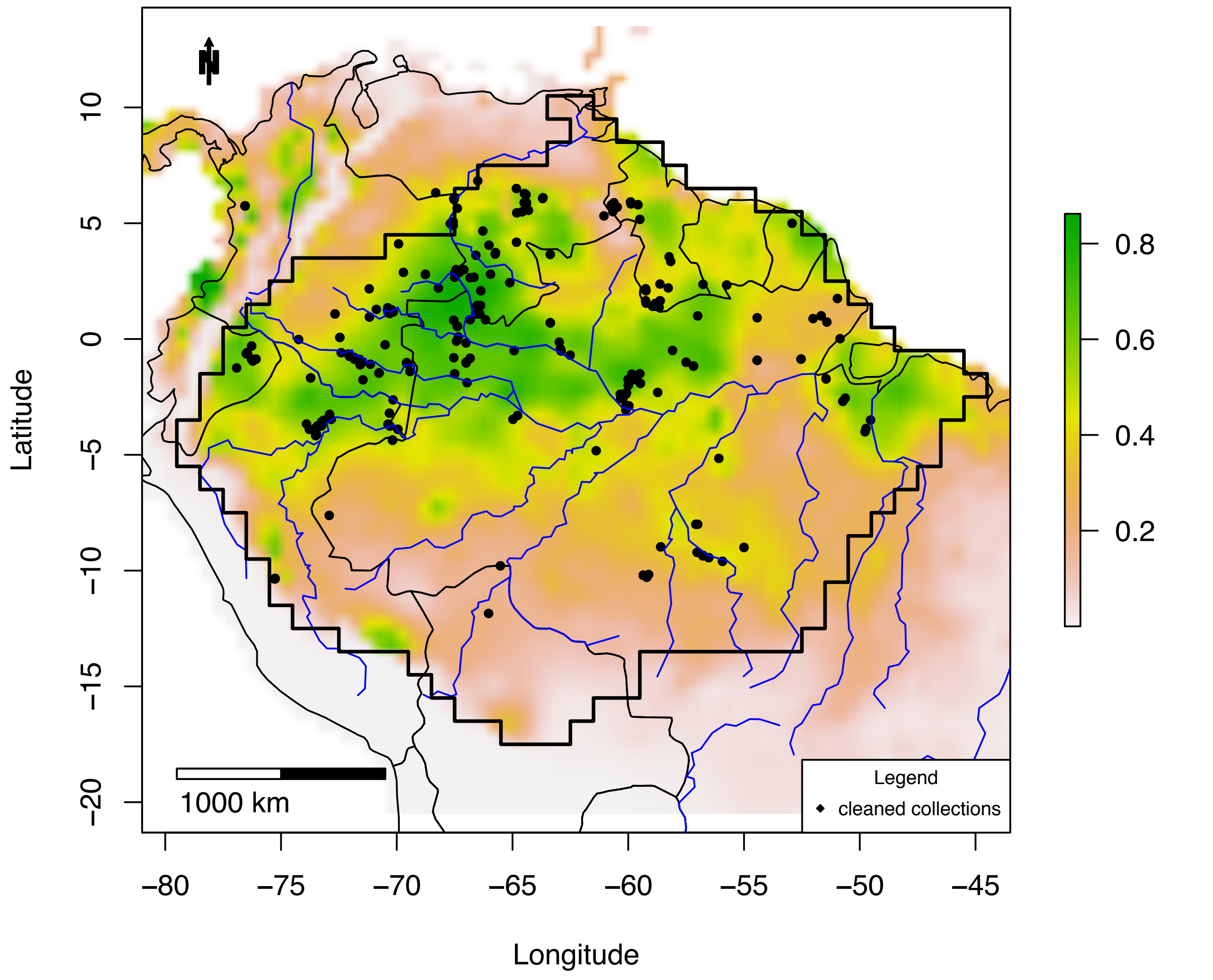

Figure S3_93C IDW map of Macrolobium_angustifolium: plots =0 – 0.23; IDW: 0 – 0.05

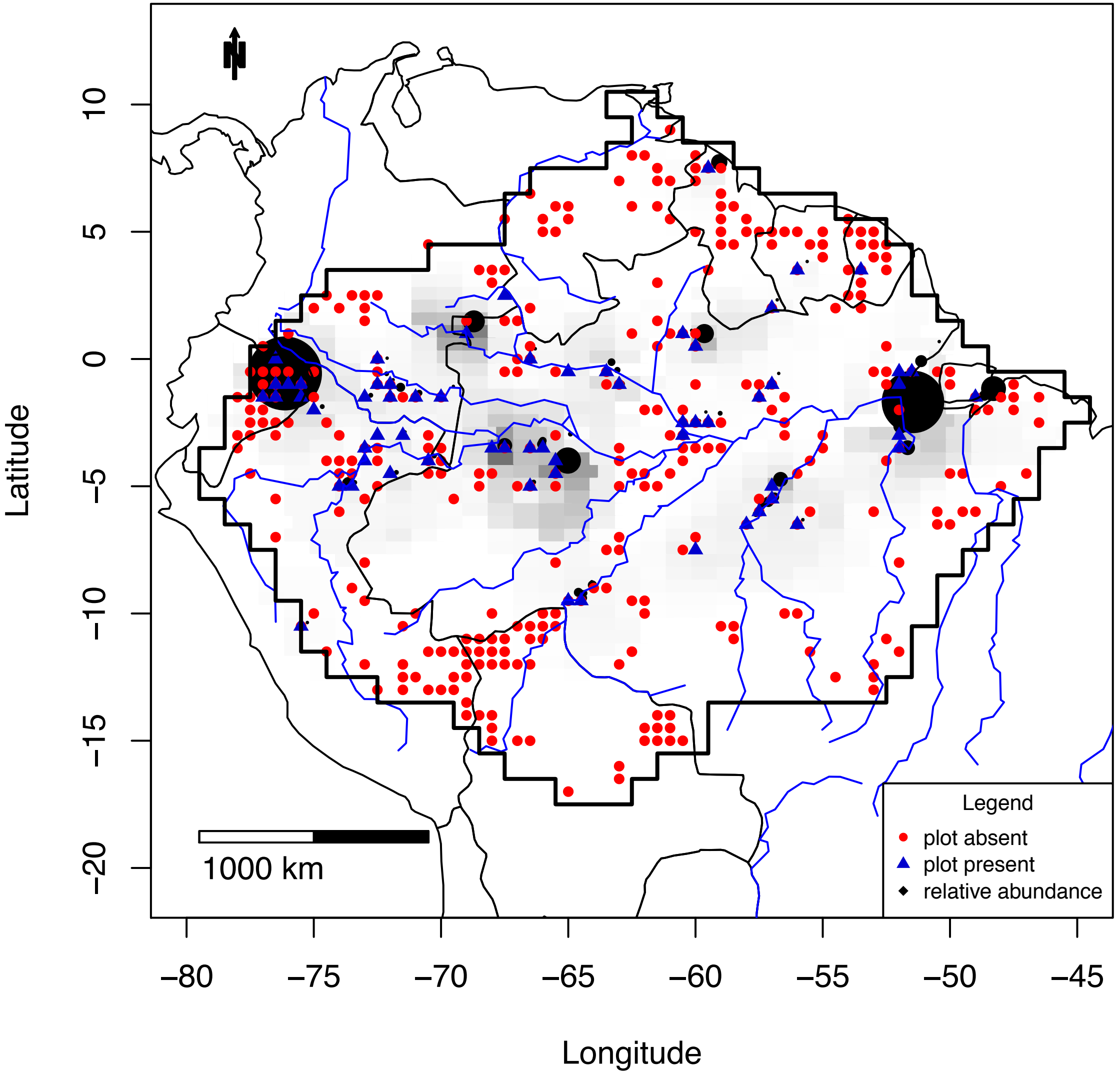

Figure S3_94A Combined map of Manilkara_bidentata

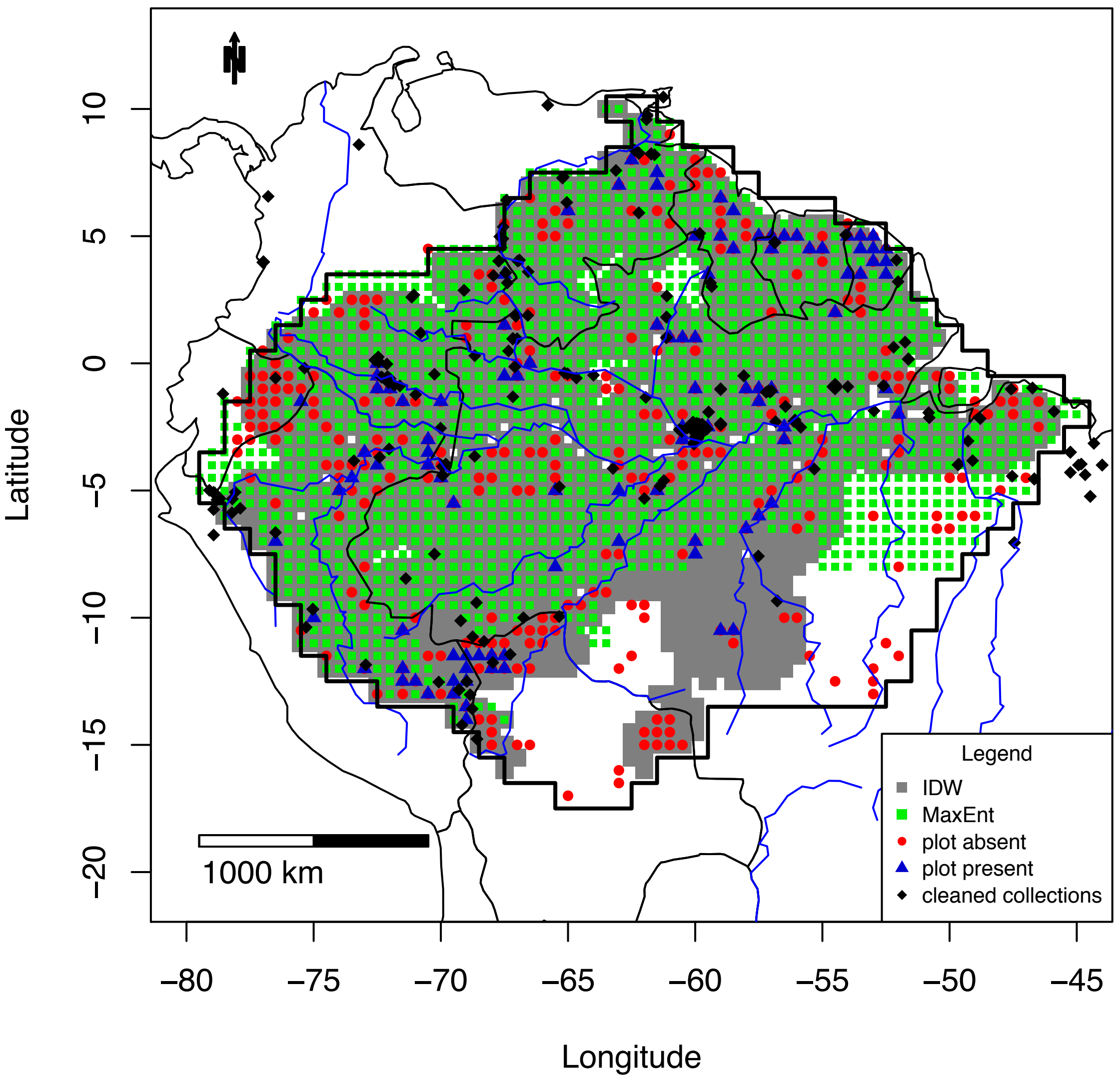

Figure S3_94B MaxEnt map of Manilkara_bidentata

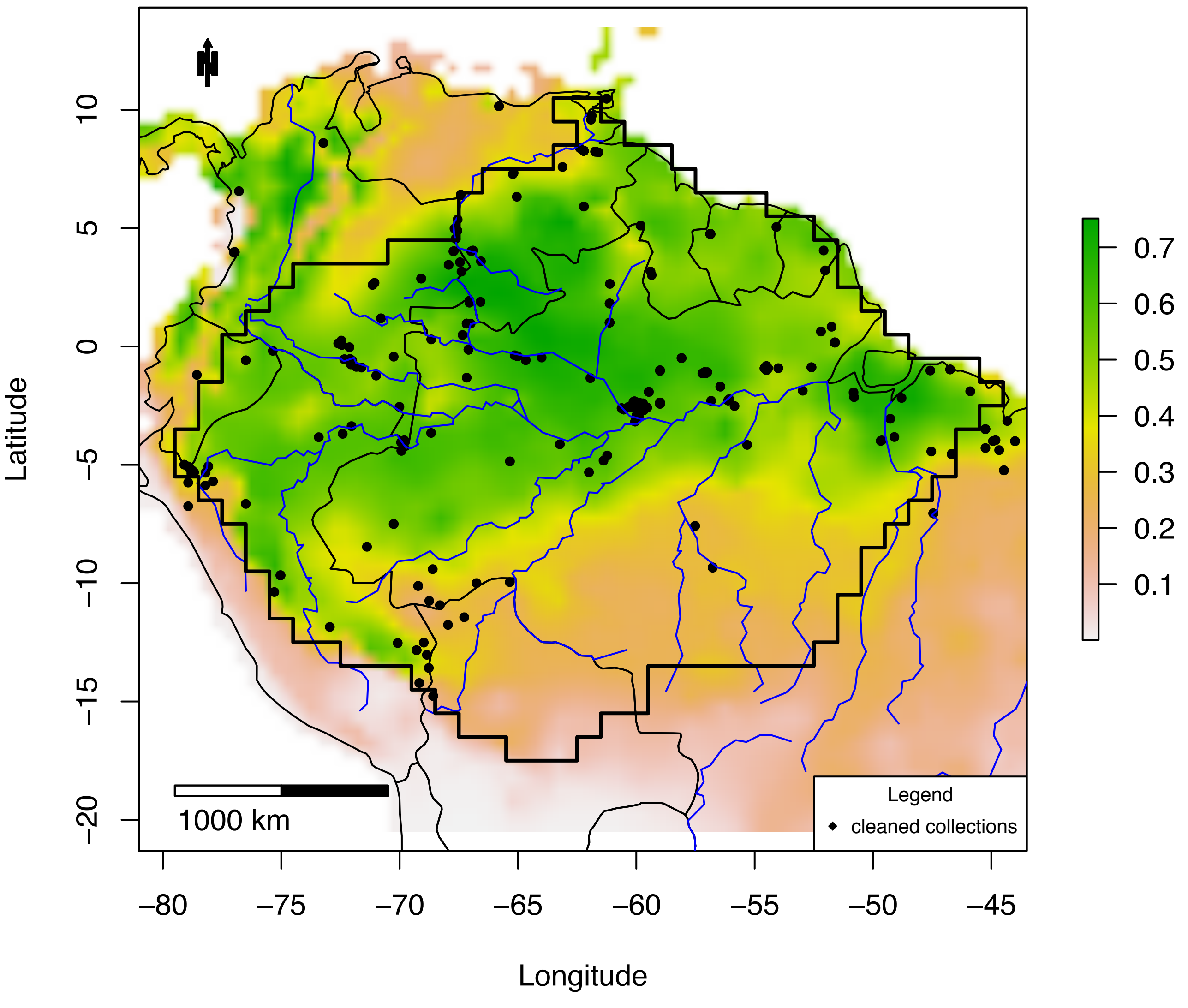

Figure S3_94C IDW map of Manilkara_bidentata: plots =0 – 0.21; IDW: 0 – 0.18

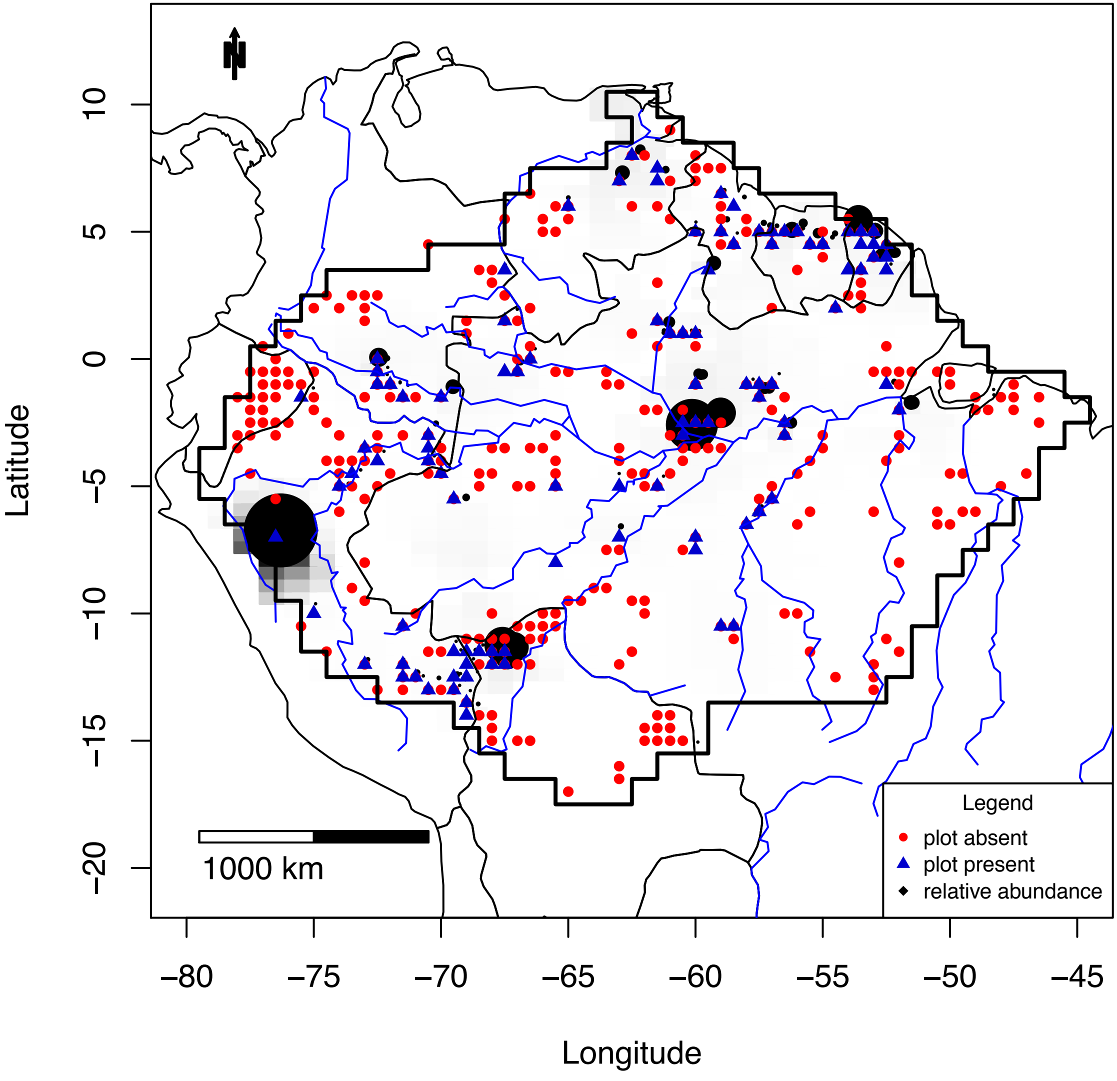

Figure S3_95A Combined map of Maquira_coriacea

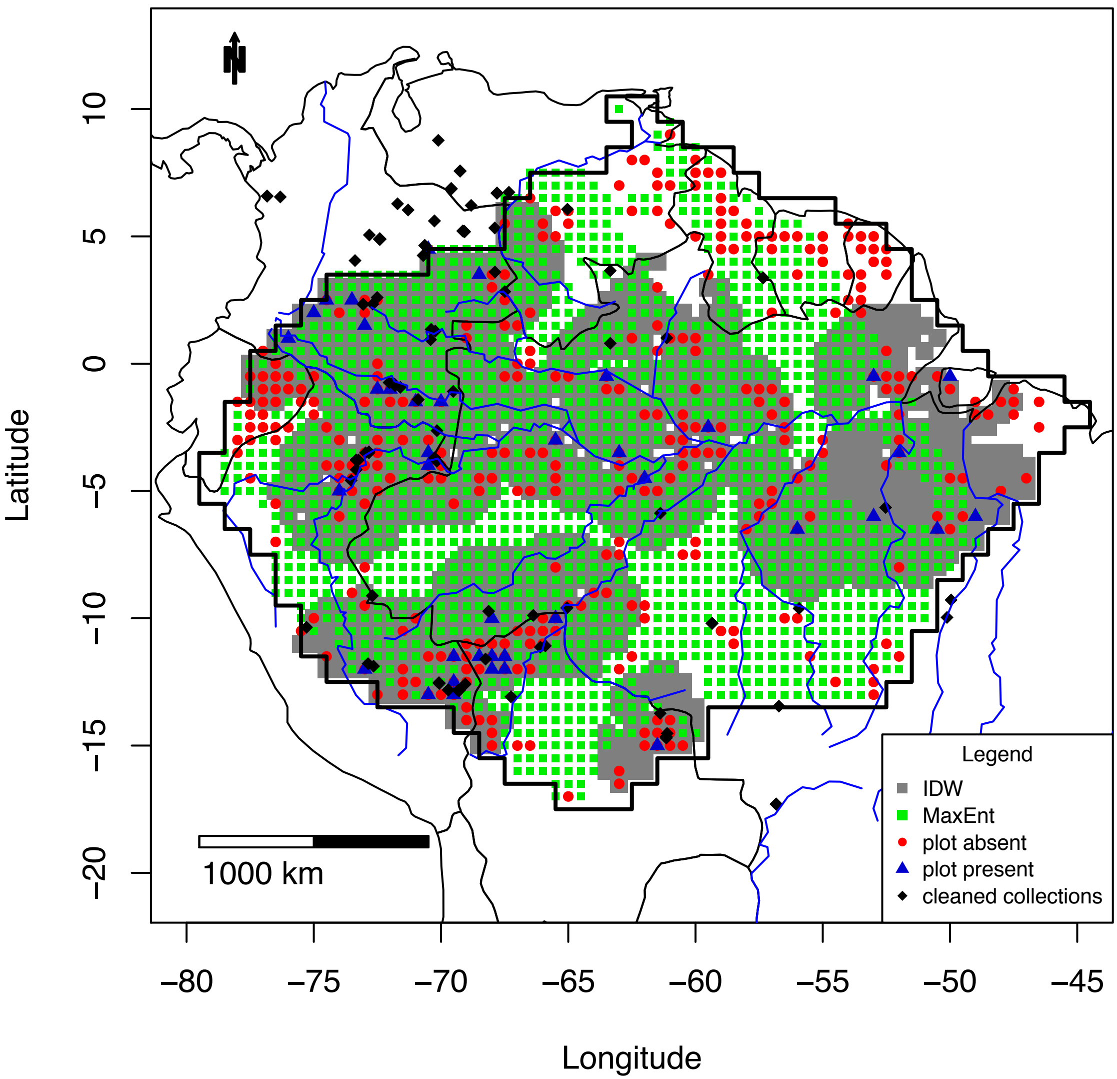

Figure S3_95B MaxEnt map of Maquira_coriacea

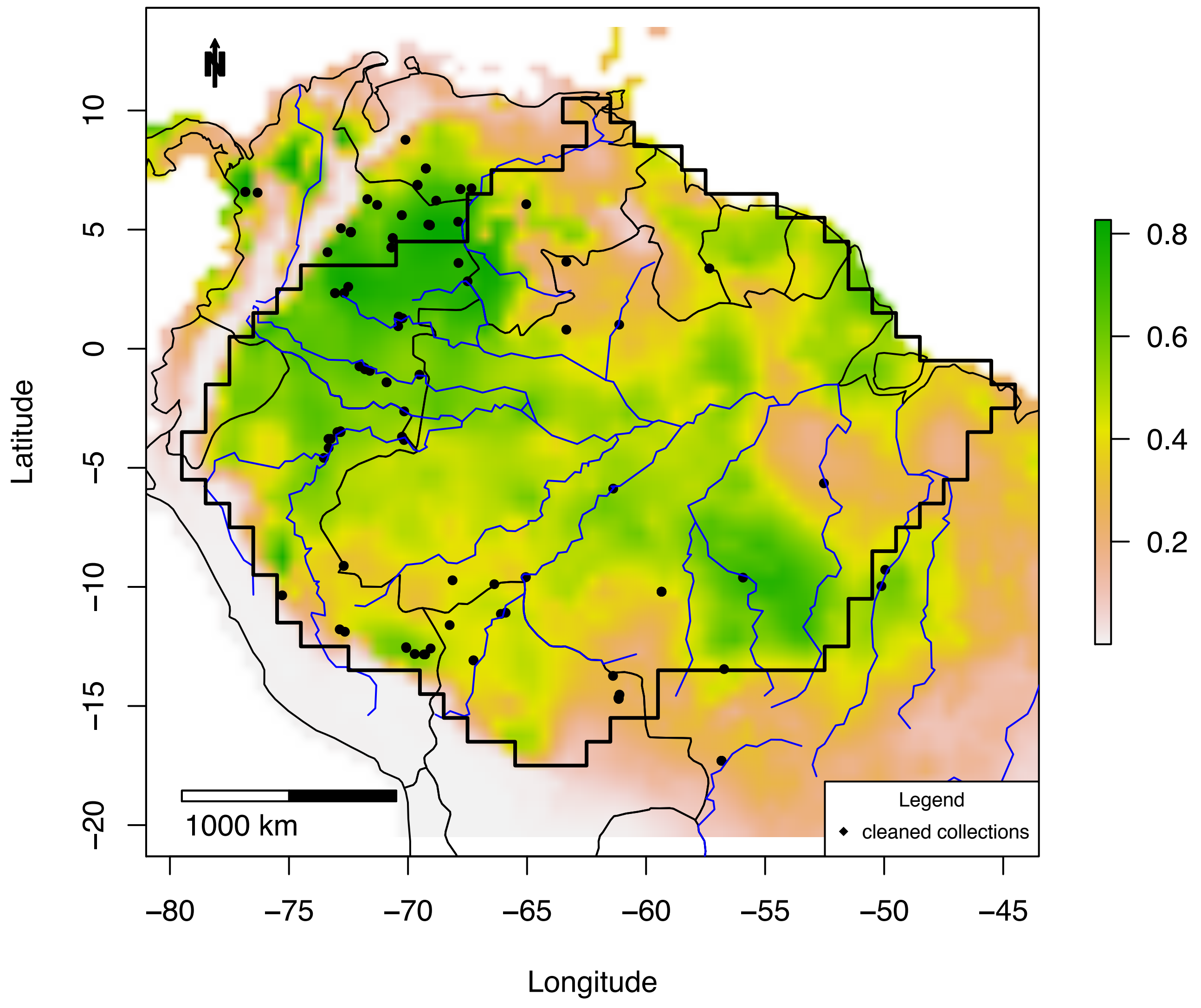

Figure S3_95C IDW map of Maquira_coriacea: plots =0 – 0.17; IDW: 0 – 0.1

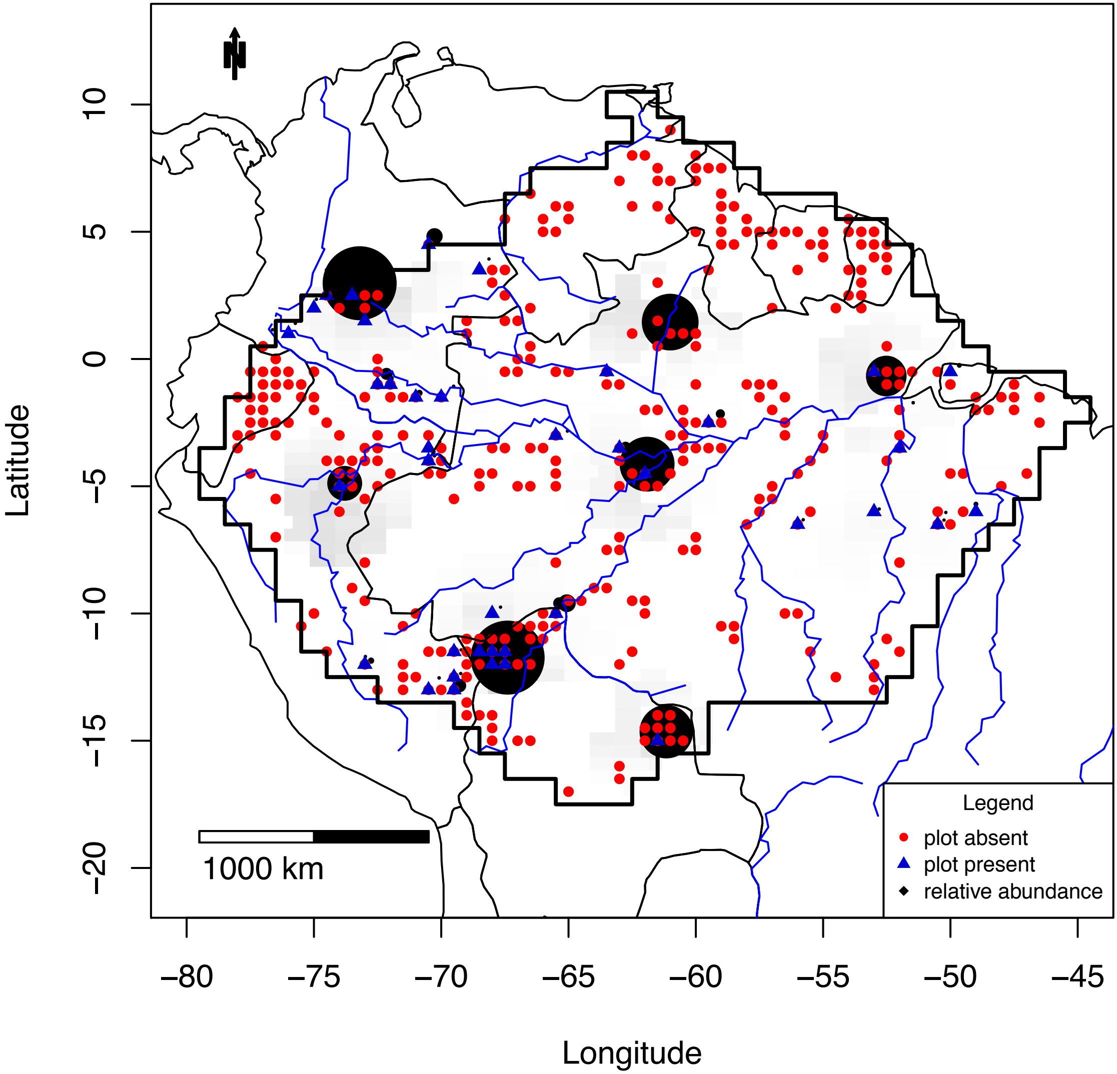

Figure S3_96A Combined map of Matisia_ochrocalyx

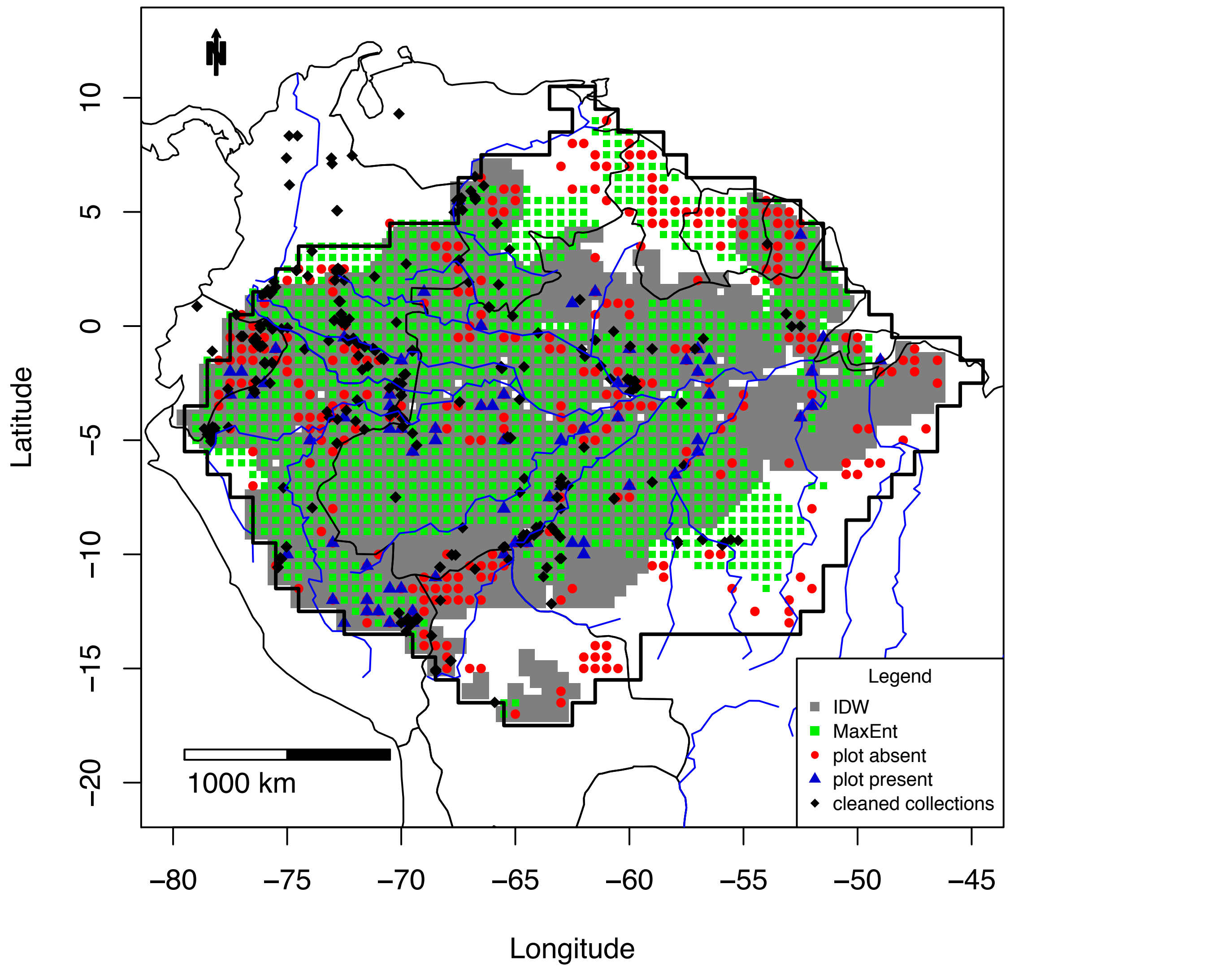

Figure S3_96B MaxEnt map of Matisia_ochrocalyx

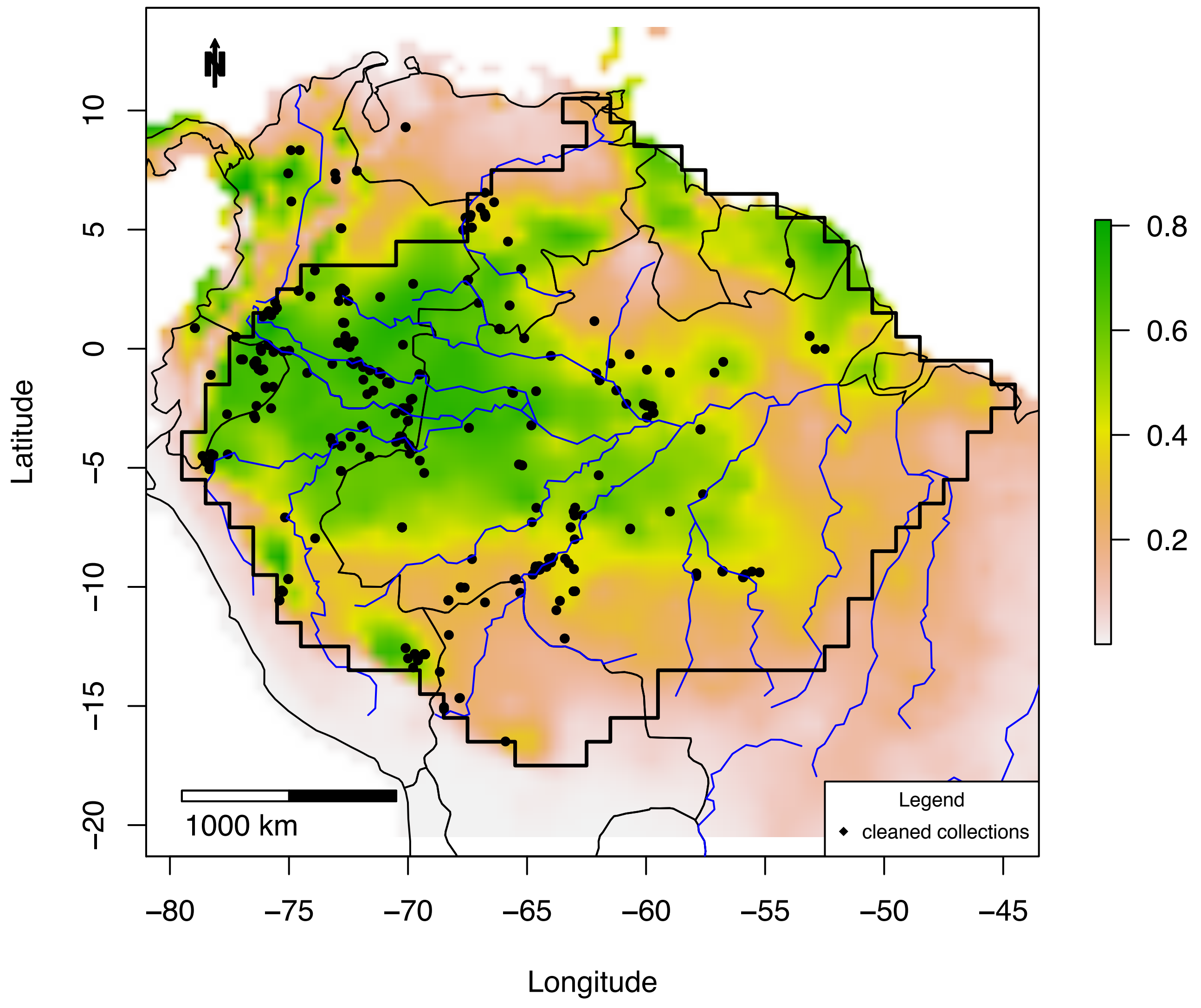

Figure S3_96C IDW map of Matisia_ochrocalyx:
plots =0 – 0.16; IDW: 0 – 0.04

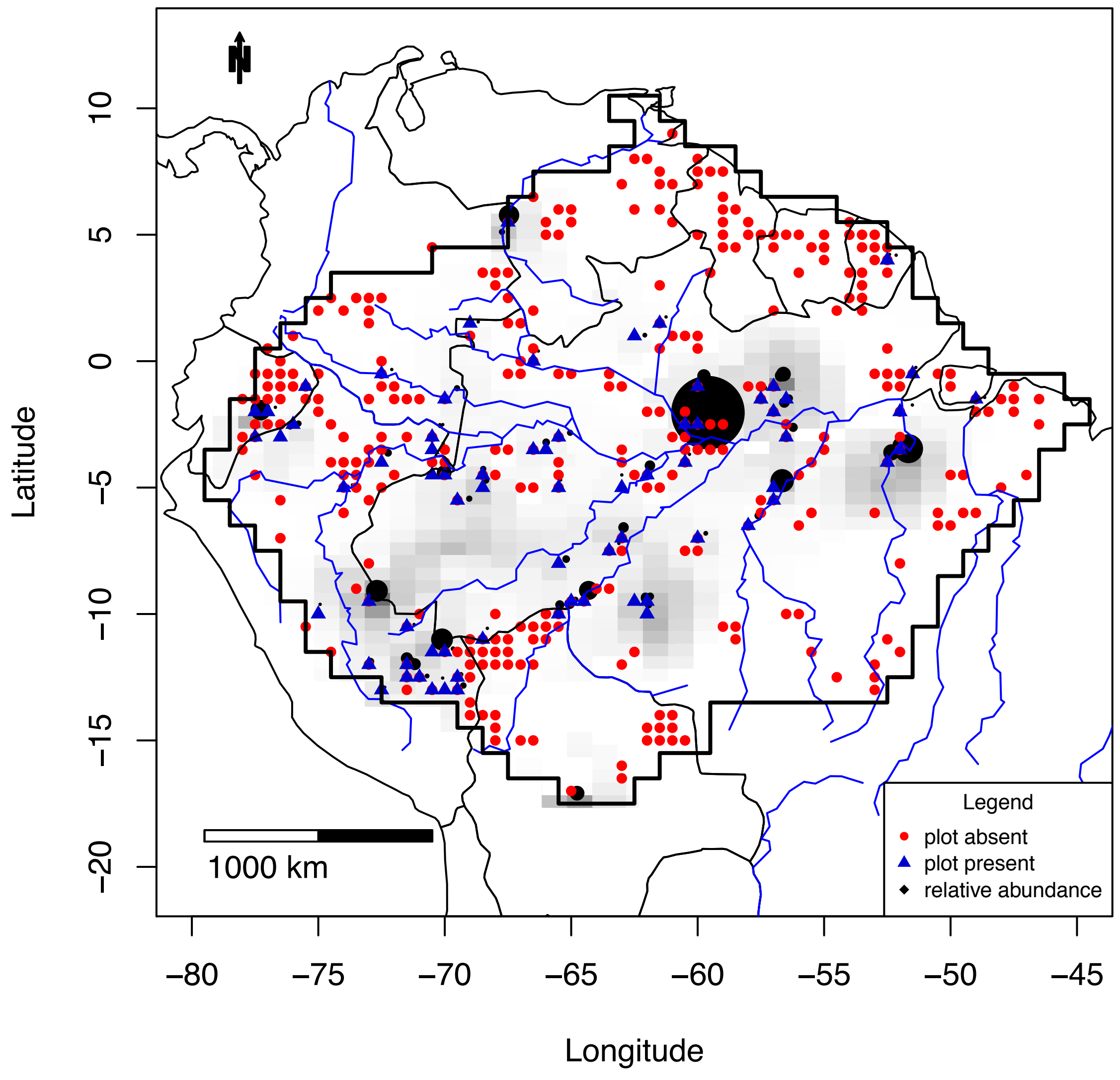

Figure S3_97A Combined map of Mauritia_flexuosa

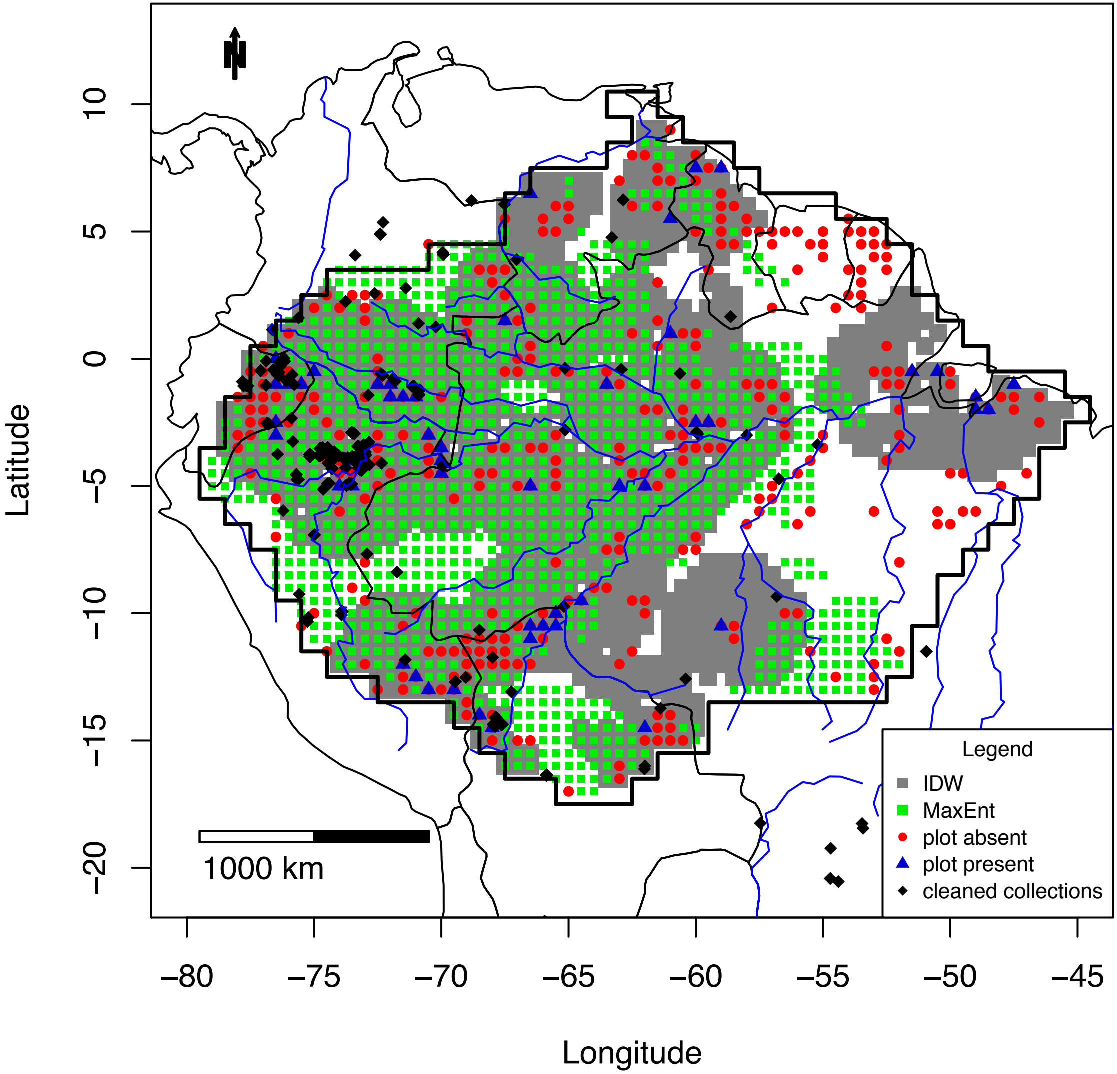

Figure S3_97B MaxEnt map of Mauritia_flexuosa

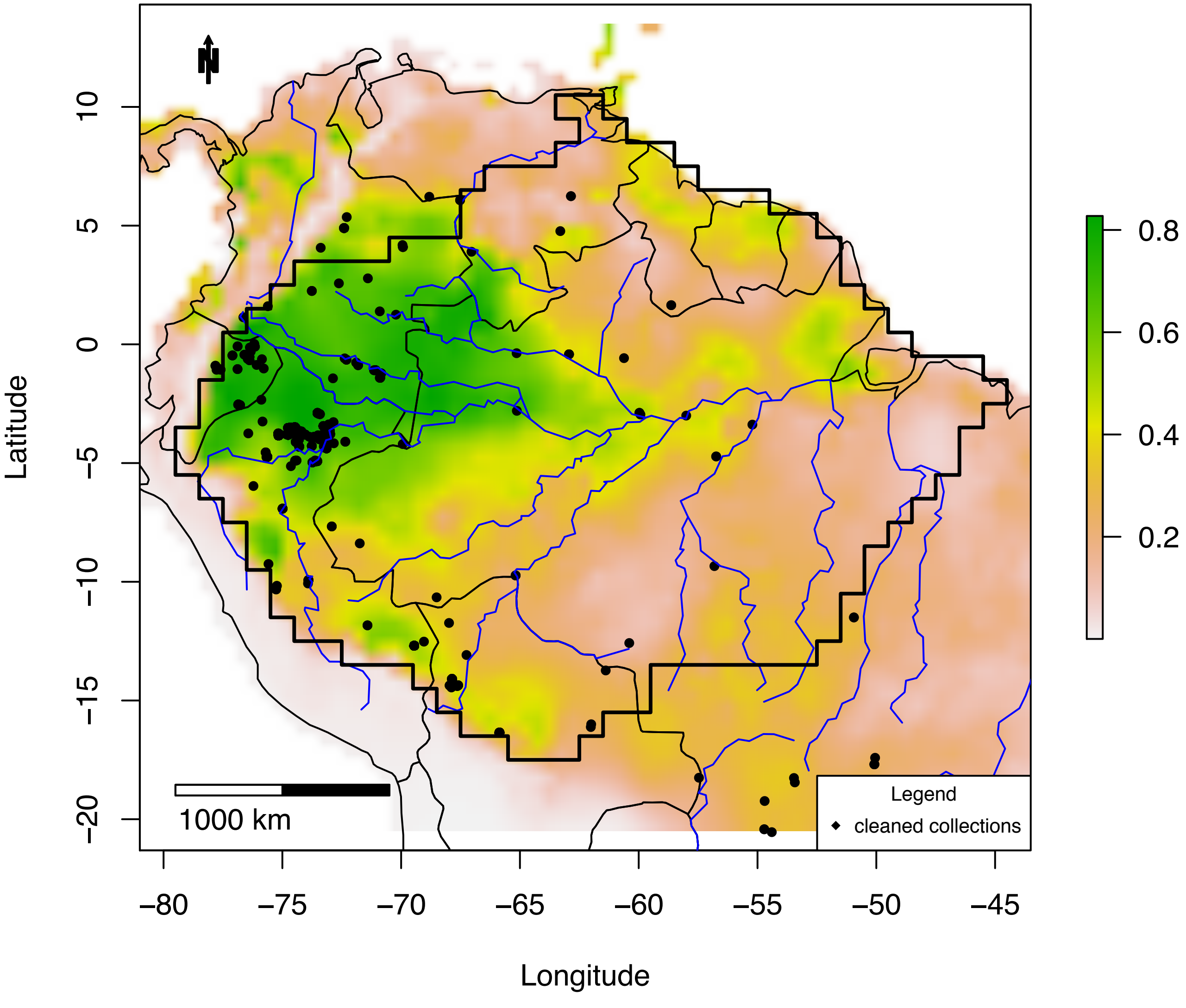

Figure S3_97C IDW map of Mauritia_flexuosa:
plots =0 – 0.63; IDW: 0 – 0.11

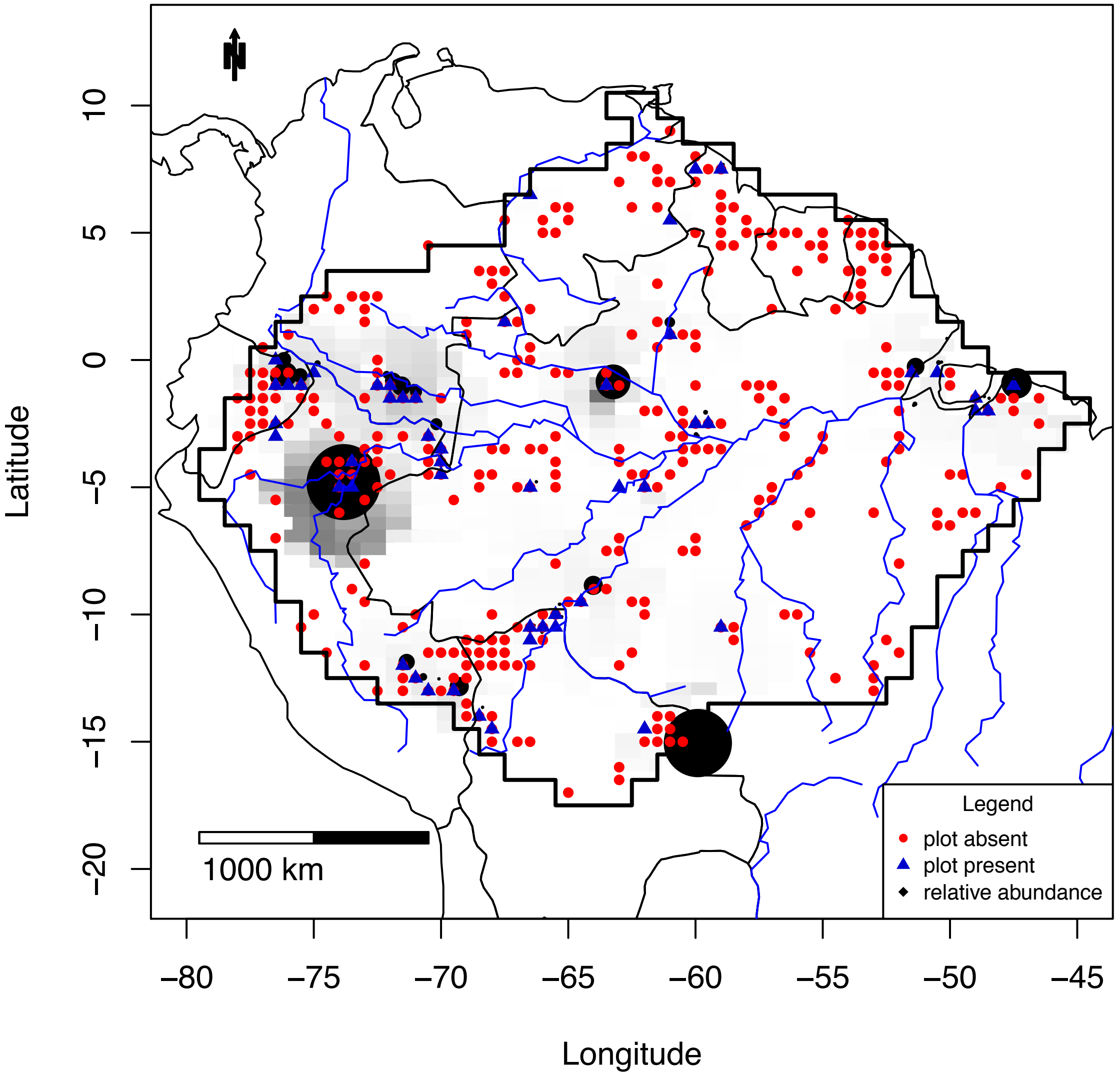

Figure S3_98A Combined map of Mauritiella_aculeata

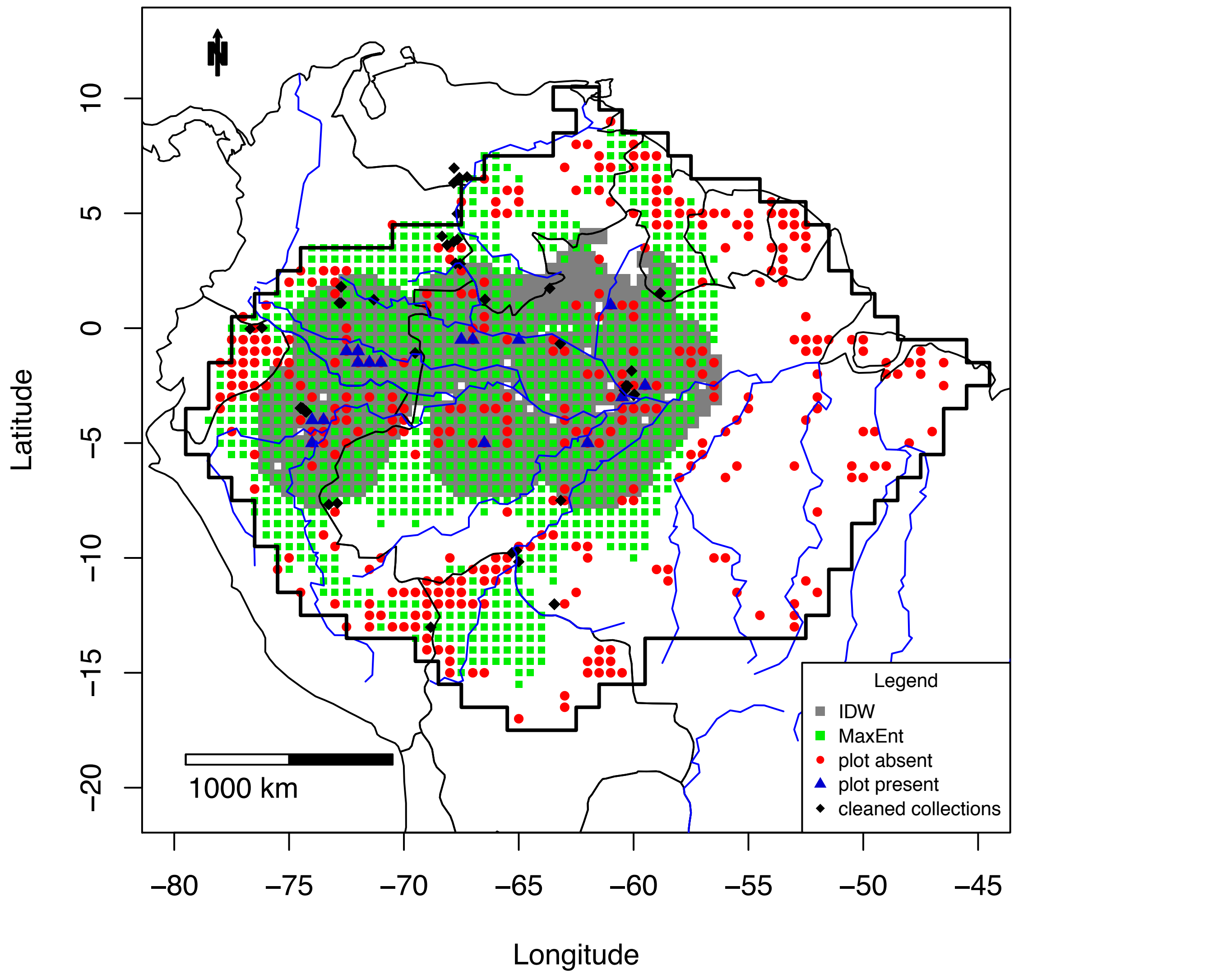

Figure S3_98B MaxEnt map of Mauritiella_aculeata

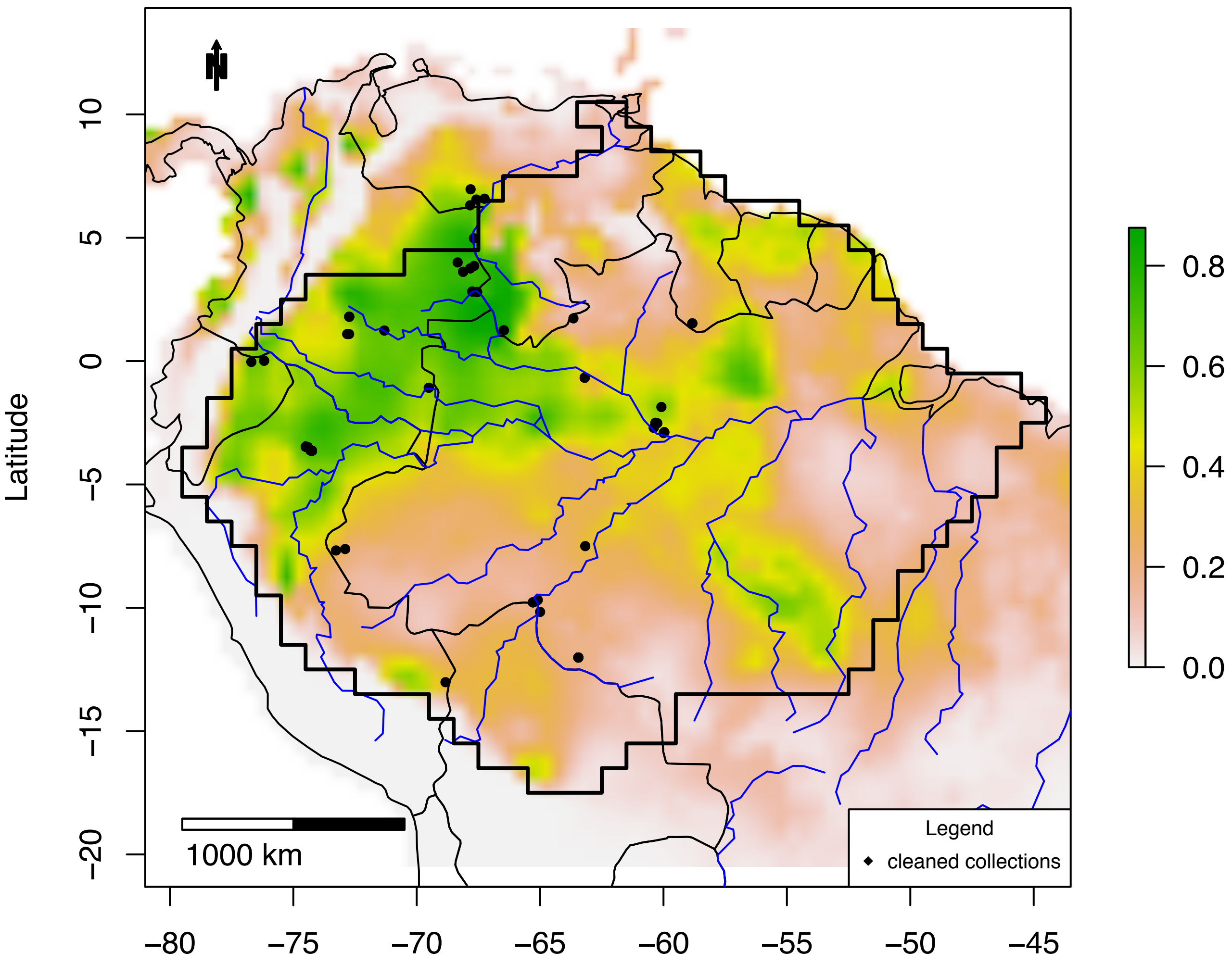

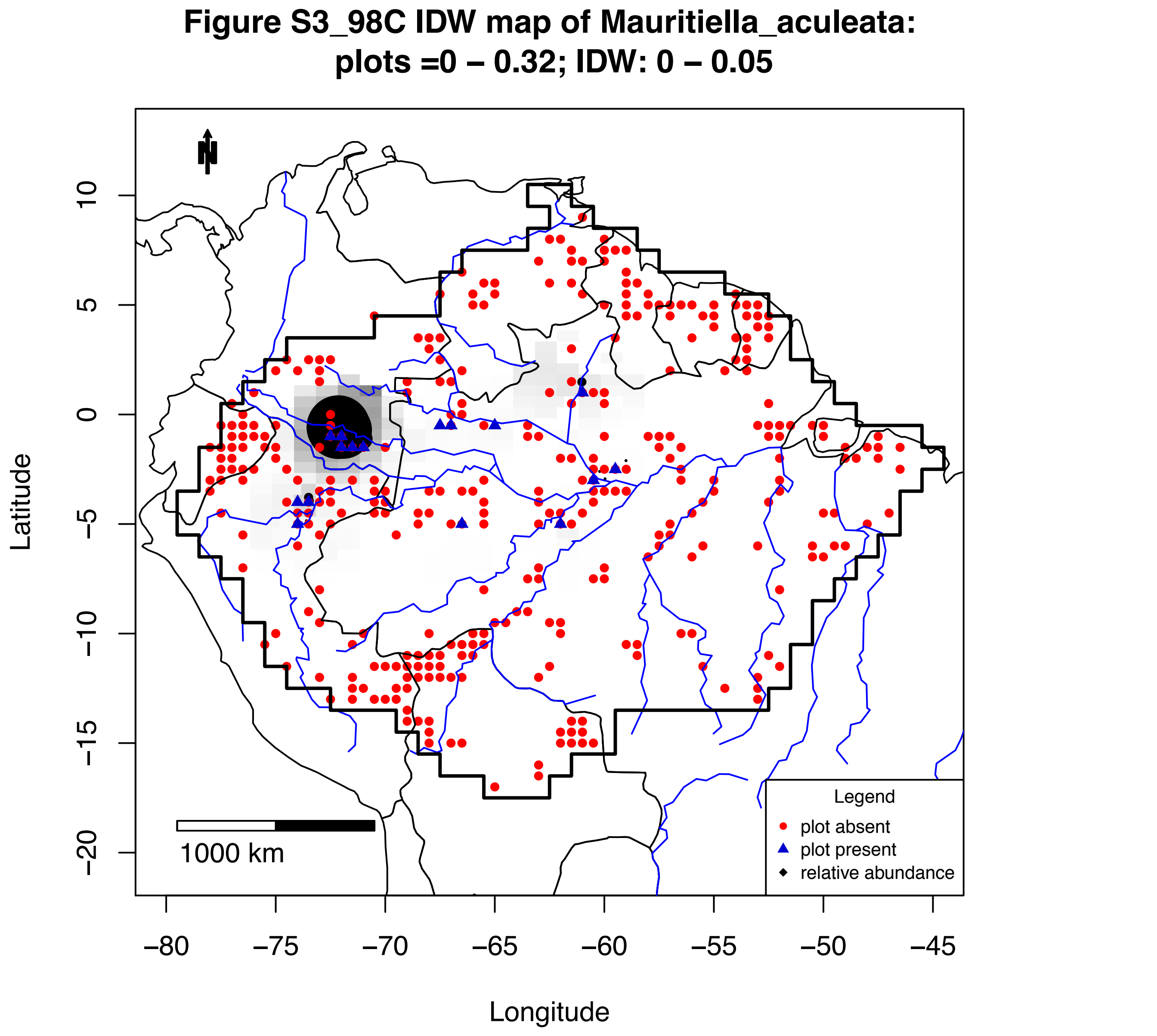
Figure S3_98C IDW map of Mauritiella_aculeata:
plots =0 – 0.32; IDW: 0 – 0.05
Latitude
Longitude
10
5
0
−5
−10
−15
−20
−80
−75
−70
−65
−60
−55
−50
−45
N
1000 km
Legend
plot absent
plot present
relative abundance

Figure S3_99A Combined map of Metrodorea_flavida

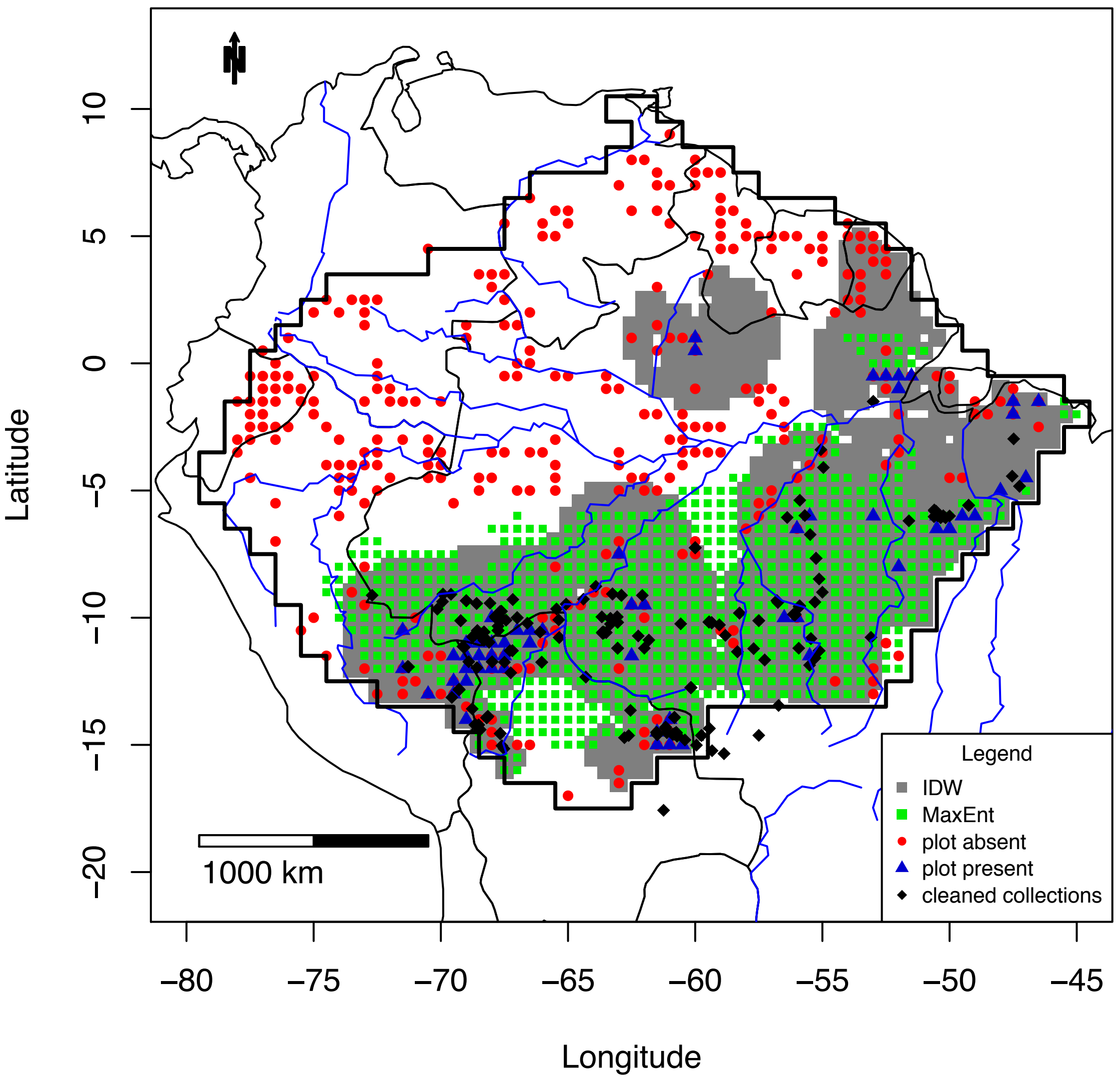

Figure S3_99B MaxEnt map of Metrodorea_flavida

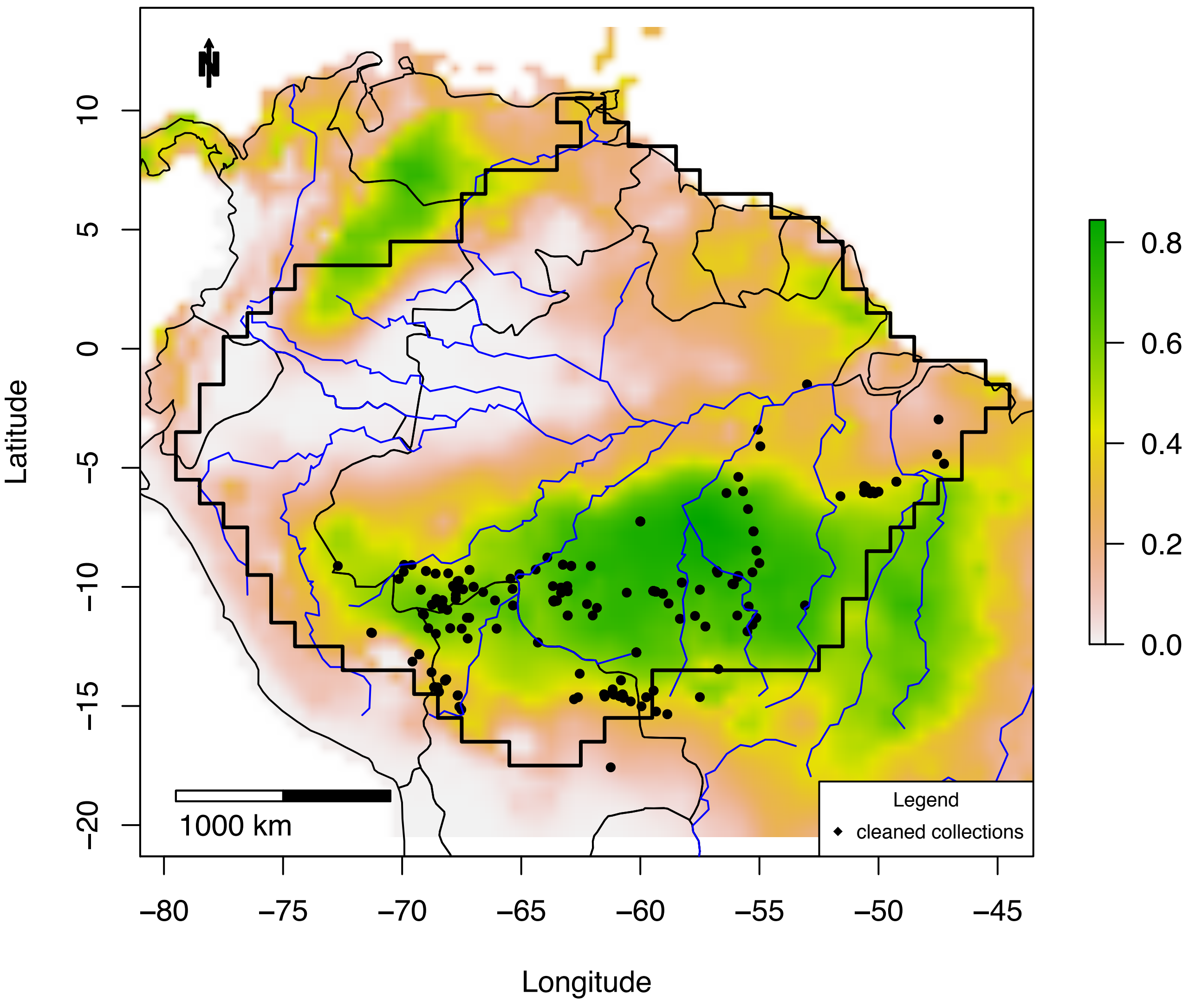

Figure S3_99C IDW map of Metrodorea_flavida: plots =0 – 0.19; IDW: 0 – 0.08

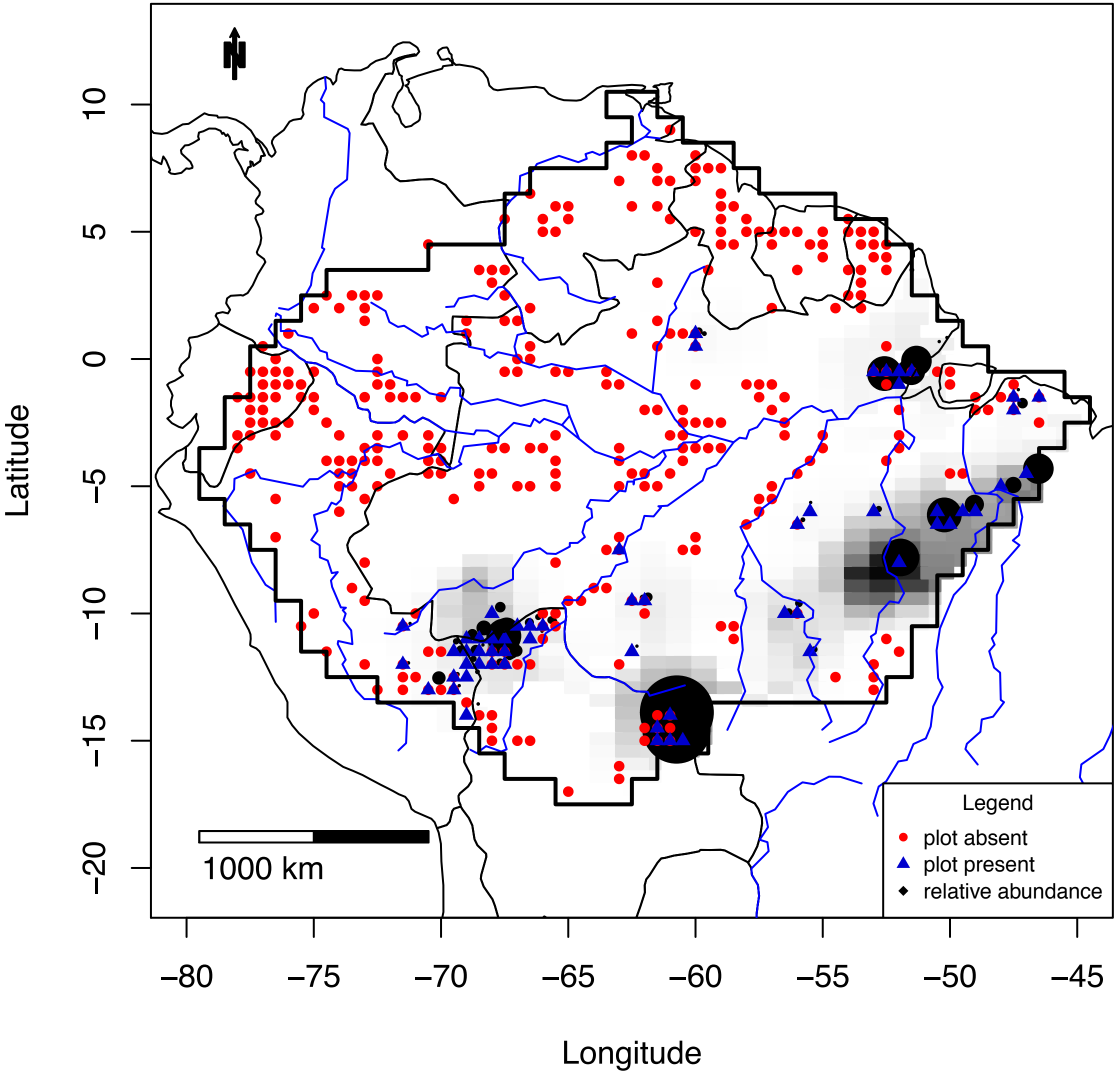

Figure S3_100A Combined map of Mezilaurus_itauba

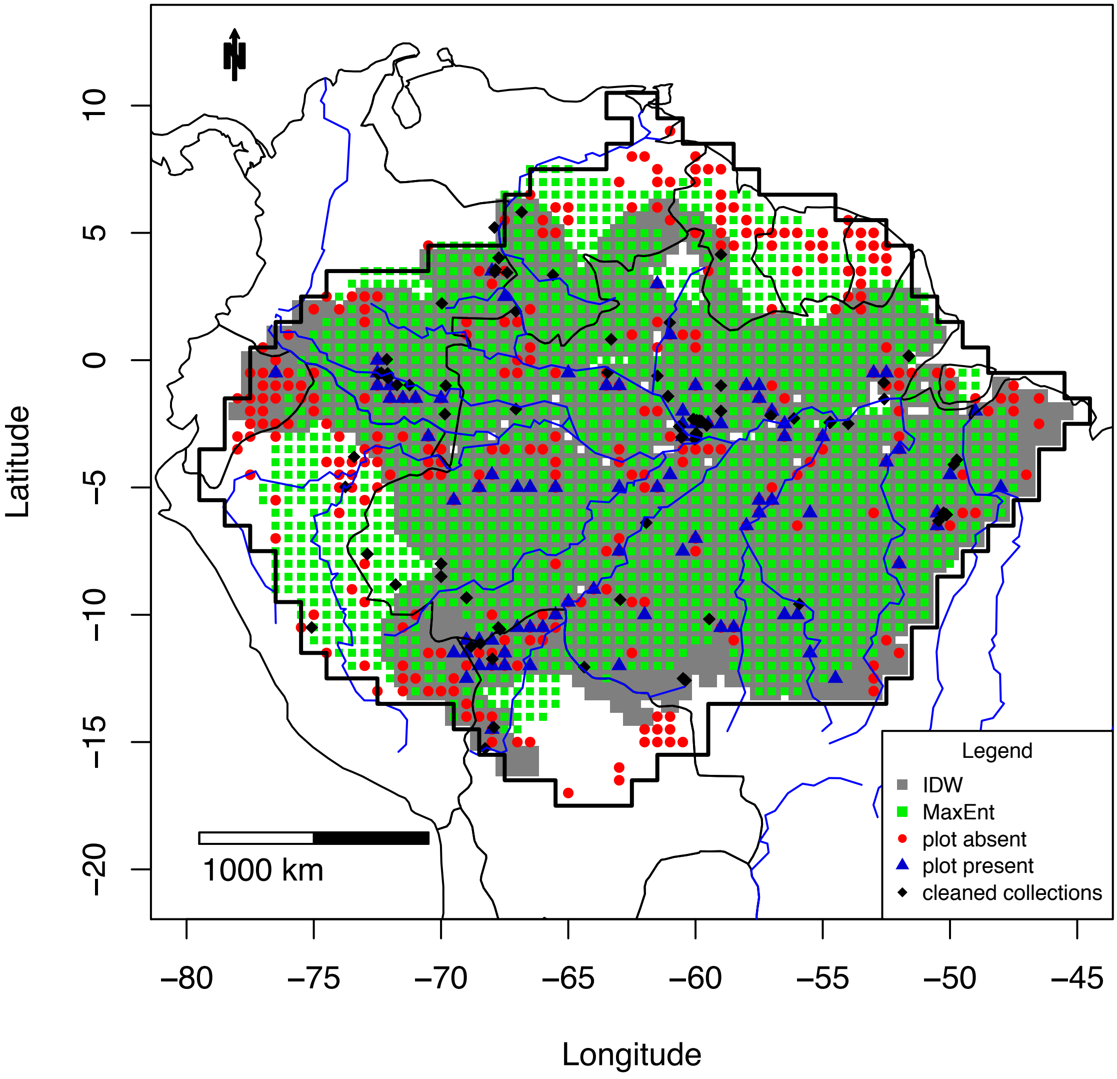

Figure S3_100B MaxEnt map of Mezilaurus_itauba

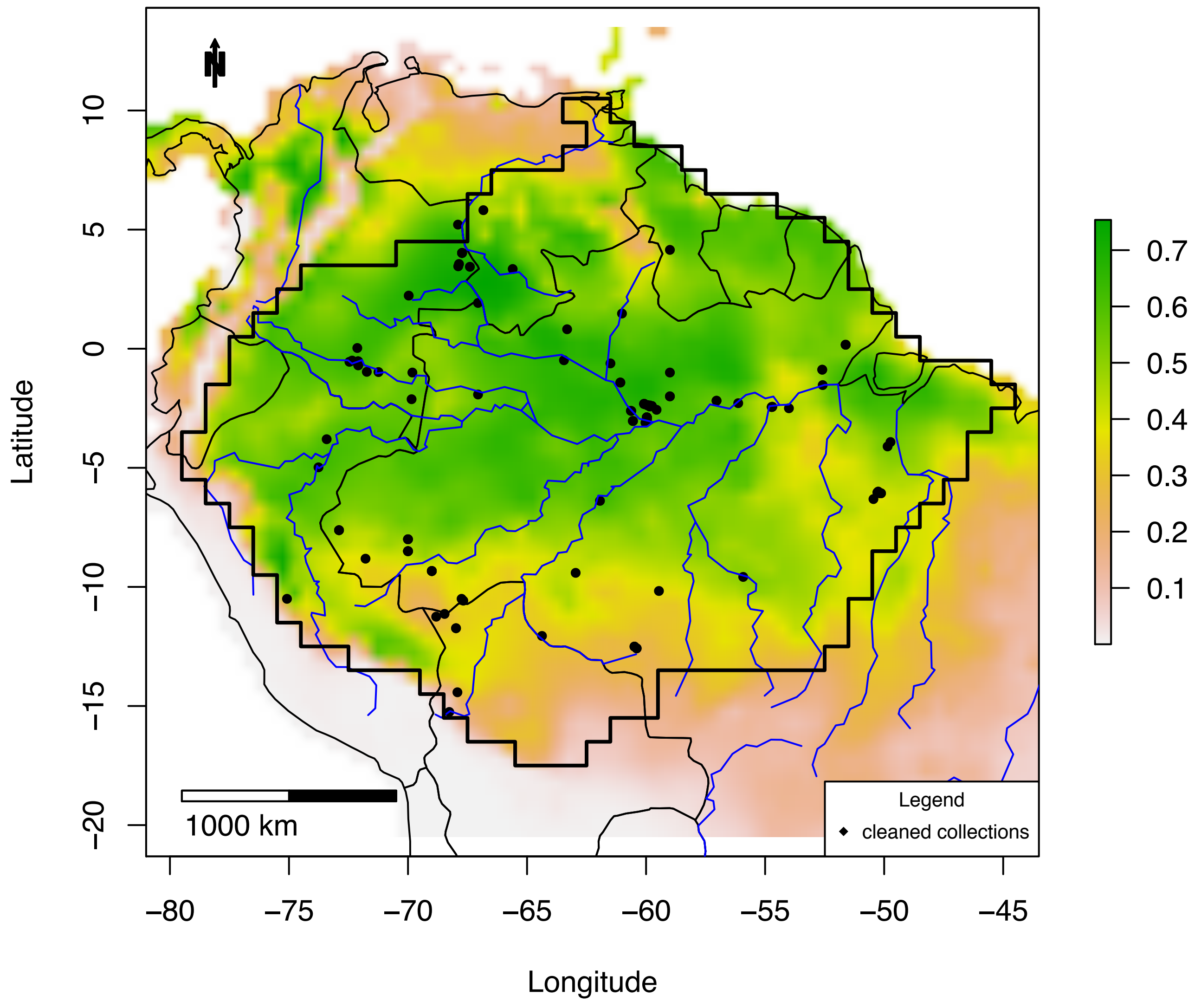

Figure S3_100C IDW map of Mezilaurus_itauba: plots =0 – 0.13; IDW: 0 – 0.03

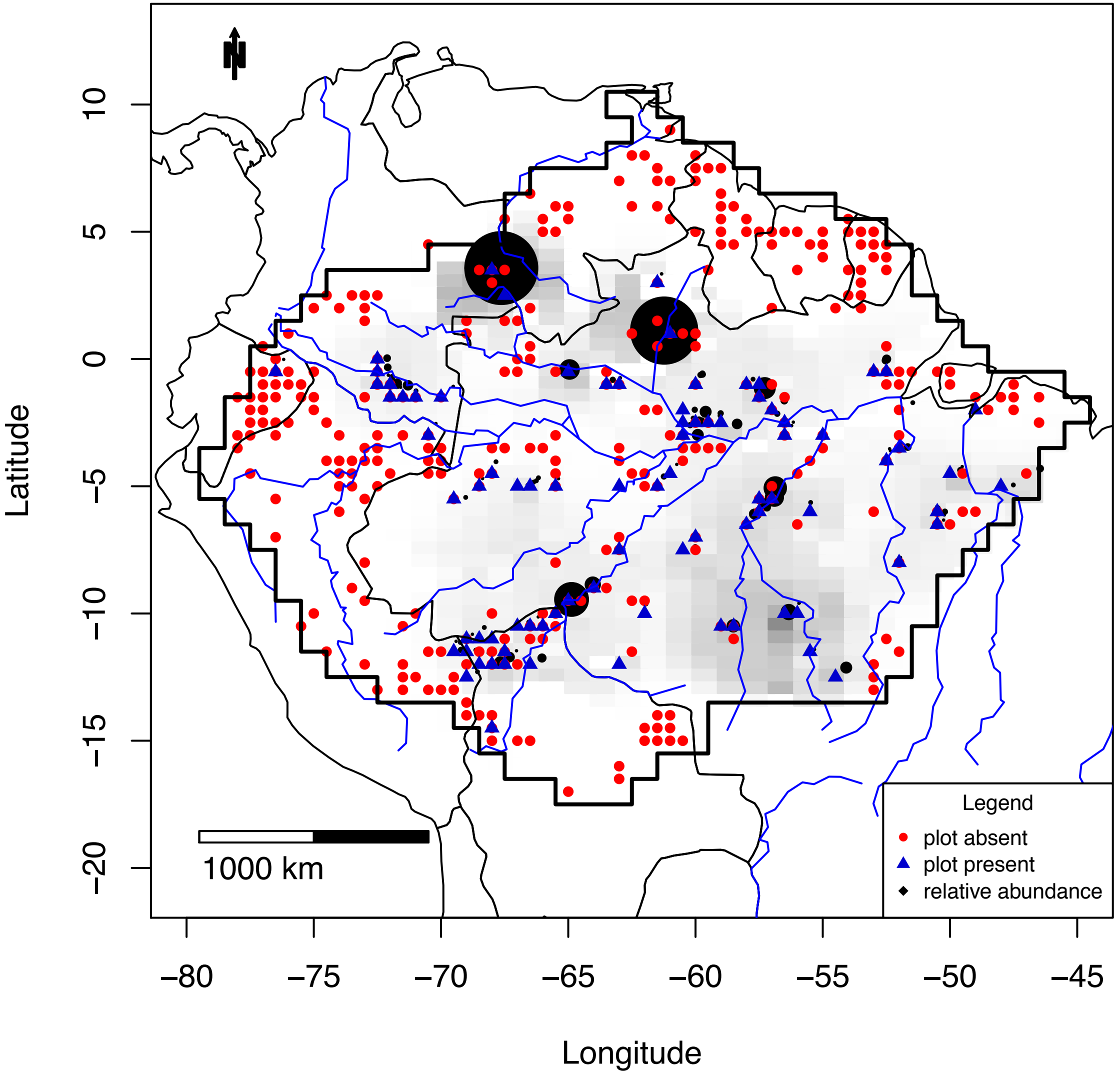

Figure S3_101A Combined map of Micrandra_elata

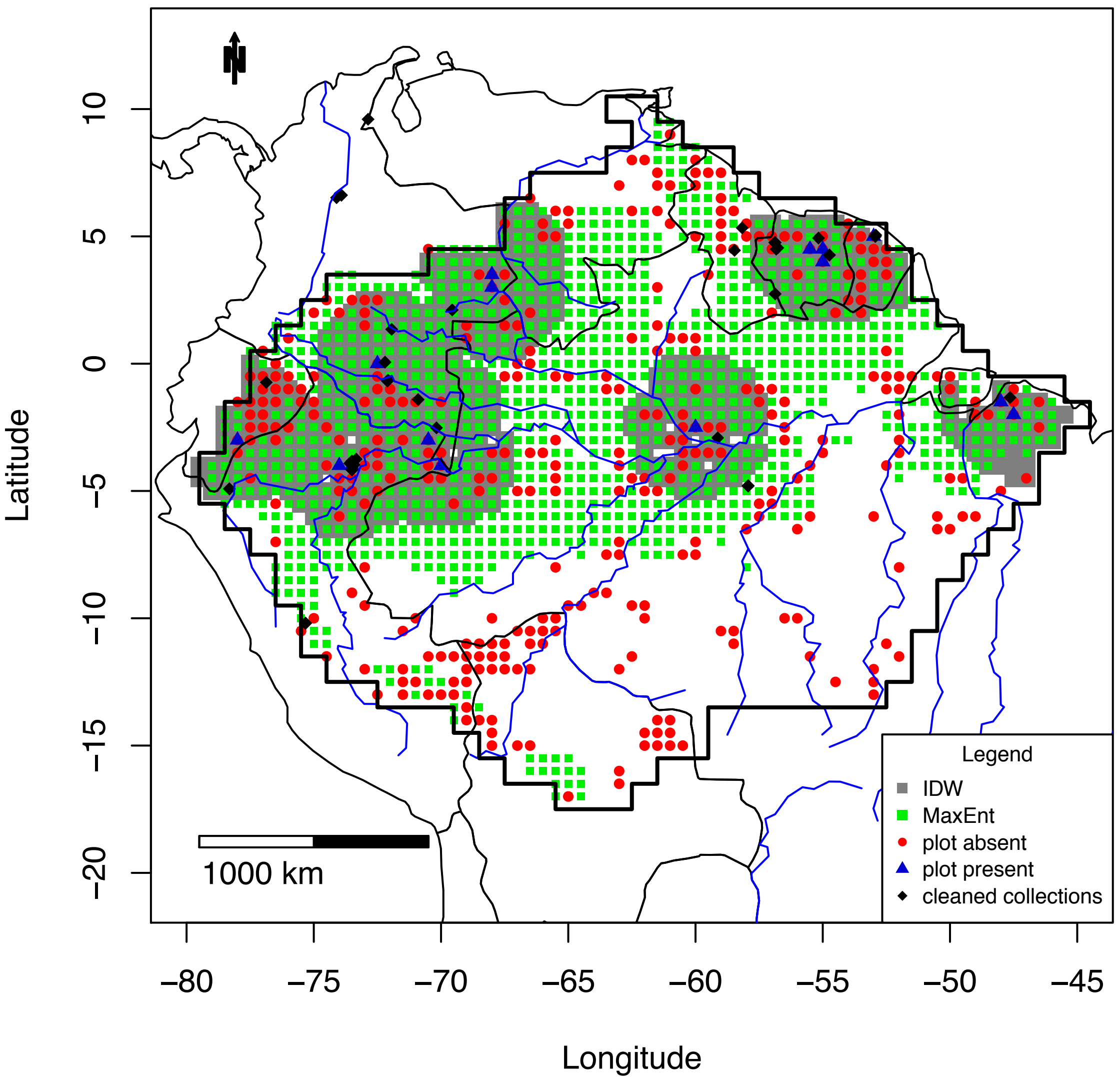

Figure S3_101B MaxEnt map of Micrandra_elata

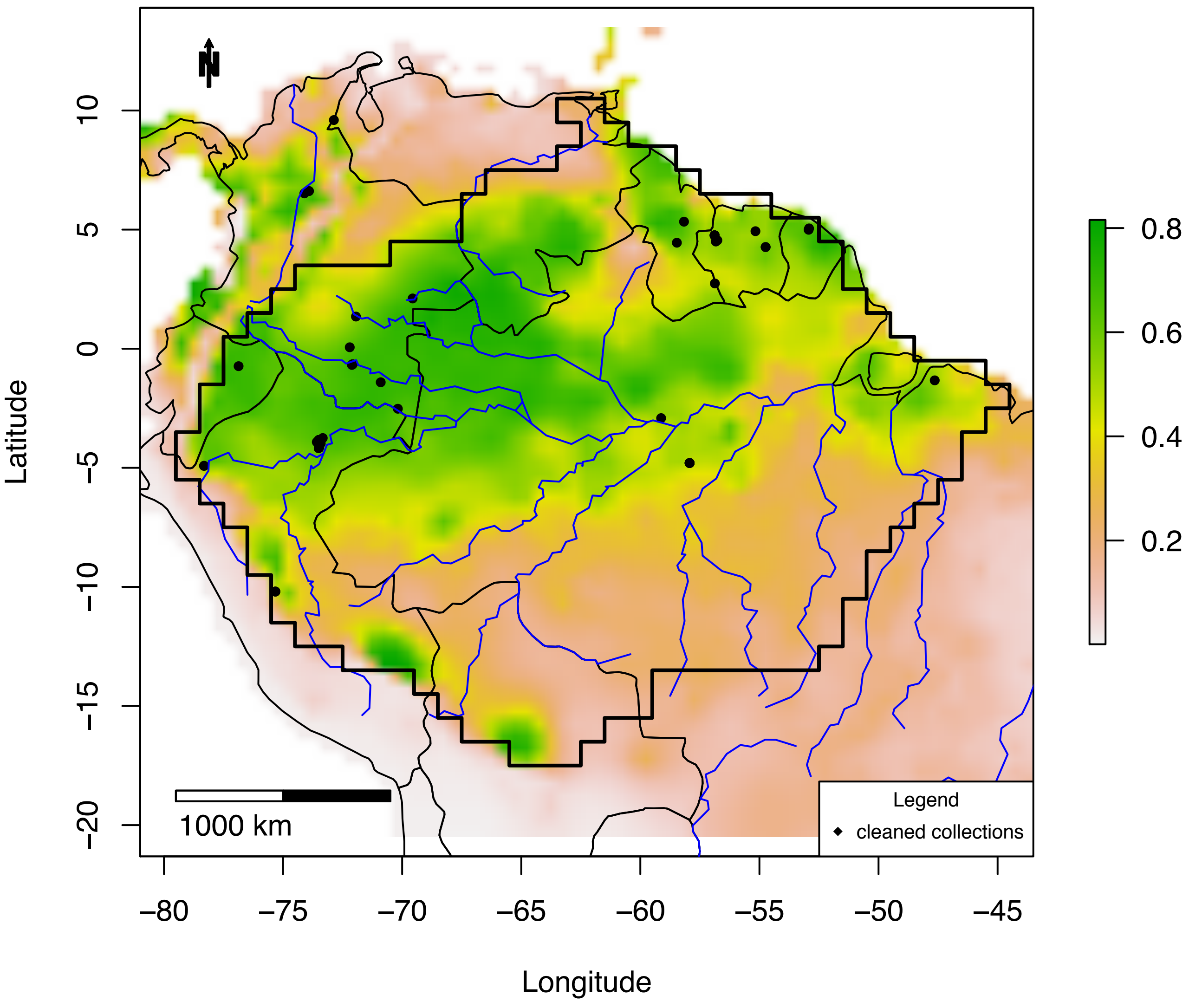

Figure S3_101C IDW map of Micrandra_elata: plots =0 – 0.29; IDW: 0 – 0.05

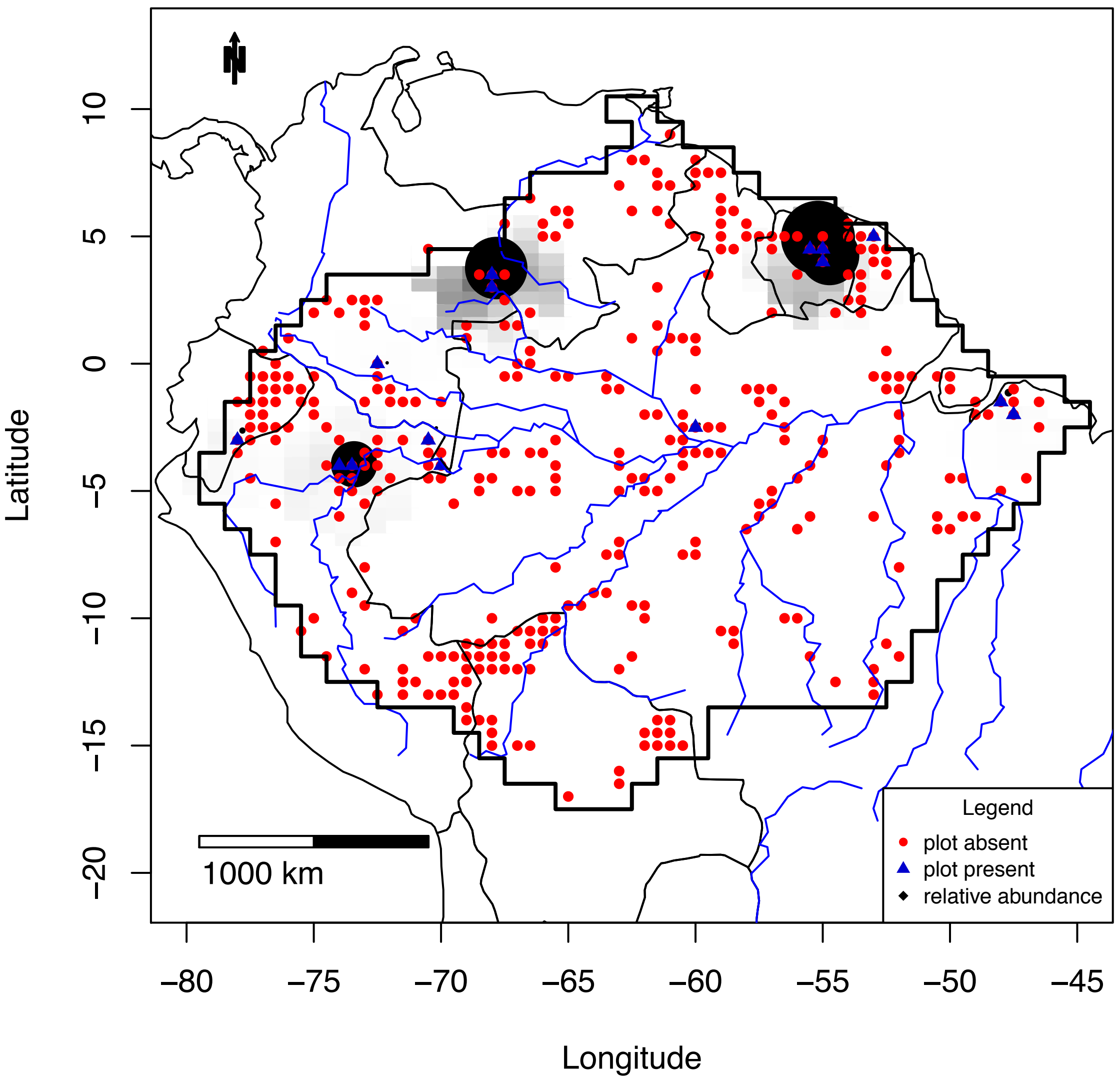

Figure S3_102A Combined map of Micrandra_glabra

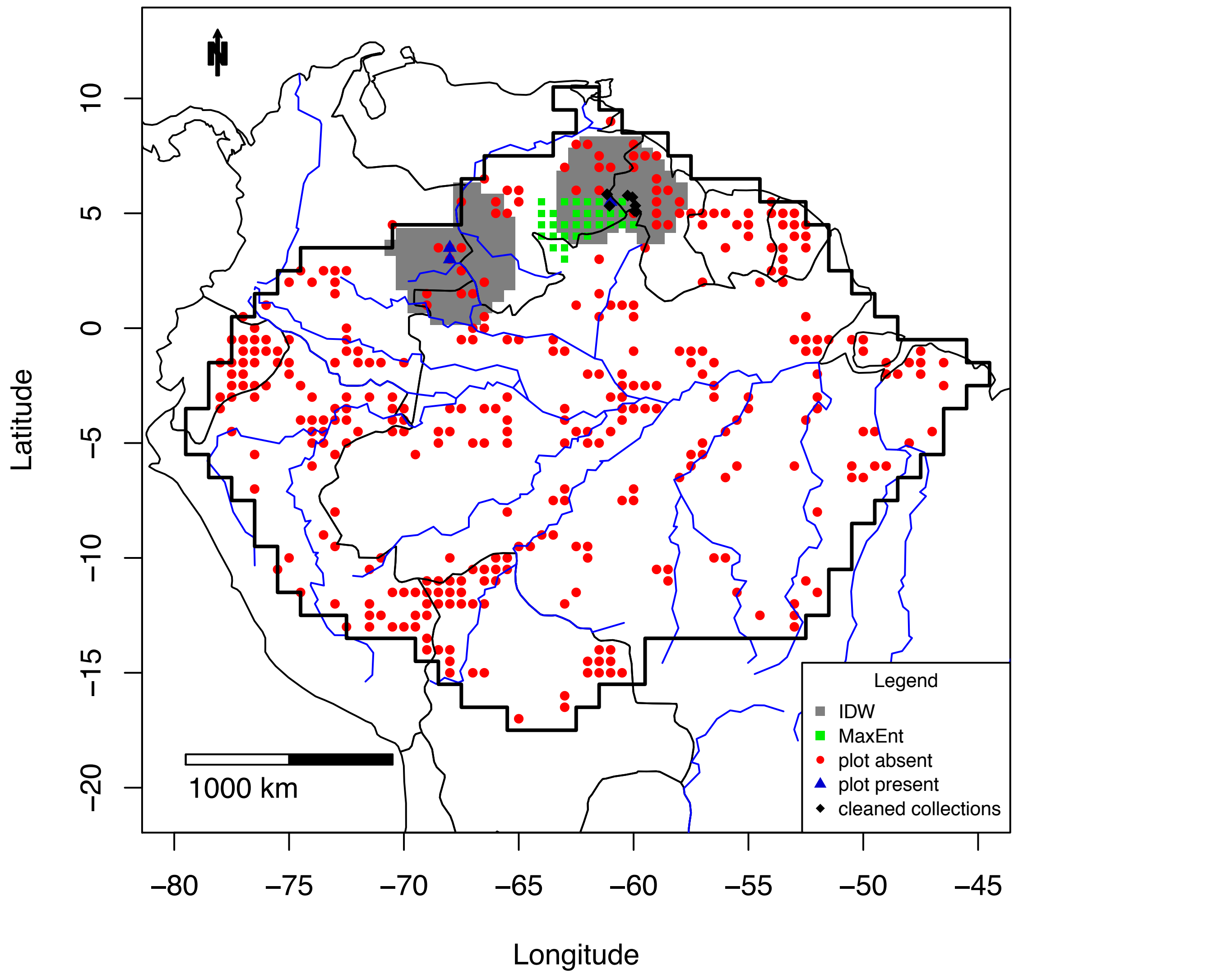

Figure S3_102B MaxEnt map of Micrandra_glabra

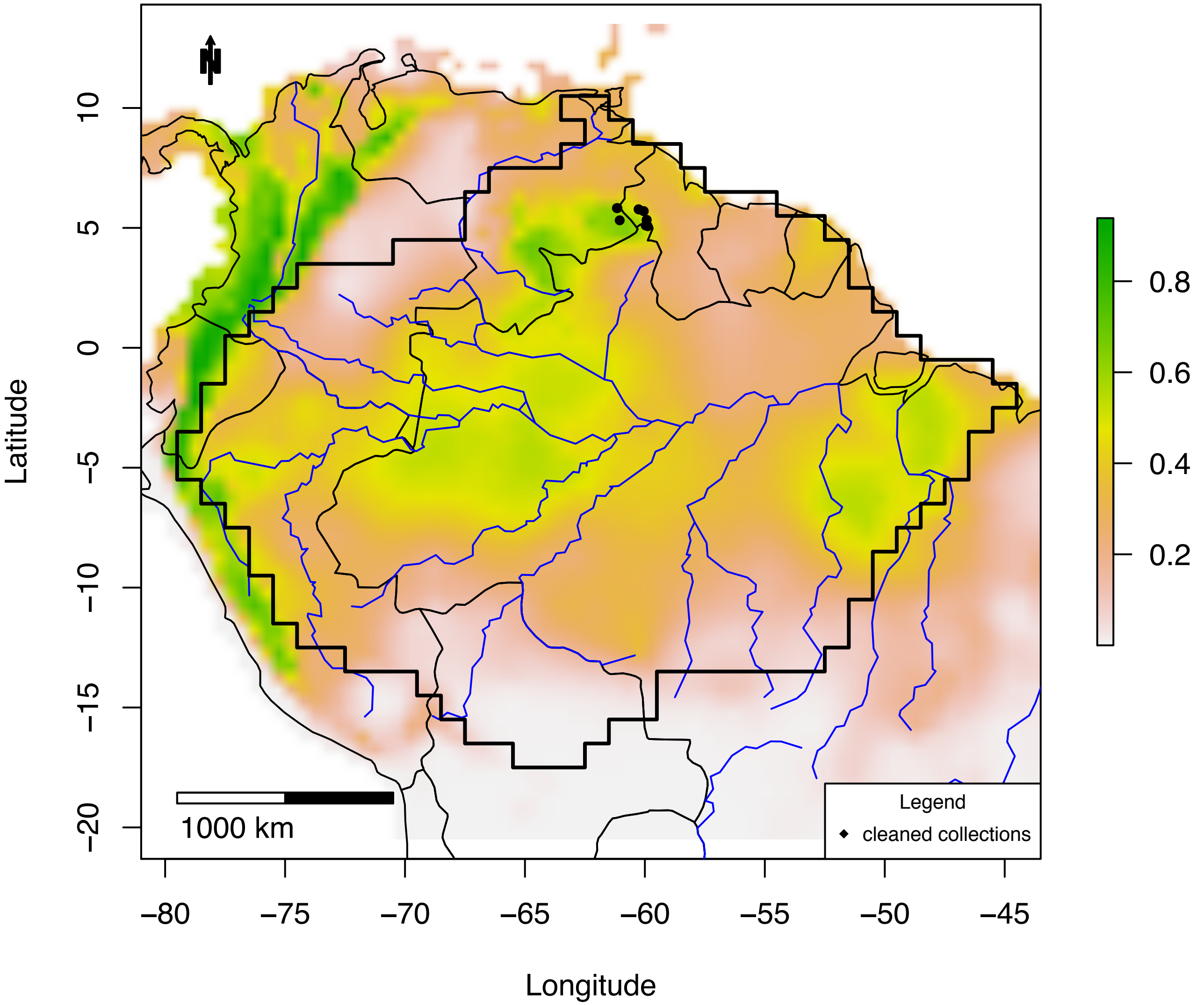

Figure S3_102C IDW map of Micrandra_glabra: plots =0 – 0.92; IDW: 0 – 0.25

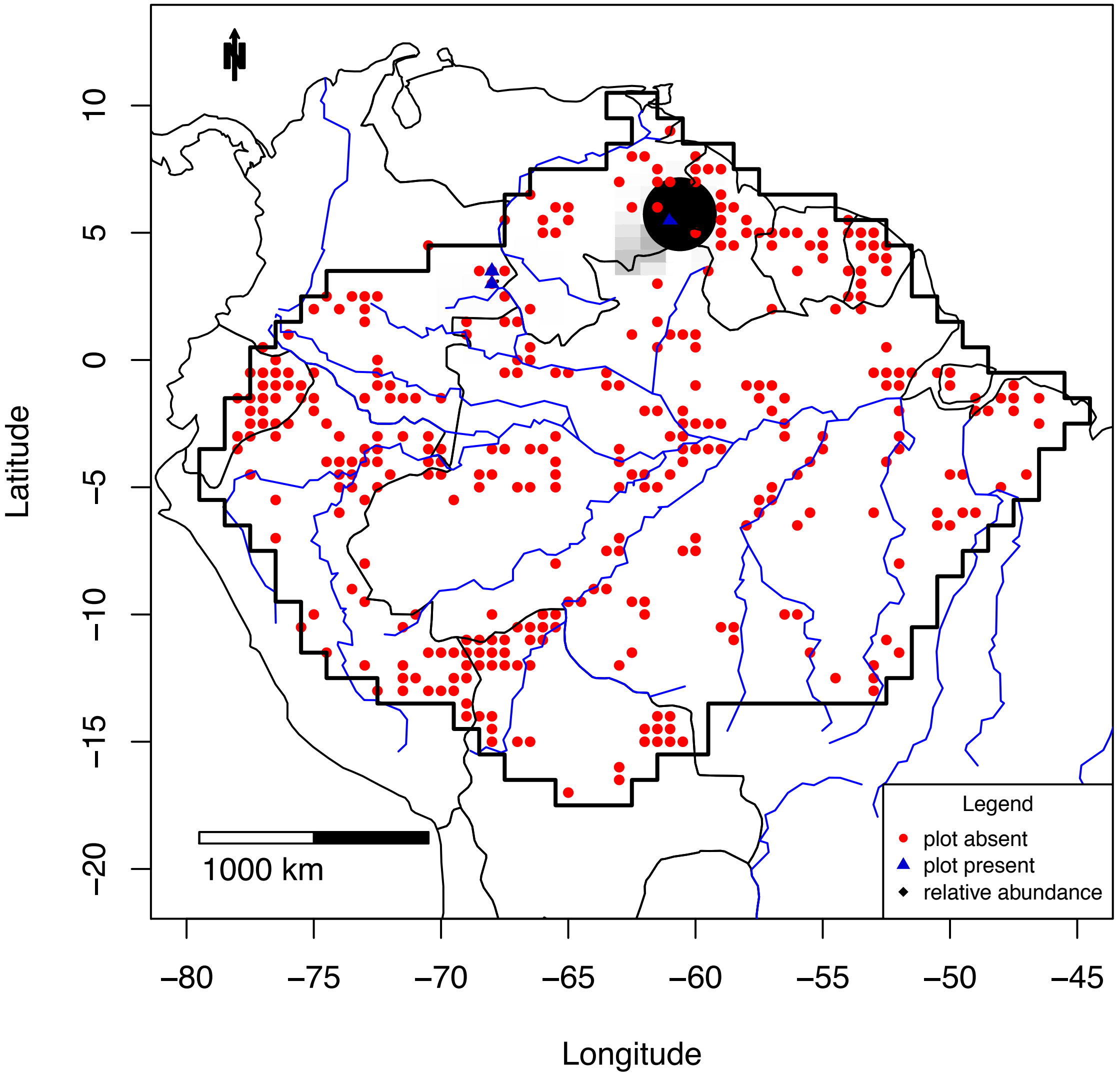

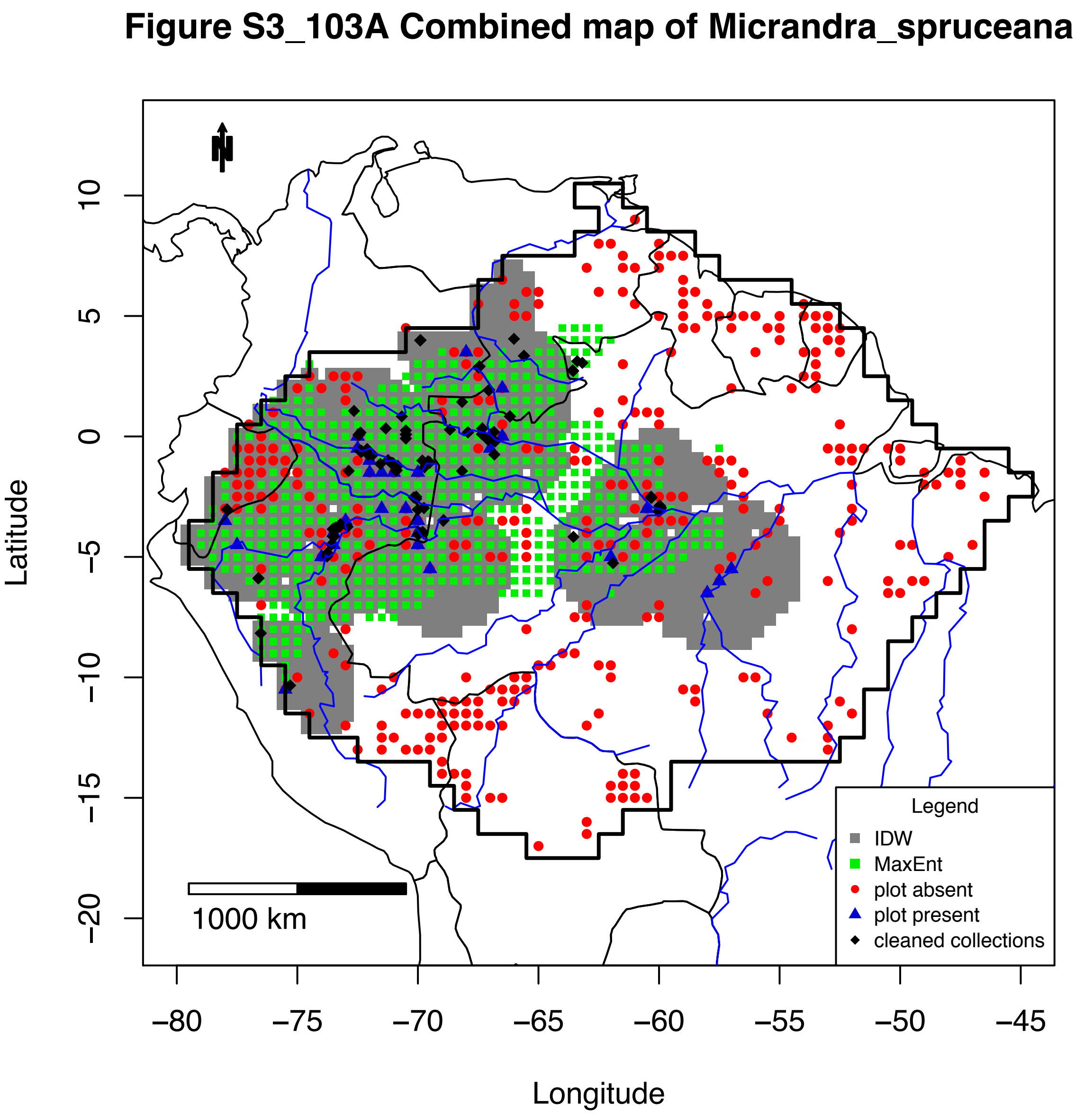
Figure S3_103A Combined map of Micrandra_spruceana
Latitude
Longitude
10
5
0
−5
−10
−15
−20
−80
−75
−70
−65
−60
−55
−50
−45
N
1000 km
Legend
IDW
MaxEnt
plot absent
plot present
cleaned collections

Figure S3_103B MaxEnt map of Micrandra_spruceana

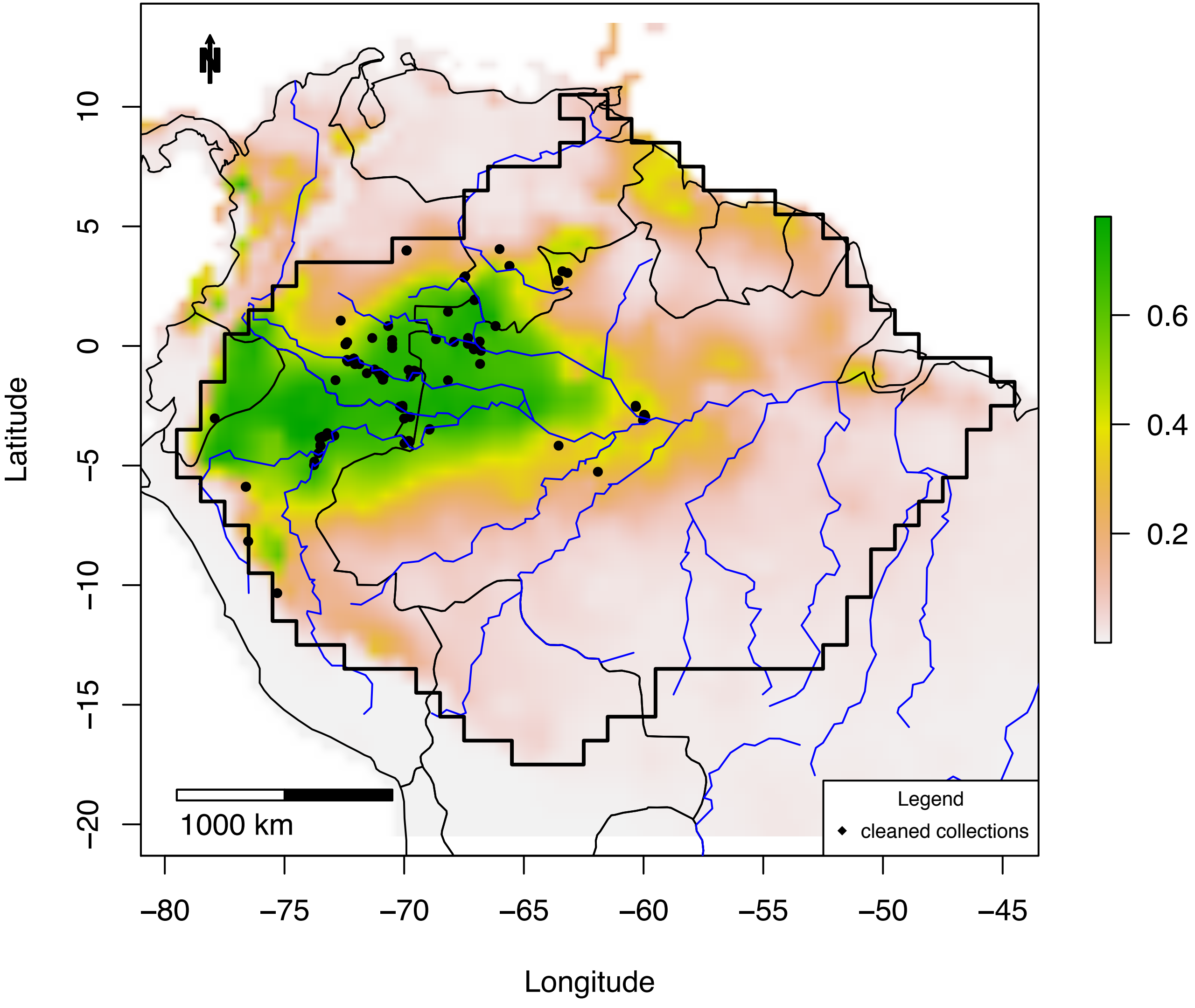

Figure S3_103C IDW map of Micrandra_spruceana: plots =0 – 0.16; IDW: 0 – 0.07

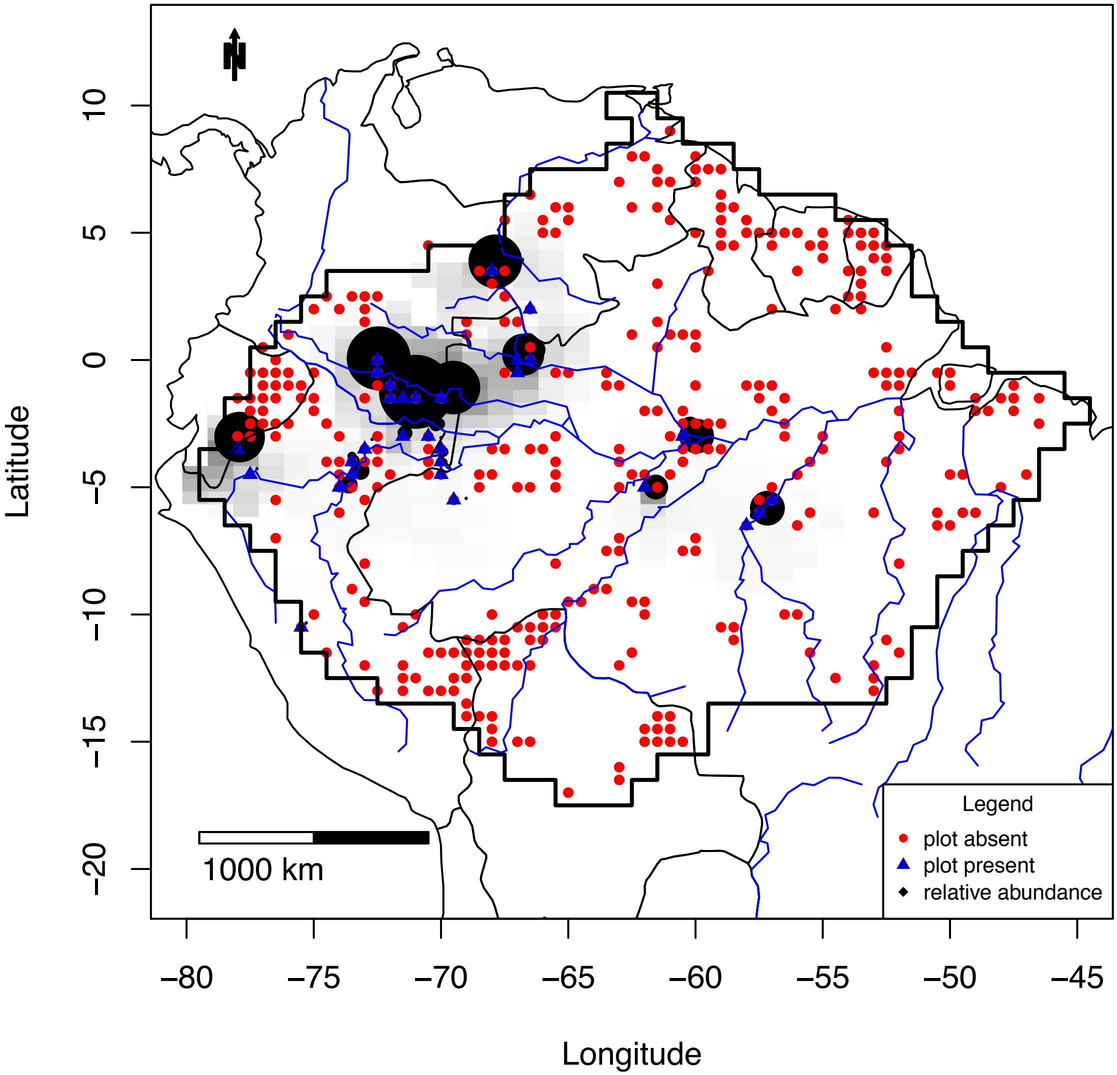

Figure S3_104A Combined map of Micrandra_sprucei

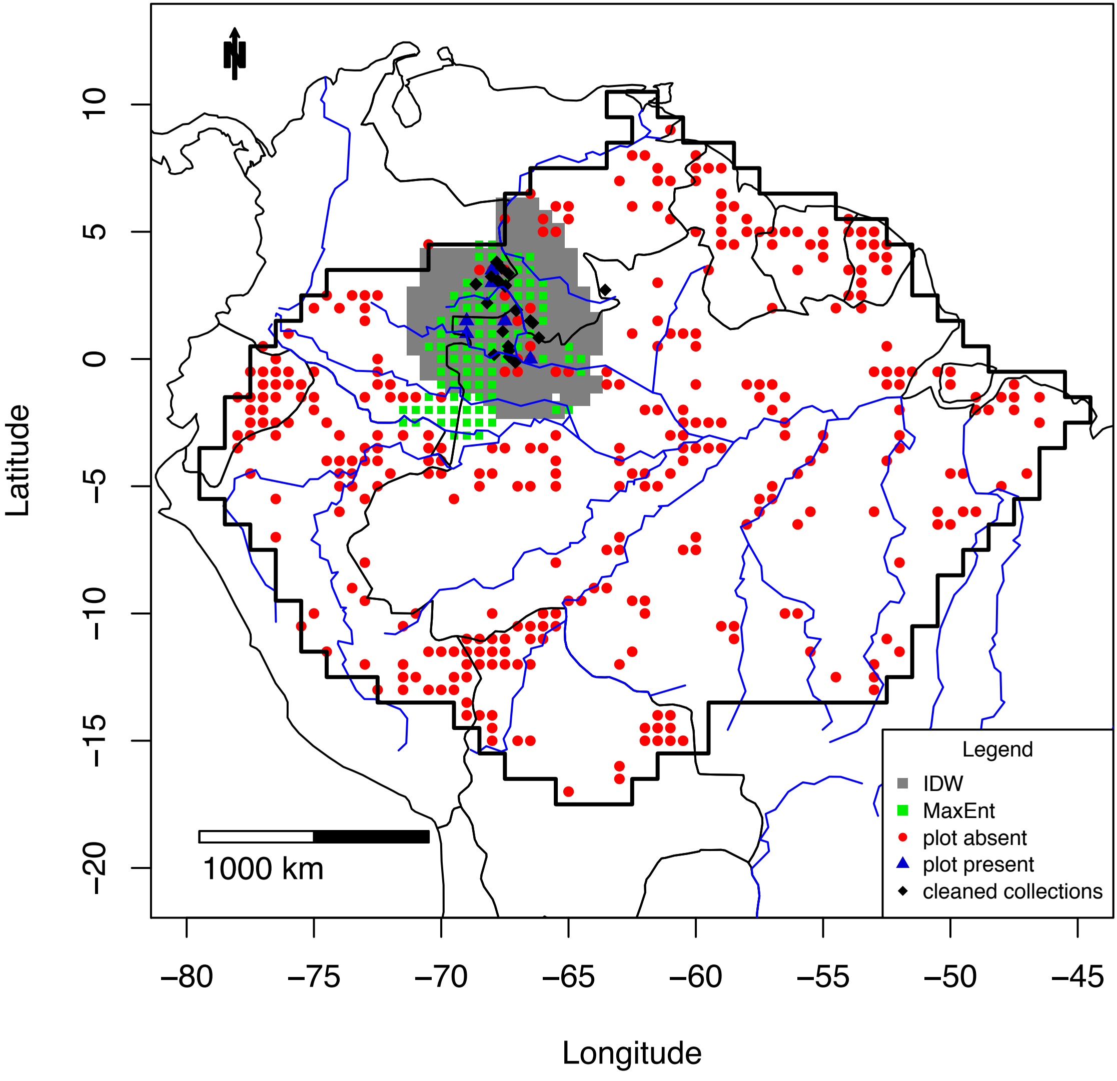

Figure S3_104B MaxEnt map of Micrandra_sprucei

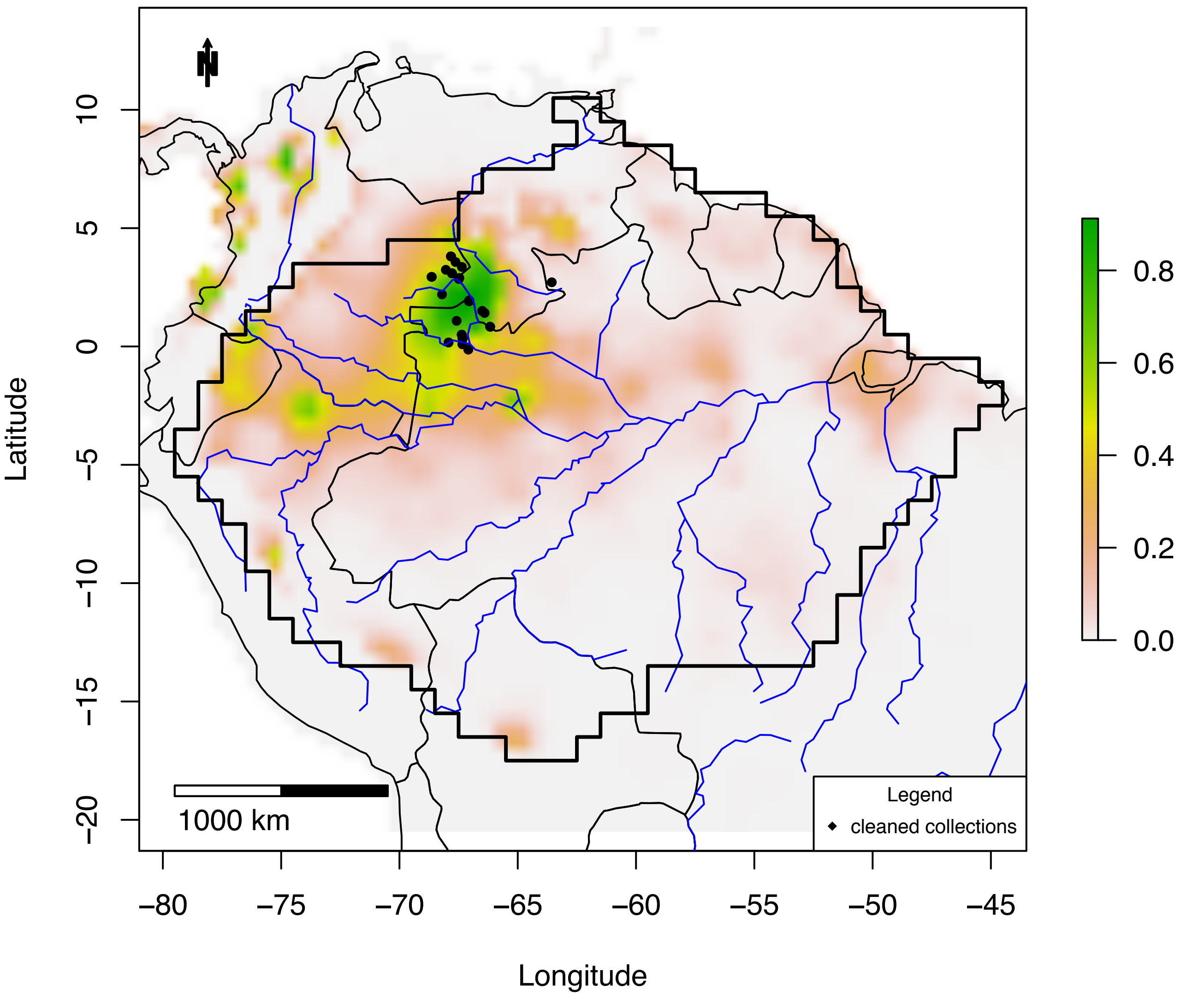

Figure S3_104C IDW map of Micrandra_sprucei: plots =0 – 0.63; IDW: 0 – 0.24

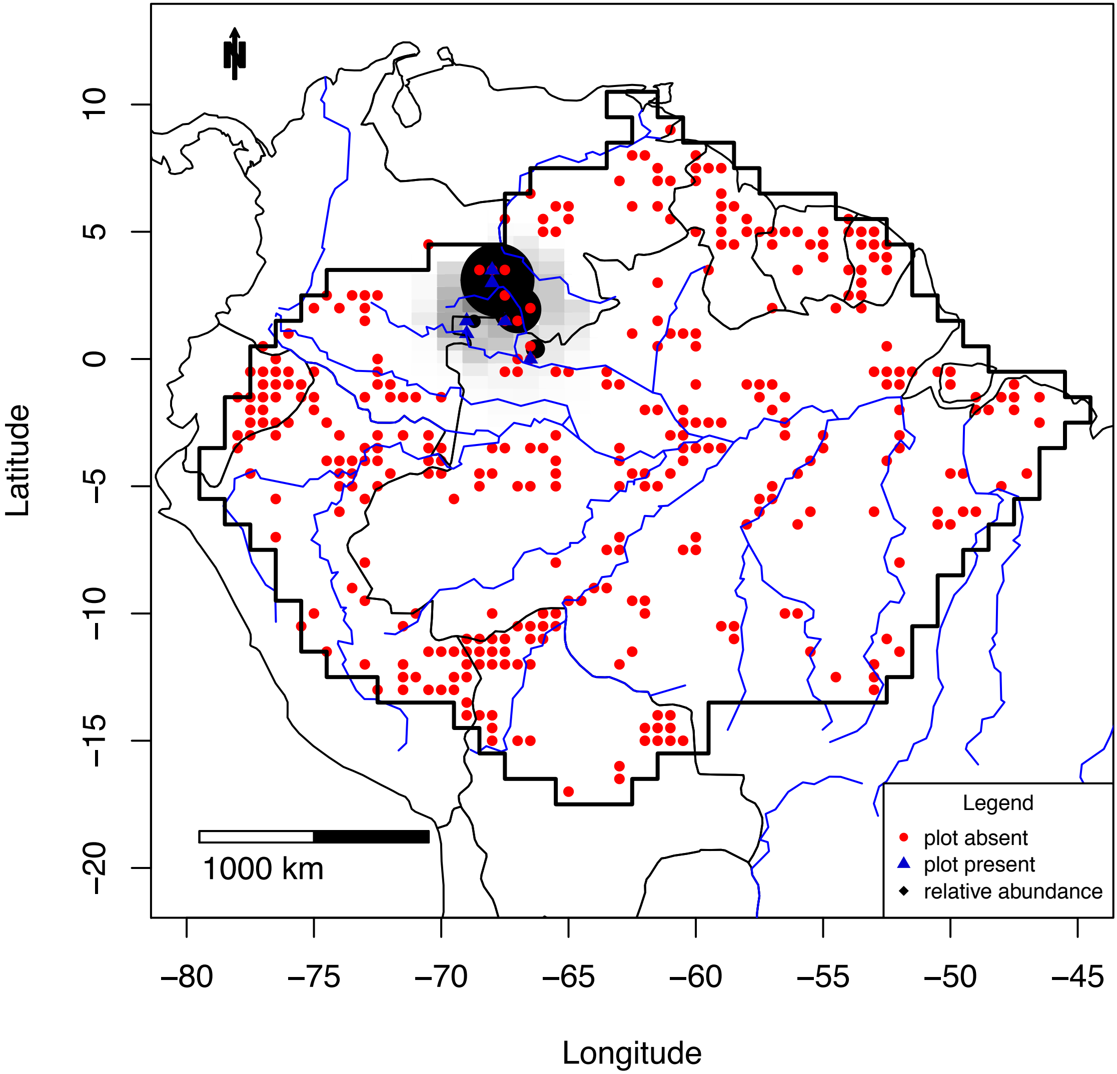

Figure S3_105A Combined map of Micropholis_gardneriana

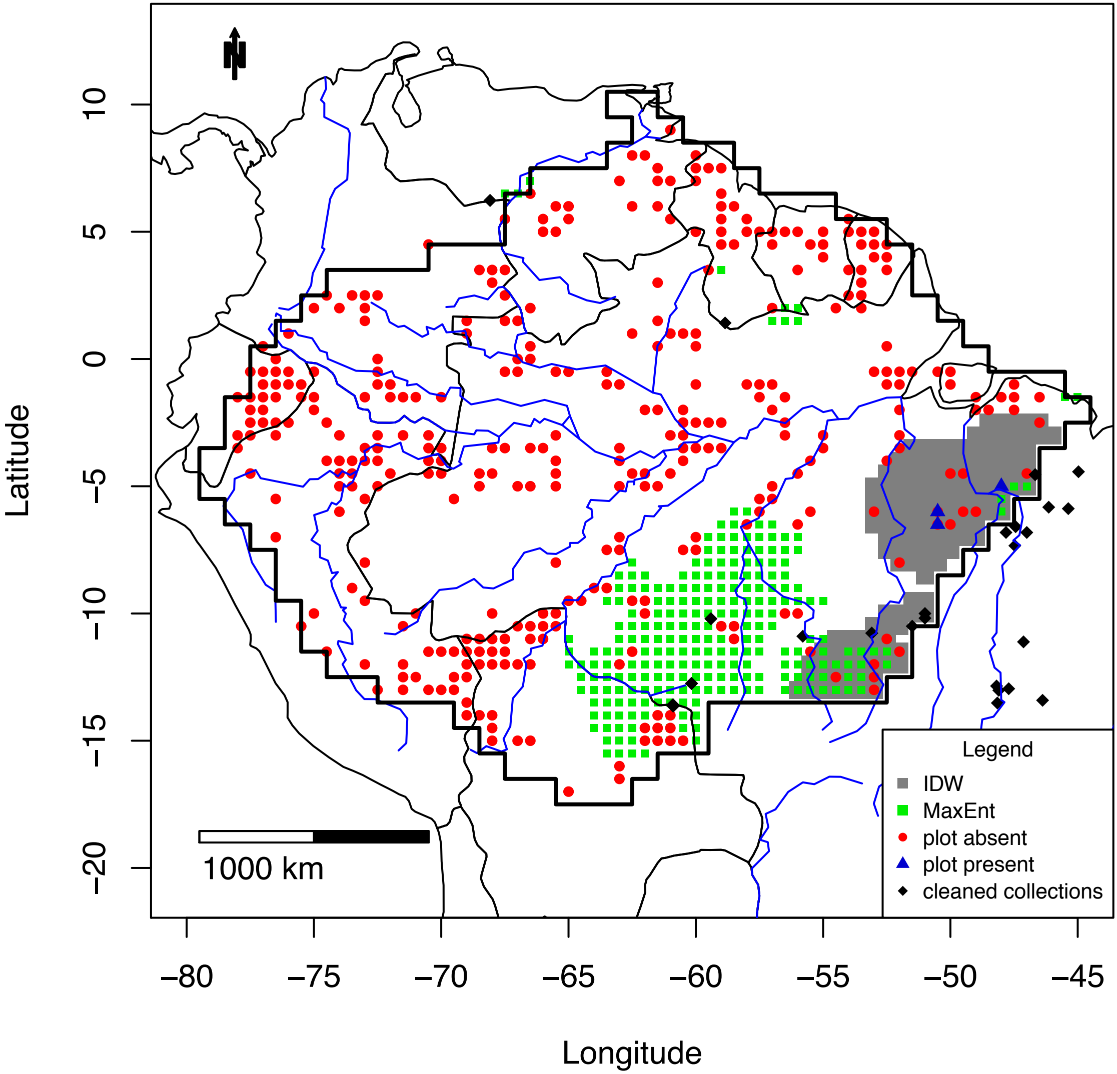

Figure S3_105B MaxEnt map of Micropholis_gardneriana

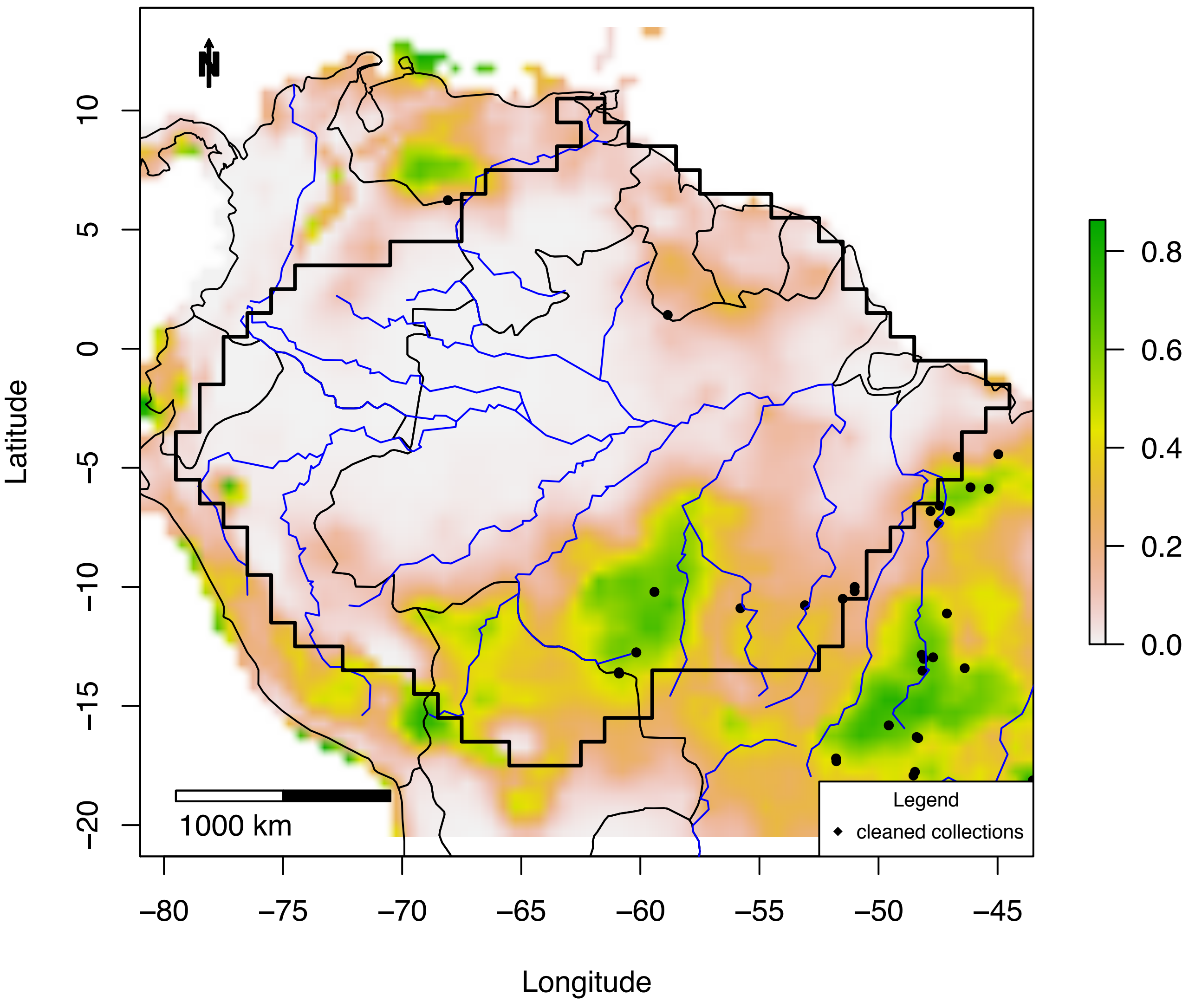

Figure S3_105C IDW map of Micropholis_gardneriana: plots =0 – 0.32; IDW: 0 – 0.06

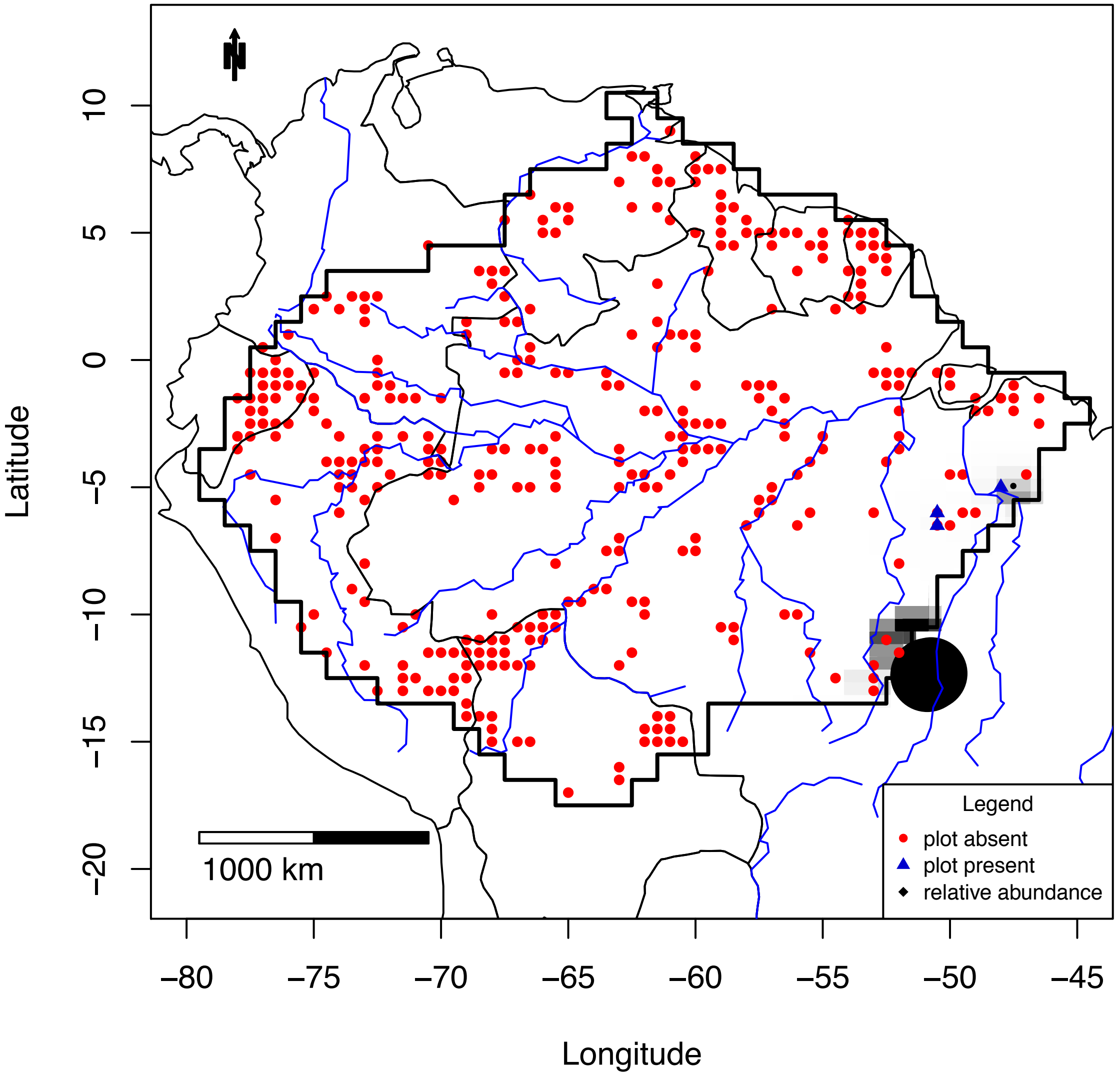

Figure S3_106A Combined map of Micropholis_guyanensis

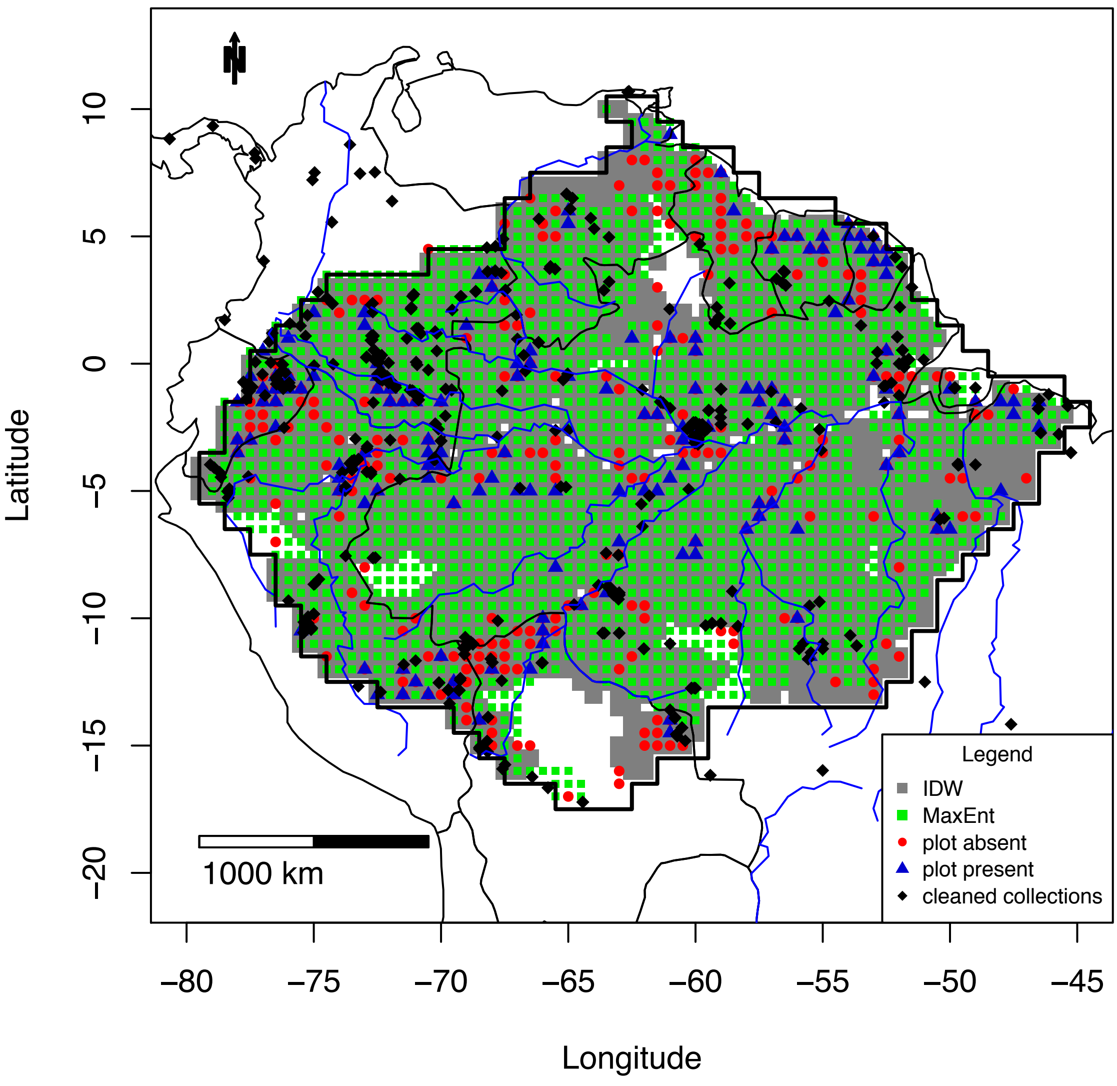

Figure S3_106B MaxEnt map of Micropholis_guyanensis

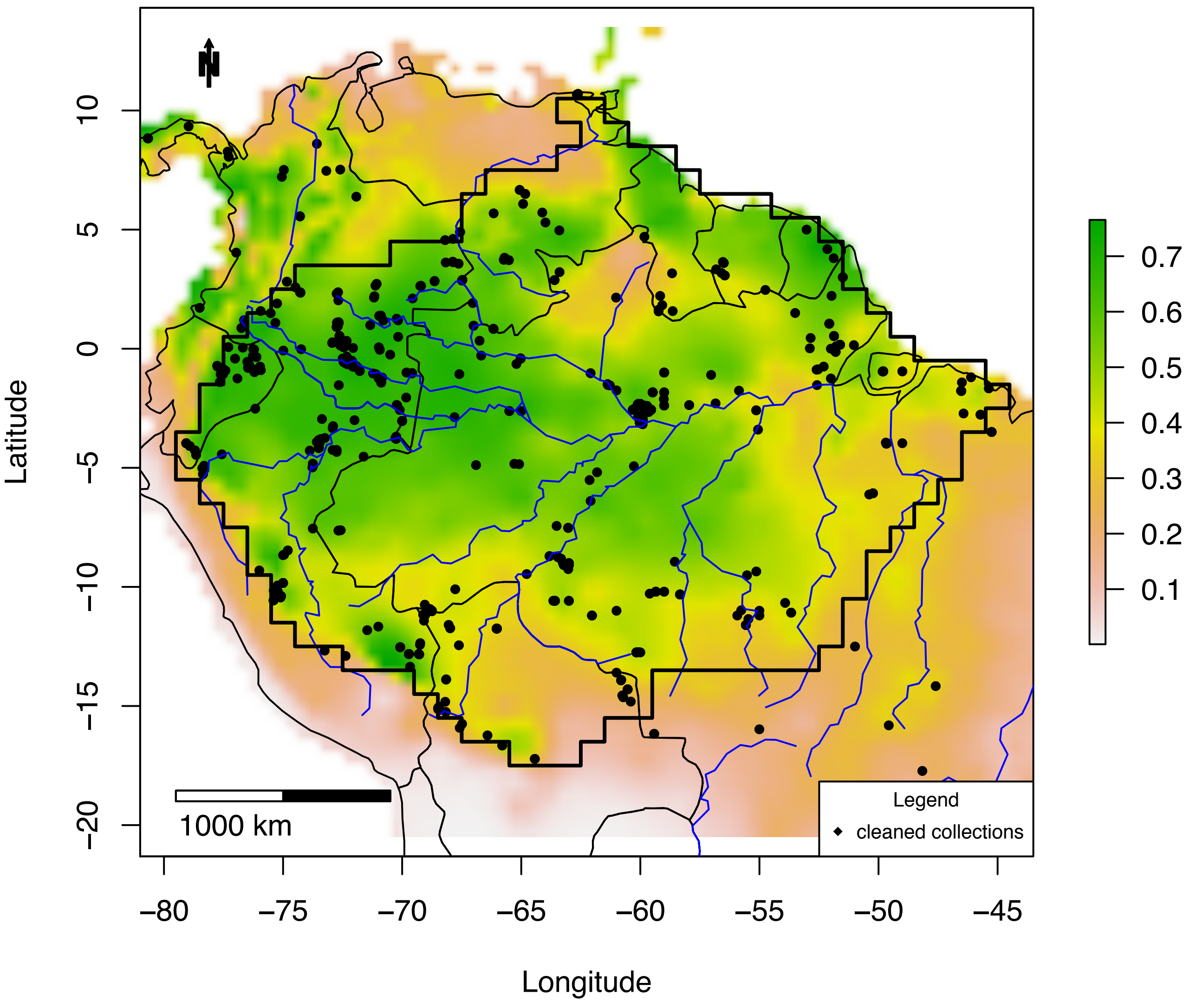

Figure S3_106C IDW map of Micropholis_guyanensis: plots =0 – 0.15; IDW: 0 – 0.06

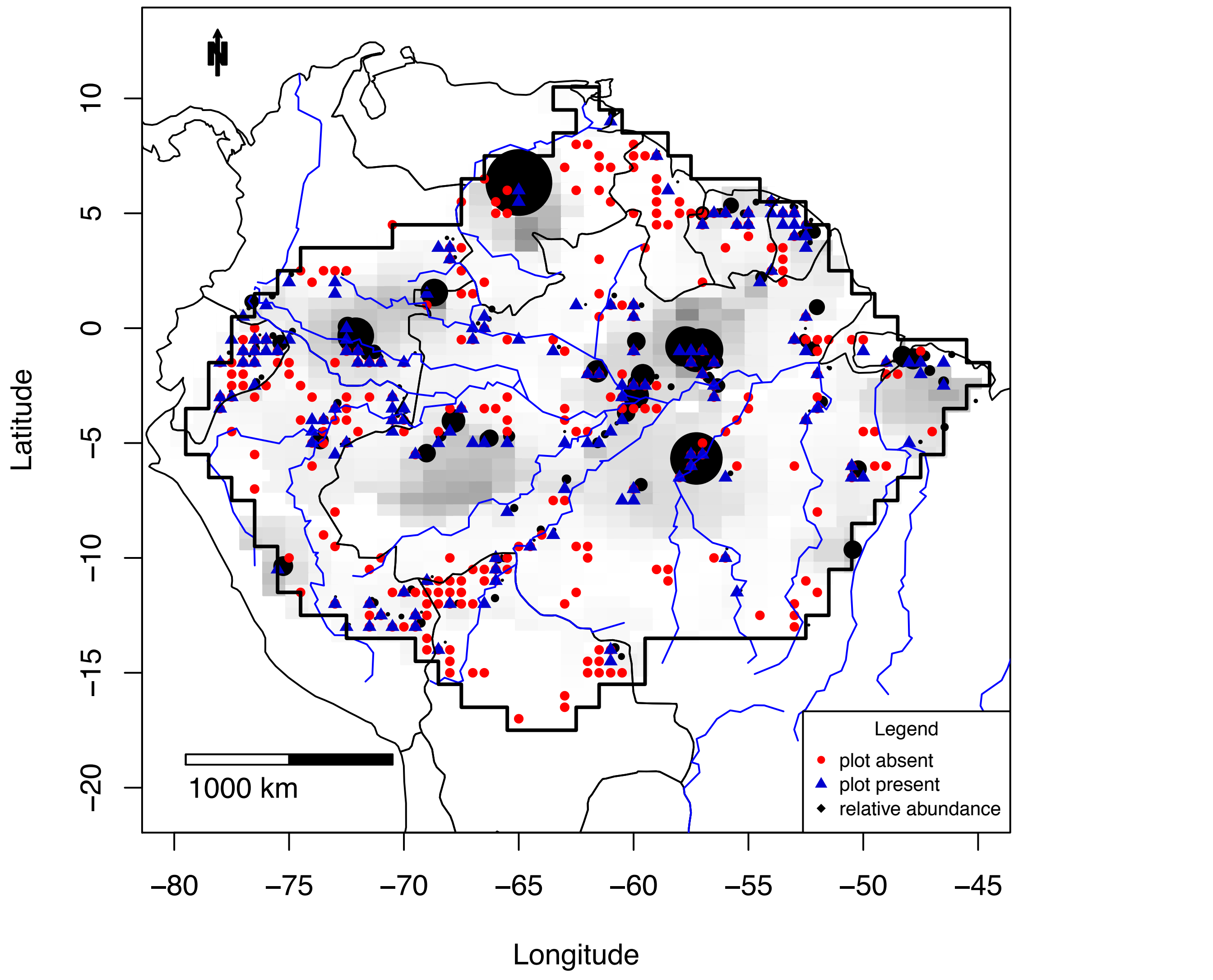

Figure S3_107A Combined map of Minquartia_guianensis

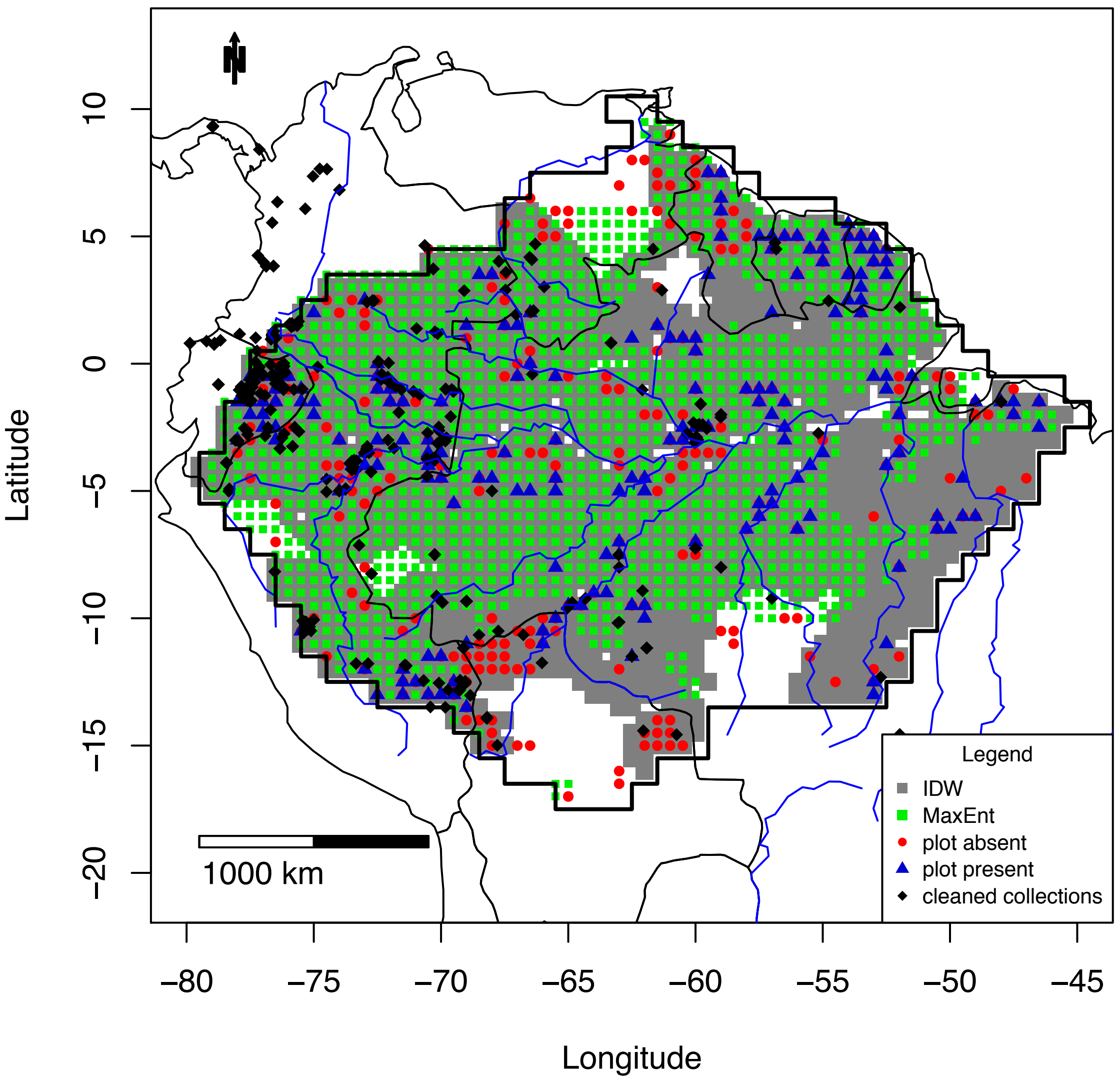

Figure S3_107B MaxEnt map of Minquartia_guianensis

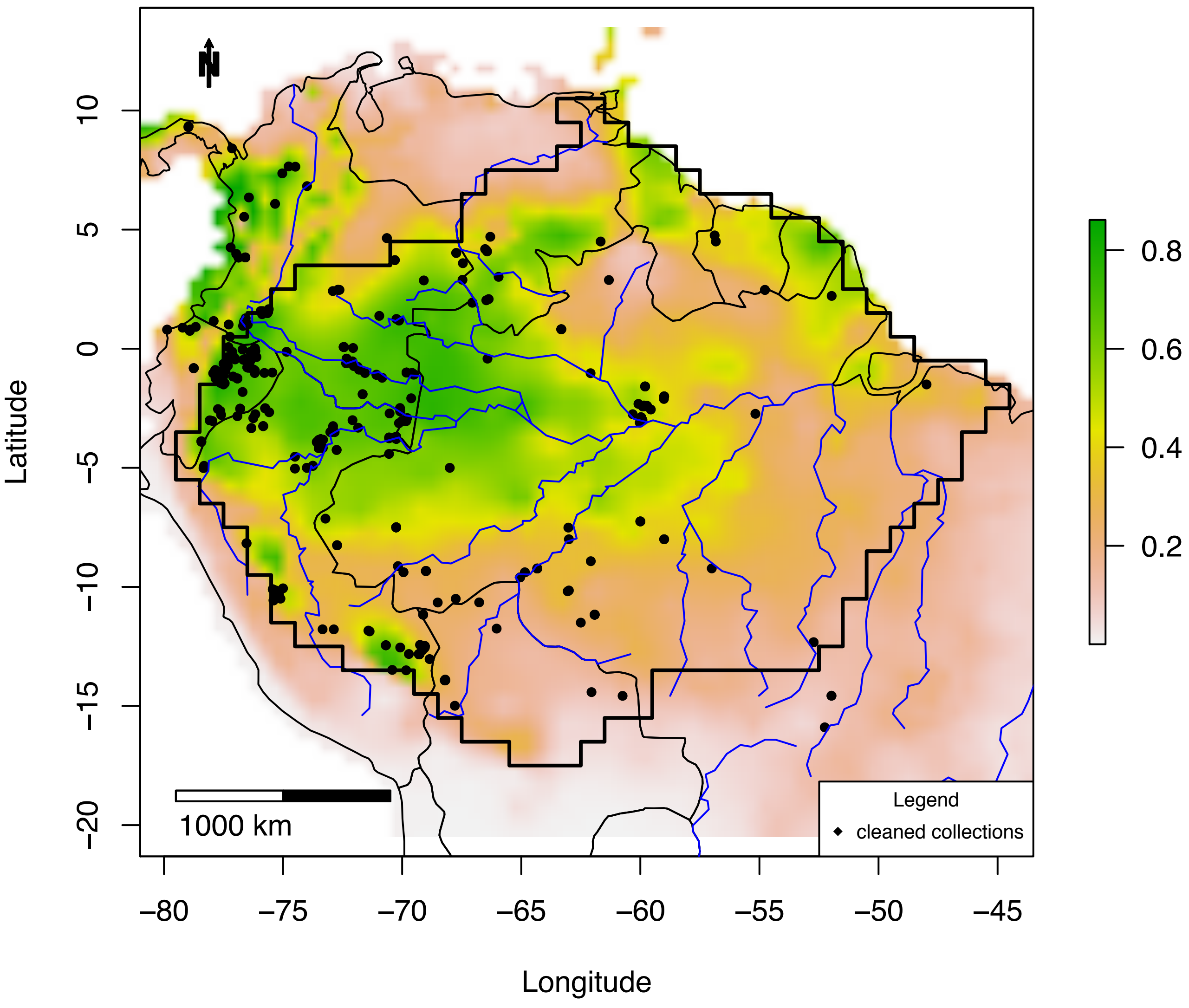

Figure S3_107C IDW map of Minquartia_guianensis:
plots =0 – 0.07; IDW: 0 – 0.03

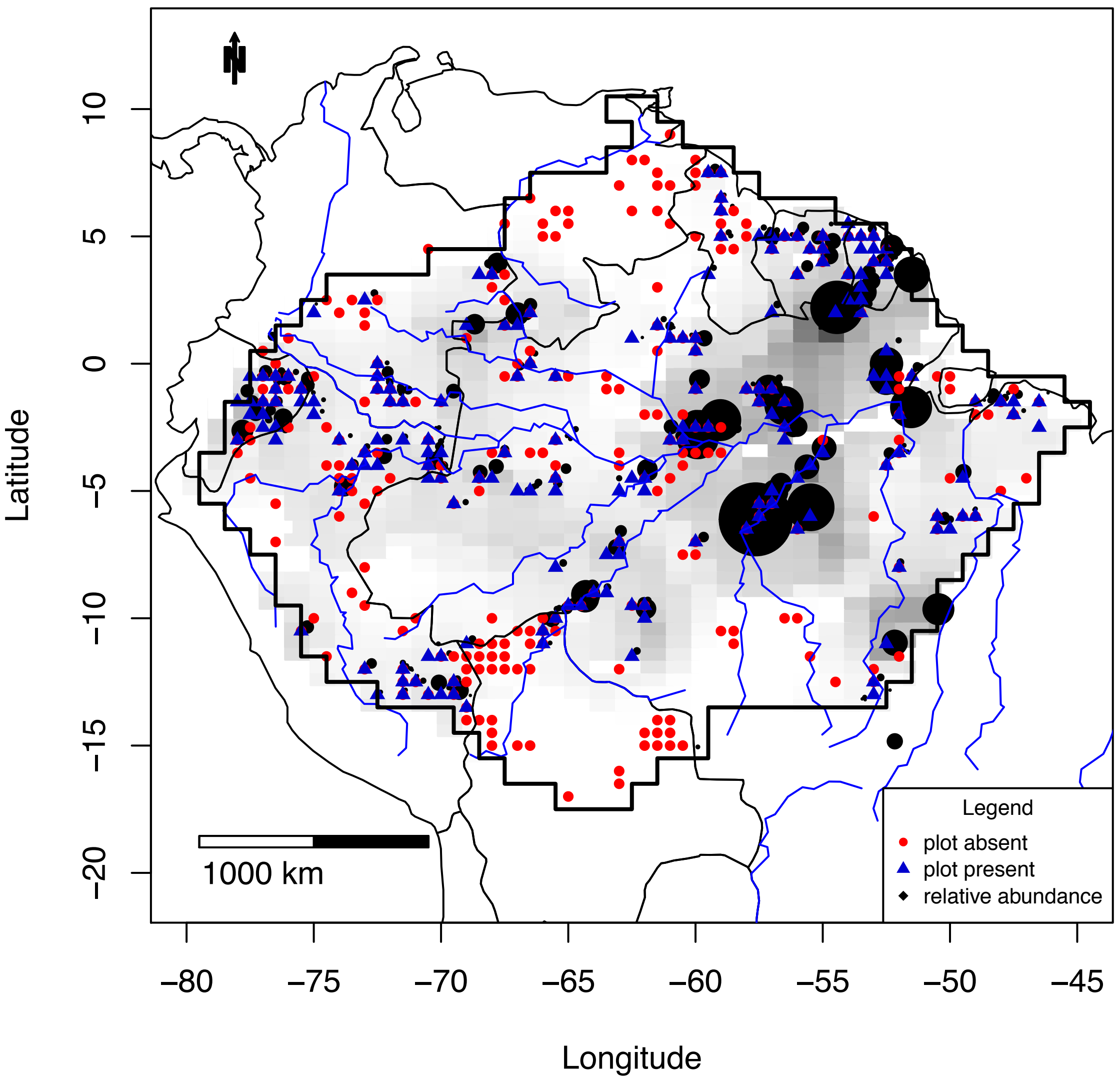

Figure S3_108A Combined map of Mollia_gracilis

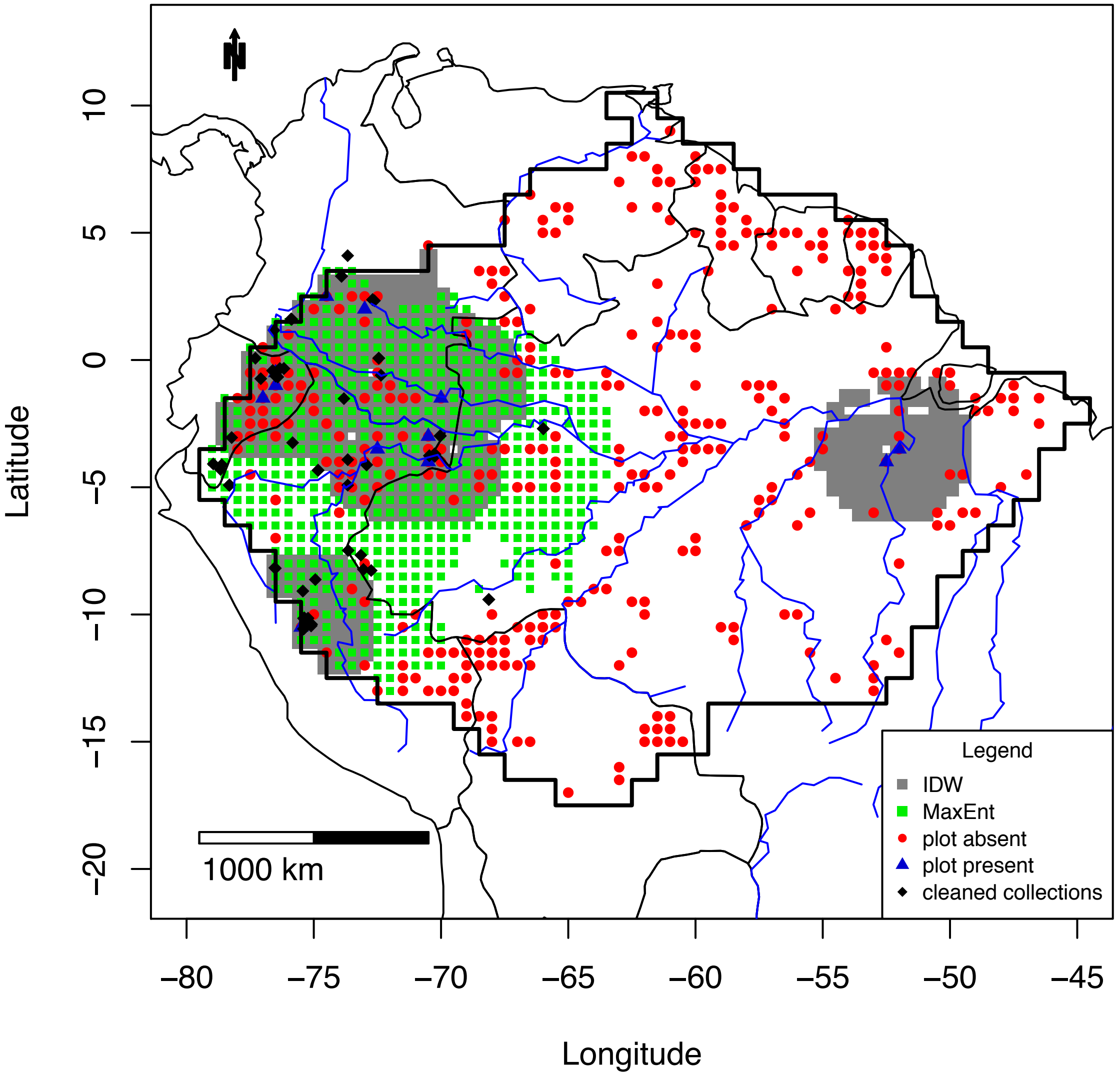

Figure S3_108B MaxEnt map of Mollia_gracilis

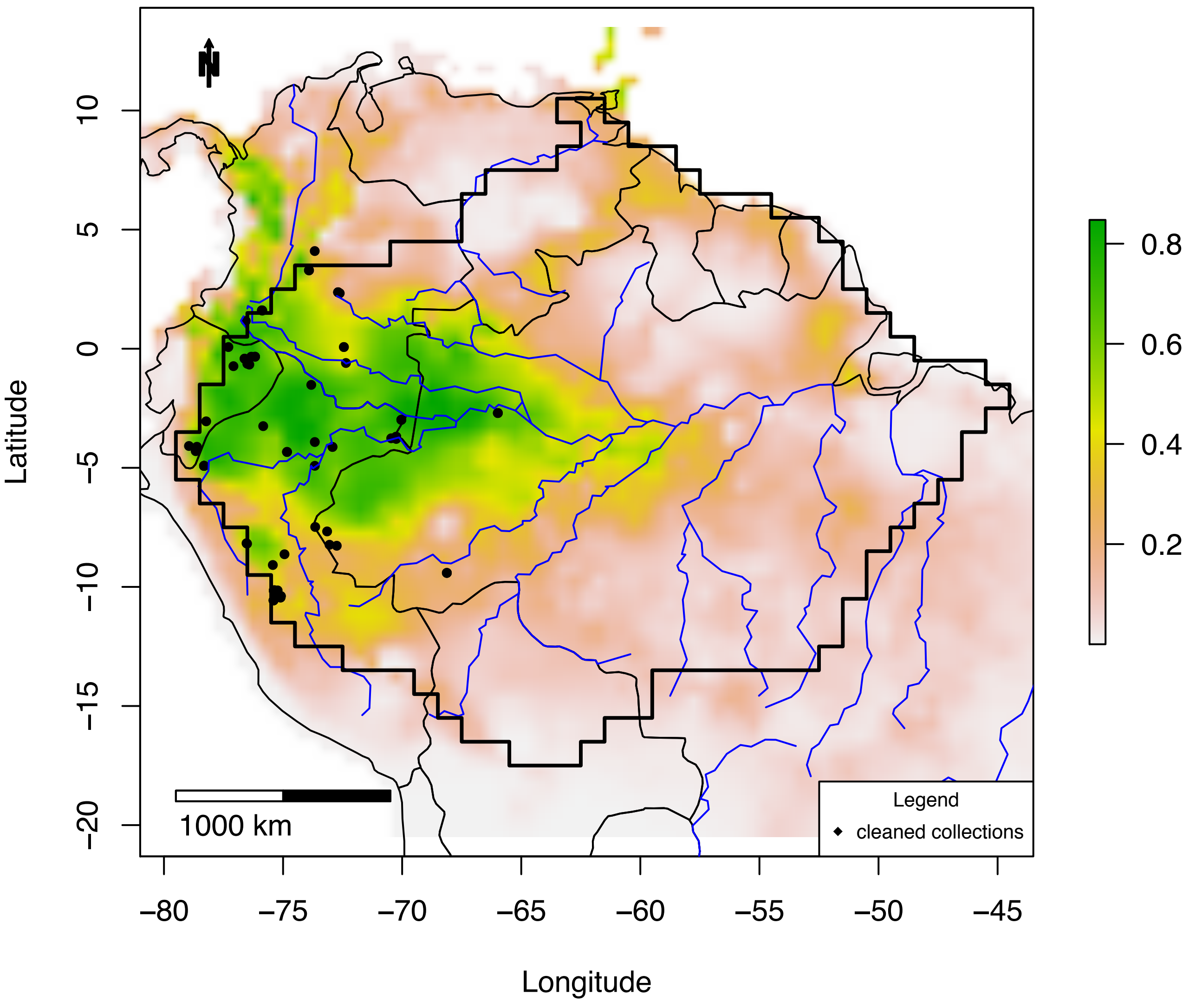

Figure S3_108C IDW map of Mollia_gracilis:
plots =0 – 0.21; IDW: 0 – 0.05

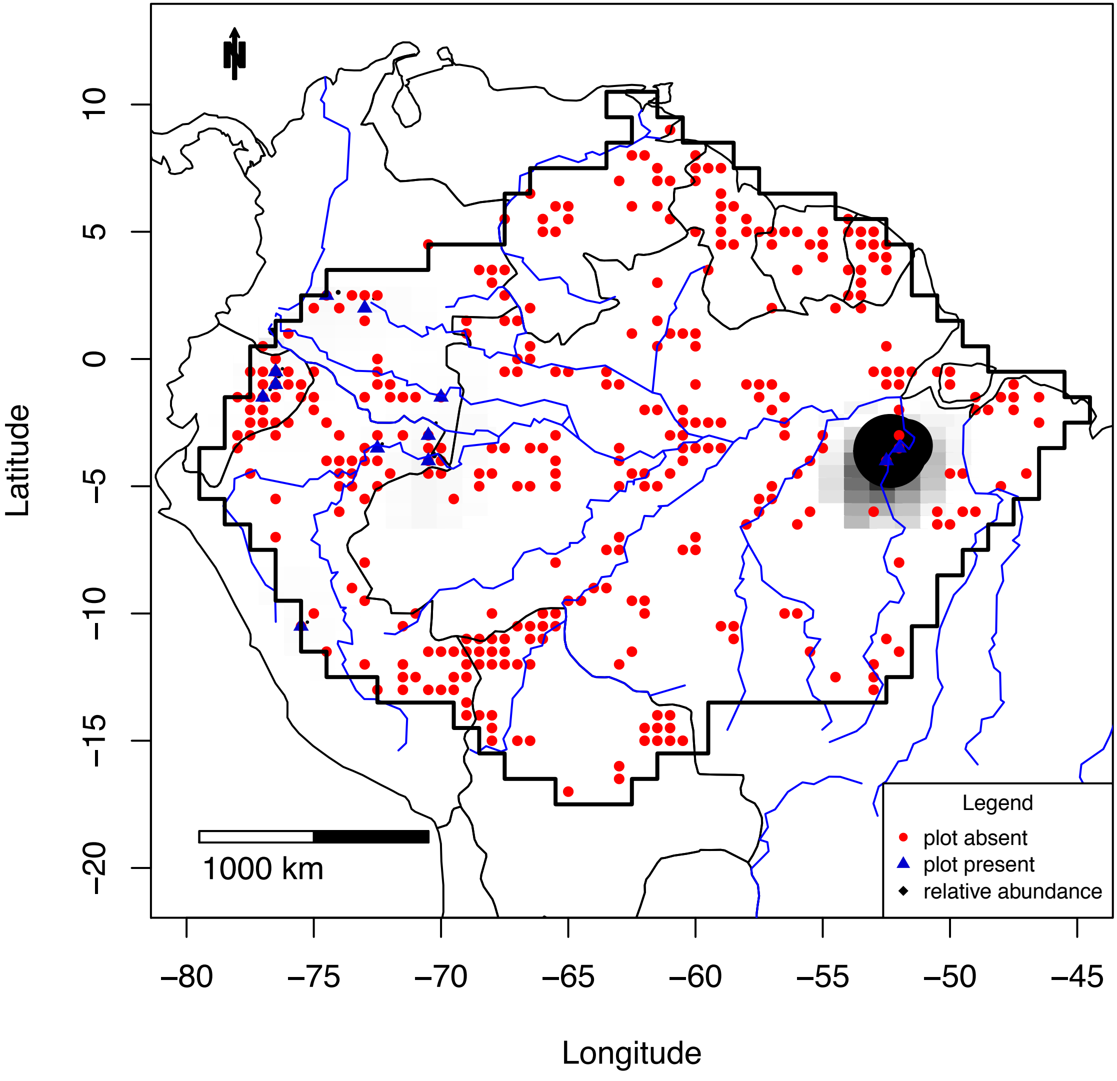

Figure S3_109A Combined map of Monopteryx_uaucu

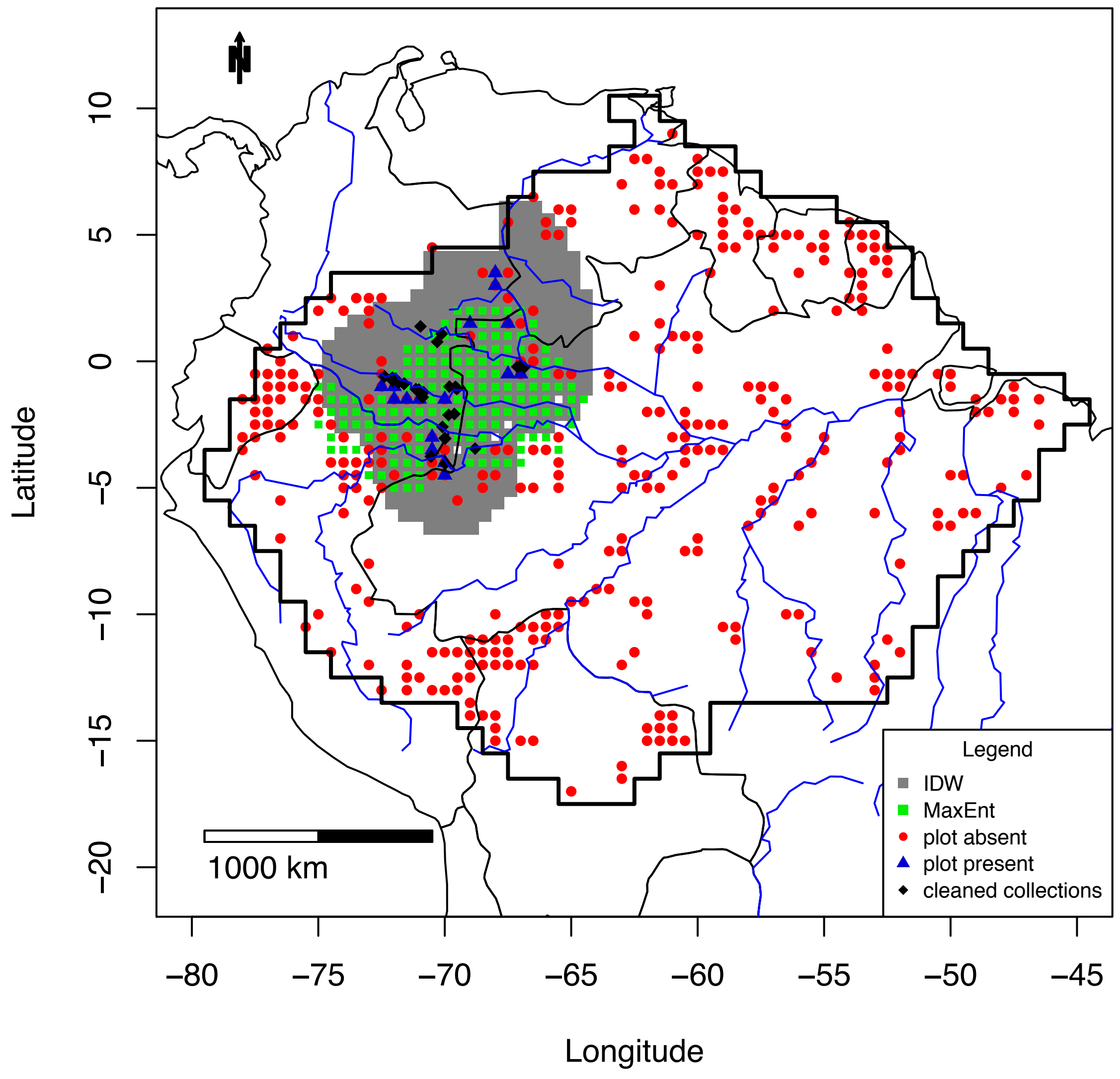

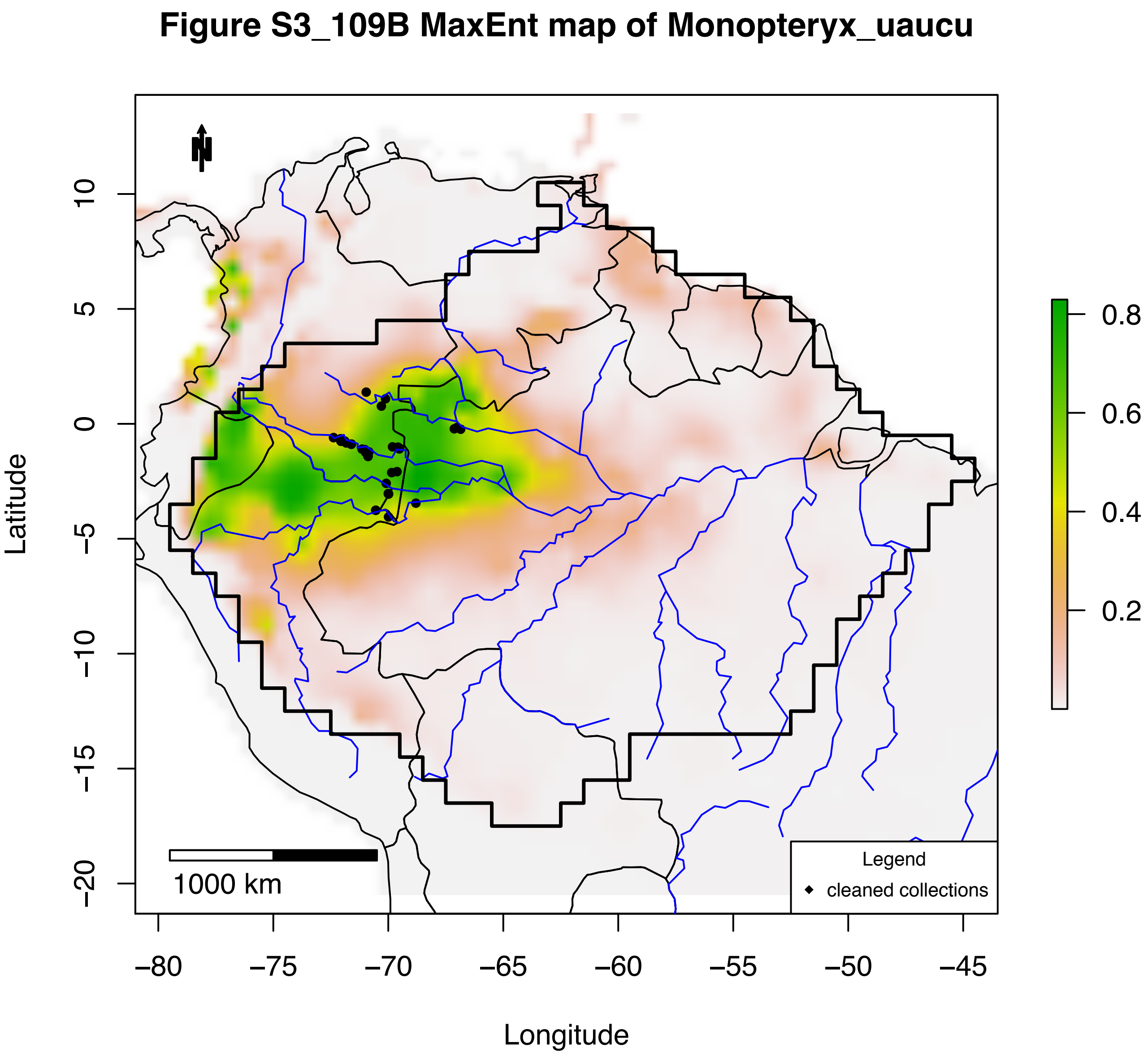

Figure S3_109B MaxEnt map of Monopteryx_uaucu
N
10
5
0
−5
−10
−15
−20
Latitude
−80
−75
−70
−65
−60
−55
−50
−45
Longitude
1000 km
Legend
cleaned collections
0.8
0.6
0.4
0.2

Figure S3_109C IDW map of Monopteryx_uaucu: plots =0 – 0.42; IDW: 0 – 0.1

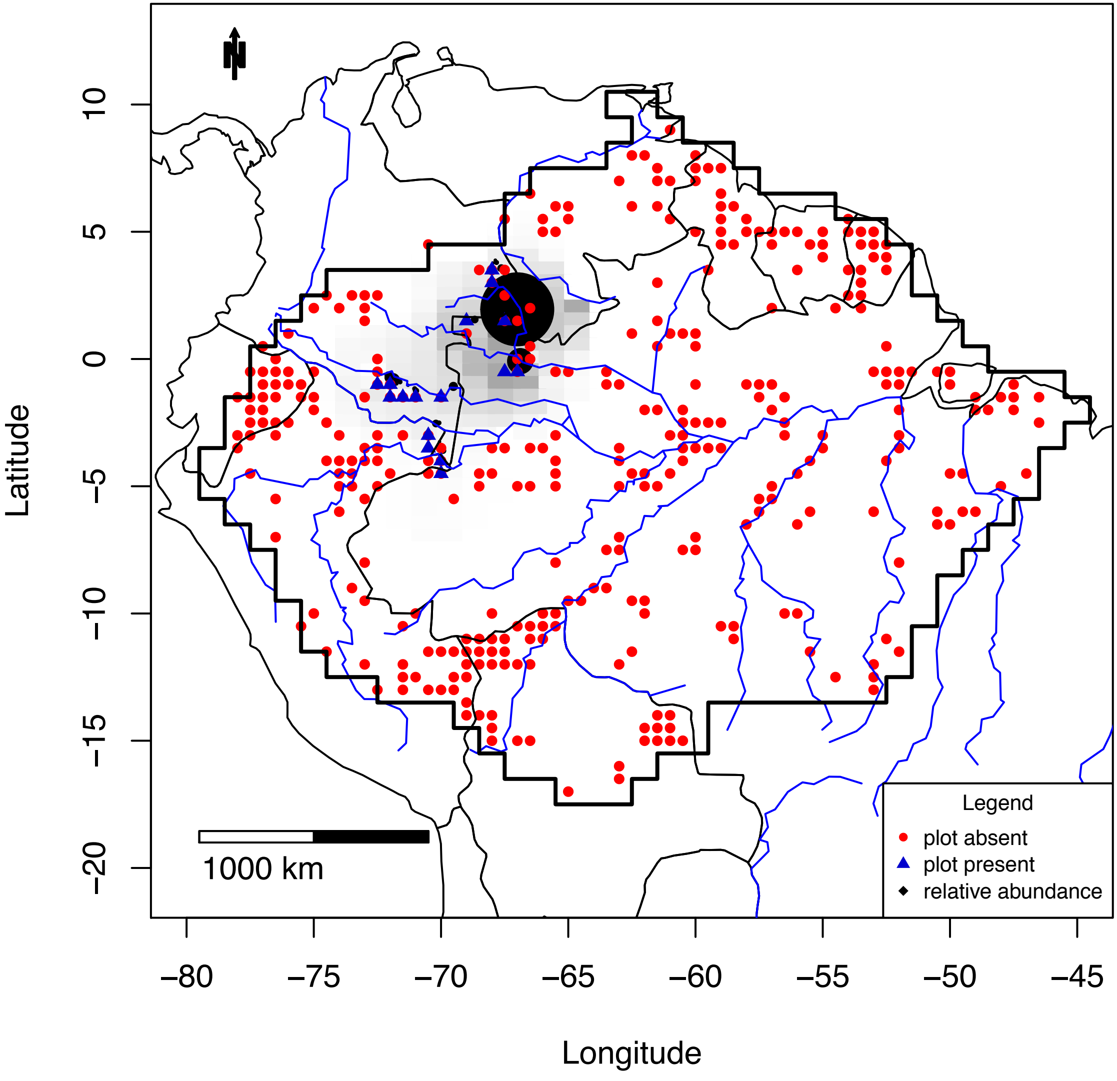

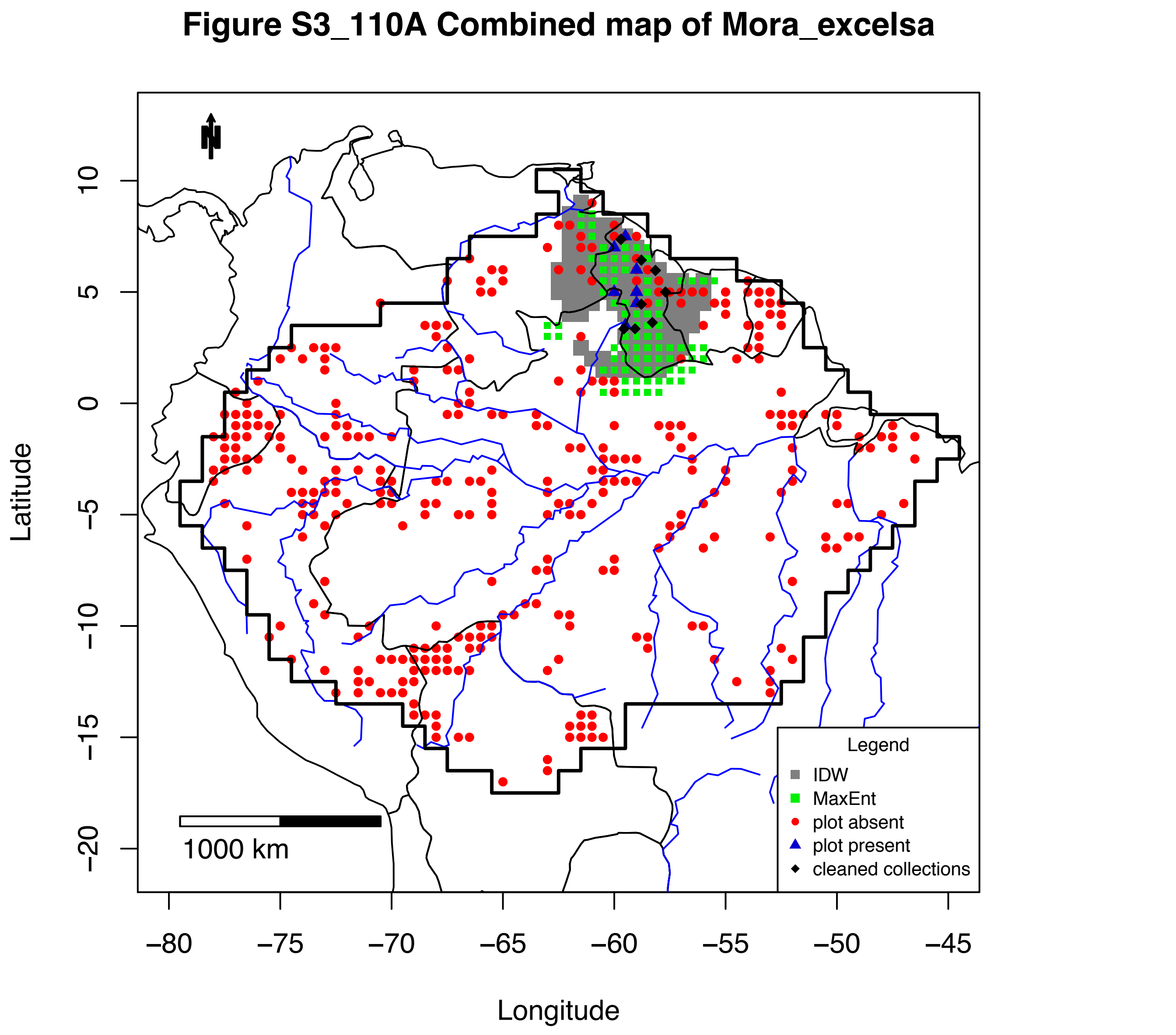
Figure S3_110A Combined map of Mora_excelsa
Latitude
Longitude
10
5
0
−5
−10
−15
−20
−80
−75
−70
−65
−60
−55
−50
−45
N
1000 km
Legend
IDW
MaxEnt
plot absent
plot present
cleaned collections

Figure S3_110B MaxEnt map of Mora_excelsa

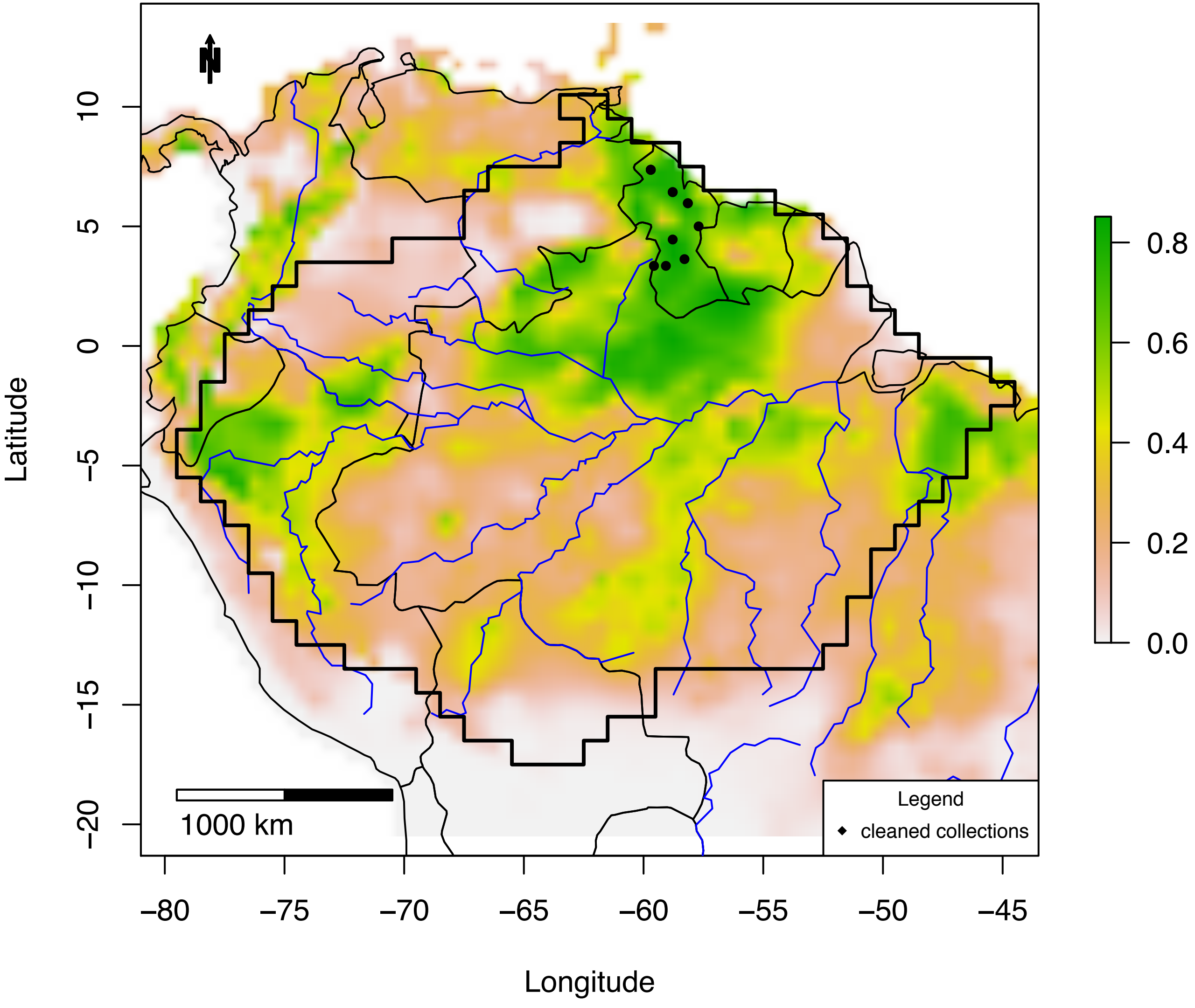

Figure S3_110C IDW map of Mora_excelsa:
plots =0 – 0.59; IDW: 0 – 0.13

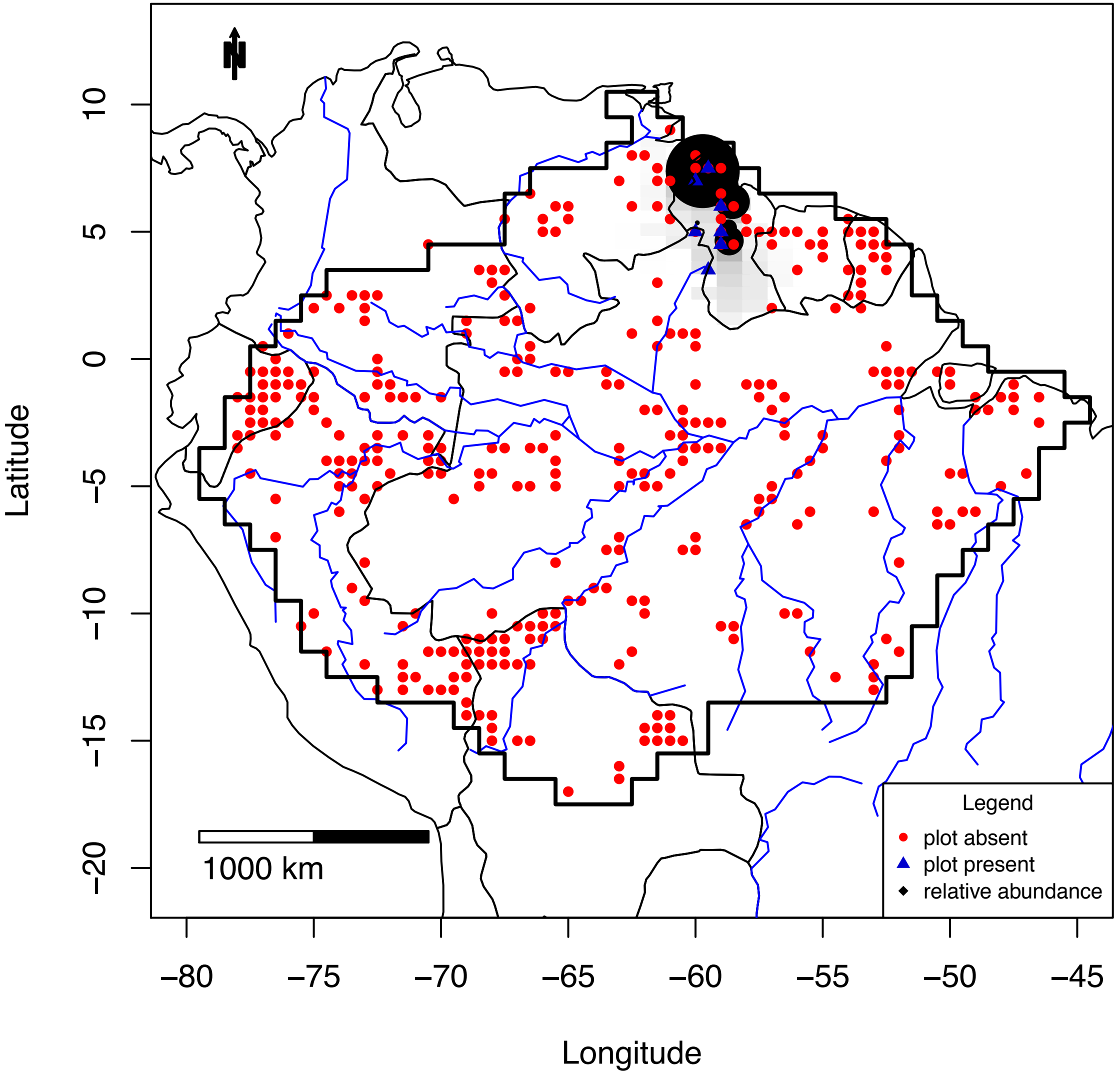

Figure S3_111A Combined map of Ocotea_aciphylla

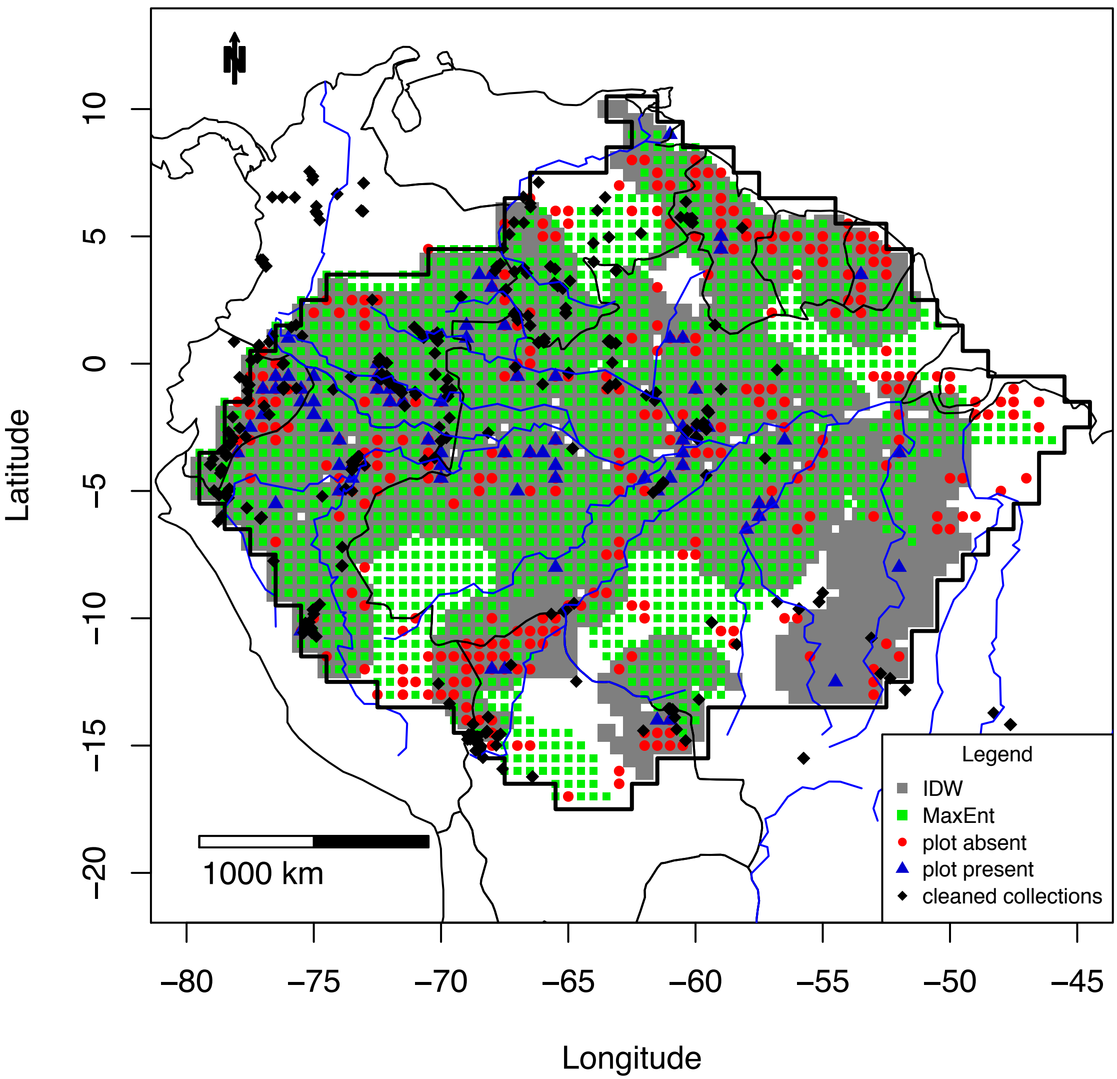

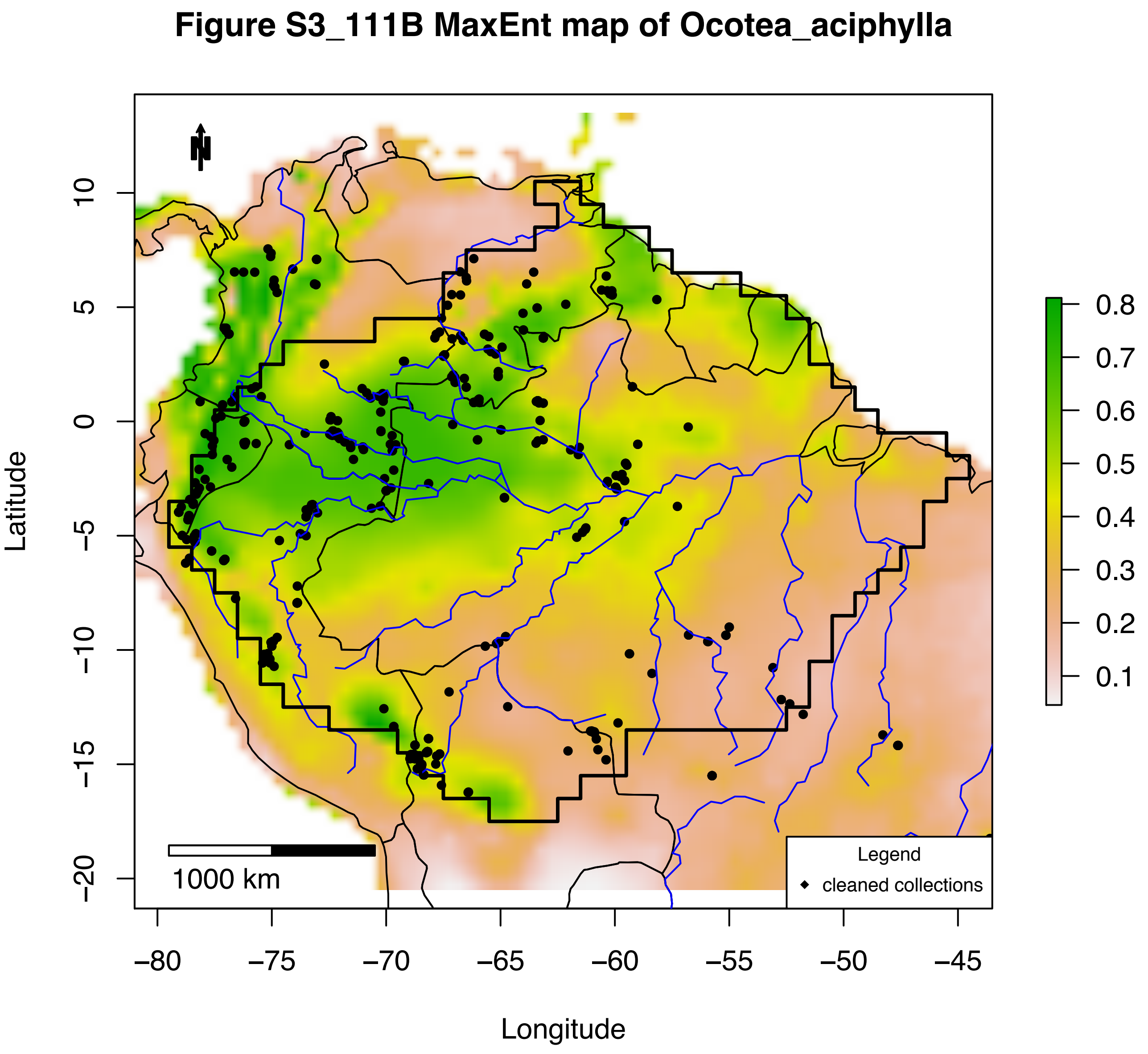
Figure S3_111B MaxEnt map of Ocotea_aciphylla
Latitude
Longitude
10
5
0
−5
−10
−15
−20
−80
−75
−70
−65
−60
−55
−50
−45
0.8
0.7
0.6
0.5
0.4
0.3
0.2
0.1
N
1000 km
Legend
cleaned collections

Figure S3_111C IDW map of Ocotea_aciphylla: plots =0 – 0.16; IDW: 0 – 0.05

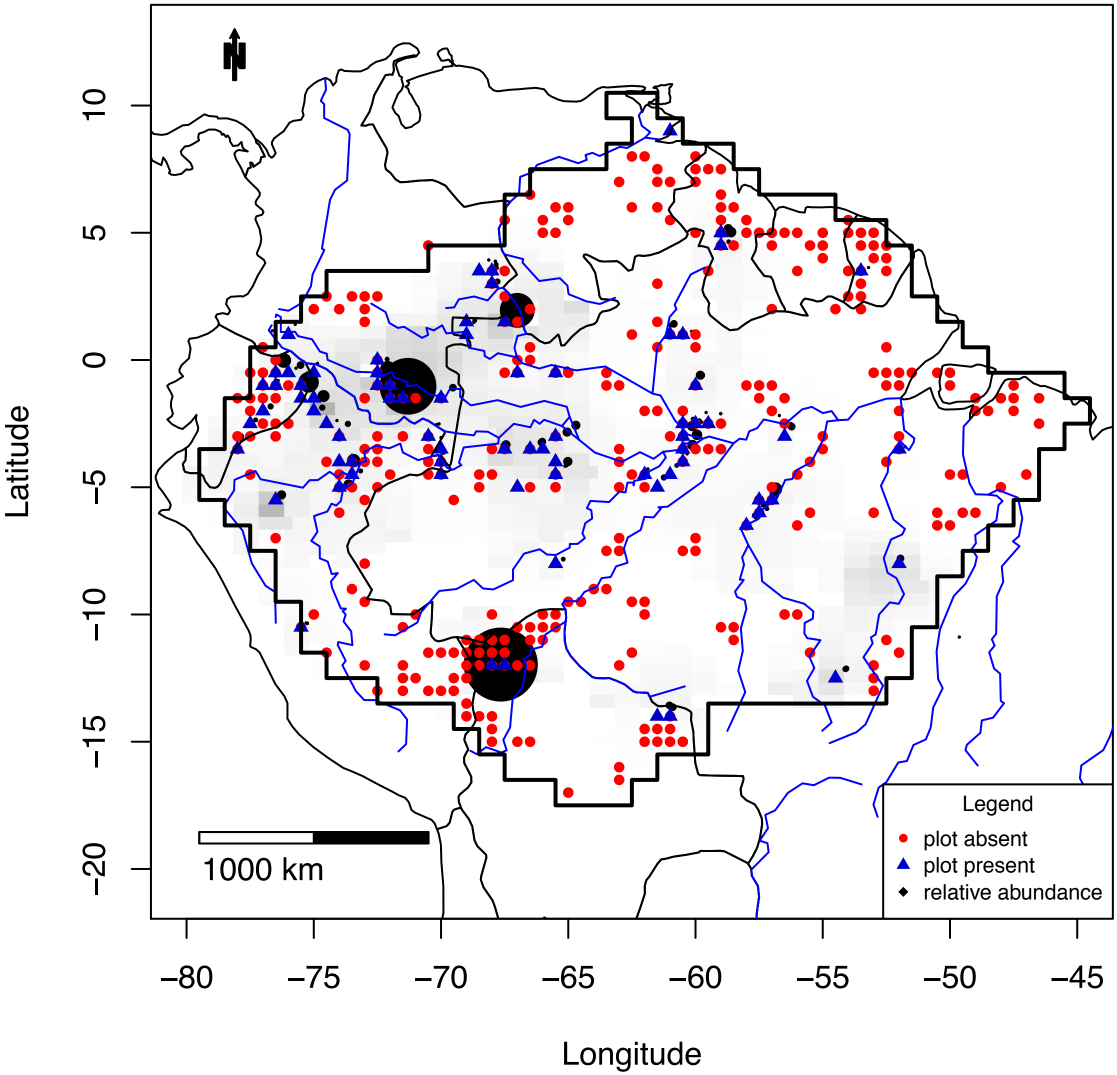

Figure S3_112A Combined map of Ocotea_cernua

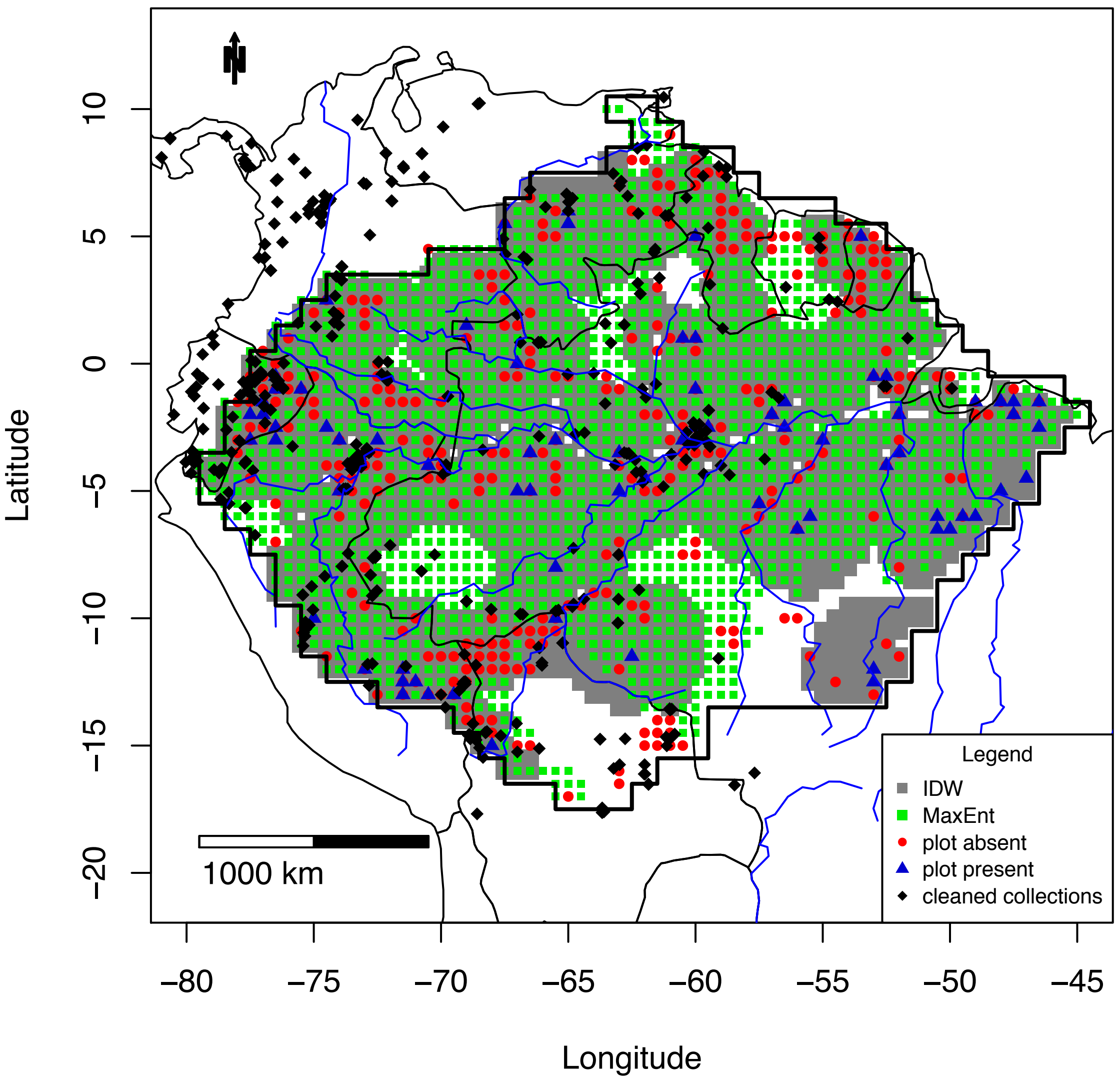

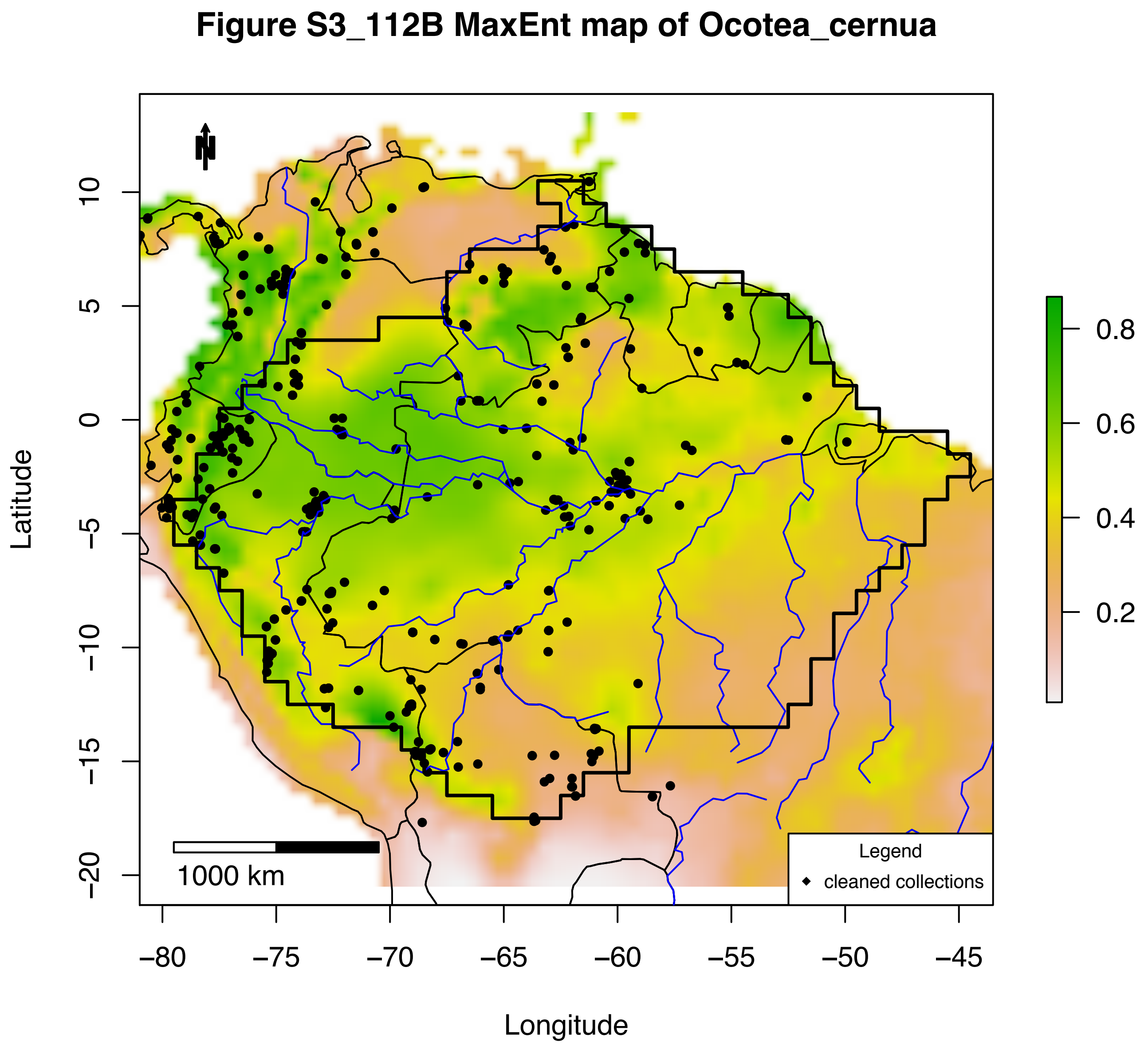

Figure S3_112B MaxEnt map of Ocotea_cernua
N
1000 km
Legend
cleaned collections
Latitude
Longitude
10
5
0
−5
−10
−15
−20
−80
−75
−70
−65
−60
−55
−50
−45
0.8
0.6
0.4
0.2

Figure S3_112C IDW map of Ocotea_cernua: plots =0 – 0.04; IDW: 0 – 0.03

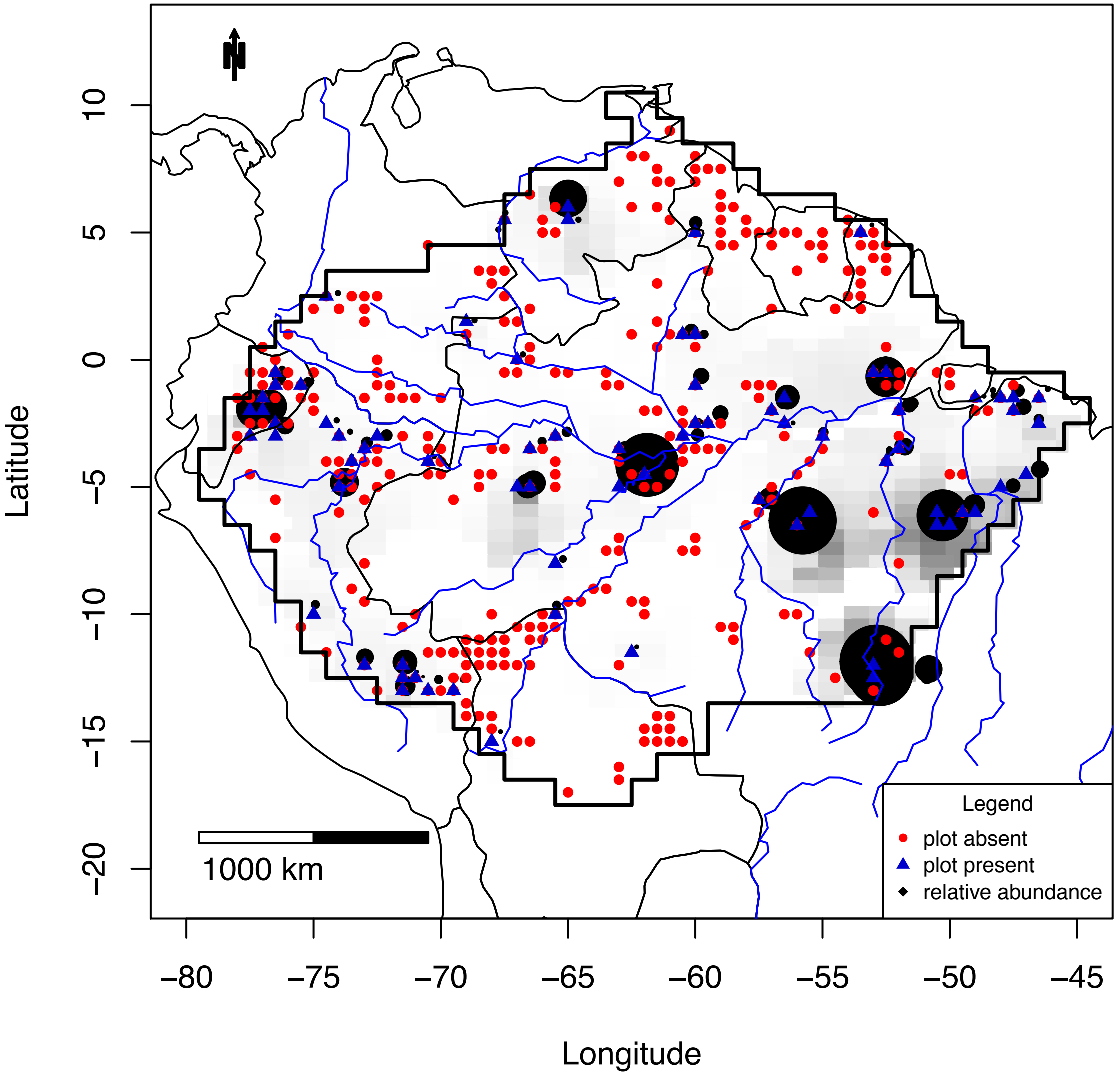

Figure S3_113A Combined map of Oenocarpus_bacaba

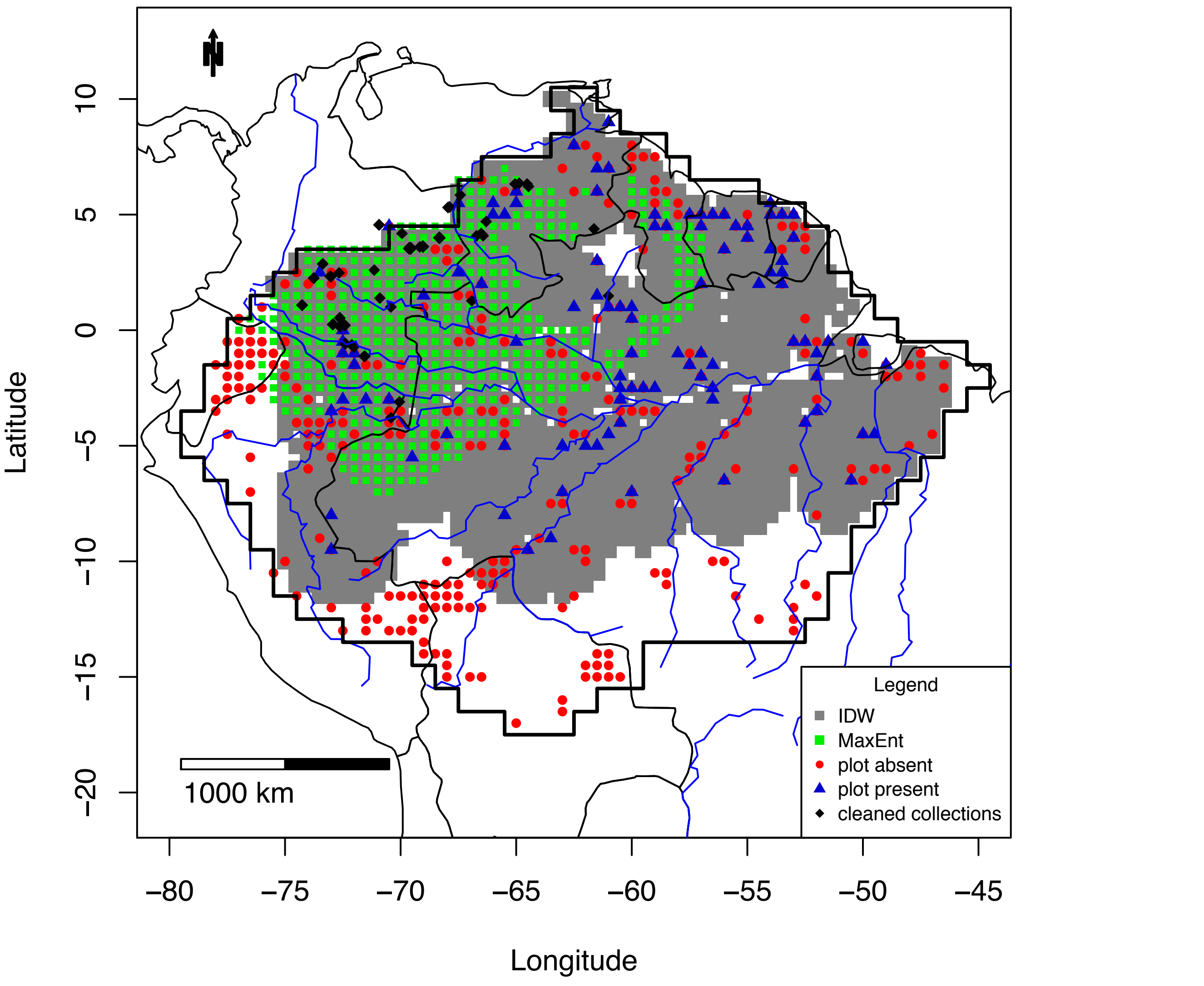

Figure S3_113B MaxEnt map of Oenocarpus_bacaba

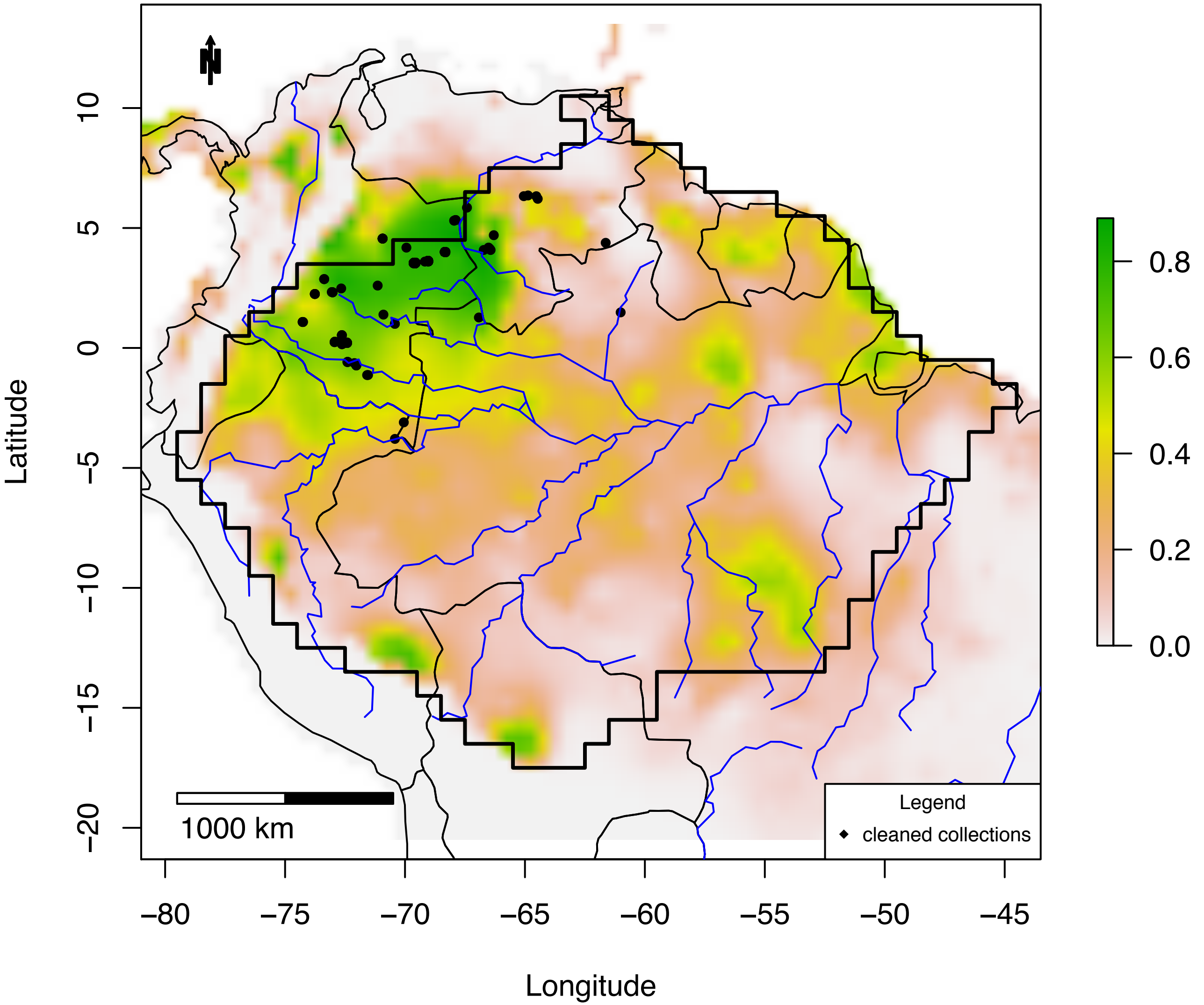

Figure S3_113C IDW map of Oenocarpus_bacaba: plots =0 – 0.16; IDW: 0 – 0.06

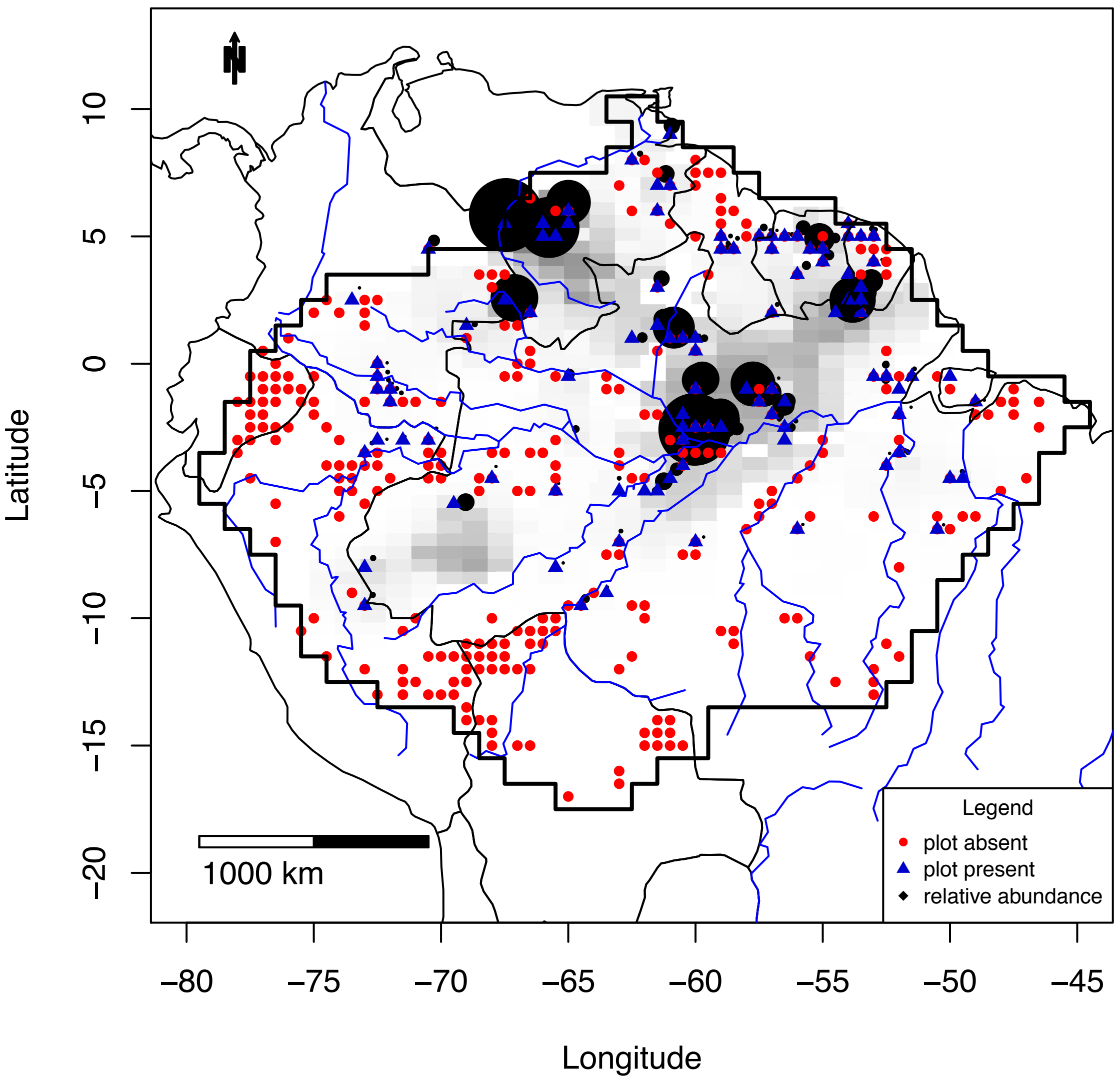

Figure S3_114A Combined map of Oenocarpus_bataua

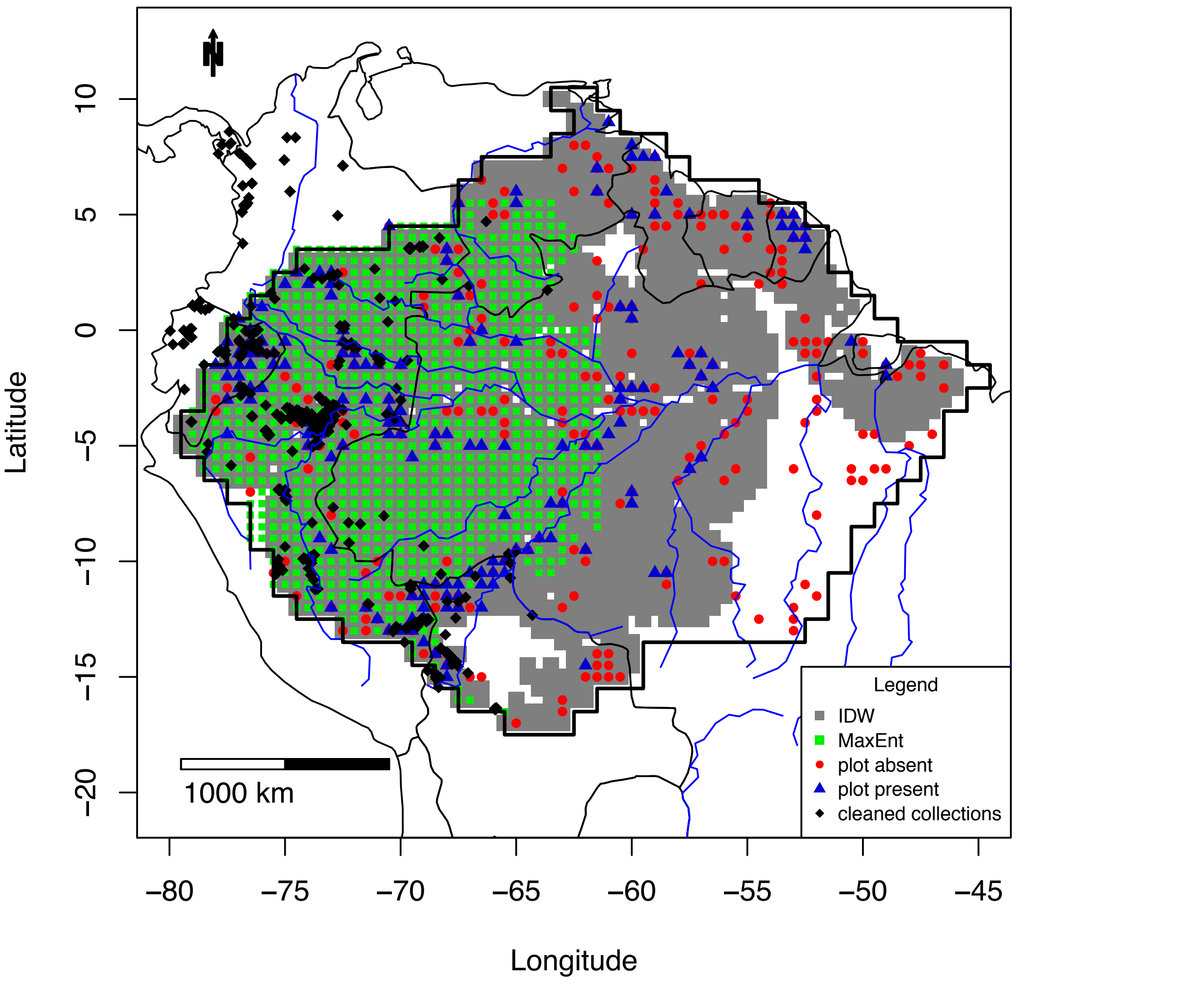

Figure S3_114B MaxEnt map of Oenocarpus_bataua

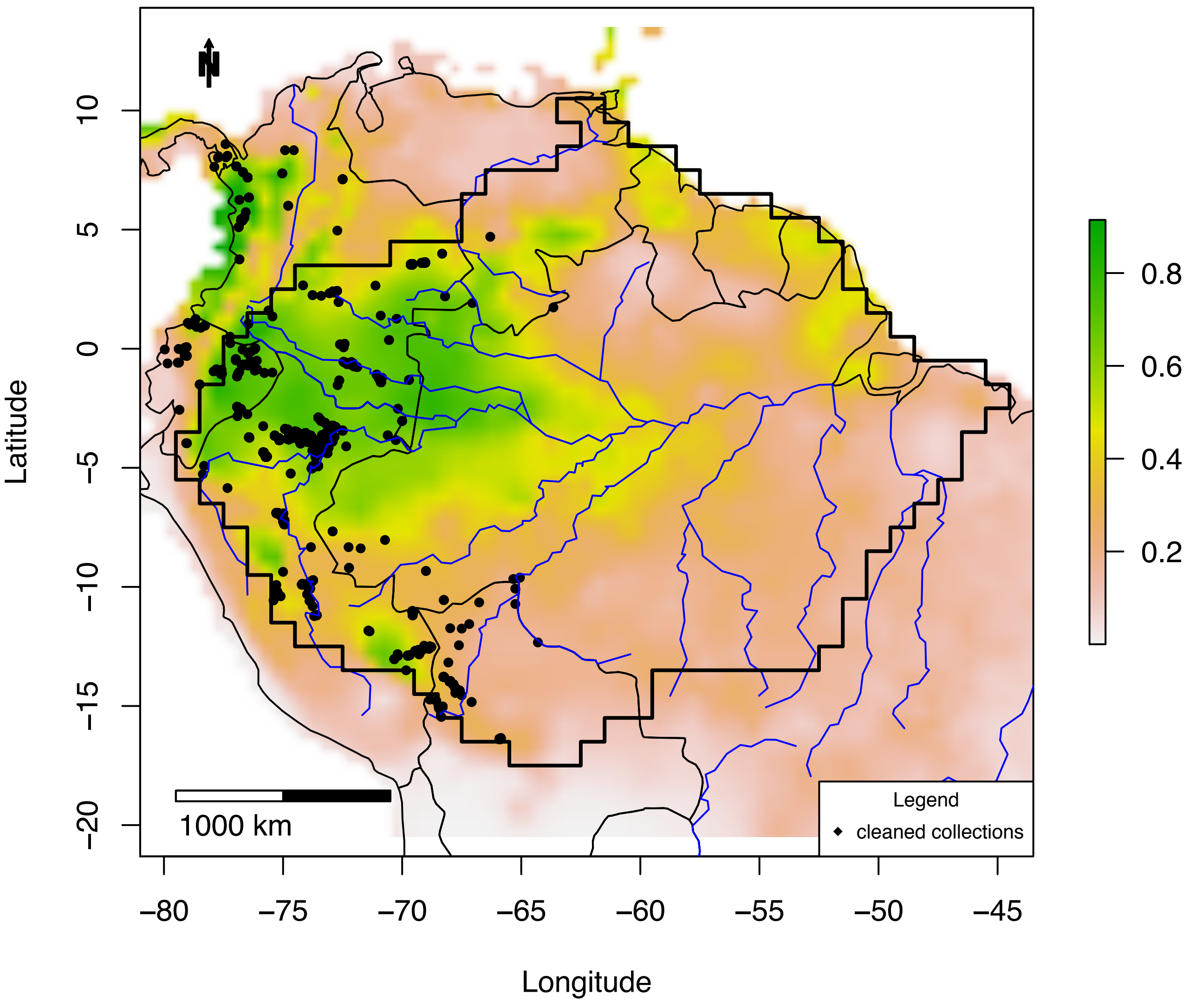

Figure S3_114C IDW map of Oenocarpus_bataua: plots =0 – 0.27; IDW: 0 – 0.22

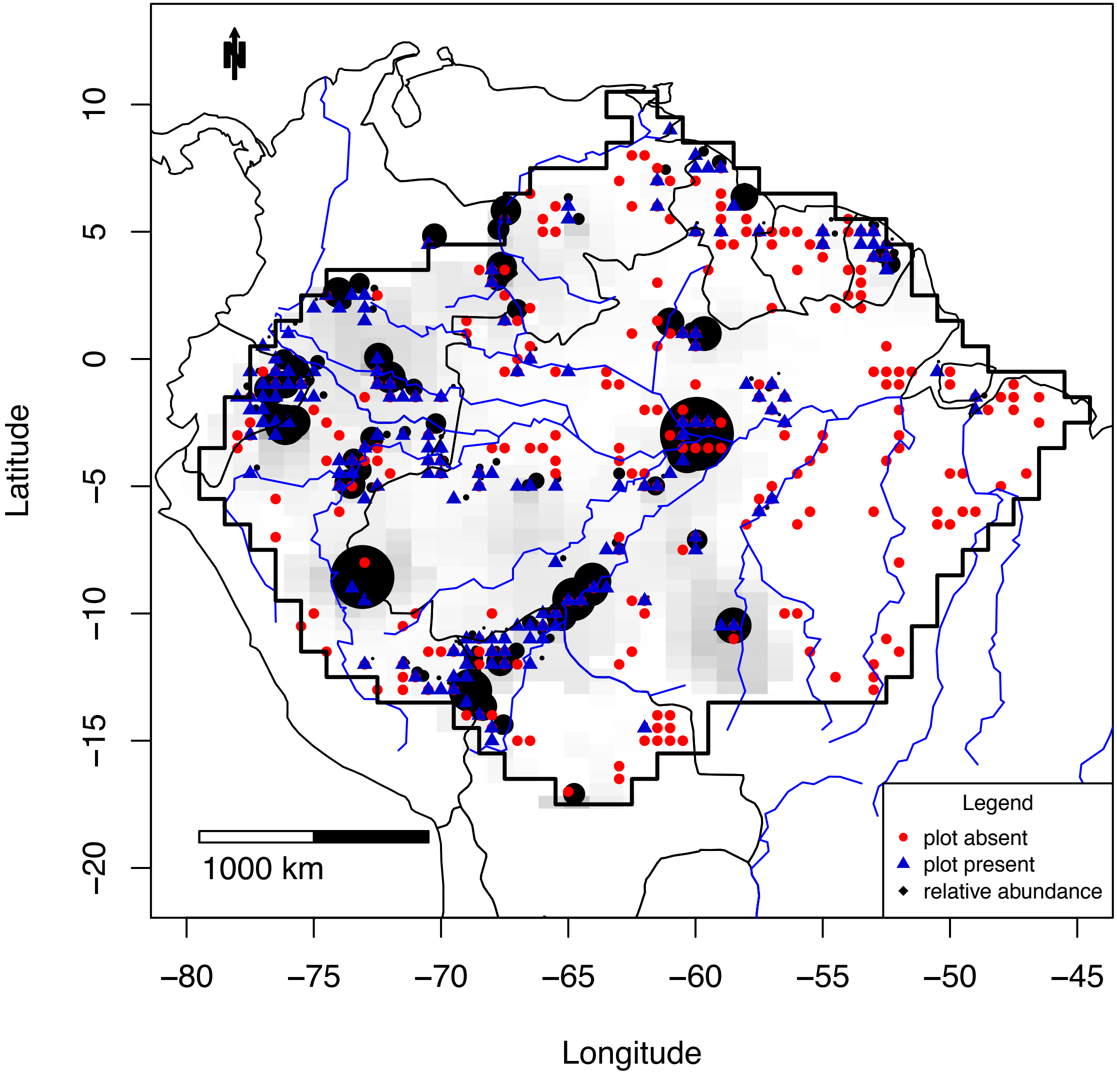

Figure S3_115A Combined map of Osteophloeum_platyspermum

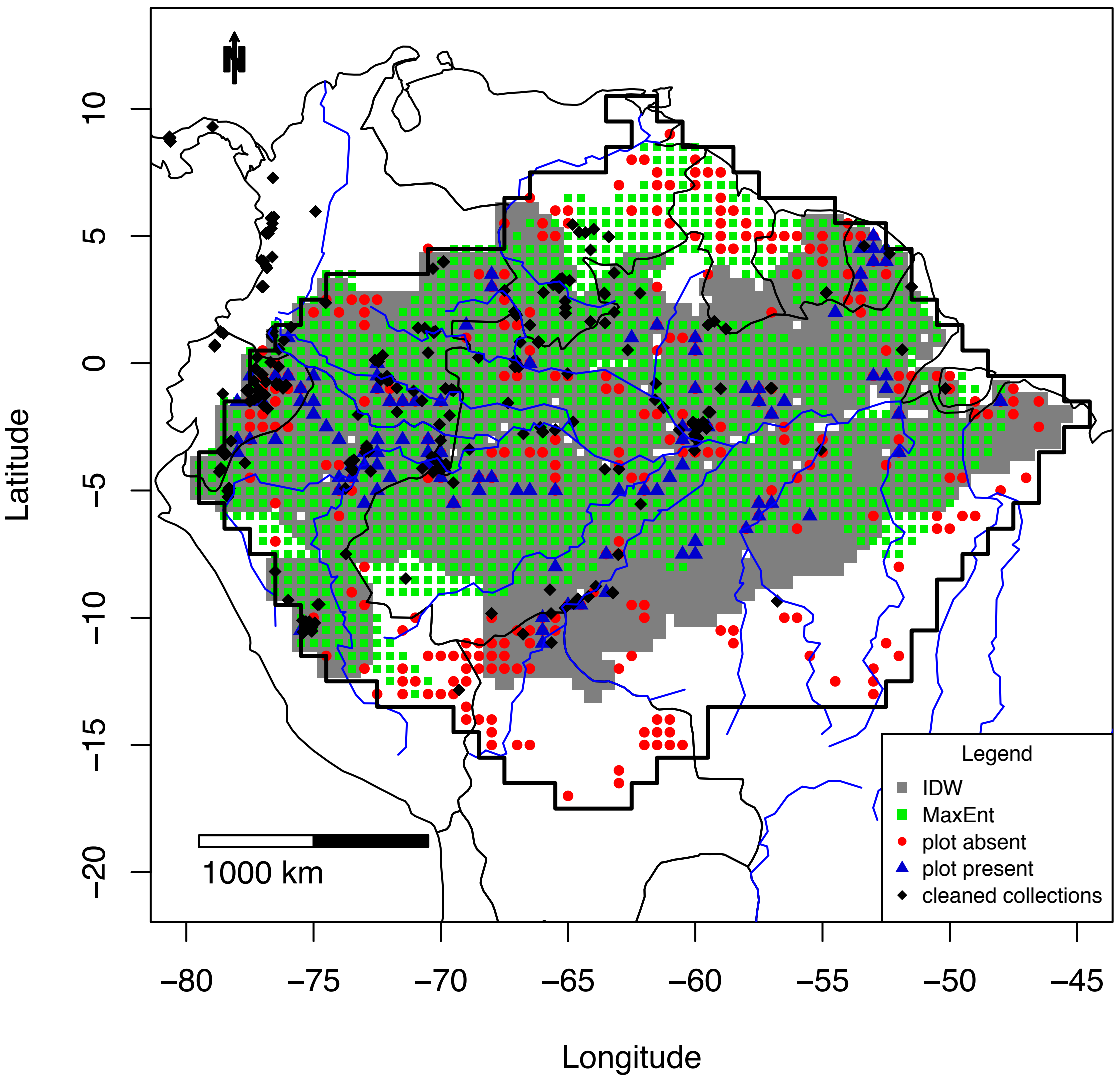

Figure S3_115B MaxEnt map of Osteophloeum_platyspermum

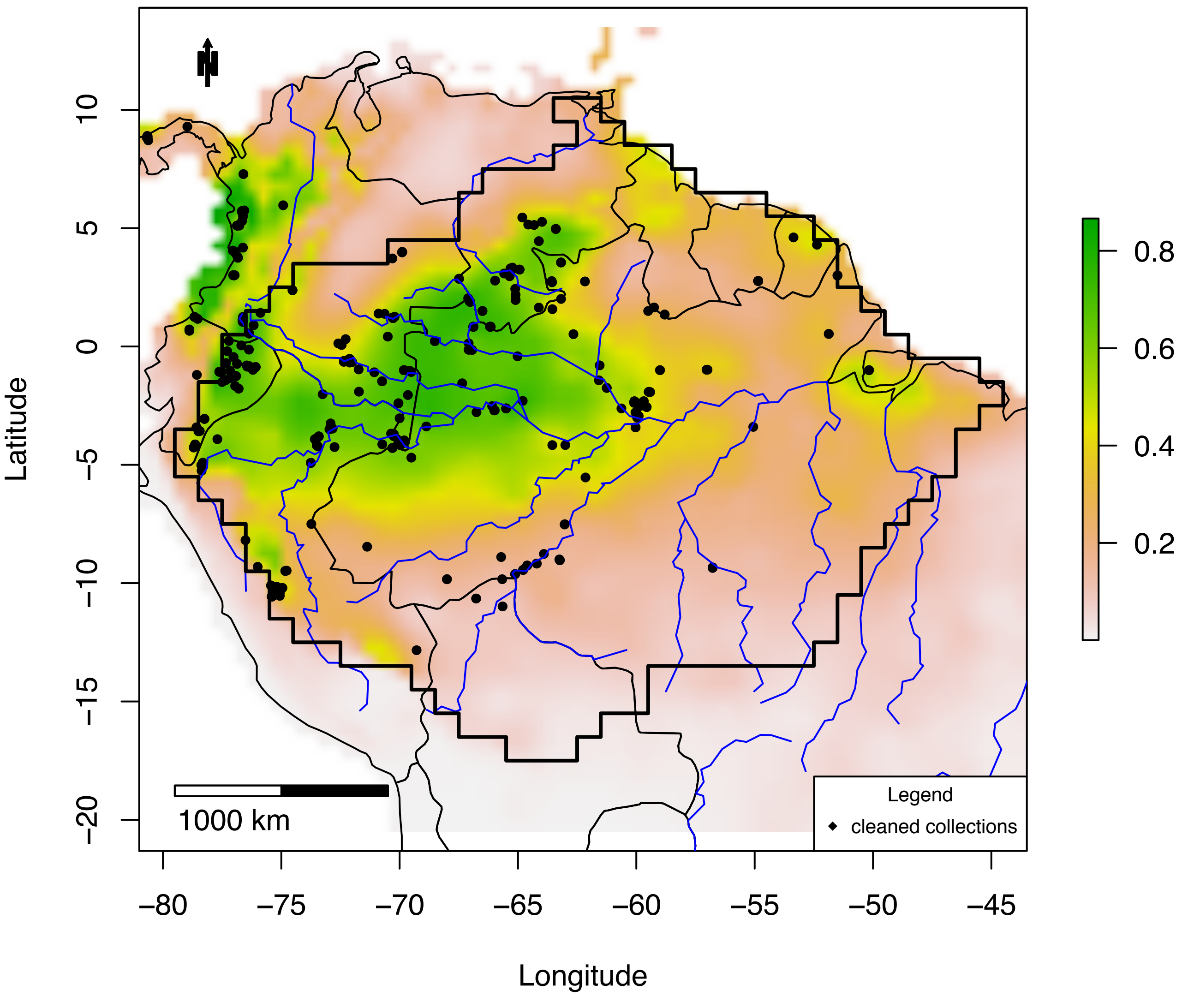

Figure S3_115C IDW map of Osteophloeum_platyspermum: plots =0 – 0.08; IDW: 0 – 0.03

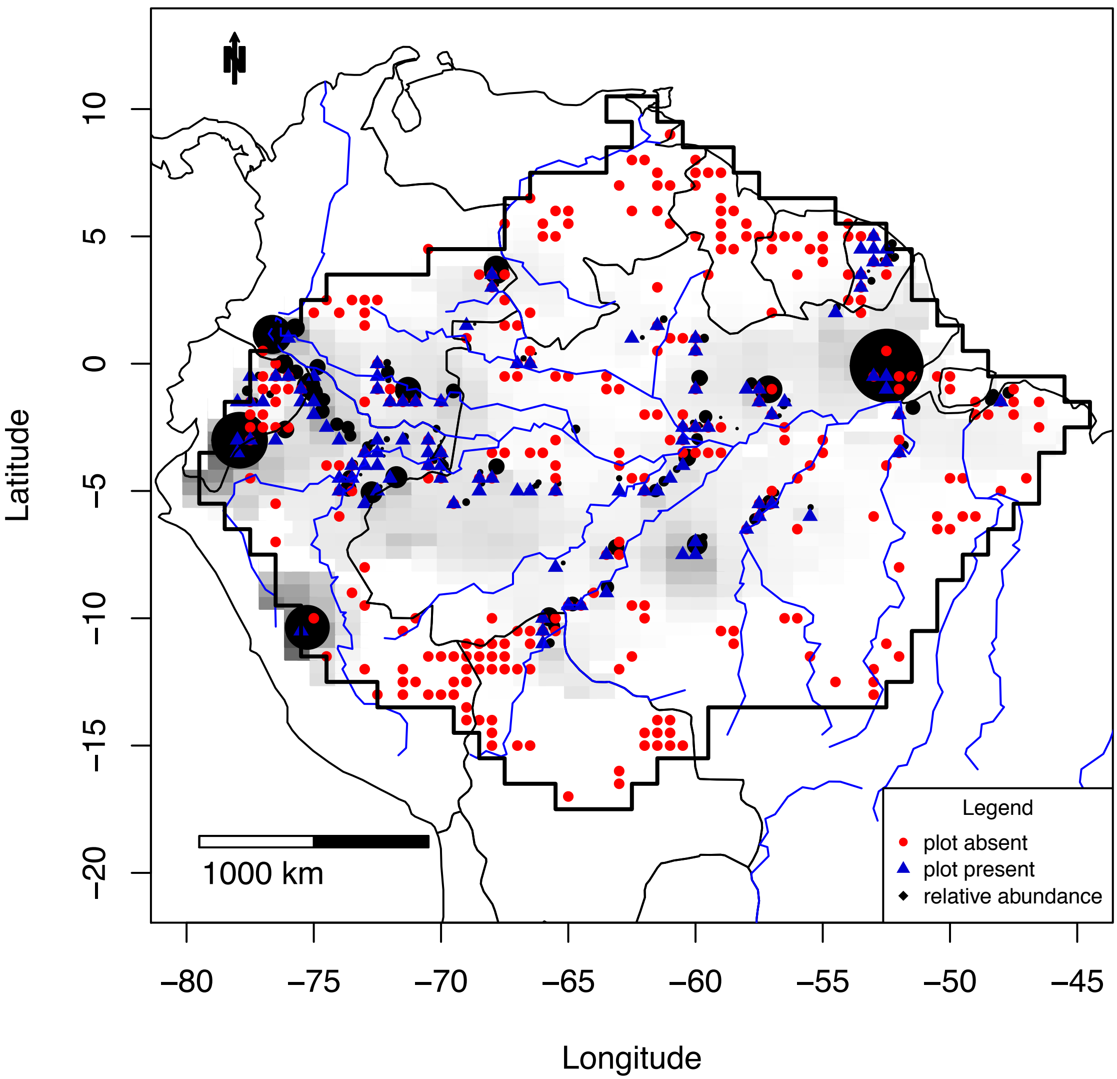

Figure S3_116A Combined map of Otoba_parvifolia

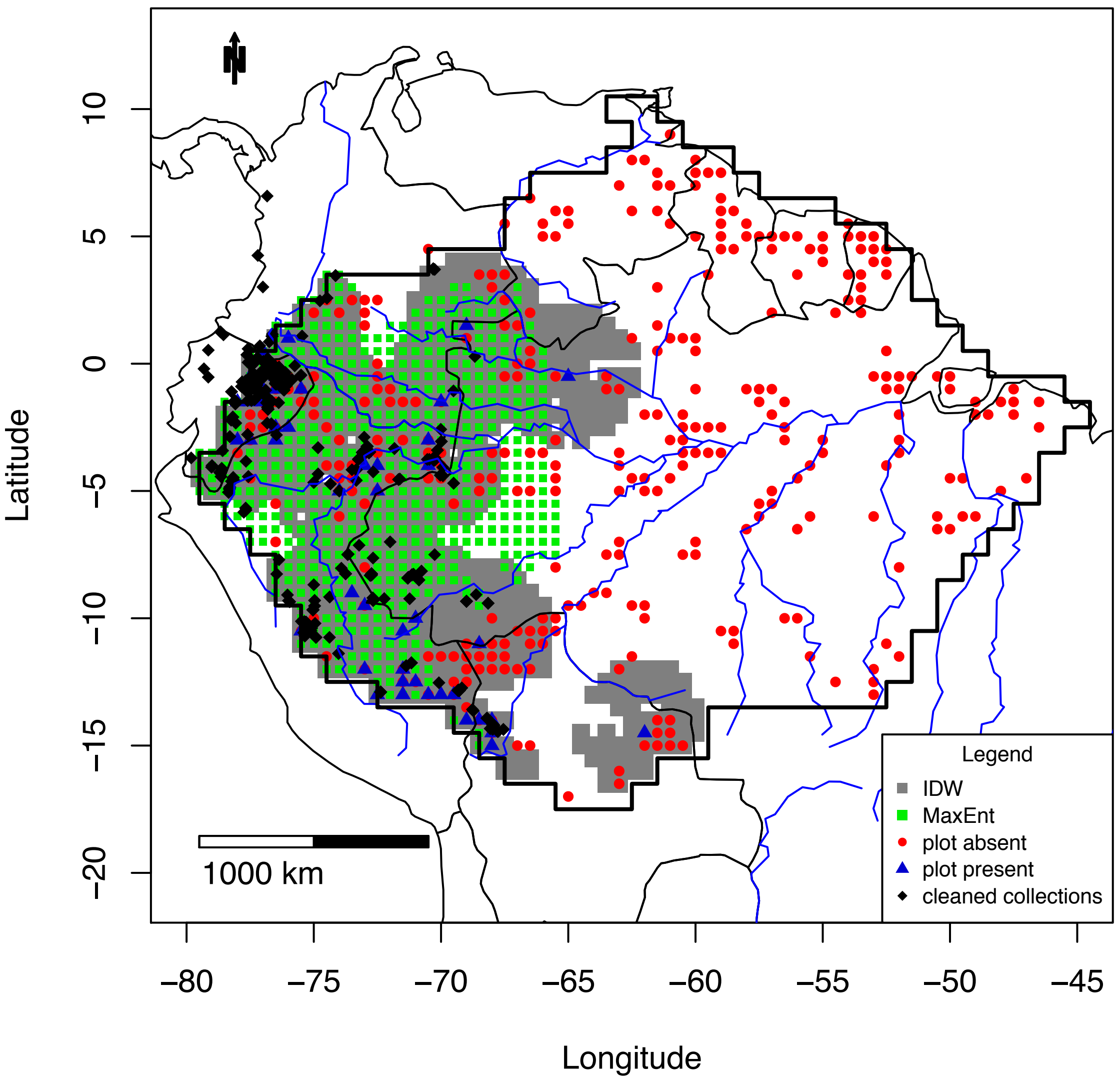

Figure S3_116B MaxEnt map of Otoba_parvifolia

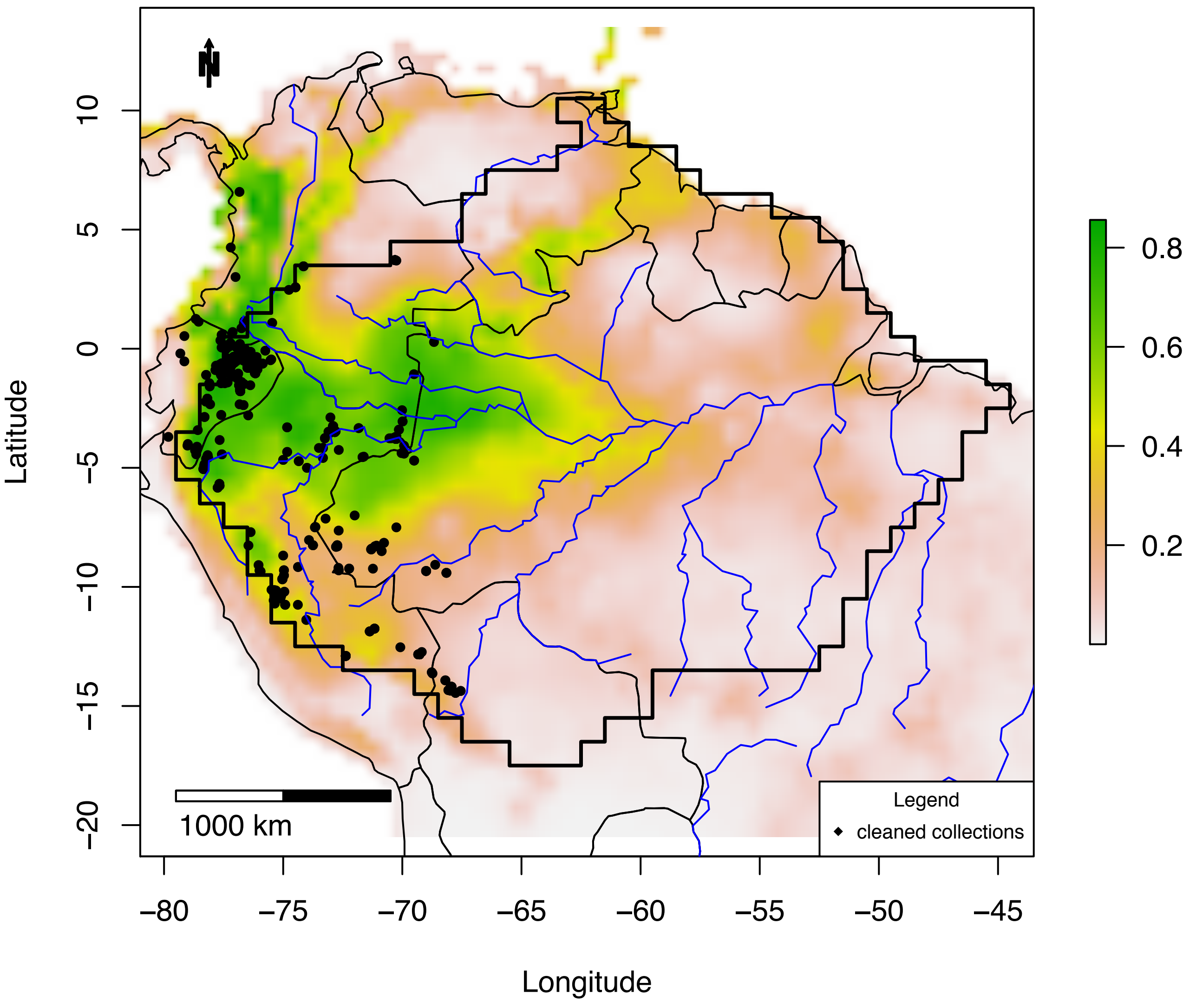

Figure S3_116C IDW map of Otoba_parvifolia:
plots =0 – 0.15; IDW: 0 – 0.05

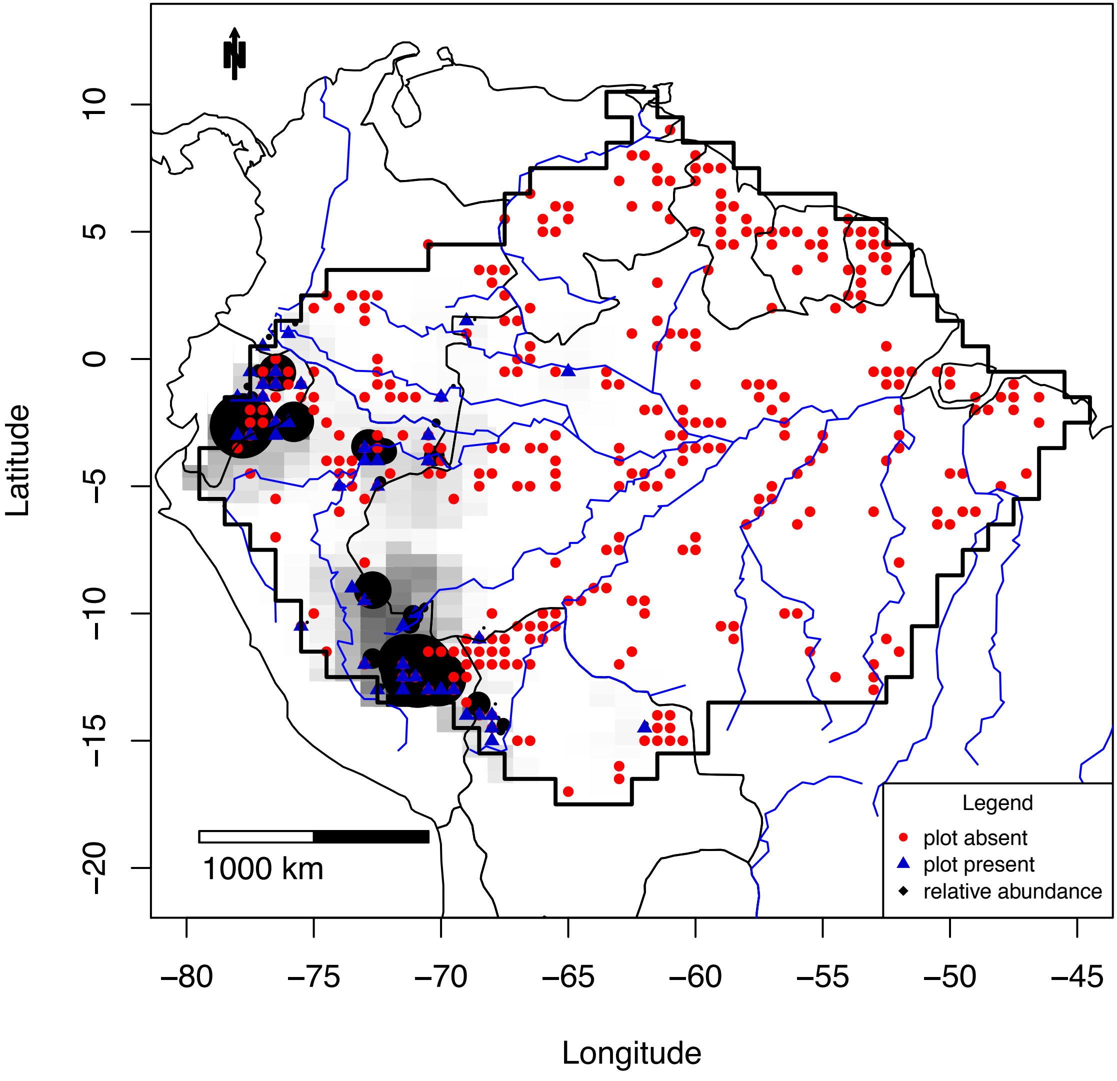

Figure S3_117A Combined map of Oxandra_polyantha

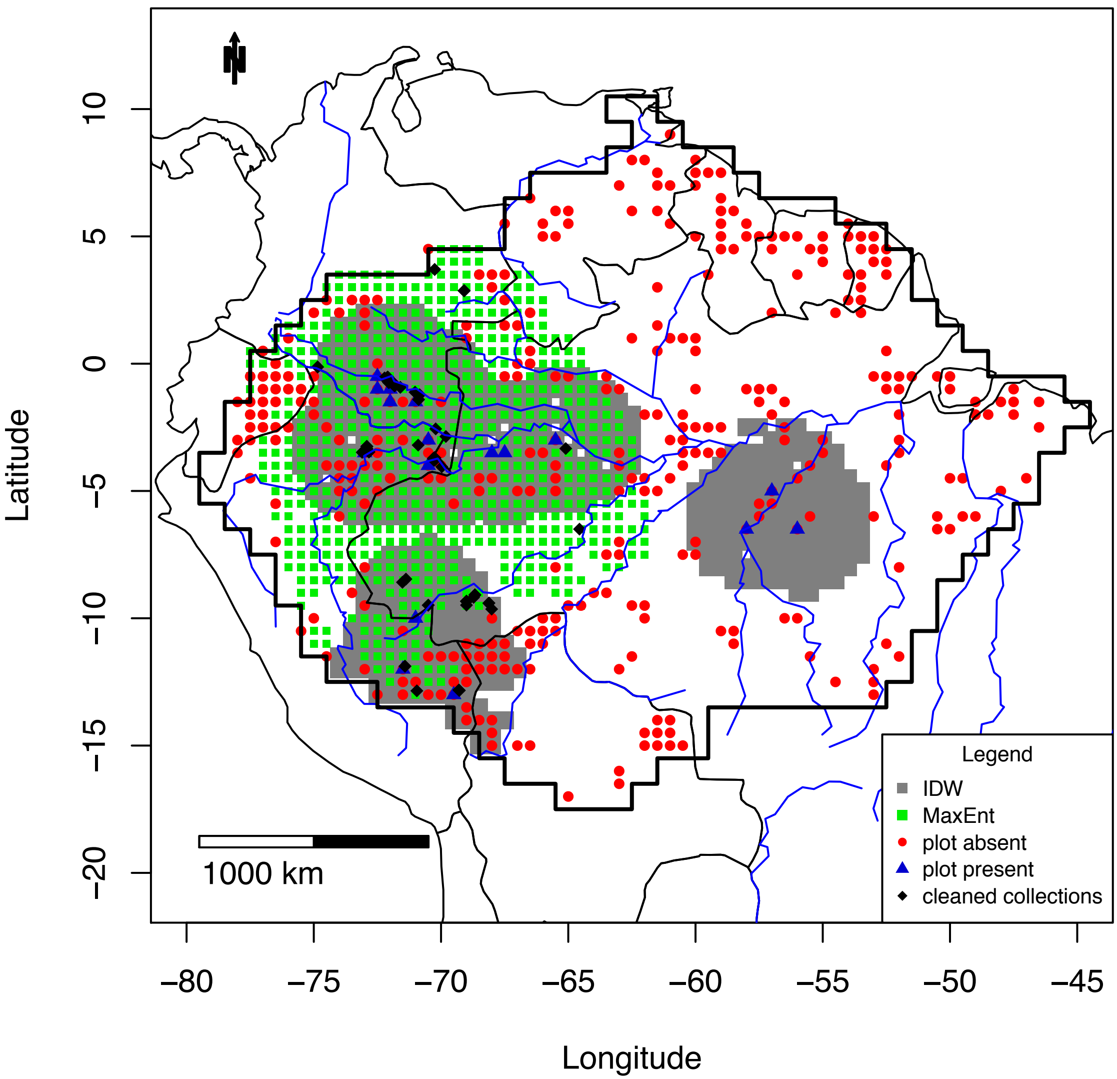

Figure S3_117B MaxEnt map of Oxandra_polyantha

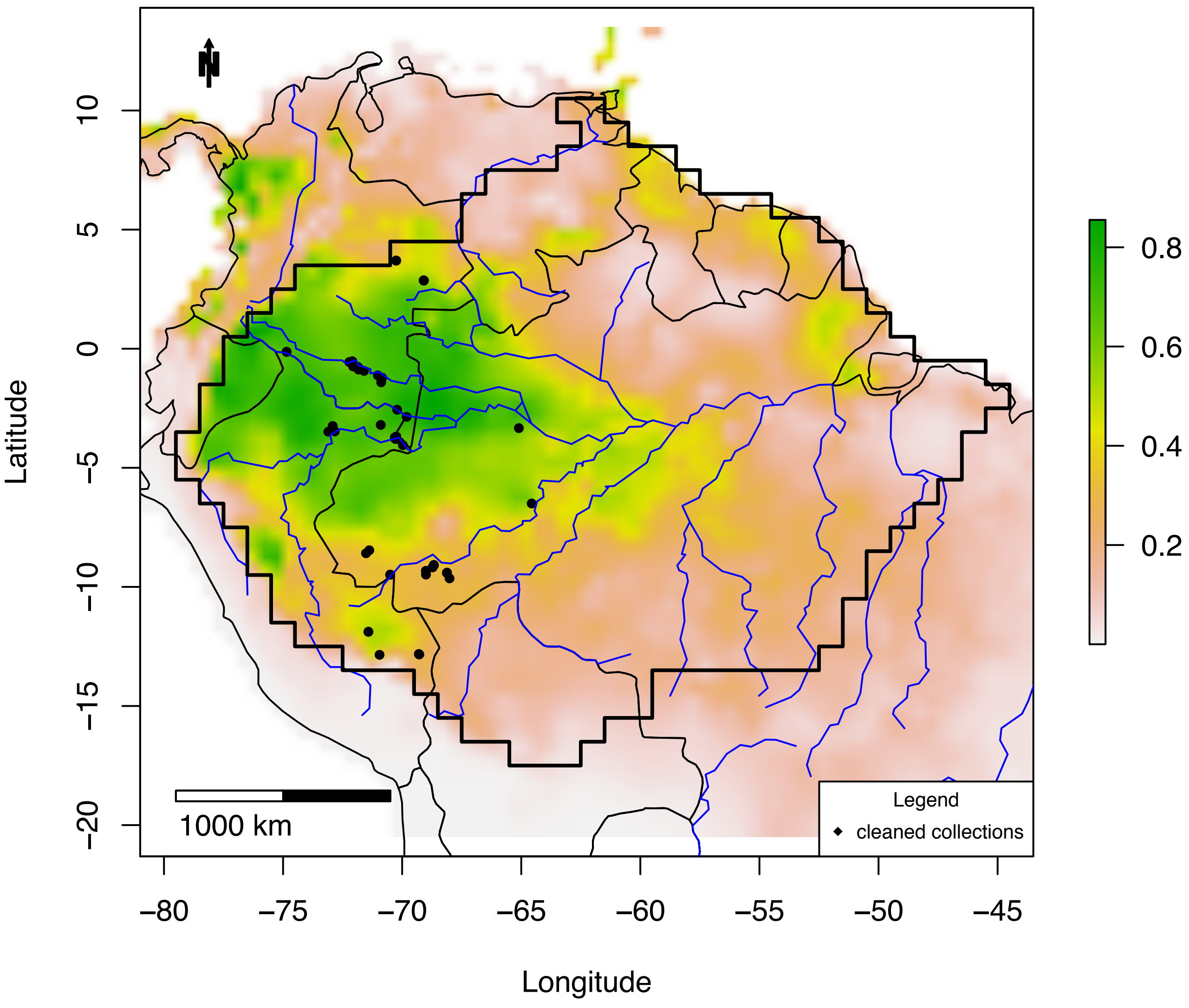

Figure S3_117C IDW map of Oxandra_polyantha: plots =0 – 0.59; IDW: 0 – 0.08

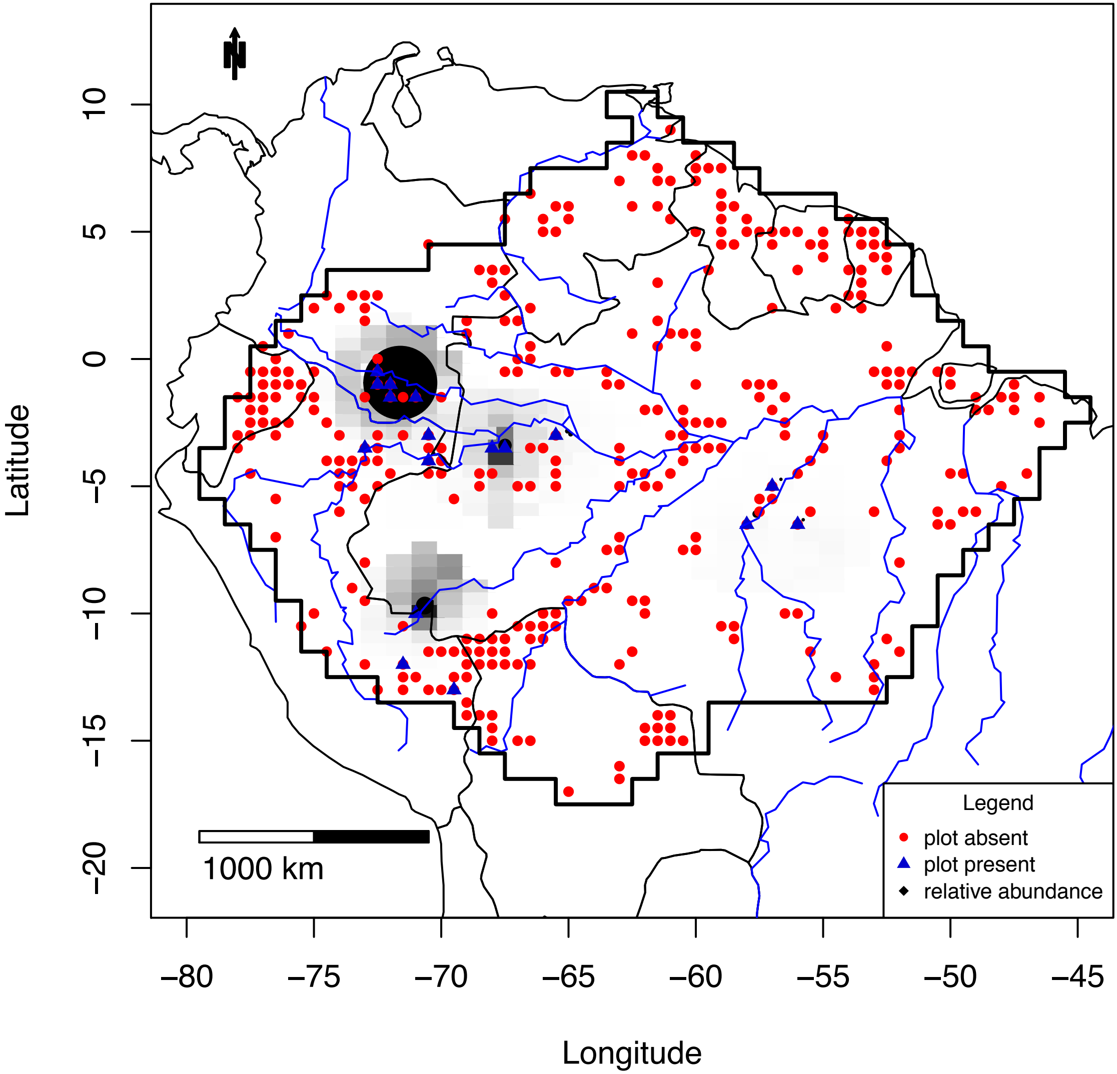

Figure S3_118A Combined map of Pachira_brevipes

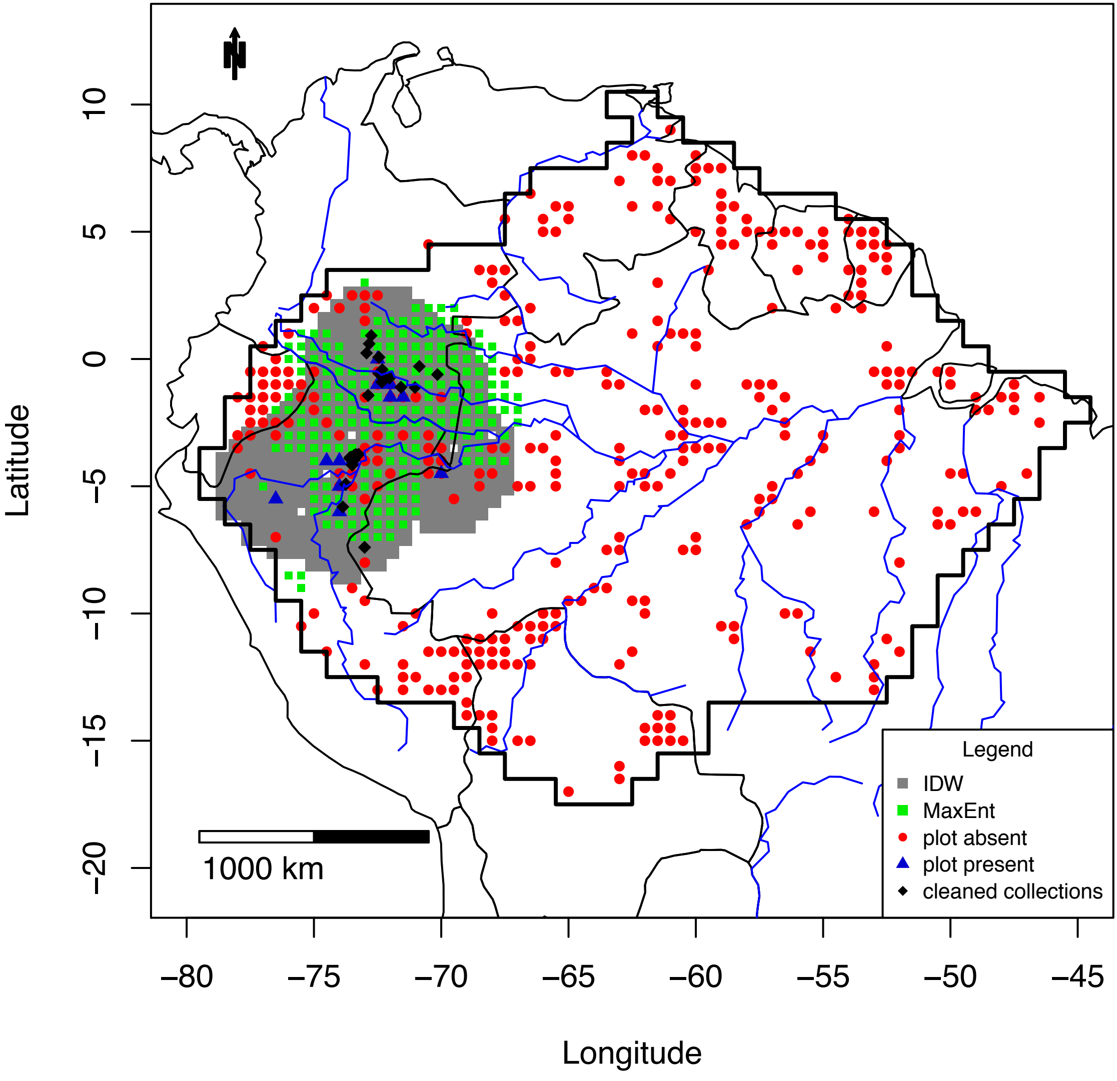

Figure S3_118B MaxEnt map of Pachira_brevipes

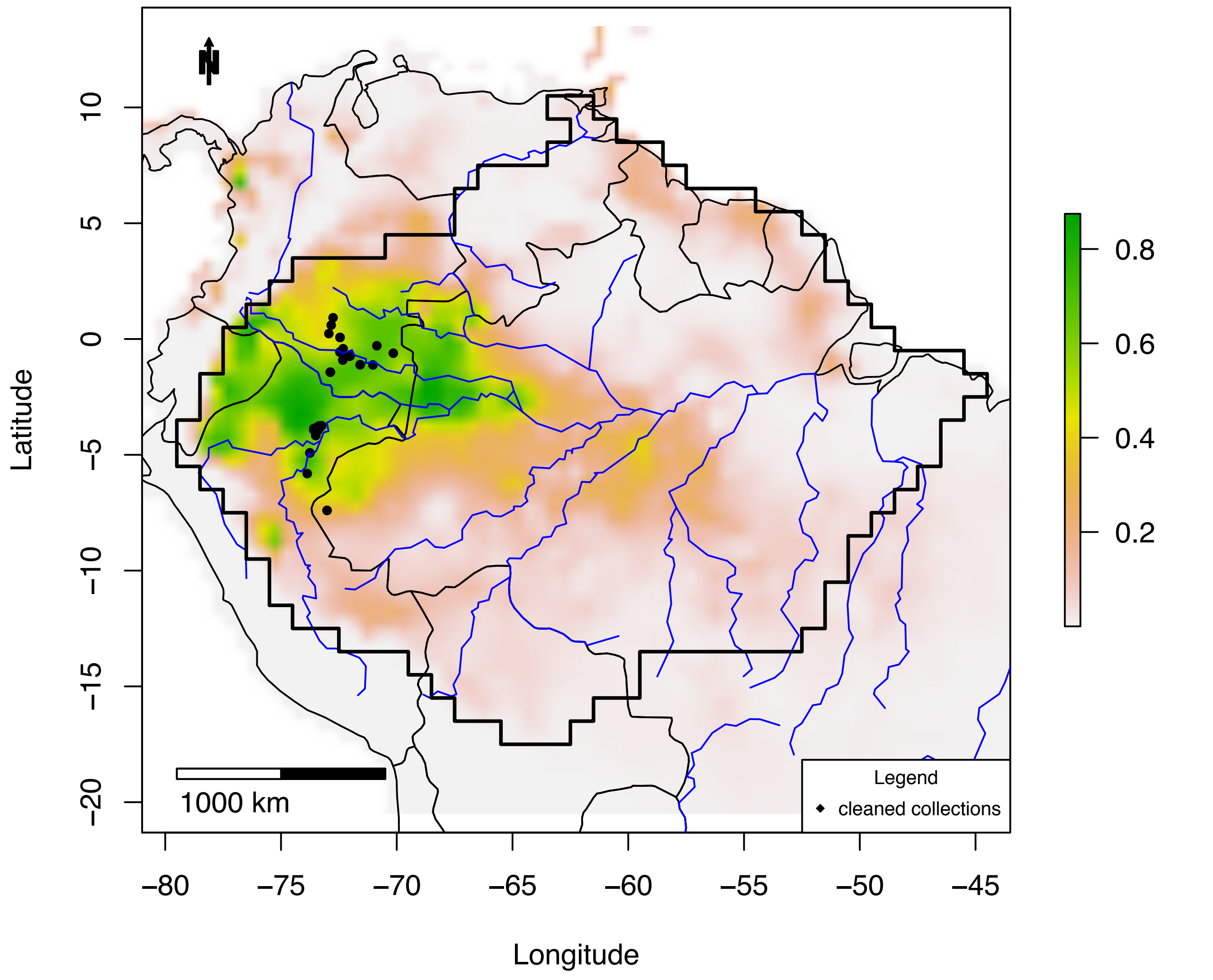

Figure S3_118C IDW map of Pachira_brevipes: plots =0 – 0.57; IDW: 0 – 0.17

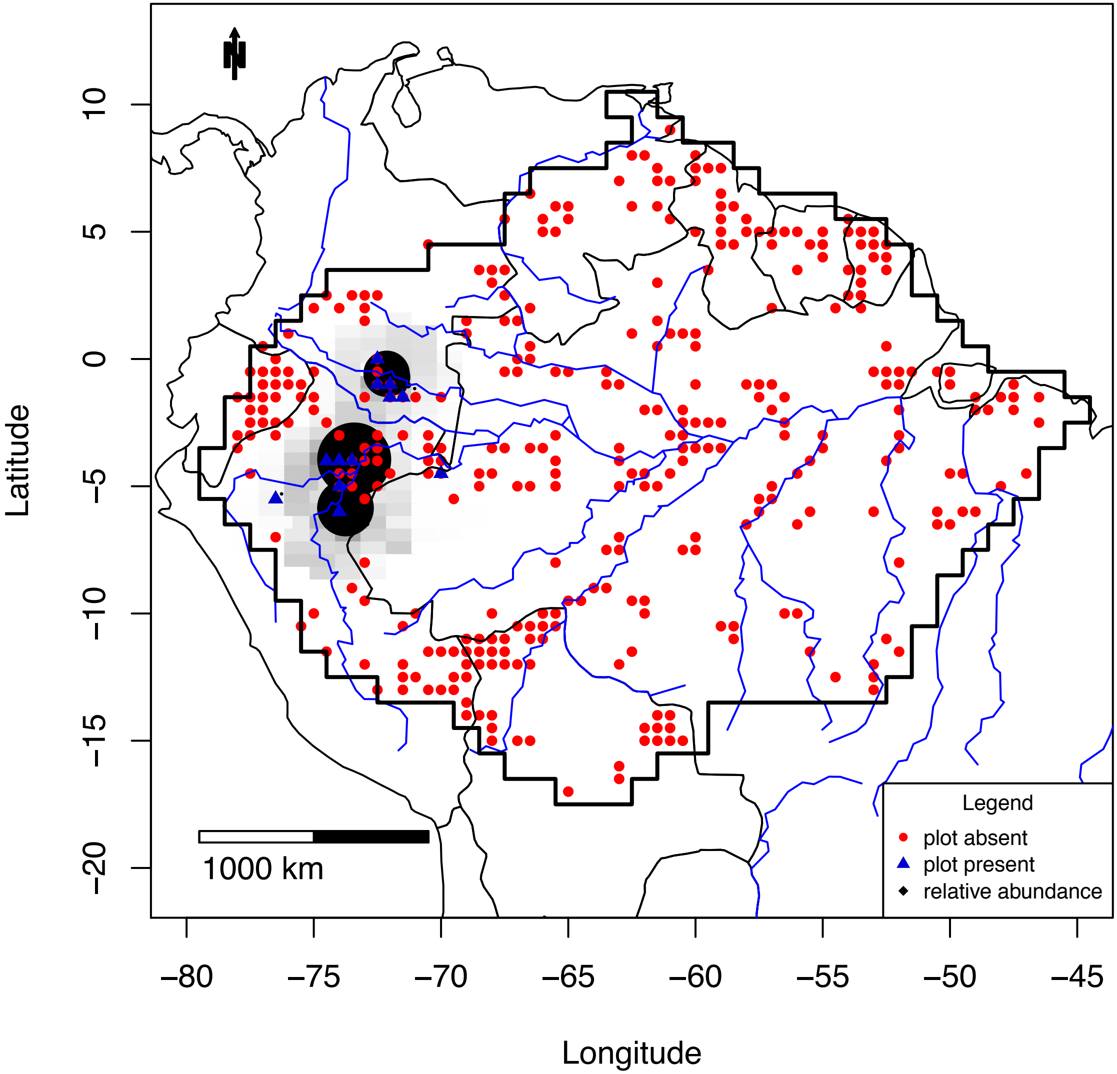

Figure S3_119A Combined map of Pentaclethra_macroloba

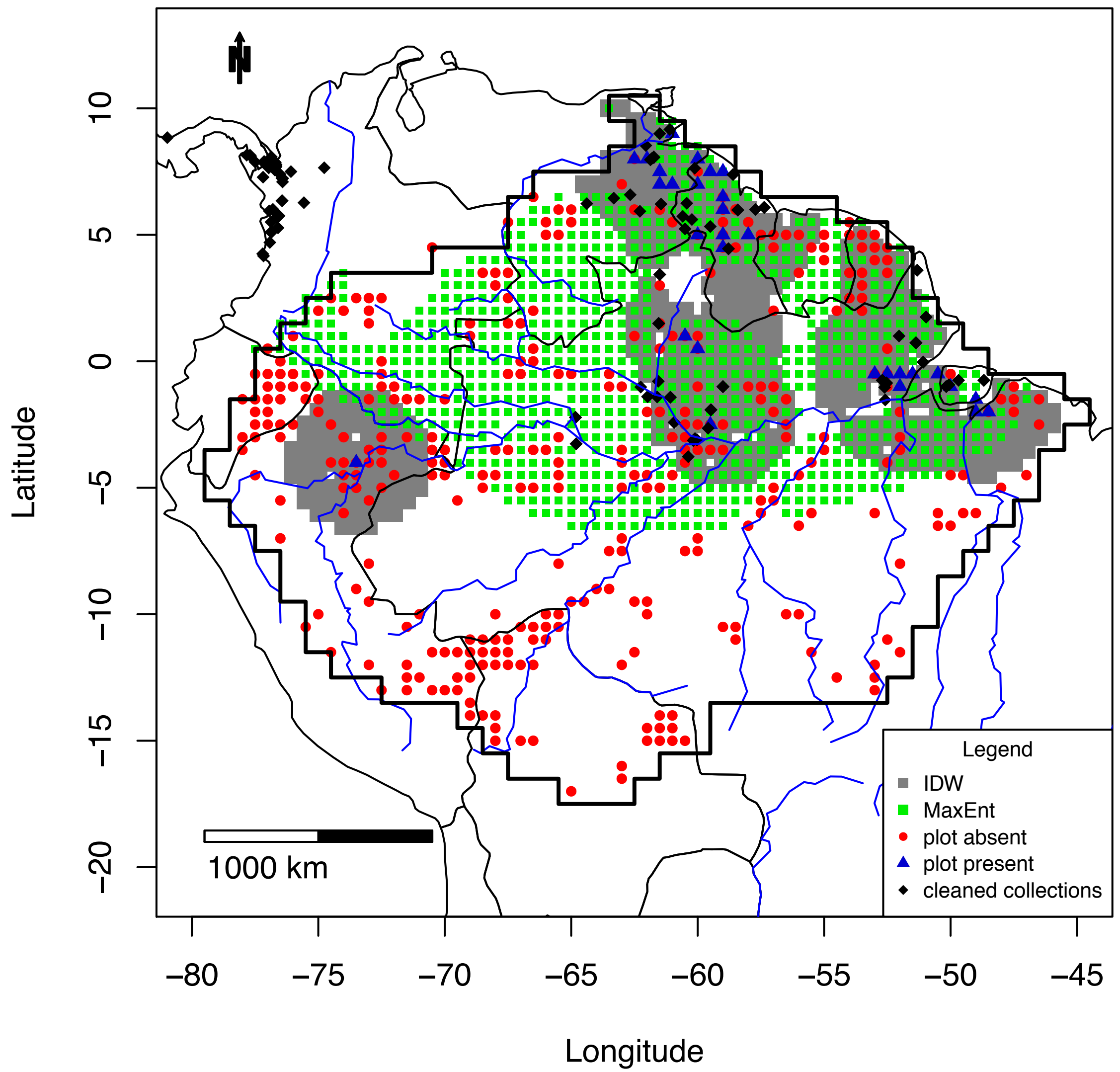

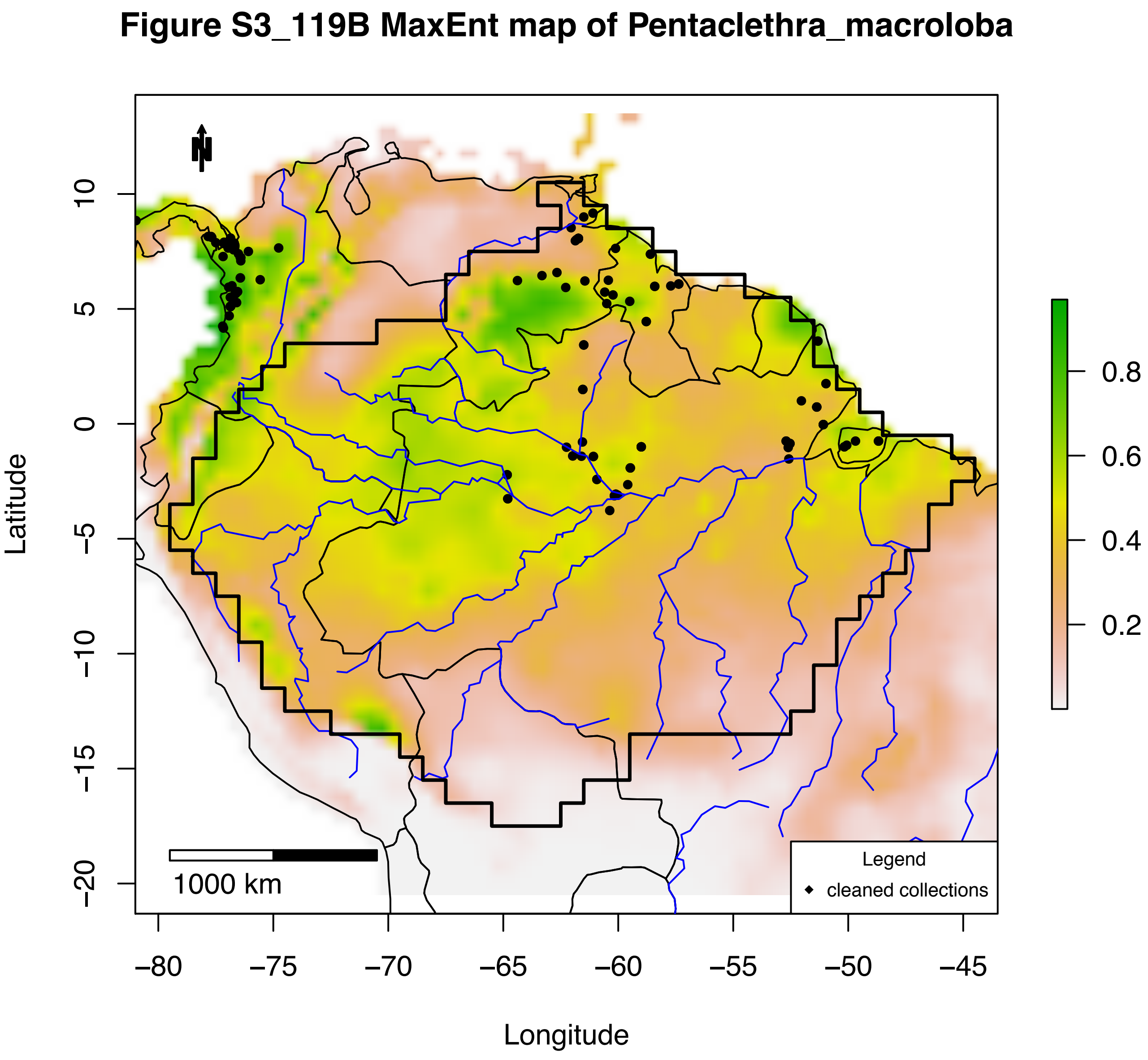

Figure S3_119B MaxEnt map of Pentaclethra_macroloba
N
Latitude
Longitude
10
5
0
−5
−10
−15
−20
−80
−75
−70
−65
−60
−55
−50
−45
0.8
0.6
0.4
0.2
1000 km
Legend
cleaned collections

Figure S3_119C IDW map of Pentaclethra_macroloba: plots =0 – 0.64; IDW: 0 – 0.24

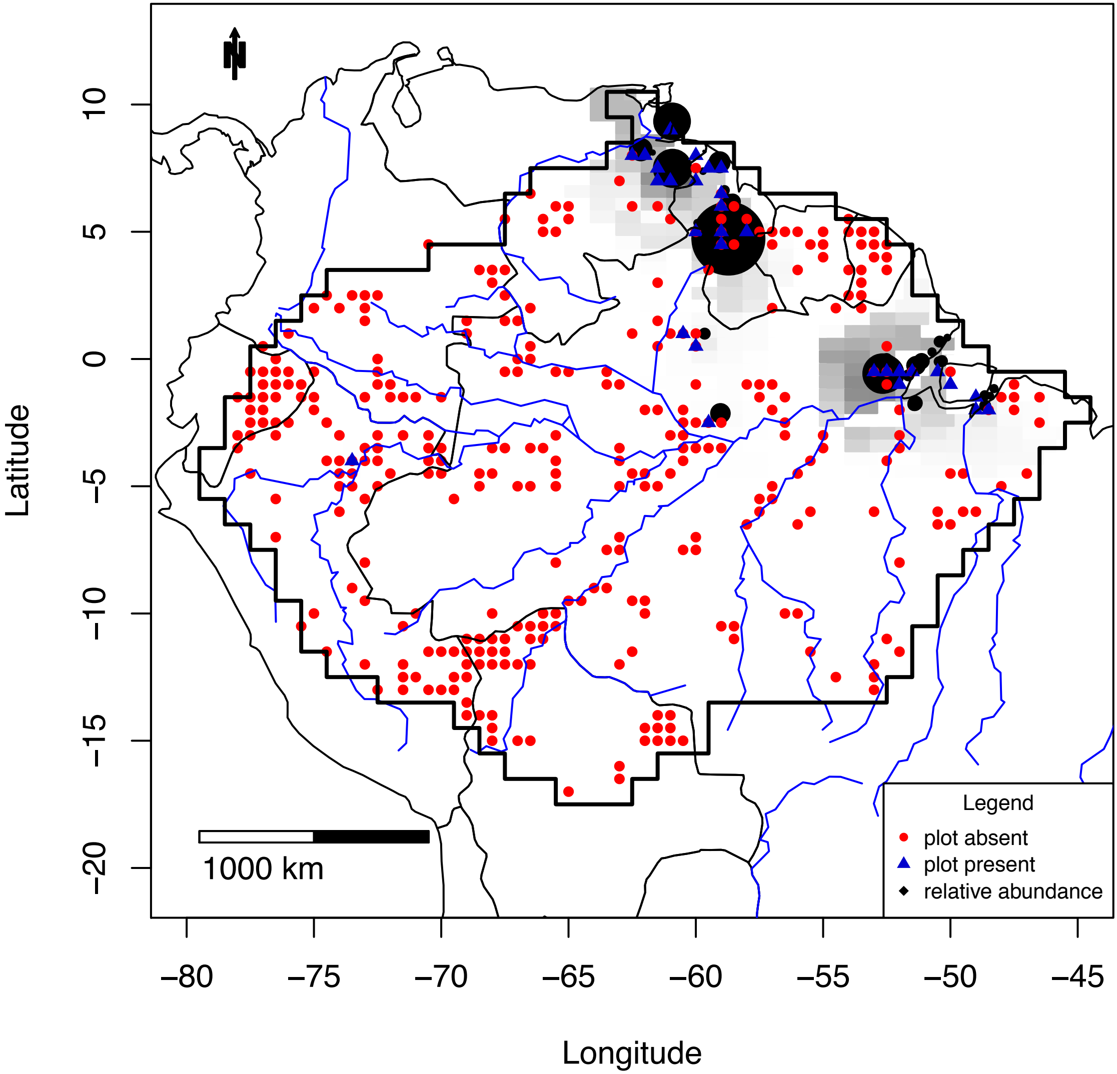

Figure S3_120A Combined map of Poulsenia_armata

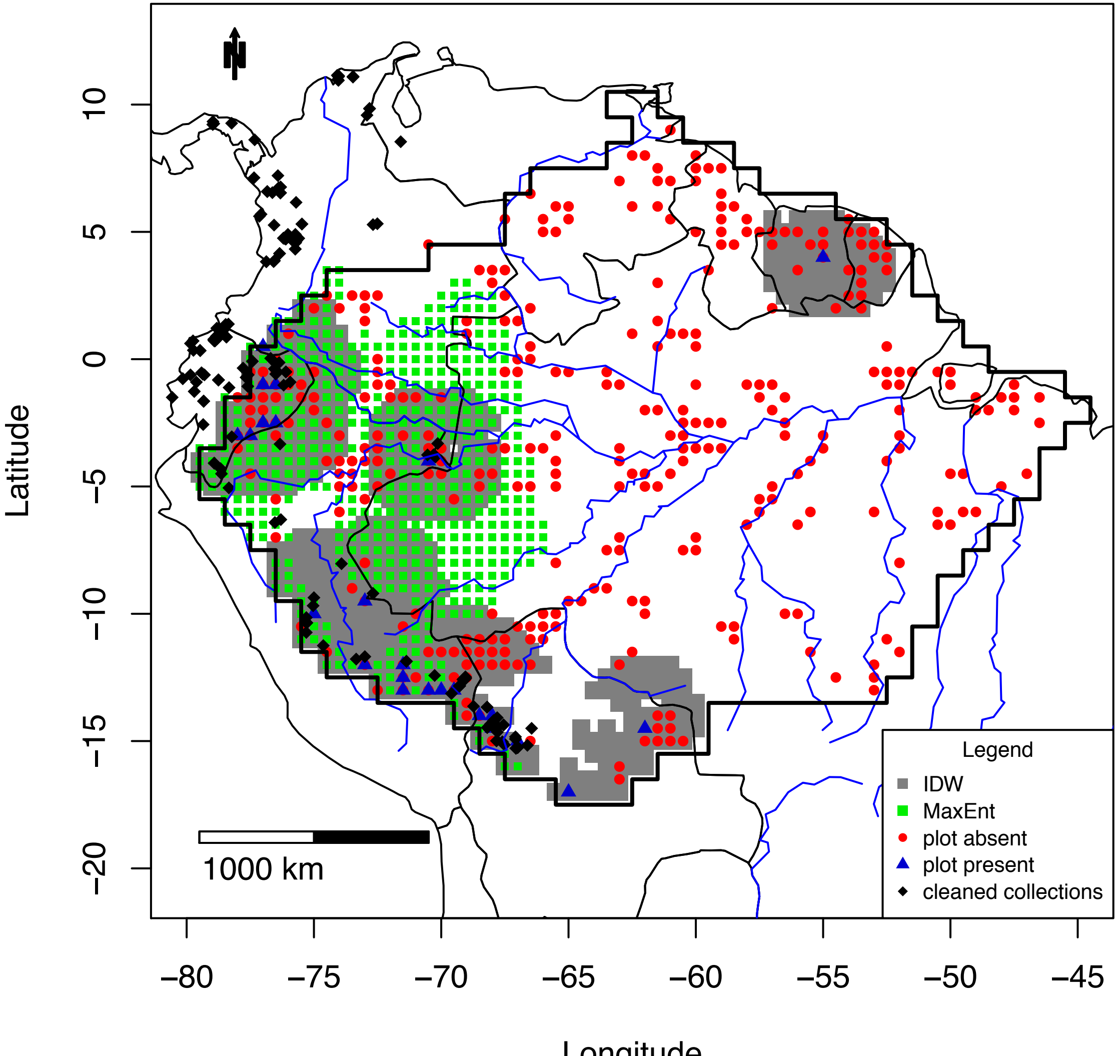

Figure S3_120B MaxEnt map of Poulsenia_armata

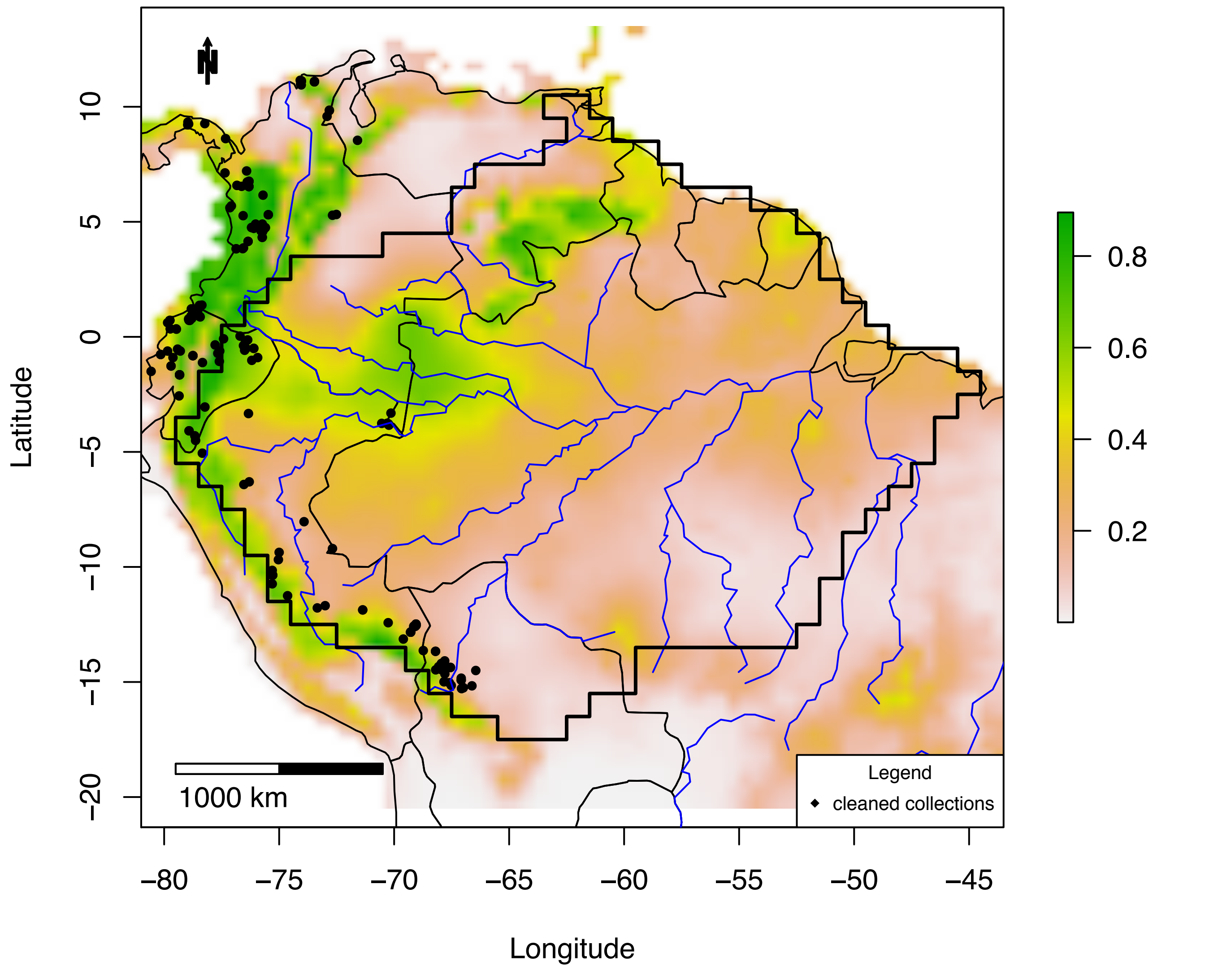

Figure S3_120C IDW map of Poulsenia_armata: plots =0 – 0.22; IDW: 0 – 0.17

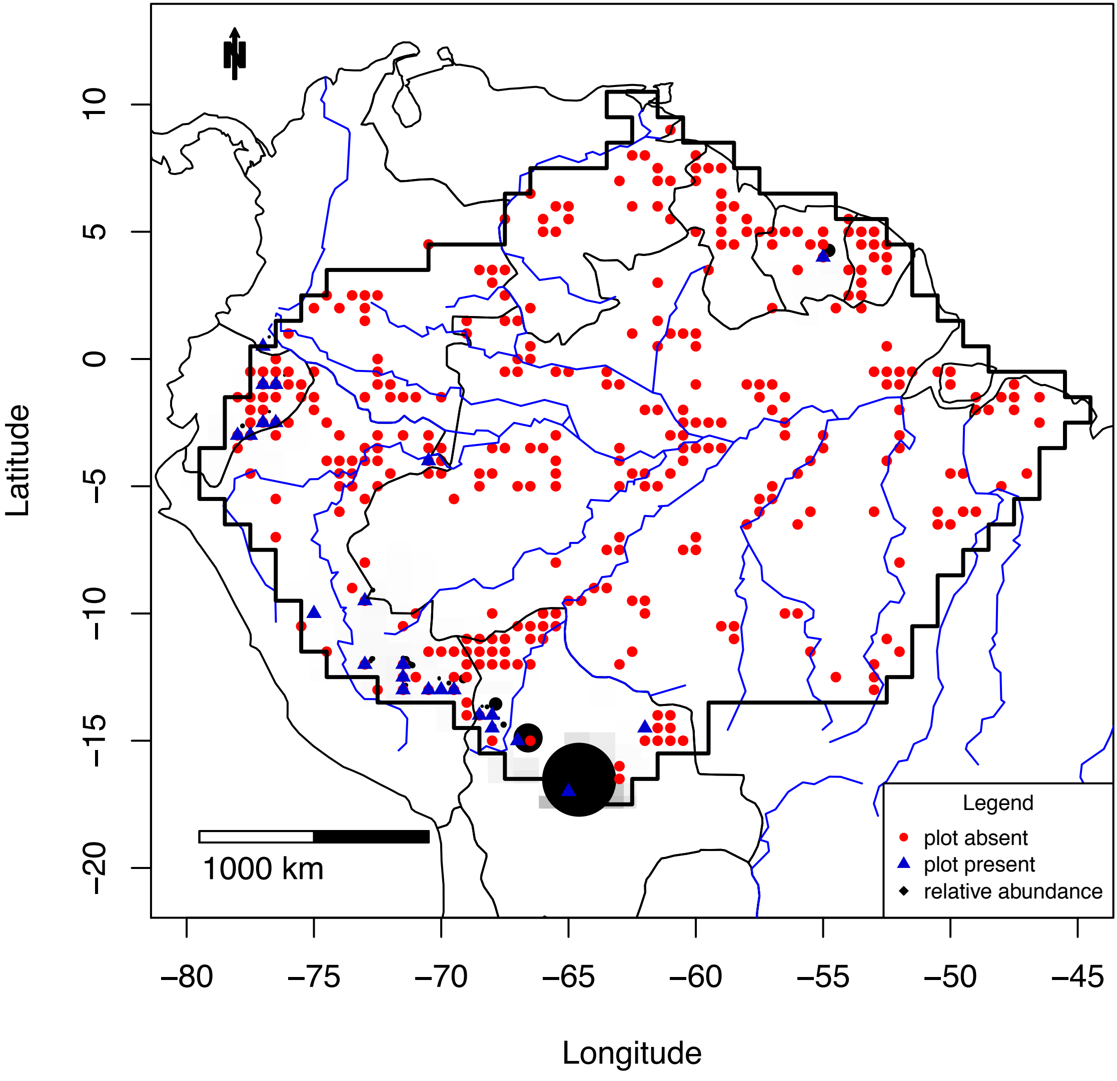

Figure S3_121A Combined map of Pourouma_bicolor

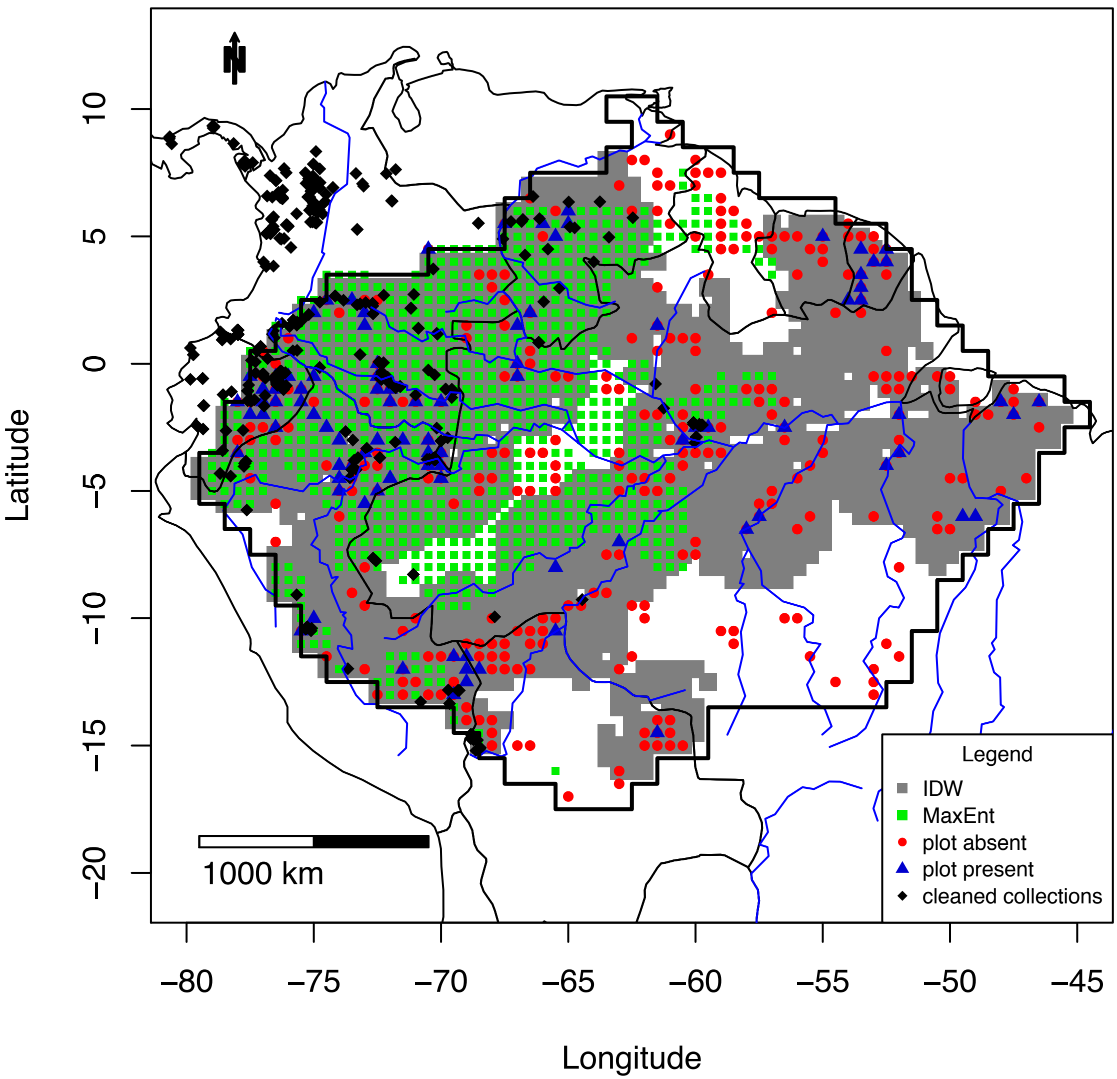

Figure S3_121B MaxEnt map of Pourouma_bicolor

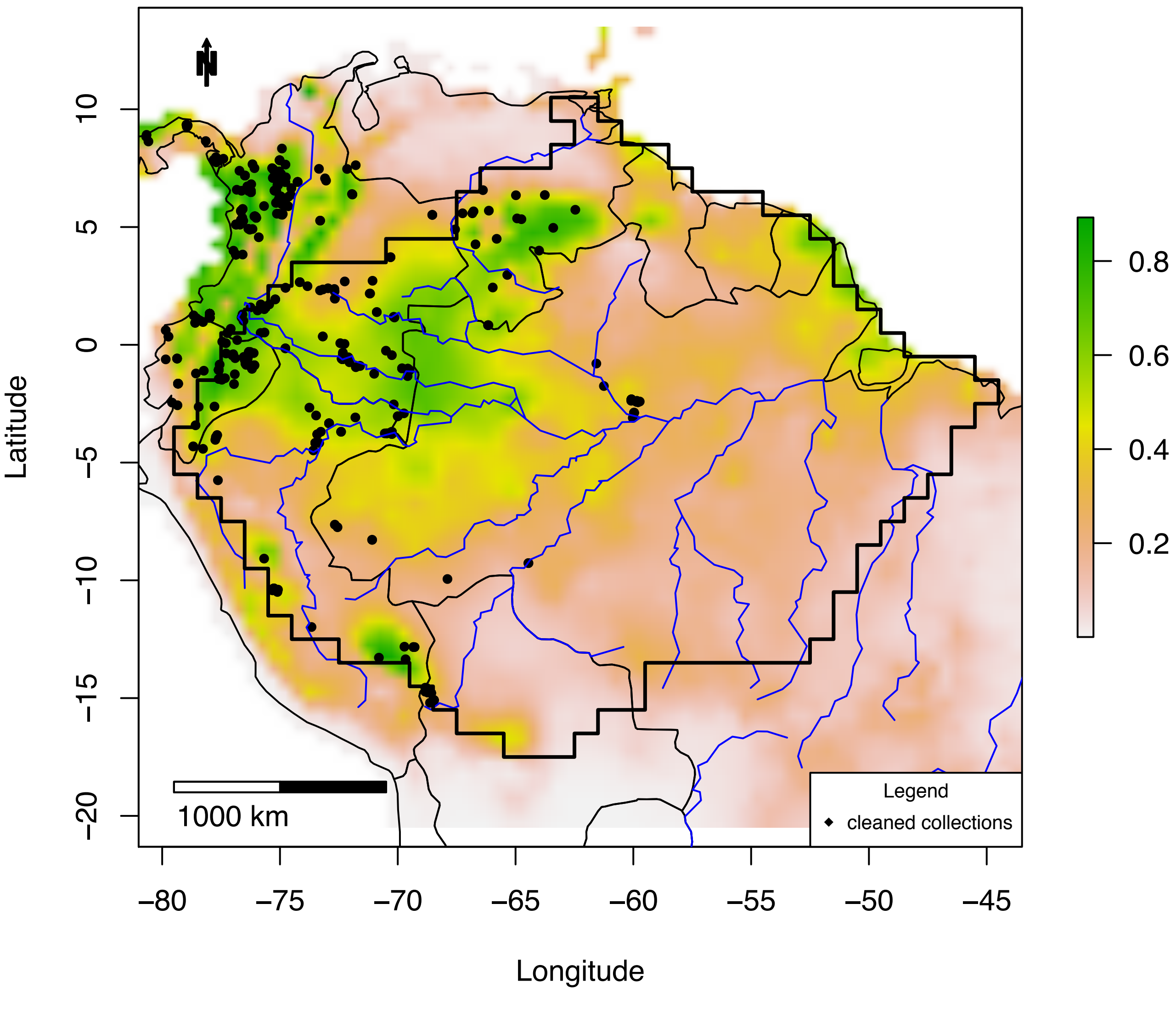

Figure S3_121C IDW map of Pourouma_bicolor: plots =0 – 0.04; IDW: 0 – 0.02

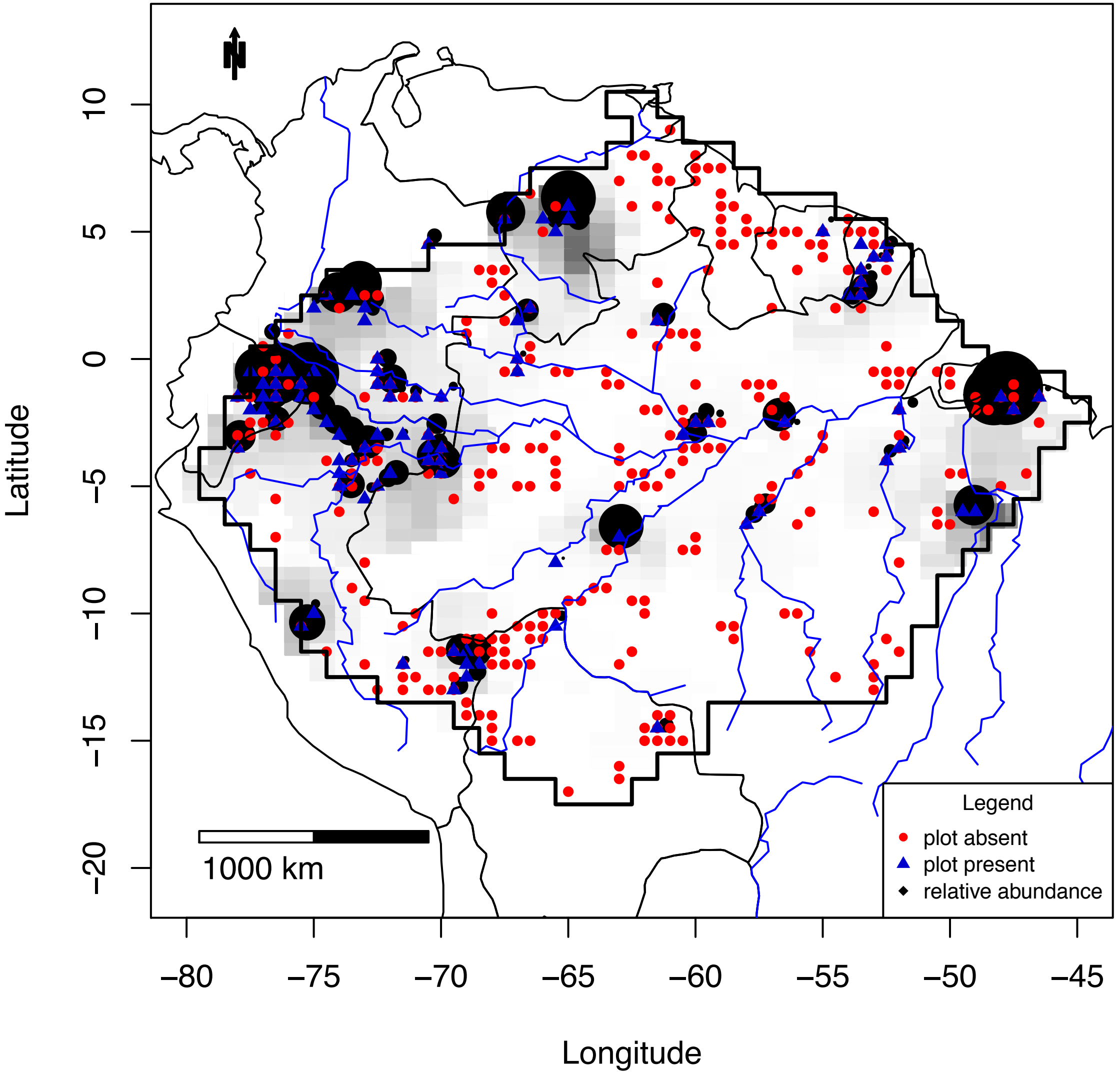

Figure S3_122A Combined map of Pourouma_cecropiifolia

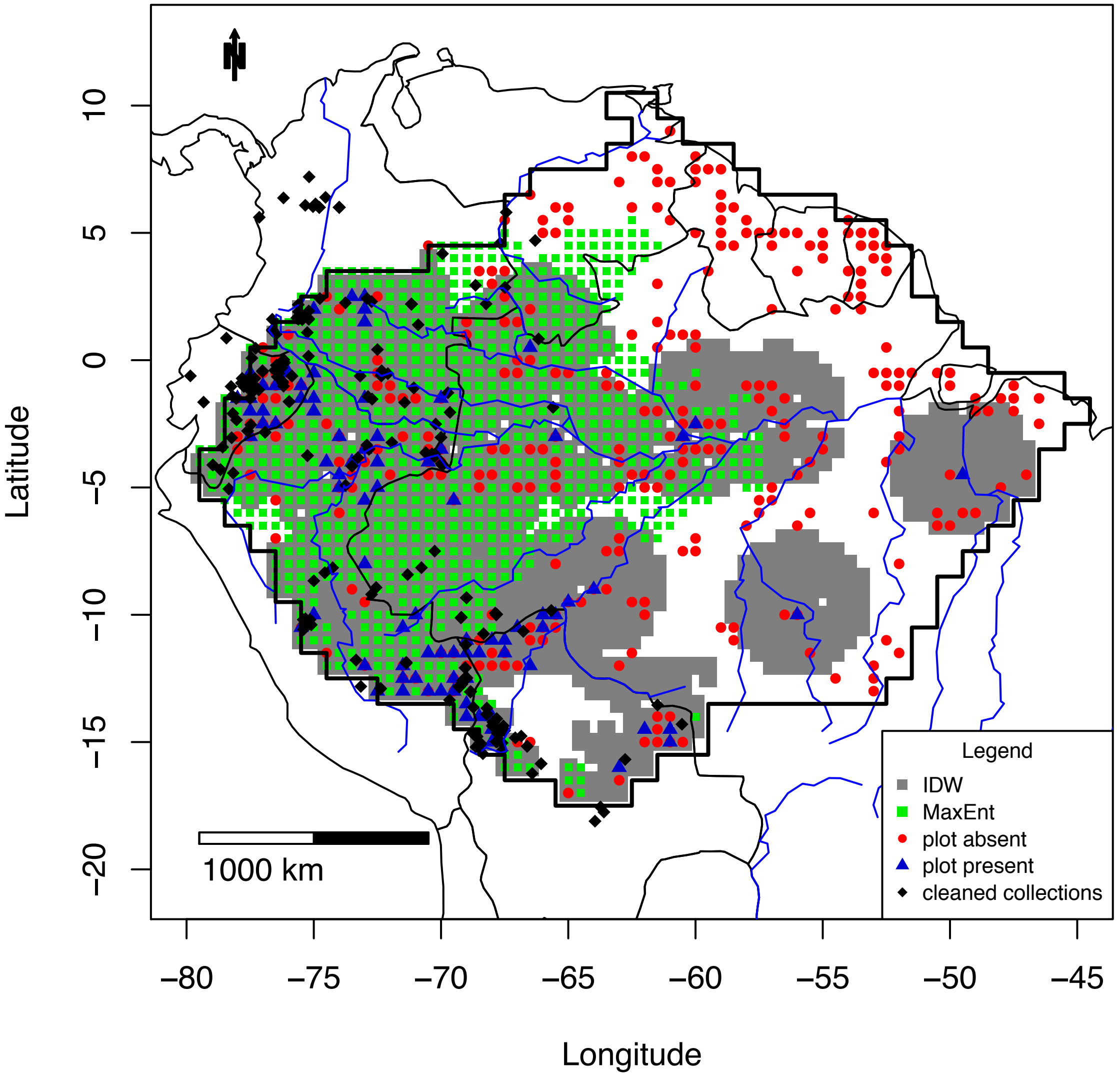

Figure S3_122B MaxEnt map of Pourouma_cecropiifolia

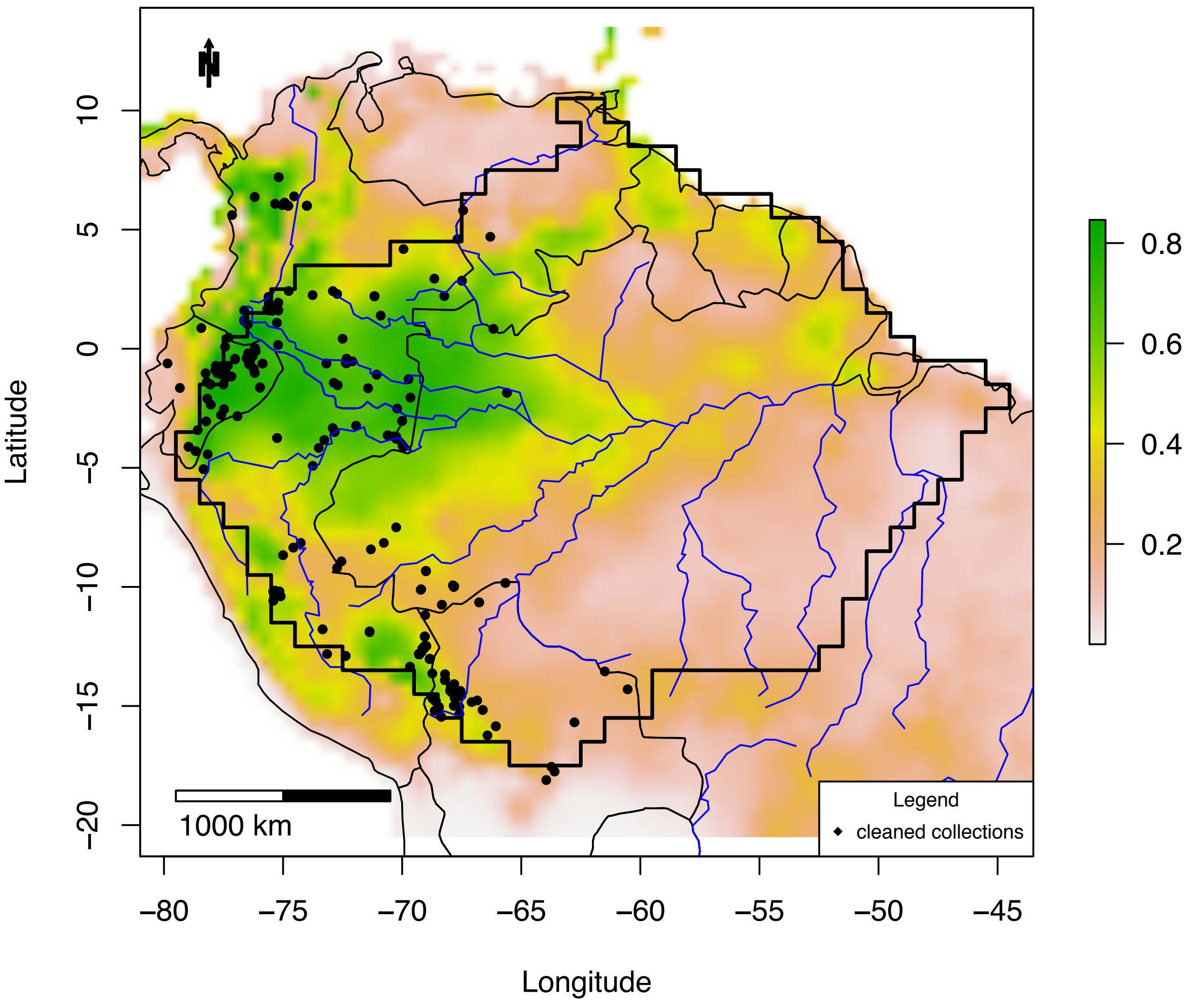

Figure S3_122C IDW map of Pourouma_cecropiifolia: plots =0 – 0.07; IDW: 0 – 0.04

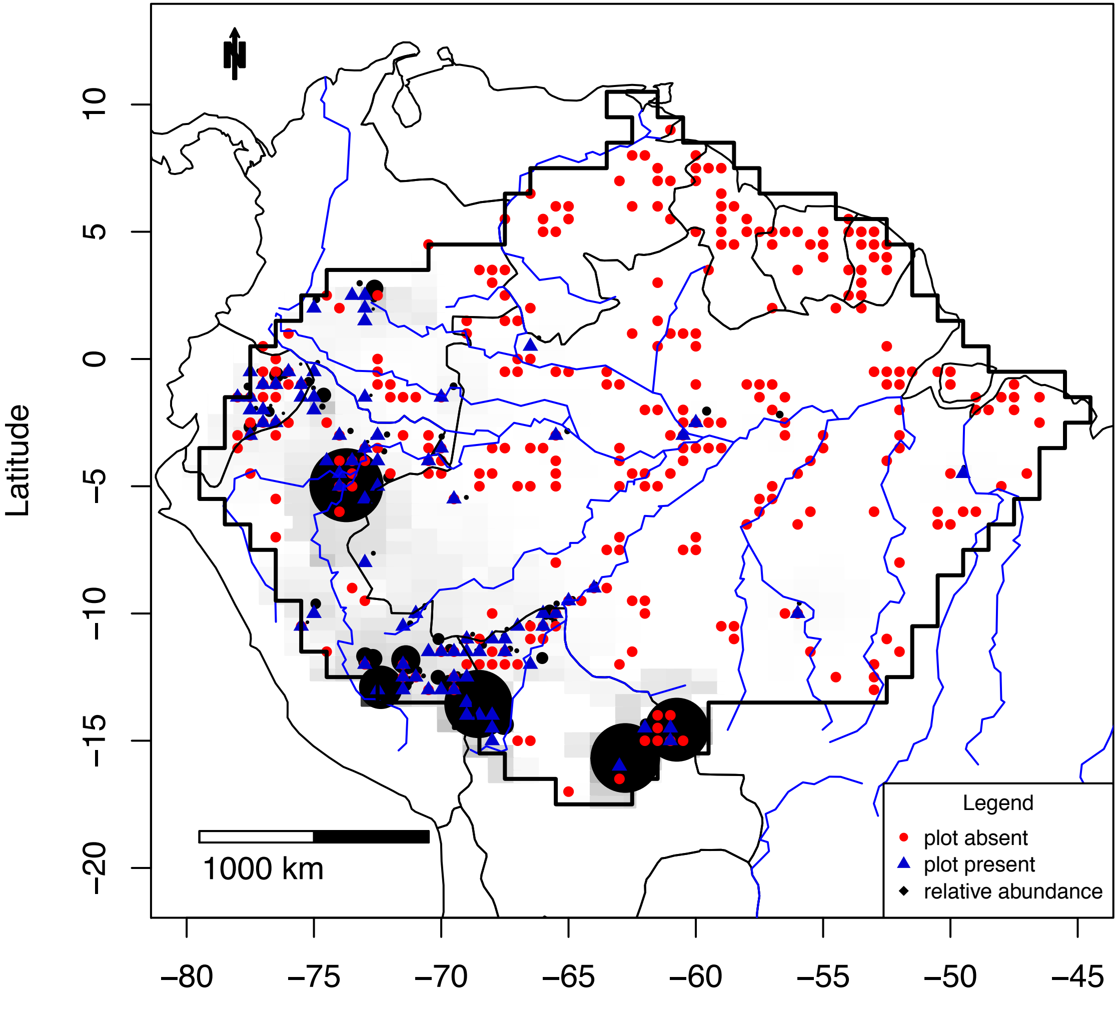

Figure S3_123A Combined map of Pourouma_minor

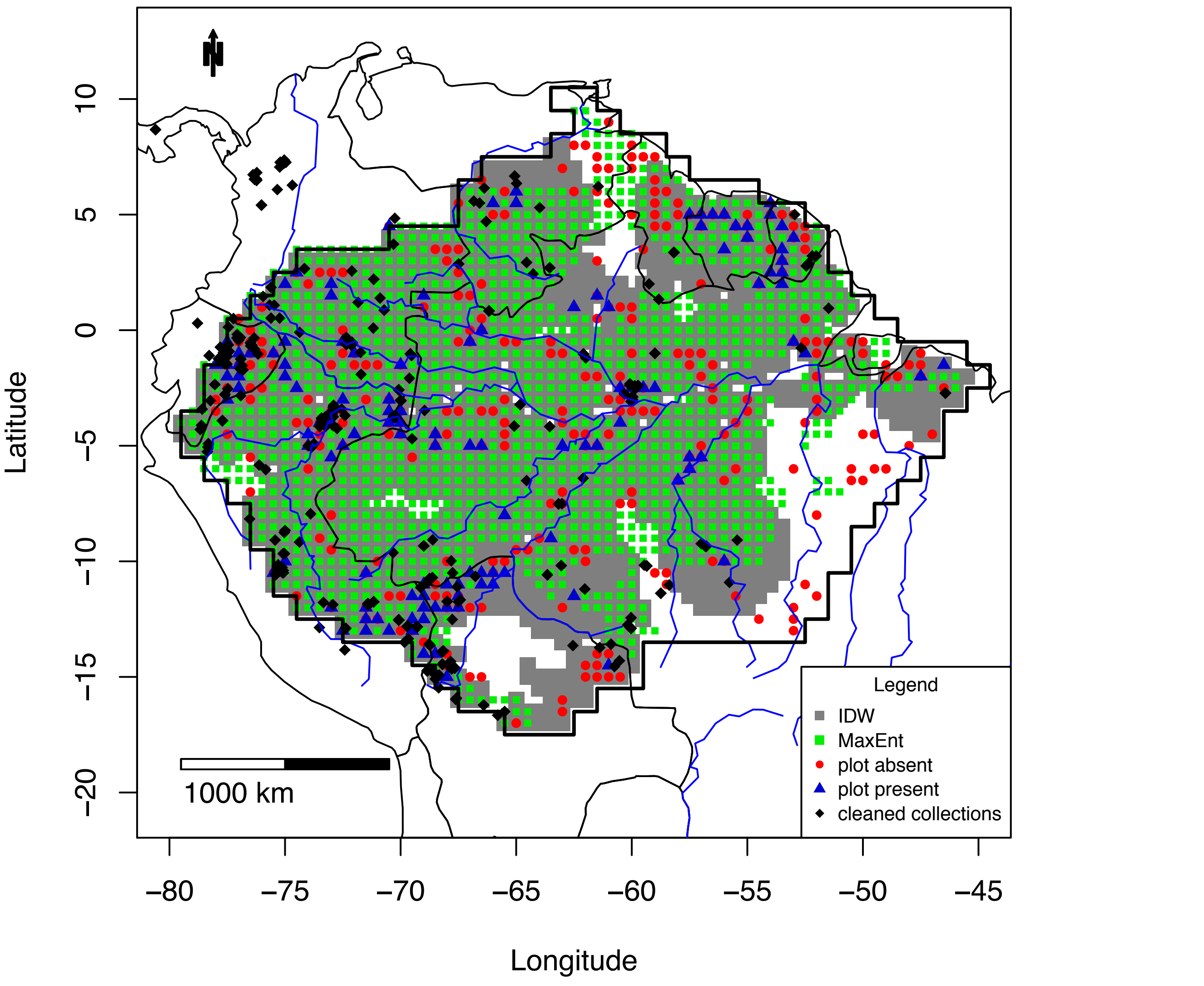

Figure S3_123B MaxEnt map of Pourouma_minor

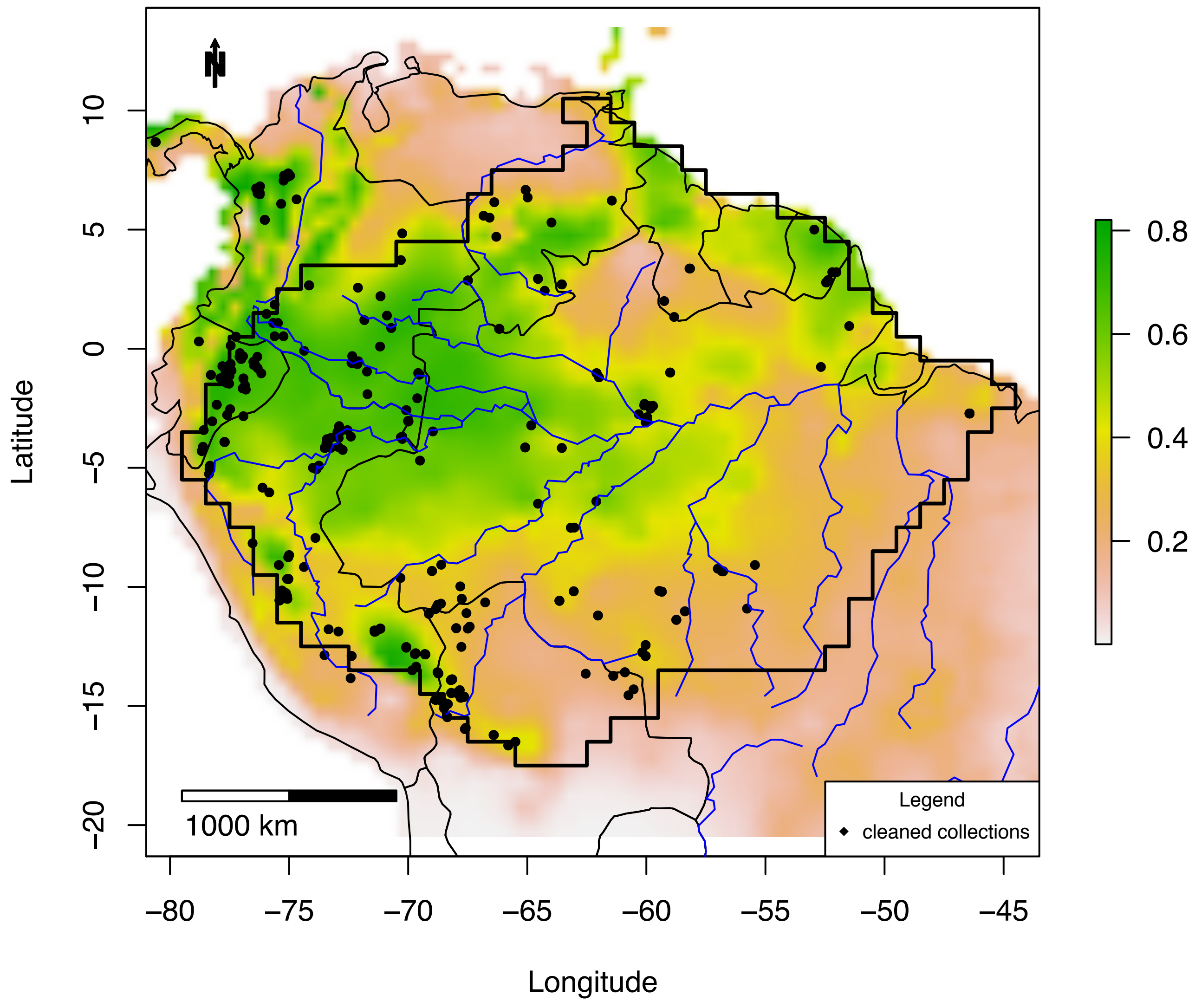

Figure S3_123C IDW map of Pourouma_minor: plots =0 – 0.17; IDW: 0 – 0.07

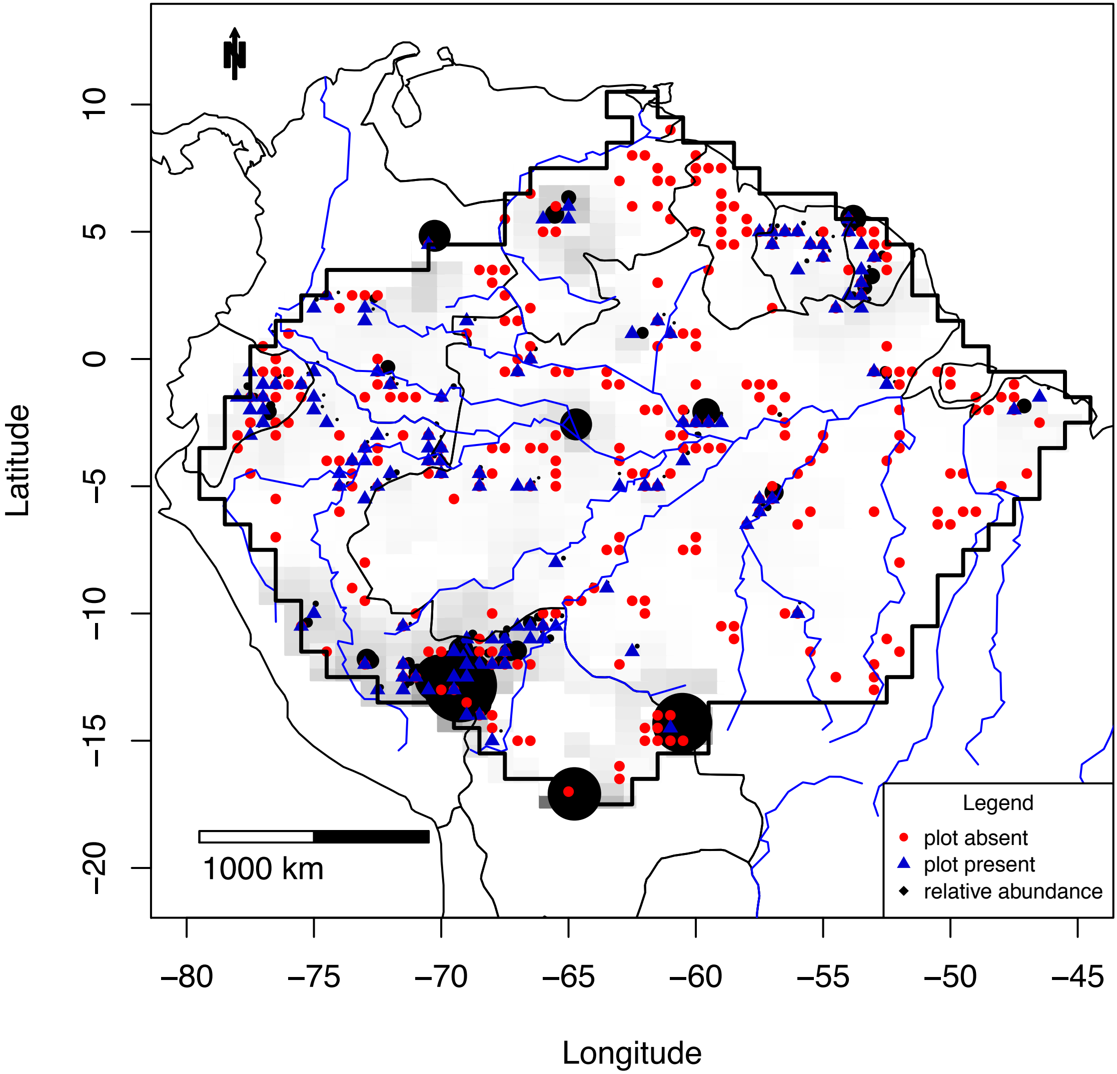

Figure S3_124A Combined map of Pouteria_cuspidata

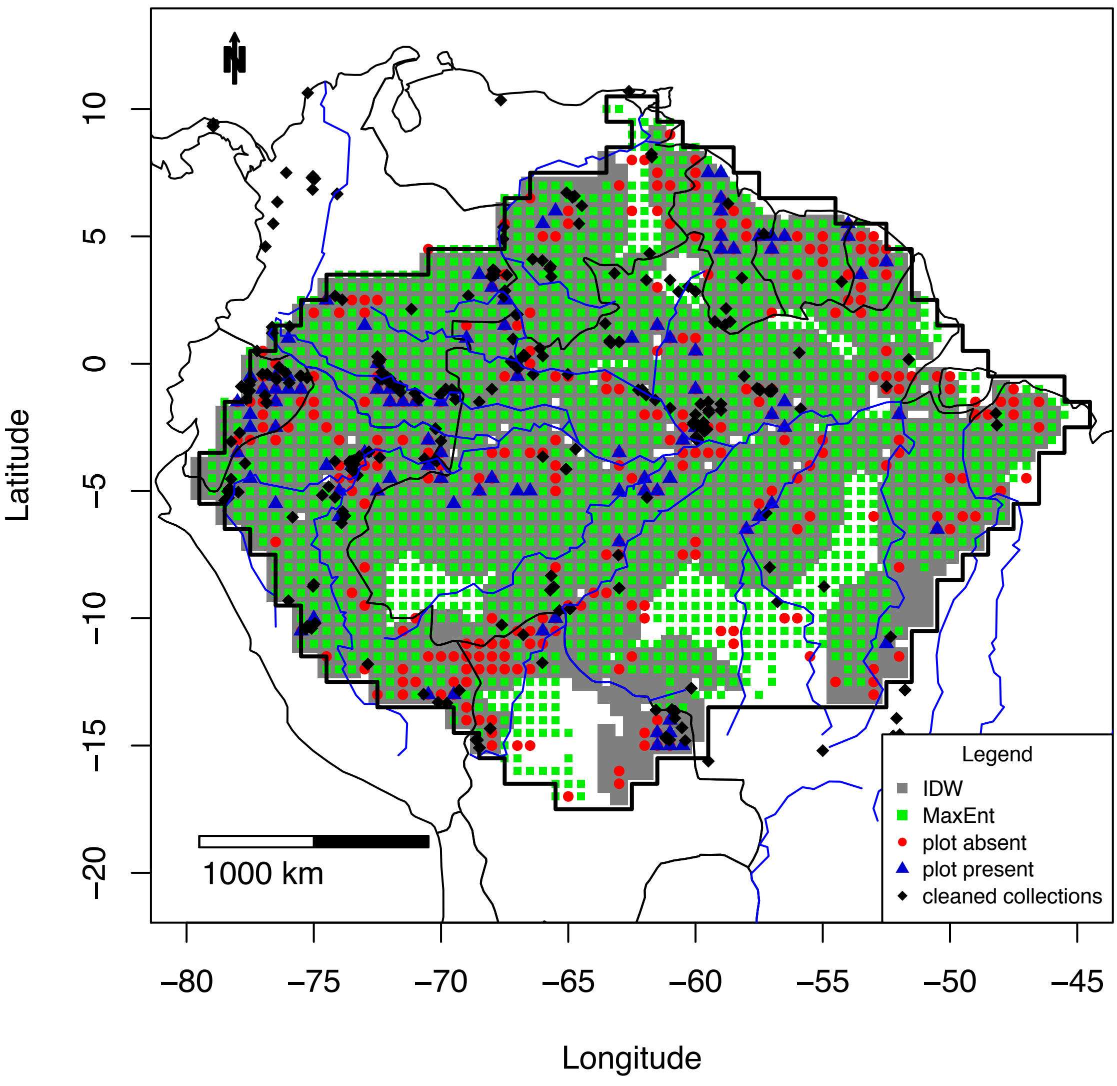

Figure S3_124B MaxEnt map of Pouteria_cuspidata

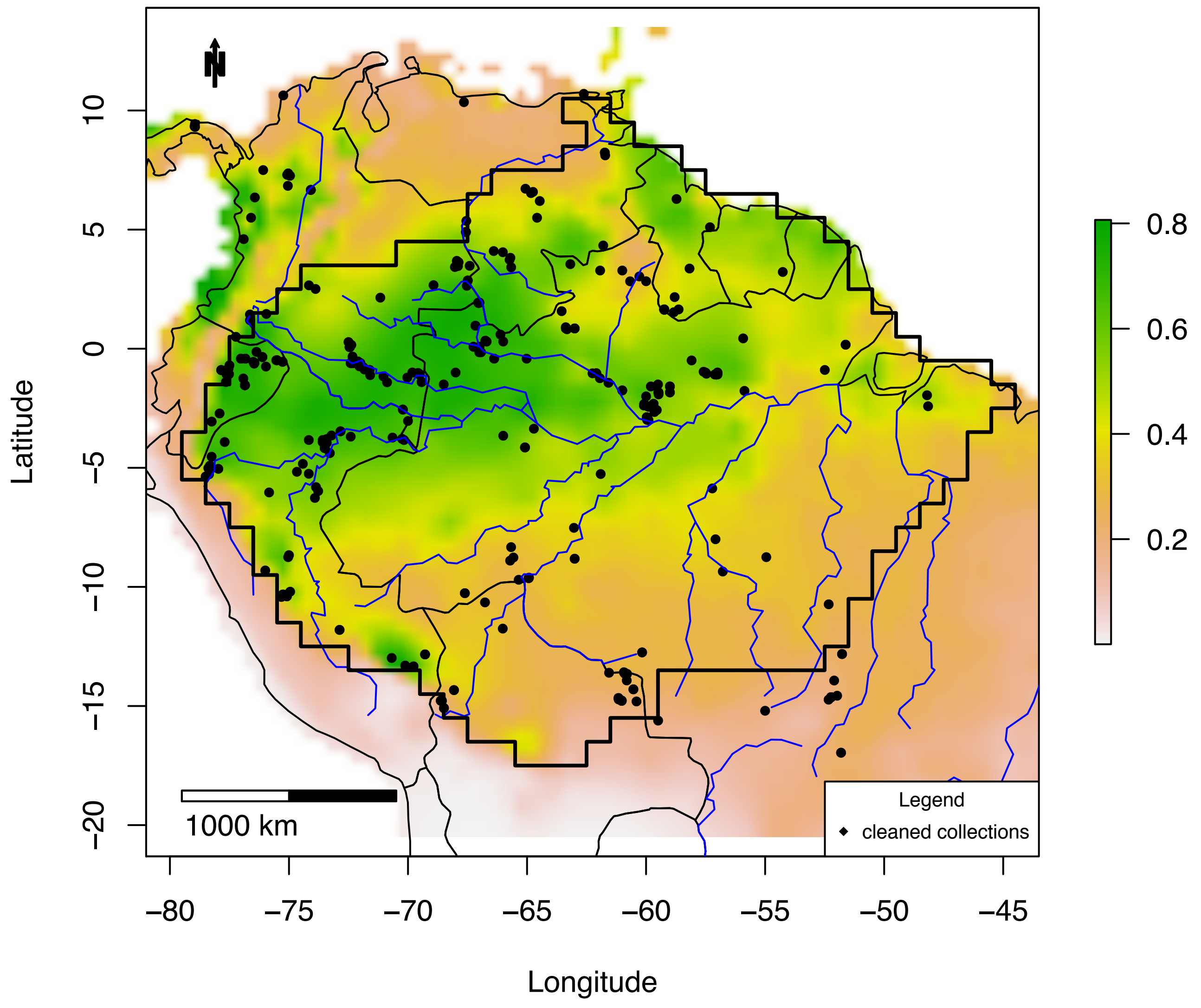

Figure S3_124C IDW map of Pouteria_cuspidata: plots =0 – 0.1; IDW: 0 – 0.04

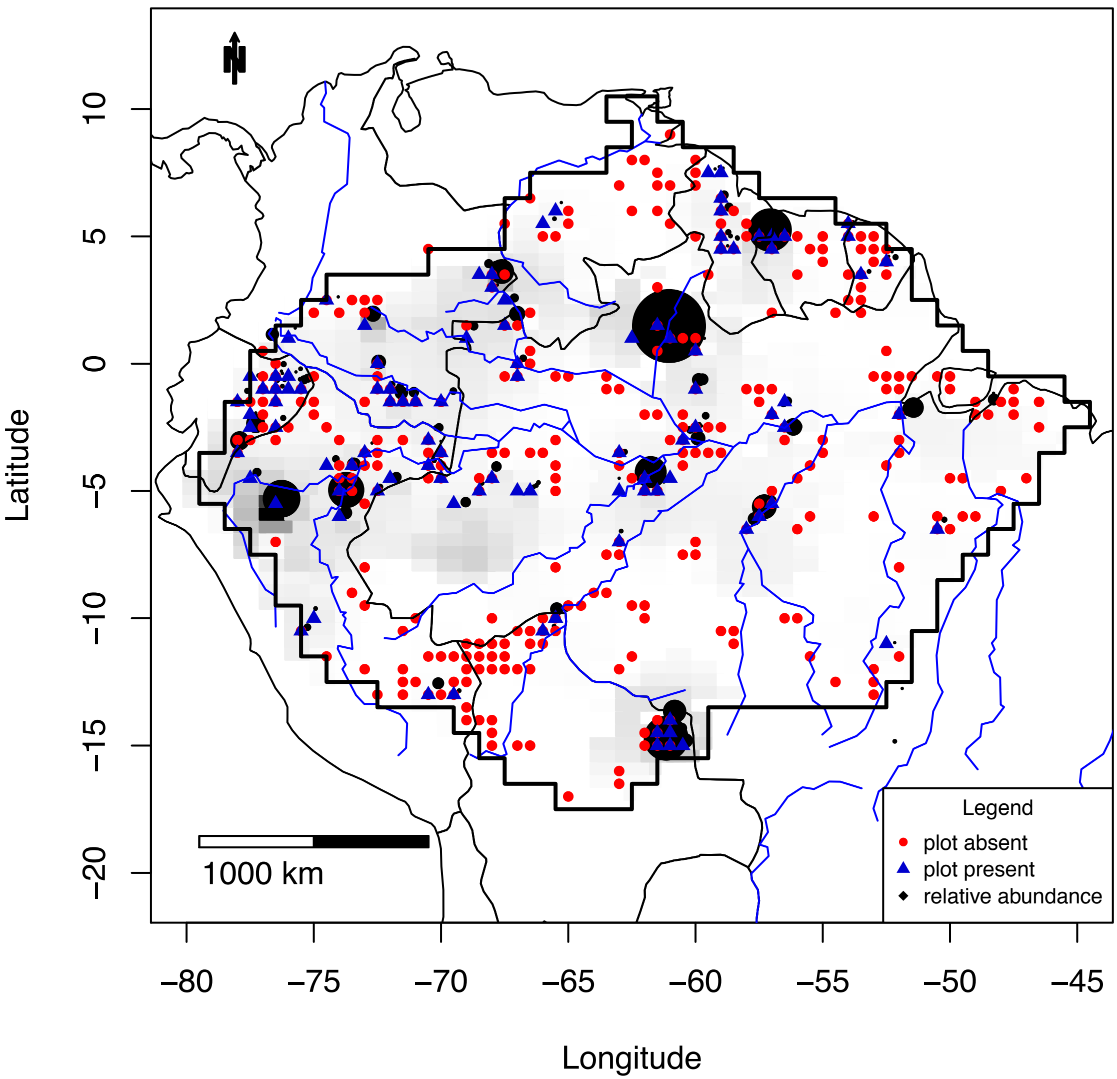

Figure S3_125A Combined map of Pouteria_elegans

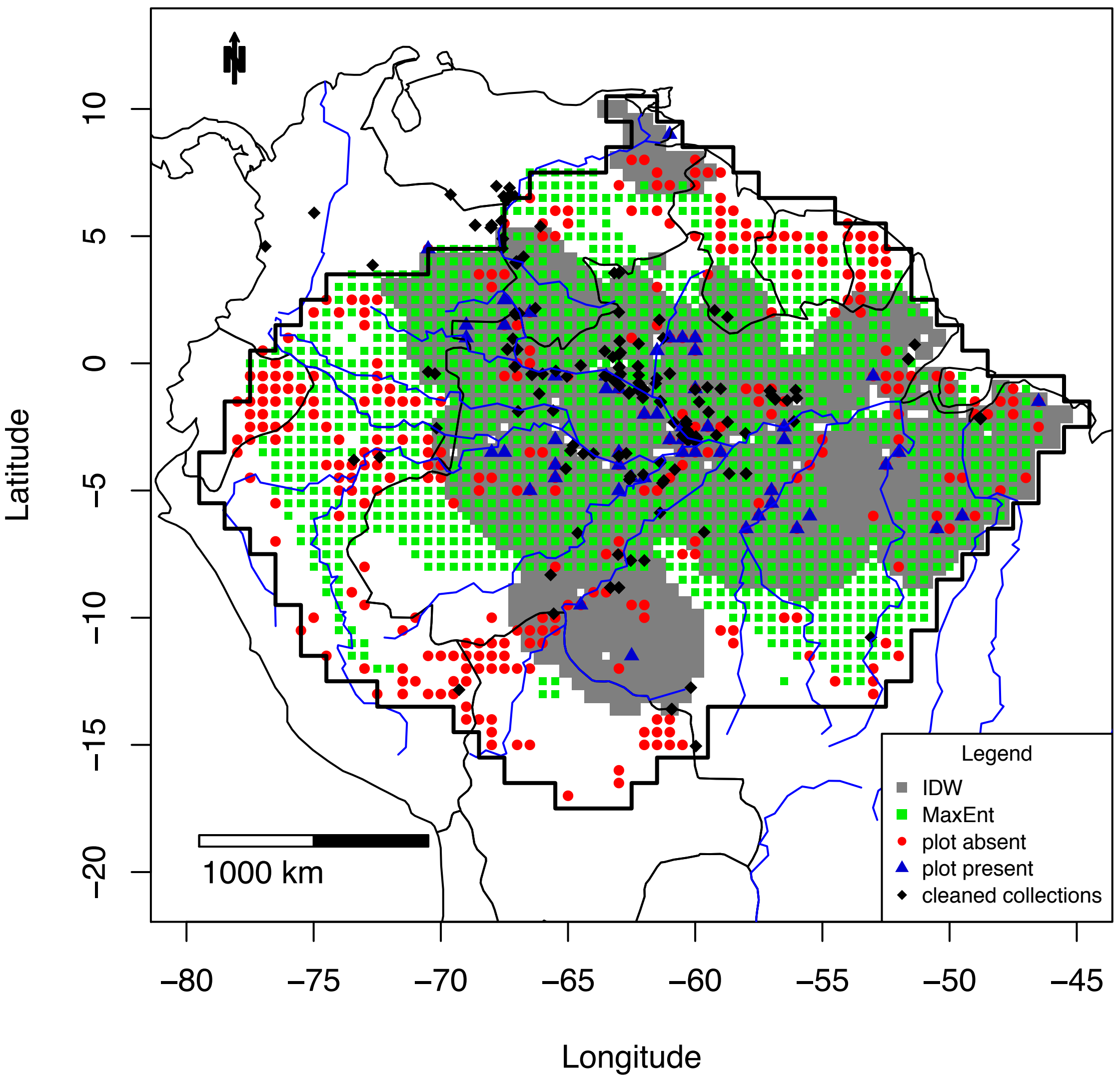

Figure S3_125B MaxEnt map of Pouteria_elegans

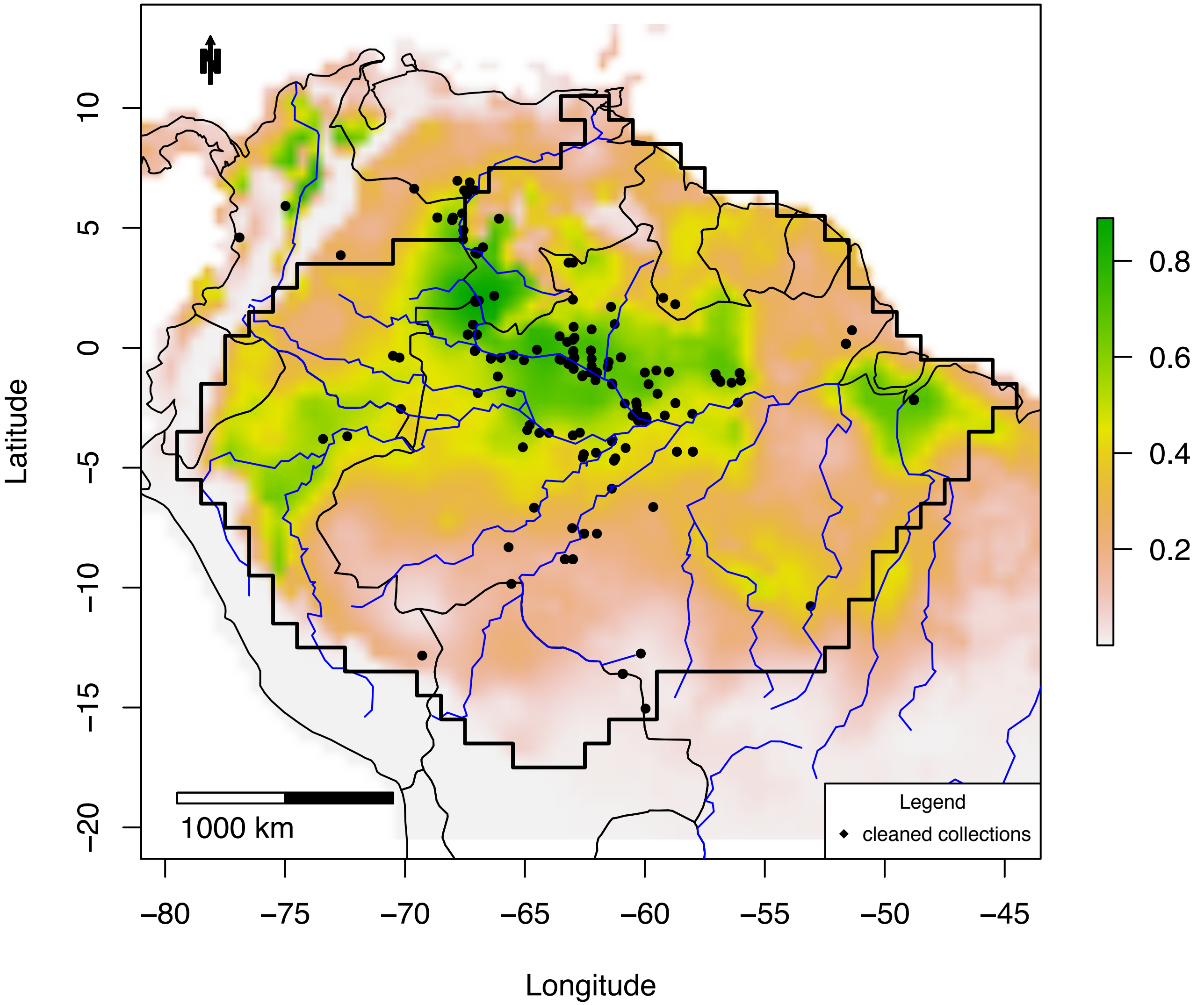

Figure S3_125C IDW map of Pouteria_elegans: plots =0 – 0.32; IDW: 0 – 0.06

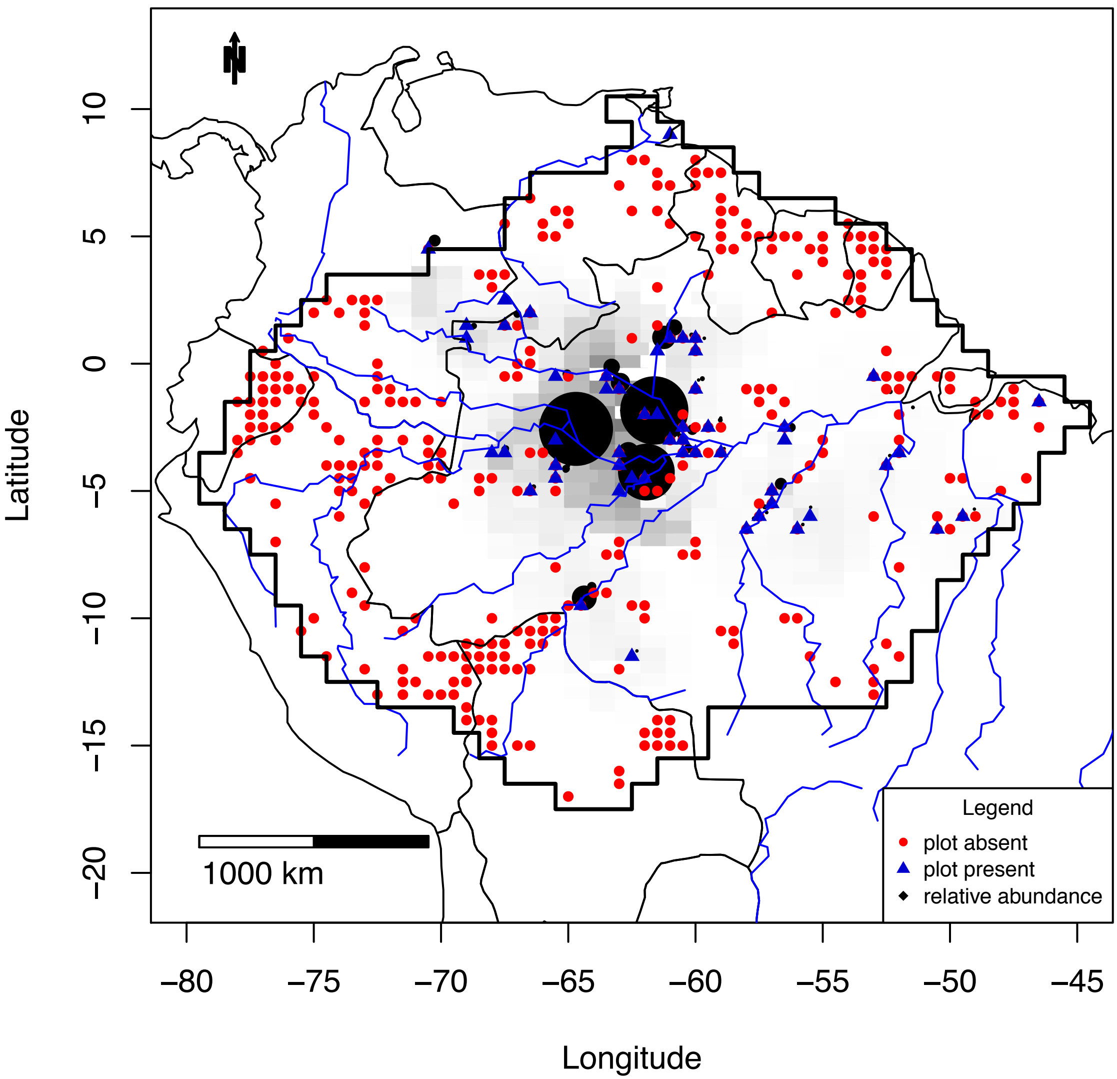

Figure S3_126A Combined map of Protium_apiculatum

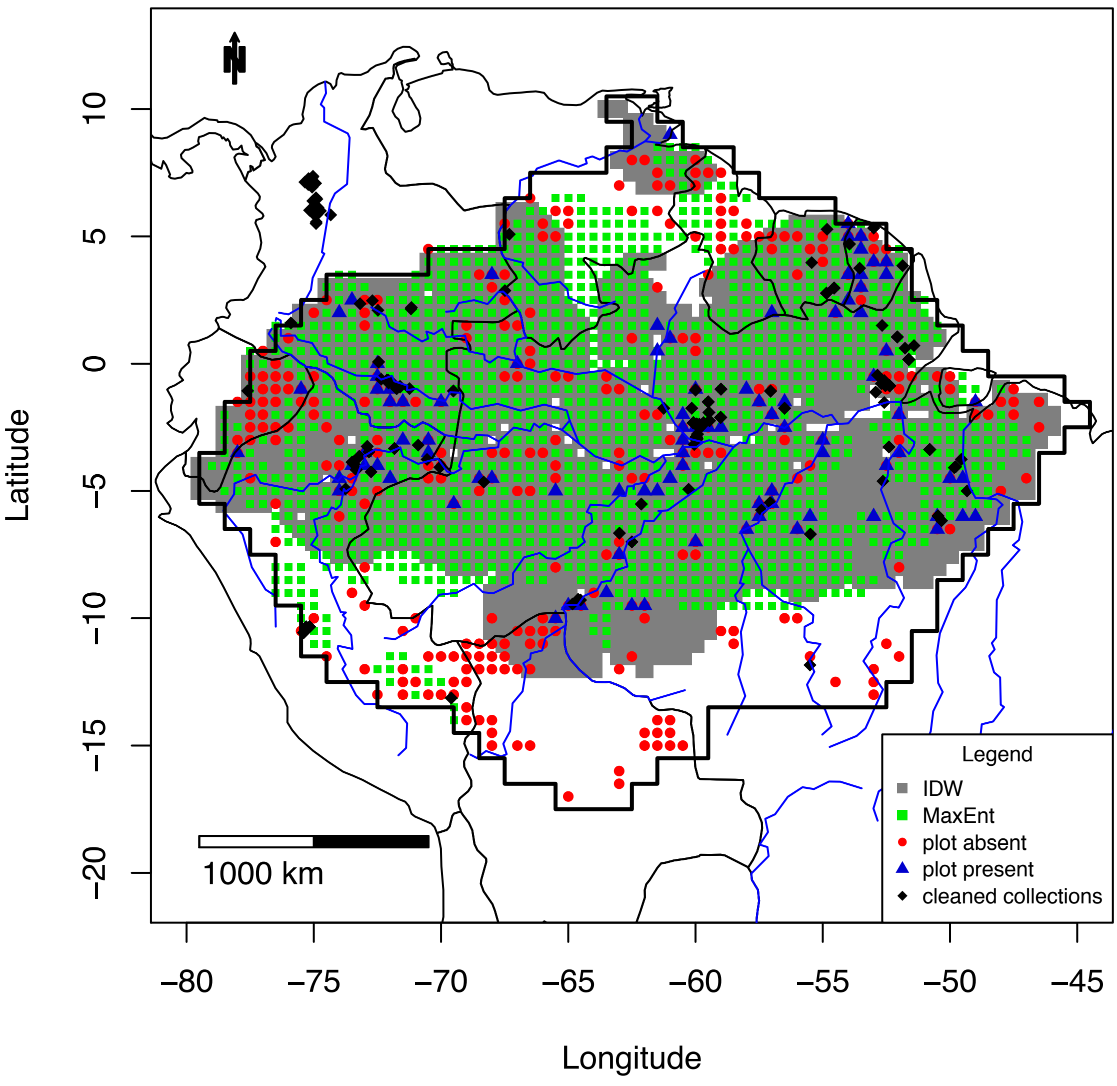

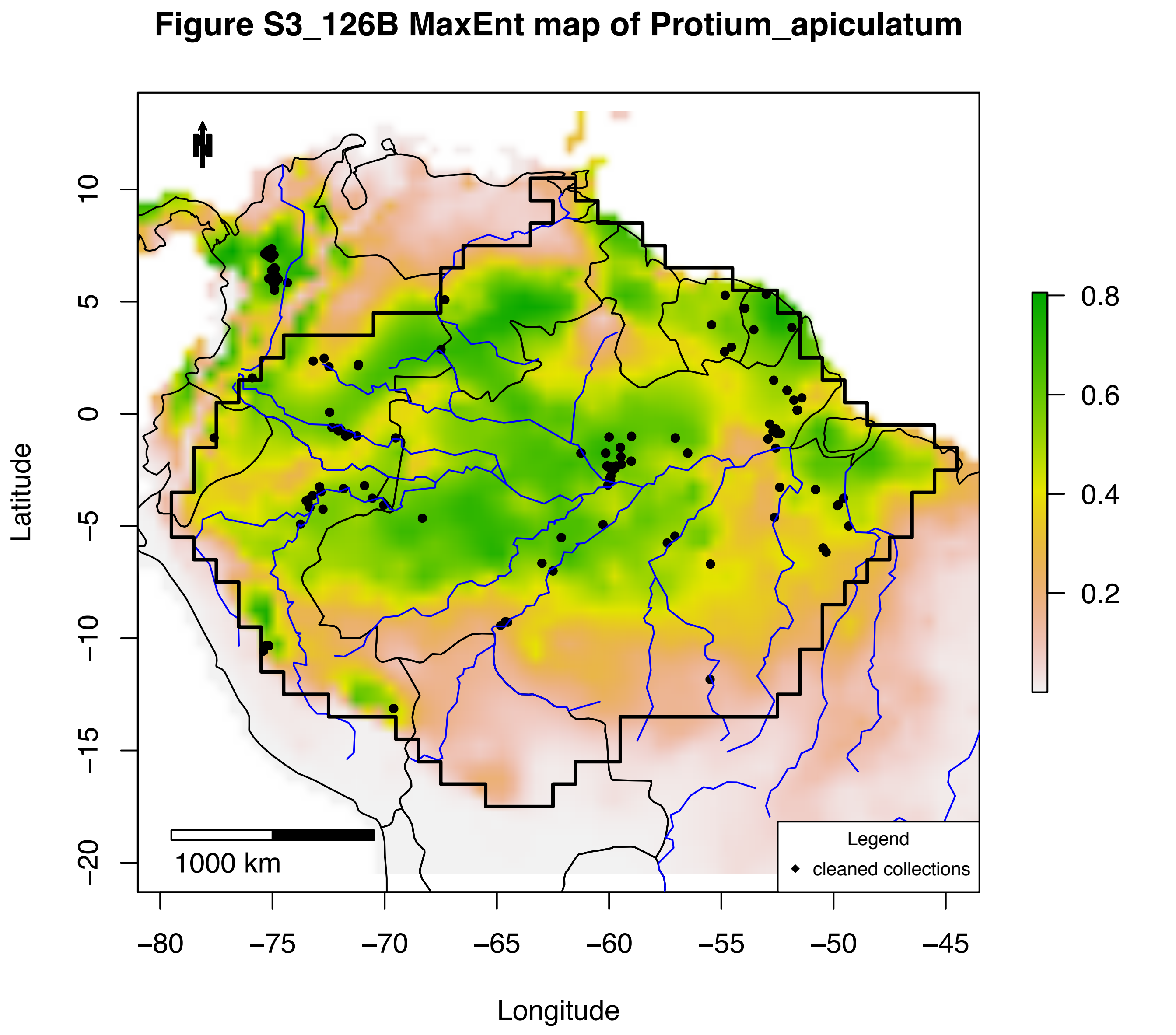
Figure S3_126B MaxEnt map of Protium_apiculatum
N
10
5
0
−5
−10
−15
−20
Latitude
−80
−75
−70
−65
−60
−55
−50
−45
Longitude
1000 km
Legend
cleaned collections
0.8
0.6
0.4
0.2

Figure S3_126C IDW map of Protium_apiculatum: plots =0 – 0.18; IDW: 0 – 0.05

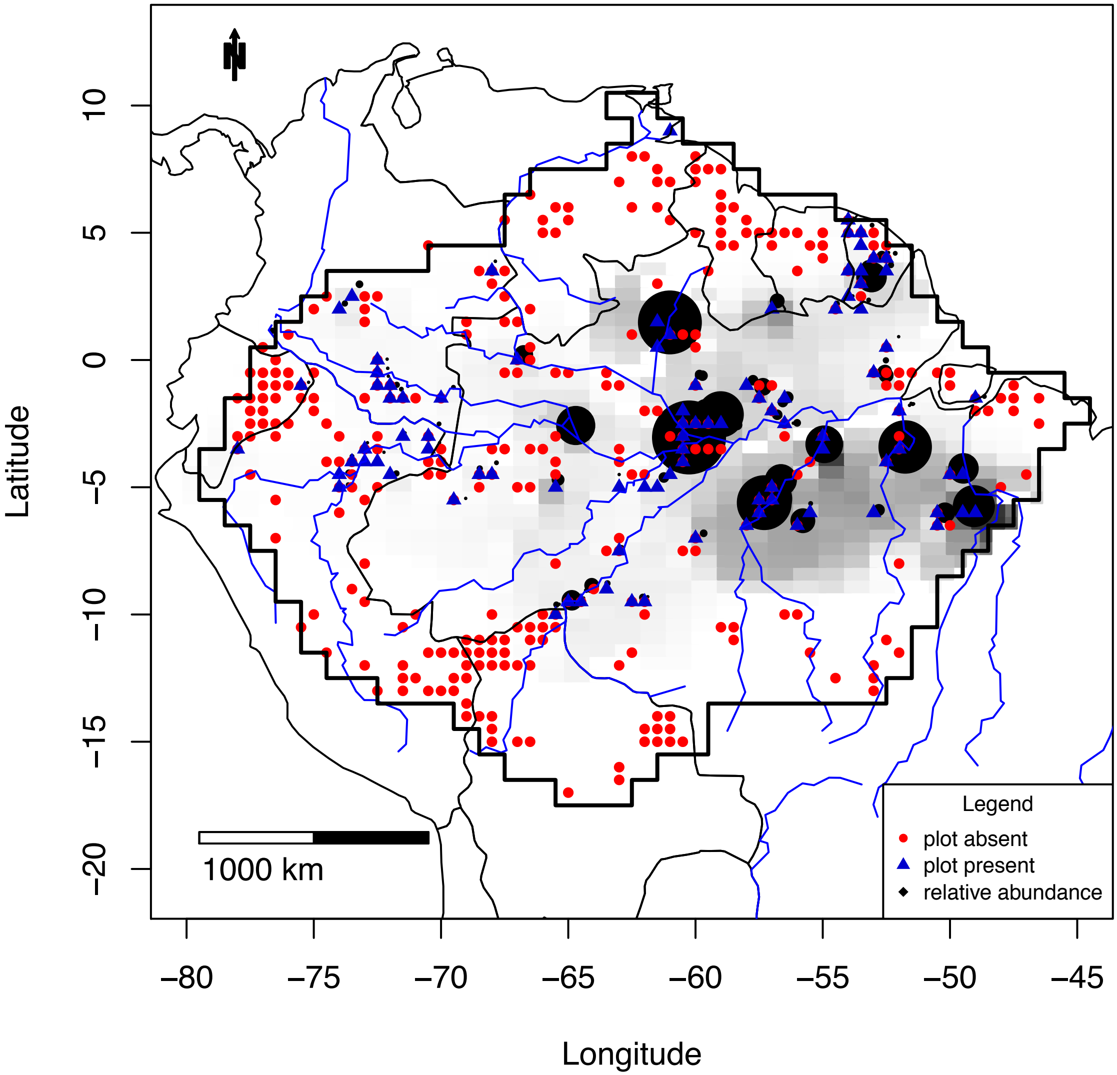

Figure S3_127A Combined map of Protium_aracouchini

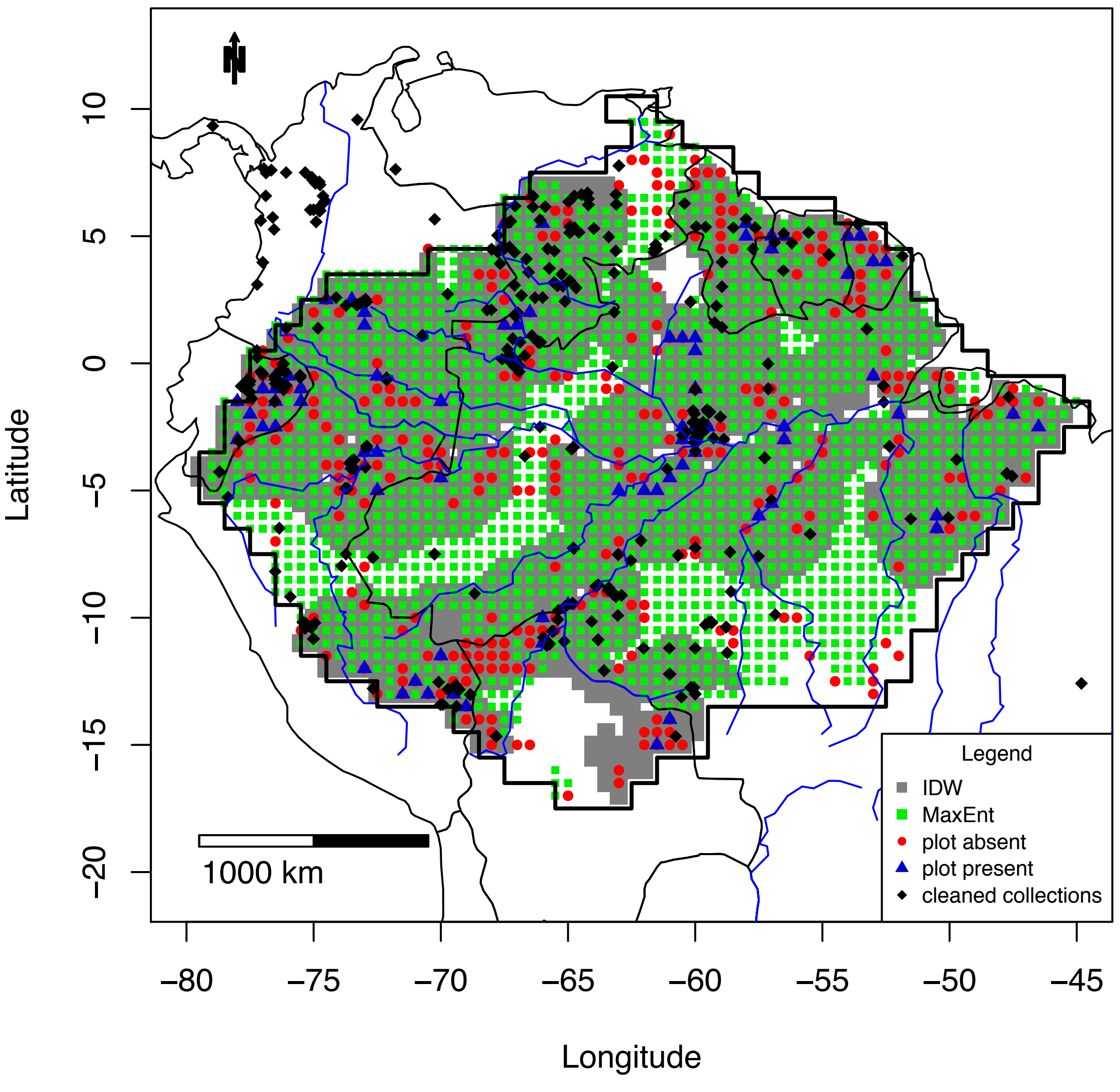

Figure S3_127B MaxEnt map of Protium_aracouchini

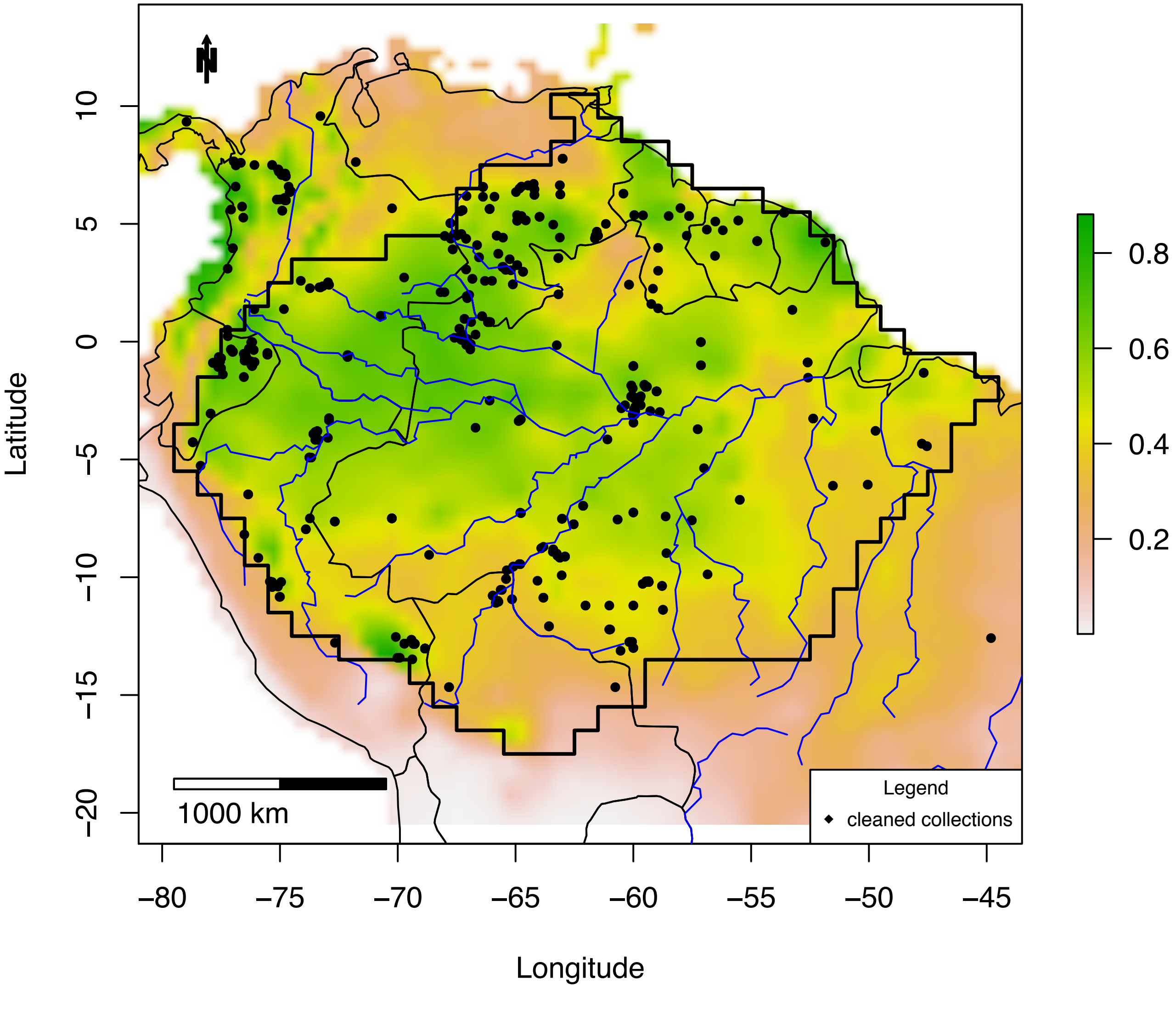

Figure S3_127C IDW map of Protium_aracouchini:
plots =0 – 0.08; IDW: 0 – 0.03

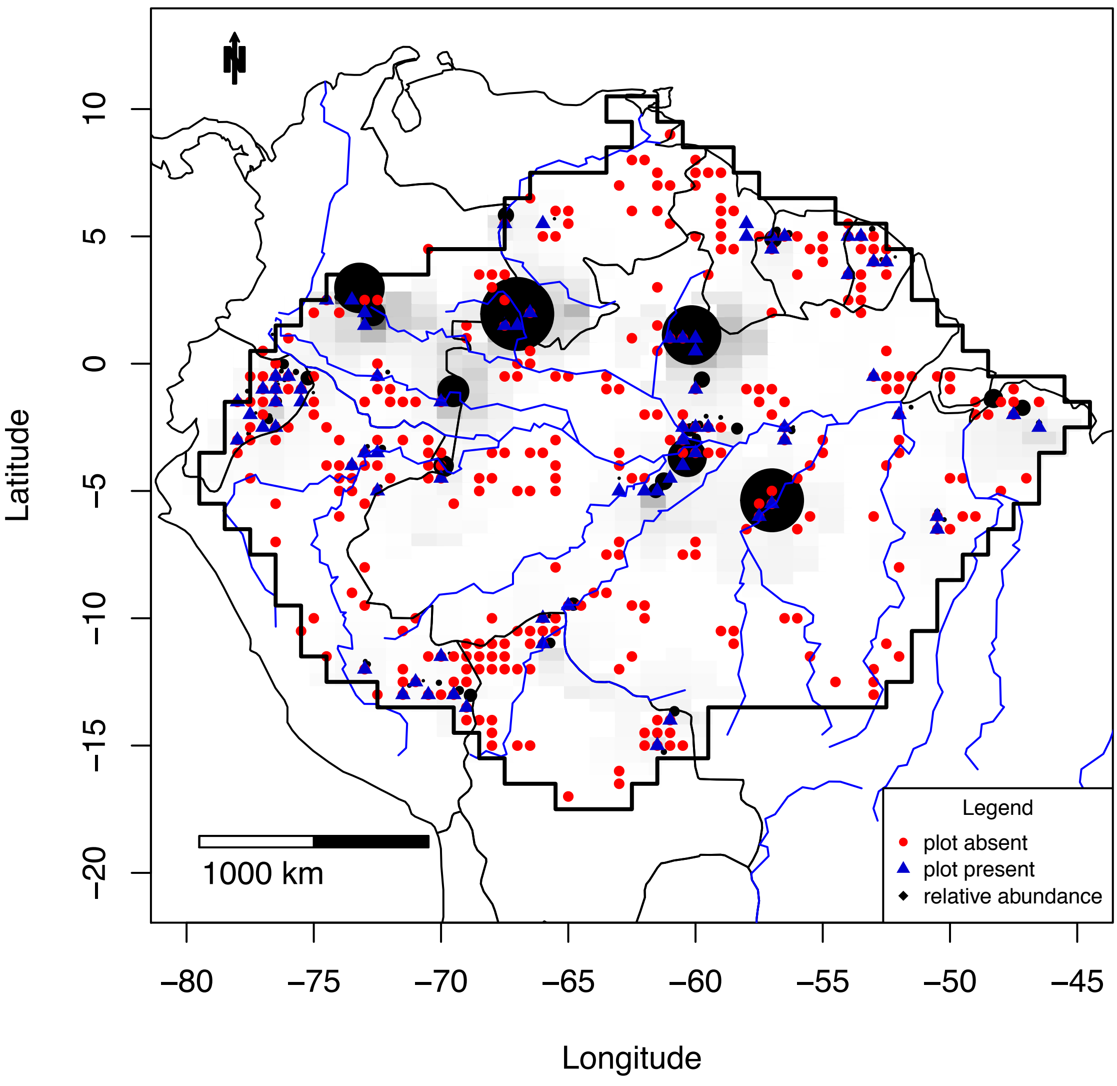

Figure S3_128A Combined map of Protium_decandrum

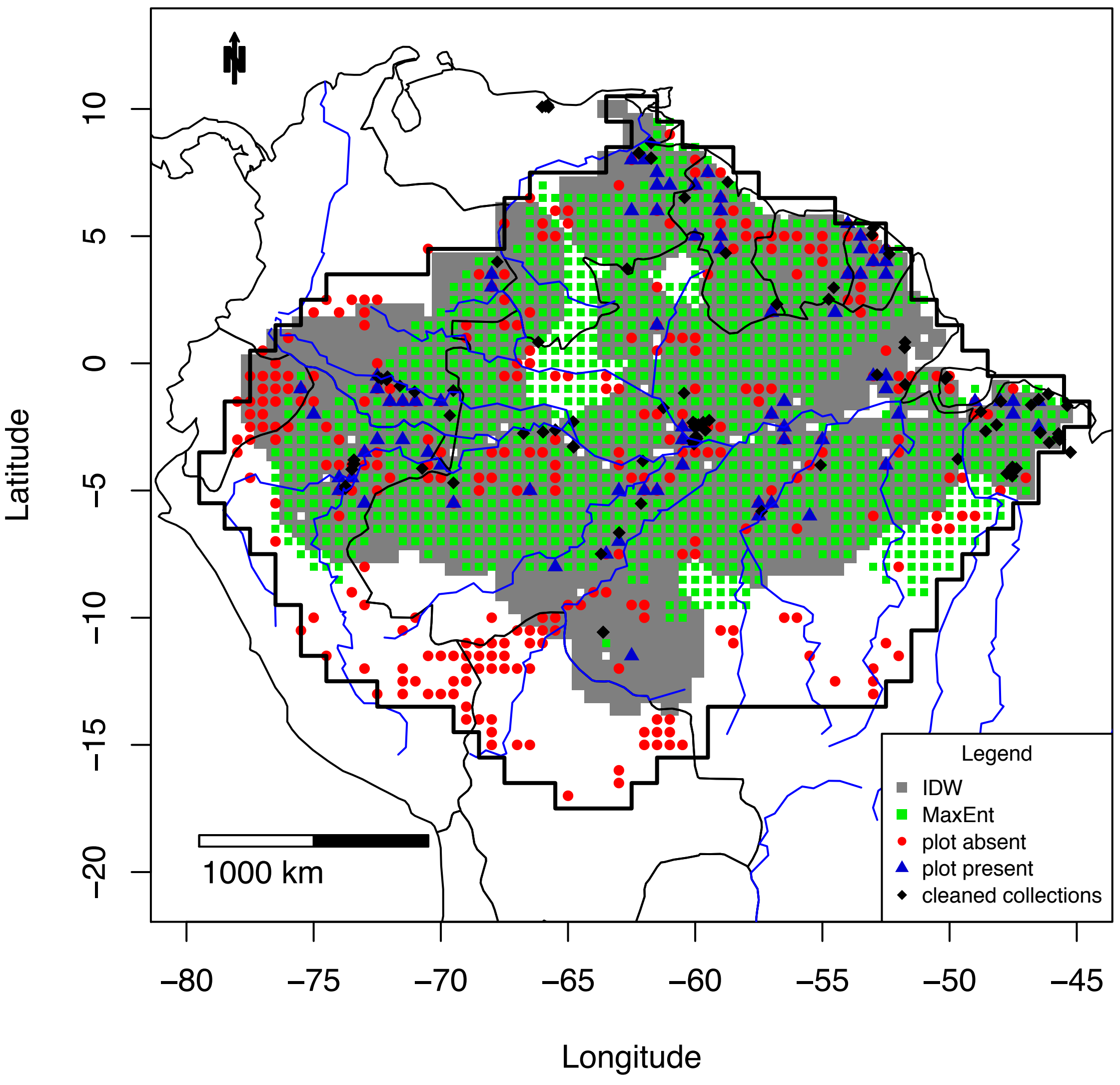

Figure S3_128B MaxEnt map of Protium_decandrum

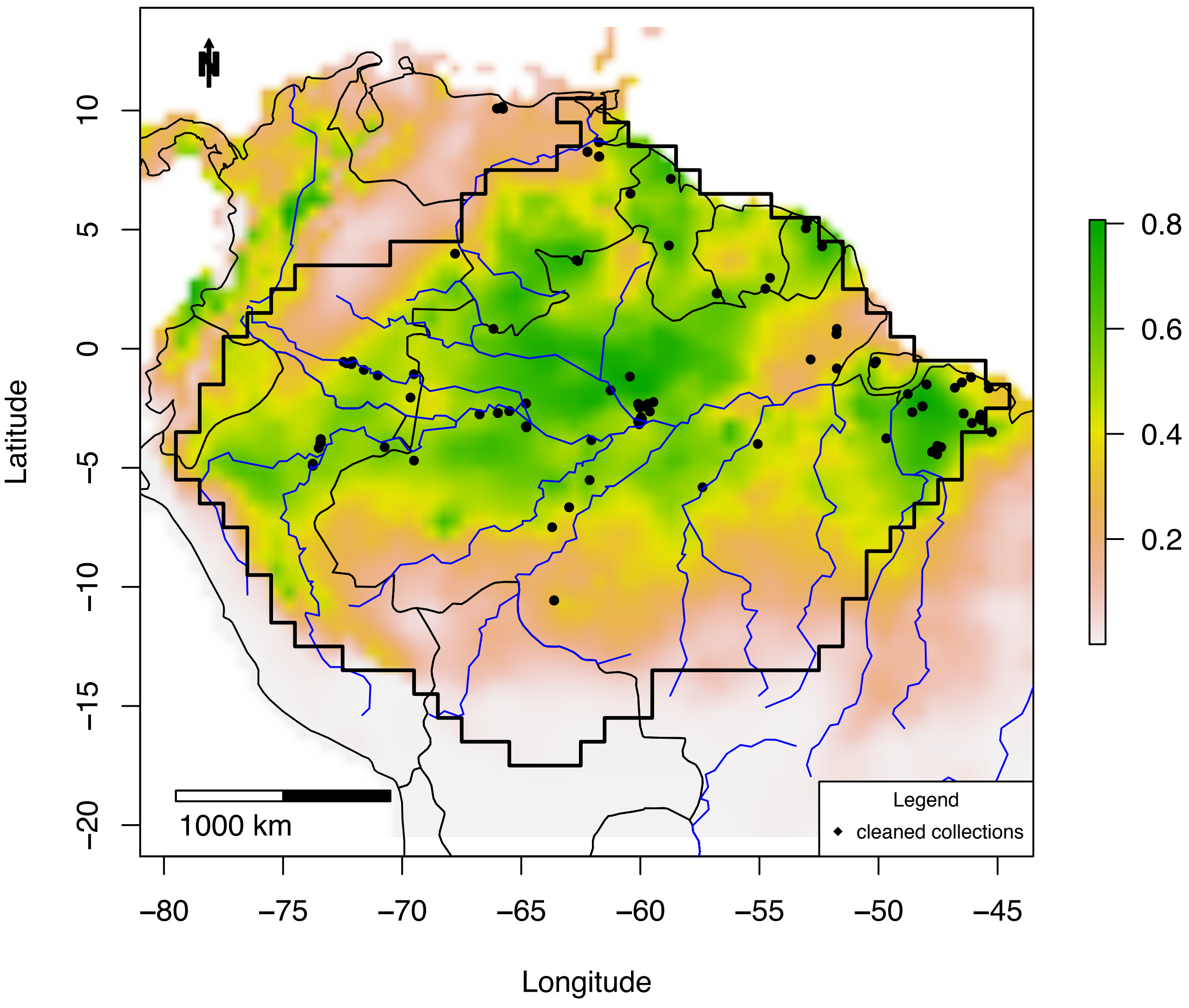

Figure S3_128C IDW map of Protium_decandrum: plots =0 – 0.16; IDW: 0 – 0.07

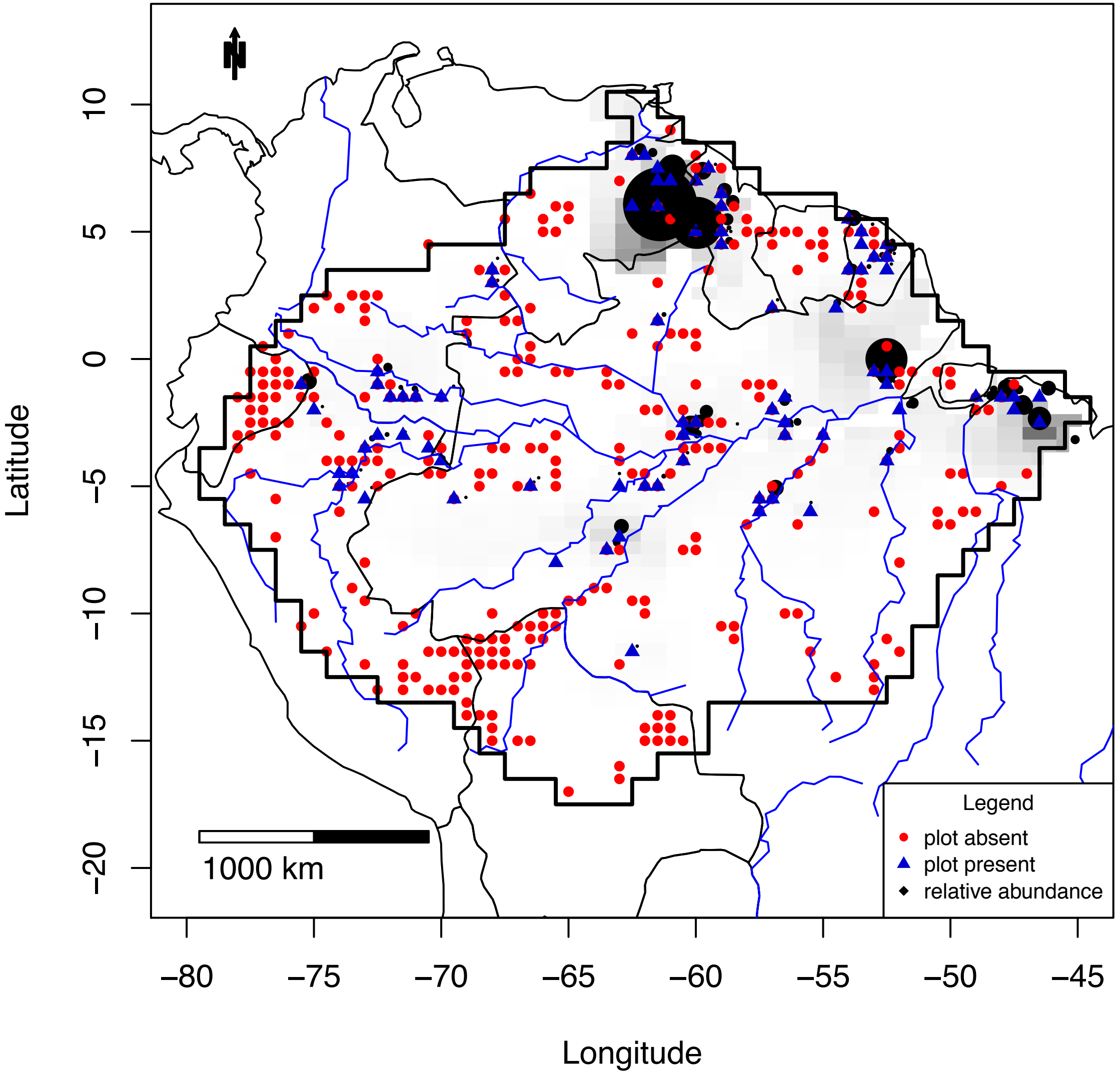

Figure S3_129A Combined map of Protium_hebetatum

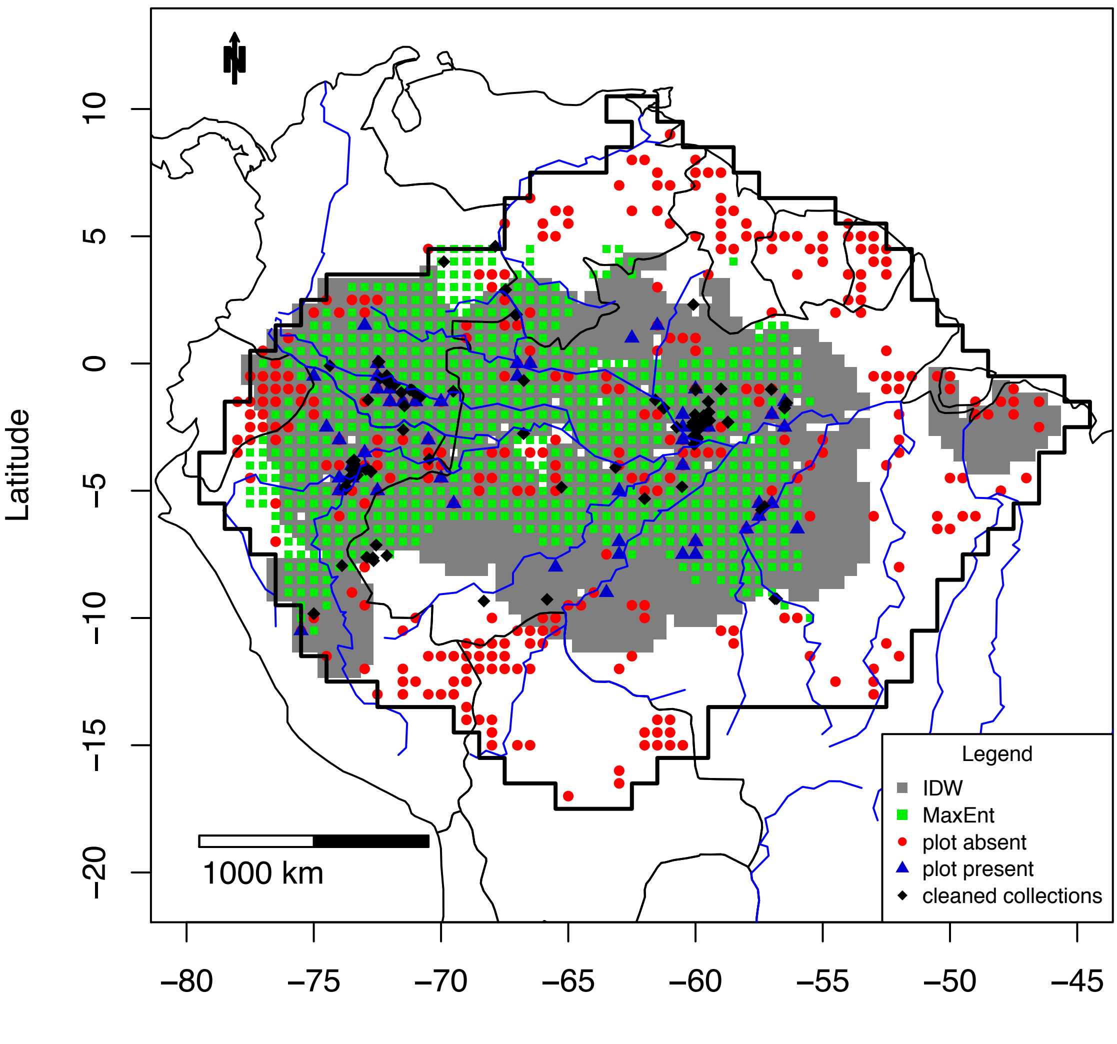

Figure S3_129B MaxEnt map of Protium_hebetatum

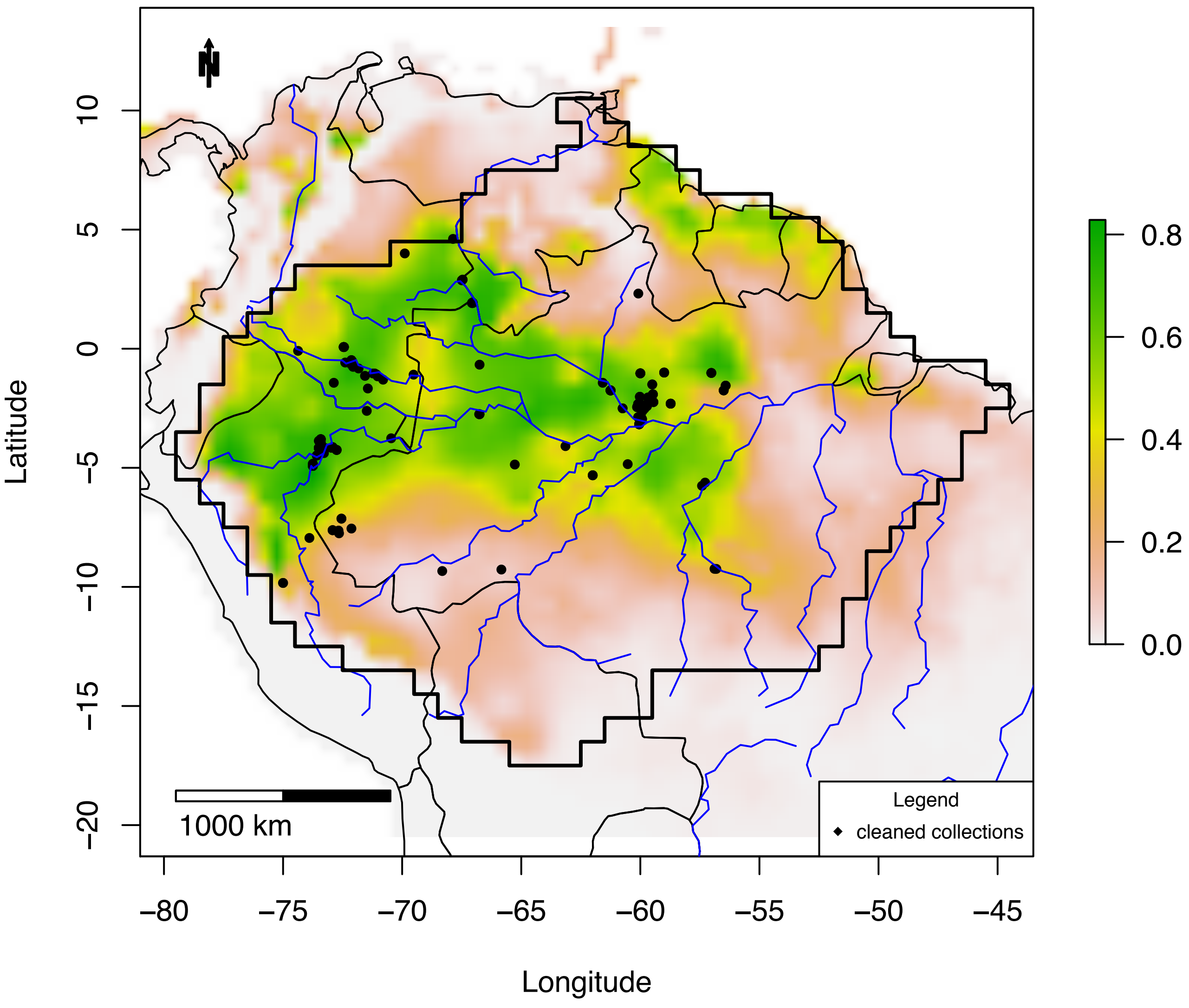

Figure S3_129C IDW map of Protium_hebetatum: plots =0 – 0.14; IDW: 0 – 0.12

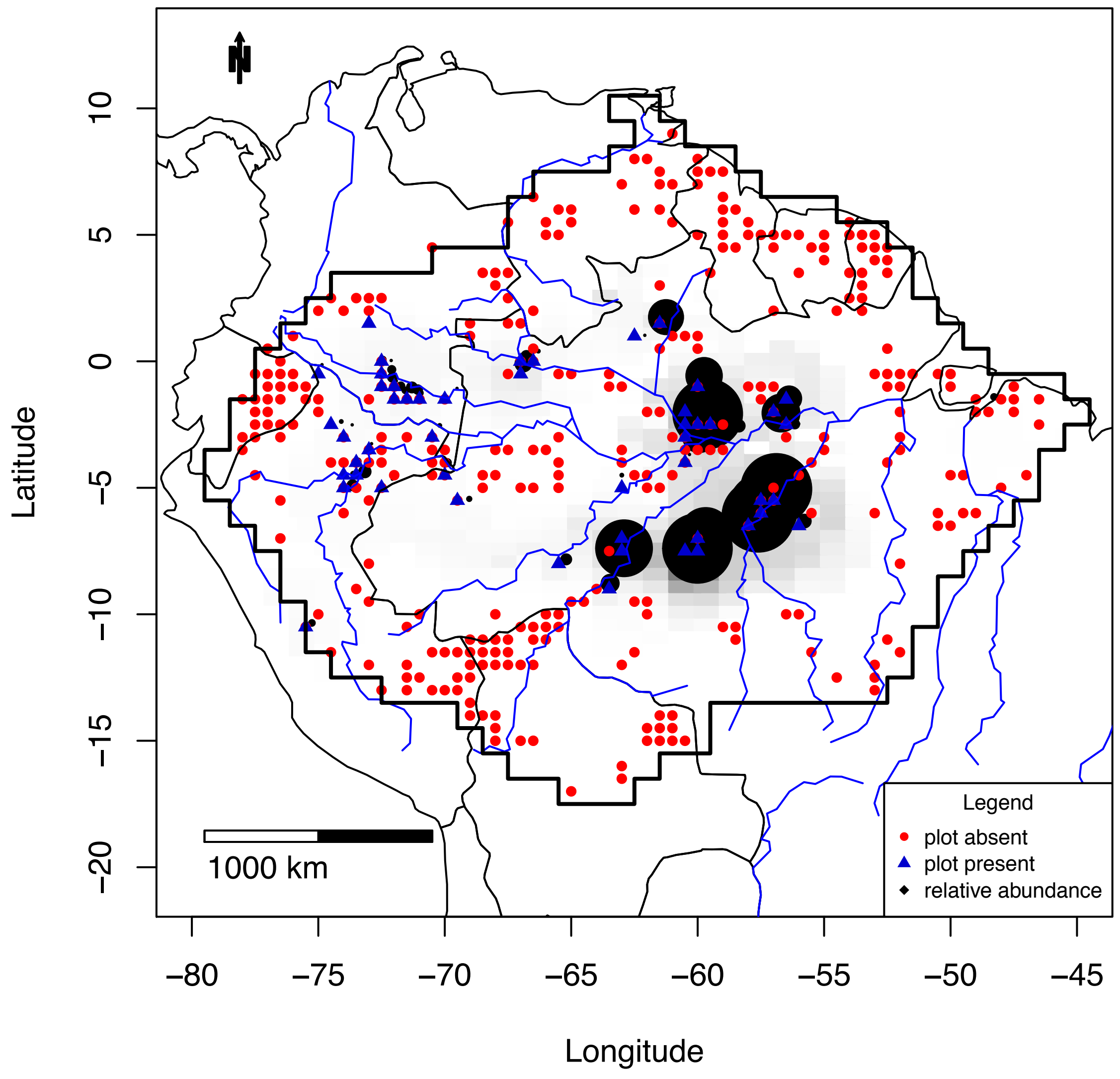

Figure S3_130A Combined map of Protium_sagotianum

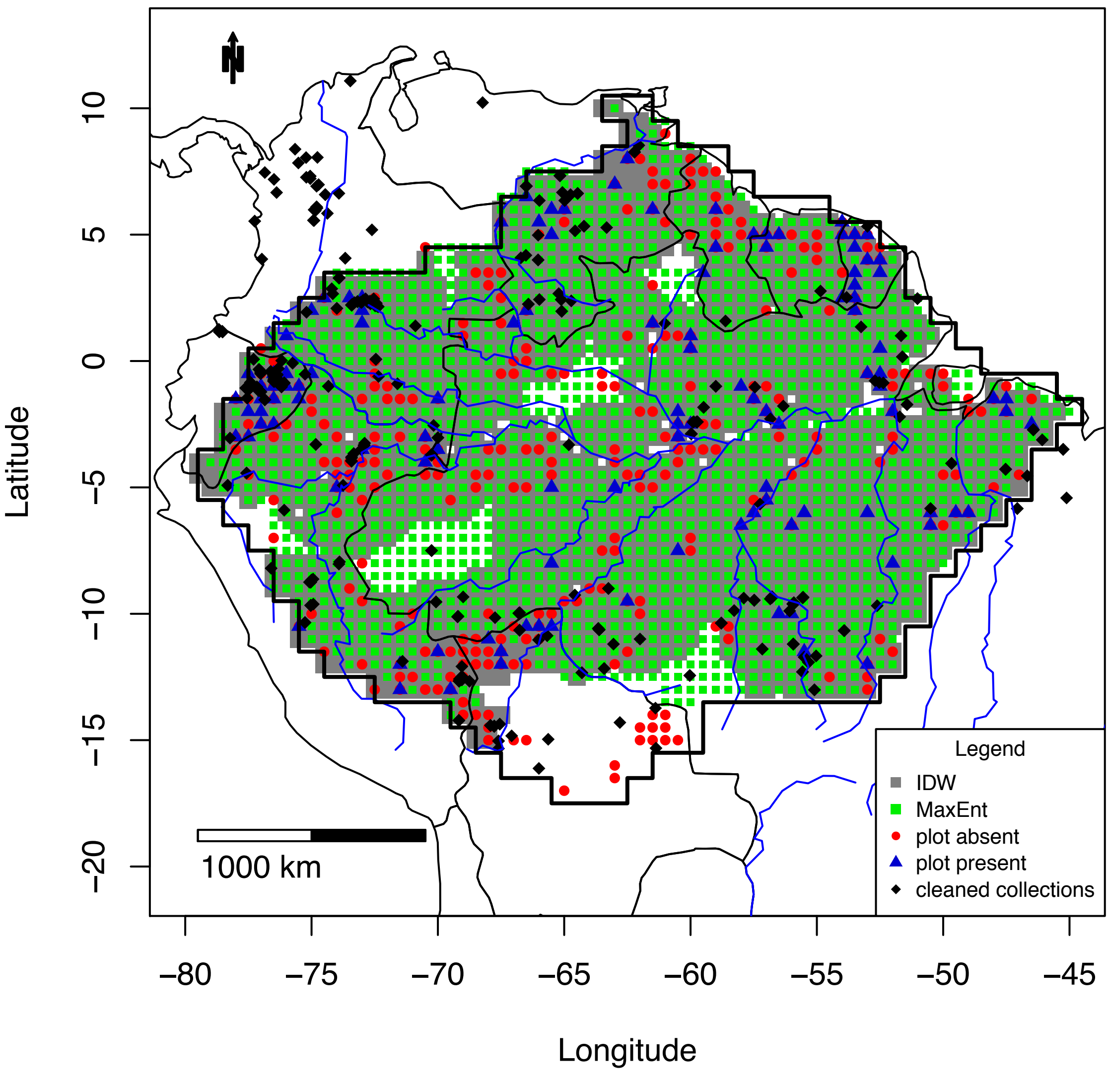

Figure S3_130B MaxEnt map of Protium_sagotianum

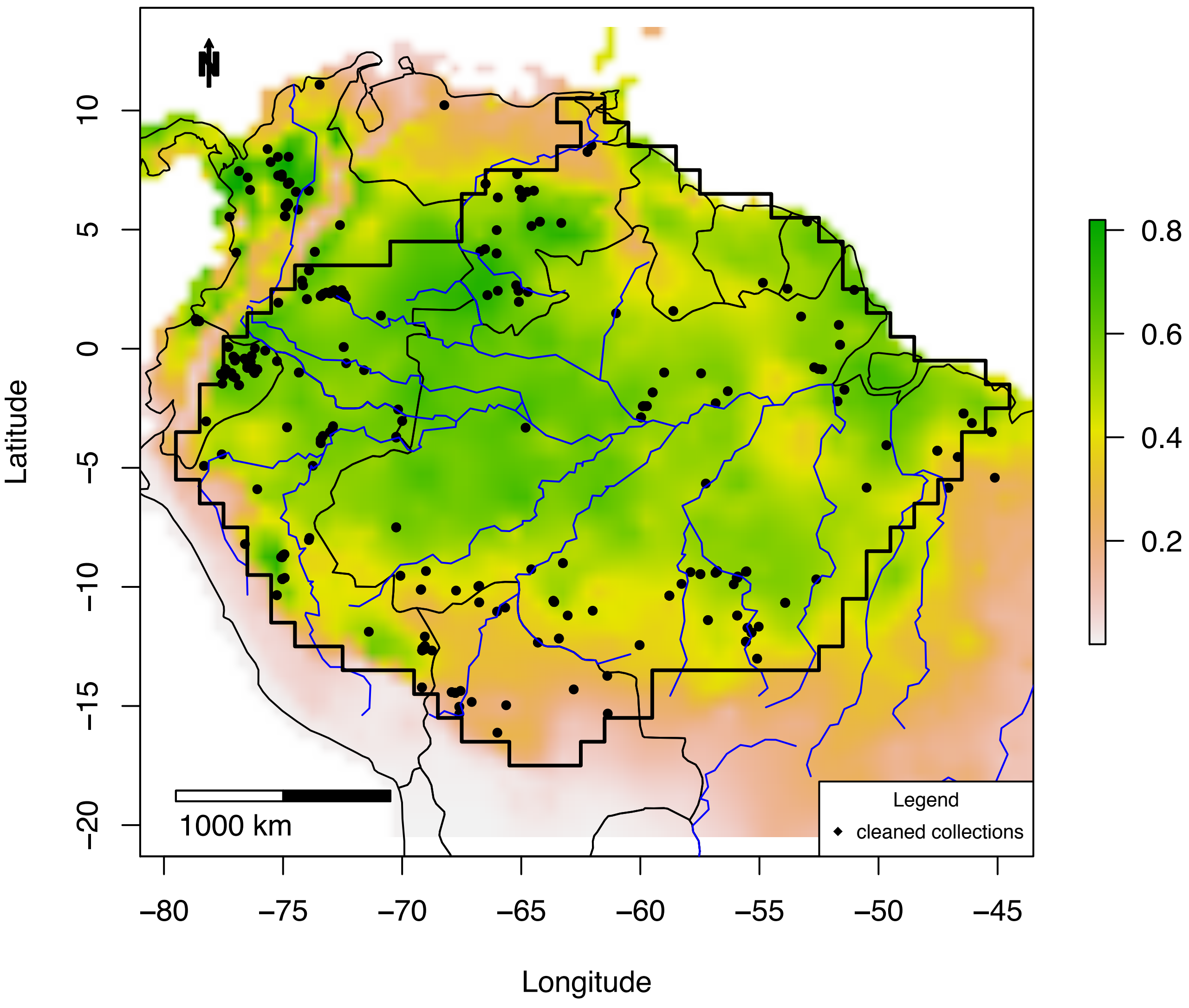

Figure S3_130C IDW map of Protium_sagotianum: plots =0 – 0.16; IDW: 0 – 0.16

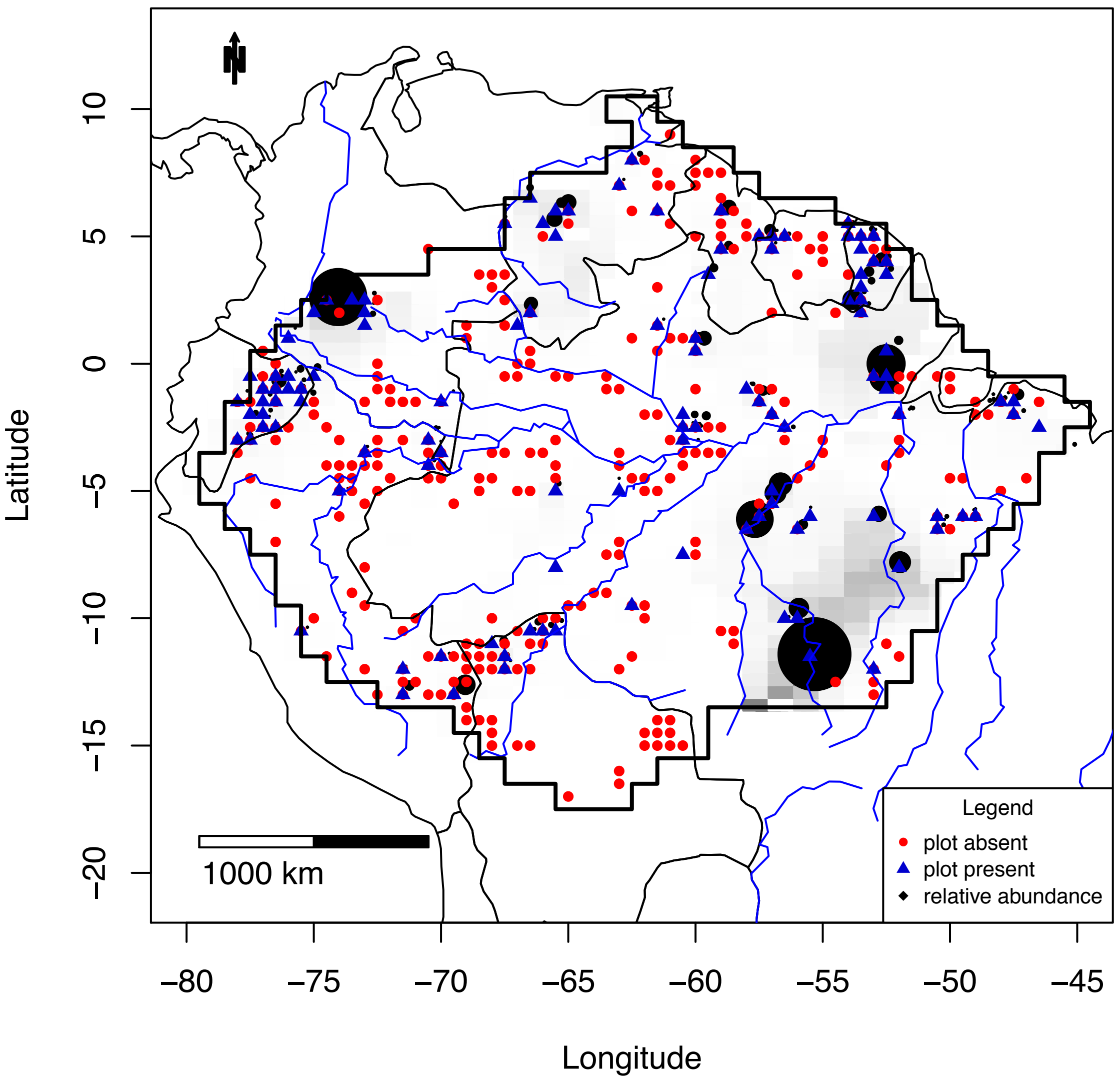

Figure S3_131A Combined map of Protium_tenuifolium

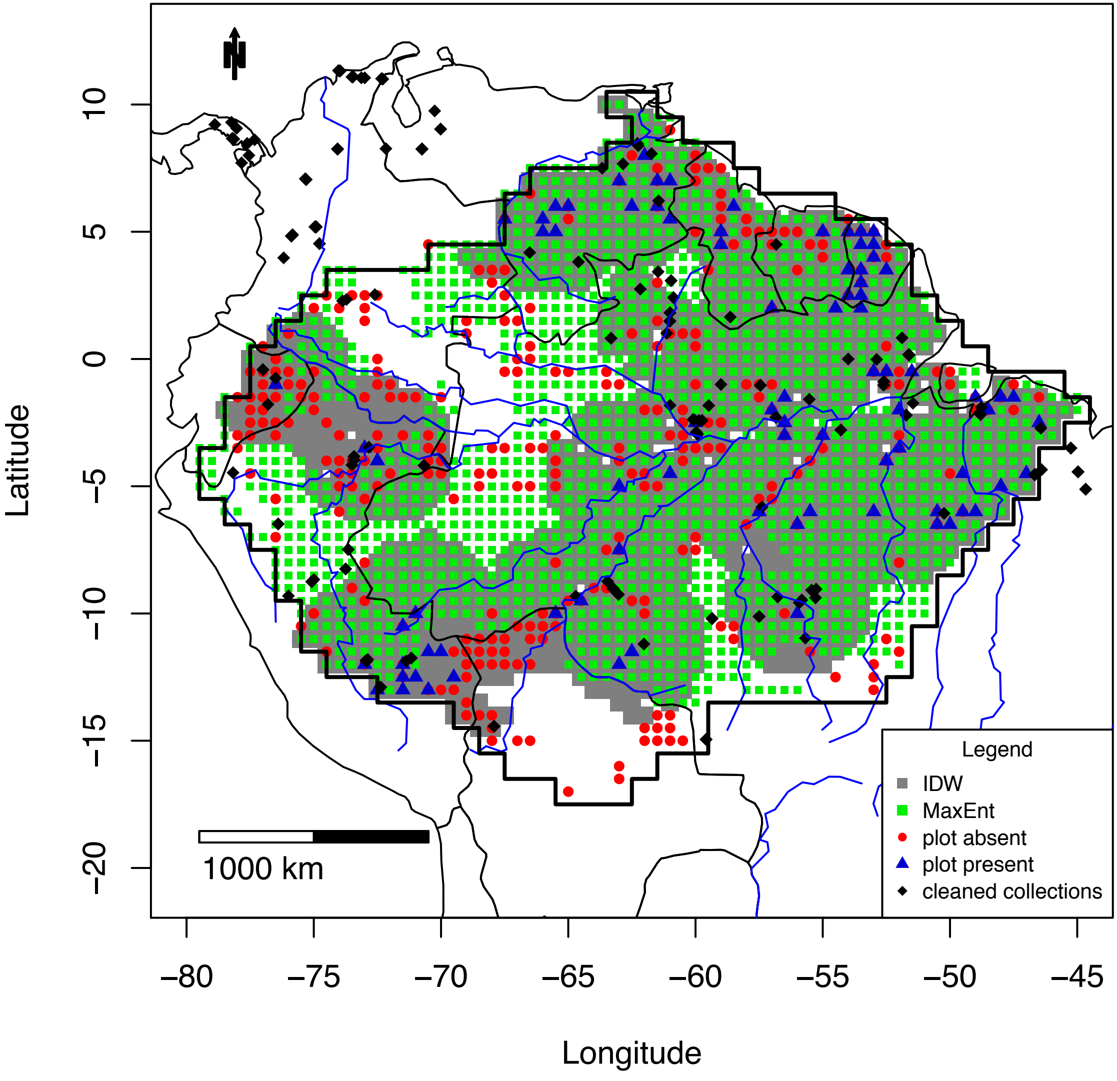

Figure S3_131B MaxEnt map of Protium_tenuifolium

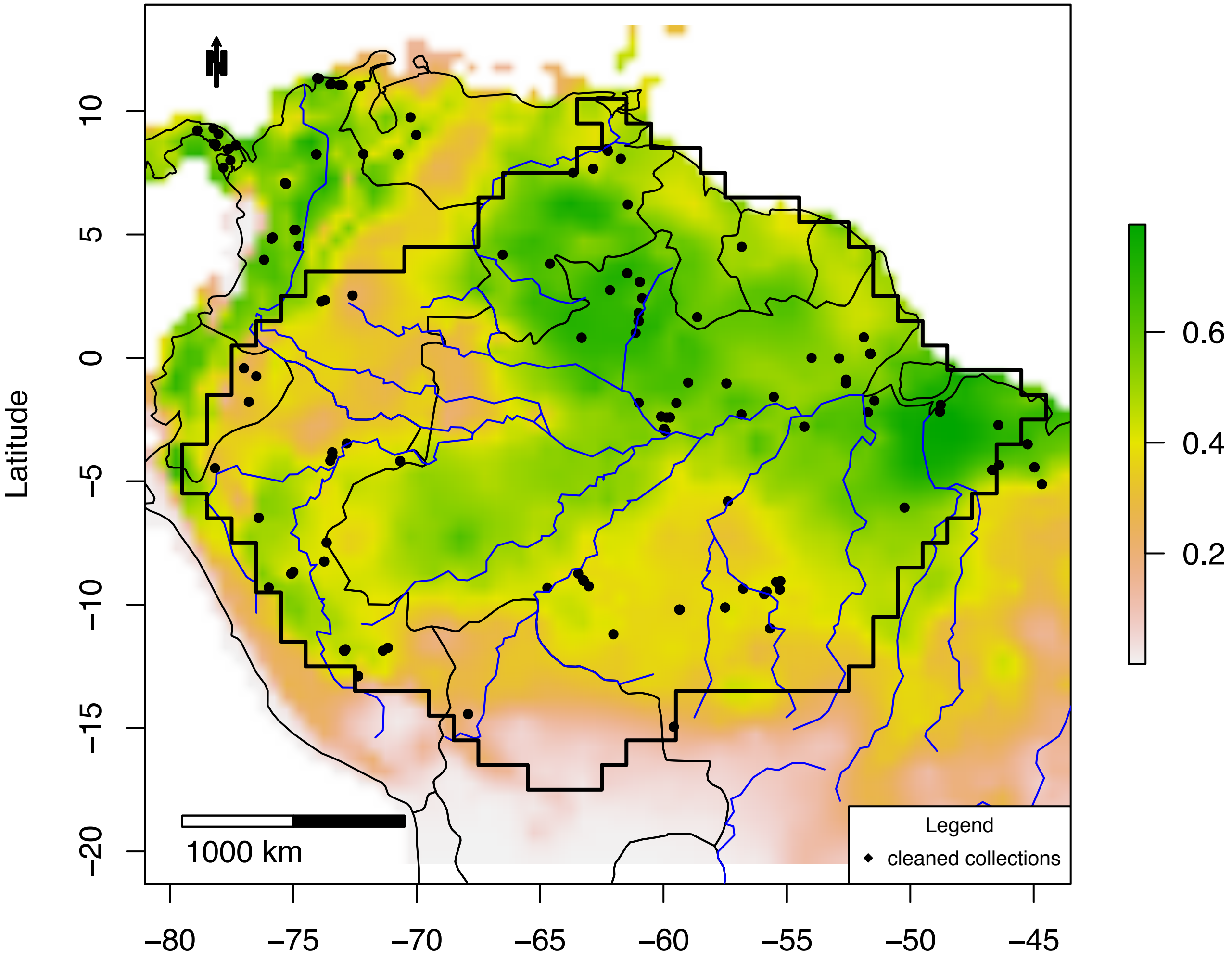

Figure S3_131C IDW map of Protium_tenuifolium: plots =0 – 0.06; IDW: 0 – 0.04

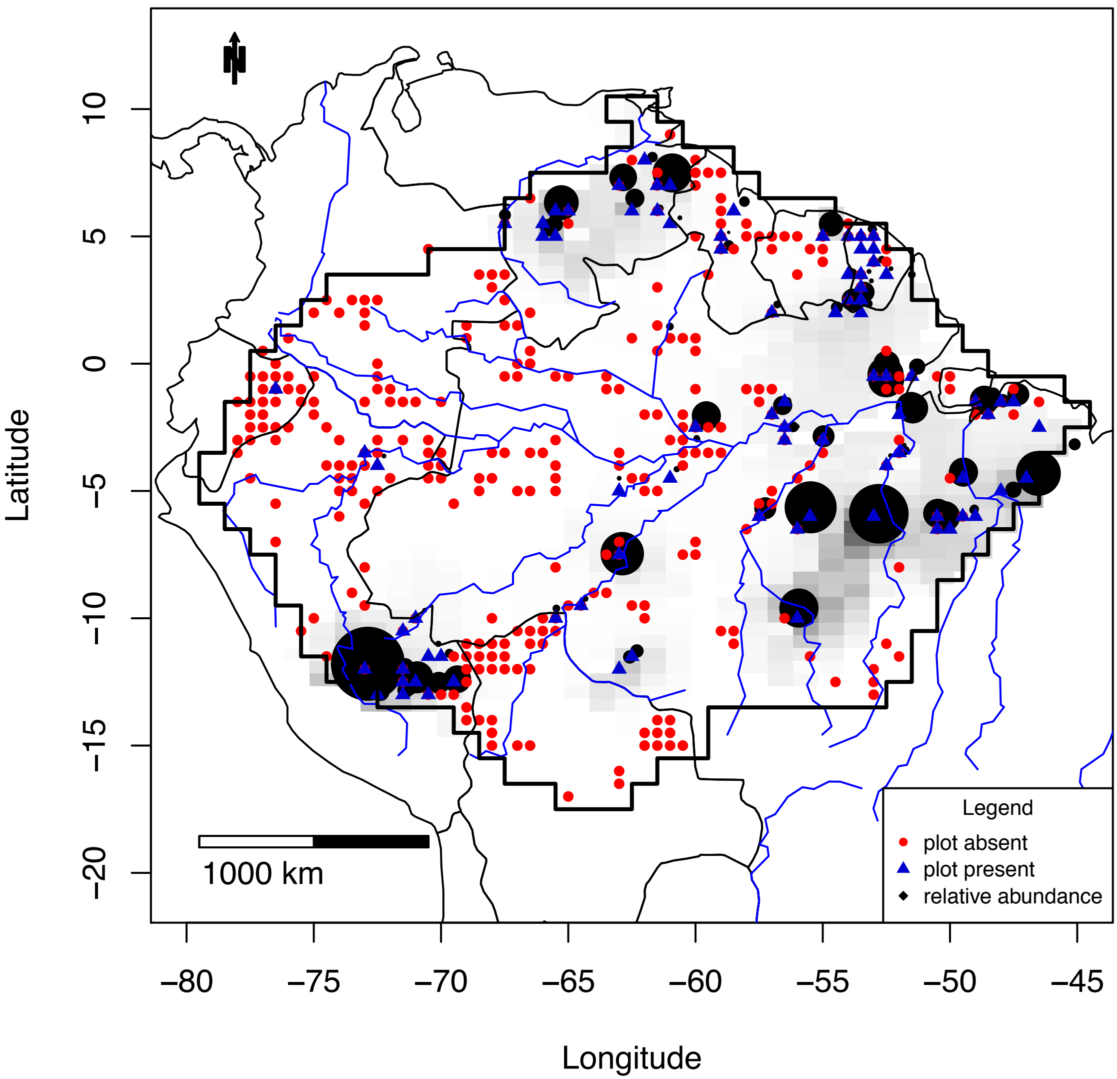

Figure S3_132A Combined map of Protium_trifoliolatum

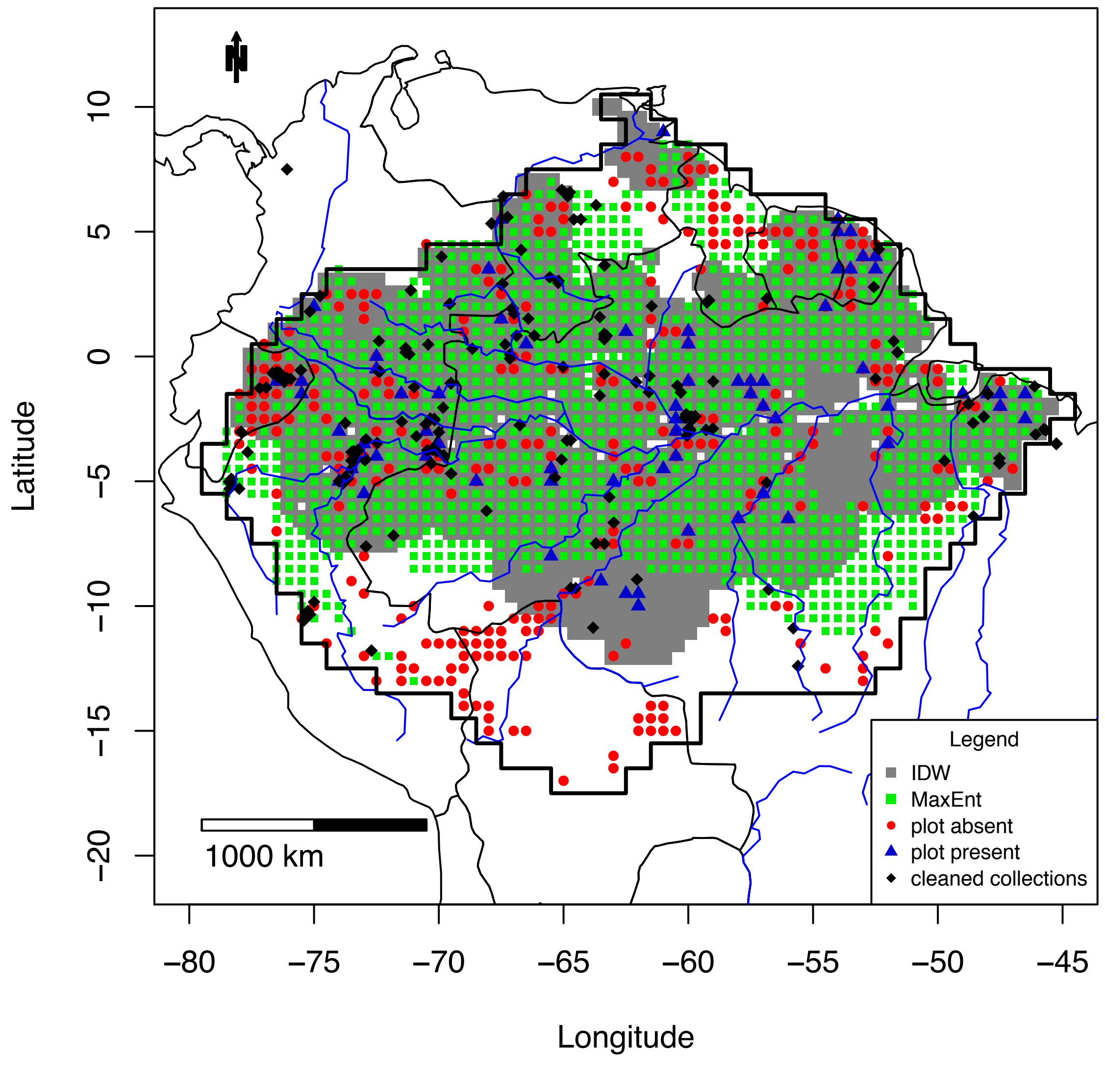

Figure S3_132B MaxEnt map of Protium_trifoliolatum

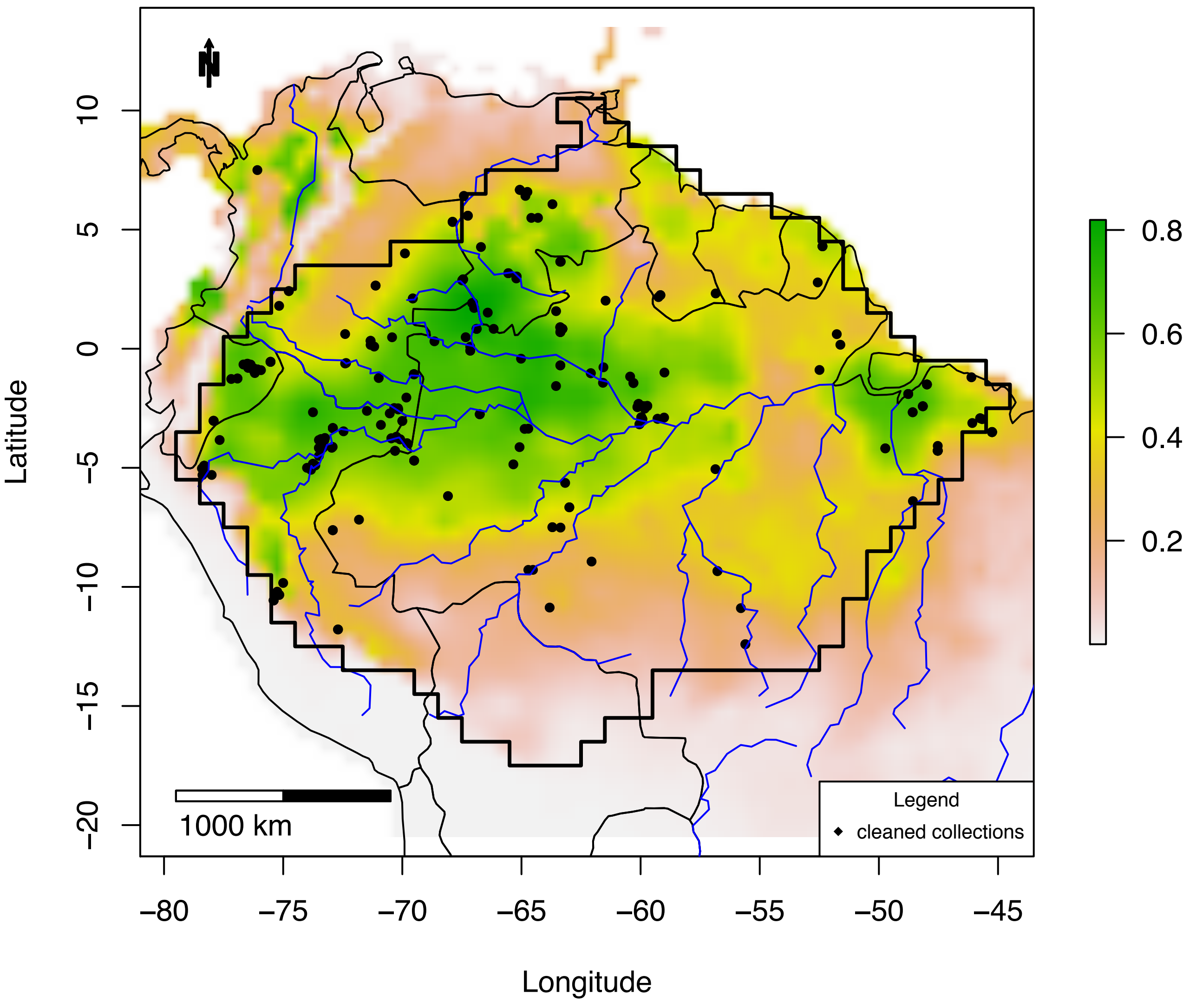

Figure S3_132C IDW map of Protium_trifoliolatum: plots =0 – 0.12; IDW: 0 – 0.04

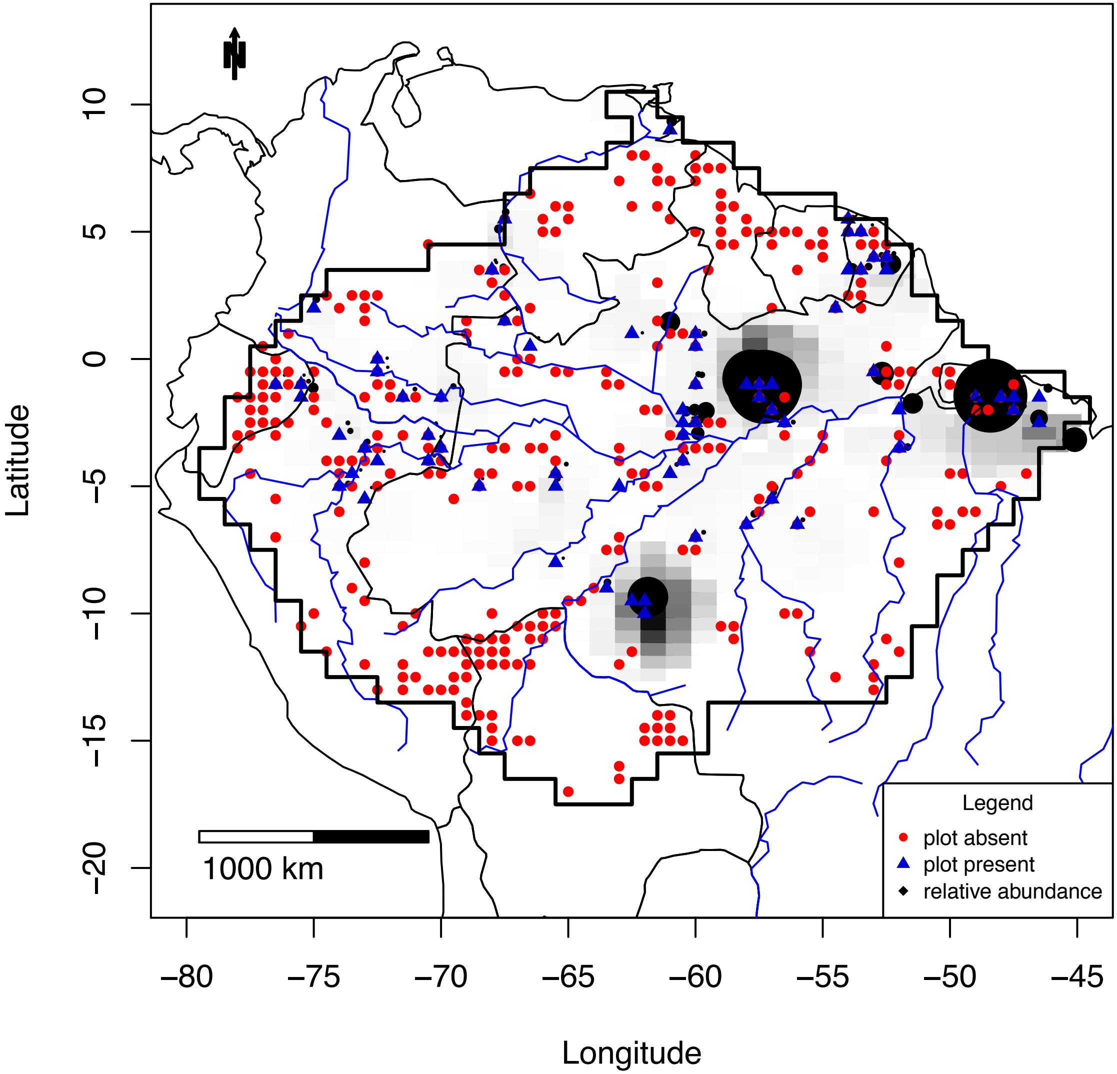

Figure S3_133A Combined map of Pseudobombax_munguba

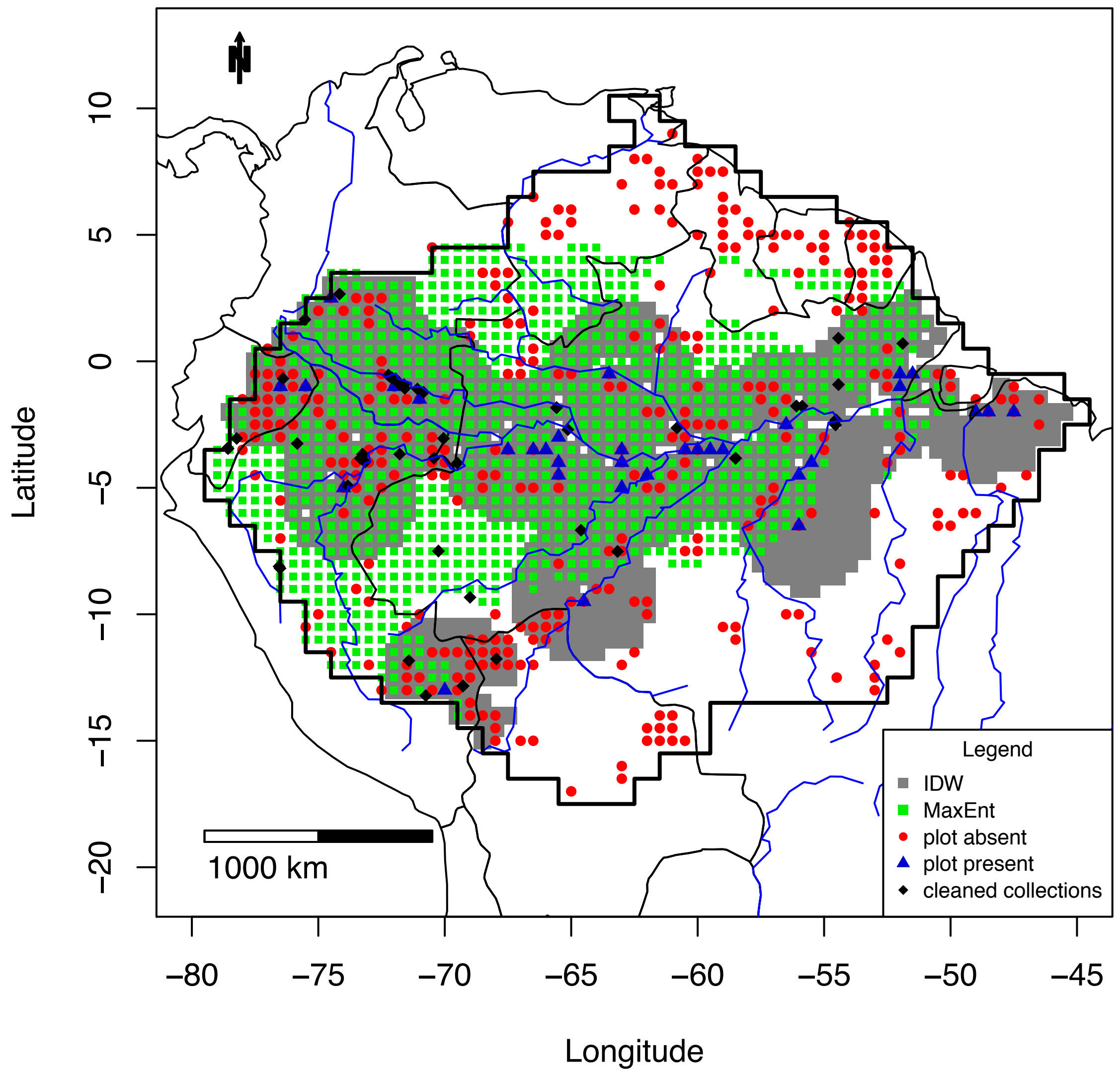

Figure S3_133B MaxEnt map of Pseudobombax_munguba

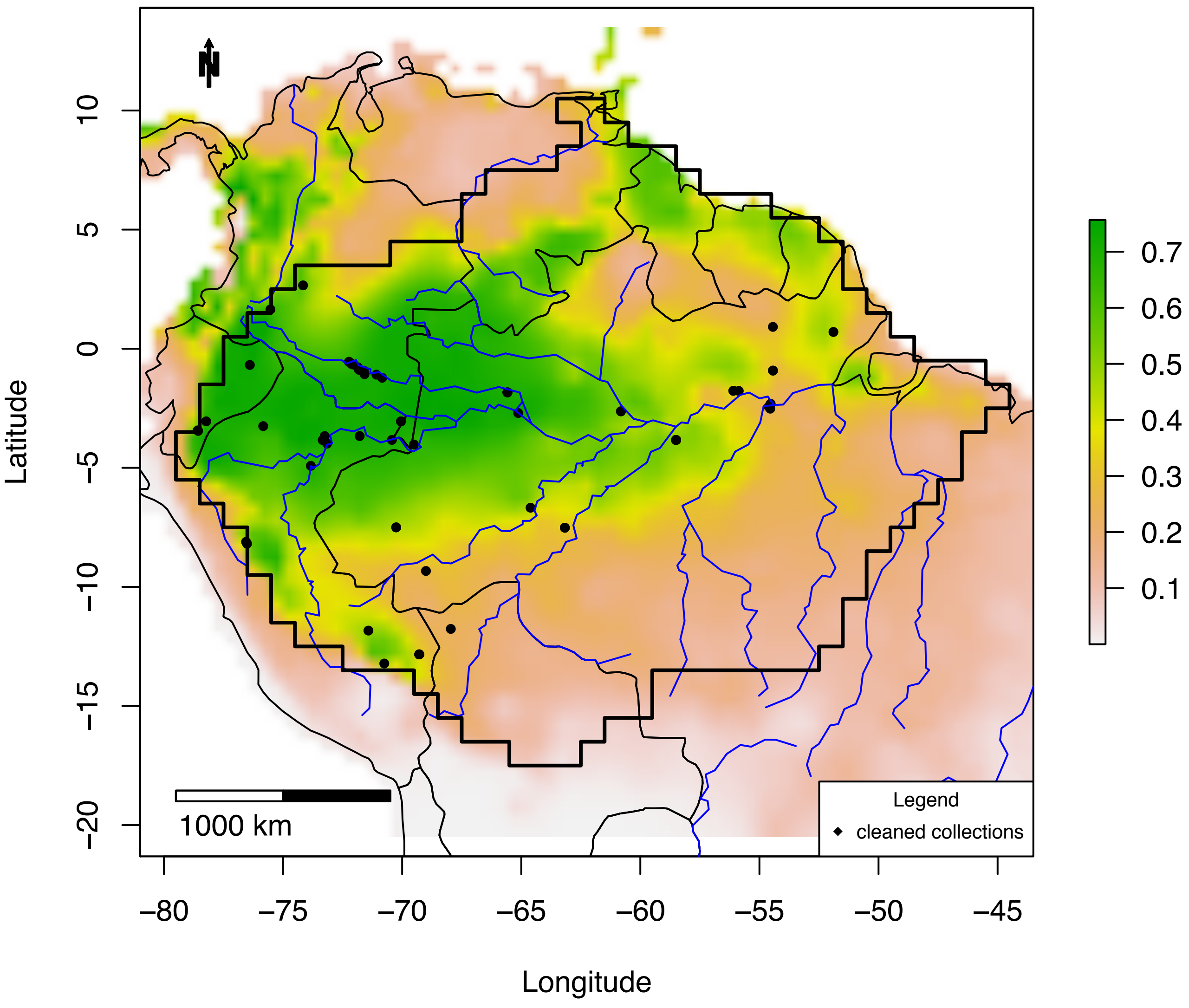

Figure S3_133C IDW map of Pseudobombax_munguba: plots =0 – 0.35; IDW: 0 – 0.06

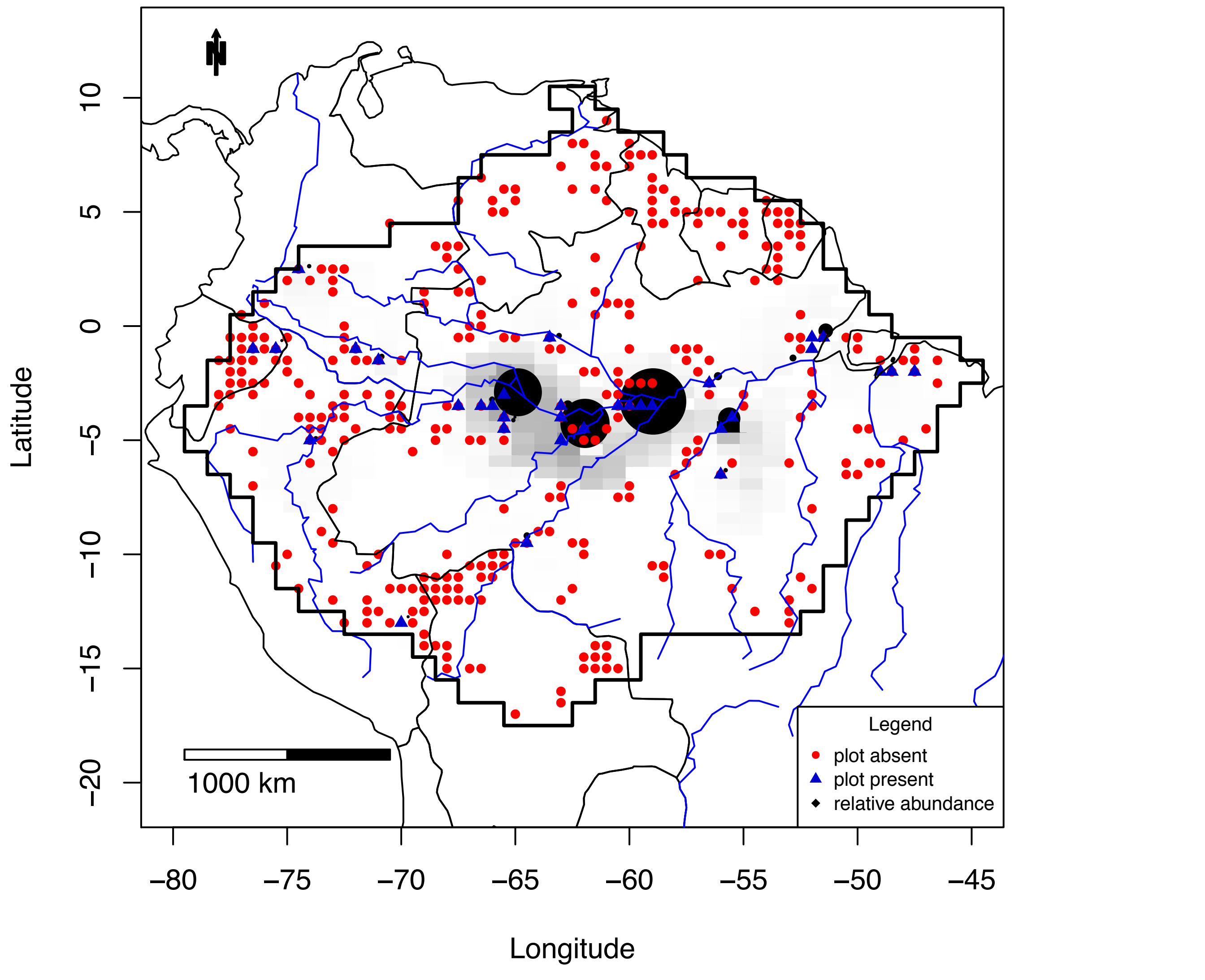

Figure S3_134A Combined map of Pseudolmedia_laevigata

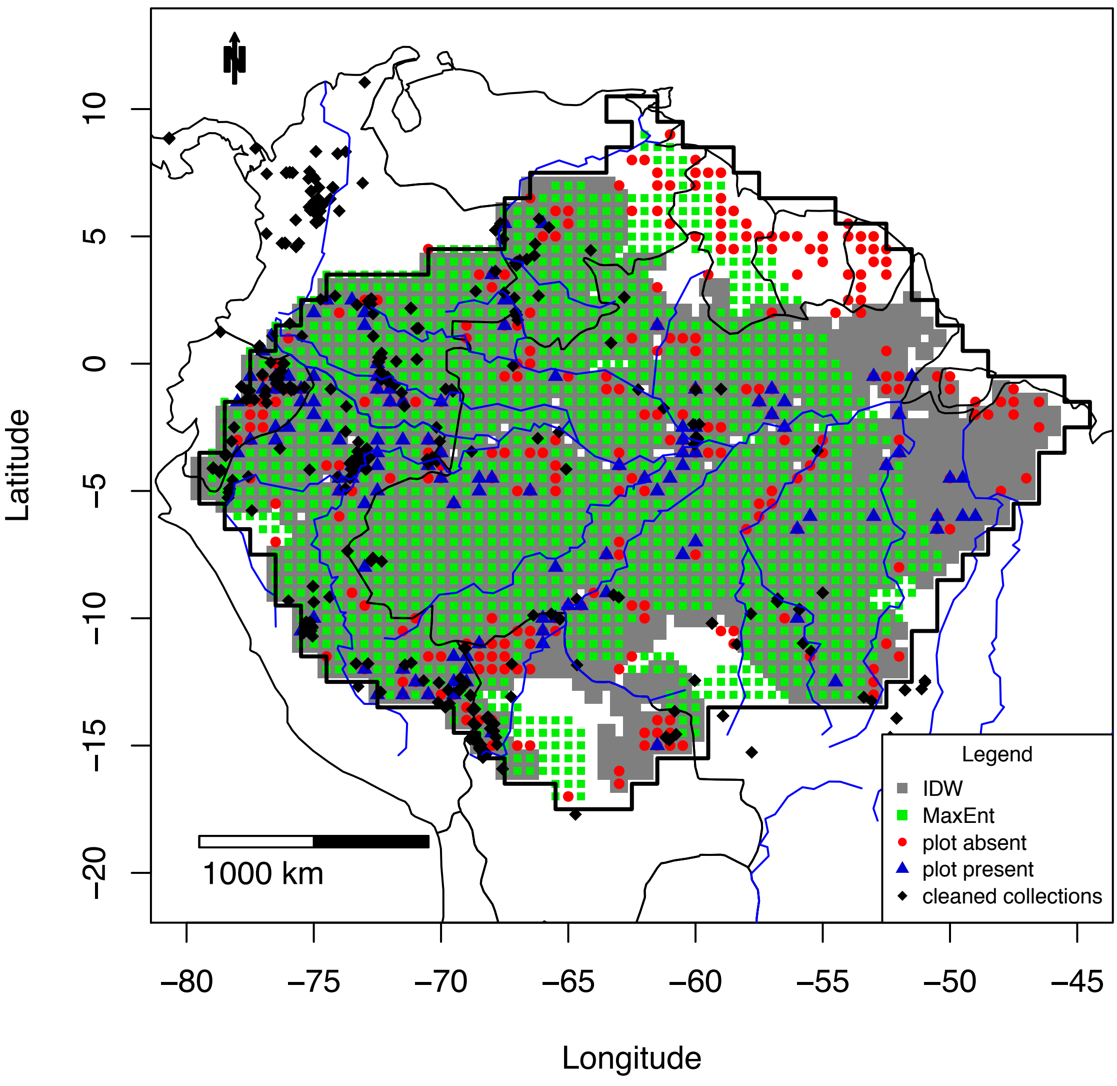

Figure S3_134B MaxEnt map of Pseudolmedia_laevigata

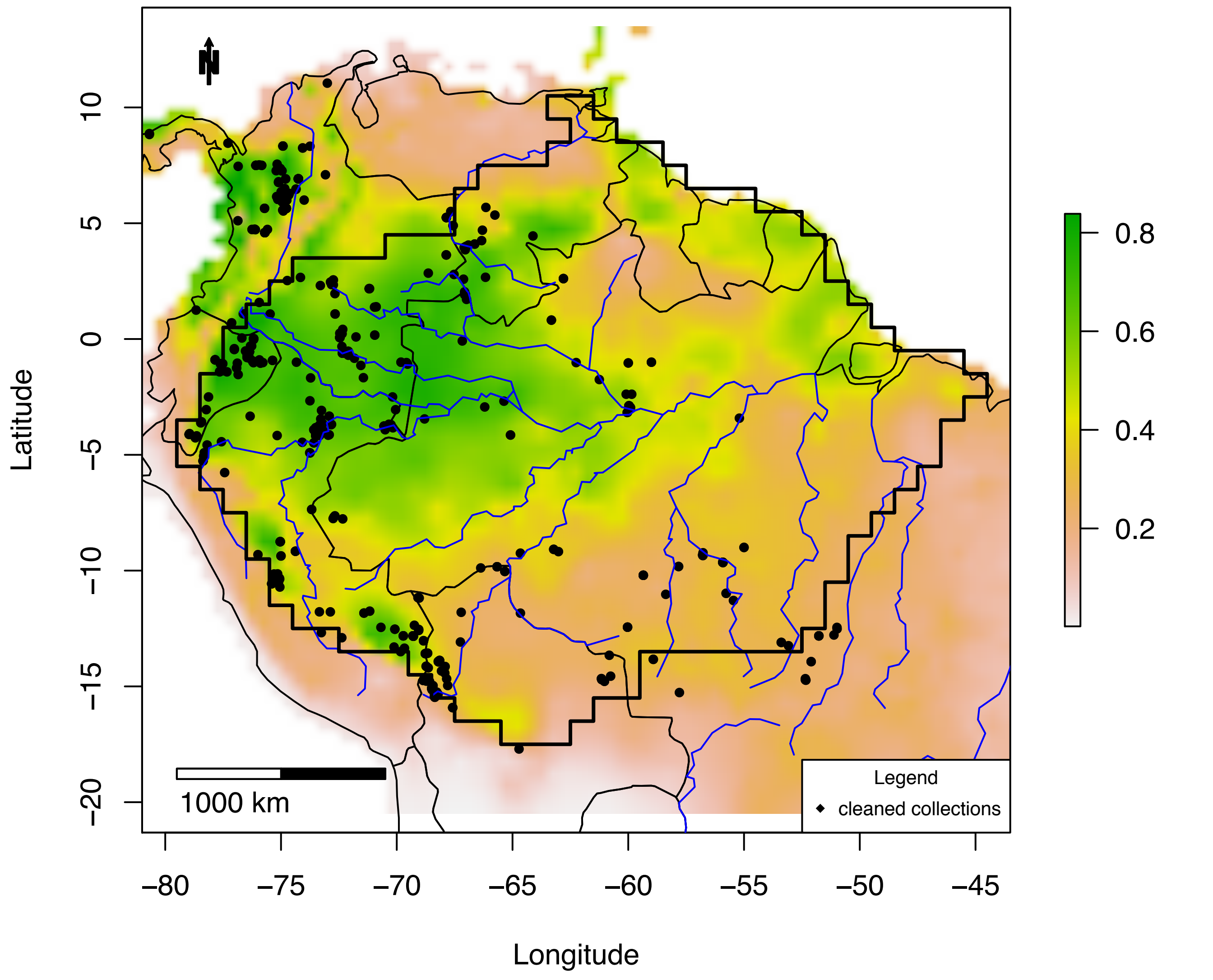

Figure S3_134C IDW map of Pseudolmedia_laevigata: plots =0 – 0.12; IDW: 0 – 0.07

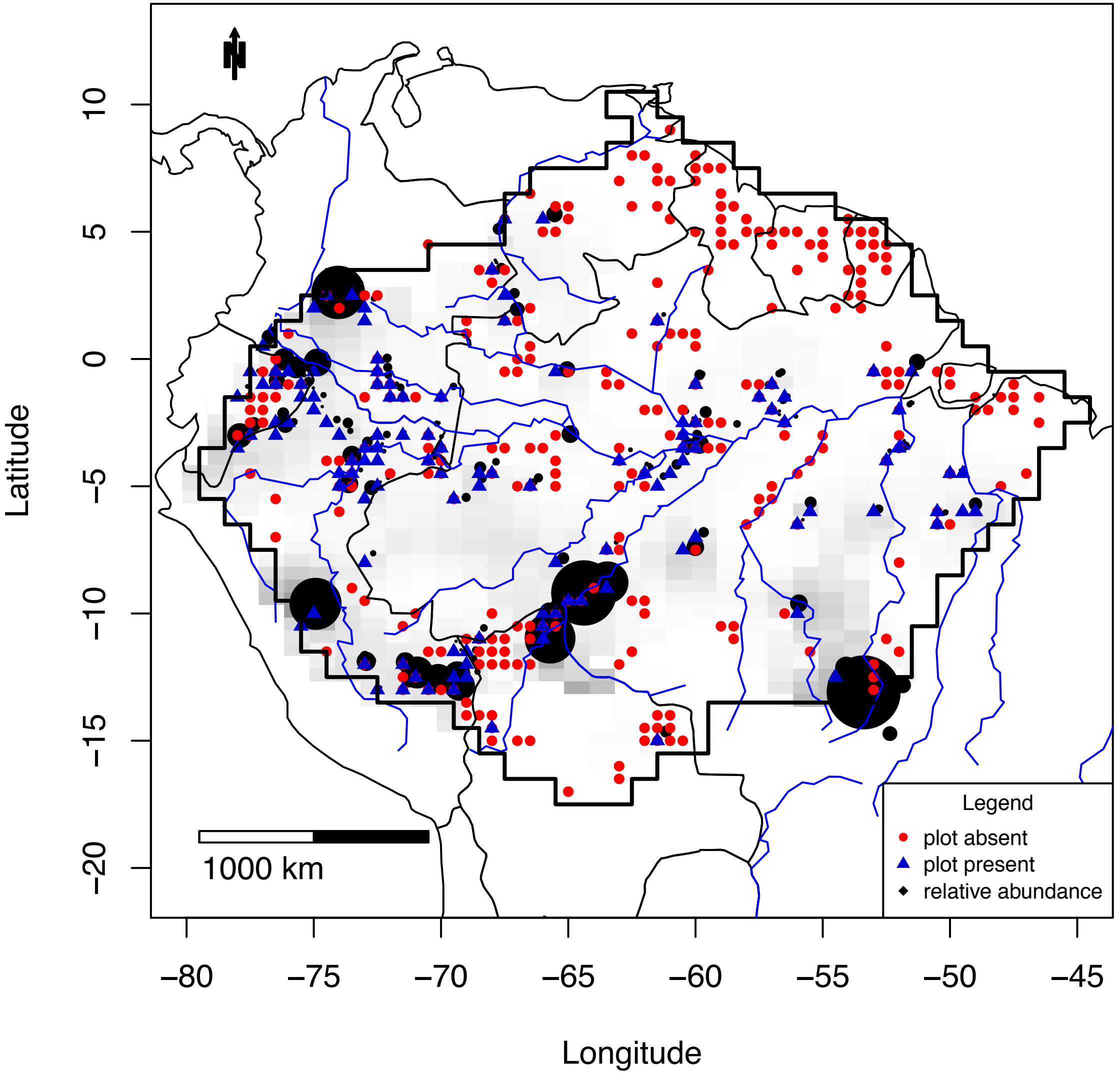

Figure S3_135A Combined map of Pseudolmedia_laevis

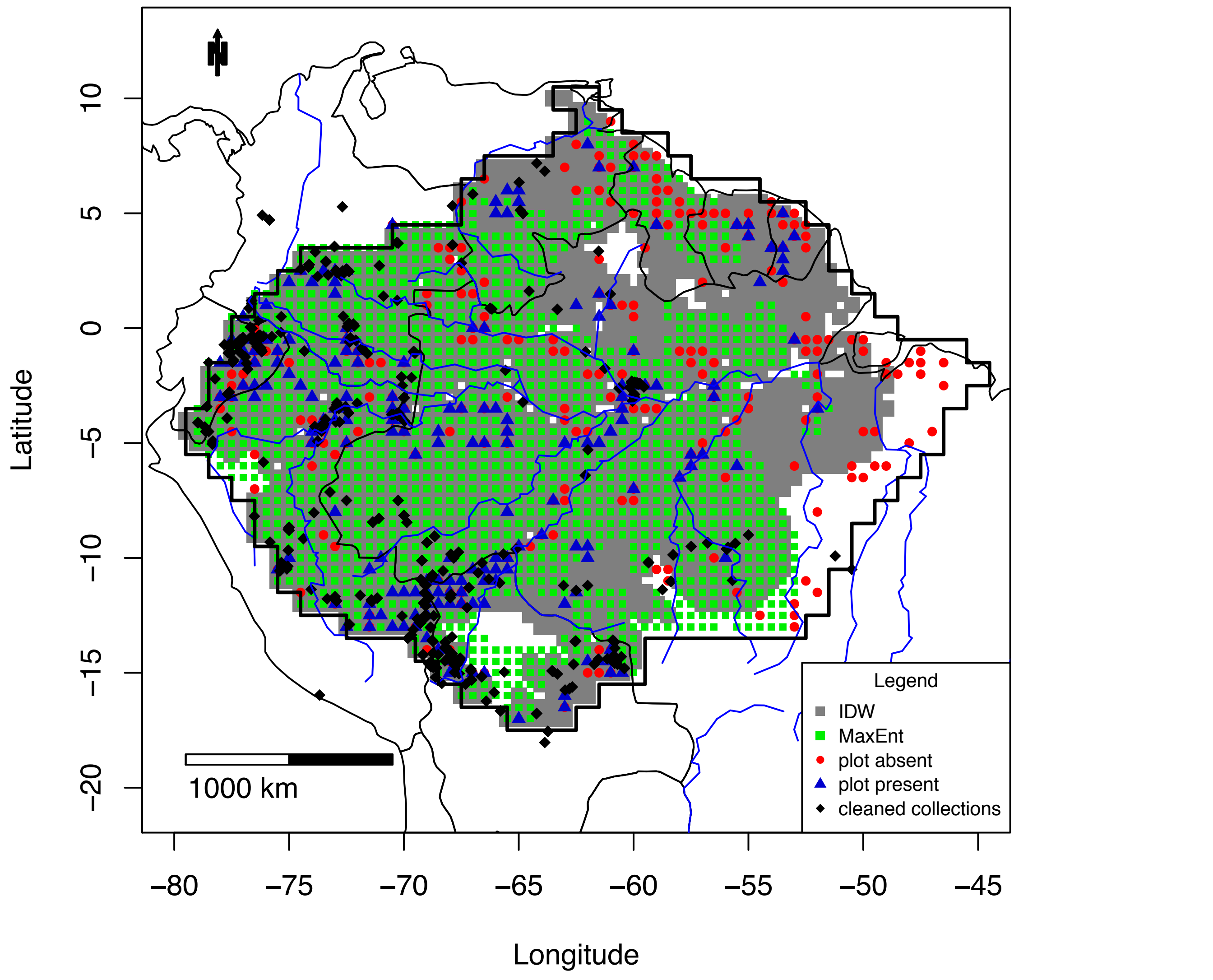

Figure S3_135B MaxEnt map of Pseudolmedia_laevis

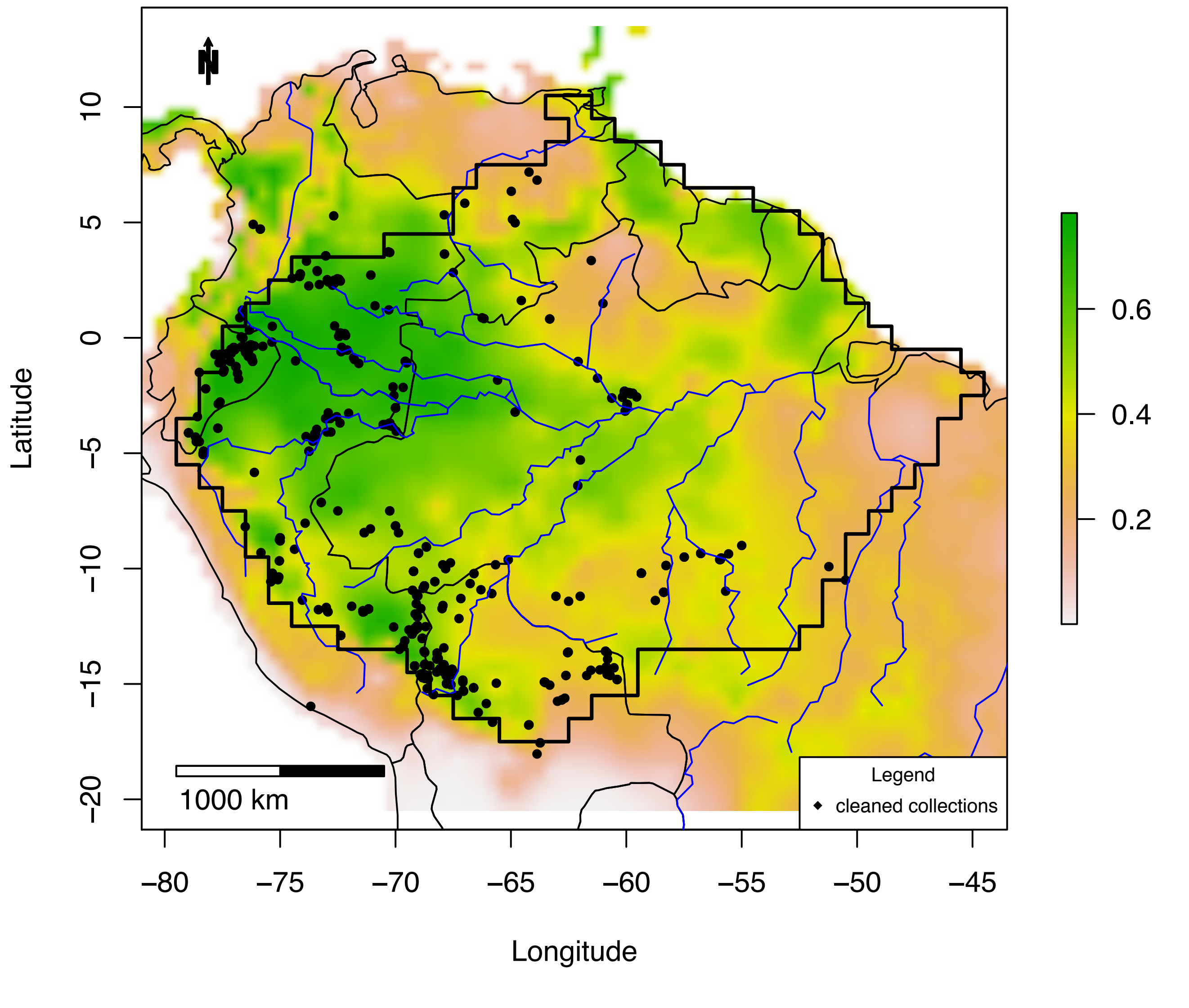

Figure S3_135C IDW map of Pseudolmedia_laevis: plots =0 – 0.36; IDW: 0 – 0.24

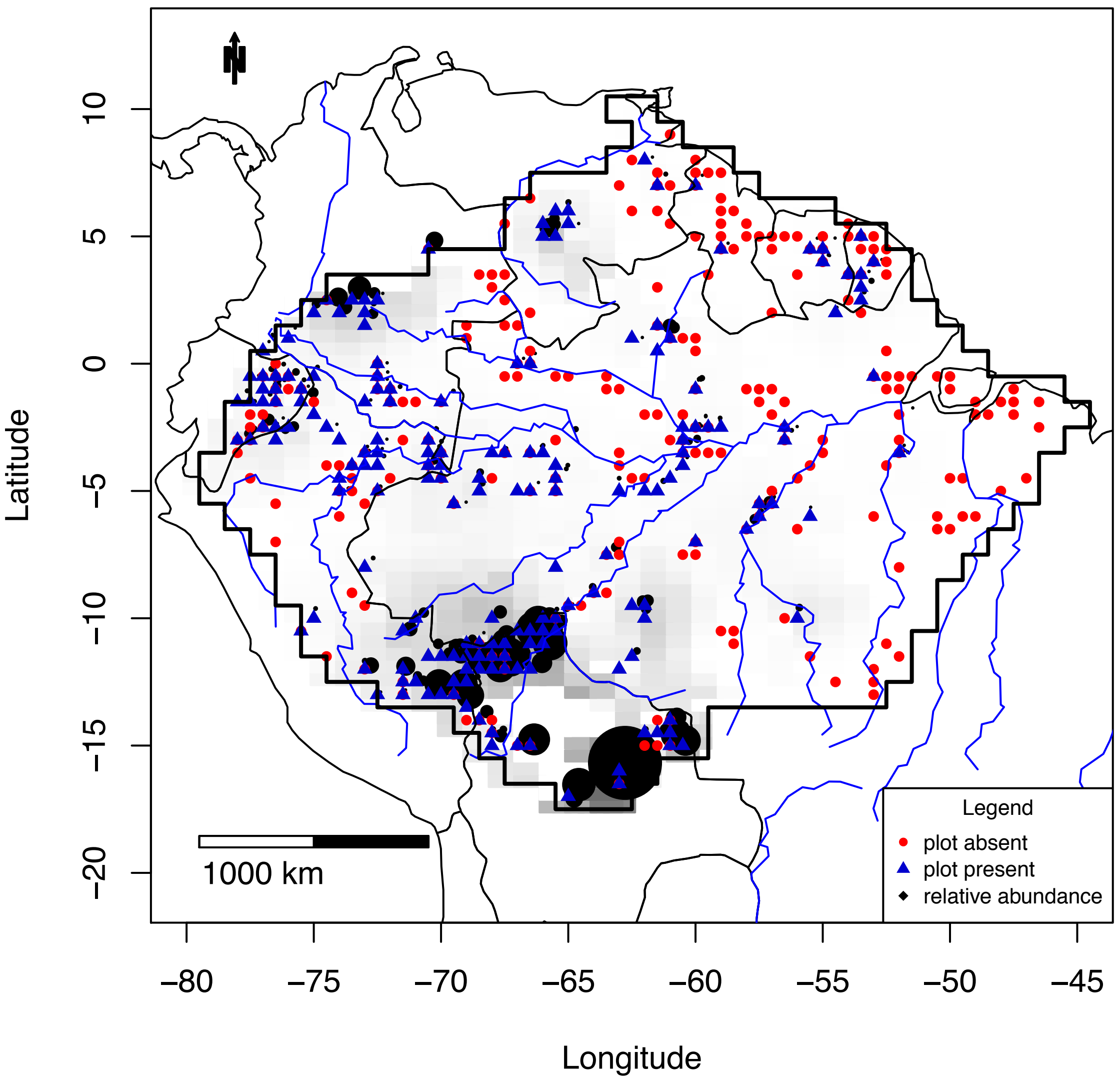

Figure S3_136A Combined map of Pseudopiptadenia_suaveolens

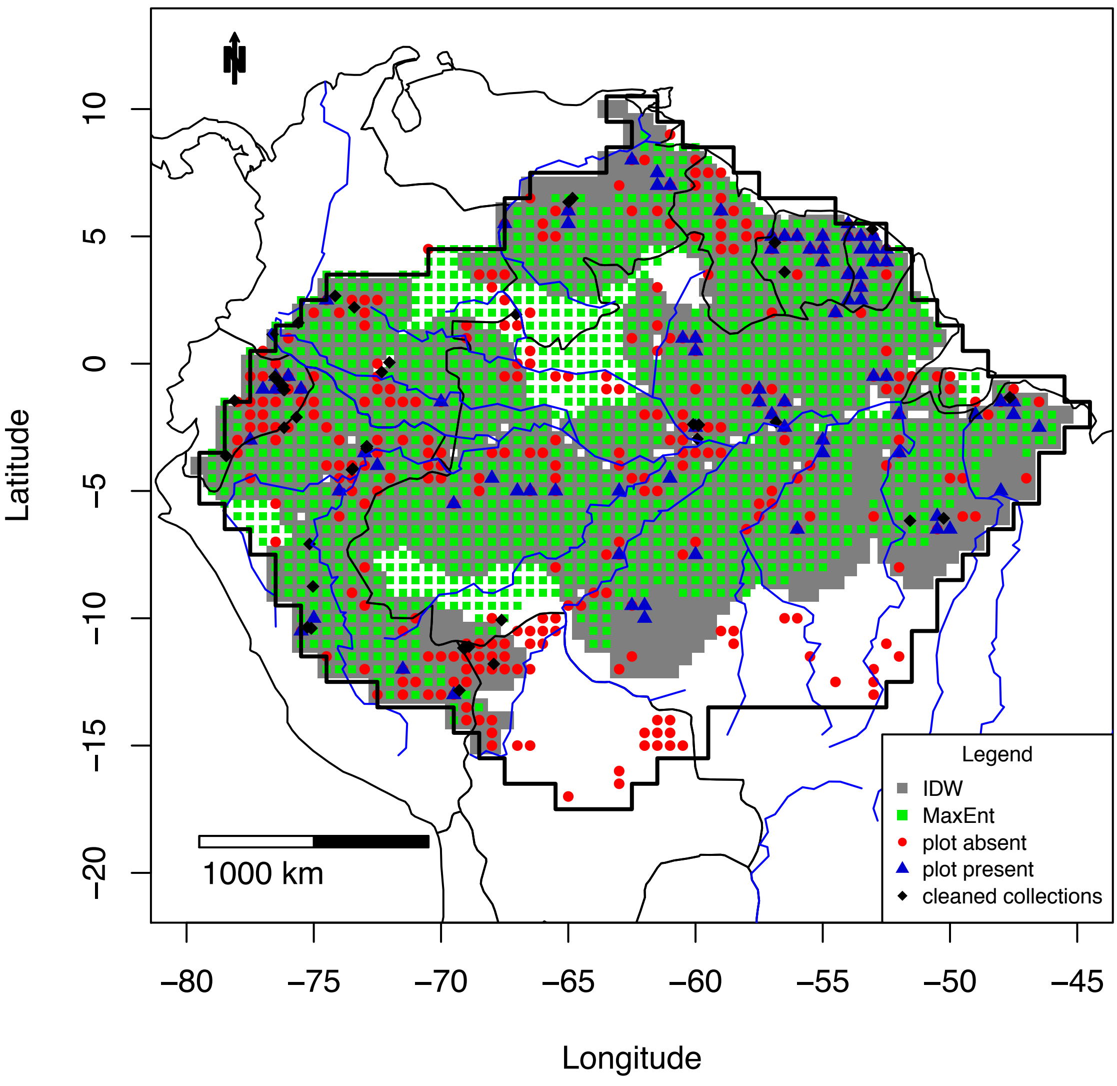

Figure S3_136B MaxEnt map of Pseudopiptadenia_suaveolens

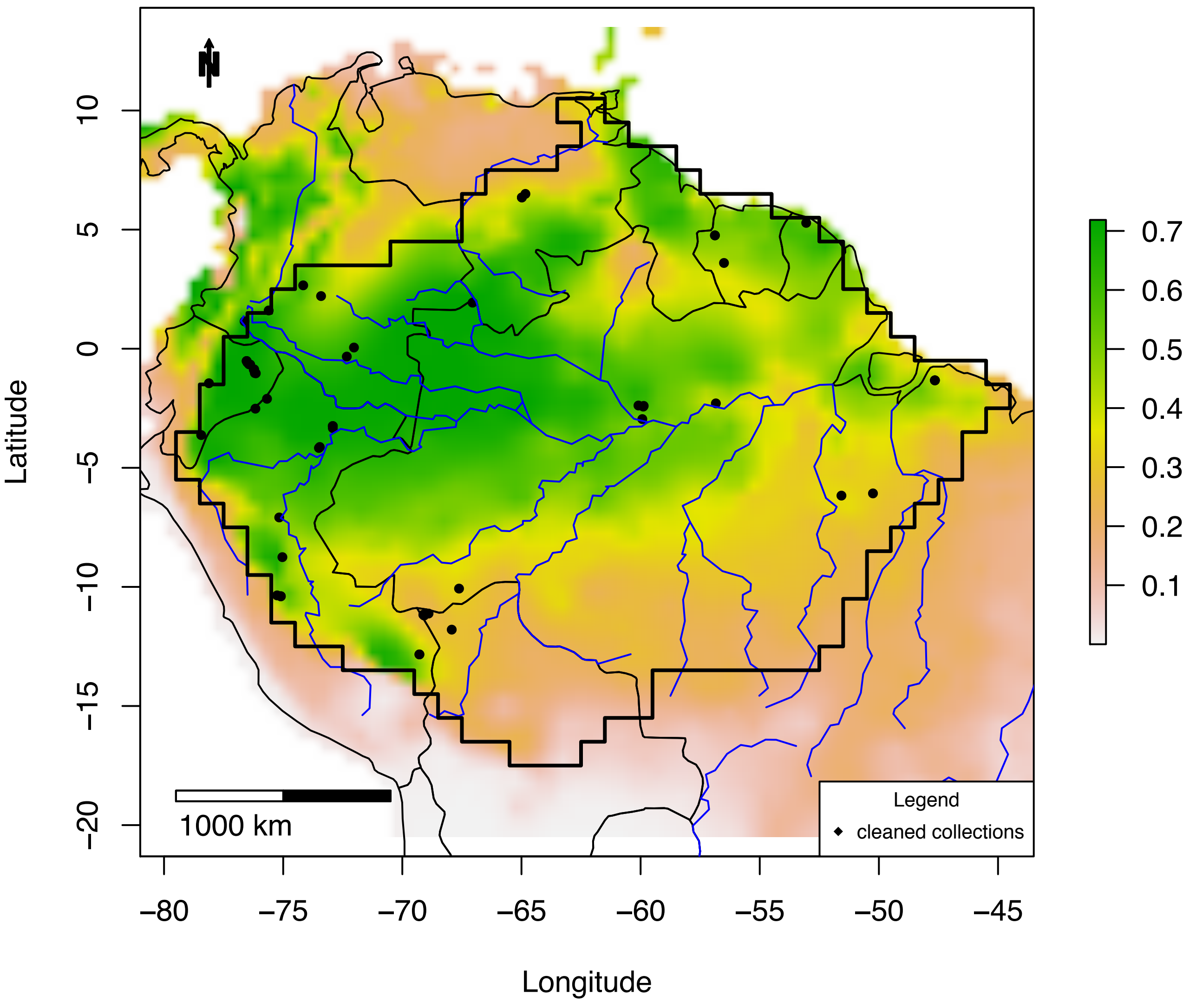

Figure S3_136C IDW map of Pseudopiptadenia_suaveolens: plots =0 – 0.03; IDW: 0 – 0.01

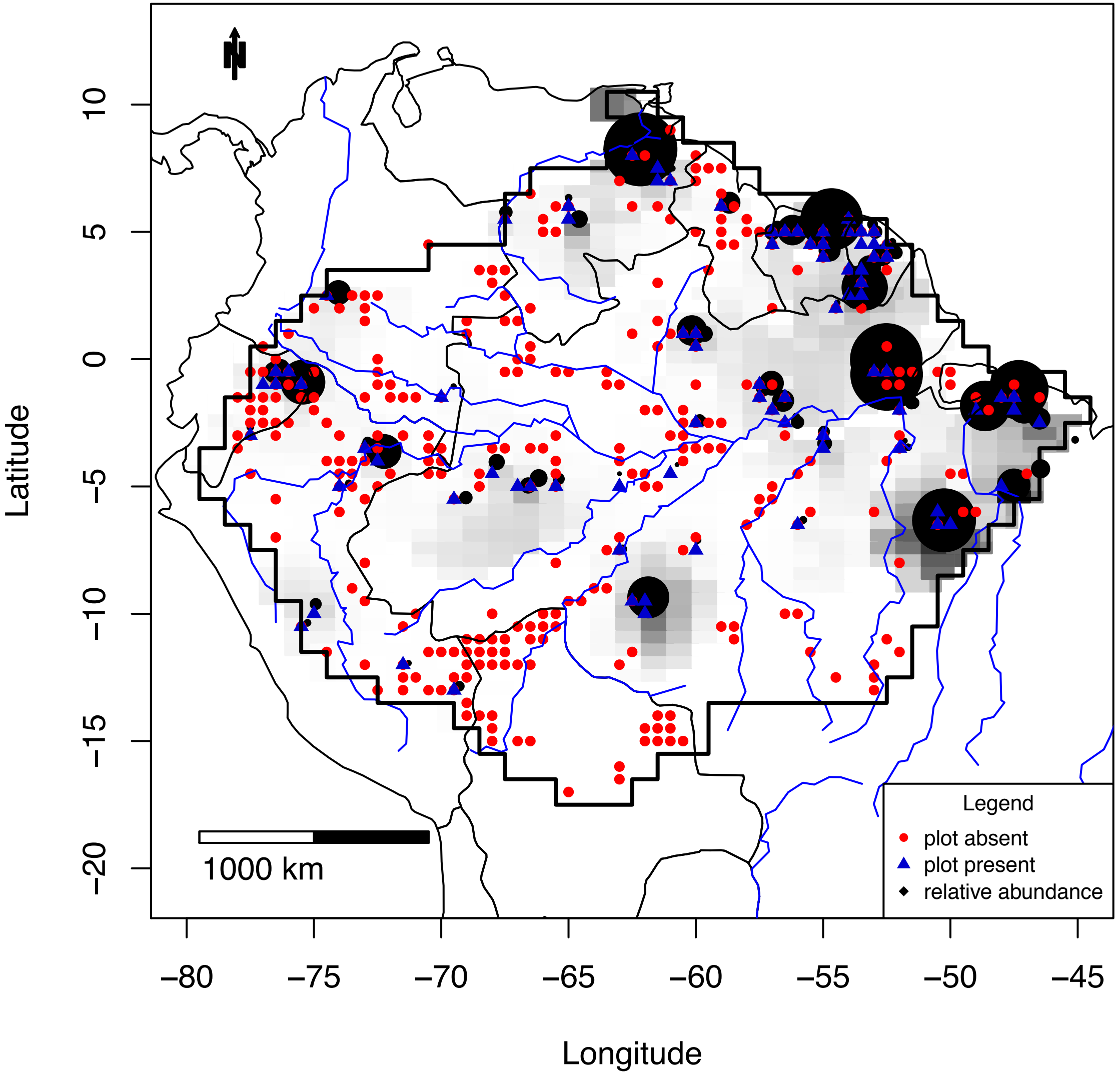

Figure S3_137A Combined map of Pterocarpus_amazonum

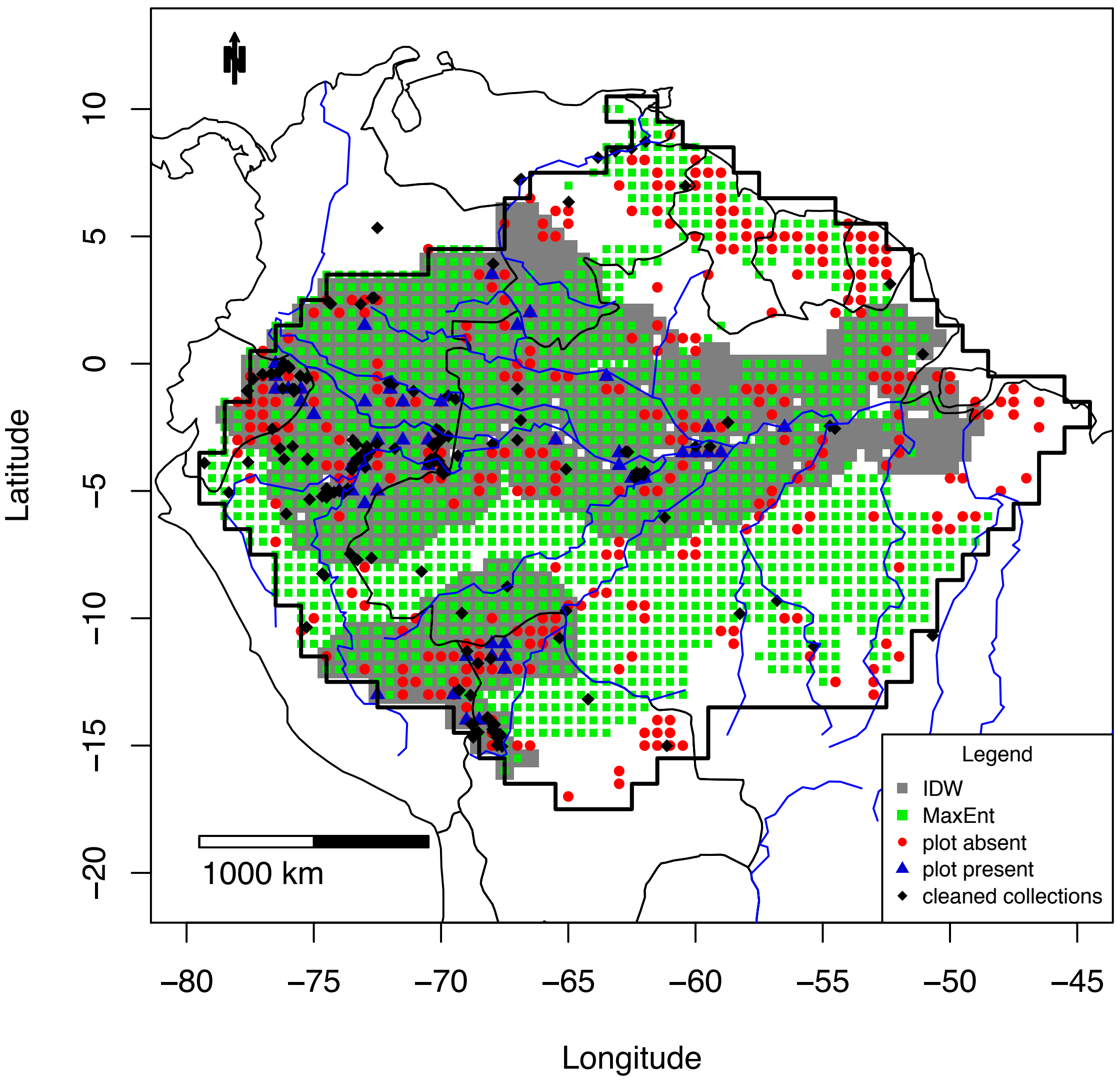

Figure S3_137B MaxEnt map of Pterocarpus_amazonum

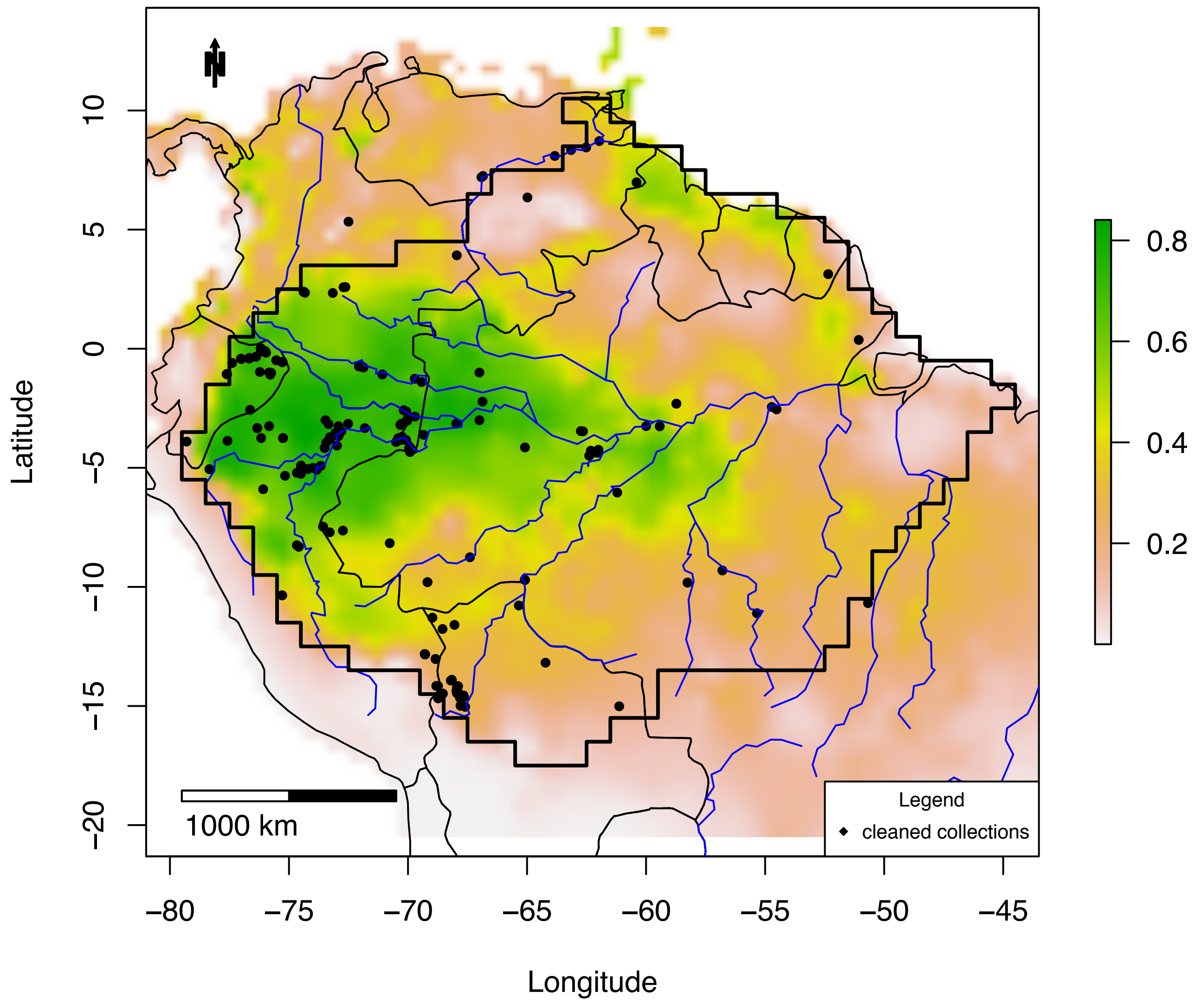

Figure S3_137C IDW map of Pterocarpus_amazonum: plots =0 – 0.22; IDW: 0 – 0.03

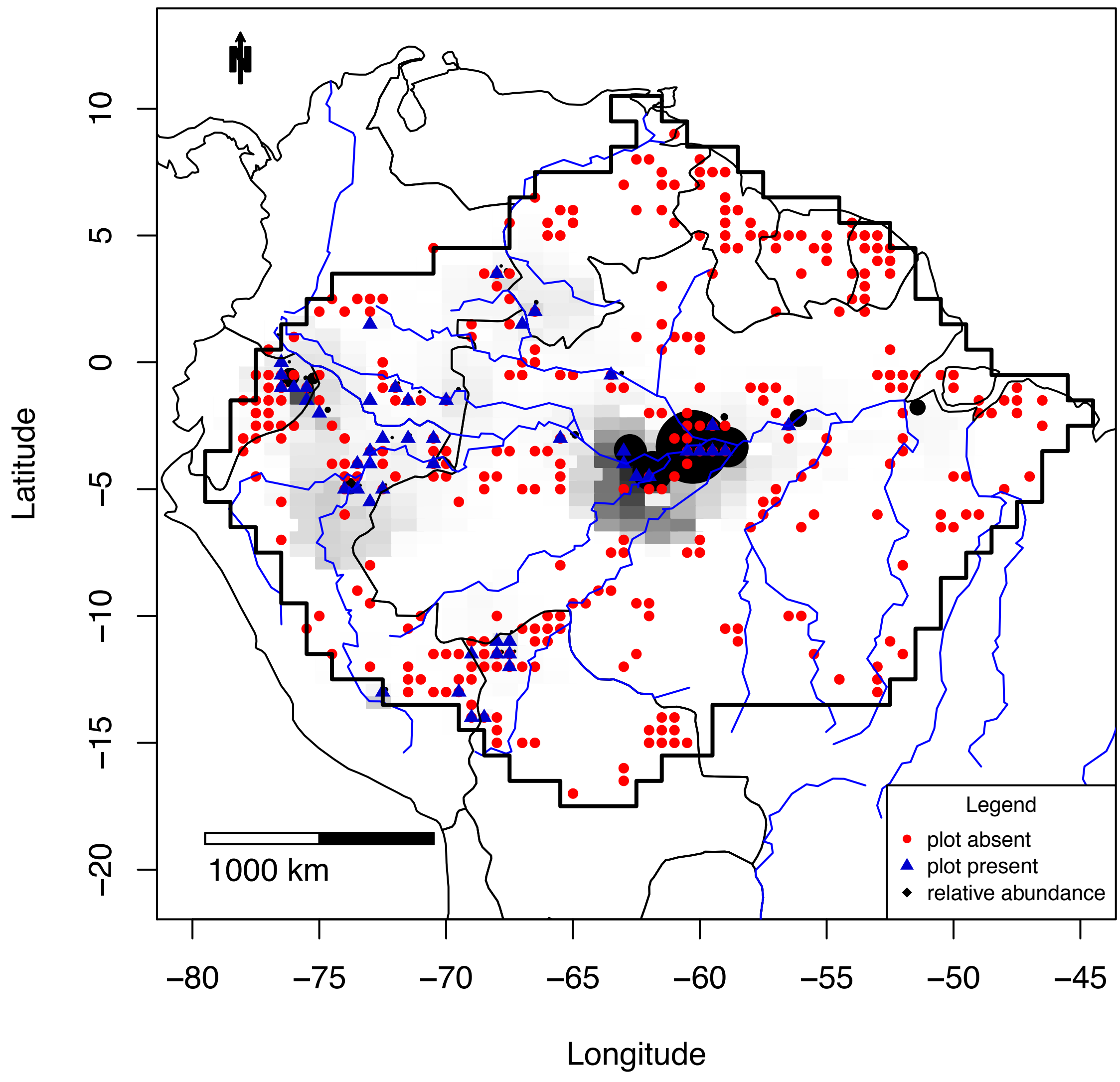

Figure S3_138A Combined map of Pterocarpus_santalinoides

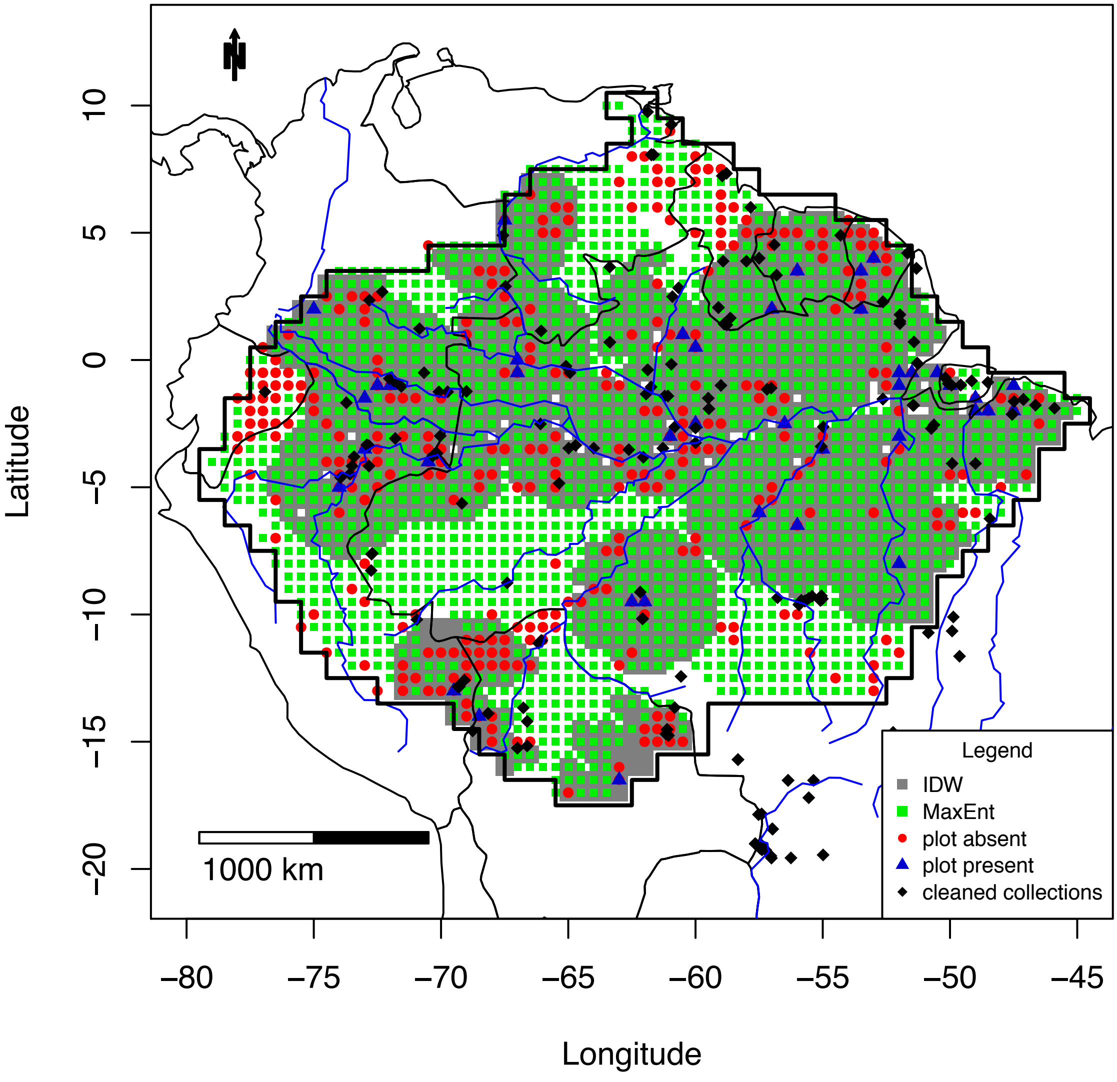

Figure S3_138B MaxEnt map of Pterocarpus_santalinoides

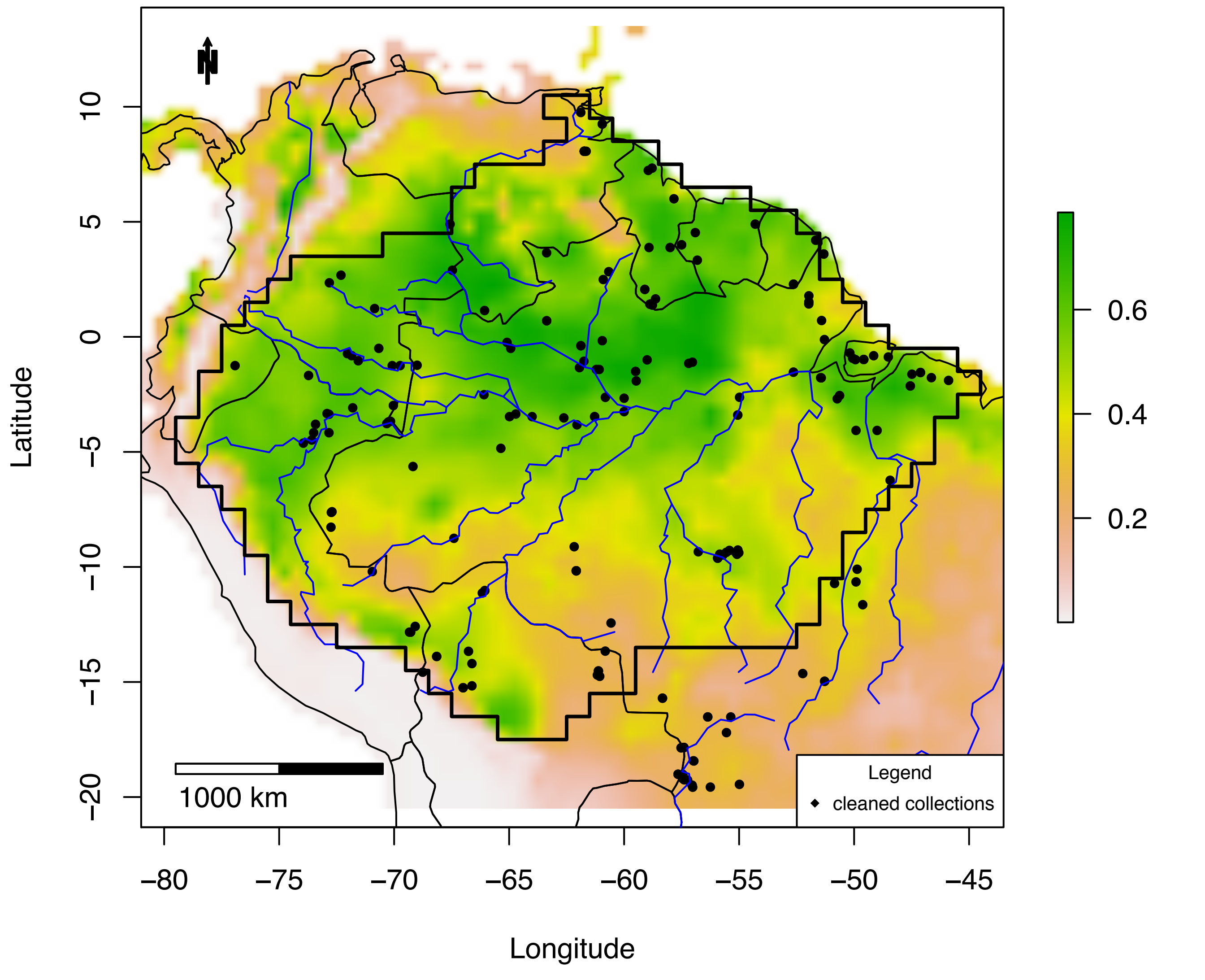

Figure S3_138C IDW map of Pterocarpus_santalinoides: plots =0 – 0.2; IDW: 0 – 0.04

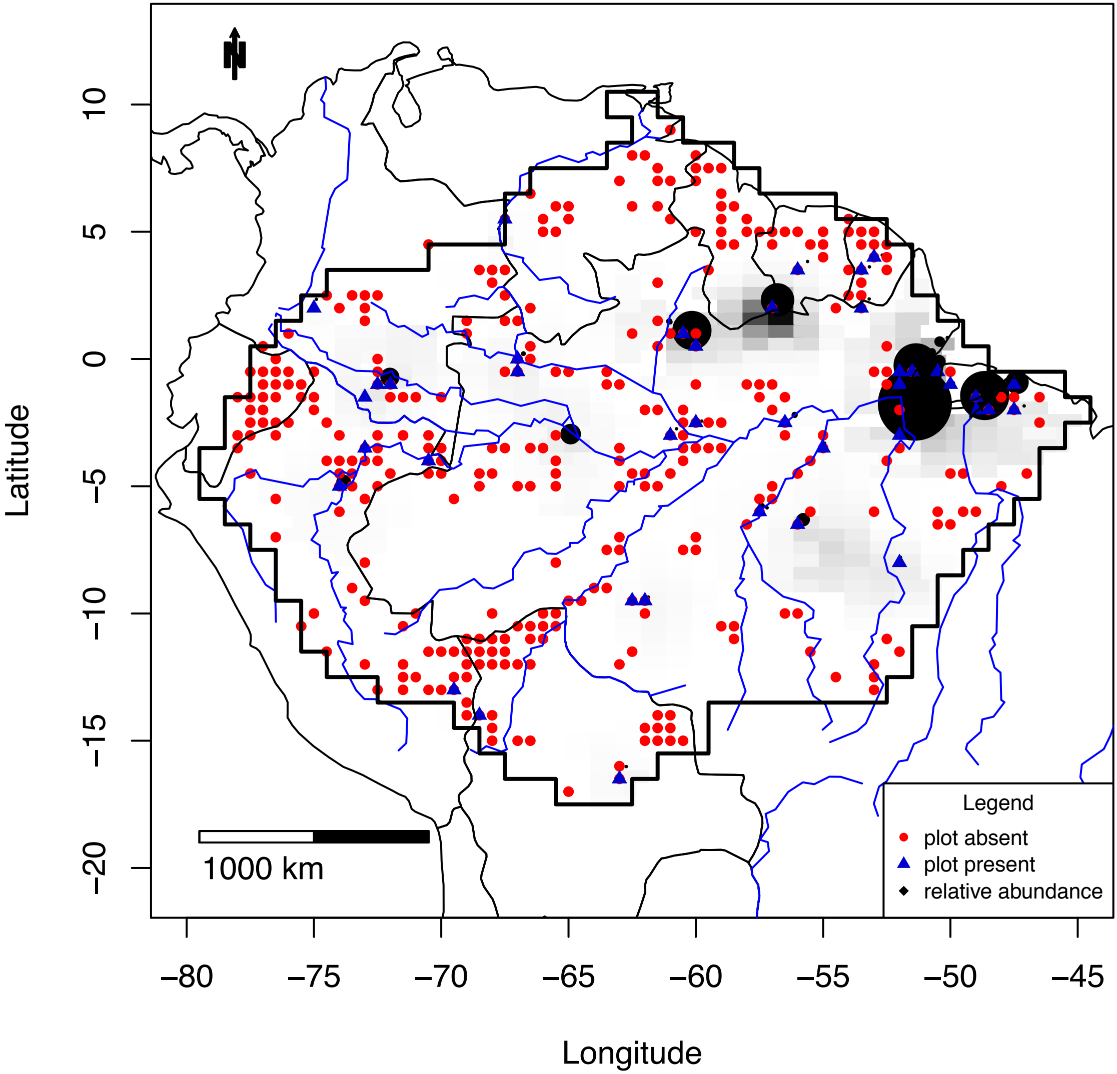

Figure S3_139A Combined map of Qualea_paraensis

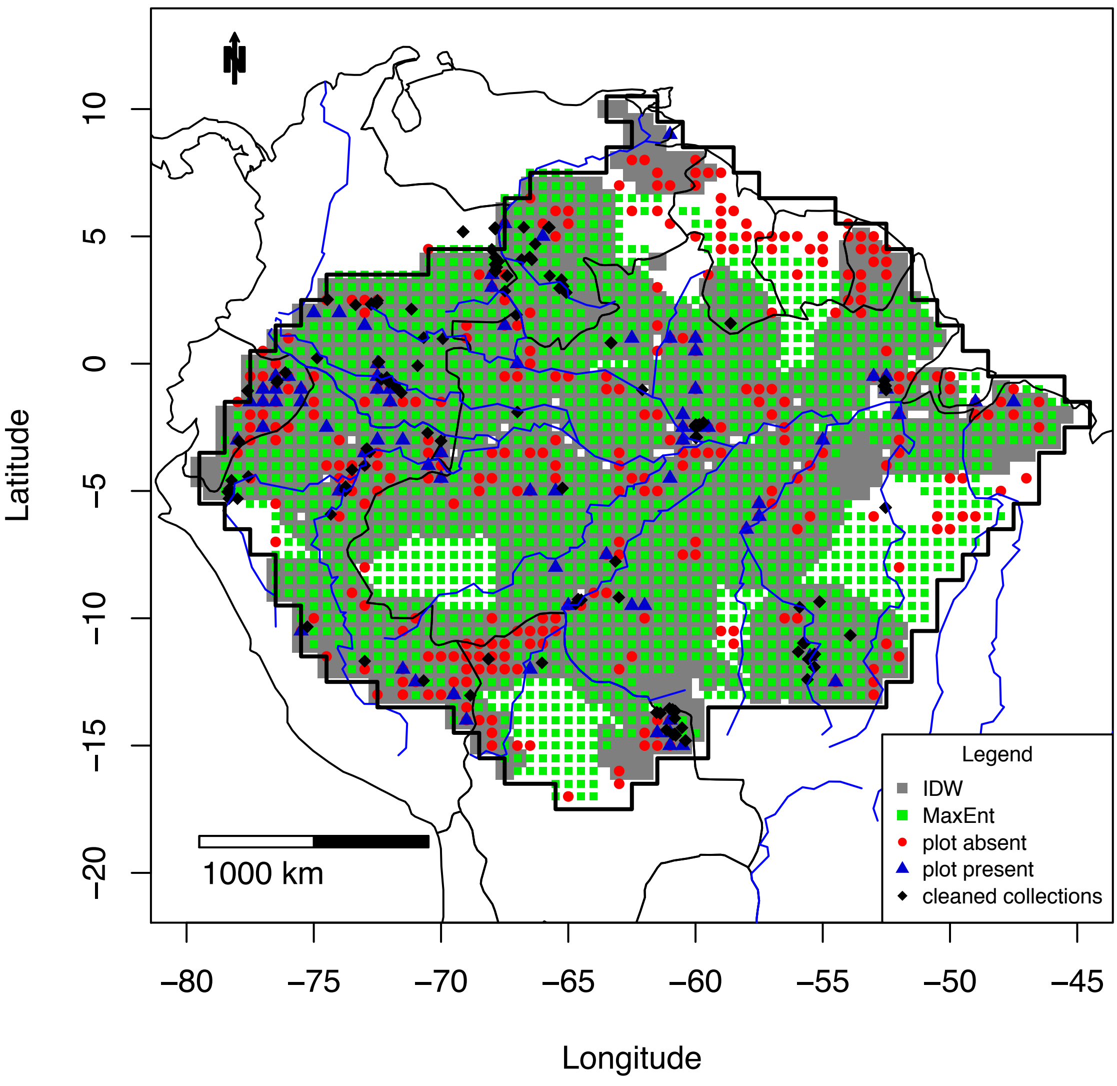

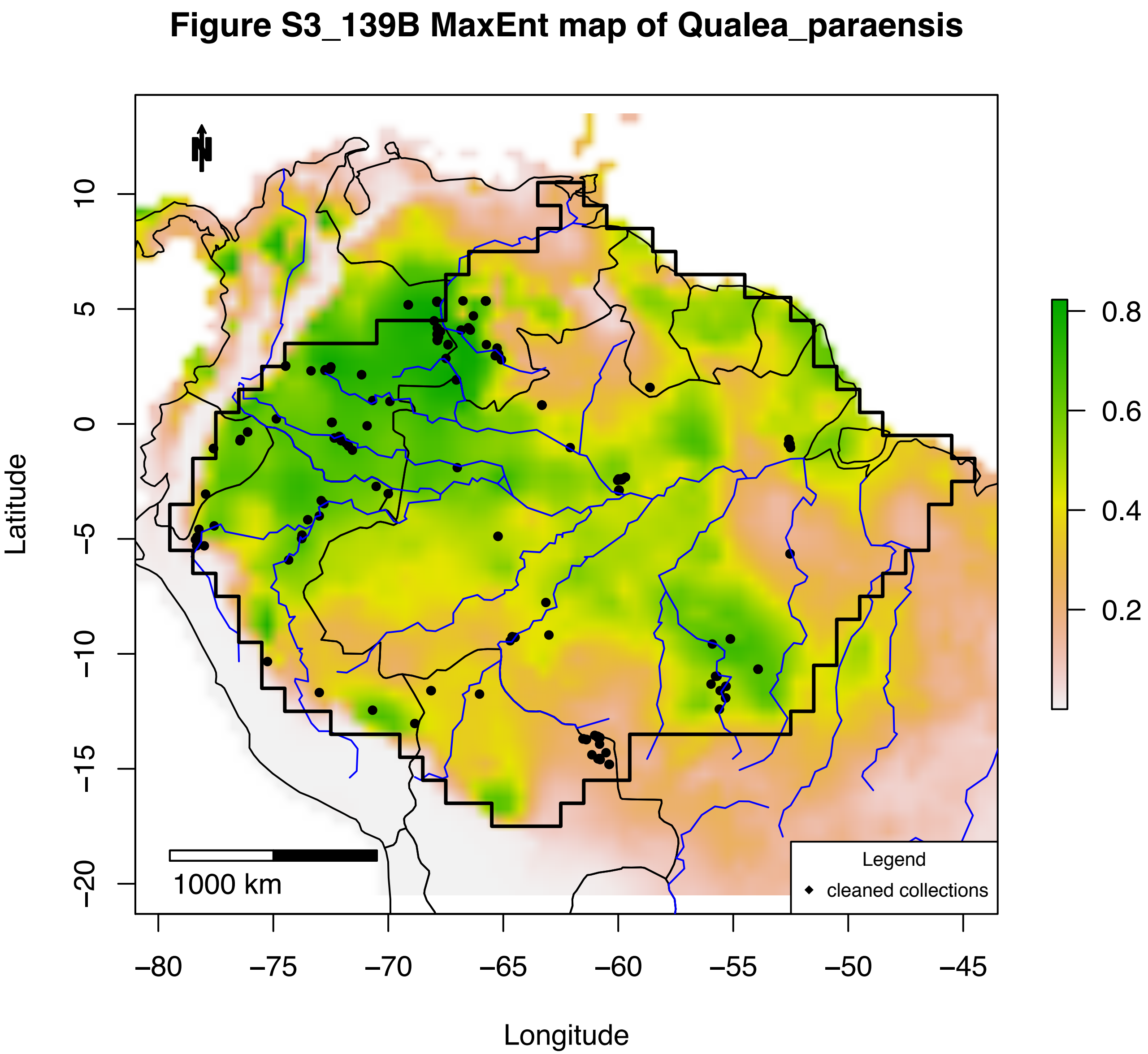

Figure S3_139B MaxEnt map of Qualea_paraensis
N
1000 km
Legend
cleaned collections
Latitude
Longitude
10
5
0
−5
−10
−15
−20
−80
−75
−70
−65
−60
−55
−50
−45
0.8
0.6
0.4
0.2

Figure S3_139C IDW map of Qualea_paraensis: plots =0 – 0.19; IDW: 0 – 0.08

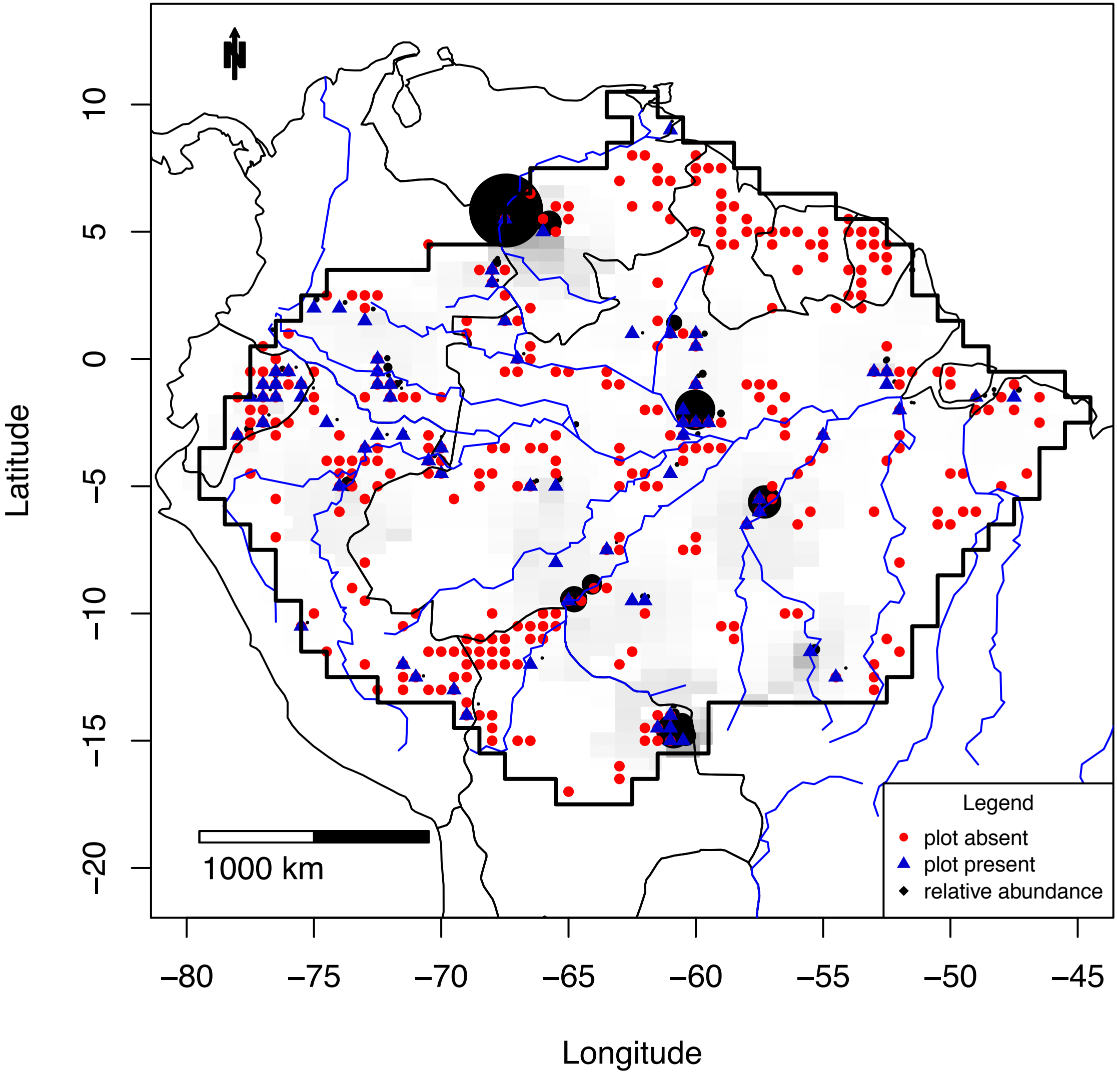

Figure S3_140A Combined map of Quararibea_guianensis

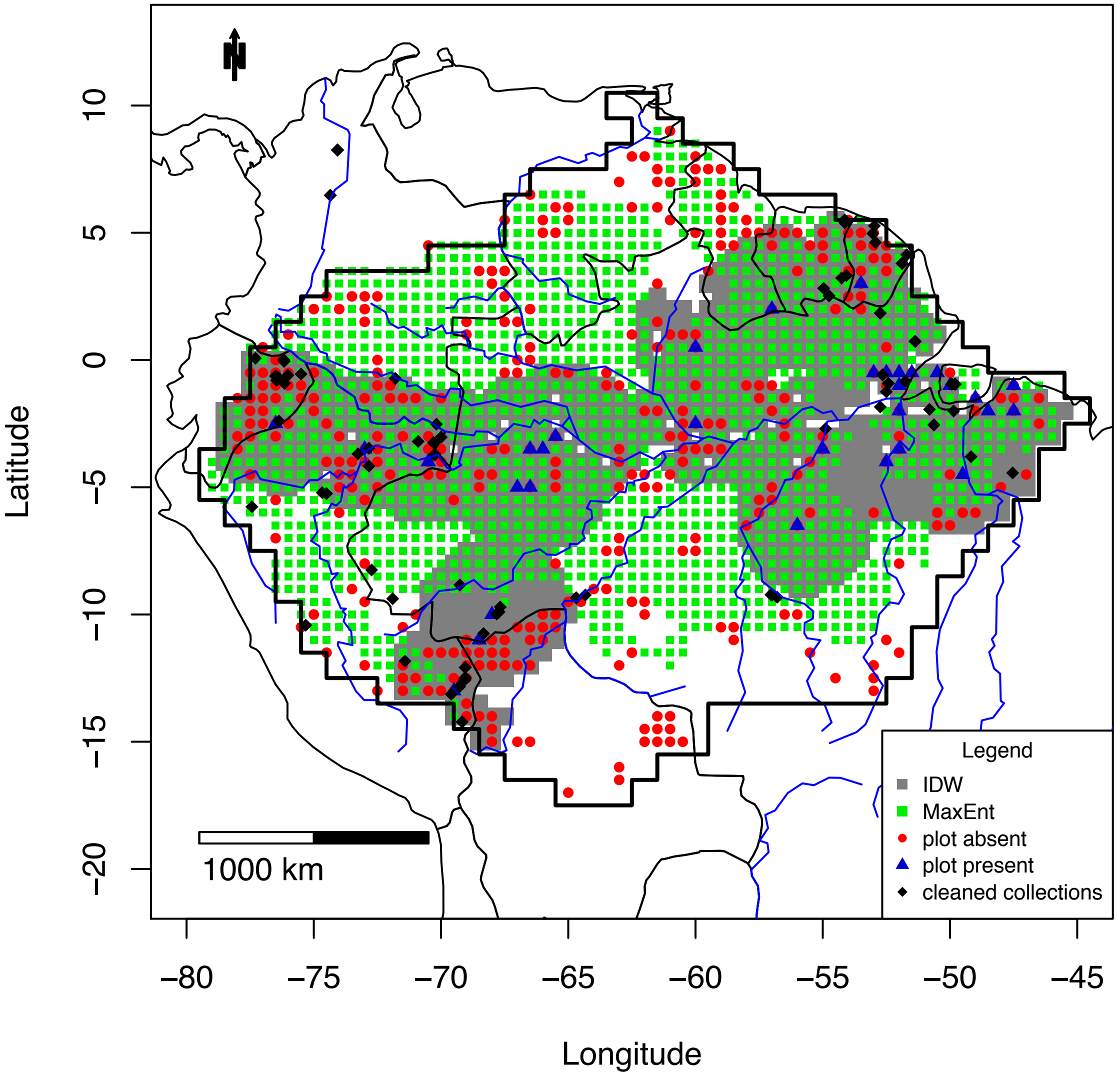

Figure S3_140B MaxEnt map of Quararibea_guianensis

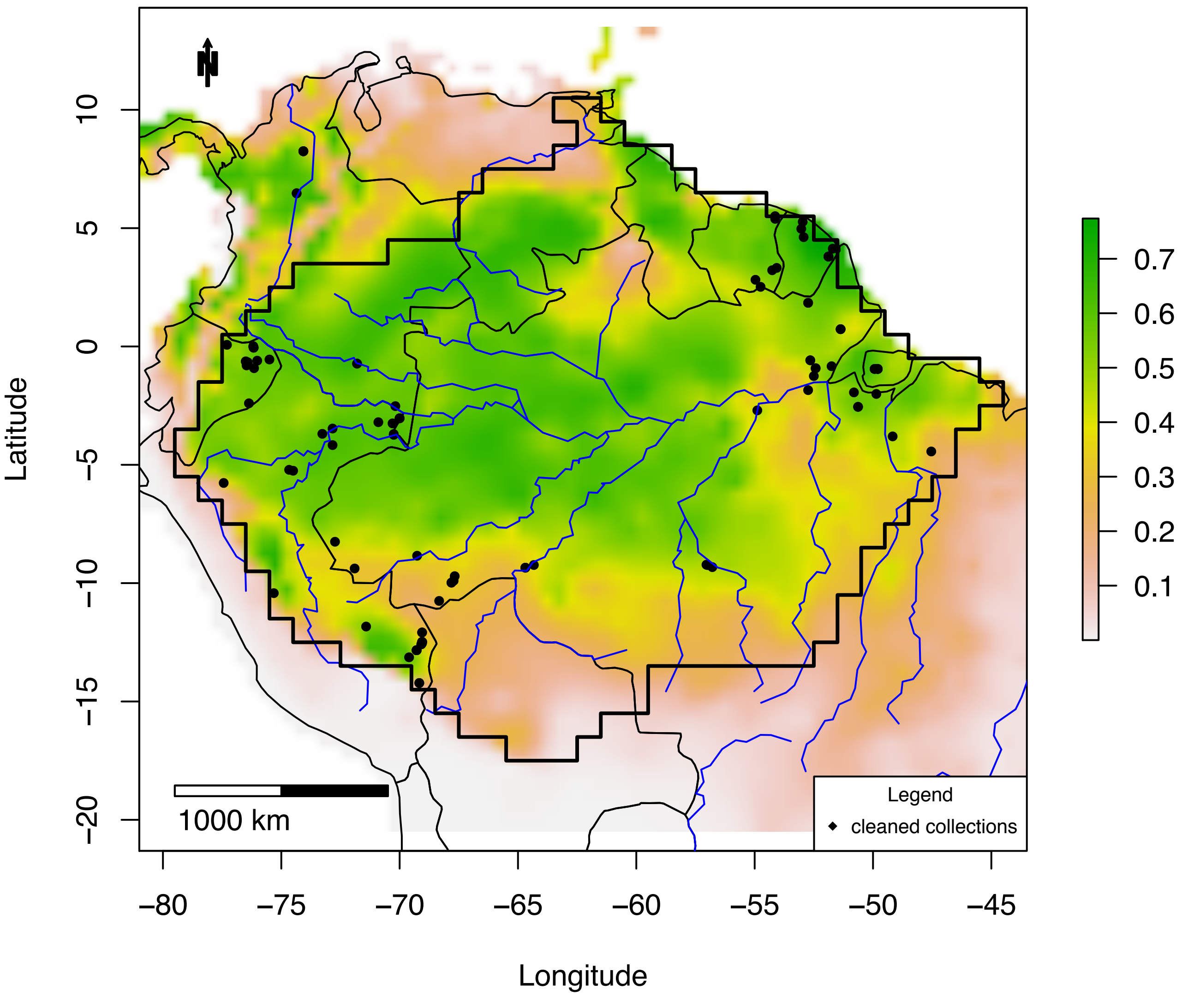

Figure S3_140C IDW map of Quararibea_guianensis: plots =0 – 0.18; IDW: 0 – 0.05

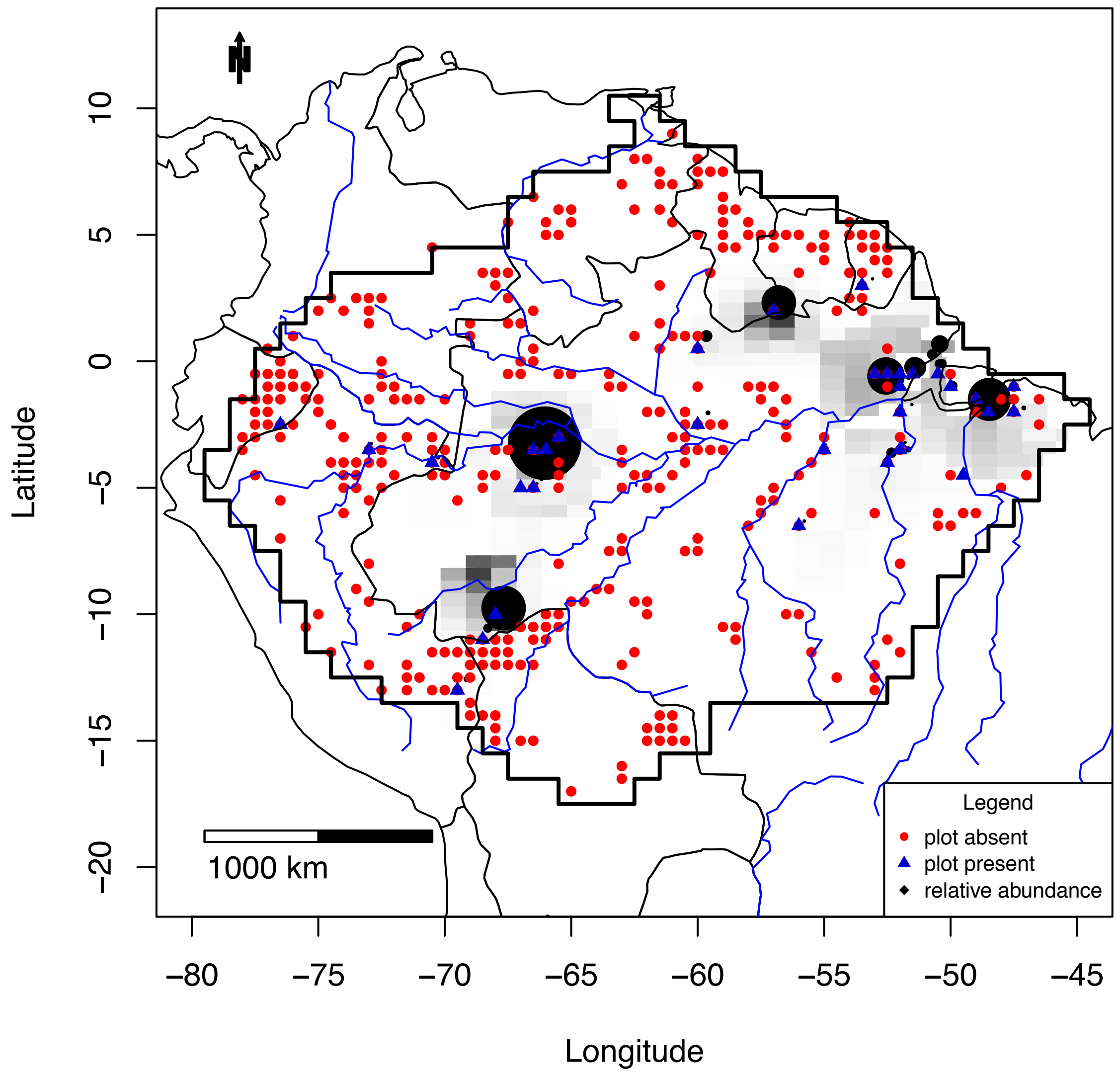

Figure S3_141A Combined map of Quararibea_wittii

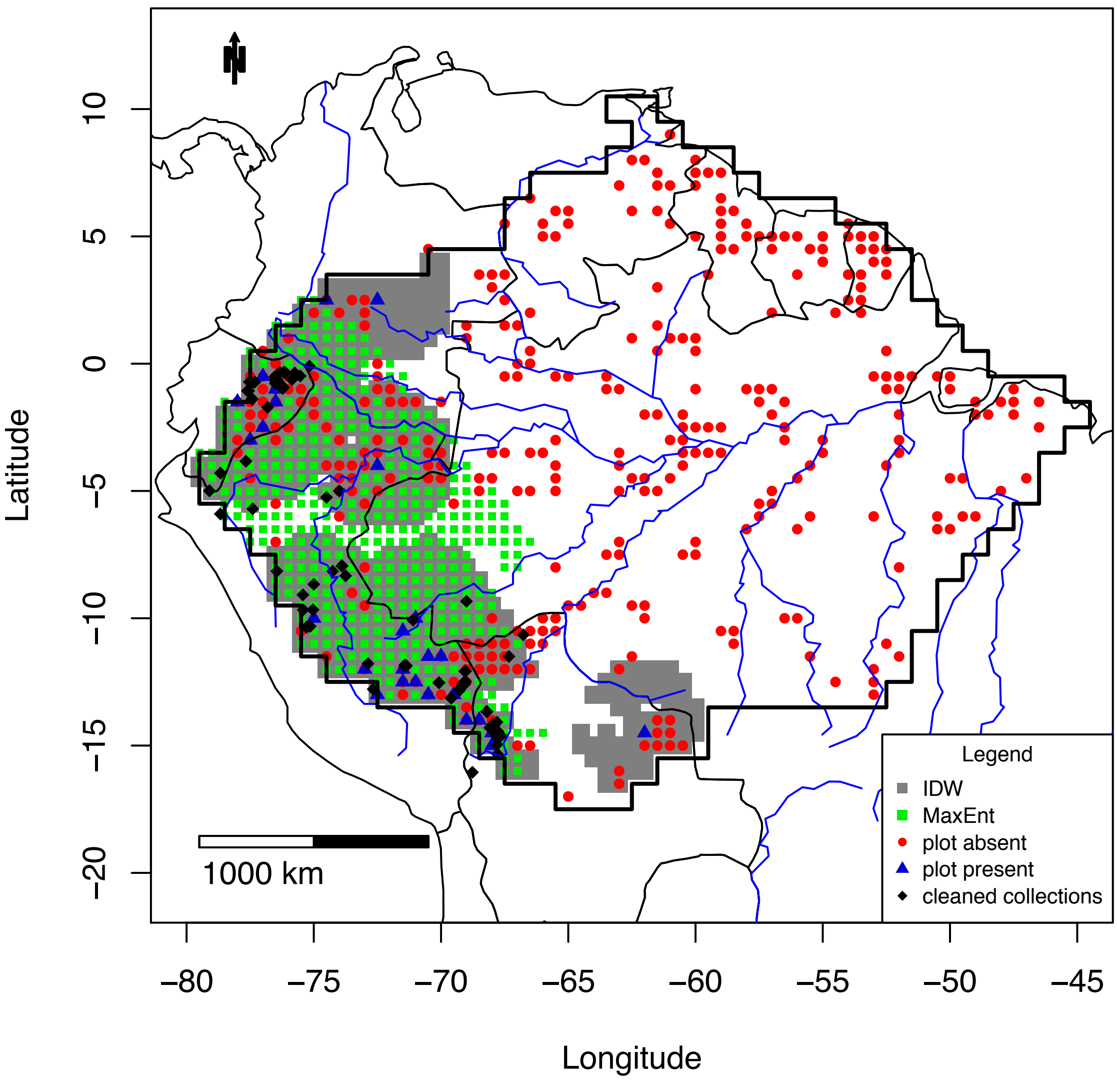

Figure S3_141B MaxEnt map of Quararibea_wittii

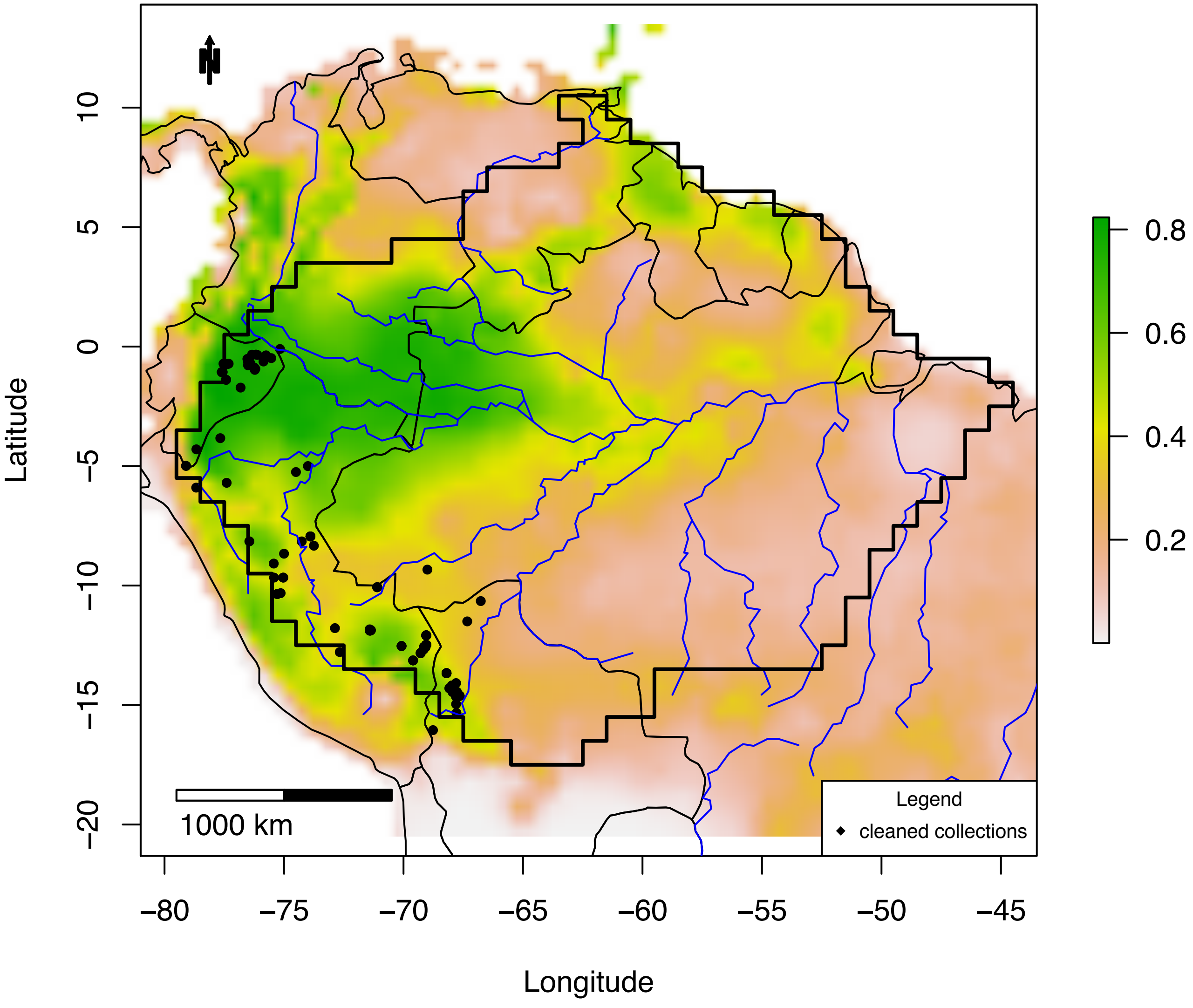

Figure S3_141C IDW map of Quararibea_wittii: plots =0 – 0.13; IDW: 0 – 0.07

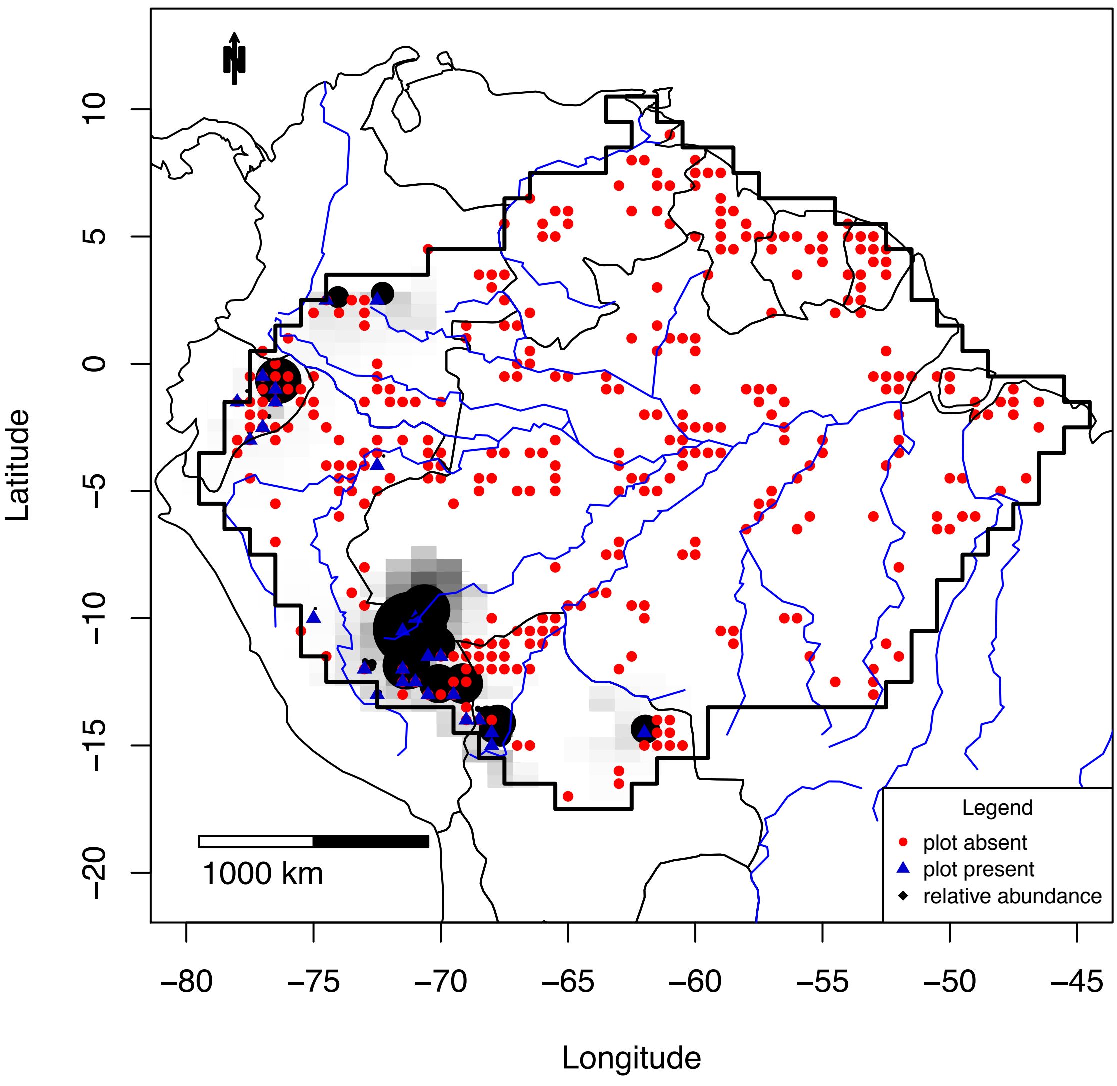

Figure S3_142A Combined map of Rinorea_racemosa

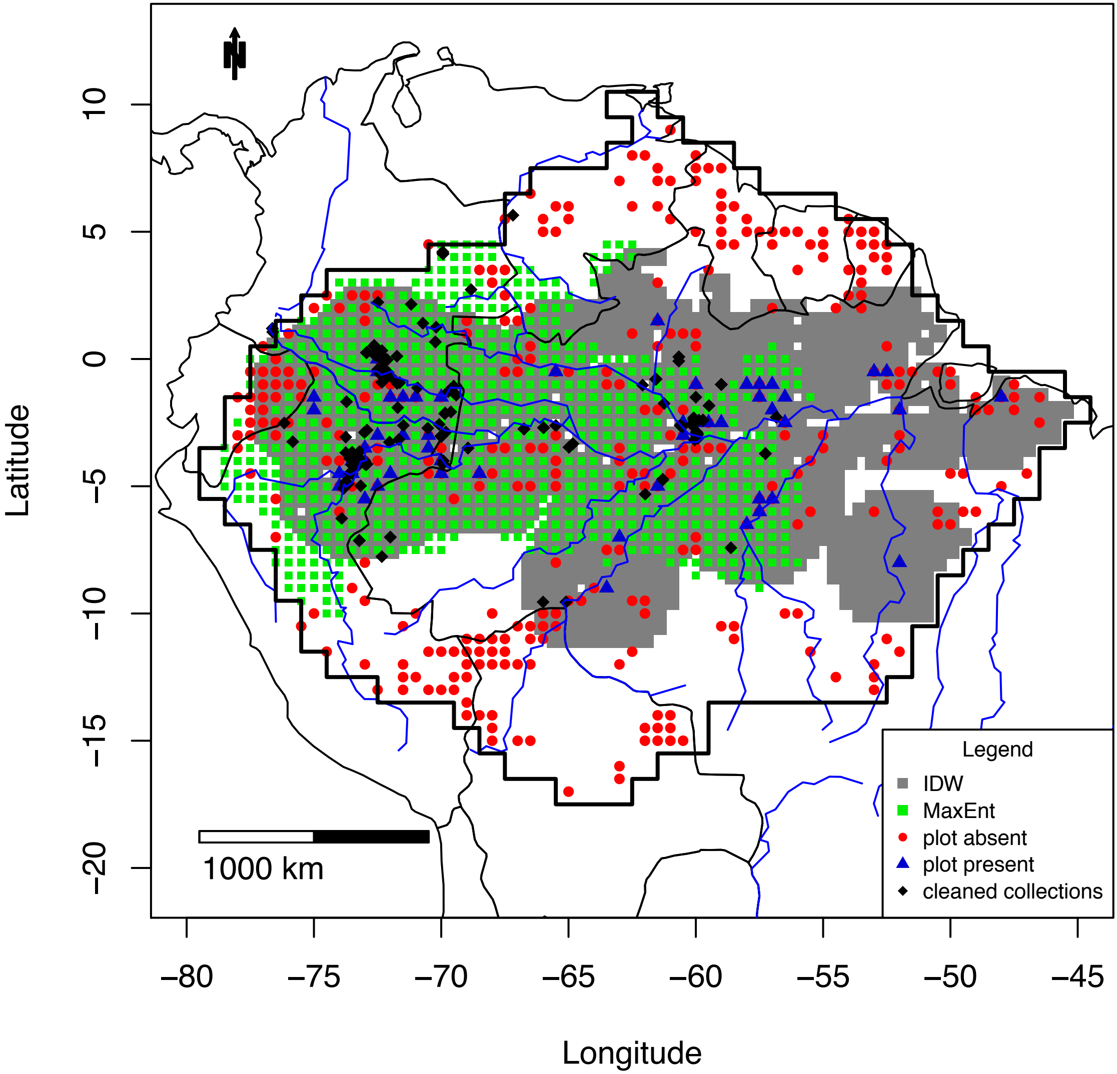

Figure S3_142B MaxEnt map of Rinorea_racemosa

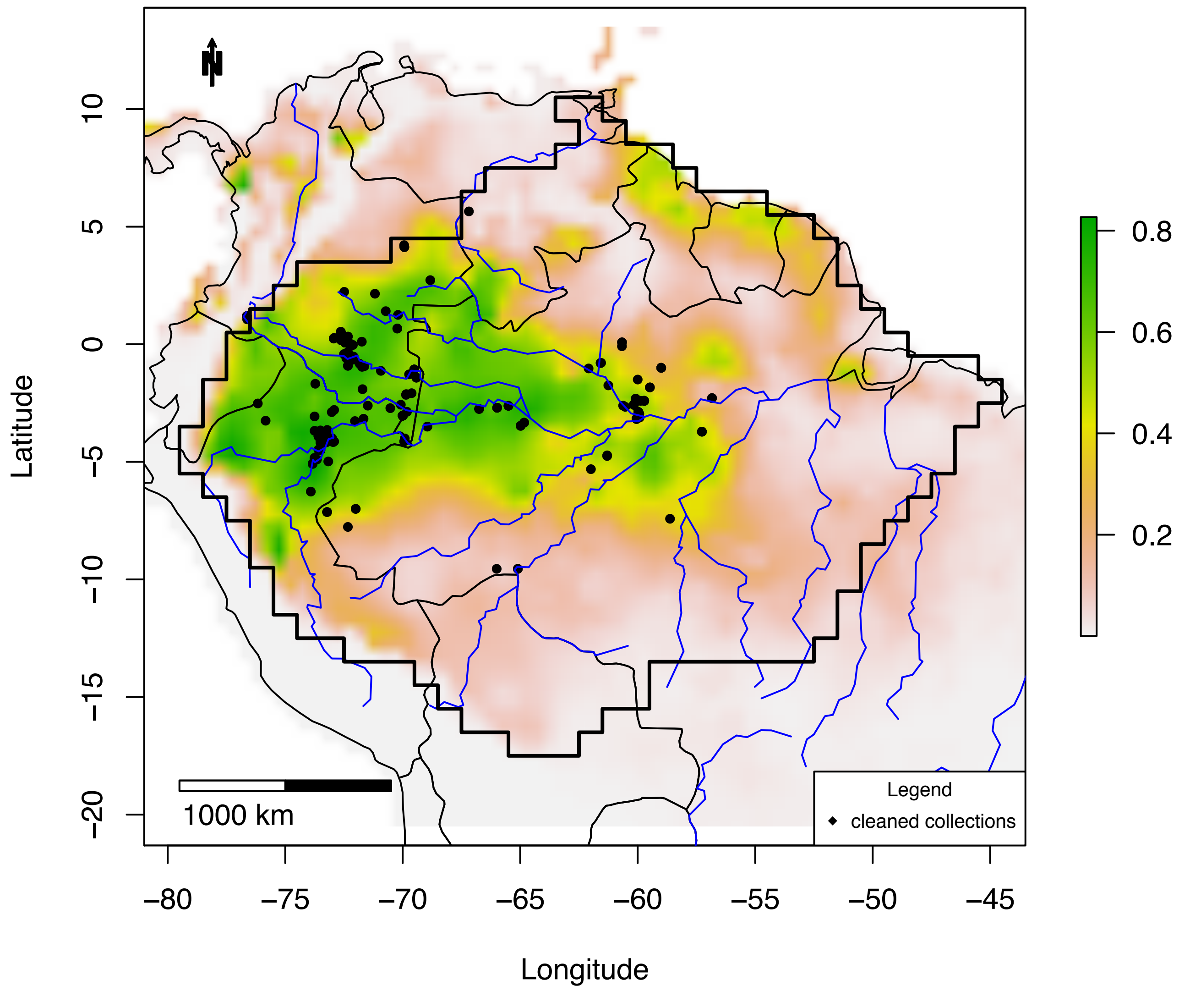

Figure S3_142C IDW map of Rinorea_racemosa:
plots =0 – 0.13; IDW: 0 – 0.05

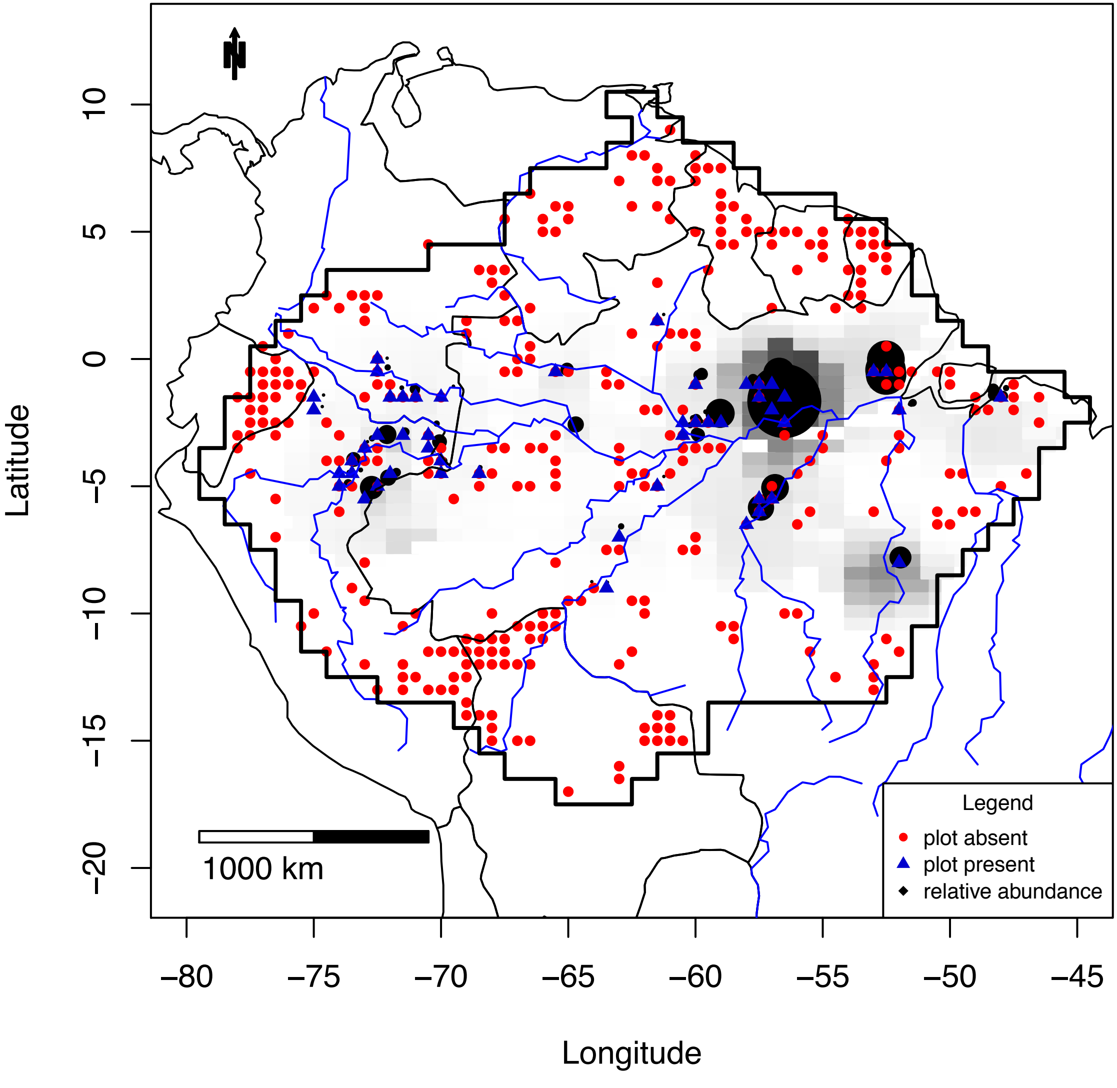

Figure S3_143A Combined map of Rinoreocarpus_ulei

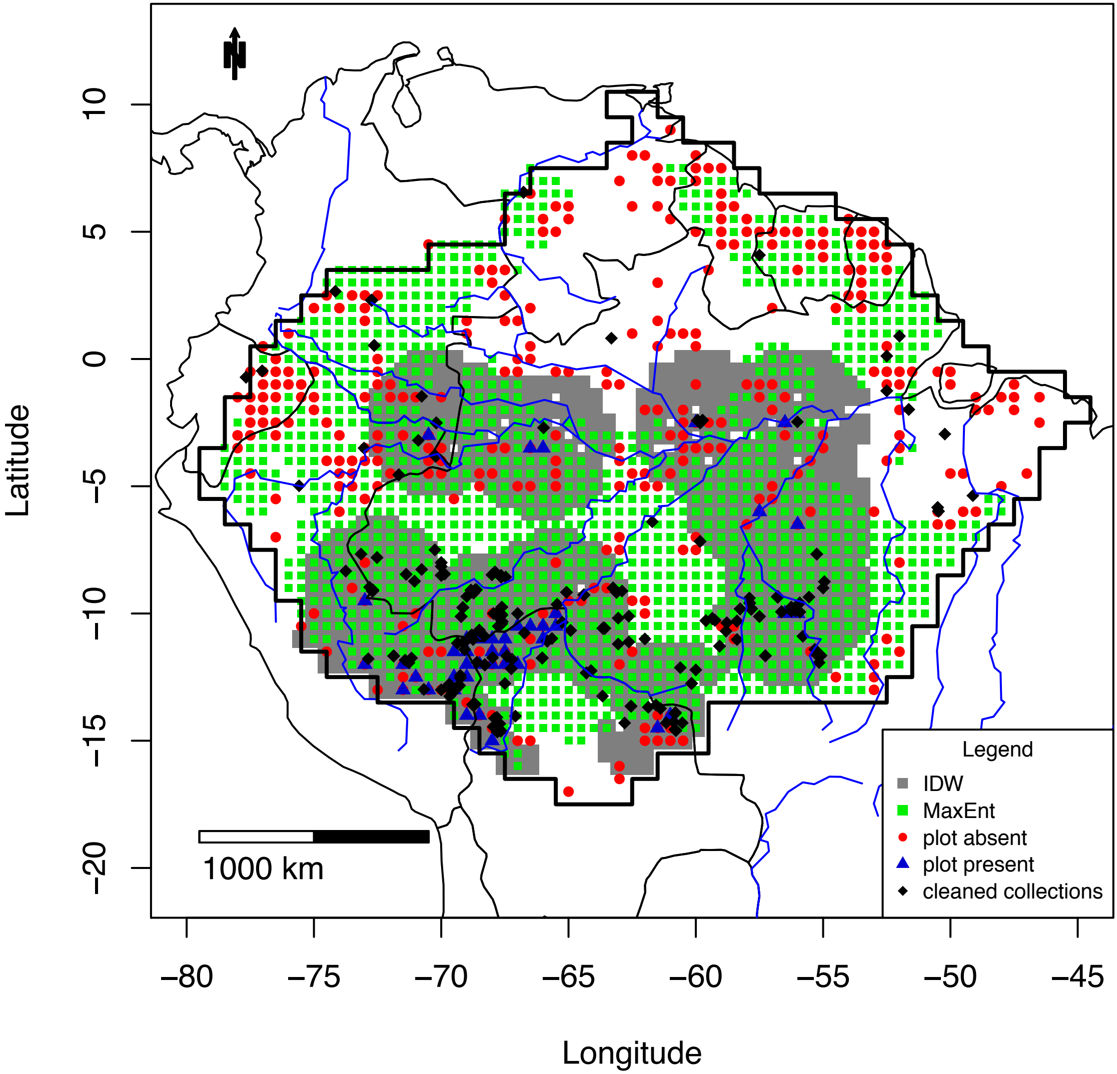

Figure S3_143B MaxEnt map of Rinoreocarpus_ulei

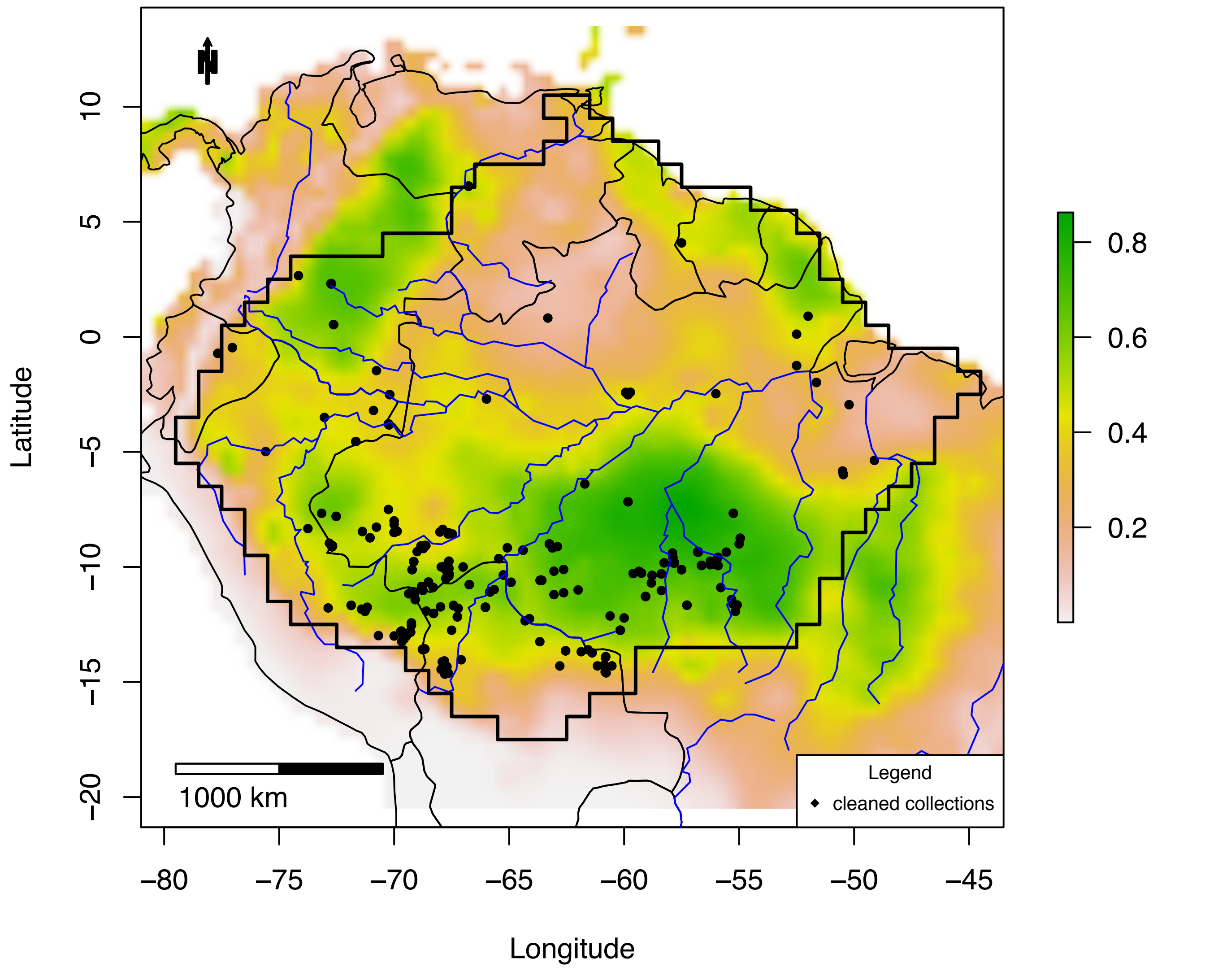

Figure S3_143C IDW map of Rinoreocarpus_ulei: plots =0 – 0.31; IDW: 0 – 0.12

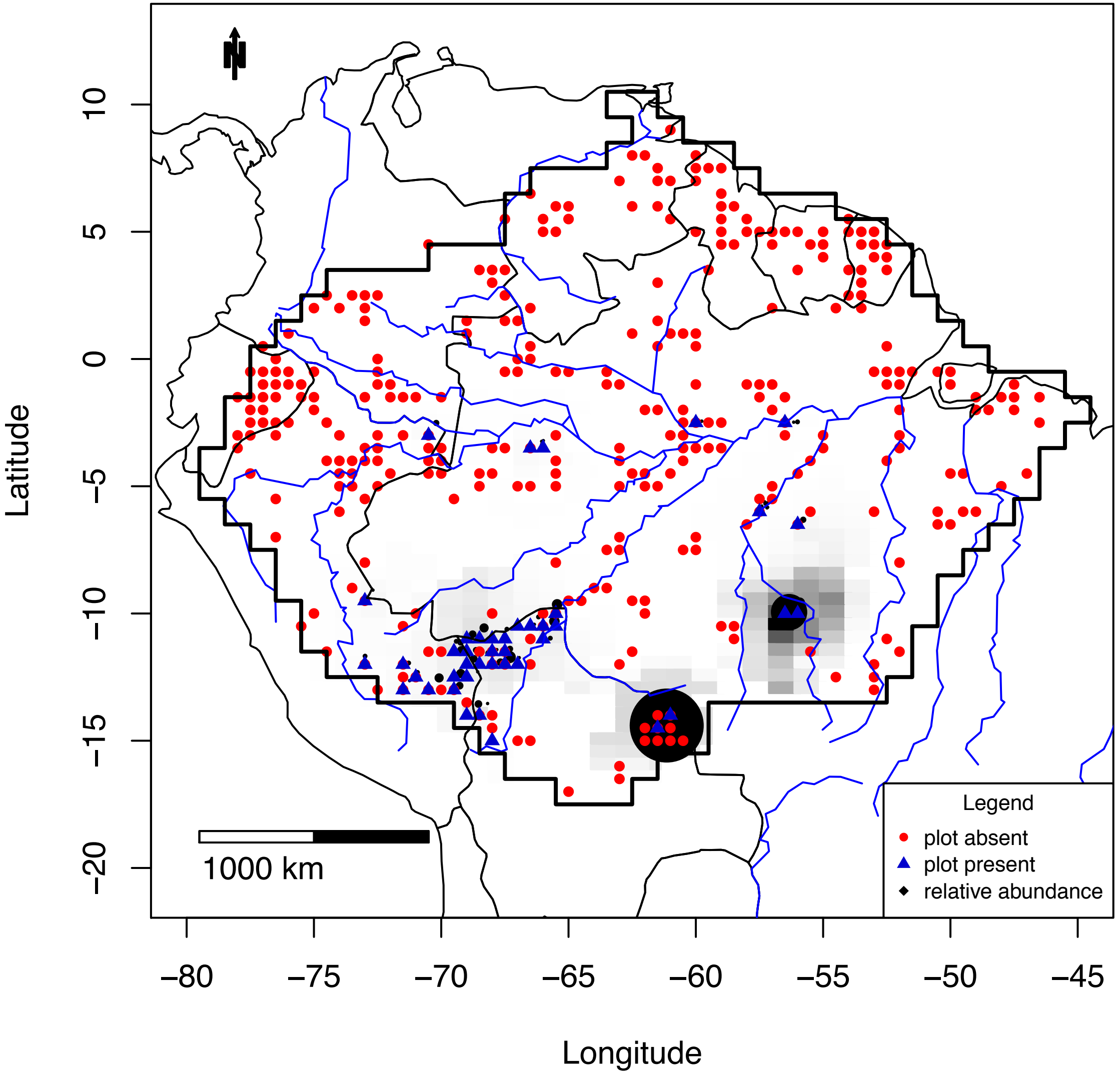

Figure S3_144A Combined map of Sacoglottis_guianensis

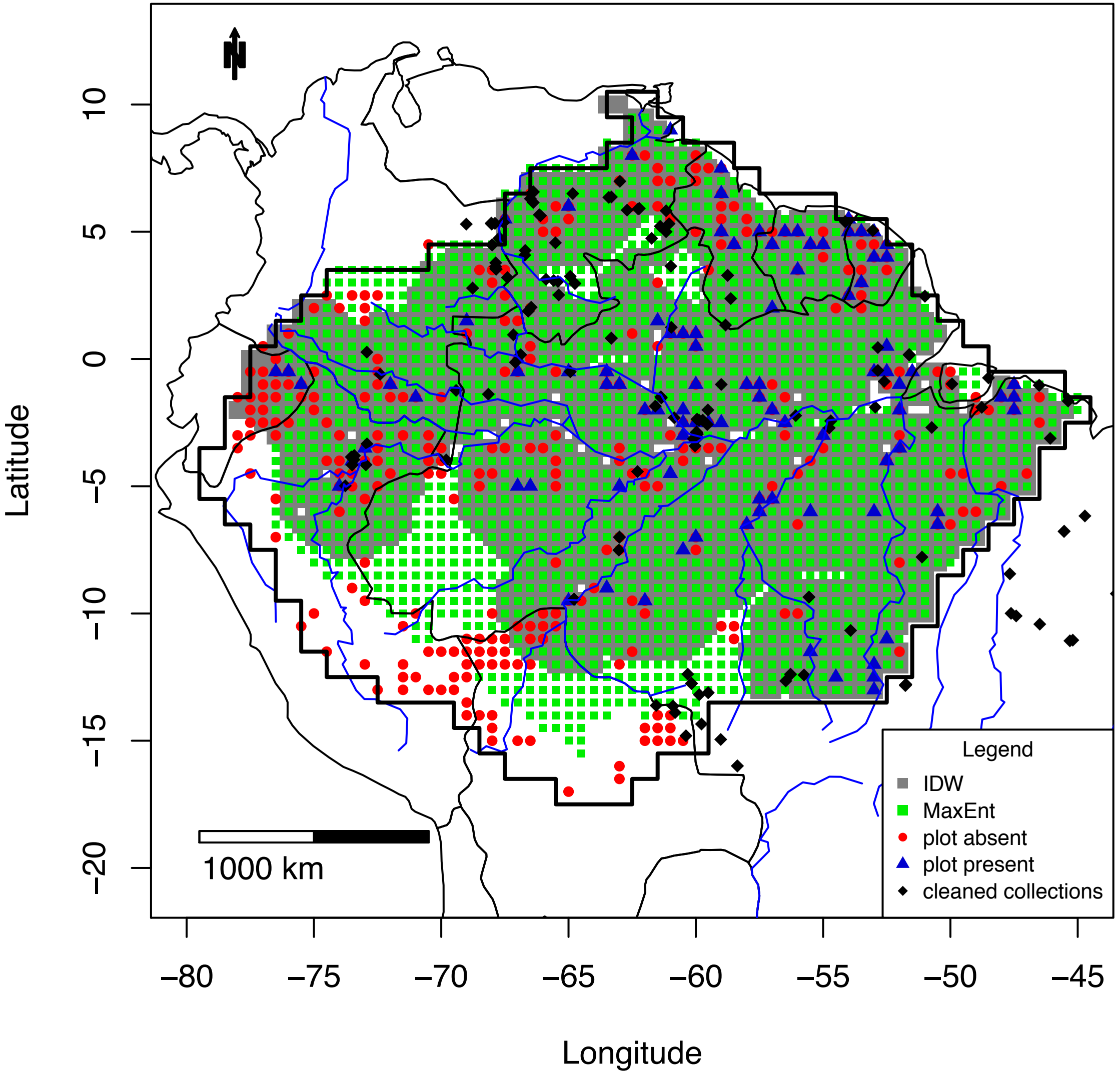

Figure S3_144B MaxEnt map of Sacoglottis_guianensis

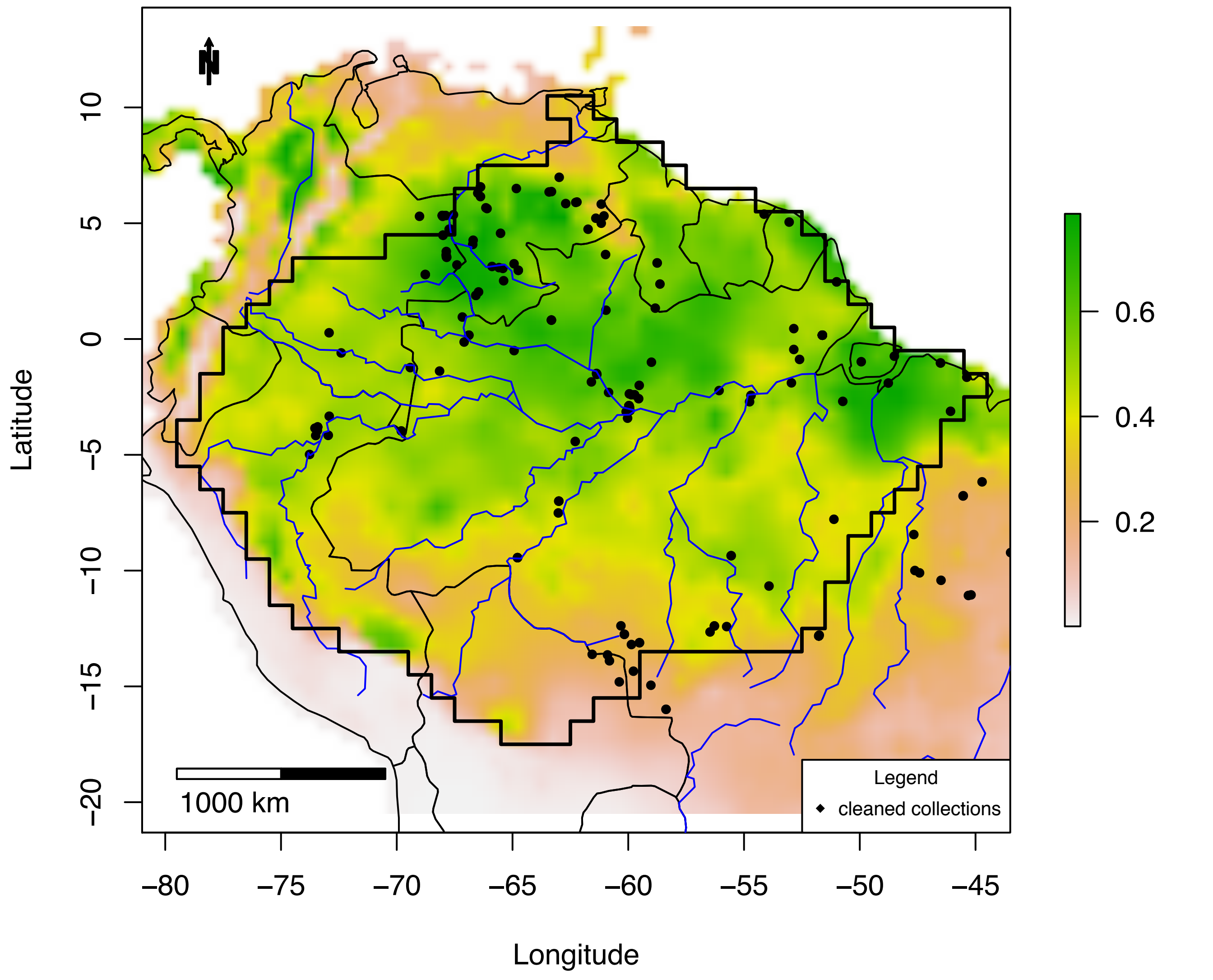

Figure S3_144C IDW map of Sacoglottis_guianensis: plots =0 – 0.28; IDW: 0 – 0.06

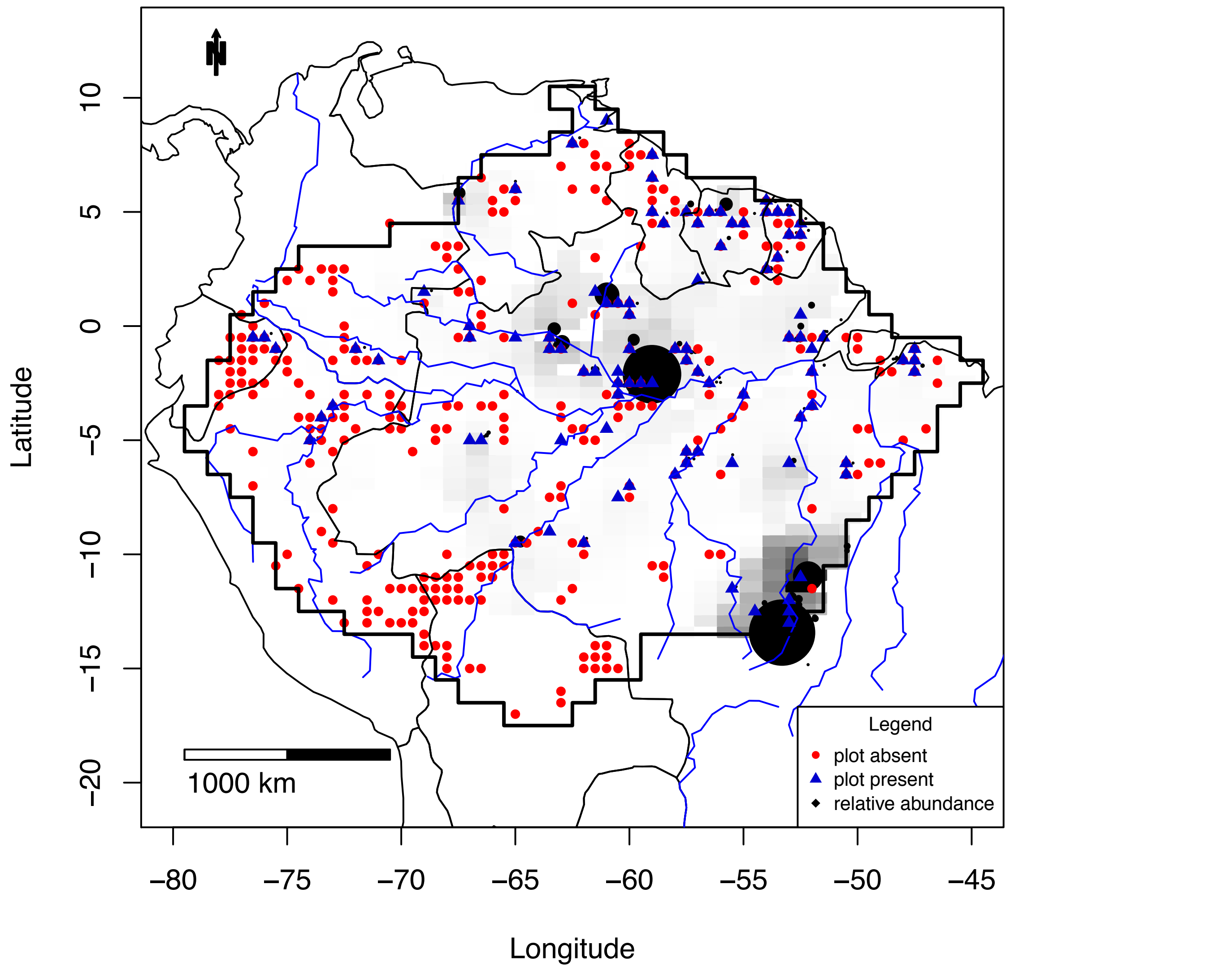

Figure S3_145A Combined map of Sagotia_brachysepala

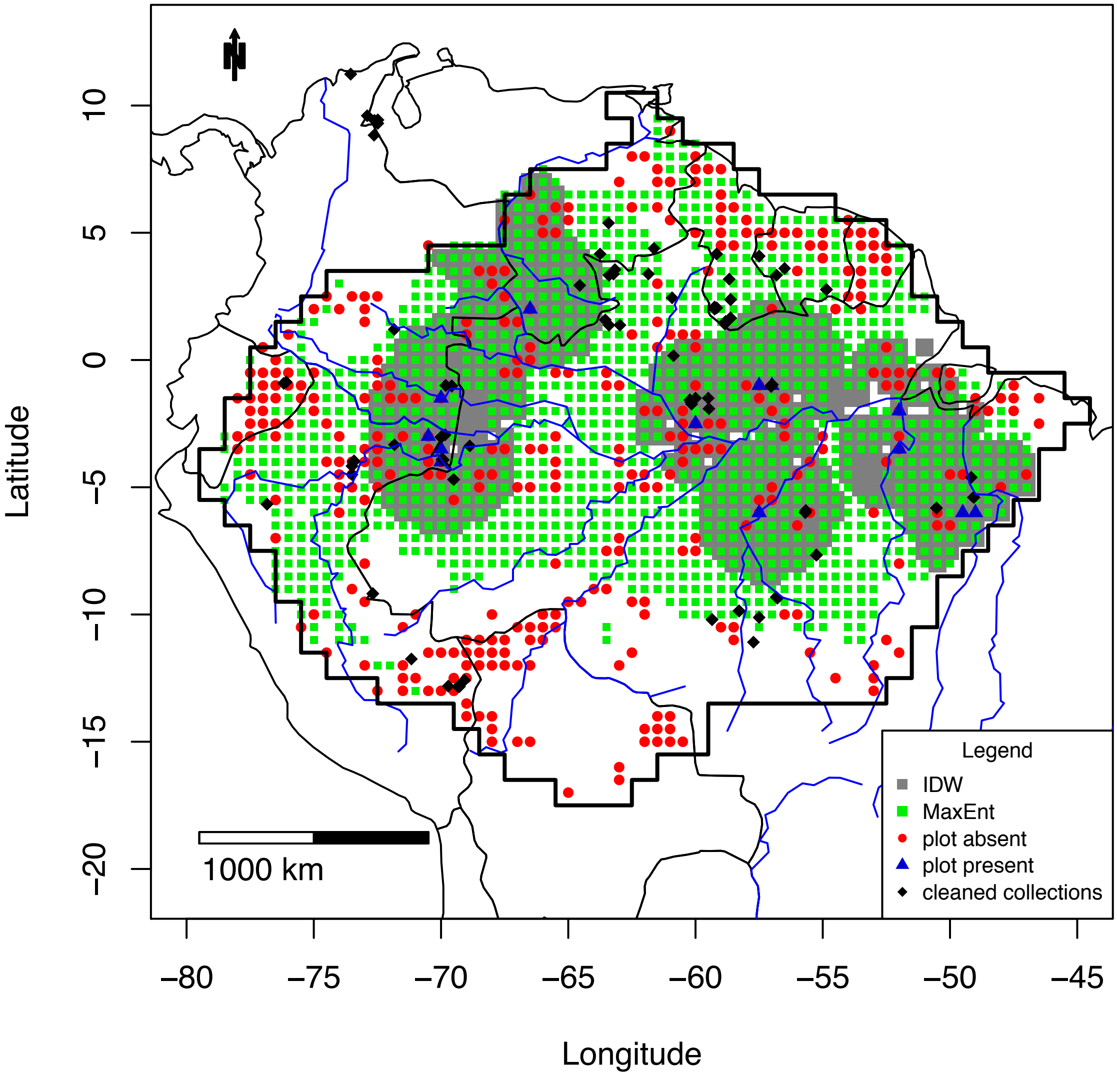

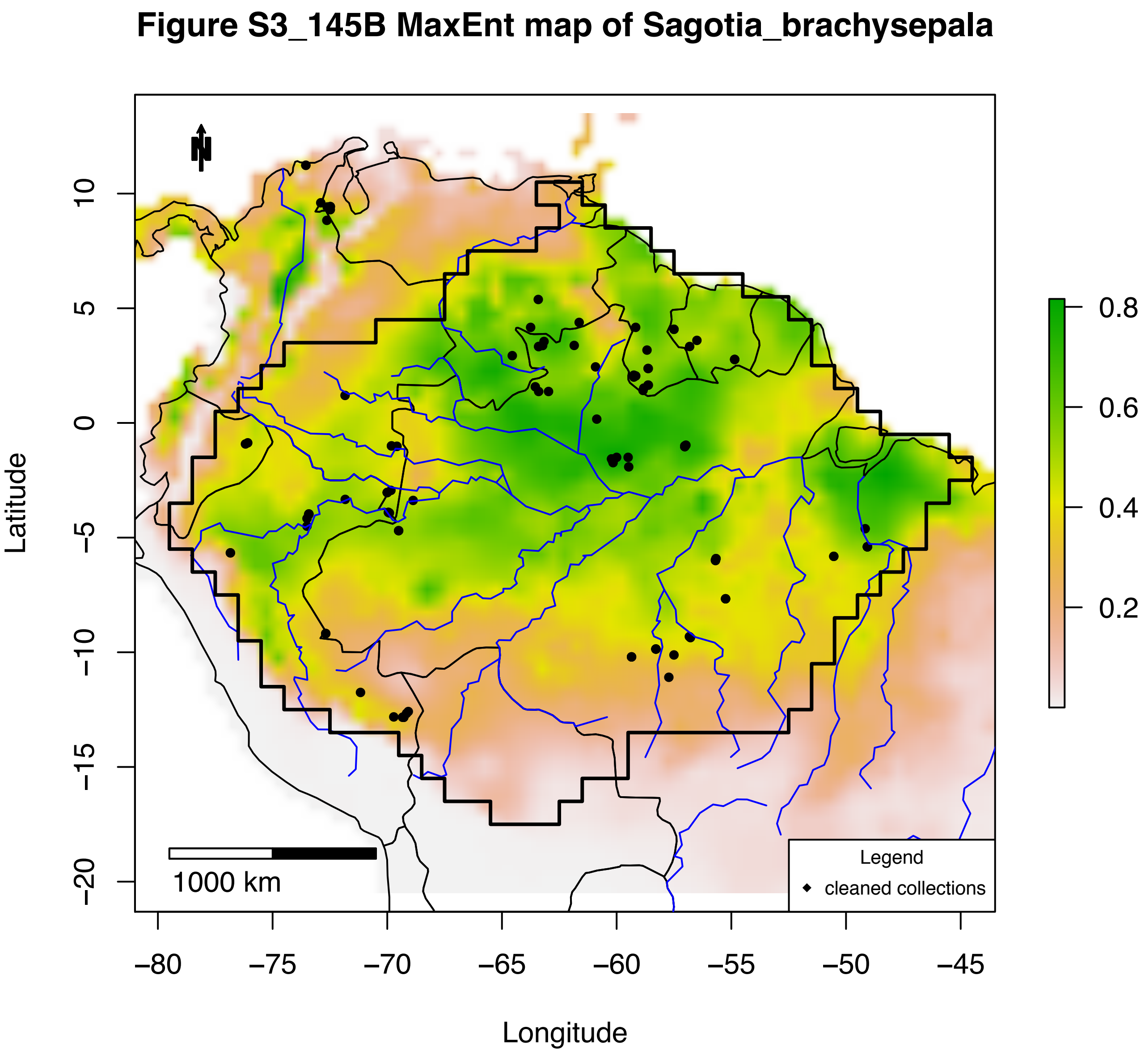
Figure S3_145B MaxEnt map of Sagotia_brachysepala
N
1000 km
Legend
cleaned collections
Latitude
Longitude
10
5
0
−5
−10
−15
−20
−80
−75
−70
−65
−60
−55
−50
−45
0.8
0.6
0.4
0.2

Figure S3_145C IDW map of Sagotia_brachysepala: plots =0 – 0.27; IDW: 0 – 0.19

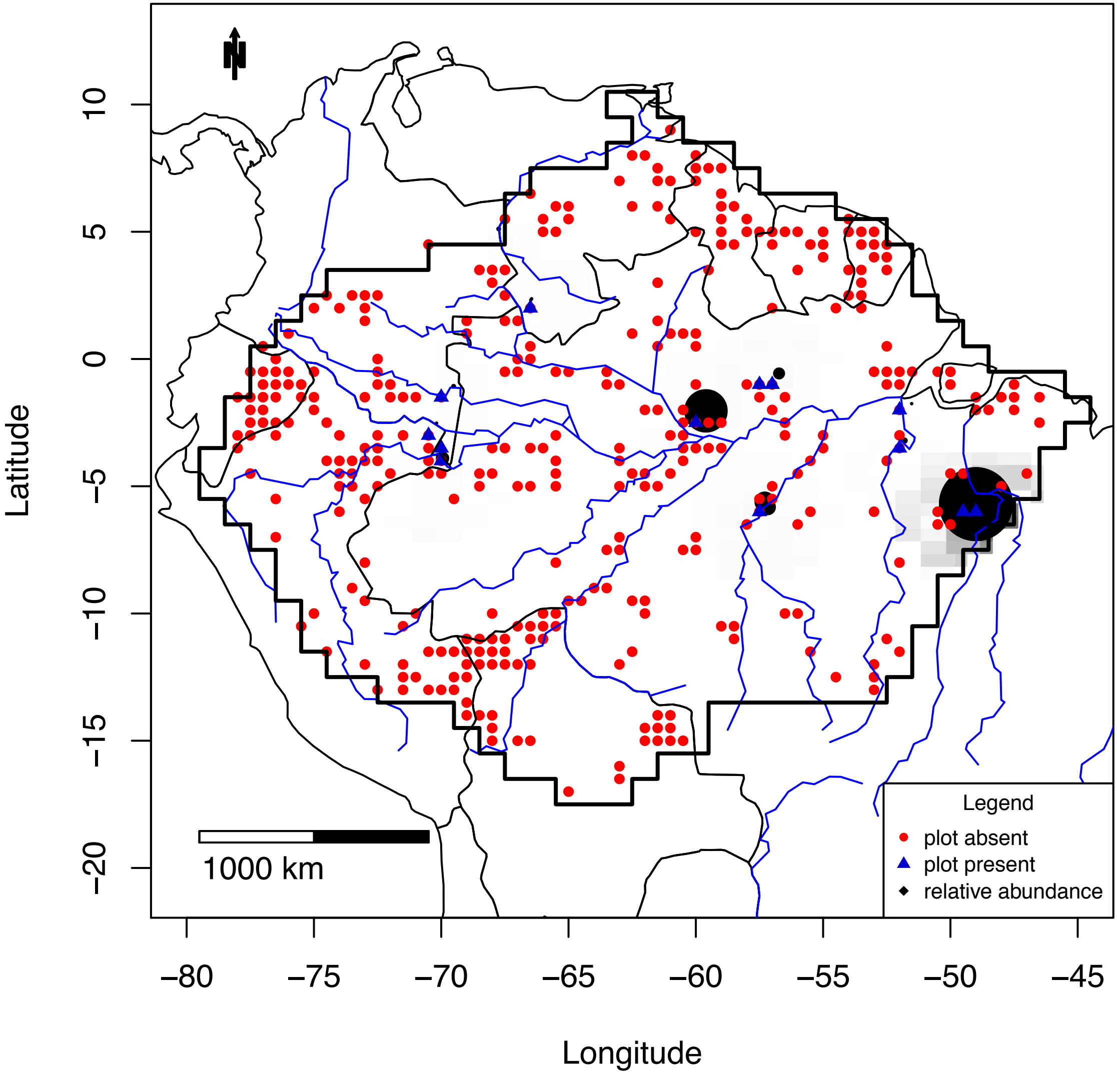

Figure S3_146A Combined map of Sagotia_racemosa

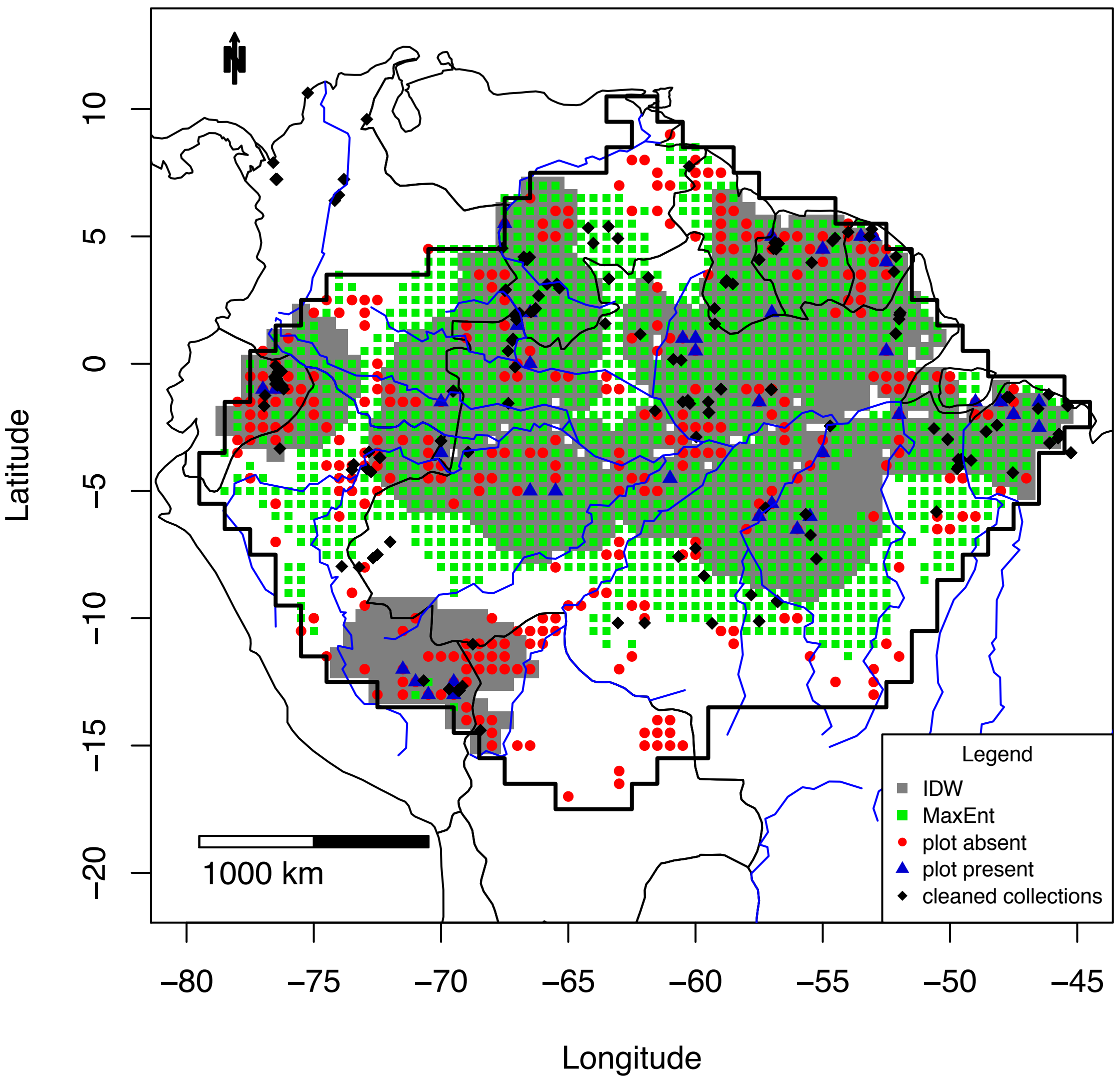

Figure S3_146B MaxEnt map of Sagotia_racemosa

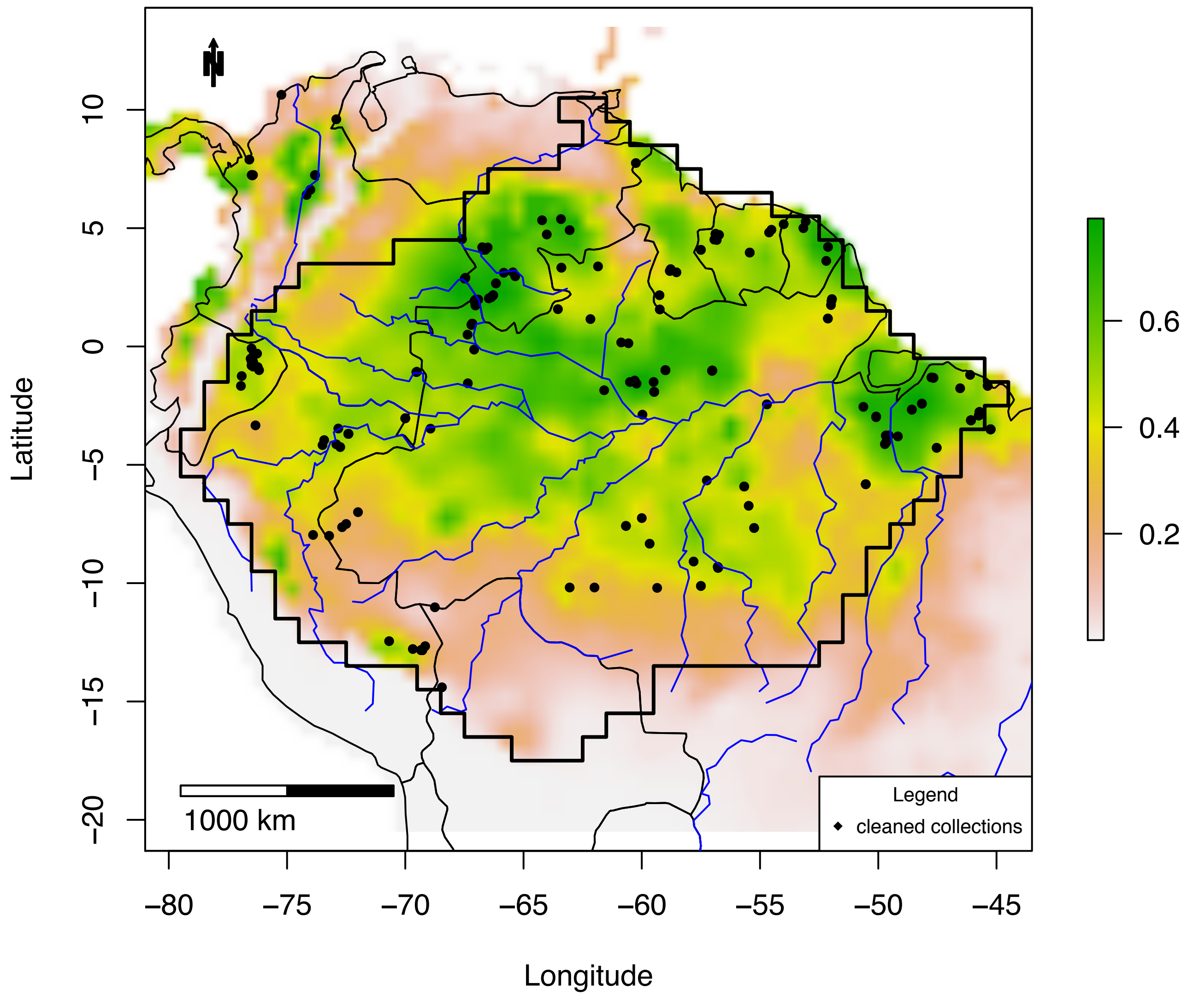

Figure S3_146C IDW map of Sagotia_racemosa: plots =0 – 0.13; IDW: 0 – 0.07

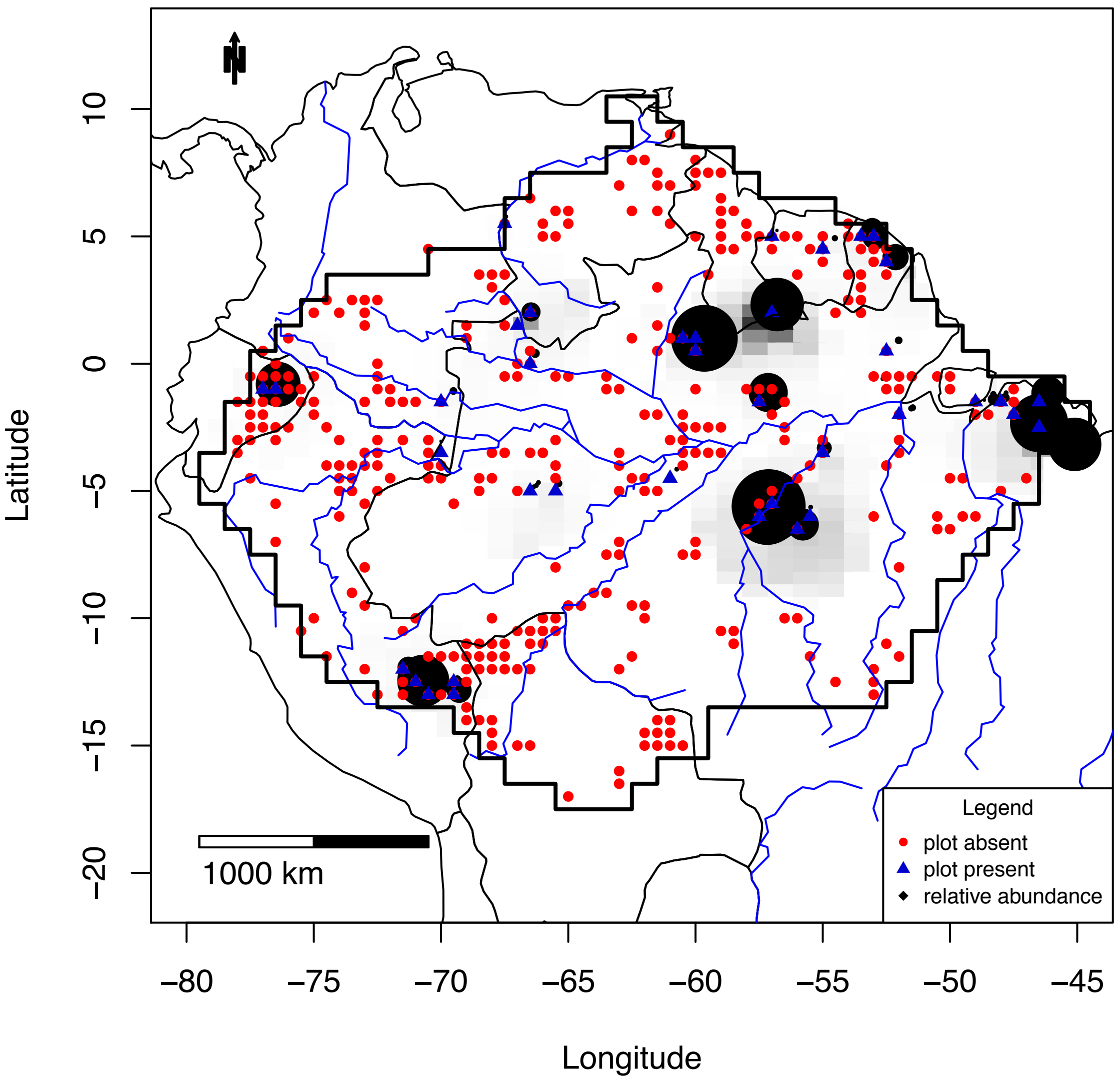

Figure S3_147A Combined map of Scleronema_micranthum

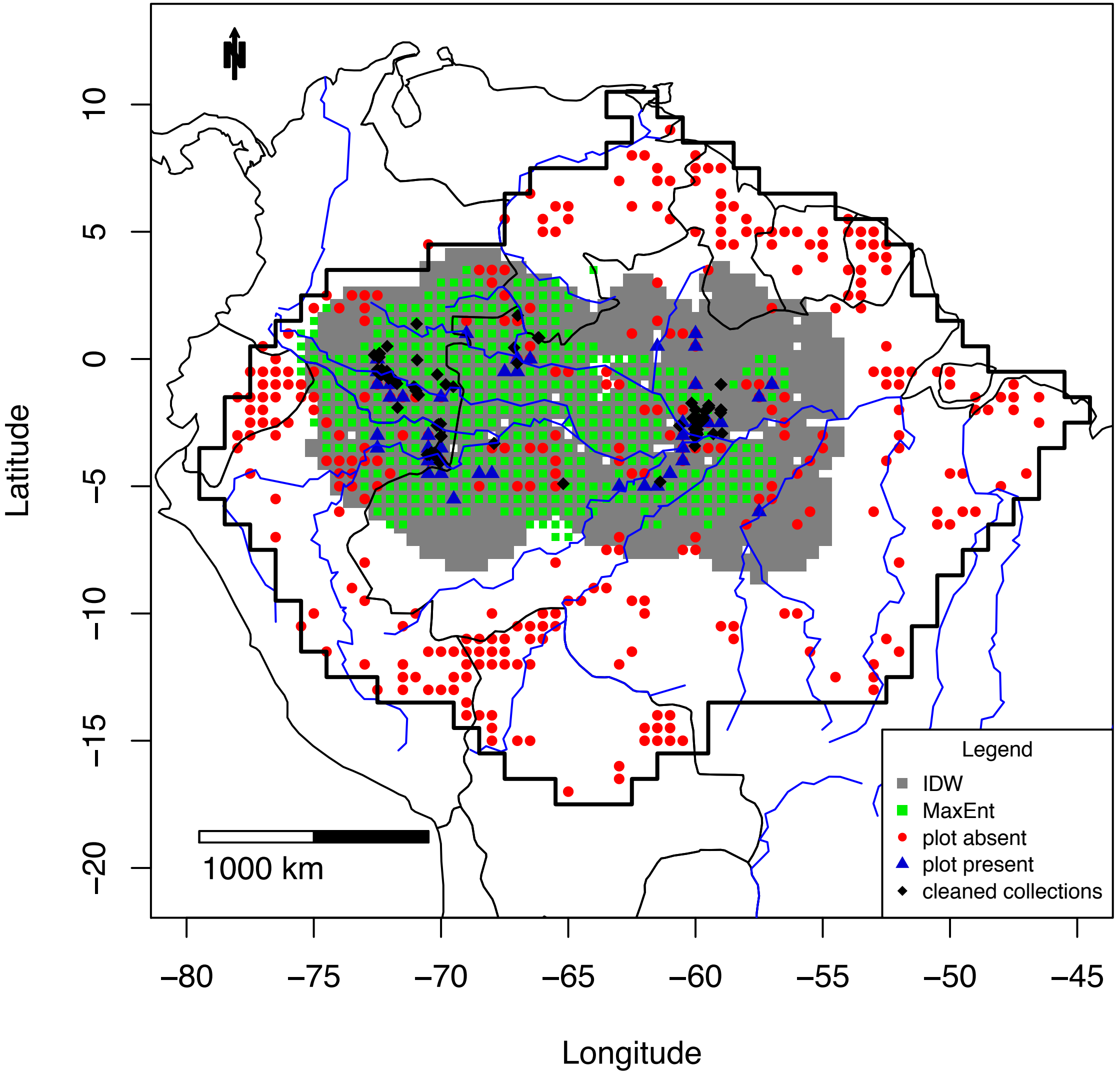

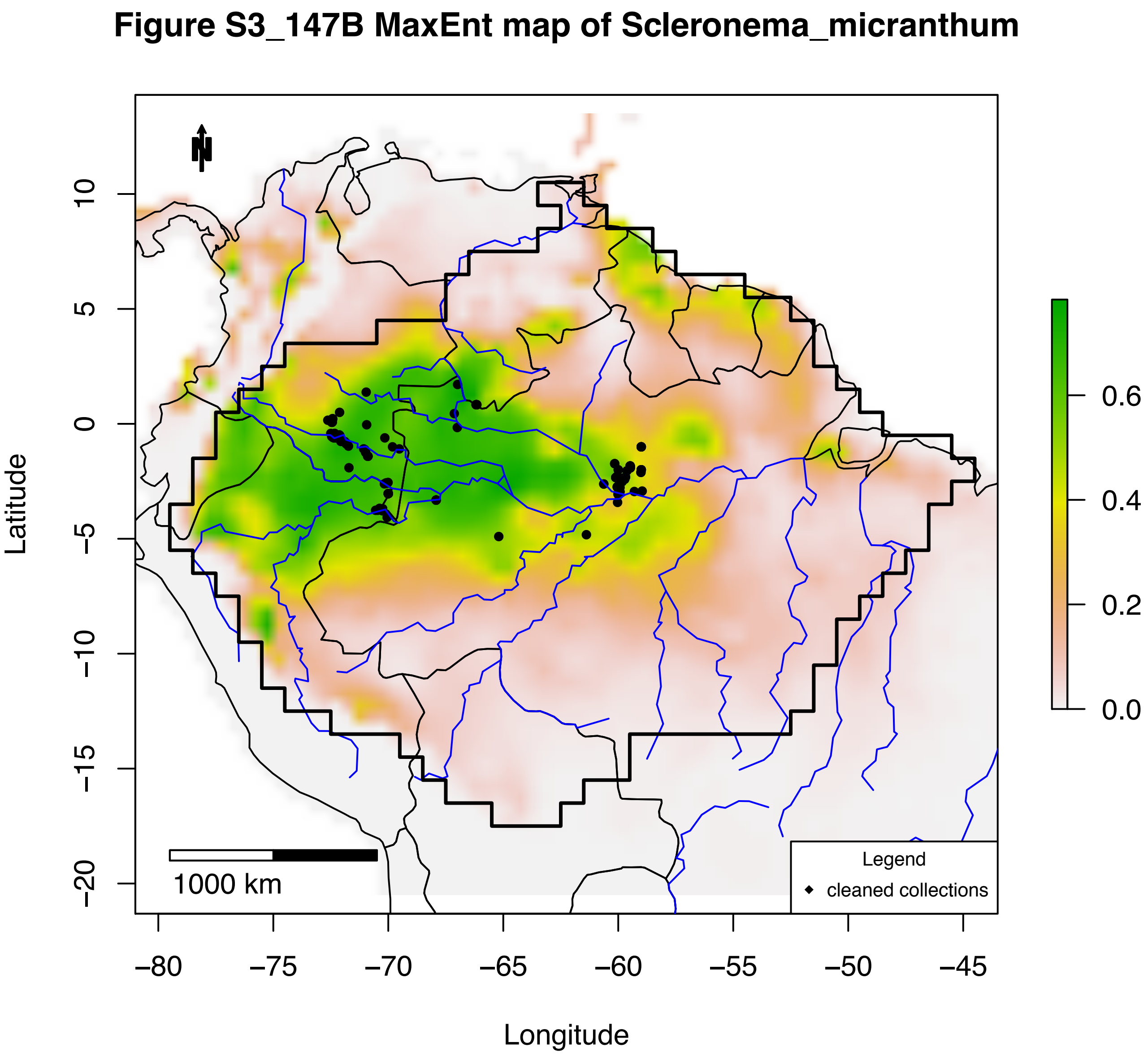

Figure S3_147B MaxEnt map of Scleronema_micranthum
N
Latitude
10
5
0
−5
−10
−15
−20
1000 km
Legend
cleaned collections
0.6
0.4
0.2
0.0
−80
−75
−70
−65
−60
−55
−50
−45
Longitude

Figure S3_147C IDW map of Scleronema_micranthum: plots =0 – 0.08; IDW: 0 – 0.02

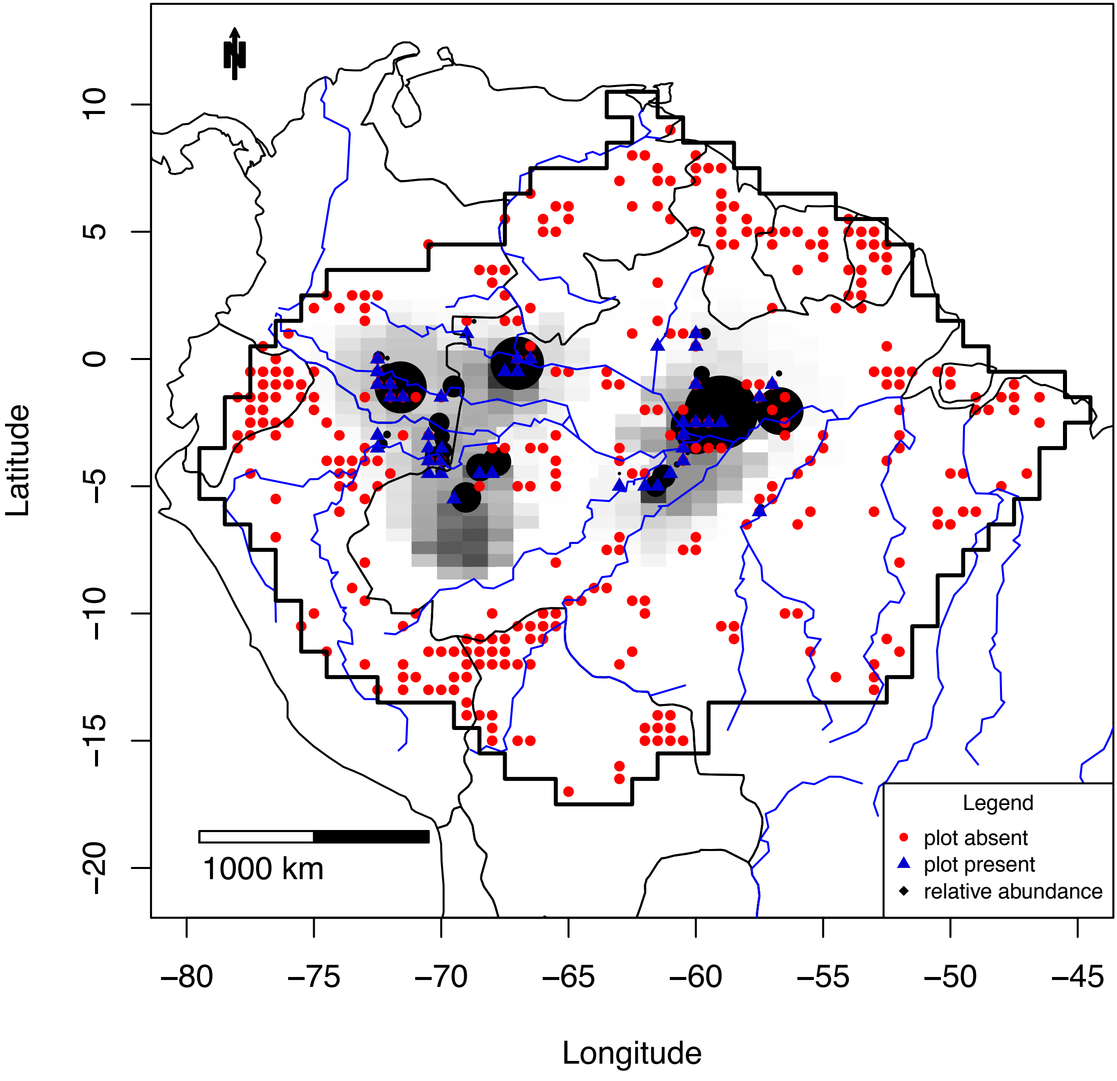

Figure S3_148A Combined map of Senefeldera_inclinata

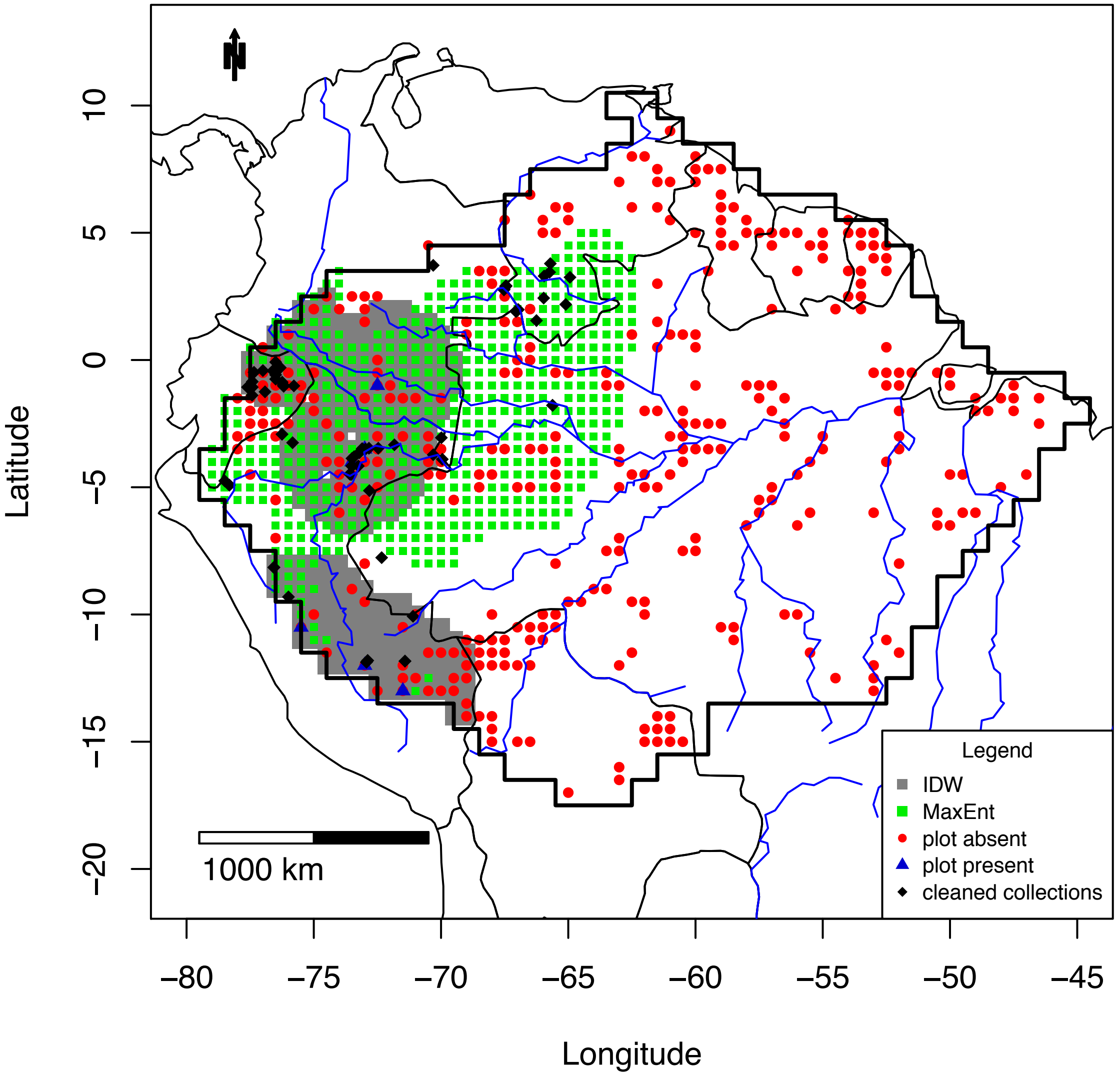

Figure S3_148B MaxEnt map of Senefeldera_inclinata

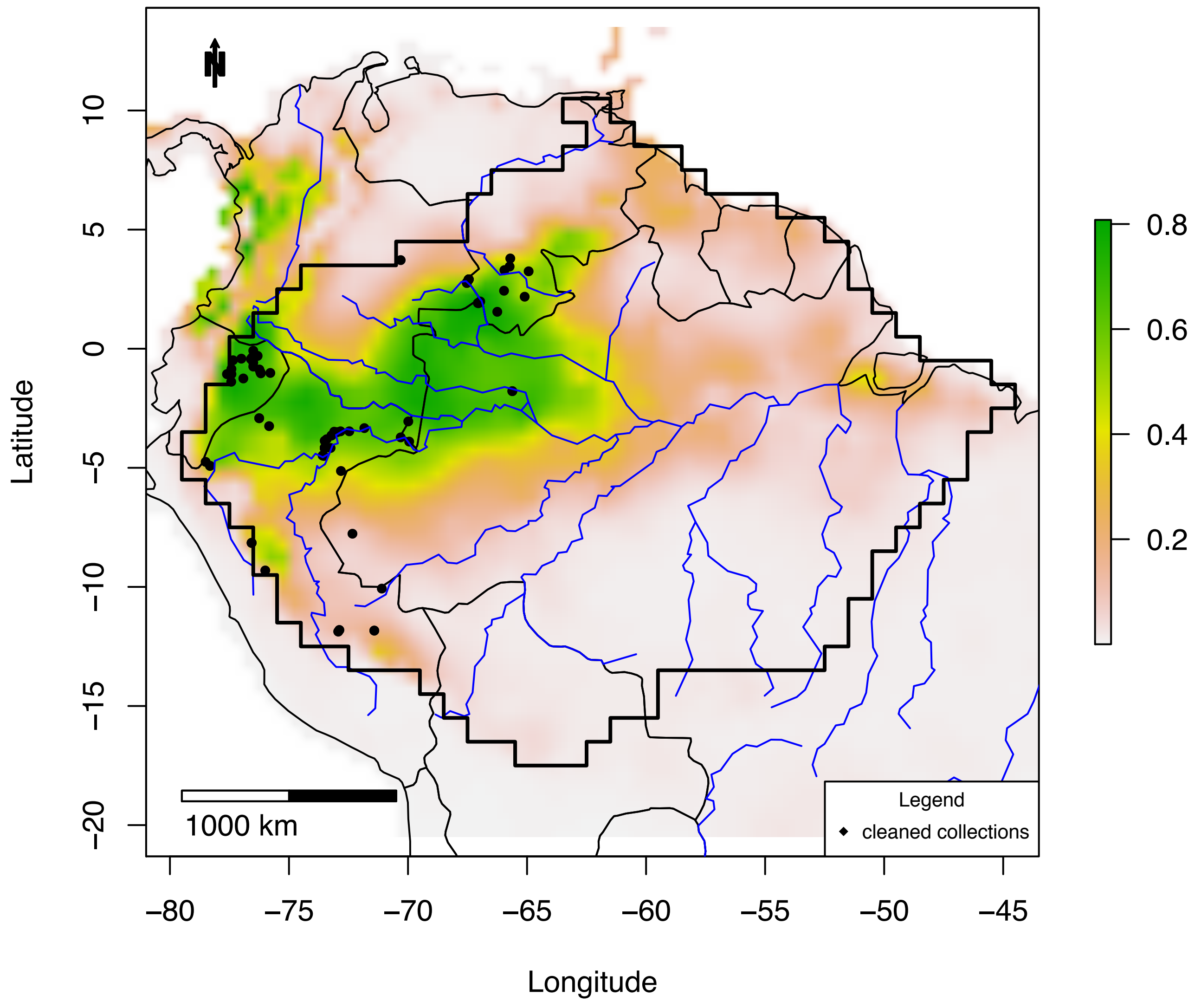

Figure S3_148C IDW map of Senefeldera_inclinata: plots =0 – 0.54; IDW: 0 – 0.15

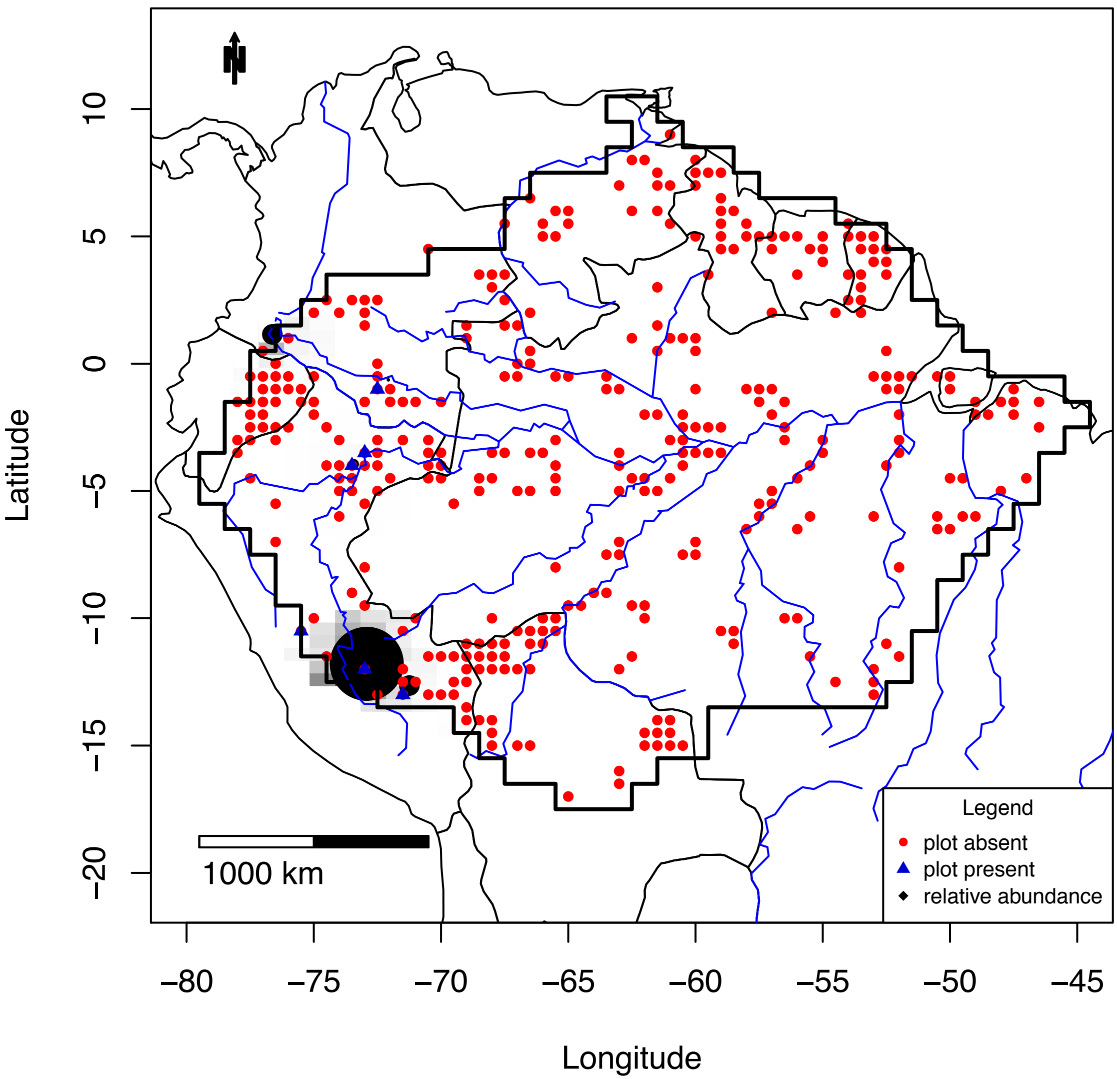

Figure S3_149A Combined map of Siparuna_decipiens

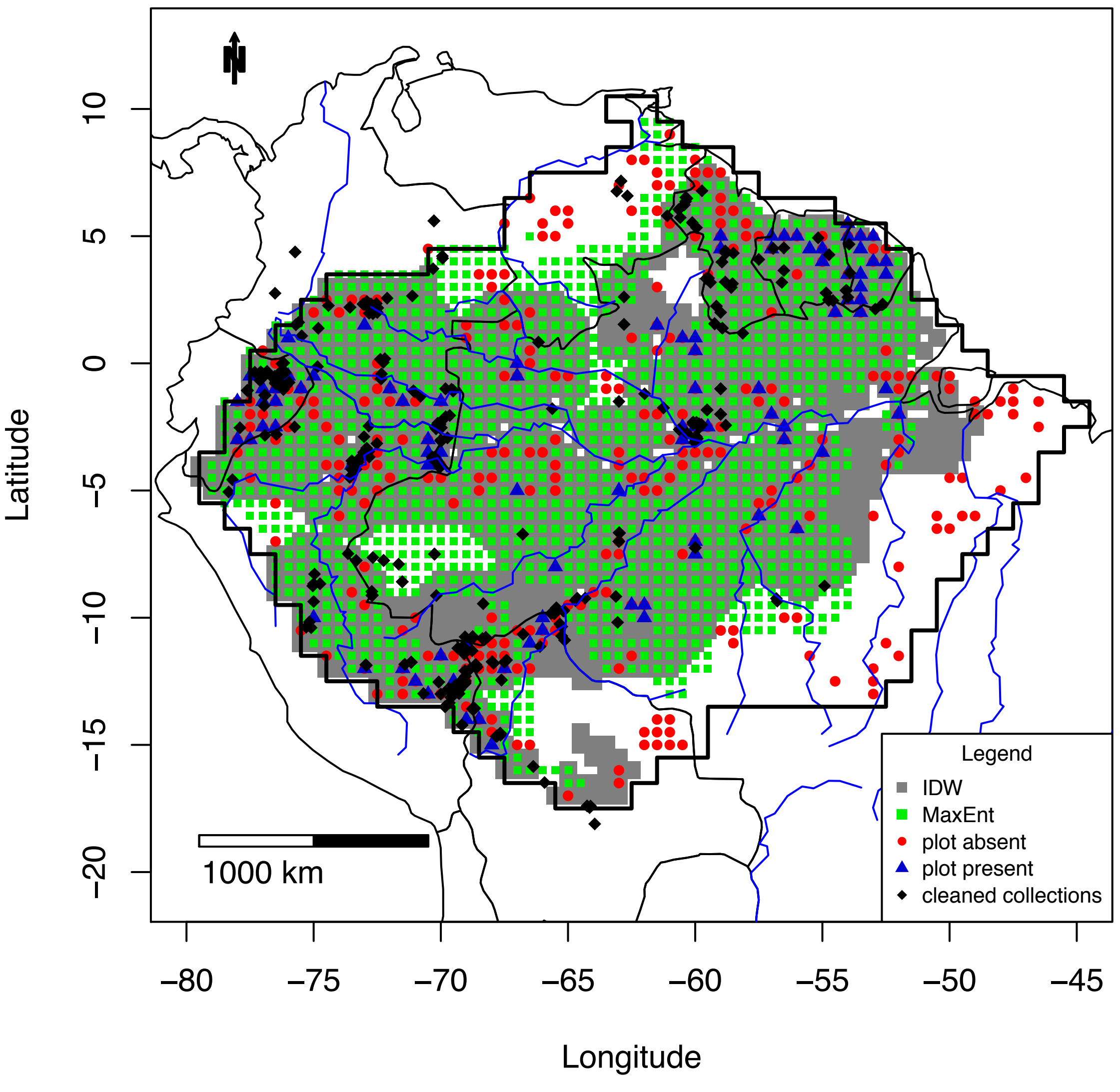

Figure S3_149B MaxEnt map of Siparuna_decipiens

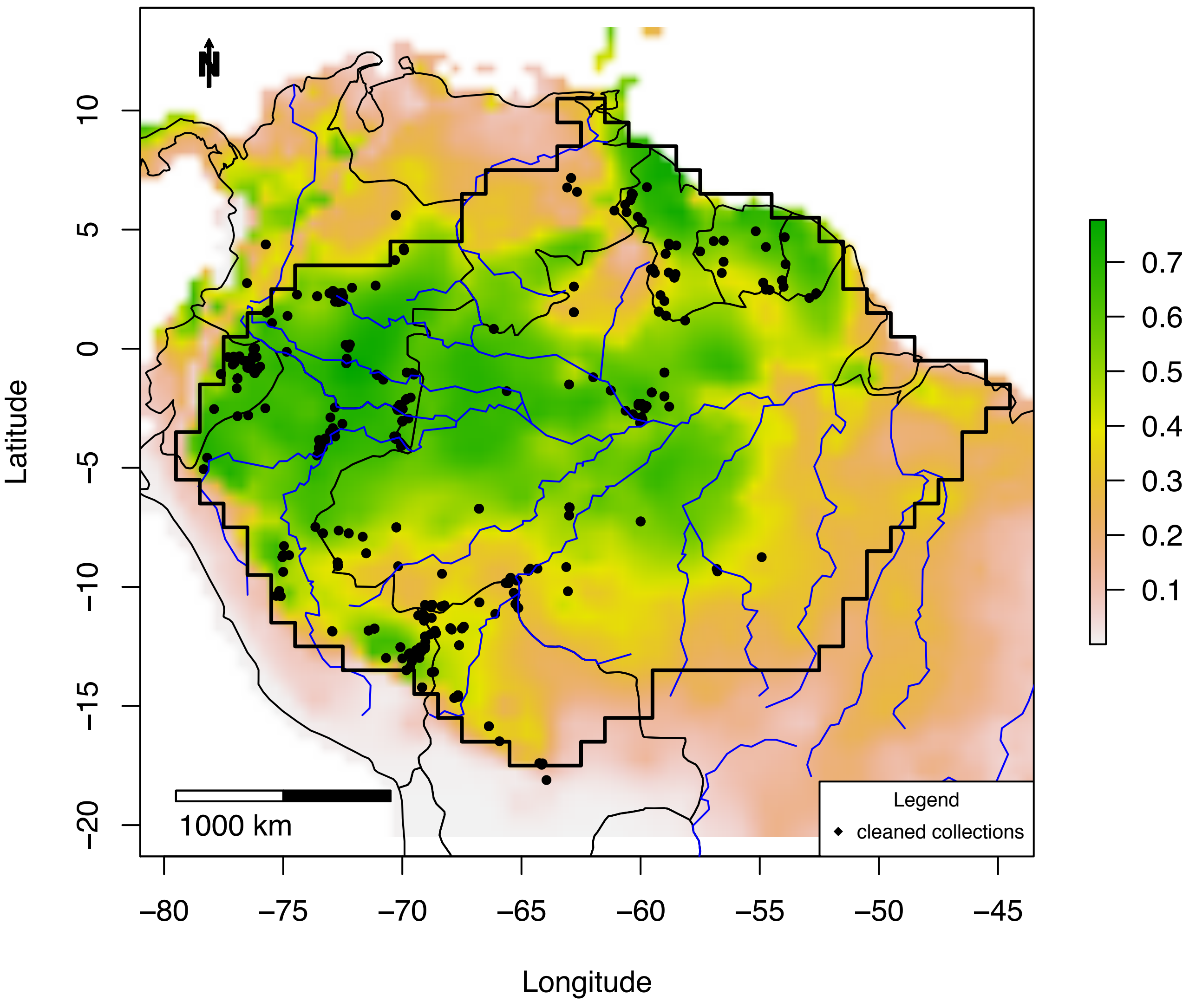

Figure S3_149C IDW map of Siparuna_decipiens: plots =0 – 0.1; IDW: 0 – 0.07

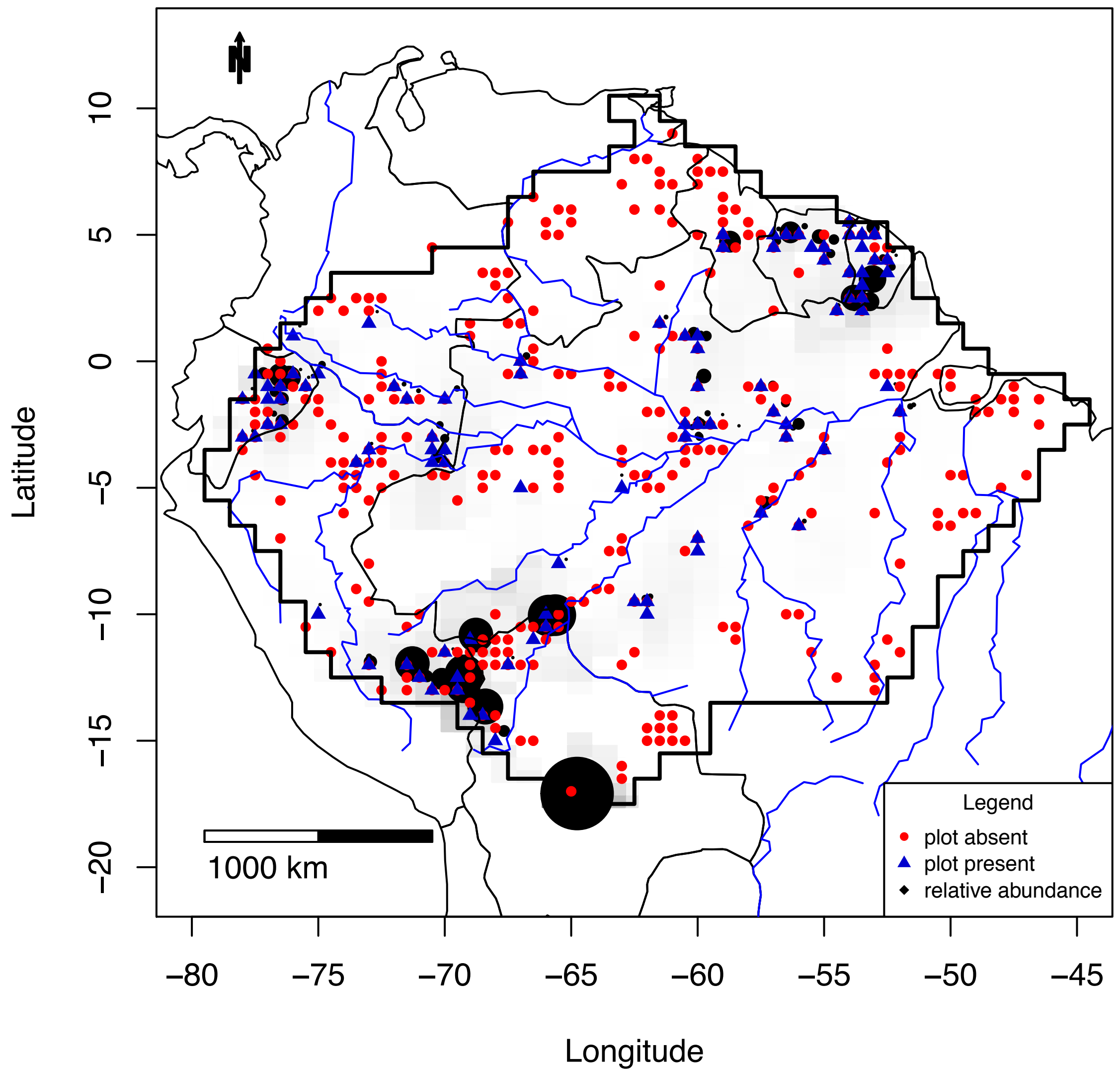

Figure S3_150A Combined map of Socratea_exorrhiza

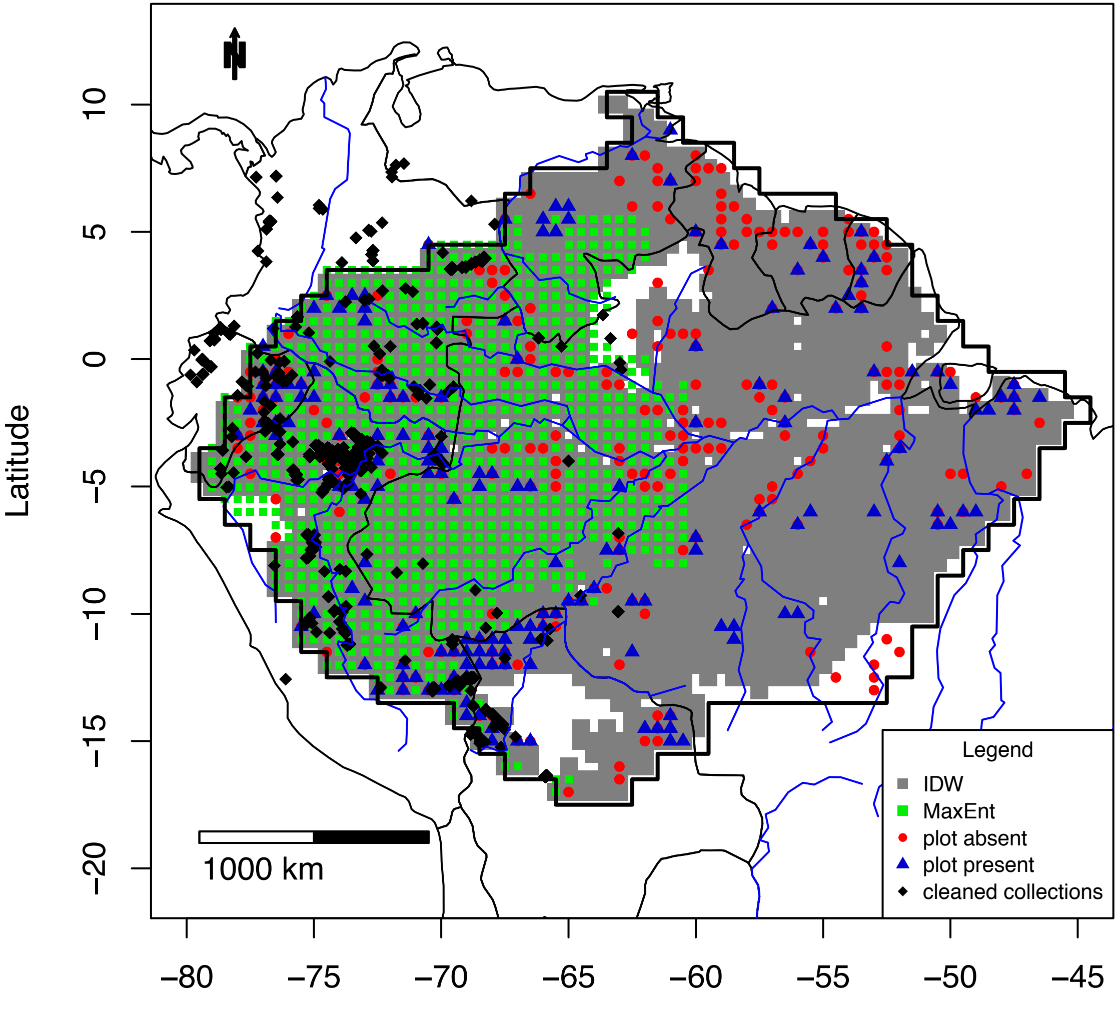

Figure S3_150B MaxEnt map of Socratea_exorrhiza

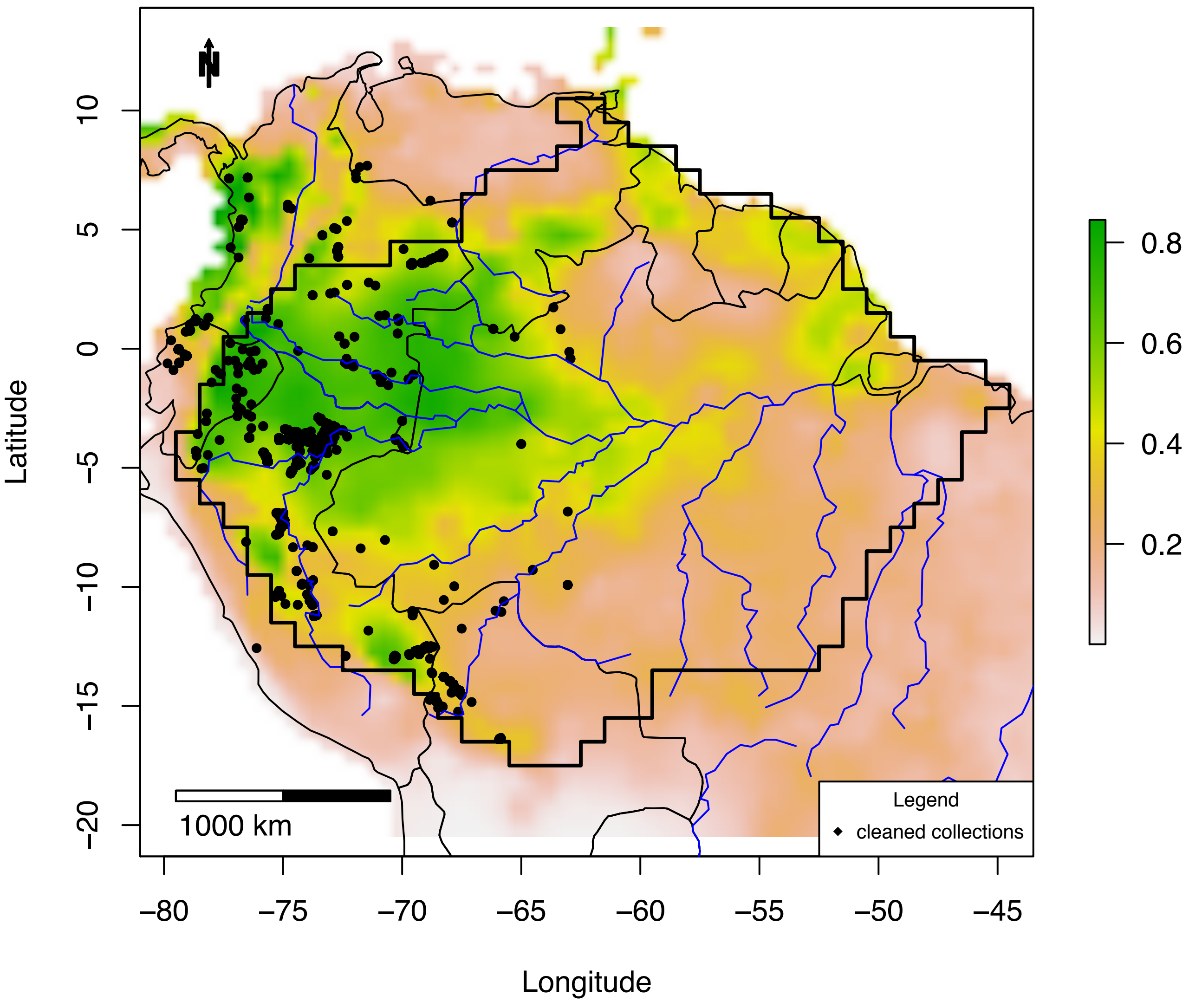

# Figure S3_150C IDW map of Socratea_exorrhiza:
plots =0 – 0.31; IDW: 0 – 0.16

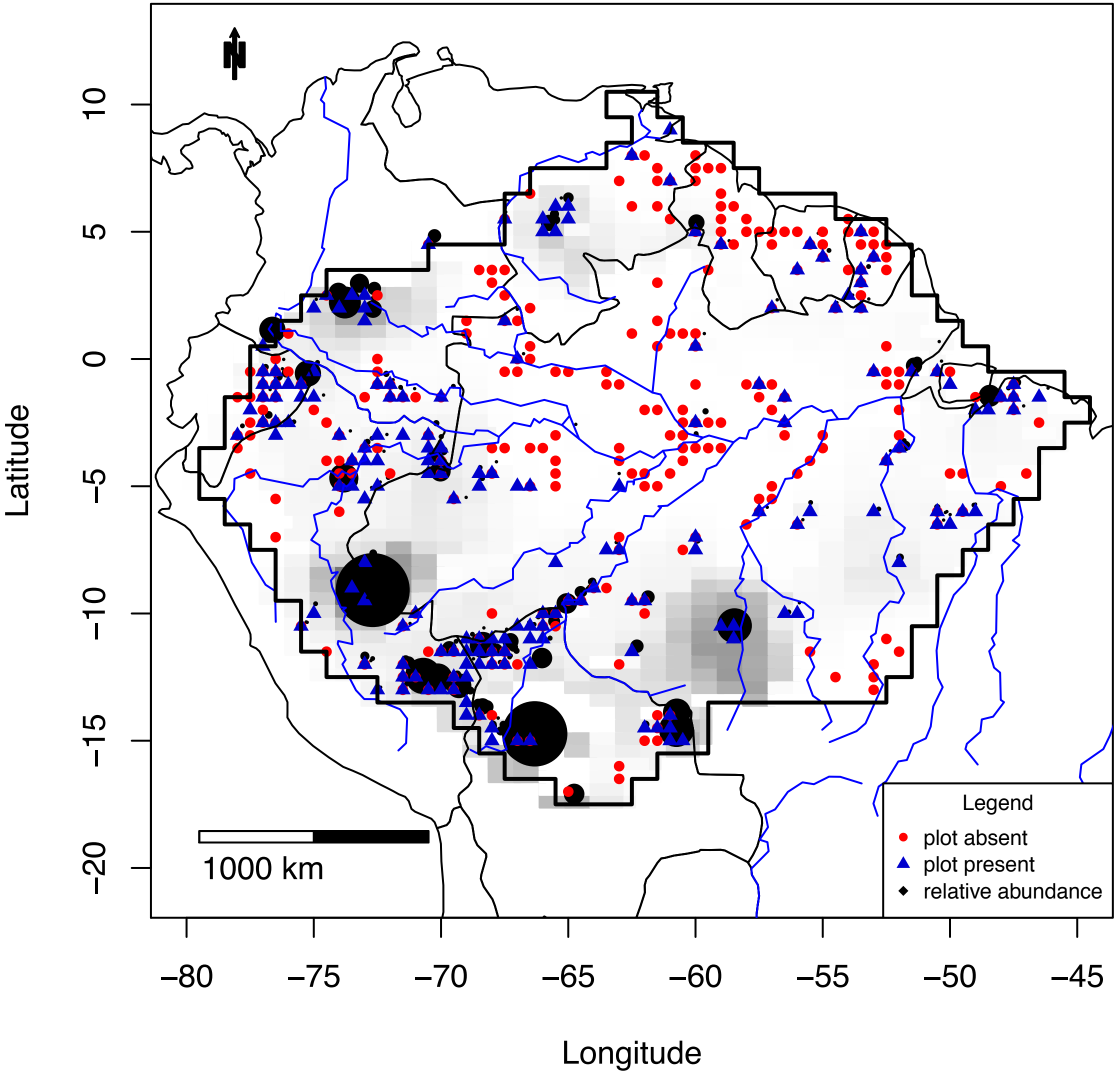

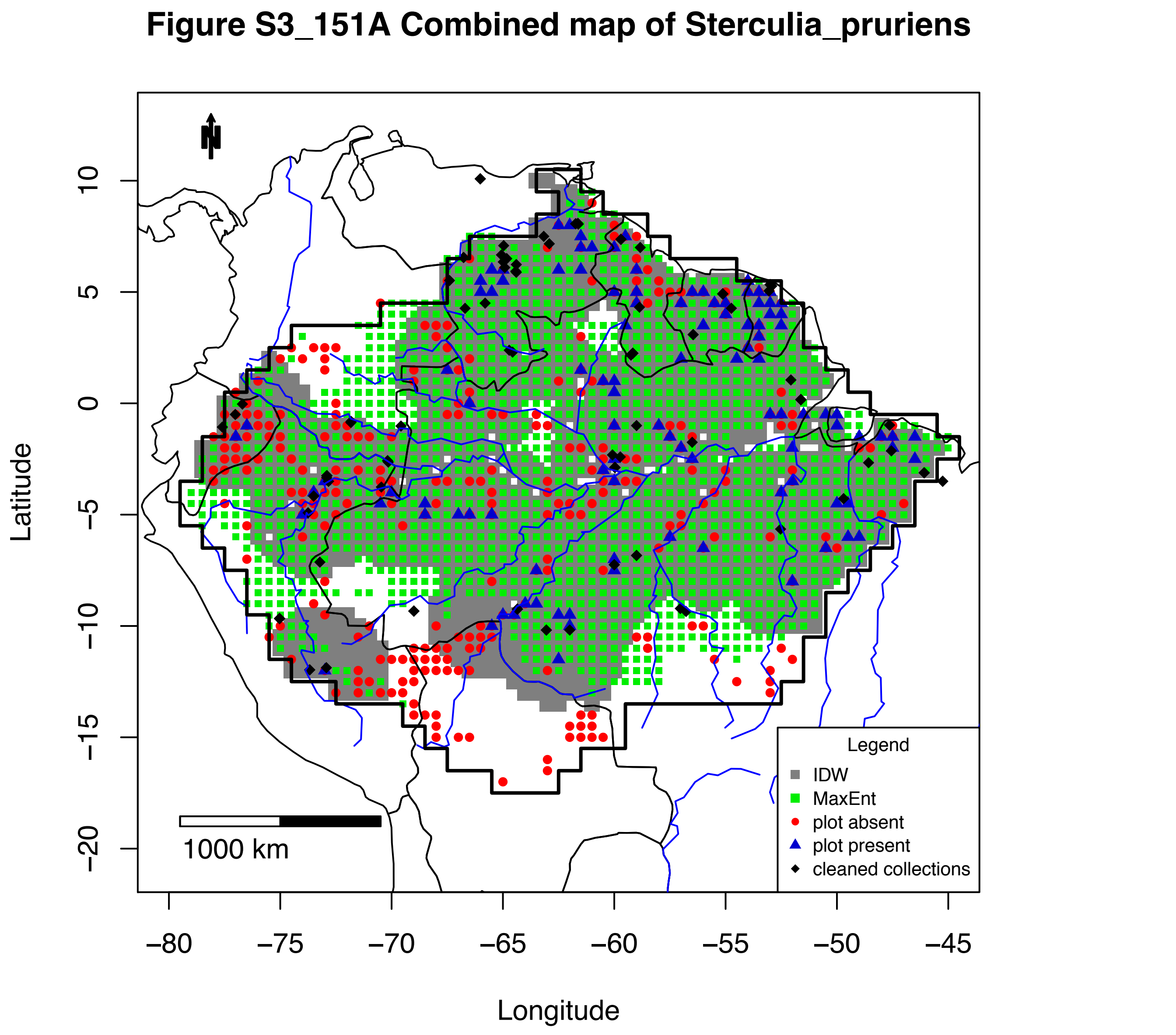
Figure S3_151A Combined map of Sterculia_pruriens
N
10
5
0
−5
−10
−15
−20
Latitude
−80
−75
−70
−65
−60
−55
−50
−45
Longitude
1000 km
Legend
IDW
MaxEnt
plot absent
plot present
cleaned collections

Figure S3_151B MaxEnt map of Sterculia_pruriens

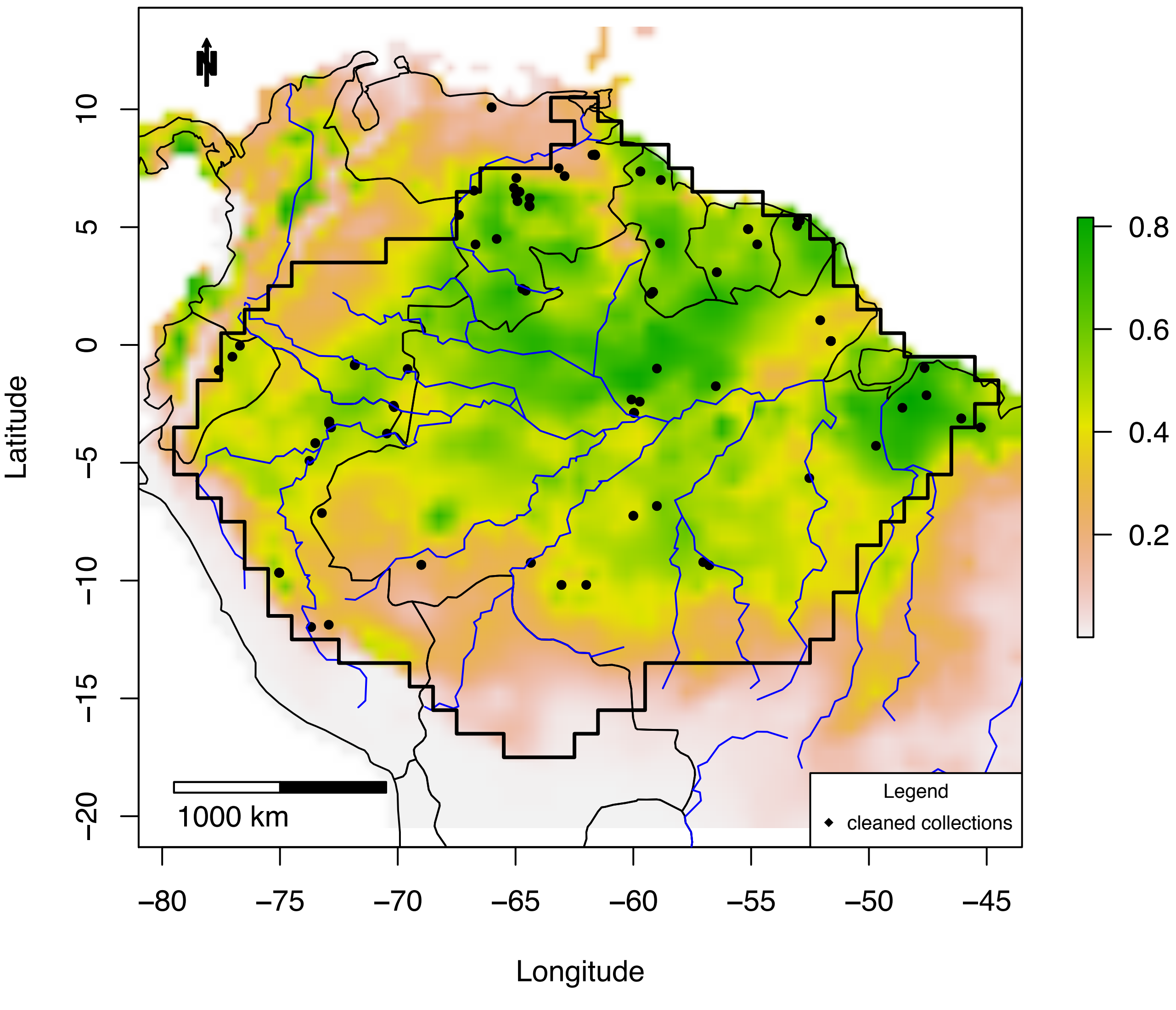

Figure S3_151C IDW map of Sterculia_pruriens: plots =0 – 0.06; IDW: 0 – 0.03

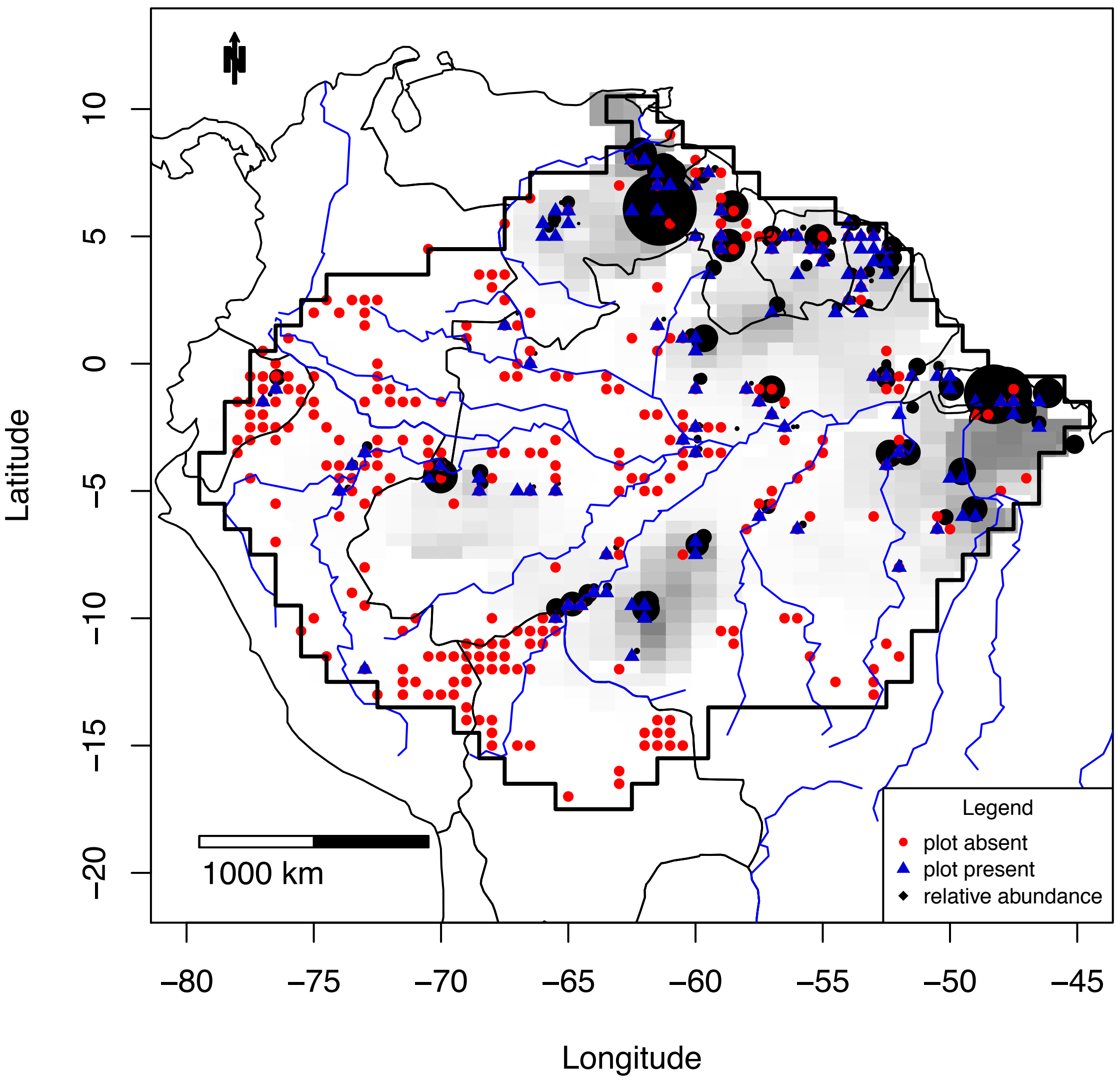

Figure S3_152A Combined map of Swartzia_polyphylla

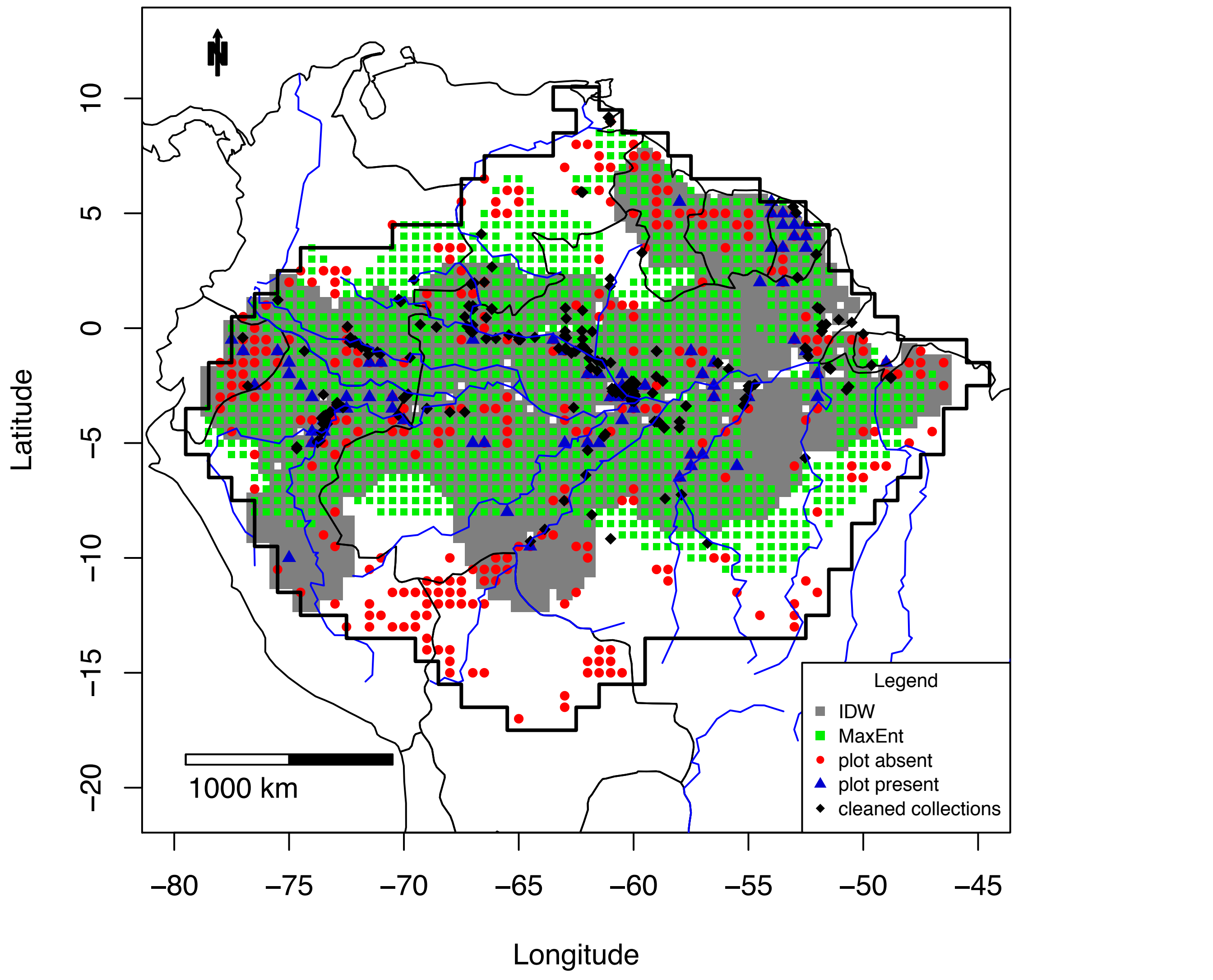

Figure S3_152B MaxEnt map of Swartzia_polyphylla

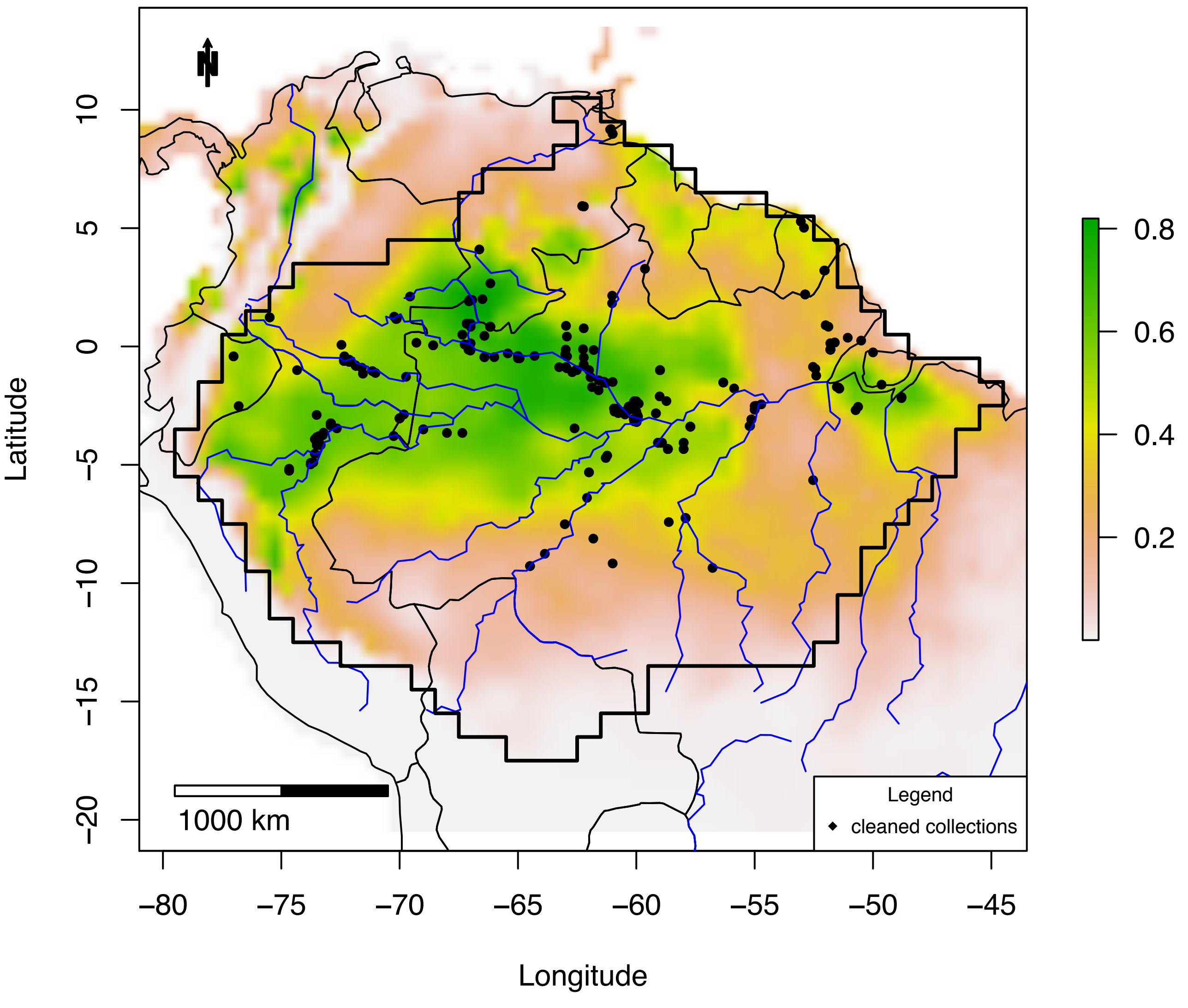

Figure S3_152C IDW map of Swartzia_polyphylla: plots =0 – 0.15; IDW: 0 – 0.04

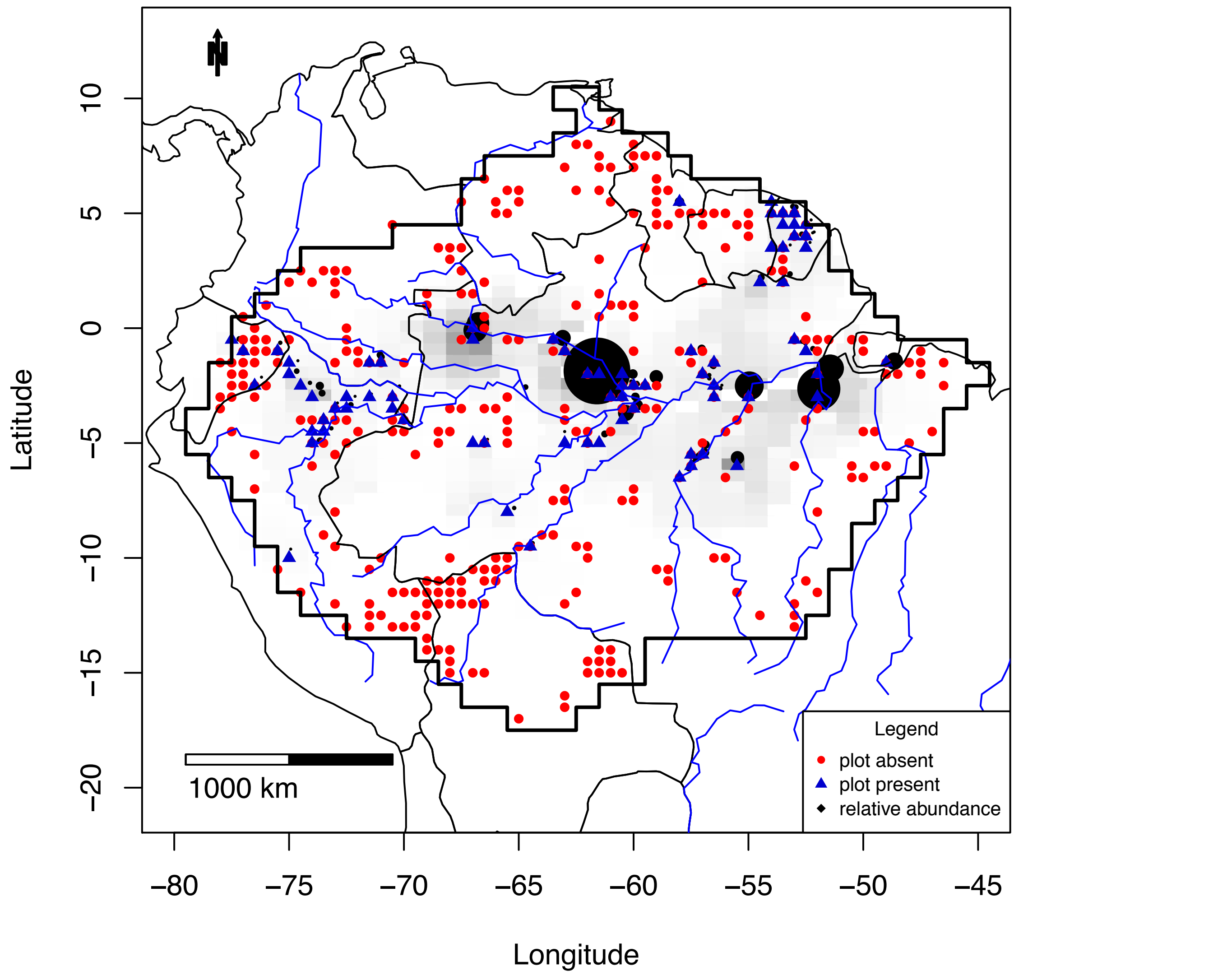

Figure S3_153A Combined map of Symphonia_globulifera

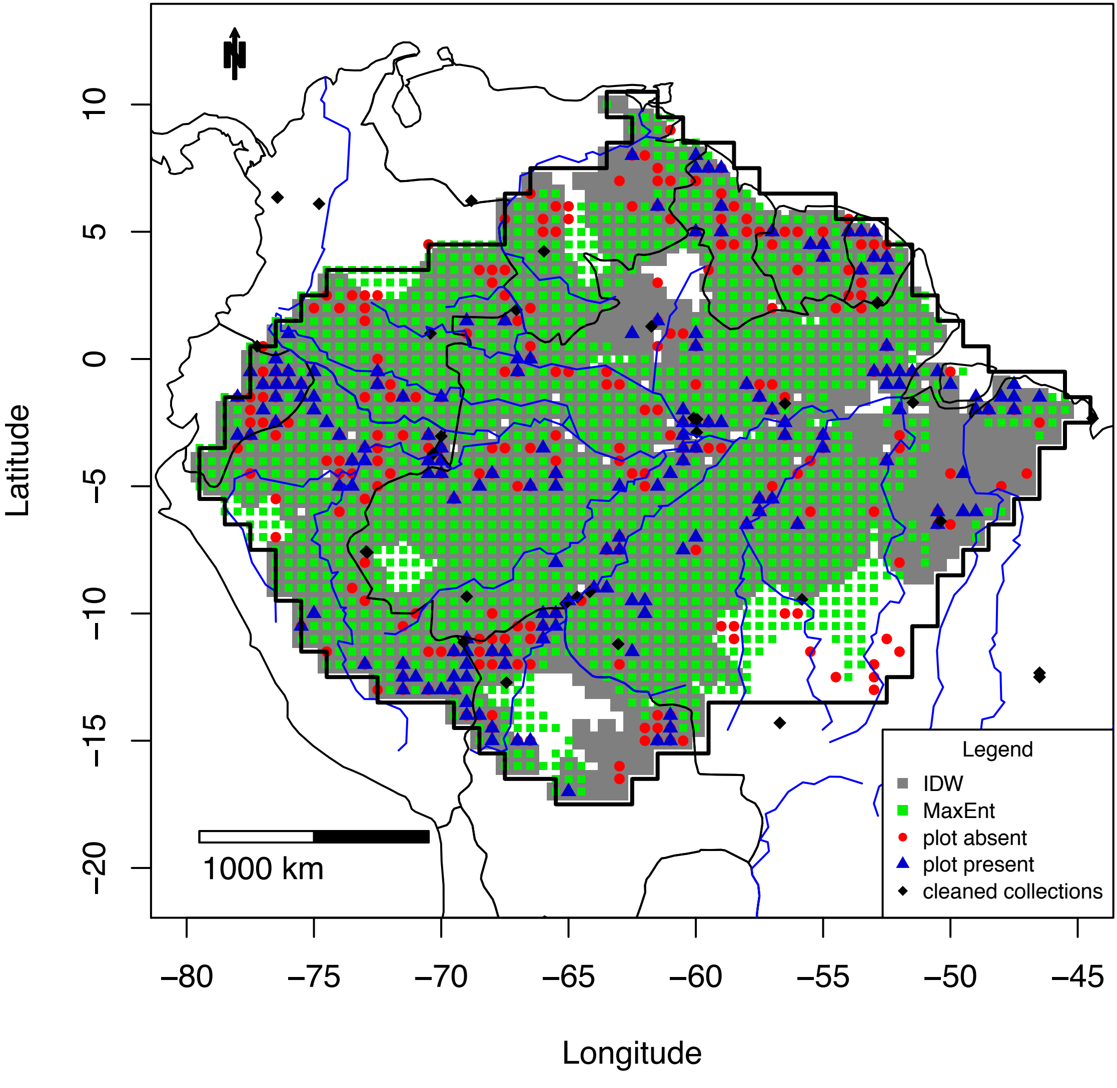

Figure S3_153B MaxEnt map of Symphonia_globulifera

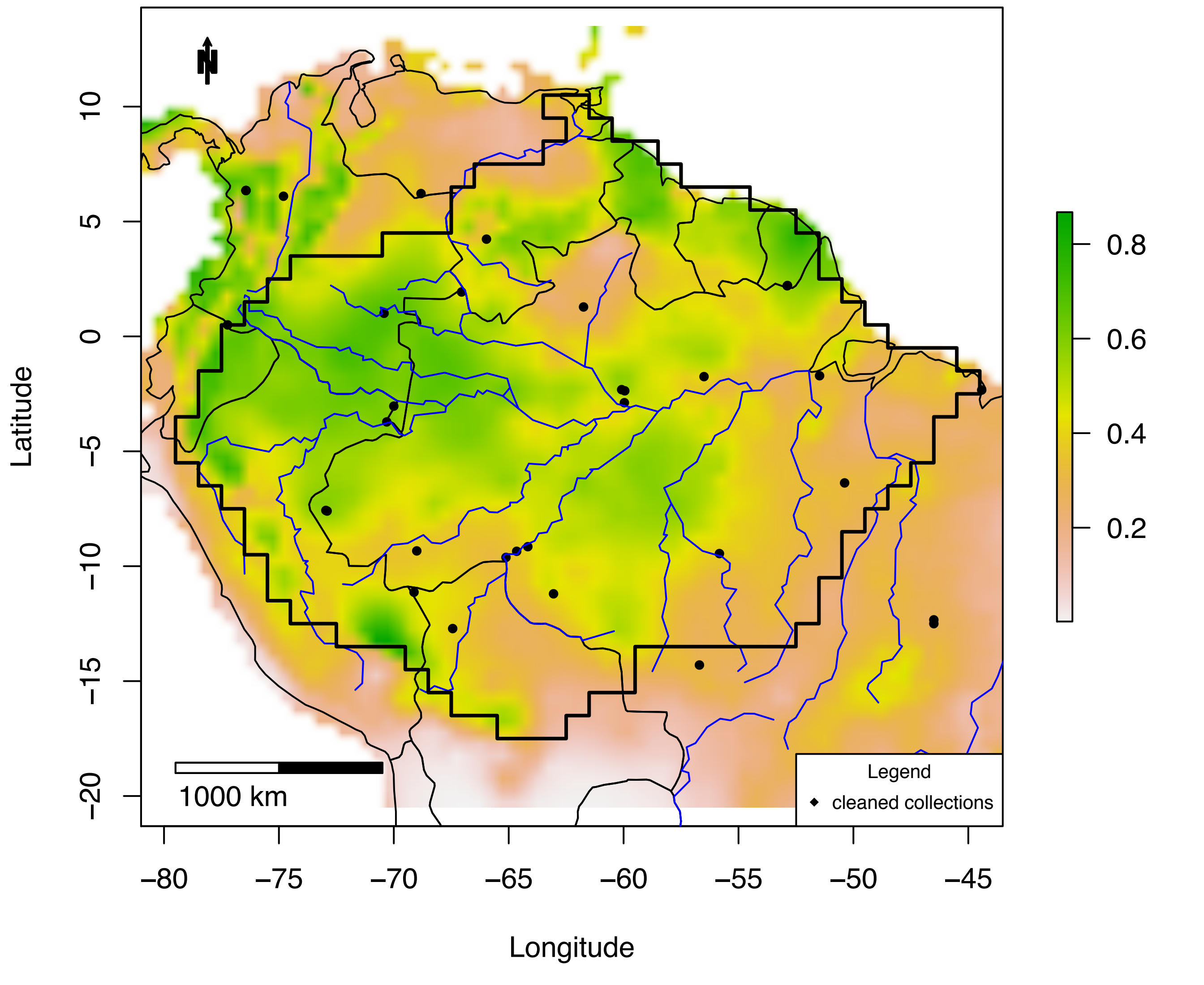

Figure S3_153C IDW map of Symphonia_globulifera: plots =0 – 0.16; IDW: 0 – 0.04

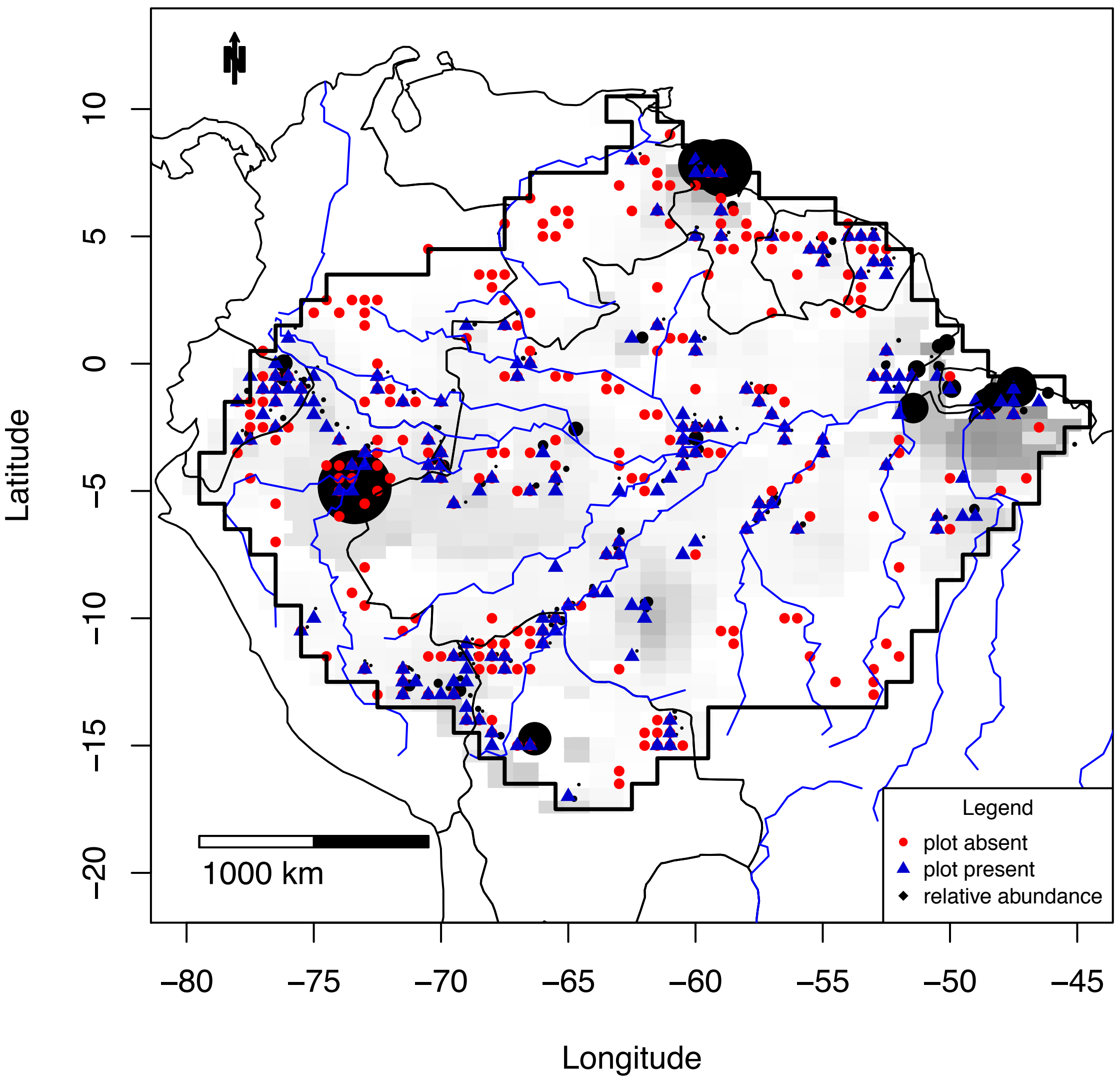

Figure S3_154A Combined map of Tachigali_paniculata

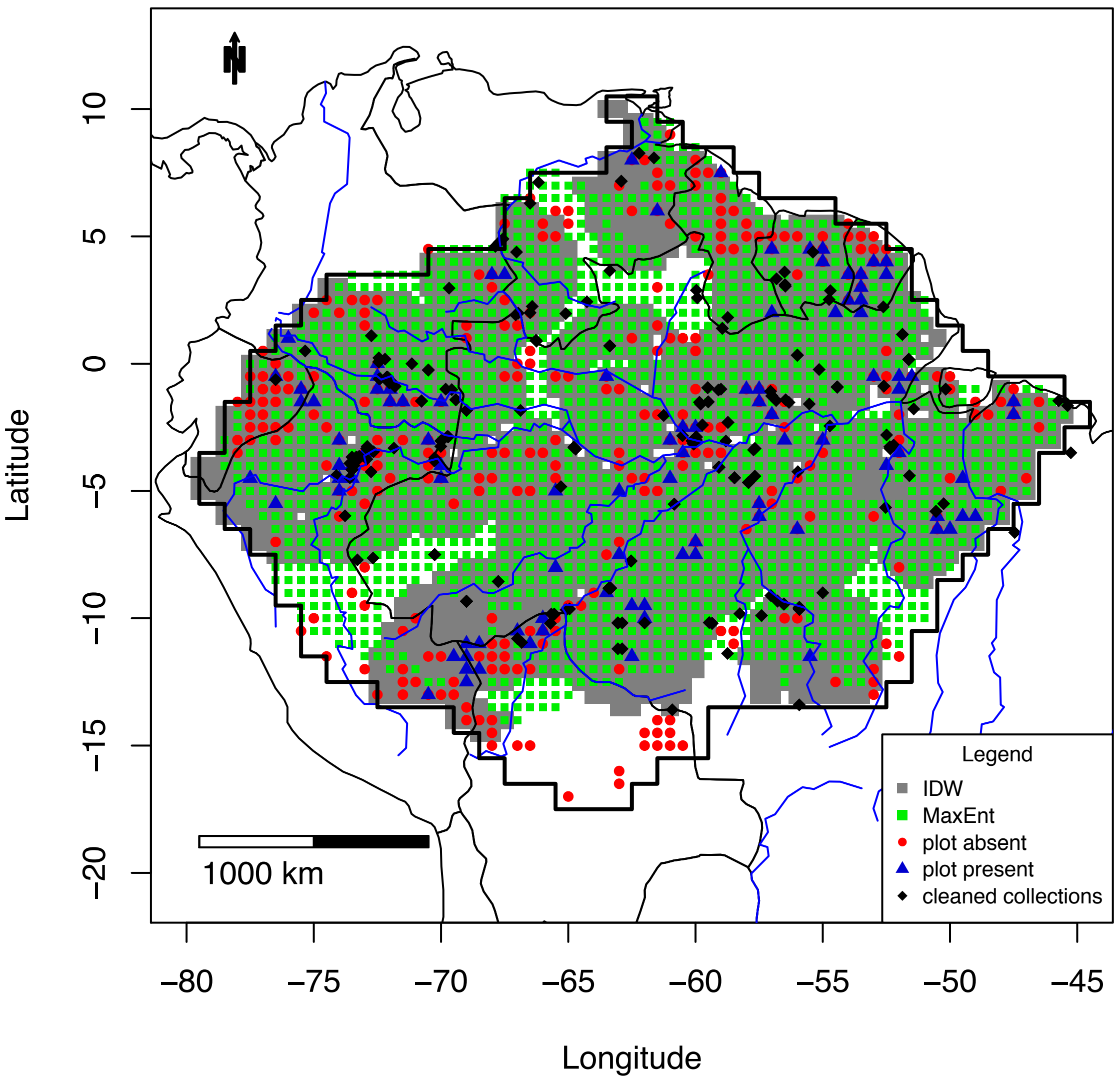

Figure S3_154B MaxEnt map of Tachigali_paniculata

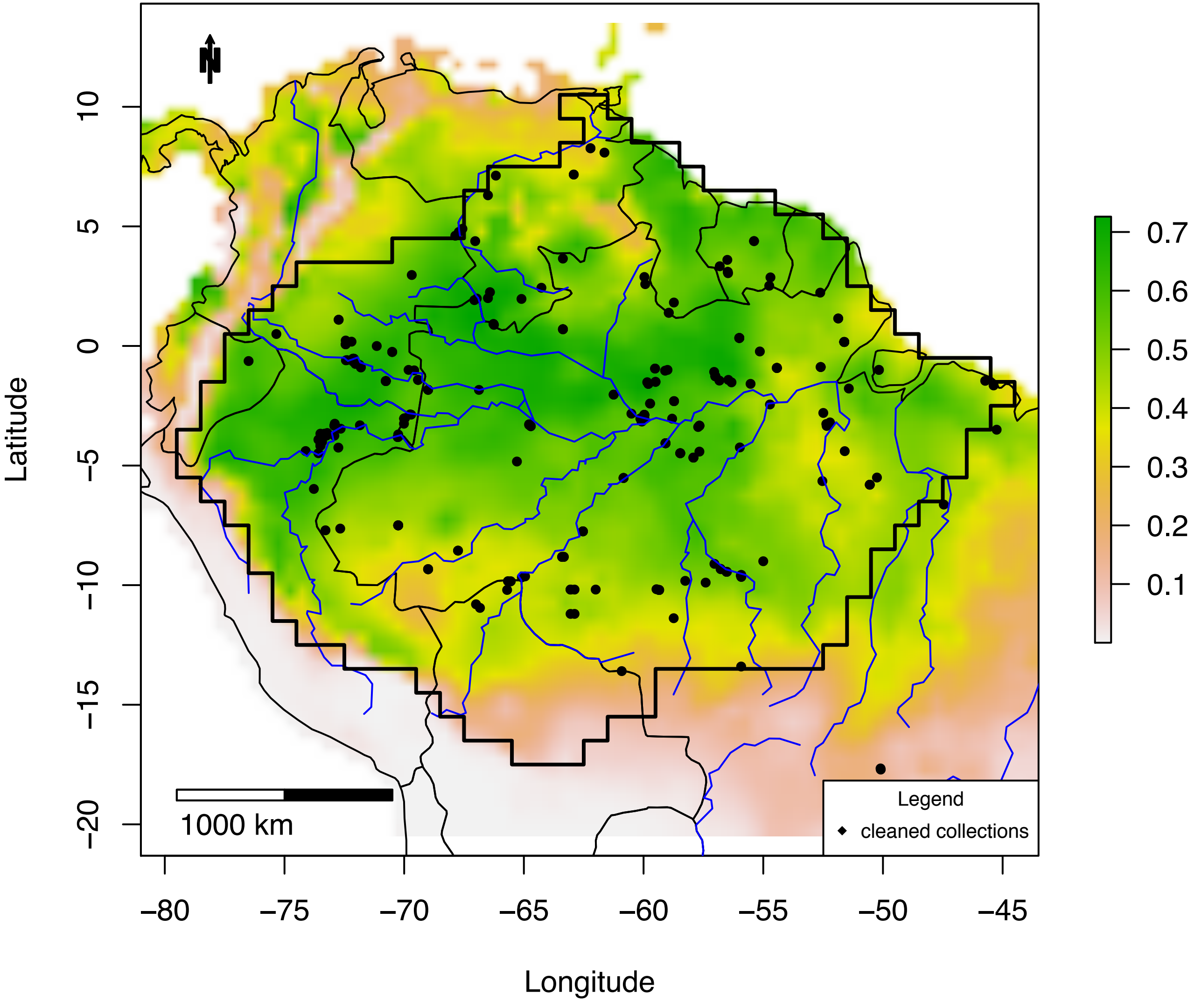

Figure S3_154C IDW map of Tachigali_paniculata: plots =0 – 0.12; IDW: 0 – 0.08

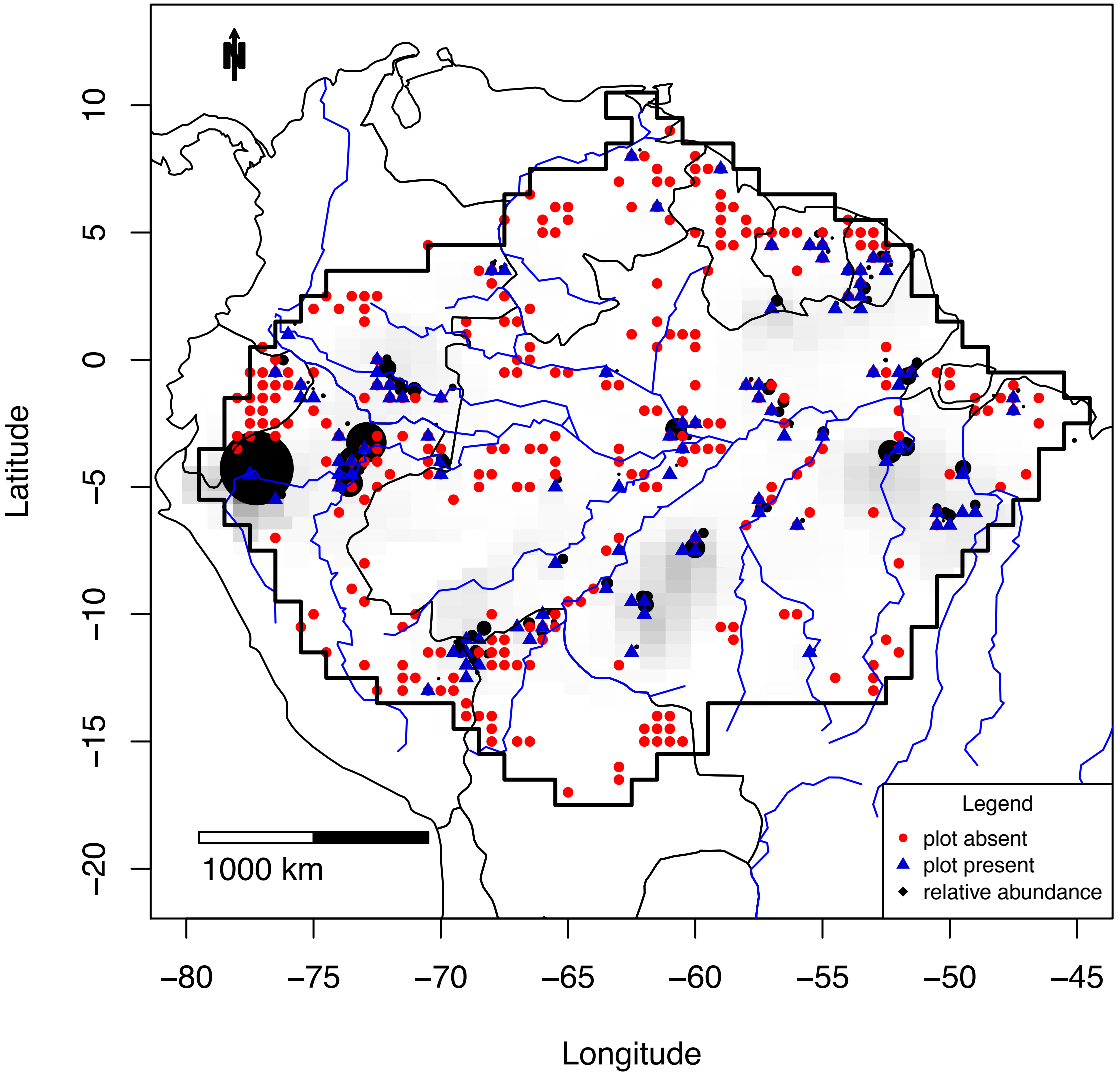

Figure S3_155A Combined map of Tetragastris_panamensis

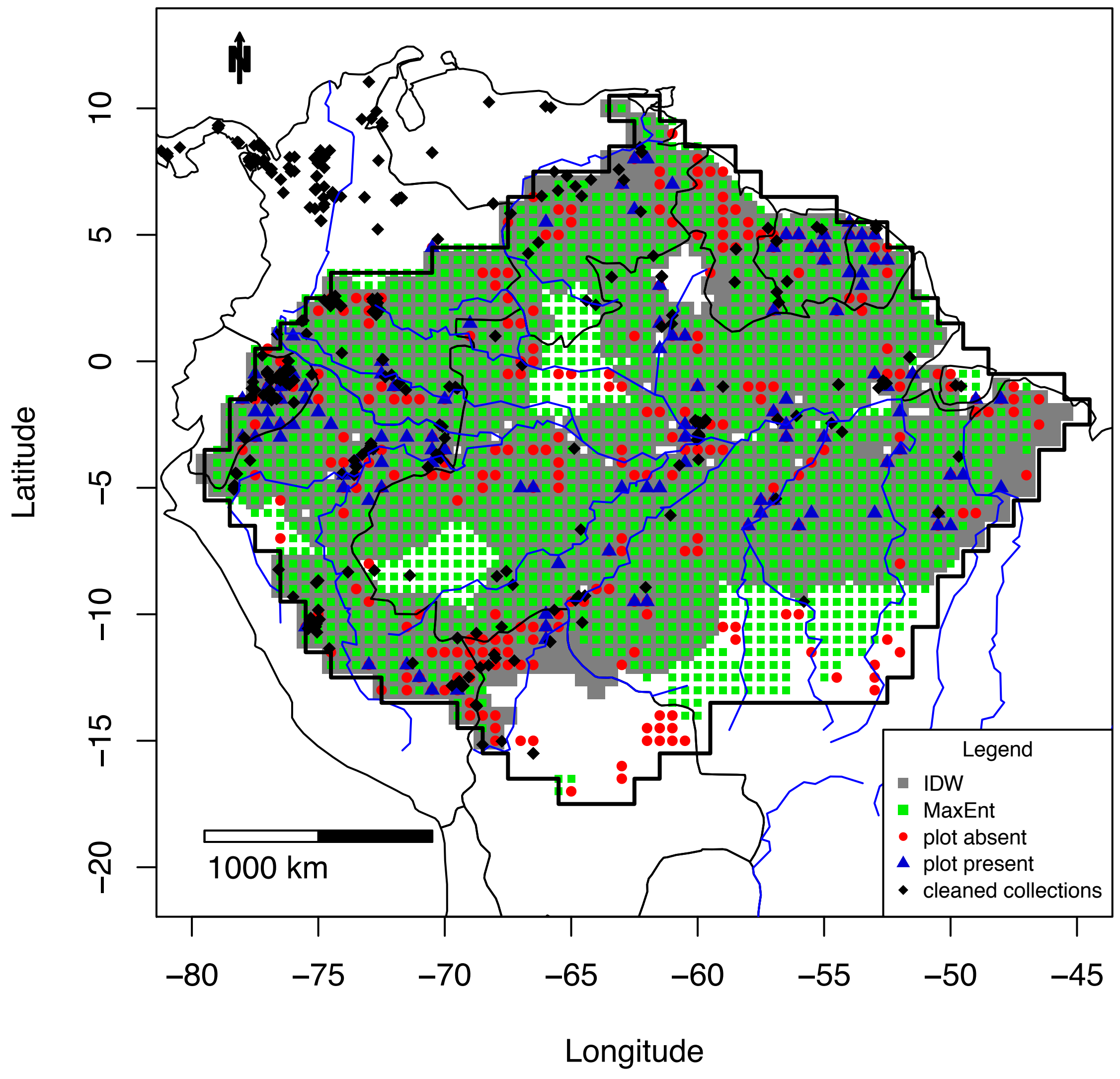

Figure S3_155B MaxEnt map of Tetragastris_panamensis

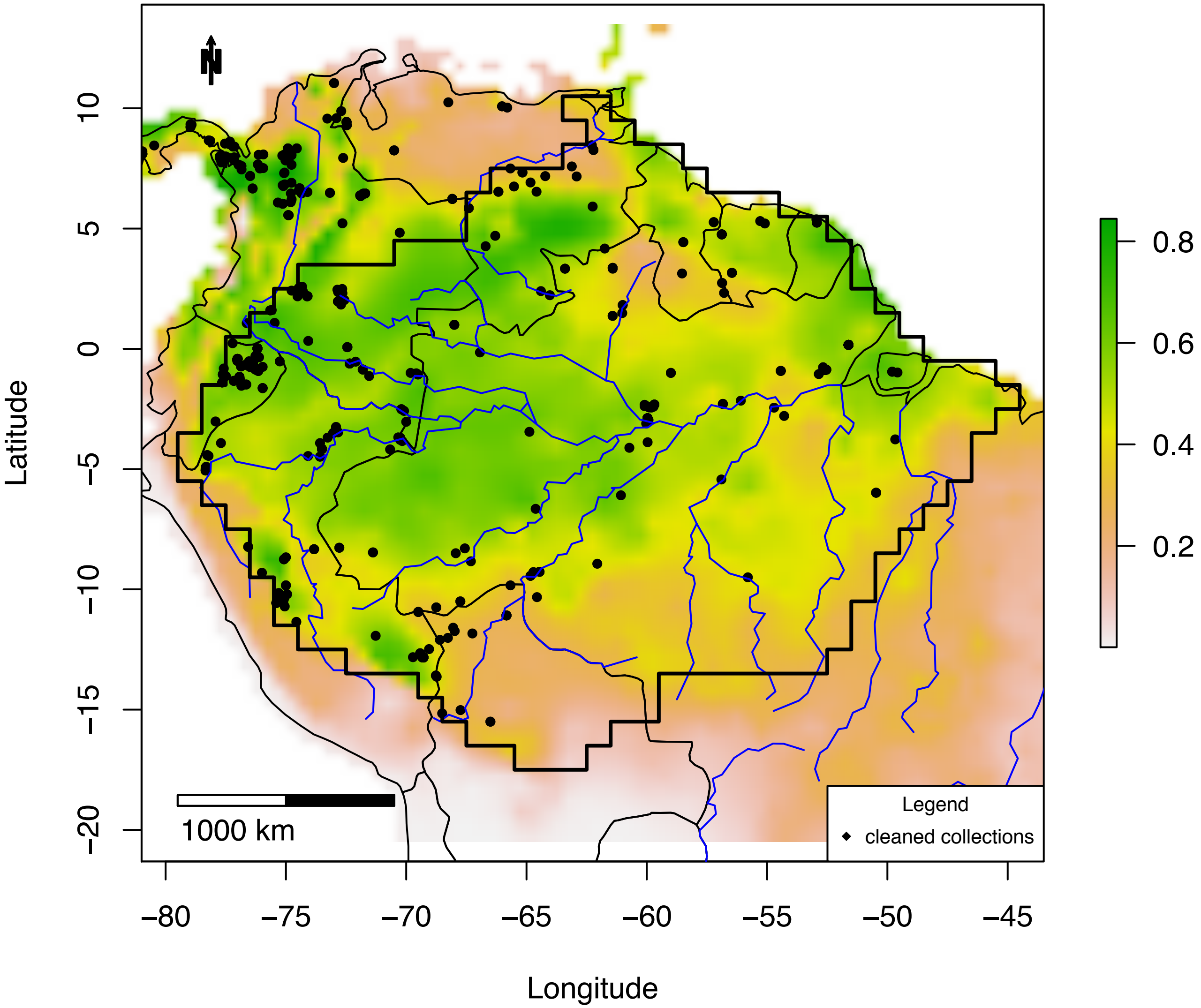

Figure S3_155C IDW map of Tetragastris_panamensis: plots =0 – 0.11; IDW: 0 – 0.05

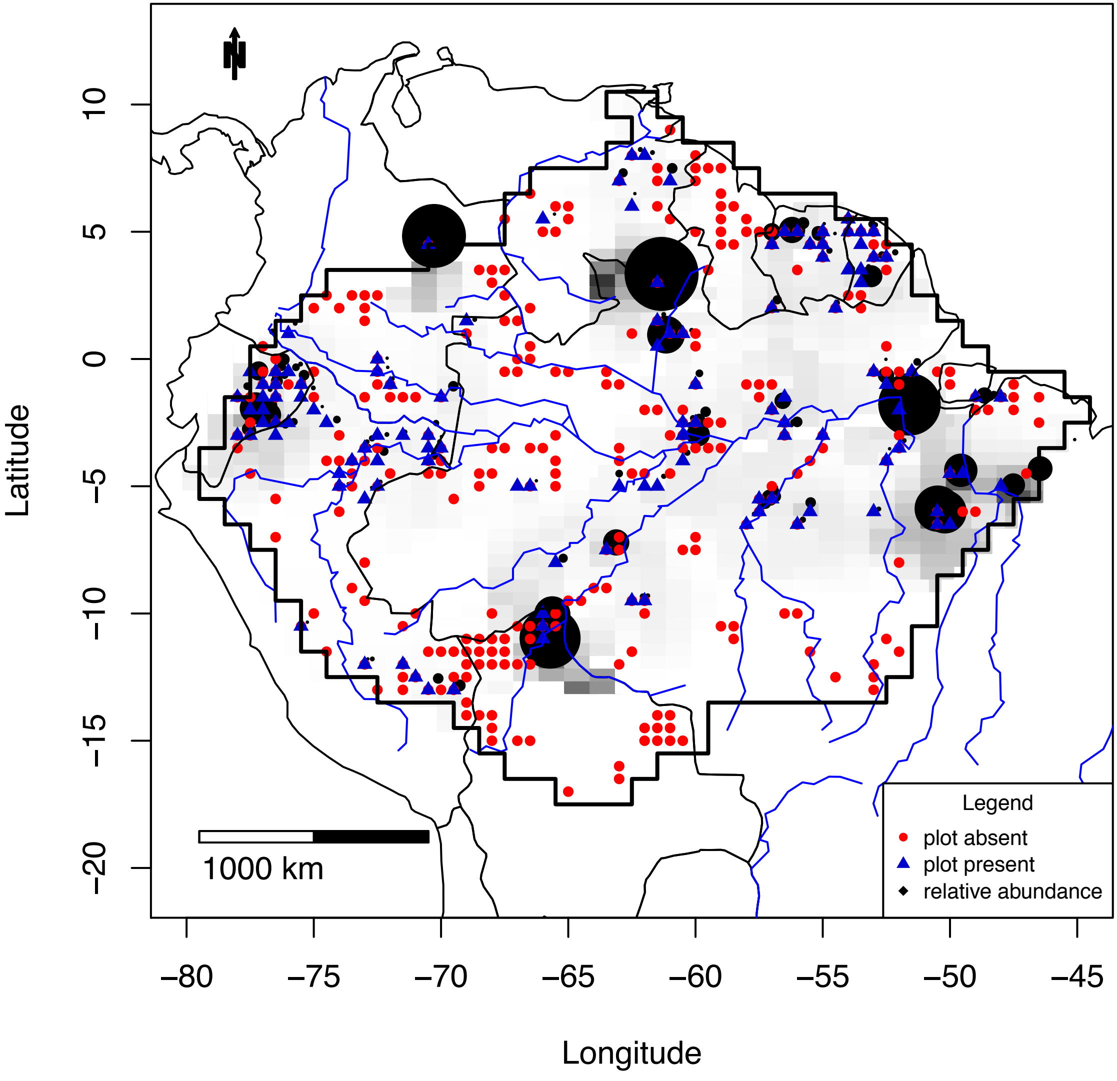

Figure S3_156A Combined map of Theobroma_cacao

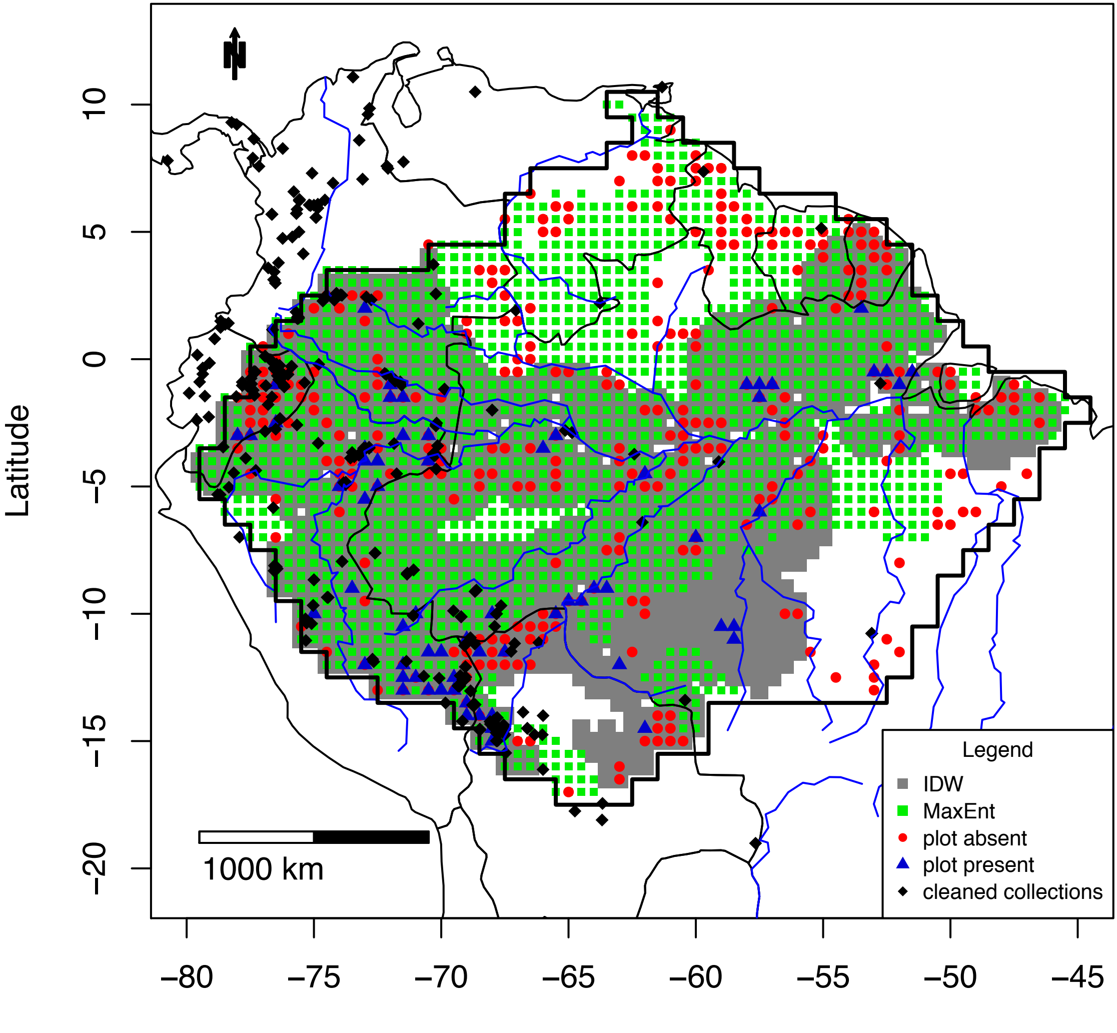

Figure S3_156B MaxEnt map of Theobroma_cacao

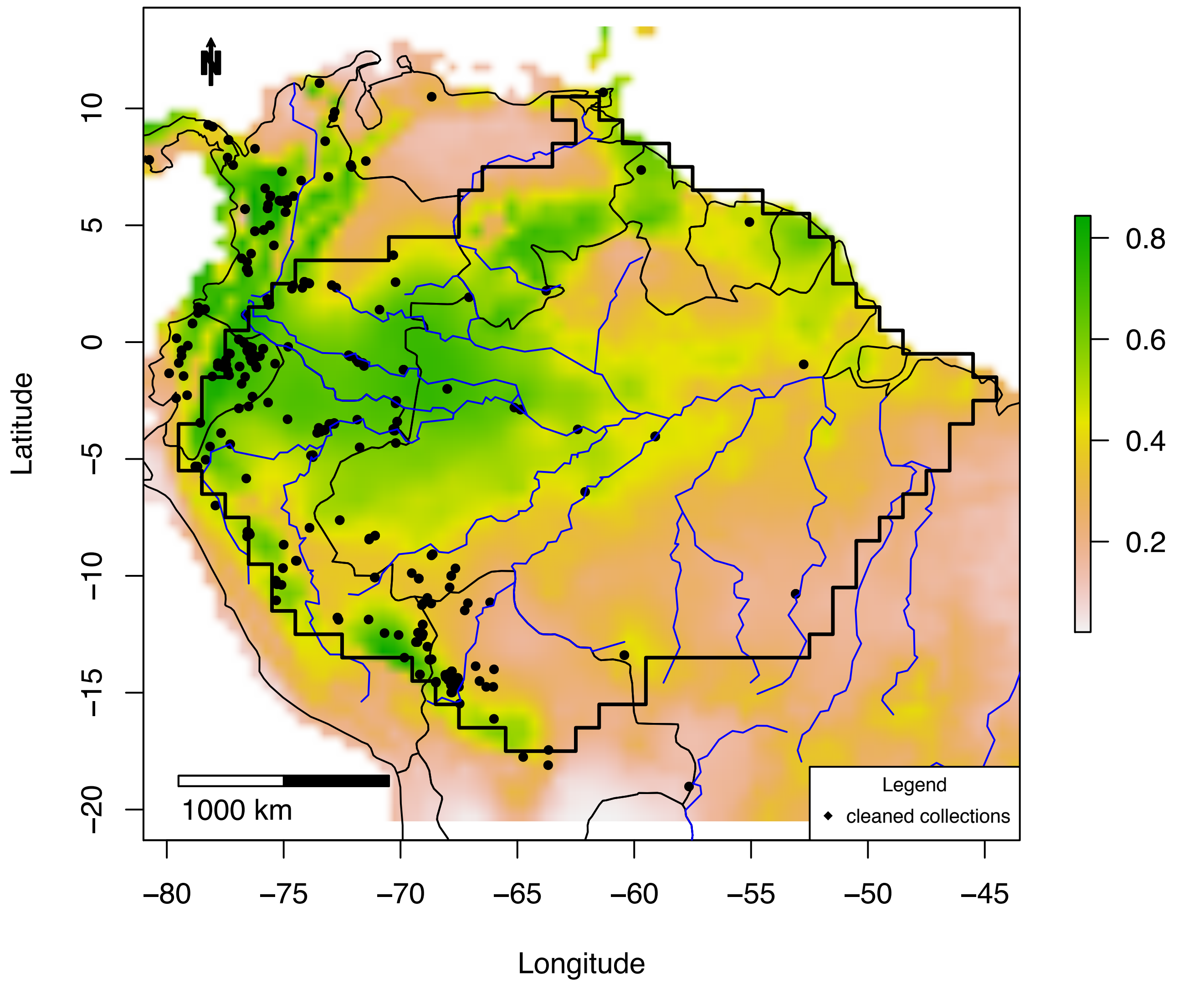

Figure S3_156C IDW map of Theobroma_cacao:
plots =0 – 0.31; IDW: 0 – 0.25

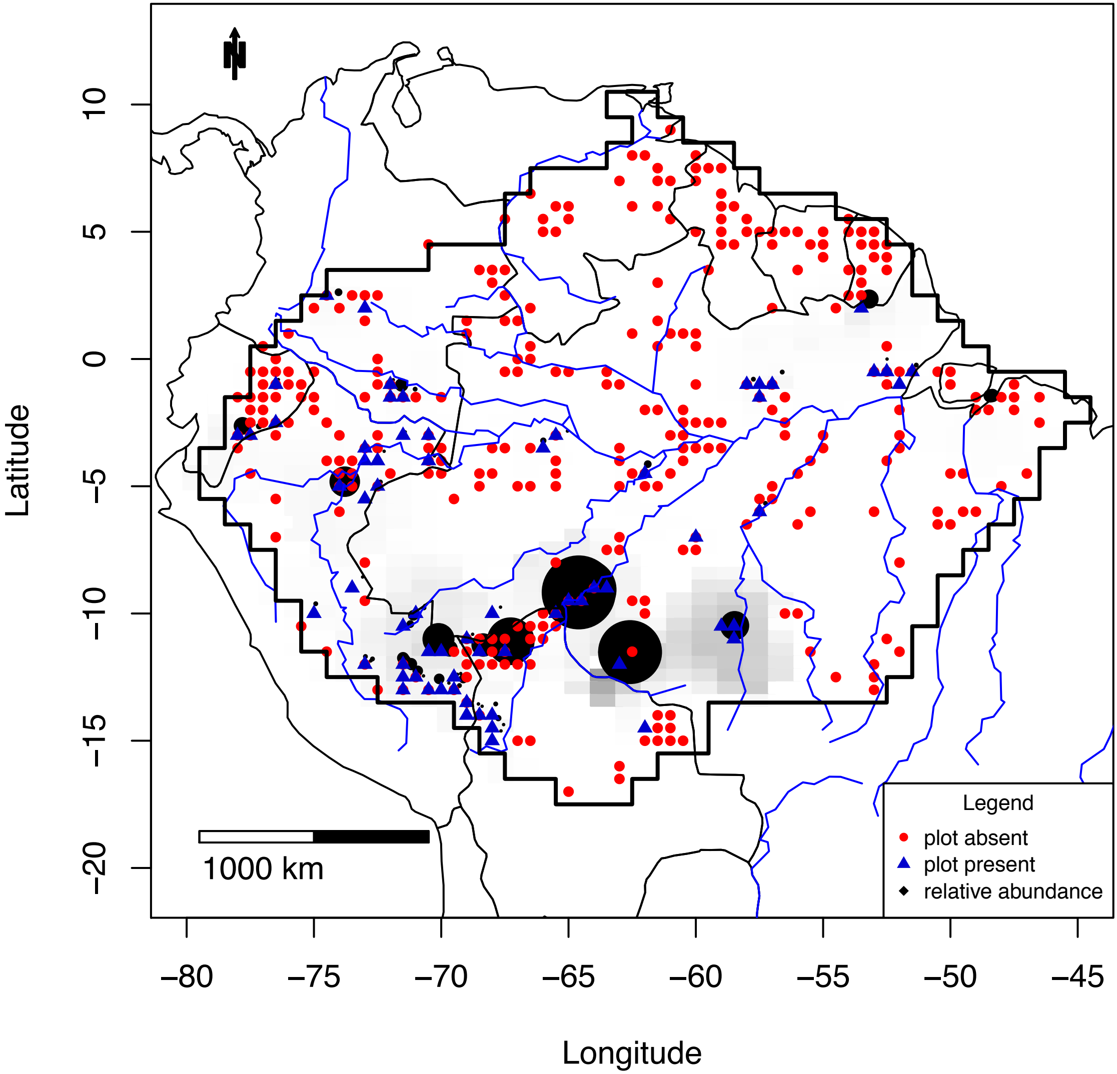

Figure S3_157A Combined map of Theobroma_speciosum

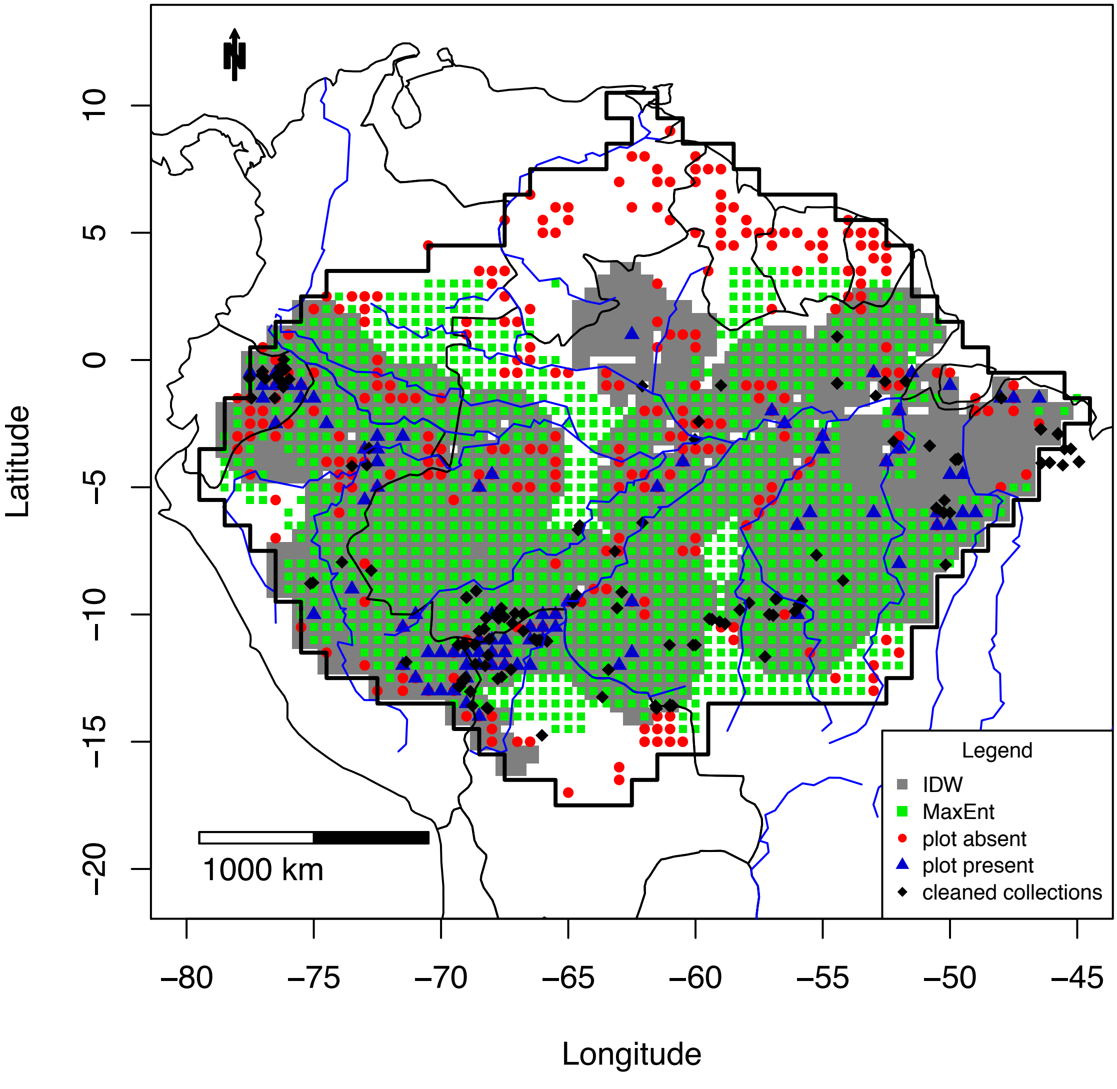

Figure S3_157B MaxEnt map of Theobroma_speciosum

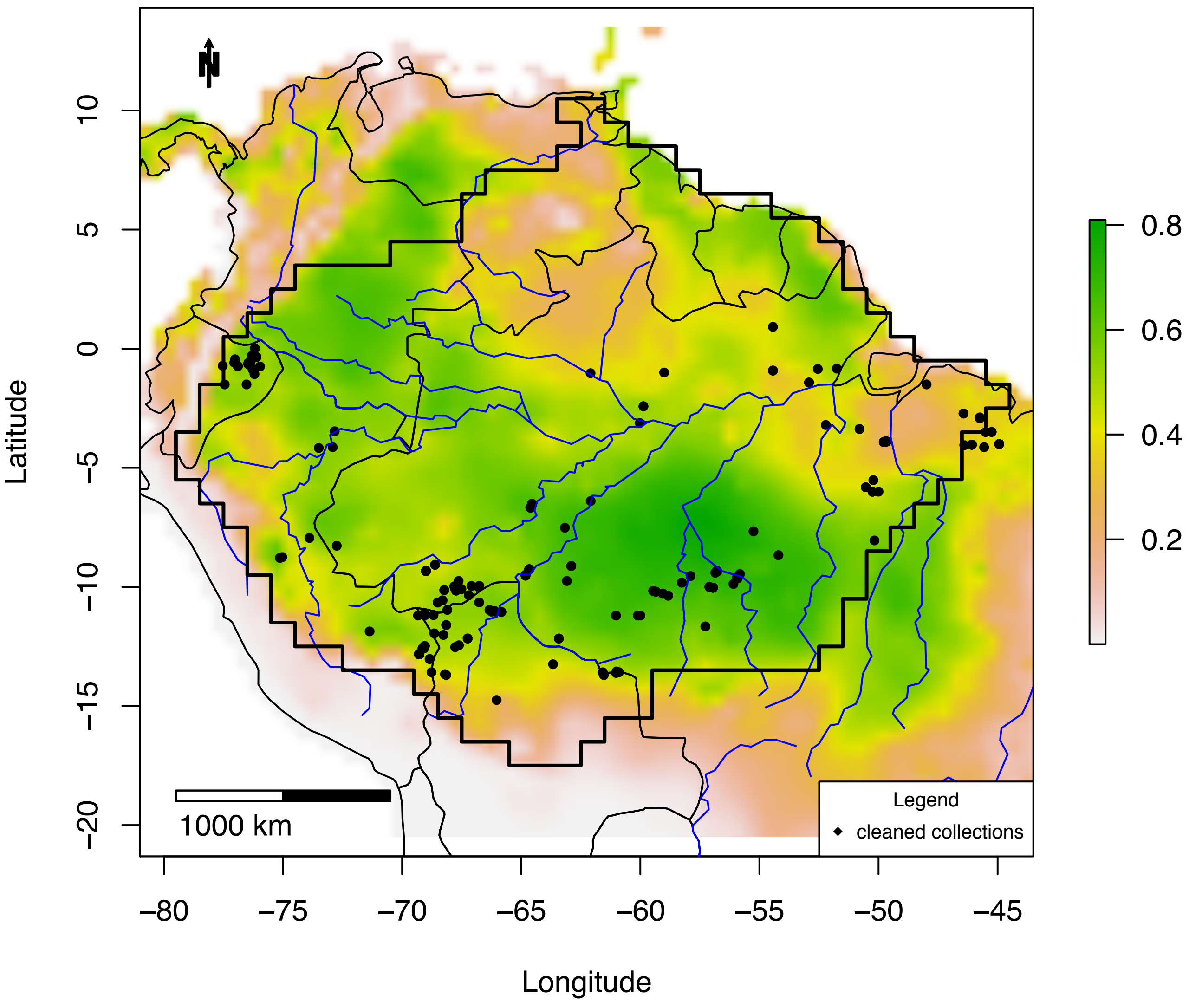

Figure S3_157C IDW map of Theobroma_speciosum:
plots =0 – 0.1; IDW: 0 – 0.07

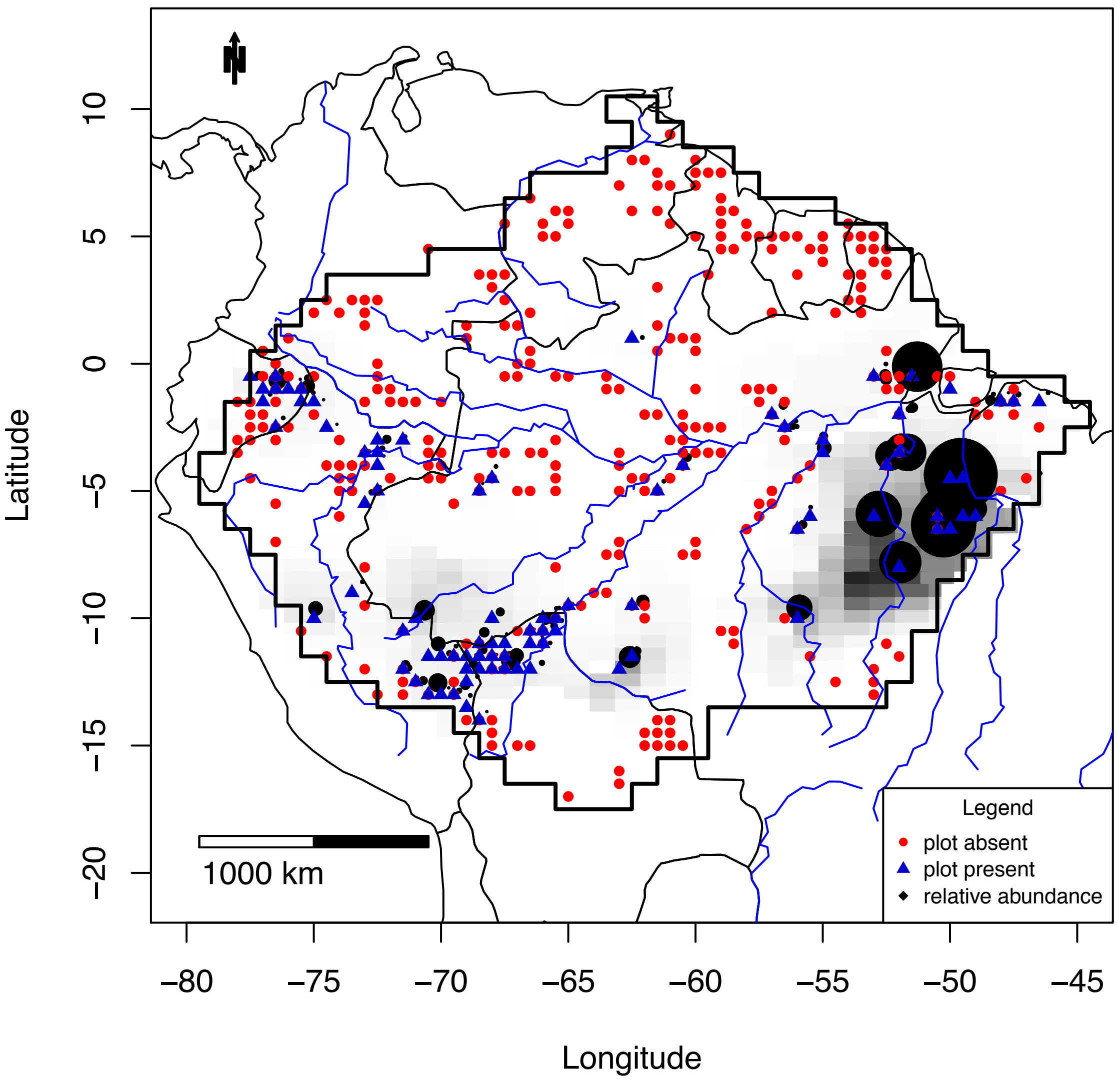

Figure S3_158A Combined map of Theobroma_subincanum

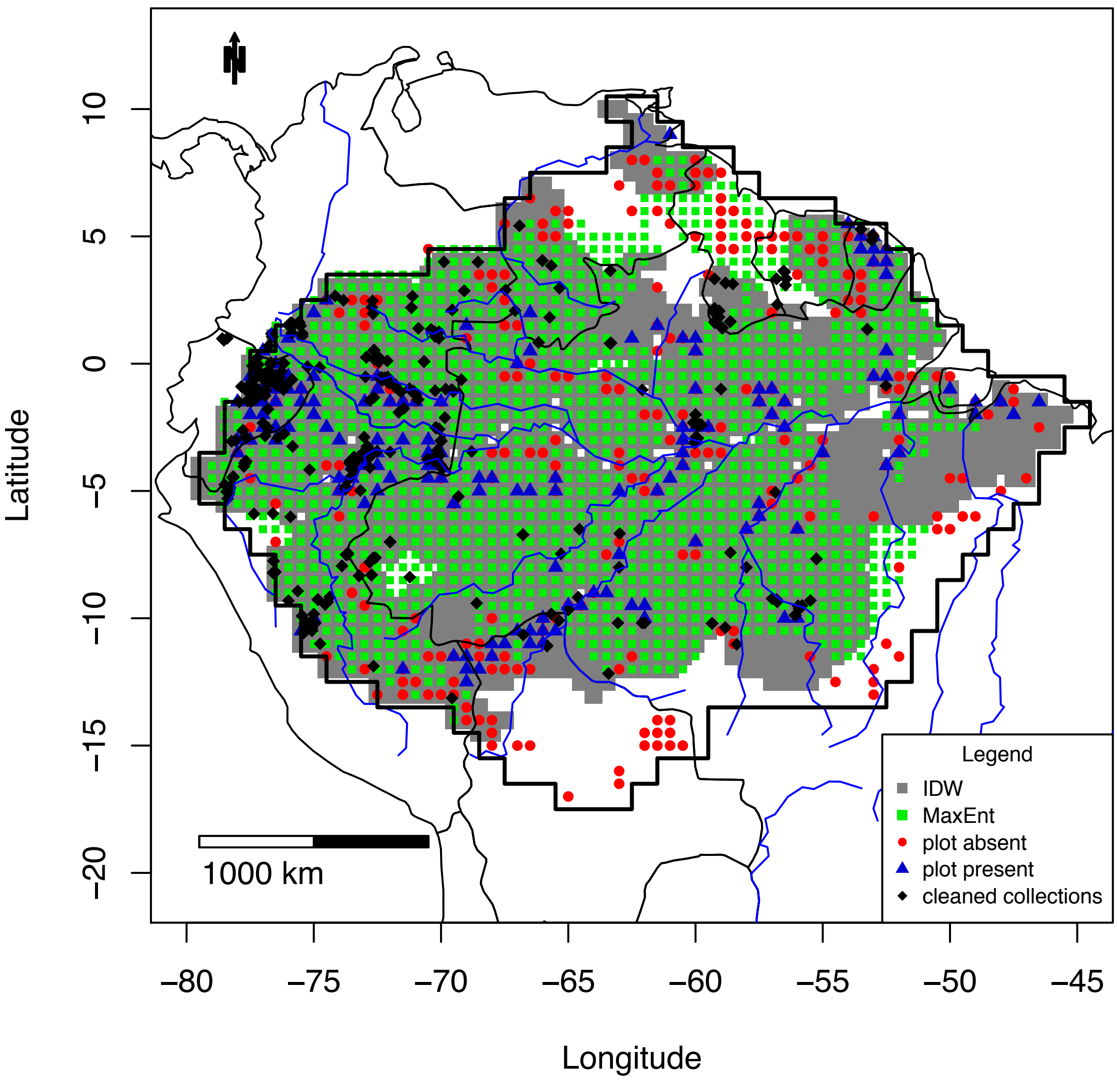

Figure S3_158B MaxEnt map of Theobroma_subincanum

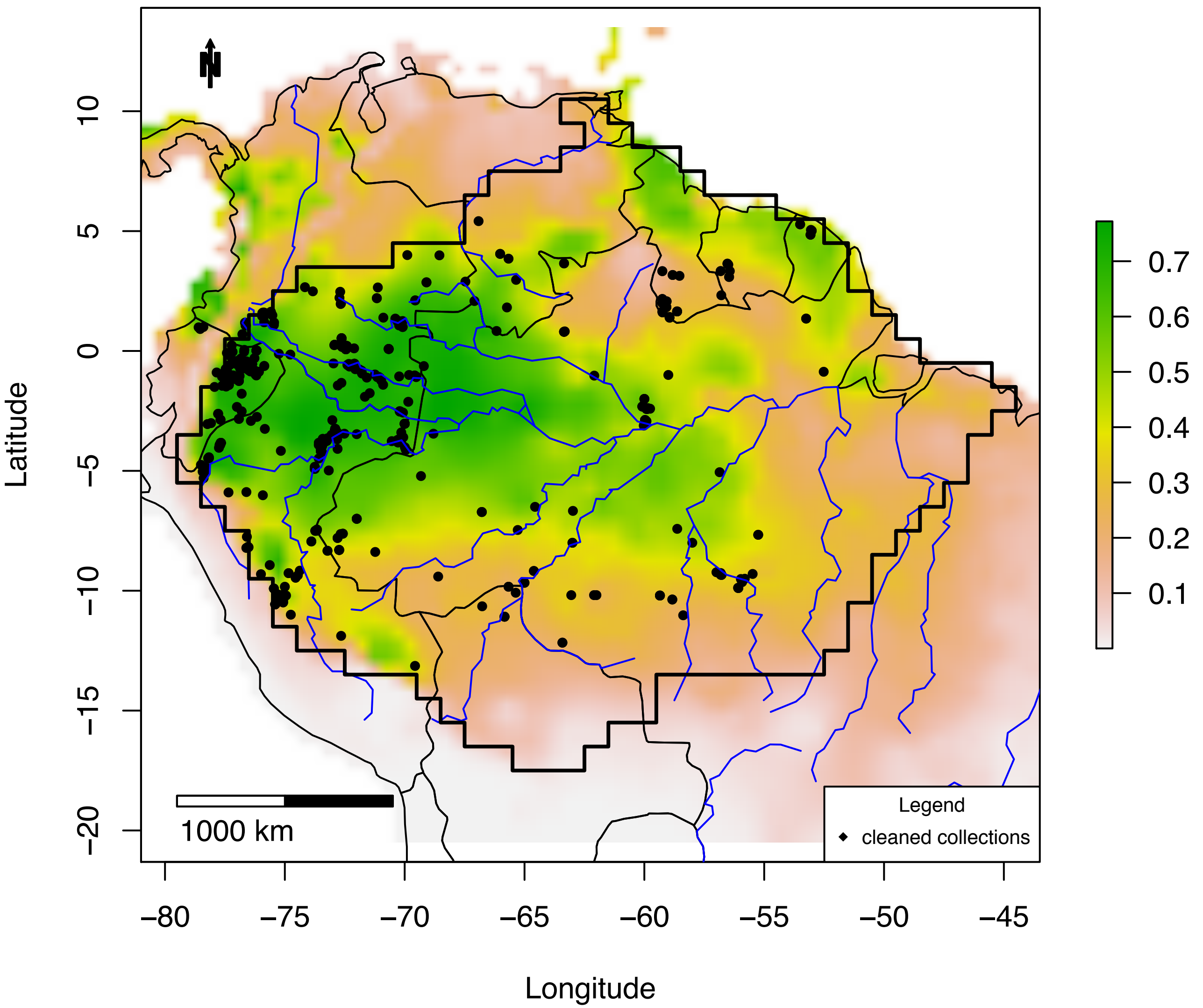

Figure S3_158C IDW map of Theobroma_subincanum: plots =0 – 0.08; IDW: 0 – 0.04

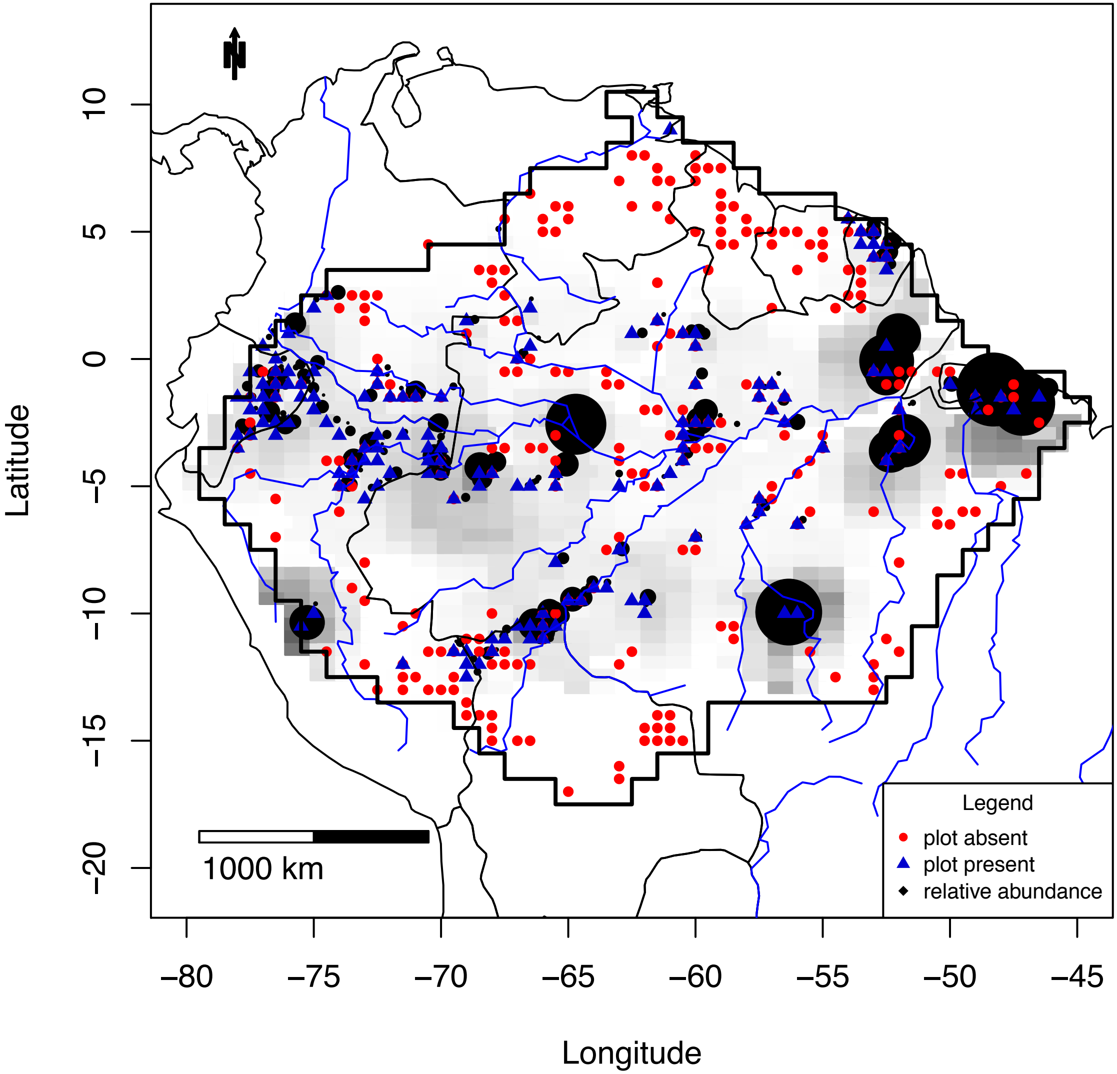

Figure S3_159A Combined map of Trattinnickia_burserifolia

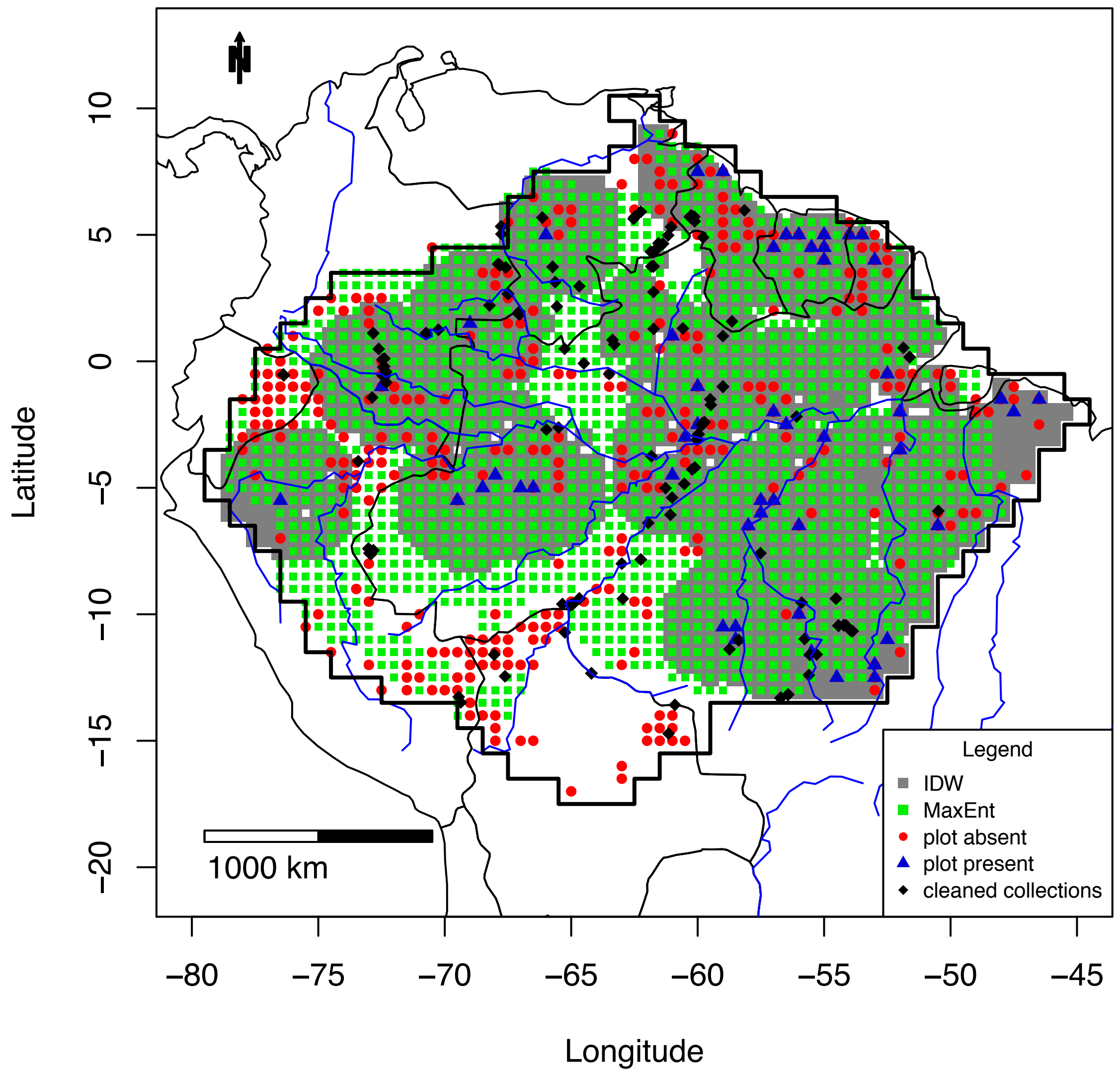

Figure S3_159B MaxEnt map of Trattinnickia_burserifolia

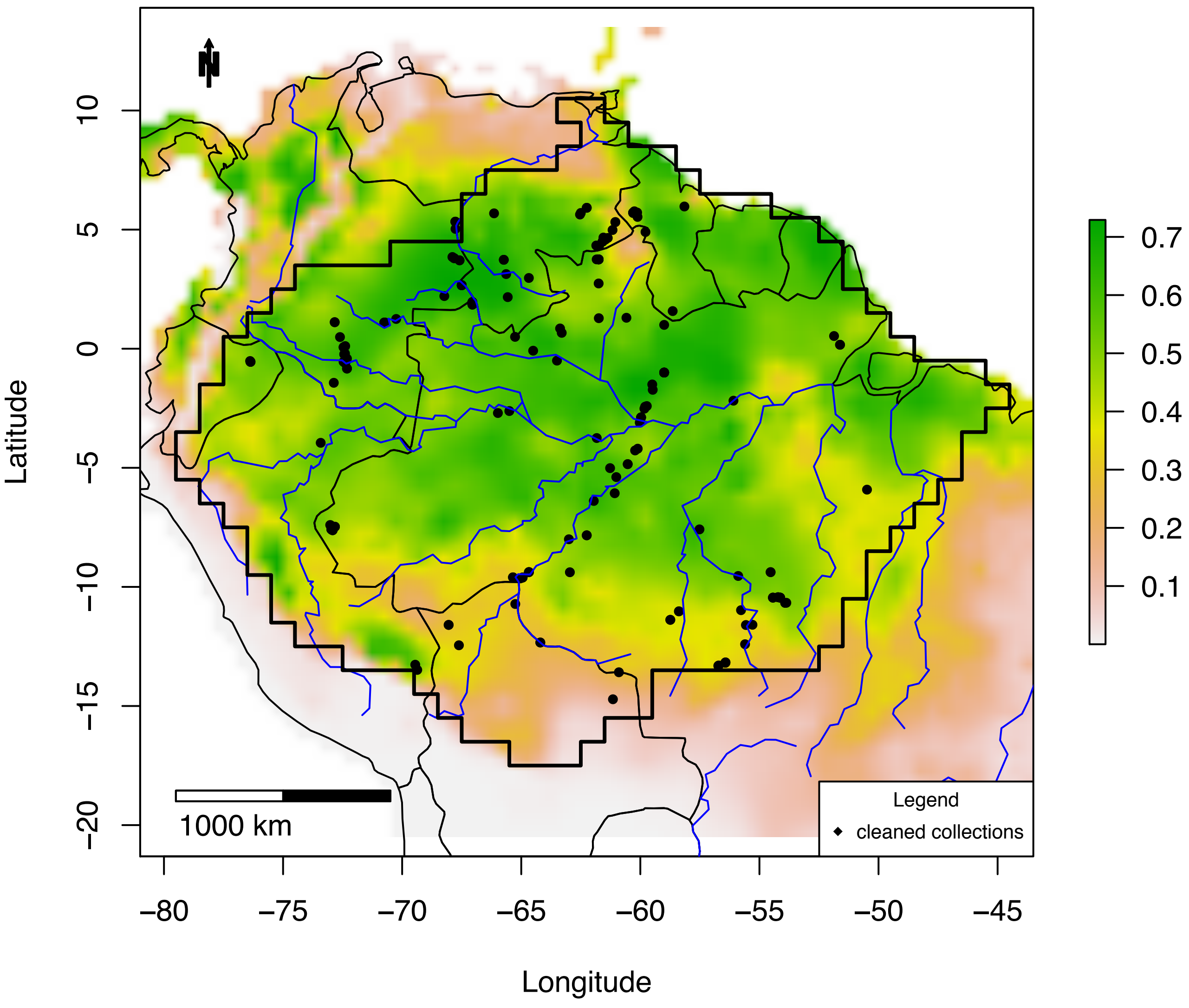

Figure S3_159C IDW map of Trattinnickia_burserifolia: plots =0 – 0.46; IDW: 0 – 0.27

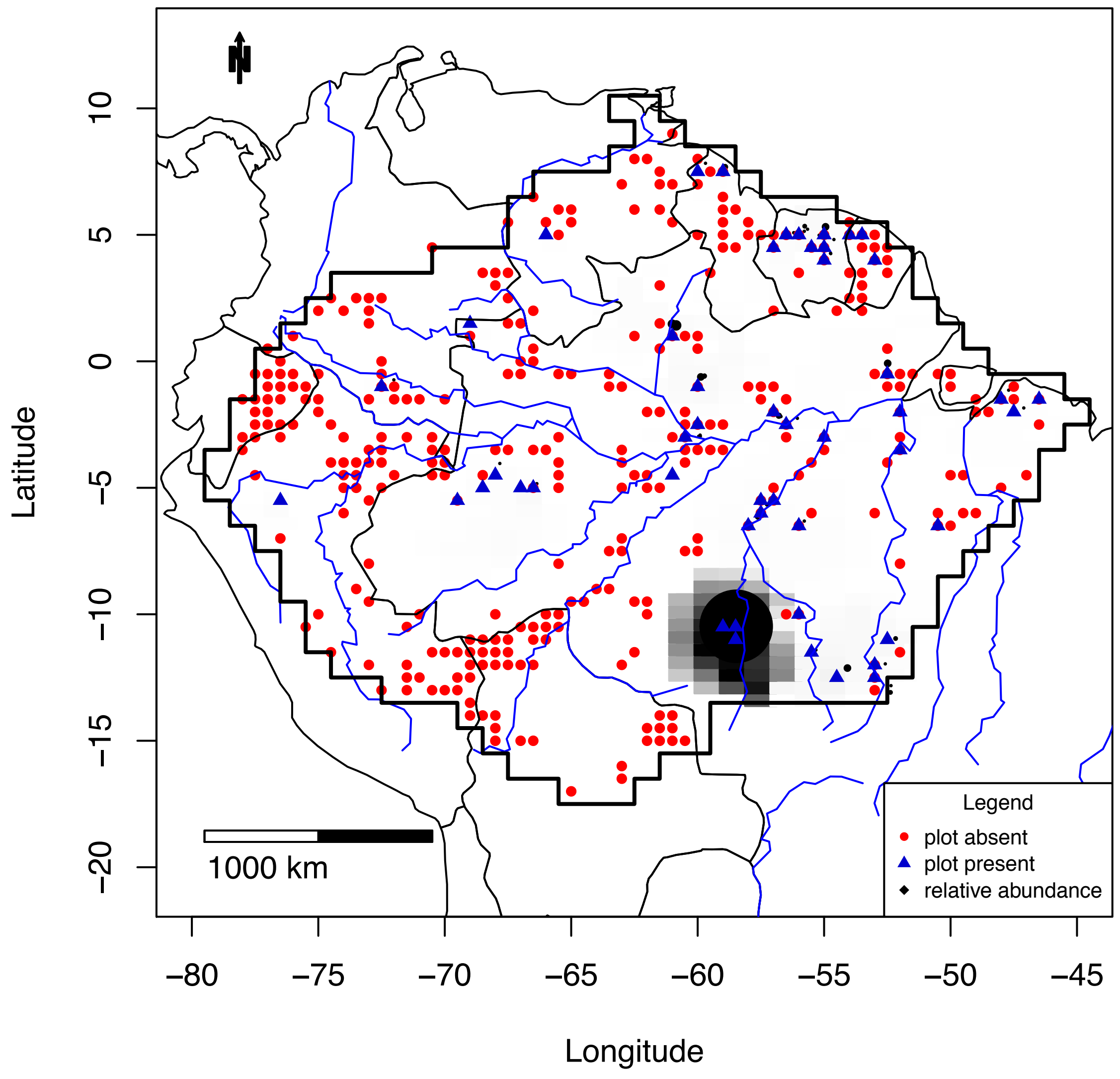

Figure S3_160A Combined map of Triplaris_weigeltiana

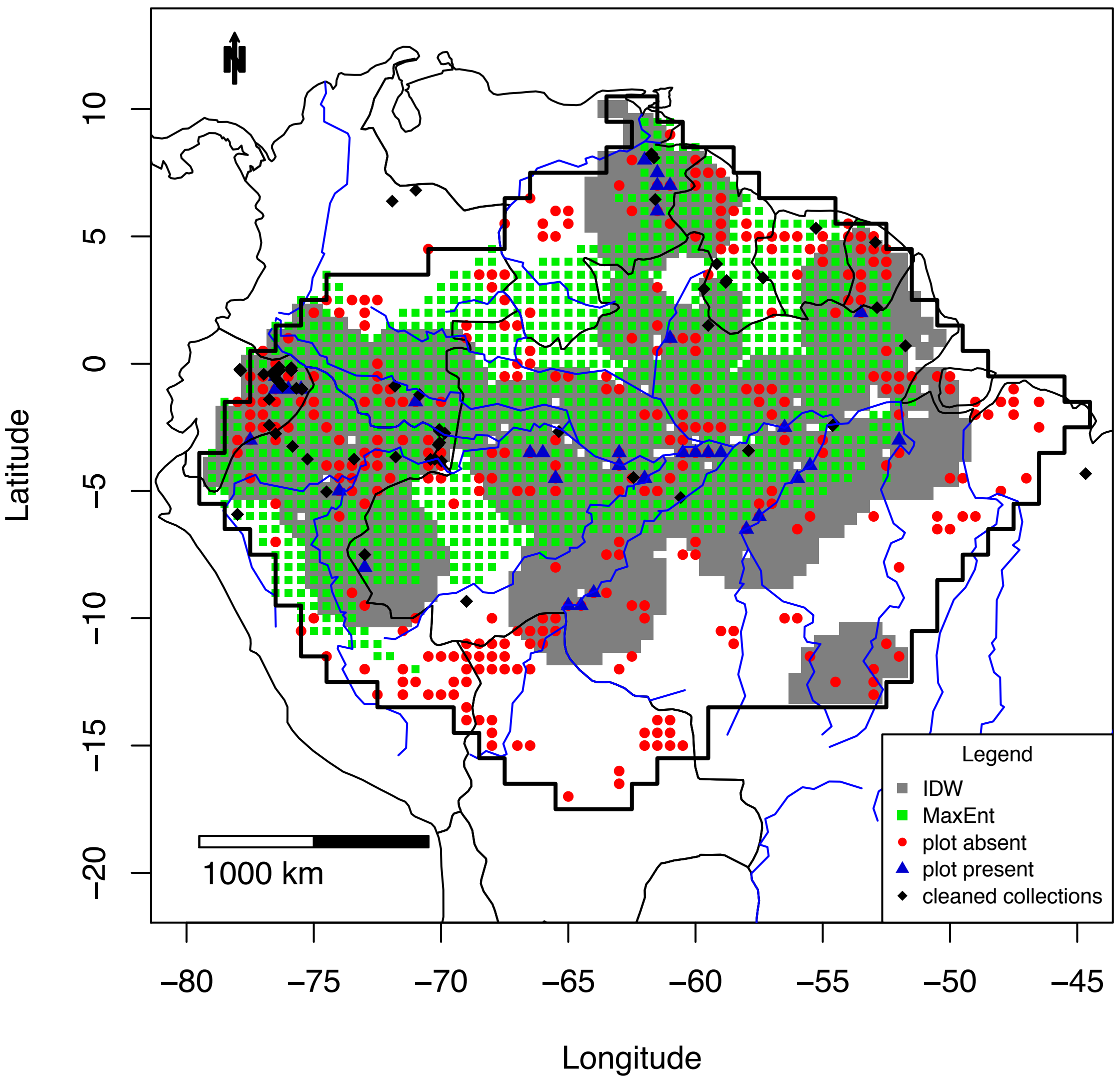

Figure S3_160B MaxEnt map of Triplaris_weigeltiana

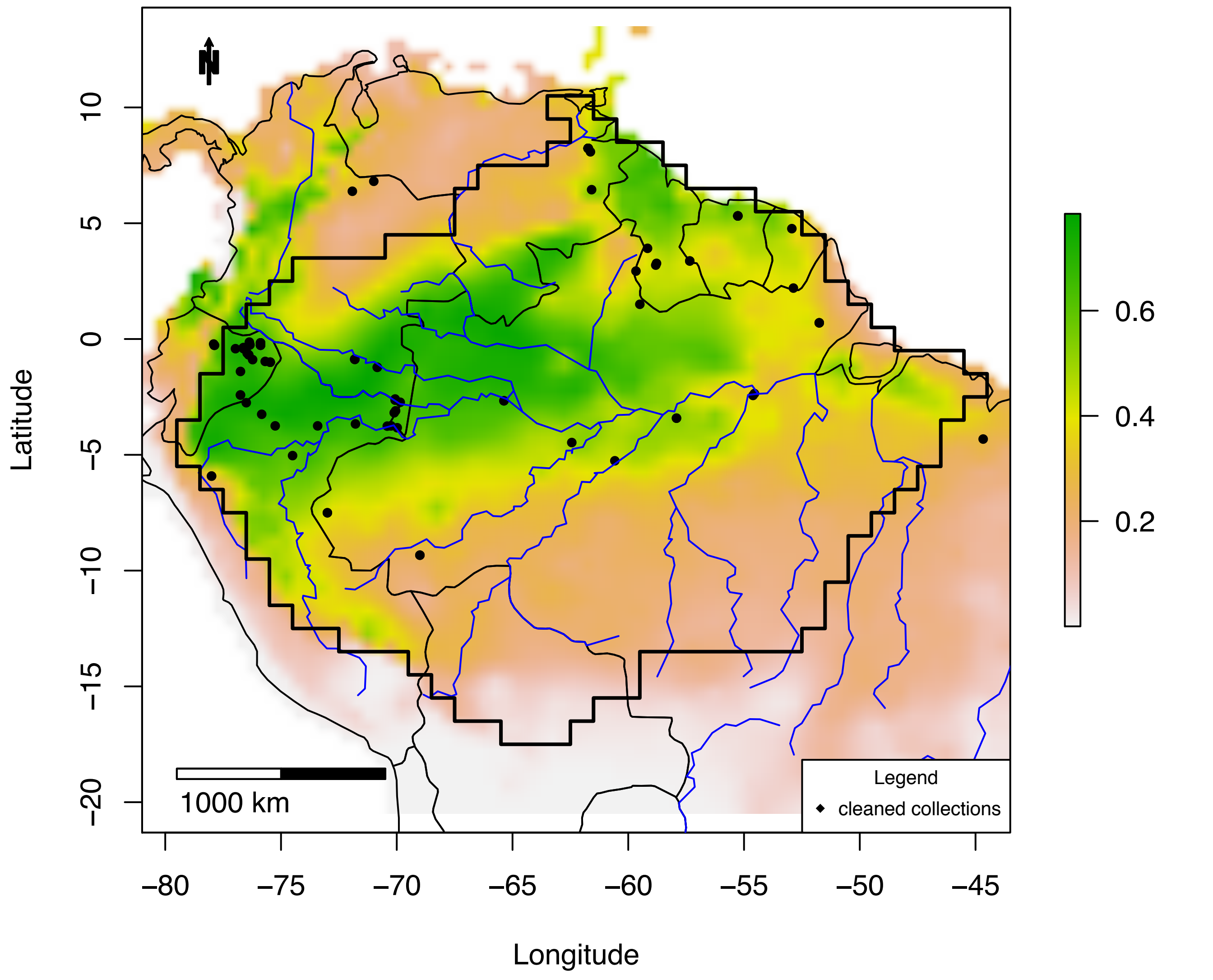

Figure S3_160C IDW map of Triplaris_weigeltiana: plots =0 – 0.61; IDW: 0 – 0.07

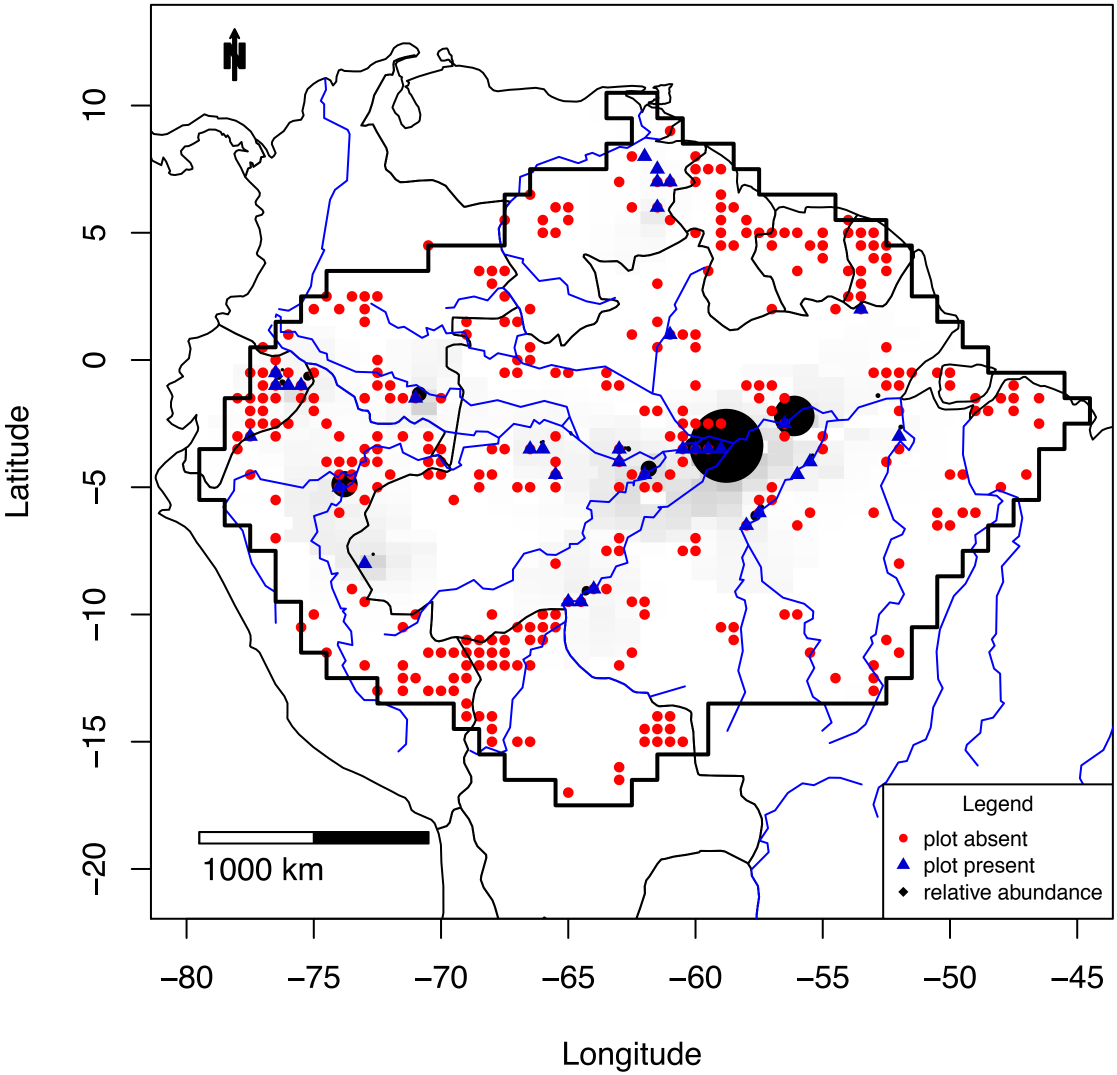

Figure S3_161A Combined map of Vatairea_guianensis

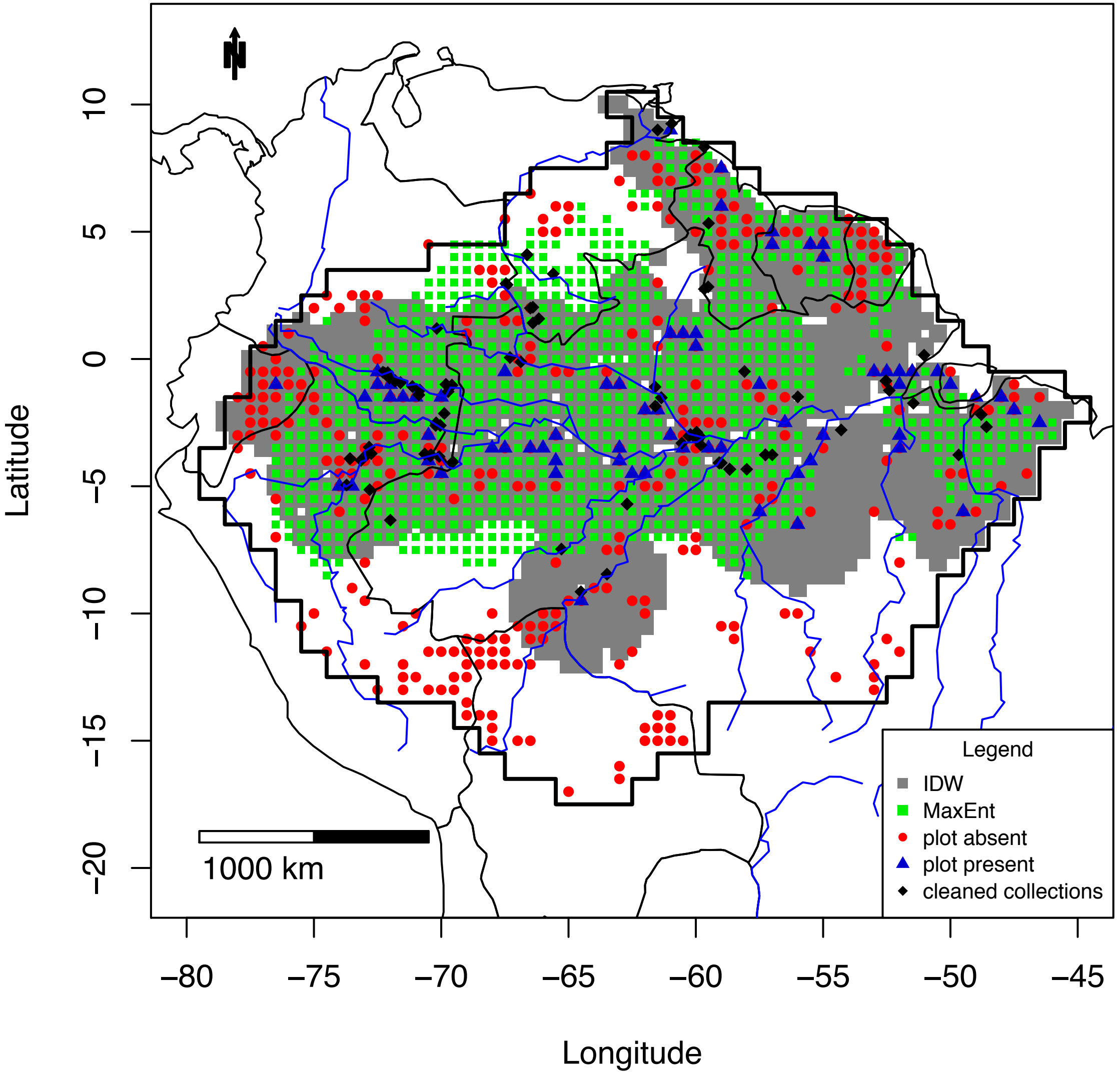

Figure S3_161B MaxEnt map of Vatairea_guianensis

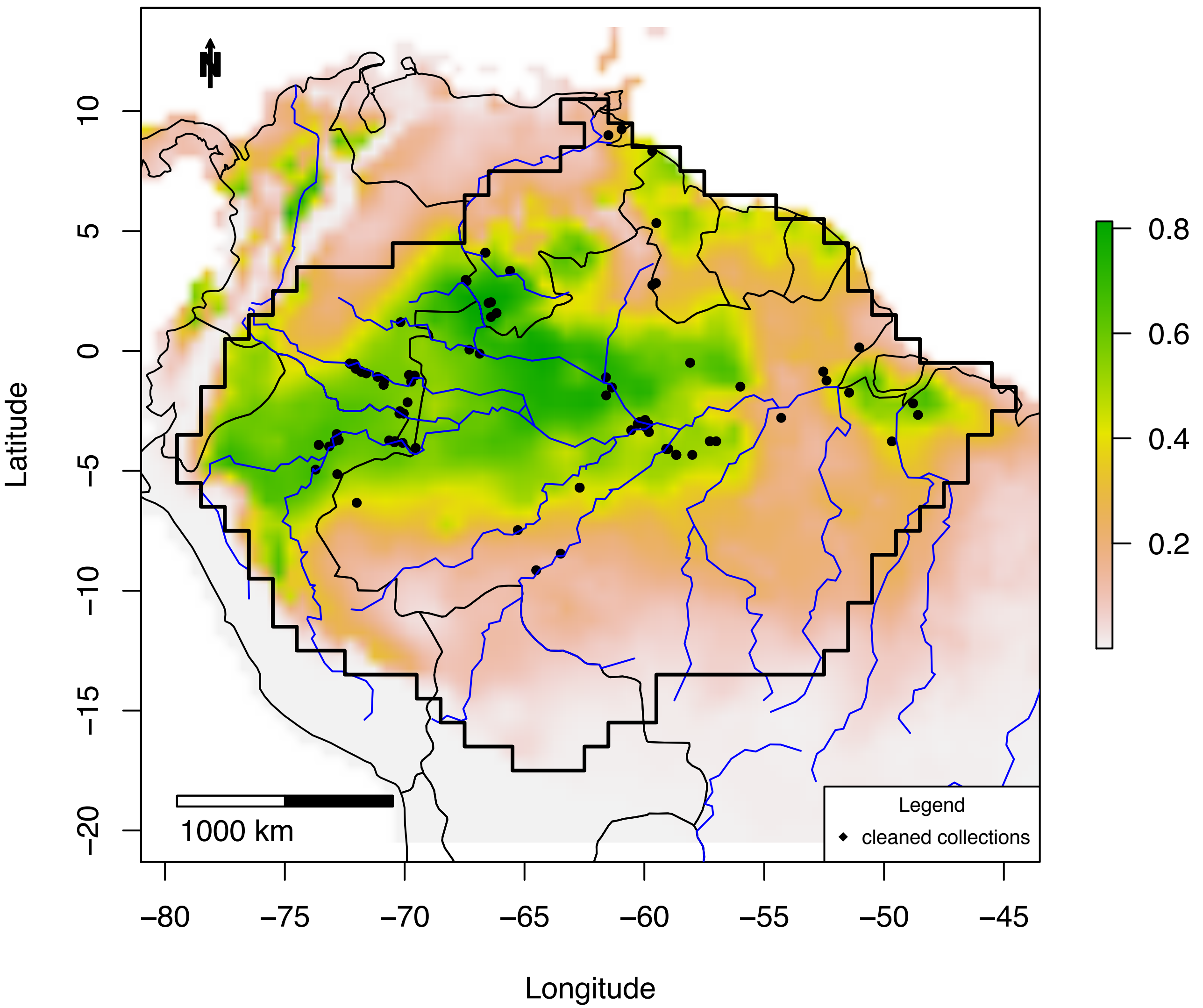

Figure S3_161C IDW map of Vatairea_guianensis: plots =0 – 0.13; IDW: 0 – 0.05

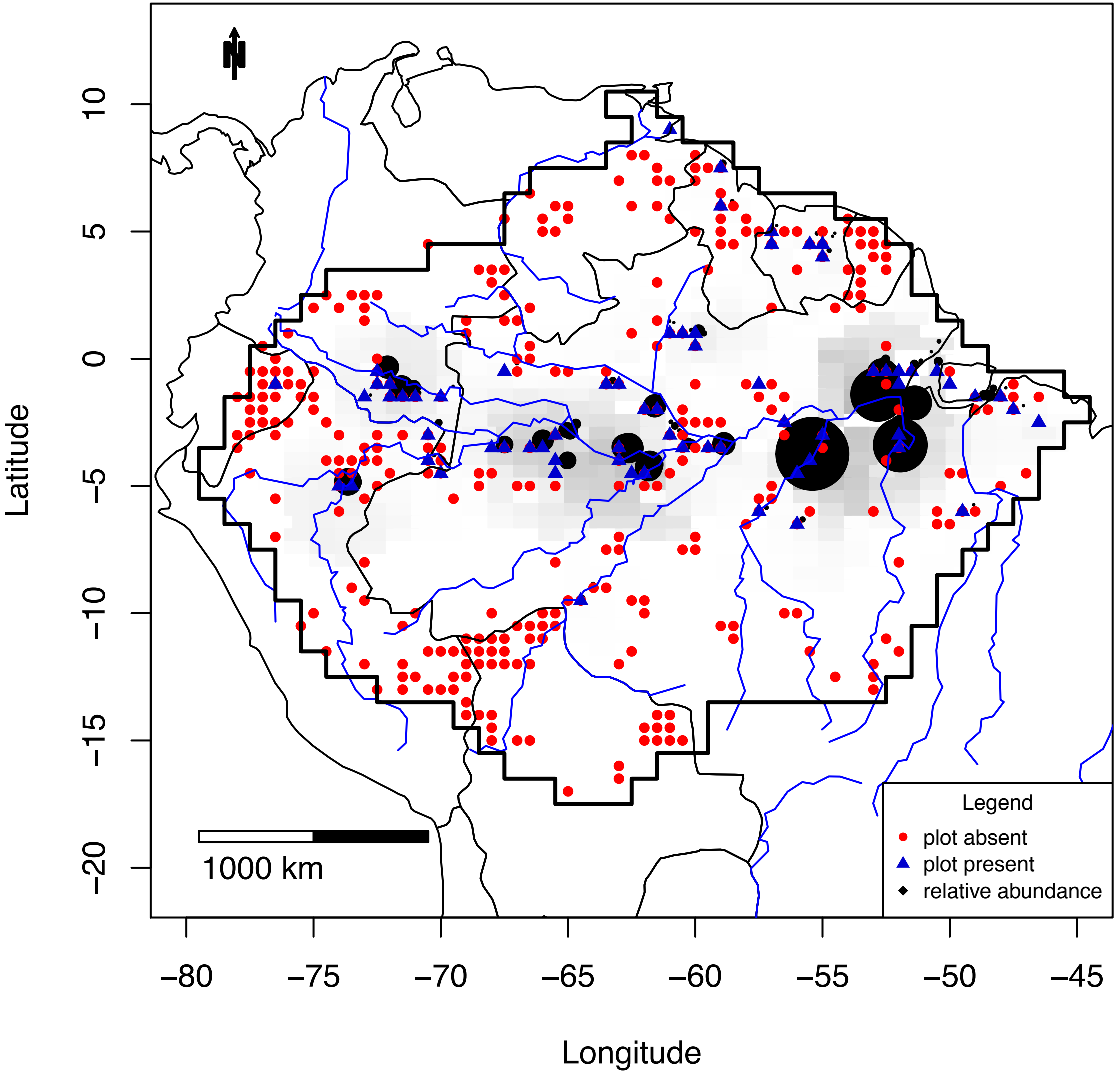

Figure S3_162A Combined map of Virola_calophylla

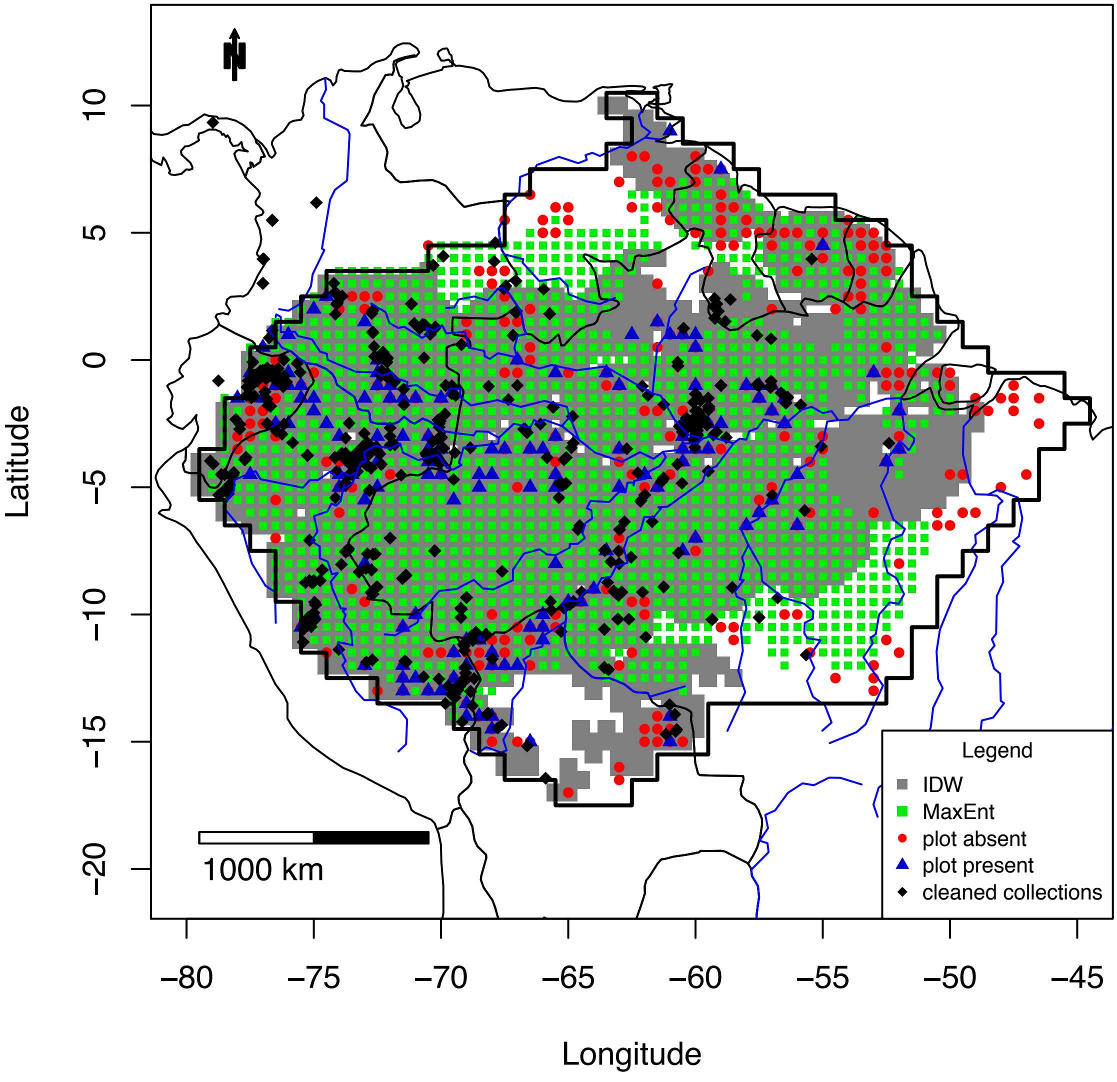

Figure S3_162B MaxEnt map of Virola_calophylla

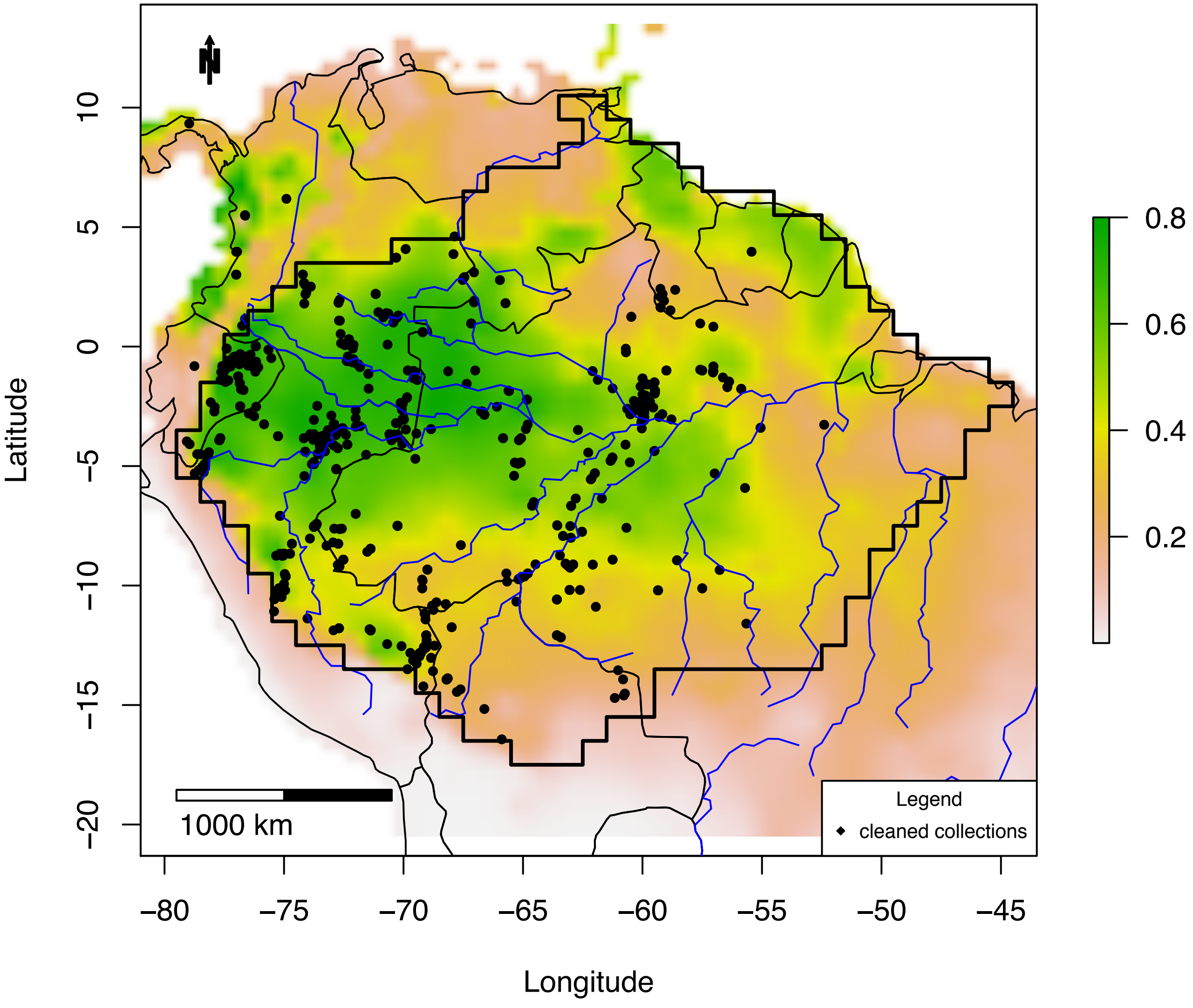

Figure S3_162C IDW map of Virola_calophylla: plots =0 – 0.2; IDW: 0 – 0.06

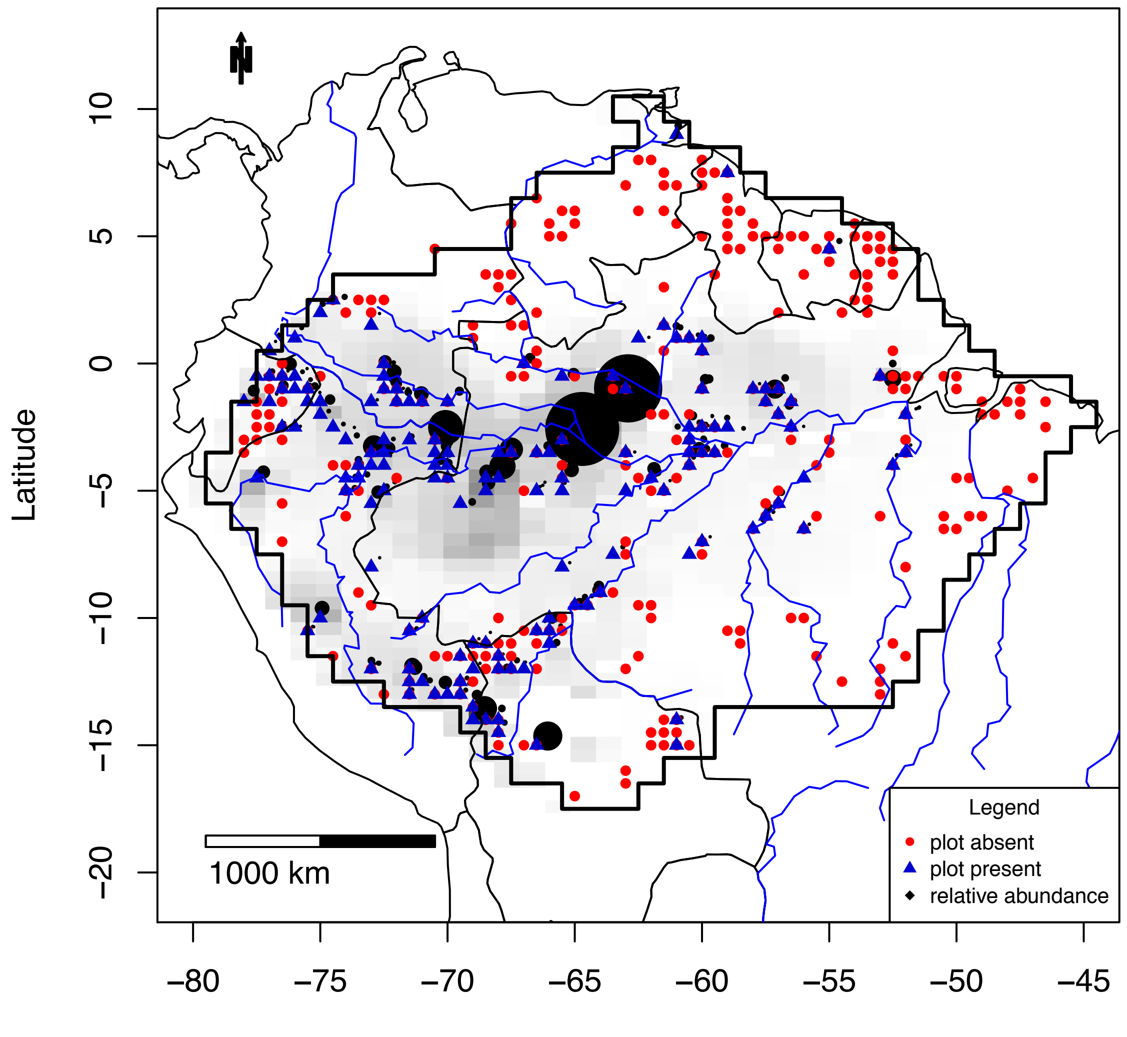

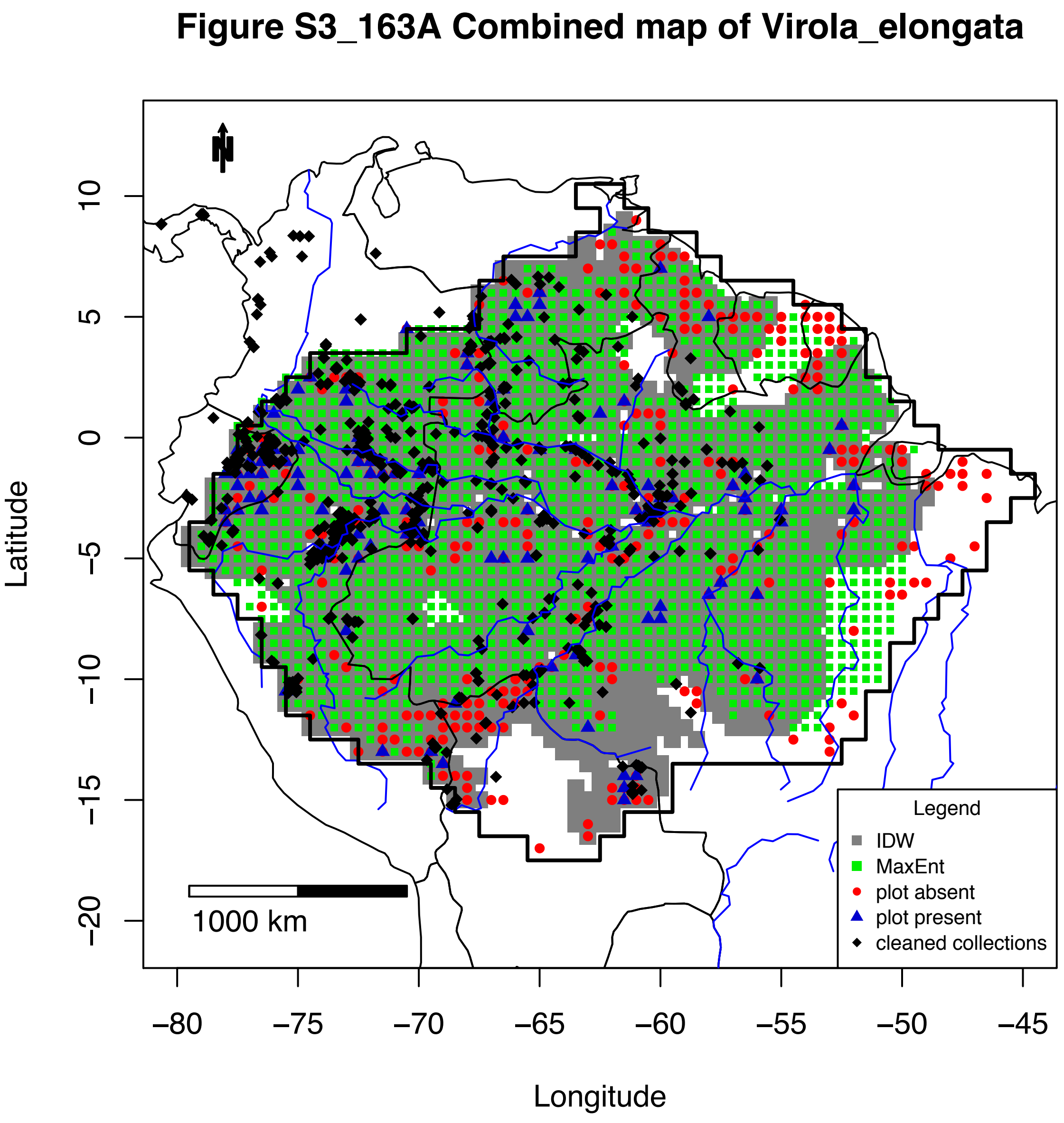
Figure S3_163A Combined map of Virola_elongata
N
Latitude
Longitude
10
5
0
−5
−10
−15
−20
−80
−75
−70
−65
−60
−55
−50
−45
1000 km
Legend
IDW
MaxEnt
plot absent
plot present
cleaned collections

Figure S3_163B MaxEnt map of Virola_elongata

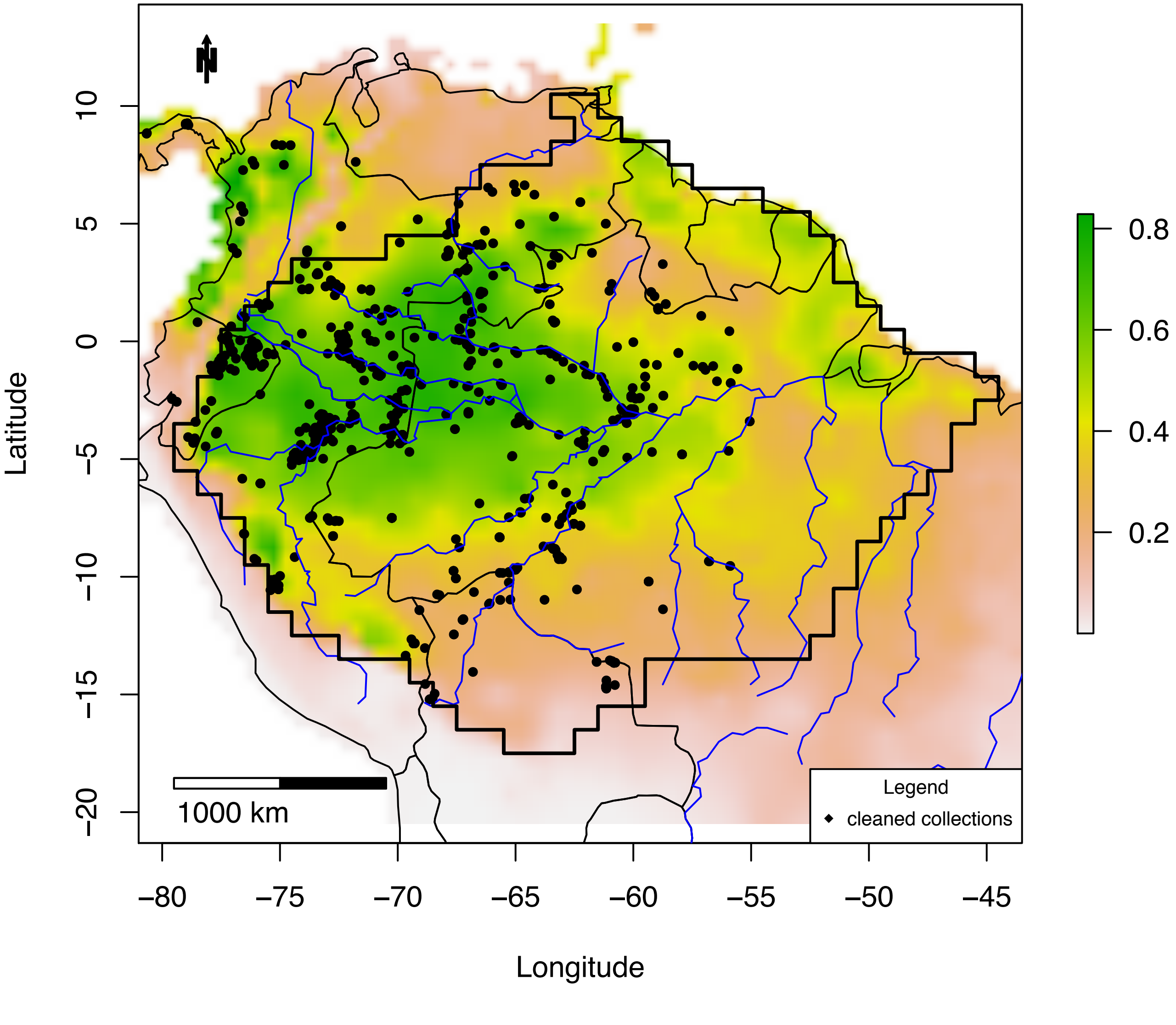

Figure S3_163C IDW map of Virola_elongata: plots =0 – 0.12; IDW: 0 – 0.05

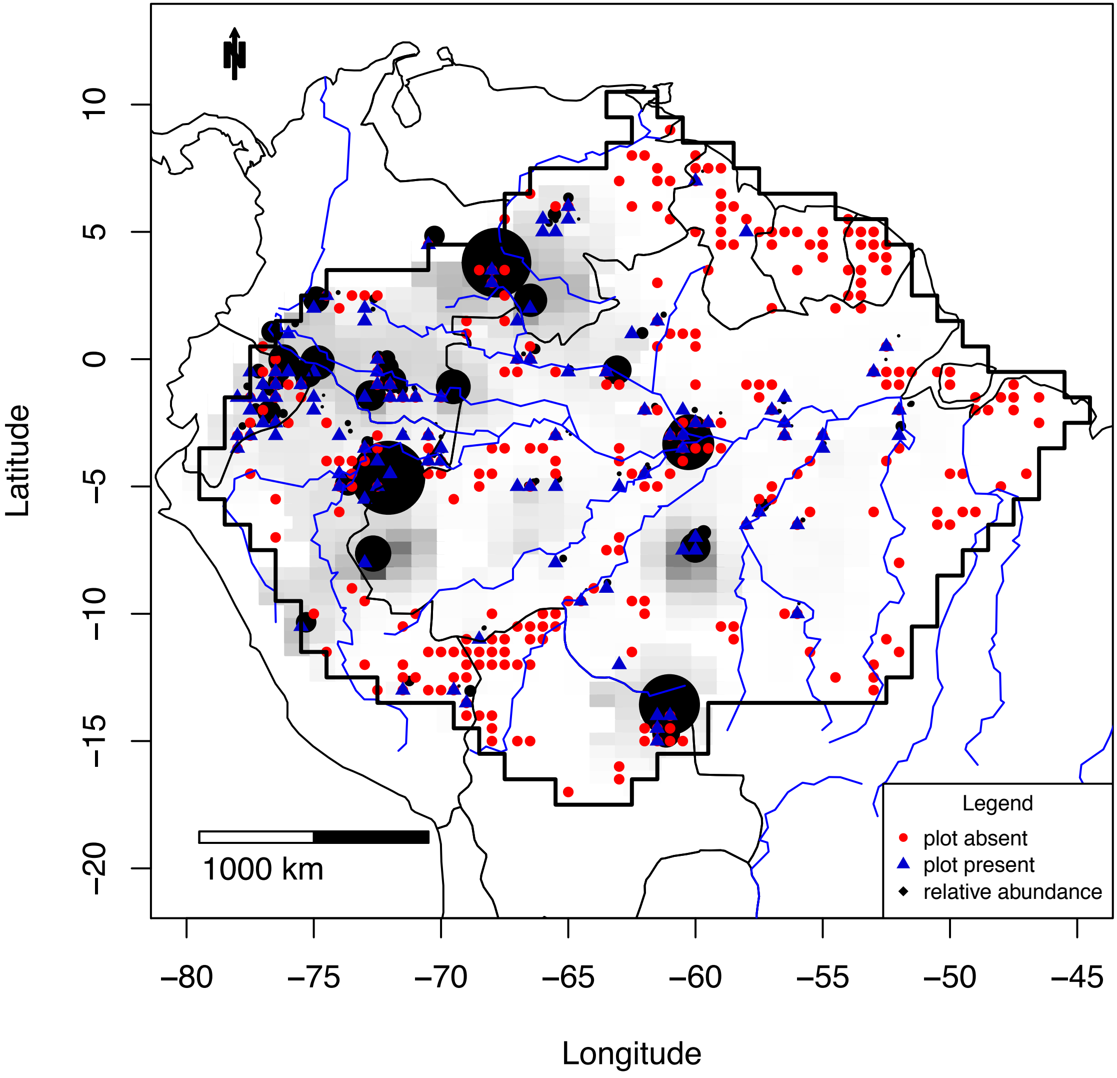

Figure S3_164A Combined map of Virola_michelii

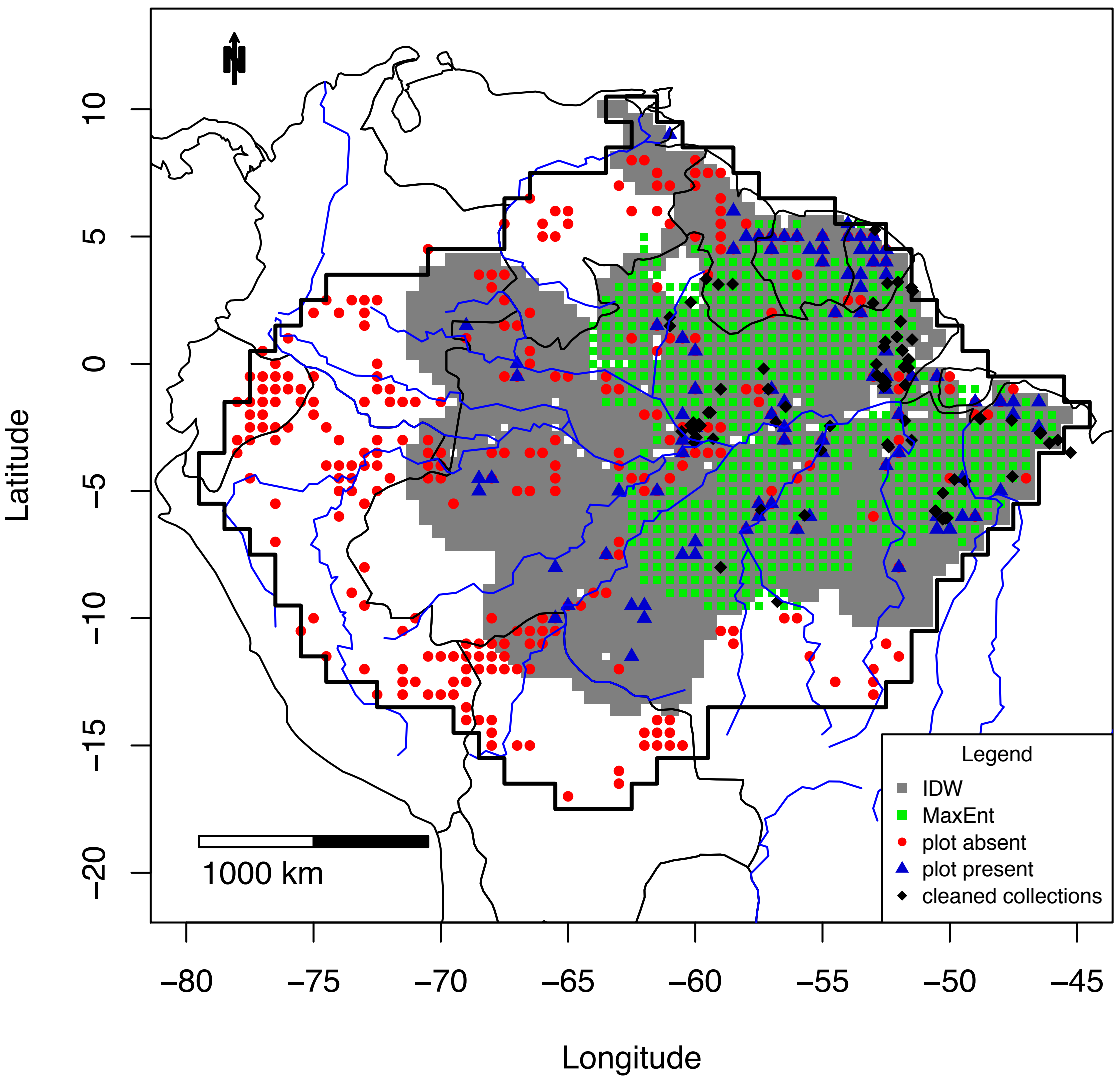

Figure S3_164B MaxEnt map of Virola_michelii

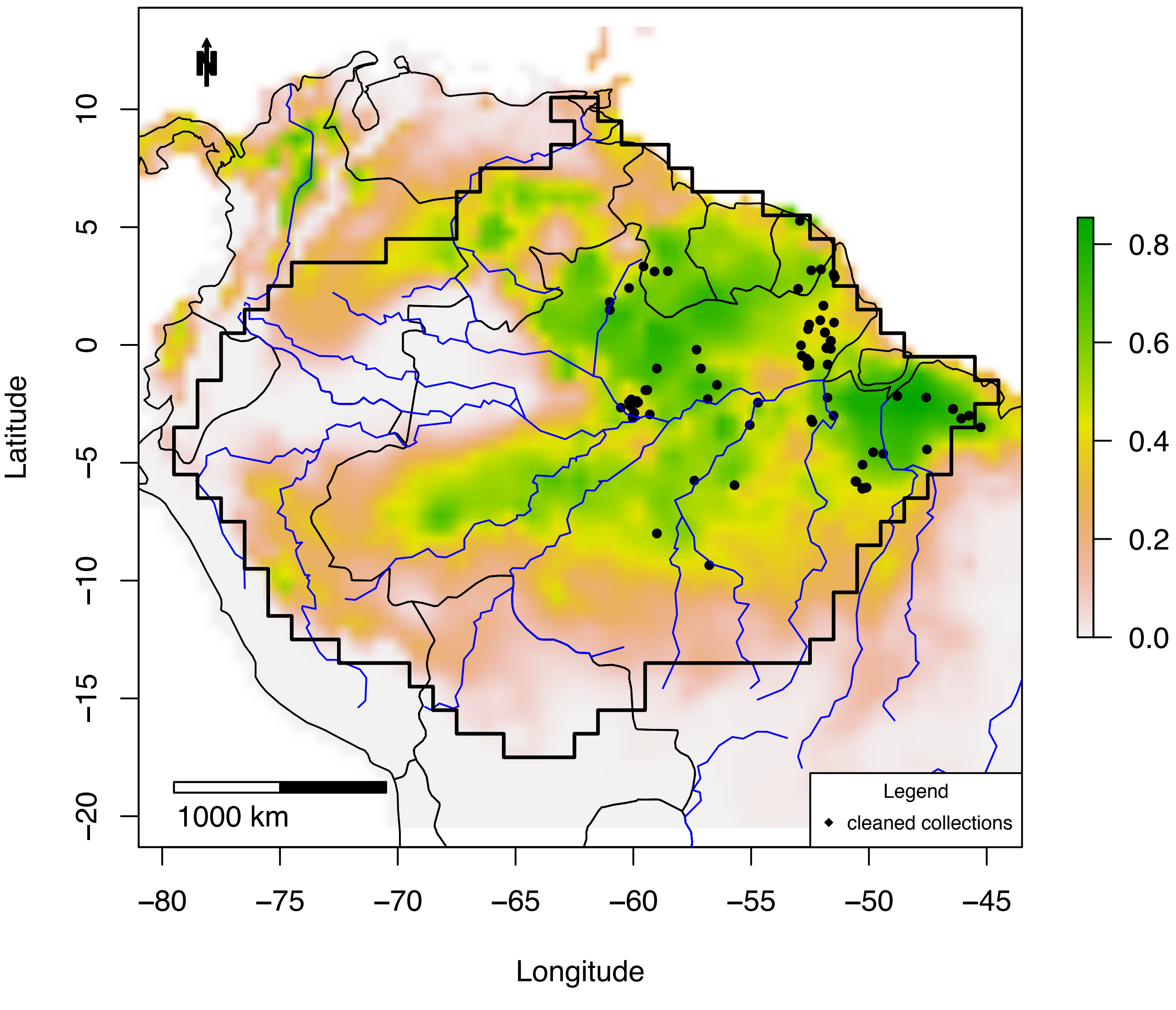

Figure S3_164C IDW map of Virola_michelii: plots =0 – 0.07; IDW: 0 – 0.04

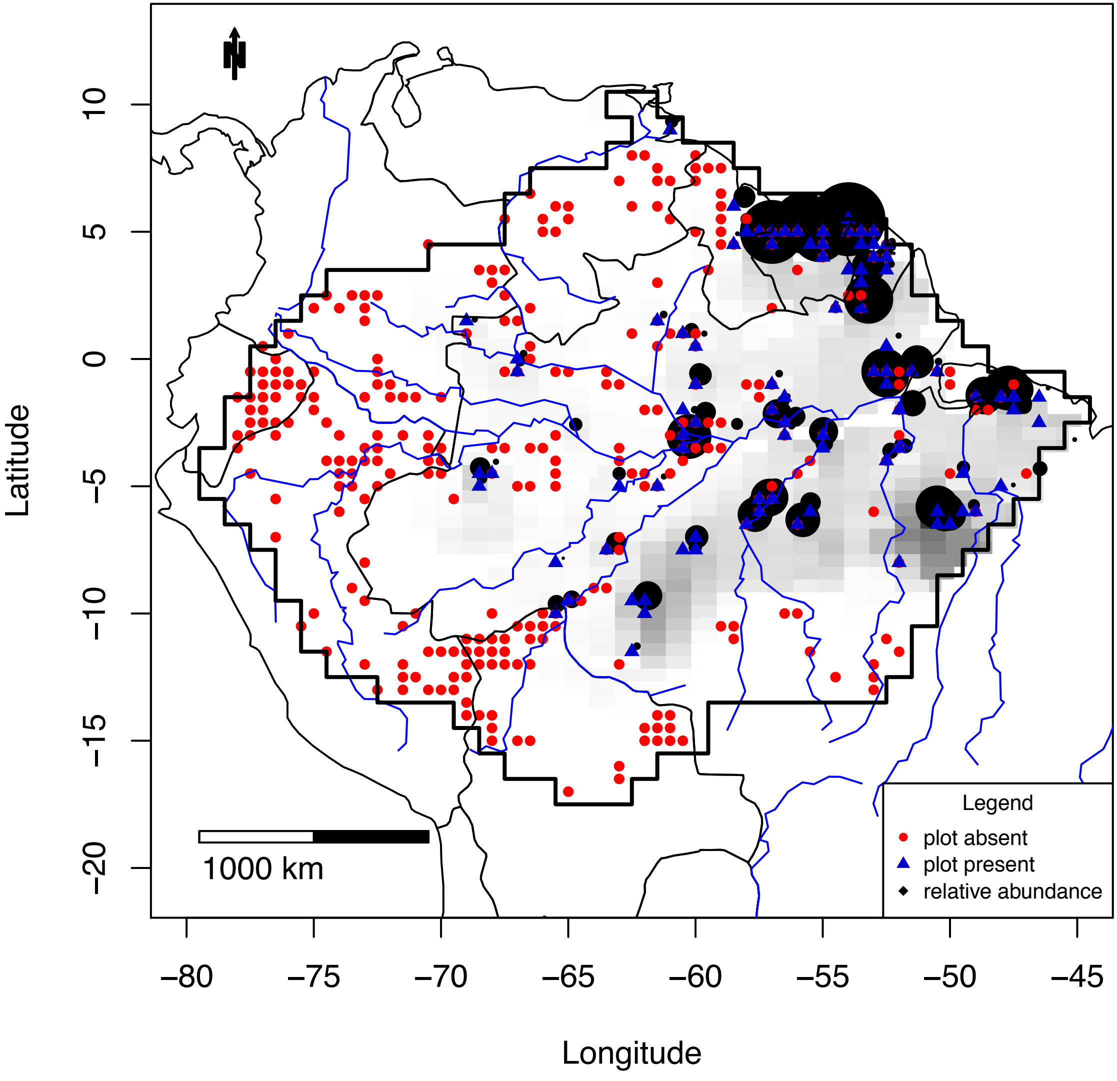

Figure S3_165A Combined map of Virola_pavonis

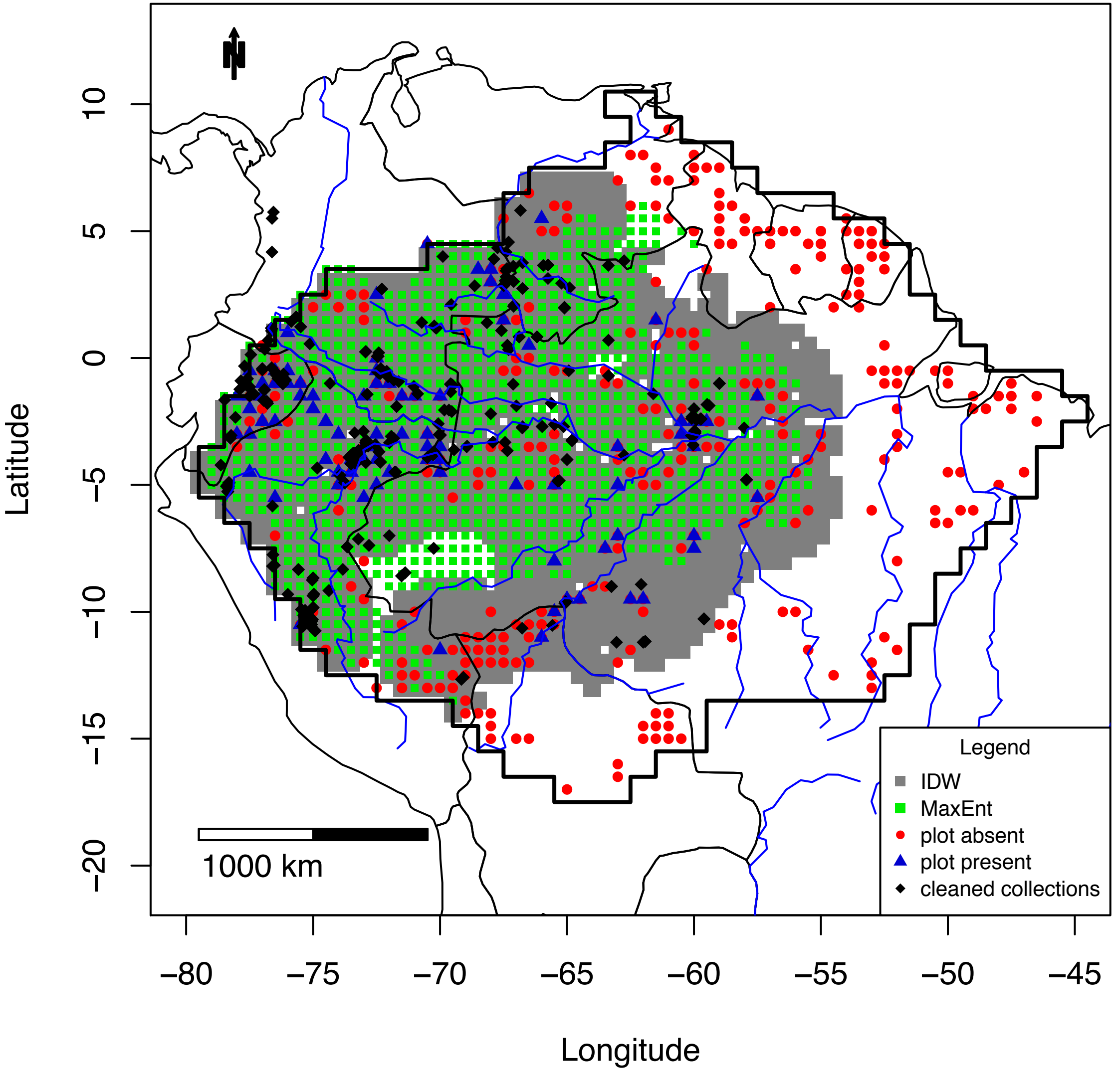

Figure S3_165B MaxEnt map of Virola_pavonis

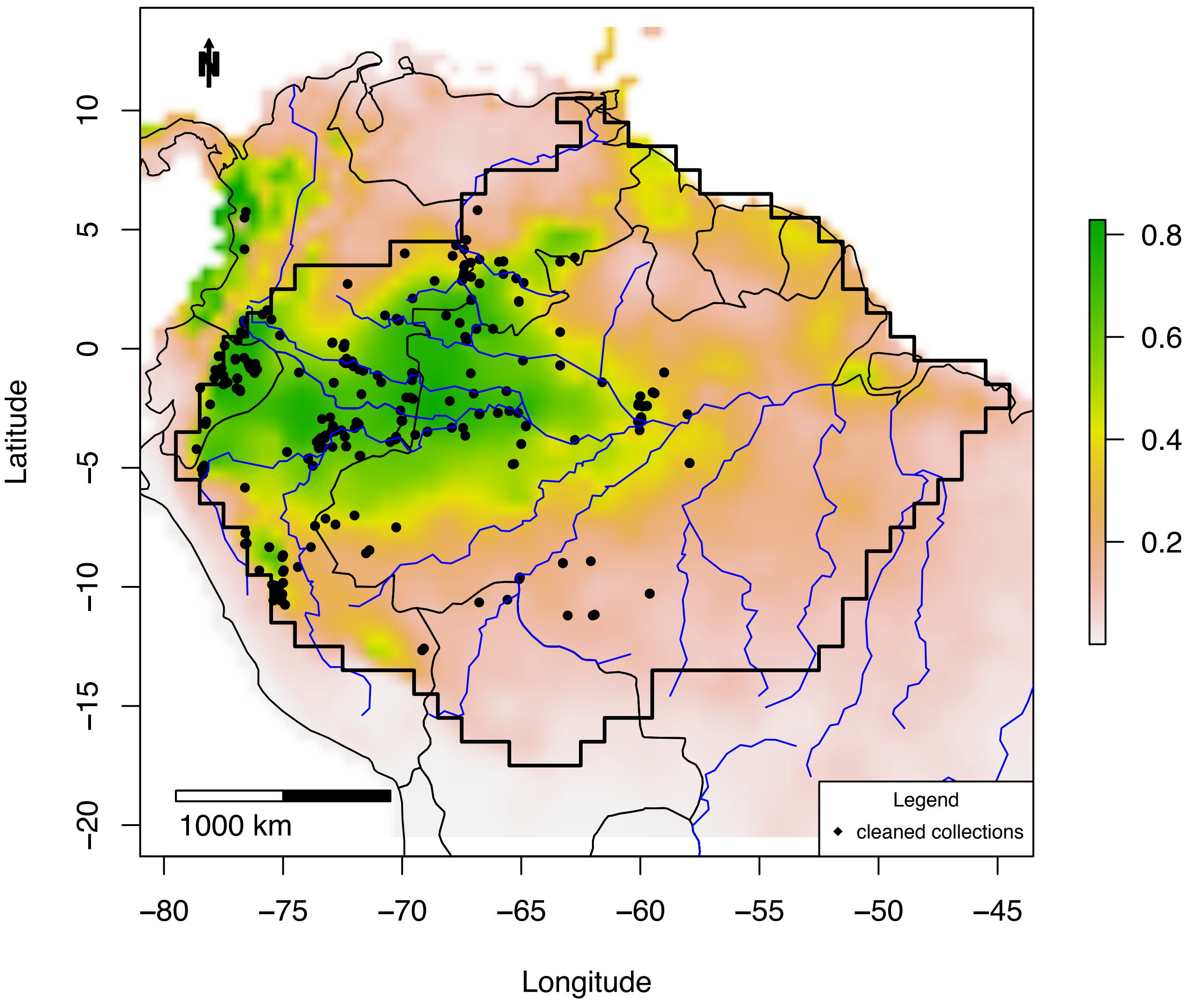

Figure S3_165C IDW map of Virola_pavonis: plots =0 – 0.12; IDW: 0 – 0.05

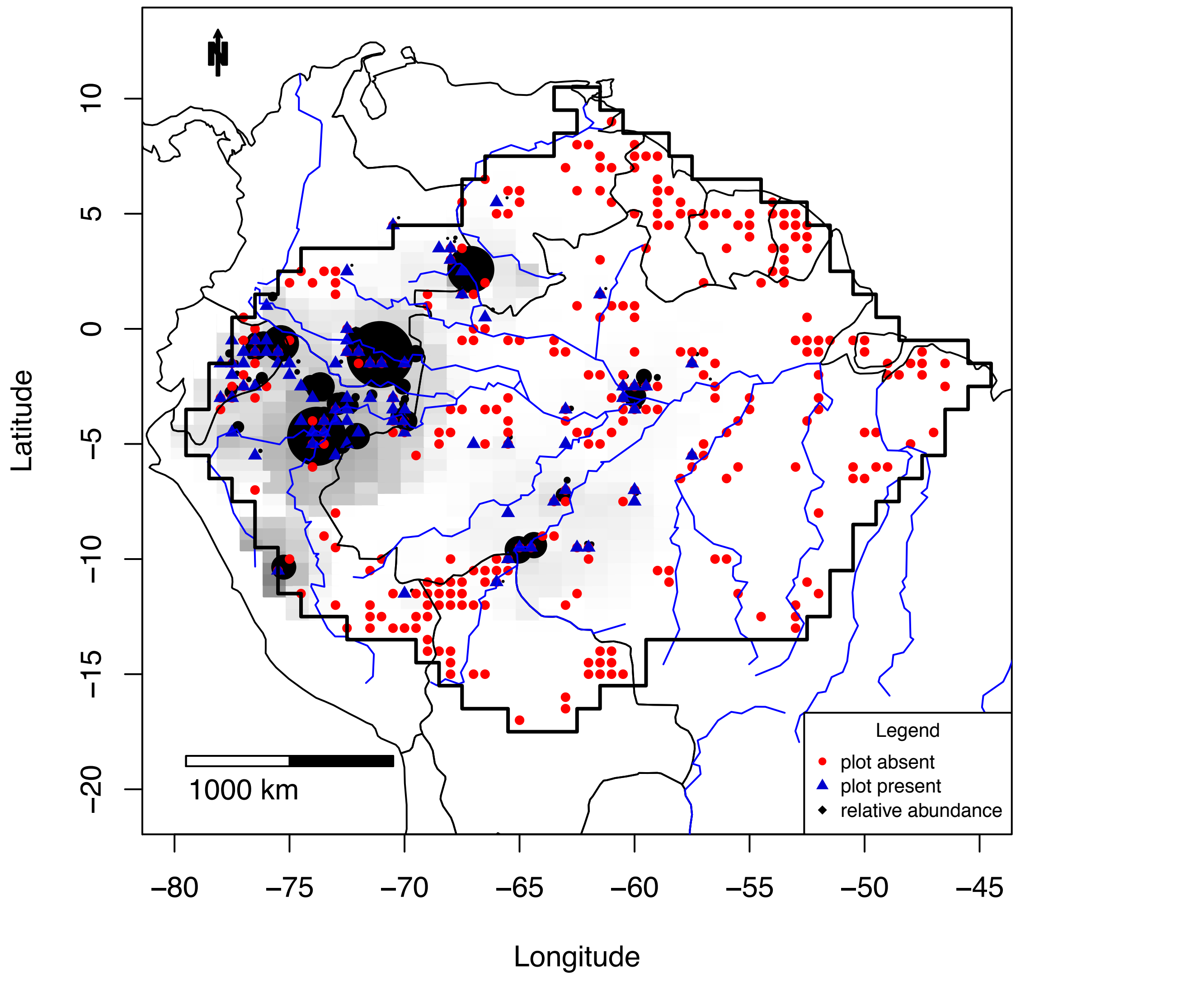

Figure S3_166A Combined map of Virola_surinamensis

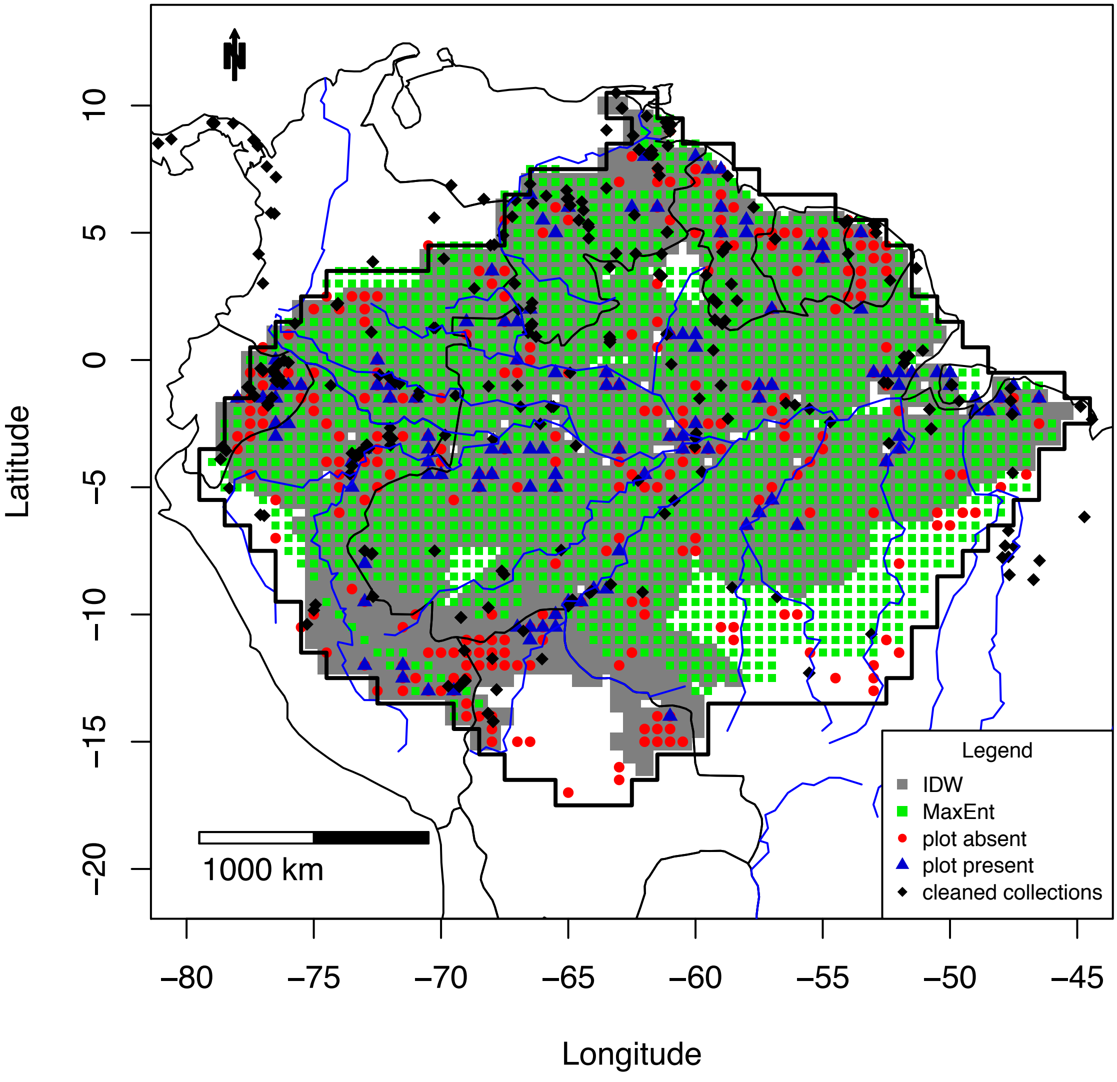

Figure S3_166B MaxEnt map of Virola_surinamensis

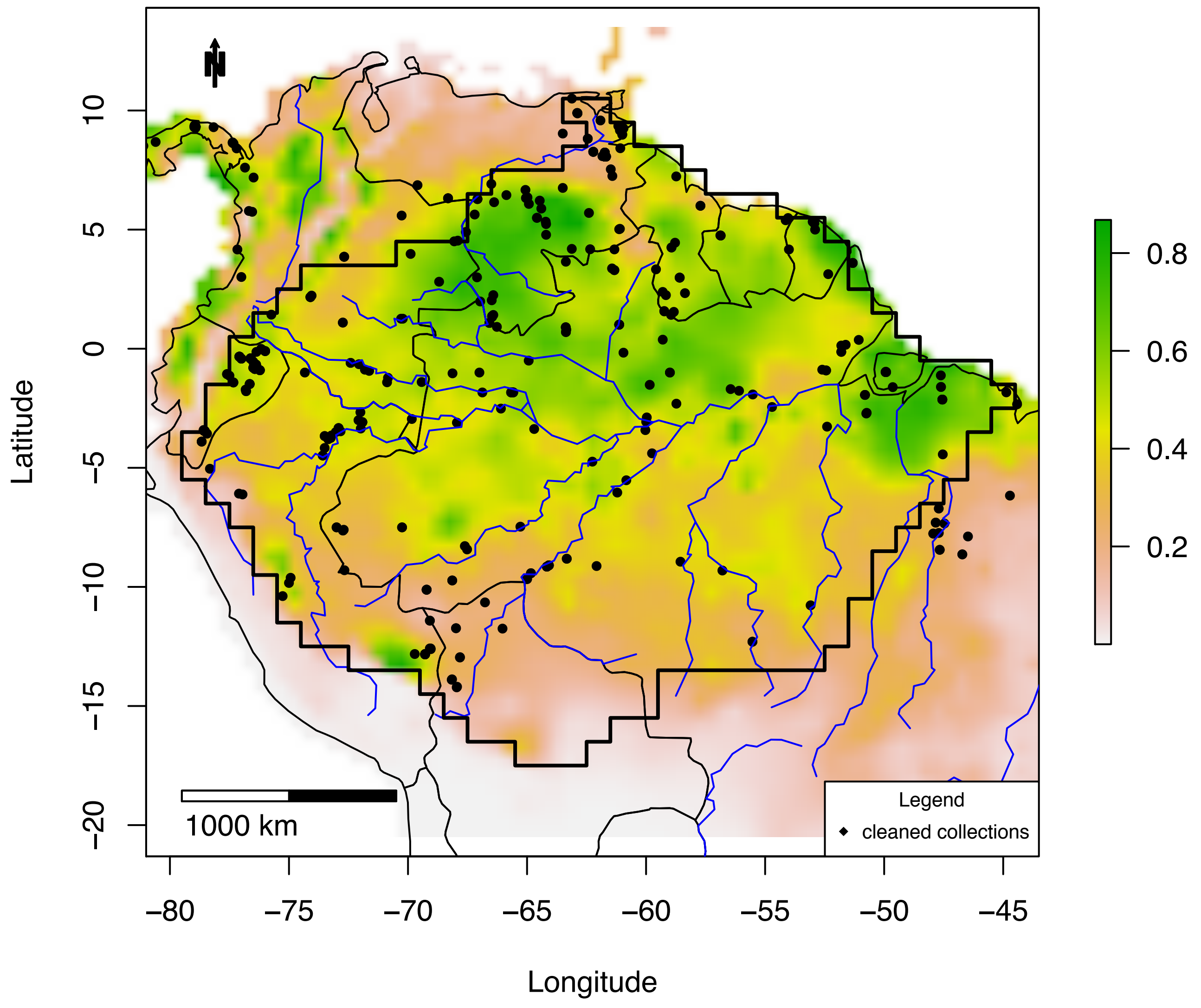

Figure S3_166C IDW map of Virola_surinamensis:
plots =0 – 0.39; IDW: 0 – 0.09

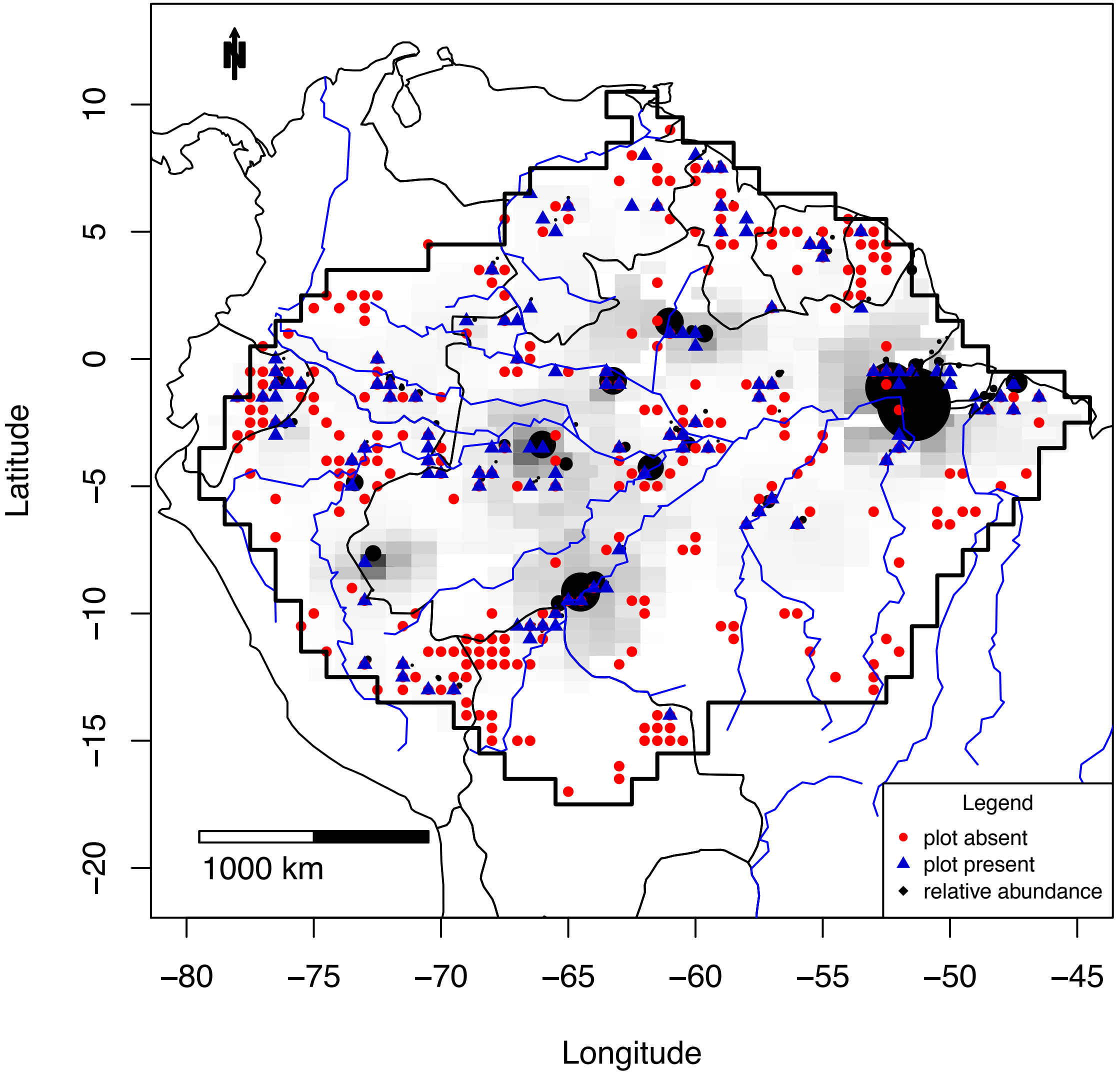

Figure S3_167A Combined map of Vouacapoua_americana

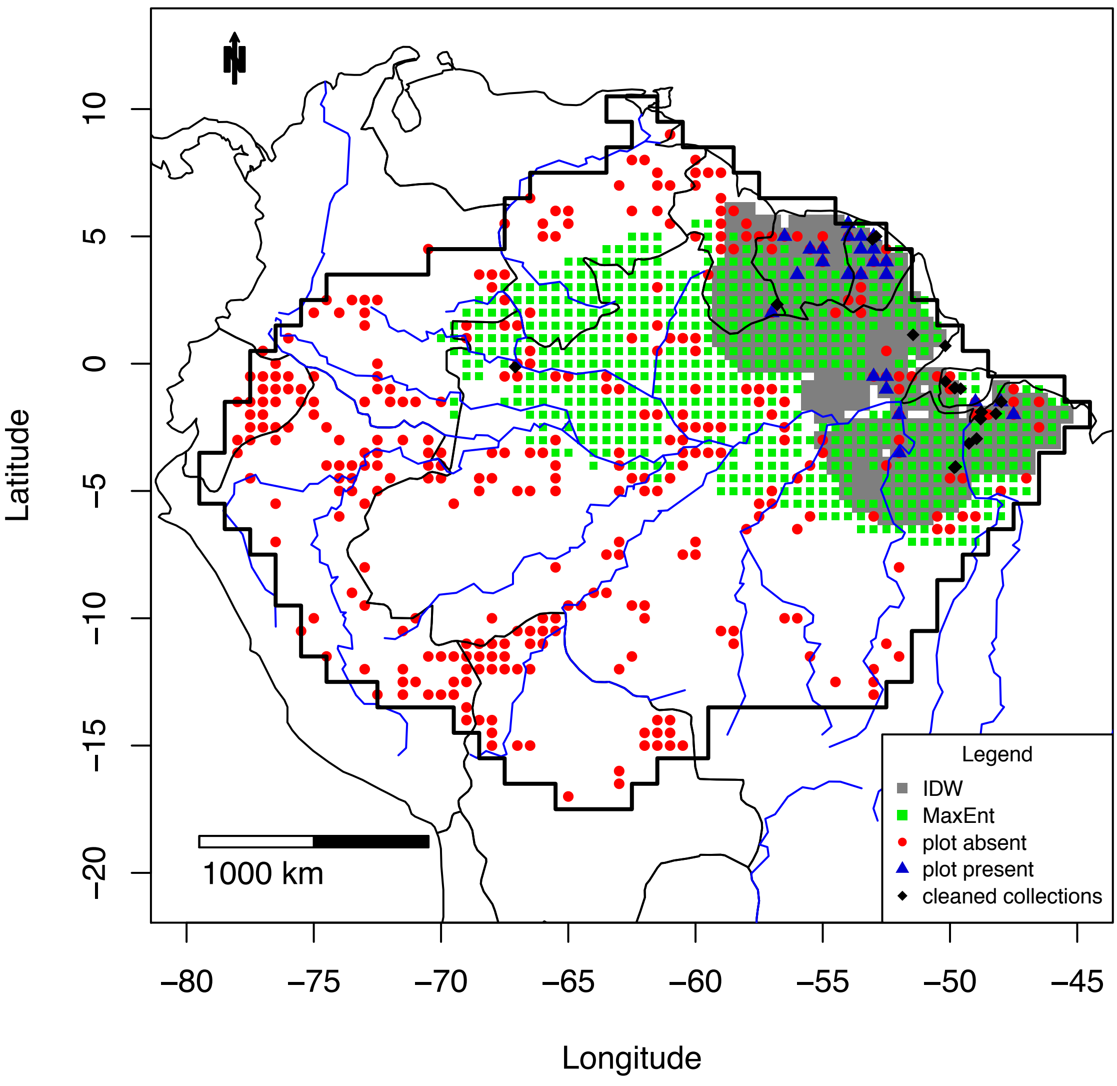

Figure S3_167B MaxEnt map of Vouacapoua_americana

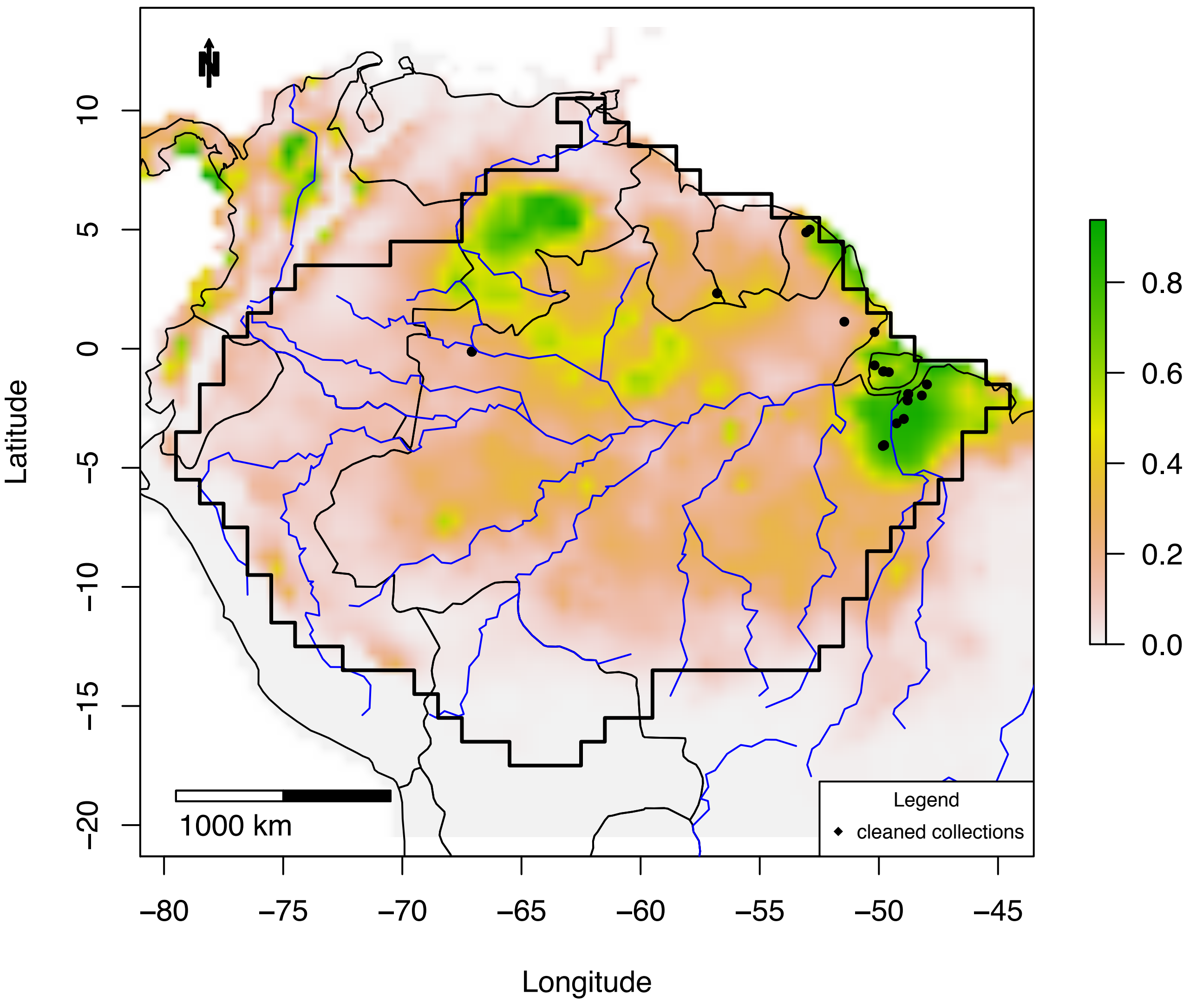

Figure S3_167C IDW map of Vouacapoua_americana: plots =0 – 0.08; IDW: 0 – 0.03

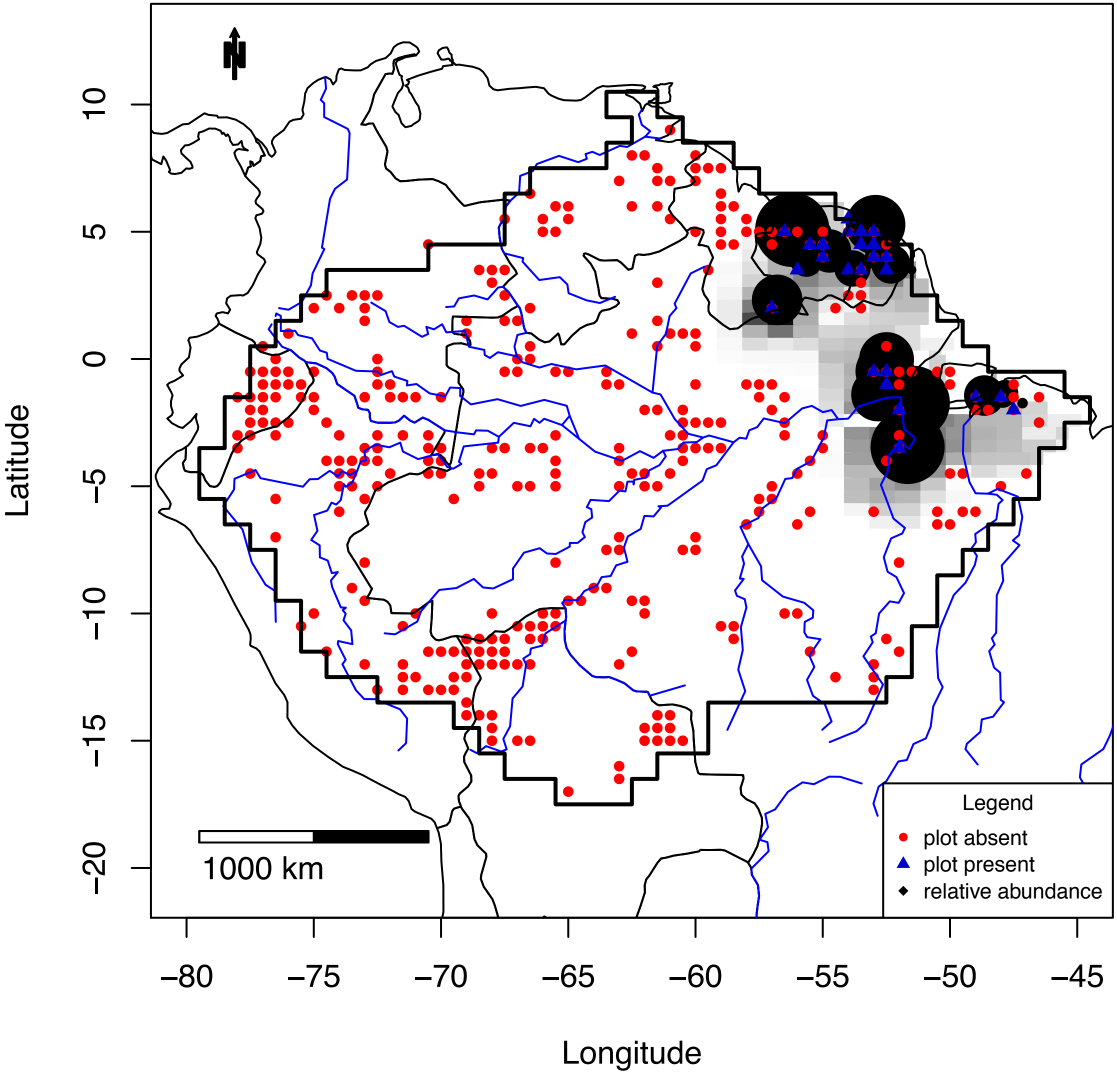

Figure S3_168A Combined map of Xylopia_amazonica

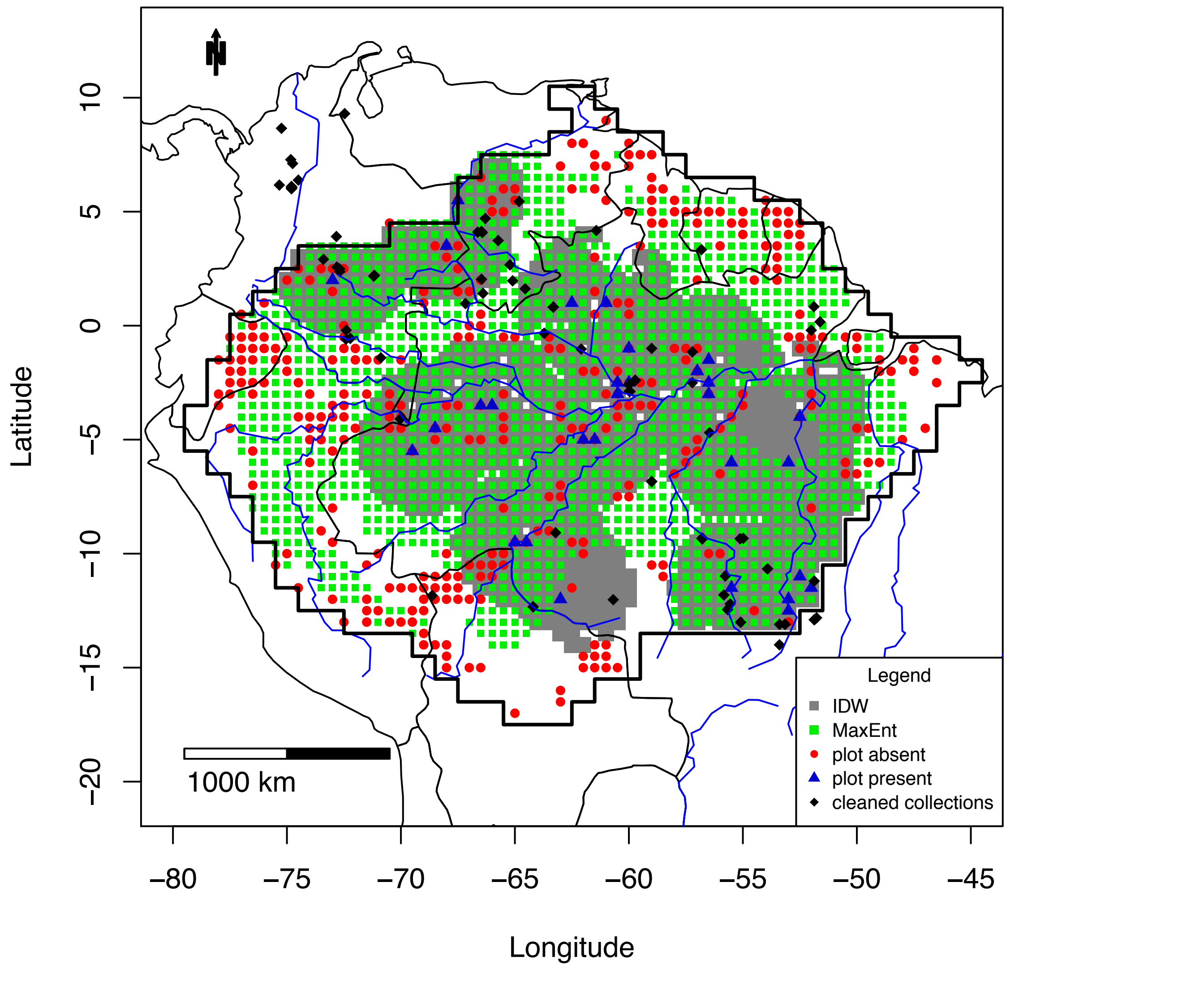

Figure S3_168B MaxEnt map of Xylopia_amazonica

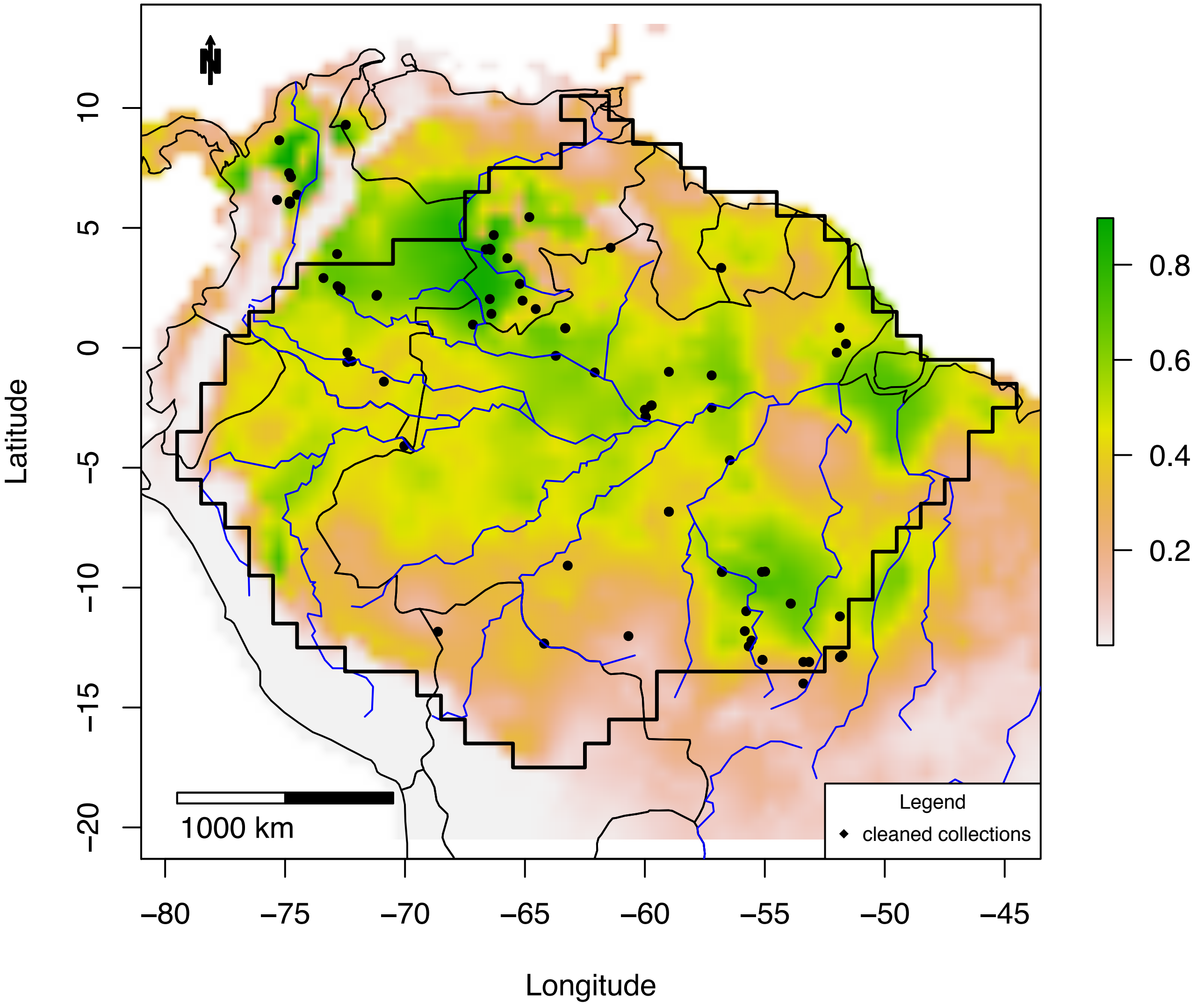

Figure S3_168C IDW map of Xylopia_amazonica: plots =0 – 0.13; IDW: 0 – 0.06

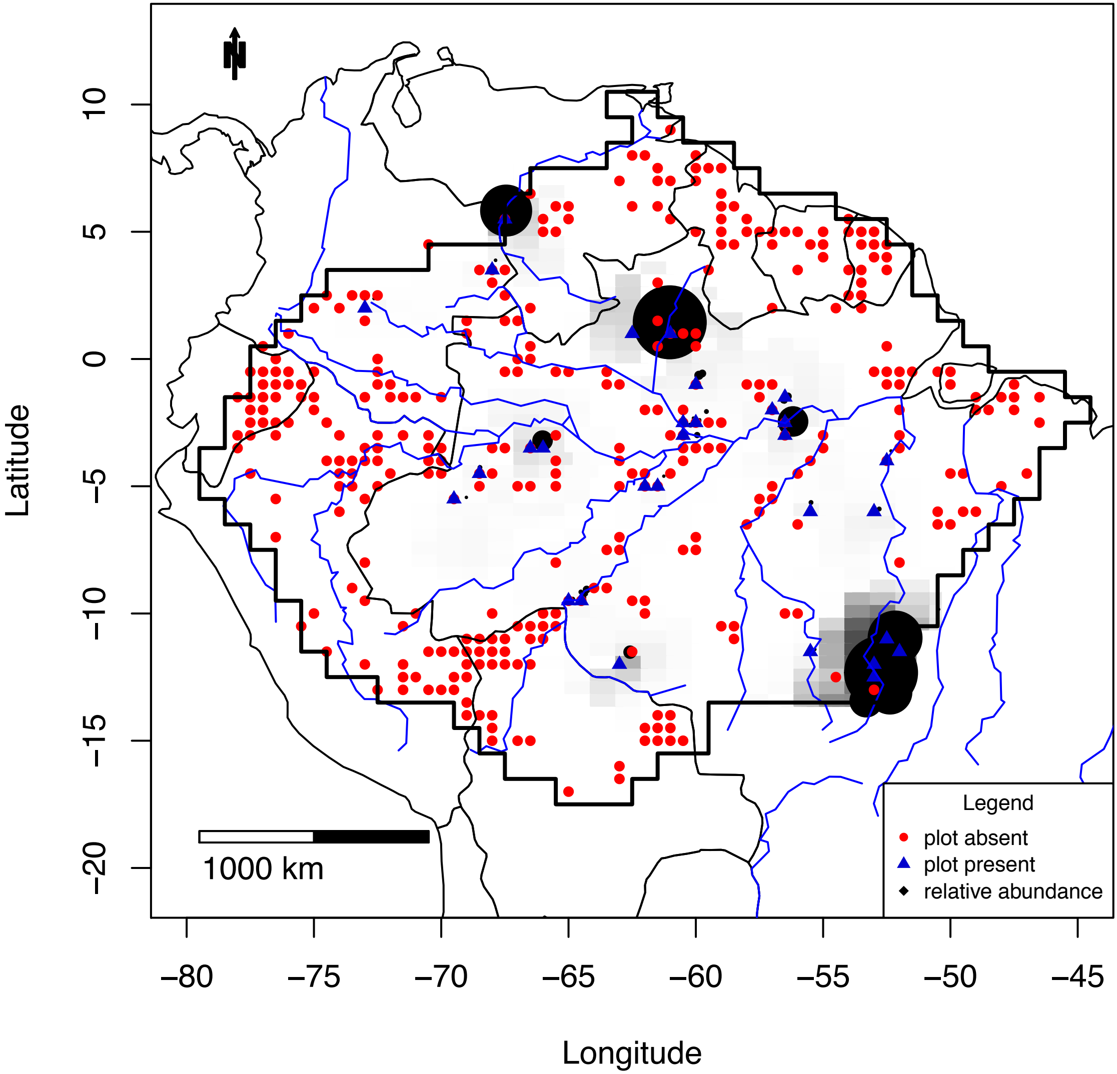

Figure S3_169A Combined map of Zygia_cataractae

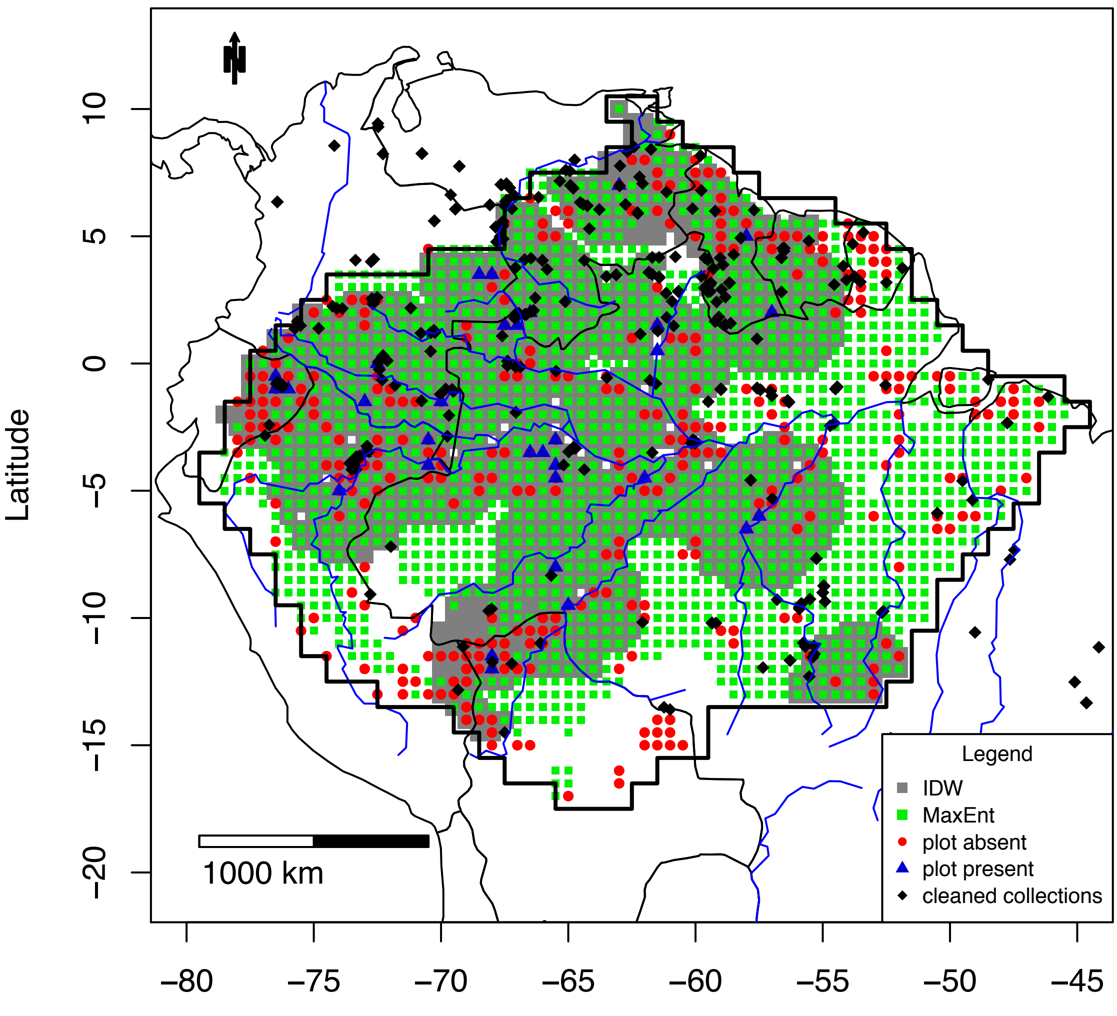

Figure S3_169B MaxEnt map of Zygia_cataractae

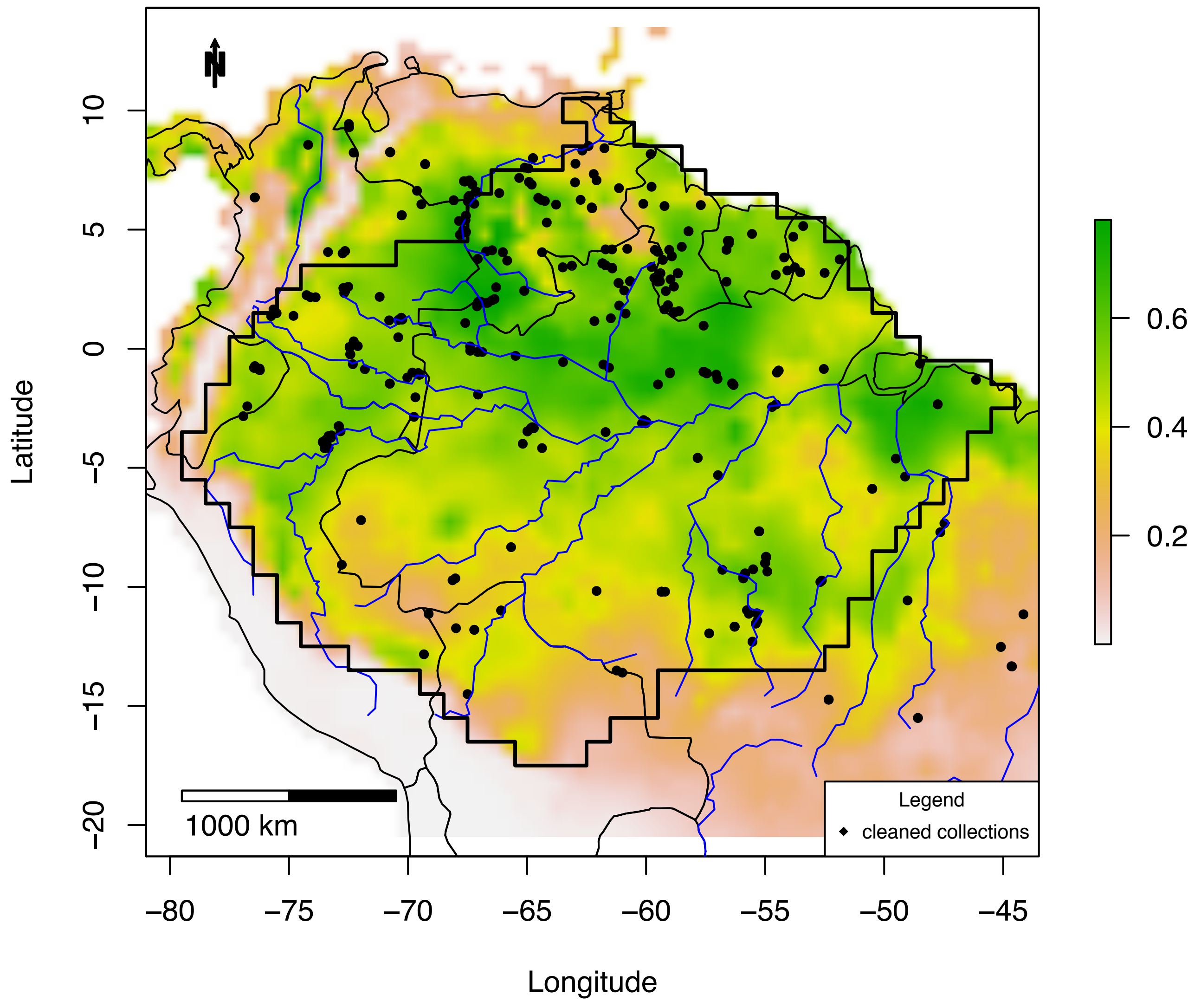

Figure S3_169C IDW map of Zygia_cataractae: plots =0 – 0.23; IDW: 0 – 0.04

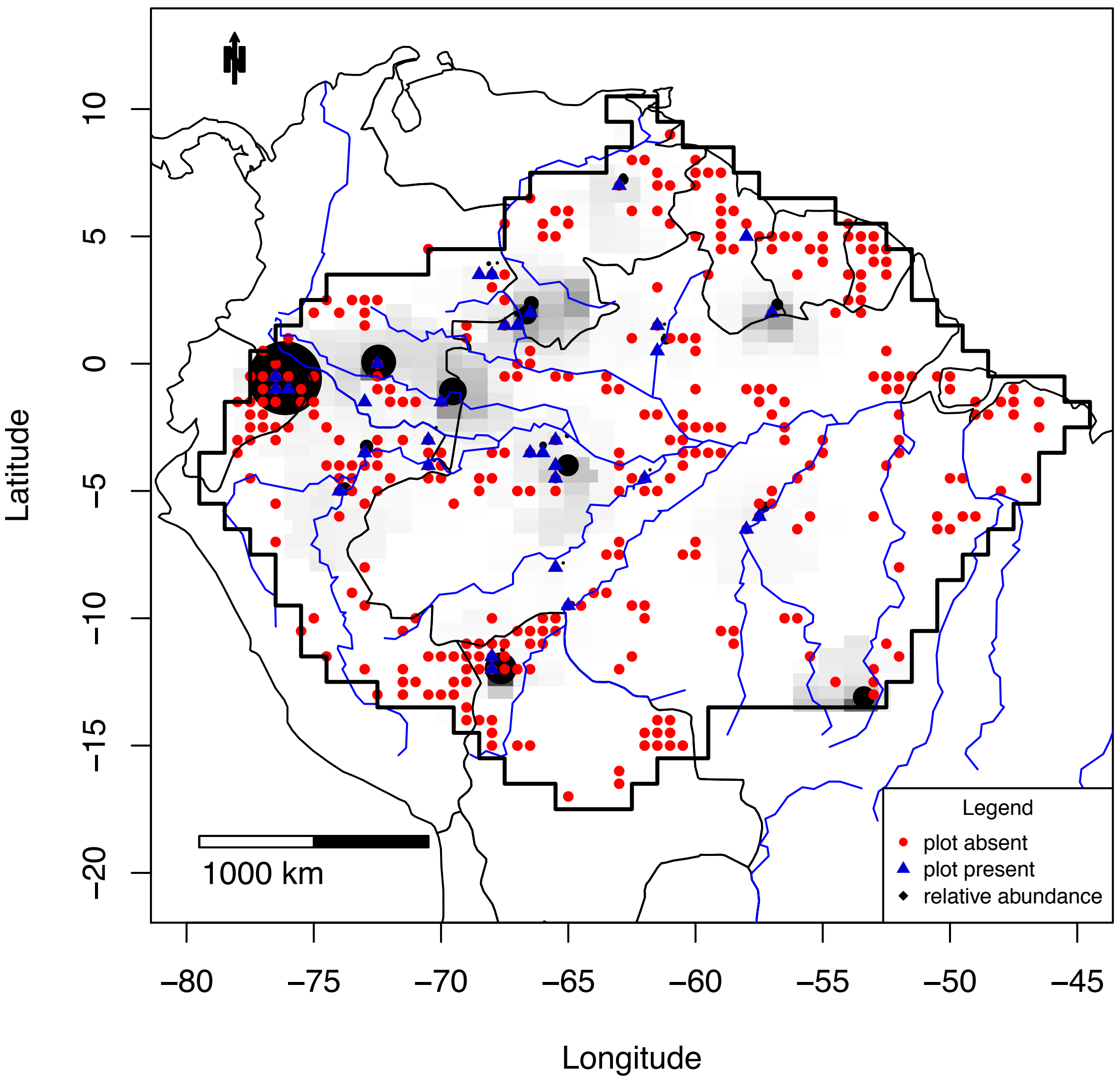

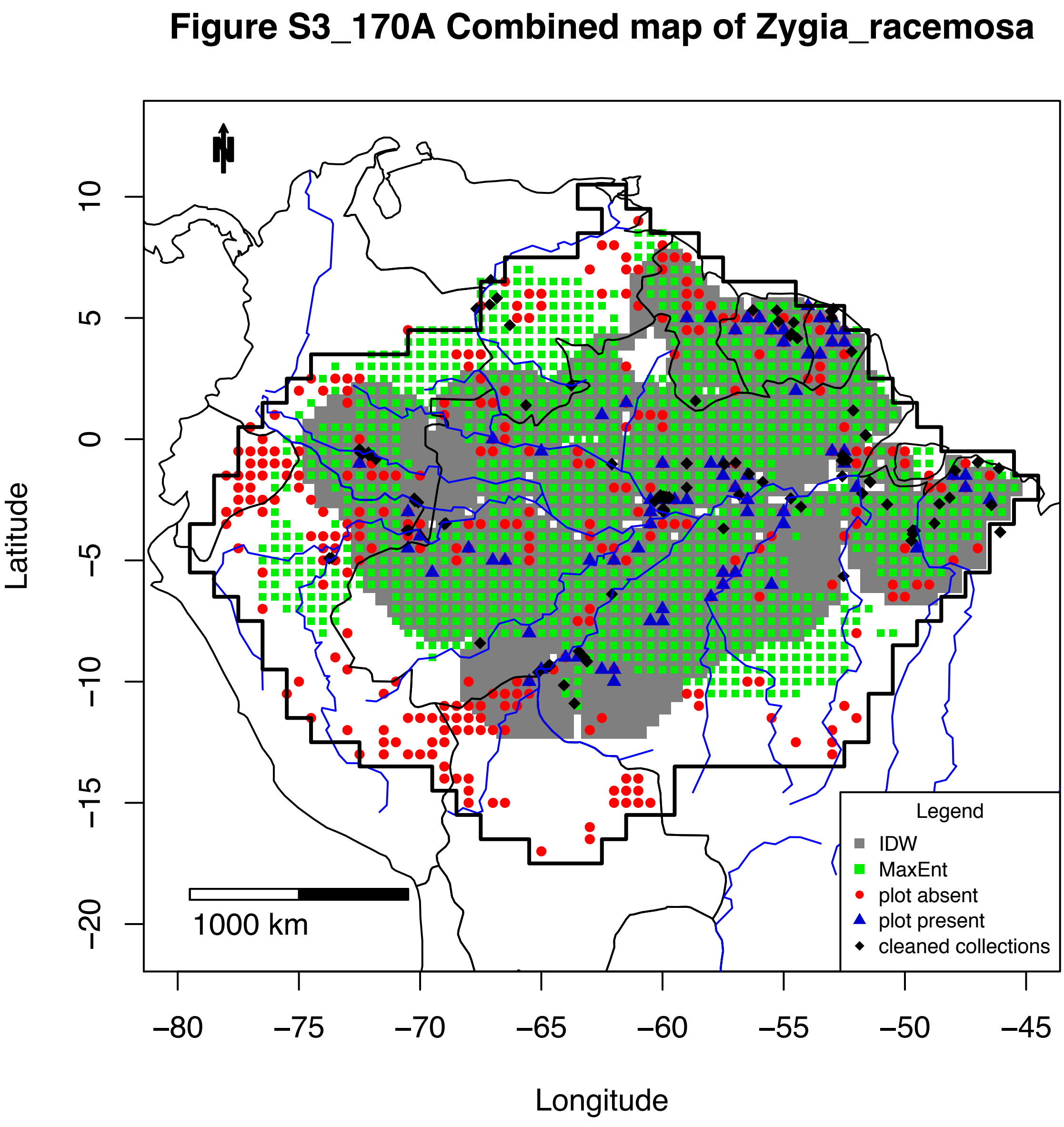

Figure S3_170A Combined map of Zygia_racemosa
N
10
5
0
−5
−10
−15
−20
Latitude
1000 km
−80
−75
−70
−65
−60
−55
−50
−45
Longitude
Legend
IDW
MaxEnt
plot absent
plot present
cleaned collections

Figure S3_170B MaxEnt map of Zygia_racemosa

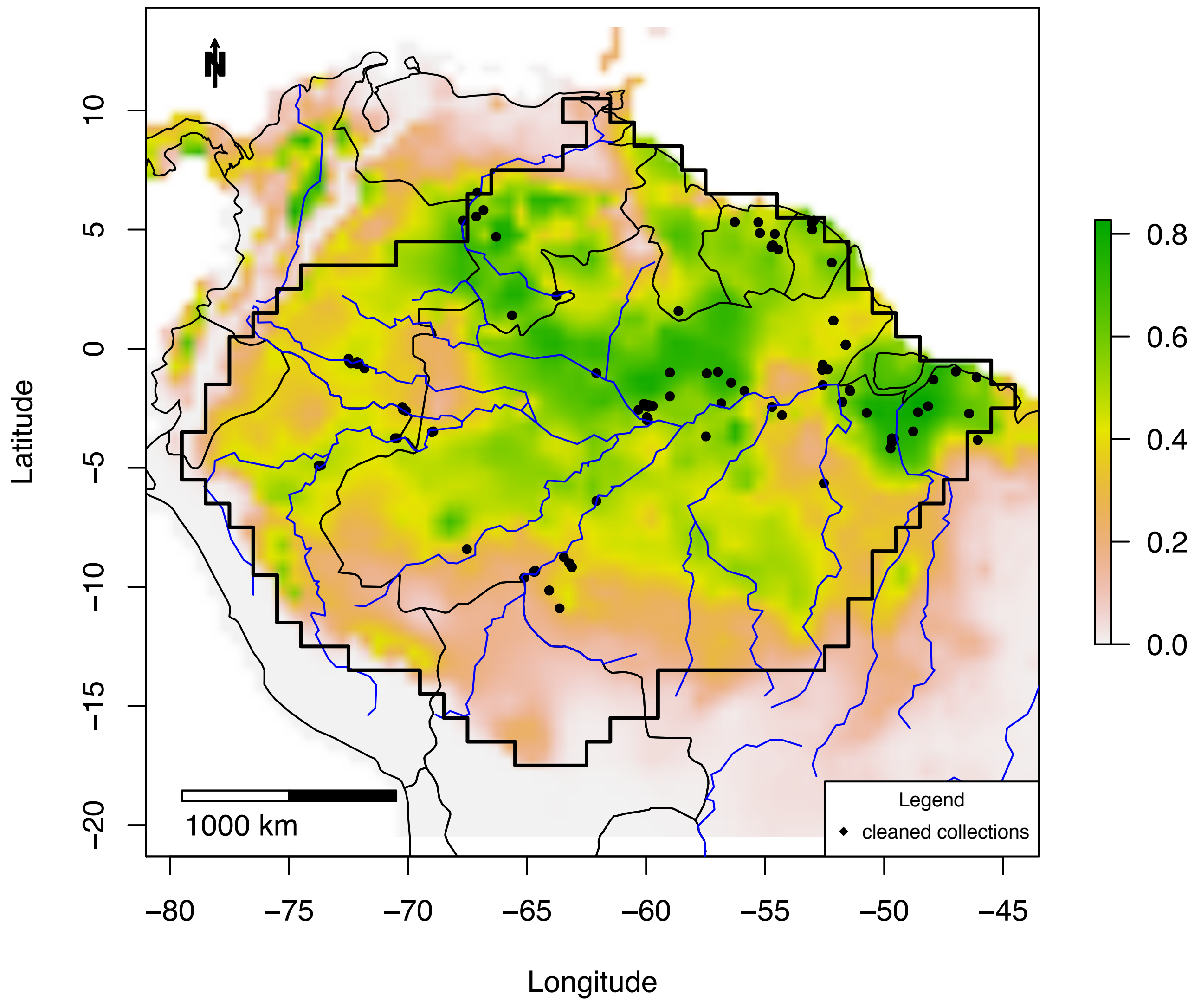

**Figure S3_170C IDW map of Zygia_racemosa:
plots =0 – 0.05; IDW: 0 – 0.03**

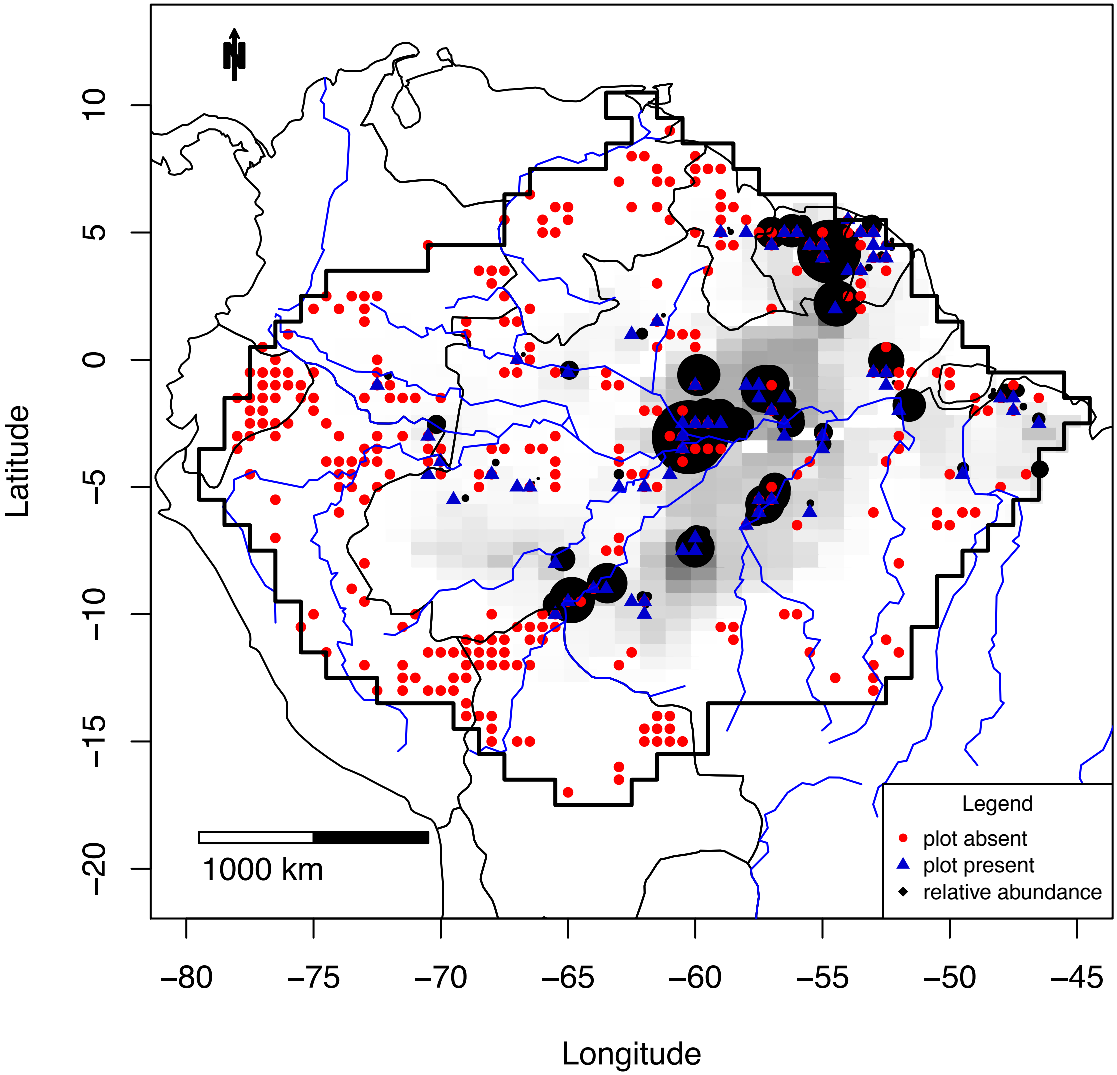

Supplement: Supplementary file 3 — Maps of combined MaxEnt IDW predictions. [file 41598_2017_18927_MOESM3_ESM.pdf]
